# Multiplex Imaging and Cellular Target Identification of Kinase Inhibitors *via* an Affinity-Based Proteome Profiling Approach

**DOI:** 10.1038/srep07724

**Published:** 2015-01-12

**Authors:** Ying Su, Sijun Pan, Zhengqiu Li, Lin Li, Xiaoyuan Wu, Piliang Hao, Siu Kwan Sze, Shao Q. Yao

**Affiliations:** 1 Department of Chemistry, National University of Singapore, 3 Science Drive 3, Singapore 117543; 2 School of Biological Sciences, Nanyang Technological University, 60 Nanyang Drive, Singapore 637551

## Abstract

MLN8237 is a highly potent and presumably selective inhibitor of Aurora kinase A (AKA) and has shown promising antitumor activities. Like other kinase inhibitors which target the ATP-binding site of kinases, MLN8237 might be expected to have potential cellular off-targets. Herein, we report the first photoaffinity-based, small molecule AKA probe capable of both live-cell imaging of AKA activities and *in situ* proteome profiling of potential off-targets of MLN8237 (including AKA-associating proteins). By using two mutually compatible, bioorthogonal reactions (copper-catalyzed azide-alkyne cycloaddition chemistry and TCO-tetrazine ligation), we demostrate small molecule-based multiplex bioimaging for simultaneous *in situ* monitoring of two important cell-cycle regulating kinases (AKA and CDK1). A broad range of proteins, as potential off-targets of MLN8237 and AKA's-interacting partners, is subsequently identified by affinity-based proteome profiling coupled with large-scale LC-MS/MS analysis. From these studies, we discover novel AKA interactions which were further validated by cell-based immunoprecipitation (IP) experiments.

Cell division (mitosis) is a tightly regulated event closely controlled by a variety of protein kinases, including Aurora kinases^1^. Aurora kinase A (AKA) is one of the three mammalian serine/threonine protein kinases belonging in the Aurora kinase family, together with Aurora B (AKB) and Aurora C (AKC). All three kinases have in recent years generated significant interest in cancer research due to their elevated expression profiles in many human cancers^2^. AKA in particular, has received much attention because of its essential role in centrosome maturation and separation, bipolar spindle assembly and chromosome alignment during mitosis^2^,^3^. A variety of small-molecule Aurora kinase inhibitors have been developed with varying degrees of success, many of which have either gone through or are at present in different stages of clinical trials^4^. VX680, the first Aurora kinase inhibitor that entered clinical trials, had promising tumor-suppressing activities in several animal models^5^. Due to its significant cross-reactivity against AKB, AKC and other protein kinases^5^,^6^, the compound showed unacceptable cardiotoxicity and was discontinued^4^. MLN8054 is a potent and selective AKA inhibitor, and possessed good antitumor activities in early clinical trials^7^. But it was discontinued due to unexpected side effects^4^. MLN8237, an improved analogue of MLN8054, is by far the most actively pursued AKA inhibitor in clinical trials^4^,^8^. The compound demonstrated promising tumor-suppressing activities in a number of phase II clinical trials^9^, and it has entered phase III recently^4^. Notwithstanding, critical off-target identification of this compound both *in cellulo* and in animal models has not been comprehensively carried out, which might eventually render it unsuitable as a drug. As an inhibitor targeting the ATP-binding site of AKA, MLN8237, like many other kinase inhibitors, might inhibit multiple cellular off-targets, as the ATP sites in most human kinases are homologous^10^. In order to study potential cellular off-targets of a kinase inhibitor, including MLN8054, recent efforts have focused on high-throughput screening (HTS) using large panels of recombinant kinases as well as mass spectrometry (MS)-based, proteome-wide chemical profiling methods^11^,^12^,^13^. Most of these methods, however, could not directly detect kinase-drug interaction *in situ* (i.e. in living cells, not lysates)^14^. Recently, small-molecule, cell-permeable probes have been developed, enabling direct target identification at the proteome-wide level inside living cells^15^. In some cases, even proteins that associate with the intended cellular target may be positively identified^16^. Such an “*in situ* drug-profiling” approach is applicable to compounds that form either irreversible or reversible complexes with their targets^17^,^18^,^19^,^20^,^21^,^22^,^23^,^24^,^25^,^26^,^27^,^28^. We previously showed that, by using a cell-based proteome profiling approach, Orlistat™ (an FDA-approved anti-obesity covalent drug) could be made “tractable” for large-scale identification of its potential cellular off-targets^17^,^18^,^19^. Similar approaches have been adopted in the study of other irreversible bioactive compounds^20^,^21^,^22^. This approach was recently extended successfully to the study of non-covalent small-molecule drugs^23^,^24^,^25^,^26^,^27^,^28^, by adopting the well-established photo-affinity labeling (PAL) strategy in the probe design^29^,^30^. In more recent studies, we found the use of so-called “minimalist” linkers in the probe design was essential for the retention of most if not all of the drug's original biological activities, because such linkers provide minimized interference to the probe upon binding to cellular targets^27^,^28^. Similarly, small molecule-based bioimaging approaches have in recent years become increasingly available for *in situ* monitoring of a variety of proteins including enzymes^31^, but chemical proteomic strategies capable of simultaneous bioimaging and target identification of noncovalent bioactive compounds in live mammalian cells, however, are still quite rare^27^,^28^.

Herein, we report the first small molecule-based AKA probe, **MLN-2**, capable of both live-cell imaging of AKA activities and cell-based proteome profiling to identify potential off-targets of MLN8237 (Fig. 1). Our research is inspired by a recent study from Weissleder *et al.* who successfully converted MLN8054 into an AKA imaging probe^32^. From our current study, we have confirmed that, as an imaging probe, **MLN-2** performed at least as well as the probe developed by Weissleder *et al.* Together with another “minimalist” probe **PU-1** which targets CDK1^27^, we show, for the first time, small molecule-based multiplex bioimaging could be conducted for simultaneous *in situ* monitoring of different cell-cycle regulating protein kinases. We further take advantage of **MLN-2**'s *in situ* proteome-profiling capability and carry out large-scale pull-down (PD) and LC-MS/MS analysis. These combined features of **MLN-2** are used to improve the confidence level of candidate protein hits obtained from the MS experiments, and enable us to successfully identify a broad range of previously unknown, putative cellular off-targets of MLN8237. Interestingly, we also find proteins that likely associate with AKA during the cell-cycle regulation. From these studies, we have successfully discovered novel AKA interactions which are further validated by *in situ* immunoprecipitation (IP) experiments.

## Results

### Design and synthesis of MLN8237 probes

**Figure 1:**
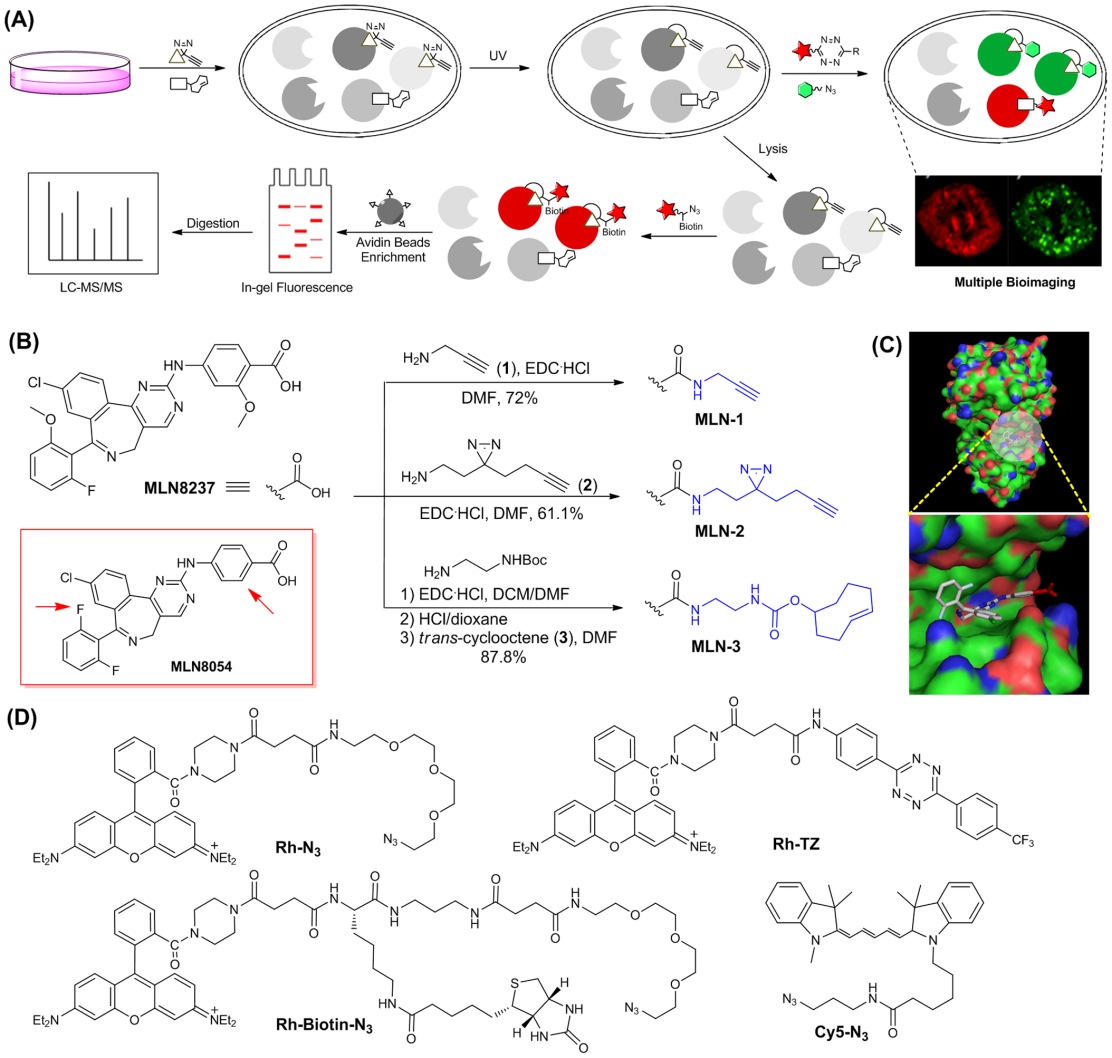
Overall workflow and structures of MLN8237 probes and reporters. (A) Overall workflow of *in situ* cell-based proteome profiling and multiplex bioimaging. (B) Synthetic scheme and structures of the three AKA probes (i.e. **MLN-1**, **MLN-2** & **MLN-3**). For comparison, the structure of MLN8054 was shown (boxed in red), in which the differences were highlighted (red arrow). (C) X-ray complex of AKA/MLN8054 (top; pdb code 2X81^33^), in which the ATP-binding pocket was highlighted (bottom). MLN8054 was shown in sticks with the CO_2_H group highlighted in red. (D) Structures of different functional reporters used in this study.

The structures of MLN8054 and MLN8237 are highly homologous, with only differences being at the two peripheral aromatic rings (i.e. arrowed in Fig. 1B). We chose MLN8237 over MLN8054 in our probe design, for the obvious reasons that (1) MLN8237 has thus far shown more favorable biological and clinical profiles^8^,^9^, and (2) MLN8237 is a more selective AKA inhibitor (thus a better candidate) for conversion into an AKA-specific imaging probe. Since the x-ray structure of the MLN8237/AKA complex was not available, we turned to MLN8054/AKA complex instead for close examination (Fig. 1C)^33^: the CO_2_H group (in red) of MLN8054 was shown to project toward the solvent-accessible surface, indicating modifications at this position with a suitable linker would minimize interference of its binding to the ATP-binding pocket of AKA. As shown in Figure 1B, **MLN-2** was obtained from MLN8237 by coupling its non-essential carboxylic acid to our recently developed “minimalist” linker (**2**), which possesses a photo-reactive diazirine moiety flanked by a terminal alkyne and a primary amine^27^. The aliphatic diazirine has advantages over other photo-reactive groups due to its small size and short UV irradiation time^29^,^30^. We used a terminal alkyne as the chemically tractable tag, as it is small thus minimizing interference of the probe upon binding to its intended protein targets. The tag is also readily amendable for subsequent cellular imaging, *in situ* proteome profiling and large-scale pull-down (PD)/target identification (by LC-MS/MS analysis) via the well-established bioorthogonal click chemistry with suitable functional reporters (Rh-N_3_, Rh-Biotin-N_3_ or Cy5-N_3_; Fig. 1D)^34^,^35^. Upon administration into live cells and photo-irradiation by UV lights, a highly reactive intermediate would be generated from **MLN-2** which would covalently capture its binding proteins in a distance-dependent manner. The chemically stable, cross-linked protein-probe complexes would thus become amenable to various downstream biochemical analyses. For comparison, we also made **MLN-1** which contains a terminal alkyne linker without the aliphatic diazirine, and **MLN-3** which contains a *trans*-cyclooctene (TCO). **MLN-3** is structurally similar to Weissleder's AKA imaging probe (Supplementary Fig. S1)^32^. and was used initially for side-by-side comparison with **MLN-2**, and subsequently for multiplex bioimaging experiments. All three probes were converted from MLN8237 through amide coupling with the corresponding linkers (**1**–**3**) in respectable yields (61–88%). A negative probe (**NP**) was also synthesized in order to remove false hits arisen from nonspecific photo-crosslinking in our LC-MS/MS experiments (Supplementary Fig. S1)^27^. The different “click” reporters (Rh-N_3_/Rh-Biotin-N_3_ & Rh-TZ; Fig. 1D) were prepared as previously described^25^,^26^,^27^,^28^, which would be conjugated to **MLN-1/2** and **MLN-3**, respectively.

### Endogenous target profiling with MLN-2 and related probes

**Figure 2:**
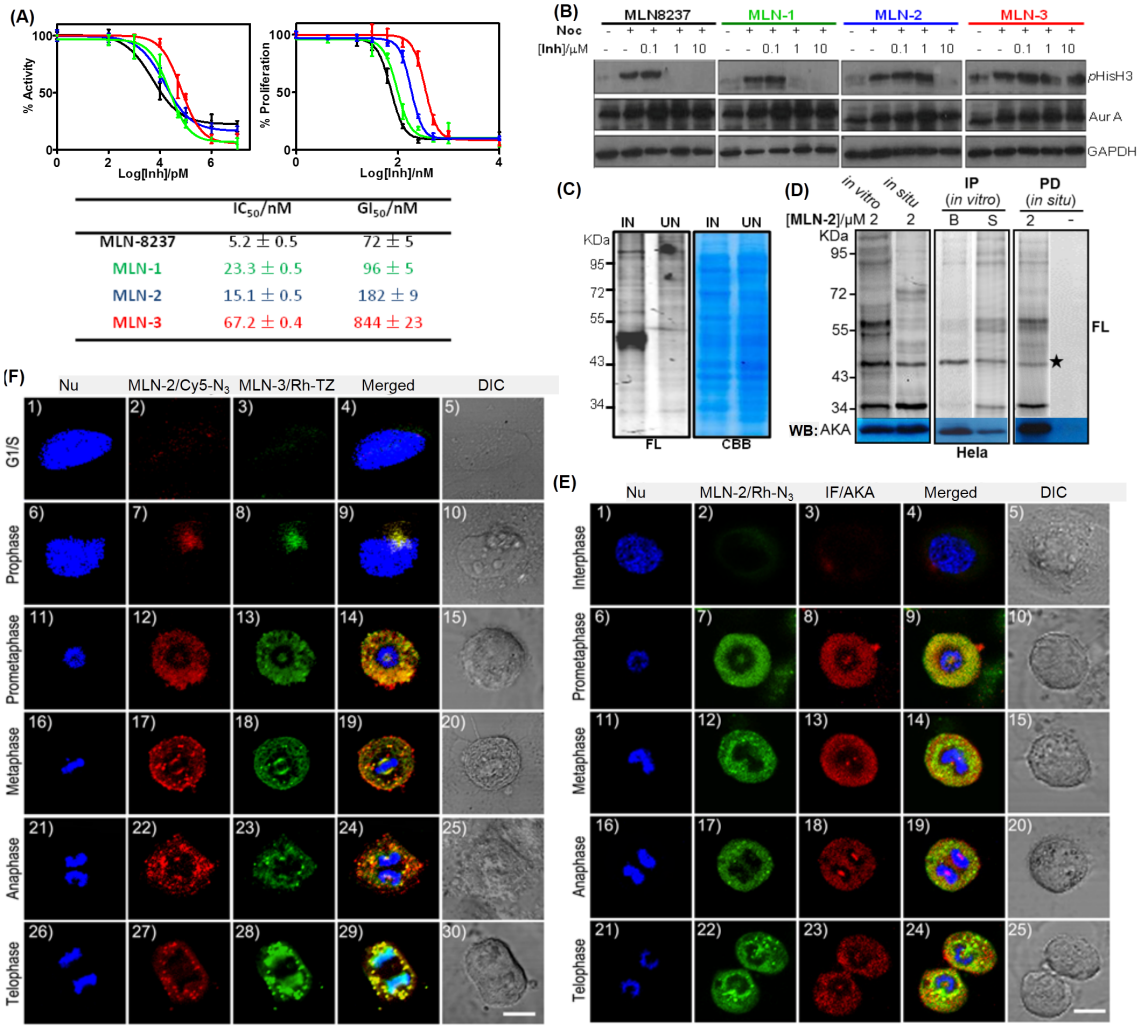
Target profiling and imaging of MLN8236 probes. (A) IC_50_ (left) and GI_50_ (right) plots and the corresponding values (bottom table) of MLN8237, **MLN-1**, **MLN-2** and **MLN-3** against recombinant AKA in a Kinase-Glo™ inhibition assay, and XTT anti-proliferation assay in HeLa cells. (B) Western blotting (WB) analysis showing dose-dependent inhibition of phosphorylation of histone H3 (Ser 10) in synchronized HeLa cells with MLN8237/**MLN-1/2/3**. Nocodazole “-”: asynchronized cells. WB of *anti*-AKA and *anti*-GADPH antibodies were used to show equal expression of AKA and equal loading in each lane. (C) Labeling of lysates from AKA-overexpressed bacterial cells (IN: IPTG-induced; UN: uninduced) using 2 μM of **MLN-2**. (left: in-gel fluorescence; right: coomassie). (D) *In vitro and in situ* labeling of synchronized HeLa cells/cell lysates with 2 μM of **MLN-2** (left), and further enriched by immunoprecipitation (IP: middle) or pull-down (PD; right). They were visualized by in-gel fluorescence scanning (FL) and Western blotting (WB) with *anti*-AKA antibody. Different reporters were used (left and middle: Rh-N_3_; right: Rh-Biotin-N_3_ The fluorescent band corresponding to the **MLN-2**-labeled endogenous AKA was detected at ~46 KD (marked with *). In the IP gels (middle), B: bead-bound fraction; S: supernatant. For negative PD, both DMSO (shown; labeled “-”) and **NP** were used in place of **MLN-2**. (E) Co-localization imaging experiments of **MLN-2**/Rh-N_3_ (green) and *anti*-AKA antibodies (red) at different stages of asynchronized mitotic HeLa cells. (F) Co-localization multiplex imaging experiments of **MLN-2**/Cy5-N_3_ (red) and **MLN-3**/Rh-TZ (green) at different stages of asynchronized mitotic HeLa cells. Scale bar = 10 μm for (E)/(F). Full-length blots are presented in Supplementary Figures.

In order to confirm the biological activities of the three probes after modification, they were compared side-by-side with their parental compound MLN8237. The inhibitory activities of MLN8237, **MLN-1**, **MLN-2** and **MLN-3** were first evaluated by standard *in vitro* kinase inhibition assays with recombinant AKA as previously reported (Fig. 2A)^33^; IC_50_ values of the three probes were measured as 23.3 ± 0.5 nM (**MLN-1**), 15.1 ± 0.5 nM (**MLN-2**) and 67.2 ± 0.4 nM (**MLN-3**), while MLN8237 showed an IC_50_ value of 5.2 ± 0.4 nM. We were particularly pleased to find that **MLN-2**, which contains a “minimalist” linker, was only 3-fold less potent than MLN8237, whereas **MLN-3** showed 13-fold increase in IC_50_ value, presumably due to its significantly bulkier TCO group. Next, the probes and MLN8237 were concurrently tested for inhibition of tumor cell growth; similar effects derived from these modifications were also observed for **MLN-2** and **MLN-3** in an XTT anti-proliferation assay with both HeLa and HCT-116 cells (Supplementary Table S1). The GI_50_ values in HeLa cells were 96 ± 5 nM (**MLN-1**), 182 ± 9 nM (**MLN-2**), and 844 ± 23 nM (**MLN-3**), while MLN8237 showed a GI_50_ value of 72 ± 5 nM. To further investigate the relative potency of **MLN-1/2/3** in inhibiting endogenous AKA activities, we tested their effects on cellular phosphorylation of histone H3 at serine 10^36^. As shown in Figure 2B, both MLN8237 and **MLN-1** completely suppressed histone H3 phosphorylation at 1 μM, while the same effect was achieved with 10 μM of **MLN-2**. For **MLN-3**, no obvious inhibition of phosphorylation was observed at 10 μM. All these lines of evidence indicated that the “minimalist” design in probe **MLN-1**/**2** could indeed effectively minimize the linker's interference to protein binding, under both *in vitro* and in-cell settings, whereas bulkier probes such as **MLN-3** should be used with caution as they might not sufficiently recapitulate genuine protein-drug interactions in native biological systems.

Next, we assessed **MLN-2**'s ability to covalently label its known endogenous cellular target, AKA (Fig. 2C & D). We started with bacteria that overexpressed AKA. Different lysates were incubated with **MLN-2** (2 μM), irradiated under UV (at 365 nm), clicked with Rh-N_3_, and analyzed by in-gel fluorescence scanning and coomassie stains. As shown in Figure 2C, **MLN-2** was able to sensitively and specifically label AKA in its over-expressed bacterial proteomes (left lane). A control bacterial proteome free of AKA activity showed no labeling (right lane). We next carried out endogenous proteome labeling by using **MLN-2** on synchronized HeLa cells under an optimal labelling concentration (2 μM; Fig. 2D); both *in vitro* (cell lysates) and *in situ* (live cells) labelling showed a strongly labeled fluorescent band at ~46 kD (left gel), which was subsequently confirmed to be endogenous AKA upon pull-down (PD) followed by western blotting (WB) analysis (bottom gels). Reciprocal experiments by first immunoprecipitation (IP) of the labeled proteome with *anti*-AKA antibody followed by in-gel fluorescence scanning clearly reaffirmed the positive labeling of endogenous AKA by **MLN-2** (right gel). Similar results were achieved with SHSY-5Y cells (Supplementary Fig. S3).

### Cellular imaging of endogenous AKA with MLN-2 and related probes

We noted that the copper-catalyzed azide-alkyne cycloaddition (CuAAC) reaction between **MLN-2** and an azide-labeled fluorophore (e.g. Rh-N_3_/Cy5-N_3_), and tetrazine ligation between **MLN-3** and Rh-TZ (Fig. 1D), are orthogonal to one another and could potentially be used in multiplex bioimaging experiments^37^,^38^. We first carried out immunofluorescence (IF) with *anti*-AKA antibody on asynchronized HeLa cells in order to visualize endogenous AKA at different stages of mitosis. We subsequently carried out IF and **MLN-3**/Rh-TZ co-localization experiments (Supplementary Fig. S4). Both results confirmed the relative expression level and localization of AKA at all five mitotic phases (e.g. interphase, prophase, metaphase, anaphase and telephase), and that the fluorescence signals obtained from the **MLN-3**/Rh-TZ channel were indeed able to colocalize reasonably with AKA at different mitotic phases, as previously reported^32^. A very low level of endogenous AKA expression during the interphase was observed.

Prolonged exposure of mammalian cells to strong UV lights is known to cause DNA damage^39^,^40^. Since **MLN-2** contains a photo-reactive group that necessitated UV activation (at 365 nm) on ice for up to 20 min, we wondered if such irradiation conditions might cause sufficient harms to cells, and lead to potential mis-translocation of AKA or even cell death. Fortunately, both XTT and FACS experiments showed that the 365-nm UV irradiation (up to 60 min) did not noticeably affect proliferation or cause apoptosis of HeLa cells (Supplementary Fig. S5A & B). It is well-known that shorter-wavelength UV lights (UVB, 280–320 nm) are much more harmful to human cells than longer-wavelength UV lights (UVA, 320–380 nm)^39^,^40^. Apparently, the UV wavelength, its power setting and the length of time used in our photo-crosslinking reactions were sufficiently mild that cell death did not occur. Mis-translocation of endogenous AKA was not detected either, and this was confirmed by co-localization imaging with **MLN-2**/Rh-N_3_ labeling and IF experiments (Fig. 2E). Briefly, asynchronized HeLa cells were seeded and incubated with **MLN-2** (2 μM) for 1 h. Upon removal of excessive probe (by washing with DMEM/PBS), the cells were UV-irradiated (at 365 nm), fixed, permeabilized, clicked with Rh-N_3_, and imaged (Fig. 2E; panels 2/7/12/17/22; pseudocolored in green). IF was subsequently performed on the same cells (panels 3/8/13/18/23; pseudocolored in red). Nuclear staining was done with Hoechest 33258 (pseudocolored in blue). Upon merging the different fluorescence channels, we were able to detect successful **MLN-2**/AKA co-localization at different phases of mitosis in HeLa cells (panels 4/9/14/19/24). Results of these co-localization experiments were also closely compared with those of IF-alone imaging results (e.g. Supplementary Fig. S4); in addition to the same low-level AKA expression during the interphase as earlier observed, AKA localization at other phases of cell cycle did not appear to change either. It should be highlighted that, although both IF and **MLN-2** were used to detect successfully endogenous AKA expression with similar resolution, they differed in some ways. As an antibody-based technique, IF offers generality and high selectivity but is only compatible with fixed cells, since antibodies rarely have adequate cell permeability. Small molecule imaging probes like **MLN-2**, on the other hand, are still not widely available and their selectivity could be limited (potential off-targets for example, *vide infra*)^25^,^26^,^27^,^32^. Nevertheless, they are drug-like molecules with excellent cell permeability, and could image cellular targets in their endogenous active state. Therefore, the entire process, from cell growth, probe treatment, click chemistry to image acquisition, could be performed under live-cell conditions if necessary. In our current protocols, we found it more convenient to use live cells up to the step before the CuAAC reaction (e.g. after the transient protein-probe interaction was permanently “fixed”). This was due to the known cytotoxicity of Cu(I) catalyst to mammalian cells, but if necessary, live cell-compatible CuAAC conditions are available and could be readily adopted^41^,^42^,^43^.

Finally, to assess whether **MLN-2** could also colocalize with **MLN-3**, and whether **MLN-2** might be suitable for multiplex bioimaging experiments, we carried out live-cell imaging experiments with **MLN-2/3** simultaneously in the same asynchronized HeLa cells by following the above-described protocol with the exception that, in this case, two orthogonal pairs of probes and reporters (e.g. Cy5-N_3_ and Rh-TZ) were added to the same live HeLa cells (Fig. 1A). This was possible because of the orthogonality between the two types of click chemistry^38^, and the use of two fluorophores that possess different excitation/emission spectra (e.g. Cy5 and Rh, respectively). As shown in Figure 2F, virtually identical colocalizations were detected at different phases of cells (panels 4/9/14/19/24/29). Again, a low level of endogenous AKA expression with both probes was detected during the interphase (panels 2/3). These results thus confirmed that, as a small-molecule AKA imaging probe, **MLN-2** performed at least as well as **MLN-3** (which is similar to Weissleder's probe^32^). Furthermore, it showed the possibility of imaging different cellular targets by using mutually compatible bioorthogonal “click” handles.

### Multiplex imaging of endogenous AKA and CDK1 activities in HeLa cells

**Figure 3:**
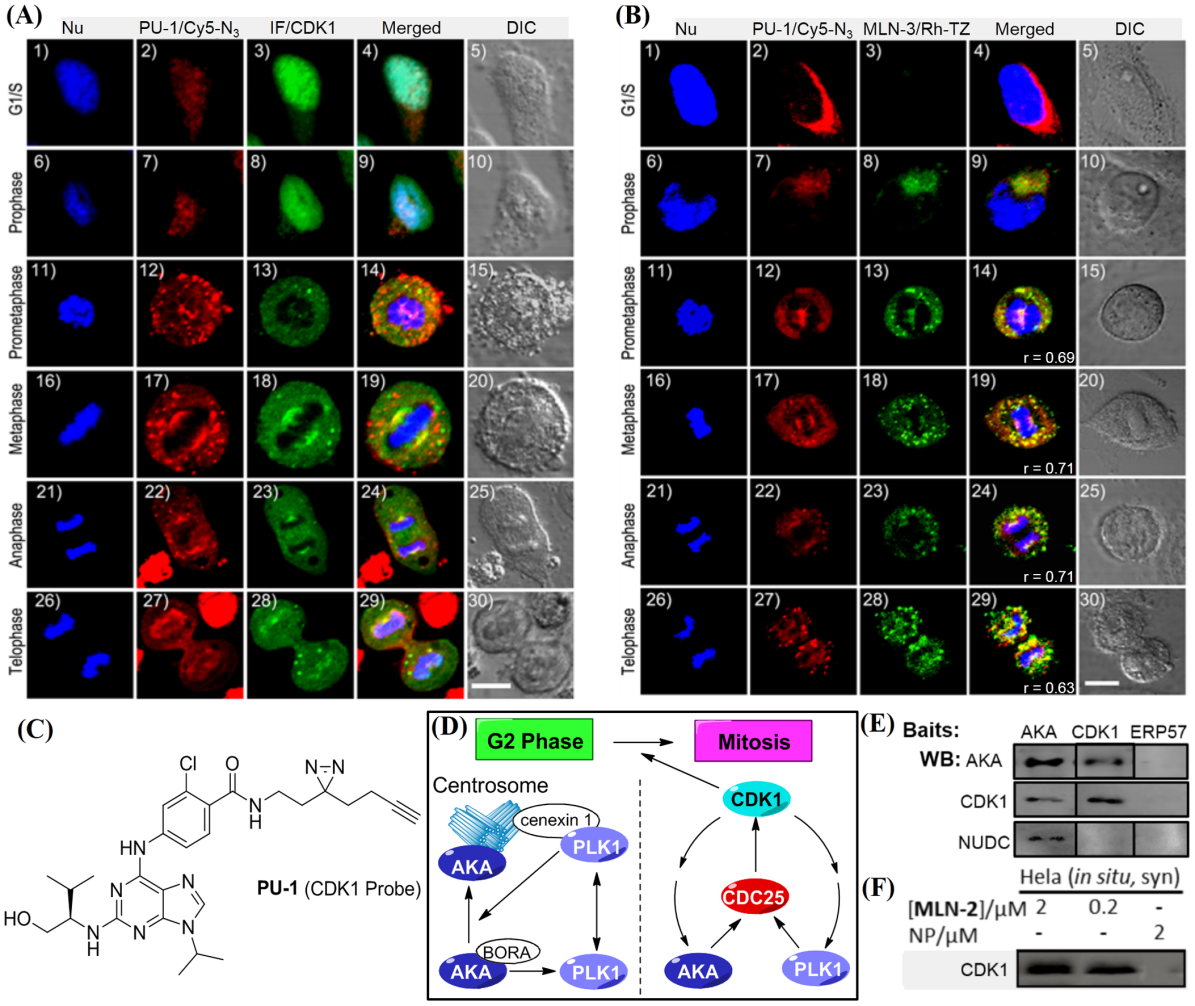
Multiplex imaging of AKA and CDK1. In (A), cells were treated with Hoechest 33258 (in blue), **PU-1**/Cy5-N_3_ (in red) and IF with anti-CDK1 antibody (in green). In (B), cells were treated with Hoechest 33258 (in blue), **PU-1**/Cy5-N_3_ (in red) and **MLN-3**/Rh-TZ (in green). Scale bar = 10 μm for (A)/(B). (C) Chemical structure of **PU-1**^27^. (D) Crosstalk network among AKA, PLK1 and CDK1 during late G2 phase or early mitosis. (E) Co-immunoprecipitation (IP) with *anti*-AKA and *anti*-CDK1 as baits to capture relevant complexes, while *anti*-ERP57 serves as negative control. Results were detected by WB. (F) *In situ* proteome labeling with synchronized HeLa cells followed by PD/WB analysis. Full-length blots are presented in Supplementary Figures.

To the best of our knowledge, few reports on small molecule-based imaging of multiple endogenous enzymatic activities are available^44^,^45^. **PU-1** is another recently reported minimalist small molecule probe, which showed affinity-based covalent labeling of endogenous CDK1 (Fig. 3C)^27^. CDK1 belongs to the family of cyclin-dependent kinases (CDKs) that play key roles in cell-cycle progression^46^. Although both AKA and CDK1 are known to be critically involved in mitosis, they perform very different tasks (Fig. 3D). As earlier mentioned, AKA is required for centrosome separation and spindle formation during mitosis, and is present on the centrosomes/spindle poles^2^,^3^. CDK1, on the other hand, is required for both G1/S and G2/M transitions upon association with cyclin A and cyclin B, respectively. In G_2_ phase, AKA-BORA initiates the activation of PLK1 (Polo-like kinase 1), which, upon being recruited to centrosome by cenexin 1 in the late G_2_ phase, promotes the localization of AKA to centrosome^47^. CDK1 is activated prior to its mitotic entry, which in turn activates both AKA and PLK1. Meanwhile, these two kinases form a positive feedback activation loop to CDK1, promoting its rapid and timely mitotic entry^48^. Since CDK1 and AKA are intimately inter-connected during mitosis, we wondered if their endogenous activities could be simultaneously imaged in a multiplex experiment, by taking advantage of the mutually compatible bioorthogonality of TCO and terminal alkyne handles in **MLN-3** and **PU-1**, respectively.

We first performed co-IF experiments (with an *anti*-CDK1 antibody) to ensure endogenous CDK1 activity could be reasonably imaged by **PU-1**/Cy5-N_3_ in HeLa cells during mitosis (Figure 3A); the **PU-1**/Cy5-N_3_ channels (pseudocolored in red) and IF channels (pseudocolored in green) colocalized fairly well in four out of five phases of mitotic cells (panels 14/19/24/29), whereas in G1/S phase and the prophase during mitosis, when chromatins condense into chromosomes and the cell nucleolus starts to disappear, we detected strong IF signals in the nucleus (panels 3/8), with comparatively weaker IF signals in the cytosol. On the other hand, **PU-1**/Cy5-N_3_ signals were mostly detected in the cytosol during these two phases (panels 2/7). This indicated that, while CDK1 was initially expressed in the cytosol, most of it was sent to the nucleus before/during the prophase to assist in mitosis. The inability of **PU-1** to accurately recapitulate the nuclear CDK1 expression in the G1/S phase and the prophase during mitosis might be caused by the poor permeability of Cy5-N_3_ in crossing the nuclear membrane. Notwithstanding, **PU-1** was able to reasonably image endogenous CDK1 activity during most of the mitotic phases.

Next, we performed multiplex imaging experiments with **PU-1**/Cy5-N_3_ and **MLN-3**/Rh-TZ combinations (Fig. 3B). Interestingly, reasonable colocalization patterns were observed for signals detected in both **PU-1** and **MLN-3** channels during all five mitotic phases (panels 9/14/19/24/29 in Fig. 3B), with Pearson's coefficient *R* values ranging from 0.63 to 0.71. This indicates that during mitosis, most endogenously active AKA and CDK1 were nuclear-localized and might be associating with each other, possibly via the formation of some higher-order macromolecular complexes that might involve other proteins. As earlier mentioned, previous studies showed the existence of a feedback loop between CDK1 and AKA during mitosis (Fig. 3D)^48^. Our imaging results show their association might be necessary in order to ensure proper cell division throughout the entire mitotic process. On the other hand, in the G1/S phase prior to cell division, when cells grow and DNAs/proteins are continuously synthesized, the association of these two kinases did not appear necessary, with each likely playing a distinctly different role. This was evident by their different endogenous expression/localization profiles (panel 4 in Fig. 3B); a low level of endogenous AKA activity was detected, whereas a much stronger CDK1 expression was shown in both the IF and **PU-1**/**MLN-3** imaging experiments. While the precise roles of AKA and CDK1 during cell cycle regulation, in terms of both expression level, sub-cellular localization and possible association, need to be further investigated (e.g. by other biological, cellular experiments or more advanced microscopic techniques), our results herein show, for the first time, the potential of small molecule probes for live-cell imaging of different endogenous kinases activities at up to sub-organelle spatial resolution, thus providing a relatively rapid and convenient means to detect the possible presence of macromolecular complexes.

### Cell-based proteome profiling of potential MLN-2 targets

The current limit in spatial resolution for most optical microscopic techniques, including confocal microscopy, is around 200 nm, due to the limit of light diffraction. Emerging techniques such as super-resolution microscopy can improve this limit by 10 folds, e.g. down to ~20 nm resolution, which is still insufficient to directly image the association of individual proteins^49^. Therefore, techniques such as Förster resonance energy transfer (FRET) are used to image protein-protein interactions (PPIs) in living cells^50^. In recent years, other *in vitro*-based methods such as affinity purification coupled to mass spectrometry have been developed for large-scale proteome-wide studies of PPIs^51^. Such techniques can be viewed as complementary to imaging-based techniques in that, instead of spatial resolution, they can achieve better resolution at molecular level by relying on affinity purification of an entire non-covalent protein complex, followed by MS deconvolution of individual proteins. As a cell-permeable, small molecule probe, the obvious advantage of **MLN-2** lies in its ability to convert a transient protein/drug interaction (via UV-irradiation) into a covalent complex in live cells, prior to subsequent *ex vivo* affinity enrichment (which could then be carried out under highly stringent conditions) and LC-MS/MS analysis^15^,^29^,^30^. In other words, the MS-enabled proteome profiling capability of **MLN-2** has equipped it with additional resolving power normally unattainable with direct imaging-based techniques. In addition to being able to image endogenous kinase activity at sub-organelle spatial resolution, **MLN-2** may be used to identify not only its potential cellular off-targets, but also the possible formation of macromolecular complexes of such proteins.

In our earlier proteome labeling profiles (Fig. 2D), additional fluorescent bands were observed in both *in vitro* and in *in situ* labeling lanes, which clearly indicates the presence of potential MLN8237 off-targets (or AKA-binding proteins). Furthermore, several strongly labeled protein bands were detected in the *in vitro* but not in the *in situ* experiments (Fig. 2D left gel), which were also observed in SHSY-5Y cells (Supplementary Fig. S3). This underscores the importance of *in situ* labeling approach for identification of potentially true cellular targets of MLN8237^25^. Further *in situ* proteome labeling followed by affinity enrichment also showed that, in addition to AKA, other cellular proteins were positively labeled, and in some cases, these labeled proteins were cell line-specific (Fig. 2D and Supplementary Fig. S3)^27^. In order to identify them, we carried out large-scale pull-down (PD)/LC-MS/MS analysis of different cell lines labeled by **MLN-2** under different proteomic conditions. Since the endogenous AKA expression levels changed significantly during different stages of cell cycle, we also carried out similar analysis with both synchronized (Syn) and asynchronized (Asyn) cells. Briefly, after probe incubation and UV irradiation, the labeled cellular proteome was collected, clicked with Rh-Biotin-N_3_, enriched with avidin beads and then denatured, separated on SDS-PAGE followed by in-gel tryptic digestion and LC-MS/MS analysis (see SI for a full list of proteomic conditions and results). Negative PD//LC-MS/MS was performed concurrently with cells labeled by **NP** under identical conditions^27^. We further took advantage of the imaging capability of **MLN-2**, which inferred only nuclear-localized proteins were likely targets of the probe^28^. Therefore the resulting candidate list was checked against Human Protein Reference Database. At the end, a total of 32 nuclear proteins were identified as the most likely off-targets of MLN8237, some of which might be potential AKA-associating proteins (Table 1). We also carried out similar large-scale PD/LC-MS/MS experiments and data analysis with **PU-1**. Interestingly, 21 out of the 32 targets identified by **MLN-2** were found by **PU-1**, lending further support to our earlier imaging results, as well as our hypothesis that, during mitosis, AKA and CDK-1 might form higher-order macromolecular complexes that involve many other proteins.

**Figure 4:**
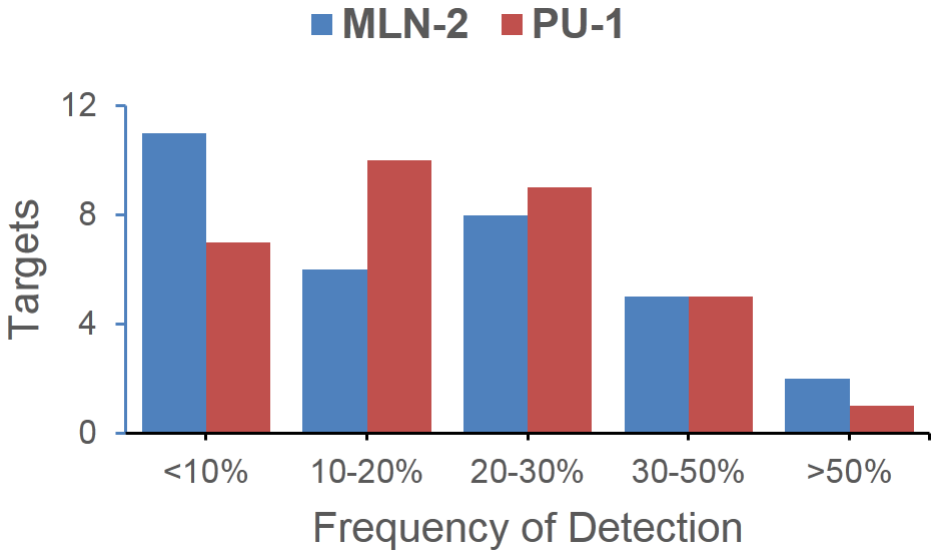
Frequency-of-Detection analysis of putative targets identified from MLN-2 and PU-1 labeled proteomes^52^. Most putative targets summarized in Table 1 were shown to be infrequently detected proteins.

Due to the intrinsic sample preparations and instrument limits, false positives from large-scale LC-MS/MS experiments could only be minimized, but not eliminated entirely^52^. We therefore further examined possible protein contaminations by cross-checking our results in Table 1 against the CRAPome database (Contaminant Repository for Affinity Purification^52^; see Figure 4); for example, of the list of **MLN-2** labeled candidate proteins, most were infrequently detected (<30%) in standard large-scale MS experiments, thus suggesting they were likely high-confidence potential off-targets of MLN8237 or AKA-associating proteins. We were particularly interested in one candidate hit, NUDC, which appeared in **MLN-2** but not **PU-1** labeled proteomes, indicating it might be specifically associated with AKA, but not CDK1. As a nuclear migration protein, NUDC plays multiple roles in mitosis and cytokinesis, including microtubule organization, spindle function and chromosome congression^53^. NUDC is also known to be phosphorylated by PLK1 and regulates PLK1's localization at kinetochore to promote chromosome congression^54^,^55^. Because of its close connection to PLK1 which is also engaged in the same mitotic network involving both AKA and CDK1 (Fig. 3D), we wondered whether NUDC, together with CDK1, might be key partners of our earlier hypothesized higher-order AKA-associating complexes formed during mitosis. We therefore carried out co-immunoprecipitation (IP) experiments with synchronized HeLa cells. Upon incubation of cell lysates immobilized separately with *anti*-AKA, *anti*-CDK1 and *anti*-ERP57 antibodies, the bead-bound protein complexes were further analyzed by WB with *anti*-AKA, *anti*-CDK1 and *anti*-NUDC antibodies, respectively (Fig. 3E); we found both AKA and CDK1 were reciprocally co-precipitated, a clear indication that these two proteins directly formed tight non-covalent complexes with each other. This result was further validated by affinity enrichment of the **MLN-2** labeled proteome followed by WB analysis with *anti*-CDK1 antibody (Fig. 3F); positive labeling of endogenous CDK1 by **MLN-2** was detected at both 2 and 0.2 μM probe concentrations. Interestingly, NUDC was co-precipitated with AKA but not with CDK1, indicating that although NUDC was part of the AKA macromolecular complex during mitosis, it directly bound to AKA but not CDK1. This result also agreed with our earlier MS data which indicated **PU-1** didn't label NUDC directly. As a negative control in the co-IP experiment, *anti*-ERP57 antibody failed to immunoprecipitate any of these three proteins.

## Discussion

In conclusion, based on a clinically important Aurora A kinase inhibitor MLN8237, we have successfully developed small molecule probes capable of both live-cell imaging of endogenous AKA activity during different phases of mitosis, and *in situ* cell-based proteome profiling for identification of potential off-targets of MLN8237 and AKA-associating proteins. From the current study, we showed the terminal alkyne-containing diazirine-based probe, **MLN-2**, could be used to image endogenous AKA activity at least as well as a TCO-based probe, **MLN-3**. By taking advantage of two mutually compatible bioorthogonal reactions (CuAAC and TCO-tetrazine ligation), we have demonstrated the first small molecule-based, multiplex bioimaging experiments for simultaneous monitoring of two key cell cycle-regulating kinases within the same mammalian cells during mitosis. Such experiments have revealed the possible formation of macromolecular complexes between AKA and CDK1 during cell division, and the results were subsequently supported by a series of studies, including cell-based proteome profiling, large-scale PD/LC-MS/MS analysis, PD/WB analysis of probe-labeled proteomes, and *in situ* co-immunoprecipitation experiments. Most of these experiments were made possible by the unique, dual capability of the probe design.

At present, we can conclude that, while some of the protein candidates identified from our putative list of potential endogenous MLN8237 targets would inevitably be false positives due to the intrinsic limits of large-scale PD/LC-MS/MS experiments^52^, many might indeed be real off-targets of MLN8237. Some of these proteins are currently under further investigation with other independent experiments, and will be reported in due course. In the current study, we had taken a particular interest in looking closely at potential AKA-associating proteins during mitosis, as revealed by the small molecule imaging and cell-based proteome profiling results. We have unequivocally confirmed that this higher-order complex likely involved CDK1 and NUDC. Their interactions with MLN8237, either directly or indirectly, might have previously evaded detection, because they were either too weak to form a stable isolable complex under *in vitro* settings, or cellular environment-dependent, that is, their interaction might be subcellular localization-dependent which is not easily recapitulated under *in vitro* conditions^51^. This again highlights the key advantage of our cell-based proteome profiling approach over other *in vitro*-based assays^11^,^12^,^13^,^51^,^52^. Based on our large-scale PD/LC-MS/MS analysis, we can now speculate that many other proteins might also take part in protein-protein interactions during the mitotic process. We foresee small molecule-based, dual-capacity probes such as **MLN-2** and **PU-1** will become increasingly valuable in future for discovery of such novel protein-interacting complexes, as they offer the unique advantages of both spatially resolved, live-cell imaging data which can be used to uncover sub-organelle organization of proteins, and complementary MS-enabled molecular resolution that may be used to study *in situ* protein-protein interactions.

Looking ahead, challenges abound with these “*in situ* drug profiling” approaches. First, while we have successfully shown the multiplex capability of such small molecule probes for live-cell imaging of kinase activities, the poor nuclear permeability of reporters used needs to be further resolved. Second, technical difficulties associated with large-scale LC-MS/MS profiling and target identification need to be improved in order to further minimize “false positives” identified from such experiments. The wider adaptation of quantitative proteomics techniques such as SILAC in future will certainly provide much improved results, as was demonstrated recently^56^. Third, the question of how faithfully the “minimalist” modification in **MLN-2** recapitulates genuine drug/target interactions remains to be more thoroughly evaluated. Forth, despite the successful discovery of a novel AKA/CDK1/NUDC macromolecular complex, extensive biological studies are needed in future to study its precise cellular mechanism, as well as other proteins involved in this mitotic network. Finally and perhaps the most pressing issue is the development of more such dual-use small molecule probes that can target other important proteins with better sensitivity and specificity. With regards to specificity, none of the existing small molecule probes (e.g. **MLN-2**/**PU-1** and those reported by others^32^), though suitable for earlier-discussed imaging experiments (which was likely due to these probes' limited spatial resolution and sensitivity), is yet able to compete with IF-based imaging experiments where high-quality antibodies are available. This is evident from our large-scale PD/LC-MS/MS results in which multiple proteins were labeled. Future higher-resolution imaging experiments with advanced spectroscopic techniques will require much higher-quality imaging probes. All of these issues are currently being addressed in our on-going research efforts.

## Methods

### Cellular inhibition assay of *p*HisH3

Determination of Phospho-Histone H3 (Ser 10) suppression in HeLa cells by WB (Fig. 2B) was done based on published protocols^36^. HeLa cells (2 × 10^5^) were seeded in each well of a 12-well plate for 24 h before synchronization by 100 ng/mL nocodazole for 16 h. The cells were then exposed to various concentrations of the compound (MLN8237/**MLN-1**/**MLN-2**/**MLN-3**; 0.1 to 10 μM) at 37°C for 3 h. Compounds were applied directly from DMSO stocks whereby the DMSO% in the final media never exceeded 0.1%. Cells were harvested by washing once with cold PBS and lysed in 50 μL of HEPES buffer. The resulting lysates were denatured and 10 μL of each sample was separated on 15% SDS-PAGE gel followed by WB analysis.

### *In vitro* and *in situ* proteome labeling

Proteome labeling was done largely based on previously published protocols^25^,^27^. For *in vitro* proteome labeling, 50 μg of fresh HeLa lysates were incubated with desired concentrations of **MLN-2**/**PU-1** for 30 min at room temperature with vigorous shaking. After UV irradiation (at 365 nm) on ice for 20 min, 4 μL of freshly mixed click cocktail (50 μM Rh-N_3_ from 2.5 mM stocks in DMSO, 100 μM TBTA from 10 mM stock in DMSO, 1 mM TCEP from 100 mM stock in H_2_O and 1 mM CuSO_4_ from 100 mM stock in H_2_O) was added and incubated at room temperature for 2 h with gentle mixing. The reaction was terminated by pre-chilled acetone (0.5 mL; −20°C for 30 min), the protein precipitation was collected by centrifugation (13,000 rpm × 10 min at 4°C) and washed twice with 200 μL pre-chilled methanol with brief sonication. The protein pellets were then resuspended in 30 μL of 1× SDS loading buffer and denatured at 95°C for 10 min, 10 μL of each sample was separated on 10% SDS-PAGE followed by In-gel fluorescence scanning and/or WB. For *in situ* labeling with asynchronized HeLa/SHSY-5Y cells, cells were grown to 80–90% confluence, then washed once with cold PBS and treated with desired concentrations of **MLN-2**/**PU-1** (final DMSO never exceeds 1%) in 0.5 mL of growth medium for 5 h at 37°C/5% CO_2_. The medium was aspirated and the cells were gently washed with DMEM once and PBS once, followed by UV irradiation for 20 min on ice. The cells were then harvested in 100 μL of HEPES buffer by cell scraper, followed by brief sonication and subsequent procedures as *in vitro* labeling described above. For *in situ* labeling with synchronized HeLa cells, cells were firstly released from thymidine block in growth medium for ~10 h before probe incubation. Subsequent procedures were exactly the same as asynchronized cells.

### Affinity enrichment/pull-down (PD) and immunoprecipitation (IP)

HeLa lysates were prepared and used as described in Supplementary Methods. Probe-labeled protein pellets (after click chemistry and methanol wash) were resolubilized with 0.5% SDS in PBS (200 μL). Primary antibody *anti*-Aurora A (1:100) was added according to manufacturer's instructions (Cell Signaling #4718) and incubated at 4°C overnight. Protein A/G beads (20 μL suspension) were added and the resulting mixture was incubated at room temperature for 3 h. The beads were then washed with PBS containing 0.5% SDS twice and PBS once, followed by addition of 1× SDS loading buffer, denaturation at 95°C for 10 min and separation on 10% SDS-PAGE gels. In-gel fluorescence scanning was used to visualize the labeled protein bands, followed by WB for further confirmation. For IP experiments, synchronized HeLa cells were detached by trypsin (1×) in PBS, quenched with DMEM, washed with PBS twice, centrifuged and gently resuspended in cold RIPA buffer (50 mM Tris pH 8, 150 mM NaCl, 1% NP-40, 0.5% deoxycholate, 0.1% SDS). Lysate was obtained by sonication on ice and centrifugation at 4°C (13200 rpm for 10 min), before being adjusted with PBS to 5 mg/mL. IP was carried out with Pierce's Co-Immunoprecipitation Kit at 4°C following the manufacturer's instructions. In brief, the lysate was pre-clarified with the control beads for 1 h, then added to antibody-bound beads in a spin column (6 μL antibody cross-linked to 40 μL beads slurry). The column was rotated for 12 h and washed with lysis/wash buffer for three times, followed by addition of conditioning buffer. The mixture was gently centrifuged at 4°C (8000 rpm for 30 s). Finally the protein complexes were eluted by elution buffer before being separated on 10% SDS-PAGE gels and visualized by WB with an appropriate antibody.

### Cellular imaging

HeLa cells were seeded in glass-bottom dishes (Mattek) and grown until 70–80% confluence. For imaging experiments with the diazirine probes (**MLN-2**/**PU-1**), cells were incubated with 2 μM of the probe in 0.5 mL of DMEM growth medium for 2 h (with medium containing 1% DMSO used as a negative control). The cells were then washed with DMEM growth medium and PBS twice each, followed by UV irradiation on ice for 15 min. The cells were then fixed with 3.7% paraformaldehyde in DMEM for 30 min at room temperature, washed with PBS twice (1 ~ 2 min with gentle agitation), and permeabilized with 0.1% Triton X-100 in PBS for 10 min at room temperature. Cells were then washed twice with PBS for 1 ~ 2 min with gentle agitation and blocked with 2% BSA in PBS (with 0.05% Tween-20) for 30 min at room temperature, and washed twice with PBS (with 0.05% Tween; ~5 min each) with gentle agitation. The cells were then treated with a freshly premixed click chemistry reaction solution of Rh-N_3_/Cy5-N_3_ (5 μM final concentration), TBTA (20 μM final concentration), TCEP (200 μM final concentration), and CuSO_4_ (200 μM final concentration), in 100 μL PBS for 2 h at room temperature. The cells were then washed with PBS (0.05% Tween-20 and 0.5 mM of EDTA) for 3 × 10 min, and with PBS for 2 × 5 min with gentle agitation. For co-localization experiments by using IF, after click reaction, cells were incubated with the antibody (1:100) overnight at 4°C, then washed twice in PBS (0.05% Tween-20; 2 × 5 min with gentle agitation), incubated with Cy5-conjugate Goat anti-Rabbit IgG (1:200) for 2 h at room temperature, washed again with PBS (0.05% Tween-20; 3 × 10 min with gentle agitation). The cells were then incubated in PBS containing 0.25 mg/mL of Hoechst for 15 min at room temperature to stain the nuclear DNA, washed with PBS (5 min) and a final wash with deionized water (1 min) before mounting. For co-localization imaging of **MLN-3** with **MLN-2**/**PU-1**, both probes were mixed (2 μM each). Click reaction of **MLN-3** by using Rh-TZ was done prior to that of **MLN-2**/**PU-1** by using Cy5-N_3_. Other conditions remained the same as above described. Subsequently images were acquired using LSM 510 Meta (Zeiss, Germany) confocal microscope equipped with an EC Plan-Neofluar 40× NA1.25 objective, or with the Leica TCS SP5X confocal microscope system equipped with Leica HCX PL APO 63×/1.20 W CORR CS, 405 nm diode laser, white laser (470–670 nm, with 1 nm increments, with eight channels AOTF for simultaneous control of eight laser lines, each excitation wavelength provides 1.5 mV), and a photomultiplier tube (PMT) detector ranging from 410 to 700 nm for steady state fluorescence. Images were processed with Leica Application Suite Advanced Fluorescence (LAS AF).

### Pull-down (PD)/LC-MS/MS analysis

To identify potential cellular targets of **MLN-2**/**PU-1**, pull-down (PD) experiments were carried out as previously described^27^. For *in vitro* PD, cellular lysates (3 mg) were supplemented with 1.5 mL 1× HEPES buffer and incubated with a desired concentration of the probe (0.2 or 2 μM) for 2 h at room temperature. The reactions were UV-irradiated for 20 min on ice, followed by CuAAC. Upon methanol precipitation, the resulting pellets were dried and resolubilized in 0.5% SDS in PBS by brief sonication, and the insoluble materials were removed by centrifugation. The supernatant was incubated with high-capacity NeturaAvidin agarose beads overnight at 4°C. Bead-bound proteins were eluted by boiling the beads in SDS loading dye, then separated on 10% SDS-PAGE gels, and visualized by in-gel fluorescence scanning and coomassie brilliant blue staining. For *in situ* PD experiments, the probe was added directly to live cells and incubation was continued for 5 h in humidified 37°C with 5% CO_2_. Subsequently, cells were washed with growth medium (2 × 10 min) and cold PBS (2 × 1 min), followed by UV irradiation on ice for 20 min, and all subsequent procedures were exactly the same as the *in vitro* experiments.

All LC-MS/MS experiments were performed as previously described^27^. Results are summarized in SI. Briefly, emPAI ratio of positive and negative PD (with **NP**) samples were calculated. Only proteins with emPAI ratio = ∞ were processed further. Data from 0.2 μM-probe PD experiments with protein scores ≥ 40 and emPAI ≥ 0.1 were considered further. Only those that appeared in synchronized live HeLa cells were considered further. Finally, results were checked against Human Protein Reference Database (www.hprd.org) and only nuclear-localized proteins were listed in Table 1.

### Other methods

Additional experimental information and results (chemistry and biology) are provided in Supplementary Methods.

## Author Contributions

Y.S. and S.Q.Y. designed the experiments. Y.S., Z.L., L.L., S.P., X.W. and P.H. performed the experiments and analyzed the data. Y.S., S.P. and S.Q.Y. wrote the manuscript. Work in NTU was done under the supervision of S.K.S., while in NUS under S.Q.Y.

## Supplementary Material

Supplementary Information SUPPLEMENTARY INFO

## Figures and Tables

**Table 1:** Putative nuclear-localized targets of MLN-2 and PU-1 identified from proteome profiling/LC-MS/MS analysis

Protein Name	Protein Type	Protein Score		Protein Name	Protein Type	Protein Score	
		MLN-2	PU-1			MLN-2	PU-1
ANXA1	Calcium binding	466	235	MDH2	Enzyme(dehydrogenase)	114	141
ARPC1B	Cytoskeletal associated	70	-	NAT10	Enzyme(acyltransferase)	-	82
CCNK	Transcription regulator	63	-	NSUN2	Enzyme(methyltransferase)	193	228
CLCC1	Ion channel	-	80	NUDC	Cell cycle control	73	-
CSTF1	RNA binding	-	52	NUP160	Transporter	-	77
CUL4B	Enzyme(deubiquitinase)	131	95	NUP214	Transporter	-	119
EIF3S9	Transcription regulator	-	56	OLA1	Enzyme(ATPase)	72	-
FEN1	Enzyme(endonuclease)	62	113	PSMC1	Enzyme(deubiquitinase)	159	110
FUBP1	Transcription regulator	211	110	RBM4	RNA binding	63	-
FUBP3	Transcription regulator	-	112	RPS2	Ribosomal subunit	60	53
HDAC1	Enzyme(deacetylase)	136	66	RPS4X	Ribosomal subunit	52	-
HDAC2	Enzyme(deacetylase)	136	170	SAMHD1	Enzyme(GTPase)	54	-
HNRNPH3	Ribonucleoprotein	44	54	SEPT9	Enzyme(GTPase)	106	158
HNRNPUL1	RNA binding	67	149	SF3B1	RNA binding	379	324
KHSRP	Transcription regulator	166	102	SQSTM1	Enzyme(deubiquitinase)	-	53
KPNA2	Transporter	256	176	STAT3	Transcription factor	52	-
LARP4	RNA binding	-	60	TGM2	Enzyme(glutamyltransferase)	150	-
LTA4H	Enzyme(hydrolase)	135	212	THRAP3	Transcription regulator	126	167
LUC7L3	Transcription regulator	-	99	TRIM25	Transcription factor	-	115
MAGED2	Unclassified	61	71	WDR12	Unclassified	99	-
MAPK1(ERK2)	Enzyme(kinase)	58	-	ZFR	Transcription regulator	69	220
MATR3	RNA binding	240	374				

“-”: not identified in the LC-MS/MS results under the defined conditions. See Methods for more details.
