# Supplementary material for: Multiplex Imaging and Cellular Target Identification of Kinase Inhibitors via an Affinity-Based Proteome Profiling Approach
Source: Sci Rep. 2015 Jan 12;5:7724. doi: 10.1038/srep07724 (PMC4290084; doi:10.1038/srep07724)
Supplement: Supplementary Information — SUPPLEMENTARY INFO [file srep07724-s1.pdf]

Supporting Information (I)

Multiplex Imaging and Cellular Target Identification of Kinase Inhibitors via an Affinity-Based Proteome Profiling Approach

Ying Su,[1,†] Sijun Pan,[1,†] Zhengqiu Li,[1] Lin Li,[1] Xiaoyuan Wu,[1] Piliang Hao,[2] Siu Kwan Sze,[2] and Shao Q. Yao[1,*]

[1] Department of Chemistry, National University of Singapore, 3 Science Drive 3, Singapore 117543

[2] School of Biological Sciences, Nanyang Technological University, 60 Nanyang Drive, Singapore 637551

[†] These authors contributed equally to this work.

* Correspondence and requests for materials should be addressed to S.Q.Y. (email: chmyaosq@nus.edu.sg)

1. Chemical synthesis.

All chemicals were purchased from commercial vendors and used without further purification, unless indicated otherwise. All reactions requiring anhydrous conditions were carried out under argon or nitrogen atmosphere using oven-dried glassware. HPLC-grade solvents were used for all reactions. Reaction progress was monitored by TCL on pre-coated silica plates (Merck 60 F_{254}, 0.25 µm) and spots were visualized by ceric ammonium molybdate, basic KMnO4, UV light or iodine stain. Flash column chromatography was carried out using Merck 60 F_{254}, 0.040-0.063 µm silica gel. All NMR spectra (^{1}H-NMR, ^{13}C-NMR) were recorded on a Bruker 300 MHz/500 MHz NMR spectrometer. Chemical shifts are reported in parts per million referenced with respect to appropriate internal standards or residual solvent peaks ($CDCl_3$ = 7.26 ppm, CD_3OD = 3.31 ppm, DMSO-d_6 = 2.50 ppm). The following abbreviations were used in reporting spectra, br s (broad singlet), s (singlet), d (doublet), t (triplet), q (quartet), m (multiplet), dd (doublet of doublets). Mass spectra were obtained on Shimadzu IT-TOF-MS or Shimadzu ESI-MS system.

MLN-1. MLN8237 (7.1 mg, 0.013 mmol) was dissolved in DMF, followed by addition of EDCI (3.1 mg, 0.02 mmol) and propargylamine (1.5 mg, 0.03 mmol). The reaction was stirred for 2 h at room temperature. 5 mL of H_2O was added and the white precipitation was collected. Upon further purification by flash chromatography, the desired product was obtained (7.1 mg; 72.0% yield). 1H-NMR (500 MHz, DMSO-d_6) δ 9.36 (s, 1H), 7.87 (s, 1H), 7.52 (t, J = 5.7 Hz, 1H), 7.45 (d, J = 8.85, 1H), 7.13 (s, 1H), 7.00-6.95 (m, 2H), 6.59-6.54 (m, 2H), 6.37 (s, 1H), 2.23 (S, 1H), 1.65-1.64 (m, 2H). ^{13}C-NMR (125 MHz, DMSO-d_6) 164.2, 160.9, 160.5, 160.23, 159.4, 158.6, 158.0, 157.8, 157.7, 157.2, 144.9, 137.7, 135.1, 134.6, 131.6, 131.2, 130.9, 130.3, 127.5, 123.5, 117.9, 114.0, 110.4, 108.0, 101.4, 81.8, 72.5, 56.3, 55.7, 49.5, 38.3, 28.5. HR-MS (ESI): m/z $[M+H]^+$ calcd: 556.1566, found 556.1546.

MLN-2. MLN8237 (13.6 mg, 0.025 mmol) was dissolved in DMF, followed by addition of EDCI (6.2 mg, 0.04 mmol) and the diazirine-containing linker (8 mg, 0.06 mmol). The reaction was stirred for 2 h at room temperature. 5 mL of H_2O was added and the white precipitation formed was collected. The crude product was further purified by flash chromatography to obtain the desired product (10.2 mg; 61.1% yield). ^{1}H-NMR (500 MHz, DMSO-d_6) δ 10.19 (s, 1H), 8.71 (s, 1H), 8.3 (d, J = 10.0, 1H), 8.11 (s, 1H), 7.97 (s, 1H), 7.81 (d, 2H), 7.64-7.53 (m, 2H), 7.41 (d, J = 10.0, 2H), 3.18 (t, J = 10.0, 2H), 2.03 (d, J = 10.0, 2H), 1.67-1.62 (m, 4H). ^{13}C-NMR (125 MHz, DMSO-d_6) 164.9, 160.9, 160.5, 160.3, 159.9, 158.6, 157.6, 154.6, 145.1, 132.8, 132.5, 131.9, 130.7, 129.2, 123.9, 115.0, 110.9, 108.5, 101.9, 83.6, 72.2, 56.8, 56.2, 49.9, 38.7, 31.9, 27.8, 13.2. HR-MS (ESI): m/z $[M+H]^+$ calcd: 638.2083, found:638.2077.

MLN-3. To MLN8237 (6.0 mg, 0.012 mmol) dissolved in a mixture of DCM/DMF (10:1; 0.8 mL in total) was added EDCI (6.0 mg, 0.031 mmol) and *tert*-butyl(2-aminoethyl)carbamate (8 mg, 0.05 mmol). The resulting mixture was stirred for 5 h at room temperature. The mixture was separated by flash chromatography to provide the intermediate which was next treated with HCl in dioxane (4 M, 1.0 mL) for 3 h. Upon solvent evaporation, the residual mixture was dissolved in DMF (1.0 ml). To this solution was added DIEA (10 mg, 0.082 mmol), (*E*)-cyclooct-4-en-1-yl(4-nitrophenyl)carbonate (8 mg, 0.027 mg). The resulting mixture was stirred at room temperature overnight. The reaction was quenched by addition of water (10 mL) in an ice-water bath. The precipitate collected was further purified by flash chromatography to give the desired product (7.2 mg; 87.8% yield). ^{1}H-NMR (500 MHz, $CDCl_3$) δ 8.55 (s, 1H), 8.24 (d, J = 8.5 Hz, 1 H), 8.21 (d, J = 8.5 Hz, 1H), 8.13 (m, 1H), 8.01 (s, 1H), 7.58 (dd, $J1$ = 8.5 Hz, $J2$ = 2.5 Hz, 2 H), 7.28-7.33 (m, 2H). 7.11 (d, J = 8.0 Hz, 1H), 5.54-5.60 (m, 2H), 5.33 (m, 1H), 4.92 (m, 1H), 4.02 (s, 3H), 3.63-3.65 (m, 3H), 3.46-3.49 (m, 3H), 2.90 (s, 1.5 H), 2.90 (s, 1.5 H), 2.20-2.32 (m, 6H), 1.15-1.82 (m, 9H), 0.83-0.91 (m, 5H); ^{13}C-NMR (125 MHz, $CDCl_3$) δ 171.2, 166.1, 162.5, 161.4, 159.2, 158.5, 156.7, 156.6, 144.1, 135.4, 133.0, 131.6, 130.9, 130.8, 130.2, 130.1, 123.9, 118.1, 114.6, 110.8, 108.3, 107.0, 101.1, 70.1, 55.8, 50.3, 41.3, 41.0, 39.8, 36.4, 34.3, 32.7, 31.4, 29.9, 29.7, 27.9; HR-MS (ESI): m/z $[M+H]^+$ calcd: 713.2650, found: 713.2649.

2. Structures of Compound 3, NP and Weissleder's AKA probe.

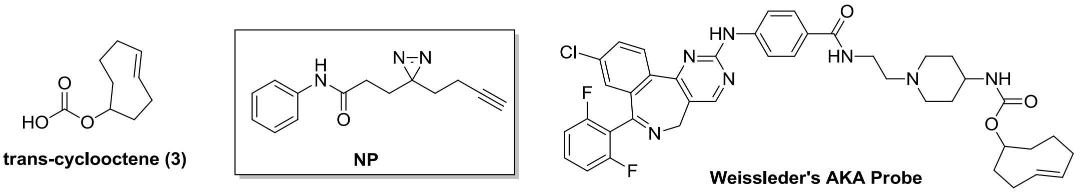

Supplementary Figure S1. Structures of *trans*-cyclooctene (**3**), control probe **NP** and Weissleder's AKA probe.[1]

3. Enzyme Inhibition Assay and IC_{50} Determination.

Recombinant kinase inhibition assays were carried out based on previously published procedures.[2] Briefly, microplate-based inhibition assay was performed with Kinase-Glo® Plus Luminescent Kinase assay kit following the manufacturer's instructions. Recombinant Aurora A, ATP and the probes were mixed in the HEPES buffer (without NP-40) at a total volume of 27.5 µL in a flat-bottom solid white 384-well plate. The incubation was allowed to continue for 20 min at 37 °C and the reaction was subsequently quenched by the addition of an equal volume of the Kinase-Glo reagent. After 5 min of incubation, the luminescence readouts from the wells were measured using Tecan microplate reader (Multimode Reader, Infinite®200) in luminescence mode with i-control TM software. The ATP and substrate peptide concentrations used in the assay were 10 µM and 50 µM, respectively. Dose-dependent inhibition assays were performed by varying the concentration of the compound/probe under optimized enzyme concentration of ~50 nM. The IC_{50} values of the compounds/probes were calculated from the percentage activity *vs* log[concentration of inhibitor] curves generated using GraphPad Prism software. Results are shown in Figure 2. Inhibition of recombinant PKA was similarly carried out.

4. Antiproliferation Assay.

The assay carried out on mammalian cells (both HeLa and HCT-116 cells) using the XTT colorimetric cell proliferation kit (Roche) following the manufacturer's guidelines. Briefly, mammalian cells were seeded at ~4000 cells in the absence of drugs in 96-well plates. The medium was aspirated, and then treated in duplicate, with 0.1 mL of the medium containing different concentrations of the compound (MLN8237/**MLN-1**/**MLN-2**/**MLN-3**; 1 nM to 10 µM). Both MLN8237 and the probes were applied from DMSO stocks whereby DMSO never exceeded 1% in the final solution. The same volume of DMSO was used as a negative control. After a total treatment time of 72 h, proliferations were assayed using reagents provided in the XTT kit (read at 450 nm). Data represent the average of at least two trials.

Supplementary Table S1. GI_{50} values of compounds with HCT-116 Cells determined by XTT Assay.

	GI_{50}/nM (HCT-116)
**MLN-8237**	128.6 ± 5.2
**MLN-1**	177.3 ± 5.2
**MLN-2**	345.6 ± 9.4
**MLN-3**	551.7 ± 17.4

5. Proteome Labeling of Aurora A-Overexpressed Bacterial Lysates.

The bacterial plasmid containing WT Aurora fused to a His-tag was transformed and expressed according to published procedures.[3] Briefly, the bacterial cultures were grown at 37 °C with shaking to reach OD_{600} = 0.6. Subsequently, 0.5 mM IPTG was added to induce the expression of Aurora A at 18 °C for ~18 h. Portions of the culture were taken out before and after the induction respectively, and harvested by centrifugation at 4000 rpm for 10 min at 4 °C. The resulting pellets were resuspended in PBS and sonicated. The supernatant was then collected by centrifugation for 10 min (13,000 rpm at 4 ºC). Total protein concentrations of these lysates were quantified by Bradford protein assay, and stored at -80°C until use. For the labeling reaction, we followed procedures similar to our previously published protocols.[2] Briefly, the same amount of lysates (50 µg in 50 µL) from both induced and uninduced cultures was incubated with 2 µM of **MLN-2** for 30 min at room temperature, followed by UV irradiation (at 365 nm) on ice for 20 min, then 2 h of click reaction with Rh-N_3 (4 µL click cocktail with final concentrations as: 50 µM Rh-N_3 from 2.5 mM stocks in DMSO, 100 µM TBTA from 10 mM stock in DMSO, 1 mM TCEP from 100 mM stock in H_2O and 1 mM $CuSO_4$ from 100 mM stock in H_2O) based on previously reported protocols.[2] The labeled samples were denatured in 6× SDS loading buffer [375 mM Tris

pH 6.8, 12% (w/v) SDS, 60% (v/v) glycerol, 0.5 M dithiothreitol (DTT) and 0.0012% (W/V) bromophenel blue] at 95 ºC for 10 min, then separated on 10% SDS-PAGE gels. In-gel fluorescence scanning was used to visualize the labeled protein bands. Total cellular proteins were visualized by staining the same gel with coomassie staining (CBB).

6. Competitive Proteome Labeling of Synchronized Hela Cells.

Different concentrations of **MLN-2** (8 µM, 2 µM, 0.2 µM from 1 mM stock) were added into synchronized HeLa Cells or lysates (50 µg in 50 µL), **NP** (2 µM) was used as negative control. Upon 1 h incubation, the reactions were UV irradiated (at 365 nm) on ice for 20 min, followed by click chemistry with 4 µL of a freshly mixed click cocktail for 2 h at room temperature with gentle mixing, then terminated by addition of 400 µL pre-chilled acetone, then further incubated at -20 °C for 30 min. The precipitation was collected by centrifugation (13,000 rpm × 10 min at 4 °C) and washed twice with pre-chilled methanol with brief sonication. The precipitated protein was then resuspended in 30 µL of 1× SDS loading buffer and denatured at 95 ºC for 10 min. 10 µL of each sample was separated on 10% SDS-PAGE gel. In-gel fluorescence scanning and WB were done where applicable. For competitive *in vitro* labeling, 50 µM of MLN8237 was firstly incubated with lysates (for 30 min) prior to the addition of various concentrations of **MLN-2**. *In situ* (live cell) competitive proteome labeling of synchronized Hela cells was carried out as described in Methods, except when MLN8237 was added simultaneously with **MLN-2**.

(A) **(B)**

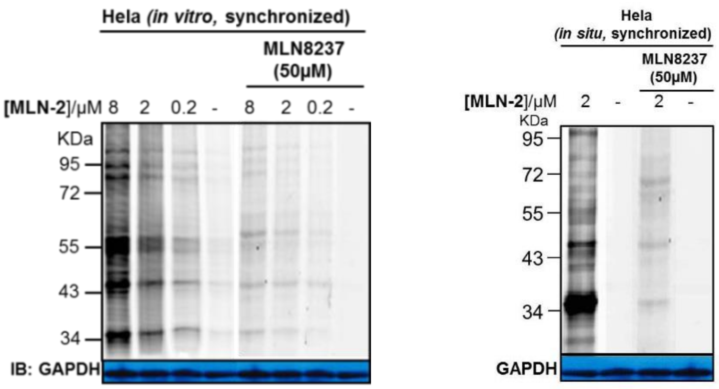

Supplementary Figure S2. (A) Competitive *in vitro* labeling of **MLN-2** with 50 µM MLN8237. WB showed equal loading of lysates. (B) Competitive *in situ* labeling of **MLN-2** with 50 µM of MLN8237.

7. *In vitro* and *In situ* labelling of SHSY-5Y cells

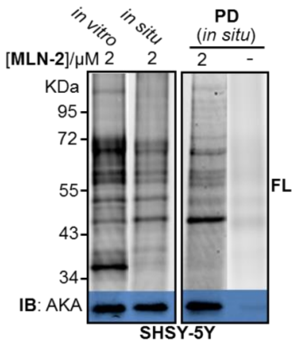

Supplementary Figure S3. Synchronized SH-SY5Y cells/cell lysates were labeled with **MLN-2** (2 µM), followed by in-gel fluorescence scanning (left gel), and pull-down (PD; right gel). Results were similarly processed as Figure 2D.

8. Cell Imaging

For immunofluorescence of Aurora A, HeLa cells were fixed with 3.7% formaldehyde and permeabilized with 0.1% Triton X-100, blocked with 2% BSA in PBS (with 0.05% Tween-20) for 30 min at room temperature,

followed by incubation with *anti*-Aurora A antibody (1:100) for 4 h at room temperature or 4 °C overnight. Upon washing with PBS (with 0.05% tween-20) twice for 5 min each and incubation with Cy5-conjugated Goat anti-Rabbit IgG (1:200) for 2 h at room temperature, the cells were then washed with PBS (with 0.05% Tween-20) three times for 5 min each, followed by staining with Hoechest 33258 (0.25 mg/mL) and washed with PBS for 5 min with gentle agitation and a final wash with deionized water (1~2 min with gentle agitation) before images were acquired. For co-localization experiments of **MLN-3**/IF (anti-Aur A), we followed published procedures closely with some necessary modifications.[1] Briefly, after incubation with 0.5 ml of DMEM growth medium containing 2 µM **MLN-3** for 1 h (medium containing 1% DMSO applied as negative control). The cells were then washed gently with growth medium twice, and incubated with 1.5 µM Rh-TZ for 1 h. After which, cells were washed with growth medium and PBS once each, followed by fixation in 3.7% paraformaldehyde in PBS for 15 min at room temperature.

(A)

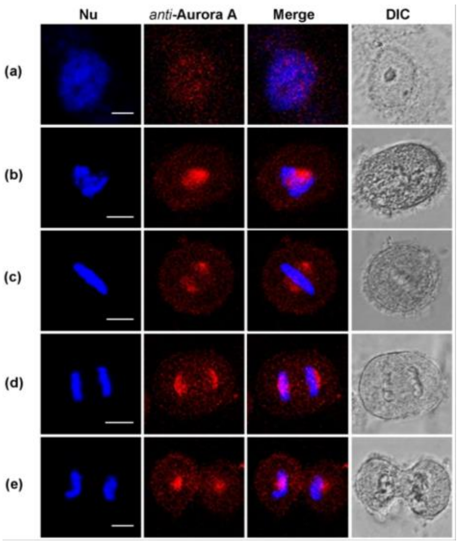

(B)

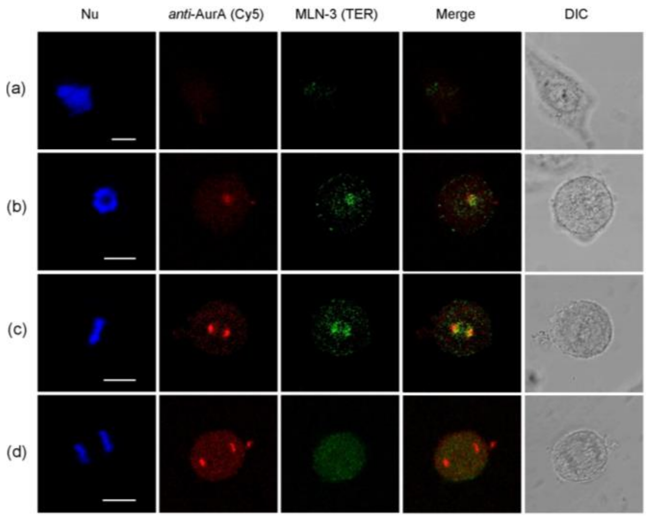

Supplementary Figure S4. (**A**) Immunofluorescence (IF) of Aurora A in HeLa cells at different phases during mitosis. (a) interphase, (b) prometaphase, (c) metaphase, (d) anaphase, (e) telephase. Scale bar = 10 µm. (**B**) Colocalization of HeLa cells at different phases during mitosis by **MLN-3** imaging and Immunofluorescence (with anti-Aurora A). Scale bar = 10 µm.

9. Effect of 365-nm UV Irradiation on HeLa Cells.

For XTT analysis, the procedure is similar to the one described above with modification. After cells were seeded and grew to ~50%, they were irradiated under 365 nm UV (UVP, UVGL-58) or dosed with STS (1 µM) as control. After incubation at 37 °C for 16h, proliferations were assayed using reagents provided in the XTT kit (read at 450 nm). For FACS analysis, we used BD Accuri™ C6 with FITC Annexin V Apoptosis Detection Kit.

Hela cells were grew in 60 mm dishes to ~50%, and irradiated under 365 nm UV (UVP, UVGL-58). After incubation at 37 ^{o}C for 16h, cells were detached by trypsin (1x) in PBS, quenched with DMEM, washed with PBS twice, centrifuged and resuspended in 200 μL of 1x buffer from the kit. To 100 μL of sample was added 5 μL PI and FITC. After incubation for 15 min, the sample was diluted with 300 μL buffer and subjected to analysis.

(A)

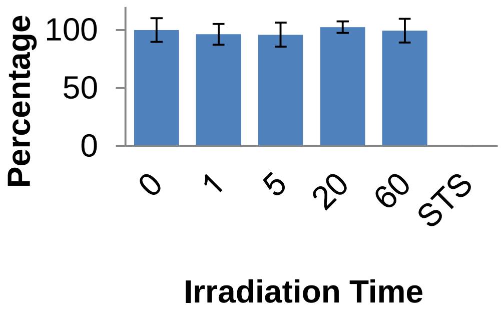

0	1	5	20	60	STS
100.0±11.4	96.3±4.0	96.0±11.8	102.5±1.2	99.5±7.8	0

(B)

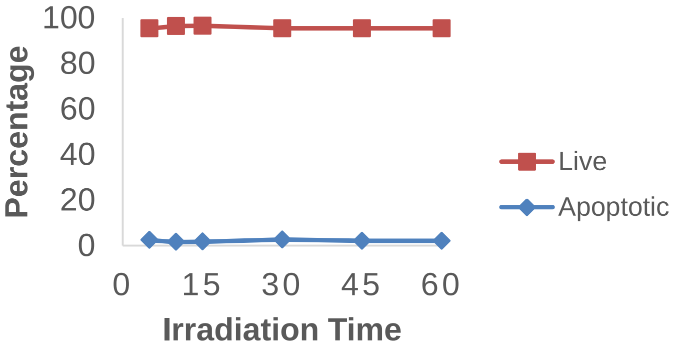

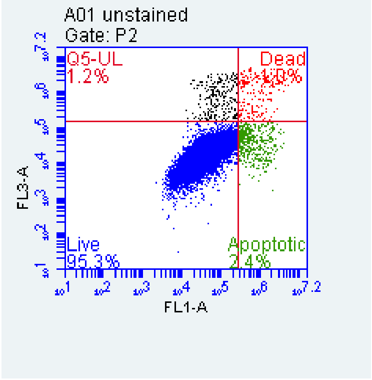

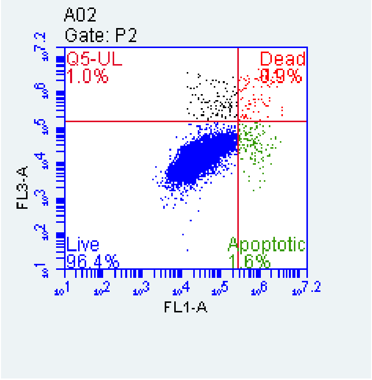

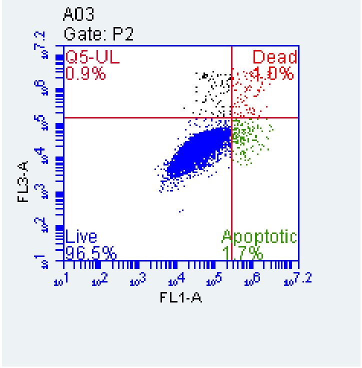

5min	10min	15min

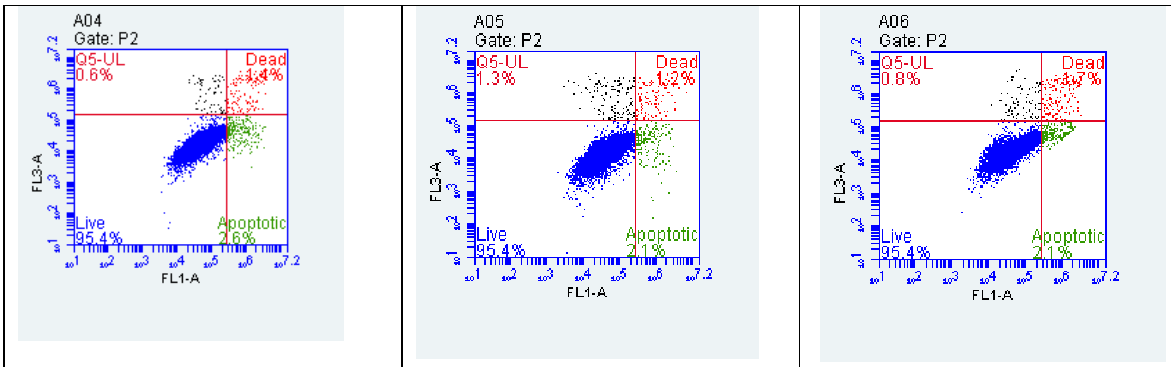

30min	45min	60min

Supplementary Figure S5. (A) XTT results showing proliferation of HeLa cells irradiated with 365 nm UV. Data represent the average of three trials ± s.e.m. (B) FACS results showing the degree of apotosis of HeLa cells irradiated with 365 nm UV. (top) summary; (bottom) original FACS plots.

10. Full-length blots.

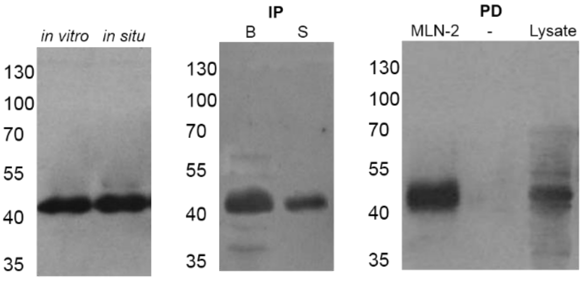

Supplementary Figure S6a. Full-length blots for WB of Fig. 3D. *In vitro* and *in situ* labeling of synchronized HeLa cells/cell lysates with 2 μM of **MLN-2** (left), and further enriched by immunoprecipitation (**IP**: middle, B:bead eluent, S:supernatant) or pull-down (**PD**; right), against *anti*-AKA.

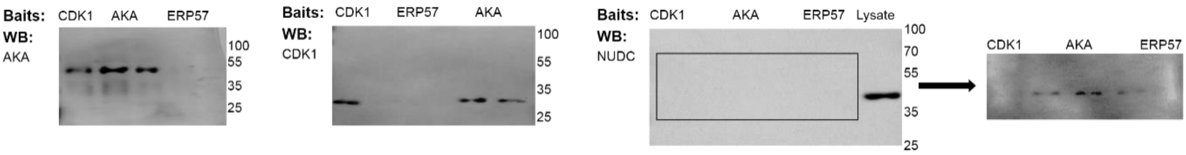

Supplementary Figure S6b. Full-length blots for Fig. 3E. Separated gel was used for each WB data (AKA, CDK1, NUDC). Co-immunoprecipitation for AKA and CDK1 was done with same samples using the same antibody baits at the same time. Samples might be analyzed with duplicate or triplicate baits. The represented bands were chopped and rearranged in maintext for comparison.

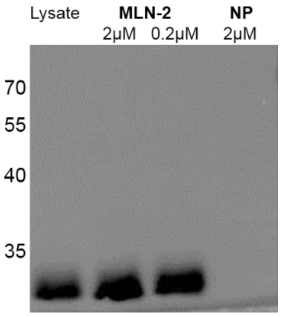

Supplementary Figure S6c. Full-length blots for Fig. 3F . PD of **MLN-2** or **NP** and visualized by WB against *anti*-CDK1.

11. Cell Culture, Cell Synchronization and Western Blotting.

Cell lines were obtained from the National Cancer Institute Developmental Therapeutics Program (NCI-60 panel). HeLa and SH-SY5Y cells were cultured in Dulbecco's Modified Eagle's Medium (DMEM, Invitrogen, Carlsbad, CA) containing 10% heat-inactivated fetal bovine serum (FBS; Gibco Invitrogen), 100 U/mL penicillin, and 100 µg/mL streptomycin (Thermo Scientific) and maintained in humidified 37 °C incubator with 5% CO_2. The cells were washed twice with cold phosphate buffered saline (PBS) before being collected by cell scraper and lysed in HEPES buffer (25 mM HEPES, 150 mM NaCl, 2 mM $MgCl_2$, 0.1% NP-40) containing protease inhibitor cocktail (Roche). Final protein concentrations in the lysates were determined using the Bradford protein assay (Bio-Rad, USA).

Synchronized HeLa lysates and live cells for *in vitro* and *in situ* proteome labeling, immunoprecipitation (IP), Co-IP and pull-down (PD) experiments were synchronized by thymidine-nocodazole or single thymidine treatment.[36-37] Briefly, for synchronized *in vitro* proteome labeling, 2.5 mM thymidine (from 100 mM stock in 1× PBS buffer) was added to HeLa cells (at ~50% confluency) and incubated for 20-24 h. The cells were then gently washed at 37 °C with growth medium once, and incubated further with 100 ng/mL nocodazole (from 1 mg/mL stock in DMSO) for 14-16 h. After washing with cold PBS once, the cells were collected by cell scraper in HEPES buffer, and used for subsequent proteome labeling. For synchronized *in situ* labeling and PD, 2.5 mM thymidine (100 mM stock in 1× PBS buffer) was added at ~50% confluency and incubation was continued for 20-24 h. The cells were subsequently released in DMEM growth medium for ~10 h before treatment with **MLN-2/PU-1** probes.

Western blotting (WB) was done where applicable. Briefly, proteome samples were separated by SDS-PAGE, and the gels were transferred to PVDF membranes. The membranes were then blocked with 4% bovine serum albumin (BSA) in PBST (0.1% of Tween-20 in PBS buffer) for 2 h at room temperature. After blocking, the membranes were incubated with the corresponding primary antibody (1:1000) for 2 h at room temperature or overnight at 4 °C. After washing with PBST for 3×10 min at room temperature, the membranes were incubated with a suitable secondary antibody (1:2000) for 1 h at room temperature. Finally, blots were washed again for 3×10 min at room temperature with PBST before being developed with SuperSignal West Dura Kit (Thermo Scientific).

12. Pull Down LCMS MS and Target Validation.

To identify potential cellular targets of **MLN-2/PU-1**, pull-down (PD) experiments were carried out (*in vitro* and *in situ*; asynchronized and synchronized; HeLa and SHSY-5Y). For *in vitro* PD, cellular lysates (3 mg) were supplemented with 1.5 mL 1× HEPES buffer and incubated with desired concentrations of **MLN-2/PU-1** (2 µM from 1 mM stock in DMSO) for 2 h at room temperature. The negative experiments (with **NP**, 2 µM) were done concurrently. The reactions were UV-irradiated for 20 min on ice, followed by click chemistry with Rh-Biotin-N_3 for 2 h. The click reactions were stopped and the pellets dried and resolubilized in 0.5% SDS in PBS by brief sonication, and the insoluble materials were removed by centrifugation. The supernatant was incubated with high-capacity NeturaAvidin agarose beads (pre-washed with PBS) overnight at 4 °C. The beads were collected and washed (3x with 1% SDS in PBS and 3x with PBS). Proteins were eluted by boiling the beads in 2×SDS loading dye at 95 °C for 15 min, then separated on 10% SDS-PAGE gels, and visualized by in-gel fluorescence scanning and coomassie brilliant blue staining. For *in situ* PD with asynchronized cells, the probe (2 and 0.2 µM) was added directly to live cells and incubation was continued for 5 h in humidified 37 °C with 5% CO_2, **NP** was used as the negative control. Subsequently, cells were washed with growth medium (2x10 min) and cold PBS (2x1 min), followed by UV irradiation on ice for 20 min. The cells were then collected by scraper, homogenized by sonication in 1× HEPES buffer, centrifuged to collect the supernatant. Final protein concentration was adjusted to 2 mg/mL. The lysates were then subjected to click reaction with Rh-Biotin-N_3, and all subsequent steps were the same as above described. For *in situ* PD with synchronized Hela cells, cells were firstly released from thymidine block in growth medium for ~10 h before probe incubation, and all subsequent procedures were exactly the same as asynchronized cells.

For LC-MS/MS experiments, enriched protein samples (e.g. after PD) were separated on 10% SDS-PAGE and stained with coomassie brilliant blue. Each gel lane was then sliced into small gel pieces and the peptides were digested using In-Gel Trypsin Digestion Kit, and extracted with 50% acetonitrile and 1% formic acid. All peptide samples were dried *in vacuo* and reconstituted with 20 µL of 0.1% TFA/H_2O. The peptides were separated and analyzed on a Shimadzu UFLC system (Shimadzu, Japan) coupled to a LTQ-FT Ultra (Thermo Electron, Germany). Mobile phase A (0.1% formic acid in H_2O) and mobile phase B (0.1% formic acid in acetonitrile) were used to establish the 60 min gradient comprising 45 min of 5-35% B, 8 min of 35-50% B, and 2 min of 80% B, followed by re-equilibrating at 5% B for 5 min. Peptides were then analyzed on LTQ-FT with an Advance CaptiveSpray Source (Michrom Bio Resources) at an electrospray potential of 1.5 kV. A gas flow

of 2 L/min, ion transfer tube temperature of 180 °C, and collision gas pressure of 0.85 mTorr were used. The LTQ-FT was set to perform data acquisition in the positive-ion mode as previously described,[4-5] except that the m/z range of 350-1600 was used in the full MS scan. The raw data were converted to mgf format. The database search was performed with an in-house Mascot server (version 2.2.07, Matrix Science) with MS tolerance of 10 ppm and MS/MS tolerance of 0.8 Da. Two missed cleavage sites of trypsin were allowed. Carbamidome-thylation (C) was set as a fixed modification, and oxidation (M) and phosphorylation (S, T, and Y) were set as variable modifications. Results are summarized in SI_2/3, and further processed as described in the maintext.

13. References

[1] (a) Yang, K. S.; Budin, G.; Reiner, T.; Vinegoni, C. & Weissleder, R. *Angew. Chem., Int. Ed.* **51**, 6598-6603 (2012); (b) Budin G., Yang K. S., Reiner T., Weissleder R. *Angew. Chem. Int. Ed.* **50**, 9378-9382 (2011).

[2] (a) Shi, H.; Cheng, X.; Sze, S. K. & Yao, S. Q. *Chem. Commun.*, **47**, 11306-11308 (2011); (b) Shi, H.; Zhang, C.-J.; Chen, G. Y. J. & Yao, S. Q. *J. Am. Chem. Soc.* **134**, 3001-3014 (2012); (c) Li, Z.; Hao, P.; Li, L.; Tan, C. Y. J.; Cheng, X.; Chen, G. Y. J.; Sze, S. K.; Shen, H.-M. & Yao, S. Q. *Angew. Chem., Int. Ed.* **52**, 8551-8556 (2013); (d) Liu, K.; Kalesh, K. A.; Ong, L. B. & Yao, S. Q. *ChemBioChem,* **9**, 1883-1888 (2008).

[3] Sloane, D. A.; Trikic, M. Z.; Chu, M. L. H.; Lamers, M. B. A. C.; Mason, C. S.; Mueller, I.; Savory, W. J.; William, D. H. & Eyers, P. A. *ACS Chem. Biol.* **5**, 563-576 (2010).

[4] Gan, C.S., Guo, T., Zhang, H., Kim, S.K., & Sze, S.K. A Comparative Study of Electrostatic Repulsion-Hydrophilic Interaction Chromatography (ERLIC) versus SCX-IMAC-Based Methods for Phosphopeptide Isolation/Enrichment. *J. Proteome Res*. **7**, 4869-4877 (2008).

[5] Hao, P., Guo, T., Li, X., Adav, S.S., Yang, J., Wei, M., & Sze S.K. Novel Application of Electrostatic Repulsion-Hydrophilic Interaction Chromatography (ERLIC) in Shotgun Proteomics: Comprehensive Profiling of Rat Kidney Proteome. *J. Proteome Res*. **9**, 3520-3526 (2010).

[6] Mellacheruvu D., Wright Z., Couzens A. L., *et al*. The CRAPome: a contaminant repository for affinity purification-mass spectrometry data. *Nat. Methods* **10**, 730-736 (2013).

1H AMX500
suy0219 3
MLN COUP NH2-1 DMSO 7.1mg

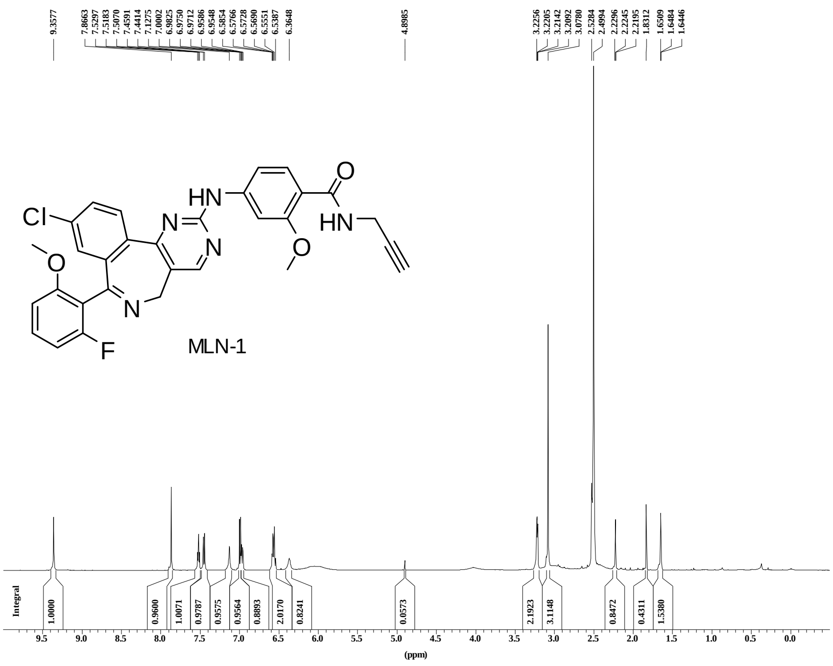

*** Current Data Parameters ***
NAME : suy0219
EXPNO : 3
PROCNO : 1
*** Acquisition Parameters ***
LOCNUC : 2H
NS : 80
NUCLEUS : off
O1 : 3088.51 Hz
PULPROG : zg30
SFO1 : 500.1330885 MHz
SOLVENT : DMSO
SW : 20.6557 ppm
TD : 32768
TE : 296.2 K
*** Processing Parameters ***
LB : 0.30 Hz
SF : 500.1304318 MHz
*** 1D NMR Plot Parameters ***
NUCLEUS : off

13C AMX500
suy0219 2
MLN COUP NH2-1 DMSO 7.1mg

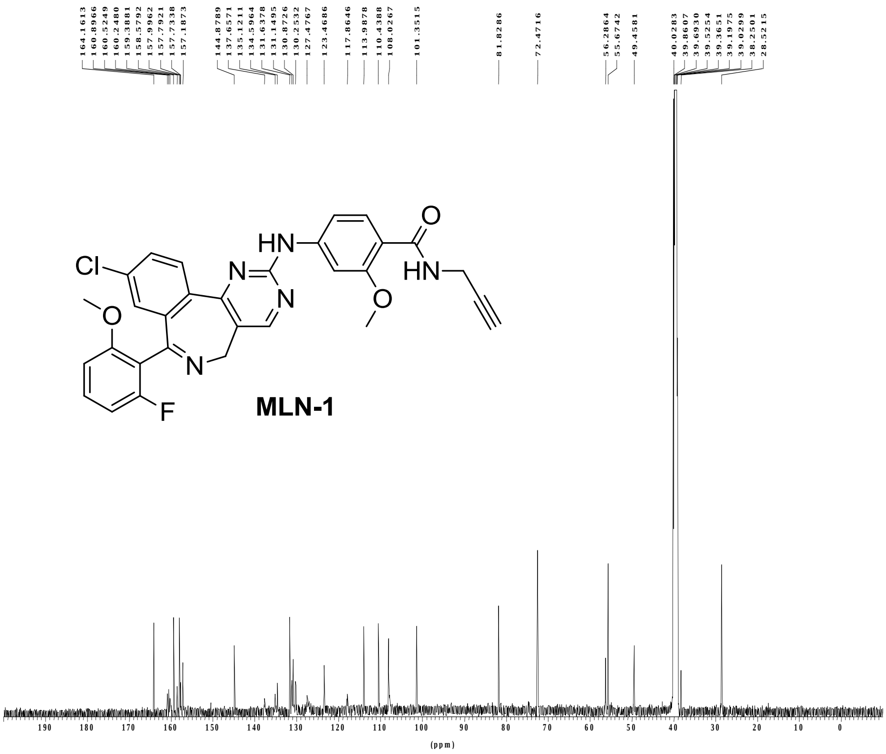

*** Current Data Parameters ***
NAME : suy0219
EXPNO : 2
PROCNO : 1
*** Acquisition Parameters ***
LOCNUC : 2H
NS : 10916
NUCLEUS : off
O1 : 13204.57 Hz
PULPROG : zgpg30
SFO1 : 125.7709936 MHz
SOLVENT : DMSO
SW : 238.7675 ppm
TD : 65536
TE : 296.4 K
*** Processing Parameters ***
LB : 1.00 Hz
SF : 125.7578473 MHz
*** 1D NMR Plot Parameters ***
NUCLEUS : off

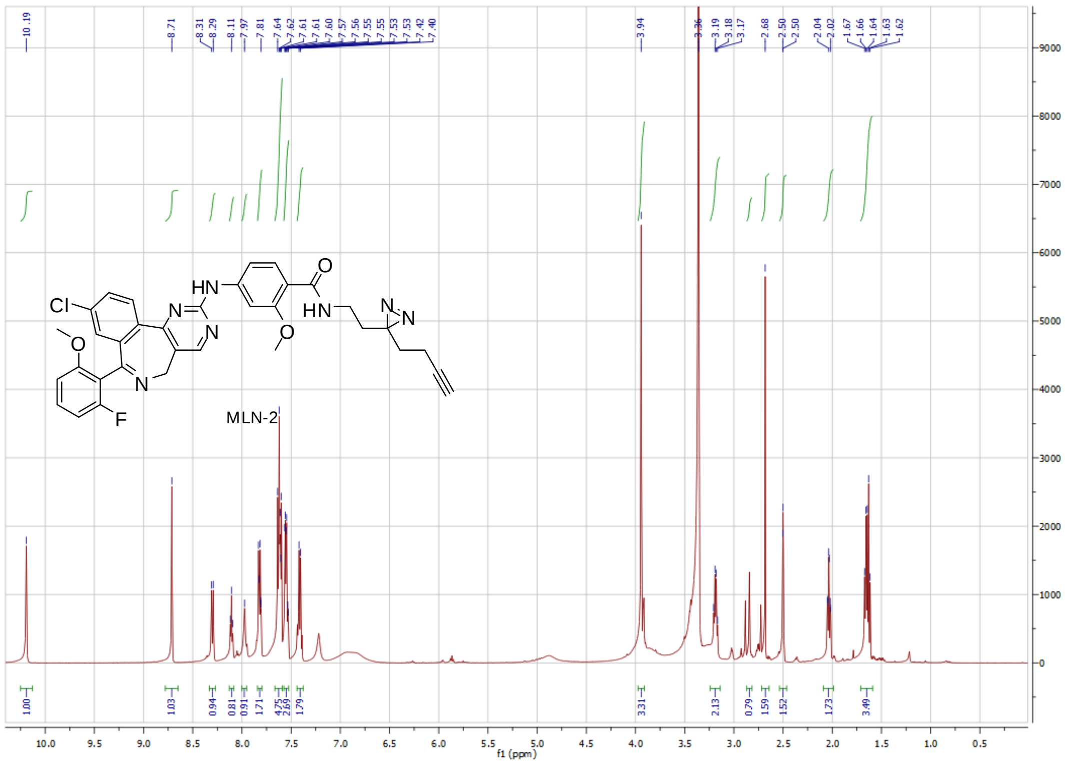

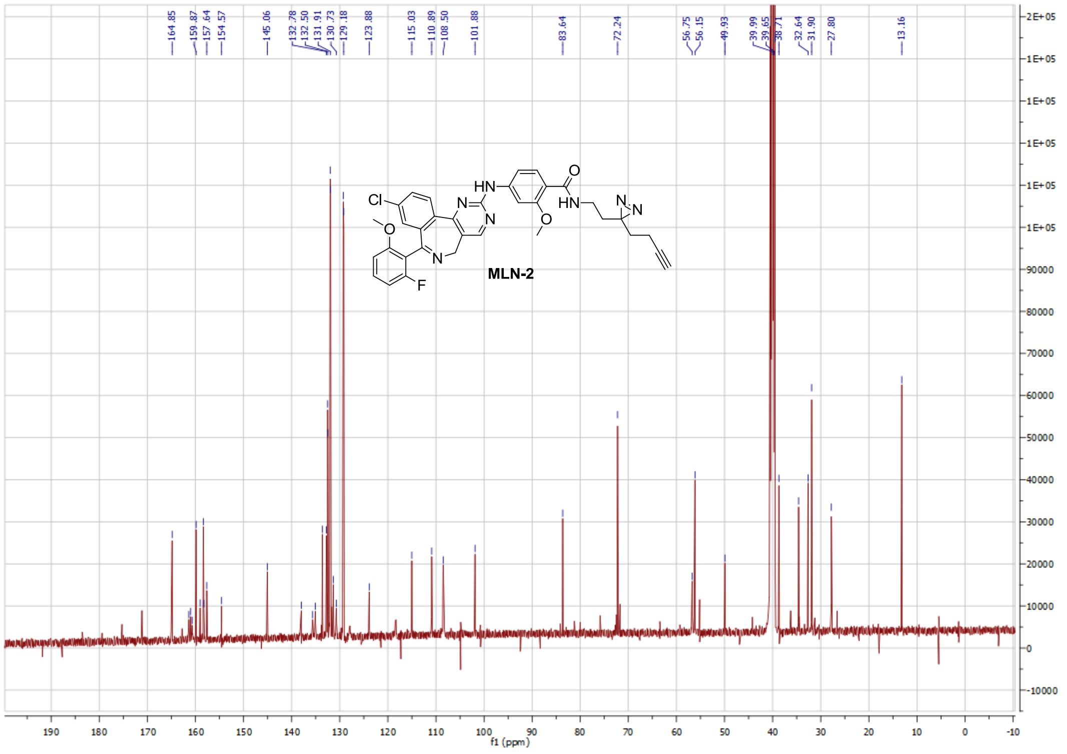

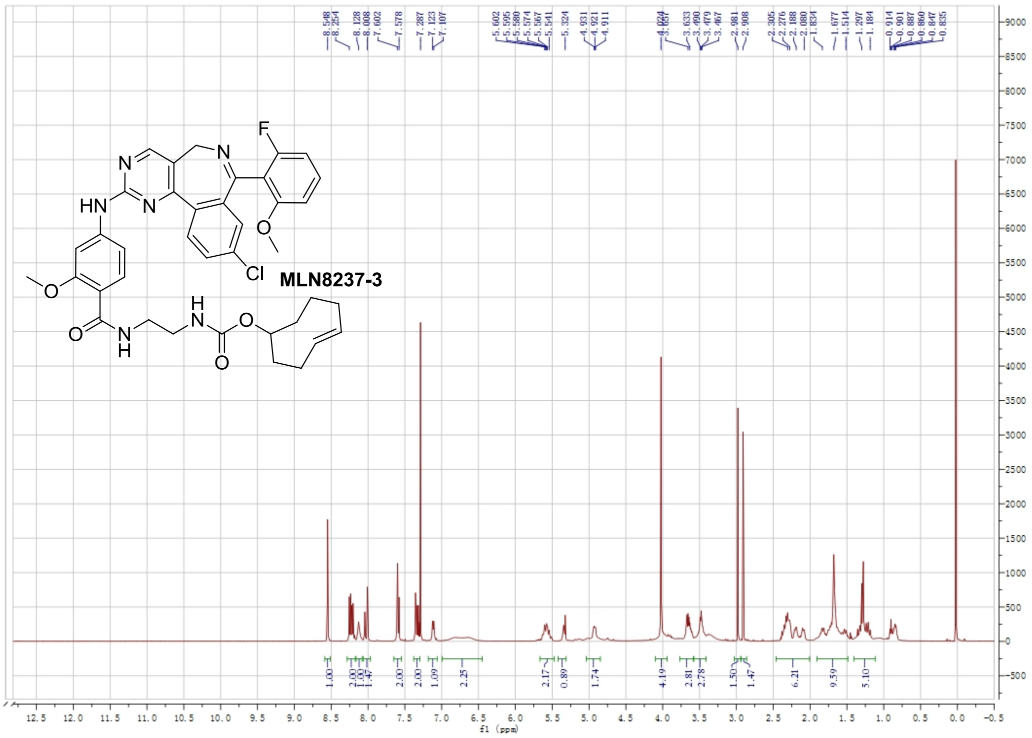

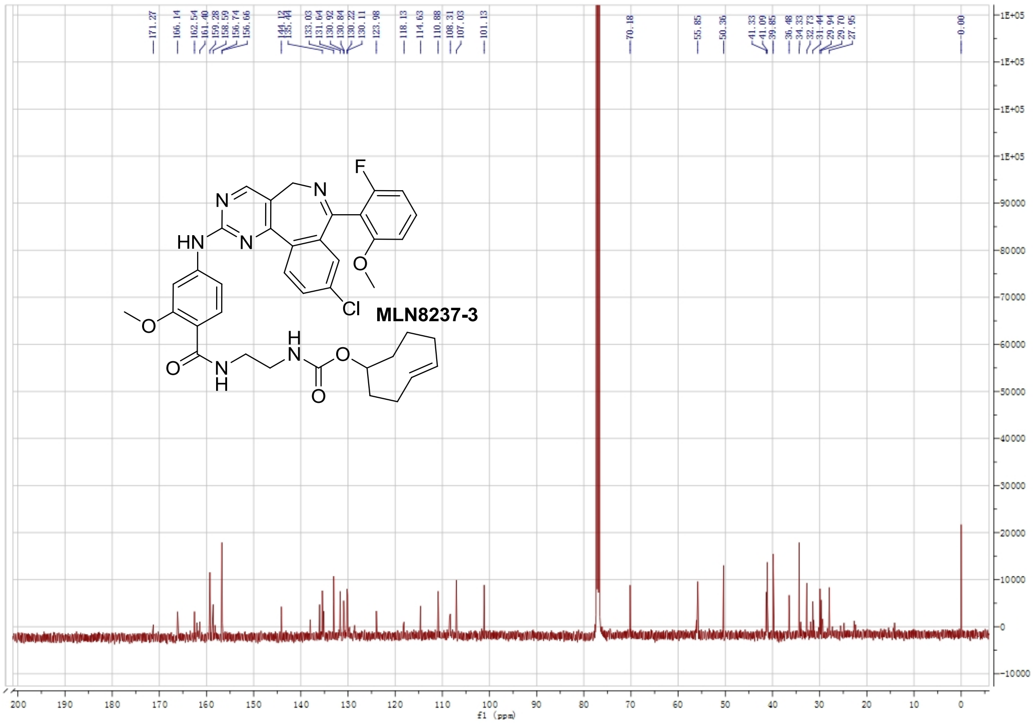

MLN-2 targets																
				Protein Score						emPAI						
				0.2 μM	2 μM					0.2 μM	2 μM					
					Hela Live		Hela Lysate		SHSY Liv		Hela Live		Hela Lysate		SHSY Live	
GS	Function	**Name**	**Nuclear localization**	Syn 12	Syn 01	Asyn 09	Syn 15	Asyn 18	Asyn 05	Syn 12	Syn 01	Asyn 09	Syn 15	Asyn 18	Asyn 05	
ANXA1	Calcium binding protein	Annexin A1	Alternate	466	187	154	163			1.66	0.92	0.63	0.5			
ARPC1B	Cytoskeletal associated protein	Actin related protein 2/3 complex, subunit 1B, 41kDa	Nucleolus as Alt	70	222	39	58	75		0.26	0.58	0.08	0.36	0.26		
CCNK	Transcription regulator	SWI/SNF complex subunit SMARCC1	Primary	63	92	63	39	35		0.1	0.08	0.08	0.08	0.08		
CUL4B	Enzyme(Ubiquitin-specific protease)	Cullin 4B	Primary	131	53	125	164	105		0.17	0.06	0.13	0.21	0.13		
FEN1	Enzyme(endonuclease)	Flap endonuclease 1	Primary	62	57	151	123	133		0.16	0.16	0.35	0.25	0.16		
FUBP1	Transcription regulator	Far upstream element-binding protein 1	Primary	211	163	237	124	183		0.26	0.2	0.44	0.15	0.48		
HDAC1	Enzyme(deacetylase)	Histone deacetylase 1	Primary	136	95	52		73		0.28	0.26	0.26		0.19		
HDAC2	Enzyme(deacetylase)	Histone deacetylase	Primary	136	105	162	45	121		0.28	0.2	0.36	0.13	0.28		
HNRNPH3	Ribonucleoprotein	Heterogeneous nuclear ribonucleoprotein H3	Primary	44	80	63	79	30		0.13	0.29	0.13	0.29	0.19		
HNRNPUL1	RNA binding protein	Heterogeneous nuclear ribonucleoprotein U-like protein 1	Primary	67	74	164	95	104		0.11	0.16	0.12	0.08	0.12		
KHSRP	Transcription regulator	Far upstream element-binding protein 2	Primary	166	86	227	118	175		0.19	0.19	0.36	0.19	0.24		
KPNA2	Transporter	Importin subunit alpha, Karyopherin alpha 2	Alternate	256	205	222	286	200		0.47	0.47	0.55	0.55	0.32		
LTA4H	Enzyme(hydrolase)	Leukotriene A-4 hydrolase	Alternate	135	98	250	104	169		0.2	0.1	0.32	0.22	0.49		
MAGED2	Unclassified	Melanoma antigen family D, 2	Primary	61	169	98	72	173		0.13	0.12	0.17	0.13	0.12		
MAPK1(ERK2)	Enzyme(kinase)	Mitogen-activated protein kinase 1	Alternate	58	34	45	30		57	0.16	0.08	0.06	0.08		0.16	
MATR3	RNA binding protein	Matrin-3	Primary	240	240	209	228	230		0.74	0.34	1.21	0.38	0.72		
MDH2	Enzyme(dehydrogenase)	Malate dehydrogenase	Mitochondrion as Pri, Nucleolus as Alt	114	59		106	223		0.98	0.29		0.63	0.98		
NSUN2	Enzyme(methyltransferase)	tRNA (cytosine(34)-C(5))-methyltransferase	Alternate, Nucleolus as Pri	193	150	215	210	190		0.34	0.16	0.39	0.29	0.34		
NUDC	Cell cycle control protein	Nuclear migration protein nudC	Alternate	73	56	47				0.18	0.18	0.18				
OLA1	Enzyme(ATPase)	Obg-like ATPase 1	Nucleolus as Pri	72	58	108	65	67		0.14	0.07	0.22	0.07	0.07		
PSMC1	Enzyme(Ubiquitin-specific protease)	26S protease regulatory subunit 4	Alternate	159	122	191	56	60		0.36	0.26	0.59	0.38	0.08		
RBM4	RNA binding protein	RNA-binding protein 4	Primary	63	93	57	98	79		0.18	0.26	0.29	0.17	0.08		
RPS2	Ribosomal subunit	40S ribosomal protein S2	Nucleolus as Alt	60	39	47	16	46		0.18	0.28	0.5	0.5	0.5		
RPS4X	Ribosomal subunit	Ribosomal protein S4	Nucleolus as Alt	52	28	62				0.11	0.24	0.24				
SAMHD1	Enzyme(GTPase)	SAM domain and HD domain-containing protein 1	Primary	54	30			36		0.17	0.05				0.05	
SEPT9	Enzyme(GTPase)	Septin 9	Primary	106	81	183	57	91		0.28	0.1	0.34	0.05	0.22		
SF3B1	RNA binding protein	Splicing factor 3B subunit 1	Primary	379	202	438	217	81		0.49	0.3	0.52	0.25	0.12		
STAT3	Transcription factor	Signal transducer and activator of transcription 3	Primary	52	75	123	126	36		0.04	0.08	0.16	0.16	0.04		
TGM2	Enzyme(glutamyltransferase)	Protein-glutamine gamma-glutamyltransferase 2	Alternate	150	47	112	67	117		0.45	0.05	0.26	0.2	0.2		
THRAP3	Transcription regulator	Thyroid hormone receptor-associated protein 3	Primary	126	67	165				0.13	0.06	0.16				
WDR12	Unclassified	Ribosome biogenesis protein WDR12	Nucleolus as Pri	99	68	91		62	18	0.22	0.07	0.14		0.07	0.07	
ZFR	Transcription regulator	Zinc finger RNA binding protein	Primary	69	188	180	116	109		0.36	0.12	0.15	0.06	0.06		

PU-1 targets															
				Protein Score						emPAI					
				0.2 µM	2 µM					0.2 µM	2 µM				
				Hela Live			Hela Lysate		SHSY Live	Hela Live			Hela Lysate		SHSY Live
GS	Function	**Name**	**Nuclear localization**	Syn 13	Syn 02	Asyn 10	Syn 16	Asyn 19	Asyn 06	Syn 13	Syn 02	Asyn 10	Syn 16	Asyn 19	Asyn 06
ANXA1	Calcium binding protein	Annexin A1	Alternate	235	156					0.92	0.93				
CUL4B	Enzyme(Ubiquitin-specific protease)	Cullin 4B	Primary	95	40					0.1	0.03				
FEN1	Enzyme(endonuclease)	Flap endonuclease 1	Primary	113	127					0.18	0.28				
FUBP1	Transcription regulator	Far upstream element-binding protein 1	Primary	79	127					0.17	0.28				
HDAC1	Enzyme(deacetylase)	Histone deacetylase 1	Primary	66	38					0.33	0.21				
HDAC2	Enzyme(deacetylase)	Histone deacetylase	Primary	170	119					0.44	0.2				
HNRNPH3	Ribonucleoprotein	Heterogeneous nuclear ribonucleoprotein H3	Primary	54	28					0.29	0.09				
HNRNPUL1	RNA binding protein	Heterogeneous nuclear ribonucleoprotein U-like protein 1	Primary	149	73					0.14	0.12				
KHSRP	Transcription regulator	Far upstream element-binding protein 2	Primary	102	213					0.14	0.14				
KPNA2	Transporter	Importin subunit alpha, Karyopherin alpha 2	Alternate	176	91					0.32	0.21				
LTA4H	Enzyme(hydrolase)	Leukotriene A-4 hydrolase	Alternate	212	228					0.32	0.26				
MAGED2	Unclassified	Melanoma antigen family D, 2	Primary	71	178					0.19	0.26				
MATR3	RNA binding protein	Matrin-3	Primary	374	173					0.96	0.24				
MDH2	Enzyme(dehydrogenase)	Malate dehydrogenase	Mitochondrion as Pri, Nucleolus as Alt	141	112					1.18	0.34				
NSUN2	Enzyme(methyltransferase)	tRNA (cytosine(34)-C(5))-methyltransferase	Alternate, Nucleolus as Pri	228	30					0.5	0.11				
PSMC1	Enzyme(Ubiquitin-specific protease)	26S protease regulatory subunit 4	Alternate	110	94					0.17	0.26				
RPS2	Ribosomal subunit	40S ribosomal protein S2	Nucleolus as Alt	53	93					0.5	0.49				
SEPT9	Enzyme(GTPase)	Septin 9	Primary	158	76					0.34	0.3				
SF3B1	RNA binding protein	Splicing factor 3B subunit 1	Primary	324	226					0.39	0.25				
THRAP3	Transcription regulator	Thyroid hormone receptor-associated protein 3	Primary	167	92					0.23	0.06				
ZFR	Transcription regulator	Zinc finger RNA binding protein	Primary	220	0.15					118	0.09				
CLCC1	Ion channel	Chloride channel CLIC-like protein 1	Alternate	80	102	117		36	72	0.11	0.11	0.11		0.05	0.05
CSTF1	RNA binding	Cleavage stimulation factor subunit 1	Primary	52	61		64	56		0.22	0.14		0.07	0.07	
EIF3S9	Transcription regulator	Eukaryotic translation initiation factor 3, subunit 9 eta	Alternate	56	113	118	67	43		0.16	0.16	0.24	0.07	0.09	
FUBP3	Transcription regulator	Far upstream element-binding protein 3	Primary	112	54	107	69	95		0.3	0.11	0.17	0.17		
LARP4	RNA binding	La-related protein 4	Primary	60	56	113	64		41	0.14	0.04	0.14	0.04		0.09
LUC7L3	Transcription regulator	Cisplatin resistance-associated overexpressed protein, isoform CRA_b	Primary	99	46	68	99	88	80	0.12	0.06	0.06	0.06	0.12	0.06
NAT10	Enzyme(Acyltransferase)	N-acetyltransferase 10	Primary	82	24	36	102	121		0.12	0.03	0.06	0.16	0.15	
NUP160	Transporter	Nuclear pore complex protein Nup160	Primary	77	39	46	180	66		0.12	0.02	0.05	0.22	0.06	
NUP214	Transporter	Nuclear pore complex protein Nup214	Primary	119	33	79	42			0.11	0.24	0.03	0.03		
SQSTM1	Enzyme(Ubiquitin-specific protease)	Sequestosome-1	Alternate	53	57		51	56		0.35	0.1		0.35	0.35	
TRIM25	Transcription factor	Tripartite motif-containing 25	Primary	115	80	81	110	84		0.19	0.07	0.07	0.39	0.3	

MDH2		Enzyme(dehydrogenase)	114	141
NAT10		Enzyme(acyltransferase)	no	82
NSUN2		Enzyme(methyltransferase)	193	228
NUDC		Cell cycle control	73	no
NUP160		Transporter	no	77
NUP214		Transporter	no	119
OLA1		Enzyme(ATPase)	72	no
PSMC1		Enzyme(deubiquitinase)	159	110
RBM4		RNA binding	63	no
RPS2		Ribosomal subunit	60	53
RPS4X		Ribosomal subunit	52	no
SAMHD1		Enzyme(GTPase)	54	no
SEPT9		Enzyme(GTPase)	106	158
SF3B1		RNA binding	379	324
SQSTM1		Enzyme(deubiquitinase)	no	53
STAT3		Transcription factor	52	no
TGM2		Enzyme(glutamyltransferase)	150	no
THRAP3		Transcription regulator	126	167
TRIM25		Transcription factor	no	115
WDR12		Unclassified	99	no
ZFR		Transcription regulator	69	220

#01	Hela live syn MLN-2_2μM																		
#04	Hela live syn NP_2μM																		
Accession	GS	Name	Pro_Mass	04_p	01_pro	04_prot	01_prot	04_em	01_em	emPAI ratio	04_No>I	01_No>I	04_No>HI	01_No>HI	04_No>20	01_No>20	04_No>All	01_No>All	Rsc
sp|Q96GD4|AURKB_HUMAN	AURKB	Aurora kinase B OS=Homo	39571	0	1048	0	26	0	0.25	#DIV/0!	0	0	0	1	0	1	0	1	1.189598
sp|P28482|MK01_HUMAN	MAPK1 (ERK2)	Mitogen-activated protein	41762	0	910	0	34	0	0.08	#DIV/0!	0	0	0	1	0	2	0	2	1.926812
sp|Q96SB4|SRPK1_HUMAN	SRPK1	SRSF protein kinase 1 OS=H	74963	0	805	0	39	0	0.04	#DIV/0!	0	1	0	1	0	1	0	1	1.189598
sp|P53350|PLK1_HUMAN	PLK1	Serine/threonine-protein ki	68953	0	864	0	36	0	0.05	#DIV/0!	0	1	0	1	0	1	0	1	1.189598
sp|Q9H4A3|WNK1_HUMAN	WNK1	Serine/threonine-protein ki	251552	0	481	0	75	0	0.03	#DIV/0!	0	1	0	2	0	2	0	3	1.926812
sp|Q9Y6E0|STK24_HUMAN	STK24	Serine/threonine-protein ki	49562	0	974	0	30	0	0.08	#DIV/0!	0	1	0	1	0	1	0	1	1.189598
sp|P51812|KS6A3_HUMAN	RPS6KA3	Ribosomal protein S6 kinas	84025	0	836	0	37	0	0.08	#DIV/0!	0	0	0	2	0	3	0	4	2.412488
sp|Q15418|KS6A1_HUMAN	RPS6KA1	Ribosomal protein S6 kinas	83070	0	600	0	59	0	0.05	#DIV/0!	0	1	0	1	0	2	0	3	1.926812
sp|Q05397|FAK1_HUMAN	PTK2 (FADK1)	Focal adhesion kinase 1 OS	119956	0	986	0	30	0	0.04	#DIV/0!	0	0	0	1	0	1	0	2	1.189598
sp|Q13418|ILK_HUMAN	ILK	Integrin-linked protein kina	51899	421	434	41	74	0.07	0.36	5.142857	1	1	1	4	1	3	1	5	0.827198
sp|P78527|PRKDC_HUMAN	PRKDC	DNA-dependent protein kin	473749	83	100	241	303	0.07	0.09	1.285714	7	9	10	12	11	15	17	17	0.035153
sp|P19367|HXK1_HUMAN	HK1	Hexokinase-1 OS=Homo sap	103561	0	853	0	36	0	0.06	#DIV/0!	0	0	0	2	0	3	0	5	2.412488
sp|Q16822|PCKGM_HUMAN	PCK2	Phosphoenolpyruvate carbo	71483	0	760	0	42	0	0.13	#DIV/0!	0	1	0	2	0	3	0	4	2.412488
tr|J3KNX3|J3KNX3_HUMAN	PFKM	6-phosphofructokinase OS=	94107	0	365	0	98	0	0.11	#DIV/0!	0	2	0	3	0	3	0	4	2.412488
sp|Q13574|DGKZ_HUMAN	DGKZ	Diacylglycerol kinase zeta C	125704	0	1087	0	23	0	0.03	#DIV/0!	0	0	0	1	0	1	0	1	1.189598
sp|P00558|PGK1_HUMAN	PGK1	Phosphoglycerate kinase 1	44985	134	55	161	490	0.76	1.9	2.5	5	13	8	16	9	16	9	18	0.401891
sp|P14618|KPYM_HUMAN	PKM	Pyruvate kinase isozymes M	58470	8	4	1820	2460	5.79	12.09	2.088083	47	61	58	85	60	85	66	94	0.104909
sp|Q01813|K6PP_HUMAN	PFKP	6-phosphofructokinase type	86454	98	127	217	259	0.4	0.51	1.275	5	7	9	11	9	13	10	14	0.111636
sp|P36873|PP1G_HUMAN	PPP1CC	Serine/threonine-protein p	37701	0	194	0	180	0	0.71	#DIV/0!	0	5	0	6	0	6	0	9	3.306321
sp|P67775|PP2AA_HUMAN	PPP2CA	Serine/threonine-protein p	36142	0	501	0	72	0	0.19	#DIV/0!	0	1	0	2	0	3	0	3	2.412488
sp|P00533|EGFR_HUMAN	EGFR	Epidermal growth factor re	137612	0	789	0	40	0	0.05	#DIV/0!	0	1	0	2	0	2	0	3	1.926812
sp|P40763|STAT3_HUMAN	STAT3	Signal transducer and activa	88810	0	480	0	75	0	0.08	#DIV/0!	0	2	0	2	0	3	0	4	2.412488
sp|O43684|BUB3_HUMAN	BUB3	Mitotic checkpoint protein	37587	681	636	20	44	0.2	0.48	2.4	0	1	1	3	0	3	1	3	2.412488
sp|Q9NSY1|BMP2K_HUMAN	BMP2K	BMP-2-inducible protein kir	129947	0	1149	0	16	0	0.03	#DIV/0!	0	0	0	1	0	0	0	1	-0.39561
sp|P55263|ADK_HUMAN	ADK	Adenosine kinase OS=Homo	40919	538	993	30	29	0.08	0.18	2.25	0	1	1	2	1	1	1	2	-0.39569
sp|O00764|PDXK_HUMAN	PDXK	Pyridoxal kinase OS=Homo	35308	566	788	29	40	0.11	0.18	1.636364	0	1	1	1	1	1	1	1	-0.39569
tr|B2RC06|B2RC06_HUMAN	tr|B2RC06|B2RC	cDNA, FLJ95791, highly sim	39540	0	1047	0	26	0	0.08	#DIV/0!	0	0	0	1	0	1	0	1	1.189598
sp|O75116|ROCK2_HUMAN	ROCK2	Rho-associated protein kina	161939	375	698	46	48	0.05	0.05	1	1	1	2	2	1	2	3	2	0.341522
sp|P51570|GALK1_HUMAN	GALK1	Galactokinase OS=Homo sa	42702	679	937	20	32	0.07	0.07	1	0	0	1	1	0	1	1	1	1.189598
sp|P12277|KCRB_HUMAN	CKB	Creatine kinase B-type OS=	42902	214	868	97	35	0.18	0.17	0.944444	2	0	2	1	2	1	2	1	-1.13299
sp|P36507|MP2K2_HUMAN	MAP2K2 (MEK2)	Dual specificity mitogen-act	44681	384	962	45	31	0.31	0.14	0.451613	1	0	2	1	2	1	2	1	-1.13299
sp|P07205|PGK2_HUMAN	PGK2	Phosphoglycerate kinase 2	45166	134	559	81	65	0.76	0.15	0.197368	1	1	3	2	4	3	4	4	-0.75875
tr|B4DRT3|B4DRT3_HUMAN	tr|B4DRT3|B4DR	Pyruvate kinase OS=Homo	56404	8	6	1475	2051	5.79	9.24	1.595855	40	52	50	71	52	73	58	80	0.090971
tr|H3BTN5|H3BTN5_HUMAN	PKM	Pyruvate kinase (Fragment)	53524	8	8	1400	1957	5.79	8.12	1.402418	38	49	49	68	50	70	56	75	0.086791
sp|Q9Y3F4|STRAP_HUMAN	STRAP	Serine-threonine kinase rec	38756	626	406	23	91	0.12	0.25	2.083333	0	2	1	2	1	2	1	2	0.341522
tr|C1PHA2|C1PHA2_HUMAN	KIF5B-ALK	Tyrosine-protein kinase rec	169283	227	265	91	129	0.04	0.19	4.75	2	2	2	5	2	5	3	6	0.74248
sp|P29966|MARCS_HUMAN	MARCKS	Myristoylated alanine-rich	31707	0	500	0	73	0	0.22	#DIV/0!	0	2	0	2	0	2	0	2	1.926812
tr|Q4VB88|Q4VB88_HUMAN	NELL1	NELL1 protein OS=Homo sa	89112	0	869	0	35	0	0.03	#DIV/0!	0	1	0	1	0	1	0	1	1.189598
sp|Q9NYJ8|TAB2_HUMAN	TAB2	TGF-beta-activated kinase 1	77017	0	682	0	50	0	0.04	#DIV/0!	0	1	0	1	0	1	0	1	1.189598
sp|Q06124|PTN11_HUMAN	PTPN11	Tyrosine-protein phosphata	68964	0	939	0	32	0	0.05	#DIV/0!	0	0	0	1	0	2	0	2	1.926812
tr|H0Y3P2|H0Y3P2_HUMAN	EIF4G2	Eukaryotic translation initia	98569	0	403	0	91	0	0.1	#DIV/0!	0	3	0	3	0	3	0	3	2.412488
sp|Q5H9R7|PP6R3_HUMAN	PPP6R3	Serine/threonine-protein p	98577	0	397	0	93	0	0.1	#DIV/0!	0	2	0	3	0	3	0	4	2.412488
sp|Q9BZG2|PPAT_HUMAN	ACPT	Testicular acid phosphatase	46459	0	1113	0	21	0	0.07	#DIV/0!	0	0	0	1	0	1	0	1	1.189598
tr|B4DSN5|B4DSN5_HUMAN	PTPN1	Tyrosine-protein phosphata	41747	0	1050	0	26	0	0.08	#DIV/0!	0	0	0	1	0	1	0	1	1.189598
sp|Q9BX95|SGPP1_HUMAN	SGPP1	Sphingosine0-phosphate ph	49703	0	942	0	32	0	0.07	#DIV/0!	0	0	0	1	0	1	0	1	1.189598
sp|Q8TAP8|PPR35_HUMAN	PPP1R35	Protein phosphatase 1 regu	28107	0	879	0	35	0	0.12	#DIV/0!	0	1	0	1	0	1	0	1	1.189598
tr|H7C292|H7C292_HUMAN	INPP5D	Phosphatidylinositol 3,4,5-t	105819	0	876	0	35	0	0.03	#DIV/0!	0	1	0	1	0	1	0	3	1.189598
tr|B2RAH5|B2RAH5_HUMAN	tr|B2RAH5|B2RA	cDNA, FLJ94919, highly sim	115658	0	800	0	39	0	0.03	#DIV/0!	0	1	0	1	0	1	0	2	1.189598
tr|J3QT22|J3QT22_HUMAN	PPME1	Protein phosphatase methy	44241	232	515	90	70	0.07	0.15	2.142857	1	2	1	2	1	2	2	2	0.341522
tr|B4DDF7|B4DDF7_HUMAN	tr|B4DDF7|B4DD	cDNA FLJ53296, highly simi	64939	443	193	39	182	0.16	0.28	1.75	1	4	3	5	3	5	3	5	0.256725
tr|C9J9S3|C9J9S3_HUMAN	PPP1CB	Serine/threonine-protein p	14484	345	176	51	31	0.24	0.65	2.708333	1	1	1	1	1	1	1	2	-0.39569
tr|A6NIT2|A6NIT2_HUMAN	PHKA1	Phosphorylase b kinase reg	133462	711	0	17	0	0.02	0	0	0	0	1	0	0	0	1	0	-0.39561
sp|O95747|OXSR1_HUMAN	OXSR1	Serine/threonine-protein ki	58271	675	0	20	0	0.06	0	0	0	0	1	0	1	0	2	0	-1.9809
tr|H0Y3Y5|H0Y3Y5_HUMAN	PPIP5K1	Inositol hexakisphosphate a	21913	632	0	22	0	0.15	0	0	0	0	1	0	1	0	2	0	-1.9809
tr|H0Y3C5|H0Y3C5_HUMAN	HCK	Tyrosine-protein kinase HC	59986	628	0	23	0	0.05	0	0	0	0	1	0	1	0	2	0	-1.9809
tr|H0YH33|H0YH33_HUMAN	STRAP	Serine-threonine kinase rec	10763	626	0	23	0	0.12	0	0	0	0	1	0	1	0	1	0	-1.9809
tr|A8K2U2|A8K2U2_HUMAN	tr|A8K2U2|A8K2	cDNA FLJ75392, highly simi	103718	599	0	26	0	0.03	0	0	0	0	1	0	1	0	2	0	-1.9809
tr|Q86U79|Q86U79_HUMAN	tr|Q86U79|Q86U	Adenosine kinase OS=Homo	39112	538	0	30	0	0.08	0	0	0	0	1	0	1	0	1	0	-1.9809
tr|B2R5T5|B2R5T5_HUMAN	PRKAR1A	Protein kinase, cAMP-depe	43183	533	0	31	0	0.08	0	0	0	0	1	0	1	0	1	0	-1.9809
sp|Q9UEW8|STK39_HUMAN	STK39	STE20/SPS1-related proline	59950	491	0	35	0	0.05	0	0	0	0	1	0	1	0	1	0	-1.9809
tr|B4E2K2|B4E2K2_HUMAN	tr|B4E2K2|B4E2	cDNA FLJ55665, highly simi	103935	246	0	79	0	0.03	0	0	1	0	1	0	1	0	1	0	-1.9809
tr|G3V4N7|G3V4N7_HUMAN	CKB	Creatine kinase B-type (Fra	24272	214	0	51	0	0.18	0	0	1	0	1	0	1	0	1	0	-1.9809
tr|Q8IWY7|Q8IWY7_HUMAN	TTBK2	Tau-tubulin kinase OS=Hom	183893	79	0	28	0	0.79	0	0	0	0	1	0	1	0	1	0	-1.9809
tr|H0YDU8|H0YDU8_HUMAN	PPP5C	Serine/threonine-protein p	55743	596	815	26	38	0.06	0.06	1	0	1	1	1	1	1	1	1	-0.39569
tr|B4DDZ8|B4DDZ8_HUMAN	tr|B4DDZ8|B4DD	cDNA FLJ55048, highly simi	55829	596	0	26	0	0.06	0	0	0	0	1	0	1	0	1	0	-1.9809
tr|B4E1Q0|B4E1Q0_HUMAN	tr|B4E1Q0|B4E1	cDNA FLJ59603, highly simi	17409	443	0	33	0	0.16	0	0	0	0	1	0	1	0	1	0	-1.9809
tr|B7Z8Z8|B7Z8Z8_HUMAN	tr|B7Z8Z8|B7Z8	cDNA FLJ61702, highly simi	181264	0	833	0	37	0	0.02	#DIV/0!	0	3	0	3	0	13	0	17	4.362515
tr|B7Z1N6|B7Z1N6_HUMAN	tr|B7Z1N6|B7Z1	cDNA FLJ52222, highly simi	35800	0	72	0	395	0	0.3	#DIV/0!	0	6	0	9	0	13	0	13	4.362515
sp|O75533|SF3B1_HUMAN	SF3B1	Splicing factor 3B subunit 1	146479	0	177	0	202	0	0.3	#DIV/0!	0	6	0	12	0	11	0	14	4.130691
tr|A8K132|A8K132_HUMAN	tr|A8K132|A8K1	cDNA FLJ75476, highly simi	74317	0	190	0	187	0	0.41	#DIV/0!	0	4	0	8	0	9	0	10	3.854557
sp|Q92626|PXDN_HUMAN	PXDN	Peroxidasin homolog OS=H	167793	0	181	0	194	0	0.17	#DIV/0!	0	5	0	8	0	9	0	10	3.854557
tr|B2RDW1|B2RDW1_HUMA	RPS27A	Ribosomal protein S27a, isc	18296	0	437	0	85	0	0.66	#DIV/0!	0	3	0	5	0	8	0	9	3.693843
sp|Q08J23|NSUN2_HUMAN	NSUN2	tRNA (cytosine(34)-C(5))-m	87214	0	251	0	150	0	0.16	#DIV/0!	0	5	0	7	0	8	0	10	3.693843
tr|Q5TZZ9|Q5TZZ9_HUMAN	ANXA1	Annexin OS=Homo sapiens	38918	0	191	0	187	0	0.92	#DIV/0!	0	5	0	8	0	8	0	9	3.693843
sp|O43776|SYNC_HUMAN	NARS	Asparagine--tRNA ligase, cy	63758	0	184	0	189	0	0.5	#DIV/0!	0	4	0	8	0	8	0	10	3.693843
tr|D6REM6|D6REM6_HUMA	MATR3	Matrin-3 OS=Homo sapiens	88647	0	144	0	240	0	0.34	#DIV/0!	0	6	0	8	0	8	0	10	3.693843
tr|Q58EY4|Q58EY4_HUMAN	SMARCC1	SWI/SNF related, matrix as	123347	0	352	0	102	0	0.14	#DIV/0!	0	3	0	5	0	7	0	8	3.513021
sp|P04899|GNAI2_HUMAN	GNAI2	Guanine nucleotide-binding	40995	0	232	0	160	0	0.47	#DIV/0!	0	4	0	5	0	7	0	7	3.513021
tr|B4DLV7|B4DLV7_HUMAN	tr|B4DLV7|B4DL	cDNA FLJ60299, highly simi	51577	0	189	0	188	0	0.45	#DIV/0!	0	5	0	6	0	7	0	7	3.513021
tr|Q6NVW7|Q6NVW7_HUM	KPNA2	Importin subunit alpha OS=	58271	0	175	0	205	0	0.47	#DIV/0!	0	5	0	7	0	7	0	8	3.513021
tr|A2A274|A2A274_HUMAN	ACO2	Aconitase 2, mitochondrial	88563	0	284	0	130	0	0.24	#DIV/0!	0	3	0	6	0	6	0	6	3.306321
tr|B4DMF5|B4DMF5_HUMA	tr|B4DMF5|B4D	Glutamate dehydrogenase	56910	0	269	0	141	0	0.32	#DIV/0!	0	3	0	5	0	6	0	6	3.306321
tr|H0Y2Y8|H0Y2Y8_HUMAN	ZYX	Zyxin (Fragment) OS=Homo	58804	0	240	0	155	0	0.39	#DIV/0!	0	5	0	6	0	6	0	7	3.306321
tr|A4D210|A4D210_HUMAN	EIF3S9	Eukaryotic translation initia	89024	0	233	0	159	0	0.24	#DIV/0!	0	5	0	6	0	6	0	6	3.306321
tr|Q5T3N1|Q5T3N1_HUMAN	ANXA1	Annexin A1 (Fragment) OS=	22741	0	202	0	177	0	1.28	#DIV/0!	0	5	0	6	0	6	0	8	3.306321
tr|B3KM58|B3KM58_HUMA	tr|B3KM58|B3K	cDNA FLJ10358 fis, clone N	41736	0	183	0	191	0	0.58	#DIV/0!	0	4	0	6	0	6	0	7	3.306321
tr|A4D275|A4D275_HUMAN	ARPC1B	Actin related protein 2/3 co	41722	0	162	0	222	0	0.58	#DIV/0!	0	5	0	6	0	6	0	8	3.306321
tr|B7Z4B8|B7Z4B8_HUMAN	HNRNPUL1	Heterogeneous nuclear ribo	86639	0	490	0	74	0	0.16	#DIV/0!	0	2	0	4	0	5	0	6	3.065064
tr|B3KY63|B3KY63_HUMAN	tr|B3KY63|B3KY	cDNA FLJ16830 fis, clone U	216574	0	474	0	77	0	0.06	#DIV/0!	0	3	0	4	0	5	0	6	3.065064
tr|E1NZA1|E1NZA1_HUMAN	PRIC295	Peroxisome proliferator act	294953	0	450	0	81	0	0.06	#DIV/0!	0	1	0	5	0	5	0	7	3.065064
sp|P01023|A2MG_HUMAN	A2M	Alpha-2-macroglobulin OS=	164613	0	343	0	104	0	0.06	#DIV/0!	0	3	0	6	0	5	0	6	3.065064
sp|Q9Y6C9|MTCH2_HUMAN	MTCH2	Mitochondrial carrier homo	33936	0	337	0	106	0	0.45	#DIV/0!	0	2	0	4	0	5	0	6	3.065064
tr|Q14400|Q14400_HUMAN	GLUD1	GLUD1 protein (Fragment)	28791	0	271	0	139	0	0.73	#DIV/0!	0	3	0	5	0	5	0	5	3.065064
tr|B4DL07|B4DL07_HUMAN	tr|B4DL07|B4DL	cDNA FLJ53353, highly simi	78980	0	236	0	157	0	0.23	#DIV/0!	0	3	0	5	0	5	0	5	3.065064
tr|B7Z4B2|B7Z4B2_HUMAN	tr|B7Z4B2|B7Z4	cDNA FLJ56108, highly simi	93434	0	235	0	157	0	0.19	#DIV/0!	0	3	0	5	0	5	0	6	3.065064
tr|B4DT31|B4DT31_HUMAN	FUBP1	Far upstream element-bind	69993	0	225	0	163	0	0.2	#DIV/0!	0	3	0	4	0	5	0	6	3.065064
tr|G3XAI2|G3XAI2_HUMAN	LAMB1	Laminin subunit beta0 OS=	207703	0	201	0	177	0	0.08	#DIV/0!	0	4	0	5	0	5	0	5	3.065064
tr|B2RNR6|B2RNR6_HUMAN	ZFR	Zinc finger RNA binding pro	118081	0	187	0	188	0	0.12	#DIV/0!	0	4	0	4	0	5	0	6	3.065064
tr|K4DI93|K4DI93_HUMAN	CUL4B	Cullin 4B, isoform CRA_e O	103318	0	659	0	53	0	0.06	#DIV/0!	0	1	0	2	0	4	0	4	2.775308
tr|B2R774|B2R774_HUMAN	tr|B2R774|B2R7	cDNA, FLJ93313, highly sim	57768	0	606	0	59	0	0.18	#DIV/0!	0	1	0	3	0	4	0	4	2.775308
sp|Q9UI26|IPO11_HUMAN	IPO11	Importin0 OS=Homo sapie	113489	0	605	0	59	0	0.09	#DIV/0!	0	1	0	3	0	4	0	5	2.775308
sp|P46013|KI67_HUMAN	MKI67	Antigen KI-67 OS=Homo sa	360698	0	550	0	66	0	0.02	#DIV/0!	0	1	0	2	0	4	0	5	2.775308
tr|A8K8U1|A8K8U1_HUMAN	tr|A8K8U1|A8K8	cDNA FLJ77762, highly simi	137941	0	530	0	69	0	0.1	#DIV/0!	0	1	0	4	0	4	0	7	2.775308
sp|Q92945|FUBP2_HUMAN	KHSRP	Far upstream element-bind	73355	0	427	0	86	0	0.19	#DIV/0!	0	1	0	4	0	4	0	4	2.775308
tr|Q59FE8|Q59FE8_HUMAN	tr|Q59FE8|Q59F	Epithelial protein lost in ne	86510	0	425	0	86	0	0.12	#DIV/0!	0	3	0	3	0	4	0	5	2.775308
tr|Q3LIF4|Q3LIF4_HUMAN	Nbla00271	NOGO-A (Fragment) OS=Ho	57162	0	423	0	87	0	0.18	#DIV/0!	0	2	0	3	0	4	0	4	2.775308
sp|Q9NTI5|PDS5B_HUMAN	PDS5B	Sister chromatid cohesion p	165818	0	388	0	95	0	0.06	#DIV/0!	0	2	0	3	0	4	0	4	2.775308
tr|B4DN25|B4DN25_HUMAN	UGDH	UDP-glucose 6-dehydrogen	44786	0	378	0	97	0	0.33	#DIV/0!	0	2	0	4	0	4	0	6	2.775308
tr|H3BPE1|H3BPE1_HUMAN	MACF1	Microtubule-actin cross-linl	861649	0	377	0	97	0	0	#DIV/0!	0	1	0	1	0	4	0	6	2.775308

sp|P30837|AL1B1_HUMAN	ALDH1B1	Aldehyde dehydrogenase X	57626	0	333	0	109	0	0.25	#DIV/0!	0	3	0	4	0	4	0	4	2.775308
sp|P52907|CAZA1_HUMAN	CAPZA1	F-actin-capping protein sub	33073	0	307	0	120	0	0.47	#DIV/0!	0	3	0	4	0	4	0	5	2.775308
tr|B2R7F8|B2R7F8_HUMAN	tr|B2R7F8|B2R7	cDNA, FLJ93426, highly sim	93217	0	295	0	127	0	0.11	#DIV/0!	0	3	0	4	0	4	0	6	2.775308
sp|Q969N2|PIGT_HUMAN	PIGT	GPI transamidase compone	66228	0	278	0	131	0	0.21	#DIV/0!	0	4	0	4	0	4	0	4	2.775308
tr|Q6NWZ1|Q6NWZ1_HUM	CKAP4	CKAP4 protein (Fragment) (	67948	0	277	0	133	0	0.21	#DIV/0!	0	4	0	4	0	4	0	5	2.775308
tr|B4DL79|B4DL79_HUMAN	KIF20A	Kinesin-like protein KIF20A	99066	0	255	0	148	0	0.14	#DIV/0!	0	4	0	4	0	4	0	4	2.775308
tr|H0YCK3|H0YCK3_HUMAN	ADAR	Double-stranded RNA-speci	133761	0	252	0	149	0	0.1	#DIV/0!	0	4	0	4	0	4	0	5	2.775308
sp|P31150|GDIA_HUMAN	GDI1	Rab GDP dissociation inhibi	51177	0	239	0	156	0	0.28	#DIV/0!	0	4	0	4	0	4	0	4	2.775308
tr|Q0VGA5|Q0VGA5_HUMA	SARS	SARS protein OS=Homo sap	58883	0	231	0	160	0	0.18	#DIV/0!	0	3	0	3	0	4	0	4	2.775308
tr|B4DY28|B4DY28_HUMAN	tr|B4DY28|B4DY	cDNA FLJ61189, highly simi	20884	0	182	0	192	0	0.82	#DIV/0!	0	4	0	4	0	4	0	5	2.775308
tr|H0YCK7|H0YCK7_HUMAN	EEF1D	Elongation factor 1-delta (F	23105	0	138	0	244	0	0.72	#DIV/0!	0	4	0	4	0	4	0	4	2.775308
sp|P46977|STT3A_HUMAN	STT3A	Dolichyl-diphosphooligosac	81104	0	927	0	33	0	0.08	#DIV/0!	0	1	0	2	0	3	0	3	2.412488
sp|Q8WX93|PALLD_HUMAN	PALLD	Palladin OS=Homo sapiens	151839	0	881	0	35	0	0.04	#DIV/0!	0	0	0	2	0	3	0	4	2.412488
tr|B4DUT7|B4DUT7_HUMAN	tr|B4DUT7|B4DU	cDNA FLJ57604, highly simi	71880	0	828	0	37	0	0.09	#DIV/0!	0	0	0	2	0	3	0	3	2.412488
tr|E9PQD7|E9PQD7_HUMAN	RPS2	40S ribosomal protein S2 O	25366	0	803	0	39	0	0.28	#DIV/0!	0	1	0	2	0	3	0	4	2.412488
tr|G3V161|G3V161_HUMAN	KBTBD3	Kelch repeat and BTB (POZ)	61834	0	763	0	42	0	0.06	#DIV/0!	0	3	0	3	0	3	0	3	2.412488
sp|P14550|AK1A1_HUMAN	AKR1A1	Alcohol dehydrogenase [NA	36892	0	743	0	44	0	0.29	#DIV/0!	0	2	0	3	0	3	0	3	2.412488
tr|B4DIT7|B4DIT7_HUMAN	TGM2	Protein-glutamine gamma-g	69632	0	709	0	47	0	0.05	#DIV/0!	0	1	0	1	0	3	0	3	2.412488
sp|P22059|OSBP1_HUMAN	OSBP	Oxysterol-binding protein 1	90220	0	670	0	51	0	0.11	#DIV/0!	0	1	0	3	0	3	0	3	2.412488
sp|O15031|PLXB2_HUMAN	PLXNB2	Plexin-B2 OS=Homo sapiens	207734	0	661	0	52	0	0.02	#DIV/0!	0	1	0	1	0	3	0	3	2.412488
tr|A8K5Y7|A8K5Y7_HUMAN	tr|A8K5Y7|A8K5	cDNA FLJ78655, highly simi	138331	0	588	0	60	0	0.05	#DIV/0!	0	2	0	2	0	3	0	3	2.412488
tr|Q5TZP7|Q5TZP7_HUMAN	APEX1	APEX nuclease (Multifuncti	35931	0	578	0	62	0	0.3	#DIV/0!	0	1	0	3	0	3	0	3	2.412488
tr|G5E9H4|G5E9H4_HUMAN	DCTN1	Dynactin 1 (P150, glued hor	127339	0	558	0	65	0	0.05	#DIV/0!	0	1	0	2	0	3	0	4	2.412488
tr|G3V1J5|G3V1J5_HUMAN	DIS3	Exosome complex exonucle	91382	0	536	0	68	0	0.11	#DIV/0!	0	2	0	3	0	3	0	5	2.412488
tr|B3KMV5|B3KMV5_HUMA	tr|B3KMV5|B3KI	cDNA FLJ12728 fis, clone N	123342	0	527	0	69	0	0.05	#DIV/0!	0	1	0	2	0	3	0	3	2.412488
tr|B0YIW5|B0YIW5_HUMAN	ARCN1	Archain 1, isoform CRA_b C	57630	0	505	0	72	0	0.18	#DIV/0!	0	2	0	3	0	3	0	4	2.412488
tr|C9J634|C9J634_HUMAN	PDHB	Pyruvate dehydrogenase E1	39064	0	489	0	74	0	0.18	#DIV/0!	0	2	0	2	0	3	0	3	2.412488
sp|Q9UHD8|SEPT9_HUMAN	Sep-09	Septin-9 OS=Homo sapiens	65646	0	452	0	81	0	0.1	#DIV/0!	0	1	0	2	0	3	0	4	2.412488
tr|A8MUD9|A8MUD9_HUM	RPL7	60S ribosomal protein L7 O	24474	0	438	0	85	0	0.47	#DIV/0!	0	2	0	3	0	3	0	3	2.412488
sp|Q8NFQ8|TOIP2_HUMAN	TOR1AIP2	Torsin0A-interacting protei	51460	0	432	0	85	0	0.2	#DIV/0!	0	2	0	3	0	3	0	3	2.412488
sp|Q9BWF3|RBM4_HUMAN	RBM4	RNA-binding protein 4 OS=I	40688	0	394	0	93	0	0.26	#DIV/0!	0	1	0	3	0	3	0	3	2.412488
tr|B4DMR3|B4DMR3_HUMA	tr|B4DMR3|B4D	cDNA FLJ51896, highly simi	37305	0	392	0	94	0	0.29	#DIV/0!	0	2	0	3	0	3	0	3	2.412488
tr|B4DUF1|B4DUF1_HUMAN	tr|B4DUF1|B4DU	cDNA FLJ59760, highly simi	76254	0	391	0	94	0	0.18	#DIV/0!	0	2	0	4	0	3	0	4	2.412488
tr|Q6IT96|Q6IT96_HUMAN	HDAC1	Histone deacetylase OS=Ho	55638	0	387	0	95	0	0.26	#DIV/0!	0	2	0	4	0	3	0	4	2.412488
tr|A8K6V7|A8K6V7_HUMAN	tr|A8K6V7|A8K6	cDNA FLJ76053, highly simi	50842	0	373	0	98	0	0.21	#DIV/0!	0	2	0	3	0	3	0	5	2.412488
tr|G3V1A4|G3V1A4_HUMAN	CFL1	Cofilin 1 (Non-muscle), isof	17029	0	349	0	103	0	0.72	#DIV/0!	0	3	0	3	0	3	0	3	2.412488
tr|B2RMN7|B2RMN7_HUMA	SPTB	Spectrin, beta, erythrocytic	247271	0	347	0	103	0	0.04	#DIV/0!	0	3	0	3	0	3	0	4	2.412488
tr|B3KRS5|B3KRS5_HUMAN	HDAC2	Histone deacetylase OS=Ho	52479	0	339	0	105	0	0.2	#DIV/0!	0	2	0	3	0	3	0	4	2.412488
tr|B3KT00|B3KT00_HUMAN	tr|B3KT00|B3KT	cDNA FLJ37368 fis, clone BI	91169	0	336	0	107	0	0.15	#DIV/0!	0	2	0	4	0	3	0	4	2.412488
sp|O96008|TOM40_HUMAN	TOMM40	Mitochondrial import recep	38211	0	332	0	110	0	0.18	#DIV/0!	0	2	0	2	0	3	0	4	2.412488
tr|Q05DF2|Q05DF2_HUMAN	SF3A2	SF3A2 protein (Fragment) (	51557	0	324	0	113	0	0.2	#DIV/0!	0	2	0	3	0	3	0	3	2.412488
tr|B4DR36|B4DR36_HUMAN	tr|B4DR36|B4DR	cDNA FLJ56414, highly simi	126101	0	318	0	116	0	0.05	#DIV/0!	0	2	0	2	0	3	0	3	2.412488
sp|P36952|SPB5_HUMAN	SERPINB5	Serpin B5 OS=Homo sapien	42530	0	310	0	119	0	0.25	#DIV/0!	0	2	0	3	0	3	0	4	2.412488
tr|B4DR63|B4DR63_HUMAN	PSMC1	26S protease regulatory sub	41255	0	302	0	122	0	0.26	#DIV/0!	0	2	0	3	0	3	0	3	2.412488
tr|B2R858|B2R858_HUMAN	tr|B2R858|B2R8	cDNA, FLJ93750, Homo sap	53582	0	300	0	123	0	0.2	#DIV/0!	0	3	0	3	0	3	0	3	2.412488
tr|E7EMC7|E7EMC7_HUMAN	SQSTM1	Sequestosome0 OS=Homo	42070	0	267	0	142	0	0.25	#DIV/0!	0	3	0	3	0	3	0	3	2.412488
sp|Q86XP3|DDX42_HUMAN	DDX42	ATP-dependent RNA helica	103197	0	242	0	154	0	0.1	#DIV/0!	0	3	0	3	0	3	0	3	2.412488
sp|Q9HC07|TM165_HUMAN	TMEM165	Transmembrane protein 16	35055	0	234	0	159	0	0.31	#DIV/0!	0	3	0	3	0	3	0	3	2.412488
tr|A1KYQ7|A1KYQ7_HUMAN	tr|A1KYQ7|A1KY	Cell migration-inducing pro	106022	0	222	0	166	0	0.1	#DIV/0!	0	2	0	3	0	3	0	4	2.412488
tr|Q5H907|Q5H907_HUMAN	MAGED2	Melanoma antigen family D	55932	0	216	0	169	0	0.12	#DIV/0!	0	3	0	3	0	3	0	3	2.412488
tr|B2RB95|B2RB95_HUMAN	tr|B2RB95|B2RB	cDNA, FLJ95381, Homo sap	64884	0	209	0	174	0	0.16	#DIV/0!	0	3	0	3	0	3	0	3	2.412488
tr|B0QY89|B0QY89_HUMAN	EIF3EIP	Eukaryotic translation initia	71085	0	185	0	189	0	0.15	#DIV/0!	0	3	0	3	0	3	0	5	2.412488
sp|P62280|RS11_HUMAN	RPS11	40S ribosomal protein S11 (	18590	0	1126	0	20	0	0.18	#DIV/0!	0	0	0	1	0	2	0	2	1.926812
tr|G5EA03|G5EA03_HUMAN	LIMCH1	LIM and calponin homology	165820	0	1096	0	23	0	0.02	#DIV/0!	0	0	0	1	0	2	0	3	1.926812
tr|B4DG89|B4DG89_HUMAN	NPLOC4	Nuclear protein localization	52144	0	1094	0	23	0	0.06	#DIV/0!	0	0	0	1	0	2	0	2	1.926812
tr|G3V529|G3V529_HUMAN	DDX24	ATP-dependent RNA helica	91994	0	1064	0	25	0	0.04	#DIV/0!	0	0	0	1	0	2	0	2	1.926812
tr|G3V140|G3V140_HUMAN	CEP57L1	Centrosomal protein CEP57	54728	0	1059	0	25	0	0.06	#DIV/0!	0	0	0	1	0	2	0	2	1.926812
tr|B3KQC5|B3KQC5_HUMAN	MAN1B1	Endoplasmic reticulum mar	46112	0	1056	0	25	0	0.07	#DIV/0!	0	0	0	1	0	2	0	2	1.926812
tr|B4DK61|B4DK61_HUMAN	tr|B4DK61|B4DK	Acyl-coenzyme A oxidase O	70622	0	1029	0	27	0	0.1	#DIV/0!	0	0	0	2	0	2	0	2	1.926812
tr|G3V1L9|G3V1L9_HUMAN	TJP1	Tight junction protein 1 (Zo	197681	0	1005	0	29	0	0.05	#DIV/0!	0	0	0	3	0	2	0	5	1.926812
tr|B4E140|B4E140_HUMAN	tr|B4E140|B4E14	Transporter OS=Homo sapi	41144	0	988	0	30	0	0.17	#DIV/0!	0	0	0	2	0	2	0	2	1.926812
tr|B3KM68|B3KM68_HUMA	tr|B3KM68|B3KI	cDNA FLJ10398 fis, clone N	48388	0	975	0	30	0	0.14	#DIV/0!	0	1	0	2	0	2	0	3	1.926812
sp|Q06210|GFPT1_HUMAN	GFPT1	Glutamine--fructose-6-phos	79555	0	972	0	30	0	0.04	#DIV/0!	0	0	0	1	0	2	0	2	1.926812
tr|Q8N5A0|Q8N5A0_HUMA	EIF5B	Eukaryotic translation initia	139172	0	950	0	31	0	0.02	#DIV/0!	0	1	0	1	0	2	0	2	1.926812
tr|H0YL72|H0YL72_HUMAN	IDH3A	Isocitrate dehydrogenase [I	36162	0	944	0	32	0	0.19	#DIV/0!	0	0	0	2	0	2	0	2	1.926812
tr|B4DPV3|B4DPV3_HUMAN	tr|B4DPV3|B4DP	cDNA FLJ58109, highly simi	117640	0	933	0	33	0	0.06	#DIV/0!	0	0	0	2	0	2	0	2	1.926812
tr|B3KN28|B3KN28_HUMAN	tr|B3KN28|B3KN	cDNA FLJ13370 fis, clone PL	60284	0	931	0	33	0	0.11	#DIV/0!	0	0	0	2	0	2	0	3	1.926812
tr|D3DUW5|D3DUW5_HUM	DNM1L	Dynamin 1-like, isoform CR	88442	0	924	0	33	0	0.08	#DIV/0!	0	0	0	2	0	2	0	2	1.926812
tr|B7ZLQ5|B7ZLQ5_HUMAN	SMARCA1	SMARCA1 protein OS=Hom	124932	0	923	0	33	0	0.05	#DIV/0!	0	0	0	2	0	2	0	3	1.926812
sp|Q5JWF2|GNAS1_HUMAN	GNAS	Guanine nucleotide-binding	111697	0	920	0	33	0	0.06	#DIV/0!	0	0	0	2	0	2	0	2	1.926812
tr|B4DZC0|B4DZC0_HUMAN	tr|B4DZC0|B4DZ	cDNA FLJ51771, highly simi	117367	0	917	0	33	0	0.06	#DIV/0!	0	0	0	2	0	2	0	4	1.926812
tr|B4DJV2|B4DJV2_HUMAN	CS	Citrate synthase OS=Homo	50628	0	916	0	33	0	0.07	#DIV/0!	0	1	0	2	0	2	0	4	1.926812
sp|Q9BS26|ERP44_HUMAN	ERP44	Endoplasmic reticulum resi	47341	0	908	0	34	0	0.14	#DIV/0!	0	0	0	2	0	2	0	5	1.926812
tr|B3KSG9|B3KSG9_HUMAN	tr|B3KSG9|B3KS	cDNA FLJ36188 fis, clone TE	66299	0	888	0	35	0	0.1	#DIV/0!	0	0	0	2	0	2	0	2	1.926812
tr|B4DX99|B4DX99_HUMAN	tr|B4DX99|B4DX	Malic enzyme OS=Homo sa	45378	0	860	0	36	0	0.15	#DIV/0!	0	0	0	2	0	2	0	2	1.926812
tr|B3KTS3|B3KTS3_HUMAN	tr|B3KTS3|B3KT	cDNA FLJ38664 fis, clone HI	35374	0	856	0	36	0	0.09	#DIV/0!	0	1	0	1	0	2	0	2	1.926812
tr|E9PDU6|E9PDU6_HUMAN	CNN3	Calponin-3 (Fragment) OS=	20309	0	852	0	36	0	0.36	#DIV/0!	0	0	0	2	0	2	0	2	1.926812
tr|B3KUL1|B3KUL1_HUMAN	tr|B3KUL1|B3KU	cDNA FLJ40132 fis, clone TE	59275	0	850	0	36	0	0.06	#DIV/0!	0	0	0	2	0	2	0	2	1.926812
tr|B4DR60|B4DR60_HUMAN	tr|B4DR60|B4DR	cDNA FLJ56882, highly simi	119789	0	848	0	36	0	0.03	#DIV/0!	0	1	0	1	0	2	0	2	1.926812
sp|O76003|GLRX3_HUMAN	GLRX3	Glutaredoxin-3 OS=Homo s	37693	0	847	0	36	0	0.09	#DIV/0!	0	1	0	1	0	2	0	3	1.926812
tr|B7Z7M6|B7Z7M6_HUMAN	KIF2C	Kinesin-like protein KIF2C C	69630	0	843	0	36	0	0.05	#DIV/0!	0	1	0	1	0	2	0	2	1.926812
tr|Q5T8U5|Q5T8U5_HUMAN	SURF4	Surfeit 4 OS=Homo sapiens	21342	0	834	0	37	0	0.16	#DIV/0!	0	1	0	1	0	2	0	2	1.926812
sp|Q9BVP2|GNL3_HUMAN	GNL3	Guanine nucleotide-binding	62468	0	825	0	37	0	0.11	#DIV/0!	0	0	0	2	0	2	0	2	1.926812
tr|F5H0J3|F5H0J3_HUMAN	NDUFA9	NADH dehydrogenase [ubic	15894	0	813	0	38	0	0.47	#DIV/0!	0	1	0	2	0	2	0	2	1.926812
tr|H7BY14|H7BY14_HUMAN	SAMD11	Sterile alpha motif domain-	63302	0	790	0	40	0	0.05	#DIV/0!	0	1	0	1	0	2	0	2	1.926812
tr|B4DXJ1|B4DXJ1_HUMAN	SEC13	Protein SEC13 homolog OS=	41405	0	781	0	40	0	0.08	#DIV/0!	0	1	0	1	0	2	0	2	1.926812
tr|Q5D1D5|Q5D1D5_HUMA	tr|Q5D1D5|Q5D	Guanylate binding protein 1	68387	0	779	0	41	0	0.05	#DIV/0!	0	1	0	1	0	2	0	2	1.926812
tr|Q56VW8|Q56VW8_HUM	tr|Q56VW8|Q56	Lung cancer-related protein	52186	0	767	0	42	0	0.13	#DIV/0!	0	0	0	2	0	2	0	2	1.926812
tr|G5EA24|G5EA24_HUMAN	MCOLN2	Mucolipin 2, isoform CRA_a	29581	0	762	0	42	0	0.12	#DIV/0!	0	1	0	1	0	2	0	2	1.926812
sp|Q92466|DDB2_HUMAN	DDB2	DNA damage-binding prote	48461	0	758	0	43	0	0.07	#DIV/0!	0	1	0	1	0	2	0	3	1.926812
tr|Q548W1|Q548W1_HUMA	OGT	HRNT1 OS=Homo sapiens G	104029	0	739	0	44	0	0.06	#DIV/0!	0	1	0	2	0	2	0	2	1.926812
sp|Q27J81|INF2_HUMAN	INF2	Inverted formin-2 OS=Hom	136851	0	737	0	45	0	0.05	#DIV/0!	0	1	0	2	0	2	0	3	1.926812
tr|D3DXC9|D3DXC9_HUMAN	SHMT1	Serine hydroxymethyltrans	49601	0	733	0	45	0	0.14	#DIV/0!	0	1	0	2	0	2	0	2	1.926812
tr|B2R894|B2R894_HUMAN	MRPL38	Mitochondrial ribosomal pr	41017	0	727	0	45	0	0.17	#DIV/0!	0	1	0	2	0	2	0	2	1.926812
tr|B2R950|B2R950_HUMAN	tr|B2R950|B2R9	cDNA, FLJ94213, highly sim	165250	0	725	0	45	0	0.04	#DIV/0!	0	1	0	3	0	2	0	4	1.926812
tr|B4DL04|B4DL04_HUMAN	tr|B4DL04|B4DL	cDNA FLJ51229, highly simi	73481	0	704	0	47	0	0.09	#DIV/0!	0	1	0	2	0	2	0	2	1.926812
tr|Q3SYK8|Q3SYK8_HUMAN	NUMA1	NUMA1 protein (Fragment)	78666	0	703	0	47	0	0.09	#DIV/0!	0	1	0	2	0	2	0	3	1.926812
tr|B2R8G6|B2R8G6_HUMAN	tr|B2R8G6|B2R8	cDNA, FLJ93891, Homo sap	38870	0	699	0	48	0	0.09	#DIV/0!	0	1	0	1	0	2	0	2	1.926812
sp|Q15043|S39AE_HUMAN	SLC39A14	Zinc transporter ZIP14 OS=I	54918	0	694	0	48	0	0.12	#DIV/0!	0	0	0	2	0	2	0	2	1.926812
tr|B3KNK5|B3KNK5_HUMAN	tr|B3KNK5|B3KN	cDNA FLJ14795 fis, clone N	70004	0	689	0	49	0	0.1	#DIV/0!	0	1	0	2	0	2	0	2	1.926812
tr|B0AZQ4|B0AZQ4_HUMAN	tr|B0AZQ4|B0AZ	cDNA, FLJ79494, highly sim	141841	0	684	0	50	0	0.05	#DIV/0!	0	1	0	2	0	2	0	4	1.926812
tr|F8W8W7|F8W8W7_HUM	TGOLN2	Trans-Golgi network integra	29554	0	678	0	50	0	0.24	#DIV/0!	0	1	0	2	0	2	0	2	1.926812
tr|Q59FA0|Q59FA0_HUMAN	tr|Q59FA0|Q59F	DNA polymerase (Fragment	113271	0	676	0	50	0	0.06	#DIV/0!	0	2	0	2	0	2	0	3	1.926812
sp|Q9UHX1|PUF60_HUMAN	PUF60	Poly(U)-binding-splicing fac	60009	0	675	0	50	0	0.11	#DIV/0!	0	1	0	2	0	2	0	2	1.926812
tr|B4DJU4|B4DJU4_HUMAN	tr|B4DJU4|B4DJ	cDNA FLJ53344, highly simi	67535	0	674	0	50	0	0.1	#DIV/0!	0	1	0	2	0	2	0	4	1.926812
sp|Q8IXI1|MIRO2_HUMAN	RHOT2	Mitochondrial Rho GTPase	69101	0	668	0	51	0	0.1	#DIV/0!	0	1	0	2	0	2	0	2	1.926812
tr|F5H740|F5H740_HUMAN	VDAC3	Voltage-dependent anion-s	31080	0	667	0	51	0	0.11	#DIV/0!	0	1	0	1	0	2	0	2	1.926812
tr|B4DM78|B4DM78_HUMA	tr|B4DM78|B4D	cDNA FLJ58199, highly simi	63280	0	662	0	52	0	0.11	#DIV/0!	0	1	0	2	0	2	0	2	1.926812
tr|B4DYY5|B4DYY5_HUMAN	tr|B4DYY5|B4DY	cDNA FLJ55671, highly simi	113662	0	646	0	55	0	0.06	#DIV/0!	0	2	0	2	0	2	0	2	1.926812
tr|H3BRE8|H3BRE8_HUMAN	RPAP1	RNA polymerase II-associat	124587	0	644	0	55	0	0.05	#DIV/0!	0	1	0	2	0	2	0	2	1.926812
tr|Q0QF37|Q0QF37_HUMAN	MDH2	Malate dehydrogenase (Fra	32405	0	641	0	55	0	0.34	#DIV/0!	0	2	0	3	0	2	0	4	1.926812
tr|H3BTZ5|H3BTZ5_HUMAN	CNN2	Calponin-2 (Fragment) OS=	21173	0	635	0	56	0	0.34	#DIV/0!	0	1	0	2	0	2	0	2	1.926812
sp|Q9Y266|NUDC_HUMAN	NUDC	Nuclear migration protein r	38276	0	630	0	56	0	0.18	#DIV/0!	0	1	0	2	0	2	0	2	1.926812

<table>
<tr><td>tr|B4E3J7|B4E3J7_HUMAN</td><td>tr|B4E3J7|B4E3J</td><td>cDNA FLJ51203, highly simi</td><td>124032</td><td>0</td><td>629</td><td>0</td><td>56</td><td>0</td><td>0.05</td><td>#DIV/0!</td><td>0</td><td>1</td><td>0</td><td>2</td><td>0</td><td>2</td><td>0</td><td>3</td><td>1.926812</td></tr>
<tr><td>tr|Q6FHX6|Q6FHX6_HUMAN</td><td>FEN1</td><td>Flap endonuclease 1 OS=Ho</td><td>42908</td><td>0</td><td>621</td><td>0</td><td>57</td><td>0</td><td>0.16</td><td>#DIV/0!</td><td>0</td><td>2</td><td>0</td><td>2</td><td>0</td><td>2</td><td>0</td><td>2</td><td>1.926812</td></tr>
<tr><td>tr|A8K4W5|A8K4W5_HUMA</td><td>tr|A8K4W5|A8K4</td><td>cDNA FLJ76813, highly simi</td><td>41866</td><td>0</td><td>611</td><td>0</td><td>58</td><td>0</td><td>0.26</td><td>#DIV/0!</td><td>0</td><td>1</td><td>0</td><td>3</td><td>0</td><td>2</td><td>0</td><td>3</td><td>1.926812</td></tr>
<tr><td>tr|E9PF58|E9PF58_HUMAN</td><td>ARPC1A</td><td>Actin-related protein 2/3 co</td><td>31268</td><td>0</td><td>610</td><td>0</td><td>58</td><td>0</td><td>0.35</td><td>#DIV/0!</td><td>0</td><td>1</td><td>0</td><td>3</td><td>0</td><td>2</td><td>0</td><td>4</td><td>1.926812</td></tr>
<tr><td>tr|G3XAL0|G3XAL0_HUMAN</td><td>MDH2</td><td>Malate dehydrogenase OS=</td><td>24921</td><td>0</td><td>602</td><td>0</td><td>59</td><td>0</td><td>0.29</td><td>#DIV/0!</td><td>0</td><td>2</td><td>0</td><td>2</td><td>0</td><td>2</td><td>0</td><td>2</td><td>1.926812</td></tr>
<tr><td>sp|P13667|PDIA4_HUMAN</td><td>PDIA4</td><td>Protein disulfide-isomerase</td><td>73229</td><td>0</td><td>598</td><td>0</td><td>59</td><td>0</td><td>0.09</td><td>#DIV/0!</td><td>0</td><td>1</td><td>0</td><td>2</td><td>0</td><td>2</td><td>0</td><td>2</td><td>1.926812</td></tr>
<tr><td>sp|Q08499|PDE4D_HUMAN</td><td>PDE4D</td><td>cAMP-specific 3~,5~-cyclic p</td><td>91628</td><td>0</td><td>597</td><td>0</td><td>59</td><td>0</td><td>0.07</td><td>#DIV/0!</td><td>0</td><td>1</td><td>0</td><td>2</td><td>0</td><td>2</td><td>0</td><td>2</td><td>1.926812</td></tr>
<tr><td>tr|Q96F88|Q96F88_HUMAN</td><td>POP1</td><td>Processing of 1, ribonucleas</td><td>116318</td><td>0</td><td>594</td><td>0</td><td>60</td><td>0</td><td>0.06</td><td>#DIV/0!</td><td>0</td><td>1</td><td>0</td><td>2</td><td>0</td><td>2</td><td>0</td><td>3</td><td>1.926812</td></tr>
<tr><td>sp|Q9UKX7|NUP50_HUMAN</td><td>NUP50</td><td>Nuclear pore complex prote</td><td>50512</td><td>0</td><td>592</td><td>0</td><td>60</td><td>0</td><td>0.13</td><td>#DIV/0!</td><td>0</td><td>1</td><td>0</td><td>2</td><td>0</td><td>2</td><td>0</td><td>2</td><td>1.926812</td></tr>
<tr><td>sp|A0AVT1|UBA6_HUMAN</td><td>UBA6</td><td>Ubiquitin-like modifier-activ</td><td>119207</td><td>0</td><td>590</td><td>0</td><td>60</td><td>0</td><td>0.06</td><td>#DIV/0!</td><td>0</td><td>2</td><td>0</td><td>2</td><td>0</td><td>2</td><td>0</td><td>2</td><td>1.926812</td></tr>
<tr><td>sp|Q32MZ4|LRRF1_HUMAN</td><td>LRRFIP1</td><td>Leucine-rich repeat flightles</td><td>89826</td><td>0</td><td>589</td><td>0</td><td>60</td><td>0</td><td>0.07</td><td>#DIV/0!</td><td>0</td><td>1</td><td>0</td><td>2</td><td>0</td><td>2</td><td>0</td><td>2</td><td>1.926812</td></tr>
<tr><td>tr|B4DF80|B4DF80_HUMAN</td><td>tr|B4DF80|B4DF</td><td>cDNA FLJ58527, highly simi</td><td>122714</td><td>0</td><td>581</td><td>0</td><td>61</td><td>0</td><td>0.05</td><td>#DIV/0!</td><td>0</td><td>1</td><td>0</td><td>2</td><td>0</td><td>2</td><td>0</td><td>2</td><td>1.926812</td></tr>
<tr><td>tr|B3KY04|B3KY04_HUMAN</td><td>tr|B3KY04|B3KY(</td><td>cDNA FLJ46506 fis, clone TH</td><td>35994</td><td>0</td><td>580</td><td>0</td><td>61</td><td>0</td><td>0.19</td><td>#DIV/0!</td><td>0</td><td>1</td><td>0</td><td>2</td><td>0</td><td>2</td><td>0</td><td>2</td><td>1.926812</td></tr>
<tr><td>sp|O43837|IDH3B_HUMAN</td><td>IDH3B</td><td>Isocitrate dehydrogenase [N</td><td>42442</td><td>0</td><td>579</td><td>0</td><td>61</td><td>0</td><td>0.16</td><td>#DIV/0!</td><td>0</td><td>2</td><td>0</td><td>2</td><td>0</td><td>2</td><td>0</td><td>2</td><td>1.926812</td></tr>
<tr><td>sp|Q96I24|FUBP3_HUMAN</td><td>FUBP3</td><td>Far upstream element-bind</td><td>61944</td><td>0</td><td>577</td><td>0</td><td>62</td><td>0</td><td>0.11</td><td>#DIV/0!</td><td>0</td><td>1</td><td>0</td><td>2</td><td>0</td><td>2</td><td>0</td><td>4</td><td>1.926812</td></tr>
<tr><td>sp|P61221|ABCE1_HUMAN</td><td>ABCE1</td><td>ATP-binding cassette sub-fa</td><td>68240</td><td>0</td><td>575</td><td>0</td><td>62</td><td>0</td><td>0.1</td><td>#DIV/0!</td><td>0</td><td>1</td><td>0</td><td>2</td><td>0</td><td>2</td><td>0</td><td>2</td><td>1.926812</td></tr>
<tr><td>sp|Q9H0A0|NAT10_HUMAN</td><td>NAT10</td><td>N-acetyltransferase 10 OS=</td><td>116569</td><td>0</td><td>573</td><td>0</td><td>63</td><td>0</td><td>0.06</td><td>#DIV/0!</td><td>0</td><td>1</td><td>0</td><td>2</td><td>0</td><td>2</td><td>0</td><td>4</td><td>1.926812</td></tr>
<tr><td>tr|B4DRK5|B4DRK5_HUMAN</td><td>tr|B4DRK5|B4DR</td><td>cDNA FLJ59584, highly simi</td><td>28622</td><td>0</td><td>570</td><td>0</td><td>63</td><td>0</td><td>0.25</td><td>#DIV/0!</td><td>0</td><td>1</td><td>0</td><td>2</td><td>0</td><td>2</td><td>0</td><td>2</td><td>1.926812</td></tr>
<tr><td>tr|D3DTY9|D3DTY9_HUMAN</td><td>TRIM25</td><td>Tripartite motif-containing</td><td>48358</td><td>0</td><td>565</td><td>0</td><td>63</td><td>0</td><td>0.07</td><td>#DIV/0!</td><td>0</td><td>1</td><td>0</td><td>1</td><td>0</td><td>2</td><td>0</td><td>2</td><td>1.926812</td></tr>
<tr><td>tr|Q6IBS5|Q6IBS5_HUMAN</td><td>DLST</td><td>DLST protein OS=Homo sap</td><td>49131</td><td>0</td><td>564</td><td>0</td><td>63</td><td>0</td><td>0.14</td><td>#DIV/0!</td><td>0</td><td>2</td><td>0</td><td>2</td><td>0</td><td>2</td><td>0</td><td>2</td><td>1.926812</td></tr>
<tr><td>tr|H0YD73|H0YD73_HUMAN</td><td>PSMD13</td><td>26S proteasome non-ATPas</td><td>25299</td><td>0</td><td>554</td><td>0</td><td>66</td><td>0</td><td>0.28</td><td>#DIV/0!</td><td>0</td><td>2</td><td>0</td><td>2</td><td>0</td><td>2</td><td>0</td><td>3</td><td>1.926812</td></tr>
<tr><td>tr|B2RBM7|B2RBM7_HUMA</td><td>tr|B2RBM7|B2RI</td><td>cDNA, FLJ95595, highly sim</td><td>43131</td><td>0</td><td>553</td><td>0</td><td>66</td><td>0</td><td>0.16</td><td>#DIV/0!</td><td>0</td><td>2</td><td>0</td><td>2</td><td>0</td><td>2</td><td>0</td><td>3</td><td>1.926812</td></tr>
<tr><td>tr|B4E1K0|B4E1K0_HUMAN</td><td>KIF23</td><td>Kinesin-like protein KIF23 O</td><td>78221</td><td>0</td><td>551</td><td>0</td><td>66</td><td>0</td><td>0.09</td><td>#DIV/0!</td><td>0</td><td>1</td><td>0</td><td>2</td><td>0</td><td>2</td><td>0</td><td>2</td><td>1.926812</td></tr>
<tr><td>sp|Q9Y2W1|TR150_HUMAN</td><td>THRAP3</td><td>Thyroid hormone receptor-</td><td>108658</td><td>0</td><td>546</td><td>0</td><td>67</td><td>0</td><td>0.06</td><td>#DIV/0!</td><td>0</td><td>2</td><td>0</td><td>2</td><td>0</td><td>2</td><td>0</td><td>4</td><td>1.926812</td></tr>
<tr><td>tr|B3KQ43|B3KQ43_HUMAN</td><td>tr|B3KQ43|B3KC</td><td>cDNA FLJ32791 fis, clone TE</td><td>47208</td><td>0</td><td>544</td><td>0</td><td>67</td><td>0</td><td>0.14</td><td>#DIV/0!</td><td>0</td><td>1</td><td>0</td><td>2</td><td>0</td><td>2</td><td>0</td><td>2</td><td>1.926812</td></tr>
<tr><td>tr|B3KQH2|B3KQH2_HUMAN</td><td>tr|B3KQH2|B3KC</td><td>cDNA FLJ90454 fis, clone NT</td><td>76247</td><td>0</td><td>539</td><td>0</td><td>68</td><td>0</td><td>0.09</td><td>#DIV/0!</td><td>0</td><td>1</td><td>0</td><td>2</td><td>0</td><td>2</td><td>0</td><td>2</td><td>1.926812</td></tr>
<tr><td>tr|B3KPA6|B3KPA6_HUMAN</td><td>ACADVL</td><td>Acyl-Coenzyme A dehydrog</td><td>62941</td><td>0</td><td>538</td><td>0</td><td>68</td><td>0</td><td>0.17</td><td>#DIV/0!</td><td>0</td><td>1</td><td>0</td><td>3</td><td>0</td><td>2</td><td>0</td><td>3</td><td>1.926812</td></tr>
<tr><td>sp|Q9GZL7|WDR12_HUMAN</td><td>WDR12</td><td>Ribosome biogenesis prote</td><td>48191</td><td>0</td><td>535</td><td>0</td><td>68</td><td>0</td><td>0.07</td><td>#DIV/0!</td><td>0</td><td>1</td><td>0</td><td>1</td><td>0</td><td>2</td><td>0</td><td>2</td><td>1.926812</td></tr>
<tr><td>sp|Q96HE7|ERO1A_HUMAN</td><td>ERO1L</td><td>ERO1-like protein alpha OS=</td><td>55213</td><td>0</td><td>533</td><td>0</td><td>69</td><td>0</td><td>0.06</td><td>#DIV/0!</td><td>0</td><td>1</td><td>0</td><td>1</td><td>0</td><td>2</td><td>0</td><td>2</td><td>1.926812</td></tr>
<tr><td>tr|B4DZ87|B4DZ87_HUMAN</td><td>tr|B4DZ87|B4DZ</td><td>cDNA FLJ57240, highly simi</td><td>57579</td><td>0</td><td>526</td><td>0</td><td>69</td><td>0</td><td>0.12</td><td>#DIV/0!</td><td>0</td><td>2</td><td>0</td><td>2</td><td>0</td><td>2</td><td>0</td><td>2</td><td>1.926812</td></tr>
<tr><td>tr|B4DSL6|B4DSL6_HUMAN</td><td>tr|B4DSL6|B4DS</td><td>cDNA FLJ57190, highly simi</td><td>111977</td><td>0</td><td>524</td><td>0</td><td>70</td><td>0</td><td>0.06</td><td>#DIV/0!</td><td>0</td><td>2</td><td>0</td><td>2</td><td>0</td><td>2</td><td>0</td><td>2</td><td>1.926812</td></tr>
<tr><td>tr|B3KST5|B3KST5_HUMAN</td><td>COPS4</td><td>COP9 constitutive photomo</td><td>40457</td><td>0</td><td>522</td><td>0</td><td>70</td><td>0</td><td>0.17</td><td>#DIV/0!</td><td>0</td><td>2</td><td>0</td><td>2</td><td>0</td><td>2</td><td>0</td><td>2</td><td>1.926812</td></tr>
<tr><td>tr|A8K4T6|A8K4T6_HUMAN</td><td>tr|A8K4T6|A8K4</td><td>cDNA FLJ76282, highly simi</td><td>56588</td><td>0</td><td>521</td><td>0</td><td>70</td><td>0</td><td>0.12</td><td>#DIV/0!</td><td>0</td><td>2</td><td>0</td><td>2</td><td>0</td><td>2</td><td>0</td><td>2</td><td>1.926812</td></tr>
<tr><td>tr|F8W6C2|F8W6C2_HUMAI</td><td>SPATS2L</td><td>SPATS2-like protein (Fragm</td><td>30163</td><td>0</td><td>510</td><td>0</td><td>72</td><td>0</td><td>0.23</td><td>#DIV/0!</td><td>0</td><td>2</td><td>0</td><td>2</td><td>0</td><td>2</td><td>0</td><td>2</td><td>1.926812</td></tr>
<tr><td>tr|I1E4Y6|I1E4Y6_HUMAN</td><td>GIGYF2</td><td>PERQ amino acid-rich with</td><td>152891</td><td>0</td><td>503</td><td>0</td><td>72</td><td>0</td><td>0.04</td><td>#DIV/0!</td><td>0</td><td>2</td><td>0</td><td>2</td><td>0</td><td>2</td><td>0</td><td>2</td><td>1.926812</td></tr>
<tr><td>tr|H7BZF2|H7BZF2_HUMAN</td><td>DKC1</td><td>H/ACA ribonucleoprotein co</td><td>8803</td><td>0</td><td>498</td><td>0</td><td>73</td><td>0</td><td>0.96</td><td>#DIV/0!</td><td>0</td><td>2</td><td>0</td><td>2</td><td>0</td><td>2</td><td>0</td><td>2</td><td>1.926812</td></tr>
<tr><td>tr|B3KMN2|B3KMN2_HUMA</td><td>tr|B3KMN2|B3KI</td><td>cDNA FLJ11695 fis, clone HI</td><td>36724</td><td>0</td><td>495</td><td>0</td><td>73</td><td>0</td><td>0.19</td><td>#DIV/0!</td><td>0</td><td>2</td><td>0</td><td>2</td><td>0</td><td>2</td><td>0</td><td>2</td><td>1.926812</td></tr>
<tr><td>tr|B4DPW5|B4DPW5_HUMA</td><td>tr|B4DPW5|B4D</td><td>cDNA FLJ59374, highly simi</td><td>57436</td><td>0</td><td>488</td><td>0</td><td>74</td><td>0</td><td>0.12</td><td>#DIV/0!</td><td>0</td><td>1</td><td>0</td><td>2</td><td>0</td><td>2</td><td>0</td><td>2</td><td>1.926812</td></tr>
<tr><td>tr|Q59GM9|Q59GM9_HUM</td><td>tr|Q59GM9|Q59</td><td>Phosphorylase (Fragment)</td><td>99508</td><td>0</td><td>485</td><td>0</td><td>75</td><td>0</td><td>0.1</td><td>#DIV/0!</td><td>0</td><td>2</td><td>0</td><td>3</td><td>0</td><td>2</td><td>0</td><td>3</td><td>1.926812</td></tr>
<tr><td>tr|B3KRR1|B3KRR1_HUMAN</td><td>tr|B3KRR1|B3KR</td><td>cDNA FLJ34725 fis, clone M</td><td>40752</td><td>0</td><td>469</td><td>0</td><td>78</td><td>0</td><td>0.17</td><td>#DIV/0!</td><td>0</td><td>2</td><td>0</td><td>2</td><td>0</td><td>2</td><td>0</td><td>2</td><td>1.926812</td></tr>
<tr><td>tr|H7BZT7|H7BZT7_HUMAN</td><td>ESD</td><td>S-formylglutathione hydrola</td><td>25806</td><td>0</td><td>468</td><td>0</td><td>78</td><td>0</td><td>0.28</td><td>#DIV/0!</td><td>0</td><td>1</td><td>0</td><td>2</td><td>0</td><td>2</td><td>0</td><td>2</td><td>1.926812</td></tr>
<tr><td>tr|B7Z556|B7Z556_HUMAN</td><td>tr|B7Z556|B7Z5</td><td>cDNA FLJ56822, highly simi</td><td>27850</td><td>0</td><td>462</td><td>0</td><td>80</td><td>0</td><td>0.12</td><td>#DIV/0!</td><td>0</td><td>2</td><td>0</td><td>2</td><td>0</td><td>2</td><td>0</td><td>2</td><td>1.926812</td></tr>
<tr><td>tr|B4DHY1|B4DHY1_HUMAN</td><td>HNRNPH3</td><td>Heterogeneous nuclear ribo</td><td>24925</td><td>0</td><td>460</td><td>0</td><td>80</td><td>0</td><td>0.29</td><td>#DIV/0!</td><td>0</td><td>2</td><td>0</td><td>2</td><td>0</td><td>2</td><td>0</td><td>2</td><td>1.926812</td></tr>
<tr><td>tr|Q6IBU0|Q6IBU0_HUMAN</td><td>EIF5</td><td>EIF5 protein OS=Homo sapi</td><td>49577</td><td>0</td><td>457</td><td>0</td><td>81</td><td>0</td><td>0.14</td><td>#DIV/0!</td><td>0</td><td>1</td><td>0</td><td>2</td><td>0</td><td>2</td><td>0</td><td>2</td><td>1.926812</td></tr>
<tr><td>sp|Q96CS3|FAF2_HUMAN</td><td>FAF2</td><td>FAS-associated factor 2 OS=</td><td>52933</td><td>0</td><td>446</td><td>0</td><td>83</td><td>0</td><td>0.13</td><td>#DIV/0!</td><td>0</td><td>2</td><td>0</td><td>2</td><td>0</td><td>2</td><td>0</td><td>3</td><td>1.926812</td></tr>
<tr><td>tr|B4DRW3|B4DRW3_HUMA</td><td>tr|B4DRW3|B4D</td><td>cDNA FLJ57180, highly simi</td><td>35596</td><td>0</td><td>440</td><td>0</td><td>84</td><td>0</td><td>0.09</td><td>#DIV/0!</td><td>0</td><td>1</td><td>0</td><td>1</td><td>0</td><td>2</td><td>0</td><td>2</td><td>1.926812</td></tr>
<tr><td>tr|Q96FS1|Q96FS1_HUMAN</td><td>CTNND1</td><td>CTNND1 protein (Fragment</td><td>92901</td><td>0</td><td>436</td><td>0</td><td>85</td><td>0</td><td>0.07</td><td>#DIV/0!</td><td>0</td><td>2</td><td>0</td><td>2</td><td>0</td><td>2</td><td>0</td><td>4</td><td>1.926812</td></tr>
<tr><td>tr|B2RDD7|B2RDD7_HUMAN</td><td>tr|B2RDD7|B2RD</td><td>cDNA, FLJ96564, highly sim</td><td>73348</td><td>0</td><td>433</td><td>0</td><td>85</td><td>0</td><td>0.09</td><td>#DIV/0!</td><td>0</td><td>2</td><td>0</td><td>2</td><td>0</td><td>2</td><td>0</td><td>2</td><td>1.926812</td></tr>
<tr><td>tr|B4E063|B4E063_HUMAN</td><td>tr|B4E063|B4E0</td><td>cDNA FLJ54292, highly simi</td><td>73571</td><td>0</td><td>430</td><td>0</td><td>85</td><td>0</td><td>0.09</td><td>#DIV/0!</td><td>0</td><td>2</td><td>0</td><td>2</td><td>0</td><td>2</td><td>0</td><td>3</td><td>1.926812</td></tr>
<tr><td>sp|Q9H3P7|GCP60_HUMAN</td><td>ACBD3</td><td>Golgi resident protein GCP6</td><td>60841</td><td>0</td><td>429</td><td>0</td><td>85</td><td>0</td><td>0.05</td><td>#DIV/0!</td><td>0</td><td>1</td><td>0</td><td>1</td><td>0</td><td>2</td><td>0</td><td>3</td><td>1.926812</td></tr>
<tr><td>tr|B4DN01|B4DN01_HUMAN</td><td>tr|B4DN01|B4DN</td><td>cDNA FLJ57332, highly simi</td><td>33950</td><td>0</td><td>424</td><td>0</td><td>86</td><td>0</td><td>0.2</td><td>#DIV/0!</td><td>0</td><td>2</td><td>0</td><td>2</td><td>0</td><td>2</td><td>0</td><td>2</td><td>1.926812</td></tr>
<tr><td>tr|B3KXH0|B3KXH0_HUMAN</td><td>tr|B3KXH0|B3KX</td><td>cDNA FLJ45395 fis, clone BF</td><td>110601</td><td>0</td><td>420</td><td>0</td><td>87</td><td>0</td><td>0.06</td><td>#DIV/0!</td><td>0</td><td>2</td><td>0</td><td>2</td><td>0</td><td>2</td><td>0</td><td>3</td><td>1.926812</td></tr>
<tr><td>tr|Q6ZVI6|Q6ZVI6_HUMAN</td><td>tr|Q6ZVI6|Q6ZV</td><td>cDNA FLJ42537 fis, clone BF</td><td>29141</td><td>0</td><td>419</td><td>0</td><td>87</td><td>0</td><td>0.24</td><td>#DIV/0!</td><td>0</td><td>2</td><td>0</td><td>2</td><td>0</td><td>2</td><td>0</td><td>2</td><td>1.926812</td></tr>
<tr><td>tr|B4DSP0|B4DSP0_HUMAN</td><td>hCG_1642748</td><td>HCG1642748, isoform CRA_</td><td>45559</td><td>0</td><td>418</td><td>0</td><td>88</td><td>0</td><td>0.15</td><td>#DIV/0!</td><td>0</td><td>2</td><td>0</td><td>2</td><td>0</td><td>2</td><td>0</td><td>2</td><td>1.926812</td></tr>
<tr><td>tr|F1D8N4|F1D8N4_HUMAN</td><td>NR3C1</td><td>Glucocorticoid nuclear rece</td><td>86745</td><td>0</td><td>413</td><td>0</td><td>89</td><td>0</td><td>0.08</td><td>#DIV/0!</td><td>0</td><td>1</td><td>0</td><td>2</td><td>0</td><td>2</td><td>0</td><td>3</td><td>1.926812</td></tr>
<tr><td>tr|E9PAU2|E9PAU2_HUMAN</td><td>RAVER1</td><td>Ribonucleoprotein PTB-binc</td><td>80042</td><td>0</td><td>410</td><td>0</td><td>89</td><td>0</td><td>0.04</td><td>#DIV/0!</td><td>0</td><td>1</td><td>0</td><td>1</td><td>0</td><td>2</td><td>0</td><td>2</td><td>1.926812</td></tr>
<tr><td>sp|Q9UJA5|TRM6_HUMAN</td><td>TRMT6</td><td>tRNA (adenine(58)-N(1))-me</td><td>56049</td><td>0</td><td>408</td><td>0</td><td>91</td><td>0</td><td>0.12</td><td>#DIV/0!</td><td>0</td><td>2</td><td>0</td><td>2</td><td>0</td><td>2</td><td>0</td><td>2</td><td>1.926812</td></tr>
<tr><td>tr|H1UBN2|H1UBN2_HUMA</td><td>CCNB1V</td><td>Cyclin B1 OS=Homo sapiens</td><td>46024</td><td>0</td><td>393</td><td>0</td><td>94</td><td>0</td><td>0.15</td><td>#DIV/0!</td><td>0</td><td>2</td><td>0</td><td>2</td><td>0</td><td>2</td><td>0</td><td>3</td><td>1.926812</td></tr>
<tr><td>tr|Q6EHZ3|Q6EHZ3_HUMAN</td><td>STRF8</td><td>Putative uncharacterized pr</td><td>41361</td><td>0</td><td>384</td><td>0</td><td>96</td><td>0</td><td>0.17</td><td>#DIV/0!</td><td>0</td><td>2</td><td>0</td><td>2</td><td>0</td><td>2</td><td>0</td><td>2</td><td>1.926812</td></tr>
<tr><td>tr|F5GZ76|F5GZ76_HUMAN</td><td>USO1</td><td>General vesicular transport</td><td>90003</td><td>0</td><td>330</td><td>0</td><td>111</td><td>0</td><td>0.07</td><td>#DIV/0!</td><td>0</td><td>1</td><td>0</td><td>2</td><td>0</td><td>2</td><td>0</td><td>3</td><td>1.926812</td></tr>
<tr><td>tr|Q4KMR3|Q4KMR3_HUMA</td><td>MYO1E</td><td>MYO1E variant protein OS=</td><td>127552</td><td>0</td><td>325</td><td>0</td><td>113</td><td>0</td><td>0.05</td><td>#DIV/0!</td><td>0</td><td>2</td><td>0</td><td>2</td><td>0</td><td>2</td><td>0</td><td>2</td><td>1.926812</td></tr>
<tr><td>tr|A8K0D2|A8K0D2_HUMAN</td><td>tr|A8K0D2|A8K0</td><td>cDNA FLJ77740, highly simi</td><td>55175</td><td>0</td><td>312</td><td>0</td><td>119</td><td>0</td><td>0.12</td><td>#DIV/0!</td><td>0</td><td>2</td><td>0</td><td>2</td><td>0</td><td>2</td><td>0</td><td>2</td><td>1.926812</td></tr>
<tr><td>tr|A8K8Z4|A8K8Z4_HUMAN</td><td>tr|A8K8Z4|A8K8</td><td>cDNA FLJ78071, highly simi</td><td>108298</td><td>0</td><td>206</td><td>0</td><td>174</td><td>0</td><td>0.06</td><td>#DIV/0!</td><td>0</td><td>2</td><td>0</td><td>2</td><td>0</td><td>2</td><td>0</td><td>2</td><td>1.926812</td></tr>
<tr><td>tr|Q4LE61|Q4LE61_HUMAN</td><td>SYMPK</td><td>SYMPK variant protein (Frag</td><td>119459</td><td>0</td><td>1157</td><td>0</td><td>15</td><td>0</td><td>0.03</td><td>#DIV/0!</td><td>0</td><td>0</td><td>0</td><td>1</td><td>0</td><td>1</td><td>0</td><td>2</td><td>1.189598</td></tr>
<tr><td>sp|O76031|CLPX_HUMAN</td><td>CLPX</td><td>ATP-dependent Clp proteas</td><td>69922</td><td>0</td><td>1140</td><td>0</td><td>17</td><td>0</td><td>0.05</td><td>#DIV/0!</td><td>0</td><td>0</td><td>0</td><td>1</td><td>0</td><td>1</td><td>0</td><td>2</td><td>1.189598</td></tr>
<tr><td>tr|A2RTY6|A2RTY6_HUMAN</td><td>ITIH2</td><td>Inter-alpha (Globulin) inhib</td><td>106826</td><td>0</td><td>1133</td><td>0</td><td>18</td><td>0</td><td>0.03</td><td>#DIV/0!</td><td>0</td><td>0</td><td>0</td><td>1</td><td>0</td><td>1</td><td>0</td><td>3</td><td>1.189598</td></tr>
<tr><td>tr|B3KY47|B3KY47_HUMAN</td><td>tr|B3KY47|B3KY4</td><td>cDNA FLJ46841 fis, clone UT</td><td>193959</td><td>0</td><td>1125</td><td>0</td><td>20</td><td>0</td><td>0.02</td><td>#DIV/0!</td><td>0</td><td>0</td><td>0</td><td>1</td><td>0</td><td>1</td><td>0</td><td>1</td><td>1.189598</td></tr>
<tr><td>sp|Q969E2|SCAM4_HUMAN</td><td>SCAMP4</td><td>Secretory carrier-associated</td><td>26053</td><td>0</td><td>1124</td><td>0</td><td>20</td><td>0</td><td>0.13</td><td>#DIV/0!</td><td>0</td><td>0</td><td>0</td><td>1</td><td>0</td><td>1</td><td>0</td><td>1</td><td>1.189598</td></tr>
<tr><td>tr|Q5H928|Q5H928_HUMAN</td><td>HSD17B10</td><td>3-hydroxyacyl-CoA dehydro</td><td>17498</td><td>0</td><td>1121</td><td>0</td><td>21</td><td>0</td><td>0.19</td><td>#DIV/0!</td><td>0</td><td>0</td><td>0</td><td>1</td><td>0</td><td>1</td><td>0</td><td>1</td><td>1.189598</td></tr>
<tr><td>tr|Q5VVC8|Q5VVC8_HUMAN</td><td>RPL11</td><td>60S ribosomal protein L11 (</td><td>20167</td><td>0</td><td>1120</td><td>0</td><td>21</td><td>0</td><td>0.17</td><td>#DIV/0!</td><td>0</td><td>0</td><td>0</td><td>1</td><td>0</td><td>1</td><td>0</td><td>1</td><td>1.189598</td></tr>
<tr><td>tr|B3KVJ5|B3KVJ5_HUMAN</td><td>tr|B3KVJ5|B3KVJ</td><td>cDNA FLJ16648 fis, clone TE</td><td>101354</td><td>0</td><td>1118</td><td>0</td><td>21</td><td>0</td><td>0.03</td><td>#DIV/0!</td><td>0</td><td>0</td><td>0</td><td>1</td><td>0</td><td>1</td><td>0</td><td>4</td><td>1.189598</td></tr>
<tr><td>tr|B4DWN1|B4DWN1_HUM</td><td>tr|B4DWN1|B4D</td><td>cDNA FLJ52285, highly simi</td><td>32842</td><td>0</td><td>1117</td><td>0</td><td>21</td><td>0</td><td>0.1</td><td>#DIV/0!</td><td>0</td><td>0</td><td>0</td><td>1</td><td>0</td><td>1</td><td>0</td><td>1</td><td>1.189598</td></tr>
<tr><td>tr|B3KN49|B3KN49_HUMAN</td><td>tr|B3KN49|B3KN</td><td>cDNA FLJ13562 fis, clone PL</td><td>40868</td><td>0</td><td>1116</td><td>0</td><td>21</td><td>0</td><td>0.08</td><td>#DIV/0!</td><td>0</td><td>0</td><td>0</td><td>1</td><td>0</td><td>1</td><td>0</td><td>1</td><td>1.189598</td></tr>
<tr><td>sp|Q8IWT6|LRC8A_HUMAN</td><td>LRRC8A</td><td>Leucine-rich repeat-contain</td><td>95051</td><td>0</td><td>1115</td><td>0</td><td>21</td><td>0</td><td>0.03</td><td>#DIV/0!</td><td>0</td><td>0</td><td>0</td><td>1</td><td>0</td><td>1</td><td>0</td><td>1</td><td>1.189598</td></tr>
<tr><td>tr|A2VCR0|A2VCR0_HUMAN</td><td>MAPRE1</td><td>MAPRE1 protein (Fragment</td><td>19510</td><td>0</td><td>1114</td><td>0</td><td>21</td><td>0</td><td>0.17</td><td>#DIV/0!</td><td>0</td><td>0</td><td>0</td><td>1</td><td>0</td><td>1</td><td>0</td><td>1</td><td>1.189598</td></tr>
<tr><td>tr|B7WP88|B7WP88_HUMA</td><td>TRANK1</td><td>TPR and ankyrin repeat-con</td><td>276902</td><td>0</td><td>1111</td><td>0</td><td>21</td><td>0</td><td>0.01</td><td>#DIV/0!</td><td>0</td><td>0</td><td>0</td><td>1</td><td>0</td><td>1</td><td>0</td><td>1</td><td>1.189598</td></tr>
<tr><td>tr|B2R7M1|B2R7M1_HUMA</td><td>tr|B2R7M1|B2R7</td><td>cDNA, FLJ93507, highly sim</td><td>40747</td><td>0</td><td>1110</td><td>0</td><td>22</td><td>0</td><td>0.08</td><td>#DIV/0!</td><td>0</td><td>0</td><td>0</td><td>1</td><td>0</td><td>1</td><td>0</td><td>1</td><td>1.189598</td></tr>
<tr><td>sp|O75886|STAM2_HUMAN</td><td>STAM2</td><td>Signal transducing adapter</td><td>58527</td><td>0</td><td>1109</td><td>0</td><td>22</td><td>0</td><td>0.06</td><td>#DIV/0!</td><td>0</td><td>0</td><td>0</td><td>1</td><td>0</td><td>1</td><td>0</td><td>1</td><td>1.189598</td></tr>
<tr><td>tr|B2R825|B2R825_HUMAN</td><td>tr|B2R825|B2R8</td><td>Phosphorylase OS=Homo sa</td><td>97405</td><td>0</td><td>1108</td><td>0</td><td>22</td><td>0</td><td>0.03</td><td>#DIV/0!</td><td>0</td><td>0</td><td>0</td><td>1</td><td>0</td><td>1</td><td>0</td><td>1</td><td>1.189598</td></tr>
<tr><td>tr|Q6MZF7|Q6MZF7_HUMA</td><td>DKFZp686E1124</td><td>Putative uncharacterized pr</td><td>29013</td><td>0</td><td>1107</td><td>0</td><td>22</td><td>0</td><td>0.11</td><td>#DIV/0!</td><td>0</td><td>0</td><td>0</td><td>1</td><td>0</td><td>1</td><td>0</td><td>1</td><td>1.189598</td></tr>
<tr><td>tr|H0Y8H0|H0Y8H0_HUMAN</td><td>DDX60L</td><td>Probable ATP-dependent R</td><td>102233</td><td>0</td><td>1106</td><td>0</td><td>22</td><td>0</td><td>0.03</td><td>#DIV/0!</td><td>0</td><td>0</td><td>0</td><td>1</td><td>0</td><td>1</td><td>0</td><td>1</td><td>1.189598</td></tr>
<tr><td>tr|A6NHU9|A6NHU9_HUMA</td><td>SHANK2</td><td>SH3 and multiple ankyrin re</td><td>202297</td><td>0</td><td>1105</td><td>0</td><td>22</td><td>0</td><td>0.02</td><td>#DIV/0!</td><td>0</td><td>0</td><td>0</td><td>1</td><td>0</td><td>1</td><td>0</td><td>2</td><td>1.189598</td></tr>
<tr><td>sp|P20594|ANPRB_HUMAN</td><td>NPR2</td><td>Atrial natriuretic peptide re</td><td>117803</td><td>0</td><td>1104</td><td>0</td><td>22</td><td>0</td><td>0.03</td><td>#DIV/0!</td><td>0</td><td>0</td><td>0</td><td>1</td><td>0</td><td>1</td><td>0</td><td>1</td><td>1.189598</td></tr>
<tr><td>sp|A9QM74|IMA8_HUMAN</td><td>KPNA7</td><td>Importin subunit alpha-8 OS</td><td>57529</td><td>0</td><td>1103</td><td>0</td><td>22</td><td>0</td><td>0.06</td><td>#DIV/0!</td><td>0</td><td>0</td><td>0</td><td>1</td><td>0</td><td>1</td><td>0</td><td>1</td><td>1.189598</td></tr>
<tr><td>sp|Q8WXI7|MUC16_HUMAN</td><td>MUC16</td><td>Mucin06 OS=Homo sapiens</td><td>2359682</td><td>0</td><td>1102</td><td>0</td><td>22</td><td>0</td><td>0</td><td>#DIV/0!</td><td>0</td><td>0</td><td>0</td><td>1</td><td>0</td><td>1</td><td>0</td><td>6</td><td>1.189598</td></tr>
<tr><td>tr|Q2L6I0|Q2L6I0_HUMAN</td><td>PPP1R10</td><td>FB19 protein OS=Homo sap</td><td>99338</td><td>0</td><td>1101</td><td>0</td><td>22</td><td>0</td><td>0.03</td><td>#DIV/0!</td><td>0</td><td>0</td><td>0</td><td>1</td><td>0</td><td>1</td><td>0</td><td>1</td><td>1.189598</td></tr>
<tr><td>tr|H0YMD2|H0YMD2_HUMA</td><td>NUSAP1</td><td>Nucleolar and spindle-asso</td><td>15923</td><td>0</td><td>1100</td><td>0</td><td>22</td><td>0</td><td>0.21</td><td>#DIV/0!</td><td>0</td><td>0</td><td>0</td><td>1</td><td>0</td><td>1</td><td>0</td><td>1</td><td>1.189598</td></tr>
<tr><td>sp|Q86YC3|LRC33_HUMAN</td><td>LRRC33</td><td>Leucine-rich repeat-contain</td><td>77457</td><td>0</td><td>1099</td><td>0</td><td>23</td><td>0</td><td>0.04</td><td>#DIV/0!</td><td>0</td><td>1</td><td>0</td><td>1</td><td>0</td><td>1</td><td>0</td><td>1</td><td>1.189598</td></tr>
<tr><td>sp|Q9BUI4|RPC3_HUMAN</td><td>POLR3C</td><td>DNA-directed RNA polymer</td><td>60972</td><td>0</td><td>1095</td><td>0</td><td>23</td><td>0</td><td>0.05</td><td>#DIV/0!</td><td>0</td><td>0</td><td>0</td><td>1</td><td>0</td><td>1</td><td>0</td><td>1</td><td>1.189598</td></tr>
<tr><td>tr|Q6DCA8|Q6DCA8_HUMAI</td><td>BCLAF1</td><td>BCLAF1 protein (Fragment)</td><td>77958</td><td>0</td><td>1093</td><td>0</td><td>23</td><td>0</td><td>0.04</td><td>#DIV/0!</td><td>0</td><td>0</td><td>0</td><td>1</td><td>0</td><td>1</td><td>0</td><td>2</td><td>1.189598</td></tr>
<tr><td>tr|B7Z396|B7Z396_HUMAN</td><td>tr|B7Z396|B7Z3</td><td>cDNA FLJ54685, highly simi</td><td>64556</td><td>0</td><td>1092</td><td>0</td><td>23</td><td>0</td><td>0.05</td><td>#DIV/0!</td><td>0</td><td>0</td><td>0</td><td>1</td><td>0</td><td>1</td><td>0</td><td>2</td><td>1.189598</td></tr>
<tr><td>sp|Q00653|NFKB2_HUMAN</td><td>NFKB2</td><td>Nuclear factor NF-kappa-B</td><td>97373</td><td>0</td><td>1091</td><td>0</td><td>23</td><td>0</td><td>0.03</td><td>#DIV/0!</td><td>0</td><td>0</td><td>0</td><td>1</td><td>0</td><td>1</td><td>0</td><td>1</td><td>1.189598</td></tr>
<tr><td>tr|B0V0F7|B0V0F7_HUMAN</td><td>DHX16</td><td>Putative pre-mRNA-splicing</td><td>21454</td><td>0</td><td>1090</td><td>0</td><td>23</td><td>0</td><td>0.16</td><td>#DIV/0!</td><td>0</td><td>0</td><td>0</td><td>1</td><td>0</td><td>1</td><td>0</td><td>1</td><td>1.189598</td></tr>
<tr><td>tr|F5H885|F5H885_HUMAN</td><td>MKS1</td><td>Meckel syndrome type 1 pr</td><td>63647</td><td>0</td><td>1089</td><td>0</td><td>23</td><td>0</td><td>0.05</td><td>#DIV/0!</td><td>0</td><td>0</td><td>0</td><td>1</td><td>0</td><td>1</td><td>0</td><td>2</td><td>1.189598</td></tr>
<tr><td>tr|G3V1U5|G3V1U5_HUMAN</td><td>GOLT1B</td><td>Golgi transport 1 homolog</td><td>8208</td><td>0</td><td>1088</td><td>0</td><td>23</td><td>0</td><td>0.43</td><td>#DIV/0!</td><td>0</td><td>1</td><td>0</td><td>1</td><td>0</td><td>1</td><td>0</td><td>1</td><td>1.189598</td></tr>
<tr><td>tr|B4E3W1|B4E3W1_HUMA</td><td>tr|B4E3W1|B4E3</td><td>cDNA FLJ57204, highly simi</td><td>51668</td><td>0</td><td>1086</td><td>0</td><td>24</td><td>0</td><td>0.06</td><td>#DIV/0!</td><td>0</td><td>0</td><td>0</td><td>1</td><td>0</td><td>1</td><td>0</td><td>1</td><td>1.189598</td></tr>
<tr><td>sp|Q8TA86|RP9_HUMAN</td><td>RP9</td><td>Retinitis pigmentosa 9 prot</td><td>26319</td><td>0</td><td>1085</td><td>0</td><td>24</td><td>0</td><td>0.13</td><td>#DIV/0!</td><td>0</td><td>0</td><td>0</td><td>1</td><td>0</td><td>1</td><td>0</td><td>1</td><td>1.189598</td></tr>
<tr><td>tr|A0JLQ5|A0JLQ5_HUMAN</td><td>BXDC2</td><td>BXDC2 protein (Fragment)</td><td>39430</td><td>0</td><td>1081</td><td>0</td><td>24</td><td>0</td><td>0.08</td><td>#DIV/0!</td><td>0</td><td>0</td><td>0</td><td>1</td><td>0</td><td>1</td><td>0</td><td>2</td><td>1.189598</td></tr>
<tr><td>tr|F5H8E5|F5H8E5_HUMAN</td><td>USP7</td><td>Ubiquitin carboxyl-terminal</td><td>117951</td><td>0</td><td>1079</td><td>0</td><td>24</td><td>0</td><td>0.03</td><td>#DIV/0!</td><td>0</td><td>0</td><td>0</td><td>1</td><td>0</td><td>1</td><td>0</td><td>1</td><td>1.189598</td></tr>
<tr><td>tr|A8K6X5|A8K6X5_HUMAN</td><td>tr|A8K6X5|A8K6</td><td>cDNA FLJ78680, highly simi</td><td>122741</td><td>0</td><td>1077</td><td>0</td><td>24</td><td>0</td><td>0.03</td><td>#DIV/0!</td><td>0</td><td>0</td><td>0</td><td>1</td><td>0</td><td>1</td><td>0</td><td>1</td><td>1.189598</td></tr>
<tr><td>tr|F6U211|F6U211_HUMAN</td><td>RPS10</td><td>40S ribosomal protein S10</td><td>19912</td><td>0</td><td>1076</td><td>0</td><td>24</td><td>0</td><td>0.17</td><td>#DIV/0!</td><td>0</td><td>0</td><td>0</td><td>1</td><td>0</td><td>1</td><td>0</td><td>1</td><td>1.189598</td></tr>
<tr><td>tr|F8W9B5|F8W9B5_HUMAI</td><td>PRC1</td><td>Protein regulator of cytokin</td><td>66790</td><td>0</td><td>1075</td><td>0</td><td>24</td><td>0</td><td>0.05</td><td>#DIV/0!</td><td>0</td><td>0</td><td>0</td><td>1</td><td>0</td><td>1</td><td>0</td><td>1</td><td>1.189598</td></tr>
<tr><td>tr|Q5IRN4|Q5IRN4_HUMAN</td><td>MEF2D/DAZAP1</td><td>Myocyte enhancer factor 2I</td><td>50610</td><td>0</td><td>1074</td><td>0</td><td>24</td><td>0</td><td>0.07</td><td>#DIV/0!</td><td>0</td><td>0</td><td>0</td><td>1</td><td>0</td><td>1</td><td>0</td><td>1</td><td>1.189598</td></tr>
<tr><td>tr|Q8TEP9|Q8TEP9_HUMAN</td><td>FLJ00144</td><td>FLJ00144 protein (Fragmen</td><td>28437</td><td>0</td><td>1073</td><td>0</td><td>24</td><td>0</td><td>0.12</td><td>#DIV/0!</td><td>0</td><td>0</td><td>0</td><td>1</td><td>0</td><td>1</td><td>0</td><td>2</td><td>1.189598</td></tr>
<tr><td>sp|Q6PKG0|LARP1_HUMAN</td><td>LARP1</td><td>La-related protein 1 OS=Ho</td><td>123833</td><td>0</td><td>1072</td><td>0</td><td>24</td><td>0</td><td>0.03</td><td>#DIV/0!</td><td>0</td><td>0</td><td>0</td><td>1</td><td>0</td><td>1</td><td>0</td><td>3</td><td>1.189598</td></tr>
<tr><td>tr|H3BMJ8|H3BMJ8_HUMAN</td><td>TCF25</td><td>Transcription factor 25 (Fra</td><td>23584</td><td>0</td><td>1071</td><td>0</td><td>24</td><td>0</td><td>0.14</td><td>#DIV/0!</td><td>0</td><td>0</td><td>0</td><td>1</td><td>0</td><td>1</td><td>0</td><td>1</td><td>1.189598</td></tr>
<tr><td>tr|H0YHA8|H0YHA8_HUMAN</td><td>NAV3</td><td>Neuron navigator 3 (Fragm</td><td>136766</td><td>0</td><td>1070</td><td>0</td><td>24</td><td>0</td><td>0.02</td><td>#DIV/0!</td><td>0</td><td>0</td><td>0</td><td>1</td><td>0</td><td>1</td><td>0</td><td>2</td><td>1.189598</td></tr>
<tr><td>tr|B4DSN3|B4DSN3_HUMAN</td><td>tr|B4DSN3|B4DS</td><td>cDNA FLJ60345, highly simi</td><td>107974</td><td>0</td><td>1069</td><td>0</td><td>25</td><td>0</td><td>0.03</td><td>#DIV/0!</td><td>0</td><td>0</td><td>0</td><td>1</td><td>0</td><td>1</td><td>0</td><td>1</td><td>1.189598</td></tr>
<tr><td>tr|B2RA56|B2RA56_HUMAN</td><td>tr|B2RA56|B2RA</td><td>cDNA, FLJ94705, highly sim</td><td>61891</td><td>0</td><td>1068</td><td>0</td><td>25</td><td>0</td><td>0.05</td><td>#DIV/0!</td><td>0</td><td>0</td><td>0</td><td>1</td><td>0</td><td>1</td><td>0</td><td>1</td><td>1.189598</td></tr>
<tr><td>tr|A0PJ61|A0PJ61_HUMAN</td><td>RSL1D1</td><td>RSL1D1 protein (Fragment)</td><td>49688</td><td>0</td><td>1066</td><td>0</td><td>25</td><td>0</td><td>0.07</td><td>#DIV/0!</td><td>0</td><td>0</td><td>0</td><td>1</td><td>0</td><td>1</td><td>0</td><td>1</td><td>1.189598</td></tr>
<tr><td>sp|Q2M389|WASH7_HUMAI</td><td>KIAA1033</td><td>WASH complex subunit 7 O</td><td>137343</td><td>0</td><td>1065</td><td>0</td><td>25</td><td>0</td><td>0.02</td><td>#DIV/0!</td><td>0</td><td>0</td><td>0</td><td>1</td><td>0</td><td>1</td><td>0</td><td>1</td><td>1.189598</td></tr>
<tr><td>tr|B4DWV9|B4DWV9_HUM</td><td>tr|B4DWV9|B4D</td><td>cDNA FLJ53108, highly simi</td><td>41291</td><td>0</td><td>1063</td><td>0</td><td>25</td><td>0</td><td>0.08</td><td>#DIV/0!</td><td>0</td><td>0</td><td>0</td><td>1</td><td>0</td><td>1</td><td>0</td><td>1</td><td>1.189598</td></tr>
<tr><td>tr|B7Z2B4|B7Z2B4_HUMAN</td><td>RABGAP1</td><td>Rab GTPase-activating prot</td><td>48660</td><td>0</td><td>1062</td><td>0</td><td>25</td><td>0</td><td>0.07</td><td>#DIV/0!</td><td>0</td><td>0</td><td>0</td><td>1</td><td>0</td><td>1</td><td>0</td><td>2</td><td>1.189598</td></tr>
</table>

tr|H0Y532|H0Y532_HUMAN	SELENBP1	Selenium-binding protein 1	34415	0	1061	0	25	0	0.1	#DIV/0!	0	0	0	1	0	1	0	1	1.189598
tr|B3KVH8|B3KVH8_HUMAN	PHF23	PHD finger protein 23 OS=H	43934	0	1058	0	25	0	0.08	#DIV/0!	0	0	0	1	0	1	0	2	1.189598
tr|H3BM98|H3BM98_HUMA	NEIL1	Endonuclease 8-like 1 (Frag	17611	0	1057	0	25	0	0.19	#DIV/0!	0	0	0	1	0	1	0	1	1.189598
tr|B7Z7Q6|B7Z7Q6_HUMAN	PRCP	Lysosomal Pro-X carboxype	44362	0	1055	0	26	0	0.07	#DIV/0!	0	0	0	1	0	1	0	1	1.189598
tr|B4DH29|B4DH29_HUMAN	tr|B4DH29|B4DH	DNA-directed RNA polymer	126564	0	1054	0	26	0	0.03	#DIV/0!	0	0	0	1	0	1	0	1	1.189598
tr|A8K9B9|A8K9B9_HUMAN	tr|A8K9B9|A8K9	cDNA FLJ77391, highly simi	61346	0	1053	0	26	0	0.05	#DIV/0!	0	0	0	1	0	1	0	2	1.189598
tr|J3QRH4|J3QRH4_HUMAN	CNDP2	Cytosolic non-specific dipep	22770	0	1051	0	26	0	0.15	#DIV/0!	0	0	0	1	0	1	0	2	1.189598
tr|H0YIK5|H0YIK5_HUMAN	RACGAP1	Rac GTPase-activating prote	31466	0	1049	0	26	0	0.11	#DIV/0!	0	0	0	1	0	1	0	1	1.189598
tr|B4DQG8|B4DQG8_HUMA	tr|B4DQG8|B4D(	cDNA FLJ54124, highly simi	52000	0	1046	0	26	0	0.06	#DIV/0!	0	0	0	1	0	1	0	1	1.189598
tr|B4E2N3|B4E2N3_HUMAN	tr|B4E2N3|B4E2	cDNA FLJ52151, highly simi	36154	0	1045	0	26	0	0.09	#DIV/0!	0	0	0	1	0	1	0	1	1.189598
tr|B4DEP6|B4DEP6_HUMAN	tr|B4DEP6|B4DE	cDNA FLJ59206, highly simi	66146	0	1044	0	26	0	0.05	#DIV/0!	0	0	0	1	0	1	0	1	1.189598
tr|B3KMN5|B3KMN5_HUMA	tr|B3KMN5|B3K	cDNA FLJ11713 fis, clone H	44787	0	1043	0	26	0	0.07	#DIV/0!	0	0	0	1	0	1	0	1	1.189598
tr|G3V1N8|G3V1N8_HUMAN	VPS26A	Vacuolar protein sorting 26	25547	0	1041	0	27	0	0.13	#DIV/0!	0	0	0	1	0	1	0	1	1.189598
tr|B4DZ55|B4DZ55_HUMAN	tr|B4DZ55|B4DZ	cDNA FLJ52097, weakly sim	104668	0	1040	0	27	0	0.03	#DIV/0!	0	0	0	1	0	1	0	1	1.189598
tr|B4DEY0|B4DEY0_HUMAN	tr|B4DEY0|B4DE	cDNA FLJ60450, highly simi	22023	0	1039	0	27	0	0.15	#DIV/0!	0	0	0	1	0	1	0	1	1.189598
tr|H0YD93|H0YD93_HUMAN	RUFY2	RUN and FYVE domain-cont	72131	0	1038	0	27	0	0.05	#DIV/0!	0	0	0	1	0	1	0	1	1.189598
tr|F5GWY5|F5GWY5_HUMA	PODXL	Podocalyxin OS=Homo sapi	59285	0	1037	0	27	0	0.06	#DIV/0!	0	0	0	1	0	1	0	1	1.189598
tr|H0Y8W5|H0Y8W5_HUMA	ARHGAP10	Rho GTPase-activating prot	46225	0	1036	0	27	0	0.07	#DIV/0!	0	0	0	1	0	1	0	1	1.189598
tr|B4DV28|B4DV28_HUMAN	tr|B4DV28|B4DV	cDNA FLJ54170, highly simi	51811	0	1035	0	27	0	0.13	#DIV/0!	0	0	0	2	0	1	0	3	1.189598
tr|B0AZR9|B0AZR9_HUMAN	GNAS	GNAS complex locus, isofor	39373	0	1034	0	27	0	0.08	#DIV/0!	0	0	0	1	0	1	0	2	1.189598
tr|H0Y6R6|H0Y6R6_HUMAN	TTC5	Tetratricopeptide repeat pr	42999	0	1031	0	27	0	0.08	#DIV/0!	0	0	0	1	0	1	0	1	1.189598
tr|D3DNS0|D3DNS0_HUMAN	ACTL6A	Actin-like 6A, isoform CRA_	25781	0	1030	0	27	0	0.13	#DIV/0!	0	0	0	1	0	1	0	1	1.189598
tr|Q05CP4|Q05CP4_HUMAN	EIF2AK2	EIF2AK2 protein (Fragment)	40940	0	1026	0	27	0	0.08	#DIV/0!	0	0	0	1	0	1	0	1	1.189598
sp|Q5K651|SAMD9_HUMAN	SAMD9	Sterile alpha motif domain-	185704	0	1025	0	27	0	0.02	#DIV/0!	0	0	0	1	0	1	0	1	1.189598
tr|B4DFN6|B4DFN6_HUMAN	CNP	2~,3~-cyclic-nucleotide 3~-	20461	0	1024	0	27	0	0.16	#DIV/0!	0	0	0	1	0	1	0	1	1.189598
tr|B3KQ73|B3KQ73_HUMAN	tr|B3KQ73|B3KC	cDNA FLJ33002 fis, clone TH	37025	0	1021	0	28	0	0.09	#DIV/0!	0	0	0	1	0	1	0	1	1.189598
sp|Q0VAR9|CTAS1_HUMAN	CTBP1-AS1	Putative uncharacterized pr	20240	0	1020	0	28	0	0.17	#DIV/0!	0	0	0	1	0	1	0	1	1.189598
tr|Q96LK9|Q96LK9_HUMAN	tr|Q96LK9|Q96L	cDNA FLJ25413 fis, clone TS	16911	0	1019	0	28	0	0.2	#DIV/0!	0	0	0	1	0	1	0	1	1.189598
tr|B2R491|B2R491_HUMAN	RPS4X	Ribosomal protein S4, X-lin	29807	0	1018	0	28	0	0.24	#DIV/0!	0	0	0	2	0	1	0	4	1.189598
tr|Q6IB29|Q6IB29_HUMAN	EBNA1BP2	EBNA1 binding protein 2 OS	34887	0	1017	0	28	0	0.1	#DIV/0!	0	0	0	1	0	1	0	1	1.189598
tr|G3V1C1|G3V1C1_HUMAN	AKR1E2	1,5-anhydro-D-fructose red	23420	0	1016	0	28	0	0.14	#DIV/0!	0	1	0	1	0	1	0	1	1.189598
tr|Q86W17|Q86W17_HUMA	PRSS1	Protease serine 1 (Fragmen	9251	0	1015	0	28	0	0.9	#DIV/0!	0	0	0	2	0	1	0	2	1.189598
tr|I3L1M4|I3L1M4_HUMAN	ALDH3A2	Fatty aldehyde dehydrogen	18870	0	1014	0	28	0	0.18	#DIV/0!	0	0	0	1	0	1	0	1	1.189598
tr|A8K4G7|A8K4G7_HUMAN	tr|A8K4G7|A8K4	cDNA FLJ78528, highly simi	49413	0	1013	0	28	0	0.07	#DIV/0!	0	0	0	1	0	1	0	1	1.189598
tr|B4DH88|B4DH88_HUMAN	tr|B4DH88|B4DH	cDNA FLJ54092, highly simi	64520	0	1012	0	28	0	0.05	#DIV/0!	0	0	0	1	0	1	0	1	1.189598
tr|B4DQR0|B4DQR0_HUMAN	tr|B4DQR0|B4D(	cDNA FLJ54377 OS=Homo s	91332	0	1010	0	28	0	0.04	#DIV/0!	0	0	0	1	0	1	0	1	1.189598
tr|B2MV13|B2MV13_HUMA	LTF	Lactoferrin OS=Homo sapie	80228	0	1009	0	28	0	0.04	#DIV/0!	0	0	0	1	0	1	0	1	1.189598
tr|B1ANE3|B1ANE3_HUMAN	MTR	5-methyltetrahydrofolate-h	92463	0	1008	0	28	0	0.04	#DIV/0!	0	0	0	1	0	1	0	1	1.189598
sp|Q14684|RRP1B_HUMAN	RRP1B	Ribosomal RNA processing	84774	0	1007	0	28	0	0.04	#DIV/0!	0	0	0	1	0	1	0	1	1.189598
tr|H3BM93|H3BM93_HUMA	NUP93	Nuclear pore complex prote	19858	0	1006	0	28	0	0.17	#DIV/0!	0	0	0	1	0	1	0	1	1.189598
sp|Q9UBY0|SL9A2_HUMAN	SLC9A2	Sodium/hydrogen exchange	92032	0	1003	0	29	0	0.04	#DIV/0!	0	0	0	1	0	1	0	1	1.189598
tr|G3V1P5|G3V1P5_HUMAN	MED15	Mediator of RNA polymeras	80007	0	1002	0	29	0	0.04	#DIV/0!	0	0	0	1	0	1	0	1	1.189598
tr|B4DPF7|B4DPF7_HUMAN	tr|B4DPF7|B4DP	cDNA FLJ60764, highly simi	71406	0	1001	0	29	0	0.05	#DIV/0!	0	0	0	1	0	1	0	1	1.189598
tr|B7ZKL3|B7ZKL3_HUMAN	EPS8L2	EPS8L2 protein OS=Homo s	82898	0	1000	0	29	0	0.04	#DIV/0!	0	0	0	1	0	1	0	1	1.189598
tr|B4E0N6|B4E0N6_HUMAN	tr|B4E0N6|B4E0	cDNA FLJ56280, highly simi	26584	0	999	0	29	0	0.13	#DIV/0!	0	0	0	1	0	1	0	1	1.189598
tr|B2R9S4|B2R9S4_HUMAN	tr|B2R9S4|B2R9	cDNA, FLJ94534, highly sim	38759	0	998	0	29	0	0.09	#DIV/0!	0	0	0	1	0	1	0	1	1.189598
tr|Q5F0I5|Q5F0I5_HUMAN	GRIN3B	NMDA receptor subunit 3B	113842	0	997	0	29	0	0.03	#DIV/0!	0	0	0	1	0	1	0	1	1.189598
tr|B3KUB9|B3KUB9_HUMAN	SWAP70	SWAP-70 protein, isoform (	62280	0	996	0	29	0	0.05	#DIV/0!	0	0	0	1	0	1	0	1	1.189598
tr|B0I1S9|B0I1S9_HUMAN	MYO1B	MYO1B variant protein OS=	132928	0	995	0	29	0	0.02	#DIV/0!	0	0	0	1	0	1	0	2	1.189598
tr|B4DTY7|B4DTY7_HUMAN	tr|B4DTY7|B4DT	cDNA FLJ57813, weakly sim	168277	0	994	0	29	0	0.02	#DIV/0!	0	0	0	1	0	1	0	1	1.189598
tr|B1AK81|B1AK81_HUMAN	PIGK	GPI-anchor transamidase O	34462	0	992	0	29	0	0.1	#DIV/0!	0	0	0	1	0	1	0	1	1.189598
sp|Q969S3|ZN622_HUMAN	ZNF622	Zinc finger protein 622 OS=	54808	0	991	0	29	0	0.06	#DIV/0!	0	0	0	1	0	1	0	2	1.189598
sp|Q0VDG4|SCRN3_HUMAN	SCRN3	Secernin-3 OS=Homo sapie	48969	0	990	0	29	0	0.07	#DIV/0!	0	0	0	1	0	1	0	1	1.189598
sp|Q9Y3Z3|SAMH1_HUMAN	SAMHD1	SAM domain and HD domai	72896	0	989	0	30	0	0.05	#DIV/0!	0	0	0	1	0	1	0	1	1.189598
tr|H3BQ23|H3BQ23_HUMAN	CIAPIN1	Anamorsin (Fragment) OS=	13738	0	985	0	30	0	0.25	#DIV/0!	0	0	0	1	0	1	0	1	1.189598
tr|H0Y8D1|H0Y8D1_HUMAN	PRSS1	Trypsin0 (Fragment) OS=Ho	15697	0	984	0	30	0	0.22	#DIV/0!	0	0	0	1	0	1	0	1	1.189598
tr|G3V198|G3V198_HUMAN	NUP160	Nuclear pore complex prote	142365	0	983	0	30	0	0.02	#DIV/0!	0	1	0	1	0	1	0	1	1.189598
tr|C0JXB8|C0JXB8_HUMAN	AIP	Aryl hydrocarbon receptor	38041	0	982	0	30	0	0.09	#DIV/0!	0	1	0	1	0	1	0	1	1.189598
tr|B4DQL0|B4DQL0_HUMAN	MTMR8	Myotubularin-related prote	78337	0	981	0	30	0	0.04	#DIV/0!	0	1	0	1	0	1	0	1	1.189598
tr|B4DLG2|B4DLG2_HUMAN	tr|B4DLG2|B4DL	cDNA FLJ58196, highly simi	83177	0	979	0	30	0	0.04	#DIV/0!	0	0	0	1	0	1	0	1	1.189598
sp|Q99581|FEV_HUMAN	FEV	Protein FEV OS=Homo sapie	25129	0	978	0	30	0	0.13	#DIV/0!	0	0	0	1	0	1	0	1	1.189598
sp|Q0VDF9|HSP7E_HUMAN	HSPA14	Heat shock 70 kDa protein	55444	0	977	0	30	0	0.06	#DIV/0!	0	0	0	1	0	1	0	1	1.189598
tr|H0YM25|H0YM25_HUMA	LOC440233	Protein LOC440233 OS=Hor	107878	0	968	0	31	0	0.03	#DIV/0!	0	1	0	1	0	1	0	1	1.189598
tr|I3L495|I3L495_HUMAN	PAFAH1B1	Platelet-activating factor ac	9626	0	967	0	31	0	0.36	#DIV/0!	0	0	0	1	0	1	0	1	1.189598
tr|H0YHL1|H0YHL1_HUMAN	DCTN2	Dynactin subunit 2 (Fragme	14255	0	965	0	31	0	0.24	#DIV/0!	0	0	0	1	0	1	0	1	1.189598
tr|E9PPY3|E9PPY3_HUMAN	RRP8	Ribosomal RNA-processing	34579	0	961	0	31	0	0.1	#DIV/0!	0	0	0	1	0	1	0	1	1.189598
tr|B4E2K4|B4E2K4_HUMAN	tr|B4E2K4|B4E2	cDNA FLJ54576, highly simi	83685	0	960	0	31	0	0.04	#DIV/0!	0	0	0	1	0	1	0	1	1.189598
sp|Q7Z5H4|VN1R5_HUMAN	VN1R5	Vomeronasal type0 recepto	41265	0	959	0	31	0	0.08	#DIV/0!	0	1	0	1	0	1	0	1	1.189598
tr|B4DDL6|B4DDL6_HUMAN	tr|B4DDL6|B4DD	cDNA FLJ54325, highly simi	36644	0	958	0	31	0	0.09	#DIV/0!	0	0	0	1	0	1	0	1	1.189598
tr|Q5T5N3|Q5T5N3_HUMAN	IDE	Insulin-degrading enzyme (	29418	0	957	0	31	0	0.11	#DIV/0!	0	0	0	1	0	1	0	1	1.189598
tr|Q6ZNZ8|Q6ZNZ8_HUMAN	tr|Q6ZNZ8|Q6ZN	cDNA FLJ26816 fis, clone PR	54459	0	956	0	31	0	0.06	#DIV/0!	0	0	0	1	0	1	0	1	1.189598
tr|J3QKW7|J3QKW7_HUMA	DRG2	Developmentally-regulated	26892	0	955	0	31	0	0.12	#DIV/0!	0	0	0	1	0	1	0	1	1.189598
sp|Q5VTR2|BRE1A_HUMAN	RNF20	E3 ubiquitin-protein ligase	114220	0	954	0	31	0	0.03	#DIV/0!	0	0	0	1	0	1	0	2	1.189598
tr|D6RGZ2|D6RGZ2_HUMAN	THOC3	THO complex subunit 3 OS=	11169	0	953	0	31	0	0.31	#DIV/0!	0	0	0	1	0	1	0	1	1.189598
sp|P01008|ANT3_HUMAN	SERPINC1	Antithrombin-III OS=Homo	53025	0	951	0	31	0	0.06	#DIV/0!	0	1	0	1	0	1	0	1	1.189598
tr|F4MHI3|F4MHI3_HUMAN	UTY	Ubiquitously transcribed te	112681	0	946	0	32	0	0.03	#DIV/0!	0	0	0	1	0	1	0	2	1.189598
sp|Q9Y512|SAM50_HUMAN	SAMM50	Sorting and assembly mach	52342	0	945	0	32	0	0.06	#DIV/0!	0	0	0	1	0	1	0	1	1.189598
sp|Q9UBK8|MTRR_HUMAN	MTRR	Methionine synthase reduc	81386	0	943	0	32	0	0.04	#DIV/0!	0	0	0	1	0	1	0	1	1.189598
sp|Q5T7P6|TMM78_HUMAN	TMEM78	Transmembrane protein 78	15468	0	941	0	32	0	0.22	#DIV/0!	0	1	0	1	0	1	0	1	1.189598
tr|B4DDT3|B4DDT3_HUMAN	tr|B4DDT3|B4DD	cDNA FLJ54622, highly simi	54084	0	940	0	32	0	0.06	#DIV/0!	0	0	0	1	0	1	0	1	1.189598
tr|B7Z871|B7Z871_HUMAN	tr|B7Z871|B7Z87	cDNA FLJ50930, highly simi	49854	0	938	0	32	0	0.07	#DIV/0!	0	0	0	1	0	1	0	1	1.189598
tr|H7C1L6|H7C1L6_HUMAN	CUL3	Cullin-3 (Fragment) OS=Hor	22753	0	926	0	33	0	0.15	#DIV/0!	0	1	0	1	0	1	0	1	1.189598
tr|B4E2A4|B4E2A4_HUMAN	tr|B4E2A4|B4E2	cDNA FLJ53275, highly simi	110910	0	925	0	33	0	0.03	#DIV/0!	0	0	0	1	0	1	0	1	1.189598
tr|J3KR44|J3KR44_HUMAN	OTUB1	Ubiquitin thioesterase OTU	35649	0	921	0	33	0	0.09	#DIV/0!	0	0	0	1	0	1	0	1	1.189598
tr|B3KSE4|B3KSE4_HUMAN	tr|B3KSE4|B3KS	cDNA FLJ36076 fis, clone TE	30924	0	919	0	33	0	0.11	#DIV/0!	0	1	0	1	0	1	0	1	1.189598
tr|A4D110|A4D110_HUMAN	LOC401309	Putative uncharacterized pr	13436	0	915	0	33	0	0.24	#DIV/0!	0	0	0	1	0	1	0	1	1.189598
sp|Q9H9V9|JMJD4_HUMAN	JMJD4	JmjC domain-containing pro	52973	0	912	0	34	0	0.06	#DIV/0!	0	1	0	1	0	1	0	1	1.189598
tr|Q5SRT3|Q5SRT3_HUMAN	CLIC1	Chloride intracellular chann	27248	0	911	0	34	0	0.12	#DIV/0!	0	0	0	1	0	1	0	1	1.189598
tr|Q4ZG72|Q4ZG72_HUMAN	DDX18	Putative uncharacterized pr	61898	0	909	0	34	0	0.05	#DIV/0!	0	1	0	1	0	1	0	3	1.189598
tr|Q6FG59|Q6FG59_HUMAN	CDC37	CDC37 protein OS=Homo sa	44938	0	907	0	34	0	0.07	#DIV/0!	0	1	0	1	0	1	0	1	1.189598
tr|H0YKH6|H0YKH6_HUMAN	CERS2	Ceramide synthase 2 (Fragr	26788	0	906	0	34	0	0.12	#DIV/0!	0	0	0	1	0	1	0	1	1.189598
tr|H0YJG6|H0YJG6_HUMAN	AHSA1	Activator of 90 kDa heat sh	13086	0	905	0	34	0	0.26	#DIV/0!	0	1	0	1	0	1	0	1	1.189598
tr|H0YE34|H0YE34_HUMAN	AMBRA1	Activating molecule in BECN	26856	0	904	0	34	0	0.12	#DIV/0!	0	0	0	1	0	1	0	1	1.189598
tr|D1LUZ0|D1LUZ0_HUMAN	R3HDM2/NFE2	NFE2 protein (Fragment) OS	1997	0	899	0	34	0	2.06	#DIV/0!	0	1	0	1	0	1	0	1	1.189598
tr|F5GWJ1|F5GWJ1_HUMAN	PCDH7	Protocadherin-7 OS=Homo	137228	0	898	0	34	0	0.02	#DIV/0!	0	1	0	1	0	1	0	2	1.189598
sp|Q92536|YLAT2_HUMAN	SLC7A6	Y+L amino acid transporter	57532	0	897	0	34	0	0.06	#DIV/0!	0	1	0	1	0	1	0	2	1.189598
tr|B3KR24|B3KR24_HUMAN	tr|B3KR24|B3KR	cDNA FLJ33515 fis, clone BR	25101	0	896	0	34	0	0.13	#DIV/0!	0	0	0	1	0	1	0	1	1.189598
tr|B4DM84|B4DM84_HUMA	tr|B4DM84|B4D	cDNA FLJ60694, highly simi	87230	0	894	0	35	0	0.04	#DIV/0!	0	0	0	1	0	1	0	1	1.189598
tr|B3KY11|B3KY11_HUMAN	tr|B3KY11|B3KY	cDNA FLJ46571 fis, clone TH	93519	0	893	0	35	0	0.04	#DIV/0!	0	0	0	1	0	1	0	1	1.189598
tr|B2RCY5|B2RCY5_HUMAN	tr|B2RCY5|B2RC	cDNA, FLJ96373, highly sim	69836	0	892	0	35	0	0.05	#DIV/0!	0	0	0	1	0	1	0	1	1.189598
tr|Q16083|Q16083_HUMAN	tr|Q16083|Q160	U1 small nuclear ribonucle	11203	0	891	0	35	0	0.31	#DIV/0!	0	1	0	1	0	1	0	1	1.189598
tr|B3KSB5|B3KSB5_HUMAN	tr|B3KSB5|B3KS	cDNA FLJ35923 fis, clone TE	104031	0	890	0	35	0	0.03	#DIV/0!	0	1	0	1	0	1	0	1	1.189598
sp|Q9UKV8|AGO2_HUMAN	EIF2C2	Protein argonaute-2 OS=Ho	98400	0	887	0	35	0	0.03	#DIV/0!	0	0	0	1	0	1	0	3	1.189598
sp|Q9BVM4|GGACT_HUMAN	GGACT	Gamma-glutamylaminecycl	17432	0	885	0	35	0	0.19	#DIV/0!	0	1	0	1	0	1	0	1	1.189598
tr|A8KAQ5|A8KAQ5_HUMAN	tr|A8KAQ5|A8KA	cDNA FLJ77404, highly simi	51522	0	884	0	35	0	0.06	#DIV/0!	0	1	0	1	0	1	0	1	1.189598
sp|Q09161|NCBP1_HUMAN	NCBP1	Nuclear cap-binding protein	92864	0	883	0	35	0	0.04	#DIV/0!	0	0	0	1	0	1	0	1	1.189598
sp|Q4G0J3|LARP7_HUMAN	LARP7	La-related protein 7 OS=Ho	67143	0	882	0	35	0	0.05	#DIV/0!	0	0	0	1	0	1	0	1	1.189598
tr|H7C0M3|H7C0M3_HUMA	BPHL	Valacyclovir hydrolase (Frag	3314	0	880	0	35	0	1.2	#DIV/0!	0	1	0	1	0	1	0	1	1.189598
tr|B4DZI8|B4DZI8_HUMAN	COPB2	Coatomer protein complex,	99839	0	878	0	35	0	0.03	#DIV/0!	0	0	0	1	0	1	0	1	1.189598
tr|B3KWW3|B3KWW3_HUM	tr|B3KWW3|B3K	cDNA FLJ44017 fis, clone TE	116458	0	877	0	35	0	0.03	#DIV/0!	0	1	0	1	0	1	0	2	1.189598
tr|H0YB22|H0YB22_HUMAN	RPS14	40S ribosomal protein S14 (	13220	0	875	0	35	0	0.26	#DIV/0!	0	1	0	1	0	1	0	1	1.189598
tr|B4E074|B4E074_HUMAN	tr|B4E074|B4E0	cDNA FLJ58655, highly simi	51211	0	873	0	35	0	0.06	#DIV/0!	0	0	0	1	0	1	0	1	1.189598
tr|G3XAL9|G3XAL9_HUMAN	SLC12A2	Solute carrier family 12 (So	125254	0	872	0	35	0	0.03	#DIV/0!	0	1	0	1	0	1	0	1	1.189598

tr|Q5TGM0|Q5TGM0_HUMA	VTA1	Vacuolar protein sorting-as	27485	0	870	0	35	0	0.12	#DIV/0!	0	0	0	1	0	1	0	1	1.189598
tr|Q5HYG2|Q5HYG2_HUMA	DKFZp686H0286	Putative uncharacterized pr	8427	0	867	0	35	0	0.44	#DIV/0!	0	1	0	1	0	1	0	1	1.189598
tr|B4DFG0|B4DFG0_HUMAN	DEK	Protein DEK OS=Homo sapi	39704	0	866	0	36	0	0.08	#DIV/0!	0	1	0	1	0	1	0	1	1.189598
tr|B3KM47|B3KM47_HUMA	tr|B3KM47|B3KI	cDNA FLJ10273 fis, clone HI	111730	0	865	0	36	0	0.03	#DIV/0!	0	0	0	1	0	1	0	1	1.189598
tr|J3KQ58|J3KQ58_HUMAN	TTC34	Tetratricopeptide repeat pr	40214	0	862	0	36	0	0.08	#DIV/0!	0	1	0	1	0	1	0	1	1.189598
tr|F5GXR6|F5GXR6_HUMAN	MYO18B	Unconventional myosin-XV	287333	0	861	0	36	0	0.01	#DIV/0!	0	1	0	1	0	1	0	1	1.189598
tr|D3DVS1|D3DVS1_HUMAN	TMEM128	Transmembrane protein 12	19038	0	859	0	36	0	0.18	#DIV/0!	0	1	0	1	0	1	0	1	1.189598
tr|Q5VZA6|Q5VZA6_HUMAN	ACO1	Cytoplasmic aconitate hydr	48888	0	858	0	36	0	0.07	#DIV/0!	0	0	0	1	0	1	0	1	1.189598
tr|A0JLT1|A0JLT1_HUMAN	CD3EAP	CD3EAP protein (Fragment)	46280	0	857	0	36	0	0.07	#DIV/0!	0	0	0	1	0	1	0	1	1.189598
tr|B4DPJ2|B4DPJ2_HUMAN	tr|B4DPJ2|B4DP.	Annexin OS=Homo sapiens	46025	0	854	0	36	0	0.07	#DIV/0!	0	1	0	1	0	1	0	2	1.189598
tr|D3DTD2|D3DTD2_HUMAN	TRIO	Triple functional domain (P	270210	0	851	0	36	0	0.01	#DIV/0!	0	1	0	1	0	1	0	1	1.189598
sp|Q5T8P6|RBM26_HUMAN	RBM26	RNA-binding protein 26 OS	113927	0	849	0	36	0	0.03	#DIV/0!	0	0	0	1	0	1	0	1	1.189598
tr|B4DZM3|B4DZM3_HUMA	tr|B4DZM3|B4D	cDNA FLJ61500, highly simi	46521	0	846	0	36	0	0.07	#DIV/0!	0	1	0	1	0	1	0	1	1.189598
tr|G3V0G3|G3V0G3_HUMAN	DDX56	DEAD (Asp-Glu-Ala-Asp) bo	41969	0	845	0	36	0	0.08	#DIV/0!	0	1	0	1	0	1	0	2	1.189598
tr|H0Y7L9|H0Y7L9_HUMAN	ABCD1	ATP-binding cassette sub-fa	24597	0	841	0	36	0	0.14	#DIV/0!	0	1	0	1	0	1	0	1	1.189598
tr|H0Y9G6|H0Y9G6_HUMAN	MRPL3	39S ribosomal protein L3, m	40898	0	839	0	37	0	0.08	#DIV/0!	0	1	0	1	0	1	0	1	1.189598
tr|H0YF92|H0YF92_HUMAN	SLC38A10	Putative sodium-coupled ne	31742	0	838	0	37	0	0.1	#DIV/0!	0	1	0	1	0	1	0	1	1.189598
tr|B2R806|B2R806_HUMAN	tr|B2R806|B2R8	Eukaryotic translation initia	52622	0	837	0	37	0	0.06	#DIV/0!	0	0	0	1	0	1	0	2	1.189598
tr|A0PJC9|A0PJC9_HUMAN	TPR	TPR protein (Fragment) OS=	53894	0	835	0	37	0	0.06	#DIV/0!	0	1	0	1	0	1	0	1	1.189598
tr|Q14730|Q14730_HUMAN	tr|Q14730|Q147	La 4.1 protein (Fragment) C	33852	0	832	0	37	0	0.1	#DIV/0!	0	0	0	1	0	1	0	1	1.189598
tr|B4DNM8|B4DNM8_HUMA	tr|B4DNM8|B4D	cDNA FLJ53395, highly simi	69572	0	829	0	37	0	0.05	#DIV/0!	0	1	0	1	0	1	0	1	1.189598
tr|Q8TBT6|Q8TBT6_HUMAN	tr|Q8TBT6|Q8TE	Putative uncharacterized pr	34810	0	827	0	37	0	0.1	#DIV/0!	0	0	0	1	0	1	0	1	1.189598
sp|Q9Y295|DRG1_HUMAN	DRG1	Developmentally-regulated	40802	0	826	0	37	0	0.08	#DIV/0!	0	1	0	1	0	1	0	1	1.189598
sp|Q8IZW8|TENS4_HUMAN	TNS4	Tensin-4 OS=Homo sapiens	77798	0	824	0	38	0	0.04	#DIV/0!	0	1	0	1	0	1	0	1	1.189598
tr|G3V1B3|G3V1B3_HUMAN	RPL21	60S ribosomal protein L21 (	9994	0	822	0	38	0	0.35	#DIV/0!	0	1	0	1	0	1	0	1	1.189598
tr|B3KM97|B3KM97_HUMA	tr|B3KM97|B3KI	cDNA FLJ10554 fis, clone NT	36429	0	821	0	38	0	0.09	#DIV/0!	0	1	0	1	0	1	0	2	1.189598
tr|Q9UNM7|Q9UNM7_HUM	tr|Q9UNM7|Q9U	26S proteasome subunit p4	18221	0	820	0	38	0	0.19	#DIV/0!	0	1	0	1	0	1	0	2	1.189598
tr|F8VQQ3|F8VQQ3_HUMAI	C12orf10	UPF0160 protein MYG1, mi	34140	0	819	0	38	0	0.1	#DIV/0!	0	1	0	1	0	1	0	1	1.189598
tr|B0QZ18|B0QZ18_HUMAN	CPNE1	Copine I OS=Homo sapiens	60307	0	818	0	38	0	0.05	#DIV/0!	0	1	0	1	0	1	0	1	1.189598
sp|O95396|MOCS3_HUMAN	MOCS3	Adenylyltransferase and su	50550	0	812	0	39	0	0.07	#DIV/0!	0	1	0	1	0	1	0	1	1.189598
sp|Q14690|RRP5_HUMAN	PDCD11	Protein RRP5 homolog OS=	209939	0	810	0	39	0	0.02	#DIV/0!	0	1	0	1	0	1	0	1	1.189598
tr|H0Y6E7|H0Y6E7_HUMAN	RBMX	RNA-binding motif protein,	31837	0	808	0	39	0	0.1	#DIV/0!	0	1	0	1	0	1	0	1	1.189598
tr|H0Y8R1|H0Y8R1_HUMAN	GRSF1	G-rich sequence factor 1 (F	47637	0	807	0	39	0	0.14	#DIV/0!	0	1	0	2	0	1	0	2	1.189598
sp|Q9BXW9|FACD2_HUMAN	FANCD2	Fanconi anemia group D2 p	168295	0	806	0	39	0	0.02	#DIV/0!	0	1	0	1	0	1	0	1	1.189598
tr|B0QZ34|B0QZ34_HUMAN	ARID4B	AT rich interactive domain	61288	0	799	0	40	0	0.05	#DIV/0!	0	1	0	1	0	1	0	1	1.189598
tr|K7EPI4|K7EPI4_HUMAN	GFAP	Glial fibrillary acidic protein	7120	0	798	0	40	0	0.5	#DIV/0!	0	1	0	1	0	1	0	1	1.189598
tr|B2R960|B2R960_HUMAN	tr|B2R960|B2R9	cDNA, FLJ94230, highly sim	32616	0	797	0	40	0	0.1	#DIV/0!	0	1	0	1	0	1	0	1	1.189598
tr|Q5VXJ5|Q5VXJ5_HUMAN	SYCP1	Synaptonemal complex pro	93629	0	796	0	40	0	0.03	#DIV/0!	0	1	0	1	0	1	0	1	1.189598
tr|B4DTS5|B4DTS5_HUMAN	tr|B4DTS5|B4DT	cDNA FLJ58882, highly simi	35374	0	795	0	40	0	0.09	#DIV/0!	0	1	0	1	0	1	0	1	1.189598
tr|B1AJQ5|B1AJQ5_HUMAN	FAM76A	Family with sequence simila	14184	0	794	0	40	0	0.25	#DIV/0!	0	1	0	1	0	1	0	2	1.189598
sp|Q7L1S5|CHST9_HUMAN	CHST9	Carbohydrate sulfotransfer	52364	0	792	0	40	0	0.06	#DIV/0!	0	1	0	1	0	1	0	2	1.189598
tr|H7C3J6|H7C3J6_HUMAN	SAMD11	Sterile alpha motif domain-	57548	0	791	0	40	0	0.06	#DIV/0!	0	1	0	1	0	1	0	2	1.189598
tr|B3KM89|B3KM89_HUMA	tr|B3KM89|B3KI	cDNA FLJ10528 fis, clone NT	101723	0	786	0	40	0	0.03	#DIV/0!	0	1	0	1	0	1	0	1	1.189598
tr|K7EM59|K7EM59_HUMAI	MRI1	Methylthioribose0-phospha	26783	0	785	0	40	0	0.12	#DIV/0!	0	1	0	1	0	1	0	1	1.189598
sp|Q9P265|DIP2B_HUMAN	DIP2B	Disco-interacting protein 2	173606	0	784	0	40	0	0.02	#DIV/0!	0	1	0	1	0	1	0	1	1.189598
tr|Q53G71|Q53G71_HUMAN	tr|Q53G71|Q53(	Calreticulin variant (Fragme	47061	0	783	0	40	0	0.15	#DIV/0!	0	1	0	2	0	1	0	3	1.189598
sp|Q96F86|EDC3_HUMAN	EDC3	Enhancer of mRNA-decappi	56784	0	778	0	41	0	0.06	#DIV/0!	0	1	0	1	0	1	0	2	1.189598
tr|B2RAG9|B2RAG9_HUMAN	tr|B2RAG9|B2RA	cDNA, FLJ94908, highly sim	167569	0	777	0	41	0	0.02	#DIV/0!	0	1	0	1	0	1	0	1	1.189598
tr|H0Y5T1|H0Y5T1_HUMAN	CLASP1	CLIP-associating protein 1 (	76997	0	775	0	41	0	0.04	#DIV/0!	0	1	0	1	0	1	0	1	1.189598
sp|Q9UHN6|TMEM2_HUMA	TMEM2	Transmembrane protein 2 (	155702	0	774	0	41	0	0.02	#DIV/0!	0	1	0	1	0	1	0	1	1.189598
tr|Q59G04|Q59G04_HUMAN	tr|Q59G04|Q59(	Dystrophin related protein	66446	0	773	0	41	0	0.05	#DIV/0!	0	1	0	1	0	1	0	1	1.189598
tr|G5EA30|G5EA30_HUMAN	CELF1	CUG triplet repeat, RNA bin	55564	0	771	0	42	0	0.06	#DIV/0!	0	1	0	1	0	1	0	1	1.189598
tr|B0V109|B0V109_HUMAN	FLOT1	Flotillin0 (Fragment) OS=Ho	39810	0	770	0	42	0	0.08	#DIV/0!	0	1	0	1	0	1	0	3	1.189598
tr|B2R6H7|B2R6H7_HUMAN	tr|B2R6H7|B2R6	cDNA, FLJ92955, highly sim	105989	0	765	0	42	0	0.03	#DIV/0!	0	1	0	1	0	1	0	1	1.189598
tr|H7C1C2|H7C1C2_HUMAN	ANLN	Actin-binding protein anillir	28113	0	761	0	42	0	0.12	#DIV/0!	0	1	0	1	0	1	0	2	1.189598
tr|Q5W1M0|Q5W1M0_HUM	HLA-C	MHC class I antigen (Fragm	10534	0	757	0	43	0	0.33	#DIV/0!	0	1	0	1	0	1	0	1	1.189598
tr|B0UY12|B0UY12_HUMAN	HLA-C	HLA class I histocompatibili	42012	0	755	0	43	0	0.08	#DIV/0!	0	1	0	1	0	1	0	2	1.189598
tr|H0Y9V9|H0Y9V9_HUMAN	RPL9	60S ribosomal protein L9 (F	21671	0	753	0	43	0	0.16	#DIV/0!	0	1	0	1	0	1	0	1	1.189598
sp|Q5BKZ1|ZN326_HUMAN	ZNF326	DBIRD complex subunit ZNI	65955	0	751	0	43	0	0.05	#DIV/0!	0	1	0	1	0	1	0	2	1.189598
sp|Q9Y4W2|LAS1L_HUMAN	LAS1L	Ribosomal biogenesis prote	83982	0	750	0	43	0	0.08	#DIV/0!	0	1	0	2	0	1	0	2	1.189598
tr|B2RE56|B2RE56_HUMAN	tr|B2RE56|B2RE	Peptidyl-prolyl cis-trans iso	18225	0	746	0	44	0	0.19	#DIV/0!	0	1	0	1	0	1	0	1	1.189598
tr|B3KNR6|B3KNR6_HUMAN	tr|B3KNR6|B3KN	cDNA FLJ30255 fis, clone BI	27615	0	745	0	44	0	0.12	#DIV/0!	0	1	0	1	0	1	0	1	1.189598
tr|Q2NL95|Q2NL95_HUMAN	DDX19B	DDX19B protein (Fragment)	51068	0	744	0	44	0	0.06	#DIV/0!	0	1	0	1	0	1	0	2	1.189598
tr|F8VX04|F8VX04_HUMAN	SLC38A1	Sodium-coupled neutral am	56900	0	742	0	44	0	0.06	#DIV/0!	0	1	0	1	0	1	0	1	1.189598
sp|Q8IWB1|IPRI_HUMAN	ITPRIP	Inositol 1,4,5-trisphosphate	62932	0	741	0	44	0	0.05	#DIV/0!	0	1	0	1	0	1	0	1	1.189598
tr|B7ZLH8|B7ZLH8_HUMAN	EVPL	EVPL protein OS=Homo sap	235047	0	740	0	44	0	0.01	#DIV/0!	0	1	0	1	0	1	0	2	1.189598
tr|A8K8N3|A8K8N3_HUMAN	tr|A8K8N3|A8K8	cDNA FLJ78740, highly simi	135680	0	738	0	44	0	0.02	#DIV/0!	0	1	0	1	0	1	0	2	1.189598
tr|B2R5U7|B2R5U7_HUMAN	tr|B2R5U7|B2R5	cDNA, FLJ92633, highly sim	114755	0	736	0	45	0	0.03	#DIV/0!	0	1	0	1	0	1	0	1	1.189598
tr|B7Z213|B7Z213_HUMAN	tr|B7Z213|B7Z2	cDNA FLJ50130, highly simi	28561	0	735	0	45	0	0.12	#DIV/0!	0	1	0	1	0	1	0	1	1.189598
tr|B4DFK7|B4DFK7_HUMAN	CBR1	Carbonyl reductase 1, isofo	19092	0	734	0	45	0	0.18	#DIV/0!	0	1	0	1	0	1	0	1	1.189598
tr|A0JLU4|A0JLU4_HUMAN	YBX1	YBX1 protein (Fragment) OS	21417	0	732	0	45	0	0.16	#DIV/0!	0	1	0	1	0	1	0	1	1.189598
tr|H0YFV6|H0YFV6_HUMAN	METTL3	N6-adenosine-methyltransf	36576	0	729	0	45	0	0.09	#DIV/0!	0	1	0	1	0	1	0	1	1.189598
tr|B4DWI8|B4DWI8_HUMAN	tr|B4DWI8|B4D\	cDNA FLJ57805, highly simi	52660	0	726	0	45	0	0.06	#DIV/0!	0	1	0	1	0	1	0	1	1.189598
tr|Q6FHF5|Q6FHF5_HUMAN	PCNA	Proliferating cell nuclear an	29029	0	724	0	45	0	0.11	#DIV/0!	0	1	0	1	0	1	0	2	1.189598
tr|B4DKA8|B4DKA8_HUMAN	USP8	Ubiquitin carboxyl-terminal	115921	0	722	0	46	0	0.03	#DIV/0!	0	1	0	1	0	1	0	1	1.189598
tr|A1A693|A1A693_HUMAN	RBM15	RNA binding motif protein	99689	0	720	0	46	0	0.07	#DIV/0!	0	1	0	2	0	1	0	3	1.189598
sp|Q9H4F8|SMOC1_HUMAN	SMOC1	SPARC-related modular calc	49443	0	717	0	46	0	0.07	#DIV/0!	0	1	0	1	0	1	0	1	1.189598
tr|Q6NSG9|Q6NSG9_HUMAI	AKAP12	AKAP12 protein (Fragment)	19461	0	716	0	46	0	0.17	#DIV/0!	0	1	0	1	0	1	0	1	1.189598
sp|Q14558|KPRA_HUMAN	PRPSAP1	Phosphoribosyl pyrophosph	39654	0	714	0	46	0	0.08	#DIV/0!	0	1	0	1	0	1	0	1	1.189598
sp|Q8NI27|THOC2_HUMAN	THOC2	THO complex subunit 2 OS=	184541	0	713	0	46	0	0.02	#DIV/0!	0	1	0	1	0	1	0	2	1.189598
tr|H0YAM1|H0YAM1_HUMA	GRSF1	G-rich sequence factor 1 (F	13410	0	711	0	47	0	0.25	#DIV/0!	0	1	0	1	0	1	0	1	1.189598
tr|A2A2Q9|A2A2Q9_HUMAN	C20orf4	Chromosome 20 open read	45575	0	710	0	47	0	0.07	#DIV/0!	0	1	0	1	0	1	0	1	1.189598
tr|D3DU01|D3DU01_HUMAI	TMEM49	Transmembrane protein 49	47923	0	702	0	47	0	0.07	#DIV/0!	0	1	0	1	0	1	0	2	1.189598
sp|Q7Z4V5|HDGR2_HUMAN	HDGFRP2	Hepatoma-derived growth	74443	0	696	0	48	0	0.04	#DIV/0!	0	1	0	1	0	1	0	2	1.189598
tr|B3KUZ7|B3KUZ7_HUMAN	tr|B3KUZ7|B3KU	cDNA FLJ40986 fis, clone U	56445	0	695	0	48	0	0.06	#DIV/0!	0	1	0	1	0	1	0	2	1.189598
tr|B4E1L0|B4E1L0_HUMAN	tr|B4E1L0|B4E1L	Adenylosuccinate syntheta	48308	0	693	0	49	0	0.07	#DIV/0!	0	1	0	1	0	1	0	1	1.189598
sp|Q9UPT8|ZC3H4_HUMAN	ZC3H4	Zinc finger CCCH domain-co	140797	0	691	0	49	0	0.02	#DIV/0!	0	1	0	1	0	1	0	1	1.189598
tr|B4DJS7|B4DJS7_HUMAN	SNX6	Sorting nexin 6, isoform CR	34196	0	688	0	49	0	0.1	#DIV/0!	0	1	0	1	0	1	0	1	1.189598
tr|B4E0V7|B4E0V7_HUMAN	tr|B4E0V7|B4E0	cDNA FLJ51709, highly simi	61918	0	687	0	49	0	0.05	#DIV/0!	0	1	0	1	0	1	0	1	1.189598
tr|H3BLU7|H3BLU7_HUMAN	AKR7A2	Aflatoxin B1 aldehyde redu	35004	0	686	0	49	0	0.09	#DIV/0!	0	1	0	1	0	1	0	1	1.189598
tr|B1AK66|B1AK66_HUMAN	SNIP1	Smad nuclear interacting pr	45807	0	685	0	49	0	0.07	#DIV/0!	0	1	0	1	0	1	0	1	1.189598
tr|Q5U043|Q5U043_HUMAN	tr|Q5U043|Q5U	Alcohol dehydrogenase 5 (	40520	0	683	0	50	0	0.08	#DIV/0!	0	1	0	1	0	1	0	1	1.189598
sp|Q8IVT2|CS021_HUMAN	C19orf21	Uncharacterized protein C1	75482	0	681	0	50	0	0.04	#DIV/0!	0	1	0	1	0	1	0	1	1.189598
tr|Q59FI9|Q59FI9_HUMAN	tr|Q59FI9|Q59FI	Ribosomal protein L12 varia	21755	0	679	0	50	0	0.15	#DIV/0!	0	1	0	1	0	1	0	1	1.189598
tr|Q6FGM0|Q6FGM0_HUMA	SH3GL1	SH3 domain GRB2-like 1 OS	41692	0	669	0	51	0	0.08	#DIV/0!	0	1	0	1	0	1	0	1	1.189598
sp|Q96LD4|TRI47_HUMAN	TRIM47	Tripartite motif-containing	70970	0	666	0	51	0	0.05	#DIV/0!	0	1	0	1	0	1	0	1	1.189598
tr|D6RAZ8|D6RAZ8_HUMAN	ANXA3	Annexin (Fragment) OS=Ho	11594	0	665	0	51	0	0.3	#DIV/0!	0	1	0	1	0	1	0	1	1.189598
tr|H0YL11|H0YL11_HUMAN	IDH2	Isocitrate dehydrogenase [I	35536	0	658	0	53	0	0.09	#DIV/0!	0	1	0	1	0	1	0	1	1.189598
tr|F5H6D0|F5H6D0_HUMAN	MOGS	Mannosyl-oligosaccharide g	80824	0	655	0	53	0	0.04	#DIV/0!	0	1	0	1	0	1	0	1	1.189598
sp|P49792|RBP2_HUMAN	RANBP2	E3 SUMO-protein ligase Ra	362365	0	654	0	53	0	0.01	#DIV/0!	0	1	0	1	0	1	0	1	1.189598
tr|H0YK42|H0YK42_HUMAN	SNX1	Sorting nexin0 OS=Homo sa	35628	0	653	0	54	0	0.09	#DIV/0!	0	1	0	1	0	1	0	1	1.189598
sp|Q14241|ELOA1_HUMAN	TCEB3	Transcription elongation fa	90309	0	648	0	55	0	0.04	#DIV/0!	0	1	0	1	0	1	0	1	1.189598
tr|E9PDG5|E9PDG5_HUMAN	LARP4	La-related protein 4 OS=Ho	50319	0	647	0	55	0	0.07	#DIV/0!	0	1	0	1	0	1	0	1	1.189598
tr|A0AV56|A0AV56_HUMAN	SAFB	SAFB protein OS=Homo sap	103249	0	645	0	55	0	0.03	#DIV/0!	0	1	0	1	0	1	0	1	1.189598
tr|B4DZP5|B4DZP5_HUMAN	tr|B4DZP5|B4DZ	cDNA FLJ51165, highly simi	92726	0	639	0	55	0	0.04	#DIV/0!	0	1	0	1	0	1	0	2	1.189598
sp|Q96IZ0|PAWR_HUMAN	PAWR	PRKC apoptosis WT1 regula	36659	0	638	0	55	0	0.09	#DIV/0!	0	1	0	1	0	1	0	1	1.189598
tr|B5BU99|B5BU99_HUMAN	YTHDF2	High glucose-regulated pro	61510	0	637	0	55	0	0.05	#DIV/0!	0	1	0	1	0	1	0	2	1.189598
tr|H0Y2P0|H0Y2P0_HUMAN	CD44	CD44 antigen (Fragment) O	31332	0	634	0	56	0	0.11	#DIV/0!	0	1	0	1	0	1	0	2	1.189598
tr|Q5JTE4|Q5JTE4_HUMAN	POLR1C	Polymerase (RNA) I polype	36043	0	632	0	56	0	0.09	#DIV/0!	0	1	0	1	0	1	0	1	1.189598
sp|Q9C0C2|TB182_HUMAN	TNKS1BP1	182 kDa tankyrase0-binding	182711	0	628	0	56	0	0.02	#DIV/0!	0	1	0	1	0	1	0	2	1.189598
sp|A2A3N6|PIPSL_HUMAN	PIPSL	Putative PIP5K1A and PSMI	95729	0	626	0	56	0	0.03	#DIV/0!	0	1	0	1	0	1	0	1	1.189598
sp|Q9UHD1|CHRD1_HUMAN	CHORDC1	Cysteine and histidine-rich	38264	0	624	0	57	0	0.09	#DIV/0!	0	1	0	1	0	1	0	1	1.189598
sp|P02748|CO9_HUMAN	C9	Complement component C	64615	0	623	0	57	0	0.05	#DIV/0!	0	1	0	1	0	1	0	1	1.189598

tr|B2R7T6|B2R7T6_HUMAN	tr|B2R7T6|B2R7	cDNA, FLJ93596, highly sim	50199	0	622	0	57	0	0.07	#DIV/0!	0	1	0	1	0	1	0	1	1.189598
tr|D3YTB1|D3YTB1_HUMAN	RPL32	60S ribosomal protein L32 (	15721	0	620	0	57	0	0.22	#DIV/0!	0	1	0	1	0	1	0	1	1.189598
tr|J3KQ32|J3KQ32_HUMAN	OLA1	Obg-like ATPase 1 OS=Hom	47136	0	619	0	58	0	0.07	#DIV/0!	0	1	0	1	0	1	0	1	1.189598
tr|B3KMQ6|B3KMQ6_HUMA	tr|B3KMQ6|B3K	cDNA FLJ12172 fis, clone M	73894	0	618	0	58	0	0.04	#DIV/0!	0	1	0	1	0	1	0	2	1.189598
tr|B2RAN1|B2RAN1_HUMAN	tr|B2RAN1|B2RA	cDNA, FLJ95012, highly sim	57042	0	617	0	58	0	0.06	#DIV/0!	0	1	0	1	0	1	0	2	1.189598
tr|G3V3B0|G3V3B0_HUMAN	ACIN1	Apoptotic chromatin conde	67683	0	616	0	58	0	0.05	#DIV/0!	0	1	0	1	0	1	0	1	1.189598
tr|Q53T70|Q53T70_HUMAN	RAB10	Putative uncharacterized pr	17970	0	614	0	58	0	0.19	#DIV/0!	0	1	0	1	0	1	0	1	1.189598
tr|B3KM42|B3KM42_HUMA	tr|B3KM42|B3KM	cDNA FLJ10219 fis, clone HI	41226	0	609	0	59	0	0.08	#DIV/0!	0	1	0	1	0	1	0	1	1.189598
sp|P35658|NU214_HUMAN	NUP214	Nuclear pore complex prote	214230	0	608	0	59	0	0.02	#DIV/0!	0	1	0	1	0	1	0	2	1.189598
tr|Q53TH1|Q53TH1_HUMAN	PSMD14	Putative uncharacterized pr	18165	0	604	0	59	0	0.19	#DIV/0!	0	1	0	1	0	1	0	1	1.189598
tr|A8K6H9|A8K6H9_HUMAN	tr|A8K6H9|A8K6	cDNA FLJ75876, highly simi	56360	0	595	0	60	0	0.06	#DIV/0!	0	1	0	1	0	1	0	1	1.189598
tr|J3KMX1|J3KMX1_HUMAN	NUP88	Nuclear pore complex prote	80029	0	591	0	60	0	0.04	#DIV/0!	0	1	0	1	0	1	0	1	1.189598
sp|P49419|AL7A1_HUMAN	ALDH7A1	Alpha-aminoadipic semiald	59020	0	584	0	61	0	0.06	#DIV/0!	0	1	0	1	0	1	0	2	1.189598
tr|H3BMZ1|H3BMZ1_HUMA	PTPLAD1	Very-long-chain (3R)-3-hydr	16267	0	576	0	62	0	0.21	#DIV/0!	0	1	0	1	0	1	0	1	1.189598
tr|I6L9B1|I6L9B1_HUMAN	EPB41L2	EPB41L2 protein OS=Homo	71998	0	561	0	65	0	0.05	#DIV/0!	0	1	0	1	0	1	0	1	1.189598
tr|Q14756|Q14756_HUMAN	LLDBP	LLDBP (Fragment) OS=Hom	58557	0	542	0	67	0	0.06	#DIV/0!	0	1	0	1	0	1	0	1	1.189598
sp|Q9Y2U8|MAN1_HUMAN	LEMD3	Inner nuclear membrane pr	100790	0	537	0	68	0	0.03	#DIV/0!	0	1	0	1	0	1	0	1	1.189598
tr|F5GY99|F5GY99_HUMAN	GALNT1	Polypeptide N-acetylgalact	58084	0	534	0	69	0	0.06	#DIV/0!	0	1	0	1	0	1	0	1	1.189598
tr|B4E136|B4E136_HUMAN	HSDL2	Hydroxysteroid dehydroger	33182	0	513	0	71	0	0.1	#DIV/0!	0	1	0	1	0	1	0	2	1.189598
tr|Q0VAB1|Q0VAB1_HUMA	TIMM50	Translocase of inner mitoch	50960	0	508	0	72	0	0.06	#DIV/0!	0	1	0	1	0	1	0	2	1.189598
tr|B2RB52|B2RB52_HUMAN	tr|B2RB52|B2RB	cDNA, FLJ95314, highly sim	50423	0	506	0	72	0	0.07	#DIV/0!	0	1	0	1	0	1	0	2	1.189598
tr|B4DQ08|B4DQ08_HUMA	tr|B4DQ08|B4D	cDNA FLJ53406, highly simi	82068	0	497	0	73	0	0.04	#DIV/0!	0	1	0	1	0	1	0	2	1.189598
tr|G5E988|G5E988_HUMAN	AP3D1	AP-3 complex subunit delta	126074	0	494	0	73	0	0.03	#DIV/0!	0	1	0	1	0	1	0	1	1.189598
tr|Q6AHZ7|Q6AHZ7_HUMA	DKFZp686A111	Putative uncharacterized pr	240988	0	493	0	74	0	0.01	#DIV/0!	0	1	0	1	0	1	0	1	1.189598
tr|A6NN60|A6NN60_HUMA	CRYZ	Quinone oxidoreductase OS	31680	0	486	0	75	0	0.11	#DIV/0!	0	1	0	1	0	1	0	1	1.189598
tr|E7EVL6|E7EVL6_HUMAN	REPIN1	Replication initiator 1 (Frag	16586	0	477	0	77	0	0.2	#DIV/0!	0	1	0	1	0	1	0	1	1.189598
tr|C9JDR0|C9JDR0_HUMAN	NSDHL	Sterol-4-alpha-carboxylate	28356	0	475	0	77	0	0.12	#DIV/0!	0	1	0	1	0	1	0	1	1.189598
sp|P98160|PGBM_HUMAN	HSPG2	Basement membrane-speci	479253	0	470	0	78	0	0.01	#DIV/0!	0	1	0	1	0	1	0	2	1.189598
tr|H0YEL7|H0YEL7_HUMAN	ACAT1	Acetyl-CoA acetyltransferas	14377	0	464	0	79	0	0.24	#DIV/0!	0	1	0	1	0	1	0	2	1.189598
tr|B4DFP1|B4DFP1_HUMAN	tr|B4DFP1|B4DF	cDNA FLJ51818, highly simi	58987	0	463	0	79	0	0.11	#DIV/0!	0	1	0	2	0	1	0	2	1.189598
tr|H0Y6K5|H0Y6K5_HUMAN	SP3	Transcription factor Sp3 OS	75780	0	458	0	80	0	0.04	#DIV/0!	0	1	0	1	0	1	0	1	1.189598
tr|E5RJ24|E5RJ24_HUMAN	tr|E5RJ24|E5RJ2	Uncharacterized protein (Fr	53738	0	454	0	81	0	0.06	#DIV/0!	0	1	0	1	0	1	0	2	1.189598
tr|J3KPP4|J3KPP4_HUMAN	LUC7L3	Cisplatin resistance-associa	58698	0	439	0	84	0	0.06	#DIV/0!	0	1	0	1	0	1	0	1	1.189598
sp|Q9NXV6|CARF_HUMAN	CDKN2AIP	CDKN2A-interacting protein	61544	0	431	0	85	0	0.05	#DIV/0!	0	1	0	1	0	1	0	1	1.189598
tr|K7EQ60|K7EQ60_HUMAN	COASY	Bifunctional coenzyme A sy	12950	0	426	0	86	0	0.26	#DIV/0!	0	1	0	1	0	1	0	1	1.189598
tr|B3KMC2|B3KMC2_HUMA	tr|B3KMC2|B3KM	cDNA FLJ10676 fis, clone NT	73526	0	422	0	87	0	0.04	#DIV/0!	0	1	0	1	0	1	0	1	1.189598
sp|Q49AR2|CE022_HUMAN	C5orf22	UPF0489 protein C5orf22 O	50506	0	416	0	88	0	0.07	#DIV/0!	0	1	0	1	0	1	0	1	1.189598
tr|H0Y5W0|H0Y5W0_HUMA	HUWE1	E3 ubiquitin-protein ligase I	376696	0	402	0	91	0	0.01	#DIV/0!	0	1	0	1	0	1	0	1	1.189598
tr|G3V5E1|G3V5E1_HUMAN	CCNK	Cyclin-K OS=Homo sapiens	43776	0	401	0	92	0	0.08	#DIV/0!	0	1	0	1	0	1	0	1	1.189598
tr|B4DM73|B4DM73_HUMA	tr|B4DM73|B4D	cDNA FLJ60078, highly simi	116274	0	383	0	96	0	0.03	#DIV/0!	0	1	0	1	0	1	0	2	1.189598
tr|B3KUI0|B3KUI0_HUMAN	tr|B3KUI0|B3KU	cDNA FLJ39945 fis, clone SF	79803	0	380	0	96	0	0.04	#DIV/0!	0	1	0	1	0	1	0	2	1.189598
tr|B0V3I9|B0V3I9_HUMAN	TSEN34	tRNA splicing endonuclease	27320	0	375	0	97	0	0.12	#DIV/0!	0	1	0	1	0	1	0	1	1.189598
sp|P09960|LKHA4_HUMAN	LTA4H	Leukotriene A-4 hydrolase	69868	0	366	0	98	0	0.1	#DIV/0!	0	1	0	2	0	1	0	2	1.189598
sp|Q14739|LBR_HUMAN	LBR	Lamin-B receptor OS=Homo	71057	0	361	0	99	0	0.05	#DIV/0!	0	1	0	1	0	1	0	1	1.189598
tr|H0Y7C0|H0Y7C0_HUMAN	USO1	General vesicular transport	72345	0	321	0	115	0	0.05	#DIV/0!	0	1	0	1	0	1	0	2	1.189598
tr|B1AKE9|B1AKE9_HUMAN	TRO	Trophinin OS=Homo sapien	103298	0	1158	0	15	0	0.03	#DIV/0!	0	0	0	1	0	0	0	1	-0.39561
sp|Q6ZQN7|SO4C1_HUMAN	SLCO4C1	Solute carrier organic anion	80379	0	1156	0	15	0	0.04	#DIV/0!	0	0	0	1	0	0	0	1	-0.39561
tr|Q6MZP3|Q6MZP3_HUMA	DKFZp686I05169	Putative uncharacterized pr	146779	0	1154	0	15	0	0.02	#DIV/0!	0	0	0	1	0	0	0	2	-0.39561
tr|Q4LE48|Q4LE48_HUMAN	STAG1	STAG1 variant protein (Frag	147272	0	1153	0	15	0	0.02	#DIV/0!	0	0	0	1	0	0	0	2	-0.39561
sp|Q8TBF8|FA81A_HUMAN	FAM81A	Protein FAM81A OS=Homo	42479	0	1151	0	16	0	0.08	#DIV/0!	0	0	0	1	0	0	0	1	-0.39561
tr|A8KA19|A8KA19_HUMAN	tr|A8KA19|A8KA	cDNA FLJ75831, highly simi	111104	0	1150	0	16	0	0.03	#DIV/0!	0	0	0	1	0	0	0	1	-0.39561
tr|B4DDV1|B4DDV1_HUMAN	IFIT5	Interferon-induced protein	50944	0	1148	0	16	0	0.06	#DIV/0!	0	0	0	1	0	0	0	1	-0.39561
tr|B7Z6S0|B7Z6S0_HUMAN	tr|B7Z6S0|B7Z6	cDNA FLJ51322, highly simi	25040	0	1146	0	16	0	0.13	#DIV/0!	0	0	0	1	0	0	0	1	-0.39561
tr|B3LDH0|B3LDH0_HUMAN	INV	Invadolysin OS=Homo sapie	69648	0	1145	0	16	0	0.05	#DIV/0!	0	0	0	1	0	0	0	2	-0.39561
tr|B4DTM6|B4DTM6_HUMA	tr|B4DTM6|B4D	cDNA FLJ61391, highly simi	130037	0	1144	0	16	0	0.03	#DIV/0!	0	0	0	1	0	0	0	1	-0.39561
tr|B3KTD5|B3KTD5_HUMAN	tr|B3KTD5|B3KT	cDNA FLJ38083 fis, clone CT	31258	0	1143	0	17	0	0.11	#DIV/0!	0	0	0	1	0	0	0	1	-0.39561
tr|B2RBH1|B2RBH1_HUMAN	tr|B2RBH1|B2RB	cDNA, FLJ95507, highly sim	45922	0	1142	0	17	0	0.07	#DIV/0!	0	0	0	1	0	0	0	1	-0.39561
tr|B4DFM1|B4DFM1_HUMA	tr|B4DFM1|B4D	cDNA FLJ53034, highly simi	44226	0	1141	0	17	0	0.08	#DIV/0!	0	0	0	1	0	0	0	1	-0.39561
tr|B3KQH4|B3KQH4_HUMAN	tr|B3KQH4|B3KQ	cDNA FLJ90464 fis, clone N	59339	0	1138	0	18	0	0.06	#DIV/0!	0	0	0	1	0	0	0	1	-0.39561
sp|P62333|PRS10_HUMAN	PSMC6	26S protease regulatory sul	44430	0	1136	0	18	0	0.07	#DIV/0!	0	0	0	1	0	0	0	1	-0.39561
tr|B3KVA0|B3KVA0_HUMAN	tr|B3KVA0|B3KV	Cytosine-specific methyltra	61027	0	1135	0	18	0	0.05	#DIV/0!	0	0	0	1	0	0	0	1	-0.39561
tr|Q1L857|Q1L857_HUMAN	tr|Q1L857|Q1L8	Ceruloplasmin (Fragment) (	116197	0	1134	0	18	0	0.03	#DIV/0!	0	0	0	1	0	0	0	1	-0.39561
tr|A8KAN5|A8KAN5_HUMAN	tr|A8KAN5|A8KA	cDNA FLJ75097, highly simi	60910	0	1132	0	19	0	0.05	#DIV/0!	0	0	0	1	0	0	0	1	-0.39561
tr|E5RIA9|E5RIA9_HUMAN	MSRA	Mitochondrial peptide met	23809	0	1131	0	19	0	0.14	#DIV/0!	0	0	0	1	0	0	0	1	-0.39561
tr|B3KV20|B3KV20_HUMAN	tr|B3KV20|B3KV	cDNA FLJ16058 fis, clone TE	64296	0	1130	0	19	0	0.05	#DIV/0!	0	0	0	1	0	0	0	1	-0.39561
tr|B4DG47|B4DG47_HUMAN	tr|B4DG47|B4D	cDNA FLJ56967, highly simi	54387	0	1129	0	19	0	0.06	#DIV/0!	0	0	0	1	0	0	0	1	-0.39561
tr|D3DP75|D3DP75_HUMAN	RAB3GAP1	RAB3 GTPase activating pro	100213	0	1128	0	19	0	0.03	#DIV/0!	0	0	0	1	0	0	0	1	-0.39561
tr|B7ZW15|B7ZW15_HUMA	tr|B7ZW15|B7ZW	Putative uncharacterized pr	14000	0	1127	0	20	0	0.24	#DIV/0!	0	0	0	1	0	0	0	1	-0.39561
tr|B2R9U2|B2R9U2_HUMAN	tr|B2R9U2|B2R9	cDNA, FLJ94557, highly sim	52087	623	158	24	228	0.06	0.63	10.5	0	6	1	8	2	8	2	9	1.371259
sp|Q09666|AHNK_HUMAN	AHNAK	Neuroblast differentiation-a	629213	401	87	43	339	0.01	0.1	10	1	9	1	19	3	21	6	25	2.227553
tr|Q2TU64|Q2TU64_HUMAN	tr|Q2TU64|Q2TU	PIG48 OS=Homo sapiens PE	61110	279	263	68	96	0.05	0.47	9.4	1	2	1	6	3	7	3	7	0.704683
tr|Q5T7Q0|Q5T7Q0_HUMAN	DNAJA1	DnaJ (Hsp40) homolog, sub	45581	703	301	18	123	0.07	0.63	9	0	4	1	7	0	10	1	11	3.999197
sp|O94906|PRP6_HUMAN	PRPF6	Pre-mRNA-processing facto	107656	492	205	35	174	0.03	0.23	7.666667	1	5	1	7	1	7	2	7	1.927731
sp|Q68EM7|RHG17_HUMAN	ARHGAP17	Rho GTPase-activating prot	95776	328	759	54	42	0.03	0.23	7.666667	1	1	1	1	2	1	2	1	-1.13299
tr|Q0VGD6|Q0VGD6_HUMA	HNRPR	HNRPR protein (Fragment)	68218	390	108	44	284	0.1	0.76	7.6	1	8	2	12	2	14	4	15	2.143275
tr|B2RE46|B2RE46_HUMAN	tr|B2RE46|B2RE	cDNA, FLJ96923, highly sim	69405	589	272	27	138	0.05	0.32	6.4	0	3	1	6	2	6	2	6	0.983737
tr|A6NKB8|A6NKB8_HUMAN	RNPEP	Aminopeptidase B OS=Hom	68752	316	196	57	178	0.05	0.32	6.4	1	5	2	6	2	6	2	6	0.983737
tr|B4DY09|B4DY09_HUMAN	tr|B4DY09|B4DY	cDNA FLJ51660, highly simi	39057	383	273	45	137	0.08	0.5	6.25	1	4	1	5	1	5	1	5	1.479773
tr|B4DT29|B4DT29_HUMAN	tr|B4DT29|B4DT	cDNA FLJ51082, highly simi	40959	548	145	30	88	0.08	0.49	6.125	0	2	1	3	1	4	1	4	1.190017
tr|A8K2Y2|A8K2Y2_HUMAN	tr|A8K2Y2|A8K2	cDNA FLJ78120, highly simi	51648	475	291	37	127	0.06	0.36	6	1	3	1	5	1	5	1	5	1.479773
sp|O14980|XPO1_HUMAN	XPO1	Exportin0 OS=Homo sapien	124447	305	405	59	91	0.03	0.17	5.666667	1	2	1	6	1	5	3	7	1.479773
tr|B2R7B5|B2R7B5_HUMAN	tr|B2R7B5|B2R7	cDNA, FLJ93365, highly sim	48309	347	224	51	164	0.07	0.39	5.571429	1	3	1	5	2	7	4	7	1.190438
tr|B2R959|B2R959_HUMAN	tr|B2R959|B2R9	cDNA, FLJ94229, highly sim	60838	182	47	117	460	0.23	1.27	5.521739	4	14	4	16	6	17	8	17	1.035503
tr|B3KW21|B3KW21_HUMA	tr|B3KW21|B3KW	cDNA FLJ41945 fis, clone PL	68674	705	303	18	103	0.04	0.22	5.5	0	2	1	5	0	4	1	5	2.775308
tr|B4DLT2|B4DLT2_HUMAN	tr|B4DLT2|B4DL	cDNA FLJ56637, highly simi	152734	489	172	35	207	0.02	0.11	5.5	0	5	1	5	1	5	1	6	1.479773
tr|J3KR97|J3KR97_HUMAN	TBCD	Tubulin-specific chaperone	138346	714	934	15	33	0.02	0.11	5.5	0	0	1	1	0	1	1	1	1.189598
tr|B2R8R5|B2R8R5_HUMAN	tr|B2R8R5|B2R8	cDNA, FLJ94025, highly sim	90271	320	113	56	276	0.07	0.38	5.428571	1	6	3	9	2	10	4	11	1.676613
tr|B3KQT2|B3KQT2_HUMAN	tr|B3KQT2|B3KQ	cDNA PSEC0148 fis, clone P	55268	546	114	30	275	0.19	1	5.263158	0	6	3	12	2	12	8	12	1.928651
tr|B2R6D0|B2R6D0_HUMAN	tr|B2R6D0|B2R6	cDNA, FLJ92896, highly sim	106797	386	142	45	242	0.06	0.31	5.166667	0	9	2	9	2	9	4	10	1.531974
tr|B2R5P6|B2R5P6_HUMAN	TXNRD1	Thioredoxin reductase 1, cy	55253	108	57	204	479	0.26	1.33	5.115385	3	10	5	15	5	16	6	18	1.1917
tr|Q96QL0|Q96QL0_HUMAN	RPL3	Ribosomal protein L3 OS=H	46376	245	58	82	438	0.32	1.62	5.0625	4	12	4	14	5	15	7	16	1.101253
tr|B2R4K7|B2R4K7_HUMAN	tr|B2R4K7|B2R4	60S ribosomal protein L6 O	32739	276	143	68	241	0.47	2.37	5.042553	2	9	4	12	3	12	5	14	1.442896
tr|H0YA24|H0YA24_HUMAN	PLRG1	Pleiotropic regulator 1 (Fra	17588	456	131	38	173	0.19	0.95	5	1	4	1	6	1	6	2	7	1.721031
sp|P01024|CO3_HUMAN	C3	Complement C3 OS=Homo	188569	370	228	47	161	0.03	0.15	5	1	5	2	8	2	10	2	11	1.676613
tr|Q59G92|Q59G92_HUMAN	tr|Q59G92|Q59G	HLA-B associated transcript	35528	394	362	44	55	0.09	0.45	5	1	1	1	3	2	3	2	3	0.089905
tr|E9PBS1|E9PBS1_HUMAN	PAICS	Phosphoribosylaminoimida	46307	270	607	69	59	0.07	0.35	5	1	2	1	3	2	4	5	4	0.452724
tr|H7BZJ3|H7BZJ3_HUMAN	PDIA3	Thioredoxin (Fragment) OS=	13739	546	415	22	88	0.19	0.94	4.947368	0	3	1	3	1	3	1	3	0.827198
tr|B4DKS0|B4DKS0_HUMAN	tr|B4DKS0|B4DK	cDNA FLJ53381, highly simi	52487	598	308	26	115	0.06	0.28	4.666667	0	3	1	3	1	4	1	4	1.190017
sp|P42330|AK1C3_HUMAN	AKR1C3	Aldo-keto reductase family	37229	203	94	102	307	0.29	1.35	4.655172	3	7	3	11	3	10	4	12	1.190858
tr|B7Z1R5|B7Z1R5_HUMAN	ATP6V1A	V-type proton ATPase catal	65037	428	306	40	120	0.05	0.22	4.4	1	2	1	4	1	4	2	7	1.190017
tr|B4DJ60|B4DJ60_HUMAN	tr|B4DJ60|B4DJ	cDNA FLJ55072, highly simi	68233	393	166	44	216	0.1	0.44	4.4	0	4	2	7	3	7	5	7	0.704683
tr|Q2Q9B7|Q2Q9B7_HUMA	G6PD	Glucose-6-phosphate 1-deh	55221	176	126	118	251	0.18	0.79	4.388889	2	6	2	9	5	14	6	14	1.004788
tr|Q59FV6|Q59FV6_HUMAN	tr|Q59FV6|Q59F	ARP3 actin-related protein	42517	548	237	30	156	0.08	0.35	4.375	0	4	1	6	1	7	1	7	1.927731
tr|B4DMU0|B4DMU0_HUM	PYCR1	Pyrroline-5-carboxylate red	36186	174	195	125	179	0.19	0.82	4.315789	2	4	2	4	3	4	3	4	-0.03303
sp|Q15645|PCH2_HUMAN	TRIP13	Pachytene checkpoint prote	48863	507	385	33	96	0.07	0.3	4.285714	0	2	1	4	2	3	2	4	0.089905
tr|B2R5M9|B2R5M9_HUMAN	tr|B2R5M9|B2R5	cDNA, FLJ92537, highly sim	84022	251	304	76	121	0.04	0.17	4.25	1	1	1	4	1	6	1	6	1.721031
tr|B1AHB0|B1AHB0_HUMAN	MCM5	MCM5 minichromosome m	83031	562	478	29	76	0.04	0.17	4.25	0	2	1	4	1	5	2	5	1.479773
tr|B2RBE0|B2RBE0_HUMAN	tr|B2RBE0|B2RB	cDNA, FLJ95462, highly sim	81266	462	404	37	91	0.04	0.17	4.25	0	2	1	4	1	4	1	6	1.190017
tr|B4DQJ8|B4DQJ8_HUMAN	PGD	6-phosphogluconate dehyd	52352	293	139	62	243	0.2	0.84	4.2	1	6	3	10	2	12	3	15	1.928651
tr|B1ANR0|B1ANR0_HUMAN	PABPC4	Poly(A) binding protein, cyt	68156	504	569	33	63	0.05	0.21	4.2	0	2	1	4	1	4	2	4	1.190017
sp|Q9NPQ8|RIC8A_HUMAN	RIC8A	Synembryn-A OS=Homo sap	60185	496	1137	34	18	0.05	0.21	4.2	0	1	2	1	0	1	2	2	-1.9809
sp|O14929|HAT1_HUMAN	HAT1	Histone acetyltransferase t	49880	343	229	51	161	0.07	0.29	4.142857	1	3	1	4	1	6	1	6	1.721031
sp|P50552|VASP_HUMAN	VASP	Vasodilator-stimulated pho	39976	363	633	48	56	0.17	0.69	4.058824	1	1	1	2	1	2	1	2	0.341522

tr|J3KR24|J3KR24_HUMAN	IARS	Isoleucine--tRNA ligase, cyt	132820	366	557	47	65	0.02	0.08	4	1	1	1	3	2	6	2	6	0.983737
tr|Q2XPP3|Q2XPP3_HUMAN	tr|Q2XPP3|Q2XF	Type II 3a-hydroxysteroid d	37201	203	198	102	177	0.29	1.16	4	3	5	3	9	3	8	4	10	0.885505
tr|B3KSH1|B3KSH1_HUMAN	EIF3F	Eukaryotic translation initia	39236	652	455	22	81	0.08	0.32	4	0	1	1	1	1	1	1	2	-0.39569
tr|B4DYA7|B4DYA7_HUMAN	tr|B4DYA7|B4DY	Glucose-6-phosphate 1-deh	58757	176	125	123	244	0.18	0.71	3.944444	2	5	3	7	6	13	7	13	0.660108
tr|F1T0B3|F1T0B3_HUMAN	DDX1	ATP-dependent RNA helicas	74838	501	208	33	174	0.09	0.35	3.888889	0	6	2	7	3	8	3	8	0.885505
tr|B4DR68|B4DR68_HUMAN	tr|B4DR68|B4DR	cDNA FLJ58608, highly simi	74408	169	105	127	287	0.09	0.35	3.888889	2	7	3	7	3	8	5	8	0.885505
tr|B2R7C5|B2R7C5_HUMAN	tr|B2R7C5|B2R7	cDNA, FLJ93378, highly sim	91579	479	502	36	72	0.04	0.15	3.75	1	2	1	4	1	4	1	4	1.190017
tr|E5KNY5|E5KNY5_HUMAN	LRPPRC	Leucine-rich PPR-motif cont	159003	329	90	53	325	0.08	0.3	3.75	1	7	4	10	5	10	7	11	0.538126
tr|B4DZ53|B4DZ53_HUMAN	tr|B4DZ53|B4DZ	cDNA FLJ59643, highly simi	96752	47	22	400	1178	0.62	2.31	3.725806	10	28	17	43	15	44	19	47	1.132002
tr|Q9NSU9|Q9NSU9_HUMA	DKFZp434G0719	Putative uncharacterized pr	41107	56	363	169	99	0.07	0.26	3.714286	4	2	5	3	5	3	5	3	-1.04858
sp|Q6P2Q9|PRP8_HUMAN	PRPF8	Pre-mRNA-processing-splic	274738	130	40	167	686	0.1	0.36	3.6	7	19	8	26	10	32	10	39	1.239149
tr|Q8TC62|Q8TC62_HUMAN	Sep-07	Septin 7 OS=Homo sapiens	48913	531	549	31	67	0.07	0.25	3.571429	1	2	1	3	1	3	2	3	0.827198
sp|O76094|SRP72_HUMAN	SRP72	Signal recognition particle 7	75130	455	355	38	101	0.04	0.14	3.5	1	2	1	3	1	4	1	4	1.190017
tr|B4DVB8|B4DVB8_HUMAN	ELAVL1	ELAV-like protein 1 OS=Hor	39200	612	509	24	72	0.08	0.28	3.5	0	1	1	3	1	3	1	4	0.827198
tr|B2R5V9|B2R5V9_HUMAN	tr|B2R5V9|B2R5	cDNA, FLJ92652, highly sim	141919	253	327	76	112	0.02	0.07	3.5	1	3	1	3	2	3	2	3	0.089905
tr|B4DYL7|B4DYL7_HUMAN	tr|B4DYL7|B4DY	cDNA FLJ58490, highly simi	140991	388	929	45	33	0.02	0.07	3.5	1	0	1	1	1	1	2	1	-0.39569
tr|A6NG51|A6NG51_HUMAN	SPTAN1	Spectrin alpha chain, non-e	285628	167	49	130	482	0.06	0.21	3.5	2	11	5	16	6	17	9	18	1.035503
tr|A4FU77|A4FU77_HUMAN	SNRNP200	SNRNP200 protein (Fragme	217585	243	165	83	205	0.05	0.17	3.4	2	4	3	11	7	12	8	12	0.342048
tr|H0UID3|H0UID3_HUMAN	AP2B1	Adaptor-related protein co	106321	648	529	22	69	0.03	0.1	3.333333	0	1	1	3	3	3	3	6	-0.39585
sp|P11021|GRP78_HUMAN	HSPA5	78 kDa glucose-regulated p	72402	129	80	170	379	0.19	0.63	3.315789	3	10	5	12	6	11	6	13	0.428283
tr|B4DHQ3|B4DHQ3_HUMA	tr|B4DHQ3|B4D	cDNA FLJ56437, highly simi	45783	552	492	30	74	0.07	0.23	3.285714	0	2	1	3	1	5	2	6	1.479773
tr|H0Y8E6|H0Y8E6_HUMAN	MCM2	DNA replication licensing fa	94792	211	315	98	117	0.07	0.23	3.285714	1	3	2	6	2	7	2	9	1.190438
tr|B4E091|B4E091_HUMAN	tr|B4E091|B4E0	cDNA FLJ55438, highly simi	77415	482	560	36	65	0.04	0.13	3.25	1	1	1	3	1	3	1	3	0.827198
tr|B5MCX3|B5MCX3_HUMA	Sep-02	Septin-2 OS=Homo sapiens	37145	543	531	30	69	0.09	0.29	3.222222	1	2	1	3	2	2	3	3	-0.39577
tr|B4DMJ6|B4DMJ6_HUMAN	tr|B4DMJ6|B4DM	cDNA FLJ50996, highly simi	46269	163	76	132	345	0.51	1.64	3.215686	4	9	6	13	8	13	9	15	0.272429
sp|O00425|IF2B3_HUMAN	IGF2BP3	Insulin-like growth factor 2	64008	556	936	29	32	0.05	0.16	3.2	0	0	1	3	2	2	2	3	-0.39577
tr|B4DJ30|B4DJ30_HUMAN	tr|B4DJ30|B4DJ	cDNA FLJ61290, highly simi	113539	47	16	446	1287	0.62	1.94	3.129032	12	32	19	45	17	49	22	53	1.111136
tr|B2RDN4|B2RDN4_HUMAN	tr|B2RDN4|B2RD	cDNA, FLJ96693, highly sim	84219	505	479	33	44	0.08	0.25	3.125	0	2	2	4	2	4	3	4	0.452724
tr|B5BU01|B5BU01_HUMAN	EIF2S2	Eukaryotic translation initia	38648	654	473	22	77	0.09	0.28	3.111111	0	2	1	3	1	3	1	3	0.827198
tr|H0YLA4|H0YLA4_HUMAN	SORD	Sorbitol dehydrogenase OS	36833	473	374	37	74	0.09	0.28	3.111111	0	1	1	2	2	1	2	3	-1.13299
tr|G3V0E8|G3V0E8_HUMAN	PCBP2	Poly(RC) binding protein 2,	33817	132	168	166	161	0.32	0.99	3.09375	3	3	3	6	3	5	5	6	0.256725
tr|B1Q2N1|B1Q2N1_HUMA	DDX39	ATP-dependent RNA helicas	30925	395	496	44	45	0.11	0.34	3.090909	1	0	1	3	1	3	2	3	0.827198
tr|I3L312|I3L312_HUMAN	P4HB	Protein disulfide-isomerase	26348	536	153	30	100	0.27	0.83	3.074074	1	3	2	4	1	5	2	5	1.479773
tr|B2R6J4|B2R6J4_HUMAN	tr|B2R6J4|B2R6J	cDNA, FLJ92975, highly sim	42940	417	317	42	37	0.16	0.49	3.0625	0	1	2	1	3	1	3	1	-1.61874
tr|B2RU06|B2RU06_HUMAN	EIF4G1	EIF4G1 protein OS=Homo s	176807	669	268	20	141	0.04	0.12	3	0	4	2	6	1	7	3	8	1.927731
tr|A8KAK1|A8KAK1_HUMAN	tr|A8KAK1|A8KA	cDNA FLJ77398, highly simi	175467	635	752	22	43	0.02	0.06	3	0	1	1	3	1	4	1	7	1.190017
sp|P41250|SYG_HUMAN	GARS	Glycine--tRNA ligase OS=Ho	83854	392	532	44	69	0.04	0.12	3	1	2	1	3	1	3	2	5	0.827198
tr|B7Z8A2|B7Z8A2_HUMAN	tr|B7Z8A2|B7Z8	cDNA FLJ51671, highly simi	54918	355	412	49	89	0.06	0.18	3	1	2	1	3	1	3	1	3	0.827198
tr|B2ZZ89|B2ZZ89_HUMAN	SPTBN1	Spectrin beta non-erythroc	275237	49	12	431	1516	0.22	0.66	3	11	36	17	43	20	46	24	49	0.790928
sp|Q14204|DYHC1_HUMAN	DYNC1H1	Cytoplasmic dynein 1 heavy	534809	168	248	129	151	0.02	0.06	3	4	4	10	4	7	6	16	0.341785
tr|Q68DB1|Q68DB1_HUMAN	DKFZp686F2281	Metastasis-associated prote	55515	676	389	20	38	0.06	0.18	3	0	1	1	2	1	2	1	2	0.341522
tr|D3DP78|D3DP78_HUMAN	DARS	Aspartyl-tRNA synthetase, i	53829	344	451	51	45	0.06	0.18	3	1	1	1	2	2	2	3	2	-0.39577
sp|O00629|IMA4_HUMAN	KPNA4	Importin subunit alpha-4 O	58364	472	562	37	65	0.06	0.18	3	0	1	1	1	2	1	2	1	-1.13299
tr|H0YD14|H0YD14_HUMAN	MYOF	Myoferlin (Fragment) OS=H	135822	220	109	95	189	0.07	0.21	3	3	4	3	9	4	10	4	10	0.82796
tr|B4DFL1|B4DFL1_HUMAN	tr|B4DFL1|B4DF	Dihydrolipoyl dehydrogena	49381	666	528	21	69	0.07	0.21	3	0	2	1	3	1	3	1	3	0.827198
tr|B1AMS2|B1AMS2_HUMA	Sep-06	Septin 6 OS=Homo sapiens	49614	532	215	31	35	0.07	0.21	3	1	1	1	1	1	1	1	2	-0.39569
tr|Q05CK9|Q05CK9_HUMAN	SYNCRIP	SYNCRIP protein (Fragment	50847	252	173	76	192	0.13	0.38	2.923077	2	5	2	6	2	8	2	9	1.371259
tr|B4DR70|B4DR70_HUMAN	tr|B4DR70|B4DR	cDNA FLJ58049, highly simi	45013	450	359	38	100	0.07	0.2	2.857143	1	3	1	3	1	3	1	3	0.827198
sp|P15121|ALDR_HUMAN	AKR1B1	Aldose reductase OS=Homo	36230	185	120	116	269	0.42	1.2	2.857143	3	6	4	10	5	12	5	12	0.790163
tr|B2RDF5|B2RDF5_HUMAN	tr|B2RDF5|B2RD	cDNA, FLJ96587, highly sim	71705	590	346	27	103	0.05	0.14	2.8	0	3	1	3	1	3	1	4	0.827198
sp|O75369|FLNB_HUMAN	FLNB	Filamin-B OS=Homo sapien	280157	45	21	463	1190	0.22	0.61	2.772727	14	30	18	43	20	47	28	58	0.821876
tr|A8K6Q8|A8K6Q8_HUMAN	tr|A8K6Q8|A8K6	cDNA FLJ75881, highly simi	85276	122	61	176	444	0.3	0.83	2.766667	6	11	7	16	7	17	8	19	0.828724
tr|B4DWJ2|B4DWJ2_HUMA	QARS	Glutamine--tRNA ligase OS=	87437	277	107	68	275	0.16	0.44	2.75	3	6	4	9	5	10	5	10	0.538126
tr|I3L0N3|I3L0N3_HUMAN	NSF	Vesicle-fusing ATPase OS=H	82553	412	1083	42	24	0.04	0.11	2.75	1	0	1	1	1	1	1	1	-0.39569
tr|B4DU00|B4DU00_HUMAN	tr|B4DU00|B4DU	cDNA FLJ54547, highly simi	89332	389	152	45	172	0.07	0.19	2.714286	0	4	2	4	2	4	4	4	0.452724
tr|Q5SU16|Q5SU16_HUMAN	TUBB	Beta 5-tubulin OS=Homo sa	50095	25	10	680	1587	3.07	8.32	2.710098	26	51	33	59	35	65	39	72	0.49283
sp|P49411|EFTU_HUMAN	TUFM	Elongation factor Tu, mitoc	49852	210	157	99	228	0.29	0.78	2.689655	3	6	4	9	4	11	5	13	0.959454
tr|B4DF96|B4DF96_HUMAN	EIF2A	Eukaryotic translation initia	58357	206	186	101	105	0.32	0.85	2.65625	2	2	5	9	4	11	5	11	0.959454
tr|B2RBR9|B2RBR9_HUMAN	tr|B2RBR9|B2RB	cDNA, FLJ95650, highly sim	98434	171	67	126	423	0.22	0.58	2.636364	4	11	6	14	9	15	9	15	0.311444
sp|Q09028|RBBP4_HUMAN	RBBP4	Histone-binding protein RB	47911	321	574	55	62	0.22	0.58	2.636364	2	1	3	3	4	3	4	3	-0.75875
tr|B4DSQ5|B4DSQ5_HUMAN	tr|B4DSQ5|B4DS	cDNA FLJ53608, highly simi	83787	474	197	37	106	0.08	0.21	2.625	0	3	2	4	2	6	3	6	0.983737
sp|O95373|IPO7_HUMAN	IPO7	Importin-7 OS=Homo sapie	120751	162	313	132	119	0.08	0.21	2.625	3	3	3	7	3	5	4	10	0.256725
tr|Q6DC98|Q6DC98_HUMAN	LMNB1	LMNB1 protein (Fragment)	38289	219	134	95	173	0.18	0.47	2.611111	2	5	2	6	2	6	4	7	0.983737
sp|O43175|SERA_HUMAN	PHGDH	D-3-phosphoglycerate dehy	57356	131	103	166	295	0.25	0.65	2.6	3	5	4	9	4	11	4	11	0.959454
tr|D6RF62|D6RF62_HUMAN	PAICS	Phosphoribosylaminoimida	37658	270	599	69	59	0.07	0.18	2.571429	1	2	1	2	2	3	4	3	0.089905
tr|Q53GX7|Q53GX7_HUMAN	tr|Q53GX7|Q53G	Threonyl-tRNA synthetase v	84304	94	56	221	487	0.64	1.64	2.5625	6	16	13	25	17	27	18	29	0.257623
tr|Q5VZU9|Q5VZU9_HUMA	TPP2	Tripeptidyl-peptidase 2 OS=	141160	693	948	19	32	0.02	0.05	2.5	0	0	1	2	0	2	1	2	1.926812
tr|B3KT66|B3KT66_HUMAN	PSMD6	Proteasome (Prosome, mac	40600	399	368	43	70	0.08	0.2	2.5	1	2	1	2	1	3	1	4	0.827198
tr|I3L1L3|I3L1L3_HUMAN	MYBBP1A	Myb-binding protein 1A (Fr	141124	698	449	18	82	0.02	0.05	2.5	0	1	1	2	1	2	2	3	0.341522
tr|G3XAD8|G3XAD8_HUMAN	STIP1	Stress-induced-phosphopro	68721	271	128	69	258	0.21	0.52	2.47619	2	8	4	9	4	9	5	10	0.683321
tr|A8K492|A8K492_HUMAN	tr|A8K492|A8K4	cDNA FLJ76789, highly simi	102277	385	285	45	129	0.07	0.17	2.428571	1	3	2	5	2	4	2	5	0.452724
tr|J3KPS3|J3KPS3_HUMAN	ALDOA	Fructose-bisphosphate aldo	40249	63	19	338	1142	1.79	4.34	2.424581	11	24	15	26	17	32	18	33	0.499883
tr|B0YJC4|B0YJC4_HUMAN	VIM	Vimentin OS=Homo sapiens	49680	66	44	312	522	1.03	2.49	2.417476	9	16	11	20	12	22	15	23	0.453936
tr|B7Z6M1|B7Z6M1_HUMA	PLS3	Plastin-3 OS=Homo sapiens	66047	282	140	66	243	0.34	0.82	2.411765	1	6	6	13	6	12	8	14	0.548827
tr|A2VCT2|A2VCT2_HUMAN	OGDH	OGDH protein (Fragment) (	72973	432	283	40	84	0.05	0.12	2.4	1	2	1	2	1	4	1	5	1.190017
tr|B2R8N0|B2R8N0_HUMAN	tr|B2R8N0|B2R8	cDNA, FLJ93975, highly sim	53129	115	112	184	276	0.62	1.46	2.354839	6	9	8	16	8	17	8	19	0.647823
tr|Q8IZ29|Q8IZ29_HUMAN	TUBB2C	Tubulin, beta 2C OS=Homo	50264	27	11	674	1558	3.04	7.13	2.345395	26	48	32	58	34	64	38	69	0.511935
sp|Q15046|SYK_HUMAN	KARS	Lysine--tRNA ligase OS=Hor	68461	265	106	71	287	0.32	0.75	2.34375	1	8	6	12	6	14	7	15	0.763451
sp|P04350|TBB4A_HUMAN	TUBB4A	Tubulin beta-4A chain OS=H	50010	42	20	509	1200	1.96	4.59	2.341837	21	40	27	49	29	55	32	60	0.52039
tr|F5H7Y0|F5H7Y0_HUMAN	PDLIM5	PDZ and LIM domain protei	52332	175	84	109	341	0.41	0.96	2.341463	2	8	5	10	5	11	6	12	0.66962
tr|B3KM77|B3KM77_HUMA	tr|B3KM77|B3KM	cDNA FLJ10435 fis, clone N	56736	356	316	49	72	0.06	0.14	2.333333	1	1	1	3	1	2	1	3	0.341522
sp|Q8TCJ2|STT3B_HUMAN	STT3B	Dolichyl-diphosphooligosac	94241	700	507	18	72	0.03	0.07	2.333333	0	2	1	2	2	3	3	5	0.089905
sp|P45974|UBP5_HUMAN	USP5	Ubiquitin carboxyl-terminal	96638	622	246	24	152	0.03	0.07	2.333333	0	3	1	3	2	3	2	3	0.089905
tr|Q05D08|Q05D08_HUMAN	PA2G4	PA2G4 protein (Fragment) (	45579	286	305	64	78	0.15	0.35	2.333333	1	2	2	3	3	3	3	4	-0.39585
tr|H0Y804|H0Y804_HUMAN	AKR1C1	Aldo-keto reductase family	28617	186	92	61	99	0.67	1.56	2.328358	2	3	4	6	4	5	6	6	-0.10617
tr|B7TY16|B7TY16_HUMAN	ACTN1	Actinin alpha 1 isoform 3 O	107644	50	25	428	997	0.77	1.79	2.324675	15	26	21	37	22	43	29	45	0.559
tr|Q6IBR0|Q6IBR0_HUMAN	RPN1	RPN1 protein OS=Homo sap	68678	170	161	127	225	0.26	0.6	2.307692	4	4	5	10	8	9	8	12	-0.23553
sp|P25490|TYY1_HUMAN	YY1	Transcriptional repressor pr	45141	630	697	23	48	0.07	0.16	2.285714	0	1	1	1	2	1	2	1	-1.13299
sp|P08238|HS90B_HUMAN	HSP90AB1	Heat shock protein HSP 90-	83554	32	15	622	1309	1.24	2.8	2.258065	18	34	28	40	29	43	35	49	0.165903
tr|B7Z8W6|B7Z8W6_HUMA	tr|B7Z8W6|B7Z8	cDNA FLJ55038, highly simi	26905	520	266	32	79	0.12	0.27	2.25	0	2	1	2	1	3	3	3	0.827198
tr|B4DH39|B4DH39_HUMAN	tr|B4DH39|B4DH	cDNA FLJ57028, highly simi	73123	529	823	31	38	0.04	0.09	2.25	0	0	1	2	1	3	3	4	0.827198
tr|A1L3A7|A1L3A7_HUMAN	NUFIP2	Nuclear fragile X mental ret	76132	301	447	59	82	0.04	0.09	2.25	1	2	1	2	1	2	1	3	0.341522
tr|Q53SS8|Q53SS8_HUMAN	PCBP1	Poly(RC) binding protein 1 (	37987	110	178	197	200	0.29	0.65	2.241379	3	5	3	6	3	6	3	6	0.497983
tr|B2R5U1|B2R5U1_HUMAN	tr|B2R5U1|B2R5	cDNA, FLJ92620, highly sim	100294	150	121	144	268	0.17	0.38	2.235294	4	7	5	10	10	12	12	14	-0.14436
sp|Q9UMS4|PRP19_HUMAN	PRPF19	Pre-mRNA-processing facto	55603	237	132	87	257	0.26	0.58	2.230769	2	7	5	8	7	9	11	11	-0.05463
sp|P00390|GSHR_HUMAN	GSR	Glutathione reductase, mito	56791	313	382	58	96	0.18	0.4	2.222222	1	3	3	6	4	6	4	6	0.135085
tr|Q9NZ23|Q9NZ23_HUMAN	YA61	Drug-sensitive protein 1 OS	14858	588	631	27	56	0.23	0.51	2.217391	0	2	1	2	1	2	1	4	0.341522
tr|B4DGQ0|B4DGQ0_HUMA	LASP1	LIM and SH3 domain protei	23167	568	358	29	100	0.14	0.31	2.214286	0	2	1	2	1	3	1	3	0.827198
tr|B0QYA5|B0QYA5_HUMAN	EIF3D	Eukaryotic translation initia	31937	710	286	17	129	0.1	0.22	2.2	0	2	1	2	0	2	2	2	1.926812
sp|Q9NZB2|F120A_HUMAN	FAM120A	Constitutive coactivator of	123008	263	261	73	145	0.05	0.11	2.2	2	4	2	4	2	5	2	6	0.74248
tr|B4DMY3|B4DMY3_HUMA	tr|B4DMY3|B4D	cDNA FLJ60713, highly simi	35101	318	244	56	152	0.2	0.44	2.2	1	3	2	4	2	4	2	4	0.452724
tr|B3KNM0|B3KNM0_HUMA	tr|B3KNM0|B3K	cDNA FLJ14916 fis, clone PL	64588	191	314	113	118	0.1	0.22	2.2	2	3	2	4	2	3	3	4	0.089905
tr|D3DTW3|D3DTW3_HUMA	IGF2BP1	Insulin-like growth factor 2	48111	556	566	29	58	0.05	0.11	2.2	0	1	1	1	1	1	1	1	-0.39569
tr|E7ENU7|E7ENU7_HUMAN	RPL15	Ribosomal protein L15 (Frag	20596	534	448	31	82	0.16	0.35	2.1875	0	2	1	3	1	3	1	5	0.827198
tr|Q8IWP6|Q8IWP6_HUMAN	tr|Q8IWP6|Q8IW	Class IVb beta tubulin OS=H	50177	28	13	654	1507	3.05	6.66	2.183607	27	47	32	56	34	63	37	68	0.489141
tr|F8WJN3|F8WJN3_HUMAN	CPSF6	Cleavage and polyadenylati	52409	379	390	46	94	0.06	0.13	2.166667	1	2	1	2	1	3	1	3	0.827198
sp|O00232|PSD12_HUMAN	PSMD12	26S proteasome non-ATPas	53270	283	369	66	98	0.06	0.13	2.166667	1	2	1	2	1	2	1	2	0.341522
sp|Q01650|LAT1_HUMAN	SLC7A5	Large neutral amino acids t	55659	239	167	85	213	0.12	0.26	2.166667	1	4	3	6	4	6	5	6	0.135085
tr|B4DDB6|B4DDB6_HUMAN	HNRPA3	Heterogeneous nuclear ribo	37234	201	360	103	99	0.19	0.41	2.157895	2	3	2	4	2	6	2	6	0.983737
tr|Q6DEN2|Q6DEN2_HUMA	DPYSL3	DPYSL3 protein OS=Homo s	74320	490	123	35	266	0.19	0.41	2.157895	0	7	4	8	3	8	8	8	0.885505
tr|B4DDR2|B4DDR2_HUMAN	tr|B4DDR2|B4DD	cDNA FLJ54588, highly simi	98526	304	322	59	105	0.07	0.15	2.142857	2	2	2	3	3	3	3	3	-0.39585
tr|Q5TZX1|Q5TZX1_HUMAN	GNAI3	Guanine nucleotide binding	41076	376	723	46	45	0.08	0.17	2.125	1	1	1	2	1	4	1	5	1.190017
sp|Q6IBS0|TWF2_HUMAN	TWF2	Twinfilin-2 OS=Homo sapier	39751	535	516	30	70	0.08	0.17	2.125	0	1	1	2	1	2	1	2	0.341522

tr|B4DUC5|B4DUC5_HUMAN	CSE1L	Exportin-2 OS=Homo sapien	86045	433	254	40	93	0.08	0.17	2.125	0	3	2	3	4	5	4	6	-0.10617
sp|O75367|H2AY_HUMAN	H2AFY	Core histone macro-H2A.1	39764	565	398	29	93	0.08	0.17	2.125	0	2	1	2	2	2	2	3	-0.39577
tr|B4DEM7|B4DEM7_HUMA	CCT8	T-complex protein 1 subuni	58179	268	171	70	210	0.18	0.38	2.111111	2	6	3	6	5	7	6	7	0.05195
tr|A6NE09|A6NE09_HUMAN	RPSAP58	Protein RPSAP58 OS=Homo	33002	521	441	31	84	0.1	0.21	2.1	0	2	1	2	1	2	1	2	0.341522
tr|Q75L23|Q75L23_HUMAN	PSMC2	Putative uncharacterized pr	46202	610	596	25	60	0.1	0.21	2.1	0	1	1	1	1	1	1	1	-0.39569
tr|J3QQM1|J3QQM1_HUMA	PSMC5	26S protease regulatory sul	29442	314	555	57	38	0.11	0.23	2.090909	1	1	1	1	1	1	2	2	-0.39569
tr|H0YAF8|H0YAF8_HUMAN	GNB2L1	Guanine nucleotide-binding	22373	216	163	96	199	0.52	1.08	2.076923	2	5	3	5	3	5	4	5	0.256725
tr|A8K4D5|A8K4D5_HUMAN	tr|A8K4D5|A8K4	Kynureninase OS=Homo sa	52819	266	275	71	64	0.13	0.27	2.076923	2	1	2	3	3	3	3	4	-0.39585
sp|O15427|MOT4_HUMAN	SLC16A3	Monocarboxylate transport	50064	362	671	48	51	0.14	0.29	2.071429	2	1	2	4	2	5	2	6	0.74248
tr|B2RBA6|B2RBA6_HUMAN	tr|B2RBA6|B2RB	cDNA, FLJ95407, highly sim	81856	374	895	46	34	0.04	0.08	2	1	0	1	2	1	4	1	5	1.190017
tr|F5H1A8|F5H1A8_HUMAN	GSN	Gelsolin OS=Homo sapiens	81776	297	281	61	131	0.04	0.08	2	1	2	1	2	1	3	2	3	0.827198
tr|K7EIG1|K7EIG1_HUMAN	CLUH	Clustered mitochondria pro	140903	438	293	39	127	0.05	0.1	2	1	3	2	4	2	5	2	5	0.74248
tr|B4E0E1|B4E0E1_HUMAN	tr|B4E0E1|B4E0I	cDNA FLJ53442, highly simi	111855	549	296	30	126	0.06	0.12	2	0	2	2	4	2	4	2	5	0.452724
sp|P54886|P5CS_HUMAN	ALDH18A1	Delta0-pyrroline-5-carboxyl	87989	658	523	21	70	0.04	0.08	2	1	2	1	2	1	2	1	3	0.341522
sp|Q96A33|CCD47_HUMAN	CCDC47	Coiled-coil domain-containi	56123	560	370	29	98	0.06	0.12	2	0	2	1	2	1	2	1	2	0.341522
tr|B2RDF2|B2RDF2_HUMAN	PES1	Pescadillo homolog 1 OS=H	68359	488	467	35	78	0.05	0.1	2	1	2	1	2	1	2	3	3	0.341522
tr|H0Y2W2|H0Y2W2_HUMA	ATAD3A	ATPase family AAA domain-	64600	281	328	68	74	0.1	0.2	2	2	1	2	3	2	3	2	3	0.089905
sp|O95782|AP2A1_HUMAN	AP2A1	AP-2 complex subunit alpha	108561	500	930	34	33	0.03	0.06	2	0	0	1	1	1	1	2	1	-0.39569
tr|H0YCG1|H0YCG1_HUMAN	SF3B2	Splicing factor 3B subunit 2	42431	440	541	39	68	0.04	0.08	2	1	1	1	1	1	1	1	1	-0.39569
tr|H3BUF6|H3BUF6_HUMAN	ATXN2L	Ataxin-2-like protein OS=Ho	112929	656	673	22	51	0.03	0.06	2	0	1	1	2	2	2	2	3	-0.39577
tr|B4DH02|B4DH02_HUMAN	tr|B4DH02|B4DH	cDNA FLJ50510, highly simi	95157	123	86	175	347	0.23	0.45	1.956522	4	8	6	11	5	12	6	12	0.790163
tr|B4DMB5|B4DMB5_HUMA	tr|B4DMB5|B4D	cDNA FLJ60075, highly simi	56542	449	280	38	110	0.19	0.37	1.947368	1	1	3	4	3	4	4	5	-0.03303
tr|J3KQL0|J3KQL0_HUMAN	CPS1	Carbamoyl-phosphate syntl	166686	204	98	102	305	0.15	0.29	1.933333	4	9	7	13	10	13	12	16	-0.03308
sp|Q15149|PLEC_HUMAN	PLEC	Plectin OS=Homo sapiens G	533462	74	27	270	860	0.11	0.21	1.909091	11	25	17	31	18	35	32	43	0.547514
sp|Q96TA1|NIBL1_HUMAN	FAM129B	Niban-like protein 1 OS=Ho	84598	155	351	137	102	0.16	0.3	1.875	4	3	4	7	4	8	4	9	0.522607
tr|B6F210|B6F210_HUMAN	GSS	Glutathione synthetase OS=	40495	551	483	30	28	0.08	0.15	1.875	0	0	1	1	1	1	1	1	-0.39569
sp|P26038|MOES_HUMAN	MSN	Moesin OS=Homo sapiens (	67892	119	77	179	383	0.46	0.85	1.847826	7	12	8	14	11	17	12	19	0.210739
tr|Q6IAV5|Q6IAV5_HUMAN	OXCT	Succinyl-CoA:3-ketoacid-co	56612	209	270	100	140	0.18	0.33	1.833333	1	4	3	5	5	5	6	5	-0.39601
sp|P22626|ROA2_HUMAN	HNRNPA2B1	Heterogeneous nuclear ribo	37464	77	65	256	435	0.97	1.76	1.814433	7	12	8	16	9	18	10	19	0.56745
tr|B4DVY1|B4DVY1_HUMAN	EIF3D	Eukaryotic translation initia	58731	710	288	17	129	0.1	0.18	1.8	0	2	1	3	0	2	2	4	1.926812
tr|A8K8N7|A8K8N7_HUMAN	PFAS	Phosphoribosylformylglycin	146286	380	340	46	105	0.05	0.09	1.8	0	2	2	4	3	5	5	6	0.256725
tr|B3KXU8|B3KXU8_HUMAN	tr|B3KXU8|B3KX	cDNA FLJ46087 fis, clone TE	70489	367	700	47	28	0.05	0.09	1.8	1	0	1	1	1	1	1	1	-0.39569
tr|B4DE27|B4DE27_HUMAN	tr|B4DE27|B4DE	cDNA FLJ58765, highly simi	33987	290	603	63	22	0.1	0.18	1.8	1	0	1	1	1	1	1	1	-0.39569
sp|O43707|ACTN4_HUMAN	ACTN4	Alpha-actinin-4 OS=Homo s	105245	19	7	849	2011	2.11	3.77	1.78673	26	54	41	64	44	69	52	78	0.250394
sp|P26599|PTBP1_HUMAN	PTBP1	Polypyrimidine tract-bindin	57357	567	130	29	258	0.18	0.32	1.777778	0	6	3	6	3	6	3	7	0.497983
sp|Q96HC4|PDLI5_HUMAN	PDLIM5	PDZ and LIM domain protei	65102	175	85	123	359	0.41	0.72	1.756098	3	9	7	11	7	12	8	14	0.342048
tr|B3KY60|B3KY60_HUMAN	tr|B3KY60|B3KY(	cDNA FLJ16777 fis, clone BI	92448	587	421	27	87	0.04	0.07	1.75	0	2	1	2	1	2	1	3	0.341522
tr|Q7Z3K9|Q7Z3K9_HUMAN	DKFZp781L0540	Putative uncharacterized pr	90081	440	543	39	67	0.04	0.07	1.75	1	1	1	2	2	1	2	3	-1.13299
sp|P49327|FAS_HUMAN	FASN	Fatty acid synthase OS=Hor	275877	55	32	394	781	0.28	0.49	1.75	12	22	21	35	23	38	29	42	0.318524
tr|Q60FE5|Q60FE5_HUMAN	FLNA	Filamin A OS=Homo sapien:	280790	9	3	1306	2899	0.86	1.5	1.744186	41	70	60	95	75	102	88	118	0.046307
tr|E9PKU4|E9PKU4_HUMAN	RPL8	60S ribosomal protein L8 (F	25806	231	407	90	65	0.28	0.48	1.714286	2	1	2	1	4	1	5	1	-1.98164
tr|Q53ET2|Q53ET2_HUMAN	tr|Q53ET2|Q53E	Dihydropyrimidinase-like 2	62688	342	155	51	224	0.23	0.39	1.695652	1	4	4	6	3	7	5	8	0.704683
tr|Q5I6Y6|Q5I6Y6_HUMAN	LMNA	Lamin A/C transcript varian	74322	95	46	221	625	0.75	1.27	1.693333	7	13	13	20	15	22	15	24	0.14261
tr|B4DUQ1|B4DUQ1_HUMA	tr|B4DUQ1|B4D(	cDNA FLJ54552, highly simi	48708	147	95	147	305	0.58	0.98	1.689655	3	9	7	10	7	9	8	10	-0.05463
tr|B2RBE5|B2RBE5_HUMAN	tr|B2RBE5|B2RB	cDNA, FLJ95468, highly sim	102490	446	708	38	47	0.06	0.1	1.666667	0	1	2	3	2	4	2	4	0.452724
tr|B2R5M8|B2R5M8_HUMA	tr|B2R5M8|B2R!	Isocitrate dehydrogenase [I	46905	555	731	29	45	0.15	0.25	1.666667	0	1	2	4	3	4	3	4	-0.03303
tr|B4DE36|B4DE36_HUMAN	tr|B4DE36|B4DE	Glucose-6-phosphate isome	60376	117	97	182	269	0.3	0.5	1.666667	4	4	5	8	7	9	7	11	-0.05463
tr|Q4J697|Q4J697_HUMAN	PMCA2	Plasma membrane Ca(2+)-A	122274	528	787	31	36	0.03	0.05	1.666667	0	1	1	1	1	1	2	1	-0.39569
tr|F5H3A1|F5H3A1_HUMAN	ATP1A1	Sodium/potassium-transpo	114239	324	220	55	166	0.09	0.15	1.666667	2	2	3	5	8	5	12	5	-1.02502
sp|P23526|SAHH_HUMAN	AHCY	Adenosylhomocysteinase O	48255	195	241	111	155	0.49	0.81	1.653061	2	5	6	9	6	10	8	11	0.296789
tr|Q6NZ55|Q6NZ55_HUMAN	RPL13	60S ribosomal protein L13 (	24308	422	913	41	34	0.14	0.23	1.642857	1	0	1	1	2	1	2	1	-1.13299
tr|B4DY32|B4DY32_HUMAN	tr|B4DY32|B4DY	Asparagine synthetase OS=	55055	141	179	156	198	0.26	0.42	1.615385	3	3	5	6	6	8	6	11	-0.00856
tr|Q2NKY5|Q2NKY5_HUMAN	TUBB6	TUBB6 protein OS=Homo sa	50514	65	51	317	512	0.88	1.42	1.613636	12	13	16	20	17	22	19	26	-0.03313
tr|Q05BW9|Q05BW9_HUMA	PAPSS1	PAPSS1 protein (Fragment)	65264	553	900	29	34	0.1	0.16	1.6	0	0	2	3	1	2	2	4	0.341522
tr|A5D906|A5D906_HUMAN	TUBB2A	TUBB2A protein (Fragment)	28128	25	14	143	603	3.07	4.89	1.592834	4	18	7	21	8	24	8	25	1.135
sp|P23381|SYWC_HUMAN	WARS	Tryptophan--tRNA ligase, cy	53474	145	230	148	160	0.27	0.43	1.592593	3	3	4	6	5	5	5	7	-0.39601
tr|B3KNM6|B3KNM6_HUMA	tr|B3KNM6|B3K	cDNA FLJ14968 fis, clone TH	27279	547	627	30	32	0.12	0.19	1.583333	1	1	1	1	1	1	1	1	-0.39569
tr|B1AHC9|B1AHC9_HUMAN	XRCC6	X-ray repair complementing	64528	116	101	184	299	0.64	1.01	1.578125	5	9	10	14	11	16	12	18	0.1256
tr|B4DTG2|B4DTG2_HUMAN	EEF1G	Elongation factor 1-gamma	56456	93	54	225	498	0.86	1.34	1.55814	5	12	15	18	14	20	18	22	0.104353
tr|B7Z8X5|B7Z8X5_HUMAN	tr|B7Z8X5|B7Z8)	cDNA FLJ61541, highly simi	53809	189	102	115	298	0.52	0.81	1.557692	2	8	7	10	6	11	8	13	0.428283
sp|Q96SI9|STRBP_HUMAN	STRBP	Spermatid perinuclear RNA	74290	471	568	37	63	0.09	0.14	1.555556	0	3	2	3	2	3	2	3	0.089905
tr|B2R6X5|B2R6X5_HUMAN	tr|B2R6X5|B2R6	cDNA, FLJ93166, highly sim	71370	193	180	112	198	0.2	0.31	1.55	3	6	5	6	6	6	6	7	-0.39609
sp|P04040|CATA_HUMAN	CAT	Catalase OS=Homo sapiens	59947	299	625	60	57	0.11	0.17	1.545455	1	1	2	3	2	3	2	3	0.089905
tr|B3KS31|B3KS31_HUMAN	TUBB6	Tubulin, beta 6, isoform CR.	42324	73	79	273	344	0.83	1.28	1.542169	10	10	14	15	17	19	20	23	-0.24034
tr|A8K2S1|A8K2S1_HUMAN	tr|A8K2S1|A8K2!	cDNA FLJ75258, highly simi	85695	369	547	47	67	0.08	0.12	1.5	1	1	2	3	2	4	5	4	0.452724
sp|P53618|COPB_HUMAN	COPB1	Coatomer subunit beta OS=	108214	303	320	59	115	0.06	0.09	1.5	1	3	2	3	2	3	3	3	0.089905
tr|B3KX72|B3KX72_HUMAN	tr|B3KX72|B3KX	cDNA FLJ44920 fis, clone BI	83774	59	71	367	333	0.58	0.87	1.5	10	12	14	15	16	17	20	20	-0.31174
tr|J3KPM9|J3KPM9_HUMAN	STAT1	Signal transducer and activa	83878	364	344	48	95	0.08	0.12	1.5	1	1	2	2	2	2	2	2	-0.39577
tr|B7Z4W3|B7Z4W3_HUMAI	tr|B7Z4W3|B7Z4	cDNA FLJ57543, highly simi	17852	553	571	19	31	0.1	0.15	1.5	0	0	1	1	0	1	1	1	1.189598
sp|P22314|UBA1_HUMAN	UBA1	Ubiquitin-like modifier-activ	118858	114	74	187	390	0.28	0.42	1.5	5	8	10	14	10	15	12	17	0.166726
sp|P13010|XRCC5_HUMAN	XRCC5	X-ray repair cross-complem	83222	128	115	172	272	0.36	0.53	1.472222	6	7	8	12	8	14	9	17	0.375772
tr|H0YBW4|H0YBW4_HUMA	PLAA	Phospholipase A-2-activatir	31645	539	247	30	139	0.11	0.16	1.454545	0	2	1	3	1	3	1	3	0.827198
tr|Q2VPA0|Q2VPA0_HUMAN	LONP1	Lon protease homolog (Frag	95436	414	326	42	112	0.11	0.16	1.454545	2	3	3	4	3	4	3	5	-0.03303
tr|B4DQX0|B4DQX0_HUMAN	tr|B4DQX0|B4D(	cDNA FLJ59219, highly simi	67239	215	212	96	154	0.33	0.48	1.454545	3	6	6	7	6	8	10	10	-0.00856
tr|B4DX08|B4DX08_HUMAN	tr|B4DX08|B4DX	cDNA FLJ58784, highly simi	57521	90	210	229	144	0.4	0.58	1.45	6	5	6	7	6	8	6	9	-0.00856
sp|P17987|TCPA_HUMAN	TCP1	T-complex protein 1 subuni	60819	197	290	106	127	0.3	0.43	1.433333	2	5	5	5	8	5	10	6	-1.02502
tr|B1ALA9|B1ALA9_HUMAN	PRPS1	Phosphoribosyl pyrophosph	24468	638	690	22	32	0.14	0.2	1.428571	0	0	1	1	1	1	2	1	-0.39569
tr|Q5TCU6|Q5TCU6_HUMAN	TLN1	Talin0 OS=Homo sapiens GI	260030	48	26	446	860	0.27	0.38	1.407407	12	20	19	26	20	28	25	30	0.080147
tr|B2R6J2|B2R6J2_HUMAN	tr|B2R6J2|B2R6J	cDNA, FLJ92973, highly sim	69472	126	151	173	189	0.32	0.45	1.40625	6	7	6	7	8	10	8	10	-0.09089
tr|E9PBU3|E9PBU3_HUMAN	ATIC	Phosphoribosylaminoimida	64996	72	69	274	405	0.64	0.9	1.40625	8	8	11	13	12	13	13	16	-0.28528
sp|Q15392|DHC24_HUMAN	DHCR24	Delta(24)-sterol reductase (	60803	442	842	39	36	0.05	0.07	1.4	1	0	1	1	2	2	3	3	-0.39577
tr|B3KMX0|B3KMX0_HUMA	tr|B3KMX0|B3KI	cDNA FLJ12837 fis, clone NT	97114	240	356	85	101	0.1	0.14	1.4	2	1	3	4	4	4	5	6	-0.39593
sp|Q7L2E3|DHX30_HUMAN	DHX30	Putative ATP-dependent RN	134938	397	886	43	35	0.05	0.07	1.4	1	1	2	3	3	1	4	4	-1.61874
tr|J3QT54|J3QT54_HUMAN	CPSF7	Cleavage and polyadenylati	22762	665	364	21	72	0.15	0.21	1.4	0	1	1	2	1	2	1	3	0.341522
tr|E0X098|E0X098_HUMAN	TES	Testin variant (Fragment) O	22009	430	556	40	0	0.15	0.21	1.4	1	0	1	0	1	1	1	1	-0.39569
tr|B3KNN7|B3KNN7_HUMAN	tr|B3KNN7|B3KN	cDNA FLJ30049 fis, clone AI	57311	273	309	69	119	0.18	0.25	1.388889	3	3	3	4	3	4	3	4	-0.03303
tr|B3GQS7|B3GQS7_HUMAN	HSPD1	Mitochondrial heat shock 6	60813	16	33	904	779	1.72	2.36	1.372093	24	19	27	26	26	27	33	30	-0.34398
tr|Q5JP02|Q5JP02_HUMAN	RBBP7	Histone-binding protein RB	19626	321	563	37	37	0.22	0.3	1.363636	1	0	1	2	2	2	2	2	-0.39577
tr|J3KPF3|J3KPF3_HUMAN	SLC3A2	4F2 cell-surface antigen hea	68230	60	38	366	736	1.33	1.81	1.360902	12	19	20	24	20	24	20	25	-0.13903
sp|P52895|AK1C2_HUMAN	AKR1C2	Aldo-keto reductase family	37111	186	249	116	151	0.67	0.91	1.358209	3	4	6	7	7	7	9	8	-0.39616
tr|A6NN80|A6NN80_HUMAI	ANXA6	Annexin OS=Homo sapiens	75571	349	245	50	152	0.14	0.19	1.357143	1	3	3	4	3	3	3	5	-0.39585
tr|Q53GN4|Q53GN4_HUMA	WDR1	WD repeat domain 1, isofor	66822	137	70	160	398	0.4	0.54	1.35	4	8	7	9	7	10	9	11	0.09001
tr|B4DGU4|B4DGU4_HUMA	CTNNB1	Catenin beta0 OS=Homo sa	85339	340	331	52	111	0.12	0.16	1.333333	2	3	3	4	3	5	3	5	0.256725
sp|Q14315|FLNC_HUMAN	FLNC	Filamin-C OS=Homo sapien:	293407	143	150	153	236	0.06	0.08	1.333333	5	8	6	10	10	11	11	13	-0.26491
tr|B3KQQ3|B3KQQ3_HUMAI	tr|B3KQQ3|B3K(	cDNA PSEC0016 fis, clone N	85161	325	299	54	84	0.12	0.16	1.333333	1	1	3	3	3	3	3	3	-0.39585
tr|B3KT93|B3KT93_HUMAN	tr|B3KT93|B3KT!	cDNA FLJ37875 fis, clone BI	70799	215	213	96	171	0.33	0.44	1.333333	3	7	6	8	5	9	9	10	0.393486
tr|B4DSX0|B4DSX0_HUMAN	tr|B4DSX0|B4DS	cDNA FLJ61380, highly simi	47819	19	31	369	807	2.11	2.79	1.322275	10	19	15	24	15	26	18	32	0.379678
tr|A2RUM7|A2RUM7_HUMA	RPL5	Ribosomal protein L5 OS=H	34569	187	211	115	173	0.44	0.58	1.318182	2	5	4	5	5	5	7	6	-0.39601
tr|B1AH89|B1AH89_HUMAN	TTLL12	Tubulin tyrosine ligase-like	74185	104	104	209	290	0.41	0.54	1.317073	6	10	8	10	10	10	11	11	-0.3964
tr|Q9BSV4|Q9BSV4_HUMAN	SFPQ	SFPQ protein (Fragment) O!	68702	228	117	91	272	0.26	0.34	1.307692	2	5	5	7	6	7	7	10	-0.18939
sp|O00410|IPO5_HUMAN	IPO5	Importin-5 OS=Homo sapie	125032	85	214	236	170	0.23	0.3	1.304348	7	4	8	10	10	10	15	11	-0.3964
sp|P53621|COPA_HUMAN	COPA	Coatomer subunit alpha OS	139797	181	188	118	188	0.18	0.23	1.277778	5	6	7	9	8	9	11	9	-0.23553
sp|P13639|EF2_HUMAN	EEF2	Elongation factor 2 OS=Hor	96246	41	41	511	660	1.23	1.55	1.260163	13	20	26	33	27	35	32	46	-0.02736
tr|A8K7F6|A8K7F6_HUMAN	tr|A8K7F6|A8K7	cDNA FLJ78244, highly simi	46321	52	73	404	394	1.62	2.01	1.240741	10	10	14	20	15	19	20	21	-0.06459
tr|Q3B7A7|Q3B7A7_HUMAN	GART	Phosphoribosylglycinamide	108909	341	459	51	80	0.13	0.16	1.230769	1	1	4	5	3	5	4	6	0.256725
tr|B4E022|B4E022_HUMAN	TKT	Transketolase OS=Homo sa	63410	35	45	578	600	1.36	1.67	1.227941	17	14	24	26	26	28	32	31	-0.2922
sp|P08729|K2C7_HUMAN	KRT7	Keratin, type II cytoskeletal	51411	259	294	74	127	0.45	0.55	1.222222	2	4	6	8	7	10	9	12	0.09001
tr|F8VRQ1|F8VRQ1_HUMAN	HNRNPA1	Heterogeneous nuclear ribo	33249	101	164	214	221	0.77	0.94	1.220779	4	5	6	7	7	9	9	10	-0.05463
tr|Q7Z5T5|Q7Z5T5_HUMAN	EIF3A	EIF3A protein (Fragment) O	97203	192	174	112	206	0.14	0.17	1.214286	2	8	4	8	5	11	8	11	0.66962
tr|Q59EG8|Q59EG8_HUMAN	tr|Q59EG8|Q59I	Proteasome 26S non-ATPas	101322	198	256	105	148	0.14	0.17	1.214286	1	3	4	5	4	6	5	9	0.135085
tr|Q53HF2|Q53HF2_HUMAN	tr|Q53HF2|Q53H	Heat shock 70kDa protein 8	53580	51	68	426	422	1.45	1.76	1.213793	12	12	17	17	19	20	19	23	-0.32471
sp|Q00610|CLH1_HUMAN	CLTC	Clathrin heavy chain 1 OS=I	193260	30	42	629	659	0.52	0.63	1.211538	19	17	25	29	24	31	28	38	-0.03319
tr|Q60FE2|Q60FE2_HUMAN	MYH9	MYH9 variant protein OS=H	227646	17	9	900	1675	0.9	1.09	1.211111	30	38	46	55	52	59	60	62	-0.21741

tr|H0YMR4|H0YMR4_HUMA	IPO4	Importin-4 (Fragment) OS=	60104	655	656	22	24	0.05	0.06	1.2	0	1	1	1	1	1	2	-0.39569
sp|Q14247|SRC8_HUMAN	CTTN	Src substrate cortactin OS=	61720	378	952	46	31	0.11	0.13	1.181818	1	0	2	1	3	1	3	2	-1.61874
tr|B2RWP9|B2RWP9_HUMA	MYH10	MYH10 protein OS=Homo s	230858	200	219	104	167	0.06	0.07	1.166667	3	4	4	5	4	6	6	7	0.135085
sp|P07355|ANXA2_HUMAN	ANXA2	Annexin A2 OS=Homo sapie	38808	100	129	214	258	1.26	1.46	1.15873	8	7	11	13	12	13	13	14	-0.28528
tr|K9JA46|K9JA46_HUMAN	EL52	Epididymis luminal secretor	85006	46	43	462	654	1.05	1.21	1.152381	15	18	25	26	24	26	29	32	-0.2838
tr|F5H6T1|F5H6T1_HUMAN	ACTR2	Actin-related protein 2 OS=	34729	244	499	82	73	0.2	0.23	1.15	2	2	2	2	2	2	4	2	-0.39577
sp|P58107|EPIPL_HUMAN	EPPK1	Epiplakin OS=Homo sapiens	557674	56	64	390	436	0.07	0.08	1.142857	10	10	12	13	12	13	13	13	-0.28528
tr|B4DUY3|B4DUY3_HUMAN	tr|B4DUY3|B4DU	cDNA FLJ56668, highly simi	16345	515	341	32	29	0.21	0.24	1.142857	0	0	1	1	1	1	1	2	-0.39569
tr|H0Y4R1|H0Y4R1_HUMAN	IMPDH2	Inosine-5~-monophosphate	51549	177	119	122	270	0.36	0.41	1.138889	2	5	5	6	5	6	6	8	-0.15475
tr|B4DNN4|B4DNN4_HUMA	tr|B4DNN4|B4DI	Ribonucleoside-diphosphat	87252	156	260	136	146	0.29	0.33	1.137931	5	5	7	7	8	7	8	9	-0.57707
tr|A8K525|A8K525_HUMAN	tr|A8K525|A8K5	cDNA FLJ76817, highly simi	54367	75	66	266	432	0.8	0.91	1.1375	10	12	11	12	12	14	14	14	-0.18194
tr|Q2TSD0|Q2TSD0_HUMAN	tr|Q2TSD0|Q2TS	Glyceraldehyde-3-phosphat	36197	18	17	891	1100	3.44	3.84	1.116279	23	23	31	33	31	35	36	38	-0.2246
sp|O00116|ADAS_HUMAN	AGPS	Alkyldihydroxyacetonephos	73664	448	601	38	59	0.09	0.1	1.111111	1	1	2	2	2	2	3	2	-0.39577
tr|H0Y9Q7|H0Y9Q7_HUMAN	CANX	Calnexin (Fragment) OS=Ho	18990	352	116	49	71	0.39	0.43	1.102564	1	1	2	1	3	3	5	3	-0.39585
sp|Q6S8J3|POTEE_HUMAN	POTEE	POTE ankyrin domain family	122882	5	5	2211	2227	0.44	0.48	1.090909	45	49	66	68	70	72	75	81	-0.36033
tr|Q96BS4|Q96BS4_HUMAN	FBL	FBL protein (Fragment) OS=	28546	423	672	41	51	0.11	0.12	1.090909	1	1	1	1	1	1	1	1	-0.39569
tr|B4DPP6|B4DPP6_HUMAN	tr|B4DPP6|B4DP	cDNA FLJ54371, highly simi	72370	222	379	93	96	0.25	0.27	1.08	3	2	5	4	6	5	10	7	-0.63734
tr|B2R6K4|B2R6K4_HUMAN	tr|B2R6K4|B2R6	cDNA, FLJ92996, highly sim	38075	199	381	105	96	0.18	0.19	1.055556	1	2	2	2	2	2	2	2	-0.39577
tr|B4DZB1|B4DZB1_HUMAN	tr|B4DZB1|B4DZ	cDNA FLJ59645, highly simi	104668	111	200	193	177	0.27	0.28	1.037037	4	5	8	8	8	10	10	12	-0.09089
tr|G3XAF4|G3XAF4_HUMAN	L1CAM	L1 cell adhesion molecule, i	139790	572	342	28	105	0.05	0.05	1	1	2	2	2	1	4	2	5	1.190017
tr|J3KN86|J3KN86_HUMAN	RPS3	40S ribosomal protein S3 O	28640	480	680	36	50	0.12	0.12	1	1	1	1	1	1	4	4	4	1.190017
tr|B4DQY2|B4DQY2_HUMAN	tr|B4DQY2|B4DQ	cDNA FLJ59388, highly simi	79300	712	519	17	70	0.04	0.04	1	0	1	1	1	0	1	1	3	1.189598
tr|H7C3A1|H7C3A1_HUMAN	SRRT	Serrate RNA effector molec	57154	701	1082	18	24	0.06	0.06	1	0	0	1	1	0	1	1	1	1.189598
tr|B3KNL2|B3KNL2_HUMAN	RUVBL2	RuvB-like 2 (E. coli), isoform	47603	584	776	28	41	0.14	0.14	1	0	1	2	2	1	3	2	3	0.827198
tr|B4DTA2|B4DTA2_HUMAN	tr|B4DTA2|B4DT	cDNA FLJ60148, highly simi	30367	229	472	39	77	0.37	0.37	1	1	2	1	3	1	3	1	3	0.827198
tr|G8JLB6|G8JLB6_HUMAN	HNRNPH1	Heterogeneous nuclear ribo	51482	207	160	101	226	0.45	0.45	1	2	6	6	7	5	10	6	11	0.538126
tr|B4DR45|B4DR45_HUMAN	tr|B4DR45|B4DR	cDNA FLJ61339, highly simi	110017	238	461	86	80	0.06	0.06	1	1	2	2	3	2	4	2	5	0.452724
sp|O43286|B4GT5_HUMAN	B4GALT5	Beta0,4-galactosyltransfera	45318	651	1097	22	23	0.07	0.07	1	0	1	1	1	1	2	1	2	0.341522
tr|B4DLB8|B4DLB8_HUMAN	tr|B4DLB8|B4DL	cDNA FLJ52205, highly simi	40269	650	428	22	85	0.08	0.08	1	0	1	1	1	1	2	1	2	0.341522
tr|Q1L838|Q1L838_HUMAN	GTBP	GTBP-ALT OS=Homo sapien	122267	559	1080	29	24	0.03	0.03	1	0	0	1	1	1	2	1	3	0.341522
sp|Q8NBJ5|GT251_HUMAN	GLT25D1	Procollagen galactosyltrans	71933	523	706	31	47	0.09	0.09	1	0	1	2	2	1	2	2	3	0.341522
tr|Q0P512|Q0P512_HUMAN	SLC2A1	Solute carrier family 2 (Faci	54361	377	970	46	30	0.06	0.06	1	1	0	1	1	1	2	1	3	0.341522
sp|Q13151|ROA0_HUMAN	HNRNPA0	Heterogeneous nuclear ribo	30993	323	417	55	88	0.36	0.36	1	2	3	3	3	3	5	3	5	0.256725
tr|B2R5W2|B2R5W2_HUMA	tr|B2R5W2|B2R5	cDNA, FLJ92657, highly sim	31986	178	642	122	55	0.35	0.35	1	3	1	3	3	3	5	4	6	0.256725
tr|B4DJW8|B4DJW8_HUMAN	tr|B4DJW8|B4DJ	cDNA FLJ55697, highly simi	85347	558	517	29	70	0.08	0.08	1	0	1	2	2	2	3	3	3	0.089905
tr|D3DVQ1|D3DVQ1_HUMA	LETM1	Leucine zipper-EF-hand con	63730	463	664	37	52	0.11	0.11	1	1	1	2	2	2	3	4	3	0.089905
tr|B5BU25|B5BU25_HUMAN	U2AF2	U2 small nuclear RNA auxili	53445	260	371	74	98	0.13	0.13	1	1	1	2	2	2	3	2	3	0.089905
tr|B3KX19|B3KX19_HUMAN	tr|B3KX19|B3KX	cDNA FLJ44500 fis, clone U	109111	111	199	193	177	0.27	0.27	1	4	5	8	8	8	10	10	12	-0.09089
tr|Q53XC0|Q53XC0_HUMAN	EIF2S1	Eukaryotic translation initia	36374	208	223	100	165	0.42	0.42	1	4	4	4	4	4	5	4	6	-0.10617
sp|P07814|SYEP_HUMAN	EPRS	Bifunctional glutamate/pro	172080	57	83	380	361	0.33	0.33	1	9	10	15	15	15	18	17	18	-0.14079
tr|Q6IBT3|Q6IBT3_HUMAN	CCT7	CCT7 protein OS=Homo sap	59804	161	250	133	150	0.38	0.38	1	2	4	6	6	5	6	7	8	-0.15475
sp|Q9NR30|DDX21_HUMAN	DDX21	Nucleolar RNA helicase 2 O	87804	148	227	147	162	0.34	0.34	1	4	5	8	8	8	9	9	9	-0.23553
tr|B0UX83|B0UX83_HUMAN	BAG6	HLA-B associated transcript	119085	704	1139	18	18	0.03	0.03	1	0	0	1	1	0	0	1	1	-0.39561
tr|B4DS66|B4DS66_HUMAN	tr|B4DS66|B4DS	cDNA FLJ54290, highly simi	73509	712	520	17	70	0.04	0.04	1	0	1	1	1	1	1	6	3	-0.39569
sp|Q9UG63|ABCF2_HUMAN	ABCF2	ATP-binding cassette sub-fa	71815	673	811	20	39	0.05	0.05	1	0	1	1	1	1	1	2	3	-0.39569
tr|Q6UWE9|Q6UWE9_HUM	UNQ2550	SFVP2550 OS=Homo sapien	11890	664	1119	21	21	0.29	0.29	1	1	1	1	1	1	1	1	1	-0.39569
tr|F1T0A5|F1T0A5_HUMAN	PRPF31	PRP31 pre-mRNA processin	55649	661	1098	21	23	0.06	0.06	1	0	0	1	1	1	1	2	1	-0.39569
tr|B4DG42|B4DG42_HUMAN	tr|B4DG42|B4DG	cDNA FLJ53753, highly simi	31889	609	491	25	74	0.1	0.1	1	0	1	1	1	1	1	1	1	-0.39569
tr|B4DKD0|B4DKD0_HUMAN	tr|B4DKD0|B4DK	DNA topoisomerase 2 (Frag	135015	606	914	25	0	0.02	0.02	1	0	0	1	0	1	1	3	2	-0.39569
tr|B2RAX6|B2RAX6_HUMAN	tr|B2RAX6|B2RA	cDNA, FLJ95176, Homo sap	58127	605	863	25	36	0.06	0.06	1	0	0	1	1	1	1	1	1	-0.39569
tr|B4DJE3|B4DJE3_HUMAN	tr|B4DJE3|B4DJE	cDNA FLJ52929, highly simi	46620	604	1078	26	24	0.07	0.07	1	0	0	1	1	1	1	2	2	-0.39569
tr|B4DNK3|B4DNK3_HUMAN	tr|B4DNK3|B4DN	cDNA FLJ52127, highly simi	29955	601	772	26	41	0.11	0.11	1	0	1	1	1	1	1	1	1	-0.39569
tr|Q8TC04|Q8TC04_HUMAN	KRT23	Keratin 23 (Histone deacety	48318	600	963	26	31	0.07	0.07	1	0	0	1	1	1	1	1	1	-0.39569
tr|H3BS19|H3BS19_HUMAN	ZNF469	Zinc finger protein 469 OS=	417347	591	969	27	31	0.01	0.01	1	0	0	1	1	1	1	1	1	-0.39569
sp|Q12792|TWF1_HUMAN	TWF1	Twinfilin0 OS=Homo sapien	40429	575	650	28	54	0.08	0.08	1	0	1	1	1	1	1	1	2	-0.39569
tr|B4DM97|B4DM97_HUMA	tr|B4DM97|B4D	cDNA FLJ55002, highly simi	38365	561	1123	29	20	0.09	0.09	1	0	0	1	1	1	1	1	1	-0.39569
tr|H3BRB3|H3BRB3_HUMAN	KIF22	Kinesin-like protein KIF22 O	67996	557	1112	29	21	0.05	0.05	1	0	0	1	1	1	1	1	1	-0.39569
tr|B2RB23|B2RB23_HUMAN	tr|B2RB23|B2RB	cDNA, FLJ95265, highly sim	42439	544	657	30	53	0.08	0.08	1	0	1	1	1	1	1	1	1	-0.39569
tr|H0Y7S3|H0Y7S3_HUMAN	ATP2B2	Plasma membrane calcium-	122272	528	844	31	36	0.03	0.03	1	0	1	1	1	1	3	2	-0.39569
tr|B3KUZ8|B3KUZ8_HUMAN	tr|B3KUZ8|B3KU	Aspartate aminotransferase	41699	522	980	31	30	0.08	0.08	1	0	0	1	1	1	1	2	2	-0.39569
tr|B2R4C0|B2R4C0_HUMAN	tr|B2R4C0|B2R4	60S ribosomal protein L18a	21024	514	769	32	42	0.16	0.16	1	0	1	1	1	1	1	2	1	-0.39569
tr|A2BDK6|A2BDK6_HUMAN	MAP1B	Microtubule-associated pro	257692	511	749	32	44	0.01	0.01	1	0	1	1	1	1	1	2	3	-0.39569
tr|Q2NKW8|Q2NKW8_HUM	AHCYL1	Adenosylhomocysteinase (I	57630	509	748	33	44	0.06	0.06	1	0	1	1	1	1	1	1	1	-0.39569
tr|A6NEM2|A6NEM2_HUMA	HCFC1	HCF N-terminal chain 5 OS=	215338	508	583	33	61	0.02	0.02	1	0	1	1	1	1	1	2	3	-0.39569
sp|Q9Y5B9|SP16H_HUMAN	SUPT16H	FACT complex subunit SPT1	120409	506	1060	33	25	0.03	0.03	1	0	0	1	1	1	1	2	1	-0.39569
tr|B3KSQ7|B3KSQ7_HUMAN	DBN1	Drebrin 1, isoform CRA_d O	66053	478	482	36	75	0.05	0.05	1	0	1	1	1	1	1	1	1	-0.39569
tr|H0Y5J4|H0Y5J4_HUMAN	DBNL	Drebrin-like protein (Fragm	40475	466	903	37	34	0.08	0.08	1	1	0	1	1	1	1	1	1	-0.39569
tr|B4DXC4|B4DXC4_HUMAN	tr|B4DXC4|B4DX	cDNA FLJ58636, moderately	59162	451	466	38	78	0.06	0.06	1	1	1	1	1	1	1	1	1	-0.39569
tr|E9PIM9|E9PIM9_HUMAN	RNH1	Ribonuclease inhibitor (Frag	22362	431	718	40	46	0.15	0.15	1	0	1	1	1	1	1	1	1	-0.39569
sp|Q9NSB4|KRT82_HUMAN	KRT82	Keratin, type II cuticular Hb	57985	411	901	43	34	0.06	0.06	1	1	1	1	1	1	1	2	2	-0.39569
tr|A6NKV8|A6NKV8_HUMAN	PAIP1	Polyadenylate-binding prot	42379	361	987	48	30	0.08	0.08	1	1	0	1	1	1	1	1	2	-0.39569
tr|B2RAR2|B2RAR2_HUMAN	tr|B2RAR2|B2RA	cDNA, FLJ95064, highly sim	47015	338	802	52	39	0.07	0.07	1	1	1	1	1	1	1	1	1	-0.39569
tr|J3KTE4|J3KTE4_HUMAN	RPL19	Ribosomal protein L19 OS=	23347	336	615	52	58	0.14	0.14	1	1	1	1	1	1	1	1	1	-0.39569
tr|B4DEX8|B4DEX8_HUMAN	MAT2A	S-adenosylmethionine synt	40027	333	652	52	54	0.08	0.08	1	1	1	1	1	1	1	1	2	-0.39569
tr|H7C1U0|H7C1U0_HUMAN	APEH	Acylamino-acid-releasing er	35132	319	768	56	42	0.09	0.09	1	1	1	1	1	1	1	1	1	-0.39569
tr|B3KV39|B3KV39_HUMAN	tr|B3KV39|B3KV	cDNA FLJ16099 fis, clone TE	50383	188	840	115	36	0.07	0.07	1	1	1	1	1	1	1	2	1	-0.39569
sp|Q96P70|IPO9_HUMAN	IPO9	Importin-9 OS=Homo sapie	116858	580	973	28	30	0.06	0.06	1	0	0	2	2	2	2	2	2	-0.39577
sp|Q9Y3I0|RTCB_HUMAN	C22orf28	tRNA-splicing ligase RtcB ho	55688	458	367	38	98	0.12	0.12	1	0	2	2	2	2	2	2	3	-0.39577
tr|B4DHG3|B4DHG3_HUMA	tr|B4DHG3|B4DI	cDNA FLJ55329, highly simi	81716	453	376	38	97	0.08	0.08	1	0	1	2	2	2	2	2	3	-0.39577
tr|B4DP08|B4DP08_HUMAN	ALG1	Chitobiosyldiphosphodolich	40938	452	705	38	47	0.17	0.17	1	0	1	2	2	2	2	2	2	-0.39577
tr|Q4ZG57|Q4ZG57_HUMAN	MCM6	Putative uncharacterized pr	89803	444	793	39	40	0.04	0.04	1	1	1	1	1	2	2	3	2	-0.39577
tr|Q58FF4|Q58FF4_HUMAN	HSP90Bf	Heat shock protein 90Bf OS	41833	407	651	43	54	0.16	0.16	1	1	1	2	2	2	2	2	2	-0.39577
sp|P53004|BIEA_HUMAN	BLVRA	Biliverdin reductase A OS=H	33692	403	692	43	49	0.21	0.21	1	1	2	2	2	2	2	3	2	-0.39577
tr|B2RCT6|B2RCT6_HUMAN	tr|B2RCT6|B2RC	cDNA, FLJ96276, highly sim	56320	391	1033	44	27	0.12	0.12	1	1	1	2	2	2	2	2	3	-0.39577
tr|B1ANK7|B1ANK7_HUMAN	FH	Fumarate hydratase OS=Ho	54773	288	345	64	103	0.12	0.12	1	1	1	2	2	2	2	5	3	-0.39577
tr|G3V1S6|G3V1S6_HUMAN	GALNT2	Polypeptide N-acetylgalacto	61440	254	512	76	71	0.11	0.11	1	2	2	2	2	2	2	2	3	-0.39577
tr|Q2TB59|Q2TB59_HUMAN	NNT	Nicotinamide nucleotide tra	114552	234	511	88	71	0.06	0.06	1	1	1	2	2	2	2	3	2	-0.39577
tr|B4DII5|B4DII5_HUMAN	tr|B4DII5|B4DII5	Importin subunit alpha OS=	60345	573	282	28	131	0.11	0.11	1	0	2	2	2	3	3	3	4	-0.39585
tr|L0R5A1|L0R5A1_HUMAN	CSF2RB	Alternative protein CSF2RB	11752	486	922	36	33	0.29	0.29	1	1	1	1	2	3	3	5	5	-0.39585
tr|B4E0S6|B4E0S6_HUMAN	tr|B4E0S6|B4E0S	cDNA FLJ55635, highly simi	90289	183	287	117	129	0.15	0.15	1	2	4	4	4	4	4	7	4	-0.39593
tr|Q4W4Y1|Q4W4Y1_HUMA	DRIP4	Dopamine receptor interact	96646	136	259	160	148	0.18	0.18	1	2	2	5	5	5	5	6	5	-0.39601
sp|P14923|PLAK_HUMAN	JUP	Junction plakoglobin OS=Ho	82434	205	396	102	93	0.26	0.26	1	3	4	6	7	6	6	7	7	-0.39609
tr|B7Z2Z8|B7Z2Z8_HUMAN	tr|B7Z2Z8|B7Z2Z	T-complex protein 1 subuni	52781	113	442	188	75	0.27	0.27	1	4	3	5	4	7	7	7	8	-0.39616
sp|P55072|TERA_HUMAN	VCP	Transitional endoplasmic re	89950	64	62	326	444	0.65	0.65	1	7	8	14	14	14	13	15	18	-0.50006
sp|P52272|HNRPM_HUMAN	HNRNPM	Heterogeneous nuclear ribo	77749	164	135	132	251	0.39	0.39	1	7	5	8	8	11	10	12	10	-0.52797
tr|B4DRH6|B4DRH6_HUMAN	tr|B4DRH6|B4DR	cDNA FLJ54509, highly simi	79007	133	311	164	119	0.28	0.28	1	5	3	6	6	7	6	7	6	-0.60287
tr|B0V043|B0V043_HUMAN	VARS	Valine--tRNA ligase OS=Hor	141632	275	159	69	227	0.15	0.15	1	2	5	6	6	6	5	8	8	-0.63734
tr|F8VPD4|F8VPD4_HUMAN	CAD	CAD protein OS=Homo sapi	238097	317	411	57	89	0.04	0.04	1	1	2	3	3	4	3	5	3	-0.75875
tr|H0Y8G5|H0Y8G5_HUMAN	HNRNPD	Heterogeneous nuclear ribo	29877	229	323	90	114	0.37	0.37	1	3	3	3	3	4	3	4	3	-0.75875
tr|A0PJ92|A0PJ92_HUMAN	NOP56	NOP56 protein (Fragment)	50068	311	640	58	55	0.14	0.14	1	1	1	2	2	3	2	3	3	-0.88153
tr|Q0IIN1|Q0IIN1_HUMAN	KRT77	Keratin 77 OS=Homo sapier	62050	76	169	264	212	0.23	0.23	1	10	7	10	7	10	7	12	7	-0.88258
tr|B1PS43|B1PS43_HUMAN	MYH11	Myosin heavy chain 11 smo	235004	142	192	155	182	0.06	0.06	1	5	4	5	5	9	6	11	7	-0.94456
sp|Q92973|TNPO1_HUMAN	TNPO1	Transportin0 OS=Homo sap	103771	139	204	158	174	0.17	0.17	1	3	3	5	5	8	5	8	8	-1.02502
tr|A8K781|A8K781_HUMAN	tr|A8K781|A8K7	cDNA FLJ75299, highly simi	47578	603	949	26	32	0.07	0.07	1	0	0	1	1	2	1	2	1	-1.13299
tr|Q0IJ56|Q0IJ56_HUMAN	ST13	ST13 protein (Fragment) OS	35164	382	444	46	83	0.09	0.09	1	1	1	1	1	2	1	3	1	-1.13299
tr|Q2QD09|Q2QD09_HUMA	tr|Q2QD09|Q2Q	Triosephosphate isomerase	27211	267	707	71	41	0.26	0.26	1	1	1	2	1	2	1	2	1	-1.13299
tr|B4DXX7|B4DXX7_HUMAN	DDX3Y	ATP-dependent RNA helicas	73342	217	264	95	84	0.19	0.19	1	2	2	5	2	6	3	6	4	-1.28992
tr|F8W930|F8W930_HUMAN	IGF2BP2	Insulin-like growth factor 2	66859	556	1152	29	15	0.05	0.05	1	0	0	1	1	1	0	1	1	-1.9809
tr|A0N4V7|A0N4V7_HUMAN	Tcr-alpha	HCG2039797 (Fragment) OS	2269	212	582	98	61	1.81	1.81	1	5	5	15	5	31	5	46	5	-2.92241
tr|F4ZW64|F4ZW64_HUMAN	tr|F4ZW64|F4ZW	NF90a OS=Homo sapiens PI	76369	154	217	137	141	0.46	0.45	0.978261	3	6	9	10	10	13	11	14	-0.03308
tr|I3L492|I3L492_HUMAN	ETF1	Eukaryotic peptide chain re	23028	274	386	69	55	0.15	0.14	0.933333	1	1	1	1	1	1	1	1	-0.39569
tr|Q75KX8|Q75KX8_HUMAN	SLC25A13	Putative uncharacterized pr	32876	353	552	0	66	0.11	0.1	0.909091	0	1	0	1	1	2	2	2	0.341522
tr|B5LY64|B5LY64_HUMAN	UMPS	Orotidine 5~-phosphate de	33258	302	804	59	0	0.1	0.09	0.9	1	0	1	0	1	1	1	1	-0.39569

tr|Q96K98|Q96K98_HUMAN	tr|Q96K98|Q96K	cDNA FLJ14414 fis, clone HE	37365	180	476	119	53	0.18	0.16	0.888889	2	1	2	2	2	2	4	2	-0.39577
tr|Q53GU8|Q53GU8_HUMA	tr|Q53GU8|Q53	Transforming growth factor	75231	358	1042	48	26	0.09	0.08	0.888889	1	0	2	1	2	1	2	3	-1.13299
tr|A4QPB0|A4QPB0_HUMAN	IQGAP1	IQ motif containing GTPase	189789	84	78	239	383	0.31	0.27	0.870968	8	9	17	14	18	15	21	20	-0.65304
tr|E9PKE3|E9PKE3_HUMAN	HSPA8	Heat shock cognate 71 kDa	68991	51	60	426	449	1.45	1.26	0.868966	12	13	17	17	19	20	19	22	-0.32471
tr|Q6I9U3|Q6I9U3_HUMAN	M6PR	M6PR protein OS=Homo sa	31469	416	817	42	38	0.29	0.25	0.862069	1	1	1	1	1	1	1	1	-0.39569
sp|P26232|CTNA2_HUMAN	CTNNA2	Catenin alpha-2 OS=Homo s	106045	389	297	31	126	0.07	0.06	0.857143	0	2	1	2	1	3	1	3	0.827198
tr|F5GWP8|F5GWP8_HUMA	JUP	Junction plakoglobin OS=Ho	66708	26	53	676	504	1.74	1.49	0.856322	20	18	27	21	31	22	34	25	-0.88574
tr|Q9UE89|Q9UE89_HUMAN	tr|Q9UE89|Q9UE	Beta-actin (Fragment) OS=H	7908	1	30	448	815	31.43	26.84	0.853961	9	18	13	25	13	26	14	29	0.579644
tr|B4DKM5|B4DKM5_HUMA	VDAC2	Voltage-dependent anion-s	27804	196	111	108	207	0.76	0.64	0.842105	1	5	5	5	5	5	5	5	-0.39601
sp|Q04695|K1C17_HUMAN	KRT17	Keratin, type I cytoskeletal	48361	24	52	683	508	2.99	2.5	0.83612	19	17	27	21	32	22	35	25	-0.93116
tr|B4DFM0|B4DFM0_HUMA	FARSLB	Phenylalanine-tRNA synthe	55353	360	766	48	42	0.06	0.05	0.833333	1	1	1	1	1	3	3	3	0.827198
sp|Q08211|DHX9_HUMAN	DHX9	ATP-dependent RNA helicas	142181	96	91	218	324	0.41	0.34	0.829268	8	9	16	13	18	13	22	15	-0.85285
tr|B4DXW6|B4DXW6_HUMA	tr|B4DXW6|B4D	cDNA FLJ50285, highly simi	52771	235	274	88	103	0.35	0.29	0.828571	3	3	5	5	6	5	7	5	-0.63734
tr|F5GX33|F5GX33_HUMAN	SLC25A13	Calcium-binding mitochond	62548	353	587	49	17	0.11	0.09	0.818182	1	0	2	1	3	1	4	2	-1.61874
sp|P68366|TBA4A_HUMAN	TUBA4A	Tubulin alpha-4A chain OS=	50634	22	28	796	840	3.01	2.45	0.813953	20	25	31	37	31	38	36	46	-0.1068
tr|Q5HYG8|Q5HYG8_HUMA	DKFZp686P09201	Putative uncharacterized pr	53831	86	238	235	156	0.61	0.49	0.803279	4	4	8	6	9	6	10	6	-0.94456
tr|H0Y9Q1|H0Y9Q1_HUMAN	LAP3	Cytosol aminopeptidase (Fr	22641	368	649	47	46	0.15	0.12	0.8	1	1	1	1	1	1	1	1	-0.39569
sp|P06733|ENOA_HUMAN	ENO1	Alpha-enolase OS=Homo sa	47481	40	118	520	271	1.39	1.09	0.784173	13	6	18	13	19	15	25	17	-0.72932
tr|B4DF68|B4DF68_HUMAN	tr|B4DF68|B4DF	cDNA FLJ55325, highly simi	86500	123	133	47	203	0.23	0.18	0.782609	1	4	1	4	1	4	1	4	1.190017
tr|Q5HYM2|Q5HYM2_HUMA	DKFZp686O2462	Putative uncharacterized pr	72627	569	435	29	85	0.09	0.07	0.777778	0	2	2	2	2	2	2	4	-0.39577
tr|D3DPU2|D3DPU2_HUMA	CAP1	Adenylyl cyclase-associated	51926	172	226	125	162	0.36	0.28	0.777778	4	3	5	4	6	5	7	6	-0.63734
sp|Q1KMD3|HNRL2_HUMAN	HNRNPUL2	Heterogeneous nuclear ribo	85622	249	319	78	116	0.21	0.16	0.761905	2	2	5	4	6	3	6	4	-1.28992
tr|G3XAP5|G3XAP5_HUMAN	LDHC	L-lactate dehydrogenase OS	26552	39	124	67	52	1.81	1.36	0.751381	1	1	1	1	1	1	1	1	-0.39569
tr|B7Z438|B7Z438_HUMAN	tr|B7Z438|B7Z43	cDNA FLJ56352, highly simi	26801	467	947	37	32	0.12	0.09	0.75	1	0	1	1	1	1	1	1	-0.39569
tr|B0I1S7|B0I1S7_HUMAN	tr|B0I1S7|B0I1S7	KIF5A variant protein OS=H	118189	227	801	46	39	0.04	0.03	0.75	1	1	1	1	1	1	1	3	-0.39569
sp|P42166|LAP2A_HUMAN	TMPO	Lamina-associated polypep	76016	241	572	84	63	0.24	0.18	0.75	1	1	5	4	5	4	5	4	-0.68576
sp|Q92621|NU205_HUMAN	NUP205	Nuclear pore complex prote	230171	415	613	42	58	0.04	0.03	0.75	0	1	3	2	3	2	5	2	-0.88153
tr|B3KTJ9|B3KTJ9_HUMAN	tr|B3KTJ9|B3KTJ	cDNA FLJ38393 fis, clone FE	103493	82	136	251	249	0.32	0.24	0.75	7	5	9	7	9	6	12	8	-0.94456
tr|B4DN87|B4DN87_HUMAN	tr|B4DN87|B4DN	cDNA FLJ52569, highly simi	44290	233	334	89	108	0.43	0.32	0.744186	4	4	5	4	5	5	5	5	-0.39601
tr|Q562M3|Q562M3_HUMA	ACT	Actin-like protein (Fragmen	11529	103	96	213	306	7.06	5.21	0.73796	10	12	14	19	17	20	20	23	-0.16794
sp|Q3ZCM7|TBB8_HUMAN	TUBB8	Tubulin beta-8 chain OS=Ho	50257	67	82	306	374	0.77	0.56	0.727273	13	14	17	18	19	21	21	24	-0.25575
tr|B7Z4V2|B7Z4V2_HUMAN	tr|B7Z4V2|B7Z4V	cDNA FLJ51907, highly simi	72641	81	221	251	166	0.42	0.3	0.714286	7	5	8	6	8	7	8	7	-0.57707
tr|H0YGW7|H0YGW7_HUMA	ABCF1	ATP-binding cassette sub-fa	68075	144	170	151	199	0.27	0.19	0.703704	4	3	5	3	5	3	7	3	-1.04858
sp|P18206|VINC_HUMAN	VCL	Vinculin OS=Homo sapiens	124292	68	149	299	236	0.37	0.26	0.702703	8	5	12	9	13	9	13	11	-0.9046
tr|B7Z4V1|B7Z4V1_HUMAN	tr|B7Z4V1|B7Z4V	cDNA FLJ55401, highly simi	32962	50	59	103	169	0.77	0.54	0.701299	3	4	4	6	4	8	4	10	0.522607
sp|P08559|ODPA_HUMAN	PDHA1	Pyruvate dehydrogenase E1	43952	516	889	32	35	0.1	0.07	0.7	0	1	1	1	1	1	1	1	-0.39569
sp|P68363|TBA1B_HUMAN	TUBA1B	Tubulin alpha0B chain OS=H	50804	13	18	1091	1209	5.58	3.8	0.681004	31	31	43	44	45	46	49	55	-0.36757
tr|B2R6N6|B2R6N6_HUMAN	tr|B2R6N6|B2R6	cDNA, FLJ93036, highly sim	28274	258	445	28	0	0.4	0.27	0.675	0	0	1	0	1	1	1	1	-0.39569
sp|Q71U36|TBA1A_HUMAN	TUBA1A	Tubulin alpha0A chain OS=H	50788	15	24	1022	1078	5.18	3.49	0.673745	30	27	41	39	43	41	46	49	-0.46743
tr|B7Z601|B7Z601_HUMAN	tr|B7Z601|B7Z60	cDNA FLJ57187, highly simi	56047	512	1155	32	0	0.06	0.04	0.666667	0	0	1	0	2	1	2	2	-1.13299
tr|B0AZS6|B0AZS6_HUMAN	YWHAZ	14-3-3 protein zeta/delta O	19174	497	484	34	35	0.38	0.25	0.657895	0	0	2	1	2	1	2	1	-1.13299
sp|P53396|ACLY_HUMAN	ACLY	ATP-citrate synthase OS=Ho	121674	20	35	845	765	1.28	0.84	0.65625	21	23	32	29	34	29	40	39	-0.62544
tr|Q5T081|Q5T081_HUMAN	RCC1	CHC1 protein OS=Homo sap	45397	269	443	70	84	0.23	0.15	0.652174	2	1	3	2	2	2	4	2	-0.39577
sp|Q9NV06|DCA13_HUMAN	DCAF13	DDB1- and CUL4-associated	51996	435	871	39	35	0.2	0.13	0.65	1	0	3	1	3	1	6	1	-1.61874
sp|Q9NY65|TBA8_HUMAN	TUBA8	Tubulin alpha-8 chain OS=H	50746	34	48	592	591	1.74	1.13	0.649425	15	20	23	28	23	27	27	35	-0.16966
tr|Q8TE01|Q8TE01_HUMAN	derp12	DERP12 (Dermal papilla der	38340	69	110	297	278	0.79	0.51	0.64557	6	6	7	7	8	8	8	8	-0.39624
tr|D2CFK9|D2CFK9_HUMAN	GTPBP4	Nucleolar GTP-binding prot	74317	264	831	72	37	0.14	0.09	0.642857	2	1	3	2	3	2	3	3	-0.88153
sp|Q9H853|TBA4B_HUMAN	TUBA4B	Putative tubulin-like protein	27819	146	292	147	127	0.4	0.25	0.625	3	4	4	6	4	5	6	9	-0.10617
tr|D0W033|D0W033_HUMA	HLA-A	MHC class I antigen (Fragm	31801	639	756	22	43	0.16	0.1	0.625	0	1	1	1	1	1	1	1	-0.39569
tr|Q4QZC0|Q4QZC0_HUMAN	HLA-A	MHC class I antigen (Fragm	31984	639	540	22	68	0.16	0.1	0.625	0	1	1	1	1	1	2	1	-0.39569
tr|K7EKB6|K7EKB6_HUMAN	THOP1	Thimet oligopeptidase (Frag	20792	278	728	68	32	0.16	0.1	0.625	1	0	1	1	1	1	1	1	-0.39569
tr|F5H5D3|F5H5D3_HUMAN	TUBA1C	Tubulin alpha0C chain OS=H	58606	21	23	809	1101	2.92	1.82	0.623288	24	29	34	39	37	41	41	52	-0.25132
tr|G3V2A3|G3V2A3_HUMAN	TUBB3	Tubulin beta-3 chain (Fragn	20929	25	39	147	337	3.07	1.91	0.62215	5	8	6	8	7	12	7	12	0.342048
sp|Q58FF8|H90B2_HUMAN	HSP90AB2P	Putative heat shock protein	44492	32	148	97	236	1.24	0.77	0.620968	3	7	5	8	5	8	6	10	0.232772
tr|H0YIE7|H0YIE7_HUMAN	HSPA1L	Heat shock 70 kDa protein	25488	51	75	132	132	1.45	0.9	0.62069	3	3	4	3	4	3	4	4	-0.75875
tr|A8K4Z4|A8K4Z4_HUMAN	tr|A8K4Z4|A8K4	cDNA FLJ75549, highly simi	34389	109	253	201	149	0.74	0.45	0.608108	6	3	6	4	6	4	6	6	-0.9271
tr|Q6IPN6|Q6IPN6_HUMAN	EEF1A1	Elongation factor 1-alpha O	50433	70	154	295	230	1.14	0.68	0.596491	11	8	13	10	18	11	21	11	-1.08467
tr|B9A041|B9A041_HUMAN	MDH1	Malate dehydrogenase, cyt	23195	309	971	58	30	0.31	0.18	0.580645	2	0	2	1	2	1	2	2	-1.13299
tr|J3KTA4|J3KTA4_HUMAN	DDX5	Probable ATP-dependent R	69557	97	207	218	174	0.45	0.26	0.577778	7	4	9	5	10	6	10	7	-1.08928
tr|B2R9K8|B2R9K8_HUMAN	tr|B2R9K8|B2R9	cDNA, FLJ94440, highly sim	58386	118	243	181	153	0.47	0.27	0.574468	5	4	7	5	9	6	10	6	-0.94456
tr|B2RAQ8|B2RAQ8_HUMAN	tr|B2RAQ8|B2RA	cDNA, FLJ95058, highly sim	88967	354	567	49	63	0.07	0.04	0.571429	1	1	2	1	3	2	3	2	-0.88153
sp|P16615|AT2A2_HUMAN	ATP2A2	Sarcoplasmic/endoplasmic	116336	149	156	145	228	0.32	0.18	0.5625	4	5	10	6	12	6	13	6	-1.34147
tr|B4DPZ3|B4DPZ3_HUMAN	tr|B4DPZ3|B4DP	cDNA FLJ53290, highly simi	70869	298	721	60	46	0.09	0.05	0.555556	2	1	2	1	2	1	2	1	-1.13299
sp|P38919|IF4A3_HUMAN	EIF4A3	Eukaryotic initiation factor	47126	262	354	74	101	0.4	0.22	0.55	1	2	5	4	4	5	6	7	-0.10617
tr|B2RCM3|B2RCM3_HUMA	tr|B2RCM3|B2RC	cDNA, FLJ96158, highly sim	80883	173	414	125	89	0.22	0.12	0.545455	3	2	7	3	7	3	8	4	-1.4967
tr|J3KSN7|J3KSN7_HUMAN	EIF4A2	Eukaryotic initiation factor	12120	52	218	116	90	1.62	0.85	0.524691	1	1	3	4	2	4	3	4	0.452724
tr|H3BLZ8|H3BLZ8_HUMAN	DDX17	Probable ATP-dependent R	81073	166	276	130	133	0.37	0.19	0.513514	3	3	9	4	9	5	10	6	-1.18582
tr|Q96HI4|Q96HI4_HUMAN	NMT1	Glycylpeptide N-tetradecan	56910	350	348	50	103	0.49	0.25	0.510204	1	2	2	4	2	4	2	4	0.452724
tr|A0PBY4|A0PBY4_HUMAN	HLA-A	MHC class I antigen (Fragm	32047	639	754	22	43	0.16	0.08	0.5	0	1	1	1	1	1	3	1	-0.39569
tr|B3KV72|B3KV72_HUMAN	tr|B3KV72|B3KV	cDNA FLJ16204 fis, clone CT	78791	579	677	28	50	0.04	0.02	0.5	0	1	1	1	1	1	1	1	-0.39569
sp|P27348|1433T_HUMAN	YWHAQ	14-3-3 protein theta OS=Ho	28032	258	816	74	38	0.4	0.2	0.5	2	1	3	1	3	2	3	2	-0.88153
tr|B2RCM2|B2RCM2_HUMA	tr|B2RCM2|B2RC	cDNA, FLJ96156, highly sim	135550	257	593	75	60	0.1	0.05	0.5	2	1	4	2	3	2	5	3	-0.88153
sp|Q9UHR4|BI2L1_HUMAN	BAIAP2L1	Brain-specific angiogenesis	57189	613	504	24	72	0.12	0.06	0.5	0	1	2	1	2	1	4	2	-1.13299
tr|B4DH52|B4DH52_HUMAN	tr|B4DH52|B4DH	cDNA FLJ55916, highly simi	112359	554	586	29	61	0.06	0.03	0.5	0	1	2	1	2	1	3	2	-1.13299
tr|G3V180|G3V180_HUMAN	DPP3	Dipeptidyl peptidase 3 OS=H	84666	335	1022	52	28	0.08	0.04	0.5	1	0	2	1	2	1	2	1	-1.13299
sp|Q9BYZ2|LDH6B_HUMAN	LDHAL6B	L-lactate dehydrogenase A-	42372	327	932	54	33	0.16	0.08	0.5	1	1	2	1	2	1	2	2	-1.13299
tr|A0MNN4|A0MNN4_HUM	SMU1	CDW3/SMU1 OS=Homo sap	58134	221	1067	94	25	0.12	0.06	0.5	1	0	2	1	2	1	2	2	-1.13299
sp|Q3SY84|K2C71_HUMAN	KRT71	Keratin, type II cytoskeletal	57769	372	902	47	34	0.12	0.06	0.5	2	1	2	1	3	1	4	2	-1.61874
tr|B0QYT3|B0QYT3_HUMAN	RANGAP1	Ran GTPase activating prote	27122	574	465	28	0	0.12	0.06	0.5	0	0	1	0	1	0	1	1	-1.9809
tr|E9PRA5|E9PRA5_HUMAN	ATP1A4	Sodium/potassium-transpo	57835	289	976	63	30	0.12	0.06	0.5	0	0	6	2	10	2	19	3	-2.46879
tr|B3KTA3|B3KTA3_HUMAN	tr|B3KTA3|B3KT	Fascin OS=Homo sapiens PE	52814	53	122	403	267	1.07	0.53	0.495327	9	7	14	9	16	9	20	12	-1.19509
tr|B0QZK4|B0QZK4_HUMAN	HP1BP3	Heterochromatin protein 1-	28725	631	809	23	39	0.25	0.12	0.48	0	1	2	1	1	1	3	1	-0.39569
tr|B3KNB4|B3KNB4_HUMAN	tr|B3KNB4|B3KN	cDNA FLJ14168 fis, clone NT	28453	541	747	30	44	0.25	0.12	0.48	0	1	2	1	2	2	2	2	-0.39577
sp|Q01546|K22O_HUMAN	KRT76	Keratin, type II cytoskeletal	66370	213	719	97	46	0.21	0.1	0.47619	4	2	4	2	5	2	9	3	-1.53426
tr|B4E102|B4E102_HUMAN	EIF4A1	Eukaryotic initiation factor	16061	52	409	146	90	1.62	0.77	0.475309	2	1	5	4	5	5	6	6	-0.39601
tr|B2R7M3|B2R7M3_HUMA	tr|B2R7M3|B2R7	cDNA, FLJ93510, highly sim	35767	308	1147	58	16	0.19	0.09	0.473684	2	0	2	1	2	0	2	1	-2.7182
tr|A0MNP2|A0MNP2_HUMA	WDR57	CDW11/WDR57 OS=Homo	39742	413	764	42	42	0.17	0.08	0.470588	1	1	2	1	2	3	3	4	0.089905
tr|B2R4P2|B2R4P2_HUMAN	tr|B2R4P2|B2R4	cDNA, FLJ92164, highly sim	22414	481	1004	36	0	0.32	0.15	0.46875	0	0	2	0	2	0	2	1	-2.7182
sp|P46087|NOP2_HUMAN	NOP2	Putative ribosomal RNA me	89589	296	471	61	77	0.15	0.07	0.466667	2	1	4	2	5	2	6	2	-1.53426
tr|B3KY95|B3KY95_HUMAN	tr|B3KY95|B3KY9	cDNA FLJ16143 fis, clone BF	53655	445	964	38	31	0.13	0.06	0.461538	0	0	2	1	2	1	2	1	-1.13299
tr|H0YDE4|H0YDE4_HUMAN	PRMT1	Protein arginine N-methyltr	27345	437	874	39	35	0.26	0.12	0.461538	1	1	2	1	2	1	2	2	-1.13299
sp|Q9NR12|PDLI7_HUMAN	PDLIM7	PDZ and LIM domain protei	50896	396	782	43	40	0.13	0.06	0.461538	0	1	2	1	2	1	4	1	-1.13299
tr|Q5U077|Q5U077_HUMAN	LDHB	L-lactate dehydrogenase OS	36900	39	147	530	237	1.81	0.83	0.458564	13	6	16	8	18	9	20	10	-1.36081
tr|B3KWR2|B3KWR2_HUMA	tr|B3KWR2|B3KW	cDNA FLJ43638 fis, clone SP	38087	573	518	22	70	0.11	0.05	0.454545	0	1	1	1	1	1	1	2	-0.39569
sp|P13645|K1C10_HUMAN	KRT10	Keratin, type I cytoskeletal	59020	12	29	1226	828	4.67	2.12	0.453961	34	19	42	23	47	25	54	25	-1.30229
sp|Q13867|BLMH_HUMAN	BLMH	Bleomycin hydrolase OS=Ho	53155	332	1122	52	20	0.2	0.09	0.45	1	0	3	1	2	2	4	2	-0.39577
sp|P19012|K1C15_HUMAN	KRT15	Keratin, type I cytoskeletal	49409	106	545	206	67	0.47	0.21	0.446809	8	2	9	3	12	3	13	6	-2.23531
tr|F2Z2U8|F2Z2U8_HUMAN	MYH14	Myosin04 OS=Homo sapien	232102	152	453	140	81	0.07	0.03	0.428571	5	2	5	2	7	3	8	3	-1.4967
tr|D3DQA6|D3DQA6_HUMA	DDX46	DEAD (Asp-Glu-Ala-Asp) bo	100958	595	1032	26	27	0.07	0.03	0.428571	0	0	2	1	3	1	3	1	-1.61874
tr|D3DTH7|D3DTH7_HUMAN	MYO1C	Myosin IC, isoform CRA_a C	99613	284	456	65	81	0.07	0.03	0.428571	1	1	3	1	3	1	4	3	-1.61874
tr|B4DW52|B4DW52_HUMA	tr|B4DW52|B4D	cDNA FLJ55253, highly simi	38950	4	2	3120	3340	27.29	11.52	0.422133	74	80	108	111	123	127	134	147	-0.35852
tr|B4DQX3|B4DQX3_HUMAN	tr|B4DQX3|B4DQ	cDNA FLJ58927, highly simi	18712	32	63	32	65	1.24	0.52	0.419355	0	1	1	1	1	2	1	2	0.341522
tr|H0YF33|H0YF33_HUMAN	NASP	Nuclear autoantigenic sperr	31883	542	1027	30	27	0.1	0.04	0.4	0	0	1	1	2	2	3	2	-0.39577
sp|P68032|ACTC_HUMAN	ACTC1	Actin, alpha cardiac muscle	42334	23	34	771	767	6.63	2.58	0.38914	25	28	40	38	48	46	55	49	-0.46068
sp|P07737|PROF1_HUMAN	PFN1	Profilin0 OS=Homo sapiens	15216	436	928	39	33	0.5	0.19	0.38	1	1	2	1	2	1	2	1	-1.13299
tr|D6RAT0|D6RAT0_HUMAN	RPS3A	40S ribosomal protein S3a (	26099	223	855	93	36	0.44	0.16	0.363636	2	1	3	1	4	1	5	1	-1.98164
sp|P78371|TCPB_HUMAN	CCT2	T-complex protein 1 subuni	57794	37	93	563	311	1.86	0.65	0.349462	14	8	20	11	20	14	23	16	-0.89826
sp|O60518|RNBP6_HUMAN	RANBP6	Ran-binding protein 6 OS=H	126173	85	935	36	32	0.23	0.08	0.347826	1	0	1	3	2	3	2	3	0.089905
tr|H0Y7N2|H0Y7N2_HUMAN	ATXN10	Ataxin00 (Fragment) OS=Ho	19107	470	289	37	0	0.38	0.13	0.342105	0	0	2	0	2	1	2	3	-1.13299
tr|K7EK77|K7EK77_HUMAN	ATP5A1	ATP synthase subunit alpha	22243	135	141	160	133	1.33	0.45	0.338346	5	3	6	4	6	4	6	4	-0.9271
tr|B3KY12|B3KY12_HUMAN	tr|B3KY12|B3KY1	cDNA FLJ46581 fis, clone TH	59082	495	643	34	55	0.18	0.06	0.333333	1	1	3	1	2	1	3	2	-1.13299
tr|Q6FHM2|Q6FHM2_HUMA	GNB2	GNB2 protein OS=Homo sap	38048	194	548	111	67	0.27	0.09	0.333333	1	1	2	1	2	1	2	1	-1.13299
tr|Q1KLZ0|Q1KLZ0_HUMAN	PS1TP5BP1	HCG15971, isoform CRA_a (	42052	1	1	3367	3408	31.43	10.25	0.326122	82	82	119	113	130	121	141	139	-0.51149

sp|Q562R1|ACTBL_HUMAN	ACTBL2	Beta-actin-like protein 2 OS	42318	61	89	349	328	1.47	0.46	0.312925	13	8	16	11	24	18	27	21	-0.80427
sp|P21796|VDAC1_HUMAN	VDAC1	Voltage-dependent anion-s	30868	250	514	78	70	0.36	0.11	0.305556	1	1	3	1	4	1	4	1	-1.98164
sp|O60884|DNJA2_HUMAN	DNAJA2	DnaJ homolog subfamily A	46344	611	1023	24	27	0.23	0.07	0.304348	0	0	1	1	1	1	1	1	-0.39569
tr|A5PLK7|A5PLK7_HUMAN	RCC2	RCC2 protein (Fragment) O	50331	165	487	131	75	0.46	0.14	0.304348	4	2	6	2	6	4	6	5	-0.9271
tr|A4ZU86|A4ZU86_HUMAN	NPM1	Truncated nucleolar phosp	30074	138	258	160	148	0.69	0.21	0.304348	4	3	5	3	5	3	5	3	-1.04858
sp|P04259|K2C6B_HUMAN	KRT6B	Keratin, type II cytoskeletal	60315	79	357	252	101	0.79	0.24	0.303797	10	4	13	5	15	5	17	5	-1.89406
tr|G3V2B8|G3V2B8_HUMAN	MTHFD1	C0-tetrahydrofolate syntha	102152	112	329	192	112	0.33	0.1	0.30303	5	3	9	3	12	5	12	6	-1.58273
tr|B3KM80|B3KM80_HUMAN	NCL	Nucleolin, isoform CRA_c O	58576	71	203	283	176	1.03	0.31	0.300971	9	4	14	5	15	6	15	8	-1.6528
sp|Q14651|PLSI_HUMAN	PLS1	Plastin0 OS=Homo sapiens	70608	282	660	57	52	0.34	0.1	0.294118	1	1	3	2	3	2	3	2	-0.88153
tr|H0YJ34|H0YJ34_HUMAN	FERMT2	Fermitin family homolog 2	73877	306	830	59	37	0.14	0.04	0.285714	1	1	3	1	3	3	3	4	-0.39585
sp|P46063|RECQ1_HUMAN	RECQL	ATP-dependent DNA helica	74436	439	780	39	41	0.14	0.04	0.285714	1	1	3	1	3	2	3	2	-0.88153
tr|F8VQP2|F8VQP2_HUMAN	ATXN2	Ataxin-2 OS=Homo sapiens	117012	157	335	135	107	0.21	0.06	0.285714	4	2	7	2	10	2	10	2	-2.46879
sp|P35527|K1C9_HUMAN	KRT9	Keratin, type I cytoskeletal	62255	14	88	1044	336	3.01	0.85	0.282392	25	7	36	12	40	15	44	17	-1.79069
sp|P11498|PYC_HUMAN	PC	Pyruvate carboxylase, mito	130293	10	81	1295	377	1.76	0.49	0.278409	40	7	52	17	56	18	67	20	-2.02032
tr|H6VRF8|H6VRF8_HUMAN	KRT1	Keratin 1 OS=Homo sapiens	66184	6	36	2032	761	5.01	1.39	0.277445	55	20	64	22	69	23	76	26	-1.97702
tr|H6VRG2|H6VRG2_HUMAN	KRT1	Keratin 1 OS=Homo sapiens	66197	7	37	2027	744	5.01	1.39	0.277445	55	19	65	22	70	23	77	26	-1.99796
tr|G5E9Q2|G5E9Q2_HUMAN	CSDE1	Cold shock domain contain	94539	312	585	58	61	0.11	0.03	0.272727	1	1	3	1	3	5	3	5	0.256725
sp|Q9NXW2|DJB12_HUMAN	DNAJB12	DnaJ homolog subfamily B	42021	124	353	174	101	0.57	0.15	0.263158	5	3	7	3	9	3	9	3	-1.83839
sp|Q05639|EF1A2_HUMAN	EEF1A2	Elongation factor 1-alpha 2	50780	70	279	238	131	1.14	0.29	0.254386	8	4	9	5	12	5	14	5	-1.58273
sp|P21281|VATB2_HUMAN	ATP6V1B2	V-type proton ATPase subu	56807	287	1028	64	27	0.65	0.16	0.246154	1	0	1	1	1	1	1	1	-0.39569
sp|Q92878|RAD50_HUMAN	RAD50	DNA repair protein RAD50	154823	563	730	29	45	0.09	0.02	0.222222	0	1	2	1	1	1	2	3	-0.39569
tr|Q05DV5|Q05DV5_HUMAN	ZC3HAV1	ZC3HAV1 protein (Fragmen	49964	224	338	92	105	0.14	0.03	0.214286	1	1	2	1	2	1	2	1	-1.13299
sp|P08779|K1C16_HUMAN	KRT16	Keratin, type I cytoskeletal	51578	62	372	342	98	1.38	0.28	0.202899	14	3	17	4	21	5	21	6	-2.3681
sp|P02533|K1C14_HUMAN	KRT14	Keratin, type I cytoskeletal	51872	33	137	604	247	2.22	0.45	0.202703	19	7	25	8	29	9	30	11	-2.03762
tr|B2RA03|B2RA03_HUMAN	tr|B2RA03|B2RA	cDNA, FLJ94640, highly sim	48000	127	715	88	46	0.36	0.07	0.194444	4	1	4	1	5	2	6	2	-1.53426
sp|Q58FF6|H90B4_HUMAN	HSP90AB4P	Putative heat shock protein	58855	32	399	77	92	1.24	0.24	0.193548	3	3	5	4	3	5	5	6	0.256725
tr|Q53G89|Q53G89_HUMAN	tr|Q53G89|Q53G	Elongation factor 1-alpha (F	47207	70	298	226	124	1.14	0.22	0.192982	7	3	8	3	13	4	16	5	-1.98385
tr|Q5U0Q1|Q5U0Q1_HUMAN	DKFZp686L1159	Putative uncharacterized pr	52189	294	712	62	47	0.58	0.11	0.189655	2	1	2	1	2	1	2	2	-1.13299
tr|Q5QTS3|Q5QTS3_HUMAN	tr|Q5QTS3|Q5QT	FWP004 OS=Homo sapiens	23704	300	814	59	38	0.74	0.14	0.189189	3	1	3	1	3	1	3	2	-1.61874
tr|H0Y5G9|H0Y5G9_HUMAN	NRD1	Nardilysin (Fragment) OS=H	62196	225	1052	92	0	0.11	0.02	0.181818	2	0	2	0	2	0	2	1	-2.7182
sp|P13647|K2C5_HUMAN	KRT5	Keratin, type II cytoskeletal	62568	58	663	378	52	0.95	0.17	0.178947	13	2	15	3	20	3	24	4	-2.95163
tr|B3KVL0|B3KVL0_HUMAN	tr|B3KVL0|B3KV	cDNA FLJ16691 fis, clone TF	65600	159	1084	133	24	0.28	0.05	0.178571	3	0	5	1	6	1	6	1	-2.51281
tr|B2R8Y4|B2R8Y4_HUMAN	tr|B2R8Y4|B2R8	cDNA, FLJ94117, highly sim	103871	19	99	99	304	2.11	0.36	0.170616	3	7	3	11	3	12	4	14	1.442896
sp|P35908|K22E_HUMAN	KRT2	Keratin, type II cytoskeletal	65678	11	50	1255	512	4.79	0.8	0.167015	41	12	48	14	49	15	57	18	-2.08318
tr|D3DTL4|D3DTL4_HUMAN	ENO3	Enolase OS=Homo sapiens	37167	40	262	112	119	1.39	0.22	0.158273	2	1	2	3	2	3	4	4	0.089905
tr|D6REX3|D6REX3_HUMAN	SEC31A	Protein transport protein S	137110	121	612	178	58	0.13	0.02	0.153846	4	1	5	1	4	1	5	2	-1.98164
tr|J3QQX0|J3QQX0_HUMAN	GPS1	COP9 signalosome complex	8703	636	525	22	69	0.4	0.06	0.15	0	1	1	1	1	1	1	1	-0.39569
tr|A8K2H9|A8K2H9_HUMAN	tr|A8K2H9|A8K2	cDNA FLJ78503, highly simi	49828	125	701	174	47	0.47	0.07	0.148936	5	1	6	1	8	1	10	2	-2.90049
tr|K0J110|K0J110_HUMAN	mcdrh	Malignant cell derived RNA	82499	148	1011	61	28	0.34	0.04	0.117647	2	0	2	1	2	1	2	1	-1.13299
tr|Q562L9|Q562L9_HUMAN	ACT	Actin-like protein (Fragmen	11549	1	146	147	239	31.43	2.69	0.085587	8	10	9	13	12	14	14	17	-0.18194
tr|E9PHF7|E9PHF7_HUMAN	MCCC1	Methylcrotonoyl-CoA carbo	68745	102	966	214	31	0.83	0.07	0.084337	7	0	13	1	11	1	16	1	-3.33757
tr|F8VP67|F8VP67_HUMAN	KRT8	Keratin, type II cytoskeletal	13580	6	395	50	83	5.01	0.41	0.081836	2	2	3	2	5	2	5	2	-1.53426
tr|Q562V5|Q562V5_HUMAN	ACT	Actin-like protein (Fragmen	11481	1	257	136	148	31.43	1.87	0.059497	6	7	6	8	7	9	8	10	-0.05463
tr|B2ZZ90|B2ZZ90_HUMAN	ACACA	Acetyl-Coenzyme A carboxy	267095	31	918	628	33	0.44	0.02	0.045455	25	0	30	2	38	2	46	2	-4.35248
sp|A6NHL2|TBAL3_HUMAN	TUBAL3	Tubulin alpha chain-like 3 C	50675	13	350	48	102	5.58	0.21	0.037634	2	4	2	4	3	5	3	5	0.256725
tr|Q562Q6|Q562Q6_HUMAN	ACT	Actin-like protein (Fragmen	11559	1	400	91	92	31.43	0.69	0.021954	2	2	5	6	5	7	6	8	0.05195
tr|F5H4R9|F5H4R9_HUMAN	DNM2	Dynamin-2 OS=Homo sapie	98218	713	0	16	0	0.03	0	0	0	0	1	0	0	0	2	0	-0.39561
sp|P54826|GAS1_HUMAN	GAS1	Growth arrest-specific prot	36867	708	0	17	0	0.09	0	0	0	0	1	0	0	0	1	0	-0.39561
tr|H0Y4R4|H0Y4R4_HUMAN	TOR1AIP1	Torsin0A-interacting protei	33844	707	0	17	0	0.1	0	0	0	0	1	0	0	0	1	0	-0.39561
tr|J3KMW7|J3KMW7_HUMAN	UBR5	E3 ubiquitin-protein ligase	312224	706	0	18	0	0.01	0	0	0	0	1	0	0	0	1	0	-0.39561
tr|Q2NLA0|Q2NLA0_HUMAN	COPG2	Coatomer subunit gamma	82400	705	0	18	0	0.04	0	0	0	0	1	0	0	0	1	0	-0.39561
tr|B0UZL4|B0UZL4_HUMAN	BAT3	HLA-B associated transcript	15422	704	0	18	0	0.03	0	0	0	0	1	0	0	0	1	0	-0.39561
tr|J3KQ96|J3KQ96_HUMAN	TCOF1	Treacle protein (Fragment)	144268	702	0	18	0	0.02	0	0	0	0	1	0	0	0	2	0	-0.39561
tr|B4DY47|B4DY47_HUMAN	PHB	Prohibitin OS=Homo sapien	17064	699	0	18	0	0.2	0	0	0	0	1	0	0	0	1	0	-0.39561
tr|I3L2H8|I3L2H8_HUMAN	MYBBP1A	Myb-binding protein 1A (Fr	22828	698	0	18	0	0.02	0	0	0	0	1	0	0	0	1	0	-0.39561
tr|F8W9S7|F8W9S7_HUMAN	GAPVD1	GTPase-activating protein a	158790	697	0	18	0	0.02	0	0	0	0	1	0	0	0	2	0	-0.39561
tr|Q5T097|Q5T097_HUMAN	UTRN	Utrophin OS=Homo sapiens	114423	696	0	19	0	0.03	0	0	0	0	1	0	0	0	1	0	-0.39561
tr|H0Y304|H0Y304_HUMAN	DMD	Dystrophin (Fragment) OS=	160176	695	0	19	0	0.02	0	0	0	0	1	0	0	0	1	0	-0.39561
tr|H0YE81|H0YE81_HUMAN	NDUFV1	NADH dehydrogenase [ubiq	14494	694	0	19	0	0.23	0	0	0	0	1	0	0	0	1	0	-0.39561
tr|B3KND7|B3KND7_HUMAN	tr|B3KND7|B3KN	cDNA FLJ14378 fis, clone HI	58221	692	0	19	0	0.06	0	0	0	0	1	0	0	0	1	0	-0.39561
tr|H7C4X2|H7C4X2_HUMAN	PCMT1	Protein-L-isoaspartate(D-as	7342	691	0	19	0	0.48	0	0	0	0	1	0	0	0	2	0	-0.39561
tr|D3DUL0|D3DUL0_HUMAN	ASCIZ	ATM/ATR-Substrate Chk2-I	79474	690	0	19	0	0.04	0	0	0	0	1	0	0	0	1	0	-0.39561
tr|G3V448|G3V448_HUMAN	TMX1	Thioredoxin-related transm	12018	689	0	19	0	0.29	0	0	0	0	1	0	0	0	1	0	-0.39561
tr|E9PQG7|E9PQG7_HUMAN	MLL	MLL cleavage product C180	436345	687	0	19	0	0.01	0	0	0	0	1	0	0	0	1	0	-0.39561
tr|G5E9Q3|G5E9Q3_HUMAN	TSEN2	tRNA splicing endonuclease	45959	686	0	19	0	0.07	0	0	0	0	1	0	0	0	1	0	-0.39561
sp|Q9Y2A7|NCKP1_HUMAN	NCKAP1	Nck-associated protein 1 O	130018	685	0	19	0	0.03	0	0	0	0	1	0	0	0	1	0	-0.39561
tr|Q9UH66|Q9UH66_HUMAN	tr|Q9UH66|Q9U	7h3 protein (Fragment) OS=	49963	684	0	19	0	0.07	0	0	0	0	1	0	0	0	2	0	-0.39561
tr|B4DZD5|B4DZD5_HUMAN	tr|B4DZD5|B4DZ	cDNA FLJ60991, highly simi	158783	683	0	20	0	0.02	0	0	0	0	1	0	0	0	2	0	-0.39561
tr|Q3MNE1|Q3MNE1_HUMAN	tr|Q3MNE1|Q3M	Adaptor-related protein co	89828	682	0	20	0	0.04	0	0	0	0	1	0	0	0	1	0	-0.39561
tr|J3KN33|J3KN33_HUMAN	CNTLN	Centlein OS=Homo sapiens	162069	680	0	20	0	0.02	0	0	0	0	1	0	0	0	2	0	-0.39561
tr|J3KTB7|J3KTB7_HUMAN	TOP2A	DNA topoisomerase 2-alph	17149	606	0	0	0	0.02	0	0	0	0	0	0	0	0	1	0	-0.39561
tr|H7C1T1|H7C1T1_HUMAN	Sep-02	Septin-2 (Fragment) OS=Ho	3094	543	0	0	0	0.09	0	0	0	0	0	0	0	0	1	0	-0.39561
tr|D6RD96|D6RD96_HUMAN	ACSL4	Long-chain-fatty-acid--CoA	16399	529	0	0	0	0.04	0	0	0	0	0	0	0	0	1	0	-0.39561
tr|B4DM94|B4DM94_HUMAN	tr|B4DM94|B4D	60S ribosomal protein L18a	17672	514	0	0	0	0.16	0	0	0	0	0	0	0	0	1	0	-0.39561
tr|H0YEU6|H0YEU6_HUMAN	PABPC4	Polyadenylate-binding prot	27445	504	0	0	0	0.05	0	0	0	0	0	0	0	0	1	0	-0.39561
tr|H7BYY8|H7BYY8_HUMAN	PES1	Pescadillo homolog OS=Hor	1501	488	0	0	0	0.05	0	0	0	0	0	0	0	0	1	0	-0.39561
tr|B8ZZ81|B8ZZ81_HUMAN	AGPS	Alkyldihydroxyacetonephos	20259	448	0	17	0	0.09	0	0	0	0	1	0	0	0	1	0	-0.39561
tr|E5RHM4|E5RHM4_HUMAN	DCAF13	DDB1- and CUL4-associated	20862	435	0	0	0	0.2	0	0	0	0	0	0	0	0	1	0	-0.39561
tr|H0Y386|H0Y386_HUMAN	PABPC1L	Polyadenylate-binding prot	16787	404	0	0	0	0.1	0	0	0	0	0	0	0	0	1	0	-0.39561
tr|A8K171|A8K171_HUMAN	tr|A8K171|A8K1	cDNA FLJ77253, highly simi	127609	401	0	0	0	0.01	0	0	0	0	0	0	0	0	1	0	-0.39561
tr|F8WF71|F8WF71_HUMAN	XPO1	Exportin0 OS=Homo sapien	9607	305	0	0	0	0.03	0	0	0	0	0	0	0	0	1	0	-0.39561
tr|I3L204|I3L204_HUMAN	MYO1C	Unconventional myosin-Ic	34968	284	0	0	0	0.07	0	0	0	0	0	0	0	0	1	0	-0.39561
tr|D6RAI5|D6RAI5_HUMAN	NNT	NAD(P) transhydrogenase,	23675	234	0	0	0	0.06	0	0	0	0	0	0	0	0	1	0	-0.39561
tr|E9PLJ0|E9PLJ0_HUMAN	IPO7	Importin-7 OS=Homo sapie	7255	162	0	0	0	0.08	0	0	0	0	0	0	0	0	1	0	-0.39561
tr|E9PQ69|E9PQ69_HUMAN	GANAB	Neutral alpha-glucosidase A	8560	47	0	0	0	0.62	0	0	0	0	0	0	0	0	1	0	-0.39561
tr|H7C2E7|H7C2E7_HUMAN	FLNA	Filamin-A (Fragment) OS=H	10032	9	0	0	0	0.86	0	0	0	0	0	0	0	0	1	0	-0.39561
tr|B4E2B5|B4E2B5_HUMAN	tr|B4E2B5|B4E2	cDNA FLJ53959, highly simi	43536	712	0	17	0	0.04	0	0	0	0	1	0	1	0	5	0	-1.9809
tr|Q8WUM5|Q8WUM5_HUMAN	GEMIN4	Gem (Nuclear organelle) as	121732	709	0	17	0	0.03	0	0	0	0	1	0	1	0	2	0	-1.9809
sp|O95159|ZFPL1_HUMAN	ZFPL1	Zinc finger protein-like 1 O	34833	678	0	20	0	0.1	0	0	0	0	1	0	1	0	1	0	-1.9809
tr|B4DE48|B4DE48_HUMAN	tr|B4DE48|B4DE	cDNA FLJ53824, highly simi	50009	677	0	20	0	0.07	0	0	0	0	1	0	1	0	1	0	-1.9809
sp|P55212|CASP6_HUMAN	CASP6	Caspase-6 OS=Homo sapier	33859	674	0	20	0	0.1	0	0	0	0	1	0	1	0	1	0	-1.9809
tr|C9JHK9|C9JHK9_HUMAN	ABCF2	ATP-binding cassette sub-fa	27027	673	0	20	0	0.05	0	0	0	0	1	0	1	0	2	0	-1.9809
tr|J3KNE5|J3KNE5_HUMAN	HARS	Histidine--tRNA ligase, cyto	50581	672	0	20	0	0.07	0	0	0	0	1	0	1	0	2	0	-1.9809
tr|H0YJL6|H0YJL6_HUMAN	EVL	Ena/VASP-like protein (Frag	10347	671	0	20	0	0.34	0	0	0	0	1	0	1	0	1	0	-1.9809
tr|A0AVG7|A0AVG7_HUMAN	BCAS1	Breast carcinoma amplified	61929	670	0	20	0	0.05	0	0	0	0	1	0	1	0	1	0	-1.9809
tr|B4DK80|B4DK80_HUMAN	TOP3A	DNA topoisomerase OS=Ho	103082	668	0	20	0	0.03	0	0	0	0	1	0	1	0	1	0	-1.9809
tr|G0ZJH6|G0ZJH6_HUMAN	Ube4B	UFD2A-III/UBE4B-III splice i	152890	667	0	21	0	0.02	0	0	0	0	1	0	1	0	1	0	-1.9809
tr|J3KN69|J3KN69_HUMAN	NCEH1	Arylacetamide deacetylase-	49431	663	0	21	0	0.07	0	0	0	0	1	0	1	0	1	0	-1.9809
tr|B3KRG8|B3KRG8_HUMAN	tr|B3KRG8|B3KR	Lipase OS=Homo sapiens PE	45736	662	0	21	0	0.07	0	0	0	0	1	0	1	0	1	0	-1.9809
tr|F1T0A4|F1T0A4_HUMAN	PRPF31	U4/U6 small nuclear ribonu	55217	661	0	21	0	0.06	0	0	0	0	1	0	1	0	1	0	-1.9809
tr|C9J935|C9J935_HUMAN	SETD9	SET domain-containing prot	5515	660	0	21	0	0.65	0	0	0	0	1	0	1	0	1	0	-1.9809
tr|Q5M7Z5|Q5M7Z5_HUMAN	GRHPR	GRHPR protein (Fragment)	37314	659	0	21	0	0.09	0	0	0	0	1	0	1	0	1	0	-1.9809
tr|H3BSK9|H3BSK9_HUMAN	ATXN2L	Ataxin-2-like protein (Fragn	37229	656	0	22	0	0.03	0	0	0	0	1	0	1	0	1	0	-1.9809
tr|B4DQ49|B4DQ49_HUMAN	tr|B4DQ49|B4DQ	cDNA FLJ54374, highly simi	31874	653	0	22	0	0.1	0	0	0	0	1	0	1	0	3	0	-1.9809
sp|Q96EK5|KBP_HUMAN	KIAA1279	KIF1-binding protein OS=Hc	72453	649	0	22	0	0.05	0	0	0	0	1	0	1	0	1	0	-1.9809
tr|E7ET15|E7ET15_HUMAN	U2SURP	U2 snRNP-associated SURP	118631	647	0	22	0	0.03	0	0	0	0	1	0	1	0	2	0	-1.9809
tr|D5FZU2|D5FZU2_HUMAN	HLA-B	MHC class I antigen (Fragm	21233	645	0	22	0	0.16	0	0	0	0	1	0	1	0	2	0	-1.9809
tr|E0YTJ8|E0YTJ8_HUMAN	HLA-B	MHC class I antigen (Fragm	21237	644	0	22	0	0.16	0	0	0	0	1	0	1	0	2	0	-1.9809
tr|E2DH89|E2DH89_HUMAN	HLA-B	MHC class I antigen (Fragm	21132	643	0	22	0	0.09	0	0	0	0	1	0	1	0	1	0	-1.9809
tr|F2X5X4|F2X5X4_HUMAN	HLA-A	MHC class I antigen (Fragm	21229	642	0	22	0	0.16	0	0	0	0	1	0	1	0	2	0	-1.9809
sp|Q9Y305|ACOT9_HUMAN	ACOT9	Acyl-coenzyme A thioester	50269	637	0	22	0	0.07	0	0	0	0	1	0	1	0	2	0	-1.9809
tr|Q5VSQ6|Q5VSQ6_HUMAN	P4HA1	Procollagen-proline, 2-oxog	61214	634	0	22	0	0.05	0	0	0	0	1	0	1	0	2	0	-1.9809
tr|B3KPR5|B3KPR5_HUMAN	tr|B3KPR5|B3KP	cDNA FLJ32094 fis, clone O	97046	633	0	22	0	0.03	0	0	0	0	1	0	1	0	1	0	-1.9809

sp|Q9Y5P6|GMPPB_HUMAN	GMPPB	Mannose0-phosphate guan	40379	629	0	23	0	0.08	0	0	0	0	1	0	1	0	1	0	-1.9809
tr|B4DFE4|B4DFE4_HUMAN	tr|B4DFE4|B4DF	cDNA FLJ59618, highly simi	61733	627	0	23	0	0.05	0	0	0	0	1	0	1	0	1	0	-1.9809
tr|K7EPB5|K7EPB5_HUMAN	ELAVL3	ELAV-like protein 3 (Fragme	9728	625	0	23	0	0.36	0	0	0	0	1	0	1	0	1	0	-1.9809
tr|B4DN13|B4DN13_HUMAN	tr|B4DN13|B4DN	cDNA FLJ59136, highly simi	29629	624	0	23	0	0.11	0	0	0	0	1	0	1	0	1	0	-1.9809
tr|B0QXZ5|B0QXZ5_HUMAN	DNAL4	Dynein light chain 4, axoner	7786	621	0	24	0	0.47	0	0	0	0	1	0	1	0	1	0	-1.9809
tr|B4DU06|B4DU06_HUMAN	tr|B4DU06|B4DU	Guanylate cyclase OS=Hom	91756	620	0	24	0	0.04	0	0	0	0	1	0	1	0	1	0	-1.9809
tr|Q2TQ40|Q2TQ40_HUMAN	TNFRSF17	B cell maturation antigen tr	15460	618	0	24	0	0.22	0	0	0	0	1	0	1	0	1	0	-1.9809
tr|Q4VAN1|Q4VAN1_HUMA	BTN1A1	BTN1A1 protein OS=Homo	55878	617	0	24	0	0.06	0	0	0	0	1	0	1	0	1	0	-1.9809
tr|B4E2P4|B4E2P4_HUMAN	tr|B4E2P4|B4E2I	cDNA FLJ58474, highly simi	50020	615	0	24	0	0.07	0	0	0	0	1	0	1	0	1	0	-1.9809
tr|H3BMW5|H3BMW5_HUM	DNAJA2	DnaJ homolog subfamily A	15134	611	0	24	0	0.23	0	0	0	0	1	0	1	0	1	0	-1.9809
tr|B7Z5E2|B7Z5E2_HUMAN	PSMC2	26S protease regulatory sub	33594	610	0	25	0	0.1	0	0	0	0	1	0	1	0	1	0	-1.9809
tr|J3KSB9|J3KSB9_HUMAN	tr|J3KSB9|J3KSB	Uncharacterized protein (Fr	8877	608	0	25	0	0.39	0	0	0	0	1	0	1	0	1	0	-1.9809
tr|J3KNP0|J3KNP0_HUMAN	RAB11FIP1	Rab11 family-interacting pr	137482	607	0	25	0	0.02	0	0	0	0	1	0	1	0	2	0	-1.9809
tr|B4E2I9|B4E2I9_HUMAN	tr|B4E2I9|B4E2I	cDNA FLJ55486, highly simi	88481	602	0	26	0	0.04	0	0	0	0	1	0	1	0	2	0	-1.9809
tr|D6R937|D6R937_HUMAN	AIMP1	Endothelial monocyte-activ	16568	601	0	26	0	0.11	0	0	0	0	1	0	1	0	1	0	-1.9809
sp|Q0VD83|APOBR_HUMAN	APOBR	Apolipoprotein B receptor (	115207	597	0	26	0	0.03	0	0	0	0	1	0	1	0	1	0	-1.9809
tr|Q5T8C6|Q5T8C6_HUMAN	CDC16	Cell division cycle protein 1	55239	594	0	26	0	0.06	0	0	0	0	1	0	1	0	1	0	-1.9809
tr|B7Z4L9|B7Z4L9_HUMAN	tr|B7Z4L9|B7Z4L	cDNA FLJ55342, highly simi	56884	593	0	26	0	0.06	0	0	0	0	1	0	1	0	1	0	-1.9809
tr|H0Y512|H0Y512_HUMAN	APMAP	Adipocyte plasma membrar	45662	592	0	27	0	0.07	0	0	0	0	1	0	1	0	1	0	-1.9809
tr|A6NE42|A6NE42_HUMAN	TGIF1	Homeobox protein TGIF1 O	27983	586	0	28	0	0.12	0	0	0	0	1	0	1	0	1	0	-1.9809
tr|H0YCU9|H0YCU9_HUMAN	TAGLN	Transgelin (Fragment) OS=H	16833	585	0	28	0	0.2	0	0	0	0	1	0	1	0	1	0	-1.9809
tr|J3KN32|J3KN32_HUMAN	RCOR1	REST corepressor 1 OS=Hor	53409	583	0	28	0	0.06	0	0	0	0	1	0	1	0	1	0	-1.9809
sp|Q14683|SMC1A_HUMAN	SMC1A	Structural maintenance of c	143771	581	0	28	0	0.02	0	0	0	0	1	0	1	0	1	0	-1.9809
sp|Q16322|KCA10_HUMAN	KCNA10	Potassium voltage-gated ch	58147	578	0	28	0	0.06	0	0	1	0	1	0	1	0	1	0	-1.9809
tr|B4DNG6|B4DNG6_HUMA	tr|B4DNG6|B4DI	Annexin OS=Homo sapiens	12870	577	0	28	0	0.27	0	0	0	0	1	0	1	0	1	0	-1.9809
tr|C9JYI4|C9JYI4_HUMAN	KPNA1	Importin subunit alpha0 (Fr	37206	573	0	22	0	0.11	0	0	0	0	1	0	1	0	1	0	-1.9809
tr|J3KQE5|J3KQE5_HUMAN	RAN	GTP-binding nuclear protei	27027	571	0	29	0	0.12	0	0	0	0	1	0	1	0	1	0	-1.9809
tr|I3L361|I3L361_HUMAN	SGSM2	Small G protein-signaling m	1642	570	0	29	0	2.38	0	0	0	0	1	0	1	0	1	0	-1.9809
sp|Q6KB66|K2C80_HUMAN	KRT80	Keratin, type II cytoskeletal	51007	564	0	29	0	0.06	0	0	0	0	1	0	1	0	1	0	-1.9809
tr|E7EN38|E7EN38_HUMAN	RAD50	DNA repair protein RAD50 (	77138	563	0	29	0	0.09	0	0	0	0	2	0	1	0	2	0	-1.9809
tr|B4DKT5|B4DKT5_HUMAN	tr|B4DKT5|B4DK	cDNA FLJ53054, highly simi	33360	556	0	29	0	0.05	0	0	0	0	1	0	1	0	1	0	-1.9809
tr|C9JJE5|C9JJE5_HUMAN	IDH1	Isocitrate dehydrogenase [I	7068	555	0	23	0	0.15	0	0	0	0	1	0	1	0	1	0	-1.9809
tr|H0YK49|H0YK49_HUMAN	ETFA	Electron transfer flavoprote	24259	545	0	30	0	0.14	0	0	0	0	1	0	1	0	1	0	-1.9809
tr|K7EKM4|K7EKM4_HUMAI	SRM	Spermidine synthase OS=H	10680	540	0	30	0	0.32	0	0	0	0	1	0	1	0	1	0	-1.9809
sp|Q96PE2|ARHGH_HUMAN	ARHGEF17	Rho guanine nucleotide exc	223645	537	0	30	0	0.01	0	0	1	0	1	0	1	0	1	0	-1.9809
tr|B7Z9W6|B7Z9W6_HUMAI	tr|B7Z9W6|B7Z9	cDNA, FLJ78980, highly sim	50776	527	0	31	0	0.06	0	0	0	0	1	0	1	0	1	0	-1.9809
tr|F2Z2J7|F2Z2J7_HUMAN	TTLL7	Tubulin polyglutamylase TT	55925	526	0	31	0	0.06	0	0	1	0	1	0	1	0	1	0	-1.9809
tr|B4E0D5|B4E0D5_HUMAN	tr|B4E0D5|B4E0	cDNA FLJ54218, highly simi	19540	525	0	31	0	0.17	0	0	0	0	1	0	1	0	1	0	-1.9809
tr|L0R4T3|L0R4T3_HUMAN	ABCF2	Alternative protein ABCF2 (	7359	524	0	31	0	0.48	0	0	1	0	1	0	1	0	1	0	-1.9809
tr|E9PNN6|E9PNN6_HUMAN	ALDH3A1	Aldehyde dehydrogenase, c	44518	519	0	32	0	0.07	0	0	0	0	1	0	1	0	1	0	-1.9809
tr|B4DUH7|B4DUH7_HUMAI	tr|B4DUH7|B4DI	cDNA FLJ60162, highly simi	52632	518	0	32	0	0.06	0	0	1	0	1	0	1	0	1	0	-1.9809
tr|J3KRB3|J3KRB3_HUMAN	RPL17	60S ribosomal protein L17 (	12356	517	0	32	0	0.28	0	0	0	0	1	0	1	0	1	0	-1.9809
tr|A5PHJ9|A5PHJ9_HUMAN	PDHA1/LOC7906	PDHA1/LOC79064 protein (	34113	516	0	32	0	0.1	0	0	0	0	1	0	1	0	1	0	-1.9809
tr|A0JLS9|A0JLS9_HUMAN	ZEB1	ZEB1 protein (Fragment) OS	118576	513	0	32	0	0.03	0	0	1	0	1	0	1	0	1	0	-1.9809
sp|Q7Z494|NPHP3_HUMAN	NPHP3	Nephrocystin-3 OS=Homo s	151967	510	0	32	0	0.02	0	0	1	0	1	0	1	0	1	0	-1.9809
sp|Q9P287|BCCIP_HUMAN	BCCIP	BRCA2 and CDKN1A-interac	36299	503	0	33	0	0.09	0	0	0	0	1	0	1	0	3	0	-1.9809
sp|Q8NFZ5|TNIP2_HUMAN	TNIP2	TNFAIP3-interacting protein	49240	502	0	33	0	0.07	0	0	1	0	1	0	1	0	2	0	-1.9809
tr|B4DXJ6|B4DXJ6_HUMAN	tr|B4DXJ6|B4DX	cDNA FLJ61453, highly simi	55511	500	0	34	0	0.03	0	0	0	0	1	0	1	0	1	0	-1.9809
tr|B4DDC9|B4DDC9_HUMAN	tr|B4DDC9|B4DI	cDNA FLJ59448, highly simi	49937	499	0	34	0	0.07	0	0	0	0	1	0	1	0	1	0	-1.9809
sp|Q6PGP7|TTC37_HUMAN	TTC37	Tetratricopeptide repeat pr	177485	498	0	34	0	0.02	0	0	1	0	1	0	1	0	1	0	-1.9809
tr|E9PI04|E9PI04_HUMAN	RIC8A	Synembryn-A (Fragment) O	16936	496	0	34	0	0.05	0	0	0	0	1	0	1	0	1	0	-1.9809
tr|C9JNW5|C9JNW5_HUMAI	RPL24	60S ribosomal protein L24 (	17646	487	0	35	0	0.19	0	0	0	0	1	0	1	0	2	0	-1.9809
tr|Q68E00|Q68E00_HUMAN	DKFZp686G2045	Putative uncharacterized pr	32787	485	0	36	0	0.1	0	0	0	0	1	0	1	0	1	0	-1.9809
tr|B7WPL0|B7WPL0_HUMAI	RIC8B	Synembryn-B OS=Homo sap	64252	483	0	36	0	0.05	0	0	1	0	1	0	1	0	1	0	-1.9809
tr|E9PJH4|E9PJH4_HUMAN	RPS3	40S ribosomal protein S3 O	13047	480	0	36	0	0.12	0	0	1	0	1	0	1	0	2	0	-1.9809
tr|D6R9W4|D6R9W4_HUMA	DBN1	Drebrin (Fragment) OS=Hor	36677	478	0	36	0	0.05	0	0	0	0	1	0	1	0	1	0	-1.9809
tr|H0YIX9|H0YIX9_HUMAN	R3HDM2	R3H domain-containing pro	31776	469	0	37	0	0.1	0	0	1	0	1	0	1	0	1	0	-1.9809
tr|C9J7L0|C9J7L0_HUMAN	RBBP7	Histone-binding protein RB	7740	468	0	0	0	0.07	0	0	0	0	0	0	1	0	1	0	-1.9809
tr|B4DDP6|B4DDP6_HUMAN	DBNL	Drebrin-like protein OS=Ho	43242	466	0	37	0	0.08	0	0	1	0	1	0	1	0	1	0	-1.9809
sp|Q6ZU64|CC108_HUMAN	CCDC108	Coiled-coil domain-contain	220247	465	0	37	0	0.01	0	0	1	0	1	0	1	0	1	0	-1.9809
tr|B3KUJ0|B3KUJ0_HUMAN	tr|B3KUJ0|B3KU	cDNA FLJ39996 fis, clone ST	44512	464	0	37	0	0.07	0	0	1	0	1	0	1	0	2	0	-1.9809
tr|H7C1M0|H7C1M0_HUMA	FANCL	E3 ubiquitin-protein ligase I	33067	461	0	37	0	0.1	0	0	1	0	1	0	1	0	1	0	-1.9809
sp|Q9H9F9|ARP5_HUMAN	ACTR5	Actin-related protein 5 OS=	68825	460	0	38	0	0.05	0	0	1	0	1	0	1	0	2	0	-1.9809
tr|B3KME2|B3KME2_HUMAI	tr|B3KME2|B3KI	cDNA FLJ10772 fis, clone NT	47101	459	0	38	0	0.07	0	0	1	0	1	0	1	0	1	0	-1.9809
tr|H0Y9L5|H0Y9L5_HUMAN	EXOSC9	Exosome complex compone	18940	457	0	38	0	0.18	0	0	1	0	1	0	1	0	1	0	-1.9809
tr|B4E363|B4E363_HUMAN	FARSA	Phenylalanine--tRNA ligase	54181	447	0	38	0	0.06	0	0	1	0	1	0	1	0	1	0	-1.9809
tr|B7Z943|B7Z943_HUMAN	tr|B7Z943|B7Z94	cDNA FLJ53131, highly simi	29093	442	0	39	0	0.05	0	0	1	0	1	0	1	0	2	0	-1.9809
tr|E9PI83|E9PI83_HUMAN	PRMT1	Protein arginine N-methyltr	12790	437	0	36	0	0.26	0	0	0	0	1	0	1	0	1	0	-1.9809
tr|K7EJ44|K7EJ44_HUMAN	PFN1	Profilin 1, isoform CRA_b O	11497	436	0	23	0	0.5	0	0	0	0	1	0	1	0	1	0	-1.9809
tr|A4D1I7|A4D1I7_HUMAN	DNCI1	Dynein, cytoplasmic 1, inte	73423	427	0	40	0	0.04	0	0	1	0	1	0	1	0	1	0	-1.9809
tr|Q5JR94|Q5JR94_HUMAN	RPS8	40S ribosomal protein S8 O	24475	426	0	41	0	0.14	0	0	1	0	1	0	1	0	2	0	-1.9809
tr|B4DPY2|B4DPY2_HUMAN	tr|B4DPY2|B4DP	cDNA FLJ59286, highly simi	93124	425	0	41	0	0.04	0	0	1	0	1	0	1	0	1	0	-1.9809
tr|B4DLD4|B4DLD4_HUMAN	tr|B4DLD4|B4DL	cDNA FLJ56738, highly simi	30279	423	0	41	0	0.11	0	0	1	0	1	0	1	0	1	0	-1.9809
tr|O60250|O60250_HUMAN	tr|O60250|O602	Ribosomal protein L13 (Frag	7385	422	0	41	0	0.14	0	0	1	0	1	0	1	0	1	0	-1.9809
tr|A0JLS5|A0JLS5_HUMAN	TBL3	TBL3 protein (Fragment) OS	64079	418	0	42	0	0.05	0	0	1	0	1	0	1	0	1	0	-1.9809
tr|H0YGE9|H0YGE9_HUMAN	M6PR	Cation-dependent mannose	11814	416	0	42	0	0.29	0	0	1	0	1	0	1	0	1	0	-1.9809
tr|B4DRG7|B4DRG7_HUMAN	tr|B4DRG7|B4DF	cDNA FLJ60336, highly simi	80877	410	0	43	0	0.04	0	0	1	0	1	0	1	0	1	0	-1.9809
tr|B3KMW5|B3KMW5_HUM	tr|B3KMW5|B3K	cDNA FLJ12796 fis, clone NT	71519	408	0	43	0	0.05	0	0	1	0	1	0	1	0	1	0	-1.9809
tr|E9PCI9|E9PCI9_HUMAN	FDPS	Farnesyl pyrophosphate syr	40849	405	0	43	0	0.08	0	0	1	0	1	0	1	0	1	0	-1.9809
tr|Q1LZN2|Q1LZN2_HUMAN	NOMO3	NOMO3 protein (Fragment	90388	400	0	43	0	0.04	0	0	1	0	1	0	1	0	1	0	-1.9809
tr|H0Y327|H0Y327_HUMAN	MTHFD1L	Monofunctional C1-tetrahy	36808	398	0	43	0	0.09	0	0	1	0	1	0	1	0	1	0	-1.9809
tr|Q6W6M7|Q6W6M7_HUM	tr|Q6W6M7|Q6W	Antigen MLAA-43 (Fragmen	23572	397	0	0	0	0.05	0	0	0	0	0	0	1	0	1	0	-1.9809
tr|D6RAN1|D6RAN1_HUMAI	PDLIM7	PDZ and LIM domain protei	9510	396	0	33	0	0.13	0	0	0	0	1	0	1	0	2	0	-1.9809
tr|B4DNB2|B4DNB2_HUMAI	tr|B4DNB2|B4DI	cDNA FLJ58919, moderately	14636	393	0	0	0	0.1	0	0	0	0	0	0	1	0	2	0	-1.9809
tr|E5RGY7|E5RGY7_HUMAN	CTNNA1	Catenin alpha0 (Fragment)	14895	389	0	35	0	0.07	0	0	0	0	1	0	1	0	1	0	-1.9809
tr|B3KPZ0|B3KPZ0_HUMAN	tr|B3KPZ0|B3KP	cDNA FLJ32485 fis, clone SK	121468	387	0	45	0	0.03	0	0	1	0	1	0	1	0	1	0	-1.9809
sp|Q8NFI4|F10A5_HUMAN	ST13P5	Putative protein FAM10A5	41579	382	0	0	0	0.09	0	0	0	0	0	0	1	0	2	0	-1.9809
tr|B4DT09|B4DT09_HUMAN	tr|B4DT09|B4DT	cDNA FLJ60145, highly simi	13697	381	0	46	0	0.25	0	0	1	0	1	0	1	0	1	0	-1.9809
tr|H0YGH1|H0YGH1_HUMAI	PFAS	Phosphoribosylformylglycin	41755	380	0	37	0	0.05	0	0	0	0	1	0	1	0	1	0	-1.9809
tr|H0YCD9|H0YCD9_HUMAN	CTTN	Src substrate cortactin (Frag	29938	378	0	0	0	0.11	0	0	0	0	0	0	1	0	1	0	-1.9809
tr|B3KU96|B3KU96_HUMAN	tr|B3KU96|B3KU	cDNA FLJ39433 fis, clone PF	17856	371	0	47	0	0.19	0	0	1	0	1	0	1	0	1	0	-1.9809
tr|Q5TCC5|Q5TCC5_HUMAN	IARS	Isoleucine--tRNA ligase, cyt	19001	366	0	47	0	0.02	0	0	1	0	1	0	1	0	1	0	-1.9809
tr|K7EM16|K7EM16_HUMAI	VASP	Vasodilator-stimulated pho	20260	363	0	48	0	0.17	0	0	1	0	1	0	1	0	1	0	-1.9809
tr|D6RJF2|D6RJF2_HUMAN	PAIP1	Polyadenylate-binding prot	22826	361	0	48	0	0.08	0	0	1	0	1	0	1	0	1	0	-1.9809
tr|D6RBX4|D6RBX4_HUMAN	TGFBI	Transforming growth factor	7268	358	0	31	0	0.09	0	0	0	0	1	0	1	0	1	0	-1.9809
tr|B3KMV8|B3KMV8_HUMA	tr|B3KMV8|B3KI	cDNA FLJ12766 fis, clone NT	75130	353	0	41	0	0.11	0	0	1	0	1	0	1	0	1	0	-1.9809
tr|B3KRS7|B3KRS7_HUMAN	tr|B3KRS7|B3KR	cDNA FLJ34844 fis, clone NT	34946	351	0	50	0	0.09	0	0	1	0	1	0	1	0	1	0	-1.9809
tr|C9JQM9|C9JQM9_HUMAI	DARS	Aspartate--tRNA ligase, cyto	15386	344	0	51	0	0.06	0	0	1	0	1	0	1	0	1	0	-1.9809
tr|E5RFU4|E5RFU4_HUMAN	DPYSL2	Dihydropyrimidinase-relate	18320	342	0	20	0	0.23	0	0	0	0	1	0	1	0	1	0	-1.9809
tr|H7C5I9|H7C5I9_HUMAN	GART	Phosphoribosylformylglycin	2640	341	0	23	0	0.13	0	0	0	0	1	0	1	0	1	0	-1.9809
tr|A8K517|A8K517_HUMAN	RPS23	Ribosomal protein S23, isof	15969	339	0	52	0	0.21	0	0	1	0	1	0	1	0	1	0	-1.9809
tr|A8KA82|A8KA82_HUMAN	DNAJC3	DnaJ (Hsp40) homolog, sub	58000	337	0	52	0	0.06	0	0	1	0	1	0	1	0	1	0	-1.9809
sp|Q9Y6M5|ZNT1_HUMAN	SLC30A1	Zinc transporter 1 OS=Hom	56290	334	0	52	0	0.06	0	0	1	0	1	0	1	0	1	0	-1.9809
tr|Q59GV0|Q59GV0_HUMAI	tr|Q59GV0|Q59(	Propionyl Coenzyme A carb	60578	331	0	53	0	0.05	0	0	1	0	1	0	1	0	2	0	-1.9809
tr|A8K3Q9|A8K3Q9_HUMAN	tr|A8K3Q9|A8K3	cDNA FLJ76611, highly simi	23554	330	0	53	0	0.14	0	0	1	0	1	0	1	0	1	0	-1.9809
tr|I3L4P6|I3L4P6_HUMAN	ARHGAP17	Rho GTPase-activating prot	8676	328	0	54	0	0.03	0	0	1	0	1	0	1	0	1	0	-1.9809
sp|O95831|AIFM1_HUMAN	AIFM1	Apoptosis-inducing factor 1	67144	326	0	54	0	0.05	0	0	1	0	1	0	1	0	2	0	-1.9809
tr|C9JIX5|C9JIX5_HUMAN	PLOD3	Procollagen-lysine,2-oxoglu	8424	325	0	41	0	0.12	0	0	1	0	1	0	1	0	1	0	-1.9809
tr|C9JPP3|C9JPP3_HUMAN	RBBP4	Histone-binding protein RB	9655	315	0	57	0	0.36	0	0	1	0	1	0	1	0	1	0	-1.9809
tr|J3KRP2|J3KRP2_HUMAN	PSMC5	26S protease regulatory sub	27292	314	0	57	0	0.11	0	0	1	0	1	0	1	0	1	0	-1.9809
sp|Q5TZA2|CROCC_HUMAN	CROCC	Rootletin OS=Homo sapiens	228787	310	0	58	0	0.01	0	0	1	0	1	0	1	0	2	0	-1.9809
tr|C9JRL4|C9JRL4_HUMAN	MDH1	Malate dehydrogenase, cyt	13207	309	0	39	0	0.31	0	0	1	0	1	0	1	0	1	0	-1.9809
tr|B3KPC1|B3KPC1_HUMAN	tr|B3KPC1|B3KP	cDNA FLJ31582 fis, clone NT	43807	307	0	59	0	0.08	0	0	1	0	1	0	1	0	1	0	-1.9809

tr|B2RAX1|B2RAX1_HUMAN	tr|B2RAX1|B2RA	cDNA, FLJ95169 OS=Homo	77979	306	0	41	0	0.14	0	0	1	0	1	0	1	0	1	0	-1.9809
tr|H3BP76|H3BP76_HUMAN	MVP	Major vault protein (Fragm	10921	304	0	38	0	0.07	0	0	1	0	1	0	1	0	1	0	-1.9809
tr|B4DX93|B4DX93_HUMAN	tr|B4DX93|B4DX	cDNA FLJ55471, highly simi	40089	298	0	40	0	0.09	0	0	1	0	1	0	1	0	1	0	-1.9809
tr|Q5T0I0|Q5T0I0_HUMAN	GSN	Gelsolin (Fragment) OS=Ho	29107	297	0	61	0	0.04	0	0	1	0	1	0	1	0	2	0	-1.9809
sp|P81605|DCD_HUMAN	DCD	Dermcidin OS=Homo sapier	11391	295	0	62	0	0.3	0	0	1	0	1	0	1	0	1	0	-1.9809
tr|H0YC45|H0YC45_HUMAN	ATP6V1B2	V-type proton ATPase subu	5569	287	0	64	0	0.65	0	0	1	0	1	0	1	0	1	0	-1.9809
tr|E5RFS9|E5RFS9_HUMAN	MAT2B	Methionine adenosyltransf	9467	280	0	68	0	0.37	0	0	1	0	1	0	1	0	1	0	-1.9809
tr|B4DXK4|B4DXK4_HUMAN	KRT72	Keratin, type II cytoskeletal	36031	259	0	0	0	0.45	0	0	0	0	0	0	1	0	1	0	-1.9809
tr|Q2HJC8|Q2HJC8_HUMAN	HDLBP	HDLBP protein (Fragment)	65655	253	0	0	0	0.02	0	0	0	0	0	0	1	0	1	0	-1.9809
tr|Q5JXB7|Q5JXB7_HUMAN	PLOD1	Procollagen-lysine,2-oxoglu	31911	251	0	76	0	0.04	0	0	1	0	1	0	1	0	1	0	-1.9809
tr|B4DE40|B4DE40_HUMAN	SMS	Spermine synthase OS=Hon	31596	248	0	78	0	0.11	0	0	1	0	1	0	1	0	1	0	-1.9809
tr|H0YGZ5|H0YGZ5_HUMAN	PRPF19	Pre-mRNA-processing facto	13367	237	0	62	0	0.26	0	0	1	0	1	0	1	0	1	0	-1.9809
tr|E9PLA6|E9PLA6_HUMAN	SERPINH1	Serpin H1 (Fragment) OS=H	8925	233	0	45	0	0.43	0	0	1	0	1	0	1	0	1	0	-1.9809
tr|H0Y960|H0Y960_HUMAN	DDX4	Probable ATP-dependent R	33292	217	0	43	0	0.19	0	0	1	0	1	0	1	0	1	0	-1.9809
sp|Q5JQF8|PAP1M_HUMAN	PABPC1L2A	Polyadenylate-binding prot	22956	215	0	43	0	0.33	0	0	1	0	1	0	1	0	1	0	-1.9809
tr|H7C2Q3|H7C2Q3_HUMAN	PSMD2	26S proteasome non-ATPas	16652	198	0	31	0	0.14	0	0	0	0	1	0	1	0	1	0	-1.9809
tr|E7ERW8|E7ERW8_HUMAN	DIAPH1	Protein diaphanous homolo	135945	184	0	116	0	0.02	0	0	1	0	1	0	1	0	2	0	-1.9809
tr|J3KSA9|J3KSA9_HUMAN	PYCR1	Pyrroline-5-carboxylate red	11860	174	0	0	0	0.19	0	0	0	0	0	0	1	0	1	0	-1.9809
tr|K7EQ75|K7EQ75_HUMAN	ILF3	Interleukin enhancer-bindir	6139	154	0	29	0	0.46	0	0	0	0	1	0	1	0	1	0	-1.9809
tr|G3V423|G3V423_HUMAN	WARS	T1-TrpRS (Fragment) OS=Ho	15836	145	0	34	0	0.27	0	0	1	0	1	0	1	0	1	0	-1.9809
tr|Q9BTI9|Q9BTI9_HUMAN	NPM1	NPM1 protein (Fragment) C	25147	138	0	46	0	0.69	0	0	1	0	1	0	1	0	1	0	-1.9809
tr|F8WDK9|F8WDK9_HUMA	PDCD6IP	Programmed cell death 6-ir	10956	136	0	61	0	0.18	0	0	1	0	1	0	1	0	1	0	-1.9809
tr|Q659G2|Q659G2_HUMAN	DKFZp586B2023	Putative uncharacterized pr	7133	124	0	41	0	0.57	0	0	1	0	1	0	1	0	1	0	-1.9809
tr|K7EIL4|K7EIL4_HUMAN	GPI	Glucose-6-phosphate isome	12790	117	0	0	0	0.3	0	0	0	0	0	0	1	0	1	0	-1.9809
tr|Q9BR23|Q9BR23_HUMAN	tr|Q9BR23|Q9BR	Putative uncharacterized pr	23788	104	0	44	0	0.41	0	0	1	0	1	0	1	0	1	0	-1.9809
tr|D6RCS6|D6RCS6_HUMAN	TARS	Threonine--tRNA ligase, cyt	6912	94	0	53	0	0.64	0	0	1	0	1	0	1	0	1	0	-1.9809
tr|H0YBG6|H0YBG6_HUMAN	HSPA9	Stress-70 protein, mitochor	18491	81	0	38	0	0.42	0	0	1	0	1	0	1	0	1	0	-1.9809
tr|C9J4X2|C9J4X2_HUMAN	NONO	Non-POU domain-containir	14519	75	0	31	0	0.8	0	0	0	0	1	0	1	0	2	0	-1.9809
tr|C9JUP7|C9JUP7_HUMAN	VCP	Transitional endoplasmic re	13400	64	0	73	0	0.65	0	0	1	0	1	0	1	0	1	0	-1.9809
tr|Q8WXT3|Q8WXT3_HUMA	FLNB	Filamin B variant 2 (Fragme	7949	45	0	0	0	0.22	0	0	0	0	0	0	1	0	2	0	-1.9809
tr|K7ENV3|K7ENV3_HUMAN	KRT16	Keratin, type I cytoskeletal	4775	33	0	48	0	2.22	0	0	1	0	1	0	1	0	1	0	-1.9809
tr|B7Z2Y4|B7Z2Y4_HUMAN	tr|B7Z2Y4|B7Z2Y	cDNA FLJ56961, highly simi	54629	30	0	60	0	0.52	0	0	1	0	1	0	1	0	2	0	-1.9809
sp|O14556|G3PT_HUMAN	GAPDHS	Glyceraldehyde-3-phosphat	44815	18	0	32	0	3.44	0	0	0	0	1	0	1	0	1	0	-1.9809
tr|A1L2Z2|A1L2Z2_HUMAN	MYH14	MYH14 protein OS=Homo s	73418	17	0	56	0	0.9	0	0	1	0	1	0	1	0	1	0	-1.9809
tr|B9VP24|B9VP24_HUMAN	HSPD1	60 kDa chaperonin (Fragme	19880	16	0	40	0	1.72	0	0	1	0	1	0	1	0	1	0	-1.9809
tr|D5FHQ7|D5FHQ7_HUMAN	HLA-A	MHC class I antigen (Fragm	21188	640	0	22	0	0.16	0	0	0	0	1	0	2	0	3	0	-2.7182
tr|F8SKU2|F8SKU2_HUMAN	HLA-A	MHC class I antigen (Fragm	21143	639	0	22	0	0.16	0	0	0	0	1	0	2	0	3	0	-2.7182
tr|D3DSQ0|D3DSQ0_HUMAN	PCM1	Pericentriolar material 1, is	230041	619	0	24	0	0.01	0	0	0	0	1	0	2	0	3	0	-2.7182
sp|Q9NRF8|PYRG2_HUMAN	CTPS2	CTP synthase 2 OS=Homo s	66320	614	0	24	0	0.05	0	0	0	0	1	0	2	0	6	0	-2.7182
tr|D6RJA6|D6RJA6_HUMAN	DDX46	DEAD (Asp-Glu-Ala-Asp) bo	54216	595	0	22	0	0.07	0	0	0	0	1	0	2	0	2	0	-2.7182
tr|H3BNV2|H3BNV2_HUMAN	NQO1	NAD(P)H dehydrogenase [q	28533	576	0	28	0	0.25	0	0	0	0	2	0	2	0	2	0	-2.7182
tr|A6NLN1|A6NLN1_HUMAN	PTBP1	Polypyrimidine tract bindin	21899	567	0	26	0	0.18	0	0	0	0	2	0	2	0	2	0	-2.7182
tr|B2RD27|B2RD27_HUMAN	tr|B2RD27|B2RD	cDNA, FLJ96428, highly sim	37046	550	0	30	0	0.19	0	0	0	0	2	0	2	0	2	0	-2.7182
tr|C1KES1|C1KES1_HUMAN	LRRC16B	LRRC16B (Fragment) OS=Ho	4125	530	0	31	0	0.94	0	0	0	0	1	0	2	0	2	0	-2.7182
tr|A0PJ76|A0PJ76_HUMAN	NCAPD2	NCAPD2 protein (Fragment	44957	494	0	34	0	0.15	0	0	0	0	2	0	2	0	2	0	-2.7182
sp|Q14432|PDE3A_HUMAN	PDE3A	cGMP-inhibited 3~,5~-cyclic	126440	493	0	35	0	0.05	0	0	1	0	2	0	2	0	2	0	-2.7182
tr|A5D904|A5D904_HUMAN	RPS9	RPS9 protein (Fragment) OS	13210	434	0	40	0	0.58	0	0	1	0	2	0	2	0	2	0	-2.7182
tr|E7EVA0|E7EVA0_HUMAN	MAP4	Microtubule-associated pro	246546	429	0	40	0	0.01	0	0	1	0	1	0	2	0	4	0	-2.7182
tr|C9J1B1|C9J1B1_HUMAN	NAP1L4	Nucleosome assembly prot	11455	417	0	31	0	0.16	0	0	0	0	1	0	2	0	2	0	-2.7182
tr|B4DE72|B4DE72_HUMAN	tr|B4DE72|B4DE	cDNA FLJ50078, highly simi	106663	415	0	41	0	0.04	0	0	0	0	2	0	2	0	4	0	-2.7182
sp|O00505|IMA3_HUMAN	KPNA3	Importin subunit alpha-3 O	58288	406	0	43	0	0.12	0	0	0	0	2	0	2	0	2	0	-2.7182
tr|H0YIG3|H0YIG3_HUMAN	KRT72	Keratin, type II cytoskeletal	21922	372	0	39	0	0.12	0	0	1	0	1	0	2	0	2	0	-2.7182
tr|B7ZB91|B7ZB91_HUMAN	TNRC6B	Trinucleotide repeat contai	81825	359	0	48	0	0.13	0	0	1	0	3	0	2	0	3	0	-2.7182
tr|B4DUL5|B4DUL5_HUMAN	tr|B4DUL5|B4DU	cDNA FLJ51625, highly simi	40917	357	0	48	0	0.17	0	0	1	0	2	0	2	0	2	0	-2.7182
tr|B7Z8J4|B7Z8J4_HUMAN	NMT1	Glycylpeptide N-tetradecan	15265	350	0	50	0	0.49	0	0	1	0	2	0	2	0	2	0	-2.7182
tr|Q6NZ52|Q6NZ52_HUMAN	RPL27A	Ribosomal protein L27a OS	16582	348	0	50	0	0.2	0	0	1	0	1	0	2	0	2	0	-2.7182
tr|E5RHF2|E5RHF2_HUMAN	COPS5	COP9 signalosome complex	16655	346	0	51	0	0.45	0	0	0	0	2	0	2	0	2	0	-2.7182
tr|F5GYR3|F5GYR3_HUMAN	NOP2	Putative ribosomal RNA me	30665	296	0	28	0	0.15	0	0	1	0	1	0	2	0	3	0	-2.7182
tr|E5RH42|E5RH42_HUMAN	G3BP1	Ras GTPase-activating prote	13338	294	0	62	0	0.58	0	0	2	0	2	0	2	0	2	0	-2.7182
tr|C9JAM8|C9JAM8_HUMAN	PLS1	Plastin0 (Fragment) OS=Hor	29616	282	0	48	0	0.34	0	0	1	0	2	0	2	0	2	0	-2.7182
tr|B4DMT8|B4DMT8_HUMA	tr|B4DMT8|B4D	cDNA FLJ52118, highly simi	24050	258	0	42	0	0.4	0	0	1	0	2	0	2	0	2	0	-2.7182
sp|Q6IS14|IF5AL_HUMAN	EIF5AL1	Eukaryotic translation initia	16990	256	0	75	0	0.44	0	0	1	0	2	0	2	0	2	0	-2.7182
sp|Q9UQ35|SRRM2_HUMAN	SRRM2	Serine/arginine repetitive n	300179	247	0	78	0	0.02	0	0	1	0	2	0	2	0	5	0	-2.7182
tr|Q9P172|Q9P172_HUMAN	tr|Q9P172|Q9P1	PRO2281 OS=Homo sapiens	37451	243	0	20	0	0.05	0	0	0	0	1	0	2	0	3	0	-2.7182
tr|Q53G58|Q53G58_HUMAN	tr|Q53G58|Q53G	Coronin (Fragment) OS=Hor	53931	242	0	83	0	0.13	0	0	1	0	2	0	2	0	4	0	-2.7182
sp|Q2NL82|TSR1_HUMAN	TSR1	Pre-rRNA-processing protei	92151	230	0	90	0	0.07	0	0	2	0	2	0	2	0	2	0	-2.7182
tr|D6RAS7|D6RAS7_HUMAN	RPS3A	40S ribosomal protein S3a (	14608	223	0	72	0	0.44	0	0	1	0	1	0	2	0	3	0	-2.7182
tr|Q71UM7|Q71UM7_HUMA	tr|Q71UM7|Q71	Beta-subunit signal transdu	14203	199	0	105	0	0.18	0	0	1	0	2	0	2	0	2	0	-2.7182
tr|B3KPU1|B3KPU1_HUMAN	GNB2	Guanine nucleotide-binding	26598	194	0	111	0	0.27	0	0	1	0	2	0	2	0	2	0	-2.7182
tr|Q5JVP3|Q5JVP3_HUMAN	PTGR1	Leukotriene B4 12-hydroxy	19441	190	0	113	0	0.38	0	0	2	0	2	0	2	0	2	0	-2.7182
tr|B4DRY6|B4DRY6_HUMAN	tr|B4DRY6|B4DR	cDNA FLJ57182, highly simi	36791	159	0	73	0	0.28	0	0	1	0	2	0	2	0	2	0	-2.7182
tr|B3KS35|B3KS35_HUMAN	tr|B3KS35|B3KS3	cDNA FLJ35372 fis, clone SK	32698	149	0	55	0	0.32	0	0	1	0	2	0	2	0	2	0	-2.7182
tr|Q53FI9|Q53FI9_HUMAN	tr|Q53FI9|Q53FI	Nucleolar protein GU2 varia	41384	148	0	61	0	0.34	0	0	2	0	2	0	2	0	2	0	-2.7182
tr|O14729|O14729_HUMAN	tr|O14729|O147	Smooth muscle myosin hea	68203	142	0	56	0	0.06	0	0	1	0	1	0	2	0	3	0	-2.7182
tr|C9J057|C9J057_HUMAN	ASNS	Asparagine synthetase [glut	24946	141	0	32	0	0.26	0	0	0	0	1	0	2	0	2	0	-2.7182
tr|J3QRQ7|J3QRQ7_HUMAN	DDX5	Probable ATP-dependent R	23758	97	0	75	0	0.45	0	0	2	0	2	0	2	0	2	0	-2.7182
tr|Q8WYB3|Q8WYB3_HUMA	tr|Q8WYB3|Q8W	Beta II spectrin-short isofor	7547	49	0	79	0	0.22	0	0	2	0	2	0	2	0	2	0	-2.7182
tr|Q7KZ86|Q7KZ86_HUMAN	tr|Q7KZ86|Q7KZ	Plakoglobin (Fragment) OS=	19007	26	0	37	0	1.74	0	0	1	0	2	0	2	0	2	0	-2.7182
sp|P35900|K1C20_HUMAN	KRT20	Keratin, type I cytoskeletal	48514	24	0	78	0	2.99	0	0	2	0	2	0	2	0	2	0	-2.7182
tr|B7Z4P8|B7Z4P8_HUMAN	ACTN2	Alpha-actinin-2 OS=Homo s	44675	19	0	51	0	2.11	0	0	1	0	2	0	2	0	2	0	-2.7182
sp|Q7Z3Y8|K1C27_HUMAN	KRT27	Keratin, type I cytoskeletal	50419	12	0	72	0	4.67	0	0	2	0	2	0	2	0	2	0	-2.7182
tr|H0YID6|H0YID6_HUMAN	KRT79	Keratin, type II cytoskeletal	13441	11	0	64	0	4.79	0	0	2	0	2	0	2	0	3	0	-2.7182
tr|B2R659|B2R659_HUMAN	tr|B2R659|B2R6	cDNA, FLJ92803, highly sim	80018	484	0	36	0	0.13	0	0	0	0	3	0	3	0	4	0	-3.20395
tr|A0AV58|A0AV58_HUMAN	STRN3	Striatin, calmodulin binding	78069	419	0	42	0	0.09	0	0	1	0	2	0	3	0	3	0	-3.20395
tr|B4DZ88|B4DZ88_HUMAN	KTN1	KTN1 protein OS=Homo sap	153179	322	0	55	0	0.04	0	0	2	0	2	0	3	0	4	0	-3.20395
tr|Q0VGL3|Q0VGL3_HUMAN	RPL13A	RPL13A protein OS=Homo s	16706	300	0	59	0	0.74	0	0	3	0	3	0	3	0	3	0	-3.20395
tr|Q5JQZ9|Q5JQZ9_HUMAN	CDC2L6	Cell division cycle 2-like 6 (	29552	285	0	65	0	0.24	0	0	2	0	2	0	3	0	3	0	-3.20395
tr|Q2TU79|Q2TU79_HUMAN	PIG44	Cell proliferation-inducing p	55601	257	0	74	0	0.1	0	0	2	0	3	0	3	0	3	0	-3.20395
tr|C9JI87|C9JI87_HUMAN	VDAC1	Voltage-dependent anion-s	20418	250	0	78	0	0.36	0	0	1	0	3	0	3	0	3	0	-3.20395
tr|C9JWY7|C9JWY7_HUMAN	CAPN2	Calpain-2 catalytic subunit (	14668	173	0	48	0	0.22	0	0	0	0	4	0	3	0	4	0	-3.20395
tr|Q5STZ7|Q5STZ7_HUMAN	ABCF1	ATP-binding cassette sub-fa	27429	144	0	112	0	0.27	0	0	2	0	3	0	3	0	3	0	-3.20395
tr|K7ENJ4|K7ENJ4_HUMAN	ATP5A1	ATP synthase subunit alpha	21383	135	0	74	0	1.33	0	0	3	0	3	0	3	0	3	0	-3.20395
sp|P35237|SPB6_HUMAN	SERPINB6	Serpin B6 OS=Homo sapien	42936	120	0	179	0	0.25	0	0	3	0	3	0	3	0	3	0	-3.20395
tr|Q5JRR9|Q5JRR9_HUMAN	UBA1	Ubiquitin-like modifier-activ	29447	114	0	73	0	0.28	0	0	2	0	3	0	3	0	3	0	-3.20395
sp|A2RTY3|CQ066_HUMAN	C17orf66	Uncharacterized protein C1	66437	477	0	36	0	0.05	0	0	0	0	1	0	4	0	8	0	-3.56685
tr|Q53RS3|Q53RS3_HUMAN	DDEF2	Putative uncharacterized pr	70046	441	0	39	0	0.05	0	0	1	0	1	0	4	0	4	0	-3.56685
tr|F5GZI8|F5GZI8_HUMAN	TCP1	T-complex protein 1 subuni	10411	197	0	64	0	0.3	0	0	1	0	2	0	4	0	4	0	-3.56685
tr|B4DL17|B4DL17_HUMAN	tr|B4DL17|B4DL	cDNA FLJ52558, highly simi	35892	88	0	116	0	0.63	0	0	3	0	3	0	4	0	4	0	-3.56685
tr|B3KT59|B3KT59_HUMAN	tr|B3KT59|B3KT5	cDNA FLJ37698 fis, clone BR	45662	157	0	91	0	0.21	0	0	3	0	4	0	6	0	6	0	-4.09802
tr|B7ZKM0|B7ZKM0_HUMAN	SART3	SART3 protein OS=Homo sa	106372	153	0	139	0	0.16	0	0	5	0	5	0	7	0	7	0	-4.3048
sp|Q96RW7|HMCN1_HUMA	HMCN1	Hemicentin0 OS=Homo sap	623265	291	0	63	0	0.01	0	0	7	0	7	0	11	0	16	0	-4.92279
sp|P05165|PCCA_HUMAN	PCCA	Propionyl-CoA carboxylase	80635	99	0	216	0	0.49	0	0	6	0	10	0	13	0	13	0	-5.15477
tr|Q59FY4|Q59FY4_HUMAN	ACACA	Acetyl-CoA carboxylase 1 (F	115059	31	0	225	0	0.44	0	0	10	0	12	0	15	0	18	0	-5.35473
tr|B3KPL5|B3KPL5_HUMAN	tr|B3KPL5|B3KPL	cDNA FLJ31917 fis, clone NT	48711	657	0	21	0	0.07	0	0	0	0	1	0	16	0	51	0	-5.44526

#12	Hela live syn MLN-2_0.2µM																		
#04	Hela live syn NP_2µM																		
Accession	GS	Name	Pro_Mass	04_pro	12_pro	04_pro	12_pro	04_emPA	12_emPA	emPAI ratio	04_No>I	12_No>I	04_No>HI	12_No>HI	04_No>20	12_No>20	04_No=All	12_No=All	Rsc
sp|P53350|PLK1_HUMAN	PLK1	Serine/threonine-protein kinase PLK1 OS=Homo sapiens GN=PLK1 PE=1 SV=1	68953	0	1169	0	20	0	0.05	#DIV/0!	0	0	0	1	0	1	0	2	0.875153
sp|P28482|MK01_HUMAN	MAPK1	Mitogen-activated protein kinase 1 OS=Homo sapiens GN=MAPK1 PE=1 SV=3	41762	0	685	0	58	0	0.16	#DIV/0!	0	1	0	2	0	2	0	2	1.612325
sp|Q9H4A3|WNK1_HUMAN	WNK1	Serine/threonine-protein kinase WNK1 OS=Homo sapiens GN=WNK1 PE=1 SV=2	251552	0	581	0	71	0	0.01	#DIV/0!	0	1	0	1	0	1	0	1	0.875153
sp|Q9Y6E0|STK24_HUMAN	STK24	Serine/threonine-protein kinase 24 OS=Homo sapiens GN=STK24 PE=1 SV=1	49562	0	1046	0	29	0	0.08	#DIV/0!	0	1	0	1	0	1	0	1	0.875153
sp|P19784|CSK22_HUMAN	CSNK2A2	Casein kinase II subunit alpha~ OS=Homo sapiens GN=CSNK2A2 PE=1 SV=1	41358	0	665	0	60	0	0.17	#DIV/0!	0	1	0	2	0	2	0	2	1.612325
sp|Q8N5S9|KKCC1_HUMAN	CAMKK1	Calcium/calmodulin-dependent protein kinase kinase 1 OS=Homo sapiens GN=CAMKK1 PE=1 SV=2	56157	0	1098	0	25	0	0.06	#DIV/0!	0	0	0	1	0	1	0	1	0.875153
sp|P43250|GRK6_HUMAN	GRK6	G protein-coupled receptor kinase 6 OS=Homo sapiens GN=GRK6 PE=1 SV=2	67203	0	962	0	34	0	0.05	#DIV/0!	0	1	0	1	0	1	0	2	0.875153
sp|Q13418|ILK_HUMAN	ILK	Integrin-linked protein kinase OS=Homo sapiens GN=ILK PE=1 SV=2	51899	421	390	41	114	0.07	0.15	2.142857143	1	2	1	2	1	2	1	2	0.027025
sp|P78527|PRKDC_HUMAN	PRKDC	DNA-dependent protein kinase catalytic subunit OS=Homo sapiens GN=PRKDC PE=1 SV=3	473749	83	68	241	506	0.07	0.14	2	7	13	10	19	11	19	17	23	0.052032
sp|Q9Y223|GLCNE_HUMAN	GNE	Bifunctional UDP-N-acetylglucosamine 2-epimerase/N-acetylmannosamine kinase OS=Homo sapiens GN=GNE PE=1 SV=1	80193	0	1068	0	27	0	0.04	#DIV/0!	0	0	0	1	0	1	0	1	0.875153
sp|Q9BTU6|P4K2A_HUMAN	PI4K2A	Phosphatidylinositol 4-kinase type 2-alpha OS=Homo sapiens GN=PI4K2A PE=1 SV=1	54388	0	992	0	32	0	0.06	#DIV/0!	0	1	0	1	0	1	0	1	0.875153
sp|P19367|HXK1_HUMAN	HK1	Hexokinase-1 OS=Homo sapiens GN=HK1 PE=1 SV=3	103561	0	975	0	33	0	0.05	#DIV/0!	0	1	0	1	0	1	0	1	0.875153
sp|P13861|KAP2_HUMAN	PRKAR2A	cAMP-dependent protein kinase type II-alpha regulatory subunit OS=Homo sapiens GN=PRKAR2A PE=1 SV=2	45832	0	940	0	35	0	0.15	#DIV/0!	0	0	0	1	0	1	0	1	0.875153
sp|Q9BRR6|ADPGK_HUMAN	ADPGK	ADP-dependent glucokinase OS=Homo sapiens GN=ADPGK PE=1 SV=1	54397	0	751	0	50	0	0.07	#DIV/0!	0	1	0	1	0	1	0	1	0.875153
sp|P12277|KCRB_HUMAN	CKB	Creatine kinase B-type OS=Homo sapiens GN=CKB PE=1 SV=1	42902	214	275	97	121	0.18	0.45	2.5	2	3	2	4	2	4	2	4	0.138131
sp|P00558|PGK1_HUMAN	PGK1	Phosphoglycerate kinase 1 OS=Homo sapiens GN=PGK1 PE=1 SV=3	44985	134	81	161	455	0.76	1.9	2.5	5	11	8	17	9	18	9	20	0.252187
sp|P55263|ADK_HUMAN	ADK	Adenosine kinase OS=Homo sapiens GN=ADK PE=1 SV=2	40919	538	694	30	57	0.08	0.18	2.25	0	1	1	2	1	2	1	2	0.027025
sp|P67775|PP2AA_HUMAN	PPP2CA	Serine/threonine-protein phosphatase 2A catalytic subunit alpha isoform OS=Homo sapiens GN=PPP2CA PE=1 SV=1	36142	0	1105	0	25	0	0.24	#DIV/0!	0	0	0	1	0	2	0	2	1.612325
sp|P40763|STAT3_HUMAN	STAT3	Signal transducer and activator of transcription 3 OS=Homo sapiens GN=STAT3 PE=1 SV=2	88810	0	735	0	52	0	0.04	#DIV/0!	0	1	0	1	0	2	0	2	1.612325
sp|Q96HS1|PGAM5_HUMAN	PGAM5	Serine/threonine-protein phosphatase PGAM5, mitochondrial OS=Homo sapiens GN=PGAM5 PE=1 SV=2	32213	0	1125	0	24	0	0.1	#DIV/0!	0	0	0	1	0	1	0	1	0.875153
sp|P62140|PP1B_HUMAN	PPP1CB	Serine/threonine-protein phosphatase PP1-beta catalytic subunit OS=Homo sapiens GN=PPP1CB PE=1 SV=3	37961	345	321	51	106	0.24	0.31	1.291666667	1	3	1	3	1	3	1	3	0.512658
tr|H0YCI6|H0YCI6_HUMAN	SCYL1 (NTKL)	N-terminal kinase-like protein (Fragment) OS=Homo sapiens GN=SCYL1 PE=4 SV=1	19601	0	1146	0	22	0	0.17	#DIV/0!	0	0	0	1	0	1	0	1	0.875153
sp|O00764|PDXK_HUMAN	PDXK	Pyridoxal kinase OS=Homo sapiens GN=PDXK PE=1 SV=1	35308	566	429	29	101	0.11	0.38	3.454545455	0	1	1	2	1	1	1	2	-0.71015
sp|P14618|KPYM_HUMAN	PKM	Pyruvate kinase isozymes M1/M2 OS=Homo sapiens GN=PKM PE=1 SV=4	58470	8	7	1820	2375	5.79	13.6	2.348877375	47	57	58	74	60	78	66	84	-0.33846
sp|P29966|MARCS_HUMAN	MARCKS	Myristoylated alanine-rich C-kinase substrate OS=Homo sapiens GN=MARCKS PE=1 SV=4	31707	0	672	0	59	0	0.51	#DIV/0!	0	1	0	2	0	2	0	2	1.612325
tr|H0YFG1|H0YFG1_HUMAN	EPS8	Epidermal growth factor receptor kinase substrate 8 (Fragment) OS=Homo sapiens GN=EPS8 PE=4 SV=1	7139	0	993	0	32	0	0.5	#DIV/0!	0	0	0	1	0	1	0	2	0.875153
tr|B0AZV0|B0AZV0_HUMAN	STRAP	Serine-threonine kinase receptor-associated protein OS=Homo sapiens GN=STRAP PE=2 SV=1	28716	626	514	23	80	0.12	0.17	1.416666667	0	2	1	2	1	3	1	3	0.512658
tr|A4D1C5|A4D1C5_HUMAN	PTPN12	Protein tyrosine phosphatase, non-receptor type 12 OS=Homo sapiens GN=PTPN12 PE=2 SV=1	88907	0	780	0	48	0	0.04	#DIV/0!	0	1	0	1	0	1	0	1	0.875153
tr|J3QSX4|J3QSX4_HUMAN	BUB3	Mitotic checkpoint protein BUB3 OS=Homo sapiens GN=BUB3 PE=4 SV=1	16498	681	601	20	67	0.2	0.1	0.5	0	1	1	1	0	1	1	1	0.875153
sp|Q01813|K6PP_HUMAN	PFKP	6-phosphofructokinase type C OS=Homo sapiens GN=PFKP PE=1 SV=2	86454	98	93	217	418	0.4	0.4	1	5	9	9	9	9	11	10	12	-0.43515
sp|O75116|ROCK2_HUMAN	ROCK2	Rho-associated protein kinase 2 OS=Homo sapiens GN=ROCK2 PE=1 SV=4	161939	375	775	46	48	0.05	0.04	0.8	1	0	2	2	1	2	3	4	0.027025
tr|A4QPA9|A4QPA9_HUMAN	MAP2K1	Mitogen-activated protein kinase kinase 1 OS=Homo sapiens GN=MAP2K1 PE=2 SV=1	43753	384	660	45	61	0.31	0.08	0.258064516	1	1	2	1	2	1	2	1	-1.44745
sp|P17858|K6PL_HUMAN	PFKL	6-phosphofructokinase, liver type OS=Homo sapiens GN=PFKL PE=1 SV=6	85762	98	459	88	92	0.4	0.08	0.2	1	1	2	2	2	2	2	3	-0.71028
sp|P07205|PGK2_HUMAN	PGK2	Phosphoglycerate kinase 2 OS=Homo sapiens GN=PGK2 PE=1 SV=3	45166	134	936	81	35	0.76	0.07	0.092105263	1	0	3	1	4	2	4	3	-1.55895
tr|B4E1G6|B4E1G6_HUMAN	GALK1	Galactokinase OS=Homo sapiens GN=GALK1 PE=2 SV=1	45956	679	0	20	0	0.07	0	0	0	0	1	0	0	0	1	0	-0.71002
tr|A6NIT2|A6NIT2_HUMAN	PHKA1	Phosphorylase b kinase regulatory subunit alpha, skeletal muscle isoform OS=Homo sapiens GN=PHKA1 PE=2 SV=1	133462	711	0	17	0	0.02	0	0	0	0	1	0	0	0	1	0	-0.71002
tr|Q8IWY7|Q8IWY7_HUMAN	TTBK2	Tau-tubulin kinase OS=Homo sapiens GN=TTBK2 PE=2 SV=1	183893	79	0	28	0	0.79	0	0	0	0	1	0	1	0	1	0	-2.29532
tr|B4E2K2|B4E2K2_HUMAN	tr|B4E2K2|B4E2	cDNA FLJ55665, highly similar to A-kinase anchor protein 2 OS=Homo sapiens PE=2 SV=1	103935	246	0	79	0	0.03	0	0	1	0	1	0	1	0	1	0	-2.29532
sp|Q9UEW8|STK39_HUMAN	STK39	STE20/SPS1-related proline-alanine-rich protein kinase OS=Homo sapiens GN=STK39 PE=1 SV=3	59950	491	0	35	0	0.05	0	0	0	0	1	0	1	0	1	0	-2.29532
tr|B2R5T5|B2R5T5_HUMAN	PRKAR1A	Protein kinase, cAMP-dependent, regulatory, type I, alpha (Tissue specific extinguisher 1), isoform CRA_a OS=Homo sapiens GN=PRKAR1A P	43183	533	0	31	0	0.08	0	0	0	0	1	0	1	0	1	0	-2.29532
tr|A8K2U2|A8K2U2_HUMAN	tr|A8K2U2|A8K	cDNA FLJ75392, highly similar to Homo sapiens hexokinase II (HKII) mRNA OS=Homo sapiens PE=2 SV=1	103718	599	0	26	0	0.03	0	0	0	0	1	0	1	0	2	0	-2.29532
tr|H0Y3C5|H0Y3C5_HUMAN	HCK	Tyrosine-protein kinase HCK OS=Homo sapiens GN=HCK PE=4 SV=1	59986	628	0	23	0	0.05	0	0	0	0	1	0	1	0	2	0	-2.29532
tr|H0Y3Y5|H0Y3Y5_HUMAN	PPIP5K1	Inositol hexakisphosphate and diphosphoinositol-pentakisphosphate kinase 1 (Fragment) OS=Homo sapiens GN=PPIP5K1 PE=4 SV=1	21913	632	0	22	0	0.15	0	0	0	0	1	0	1	0	2	0	-2.29532
sp|O95747|OXSR1_HUMAN	OXSR1	Serine/threonine-protein kinase OSR1 OS=Homo sapiens GN=OXSR1 PE=1 SV=1	58271	675	0	20	0	0.06	0	0	0	0	1	0	1	0	2	0	-2.29532
tr|B3KS97|B3KS97_HUMAN	MAP2K2	Mitogen-activated protein kinase kinase 2, isoform CRA_d OS=Homo sapiens GN=MAP2K2 PE=2 SV=1	23450	384	0	45	0	0.31	0	0	1	0	2	0	2	0	2	0	-3.03262
tr|H0YJ75|H0YJ75_HUMAN	PPP2R5C	Serine/threonine-protein phosphatase 2A 56 kDa regulatory subunit gamma isoform (Fragment) OS=Homo sapiens GN=PPP2R5C PE=4 SV=1	64365	0	1012	0	31	0	0.05	#DIV/0!	0	0	0	1	0	2	0	2	1.612325
tr|B2R665|B2R665_HUMAN	tr|B2R665|B2R	cDNA, FLJ92810, highly similar to Homo sapiens protein phosphatase 1G (formerly 2C), magnesium-dependent, gamma isoform (PPM1G), m	59889	0	510	0	80	0	0.11	#DIV/0!	0	2	0	2	0	2	0	3	1.612325
tr|B2RAH5|B2RAH5_HUMAN	tr|B2RAH5|B2R	cDNA, FLJ94919, highly similar to Homo sapiens protein phosphatase 1, regulatory (inhibitor) subunit 12A (PPP1R12A), mRNA OS=Homo sap	115658	0	1128	0	23	0	0.08	#DIV/0!	0	0	0	1	0	1	0	1	0.875153
sp|Q8TAP8|PPR35_HUMAN	PPP1R35	Protein phosphatase 1 regulatory subunit 35 OS=Homo sapiens GN=PPP1R35 PE=1 SV=1	28107	0	732	0	52	0	0.12	#DIV/0!	0	1	0	1	0	1	0	1	0.875153
tr|B4DDF7|B4DDF7_HUMAN	tr|B4DDF7|B4D	cDNA FLJ53296, highly similar to Serine/threonine-protein phosphatase 2A 65 kDa regulatory subunit A alpha isoform OS=Homo sapiens PE	64939	443	137	39	302	0.16	0.48	3	1	7	3	8	3	8	3	9	0.57073
tr|H0YDU8|H0YDU8_HUMAN	PPP5C	Serine/threonine-protein phosphatase (Fragment) OS=Homo sapiens GN=PPP5C PE=3 SV=1	55743	596	701	26	56	0.06	0.06	1	0	1	1	1	1	1	1	1	-0.71015
tr|J3QT22|J3QT22_HUMAN	PPME1	Protein phosphatase methylesterase 1 OS=Homo sapiens GN=PPME1 PE=3 SV=1	44241	232	0	90	0	0.07	0	0	1	0	1	0	1	0	2	0	-2.29532
tr|B4DDZ8|B4DDZ8_HUMAN	tr|B4DDZ8|B4D	cDNA FLJ55048, highly similar to Serine/threonine-protein phosphatase 5 (EC 3.1.3.16) OS=Homo sapiens PE=2 SV=1	55829	596	0	26	0	0.06	0	0	0	0	1	0	1	0	1	0	-2.29532
tr|C1PHA2|C1PHA2_HUMAN	KIF5B-ALK	Tyrosine-protein kinase receptor OS=Homo sapiens GN=KIF5B-ALK PE=2 SV=1	169283	227	341	91	134	0.04	0.04	1	2	2	2	2	2	2	3	3	-0.71028
tr|H0YH33|H0YH33_HUMAN	STRAP	Serine-threonine kinase receptor-associated protein (Fragment) OS=Homo sapiens GN=STRAP PE=4 SV=1	10763	626	0	23	0	0.12	0	0	0	0	1	0	1	0	1	0	-2.29532
sp|O75533|SF3B1_HUMAN	SF3B1	Splicing factor 3B subunit 1 OS=Homo sapiens GN=SF3B1 PE=1 SV=3	146479	0	109	0	379	0	0.49	#DIV/0!	0	10	0	18	0	15	0	19	4.247275
tr|A8K8U1|A8K8U1_HUMAN	tr|A8K8U1|A8K	cDNA FLJ77762, highly similar to Homo sapiens cullin-associated and neddylation-dissociated 1 (CAND1), mRNA OS=Homo sapiens PE=2 SV=	137941	0	157	0	273	0	0.32	#DIV/0!	0	8	0	12	0	14	0	15	4.150853
tr|B7Z8Z8|B7Z8Z8_HUMAN	tr|B7Z8Z8|B7Z8	cDNA FLJ61702, highly similar to 80 kDa MCM3-associated protein OS=Homo sapiens PE=2 SV=1	181264	0	938	0	35	0	0.02	#DIV/0!	0	1	0	1	0	13	0	13	4.047553
sp|Q08J23|NSUN2_HUMAN	NSUN2	tRNA (cytosine(34)-C(5))-methyltransferase OS=Homo sapiens GN=NSUN2 PE=1 SV=2	87214	0	230	0	193	0	0.34	#DIV/0!	0	6	0	9	0	13	0	15	4.047553
tr|D6REM6|D6REM6_HUMAN	MATR3	Matrin-3 OS=Homo sapiens GN=MATR3 PE=2 SV=1	88647	0	179	0	240	0	0.47	#DIV/0!	0	7	0	11	0	12	0	13	3.936316
tr|Q5TZZ9|Q5TZZ9_HUMAN	ANXA1	Annexin OS=Homo sapiens GN=ANXA1 PE=2 SV=1	38918	0	79	0	466	0	1.66	#DIV/0!	0	9	0	12	0	12	0	12	3.936316
tr|B4DMR3|B4DMR3_HUMAN	tr|B4DMR3|B4D	cDNA FLJ51896, highly similar to Glia-derived nexin OS=Homo sapiens PE=2 SV=1	37305	0	206	0	219	0	1.55	#DIV/0!	0	7	0	11	0	11	0	12	3.815815
sp|O43776|SYNC_HUMAN	NARS	Asparagine--tRNA ligase, cytoplasmic OS=Homo sapiens GN=NARS PE=1 SV=1	63758	0	187	0	236	0	0.65	#DIV/0!	0	7	0	10	0	11	0	11	3.815815
sp|Q92626|PXDN_HUMAN	PXDN	Peroxidasin homolog OS=Homo sapiens GN=PXDN PE=1 SV=2	167793	0	148	0	294	0	0.21	#DIV/0!	0	8	0	11	0	11	0	12	3.815815
sp|P54577|SYYC_HUMAN	YARS	Tyrosine--tRNA ligase, cytoplasmic OS=Homo sapiens GN=YARS PE=1 SV=4	59448	0	240	0	188	0	0.46	#DIV/0!	0	4	0	7	0	10	0	11	3.684364
sp|P07093|GDN_HUMAN	SERPINE2	Glia-derived nexin OS=Homo sapiens GN=SERPINE2 PE=1 SV=1	44202	0	208	0	217	0	1.06	#DIV/0!	0	7	0	10	0	10	0	10	3.684364
tr|Q6NWZ1|Q6NWZ1_HUMAN	CKAP4	CKAP4 protein (Fragment) OS=Homo sapiens GN=CKAP4 PE=2 SV=1	67948	0	162	0	272	0	0.6	#DIV/0!	0	6	0	10	0	10	0	12	3.684364
tr|B7Z1N6|B7Z1N6_HUMAN	tr|B7Z1N6|B7Z	cDNA FLJ52222, highly similar to Fructose-bisphosphate aldolase C (EC4.1.2.13) OS=Homo sapiens PE=2 SV=1	35800	0	114	0	367	0	0.42	#DIV/0!	0	6	0	9	0	10	0	12	3.684364
tr|B2RDW1|B2RDW1_HUMAN	RPS27A	Ribosomal protein S27a, isoform CRA_c OS=Homo sapiens GN=RPS27A PE=2 SV=1	18296	0	244	0	186	0	0.4	#DIV/0!	0	6	0	8	0	9	0	9	3.539768
tr|B4DMF5|B4DMF5_HUMAN	tr|B4DMF5|B4D	Glutamate dehydrogenase OS=Homo sapiens PE=2 SV=1	56910	0	202	0	221	0	0.66	#DIV/0!	0	5	0	9	0	9	0	10	3.539768
tr|Q6NVW7|Q6NVW7_HUMAN	KPNA2	Importin subunit alpha OS=Homo sapiens GN=KPNA2 PE=2 SV=1	58271	0	170	0	256	0	0.47	#DIV/0!	0	5	0	7	0	9	0	12	3.539768
tr|B4DLV7|B4DLV7_HUMAN	tr|B4DLV7|B4D	cDNA FLJ60299, highly similar to Rab GDP dissociation inhibitor beta OS=Homo sapiens PE=2 SV=1	51577	0	156	0	275	0	0.75	#DIV/0!	0	7	0	9	0	9	0	10	3.539768
tr|E1NZA1|E1NZA1_HUMAN	PRIC295	Peroxisome proliferator activated receptor interacting complex protein OS=Homo sapiens GN=PRIC295 PE=2 SV=1	294953	0	287	0	159	0	0.08	#DIV/0!	0	4	0	7	0	8	0	9	3.379097
tr|Q53G71|Q53G71_HUMAN	tr|Q53G71|Q53	Calreticulin variant (Fragment) OS=Homo sapiens PE=2 SV=1	47061	0	283	0	161	0	0.61	#DIV/0!	0	4	0	8	0	8	0	8	3.379097
tr|B7Z4B2|B7Z4B2_HUMAN	tr|B7Z4B2|B7Z4	cDNA FLJ56108, highly similar to Puromycin-sensitive aminopeptidase (EC 3.4.11.-) OS=Homo sapiens PE=2 SV=1	93434	0	272	0	168	0	0.32	#DIV/0!	0	4	0	8	0	8	0	9	3.379097
sp|P98160|PGBM_HUMAN	HSPG2	Basement membrane-specific heparan sulfate proteoglycan core protein OS=Homo sapiens GN=HSPG2 PE=1 SV=4	479253	0	227	0	193	0	0.04	#DIV/0!	0	6	0	6	0	8	0	8	3.379097
tr|G3XAI2|G3XAI2_HUMAN	LAMB1	Laminin subunit beta-1 OS=Homo sapiens GN=LAMB1 PE=2 SV=1	207703	0	196	0	226	0	0.13	#DIV/0!	0	6	0	8	0	8	0	9	3.379097
tr|B3KPA6|B3KPA6_HUMAN	ACADVL	Acyl-Coenzyme A dehydrogenase, very long chain, isoform CRA_e OS=Homo sapiens GN=ACADVL PE=2 SV=1	62941	0	307	0	149	0	0.29	#DIV/0!	0	3	0	5	0	7	0	8	3.198318
tr|B4DIT7|B4DIT7_HUMAN	TGM2	Protein-glutamine gamma-glutamyltransferase 2 OS=Homo sapiens GN=TGM2 PE=2 SV=1	69632	0	303	0	150	0	0.45	#DIV/0!	0	4	0	8	0	7	0	10	3.198318
tr|B2R806|B2R806_HUMAN	tr|B2R806|B2R	Eukaryotic translation initiation factor 3 subunit E OS=Homo sapiens PE=2 SV=1	52622	0	284	0	97	0	0.53	#DIV/0!	0	3	0	7	0	7	0	8	3.198318
tr|B3KNK5|B3KNK5_HUMAN	tr|B3KNK5|B3K	cDNA FLJ14795 fis, clone NT2RP4001219, highly similar to Homo sapiens DnaJ (Hsp40) homolog, subfamily C, member 10 (DNAJC10), mRNA	70004	0	210	0	124	0	0.37	#DIV/0!	0	2	0	5	0	7	0	9	3.198318
tr|A2A274|A2A274_HUMAN	ACO2	Aconitase 2, mitochondrial OS=Homo sapiens GN=ACO2 PE=4 SV=1	88563	0	182	0	245	0	0.29	#DIV/0!	0	7	0	7	0	7	0	11	3.198318
sp|Q9UHD8|SEPT9_HUMAN	Sep-09	Septin-9 OS=Homo sapiens GN=SEPT9 PE=1 SV=2	65646	0	410	0	106	0	0.28	#DIV/0!	0	2	0	5	0	6	0	6	2.991661
tr|B2R774|B2R774_HUMAN	tr|B2R774|B2R	cDNA, FLJ93313, highly similar to Homo sapiens lectin, mannose-binding, 1 (LMAN1), mRNA OS=Homo sapiens PE=2 SV=1	57768	0	391	0	114	0	0.32	#DIV/0!	0	3	0	5	0	6	0	6	2.991661
tr|K4DI93|K4DI93_HUMAN	CUL4B	Cullin 4B, isoform CRA_e OS=Homo sapiens GN=CUL4B PE=3 SV=1	103318	0	349	0	131	0	0.17	#DIV/0!	0	4	0	5	0	6	0	8	2.991661
sp|Q9Y6C9|MTCH2_HUMAN	MTCH2	Mitochondrial carrier homolog 2 OS=Homo sapiens GN=MTCH2 PE=1 SV=1	33936	0	296	0	153	0	0.75	#DIV/0!	0	5	0	6	0	6	0	7	2.991661
tr|B4E3J7|B4E3J7_HUMAN	tr|B4E3J7|B4E3	cDNA FLJ51203, highly similar to Thrombospondin-1 OS=Homo sapiens PE=2 SV=1	124032	0	253	0	180	0	0.17	#DIV/0!	0	4	0	6	0	6	0	6	2.991661
tr|Q14400|Q14400_HUMAN	GLUD1	GLUD1 protein (Fragment) OS=Homo sapiens GN=GLUD1 PE=2 SV=1	28791	0	249	0	184	0	0.73	#DIV/0!	0	3	0	5	0	6	0	6	2.991661
tr|Q71UF1|Q71UF1_HUMAN	ACO2	Aconitase OS=Homo sapiens GN=ACO2 PE=2 SV=1	86304	0	239	0	189	0	0.25	#DIV/0!	0	6	0	6	0	6	0	10	2.991661
tr|B0QY89|B0QY89_HUMAN	EIF3EIP	Eukaryotic translation initiation factor 3 subunit L OS=Homo sapiens GN=EIF3EIP PE=4 SV=1	71085	0	232	0	192	0	0.25	#DIV/0!	0	4	0	5	0	6	0	6	2.991661
tr|Q6ZVI6|Q6ZVI6_HUMAN	tr|Q6ZVI6|Q6Z\	cDNA FLJ42537 fis, clone BRACE3004058, highly similar to NADH-cytochrome B5 reductase (EC 1.6.2.2) OS=Homo sapiens PE=2 SV=1	29141	0	224	0	174	0	0.74	#DIV/0!	0	3	0	5	0	6	0	6	2.991661
tr|B4DT31|B4DT31_HUMAN	FUBP1	Far upstream element-binding protein 1 OS=Homo sapiens GN=FUBP1 PE=2 SV=1	69993	0	213	0	211	0	0.26	#DIV/0!	0	5	0	5	0	6	0	6	2.991661
sp|Q969N2|PIGT_HUMAN	PIGT	GPI transamidase component PIG-T OS=Homo sapiens GN=PIGT PE=1 SV=1	66228	0	743	0	51	0	0.16	#DIV/0!	0	1	0	3	0	5	0	7	2.750447
tr|B4DL07|B4DL07_HUMAN	tr|B4DL07|B4D	cDNA FLJ53353, highly similar to ATP-binding cassette sub-family D member 3 OS=Homo sapiens PE=2 SV=1	78980	0	729	0	52	0	0.18	#DIV/0!	0	2	0	4	0	5	0	5	2.750447
tr|B4DZC0|B4DZC0_HUMAN	tr|B4DZC0|B4D	cDNA FLJ51771, highly similar to SWI/SNF-related matrix-associatedactin-dependent regulator of chromatin subfamily A member5 (EC 3.6.1	117367	0	628	0	63	0	0.12	#DIV/0!	0	3	0	4	0	5	0	7	2.750447
tr|B4DJV2|B4DJV2_HUMAN	CS	Citrate synthase OS=Homo sapiens GN=CS PE=2 SV=1	50628	0	599	0	68	0	0.29	#DIV/0!	0	3	0	4	0	5	0	6	2.750447
sp|P13667|PDIA4_HUMAN	PDIA4	Protein disulfide-isomerase A4 OS=Homo sapiens GN=PDIA4 PE=1 SV=2	73229	0	424	0	102	0	0.24	#DIV/0!	0	3	0	5	0	5	0	6	2.750447
tr|Q0VGA5|Q0VGA5_HUMAN	SARS	SARS protein OS=Homo sapiens GN=SARS PE=2 SV=1	58883	0	423	0	103	0	0.31	#DIV/0!	0	3	0	5	0	5	0	5	2.750447
tr|Q6YPB0|Q6YPB0_HUMAN	tr|Q6YPB0|Q6Y	Endoplasmic reticulum DnaJ/PDI fusion protein 3 OS=Homo sapiens PE=4 SV=1	38170	0	389	0	114	0	0.52	#DIV/0!	0	2	0	5	0	5	0	6	2.750447
tr|D9UAZ3|D9UAZ3_HUMAN	HLA-C	MHC class I antigen (Fragment) OS=Homo sapiens GN=HLA-C PE=3 SV=1	34263	0	384	0	115	0	0.32	#DIV/0!	0	3	0	3	0	5	0	6	2.750447
tr|Q59FE8|Q59FE8_HUMAN	tr|Q59FE8|Q59	Epithelial protein lost in neoplasm beta variant (Fragment) OS=Homo sapiens PE=2 SV=1	86510	0	367	0	119	0	0.23	#DIV/0!	0	2	0	5	0	5	0	5	2.750447
tr|A1KYQ7|A1KYQ7_HUMAN	tr|A1KYQ7|A1K	Cell migration-inducing protein 17 OS=Homo sapiens PE=2 SV=1	106022	0	299	0	152	0	0.13	#DIV/0!	0	3	0	4	0	5	0	5	2.750447
tr|H0YH81|H0YH81_HUMAN	ATP5B	ATP synthase subunit beta (Fragment) OS=Homo sapiens GN=ATP5B PE=3 SV=1	38226	0	293	0	154	0	0.52	#DIV/0!	0	4	0	5	0	5	0	6	2.750447
tr|G3XAL0|G3XAL0_HUMAN	MDH2	Malate dehydrogenase OS=Homo sapiens GN=MDH2 PE=2 SV=1	24921	0	231	0	114	0	0.98	#DIV/0!	0	3	0	5	0	5	0	5	2.750447
tr|H0YCK7|H0YCK7_HUMAN	EEF1D	Elongation factor 1-delta (Fragment) OS=Homo sapiens GN=EEF1D PE=4 SV=1	23105	0	190	0	234	0	0.72	#DIV/0!	0	4	0	4	0	5	0	6	2.750447
tr|B2RMN7|B2RMN7_HUMAN	SPTB	Spectrin, beta, erythrocytic OS=Homo sapiens GN=SPTB PE=2 SV=1	247271	0	942	0	35	0	0.01	#DIV/0!	0	1	0	1	0	4	0	5	2.460734
sp|A0AVT1|UBA6_HUMAN	UBA6	Ubiquitin-like modifier-activating enzyme 6 OS=Homo sapiens GN=UBA6 PE=1 SV=1	119207	0	912	0	37	0	0.08	#DIV/0!	0	0	0	3	0	4	0	4	2.460734
tr|A0PJ61|A0PJ61_HUMAN	RSL1D1	RSL1D1 protein (Fragment) OS=Homo sapiens GN=RSL1D1 PE=2 SV=1	49688	0	808	0	45	0	0.21	#DIV/0!	0	1	0	3	0	4	0	5	2.460734
tr|Q149P0|Q149P0_HUMAN	GBF1	GBF1 protein OS=Homo sapiens GN=GBF1 PE=2 SV=1	207938	0	683	0	58	0	0.06	#DIV/0!	0	2	0	4	0	4	0	4	2.460734
tr|Q6FHX6|Q6FHX6_HUMAN	FEN1	Flap endonuclease 1 OS=Homo sapiens GN=FEN1 PE=2 SV=1	42908	0	641	0	62	0	0.16	#DIV/0!	0	2	0	2	0	4	0	4	2.460734
tr|B4DNM8|B4DNM8_HUMAN	tr|B4DNM8|B4	cDNA FLJ53395, highly similar to Prolyl 3-hydroxylase 1 (EC 1.14.11.7) OS=Homo sapiens PE=2 SV=1	69572	0	632	0	63	0	0.15	#DIV/0!	0	2	0	3	0	4	0	4	2.460734
tr|B7ZMF2|B7ZMF2_HUMAN	FANCI	Fanconi anemia, complementation group I OS=Homo sapiens GN=FANCI PE=2 SV=1	150724	0	559	0	73	0	0.09	#DIV/0!	0	1	0	4	0	4	0	6	2.460734
tr|B0AZQ4|B0AZQ4_HUMAN	tr|B0AZQ4|B0A	cDNA, FLJ79494, highly similar to Structural maintenance of chromosome 3 OS=Homo sapiens PE=2 SV=1	141841	0	548	0	75	0	0.07	#DIV/0!	0	2	0	3	0	4	0	4	2.460734
tr|B4E2K4|B4E2K4_HUMAN	tr|B4E2K4|B4E2	cDNA FLJ54576, highly similar to Aspartyl/asparaginyl beta-hydroxylase (EC 1.14.11.16) OS=Homo sapiens PE=2 SV=1	83685	0	501	0	83	0	0.17	#DIV/0!	0	2	0	4	0	4	0	4	2.460734
tr|B3KMV5|B3KMV5_HUMAN	tr|B3KMV5|B3K	cDNA FLJ12728 fis, clone NT2RP2000040, highly similar to Protein FAM62A OS=Homo sapiens PE=2 SV=1	123342	0	495	0	85	0	0.11	#DIV/0!	0	2	0	4	0	4	0	4	2.460734
tr|B4DQK8|B4DQK8_HUMAN	tr|B4DQK8|B4D	cDNA FLJ54750, moderately similar to Pyrroline-5-carboxylate reductase 2 (EC 1.5.1.2) OS=Homo sapiens PE=2 SV=1	37009	0	481	0	88	0	0.29	#DIV/0!	0	2	0	3	0	4	0	5	2.460734
tr|Q4LE64|Q4LE64_HUMAN	NUMA1	NUMA1 variant protein (Fragment) OS=Homo sapiens GN=NUMA1 variant protein PE=2 SV=1	239798	0	473	0	90	0	0.03	#DIV/0!	0	1	0	2	0	4	0	5	2.460734
sp|Q6UXN9|WDR82_HUMAN	WDR82	WD repeat-containing protein 82 OS=Homo sapiens GN=WDR82 PE=1 SV=1	35456	0	451	0	97	0	0.2	#DIV/0!	0	2	0	2	0	4	0	5	2.460734
sp|P31150|GDIA_HUMAN	GDI1	Rab GDP dissociation inhibitor alpha OS=Homo sapiens GN=GDI1 PE=1 SV=2	51177	0	443	0	98	0	0.31	#DIV/0!	0	2	0	4	0	4	0	5	2.460734
tr|Q5TZP7|Q5TZP7_HUMAN	APEX1	APEX nuclease (Multifunctional DNA repair enzyme) 1 OS=Homo sapiens GN=APEX1 PE=2 SV=1	35931	0	434	0	99	0	0.55	#DIV/0!	0	3	0	5	0	4	0	6	2.460734
tr|F5H6D0|F5H6D0_HUMAN	MOGS	Mannosyl-oligosaccharide glucosidase OS=Homo sapiens GN=MOGS PE=2 SV=1	80824	0	413	0	106	0	0.27	#DIV/0!	0	1	0	6	0	4	0	7	2.460734
tr|B6V6K3|B6V6K3_HUMAN	HLA-Cw	MHC class I antigen (Fragment) OS=Homo sapiens GN=HLA-Cw PE=3 SV=1	21276	0	387	0	115	0	0.55	#DIV/0!	0	3	0	3	0	4	0	5	2.460734
tr|E0WMV4|E0WMV4_HUMAN	HLA-C	MHC class I antigen (Fragment) OS=Homo sapiens GN=HLA-C PE=3 SV=1	31992	0	386	0	115	0	0.35	#DIV/0!	0	3	0	3	0	4	0	5	2.460734
tr|C6K4M2|C6K4M2_HUMAN	HLA-C	MHC class I antigen (Fragment) OS=Homo sapiens GN=HLA-C PE=3 SV=1	20827	0	385	0	115	0	0.56	#DIV/0!	0	3	0	3	0	4	0	6	2.460734
sp|P55268|LAMB2_HUMAN	LAMB2	Laminin subunit beta-2 OS=Homo sapiens GN=LAMB2 PE=1 SV=2	202982	0	372	0	118	0	0.07	#DIV/0!	0	2	0	4	0	4	0	4	2.460734
sp|Q9Y2W1|TR150_HUMAN	THRAP3	Thyroid hormone receptor-associated protein 3 OS=Homo sapiens GN=THRAP3 PE=1 SV=2	108658	0	359	0	126	0	0.13	#DIV/0!	0	3	0	4	0	4	0	5	2.460734
sp|P09960|LKHA4_HUMAN	LTA4H	Leukotriene A-4 hydrolase OS=Homo sapiens GN=LTA4H PE=1 SV=2	69868	0	340	0	135	0	0.2	#DIV/0!	0	2	0	4	0	4	0	4	2.460734
tr|B3KRS5|B3KRS5_HUMAN	HDAC2	Histone deacetylase OS=Homo sapiens GN=HDAC2 PE=2 SV=1	52479	0	335	0	136	0	0.28	#DIV/0!	0	3	0	4	0	4	0	4	2.460734
tr|Q5SX91|Q5SX91_HUMAN	GDI2	Rab GDP dissociation inhibitor beta (Fragment) OS=Homo sapiens GN=GDI2 PE=4 SV=1	23385	0	331	0	137	0	0.71	#DIV/0!	0	3	0	4	0	4	0	5	2.460734
sp|P30837|AL1B1_HUMAN	ALDH1B1	Aldehyde dehydrogenase X, mitochondrial OS=Homo sapiens GN=ALDH1B1 PE=1 SV=3	57626	0	325	0	139	0	0.32	#DIV/0!	0	4	0	5	0	4	0	5	2.460734
tr|D3DUW5|D3DUW5_HUMAN	DNM1L	Dynamin 1-like, isoform CRA_c OS=Homo sapiens GN=DNM1L PE=3 SV=1	88442	0	322	0	140	0	0.16	#DIV/0!	0	3	0	4	0	4	0	5	2.460734
tr|B2R7F8|B2R7F8_HUMAN	tr|B2R7F8|B2R	cDNA, FLJ93426, highly similar to Homo sapiens plasminogen (PLG), mRNA OS=Homo sapiens PE=2 SV=1	93217	0	317	0	143	0	0.11	#DIV/0!	0	2	0	3	0	4	0	4	2.460734
sp|Q92945|FUBP2_HUMAN	KHSRP	Far upstream element-binding protein 2 OS=Homo sapiens GN=KHSRP PE=1 SV=4	73355	0	273	0	166	0	0.19	#DIV/0!	0	3	0	4	0	4	0	4	2.460734
tr|Q05DF2|Q05DF2_HUMAN	SF3A2	SF3A2 protein (Fragment) OS=Homo sapiens GN=SF3A2 PE=2 SV=1	51557	0	270	0	169	0	0.28	#DIV/0!	0	3	0	4	0	4	0	4	2.460734
tr|H3BPX2|H3BPX2_HUMAN	OBSCN	Obscurin OS=Homo sapiens GN=OBSCN PE=4 SV=1	958745	0	1130	0	23	0	0	#DIV/0!	0	0	0	1	0	3	0	3	2.097958
tr|Q6MZM7|Q6MZM7_HUMAN	DKFZp686O121	Putative uncharacterized protein DKFZp686O12165 (Fragment) OS=Homo sapiens GN=DKFZp686O12165 PE=1 SV=1	242944	0	1063	0	28	0	0.03	#DIV/0!	0	0	0	2	0	3	0	4	2.097958
tr|Q56VW8|Q56VW8_HUMAN	tr|Q56VW8|Q5	Lung cancer-related protein 9 OS=Homo sapiens PE=2 SV=1	52186	0	1045	0	29	0	0.06	#DIV/0!	0	0	0	1	0	3	0	3	2.097958
sp|P14550|AK1A1_HUMAN	AKR1A1	Alcohol dehydrogenase [NADP(+)] OS=Homo sapiens GN=AKR1A1 PE=1 SV=3	36892	0	1035	0	30	0	0.19	#DIV/0!	0	0	0	2	0	3	0	3	2.097958
tr|B3KQ43|B3KQ43_HUMAN	tr|B3KQ43|B3K	cDNA FLJ32791 fis, clone TESTI2002365, highly similar to helicase MOV-10 (EC 3.6.1.-) OS=Homo sapiens PE=2 SV=1	47208	0	937	0	35	0	0.14	#DIV/0!	0	0	0	2	0	3	0	3	2.097958
sp|Q8WX93|PALLD_HUMAN	PALLD	Palladin OS=Homo sapiens GN=PALLD PE=1 SV=3	151839	0	923	0	36	0	0.04	#DIV/0!	0	0	0	2	0	3	0	4	2.097958
sp|Q9Y4G6|TLN2_HUMAN	TLN2	Talin-2 OS=Homo sapiens GN=TLN2 PE=1 SV=4	273781	0	894	0	39	0	0.01	#DIV/0!	0	1	0	1	0	3	0	3	2.097958
tr|B3KUZ7|B3KUZ7_HUMAN	tr|B3KUZ7|B3K	cDNA FLJ40986 fis, clone UTERU2014898, highly similar to Vacuolar ATP synthase subunit H (EC 3.6.3.14) OS=Homo sapiens PE=2 SV=1	56445	0	887	0	39	0	0.12	#DIV/0!	0	1	0	2	0	3	0	3	2.097958
tr|B3KY04|B3KY04_HUMAN	tr|B3KY04|B3K	cDNA FLJ46506 fis, clone THYMU3030752, highly similar to BTB/POZ domain-containing protein KCTD12 OS=Homo sapiens PE=2 SV=1	35994	0	836	0	42	0	0.3	#DIV/0!	0	0	0	3	0	3	0	4	2.097958
tr|B2R6H7|B2R6H7_HUMAN	tr|B2R6H7|B2R	cDNA, FLJ92955, highly similar to Homo sapiens transportin-SR (TRN-SR), mRNA OS=Homo sapiens PE=2 SV=1	105989	0	820	0	44	0	0.06	#DIV/0!	0	2	0	2	0	3	0	3	2.097958
tr|G5E9H4|G5E9H4_HUMAN	DCTN1	Dynactin 1 (P150, glued homolog, Drosophila), isoform CRA_a OS=Homo sapiens GN=DCTN1 PE=2 SV=1	127339	0	720	0	54	0	0.05	#DIV/0!	0	1	0	2	0	3	0	3	2.097958
tr|Q6FG59|Q6FG59_HUMAN	CDC37	CDC37 protein OS=Homo sapiens GN=CDC37 PE=2 SV=1	44938	0	671	0	59	0	0.15	#DIV/0!	0	1	0	2	0	3	0	5	2.097958
sp|P52907|CAZA1_HUMAN	CAPZA1	F-actin-capping protein subunit alpha-1 OS=Homo sapiens GN=CAPZA1 PE=1 SV=3	33073	0	658	0	61	0	0.47	#DIV/0!	0	1	0	4	0	3	0	4	2.097958
tr|A0MNN5|A0MNN5_HUMAN	GRWD1	CDW4/GRWD1 OS=Homo sapiens GN=GRWD1 PE=2 SV=1	49787	0	656	0	61	0	0.07	#DIV/0!	0	1	0	1	0	3	0	4	2.097958
tr|Q9BXV5|Q9BXV5_HUMAN	tr|Q9BXV5|Q9E	PNAS-139 OS=Homo sapiens PE=2 SV=1	22952	0	630	0	63	0	0.5	#DIV/0!	0	1	0	3	0	3	0	3	2.097958
sp|Q9Y295|DRG1_HUMAN	DRG1	Developmentally-regulated GTP-binding protein 1 OS=Homo sapiens GN=DRG1 PE=1 SV=1	40802	0	625	0	64	0	0.26	#DIV/0!	0	1	0	3	0	3	0	3	2.097958
tr|F5H740|F5H740_HUMAN	VDAC3	Voltage-dependent anion-selective channel protein 3 OS=Homo sapiens GN=VDAC3 PE=2 SV=1	31080	0	620	0	64	0	0.11	#DIV/0!	0	1	0	1	0	3	0	3	2.097958
tr|A8K4T6|A8K4T6_HUMAN	tr|A8K4T6|A8K	cDNA FLJ76282, highly similar to Homo sapiens proteasome (prosome, macropain) 26S subunit, non-ATPase, 5 (PSMD5), mRNA OS=Homo s	56588	0	615	0	65	0	0.18	#DIV/0!	0	2	0	3	0	3	0	3	2.097958
tr|A0JLQ5|A0JLQ5_HUMAN	BXDC2	BXDC2 protein (Fragment) OS=Homo sapiens GN=BXDC2 PE=2 SV=1	39430	0	613	0	66	0	0.17	#DIV/0!	0	1	0	2	0	3	0	3	2.097958
tr|A8K2M0|A8K2M0_HUMAN	PSMC4	Proteasome (Prosome, macropain) 26S subunit, ATPase, 4, isoform CRA_b OS=Homo sapiens GN=PSMC4 PE=2 SV=1	47451	0	604	0	67	0	0.22	#DIV/0!	0	2	0	3	0	3	0	3	2.097958
tr|B7Z4B8|B7Z4B8_HUMAN	HNRNPUL1	Heterogeneous nuclear ribonucleoprotein U-like protein 1 OS=Homo sapiens GN=HNRNPUL1 PE=2 SV=1	86639	0	602	0	67	0	0.11	#DIV/0!	0	1	0	3	0	3	0	4	2.097958
tr|B4DUT7|B4DUT7_HUMAN	tr|B4DUT7|B4D	cDNA FLJ57604, highly similar to GMP synthase (glutamine-hydrolyzing) (EC 6.3.5.2) OS=Homo sapiens PE=2 SV=1	71880	0	595	0	60	0	0.18	#DIV/0!	0	2	0	3	0	3	0	4	2.097958
tr|B4DZP5|B4DZP5_HUMAN	tr|B4DZP5|B4D	cDNA FLJ51165, highly similar to DNA damage-binding protein 1 OS=Homo sapiens PE=2 SV=1	92726	0	594	0	68	0	0.11	#DIV/0!	0	2	0	3	0	3	0	4	2.097958
tr|A4D275|A4D275_HUMAN	ARPC1B	Actin related protein 2/3 complex, subunit 1B, 41kDa OS=Homo sapiens GN=ARPC1B PE=2 SV=1	41722	0	584	0	70	0	0.26	#DIV/0!	0	2	0	3	0	3	0	3	2.097958
tr|B4DGD5|B4DGD5_HUMAN	DNAJC11	DnaJ homolog subfamily C member 11 OS=Homo sapiens GN=DNAJC11 PE=2 SV=1	52854	0	578	0	71	0	0.13	#DIV/0!	0	1	0	2	0	3	0	3	2.097958
tr|A8KAQ5|A8KAQ5_HUMAN	tr|A8KAQ5|A8K	cDNA FLJ77404, highly similar to Homo sapiens small nuclear ribonucleoprotein 70kDa polypeptide (RNP antigen) (SNRP70), transcript varia	51522	0	576	0	71	0	0.2	#DIV/0!	0	2	0	3	0	3	0	3	2.097958
sp|O96008|TOM40_HUMAN	TOMM40	Mitochondrial import receptor subunit TOM40 homolog OS=Homo sapiens GN=TOMM40 PE=1 SV=1	38211	0	573	0	72	0	0.28	#DIV/0!	0	3	0	3	0	3	0	4	2.097958
tr|Q29945|Q29945_HUMAN	tr|Q29945|Q29	MHC class I HLA-A cell surface antigen (Fragment) OS=Homo sapiens PE=2 SV=1	27845	0	570	0	72	0	0.25	#DIV/0!	0	1	0	2	0	3	0	3	2.097958
sp|Q9Y266|NUDC_HUMAN	NUDC	Nuclear migration protein nudC OS=Homo sapiens GN=NUDC PE=1 SV=1	38276	0	564	0	73	0	0.18	#DIV/0!	0	2	0	2	0	3	0	3	2.097958
tr|B4E0N6|B4E0N6_HUMAN	tr|B4E0N6|B4E0	cDNA FLJ56280, highly similar to Endoplasmic reticulum-Golgi intermediate compartment protein 1 OS=Homo sapiens PE=2 SV=1	26584	0	554	0	74	0	0.27	#DIV/0!	0	2	0	2	0	3	0	3	2.097958
tr|F5H0J3|F5H0J3_HUMAN	NDUFA9	NADH dehydrogenase [ubiquinone] 1 alpha subcomplex subunit 9, mitochondrial OS=Homo sapiens GN=NDUFA9 PE=2 SV=1	15894	0	538	0	77	0	0.35	#DIV/0!	0	2	0	3	0	3	0	3	2.097958
tr|B2RBM7|B2RBM7_HUMAN	tr|B2RBM7|B2R	cDNA, FLJ95595, highly similar to Homo sapiens proteasome (prosome, macropain) 26S subunit, non-ATPase, 13 (PSMD13), mRNA OS=Hom	43131	0	516	0	79	0	0.25	#DIV/0!	0	1	0	3	0	3	0	3	2.097958
tr|B4DZ87|B4DZ87_HUMAN	tr|B4DZ87|B4D	cDNA FLJ57240, highly similar to Mitochondrial proteins import receptor OS=Homo sapiens PE=2 SV=1	57579	0	509	0	81	0	0.12	#DIV/0!	0	1	0	2	0	3	0	4	2.097958
sp|P11047|LAMC1_HUMAN	LAMC1	Laminin subunit gamma-1 OS=Homo sapiens GN=LAMC1 PE=1 SV=3	183191	0	497	0	84	0	0.05	#DIV/0!	0	2	0	3	0	3	0	4	2.097958
tr|B3KXH0|B3KXH0_HUMAN	tr|B3KXH0|B3K	cDNA FLJ45395 fis, clone BRHIP3027191, highly similar to 150 kDa oxygen-regulated protein (Orp150) OS=Homo sapiens PE=2 SV=1	110601	0	488	0	87	0	0.09	#DIV/0!	0	2	0	3	0	3	0	6	2.097958
tr|H3BRG4|H3BRG4_HUMAN	UQCRC2	Cytochrome b-c1 complex subunit 2, mitochondrial OS=Homo sapiens GN=UQCRC2 PE=3 SV=1	44778	0	483	0	88	0	0.24	#DIV/0!	0	1	0	3	0	3	0	4	2.097958
sp|Q96PK6|RBM14_HUMAN	RBM14	RNA-binding protein 14 OS=Homo sapiens GN=RBM14 PE=1 SV=2	69620	0	479	0	89	0	0.1	#DIV/0!	0	2	0	2	0	3	0	3	2.097958
tr|A4D210|A4D210_HUMAN	EIF3S9	Eukaryotic translation initiation factor 3, subunit 9 eta, 116kDa OS=Homo sapiens GN=EIF3S9 PE=4 SV=1	89024	0	474	0	90	0	0.07	#DIV/0!	0	1	0	2	0	3	0	3	2.097958
tr|J7F652|J7F652_HUMAN	HLA-B	MHC class I antigen (Fragment) OS=Homo sapiens GN=HLA-B PE=3 SV=1	21264	0	456	0	94	0	0.34	#DIV/0!	0	2	0	2	0	3	0	4	2.097958
sp|Q9NUL7|DDX28_HUMAN	DDX28	Probable ATP-dependent RNA helicase DDX28 OS=Homo sapiens GN=DDX28 PE=1 SV=2	59773	0	453	0	96	0	0.17	#DIV/0!	0	3	0	3	0	3	0	4	2.097958
tr|B4DPJ2|B4DPJ2_HUMAN	tr|B4DPJ2|B4DI	Annexin OS=Homo sapiens PE=2 SV=1	46025	0	446	0	97	0	0.15	#DIV/0!	0	2	0	2	0	3	0	3	2.097958

tr|F4NBX2|F4NBX2_HUMAN	HLA-B	MHC class I antigen (Fragment) OS=Homo sapiens GN=HLA-B PE=3 SV=1	38566	0	432	0	100	0	0.18	#DIV/0!	0	2	0	2	0	3	0	4	2.097958
tr|B4DN01|B4DN01_HUMAN	tr|B4DN01|B4D	cDNA FLJ57332, highly similar to COP9 signalosome complex subunit 3 OS=Homo sapiens PE=2 SV=1	33950	0	415	0	105	0	0.32	#DIV/0!	0	3	0	3	0	3	0	3	2.097958
tr|A8K9K4|A8K9K4_HUMAN	tr|A8K9K4|A8K9	cDNA FLJ75504, highly similar to Homo sapiens glucosidase I, mRNA OS=Homo sapiens PE=2 SV=1	62314	0	412	0	104	0	0.23	#DIV/0!	0	1	0	5	0	3	0	6	2.097958
tr|A9LSU1|A9LSU1_HUMAN	tr|A9LSU1|A9LS	Type IV collagen alpha 1 (Fragment) OS=Homo sapiens PE=2 SV=1	25998	0	393	0	113	0	0.44	#DIV/0!	0	2	0	3	0	3	0	4	2.097958
tr|E3Q0X1|E3Q0X1_HUMAN	HLA-B	MHC class I antigen (Fragment) OS=Homo sapiens GN=HLA-B PE=3 SV=1	31939	0	371	0	118	0	0.35	#DIV/0!	0	3	0	3	0	3	0	3	2.097958
tr|G0KXF8|G0KXF8_HUMAN	HLA-B	MHC class I antigen (Fragment) OS=Homo sapiens GN=HLA-B PE=3 SV=1	21235	0	370	0	118	0	0.55	#DIV/0!	0	3	0	3	0	3	0	3	2.097958
tr|G1ENK8|G1ENK8_HUMAN	HLA-B	MHC class I antigen (Fragment) OS=Homo sapiens GN=HLA-B PE=3 SV=1	21236	0	369	0	118	0	0.55	#DIV/0!	0	3	0	3	0	3	0	3	2.097958
sp|Q9BS26|ERP44_HUMAN	ERP44	Endoplasmic reticulum resident protein 44 OS=Homo sapiens GN=ERP44 PE=1 SV=1	47341	0	338	0	135	0	0.22	#DIV/0!	0	3	0	3	0	3	0	3	2.097958
tr|H3BLU7|H3BLU7_HUMAN	AKR7A2	Aflatoxin B1 aldehyde reductase member 2 (Fragment) OS=Homo sapiens GN=AKR7A2 PE=4 SV=1	35004	0	334	0	104	0	0.37	#DIV/0!	0	3	0	3	0	3	0	3	2.097958
tr|Q6EHZ3|Q6EHZ3_HUMAN	STRF8	Putative uncharacterized protein STRF8 OS=Homo sapiens GN=STRF8 PE=2 SV=1	41361	0	324	0	139	0	0.26	#DIV/0!	0	3	0	3	0	3	0	4	2.097958
tr|B4DR63|B4DR63_HUMAN	PSMC1	26S protease regulatory subunit 4 OS=Homo sapiens GN=PSMC1 PE=2 SV=1	41255	0	288	0	159	0	0.36	#DIV/0!	0	3	0	4	0	3	0	5	2.097958
tr|B3KM58|B3KM58_HUMAN	tr|B3KM58|B3K	cDNA FLJ10358 fis, clone NT2RM2001238, highly similar to Glutaminase kidney isoform, mitochondrial (Fragment) OS=Homo sapiens PE=2 S	41736	0	200	0	222	0	0.26	#DIV/0!	0	3	0	3	0	3	0	3	2.097958
tr|G3V1P5|G3V1P5_HUMAN	MED15	Mediator of RNA polymerase II transcription subunit 15 OS=Homo sapiens GN=MED15 PE=4 SV=1	80007	0	1124	0	24	0	0.04	#DIV/0!	0	0	0	1	0	2	0	2	1.612325
tr|Q2I0A3|Q2I0A3_HUMAN	CFTR	Cystic fibrosis transmembrane conductance regulator ATP-binding cassette sub-family C member 7 OS=Homo sapiens GN=CFTR PE=3 SV=1	168854	0	1111	0	24	0	0.02	#DIV/0!	0	0	0	1	0	2	0	2	1.612325
tr|B2RB06|B2RB06_HUMAN	tr|B2RB06|B2R	cDNA, FLJ95242, highly similar to Homo sapiens L-3-hydroxyacyl-Coenzyme A dehydrogenase, short chain (HADHSC), mRNA OS=Homo sapie	34297	0	1091	0	26	0	0.1	#DIV/0!	0	0	0	1	0	2	0	2	1.612325
sp|O14647|CHD2_HUMAN	CHD2	Chromodomain-helicase-DNA-binding protein 2 OS=Homo sapiens GN=CHD2 PE=1 SV=2	212183	0	1079	0	27	0	0.02	#DIV/0!	0	0	0	1	0	2	0	3	1.612325
sp|Q27J81|INF2_HUMAN	INF2	Inverted formin-2 OS=Homo sapiens GN=INF2 PE=1 SV=2	136851	0	1073	0	27	0	0.02	#DIV/0!	0	0	0	1	0	2	0	2	1.612325
tr|B2RAN1|B2RAN1_HUMAN	tr|B2RAN1|B2R	cDNA, FLJ95012, highly similar to Homo sapiens UDP-glucose pyrophosphorylase 2 (UGP2), mRNA OS=Homo sapiens PE=2 SV=1	57042	0	1060	0	28	0	0.06	#DIV/0!	0	0	0	1	0	2	0	2	1.612325
tr|Q6IBU0|Q6IBU0_HUMAN	EIF5	EIF5 protein OS=Homo sapiens GN=EIF5 PE=2 SV=1	49577	0	1049	0	29	0	0.07	#DIV/0!	0	0	0	1	0	2	0	2	1.612325
sp|Q9UBG0|MRC2_HUMAN	MRC2	C-type mannose receptor 2 OS=Homo sapiens GN=MRC2 PE=1 SV=2	169761	0	1032	0	30	0	0.04	#DIV/0!	0	0	0	2	0	2	0	2	1.612325
tr|B4DPW5|B4DPW5_HUMAN	tr|B4DPW5|B4D	cDNA FLJ59374, highly similar to Homo sapiens caldesmon 1 (CALD1), transcript variant 2, mRNA OS=Homo sapiens PE=2 SV=1	57436	0	1027	0	30	0	0.12	#DIV/0!	0	0	0	2	0	2	0	2	1.612325
sp|Q08945|SSRP1_HUMAN	SSRP1	FACT complex subunit SSRP1 OS=Homo sapiens GN=SSRP1 PE=1 SV=1	81367	0	1023	0	30	0	0.04	#DIV/0!	0	0	0	1	0	2	0	2	1.612325
sp|Q9UK61|F208A_HUMAN	FAM208A	Protein FAM208A OS=Homo sapiens GN=FAM208A PE=1 SV=3	190453	0	1022	0	30	0	0.02	#DIV/0!	0	1	0	1	0	2	0	2	1.612325
tr|B0AZM4|B0AZM4_HUMAN	tr|B0AZM4|B0A	cDNA, FLJ79464, highly similar to Homo sapiens aquarius (Fragment) OS=Homo sapiens PE=2 SV=1	157764	0	1002	0	31	0	0.02	#DIV/0!	0	0	0	1	0	2	0	2	1.612325
tr|Q5T621|Q5T621_HUMAN	AKR1A1	Alcohol dehydrogenase [NADP(+)] (Fragment) OS=Homo sapiens GN=AKR1A1 PE=4 SV=1	16677	0	986	0	33	0	0.2	#DIV/0!	0	0	0	1	0	2	0	2	1.612325
tr|B4DN27|B4DN27_HUMAN	tr|B4DN27|B4D	cDNA FLJ57057, highly similar to Small subunit processome component 20 homolog OS=Homo sapiens PE=2 SV=1	62656	0	955	0	35	0	0.05	#DIV/0!	0	1	0	1	0	2	0	2	1.612325
tr|G3V161|G3V161_HUMAN	KBTBD3	Kelch repeat and BTB (POZ) domain containing 3, isoform CRA_a OS=Homo sapiens GN=KBTBD3 PE=2 SV=1	61834	0	948	0	35	0	0.05	#DIV/0!	0	1	0	1	0	2	0	4	1.612325
tr|Q5U043|Q5U043_HUMAN	tr|Q5U043|Q5U	Alcohol dehydrogenase 5 (Class III), chi polypeptide OS=Homo sapiens PE=2 SV=1	40520	0	945	0	35	0	0.08	#DIV/0!	0	0	0	1	0	2	0	2	1.612325
tr|Q2VIM7|Q2VIM7_HUMAN	tr|Q2VIM7|Q2V	RcADH5 (Fragment) OS=Homo sapiens PE=2 SV=1	39774	0	944	0	35	0	0.08	#DIV/0!	0	0	0	1	0	2	0	2	1.612325
sp|Q9BVM4|GGACT_HUMAN	GGACT	Gamma-glutamylaminecyclotransferase OS=Homo sapiens GN=GGACT PE=1 SV=2	17432	0	943	0	35	0	0.2	#DIV/0!	0	1	0	1	0	2	0	2	1.612325
sp|Q9UHX1|PUF60_HUMAN	PUF60	Poly(U)-binding-splicing factor PUF60 OS=Homo sapiens GN=PUF60 PE=1 SV=1	60009	0	925	0	36	0	0.11	#DIV/0!	0	0	0	2	0	2	0	2	1.612325
tr|E9PDU6|E9PDU6_HUMAN	CNN3	Calponin-3 (Fragment) OS=Homo sapiens GN=CNN3 PE=2 SV=1	20309	0	924	0	36	0	0.1	#DIV/0!	0	0	0	1	0	2	0	2	1.612325
tr|G1UI16|G1UI16_HUMAN	PDS5A	SCC-112 protein, isoform CRA_b OS=Homo sapiens GN=PDS5A PE=2 SV=1	152274	0	919	0	37	0	0.04	#DIV/0!	0	1	0	2	0	2	0	2	1.612325
tr|Q4ZG72|Q4ZG72_HUMAN	DDX18	Putative uncharacterized protein DDX18 (Fragment) OS=Homo sapiens GN=DDX18 PE=2 SV=1	61898	0	914	0	37	0	0.17	#DIV/0!	0	0	0	3	0	2	0	5	1.612325
tr|H0Y2Y8|H0Y2Y8_HUMAN	ZYX	Zyxin (Fragment) OS=Homo sapiens GN=ZYX PE=4 SV=1	58804	0	907	0	38	0	0.11	#DIV/0!	0	0	0	2	0	2	0	2	1.612325
tr|J3QR89|J3QR89_HUMAN	tr|J3QR89|J3QR	Uncharacterized protein (Fragment) OS=Homo sapiens PE=4 SV=1	6611	0	906	0	38	0	0.54	#DIV/0!	0	1	0	1	0	2	0	2	1.612325
tr|G5E9P1|G5E9P1_HUMAN	ITPR1	Inositol 1,4,5-triphosphate receptor, type 1, isoform CRA_d OS=Homo sapiens GN=ITPR1 PE=2 SV=1	311832	0	893	0	39	0	0.02	#DIV/0!	0	1	0	2	0	2	0	2	1.612325
sp|Q9NP92|RT30_HUMAN	MRPS30	28S ribosomal protein S30, mitochondrial OS=Homo sapiens GN=MRPS30 PE=1 SV=2	50960	0	889	0	39	0	0.13	#DIV/0!	0	0	0	2	0	2	0	2	1.612325
tr|H0YCK3|H0YCK3_HUMAN	ADAR	Double-stranded RNA-specific adenosine deaminase (Fragment) OS=Homo sapiens GN=ADAR PE=4 SV=1	133761	0	883	0	39	0	0.02	#DIV/0!	0	1	0	1	0	2	0	2	1.612325
tr|B4DV28|B4DV28_HUMAN	tr|B4DV28|B4D	cDNA FLJ54170, highly similar to Cytosolic nonspecific dipeptidase OS=Homo sapiens PE=2 SV=1	51811	0	868	0	40	0	0.25	#DIV/0!	0	0	0	3	0	2	0	3	1.612325
tr|Q6FHH6|Q6FHH6_HUMAN	LANCL1	LANCL1 protein (Fragment) OS=Homo sapiens GN=LANCL1 PE=2 SV=1	45979	0	850	0	41	0	0.07	#DIV/0!	0	1	0	1	0	2	0	2	1.612325
tr|B3KM73|B3KM73_HUMAN	tr|B3KM73|B3K	cDNA FLJ10419 fis, clone NT2RP1000163, highly similar to Homo sapiens transforming growth factor beta regulator 4 (TBRG4), transcript va	55418	0	826	0	43	0	0.12	#DIV/0!	0	1	0	2	0	2	0	2	1.612325
tr|B3KNV9|B3KNV9_HUMAN	tr|B3KNV9|B3K	cDNA FLJ30554 fis, clone BRAWH2003693 OS=Homo sapiens PE=2 SV=1	112670	0	818	0	44	0	0.06	#DIV/0!	0	1	0	2	0	2	0	2	1.612325
tr|H7C4A8|H7C4A8_HUMAN	APEX1	DNA-(apurinic or apyrimidinic site) lyase (Fragment) OS=Homo sapiens GN=APEX1 PE=4 SV=1	17122	0	806	0	45	0	0.43	#DIV/0!	0	1	0	2	0	2	0	4	1.612325
sp|O76003|GLRX3_HUMAN	GLRX3	Glutaredoxin-3 OS=Homo sapiens GN=GLRX3 PE=1 SV=2	37693	0	789	0	47	0	0.18	#DIV/0!	0	1	0	2	0	2	0	2	1.612325
sp|Q09161|NCBP1_HUMAN	NCBP1	Nuclear cap-binding protein subunit 1 OS=Homo sapiens GN=NCBP1 PE=1 SV=1	92864	0	773	0	48	0	0.07	#DIV/0!	0	2	0	2	0	2	0	2	1.612325
tr|Q05DA4|Q05DA4_HUMAN	P4HA2	P4HA2 protein OS=Homo sapiens GN=P4HA2 PE=2 SV=1	57684	0	768	0	49	0	0.12	#DIV/0!	0	1	0	2	0	2	0	3	1.612325
sp|Q96ER9|CCD51_HUMAN	CCDC51	Coiled-coil domain-containing protein 51 OS=Homo sapiens GN=CCDC51 PE=1 SV=2	46011	0	764	0	49	0	0.15	#DIV/0!	0	1	0	2	0	2	0	2	1.612325
tr|F5GZS0|F5GZS0_HUMAN	DHX36	Probable ATP-dependent RNA helicase DHX36 OS=Homo sapiens GN=DHX36 PE=2 SV=1	113928	0	758	0	50	0	0.06	#DIV/0!	0	1	0	2	0	2	0	2	1.612325
tr|B2RDE1|B2RDE1_HUMAN	tr|B2RDE1|B2R	cDNA, FLJ96568, highly similar to Homo sapiens tropomyosin 3 (TPM3), mRNA OS=Homo sapiens PE=2 SV=1	29228	0	755	0	50	0	0.11	#DIV/0!	0	1	0	1	0	2	0	2	1.612325
sp|Q8N1G4|LRC47_HUMAN	LRRC47	Leucine-rich repeat-containing protein 47 OS=Homo sapiens GN=LRRC47 PE=1 SV=1	64004	0	750	0	50	0	0.11	#DIV/0!	0	2	0	2	0	2	0	2	1.612325
tr|B3KWV6|B3KWV6_HUMAN	tr|B3KWV6|B3K	cDNA FLJ43948 fis, clone TESTI4014924, highly similar to Homo sapiens cytoplasmic FMR1 interacting protein 1 (CYFIP1), transcript variant 1	147082	0	740	0	51	0	0.04	#DIV/0!	0	1	0	2	0	2	0	2	1.612325
tr|G1UI17|G1UI17_HUMAN	AGL	Glycogen debranching enzyme (Fragment) OS=Homo sapiens GN=AGL PE=2 SV=1	145569	0	738	0	52	0	0.05	#DIV/0!	0	1	0	2	0	2	0	2	1.612325
tr|B2R491|B2R491_HUMAN	RPS4X	Ribosomal protein S4, X-linked, isoform CRA_c OS=Homo sapiens GN=RPS4X PE=2 SV=1	29807	0	736	0	52	0	0.11	#DIV/0!	0	1	0	1	0	2	0	2	1.612325
tr|B3KY63|B3KY63_HUMAN	tr|B3KY63|B3KY	cDNA FLJ16830 fis, clone UTERU3022536, highly similar to Chromodomain helicase-DNA-binding protein 4 (EC 3.6.1.-) OS=Homo sapiens PE-	216574	0	724	0	53	0	0.03	#DIV/0!	0	2	0	2	0	2	0	4	1.612325
tr|Q0PHS4|Q0PHS4_HUMAN	G6PD	Glucose-6-phosphate dehydrogenase (Fragment) OS=Homo sapiens GN=G6PD PE=2 SV=1	7182	0	718	0	54	0	1.23	#DIV/0!	0	1	0	2	0	2	0	2	1.612325
sp|Q9NR45|SIAS_HUMAN	NANS	Sialic acid synthase OS=Homo sapiens GN=NANS PE=1 SV=2	40738	0	713	0	54	0	0.08	#DIV/0!	0	1	0	1	0	2	0	2	1.612325
sp|Q14558|KPRA_HUMAN	PRPSAP1	Phosphoribosyl pyrophosphate synthase-associated protein 1 OS=Homo sapiens GN=PRPSAP1 PE=1 SV=2	39654	0	696	0	56	0	0.35	#DIV/0!	0	1	0	3	0	2	0	3	1.612325
tr|G3V1K3|G3V1K3_HUMAN	PON2	Paraoxonase 2, isoform CRA_a OS=Homo sapiens GN=PON2 PE=2 SV=1	41672	0	691	0	57	0	0.17	#DIV/0!	0	2	0	2	0	2	0	2	1.612325
tr|D3DTY9|D3DTY9_HUMAN	TRIM25	Tripartite motif-containing 25, isoform CRA_a OS=Homo sapiens GN=TRIM25 PE=2 SV=1	48358	0	689	0	57	0	0.09	#DIV/0!	0	1	0	2	0	2	0	2	1.612325
tr|Q58EY4|Q58EY4_HUMAN	SMARCC1	SWI/SNF related, matrix associated, actin dependent regulator of chromatin, subfamily c, member 1 OS=Homo sapiens GN=SMARCC1 PE=2	123347	0	681	0	58	0	0.07	#DIV/0!	0	1	0	2	0	2	0	3	1.612325
tr|B7Z653|B7Z653_HUMAN	tr|B7Z653|B7Z6	cDNA FLJ61657, highly similar to Band 4.1-like protein 1 OS=Homo sapiens PE=2 SV=1	90347	0	680	0	58	0	0.04	#DIV/0!	0	1	0	1	0	2	0	3	1.612325
tr|A6NN60|A6NN60_HUMAN	CRYZ	Quinone oxidoreductase OS=Homo sapiens GN=CRYZ PE=2 SV=1	31680	0	679	0	58	0	0.22	#DIV/0!	0	2	0	2	0	2	0	3	1.612325
tr|D6REA1|D6REA1_HUMAN	SIL1	Nucleotide exchange factor SIL1 OS=Homo sapiens GN=SIL1 PE=2 SV=1	53000	0	668	0	59	0	0.13	#DIV/0!	0	1	0	2	0	2	0	3	1.612325
sp|Q8IYS2|K2013_HUMAN	KIAA2013	Uncharacterized protein KIAA2013 OS=Homo sapiens GN=KIAA2013 PE=2 SV=1	69684	0	667	0	60	0	0.1	#DIV/0!	0	1	0	2	0	2	0	2	1.612325
tr|B7Z213|B7Z213_HUMAN	tr|B7Z213|B7Z2	cDNA FLJ50130, highly similar to Heterogeneous nuclear ribonucleoprotein Q OS=Homo sapiens PE=2 SV=1	28561	0	666	0	60	0	0.25	#DIV/0!	0	1	0	2	0	2	0	2	1.612325
tr|H0YE29|H0YE29_HUMAN	ARHGAP1	Rho GTPase-activating protein 1 (Fragment) OS=Homo sapiens GN=ARHGAP1 PE=4 SV=1	38420	0	659	0	61	0	0.18	#DIV/0!	0	1	0	2	0	2	0	3	1.612325
tr|Q5H907|Q5H907_HUMAN	MAGED2	Melanoma antigen family D, 2, isoform CRA_d OS=Homo sapiens GN=MAGED2 PE=4 SV=1	55932	0	657	0	61	0	0.13	#DIV/0!	0	1	0	1	0	2	0	2	1.612325
sp|Q5JVF3|PCID2_HUMAN	PCID2	PCI domain-containing protein 2 OS=Homo sapiens GN=PCID2 PE=1 SV=2	46627	0	653	0	61	0	0.15	#DIV/0!	0	1	0	2	0	2	0	2	1.612325
tr|B3KM89|B3KM89_HUMAN	tr|B3KM89|B3K	cDNA FLJ10528 fis, clone NT2RP2000943, highly similar to Protein transport protein Sec24D OS=Homo sapiens PE=2 SV=1	101723	0	642	0	62	0	0.15	#DIV/0!	0	1	0	2	0	2	0	2	1.612325
tr|B1AK81|B1AK81_HUMAN	PIGK	GPI-anchor transamidase OS=Homo sapiens GN=PIGK PE=4 SV=1	34462	0	640	0	62	0	0.2	#DIV/0!	0	2	0	2	0	2	0	2	1.612325
tr|B3KQH2|B3KQH2_HUMAN	tr|B3KQH2|B3K	cDNA FLJ90454 fis, clone NT2RP3001560, highly similar to Cleft lip and palate transmembrane protein 1 OS=Homo sapiens PE=2 SV=1	76247	0	636	0	63	0	0.09	#DIV/0!	0	2	0	2	0	2	0	2	1.612325
tr|Q3LIF4|Q3LIF4_HUMAN	Nbla00271	NOGO-A (Fragment) OS=Homo sapiens GN=Nbla00271 PE=2 SV=1	57162	0	635	0	49	0	0.29	#DIV/0!	0	0	0	2	0	2	0	2	1.612325
sp|O15230|LAMA5_HUMAN	LAMA5	Laminin subunit alpha-5 OS=Homo sapiens GN=LAMA5 PE=1 SV=8	412023	0	634	0	63	0	0.02	#DIV/0!	0	1	0	2	0	2	0	3	1.612325
tr|H3BM93|H3BM93_HUMAN	NUP93	Nuclear pore complex protein Nup93 (Fragment) OS=Homo sapiens GN=NUP93 PE=4 SV=1	19858	0	633	0	55	0	0.07	#DIV/0!	0	1	0	1	0	2	0	3	1.612325
sp|Q9H9J2|RM44_HUMAN	MRPL44	39S ribosomal protein L44, mitochondrial OS=Homo sapiens GN=MRPL44 PE=1 SV=1	37854	0	626	0	64	0	0.09	#DIV/0!	0	1	0	1	0	2	0	2	1.612325
tr|B4DH43|B4DH43_HUMAN	tr|B4DH43|B4D	cDNA FLJ56433, highly similar to Collagen alpha-2(IV) chain OS=Homo sapiens PE=2 SV=1	83428	0	624	0	64	0	0.08	#DIV/0!	0	1	0	2	0	2	0	2	1.612325
tr|B2R825|B2R825_HUMAN	tr|B2R825|B2R	Phosphorylase OS=Homo sapiens PE=2 SV=1	97405	0	612	0	48	0	0.11	#DIV/0!	0	1	0	2	0	2	0	4	1.612325
sp|Q6PKG0|LARP1_HUMAN	LARP1	La-related protein 1 OS=Homo sapiens GN=LARP1 PE=1 SV=2	123833	0	600	0	67	0	0.05	#DIV/0!	0	1	0	2	0	2	0	3	1.612325
tr|B2R6E5|B2R6E5_HUMAN	tr|B2R6E5|B2R	NADPH--cytochrome P450 reductase OS=Homo sapiens PE=2 SV=1	77429	0	597	0	68	0	0.09	#DIV/0!	0	2	0	2	0	2	0	4	1.612325
tr|F5GY99|F5GY99_HUMAN	GALNT1	Polypeptide N-acetylgalactosaminyltransferase 1 soluble form OS=Homo sapiens GN=GALNT1 PE=2 SV=1	58084	0	596	0	68	0	0.18	#DIV/0!	0	1	0	3	0	2	0	3	1.612325
tr|B2RNR6|B2RNR6_HUMAN	ZFR	Zinc finger RNA binding protein OS=Homo sapiens GN=ZFR PE=2 SV=1	118081	0	588	0	69	0	0.36	#DIV/0!	0	1	0	2	0	2	0	3	1.612325
sp|Q13619|CUL4A_HUMAN	CUL4A	Cullin-4A OS=Homo sapiens GN=CUL4A PE=1 SV=3	88138	0	582	0	71	0	0.13	#DIV/0!	0	2	0	3	0	2	0	3	1.612325
tr|B2RA56|B2RA56_HUMAN	tr|B2RA56|B2R	cDNA, FLJ94705, highly similar to Homo sapiens nicalin (LOC56926), mRNA OS=Homo sapiens PE=2 SV=1	61891	0	575	0	71	0	0.11	#DIV/0!	0	2	0	2	0	2	0	3	1.612325
tr|J3KQ32|J3KQ32_HUMAN	OLA1	Obg-like ATPase 1 OS=Homo sapiens GN=OLA1 PE=4 SV=1	47136	0	572	0	72	0	0.14	#DIV/0!	0	2	0	2	0	2	0	2	1.612325
sp|Q9NTI5|PDS5B_HUMAN	PDS5B	Sister chromatid cohesion protein PDS5 homolog B OS=Homo sapiens GN=PDS5B PE=1 SV=1	165818	0	563	0	73	0	0.04	#DIV/0!	0	1	0	2	0	2	0	2	1.612325
sp|P04899|GNAI2_HUMAN	GNAI2	Guanine nucleotide-binding protein G(i) subunit alpha-2 OS=Homo sapiens GN=GNAI2 PE=1 SV=3	40995	0	555	0	74	0	0.18	#DIV/0!	0	2	0	2	0	2	0	2	1.612325
sp|A1L0T0|ILVBL_HUMAN	ILVBL	Acetolactate synthase-like protein OS=Homo sapiens GN=ILVBL PE=1 SV=2	68452	0	542	0	76	0	0.1	#DIV/0!	0	2	0	2	0	2	0	2	1.612325
tr|Q4KMR3|Q4KMR3_HUMAN	MYO1E	MYO1E variant protein OS=Homo sapiens GN=MYO1E PE=2 SV=1	127552	0	539	0	77	0	0.05	#DIV/0!	0	2	0	2	0	2	0	2	1.612325
sp|P36952|SPB5_HUMAN	SERPINB5	Serpin B5 OS=Homo sapiens GN=SERPINB5 PE=1 SV=2	42530	0	532	0	77	0	0.16	#DIV/0!	0	2	0	2	0	2	0	2	1.612325
tr|B0QZ18|B0QZ18_HUMAN	CPNE1	Copine I OS=Homo sapiens GN=CPNE1 PE=2 SV=1	60307	0	530	0	78	0	0.11	#DIV/0!	0	2	0	2	0	2	0	2	1.612325
sp|Q00653|NFKB2_HUMAN	NFKB2	Nuclear factor NF-kappa-B p100 subunit OS=Homo sapiens GN=NFKB2 PE=1 SV=4	97373	0	527	0	78	0	0.07	#DIV/0!	0	2	0	2	0	2	0	2	1.612325
tr|H0Y3P2|H0Y3P2_HUMAN	EIF4G2	Eukaryotic translation initiation factor 4 gamma 2 OS=Homo sapiens GN=EIF4G2 PE=4 SV=1	98569	0	518	0	79	0	0.07	#DIV/0!	0	2	0	2	0	2	0	2	1.612325
tr|B2R8G6|B2R8G6_HUMAN	tr|B2R8G6|B2R	cDNA, FLJ93891, Homo sapiens FK506 binding protein 8, 38kDa (FKBP8), mRNA OS=Homo sapiens PE=2 SV=1	38870	0	506	0	81	0	0.18	#DIV/0!	0	1	0	2	0	2	0	2	1.612325
tr|B2RBB2|B2RBB2_HUMAN	tr|B2RBB2|B2R	cDNA, FLJ95416, highly similar to Homo sapiens phosphatidylinositol glycan, class S (PIGS), mRNA OS=Homo sapiens PE=2 SV=1	61818	0	489	0	86	0	0.11	#DIV/0!	0	2	0	2	0	2	0	2	1.612325
sp|Q96CS3|FAF2_HUMAN	FAF2	FAS-associated factor 2 OS=Homo sapiens GN=FAF2 PE=1 SV=2	52933	0	482	0	88	0	0.13	#DIV/0!	0	1	0	2	0	2	0	3	1.612325
tr|G3V1L9|G3V1L9_HUMAN	TJP1	Tight junction protein 1 (Zona occludens 1), isoform CRA_a OS=Homo sapiens GN=TJP1 PE=4 SV=1	197681	0	467	0	90	0	0.03	#DIV/0!	0	2	0	2	0	2	0	3	1.612325
tr|B3KRR1|B3KRR1_HUMAN	tr|B3KRR1|B3K	cDNA FLJ34725 fis, clone MESAN2005958, highly similar to RNA-binding protein Luc7-like 2 OS=Homo sapiens PE=2 SV=1	40752	0	462	0	91	0	0.08	#DIV/0!	0	1	0	1	0	2	0	2	1.612325
tr|B5BU61|B5BU61_HUMAN	HDAC1	Histone deacetylase OS=Homo sapiens GN=HDAC1 PE=2 SV=1	55611	0	460	0	48	0	0.19	#DIV/0!	0	1	0	1	0	2	0	2	1.612325
tr|A8KA19|A8KA19_HUMAN	tr|A8KA19|A8K	cDNA FLJ75831, highly similar to Homo sapiens exportin, tRNA (nuclear export receptor for tRNAs) (XPOT), mRNA OS=Homo sapiens PE=2 SV	111104	0	450	0	97	0	0.06	#DIV/0!	0	2	0	2	0	2	0	2	1.612325
sp|Q9GZL7|WDR12_HUMAN	WDR12	Ribosome biogenesis protein WDR12 OS=Homo sapiens GN=WDR12 PE=1 SV=2	48191	0	438	0	99	0	0.22	#DIV/0!	0	2	0	3	0	2	0	3	1.612325
tr|F5GZ76|F5GZ76_HUMAN	USO1	General vesicular transport factor p115 OS=Homo sapiens GN=USO1 PE=2 SV=1	90003	0	419	0	104	0	0.07	#DIV/0!	0	1	0	2	0	2	0	2	1.612325
tr|A8K0D2|A8K0D2_HUMAN	tr|A8K0D2|A8K	cDNA FLJ77740, highly similar to Homo sapiens 7-dehydrocholesterol reductase, mRNA OS=Homo sapiens PE=2 SV=1	55175	0	416	0	105	0	0.12	#DIV/0!	0	2	0	2	0	2	0	2	1.612325
tr|B4DFP1|B4DFP1_HUMAN	tr|B4DFP1|B4D	cDNA FLJ51818, highly similar to Phosphoglucomutase-1 (EC 5.4.2.2) OS=Homo sapiens PE=2 SV=1	58987	0	414	0	78	0	0.17	#DIV/0!	0	2	0	2	0	2	0	2	1.612325
sp|Q9H3P7|GCP60_HUMAN	ACBD3	Golgi resident protein GCP60 OS=Homo sapiens GN=ACBD3 PE=1 SV=4	60841	0	405	0	108	0	0.11	#DIV/0!	0	2	0	2	0	2	0	2	1.612325
sp|P02748|CO9_HUMAN	C9	Complement component C9 OS=Homo sapiens GN=C9 PE=1 SV=2	64615	0	403	0	109	0	0.1	#DIV/0!	0	2	0	2	0	2	0	2	1.612325
tr|Q0VAB1|Q0VAB1_HUMAN	TIMM50	Translocase of inner mitochondrial membrane 50 homolog (S. cerevisiae) OS=Homo sapiens GN=TIMM50 PE=2 SV=1	50960	0	375	0	118	0	0.13	#DIV/0!	0	2	0	2	0	2	0	2	1.612325
tr|B4DUF1|B4DUF1_HUMAN	tr|B4DUF1|B4D	cDNA FLJ59760, highly similar to 1,4-alpha-glucan branching enzyme (EC 2.4.1.18) OS=Homo sapiens PE=2 SV=1	76254	0	366	0	120	0	0.08	#DIV/0!	0	2	0	2	0	2	0	2	1.612325
tr|H0YHX9|H0YHX9_HUMAN	NACA	Nascent polypeptide-associated complex subunit alpha (Fragment) OS=Homo sapiens GN=NACA PE=4 SV=1	22930	0	348	0	132	0	0.31	#DIV/0!	0	2	0	2	0	2	0	2	1.612325
tr|B3KMB7|B3KMB7_HUMAN	PYCR2	Pyrroline-5-carboxylate reductase family, member 2, isoform CRA_a OS=Homo sapiens GN=PYCR2 PE=2 SV=1	22559	0	343	0	87	0	0.59	#DIV/0!	0	2	0	3	0	2	0	3	1.612325
tr|A8K132|A8K132_HUMAN	tr|A8K132|A8K	cDNA FLJ75476, highly similar to Homo sapiens glutaminase (GLS), mRNA OS=Homo sapiens PE=2 SV=1	74317	0	339	0	135	0	0.14	#DIV/0!	0	2	0	3	0	2	0	5	1.612325
tr|Q5VZA6|Q5VZA6_HUMAN	ACO1	Cytoplasmic aconitate hydratase OS=Homo sapiens GN=ACO1 PE=4 SV=1	48888	0	268	0	113	0	0.18	#DIV/0!	0	2	0	2	0	2	0	2	1.612325
sp|Q9BVP2|GNL3_HUMAN	GNL3	Guanine nucleotide-binding protein-like 3 OS=Homo sapiens GN=GNL3 PE=1 SV=2	62468	0	1190	0	17	0	0.05	#DIV/0!	0	0	0	1	0	1	0	2	0.875153
tr|B4E2N1|B4E2N1_HUMAN	tr|B4E2N1|B4E2	cDNA FLJ56560, highly similar to Caspase recruitment domain-containing protein 6 OS=Homo sapiens PE=2 SV=1	55261	0	1188	0	17	0	0.06	#DIV/0!	0	1	0	1	0	1	0	2	0.875153
sp|Q96GA3|LTV1_HUMAN	LTV1	Protein LTV1 homolog OS=Homo sapiens GN=LTV1 PE=1 SV=1	55049	0	1181	0	18	0	0.06	#DIV/0!	0	0	0	1	0	1	0	2	0.875153
tr|B4DYU9|B4DYU9_HUMAN	tr|B4DYU9|B4D	cDNA FLJ60060, highly similar to Origin recognition complex subunit 2 OS=Homo sapiens PE=2 SV=1	58631	0	1168	0	20	0	0.06	#DIV/0!	0	0	0	1	0	1	0	1	0.875153
tr|B4DYX0|B4DYX0_HUMAN	tr|B4DYX0|B4D	cDNA FLJ58344 OS=Homo sapiens PE=2 SV=1	67806	0	1167	0	20	0	0.05	#DIV/0!	0	0	0	1	0	1	0	1	0.875153
sp|Q32MZ4|LRRF1_HUMAN	LRRFIP1	Leucine-rich repeat flightless-interacting protein 1 OS=Homo sapiens GN=LRRFIP1 PE=1 SV=2	89826	0	1166	0	20	0	0.04	#DIV/0!	0	0	0	1	0	1	0	1	0.875153
sp|Q6NVY1|HIBCH_HUMAN	HIBCH	3-hydroxyisobutyryl-CoA hydrolase, mitochondrial OS=Homo sapiens GN=HIBCH PE=1 SV=2	43797	0	1165	0	20	0	0.08	#DIV/0!	0	0	0	1	0	1	0	1	0.875153
tr|H0Y580|H0Y580_HUMAN	EIF2B3	Translation initiation factor eIF-2B subunit gamma (Fragment) OS=Homo sapiens GN=EIF2B3 PE=4 SV=1	25394	0	1163	0	20	0	0.13	#DIV/0!	0	0	0	1	0	1	0	1	0.875153
tr|B4DSY3|B4DSY3_HUMAN	tr|B4DSY3|B4D	cDNA FLJ54309, highly similar to Krueppel-related zinc finger protein 1 OS=Homo sapiens PE=2 SV=1	75017	0	1162	0	21	0	0.04	#DIV/0!	0	0	0	1	0	1	0	1	0.875153
sp|Q9NX58|LYAR_HUMAN	LYAR	Cell growth-regulating nucleolar protein OS=Homo sapiens GN=LYAR PE=1 SV=2	44044	0	1160	0	21	0	0.07	#DIV/0!	0	0	0	1	0	1	0	1	0.875153
tr|E5RK48|E5RK48_HUMAN	RDH10	Retinol dehydrogenase 10 OS=Homo sapiens GN=RDH10 PE=2 SV=1	20236	0	1159	0	21	0	0.17	#DIV/0!	0	0	0	1	0	1	0	1	0.875153
tr|B4E3R2|B4E3R2_HUMAN	tr|B4E3R2|B4E3	cDNA FLJ59085, highly similar to Polycystin-1 OS=Homo sapiens PE=2 SV=1	65161	0	1158	0	21	0	0.05	#DIV/0!	0	0	0	1	0	1	0	1	0.875153
sp|Q8IW19|APLF_HUMAN	APLF	Aprataxin and PNK-like factor OS=Homo sapiens GN=APLF PE=1 SV=1	57605	0	1157	0	21	0	0.06	#DIV/0!	0	0	0	1	0	1	0	1	0.875153
tr|Q5SRT3|Q5SRT3_HUMAN	CLIC1	Chloride intracellular channel 1, isoform CRA_a OS=Homo sapiens GN=CLIC1 PE=2 SV=2	27248	0	1154	0	21	0	0.12	#DIV/0!	0	0	0	1	0	1	0	1	0.875153
tr|B4DYF0|B4DYF0_HUMAN	tr|B4DYF0|B4D	cDNA FLJ61469, highly similar to Zinc finger protein 694 OS=Homo sapiens PE=2 SV=1	89134	0	1153	0	21	0	0.04	#DIV/0!	0	0	0	1	0	1	0	1	0.875153
tr|A0AV56|A0AV56_HUMAN	SAFB	SAFB protein OS=Homo sapiens GN=SAFB PE=2 SV=1	103249	0	1152	0	21	0	0.03	#DIV/0!	0	0	0	1	0	1	0	1	0.875153
tr|B4DL26|B4DL26_HUMAN	tr|B4DL26|B4D	cDNA FLJ60289, highly similar to Zinc finger protein 561 OS=Homo sapiens PE=2 SV=1	40733	0	1151	0	21	0	0.08	#DIV/0!	0	0	0	1	0	1	0	1	0.875153
sp|P01008|ANT3_HUMAN	SERPINC1	Antithrombin-III OS=Homo sapiens GN=SERPINC1 PE=1 SV=1	53025	0	1147	0	22	0	0.06	#DIV/0!	0	0	0	1	0	1	0	1	0.875153
sp|O43815|STRN_HUMAN	STRN	Striatin OS=Homo sapiens GN=STRN PE=1 SV=4	86535	0	1145	0	22	0	0.04	#DIV/0!	0	0	0	1	0	1	0	2	0.875153
sp|Q5T5B0|LCE3E_HUMAN	LCE3E	Late cornified envelope protein 3E OS=Homo sapiens GN=LCE3E PE=2 SV=1	10298	0	1144	0	22	0	0.34	#DIV/0!	0	0	0	1	0	1	0	1	0.875153
tr|B4DT66|B4DT66_HUMAN	tr|B4DT66|B4D	cDNA FLJ55367, highly similar to Rattus norvegicus SDA1 domain containing 1 (Sdad1), mRNA OS=Homo sapiens PE=2 SV=1	75834	0	1143	0	22	0	0.04	#DIV/0!	0	0	0	1	0	1	0	1	0.875153
sp|Q8WWL7|CCNB3_HUMAN	CCNB3	G2/mitotic-specific cyclin-B3 OS=Homo sapiens GN=CCNB3 PE=1 SV=2	159013	0	1142	0	22	0	0.02	#DIV/0!	0	0	0	1	0	1	0	2	0.875153
tr|Q5JPD9|Q5JPD9_HUMAN	DKFZp313I2429	Coiled-coil domain-containing protein 132 OS=Homo sapiens GN=DKFZp313I2429 PE=2 SV=1	68615	0	1141	0	22	0	0.05	#DIV/0!	0	0	0	1	0	1	0	1	0.875153
sp|Q14500|IRK12_HUMAN	KCNJ12	ATP-sensitive inward rectifier potassium channel 12 OS=Homo sapiens GN=KCNJ12 PE=2 SV=2	49540	0	1140	0	22	0	0.07	#DIV/0!	0	0	0	1	0	1	0	1	0.875153
tr|H0YD93|H0YD93_HUMAN	RUFY2	RUN and FYVE domain-containing protein 2 (Fragment) OS=Homo sapiens GN=RUFY2 PE=4 SV=1	72131	0	1139	0	22	0	0.05	#DIV/0!	0	0	0	1	0	1	0	1	0.875153
sp|Q96IJ6|GMPPA_HUMAN	GMPPA	Mannose-1-phosphate guanyltransferase alpha OS=Homo sapiens GN=GMPPA PE=1 SV=1	46604	0	1138	0	22	0	0.07	#DIV/0!	0	1	0	1	0	1	0	2	0.875153
sp|Q9BTC0|DIDO1_HUMAN	DIDO1	Death-inducer obliterator 1 OS=Homo sapiens GN=DIDO1 PE=1 SV=5	245434	0	1137	0	22	0	0.01	#DIV/0!	0	0	0	1	0	1	0	1	0.875153
sp|Q08499|PDE4D_HUMAN	PDE4D	cAMP-specific 3~,5~-cyclic phosphodiesterase 4D OS=Homo sapiens GN=PDE4D PE=1 SV=2	91628	0	1136	0	23	0	0.07	#DIV/0!	0	0	0	1	0	1	0	1	0.875153
tr|Q6IPJ9|Q6IPJ9_HUMAN	LAD1	Ladinin 1 OS=Homo sapiens GN=LAD1 PE=2 SV=1	57125	0	1133	0	23	0	0.06	#DIV/0!	0	0	0	1	0	1	0	1	0.875153
tr|H0YJ41|H0YJ41_HUMAN	BTBD7	BTB/POZ domain-containing protein 7 (Fragment) OS=Homo sapiens GN=BTBD7 PE=4 SV=1	83236	0	1132	0	23	0	0.04	#DIV/0!	0	0	0	1	0	1	0	1	0.875153
tr|Q8NEQ1|Q8NEQ1_HUMAN	LOC100132686	LOC100132686 protein OS=Homo sapiens GN=LOC100132686 PE=2 SV=1	4430	0	1131	0	23	0	0.84	#DIV/0!	0	0	0	1	0	1	0	1	0.875153
sp|Q9NQN1|OR2S1_HUMAN	OR2S2	Olfactory receptor 2S2 OS=Homo sapiens GN=OR2S2 PE=2 SV=2	35605	0	1127	0	23	0	0.09	#DIV/0!	0	0	0	1	0	1	0	1	0.875153
sp|Q96N38|ZN714_HUMAN	ZNF714	Zinc finger protein 714 OS=Homo sapiens GN=ZNF714 PE=2 SV=2	65951	0	1126	0	23	0	0.05	#DIV/0!	0	1	0	1	0	1	0	2	0.875153
sp|Q8TCS8|PNPT1_HUMAN	PNPT1	Polyribonucleotide nucleotidyltransferase 1, mitochondrial OS=Homo sapiens GN=PNPT1 PE=1 SV=2	86524	0	1123	0	24	0	0.04	#DIV/0!	0	0	0	1	0	1	0	1	0.875153
sp|Q6DKJ4|NXN_HUMAN	NXN	Nucleoredoxin OS=Homo sapiens GN=NXN PE=1 SV=2	48761	0	1120	0	24	0	0.07	#DIV/0!	0	0	0	1	0	1	0	1	0.875153
tr|B2RBQ0|B2RBQ0_HUMAN	tr|B2RBQ0|B2R	cDNA, FLJ95627, highly similar to Homo sapiens heparanase 2 (HPSE2), mRNA OS=Homo sapiens PE=2 SV=1	66940	0	1118	0	24	0	0.05	#DIV/0!	0	0	0	1	0	1	0	1	0.875153
tr|J3KNY9|J3KNY9_HUMAN	RIMBP3	RIMS-binding protein 3A OS=Homo sapiens GN=RIMBP3 PE=4 SV=1	171916	0	1116	0	24	0	0.02	#DIV/0!	0	0	0	1	0	1	0	1	0.875153
tr|G3V438|G3V438_HUMAN	AHSA1	Activator of 90 kDa heat shock protein ATPase homolog 1 (Fragment) OS=Homo sapiens GN=AHSA1 PE=2 SV=1	23078	0	1115	0	24	0	0.14	#DIV/0!	0	0	0	1	0	1	0	1	0.875153
tr|B9A6K1|B9A6K1_HUMAN	TBC1D5	TBC1 domain family, member 5 OS=Homo sapiens GN=TBC1D5 PE=2 SV=1	89519	0	1113	0	24	0	0.04	#DIV/0!	0	0	0	1	0	1	0	1	0.875153
sp|Q5TFE4|NT5D1_HUMAN	NT5DC1	5~-nucleotidase domain-containing protein 1 OS=Homo sapiens GN=NT5DC1 PE=1 SV=1	52439	0	1112	0	24	0	0.07	#DIV/0!	0	0	0	1	0	1	0	1	0.875153
sp|P27694|RFA1_HUMAN	RPA1	Replication protein A 70 kDa DNA-binding subunit OS=Homo sapiens GN=RPA1 PE=1 SV=2	68723	0	1110	0	24	0	0.05	#DIV/0!	0	0	0	1	0	1	0	1	0.875153
tr|B4E063|B4E063_HUMAN	tr|B4E063|B4E0	cDNA FLJ54292, highly similar to Kinesin-like protein KIFC1 OS=Homo sapiens PE=2 SV=1	73571	0	1109	0	25	0	0.15	#DIV/0!	0	0	0	1	0	1	0	1	0.875153
tr|B3KNJ4|B3KNJ4_HUMAN	SAE1	SUMO-1 activating enzyme subunit 1, isoform CRA_a OS=Homo sapiens GN=SAE1 PE=2 SV=1	33705	0	1108	0	24	0	0.18	#DIV/0!	0	1	0	1	0	1	0	1	0.875153
tr|G3V529|G3V529_HUMAN	DDX24	ATP-dependent RNA helicase DDX24 OS=Homo sapiens GN=DDX24 PE=2 SV=1	91994	0	1107	0	25	0	0.04	#DIV/0!	0	0	0	1	0	1	0	1	0.875153
tr|H0YL72|H0YL72_HUMAN	IDH3A	Isocitrate dehydrogenase [NAD] subunit alpha, mitochondrial OS=Homo sapiens GN=IDH3A PE=3 SV=1	36162	0	1106	0	25	0	0.38	#DIV/0!	0	0	0	2	0	1	0	2	0.875153
tr|B4DSJ1|B4DSJ1_HUMAN	tr|B4DSJ1|B4DS	cDNA FLJ56677, highly similar to RNA-binding protein Raly OS=Homo sapiens PE=2 SV=1	14125	0	1104	0	25	0	0.24	#DIV/0!	0	0	0	1	0	1	0	1	0.875153
tr|B3KSX3|B3KSX3_HUMAN	tr|B3KSX3|B3KS	cDNA FLJ37224 fis, clone BRAMY2000052, moderately similar to RNA-binding protein Raly OS=Homo sapiens PE=2 SV=1	32441	0	1103	0	25	0	0.1	#DIV/0!	0	0	0	1	0	1	0	1	0.875153
tr|B4DHS5|B4DHS5_HUMAN	tr|B4DHS5|B4D	cDNA FLJ54678, highly similar to N(2),N(2)-dimethylguanosine tRNA methyltransferase (EC 2.1.1.32) OS=Homo sapiens PE=2 SV=1	59256	0	1102	0	25	0	0.06	#DIV/0!	0	0	0	1	0	1	0	1	0.875153
tr|Q2VIM1|Q2VIM1_HUMAN	tr|Q2VIM1|Q2V	RcCDC20 (Fragment) OS=Homo sapiens PE=2 SV=1	50517	0	1101	0	25	0	0.07	#DIV/0!	0	0	0	1	0	1	0	1	0.875153
tr|H0YKU5|H0YKU5_HUMAN	COPS2	COP9 signalosome complex subunit 2 (Fragment) OS=Homo sapiens GN=COPS2 PE=4 SV=1	31061	0	1100	0	25	0	0.11	#DIV/0!	0	0	0	1	0	1	0	1	0.875153
tr|O43245|O43245_HUMAN	p65	Protein p65 OS=Homo sapiens GN=p65 PE=2 SV=2	69659	0	1099	0	25	0	0.05	#DIV/0!	0	0	0	1	0	1	0	1	0.875153
tr|B3KWW3|B3KWW3_HUMAN	tr|B3KWW3|B3	cDNA FLJ44017 fis, clone TESTI4025920, weakly similar to Homo sapiens maestro (MRO), mRNA OS=Homo sapiens PE=2 SV=1	116458	0	1096	0	25	0	0.03	#DIV/0!	0	1	0	1	0	1	0	2	0.875153
tr|B4E2S2|B4E2S2_HUMAN	tr|B4E2S2|B4E2	cDNA FLJ61556, highly similar to Poly (ADP-ribose) polymerase 4 (EC 2.4.2.30) OS=Homo sapiens PE=2 SV=1	113804	0	1095	0	26	0	0.03	#DIV/0!	0	0	0	1	0	1	0	1	0.875153
tr|E9PCX8|E9PCX8_HUMAN	TNS3	Tensin-3 (Fragment) OS=Homo sapiens GN=TNS3 PE=2 SV=1	135142	0	1094	0	26	0	0.05	#DIV/0!	0	0	0	1	0	1	0	1	0.875153
tr|E9PRF4|E9PRF4_HUMAN	SETDB1	Histone-lysine N-methyltransferase SETDB1 (Fragment) OS=Homo sapiens GN=SETDB1 PE=2 SV=1	141124	0	1093	0	26	0	0.02	#DIV/0!	0	0	0	1	0	1	0	1	0.875153
tr|B2R894|B2R894_HUMAN	MRPL38	Mitochondrial ribosomal protein L38, isoform CRA_b OS=Homo sapiens GN=MRPL38 PE=2 SV=1	41017	0	1092	0	26	0	0.09	#DIV/0!	0	0	0	1	0	1	0	2	0.875153
tr|H7C4G8|H7C4G8_HUMAN	NT5DC2	5~-nucleotidase domain-containing protein 2 (Fragment) OS=Homo sapiens GN=NT5DC2 PE=4 SV=1	30207	0	1090	0	26	0	0.11	#DIV/0!	0	0	0	1	0	1	0	1	0.875153
tr|B4DWN1|B4DWN1_HUMAN	tr|B4DWN1|B4	cDNA FLJ52285, highly similar to Vesicular integral-membrane protein VIP36 OS=Homo sapiens PE=2 SV=1	32842	0	1088	0	26	0	0.1	#DIV/0!	0	0	0	1	0	1	0	2	0.875153
tr|G3V2E7|G3V2E7_HUMAN	KLC1	Kinesin light chain 1 OS=Homo sapiens GN=KLC1 PE=2 SV=1	63133	0	1087	0	26	0	0.11	#DIV/0!	0	0	0	1	0	1	0	1	0.875153
tr|B4DYY5|B4DYY5_HUMAN	tr|B4DYY5|B4D	cDNA FLJ55671, highly similar to Ubiquitin-associated protein 2-like OS=Homo sapiens PE=2 SV=1	113662	0	1086	0	26	0	0.08	#DIV/0!	0	0	0	1	0	1	0	1	0.875153
sp|O00487|PSDE_HUMAN	PSMD14	26S proteasome non-ATPase regulatory subunit 14 OS=Homo sapiens GN=PSMD14 PE=1 SV=1	34726	0	1085	0	26	0	0.2	#DIV/0!	0	0	0	1	0	1	0	1	0.875153
tr|Q6LER7|Q6LER7_HUMAN	alpha-GalA	Alpha-galactosidase A (Fragment) OS=Homo sapiens GN=alpha-GalA PE=2 SV=1	46459	0	1084	0	26	0	0.07	#DIV/0!	0	0	0	1	0	1	0	1	0.875153
sp|Q04941|PLP2_HUMAN	PLP2	Proteolipid protein 2 OS=Homo sapiens GN=PLP2 PE=1 SV=1	17022	0	1083	0	26	0	0.2	#DIV/0!	0	0	0	1	0	1	0	1	0.875153
tr|B0YIW5|B0YIW5_HUMAN	ARCN1	Archain 1, isoform CRA_b OS=Homo sapiens GN=ARCN1 PE=2 SV=1	57630	0	1081	0	27	0	0.06	#DIV/0!	0	0	0	1	0	1	0	1	0.875153
tr|Q6DCA8|Q6DCA8_HUMAN	BCLAF1	BCLAF1 protein (Fragment) OS=Homo sapiens GN=BCLAF1 PE=2 SV=1	77958	0	1080	0	27	0	0.03	#DIV/0!	0	0	0	1	0	1	0	1	0.875153
tr|B4DR36|B4DR36_HUMAN	tr|B4DR36|B4D	cDNA FLJ56414, highly similar to Homo sapiens proline-, glutamic acid-, leucine-rich protein 1 (PELP1), mRNA OS=Homo sapiens PE=2 SV=1	126101	0	1077	0	27	0	0.03	#DIV/0!	0	0	0	1	0	1	0	1	0.875153
tr|Q96F88|Q96F88_HUMAN	POP1	Processing of 1, ribonuclease P/MRP subunit (S. cerevisiae) OS=Homo sapiens GN=POP1 PE=2 SV=1	116318	0	1076	0	27	0	0.03	#DIV/0!	0	0	0	1	0	1	0	1	0.875153

sp|Q9P0J7|KCMF1_HUMAN	KCMF1	E3 ubiquitin-protein ligase KCMF1 OS=Homo sapiens GN=KCMF1 PE=1 SV=2	42603	0	1074	0	27	0	0.08	#DIV/0!	0	0	0	1	0	1	0	1	0.875153
sp|Q6L8Q7|PDE12_HUMAN	PDE12	2~,5~-phosphodiesterase 12 OS=Homo sapiens GN=PDE12 PE=1 SV=2	68221	0	1072	0	27	0	0.05	#DIV/0!	0	0	0	1	0	1	0	1	0.875153
sp|Q14684|RRP1B_HUMAN	RRP1B	Ribosomal RNA processing protein 1 homolog B OS=Homo sapiens GN=RRP1B PE=1 SV=3	84774	0	1071	0	27	0	0.04	#DIV/0!	0	0	0	1	0	1	0	1	0.875153
sp|Q5VW32|BROX_HUMAN	BROX	BRO1 domain-containing protein BROX OS=Homo sapiens GN=BROX PE=1 SV=1	46960	0	1070	0	27	0	0.07	#DIV/0!	0	0	0	1	0	1	0	2	0.875153
tr|B4DQV4|B4DQV4_HUMAN	SNX4	Sorting nexin-4 OS=Homo sapiens GN=SNX4 PE=2 SV=1	35873	0	1069	0	27	0	0.09	#DIV/0!	0	0	0	1	0	1	0	1	0.875153
sp|Q5SRE5|NU188_HUMAN	NUP188	Nucleoporin NUP188 homolog OS=Homo sapiens GN=NUP188 PE=1 SV=1	198369	0	1067	0	27	0	0.02	#DIV/0!	0	0	0	1	0	1	0	1	0.875153
tr|D6RCD0|D6RCD0_HUMAN	HSD17B11	Estradiol 17-beta-dehydrogenase 11 OS=Homo sapiens GN=HSD17B11 PE=2 SV=1	28398	0	1066	0	27	0	0.12	#DIV/0!	0	0	0	1	0	1	0	1	0.875153
tr|E7EPN9|E7EPN9_HUMAN	PRRC2C	Protein PRRC2C OS=Homo sapiens GN=PRRC2C PE=2 SV=1	309159	0	1065	0	28	0	0.03	#DIV/0!	0	0	0	1	0	1	0	1	0.875153
tr|A6NJ10|A6NJ10_HUMAN	CYP2W1	Cytochrome P450 2W1 OS=Homo sapiens GN=CYP2W1 PE=2 SV=1	44988	0	1064	0	28	0	0.07	#DIV/0!	0	1	0	1	0	1	0	2	0.875153
tr|E9PC74|E9PC74_HUMAN	EIF2B5	Translation initiation factor eIF-2B subunit epsilon OS=Homo sapiens GN=EIF2B5 PE=2 SV=1	79096	0	1059	0	28	0	0.04	#DIV/0!	0	0	0	1	0	1	0	1	0.875153
tr|Q8TCT3|Q8TCT3_HUMAN	NOX1	Nox1 NAD(P)H-oxidase subunit OS=Homo sapiens GN=NOX1 PE=2 SV=1	59779	0	1058	0	28	0	0.05	#DIV/0!	0	0	0	1	0	1	0	1	0.875153
tr|B4DYP7|B4DYP7_HUMAN	tr|B4DYP7|B4D	cDNA FLJ55435, highly similar to Gamma-tubulin complex component 3 OS=Homo sapiens PE=2 SV=1	103306	0	1057	0	28	0	0.03	#DIV/0!	0	0	0	1	0	1	0	1	0.875153
tr|B4DX99|B4DX99_HUMAN	tr|B4DX99|B4D	Malic enzyme OS=Homo sapiens PE=2 SV=1	45378	0	1055	0	28	0	0.07	#DIV/0!	0	0	0	1	0	1	0	1	0.875153
tr|Q5IRN4|Q5IRN4_HUMAN	MEF2D/DAZAP1	Myocyte enhancer factor 2D/deleted in azoospermia associated protein 1 fusion protein OS=Homo sapiens GN=MEF2D/DAZAP1 fusion PE=2	50610	0	1054	0	28	0	0.07	#DIV/0!	0	0	0	1	0	1	0	1	0.875153
tr|Q59F22|Q59F22_HUMAN	tr|Q59F22|Q59	Interferon stimulated gene 20kDa variant (Fragment) OS=Homo sapiens PE=2 SV=1	21443	0	1052	0	29	0	0.16	#DIV/0!	0	0	0	1	0	1	0	1	0.875153
tr|D0PQI2|D0PQI2_HUMAN	ENAH	Enabled-like protein variant hMenaDv6 OS=Homo sapiens GN=ENAH PE=2 SV=1	60426	0	1051	0	29	0	0.05	#DIV/0!	0	0	0	1	0	1	0	1	0.875153
tr|G1UI18|G1UI18_HUMAN	PSME4	Proteasome activator complex subunit 4 (Fragment) OS=Homo sapiens GN=PSME4 PE=2 SV=1	130054	0	1050	0	29	0	0.03	#DIV/0!	0	1	0	1	0	1	0	1	0.875153
tr|B4DSE9|B4DSE9_HUMAN	tr|B4DSE9|B4D	cDNA FLJ58278, highly similar to Homo sapiens WD repeat domain 44 (WDR44), mRNA OS=Homo sapiens PE=2 SV=1	91610	0	1047	0	29	0	0.04	#DIV/0!	0	0	0	1	0	1	0	1	0.875153
sp|Q9UHD1|CHRD1_HUMAN	CHORDC1	Cysteine and histidine-rich domain-containing protein 1 OS=Homo sapiens GN=CHORDC1 PE=1 SV=2	38264	0	1043	0	29	0	0.09	#DIV/0!	0	0	0	1	0	1	0	1	0.875153
tr|Q7Z4Z1|Q7Z4Z1_HUMAN	tr|Q7Z4Z1|Q7Z	Epsilon-COP OS=Homo sapiens PE=2 SV=1	34608	0	1040	0	29	0	0.1	#DIV/0!	0	0	0	1	0	1	0	1	0.875153
tr|B4DJU4|B4DJU4_HUMAN	tr|B4DJU4|B4D	cDNA FLJ53344, highly similar to Splicing factor 1 OS=Homo sapiens PE=2 SV=1	67535	0	1039	0	29	0	0.05	#DIV/0!	0	0	0	1	0	1	0	1	0.875153
tr|H0YK42|H0YK42_HUMAN	SNX1	Sorting nexin-1 OS=Homo sapiens GN=SNX1 PE=4 SV=1	35628	0	1038	0	30	0	0.06	#DIV/0!	0	0	0	1	0	1	0	1	0.875153
tr|B4DI61|B4DI61_HUMAN	tr|B4DI61|B4DI	cDNA FLJ58182, highly similar to Protein CYR61 OS=Homo sapiens PE=2 SV=1	41592	0	1036	0	30	0	0.08	#DIV/0!	0	0	0	1	0	1	0	1	0.875153
tr|A8K8Z4|A8K8Z4_HUMAN	tr|A8K8Z4|A8K	cDNA FLJ78071, highly similar to Human MHC class III complement component C6 mRNA OS=Homo sapiens PE=2 SV=1	108298	0	1034	0	30	0	0.03	#DIV/0!	0	0	0	1	0	1	0	1	0.875153
tr|B7Z1V5|B7Z1V5_HUMAN	tr|B7Z1V5|B7Z1	cDNA FLJ55678, highly similar to Syntaxin-binding protein 1 OS=Homo sapiens PE=2 SV=1	63779	0	1031	0	30	0	0.05	#DIV/0!	0	0	0	1	0	1	0	1	0.875153
sp|Q9BXW9|FACD2_HUMAN	FANCD2	Fanconi anemia group D2 protein OS=Homo sapiens GN=FANCD2 PE=1 SV=1	168295	0	1030	0	30	0	0.02	#DIV/0!	0	0	0	1	0	1	0	2	0.875153
tr|Q6ZWH1|Q6ZWH1_HUMAN	tr|Q6ZWH1|Q6	cDNA FLJ41081 fis, clone ADRGL2009755, highly similar to Homo sapiens brain and reproductive organ-expressed protein (BRE) mRNA OS=H	45824	0	1029	0	30	0	0.07	#DIV/0!	0	0	0	1	0	1	0	1	0.875153
tr|H0Y8S8|H0Y8S8_HUMAN	UBA6	Ubiquitin-like modifier-activating enzyme 6 (Fragment) OS=Homo sapiens GN=UBA6 PE=4 SV=1	31255	0	1028	0	30	0	0.11	#DIV/0!	0	0	0	1	0	1	0	2	0.875153
tr|H7C086|H7C086_HUMAN	ATP5O	ATP synthase subunit O, mitochondrial (Fragment) OS=Homo sapiens GN=ATP5O PE=4 SV=1	8242	0	1026	0	30	0	0.43	#DIV/0!	0	0	0	1	0	1	0	1	0.875153
tr|B3KVJ5|B3KVJ5_HUMAN	tr|B3KVJ5|B3KV	cDNA FLJ16648 fis, clone TESTI4035508, highly similar to Structural maintenance of chromosomes 4-like 1 protein OS=Homo sapiens PE=2 S	101354	0	1025	0	30	0	0.03	#DIV/0!	0	0	0	1	0	1	0	1	0.875153
tr|B3KVY3|B3KVY3_HUMAN	tr|B3KVY3|B3K	cDNA FLJ41723 fis, clone HLUNG2013851 OS=Homo sapiens PE=2 SV=1	20460	0	1021	0	30	0	0.16	#DIV/0!	0	1	0	1	0	1	0	1	0.875153
tr|B3KUL1|B3KUL1_HUMAN	tr|B3KUL1|B3K	cDNA FLJ40132 fis, clone TESTI2012155, highly similar to NUCLEOPORIN-LIKE PROTEIN RIP OS=Homo sapiens PE=2 SV=1	59275	0	1020	0	30	0	0.06	#DIV/0!	0	0	0	1	0	1	0	1	0.875153
tr|B3KSS4|B3KSS4_HUMAN	tr|B3KSS4|B3KS	cDNA FLJ36858 fis, clone ASTRO2015185, highly similar to POLIOVIRUS RECEPTOR OS=Homo sapiens PE=2 SV=1	40011	0	1019	0	30	0	0.08	#DIV/0!	0	0	0	1	0	1	0	1	0.875153
sp|Q96I24|FUBP3_HUMAN	FUBP3	Far upstream element-binding protein 3 OS=Homo sapiens GN=FUBP3 PE=1 SV=2	61944	0	1018	0	30	0	0.05	#DIV/0!	0	0	0	1	0	1	0	2	0.875153
tr|Q6FIA3|Q6FIA3_HUMAN	PACSIN2	PACSIN2 protein OS=Homo sapiens GN=PACSIN2 PE=2 SV=1	51663	0	1017	0	30	0	0.06	#DIV/0!	0	0	0	1	0	1	0	1	0.875153
tr|H0YLC7|H0YLC7_HUMAN	FAH	Fumarylacetoacetase (Fragment) OS=Homo sapiens GN=FAH PE=4 SV=1	18072	0	1016	0	30	0	0.19	#DIV/0!	0	0	0	1	0	1	0	1	0.875153
tr|B4DYQ7|B4DYQ7_HUMAN	tr|B4DYQ7|B4D	cDNA FLJ57822, highly similar to Integrin alpha-V OS=Homo sapiens PE=2 SV=1	53051	0	1014	0	31	0	0.06	#DIV/0!	0	0	0	1	0	1	0	1	0.875153
tr|G3V1J5|G3V1J5_HUMAN	DIS3	Exosome complex exonuclease RRP44 OS=Homo sapiens GN=DIS3 PE=2 SV=1	91382	0	1013	0	31	0	0.22	#DIV/0!	0	0	0	1	0	1	0	1	0.875153
tr|B0QYK0|B0QYK0_HUMAN	EWSR1	Ewing sarcoma breakpoint region 1 OS=Homo sapiens GN=EWSR1 PE=4 SV=1	65174	0	1010	0	31	0	0.05	#DIV/0!	0	0	0	1	0	1	0	1	0.875153
tr|B4DU69|B4DU69_HUMAN	tr|B4DU69|B4D	cDNA FLJ56583, highly similar to Argininosuccinate lyase (EC 4.3.2.1) OS=Homo sapiens PE=2 SV=1	24664	0	1009	0	31	0	0.14	#DIV/0!	0	0	0	1	0	1	0	1	0.875153
tr|B4DK61|B4DK61_HUMAN	tr|B4DK61|B4D	Acyl-coenzyme A oxidase OS=Homo sapiens PE=2 SV=1	70622	0	1008	0	31	0	0.05	#DIV/0!	0	0	0	1	0	1	0	1	0.875153
sp|Q15050|RRS1_HUMAN	RRS1	Ribosome biogenesis regulatory protein homolog OS=Homo sapiens GN=RRS1 PE=1 SV=2	41225	0	1007	0	31	0	0.08	#DIV/0!	0	0	0	1	0	1	0	1	0.875153
tr|H0YKD0|H0YKD0_HUMAN	IDH3A	Isocitrate dehydrogenase [NAD] subunit alpha, mitochondrial (Fragment) OS=Homo sapiens GN=IDH3A PE=3 SV=1	12184	0	1006	0	31	0	0.28	#DIV/0!	0	0	0	1	0	1	0	1	0.875153
tr|I1E4Y6|I1E4Y6_HUMAN	GIGYF2	PERQ amino acid-rich with GYF domain-containing protein 2 OS=Homo sapiens GN=GIGYF2 PE=4 SV=1	152891	0	1005	0	31	0	0.02	#DIV/0!	0	0	0	1	0	1	0	3	0.875153
tr|B2RE88|B2RE88_HUMAN	tr|B2RE88|B2R	cDNA, FLJ96465, highly similar to Homo sapiens solute carrier family 25 (mitochondrial carrier;phosphate carrier), member 3 (SLC25A3), nuc	40379	0	1004	0	31	0	0.08	#DIV/0!	0	0	0	1	0	1	0	1	0.875153
tr|B4DGP9|B4DGP9_HUMAN	tr|B4DGP9|B4D	cDNA FLJ54102, highly similar to Beta-soluble NSF attachment protein OS=Homo sapiens PE=2 SV=1	29061	0	1001	0	31	0	0.11	#DIV/0!	0	0	0	1	0	1	0	1	0.875153
tr|Q53G42|Q53G42_HUMAN	tr|Q53G42|Q53	mRNA decapping enzyme variant (Fragment) OS=Homo sapiens PE=2 SV=1	38757	0	1000	0	32	0	0.09	#DIV/0!	0	0	0	1	0	1	0	1	0.875153
tr|J3KPH8|J3KPH8_HUMAN	HDAC7	Histone deacetylase 7 OS=Homo sapiens GN=HDAC7 PE=4 SV=1	109925	0	998	0	32	0	0.03	#DIV/0!	0	1	0	1	0	1	0	2	0.875153
tr|J3KS45|J3KS45_HUMAN	TMCO1	Transmembrane and coiled-coil domain-containing protein 1 (Fragment) OS=Homo sapiens GN=TMCO1 PE=4 SV=1	16741	0	996	0	32	0	0.2	#DIV/0!	0	0	0	1	0	1	0	1	0.875153
tr|B4DLG2|B4DLG2_HUMAN	tr|B4DLG2|B4D	cDNA FLJ58196, highly similar to Zinc finger CCCH domain-containing protein 11A OS=Homo sapiens PE=2 SV=1	83177	0	995	0	32	0	0.04	#DIV/0!	0	0	0	1	0	1	0	1	0.875153
tr|A8KA66|A8KA66_HUMAN	tr|A8KA66|A8K	cDNA FLJ78485, highly similar to Homo sapiens epidermal growth factor receptor pathway substrate 8 (EPS8), mRNA OS=Homo sapiens PE=	92199	0	994	0	32	0	0.04	#DIV/0!	0	0	0	1	0	1	0	2	0.875153
tr|B3KXW5|B3KXW5_HUMAN	AP1G1	AP-1 complex subunit gamma-1 OS=Homo sapiens GN=AP1G1 PE=2 SV=1	94992	0	991	0	32	0	0.03	#DIV/0!	0	1	0	1	0	1	0	1	0.875153
sp|Q96AG4|LRC59_HUMAN	LRRC59	Leucine-rich repeat-containing protein 59 OS=Homo sapiens GN=LRRC59 PE=1 SV=1	35308	0	990	0	32	0	0.09	#DIV/0!	0	1	0	1	0	1	0	2	0.875153
tr|Q14730|Q14730_HUMAN	tr|Q14730|Q14	La 4.1 protein (Fragment) OS=Homo sapiens PE=2 SV=1	33852	0	988	0	32	0	0.1	#DIV/0!	0	0	0	1	0	1	0	1	0.875153
tr|E5RGX5|E5RGX5_HUMAN	STMN2	Stathmin OS=Homo sapiens GN=STMN2 PE=2 SV=1	19676	0	987	0	33	0	0.17	#DIV/0!	0	1	0	1	0	1	0	1	0.875153
tr|B2R642|B2R642_HUMAN	tr|B2R642|B2R	cDNA, FLJ92775, highly similar to Homo sapiens melanoma cell adhesion molecule (MCAM), mRNA OS=Homo sapiens PE=2 SV=1	72560	0	985	0	33	0	0.05	#DIV/0!	0	0	0	1	0	1	0	3	0.875153
sp|Q2M389|WASH7_HUMAN	KIAA1033	WASH complex subunit 7 OS=Homo sapiens GN=KIAA1033 PE=1 SV=2	137343	0	984	0	33	0	0.02	#DIV/0!	0	0	0	1	0	1	0	2	0.875153
sp|Q9H488|OFUT1_HUMAN	POFUT1	GDP-fucose protein O-fucosyltransferase 1 OS=Homo sapiens GN=POFUT1 PE=1 SV=1	44383	0	980	0	33	0	0.07	#DIV/0!	0	0	0	1	0	1	0	1	0.875153
tr|B3KQI5|B3KQI5_HUMAN	tr|B3KQI5|B3K	cDNA FLJ90522 fis, clone NT2RP4000108, highly similar to Neurofilament triplet L protein OS=Homo sapiens PE=2 SV=1	61508	0	979	0	33	0	0.08	#DIV/0!	0	0	0	1	0	1	0	2	0.875153
tr|H0Y9G6|H0Y9G6_HUMAN	MRPL3	39S ribosomal protein L3, mitochondrial (Fragment) OS=Homo sapiens GN=MRPL3 PE=3 SV=1	40898	0	977	0	33	0	0.08	#DIV/0!	0	0	0	1	0	1	0	1	0.875153
sp|Q9HD26|GOPC_HUMAN	GOPC	Golgi-associated PDZ and coiled-coil motif-containing protein OS=Homo sapiens GN=GOPC PE=1 SV=1	50888	0	976	0	33	0	0.06	#DIV/0!	0	1	0	1	0	1	0	2	0.875153
sp|Q9Y2T3|GUAD_HUMAN	GDA	Guanine deaminase OS=Homo sapiens GN=GDA PE=1 SV=1	51484	0	974	0	33	0	0.13	#DIV/0!	0	1	0	2	0	1	0	2	0.875153
sp|Q96BN8|F105B_HUMAN	FAM105B	Protein FAM105B OS=Homo sapiens GN=FAM105B PE=1 SV=3	40636	0	973	0	33	0	0.08	#DIV/0!	0	0	0	1	0	1	0	1	0.875153
tr|H0Y892|H0Y892_HUMAN	ZNF782	Zinc finger protein 782 (Fragment) OS=Homo sapiens GN=ZNF782 PE=4 SV=1	81419	0	972	0	33	0	0.04	#DIV/0!	0	1	0	1	0	1	0	1	0.875153
tr|B4DXJ1|B4DXJ1_HUMAN	SEC13	Protein SEC13 homolog OS=Homo sapiens GN=SEC13 PE=2 SV=1	41405	0	971	0	34	0	0.17	#DIV/0!	0	1	0	2	0	1	0	2	0.875153
tr|B7ZW05|B7ZW05_HUMAN	AKAP13	AKAP13 protein (Fragment) OS=Homo sapiens GN=AKAP13 PE=2 SV=1	124144	0	970	0	34	0	0.03	#DIV/0!	0	0	0	1	0	1	0	1	0.875153
tr|B4DM41|B4DM41_HUMAN	RFC4	Replication factor C (Activator 1) 4, 37kDa, isoform CRA_b OS=Homo sapiens GN=RFC4 PE=2 SV=1	33996	0	968	0	34	0	0.1	#DIV/0!	0	0	0	1	0	1	0	1	0.875153
tr|Q13380|Q13380_HUMAN	RBM	RNA binding motif (Fragment) OS=Homo sapiens GN=RBM PE=4 SV=1	4015	0	967	0	34	0	0.97	#DIV/0!	0	1	0	1	0	1	0	1	0.875153
tr|B4E1K7|B4E1K7_HUMAN	STOML2	Stomatin-like protein 2 OS=Homo sapiens GN=STOML2 PE=2 SV=1	33317	0	966	0	34	0	0.1	#DIV/0!	0	0	0	1	0	1	0	1	0.875153
tr|A0JLU4|A0JLU4_HUMAN	YBX1	YBX1 protein (Fragment) OS=Homo sapiens GN=YBX1 PE=2 SV=1	21417	0	965	0	34	0	0.16	#DIV/0!	0	1	0	1	0	1	0	1	0.875153
sp|Q5T2S8|ARMC4_HUMAN	ARMC4	Armadillo repeat-containing protein 4 OS=Homo sapiens GN=ARMC4 PE=2 SV=1	117146	0	964	0	34	0	0.03	#DIV/0!	0	1	0	1	0	1	0	1	0.875153
sp|P23368|MAOM_HUMAN	ME2	NAD-dependent malic enzyme, mitochondrial OS=Homo sapiens GN=ME2 PE=1 SV=1	66029	0	961	0	34	0	0.05	#DIV/0!	0	1	0	1	0	1	0	1	0.875153
tr|B4DI19|B4DI19_HUMAN	tr|B4DI19|B4DI	cDNA FLJ59524, highly similar to Cysteinyl-tRNA synthetase (EC 6.1.1.16) OS=Homo sapiens PE=2 SV=1	76209	0	960	0	34	0	0.04	#DIV/0!	0	1	0	1	0	1	0	2	0.875153
tr|B3KQC5|B3KQC5_HUMAN	MAN1B1	Endoplasmic reticulum mannosyl-oligosaccharide 1,2-alpha-mannosidase OS=Homo sapiens GN=MAN1B1 PE=2 SV=1	46112	0	959	0	34	0	0.15	#DIV/0!	0	0	0	2	0	1	0	3	0.875153
tr|Q53S54|Q53S54_HUMAN	CUL3	Putative uncharacterized protein CUL3 (Fragment) OS=Homo sapiens GN=CUL3 PE=2 SV=1	86981	0	958	0	34	0	0.05	#DIV/0!	0	0	0	1	0	1	0	1	0.875153
tr|H0YBI0|H0YBI0_HUMAN	SHPRH	E3 ubiquitin-protein ligase SHPRH (Fragment) OS=Homo sapiens GN=SHPRH PE=4 SV=1	10967	0	957	0	35	0	0.31	#DIV/0!	0	1	0	1	0	1	0	1	0.875153
tr|K7EKI4|K7EKI4_HUMAN	MRPL4	39S ribosomal protein L4, mitochondrial OS=Homo sapiens GN=MRPL4 PE=4 SV=1	14161	0	956	0	35	0	0.24	#DIV/0!	0	0	0	1	0	1	0	1	0.875153
tr|E1P5S2|E1P5S2_HUMAN	RBM39	RNA-binding protein 39 OS=Homo sapiens GN=RBM39 PE=4 SV=1	40744	0	954	0	35	0	0.09	#DIV/0!	0	1	0	1	0	1	0	1	0.875153
tr|B4DF22|B4DF22_HUMAN	tr|B4DF22|B4D	cDNA FLJ55615, highly similar to SWI/SNF-related matrix-associatedactin-dependent regulator of chromatin subfamily C member 2 OS=Hom	112428	0	952	0	35	0	0.03	#DIV/0!	0	0	0	1	0	1	0	1	0.875153
sp|Q5BKZ1|ZN326_HUMAN	ZNF326	DBIRD complex subunit ZNF326 OS=Homo sapiens GN=ZNF326 PE=1 SV=2	65955	0	951	0	35	0	0.05	#DIV/0!	0	1	0	1	0	1	0	1	0.875153
tr|J3QSY3|J3QSY3_HUMAN	GDA	Guanine deaminase OS=Homo sapiens GN=GDA PE=4 SV=1	46321	0	949	0	35	0	0.07	#DIV/0!	0	1	0	1	0	1	0	1	0.875153
sp|Q96T76|MMS19_HUMAN	MMS19	MMS19 nucleotide excision repair protein homolog OS=Homo sapiens GN=MMS19 PE=1 SV=2	114928	0	947	0	35	0	0.1	#DIV/0!	0	1	0	1	0	1	0	1	0.875153
tr|Q96LK9|Q96LK9_HUMAN	tr|Q96LK9|Q96	cDNA FLJ25413 fis, clone TST03402, highly similar to 3-OXO-5-BETA-STEROID 4-DEHYDROGENASE (EC 1.3.99.6) OS=Homo sapiens PE=2 SV=1	16911	0	946	0	35	0	0.2	#DIV/0!	0	0	0	1	0	1	0	2	0.875153
sp|Q2NL98|VMAC_HUMAN	VMAC	Vimentin-type intermediate filament-associated coiled-coil protein OS=Homo sapiens GN=VMAC PE=2 SV=1	18394	0	941	0	35	0	0.18	#DIV/0!	0	1	0	1	0	1	0	1	0.875153
tr|Q5D1D5|Q5D1D5_HUMAN	tr|Q5D1D5|Q5D	Guanylate binding protein 1 OS=Homo sapiens PE=2 SV=1	68387	0	939	0	35	0	0.13	#DIV/0!	0	1	0	1	0	1	0	1	0.875153
sp|Q92624|APBP2_HUMAN	APPBP2	Amyloid protein-binding protein 2 OS=Homo sapiens GN=APPBP2 PE=1 SV=2	67666	0	935	0	35	0	0.05	#DIV/0!	0	1	0	1	0	1	0	1	0.875153
tr|A8K6V7|A8K6V7_HUMAN	tr|A8K6V7|A8K	cDNA FLJ76053, highly similar to Homo sapiens Ras-GTPase activating protein SH3 domain-binding protein 2 (G3BP2), transcript variant 3, m	50842	0	934	0	35	0	0.06	#DIV/0!	0	0	0	1	0	1	0	1	0.875153
tr|Q6NUR1|Q6NUR1_HUMAN	NCAPG	Non-SMC condensin I complex, subunit G OS=Homo sapiens GN=NCAPG PE=2 SV=1	115205	0	931	0	36	0	0.11	#DIV/0!	0	0	0	1	0	1	0	1	0.875153
tr|B2R7I0|B2R7I0_HUMAN	tr|B2R7I0|B2R7	cDNA, FLJ93451, highly similar to Homo sapiens thioredoxin domain containing 14 (TXNDC14), mRNA OS=Homo sapiens PE=2 SV=1	34344	0	928	0	36	0	0.1	#DIV/0!	0	1	0	1	0	1	0	1	0.875153
sp|P62333|PRS10_HUMAN	PSMC6	26S protease regulatory subunit 10B OS=Homo sapiens GN=PSMC6 PE=1 SV=1	44430	0	927	0	36	0	0.15	#DIV/0!	0	1	0	1	0	1	0	1	0.875153
tr|K7EQF6|K7EQF6_HUMAN	DAND5	DAN domain family member 5 OS=Homo sapiens GN=DAND5 PE=4 SV=1	17549	0	926	0	36	0	0.19	#DIV/0!	0	1	0	1	0	1	0	1	0.875153
tr|B7Z2D6|B7Z2D6_HUMAN	tr|B7Z2D6|B7Z2	cDNA FLJ53863, highly similar to Cystathionine beta-synthase (EC 4.2.1.22) OS=Homo sapiens PE=2 SV=1	56409	0	920	0	37	0	0.06	#DIV/0!	0	1	0	1	0	1	0	1	0.875153
tr|B1ANM7|B1ANM7_HUMAN	FAF1	FAS-associated factor 1 OS=Homo sapiens GN=FAF1 PE=4 SV=1	56305	0	918	0	37	0	0.06	#DIV/0!	0	1	0	1	0	1	0	1	0.875153
tr|Q6NT15|Q6NT15_HUMAN	STON2	STON2 protein (Fragment) OS=Homo sapiens GN=STON2 PE=2 SV=1	101619	0	917	0	37	0	0.03	#DIV/0!	0	1	0	1	0	1	0	1	0.875153
tr|A8MXL6|A8MXL6_HUMAN	SEC13	Protein SEC13 homolog OS=Homo sapiens GN=SEC13 PE=2 SV=1	32031	0	916	0	37	0	0.1	#DIV/0!	0	1	0	1	0	1	0	1	0.875153
tr|A8K4W5|A8K4W5_HUMAN	tr|A8K4W5|A8K	cDNA FLJ76813, highly similar to Homo sapiens acetyl-Coenzyme A acetyltransferase 2 (acetoacetyl Coenzyme A thiolase), mRNA OS=Homo	41866	0	915	0	37	0	0.07	#DIV/0!	0	1	0	1	0	1	0	1	0.875153
tr|F6U211|F6U211_HUMAN	RPS10	40S ribosomal protein S10 OS=Homo sapiens GN=RPS10 PE=2 SV=1	19912	0	913	0	37	0	0.17	#DIV/0!	0	1	0	1	0	1	0	1	0.875153
tr|B4DVM9|B4DVM9_HUMAN	tr|B4DVM9|B4D	cDNA FLJ50247, highly similar to Jumonji/ARID domain-containing protein 1A (Fragment) OS=Homo sapiens PE=2 SV=1	121122	0	911	0	37	0	0.03	#DIV/0!	0	1	0	1	0	1	0	1	0.875153
sp|Q96G03|PGM2_HUMAN	PGM2	Phosphoglucomutase-2 OS=Homo sapiens GN=PGM2 PE=1 SV=4	68754	0	910	0	38	0	0.06	#DIV/0!	0	1	0	1	0	1	0	1	0.875153
sp|Q16880|CGT_HUMAN	UGT8	2-hydroxyacylsphingosine 1-beta-galactosyltransferase OS=Homo sapiens GN=UGT8 PE=2 SV=2	61627	0	909	0	38	0	0.05	#DIV/0!	0	1	0	1	0	1	0	1	0.875153
sp|P82673|RT35_HUMAN	MRPS35	28S ribosomal protein S35, mitochondrial OS=Homo sapiens GN=MRPS35 PE=1 SV=1	37106	0	908	0	38	0	0.09	#DIV/0!	0	1	0	1	0	1	0	1	0.875153
sp|Q8IVT2|CS021_HUMAN	C19orf21	Uncharacterized protein C19orf21 OS=Homo sapiens GN=C19orf21 PE=1 SV=1	75482	0	905	0	38	0	0.04	#DIV/0!	0	1	0	1	0	1	0	1	0.875153
tr|H0Y7L9|H0Y7L9_HUMAN	ABCD1	ATP-binding cassette sub-family D member 1 (Fragment) OS=Homo sapiens GN=ABCD1 PE=4 SV=1	24597	0	904	0	38	0	0.04	#DIV/0!	0	1	0	1	0	1	0	1	0.875153
tr|F8VV56|F8VV56_HUMAN	CD63	CD63 antigen OS=Homo sapiens GN=CD63 PE=4 SV=1	16466	0	903	0	38	0	0.23	#DIV/0!	0	1	0	1	0	1	0	1	0.875153
tr|B4DSN3|B4DSN3_HUMAN	tr|B4DSN3|B4D	cDNA FLJ60345, highly similar to Protein transport protein Sec24C OS=Homo sapiens PE=2 SV=1	107974	0	902	0	38	0	0.03	#DIV/0!	0	1	0	1	0	1	0	3	0.875153
sp|P10768|ESTD_HUMAN	ESD	S-formylglutathione hydrolase OS=Homo sapiens GN=ESD PE=1 SV=2	31956	0	900	0	38	0	0.1	#DIV/0!	0	1	0	1	0	1	0	1	0.875153
sp|O15397|IPO8_HUMAN	IPO8	Importin-8 OS=Homo sapiens GN=IPO8 PE=1 SV=2	120945	0	899	0	38	0	0.03	#DIV/0!	0	1	0	1	0	1	0	2	0.875153
sp|Q96LW7|BINCA_HUMAN	C9orf89	Bcl10-interacting CARD protein OS=Homo sapiens GN=C9orf89 PE=1 SV=1	26029	0	896	0	38	0	0.13	#DIV/0!	0	1	0	1	0	1	0	1	0.875153
tr|B2R686|B2R686_HUMAN	TGOLN2	Trans-golgi network protein 2, isoform CRA_a OS=Homo sapiens GN=TGOLN2 PE=2 SV=1	45882	0	892	0	39	0	0.07	#DIV/0!	0	1	0	1	0	1	0	1	0.875153
tr|B3KPF2|B3KPF2_HUMAN	tr|B3KPF2|B3K	cDNA FLJ31717 fis, clone NT2RI2006640, highly similar to Dynamin-3 (EC 3.6.5.5) OS=Homo sapiens PE=2 SV=1	63057	0	891	0	39	0	0.05	#DIV/0!	0	1	0	1	0	1	0	1	0.875153
tr|H3BUQ2|H3BUQ2_HUMAN	OGFOD1	2-oxoglutarate and iron-dependent oxygenase domain-containing protein 1 OS=Homo sapiens GN=OGFOD1 PE=4 SV=1	58798	0	890	0	39	0	0.06	#DIV/0!	0	1	0	1	0	1	0	1	0.875153
tr|H7C0M3|H7C0M3_HUMAN	BPHL	Valacyclovir hydrolase (Fragment) OS=Homo sapiens GN=BPHL PE=4 SV=1	3314	0	886	0	39	0	1.2	#DIV/0!	0	1	0	1	0	1	0	1	0.875153
tr|B4DN25|B4DN25_HUMAN	UGDH	UDP-glucose 6-dehydrogenase OS=Homo sapiens GN=UGDH PE=2 SV=1	44786	0	885	0	39	0	0.07	#DIV/0!	0	1	0	1	0	1	0	1	0.875153
sp|P41214|EIF2D_HUMAN	EIF2D	Eukaryotic translation initiation factor 2D OS=Homo sapiens GN=EIF2D PE=1 SV=3	65293	0	881	0	40	0	0.07	#DIV/0!	0	1	0	1	0	1	0	1	0.875153
sp|Q8IWB1|IPRI_HUMAN	ITPRIP	Inositol 1,4,5-trisphosphate receptor-interacting protein OS=Homo sapiens GN=ITPRIP PE=1 SV=1	62932	0	879	0	40	0	0.05	#DIV/0!	0	1	0	1	0	1	0	1	0.875153
tr|B3KSG9|B3KSG9_HUMAN	tr|B3KSG9|B3K	cDNA FLJ36188 fis, clone TESTI2027179, highly similar to Transmembrane 9 superfamily protein member 2 OS=Homo sapiens PE=2 SV=1	66299	0	876	0	40	0	0.05	#DIV/0!	0	1	0	1	0	1	0	1	0.875153
tr|B4DUS9|B4DUS9_HUMAN	BPNT1	3~(2~),5~-bisphosphate nucleotidase 1 OS=Homo sapiens GN=BPNT1 PE=2 SV=1	29455	0	874	0	40	0	0.12	#DIV/0!	0	1	0	1	0	1	0	1	0.875153
sp|Q9NTX7|RN146_HUMAN	RNF146	E3 ubiquitin-protein ligase RNF146 OS=Homo sapiens GN=RNF146 PE=1 SV=1	39610	0	873	0	40	0	0.08	#DIV/0!	0	1	0	1	0	1	0	1	0.875153
tr|Q6FHF5|Q6FHF5_HUMAN	PCNA	Proliferating cell nuclear antigen (Fragment) OS=Homo sapiens GN=PCNA PE=2 SV=1	29029	0	872	0	40	0	0.11	#DIV/0!	0	1	0	1	0	1	0	1	0.875153
tr|Q7Z6C8|Q7Z6C8_HUMAN	CPNE5	CPNE5 protein OS=Homo sapiens GN=CPNE5 PE=2 SV=1	32778	0	870	0	40	0	0.1	#DIV/0!	0	1	0	1	0	1	0	2	0.875153
sp|O75131|CPNE3_HUMAN	CPNE3	Copine-3 OS=Homo sapiens GN=CPNE3 PE=1 SV=1	60947	0	869	0	40	0	0.05	#DIV/0!	0	1	0	1	0	1	0	1	0.875153
tr|G0XQ39|G0XQ39_HUMAN	STIM1	STIM1L OS=Homo sapiens GN=STIM1 PE=2 SV=1	88933	0	867	0	40	0	0.04	#DIV/0!	0	1	0	1	0	1	0	1	0.875153
tr|E7ERK9|E7ERK9_HUMAN	EIF2B4	Translation initiation factor eIF-2B subunit delta OS=Homo sapiens GN=EIF2B4 PE=2 SV=1	60248	0	866	0	41	0	0.09	#DIV/0!	0	1	0	1	0	1	0	1	0.875153
tr|B3KY55|B3KY55_HUMAN	tr|B3KY55|B3K	cDNA FLJ46894 fis, clone UTERU3019708, highly similar to Regulator of nonsense transcripts 1 OS=Homo sapiens PE=2 SV=1	41002	0	865	0	0	0	0.08	#DIV/0!	0	0	0	0	0	1	0	2	0.875153
tr|H7BZF2|H7BZF2_HUMAN	DKC1	H/ACA ribonucleoprotein complex subunit 4 (Fragment) OS=Homo sapiens GN=DKC1 PE=4 SV=1	8803	0	864	0	41	0	0.06	#DIV/0!	0	1	0	1	0	1	0	2	0.875153
tr|A4D1I0|A4D1I0_HUMAN	PPP1R9A	HCG1741805, isoform CRA_c OS=Homo sapiens GN=PPP1R9A PE=2 SV=1	148567	0	863	0	41	0	0.02	#DIV/0!	0	1	0	1	0	1	0	2	0.875153
tr|B3KT00|B3KT00_HUMAN	tr|B3KT00|B3K	cDNA FLJ37368 fis, clone BRAMY2024530, highly similar to Zinc phosphodiesterase ELAC protein 2 (EC 3.1.26.11) OS=Homo sapiens PE=2 SV	91169	0	862	0	41	0	0.04	#DIV/0!	0	1	0	1	0	1	0	1	0.875153
tr|B3KNE7|B3KNE7_HUMAN	tr|B3KNE7|B3K	cDNA FLJ14468 fis, clone MAMMA1000734, highly similar to Translocation protein SEC63 homolog OS=Homo sapiens PE=2 SV=1	48242	0	861	0	41	0	0.07	#DIV/0!	0	1	0	1	0	1	0	1	0.875153
tr|J3KQL8|J3KQL8_HUMAN	APOL2	Apolipoprotein L2 OS=Homo sapiens GN=APOL2 PE=4 SV=1	49070	0	859	0	41	0	0.07	#DIV/0!	0	1	0	1	0	1	0	1	0.875153
tr|H0YMD2|H0YMD2_HUMAN	NUSAP1	Nucleolar and spindle-associated protein 1 (Fragment) OS=Homo sapiens GN=NUSAP1 PE=4 SV=1	15923	0	858	0	26	0	0.14	#DIV/0!	0	1	0	1	0	1	0	1	0.875153
tr|Q2TB12|Q2TB12_HUMAN	LIG1	DNA ligase OS=Homo sapiens GN=LIG1 PE=2 SV=1	88975	0	855	0	41	0	0.04	#DIV/0!	0	1	0	1	0	1	0	1	0.875153
tr|H0YGH7|H0YGH7_HUMAN	PLCZ1	1-phosphatidylinositol 4,5-bisphosphate phosphodiesterase zeta-1 (Fragment) OS=Homo sapiens GN=PLCZ1 PE=4 SV=1	47240	0	854	0	41	0	0.07	#DIV/0!	0	1	0	1	0	1	0	1	0.875153
tr|H0Y360|H0Y360_HUMAN	AMPD2	AMP deaminase 2 (Fragment) OS=Homo sapiens GN=AMPD2 PE=4 SV=1	99192	0	851	0	41	0	0.03	#DIV/0!	0	1	0	1	0	1	0	1	0.875153
tr|B0QYV7|B0QYV7_HUMAN	ARFGAP3	ADP-ribosylation factor GTPase activating protein 3 (Fragment) OS=Homo sapiens GN=ARFGAP3 PE=2 SV=1	30052	0	849	0	41	0	0.1	#DIV/0!	0	1	0	1	0	1	0	1	0.875153
tr|B2R858|B2R858_HUMAN	tr|B2R858|B2R8	cDNA, FLJ93750, Homo sapiens DEAD (Asp-Glu-Ala-Asp) box polypeptide 6 (DDX6), mRNA OS=Homo sapiens PE=2 SV=1	53582	0	848	0	41	0	0.06	#DIV/0!	0	1	0	1	0	1	0	2	0.875153
sp|Q03169|TNAP2_HUMAN	TNFAIP2	Tumor necrosis factor alpha-induced protein 2 OS=Homo sapiens GN=TNFAIP2 PE=1 SV=2	73015	0	846	0	42	0	0.08	#DIV/0!	0	1	0	1	0	1	0	1	0.875153
tr|I3L3B0|I3L3B0_HUMAN	C1QBP	Complement component 1 Q subcomponent-binding protein, mitochondrial OS=Homo sapiens GN=C1QBP PE=4 SV=1	20179	0	844	0	42	0	0.17	#DIV/0!	0	1	0	1	0	1	0	1	0.875153
tr|A2A306|A2A306_HUMAN	UBAP2	Ubiquitin-associated protein 2 OS=Homo sapiens GN=UBAP2 PE=4 SV=1	34828	0	841	0	42	0	0.1	#DIV/0!	0	1	0	1	0	1	0	1	0.875153
tr|H0YLI9|H0YLI9_HUMAN	SHOX	Short stature homeobox protein OS=Homo sapiens GN=SHOX PE=3 SV=1	25703	0	840	0	42	0	0.13	#DIV/0!	0	1	0	1	0	1	0	1	0.875153
tr|H3BSD9|H3BSD9_HUMAN	MYO7B	Unconventional myosin-VIIb (Fragment) OS=Homo sapiens GN=MYO7B PE=4 SV=1	10022	0	838	0	42	0	0.35	#DIV/0!	0	1	0	1	0	1	0	1	0.875153
sp|Q8TC12|RDH11_HUMAN	RDH11	Retinol dehydrogenase 11 OS=Homo sapiens GN=RDH11 PE=1 SV=2	35763	0	835	0	42	0	0.18	#DIV/0!	0	1	0	1	0	1	0	1	0.875153
tr|B3KQ39|B3KQ39_HUMAN	tr|B3KQ39|B3K	cDNA FLJ32740 fis, clone TESTI2001339, highly similar to UDP-GLUCOSE 4-EPIMERASE (EC 5.1.3.2) OS=Homo sapiens PE=2 SV=1	30550	0	834	0	42	0	0.13	#DIV/0!	0	1	0	1	0	1	0	1	0.875153
tr|B4DQR0|B4DQR0_HUMAN	tr|B4DQR0|B4D	cDNA FLJ54377 OS=Homo sapiens PE=2 SV=1	91332	0	833	0	29	0	0.03	#DIV/0!	0	0	0	1	0	1	0	1	0.875153
tr|Q6IAM0|Q6IAM0_HUMAN	EIF3S4	Eukaryotic translation initiation factor 3 subunit G OS=Homo sapiens GN=EIF3S4 PE=2 SV=1	35864	0	831	0	43	0	0.09	#DIV/0!	0	1	0	1	0	1	0	1	0.875153
sp|Q8IVM0|CCD50_HUMAN	CCDC50	Coiled-coil domain-containing protein 50 OS=Homo sapiens GN=CCDC50 PE=1 SV=1	35914	0	828	0	43	0	0.09	#DIV/0!	0	1	0	1	0	1	0	2	0.875153
tr|H7C4F9|H7C4F9_HUMAN	PDIA5	Protein disulfide-isomerase A5 (Fragment) OS=Homo sapiens GN=PDIA5 PE=4 SV=1	9821	0	827	0	43	0	0.36	#DIV/0!	0	1	0	1	0	1	0	1	0.875153
tr|B2RE56|B2RE56_HUMAN	tr|B2RE56|B2R	Peptidyl-prolyl cis-trans isomerase OS=Homo sapiens PE=2 SV=1	18225	0	825	0	43	0	0.19	#DIV/0!	0	1	0	1	0	1	0	1	0.875153
tr|D3YTB1|D3YTB1_HUMAN	RPL32	60S ribosomal protein L32 (Fragment) OS=Homo sapiens GN=RPL32 PE=2 SV=1	15721	0	823	0	43	0	0.21	#DIV/0!	0	1	0	1	0	1	0	1	0.875153
tr|G3V198|G3V198_HUMAN	NUP160	Nuclear pore complex protein Nup160 OS=Homo sapiens GN=NUP160 PE=2 SV=1	142365	0	822	0	43	0	0.07	#DIV/0!	0	1	0	3	0	1	0	3	0.875153
tr|Q969H1|Q969H1_HUMAN	hCG_2036967	HCG2036967 OS=Homo sapiens GN=hCG_2036967 PE=2 SV=1	14112	0	821	0	44	0	0.25	#DIV/0!	0	1	0	1	0	1	0	1	0.875153
tr|B2R960|B2R960_HUMAN	tr|B2R960|B2R9	cDNA, FLJ94230, highly similar to Homo sapiens thioredoxin-like 1 (TXNL1), mRNA OS=Homo sapiens PE=2 SV=1	32616	0	819	0	44	0	0.1	#DIV/0!	0	1	0	1	0	1	0	1	0.875153
tr|J3KTL8|J3KTL8_HUMAN	SMCHD1	Structural maintenance of chromosomes flexible hinge domain-containing protein 1 (Fragment) OS=Homo sapiens GN=SMCHD1 PE=4 SV=1	156690	0	817	0	44	0	0.01	#DIV/0!	0	1	0	1	0	1	0	1	0.875153
tr|G5E977|G5E977_HUMAN	NAPRT1	Nicotinate phosphoribosyltransferase (Fragment) OS=Homo sapiens GN=NAPRT1 PE=2 SV=1	62817	0	816	0	44	0	0.05	#DIV/0!	0	1	0	1	0	1	0	2	0.875153
tr|B4DHY1|B4DHY1_HUMAN	HNRNPH3	Heterogeneous nuclear ribonucleoprotein H3 OS=Homo sapiens GN=HNRNPH3 PE=2 SV=1	24925	0	815	0	44	0	0.13	#DIV/0!	0	1	0	1	0	1	0	1	0.875153
tr|B3KTS3|B3KTS3_HUMAN	tr|B3KTS3|B3K	cDNA FLJ38664 fis, clone HLUNG2002334, highly similar to Homo sapiens KH domain containing, RNA binding, signal transduction associated	35374	0	814	0	44	0	0.09	#DIV/0!	0	1	0	1	0	1	0	2	0.875153
sp|Q16222|UAP1_HUMAN	UAP1	UDP-N-acetylhexosamine pyrophosphorylase OS=Homo sapiens GN=UAP1 PE=1 SV=3	59131	0	813	0	44	0	0.06	#DIV/0!	0	1	0	1	0	1	0	1	0.875153
tr|Q9NP01|Q9NP01_HUMAN	tr|Q9NP01|Q9N	Fibrillin 15 (Fragment) OS=Homo sapiens PE=2 SV=1	60367	0	812	0	44	0	0.05	#DIV/0!	0	1	0	1	0	1	0	1	0.875153
sp|P24534|EF1B_HUMAN	EEF1B2	Elongation factor 1-beta OS=Homo sapiens GN=EEF1B2 PE=1 SV=3	24919	0	805	0	45	0	0.46	#DIV/0!	0	1	0	1	0	1	0	1	0.875153
sp|Q9NYK5|RM39_HUMAN	MRPL39	39S ribosomal protein L39, mitochondrial OS=Homo sapiens GN=MRPL39 PE=1 SV=3	39200	0	804	0	45	0	0.08	#DIV/0!	0	1	0	1	0	1	0	1	0.875153
tr|B3KRC6|B3KRC6_HUMAN	tr|B3KRC6|B3K	cDNA FLJ34004 fis, clone FCBBF1000232, highly similar to Cytochrome P450 51A1 (EC 1.14.13.70) OS=Homo sapiens PE=2 SV=1	51732	0	801	0	45	0	0.06	#DIV/0!	0	1	0	1	0	1	0	1	0.875153
tr|J3QRD1|J3QRD1_HUMAN	ALDH3A2	Fatty aldehyde dehydrogenase OS=Homo sapiens GN=ALDH3A2 PE=3 SV=1	45019	0	798	0	46	0	0.07	#DIV/0!	0	1	0	1	0	1	0	1	0.875153
tr|B4DNX5|B4DNX5_HUMAN	tr|B4DNX5|B4D	cDNA FLJ59941, highly similar to Drebrin-like protein OS=Homo sapiens PE=2 SV=1	15569	0	795	0	46	0	0.22	#DIV/0!	0	1	0	1	0	1	0	1	0.875153
tr|H0UI76|H0UI76_HUMAN	NPEPL1	Aminopeptidase-like 1, isoform CRA_d OS=Homo sapiens GN=NPEPL1 PE=4 SV=1	44188	0	794	0	46	0	0.07	#DIV/0!	0	1	0	1	0	1	0	1	0.875153
tr|B4DW54|B4DW54_HUMAN	tr|B4DW54|B4D	cDNA FLJ50888, highly similar to Aldehyde dehydrogenase, mitochondrial (EC 1.2.1.3) OS=Homo sapiens PE=2 SV=1	51467	0	793	0	46	0	0.06	#DIV/0!	0	1	0	1	0	1	0	1	0.875153
tr|D3DUE6|D3DUE6_HUMAN	N-PAC	Cytokine-like nuclear factor n-pac, isoform CRA_c OS=Homo sapiens GN=N-PAC PE=2 SV=1	61517	0	792	0	46	0	0.05	#DIV/0!	0	1	0	1	0	1	0	1	0.875153
tr|E9PDG5|E9PDG5_HUMAN	LARP4	La-related protein 4 OS=Homo sapiens GN=LARP4 PE=2 SV=1	50319	0	790	0	47	0	0.04	#DIV/0!	0	1	0	1	0	1	0	1	0.875153
tr|G3V1C3|G3V1C3_HUMAN	API5	Apoptosis inhibitor 5 OS=Homo sapiens GN=API5 PE=2 SV=1	57867	0	788	0	47	0	0.06	#DIV/0!	0	1	0	1	0	1	0	1	0.875153
tr|A8K4H7|A8K4H7_HUMAN	PPAT	Amidophosphoribosyltransferase OS=Homo sapiens GN=PPAT PE=2 SV=1	58161	0	786	0	47	0	0.06	#DIV/0!	0	1	0	1	0	1	0	1	0.875153
sp|P46013|KI67_HUMAN	MKI67	Antigen KI-67 OS=Homo sapiens GN=MKI67 PE=1 SV=2	360698	0	785	0	47	0	0.01	#DIV/0!	0	1	0	1	0	1	0	3	0.875153
sp|P04181|OAT_HUMAN	OAT	Ornithine aminotransferase, mitochondrial OS=Homo sapiens GN=OAT PE=1 SV=1	48846	0	784	0	47	0	0.07	#DIV/0!	0	1	0	1	0	1	0	1	0.875153
tr|A8K5Y7|A8K5Y7_HUMAN	tr|A8K5Y7|A8K5	cDNA FLJ78655, highly similar to Homo sapiens exportin 5 (XPO5), mRNA OS=Homo sapiens PE=2 SV=1	138331	0	783	0	47	0	0.02	#DIV/0!	0	1	0	1	0	1	0	2	0.875153
tr|B2RS14|B2RS14_HUMAN	tr|B2RS14|B2RS	cDNA, FLJ92300, Homo sapiens COP9 subunit 6 (MOV34 homolog, 34 kD) (COPS6), mRNA OS=Homo sapiens PE=2 SV=1	33896	0	779	0	48	0	0.1	#DIV/0!	0	1	0	1	0	1	0	1	0.875153
tr|B2R704|B2R704_HUMAN	tr|B2R704|B2R7	cDNA, FLJ93207, highly similar to Homo sapiens microtubule-associated protein 7 (MAP7), mRNA OS=Homo sapiens PE=2 SV=1	84046	0	778	0	48	0	0.04	#DIV/0!	0	1	0	1	0	1	0	1	0.875153
tr|F5GWY5|F5GWY5_HUMAN	PODXL	Podocalyxin OS=Homo sapiens GN=PODXL PE=2 SV=1	59285	0	770	0	48	0	0.06	#DIV/0!	0	1	0	1	0	1	0	1	0.875153
tr|B1AH49|B1AH49_HUMAN	MPST	Sulfurtransferase OS=Homo sapiens GN=MPST PE=4 SV=1	25081	0	769	0	49	0	0.13	#DIV/0!	0	1	0	1	0	1	0	1	0.875153
tr|B4DVG5|B4DVG5_HUMAN	tr|B4DVG5|B4D	cDNA FLJ53214, highly similar to Tubulointerstitial nephritis antigen-like OS=Homo sapiens PE=2 SV=1	45299	0	767	0	49	0	0.07	#DIV/0!	0	1	0	1	0	1	0	1	0.875153
tr|B2C8Z3|B2C8Z3_HUMAN	TGFB1	Transforming growth factor beta 1 (Fragment) OS=Homo sapiens GN=TGFB1 PE=2 SV=1	12965	0	766	0	49	0	0.07	#DIV/0!	0	1	0	1	0	1	0	1	0.875153
sp|P61221|ABCE1_HUMAN	ABCE1	ATP-binding cassette sub-family E member 1 OS=Homo sapiens GN=ABCE1 PE=1 SV=1	68240	0	763	0	49	0	0.07	#DIV/0!	0	1	0	1	0	1	0	1	0.875153
sp|Q86XP3|DDX42_HUMAN	DDX42	ATP-dependent RNA helicase DDX42 OS=Homo sapiens GN=DDX42 PE=1 SV=1	103197	0	762	0	49	0	0.03	#DIV/0!	0	1	0	1	0	1	0	3	0.875153

sp|O14776|TCERG1_HUMAN	TCERG1	Transcription elongation regulator 1 OS=Homo sapiens GN=TCERG1 PE=1 SV=2	124051	0	761	0	49	0	0.03	#DIV/0!	0	1	0	1	0	1	0	1	0.875153
tr|B3KN28|B3KN28_HUMAN	tr|B3KN28|B3K	cDNA FLJ13370 fis, clone PLACE1000653, highly similar to Phosphoacetylglucosamine mutase (EC 5.4.2.3) OS=Homo sapiens PE=2 SV=1	60284	0	760	0	50	0	0.05	#DIV/0!	0	1	0	1	0	1	0	1	0.875153
tr|B4DV94|B4DV94_HUMAN	tr|B4DV94|B4D	cDNA FLJ58285, highly similar to Homo sapiens pre-B-cell leukemia transcription factor interacting protein 1 (PBXIP1), mRNA OS=Homo sapi	57783	0	756	0	50	0	0.06	#DIV/0!	0	1	0	1	0	1	0	1	0.875153
tr|H0YKX5|H0YKX5_HUMAN	TPM1	Tropomyosin alpha-1 chain (Fragment) OS=Homo sapiens GN=TPM1 PE=4 SV=1	16412	0	754	0	0	0	0.13	#DIV/0!	0	0	0	0	0	1	0	1	0.875153
tr|A0JNT2|A0JNT2_HUMAN	KRT83	KRT83 protein OS=Homo sapiens GN=KRT83 PE=2 SV=1	50978	0	753	0	50	0	0.06	#DIV/0!	0	1	0	1	0	1	0	1	0.875153
tr|B2R694|B2R694_HUMAN	tr|B2R694|B2R	cDNA, FLJ92849, highly similar to Homo sapiens lanosterol synthase (2,3-oxidosqualene-lanosterol cyclase) (LSS), mRNA OS=Homo sapiens P	84568	0	747	0	51	0	0.04	#DIV/0!	0	1	0	1	0	1	0	2	0.875153
tr|B2R9S4|B2R9S4_HUMAN	tr|B2R9S4|B2R	cDNA, FLJ94534, highly similar to Homo sapiens capping protein (actin filament), gelsolin-like(CAPG), mRNA OS=Homo sapiens PE=2 SV=1	38759	0	742	0	51	0	0.09	#DIV/0!	0	1	0	1	0	1	0	1	0.875153
tr|Q6FHJ5|Q6FHJ5_HUMAN	SCAMP3	SCAMP3 protein (Fragment) OS=Homo sapiens GN=SCAMP3 PE=2 SV=1	38674	0	741	0	51	0	0.09	#DIV/0!	0	1	0	1	0	1	0	1	0.875153
sp|O14746|TERT_HUMAN	TERT	Telomerase reverse transcriptase OS=Homo sapiens GN=TERT PE=1 SV=1	128570	0	739	0	51	0	0.03	#DIV/0!	0	1	0	1	0	1	0	1	0.875153
sp|Q96QR8|PURB_HUMAN	PURB	Transcriptional activator protein Pur-beta OS=Homo sapiens GN=PURB PE=1 SV=3	33392	0	737	0	52	0	0.1	#DIV/0!	0	1	0	1	0	1	0	1	0.875153
tr|Q6IPI1|Q6IPI1_HUMAN	RPL29	Ribosomal protein L29 OS=Homo sapiens GN=RPL29 PE=2 SV=1	17997	0	728	0	52	0	0.19	#DIV/0!	0	1	0	1	0	1	0	1	0.875153
tr|F5H8E5|F5H8E5_HUMAN	USP7	Ubiquitin carboxyl-terminal hydrolase OS=Homo sapiens GN=USP7 PE=2 SV=1	117951	0	727	0	52	0	0.03	#DIV/0!	0	1	0	1	0	1	0	1	0.875153
sp|Q6ZRP7|QSOX2_HUMAN	QSOX2	Sulfhydryl oxidase 2 OS=Homo sapiens GN=QSOX2 PE=1 SV=3	78221	0	725	0	53	0	0.04	#DIV/0!	0	1	0	1	0	1	0	3	0.875153
tr|K7EQ60|K7EQ60_HUMAN	COASY	Bifunctional coenzyme A synthase (Fragment) OS=Homo sapiens GN=COASY PE=4 SV=1	12950	0	723	0	52	0	0.11	#DIV/0!	0	1	0	1	0	1	0	1	0.875153
sp|Q06210|GFPT1_HUMAN	GFPT1	Glutamine--fructose-6-phosphate aminotransferase [isomerizing] 1 OS=Homo sapiens GN=GFPT1 PE=1 SV=3	79555	0	721	0	53	0	0.04	#DIV/0!	0	1	0	1	0	1	0	2	0.875153
sp|Q9Y3Z3|SAMH1_HUMAN	SAMHD1	SAM domain and HD domain-containing protein 1 OS=Homo sapiens GN=SAMHD1 PE=1 SV=2	72896	0	716	0	54	0	0.17	#DIV/0!	0	1	0	1	0	1	0	1	0.875153
tr|H0Y4J2|H0Y4J2_HUMAN	MRPL37	39S ribosomal protein L37, mitochondrial (Fragment) OS=Homo sapiens GN=MRPL37 PE=4 SV=1	29806	0	712	0	54	0	0.11	#DIV/0!	0	1	0	1	0	1	0	1	0.875153
tr|K7EIS0|K7EIS0_HUMAN	TUBG1	Tubulin gamma chain (Fragment) OS=Homo sapiens GN=TUBG1 PE=3 SV=1	29797	0	711	0	55	0	0.11	#DIV/0!	0	1	0	1	0	1	0	1	0.875153
sp|P35251|RFC1_HUMAN	RFC1	Replication factor C subunit 1 OS=Homo sapiens GN=RFC1 PE=1 SV=4	128688	0	709	0	55	0	0.03	#DIV/0!	0	1	0	1	0	1	0	1	0.875153
tr|B3KST5|B3KST5_HUMAN	COPS4	COP9 constitutive photomorphogenic homolog subunit 4 (Arabidopsis), isoform CRA_a OS=Homo sapiens GN=COPS4 PE=2 SV=1	40457	0	702	0	56	0	0.07	#DIV/0!	0	1	0	1	0	1	0	1	0.875153
tr|Q7Z3H5|Q7Z3H5_HUMAN	DKFZp686O021!	Putative uncharacterized protein DKFZp686O0215 (Fragment) OS=Homo sapiens GN=DKFZp686O0215 PE=2 SV=1	37820	0	700	0	56	0	0.34	#DIV/0!	0	1	0	1	0	1	0	1	0.875153
tr|B2RAH7|B2RAH7_HUMAN	tr|B2RAH7|B2R	cDNA, FLJ94921, highly similar to Homo sapiens prolyl endopeptidase (PREP), mRNA OS=Homo sapiens PE=2 SV=1	81594	0	698	0	56	0	0.04	#DIV/0!	0	1	0	1	0	1	0	1	0.875153
tr|B7Z4U7|B7Z4U7_HUMAN	SCFD1	Sec1 family domain containing 1, isoform CRA_b OS=Homo sapiens GN=SCFD1 PE=2 SV=1	65362	0	688	0	57	0	0.05	#DIV/0!	0	1	0	1	0	1	0	2	0.875153
tr|H0YEL7|H0YEL7_HUMAN	ACAT1	Acetyl-CoA acetyltransferase, mitochondrial (Fragment) OS=Homo sapiens GN=ACAT1 PE=3 SV=1	14377	0	687	0	57	0	0.24	#DIV/0!	0	1	0	1	0	1	0	1	0.875153
tr|B3KVR1|B3KVR1_HUMAN	SNRPN	Small nuclear ribonucleoprotein-associated protein OS=Homo sapiens GN=SNRPN PE=2 SV=1	25230	0	686	0	58	0	0.13	#DIV/0!	0	1	0	1	0	1	0	1	0.875153
sp|Q8NI27|THOC2_HUMAN	THOC2	THO complex subunit 2 OS=Homo sapiens GN=THOC2 PE=1 SV=2	184541	0	677	0	59	0	0.03	#DIV/0!	0	1	0	1	0	1	0	1	0.875153
tr|Q58F23|Q58F23_HUMAN	TPR	TPR protein (Fragment) OS=Homo sapiens GN=TPR PE=2 SV=1	79452	0	676	0	0	0	0.01	#DIV/0!	0	0	0	0	0	1	0	2	0.875153
tr|Q2TSD2|Q2TSD2_HUMAN	tr|Q2TSD2|Q2T	Aging-associated gene 7 protein OS=Homo sapiens PE=2 SV=1	41778	0	673	0	59	0	0.08	#DIV/0!	0	1	0	1	0	1	0	1	0.875153
tr|Q6FI30|Q6FI30_HUMAN	NFIC	Nuclear factor 1 OS=Homo sapiens GN=NFIC PE=2 SV=1	55019	0	669	0	59	0	0.06	#DIV/0!	0	1	0	1	0	1	0	1	0.875153
tr|E9PQD7|E9PQD7_HUMAN	RPS2	40S ribosomal protein S2 OS=Homo sapiens GN=RPS2 PE=2 SV=1	25366	0	664	0	60	0	0.18	#DIV/0!	0	1	0	1	0	1	0	2	0.875153
tr|F8WE13|F8WE13_HUMAN	SMARCC1	SWI/SNF complex subunit SMARCC1 OS=Homo sapiens GN=SMARCC1 PE=2 SV=1	13945	0	663	0	60	0	0.24	#DIV/0!	0	1	0	1	0	1	0	1	0.875153
sp|A9QM74|IMA8_HUMAN	KPNA7	Importin subunit alpha-8 OS=Homo sapiens GN=KPNA7 PE=1 SV=1	57529	0	661	0	60	0	0.06	#DIV/0!	0	1	0	1	0	1	0	1	0.875153
tr|Q6ZPD0|Q6ZPD0_HUMAN	tr|Q6ZPD0|Q6Z	cDNA FLJ26027 fis, clone PNC04328, highly similar to Homo sapiens translocase of outer mitochondrial membrane 34 (TOMM34) OS=Homo	20621	0	652	0	61	0	0.16	#DIV/0!	0	1	0	1	0	1	0	1	0.875153
tr|B4E136|B4E136_HUMAN	HSDL2	Hydroxysteroid dehydrogenase-like protein 2 OS=Homo sapiens GN=HSDL2 PE=2 SV=1	33182	0	650	0	61	0	0.09	#DIV/0!	0	1	0	1	0	1	0	1	0.875153
tr|E9PJ81|E9PJ81_HUMAN	UBXN1	UBX domain-containing protein 1 (Fragment) OS=Homo sapiens GN=UBXN1 PE=2 SV=1	32649	0	647	0	61	0	0.15	#DIV/0!	0	1	0	1	0	1	0	1	0.875153
tr|E7EVL6|E7EVL6_HUMAN	REPIN1	Replication initiator 1 (Fragment) OS=Homo sapiens GN=REPIN1 PE=2 SV=1	16586	0	646	0	62	0	0.2	#DIV/0!	0	1	0	1	0	1	0	1	0.875153
tr|J3KMX1|J3KMX1_HUMAN	NUP88	Nuclear pore complex protein Nup88 (Fragment) OS=Homo sapiens GN=NUP88 PE=4 SV=1	80029	0	627	0	64	0	0.2	#DIV/0!	0	1	0	1	0	1	0	1	0.875153
tr|B4DRK5|B4DRK5_HUMAN	tr|B4DRK5|B4D	cDNA FLJ59584, highly similar to Mitochondrial-processing peptidase alpha subunit, mitochondrial (EC 3.4.24.64) OS=Homo sapiens PE=2 SV	28622	0	621	0	64	0	0.12	#DIV/0!	0	1	0	1	0	1	0	1	0.875153
tr|G3V5E1|G3V5E1_HUMAN	CCNK	Cyclin-K OS=Homo sapiens GN=CCNK PE=2 SV=1	43776	0	611	0	63	0	0.1	#DIV/0!	0	1	0	1	0	1	0	1	0.875153
tr|B4DX52|B4DX52_HUMAN	DNAJB1	DnaJ homolog subfamily B member 1 OS=Homo sapiens GN=DNAJB1 PE=2 SV=1	27170	0	609	0	54	0	0.18	#DIV/0!	0	1	0	1	0	1	0	2	0.875153
tr|B2R657|B2R657_HUMAN	tr|B2R657|B2R	Annexin OS=Homo sapiens PE=2 SV=1	52892	0	608	0	66	0	0.06	#DIV/0!	0	1	0	1	0	1	0	1	0.875153
tr|B4E1L0|B4E1L0_HUMAN	tr|B4E1L0|B4E1	Adenylosuccinate synthetase OS=Homo sapiens PE=2 SV=1	48308	0	607	0	67	0	0.07	#DIV/0!	0	1	0	1	0	1	0	1	0.875153
tr|H3BTZ5|H3BTZ5_HUMAN	CNN2	Calponin-2 (Fragment) OS=Homo sapiens GN=CNN2 PE=4 SV=1	21173	0	591	0	66	0	0.21	#DIV/0!	0	1	0	1	0	1	0	1	0.875153
tr|B2RB52|B2RB52_HUMAN	tr|B2RB52|B2R	cDNA, FLJ95314, highly similar to Homo sapiens transducin (beta)-like 2 (TBL2), transcript variant 1, mRNA OS=Homo sapiens PE=2 SV=1	50423	0	587	0	69	0	0.07	#DIV/0!	0	1	0	1	0	1	0	1	0.875153
tr|B3KUI0|B3KUI0_HUMAN	tr|B3KUI0|B3K	cDNA FLJ39945 fis, clone SPLEN2023977, highly similar to Homo sapiens CD97 antigen (CD97), transcript variant 2, mRNA OS=Homo sapiens	79803	0	583	0	71	0	0.04	#DIV/0!	0	1	0	1	0	1	0	1	0.875153
sp|P43034|LIS1_HUMAN	PAFAH1B1	Platelet-activating factor acetylhydrolase IB subunit alpha OS=Homo sapiens GN=PAFAH1B1 PE=1 SV=2	47178	0	569	0	72	0	0.08	#DIV/0!	0	1	0	1	0	1	0	1	0.875153
sp|P49419|AL7A1_HUMAN	ALDH7A1	Alpha-aminoadipic semialdehyde dehydrogenase OS=Homo sapiens GN=ALDH7A1 PE=1 SV=5	59020	0	567	0	72	0	0.27	#DIV/0!	0	1	0	2	0	1	0	2	0.875153
sp|Q96HE7|ERO1A_HUMAN	ERO1L	ERO1-like protein alpha OS=Homo sapiens GN=ERO1L PE=1 SV=2	55213	0	553	0	74	0	0.06	#DIV/0!	0	1	0	1	0	1	0	2	0.875153
sp|Q14739|LBR_HUMAN	LBR	Lamin-B receptor OS=Homo sapiens GN=LBR PE=1 SV=2	71057	0	551	0	75	0	0.05	#DIV/0!	0	1	0	1	0	1	0	1	0.875153
tr|C7FDR3|C7FDR3_HUMAN	HLA-B	MHC class I antigen (Fragment) OS=Homo sapiens GN=HLA-B PE=3 SV=1	21132	0	550	0	75	0	0.16	#DIV/0!	0	1	0	1	0	1	0	2	0.875153
tr|H3BQC4|H3BQC4_HUMAN	TGFB1I1	Transforming growth factor beta-1-induced transcript 1 protein (Fragment) OS=Homo sapiens GN=TGFB1I1 PE=4 SV=1	23016	0	545	0	76	0	0.15	#DIV/0!	0	1	0	1	0	1	0	1	0.875153
tr|B3KQ95|B3KQ95_HUMAN	FDFT1	Squalene synthase OS=Homo sapiens GN=FDFT1 PE=2 SV=1	35421	0	544	0	76	0	0.09	#DIV/0!	0	1	0	1	0	1	0	1	0.875153
tr|B5ME97|B5ME97_HUMAN	Sep-10	Septin 10, isoform CRA_c OS=Homo sapiens GN=SEPT10 PE=3 SV=2	63703	0	541	0	76	0	0.05	#DIV/0!	0	1	0	1	0	1	0	1	0.875153
tr|B4DG89|B4DG89_HUMAN	NPLOC4	Nuclear protein localization protein 4 homolog OS=Homo sapiens GN=NPLOC4 PE=2 SV=1	52144	0	540	0	0	0	0.05	#DIV/0!	0	0	0	0	0	1	0	1	0.875153
tr|B7Z553|B7Z553_HUMAN	tr|B7Z553|B7Z5	cDNA FLJ51266, highly similar to Vitronectin OS=Homo sapiens PE=2 SV=1	23761	0	534	0	77	0	0.14	#DIV/0!	0	1	0	1	0	1	0	1	0.875153
tr|B3KQ73|B3KQ73_HUMAN	tr|B3KQ73|B3K	cDNA FLJ33002 fis, clone THYMU1000217, highly similar to Homo sapiens ubiquitin associated protein 2 (UBAP2), transcript variant 1, mRNA	37025	0	528	0	37	0	0.09	#DIV/0!	0	1	0	1	0	1	0	1	0.875153
tr|J3KPP4|J3KPP4_HUMAN	LUC7L3	Cisplatin resistance-associated overexpressed protein, isoform CRA_b OS=Homo sapiens GN=LUC7L3 PE=4 SV=1	58698	0	521	0	79	0	0.06	#DIV/0!	0	1	0	1	0	1	0	1	0.875153
tr|H0Y2P0|H0Y2P0_HUMAN	CD44	CD44 antigen (Fragment) OS=Homo sapiens GN=CD44 PE=4 SV=1	31332	0	520	0	79	0	0.11	#DIV/0!	0	1	0	1	0	1	0	1	0.875153
sp|Q9UJA5|TRM6_HUMAN	TRMT6	tRNA (adenine(58)-N(1))-methyltransferase non-catalytic subunit TRM6 OS=Homo sapiens GN=TRMT6 PE=1 SV=1	56049	0	511	0	80	0	0.09	#DIV/0!	0	1	0	1	0	1	0	1	0.875153
tr|C9JDR0|C9JDR0_HUMAN	NSDHL	Sterol-4-alpha-carboxylate 3-dehydrogenase, decarboxylating (Fragment) OS=Homo sapiens GN=NSDHL PE=2 SV=1	28356	0	504	0	72	0	0.16	#DIV/0!	0	1	0	1	0	1	0	1	0.875153
tr|Q6IBS5|Q6IBS5_HUMAN	DLST	DLST protein OS=Homo sapiens GN=DLST PE=2 SV=1	49131	0	500	0	84	0	0.4	#DIV/0!	0	1	0	1	0	1	0	1	0.875153
sp|Q9H009|NACA2_HUMAN	NACA2	Nascent polypeptide-associated complex subunit alpha-2 OS=Homo sapiens GN=NACA2 PE=1 SV=1	23209	0	499	0	84	0	0.14	#DIV/0!	0	1	0	1	0	1	0	1	0.875153
sp|Q5F1R6|DJC21_HUMAN	DNAJC21	DnaJ homolog subfamily C member 21 OS=Homo sapiens GN=DNAJC21 PE=1 SV=2	62445	0	496	0	85	0	0.05	#DIV/0!	0	1	0	1	0	1	0	1	0.875153
sp|Q49AR2|CE022_HUMAN	C5orf22	UPF0489 protein C5orf22 OS=Homo sapiens GN=C5orf22 PE=1 SV=2	50506	0	494	0	85	0	0.07	#DIV/0!	0	1	0	1	0	1	0	1	0.875153
sp|Q70J99|UN13D_HUMAN	UNC13D	Protein unc-13 homolog D OS=Homo sapiens GN=UNC13D PE=1 SV=1	124288	0	487	0	87	0	0.03	#DIV/0!	0	1	0	1	0	1	0	2	0.875153
sp|O43837|IDH3B_HUMAN	IDH3B	Isocitrate dehydrogenase [NAD] subunit beta, mitochondrial OS=Homo sapiens GN=IDH3B PE=1 SV=2	42442	0	486	0	87	0	0.2	#DIV/0!	0	1	0	1	0	1	0	1	0.875153
sp|Q9H0A0|NAT10_HUMAN	NAT10	N-acetyltransferase 10 OS=Homo sapiens GN=NAT10 PE=1 SV=2	116569	0	477	0	89	0	0.03	#DIV/0!	0	1	0	1	0	1	0	1	0.875153
tr|B0I1S9|B0I1S9_HUMAN	MYO1B	MYO1B variant protein OS=Homo sapiens GN=MYO1B PE=2 SV=1	132928	0	476	0	89	0	0.05	#DIV/0!	0	1	0	2	0	1	0	3	0.875153
tr|C9J634|C9J634_HUMAN	PDHB	Pyruvate dehydrogenase E1 component subunit beta, mitochondrial OS=Homo sapiens GN=PDHB PE=2 SV=1	39064	0	475	0	60	0	0.17	#DIV/0!	0	1	0	1	0	1	0	1	0.875153
tr|D6RFG5|D6RFG5_HUMAN	ANXA3	Annexin (Fragment) OS=Homo sapiens GN=ANXA3 PE=2 SV=1	17135	0	469	0	60	0	0.22	#DIV/0!	0	1	0	1	0	1	0	1	0.875153
sp|Q9BWF3|RBM4_HUMAN	RBM4	RNA-binding protein 4 OS=Homo sapiens GN=RBM4 PE=1 SV=1	40688	0	468	0	63	0	0.18	#DIV/0!	0	1	0	1	0	1	0	1	0.875153
tr|I6L9E8|I6L9E8_HUMAN	FAM98A	Family with sequence similarity 98, member A OS=Homo sapiens GN=FAM98A PE=2 SV=1	55595	0	464	0	91	0	0.1	#DIV/0!	0	1	0	1	0	1	0	2	0.875153
tr|Q5W1M0|Q5W1M0_HUMAN	HLA-C	MHC class I antigen (Fragment) OS=Homo sapiens GN=HLA-C PE=2 SV=1	10534	0	439	0	0	0	0.17	#DIV/0!	0	0	0	0	0	1	0	1	0.875153
tr|H0Y7C0|H0Y7C0_HUMAN	USO1	General vesicular transport factor p115 (Fragment) OS=Homo sapiens GN=USO1 PE=4 SV=1	72345	0	406	0	108	0	0.05	#DIV/0!	0	1	0	1	0	1	0	1	0.875153
sp|P05556|ITB1_HUMAN	ITGB1	Integrin beta-1 OS=Homo sapiens GN=ITGB1 PE=1 SV=2	91664	0	1196	0	15	0	0.22	#DIV/0!	0	0	0	1	0	0	0	1	-0.71002
tr|B4E3H4|B4E3H4_HUMAN	tr|B4E3H4|B4E	cDNA FLJ54470, highly similar to Puratrophin-1 OS=Homo sapiens PE=2 SV=1	112357	0	1195	0	15	0	0.03	#DIV/0!	0	0	0	1	0	0	0	1	-0.71002
tr|B2R7V4|B2R7V4_HUMAN	tr|B2R7V4|B2R	cDNA, FLJ93619, highly similar to Homo sapiens PRP4 pre-mRNA processing factor 4 homolog (yeast) (PRPF4), mRNA OS=Homo sapiens PE=	59097	0	1194	0	16	0	0.06	#DIV/0!	0	0	0	1	0	0	0	1	-0.71002
tr|I3L301|I3L301_HUMAN	TFAP4	Transcription factor AP-4 OS=Homo sapiens GN=TFAP4 PE=4 SV=1	18073	0	1193	0	16	0	0.19	#DIV/0!	0	0	0	1	0	0	0	1	-0.71002
tr|B2RDZ9|B2RDZ9_HUMAN	tr|B2RDZ9|B2R	cDNA, FLJ96850 OS=Homo sapiens PE=2 SV=1	32424	0	1192	0	16	0	0.1	#DIV/0!	0	0	0	1	0	0	0	2	-0.71002
tr|B3KM40|B3KM40_HUMAN	tr|B3KM40|B3K	cDNA FLJ10185 fis, clone HEMBA1004509, highly similar to U4/U6.U5 tri-snRNP-associated protein 2 OS=Homo sapiens PE=2 SV=1	56618	0	1191	0	16	0	0.06	#DIV/0!	0	0	0	1	0	0	0	2	-0.71002
sp|Q9UMW8|UBP18_HUMAN	USP18	Ubl carboxyl-terminal hydrolase 18 OS=Homo sapiens GN=USP18 PE=1 SV=1	44009	0	1189	0	17	0	0.08	#DIV/0!	0	0	0	1	0	0	0	1	-0.71002
tr|A1A564|A1A564_HUMAN	BCOR	BCOR protein OS=Homo sapiens GN=BCOR PE=2 SV=1	44805	0	1187	0	17	0	0.07	#DIV/0!	0	0	0	1	0	0	0	1	-0.71002
tr|H0YHM7|H0YHM7_HUMAN	FKBP11	Peptidyl-prolyl cis-trans isomerase FKBP11 (Fragment) OS=Homo sapiens GN=FKBP11 PE=4 SV=1	6984	0	1186	0	17	0	0.51	#DIV/0!	0	0	0	1	0	0	0	1	-0.71002
tr|B4DTF3|B4DTF3_HUMAN	tr|B4DTF3|B4D	cDNA FLJ54338, highly similar to Golgin subfamily A member 4 (Fragment) OS=Homo sapiens PE=2 SV=1	62263	0	1185	0	17	0	0.05	#DIV/0!	0	0	0	1	0	0	0	2	-0.71002
tr|B4E3M6|B4E3M6_HUMAN	tr|B4E3M6|B4E	cDNA FLJ55446, highly similar to Superkiller viralicidic activity 2-like 2 (EC 3.6.1.-) OS=Homo sapiens PE=2 SV=1	107714	0	1184	0	17	0	0.03	#DIV/0!	0	0	0	1	0	0	0	2	-0.71002
tr|L0R6G9|L0R6G9_HUMAN	FUNDC2	Alternative protein FUNDC2 OS=Homo sapiens GN=FUNDC2 PE=4 SV=1	8134	0	1183	0	18	0	0.43	#DIV/0!	0	0	0	1	0	0	0	1	-0.71002
tr|Q8NGU3|Q8NGU3_HUMAN	tr|Q8NGU3|Q8	Seven transmembrane helix receptor OS=Homo sapiens PE=4 SV=1	39097	0	1180	0	18	0	0.18	#DIV/0!	0	0	0	2	0	0	0	2	-0.71002
tr|H7BZ11|H7BZ11_HUMAN	tr|H7BZ11|H7B	Uncharacterized protein (Fragment) OS=Homo sapiens PE=3 SV=1	15266	0	1179	0	18	0	0.22	#DIV/0!	0	0	0	1	0	0	0	1	-0.71002
tr|B2R7R5|B2R7R5_HUMAN	tr|B2R7R5|B2R	cDNA, FLJ93570, highly similar to Homo sapiens phosphoribosyl pyrophosphate synthetase-associated protein 2 (PRPSAP2), mRNA OS=Hom	41256	0	1178	0	18	0	0.08	#DIV/0!	0	0	0	1	0	0	0	1	-0.71002
tr|D6R918|D6R918_HUMAN	OCIAD1	OCIA domain-containing protein 1 (Fragment) OS=Homo sapiens GN=OCIAD1 PE=2 SV=1	8339	0	1177	0	18	0	0.42	#DIV/0!	0	0	0	1	0	0	0	1	-0.71002
tr|Q8TEP9|Q8TEP9_HUMAN	FLJ00144	FLJ00144 protein (Fragment) OS=Homo sapiens GN=FLJ00144 PE=2 SV=1	28437	0	1176	0	0	0	0.07	#DIV/0!	0	0	0	0	0	0	0	2	-0.71002
sp|Q7Z3U7|MON2_HUMAN	MON2	Protein MON2 homolog OS=Homo sapiens GN=MON2 PE=1 SV=2	192475	0	1175	0	19	0	0.02	#DIV/0!	0	1	0	1	0	0	0	1	-0.71002
tr|B2R829|B2R829_HUMAN	tr|B2R829|B2R	cDNA, FLJ93711, highly similar to Homo sapiens myeloid cell nuclear differentiation antigen (MNDA), mRNA OS=Homo sapiens PE=2 SV=1	46106	0	1174	0	19	0	0.07	#DIV/0!	0	0	0	1	0	0	0	1	-0.71002
tr|B3KSE4|B3KSE4_HUMAN	tr|B3KSE4|B3K	cDNA FLJ36076 fis, clone TESTI2019760, highly similar to TRANSCRIPTIONAL REPRESSOR CTCF OS=Homo sapiens PE=2 SV=1	30924	0	1173	0	19	0	0.11	#DIV/0!	0	0	0	1	0	0	0	1	-0.71002
tr|A4D0U1|A4D0U1_HUMAN	IFRD1	Interferon-related developmental regulator 1 OS=Homo sapiens GN=IFRD1 PE=2 SV=1	51035	0	1172	0	19	0	0.06	#DIV/0!	0	0	0	1	0	0	0	1	-0.71002
tr|A4UCU0|A4UCU0_HUMAN	tr|A4UCU0|A4U	DEAD box polypeptide 47 isoform 1 variant (Fragment) OS=Homo sapiens PE=2 SV=1	19904	0	1171	0	19	0	0.17	#DIV/0!	0	0	0	1	0	0	0	1	-0.71002
tr|D6RGD1|D6RGD1_HUMAN	MYO10	Unconventional myosin-X (Fragment) OS=Homo sapiens GN=MYO10 PE=2 SV=1	96861	0	1170	0	20	0	0.03	#DIV/0!	0	0	0	1	0	0	0	1	-0.71002
tr|A8K9B9|A8K9B9_HUMAN	tr|A8K9B9|A8K	cDNA FLJ77391, highly similar to Homo sapiens EH-domain containing 4 (EHD4), mRNA OS=Homo sapiens PE=2 SV=1	61346	0	1149	0	0	0	0.05	#DIV/0!	0	0	0	0	0	0	0	1	-0.71002
sp|O00507|USP9Y_HUMAN	USP9Y	Probable ubiquitin carboxyl-terminal hydrolase FAF-Y OS=Homo sapiens GN=USP9Y PE=1 SV=2	294370	0	989	0	18	0	0.02	#DIV/0!	0	0	0	1	0	0	0	2	-0.71002
tr|B2R5M9|B2R5M9_HUMAN	tr|B2R5M9|B2R	cDNA, FLJ92537, highly similar to Homo sapiens procollagen-lysine, 2-oxoglutarate 5-dioxygenase (lysine hydroxylase, Ehlers-Danlos syndror	84022	251	105	76	386	0.04	0.64	16	1	10	1	13	1	14	1	16	2.565554
tr|B7ZAW0|B7ZAW0_HUMAN	tr|B7ZAW0|B7Z	cDNA, FLJ79324, highly similar to Carbamoyl-phosphate synthase (ammonia), mitochondrial (EC 6.3.4.16) OS=Homo sapiens PE=2 SV=1	20368	204	164	44	267	0.15	2.4	16	1	6	1	8	2	7	2	9	0.875716
tr|Q2TU64|Q2TU64_HUMAN	tr|Q2TU64|Q2T	PIG48 OS=Homo sapiens PE=2 SV=1	61110	279	193	68	183	0.05	0.73	14.6	1	5	1	8	3	10	3	10	0.875997
tr|B2R4K7|B2R4K7_HUMAN	tr|B2R4K7|B2R	60S ribosomal protein L6 OS=Homo sapiens PE=2 SV=1	32739	276	64	68	512	0.47	5.18	11.0212766	2	15	4	17	3	17	5	20	1.614408
tr|B2R9U2|B2R9U2_HUMAN	tr|B2R9U2|B2R	cDNA, FLJ94557, highly similar to Homo sapiens FK506 binding protein 4, 59kDa (FKBP4), mRNA OS=Homo sapiens PE=2 SV=1	52087	623	181	24	234	0.06	0.63	10.5	0	4	1	7	2	8	2	8	1.056494
tr|B4DT29|B4DT29_HUMAN	tr|B4DT29|B4D	cDNA FLJ51082, highly similar to Actin-like protein 3 OS=Homo sapiens PE=2 SV=1	40959	548	191	30	94	0.08	0.82	10.25	0	4	1	6	1	5	1	6	1.165147
tr|H0UID3|H0UID3_HUMAN	AP2B1	Adaptor-related protein complex 2, beta 1 subunit, isoform CRA_d OS=Homo sapiens GN=AP2B1 PE=4 SV=1	106321	648	174	22	252	0.03	0.3	10	0	5	1	8	3	9	3	9	0.731401
tr|B3KQT2|B3KQT2_HUMAN	tr|B3KQT2|B3K	cDNA PSEC0148 fis, clone PLACE1007202, highly similar to Protein disulfide-isomerase A3 (EC 5.3.4.1) OS=Homo sapiens PE=2 SV=1	55268	546	116	30	333	0.19	1.71	9	0	11	3	16	2	17	8	21	2.100173
tr|Q59FV6|Q59FV6_HUMAN	tr|Q59FV6|Q59	ARP3 actin-related protein 3 homolog variant (Fragment) OS=Homo sapiens PE=2 SV=1	42517	548	280	30	163	0.08	0.69	8.625	0	6	1	9	1	8	1	9	1.793797
tr|B4DJ30|B4DJ30_HUMAN	tr|B4DJ30|B4D	cDNA FLJ61290, highly similar to Neutral alpha-glucosidase AB OS=Homo sapiens PE=2 SV=1	113539	47	9	446	2144	0.62	4.98	8.032258065	12	55	19	83	17	90	22	101	1.673473
tr|B4DVJ0|B4DVJ0_HUMAN	tr|B4DVJ0|B4D	Glucose-6-phosphate isomerase OS=Homo sapiens PE=2 SV=1	60181	117	36	182	841	0.3	2.4	8	4	18	5	27	6	27	6	28	1.374455
tr|Q96QL0|Q96QL0_HUMAN	RPL3	Ribosomal protein L3 OS=Homo sapiens GN=RPL3 PE=2 SV=1	46376	245	110	82	377	0.32	2.55	7.96875	4	10	4	18	5	18	7	20	1.042034
tr|B4DE36|B4DE36_HUMAN	tr|B4DE36|B4D	Glucose-6-phosphate isomerase OS=Homo sapiens PE=2 SV=1	60376	117	35	182	841	0.3	2.39	7.966666667	4	18	5	27	7	28	7	30	1.219404
tr|Q9NZ23|Q9NZ23_HUMAN	YA61	Drug-sensitive protein 1 OS=Homo sapiens GN=YA61 PE=2 SV=1	14858	588	278	27	164	0.23	1.8	7.826086957	0	5	1	5	1	6	1	6	1.406361
sp|O14980|XPO1_HUMAN	XPO1	Exportin-1 OS=Homo sapiens GN=XPO1 PE=1 SV=1	124447	305	173	59	253	0.03	0.23	7.666666667	1	6	1	8	1	10	3	13	2.099064
tr|H7BZJ3|H7BZJ3_HUMAN	PDIA3	Thioredoxin (Fragment) OS=Homo sapiens GN=PDIA3 PE=3 SV=1	13739	546	400	22	111	0.19	1.43	7.526315789	0	3	1	4	1	3	1	4	0.512658
tr|I3L1L3|I3L1L3_HUMAN	MYBBP1A	Myb-binding protein 1A (Fragment) OS=Homo sapiens GN=MYBBP1A PE=4 SV=1	141124	698	437	18	99	0.02	0.15	7.5	0	2	1	6	1	5	2	6	1.165147
tr|B4DZ53|B4DZ53_HUMAN	tr|B4DZ53|B4D	cDNA FLJ59643, highly similar to Neutral alpha-glucosidase AB OS=Homo sapiens PE=2 SV=1	96752	47	11	400	1824	0.62	4.64	7.483870968	10	46	17	68	15	71	19	81	1.5053
tr|A2VCT2|A2VCT2_HUMAN	OGDH	OGDH protein (Fragment) OS=Homo sapiens GN=OGDH PE=2 SV=1	72973	432	192	40	132	0.05	0.37	7.4	1	3	1	5	1	6	1	7	1.406361
tr|B4DTV0|B4DTV0_HUMAN	tr|B4DTV0|B4D	cDNA FLJ57801, highly similar to Homo sapiens AHNAK nucleoprotein (desmoyokin) (AHNAK), transcript variant 1, mRNA (Fragment) OS=Ho	114179	454	259	38	176	0.03	0.22	7.333333333	1	5	1	7	3	8	3	11	0.57073
tr|B2R7B5|B2R7B5_HUMAN	tr|B2R7B5|B2R	cDNA, FLJ93365, highly similar to Homo sapiens KH domain containing, RNA binding, signal transduction associated 1 (KHDRBS1), mRNA OS=	48309	347	233	51	152	0.07	0.51	7.285714286	1	4	1	5	2	5	4	7	0.427844
tr|B4DSQ5|B4DSQ5_HUMAN	tr|B4DSQ5|B4D	cDNA FLJ53608, highly similar to Protein transport protein Sec23A OS=Homo sapiens PE=2 SV=1	83787	474	211	37	181	0.08	0.57	7.125	0	4	2	9	2	8	3	10	1.056494
tr|B2R8N0|B2R8N0_HUMAN	tr|B2R8N0|B2R	cDNA, FLJ93975, highly similar to Homo sapiens epoxide hydrolase 1, microsomal (xenobiotic) (EPHX1), mRNA OS=Homo sapiens PE=2 SV=1	53129	115	40	184	788	0.62	4.38	7.064516129	6	26	8	33	8	38	8	40	1.474461
sp|Q09666|AHNK_HUMAN	AHNAK	Neuroblast differentiation-associated protein AHNAK OS=Homo sapiens GN=AHNAK PE=1 SV=2	629213	401	133	43	307	0.01	0.07	7	1	10	1	14	3	19	6	23	1.770941
tr|B4DKD0|B4DKD0_HUMAN	tr|B4DKD0|B4D	DNA topoisomerase 2 (Fragment) OS=Homo sapiens PE=2 SV=1	135015	606	401	25	33	0.02	0.14	7	0	0	1	1	1	1	3	4	-0.71015
sp|O00425|IF2B3_HUMAN	IGF2BP3	Insulin-like growth factor 2 mRNA-binding protein 3 OS=Homo sapiens GN=IGF2BP3 PE=1 SV=2	64008	556	377	29	116	0.05	0.35	7	0	2	1	6	2	6	2	6	0.669059
sp|P11021|GRP78_HUMAN	HSPA5	78 kDa glucose-regulated protein OS=Homo sapiens GN=HSPA5 PE=1 SV=2	72402	129	46	170	694	0.19	1.32	6.947368421	3	15	5	21	6	23	6	26	1.146857
tr|Q0VGD6|Q0VGD6_HUMAN	HNRPR	HNRPR protein (Fragment) OS=Homo sapiens GN=HNRPR PE=2 SV=1	68218	390	166	44	260	0.1	0.68	6.8	1	7	2	11	2	12	4	12	1.613713
tr|B4DMU0|B4DMU0_HUMAN	PYCR1	Pyrroline-5-carboxylate reductase OS=Homo sapiens GN=PYCR1 PE=2 SV=1	36186	174	177	125	250	0.19	1.29	6.789473684	2	6	2	8	3	8	3	10	0.57073
tr|H0YLA4|H0YLA4_HUMAN	SORD	Sorbitol dehydrogenase OS=Homo sapiens GN=SORD PE=3 SV=1	36833	473	392	37	90	0.09	0.61	6.777777778	0	1	1	1	2	1	2	2	-1.44745
tr|B4DGQ0|B4DGQ0_HUMAN	LASP1	LIM and SH3 domain protein 1 OS=Homo sapiens GN=LASP1 PE=2 SV=1	23167	568	484	29	68	0.14	0.92	6.571428571	0	1	1	3	1	5	1	5	1.165147
tr|J3KR24|J3KR24_HUMAN	IARS	Isoleucine--tRNA ligase, cytoplasmic OS=Homo sapiens GN=IARS PE=4 SV=1	132820	366	269	47	169	0.02	0.13	6.5	1	3	1	5	2	7	2	7	0.875716
tr|B4DJ60|B4DJ60_HUMAN	tr|B4DJ60|B4D	cDNA FLJ55072, highly similar to Succinate dehydrogenase (ubiquinone) flavoprotein subunit, mitochondrial (EC1.3.5.1) OS=Homo sapiens P	68233	393	152	44	281	0.1	0.64	6.4	0	8	2	9	3	9	5	9	0.731401
tr|A6NN80|A6NN80_HUMAN	ANXA6	Annexin OS=Homo sapiens GN=ANXA6 PE=3 SV=3	75571	349	102	50	388	0.14	0.89	6.357142857	1	11	3	15	3	17	3	19	1.614408
tr|B3KRS7|B3KRS7_HUMAN	tr|B3KRS7|B3K	cDNA FLJ34844 fis, clone NT2NE2011154, highly similar to RuvB-like 1 OS=Homo sapiens PE=2 SV=1	34946	351	787	50	47	0.09	0.57	6.333333333	1	1	1	5	1	3	1	7	0.512658
tr|Q05CK9|Q05CK9_HUMAN	SYNCRIP	SYNCRIP protein (Fragment) OS=Homo sapiens GN=SYNCRIP PE=2 SV=1	50847	252	112	76	349	0.13	0.82	6.307692308	2	9	2	12	2	13	2	13	1.724951
tr|Q2F839|Q2F839_HUMAN	tr|Q2F839|Q2F	Heat shock 70 kDa protein 9B (Fragment) OS=Homo sapiens PE=2 SV=1	19208	81	149	59	292	0.42	2.64	6.285714286	2	6	2	8	2	7	2	10	0.875716
tr|B4DY09|B4DY09_HUMAN	tr|B4DY09|B4D	cDNA FLJ51660, highly similar to Interleukin enhancer-binding factor 2 OS=Homo sapiens PE=2 SV=1	39057	383	260	45	176	0.08	0.5	6.25	1	4	1	5	1	5	1	5	1.165147
tr|Q2Q9B7|Q2Q9B7_HUMAN	G6PD	Glucose-6-phosphate 1-dehydrogenase (Fragment) OS=Homo sapiens GN=G6PD PE=2 SV=1	55221	176	142	118	289	0.18	1.12	6.222222222	2	6	2	12	5	15	6	15	0.786157
tr|B4DYA7|B4DYA7_HUMAN	tr|B4DYA7|B4D	Glucose-6-phosphate 1-dehydrogenase OS=Homo sapiens PE=2 SV=1	58757	176	143	123	265	0.18	1.12	6.222222222	2	5	3	11	6	14	7	14	0.44839
tr|F8W9S7|F8W9S7_HUMAN	GAPVD1	GTPase-activating protein and VPS9 domain-containing protein 1 OS=Homo sapiens GN=GAPVD1 PE=2 SV=1	158790	697	803	18	45	0.02	0.12	6	0	1	1	2	0	2	2	3	1.612325
tr|E9PBS1|E9PBS1_HUMAN	PAICS	Phosphoribosylaminoimidazole carboxylase (Fragment) OS=Homo sapiens GN=PAICS PE=2 SV=1	46307	270	304	69	149	0.07	0.41	5.857142857	1	4	1	5	2	6	5	6	0.669059
sp|P52597|HNRPF_HUMAN	HNRNPF	Heterogeneous nuclear ribonucleoprotein F OS=Homo sapiens GN=HNRNPF PE=1 SV=3	45985	226	292	91	155	0.07	0.4	5.714285714	1	3	1	5	2	5	3	5	0.427844
sp|P04040|CATA_HUMAN	CAT	Catalase OS=Homo sapiens GN=CAT PE=1 SV=3	59947	299	205	60	221	0.11	0.62	5.636363636	1	3	2	9	2	11	2	12	1.493212
tr|B2R959|B2R959_HUMAN	tr|B2R959|B2R	cDNA, FLJ94229, highly similar to Homo sapiens heterogeneous nuclear ribonucleoprotein L (HNRPL),mRNA OS=Homo sapiens PE=2 SV=1	60838	182	103	117	317	0.23	1.27	5.52173913	4	10	4	15	6	15	8	18	0.544811
sp|P07355|ANXA2_HUMAN	ANXA2	Annexin A2 OS=Homo sapiens GN=ANXA2 PE=1 SV=2	38808	100	42	214	761	1.26	6.72	5.333333333	8	23	11	30	12	31	13	31	0.62576
sp|P41250|SYG_HUMAN	GARS	Glycine--tRNA ligase OS=Homo sapiens GN=GARS PE=1 SV=3	83854	392	274	44	165	0.04	0.21	5.25	1	4	1	5	1	6	2	7	1.406361
tr|B4DR68|B4DR68_HUMAN	tr|B4DR68|B4D	cDNA FLJ58608, highly similar to Heat shock protein 75 kDa, mitochondrial OS=Homo sapiens PE=2 SV=1	74408	169	99	127	401	0.09	0.47	5.222222222	2	9	3	10	3	10	5	11	0.875997
tr|G3XAF4|G3XAF4_HUMAN	L1CAM	L1 cell adhesion molecule, isoform CRA_a OS=Homo sapiens GN=L1CAM PE=4 SV=1	139790	572	155	28	278	0.05	0.26	5.2	1	7	2	10	1	12	2	13	2.351016
tr|B4DMJ6|B4DMJ6_HUMAN	tr|B4DMJ6|B4D	cDNA FLJ50996, highly similar to 60S ribosomal protein L4 OS=Homo sapiens PE=2 SV=1	46269	163	67	132	463	0.51	2.65	5.196078431	4	12	6	16	8	17	9	19	0.332613
tr|E7ENU7|E7ENU7_HUMAN	RPL15	Ribosomal protein L15 (Fragment) OS=Homo sapiens GN=RPL15 PE=2 SV=1	20596	534	674	31	47	0.16	0.83	5.1875	0	0	1	3	1	3	1	3	0.512658
tr|B5MCX3|B5MCX3_HUMAN	Sep-02	Septin-2 OS=Homo sapiens GN=SEPT2 PE=2 SV=1	37145	543	279	30	128	0.09	0.46	5.111111111	1	3	1	3	1	3	1	3	0.427844
sp|Q6P2Q9|PRP8_HUMAN	PRPF8	Pre-mRNA-processing-splicing factor 8 OS=Homo sapiens GN=PRPF8 PE=1 SV=2	274738	130	33	167	910	0.1	0.51	5.1	7	26	8	35	10	36	10	43	1.091554
tr|B0ZSE7|B0ZSE7_HUMAN	HLA-A	MHC class I antigen (Fragment) OS=Homo sapiens GN=HLA-A PE=3 SV=1	21069	639	294	22	75	0.16	0.81	5.0625	0	1	1	1	1	2	1	3	0.027025
tr|A8KAK1|A8KAK1_HUMAN	tr|A8KAK1|A8K	cDNA FLJ77398, highly similar to Homo sapiens UDP-glucose ceramide glucosyltransferase-like 1, transcript variant 2, mRNA (Fragment) OS=Homo sapie	175467	635	395	22	112	0.02	0.11	5	0	1	1	2	1	2	1	2	0.027025
sp|Q8TCJ2|STT3B_HUMAN	STT3B	Dolichyl-diphosphooligosaccharide--protein glycosyltransferase subunit STT3B OS=Homo sapiens GN=STT3B PE=1 SV=1	94241	700	351	18	131	0.03	0.15	5	0	3	1	4	1	7	1	8	1.613019
tr|D5FHQ7|D5FHQ7_HUMAN	HLA-A	MHC class I antigen (Fragment) OS=Homo sapiens GN=HLA-A PE=3 SV=1	21188	640	337	22	135	0.16	0.8	5	0	3	1	4	2	6	3	7	0.669059
tr|B2R5V9|B2R5V9_HUMAN	tr|B2R5V9|B2R	cDNA, FLJ92652, highly similar to Homo sapiens high density lipoprotein binding protein (vigilin)(HDLBP), mRNA OS=Homo sapiens PE=2 SV=1	141919	253	282	76	163	0.02	0.1	5	1	4	1	4	2	4	2	5	0.138131
tr|Q6ZS03|Q6ZS03_HUMAN	tr|Q6ZS03|Q6Z	cDNA FLJ45936 fis, clone PLACE7004103, highly similar to Vigilin OS=Homo sapiens PE=2 SV=1	95244	253	305	0	68	0.02	0.1	5	0	1	0	2	1	2	1	2	0.027025
tr|A0EVK0|A0EVK0_HUMAN	HLA-A	MHC class I antigen (Fragment) OS=Homo sapiens GN=HLA-A PE=3 SV=1	21359	639	295	22	75	0.16	0.8	5	0	1	1	1	1	2	1	3	0.027025
tr|A6NKB8|A6NKB8_HUMAN	RNPEP	Aminopeptidase B OS=Homo sapiens GN=RNPEP PE=2 SV=1	68752	316	360	57	120	0.05	0.24	4.8	1	3	2	4	2	4	2	7	0.138131
tr|A8K4D5|A8K4D5_HUMAN	tr|A8K4D5|A8K	Kynureninase OS=Homo sapiens PE=2 SV=1	52819	266	261	71	149	0.13	0.62	4.769230769	2	5	2	7	3	6	3	8	0.183294
tr|B3KY60|B3KY60_HUMAN	tr|B3KY60|B3K	cDNA FLJ16777 fis, clone BRHIP2029567, highly similar to Cell division cycle 5-like protein OS=Homo sapiens PE=2 SV=1	92448	587	319	27	142	0.04	0.19	4.75	0	2	1	4	1	5	1	7	1.165147
sp|O75367|H2AY_HUMAN	H2AFY	Core histone macro-H2A.1 OS=Homo sapiens GN=H2AFY PE=1 SV=4	39764	565	289	29	158	0.08	0.38	4.75	0	4	1	4	2	5	2	6	0.138131
tr|A8K781|A8K781_HUMAN	tr|A8K781|A8K	cDNA FLJ75299, highly similar to Xenopus laevis proteasome (prosome, macropain) 26S subunit, ATPase 3, mRNA OS=Homo sapiens PE=2 SV	47578	603	616	26	32	0.07	0.33	4.714285714	0	1	0	2	2	4	2	4	0.138131
tr|A6NG51|A6NG51_HUMAN	SPTAN1	Spectrin alpha chain, non-erythrocytic 1 OS=Homo sapiens GN=SPTAN1 PE=2 SV=2	285628	167	60	130	501	0.06	0.28	4.666666667	2	14	5	21	6	21	9	27	1.018119
tr|F8WJN3|F8WJN3_HUMAN	CPSF6	Cleavage and polyadenylation-specificity factor subunit 6 OS=Homo sapiens GN=CPSF6 PE=2 SV=1	52409	379	308	46	148	0.06	0.28	4.666666667	1	4	1	4	1	4	1	4	0.875434
tr|B4DKS0|B4DKS0_HUMAN	tr|B4DKS0|B4D	cDNA FLJ53381, highly similar to Monocarboxylate transporter 1 OS=Homo sapiens PE=2 SV=1	52487	598	248	26	154	0.06	0.28	4.666666667	0	2	1	3	1	4	1	4	0.512658
sp|P45974|UBP5_HUMAN	USP5	Ubiquitin carboxyl-terminal hydrolase 5 OS=Homo sapiens GN=USP5 PE=1 SV=2	96638	622	353	24	130	0.03	0.14	4.666666667	0	4	1	4	2	5	2	6	0.427844
tr|J3KPS3|J3KPS3_HUMAN	ALDOA	Fructose-bisphosphate aldolase OS=Homo sapiens GN=ALDOA PE=3 SV=1	40249	63	20	338	1199	1.79	8.33	4.653631285	11	25	15	32	17	36	18	39	0.352222
tr|H0YD14|H0YD14_HUMAN	MYOF	Myoferlin (Fragment) OS=Homo sapiens GN=MYOF PE=4 SV=1	135822	220	72	95	324	0.07	0.32	4.571428571	3	9	3	12	4	15	4	16	1.076001
tr|B4E091|B4E091_HUMAN	tr|B4E091|B4E	cDNA FLJ55438, highly similar to Splicing factor 3 subunit 1 OS=Homo sapiens PE=2 SV=1	77415	482	502	36	83	0.04	0.18	4.5	1	2	1	4	1	4	1	4	0.875434
tr|E5KNY5|E5KNY5_HUMAN	LRPPRC	Leucine-rich PPR-motif containing OS=Homo sapiens GN=LRPPRC PE=2 SV=1	159003	329	153	53	282	0.08	0.36	4.5	1	10	4	15	5	15	7	19	0.786157
tr|B4DJE3|B4DJE3_HUMAN	tr|B4DJE3|B4D	cDNA FLJ52929, highly similar to Dolichyl-diphosphooligosaccharide--proteinglycosyltransferase 48 kDa subunit (EC2.4.1.119) OS=Homo sap	46620	604	454	26	95	0.07	0.31	4.428571429	0	3	1	4	1	4	2	4	0.875434
tr|F5H6T1|F5H6T1_HUMAN	ACTR2	Actin-related protein 2 OS=Homo sapiens GN=ACTR2 PE=2 SV=1	34729	244	201	82	210	0.2	0.88	4.4	2	5	2	6	2	8	4	9	1.056494
sp|Q96A33|CCD47_HUMAN	CCDC47	Coiled-coil domain-containing protein 47 OS=Homo sapiens GN=CCDC47 PE=1 SV=1	56123	560	492	29	86	0.06	0.26	4.333333333	0	2	1	4	1	5	1	5	1.165147
sp|Q15645|PCH2_HUMAN	TRIP13	Pachytene checkpoint protein 2 homolog OS=Homo sapiens GN=TRIP13 PE=1 SV=2	48863	507	505	33	82	0.07	0.3	4.285714286	1	2	1	4	1	4	1	4	-0.22464
tr|B1AMS2|B1AMS2_HUMAN	Sep-06	Septin 6 OS=Homo sapiens GN=SEPT6 PE=3 SV=1	49614	532	402	31	22	0.07	0.3	4.285714286	0	1	1	2	1	3	1	3	-0.71015
tr|B2RU06|B2RU06_HUMAN	EIF4G1	EIF4G1 protein OS=Homo sapiens GN=EIF4G1 PE=2 SV=1	176807	669	422	20	103	0.04	0.17	4.25	0	4	2	4	1	4	3	4	0.875434
tr|B2RBE0|B2RBE0_HUMAN	tr|B2RBE0|B2R	cDNA, FLJ95462, highly similar to Homo sapiens fatty-acid-Coenzyme A ligase, long-chain 3 (FACL3),mRNA OS=Homo sapiens PE=2 SV=1	81266	462	373	37	118	0.04	0.17	4.25	0	2	1	4	1	4	1	4	0.875434

tr|B4DS66|B4DS66_HUMAN	tr|B4DS66|B4D	cDNA FLJ54290, highly similar to Mitochondrial inner membrane protein OS=Homo sapiens PE=2 SV=1	73509	712	347	17	133	0.04	0.17	4.25	0	2	1	4	1	4	6	4	0.875434
tr|F5H1A8|F5H1A8_HUMAN	GSN	Gelsolin OS=Homo sapiens GN=GSN PE=2 SV=1	81776	297	344	61	134	0.04	0.17	4.25	1	2	1	4	1	4	2	5	0.875434
tr|B4DUC5|B4DUC5_HUMAN	CSE1L	Exportin-2 OS=Homo sapiens GN=CSE1L PE=2 SV=1	86045	433	113	40	161	0.08	0.34	4.25	0	3	2	5	4	5	4	6	-0.42083
tr|B2R5P6|B2R5P6_HUMAN	TXNRD1	Thioredoxin reductase 1, cytoplasmic OS=Homo sapiens GN=TXNRD1 PE=2 SV=1	55253	108	89	204	389	0.26	1.09	4.192307692	3	10	5	13	5	13	6	16	0.586435
tr|B6F210|B6F210_HUMAN	GSS	Glutathione synthetase OS=Homo sapiens GN=GSS PE=2 SV=1	40495	551	470	30	40	0.08	0.33	4.125	0	1	1	2	1	1	1	2	-0.71015
tr|B2R5M8|B2R5M8_HUMAN	tr|B2R5M8|B2R	Isocitrate dehydrogenase [NADP] OS=Homo sapiens PE=2 SV=1	46905	555	250	29	184	0.15	0.61	4.066666667	0	5	2	7	3	7	3	9	0.389952
sp|P54886|P5CS_HUMAN	ALDH18A1	Delta-1-pyrroline-5-carboxylate synthase OS=Homo sapiens GN=ALDH18A1 PE=1 SV=2	87989	658	346	21	134	0.04	0.16	4	1	4	1	4	1	6	1	7	1.406361
sp|O15427|MOT4_HUMAN	SLC16A3	Monocarboxylate transporter 4 OS=Homo sapiens GN=SLC16A3 PE=1 SV=1	50064	362	352	48	130	0.14	0.56	4	2	4	2	7	2	8	2	8	1.056494
sp|O76094|SRP72_HUMAN	SRP72	Signal recognition particle 72 kDa protein OS=Homo sapiens GN=SRP72 PE=1 SV=3	75130	455	368	38	119	0.04	0.16	4	1	3	1	3	1	3	1	3	0.512658
tr|B2RE46|B2RE46_HUMAN	tr|B2RE46|B2R	cDNA, FLJ96923, highly similar to Homo sapiens ribophorin II (RPN2), mRNA OS=Homo sapiens PE=2 SV=1	69405	589	326	27	138	0.05	0.2	4	0	3	1	4	2	3	2	4	-0.22464
tr|A2RUM7|A2RUM7_HUMAN	RPL5	Ribosomal protein L5 OS=Homo sapiens GN=RPL5 PE=2 SV=1	34569	187	136	115	304	0.44	1.74	3.954545455	2	8	4	12	5	12	7	13	0.475197
tr|Q53GU8|Q53GU8_HUMAN	tr|Q53GU8|Q53	Transforming growth factor, beta-induced, 68kDa variant (Fragment) OS=Homo sapiens PE=2 SV=1	75231	358	379	48	116	0.09	0.35	3.888888889	1	4	2	7	2	7	2	11	0.875716
sp|O43175|SERA_HUMAN	PHGDH	D-3-phosphoglycerate dehydrogenase OS=Homo sapiens GN=PHGDH PE=1 SV=4	57356	131	122	166	342	0.25	0.95	3.8	3	8	4	12	4	12	4	13	0.765042
tr|A8K8N7|A8K8N7_HUMAN	PFAS	Phosphoribosylformylglycinamidine synthase (FGAR amidotransferase), isoform CRA_b OS=Homo sapiens GN=PFAS PE=2 SV=1	146286	380	186	46	240	0.05	0.19	3.8	0	5	2	8	3	6	5	8	0.183294
tr|Q6DC98|Q6DC98_HUMAN	LMNB1	LMNB1 protein (Fragment) OS=Homo sapiens GN=LMNB1 PE=2 SV=1	38289	219	176	95	179	0.18	0.68	3.777777778	2	4	2	4	2	5	4	5	0.427844
tr|Q4ZG57|Q4ZG57_HUMAN	MCM6	Putative uncharacterized protein MCM6 (Fragment) OS=Homo sapiens GN=MCM6 PE=2 SV=1	89803	444	445	39	97	0.04	0.15	3.75	1	4	1	4	2	4	3	4	0.138131
tr|J3KQL0|J3KQL0_HUMAN	CPS1	Carbamoyl-phosphate synthase [ammonia], mitochondrial OS=Homo sapiens GN=CPS1 PE=3 SV=1	166686	204	59	102	539	0.15	0.56	3.733333333	4	15	7	23	10	24	12	26	0.513956
tr|B2ZZ89|B2ZZ89_HUMAN	SPTBN1	Spectrin beta non-erythrocytic 1 OS=Homo sapiens GN=SPTBN1 PE=2 SV=1	275237	49	10	431	1901	0.22	0.82	3.727272727	11	42	17	55	20	60	24	69	0.856956
tr|B4E0E1|B4E0E1_HUMAN	tr|B4E0E1|B4E0	cDNA FLJ53442, highly similar to Poly (ADP-ribose) polymerase 1 (EC 2.4.2.30) OS=Homo sapiens PE=2 SV=1	111855	549	161	30	272	0.06	0.22	3.666666667	0	5	2	7	2	8	2	9	1.056494
sp|Q9P287|BCCIP_HUMAN	BCCIP	BRCA2 and CDKN1A-interacting protein OS=Homo sapiens GN=BCCIP PE=1 SV=1	36299	503	603	33	67	0.09	0.33	3.666666667	0	2	1	3	1	3	3	3	0.512658
tr|B7Z8A2|B7Z8A2_HUMAN	tr|B7Z8A2|B7Z8	cDNA FLJ51671, highly similar to Prenylcysteine oxidase (EC 1.8.3.5) OS=Homo sapiens PE=2 SV=1	54918	355	436	49	99	0.06	0.22	3.666666667	1	3	1	3	1	3	1	4	0.512658
tr|B4DQJ8|B4DQJ8_HUMAN	PGD	6-phosphogluconate dehydrogenase, decarboxylating OS=Homo sapiens GN=PGD PE=2 SV=1	52352	293	125	62	335	0.2	0.73	3.65	1	6	3	10	2	11	3	13	1.493212
tr|B2RBR9|B2RBR9_HUMAN	tr|B2RBR9|B2R	cDNA, FLJ95650, highly similar to Homo sapiens karyopherin (importin) beta 1 (KPNB1), mRNA OS=Homo sapiens PE=2 SV=1	98434	171	57	126	558	0.22	0.8	3.636363636	4	15	6	21	9	24	9	27	0.658684
tr|F8VRQ1|F8VRQ1_HUMAN	HNRNPA1	Heterogeneous nuclear ribonucleoprotein A1 OS=Homo sapiens GN=HNRNPA1 PE=2 SV=1	33249	101	65	214	519	0.77	2.78	3.61038961	4	10	6	15	7	15	9	16	0.338023
tr|Q8TC62|Q8TC62_HUMAN	Sep-07	Septin 7 OS=Homo sapiens GN=SEPT7 PE=2 SV=3	48913	531	503	31	82	0.07	0.25	3.571428571	1	2	1	3	1	3	2	4	0.512658
tr|B4DDR2|B4DDR2_HUMAN	tr|B4DDR2|B4D	cDNA FLJ54588, highly similar to Major vault protein OS=Homo sapiens PE=2 SV=1	98526	304	236	59	157	0.07	0.25	3.571428571	2	4	2	4	3	5	3	5	-0.05792
tr|Q6IBR0|Q6IBR0_HUMAN	RPN1	RPN1 protein OS=Homo sapiens GN=RPN1 PE=2 SV=1	68678	170	86	127	440	0.26	0.92	3.538461538	4	10	5	15	8	16	8	18	0.247517
sp|P49411|EFTU_HUMAN	TUFM	Elongation factor Tu, mitochondrial OS=Homo sapiens GN=TUFM PE=1 SV=2	49852	210	160	99	272	0.29	1.02	3.517241379	3	7	4	11	4	11	5	11	0.644541
tr|E7ERW8|E7ERW8_HUMAN	DIAPH1	Protein diaphanous homolog 1 OS=Homo sapiens GN=DIAPH1 PE=2 SV=1	135945	184	585	116	70	0.02	0.07	3.5	1	2	1	3	1	3	2	3	0.512658
tr|B4DLT2|B4DLT2_HUMAN	tr|B4DLT2|B4D	cDNA FLJ56637, highly similar to Nuclear pore complex protein Nup155 OS=Homo sapiens PE=2 SV=1	152734	489	485	35	87	0.02	0.07	3.5	0	1	1	3	1	3	1	3	0.512658
tr|B5BU25|B5BU25_HUMAN	U2AF2	U2 small nuclear RNA auxiliary factor 2 isoform b OS=Homo sapiens GN=U2AF2 PE=2 SV=1	53445	260	714	74	54	0.13	0.45	3.461538462	1	0	2	4	2	4	2	6	0.138131
tr|Q0IJ56|Q0IJ56_HUMAN	ST13	ST13 protein (Fragment) OS=Homo sapiens GN=ST13 PE=2 SV=1	35164	382	466	46	90	0.09	0.31	3.444444444	1	2	1	3	2	3	3	3	-0.22464
tr|B7Z6M1|B7Z6M1_HUMAN	PLS3	Plastin-3 OS=Homo sapiens GN=PLS3 PE=2 SV=1	66047	282	77	66	468	0.34	1.17	3.441176471	1	11	6	16	6	19	8	23	0.876844
tr|G1EP52|G1EP52_HUMAN	HLA-A	MHC class I antigen (Fragment) OS=Homo sapiens GN=HLA-A PE=3 SV=1	21260	639	537	22	77	0.16	0.55	3.4375	0	1	1	3	1	4	2	6	0.875434
sp|P06733|ENOA_HUMAN	ENO1	Alpha-enolase OS=Homo sapiens GN=ENO1 PE=1 SV=2	47481	40	39	520	800	1.39	4.73	3.402877698	13	23	18	30	19	37	25	40	0.234627
sp|Q9NZB2|F120A_HUMAN	FAM120A	Constitutive coactivator of PPAR-gamma-like protein 1 OS=Homo sapiens GN=FAM120A PE=1 SV=2	123008	263	198	73	223	0.05	0.17	3.4	2	5	2	6	2	6	2	7	0.669059
sp|O95373|IPO7_HUMAN	IPO7	Importin-7 OS=Homo sapiens GN=IPO7 PE=1 SV=1	120751	162	226	132	193	0.08	0.27	3.375	3	5	3	10	3	11	4	16	1.007448
tr|B4DEX8|B4DEX8_HUMAN	MAT2A	S-adenosylmethionine synthase OS=Homo sapiens GN=MAT2A PE=2 SV=1	40027	333	452	52	96	0.08	0.27	3.375	1	2	1	3	1	3	1	3	0.512658
sp|Q6IBS0|TWF2_HUMAN	TWF2	Twinfilin-2 OS=Homo sapiens GN=TWF2 PE=1 SV=2	39751	535	404	30	109	0.08	0.27	3.375	0	2	1	3	1	3	1	3	0.512658
tr|B2R5U1|B2R5U1_HUMAN	tr|B2R5U1|B2R	cDNA, FLJ92620, highly similar to Homo sapiens staphylococcal nuclease domain containing 1 (SND1),mRNA OS=Homo sapiens PE=2 SV=1	100294	150	84	144	441	0.17	0.57	3.352941176	4	11	5	15	10	16	12	19	-0.05801
tr|Q8IWP6|Q8IWP6_HUMAN	tr|Q8IWP6|Q8I	Class IVb beta tubulin OS=Homo sapiens PE=2 SV=1	50177	28	17	654	1471	3.05	10.22	3.350819672	27	48	32	60	34	71	37	76	0.34453
tr|Q5SU16|Q5SU16_HUMAN	TUBB	Beta 5-tubulin OS=Homo sapiens GN=TUBB PE=2 SV=1	50095	25	12	680	1584	3.07	10.28	3.348534202	26	49	33	62	35	72	39	78	0.323214
tr|A8K2Y2|A8K2Y2_HUMAN	tr|A8K2Y2|A8K2	cDNA FLJ78120, highly similar to Homo sapiens eukaryotic translation initiation factor 2, subunit 3 gamma, 52kDa (EIF2S3), mRNA OS=Homo	51648	475	574	37	72	0.06	0.2	3.333333333	1	2	1	3	1	3	1	5	0.512658
sp|P22626|ROA2_HUMAN	HNRNPA2B1	Heterogeneous nuclear ribonucleoproteins A2/B1 OS=Homo sapiens GN=HNRNPA2B1 PE=1 SV=2	37464	77	88	256	435	0.97	3.21	3.309278351	7	15	8	18	9	22	10	23	0.535414
tr|Q5T7Q0|Q5T7Q0_HUMAN	DNAJA1	DnaJ (Hsp40) homolog, subfamily A, member 1, isoform CRA_c OS=Homo sapiens GN=DNAJA1 PE=2 SV=1	45581	703	552	18	74	0.07	0.23	3.285714286	0	1	1	3	0	2	1	5	1.612325
tr|B4DHQ3|B4DHQ3_HUMAN	tr|B4DHQ3|B4D	cDNA FLJ56437, highly similar to Phosphoserine aminotransferase (EC 2.6.1.52) OS=Homo sapiens PE=2 SV=1	45783	552	557	30	74	0.07	0.23	3.285714286	0	2	1	3	1	4	2	4	0.875434
tr|Q53SS8|Q53SS8_HUMAN	PCBP1	Poly(RC) binding protein 1 OS=Homo sapiens GN=PCBP1 PE=2 SV=1	37987	110	255	197	179	0.29	0.95	3.275862069	3	6	3	8	3	7	3	8	0.389952
sp|O75369|FLNB_HUMAN	FLNB	Filamin-B OS=Homo sapiens GN=FLNB PE=1 SV=2	280157	45	23	463	1127	0.22	0.72	3.272727273	14	34	18	48	20	53	28	61	0.678105
sp|P08238|HS90B_HUMAN	HSP90AB1	Heat shock protein HSP 90-beta OS=Homo sapiens GN=HSP90AB1 PE=1 SV=4	83554	32	15	622	1495	1.24	4.03	3.25	18	41	28	51	29	58	35	62	0.279899
tr|J3KN86|J3KN86_HUMAN	RPS3	40S ribosomal protein S3 OS=Homo sapiens GN=RPS3 PE=3 SV=1	28640	480	706	36	55	0.12	0.39	3.25	1	1	1	2	1	2	4	3	0.027025
tr|B4E2U0|B4E2U0_HUMAN	tr|B4E2U0|B4E2	6-phosphogluconate dehydrogenase, decarboxylating OS=Homo sapiens PE=2 SV=1	51273	293	218	62	199	0.2	0.65	3.25	1	4	3	8	2	9	3	11	1.217165
tr|A1L3A7|A1L3A7_HUMAN	NUFIP2	Nuclear fragile X mental retardation protein interacting protein 2 OS=Homo sapiens GN=NUFIP2 PE=2 SV=1	76132	301	610	59	66	0.04	0.13	3.25	1	1	1	3	1	2	1	3	0.027025
tr|B7TY16|B7TY16_HUMAN	ACTN1	Actinin alpha 1 isoform 3 OS=Homo sapiens GN=ACTN1 PE=2 SV=1	107644	50	13	428	1502	0.77	2.47	3.207792208	15	35	21	45	22	50	29	54	0.459248
tr|Q9BSV4|Q9BSV4_HUMAN	SFPQ	SFPQ protein (Fragment) OS=Homo sapiens GN=SFPQ PE=2 SV=2	68702	228	94	91	414	0.26	0.83	3.192307692	2	12	5	14	6	14	7	14	0.44839
sp|P04350|TBB4A_HUMAN	TUBB4A	Tubulin beta-4A chain OS=Homo sapiens GN=TUBB4A PE=1 SV=2	50010	42	22	509	1180	1.96	6.22	3.173469388	21	40	27	53	29	63	32	67	0.399258
tr|B7Z528|B7Z528_HUMAN	OXCT1	Succinyl-CoA:3-ketoacid coenzyme A transferase 1, mitochondrial OS=Homo sapiens GN=OXCT1 PE=2 SV=1	13520	209	639	0	62	0.18	0.57	3.166666667	0	3	0	3	1	3	1	4	0.512658
tr|B2RB23|B2RB23_HUMAN	tr|B2RB23|B2R	cDNA, FLJ95265, highly similar to Homo sapiens acetyl-Coenzyme A acyltransferase 2 (mitochondrial 3-oxoacyl-Coenzyme A thiolase) (ACAA	42439	544	332	30	137	0.08	0.25	3.125	0	3	1	3	1	3	1	3	0.512658
tr|Q8IZ29|Q8IZ29_HUMAN	TUBB2C	Tubulin, beta 2C OS=Homo sapiens GN=TUBB2C PE=2 SV=1	50264	27	16	674	1494	3.04	9.48	3.118421053	26	48	32	61	34	71	38	77	0.34453
tr|Q59G92|Q59G92_HUMAN	tr|Q59G92|Q59	HLA-B associated transcript 1 variant (Fragment) OS=Homo sapiens PE=2 SV=1	35528	394	535	44	49	0.09	0.28	3.111111111	1	1	1	2	2	4	2	4	0.138131
tr|I3L312|I3L312_HUMAN	P4HB	Protein disulfide-isomerase (Fragment) OS=Homo sapiens GN=P4HB PE=4 SV=1	26348	536	216	30	96	0.27	0.84	3.111111111	1	3	2	6	1	7	2	10	1.613019
sp|O43707|ACTN4_HUMAN	ACTN4	Alpha-actinin-4 OS=Homo sapiens GN=ACTN4 PE=1 SV=2	105245	19	8	849	2351	2.11	6.55	3.104265403	26	61	41	81	44	89	52	98	0.301616
tr|H0Y2W2|H0Y2W2_HUMAN	ATAD3A	ATPase family AAA domain-containing protein 3A (Fragment) OS=Homo sapiens GN=ATAD3A PE=4 SV=1	64600	281	199	68	174	0.1	0.31	3.1	2	4	2	4	2	6	2	6	0.669059
tr|Q2TSD0|Q2TSD0_HUMAN	tr|Q2TSD0|Q2T	Glyceraldehyde-3-phosphate dehydrogenase OS=Homo sapiens PE=2 SV=1	36197	18	14	891	1307	3.44	10.63	3.090116279	23	30	31	36	31	38	36	44	-0.42315
tr|B7XGC2|B7XGC2_HUMAN	HLA-A	MHC class I antigen (Fragment) OS=Homo sapiens GN=HLA-A PE=3 SV=1	31846	639	448	0	97	0.16	0.49	3.0625	0	2	0	4	0	6	1	7	2.991661
tr|H0YH88|H0YH88_HUMAN	NAP1L1	Nucleosome assembly protein 1-like 1 (Fragment) OS=Homo sapiens GN=NAP1L1 PE=3 SV=1	21179	417	342	33	112	0.16	0.49	3.0625	0	2	1	2	1	3	1	3	0.512658
tr|Q2TB59|Q2TB59_HUMAN	NNT	Nicotinamide nucleotide transhydrogenase OS=Homo sapiens GN=NNT PE=2 SV=1	114552	234	358	88	128	0.06	0.18	3	1	3	2	6	2	7	3	7	0.875716
sp|O94906|PRP6_HUMAN	PRPF6	Pre-mRNA-processing factor 6 OS=Homo sapiens GN=PRPF6 PE=1 SV=1	107656	492	491	35	86	0.03	0.09	3	1	3	1	3	1	3	2	3	0.512658
sp|Q14204|DYHC1_HUMAN	DYNC1H1	Cytoplasmic dynein 1 heavy chain 1 OS=Homo sapiens GN=DYNC1H1 PE=1 SV=5	534809	168	328	129	137	0.02	0.06	3	3	3	4	9	4	9	6	14	0.368494
tr|B4DXC4|B4DXC4_HUMAN	tr|B4DXC4|B4D	cDNA FLJ58636, moderately similar to Atlastin OS=Homo sapiens PE=2 SV=1	59162	451	465	38	91	0.06	0.18	3	1	2	1	3	1	2	1	3	0.027025
sp|Q68EM7|RHG17_HUMAN	ARHGAP17	Rho GTPase-activating protein 17 OS=Homo sapiens GN=ARHGAP17 PE=1 SV=1	95776	328	752	54	50	0.03	0.09	3	1	1	1	2	2	2	2	2	-0.71028
sp|O00629|IMA4_HUMAN	KPNA4	Importin subunit alpha-4 OS=Homo sapiens GN=KPNA4 PE=1 SV=1	58364	472	662	37	60	0.06	0.18	3	0	1	1	1	2	1	2	1	-1.44745
sp|O14929|HAT1_HUMAN	HAT1	Histone acetyltransferase type B catalytic subunit OS=Homo sapiens GN=HAT1 PE=1 SV=1	49880	343	221	51	199	0.07	0.21	3	1	3	1	3	1	4	1	4	0.875434
tr|J3KNE5|J3KNE5_HUMAN	HARS	Histidine--tRNA ligase, cytoplasmic OS=Homo sapiens GN=HARS PE=4 SV=1	50581	672	697	20	38	0.07	0.21	3	0	1	1	2	1	2	2	2	0.027025
sp|P13639|EF2_HUMAN	EEF2	Elongation factor 2 OS=Homo sapiens GN=EEF2 PE=1 SV=4	96246	41	18	511	1288	1.23	3.66	2.975609756	13	42	26	56	27	57	32	64	0.356779
tr|H0YAF8|H0YAF8_HUMAN	GNB2L1	Guanine nucleotide-binding protein subunit beta-2-like 1 (Fragment) OS=Homo sapiens GN=GNB2L1 PE=4 SV=1	22373	216	108	96	360	0.52	1.54	2.961538462	2	6	3	7	3	7	4	7	0.389952
tr|Q60FE2|Q60FE2_HUMAN	MYH9	MYH9 variant protein OS=Homo sapiens GN=MYH9 PE=2 SV=1	227646	17	3	900	3142	0.9	2.65	2.944444444	30	74	46	94	52	98	60	108	0.200492
tr|F8SKU2|F8SKU2_HUMAN	HLA-A	MHC class I antigen (Fragment) OS=Homo sapiens GN=HLA-A PE=3 SV=1	21143	639	315	22	75	0.16	0.47	2.9375	0	1	1	1	2	3	3	4	-0.22464
tr|B4DNK3|B4DNK3_HUMAN	tr|B4DNK3|B4D	cDNA FLJ52127, highly similar to Multisynthetase complex auxiliary component p43 OS=Homo sapiens PE=2 SV=1	29955	601	897	26	38	0.11	0.32	2.909090909	0	0	1	3	1	3	1	3	0.512658
sp|Q96TA1|NIBL1_HUMAN	FAM129B	Niban-like protein 1 OS=Homo sapiens GN=FAM129B PE=1 SV=3	84598	155	185	137	241	0.16	0.46	2.875	4	5	4	10	4	11	4	13	0.644541
tr|B2R8R5|B2R8R5_HUMAN	tr|B2R8R5|B2R	cDNA, FLJ94025, highly similar to Homo sapiens tripartite motif-containing 28 (TRIM28), mRNA OS=Homo sapiens PE=2 SV=1	90271	320	396	56	112	0.07	0.2	2.857142857	1	3	3	5	2	5	4	5	0.427844
tr|B3KM77|B3KM77_HUMAN	tr|B3KM77|B3K	cDNA FLJ10435 fis, clone NT2RP1000493, highly similar to Splicing factor 3B subunit 3 OS=Homo sapiens PE=2 SV=1	56736	356	290	49	128	0.06	0.17	2.833333333	1	3	1	3	1	3	1	3	0.512658
sp|O00505|IMA3_HUMAN	KPNA3	Importin subunit alpha-3 OS=Homo sapiens GN=KPNA3 PE=1 SV=2	58288	406	1182	43	18	0.12	0.34	2.833333333	0	0	2	1	2	0	2	1	-3.03262
tr|Q2XPP3|Q2XPP3_HUMAN	tr|Q2XPP3|Q2X	Type II 3a-hydroxysteroid dehydrogenase variant OS=Homo sapiens PE=2 SV=1	37201	203	194	102	109	0.29	0.82	2.827586207	3	4	3	5	3	5	4	5	-0.05792
tr|D3DTW3|D3DTW3_HUMAN	IGF2BP1	Insulin-like growth factor 2 mRNA binding protein 1, isoform CRA_a OS=Homo sapiens GN=IGF2BP1 PE=2 SV=1	48111	556	871	29	40	0.05	0.14	2.8	0	1	1	2	1	4	1	5	0.875434
tr|Q05D08|Q05D08_HUMAN	PA2G4	PA2G4 protein (Fragment) OS=Homo sapiens GN=PA2G4 PE=2 SV=1	45579	286	498	64	84	0.15	0.42	2.8	1	3	2	5	3	5	3	6	-0.05792
sp|Q8NBJ5|GT251_HUMAN	GLT25D1	Procollagen galactosyltransferase 1 OS=Homo sapiens GN=GLT25D1 PE=1 SV=1	71933	523	490	31	86	0.09	0.25	2.777777778	0	1	2	5	1	6	2	7	1.406361
sp|P13010|XRCC5_HUMAN	XRCC5	X-ray repair cross-complementing protein 5 OS=Homo sapiens GN=XRCC5 PE=1 SV=3	83222	128	74	172	473	0.36	1	2.777777778	6	12	8	19	8	23	9	26	0.759159
tr|B4DFN9|B4DFN9_HUMAN	tr|B4DFN9|B4D	cDNA FLJ54303, highly similar to Heat shock 70 kDa protein 1 OS=Homo sapiens PE=2 SV=1	62699	80	61	252	465	0.58	1.61	2.775862069	7	12	10	21	10	21	10	27	0.324891
tr|B2R7C5|B2R7C5_HUMAN	tr|B2R7C5|B2R	cDNA, FLJ93378, highly similar to Homo sapiens MCM3 minichromosome maintenance deficient 3 (S. cerevisiae) (MCM3), mRNA OS=Homo	91579	479	420	36	104	0.04	0.11	2.75	1	2	1	3	1	3	1	3	0.512658
tr|Q7Z3K9|Q7Z3K9_HUMAN	DKFZp781L0540	Putative uncharacterized protein DKFZp781L0540 (Fragment) OS=Homo sapiens GN=DKFZp781L0540 PE=2 SV=1	90081	440	357	39	128	0.04	0.11	2.75	1	3	1	3	2	5	2	5	0.427844
tr|B4DHG3|B4DHG3_HUMAN	tr|B4DHG3|B4D	cDNA FLJ55329, highly similar to Procollagen-lysine,2-oxoglutarate5-dioxygenase 2 (EC 1.14.11.4) OS=Homo sapiens PE=2 SV=1	81716	453	361	38	125	0.08	0.22	2.75	0	3	2	5	2	4	2	5	0.138131
tr|Q6IAV5|Q6IAV5_HUMAN	OXCT	Succinyl-CoA:3-ketoacid-coenzyme A transferase OS=Homo sapiens GN=OXCT PE=2 SV=1	56612	209	188	100	222	0.18	0.49	2.722222222	1	7	3	7	5	9	6	10	0.07865
tr|H0Y8E6|H0Y8E6_HUMAN	MCM2	DNA replication licensing factor MCM2 (Fragment) OS=Homo sapiens GN=MCM2 PE=3 SV=1	94792	211	417	98	105	0.07	0.19	2.714285714	1	3	2	5	2	6	2	6	0.669059
tr|B4DU00|B4DU00_HUMAN	tr|B4DU00|B4D	cDNA FLJ54547, highly similar to Alpha-1 catenin (Cadherin-associated protein) OS=Homo sapiens PE=2 SV=1	89332	389	354	45	67	0.07	0.19	2.714285714	0	1	2	4	2	4	4	4	0.138131
tr|A0PJ92|A0PJ92_HUMAN	NOP56	NOP56 protein (Fragment) OS=Homo sapiens GN=NOP56 PE=2 SV=1	50068	311	309	58	146	0.14	0.38	2.714285714	1	4	2	5	3	5	3	5	-0.05792
tr|B3KY95|B3KY95_HUMAN	tr|B3KY95|B3KY	cDNA FLJ16143 fis, clone BRAMY2038516, highly similar to Protein disulfide-isomerase A6 (EC 5.3.4.1) OS=Homo sapiens PE=2 SV=1	53655	445	265	38	174	0.13	0.35	2.692307692	0	4	2	5	2	6	2	6	0.669059
tr|B3GQS7|B3GQS7_HUMAN	HSPD1	Mitochondrial heat shock 60kD protein 1 variant 1 OS=Homo sapiens GN=HSPD1 PE=2 SV=1	60813	16	28	904	878	1.72	4.63	2.691860465	24	20	27	33	26	34	33	40	-0.33118
tr|B2R6D0|B2R6D0_HUMAN	tr|B2R6D0|B2R	cDNA, FLJ92896, highly similar to Homo sapiens proteasome (prosome, macropain) 26S subunit, non-ATPase, 1 (PSMD1), mRNA OS=Homo s	106797	386	306	45	149	0.06	0.16	2.666666667	0	3	2	5	2	5	4	7	0.427844
sp|P26599|PTBP1_HUMAN	PTBP1	Polypyrimidine tract-binding protein 1 OS=Homo sapiens GN=PTBP1 PE=1 SV=1	57357	567	132	29	310	0.18	0.48	2.666666667	0	5	3	7	3	7	3	8	0.389952
tr|F8W8D8|F8W8D8_HUMAN	PLS3	Plastin-3 OS=Homo sapiens GN=PLS3 PE=2 SV=1	67051	282	82	66	449	0.34	0.9	2.647058824	1	11	6	14	6	17	8	22	0.720311
tr|Q59EG8|Q59EG8_HUMAN	tr|Q59EG8|Q59	Proteasome 26S non-ATPase subunit 2 variant (Fragment) OS=Homo sapiens PE=2 SV=1	101322	198	147	105	295	0.14	0.37	2.642857143	1	9	4	10	4	10	5	11	0.51309
tr|B4DN87|B4DN87_HUMAN	tr|B4DN87|B4D	cDNA FLJ52569, highly similar to Collagen-binding protein 2 OS=Homo sapiens PE=2 SV=1	44290	233	123	89	295	0.43	1.13	2.627906977	4	8	5	11	5	12	5	14	0.475197
sp|Q12792|TWF1_HUMAN	TWF1	Twinfilin-1 OS=Homo sapiens GN=TWF1 PE=1 SV=3	40429	575	433	28	100	0.08	0.21	2.625	0	2	1	2	1	2	1	2	0.027025
tr|D3DTH7|D3DTH7_HUMAN	MYO1C	Myosin IC, isoform CRA_a OS=Homo sapiens GN=MYO1C PE=2 SV=1	99613	284	300	65	124	0.07	0.18	2.571428571	1	3	3	4	3	5	4	5	-0.05792
tr|B4DFK9|B4DFK9_HUMAN	tr|B4DFK9|B4D	cDNA FLJ57964, highly similar to Heterogeneous nuclear ribonucleoprotein H~ OS=Homo sapiens PE=2 SV=1	44637	272	252	69	154	0.15	0.38	2.533333333	1	4	2	5	1	5	2	5	1.165147
tr|G3V0E8|G3V0E8_HUMAN	PCBP2	Poly(RC) binding protein 2, isoform CRA_f OS=Homo sapiens GN=PCBP2 PE=2 SV=1	33817	132	302	166	138	0.32	0.81	2.53125	3	3	3	5	3	5	5	5	-0.05792
sp|Q6ZMR3|LDH6A_HUMAN	LDHAL6A	L-lactate dehydrogenase A-like 6A OS=Homo sapiens GN=LDHAL6A PE=2 SV=1	36826	39	51	67	72	1.81	4.57	2.524861878	1	2	1	2	1	3	1	3	0.512658
tr|B3KS31|B3KS31_HUMAN	TUBB6	Tubulin, beta 6, isoform CRA_a OS=Homo sapiens GN=TUBB6 PE=2 SV=1	42324	73	97	273	406	0.83	2.09	2.518072289	10	17	14	21	17	27	20	31	-0.05811
tr|J3KR97|J3KR97_HUMAN	TBCD	Tubulin-specific chaperone D OS=Homo sapiens GN=TBCD PE=4 SV=1	138346	714	860	15	41	0.02	0.05	2.5	0	0	1	2	0	2	1	2	1.612325
tr|A6NEM2|A6NEM2_HUMAN	HCFC1	HCF N-terminal chain 5 OS=Homo sapiens GN=HCFC1 PE=2 SV=2	215338	508	571	33	72	0.02	0.05	2.5	0	1	1	3	1	3	2	3	0.512658
tr|F6IQT5|F6IQT5_HUMAN	HLA-A	MHC class I antigen (Fragment) OS=Homo sapiens GN=HLA-A PE=2 SV=1	38400	639	507	0	81	0.16	0.39	2.4375	0	2	0	4	0	6	1	7	2.991661
tr|B0YJC4|B0YJC4_HUMAN	VIM	Vimentin OS=Homo sapiens GN=VIM PE=3 SV=1	49680	66	66	312	429	1.03	2.49	2.417475728	9	15	11	22	12	27	15	28	0.429012
sp|P22314|UBA1_HUMAN	UBA1	Ubiquitin-like modifier-activating enzyme 1 OS=Homo sapiens GN=UBA1 PE=1 SV=3	118858	114	71	187	503	0.28	0.67	2.392857143	5	11	10	19	10	18	12	24	0.107459
tr|H0Y9Q7|H0Y9Q7_HUMAN	CANX	Calnexin (Fragment) OS=Homo sapiens GN=CANX PE=3 SV=1	18990	352	256	49	177	0.39	0.92	2.358974359	1	4	2	5	3	5	5	6	-0.05792
tr|F5H4R9|F5H4R9_HUMAN	DNM2	Dynamin-2 OS=Homo sapiens GN=DNM2 PE=2 SV=1	98218	713	643	16	62	0.03	0.07	2.333333333	0	2	1	2	0	2	2	2	1.612325
tr|B2RAQ8|B2RAQ8_HUMAN	tr|B2RAQ8|B2R	cDNA, FLJ95058, highly similar to Homo sapiens carnitine palmitoyltransferase 1A (liver) (CPT1A),nuclear gene encoding mitochondrial prote	88967	354	678	49	58	0.07	0.16	2.285714286	1	1	2	4	3	2	3	4	-1.19604
tr|B3KV72|B3KV72_HUMAN	tr|B3KV72|B3K	cDNA FLJ16204 fis, clone CTONG2019232, highly similar to Proteasome-associated protein ECM29 homolog (Fragment) OS=Homo sapiens P	78791	579	318	28	68	0.04	0.09	2.25	0	1	1	2	1	2	1	2	0.027025
tr|B7Z481|B7Z481_HUMAN	tr|B7Z481|B7Z4	cDNA FLJ54018, highly similar to PDZ and LIM domain protein 5 OS=Homo sapiens PE=2 SV=1	52359	175	165	109	251	0.41	0.92	2.243902439	2	6	5	10	5	10	6	11	0.223246
tr|B7Z4V2|B7Z4V2_HUMAN	tr|B7Z4V2|B7Z4	cDNA FLJ51907, highly similar to Stress-70 protein, mitochondrial OS=Homo sapiens PE=2 SV=1	72641	81	73	251	476	0.42	0.94	2.238095238	7	12	8	15	8	14	8	15	0.060691
sp|Q9UMS4|PRP19_HUMAN	PRPF19	Pre-mRNA-processing factor 19 OS=Homo sapiens GN=PRPF19 PE=1 SV=1	55603	237	238	87	190	0.26	0.58	2.230769231	2	5	5	8	7	9	11	11	-0.36948
sp|P00390|GSHR_HUMAN	GSR	Glutathione reductase, mitochondrial OS=Homo sapiens GN=GSR PE=1 SV=2	56791	313	327	58	138	0.18	0.4	2.222222222	1	3	3	6	4	7	4	8	0.027044
sp|O00410|IPO5_HUMAN	IPO5	Importin-5 OS=Homo sapiens GN=IPO5 PE=1 SV=4	125032	85	80	236	461	0.23	0.51	2.217391304	7	13	8	16	10	18	15	24	0.107459
tr|B0QYA5|B0QYA5_HUMAN	EIF3D	Eukaryotic translation initiation factor 3 subunit D (Fragment) OS=Homo sapiens GN=EIF3D PE=4 SV=1	31937	710	463	17	91	0.1	0.22	2.2	0	2	1	2	0	2	2	3	1.612325
tr|B4DMY3|B4DMY3_HUMAN	tr|B4DMY3|B4D	cDNA FLJ60713, highly similar to Homo sapiens heterogeneous nuclear ribonucleoprotein A/B (HNRPAB), transcript variant 1, mRNA OS=Ho	35101	318	380	56	116	0.2	0.44	2.2	1	5	2	5	2	6	2	6	0.669059
tr|A4FU77|A4FU77_HUMAN	SNRNP200	SNRNP200 protein (Fragment) OS=Homo sapiens GN=SNRNP200 PE=2 SV=1	217585	243	154	83	256	0.05	0.11	2.2	2	6	3	7	7	8	8	11	-0.53016
sp|P53004|BIEA_HUMAN	BLVRA	Biliverdin reductase A OS=Homo sapiens GN=BLVRA PE=1 SV=2	33692	403	363	43	123	0.21	0.46	2.19047619	1	3	2	4	2	4	3	7	0.138131
tr|K4TZQ2|K4TZQ2_HUMAN	HLA-A	MHC class I antigen (Fragment) OS=Homo sapiens GN=HLA-A PE=3 SV=1	31799	639	536	22	77	0.16	0.35	2.1875	0	1	1	3	1	5	1	6	1.165147
tr|B4DGP8|B4DGP8_HUMAN	CANX	Calnexin OS=Homo sapiens GN=CANX PE=2 SV=1	71971	352	98	49	403	0.39	0.85	2.179487179	1	10	2	15	3	16	4	17	1.529313
sp|Q01650|LAT1_HUMAN	SLC7A5	Large neutral amino acids transporter small subunit 1 OS=Homo sapiens GN=SLC7A5 PE=1 SV=2	55659	239	151	85	288	0.12	0.26	2.166666667	1	6	3	6	4	6	5	6	-0.17961
tr|B7Z601|B7Z601_HUMAN	tr|B7Z601|B7Z6	cDNA FLJ57187, highly similar to Glycerol-3-phosphate dehydrogenase, mitochondrial (EC 1.1.99.5) OS=Homo sapiens PE=2 SV=1	56047	512	777	32	48	0.06	0.13	2.166666667	0	1	1	3	2	3	2	4	-0.22464
sp|P53618|COPB_HUMAN	COPB1	Coatomer subunit beta OS=Homo sapiens GN=COPB1 PE=1 SV=3	108214	303	444	59	97	0.06	0.13	2.166666667	1	3	2	4	2	3	3	4	-0.22464
tr|B7Z8W6|B7Z8W6_HUMAN	tr|B7Z8W6|B7Z	cDNA FLJ55038, highly similar to Nicotinamide phosphoribosyltransferase (EC 2.4.2.12) OS=Homo sapiens PE=2 SV=1	26905	520	246	32	47	0.12	0.26	2.166666667	0	1	1	1	1	1	3	2	-0.71015
tr|B4DDB6|B4DDB6_HUMAN	HNRPA3	Heterogeneous nuclear ribonucleoprotein A3, isoform CRA_a OS=Homo sapiens GN=HNRPA3 PE=2 SV=1	37234	201	345	103	134	0.19	0.41	2.157894737	2	4	2	4	2	5	2	5	0.427844
tr|B2R6X5|B2R6X5_HUMAN	tr|B2R6X5|B2R	cDNA, FLJ93166, highly similar to Homo sapiens heat shock 70kDa protein 6 (HSP70B~) (HSPA6), mRNA OS=Homo sapiens PE=2 SV=1	71370	193	167	112	257	0.2	0.43	2.15	3	9	5	10	6	11	6	12	0.113351
tr|G3XAD8|G3XAD8_HUMAN	STIP1	Stress-induced-phosphoprotein 1 OS=Homo sapiens GN=STIP1 PE=2 SV=1	68721	271	257	69	177	0.21	0.45	2.142857143	2	5	4	8	4	9	5	11	0.368494
sp|O43286|B4GT5_HUMAN	B4GALT5	Beta-1,4-galactosyltransferase 5 OS=Homo sapiens GN=B4GALT5 PE=2 SV=1	45318	651	715	22	54	0.07	0.15	2.142857143	0	2	1	3	1	2	1	3	0.027025
tr|B3KUJ0|B3KUJ0_HUMAN	tr|B3KUJ0|B3KU	cDNA FLJ39996 fis, clone STOMA2002166, highly similar to Splicing factor 3B subunit 4 OS=Homo sapiens PE=2 SV=1	44512	464	895	37	38	0.07	0.15	2.142857143	1	1	1	2	1	1	2	2	-0.71015
tr|C5IZQ1|C5IZQ1_HUMAN	HLA-A	MHC class I antigen (Fragment) OS=Homo sapiens GN=HLA-A PE=3 SV=1	21127	639	440	22	98	0.16	0.34	2.125	0	2	1	2	1	4	2	5	0.875434
tr|D2DKJ6|D2DKJ6_HUMAN	HLA-C	MHC class I antigen (Fragment) OS=Homo sapiens GN=HLA-C PE=3 SV=1	21319	639	441	22	98	0.16	0.34	2.125	0	2	1	2	1	3	1	4	0.512658
tr|H0Y5J4|H0Y5J4_HUMAN	DBNL	Drebrin-like protein (Fragment) OS=Homo sapiens GN=DBNL PE=4 SV=1	40475	466	884	37	39	0.08	0.17	2.125	1	1	1	2	1	2	1	2	0.027025
tr|B4DLB8|B4DLB8_HUMAN	tr|B4DLB8|B4D	cDNA FLJ52205, highly similar to Beta-1,4-galactosyltransferase 1 (EC 2.4.1.-) OS=Homo sapiens PE=2 SV=1	40269	650	356	22	128	0.08	0.17	2.125	0	2	1	3	1	2	1	3	0.027025
tr|Q96HI4|Q96HI4_HUMAN	NMT1	Glycylpeptide N-tetradecanoyltransferase (Fragment) OS=Homo sapiens GN=NMT1 PE=2 SV=2	56910	350	845	50	42	0.49	1.04	2.12244898	1	0	2	2	2	2	2	2	-0.71028
sp|Q92973|TNPO1_HUMAN	TNPO1	Transportin-1 OS=Homo sapiens GN=TNPO1 PE=1 SV=2	103771	139	219	158	199	0.17	0.36	2.117647059	3	5	5	10	8	12	8	15	-0.15385
tr|B4DEM7|B4DEM7_HUMAN	CCT8	T-complex protein 1 subunit theta OS=Homo sapiens GN=CCT8 PE=2 SV=1	58179	268	298	70	143	0.18	0.38	2.111111111	2	3	3	5	5	6	6	6	-0.46946
tr|Q5TCU6|Q5TCU6_HUMAN	TLN1	Talin-1 OS=Homo sapiens GN=TLN1 PE=4 SV=1	260030	48	19	446	1193	0.27	0.57	2.111111111	12	25	19	34	20	38	25	44	0.200313
tr|F5GX33|F5GX33_HUMAN	SLC25A13	Calcium-binding mitochondrial carrier protein Aralar2 OS=Homo sapiens GN=SLC25A13 PE=2 SV=1	62548	353	457	49	94	0.11	0.23	2.090909091	1	3	2	4	3	4	4	4	-0.34763
tr|H0Y4R1|H0Y4R1_HUMAN	IMPDH2	Inosine-5~-monophosphate dehydrogenase 2 (Fragment) OS=Homo sapiens GN=IMPDH2 PE=3 SV=1	51549	177	120	122	344	0.36	0.75	2.083333333	2	8	5	10	5	10	6	11	0.223246
tr|A4QPB0|A4QPB0_HUMAN	IQGAP1	IQ motif containing GTPase activating protein 1 OS=Homo sapiens GN=IQGAP1 PE=2 SV=1	189789	84	32	239	924	0.31	0.64	2.064516129	8	21	17	29	18	33	21	37	0.147356
tr|B4DUL5|B4DUL5_HUMAN	tr|B4DUL5|B4D	cDNA FLJ51625, highly similar to Ubiquinol-cytochrome-c reductase complex core protein I, mitochondrial (EC 1.10.2.2) OS=Homo sapiens PE	40917	357	214	48	156	0.17	0.35	2.058823529	1	4	2	4	2	4	2	5	0.138131
tr|B1AH89|B1AH89_HUMAN	TTLL12	Tubulin tyrosine ligase-like family, member 12 OS=Homo sapiens GN=TTLL12 PE=4 SV=1	74185	104	75	209	472	0.41	0.83	2.024390244	6	8	8	14	10	17	11	20	0.027082
sp|P15121|ALDR_HUMAN	AKR1B1	Aldose reductase OS=Homo sapiens GN=AKR1B1 PE=1 SV=3	36230	185	229	116	193	0.42	0.85	2.023809524	3	4	4	7	5	7	5	7	-0.2628
tr|Q5HY54|Q5HY54_HUMAN	FLNA	Filamin-A OS=Homo sapiens GN=FLNA PE=2 SV=1	279115	9	4	1304	2686	0.86	1.74	2.023255814	41	68	59	96	74	105	87	116	-0.21145
tr|H0Y8W2|H0Y8W2_HUMAN	GNB2L1	Guanine nucleotide-binding protein subunit beta-2-like 1 (Fragment) OS=Homo sapiens GN=GNB2L1 PE=4 SV=1	30871	216	118	67	349	0.52	1.05	2.019230769	1	6	2	7	2	8	3	9	1.056494
sp|P68366|TBA4A_HUMAN	TUBA4A	Tubulin alpha-4A chain OS=Homo sapiens GN=TUBA4A PE=1 SV=1	50634	22	25	796	1075	3.01	6.07	2.016611296	20	25	31	40	31	42	36	46	-0.27971
tr|Q60FE5|Q60FE5_HUMAN	FLNA	Filamin A OS=Homo sapiens GN=FLNA PE=2 SV=1	280790	9	5	1306	2685	0.86	1.73	2.011627907	41	67	60	96	75	105	88	116	-0.23103
tr|K9JA46|K9JA46_HUMAN	EL52	Epididymis luminal secretory protein 52 OS=Homo sapiens GN=EL52 PE=2 SV=1	85006	46	31	462	932	1.05	2.11	2.00952381	15	23	25	37	24	41	29	44	0.050675
tr|B4DZD5|B4DZD5_HUMAN	tr|B4DZD5|B4D	cDNA FLJ60991, highly similar to FKBP12-rapamycin complex-associated protein OS=Homo sapiens PE=2 SV=1	158783	683	517	20	79	0.02	0.04	2	0	2	1	2	0	3	2	3	2.097958
tr|Q5TB52|Q5TB52_HUMAN	PAPSS2	3~-phosphoadenosine 5~-phosphosulfate synthase 2, isoform CRA_b OS=Homo sapiens GN=PAPSS2 PE=2 SV=1	70027	688	560	19	73	0.05	0.1	2	0	2	1	2	0	2	4	2	1.612325
tr|B4DFM0|B4DFM0_HUMAN	FARSLB	Phenylalanine-tRNA synthetase-like, beta subunit, isoform CRA_c OS=Homo sapiens GN=FARSLB PE=2 SV=1	55353	360	670	48	59	0.06	0.12	2	1	1	1	2	1	3	3	3	0.512658
tr|Q13727|Q13727_HUMAN	tr|Q13727|Q13	AHNAK-related protein (Fragment) OS=Homo sapiens PE=2 SV=1	57703	402	726	43	53	0.06	0.12	2	1	2	1	2	2	5	3	6	0.427844
tr|B4DFL1|B4DFL1_HUMAN	tr|B4DFL1|B4D	Dihydrolipoyl dehydrogenase OS=Homo sapiens PE=2 SV=1	49381	666	875	21	40	0.07	0.14	2	0	0	1	3	1	2	1	4	0.027025
tr|A6NKV8|A6NKV8_HUMAN	PAIP1	Polyadenylate-binding protein-interacting protein 1 OS=Homo sapiens GN=PAIP1 PE=2 SV=1	42379	361	811	48	44	0.08	0.16	2	1	0	1	2	1	2	1	2	0.027025
sp|Q9Y6M5|ZNT1_HUMAN	SLC30A1	Zinc transporter 1 OS=Homo sapiens GN=SLC30A1 PE=1 SV=3	56290	334	782	52	48	0.06	0.12	2	1	1	1	2	1	2	1	2	0.027025
tr|B4DYL7|B4DYL7_HUMAN	tr|B4DYL7|B4D	cDNA FLJ58490, highly similar to Homo sapiens CCR4-NOT transcription complex, subunit 1 (CNOT1), transcript variant 1, mRNA OS=Homo s	140991	388	707	45	50	0.02	0.04	2	1	1	1	2	1	2	2	2	0.027025
sp|Q9Y305|ACOT9_HUMAN	ACOT9	Acyl-coenzyme A thioesterase 9, mitochondrial OS=Homo sapiens GN=ACOT9 PE=1 SV=2	50269	637	592	22	68	0.07	0.14	2	0	1	1	2	1	2	2	2	0.027025
tr|B4DM97|B4DM97_HUMAN	tr|B4DM97|B4D	cDNA FLJ55002, highly similar to Alpha-centractin OS=Homo sapiens PE=2 SV=1	38365	561	531	29	77	0.09	0.18	2	0	2	1	2	1	2	2	2	0.027025
sp|O95782|AP2A1_HUMAN	AP2A1	AP-2 complex subunit alpha-1 OS=Homo sapiens GN=AP2A1 PE=1 SV=3	108561	500	1156	34	21	0.03	0.06	2	0	0	1	1	1	1	2	1	-0.71015
tr|B5BU01|B5BU01_HUMAN	EIF2S2	Eukaryotic translation initiation factor 2 beta OS=Homo sapiens GN=EIF2S2 PE=2 SV=1	38648	654	1024	22	30	0.09	0.18	2	0	0	1	2	1	1	1	3	-0.71015
tr|H0YK49|H0YK49_HUMAN	ETFA	Electron transfer flavoprotein subunit alpha, mitochondrial OS=Homo sapiens GN=ETFA PE=4 SV=1	24259	545	704	30	55	0.14	0.28	2	0	1	1	1	1	1	1	1	-0.71015
tr|H3BUF6|H3BUF6_HUMAN	ATXN2L	Ataxin-2-like protein OS=Homo sapiens GN=ATXN2L PE=4 SV=1	112929	656	800	22	46	0.03	0.06	2	0	1	1	2	2	2	2	2	-0.71028
tr|Q2NKY5|Q2NKY5_HUMAN	TUBB6	TUBB6 protein OS=Homo sapiens GN=TUBB6 PE=2 SV=1	50514	65	58	317	547	0.88	1.75	1.988636364	12	19	16	22	17	26	19	31	-0.11175
tr|B4E022|B4E022_HUMAN	TKT	Transketolase OS=Homo sapiens GN=TKT PE=2 SV=1	63410	35	30	578	955	1.36	2.7	1.985294118	17	23	24	33	26	37	32	39	-0.21026
tr|Q9UJZ2|Q9UJZ2_HUMAN	VIL2	Cytovillin 2 (Fragment) OS=Homo sapiens GN=VIL2 PE=2 SV=1	18464	119	242	88	156	0.46	0.9	1.956521739	2	4	2	4	2	5	2	6	0.427844
sp|Q96HC4|PDLI5_HUMAN	PDLIM5	PDZ and LIM domain protein 5 OS=Homo sapiens GN=PDLIM5 PE=1 SV=5	65102	175	135	123	304	0.41	0.8	1.951219512	3	7	7	12	7	12	8	14	0.027063
tr|B4DL86|B4DL86_HUMAN	tr|B4DL86|B4D	6-phosphogluconate dehydrogenase, decarboxylating OS=Homo sapiens PE=2 SV=1	47901	293	172	38	253	0.2	0.39	1.95	0	4	2	6	1	7	2	8	1.613019
tr|B4DMB5|B4DMB5_HUMAN	tr|B4DMB5|B4D	cDNA FLJ60075, highly similar to CTP synthase 1 (EC 6.3.4.2) OS=Homo sapiens PE=2 SV=1	56542	449	241	38	157	0.19	0.37	1.947368421	1	4	3	4	3	5	4	5	-0.05792
tr|Q53GX7|Q53GX7_HUMAN	tr|Q53GX7|Q53	Threonyl-tRNA synthetase variant (Fragment) OS=Homo sapiens PE=2 SV=1	84304	94	70	221	503	0.64	1.23	1.921875	6	16	13	24	17	26	18	30	-0.11175
tr|Q7Z759|Q7Z759_HUMAN	CCT8	CCT8 protein OS=Homo sapiens GN=CCT8 PE=2 SV=1	54585	268	316	70	143	0.18	0.34	1.888888889	2	3	3	5	5	7	6	7	-0.2628
tr|B3KX19|B3KX19_HUMAN	tr|B3KX19|B3K	cDNA FLJ44500 fis, clone UTERU3000828, highly similar to 116 kDa U5 small nuclear ribonucleoprotein component OS=Homo sapiens PE=2	109111	111	85	193	441	0.27	0.51	1.888888889	4	11	8	14	8	16	10	19	0.247517

tr|B4DTG2|B4DTG2_HUMAN	EEF1G	Elongation factor 1-gamma OS=Homo sapiens GN=EEF1G PE=2 SV=1	56456	93	69	225	505	0.86	1.62	1.88372093	5	16	15	22	14	23	18	27	-0.01339
tr|E9PBU3|E9PBU3_HUMAN	ATIC	Phosphoribosylaminoimidazolecarboxamide formyltransferase OS=Homo sapiens GN=ATIC PE=2 SV=1	64996	72	100	274	400	0.64	1.2	1.875	8	11	11	16	12	17	13	18	-0.22513
tr|B3KNL2|B3KNL2_HUMAN	RUVBL2	RuvB-like 2 (E. coli), isoform CRA_d OS=Homo sapiens GN=RUVBL2 PE=2 SV=1	47603	584	590	28	42	0.14	0.26	1.857142857	0	1	2	1	1	2	2	2	0.027025
tr|B4DQX0|B4DQX0_HUMAN	tr|B4DQX0|B4D	cDNA FLJ59219, highly similar to Poly(A)-binding protein 1 OS=Homo sapiens PE=2 SV=1	67239	215	158	96	272	0.33	0.61	1.848484848	3	5	6	10	6	12	10	13	0.233852
sp|P26038|MOES_HUMAN	MSN	Moesin OS=Homo sapiens GN=MSN PE=1 SV=3	67892	119	90	179	425	0.46	0.85	1.847826087	7	14	8	16	11	19	12	21	0.052032
tr|H0Y7N2|H0Y7N2_HUMAN	ATXN10	Ataxin-10 (Fragment) OS=Homo sapiens GN=ATXN10 PE=4 SV=1	19107	470	258	37	47	0.38	0.7	1.842105263	0	1	2	2	2	4	2	4	0.138131
tr|B4DUQ1|B4DUQ1_HUMAN	tr|B4DUQ1|B4D	cDNA FLJ54552, highly similar to Heterogeneous nuclear ribonucleoprotein K OS=Homo sapiens PE=2 SV=1	48708	147	128	147	314	0.58	1.06	1.827586207	3	9	7	11	7	12	8	13	0.027063
tr|Q9UK20|Q9UK20_HUMAN	tr|Q9UK20|Q9U	Ezrin (Fragment) OS=Homo sapiens PE=2 SV=2	19595	119	111	88	187	0.46	0.83	1.804347826	2	5	2	5	2	6	2	7	0.669059
tr|B3KQQ3|B3KQQ3_HUMAN	tr|B3KQQ3|B3K	cDNA PSEC0016 fis, clone NT2RM1001076, highly similar to Procollagen-lysine,2-oxoglutarate 5-dioxygenase 3 (EC 1.14.11.4) OS=Homo sapi	85161	325	235	54	191	0.12	0.21	1.75	1	4	3	6	3	8	3	9	0.57073
tr|B4DGU4|B4DGU4_HUMAN	CTNNB1	Catenin beta-1 OS=Homo sapiens GN=CTNNB1 PE=2 SV=1	85339	340	421	52	103	0.12	0.21	1.75	2	4	3	5	3	5	3	6	-0.05792
tr|H0Y804|H0Y804_HUMAN	AKR1C1	Aldo-keto reductase family 1 member C1 (Fragment) OS=Homo sapiens GN=AKR1C1 PE=4 SV=1	28617	186	127	61	145	0.67	1.16	1.731343284	2	4	4	5	4	5	6	6	-0.42083
tr|B3KT93|B3KT93_HUMAN	tr|B3KT93|B3KT	cDNA FLJ37875 fis, clone BRSSN2018771, highly similar to Poly(A)-binding protein 1 OS=Homo sapiens PE=2 SV=1	70799	215	159	96	272	0.33	0.57	1.727272727	3	5	6	10	5	11	9	12	0.354697
tr|B4DPP6|B4DPP6_HUMAN	tr|B4DPP6|B4D	cDNA FLJ54371, highly similar to Serum albumin OS=Homo sapiens PE=2 SV=1	72370	222	145	93	298	0.25	0.43	1.72	3	9	5	13	6	15	10	16	0.544811
tr|Q53XC0|Q53XC0_HUMAN	EIF2S1	Eukaryotic translation initiation factor 2, subunit 1 alpha, 35kDa, isoform CRA_a OS=Homo sapiens GN=EIF2S1 PE=2 SV=1	36374	208	355	100	129	0.42	0.72	1.714285714	4	4	4	5	4	5	4	8	-0.42083
tr|Q7Z5T5|Q7Z5T5_HUMAN	EIF3A	EIF3A protein (Fragment) OS=Homo sapiens GN=EIF3A PE=2 SV=1	97203	192	141	112	300	0.14	0.24	1.714285714	2	7	4	11	5	11	8	13	0.354697
tr|Q6IPN6|Q6IPN6_HUMAN	EEF1A1	Elongation factor 1-alpha OS=Homo sapiens GN=EEF1A1 PE=2 SV=1	50433	70	96	295	408	1.14	1.94	1.701754386	11	10	13	20	18	19	21	25	-0.63623
tr|H0YA24|H0YA24_HUMAN	PLRG1	Pleiotropic regulator 1 (Fragment) OS=Homo sapiens GN=PLRG1 PE=4 SV=1	17588	456	277	38	73	0.19	0.32	1.684210526	1	1	1	1	1	1	2	1	-0.71015
sp|P01024|CO3_HUMAN	C3	Complement C3 OS=Homo sapiens GN=C3 PE=1 SV=2	188569	370	449	47	97	0.03	0.05	1.666666667	1	2	2	2	2	2	2	3	-0.71028
sp|P23526|SAHH_HUMAN	AHCY	Adenosylhomocysteinase OS=Homo sapiens GN=AHCY PE=1 SV=4	48255	195	203	111	221	0.49	0.81	1.653061224	2	5	6	11	6	13	8	13	0.34509
tr|Q8TE01|Q8TE01_HUMAN	derp12	DERP12 (Dermal papilla derived protein 12) OS=Homo sapiens GN=derp12 PE=2 SV=1	38340	69	119	297	347	0.79	1.29	1.632911392	6	9	7	10	8	10	8	12	-0.4058
tr|B0S7Z4|B0S7Z4_HUMAN	HLA-C	HLA class I histocompatibility antigen, Cw-14 alpha chain OS=Homo sapiens GN=HLA-C PE=4 SV=1	38649	639	383	22	98	0.16	0.26	1.625	0	2	1	2	1	4	1	5	0.875434
tr|A5PHJ9|A5PHJ9_HUMAN	PDHA1/LOC790(	PDHA1/LOC79064 protein (Fragment) OS=Homo sapiens GN=PDHA1/LOC79064 PE=2 SV=1	34113	516	442	32	80	0.1	0.16	1.6	0	1	1	1	1	1	1	1	-0.71015
tr|B3KNM6|B3KNM6_HUMAN	tr|B3KNM6|B3K	cDNA FLJ14968 fis, clone THYRO1000288, highly similar to CAAX prenyl protease 1 homolog (EC 3.4.24.84) OS=Homo sapiens PE=2 SV=1	27279	547	513	30	74	0.12	0.19	1.583333333	1	2	1	2	1	2	1	3	0.027025
sp|Q9Y3I0|RTCB_HUMAN	C22orf28	tRNA-splicing ligase RtcB homolog OS=Homo sapiens GN=C22orf28 PE=1 SV=1	55688	458	472	38	90	0.12	0.19	1.583333333	0	2	2	3	2	3	2	3	-0.22464
sp|P49327|FAS_HUMAN	FASN	Fatty acid synthase OS=Homo sapiens GN=FASN PE=1 SV=3	275877	55	38	394	812	0.28	0.44	1.571428571	12	20	21	31	23	39	29	44	0.039461
tr|B4DH02|B4DH02_HUMAN	tr|B4DH02|B4D	cDNA FLJ50510, highly similar to Heat shock 70 kDa protein 4 OS=Homo sapiens PE=2 SV=1	95157	123	144	175	298	0.23	0.36	1.565217391	4	7	6	9	5	11	6	11	0.354697
sp|Q9BYZ2|LDH6B_HUMAN	LDHAL6B	L-lactate dehydrogenase A-like 6B OS=Homo sapiens GN=LDHAL6B PE=1 SV=3	42372	327	426	54	101	0.16	0.25	1.5625	1	3	2	3	2	5	2	5	0.427844
tr|B2R9K8|B2R9K8_HUMAN	tr|B2R9K8|B2R9	cDNA, FLJ94440, highly similar to Homo sapiens chaperonin containing TCP1, subunit 6A (zeta 1)(CCT6A), mRNA OS=Homo sapiens PE=2 SV=	58386	118	247	181	185	0.47	0.73	1.553191489	5	4	7	10	9	11	10	13	-0.43515
tr|G3V1S6|G3V1S6_HUMAN	GALNT2	Polypeptide N-acetylgalactosaminyltransferase 2 soluble form OS=Homo sapiens GN=GALNT2 PE=2 SV=1	61440	254	243	76	187	0.11	0.17	1.545454545	2	3	2	3	2	3	2	5	-0.22464
sp|Q14247|SRC8_HUMAN	CTTN	Src substrate cortactin OS=Homo sapiens GN=CTTN PE=1 SV=2	61720	378	237	46	190	0.11	0.17	1.545454545	1	2	2	3	3	3	3	3	-0.71041
tr|B0V043|B0V043_HUMAN	VARS	Valine--tRNA ligase OS=Homo sapiens GN=VARS PE=3 SV=1	141632	275	209	69	216	0.15	0.23	1.533333333	2	7	6	9	6	9	8	12	-0.1627
tr|A8K6Q8|A8K6Q8_HUMAN	tr|A8K6Q8|A8K	cDNA FLJ75881, highly similar to Homo sapiens transferrin receptor (p90, CD71) (TFRC), mRNA OS=Homo sapiens PE=2 SV=1	85276	122	134	176	280	0.3	0.46	1.533333333	6	7	7	9	7	9	8	13	-0.36948
tr|J3KPM9|J3KPM9_HUMAN	STAT1	Signal transducer and activator of transcription 1-alpha/beta OS=Homo sapiens GN=STAT1 PE=4 SV=1	83878	364	397	48	112	0.08	0.12	1.5	1	3	2	3	2	3	2	3	-0.22464
tr|B4DR45|B4DR45_HUMAN	tr|B4DR45|B4D	cDNA FLJ61339, highly similar to Alanyl-tRNA synthetase (EC 6.1.1.7) OS=Homo sapiens PE=2 SV=1	110017	238	330	86	137	0.06	0.09	1.5	1	2	2	3	2	3	2	5	-0.22464
tr|B1PS43|B1PS43_HUMAN	MYH11	Myosin heavy chain 11 smooth muscle isoform OS=Homo sapiens GN=MYH11 PE=2 SV=1	235004	142	180	155	247	0.06	0.09	1.5	5	7	5	7	9	11	11	13	-0.43515
tr|B3KX72|B3KX72_HUMAN	tr|B3KX72|B3K	cDNA FLJ44920 fis, clone BRAMY3011501, highly similar to Heterogeneous nuclear ribonucleoprotein U OS=Homo sapiens PE=2 SV=1	83774	59	63	367	434	0.58	0.87	1.5	10	11	14	17	16	18	20	20	-0.54665
sp|P17987|TCPA_HUMAN	TCP1	T-complex protein 1 subunit alpha OS=Homo sapiens GN=TCP1 PE=1 SV=1	60819	197	313	106	144	0.3	0.45	1.5	2	3	5	7	8	6	10	8	-1.0985
tr|Q6IBT3|Q6IBT3_HUMAN	CCT7	CCT7 protein OS=Homo sapiens GN=CCT7 PE=2 SV=1	59804	161	267	133	170	0.38	0.57	1.5	2	6	6	7	5	8	7	8	-0.08202
tr|B7Z5E2|B7Z5E2_HUMAN	PSMC2	26S protease regulatory subunit 7 OS=Homo sapiens GN=PSMC2 PE=2 SV=1	33594	610	618	25	0	0.1	0.15	1.5	0	0	1	0	1	0	1	2	-2.29532
tr|O43209|O43209_HUMAN	tr|O43209|O43	Homo sapiens clone 23678 (Fragment) OS=Homo sapiens PE=2 SV=1	38945	27	24	440	841	3.04	4.53	1.490131579	17	30	22	37	25	46	28	51	0.157776
sp|Q3ZCM7|TBB8_HUMAN	TUBB8	Tubulin beta-8 chain OS=Homo sapiens GN=TUBB8 PE=1 SV=2	50257	67	78	306	467	0.77	1.14	1.480519481	13	20	17	26	19	33	21	35	0.071069
tr|I3L492|I3L492_HUMAN	ETF1	Eukaryotic peptide chain release factor subunit 1 (Fragment) OS=Homo sapiens GN=ETF1 PE=4 SV=1	23028	274	508	69	37	0.15	0.22	1.466666667	1	1	1	1	1	1	1	1	-0.71015
tr|J3KPF3|J3KPF3_HUMAN	SLC3A2	4F2 cell-surface antigen heavy chain OS=Homo sapiens GN=SLC3A2 PE=4 SV=1	68230	60	37	366	826	1.33	1.95	1.466165414	12	21	20	25	20	27	20	30	-0.28739
tr|Q3B7A7|Q3B7A7_HUMAN	GART	Phosphoribosylglycinamide formyltransferase, phosphoribosylglycinamide synthetase, phosphoribosylaminoimidazole synthetase OS=Homo	108909	341	336	51	135	0.13	0.19	1.461538462	1	4	4	6	3	6	4	6	0.183294
tr|Q5U077|Q5U077_HUMAN	LDHB	L-lactate dehydrogenase OS=Homo sapiens GN=LDHB PE=2 SV=1	36900	39	62	530	529	1.81	2.64	1.458563536	13	15	16	19	18	22	20	25	-0.42916
tr|Q5BKZ2|Q5BKZ2_HUMAN	KPNA1	Importin subunit alpha OS=Homo sapiens GN=KPNA1 PE=2 SV=1	60955	573	699	22	56	0.11	0.16	1.454545455	0	1	1	1	1	1	1	2	-0.71015
tr|A8K525|A8K525_HUMAN	tr|A8K525|A8K	cDNA FLJ76817, highly similar to Homo sapiens non-POU domain containing, octamer-binding (NONO), mRNA OS=Homo sapiens PE=2 SV=1	54367	75	150	266	291	0.8	1.15	1.4375	10	9	11	13	12	13	14	13	-0.60035
tr|Q59GP7|Q59GP7_HUMAN	tr|Q59GP7|Q59	Interleukin enhancer binding factor 3 isoform c variant (Fragment) OS=Homo sapiens PE=2 SV=1	50473	154	262	65	176	0.46	0.66	1.434782609	1	7	6	8	7	9	8	10	-0.36948
sp|P26232|CTNA2_HUMAN	CTNNA2	Catenin alpha-2 OS=Homo sapiens GN=CTNNA2 PE=1 SV=5	106045	389	409	31	107	0.07	0.1	1.428571429	0	2	1	3	1	3	1	3	0.512658
sp|Q1KMD3|HNRL2_HUMAN	HNRNPUL2	Heterogeneous nuclear ribonucleoprotein U-like protein 2 OS=Homo sapiens GN=HNRNPUL2 PE=1 SV=1	85622	249	281	78	163	0.21	0.3	1.428571429	2	5	5	7	6	7	6	8	-0.50415
sp|Q9H853|TBA4B_HUMAN	TUBA4B	Putative tubulin-like protein alpha-4B OS=Homo sapiens GN=TUBA4B PE=5 SV=2	27819	146	195	147	228	0.4	0.57	1.425	3	4	4	6	4	6	6	6	-0.17961
sp|P68363|TBA1B_HUMAN	TUBA1B	Tubulin alpha-1B chain OS=Homo sapiens GN=TUBA1B PE=1 SV=1	50804	13	21	1091	1250	5.58	7.88	1.41218638	31	31	43	44	45	46	49	51	-0.68439
sp|Q15392|DHC24_HUMAN	DHCR24	Delta(24)-sterol reductase OS=Homo sapiens GN=DHCR24 PE=1 SV=2	60803	442	1044	39	29	0.05	0.07	1.4	1	0	1	2	2	2	3	3	-0.71028
tr|B3KNN7|B3KNN7_HUMAN	tr|B3KNN7|B3K	cDNA FLJ30049 fis, clone ADRGL1000033, highly similar to 26S proteasome non-ATPase regulatory subunit 3 OS=Homo sapiens PE=2 SV=1	57311	273	365	69	112	0.18	0.25	1.388888889	3	3	3	3	3	5	3	6	-0.05792
sp|Q13151|ROA0_HUMAN	HNRNPA0	Heterogeneous nuclear ribonucleoprotein A0 OS=Homo sapiens GN=HNRNPA0 PE=1 SV=1	30993	323	350	55	131	0.36	0.5	1.388888889	2	4	3	4	3	4	3	4	-0.34763
sp|P18206|VINC_HUMAN	VCL	Vinculin OS=Homo sapiens GN=VCL PE=1 SV=4	124292	68	55	299	567	0.37	0.51	1.378378378	8	11	12	16	13	20	13	23	-0.10761
tr|H0Y8G5|H0Y8G5_HUMAN	HNRNPD	Heterogeneous nuclear ribonucleoprotein D0 (Fragment) OS=Homo sapiens GN=HNRNPD PE=4 SV=1	29877	229	276	90	156	0.37	0.51	1.378378378	3	5	3	5	4	6	4	6	-0.17961
tr|E3Q1J2|E3Q1J2_HUMAN	HLA-B	MHC class I antigen (Fragment) OS=Homo sapiens GN=HLA-B PE=3 SV=1	31790	639	512	22	80	0.16	0.22	1.375	0	2	1	2	1	3	1	4	0.512658
tr|B7XH59|B7XH59_HUMAN	HLA-B	MHC class I antigen (Fragment) OS=Homo sapiens GN=HLA-B PE=3 SV=1	31924	639	394	22	113	0.16	0.22	1.375	0	2	1	2	1	3	1	4	0.512658
tr|A8K7F6|A8K7F6_HUMAN	tr|A8K7F6|A8K	cDNA FLJ78244, highly similar to Homo sapiens eukaryotic translation initiation factor 4A, isoform 1 (EIF4A1), mRNA OS=Homo sapiens PE=2	46321	52	87	404	435	1.62	2.22	1.37037037	10	13	14	18	15	20	20	21	-0.3076
tr|Q53HF2|Q53HF2_HUMAN	tr|Q53HF2|Q53	Heat shock 70kDa protein 8 isoform 2 variant (Fragment) OS=Homo sapiens PE=2 SV=1	53580	51	45	426	630	1.45	1.96	1.351724138	12	17	17	26	19	24	19	27	-0.38217
tr|B4DNN4|B4DNN4_HUMAN	tr|B4DNN4|B4D	Ribonucleoside-diphosphate reductase OS=Homo sapiens PE=2 SV=1	87252	156	215	136	201	0.29	0.39	1.344827586	5	5	7	7	8	7	8	8	-0.89184
sp|Q14315|FLNC_HUMAN	FLNC	Filamin-C OS=Homo sapiens GN=FLNC PE=1 SV=3	293407	143	301	153	152	0.06	0.08	1.333333333	5	5	6	9	10	12	11	12	-0.45938
tr|Q5I6Y6|Q5I6Y6_HUMAN	LMNA	Lamin A/C transcript variant 1 OS=Homo sapiens GN=LMNA PE=2 SV=1	74322	95	54	221	570	0.75	1	1.333333333	7	14	13	16	15	16	15	19	-0.62158
tr|F5H3A1|F5H3A1_HUMAN	ATP1A1	Sodium/potassium-transporting ATPase subunit alpha-1 OS=Homo sapiens GN=ATP1A1 PE=2 SV=1	114239	324	430	55	100	0.09	0.12	1.333333333	2	3	3	5	8	6	12	7	-1.0985
tr|Q5HYG8|Q5HYG8_HUMAN	DKFZp686P0920	Putative uncharacterized protein DKFZp686P09201 OS=Homo sapiens GN=DKFZp686P09201 PE=2 SV=1	53831	86	140	235	301	0.61	0.81	1.327868852	4	7	8	10	9	12	10	13	-0.31465
tr|B4DXW6|B4DXW6_HUMAN	tr|B4DXW6|B4D	cDNA FLJ50285, highly similar to Arginyl-tRNA synthetase (EC 6.1.1.19) OS=Homo sapiens PE=2 SV=1	52771	235	220	88	118	0.35	0.46	1.314285714	3	5	5	6	6	8	7	8	-0.32337
tr|B4DWJ2|B4DWJ2_HUMAN	QARS	Glutamine--tRNA ligase OS=Homo sapiens GN=QARS PE=2 SV=1	87437	277	374	68	118	0.16	0.21	1.3125	3	4	4	5	5	6	5	8	-0.46946
tr|B4DKM5|B4DKM5_HUMAN	VDAC2	Voltage-dependent anion-selective channel protein 2 OS=Homo sapiens GN=VDAC2 PE=2 SV=1	27804	196	163	108	191	0.76	0.99	1.302631579	1	6	5	7	5	9	5	9	0.07865
tr|B7Z2Z8|B7Z2Z8_HUMAN	tr|B7Z2Z8|B7Z2	T-complex protein 1 subunit delta OS=Homo sapiens PE=2 SV=1	52781	113	263	188	169	0.27	0.35	1.296296296	4	4	5	4	7	6	7	6	-0.91759
tr|F4ZW64|F4ZW64_HUMAN	tr|F4ZW64|F4Z	NF90a OS=Homo sapiens PE=2 SV=1	76369	154	168	137	257	0.46	0.59	1.282608696	3	10	9	11	10	11	11	12	-0.57988
sp|Q15149|PLEC_HUMAN	PLEC	Plectin OS=Homo sapiens GN=PLEC PE=1 SV=3	533462	74	52	270	578	0.11	0.14	1.272727273	11	15	17	21	18	25	32	31	-0.24796
tr|Q2VPA0|Q2VPA0_HUMAN	LONP1	Lon protease homolog (Fragment) OS=Homo sapiens GN=LONP1 PE=2 SV=1	95436	414	407	42	107	0.11	0.14	1.272727273	2	2	3	4	3	4	3	4	-0.34763
tr|Q6DEN2|Q6DEN2_HUMAN	DPYSL3	DPYSL3 protein OS=Homo sapiens GN=DPYSL3 PE=2 SV=1	74320	490	212	35	214	0.19	0.24	1.263157895	0	5	4	5	3	8	8	9	0.57073
tr|B3KU96|B3KU96_HUMAN	tr|B3KU96|B3K	cDNA FLJ39433 fis, clone PROST2004142, highly similar to GDP-L-fucose synthetase (EC 1.1.1.271) OS=Homo sapiens PE=2 SV=1	17856	371	933	47	30	0.19	0.24	1.263157895	1	0	1	1	1	1	1	1	-0.71015
tr|B4DXX7|B4DXX7_HUMAN	DDX3Y	ATP-dependent RNA helicase DDX3Y OS=Homo sapiens GN=DDX3Y PE=2 SV=1	73342	217	311	95	79	0.19	0.24	1.263157895	2	2	5	4	6	5	6	5	-0.95202
tr|B3KW21|B3KW21_HUMAN	tr|B3KW21|B3K	cDNA FLJ41945 fis, clone PLACE6019676, highly similar to Coatomer subunit gamma OS=Homo sapiens PE=2 SV=1	68674	705	1075	18	27	0.04	0.05	1.25	0	0	1	1	0	1	1	1	0.875153
tr|D3DPU2|D3DPU2_HUMAN	CAP1	Adenylyl cyclase-associated protein OS=Homo sapiens GN=CAP1 PE=3 SV=1	51926	172	204	125	221	0.36	0.45	1.25	4	5	5	6	6	5	7	8	-0.95202
tr|G3V2A3|G3V2A3_HUMAN	TUBB3	Tubulin beta-3 chain (Fragment) OS=Homo sapiens GN=TUBB3 PE=2 SV=1	20929	25	34	147	379	3.07	3.81	1.241042345	5	10	6	10	7	14	7	14	0.241601
tr|F5H5D3|F5H5D3_HUMAN	TUBA1C	Tubulin alpha-1C chain OS=Homo sapiens GN=TUBA1C PE=2 SV=1	58606	21	26	809	1071	2.92	3.61	1.23630137	24	29	34	38	37	40	41	45	-0.60324
tr|Q53GN4|Q53GN4_HUMAN	WDR1	WD repeat domain 1, isoform CRA_a (Fragment) OS=Homo sapiens GN=WDR1 PE=2 SV=1	66822	137	115	160	364	0.4	0.47	1.175	4	6	7	9	7	12	9	14	0.027063
tr|H3BLZ8|H3BLZ8_HUMAN	DDX17	Probable ATP-dependent RNA helicase DDX17 OS=Homo sapiens GN=DDX17 PE=3 SV=1	81073	166	129	130	314	0.37	0.43	1.162162162	3	9	9	9	9	11	10	11	-0.43515
sp|P07814|SYEP_HUMAN	EPRS	Bifunctional glutamate/proline--tRNA ligase OS=Homo sapiens GN=EPRS PE=1 SV=5	172080	57	76	380	470	0.33	0.38	1.151515152	9	12	15	18	15	19	17	22	-0.37995
tr|B1AHC9|B1AHC9_HUMAN	XRCC6	X-ray repair complementing defective repair in Chinese hamster cells 6 (Ku autoantigen, 70kDa) OS=Homo sapiens GN=XRCC6 PE=4 SV=1	64528	116	106	184	374	0.64	0.73	1.140625	5	9	10	11	11	12	12	17	-0.59096
tr|G9FTK0|G9FTK0_HUMAN	HLA-A	MHC class I antigen (Fragment) OS=Homo sapiens GN=HLA-A PE=3 SV=2	38446	639	431	22	100	0.16	0.18	1.125	0	2	1	2	1	4	2	5	0.875434
tr|B4DZI4|B4DZI4_HUMAN	tr|B4DZI4|B4DZ	cDNA FLJ51413, highly similar to Cytosolic phospholipase A2 OS=Homo sapiens PE=2 SV=1	78553	369	580	47	71	0.08	0.09	1.125	1	2	2	2	2	3	2	4	-0.22464
tr|E9PIF8|E9PIF8_HUMAN	BOP1	Ribosome biogenesis protein BOP1 (Fragment) OS=Homo sapiens GN=BOP1 PE=2 SV=1	36286	505	568	0	72	0.08	0.09	1.125	0	1	0	1	1	1	1	1	-0.71015
tr|B2RDN4|B2RDN4_HUMAN	tr|B2RDN4|B2R	cDNA, FLJ96693, highly similar to Homo sapiens block of proliferation 1 (BOP1), mRNA OS=Homo sapiens PE=2 SV=1	84219	505	577	33	20	0.08	0.09	1.125	0	0	2	1	2	1	3	1	-1.44745
tr|B4DQX3|B4DQX3_HUMAN	tr|B4DQX3|B4D	cDNA FLJ58927, highly similar to Endoplasmin (Heat shock protein 90 kDa beta member 1) OS=Homo sapiens PE=2 SV=1	18712	32	56	32	104	1.24	1.39	1.120967742	0	3	1	3	1	3	1	3	0.512658
tr|H7C393|H7C393_HUMAN	APEH	Acylamino-acid-releasing enzyme (Fragment) OS=Homo sapiens GN=APEH PE=4 SV=1	32397	319	824	56	43	0.09	0.1	1.111111111	1	1	1	1	1	1	1	1	-0.71015
sp|P55072|TERA_HUMAN	VCP	Transitional endoplasmic reticulum ATPase OS=Homo sapiens GN=VCP PE=1 SV=4	89950	64	50	326	579	0.65	0.71	1.092307692	7	13	14	16	14	17	15	18	-0.43993
sp|Q6S8J3|POTEE_HUMAN	POTEE	POTE ankyrin domain family member E OS=Homo sapiens GN=POTEE PE=1 SV=3	122882	5	6	2211	2425	0.44	0.48	1.090909091	45	51	66	71	70	74	75	84	-0.63886
tr|B4DII5|B4DII5_HUMAN	tr|B4DII5|B4DII	Importin subunit alpha OS=Homo sapiens PE=2 SV=1	60345	573	842	28	42	0.11	0.12	1.090909091	0	1	2	1	3	1	3	2	-1.93321
tr|B2R6K4|B2R6K4_HUMAN	tr|B2R6K4|B2R	cDNA, FLJ92996, highly similar to Homo sapiens guanine nucleotide binding protein (G protein), beta polypeptide 1 (GNB1), mRNA OS=Hom	38075	199	478	105	89	0.18	0.19	1.055555556	1	1	2	2	2	3	2	3	-0.22464
sp|P53396|ACLY_HUMAN	ACLY	ATP-citrate synthase OS=Homo sapiens GN=ACLY PE=1 SV=3	121674	20	41	845	768	1.28	1.34	1.046875	21	21	32	34	34	34	40	42	-0.7145
tr|B3KPU1|B3KPU1_HUMAN	GNB2	Guanine nucleotide-binding protein G(I)/G(S)/G(T) subunit beta-2 OS=Homo sapiens GN=GNB2 PE=2 SV=1	26598	194	455	111	33	0.27	0.28	1.037037037	1	0	2	2	2	2	2	2	-0.71028
tr|B4DTA2|B4DTA2_HUMAN	tr|B4DTA2|B4D	cDNA FLJ60148, highly similar to Homo sapiens heterogeneous nuclear ribonucleoprotein D-like (HNRPDL), transcript variant 2, mRNA OS=H	30367	229	428	39	101	0.37	0.37	1	1	4	1	4	1	4	1	4	0.875434
tr|Q5VZU9|Q5VZU9_HUMAN	TPP2	Tripeptidyl-peptidase 2 OS=Homo sapiens GN=TPP2 PE=4 SV=1	141160	693	999	19	32	0.02	0.02	1	0	0	1	1	0	1	1	1	0.875153
tr|B0UX83|B0UX83_HUMAN	BAG6	HLA-B associated transcript 3 OS=Homo sapiens GN=BAG6 PE=4 SV=1	119085	704	796	18	46	0.03	0.03	1	0	1	1	1	0	1	1	2	0.875153
sp|Q9Y2A7|NCKP1_HUMAN	NCKAP1	Nck-associated protein 1 OS=Homo sapiens GN=NCKAP1 PE=1 SV=1	130018	685	605	19	67	0.03	0.03	1	0	1	1	1	0	1	1	2	0.875153
tr|Q6UWE9|Q6UWE9_HUMAN	UNQ2550	SFVP2550 OS=Homo sapiens GN=UNQ2550 PE=4 SV=1	11890	664	853	21	41	0.29	0.29	1	1	3	1	3	1	3	1	3	0.512658
tr|Q53ET2|Q53ET2_HUMAN	tr|Q53ET2|Q53	Dihydropyrimidinase-like 2 variant (Fragment) OS=Homo sapiens PE=2 SV=1	62688	342	266	51	172	0.23	0.23	1	1	4	4	4	3	6	5	7	0.183294
tr|A8K2S1|A8K2S1_HUMAN	tr|A8K2S1|A8K2	cDNA FLJ75258, highly similar to Homo sapiens phospholipase A2, group IVA (cytosolic, calcium-dependent) (PLA2G4A), mRNA OS=Homo sa	85695	369	579	47	71	0.08	0.08	1	1	2	2	2	2	4	5	4	0.138131
tr|B2RBA6|B2RBA6_HUMAN	tr|B2RBA6|B2R	cDNA, FLJ95407, highly similar to Homo sapiens MCM7 minichromosome maintenance deficient 7 (S. cerevisiae) (MCM7), mRNA OS=Homo	81856	374	1129	46	23	0.04	0.04	1	1	0	1	1	1	2	1	3	0.027025
tr|E3SWF7|E3SWF7_HUMAN	HLA-A	MHC class I antigen (Fragment) OS=Homo sapiens GN=HLA-A PE=3 SV=1	21021	639	1041	22	29	0.16	0.16	1	0	0	1	1	1	2	1	3	0.027025
tr|Q5M7Z5|Q5M7Z5_HUMAN	GRHPR	GRHPR protein (Fragment) OS=Homo sapiens GN=GRHPR PE=2 SV=1	37314	659	1033	21	30	0.09	0.09	1	0	1	1	1	1	2	1	3	0.027025
tr|B4DH39|B4DH39_HUMAN	tr|B4DH39|B4D	cDNA FLJ57028, highly similar to Long-chain-fatty-acid--CoA ligase 4 (EC 6.2.1.3) OS=Homo sapiens PE=2 SV=1	73123	529	1015	31	31	0.04	0.04	1	0	0	1	1	1	2	3	3	0.027025
tr|B4DG42|B4DG42_HUMAN	tr|B4DG42|B4D	cDNA FLJ53753, highly similar to Myeloid-associated differentiation marker OS=Homo sapiens PE=2 SV=1	31889	609	983	25	33	0.1	0.1	1	0	0	1	1	1	2	1	2	0.027025
tr|B2RDF2|B2RDF2_HUMAN	PES1	Pescadillo homolog 1 OS=Homo sapiens GN=PES1 PE=2 SV=1	68359	488	921	35	37	0.05	0.05	1	1	1	1	1	1	2	3	2	0.027025
tr|Q8TC04|Q8TC04_HUMAN	KRT23	Keratin 23 (Histone deacetylase inducible) OS=Homo sapiens GN=KRT23 PE=2 SV=1	48318	600	877	26	40	0.07	0.07	1	0	1	1	1	1	2	1	2	0.027025
tr|B7Z9W6|B7Z9W6_HUMAN	tr|B7Z9W6|B7Z	cDNA, FLJ78980, highly similar to DnaJ homolog subfamily C member 7 OS=Homo sapiens PE=2 SV=1	50776	527	772	31	48	0.06	0.06	1	0	1	1	1	1	2	1	2	0.027025
sp|Q14683|SMC1A_HUMAN	SMC1A	Structural maintenance of chromosomes protein 1A OS=Homo sapiens GN=SMC1A PE=1 SV=2	143771	581	746	28	51	0.02	0.02	1	0	1	1	1	1	2	1	5	0.027025
tr|B0QYT3|B0QYT3_HUMAN	RANGAP1	Ran GTPase activating protein 1 (Fragment) OS=Homo sapiens GN=RANGAP1 PE=2 SV=1	27122	574	645	28	28	0.12	0.12	1	0	0	1	1	1	2	1	2	0.027025
tr|E0YTJ7|E0YTJ7_HUMAN	HLA-B	MHC class I antigen (Fragment) OS=Homo sapiens GN=HLA-B PE=3 SV=1	21175	639	549	22	75	0.16	0.16	1	0	1	1	1	1	2	1	3	0.027025
tr|Q5QPE2|Q5QPE2_HUMAN	SEC23B	Protein transport protein Sec23B (Fragment) OS=Homo sapiens GN=SEC23B PE=4 SV=1	37575	474	529	24	78	0.08	0.08	1	0	1	1	2	1	2	1	2	0.027025
tr|Q05BW9|Q05BW9_HUMAN	PAPSS1	PAPSS1 protein (Fragment) OS=Homo sapiens GN=PAPSS1 PE=2 SV=1	65264	553	522	29	79	0.1	0.1	1	0	2	2	2	1	2	2	2	0.027025
tr|B2RCM2|B2RCM2_HUMAN	tr|B2RCM2|B2R	cDNA, FLJ96156, highly similar to Homo sapiens leucyl-tRNA synthetase (LARS), mRNA OS=Homo sapiens PE=2 SV=1	135550	257	703	75	56	0.1	0.1	1	2	1	4	4	3	5	5	5	-0.05792
tr|Q4W4Y1|Q4W4Y1_HUMAN	DRIP4	Dopamine receptor interacting protein 4 OS=Homo sapiens GN=DRIP4 PE=2 SV=1	96646	136	264	160	175	0.18	0.18	1	2	4	5	5	5	8	6	8	-0.08202
sp|P68133|ACTS_HUMAN	ACTA1	Actin, alpha skeletal muscle OS=Homo sapiens GN=ACTA1 PE=1 SV=1	42366	29	29	643	960	4.62	4.62	1	23	37	35	55	45	65	50	76	-0.18618
tr|Q5T081|Q5T081_HUMAN	RCC1	CHC1 protein OS=Homo sapiens GN=RCC1 PE=2 SV=1	45397	269	719	70	54	0.23	0.23	1	2	1	3	3	2	3	4	3	-0.22464
tr|Q5JP02|Q5JP02_HUMAN	RBBP7	Histone-binding protein RBBP7 (Fragment) OS=Homo sapiens GN=RBBP7 PE=4 SV=1	19626	321	493	37	47	0.22	0.22	1	1	1	1	2	2	3	2	3	-0.22464
tr|B2R5W2|B2R5W2_HUMAN	tr|B2R5W2|B2R	cDNA, FLJ92657, highly similar to Homo sapiens heterogeneous nuclear ribonucleoprotein C (C1/C2) (HNRPC), transcript variant 2, mRNA OS	31986	178	378	122	116	0.35	0.35	1	3	3	3	3	3	4	4	4	-0.34763
sp|Q09028|RBBP4_HUMAN	RBBP4	Histone-binding protein RBBP4 OS=Homo sapiens GN=RBBP4 PE=1 SV=3	47911	321	598	55	68	0.22	0.22	1	2	2	3	3	4	5	4	5	-0.42083
tr|B4E0S6|B4E0S6_HUMAN	tr|B4E0S6|B4E0	cDNA FLJ55635, highly similar to pre-mRNA-splicing factorATP-dependent RNA helicase DHX15 (EC 3.6.1.-) OS=Homo sapiens PE=2 SV=1	90289	183	333	117	136	0.15	0.15	1	2	3	4	4	4	5	7	7	-0.42083
sp|Q15046|SYK_HUMAN	KARS	Lysine--tRNA ligase OS=Homo sapiens GN=KARS PE=1 SV=3	68461	265	223	71	197	0.32	0.32	1	1	6	6	6	6	7	7	9	-0.50415
sp|P08729|K2C7_HUMAN	KRT7	Keratin, type II cytoskeletal 7 OS=Homo sapiens GN=KRT7 PE=1 SV=5	51411	259	171	74	255	0.45	0.45	1	2	6	6	7	7	8	9	8	-0.53016
sp|Q00610|CLH1_HUMAN	CLTC	Clathrin heavy chain 1 OS=Homo sapiens GN=CLTC PE=1 SV=5	193260	30	43	629	733	0.52	0.52	1	19	17	25	25	24	27	28	31	-0.5459
tr|B4E2I9|B4E2I9_HUMAN	tr|B4E2I9|B4E2	cDNA FLJ55486, highly similar to Semaphorin-3C OS=Homo sapiens PE=2 SV=1	88481	602	1135	26	23	0.04	0.04	1	0	0	1	1	1	1	2	1	-0.71015
tr|B4DDP6|B4DDP6_HUMAN	DBNL	Drebrin-like protein OS=Homo sapiens GN=DBNL PE=2 SV=1	43242	466	1134	37	23	0.08	0.08	1	1	0	1	1	1	1	1	2	-0.71015
tr|B3KUZ8|B3KUZ8_HUMAN	tr|B3KUZ8|B3K	Aspartate aminotransferase OS=Homo sapiens PE=2 SV=1	41699	522	1122	31	24	0.08	0.08	1	0	0	1	1	1	1	2	1	-0.71015
tr|Q5VSQ6|Q5VSQ6_HUMAN	P4HA1	Procollagen-proline, 2-oxoglutarate 4-dioxygenase (Proline 4-hydroxylase), alpha polypeptide I variant (Fragment) OS=Homo sapiens GN=P4	61214	634	1121	22	24	0.05	0.05	1	0	0	1	1	1	1	2	1	-0.71015
tr|F8W930|F8W930_HUMAN	IGF2BP2	Insulin-like growth factor 2 mRNA-binding protein 2 OS=Homo sapiens GN=IGF2BP2 PE=2 SV=1	66859	556	1117	29	24	0.05	0.05	1	0	0	1	1	1	1	1	1	-0.71015
tr|L0R4T3|L0R4T3_HUMAN	ABCF2	Alternative protein ABCF2 OS=Homo sapiens GN=ABCF2 PE=4 SV=1	7359	524	1056	31	28	0.48	0.48	1	1	1	1	1	1	1	1	1	-0.71015
tr|G1ENZ4|G1ENZ4_HUMAN	HLA-A	MHC class I antigen (Fragment) OS=Homo sapiens GN=HLA-A PE=3 SV=1	21022	639	1042	22	29	0.16	0.16	1	0	0	1	1	1	1	1	2	-0.71015
sp|Q9Y5B9|SP16H_HUMAN	SUPT16H	FACT complex subunit SPT16 OS=Homo sapiens GN=SUPT16H PE=1 SV=1	120409	506	1037	33	30	0.03	0.03	1	0	1	1	1	1	2	1	1	-0.71015
tr|E0X098|E0X098_HUMAN	TES	Testin variant (Fragment) OS=Homo sapiens GN=TES PE=2 SV=1	22009	430	1011	40	31	0.15	0.15	1	1	0	1	1	1	1	1	1	-0.71015
tr|H0Y512|H0Y512_HUMAN	APMAP	Adipocyte plasma membrane-associated protein (Fragment) OS=Homo sapiens GN=APMAP PE=4 SV=1	45662	592	1003	27	31	0.07	0.07	1	0	0	1	1	1	1	1	1	-0.71015
tr|H0YMR4|H0YMR4_HUMAN	IPO4	Importin-4 (Fragment) OS=Homo sapiens GN=IPO4 PE=4 SV=1	60104	655	982	22	33	0.05	0.05	1	0	1	1	1	1	1	1	1	-0.71015
tr|Q2NKW8|Q2NKW8_HUMAN	AHCYL1	Adenosylhomocysteinase (Fragment) OS=Homo sapiens GN=AHCYL1 PE=2 SV=1	57630	509	969	33	34	0.06	0.06	1	0	1	1	1	1	1	1	1	-0.71015
tr|B1AHB0|B1AHB0_HUMAN	MCM5	MCM5 minichromosome maintenance deficient 5, cell division cycle 46 (S. cerevisiae), isoform CRA_a OS=Homo sapiens GN=MCM5 PE=2 S\	83031	562	930	29	36	0.04	0.04	1	0	0	1	1	1	2	2	2	-0.71015
tr|B3KSH1|B3KSH1_HUMAN	EIF3F	Eukaryotic translation initiation factor 3 subunit F OS=Homo sapiens GN=EIF3F PE=2 SV=1	39236	652	878	22	40	0.08	0.08	1	0	1	1	1	1	1	1	1	-0.71015
tr|B3KT66|B3KT66_HUMAN	PSMD6	Proteasome (Prosome, macropain) 26S subunit, non-ATPase, 6, isoform CRA_c OS=Homo sapiens GN=PSMD6 PE=2 SV=1	40600	399	857	43	41	0.08	0.08	1	1	1	1	1	1	1	1	2	-0.71015
tr|A6NE09|A6NE09_HUMAN	RPSAP58	Protein RPSAP58 OS=Homo sapiens GN=RPSAP58 PE=3 SV=1	33002	521	837	31	42	0.1	0.1	1	0	1	1	1	1	1	1	1	-0.71015
tr|B4DVB8|B4DVB8_HUMAN	ELAVL1	ELAV-like protein 1 OS=Homo sapiens GN=ELAVL1 PE=2 SV=1	39200	612	832	24	43	0.08	0.08	1	0	1	1	1	1	1	1	2	-0.71015
tr|Q68E00|Q68E00_HUMAN	DKFZp686G204!	Putative uncharacterized protein DKFZp686G2045 (Fragment) OS=Homo sapiens GN=DKFZp686G2045 PE=2 SV=2	32787	485	829	36	43	0.1	0.1	1	0	1	1	1	1	1	1	1	-0.71015
tr|B3KXU8|B3KXU8_HUMAN	tr|B3KXU8|B3K	cDNA FLJ46087 fis, clone TESTI2009497, highly similar to GPI-anchored protein p137 OS=Homo sapiens PE=2 SV=1	70489	367	781	47	48	0.05	0.05	1	1	1	1	1	1	1	1	1	-0.71015
sp|Q9UG63|ABCF2_HUMAN	ABCF2	ATP-binding cassette sub-family F member 2 OS=Homo sapiens GN=ABCF2 PE=1 SV=2	71815	673	771	20	48	0.05	0.05	1	0	1	1	1	1	1	2	1	-0.71015
tr|Q5JR94|Q5JR94_HUMAN	RPS8	40S ribosomal protein S8 OS=Homo sapiens GN=RPS8 PE=2 SV=1	24475	426	759	41	50	0.14	0.14	1	1	1	1	1	1	1	2	1	-0.71015
sp|O00232|PSD12_HUMAN	PSMD12	26S proteasome non-ATPase regulatory subunit 12 OS=Homo sapiens GN=PSMD12 PE=1 SV=3	53270	283	757	66	50	0.06	0.06	1	1	1	1	1	1	1	1	2	-0.71015
tr|J3QQX0|J3QQX0_HUMAN	GPS1	COP9 signalosome complex subunit 1 (Fragment) OS=Homo sapiens GN=GPS1 PE=4 SV=1	8703	636	749	22	50	0.4	0.4	1	0	1	1	1	1	1	1	1	-0.71015
tr|I3L0N3|I3L0N3_HUMAN	NSF	Vesicle-fusing ATPase OS=Homo sapiens GN=NSF PE=3 SV=1	82553	412	748	42	50	0.04	0.04	1	1	1	1	1	1	1	1	2	-0.71015
tr|A8KA82|A8KA82_HUMAN	DNAJC3	DnaJ (Hsp40) homolog, subfamily C, member 3 OS=Homo sapiens GN=DNAJC3 PE=2 SV=1	58000	337	722	52	53	0.06	0.06	1	1	1	1	1	1	1	1	1	-0.71015
tr|K7EM16|K7EM16_HUMAN	VASP	Vasodilator-stimulated phosphoprotein (Fragment) OS=Homo sapiens GN=VASP PE=4 SV=1	20260	363	654	48	53	0.17	0.17	1	1	1	1	1	1	1	1	2	-0.71015
tr|H0YBW4|H0YBW4_HUMAN	PLAA	Phospholipase A-2-activating protein (Fragment) OS=Homo sapiens GN=PLAA PE=4 SV=1	31645	539	648	30	61	0.11	0.11	1	0	1	1	1	1	1	1	1	-0.71015
tr|B7Z1R5|B7Z1R5_HUMAN	ATP6V1A	V-type proton ATPase catalytic subunit A OS=Homo sapiens GN=ATP6V1A PE=2 SV=1	65037	428	637	40	62	0.05	0.05	1	1	1	1	1	1	2	1	1	-0.71015
tr|Q1L838|Q1L838_HUMAN	GTBP	GTBP-ALT OS=Homo sapiens GN=GTBP PE=2 SV=1	122267	559	586	29	70	0.03	0.03	1	0	1	1	1	1	1	1	1	-0.71015
tr|A2BDK6|A2BDK6_HUMAN	MAP1B	Microtubule-associated protein 1B OS=Homo sapiens GN=MAP1B PE=2 SV=1	257692	511	526	32	78	0.01	0.01	1	0	1	1	1	1	2	2	2	-0.71015
tr|H0YF33|H0YF33_HUMAN	NASP	Nuclear autoantigenic sperm protein (Fragment) OS=Homo sapiens GN=NASP PE=4 SV=1	31883	542	1097	30	25	0.1	0.1	1	0	0	1	1	2	2	3	2	-0.71028
sp|Q9NRF8|PYRG2_HUMAN	CTPS2	CTP synthase 2 OS=Homo sapiens GN=CTPS2 PE=1 SV=1	66320	614	888	24	39	0.05	0.05	1	0	1	1	1	2	2	6	2	-0.71028
sp|Q96SI9|STRBP_HUMAN	STRBP	Spermatid perinuclear RNA-binding protein OS=Homo sapiens GN=STRBP PE=1 SV=1	74290	471	631	37	63	0.09	0.09	1	0	1	2	2	2	2	2	3	-0.71028
sp|Q96P70|IPO9_HUMAN	IPO9	Importin-9 OS=Homo sapiens GN=IPO9 PE=1 SV=3	116858	580	619	28	65	0.06	0.06	1	0	1	2	2	2	2	2	2	-0.71028
tr|B4DP08|B4DP08_HUMAN	ALG1	Chitobiosyldiphosphodolichol beta-mannosyltransferase OS=Homo sapiens GN=ALG1 PE=2 SV=1	40938	452	461	38	91	0.17	0.17	1	0	1	2	2	2	2	2	2	-0.71028
tr|B9A041|B9A041_HUMAN	MDH1	Malate dehydrogenase, cytoplasmic OS=Homo sapiens GN=MDH1 PE=2 SV=1	23195	309	447	58	97	0.31	0.31	1	2	2	2	2	2	2	2	2	-0.71028
tr|F2Z2U8|F2Z2U8_HUMAN	MYH14	Myosin-14 OS=Homo sapiens GN=MYH14 PE=2 SV=1	232102	152	314	140	143	0.07	0.07	1	5	4	5	5	7	7	8	11	-0.71093
sp|P58107|EPIPL_HUMAN	EPPK1	Epiplakin OS=Homo sapiens GN=EPPK1 PE=1 SV=2	557674	56	131	390	312	0.07	0.07	1	10	8	12	11	12	11	13	12	-0.83209
sp|P52895|AK1C2_HUMAN	AKR1C2	Aldo-keto reductase family 1 member C2 OS=Homo sapiens GN=AKR1C2 PE=1 SV=3	37111	186	245	116	186	0.67	0.67	1	3	6	6	6	7	6	9	7	-0.91759
sp|P23381|SYWC_HUMAN	WARS	Tryptophan--tRNA ligase, cytoplasmic OS=Homo sapiens GN=WARS PE=1 SV=2	53474	145	320	148	141	0.27	0.27	1	3	4	4	4	5	4	5	4	-1.00038
tr|B3KMX0|B3KMX0_HUMAN	tr|B3KMX0|B3K	cDNA FLJ12837 fis, clone NT2RP2003228, highly similar to DNA replication licensing factor MCM4 OS=Homo sapiens PE=2 SV=1	97114	240	655	85	61	0.1	0.1	1	2	1	3	3	4	3	5	4	-1.07332
sp|P21796|VDAC1_HUMAN	VDAC1	Voltage-dependent anion-selective channel protein 1 OS=Homo sapiens GN=VDAC1 PE=1 SV=2	30868	250	411	78	106	0.36	0.36	1	1	3	3	3	4	3	4	3	-1.07332
tr|B4DZ88|B4DZ88_HUMAN	KTN1	KTN1 protein OS=Homo sapiens GN=KTN1 PE=2 SV=1	153179	322	830	55	43	0.04	0.04	1	2	0	2	2	3	2	4	2	-1.19604
tr|F1T0B3|F1T0B3_HUMAN	DDX1	ATP-dependent RNA helicase DDX1 OS=Homo sapiens GN=DDX1 PE=2 SV=1	74838	501	730	33	52	0.09	0.09	1	0	1	2	2	3	2	3	3	-1.19604
tr|J3KTA4|J3KTA4_HUMAN	DDX5	Probable ATP-dependent RNA helicase DDX5 OS=Homo sapiens GN=DDX5 PE=3 SV=1	69557	97	183	218	245	0.45	0.45	1	7	7	9	8	10	7	10	9	-1.19737
tr|Q6NZ55|Q6NZ55_HUMAN	RPL13	60S ribosomal protein L13 OS=Homo sapiens GN=RPL13 PE=2 SV=1	24308	422	1164	41	20	0.14	0.14	1	1	0	1	1	2	1	2	2	-1.44745
tr|G3V340|G3V340_HUMAN	STRN3	Striatin-3 OS=Homo sapiens GN=STRN3 PE=2 SV=1	36306	419	953	31	35	0.09	0.09	1	0	0	1	1	2	1	2	1	-1.44745
tr|E7EVA0|E7EVA0_HUMAN	MAP4	Microtubule-associated protein OS=Homo sapiens GN=MAP4 PE=2 SV=1	246546	429	922	40	37	0.01	0.01	1	1	1	1	1	2	1	4	3	-1.44745
tr|A0AV58|A0AV58_HUMAN	STRN3	Striatin, calmodulin binding protein 3 OS=Homo sapiens GN=STRN3 PE=2 SV=1	78069	419	963	42	34	0.09	0.09	1	1	0	2	2	3	1	3	2	-1.93321
sp|Q96RW7|HMCN1_HUMAN	HMCN1	Hemicentin-1 OS=Homo sapiens GN=HMCN1 PE=1 SV=2	623265	291	774	63	48	0.01	0.01	1	7	2	7	2	11	3	16	3	-2.42932
tr|A0N4V7|A0N4V7_HUMAN	Tcr-alpha	HCG2039797 (Fragment) OS=Homo sapiens GN=Tcr-alpha PE=4 SV=1	2269	212	797	98	46	1.81	1.81	1	5	3	15	3	31	4	46	4	-3.52703
tr|B4DX08|B4DX08_HUMAN	tr|B4DX08|B4D	cDNA FLJ58784, highly similar to T-complex protein 1 subunit epsilon OS=Homo sapiens PE=2 SV=1	57521	90	286	229	159	0.4	0.38	0.95	6	5	6	5	6	6	6	8	-0.7108

tr|B3KM80|B3KM80_HUMAN	NCL	Nucleolin, isoform CRA_c OS=Homo sapiens GN=NCL PE=2 SV=1	58576	71	104	283	387	1.03	0.93	0.902912621	9	9	14	13	15	13	15	14	-0.91171
tr|A4ZU86|A4ZU86_HUMAN	NPM1	Truncated nucleolar phosphoprotein B23 OS=Homo sapiens GN=NPM1 PE=2 SV=1	30074	138	197	160	186	0.69	0.62	0.898550725	4	3	5	3	5	4	5	5	-1.00038
tr|B7Z4V1|B7Z4V1_HUMAN	tr|B7Z4V1|B7Z4	cDNA FLJ55401, highly similar to Alpha-actinin-2 OS=Homo sapiens PE=2 SV=1	32962	50	83	103	201	0.77	0.69	0.896103896	3	5	4	8	4	10	4	10	0.51309
tr|K7EK77|K7EK77_HUMAN	ATP5A1	ATP synthase subunit alpha, mitochondrial (Fragment) OS=Homo sapiens GN=ATP5A1 PE=3 SV=1	22243	135	101	160	185	1.33	1.19	0.894736842	5	5	6	6	6	6	6	7	-0.7108
tr|C9JIF9|C9JIF9_HUMAN	APEH	Acylamino-acid-releasing enzyme OS=Homo sapiens GN=APEH PE=2 SV=1	82705	319	932	56	36	0.09	0.08	0.888888889	1	1	1	2	1	3	1	3	0.512658
tr|G3V2B8|G3V2B8_HUMAN	MTHFD1	C-1-tetrahydrofolate synthase, cytoplasmic OS=Homo sapiens GN=MTHFD1 PE=2 SV=1	102152	112	189	192	235	0.33	0.29	0.878787879	5	6	9	8	12	8	12	11	-1.26881
tr|B3KTJ9|B3KTJ9_HUMAN	tr|B3KTJ9|B3KT	cDNA FLJ38393 fis, clone FEBRA2007212 OS=Homo sapiens PE=2 SV=1	103493	82	169	251	256	0.32	0.28	0.875	7	4	9	8	9	7	12	8	-1.05265
tr|A8K7A4|A8K7A4_HUMAN	tr|A8K7A4|A8K	cDNA FLJ76904, highly similar to Homo sapiens methionine adenosyltransferase II, beta (MAT2B), transcript variant 2, mRNA OS=Homo sapi	36692	280	799	68	46	0.37	0.32	0.864864865	1	1	1	2	1	2	1	2	0.027025
tr|Q6I9U3|Q6I9U3_HUMAN	M6PR	M6PR protein OS=Homo sapiens GN=M6PR PE=2 SV=1	31469	416	684	42	58	0.29	0.25	0.862068966	1	1	1	1	1	1	1	1	-0.71015
tr|B2R7G6|B2R7G6_HUMAN	tr|B2R7G6|B2R	cDNA, FLJ93437, highly similar to Homo sapiens histidyl-tRNA synthetase-like (HARSL), mRNA OS=Homo sapiens PE=2 SV=1	57594	672	929	0	36	0.07	0.06	0.857142857	0	1	0	1	0	1	1	2	0.875153
sp|Q9NR30|DDX21_HUMAN	DDX21	Nucleolar RNA helicase 2 OS=Homo sapiens GN=DDX21 PE=1 SV=5	87804	148	519	147	79	0.34	0.29	0.852941176	4	2	8	7	8	5	9	8	-1.33972
tr|B4DZY9|B4DZY9_HUMAN	tr|B4DZY9|B4D	cDNA FLJ59103, highly similar to T-complex protein 1 subunit epsilon OS=Homo sapiens PE=2 SV=1	32544	90	376	126	117	0.4	0.34	0.85	3	3	3	3	3	4	3	5	-0.34763
tr|B4DY32|B4DY32_HUMAN	tr|B4DY32|B4D	Asparagine synthetase OS=Homo sapiens PE=2 SV=1	55055	141	362	156	111	0.26	0.22	0.846153846	3	2	5	3	6	3	6	3	-1.60451
tr|B4DW52|B4DW52_HUMAN	tr|B4DW52|B4D	cDNA FLJ55253, highly similar to Actin, cytoplasmic 1 OS=Homo sapiens PE=2 SV=1	38950	4	2	3120	3862	27.29	23.03	0.843898864	74	93	108	127	123	139	134	163	-0.5474
sp|P53621|COPA_HUMAN	COPA	Coatomer subunit alpha OS=Homo sapiens GN=COPA PE=1 SV=2	139797	181	291	118	156	0.18	0.15	0.833333333	5	4	7	6	8	6	11	8	-1.0985
sp|Q08211|DHX9_HUMAN	DHX9	ATP-dependent RNA helicase A OS=Homo sapiens GN=DHX9 PE=1 SV=4	142181	96	124	218	337	0.41	0.34	0.829268293	8	11	16	14	18	16	22	19	-0.87785
tr|B3KMV8|B3KMV8_HUMAN	tr|B3KMV8|B3K	cDNA FLJ12766 fis, clone NT2RP2001520, highly similar to Calcium-binding mitochondrial carrier protein Aralar1 OS=Homo sapiens PE=2 SV	75130	353	692	41	57	0.11	0.09	0.818181818	1	2	1	2	1	2	1	2	0.027025
tr|G8JLB6|G8JLB6_HUMAN	HNRNPH1	Heterogeneous nuclear ribonucleoprotein H OS=Homo sapiens GN=HNRNPH1 PE=4 SV=1	51482	207	175	101	251	0.45	0.36	0.8	2	5	6	7	5	7	6	7	-0.2628
tr|Q1KLZ0|Q1KLZ0_HUMAN	PS1TP5BP1	HCG15971, isoform CRA_a OS=Homo sapiens GN=PS1TP5BP1 PE=2 SV=1	42052	1	1	3367	4086	31.43	24.84	0.790327712	82	100	119	136	130	143	141	163	-0.58774
tr|B4DF96|B4DF96_HUMAN	EIF2A	Eukaryotic translation initiation factor 2A OS=Homo sapiens GN=EIF2A PE=2 SV=1	58357	206	364	101	122	0.32	0.25	0.78125	2	3	5	4	4	4	5	6	-0.71054
sp|P38919|IF4A3_HUMAN	EIF4A3	Eukaryotic initiation factor 4A-III OS=Homo sapiens GN=EIF4A3 PE=1 SV=4	47126	262	228	74	193	0.4	0.31	0.775	1	4	5	5	4	7	6	9	0.027044
sp|Q05639|EF1A2_HUMAN	EEF1A2	Elongation factor 1-alpha 2 OS=Homo sapiens GN=EEF1A2 PE=1 SV=1	50780	70	234	238	192	1.14	0.88	0.771929825	8	3	9	11	12	11	14	12	-0.83209
sp|P68032|ACTC_HUMAN	ACTC1	Actin, alpha cardiac muscle 1 OS=Homo sapiens GN=ACTC1 PE=1 SV=1	42334	23	27	771	1016	6.63	5.08	0.766214178	25	38	40	56	48	66	55	78	-0.25725
sp|Q9BTT0|AN32E_HUMAN	ANP32E	Acidic leucine-rich nuclear phosphoprotein 32 family member E OS=Homo sapiens GN=ANP32E PE=1 SV=1	30902	525	807	31	45	0.17	0.13	0.764705882	0	1	1	1	1	1	1	1	-0.71015
sp|P78371|TCPB_HUMAN	CCT2	T-complex protein 1 subunit beta OS=Homo sapiens GN=CCT2 PE=1 SV=4	57794	37	49	563	599	1.86	1.42	0.76344086	14	14	20	18	20	20	23	21	-0.71265
tr|Q3MNE1|Q3MNE1_HUMAN	tr|Q3MNE1|Q3	Adaptor-related protein complex 3 beta 1 subunit (Fragment) OS=Homo sapiens PE=2 SV=1	89828	682	1161	20	21	0.04	0.03	0.75	0	0	1	1	0	1	1	1	0.875153
tr|F8VPD4|F8VPD4_HUMAN	CAD	CAD protein OS=Homo sapiens GN=CAD PE=2 SV=1	238097	317	791	57	46	0.04	0.03	0.75	1	2	3	2	4	2	5	2	-1.55895
tr|B4DRH6|B4DRH6_HUMAN	tr|B4DRH6|B4D	cDNA FLJ54509, highly similar to Trifunctional enzyme subunit alpha, mitochondrial OS=Homo sapiens PE=2 SV=1	79007	133	398	164	63	0.28	0.21	0.75	5	2	6	3	7	3	7	4	-1.81129
tr|Q0IIN1|Q0IIN1_HUMAN	KRT77	Keratin 77 OS=Homo sapiens GN=KRT77 PE=2 SV=1	62050	76	285	264	160	0.23	0.17	0.739130435	10	5	10	5	10	5	12	5	-1.64525
tr|Q562M3|Q562M3_HUMAN	ACT	Actin-like protein (Fragment) OS=Homo sapiens GN=ACT PE=2 SV=1	11529	103	107	213	383	7.06	5.21	0.73796034	10	14	14	20	17	25	20	30	-0.16745
sp|P46087|NOP2_HUMAN	NOP2	Putative ribosomal RNA methyltransferase NOP2 OS=Homo sapiens GN=NOP2 PE=1 SV=2	89589	296	427	61	101	0.15	0.11	0.733333333	2	3	4	3	5	3	6	3	-1.36316
sp|Q9Y263|PLAP_HUMAN	PLAA	Phospholipase A-2-activating protein OS=Homo sapiens GN=PLAA PE=1 SV=2	88641	539	649	30	61	0.11	0.08	0.727272727	1	1	2	1	1	1	1	2	-0.71015
tr|D3DVQ1|D3DVQ1_HUMAN	LETM1	Leucine zipper-EF-hand containing transmembrane protein 1, isoform CRA_a OS=Homo sapiens GN=LETM1 PE=2 SV=1	63730	463	675	37	59	0.11	0.08	0.727272727	1	1	2	2	2	1	4	2	-1.44745
sp|P52272|HNRPM_HUMAN	HNRNPM	Heterogeneous nuclear ribonucleoprotein M OS=Homo sapiens GN=HNRNPM PE=1 SV=3	77749	164	425	132	102	0.39	0.28	0.717948718	7	2	8	6	11	10	12	10	-0.84291
tr|B4E2P4|B4E2P4_HUMAN	tr|B4E2P4|B4E2	cDNA FLJ58474, highly similar to Xaa-Pro aminopeptidase 1 (EC 3.4.11.9) OS=Homo sapiens PE=2 SV=1	50020	615	1062	24	28	0.07	0.05	0.714285714	0	0	1	1	1	1	1	1	-0.71015
tr|A8K492|A8K492_HUMAN	tr|A8K492|A8K4	cDNA FLJ76789, highly similar to Homo sapiens methionine-tRNA synthetase (MARS), mRNA OS=Homo sapiens PE=2 SV=1	102277	385	708	45	55	0.07	0.05	0.714285714	1	1	2	1	2	1	2	3	-1.44745
sp|P08727|K1C19_HUMAN	KRT19	Keratin, type I cytoskeletal 19 OS=Homo sapiens GN=KRT19 PE=1 SV=4	44079	158	629	135	63	0.34	0.24	0.705882353	6	2	7	3	11	6	12	7	-1.53561
tr|H0YGW7|H0YGW7_HUMAN	ABCF1	ATP-binding cassette sub-family F member 1 (Fragment) OS=Homo sapiens GN=ABCF1 PE=3 SV=1	68075	144	271	151	169	0.27	0.19	0.703703704	4	4	5	5	5	6	7	6	-0.46946
tr|B4DNG6|B4DNG6_HUMAN	tr|B4DNG6|B4D	Annexin OS=Homo sapiens PE=3 SV=1	12870	577	408	28	106	0.27	0.19	0.703703704	0	1	1	1	1	1	1	1	-0.71015
tr|B4DF68|B4DF68_HUMAN	tr|B4DF68|B4D	cDNA FLJ55325, highly similar to Heat-shock protein 105 kDa OS=Homo sapiens PE=2 SV=1	86500	123	558	47	73	0.23	0.16	0.695652174	1	1	1	4	1	5	1	5	1.165147
sp|P13489|RINI_HUMAN	RNH1	Ribonuclease inhibitor OS=Homo sapiens GN=RNH1 PE=1 SV=2	51766	431	682	40	58	0.15	0.1	0.666666667	0	1	1	1	1	1	1	1	-0.71015
tr|Q68DB1|Q68DB1_HUMAN	DKFZp686F2281	Metastasis-associated protein MTA2 OS=Homo sapiens GN=DKFZp686F2281 PE=2 SV=1	55515	676	810	20	0	0.06	0.04	0.666666667	0	0	1	0	1	0	1	2	-2.29532
tr|H0YC69|H0YC69_HUMAN	KIAA1967	DBIRD complex subunit KIAA1967 (Fragment) OS=Homo sapiens GN=KIAA1967 PE=4 SV=1	16355	82	1089	45	26	0.32	0.21	0.65625	1	0	1	1	1	1	2	2	-0.71015
tr|B4E3C3|B4E3C3_HUMAN	tr|B4E3C3|B4E3	cDNA FLJ55610, highly similar to Protein arginine N-methyltransferase 1 (EC 2.1.1.-) OS=Homo sapiens PE=2 SV=1	41150	437	651	39	61	0.26	0.17	0.653846154	1	2	2	2	2	2	2	2	-0.71028
tr|H0YJ34|H0YJ34_HUMAN	FERMT2	Fermitin family homolog 2 (Fragment) OS=Homo sapiens GN=FERMT2 PE=4 SV=1	73877	306	705	59	55	0.14	0.09	0.642857143	2	1	3	2	3	3	3	3	-0.71041
tr|B4DE59|B4DE59_HUMAN	tr|B4DE59|B4D	cDNA FLJ60424, highly similar to Junction plakoglobin OS=Homo sapiens PE=2 SV=1	62862	127	565	172	72	0.36	0.23	0.638888889	8	3	9	4	12	6	14	7	-1.65625
tr|Q59GS3|Q59GS3_HUMAN	tr|Q59GS3|Q59	Proteasome 26S ATPase subunit 5 variant (Fragment) OS=Homo sapiens PE=2 SV=1	38907	314	617	57	65	0.11	0.07	0.636363636	1	1	1	1	1	1	2	1	-0.71015
tr|G5E9Q2|G5E9Q2_HUMAN	CSDE1	Cold shock domain containing E1, RNA-binding, isoform CRA_d OS=Homo sapiens GN=CSDE1 PE=2 SV=1	94539	312	606	58	67	0.11	0.07	0.636363636	1	2	3	2	3	3	3	3	-0.71041
tr|H0Y5G9|H0Y5G9_HUMAN	NRD1	Nardilysin (Fragment) OS=Homo sapiens GN=NRD1 PE=3 SV=1	62196	225	458	92	51	0.11	0.07	0.636363636	2	1	2	1	2	1	2	1	-1.44745
tr|B3KTA3|B3KTA3_HUMAN	tr|B3KTA3|B3KT	Fascin OS=Homo sapiens PE=2 SV=1	52814	53	146	403	259	1.07	0.68	0.635514019	9	7	14	8	16	9	20	10	-1.51003
tr|A5PLK7|A5PLK7_HUMAN	RCC2	RCC2 protein (Fragment) OS=Homo sapiens GN=RCC2 PE=2 SV=1	50331	165	382	131	115	0.46	0.29	0.630434783	4	3	6	4	6	5	6	5	-0.95202
tr|K7W4U9|K7W4U9_HUMAN	tr|K7W4U9|K7W	MHC class I antigen (Fragment) OS=Homo sapiens PE=3 SV=1	31851	639	695	0	56	0.16	0.1	0.625	0	1	0	1	0	4	1	5	2.460734
tr|D0W033|D0W033_HUMAN	HLA-A	MHC class I antigen (Fragment) OS=Homo sapiens GN=HLA-A PE=3 SV=1	31801	639	547	22	75	0.16	0.1	0.625	0	1	1	1	1	3	1	4	0.512658
tr|Q96CV8|Q96CV8_HUMAN	THOP1	Thimet oligopeptidase 1 OS=Homo sapiens GN=THOP1 PE=2 SV=1	79686	278	471	68	90	0.16	0.1	0.625	1	2	1	2	1	2	1	2	0.027025
tr|A2T926|A2T926_HUMAN	TMPO	Thymopentin OS=Homo sapiens GN=TMPO PE=2 SV=1	27428	241	765	41	30	0.24	0.15	0.625	0	0	3	1	3	1	3	2	-1.93321
sp|Q58FF8|H90B2_HUMAN	HSP90AB2P	Putative heat shock protein HSP 90-beta 2 OS=Homo sapiens GN=HSP90AB2P PE=1 SV=2	44492	32	178	97	250	1.24	0.77	0.620967742	3	7	5	10	5	11	6	12	0.354697
tr|E5RH42|E5RH42_HUMAN	G3BP1	Ras GTPase-activating protein-binding protein 1 (Fragment) OS=Homo sapiens GN=G3BP1 PE=2 SV=1	13338	294	225	62	84	0.58	0.36	0.620689655	2	2	2	3	2	4	2	5	0.138131
sp|P19012|K1C15_HUMAN	KRT15	Keratin, type I cytoskeletal 15 OS=Homo sapiens GN=KRT15 PE=1 SV=3	49409	106	561	206	73	0.47	0.29	0.617021277	8	2	9	4	12	6	13	8	-1.65625
tr|B7ZB91|B7ZB91_HUMAN	TNRC6B	Trinucleotide repeat containing 6B (Fragment) OS=Homo sapiens GN=TNRC6B PE=4 SV=1	81825	359	710	48	55	0.13	0.08	0.615384615	1	2	3	2	2	2	3	3	-0.71028
tr|B3KY12|B3KY12_HUMAN	tr|B3KY12|B3KY	cDNA FLJ46581 fis, clone THYMU3043200, highly similar to Splicing factor 3A subunit 3 OS=Homo sapiens PE=2 SV=1	59082	495	524	34	79	0.18	0.11	0.611111111	1	2	3	2	2	2	3	3	-0.71028
tr|F5GWP8|F5GWP8_HUMAN	JUP	Junction plakoglobin OS=Homo sapiens GN=JUP PE=2 SV=1	66708	26	95	676	412	1.74	1.06	0.609195402	20	12	27	15	31	18	34	19	-1.48462
tr|Q59ES3|Q59ES3_HUMAN	tr|Q59ES3|Q59	Solute carrier family 1 (Neutral amino acid transporter), member 5 variant (Fragment) OS=Homo sapiens PE=2 SV=1	57810	290	1155	63	21	0.1	0.06	0.6	1	0	1	1	1	1	1	1	-0.71015
tr|B2RCM3|B2RCM3_HUMAN	tr|B2RCM3|B2R	cDNA, FLJ96158, highly similar to Homo sapiens calpain 2, (m/II) large subunit (CAPN2), mRNA OS=Homo sapiens PE=2 SV=1	80883	173	543	125	76	0.22	0.13	0.590909091	3	1	7	3	7	3	8	3	-1.81129
sp|Q14651|PLSI_HUMAN	PLS1	Plastin-1 OS=Homo sapiens GN=PLS1 PE=1 SV=2	70608	282	323	57	139	0.34	0.2	0.588235294	1	4	3	4	3	5	3	5	-0.05792
sp|Q2NL82|TSR1_HUMAN	TSR1	Pre-rRNA-processing protein TSR1 homolog OS=Homo sapiens GN=TSR1 PE=1 SV=1	92151	230	1119	90	24	0.07	0.04	0.571428571	2	0	2	1	2	1	2	1	-1.44745
tr|F5H1A7|F5H1A7_HUMAN	RDX	Radixin OS=Homo sapiens GN=RDX PE=2 SV=1	52703	119	251	88	168	0.46	0.26	0.565217391	2	5	2	5	2	6	2	7	0.669059
sp|Q04695|K1C17_HUMAN	KRT17	Keratin, type I cytoskeletal 17 OS=Homo sapiens GN=KRT17 PE=1 SV=2	48361	24	91	683	422	2.99	1.69	0.565217391	19	12	27	15	32	19	35	20	-1.45389
sp|Q562R1|ACTBL_HUMAN	ACTBL2	Beta-actin-like protein 2 OS=Homo sapiens GN=ACTBL2 PE=1 SV=2	42318	61	126	349	330	1.47	0.83	0.56462585	13	10	16	17	24	22	27	31	-0.83645
tr|E2FIL8|E2FIL8_HUMAN	HLA-A	MHC class I antigen (Fragment) OS=Homo sapiens GN=HLA-A PE=3 SV=1	38417	639	546	22	75	0.16	0.09	0.5625	0	1	1	1	1	3	2	4	0.512658
tr|B4DPZ3|B4DPZ3_HUMAN	tr|B4DPZ3|B4D	cDNA FLJ53290, highly similar to Cytoplasmic dynein 1 intermediate chain 2 OS=Homo sapiens PE=2 SV=1	70869	298	644	60	62	0.09	0.05	0.555555556	2	1	2	1	2	1	2	1	-1.44745
tr|Q5HYM2|Q5HYM2_HUMAN	DKFZp686O2462	Putative uncharacterized protein DKFZp686O2462 (Fragment) OS=Homo sapiens GN=DKFZp686O2462 PE=2 SV=1	72627	569	562	29	73	0.09	0.05	0.555555556	0	1	2	1	2	1	2	2	-1.44745
tr|Q96K98|Q96K98_HUMAN	tr|Q96K98|Q96	cDNA FLJ14414 fis, clone HEMBA1004847, highly similar to SIGNAL RECOGNITION PARTICLE 68 KD PROTEIN OS=Homo sapiens PE=2 SV=1	37365	180	515	119	46	0.18	0.1	0.555555556	2	1	2	1	2	1	4	2	-1.44745
tr|A8K4Z4|A8K4Z4_HUMAN	tr|A8K4Z4|A8K4	cDNA FLJ75549, highly similar to Homo sapiens ribosomal protein, large, P0 (RPLP0), transcript variant 1, mRNA OS=Homo sapiens PE=2 SV=	34389	109	525	201	78	0.74	0.4	0.540540541	6	2	6	2	6	4	6	4	-1.24173
tr|D6REX3|D6REX3_HUMAN	SEC31A	Protein transport protein Sec31A OS=Homo sapiens GN=SEC31A PE=2 SV=1	137110	121	622	178	64	0.13	0.07	0.538461538	4	3	5	3	4	3	5	3	-1.07332
tr|B4DUY3|B4DUY3_HUMAN	tr|B4DUY3|B4D	cDNA FLJ56668, highly similar to Nucleolar protein NOP5 OS=Homo sapiens PE=2 SV=1	16345	515	480	32	53	0.21	0.11	0.523809524	0	1	1	1	1	1	1	2	-0.71015
sp|Q9H3N1|TMX1_HUMAN	TMX1	Thioredoxin-related transmembrane protein 1 OS=Homo sapiens GN=TMX1 PE=1 SV=1	32170	689	882	19	39	0.29	0.15	0.517241379	0	1	1	0	1	1	1	1	0.875153
tr|B1ANK7|B1ANK7_HUMAN	FH	Fumarate hydratase OS=Homo sapiens GN=FH PE=2 SV=1	54773	288	997	64	32	0.12	0.06	0.5	1	0	2	1	2	2	5	4	-0.71028
tr|B2RBE5|B2RBE5_HUMAN	tr|B2RBE5|B2R	cDNA, FLJ95468, highly similar to Homo sapiens transcriptional coactivator tubedown-100 (TBDN100),transcript variant 1, mRNA OS=Homo	102490	446	734	38	52	0.06	0.03	0.5	0	1	2	1	2	2	2	3	-0.71028
tr|Q58F05|Q58F05_HUMAN	NARG1	NARG1 protein (Fragment) OS=Homo sapiens GN=NARG1 PE=2 SV=1	73310	446	733	38	52	0.06	0.03	0.5	0	1	2	1	2	2	2	2	-0.71028
sp|Q9UHR4|BI2L1_HUMAN	BAIAP2L1	Brain-specific angiogenesis inhibitor 1-associated protein 2-like protein 1 OS=Homo sapiens GN=BAIAP2L1 PE=1 SV=2	57189	613	693	24	57	0.12	0.06	0.5	0	1	2	1	2	2	4	2	-0.71028
tr|A0MNN4|A0MNN4_HUMAN	SMU1	CDW3/SMU1 OS=Homo sapiens GN=SMU1 PE=2 SV=1	58134	221	1082	94	26	0.12	0.06	0.5	1	0	2	1	2	1	2	1	-1.44745
tr|E9PRA5|E9PRA5_HUMAN	ATP1A4	Sodium/potassium-transporting ATPase subunit alpha-4 OS=Homo sapiens GN=ATP1A4 PE=2 SV=1	57835	289	593	63	55	0.12	0.06	0.5	0	0	6	5	10	4	19	6	-1.93496
tr|B0QZK4|B0QZK4_HUMAN	HP1BP3	Heterochromatin protein 1-binding protein 3 (Fragment) OS=Homo sapiens GN=HP1BP3 PE=4 SV=1	28725	631	839	23	42	0.25	0.12	0.48	0	1	2	1	1	1	3	1	-0.71015
tr|H3BNV2|H3BNV2_HUMAN	NQO1	NAD(P)H dehydrogenase [quinone] 1 OS=Homo sapiens GN=NQO1 PE=4 SV=1	28533	576	731	28	52	0.25	0.12	0.48	0	1	2	1	2	1	2	1	-1.44745
tr|B2R7M3|B2R7M3_HUMAN	tr|B2R7M3|B2R	cDNA, FLJ93510, highly similar to Homo sapiens JTV1 gene (JTV1), mRNA OS=Homo sapiens PE=2 SV=1	35767	308	1061	58	28	0.19	0.09	0.473684211	2	0	2	1	2	1	2	3	-1.44745
tr|A0MNP2|A0MNP2_HUMAN	WDR57	CDW11/WDR57 OS=Homo sapiens GN=WDR57 PE=2 SV=1	39742	413	1150	42	21	0.17	0.08	0.470588235	1	0	2	1	2	3	3	5	-0.22464
tr|Q53G58|Q53G58_HUMAN	tr|Q53G58|Q53	Coronin (Fragment) OS=Homo sapiens PE=2 SV=1	53931	242	717	83	54	0.13	0.06	0.461538462	1	1	2	1	2	3	4	3	-0.22464
sp|Q9NR12|PDLI7_HUMAN	PDLIM7	PDZ and LIM domain protein 7 OS=Homo sapiens GN=PDLIM7 PE=1 SV=1	50896	396	856	43	41	0.13	0.06	0.461538462	0	1	2	1	2	2	4	2	-0.71028
sp|Q6IS14|IF5AL_HUMAN	EIF5AL1	Eukaryotic translation initiation factor 5A-1-like OS=Homo sapiens GN=EIF5AL1 PE=1 SV=2	16990	256	1078	75	27	0.44	0.2	0.454545455	1	0	2	1	2	1	2	1	-1.44745
tr|H0YIE7|H0YIE7_HUMAN	HSPA1L	Heat shock 70 kDa protein 1-like OS=Homo sapiens GN=HSPA1L PE=3 SV=1	25488	51	130	132	204	1.45	0.65	0.448275862	3	5	4	6	4	6	4	7	-0.17961
sp|P35237|SPB6_HUMAN	SERPINB6	Serpin B6 OS=Homo sapiens GN=SERPINB6 PE=1 SV=3	42936	120	589	179	69	0.25	0.11	0.44	3	1	3	1	3	1	3	2	-1.93321
tr|F8VQP2|F8VQP2_HUMAN	ATXN2	Ataxin-2 OS=Homo sapiens GN=ATXN2 PE=2 SV=1	117012	157	329	135	137	0.21	0.09	0.428571429	4	3	7	3	10	3	10	3	-2.29773
tr|D3DQA6|D3DQA6_HUMAN	DDX46	DEAD (Asp-Glu-Ala-Asp) box polypeptide 46, isoform CRA_a OS=Homo sapiens GN=DDX46 PE=3 SV=1	100958	595	901	26	38	0.07	0.03	0.428571429	0	1	2	1	3	1	3	1	-1.93321
sp|P16615|AT2A2_HUMAN	ATP2A2	Sarcoplasmic/endoplasmic reticulum calcium ATPase 2 OS=Homo sapiens GN=ATP2A2 PE=1 SV=1	116336	149	279	145	164	0.32	0.13	0.40625	4	4	10	4	12	4	13	4	-2.18717
sp|Q9NXW2|DJB12_HUMAN	DNAJB12	DnaJ homolog subfamily B member 12 OS=Homo sapiens GN=DNAJB12 PE=1 SV=4	42021	124	435	174	99	0.57	0.23	0.403508772	5	3	7	3	9	3	9	3	-2.15301
tr|K7EIG1|K7EIG1_HUMAN	CLUH	Clustered mitochondria protein homolog OS=Homo sapiens GN=CLUH PE=3 SV=1	140903	438	776	39	48	0.05	0.02	0.4	1	1	2	1	2	2	2	3	-0.71028
sp|Q7L2E3|DHX30_HUMAN	DHX30	Putative ATP-dependent RNA helicase DHX30 OS=Homo sapiens GN=DHX30 PE=1 SV=1	134938	397	802	43	45	0.05	0.02	0.4	1	1	2	1	3	2	4	3	-1.19604
sp|P42166|LAP2A_HUMAN	TMPO	Lamina-associated polypeptide 2, isoform alpha OS=Homo sapiens GN=TMPO PE=1 SV=2	76016	241	809	84	45	0.24	0.09	0.375	1	1	5	2	5	3	5	4	-1.36316
sp|Q5XKE5|K2C79_HUMAN	KRT79	Keratin, type II cytoskeletal 79 OS=Homo sapiens GN=KRT79 PE=1 SV=2	58085	160	623	133	64	0.32	0.12	0.375	5	1	6	2	6	3	8	3	-1.60451
tr|D6RAT0|D6RAT0_HUMAN	RPS3A	40S ribosomal protein S3a OS=Homo sapiens GN=RPS3A PE=3 SV=1	26099	223	950	93	35	0.44	0.16	0.363636364	2	1	3	1	4	1	5	1	-2.29612
tr|J3KSN7|J3KSN7_HUMAN	EIF4A2	Eukaryotic initiation factor 4A-II (Fragment) OS=Homo sapiens GN=EIF4A2 PE=4 SV=1	12120	52	254	116	116	1.62	0.58	0.358024691	1	2	3	2	2	3	3	3	-0.22464
sp|P13645|K1C10_HUMAN	KRT10	Keratin, type I cytoskeletal 10 OS=Homo sapiens GN=KRT10 PE=1 SV=6	59020	12	44	1226	703	4.67	1.65	0.353319058	34	16	42	19	47	23	54	23	-1.73647
tr|H6VRF8|H6VRF8_HUMAN	KRT1	Keratin 1 OS=Homo sapiens GN=KRT1 PE=3 SV=1	66184	6	47	2032	677	5.01	1.77	0.353293413	55	18	64	26	69	30	76	31	-1.91547
tr|H6VRG2|H6VRG2_HUMAN	KRT1	Keratin 1 OS=Homo sapiens GN=KRT1 PE=3 SV=1	66197	7	48	2027	611	5.01	1.77	0.353293413	55	17	65	24	70	27	77	28	-2.08643
tr|A1L196|A1L196_HUMAN	tr|A1L196|A1L1	Putative uncharacterized protein (Fragment) OS=Homo sapiens PE=2 SV=1	16876	25	207	64	218	3.07	1.08	0.351791531	2	7	3	8	4	11	4	12	0.644541
tr|H0Y327|H0Y327_HUMAN	MTHFD1L	Monofunctional C1-tetrahydrofolate synthase, mitochondrial (Fragment) OS=Homo sapiens GN=MTHFD1L PE=4 SV=1	36808	398	1114	43	0	0.09	0.03	0.333333333	1	0	1	0	1	1	1	1	-0.71015
sp|P02533|K1C14_HUMAN	KRT14	Keratin, type I cytoskeletal 14 OS=Homo sapiens GN=KRT14 PE=1 SV=4	51872	33	222	604	198	2.22	0.74	0.333333333	19	7	25	9	29	12	30	13	-1.95614
sp|P53675|CLH2_HUMAN	CLTCL1	Clathrin heavy chain 2 OS=Homo sapiens GN=CLTCL1 PE=1 SV=2	189020	30	217	107	200	0.52	0.17	0.326923077	2	4	2	7	2	8	4	9	1.056494
sp|P35527|K1C9_HUMAN	KRT9	Keratin, type I cytoskeletal 9 OS=Homo sapiens GN=KRT9 PE=1 SV=3	62255	14	121	1044	343	3.01	0.95	0.315614618	25	10	36	13	40	13	44	15	-2.30584
tr|B7ZKM0|B7ZKM0_HUMAN	SART3	SART3 protein OS=Homo sapiens GN=SART3 PE=2 SV=1	106372	153	847	139	42	0.16	0.05	0.3125	5	1	5	1	7	2	7	3	-2.29693
tr|B2R659|B2R659_HUMAN	tr|B2R659|B2R	cDNA, FLJ92803, highly similar to Homo sapiens hydroxysteroid (17-beta) dehydrogenase 4 (HSD17B4), mRNA OS=Homo sapiens PE=2 SV=1	80018	484	556	36	74	0.13	0.04	0.307692308	0	1	3	1	3	1	4	3	-1.93321
sp|P27348|1433T_HUMAN	YWHAQ	14-3-3 protein theta OS=Homo sapiens GN=YWHAQ PE=1 SV=1	28032	258	744	74	0	0.4	0.12	0.3	2	0	3	0	3	0	3	1	-3.51838
tr|D3DTL4|D3DTL4_HUMAN	ENO3	Enolase OS=Homo sapiens GN=ENO3 PE=2 SV=1	37167	40	184	112	168	1.39	0.4	0.287769784	2	4	2	5	2	6	4	7	0.669059
tr|D2CFK9|D2CFK9_HUMAN	GTPBP4	Nucleolar GTP-binding protein 1 OS=Homo sapiens GN=GTPBP4 PE=2 SV=1	74317	264	1148	72	21	0.14	0.04	0.285714286	2	0	3	1	3	2	3	3	-1.19604
sp|P46063|RECQ1_HUMAN	RECQL	ATP-dependent DNA helicase Q1 OS=Homo sapiens GN=RECQL PE=1 SV=3	74436	439	981	39	33	0.14	0.04	0.285714286	1	0	3	1	3	1	3	2	-1.93321
sp|Q58FF6|H90B4_HUMAN	HSP90AB4P	Putative heat shock protein HSP 90-beta 4 OS=Homo sapiens GN=HSP90AB4P PE=5 SV=1	58855	32	388	77	115	1.24	0.31	0.25	3	3	5	5	3	7	5	8	0.389952
sp|Q92621|NU205_HUMAN	NUP205	Nuclear pore complex protein Nup205 OS=Homo sapiens GN=NUP205 PE=1 SV=3	230171	415	614	42	66	0.04	0.01	0.25	0	1	3	1	3	1	5	3	-1.93321
tr|F5H0C8|F5H0C8_HUMAN	ENO2	Enolase OS=Homo sapiens GN=ENO2 PE=2 SV=1	35026	40	399	112	111	1.39	0.31	0.223021583	2	1	2	3	2	5	4	6	0.427844
tr|E7EN38|E7EN38_HUMAN	RAD50	DNA repair protein RAD50 (Fragment) OS=Homo sapiens GN=RAD50 PE=2 SV=1	77138	563	1053	29	29	0.09	0.02	0.222222222	0	0	2	1	2	1	2	1	-0.71015
tr|B2RA03|B2RA03_HUMAN	tr|B2RA03|B2R	cDNA, FLJ94640, highly similar to Homo sapiens keratin 18 (KRT18), mRNA OS=Homo sapiens PE=2 SV=1	48000	127	843	88	42	0.36	0.08	0.222222222	4	1	4	1	5	2	6	3	-1.84879
sp|P11498|PYC_HUMAN	PC	Pyruvate carboxylase, mitochondrial OS=Homo sapiens GN=PC PE=1 SV=2	130293	10	92	1295	420	1.76	0.38	0.215909091	40	10	52	13	56	15	67	21	-2.59191
tr|E7ERA5|E7ERA5_HUMAN	MYH10	Myosin-10 (Fragment) OS=Homo sapiens GN=MYH10 PE=2 SV=1	21768	17	139	73	84	0.9	0.18	0.2	1	2	1	3	1	3	1	3	0.512658
tr|B2R8Y4|B2R8Y4_HUMAN	tr|B2R8Y4|B2R8	cDNA, FLJ94117, highly similar to Homo sapiens actinin, alpha 3 (ACTN3), mRNA OS=Homo sapiens PE=2 SV=1	103871	19	117	99	350	2.11	0.41	0.194312796	3	8	3	11	3	12	4	13	1.127949
sp|P13647|K2C5_HUMAN	KRT5	Keratin, type II cytoskeletal 5 OS=Homo sapiens GN=KRT5 PE=1 SV=3	62568	58	310	378	146	0.95	0.17	0.178947368	13	3	15	3	20	4	24	6	-2.90358
tr|B3KVL0|B3KVL0_HUMAN	tr|B3KVL0|B3KV	cDNA FLJ16691 fis, clone TRACH3000692, highly similar to Paxillin OS=Homo sapiens PE=2 SV=1	65600	159	690	133	57	0.28	0.05	0.178571429	3	1	5	1	6	1	6	1	-2.82731
sp|O60518|RNBP6_HUMAN	RANBP6	Ran-binding protein 6 OS=Homo sapiens GN=RANBP6 PE=1 SV=2	126173	85	638	36	62	0.23	0.04	0.173913043	1	1	1	1	2	2	2	3	-0.71028
sp|P35908|K22E_HUMAN	KRT2	Keratin, type II cytoskeletal 2 epidermal OS=Homo sapiens GN=KRT2 PE=1 SV=2	65678	11	53	1255	575	4.79	0.8	0.167014614	41	11	48	14	49	15	57	16	-2.3987
tr|J3KR91|J3KR91_HUMAN	IQGAP2	Ras GTPase-activating-like protein IQGAP2 OS=Homo sapiens GN=IQGAP2 PE=4 SV=1	123072	84	745	32	51	0.31	0.05	0.161290323	0	1	1	2	1	2	1	3	0.027025
tr|C1IK54|C1IK54_HUMAN	DDX5-ETV4	DEAD box polypeptide 5/ets variant protein 4 fusion protein OS=Homo sapiens GN=DDX5-ETV4 fusion PE=2 SV=1	58580	97	852	51	41	0.45	0.06	0.133333333	1	1	1	1	1	1	1	2	-0.71015
tr|A2RRC9|A2RRC9_HUMAN	IQGAP3	IQ motif containing GTPase activating protein 3 OS=Homo sapiens GN=IQGAP3 PE=2 SV=1	185145	84	1048	32	29	0.31	0.04	0.129032258	0	0	1	2	1	2	1	3	0.027025
tr|B4DX20|B4DX20_HUMAN	CCT6B	T-complex protein 1 subunit zeta-2 OS=Homo sapiens GN=CCT6B PE=2 SV=1	54066	118	880	46	40	0.47	0.06	0.127659574	1	1	1	1	2	1	3	2	-1.44745
tr|Q7KZ86|Q7KZ86_HUMAN	tr|Q7KZ86|Q7K	Plakoglobin (Fragment) OS=Homo sapiens PE=2 SV=1	19007	26	418	37	45	1.74	0.22	0.126436782	1	1	2	1	2	1	2	2	-1.44745
tr|F8VP67|F8VP67_HUMAN	KRT8	Keratin, type II cytoskeletal 8 (Fragment) OS=Homo sapiens GN=KRT8 PE=2 SV=1	13580	6	381	50	71	5.01	0.51	0.101796407	2	1	3	1	5	1	5	1	-2.58597
tr|B4DFM5|B4DFM5_HUMAN	tr|B4DFM5|B4D	cDNA FLJ51779, highly similar to Vacuolar ATP synthase subunit B, brain isoform (EC 3.6.3.14) OS=Homo sapiens PE=2 SV=1	43446	287	898	64	38	0.65	0.06	0.092307692	1	1	1	1	1	1	1	1	-0.71015
tr|K4ENJ5|K4ENJ5_HUMAN	tr|K4ENJ5|K4EN	Beta actin (Fragment) OS=Homo sapiens PE=4 SV=1	11129	1	138	103	301	31.43	2.87	0.091314031	3	8	3	14	6	18	7	20	0.800688
tr|G3XAP5|G3XAP5_HUMAN	LDHC	L-lactate dehydrogenase OS=Homo sapiens GN=LDHC PE=2 SV=1	26552	39	566	67	72	1.81	0.13	0.071823204	1	2	1	2	1	2	1	3	0.027025
tr|Q562V5|Q562V5_HUMAN	ACT	Actin-like protein (Fragment) OS=Homo sapiens GN=ACT PE=2 SV=1	11481	1	312	136	144	31.43	1.2	0.038180083	6	5	6	6	7	10	8	12	-0.22489
sp|O95678|K2C75_HUMAN	KRT75	Keratin, type II cytoskeletal 75 OS=Homo sapiens GN=KRT75 PE=1 SV=2	59809	11	533	94	54	4.79	0.17	0.035490605	2	1	3	2	3	3	5	3	-0.71041
sp|P12036|NFH_HUMAN	NEFH	Neurofilament heavy polypeptide OS=Homo sapiens GN=NEFH PE=1 SV=4	112639	11	978	0	33	4.79	0.03	0.006263048	0	0	0	1	0	2	1	2	1.612325
sp|Q9H254|SPTN4_HUMAN	SPTBN4	Spectrin beta chain, non-erythrocytic 4 OS=Homo sapiens GN=SPTBN4 PE=1 SV=2	290005	19	523	29	79	2.11	0.01	0.004739336	1	2	2	2	3	2	2	6	-0.22464
tr|H7C2E7|H7C2E7_HUMAN	FLNA	Filamin-A (Fragment) OS=Homo sapiens GN=FLNA PE=4 SV=1	10032	9	0	0	0	0.86	0	0	0	0	0	0	0	0	1	0	-0.71002
tr|K7ELP7|K7ELP7_HUMAN	KRT17	Keratin, type I cytoskeletal 17 (Fragment) OS=Homo sapiens GN=KRT17 PE=4 SV=1	5508	24	0	20	0	2.99	0	0	0	0	1	0	0	0	1	0	-0.71002
tr|E9PQ69|E9PQ69_HUMAN	GANAB	Neutral alpha-glucosidase AB OS=Homo sapiens GN=GANAB PE=2 SV=1	8560	47	0	0	0	0.62	0	0	0	0	0	0	0	0	1	0	-0.71002
tr|F8VU69|F8VU69_HUMAN	KRT5	Keratin, type II cytoskeletal 5 (Fragment) OS=Homo sapiens GN=KRT5 PE=2 SV=1	14387	58	0	0	0	0.95	0	0	0	0	0	0	0	0	2	0	-0.71002
tr|E9PLJ0|E9PLJ0_HUMAN	IPO7	Importin-7 OS=Homo sapiens GN=IPO7 PE=2 SV=1	7255	162	0	0	0	0.08	0	0	0	0	0	0	0	0	1	0	-0.71002
tr|F8WAT3|F8WAT3_HUMAN	EIF2A	Eukaryotic translation initiation factor 2A OS=Homo sapiens GN=EIF2A PE=2 SV=1	7970	206	0	18	0	0.32	0	0	0	0	1	0	0	0	1	0	-0.71002
tr|Q1XBU7|Q1XBU7_HUMAN	tr|Q1XBU7|Q1X	Aging-associated protein 14a OS=Homo sapiens PE=2 SV=1	14000	382	0	0	0	0.09	0	0	0	0	0	0	0	0	1	0	-0.71002
tr|B7Z6J5|B7Z6J5_HUMAN	tr|B7Z6J5|B7Z6	cDNA FLJ56858, highly similar to Succinate dehydrogenase (ubiquinone) flavoprotein subunit, mitochondrial (EC 1.3.5.1) OS=Homo sapiens	28776	393	0	0	0	0.1	0	0	0	0	0	0	0	0	1	0	-0.71002
tr|A8K171|A8K171_HUMAN	tr|A8K171|A8K	cDNA FLJ77253, highly similar to Homo sapiens nuclear receptor interacting protein 1 (NRIP1), mRNA OS=Homo sapiens PE=2 SV=1	127609	401	0	0	0	0.01	0	0	0	0	0	0	0	0	1	0	-0.71002
tr|E9PQX2|E9PQX2_HUMAN	RPS3	40S ribosomal protein S3 OS=Homo sapiens GN=RPS3 PE=2 SV=1	5066	480	0	0	0	0.12	0	0	0	0	0	0	0	0	1	0	-0.71002
tr|H7BYY8|H7BYY8_HUMAN	PES1	Pescadillo homolog OS=Homo sapiens GN=PES1 PE=4 SV=1	1501	488	0	0	0	0.05	0	0	0	0	0	0	0	0	1	0	-0.71002
tr|H0YEU6|H0YEU6_HUMAN	PABPC4	Polyadenylate-binding protein 4 (Fragment) OS=Homo sapiens GN=PABPC4 PE=4 SV=1	27445	504	0	0	0	0.05	0	0	0	0	0	0	0	0	1	0	-0.71002
tr|D6RF95|D6RF95_HUMAN	ACSL4	Long-chain-fatty-acid--CoA ligase 4 (Fragment) OS=Homo sapiens GN=ACSL4 PE=2 SV=1	17345	529	0	0	0	0.04	0	0	0	0	0	0	0	0	1	0	-0.71002
tr|H7C1T1|H7C1T1_HUMAN	Sep-02	Septin-2 (Fragment) OS=Homo sapiens GN=SEPT2 PE=4 SV=1	3094	543	0	0	0	0.09	0	0	0	0	0	0	0	0	1	0	-0.71002
tr|B7Z4W3|B7Z4W3_HUMAN	tr|B7Z4W3|B7Z	cDNA FLJ57543, highly similar to Bifunctional 3~-phosphoadenosine5~-phosphosulfate synthetase 1 OS=Homo sapiens PE=2 SV=1	17852	553	0	19	0	0.1	0	0	0	0	1	0	0	0	1	0	-0.71002
tr|Q5SWC8|Q5SWC8_HUMAN	HP1BP3	Heterochromatin protein 1-binding protein 3 (Fragment) OS=Homo sapiens GN=HP1BP3 PE=4 SV=1	31566	631	0	0	0	0.25	0	0	0	0	0	0	0	0	1	0	-0.71002
tr|Q96I65|Q96I65_HUMAN	EIF4G1	EIF4G1 protein OS=Homo sapiens GN=EIF4G1 PE=2 SV=1	72441	669	0	20	0	0.04	0	0	0	0	2	0	0	0	2	0	-0.71002
tr|J3KN33|J3KN33_HUMAN	CNTLN	Centlein OS=Homo sapiens GN=CNTLN PE=4 SV=1	162069	680	0	20	0	0.02	0	0	0	0	1	0	0	0	2	0	-0.71002
tr|B4E0B2|B4E0B2_HUMAN	tr|B4E0B2|B4E0	cDNA FLJ51426, highly similar to Adapter-related protein complex 3 beta-1 subunit OS=Homo sapiens PE=2 SV=1	64073	682	0	20	0	0.04	0	0	0	0	1	0	0	0	1	0	-0.71002
tr|Q9UH66|Q9UH66_HUMAN	tr|Q9UH66|Q9U	7h3 protein (Fragment) OS=Homo sapiens PE=2 SV=1	49963	684	0	19	0	0.07	0	0	0	0	1	0	0	0	2	0	-0.71002
tr|G5E9Q3|G5E9Q3_HUMAN	TSEN2	tRNA splicing endonuclease 2 homolog (S. cerevisiae), isoform CRA_b OS=Homo sapiens GN=TSEN2 PE=2 SV=1	45959	686	0	19	0	0.07	0	0	0	0	1	0	0	0	1	0	-0.71002
tr|E9PQG7|E9PQG7_HUMAN	MLL	MLL cleavage product C180 OS=Homo sapiens GN=MLL PE=2 SV=1	436345	687	0	19	0	0.01	0	0	0	0	1	0	0	0	1	0	-0.71002
tr|G3V448|G3V448_HUMAN	TMX1	Thioredoxin-related transmembrane protein 1 OS=Homo sapiens GN=TMX1 PE=2 SV=1	12018	689	0	19	0	0.29	0	0	0	0	1	0	0	0	1	0	-0.71002
tr|D3DUL0|D3DUL0_HUMAN	ASCIZ	ATM/ATR-Substrate Chk2-Interacting Zn2+-finger protein, isoform CRA_b OS=Homo sapiens GN=ASCIZ PE=2 SV=1	79474	690	0	19	0	0.04	0	0	0	0	1	0	0	0	1	0	-0.71002
tr|H7C4X2|H7C4X2_HUMAN	PCMT1	Protein-L-isoaspartate(D-aspartate) O-methyltransferase (Fragment) OS=Homo sapiens GN=PCMT1 PE=4 SV=1	7342	691	0	19	0	0.48	0	0	0	0	1	0	0	0	2	0	-0.71002
tr|B3KND7|B3KND7_HUMAN	tr|B3KND7|B3K	cDNA FLJ14378 fis, clone HEMBA1001804, highly similar to Zinc finger protein 281 (Fragment) OS=Homo sapiens PE=2 SV=1	58221	692	0	19	0	0.06	0	0	0	0	1	0	0	0	1	0	-0.71002
tr|H0YE81|H0YE81_HUMAN	NDUFV1	NADH dehydrogenase [ubiquinone] flavoprotein 1, mitochondrial (Fragment) OS=Homo sapiens GN=NDUFV1 PE=4 SV=1	14494	694	0	19	0	0.23	0	0	0	0	1	0	0	0	1	0	-0.71002
tr|H0Y304|H0Y304_HUMAN	DMD	Dystrophin (Fragment) OS=Homo sapiens GN=DMD PE=4 SV=1	160176	695	0	19	0	0.02	0	0	0	0	1	0	0	0	1	0	-0.71002
tr|Q5T097|Q5T097_HUMAN	UTRN	Utrophin OS=Homo sapiens GN=UTRN PE=4 SV=1	114423	696	0	19	0	0.03	0	0	0	0	1	0	0	0	1	0	-0.71002
tr|B3KMM3|B3KMM3_HUMAN	tr|B3KMM3|B3	cDNA FLJ11471 fis, clone HEMBA1001675, highly similar to Mus musculus GTPase activating protein and VPS9 domains 1 (Gapvd1), mRNA O	19935	697	0	0	0	0.02	0	0	0	0	0	0	0	0	1	0	-0.71002
tr|B4DY47|B4DY47_HUMAN	PHB	Prohibitin OS=Homo sapiens GN=PHB PE=2 SV=1	17064	699	0	18	0	0.2	0	0	0	0	1	0	0	0	1	0	-0.71002
tr|H7C3A1|H7C3A1_HUMAN	SRRT	Serrate RNA effector molecule homolog (Fragment) OS=Homo sapiens GN=SRRT PE=4 SV=1	57154	701	0	18	0	0.06	0	0	0	0	1	0	0	0	1	0	-0.71002
tr|J3KQ96|J3KQ96_HUMAN	TCOF1	Treacle protein (Fragment) OS=Homo sapiens GN=TCOF1 PE=4 SV=1	144268	702	0	18	0	0.02	0	0	0	0	1	0	0	0	2	0	-0.71002
tr|B0UZL4|B0UZL4_HUMAN	BAT3	HLA-B associated transcript 3 (Fragment) OS=Homo sapiens GN=BAT3 PE=2 SV=1	15422	704	0	18	0	0.03	0	0	0	0	1	0	0	0	1	0	-0.71002
tr|Q2NLA0|Q2NLA0_HUMAN	COPG2	Coatomer subunit gamma OS=Homo sapiens GN=COPG2 PE=2 SV=1	82400	705	0	18	0	0.04	0	0	0	0	1	0	0	0	1	0	-0.71002
tr|J3KMW7|J3KMW7_HUMAN	UBR5	E3 ubiquitin-protein ligase UBR5 OS=Homo sapiens GN=UBR5 PE=4 SV=1	312224	706	0	18	0	0.01	0	0	0	0	1	0	0	0	1	0	-0.71002
tr|H0Y4R4|H0Y4R4_HUMAN	TOR1AIP1	Torsin-1A-interacting protein 1 (Fragment) OS=Homo sapiens GN=TOR1AIP1 PE=4 SV=1	33844	707	0	17	0	0.1	0	0	0	0	1	0	0	0	1	0	-0.71002
sp|P54826|GAS1_HUMAN	GAS1	Growth arrest-specific protein 1 OS=Homo sapiens GN=GAS1 PE=1 SV=2	36867	708	0	17	0	0.09	0	0	0	0	1	0	0	0	1	0	-0.71002
tr|D6RAW4|D6RAW4_HUMAN	IMMT	Mitochondrial inner membrane protein OS=Homo sapiens GN=IMMT PE=2 SV=1	15918	712	0	0	0	0.04	0	0	0	0	0	0	0	0	1	0	-0.71002

tr|B7Z2Y4|B7Z2Y4_HUMAN	tr|B7Z2Y4|B7Z2	cDNA FLJ56961, highly similar to Clathrin heavy chain 2 (CLH-22) OS=Homo sapiens PE=2 SV=1	54629	30	0	60	0	0.52	0	0	1	0	1	0	1	0	2	0	-2.29532
tr|K7ENV3|K7ENV3_HUMAN	KRT16	Keratin, type I cytoskeletal 16 (Fragment) OS=Homo sapiens GN=KRT16 PE=4 SV=1	4775	33	0	48	0	2.22	0	0	1	0	1	0	1	0	1	0	-2.29532
tr|F8VS61|F8VS61_HUMAN	KRT77	Keratin, type II cytoskeletal 1b OS=Homo sapiens GN=KRT77 PE=2 SV=1	25704	76	0	43	0	0.23	0	0	1	0	1	0	1	0	3	0	-2.29532
tr|Q3MHV2|Q3MHV2_HUMAN	RPLP0	RPLP0 protein (Fragment) OS=Homo sapiens GN=RPLP0 PE=2 SV=1	16571	109	0	75	0	0.74	0	0	1	0	1	0	1	0	1	0	-2.29532
tr|D6RCQ9|D6RCQ9_HUMAN	SEC31A	Protein transport protein Sec31A (Fragment) OS=Homo sapiens GN=SEC31A PE=2 SV=1	18137	121	0	95	0	0.13	0	0	1	0	1	0	1	0	1	0	-2.29532
tr|F8WBE5|F8WBE5_HUMAN	TFRC	Transferrin receptor protein 1, serum form OS=Homo sapiens GN=TFRC PE=2 SV=1	9046	122	0	37	0	0.3	0	0	1	0	1	0	1	0	1	0	-2.29532
tr|Q659G2|Q659G2_HUMAN	DKFZp586B2023	Putative uncharacterized protein DKFZp586B2023 (Fragment) OS=Homo sapiens GN=DKFZp586B2023 PE=2 SV=1	7133	124	0	41	0	0.57	0	0	1	0	1	0	1	0	1	0	-2.29532
tr|H3BND9|H3BND9_HUMAN	PCBP2	Poly(rC)-binding protein 2 (Fragment) OS=Homo sapiens GN=PCBP2 PE=4 SV=1	12425	132	0	35	0	0.32	0	0	1	0	1	0	1	0	2	0	-2.29532
tr|K7ESA0|K7ESA0_HUMAN	ATP5A1	ATP synthase subunit alpha, mitochondrial OS=Homo sapiens GN=ATP5A1 PE=4 SV=1	8259	135	0	54	0	1.33	0	0	1	0	1	0	1	0	1	0	-2.29532
tr|Q9BZT4|Q9BZT4_HUMAN	tr|Q9BZT4|Q9B	PNAS-29 OS=Homo sapiens PE=4 SV=1	8036	137	0	59	0	0.4	0	0	1	0	1	0	1	0	1	0	-2.29532
tr|Q8NI92|Q8NI92_HUMAN	OK/SW-cl.65	DEAD/H(Asp-Glu-Ala-Asp/His)box polypeptide 21 OS=Homo sapiens GN=OK/SW-cl.65 PE=2 SV=1	16499	148	0	21	0	0.34	0	0	0	0	1	0	1	0	1	0	-2.29532
tr|H0YHU8|H0YHU8_HUMAN	SART3	Squamous cell carcinoma antigen recognized by T-cells 3 (Fragment) OS=Homo sapiens GN=SART3 PE=4 SV=1	16246	153	0	45	0	0.16	0	0	1	0	1	0	1	0	1	0	-2.29532
tr|K7EJ09|K7EJ09_HUMAN	ILF3	Interleukin enhancer-binding factor 3 (Fragment) OS=Homo sapiens GN=ILF3 PE=4 SV=1	13696	154	0	29	0	0.46	0	0	0	0	1	0	1	0	2	0	-2.29532
tr|F8VZQ7|F8VZQ7_HUMAN	ATXN2	Ataxin-2 (Fragment) OS=Homo sapiens GN=ATXN2 PE=2 SV=1	16188	157	0	31	0	0.21	0	0	0	0	1	0	1	0	1	0	-2.29532
tr|F8WAM2|F8WAM2_HUMAN	CCT7	T-complex protein 1 subunit eta (Fragment) OS=Homo sapiens GN=CCT7 PE=2 SV=1	10488	161	0	47	0	0.38	0	0	1	0	1	0	1	0	1	0	-2.29532
tr|J3KSA9|J3KSA9_HUMAN	PYCR1	Pyrroline-5-carboxylate reductase 1, mitochondrial (Fragment) OS=Homo sapiens GN=PYCR1 PE=4 SV=1	11860	174	0	0	0	0.19	0	0	0	0	0	0	1	0	1	0	-2.29532
tr|Q6RUP9|Q6RUP9_HUMAN	IMPDH2	Inosine monophosphate dehydrogenase type II (Fragment) OS=Homo sapiens GN=IMPDH2 PE=2 SV=1	7313	177	0	25	0	0.36	0	0	0	0	1	0	1	0	1	0	-2.29532
tr|B2RXH8|B2RXH8_HUMAN	LOC440563	Heterogeneous nuclear ribonucleoprotein C-like OS=Homo sapiens GN=LOC440563 PE=2 SV=1	32110	178	0	37	0	0.35	0	0	1	0	1	0	1	0	1	0	-2.29532
tr|A4D1P0|A4D1P0_HUMAN	LOC402299	Uncharacterized protein OS=Homo sapiens GN=LOC402299 PE=4 SV=1	42109	185	0	40	0	0.42	0	0	1	0	1	0	1	0	1	0	-2.29532
tr|B3KV39|B3KV39_HUMAN	tr|B3KV39|B3K\	cDNA FLJ16099 fis, clone TESTI2008278, highly similar to Homo sapiens forkhead box K1 (FOXK1), mRNA OS=Homo sapiens PE=2 SV=1	50383	188	0	115	0	0.07	0	0	1	0	1	0	1	0	2	0	-2.29532
tr|D6R9D3|D6R9D3_HUMAN	HNRNPH1	Heterogeneous nuclear ribonucleoprotein H OS=Homo sapiens GN=HNRNPH1 PE=4 SV=1	4404	207	0	23	0	0.45	0	0	0	0	1	0	1	0	1	0	-2.29532
tr|Q4VY17|Q4VY17_HUMAN	C20orf119	Chromosome 20 open reading frame 119 OS=Homo sapiens GN=C20orf119 PE=2 SV=1	37941	215	0	43	0	0.33	0	0	1	0	2	0	1	0	2	0	-2.29532
tr|A1L179|A1L179_HUMAN	KIF5C	KIF5C protein (Fragment) OS=Homo sapiens GN=KIF5C PE=2 SV=1	99437	227	0	46	0	0.04	0	0	1	0	1	0	1	0	2	0	-2.29532
tr|E9PLA6|E9PLA6_HUMAN	SERPINH1	Serpin H1 (Fragment) OS=Homo sapiens GN=SERPINH1 PE=2 SV=1	8925	233	0	45	0	0.43	0	0	1	0	1	0	1	0	1	0	-2.29532
tr|B4DE40|B4DE40_HUMAN	SMS	Spermine synthase OS=Homo sapiens GN=SMS PE=2 SV=1	31596	248	0	78	0	0.11	0	0	1	0	1	0	1	0	1	0	-2.29532
tr|B4DXK4|B4DXK4_HUMAN	KRT72	Keratin, type II cytoskeletal 72 OS=Homo sapiens GN=KRT72 PE=2 SV=1	36031	259	0	0	0	0.45	0	0	0	0	0	0	1	0	1	0	-2.29532
tr|K7EKB6|K7EKB6_HUMAN	THOP1	Thimet oligopeptidase (Fragment) OS=Homo sapiens GN=THOP1 PE=4 SV=1	20792	278	0	68	0	0.16	0	0	1	0	1	0	1	0	1	0	-2.29532
tr|E5RFS9|E5RFS9_HUMAN	MAT2B	Methionine adenosyltransferase 2 subunit beta OS=Homo sapiens GN=MAT2B PE=2 SV=1	9467	280	0	68	0	0.37	0	0	1	0	1	0	1	0	1	0	-2.29532
tr|H0YC45|H0YC45_HUMAN	ATP6V1B2	V-type proton ATPase subunit B, brain isoform (Fragment) OS=Homo sapiens GN=ATP6V1B2 PE=4 SV=1	5569	287	0	64	0	0.65	0	0	1	0	1	0	1	0	1	0	-2.29532
tr|B4DE27|B4DE27_HUMAN	tr|B4DE27|B4D	cDNA FLJ58765, highly similar to Neutral amino acid transporter B(0) OS=Homo sapiens PE=2 SV=1	33987	290	0	63	0	0.1	0	0	1	0	1	0	1	0	1	0	-2.29532
sp|P81605|DCD_HUMAN	DCD	Dermcidin OS=Homo sapiens GN=DCD PE=1 SV=2	11391	295	0	62	0	0.3	0	0	1	0	1	0	1	0	1	0	-2.29532
tr|E7EMU4|E7EMU4_HUMAN	DYNC1I2	Cytoplasmic dynein 1 intermediate chain 2 (Fragment) OS=Homo sapiens GN=DYNC1I2 PE=2 SV=1	24071	298	0	48	0	0.09	0	0	1	0	1	0	1	0	1	0	-2.29532
tr|B5LY64|B5LY64_HUMAN	UMPS	Orotidine 5~-phosphate decarboxylase OS=Homo sapiens GN=UMPS PE=2 SV=1	33258	302	0	59	0	0.1	0	0	1	0	1	0	1	0	1	0	-2.29532
tr|B2RAX1|B2RAX1_HUMAN	tr|B2RAX1|B2R	cDNA, FLJ95169 OS=Homo sapiens PE=2 SV=1	77979	306	0	41	0	0.14	0	0	1	0	1	0	1	0	1	0	-2.29532
tr|B3KPC1|B3KPC1_HUMAN	tr|B3KPC1|B3K	cDNA FLJ31582 fis, clone NT2RI2002117, highly similar to Protein pelota homolog OS=Homo sapiens PE=2 SV=1	43807	307	0	59	0	0.08	0	0	1	0	1	0	1	0	1	0	-2.29532
sp|Q5TZA2|CROCC_HUMAN	CROCC	Rootletin OS=Homo sapiens GN=CROCC PE=1 SV=1	228787	310	0	58	0	0.01	0	0	1	0	1	0	1	0	2	0	-2.29532
tr|J3QQM1|J3QQM1_HUMAN	PSMC5	26S protease regulatory subunit 8 (Fragment) OS=Homo sapiens GN=PSMC5 PE=4 SV=1	29442	314	0	57	0	0.11	0	0	1	0	1	0	1	0	2	0	-2.29532
tr|C9JPP3|C9JPP3_HUMAN	RBBP4	Histone-binding protein RBBP4 (Fragment) OS=Homo sapiens GN=RBBP4 PE=2 SV=2	9655	315	0	57	0	0.36	0	0	1	0	1	0	1	0	1	0	-2.29532
tr|H7C2E4|H7C2E4_HUMAN	CAD	CAD protein (Fragment) OS=Homo sapiens GN=CAD PE=3 SV=1	32547	317	0	40	0	0.04	0	0	1	0	1	0	1	0	1	0	-2.29532
tr|H7C1U0|H7C1U0_HUMAN	APEH	Acylamino-acid-releasing enzyme (Fragment) OS=Homo sapiens GN=APEH PE=4 SV=1	35132	319	0	56	0	0.09	0	0	1	0	1	0	1	0	1	0	-2.29532
tr|C9JIX5|C9JIX5_HUMAN	PLOD3	Procollagen-lysine,2-oxoglutarate 5-dioxygenase 3 (Fragment) OS=Homo sapiens GN=PLOD3 PE=2 SV=1	8424	325	0	41	0	0.12	0	0	1	0	1	0	1	0	1	0	-2.29532
sp|O95831|AIFM1_HUMAN	AIFM1	Apoptosis-inducing factor 1, mitochondrial OS=Homo sapiens GN=AIFM1 PE=1 SV=1	67144	326	0	54	0	0.05	0	0	1	0	1	0	1	0	2	0	-2.29532
tr|I3L4P6|I3L4P6_HUMAN	ARHGAP17	Rho GTPase-activating protein 17 (Fragment) OS=Homo sapiens GN=ARHGAP17 PE=4 SV=1	8676	328	0	54	0	0.03	0	0	1	0	1	0	1	0	1	0	-2.29532
tr|A8K3Q9|A8K3Q9_HUMAN	tr|A8K3Q9|A8K	cDNA FLJ76611, highly similar to Homo sapiens ribosomal protein L14 (RPL14), mRNA OS=Homo sapiens PE=2 SV=1	23554	330	0	53	0	0.14	0	0	1	0	1	0	1	0	1	0	-2.29532
tr|Q59GV0|Q59GV0_HUMAN	tr|Q59GV0|Q59	Propionyl Coenzyme A carboxylase, beta polypeptide variant (Fragment) OS=Homo sapiens PE=2 SV=1	60578	331	0	53	0	0.05	0	0	1	0	1	0	1	0	2	0	-2.29532
tr|J3KTE4|J3KTE4_HUMAN	RPL19	Ribosomal protein L19 OS=Homo sapiens GN=RPL19 PE=3 SV=1	23347	336	0	52	0	0.14	0	0	1	0	1	0	1	0	1	0	-2.29532
tr|B2RAR2|B2RAR2_HUMAN	tr|B2RAR2|B2R	cDNA, FLJ95064, highly similar to Homo sapiens nin one binding protein (NOB1P), mRNA OS=Homo sapiens PE=2 SV=1	47015	338	0	52	0	0.07	0	0	1	0	1	0	1	0	1	0	-2.29532
tr|A8K517|A8K517_HUMAN	RPS23	Ribosomal protein S23, isoform CRA_a OS=Homo sapiens GN=RPS23 PE=2 SV=1	15969	339	0	52	0	0.21	0	0	1	0	1	0	1	0	1	0	-2.29532
tr|H7C5I9|H7C5I9_HUMAN	GART	Phosphoribosylformylglycinamidine cyclo-ligase (Fragment) OS=Homo sapiens GN=GART PE=4 SV=1	2640	341	0	23	0	0.13	0	0	0	0	1	0	1	0	1	0	-2.29532
tr|E5RFU4|E5RFU4_HUMAN	DPYSL2	Dihydropyrimidinase-related protein 2 (Fragment) OS=Homo sapiens GN=DPYSL2 PE=2 SV=1	18320	342	0	20	0	0.23	0	0	0	0	1	0	1	0	1	0	-2.29532
tr|Q53ST0|Q53ST0_HUMAN	PCYOX1	Putative uncharacterized protein PCYOX1 (Fragment) OS=Homo sapiens GN=PCYOX1 PE=2 SV=1	11626	355	0	49	0	0.06	0	0	1	0	1	0	1	0	1	0	-2.29532
tr|D6RJF2|D6RJF2_HUMAN	PAIP1	Polyadenylate-binding protein-interacting protein 1 (Fragment) OS=Homo sapiens GN=PAIP1 PE=2 SV=1	22826	361	0	48	0	0.08	0	0	1	0	1	0	1	0	1	0	-2.29532
tr|H0Y9Q1|H0Y9Q1_HUMAN	LAP3	Cytosol aminopeptidase (Fragment) OS=Homo sapiens GN=LAP3 PE=4 SV=1	22641	368	0	47	0	0.15	0	0	1	0	1	0	1	0	1	0	-2.29532
tr|Q5TZX1|Q5TZX1_HUMAN	GNAI3	Guanine nucleotide binding protein (G protein), alpha inhibiting activity polypeptide 3 OS=Homo sapiens GN=GNAI3 PE=2 SV=1	41076	376	0	46	0	0.08	0	0	1	0	1	0	1	0	1	0	-2.29532
tr|Q0P512|Q0P512_HUMAN	SLC2A1	Solute carrier family 2 (Facilitated glucose transporter), member 1 OS=Homo sapiens GN=SLC2A1 PE=2 SV=1	54361	377	0	46	0	0.06	0	0	1	0	1	0	1	0	1	0	-2.29532
tr|H0YGH1|H0YGH1_HUMAN	PFAS	Phosphoribosylformylglycinamidine synthase (Fragment) OS=Homo sapiens GN=PFAS PE=4 SV=2	41755	380	0	37	0	0.05	0	0	0	0	1	0	1	0	1	0	-2.29532
tr|B4DT09|B4DT09_HUMAN	tr|B4DT09|B4D	cDNA FLJ60145, highly similar to Homo sapiens dendritic cell protein (hfl-B5), mRNA OS=Homo sapiens PE=2 SV=1	13697	381	0	46	0	0.25	0	0	1	0	1	0	1	0	1	0	-2.29532
tr|H0YHL6|H0YHL6_HUMAN	MARS	Methionine--tRNA ligase, cytoplasmic (Fragment) OS=Homo sapiens GN=MARS PE=4 SV=1	8869	385	0	40	0	0.07	0	0	1	0	1	0	1	0	1	0	-2.29532
tr|B3KPZ0|B3KPZ0_HUMAN	tr|B3KPZ0|B3K	cDNA FLJ32485 fis, clone SKNMC2001596, highly similar to Ankyrin repeat and FYVE domain-containing protein 1 (Fragment) OS=Homo sapi	121468	387	0	45	0	0.03	0	0	1	0	1	0	1	0	1	0	-2.29532
tr|E5RGY7|E5RGY7_HUMAN	CTNNA1	Catenin alpha-1 (Fragment) OS=Homo sapiens GN=CTNNA1 PE=2 SV=1	14895	389	0	35	0	0.07	0	0	0	0	1	0	1	0	1	0	-2.29532
tr|D6RAN1|D6RAN1_HUMAN	PDLIM7	PDZ and LIM domain protein 7 (Fragment) OS=Homo sapiens GN=PDLIM7 PE=2 SV=1	9510	396	0	33	0	0.13	0	0	0	0	1	0	1	0	2	0	-2.29532
tr|Q6W6M7|Q6W6M7_HUMAN	tr|Q6W6M7|Q6	Antigen MLAA-43 (Fragment) OS=Homo sapiens PE=2 SV=1	23572	397	0	0	0	0.05	0	0	0	0	0	0	1	0	1	0	-2.29532
tr|Q1LZN2|Q1LZN2_HUMAN	NOMO3	NOMO3 protein (Fragment) OS=Homo sapiens GN=NOMO3 PE=2 SV=1	90388	400	0	43	0	0.04	0	0	1	0	1	0	1	0	1	0	-2.29532
sp|Q4VXU2|PAP1L_HUMAN	PABPC1L	Polyadenylate-binding protein 1-like OS=Homo sapiens GN=PABPC1L PE=2 SV=1	68976	404	0	43	0	0.1	0	0	1	0	2	0	1	0	3	0	-2.29532
tr|E9PCI9|E9PCI9_HUMAN	FDPS	Farnesyl pyrophosphate synthase OS=Homo sapiens GN=FDPS PE=2 SV=1	40849	405	0	43	0	0.08	0	0	1	0	1	0	1	0	1	0	-2.29532
tr|B3KMW5|B3KMW5_HUMAN	tr|B3KMW5|B3	cDNA FLJ12796 fis, clone NT2RP2002058, highly similar to WD repeat protein 3 OS=Homo sapiens PE=2 SV=1	71519	408	0	43	0	0.05	0	0	1	0	1	0	1	0	1	0	-2.29532
sp|Q9NSB2|KRT84_HUMAN	KRT84	Keratin, type II cuticular Hb4 OS=Homo sapiens GN=KRT84 PE=2 SV=2	65942	409	0	43	0	0.05	0	0	1	0	1	0	1	0	3	0	-2.29532
tr|B4DRG7|B4DRG7_HUMAN	tr|B4DRG7|B4D	cDNA FLJ60336, highly similar to Condensin complex subunit 2 OS=Homo sapiens PE=2 SV=1	80877	410	0	43	0	0.04	0	0	1	0	1	0	1	0	1	0	-2.29532
sp|Q9NSB4|KRT82_HUMAN	KRT82	Keratin, type II cuticular Hb2 OS=Homo sapiens GN=KRT82 PE=1 SV=3	57985	411	0	43	0	0.06	0	0	1	0	1	0	1	0	2	0	-2.29532
tr|H0YGE9|H0YGE9_HUMAN	M6PR	Cation-dependent mannose-6-phosphate receptor (Fragment) OS=Homo sapiens GN=M6PR PE=4 SV=1	11814	416	0	42	0	0.29	0	0	1	0	1	0	1	0	1	0	-2.29532
tr|A0JLS5|A0JLS5_HUMAN	TBL3	TBL3 protein (Fragment) OS=Homo sapiens GN=TBL3 PE=2 SV=1	64079	418	0	42	0	0.05	0	0	1	0	1	0	1	0	1	0	-2.29532
tr|B4DLD4|B4DLD4_HUMAN	tr|B4DLD4|B4D	cDNA FLJ56738, highly similar to Fibrillarin OS=Homo sapiens PE=2 SV=1	30279	423	0	41	0	0.11	0	0	1	0	1	0	1	0	1	0	-2.29532
tr|B4DPY2|B4DPY2_HUMAN	tr|B4DPY2|B4D	cDNA FLJ59286, highly similar to Pre-mRNA-processing factor 40 homolog A (Fragment) OS=Homo sapiens PE=2 SV=1	93124	425	0	41	0	0.04	0	0	1	0	1	0	1	0	1	0	-2.29532
tr|A4D1I7|A4D1I7_HUMAN	DNCI1	Dynein, cytoplasmic 1, intermediate chain 1, isoform CRA_e OS=Homo sapiens GN=DNCI1 PE=2 SV=1	73423	427	0	40	0	0.04	0	0	1	0	1	0	1	0	1	0	-2.29532
tr|B7Z5L5|B7Z5L5_HUMAN	tr|B7Z5L5|B7Z5	cDNA FLJ59160, highly similar to Testin OS=Homo sapiens PE=2 SV=1	17178	430	0	40	0	0.15	0	0	1	0	1	0	1	0	1	0	-2.29532
tr|E9PIM9|E9PIM9_HUMAN	RNH1	Ribonuclease inhibitor (Fragment) OS=Homo sapiens GN=RNH1 PE=2 SV=1	22362	431	0	40	0	0.15	0	0	0	0	1	0	1	0	1	0	-2.29532
tr|B7Z943|B7Z943_HUMAN	tr|B7Z943|B7Z9	cDNA FLJ53131, highly similar to 24-dehydrocholesterol reductase (EC 1.3.1.-) OS=Homo sapiens PE=2 SV=1	29093	442	0	39	0	0.05	0	0	1	0	1	0	1	0	2	0	-2.29532
tr|B4E363|B4E363_HUMAN	FARSA	Phenylalanine--tRNA ligase alpha subunit OS=Homo sapiens GN=FARSA PE=2 SV=1	54181	447	0	38	0	0.06	0	0	1	0	1	0	1	0	1	0	-2.29532
tr|B4DR70|B4DR70_HUMAN	tr|B4DR70|B4D	cDNA FLJ58049, highly similar to RNA-binding protein FUS OS=Homo sapiens PE=2 SV=1	45013	450	0	38	0	0.07	0	0	1	0	1	0	1	0	1	0	-2.29532
tr|H0Y9L5|H0Y9L5_HUMAN	EXOSC9	Exosome complex component RRP45 (Fragment) OS=Homo sapiens GN=EXOSC9 PE=4 SV=1	18940	457	0	38	0	0.18	0	0	1	0	1	0	1	0	1	0	-2.29532
tr|B3KME2|B3KME2_HUMAN	tr|B3KME2|B3K	cDNA FLJ10772 fis, clone NT2RP4000243, highly similar to Cartilage-associated protein OS=Homo sapiens PE=2 SV=1	47101	459	0	38	0	0.07	0	0	1	0	1	0	1	0	1	0	-2.29532
sp|Q9H9F9|ARP5_HUMAN	ACTR5	Actin-related protein 5 OS=Homo sapiens GN=ACTR5 PE=1 SV=2	68825	460	0	38	0	0.05	0	0	1	0	1	0	1	0	2	0	-2.29532
tr|H7C1M0|H7C1M0_HUMAN	FANCL	E3 ubiquitin-protein ligase FANCL (Fragment) OS=Homo sapiens GN=FANCL PE=4 SV=1	33067	461	0	37	0	0.1	0	0	1	0	1	0	1	0	1	0	-2.29532
tr|Q5SZ64|Q5SZ64_HUMAN	SF3B4	Splicing factor 3B subunit 4 (Fragment) OS=Homo sapiens GN=SF3B4 PE=4 SV=1	20733	464	0	37	0	0.07	0	0	1	0	1	0	1	0	1	0	-2.29532
sp|Q6ZU64|CC108_HUMAN	CCDC108	Coiled-coil domain-containing protein 108 OS=Homo sapiens GN=CCDC108 PE=2 SV=2	220247	465	0	37	0	0.01	0	0	1	0	1	0	1	0	1	0	-2.29532
tr|B7Z438|B7Z438_HUMAN	tr|B7Z438|B7Z4	cDNA FLJ56352, highly similar to Succinyl-CoA ligase (GDP-forming) subunit alpha, mitochondrial (EC 6.2.1.4) OS=Homo sapiens PE=2 SV=1	26801	467	0	37	0	0.12	0	0	1	0	1	0	1	0	1	0	-2.29532
tr|H0YIX9|H0YIX9_HUMAN	R3HDM2	R3H domain-containing protein 2 (Fragment) OS=Homo sapiens GN=R3HDM2 PE=4 SV=1	31776	469	0	37	0	0.1	0	0	1	0	1	0	1	0	1	0	-2.29532
tr|B4DKI2|B4DKI2_HUMAN	tr|B4DKI2|B4D	cDNA FLJ60282, highly similar to Sorbitol dehydrogenase (EC 1.1.1.14) OS=Homo sapiens PE=2 SV=1	30315	473	0	37	0	0.09	0	0	0	0	1	0	1	0	1	0	-2.29532
tr|G3V1W4|G3V1W4_HUMAN	SEC23A	Protein transport protein Sec23A OS=Homo sapiens GN=SEC23A PE=2 SV=1	12904	474	0	24	0	0.08	0	0	0	0	1	0	1	0	1	0	-2.29532
tr|B3KSQ7|B3KSQ7_HUMAN	DBN1	Drebrin 1, isoform CRA_d OS=Homo sapiens GN=DBN1 PE=2 SV=1	66053	478	0	36	0	0.05	0	0	0	0	1	0	1	0	1	0	-2.29532
tr|B7WPL0|B7WPL0_HUMAN	RIC8B	Synembryn-B OS=Homo sapiens GN=RIC8B PE=2 SV=1	64252	483	0	36	0	0.05	0	0	1	0	1	0	1	0	1	0	-2.29532
tr|C9JNW5|C9JNW5_HUMAN	RPL24	60S ribosomal protein L24 OS=Homo sapiens GN=RPL24 PE=4 SV=1	17646	487	0	35	0	0.19	0	0	0	0	1	0	1	0	2	0	-2.29532
sp|Q9NPQ8|RIC8A_HUMAN	RIC8A	Synembryn-A OS=Homo sapiens GN=RIC8A PE=1 SV=3	60185	496	0	34	0	0.05	0	0	0	0	1	0	1	0	1	0	-2.29532
sp|Q6PGP7|TTC37_HUMAN	TTC37	Tetratricopeptide repeat protein 37 OS=Homo sapiens GN=TTC37 PE=1 SV=1	177485	498	0	34	0	0.02	0	0	1	0	1	0	1	0	1	0	-2.29532
tr|B4DDC9|B4DDC9_HUMAN	tr|B4DDC9|B4D	cDNA FLJ59448, highly similar to Trifunctional enzyme subunit beta, mitochondrial OS=Homo sapiens PE=2 SV=1	49937	499	0	34	0	0.07	0	0	0	0	1	0	1	0	1	0	-2.29532
tr|B4DXJ6|B4DXJ6_HUMAN	tr|B4DXJ6|B4D	cDNA FLJ61453, highly similar to Adapter-relatedprotein complex 2 alpha- 1 subunit OS=Homo sapiens PE=2 SV=1	55511	500	0	34	0	0.03	0	0	0	0	1	0	1	0	1	0	-2.29532
sp|Q8NFZ5|TNIP2_HUMAN	TNIP2	TNFAIP3-interacting protein 2 OS=Homo sapiens GN=TNIP2 PE=1 SV=1	49240	502	0	33	0	0.07	0	0	1	0	1	0	1	0	2	0	-2.29532
sp|Q7Z494|NPHP3_HUMAN	NPHP3	Nephrocystin-3 OS=Homo sapiens GN=NPHP3 PE=1 SV=1	151967	510	0	32	0	0.02	0	0	1	0	1	0	1	0	1	0	-2.29532
tr|Q5H9P1|Q5H9P1_HUMAN	DKFZp686F1345	Putative uncharacterized protein DKFZp686F1345 (Fragment) OS=Homo sapiens GN=DKFZp686F1345 PE=2 SV=1	193061	511	0	32	0	0.01	0	0	0	0	1	0	1	0	1	0	-2.29532
tr|A0JLS9|A0JLS9_HUMAN	ZEB1	ZEB1 protein (Fragment) OS=Homo sapiens GN=ZEB1 PE=2 SV=1	118576	513	0	32	0	0.03	0	0	1	0	1	0	1	0	1	0	-2.29532
tr|B2R4C0|B2R4C0_HUMAN	tr|B2R4C0|B2R	60S ribosomal protein L18a OS=Homo sapiens PE=2 SV=1	21024	514	0	32	0	0.16	0	0	0	0	1	0	1	0	2	0	-2.29532
tr|J3KRB3|J3KRB3_HUMAN	RPL17	60S ribosomal protein L17 (Fragment) OS=Homo sapiens GN=RPL17 PE=3 SV=1	12356	517	0	32	0	0.28	0	0	0	0	1	0	1	0	1	0	-2.29532
tr|B4DUH7|B4DUH7_HUMAN	tr|B4DUH7|B4D	cDNA FLJ60162, highly similar to Chloride anion exchanger OS=Homo sapiens PE=2 SV=1	52632	518	0	32	0	0.06	0	0	1	0	1	0	1	0	1	0	-2.29532
tr|E9PNN6|E9PNN6_HUMAN	ALDH3A1	Aldehyde dehydrogenase, dimeric NADP-preferring OS=Homo sapiens GN=ALDH3A1 PE=2 SV=1	44518	519	0	32	0	0.07	0	0	0	0	1	0	1	0	1	0	-2.29532
tr|B4E0D5|B4E0D5_HUMAN	tr|B4E0D5|B4E	cDNA FLJ54218, highly similar to Acidic leucine-rich nuclear phosphoprotein 32 family member E OS=Homo sapiens PE=2 SV=1	19540	525	0	31	0	0.17	0	0	0	0	1	0	1	0	1	0	-2.29532
tr|F2Z2J7|F2Z2J7_HUMAN	TTLL7	Tubulin polyglutamylase TTLL7 OS=Homo sapiens GN=TTLL7 PE=2 SV=1	55925	526	0	31	0	0.06	0	0	1	0	1	0	1	0	1	0	-2.29532
tr|H0Y7S3|H0Y7S3_HUMAN	ATP2B2	Plasma membrane calcium-transporting ATPase 2 (Fragment) OS=Homo sapiens GN=ATP2B2 PE=3 SV=1	122272	528	0	31	0	0.03	0	0	0	0	1	0	1	0	3	0	-2.29532
sp|Q96PE2|ARHGH_HUMAN	ARHGEF17	Rho guanine nucleotide exchange factor 17 OS=Homo sapiens GN=ARHGEF17 PE=1 SV=1	223645	537	0	30	0	0.01	0	0	1	0	1	0	1	0	1	0	-2.29532
tr|K7EKM4|K7EKM4_HUMAN	SRM	Spermidine synthase OS=Homo sapiens GN=SRM PE=4 SV=1	10680	540	0	30	0	0.32	0	0	0	0	1	0	1	0	1	0	-2.29532
tr|B4DKT5|B4DKT5_HUMAN	tr|B4DKT5|B4D	cDNA FLJ53054, highly similar to Insulin-like growth factor 2 mRNA-binding protein 2 OS=Homo sapiens PE=2 SV=1	33360	556	0	29	0	0.05	0	0	0	0	1	0	1	0	1	0	-2.29532
tr|H3BRB3|H3BRB3_HUMAN	KIF22	Kinesin-like protein KIF22 OS=Homo sapiens GN=KIF22 PE=3 SV=1	67996	557	0	29	0	0.05	0	0	0	0	1	0	1	0	1	0	-2.29532
tr|B4DS04|B4DS04_HUMAN	tr|B4DS04|B4D	cDNA FLJ54142, highly similar to Protein transport protein Sec23B OS=Homo sapiens PE=2 SV=1	41720	558	0	27	0	0.08	0	0	0	0	1	0	1	0	1	0	-2.29532
tr|E7ESD9|E7ESD9_HUMAN	RAD50	DNA repair protein RAD50 OS=Homo sapiens GN=RAD50 PE=2 SV=1	64414	563	0	28	0	0.09	0	0	0	0	1	0	1	0	1	0	-2.29532
sp|Q6KB66|K2C80_HUMAN	KRT80	Keratin, type II cytoskeletal 80 OS=Homo sapiens GN=KRT80 PE=1 SV=2	51007	564	0	29	0	0.06	0	0	0	0	1	0	1	0	1	0	-2.29532
tr|I3L361|I3L361_HUMAN	SGSM2	Small G protein-signaling modulator 2 (Fragment) OS=Homo sapiens GN=SGSM2 PE=4 SV=2	1642	570	0	29	0	2.38	0	0	0	0	1	0	1	0	1	0	-2.29532
tr|J3KQE5|J3KQE5_HUMAN	RAN	GTP-binding nuclear protein Ran (Fragment) OS=Homo sapiens GN=RAN PE=4 SV=1	27027	571	0	29	0	0.12	0	0	0	0	1	0	1	0	1	0	-2.29532
tr|Q53G81|Q53G81_HUMAN	tr|Q53G81|Q53	NAD(P)H menadione oxidoreductase 1, dioxin-inducible variant (Fragment) OS=Homo sapiens PE=2 SV=1	30857	576	0	22	0	0.25	0	0	0	0	1	0	1	0	1	0	-2.29532
sp|Q16322|KCA10_HUMAN	KCNA10	Potassium voltage-gated channel subfamily A member 10 OS=Homo sapiens GN=KCNA10 PE=1 SV=2	58147	578	0	28	0	0.06	0	0	1	0	1	0	1	0	1	0	-2.29532
tr|J3KN32|J3KN32_HUMAN	RCOR1	REST corepressor 1 OS=Homo sapiens GN=RCOR1 PE=4 SV=1	53409	583	0	28	0	0.06	0	0	0	0	1	0	1	0	1	0	-2.29532
tr|H0YCU9|H0YCU9_HUMAN	TAGLN	Transgelin (Fragment) OS=Homo sapiens GN=TAGLN PE=4 SV=1	16833	585	0	28	0	0.2	0	0	0	0	1	0	1	0	1	0	-2.29532
tr|A6NE42|A6NE42_HUMAN	TGIF1	Homeobox protein TGIF1 OS=Homo sapiens GN=TGIF1 PE=3 SV=2	27983	586	0	28	0	0.12	0	0	0	0	1	0	1	0	1	0	-2.29532
tr|B2RDF5|B2RDF5_HUMAN	tr|B2RDF5|B2R	cDNA, FLJ96587, highly similar to Homo sapiens SUMO-1 activating enzyme subunit 2 (UBA2), mRNA OS=Homo sapiens PE=2 SV=1	71705	590	0	27	0	0.05	0	0	0	0	1	0	1	0	1	0	-2.29532
tr|H3BS19|H3BS19_HUMAN	ZNF469	Zinc finger protein 469 OS=Homo sapiens GN=ZNF469 PE=4 SV=1	417347	591	0	27	0	0.01	0	0	0	0	1	0	1	0	1	0	-2.29532
tr|B7Z4L9|B7Z4L9_HUMAN	tr|B7Z4L9|B7Z4	cDNA FLJ55342, highly similar to Kelch-like protein 13 OS=Homo sapiens PE=2 SV=1	56884	593	0	26	0	0.06	0	0	0	0	1	0	1	0	1	0	-2.29532
tr|Q5T8C6|Q5T8C6_HUMAN	CDC16	Cell division cycle protein 16 homolog OS=Homo sapiens GN=CDC16 PE=4 SV=1	55239	594	0	26	0	0.06	0	0	0	0	1	0	1	0	1	0	-2.29532
sp|Q0VD83|APOBR_HUMAN	APOBR	Apolipoprotein B receptor OS=Homo sapiens GN=APOBR PE=1 SV=1	115207	597	0	26	0	0.03	0	0	0	0	1	0	1	0	1	0	-2.29532
tr|D6R937|D6R937_HUMAN	AIMP1	Endothelial monocyte-activating polypeptide 2 (Fragment) OS=Homo sapiens GN=AIMP1 PE=2 SV=1	16568	601	0	26	0	0.11	0	0	0	0	1	0	1	0	1	0	-2.29532
tr|B4DRL8|B4DRL8_HUMAN	SEMA3C	Semaphorin-3C OS=Homo sapiens GN=SEMA3C PE=2 SV=1	26580	602	0	26	0	0.04	0	0	0	0	1	0	1	0	1	0	-2.29532
tr|B2RAX6|B2RAX6_HUMAN	tr|B2RAX6|B2R	cDNA, FLJ95176, Homo sapiens CGI-48 protein (CGI-48), mRNA OS=Homo sapiens PE=2 SV=1	58127	605	0	25	0	0.06	0	0	0	0	1	0	1	0	1	0	-2.29532
tr|J3KNP0|J3KNP0_HUMAN	RAB11FIP1	Rab11 family-interacting protein 1 OS=Homo sapiens GN=RAB11FIP1 PE=4 SV=1	137482	607	0	25	0	0.02	0	0	0	0	1	0	1	0	2	0	-2.29532
tr|J3KSB9|J3KSB9_HUMAN	tr|J3KSB9|J3KSI	Uncharacterized protein (Fragment) OS=Homo sapiens PE=4 SV=1	8877	608	0	25	0	0.39	0	0	0	0	1	0	1	0	1	0	-2.29532
tr|H3BMW5|H3BMW5_HUMAN	DNAJA2	DnaJ homolog subfamily A member 2 (Fragment) OS=Homo sapiens GN=DNAJA2 PE=4 SV=1	15134	611	0	24	0	0.23	0	0	0	0	1	0	1	0	1	0	-2.29532
tr|Q4VAN1|Q4VAN1_HUMAN	BTN1A1	BTN1A1 protein OS=Homo sapiens GN=BTN1A1 PE=2 SV=1	55878	617	0	24	0	0.06	0	0	0	0	1	0	1	0	1	0	-2.29532
tr|Q2TQ40|Q2TQ40_HUMAN	TNFRSF17	B cell maturation antigen transcript variant 4 OS=Homo sapiens GN=TNFRSF17 PE=2 SV=1	15460	618	0	24	0	0.22	0	0	0	0	1	0	1	0	1	0	-2.29532
tr|B4DU06|B4DU06_HUMAN	tr|B4DU06|B4D	Guanylate cyclase OS=Homo sapiens PE=2 SV=1	91756	620	0	24	0	0.04	0	0	0	0	1	0	1	0	1	0	-2.29532
tr|B0QXZ5|B0QXZ5_HUMAN	DNAL4	Dynein light chain 4, axonemal OS=Homo sapiens GN=DNAL4 PE=4 SV=1	7786	621	0	24	0	0.47	0	0	0	0	1	0	1	0	1	0	-2.29532
tr|F5H120|F5H120_HUMAN	FKBP4	Peptidyl-prolyl cis-trans isomerase FKBP4, N-terminally processed OS=Homo sapiens GN=FKBP4 PE=2 SV=1	5528	623	0	24	0	0.06	0	0	0	0	1	0	1	0	1	0	-2.29532
tr|B4DN13|B4DN13_HUMAN	tr|B4DN13|B4D	cDNA FLJ59136, highly similar to Transmembrane 9 superfamily protein member 3 OS=Homo sapiens PE=2 SV=1	29629	624	0	23	0	0.11	0	0	0	0	1	0	1	0	1	0	-2.29532
tr|K7EPB5|K7EPB5_HUMAN	ELAVL3	ELAV-like protein 3 (Fragment) OS=Homo sapiens GN=ELAVL3 PE=4 SV=1	9728	625	0	23	0	0.36	0	0	0	0	1	0	1	0	1	0	-2.29532
tr|B4DFE4|B4DFE4_HUMAN	tr|B4DFE4|B4D	cDNA FLJ59618, highly similar to Exosome component 10 OS=Homo sapiens PE=2 SV=1	61733	627	0	23	0	0.05	0	0	0	0	1	0	1	0	1	0	-2.29532
sp|Q9Y5P6|GMPPB_HUMAN	GMPPB	Mannose-1-phosphate guanyltransferase beta OS=Homo sapiens GN=GMPPB PE=1 SV=2	40379	629	0	23	0	0.08	0	0	0	0	1	0	1	0	1	0	-2.29532
tr|B3KPR5|B3KPR5_HUMAN	tr|B3KPR5|B3K	cDNA FLJ32094 fis, clone OCBBF2000986, highly similar to Homo sapiens elongation factor Tu GTP binding domain containing 1, transcript v	97046	633	0	22	0	0.03	0	0	0	0	1	0	1	0	1	0	-2.29532
tr|B1ALA9|B1ALA9_HUMAN	PRPS1	Phosphoribosyl pyrophosphate synthetase 1 OS=Homo sapiens GN=PRPS1 PE=3 SV=1	24468	638	0	22	0	0.14	0	0	0	0	1	0	1	0	2	0	-2.29532
tr|I0BW36|I0BW36_HUMAN	HLA-A	MHC class I antigen (Fragment) OS=Homo sapiens GN=HLA-A PE=4 SV=1	8588	639	0	22	0	0.16	0	0	0	0	1	0	1	0	2	0	-2.29532
tr|E7ET15|E7ET15_HUMAN	U2SURP	U2 snRNP-associated SURP motif-containing protein OS=Homo sapiens GN=U2SURP PE=2 SV=1	118631	647	0	22	0	0.03	0	0	0	0	1	0	1	0	2	0	-2.29532
sp|Q96EK5|KBP_HUMAN	KIAA1279	KIF1-binding protein OS=Homo sapiens GN=KIAA1279 PE=1 SV=1	72453	649	0	22	0	0.05	0	0	0	0	1	0	1	0	1	0	-2.29532
tr|Q96I16|Q96I16_HUMAN	tr|Q96I16|Q96I	EIF2S2 protein OS=Homo sapiens PE=2 SV=1	24902	654	0	22	0	0.09	0	0	0	0	1	0	1	0	1	0	-2.29532
tr|C9J935|C9J935_HUMAN	SETD9	SET domain-containing protein 9 (Fragment) OS=Homo sapiens GN=SETD9 PE=2 SV=1	5515	660	0	21	0	0.65	0	0	0	0	1	0	1	0	1	0	-2.29532
tr|F1T0A5|F1T0A5_HUMAN	PRPF31	PRP31 pre-mRNA processing factor 31 homolog (Yeast), isoform CRA_a OS=Homo sapiens GN=PRPF31 PE=2 SV=1	55649	661	0	21	0	0.06	0	0	0	0	1	0	1	0	2	0	-2.29532
tr|B3KRG8|B3KRG8_HUMAN	tr|B3KRG8|B3K	Lipase OS=Homo sapiens PE=2 SV=1	45736	662	0	21	0	0.07	0	0	0	0	1	0	1	0	1	0	-2.29532
tr|J3KN69|J3KN69_HUMAN	NCEH1	Arylacetamide deacetylase-like 1 OS=Homo sapiens GN=NCEH1 PE=4 SV=1	49431	663	0	21	0	0.07	0	0	0	0	1	0	1	0	1	0	-2.29532
tr|J3QT54|J3QT54_HUMAN	CPSF7	Cleavage and polyadenylation-specificity factor subunit 7 (Fragment) OS=Homo sapiens GN=CPSF7 PE=4 SV=1	22762	665	0	21	0	0.15	0	0	0	0	1	0	1	0	1	0	-2.29532
tr|G0ZJH6|G0ZJH6_HUMAN	Ube4B	UFD2A-III/UBE4B-III splice isoform OS=Homo sapiens GN=Ube4B PE=2 SV=1	152890	667	0	21	0	0.02	0	0	0	0	1	0	1	0	1	0	-2.29532
tr|B4DK80|B4DK80_HUMAN	TOP3A	DNA topoisomerase OS=Homo sapiens GN=TOP3A PE=2 SV=1	103082	668	0	20	0	0.03	0	0	0	0	1	0	1	0	1	0	-2.29532
tr|A0AVG7|A0AVG7_HUMAN	BCAS1	Breast carcinoma amplified sequence 1 OS=Homo sapiens GN=BCAS1 PE=2 SV=1	61929	670	0	20	0	0.05	0	0	0	0	1	0	1	0	1	0	-2.29532
tr|H0YJL6|H0YJL6_HUMAN	EVL	Ena/VASP-like protein (Fragment) OS=Homo sapiens GN=EVL PE=4 SV=1	10347	671	0	20	0	0.34	0	0	0	0	1	0	1	0	1	0	-2.29532
tr|D6RF05|D6RF05_HUMAN	HARS	Histidine--tRNA ligase, cytoplasmic OS=Homo sapiens GN=HARS PE=2 SV=1	6740	672	0	20	0	0.07	0	0	0	0	1	0	1	0	1	0	-2.29532
tr|C9JHK9|C9JHK9_HUMAN	ABCF2	ATP-binding cassette sub-family F member 2 (Fragment) OS=Homo sapiens GN=ABCF2 PE=2 SV=1	27027	673	0	20	0	0.05	0	0	0	0	1	0	1	0	2	0	-2.29532
sp|P55212|CASP6_HUMAN	CASP6	Caspase-6 OS=Homo sapiens GN=CASP6 PE=1 SV=2	33859	674	0	20	0	0.1	0	0	0	0	1	0	1	0	1	0	-2.29532
tr|B4DE48|B4DE48_HUMAN	tr|B4DE48|B4D	cDNA FLJ53824, highly similar to Sorting nexin-17 OS=Homo sapiens PE=2 SV=1	50009	677	0	20	0	0.07	0	0	0	0	1	0	1	0	1	0	-2.29532
sp|O95159|ZFPL1_HUMAN	ZFPL1	Zinc finger protein-like 1 OS=Homo sapiens GN=ZFPL1 PE=1 SV=2	34833	678	0	20	0	0.1	0	0	0	0	1	0	1	0	1	0	-2.29532
tr|Q8WUM5|Q8WUM5_HUMAN	GEMIN4	Gem (Nuclear organelle) associated protein 4 OS=Homo sapiens GN=GEMIN4 PE=2 SV=1	121732	709	0	17	0	0.03	0	0	0	0	1	0	1	0	2	0	-2.29532
tr|H0YID6|H0YID6_HUMAN	KRT79	Keratin, type II cytoskeletal 79 (Fragment) OS=Homo sapiens GN=KRT79 PE=3 SV=1	13441	11	0	64	0	4.79	0	0	2	0	2	0	2	0	3	0	-3.03262
tr|Q8WYB3|Q8WYB3_HUMAN	tr|Q8WYB3|Q8	Beta II spectrin-short isoform (Fragment) OS=Homo sapiens PE=2 SV=1	7547	49	0	79	0	0.22	0	0	2	0	2	0	2	0	2	0	-3.03262
tr|B4DKJ0|B4DKJ0_HUMAN	tr|B4DKJ0|B4D	cDNA FLJ58539, highly similar to Keratin, type II cytoskeletal 4 OS=Homo sapiens PE=2 SV=1	55657	105	0	61	0	0.7	0	0	2	0	2	0	2	0	4	0	-3.03262
tr|B4DRY6|B4DRY6_HUMAN	tr|B4DRY6|B4D	cDNA FLJ57182, highly similar to Paxillin OS=Homo sapiens PE=2 SV=1	36791	159	0	73	0	0.28	0	0	1	0	2	0	2	0	2	0	-3.03262
tr|Q5JVP3|Q5JVP3_HUMAN	PTGR1	Leukotriene B4 12-hydroxydehydrogenase, isoform CRA_d OS=Homo sapiens GN=PTGR1 PE=4 SV=1	19441	190	0	113	0	0.38	0	0	2	0	2	0	2	0	2	0	-3.03262
tr|D6RAS7|D6RAS7_HUMAN	RPS3A	40S ribosomal protein S3a (Fragment) OS=Homo sapiens GN=RPS3A PE=2 SV=1	14608	223	0	72	0	0.44	0	0	1	0	1	0	2	0	3	0	-3.03262
tr|Q05DV5|Q05DV5_HUMAN	ZC3HAV1	ZC3HAV1 protein (Fragment) OS=Homo sapiens GN=ZC3HAV1 PE=2 SV=1	49964	224	0	92	0	0.14	0	0	2	0	2	0	2	0	2	0	-3.03262
sp|Q9UQ35|SRRM2_HUMAN	SRRM2	Serine/arginine repetitive matrix protein 2 OS=Homo sapiens GN=SRRM2 PE=1 SV=2	300179	247	0	78	0	0.02	0	0	1	0	2	0	2	0	5	0	-3.03262
tr|B3KXA9|B3KXA9_HUMAN	tr|B3KXA9|B3K	cDNA FLJ45065 fis, clone BRAWH3024506, highly similar to Leucyl-tRNA synthetase, cytoplasmic (EC 6.1.1.4) OS=Homo sapiens PE=2 SV=1	38658	257	0	49	0	0.1	0	0	1	0	2	0	2	0	2	0	-3.03262
tr|E9PG15|E9PG15_HUMAN	YWHAQ	14-3-3 protein theta (Fragment) OS=Homo sapiens GN=YWHAQ PE=2 SV=1	17209	258	0	73	0	0.4	0	0	2	0	2	0	2	0	2	0	-3.03262
tr|Q2QD09|Q2QD09_HUMAN	tr|Q2QD09|Q2C	Triosephosphate isomerase (Fragment) OS=Homo sapiens PE=2 SV=1	27211	267	0	71	0	0.26	0	0	1	0	2	0	2	0	2	0	-3.03262
tr|F5GYR3|F5GYR3_HUMAN	NOP2	Putative ribosomal RNA methyltransferase NOP2 (Fragment) OS=Homo sapiens GN=NOP2 PE=2 SV=1	30665	296	0	28	0	0.15	0	0	1	0	1	0	2	0	3	0	-3.03262
sp|Q13867|BLMH_HUMAN	BLMH	Bleomycin hydrolase OS=Homo sapiens GN=BLMH PE=1 SV=1	53155	332	0	52	0	0.2	0	0	1	0	3	0	2	0	4	0	-3.03262
tr|G3V180|G3V180_HUMAN	DPP3	Dipeptidyl peptidase 3 OS=Homo sapiens GN=DPP3 PE=2 SV=1	84666	335	0	52	0	0.08	0	0	1	0	2	0	2	0	2	0	-3.03262
tr|D3DP78|D3DP78_HUMAN	DARS	Aspartyl-tRNA synthetase, isoform CRA_b OS=Homo sapiens GN=DARS PE=2 SV=1	53829	344	0	51	0	0.06	0	0	1	0	1	0	2	0	3	0	-3.03262
tr|E5RHF2|E5RHF2_HUMAN	COPS5	COP9 signalosome complex subunit 5 (Fragment) OS=Homo sapiens GN=COPS5 PE=2 SV=1	16655	346	0	51	0	0.45	0	0	0	0	2	0	2	0	2	0	-3.03262
tr|Q6NZ52|Q6NZ52_HUMAN	RPL27A	Ribosomal protein L27a OS=Homo sapiens GN=RPL27A PE=2 SV=1	16582	348	0	50	0	0.2	0	0	1	0	1	0	2	0	2	0	-3.03262

tr|B7Z8J4|B7Z8J4_HUMAN	NMT1	Glycylpeptide N-tetradecanoyltransferase 1 OS=Homo sapiens GN=NMT1 PE=2 SV=1	15265	350	0	50	0	0.49	0	0	1	0	2	0	2	0	2	0	-3.03262
tr|H0Y9H1|H0Y9H1_HUMAN	CANX	Calnexin (Fragment) OS=Homo sapiens GN=CANX PE=3 SV=1	21748	352	0	49	0	0.39	0	0	1	0	2	0	2	0	3	0	-3.03262
tr|H7C314|H7C314_HUMAN	CTTN	Src substrate cortactin (Fragment) OS=Homo sapiens GN=CTTN PE=4 SV=1	25136	378	0	46	0	0.11	0	0	1	0	2	0	2	0	2	0	-3.03262
tr|B2RCT6|B2RCT6_HUMAN	tr|B2RCT6|B2R(	cDNA, FLJ96276, highly similar to Homo sapiens G1 to S phase transition 1 (GSPT1), mRNA OS=Homo sapiens PE=2 SV=1	56320	391	0	44	0	0.12	0	0	1	0	2	0	2	0	2	0	-3.03262
tr|Q58FF4|Q58FF4_HUMAN	HSP90Bf	Heat shock protein 90Bf OS=Homo sapiens GN=HSP90Bf PE=1 SV=1	41833	407	0	43	0	0.16	0	0	1	0	2	0	2	0	2	0	-3.03262
tr|B2R7Y3|B2R7Y3_HUMAN	tr|B2R7Y3|B2R7	cDNA, FLJ93657, highly similar to Homo sapiens ribosomal protein L3-like (RPL3L), mRNA OS=Homo sapiens PE=2 SV=1	46581	420	0	42	0	0.07	0	0	1	0	1	0	2	0	3	0	-3.03262
tr|A5D904|A5D904_HUMAN	RPS9	RPS9 protein (Fragment) OS=Homo sapiens GN=RPS9 PE=2 SV=1	13210	434	0	40	0	0.58	0	0	1	0	2	0	2	0	2	0	-3.03262
sp|P07737|PROF1_HUMAN	PFN1	Profilin-1 OS=Homo sapiens GN=PFN1 PE=1 SV=2	15216	436	0	39	0	0.5	0	0	1	0	2	0	2	0	2	0	-3.03262
tr|H0YDE4|H0YDE4_HUMAN	PRMT1	Protein arginine N-methyltransferase 1 (Fragment) OS=Homo sapiens GN=PRMT1 PE=4 SV=1	27345	437	0	39	0	0.26	0	0	1	0	2	0	2	0	2	0	-3.03262
sp|O00116|ADAS_HUMAN	AGPS	Alkyldihydroxyacetonephosphate synthase, peroxisomal OS=Homo sapiens GN=AGPS PE=1 SV=1	73664	448	0	38	0	0.09	0	0	1	0	2	0	2	0	3	0	-3.03262
tr|B2R4P2|B2R4P2_HUMAN	tr|B2R4P2|B2R4	cDNA, FLJ92164, highly similar to Homo sapiens peroxiredoxin 1 (PRDX1), mRNA OS=Homo sapiens PE=2 SV=1	22414	481	0	36	0	0.32	0	0	0	0	2	0	2	0	2	0	-3.03262
sp|Q14432|PDE3A_HUMAN	PDE3A	cGMP-inhibited 3~,5~-cyclic phosphodiesterase A OS=Homo sapiens GN=PDE3A PE=1 SV=3	126440	493	0	35	0	0.05	0	0	1	0	2	0	2	0	2	0	-3.03262
tr|A0PJ76|A0PJ76_HUMAN	NCAPD2	NCAPD2 protein (Fragment) OS=Homo sapiens GN=NCAPD2 PE=2 SV=1	44957	494	0	34	0	0.15	0	0	0	0	2	0	2	0	2	0	-3.03262
tr|C1KES1|C1KES1_HUMAN	LRRC16B	LRRC16B (Fragment) OS=Homo sapiens GN=LRRC16B PE=4 SV=1	4125	530	0	31	0	0.94	0	0	0	0	1	0	2	0	2	0	-3.03262
tr|B2RD27|B2RD27_HUMAN	tr|B2RD27|B2R	cDNA, FLJ96428, highly similar to Homo sapiens proteasome (prosome, macropain) 26S subunit, non-ATPase, 7 (Mov34 homolog) (PSMD7),	37046	550	0	30	0	0.19	0	0	0	0	2	0	2	0	2	0	-3.03262
tr|B4DH52|B4DH52_HUMAN	tr|B4DH52|B4D	cDNA FLJ55916, highly similar to General transcription factor II-I OS=Homo sapiens PE=2 SV=1	112359	554	0	29	0	0.06	0	0	0	0	2	0	2	0	3	0	-3.03262
sp|O15131|IMA5_HUMAN	KPNA5	Importin subunit alpha-6 OS=Homo sapiens GN=KPNA5 PE=1 SV=2	61052	573	0	28	0	0.11	0	0	0	0	2	0	2	0	2	0	-3.03262
tr|D3DSQ0|D3DSQ0_HUMAN	PCM1	Pericentriolar material 1, isoform CRA_a OS=Homo sapiens GN=PCM1 PE=2 SV=1	230041	619	0	24	0	0.01	0	0	0	0	1	0	2	0	3	0	-3.03262
sp|P25490|TYY1_HUMAN	YY1	Transcriptional repressor protein YY1 OS=Homo sapiens GN=YY1 PE=1 SV=2	45141	630	0	23	0	0.07	0	0	0	0	1	0	2	0	2	0	-3.03262
tr|I0BW39|I0BW39_HUMAN	HLA-A	MHC class I antigen (Fragment) OS=Homo sapiens GN=HLA-A PE=4 SV=1	8492	640	0	22	0	0.16	0	0	0	0	1	0	2	0	2	0	-3.03262
tr|B1AHM2|B1AHM2_HUMAN	DDX17	DEAD (Asp-Glu-Ala-Asp) box polypeptide 17 (Fragment) OS=Homo sapiens GN=DDX17 PE=2 SV=1	19700	166	0	64	0	0.37	0	0	1	0	3	0	3	0	3	0	-3.51838
tr|Q6ZVC6|Q6ZVC6_HUMAN	tr|Q6ZVC6|Q6Z	cDNA FLJ42761 fis, clone BRAWH3002574, highly similar to Calpain 2, large OS=Homo sapiens PE=2 SV=1	24572	173	0	82	0	0.22	0	0	2	0	2	0	3	0	3	0	-3.51838
tr|C9JI87|C9JI87_HUMAN	VDAC1	Voltage-dependent anion-selective channel protein 1 (Fragment) OS=Homo sapiens GN=VDAC1 PE=2 SV=1	20418	250	0	78	0	0.36	0	0	1	0	3	0	3	0	3	0	-3.51838
tr|J3QST3|J3QST3_HUMAN	KRT3	Keratin, type II cytoskeletal 3 OS=Homo sapiens GN=KRT3 PE=3 SV=1	64636	255	0	76	0	0.16	0	0	3	0	3	0	3	0	6	0	-3.51838
tr|Q5T3R7|Q5T3R7_HUMAN	GTPBP4	Nucleolar GTP-binding protein 1 (Fragment) OS=Homo sapiens GN=GTPBP4 PE=4 SV=1	16641	264	0	72	0	0.14	0	0	2	0	3	0	3	0	3	0	-3.51838
tr|Q5JQZ9|Q5JQZ9_HUMAN	CDC2L6	Cell division cycle 2-like 6 (CDK8-like) (Fragment) OS=Homo sapiens GN=CDC2L6 PE=4 SV=1	29552	285	0	65	0	0.24	0	0	2	0	2	0	3	0	3	0	-3.51838
tr|Q0VGL3|Q0VGL3_HUMAN	RPL13A	RPL13A protein OS=Homo sapiens GN=RPL13A PE=2 SV=1	16706	300	0	59	0	0.74	0	0	3	0	3	0	3	0	3	0	-3.51838
tr|B2R6J4|B2R6J4_HUMAN	tr|B2R6J4|B2R6	cDNA, FLJ92975, highly similar to Homo sapiens nucleosome assembly protein 1-like 4 (NAP1L4), mRNA OS=Homo sapiens PE=2 SV=1	42940	417	0	42	0	0.16	0	0	0	0	2	0	3	0	3	0	-3.51838
sp|Q9NV06|DCA13_HUMAN	DCAF13	DDB1- and CUL4-associated factor 13 OS=Homo sapiens GN=DCAF13 PE=1 SV=2	51996	435	0	39	0	0.2	0	0	1	0	3	0	3	0	6	0	-3.51838
tr|L0R5A1|L0R5A1_HUMAN	CSF2RB	Alternative protein CSF2RB OS=Homo sapiens GN=CSF2RB PE=4 SV=1	11752	486	0	36	0	0.29	0	0	1	0	1	0	3	0	5	0	-3.51838
tr|H7BYX5|H7BYX5_HUMAN	FSCN1	Fascin (Fragment) OS=Homo sapiens GN=FSCN1 PE=4 SV=1	15688	53	0	188	0	1.07	0	0	4	0	5	0	4	0	7	0	-3.88129
tr|F5GZI8|F5GZI8_HUMAN	TCP1	T-complex protein 1 subunit alpha OS=Homo sapiens GN=TCP1 PE=2 SV=1	10411	197	0	64	0	0.3	0	0	1	0	2	0	4	0	4	0	-3.88129
tr|E9PKU4|E9PKU4_HUMAN	RPL8	60S ribosomal protein L8 (Fragment) OS=Homo sapiens GN=RPL8 PE=2 SV=1	25806	231	0	90	0	0.28	0	0	2	0	2	0	4	0	5	0	-3.88129
sp|Q14CN4|K2C72_HUMAN	KRT72	Keratin, type II cytoskeletal 72 OS=Homo sapiens GN=KRT72 PE=1 SV=2	56470	372	0	47	0	0.12	0	0	2	0	2	0	4	0	5	0	-3.88129
tr|Q53RS3|Q53RS3_HUMAN	DDEF2	Putative uncharacterized protein DDEF2 (Fragment) OS=Homo sapiens GN=DDEF2 PE=2 SV=1	70046	441	0	39	0	0.05	0	0	1	0	1	0	4	0	4	0	-3.88129
sp|A2RTY3|CQ066_HUMAN	C17orf66	Uncharacterized protein C17orf66 OS=Homo sapiens GN=C17orf66 PE=2 SV=2	66437	477	0	36	0	0.05	0	0	0	0	1	0	4	0	8	0	-3.88129
tr|K7ENW6|K7ENW6_HUMAN	KRT16	Keratin, type I cytoskeletal 16 (Fragment) OS=Homo sapiens GN=KRT16 PE=4 SV=1	18735	62	0	109	0	1.38	0	0	3	0	4	0	5	0	5	0	-4.17113
tr|B3KY17|B3KY17_HUMAN	ATP2A1	Sarcoplasmic/endoplasmic reticulum calcium ATPase 1 OS=Homo sapiens GN=ATP2A1 PE=2 SV=1	96392	149	0	86	0	0.32	0	0	2	0	4	0	5	0	5	0	-4.17113
tr|E9PHF7|E9PHF7_HUMAN	MCCC1	Methylcrotonoyl-CoA carboxylase subunit alpha, mitochondrial OS=Homo sapiens GN=MCCC1 PE=2 SV=1	68745	102	0	214	0	0.83	0	0	7	0	13	0	11	0	16	0	-5.23729
sp|P05165|PCCA_HUMAN	PCCA	Propionyl-CoA carboxylase alpha chain, mitochondrial OS=Homo sapiens GN=PCCA PE=1 SV=4	80635	99	0	216	0	0.49	0	0	6	0	10	0	13	0	13	0	-5.46929
tr|B3KPL5|B3KPL5_HUMAN	tr|B3KPL5|B3KP	cDNA FLJ31917 fis, clone NT2RP7004925, highly similar to Sprouty-related, EVH1 domain-containing protein 2 OS=Homo sapiens PE=2 SV=1	48711	657	0	21	0	0.07	0	0	0	0	1	0	16	0	51	0	-5.75981
tr|B2ZZ90|B2ZZ90_HUMAN	ACACA	Acetyl-Coenzyme A carboxylase alpha OS=Homo sapiens GN=ACACA PE=2 SV=1	267095	31	0	628	0	0.44	0	0	25	0	30	0	38	0	46	0	-6.98967

Accession	GS	Name	Pro_Ma	04_prot	09_prot	04_pr	09_prot_score	04_emP	09_emPAI	emPAI ra	04_No>I	09_No>I	04_No>H	09_No>H	04_No>2	09_No>2	04_No=A	09_No=A	Rsc
**#09**	**Hela live unsyn MLN-2_2µM**																		
#04	Hela live syn NP_2µM																		
sp|Q8IWQ3|BRSK2_H	BRSK2	Serine/threonine-protein kinase BRSK2 OS=Homo sapiens GN=BRSK2 PE=1 SV=3	82209	0	1207	0	24	0	0.04	#DIV/0!	0	0	0	1	0	1	0	1	0.736677
sp|P28482|MK01_HU	MAPK1 (ERK2)	Mitogen-activated protein kinase 1 OS=Homo sapiens GN=MAPK1 PE=1 SV=3	41762	0	859	0	45	0	0.06	#DIV/0!	0	1	0	1	0	1	0	1	0.736677
sp|Q16539|MK14_HU	MAPK14 (p38 alpha)	Mitogen-activated protein kinase 14 OS=Homo sapiens GN=MAPK14 PE=1 SV=3	41495	0	1250	0	22	0	0.1	#DIV/0!	0	0	0	1	0	1	0	2	0.736677
sp|Q8TD08|MK15_HU	MAPK15	Mitogen-activated protein kinase 15 OS=Homo sapiens GN=MAPK15 PE=1 SV=1	60251	0	858	0	45	0	0.05	#DIV/0!	0	1	0	1	0	1	0	2	0.736677
sp|Q13976|KGP1_HU	PRKG1	cGMP-dependent protein kinase 1 OS=Homo sapiens GN=PRKG1 PE=1 SV=3	76943	0	1055	0	33	0	0.04	#DIV/0!	0	1	0	1	0	1	0	2	0.736677
sp|Q9Y6E0|STK24_HU	STK24	Serine/threonine-protein kinase 24 OS=Homo sapiens GN=STK24 PE=1 SV=1	49562	0	1006	0	36	0	0.08	#DIV/0!	0	1	0	1	0	1	0	1	0.736677
sp|O14578|CTRO_HU	CIT (STK21)	Citron Rho-interacting kinase OS=Homo sapiens GN=CIT PE=1 SV=2	233339	0	1185	0	26	0	0.02	#DIV/0!	0	0	0	1	0	1	0	2	0.736677
sp|P17612|KAPCA_HU	PRKACA (PKA-C alpha)	cAMP-dependent protein kinase catalytic subunit alpha OS=Homo sapiens GN=PRKACA P	40678	0	1163	0	27	0	0.08	#DIV/0!	0	0	0	1	0	2	0	3	1.473823
sp|Q15418|KS6A1_HU	RPS6KA1 (S6K-alpha-1,F	Ribosomal protein S6 kinase alpha-1 OS=Homo sapiens GN=RPS6KA1 PE=1 SV=2	83070	0	662	0	68	0	0.15	#DIV/0!	0	2	0	3	0	3	0	3	1.95943
sp|P51812|KS6A3_HU	RPS6KA3 (S6K-alpha-3,F	Ribosomal protein S6 kinase alpha-3 OS=Homo sapiens GN=RPS6KA3 PE=1 SV=1	84025	0	585	0	73	0	0.13	#DIV/0!	0	1	0	2	0	2	0	2	1.473823
sp|Q9H4A3|WNK1_HU	WNK1	Serine/threonine-protein kinase WNK1 OS=Homo sapiens GN=WNK1 PE=1 SV=2	251552	0	647	0	70	0	0.03	#DIV/0!	0	2	0	2	0	2	0	3	1.473823
sp|P19784|CSK22_HU	CSNK2A2 (CK2-alpha-2)	Casein kinase II subunit alpha~ OS=Homo sapiens GN=CSNK2A2 PE=1 SV=1	41358	0	714	0	60	0	0.12	#DIV/0!	0	1	0	1	0	1	0	1	0.736677
sp|Q9BVS4|RIOK2_HU	RIOK2	Serine/threonine-protein kinase RIO2 OS=Homo sapiens GN=RIOK2 PE=1 SV=2	63699	0	1114	0	30	0	0.06	#DIV/0!	0	0	0	1	0	1	0	1	0.736677
sp|P78527|PRKDC_HU	PRKDC	DNA-dependent protein kinase catalytic subunit OS=Homo sapiens GN=PRKDC PE=1 SV=	473749	83	75	241	542	0.07	0.19	2.71429	7	16	10	25	11	27	17	32	0.410632
sp|P12277|KCRB_HUN	CKB	Creatine kinase B-type OS=Homo sapiens GN=CKB PE=1 SV=1	42902	214	282	97	187	0.18	0.68	3.77778	2	5	2	7	2	9	2	9	1.078507
sp|P00558|PGK1_HUN	PGK1	Phosphoglycerate kinase 1 OS=Homo sapiens GN=PGK1 PE=1 SV=3	44985	134	62	161	621	0.76	1.9	2.5	5	13	8	16	9	17	9	17	0.033034
tr|J3KNX3|J3KNX3_HU	PFKM	6-phosphofructokinase OS=Homo sapiens GN=PFKM PE=3 SV=1	94107	0	400	0	132	0	0.11	#DIV/0!	0	2	0	3	0	4	0	4	2.32218
sp|P21108|PRPS3_HU	PRPS1L1	Ribose-phosphate pyrophosphokinase 3 OS=Homo sapiens GN=PRPS1L1 PE=1 SV=2	35387	0	997	0	36	0	0.09	#DIV/0!	0	1	0	1	0	2	0	2	1.473823
sp|Q53H12|AGK_HUN	AGK	Acylglycerol kinase, mitochondrial OS=Homo sapiens GN=AGK PE=1 SV=2	47564	0	871	0	44	0	0.07	#DIV/0!	0	1	0	1	0	1	0	1	0.736677
sp|P55263|ADK_HUM	ADK	Adenosine kinase OS=Homo sapiens GN=ADK PE=1 SV=2	40919	538	1029	30	34	0.08	0.18	2.25	0	0	1	2	1	3	1	4	0.374143
sp|P40763|STAT3_HU	STAT3	Signal transducer and activator of transcription 3 OS=Homo sapiens GN=STAT3 PE=1 SV=	88810	0	421	0	123	0	0.16	#DIV/0!	0	2	0	4	0	4	0	5	2.32218
sp|P67775|PP2AA_HU	PPP2CA	Serine/threonine-protein phosphatase 2A catalytic subunit alpha isoform OS=Homo sapi	36142	0	490	0	101	0	0.39	#DIV/0!	0	2	0	3	0	4	0	4	2.32218
sp|P62140|PP1B_HUN	PPP1CB	Serine/threonine-protein phosphatase PP1-beta catalytic subunit OS=Homo sapiens GN=	37961	345	395	51	71	0.24	0.58	2.41667	1	2	1	4	1	4	1	5	0.736894
sp|O00743|PPP6_HUN	PPP6C	Serine/threonine-protein phosphatase 6 catalytic subunit OS=Homo sapiens GN=PPP6C I	35806	0	1107	0	30	0	0.18	#DIV/0!	0	0	0	1	0	1	0	1	0.736677
tr|J3QSX4|J3QSX4_HU	BUB3	Mitotic checkpoint protein BUB3 OS=Homo sapiens GN=BUB3 PE=4 SV=1	16498	681	512	20	67	0.2	0.52	2.6	0	1	1	3	0	3	1	3	1.95943
sp|P53779|MK10_HU	MAPK10 (JNK3)	Mitogen-activated protein kinase 10 OS=Homo sapiens GN=MAPK10 PE=1 SV=2	53179	0	1313	0	15	0	0.09	#DIV/0!	0	0	0	1	0	0	0	1	-0.84847
sp|Q8N4C8|MINK1_H	MINK1	Misshapen-like kinase 1 OS=Homo sapiens GN=MINK1 PE=1 SV=2	150413	0	892	0	43	0	0.02	#DIV/0!	0	0	0	0	0	0	0	2	-0.84847
sp|Q13418|ILK_HUM	ILK	Integrin-linked protein kinase OS=Homo sapiens GN=ILK PE=1 SV=2	51899	421	566	41	71	0.07	0.2	2.85714	1	1	1	2	1	2	1	2	-0.11146
sp|P60891|PRPS1_HU	PRPS1	Ribose-phosphate pyrophosphokinase 1 OS=Homo sapiens GN=PRPS1 PE=1 SV=2	35325	638	910	22	41	0.14	0.39	2.78571	0	1	1	2	1	2	2	2	-0.11146
sp|P14618|KPYM_HU	PKM	Pyruvate kinase isozymes M1/M2 OS=Homo sapiens GN=PKM PE=1 SV=4	58470	8	7	1820	2837	5.79	18.2	3.14335	47	63	58	84	60	90	66	97	-0.27077
sp|Q01813|K6PP_HUI	PFKP (PFK-C)	6-phosphofructokinase type C OS=Homo sapiens GN=PFKP PE=1 SV=2	86454	98	74	217	546	0.4	0.68	1.7	5	10	9	15	9	15	10	16	-0.14241
sp|O00764|PDXK_HU	PDXK	Pyridoxal kinase OS=Homo sapiens GN=PDXK PE=1 SV=1	35308	566	1296	29		0.11	0.38	3.45455	0	0	1	2	1	0	1	2	-2.43375
sp|Q05209|PTN12_HU	PTPN12	Tyrosine-protein phosphatase non-receptor type 12 OS=Homo sapiens GN=PTPN12 PE=1	88907	0	771	0	54	0	0.04	#DIV/0!	0	1	0	1	0	1	0	2	0.736677
sp|Q9UKS6|PACN3_H	PACSIN3 (SH3 domain-c	Protein kinase C and casein kinase substrate in neurons protein 3 OS=Homo sapiens GN=	48799	0	903	0	42	0	0.07	#DIV/0!	0	1	0	1	0	1	0	2	0.736677
sp|Q9NYJ8|TAB2_HUI	TAB2	TGF-beta-activated kinase 1 and MAP3K7-binding protein 2 OS=Homo sapiens GN=TAB2	77017	0	754	0	56	0	0.04	#DIV/0!	0	1	0	1	0	1	0	1	0.736677
tr|H0YFG1|H0YFG1_H	EPS8	Epidermal growth factor receptor kinase substrate 8 (Fragment) OS=Homo sapiens GN=E	7139	0	1039	0	34	0	0.5	#DIV/0!	0	0	0	1	0	1	0	1	0.736677
tr|D6RAR6|D6RAR6_H	FASTKD3	FAST kinase domain-containing protein 3 OS=Homo sapiens GN=FASTKD3 PE=2 SV=1	70592	0	684	0	65	0	0.05	#DIV/0!	0	1	0	1	0	1	0	1	0.736677
sp|P29966|MARCS_H	MARCKS	Myristoylated alanine-rich C-kinase substrate OS=Homo sapiens GN=MARCKS PE=1 SV=4	31707	0	374	0	141	0	0.64	#DIV/0!	0	3	0	5	0	5	0	5	2.611867
sp|O15357|SHIP2_HU	INPPL1	Phosphatidylinositol 3,4,5-trisphosphate 5-phosphatase 2 OS=Homo sapiens GN=INPPL1	139311	0	1247	0	22	0	0.02	#DIV/0!	0	0	0	1	0	3	0	3	1.95943
tr|B2R665|B2R665_HU	tr|B2R665|B2R665_HU	cDNA, FLJ92810, highly similar to Homo sapiens protein phosphatase 1G (formerly 2C), m	59889	0	709	0	61	0	0.11	#DIV/0!	0	2	0	2	0	2	0	2	1.473823
tr|H0YJ75|H0YJ75_HU	PPP2R5C	Serine/threonine-protein phosphatase 2A 56 kDa regulatory subunit gamma isoform (Fra	64365	0	1190	0	26	0	0.05	#DIV/0!	0	0	0	1	0	1	0	2	0.736677
tr|A8K3M3|A8K3M3_	PTPN1	Tyrosine-protein phosphatase non-receptor type OS=Homo sapiens GN=PTPN1 PE=2 SV=	50505	0	1066	0	32	0	0.07	#DIV/0!	0	0	0	1	0	1	0	1	0.736677
sp|Q8TAP8|PPR35_HU	PPP1R35	Protein phosphatase 1 regulatory subunit 35 OS=Homo sapiens GN=PPP1R35 PE=1 SV=1	28107	0	834	0	47	0	0.12	#DIV/0!	0	1	0	1	0	1	0	1	0.736677
tr|B4DDF7|B4DDF7_H	tr|B4DDF7|B4DDF7_HU	cDNA FLJ53296, highly similar to Serine/threonine-protein phosphatase 2A 65 kDa regula	64939	443	187	39	267	0.16	0.72	4.5	1	5	3	11	3	13	3	18	1.100437
tr|B4E1G6|B4E1G6_H	GALK1	Galactokinase OS=Homo sapiens GN=GALK1 PE=2 SV=1	45956	679	1153	20	28	0.07	0.07	1	0	0	1	1	0	1	1	1	0.736677
tr|C1PHA2|C1PHA2_H	KIF5B-ALK	Tyrosine-protein kinase receptor OS=Homo sapiens GN=KIF5B-ALK PE=2 SV=1	169283	227	431	91	118	0.04	0.04	1	2	2	2	2	2	2	3	4	-0.84875
tr|H0Y3C5|H0Y3C5_H	HCK	Tyrosine-protein kinase HCK OS=Homo sapiens GN=HCK PE=4 SV=1	59986	628	1023	23	34	0.05	0.05	1	0	1	1	1	1	1	2	1	-0.84861
sp|Q9UEW8|STK39_H	STK39	STE20/SPS1-related proline-alanine-rich protein kinase OS=Homo sapiens GN=STK39 PE=	59950	491	886	35	43	0.05	0.05	1	0	1	1	1	1	1	1	1	-0.84861
tr|E9PF63|E9PF63_HU	ROCK2	Rho-associated protein kinase 2 OS=Homo sapiens GN=ROCK2 PE=2 SV=1	134266	375	854	46	45	0.05	0.04	0.8	1	1	2	1	1	1	3	2	-0.84861
tr|B3KS97|B3KS97_HU	MAP2K2	Mitogen-activated protein kinase kinase 2, isoform CRA_d OS=Homo sapiens GN=MAP2K	23450	384	701	45	29	0.31	0.15	0.48387	1	0	2	1	2	1	2	1	-1.5859
tr|A4QPA9|A4QPA9_I	MAP2K1	Mitogen-activated protein kinase kinase 1 OS=Homo sapiens GN=MAP2K1 PE=2 SV=1	43753	384	1225	45	23	0.31	0.08	0.25806	1	0	2	1	2	1	2	2	-1.5859
sp|P17858|K6PL_HUN	PFKL	6-phosphofructokinase, liver type OS=Homo sapiens GN=PFKL PE=1 SV=6	85762	98	511	88	97	0.4	0.08	0.2	1	1	2	2	2	2	2	3	-0.84875
tr|A6NIT2|A6NIT2_HU	PHKA1	Phosphorylase b kinase regulatory subunit alpha, skeletal muscle isoform OS=Homo sapi	133462	711	0	17	0	0.02	0	0	0	0	1	0	0	0	1	0	-0.84847
tr|H0Y3Y5|H0Y3Y5_HU	PPIP5K1	Inositol hexakisphosphate and diphosphoinositol-pentakisphosphate kinase 1 (Fragment	21913	632	0	22	0	0.15	0	0	0	0	1	0	1	0	2	0	-2.43375
tr|A8K2U2|A8K2U2_H	tr|A8K2U2|A8K2U2_HU	cDNA FLJ75392, highly similar to Homo sapiens hexokinase II (HKII) mRNA OS=Homo sap	103718	599	0	26	0	0.03	0	0	0	0	1	0	1	0	2	0	-2.43375
tr|B2R5T5|B2R5T5_HU	PRKAR1A	Protein kinase, cAMP-dependent, regulatory, type I, alpha (Tissue specific extinguisher 1	43183	533	0	31	0	0.08	0	0	0	0	1	0	1	0	1	0	-2.43375
tr|B4E2K2|B4E2K2_HU	tr|B4E2K2|B4E2K2_HU	cDNA FLJ55665, highly similar to A-kinase anchor protein 2 OS=Homo sapiens PE=2 SV=1	103935	246	0	79	0	0.03	0	0	1	0	1	0	1	0	1	0	-2.43375
tr|Q8IWY7|Q8IWY7_H	TTBK2	Tau-tubulin kinase OS=Homo sapiens GN=TTBK2 PE=2 SV=1	183893	79	0	28	0	0.79	0	0	0	0	1	0	1	0	1	0	-2.43375
tr|H3BRW9|H3BRW9_	MAP2K1	Dual-specificity mitogen-activated protein kinase kinase 1 OS=Homo sapiens GN=MAP2K	24160	384	0	45	0	0.31	0	0	1	0	2	0	2	0	2	0	-3.17104
tr|H0YDU8|H0YDU8_I	PPP5C	Serine/threonine-protein phosphatase (Fragment) OS=Homo sapiens GN=PPP5C PE=3 SV	55743	596	619	26	75	0.06	0.06	1	0	1	1	1	1	1	1	1	-0.84861
tr|J3QT22|J3QT22_HU	PPME1	Protein phosphatase methylesterase 1 OS=Homo sapiens GN=PPME1 PE=3 SV=1	44241	232	1226	90	23	0.07	0.07	1	1	0	1	1	1	1	2	1	-0.84861
tr|B4DDZ8|B4DDZ8_H	tr|B4DDZ8|B4DDZ8_HU	cDNA FLJ55048, highly similar to Serine/threonine-protein phosphatase 5 (EC 3.1.3.16) C	55829	596	0	26	0	0.06	0	0	0	0	1	0	1	0	1	0	-2.43375
tr|B0AZV0|B0AZV0_H	STRAP	Serine-threonine kinase receptor-associated protein OS=Homo sapiens GN=STRAP PE=2	28716	626	286	23	183	0.12	0.37	3.08333	0	3	1	4	1	4	1	4	0.736894
tr|B3KPZ7|B3KPZ7_HU	tr|B3KPZ7|B3KPZ7_HU	cDNA FLJ32517 fis, clone SMINT1000117, highly similar to Pyruvate dehydrogenase (lipo	61329	0	986	0	37	0	0.05	#DIV/0!	0	1	0	1	0	1	0	1	0.736677
sp|O75533|SF3B1_HU	SF3B1	Splicing factor 3B subunit 1 OS=Homo sapiens GN=SF3B1 PE=1 SV=3	146479	0	101	0	438	0	0.52	#DIV/0!	0	11	0	19	0	19	0	23	4.440362
sp|Q92626|PXDN_HU	PXDN	Peroxidasin homolog OS=Homo sapiens GN=PXDN PE=1 SV=2	167793	0	64	0	601	0	0.39	#DIV/0!	0	14	0	18	0	19	0	20	4.440362
tr|E1NZA1|E1NZA1_H	PRIC295	Peroxisome proliferator activated receptor interacting complex protein OS=Homo sapien	294953	0	129	0	357	0	0.19	#DIV/0!	0	9	0	16	0	14	0	17	4.012039
tr|D6REM6|D6REM6_	MATR3	Matrin-3 OS=Homo sapiens GN=MATR3 PE=2 SV=1	88647	0	249	0	209	0	1.21	#DIV/0!	0	3	0	12	0	13	0	14	3.908765
tr|Q6NVW7|Q6NVW7	KPNA2	Importin subunit alpha OS=Homo sapiens GN=KPNA2 PE=2 SV=1	58271	0	223	0	227	0	0.55	#DIV/0!	0	4	0	8	0	12	0	13	3.797553
tr|A8K8U1|A8K8U1_H	tr|A8K8U1|A8K8U1_HU	cDNA FLJ77762, highly similar to Homo sapiens cullin-associated and neddylation-dissoci	137941	0	150	0	323	0	0.26	#DIV/0!	0	9	0	11	0	12	0	15	3.797553
sp|O43776|SYNC_HUI	NARS	Asparagine--tRNA ligase, cytoplasmic OS=Homo sapiens GN=NARS PE=1 SV=1	63758	0	113	0	407	0	0.83	#DIV/0!	0	10	0	12	0	12	0	13	3.797553
tr|B7Z1N6|B7Z1N6_H	tr|B7Z1N6|B7Z1N6_HU	cDNA FLJ52222, highly similar to Fructose-bisphosphate aldolase C (EC4.1.2.13) OS=Hom	35800	0	103	0	432	0	0.42	#DIV/0!	0	9	0	10	0	12	0	14	3.797553
tr|B4DLV7|B4DLV7_H	tr|B4DLV7|B4DLV7_HU	cDNA FLJ60299, highly similar to Rab GDP dissociation inhibitor beta OS=Homo sapiens P	51577	0	168	0	293	0	0.98	#DIV/0!	0	6	0	11	0	11	0	12	3.677079
tr|Q53G71|Q53G71_H	tr|Q53G71|Q53G71_HU	Calreticulin variant (Fragment) OS=Homo sapiens PE=2 SV=1	47061	0	266	0	201	0	0.97	#DIV/0!	0	5	0	12	0	10	0	13	3.545654
sp|Q08J23|NSUN2_HU	NSUN2	tRNA (cytosine(34)-C(5))-methyltransferase OS=Homo sapiens GN=NSUN2 PE=1 SV=2	87214	0	242	0	215	0	0.39	#DIV/0!	0	4	0	9	0	10	0	15	3.545654
tr|B4DT31|B4DT31_H	FUBP1	Far upstream element-binding protein 1 OS=Homo sapiens GN=FUBP1 PE=2 SV=1	69993	0	213	0	237	0	0.44	#DIV/0!	0	4	0	8	0	10	0	10	3.545654
tr|A2A274|A2A274_H	ACO2	Aconitase 2, mitochondrial OS=Homo sapiens GN=ACO2 PE=4 SV=1	88563	0	194	0	260	0	0.39	#DIV/0!	0	4	0	9	0	10	0	11	3.545654
tr|Q0VGA5|Q0VGA5_	SARS	SARS protein OS=Homo sapiens GN=SARS PE=2 SV=1	58883	0	183	0	274	0	0.55	#DIV/0!	0	7	0	8	0	10	0	11	3.545654
sp|Q9UHD8|SEPT9_H	Sep-09	Septin-9 OS=Homo sapiens GN=SEPT9 PE=1 SV=2	65646	0	287	0	183	0	0.34	#DIV/0!	0	3	0	6	0	9	0	10	3.401084
tr|B3KPA6|B3KPA6_H	ACADVL	Acyl-Coenzyme A dehydrogenase, very long chain, isoform CRA_e OS=Homo sapiens GN=	62941	0	279	0	189	0	0.5	#DIV/0!	0	5	0	8	0	9	0	9	3.401084
tr|B4DMF5|B4DMF5_	tr|B4DMF5|B4DMF5_H	Glutamate dehydrogenase OS=Homo sapiens PE=2 SV=1	56910	0	277	0	191	0	0.8	#DIV/0!	0	4	0	8	0	9	0	10	3.401084
tr|B2RDW1|B2RDW1_	RPS27A	Ribosomal protein S27a, isoform CRA_c OS=Homo sapiens GN=RPS27A PE=2 SV=1	18296	0	271	0	196	0	0.66	#DIV/0!	0	5	0	8	0	9	0	10	3.401084
tr|B4DMR3|B4DMR3_	tr|B4DMR3|B4DMR3_I	cDNA FLJ51896, highly similar to Glia-derived nexin OS=Homo sapiens PE=2 SV=1	37305	0	233	0	221	0	1.15	#DIV/0!	0	7	0	9	0	9	0	9	3.401084
tr|Q6NWZ1|Q6NWZ1	CKAP4	CKAP4 protein (Fragment) OS=Homo sapiens GN=CKAP4 PE=2 SV=1	67948	0	151	0	317	0	0.62	#DIV/0!	0	6	0	9	0	9	0	9	3.401084
tr|B7Z8Z8|B7Z8Z8_HU	tr|B7Z8Z8|B7Z8Z8_HU	cDNA FLJ61702, highly similar to 80 kDa MCM3-associated protein OS=Homo sapiens PE	181264	0	1031	0	34	0	0.02	#DIV/0!	0	1	0	1	0	8	0	10	3.240439
sp|Q969N2|PIGT_HUI	PIGT	GPI transamidase component PIG-T OS=Homo sapiens GN=PIGT PE=1 SV=1	66228	0	362	0	144	0	0.47	#DIV/0!	0	4	0	8	0	8	0	10	3.240439
tr|B4E0N9|B4E0N9_H	tr|B4E0N9|B4E0N9_HU	Glutamate dehydrogenase OS=Homo sapiens PE=2 SV=1	54567	0	276	0	191	0	0.51	#DIV/0!	0	4	0	7	0	8	0	9	3.240439
tr|Q71UF1|Q71UF1_H	ACO2	Aconitase OS=Homo sapiens GN=ACO2 PE=2 SV=1	86304	0	269	0	197	0	0.3	#DIV/0!	0	3	0	7	0	8	0	9	3.240439
tr|F6IQG9|F6IQG9_HU	HLA-C	MHC class I antigen (Fragment) OS=Homo sapiens GN=HLA-C PE=3 SV=1	39693	0	914	0	41	0	0.17	#DIV/0!	0	1	0	2	0	7	0	7	3.059686
tr|B7ZMF2|B7ZMF2_I	FANCI	Fanconi anemia, complementation group I OS=Homo sapiens GN=FANCI PE=2 SV=1	150724	0	388	0	136	0	0.16	#DIV/0!	0	4	0	7	0	7	0	9	3.059686
tr|A8K4T6|A8K4T6_HU	tr|A8K4T6|A8K4T6_HU	cDNA FLJ76282, highly similar to Homo sapiens proteasome (prosome, macropain) 26S s	56588	0	350	0	149	0	0.33	#DIV/0!	0	4	0	5	0	7	0	7	3.059686
tr|A8K132|A8K132_H	tr|A8K132|A8K132_HU	cDNA FLJ75476, highly similar to Homo sapiens glutaminase (GLS), mRNA OS=Homo sapi	74317	0	312	0	167	0	0.3	#DIV/0!	0	3	0	6	0	7	0	8	3.059686
sp|P30837|AL1B1_HU	ALDH1B1	Aldehyde dehydrogenase X, mitochondrial OS=Homo sapiens GN=ALDH1B1 PE=1 SV=3	57626	0	283	0	184	0	0.4	#DIV/0!	0	4	0	6	0	7	0	7	3.059686
tr|Q05DF2|Q05DF2_H	SF3A2	SF3A2 protein (Fragment) OS=Homo sapiens GN=SF3A2 PE=2 SV=1	51557	0	228	0	225	0	0.54	#DIV/0!	0	5	0	7	0	7	0	7	3.059686
sp|Q92945|FUBP2_HU	KHSRP	Far upstream element-binding protein 2 OS=Homo sapiens GN=KHSRP PE=1 SV=4	73355	0	225	0	227	0	0.36	#DIV/0!	0	6	0	7	0	7	0	8	3.059686
tr|D3DUW5|D3DUW5	DNM1L	Dynamin 1-like, isoform CRA_c OS=Homo sapiens GN=DNM1L PE=3 SV=1	88442	0	167	0	295	0	0.29	#DIV/0!	0	5	0	7	0	7	0	7	3.059686
tr|F4NBX2|F4NBX2_H	HLA-B	MHC class I antigen (Fragment) OS=Homo sapiens GN=HLA-B PE=3 SV=1	38566	0	572	0	82	0	0.39	#DIV/0!	0	3	0	4	0	6	0	6	2.853055
tr|B4DZC0|B4DZC0_H	tr|B4DZC0|B4DZC0_HU	cDNA FLJ51771, highly similar to SWI/SNF-related matrix-associatedactin-dependent reg	117367	0	475	0	105	0	0.12	#DIV/0!	0	4	0	4	0	6	0	7	2.853055
tr|F6SBX2|F6SBX2_HU	IARS2	Isoleucine--tRNA ligase, mitochondrial OS=Homo sapiens GN=IARS2 PE=2 SV=1	106884	0	433	0	118	0	0.13	#DIV/0!	0	3	0	4	0	6	0	6	2.853055
tr|B7Z4B2|B7Z4B2_HU	tr|B7Z4B2|B7Z4B2_HU	cDNA FLJ56108, highly similar to Puromycin-sensitive aminopeptidase (EC 3.4.11.-) OS=H	93434	0	383	0	122	0	0.28	#DIV/0!	0	4	0	7	0	6	0	9	2.853055
tr|G3XAI2|G3XAI2_HU	LAMB1	Laminin subunit beta-1 OS=Homo sapiens GN=LAMB1 PE=2 SV=1	207703	0	359	0	145	0	0.22	#DIV/0!	0	4	0	6	0	6	0	6	2.853055
tr|Q14400|Q14400_H	GLUD1	GLUD1 protein (Fragment) OS=Homo sapiens GN=GLUD1 PE=2 SV=1	28791	0	315	0	165	0	0.93	#DIV/0!	0	4	0	6	0	6	0	7	2.853055
sp|Q9Y2W1|TR150_H	THRAP3	Thyroid hormone receptor-associated protein 3 OS=Homo sapiens GN=THRAP3 PE=1 SV=	108658	0	314	0	165	0	0.16	#DIV/0!	0	3	0	5	0	6	0	9	2.853055
sp|Q86XP3|DDX42_HU	DDX42	ATP-dependent RNA helicase DDX42 OS=Homo sapiens GN=DDX42 PE=1 SV=1	103197	0	305	0	170	0	0.21	#DIV/0!	0	3	0	6	0	6	0	7	2.853055
tr|A8K5Y7|A8K5Y7_HU	tr|A8K5Y7|A8K5Y7_HU	cDNA FLJ78655, highly similar to Homo sapiens exportin 5 (XPO5), mRNA OS=Homo sapi	138331	0	300	0	175	0	0.22	#DIV/0!	0	4	0	6	0	6	0	6	2.853055
tr|Q53HB3|Q53HB3_I	tr|Q53HB3|Q53HB3_H	Proteasome 26S ATPase subunit 1 variant (Fragment) OS=Homo sapiens PE=2 SV=1	49353	0	275	0	191	0	0.57	#DIV/0!	0	3	0	7	0	6	0	7	2.853055
tr|B3KM58|B3KM58_	tr|B3KM58|B3KM58_H	cDNA FLJ10358 fis, clone NT2RM2001238, highly similar to Glutaminase kidney isoform,	41736	0	264	0	203	0	0.46	#DIV/0!	0	3	0	5	0	6	0	7	2.853055
sp|P09960|LKHA4_HU	LTA4H	Leukotriene A-4 hydrolase OS=Homo sapiens GN=LTA4H PE=1 SV=2	69868	0	203	0	250	0	0.32	#DIV/0!	0	6	0	6	0	6	0	7	2.853055
tr|Q9MY63|Q9MY63_	HLA-Cw	Leucocyte antigen (Fragment) OS=Homo sapiens GN=HLA-Cw PE=3 SV=1	26578	0	1169	0	27	0	0.13	#DIV/0!	0	0	0	1	0	5	0	5	2.611867
tr|C0M122|C0M122_	HLA-Cw	MHC class I antigen (Fragment) OS=Homo sapiens GN=HLA-Cw PE=3 SV=1	20987	0	919	0	41	0	0.35	#DIV/0!	0	1	0	2	0	5	0	5	2.611867
tr|E0WMV4|E0WMV4	HLA-C	MHC class I antigen (Fragment) OS=Homo sapiens GN=HLA-C PE=3 SV=1	31992	0	918	0	41	0	0.22	#DIV/0!	0	1	0	2	0	5	0	5	2.611867
tr|C6K4M2|C6K4M2_	HLA-C	MHC class I antigen (Fragment) OS=Homo sapiens GN=HLA-C PE=3 SV=1	20827	0	917	0	41	0	0.35	#DIV/0!	0	1	0	2	0	5	0	6	2.611867
tr|E0WBR2|E0WBR2_	HLA-C	MHC class I antigen (Fragment) OS=Homo sapiens GN=HLA-C PE=3 SV=1	21018	0	916	0	41	0	0.35	#DIV/0!	0	1	0	2	0	5	0	7	2.611867
tr|C0KK01|C0KK01_H	HLA-C	MHC class I antigen (Fragment) OS=Homo sapiens GN=HLA-C PE=3 SV=1	20940	0	690	0	64	0	0.56	#DIV/0!	0	2	0	3	0	5	0	5	2.611867
tr|B2R774|B2R774_H	tr|B2R774|B2R774_HU	cDNA, FLJ93313, highly similar to Homo sapiens lectin, mannose-binding, 1 (LMAN1), mR	57768	0	579	0	81	0	0.18	#DIV/0!	0	2	0	3	0	5	0	5	2.611867
tr|H0YE29|H0YE29_H	ARHGAP1	Rho GTPase-activating protein 1 (Fragment) OS=Homo sapiens GN=ARHGAP1 PE=4 SV=1	38420	0	529	0	75	0	0.37	#DIV/0!	0	2	0	4	0	5	0	5	2.611867
tr|B4DYY5|B4DYY5_H	tr|B4DYY5|B4DYY5_HU	cDNA FLJ55671, highly similar to Ubiquitin-associated protein 2-like OS=Homo sapiens PI	113662	0	467	0	108	0	0.12	#DIV/0!	0	3	0	4	0	5	0	5	2.611867
sp|Q9NTI5|PDS5B_HU	PDS5B	Sister chromatid cohesion protein PDS5 homolog B OS=Homo sapiens GN=PDS5B PE=1 S	165818	0	447	0	113	0	0.09	#DIV/0!	0	2	0	4	0	5	0	6	2.611867
tr|G3V1L9|G3V1L9_H	TJP1	Tight junction protein 1 (Zona occludens 1), isoform CRA_a OS=Homo sapiens GN=TJP1 P	197681	0	438	0	116	0	0.05	#DIV/0!	0	3	0	3	0	5	0	6	2.611867
tr|K4DI93|K4DI93_HU	CUL4B	Cullin 4B, isoform CRA_e OS=Homo sapiens GN=CUL4B PE=3 SV=1	103318	0	415	0	125	0	0.13	#DIV/0!	0	4	0	4	0	5	0	7	2.611867
sp|P12270|TPR_HUM	TPR	Nucleoprotein TPR OS=Homo sapiens GN=TPR PE=1 SV=3	267530	0	411	0	127	0	0.05	#DIV/0!	0	2	0	4	0	5	0	5	2.611867
tr|Q6EHZ3|Q6EHZ3_H	STRF8	Putative uncharacterized protein STRF8 OS=Homo sapiens GN=STRF8 PE=2 SV=1	41361	0	375	0	141	0	0.36	#DIV/0!	0	3	0	4	0	5	0	5	2.611867
tr|Q6FHX6|Q6FHX6_H	FEN1	Flap endonuclease 1 OS=Homo sapiens GN=FEN1 PE=2 SV=1	42908	0	348	0	151	0	0.35	#DIV/0!	0	2	0	4	0	5	0	5	2.611867
tr|A1KYQ7|A1KYQ7_H	tr|A1KYQ7|A1KYQ7_HU	Cell migration-inducing protein 17 OS=Homo sapiens PE=2 SV=1	106022	0	342	0	154	0	0.2	#DIV/0!	0	3	0	6	0	5	0	7	2.611867
tr|Q5TZZ9|Q5TZZ9_HU	ANXA1	Annexin OS=Homo sapiens GN=ANXA1 PE=2 SV=1	38918	0	340	0	154	0	0.63	#DIV/0!	0	4	0	6	0	5	0	6	2.611867
tr|B2R858|B2R858_HU	tr|B2R858|B2R858_HU	cDNA, FLJ93750, Homo sapiens DEAD (Asp-Glu-Ala-Asp) box polypeptide 6 (DDX6), mRN	53582	0	334	0	109	0	0.34	#DIV/0!	0	3	0	4	0	5	0	5	2.611867
tr|Q5H907|Q5H907_H	MAGED2	Melanoma antigen family D, 2, isoform CRA_d OS=Homo sapiens GN=MAGED2 PE=4 SV=	55932	0	320	0	98	0	0.17	#DIV/0!	0	3	0	3	0	5	0	5	2.611867
tr|Q5TZP7|Q5TZP7_H	APEX1	APEX nuclease (Multifunctional DNA repair enzyme) 1 OS=Homo sapiens GN=APEX1 PE=	35931	0	316	0	164	0	0.55	#DIV/0!	0	3	0	5	0	5	0	6	2.611867
tr|B0AZQ4|B0AZQ4_H	tr|B0AZQ4|B0AZQ4_HU	cDNA, FLJ79494, highly similar to Structural maintenance of chromosome 3 OS=Homo sa	141841	0	301	0	174	0	0.1	#DIV/0!	0	4	0	4	0	5	0	7	2.611867
tr|B2RNR6|B2RNR6_H	ZFR	Zinc finger RNA binding protein OS=Homo sapiens GN=ZFR PE=2 SV=1	118081	0	294	0	180	0	0.15	#DIV/0!	0	5	0	5	0	5	0	6	2.611867
tr|B4DR63|B4DR63_H	PSMC1	26S protease regulatory subunit 4 OS=Homo sapiens GN=PSMC1 PE=2 SV=1	41255	0	274	0	191	0	0.59	#DIV/0!	0	3	0	6	0	5	0	6	2.611867
tr|B0QY89|B0QY89_H	EIF3EIP	Eukaryotic translation initiation factor 3 subunit L OS=Homo sapiens GN=EIF3EIP PE=4 SV	71085	0	258	0	206	0	0.31	#DIV/0!	0	5	0	6	0	5	0	6	2.611867
tr|A8KAQ5|A8KAQ5_I	tr|A8KAQ5|A8KAQ5_H	cDNA FLJ77404, highly similar to Homo sapiens small nuclear ribonucleoprotein 70kDa p	51522	0	791	0	51	0	0.28	#DIV/0!	0	0	0	4	0	4	0	4	2.32218
tr|Q6MZM7|Q6MZM7	DKFZp686O12165	Putative uncharacterized protein DKFZp686O12165 (Fragment) OS=Homo sapiens GN=D	242944	0	786	0	52	0	0.04	#DIV/0!	0	1	0	3	0	4	0	4	2.32218
tr|B4E3M6|B4E3M6_I	tr|B4E3M6|B4E3M6_H	cDNA FLJ55446, highly similar to Superkiller viralicidic activity 2-like 2 (EC 3.6.1.-) OS=Ho	107714	0	768	0	54	0	0.09	#DIV/0!	0	1	0	3	0	4	0	5	2.32218
tr|A0PJ61|A0PJ61_HU	RSL1D1	RSL1D1 protein (Fragment) OS=Homo sapiens GN=RSL1D1 PE=2 SV=1	49688	0	759	0	55	0	0.21	#DIV/0!	0	2	0	3	0	4	0	6	2.32218

tr|F4NBS4|F4NBS4_H	HLA-B	MHC class I antigen (Fragment) OS=Homo sapiens GN=HLA-B PE=3 SV=1	38434	0	706	0	62	0	0.18	#DIV/0!	0	2	0	2	0	4	0	5	2.32218
tr|B4DH17|B4DH17_H	tr|B4DH17|B4DH17_H	cDNA FLJ59298, highly similar to Eukaryotic translation initiation factor 4 gamma 3 OS=H	134921	0	677	0	66	0	0.03	#DIV/0!	0	2	0	2	0	4	0	4	2.32218
sp|Q13619|CUL4A_HU	CUL4A	Cullin-4A OS=Homo sapiens GN=CUL4A PE=1 SV=3	88138	0	610	0	76	0	0.18	#DIV/0!	0	3	0	4	0	4	0	5	2.32218
tr|Q149P0|Q149P0_H	GBF1	GBF1 protein OS=Homo sapiens GN=GBF1 PE=2 SV=1	207938	0	583	0	80	0	0.05	#DIV/0!	0	2	0	3	0	4	0	5	2.32218
sp|Q96I24|FUBP3_HU	FUBP3	Far upstream element-binding protein 3 OS=Homo sapiens GN=FUBP3 PE=1 SV=2	61944	0	575	0	81	0	0.17	#DIV/0!	0	1	0	3	0	4	0	5	2.32218
tr|J3K000|J3K000_HU	PEPD	PEPD protein OS=Homo sapiens GN=PEPD PE=2 SV=1	55345	0	573	0	81	0	0.19	#DIV/0!	0	2	0	3	0	4	0	4	2.32218
tr|Q59FE8|Q59FE8_H	tr|Q59FE8|Q59FE8_HU	Epithelial protein lost in neoplasm beta variant (Fragment) OS=Homo sapiens PE=2 SV=1	86510	0	570	0	82	0	0.2	#DIV/0!	0	2	0	3	0	4	0	4	2.32218
sp|P13667|PDIA4_HU	PDIA4	Protein disulfide-isomerase A4 OS=Homo sapiens GN=PDIA4 PE=1 SV=2	73229	0	554	0	85	0	0.19	#DIV/0!	0	2	0	4	0	4	0	4	2.32218
tr|B4E2K4|B4E2K4_H	tr|B4E2K4|B4E2K4_HU	cDNA FLJ54576, highly similar to Aspartyl/asparaginyl beta-hydroxylase (EC 1.14.11.16) (	83685	0	534	0	91	0	0.17	#DIV/0!	0	3	0	4	0	4	0	4	2.32218
tr|B3KM89|B3KM89_	tr|B3KM89|B3KM89_H	cDNA FLJ10528 fis, clone NT2RP2000943, highly similar to Protein transport protein Sec2	101723	0	515	0	97	0	0.07	#DIV/0!	0	2	0	3	0	4	0	5	2.32218
tr|B2R6H7|B2R6H7_H	tr|B2R6H7|B2R6H7_HU	cDNA, FLJ92955, highly similar to Homo sapiens transportin-SR (TRN-SR), mRNA OS=Hom	105989	0	509	0	98	0	0.1	#DIV/0!	0	3	0	3	0	4	0	4	2.32218
tr|B4DPJ2|B4DPJ2_HU	tr|B4DPJ2|B4DPJ2_HU	Annexin OS=Homo sapiens PE=2 SV=1	46025	0	504	0	98	0	0.23	#DIV/0!	0	2	0	3	0	4	0	4	2.32218
tr|B4DNM8|B4DNM8	tr|B4DNM8|B4DNM8_	cDNA FLJ53395, highly similar to Prolyl 3-hydroxylase 1 (EC 1.14.11.7) OS=Homo sapiens	69572	0	493	0	101	0	0.2	#DIV/0!	0	2	0	4	0	4	0	5	2.32218
tr|B2R825|B2R825_H	tr|B2R825|B2R825_HU	Phosphorylase OS=Homo sapiens PE=2 SV=1	97405	0	462	0	96	0	0.23	#DIV/0!	0	3	0	5	0	4	0	5	2.32218
tr|B4DIT7|B4DIT7_HU	TGM2	Protein-glutamine gamma-glutamyltransferase 2 OS=Homo sapiens GN=TGM2 PE=2 SV=	69632	0	451	0	112	0	0.26	#DIV/0!	0	3	0	5	0	4	0	5	2.32218
tr|Q96F88|Q96F88_H	POP1	Processing of 1, ribonuclease P/MRP subunit (S. cerevisiae) OS=Homo sapiens GN=POP1	116318	0	449	0	113	0	0.12	#DIV/0!	0	3	0	4	0	4	0	4	2.32218
tr|B4DTN7|B4DTN7_H	tr|B4DTN7|B4DTN7_H	cDNA FLJ57198, highly similar to Protein-glutamine gamma-glutamyltransferase 2 (EC 2.3	71299	0	445	0	114	0	0.2	#DIV/0!	0	3	0	4	0	4	0	4	2.32218
tr|B2R7F8|B2R7F8_H	tr|B2R7F8|B2R7F8_HU	cDNA, FLJ93426, highly similar to Homo sapiens plasminogen (PLG), mRNA OS=Homo sa	93217	0	435	0	116	0	0.07	#DIV/0!	0	2	0	2	0	4	0	4	2.32218
tr|Q5VZA6|Q5VZA6_H	ACO1	Cytoplasmic aconitate hydratase OS=Homo sapiens GN=ACO1 PE=4 SV=1	48888	0	410	0	104	0	0.22	#DIV/0!	0	2	0	3	0	4	0	4	2.32218
sp|P52907|CAZA1_HU	CAPZA1	F-actin-capping protein subunit alpha-1 OS=Homo sapiens GN=CAPZA1 PE=1 SV=3	33073	0	404	0	130	0	0.33	#DIV/0!	0	3	0	4	0	4	0	4	2.32218
sp|Q96PK6|RBM14_H	RBM14	RNA-binding protein 14 OS=Homo sapiens GN=RBM14 PE=1 SV=2	69620	0	393	0	134	0	0.2	#DIV/0!	0	2	0	4	0	4	0	5	2.32218
sp|A0AVT1|UBA6_HU	UBA6	Ubiquitin-like modifier-activating enzyme 6 OS=Homo sapiens GN=UBA6 PE=1 SV=1	119207	0	392	0	134	0	0.08	#DIV/0!	0	3	0	3	0	4	0	4	2.32218
tr|B4DN25|B4DN25_H	UGDH	UDP-glucose 6-dehydrogenase OS=Homo sapiens GN=UGDH PE=2 SV=1	44786	0	385	0	92	0	0.26	#DIV/0!	0	3	0	3	0	4	0	4	2.32218
tr|B7Z8P5|B7Z8P5_H	tr|B7Z8P5|B7Z8P5_HU	cDNA FLJ51438, highly similar to Probable ATP-dependent RNA helicase DHX36 (EC 3.6.1	100935	0	382	0	138	0	0.19	#DIV/0!	0	4	0	5	0	4	0	5	2.32218
tr|F5GZS0|F5GZS0_H	DHX36	Probable ATP-dependent RNA helicase DHX36 OS=Homo sapiens GN=DHX36 PE=2 SV=1	113928	0	377	0	140	0	0.12	#DIV/0!	0	4	0	4	0	4	0	4	2.32218
tr|B4DUT7|B4DUT7_H	tr|B4DUT7|B4DUT7_H	cDNA FLJ57604, highly similar to GMP synthase (glutamine-hydrolyzing) (EC 6.3.5.2) OS=	71880	0	368	0	126	0	0.23	#DIV/0!	0	4	0	4	0	4	0	6	2.32218
tr|B3KNK5|B3KNK5_H	tr|B3KNK5|B3KNK5_HU	cDNA FLJ14795 fis, clone NT2RP4001219, highly similar to Homo sapiens DnaJ (Hsp40) h	70004	0	345	0	104	0	0.19	#DIV/0!	0	2	0	4	0	4	0	4	2.32218
sp|Q96CS3|FAF2_HUN	FAF2	FAS-associated factor 2 OS=Homo sapiens GN=FAF2 PE=1 SV=2	52933	0	344	0	152	0	0.35	#DIV/0!	0	4	0	5	0	4	0	6	2.32218
tr|B3KRS5|B3KRS5_H	HDAC2	Histone deacetylase OS=Homo sapiens GN=HDAC2 PE=2 SV=1	52479	0	322	0	162	0	0.36	#DIV/0!	0	3	0	5	0	4	0	6	2.32218
tr|B7Z4B8|B7Z4B8_H	HNRNPUL1	Heterogeneous nuclear ribonucleoprotein U-like protein 1 OS=Homo sapiens GN=HNRNI	86639	0	317	0	164	0	0.12	#DIV/0!	0	3	0	3	0	4	0	6	2.32218
tr|Q6ZVI6|Q6ZVI6_HU	tr|Q6ZVI6|Q6ZVI6_HU	cDNA FLJ42537 fis, clone BRACE3004058, highly similar to NADH-cytochrome B5 reducta	29141	0	299	0	123	0	0.45	#DIV/0!	0	2	0	3	0	4	0	4	2.32218
tr|B2R806|B2R806_H	tr|B2R806|B2R806_HU	Eukaryotic translation initiation factor 3 subunit E OS=Homo sapiens PE=2 SV=1	52622	0	289	0	105	0	0.44	#DIV/0!	0	4	0	4	0	4	0	4	2.32218
sp|P46013|KI67_HUM	MKI67	Antigen KI-67 OS=Homo sapiens GN=MKI67 PE=1 SV=2	360698	0	1160	0	28	0	0.02	#DIV/0!	0	0	0	2	0	3	0	4	1.95943
tr|B4DI19|B4DI19_HU	tr|B4DI19|B4DI19_HU	cDNA FLJ59524, highly similar to Cysteinyl-tRNA synthetase (EC 6.1.1.16) OS=Homo sapie	76209	0	1130	0	29	0	0.09	#DIV/0!	0	0	0	2	0	3	0	3	1.95943
sp|Q08945|SSRP1_HU	SSRP1	FACT complex subunit SSRP1 OS=Homo sapiens GN=SSRP1 PE=1 SV=1	81367	0	1106	0	30	0	0.08	#DIV/0!	0	0	0	2	0	3	0	3	1.95943
tr|Q5VWC4|Q5VWC4	PSMD4	26S proteasome non-ATPase regulatory subunit 4 OS=Homo sapiens GN=PSMD4 PE=4 SV	41281	0	957	0	39	0	0.17	#DIV/0!	0	1	0	2	0	3	0	3	1.95943
sp|Q9BVP2|GNL3_HU	GNL3	Guanine nucleotide-binding protein-like 3 OS=Homo sapiens GN=GNL3 PE=1 SV=2	62468	0	925	0	41	0	0.11	#DIV/0!	0	1	0	2	0	3	0	5	1.95943
tr|A0MNN5|A0MNN5	GRWD1	CDW4/GRWD1 OS=Homo sapiens GN=GRWD1 PE=2 SV=1	49787	0	889	0	43	0	0.21	#DIV/0!	0	1	0	3	0	3	0	4	1.95943
tr|B3KWV6|B3KWV6_	tr|B3KWV6|B3KWV6_H	cDNA FLJ43948 fis, clone TESTI4014924, highly similar to Homo sapiens cytoplasmic FMR	147082	0	882	0	44	0	0.02	#DIV/0!	0	1	0	1	0	3	0	4	1.95943
tr|Q56VW8|Q56VW8_	tr|Q56VW8|Q56VW8_	Lung cancer-related protein 9 OS=Homo sapiens PE=2 SV=1	52186	0	848	0	46	0	0.13	#DIV/0!	0	0	0	2	0	3	0	4	1.95943
tr|F2YGG7|F2YGG7_H	tr|F2YGG7|F2YGG7_HU	Epidermal growth factor receptor OS=Homo sapiens PE=2 SV=1	132588	0	842	0	47	0	0.08	#DIV/0!	0	0	0	3	0	3	0	3	1.95943
sp|Q92896|GSLG1_HU	GLG1	Golgi apparatus protein 1 OS=Homo sapiens GN=GLG1 PE=1 SV=2	138341	0	832	0	47	0	0.02	#DIV/0!	0	1	0	1	0	3	0	5	1.95943
tr|Q59FA0|Q59FA0_H	tr|Q59FA0|Q59FA0_HU	DNA polymerase (Fragment) OS=Homo sapiens PE=2 SV=1	113271	0	824	0	48	0	0.06	#DIV/0!	0	1	0	2	0	3	0	4	1.95943
tr|E2GJL3|E2GJL3_HU	HLA-B	MHC class I antigen (Fragment) OS=Homo sapiens GN=HLA-B PE=3 SV=1	21222	0	780	0	53	0	0.34	#DIV/0!	0	1	0	2	0	3	0	4	1.95943
tr|H0Y4L0|H0Y4L0_HU	HLA-B	HLA class I histocompatibility antigen, B-35 alpha chain (Fragment) OS=Homo sapiens GN	26083	0	779	0	53	0	0.27	#DIV/0!	0	1	0	2	0	3	0	3	1.95943
tr|Q6FHH6|Q6FHH6_	LANCL1	LANCL1 protein (Fragment) OS=Homo sapiens GN=LANCL1 PE=2 SV=1	45979	0	774	0	53	0	0.15	#DIV/0!	0	1	0	2	0	3	0	3	1.95943
sp|P35658|NU214_HU	NUP214	Nuclear pore complex protein Nup214 OS=Homo sapiens GN=NUP214 PE=1 SV=2	214230	0	767	0	55	0	0.05	#DIV/0!	0	1	0	3	0	3	0	5	1.95943
sp|Q9NUL7|DDX28_H	DDX28	Probable ATP-dependent RNA helicase DDX28 OS=Homo sapiens GN=DDX28 PE=1 SV=2	59773	0	742	0	57	0	0.11	#DIV/0!	0	1	0	2	0	3	0	3	1.95943
tr|B2R6E5|B2R6E5_H	tr|B2R6E5|B2R6E5_HU	NADPH--cytochrome P450 reductase OS=Homo sapiens PE=2 SV=1	77429	0	735	0	58	0	0.17	#DIV/0!	0	1	0	4	0	3	0	4	1.95943
tr|B3KMV5|B3KMV5_	tr|B3KMV5|B3KMV5_H	cDNA FLJ12728 fis, clone NT2RP2000040, highly similar to Protein FAM62A OS=Homo sa	123342	0	734	0	58	0	0.08	#DIV/0!	0	1	0	3	0	3	0	4	1.95943
tr|B7Z2D6|B7Z2D6_H	tr|B7Z2D6|B7Z2D6_HU	cDNA FLJ53863, highly similar to Cystathionine beta-synthase (EC 4.2.1.22) OS=Homo sa	56409	0	733	0	58	0	0.19	#DIV/0!	0	1	0	3	0	3	0	3	1.95943
tr|B2RDD7|B2RDD7_H	tr|B2RDD7|B2RDD7_H	cDNA, FLJ96564, highly similar to Homo sapiens SKB1 homolog (S. pombe) (SKB1), mRNA	73348	0	730	0	59	0	0.22	#DIV/0!	0	2	0	2	0	3	0	3	1.95943
tr|B3KY63|B3KY63_H	tr|B3KY63|B3KY63_HU	cDNA FLJ16830 fis, clone UTERU3022536, highly similar to Chromodomain helicase-DNA-	216574	0	725	0	59	0	0.05	#DIV/0!	0	2	0	3	0	3	0	4	1.95943
tr|E9PDG5|E9PDG5_H	LARP4	La-related protein 4 OS=Homo sapiens GN=LARP4 PE=2 SV=1	50319	0	702	0	62	0	0.13	#DIV/0!	0	2	0	3	0	3	0	3	1.95943
tr|C7FDR3|C7FDR3_H	HLA-B	MHC class I antigen (Fragment) OS=Homo sapiens GN=HLA-B PE=3 SV=1	21132	0	686	0	64	0	0.34	#DIV/0!	0	2	0	2	0	3	0	3	1.95943
tr|G1UI18|G1UI18_HU	PSME4	Proteasome activator complex subunit 4 (Fragment) OS=Homo sapiens GN=PSME4 PE=2	130054	0	666	0	64	0	0.06	#DIV/0!	0	1	0	3	0	3	0	5	1.95943
sp|P54577|SYYC_HUN	YARS	Tyrosine--tRNA ligase, cytoplasmic OS=Homo sapiens GN=YARS PE=1 SV=4	59448	0	658	0	68	0	0.11	#DIV/0!	0	1	0	2	0	3	0	4	1.95943
tr|B4DUS9|B4DUS9_H	BPNT1	3~(2~),5~-bisphosphate nucleotidase 1 OS=Homo sapiens GN=BPNT1 PE=2 SV=1	29455	0	648	0	70	0	0.24	#DIV/0!	0	1	0	2	0	3	0	4	1.95943
tr|E3Q0X1|E3Q0X1_H	HLA-B	MHC class I antigen (Fragment) OS=Homo sapiens GN=HLA-B PE=3 SV=1	31939	0	627	0	73	0	0.35	#DIV/0!	0	2	0	3	0	3	0	3	1.95943
sp|Q9Y295|DRG1_HU	DRG1	Developmentally-regulated GTP-binding protein 1 OS=Homo sapiens GN=DRG1 PE=1 SV=	40802	0	621	0	75	0	0.26	#DIV/0!	0	3	0	3	0	3	0	3	1.95943
sp|P61221|ABCE1_HU	ABCE1	ATP-binding cassette sub-family E member 1 OS=Homo sapiens GN=ABCE1 PE=1 SV=1	68240	0	620	0	75	0	0.22	#DIV/0!	0	2	0	3	0	3	0	3	1.95943
tr|F5GY99|F5GY99_H	GALNT1	Polypeptide N-acetylgalactosaminyltransferase 1 soluble form OS=Homo sapiens GN=GA	58084	0	602	0	77	0	0.12	#DIV/0!	0	1	0	2	0	3	0	5	1.95943
tr|Q6IBU0|Q6IBU0_H	EIF5	EIF5 protein OS=Homo sapiens GN=EIF5 PE=2 SV=1	49577	0	599	0	78	0	0.14	#DIV/0!	0	1	0	2	0	3	0	3	1.95943
tr|A8K2M0|A8K2M0_	PSMC4	Proteasome (Prosome, macropain) 26S subunit, ATPase, 4, isoform CRA_b OS=Homo sap	47451	0	596	0	78	0	0.22	#DIV/0!	0	2	0	3	0	3	0	3	1.95943
sp|P11047|LAMC1_HU	LAMC1	Laminin subunit gamma-1 OS=Homo sapiens GN=LAMC1 PE=1 SV=3	183191	0	590	0	79	0	0.05	#DIV/0!	0	2	0	3	0	3	0	4	1.95943
sp|P31150|GDIA_HUN	GDI1	Rab GDP dissociation inhibitor alpha OS=Homo sapiens GN=GDI1 PE=1 SV=2	51177	0	582	0	80	0	0.14	#DIV/0!	0	2	0	2	0	3	0	4	1.95943
tr|Q6DCA8|Q6DCA8_	BCLAF1	BCLAF1 protein (Fragment) OS=Homo sapiens GN=BCLAF1 PE=2 SV=1	77958	0	580	0	80	0	0.13	#DIV/0!	0	3	0	3	0	3	0	3	1.95943
tr|B0YIW5|B0YIW5_H	ARCN1	Archain 1, isoform CRA_b OS=Homo sapiens GN=ARCN1 PE=2 SV=1	57630	0	576	0	81	0	0.18	#DIV/0!	0	3	0	3	0	3	0	3	1.95943
tr|A0JLQ5|A0JLQ5_H	BXDC2	BXDC2 protein (Fragment) OS=Homo sapiens GN=BXDC2 PE=2 SV=1	39430	0	574	0	61	0	0.47	#DIV/0!	0	1	0	3	0	3	0	4	1.95943
tr|B4DZ87|B4DZ87_H	tr|B4DZ87|B4DZ87_HU	cDNA FLJ57240, highly similar to Mitochondrial proteins import receptor OS=Homo sapie	57579	0	561	0	84	0	0.12	#DIV/0!	0	2	0	2	0	3	0	3	1.95943
tr|H0Y2Y8|H0Y2Y8_H	ZYX	Zyxin (Fragment) OS=Homo sapiens GN=ZYX PE=4 SV=1	58804	0	558	0	85	0	0.18	#DIV/0!	0	1	0	3	0	3	0	4	1.95943
tr|B4DWI8|B4DWI8_H	tr|B4DWI8|B4DWI8_H	cDNA FLJ57805, highly similar to Homo sapiens paraspeckle component 1 (PSPC1), transc	52660	0	528	0	91	0	0.2	#DIV/0!	0	2	0	3	0	3	0	3	1.95943
tr|B2RBM7|B2RBM7_	tr|B2RBM7|B2RBM7_H	cDNA, FLJ95595, highly similar to Homo sapiens proteasome (prosome, macropain) 26S s	43131	0	519	0	95	0	0.45	#DIV/0!	0	3	0	3	0	3	0	3	1.95943
tr|A1A5C4|A1A5C4_H	RRBP1	RRBP1 protein OS=Homo sapiens GN=RRBP1 PE=2 SV=1	103162	0	516	0	96	0	0.1	#DIV/0!	0	1	0	3	0	3	0	3	1.95943
tr|H3BTZ5|H3BTZ5_H	CNN2	Calponin-2 (Fragment) OS=Homo sapiens GN=CNN2 PE=4 SV=1	21173	0	491	0	101	0	0.55	#DIV/0!	0	2	0	3	0	3	0	3	1.95943
tr|H0Y3P2|H0Y3P2_H	EIF4G2	Eukaryotic translation initiation factor 4 gamma 2 OS=Homo sapiens GN=EIF4G2 PE=4 SV	98569	0	486	0	102	0	0.1	#DIV/0!	0	3	0	3	0	3	0	5	1.95943
tr|B4DL07|B4DL07_H	tr|B4DL07|B4DL07_HU	cDNA FLJ53353, highly similar to ATP-binding cassette sub-family D member 3 OS=Homo	78980	0	485	0	102	0	0.13	#DIV/0!	0	2	0	3	0	3	0	5	1.95943
tr|B3KRR1|B3KRR1_H	tr|B3KRR1|B3KRR1_HU	cDNA FLJ34725 fis, clone MESAN2005958, highly similar to RNA-binding protein Luc7-like	40752	0	484	0	67	0	0.19	#DIV/0!	0	1	0	2	0	3	0	3	1.95943
sp|Q09161|NCBP1_H	NCBP1	Nuclear cap-binding protein subunit 1 OS=Homo sapiens GN=NCBP1 PE=1 SV=1	92864	0	472	0	106	0	0.11	#DIV/0!	0	3	0	3	0	3	0	4	1.95943
tr|B4DH43|B4DH43_H	tr|B4DH43|B4DH43_H	cDNA FLJ56433, highly similar to Collagen alpha-2(IV) chain OS=Homo sapiens PE=2 SV=1	83428	0	471	0	107	0	0.12	#DIV/0!	0	2	0	3	0	3	0	3	1.95943
tr|A9LSU1|A9LSU1_H	tr|A9LSU1|A9LSU1_HU	Type IV collagen alpha 1 (Fragment) OS=Homo sapiens PE=2 SV=1	25998	0	470	0	107	0	0.44	#DIV/0!	0	2	0	3	0	3	0	4	1.95943
tr|J3KQ32|J3KQ32_HU	OLA1	Obg-like ATPase 1 OS=Homo sapiens GN=OLA1 PE=4 SV=1	47136	0	466	0	108	0	0.22	#DIV/0!	0	3	0	3	0	3	0	3	1.95943
tr|Q5T0F3|Q5T0F3_H	GNL2	Guanine nucleotide binding protein-like 2 (Nucleolar) OS=Homo sapiens GN=GNL2 PE=2	83831	0	458	0	110	0	0.12	#DIV/0!	0	3	0	3	0	3	0	3	1.95943
tr|H0YDD4|H0YDD4_	DLAT	Dihydrolipoyllysine-residue acetyltransferase component of pyruvate dehydrogenase co	51511	0	442	0	114	0	0.2	#DIV/0!	0	1	0	3	0	3	0	3	1.95943
tr|A8K0D2|A8K0D2_H	tr|A8K0D2|A8K0D2_HU	cDNA FLJ77740, highly similar to Homo sapiens 7-dehydrocholesterol reductase, mRNA (	55175	0	437	0	116	0	0.12	#DIV/0!	0	2	0	2	0	3	0	3	1.95943
sp|P04899|GNAI2_HU	GNAI2	Guanine nucleotide-binding protein G(i) subunit alpha-2 OS=Homo sapiens GN=GNAI2 PI	40995	0	414	0	126	0	0.26	#DIV/0!	0	2	0	3	0	3	0	4	1.95943
tr|B7Z4U7|B7Z4U7_H	SCFD1	Sec1 family domain containing 1, isoform CRA_b OS=Homo sapiens GN=SCFD1 PE=2 SV=	65362	0	403	0	130	0	0.16	#DIV/0!	0	3	0	3	0	3	0	3	1.95943
tr|H3BLU7|H3BLU7_H	AKR7A2	Aflatoxin B1 aldehyde reductase member 2 (Fragment) OS=Homo sapiens GN=AKR7A2 P	35004	0	397	0	98	0	0.37	#DIV/0!	0	3	0	3	0	3	0	4	1.95943
tr|B4DK35|B4DK35_H	tr|B4DK35|B4DK35_HU	cDNA FLJ61591, highly similar to Probable global transcription activator SNF2L2 (EC 3.6.1	112636	0	380	0	89	0	0.09	#DIV/0!	0	2	0	3	0	3	0	3	1.95943
tr|B5BU61|B5BU61_H	HDAC1	Histone deacetylase OS=Homo sapiens GN=HDAC1 PE=2 SV=1	55611	0	372	0	52	0	0.26	#DIV/0!	0	1	0	2	0	3	0	4	1.95943
tr|A8K6V7|A8K6V7_H	tr|A8K6V7|A8K6V7_HU	cDNA FLJ76053, highly similar to Homo sapiens Ras-GTPase activating protein SH3 doma	50842	0	370	0	142	0	0.21	#DIV/0!	0	3	0	3	0	3	0	3	1.95943
tr|B7Z213|B7Z213_HU	tr|B7Z213|B7Z213_HU	cDNA FLJ50130, highly similar to Heterogeneous nuclear ribonucleoprotein Q OS=Homo	28561	0	365	0	144	0	0.39	#DIV/0!	0	3	0	3	0	3	0	3	1.95943
tr|H0YCK7|H0YCK7_H	EEF1D	Elongation factor 1-delta (Fragment) OS=Homo sapiens GN=EEF1D PE=4 SV=1	23105	0	335	0	158	0	0.15	#DIV/0!	0	2	0	3	0	3	0	3	1.95943
tr|Q58EY4|Q58EY4_H	SMARCC1	SWI/SNF related, matrix associated, actin dependent regulator of chromatin, subfamily c	123347	0	1274	0	20	0	0.03	#DIV/0!	0	0	0	1	0	2	0	5	1.473823
tr|D3DU41|D3DU41_	LUC7L	LUC7-like (S. cerevisiae), isoform CRA_a OS=Homo sapiens GN=LUC7L PE=2 SV=1	34110	0	1271	0	20	0	0.1	#DIV/0!	0	0	0	1	0	2	0	2	1.473823
tr|H0Y659|H0Y659_H	HUWE1	E3 ubiquitin-protein ligase HUWE1 (Fragment) OS=Homo sapiens GN=HUWE1 PE=4 SV=1	134352	0	1268	0	20	0	0.02	#DIV/0!	0	0	0	1	0	2	0	2	1.473823
sp|Q5JWF2|GNAS1_H	GNAS	Guanine nucleotide-binding protein G(s) subunit alpha isoforms XLas OS=Homo sapiens (	111697	0	1252	0	22	0	0.03	#DIV/0!	0	0	0	1	0	2	0	3	1.473823
tr|Q59GM9|Q59GM9	tr|Q59GM9|Q59GM9_	Phosphorylase (Fragment) OS=Homo sapiens PE=2 SV=1	99508	0	1237	0	22	0	0.07	#DIV/0!	0	0	0	1	0	2	0	2	1.473823
sp|O95714|HERC2_HU	HERC2	E3 ubiquitin-protein ligase HERC2 OS=Homo sapiens GN=HERC2 PE=1 SV=2	533510	0	1229	0	23	0	0.01	#DIV/0!	0	0	0	1	0	2	0	2	1.473823
sp|Q5VW32|BROX_H	BROX	BRO1 domain-containing protein BROX OS=Homo sapiens GN=BROX PE=1 SV=1	46960	0	1222	0	23	0	0.07	#DIV/0!	0	0	0	1	0	2	0	2	1.473823
tr|B4DRN6|B4DRN6_	tr|B4DRN6|B4DRN6_H	cDNA FLJ51284, highly similar to Adapter-relatedprotein complex 3 mu-1 subunit OS=Ho	41285	0	1218	0	23	0	0.08	#DIV/0!	0	0	0	1	0	2	0	3	1.473823
sp|O95171|SCEL_HUN	SCEL	Sciellin OS=Homo sapiens GN=SCEL PE=1 SV=2	78018	0	1212	0	24	0	0.04	#DIV/0!	0	0	0	1	0	2	0	2	1.473823
tr|I1E4Y6|I1E4Y6_HU	GIGYF2	PERQ amino acid-rich with GYF domain-containing protein 2 OS=Homo sapiens GN=GIGY	152891	0	1188	0	26	0	0.02	#DIV/0!	0	0	0	1	0	2	0	3	1.473823
tr|H6A2E0|H6A2E0_H	HLA-B	MHC class I antigen (Fragment) OS=Homo sapiens GN=HLA-B PE=3 SV=1	21181	0	1170	0	27	0	0.16	#DIV/0!	0	0	0	1	0	2	0	2	1.473823
tr|B4DWN1|B4DWN1	tr|B4DWN1|B4DWN1_	cDNA FLJ52285, highly similar to Vesicular integral-membrane protein VIP36 OS=Homo s	32842	0	1149	0	28	0	0.21	#DIV/0!	0	0	0	2	0	2	0	2	1.473823
tr|Q59F65|Q59F65_H	tr|Q59F65|Q59F65_HU	G protein pathway suppressor 1 isoform 2 variant (Fragment) OS=Homo sapiens PE=2 SV	30821	0	1144	0	28	0	0.11	#DIV/0!	0	0	0	1	0	2	0	2	1.473823
sp|P40222|TXLNA_HU	TXLNA	Alpha-taxilin OS=Homo sapiens GN=TXLNA PE=1 SV=3	62195	0	1134	0	29	0	0.05	#DIV/0!	0	0	0	1	0	2	0	3	1.473823
sp|P07384|CAN1_HUI	CAPN1	Calpain-1 catalytic subunit OS=Homo sapiens GN=CAPN1 PE=1 SV=1	82465	0	1128	0	29	0	0.13	#DIV/0!	0	0	0	3	0	2	0	3	1.473823
sp|Q9NQH7|XPP3_HL	XPNPEP3	Probable Xaa-Pro aminopeptidase 3 OS=Homo sapiens GN=XPNPEP3 PE=1 SV=1	57624	0	1124	0	30	0	0.06	#DIV/0!	0	0	0	1	0	2	0	2	1.473823
tr|H0YNU5|H0YNU5_	BLM	Bloom syndrome protein OS=Homo sapiens GN=BLM PE=4 SV=1	145978	0	1123	0	30	0	0.02	#DIV/0!	0	0	0	1	0	2	0	2	1.473823
sp|Q9P2D7|DYH1_HU	DNAH1	Dynein heavy chain 1, axonemal OS=Homo sapiens GN=DNAH1 PE=2 SV=4	497685	0	1121	0	30	0	0.01	#DIV/0!	0	0	0	1	0	2	0	3	1.473823
tr|Q96LK9|Q96LK9_H	tr|Q96LK9|Q96LK9_HU	cDNA FLJ25413 fis, clone TST03402, highly similar to 3-OXO-5-BETA-STEROID 4-DEHYDRO	16911	0	1115	0	30	0	0.2	#DIV/0!	0	0	0	1	0	2	0	2	1.473823
tr|B3KVJ5|B3KVJ5_HU	tr|B3KVJ5|B3KVJ5_HU	cDNA FLJ16648 fis, clone TESTI4035508, highly similar to Structural maintenance of chro	101354	0	1110	0	30	0	0.03	#DIV/0!	0	0	0	1	0	2	0	2	1.473823
tr|Q548W1|Q548W1_	OGT	HRNT1 OS=Homo sapiens GN=OGT PE=2 SV=1	104029	0	1103	0	30	0	0.03	#DIV/0!	0	0	0	1	0	2	0	2	1.473823
tr|B2RDI5|B2RDI5_HU	tr|B2RDI5|B2RDI5_HU	cDNA, FLJ96627, highly similar to Homo sapiens calpain 1, (mu/I) large subunit (CAPN1),	82529	0	1095	0	30	0	0.08	#DIV/0!	0	0	0	2	0	2	0	2	1.473823
tr|H3BRG4|H3BRG4_	UQCRC2	Cytochrome b-c1 complex subunit 2, mitochondrial OS=Homo sapiens GN=UQCRC2 PE=3	44778	0	1089	0	31	0	0.24	#DIV/0!	0	0	0	3	0	2	0	4	1.473823
sp|Q9UHX1|PUF60_H	PUF60	Poly(U)-binding-splicing factor PUF60 OS=Homo sapiens GN=PUF60 PE=1 SV=1	60009	0	1087	0	31	0	0.11	#DIV/0!	0	0	0	2	0	2	0	2	1.473823
tr|B4DH53|B4DH53_H	MAP1S	MAP1S light chain OS=Homo sapiens GN=MAP1S PE=2 SV=1	110689	0	1085	0	31	0	0.06	#DIV/0!	0	0	0	2	0	2	0	3	1.473823
sp|Q8IXI1|MIRO2_HU	RHOT2	Mitochondrial Rho GTPase 2 OS=Homo sapiens GN=RHOT2 PE=1 SV=2	69101	0	1082	0	32	0	0.3	#DIV/0!	0	1	0	2	0	2	0	2	1.473823
tr|G3V161|G3V161_H	KBTBD3	Kelch repeat and BTB (POZ) domain containing 3, isoform CRA_a OS=Homo sapiens GN=I	61834	0	1077	0	32	0	0.06	#DIV/0!	0	1	0	1	0	2	0	3	1.473823
sp|Q6P1J9|CDC73_HL	CDC73	Parafibromin OS=Homo sapiens GN=CDC73 PE=1 SV=1	60653	0	1072	0	32	0	0.05	#DIV/0!	0	1	0	1	0	2	0	2	1.473823
sp|Q8NB90|SPAT5_H	SPATA5	Spermatogenesis-associated protein 5 OS=Homo sapiens GN=SPATA5 PE=1 SV=3	98470	0	1044	0	34	0	0.03	#DIV/0!	0	1	0	1	0	2	0	2	1.473823
tr|B4DJU4|B4DJU4_H	tr|B4DJU4|B4DJU4_HU	cDNA FLJ53344, highly similar to Splicing factor 1 OS=Homo sapiens PE=2 SV=1	67535	0	1043	0	34	0	0.05	#DIV/0!	0	0	0	1	0	2	0	3	1.473823
sp|P22059|OSBP1_HU	OSBP	Oxysterol-binding protein 1 OS=Homo sapiens GN=OSBP PE=1 SV=1	90220	0	1038	0	34	0	0.04	#DIV/0!	0	0	0	1	0	2	0	2	1.473823
tr|F8VV56|F8VV56_H	CD63	CD63 antigen OS=Homo sapiens GN=CD63 PE=4 SV=1	16466	0	1035	0	34	0	0.21	#DIV/0!	0	1	0	1	0	2	0	2	1.473823
tr|B4DRY5|B4DRY5_H	tr|B4DRY5|B4DRY5_HU	cDNA FLJ54538, highly similar to Transportin-2 OS=Homo sapiens PE=2 SV=1	101141	0	1027	0	34	0	0.07	#DIV/0!	0	1	0	2	0	2	0	4	1.473823
tr|A8MUD9|A8MUD9	RPL7	60S ribosomal protein L7 OS=Homo sapiens GN=RPL7 PE=3 SV=1	24474	0	1018	0	34	0	0.14	#DIV/0!	0	0	0	1	0	2	0	2	1.473823
tr|B4DEX7|B4DEX7_H	tr|B4DEX7|B4DEX7_HU	cDNA FLJ51806, highly similar to Homo sapiens proline-, glutamic acid-, leucine-rich prot	97913	0	1017	0	34	0	0.31	#DIV/0!	0	0	0	1	0	2	0	2	1.473823
tr|Q6NUR1|Q6NUR1_	NCAPG	Non-SMC condensin I complex, subunit G OS=Homo sapiens GN=NCAPG PE=2 SV=1	115205	0	1007	0	36	0	0.03	#DIV/0!	0	0	0	1	0	2	0	2	1.473823
tr|H0YMD2|H0YMD2_	NUSAP1	Nucleolar and spindle-associated protein 1 (Fragment) OS=Homo sapiens GN=NUSAP1 P	15923	0	1002	0	36	0	0.47	#DIV/0!	0	0	0	2	0	2	0	2	1.473823
sp|Q86W24|NAL14_H	NLRP14	NACHT, LRR and PYD domains-containing protein 14 OS=Homo sapiens GN=NLRP14 PE=2	127617	0	983	0	37	0	0.03	#DIV/0!	0	1	0	1	0	2	0	2	1.473823
tr|G3V3B0|G3V3B0_H	ACIN1	Apoptotic chromatin condensation inducer in the nucleus OS=Homo sapiens GN=ACIN1	67683	0	978	0	37	0	0.1	#DIV/0!	0	0	0	1	0	2	0	2	1.473823
sp|Q9NR45|SIAS_HUN	NANS	Sialic acid synthase OS=Homo sapiens GN=NANS PE=1 SV=2	40738	0	973	0	38	0	0.17	#DIV/0!	0	0	0	2	0	2	0	3	1.473823
tr|B3KNJ4|B3KNJ4_H	SAE1	SUMO-1 activating enzyme subunit 1, isoform CRA_a OS=Homo sapiens GN=SAE1 PE=2 S	33705	0	967	0	38	0	0.21	#DIV/0!	0	1	0	2	0	2	0	2	1.473823
tr|B3KM73|B3KM73_	tr|B3KM73|B3KM73_H	cDNA FLJ10419 fis, clone NT2RP1000163, highly similar to Homo sapiens transforming gr	55418	0	964	0	38	0	0.06	#DIV/0!	0	1	0	1	0	2	0	3	1.473823
tr|Q59EZ3|Q59EZ3_H	tr|Q59EZ3|Q59EZ3_HU	Insulin-like growth factor 2 receptor variant (Fragment) OS=Homo sapiens PE=2 SV=1	272672	0	960	0	38	0	0.02	#DIV/0!	0	0	0	2	0	2	0	4	1.473823
tr|G3V1J5|G3V1J5_HL	DIS3	Exosome complex exonuclease RRP44 OS=Homo sapiens GN=DIS3 PE=2 SV=1	91382	0	959	0	39	0	0.07	#DIV/0!	0	0	0	2	0	2	0	2	1.473823
tr|A4D275|A4D275_H	ARPC1B	Actin related protein 2/3 complex, subunit 1B, 41kDa OS=Homo sapiens GN=ARPC1B PE=	41722	0	956	0	39	0	0.08	#DIV/0!	0	1	0	1	0	2	0	2	1.473823
tr|B4DGD5|B4DGD5_	DNAJC11	DnaJ homolog subfamily C member 11 OS=Homo sapiens GN=DNAJC11 PE=2 SV=1	52854	0	954	0	39	0	0.13	#DIV/0!	0	0	0	2	0	2	0	2	1.473823
tr|B0AZM4|B0AZM4_	tr|B0AZM4|B0AZM4_H	cDNA, FLJ79464, highly similar to Homo sapiens aquarius (Fragment) OS=Homo sapiens I	157764	0	940	0	40	0	0.02	#DIV/0!	0	1	0	1	0	2	0	2	1.473823

tr|B3KMR5|B3KMR5_	tr|B3KMR5|B3KMR5_H	cDNA FLJ12434 fis, clone NT2RM1000037, highly similar to Homo sapiens KIAA0690 prot	145007	0	932	0	40	0	0.06	#DIV/0!	0	1	0	2	0	2	0	5	1.473823
tr|B2RD79|B2RD79_H	tr|B2RD79|B2RD79_HU	Ubiquitin carboxyl-terminal hydrolase OS=Homo sapiens PE=2 SV=1	56462	0	930	0	40	0	0.12	#DIV/0!	0	1	0	2	0	2	0	3	1.473823
sp|P02748|CO9_HUM	C9	Complement component C9 OS=Homo sapiens GN=C9 PE=1 SV=2	64615	0	926	0	41	0	0.1	#DIV/0!	0	1	0	2	0	2	0	2	1.473823
tr|A1A693|A1A693_H	RBM15	RNA binding motif protein 15 isoform L2 OS=Homo sapiens GN=RBM15 PE=2 SV=1	99689	0	913	0	41	0	0.07	#DIV/0!	0	1	0	2	0	2	0	2	1.473823
tr|B3KNE7|B3KNE7_H	tr|B3KNE7|B3KNE7_HU	cDNA FLJ14468 fis, clone MAMMA1000734, highly similar to Translocation protein SEC63	48242	0	907	0	41	0	0.04	#DIV/0!	0	1	0	1	0	2	0	2	1.473823
sp|Q14558|KPRA_HU	PRPSAP1	Phosphoribosyl pyrophosphate synthase-associated protein 1 OS=Homo sapiens GN=PRI	39654	0	893	0	43	0	0.11	#DIV/0!	0	1	0	1	0	2	0	2	1.473823
tr|B2RAN1|B2RAN1_H	tr|B2RAN1|B2RAN1_H	cDNA, FLJ95012, highly similar to Homo sapiens UDP-glucose pyrophosphorylase 2 (UGP	57042	0	885	0	43	0	0.12	#DIV/0!	0	0	0	2	0	2	0	2	1.473823
tr|B3KM40|B3KM40_	tr|B3KM40|B3KM40_H	cDNA FLJ10185 fis, clone HEMBA1004509, highly similar to U4/U6.U5 tri-snRNP-associat	56618	0	878	0	44	0	0.12	#DIV/0!	0	1	0	2	0	2	0	2	1.473823
sp|Q14690|RRP5_HU	PDCD11	Protein RRP5 homolog OS=Homo sapiens GN=PDCD11 PE=1 SV=3	209939	0	867	0	45	0	0.03	#DIV/0!	0	1	0	2	0	2	0	3	1.473823
tr|B3KT00|B3KT00_H	tr|B3KT00|B3KT00_HU	cDNA FLJ37368 fis, clone BRAMY2024530, highly similar to Zinc phosphodiesterase ELAC	91169	0	864	0	45	0	0.05	#DIV/0!	0	1	0	1	0	2	0	2	1.473823
sp|Q9NYU1|UGGG2_H	UGGT2	UDP-glucose:glycoprotein glucosyltransferase 2 OS=Homo sapiens GN=UGGT2 PE=1 SV=	175251	0	856	0	45	0	0.02	#DIV/0!	0	1	0	1	0	2	0	3	1.473823
tr|B7Z475|B7Z475_H	tr|B7Z475|B7Z475_HU	cDNA FLJ55712, highly similar to F-box-like/WD repeat protein TBL1XR1 OS=Homo sapie	41301	0	853	0	46	0	0.17	#DIV/0!	0	1	0	2	0	2	0	2	1.473823
tr|F5H8E5|F5H8E5_H	USP7	Ubiquitin carboxyl-terminal hydrolase OS=Homo sapiens GN=USP7 PE=2 SV=1	117951	0	851	0	46	0	0.06	#DIV/0!	0	1	0	2	0	2	0	4	1.473823
tr|H0Y650|H0Y650_H	HEATR2	HEAT repeat-containing protein 2 (Fragment) OS=Homo sapiens GN=HEATR2 PE=4 SV=1	73507	0	850	0	46	0	0.09	#DIV/0!	0	1	0	2	0	2	0	2	1.473823
sp|Q03169|TNAP2_H	TNFAIP2	Tumor necrosis factor alpha-induced protein 2 OS=Homo sapiens GN=TNFAIP2 PE=1 SV=	73015	0	846	0	46	0	0.09	#DIV/0!	0	1	0	2	0	2	0	2	1.473823
sp|Q2M389|WASH7_	KIAA1033	WASH complex subunit 7 OS=Homo sapiens GN=KIAA1033 PE=1 SV=2	137343	0	837	0	47	0	0.05	#DIV/0!	0	0	0	2	0	2	0	2	1.473823
sp|Q5SRE5|NU188_H	NUP188	Nucleoporin NUP188 homolog OS=Homo sapiens GN=NUP188 PE=1 SV=1	198369	0	822	0	48	0	0.02	#DIV/0!	0	1	0	1	0	2	0	3	1.473823
sp|Q96T76|MMS19_H	MMS19	MMS19 nucleotide excision repair protein homolog OS=Homo sapiens GN=MMS19 PE=1	114928	0	818	0	49	0	0.06	#DIV/0!	0	1	0	2	0	2	0	3	1.473823
tr|Q4ZG72|Q4ZG72_H	DDX18	Putative uncharacterized protein DDX18 (Fragment) OS=Homo sapiens GN=DDX18 PE=2	61898	0	814	0	49	0	0.09	#DIV/0!	0	1	0	2	0	2	0	4	1.473823
tr|B4DSN3|B4DSN3_H	tr|B4DSN3|B4DSN3_H	cDNA FLJ60345, highly similar to Protein transport protein Sec24C OS=Homo sapiens PE=	107974	0	810	0	49	0	0.06	#DIV/0!	0	1	0	2	0	2	0	3	1.473823
sp|P01023|A2MG_HU	A2M	Alpha-2-macroglobulin OS=Homo sapiens GN=A2M PE=1 SV=3	164613	0	804	0	50	0	0.02	#DIV/0!	0	2	0	2	0	2	0	2	1.473823
sp|O00625|PIR_HUM	PIR	Pirin OS=Homo sapiens GN=PIR PE=1 SV=1	32207	0	800	0	50	0	0.22	#DIV/0!	0	1	0	2	0	2	0	2	1.473823
tr|B0V0B9|B0V0B9_H	HLA-C	HLA class I histocompatibility antigen, Cw-14 alpha chain OS=Homo sapiens GN=HLA-C P	41847	0	798	0	27	0	0.22	#DIV/0!	0	0	0	1	0	2	0	2	1.473823
tr|Q8N254|Q8N254_H	tr|Q8N254|Q8N254_H	cDNA FLJ33908 fis, clone CTONG2008518, highly similar to DEAD/H (Asp-Glu-Ala-Asp/His	46899	0	795	0	51	0	0.07	#DIV/0!	0	1	0	1	0	2	0	3	1.473823
tr|B4DL79|B4DL79_H	KIF20A	Kinesin-like protein KIF20A OS=Homo sapiens GN=KIF20A PE=2 SV=1	99066	0	787	0	52	0	0.07	#DIV/0!	0	1	0	2	0	2	0	2	1.473823
tr|A2TEM8|A2TEM8_	HLA-B	MHC class I antigen (Fragment) OS=Homo sapiens GN=HLA-B PE=4 SV=1	10623	0	785	0	52	0	0.32	#DIV/0!	0	1	0	1	0	2	0	2	1.473823
tr|G1ENK8|G1ENK8_H	HLA-B	MHC class I antigen (Fragment) OS=Homo sapiens GN=HLA-B PE=3 SV=1	21236	0	781	0	53	0	0.34	#DIV/0!	0	1	0	2	0	2	0	2	1.473823
tr|G0KXF8|G0KXF8_H	HLA-B	MHC class I antigen (Fragment) OS=Homo sapiens GN=HLA-B PE=3 SV=1	21235	0	773	0	54	0	0.34	#DIV/0!	0	1	0	2	0	2	0	2	1.473823
tr|J3KPP4|J3KPP4_HU	LUC7L3	Cisplatin resistance-associated overexpressed protein, isoform CRA_b OS=Homo sapiens	58698	0	761	0	55	0	0.12	#DIV/0!	0	1	0	2	0	2	0	2	1.473823
tr|H0YL11|H0YL11_H	IDH2	Isocitrate dehydrogenase [NADP], mitochondrial OS=Homo sapiens GN=IDH2 PE=3 SV=1	35536	0	747	0	56	0	0.07	#DIV/0!	0	1	0	1	0	2	0	3	1.473823
tr|G3V1K3|G3V1K3_H	PON2	Paraoxonase 2, isoform CRA_a OS=Homo sapiens GN=PON2 PE=2 SV=1	41672	0	744	0	57	0	0.08	#DIV/0!	0	1	0	2	0	2	0	2	1.473823
tr|A4FUT8|A4FUT8_H	JMJD1B	JMJD1B protein (Fragment) OS=Homo sapiens GN=JMJD1B PE=2 SV=1	170603	0	741	0	57	0	0.04	#DIV/0!	0	2	0	2	0	2	0	3	1.473823
tr|Q6FHF5|Q6FHF5_H	PCNA	Proliferating cell nuclear antigen (Fragment) OS=Homo sapiens GN=PCNA PE=2 SV=1	29029	0	740	0	57	0	0.24	#DIV/0!	0	1	0	2	0	2	0	2	1.473823
sp|Q9NYK5|RM39_HU	MRPL39	39S ribosomal protein L39, mitochondrial OS=Homo sapiens GN=MRPL39 PE=1 SV=3	39200	0	727	0	59	0	0.08	#DIV/0!	0	1	0	1	0	2	0	2	1.473823
tr|C9JDR0|C9JDR0_H	NSDHL	Sterol-4-alpha-carboxylate 3-dehydrogenase, decarboxylating (Fragment) OS=Homo sapi	28356	0	722	0	60	0	0.25	#DIV/0!	0	1	0	2	0	2	0	2	1.473823
tr|G5E9H4|G5E9H4_H	DCTN1	Dynactin 1 (P150, glued homolog, Drosophila), isoform CRA_a OS=Homo sapiens GN=DC	127339	0	710	0	61	0	0.05	#DIV/0!	0	1	0	2	0	2	0	2	1.473823
tr|G3V198|G3V198_H	NUP160	Nuclear pore complex protein Nup160 OS=Homo sapiens GN=NUP160 PE=2 SV=1	142365	0	707	0	62	0	0.05	#DIV/0!	0	1	0	2	0	2	0	2	1.473823
sp|P14550|AK1A1_HU	AKR1A1	Alcohol dehydrogenase [NADP(+)] OS=Homo sapiens GN=AKR1A1 PE=1 SV=3	36892	0	705	0	62	0	0.19	#DIV/0!	0	2	0	2	0	2	0	2	1.473823
tr|B2R491|B2R491_H	RPS4X	Ribosomal protein S4, X-linked, isoform CRA_c OS=Homo sapiens GN=RPS4X PE=2 SV=1	29807	0	704	0	62	0	0.24	#DIV/0!	0	2	0	2	0	2	0	2	1.473823
tr|I6L9E8|I6L9E8_HUN	FAM98A	Family with sequence similarity 98, member A OS=Homo sapiens GN=FAM98A PE=2 SV=	55595	0	703	0	62	0	0.06	#DIV/0!	0	1	0	1	0	2	0	2	1.473823
tr|B3KQ73|B3KQ73_H	tr|B3KQ73|B3KQ73_H	cDNA FLJ33002 fis, clone THYMU1000217, highly similar to Homo sapiens ubiquitin asso	37025	0	691	0	37	0	0.06	#DIV/0!	0	1	0	1	0	2	0	2	1.473823
tr|E1P5S2|E1P5S2_H	RBM39	RNA-binding protein 39 OS=Homo sapiens GN=RBM39 PE=4 SV=1	40744	0	689	0	64	0	0.17	#DIV/0!	0	2	0	2	0	2	0	2	1.473823
tr|B4DW54|B4DW54_	tr|B4DW54|B4DW54_H	cDNA FLJ50888, highly similar to Aldehyde dehydrogenase, mitochondrial (EC 1.2.1.3) O	51467	0	685	0	65	0	0.13	#DIV/0!	0	1	0	2	0	2	0	3	1.473823
tr|B4DJV2|B4DJV2_H	CS	Citrate synthase OS=Homo sapiens GN=CS PE=2 SV=1	50628	0	680	0	66	0	0.21	#DIV/0!	0	2	0	3	0	2	0	4	1.473823
tr|Q14730|Q14730_H	tr|Q14730|Q14730_HU	La 4.1 protein (Fragment) OS=Homo sapiens PE=2 SV=1	33852	0	674	0	66	0	0.21	#DIV/0!	0	2	0	2	0	2	0	2	1.473823
tr|Q6IAM0|Q6IAM0_	EIF3S4	Eukaryotic translation initiation factor 3 subunit G OS=Homo sapiens GN=EIF3S4 PE=2 SV	35864	0	673	0	66	0	0.19	#DIV/0!	0	2	0	2	0	2	0	2	1.473823
sp|Q8TDD1|DDX54_H	DDX54	ATP-dependent RNA helicase DDX54 OS=Homo sapiens GN=DDX54 PE=1 SV=2	98819	0	661	0	68	0	0.07	#DIV/0!	0	2	0	2	0	2	0	3	1.473823
tr|B7Z592|B7Z592_H	tr|B7Z592|B7Z592_HU	cDNA FLJ61635, highly similar to Homo sapiens likely ortholog of mouse immediate early	45262	0	649	0	70	0	0.15	#DIV/0!	0	1	0	2	0	2	0	2	1.473823
sp|Q9H9J2|RM44_HU	MRPL44	39S ribosomal protein L44, mitochondrial OS=Homo sapiens GN=MRPL44 PE=1 SV=1	37854	0	646	0	70	0	0.18	#DIV/0!	0	1	0	2	0	2	0	2	1.473823
tr|D3DWB6|D3DWB6	USP9X	Ubiquitin carboxyl-terminal hydrolase OS=Homo sapiens GN=USP9X PE=2 SV=1	274331	0	642	0	71	0	0.02	#DIV/0!	0	1	0	2	0	2	0	4	1.473823
sp|Q07954|LRP1_HU	LRP1	Prolow-density lipoprotein receptor-related protein 1 OS=Homo sapiens GN=LRP1 PE=1	523150	0	641	0	72	0	0.01	#DIV/0!	0	2	0	2	0	2	0	4	1.473823
tr|G3V438|G3V438_H	AHSA1	Activator of 90 kDa heat shock protein ATPase homolog 1 (Fragment) OS=Homo sapiens	23078	0	632	0	59	0	0.48	#DIV/0!	0	1	0	2	0	2	0	2	1.473823
tr|B3KUZ7|B3KUZ7_H	tr|B3KUZ7|B3KUZ7_HU	cDNA FLJ40986 fis, clone UTERU2014898, highly similar to Vacuolar ATP synthase subuni	56445	0	631	0	73	0	0.12	#DIV/0!	0	2	0	2	0	2	0	2	1.473823
tr|F4NBW3|F4NBW3_	HLA-B	MHC class I antigen (Fragment) OS=Homo sapiens GN=HLA-B PE=3 SV=1	38308	0	624	0	74	0	0.18	#DIV/0!	0	2	0	2	0	2	0	4	1.473823
tr|B4DGX9|B4DGX9_H	tr|B4DGX9|B4DGX9_H	cDNA FLJ60509, highly similar to Probable ubiquitin carboxyl-terminalhydrolase FAF-X (E	66195	0	623	0	74	0	0.05	#DIV/0!	0	1	0	1	0	2	0	3	1.473823
sp|O00507|USP9Y_HU	USP9Y	Probable ubiquitin carboxyl-terminal hydrolase FAF-Y OS=Homo sapiens GN=USP9Y PE=1	294370	0	622	0	74	0	0.01	#DIV/0!	0	1	0	1	0	2	0	3	1.473823
sp|P98160|PGBM_HU	HSPG2	Basement membrane-specific heparan sulfate proteoglycan core protein OS=Homo sapie	479253	0	611	0	76	0	0.01	#DIV/0!	0	2	0	2	0	2	0	3	1.473823
tr|B3KY11|B3KY11_H	tr|B3KY11|B3KY11_HU	cDNA FLJ46571 fis, clone THYMU3041428, highly similar to Probable ATP-dependent RN	93519	0	608	0	77	0	0.07	#DIV/0!	0	1	0	2	0	2	0	2	1.473823
sp|Q8N1G4|LRC47_H	LRRC47	Leucine-rich repeat-containing protein 47 OS=Homo sapiens GN=LRRC47 PE=1 SV=1	64004	0	607	0	77	0	0.11	#DIV/0!	0	1	0	2	0	2	0	5	1.473823
tr|B4DMA4|B4DMA4_	tr|B4DMA4|B4DMA4_	cDNA FLJ58400, highly similar to Cleavage stimulation factor 77 kDa subunit OS=Homo s	75580	0	595	0	78	0	0.09	#DIV/0!	0	2	0	2	0	2	0	2	1.473823
tr|B0QZ18|B0QZ18_H	CPNE1	Copine I OS=Homo sapiens GN=CPNE1 PE=2 SV=1	60307	0	593	0	79	0	0.11	#DIV/0!	0	2	0	2	0	2	0	2	1.473823
sp|Q8N0X7|SPG20_H	SPG20	Spartin OS=Homo sapiens GN=SPG20 PE=1 SV=1	73358	0	569	0	83	0	0.09	#DIV/0!	0	2	0	2	0	2	0	2	1.473823
sp|Q9BWF3|RBM4_H	RBM4	RNA-binding protein 4 OS=Homo sapiens GN=RBM4 PE=1 SV=1	40688	0	567	0	57	0	0.29	#DIV/0!	0	2	0	2	0	2	0	2	1.473823
tr|C9J634|C9J634_HU	PDHB	Pyruvate dehydrogenase E1 component subunit beta, mitochondrial OS=Homo sapiens (	39064	0	552	0	79	0	0.27	#DIV/0!	0	1	0	2	0	2	0	3	1.473823
sp|O15031|PLXB2_HU	PLXNB2	Plexin-B2 OS=Homo sapiens GN=PLXNB2 PE=1 SV=3	207734	0	551	0	86	0	0.06	#DIV/0!	0	2	0	2	0	2	0	2	1.473823
tr|F5GYR8|F5GYR8_H	USO1	General vesicular transport factor p115 OS=Homo sapiens GN=USO1 PE=2 SV=1	109394	0	546	0	88	0	0.06	#DIV/0!	0	1	0	2	0	2	0	3	1.473823
sp|Q9H3U1|UN45A_H	UNC45A	Protein unc-45 homolog A OS=Homo sapiens GN=UNC45A PE=1 SV=1	104266	0	545	0	88	0	0.06	#DIV/0!	0	1	0	2	0	2	0	2	1.473823
sp|Q6ZRP7|QSOX2_H	QSOX2	Sulfhydryl oxidase 2 OS=Homo sapiens GN=QSOX2 PE=1 SV=3	78221	0	540	0	89	0	0.13	#DIV/0!	0	2	0	3	0	2	0	3	1.473823
tr|B4DG89|B4DG89_H	NPLOC4	Nuclear protein localization protein 4 homolog OS=Homo sapiens GN=NPLOC4 PE=2 SV=	52144	0	537	0	39	0	0.1	#DIV/0!	0	1	0	1	0	2	0	2	1.473823
tr|H0YCK3|H0YCK3_H	ADAR	Double-stranded RNA-specific adenosine deaminase (Fragment) OS=Homo sapiens GN=/	133761	0	533	0	91	0	0.06	#DIV/0!	0	2	0	2	0	2	0	3	1.473823
sp|Q9GZL7|WDR12_H	WDR12	Ribosome biogenesis protein WDR12 OS=Homo sapiens GN=WDR12 PE=1 SV=2	48191	0	532	0	91	0	0.14	#DIV/0!	0	2	0	2	0	2	0	2	1.473823
tr|F5H6D0|F5H6D0_H	MOGS	Mannosyl-oligosaccharide glucosidase OS=Homo sapiens GN=MOGS PE=2 SV=1	80824	0	531	0	91	0	0.08	#DIV/0!	0	1	0	2	0	2	0	2	1.473823
sp|P55268|LAMB2_H	LAMB2	Laminin subunit beta-2 OS=Homo sapiens GN=LAMB2 PE=1 SV=2	202982	0	522	0	93	0	0.03	#DIV/0!	0	2	0	2	0	2	0	2	1.473823
tr|Q6FG59|Q6FG59_H	CDC37	CDC37 protein OS=Homo sapiens GN=CDC37 PE=2 SV=1	44938	0	521	0	93	0	0.15	#DIV/0!	0	2	0	2	0	2	0	2	1.473823
tr|B4E3J7|B4E3J7_HU	tr|B4E3J7|B4E3J7_HUN	cDNA FLJ51203, highly similar to Thrombospondin-1 OS=Homo sapiens PE=2 SV=1	124032	0	520	0	93	0	0.05	#DIV/0!	0	2	0	2	0	2	0	2	1.473823
tr|B3KXH0|B3KXH0_H	tr|B3KXH0|B3KXH0_H	cDNA FLJ45395 fis, clone BRHIP3027191, highly similar to 150 kDa oxygen-regulated prot	110601	0	510	0	97	0	0.06	#DIV/0!	0	2	0	2	0	2	0	2	1.473823
tr|B4E1L0|B4E1L0_H	tr|B4E1L0|B4E1L0_HU	Adenylosuccinate synthetase OS=Homo sapiens PE=2 SV=1	48308	0	507	0	98	0	0.07	#DIV/0!	0	1	0	1	0	2	0	2	1.473823
tr|B4DF80|B4DF80_H	tr|B4DF80|B4DF80_HU	cDNA FLJ58527, highly similar to Nucleolar protein 6 OS=Homo sapiens PE=2 SV=1	122714	0	505	0	34	0	0.05	#DIV/0!	0	0	0	1	0	2	0	3	1.473823
tr|B4DM73|B4DM73_	tr|B4DM73|B4DM73_H	cDNA FLJ60078, highly similar to CLIP-associating protein 2 (Fragment) OS=Homo sapien	116274	0	499	0	88	0	0.04	#DIV/0!	0	1	0	1	0	2	0	2	1.473823
tr|B4DFP1|B4DFP1_H	tr|B4DFP1|B4DFP1_HU	cDNA FLJ51818, highly similar to Phosphoglucomutase-1 (EC 5.4.2.2) OS=Homo sapiens F	58987	0	478	0	81	0	0.23	#DIV/0!	0	2	0	3	0	2	0	3	1.473823
tr|B4DUF1|B4DUF1_H	tr|B4DUF1|B4DUF1_HU	cDNA FLJ59760, highly similar to 1,4-alpha-glucan branching enzyme (EC 2.4.1.18) OS=H	76254	0	476	0	105	0	0.08	#DIV/0!	0	2	0	2	0	2	0	2	1.473823
tr|H0Y4J2|H0Y4J2_HU	MRPL37	39S ribosomal protein L37, mitochondrial (Fragment) OS=Homo sapiens GN=MRPL37 PE	29806	0	450	0	61	0	0.3	#DIV/0!	0	1	0	2	0	2	0	2	1.473823
tr|A6NN60|A6NN60_	CRYZ	Quinone oxidoreductase OS=Homo sapiens GN=CRYZ PE=2 SV=1	31680	0	444	0	104	0	0.43	#DIV/0!	0	2	0	3	0	2	0	3	1.473823
tr|B3KRC6|B3KRC6_H	tr|B3KRC6|B3KRC6_HU	cDNA FLJ34004 fis, clone FCBBF1000232, highly similar to Cytochrome P450 51A1 (EC 1.1	51732	0	443	0	114	0	0.13	#DIV/0!	0	2	0	2	0	2	0	3	1.473823
tr|B4E1K7|B4E1K7_H	STOML2	Stomatin-like protein 2 OS=Homo sapiens GN=STOML2 PE=2 SV=1	33317	0	430	0	118	0	0.21	#DIV/0!	0	2	0	2	0	2	0	3	1.473823
tr|B2RAH7|B2RAH7_H	tr|B2RAH7|B2RAH7_H	cDNA, FLJ94921, highly similar to Homo sapiens prolyl endopeptidase (PREP), mRNA OS=	81594	0	424	0	122	0	0.08	#DIV/0!	0	2	0	2	0	2	0	2	1.473823
sp|Q96S66|CLCC1_H	CLCC1	Chloride channel CLIC-like protein 1 OS=Homo sapiens GN=CLCC1 PE=1 SV=1	62667	0	417	0	124	0	0.11	#DIV/0!	0	2	0	2	0	2	0	2	1.473823
tr|K7EQ60|K7EQ60_H	COASY	Bifunctional coenzyme A synthase (Fragment) OS=Homo sapiens GN=COASY PE=4 SV=1	12950	0	407	0	85	0	0.23	#DIV/0!	0	1	0	2	0	2	0	2	1.473823
sp|Q9H3P7|GCP60_H	ACBD3	Golgi resident protein GCP60 OS=Homo sapiens GN=ACBD3 PE=1 SV=4	60841	0	399	0	132	0	0.11	#DIV/0!	0	2	0	2	0	2	0	2	1.473823
sp|P36952|SPB5_HUN	SERPINB5	Serpin B5 OS=Homo sapiens GN=SERPINB5 PE=1 SV=2	42530	0	379	0	140	0	0.16	#DIV/0!	0	2	0	2	0	2	0	2	1.473823
tr|B4DN01|B4DN01_H	tr|B4DN01|B4DN01_H	cDNA FLJ57332, highly similar to COP9 signalosome complex subunit 3 OS=Homo sapien	33950	0	313	0	99	0	0.27	#DIV/0!	0	2	0	2	0	2	0	2	1.473823
tr|H0YEL7|H0YEL7_H	ACAT1	Acetyl-CoA acetyltransferase, mitochondrial (Fragment) OS=Homo sapiens GN=ACAT1 PE	14377	0	309	0	108	0	0.32	#DIV/0!	0	2	0	2	0	2	0	2	1.473823
tr|Q3SYK8|Q3SYK8_H	NUMA1	NUMA1 protein (Fragment) OS=Homo sapiens GN=NUMA1 PE=2 SV=1	78666	0	307	0	61	0	0.07	#DIV/0!	0	1	0	2	0	2	0	2	1.473823
tr|B2CKC5|B2CKC5_H	EFHC1	EF-hand domain (C-terminal) containing 1, isoform CRA_a OS=Homo sapiens GN=EFHC1	74400	0	1285	0	19	0	0.04	#DIV/0!	0	0	0	1	0	1	0	2	0.736677
sp|Q6UXN9|WDR82_	WDR82	WD repeat-containing protein 82 OS=Homo sapiens GN=WDR82 PE=1 SV=1	35456	0	1280	0	20	0	0.09	#DIV/0!	0	0	0	1	0	1	0	2	0.736677
sp|Q7RTS9|DYM_HUN	DYM	Dymeclin OS=Homo sapiens GN=DYM PE=1 SV=1	76571	0	1275	0	20	0	0.04	#DIV/0!	0	0	0	1	0	1	0	1	0.736677
tr|B7Z553|B7Z553_H	tr|B7Z553|B7Z553_HU	cDNA FLJ51266, highly similar to Vitronectin OS=Homo sapiens PE=2 SV=1	23761	0	1273	0	20	0	0.14	#DIV/0!	0	0	0	1	0	1	0	1	0.736677
tr|Q4LE61|Q4LE61_H	SYMPK	SYMPK variant protein (Fragment) OS=Homo sapiens GN=SYMPK variant protein PE=2 SV	119459	0	1272	0	20	0	0.03	#DIV/0!	0	0	0	1	0	1	0	3	0.736677
tr|B3KUL1|B3KUL1_H	tr|B3KUL1|B3KUL1_HU	cDNA FLJ40132 fis, clone TESTI2012155, highly similar to NUCLEOPORIN-LIKE PROTEIN R	59275	0	1270	0	20	0	0.06	#DIV/0!	0	0	0	1	0	1	0	1	0.736677
tr|B7Z7Q6|B7Z7Q6_H	PRCP	Lysosomal Pro-X carboxypeptidase OS=Homo sapiens GN=PRCP PE=2 SV=1	44362	0	1269	0	20	0	0.23	#DIV/0!	0	0	0	1	0	1	0	1	0.736677
tr|H0Y5W0|H0Y5W0_	HUWE1	E3 ubiquitin-protein ligase HUWE1 (Fragment) OS=Homo sapiens GN=HUWE1 PE=4 SV=1	376696	0	1267	0	20	0	0.01	#DIV/0!	0	0	0	1	0	1	0	2	0.736677
tr|Q6IB29|Q6IB29_H	EBNA1BP2	EBNA1 binding protein 2 OS=Homo sapiens GN=EBNA1BP2 PE=2 SV=1	34887	0	1266	0	21	0	0.1	#DIV/0!	0	0	0	1	0	1	0	1	0.736677
sp|Q9H9A6|LRC40_H	LRRC40	Leucine-rich repeat-containing protein 40 OS=Homo sapiens GN=LRRC40 PE=1 SV=1	68720	0	1265	0	21	0	0.05	#DIV/0!	0	0	0	1	0	1	0	1	0.736677
tr|B2R862|B2R862_H	tr|B2R862|B2R862_HU	cDNA, FLJ93754, highly similar to Homo sapiens tripartite motif-containing 38 (TRIM38),	54620	0	1263	0	21	0	0.06	#DIV/0!	0	0	0	1	0	1	0	1	0.736677
sp|O15020|SPTN2_H	SPTBN2	Spectrin beta chain, non-erythrocytic 2 OS=Homo sapiens GN=SPTBN2 PE=1 SV=3	272526	0	1262	0	21	0	0.01	#DIV/0!	0	0	0	1	0	1	0	2	0.736677
tr|B3KVG3|B3KVG3_H	tr|B3KVG3|B3KVG3_H	cDNA FLJ16519 fis, clone NT2RI3007684, highly similar to Prickle-like protein 1 OS=Homo	96121	0	1261	0	21	0	0.03	#DIV/0!	0	0	0	1	0	1	0	1	0.736677
tr|B4DKA8|B4DKA8_H	USP8	Ubiquitin carboxyl-terminal hydrolase OS=Homo sapiens GN=USP8 PE=2 SV=1	115921	0	1260	0	21	0	0.03	#DIV/0!	0	0	0	1	0	1	0	1	0.736677
tr|K7EK31|K7EK31_H	HEATR6	HEAT repeat-containing protein 6 (Fragment) OS=Homo sapiens GN=HEATR6 PE=4 SV=1	20572	0	1259	0	21	0	0.16	#DIV/0!	0	0	0	1	0	1	0	2	0.736677
tr|F1T0I5|F1T0I5_HU	AANAT	Arylalkylamine N-acetyltransferase OS=Homo sapiens GN=AANAT PE=2 SV=1	23842	0	1258	0	21	0	0.14	#DIV/0!	0	0	0	1	0	1	0	1	0.736677
tr|J3QTL0|J3QTL0_HU	TTC40	Tetratricopeptide repeat protein 40 OS=Homo sapiens GN=TTC40 PE=4 SV=1	175138	0	1257	0	21	0	0.02	#DIV/0!	0	0	0	1	0	1	0	1	0.736677
tr|B4E0N6|B4E0N6_H	tr|B4E0N6|B4E0N6_H	cDNA FLJ56280, highly similar to Endoplasmic reticulum-Golgi intermediate compartmer	26584	0	1256	0	21	0	0.18	#DIV/0!	0	0	0	1	0	1	0	1	0.736677
tr|A1YBP1|A1YBP1_H	BRCA2	Breast and ovarian cancer susceptibility protein 2 truncated variant OS=Homo sapiens G	300478	0	1254	0	22	0	0.01	#DIV/0!	0	0	0	1	0	1	0	1	0.736677
tr|B2RTX8|B2RTX8_H	WAPAL	WAPAL protein OS=Homo sapiens GN=WAPAL PE=2 SV=1	133797	0	1253	0	22	0	0.02	#DIV/0!	0	0	0	1	0	1	0	1	0.736677
sp|Q92995|UBP13_H	USP13	Ubiquitin carboxyl-terminal hydrolase 13 OS=Homo sapiens GN=USP13 PE=1 SV=2	98006	0	1251	0	22	0	0.03	#DIV/0!	0	0	0	1	0	1	0	2	0.736677
tr|A1A528|A1A528_H	ZW10	Centromere/kinetochore protein zw10 homolog OS=Homo sapiens GN=ZW10 PE=2 SV=1	77556	0	1249	0	22	0	0.04	#DIV/0!	0	0	0	1	0	1	0	1	0.736677
sp|Q06210|GFPT1_H	GFPT1	Glutamine--fructose-6-phosphate aminotransferase [isomerizing] 1 OS=Homo sapiens G	79555	0	1246	0	22	0	0.04	#DIV/0!	0	0	0	1	0	1	0	1	0.736677
sp|Q86YC3|LRC33_H	LRRC33	Leucine-rich repeat-containing protein 33 OS=Homo sapiens GN=LRRC33 PE=2 SV=1	77457	0	1244	0	22	0	0.04	#DIV/0!	0	1	0	1	0	1	0	1	0.736677
tr|H0YLV5|H0YLV5_H	GMPR2	GMP reductase 2 OS=Homo sapiens GN=GMPR2 PE=3 SV=1	36342	0	1243	0	22	0	0.56	#DIV/0!	0	0	0	1	0	1	0	1	0.736677
tr|B2RAM6|B2RAM6_	tr|B2RAM6|B2RAM6_	cDNA, FLJ95005, highly similar to Homo sapiens kinesin family member 11 (KIF11), mRN	120084	0	1242	0	22	0	0.03	#DIV/0!	0	0	0	1	0	1	0	1	0.736677
sp|B2RPK0|HGB1A_H	HMGB1P1	Putative high mobility group protein B1-like 1 OS=Homo sapiens GN=HMGB1P1 PE=5 SV	24394	0	1241	0	22	0	0.14	#DIV/0!	0	0	0	1	0	1	0	1	0.736677
tr|B3KNR3|B3KNR3_H	tr|B3KNR3|B3KNR3_H	Glutathione S-transferase OS=Homo sapiens PE=2 SV=1	19194	0	1238	0	23	0	0.18	#DIV/0!	0	0	0	1	0	1	0	1	0.736677
tr|A8K8Z4|A8K8Z4_H	tr|A8K8Z4|A8K8Z4_HU	cDNA FLJ78071, highly similar to Human MHC class III complement component C6 mRNA	108298	0	1236	0	23	0	0.03	#DIV/0!	0	0	0	1	0	1	0	1	0.736677
tr|H3BQP1|H3BQP1_	USP10	Ubiquitin carboxyl-terminal hydrolase 10 (Fragment) OS=Homo sapiens GN=USP10 PE=4	12544	0	1235	0	23	0	0.27	#DIV/0!	0	1	0	1	0	1	0	1	0.736677
tr|F5H3P6|F5H3P6_H	FADS1	Fatty acid desaturase 1 OS=Homo sapiens GN=FADS1 PE=2 SV=1	17375	0	1234	0	23	0	0.19	#DIV/0!	0	0	0	1	0	1	0	1	0.736677
tr|B0V109|B0V109_H	FLOT1	Flotillin-1 (Fragment) OS=Homo sapiens GN=FLOT1 PE=4 SV=1	39810	0	1232	0	23	0	0.08	#DIV/0!	0	0	0	1	0	1	0	1	0.736677
tr|B3KN37|B3KN37_H	tr|B3KN37|B3KN37_H	cDNA FLJ13414 fis, clone PLACE1001748, highly similar to Homo sapiens pitrilysin metall	68281	0	1230	0	23	0	0.13	#DIV/0!	0	0	0	1	0	1	0	1	0.736677
tr|B2R5U3|B2R5U3_H	EHD1	EH-domain containing 1, isoform CRA_b OS=Homo sapiens GN=EHD1 PE=2 SV=1	60646	0	1228	0	23	0	0.05	#DIV/0!	0	0	0	1	0	1	0	1	0.736677
tr|F8W8W7|F8W8W7	TGOLN2	Trans-Golgi network integral membrane protein 2 OS=Homo sapiens GN=TGOLN2 PE=2 S	29554	0	1227	0	23	0	0.11	#DIV/0!	0	0	0	1	0	1	0	1	0.736677
sp|Q8IYU8|EFHA1_H	EFHA1	EF-hand domain-containing family member A1 OS=Homo sapiens GN=EFHA1 PE=1 SV=2	49919	0	1224	0	23	0	0.07	#DIV/0!	0	0	0	1	0	1	0	1	0.736677
sp|A9QM74|IMA8_HU	KPNA7	Importin subunit alpha-8 OS=Homo sapiens GN=KPNA7 PE=1 SV=1	57529	0	1223	0	23	0	0.06	#DIV/0!	0	0	0	1	0	1	0	1	0.736677
tr|B2RDU4|B2RDU4_	tr|B2RDU4|B2RDU4_H	cDNA, FLJ96770 OS=Homo sapiens PE=2 SV=1	42200	0	1221	0	23	0	0.08	#DIV/0!	0	0	0	1	0	1	0	2	0.736677
tr|C9K0J5|C9K0J5_HU	RAPH1	Ras association (RalGDS/AF-6) and pleckstrin homology domains 1, isoform CRA_b OS=H	141776	0	1220	0	23	0	0.02	#DIV/0!	0	0	0	1	0	1	0	2	0.736677
tr|B7Z1G2|B7Z1G2_H	tr|B7Z1G2|B7Z1G2_HU	cDNA FLJ56017, highly similar to Pancreatic secretory granule membrane major glycopro	58875	0	1219	0	23	0	0.06	#DIV/0!	0	0	0	1	0	1	0	2	0.736677
sp|Q9H0A0|NAT10_H	NAT10	N-acetyltransferase 10 OS=Homo sapiens GN=NAT10 PE=1 SV=2	116569	0	1215	0	24	0	0.03	#DIV/0!	0	0	0	1	0	1	0	1	0.736677
tr|F5GZI5|F5GZI5_HU	ZAN	Zonadhesin OS=Homo sapiens GN=ZAN PE=2 SV=1	299445	0	1214	0	24	0	0.01	#DIV/0!	0	0	0	1	0	1	0	5	0.736677
tr|B2RBM8|B2RBM8_	tr|B2RBM8|B2RBM8_H	cDNA, FLJ95596, highly similar to Homo sapiens activity-dependent neuroprotector (ADN	124850	0	1213	0	24	0	0.03	#DIV/0!	0	0	0	1	0	1	0	1	0.736677
sp|Q9BR76|COR1B_H	CORO1B	Coronin-1B OS=Homo sapiens GN=CORO1B PE=1 SV=1	54885	0	1211	0	24	0	0.06	#DIV/0!	0	0	0	1	0	1	0	1	0.736677
tr|E9PIA2|E9PIA2_HU	PLEC	Plectin (Fragment) OS=Homo sapiens GN=PLEC PE=2 SV=1	20169	0	1210	0	24	0	0.17	#DIV/0!	0	0	0	1	0	1	0	2	0.736677
tr|L0R804|L0R804_HU	AAK1	Alternative protein AAK1 OS=Homo sapiens GN=AAK1 PE=4 SV=1	32517	0	1209	0	24	0	0.1	#DIV/0!	0	0	0	1	0	1	0	1	0.736677
tr|H3BND4|H3BND4_	PDXDC1	Pyridoxal-dependent decarboxylase domain-containing protein 1 OS=Homo sapiens GN=	89733	0	1208	0	24	0	0.07	#DIV/0!	0	0	0	2	0	1	0	2	0.736677

sp|Q6UWF7|NXPE4_H	NXPE4	NXPE family member 4 OS=Homo sapiens GN=NXPE4 PE=2 SV=1	62850	0	1206	0	25	0	0.05	#DIV/0!	0	0	0	1	0	1	0	1	0.736677
tr|B0QZ36|B0QZ36_H	RAE1	RAE1 RNA export 1 homolog (S. pombe) (Fragment) OS=Homo sapiens GN=RAE1 PE=4 SV	14945	0	1205	0	25	0	0.23	#DIV/0!	0	0	0	1	0	1	0	1	0.736677
tr|H7BZT7|H7BZT7_H	ESD	S-formylglutathione hydrolase (Fragment) OS=Homo sapiens GN=ESD PE=4 SV=1	25806	0	1204	0	25	0	0.13	#DIV/0!	0	0	0	1	0	1	0	1	0.736677
sp|Q9Y3L5|RAP2C_HU	RAP2C	Ras-related protein Rap-2c OS=Homo sapiens GN=RAP2C PE=1 SV=1	20959	0	1203	0	25	0	0.16	#DIV/0!	0	1	0	1	0	1	0	1	0.736677
tr|Q6ZWH1|Q6ZWH1_	tr|Q6ZWH1|Q6ZWH1_	cDNA FLJ41081 fis, clone ADRGL2009755, highly similar to Homo sapiens brain and repro	45824	0	1202	0	25	0	0.07	#DIV/0!	0	0	0	1	0	1	0	1	0.736677
tr|F6U211|F6U211_H	RPS10	40S ribosomal protein S10 OS=Homo sapiens GN=RPS10 PE=2 SV=1	19912	0	1201	0	25	0	0.17	#DIV/0!	0	0	0	1	0	1	0	1	0.736677
tr|B4DH29|B4DH29_H	tr|B4DH29|B4DH29_HU	DNA-directed RNA polymerase OS=Homo sapiens PE=2 SV=1	126564	0	1200	0	25	0	0.03	#DIV/0!	0	0	0	1	0	1	0	1	0.736677
tr|B4DH22|B4DH22_H	tr|B4DH22|B4DH22_HU	cDNA FLJ59884, highly similar to Secernin-1 OS=Homo sapiens PE=2 SV=1	25067	0	1199	0	25	0	0.13	#DIV/0!	0	0	0	1	0	1	0	1	0.736677
tr|H0YD93|H0YD93_H	RUFY2	RUN and FYVE domain-containing protein 2 (Fragment) OS=Homo sapiens GN=RUFY2 PE	72131	0	1198	0	25	0	0.05	#DIV/0!	0	0	0	1	0	1	0	1	0.736677
tr|B4E2N1|B4E2N1_H	tr|B4E2N1|B4E2N1_HU	cDNA FLJ56560, highly similar to Caspase recruitment domain-containing protein 6 OS=H	55261	0	1197	0	25	0	0.06	#DIV/0!	0	1	0	1	0	1	0	1	0.736677
tr|H0YH81|H0YH81_H	ATP5B	ATP synthase subunit beta (Fragment) OS=Homo sapiens GN=ATP5B PE=3 SV=1	38226	0	1196	0	25	0	0.09	#DIV/0!	0	0	0	1	0	1	0	1	0.736677
sp|Q99797|MIPEP_HU	MIPEP	Mitochondrial intermediate peptidase OS=Homo sapiens GN=MIPEP PE=1 SV=2	81387	0	1195	0	25	0	0.04	#DIV/0!	0	0	0	1	0	1	0	2	0.736677
sp|Q6IF42|OR2A2_HU	OR2A2	Olfactory receptor 2A2 OS=Homo sapiens GN=OR2A2 PE=2 SV=2	36594	0	1194	0	25	0	0.09	#DIV/0!	0	0	0	1	0	1	0	1	0.736677
tr|G5E9V1|G5E9V1_H	TFG	Protein TFG OS=Homo sapiens GN=TFG PE=4 SV=1	43051	0	1191	0	25	0	0.08	#DIV/0!	0	0	0	1	0	1	0	1	0.736677
sp|Q8IYA2|C144C_HU	CCDC144C	Coiled-coil domain-containing protein 144C OS=Homo sapiens GN=CCDC144C PE=2 SV=3	144106	0	1189	0	26	0	0.02	#DIV/0!	0	0	0	1	0	1	0	2	0.736677
tr|H3BSH7|H3BSH7_H	CIRH1A	Cirhin (Fragment) OS=Homo sapiens GN=CIRH1A PE=4 SV=1	78578	0	1186	0	26	0	0.04	#DIV/0!	0	0	0	1	0	1	0	1	0.736677
sp|Q9Y512|SAM50_H	SAMM50	Sorting and assembly machinery component 50 homolog OS=Homo sapiens GN=SAMM5	52342	0	1184	0	26	0	0.06	#DIV/0!	0	0	0	1	0	1	0	2	0.736677
tr|B2RDG1|B2RDG1_H	tr|B2RDG1|B2RDG1_H	cDNA, FLJ96593 OS=Homo sapiens PE=2 SV=1	59687	0	1183	0	26	0	0.06	#DIV/0!	0	0	0	1	0	1	0	1	0.736677
tr|B4DN44|B4DN44_H	tr|B4DN44|B4DN44_HU	cDNA FLJ53581, highly similar to Ras and Rab interactor 1 OS=Homo sapiens PE=2 SV=1	65995	0	1182	0	26	0	0.05	#DIV/0!	0	1	0	1	0	1	0	1	0.736677
tr|Q2VIM1|Q2VIM1_H	tr|Q2VIM1|Q2VIM1_H	RcCDC20 (Fragment) OS=Homo sapiens PE=2 SV=1	50517	0	1181	0	26	0	0.07	#DIV/0!	0	0	0	1	0	1	0	1	0.736677
tr|Q8WW55|Q8WW5	tr|Q8WW55|Q8WW55	STAT5B protein OS=Homo sapiens PE=2 SV=1	45148	0	1180	0	26	0	0.07	#DIV/0!	0	0	0	1	0	1	0	1	0.736677
tr|B4E3I3|B4E3I3_HU	tr|B4E3I3|B4E3I3_HUM	cDNA FLJ59614, highly similar to Ubiquitin fusion degradation protein 1 homolog OS=Ho	35730	0	1178	0	26	0	0.09	#DIV/0!	0	0	0	1	0	1	0	1	0.736677
tr|F5H3M2|F5H3M2_	KIFC3	Kinesin-like protein KIFC3 OS=Homo sapiens GN=KIFC3 PE=2 SV=1	81892	0	1176	0	26	0	0.04	#DIV/0!	0	0	0	1	0	1	0	1	0.736677
sp|Q6ZST2|ZCH23_HU	ZCCHC23	Zinc finger CCHC domain-containing protein 23 OS=Homo sapiens GN=ZCCHC23 PE=2 SV	14913	0	1175	0	26	0	0.23	#DIV/0!	0	0	0	1	0	1	0	1	0.736677
tr|B0I1S1|B0I1S1_HU	tr|B0I1S1|B0I1S1_HUM	DNAH10 variant protein (Fragment) OS=Homo sapiens PE=2 SV=1	382946	0	1174	0	26	0	0.01	#DIV/0!	0	0	0	1	0	1	0	2	0.736677
tr|B4DK61|B4DK61_H	tr|B4DK61|B4DK61_HU	Acyl-coenzyme A oxidase OS=Homo sapiens PE=2 SV=1	70622	0	1173	0	23	0	0.1	#DIV/0!	0	0	0	1	0	1	0	1	0.736677
tr|H0YJV2|H0YJV2_HU	SPTLC2	Serine palmitoyltransferase 2 (Fragment) OS=Homo sapiens GN=SPTLC2 PE=3 SV=1	46191	0	1171	0	26	0	0.07	#DIV/0!	0	0	0	1	0	1	0	1	0.736677
sp|Q86UE4|LYRIC_HU	MTDH	Protein LYRIC OS=Homo sapiens GN=MTDH PE=1 SV=2	63856	0	1168	0	27	0	0.05	#DIV/0!	0	0	0	1	0	1	0	1	0.736677
tr|G3V335|G3V335_H	CCDC176	Coiled-coil domain-containing protein 176 OS=Homo sapiens GN=CCDC176 PE=2 SV=1	12240	0	1167	0	27	0	0.28	#DIV/0!	0	0	0	1	0	1	0	1	0.736677
sp|Q9BVI4|NOC4L_HU	NOC4L	Nucleolar complex protein 4 homolog OS=Homo sapiens GN=NOC4L PE=1 SV=1	58830	0	1166	0	27	0	0.06	#DIV/0!	0	0	0	1	0	1	0	2	0.736677
sp|P60880|SNP25_HU	SNAP25	Synaptosomal-associated protein 25 OS=Homo sapiens GN=SNAP25 PE=1 SV=1	23528	0	1165	0	27	0	0.14	#DIV/0!	0	0	0	1	0	1	0	1	0.736677
tr|Q96FS1|Q96FS1_H	CTNND1	CTNND1 protein (Fragment) OS=Homo sapiens GN=CTNND1 PE=2 SV=2	92901	0	1164	0	27	0	0.03	#DIV/0!	0	0	0	1	0	1	0	1	0.736677
tr|Q5JTE4|Q5JTE4_HU	POLR1C	Polymerase (RNA) I polypeptide C, 30kDa OS=Homo sapiens GN=POLR1C PE=2 SV=1	36043	0	1162	0	27	0	0.15	#DIV/0!	0	0	0	1	0	1	0	1	0.736677
tr|B4DSE9|B4DSE9_H	tr|B4DSE9|B4DSE9_HU	cDNA FLJ58278, highly similar to Homo sapiens WD repeat domain 44 (WDR44), mRNA C	91610	0	1161	0	27	0	0.04	#DIV/0!	0	0	0	1	0	1	0	1	0.736677
tr|G3V1B3|G3V1B3_H	RPL21	60S ribosomal protein L21 OS=Homo sapiens GN=RPL21 PE=2 SV=1	9994	0	1159	0	28	0	0.35	#DIV/0!	0	0	0	1	0	1	0	1	0.736677
tr|C9JP81|C9JP81_HU	MAD1L1	Mitotic spindle assembly checkpoint protein MAD1 (Fragment) OS=Homo sapiens GN=M	30913	0	1157	0	28	0	0.11	#DIV/0!	0	0	0	1	0	1	0	1	0.736677
tr|B7Z7U0|B7Z7U0_H	tr|B7Z7U0|B7Z7U0_HU	cDNA FLJ61739, highly similar to Serine/arginine repetitive matrix protein 1 OS=Homo sa	103792	0	1156	0	28	0	0.03	#DIV/0!	0	0	0	1	0	1	0	1	0.736677
tr|H0YJ30|H0YJ30_HU	GPHN	Molybdopterin molybdenumtransferase (Fragment) OS=Homo sapiens GN=GPHN PE=4 S	30436	0	1155	0	28	0	0.11	#DIV/0!	0	0	0	1	0	1	0	1	0.736677
sp|Q7Z5H4|VN1R5_H	VN1R5	Vomeronasal type-1 receptor 5 OS=Homo sapiens GN=VN1R5 PE=2 SV=2	41265	0	1154	0	28	0	0.08	#DIV/0!	0	0	0	1	0	1	0	1	0.736677
tr|B2R642|B2R642_H	tr|B2R642|B2R642_HU	cDNA, FLJ92775, highly similar to Homo sapiens melanoma cell adhesion molecule (MCA	72560	0	1152	0	28	0	0.05	#DIV/0!	0	0	0	1	0	1	0	1	0.736677
tr|B4DEP6|B4DEP6_H	tr|B4DEP6|B4DEP6_HU	cDNA FLJ59206, highly similar to Eukaryotic translation initiation factor 4B OS=Homo sap	66146	0	1150	0	28	0	0.05	#DIV/0!	0	0	0	1	0	1	0	1	0.736677
tr|A4D0U1|A4D0U1_H	IFRD1	Interferon-related developmental regulator 1 OS=Homo sapiens GN=IFRD1 PE=2 SV=1	51035	0	1147	0	28	0	0.06	#DIV/0!	0	0	0	1	0	1	0	1	0.736677
tr|B4DFU8|B4DFU8_H	tr|B4DFU8|B4DFU8_HU	cDNA FLJ54647, highly similar to Liprin-beta-1 (Fragment) OS=Homo sapiens PE=2 SV=1	84347	0	1146	0	28	0	0.04	#DIV/0!	0	1	0	1	0	1	0	1	0.736677
tr|B4DRJ4|B4DRJ4_HU	tr|B4DRJ4|B4DRJ4_HU	cDNA FLJ55392, highly similar to Telomere-associated protein RIF1 (Fragment) OS=Homo	113092	0	1145	0	28	0	0.03	#DIV/0!	0	0	0	1	0	1	0	1	0.736677
tr|A8K4H7|A8K4H7_H	PPAT	Amidophosphoribosyltransferase OS=Homo sapiens GN=PPAT PE=2 SV=1	58161	0	1143	0	28	0	0.06	#DIV/0!	0	0	0	1	0	1	0	1	0.736677
sp|Q96HE7|ERO1A_H	ERO1L	ERO1-like protein alpha OS=Homo sapiens GN=ERO1L PE=1 SV=2	55213	0	1142	0	28	0	0.39	#DIV/0!	0	0	0	1	0	1	0	1	0.736677
tr|G3V0J0|G3V0J0_HU	FMR1	Fragile X mental retardation 1, isoform CRA_e OS=Homo sapiens GN=FMR1 PE=2 SV=1	67030	0	1141	0	28	0	0.05	#DIV/0!	0	0	0	1	0	1	0	1	0.736677
tr|Q8N6B4|Q8N6B4_H	MLL/CALM	Myeloid/lymphoid or mixed-lineage leukemia/clathrin assembly protein fusion protein (F	63408	0	1140	0	28	0	0.05	#DIV/0!	0	0	0	1	0	1	0	3	0.736677
tr|Q6MZF7|Q6MZF7_	DKFZp686E11248	Putative uncharacterized protein DKFZp686E11248 (Fragment) OS=Homo sapiens GN=DI	29013	0	1139	0	28	0	0.11	#DIV/0!	0	1	0	1	0	1	0	1	0.736677
tr|H0Y8H0|H0Y8H0_H	DDX60L	Probable ATP-dependent RNA helicase DDX60-like (Fragment) OS=Homo sapiens GN=DD	102233	0	1138	0	28	0	0.03	#DIV/0!	0	1	0	1	0	1	0	1	0.736677
tr|B1AMX9|B1AMX9_	TRAF2	TNF receptor-associated factor 2 OS=Homo sapiens GN=TRAF2 PE=4 SV=1	47848	0	1137	0	29	0	0.07	#DIV/0!	0	0	0	1	0	1	0	1	0.736677
tr|B3KU60|B3KU60_H	tr|B3KU60|B3KU60_HU	cDNA FLJ39235 fis, clone OCBBF2007829, highly similar to Mus musculus fatso protein O	59071	0	1136	0	29	0	0.06	#DIV/0!	0	0	0	1	0	1	0	1	0.736677
tr|B4DWS5|B4DWS5_	tr|B4DWS5|B4DWS5_H	cDNA FLJ57639, highly similar to Brix domain-containing protein 1 OS=Homo sapiens PE=	29109	0	1135	0	29	0	0.14	#DIV/0!	0	0	0	1	0	1	0	1	0.736677
sp|P49756|RBM25_HU	RBM25	RNA-binding protein 25 OS=Homo sapiens GN=RBM25 PE=1 SV=3	100467	0	1133	0	29	0	0.03	#DIV/0!	0	1	0	1	0	1	0	3	0.736677
tr|Q5HYG2|Q5HYG2_	DKFZp686H0286	Putative uncharacterized protein DKFZp686H0286 OS=Homo sapiens GN=DKFZp686H028	8427	0	1132	0	29	0	0.03	#DIV/0!	0	0	0	1	0	1	0	1	0.736677
tr|H0YKU5|H0YKU5_H	COPS2	COP9 signalosome complex subunit 2 (Fragment) OS=Homo sapiens GN=COPS2 PE=4 SV=	31061	0	1131	0	29	0	0.11	#DIV/0!	0	0	0	1	0	1	0	1	0.736677
tr|G3V0G3|G3V0G3_H	DDX56	DEAD (Asp-Glu-Ala-Asp) box polypeptide 56, isoform CRA_a OS=Homo sapiens GN=DDX5	41969	0	1127	0	29	0	0.08	#DIV/0!	0	0	0	1	0	1	0	1	0.736677
tr|Q9P0D3|Q9P0D3_H	tr|Q9P0D3|Q9P0D3_HU	HSPC103 (Fragment) OS=Homo sapiens PE=2 SV=1	12624	0	1126	0	29	0	0.27	#DIV/0!	0	1	0	1	0	1	0	1	0.736677
tr|E7EMC7|E7EMC7_H	SQSTM1	Sequestosome-1 OS=Homo sapiens GN=SQSTM1 PE=2 SV=1	42070	0	1125	0	0	0	0.1	#DIV/0!	0	0	0	0	0	1	0	1	0.736677
tr|O00373|O00373_H	tr|O00373|O00373_HU	P40 OS=Homo sapiens PE=4 SV=1	40116	0	1120	0	30	0	0.08	#DIV/0!	0	0	0	1	0	1	0	1	0.736677
tr|Q13380|Q13380_H	RBM	RNA binding motif (Fragment) OS=Homo sapiens GN=RBM PE=4 SV=1	4015	0	1119	0	30	0	0.97	#DIV/0!	0	1	0	1	0	1	0	1	0.736677
sp|Q9UN73|PCDA6_H	PCDHA6	Protocadherin alpha-6 OS=Homo sapiens GN=PCDHA6 PE=2 SV=1	103336	0	1118	0	30	0	0.03	#DIV/0!	0	1	0	1	0	1	0	1	0.736677
tr|G3V529|G3V529_H	DDX24	ATP-dependent RNA helicase DDX24 OS=Homo sapiens GN=DDX24 PE=2 SV=1	91994	0	1116	0	30	0	0.04	#DIV/0!	0	1	0	1	0	1	0	1	0.736677
tr|A1L172|A1L172_HU	ACOT1	Acyl-CoA thioesterase 1 OS=Homo sapiens GN=ACOT1 PE=2 SV=1	46666	0	1113	0	30	0	0.07	#DIV/0!	0	0	0	1	0	1	0	1	0.736677
sp|P49750|YLPM1_HU	YLPM1	YLP motif-containing protein 1 OS=Homo sapiens GN=YLPM1 PE=1 SV=3	220077	0	1112	0	30	0	0.02	#DIV/0!	0	0	0	1	0	1	0	1	0.736677
sp|P82673|RT35_HUM	MRPS35	28S ribosomal protein S35, mitochondrial OS=Homo sapiens GN=MRPS35 PE=1 SV=1	37106	0	1109	0	30	0	0.09	#DIV/0!	0	0	0	1	0	1	0	1	0.736677
tr|Q9UFD5|Q9UFD5_	DKFZp434N181	Putative uncharacterized protein DKFZp434N181 (Fragment) OS=Homo sapiens GN=DKF	49392	0	1108	0	30	0	0.07	#DIV/0!	0	1	0	1	0	1	0	1	0.736677
sp|Q9UKV8|AGO2_HU	EIF2C2	Protein argonaute-2 OS=Homo sapiens GN=EIF2C2 PE=1 SV=3	98400	0	1105	0	30	0	0.04	#DIV/0!	0	0	0	1	0	1	0	1	0.736677
tr|F1D8N4|F1D8N4_H	NR3C1	Glucocorticoid nuclear receptor variant 1 OS=Homo sapiens GN=NR3C1 PE=2 SV=1	86745	0	1102	0	30	0	0.04	#DIV/0!	0	0	0	1	0	1	0	2	0.736677
tr|Q6FGM0|Q6FGM0_	SH3GL1	SH3 domain GRB2-like 1 OS=Homo sapiens GN=SH3GL1 PE=2 SV=1	41692	0	1101	0	30	0	0.09	#DIV/0!	0	0	0	1	0	1	0	1	0.736677
tr|B4DFR1|B4DFR1_H	COX10	Protoheme IX farnesyltransferase, mitochondrial OS=Homo sapiens GN=COX10 PE=2 SV=	25037	0	1099	0	30	0	0.13	#DIV/0!	0	1	0	1	0	1	0	1	0.736677
tr|B4E205|B4E205_HU	tr|B4E205|B4E205_HU	cDNA FLJ61651, highly similar to Protein transport protein Sec24A OS=Homo sapiens PE=	96953	0	1098	0	30	0	0.03	#DIV/0!	0	0	0	1	0	1	0	1	0.736677
tr|B4DTM6|B4DTM6_	tr|B4DTM6|B4DTM6_H	cDNA FLJ61391, highly similar to Protein transport protein Sec24B OS=Homo sapiens PE=	130037	0	1097	0	30	0	0.03	#DIV/0!	0	0	0	1	0	1	0	1	0.736677
tr|B4DY77|B4DY77_H	ALLC	Allantoicase, isoform CRA_a OS=Homo sapiens GN=ALLC PE=2 SV=1	43930	0	1096	0	30	0	0.08	#DIV/0!	0	1	0	1	0	1	0	1	0.736677
tr|E9PDU6|E9PDU6_H	CNN3	Calponin-3 (Fragment) OS=Homo sapiens GN=CNN3 PE=2 SV=1	20309	0	1092	0	31	0	0.17	#DIV/0!	0	0	0	1	0	1	0	1	0.736677
tr|D3DTC2|D3DTC2_H	AYTL2	Acyltransferase like 2, isoform CRA_a OS=Homo sapiens GN=AYTL2 PE=2 SV=1	54725	0	1091	0	31	0	0.06	#DIV/0!	0	0	0	1	0	1	0	2	0.736677
tr|B3KSE4|B3KSE4_HU	tr|B3KSE4|B3KSE4_HU	cDNA FLJ36076 fis, clone TESTI2019760, highly similar to TRANSCRIPTIONAL REPRESSOR	30924	0	1090	0	31	0	0.11	#DIV/0!	0	1	0	2	0	1	0	2	0.736677
tr|K7EIS0|K7EIS0_HUM	TUBG1	Tubulin gamma chain (Fragment) OS=Homo sapiens GN=TUBG1 PE=3 SV=1	29797	0	1088	0	31	0	0.11	#DIV/0!	0	0	0	1	0	1	0	1	0.736677
sp|Q8IYS2|K2013_HU	KIAA2013	Uncharacterized protein KIAA2013 OS=Homo sapiens GN=KIAA2013 PE=2 SV=1	69684	0	1086	0	31	0	0.05	#DIV/0!	0	0	0	1	0	1	0	1	0.736677
sp|P27694|RFA1_HUM	RPA1	Replication protein A 70 kDa DNA-binding subunit OS=Homo sapiens GN=RPA1 PE=1 SV=	68723	0	1084	0	31	0	0.05	#DIV/0!	0	0	0	1	0	1	0	1	0.736677
tr|A0JLT1|A0JLT1_HU	CD3EAP	CD3EAP protein (Fragment) OS=Homo sapiens GN=CD3EAP PE=2 SV=1	46280	0	1083	0	31	0	0.07	#DIV/0!	0	0	0	1	0	1	0	1	0.736677
tr|Q2TAM4|Q2TAM4_	POLR1B	DNA-directed RNA polymerase OS=Homo sapiens GN=POLR1B PE=2 SV=1	108996	0	1081	0	32	0	0.03	#DIV/0!	0	1	0	1	0	1	0	1	0.736677
tr|B4DHJ2|B4DHJ2_H	tr|B4DHJ2|B4DHJ2_HU	cDNA FLJ59326, highly similar to Protein FAM21C OS=Homo sapiens PE=2 SV=1	65820	0	1080	0	32	0	0.05	#DIV/0!	0	1	0	1	0	1	0	1	0.736677
tr|E7ERH3|E7ERH3_H	TNS3	Tensin-3 OS=Homo sapiens GN=TNS3 PE=2 SV=1	130127	0	1076	0	32	0	0.03	#DIV/0!	0	0	0	1	0	1	0	1	0.736677
tr|Q4LE49|Q4LE49_H	ARID1A	ARID1A variant protein (Fragment) OS=Homo sapiens GN=ARID1A variant protein PE=2 S	147154	0	1075	0	32	0	0.02	#DIV/0!	0	0	0	1	0	1	0	1	0.736677
sp|Q14684|RRP1B_HU	RRP1B	Ribosomal RNA processing protein 1 homolog B OS=Homo sapiens GN=RRP1B PE=1 SV=3	84774	0	1071	0	32	0	0.04	#DIV/0!	0	0	0	1	0	1	0	1	0.736677
tr|J3KT29|J3KT29_HU	RPL23	60S ribosomal protein L23 OS=Homo sapiens GN=RPL23 PE=3 SV=1	13241	0	1070	0	32	0	0.26	#DIV/0!	0	0	0	1	0	1	0	1	0.736677
sp|Q14677|EPN4_HU	CLINT1	Clathrin interactor 1 OS=Homo sapiens GN=CLINT1 PE=1 SV=1	68273	0	1069	0	32	0	0.05	#DIV/0!	0	0	0	1	0	1	0	2	0.736677
sp|P51398|RT29_HUM	DAP3	28S ribosomal protein S29, mitochondrial OS=Homo sapiens GN=DAP3 PE=1 SV=1	45880	0	1068	0	32	0	0.07	#DIV/0!	0	0	0	1	0	1	0	2	0.736677
tr|B4DR36|B4DR36_H	tr|B4DR36|B4DR36_HU	cDNA FLJ56414, highly similar to Homo sapiens proline-, glutamic acid-, leucine-rich prot	126101	0	1067	0	32	0	0.05	#DIV/0!	0	0	0	2	0	1	0	2	0.736677
tr|Q6NSG9|Q6NSG9_	AKAP12	AKAP12 protein (Fragment) OS=Homo sapiens GN=AKAP12 PE=2 SV=1	19461	0	1065	0	33	0	0.17	#DIV/0!	0	0	0	1	0	1	0	1	0.736677
sp|Q9BTV4|TMM43_H	TMEM43	Transmembrane protein 43 OS=Homo sapiens GN=TMEM43 PE=1 SV=1	44904	0	1063	0	33	0	0.07	#DIV/0!	0	0	0	1	0	1	0	1	0.736677
tr|B4DGG1|B4DGG1_	ITFG3	Protein ITFG3 OS=Homo sapiens GN=ITFG3 PE=2 SV=1	58252	0	1062	0	33	0	0.06	#DIV/0!	0	0	0	1	0	1	0	1	0.736677
sp|A1L0T0|ILVBL_HUM	ILVBL	Acetolactate synthase-like protein OS=Homo sapiens GN=ILVBL PE=1 SV=2	68452	0	1061	0	33	0	0.05	#DIV/0!	0	0	0	1	0	1	0	1	0.736677
sp|Q9UBG0|MRC2_HU	MRC2	C-type mannose receptor 2 OS=Homo sapiens GN=MRC2 PE=1 SV=2	169761	0	1060	0	33	0	0.02	#DIV/0!	0	0	0	1	0	1	0	1	0.736677
tr|H0YEG8|H0YEG8_H	NUCB2	Nefastin-1 (Fragment) OS=Homo sapiens GN=NUCB2 PE=4 SV=1	23746	0	1058	0	33	0	0.14	#DIV/0!	0	0	0	1	0	1	0	1	0.736677
sp|Q14126|DSG2_HU	DSG2	Desmoglein-2 OS=Homo sapiens GN=DSG2 PE=1 SV=2	123016	0	1057	0	33	0	0.03	#DIV/0!	0	0	0	1	0	1	0	2	0.736677
tr|Q5U608|Q5U608_H	SUPT3H	SUPT3H protein (Fragment) OS=Homo sapiens GN=SUPT3H PE=2 SV=1	41807	0	1056	0	33	0	0.08	#DIV/0!	0	0	0	1	0	1	0	1	0.736677
tr|G5EA24|G5EA24_H	MCOLN2	Mucolipin 2, isoform CRA_a OS=Homo sapiens GN=MCOLN2 PE=2 SV=1	29581	0	1052	0	33	0	0.12	#DIV/0!	0	1	0	1	0	1	0	2	0.736677
sp|O76003|GLRX3_HU	GLRX3	Glutaredoxin-3 OS=Homo sapiens GN=GLRX3 PE=1 SV=2	37693	0	1051	0	33	0	0.09	#DIV/0!	0	0	0	1	0	1	0	1	0.736677
tr|B3KUH4|B3KUH4_H	tr|B3KUH4|B3KUH4_H	cDNA FLJ39909 fis, clone SPLEN2017918, highly similar to EH-domain-containing protein	38714	0	1050	0	33	0	0.09	#DIV/0!	0	0	0	1	0	1	0	1	0.736677
tr|Q5QJ99|Q5QJ99_H	CACNB2	Voltage-gated calcium channel beta 2 subunit splice variant CavB2aN4 OS=Homo sapiens	68348	0	1049	0	33	0	0.05	#DIV/0!	0	1	0	1	0	1	0	1	0.736677
tr|B3KSB5|B3KSB5_H	tr|B3KSB5|B3KSB5_HU	cDNA FLJ35923 fis, clone TESTI2010724 OS=Homo sapiens PE=2 SV=1	104031	0	1048	0	33	0	0.03	#DIV/0!	0	1	0	1	0	1	0	1	0.736677
tr|Q2TSD2|Q2TSD2_H	tr|Q2TSD2|Q2TSD2_HU	Aging-associated gene 7 protein OS=Homo sapiens PE=2 SV=1	41778	0	1045	0	33	0	0.08	#DIV/0!	0	0	0	1	0	1	0	1	0.736677
tr|B3KMK2|B3KMK2_	tr|B3KMK2|B3KMK2_H	cDNA FLJ11229 fis, clone PLACE1008356, highly similar to Homo sapiens meningioma ex	54835	0	1042	0	34	0	0.06	#DIV/0!	0	0	0	1	0	1	0	1	0.736677
sp|Q8IX12|CCAR1_HU	CCAR1	Cell division cycle and apoptosis regulator protein 1 OS=Homo sapiens GN=CCAR1 PE=1 S	133423	0	1041	0	34	0	0.02	#DIV/0!	0	0	0	1	0	1	0	2	0.736677
tr|D3DW85|D3DW85_	IREB2	Iron-responsive element binding protein 2, isoform CRA_a OS=Homo sapiens GN=IREB2	106006	0	1034	0	34	0	0.03	#DIV/0!	0	0	0	1	0	1	0	1	0.736677
tr|B1AH49|B1AH49_H	MPST	Sulfurtransferase OS=Homo sapiens GN=MPST PE=4 SV=1	25081	0	1033	0	34	0	0.13	#DIV/0!	0	0	0	1	0	1	0	1	0.736677
tr|B3KNV9|B3KNV9_H	tr|B3KNV9|B3KNV9_HU	cDNA FLJ30554 fis, clone BRAWH2003693 OS=Homo sapiens PE=2 SV=1	112670	0	1032	0	34	0	0.03	#DIV/0!	0	1	0	1	0	1	0	1	0.736677
tr|Q3B768|Q3B768_H	KIAA0406	KIAA0406 protein (Fragment) OS=Homo sapiens GN=KIAA0406 PE=2 SV=1	91343	0	1028	0	34	0	0.04	#DIV/0!	0	1	0	1	0	1	0	1	0.736677
tr|B2RDE1|B2RDE1_H	tr|B2RDE1|B2RDE1_HU	cDNA, FLJ96568, highly similar to Homo sapiens tropomyosin 3 (TPM3), mRNA OS=Homo	29228	0	1026	0	34	0	0.11	#DIV/0!	0	0	0	1	0	1	0	2	0.736677
tr|H0YK48|H0YK48_H	TPM1	Tropomyosin alpha-1 chain OS=Homo sapiens GN=TPM1 PE=3 SV=1	28620	0	1025	0	34	0	0.12	#DIV/0!	0	0	0	1	0	1	0	2	0.736677
tr|Q5T9B9|Q5T9B9_H	ENG	Endoglin (Osler-Rendu-Weber syndrome 1) OS=Homo sapiens GN=ENG PE=4 SV=1	68468	0	1024	0	34	0	0.05	#DIV/0!	0	0	0	1	0	1	0	1	0.736677
tr|F5GZI3|F5GZI3_HU	PARD3	Partitioning defective 3 homolog OS=Homo sapiens GN=PARD3 PE=2 SV=1	82242	0	1021	0	34	0	0.04	#DIV/0!	0	1	0	1	0	1	0	3	0.736677
tr|B4DM41|B4DM41_	RFC4	Replication factor C (Activator 1) 4, 37kDa, isoform CRA_b OS=Homo sapiens GN=RFC4 P	33996	0	1020	0	34	0	0.1	#DIV/0!	0	0	0	1	0	1	0	1	0.736677
tr|O95036|O95036_H	WUGSC:H_RG054D04.1	Similar to 60S ribosomal protein L7; similar to P18124 (PID:d133021) OS=Homo sapiens (	29075	0	1019	0	34	0	0.11	#DIV/0!	0	0	0	1	0	1	0	3	0.736677
tr|Q4LE48|Q4LE48_HU	STAG1	STAG1 variant protein (Fragment) OS=Homo sapiens GN=STAG1 variant protein PE=2 SV=	147272	0	1016	0	0	0	0.02	#DIV/0!	0	0	0	0	0	1	0	1	0.736677
tr|H0Y9G6|H0Y9G6_H	MRPL3	39S ribosomal protein L3, mitochondrial (Fragment) OS=Homo sapiens GN=MRPL3 PE=3	40898	0	1015	0	35	0	0.08	#DIV/0!	0	1	0	1	0	1	0	1	0.736677
tr|B1AVV4|B1AVV4_H	SH2D4B	SH2 domain-containing protein 4B OS=Homo sapiens GN=SH2D4B PE=4 SV=1	26235	0	1014	0	35	0	0.14	#DIV/0!	0	1	0	1	0	1	0	1	0.736677
tr|H7C0M3|H7C0M3_	BPHL	Valacyclovir hydrolase (Fragment) OS=Homo sapiens GN=BPHL PE=4 SV=1	3314	0	1013	0	35	0	1.2	#DIV/0!	0	1	0	1	0	1	0	1	0.736677
tr|A4UCU0|A4UCU0_	tr|A4UCU0|A4UCU0_H	DEAD box polypeptide 47 isoform 1 variant (Fragment) OS=Homo sapiens PE=2 SV=1	19904	0	1011	0	35	0	0.17	#DIV/0!	0	1	0	1	0	1	0	1	0.736677
sp|Q5JVF3|PCID2_HU	PCID2	PCI domain-containing protein 2 OS=Homo sapiens GN=PCID2 PE=1 SV=2	46627	0	1005	0	36	0	0.07	#DIV/0!	0	1	0	1	0	1	0	1	0.736677
tr|A0JNT2|A0JNT2_HU	KRT83	KRT83 protein OS=Homo sapiens GN=KRT83 PE=2 SV=1	50978	0	1004	0	36	0	0.06	#DIV/0!	0	1	0	1	0	1	0	1	0.736677
tr|J9JID7|J9JID7_HUM	LMNB2	Lamin B2, isoform CRA_a OS=Homo sapiens GN=LMNB2 PE=3 SV=1	70020	0	1003	0	36	0	0.05	#DIV/0!	0	1	0	1	0	1	0	2	0.736677
sp|P23368|MAOM_HU	ME2	NAD-dependent malic enzyme, mitochondrial OS=Homo sapiens GN=ME2 PE=1 SV=1	66029	0	1001	0	36	0	0.05	#DIV/0!	0	1	0	1	0	1	0	1	0.736677
tr|E9PC74|E9PC74_HU	EIF2B5	Translation initiation factor eIF-2B subunit epsilon OS=Homo sapiens GN=EIF2B5 PE=2 SV	79096	0	1000	0	36	0	0.04	#DIV/0!	0	1	0	1	0	1	0	1	0.736677
tr|Q6IPI1|Q6IPI1_HUM	RPL29	Ribosomal protein L29 OS=Homo sapiens GN=RPL29 PE=2 SV=1	17997	0	995	0	36	0	0.19	#DIV/0!	0	1	0	1	0	1	0	1	0.736677
sp|P80723|BASP1_HU	BASP1	Brain acid soluble protein 1 OS=Homo sapiens GN=BASP1 PE=1 SV=2	22680	0	994	0	36	0	0.15	#DIV/0!	0	1	0	1	0	1	0	1	0.736677
tr|B4DN88|B4DN88_H	RBMS1	RNA-binding motif, single-stranded-interacting protein 1 OS=Homo sapiens GN=RBMS1 F	46168	0	993	0	36	0	0.17	#DIV/0!	0	0	0	1	0	1	0	1	0.736677
tr|B4DI28|B4DI28_HU	PTBP2	Polypyrimidine tract-binding protein 2 OS=Homo sapiens GN=PTBP2 PE=2 SV=1	34947	0	992	0	37	0	0.09	#DIV/0!	0	1	0	1	0	1	0	1	0.736677
tr|H7BZF2|H7BZF2_H	DKC1	H/ACA ribonucleoprotein complex subunit 4 (Fragment) OS=Homo sapiens GN=DKC1 PE=	8803	0	991	0	37	0	0.4	#DIV/0!	0	1	0	1	0	1	0	1	0.736677
sp|Q9NP92|RT30_HU	MRPS30	28S ribosomal protein S30, mitochondrial OS=Homo sapiens GN=MRPS30 PE=1 SV=2	50960	0	989	0	37	0	0.06	#DIV/0!	0	1	0	1	0	1	0	2	0.736677
tr|Q5SRT3|Q5SRT3_H	CLIC1	Chloride intracellular channel 1, isoform CRA_a OS=Homo sapiens GN=CLIC1 PE=2 SV=2	27248	0	987	0	37	0	0.12	#DIV/0!	0	0	0	1	0	1	0	1	0.736677
sp|Q9H2M9|RBGPR_H	RAB3GAP2	Rab3 GTPase-activating protein non-catalytic subunit OS=Homo sapiens GN=RAB3GAP2 (	157482	0	985	0	37	0	0.02	#DIV/0!	0	1	0	1	0	1	0	2	0.736677
tr|B3KQ95|B3KQ95_H	FDFT1	Squalene synthase OS=Homo sapiens GN=FDFT1 PE=2 SV=1	35421	0	984	0	37	0	0.1	#DIV/0!	0	1	0	1	0	1	0	1	0.736677
tr|H0Y448|H0Y448_H	CTTNBP2	Cortactin-binding protein 2 (Fragment) OS=Homo sapiens GN=CTTNBP2 PE=4 SV=1	127487	0	982	0	37	0	0.03	#DIV/0!	0	1	0	1	0	1	0	1	0.736677
sp|O14746|TERT_HUM	TERT	Telomerase reverse transcriptase OS=Homo sapiens GN=TERT PE=1 SV=1	128570	0	981	0	37	0	0.03	#DIV/0!	0	1	0	1	0	1	0	2	0.736677
tr|B4DL39|B4DL39_H	tr|B4DL39|B4DL39_HU	cDNA FLJ57316, highly similar to DNA mismatch repair protein Msh2 OS=Homo sapiens F	77368	0	979	0	37	0	0.09	#DIV/0!	0	1	0	2	0	1	0	3	0.736677
tr|A8K2I7|A8K2I7_HU	tr|A8K2I7|A8K2I7_HUM	cDNA FLJ76072, highly similar to Homo sapiens GIPC PDZ domain containing family, men	36131	0	977	0	37	0	0.24	#DIV/0!	0	0	0	1	0	1	0	1	0.736677
tr|Q5D1D5|Q5D1D5_	tr|Q5D1D5|Q5D1D5_H	Guanylate binding protein 1 OS=Homo sapiens PE=2 SV=1	68387	0	975	0	38	0	0.08	#DIV/0!	0	1	0	1	0	1	0	2	0.736677

tr|B3KVY3|B3KVY3_H	tr|B3KVY3|B3KVY3_HU	cDNA FLJ41723 fis, clone HLUNG2013851 OS=Homo sapiens PE=2 SV=1	20460	0	974	0	38	0	0.16	#DIV/0!	0	1	0	1	0	1	0	1	0.736677
tr|B4DTA7|B4DTA7_H	tr|B4DTA7|B4DTA7_HU	cDNA FLJ50207, highly similar to Pappalysin-1 (EC 3.4.24.79) OS=Homo sapiens PE=2 SV=	64336	0	972	0	38	0	0.05	#DIV/0!	0	1	0	1	0	1	0	1	0.736677
sp|P27635|RL10_HUN	RPL10	60S ribosomal protein L10 OS=Homo sapiens GN=RPL10 PE=1 SV=4	25044	0	971	0	38	0	0.16	#DIV/0!	0	1	0	1	0	1	0	1	0.736677
tr|H3BM93|H3BM93_	NUP93	Nuclear pore complex protein Nup93 (Fragment) OS=Homo sapiens GN=NUP93 PE=4 SV	19858	0	970	0	38	0	0.03	#DIV/0!	0	1	0	1	0	1	0	1	0.736677
tr|H7BY14|H7BY14_H	SAMD11	Sterile alpha motif domain-containing protein 11 (Fragment) OS=Homo sapiens GN=SAM	63302	0	968	0	38	0	0.05	#DIV/0!	0	1	0	1	0	1	0	1	0.736677
tr|B4DYU9|B4DYU9_H	tr|B4DYU9|B4DYU9_H	cDNA FLJ60060, highly similar to Origin recognition complex subunit 2 OS=Homo sapiens	58631	0	966	0	38	0	0.06	#DIV/0!	0	1	0	1	0	1	0	1	0.736677
tr|Q2VIK8|Q2VIK8_HU	tr|Q2VIK8|Q2VIK8_HU	RcNSEP1 (Fragment) OS=Homo sapiens PE=2 SV=1	35435	0	963	0	38	0	0.09	#DIV/0!	0	1	0	1	0	1	0	2	0.736677
tr|A0JLU4|A0JLU4_HU	YBX1	YBX1 protein (Fragment) OS=Homo sapiens GN=YBX1 PE=2 SV=1	21417	0	962	0	38	0	0.08	#DIV/0!	0	1	0	1	0	1	0	1	0.736677
sp|P78357|CNTP1_HU	CNTNAP1	Contactin-associated protein 1 OS=Homo sapiens GN=CNTNAP1 PE=1 SV=1	158220	0	958	0	39	0	0.02	#DIV/0!	0	1	0	1	0	1	0	1	0.736677
sp|Q16222|UAP1_HU	UAP1	UDP-N-acetylhexosamine pyrophosphorylase OS=Homo sapiens GN=UAP1 PE=1 SV=3	59131	0	953	0	39	0	0.06	#DIV/0!	0	1	0	1	0	1	0	1	0.736677
sp|Q6NVY1|HIBCH_H	HIBCH	3-hydroxyisobutyryl-CoA hydrolase, mitochondrial OS=Homo sapiens GN=HIBCH PE=1 SV	43797	0	951	0	39	0	0.08	#DIV/0!	0	1	0	1	0	1	0	1	0.736677
tr|G5EA03|G5EA03_H	LIMCH1	LIM and calponin homology domains-containing protein 1 OS=Homo sapiens GN=LIMCH	165820	0	949	0	39	0	0.03	#DIV/0!	0	1	0	1	0	1	0	2	0.736677
tr|A8K9B9|A8K9B9_H	tr|A8K9B9|A8K9B9_HU	cDNA FLJ77391, highly similar to Homo sapiens EH-domain containing 4 (EHD4), mRNA C	61346	0	948	0	39	0	0.05	#DIV/0!	0	1	0	1	0	1	0	1	0.736677
tr|B4DKE6|B4DKE6_H	tr|B4DKE6|B4DKE6_HU	cDNA FLJ60629, highly similar to Replication factor C subunit 3 OS=Homo sapiens PE=2 S	35551	0	947	0	39	0	0.09	#DIV/0!	0	1	0	1	0	1	0	1	0.736677
tr|B4DG70|B4DG70_H	tr|B4DG70|B4DG70_H	cDNA FLJ52624, highly similar to 2-hydroxyacylsphingosine1-beta-galactosyltransferase (	33947	0	946	0	39	0	0.1	#DIV/0!	0	1	0	1	0	1	0	1	0.736677
tr|Q59G04|Q59G04_H	tr|Q59G04|Q59G04_H	Dystrophin related protein 2 variant (Fragment) OS=Homo sapiens PE=2 SV=1	66446	0	945	0	39	0	0.03	#DIV/0!	0	1	0	1	0	1	0	1	0.736677
tr|B4DIS0|B4DIS0_HU	tr|B4DIS0|B4DIS0_HUN	cDNA FLJ57840, highly similar to Chromodomain-helicase-DNA-binding protein 7 (EC 3.6	139212	0	943	0	39	0	0.02	#DIV/0!	0	1	0	1	0	1	0	1	0.736677
tr|B2R646|B2R646_H	tr|B2R646|B2R646_HU	cDNA, FLJ92780, highly similar to Homo sapiens phosphodiesterase 2A, cGMP-stimulated	107379	0	941	0	40	0	0.03	#DIV/0!	0	1	0	1	0	1	0	1	0.736677
tr|H0Y3Y9|H0Y3Y9_H	PSMD4	26S proteasome non-ATPase regulatory subunit 4 (Fragment) OS=Homo sapiens GN=PSN	21105	0	939	0	40	0	0.16	#DIV/0!	0	1	0	1	0	1	0	1	0.736677
tr|B4DJ44|B4DJ44_HU	tr|B4DJ44|B4DJ44_HU	cDNA FLJ54716, highly similar to Target of Myb protein 1 OS=Homo sapiens PE=2 SV=1	49775	0	938	0	40	0	0.07	#DIV/0!	0	1	0	1	0	1	0	2	0.736677
tr|Q5SQP8|Q5SQP8_H	CTBP2	C-terminal-binding protein 2 OS=Homo sapiens GN=CTBP2 PE=4 SV=1	56808	0	937	0	40	0	0.06	#DIV/0!	0	1	0	1	0	1	0	2	0.736677
tr|H0YCY6|H0YCY6_H	DAK	FAD-AMP lyase (cyclizing) (Fragment) OS=Homo sapiens GN=DAK PE=4 SV=1	55026	0	936	0	40	0	0.06	#DIV/0!	0	1	0	1	0	1	0	1	0.736677
tr|J3QRD1|J3QRD1_H	ALDH3A2	Fatty aldehyde dehydrogenase OS=Homo sapiens GN=ALDH3A2 PE=3 SV=1	45019	0	934	0	40	0	0.07	#DIV/0!	0	1	0	1	0	1	0	1	0.736677
tr|B3KY55|B3KY55_HU	tr|B3KY55|B3KY55_HU	cDNA FLJ46894 fis, clone UTERU3019708, highly similar to Regulator of nonsense transcr	41002	0	931	0	40	0	0.08	#DIV/0!	0	1	0	1	0	1	0	1	0.736677
tr|Q53G42|Q53G42_H	tr|Q53G42|Q53G42_H	mRNA decapping enzyme variant (Fragment) OS=Homo sapiens PE=2 SV=1	38757	0	929	0	40	0	0.09	#DIV/0!	0	1	0	1	0	1	0	1	0.736677
sp|A2VCL2|CC162_HU	CCDC162P	Coiled-coil domain-containing protein 162 OS=Homo sapiens GN=CCDC162P PE=2 SV=3	104860	0	928	0	40	0	0.03	#DIV/0!	0	1	0	1	0	1	0	1	0.736677
sp|O00487|PSDE_HUI	PSMD14	26S proteasome non-ATPase regulatory subunit 14 OS=Homo sapiens GN=PSMD14 PE=1	34726	0	924	0	41	0	0.2	#DIV/0!	0	1	0	1	0	1	0	1	0.736677
sp|Q8IWB1|IPRI_HUM	ITPRIP	Inositol 1,4,5-trisphosphate receptor-interacting protein OS=Homo sapiens GN=ITPRIP P	62932	0	923	0	41	0	0.05	#DIV/0!	0	1	0	1	0	1	0	1	0.736677
tr|D3DTD2|D3DTD2_H	TRIO	Triple functional domain (PTPRF interacting), isoform CRA_a OS=Homo sapiens GN=TRIO	270210	0	922	0	41	0	0.01	#DIV/0!	0	1	0	1	0	1	0	1	0.736677
tr|B7Z6M0|B7Z6M0_	tr|B7Z6M0|B7Z6M0_H	cDNA FLJ56370, highly similar to Homo sapiens FK506 binding protein 8, 38kDa (FKBP8),	47655	0	920	0	41	0	0.07	#DIV/0!	0	1	0	1	0	1	0	1	0.736677
tr|Q5W1M0|Q5W1M	HLA-C	MHC class I antigen (Fragment) OS=Homo sapiens GN=HLA-C PE=2 SV=1	10534	0	915	0	0	0	0.17	#DIV/0!	0	0	0	0	0	1	0	2	0.736677
tr|H0YB10|H0YB10_H	TTC13	Tetratricopeptide repeat protein 13 (Fragment) OS=Homo sapiens GN=TTC13 PE=4 SV=1	14612	0	912	0	41	0	0.23	#DIV/0!	0	1	0	1	0	1	0	1	0.736677
tr|G1UI17|G1UI17_HU	AGL	Glycogen debranching enzyme (Fragment) OS=Homo sapiens GN=AGL PE=2 SV=1	145569	0	909	0	41	0	0.02	#DIV/0!	0	1	0	1	0	1	0	1	0.736677
tr|B7Z653|B7Z653_HU	tr|B7Z653|B7Z653_HU	cDNA FLJ61657, highly similar to Band 4.1-like protein 1 OS=Homo sapiens PE=2 SV=1	90347	0	908	0	41	0	0.04	#DIV/0!	0	1	0	1	0	1	0	2	0.736677
sp|O96008|TOM40_H	TOMM40	Mitochondrial import receptor subunit TOM40 homolog OS=Homo sapiens GN=TOMM4	38211	0	906	0	41	0	0.09	#DIV/0!	0	1	0	1	0	1	0	2	0.736677
tr|H0YGH7|H0YGH7_	PLCZ1	1-phosphatidylinositol 4,5-bisphosphate phosphodiesterase zeta-1 (Fragment) OS=Homo	47240	0	905	0	41	0	0.07	#DIV/0!	0	1	0	1	0	1	0	1	0.736677
tr|B3KM47|B3KM47_	tr|B3KM47|B3KM47_H	cDNA FLJ10273 fis, clone HEMBB1001137, highly similar to SEC23-interacting protein OS	111730	0	901	0	42	0	0.03	#DIV/0!	0	1	0	1	0	1	0	1	0.736677
tr|H0UI06|H0UI06_HU	COX7A2	Cytochrome c oxidase subunit 7A2, mitochondrial OS=Homo sapiens GN=COX7A2 PE=4 S	12950	0	900	0	42	0	0.27	#DIV/0!	0	1	0	1	0	1	0	1	0.736677
tr|B4E0H3|B4E0H3_H	tr|B4E0H3|B4E0H3_HU	cDNA FLJ53386, highly similar to Signal recognition particle receptor subunit alpha OS=H	66987	0	897	0	42	0	0.05	#DIV/0!	0	1	0	1	0	1	0	2	0.736677
sp|Q9UPT8|ZC3H4_H	ZC3H4	Zinc finger CCCH domain-containing protein 4 OS=Homo sapiens GN=ZC3H4 PE=1 SV=3	140797	0	895	0	42	0	0.02	#DIV/0!	0	1	0	1	0	1	0	1	0.736677
tr|Q6IPN0|Q6IPN0_H	RTN4	RTN4 protein OS=Homo sapiens GN=RTN4 PE=2 SV=1	36952	0	894	0	43	0	0.19	#DIV/0!	0	1	0	2	0	1	0	2	0.736677
sp|Q8IVF2|AHNK2_HU	AHNAK2	Protein AHNAK2 OS=Homo sapiens GN=AHNAK2 PE=1 SV=2	617383	0	891	0	43	0	0.01	#DIV/0!	0	1	0	1	0	1	0	1	0.736677
tr|G3V1P5|G3V1P5_H	MED15	Mediator of RNA polymerase II transcription subunit 15 OS=Homo sapiens GN=MED15 P	80007	0	890	0	43	0	0.04	#DIV/0!	0	1	0	1	0	1	0	1	0.736677
tr|G3V1C3|G3V1C3_H	API5	Apoptosis inhibitor 5 OS=Homo sapiens GN=API5 PE=2 SV=1	57867	0	888	0	43	0	0.06	#DIV/0!	0	1	0	1	0	1	0	1	0.736677
tr|B4DZI8|B4DZI8_HU	COPB2	Coatomer protein complex, subunit beta 2 (Beta prime), isoform CRA_b OS=Homo sapie	99839	0	884	0	43	0	0.03	#DIV/0!	0	1	0	1	0	1	0	1	0.736677
tr|B5BU08|B5BU08_H	U2AF1	U2 small nuclear RNA auxillary factor 1 isoform a OS=Homo sapiens GN=U2AF1 PE=2 SV	28369	0	881	0	44	0	0.12	#DIV/0!	0	1	0	1	0	1	0	1	0.736677
tr|J3QRR3|J3QRR3_H	PSMC5	26S protease regulatory subunit 8 (Fragment) OS=Homo sapiens GN=PSMC5 PE=4 SV=1	13944	0	880	0	44	0	0.24	#DIV/0!	0	1	0	1	0	1	0	2	0.736677
tr|B7ZW05|B7ZW05_	AKAP13	AKAP13 protein (Fragment) OS=Homo sapiens GN=AKAP13 PE=2 SV=1	124144	0	879	0	44	0	0.03	#DIV/0!	0	1	0	1	0	1	0	1	0.736677
tr|B2RB52|B2RB52_H	tr|B2RB52|B2RB52_HU	cDNA, FLJ95314, highly similar to Homo sapiens transducin (beta)-like 2 (TBL2), transcrip	50423	0	877	0	44	0	0.07	#DIV/0!	0	1	0	1	0	1	0	1	0.736677
tr|A7VJC9|A7VJC9_HU	STP	Salt-tolerant protein OS=Homo sapiens GN=STP PE=2 SV=1	62769	0	876	0	44	0	0.11	#DIV/0!	0	1	0	2	0	1	0	2	0.736677
sp|Q8TAA9|VANG1_H	VANGL1	Vang-like protein 1 OS=Homo sapiens GN=VANGL1 PE=1 SV=1	60222	0	875	0	44	0	0.05	#DIV/0!	0	1	0	1	0	1	0	1	0.736677
tr|B4DW13|B4DW13_	hCG_23341	HCG23341, isoform CRA_d OS=Homo sapiens GN=hCG_23341 PE=2 SV=1	51824	0	874	0	44	0	0.06	#DIV/0!	0	1	0	1	0	1	0	1	0.736677
tr|B2RE56|B2RE56_H	tr|B2RE56|B2RE56_HU	Peptidyl-prolyl cis-trans isomerase OS=Homo sapiens PE=2 SV=1	18225	0	868	0	45	0	0.19	#DIV/0!	0	1	0	1	0	1	0	1	0.736677
tr|B1AK81|B1AK81_H	PIGK	GPI-anchor transamidase OS=Homo sapiens GN=PIGK PE=4 SV=1	34462	0	865	0	45	0	0.09	#DIV/0!	0	1	0	1	0	1	0	1	0.736677
tr|E7EVL6|E7EVL6_HU	REPIN1	Replication initiator 1 (Fragment) OS=Homo sapiens GN=REPIN1 PE=2 SV=1	16586	0	863	0	45	0	0.2	#DIV/0!	0	1	0	1	0	1	0	2	0.736677
sp|P49792|RBP2_HUN	RANBP2	E3 SUMO-protein ligase RanBP2 OS=Homo sapiens GN=RANBP2 PE=1 SV=2	362365	0	857	0	45	0	0.01	#DIV/0!	0	1	0	1	0	1	0	2	0.736677
tr|B3KMC9|B3KMC9_	tr|B3KMC9|B3KMC9_H	cDNA FLJ10711 fis, clone NT2RP3000917, highly similar to 5~-3~ exoribonuclease 2 (EC 3	109456	0	855	0	45	0	0.03	#DIV/0!	0	1	0	1	0	1	0	1	0.736677
tr|Q3LIF4|Q3LIF4_HU	Nbla00271	NOGO-A (Fragment) OS=Homo sapiens GN=Nbla00271 PE=2 SV=1	57162	0	852	0	46	0	0.06	#DIV/0!	0	1	0	1	0	1	0	1	0.736677
tr|D0PQI2|D0PQI2_H	ENAH	Enabled-like protein variant hMenaDv6 OS=Homo sapiens GN=ENAH PE=2 SV=1	60426	0	847	0	46	0	0.05	#DIV/0!	0	1	0	1	0	1	0	1	0.736677
sp|O15397|IPO8_HUN	IPO8	Importin-8 OS=Homo sapiens GN=IPO8 PE=1 SV=2	120945	0	845	0	46	0	0.06	#DIV/0!	0	1	0	1	0	1	0	1	0.736677
tr|J3QSY3|J3QSY3_HU	GDA	Guanine deaminase OS=Homo sapiens GN=GDA PE=4 SV=1	46321	0	843	0	47	0	0.07	#DIV/0!	0	1	0	1	0	1	0	1	0.736677
tr|B3KTC7|B3KTC7_H	MRE11A	Double-strand break repair protein MRE11A OS=Homo sapiens GN=MRE11A PE=2 SV=1	81362	0	841	0	47	0	0.04	#DIV/0!	0	1	0	1	0	1	0	1	0.736677
tr|E9PQD7|E9PQD7_H	RPS2	40S ribosomal protein S2 OS=Homo sapiens GN=RPS2 PE=2 SV=1	25366	0	840	0	47	0	0.5	#DIV/0!	0	1	0	1	0	1	0	1	0.736677
sp|Q32MZ4|LRRF1_H	LRRFIP1	Leucine-rich repeat flightless-interacting protein 1 OS=Homo sapiens GN=LRRFIP1 PE=1 S	89826	0	836	0	47	0	0.04	#DIV/0!	0	1	0	1	0	1	0	1	0.736677
sp|Q9Y266|NUDC_HU	NUDC	Nuclear migration protein nudC OS=Homo sapiens GN=NUDC PE=1 SV=1	38276	0	833	0	47	0	0.18	#DIV/0!	0	1	0	2	0	1	0	2	0.736677
tr|B2R9S4|B2R9S4_H	tr|B2R9S4|B2R9S4_HU	cDNA, FLJ94534, highly similar to Homo sapiens capping protein (actin filament), gelsolin	38759	0	831	0	47	0	0.09	#DIV/0!	0	1	0	1	0	1	0	1	0.736677
tr|I6L9B1|I6L9B1_HUI	EPB41L2	EPB41L2 protein OS=Homo sapiens GN=EPB41L2 PE=2 SV=1	71998	0	828	0	48	0	0.05	#DIV/0!	0	1	0	1	0	1	0	3	0.736677
tr|F8W6C2|F8W6C2_	SPATS2L	SPATS2-like protein (Fragment) OS=Homo sapiens GN=SPATS2L PE=2 SV=1	30163	0	827	0	48	0	0.05	#DIV/0!	0	1	0	1	0	1	0	2	0.736677
tr|H0Y7L9|H0Y7L9_HU	ABCD1	ATP-binding cassette sub-family D member 1 (Fragment) OS=Homo sapiens GN=ABCD1 F	24597	0	826	0	48	0	0.14	#DIV/0!	0	1	0	1	0	1	0	1	0.736677
tr|A8K8X0|A8K8X0_H	tr|A8K8X0|A8K8X0_HU	cDNA FLJ75187, highly similar to Homo sapiens nap1 P120 OS=Homo sapiens PE=2 SV=1	113035	0	819	0	48	0	0.03	#DIV/0!	0	1	0	1	0	1	0	2	0.736677
tr|B4DQR0|B4DQR0_	tr|B4DQR0|B4DQR0_H	cDNA FLJ54377 OS=Homo sapiens PE=2 SV=1	91332	0	815	0	49	0	0.04	#DIV/0!	0	1	0	1	0	1	0	2	0.736677
sp|P14735|IDE_HUM/	IDE	Insulin-degrading enzyme OS=Homo sapiens GN=IDE PE=1 SV=4	118692	0	813	0	49	0	0.05	#DIV/0!	0	1	0	1	0	1	0	1	0.736677
sp|Q8IVT2|CS021_HU	C19orf21	Uncharacterized protein C19orf21 OS=Homo sapiens GN=C19orf21 PE=1 SV=1	75482	0	809	0	49	0	0.04	#DIV/0!	0	1	0	1	0	1	0	1	0.736677
tr|B4DRW3|B4DRW3_	tr|B4DRW3|B4DRW3_	cDNA FLJ57180, highly similar to TAR DNA-binding protein 43 OS=Homo sapiens PE=2 SV	35596	0	808	0	49	0	0.1	#DIV/0!	0	1	0	1	0	1	0	1	0.736677
tr|G3V1A4|G3V1A4_H	CFL1	Cofilin 1 (Non-muscle), isoform CRA_a OS=Homo sapiens GN=CFL1 PE=4 SV=1	17029	0	807	0	49	0	0.2	#DIV/0!	0	1	0	1	0	1	0	1	0.736677
sp|Q6PKG0|LARP1_H	LARP1	La-related protein 1 OS=Homo sapiens GN=LARP1 PE=1 SV=2	123833	0	805	0	50	0	0.03	#DIV/0!	0	1	0	1	0	1	0	4	0.736677
tr|J3KMX1|J3KMX1_H	NUP88	Nuclear pore complex protein Nup88 (Fragment) OS=Homo sapiens GN=NUP88 PE=4 SV	80029	0	801	0	50	0	0.2	#DIV/0!	0	1	0	1	0	1	0	1	0.736677
tr|B4DEE5|B4DEE5_H	tr|B4DEE5|B4DEE5_HU	cDNA FLJ60230, highly similar to Arsenical pump-driving ATPase (EC 3.6.3.16) OS=Homo	36162	0	796	0	51	0	0.09	#DIV/0!	0	1	0	1	0	1	0	1	0.736677
sp|Q9H1C3|GL8D2_H	GLT8D2	Glycosyltransferase 8 domain-containing protein 2 OS=Homo sapiens GN=GLT8D2 PE=2 S	40229	0	793	0	51	0	0.08	#DIV/0!	0	1	0	1	0	1	0	1	0.736677
sp|Q15043|S39AE_HU	SLC39A14	Zinc transporter ZIP14 OS=Homo sapiens GN=SLC39A14 PE=1 SV=3	54918	0	790	0	51	0	0.06	#DIV/0!	0	1	0	1	0	1	0	1	0.736677
tr|B4E0X4|B4E0X4_HU	tr|B4E0X4|B4E0X4_HU	cDNA FLJ51433 OS=Homo sapiens PE=2 SV=1	10805	0	788	0	52	0	0.32	#DIV/0!	0	1	0	1	0	1	0	1	0.736677
tr|Q05DA4|Q05DA4_	P4HA2	P4HA2 protein OS=Homo sapiens GN=P4HA2 PE=2 SV=1	57684	0	784	0	52	0	0.12	#DIV/0!	0	1	0	2	0	1	0	2	0.736677
sp|O75475|PSIP1_HU	PSIP1	PC4 and SFRS1-interacting protein OS=Homo sapiens GN=PSIP1 PE=1 SV=1	60181	0	776	0	53	0	0.05	#DIV/0!	0	1	0	1	0	1	0	1	0.736677
tr|B3KMQ6|B3KMQ6_	tr|B3KMQ6|B3KMQ6_	cDNA FLJ12172 fis, clone MAMMA1000684, highly similar to Opioid growth factor recept	73894	0	775	0	53	0	0.04	#DIV/0!	0	1	0	1	0	1	0	1	0.736677
tr|F5GWY5|F5GWY5_	PODXL	Podocalyxin OS=Homo sapiens GN=PODXL PE=2 SV=1	59285	0	772	0	54	0	0.06	#DIV/0!	0	1	0	1	0	1	0	1	0.736677
tr|H0Y6N5|H0Y6N5_H	SUN1	SUN domain-containing protein 1 (Fragment) OS=Homo sapiens GN=SUN1 PE=4 SV=1	71569	0	770	0	54	0	0.05	#DIV/0!	0	1	0	1	0	1	0	2	0.736677
tr|G3V346|G3V346_H	SRP54	Signal recognition particle 54 kDa protein (Fragment) OS=Homo sapiens GN=SRP54 PE=2	4785	0	769	0	54	0	0.77	#DIV/0!	0	1	0	1	0	1	0	1	0.736677
sp|Q9UHN6|TMEM2_	TMEM2	Transmembrane protein 2 OS=Homo sapiens GN=TMEM2 PE=1 SV=1	155702	0	766	0	55	0	0.02	#DIV/0!	0	1	0	1	0	1	0	2	0.736677
tr|B3KN28|B3KN28_H	tr|B3KN28|B3KN28_HU	cDNA FLJ13370 fis, clone PLACE1000653, highly similar to Phosphoacetylglucosamine mu	60284	0	758	0	55	0	0.05	#DIV/0!	0	1	0	1	0	1	0	1	0.736677
sp|Q49AR2|CE022_HU	C5orf22	UPF0489 protein C5orf22 OS=Homo sapiens GN=C5orf22 PE=1 SV=2	50506	0	756	0	56	0	0.07	#DIV/0!	0	1	0	1	0	1	0	1	0.736677
tr|B3KMS9|B3KMS9_	tr|B3KMS9|B3KMS9_H	cDNA FLJ12495 fis, clone NT2RM2001652, highly similar to Brefeldin A-inhibited guanine	78721	0	753	0	56	0	0.04	#DIV/0!	0	1	0	1	0	1	0	1	0.736677
tr|B3KST5|B3KST5_HU	COPS4	COP9 constitutive photomorphogenic homolog subunit 4 (Arabidopsis), isoform CRA_a C	40457	0	749	0	56	0	0.07	#DIV/0!	0	1	0	1	0	1	0	1	0.736677
tr|B4DTS5|B4DTS5_H	tr|B4DTS5|B4DTS5_HU	cDNA FLJ58882, highly similar to 26S proteasome non-ATPase regulatory subunit 11 OS=	35374	0	748	0	56	0	0.2	#DIV/0!	0	1	0	2	0	1	0	3	0.736677
tr|D6REA1|D6REA1_H	SIL1	Nucleotide exchange factor SIL1 OS=Homo sapiens GN=SIL1 PE=2 SV=1	53000	0	743	0	57	0	0.06	#DIV/0!	0	1	0	1	0	1	0	2	0.736677
tr|B4DDL6|B4DDL6_H	tr|B4DDL6|B4DDL6_HU	cDNA FLJ54325, highly similar to Myotubularin-related protein 2 (EC 3.1.3.-) OS=Homo sa	36644	0	739	0	57	0	0.09	#DIV/0!	0	1	0	1	0	1	0	1	0.736677
sp|P49419|AL7A1_HU	ALDH7A1	Alpha-aminoadipic semialdehyde dehydrogenase OS=Homo sapiens GN=ALDH7A1 PE=1	59020	0	738	0	57	0	0.13	#DIV/0!	0	1	0	1	0	1	0	1	0.736677
tr|D6RDI2|D6RDI2_HU	LUC7L3	Luc7-like protein 3 (Fragment) OS=Homo sapiens GN=LUC7L3 PE=2 SV=1	21007	0	736	0	58	0	0.16	#DIV/0!	0	1	0	1	0	1	0	1	0.736677
tr|Q2NLC9|Q2NLC9_H	PURA	PURA protein (Fragment) OS=Homo sapiens GN=PURA PE=2 SV=1	34316	0	729	0	59	0	0.1	#DIV/0!	0	1	0	1	0	1	0	1	0.736677
tr|B4DSP0|B4DSP0_H	hCG_1642748	HCG1642748, isoform CRA_b OS=Homo sapiens GN=hCG_1642748 PE=2 SV=1	45559	0	728	0	59	0	0.07	#DIV/0!	0	1	0	1	0	1	0	1	0.736677
tr|E9PJ81|E9PJ81_HU	UBXN1	UBX domain-containing protein 1 (Fragment) OS=Homo sapiens GN=UBXN1 PE=2 SV=1	32649	0	724	0	60	0	0.15	#DIV/0!	0	1	0	1	0	1	0	1	0.736677
tr|Q6AHX7|Q6AHX7_	DKFZp686F20131	Putative uncharacterized protein DKFZp686F20131 (Fragment) OS=Homo sapiens GN=DI	26715	0	720	0	60	0	0.12	#DIV/0!	0	1	0	1	0	1	0	1	0.736677
tr|B4DI59|B4DI59_HU	tr|B4DI59|B4DI59_HUN	cDNA FLJ59527 OS=Homo sapiens PE=2 SV=1	39360	0	718	0	60	0	0.08	#DIV/0!	0	1	0	1	0	1	0	1	0.736677
sp|Q9UKX7|NUP50_H	NUP50	Nuclear pore complex protein Nup50 OS=Homo sapiens GN=NUP50 PE=1 SV=2	50512	0	716	0	60	0	0.07	#DIV/0!	0	1	0	1	0	1	0	2	0.736677
sp|Q8NI27|THOC2_H	THOC2	THO complex subunit 2 OS=Homo sapiens GN=THOC2 PE=1 SV=2	184541	0	715	0	60	0	0.02	#DIV/0!	0	1	0	1	0	1	0	2	0.736677
tr|B3KMC2|B3KMC2_	tr|B3KMC2|B3KMC2_H	cDNA FLJ10676 fis, clone NT2RP2006464, highly similar to WD repeat and HMG-box DNA	73526	0	712	0	61	0	0.04	#DIV/0!	0	1	0	1	0	1	0	1	0.736677
tr|Q0VAB1|Q0VAB1_	TIMM50	Translocase of inner mitochondrial membrane 50 homolog (S. cerevisiae) OS=Homo sapi	50960	0	700	0	62	0	0.06	#DIV/0!	0	1	0	1	0	1	0	1	0.736677
tr|B4DHY1|B4DHY1_H	HNRNPH3	Heterogeneous nuclear ribonucleoprotein H3 OS=Homo sapiens GN=HNRNPH3 PE=2 SV	24925	0	698	0	63	0	0.13	#DIV/0!	0	1	0	1	0	1	0	1	0.736677
tr|B2R657|B2R657_H	tr|B2R657|B2R657_HU	Annexin OS=Homo sapiens PE=2 SV=1	52892	0	697	0	63	0	0.06	#DIV/0!	0	1	0	1	0	1	0	1	0.736677
tr|A8K4W5|A8K4W5_	tr|A8K4W5|A8K4W5_H	cDNA FLJ76813, highly similar to Homo sapiens acetyl-Coenzyme A acetyltransferase 2 (a	41866	0	696	0	63	0	0.07	#DIV/0!	0	1	0	1	0	1	0	1	0.736677
tr|G3V5E1|G3V5E1_H	CCNK	Cyclin-K OS=Homo sapiens GN=CCNK PE=2 SV=1	43776	0	694	0	63	0	0.08	#DIV/0!	0	1	0	1	0	1	0	1	0.736677
tr|J3KTL8|J3KTL8_HUI	SMCHD1	Structural maintenance of chromosomes flexible hinge domain-containing protein 1 (Fra	156690	0	693	0	63	0	0.02	#DIV/0!	0	1	0	1	0	1	0	1	0.736677
tr|H7C4G8|H7C4G8_H	NT5DC2	5~-nucleotidase domain-containing protein 2 (Fragment) OS=Homo sapiens GN=NT5DC2	30207	0	692	0	63	0	0.11	#DIV/0!	0	1	0	1	0	1	0	1	0.736677
sp|Q9NX58|LYAR_HU	LYAR	Cell growth-regulating nucleolar protein OS=Homo sapiens GN=LYAR PE=1 SV=2	44044	0	683	0	65	0	0.07	#DIV/0!	0	1	0	1	0	1	0	1	0.736677
tr|H0YLC7|H0YLC7_H	FAH	Fumarylacetoacetase (Fragment) OS=Homo sapiens GN=FAH PE=4 SV=1	18072	0	681	0	65	0	0.19	#DIV/0!	0	1	0	1	0	1	0	1	0.736677
tr|J3KMX2|J3KMX2_H	SMARCD2	SWI/SNF-related matrix-associated actin-dependent regulator of chromatin subfamily D	52433	0	679	0	66	0	0.06	#DIV/0!	0	1	0	1	0	1	0	1	0.736677
tr|F8VP03|F8VP03_H	tr|F8VP03|F8VP03_HU	Uncharacterized protein (Fragment) OS=Homo sapiens PE=2 SV=1	11638	0	676	0	33	0	0.45	#DIV/0!	0	1	0	1	0	1	0	1	0.736677
sp|Q9UBV2|SE1L1_H	SEL1L	Protein sel-1 homolog 1 OS=Homo sapiens GN=SEL1L PE=1 SV=3	89212	0	672	0	67	0	0.04	#DIV/0!	0	1	0	1	0	1	0	1	0.736677
tr|Q4KMR3|Q4KMR3_	MYO1E	MYO1E variant protein OS=Homo sapiens GN=MYO1E PE=2 SV=1	127552	0	670	0	67	0	0.03	#DIV/0!	0	1	0	1	0	1	0	1	0.736677
tr|B4DXJ1|B4DXJ1_HU	SEC13	Protein SEC13 homolog OS=Homo sapiens GN=SEC13 PE=2 SV=1	41405	0	669	0	67	0	0.08	#DIV/0!	0	1	0	1	0	1	0	1	0.736677
tr|B4DRK5|B4DRK5_H	tr|B4DRK5|B4DRK5_HU	cDNA FLJ59584, highly similar to Mitochondrial-processing peptidase alpha subunit, mito	28622	0	660	0	58	0	0.12	#DIV/0!	0	1	0	1	0	1	0	1	0.736677
tr|B4DX52|B4DX52_H	DNAJB1	DnaJ homolog subfamily B member 1 OS=Homo sapiens GN=DNAJB1 PE=2 SV=1	27170	0	656	0	69	0	0.12	#DIV/0!	0	1	0	1	0	1	0	1	0.736677
sp|Q9Y4F1|FARP1_HU	FARP1	FERM, RhoGEF and pleckstrin domain-containing protein 1 OS=Homo sapiens GN=FARP1	119300	0	655	0	69	0	0.03	#DIV/0!	0	1	0	1	0	1	0	1	0.736677
sp|Q9UI26|IPO11_HU	IPO11	Importin-11 OS=Homo sapiens GN=IPO11 PE=1 SV=1	113489	0	653	0	70	0	0.06	#DIV/0!	0	1	0	1	0	1	0	1	0.736677
tr|B4DM78|B4DM78_	tr|B4DM78|B4DM78_H	cDNA FLJ58199, highly similar to Fragile X mental retardation syndrome-related protein	63280	0	652	0	70	0	0.06	#DIV/0!	0	1	0	1	0	1	0	1	0.736677
tr|A8KA19|A8KA19_H	tr|A8KA19|A8KA19_HU	cDNA FLJ75831, highly similar to Homo sapiens exportin, tRNA (nuclear export receptor	111104	0	650	0	70	0	0.03	#DIV/0!	0	1	0	1	0	1	0	1	0.736677
tr|B0I1S9|B0I1S9_HUI	MYO1B	MYO1B variant protein OS=Homo sapiens GN=MYO1B PE=2 SV=1	132928	0	645	0	71	0	0.02	#DIV/0!	0	1	0	1	0	1	0	1	0.736677
tr|B7Z2R8|B7Z2R8_H	tr|B7Z2R8|B7Z2R8_HU	cDNA FLJ53324, highly similar to Tight junction protein ZO-2 OS=Homo sapiens PE=2 SV=	137474	0	644	0	71	0	0.02	#DIV/0!	0	1	0	1	0	1	0	3	0.736677
sp|Q6PI48|SYDM_HU	DARS2	Aspartate--tRNA ligase, mitochondrial OS=Homo sapiens GN=DARS2 PE=1 SV=1	74086	0	639	0	72	0	0.04	#DIV/0!	0	1	0	1	0	1	0	1	0.736677
sp|Q9UJA5|TRM6_HU	TRMT6	tRNA (adenine(58)-N(1))-methyltransferase non-catalytic subunit TRM6 OS=Homo sapie	56049	0	635	0	72	0	0.09	#DIV/0!	0	1	0	1	0	1	0	1	0.736677
sp|Q96IJ6|GMPPA_HU	GMPPA	Mannose-1-phosphate guanyltransferase alpha OS=Homo sapiens GN=GMPPA PE=1 SV=	46604	0	633	0	72	0	0.15	#DIV/0!	0	1	0	2	0	1	0	3	0.736677
tr|Q5VU21|Q5VU21_	SERBP1	PAI-1 mRNA-binding protein variant OS=Homo sapiens GN=SERBP1 PE=2 SV=1	42458	0	612	0	76	0	0.07	#DIV/0!	0	1	0	1	0	1	0	1	0.736677
tr|Q6FI97|Q6FI97_HU	BAF53A	BAF53A protein OS=Homo sapiens GN=BAF53A PE=2 SV=1	47864	0	604	0	77	0	0.07	#DIV/0!	0	1	0	1	0	1	0	1	0.736677
tr|B2C8Z3|B2C8Z3_HU	TGFB1	Transforming growth factor beta 1 (Fragment) OS=Homo sapiens GN=TGFB1 PE=2 SV=1	12965	0	600	0	55	0	0.15	#DIV/0!	0	1	0	1	0	1	0	1	0.736677
tr|B4DEN3|B4DEN3_H	tr|B4DEN3|B4DEN3_H	cDNA FLJ61101, highly similar to Eukaryotic translation initiation factor 5 OS=Homo sapie	22639	0	597	0	78	0	0.15	#DIV/0!	0	1	0	1	0	1	0	1	0.736677
tr|B4DU69|B4DU69_H	tr|B4DU69|B4DU69_H	cDNA FLJ56583, highly similar to Argininosuccinate lyase (EC 4.3.2.1) OS=Homo sapiens F	24664	0	594	0	79	0	0.14	#DIV/0!	0	1	0	1	0	1	0	2	0.736677
tr|Q6IBS5|Q6IBS5_HU	DLST	DLST protein OS=Homo sapiens GN=DLST PE=2 SV=1	49131	0	588	0	79	0	0.4	#DIV/0!	0	1	0	1	0	1	0	1	0.736677
sp|O43837|IDH3B_HU	IDH3B	Isocitrate dehydrogenase [NAD] subunit beta, mitochondrial OS=Homo sapiens GN=IDH3	42442	0	557	0	85	0	0.2	#DIV/0!	0	1	0	1	0	1	0	1	0.736677
sp|P51116|FXR2_HUN	FXR2	Fragile X mental retardation syndrome-related protein 2 OS=Homo sapiens GN=FXR2 PE	74520	0	556	0	85	0	0.04	#DIV/0!	0	1	0	1	0	1	0	2	0.736677
tr|G3V1T4|G3V1T4_H	NUP107	Nuclear pore complex protein Nup107 OS=Homo sapiens GN=NUP107 PE=2 SV=1	11398	0	542	0	88	0	0.3	#DIV/0!	0	1	0	1	0	1	0	1	0.736677
tr|B2RBB2|B2RBB2_H	tr|B2RBB2|B2RBB2_HU	cDNA, FLJ95416, highly similar to Homo sapiens phosphatidylinositol glycan, class S (PIGS	61818	0	524	0	92	0	0.05	#DIV/0!	0	1	0	1	0	1	0	1	0.736677

sp|Q5F1R6|DJC21_HU	DNAJC21	DnaJ homolog subfamily C member 21 OS=Homo sapiens GN=DNAJC21 PE=1 SV=2	62445	0	513	0	97	0	0.05	#DIV/0!	0	1	0	1	0	1	0	1	0.736677
tr|B3KMB7|B3KMB7_	PYCR2	Pyrroline-5-carboxylate reductase family, member 2, isoform CRA_a OS=Homo sapiens G	22559	0	506	0	56	0	0.2	#DIV/0!	0	1	0	1	0	1	0	2	0.736677
tr|B4DX99|B4DX99_H	tr|B4DX99|B4DX99_HU	Malic enzyme OS=Homo sapiens PE=2 SV=1	45378	0	473	0	66	0	0.16	#DIV/0!	0	1	0	2	0	1	0	2	0.736677
tr|B3KMN2|B3KMN2_	tr|B3KMN2|B3KMN2_H	cDNA FLJ11695 fis, clone HEMBA1005019, highly similar to Homo sapiens SCC-112 prote	36724	0	427	0	27	0	0.09	#DIV/0!	0	0	0	1	0	1	0	1	0.736677
tr|H0Y5T1|H0Y5T1_H	CLASP1	CLIP-associating protein 1 (Fragment) OS=Homo sapiens GN=CLASP1 PE=4 SV=1	76997	0	425	0	112	0	0.04	#DIV/0!	0	1	0	1	0	1	0	1	0.736677
tr|H0Y7C0|H0Y7C0_H	USO1	General vesicular transport factor p115 (Fragment) OS=Homo sapiens GN=USO1 PE=4 SV	72345	0	416	0	84	0	0.09	#DIV/0!	0	1	0	1	0	1	0	1	0.736677
tr|H7C5K0|H7C5K0_H	HLTF	Helicase-like transcription factor (Fragment) OS=Homo sapiens GN=HLTF PE=4 SV=1	48801	0	1315	0	15	0	0.07	#DIV/0!	0	0	0	1	0	0	0	1	-0.84847
sp|Q6ZVN7|CG076_H	C7orf76	Putative uncharacterized protein C7orf76 OS=Homo sapiens GN=C7orf76 PE=2 SV=2	14532	0	1314	0	15	0	0.24	#DIV/0!	0	0	0	1	0	0	0	1	-0.84847
sp|Q5T3J3|LRIF1_HUM	LRIF1	Ligand-dependent nuclear receptor-interacting factor 1 OS=Homo sapiens GN=LRIF1 PE=	84744	0	1312	0	15	0	0.04	#DIV/0!	0	0	0	1	0	0	0	1	-0.84847
tr|E7ERK9|E7ERK9_HU	EIF2B4	Translation initiation factor eIF-2B subunit delta OS=Homo sapiens GN=EIF2B4 PE=2 SV=	60248	0	1311	0	16	0	0.05	#DIV/0!	0	0	0	1	0	0	0	1	-0.84847
tr|H3BP20|H3BP20_H	HEXA	Beta-hexosaminidase subunit alpha OS=Homo sapiens GN=HEXA PE=4 SV=1	62416	0	1310	0	16	0	0.05	#DIV/0!	0	0	0	1	0	0	0	1	-0.84847
tr|E9PAZ2|E9PAZ2_HU	NAF1	H/ACA ribonucleoprotein complex non-core subunit NAF1 OS=Homo sapiens GN=NAF1 P	42154	0	1309	0	16	0	0.08	#DIV/0!	0	0	0	1	0	0	0	1	-0.84847
sp|Q14008|CKAP5_HU	CKAP5	Cytoskeleton-associated protein 5 OS=Homo sapiens GN=CKAP5 PE=1 SV=3	227062	0	1308	0	16	0	0.01	#DIV/0!	0	1	0	1	0	0	0	3	-0.84847
tr|Q5W9H1|Q5W9H1	KIAA0142	KIAA0142 splice variant 1 (Fragment) OS=Homo sapiens GN=KIAA0142 PE=2 SV=1	78539	0	1307	0	16	0	0.04	#DIV/0!	0	0	0	1	0	0	0	2	-0.84847
tr|J3KS45|J3KS45_HU	TMCO1	Transmembrane and coiled-coil domain-containing protein 1 (Fragment) OS=Homo sapie	16741	0	1306	0	16	0	0.2	#DIV/0!	0	0	0	1	0	0	0	1	-0.84847
sp|Q15061|WDR43_H	WDR43	WD repeat-containing protein 43 OS=Homo sapiens GN=WDR43 PE=1 SV=3	75813	0	1305	0	16	0	0.04	#DIV/0!	0	0	0	1	0	0	0	2	-0.84847
tr|G3V236|G3V236_H	RPGRIP1	X-linked retinitis pigmentosa GTPase regulator-interacting protein 1 OS=Homo sapiens G	108308	0	1304	0	17	0	0.03	#DIV/0!	0	0	0	1	0	0	0	1	-0.84847
tr|Q7Z586|Q7Z586_H	tr|Q7Z586|Q7Z586_HU	Putative uncharacterized protein (Fragment) OS=Homo sapiens PE=2 SV=1	105456	0	1303	0	17	0	0.03	#DIV/0!	0	0	0	1	0	0	0	3	-0.84847
sp|Q86YD7|F90A1_HU	FAM90A1	Protein FAM90A1 OS=Homo sapiens GN=FAM90A1 PE=2 SV=3	50333	0	1302	0	17	0	0.07	#DIV/0!	0	0	0	1	0	0	0	1	-0.84847
tr|B4DV28|B4DV28_H	tr|B4DV28|B4DV28_HU	cDNA FLJ54170, highly similar to Cytosolic nonspecific dipeptidase OS=Homo sapiens PE=	51811	0	1301	0	17	0	0.18	#DIV/0!	0	0	0	1	0	0	0	1	-0.84847
tr|Q6FI30|Q6FI30_HU	NFIC	Nuclear factor 1 OS=Homo sapiens GN=NFIC PE=2 SV=1	55019	0	1300	0	17	0	0.06	#DIV/0!	0	0	0	1	0	0	0	1	-0.84847
tr|K7EJ20|K7EJ20_HU	GPX4	Glutathione peroxidase (Fragment) OS=Homo sapiens GN=GPX4 PE=3 SV=1	24625	0	1299	0	17	0	0.14	#DIV/0!	0	0	0	1	0	0	0	1	-0.84847
tr|B3KVM6|B3KVM6_	tr|B3KVM6|B3KVM6_H	cDNA FLJ16752 fis, clone BRACE2013010, highly similar to Homo sapiens human immunc	67043	0	1298	0	18	0	0.05	#DIV/0!	0	0	0	1	0	0	0	1	-0.84847
tr|B4DFG0|B4DFG0_H	DEK	Protein DEK OS=Homo sapiens GN=DEK PE=2 SV=1	39704	0	1297	0	18	0	0.08	#DIV/0!	0	0	0	1	0	0	0	1	-0.84847
tr|B3KQC5|B3KQC5_H	MAN1B1	Endoplasmic reticulum mannosyl-oligosaccharide 1,2-alpha-mannosidase OS=Homo sapi	46112	0	1295	0	18	0	0.04	#DIV/0!	0	0	0	1	0	0	0	1	-0.84847
sp|Q6GQQ9|OTU7B_H	OTUD7B	OTU domain-containing protein 7B OS=Homo sapiens GN=OTUD7B PE=1 SV=1	93152	0	1294	0	18	0	0.04	#DIV/0!	0	0	0	1	0	0	0	1	-0.84847
tr|A5PKW6|A5PKW6_	ZNF609	ZNF609 protein OS=Homo sapiens GN=ZNF609 PE=2 SV=1	34779	0	1293	0	18	0	0.09	#DIV/0!	0	0	0	1	0	0	0	1	-0.84847
sp|Q6P2E9|EDC4_HU	EDC4	Enhancer of mRNA-decapping protein 4 OS=Homo sapiens GN=EDC4 PE=1 SV=1	152992	0	1292	0	18	0	0.02	#DIV/0!	0	0	0	1	0	0	0	1	-0.84847
tr|F6S0T5|F6S0T5_HU	KDM1A	Lysine-specific histone demethylase 1A OS=Homo sapiens GN=KDM1A PE=2 SV=1	95677	0	1291	0	18	0	0.03	#DIV/0!	0	0	0	1	0	0	0	2	-0.84847
sp|Q9BTC0|DIDO1_HU	DIDO1	Death-inducer obliterator 1 OS=Homo sapiens GN=DIDO1 PE=1 SV=5	245434	0	1290	0	18	0	0.01	#DIV/0!	0	0	0	1	0	0	0	1	-0.84847
tr|H0Y580|H0Y580_H	EIF2B3	Translation initiation factor eIF-2B subunit gamma (Fragment) OS=Homo sapiens GN=EIF	25394	0	1287	0	19	0	0.13	#DIV/0!	0	0	0	1	0	0	0	1	-0.84847
sp|P04181|OAT_HUM	OAT	Ornithine aminotransferase, mitochondrial OS=Homo sapiens GN=OAT PE=1 SV=1	48846	0	1286	0	19	0	0.07	#DIV/0!	0	0	0	1	0	0	0	3	-0.84847
tr|E5KLJ5|E5KLJ5_HU	OPA1	Dynamin-like 120 kDa protein, mitochondrial OS=Homo sapiens GN=OPA1 PE=3 SV=1	118240	0	1284	0	19	0	0.03	#DIV/0!	0	0	0	1	0	0	0	1	-0.84847
tr|G3V1P4|G3V1P4_H	HMBS	Hydroxymethylbilane synthase, isoform CRA_c OS=Homo sapiens GN=HMBS PE=2 SV=1	33415	0	1283	0	19	0	0.1	#DIV/0!	0	0	0	1	0	0	0	1	-0.84847
sp|Q9Y2U8|MAN1_HU	LEMD3	Inner nuclear membrane protein Man1 OS=Homo sapiens GN=LEMD3 PE=1 SV=2	100790	0	1282	0	19	0	0.03	#DIV/0!	0	0	0	1	0	0	0	1	-0.84847
tr|B4DN85|B4DN85_H	tr|B4DN85|B4DN85_HU	E3 ubiquitin-protein ligase OS=Homo sapiens PE=2 SV=1	93815	0	1281	0	19	0	0.03	#DIV/0!	0	0	0	1	0	0	0	1	-0.84847
sp|O14776|TCRG1_HU	TCERG1	Transcription elongation regulator 1 OS=Homo sapiens GN=TCERG1 PE=1 SV=2	124051	0	1279	0	20	0	0.03	#DIV/0!	0	0	0	1	0	0	0	1	-0.84847
sp|Q96DV4|RM38_HU	MRPL38	39S ribosomal protein L38, mitochondrial OS=Homo sapiens GN=MRPL38 PE=1 SV=2	44968	0	1278	0	20	0	0.07	#DIV/0!	0	0	0	1	0	0	0	1	-0.84847
tr|Q53GQ5|Q53GQ5_	tr|Q53GQ5|Q53GQ5_H	Ubiquitin conjugating enzyme 7 interacting protein 5 isoform a variant (Fragment) OS=H	60040	0	1277	0	20	0	0.05	#DIV/0!	0	0	0	1	0	0	0	1	-0.84847
tr|I3L495|I3L495_HUM	PAFAH1B1	Platelet-activating factor acetylhydrolase IB subunit alpha (Fragment) OS=Homo sapiens	9626	0	825	0	0	0	0.07	#DIV/0!	0	0	0	0	0	0	0	1	-0.84847
tr|Q59H95|Q59H95_H	tr|Q59H95|Q59H95_H	Flightless I homolog variant (Fragment) OS=Homo sapiens PE=2 SV=1	126396	0	816	0	0	0	0.02	#DIV/0!	0	0	0	0	0	0	0	1	-0.84847
tr|B4DJR0|B4DJR0_HU	tr|B4DJR0|B4DJR0_HU	cDNA FLJ56917, moderately similar to Homo sapiens GDP-mannose pyrophosphorylase A	14089	0	618	0	0	0	0.1	#DIV/0!	0	0	0	0	0	0	0	1	-0.84847
tr|Q8N274|Q8N274_H	tr|Q8N274|Q8N274_H	cDNA FLJ33834 fis, clone CTONG2004264, moderately similar to NEUROBLAST DIFFEREN	86111	401	219	43	228	0.01	0.4	40	1	6	1	9	2	12	4	13	1.474977
tr|B2R5M9|B2R5M9_	tr|B2R5M9|B2R5M9_H	cDNA, FLJ92537, highly similar to Homo sapiens procollagen-lysine, 2-oxoglutarate 5-dio	84022	251	84	76	490	0.04	0.9	22.5	1	12	1	15	1	15	1	16	2.523148
sp|Q09666|AHNK_HU	AHNAK	Neuroblast differentiation-associated protein AHNAK OS=Homo sapiens GN=AHNAK PE=	629213	401	48	43	770	0.01	0.2	20	1	21	1	35	3	45	6	54	2.859126
tr|Q6ZQN2|Q6ZQN2_	tr|Q6ZQN2|Q6ZQN2_H	cDNA FLJ46846 fis, clone UTERU3004635, moderately similar to Neuroblast differentiatio	180689	401	252	43	208	0.01	0.2	20	1	6	1	10	2	13	3	16	1.586188
sp|O14980|XPO1_HU	XPO1	Exportin-1 OS=Homo sapiens GN=XPO1 PE=1 SV=1	124447	305	102	59	433	0.03	0.48	16	1	11	1	15	1	18	3	19	2.778946
tr|J3KR24|J3KR24_HU	IARS	Isoleucine--tRNA ligase, cytoplasmic OS=Homo sapiens GN=IARS PE=4 SV=1	132820	366	220	47	228	0.02	0.31	15.5	1	6	1	11	2	12	2	13	1.474977
tr|Q2TU64|Q2TU64_H	tr|Q2TU64|Q2TU64_H	PIG48 OS=Homo sapiens PE=2 SV=1	61110	279	140	68	263	0.05	0.73	14.6	1	8	1	9	3	10	3	11	0.737326
sp|P41250|SYG_HUM	GARS	Glycine--tRNA ligase OS=Homo sapiens GN=GARS PE=1 SV=3	83854	392	238	44	219	0.04	0.58	14.5	1	7	1	12	1	12	2	13	2.212266
tr|B2R9U2|B2R9U2_H	tr|B2R9U2|B2R9U2_HU	cDNA, FLJ94557, highly similar to Homo sapiens FK506 binding protein 4, 59kDa (FKBP4),	52087	623	189	24	253	0.06	0.74	12.3333	0	6	1	9	2	10	2	11	1.223077
tr|B2R7B5|B2R7B5_H	tr|B2R7B5|B2R7B5_HU	cDNA, FLJ93365, highly similar to Homo sapiens KH domain containing, RNA binding, sigr	48309	347	198	51	245	0.07	0.85	12.1429	1	6	1	8	2	10	4	10	1.223077
tr|A8KAK1|A8KAK1_H	tr|A8KAK1|A8KAK1_HU	cDNA FLJ77398, highly similar to Homo sapiens UDP-glucose ceramide glucosyltransferas	175467	635	201	22	254	0.02	0.22	11	0	7	1	11	1	13	1	16	2.323478
tr|B1AHB0|B1AHB0_H	MCM5	MCM5 minichromosome maintenance deficient 5, cell division cycle 46 (S. cerevisiae), is	83031	562	1094	29	31	0.04	0.43	10.75	0	0	1	2	1	1	2	2	-0.84861
sp|P45974|UBP5_HU	USP5	Ubiquitin carboxyl-terminal hydrolase 5 OS=Homo sapiens GN=USP5 PE=1 SV=2	96638	622	254	24	207	0.03	0.31	10.3333	0	5	1	8	2	7	2	8	0.73711
tr|Q59FV6|Q59FV6_H	tr|Q59FV6|Q59FV6_HU	ARP3 actin-related protein 3 homolog variant (Fragment) OS=Homo sapiens PE=2 SV=1	42517	548	171	30	290	0.08	0.82	10.25	0	8	1	10	1	11	1	11	2.091792
tr|B4DT29|B4DT29_H	tr|B4DT29|B4DT29_HU	cDNA FLJ51082, highly similar to Actin-like protein 3 OS=Homo sapiens PE=2 SV=1	40959	548	130	30	189	0.08	0.82	10.25	0	5	1	7	1	8	1	8	1.655152
sp|Q15459|SF3A1_HU	SF3A1	Splicing factor 3A subunit 1 OS=Homo sapiens GN=SF3A1 PE=1 SV=1	88888	482	295	36	179	0.04	0.39	9.75	1	5	1	9	1	8	1	9	1.655152
tr|B4E091|B4E091_HU	tr|B4E091|B4E091_HU	cDNA FLJ55438, highly similar to Splicing factor 3 subunit 1 OS=Homo sapiens PE=2 SV=1	77415	482	292	36	180	0.04	0.39	9.75	1	5	1	8	1	8	1	8	1.655152
tr|B4DY09|B4DY09_H	tr|B4DY09|B4DY09_HU	cDNA FLJ51660, highly similar to Interleukin enhancer-binding factor 2 OS=Homo sapien	39057	383	236	45	221	0.08	0.77	9.625	1	6	1	7	1	7	1	7	1.4744
tr|Q8TC62|Q8TC62_H	Sep-07	Septin 7 OS=Homo sapiens GN=SEPT7 PE=2 SV=3	48913	531	406	31	129	0.07	0.67	9.57143	1	4	1	7	1	7	2	8	1.4744
tr|B2RBA6|B2RBA6_H	tr|B2RBA6|B2RBA6_HU	cDNA, FLJ95407, highly similar to Homo sapiens MCM7 minichromosome maintenance d	81856	374	291	46	182	0.04	0.37	9.25	1	4	1	8	1	8	1	10	1.655152
tr|E9PBS1|E9PBS1_HU	PAICS	Phosphoribosylaminoimidazole carboxylase (Fragment) OS=Homo sapiens GN=PAICS PE=	46307	270	247	69	211	0.07	0.62	8.85714	1	3	1	7	2	7	5	7	0.73711
tr|A2VCT2|A2VCT2_H	OGDH	OGDH protein (Fragment) OS=Homo sapiens GN=OGDH PE=2 SV=1	72973	432	214	40	146	0.05	0.43	8.6	1	3	1	5	1	6	1	6	1.267769
tr|B7Z1R5|B7Z1R5_HU	ATP6V1A	V-type proton ATPase catalytic subunit A OS=Homo sapiens GN=ATP6V1A PE=2 SV=1	65037	428	352	40	148	0.05	0.41	8.2	1	3	1	7	1	9	2	9	1.815797
sp|O43175|SERA_HUM	PHGDH	D-3-phosphoglycerate dehydrogenase OS=Homo sapiens GN=PHGDH PE=1 SV=4	57356	131	71	166	570	0.25	2.05	8.2	3	15	4	22	4	22	4	24	1.476132
tr|B2RE46|B2RE46_HU	tr|B2RE46|B2RE46_HU	cDNA, FLJ96923, highly similar to Homo sapiens ribophorin II (RPN2), mRNA OS=Homo sa	69405	589	1104	27	30	0.05	0.41	8.2	0	0	1	2	2	1	2	2	-1.5859
tr|I3L1L3|I3L1L3_HUM	MYBBP1A	Myb-binding protein 1A (Fragment) OS=Homo sapiens GN=MYBBP1A PE=4 SV=1	141124	698	349	18	144	0.02	0.16	8	0	3	1	5	1	9	2	10	1.815797
tr|B4DVJ0|B4DVJ0_HU	tr|B4DVJ0|B4DVJ0_HU	Glucose-6-phosphate isomerase OS=Homo sapiens PE=2 SV=1	60181	117	42	182	860	0.3	2.4	8	4	19	5	27	6	28	6	32	1.287089
tr|Q13727|Q13727_H	tr|Q13727|Q13727_HU	AHNAK-related protein (Fragment) OS=Homo sapiens PE=2 SV=1	57703	402	351	43	148	0.06	0.47	7.83333	1	4	1	7	2	9	3	9	1.078507
tr|B1ANR0|B1ANR0_H	PABPC4	Poly(A) binding protein, cytoplasmic 4 (Inducible form) OS=Homo sapiens GN=PABPC4 PI	68156	504	391	33	135	0.05	0.39	7.8	0	3	1	7	1	7	2	11	1.4744
tr|Q2Q9B7|Q2Q9B7_	G6PD	Glucose-6-phosphate 1-dehydrogenase (Fragment) OS=Homo sapiens GN=G6PD PE=2 SV	55221	176	107	118	428	0.18	1.39	7.72222	2	9	2	15	5	19	6	20	0.979308
tr|Q96QL0|Q96QL0_H	RPL3	Ribosomal protein L3 OS=Homo sapiens GN=RPL3 PE=2 SV=1	46376	245	63	82	602	0.32	2.44	7.625	4	14	4	19	5	20	7	21	1.051639
tr|Q0VGD6|Q0VGD6_	HNRPR	HNRPR protein (Fragment) OS=Homo sapiens GN=HNRPR PE=2 SV=1	68218	390	156	44	316	0.1	0.76	7.6	1	9	2	12	2	12	4	14	1.474977
tr|H0YC10|H0YC10_H	PABPC1	Polyadenylate-binding protein 1 (Fragment) OS=Homo sapiens GN=PABPC1 PE=4 SV=1	4370	215	460	39	109	0.33	2.46	7.45455	1	2	1	2	1	3	1	3	0.374143
tr|B4DE36|B4DE36_H	tr|B4DE36|B4DE36_HU	Glucose-6-phosphate isomerase OS=Homo sapiens PE=2 SV=1	60376	117	41	182	835	0.3	2.2	7.33333	4	18	5	26	7	27	7	32	1.028602
tr|B4DJ60|B4DJ60_HU	tr|B4DJ60|B4DJ60_HU	cDNA FLJ55072, highly similar to Succinate dehydrogenase (ubiquinone) flavoprotein sub	68233	393	137	44	346	0.1	0.73	7.3	0	8	2	11	3	11	5	13	0.868751
tr|B4DR68|B4DR68_H	tr|B4DR68|B4DR68_HU	cDNA FLJ58608, highly similar to Heat shock protein 75 kDa, mitochondrial OS=Homo sap	74408	169	153	127	321	0.09	0.65	7.22222	2	8	3	10	3	10	5	12	0.737326
tr|A6NKB8|A6NKB8_H	RNPEP	Aminopeptidase B OS=Homo sapiens GN=RNPEP PE=2 SV=1	68752	316	257	57	206	0.05	0.36	7.2	1	5	2	7	2	6	2	8	0.530479
sp|P11021|GRP78_HU	HSPA5	78 kDa glucose-regulated protein OS=Homo sapiens GN=HSPA5 PE=1 SV=2	72402	129	49	170	756	0.19	1.32	6.94737	3	17	5	21	6	22	6	22	0.944968
tr|Q05CK9|Q05CK9_H	SYNCRIP	SYNCRIP protein (Fragment) OS=Homo sapiens GN=SYNCRIP PE=2 SV=1	50847	252	117	76	371	0.13	0.9	6.92308	2	11	2	12	2	13	2	13	1.586188
tr|B4DZ53|B4DZ53_H	tr|B4DZ53|B4DZ53_HU	cDNA FLJ59643, highly similar to Neutral alpha-glucosidase AB OS=Homo sapiens PE=2 S	96752	47	14	400	1892	0.62	4.28	6.90323	10	49	17	70	15	74	19	84	1.425021
tr|B4DYA7|B4DYA7_H	tr|B4DYA7|B4DYA7_HU	Glucose-6-phosphate 1-dehydrogenase OS=Homo sapiens PE=2 SV=1	58757	176	106	123	427	0.18	1.23	6.83333	2	9	3	14	6	18	7	19	0.661847
tr|B4DJ30|B4DJ30_HU	tr|B4DJ30|B4DJ30_HU	cDNA FLJ61290, highly similar to Neutral alpha-glucosidase AB OS=Homo sapiens PE=2 S	113539	47	10	446	2102	0.62	4.19	6.75806	12	57	19	81	17	89	22	100	1.516659
tr|B3KQT2|B3KQT2_H	tr|B3KQT2|B3KQT2_HU	cDNA PSEC0148 fis, clone PLACE1007202, highly similar to Protein disulfide-isomerase A	55268	546	114	30	370	0.19	1.27	6.68421	0	11	3	14	2	16	8	17	1.876236
sp|O94906|PRP6_HU	PRPF6	Pre-mRNA-processing factor 6 OS=Homo sapiens GN=PRPF6 PE=1 SV=1	107656	492	419	35	123	0.03	0.2	6.66667	1	3	1	6	1	6	2	6	1.267769
tr|B5MCX3|B5MCX3_	Sep-02	Septin-2 OS=Homo sapiens GN=SEPT2 PE=2 SV=1	37145	543	273	30	178	0.09	0.58	6.44444	1	5	1	5	2	5	3	6	0.28929
tr|Q2F839|Q2F839_H	tr|Q2F839|Q2F839_HU	Heat shock 70 kDa protein 9B (Fragment) OS=Homo sapiens PE=2 SV=1	19208	81	155	59	320	0.42	2.64	6.28571	2	7	2	8	2	8	2	10	0.917862
tr|B4DH39|B4DH39_H	tr|B4DH39|B4DH39_HU	cDNA FLJ57028, highly similar to Long-chain-fatty-acid--CoA ligase 4 (EC 6.2.1.3) OS=Hom	73123	529	241	31	216	0.04	0.25	6.25	0	5	1	5	1	6	3	7	1.267769
tr|E2DH89|E2DH89_H	HLA-B	MHC class I antigen (Fragment) OS=Homo sapiens GN=HLA-B PE=3 SV=1	21132	643	598	22	78	0.09	0.56	6.22222	0	3	1	3	1	5	1	5	1.02658
tr|A8K781|A8K781_H	tr|A8K781|A8K781_HU	cDNA FLJ75299, highly similar to Xenopus laevis proteasome (prosome, macropain) 26S s	47578	603	422	26	86	0.07	0.43	6.14286	0	3	1	4	2	6	2	6	0.530479
sp|Q6IBS0|TWF2_HU	TWF2	Twinfilin-2 OS=Homo sapiens GN=TWF2 PE=1 SV=2	39751	535	250	30	209	0.08	0.49	6.125	0	5	1	5	1	5	1	6	1.02658
tr|B2R5V9|B2R5V9_H	tr|B2R5V9|B2R5V9_HU	cDNA, FLJ92652, highly similar to Homo sapiens high density lipoprotein binding protein	141919	253	357	76	145	0.02	0.12	6	1	3	1	5	2	4	2	6	-0.0004
sp|P52597|HNRPF_HU	HNRNPF	Heterogeneous nuclear ribonucleoprotein F OS=Homo sapiens GN=HNRNPF PE=1 SV=3	45985	226	373	91	141	0.07	0.4	5.71429	1	5	1	5	2	5	3	5	0.28929
tr|B7Z601|B7Z601_HU	tr|B7Z601|B7Z601_HU	cDNA FLJ57187, highly similar to Glycerol-3-phosphate dehydrogenase, mitochondrial (E	56047	512	327	32	135	0.06	0.34	5.66667	0	3	1	6	2	7	2	8	0.73711
tr|F8SKU2|F8SKU2_HU	HLA-A	MHC class I antigen (Fragment) OS=Homo sapiens GN=HLA-A PE=3 SV=1	21143	639	394	22	48	0.16	0.9	5.625	0	2	1	2	2	4	3	4	-0.0004
sp|O00425|IF2B3_HU	IGF2BP3	Insulin-like growth factor 2 mRNA-binding protein 3 OS=Homo sapiens GN=IGF2BP3 PE=	64008	556	268	29	200	0.05	0.28	5.6	0	5	1	5	2	5	2	6	0.28929
tr|B2R5P6|B2R5P6_H	TXNRD1	Thioredoxin reductase 1, cytoplasmic OS=Homo sapiens GN=TXNRD1 PE=2 SV=1	55253	108	87	204	449	0.26	1.45	5.57692	3	11	5	17	5	17	6	19	0.822828
tr|Q9NZ23|Q9NZ23_H	YA61	Drug-sensitive protein 1 OS=Homo sapiens GN=YA61 PE=2 SV=1	14858	588	613	27	76	0.23	1.28	5.56522	0	2	1	4	1	4	1	4	0.736894
tr|H0YLA4|H0YLA4_H	SORD	Sorbitol dehydrogenase OS=Homo sapiens GN=SORD PE=3 SV=1	36833	473	336	37	140	0.09	0.5	5.55556	0	2	1	4	2	4	2	4	-0.0004
sp|Q6P2Q9|PRP8_HU	PRPF8	Pre-mRNA-processing-splicing factor 8 OS=Homo sapiens GN=PRPF8 PE=1 SV=2	274738	130	44	167	853	0.1	0.55	5.5	7	24	8	37	10	40	10	44	1.103042
tr|B2RBE0|B2RBE0_H	tr|B2RBE0|B2RBE0_HU	cDNA, FLJ95462, highly similar to Homo sapiens fatty-acid-Coenzyme A ligase, long-chair	81266	462	369	37	142	0.04	0.22	5.5	0	5	1	5	1	5	1	5	1.02658
tr|Q9BSV4|Q9BSV4_H	SFPQ	SFPQ protein (Fragment) OS=Homo sapiens GN=SFPQ PE=2 SV=2	68702	228	57	91	626	0.26	1.42	5.46154	2	17	5	22	6	23	7	26	1.007884
tr|F6IR35|F6IR35_HU	HLA-A	MHC class I antigen (Fragment) OS=Homo sapiens GN=HLA-A PE=2 SV=1	38417	643	606	22	74	0.09	0.49	5.44444	0	3	1	3	1	6	2	6	1.267769
tr|B4DFL1|B4DFL1_HU	tr|B4DFL1|B4DFL1_HU	Dihydrolipoyl dehydrogenase OS=Homo sapiens PE=2 SV=1	49381	666	366	21	143	0.07	0.38	5.42857	0	3	1	6	1	6	1	6	1.267769
tr|B4DQJ8|B4DQJ8_H	PGD	6-phosphogluconate dehydrogenase, decarboxylating OS=Homo sapiens GN=PGD PE=2 S	52352	293	123	62	385	0.2	1.08	5.4	1	10	3	12	2	14	3	14	1.689462
tr|A8K8N7|A8K8N7_H	PFAS	Phosphoribosylformylglycinamidine synthase (FGAR amidotransferase), isoform CRA_b C	146286	380	174	46	288	0.05	0.27	5.4	0	4	2	12	3	12	5	13	0.989225
tr|B2ZZ89|B2ZZ89_HU	SPTBN1	Spectrin beta non-erythrocytic 1 OS=Homo sapiens GN=SPTBN1 PE=2 SV=1	275237	49	8	431	2336	0.22	1.15	5.22727	11	48	17	68	20	70	24	84	0.939694
tr|B2R8N0|B2R8N0_H	tr|B2R8N0|B2R8N0_HU	cDNA, FLJ93975, highly similar to Homo sapiens epoxide hydrolase 1, microsomal (xenob	53129	115	60	184	635	0.62	3.23	5.20968	6	17	8	28	8	30	8	36	0.997625
tr|B2R6D0|B2R6D0_H	tr|B2R6D0|B2R6D0_HU	cDNA, FLJ92896, highly similar to Homo sapiens proteasome (prosome, macropain) 26S s	106797	386	261	45	205	0.06	0.31	5.16667	0	6	2	9	2	9	4	10	1.078507
tr|B7Z5E2|B7Z5E2_HU	PSMC2	26S protease regulatory subunit 7 OS=Homo sapiens GN=PSMC2 PE=2 SV=1	33594	610	360	25	107	0.1	0.51	5.1	0	2	1	4	1	5	1	6	1.02658
tr|Q5SU16|Q5SU16_H	TUBB	Beta 5-tubulin OS=Homo sapiens GN=TUBB PE=2 SV=1	50095	25	12	680	1969	3.07	15.54	5.06189	26	62	33	74	35	78	39	85	0.299129
tr|B2R959|B2R959_HU	tr|B2R959|B2R959_HU	cDNA, FLJ94229, highly similar to Homo sapiens heterogeneous nuclear ribonucleoprote	60838	182	88	117	373	0.23	1.16	5.04348	4	12	4	15	6	16	8	18	0.496426
tr|B4DR45|B4DR45_H	tr|B4DR45|B4DR45_HU	cDNA FLJ61339, highly similar to Alanyl-tRNA synthetase (EC 6.1.1.7) OS=Homo sapiens F	110017	238	188	86	264	0.06	0.3	5	1	5	2	9	2	11	2	11	1.354502
tr|F2X5X4|F2X5X4_HU	HLA-A	MHC class I antigen (Fragment) OS=Homo sapiens GN=HLA-A PE=3 SV=1	21229	642	711	22	61	0.16	0.8	5	0	2	1	4	1	6	2	6	1.267769
sp|P11388|TOP2A_HU	TOP2A	DNA topoisomerase 2-alpha OS=Homo sapiens GN=TOP2A PE=1 SV=3	175017	606	440	25	115	0.02	0.1	5	0	3	1	5	1	6	2	7	1.267769
sp|P54886|P5CS_HUM	ALDH18A1	Delta-1-pyrroline-5-carboxylate synthase OS=Homo sapiens GN=ALDH18A1 PE=1 SV=2	87989	658	304	21	173	0.04	0.2	5	1	4	1	5	1	5	1	6	1.02658
sp|Q14204|DYHC1_HU	DYNC1H1	Cytoplasmic dynein 1 heavy chain 1 OS=Homo sapiens GN=DYNC1H1 PE=1 SV=5	534809	168	159	129	313	0.02	0.1	5	3	8	4	16	4	14	6	26	0.840816
tr|I3L239|I3L239_HUM	TRAP1	Heat shock protein 75 kDa, mitochondrial (Fragment) OS=Homo sapiens GN=TRAP1 PE=4	24972	179	303	121	173	0.13	0.65	5	2	3	2	5	2	5	4	7	0.28929
sp|P07355|ANXA2_HU	ANXA2	Annexin A2 OS=Homo sapiens GN=ANXA2 PE=1 SV=2	38808	100	51	214	744	1.26	6.11	4.84921	8	20	11	30	12	31	13	32	0.486655
tr|A6NN80|A6NN80_H	ANXA6	Annexin OS=Homo sapiens GN=ANXA6 PE=3 SV=3	75571	349	172	50	290	0.14	0.67	4.78571	1	9	3	12	3	12	3	14	0.989225
tr|Q59G92|Q59G92_H	tr|Q59G92|Q59G92_H	HLA-B associated transcript 1 variant (Fragment) OS=Homo sapiens PE=2 SV=1	35528	394	654	44	70	0.09	0.43	4.77778	1	2	1	4	2	4	2	6	-0.0004
tr|B2R7C5|B2R7C5_H	tr|B2R7C5|B2R7C5_HU	cDNA, FLJ93378, highly similar to Homo sapiens MCM3 minichromosome maintenance d	91579	479	465	36	108	0.04	0.19	4.75	1	3	1	5	1	5	1	6	1.02658
sp|O95373|IPO7_HUM	IPO7	Importin-7 OS=Homo sapiens GN=IPO7 PE=1 SV=1	120751	162	262	132	203	0.08	0.38	4.75	3	5	3	12	3	11	4	14	0.868751
sp|O75367|H2AY_HU	H2AFY	Core histone macro-H2A.1 OS=Homo sapiens GN=H2AFY PE=1 SV=4	39764	565	387	29	136	0.08	0.38	4.75	0	2	1	4	2	6	2	6	0.530479
tr|B4DUC5|B4DUC5_H	CSE1L	Exportin-2 OS=Homo sapiens GN=CSE1L PE=2 SV=1	86045	433	111	40	160	0.08	0.38	4.75	0	3	2	6	4	6	4	6	-0.31817
tr|I3L0N3|I3L0N3_HU	NSF	Vesicle-fusing ATPase OS=Homo sapiens GN=NSF PE=3 SV=1	82553	412	1289	42	18	0.04	0.19	4.75	1	0	1	1	1	0	1	1	-2.43375
tr|B4DMJ6|B4DMJ6_H	tr|B4DMJ6|B4DMJ6_H	cDNA FLJ50996, highly similar to 60S ribosomal protein L4 OS=Homo sapiens PE=2 SV=1	46269	163	56	132	597	0.51	2.42	4.7451	4	16	6	19	8	22	9	25	0.557296
sp|P26599|PTBP1_HU	PTBP1	Polypyrimidine tract-binding protein 1 OS=Homo sapiens GN=PTBP1 PE=1 SV=1	57357	567	95	29	464	0.18	0.85	4.72222	0	9	3	12	3	14	3	14	1.203711
tr|B7Z582|B7Z582_HU	tr|B7Z582|B7Z582_HU	cDNA FLJ53638, highly similar to Annexin A6 OS=Homo sapiens PE=2 SV=1	61985	349	164	50	250	0.14	0.66	4.71429	1	7	3	9	3	10	3	11	0.737326
tr|F1T0B3|F1T0B3_HU	DDX1	ATP-dependent RNA helicase DDX1 OS=Homo sapiens GN=DDX1 PE=2 SV=1	74838	501	332	33	156	0.09	0.42	4.66667	0	5	2	9	3	9	3	11	0.592756
tr|B4DKS0|B4DKS0_H	tr|B4DKS0|B4DKS0_HU	cDNA FLJ53381, highly similar to Monocarboxylate transporter 1 OS=Homo sapiens PE=2	52487	598	432	26	94	0.06	0.28	4.66667	0	2	1	3	1	3	1	3	0.374143
tr|Q05D08|Q05D08_H	PA2G4	PA2G4 protein (Fragment) OS=Homo sapiens GN=PA2G4 PE=2 SV=1	45579	286	384	64	116	0.15	0.7	4.66667	1	3	2	5	3	5	3	5	-0.19646
tr|B2R4K7|B2R4K7_H	tr|B2R4K7|B2R4K7_HU	60S ribosomal protein L6 OS=Homo sapiens PE=2 SV=1	32739	276	127	68	370	0.47	2.19	4.65957	2	11	4	16	3	18	5	20	1.555905
tr|G3XAF4|G3XAF4_H	L1CAM	L1 cell adhesion molecule, isoform CRA_a OS=Homo sapiens GN=L1CAM PE=4 SV=1	139790	572	141	28	342	0.05	0.23	4.6	1	7	2	9	1	8	2	12	1.655152
tr|B3KXU8|B3KXU8_H	tr|B3KXU8|B3KXU8_HU	cDNA FLJ46087 fis, clone TESTI2009497, highly similar to GPI-anchored protein p137 OS=	70489	367	346	47	120	0.05	0.23	4.6	1	3	1	3	1	3	1	3	0.374143
tr|B4DHQ3|B4DHQ3_	tr|B4DHQ3|B4DHQ3_H	cDNA FLJ56437, highly similar to Phosphoserine aminotransferase (EC 2.6.1.52) OS=Hom	45783	552	429	30	118	0.07	0.32	4.57143	0	3	1	4	1	5	2	6	1.02658
sp|O75369|FLNB_HU	FLNB	Filamin-B OS=Homo sapiens GN=FLNB PE=1 SV=2	280157	45	16	463	1845	0.22	1	4.54545	14	46	18	63	20	62	28	73	0.764475
tr|B4DLT2|B4DLT2_H	tr|B4DLT2|B4DLT2_HU	cDNA FLJ56637, highly similar to Nuclear pore complex protein Nup155 OS=Homo sapien	152734	489	517	35	96	0.02	0.09	4.5	0	2	1	4	1	5	1	5	1.02658
tr|B4DSQ5|B4DSQ5_H	tr|B4DSQ5|B4DSQ5_H	cDNA FLJ53608, highly similar to Protein transport protein Sec23A OS=Homo sapiens PE=	83787	474	218	37	230	0.08	0.36	4.5	0	6	2	8	2	8	3	9	0.917862

tr|B2RBR9|B2RBR9_H	tr|B2RBR9|B2RBR9_HU	cDNA, FLJ95650, highly similar to Homo sapiens karyopherin (importin) beta 1 (KPNB1), r	98434	171	92	126	473	0.22	0.99	4.5	4	11	6	23	9	23	9	25	0.459423
tr|B4DM97|B4DM97_	tr|B4DM97|B4DM97_H	cDNA FLJ55002, highly similar to Alpha-centractin OS=Homo sapiens PE=2 SV=1	38365	561	311	29	166	0.09	0.4	4.44444	0	3	1	3	1	3	1	3	0.374143
sp|P53004|BIEA_HUM	BLVRA	Biliverdin reductase A OS=Homo sapiens GN=BLVRA PE=1 SV=2	33692	403	446	43	113	0.21	0.93	4.42857	1	4	2	7	2	6	3	9	0.530479
tr|H0Y8E6|H0Y8E6_H	MCM2	DNA replication licensing factor MCM2 (Fragment) OS=Homo sapiens GN=MCM2 PE=3 S	94792	211	272	98	196	0.07	0.31	4.42857	1	6	2	8	2	8	2	8	0.917862
tr|A4FU77|A4FU77_H	SNRNP200	SNRNP200 protein (Fragment) OS=Homo sapiens GN=SNRNP200 PE=2 SV=1	217585	243	109	83	369	0.05	0.22	4.4	2	7	3	14	7	15	8	18	0.199273
sp|Q96A33|CCD47_H	CCDC47	Coiled-coil domain-containing protein 47 OS=Homo sapiens GN=CCDC47 PE=1 SV=1	56123	560	408	29	128	0.06	0.26	4.33333	0	3	1	4	1	5	1	5	1.02658
tr|A6NG51|A6NG51_	SPTAN1	Spectrin alpha chain, non-erythrocytic 1 OS=Homo sapiens GN=SPTAN1 PE=2 SV=2	285628	167	59	130	578	0.06	0.26	4.33333	2	14	5	19	6	21	9	26	0.879199
tr|B5BU01|B5BU01_H	EIF2S2	Eukaryotic translation initiation factor 2 beta OS=Homo sapiens GN=EIF2S2 PE=2 SV=1	38648	654	409	22	127	0.09	0.39	4.33333	0	3	1	4	1	4	1	5	0.736894
tr|H0UID3|H0UID3_H	AP2B1	Adaptor-related protein complex 2, beta 1 subunit, isoform CRA_d OS=Homo sapiens GN	106321	648	328	22	160	0.03	0.13	4.33333	0	3	1	4	3	5	3	5	-0.19646
tr|B2R5U1|B2R5U1_H	tr|B2R5U1|B2R5U1_HU	cDNA, FLJ92620, highly similar to Homo sapiens staphylococcal nuclease domain contain	100294	150	116	144	405	0.17	0.73	4.29412	4	11	5	17	10	21	12	22	0.186023
tr|A8K492|A8K492_H	tr|A8K492|A8K492_HU	cDNA FLJ76789, highly similar to Homo sapiens methionine-tRNA synthetase (MARS), mR	102277	385	364	45	144	0.07	0.3	4.28571	1	4	2	6	2	6	2	7	0.530479
tr|F5GX33|F5GX33_H	SLC25A13	Calcium-binding mitochondrial carrier protein Aralar2 OS=Homo sapiens GN=SLC25A13 P	62548	353	196	49	212	0.11	0.47	4.27273	1	4	2	7	3	7	4	8	0.251359
tr|Q6IBR0|Q6IBR0_HU	RPN1	RPN1 protein OS=Homo sapiens GN=RPN1 PE=2 SV=1	68678	170	96	127	459	0.26	1.11	4.26923	4	13	5	17	8	19	8	20	0.350303
sp|P04350|TBB4A_HU	TUBB4A	Tubulin beta-4A chain OS=Homo sapiens GN=TUBB4A PE=1 SV=2	50010	42	18	509	1598	1.96	8.32	4.2449	21	51	27	60	29	64	32	69	0.282256
tr|B0UZ98|B0UZ98_H	BAT1	HLA-B associated transcript 1 (Fragment) OS=Homo sapiens GN=BAT1 PE=2 SV=1	28623	394	495	44	41	0.09	0.38	4.22222	1	1	1	2	1	1	1	2	-0.84861
tr|B4DFN9|B4DFN9_H	tr|B4DFN9|B4DFN9_HU	cDNA FLJ54303, highly similar to Heat shock 70 kDa protein 1 OS=Homo sapiens PE=2 SV	62699	80	43	252	768	0.58	2.43	4.18966	7	21	10	29	10	30	10	30	0.692121
sp|O14929|HAT1_HU	HAT1	Histone acetyltransferase type B catalytic subunit OS=Homo sapiens GN=HAT1 PE=1 SV=	49880	343	293	51	180	0.07	0.29	4.14286	1	3	1	4	1	5	1	5	1.02658
tr|Q8IWP6|Q8IWP6_H	tr|Q8IWP6|Q8IWP6_H	Class IVb beta tubulin OS=Homo sapiens PE=2 SV=1	50177	28	15	654	1858	3.05	12.59	4.12787	27	58	32	68	34	73	37	79	0.244822
tr|Q8IZ29|Q8IZ29_HU	TUBB2C	Tubulin, beta 2C OS=Homo sapiens GN=TUBB2C PE=2 SV=1	50264	27	13	674	1938	3.04	12.51	4.11513	26	59	32	69	34	73	38	79	0.244822
sp|P08238|HS90B_HU	HSP90AB1	Heat shock protein HSP 90-beta OS=Homo sapiens GN=HSP90AB1 PE=1 SV=4	83554	32	11	622	1982	1.24	5.1	4.1129	18	50	28	63	29	72	35	81	0.45239
sp|Q8NBJ5|GT251_HU	GLT25D1	Procollagen galactosyltransferase 1 OS=Homo sapiens GN=GLT25D1 PE=1 SV=1	71933	523	371	31	142	0.09	0.37	4.11111	0	5	2	7	1	8	2	9	1.655152
sp|P04040|CATA_HUM	CAT	Catalase OS=Homo sapiens GN=CAT PE=1 SV=3	59947	299	205	60	243	0.11	0.45	4.09091	1	5	2	7	2	7	2	8	0.73711
tr|B4E386|B4E386_HU	tr|B4E386|B4E386_HU	cDNA FLJ52847, highly similar to Tubulin beta-6 chain OS=Homo sapiens PE=2 SV=1	34289	73	76	273	537	0.83	3.38	4.07229	10	18	14	20	17	24	20	26	-0.36423
tr|B2RU06|B2RU06_H	EIF4G1	EIF4G1 protein OS=Homo sapiens GN=EIF4G1 PE=2 SV=1	176807	669	281	20	187	0.04	0.16	4	0	5	2	9	1	10	3	12	1.960367
tr|E5BBI6|E5BBI6_HU	HLA-A	MHC class I antigen (Fragment) OS=Homo sapiens GN=HLA-A PE=3 SV=1	31828	639	638	22	72	0.16	0.64	4	0	3	1	5	1	8	2	8	1.655152
tr|K4TZQ2|K4TZQ2_H	HLA-A	MHC class I antigen (Fragment) OS=Homo sapiens GN=HLA-A PE=3 SV=1	31799	639	637	22	72	0.16	0.64	4	0	3	1	5	1	8	1	8	1.655152
tr|A2BDK6|A2BDK6_H	MAP1B	Microtubule-associated protein 1B OS=Homo sapiens GN=MAP1B PE=2 SV=1	257692	511	603	32	77	0.01	0.04	4	0	2	1	3	1	4	2	5	0.736894
tr|B2R8R5|B2R8R5_H	tr|B2R8R5|B2R8R5_HU	cDNA, FLJ94025, highly similar to Homo sapiens tripartite motif-containing 28 (TRIM28),	90271	320	381	56	139	0.07	0.28	4	1	4	3	7	2	6	4	9	0.530479
tr|H0Y5J4|H0Y5J4_HU	DBNL	Drebrin-like protein (Fragment) OS=Homo sapiens GN=DBNL PE=4 SV=1	40475	466	439	37	94	0.08	0.32	4	1	2	1	3	1	2	1	4	-0.11146
tr|B4DDB6|B4DDB6_H	HNRPA3	Heterogeneous nuclear ribonucleoprotein A3, isoform CRA_a OS=Homo sapiens GN=HNI	37234	201	461	103	99	0.19	0.75	3.94737	2	4	2	6	2	6	2	9	0.530479
tr|B4DU00|B4DU00_H	tr|B4DU00|B4DU00_HU	cDNA FLJ54547, highly similar to Alpha-1 catenin (Cadherin-associated protein) OS=Hom	89332	389	237	45	164	0.07	0.27	3.85714	0	3	2	5	2	6	4	9	0.530479
tr|F2Z3K5|F2Z3K5_HU	RPN2	Dolichyl-diphosphooligosaccharide--protein glycosyltransferase subunit 2 (Fragment) OS	17797	589	1012	27	35	0.05	0.19	3.8	0	0	1	1	1	1	1	1	-0.84861
tr|B3KY60|B3KY60_HU	tr|B3KY60|B3KY60_HU	cDNA FLJ16777 fis, clone BRHIP2029567, highly similar to Cell division cycle 5-like protein	92448	587	248	27	209	0.04	0.15	3.75	0	4	1	4	1	4	1	4	0.736894
tr|Q7Z3K9|Q7Z3K9_H	DKFZp781L0540	Putative uncharacterized protein DKFZp781L0540 (Fragment) OS=Homo sapiens GN=DKF	90081	440	468	39	107	0.04	0.15	3.75	1	4	1	4	2	4	2	4	-0.0004
tr|B2R6L0|B2R6L0_HU	tr|B2R6L0|B2R6L0_HU	cDNA, FLJ93005, highly similar to Homo sapiens tubulin, beta polypeptide (TUBB), mRNA	50264	43	21	507	1459	1.76	6.59	3.74432	19	47	26	55	29	60	33	66	0.189166
tr|B4DH02|B4DH02_H	tr|B4DH02|B4DH02_HU	cDNA FLJ50510, highly similar to Heat shock 70 kDa protein 4 OS=Homo sapiens PE=2 SV	95157	123	61	175	628	0.23	0.85	3.69565	4	16	6	16	5	17	6	17	0.822828
tr|B4E0E1|B4E0E1_HU	tr|B4E0E1|B4E0E1_HU	cDNA FLJ53442, highly similar to Poly (ADP-ribose) polymerase 1 (EC 2.4.2.30) OS=Homo	111855	549	197	30	255	0.06	0.22	3.66667	0	5	2	7	2	8	2	9	0.917862
tr|B4DDR2|B4DDR2_H	tr|B4DDR2|B4DDR2_HU	cDNA FLJ54588, highly similar to Major vault protein OS=Homo sapiens PE=2 SV=1	98526	304	161	59	285	0.07	0.25	3.57143	2	5	2	6	3	7	3	9	0.251359
tr|B4DUQ1|B4DUQ1_	tr|B4DUQ1|B4DUQ1_H	cDNA FLJ54552, highly similar to Heterogeneous nuclear ribonucleoprotein K OS=Homo s	48708	147	72	147	567	0.58	2.04	3.51724	3	17	7	19	7	20	8	21	0.603531
tr|E3SWF7|E3SWF7_H	HLA-A	MHC class I antigen (Fragment) OS=Homo sapiens GN=HLA-A PE=3 SV=1	21021	639	803	22	50	0.16	0.56	3.5	0	2	1	3	1	5	1	5	1.02658
sp|O76094|SRP72_HU	SRP72	Signal recognition particle 72 kDa protein OS=Homo sapiens GN=SRP72 PE=1 SV=3	75130	455	487	38	102	0.04	0.14	3.5	1	3	1	3	1	3	1	3	0.374143
tr|B4DYL7|B4DYL7_HU	tr|B4DYL7|B4DYL7_HU	cDNA FLJ58490, highly similar to Homo sapiens CCR4-NOT transcription complex, subuni	140991	388	1040	45	34	0.02	0.07	3.5	1	1	1	3	1	3	2	4	0.374143
tr|E5KNY5|E5KNY5_H	LRPPRC	Leucine-rich PPR-motif containing OS=Homo sapiens GN=LRPPRC PE=2 SV=1	159003	329	169	53	292	0.08	0.28	3.5	1	10	4	12	5	12	7	14	0.3365
tr|B4DFM0|B4DFM0_	FARSLB	Phenylalanine-tRNA synthetase-like, beta subunit, isoform CRA_c OS=Homo sapiens GN=	55353	360	523	48	84	0.06	0.21	3.5	1	2	1	3	1	3	3	4	0.374143
sp|Q92973|TNPO1_H	TNPO1	Transportin-1 OS=Homo sapiens GN=TNPO1 PE=1 SV=2	103771	139	136	158	347	0.17	0.59	3.47059	3	9	5	15	8	13	8	19	-0.18129
tr|B4DQX0|B4DQX0_H	tr|B4DQX0|B4DQX0_H	cDNA FLJ59219, highly similar to Poly(A)-binding protein 1 OS=Homo sapiens PE=2 SV=1	67239	215	163	96	304	0.33	1.14	3.45455	3	9	6	16	6	15	10	18	0.406048
tr|B3KRS7|B3KRS7_HU	tr|B3KRS7|B3KRS7_HU	cDNA FLJ34844 fis, clone NT2NE2011154, highly similar to RuvB-like 1 OS=Homo sapiens	34946	351	899	50	42	0.09	0.31	3.44444	1	0	1	3	1	3	1	4	0.374143
tr|B4DEM7|B4DEM7_	CCT8	T-complex protein 1 subunit theta OS=Homo sapiens GN=CCT8 PE=2 SV=1	58179	268	319	70	160	0.18	0.62	3.44444	2	4	3	8	5	9	6	11	-0.05997
tr|Q6UJZ6|Q6UJZ6_H	HLA-C	MHC class I antigen (Fragment) OS=Homo sapiens GN=HLA-C PE=2 SV=1	21221	639	862	22	45	0.16	0.55	3.4375	0	2	1	3	1	5	1	6	1.02658
tr|E0YTJ7|E0YTJ7_HU	HLA-B	MHC class I antigen (Fragment) OS=Homo sapiens GN=HLA-B PE=3 SV=1	21175	639	659	22	68	0.16	0.55	3.4375	0	3	1	3	1	5	1	6	1.02658
sp|Q9NZB2|F120A_HU	FAM120A	Constitutive coactivator of PPAR-gamma-like protein 1 OS=Homo sapiens GN=FAM120A	123008	263	217	73	232	0.05	0.17	3.4	2	5	2	6	2	5	2	8	0.28929
tr|B7Z6M1|B7Z6M1_	PLS3	Plastin-3 OS=Homo sapiens GN=PLS3 PE=2 SV=1	66047	282	105	66	384	0.34	1.15	3.38235	1	11	6	16	6	18	8	24	0.661847
tr|E9PCI9|E9PCI9_HU	FDPS	Farnesyl pyrophosphate synthase OS=Homo sapiens GN=FDPS PE=2 SV=1	40849	405	1187	43	26	0.08	0.27	3.375	1	0	1	1	1	1	1	1	-0.84861
tr|F5H4R9|F5H4R9_H	DNM2	Dynamin-2 OS=Homo sapiens GN=DNM2 PE=2 SV=1	98218	713	601	16	77	0.03	0.1	3.33333	0	2	1	3	0	3	2	4	1.95943
tr|A8K2Y2|A8K2Y2_HU	tr|A8K2Y2|A8K2Y2_HU	cDNA FLJ78120, highly similar to Homo sapiens eukaryotic translation initiation factor 2,	51648	475	355	37	146	0.06	0.2	3.33333	1	3	1	3	1	4	1	4	0.736894
tr|F8WJN3|F8WJN3_H	CPSF6	Cleavage and polyadenylation-specificity factor subunit 6 OS=Homo sapiens GN=CPSF6 P	52409	379	671	46	67	0.06	0.2	3.33333	1	2	1	3	1	3	1	3	0.374143
tr|Q5M7Z5|Q5M7Z5_	GRHPR	GRHPR protein (Fragment) OS=Homo sapiens GN=GRHPR PE=2 SV=1	37314	659	663	21	48	0.09	0.3	3.33333	0	1	1	2	1	2	1	3	-0.11146
sp|O00232|PSD12_HU	PSMD12	26S proteasome non-ATPase regulatory subunit 12 OS=Homo sapiens GN=PSMD12 PE=1	53270	283	308	66	169	0.06	0.2	3.33333	1	2	1	3	1	2	1	3	-0.11146
tr|Q5T7Q0|Q5T7Q0_	DNAJA1	DnaJ (Hsp40) homolog, subfamily A, member 1, isoform CRA_c OS=Homo sapiens GN=DN	45581	703	544	18	88	0.07	0.23	3.28571	0	3	1	3	0	5	1	6	2.611867
tr|Q59EG8|Q59EG8_H	tr|Q59EG8|Q59EG8_HU	Proteasome 26S non-ATPase subunit 2 variant (Fragment) OS=Homo sapiens PE=2 SV=1	101322	198	180	105	276	0.14	0.46	3.28571	1	6	4	12	4	13	5	16	0.737543
tr|A1L3A7|A1L3A7_H	NUFIP2	Nuclear fragile X mental retardation protein interacting protein 2 OS=Homo sapiens GN=	76132	301	617	59	75	0.04	0.13	3.25	1	2	1	3	1	3	1	3	0.374143
tr|F5H1A8|F5H1A8_H	GSN	Gelsolin OS=Homo sapiens GN=GSN PE=2 SV=1	81776	297	356	61	145	0.04	0.13	3.25	1	3	1	3	1	3	2	3	0.374143
tr|B4E2I9|B4E2I9_HU	tr|B4E2I9|B4E2I9_HUM	cDNA FLJ55486, highly similar to Semaphorin-3C OS=Homo sapiens PE=2 SV=1	88481	602	1117	26	30	0.04	0.13	3.25	0	0	1	1	1	1	2	1	-0.84861
sp|P13639|EF2_HUM	EEF2	Elongation factor 2 OS=Homo sapiens GN=EEF2 PE=1 SV=4	96246	41	17	511	1725	1.23	3.98	3.23577	13	46	26	66	27	76	32	83	0.632532
tr|Q53GX7|Q53GX7_H	tr|Q53GX7|Q53GX7_H	Threonyl-tRNA synthetase variant (Fragment) OS=Homo sapiens PE=2 SV=1	84304	94	68	221	583	0.64	2.07	3.23438	6	19	13	31	17	32	18	35	0.044871
tr|C5IZQ1|C5IZQ1_HU	HLA-A	MHC class I antigen (Fragment) OS=Homo sapiens GN=HLA-A PE=3 SV=1	21127	639	555	22	45	0.16	0.51	3.1875	0	2	1	3	1	5	2	5	1.02658
tr|I3L312|I3L312_HUM	P4HB	Protein disulfide-isomerase (Fragment) OS=Homo sapiens GN=P4HB PE=4 SV=1	26348	536	251	30	99	0.27	0.86	3.18519	1	4	2	5	1	5	2	7	1.02658
tr|F5H6T1|F5H6T1_HU	ACTR2	Actin-related protein 2 OS=Homo sapiens GN=ACTR2 PE=2 SV=1	34729	244	260	82	205	0.2	0.63	3.15	2	4	2	6	2	6	4	8	0.530479
sp|Q15645|PCH2_HU	TRIP13	Pachytene checkpoint protein 2 homolog OS=Homo sapiens GN=TRIP13 PE=1 SV=2	48863	507	396	33	133	0.07	0.22	3.14286	0	3	1	3	2	4	2	5	-0.0004
tr|B4DWJ2|B4DWJ2_	QARS	Glutamine--tRNA ligase OS=Homo sapiens GN=QARS PE=2 SV=1	87437	277	216	68	234	0.16	0.5	3.125	3	6	4	11	5	12	5	12	0.3365
tr|B3KS31|B3KS31_HU	TUBB6	Tubulin, beta 6, isoform CRA_a OS=Homo sapiens GN=TUBB6 PE=2 SV=1	42324	73	77	273	537	0.83	2.59	3.12048	10	18	14	21	17	24	20	27	-0.36423
sp|P49411|EFTU_HUM	TUFM	Elongation factor Tu, mitochondrial OS=Homo sapiens GN=TUFM PE=1 SV=2	49852	210	177	99	282	0.29	0.9	3.10345	3	8	4	10	4	11	5	12	0.505856
tr|I1U8N0|I1U8N0_HU	HLA-A	MHC class I antigen (Fragment) OS=Homo sapiens GN=HLA-A PE=3 SV=1	31658	642	625	22	74	0.16	0.49	3.0625	0	3	1	4	1	7	1	7	1.4744
tr|E9PBU3|E9PBU3_H	ATIC	Phosphoribosylaminoimidazolecarboxamide formyltransferase OS=Homo sapiens GN=AT	64996	72	80	274	508	0.64	1.96	3.0625	8	16	11	21	12	21	13	24	-0.06617
tr|B3KNN7|B3KNN7_	tr|B3KNN7|B3KNN7_H	cDNA FLJ30049 fis, clone ADRGL1000033, highly similar to 26S proteasome non-ATPase r	57311	273	209	69	230	0.18	0.55	3.05556	3	7	3	7	3	8	3	8	0.432111
sp|Q96P70|IPO9_HUM	IPO9	Importin-9 OS=Homo sapiens GN=IPO9 PE=1 SV=3	116858	580	211	28	238	0.06	0.18	3	0	5	2	6	2	7	2	7	0.73711
tr|F8W9S7|F8W9S7_H	GAPVD1	GTPase-activating protein and VPS9 domain-containing protein 1 OS=Homo sapiens GN=	158790	697	695	18	63	0.02	0.06	3	0	1	1	1	0	1	2	1	0.736677
tr|H3BUF6|H3BUF6_H	ATXN2L	Ataxin-2-like protein OS=Homo sapiens GN=ATXN2L PE=4 SV=1	112929	656	500	22	100	0.03	0.09	3	0	2	1	3	2	6	2	6	0.530479
tr|J3KQL0|J3KQL0_HU	CPS1	Carbamoyl-phosphate synthase [ammonia], mitochondrial OS=Homo sapiens GN=CPS1 P	166686	204	134	102	351	0.15	0.45	3	4	13	7	19	10	19	12	22	0.044799
tr|B6F210|B6F210_HU	GSS	Glutathione synthetase OS=Homo sapiens GN=GSS PE=2 SV=1	40495	551	434	30	22	0.08	0.24	3	0	0	1	1	1	1	1	1	-0.84861
sp|O00629|IMA4_HU	KPNA4	Importin subunit alpha-4 OS=Homo sapiens GN=KPNA4 PE=1 SV=1	58364	472	1231	37	23	0.06	0.18	3	0	0	1	1	2	2	2	2	-0.84875
tr|E7EVA0|E7EVA0_H	MAP4	Microtubule-associated protein OS=Homo sapiens GN=MAP4 PE=2 SV=1	246546	429	802	40	50	0.01	0.03	3	1	1	1	2	2	2	4	4	-0.84875
tr|B2RDF2|B2RDF2_H	PES1	Pescadillo homolog 1 OS=Homo sapiens GN=PES1 PE=2 SV=1	68359	488	592	35	79	0.05	0.15	3	1	3	1	3	1	4	3	4	0.736894
tr|B1AMS2|B1AMS2_	Sep-06	Septin 6 OS=Homo sapiens GN=SEPT6 PE=3 SV=1	49614	532	428	31	32	0.07	0.21	3	1	1	1	1	1	1	1	1	-0.84861
tr|H0YA24|H0YA24_H	PLRG1	Pleiotropic regulator 1 (Fragment) OS=Homo sapiens GN=PLRG1 PE=4 SV=1	17588	456	210	38	92	0.19	0.56	2.94737	1	1	1	3	1	3	2	3	0.374143
tr|J3KPS3|J3KPS3_HU	ALDOA	Fructose-bisphosphate aldolase OS=Homo sapiens GN=ALDOA PE=3 SV=1	40249	63	28	338	1044	1.79	5.26	2.93855	11	24	15	31	17	36	18	43	0.213051
tr|B7TY16|B7TY16_HU	ACTN1	Actinin alpha 1 isoform 3 OS=Homo sapiens GN=ACTN1 PE=2 SV=1	107644	50	24	428	1326	0.77	2.26	2.93506	15	37	21	44	22	47	29	50	0.230875
tr|J3QQM1|J3QQM1_	PSMC5	26S protease regulatory subunit 8 (Fragment) OS=Homo sapiens GN=PSMC5 PE=4 SV=1	29442	314	341	57	104	0.11	0.32	2.90909	1	2	1	3	1	3	2	3	0.374143
sp|P06733|ENOA_HU	ENO1	Alpha-enolase OS=Homo sapiens GN=ENO1 PE=1 SV=2	47481	40	38	520	970	1.39	4.01	2.88489	13	26	18	33	19	34	25	38	-0.02538
tr|K7EM16|K7EM16_	VASP	Vasodilator-stimulated phosphoprotein (Fragment) OS=Homo sapiens GN=VASP PE=4 SV	20260	363	482	48	79	0.17	0.49	2.88235	1	1	1	1	1	1	1	1	-0.84861
sp|P15121|ALDR_HUM	AKR1B1	Aldose reductase OS=Homo sapiens GN=AKR1B1 PE=1 SV=3	36230	185	259	116	206	0.42	1.2	2.85714	3	5	4	9	5	9	5	11	-0.05997
tr|B2RAQ8|B2RAQ8_	tr|B2RAQ8|B2RAQ8_H	cDNA, FLJ95058, highly similar to Homo sapiens carnitine palmitoyltransferase 1A (liver)	88967	354	296	49	178	0.07	0.2	2.85714	1	4	2	5	3	5	3	5	-0.19646
tr|B4DXC4|B4DXC4_H	tr|B4DXC4|B4DXC4_HU	cDNA FLJ58636, moderately similar to Atlastin OS=Homo sapiens PE=2 SV=1	59162	451	721	38	44	0.06	0.17	2.83333	1	1	1	1	1	1	1	1	-0.84861
sp|O00505|IMA3_HU	KPNA3	Importin subunit alpha-3 OS=Homo sapiens GN=KPNA3 PE=1 SV=2	58288	406	1172	43	26	0.12	0.34	2.83333	0	0	2	1	2	1	2	1	-1.5859
tr|B2RDF5|B2RDF5_H	tr|B2RDF5|B2RDF5_HU	cDNA, FLJ96587, highly similar to Homo sapiens SUMO-1 activating enzyme subunit 2 (U	71705	590	999	27	36	0.05	0.14	2.8	0	0	1	3	1	2	1	3	-0.11146
sp|O43707|ACTN4_H	ACTN4	Alpha-actinin-4 OS=Homo sapiens GN=ACTN4 PE=1 SV=2	105245	19	9	849	2182	2.11	5.89	2.79147	26	59	41	83	44	92	52	98	0.209508
tr|B4DGQ0|B4DGQ0_	LASP1	LIM and SH3 domain protein 1 OS=Homo sapiens GN=LASP1 PE=2 SV=1	23167	568	565	29	73	0.14	0.39	2.78571	0	1	1	1	1	2	1	2	-0.11146
tr|B3KX19|B3KX19_H	tr|B3KX19|B3KX19_HU	cDNA FLJ44500 fis, clone UTERU3000828, highly similar to 116 kDa U5 small nuclear ribo	109111	111	73	193	537	0.27	0.75	2.77778	4	14	8	18	8	19	10	21	0.350303
tr|Q53SS8|Q53SS8_H	PCBP1	Poly(RC) binding protein 1 OS=Homo sapiens GN=PCBP1 PE=2 SV=1	37987	110	231	197	224	0.29	0.8	2.75862	3	6	3	8	3	9	3	9	0.592756
tr|B4DHG3|B4DHG3_	tr|B4DHG3|B4DHG3_H	cDNA FLJ55329, highly similar to Procollagen-lysine,2-oxoglutarate5-dioxygenase 2 (EC 1	81716	453	452	38	112	0.08	0.22	2.75	0	3	2	5	2	5	2	6	0.28929
tr|Q4ZG57|Q4ZG57_H	MCM6	Putative uncharacterized protein MCM6 (Fragment) OS=Homo sapiens GN=MCM6 PE=2	89803	444	502	39	98	0.04	0.11	2.75	1	2	1	3	2	4	3	4	-0.0004
sp|O00410|IPO5_HUM	IPO5	Importin-5 OS=Homo sapiens GN=IPO5 PE=1 SV=4	125032	85	67	236	590	0.23	0.63	2.73913	7	13	8	19	10	23	15	25	0.314708
sp|Q14247|SRC8_HUM	CTTN	Src substrate cortactin OS=Homo sapiens GN=CTTN PE=1 SV=2	61720	378	160	46	312	0.11	0.3	2.72727	1	4	2	5	3	5	3	6	-0.19646
tr|Q6IAV5|Q6IAV5_H	OXCT	Succinyl-CoA:3-ketoacid-coenzyme A transferase OS=Homo sapiens GN=OXCT PE=2 SV=1	56612	209	206	100	240	0.18	0.49	2.72222	1	4	3	6	5	6	6	8	-0.608
sp|O15427|MOT4_HU	SLC16A3	Monocarboxylate transporter 4 OS=Homo sapiens GN=SLC16A3 PE=1 SV=1	50064	362	401	48	132	0.14	0.38	2.71429	2	4	2	5	2	5	2	5	0.28929
tr|A8K4D5|A8K4D5_H	tr|A8K4D5|A8K4D5_HU	Kynureninase OS=Homo sapiens PE=2 SV=1	52819	266	494	71	91	0.13	0.35	2.69231	2	1	2	4	3	5	3	5	-0.19646
tr|B3GQS7|B3GQS7_H	HSPD1	Mitochondrial heat shock 60kD protein 1 variant 1 OS=Homo sapiens GN=HSPD1 PE=2 S	60813	16	23	904	1241	1.72	4.63	2.69186	24	30	27	35	26	36	33	42	-0.38852
tr|E9PKT8|E9PKT8_HU	NAP1L4	Nucleosome assembly protein 1-like 4 (Fragment) OS=Homo sapiens GN=NAP1L4 PE=2 S	19202	417	496	42	32	0.16	0.43	2.6875	0	0	2	1	3	1	3	1	-2.07165
tr|B0UX83|B0UX83_H	BAG6	HLA-B associated transcript 3 OS=Homo sapiens GN=BAG6 PE=4 SV=1	119085	704	527	18	92	0.03	0.08	2.66667	0	1	1	3	0	4	1	5	2.32218
tr|B1PS43|B1PS43_HU	MYH11	Myosin heavy chain 11 smooth muscle isoform OS=Homo sapiens GN=MYH11 PE=2 SV=1	235004	142	239	155	219	0.06	0.16	2.66667	5	6	5	11	9	10	11	14	-0.70519
tr|B7Z2Z8|B7Z2Z8_HU	tr|B7Z2Z8|B7Z2Z8_HU	T-complex protein 1 subunit delta OS=Homo sapiens PE=2 SV=1	52781	113	232	188	218	0.27	0.72	2.66667	4	5	5	8	7	8	7	9	-0.66872
sp|P23381|SYWC_HU	WARS	Tryptophan--tRNA ligase, cytoplasmic OS=Homo sapiens GN=WARS PE=1 SV=2	53474	145	119	148	396	0.27	0.71	2.62963	3	7	4	10	5	8	5	10	-0.22061
tr|K9JA46|K9JA46_HU	EL52	Epididymis luminal secretory protein 52 OS=Homo sapiens GN=EL52 PE=2 SV=1	85006	46	19	462	1467	1.05	2.76	2.62857	15	37	25	51	24	58	29	63	0.409869
tr|B4DN87|B4DN87_H	tr|B4DN87|B4DN87_HU	cDNA FLJ52569, highly similar to Collagen-binding protein 2 OS=Homo sapiens PE=2 SV=1	44290	233	142	89	301	0.43	1.13	2.62791	4	8	5	9	5	11	5	14	0.216025
tr|A8K2S1|A8K2S1_HU	tr|A8K2S1|A8K2S1_HU	cDNA FLJ75258, highly similar to Homo sapiens phospholipase A2, group IVA (cytosolic, c	85695	369	363	47	144	0.08	0.21	2.625	1	4	2	6	2	5	5	8	0.28929
tr|Q6DC98|Q6DC98_H	LMNB1	LMNB1 protein (Fragment) OS=Homo sapiens GN=LMNB1 PE=2 SV=1	38289	219	184	95	146	0.18	0.47	2.61111	2	2	2	3	2	3	4	3	-0.36315
tr|Q53ET2|Q53ET2_H	tr|Q53ET2|Q53ET2_HU	Dihydropyrimidinase-like 2 variant (Fragment) OS=Homo sapiens PE=2 SV=1	62688	342	144	51	338	0.23	0.6	2.6087	1	7	4	9	3	10	5	10	0.737326
tr|B4DP54|B4DP54_H	tr|B4DP54|B4DP54_HU	cDNA FLJ52712, highly similar to Tubulin beta-6 chain OS=Homo sapiens PE=2 SV=1	47014	73	78	273	536	0.83	2.14	2.57831	10	18	14	21	17	24	20	27	-0.36423
tr|D3DTH7|D3DTH7_	MYO1C	Myosin IC, isoform CRA_a OS=Homo sapiens GN=MYO1C PE=2 SV=1	99613	284	256	65	189	0.07	0.18	2.57143	1	4	3	5	3	5	4	6	-0.19646
tr|G3V1S6|G3V1S6_H	GALNT2	Polypeptide N-acetylgalactosaminyltransferase 2 soluble form OS=Homo sapiens GN=GA	61440	254	526	76	88	0.11	0.28	2.54545	2	2	2	4	2	5	2	5	0.28929
tr|B4DFK9|B4DFK9_H	tr|B4DFK9|B4DFK9_HU	cDNA FLJ57964, highly similar to Heterogeneous nuclear ribonucleoprotein H~ OS=Homo	44637	272	226	69	172	0.15	0.38	2.53333	1	4	2	6	1	6	2	7	1.267769
tr|Q60FE2|Q60FE2_H	MYH9	MYH9 variant protein OS=Homo sapiens GN=MYH9 PE=2 SV=1	227646	17	6	900	2852	0.9	2.26	2.51111	30	62	46	86	52	92	60	101	-0.03163
tr|J3KR97|J3KR97_HU	TBCD	Tubulin-specific chaperone D OS=Homo sapiens GN=TBCD PE=4 SV=1	138346	714	896	15	42	0.02	0.05	2.5	0	1	1	2	0	3	1	3	1.95943
tr|B3KW21|B3KW21_	tr|B3KW21|B3KW21_H	cDNA FLJ41945 fis, clone PLACE6019676, highly similar to Coatomer subunit gamma OS=	68674	705	1073	18	32	0.04	0.1	2.5	0	0	1	2	0	2	1	2	1.473823
tr|B4DZD5|B4DZD5_H	tr|B4DZD5|B4DZD5_HU	cDNA FLJ60991, highly similar to FKBP12-rapamycin complex-associated protein OS=Hom	158783	683	503	20	38	0.02	0.05	2.5	0	0	1	2	0	2	2	4	1.473823
tr|B4DGU4|B4DGU4_	CTNNB1	Catenin beta-1 OS=Homo sapiens GN=CTNNB1 PE=2 SV=1	85339	340	347	52	151	0.12	0.3	2.5	2	6	3	7	3	9	3	9	0.592756
tr|A6NEM2|A6NEM2_	HCFC1	HCF N-terminal chain 5 OS=Homo sapiens GN=HCFC1 PE=2 SV=2	215338	508	518	33	95	0.02	0.05	2.5	0	2	1	3	1	3	2	4	0.374143
tr|B3KQQ3|B3KQQ3_	tr|B3KQQ3|B3KQQ3_H	cDNA PSEC0016 fis, clone NT2RM1001076, highly similar to Procollagen-lysine,2-oxoglut	85161	325	285	54	180	0.12	0.3	2.5	1	4	3	6	3	6	3	6	0.044728
tr|G3XAD8|G3XAD8_	STIP1	Stress-induced-phosphoprotein 1 OS=Homo sapiens GN=STIP1 PE=2 SV=1	68721	271	165	69	297	0.21	0.52	2.47619	2	8	4	9	4	9	5	12	0.229861
tr|J3KPF3|J3KPF3_HU	SLC3A2	4F2 cell-surface antigen heavy chain OS=Homo sapiens GN=SLC3A2 PE=4 SV=1	68230	60	29	366	1127	1.33	3.29	2.47368	12	29	20	42	20	43	20	46	0.238191
tr|Q5HY54|Q5HY54_H	FLNA	Filamin-A OS=Homo sapiens GN=FLNA PE=2 SV=1	279115	9	4	1304	3440	0.86	2.1	2.44186	41	90	59	115	74	129	87	150	-0.0516
tr|Q60FE5|Q60FE5_H	FLNA	Filamin A OS=Homo sapiens GN=FLNA PE=2 SV=1	280790	9	3	1306	3486	0.86	2.1	2.44186	41	91	60	116	75	130	88	151	-0.05989
tr|B3KX72|B3KX72_HU	tr|B3KX72|B3KX72_HU	cDNA FLJ44920 fis, clone BRAMY3011501, highly similar to Heterogeneous nuclear ribon	83774	59	46	367	729	0.58	1.41	2.43103	10	17	14	25	16	29	20	32	-0.01018
tr|Q9NSU9|Q9NSU9_	DKFZp434G0719	Putative uncharacterized protein DKFZp434G0719 (Fragment) OS=Homo sapiens GN=DK	41107	56	564	169	83	0.07	0.17	2.42857	4	1	5	2	5	2	5	2	-1.98723
tr|H0Y9Q7|H0Y9Q7_	CANX	Calnexin (Fragment) OS=Homo sapiens GN=CANX PE=3 SV=1	18990	352	126	49	125	0.39	0.94	2.41026	1	3	2	5	3	6	5	6	0.044728
tr|G3V0E8|G3V0E8_H	PCBP2	Poly(RC) binding protein 2, isoform CRA_f OS=Homo sapiens GN=PCBP2 PE=2 SV=1	33817	132	227	166	182	0.32	0.77	2.40625	3	5	3	6	3	6	5	6	0.044728
tr|B0QYA5|B0QYA5_H	EIF3D	Eukaryotic translation initiation factor 3 subunit D (Fragment) OS=Homo sapiens GN=EIF	31937	710	367	17	140	0.1	0.24	2.4	0	3	1	3	0	3	2	3	1.95943
tr|A5PHJ9|A5PHJ9_HU	PDHA1/LOC79064	PDHA1/LOC79064 protein (Fragment) OS=Homo sapiens GN=PDHA1/LOC79064 PE=2 SV	34113	516	651	32	53	0.1	0.24	2.4	0	1	1	1	1	1	1	1	-0.84861
tr|H0YF33|H0YF33_HU	NASP	Nuclear autoantigenic sperm protein (Fragment) OS=Homo sapiens GN=NASP PE=4 SV=1	31883	542	1036	30	23	0.1	0.24	2.4	0	0	1	1	2	1	3	1	-1.5859
tr|B4E022|B4E022_HU	TKT	Transketolase OS=Homo sapiens GN=TKT PE=2 SV=1	63410	35	27	578	1181	1.36	3.26	2.39706	17	32	24	42	26	44	32	50	-0.10116

tr|B1AH89|B1AH89_H	TTLL12	Tubulin tyrosine ligase-like family, member 12 OS=Homo sapiens GN=TTLL12 PE=4 SV=1	74185	104	93	209	466	0.41	0.98	2.39024	6	12	8	15	10	16	11	18	-0.19675
tr|H0Y8G5|H0Y8G5_H	HNRNPD	Heterogeneous nuclear ribonucleoprotein D0 (Fragment) OS=Homo sapiens GN=HNRNPI	29877	229	224	90	227	0.37	0.88	2.37838	3	7	3	7	4	8	4	10	0.069217
tr|H7C3A1|H7C3A1_H	SRRT	Serrate RNA effector molecule homolog (Fragment) OS=Homo sapiens GN=SRRT PE=4 SV	57154	701	584	18	49	0.06	0.14	2.33333	0	0	1	3	0	4	1	5	2.32218
tr|B7Z8A2|B7Z8A2_H	tr|B7Z8A2|B7Z8A2_HU	cDNA FLJ51671, highly similar to Prenylcysteine oxidase (EC 1.8.3.5) OS=Homo sapiens P	54918	355	737	49	57	0.06	0.14	2.33333	1	1	1	2	1	3	1	4	0.374143
sp|Q9P287|BCCIP_HU	BCCIP	BRCA2 and CDKN1A-interacting protein OS=Homo sapiens GN=BCCIP PE=1 SV=1	36299	503	751	33	56	0.09	0.21	2.33333	0	1	1	2	1	2	3	2	-0.11146
tr|B2RBE5|B2RBE5_H	tr|B2RBE5|B2RBE5_HU	cDNA, FLJ95468, highly similar to Homo sapiens transcriptional coactivator tubedown-10	102490	446	849	38	46	0.06	0.14	2.33333	0	1	2	3	2	3	2	3	-0.36315
sp|Q68EM7|RHG17_H	ARHGAP17	Rho GTPase-activating protein 17 OS=Homo sapiens GN=ARHGAP17 PE=1 SV=1	95776	328	835	54	47	0.03	0.07	2.33333	1	1	1	2	2	2	2	2	-0.84875
sp|Q5T2N8|ATD3C_H	ATAD3C	ATPase family AAA domain-containing protein 3C OS=Homo sapiens GN=ATAD3C PE=2 S	46635	281	353	68	147	0.1	0.23	2.3	2	3	2	3	2	4	2	4	-0.0004
tr|B2R6K4|B2R6K4_H	tr|B2R6K4|B2R6K4_HU	cDNA, FLJ92996, highly similar to Homo sapiens guanine nucleotide binding protein (G pi	38075	199	325	105	161	0.18	0.41	2.27778	1	3	2	4	2	4	2	4	-0.0004
tr|B7Z609|B7Z609_H	tr|B7Z609|B7Z609_HU	cDNA FLJ51747, highly similar to Succinyl-CoA:3-ketoacid-coenzyme A transferase1, mitc	36865	209	634	40	72	0.18	0.41	2.27778	0	2	2	4	3	4	4	6	-0.48615
tr|B1AHC9|B1AHC9_H	XRCC6	X-ray repair complementing defective repair in Chinese hamster cells 6 (Ku autoantigen,	64528	116	122	184	384	0.64	1.45	2.26563	5	10	10	17	11	20	12	23	-0.01444
sp|P22314|UBA1_HU	UBA1	Ubiquitin-like modifier-activating enzyme 1 OS=Homo sapiens GN=UBA1 PE=1 SV=3	118858	114	79	187	536	0.28	0.63	2.25	5	11	10	19	10	24	12	25	0.375009
tr|B3KSH1|B3KSH1_H	EIF3F	Eukaryotic translation initiation factor 3 subunit F OS=Homo sapiens GN=EIF3F PE=2 SV=	39236	652	548	22	88	0.08	0.18	2.25	0	1	1	2	1	3	1	3	0.374143
sp|Q96TA1|NIBL1_HU	FAM129B	Niban-like protein 1 OS=Homo sapiens GN=FAM129B PE=1 SV=3	84598	155	204	137	248	0.16	0.36	2.25	4	6	4	8	4	8	4	12	0.069217
tr|B3KV72|B3KV72_H	tr|B3KV72|B3KV72_HU	cDNA FLJ16204 fis, clone CTONG2019232, highly similar to Proteasome-associated prote	78791	579	337	28	50	0.04	0.09	2.25	0	1	1	2	1	2	1	2	-0.11146
tr|Q5JP02|Q5JP02_HU	RBBP7	Histone-binding protein RBBP7 (Fragment) OS=Homo sapiens GN=RBBP7 PE=4 SV=1	19626	321	464	37	74	0.22	0.49	2.22727	1	2	1	4	2	4	2	4	-0.0004
tr|Q0IJ56|Q0IJ56_HU	ST13	ST13 protein (Fragment) OS=Homo sapiens GN=ST13 PE=2 SV=1	35164	382	745	46	57	0.09	0.2	2.22222	1	1	1	3	2	2	3	3	-0.84875
tr|J3KTE4|J3KTE4_HU	RPL19	Ribosomal protein L19 OS=Homo sapiens GN=RPL19 PE=3 SV=1	23347	336	904	52	42	0.14	0.31	2.21429	1	1	1	2	1	2	1	2	-0.11146
tr|B4DMY3|B4DMY3_	tr|B4DMY3|B4DMY3_H	cDNA FLJ60713, highly similar to Homo sapiens heterogeneous nuclear ribonucleoprotei	35101	318	324	56	161	0.2	0.44	2.2	1	5	2	5	2	5	2	6	0.28929
tr|H0YMR4|H0YMR4_	IPO4	Importin-4 (Fragment) OS=Homo sapiens GN=IPO4 PE=4 SV=1	60104	655	535	22	70	0.05	0.11	2.2	0	2	1	2	1	2	1	2	-0.11146
tr|D3DTW3|D3DTW3_	IGF2BP1	Insulin-like growth factor 2 mRNA binding protein 1, isoform CRA_a OS=Homo sapiens G	48111	556	708	29	62	0.05	0.11	2.2	0	2	1	2	1	2	1	3	-0.11146
tr|B3KMX0|B3KMX0_	tr|B3KMX0|B3KMX0_H	cDNA FLJ12837 fis, clone NT2RP2003228, highly similar to DNA replication licensing facto	97114	240	457	85	110	0.1	0.22	2.2	2	2	3	6	4	6	5	7	-0.31817
tr|Q2NKY5|Q2NKY5_H	TUBB6	TUBB6 protein OS=Homo sapiens GN=TUBB6 PE=2 SV=1	50514	65	58	317	643	0.88	1.93	2.19318	12	19	16	23	17	26	19	30	-0.25066
tr|Q5CAQ5|Q5CAQ5_	TRA1	Tumor rejection antigen (Gp96) 1 OS=Homo sapiens GN=TRA1 PE=2 SV=1	92567	140	100	157	450	0.37	0.81	2.18919	4	14	9	18	10	19	11	20	0.044799
tr|Q09GE6|Q09GE6_H	HLA-Cw	MHC class I antigen (Fragment) OS=Homo sapiens GN=HLA-Cw PE=3 SV=1	31840	639	861	22	45	0.16	0.35	2.1875	0	2	1	3	1	7	1	7	1.4744
tr|D0W033|D0W033_	HLA-A	MHC class I antigen (Fragment) OS=Homo sapiens GN=HLA-A PE=3 SV=1	31801	639	688	22	64	0.16	0.35	2.1875	0	3	1	3	1	6	1	6	1.267769
tr|E7ENU7|E7ENU7_H	RPL15	Ribosomal protein L15 (Fragment) OS=Homo sapiens GN=RPL15 PE=2 SV=1	20596	534	778	31	53	0.16	0.35	2.1875	0	1	1	2	1	2	1	2	-0.11146
sp|P13010|XRCC5_HU	XRCC5	X-ray repair cross-complementing protein 5 OS=Homo sapiens GN=XRCC5 PE=1 SV=3	83222	128	112	172	409	0.36	0.78	2.16667	6	9	8	16	8	19	9	21	0.350303
sp|Q01650|LAT1_HUI	SLC7A5	Large neutral amino acids transporter small subunit 1 OS=Homo sapiens GN=SLC7A5 PE=	55659	239	339	85	156	0.12	0.26	2.16667	1	3	3	6	4	5	5	7	-0.55936
tr|B7Z8W6|B7Z8W6_	tr|B7Z8W6|B7Z8W6_H	cDNA FLJ55038, highly similar to Nicotinamide phosphoribosyltransferase (EC 2.4.2.12) (	26905	520	290	32	55	0.12	0.26	2.16667	0	1	1	1	1	1	3	1	-0.84861
tr|Q6DEN2|Q6DEN2_	DPYSL3	DPYSL3 protein OS=Homo sapiens GN=DPYSL3 PE=2 SV=1	74320	490	178	35	280	0.19	0.41	2.15789	0	6	4	8	3	9	8	9	0.592756
tr|H0YD14|H0YD14_H	MYOF	Myoferlin (Fragment) OS=Homo sapiens GN=MYOF PE=4 SV=1	135822	220	200	95	216	0.07	0.15	2.14286	3	6	3	7	4	8	4	9	0.069217
sp|O43286|B4GT5_HU	B4GALT5	Beta-1,4-galactosyltransferase 5 OS=Homo sapiens GN=B4GALT5 PE=2 SV=1	45318	651	1053	22	33	0.07	0.15	2.14286	0	2	1	2	1	2	1	2	-0.11146
tr|B0ZSE7|B0ZSE7_HU	HLA-A	MHC class I antigen (Fragment) OS=Homo sapiens GN=HLA-A PE=3 SV=1	21069	639	821	22	48	0.16	0.34	2.125	0	2	1	2	1	4	3	5	0.736894
tr|B4DLB8|B4DLB8_H	tr|B4DLB8|B4DLB8_HU	cDNA FLJ52205, highly similar to Beta-1,4-galactosyltransferase 1 (EC 2.4.1.-) OS=Homo s	40269	650	614	22	76	0.08	0.17	2.125	0	2	1	2	1	2	1	2	-0.11146
tr|Q5TZX1|Q5TZX1_H	GNAI3	Guanine nucleotide binding protein (G protein), alpha inhibiting activity polypeptide 3 OS	41076	376	976	46	37	0.08	0.17	2.125	1	1	1	2	1	2	1	3	-0.11146
tr|B4DEX8|B4DEX8_H	MAT2A	S-adenosylmethionine synthase OS=Homo sapiens GN=MAT2A PE=2 SV=1	40027	333	498	52	100	0.08	0.17	2.125	1	2	1	2	1	2	1	2	-0.11146
sp|Q96SI9|STRBP_HU	STRBP	Spermatid perinuclear RNA-binding protein OS=Homo sapiens GN=STRBP PE=1 SV=1	74290	471	412	37	127	0.09	0.19	2.11111	0	3	2	4	2	4	2	6	-0.0004
tr|H0Y7N2|H0Y7N2_H	ATXN10	Ataxin-10 (Fragment) OS=Homo sapiens GN=ATXN10 PE=4 SV=1	19107	470	207	37	50	0.38	0.8	2.10526	0	1	2	1	2	1	2	2	-1.5859
tr|A6NE09|A6NE09_H	RPSAP58	Protein RPSAP58 OS=Homo sapiens GN=RPSAP58 PE=3 SV=1	33002	521	794	31	51	0.1	0.21	2.1	0	1	1	2	1	2	1	2	-0.11146
tr|B0QYT3|B0QYT3_H	RANGAP1	Ran GTPase activating protein 1 (Fragment) OS=Homo sapiens GN=RANGAP1 PE=2 SV=1	27122	574	525	28	0	0.12	0.25	2.08333	0	0	1	1	1	0	1	2	-2.43375
tr|Q53G58|Q53G58_H	tr|Q53G58|Q53G58_H	Coronin (Fragment) OS=Homo sapiens PE=2 SV=1	53931	242	463	83	109	0.13	0.27	2.07692	1	3	2	4	2	4	4	4	-0.0004
tr|B0V043|B0V043_H	VARS	Valine--tRNA ligase OS=Homo sapiens GN=VARS PE=3 SV=1	141632	275	146	69	333	0.15	0.31	2.06667	2	8	6	12	6	12	8	15	0.095167
tr|B2R5M8|B2R5M8_	tr|B2R5M8|B2R5M8_H	Isocitrate dehydrogenase [NADP] OS=Homo sapiens PE=2 SV=1	46905	555	426	29	120	0.15	0.31	2.06667	0	3	2	4	3	5	3	7	-0.19646
sp|P26038|MOES_HU	MSN	Moesin OS=Homo sapiens GN=MSN PE=1 SV=3	67892	119	124	179	381	0.46	0.94	2.04348	7	12	8	15	11	13	12	15	-0.61837
tr|H0Y804|H0Y804_H	AKR1C1	Aldo-keto reductase family 1 member C1 (Fragment) OS=Homo sapiens GN=AKR1C1 PE=	28617	186	128	61	147	0.67	1.35	2.01493	2	4	4	6	4	7	6	7	-0.11154
tr|A8K7F6|A8K7F6_H	tr|A8K7F6|A8K7F6_HU	cDNA FLJ78244, highly similar to Homo sapiens eukaryotic translation initiation factor 4A	46321	52	50	404	751	1.62	3.25	2.00617	10	20	14	23	15	24	20	27	-0.18849
sp|Q15149|PLEC_HUI	PLEC	Plectin OS=Homo sapiens GN=PLEC PE=1 SV=3	533462	74	36	270	979	0.11	0.22	2	11	20	17	33	18	40	32	45	0.283304
tr|Q2TB59|Q2TB59_H	NNT	Nicotinamide nucleotide transhydrogenase OS=Homo sapiens GN=NNT PE=2 SV=1	114552	234	358	88	145	0.06	0.12	2	1	4	2	4	2	4	3	4	-0.0004
tr|B2RB23|B2RB23_H	tr|B2RB23|B2RB23_HU	cDNA, FLJ95265, highly similar to Homo sapiens acetyl-Coenzyme A acyltransferase 2 (m	42439	544	586	30	80	0.08	0.16	2	0	2	1	2	1	2	1	2	-0.11146
tr|H0YBW4|H0YBW4_	PLAA	Phospholipase A-2-activating protein (Fragment) OS=Homo sapiens GN=PLAA PE=4 SV=1	31645	539	615	30	75	0.11	0.22	2	0	2	1	2	1	2	1	2	-0.11146
tr|A6NKV8|A6NKV8_H	PAIP1	Polyadenylate-binding protein-interacting protein 1 OS=Homo sapiens GN=PAIP1 PE=2 S	42379	361	508	48	98	0.08	0.16	2	1	2	1	2	1	2	1	3	-0.11146
sp|Q7L2E3|DHX30_HU	DHX30	Putative ATP-dependent RNA helicase DHX30 OS=Homo sapiens GN=DHX30 PE=1 SV=1	134938	397	746	43	56	0.05	0.1	2	1	2	2	4	3	4	4	4	-0.48615
tr|G3V2B8|G3V2B8_H	MTHFD1	C-1-tetrahydrofolate synthase, cytoplasmic OS=Homo sapiens GN=MTHFD1 PE=2 SV=1	102152	112	182	192	275	0.33	0.66	2	5	10	9	16	12	15	12	20	-0.53932
sp|Q9NPQ8|RIC8A_HU	RIC8A	Synembryn-A OS=Homo sapiens GN=RIC8A PE=1 SV=3	60185	496	717	34	60	0.05	0.1	2	0	1	1	1	1	1	1	1	-0.84861
tr|B4DXX7|B4DXX7_H	DDX3Y	ATP-dependent RNA helicase DDX3Y OS=Homo sapiens GN=DDX3Y PE=2 SV=1	73342	217	455	95	100	0.19	0.38	2	2	3	5	4	6	4	6	6	-1.38021
tr|Q53HF2|Q53HF2_H	tr|Q53HF2|Q53HF2_HU	Heat shock 70kDa protein 8 isoform 2 variant (Fragment) OS=Homo sapiens PE=2 SV=1	53580	51	34	426	898	1.45	2.88	1.98621	12	22	17	35	19	36	19	37	0.056281
tr|B2R9K8|B2R9K8_H	tr|B2R9K8|B2R9K8_HU	cDNA, FLJ94440, highly similar to Homo sapiens chaperonin containing TCP1, subunit 6A	58386	118	166	181	296	0.47	0.93	1.97872	5	7	7	12	9	13	10	14	-0.34208
tr|A5PLK7|A5PLK7_HU	RCC2	RCC2 protein (Fragment) OS=Homo sapiens GN=RCC2 PE=2 SV=1	50331	165	193	131	262	0.46	0.89	1.93478	4	7	6	10	6	11	6	13	-0.02531
tr|Q7Z5T5|Q7Z5T5_H	EIF3A	EIF3A protein (Fragment) OS=Homo sapiens GN=EIF3A PE=2 SV=1	97203	192	121	112	383	0.14	0.27	1.92857	2	7	4	11	5	15	8	18	0.647381
sp|P49327|FAS_HUM	FASN	Fatty acid synthase OS=Homo sapiens GN=FASN PE=1 SV=3	275877	55	32	394	1063	0.28	0.54	1.92857	12	31	21	38	23	41	29	47	-0.02809
tr|Q5TCU6|Q5TCU6_H	TLN1	Talin-1 OS=Homo sapiens GN=TLN1 PE=4 SV=1	260030	48	30	446	1002	0.27	0.52	1.92593	12	22	19	33	20	32	25	38	-0.18437
sp|Q9UMS4|PRP19_H	PRPF19	Pre-mRNA-processing factor 19 OS=Homo sapiens GN=PRPF19 PE=1 SV=1	55603	237	297	87	177	0.26	0.5	1.92308	2	5	5	7	7	7	11	8	-0.84948
sp|Q5T9A4|ATD3B_H	ATAD3B	ATPase family AAA domain-containing protein 3B OS=Homo sapiens GN=ATAD3B PE=1 S	73098	281	298	68	176	0.1	0.19	1.9	2	5	2	6	2	6	2	6	0.530479
tr|B4DMB5|B4DMB5_	tr|B4DMB5|B4DMB5_	cDNA FLJ60075, highly similar to CTP synthase 1 (EC 6.3.4.2) OS=Homo sapiens PE=2 SV=	56542	449	456	38	102	0.19	0.36	1.89474	1	2	3	2	3	2	4	2	-1.3345
tr|B4DX08|B4DX08_H	tr|B4DX08|B4DX08_HU	cDNA FLJ58784, highly similar to T-complex protein 1 subunit epsilon OS=Homo sapiens	57521	90	240	229	169	0.4	0.75	1.875	6	5	6	8	6	7	6	11	-0.6427
tr|B7Z4V2|B7Z4V2_H	tr|B7Z4V2|B7Z4V2_HU	cDNA FLJ51907, highly similar to Stress-70 protein, mitochondrial OS=Homo sapiens PE=	72641	81	91	251	477	0.42	0.78	1.85714	7	11	8	15	8	13	8	16	-0.18129
sp|Q3ZCM7|TBB8_HU	TUBB8	Tubulin beta-8 chain OS=Homo sapiens GN=TUBB8 PE=1 SV=2	50257	67	54	306	698	0.77	1.43	1.85714	13	24	17	28	19	32	21	34	-0.1119
tr|B3KNL2|B3KNL2_H	RUVBL2	RuvB-like 2 (E. coli), isoform CRA_d OS=Homo sapiens GN=RUVBL2 PE=2 SV=1	47603	584	869	28	37	0.14	0.26	1.85714	0	1	2	1	1	1	2	1	-0.84861
tr|B2R6X5|B2R6X5_H	tr|B2R6X5|B2R6X5_HU	cDNA, FLJ93166, highly similar to Homo sapiens heat shock 70kDa protein 6 (HSP70B~) (I	71370	193	162	112	306	0.2	0.37	1.85	3	8	5	9	6	9	6	11	-0.3013
sp|P00390|GSHR_HU	GSR	Glutathione reductase, mitochondrial OS=Homo sapiens GN=GSR PE=1 SV=2	56791	313	420	58	123	0.18	0.33	1.83333	1	3	3	5	4	6	4	6	-0.31817
tr|J3KTA4|J3KTA4_HU	DDX5	Probable ATP-dependent RNA helicase DDX5 OS=Homo sapiens GN=DDX5 PE=3 SV=1	69557	97	131	218	356	0.45	0.82	1.82222	7	12	9	13	10	14	10	17	-0.38352
sp|P22626|ROA2_HU	HNRNPA2B1	Heterogeneous nuclear ribonucleoproteins A2/B1 OS=Homo sapiens GN=HNRNPA2B1 PI	37464	77	65	256	595	0.97	1.76	1.81443	7	12	8	13	9	18	10	21	0.113385
tr|A4QPB0|A4QPB0_H	IQGAP1	IQ motif containing GTPase activating protein 1 OS=Homo sapiens GN=IQGAP1 PE=2 SV=	189789	84	45	239	839	0.31	0.56	1.80645	8	20	17	26	18	25	21	31	-0.38683
sp|P17987|TCPA_HUI	TCP1	T-complex protein 1 subunit alpha OS=Homo sapiens GN=TCP1 PE=1 SV=1	60819	197	186	106	268	0.3	0.54	1.8	2	7	5	7	8	7	10	7	-1.03037
tr|B2RCM2|B2RCM2_	tr|B2RCM2|B2RCM2_H	cDNA, FLJ96156, highly similar to Homo sapiens leucyl-tRNA synthetase (LARS), mRNA O	135550	257	321	75	162	0.1	0.18	1.8	2	5	4	7	3	10	5	12	0.737326
tr|B3KMZ6|B3KMZ6_	tr|B3KMZ6|B3KMZ6_H	cDNA FLJ13058 fis, clone NT2RP3001587, highly similar to Ubiquitin-like 1-activating enz	71688	590	998	27	36	0.05	0.09	1.8	0	0	1	2	1	2	1	2	-0.11146
tr|B4DW52|B4DW52_	tr|B4DW52|B4DW52_H	cDNA FLJ55253, highly similar to Actin, cytoplasmic 1 OS=Homo sapiens PE=2 SV=1	38950	4	2	3120	4358	27.29	49.06	1.79773	74	101	108	142	123	152	134	169	-0.55697
sp|Q09028|RBBP4_HU	RBBP4	Histone-binding protein RBBP4 OS=Homo sapiens GN=RBBP4 PE=1 SV=3	47911	321	591	55	79	0.22	0.39	1.77273	2	2	3	5	4	5	4	7	-0.55936
tr|F2VNS8|F2VNS8_H	HLA-C	MHC class I antigen (Fragment) OS=Homo sapiens GN=HLA-C PE=3 SV=1	21240	639	860	22	45	0.16	0.28	1.75	0	2	1	3	2	6	2	6	0.530479
tr|Q53GN4|Q53GN4_	WDR1	WD repeat domain 1, isoform CRA_a (Fragment) OS=Homo sapiens GN=WDR1 PE=2 SV=	66822	137	149	160	325	0.4	0.7	1.75	4	7	7	11	7	11	9	12	-0.23208
tr|F5H7Y0|F5H7Y0_H	PDLIM5	PDZ and LIM domain protein 5 OS=Homo sapiens GN=PDLIM5 PE=2 SV=1	52332	175	195	109	219	0.41	0.71	1.73171	2	6	5	7	5	7	6	13	-0.40137
sp|P68366|TBA4A_HU	TUBA4A	Tubulin alpha-4A chain OS=Homo sapiens GN=TUBA4A PE=1 SV=1	50634	22	37	796	971	3.01	5.21	1.7309	20	28	31	44	31	48	36	54	-0.22725
sp|P18206|VINC_HUN	VCL	Vinculin OS=Homo sapiens GN=VCL PE=1 SV=4	124292	68	70	299	578	0.37	0.64	1.72973	8	13	12	19	13	22	13	25	-0.11175
tr|Q2TSD0|Q2TSD0_H	tr|Q2TSD0|Q2TSD0_HU	Glyceraldehyde-3-phosphate dehydrogenase OS=Homo sapiens PE=2 SV=1	36197	18	26	891	1110	3.44	5.87	1.7064	23	22	31	30	31	32	36	37	-0.80769
tr|Q1L838|Q1L838_H	GTBP	GTBP-ALT OS=Homo sapiens GN=GTBP PE=2 SV=1	122267	559	640	29	72	0.03	0.05	1.66667	0	2	1	2	1	2	1	2	-0.11146
tr|B3KM77|B3KM77_	tr|B3KM77|B3KM77_H	cDNA FLJ10435 fis, clone NT2RP1000493, highly similar to Splicing factor 3B subunit 3 OS	56736	356	378	49	93	0.06	0.1	1.66667	1	2	1	2	1	2	1	2	-0.11146
tr|Q5HYM2|Q5HYM2_	DKFZp686O2462	Putative uncharacterized protein DKFZp686O2462 (Fragment) OS=Homo sapiens GN=DK	72627	569	361	29	122	0.09	0.15	1.66667	0	2	2	3	2	3	2	3	-0.36315
sp|P23526|SAHH_HU	AHCY	Adenosylhomocysteinase OS=Homo sapiens GN=AHCY PE=1 SV=4	48255	195	253	111	208	0.49	0.81	1.65306	2	8	6	10	6	10	8	11	-0.15673
tr|A8K4Z4|A8K4Z4_H	tr|A8K4Z4|A8K4Z4_HU	cDNA FLJ75549, highly similar to Homo sapiens ribosomal protein, large, P0 (RPLP0), trar	34389	109	343	201	154	0.74	1.22	1.64865	6	4	6	7	6	6	6	7	-0.84933
tr|C0KJZ0|C0KJZ0_HU	HLA-C	MHC class I antigen (Fragment) OS=Homo sapiens GN=HLA-C PE=3 SV=1	21321	639	687	22	42	0.16	0.26	1.625	0	1	1	1	1	2	1	2	-0.11146
tr|B3KT66|B3KT66_H	PSMD6	Proteasome (Prosome, macropain) 26S subunit, non-ATPase, 6, isoform CRA_c OS=Homo	40600	399	760	43	55	0.08	0.13	1.625	1	2	1	2	1	2	1	2	-0.11146
tr|Q58FF4|Q58FF4_H	HSP90Bf	Heat shock protein 90Bf OS=Homo sapiens GN=HSP90Bf PE=1 SV=1	41833	407	578	43	81	0.16	0.26	1.625	1	2	2	3	2	3	2	3	-0.36315
tr|E7EQG2|E7EQG2_H	EIF4A2	Eukaryotic initiation factor 4A-II OS=Homo sapiens GN=EIF4A2 PE=2 SV=1	41492	78	110	253	412	0.71	1.15	1.61972	4	9	7	11	6	11	11	12	-0.02531
tr|Q96T67|Q96T67_H	tr|Q96T67|Q96T67_HU	TOB3 OS=Homo sapiens PE=2 SV=1	65303	281	280	68	188	0.1	0.16	1.6	2	4	2	5	2	5	2	5	0.28929
tr|H0Y2W2|H0Y2W2_	ATAD3A	ATPase family AAA domain-containing protein 3A (Fragment) OS=Homo sapiens GN=ATA	64600	281	338	68	156	0.1	0.16	1.6	2	3	2	3	2	4	2	4	-0.0004
sp|Q14432|PDE3A_HU	PDE3A	cGMP-inhibited 3~,5~-cyclic phosphodiesterase A OS=Homo sapiens GN=PDE3A PE=1 SV	126440	493	481	35	103	0.05	0.08	1.6	1	2	2	3	2	3	2	3	-0.36315
tr|Q1KLZ0|Q1KLZ0_H	PS1TP5BP1	HCG15971, isoform CRA_a OS=Homo sapiens GN=PS1TP5BP1 PE=2 SV=1	42052	1	1	3367	4598	31.43	50.05	1.59243	82	106	119	149	130	154	141	168	-0.61968
tr|B3W6H4|B3W6H4_	MLL-ACTN4	Putative uncharacterized protein MLL-ACTN4 (Fragment) OS=Homo sapiens GN=MLL-AC	14500	19	230	55	224	2.11	3.36	1.59242	1	6	1	9	1	9	1	11	1.815797
sp|Q9Y305|ACOT9_H	ACOT9	Acyl-coenzyme A thioesterase 9, mitochondrial OS=Homo sapiens GN=ACOT9 PE=1 SV=2	50269	637	1148	22	28	0.07	0.11	1.57143	0	0	1	1	1	2	2	2	-0.11146
tr|B2R6J4|B2R6J4_HU	tr|B2R6J4|B2R6J4_HUI	cDNA, FLJ92975, highly similar to Homo sapiens nucleosome assembly protein 1-like 4 (N	42940	417	731	42	58	0.16	0.25	1.5625	0	0	2	3	3	3	3	4	-0.8489
tr|B4DTG2|B4DTG2_H	EEF1G	Elongation factor 1-gamma OS=Homo sapiens GN=EEF1G PE=2 SV=1	56456	93	82	225	522	0.86	1.34	1.55814	5	17	15	18	14	19	18	21	-0.42216
tr|Q53GU8|Q53GU8_	tr|Q53GU8|Q53GU8_H	Transforming growth factor, beta-induced, 68kDa variant (Fragment) OS=Homo sapiens	75231	358	497	48	100	0.09	0.14	1.55556	1	2	2	3	2	4	2	5	-0.0004
sp|O00116|ADAS_HU	AGPS	Alkyldihydroxyacetonephosphate synthase, peroxisomal OS=Homo sapiens GN=AGPS PE	73664	448	423	38	122	0.09	0.14	1.55556	1	3	2	3	2	3	3	3	-0.36315
tr|Q9HB74|Q9HB74_H	tr|Q9HB74|Q9HB74_H	PNAS-102 OS=Homo sapiens PE=2 SV=1	19284	90	678	127	66	0.4	0.62	1.55	3	2	3	3	3	3	3	5	-0.8489
tr|B5BU25|B5BU25_H	U2AF2	U2 small nuclear RNA auxiliary factor 2 isoform b OS=Homo sapiens GN=U2AF2 PE=2 SV=	53445	260	413	74	127	0.13	0.2	1.53846	1	3	2	3	2	5	2	5	0.28929
sp|P68363|TBA1B_HU	TUBA1B	Tubulin alpha-1B chain OS=Homo sapiens GN=TUBA1B PE=1 SV=1	50804	13	22	1091	1407	5.58	8.5	1.5233	31	36	43	55	45	59	49	66	-0.46544
sp|Q71U36|TBA1A_H	TUBA1A	Tubulin alpha-1A chain OS=Homo sapiens GN=TUBA1A PE=1 SV=1	50788	15	25	1022	1312	5.18	7.88	1.52124	30	34	41	52	43	56	46	63	-0.47512
tr|H0Y4R1|H0Y4R1_H	IMPDH2	Inosine-5~-monophosphate dehydrogenase 2 (Fragment) OS=Homo sapiens GN=IMPDH2	51549	177	152	122	322	0.36	0.54	1.5	2	6	5	7	5	7	6	9	-0.40137
tr|Q5QPE2|Q5QPE2_H	SEC23B	Protein transport protein Sec23B (Fragment) OS=Homo sapiens GN=SEC23B PE=4 SV=1	37575	474	530	24	91	0.08	0.12	1.5	0	2	1	3	1	3	1	4	0.374143
sp|Q14683|SMC1A_H	SMC1A	Structural maintenance of chromosomes protein 1A OS=Homo sapiens GN=SMC1A PE=1	143771	581	927	28	41	0.02	0.03	1.5	0	1	1	1	1	1	1	1	-0.84861
sp|Q14315|FLNC_HUI	FLNC	Filamin-C OS=Homo sapiens GN=FLNC PE=1 SV=3	293407	143	245	153	213	0.06	0.09	1.5	5	8	6	10	10	10	11	15	-0.84991
tr|A0PJ92|A0PJ92_HU	NOP56	NOP56 protein (Fragment) OS=Homo sapiens GN=NOP56 PE=2 SV=1	50068	311	562	58	84	0.14	0.21	1.5	1	2	2	3	3	3	3	3	-0.8489
sp|P55072|TERA_HUI	VCP	Transitional endoplasmic reticulum ATPase OS=Homo sapiens GN=VCP PE=1 SV=4	89950	64	69	326	580	0.65	0.97	1.49231	7	14	14	19	14	19	15	20	-0.42216
sp|Q08211|DHX9_HU	DHX9	ATP-dependent RNA helicase A OS=Homo sapiens GN=DHX9 PE=1 SV=4	142181	96	81	218	527	0.41	0.61	1.4878	8	14	16	22	18	25	22	29	-0.38683
sp|P52272|HNRPM_H	HNRNPM	Heterogeneous nuclear ribonucleoprotein M OS=Homo sapiens GN=HNRNPM PE=1 SV=3	77749	164	115	132	406	0.39	0.58	1.48718	7	7	8	11	11	14	12	17	-0.51509
tr|F8VRQ1|F8VRQ1_H	HNRNPA1	Heterogeneous nuclear ribonucleoprotein A1 OS=Homo sapiens GN=HNRNPA1 PE=2 SV=	33249	101	139	214	344	0.77	1.14	1.48052	4	9	6	9	7	10	9	13	-0.36351
tr|B4DPP6|B4DPP6_H	tr|B4DPP6|B4DPP6_HU	cDNA FLJ54371, highly similar to Serum albumin OS=Homo sapiens PE=2 SV=1	72370	222	243	93	215	0.25	0.37	1.48	3	5	5	10	6	11	10	14	-0.02531
tr|I3L492|I3L492_HUI	ETF1	Eukaryotic peptide chain release factor subunit 1 (Fragment) OS=Homo sapiens GN=ETF1	23028	274	675	69	25	0.15	0.22	1.46667	1	0	1	1	1	1	1	1	-0.84861
tr|B4DKT3|B4DKT3_H	tr|B4DKT3|B4DKT3_HU	cDNA FLJ58390, highly similar to Aldo-keto reductase family 1 member C3homolog (EC 1	15755	186	157	42	252	0.67	0.98	1.46269	1	6	1	6	1	6	1	6	1.267769
sp|P07814|SYEP_HUN	EPRS	Bifunctional glutamate/proline--tRNA ligase OS=Homo sapiens GN=EPRS PE=1 SV=5	172080	57	97	380	459	0.33	0.48	1.45455	9	11	15	21	15	23	17	28	-0.2488
sp|Q8WUM4|PDC6I_H	PDCD6IP	Programmed cell death 6-interacting protein OS=Homo sapiens GN=PDCD6IP PE=1 SV=1	96590	136	330	160	159	0.18	0.26	1.44444	2	5	5	7	5	6	6	8	-0.608
tr|Q5I6Y6|Q5I6Y6_HU	LMNA	Lamin A/C transcript variant 1 OS=Homo sapiens GN=LMNA PE=2 SV=1	74322	95	85	221	506	0.75	1.08	1.44	7	12	13	17	15	19	15	21	-0.51871
sp|Q1KMD3|HNRL2_H	HNRNPUL2	Heterogeneous nuclear ribonucleoprotein U-like protein 2 OS=Homo sapiens GN=HNRNI	85622	249	278	78	189	0.21	0.3	1.42857	2	4	5	7	6	8	6	8	-0.46195
tr|D3DQA6|D3DQA6_	DDX46	DEAD (Asp-Glu-Ala-Asp) box polypeptide 46, isoform CRA_a OS=Homo sapiens GN=DDX4	100958	595	547	26	88	0.07	0.1	1.42857	0	2	2	3	3	3	3	3	-0.8489
sp|P62917|RL8_HUM	RPL8	60S ribosomal protein L8 OS=Homo sapiens GN=RPL8 PE=1 SV=2	28235	231	581	90	80	0.28	0.4	1.42857	2	2	2	2	4	2	5	2	-1.6974
sp|P68133|ACTS_HUN	ACTA1	Actin, alpha skeletal muscle OS=Homo sapiens GN=ACTA1 PE=1 SV=1	42366	29	33	643	1026	4.62	6.59	1.42641	23	38	35	52	45	59	50	65	-0.46544
tr|F4ZW64|F4ZW64_H	tr|F4ZW64|F4ZW64_H	NF90a OS=Homo sapiens PE=2 SV=1	76369	154	170	137	286	0.46	0.65	1.41304	3	10	9	14	10	14	11	19	-0.38352
sp|Q15046|SYK_HUM	KARS	Lysine--tRNA ligase OS=Homo sapiens GN=KARS PE=1 SV=3	68461	265	235	71	221	0.32	0.45	1.40625	1	7	6	8	6	11	7	16	-0.02531
sp|Q9NVI7|ATD3A_H	ATAD3A	ATPase family AAA domain-containing protein 3A OS=Homo sapiens GN=ATAD3A PE=1 S	71610	281	288	68	182	0.1	0.14	1.4	2	4	2	5	2	6	2	6	0.530479
tr|B3KNM0|B3KNM0_	tr|B3KNM0|B3KNM0_H	cDNA FLJ14916 fis, clone PLACE1006958, highly similar to Heat shock 70 kDa protein 4L (	64588	191	402	113	123	0.1	0.14	1.4	2	2	2	3	2	3	3	3	-0.36315
sp|Q15392|DHC24_H	DHCR24	Delta(24)-sterol reductase OS=Homo sapiens GN=DHCR24 PE=1 SV=2	60803	442	1255	39	21	0.05	0.07	1.4	1	0	1	1	2	1	3	1	-1.5859
tr|Q6IBT3|Q6IBT3_HU	CCT7	CCT7 protein OS=Homo sapiens GN=CCT7 PE=2 SV=1	59804	161	147	133	330	0.38	0.53	1.39474	2	8	6	8	5	8	7	8	-0.22061
tr|E0WMN0|E0WMN0	HLA-C	MHC class I antigen (Fragment) OS=Homo sapiens GN=HLA-C PE=3 SV=1	31801	639	820	22	48	0.16	0.22	1.375	0	2	1	2	1	6	1	6	1.267769
sp|Q96HC4|PDLI5_HU	PDLIM5	PDZ and LIM domain protein 5 OS=Homo sapiens GN=PDLIM5 PE=1 SV=5	65102	175	212	123	238	0.41	0.56	1.36585	3	7	7	9	7	9	8	16	-0.50808
tr|H0YAF8|H0YAF8_H	GNB2L1	Guanine nucleotide-binding protein subunit beta-2-like 1 (Fragment) OS=Homo sapiens (	22373	216	244	96	212	0.52	0.71	1.36538	2	4	3	5	3	6	4	6	0.044728
sp|Q6S8J3|POTEE_HU	POTEE	POTE ankyrin domain family member E OS=Homo sapiens GN=POTEE PE=1 SV=3	122882	5	5	2211	2945	0.44	0.6	1.36364	45	58	66	81	70	81	75	87	-0.6475
tr|B4DNN4|B4DNN4_	tr|B4DNN4|B4DNN4_H	Ribonucleoside-diphosphate reductase OS=Homo sapiens PE=2 SV=1	87252	156	190	136	263	0.29	0.39	1.34483	5	6	7	9	8	10	8	13	-0.5444
tr|B4DF96|B4DF96_H	EIF2A	Eukaryotic translation initiation factor 2A OS=Homo sapiens GN=EIF2A PE=2 SV=1	58357	206	454	101	106	0.32	0.43	1.34375	2	1	5	6	4	9	5	10	0.229861
tr|B4E0S6|B4E0S6_H	tr|B4E0S6|B4E0S6_HU	cDNA FLJ55635, highly similar to pre-mRNA-splicing factorATP-dependent RNA helicase I	90289	183	441	117	115	0.15	0.2	1.33333	2	2	4	5	4	5	7	6	-0.55936

tr|A2T926|A2T926_H	TMPO	Thymopentin OS=Homo sapiens GN=TMPO PE=2 SV=1	27428	241	390	41	82	0.24	0.32	1.33333	0	2	3	3	3	3	3	3	-0.8489
tr|H7C1U0|H7C1U0_H	APEH	Acylamino-acid-releasing enzyme (Fragment) OS=Homo sapiens GN=APEH PE=4 SV=1	35132	319	549	56	54	0.09	0.12	1.33333	1	1	1	1	1	1	1	2	-0.84861
tr|A2RUM7|A2RUM7	RPL5	Ribosomal protein L5 OS=Homo sapiens GN=RPL5 PE=2 SV=1	34569	187	318	115	163	0.44	0.58	1.31818	2	4	4	5	5	6	7	6	-0.608
tr|B4DXW6|B4DXW6_	tr|B4DXW6|B4DXW6_	cDNA FLJ50285, highly similar to Arginyl-tRNA synthetase (EC 6.1.1.19) OS=Homo sapien	52771	235	202	88	170	0.35	0.46	1.31429	3	5	5	6	6	8	7	10	-0.46195
tr|B4DY32|B4DY32_H	tr|B4DY32|B4DY32_HU	Asparagine synthetase OS=Homo sapiens PE=2 SV=1	55055	141	302	156	173	0.26	0.34	1.30769	3	4	5	5	6	7	6	7	-0.6427
tr|F8WEJ5|F8WEJ5_H	ASNS	Asparagine synthetase [glutamine-hydrolyzing] OS=Homo sapiens GN=ASNS PE=2 SV=1	46395	141	263	156	133	0.26	0.34	1.30769	3	3	5	3	6	3	6	3	-1.74296
tr|B4DUY3|B4DUY3_H	tr|B4DUY3|B4DUY3_H	cDNA FLJ56668, highly similar to Nucleolar protein NOP5 OS=Homo sapiens PE=2 SV=1	16345	515	474	32	32	0.21	0.27	1.28571	0	0	1	1	1	1	1	1	-0.84861
tr|A8K525|A8K525_H	tr|A8K525|A8K525_HU	cDNA FLJ76817, highly similar to Homo sapiens non-POU domain containing, octamer-bi	54367	75	98	266	451	0.8	1.02	1.275	10	11	11	13	12	13	14	18	-0.73899
tr|B3KMV8|B3KMV8_	tr|B3KMV8|B3KMV8_H	cDNA FLJ12766 fis, clone NT2RP2001520, highly similar to Calcium-binding mitochondria	75130	353	479	41	104	0.11	0.14	1.27273	1	3	1	3	1	3	1	3	0.374143
sp|Q12792|TWF1_HU	TWF1	Twinfilin-1 OS=Homo sapiens GN=TWF1 PE=1 SV=3	40429	575	563	28	83	0.08	0.1	1.25	0	1	1	1	1	1	1	2	-0.84861
sp|P52895|AK1C2_HU	AKR1C2	Aldo-keto reductase family 1 member C2 OS=Homo sapiens GN=AKR1C2 PE=1 SV=3	37111	186	208	116	241	0.67	0.82	1.22388	3	6	6	7	7	8	9	8	-0.66872
tr|Q4W4Y1|Q4W4Y1_	DRIP4	Dopamine receptor interacting protein 4 OS=Homo sapiens GN=DRIP4 PE=2 SV=1	96646	136	329	160	159	0.18	0.22	1.22222	2	5	5	6	5	6	6	7	-0.608
sp|Q9NY65|TBA8_HU	TUBA8	Tubulin alpha-8 chain OS=Homo sapiens GN=TUBA8 PE=1 SV=1	50746	34	53	592	712	1.74	2.11	1.21264	15	22	23	30	23	33	27	37	-0.33849
tr|Q59ES3|Q59ES3_H	tr|Q59ES3|Q59ES3_HU	Solute carrier family 1 (Neutral amino acid transporter), member 5 variant (Fragment) O	57810	290	571	63	82	0.1	0.12	1.2	1	2	1	2	1	2	1	2	-0.11146
sp|P14923|PLAK_HUN	JUP	Junction plakoglobin OS=Homo sapiens GN=JUP PE=1 SV=3	82434	205	267	102	200	0.26	0.31	1.19231	3	7	6	8	6	9	7	9	-0.3013
sp|P68032|ACTC_HUN	ACTC1	Actin, alpha cardiac muscle 1 OS=Homo sapiens GN=ACTC1 PE=1 SV=1	42334	23	31	771	1066	6.63	7.81	1.17798	25	39	40	54	48	61	55	67	-0.51048
tr|A8K6Q8|A8K6Q8_H	tr|A8K6Q8|A8K6Q8_H	cDNA FLJ75881, highly similar to Homo sapiens transferrin receptor (p90, CD71) (TFRC),	85276	122	148	176	327	0.3	0.35	1.16667	6	8	7	8	7	9	8	10	-0.50808
tr|H3BLZ8|H3BLZ8_H	DDX17	Probable ATP-dependent RNA helicase DDX17 OS=Homo sapiens GN=DDX17 PE=3 SV=1	81073	166	181	130	275	0.37	0.43	1.16216	3	7	9	9	9	10	10	13	-0.70519
tr|B3KTJ9|B3KTJ9_HU	tr|B3KTJ9|B3KTJ9_HUN	cDNA FLJ38393 fis, clone FEBRA2007212 OS=Homo sapiens PE=2 SV=1	103493	82	118	251	397	0.32	0.37	1.15625	7	8	9	10	9	11	12	11	-0.57377
sp|Q9NR30|DDX21_H	DDX21	Nucleolar RNA helicase 2 OS=Homo sapiens GN=DDX21 PE=1 SV=5	87804	148	265	147	202	0.34	0.39	1.14706	4	6	8	9	8	11	9	11	-0.41298
tr|A4ZU86|A4ZU86_H	NPM1	Truncated nucleolar phosphoprotein B23 OS=Homo sapiens GN=NPM1 PE=2 SV=1	30074	138	215	160	224	0.69	0.79	1.14493	4	4	5	5	5	6	5	8	-0.608
tr|K7EKB6|K7EKB6_H	THOP1	Thimet oligopeptidase (Fragment) OS=Homo sapiens GN=THOP1 PE=4 SV=1	20792	278	577	68	40	0.16	0.18	1.125	1	1	1	1	1	1	1	1	-0.84861
tr|Q6IPN6|Q6IPN6_H	EEF1A1	Elongation factor 1-alpha OS=Homo sapiens GN=EEF1A1 PE=2 SV=1	50433	70	221	295	227	1.14	1.28	1.12281	11	7	13	14	18	17	21	20	-0.93142
tr|B7ZB91|B7ZB91_H	TNRC6B	Trinucleotide repeat containing 6B (Fragment) OS=Homo sapiens GN=TNRC6B PE=4 SV=1	81825	359	797	48	50	0.13	0.14	1.07692	1	2	3	2	2	2	3	3	-0.84875
tr|J3KQ96|J3KQ96_H	TCOF1	Treacle protein (Fragment) OS=Homo sapiens GN=TCOF1 PE=4 SV=1	144268	702	921	18	41	0.02	0.02	1	0	1	1	1	0	2	2	2	1.473823
tr|B4DTA2|B4DTA2_H	tr|B4DTA2|B4DTA2_HU	cDNA FLJ60148, highly similar to Homo sapiens heterogeneous nuclear ribonucleoprotei	30367	229	501	39	99	0.37	0.37	1	1	3	1	4	1	4	1	5	0.736894
tr|Q3MNE1|Q3MNE1	tr|Q3MNE1|Q3MNE1_	Adaptor-related protein complex 3 beta 1 subunit (Fragment) OS=Homo sapiens PE=2 SV	89828	682	1151	20	28	0.04	0.04	1	0	1	1	1	0	1	1	1	0.736677
tr|B4DKD0|B4DKD0_H	tr|B4DKD0|B4DKD0_H	DNA topoisomerase 2 (Fragment) OS=Homo sapiens PE=2 SV=1	135015	606	955	25	39	0.02	0.02	1	0	1	1	1	1	3	3	4	0.374143
tr|Q2VPA0|Q2VPA0_H	LONP1	Lon protease homolog (Fragment) OS=Homo sapiens GN=LONP1 PE=2 SV=1	95436	414	469	42	107	0.11	0.11	1	2	3	3	3	3	6	3	6	0.044728
tr|Q3B7A7|Q3B7A7_H	GART	Phosphoribosylglycinamide formyltransferase, phosphoribosylglycinamide synthetase, p	108909	341	222	51	227	0.13	0.13	1	1	4	4	4	3	6	4	8	0.044728
tr|G0ZJH6|G0ZJH6_H	Ube4B	UFD2A-III/UBE4B-III splice isoform OS=Homo sapiens GN=Ube4B PE=2 SV=1	152890	667	1158	21	28	0.02	0.02	1	0	0	1	1	1	2	1	2	-0.11146
tr|Q6UWE9|Q6UWE9	UNQ2550	SFVP2550 OS=Homo sapiens GN=UNQ2550 PE=4 SV=1	11890	664	1046	21	33	0.29	0.29	1	1	2	1	2	1	2	1	2	-0.11146
tr|Q8TC04|Q8TC04_H	KRT23	Keratin 23 (Histone deacetylase inducible) OS=Homo sapiens GN=KRT23 PE=2 SV=1	48318	600	952	26	39	0.07	0.07	1	0	1	1	1	1	2	1	3	-0.11146
tr|Q05BW9|Q05BW9	PAPSS1	PAPSS1 protein (Fragment) OS=Homo sapiens GN=PAPSS1 PE=2 SV=1	65264	553	398	29	132	0.1	0.1	1	0	2	2	2	1	2	2	3	-0.11146
sp|O95831|AIFM1_H	AIFM1	Apoptosis-inducing factor 1, mitochondrial OS=Homo sapiens GN=AIFM1 PE=1 SV=1	67144	326	1079	54	32	0.05	0.05	1	1	0	1	1	1	2	2	2	-0.11146
tr|Q562M3|Q562M3_	ACT	Actin-like protein (Fragment) OS=Homo sapiens GN=ACT PE=2 SV=1	11529	103	120	213	393	7.06	7.06	1	10	16	14	22	17	25	20	28	-0.30634
sp|P38919|IF4A3_HU	EIF4A3	Eukaryotic initiation factor 4A-III OS=Homo sapiens GN=EIF4A3 PE=1 SV=4	47126	262	179	74	277	0.4	0.4	1	1	6	5	6	4	6	6	8	-0.31817
tr|B3KY95|B3KY95_H	tr|B3KY95|B3KY95_HU	cDNA FLJ16143 fis, clone BRAMY2038516, highly similar to Protein disulfide-isomerase A	53655	445	643	38	71	0.13	0.13	1	0	1	2	2	2	3	2	4	-0.36315
sp|Q9NR12|PDLI7_HU	PDLIM7	PDZ and LIM domain protein 7 OS=Homo sapiens GN=PDLIM7 PE=1 SV=1	50896	396	755	43	56	0.13	0.13	1	0	2	2	2	2	3	4	4	-0.36315
sp|Q9H853|TBA4B_H	TUBA4B	Putative tubulin-like protein alpha-4B OS=Homo sapiens GN=TUBA4B PE=5 SV=2	27819	146	331	147	159	0.4	0.4	1	3	4	4	5	4	5	6	5	-0.55936
sp|P42166|LAP2A_HU	TMPO	Lamina-associated polypeptide 2, isoform alpha OS=Homo sapiens GN=TMPO PE=1 SV=2	76016	241	477	84	105	0.24	0.24	1	1	2	5	5	5	6	5	8	-0.608
sp|P53396|ACLY_HUN	ACLY	ATP-citrate synthase OS=Homo sapiens GN=ACLY PE=1 SV=3	121674	20	39	845	957	1.28	1.28	1	21	27	32	34	34	35	40	41	-0.81199
tr|B4DS66|B4DS66_H	tr|B4DS66|B4DS66_HU	cDNA FLJ54290, highly similar to Mitochondrial inner membrane protein OS=Homo sapie	73509	712	1264	17	21	0.04	0.04	1	0	0	1	1	1	1	6	3	-0.84861
sp|Q9UG63|ABCF2_H	ABCF2	ATP-binding cassette sub-family F member 2 OS=Homo sapiens GN=ABCF2 PE=1 SV=2	71815	673	750	20	56	0.05	0.05	1	0	1	1	1	1	1	2	1	-0.84861
tr|J3KNE5|J3KNE5_H	HARS	Histidine--tRNA ligase, cytoplasmic OS=Homo sapiens GN=HARS PE=4 SV=1	50581	672	1074	20	32	0.07	0.07	1	0	0	1	1	1	1	2	1	-0.84861
tr|J3QT54|J3QT54_H	CPSF7	Cleavage and polyadenylation-specificity factor subunit 7 (Fragment) OS=Homo sapiens	22762	665	1245	21	22	0.15	0.15	1	0	0	1	1	1	1	1	1	-0.84861
sp|Q96EK5|KBP_HUM	KIAA1279	KIF1-binding protein OS=Homo sapiens GN=KIAA1279 PE=1 SV=1	72453	649	792	22	51	0.05	0.05	1	0	1	1	1	1	1	1	1	-0.84861
tr|Q5VSQ6|Q5VSQ6_	P4HA1	Procollagen-proline, 2-oxoglutarate 4-dioxygenase (Proline 4-hydroxylase), alpha polype	61214	634	1047	22	33	0.05	0.05	1	0	0	1	1	1	1	2	1	-0.84861
tr|B0QZK4|B0QZK4_H	HP1BP3	Heterochromatin protein 1-binding protein 3 (Fragment) OS=Homo sapiens GN=HP1BP3	28725	631	1010	23	36	0.25	0.25	1	0	0	2	2	1	1	3	2	-0.84861
tr|B4DFE4|B4DFE4_H	tr|B4DFE4|B4DFE4_HU	cDNA FLJ59618, highly similar to Exosome component 10 OS=Homo sapiens PE=2 SV=1	61733	627	839	23	47	0.05	0.05	1	0	1	1	1	1	1	1	1	-0.84861
tr|F8W930|F8W930_	IGF2BP2	Insulin-like growth factor 2 mRNA-binding protein 2 OS=Homo sapiens GN=IGF2BP2 PE=	66859	556	866	29	45	0.05	0.05	1	0	1	1	1	1	1	1	1	-0.84861
tr|B3KNM6|B3KNM6_	tr|B3KNM6|B3KNM6_	cDNA FLJ14968 fis, clone THYRO1000288, highly similar to CAAX prenyl protease 1 homo	27279	547	752	30	46	0.12	0.12	1	1	1	1	1	1	1	1	1	-0.84861
tr|B7Z9W6|B7Z9W6_	tr|B7Z9W6|B7Z9W6_H	cDNA, FLJ78980, highly similar to DnaJ homolog subfamily C member 7 OS=Homo sapien	50776	527	870	31	44	0.06	0.06	1	0	1	1	1	1	1	1	1	-0.84861
tr|B3KUZ8|B3KUZ8_H	tr|B3KUZ8|B3KUZ8_HU	Aspartate aminotransferase OS=Homo sapiens PE=2 SV=1	41699	522	996	31	36	0.08	0.08	1	0	1	1	1	1	1	2	1	-0.84861
tr|Q96RS2|Q96RS2_H	LAMR1P15	Laminin receptor-like protein LAMRL5 OS=Homo sapiens GN=LAMR1P15 PE=2 SV=1	33089	521	782	31	53	0.1	0.1	1	0	1	1	1	1	1	1	1	-0.84861
sp|O95782|AP2A1_H	AP2A1	AP-2 complex subunit alpha-1 OS=Homo sapiens GN=AP2A1 PE=1 SV=3	108561	500	1177	34	26	0.03	0.03	1	0	0	1	1	1	1	2	2	-0.84861
tr|B4DDC9|B4DDC9_H	tr|B4DDC9|B4DDC9_H	cDNA FLJ59448, highly similar to Trifunctional enzyme subunit beta, mitochondrial OS=H	49937	499	950	34	39	0.07	0.07	1	0	1	1	1	1	1	1	1	-0.84861
tr|B3KSQ7|B3KSQ7_H	DBN1	Drebrin 1, isoform CRA_d OS=Homo sapiens GN=DBN1 PE=2 SV=1	66053	478	626	36	73	0.05	0.05	1	0	1	1	1	1	1	1	1	-0.84861
tr|B3KUJ0|B3KUJ0_H	tr|B3KUJ0|B3KUJ0_HU	cDNA FLJ39996 fis, clone STOMA2002166, highly similar to Splicing factor 3B subunit 4 O	44512	464	1233	37	23	0.07	0.07	1	1	0	1	1	1	1	2	2	-0.84861
tr|B4E363|B4E363_H	FARSA	Phenylalanine--tRNA ligase alpha subunit OS=Homo sapiens GN=FARSA PE=2 SV=1	54181	447	1022	38	34	0.06	0.06	1	1	0	1	1	1	1	1	1	-0.84861
tr|Q5JR94|Q5JR94_H	RPS8	40S ribosomal protein S8 OS=Homo sapiens GN=RPS8 PE=2 SV=1	24475	426	873	41	44	0.14	0.14	1	1	1	1	1	1	1	2	1	-0.84861
tr|B4DPY2|B4DPY2_H	tr|B4DPY2|B4DPY2_HU	cDNA FLJ59286, highly similar to Pre-mRNA-processing factor 40 homolog A (Fragment)	93124	425	1239	41	22	0.04	0.04	1	1	0	1	1	1	1	1	1	-0.84861
tr|B3KPZ0|B3KPZ0_H	tr|B3KPZ0|B3KPZ0_HU	cDNA FLJ32485 fis, clone SKNMC2001596, highly similar to Ankyrin repeat and FYVE dom	121468	387	887	45	43	0.03	0.03	1	1	1	1	1	1	1	1	2	-0.84861
sp|Q9Y6M5|ZNT1_HU	SLC30A1	Zinc transporter 1 OS=Homo sapiens GN=SLC30A1 PE=1 SV=3	56290	334	587	52	79	0.06	0.06	1	1	1	1	1	1	1	1	2	-0.84861
tr|E7ERW8|E7ERW8_	DIAPH1	Protein diaphanous homolog 1 OS=Homo sapiens GN=DIAPH1 PE=2 SV=1	135945	184	757	116	55	0.02	0.02	1	1	1	1	1	1	1	2	3	-0.84861
sp|Q9Y3I0|RTCB_HUN	C22orf28	tRNA-splicing ligase RtcB homolog OS=Homo sapiens GN=C22orf28 PE=1 SV=1	55688	458	448	38	113	0.12	0.12	1	0	2	2	2	2	2	2	2	-0.84875
tr|B4DUL5|B4DUL5_H	tr|B4DUL5|B4DUL5_H	cDNA FLJ51625, highly similar to Ubiquinol-cytochrome-c reductase complex coreprotei	40917	357	630	48	73	0.17	0.17	1	1	2	2	2	2	2	2	2	-0.84875
sp|P53618|COPB_HU	COPB1	Coatomer subunit beta OS=Homo sapiens GN=COPB1 PE=1 SV=3	108214	303	541	59	89	0.06	0.06	1	1	2	2	2	2	2	3	2	-0.84875
tr|B1ANK7|B1ANK7_	FH	Fumarate hydratase OS=Homo sapiens GN=FH PE=2 SV=1	54773	288	657	64	69	0.12	0.12	1	1	2	2	2	2	2	5	2	-0.84875
tr|B4DII5|B4DII5_HUN	tr|B4DII5|B4DII5_HUM	Importin subunit alpha OS=Homo sapiens PE=2 SV=1	60345	573	699	28	63	0.11	0.11	1	0	1	2	2	3	3	3	3	-0.8489
tr|D2CFK9|D2CFK9_H	GTPBP4	Nucleolar GTP-binding protein 1 OS=Homo sapiens GN=GTPBP4 PE=2 SV=1	74317	264	616	72	75	0.14	0.14	1	2	1	3	3	3	3	3	3	-0.8489
sp|P35237|SPB6_HUN	SERPINB6	Serpin B6 OS=Homo sapiens GN=SERPINB6 PE=1 SV=3	42936	120	492	179	101	0.25	0.25	1	3	2	3	3	3	3	3	3	-0.8489
tr|B4DKM5|B4DKM5_	VDAC2	Voltage-dependent anion-selective channel protein 2 OS=Homo sapiens GN=VDAC2 PE=	27804	196	326	108	160	0.76	0.76	1	1	4	5	5	5	5	5	5	-0.84919
tr|B3KM80|B3KM80_	NCL	Nucleolin, isoform CRA_c OS=Homo sapiens GN=NCL PE=2 SV=1	58576	71	83	283	521	1.03	1.03	1	9	13	14	14	15	14	15	15	-0.94703
tr|Q5HYG8|Q5HYG8_	DKFZp686P09201	Putative uncharacterized protein DKFZp686P09201 OS=Homo sapiens GN=DKFZp686P09	53831	86	185	235	269	0.61	0.61	1	4	7	8	8	9	8	10	8	-1.01041
sp|P58107|EPIPL_HU	EPPK1	Epiplakin OS=Homo sapiens GN=EPPK1 PE=1 SV=2	557674	56	135	390	348	0.07	0.07	1	10	5	12	12	12	10	13	14	-1.1021
sp|P46087|NOP2_HU	NOP2	Putative ribosomal RNA methyltransferase NOP2 OS=Homo sapiens GN=NOP2 PE=1 SV=	89589	296	550	61	86	0.15	0.15	1	2	1	4	4	5	4	6	4	-1.13887
tr|F2Z2U8|F2Z2U8_H	MYH14	Myosin-14 OS=Homo sapiens GN=MYH14 PE=2 SV=1	232102	152	418	140	123	0.07	0.07	1	5	3	5	5	7	5	8	5	-1.29729
tr|L0R5A1|L0R5A1_H	CSF2RB	Alternative protein CSF2RB OS=Homo sapiens GN=CSF2RB PE=4 SV=1	11752	486	961	36	38	0.29	0.29	1	1	1	1	1	3	2	5	2	-1.3345
tr|A0AV58|A0AV58_H	STRN3	Striatin, calmodulin binding protein 3 OS=Homo sapiens GN=STRN3 PE=2 SV=1	78069	419	732	42	58	0.09	0.09	1	1	1	2	2	3	2	3	3	-1.3345
sp|Q92621|NU205_H	NUP205	Nuclear pore complex protein Nup205 OS=Homo sapiens GN=NUP205 PE=1 SV=3	230171	415	980	42	37	0.04	0.04	1	0	1	3	3	3	2	5	4	-1.3345
sp|Q13151|ROA0_HU	HNRNPA0	Heterogeneous nuclear ribonucleoprotein A0 OS=Homo sapiens GN=HNRNPA0 PE=1 SV=	30993	323	1054	55	33	0.36	0.36	1	2	2	3	3	3	2	3	5	-1.3345
sp|Q9UHR4|BI2L1_HU	BAIAP2L1	Brain-specific angiogenesis inhibitor 1-associated protein 2-like protein 1 OS=Homo sapi	57189	613	665	24	68	0.12	0.12	1	0	1	2	2	2	1	4	2	-1.5859
tr|Q53RS3|Q53RS3_H	DDEF2	Putative uncharacterized protein DDEF2 (Fragment) OS=Homo sapiens GN=DDEF2 PE=2	70046	441	988	39	37	0.05	0.05	1	1	1	1	1	4	2	4	2	-1.6974
tr|F5H3A1|F5H3A1_H	ATP1A1	Sodium/potassium-transporting ATPase subunit alpha-1 OS=Homo sapiens GN=ATP1A1	114239	324	713	55	61	0.09	0.09	1	2	0	3	3	8	4	12	5	-1.76788
tr|A0N4V7|A0N4V7_H	Tcr-alpha	HCG2039797 (Fragment) OS=Homo sapiens GN=Tcr-alpha PE=4 SV=1	2269	212	817	98	49	1.81	1.81	1	5	2	15	2	31	3	46	3	-4.02793
tr|Q9UJZ2|Q9UJZ2_H	VIL2	Cytovillin 2 (Fragment) OS=Homo sapiens GN=VIL2 PE=2 SV=1	18464	119	310	88	95	0.46	0.45	0.97826	2	3	2	3	2	3	2	3	-0.36315
sp|Q58FF8|H90B2_HU	HSP90AB2P	Putative heat shock protein HSP 90-beta 2 OS=Homo sapiens GN=HSP90AB2P PE=1 SV=2	44492	32	158	97	314	1.24	1.2	0.96774	3	8	5	12	5	13	6	14	0.447711
tr|H0YGW7|H0YGW7	ABCF1	ATP-binding cassette sub-family F member 1 (Fragment) OS=Homo sapiens GN=ABCF1 P	68075	144	229	151	205	0.27	0.26	0.96296	4	5	5	6	5	6	7	7	-0.608
sp|Q00610|CLH1_HU	CLTC	Clathrin heavy chain 1 OS=Homo sapiens GN=CLTC PE=1 SV=5	193260	30	47	629	787	0.52	0.49	0.94231	19	17	25	25	24	24	28	31	-0.85194
tr|B9A041|B9A041_H	MDH1	Malate dehydrogenase, cytoplasmic OS=Homo sapiens GN=MDH1 PE=2 SV=1	23195	309	811	58	45	0.31	0.29	0.93548	2	1	2	1	2	2	2	2	-0.84875
tr|E5RH42|E5RH42_H	G3BP1	Ras GTPase-activating protein-binding protein 1 (Fragment) OS=Homo sapiens GN=G3BP	13338	294	234	62	103	0.58	0.54	0.93103	2	1	2	2	2	3	2	3	-0.36315
tr|B4DRH6|B4DRH6_	tr|B4DRH6|B4DRH6_H	cDNA FLJ54509, highly similar to Trifunctional enzyme subunit alpha, mitochondrial OS=	79007	133	459	164	50	0.28	0.26	0.92857	5	1	6	4	7	5	7	6	-1.29729
sp|Q12904|AIMP1_H	AIMP1	Aminoacyl tRNA synthase complex-interacting multifunctional protein 1 OS=Homo sapie	34616	601	1217	26	24	0.11	0.1	0.90909	0	0	1	1	1	1	1	1	-0.84861
tr|Q53XC0|Q53XC0_H	EIF2S1	Eukaryotic translation initiation factor 2, subunit 1 alpha, 35kDa, isoform CRA_a OS=Hom	36374	208	726	100	59	0.42	0.38	0.90476	4	2	4	3	4	3	4	5	-1.21179
sp|Q13867|BLMH_HU	BLMH	Bleomycin hydrolase OS=Homo sapiens GN=BLMH PE=1 SV=1	53155	332	664	52	68	0.2	0.18	0.9	1	1	3	2	2	2	4	3	-0.84875
tr|G3V2A3|G3V2A3_H	TUBB3	Tubulin beta-3 chain (Fragment) OS=Homo sapiens GN=TUBB3 PE=2 SV=1	20929	25	40	147	365	3.07	2.74	0.89251	5	9	6	10	7	15	7	17	0.199273
tr|F8VVB9|F8VVB9_H	TUBA1B	Tubulin alpha-1B chain (Fragment) OS=Homo sapiens GN=TUBA1B PE=2 SV=1	27877	13	20	423	914	5.58	4.74	0.84946	11	21	15	30	17	31	20	37	-0.0004
tr|D2JYH4|D2JYH4_H	ACTA2	Actin, alpha 2, smooth muscle, aorta OS=Homo sapiens GN=ACTA2 PE=2 SV=1	42381	23	35	702	980	6.63	5.53	0.83409	23	33	35	47	43	51	49	56	-0.6097
sp|P53621|COPA_HU	COPA	Coatomer subunit alpha OS=Homo sapiens GN=COPA PE=1 SV=2	139797	181	386	118	136	0.18	0.15	0.83333	5	5	7	6	8	7	11	8	-1.03037
tr|Q8TE01|Q8TE01_H	derp12	DERP12 (Dermal papilla derived protein 12) OS=Homo sapiens GN=derp12 PE=2 SV=1	38340	69	199	297	254	0.79	0.64	0.81013	6	6	7	6	8	6	8	6	-1.237
tr|H0Y9Q1|H0Y9Q1_H	LAP3	Cytosol aminopeptidase (Fragment) OS=Homo sapiens GN=LAP3 PE=4 SV=1	22641	368	723	47	0	0.15	0.12	0.8	1	0	1	0	1	1	1	1	-0.84861
tr|G8JLB6|G8JLB6_HU	HNRNPH1	Heterogeneous nuclear ribonucleoprotein H OS=Homo sapiens GN=HNRNPH1 PE=4 SV=1	51482	207	192	101	262	0.45	0.36	0.8	2	7	6	7	5	7	6	8	-0.40137
sp|P08729|K2C7_HUN	KRT7	Keratin, type II cytoskeletal 7 OS=Homo sapiens GN=KRT7 PE=1 SV=5	51411	259	284	74	183	0.45	0.36	0.8	2	5	6	5	7	5	9	7	-1.29729
tr|B4DF68|B4DF68_H	tr|B4DF68|B4DF68_HU	cDNA FLJ55325, highly similar to Heat-shock protein 105 kDa OS=Homo sapiens PE=2 SV	86500	123	246	47	148	0.23	0.18	0.78261	1	3	1	4	1	5	1	5	1.02658
sp|P12036|NFH_HUM	NEFH	Neurofilament heavy polypeptide OS=Homo sapiens GN=NEFH PE=1 SV=4	112639	11	52	0	44	4.79	3.7	0.77244	0	1	0	1	0	1	1	1	0.736677
tr|D6REX3|D6REX3_H	SEC31A	Protein transport protein Sec31A OS=Homo sapiens GN=SEC31A PE=2 SV=1	137110	121	323	178	162	0.13	0.1	0.76923	4	3	5	4	4	5	5	5	-0.55936
tr|B7Z4V1|B7Z4V1_H	tr|B7Z4V1|B7Z4V1_HU	cDNA FLJ55401, highly similar to Alpha-actinin-2 OS=Homo sapiens PE=2 SV=1	32962	50	99	103	269	0.77	0.59	0.76623	3	9	4	10	4	12	4	12	0.626331
tr|B4DP08|B4DP08_H	ALG1	Chitobiosyldiphosphodolichol beta-mannosyltransferase OS=Homo sapiens GN=ALG1 PE	40938	452	538	38	77	0.17	0.13	0.76471	0	1	2	1	2	1	2	1	-1.5859
sp|Q562R1|ACTBL_HU	ACTBL2	Beta-actin-like protein 2 OS=Homo sapiens GN=ACTBL2 PE=1 SV=2	42318	61	90	349	483	1.47	1.12	0.7619	13	14	16	20	24	26	27	29	-0.73837
tr|B4DJ63|B4DJ63_H	tr|B4DJ63|B4DJ63_HU	Serine hydroxymethyltransferase OS=Homo sapiens PE=2 SV=1	41859	86	389	192	135	0.61	0.46	0.7541	3	4	6	5	7	5	7	6	-1.29729
tr|Q5U077|Q5U077_H	LDHB	L-lactate dehydrogenase OS=Homo sapiens GN=LDHB PE=2 SV=1	36900	39	145	530	334	1.81	1.36	0.75138	13	10	16	12	18	14	20	16	-1.20326
tr|B7Z438|B7Z438_H	tr|B7Z438|B7Z438_HU	cDNA FLJ56352, highly similar to Succinyl-CoA ligase (GDP-forming) subunit alpha, mitoc	26801	467	1240	37	22	0.12	0.09	0.75	1	0	1	1	1	1	1	1	-0.84861
tr|B4E2P4|B4E2P4_H	tr|B4E2P4|B4E2P4_HU	cDNA FLJ58474, highly similar to Xaa-Pro aminopeptidase 1 (EC 3.4.11.9) OS=Homo sapie	50020	615	933	24	40	0.07	0.05	0.71429	0	1	1	1	1	1	1	1	-0.84861
tr|Q8NFH9|Q8NFH9_	tr|Q8NFH9|Q8NFH9_H	MLL/SEPTIN6 fusion protein (Fragment) OS=Homo sapiens PE=2 SV=1	63617	532	1078	31	32	0.07	0.05	0.71429	1	1	1	1	1	1	1	2	-0.84861
sp|P78371|TCPB_HUN	CCT2	T-complex protein 1 subunit beta OS=Homo sapiens GN=CCT2 PE=1 SV=4	57794	37	66	563	592	1.86	1.29	0.69355	14	11	20	17	20	17	23	20	-1.08017
tr|B7ZKM0|B7ZKM0_	SART3	SART3 protein OS=Homo sapiens GN=SART3 PE=2 SV=1	106372	153	942	139	39	0.16	0.11	0.6875	5	1	5	2	7	2	7	2	-2.43534
sp|P01024|CO3_HUM	C3	Complement C3 OS=Homo sapiens GN=C3 PE=1 SV=2	188569	370	589	47	79	0.03	0.02	0.66667	1	1	2	1	2	2	2	2	-0.84875
tr|Q68DB1|Q68DB1_	DKFZp686F2281	Metastasis-associated protein MTA2 OS=Homo sapiens GN=DKFZp686F2281 PE=2 SV=1	55515	676	765	20	0	0.06	0.04	0.66667	0	0	1	0	1	0	1	2	-2.43375
tr|H0YC69|H0YC69_H	KIAA1967	DBIRD complex subunit KIAA1967 (Fragment) OS=Homo sapiens GN=KIAA1967 PE=4 SV=	16355	82	902	45	42	0.32	0.21	0.65625	1	1	1	1	1	1	2	2	-0.84861
tr|Q5T081|Q5T081_H	RCC1	CHC1 protein OS=Homo sapiens GN=RCC1 PE=2 SV=1	45397	269	830	70	47	0.23	0.15	0.65217	2	1	3	2	2	2	4	3	-0.84875
sp|Q9NV06|DCA13_H	DCAF13	DDB1- and CUL4-associated factor 13 OS=Homo sapiens GN=DCAF13 PE=1 SV=2	51996	435	990	39	37	0.2	0.13	0.65	1	1	3	1	3	1	6	1	-2.07165
tr|H0YJ34|H0YJ34_H	FERMT2	Fermitin family homolog 2 (Fragment) OS=Homo sapiens GN=FERMT2 PE=4 SV=1	73877	306	605	59	77	0.14	0.09	0.64286	2	2	3	2	3	2	3	2	-1.3345
tr|G5E9Q2|G5E9Q2_H	CSDE1	Cold shock domain containing E1, RNA-binding, isoform CRA_d OS=Homo sapiens GN=C	94539	312	539	58	89	0.11	0.07	0.63636	1	2	3	2	3	3	3	4	-0.8489
tr|J3QT27|J3QT27_H	PCBP3	Poly(rC)-binding protein 3 (Fragment) OS=Homo sapiens GN=PCBP3 PE=4 SV=1	34649	132	480	35	104	0.32	0.2	0.625	1	3	1	3	1	3	1	4	0.374143
tr|B4DEN9|B4DEN9_H	tr|B4DEN9|B4DEN9_H	cDNA FLJ56617, highly similar to Ribosome biogenesis protein BOP1 OS=Homo sapiens P	72443	505	628	33	73	0.08	0.05	0.625	0	1	2	1	2	1	3	1	-1.5859
tr|B2R659|B2R659_H	tr|B2R659|B2R659_HU	cDNA, FLJ92803, highly similar to Homo sapiens hydroxysteroid (17-beta) dehydrogenase	80018	484	883	36	43	0.13	0.08	0.61538	0	0	3	2	3	2	4	2	-1.3345
tr|B3KY12|B3KY12_H	tr|B3KY12|B3KY12_HU	cDNA FLJ46581 fis, clone THYMU3043200, highly similar to Splicing factor 3A subunit 3 O	59082	495	829	34	47	0.18	0.11	0.61111	1	1	3	2	2	2	3	2	-0.84875
tr|B2RCM3|B2RCM3_	tr|B2RCM3|B2RCM3_H	cDNA, FLJ96158, highly similar to Homo sapiens calpain 2, (m/II) large subunit (CAPN2),	80883	173	488	125	102	0.22	0.13	0.59091	3	3	7	3	7	3	8	4	-1.94973
tr|E9PKL9|E9PKL9_H	TSTA3	GDP-L-fucose synthase (Fragment) OS=Homo sapiens GN=TSTA3 PE=2 SV=1	29819	371	682	47	65	0.19	0.11	0.57895	1	1	1	1	1	1	1	1	-0.84861
sp|Q2NL82|TSR1_HU	TSR1	Pre-rRNA-processing protein TSR1 homolog OS=Homo sapiens GN=TSR1 PE=1 SV=1	92151	230	969	90	38	0.07	0.04	0.57143	2	1	2	1	2	1	2	1	-1.5859
tr|Q96K98|Q96K98_H	tr|Q96K98|Q96K98_HU	cDNA FLJ14414 fis, clone HEMBA1004847, highly similar to SIGNAL RECOGNITION PARTI	37365	180	719	119	38	0.18	0.1	0.55556	2	1	2	1	2	2	4	4	-0.84875
tr|B4DPZ3|B4DPZ3_H	tr|B4DPZ3|B4DPZ3_HU	cDNA FLJ53290, highly similar to Cytoplasmic dynein 1 intermediate chain 2 OS=Homo sa	70869	298	1248	60	22	0.09	0.05	0.55556	2	0	2	1	2	1	2	2	-1.5859
tr|D3DPU2|D3DPU2_	CAP1	Adenylyl cyclase-associated protein OS=Homo sapiens GN=CAP1 PE=3 SV=1	51926	172	306	125	169	0.36	0.2	0.55556	4	3	5	3	6	3	7	5	-1.74296
tr|H0YIE7|H0YIE7_HU	HSPA1L	Heat shock 70 kDa protein 1-like OS=Homo sapiens GN=HSPA1L PE=3 SV=1	25488	51	89	132	204	1.45	0.8	0.55172	3	5	4	6	4	6	4	6	-0.31817
tr|B3KTA3|B3KTA3_H	tr|B3KTA3|B3KTA3_HU	Fascin OS=Homo sapiens PE=2 SV=1	52814	53	133	403	292	1.07	0.58	0.54206	9	8	14	9	16	9	20	10	-1.64851
tr|K7EK77|K7EK77_H	ATP5A1	ATP synthase subunit alpha, mitochondrial (Fragment) OS=Homo sapiens GN=ATP5A1 PE	22243	135	173	160	126	1.33	0.71	0.53383	5	4	6	5	6	5	6	5	-1.09052
sp|P00338|LDHA_HU	LDHA	L-lactate dehydrogenase A chain OS=Homo sapiens GN=LDHA PE=1 SV=2	36950	44	176	481	283	2.33	1.17	0.50215	10	6	19	9	19	12	24	12	-1.49402
tr|B4DH52|B4DH52_H	tr|B4DH52|B4DH52_H	cDNA FLJ55916, highly similar to General transcription factor II-I OS=Homo sapiens PE=2	112359	554	806	29	49	0.06	0.03	0.5	0	1	2	1	2	2	3	2	-0.84875
tr|B4DZ88|B4DZ88_H	KTN1	KTN1 protein OS=Homo sapiens GN=KTN1 PE=2 SV=1	153179	322	783	55	53	0.04	0.02	0.5	2	1	2	1	3	3	4	4	-0.8489

tr|J3KPM9|J3KPM9_H	STAT1	Signal transducer and activator of transcription 1-alpha/beta OS=Homo sapiens GN=STAT	83878	364	789	48	52	0.08	0.04	0.5	1	1	2	1	2	1	2	1	-1.5859
sp|Q9BYZ2|LDH6B_HU	LDHAL6B	L-lactate dehydrogenase A-like 6B OS=Homo sapiens GN=LDHAL6B PE=1 SV=3	42372	327	844	54	46	0.16	0.08	0.5	1	1	2	1	2	1	2	1	-1.5859
sp|P27348|1433T_HU	YWHAQ	14-3-3 protein theta OS=Homo sapiens GN=YWHAQ PE=1 SV=1	28032	258	636	74	72	0.4	0.2	0.5	2	1	3	1	3	1	3	1	-2.07165
tr|A1L196|A1L196_HU	tr|A1L196|A1L196_HU	Putative uncharacterized protein (Fragment) OS=Homo sapiens PE=2 SV=1	16876	25	175	64	286	3.07	1.49	0.48534	2	8	3	10	4	13	4	15	0.737543
sp|Q05639|EF1A2_HU	EEF1A2	Elongation factor 1-alpha 2 OS=Homo sapiens GN=EEF1A2 PE=1 SV=1	50780	70	543	238	88	1.14	0.55	0.48246	8	2	9	7	12	7	14	10	-1.58807
tr|Q0IIN1|Q0IIN1_HU	KRT77	Keratin 77 OS=Homo sapiens GN=KRT77 PE=2 SV=1	62050	76	405	264	129	0.23	0.11	0.47826	10	3	10	3	10	4	12	5	-2.07338
tr|A0MNP2|A0MNP2_	WDR57	CDW11/WDR57 OS=Homo sapiens GN=WDR57 PE=2 SV=1	39742	413	1179	42	26	0.17	0.08	0.47059	1	0	2	1	2	2	3	2	-0.84875
tr|B2R4P2|B2R4P2_H	tr|B2R4P2|B2R4P2_HU	cDNA, FLJ92164, highly similar to Homo sapiens peroxiredoxin 1 (PRDX1), mRNA OS=Hor	22414	481	1288	36	19	0.32	0.15	0.46875	0	0	2	1	2	0	2	1	-3.17104
tr|H0YDE4|H0YDE4_H	PRMT1	Protein arginine N-methyltransferase 1 (Fragment) OS=Homo sapiens GN=PRMT1 PE=4 S	27345	437	1008	39	36	0.26	0.12	0.46154	1	0	2	1	2	1	2	1	-1.5859
tr|Q53HE2|Q53HE2_H	tr|Q53HE2|Q53HE2_HU	Triosephosphate isomerase (Fragment) OS=Homo sapiens PE=2 SV=1	26981	267	1093	71	31	0.26	0.12	0.46154	1	0	2	1	2	1	2	1	-1.5859
tr|B1AKJ5|B1AKJ5_HU	NRD1	Nardilysin OS=Homo sapiens GN=NRD1 PE=3 SV=1	140352	225	1009	92	36	0.11	0.05	0.45455	2	1	2	2	2	3	2	4	-0.36315
tr|H0Y5G9|H0Y5G9_H	NRD1	Nardilysin (Fragment) OS=Homo sapiens GN=NRD1 PE=3 SV=1	62196	225	935	92	40	0.11	0.05	0.45455	2	1	2	1	2	2	2	2	-0.84875
tr|B3KMI9|B3KMI9_H	tr|B3KMI9|B3KMI9_HU	cDNA FLJ11140 fis, clone PLACE1006488, highly similar to Signal recognition particle 68 k	41632	180	872	119	44	0.18	0.08	0.44444	2	1	2	1	2	3	3	3	-0.36315
tr|E7EN38|E7EN38_H	RAD50	DNA repair protein RAD50 (Fragment) OS=Homo sapiens GN=RAD50 PE=2 SV=1	77138	563	1059	29	33	0.09	0.04	0.44444	0	0	2	1	1	1	2	1	-0.84861
tr|B2RD24|B2RD24_H	tr|B2RD24|B2RD24_HU	cDNA, FLJ96424 OS=Homo sapiens PE=2 SV=1	80216	398	944	43	39	0.09	0.04	0.44444	1	1	1	1	1	1	1	3	-0.84861
sp|P11498|PYC_HUM	PC	Pyruvate carboxylase, mitochondrial OS=Homo sapiens GN=PC PE=1 SV=2	130293	10	55	1295	692	1.76	0.77	0.4375	40	17	52	23	56	24	67	27	-2.06788
sp|P26232|CTNA2_HU	CTNNA2	Catenin alpha-2 OS=Homo sapiens GN=CTNNA2 PE=1 SV=5	106045	389	777	31	53	0.07	0.03	0.42857	0	1	1	1	1	1	1	2	-0.84861
tr|J3QQX0|J3QQX0_H	GPS1	COP9 signalosome complex subunit 1 (Fragment) OS=Homo sapiens GN=GPS1 PE=4 SV=1	8703	636	609	22	73	0.4	0.17	0.425	0	1	1	2	1	1	1	2	-0.84861
sp|Q9NXW2|DJB12_H	DNAJB12	DnaJ homolog subfamily B member 12 OS=Homo sapiens GN=DNAJB12 PE=1 SV=4	42021	124	453	174	112	0.57	0.23	0.40351	5	2	7	4	9	4	9	4	-1.92867
tr|E9PIM9|E9PIM9_H	RNH1	Ribonuclease inhibitor (Fragment) OS=Homo sapiens GN=RNH1 PE=2 SV=1	22362	431	799	40	50	0.15	0.06	0.4	0	1	1	1	1	1	1	1	-0.84861
sp|P54652|HSP72_HU	HSPA2	Heat shock-related 70 kDa protein 2 OS=Homo sapiens GN=HSPA2 PE=1 SV=1	70263	51	132	196	353	1.45	0.58	0.4	6	9	7	11	8	12	8	12	-0.29251
tr|K7EIG1|K7EIG1_HU	CLUH	Clustered mitochondria protein homolog OS=Homo sapiens GN=CLUH PE=3 SV=1	140903	438	668	39	67	0.05	0.02	0.4	1	1	2	1	2	1	2	3	-1.5859
tr|H0Y9H1|H0Y9H1_H	CANX	Calnexin (Fragment) OS=Homo sapiens GN=CANX PE=3 SV=1	21748	352	1037	49	34	0.39	0.15	0.38462	1	0	2	1	2	1	3	2	-1.5859
tr|Q96HI4|Q96HI4_HU	NMT1	Glycylpeptide N-tetradecanoyltransferase (Fragment) OS=Homo sapiens GN=NMT1 PE=2	56910	350	436	50	116	0.49	0.18	0.36735	1	2	2	3	2	3	2	3	-0.36315
tr|D3DVQ1|D3DVQ1_	LETM1	Leucine zipper-EF-hand containing transmembrane protein 1, isoform CRA_a OS=Homo s	63730	463	764	37	55	0.11	0.04	0.36364	1	1	2	1	2	2	4	2	-0.84875
sp|Q04695|K1C17_HU	KRT17	Keratin, type I cytoskeletal 17 OS=Homo sapiens GN=KRT17 PE=1 SV=2	48361	24	138	683	344	2.99	1.07	0.35786	19	11	27	13	32	15	35	16	-1.92434
tr|A8K7A4|A8K7A4_H	tr|A8K7A4|A8K7A4_HU	cDNA FLJ76904, highly similar to Homo sapiens methionine adenosyltransferase II, beta (	36692	280	1064	68	33	0.37	0.13	0.35135	1	0	1	1	1	1	1	1	-0.84861
tr|F8VQP2|F8VQP2_H	ATXN2	Ataxin-2 OS=Homo sapiens GN=ATXN2 PE=2 SV=1	117012	157	483	135	103	0.21	0.07	0.33333	4	2	7	2	10	2	10	2	-2.92174
tr|B3KPU1|B3KPU1_H	GNB2	Guanine nucleotide-binding protein G(I)/G(S)/G(T) subunit beta-2 OS=Homo sapiens GN=	26598	194	559	111	0	0.27	0.09	0.33333	1	0	2	0	2	1	2	1	-1.5859
tr|Q53FM3|Q53FM3_H	tr|Q53FM3|Q53FM3_H	ATPase, H+/K+ exchanging, alpha polypeptide variant (Fragment) OS=Homo sapiens PE=2	115678	324	1030	33	34	0.09	0.03	0.33333	1	0	2	1	6	2	9	4	-2.22856
tr|Q14666|Q14666_H	tr|Q14666|Q14666_HU	Radiated keratinocyte mRNA 266 (keratin-related protein) (Fragment) OS=Homo sapiens	30933	24	108	451	310	2.99	0.96	0.32107	12	8	17	10	20	11	21	12	-1.68697
tr|D0PNI1|D0PNI1_HU	YWHAZ	Tyrosine 3-monooxygenase/tryptophan 5-monooxygenase activation protein zeta polype	27899	497	1100	34	30	0.38	0.12	0.31579	0	0	2	1	2	1	2	1	-1.5859
sp|P21796|VDAC1_HU	VDAC1	Voltage-dependent anion-selective channel protein 1 OS=Homo sapiens GN=VDAC1 PE=1	30868	250	1129	78	29	0.36	0.11	0.30556	1	0	3	1	4	1	4	2	-2.43454
sp|O60884|DNJA2_HU	DNAJA2	DnaJ homolog subfamily A member 2 OS=Homo sapiens GN=DNAJA2 PE=1 SV=1	46344	611	1276	24	20	0.23	0.07	0.30435	0	0	1	1	1	1	1	1	-0.84861
tr|D3DTL4|D3DTL4_H	ENO3	Enolase OS=Homo sapiens GN=ENO3 PE=2 SV=1	37167	40	125	112	300	1.39	0.4	0.28777	2	5	2	5	2	6	4	6	0.530479
tr|B3KV39|B3KV39_H	tr|B3KV39|B3KV39_HU	cDNA FLJ16099 fis, clone TESTI2008278, highly similar to Homo sapiens forkhead box K1	50383	188	1111	115	30	0.07	0.02	0.28571	1	0	1	1	1	1	2	1	-0.84861
tr|B4DFM5|B4DFM5_	tr|B4DFM5|B4DFM5_H	cDNA FLJ51779, highly similar to Vacuolar ATP synthase subunit B, brain isoform (EC 3.6.	43446	287	560	64	84	0.65	0.18	0.27692	1	3	1	3	1	3	1	3	0.374143
sp|Q58FG0|HS905_HU	HSP90AA5P	Putative heat shock protein HSP 90-alpha A5 OS=Homo sapiens GN=HSP90AA5P PE=1 SV	38942	46	354	36	146	1.05	0.28	0.26667	0	3	2	7	2	7	2	10	0.73711
tr|B3KMS0|B3KMS0_	tr|B3KMS0|B3KMS0_H	cDNA FLJ12449 fis, clone NT2RM1000314, highly similar to Condensin complex subunit 1	158464	494	514	34	97	0.15	0.04	0.26667	0	2	2	2	2	2	2	2	-0.84875
tr|E9PHF7|E9PHF7_HU	MCCC1	Methylcrotonoyl-CoA carboxylase subunit alpha, mitochondrial OS=Homo sapiens GN=M	68745	102	812	214	49	0.83	0.22	0.26506	7	1	13	3	11	3	16	3	-2.5677
tr|B4DRG9|B4DRG9_	tr|B4DRG9|B4DRG9_H	cDNA FLJ51951, highly similar to Squamous cell carcinoma antigen recognized by T-cells	74870	153	898	82	42	0.16	0.04	0.25	2	1	2	1	4	1	4	1	-2.43454
tr|H6VRF8|H6VRF8_H	KRT1	Keratin 1 OS=Homo sapiens GN=KRT1 PE=3 SV=1	66184	6	86	2032	503	5.01	1.17	0.23353	55	15	64	19	69	20	76	22	-2.62879
tr|H6VRG2|H6VRG2_	KRT1	Keratin 1 OS=Homo sapiens GN=KRT1 PE=3 SV=1	66197	7	94	2027	468	5.01	1.17	0.23353	55	14	65	18	70	19	77	21	-2.72206
tr|F5H0C8|F5H0C8_H	ENO2	Enolase OS=Homo sapiens GN=ENO2 PE=2 SV=1	35026	40	270	112	196	1.39	0.31	0.22302	2	3	2	4	2	6	4	6	0.530479
tr|C9J6P4|C9J6P4_HU	ZC3HAV1	Zinc finger CCCH-type antiviral protein 1 OS=Homo sapiens GN=ZC3HAV1 PE=2 SV=1	115838	224	1216	92	24	0.14	0.03	0.21429	1	0	2	1	2	1	2	1	-1.5859
tr|B2R8Y4|B2R8Y4_H	tr|B2R8Y4|B2R8Y4_HU	cDNA, FLJ94117, highly similar to Homo sapiens actinin, alpha 3 (ACTN3), mRNA OS=Hon	103871	19	143	99	339	2.11	0.45	0.21327	3	12	3	14	3	16	4	17	1.390485
sp|Q58FG1|HS904_HU	HSP90AA4P	Putative heat shock protein HSP 90-alpha A4 OS=Homo sapiens GN=HSP90AA4P PE=5 SV	47796	46	333	103	158	1.05	0.22	0.20952	3	3	4	4	3	5	5	7	-0.19646
tr|E7ERA5|E7ERA5_H	MYH10	Myosin-10 (Fragment) OS=Homo sapiens GN=MYH10 PE=2 SV=1	21768	17	255	73	34	0.9	0.18	0.2	1	0	1	2	1	3	1	3	0.374143
sp|P16615|AT2A2_HU	ATP2A2	Sarcoplasmic/endoplasmic reticulum calcium ATPase 2 OS=Homo sapiens GN=ATP2A2 P	116336	149	536	145	91	0.32	0.06	0.1875	4	2	10	2	12	2	13	2	-3.17393
sp|O60518|RNBP6_HU	RANBP6	Ran-binding protein 6 OS=Homo sapiens GN=RANBP6 PE=1 SV=2	126173	85	1122	36	30	0.23	0.04	0.17391	1	0	1	1	2	3	2	4	-0.36315
sp|P13645|K1C10_HU	KRT10	Keratin, type I cytoskeletal 10 OS=Homo sapiens GN=KRT10 PE=1 SV=6	59020	12	154	1226	321	4.67	0.72	0.15418	34	6	42	10	47	12	54	14	-2.78762
tr|G3XAP5|G3XAP5_H	LDHC	L-lactate dehydrogenase OS=Homo sapiens GN=LDHC PE=2 SV=1	26552	39	965	67	38	1.81	0.27	0.14917	1	1	1	2	1	1	1	2	-0.84861
sp|Q14651|PLSI_HUM	PLS1	Plastin-1 OS=Homo sapiens GN=PLS1 PE=1 SV=2	70608	282	629	57	73	0.34	0.05	0.14706	1	1	3	1	3	3	3	5	-0.8489
sp|P08779|K1C16_HU	KRT16	Keratin, type I cytoskeletal 16 OS=Homo sapiens GN=KRT16 PE=1 SV=4	51578	62	667	342	67	1.38	0.2	0.14493	14	2	17	3	21	4	21	5	-3.11091
tr|K0J110|K0J110_HU	mcdrh	Malignant cell derived RNA helicase OS=Homo sapiens GN=mcdrh PE=2 SV=1	82499	148	838	61	47	0.34	0.04	0.11765	2	1	2	1	2	1	2	2	-1.5859
tr|K7ERE3|K7ERE3_HU	KRT13	Keratin, type I cytoskeletal 13 OS=Homo sapiens GN=KRT13 PE=3 SV=1	45574	88	1192	234	25	0.63	0.07	0.11111	9	0	10	1	11	1	12	3	-3.79045
tr|K4ENJ5|K4ENJ5_HU	tr|K4ENJ5|K4ENJ5_HU	Beta actin (Fragment) OS=Homo sapiens PE=4 SV=1	11129	1	104	103	431	31.43	2.87	0.09131	3	11	3	14	6	17	7	20	0.581496
tr|B2ZZ90|B2ZZ90_HU	ACACA	Acetyl-Coenzyme A carboxylase alpha OS=Homo sapiens GN=ACACA PE=2 SV=1	267095	31	911	628	41	0.44	0.04	0.09091	25	0	30	3	38	3	46	4	-4.31973
sp|Q58FF6|H90B4_HU	HSP90AB4P	Putative heat shock protein HSP 90-beta 4 OS=Homo sapiens GN=HSP90AB4P PE=5 SV=1	58855	32	489	77	101	1.24	0.11	0.08871	3	3	5	3	3	4	5	5	-0.48615
tr|F8VP67|F8VP67_HU	KRT8	Keratin, type II cytoskeletal 8 (Fragment) OS=Homo sapiens GN=KRT8 PE=2 SV=1	13580	6	823	50	48	5.01	0.39	0.07784	2	1	3	1	5	1	5	1	-2.72438
sp|P35908|K22E_HUN	KRT2	Keratin, type II cytoskeletal 2 epidermal OS=Homo sapiens GN=KRT2 PE=1 SV=2	65678	11	191	1255	263	4.79	0.28	0.05846	41	6	48	6	49	7	57	11	-3.58564
sp|P35527|K1C9_HUN	KRT9	Keratin, type I cytoskeletal 9 OS=Homo sapiens GN=KRT9 PE=1 SV=3	62255	14	568	1044	83	3.01	0.17	0.05648	25	2	36	3	40	4	44	4	-4.03069
sp|P13647|K2C5_HUN	KRT5	Keratin, type II cytoskeletal 5 OS=Homo sapiens GN=KRT5 PE=1 SV=3	62568	58	762	378	55	0.95	0.05	0.05263	13	1	15	1	20	2	24	2	-3.89023
tr|Q562V5|Q562V5_H	ACT	Actin-like protein (Fragment) OS=Homo sapiens GN=ACT PE=2 SV=1	11481	1	376	136	141	31.43	1.2	0.03818	6	7	6	9	7	11	8	12	-0.23208
sp|Q8N1A0|KT222_HU	KRT222	Keratin-like protein KRT222 OS=Homo sapiens GN=KRT222 PE=2 SV=1	34308	24	1193	47	25	2.99	0.1	0.03344	1	0	1	1	1	1	1	2	-0.84861
sp|O95678|K2C75_HU	KRT75	Keratin, type II cytoskeletal 75 OS=Homo sapiens GN=KRT75 PE=1 SV=2	59809	11	763	94	55	4.79	0.05	0.01044	2	1	3	1	3	1	5	2	-2.07165
sp|Q9H254|SPTN4_HU	SPTBN4	Spectrin beta chain, non-erythrocytic 4 OS=Homo sapiens GN=SPTBN4 PE=1 SV=2	290005	19	553	29	85	2.11	0.02	0.00948	0	2	1	4	2	5	2	7	0.28929
tr|D6RAW4|D6RAW4_	IMMT	Mitochondrial inner membrane protein OS=Homo sapiens GN=IMMT PE=2 SV=1	15918	712	0	0	0	0.04	0	0	0	0	0	0	0	0	1	0	-0.84847
sp|P54826|GAS1_HUM	GAS1	Growth arrest-specific protein 1 OS=Homo sapiens GN=GAS1 PE=1 SV=2	36867	708	0	17	0	0.09	0	0	0	0	1	0	0	0	1	0	-0.84847
tr|H0Y4R4|H0Y4R4_H	TOR1AIP1	Torsin-1A-interacting protein 1 (Fragment) OS=Homo sapiens GN=TOR1AIP1 PE=4 SV=1	33844	707	0	17	0	0.1	0	0	0	0	1	0	0	0	1	0	-0.84847
tr|J3KMW7|J3KMW7_	UBR5	E3 ubiquitin-protein ligase UBR5 OS=Homo sapiens GN=UBR5 PE=4 SV=1	312224	706	0	18	0	0.01	0	0	0	0	1	0	0	0	1	0	-0.84847
tr|Q2NLA0|Q2NLA0_H	COPG2	Coatomer subunit gamma OS=Homo sapiens GN=COPG2 PE=2 SV=1	82400	705	0	18	0	0.04	0	0	0	0	1	0	0	0	1	0	-0.84847
tr|B0UZL4|B0UZL4_HU	BAT3	HLA-B associated transcript 3 (Fragment) OS=Homo sapiens GN=BAT3 PE=2 SV=1	15422	704	0	18	0	0.03	0	0	0	0	1	0	0	0	1	0	-0.84847
tr|B4DY47|B4DY47_H	PHB	Prohibitin OS=Homo sapiens GN=PHB PE=2 SV=1	17064	699	0	18	0	0.2	0	0	0	0	1	0	0	0	1	0	-0.84847
tr|B3KMM3|B3KMM3	tr|B3KMM3|B3KMM3_	cDNA FLJ11471 fis, clone HEMBA1001675, highly similar to Mus musculus GTPase activat	19935	697	0	0	0	0.02	0	0	0	0	0	0	0	0	1	0	-0.84847
tr|Q5T097|Q5T097_H	UTRN	Utrophin OS=Homo sapiens GN=UTRN PE=4 SV=1	114423	696	0	19	0	0.03	0	0	0	0	1	0	0	0	1	0	-0.84847
tr|H0Y304|H0Y304_H	DMD	Dystrophin (Fragment) OS=Homo sapiens GN=DMD PE=4 SV=1	160176	695	0	19	0	0.02	0	0	0	0	1	0	0	0	1	0	-0.84847
tr|H0YE81|H0YE81_H	NDUFV1	NADH dehydrogenase [ubiquinone] flavoprotein 1, mitochondrial (Fragment) OS=Homo	14494	694	0	19	0	0.23	0	0	0	0	1	0	0	0	1	0	-0.84847
tr|Q5VZU9|Q5VZU9_	TPP2	Tripeptidyl-peptidase 2 OS=Homo sapiens GN=TPP2 PE=4 SV=1	141160	693	0	19	0	0.02	0	0	0	0	1	0	0	0	1	0	-0.84847
tr|B3KND7|B3KND7_	tr|B3KND7|B3KND7_H	cDNA FLJ14378 fis, clone HEMBA1001804, highly similar to Zinc finger protein 281 (Fragr	58221	692	0	19	0	0.06	0	0	0	0	1	0	0	0	1	0	-0.84847
tr|H7C4X2|H7C4X2_H	PCMT1	Protein-L-isoaspartate(D-aspartate) O-methyltransferase (Fragment) OS=Homo sapiens (	7342	691	0	19	0	0.48	0	0	0	0	1	0	0	0	2	0	-0.84847
tr|D3DUL0|D3DUL0_H	ASCIZ	ATM/ATR-Substrate Chk2-Interacting Zn2+-finger protein, isoform CRA_b OS=Homo sapi	79474	690	0	19	0	0.04	0	0	0	0	1	0	0	0	1	0	-0.84847
tr|G3V448|G3V448_H	TMX1	Thioredoxin-related transmembrane protein 1 OS=Homo sapiens GN=TMX1 PE=2 SV=1	12018	689	0	19	0	0.29	0	0	0	0	1	0	0	0	1	0	-0.84847
tr|B4DWB5|B4DWB5_	tr|B4DWB5|B4DWB5_	cDNA FLJ53931, highly similar to Bifunctional 3~-phosphoadenosine5~-phosphosulfate s	70558	688	0	0	0	0.05	0	0	0	0	0	0	0	0	3	0	-0.84847
tr|E9PQG7|E9PQG7_H	MLL	MLL cleavage product C180 OS=Homo sapiens GN=MLL PE=2 SV=1	436345	687	0	19	0	0.01	0	0	0	0	1	0	0	0	1	0	-0.84847
tr|G5E9Q3|G5E9Q3_H	TSEN2	tRNA splicing endonuclease 2 homolog (S. cerevisiae), isoform CRA_b OS=Homo sapiens	45959	686	0	19	0	0.07	0	0	0	0	1	0	0	0	1	0	-0.84847
sp|Q9Y2A7|NCKP1_HU	NCKAP1	Nck-associated protein 1 OS=Homo sapiens GN=NCKAP1 PE=1 SV=1	130018	685	0	19	0	0.03	0	0	0	0	1	0	0	0	1	0	-0.84847
tr|Q9UH66|Q9UH66_	tr|Q9UH66|Q9UH66_H	7h3 protein (Fragment) OS=Homo sapiens PE=2 SV=1	49963	684	0	19	0	0.07	0	0	0	0	1	0	0	0	2	0	-0.84847
tr|B4E0B2|B4E0B2_HU	tr|B4E0B2|B4E0B2_HU	cDNA FLJ51426, highly similar to Adapter-related protein complex 3 beta-1 subunit OS=H	64073	682	0	20	0	0.04	0	0	0	0	1	0	0	0	1	0	-0.84847
tr|J3KN33|J3KN33_HU	CNTLN	Centlein OS=Homo sapiens GN=CNTLN PE=4 SV=1	162069	680	0	20	0	0.02	0	0	0	0	1	0	0	0	2	0	-0.84847
tr|G0Z8H5|G0Z8H5_H	HLA-A	MHC class I antigen (Fragment) OS=Homo sapiens GN=HLA-A PE=2 SV=1	10370	639	0	0	0	0.16	0	0	0	0	0	0	0	0	1	0	-0.84847
tr|Q5SWC8|Q5SWC8_	HP1BP3	Heterochromatin protein 1-binding protein 3 (Fragment) OS=Homo sapiens GN=HP1BP3	31566	631	0	0	0	0.25	0	0	0	0	0	0	0	0	1	0	-0.84847
tr|J3KTB7|J3KTB7_HU	TOP2A	DNA topoisomerase 2-alpha (Fragment) OS=Homo sapiens GN=TOP2A PE=4 SV=1	17149	606	0	0	0	0.02	0	0	0	0	0	0	0	0	1	0	-0.84847
tr|Q59F49|Q59F49_H	tr|Q59F49|Q59F49_HU	Minichromosome maintenance deficient protein 5 variant (Fragment) OS=Homo sapiens	46288	562	0	0	0	0.04	0	0	0	0	0	0	0	0	1	0	-0.84847
tr|H7C1T1|H7C1T1_H	Sep-02	Septin-2 (Fragment) OS=Homo sapiens GN=SEPT2 PE=4 SV=1	3094	543	0	0	0	0.09	0	0	0	0	0	0	0	0	1	0	-0.84847
tr|H0YDS9|H0YDS9_H	NASP	Nuclear autoantigenic sperm protein (Fragment) OS=Homo sapiens GN=NASP PE=4 SV=1	18974	542	0	0	0	0.1	0	0	0	0	0	0	0	0	1	0	-0.84847
tr|I3L3P5|I3L3P5_HUI	P4HB	Protein disulfide-isomerase (Fragment) OS=Homo sapiens GN=P4HB PE=4 SV=1	16923	536	0	19	0	0.27	0	0	0	0	1	0	0	0	1	0	-0.84847
tr|D6RF95|D6RF95_H	ACSL4	Long-chain-fatty-acid--CoA ligase 4 (Fragment) OS=Homo sapiens GN=ACSL4 PE=2 SV=1	17345	529	0	0	0	0.04	0	0	0	0	0	0	0	0	1	0	-0.84847
tr|A8K482|A8K482_H	tr|A8K482|A8K482_HU	Aspartate aminotransferase OS=Homo sapiens PE=2 SV=1	47885	522	0	0	0	0.08	0	0	0	0	0	0	0	0	1	0	-0.84847
tr|H7BYY8|H7BYY8_H	PES1	Pescadillo homolog OS=Homo sapiens GN=PES1 PE=4 SV=1	1501	488	0	0	0	0.05	0	0	0	0	0	0	0	0	1	0	-0.84847
tr|E5RHM4|E5RHM4_	DCAF13	DDB1- and CUL4-associated factor 13 OS=Homo sapiens GN=DCAF13 PE=2 SV=1	20862	435	0	0	0	0.2	0	0	0	0	0	0	0	0	1	0	-0.84847
tr|H0Y386|H0Y386_H	PABPC1L	Polyadenylate-binding protein 1-like (Fragment) OS=Homo sapiens GN=PABPC1L PE=4 S\	16787	404	0	0	0	0.1	0	0	0	0	0	0	0	0	1	0	-0.84847
tr|A8K171|A8K171_H	tr|A8K171|A8K171_HU	cDNA FLJ77253, highly similar to Homo sapiens nuclear receptor interacting protein 1 (N	127609	401	0	0	0	0.01	0	0	0	0	0	0	0	0	1	0	-0.84847
tr|H0Y8W6|H0Y8W6_	PDLIM7	PDZ and LIM domain protein 7 (Fragment) OS=Homo sapiens GN=PDLIM7 PE=4 SV=1	12898	396	0	0	0	0.13	0	0	0	0	0	0	0	0	1	0	-0.84847
tr|B7Z6J5|B7Z6J5_HU	tr|B7Z6J5|B7Z6J5_HUN	cDNA FLJ56858, highly similar to Succinate dehydrogenase (ubiquinone) flavoprotein sub	28776	393	0	0	0	0.1	0	0	0	0	0	0	0	0	1	0	-0.84847
tr|Q1XBU7|Q1XBU7_	tr|Q1XBU7|Q1XBU7_H	Aging-associated protein 14a OS=Homo sapiens PE=2 SV=1	14000	382	0	0	0	0.09	0	0	0	0	0	0	0	0	1	0	-0.84847
tr|F8WAT3|F8WAT3_	EIF2A	Eukaryotic translation initiation factor 2A OS=Homo sapiens GN=EIF2A PE=2 SV=1	7970	206	0	18	0	0.32	0	0	0	0	1	0	0	0	1	0	-0.84847
tr|E9PQ69|E9PQ69_H	GANAB	Neutral alpha-glucosidase AB OS=Homo sapiens GN=GANAB PE=2 SV=1	8560	47	0	0	0	0.62	0	0	0	0	0	0	0	0	1	0	-0.84847
tr|K7ELP7|K7ELP7_HU	KRT17	Keratin, type I cytoskeletal 17 (Fragment) OS=Homo sapiens GN=KRT17 PE=4 SV=1	5508	24	0	20	0	2.99	0	0	0	0	1	0	0	0	1	0	-0.84847
tr|Q8WUM5|Q8WUM	GEMIN4	Gem (Nuclear organelle) associated protein 4 OS=Homo sapiens GN=GEMIN4 PE=2 SV=1	121732	709	0	17	0	0.03	0	0	0	0	1	0	1	0	2	0	-2.43375
sp|O95159|ZFPL1_HU	ZFPL1	Zinc finger protein-like 1 OS=Homo sapiens GN=ZFPL1 PE=1 SV=2	34833	678	0	20	0	0.1	0	0	0	0	1	0	1	0	1	0	-2.43375
tr|B4DE48|B4DE48_H	tr|B4DE48|B4DE48_HU	cDNA FLJ53824, highly similar to Sorting nexin-17 OS=Homo sapiens PE=2 SV=1	50009	677	0	20	0	0.07	0	0	0	0	1	0	1	0	1	0	-2.43375
sp|P55212|CASP6_HU	CASP6	Caspase-6 OS=Homo sapiens GN=CASP6 PE=1 SV=2	33859	674	0	20	0	0.1	0	0	0	0	1	0	1	0	1	0	-2.43375
tr|Q52NV4|Q52NV4_	HRS	Histidyl-tRNA synthetase OS=Homo sapiens GN=HRS PE=2 SV=1	57930	672	0	20	0	0.07	0	0	0	0	1	0	1	0	2	0	-2.43375
tr|H0YJL6|H0YJL6_HU	EVL	Ena/VASP-like protein (Fragment) OS=Homo sapiens GN=EVL PE=4 SV=1	10347	671	0	20	0	0.34	0	0	0	0	1	0	1	0	1	0	-2.43375
tr|A0AVG7|A0AVG7_	BCAS1	Breast carcinoma amplified sequence 1 OS=Homo sapiens GN=BCAS1 PE=2 SV=1	61929	670	0	20	0	0.05	0	0	0	0	1	0	1	0	1	0	-2.43375
tr|B4DK80|B4DK80_H	TOP3A	DNA topoisomerase OS=Homo sapiens GN=TOP3A PE=2 SV=1	103082	668	0	20	0	0.03	0	0	0	0	1	0	1	0	1	0	-2.43375
tr|J3KN69|J3KN69_HU	NCEH1	Arylacetamide deacetylase-like 1 OS=Homo sapiens GN=NCEH1 PE=4 SV=1	49431	663	0	21	0	0.07	0	0	0	0	1	0	1	0	1	0	-2.43375
tr|B3KRG8|B3KRG8_H	tr|B3KRG8|B3KRG8_HU	Lipase OS=Homo sapiens PE=2 SV=1	45736	662	0	21	0	0.07	0	0	0	0	1	0	1	0	1	0	-2.43375
tr|F1T0A5|F1T0A5_HU	PRPF31	PRP31 pre-mRNA processing factor 31 homolog (Yeast), isoform CRA_a OS=Homo sapien	55649	661	0	21	0	0.06	0	0	0	0	1	0	1	0	2	0	-2.43375
tr|C9J935|C9J935_HU	SETD9	SET domain-containing protein 9 (Fragment) OS=Homo sapiens GN=SETD9 PE=2 SV=1	5515	660	0	21	0	0.65	0	0	0	0	1	0	1	0	1	0	-2.43375
tr|B4DQ49|B4DQ49_	tr|B4DQ49|B4DQ49_H	cDNA FLJ54374, highly similar to Homo sapiens keratin 78 (KRT78), mRNA OS=Homo sap	31874	653	0	22	0	0.1	0	0	0	0	1	0	1	0	3	0	-2.43375
tr|E7ET15|E7ET15_HU	U2SURP	U2 snRNP-associated SURP motif-containing protein OS=Homo sapiens GN=U2SURP PE=	118631	647	0	22	0	0.03	0	0	0	0	1	0	1	0	2	0	-2.43375
tr|B1ALA9|B1ALA9_H	PRPS1	Phosphoribosyl pyrophosphate synthetase 1 OS=Homo sapiens GN=PRPS1 PE=3 SV=1	24468	638	0	22	0	0.14	0	0	0	0	1	0	1	0	2	0	-2.43375
tr|B3KPR5|B3KPR5_H	tr|B3KPR5|B3KPR5_HU	cDNA FLJ32094 fis, clone OCBBF2000986, highly similar to Homo sapiens elongation fact	97046	633	0	22	0	0.03	0	0	0	0	1	0	1	0	1	0	-2.43375
sp|Q9Y5P6|GMPPB_H	GMPPB	Mannose-1-phosphate guanyltransferase beta OS=Homo sapiens GN=GMPPB PE=1 SV=2	40379	629	0	23	0	0.08	0	0	0	0	1	0	1	0	1	0	-2.43375
tr|K7EJ37|K7EJ37_HU	EXOSC10	Exosome component 10 OS=Homo sapiens GN=EXOSC10 PE=4 SV=1	13869	627	0	23	0	0.05	0	0	0	0	1	0	1	0	1	0	-2.43375
tr|K7EPB5|K7EPB5_HU	ELAVL3	ELAV-like protein 3 (Fragment) OS=Homo sapiens GN=ELAVL3 PE=4 SV=1	9728	625	0	23	0	0.36	0	0	0	0	1	0	1	0	1	0	-2.43375
tr|B4DN13|B4DN13_	tr|B4DN13|B4DN13_H	cDNA FLJ59136, highly similar to Transmembrane 9 superfamily protein member 3 OS=H	29629	624	0	23	0	0.11	0	0	0	0	1	0	1	0	1	0	-2.43375
tr|B0QXZ5|B0QXZ5_H	DNAL4	Dynein light chain 4, axonemal OS=Homo sapiens GN=DNAL4 PE=4 SV=1	7786	621	0	24	0	0.47	0	0	0	0	1	0	1	0	1	0	-2.43375
tr|B4DU06|B4DU06_	tr|B4DU06|B4DU06_H	Guanylate cyclase OS=Homo sapiens PE=2 SV=1	91756	620	0	24	0	0.04	0	0	0	0	1	0	1	0	1	0	-2.43375
tr|Q2TQ40|Q2TQ40_	TNFRSF17	B cell maturation antigen transcript variant 4 OS=Homo sapiens GN=TNFRSF17 PE=2 SV=	15460	618	0	24	0	0.22	0	0	0	0	1	0	1	0	1	0	-2.43375
tr|Q4VAN1|Q4VAN1_	BTN1A1	BTN1A1 protein OS=Homo sapiens GN=BTN1A1 PE=2 SV=1	55878	617	0	24	0	0.06	0	0	0	0	1	0	1	0	1	0	-2.43375
tr|B4DVB8|B4DVB8_H	ELAVL1	ELAV-like protein 1 OS=Homo sapiens GN=ELAVL1 PE=2 SV=1	39200	612	0	24	0	0.08	0	0	0	0	1	0	1	0	1	0	-2.43375
tr|H3BMW5|H3BMW	DNAJA2	DnaJ homolog subfamily A member 2 (Fragment) OS=Homo sapiens GN=DNAJA2 PE=4 S\	15134	611	0	24	0	0.23	0	0	0	0	1	0	1	0	1	0	-2.43375
tr|B4DG42|B4DG42_H	tr|B4DG42|B4DG42_H	cDNA FLJ53753, highly similar to Myeloid-associated differentiation marker OS=Homo sa	31889	609	0	25	0	0.1	0	0	0	0	1	0	1	0	1	0	-2.43375
tr|J3KSB9|J3KSB9_HU	tr|J3KSB9|J3KSB9_HUN	Uncharacterized protein (Fragment) OS=Homo sapiens PE=4 SV=1	8877	608	0	25	0	0.39	0	0	0	0	1	0	1	0	1	0	-2.43375
tr|J3KNP0|J3KNP0_HU	RAB11FIP1	Rab11 family-interacting protein 1 OS=Homo sapiens GN=RAB11FIP1 PE=4 SV=1	137482	607	0	25	0	0.02	0	0	0	0	1	0	1	0	2	0	-2.43375
tr|B2RAX6|B2RAX6_H	tr|B2RAX6|B2RAX6_HU	cDNA, FLJ95176, Homo sapiens CGI-48 protein (CGI-48), mRNA OS=Homo sapiens PE=2 S	58127	605	0	25	0	0.06	0	0	0	0	1	0	1	0	1	0	-2.43375
tr|B4DJE3|B4DJE3_HU	tr|B4DJE3|B4DJE3_HUI	cDNA FLJ52929, highly similar to Dolichyl-diphosphooligosaccharide--proteinglycosyltran	46620	604	0	26	0	0.07	0	0	0	0	1	0	1	0	2	0	-2.43375

tr|B4DNK3|B4DNK3_H	tr|B4DNK3|B4DNK3_H	cDNA FLJ52127, highly similar to Multisynthetase complex auxiliary component p43 OS=	29955	601	0	26	0	0.11	0	0	0	0	1	0	1	0	1	0	-2.43375
sp|Q0VD83|APOBR_H	APOBR	Apolipoprotein B receptor OS=Homo sapiens GN=APOBR PE=1 SV=1	115207	597	0	26	0	0.03	0	0	0	0	1	0	1	0	1	0	-2.43375
tr|Q5T8C6|Q5T8C6_H	CDC16	Cell division cycle protein 16 homolog OS=Homo sapiens GN=CDC16 PE=4 SV=1	55239	594	0	26	0	0.06	0	0	0	0	1	0	1	0	1	0	-2.43375
tr|B7Z4L9|B7Z4L9_HU	tr|B7Z4L9|B7Z4L9_HUI	cDNA FLJ55342, highly similar to Kelch-like protein 13 OS=Homo sapiens PE=2 SV=1	56884	593	0	26	0	0.06	0	0	0	0	1	0	1	0	1	0	-2.43375
tr|H0Y512|H0Y512_H	APMAP	Adipocyte plasma membrane-associated protein (Fragment) OS=Homo sapiens GN=APM	45662	592	0	27	0	0.07	0	0	0	0	1	0	1	0	1	0	-2.43375
tr|H3BS19|H3BS19_H	ZNF469	Zinc finger protein 469 OS=Homo sapiens GN=ZNF469 PE=4 SV=1	417347	591	0	27	0	0.01	0	0	0	0	1	0	1	0	1	0	-2.43375
tr|A6NE42|A6NE42_H	TGIF1	Homeobox protein TGIF1 OS=Homo sapiens GN=TGIF1 PE=3 SV=2	27983	586	0	28	0	0.12	0	0	0	0	1	0	1	0	1	0	-2.43375
tr|H0YCU9|H0YCU9_H	TAGLN	Transgelin (Fragment) OS=Homo sapiens GN=TAGLN PE=4 SV=1	16833	585	0	28	0	0.2	0	0	0	0	1	0	1	0	1	0	-2.43375
tr|J3KN32|J3KN32_HU	RCOR1	REST corepressor 1 OS=Homo sapiens GN=RCOR1 PE=4 SV=1	53409	583	0	28	0	0.06	0	0	0	0	1	0	1	0	1	0	-2.43375
sp|Q16322|KCA10_HU	KCNA10	Potassium voltage-gated channel subfamily A member 10 OS=Homo sapiens GN=KCNA1	58147	578	0	28	0	0.06	0	0	1	0	1	0	1	0	1	0	-2.43375
tr|B4DNG6|B4DNG6_	tr|B4DNG6|B4DNG6_H	Annexin OS=Homo sapiens PE=3 SV=1	12870	577	0	28	0	0.27	0	0	0	0	1	0	1	0	1	0	-2.43375
tr|B3KWR2|B3KWR2_	tr|B3KWR2|B3KWR2_H	cDNA FLJ43638 fis, clone SPLEN2039845, highly similar to IMPORTIN ALPHA-1 SUBUNIT	38087	573	0	22	0	0.11	0	0	0	0	1	0	1	0	1	0	-2.43375
tr|J3KQE5|J3KQE5_HU	RAN	GTP-binding nuclear protein Ran (Fragment) OS=Homo sapiens GN=RAN PE=4 SV=1	27027	571	0	29	0	0.12	0	0	0	0	1	0	1	0	1	0	-2.43375
tr|I3L361|I3L361_HUI	SGSM2	Small G protein-signaling modulator 2 (Fragment) OS=Homo sapiens GN=SGSM2 PE=4 S	1642	570	0	29	0	2.38	0	0	0	0	1	0	1	0	1	0	-2.43375
sp|Q6KB66|K2C80_HU	KRT80	Keratin, type II cytoskeletal 80 OS=Homo sapiens GN=KRT80 PE=1 SV=2	51007	564	0	29	0	0.06	0	0	0	0	1	0	1	0	1	0	-2.43375
tr|E7ESD9|E7ESD9_HU	RAD50	DNA repair protein RAD50 OS=Homo sapiens GN=RAD50 PE=2 SV=1	64414	563	0	28	0	0.09	0	0	0	0	1	0	1	0	1	0	-2.43375
tr|B4DS04|B4DS04_H	tr|B4DS04|B4DS04_HU	cDNA FLJ54142, highly similar to Protein transport protein Sec23B OS=Homo sapiens PE=	41720	558	0	27	0	0.08	0	0	0	0	1	0	1	0	1	0	-2.43375
tr|H3BRB3|H3BRB3_H	KIF22	Kinesin-like protein KIF22 OS=Homo sapiens GN=KIF22 PE=3 SV=1	67996	557	0	29	0	0.05	0	0	0	0	1	0	1	0	1	0	-2.43375
tr|B4DKT5|B4DKT5_H	tr|B4DKT5|B4DKT5_HU	cDNA FLJ53054, highly similar to Insulin-like growth factor 2 mRNA-binding protein 2 OS	33360	556	0	29	0	0.05	0	0	0	0	1	0	1	0	1	0	-2.43375
tr|H0YK49|H0YK49_H	ETFA	Electron transfer flavoprotein subunit alpha, mitochondrial OS=Homo sapiens GN=ETFA	24259	545	0	30	0	0.14	0	0	0	0	1	0	1	0	1	0	-2.43375
tr|K7EKM4|K7EKM4_	SRM	Spermidine synthase OS=Homo sapiens GN=SRM PE=4 SV=1	10680	540	0	30	0	0.32	0	0	0	0	1	0	1	0	1	0	-2.43375
sp|Q96PE2|ARHGH_H	ARHGEF17	Rho guanine nucleotide exchange factor 17 OS=Homo sapiens GN=ARHGEF17 PE=1 SV=1	223645	537	0	30	0	0.01	0	0	1	0	1	0	1	0	1	0	-2.43375
tr|H0Y7S3|H0Y7S3_HU	ATP2B2	Plasma membrane calcium-transporting ATPase 2 (Fragment) OS=Homo sapiens GN=AT	122272	528	0	31	0	0.03	0	0	0	0	1	0	1	0	3	0	-2.43375
tr|F2Z2J7|F2Z2J7_HUI	TTLL7	Tubulin polyglutamylase TTLL7 OS=Homo sapiens GN=TTLL7 PE=2 SV=1	55925	526	0	31	0	0.06	0	0	1	0	1	0	1	0	1	0	-2.43375
tr|B4E0D5|B4E0D5_H	tr|B4E0D5|B4E0D5_HU	cDNA FLJ54218, highly similar to Acidic leucine-rich nuclear phosphoprotein 32 family m	19540	525	0	31	0	0.17	0	0	0	0	1	0	1	0	1	0	-2.43375
tr|L0R4T3|L0R4T3_HU	ABCF2	Alternative protein ABCF2 OS=Homo sapiens GN=ABCF2 PE=4 SV=1	7359	524	0	31	0	0.48	0	0	1	0	1	0	1	0	1	0	-2.43375
tr|E9PNN6|E9PNN6_H	ALDH3A1	Aldehyde dehydrogenase, dimeric NADP-preferring OS=Homo sapiens GN=ALDH3A1 PE=	44518	519	0	32	0	0.07	0	0	0	0	1	0	1	0	1	0	-2.43375
tr|B4DUH7|B4DUH7_	tr|B4DUH7|B4DUH7_H	cDNA FLJ60162, highly similar to Chloride anion exchanger OS=Homo sapiens PE=2 SV=1	52632	518	0	32	0	0.06	0	0	1	0	1	0	1	0	1	0	-2.43375
tr|J3KRB3|J3KRB3_HU	RPL17	60S ribosomal protein L17 (Fragment) OS=Homo sapiens GN=RPL17 PE=3 SV=1	12356	517	0	32	0	0.28	0	0	0	0	1	0	1	0	1	0	-2.43375
tr|B2R4C0|B2R4C0_H	tr|B2R4C0|B2R4C0_HU	60S ribosomal protein L18a OS=Homo sapiens PE=2 SV=1	21024	514	0	32	0	0.16	0	0	0	0	1	0	1	0	2	0	-2.43375
tr|A0JLS9|A0JLS9_HUI	ZEB1	ZEB1 protein (Fragment) OS=Homo sapiens GN=ZEB1 PE=2 SV=1	118576	513	0	32	0	0.03	0	0	1	0	1	0	1	0	1	0	-2.43375
sp|Q7Z494|NPHP3_HU	NPHP3	Nephrocystin-3 OS=Homo sapiens GN=NPHP3 PE=1 SV=1	151967	510	0	32	0	0.02	0	0	1	0	1	0	1	0	1	0	-2.43375
tr|Q2NKW8|Q2NKW8	AHCYL1	Adenosylhomocysteinase (Fragment) OS=Homo sapiens GN=AHCYL1 PE=2 SV=1	57630	509	0	33	0	0.06	0	0	0	0	1	0	1	0	1	0	-2.43375
sp|Q9Y5B9|SP16H_HU	SUPT16H	FACT complex subunit SPT16 OS=Homo sapiens GN=SUPT16H PE=1 SV=1	120409	506	0	33	0	0.03	0	0	0	0	1	0	1	0	2	0	-2.43375
sp|Q8NFZ5|TNIP2_HU	TNIP2	TNFAIP3-interacting protein 2 OS=Homo sapiens GN=TNIP2 PE=1 SV=1	49240	502	0	33	0	0.07	0	0	1	0	1	0	1	0	2	0	-2.43375
sp|Q6PGP7|TTC37_HU	TTC37	Tetratricopeptide repeat protein 37 OS=Homo sapiens GN=TTC37 PE=1 SV=1	177485	498	0	34	0	0.02	0	0	1	0	1	0	1	0	1	0	-2.43375
tr|E9PI04|E9PI04_HUI	RIC8A	Synembryn-A (Fragment) OS=Homo sapiens GN=RIC8A PE=2 SV=1	16936	496	0	34	0	0.05	0	0	0	0	1	0	1	0	1	0	-2.43375
tr|C9JNW5|C9JNW5_	RPL24	60S ribosomal protein L24 OS=Homo sapiens GN=RPL24 PE=4 SV=1	17646	487	0	35	0	0.19	0	0	0	0	1	0	1	0	2	0	-2.43375
tr|Q68E00|Q68E00_H	DKFZp686G2045	Putative uncharacterized protein DKFZp686G2045 (Fragment) OS=Homo sapiens GN=DK	32787	485	0	36	0	0.1	0	0	0	0	1	0	1	0	1	0	-2.43375
tr|B7WPL0|B7WPL0_	RIC8B	Synembryn-B OS=Homo sapiens GN=RIC8B PE=2 SV=1	64252	483	0	36	0	0.05	0	0	1	0	1	0	1	0	1	0	-2.43375
tr|J3KN86|J3KN86_HU	RPS3	40S ribosomal protein S3 OS=Homo sapiens GN=RPS3 PE=3 SV=1	28640	480	0	36	0	0.12	0	0	1	0	1	0	1	0	4	0	-2.43375
tr|H0YIX9|H0YIX9_HU	R3HDM2	R3H domain-containing protein 2 (Fragment) OS=Homo sapiens GN=R3HDM2 PE=4 SV=1	31776	469	0	37	0	0.1	0	0	1	0	1	0	1	0	1	0	-2.43375
sp|Q6ZU64|CC108_HU	CCDC108	Coiled-coil domain-containing protein 108 OS=Homo sapiens GN=CCDC108 PE=2 SV=2	220247	465	0	37	0	0.01	0	0	1	0	1	0	1	0	1	0	-2.43375
tr|Q5SZ64|Q5SZ64_H	SF3B4	Splicing factor 3B subunit 4 (Fragment) OS=Homo sapiens GN=SF3B4 PE=4 SV=1	20733	464	0	37	0	0.07	0	0	1	0	1	0	1	0	1	0	-2.43375
tr|H7C1M0|H7C1M0_	FANCL	E3 ubiquitin-protein ligase FANCL (Fragment) OS=Homo sapiens GN=FANCL PE=4 SV=1	33067	461	0	37	0	0.1	0	0	1	0	1	0	1	0	1	0	-2.43375
sp|Q9H9F9|ARP5_HU	ACTR5	Actin-related protein 5 OS=Homo sapiens GN=ACTR5 PE=1 SV=2	68825	460	0	38	0	0.05	0	0	1	0	1	0	1	0	2	0	-2.43375
tr|B3KME2|B3KME2_	tr|B3KME2|B3KME2_H	cDNA FLJ10772 fis, clone NT2RP4000243, highly similar to Cartilage-associated protein O	47101	459	0	38	0	0.07	0	0	1	0	1	0	1	0	1	0	-2.43375
tr|H0Y9L5|H0Y9L5_HU	EXOSC9	Exosome complex component RRP45 (Fragment) OS=Homo sapiens GN=EXOSC9 PE=4 S	18940	457	0	38	0	0.18	0	0	1	0	1	0	1	0	1	0	-2.43375
tr|K7EID2|K7EID2_HU	ALG1	Chitobiosyldiphosphodolichol beta-mannosyltransferase (Fragment) OS=Homo sapiens G	19593	452	0	31	0	0.17	0	0	0	0	1	0	1	0	1	0	-2.43375
tr|B4DR70|B4DR70_H	tr|B4DR70|B4DR70_HU	cDNA FLJ58049, highly similar to RNA-binding protein FUS OS=Homo sapiens PE=2 SV=1	45013	450	0	38	0	0.07	0	0	1	0	1	0	1	0	1	0	-2.43375
tr|B7Z9C4|B7Z9C4_HU	CTPS1	CTP synthase 1 OS=Homo sapiens GN=CTPS1 PE=2 SV=1	35687	449	0	28	0	0.19	0	0	0	0	1	0	1	0	1	0	-2.43375
tr|K7EK06|K7EK06_HU	FARSA	Phenylalanine--tRNA ligase alpha subunit (Fragment) OS=Homo sapiens GN=FARSA PE=4	18547	447	0	38	0	0.06	0	0	1	0	1	0	1	0	1	0	-2.43375
tr|H0YCG1|H0YCG1_H	SF3B2	Splicing factor 3B subunit 2 (Fragment) OS=Homo sapiens GN=SF3B2 PE=4 SV=1	42431	440	0	39	0	0.04	0	0	1	0	1	0	1	0	1	0	-2.43375
tr|I3L2B0|I3L2B0_HUI	CLUH	Clustered mitochondria protein homolog OS=Homo sapiens GN=CLUH PE=3 SV=1	86269	438	0	21	0	0.05	0	0	0	0	1	0	1	0	1	0	-2.43375
tr|E9PI83|E9PI83_HUI	PRMT1	Protein arginine N-methyltransferase 1 (Fragment) OS=Homo sapiens GN=PRMT1 PE=2 S	12790	437	0	36	0	0.26	0	0	0	0	1	0	1	0	1	0	-2.43375
tr|E0X098|E0X098_HU	TES	Testin variant (Fragment) OS=Homo sapiens GN=TES PE=2 SV=1	22009	430	0	40	0	0.15	0	0	1	0	1	0	1	0	1	0	-2.43375
tr|A4D1I7|A4D1I7_HU	DNCI1	Dynein, cytoplasmic 1, intermediate chain 1, isoform CRA_e OS=Homo sapiens GN=DNCI	73423	427	0	40	0	0.04	0	0	1	0	1	0	1	0	1	0	-2.43375
tr|F5H578|F5H578_HU	PRPF40A	Pre-mRNA-processing factor 40 homolog A (Fragment) OS=Homo sapiens GN=PRPF40A	48302	425	0	41	0	0.04	0	0	1	0	1	0	1	0	1	0	-2.43375
tr|B4DLD4|B4DLD4_H	tr|B4DLD4|B4DLD4_HU	cDNA FLJ56738, highly similar to Fibrillarin OS=Homo sapiens PE=2 SV=1	30279	423	0	41	0	0.11	0	0	1	0	1	0	1	0	1	0	-2.43375
tr|A0JLS5|A0JLS5_HUI	TBL3	TBL3 protein (Fragment) OS=Homo sapiens GN=TBL3 PE=2 SV=1	64079	418	0	42	0	0.05	0	0	1	0	1	0	1	0	1	0	-2.43375
tr|H0YGE9|H0YGE9_H	M6PR	Cation-dependent mannose-6-phosphate receptor (Fragment) OS=Homo sapiens GN=M	11814	416	0	42	0	0.29	0	0	1	0	1	0	1	0	1	0	-2.43375
tr|F2Z2D0|F2Z2D0_HU	NUP205	Nuclear pore complex protein Nup205 OS=Homo sapiens GN=NUP205 PE=4 SV=1	23590	415	0	21	0	0.04	0	0	0	0	1	0	1	0	1	0	-2.43375
tr|B4DGR3|B4DGR3_	tr|B4DGR3|B4DGR3_H	cDNA FLJ54349, highly similar to Vesicle-fusing ATPase (EC 3.6.4.6) OS=Homo sapiens PE	78398	412	0	42	0	0.04	0	0	1	0	1	0	1	0	1	0	-2.43375
sp|Q9NSB4|KRT82_HU	KRT82	Keratin, type II cuticular Hb2 OS=Homo sapiens GN=KRT82 PE=1 SV=3	57985	411	0	43	0	0.06	0	0	1	0	1	0	1	0	2	0	-2.43375
tr|B4DRG7|B4DRG7_	tr|B4DRG7|B4DRG7_H	cDNA FLJ60336, highly similar to Condensin complex subunit 2 OS=Homo sapiens PE=2 S	80877	410	0	43	0	0.04	0	0	1	0	1	0	1	0	1	0	-2.43375
sp|Q9NSB2|KRT84_HU	KRT84	Keratin, type II cuticular Hb4 OS=Homo sapiens GN=KRT84 PE=2 SV=2	65942	409	0	43	0	0.05	0	0	1	0	1	0	1	0	3	0	-2.43375
tr|B3KMW5|B3KMW5	tr|B3KMW5|B3KMW5_	cDNA FLJ12796 fis, clone NT2RP2002058, highly similar to WD repeat protein 3 OS=Hom	71519	408	0	43	0	0.05	0	0	1	0	1	0	1	0	1	0	-2.43375
tr|Q1LZN2|Q1LZN2_H	NOMO3	NOMO3 protein (Fragment) OS=Homo sapiens GN=NOMO3 PE=2 SV=1	90388	400	0	43	0	0.04	0	0	1	0	1	0	1	0	1	0	-2.43375
tr|H0Y327|H0Y327_H	MTHFD1L	Monofunctional C1-tetrahydrofolate synthase, mitochondrial (Fragment) OS=Homo sapi	36808	398	0	43	0	0.09	0	0	1	0	1	0	1	0	1	0	-2.43375
tr|Q6W6M7|Q6W6M	tr|Q6W6M7|Q6W6M7	Antigen MLAA-43 (Fragment) OS=Homo sapiens PE=2 SV=1	23572	397	0	0	0	0.05	0	0	0	0	0	0	1	0	1	0	-2.43375
tr|K7EQN7|K7EQN7_H	DDX39A	ATP-dependent RNA helicase DDX39A (Fragment) OS=Homo sapiens GN=DDX39A PE=4 S	29270	395	0	44	0	0.11	0	0	1	0	1	0	1	0	2	0	-2.43375
tr|E5RGY7|E5RGY7_H	CTNNA1	Catenin alpha-1 (Fragment) OS=Homo sapiens GN=CTNNA1 PE=2 SV=1	14895	389	0	35	0	0.07	0	0	0	0	1	0	1	0	1	0	-2.43375
tr|H0YHL6|H0YHL6_H	MARS	Methionine--tRNA ligase, cytoplasmic (Fragment) OS=Homo sapiens GN=MARS PE=4 SV=	8869	385	0	40	0	0.07	0	0	1	0	1	0	1	0	1	0	-2.43375
tr|B4DT09|B4DT09_H	tr|B4DT09|B4DT09_HU	cDNA FLJ60145, highly similar to Homo sapiens dendritic cell protein (hfl-B5), mRNA OS=	13697	381	0	46	0	0.25	0	0	1	0	1	0	1	0	1	0	-2.43375
tr|J3KTL4|J3KTL4_HUI	PFAS	Phosphoribosylformylglycinamidine synthase (Fragment) OS=Homo sapiens GN=PFAS PE	11552	380	0	0	0	0.05	0	0	0	0	0	0	1	0	1	0	-2.43375
tr|Q0P512|Q0P512_H	SLC2A1	Solute carrier family 2 (Facilitated glucose transporter), member 1 OS=Homo sapiens GN	54361	377	0	46	0	0.06	0	0	1	0	1	0	1	0	1	0	-2.43375
tr|B3KU96|B3KU96_H	tr|B3KU96|B3KU96_HU	cDNA FLJ39433 fis, clone PROST2004142, highly similar to GDP-L-fucose synthetase (EC 1	17856	371	0	47	0	0.19	0	0	1	0	1	0	1	0	1	0	-2.43375
tr|D2KFR9|D2KFR9_H	STAT1	Signal transducer and activator of transcription 1-alpha/beta OS=Homo sapiens GN=STA	23360	364	0	42	0	0.08	0	0	1	0	1	0	1	0	1	0	-2.43375
tr|D6RJF2|D6RJF2_HU	PAIP1	Polyadenylate-binding protein-interacting protein 1 (Fragment) OS=Homo sapiens GN=P	22826	361	0	48	0	0.08	0	0	1	0	1	0	1	0	1	0	-2.43375
tr|Q53ST0|Q53ST0_H	PCYOX1	Putative uncharacterized protein PCYOX1 (Fragment) OS=Homo sapiens GN=PCYOX1 PE	11626	355	0	49	0	0.06	0	0	1	0	1	0	1	0	1	0	-2.43375
tr|E5RFU4|E5RFU4_H	DPYSL2	Dihydropyrimidinase-related protein 2 (Fragment) OS=Homo sapiens GN=DPYSL2 PE=2 S	18320	342	0	20	0	0.23	0	0	0	0	1	0	1	0	1	0	-2.43375
tr|H7C5I9|H7C5I9_HU	GART	Phosphoribosylformylglycinamidine cyclo-ligase (Fragment) OS=Homo sapiens GN=GART	2640	341	0	23	0	0.13	0	0	0	0	1	0	1	0	1	0	-2.43375
tr|A8K517|A8K517_HU	RPS23	Ribosomal protein S23, isoform CRA_a OS=Homo sapiens GN=RPS23 PE=2 SV=1	15969	339	0	52	0	0.21	0	0	1	0	1	0	1	0	1	0	-2.43375
tr|B2RAR2|B2RAR2_H	tr|B2RAR2|B2RAR2_HU	cDNA, FLJ95064, highly similar to Homo sapiens nin one binding protein (NOB1P), mRNA	47015	338	0	52	0	0.07	0	0	1	0	1	0	1	0	1	0	-2.43375
tr|A8KA82|A8KA82_H	DNAJC3	DnaJ (Hsp40) homolog, subfamily C, member 3 OS=Homo sapiens GN=DNAJC3 PE=2 SV=	58000	337	0	52	0	0.06	0	0	1	0	1	0	1	0	1	0	-2.43375
tr|Q59GV0|Q59GV0_	tr|Q59GV0|Q59GV0_H	Propionyl Coenzyme A carboxylase, beta polypeptide variant (Fragment) OS=Homo sapie	60578	331	0	53	0	0.05	0	0	1	0	1	0	1	0	2	0	-2.43375
tr|A8K3Q9|A8K3Q9_H	tr|A8K3Q9|A8K3Q9_HU	cDNA FLJ76611, highly similar to Homo sapiens ribosomal protein L14 (RPL14), mRNA OS	23554	330	0	53	0	0.14	0	0	1	0	1	0	1	0	1	0	-2.43375
tr|I3L4P6|I3L4P6_HUI	ARHGAP17	Rho GTPase-activating protein 17 (Fragment) OS=Homo sapiens GN=ARHGAP17 PE=4 SV	8676	328	0	54	0	0.03	0	0	1	0	1	0	1	0	1	0	-2.43375
tr|Q5RZ99|Q5RZ99_H	tr|Q5RZ99|Q5RZ99_HU	Apoptosis-inducing factor (Fragment) OS=Homo sapiens PE=2 SV=1	24524	326	0	54	0	0.05	0	0	1	0	1	0	1	0	1	0	-2.43375
tr|C9JIX5|C9JIX5_HUI	PLOD3	Procollagen-lysine,2-oxoglutarate 5-dioxygenase 3 (Fragment) OS=Homo sapiens GN=PL	8424	325	0	41	0	0.12	0	0	1	0	1	0	1	0	1	0	-2.43375
tr|C9JPP3|C9JPP3_HU	RBBP4	Histone-binding protein RBBP4 (Fragment) OS=Homo sapiens GN=RBBP4 PE=2 SV=2	9655	315	0	57	0	0.36	0	0	1	0	1	0	1	0	1	0	-2.43375
sp|Q5TZA2|CROCC_H	CROCC	Rootletin OS=Homo sapiens GN=CROCC PE=1 SV=1	228787	310	0	58	0	0.01	0	0	1	0	1	0	1	0	2	0	-2.43375
tr|B3KPC1|B3KPC1_H	tr|B3KPC1|B3KPC1_HU	cDNA FLJ31582 fis, clone NT2RI2002117, highly similar to Protein pelota homolog OS=Ho	43807	307	0	59	0	0.08	0	0	1	0	1	0	1	0	1	0	-2.43375
tr|B2RAX1|B2RAX1_H	tr|B2RAX1|B2RAX1_HU	cDNA, FLJ95169 OS=Homo sapiens PE=2 SV=1	77979	306	0	41	0	0.14	0	0	1	0	1	0	1	0	1	0	-2.43375
tr|B5LY64|B5LY64_HU	UMPS	Orotidine 5~-phosphate decarboxylase OS=Homo sapiens GN=UMPS PE=2 SV=1	33258	302	0	59	0	0.1	0	0	1	0	1	0	1	0	1	0	-2.43375
tr|E7EMU4|E7EMU4_	DYNC1I2	Cytoplasmic dynein 1 intermediate chain 2 (Fragment) OS=Homo sapiens GN=DYNC1I2 P	24071	298	0	48	0	0.09	0	0	1	0	1	0	1	0	1	0	-2.43375
tr|Q5T0I0|Q5T0I0_HU	GSN	Gelsolin (Fragment) OS=Homo sapiens GN=GSN PE=4 SV=1	29107	297	0	61	0	0.04	0	0	1	0	1	0	1	0	2	0	-2.43375
sp|P81605|DCD_HUM	DCD	Dermcidin OS=Homo sapiens GN=DCD PE=1 SV=2	11391	295	0	62	0	0.3	0	0	1	0	1	0	1	0	1	0	-2.43375
tr|B4DE27|B4DE27_H	tr|B4DE27|B4DE27_HU	cDNA FLJ58765, highly similar to Neutral amino acid transporter B(0) OS=Homo sapiens	33987	290	0	63	0	0.1	0	0	1	0	1	0	1	0	1	0	-2.43375
tr|H0YC45|H0YC45_H	ATP6V1B2	V-type proton ATPase subunit B, brain isoform (Fragment) OS=Homo sapiens GN=ATP6V	5569	287	0	64	0	0.65	0	0	1	0	1	0	1	0	1	0	-2.43375
tr|E5RFS9|E5RFS9_HU	MAT2B	Methionine adenosyltransferase 2 subunit beta OS=Homo sapiens GN=MAT2B PE=2 SV=	9467	280	0	68	0	0.37	0	0	1	0	1	0	1	0	1	0	-2.43375
tr|B4DXK4|B4DXK4_H	KRT72	Keratin, type II cytoskeletal 72 OS=Homo sapiens GN=KRT72 PE=2 SV=1	36031	259	0	0	0	0.45	0	0	0	0	0	0	1	0	1	0	-2.43375
tr|B4DE40|B4DE40_H	SMS	Spermine synthase OS=Homo sapiens GN=SMS PE=2 SV=1	31596	248	0	78	0	0.11	0	0	1	0	1	0	1	0	1	0	-2.43375
tr|E5RFJ8|E5RFJ8_HU	MCM4	DNA replication licensing factor MCM4 (Fragment) OS=Homo sapiens GN=MCM4 PE=2 S	23630	240	0	65	0	0.1	0	0	1	0	1	0	1	0	2	0	-2.43375
tr|H0YGZ5|H0YGZ5_H	PRPF19	Pre-mRNA-processing factor 19 (Fragment) OS=Homo sapiens GN=PRPF19 PE=4 SV=1	13367	237	0	62	0	0.26	0	0	1	0	1	0	1	0	1	0	-2.43375
tr|E9PLA6|E9PLA6_HU	SERPINH1	Serpin H1 (Fragment) OS=Homo sapiens GN=SERPINH1 PE=2 SV=1	8925	233	0	45	0	0.43	0	0	1	0	1	0	1	0	1	0	-2.43375
tr|A1L179|A1L179_HU	KIF5C	KIF5C protein (Fragment) OS=Homo sapiens GN=KIF5C PE=2 SV=1	99437	227	0	46	0	0.04	0	0	1	0	1	0	1	0	2	0	-2.43375
tr|H0Y960|H0Y960_H	DDX4	Probable ATP-dependent RNA helicase DDX4 (Fragment) OS=Homo sapiens GN=DDX4 PE	33292	217	0	43	0	0.19	0	0	1	0	1	0	1	0	1	0	-2.43375
tr|D6R9D3|D6R9D3_H	HNRNPH1	Heterogeneous nuclear ribonucleoprotein H OS=Homo sapiens GN=HNRNPH1 PE=4 SV=1	4404	207	0	23	0	0.45	0	0	0	0	1	0	1	0	1	0	-2.43375
tr|A4D1P0|A4D1P0_H	LOC402299	Uncharacterized protein OS=Homo sapiens GN=LOC402299 PE=4 SV=1	42109	185	0	40	0	0.42	0	0	1	0	1	0	1	0	1	0	-2.43375
tr|Q6RUP9|Q6RUP9_	IMPDH2	Inosine monophosphate dehydrogenase type II (Fragment) OS=Homo sapiens GN=IMPD	7313	177	0	25	0	0.36	0	0	0	0	1	0	1	0	1	0	-2.43375
tr|K7EQ75|K7EQ75_H	ILF3	Interleukin enhancer-binding factor 3 (Fragment) OS=Homo sapiens GN=ILF3 PE=4 SV=1	6139	154	0	29	0	0.46	0	0	0	0	1	0	1	0	1	0	-2.43375
tr|H0YHU8|H0YHU8_	SART3	Squamous cell carcinoma antigen recognized by T-cells 3 (Fragment) OS=Homo sapiens	16246	153	0	45	0	0.16	0	0	1	0	1	0	1	0	1	0	-2.43375
tr|Q8NI92|Q8NI92_HU	OK/SW-cl.65	DEAD/H(Asp-Glu-Ala-Asp/His)box polypeptide 21 OS=Homo sapiens GN=OK/SW-cl.65 PE	16499	148	0	21	0	0.34	0	0	0	0	1	0	1	0	1	0	-2.43375
tr|B4DZX5|B4DZX5_H	tr|B4DZX5|B4DZX5_HU	cDNA FLJ59130, moderately similar to Homo sapiens WD repeat domain 1 (WDR1), trans	19688	137	0	27	0	0.4	0	0	0	0	1	0	1	0	1	0	-2.43375
tr|F8WDK9|F8WDK9_	PDCD6IP	Programmed cell death 6-interacting protein OS=Homo sapiens GN=PDCD6IP PE=2 SV=1	10956	136	0	61	0	0.18	0	0	1	0	1	0	1	0	1	0	-2.43375
tr|H3BND9|H3BND9_	PCBP2	Poly(rC)-binding protein 2 (Fragment) OS=Homo sapiens GN=PCBP2 PE=4 SV=1	12425	132	0	35	0	0.32	0	0	1	0	1	0	1	0	2	0	-2.43375
tr|Q659G2|Q659G2_H	DKFZp586B2023	Putative uncharacterized protein DKFZp586B2023 (Fragment) OS=Homo sapiens GN=DK	7133	124	0	41	0	0.57	0	0	1	0	1	0	1	0	1	0	-2.43375
tr|F8WBE5|F8WBE5_	TFRC	Transferrin receptor protein 1, serum form OS=Homo sapiens GN=TFRC PE=2 SV=1	9046	122	0	37	0	0.3	0	0	1	0	1	0	1	0	1	0	-2.43375
tr|K7EIL4|K7EIL4_HUI	GPI	Glucose-6-phosphate isomerase (Fragment) OS=Homo sapiens GN=GPI PE=4 SV=1	12790	117	0	0	0	0.3	0	0	0	0	0	0	1	0	1	0	-2.43375
tr|D6RCS6|D6RCS6_H	TARS	Threonine--tRNA ligase, cytoplasmic OS=Homo sapiens GN=TARS PE=2 SV=1	6912	94	0	53	0	0.64	0	0	1	0	1	0	1	0	1	0	-2.43375
tr|A2RRC9|A2RRC9_H	IQGAP3	IQ motif containing GTPase activating protein 3 OS=Homo sapiens GN=IQGAP3 PE=2 SV=	185145	84	0	32	0	0.31	0	0	0	0	1	0	1	0	1	0	-2.43375
tr|F8VS61|F8VS61_HU	KRT77	Keratin, type II cytoskeletal 1b OS=Homo sapiens GN=KRT77 PE=2 SV=1	25704	76	0	43	0	0.23	0	0	1	0	1	0	1	0	3	0	-2.43375
tr|F5GXU1|F5GXU1_H	LDHA	L-lactate dehydrogenase A chain OS=Homo sapiens GN=LDHA PE=2 SV=1	5003	44	0	0	0	2.33	0	0	0	0	0	0	1	0	1	0	-2.43375
tr|K7ENV3|K7ENV3_H	KRT16	Keratin, type I cytoskeletal 16 (Fragment) OS=Homo sapiens GN=KRT16 PE=4 SV=1	4775	33	0	48	0	2.22	0	0	1	0	1	0	1	0	1	0	-2.43375
tr|A1L2Z2|A1L2Z2_HU	MYH14	MYH14 protein OS=Homo sapiens GN=MYH14 PE=2 SV=1	73418	17	0	56	0	0.9	0	0	1	0	1	0	1	0	1	0	-2.43375
tr|B9VP24|B9VP24_H	HSPD1	60 kDa chaperonin (Fragment) OS=Homo sapiens GN=HSPD1 PE=4 SV=1	19880	16	0	40	0	1.72	0	0	1	0	1	0	1	0	1	0	-2.43375
sp|Q8TCJ2|STT3B_HU	STT3B	Dolichyl-diphosphooligosaccharide--protein glycosyltransferase subunit STT3B OS=Hom	94241	700	0	18	0	0.03	0	0	0	0	1	0	2	0	3	0	-3.17104
sp|P25490|TYY1_HUN	YY1	Transcriptional repressor protein YY1 OS=Homo sapiens GN=YY1 PE=1 SV=2	45141	630	0	23	0	0.07	0	0	0	0	1	0	2	0	2	0	-3.17104
tr|D3DSQ0|D3DSQ0_	PCM1	Pericentriolar material 1, isoform CRA_a OS=Homo sapiens GN=PCM1 PE=2 SV=1	230041	619	0	24	0	0.01	0	0	0	0	1	0	2	0	3	0	-3.17104
sp|Q9NRF8|PYRG2_H	CTPS2	CTP synthase 2 OS=Homo sapiens GN=CTPS2 PE=1 SV=1	66320	614	0	24	0	0.05	0	0	0	0	1	0	2	0	6	0	-3.17104
tr|B7ZA86|B7ZA86_H	tr|B7ZA86|B7ZA86_HU	cDNA, FLJ79100, highly similar to 14-3-3 protein epsilon (14-3-3E) OS=Homo sapiens PE=	19138	582	0	28	0	0.18	0	0	0	0	1	0	2	0	2	0	-3.17104
tr|H3BNV2|H3BNV2_	NQO1	NAD(P)H dehydrogenase [quinone] 1 OS=Homo sapiens GN=NQO1 PE=4 SV=1	28533	576	0	28	0	0.25	0	0	0	0	2	0	2	0	2	0	-3.17104
tr|B2RD27|B2RD27_H	tr|B2RD27|B2RD27_HU	cDNA, FLJ96428, highly similar to Homo sapiens proteasome (prosome, macropain) 26S	37046	550	0	30	0	0.19	0	0	0	0	2	0	2	0	2	0	-3.17104
tr|B3KNB4|B3KNB4_H	tr|B3KNB4|B3KNB4_HU	cDNA FLJ14168 fis, clone NT2RP2001440, highly similar to 14-3-3 protein gamma OS=Ho	28453	541	0	30	0	0.25	0	0	0	0	2	0	2	0	2	0	-3.17104
tr|C1KES1|C1KES1_HU	LRRC16B	LRRC16B (Fragment) OS=Homo sapiens GN=LRRC16B PE=4 SV=1	4125	530	0	31	0	0.94	0	0	0	0	1	0	2	0	2	0	-3.17104
tr|B2RDN4|B2RDN4_	tr|B2RDN4|B2RDN4_H	cDNA, FLJ96693, highly similar to Homo sapiens block of proliferation 1 (BOP1), mRNA O	84219	505	0	33	0	0.08	0	0	0	0	2	0	2	0	3	0	-3.17104
tr|B0AZS6|B0AZS6_HU	YWHAZ	14-3-3 protein zeta/delta OS=Homo sapiens GN=YWHAZ PE=2 SV=1	19174	497	0	34	0	0.38	0	0	0	0	2	0	2	0	2	0	-3.17104
tr|A0PJ76|A0PJ76_HU	NCAPD2	NCAPD2 protein (Fragment) OS=Homo sapiens GN=NCAPD2 PE=2 SV=1	44957	494	0	34	0	0.15	0	0	0	0	2	0	2	0	2	0	-3.17104
tr|E7EPL9|E7EPL9_HU	HSD17B4	(3R)-hydroxyacyl-CoA dehydrogenase OS=Homo sapiens GN=HSD17B4 PE=2 SV=1	51239	484	0	26	0	0.13	0	0	0	0	2	0	2	0	3	0	-3.17104

sp|P07737|PROF1_HU	PFN1	Profilin-1 OS=Homo sapiens GN=PFN1 PE=1 SV=2	15216	436	0	39	0	0.5	0	0	1	0	2	0	2	0	2	0	-3.17104
tr|A5D904|A5D904_H	RPS9	RPS9 protein (Fragment) OS=Homo sapiens GN=RPS9 PE=2 SV=1	13210	434	0	40	0	0.58	0	0	1	0	2	0	2	0	2	0	-3.17104
tr|Q6NZ55|Q6NZ55_H	RPL13	60S ribosomal protein L13 OS=Homo sapiens GN=RPL13 PE=2 SV=1	24308	422	0	41	0	0.14	0	0	1	0	1	0	2	0	2	0	-3.17104
tr|C9J1B1|C9J1B1_HU	NAP1L4	Nucleosome assembly protein 1-like 4 (Fragment) OS=Homo sapiens GN=NAP1L4 PE=2 S	11455	417	0	31	0	0.16	0	0	0	0	1	0	2	0	2	0	-3.17104
tr|B4DQJ1|B4DQJ1_H	SNRNP40	U5 small nuclear ribonucleoprotein 40 kDa protein OS=Homo sapiens GN=SNRNP40 PE=2	45001	413	0	42	0	0.17	0	0	1	0	2	0	2	0	3	0	-3.17104
tr|B2RCT6|B2RCT6_H	tr|B2RCT6|B2RCT6_HU	cDNA, FLJ96276, highly similar to Homo sapiens G1 to S phase transition 1 (GSPT1), mRN	56320	391	0	44	0	0.12	0	0	1	0	2	0	2	0	2	0	-3.17104
tr|B7Z8J4|B7Z8J4_HU	NMT1	Glycylpeptide N-tetradecanoyltransferase 1 OS=Homo sapiens GN=NMT1 PE=2 SV=1	15265	350	0	50	0	0.49	0	0	1	0	2	0	2	0	2	0	-3.17104
tr|Q6NZ52|Q6NZ52_H	RPL27A	Ribosomal protein L27a OS=Homo sapiens GN=RPL27A PE=2 SV=1	16582	348	0	50	0	0.2	0	0	1	0	1	0	2	0	2	0	-3.17104
tr|E5RHF2|E5RHF2_H	COPS5	COP9 signalosome complex subunit 5 (Fragment) OS=Homo sapiens GN=COPS5 PE=2 SV=	16655	346	0	51	0	0.45	0	0	0	0	2	0	2	0	2	0	-3.17104
tr|D3DP78|D3DP78_H	DARS	Aspartyl-tRNA synthetase, isoform CRA_b OS=Homo sapiens GN=DARS PE=2 SV=1	53829	344	0	51	0	0.06	0	0	1	0	1	0	2	0	3	0	-3.17104
tr|G3V180|G3V180_H	DPP3	Dipeptidyl peptidase 3 OS=Homo sapiens GN=DPP3 PE=2 SV=1	84666	335	0	52	0	0.08	0	0	1	0	2	0	2	0	2	0	-3.17104
tr|B2R7M3|B2R7M3_	tr|B2R7M3|B2R7M3_H	cDNA, FLJ93510, highly similar to Homo sapiens JTV1 gene (JTV1), mRNA OS=Homo sapie	35767	308	0	58	0	0.19	0	0	2	0	2	0	2	0	2	0	-3.17104
tr|F5GYR3|F5GYR3_H	NOP2	Putative ribosomal RNA methyltransferase NOP2 (Fragment) OS=Homo sapiens GN=NOP	30665	296	0	28	0	0.15	0	0	1	0	1	0	2	0	3	0	-3.17104
tr|Q2QD09|Q2QD09_	tr|Q2QD09|Q2QD09_H	Triosephosphate isomerase (Fragment) OS=Homo sapiens PE=2 SV=1	27211	267	0	71	0	0.26	0	0	1	0	2	0	2	0	2	0	-3.17104
tr|B4DMT8|B4DMT8_	tr|B4DMT8|B4DMT8_H	cDNA FLJ52118, highly similar to 14-3-3 protein theta OS=Homo sapiens PE=2 SV=1	24050	258	0	42	0	0.4	0	0	1	0	2	0	2	0	2	0	-3.17104
sp|Q6IS14|IF5AL_HUN	EIF5AL1	Eukaryotic translation initiation factor 5A-1-like OS=Homo sapiens GN=EIF5AL1 PE=1 SV=	16990	256	0	75	0	0.44	0	0	1	0	2	0	2	0	2	0	-3.17104
sp|Q9UQ35|SRRM2_H	SRRM2	Serine/arginine repetitive matrix protein 2 OS=Homo sapiens GN=SRRM2 PE=1 SV=2	300179	247	0	78	0	0.02	0	0	1	0	2	0	2	0	5	0	-3.17104
tr|Q05DV5|Q05DV5_H	ZC3HAV1	ZC3HAV1 protein (Fragment) OS=Homo sapiens GN=ZC3HAV1 PE=2 SV=1	49964	224	0	92	0	0.14	0	0	1	0	2	0	2	0	2	0	-3.17104
tr|A0MNN4|A0MNN4	SMU1	CDW3/SMU1 OS=Homo sapiens GN=SMU1 PE=2 SV=1	58134	221	0	94	0	0.12	0	0	1	0	2	0	2	0	2	0	-3.17104
tr|Q5JVP3|Q5JVP3_HU	PTGR1	Leukotriene B4 12-hydroxydehydrogenase, isoform CRA_d OS=Homo sapiens GN=PTGR1	19441	190	0	113	0	0.38	0	0	2	0	2	0	2	0	2	0	-3.17104
tr|F8VQS0|F8VQS0_H	ATXN2	Ataxin-2 (Fragment) OS=Homo sapiens GN=ATXN2 PE=2 SV=1	17594	157	0	54	0	0.21	0	0	1	0	1	0	2	0	2	0	-3.17104
tr|B4DKJ0|B4DKJ0_HU	tr|B4DKJ0|B4DKJ0_HU	cDNA FLJ58539, highly similar to Keratin, type II cytoskeletal 4 OS=Homo sapiens PE=2 SV	55657	105	0	61	0	0.7	0	0	2	0	2	0	2	0	4	0	-3.17104
tr|J3KSQ2|J3KSQ2_HU	CLTC	Clathrin heavy chain 1 (Fragment) OS=Homo sapiens GN=CLTC PE=4 SV=1	13791	30	0	66	0	0.52	0	0	2	0	2	0	2	0	2	0	-3.17104
tr|B7Z4P8|B7Z4P8_HU	ACTN2	Alpha-actinin-2 OS=Homo sapiens GN=ACTN2 PE=2 SV=1	44675	19	0	51	0	2.11	0	0	1	0	2	0	2	0	2	0	-3.17104
tr|H0YID6|H0YID6_HU	KRT79	Keratin, type II cytoskeletal 79 (Fragment) OS=Homo sapiens GN=KRT79 PE=3 SV=1	13441	11	0	64	0	4.79	0	0	2	0	2	0	2	0	3	0	-3.17104
sp|P46063|RECQ1_HU	RECQL	ATP-dependent DNA helicase Q1 OS=Homo sapiens GN=RECQL PE=1 SV=3	74436	439	0	39	0	0.14	0	0	1	0	3	0	3	0	3	0	-3.65679
sp|Q7RTS7|K2C74_HU	KRT74	Keratin, type II cytoskeletal 74 OS=Homo sapiens GN=KRT74 PE=1 SV=2	58229	373	0	47	0	0.12	0	0	2	0	2	0	3	0	5	0	-3.65679
tr|Q0VGL3|Q0VGL3_H	RPL13A	RPL13A protein OS=Homo sapiens GN=RPL13A PE=2 SV=1	16706	300	0	59	0	0.74	0	0	3	0	3	0	3	0	3	0	-3.65679
tr|Q5JQZ9|Q5JQZ9_H	CDC2L6	Cell division cycle 2-like 6 (CDK8-like) (Fragment) OS=Homo sapiens GN=CDC2L6 PE=4 SV	29552	285	0	65	0	0.24	0	0	2	0	2	0	3	0	3	0	-3.65679
tr|J3QST3|J3QST3_HU	KRT3	Keratin, type II cytoskeletal 3 OS=Homo sapiens GN=KRT3 PE=3 SV=1	64636	255	0	76	0	0.16	0	0	3	0	3	0	3	0	6	0	-3.65679
tr|C9JI87|C9JI87_HUN	VDAC1	Voltage-dependent anion-selective channel protein 1 (Fragment) OS=Homo sapiens GN=	20418	250	0	78	0	0.36	0	0	1	0	3	0	3	0	3	0	-3.65679
tr|B2R5W2|B2R5W2_	tr|B2R5W2|B2R5W2_H	cDNA, FLJ92657, highly similar to Homo sapiens heterogeneous nuclear ribonucleoprote	31986	178	0	122	0	0.35	0	0	3	0	3	0	3	0	4	0	-3.65679
tr|B4DMU0|B4DMU0	PYCR1	Pyrroline-5-carboxylate reductase OS=Homo sapiens GN=PYCR1 PE=2 SV=1	36186	174	0	125	0	0.19	0	0	2	0	2	0	3	0	3	0	-3.65679
tr|Q6ZVC6|Q6ZVC6_H	tr|Q6ZVC6|Q6ZVC6_HU	cDNA FLJ42761 fis, clone BRAWH3002574, highly similar to Calpain 2, large OS=Homo sa	24572	173	0	82	0	0.22	0	0	2	0	2	0	3	0	3	0	-3.65679
sp|A2RTY3|CQ066_HU	C17orf66	Uncharacterized protein C17orf66 OS=Homo sapiens GN=C17orf66 PE=2 SV=2	66437	477	0	36	0	0.05	0	0	0	0	1	0	4	0	8	0	-4.01969
sp|Q14CN4|K2C72_HU	KRT72	Keratin, type II cytoskeletal 72 OS=Homo sapiens GN=KRT72 PE=1 SV=2	56470	372	0	47	0	0.12	0	0	2	0	2	0	4	0	5	0	-4.01969
tr|F8VPD4|F8VPD4_H	CAD	CAD protein OS=Homo sapiens GN=CAD PE=2 SV=1	238097	317	0	57	0	0.04	0	0	1	0	3	0	4	0	5	0	-4.01969
tr|E9PKU4|E9PKU4_H	RPL8	60S ribosomal protein L8 (Fragment) OS=Homo sapiens GN=RPL8 PE=2 SV=1	25806	231	0	90	0	0.28	0	0	2	0	2	0	4	0	5	0	-4.01969
tr|D6RAT0|D6RAT0_H	RPS3A	40S ribosomal protein S3a OS=Homo sapiens GN=RPS3A PE=3 SV=1	26099	223	0	93	0	0.44	0	0	2	0	3	0	4	0	5	0	-4.01969
tr|F5GZI8|F5GZI8_HU	TCP1	T-complex protein 1 subunit alpha OS=Homo sapiens GN=TCP1 PE=2 SV=1	10411	197	0	64	0	0.3	0	0	1	0	2	0	4	0	4	0	-4.01969
tr|H7BYX5|H7BYX5_H	FSCN1	Fascin (Fragment) OS=Homo sapiens GN=FSCN1 PE=4 SV=1	15688	53	0	188	0	1.07	0	0	4	0	5	0	4	0	7	0	-4.01969
sp|Q01546|K22O_HU	KRT76	Keratin, type II cytoskeletal 2 oral OS=Homo sapiens GN=KRT76 PE=1 SV=2	66370	213	0	97	0	0.21	0	0	4	0	4	0	5	0	9	0	-4.30952
tr|B3KY17|B3KY17_HU	ATP2A1	Sarcoplasmic/endoplasmic reticulum calcium ATPase 1 OS=Homo sapiens GN=ATP2A1 PI	96392	149	0	86	0	0.32	0	0	2	0	4	0	5	0	5	0	-4.30952
tr|B2RA03|B2RA03_H	tr|B2RA03|B2RA03_HU	cDNA, FLJ94640, highly similar to Homo sapiens keratin 18 (KRT18), mRNA OS=Homo sap	48000	127	0	88	0	0.36	0	0	4	0	4	0	5	0	6	0	-4.30952
tr|K7ENW6|K7ENW6_	KRT16	Keratin, type I cytoskeletal 16 (Fragment) OS=Homo sapiens GN=KRT16 PE=4 SV=1	18735	62	0	109	0	1.38	0	0	3	0	4	0	5	0	5	0	-4.30952
tr|B3KVL0|B3KVL0_HU	tr|B3KVL0|B3KVL0_HU	cDNA FLJ16691 fis, clone TRACH3000692, highly similar to Paxillin OS=Homo sapiens PE=	65600	159	0	133	0	0.28	0	0	3	0	5	0	6	0	6	0	-4.55085
sp|Q7Z3Y7|K1C28_HU	KRT28	Keratin, type I cytoskeletal 28 OS=Homo sapiens GN=KRT28 PE=1 SV=2	51163	12	0	131	0	4.67	0	0	6	0	6	0	7	0	7	0	-4.75763
tr|E9PRA5|E9PRA5_H	ATP1A4	Sodium/potassium-transporting ATPase subunit alpha-4 OS=Homo sapiens GN=ATP1A4 I	57835	289	0	63	0	0.12	0	0	0	0	6	0	10	0	19	0	-5.24403
tr|F8W0C6|F8W0C6_I	KRT5	Keratin, type II cytoskeletal 5 (Fragment) OS=Homo sapiens GN=KRT5 PE=2 SV=1	20701	58	0	93	0	0.95	0	0	5	0	6	0	10	0	12	0	-5.24403
sp|Q96RW7|HMCN1_	HMCN1	Hemicentin-1 OS=Homo sapiens GN=HMCN1 PE=1 SV=2	623265	291	0	63	0	0.01	0	0	7	0	7	0	11	0	16	0	-5.3756
sp|P05165|PCCA_HUN	PCCA	Propionyl-CoA carboxylase alpha chain, mitochondrial OS=Homo sapiens GN=PCCA PE=1	80635	99	0	216	0	0.49	0	0	6	0	10	0	13	0	13	0	-5.60757
tr|Q59FY4|Q59FY4_H	ACACA	Acetyl-CoA carboxylase 1 (Fragment) OS=Homo sapiens GN=ACACA PE=2 SV=1	115059	31	0	225	0	0.44	0	0	10	0	12	0	15	0	18	0	-5.80753
tr|B3KPL5|B3KPL5_HU	tr|B3KPL5|B3KPL5_HU	cDNA FLJ31917 fis, clone NT2RP7004925, highly similar to Sprouty-related, EVH1 domair	48711	657	0	21	0	0.07	0	0	0	0	1	0	16	0	51	0	-5.89806

#15	Hela lysate syn MLN-2_2μM																		
#04	Hela live syn NP_2μM																		
Accession	GS	Name	Pro_Mass	04_pro	15_prot_hi	04_pro	15_prot	04_emI	15_emI	emPAI ratio	04_No>I	15_No>I	04_No>HI	15_No>HI	04_No>20	15_No>20	04_No=All	15_No=All	Rsc
sp|O14965|AURKA_HUMAN	AURKA	Aurora kinase A OS=Homo sapiens GN=AURKA PE=1 SV=2	46180	0	793	0	50	0	0.15	#DIV/0!	0	2	0	2	0	2	0	2	1.430154
sp|Q96SB4|SRPK1_HUMAN	SRPK1	SRSF protein kinase 1 OS=Homo sapiens GN=SRPK1 PE=1 SV=2	74963	0	1008	0	35	0	0.13	#DIV/0!	0	1	0	3	0	3	0	4	1.915761
sp|P28482|MK01_HUMAN	MAPK1 (ERK2)	Mitogen-activated protein kinase 1 OS=Homo sapiens GN=MAPK1 PE=1 SV=3	41762	0	1073	0	30	0	0.08	#DIV/0!	0	0	0	1	0	1	0	2	0.693008
sp|Q13555|KCC2G_HUMAN	CAMK2G	Calcium/calmodulin-dependent protein kinase type II subunit gamma OS=Homo sapiens GN=CAMK2G PE=1 S\	63311	0	1014	0	34	0	0.05	#DIV/0!	0	0	0	1	0	1	0	2	0.693008
sp|P49761|CLK3_HUMAN	CLK3	Dual specificity protein kinase CLK3 OS=Homo sapiens GN=CLK3 PE=1 SV=3	74267	0	953	0	38	0	0.04	#DIV/0!	0	1	0	1	0	1	0	1	0.693008
tr|B4DR80|B4DR80_HUMAN	STK24	Serine/threonine-protein kinase 24 12 kDa subunit OS=Homo sapiens GN=STK24 PE=2 SV=1	46093	0	947	0	38	0	0.09	#DIV/0!	0	1	0	1	0	1	0	1	0.693008
sp|Q96KB5|TOPK_HUMAN	PBK	Lymphokine-activated killer T-cell-originated protein kinase OS=Homo sapiens GN=PBK PE=1 SV=3	36404	0	799	0	48	0	0.09	#DIV/0!	0	1	0	1	0	1	0	1	0.693008
sp|Q13418|ILK_HUMAN	ILK	Integrin-linked protein kinase OS=Homo sapiens GN=ILK PE=1 SV=2	51899	421	436	41	98	0.07	0.28	4	1	1	1	3	1	4	1	4	0.693214
sp|O75116|ROCK2_HUMAN	ROCK2	Rho-associated protein kinase 2 OS=Homo sapiens GN=ROCK2 PE=1 SV=4	161939	375	585	46	68	0.05	0.1	2	1	2	2	4	1	3	3	5	0.330464
sp|P53350|PLK1_HUMAN	PLK1	Serine/threonine-protein kinase PLK1 OS=Homo sapiens GN=PLK1 PE=1 SV=1	68953	0	690	0	58	0	0.1	#DIV/0!	0	2	0	2	0	2	0	3	1.430154
sp|O60566|BUB1B_HUMAN	BUB1B	Mitotic checkpoint serine/threonine-protein KINASE BUB1 beta OS=Homo sapiens GN=BUB1B PE=1 SV=3	120781	0	607	0	67	0	0.08	#DIV/0!	0	1	0	3	0	2	0	3	1.430154
sp|P48729|KC1A_HUMAN	CSNK1A1	Casein kinase I isoform alpha OS=Homo sapiens GN=CSNK1A1 PE=1 SV=2	39118	0	767	0	51	0	0.17	#DIV/0!	0	1	0	1	0	1	0	1	0.693008
sp|P78527|PRKDC_HUMAN	PRKDC	DNA-dependent protein kinase catalytic subunit OS=Homo sapiens GN=PRKDC PE=1 SV=3	473749	83	20	241	1470	0.07	0.46	6.57142857	7	37	10	53	11	61	17	72	1.534158
sp|Q8N5S9|KKCC1_HUMAN	CAMKK1	Calcium/calmodulin-dependent protein kinase kinase 1 OS=Homo sapiens GN=CAMKK1 PE=1 SV=2	56157	0	1129	0	26	0	0.06	#DIV/0!	0	0	0	1	0	1	0	1	0.693008
sp|Q99570|PI3R4_HUMAN	PIK3R4	Phosphoinositide 3-kinase regulatory subunit 4 OS=Homo sapiens GN=PIK3R4 PE=1 SV=3	154318	0	641	0	63	0	0.06	#DIV/0!	0	2	0	3	0	3	0	3	1.915761
tr|J3KNX3|J3KNX3_HUMAN	PFKM	6-phosphofructoKINASE OS=Homo sapiens GN=PFKM PE=3 SV=1	94107	0	357	0	126	0	0.23	#DIV/0!	0	4	0	6	0	5	0	8	2.568197
sp|Q16822|PCKGM_HUMAN	PCK2	Phosphoenolpyruvate carboxykinase [GTP], mitochondrial OS=Homo sapiens GN=PCK2 PE=1 SV=3	71483	0	417	0	91	0	0.14	#DIV/0!	0	2	0	2	0	3	0	4	1.915761
sp|Q53H12|AGK_HUMAN	AGK	Acylglycerol kinase, mitochondrial OS=Homo sapiens GN=AGK PE=1 SV=2	47564	0	1169	0	23	0	0.07	#DIV/0!	0	0	0	1	0	1	0	1	0.693008
sp|P42356|PI4KA_HUMAN	PI4KA	Phosphatidylinositol 4-kinase alpha OS=Homo sapiens GN=PI4KA PE=1 SV=3	233622	0	719	0	55	0	0.05	#DIV/0!	0	1	0	2	0	1	0	3	0.693008
sp|P52789|HXK2_HUMAN	HK2	Hexokinase-2 OS=Homo sapiens GN=HK2 PE=1 SV=2	103739	599	695	26	57	0.03	0.1	3.33333333	0	1	1	3	1	3	2	3	0.330464
sp|Q01813|K6PP_HUMAN	PFKP	6-phosphofructoKINASE type C OS=Homo sapiens GN=PFKP PE=1 SV=2	86454	98	53	217	733	0.4	1.1	2.75	5	16	9	21	9	23	10	25	0.415663
sp|P00558|PGK1_HUMAN	PGK1	Phosphoglycerate KINASE 1 OS=Homo sapiens GN=PGK1 PE=1 SV=3	44985	134	72	161	545	0.76	1.9	2.5	5	12	8	15	9	19	9	21	0.145755
sp|P67775|PP2AA_HUMAN	PPP2CA	Serine/threonine-protein phosphatase 2A catalytic subunit alpha isoform OS=Homo sapiens GN=PPP2CA PE=1	36142	0	510	0	82	0	0.3	#DIV/0!	0	2	0	3	0	3	0	3	1.915761
sp|P40763|STAT3_HUMAN	STAT3	Signal transducer and activator of transcription 3 OS=Homo sapiens GN=STAT3 PE=1 SV=2	88810	0	356	0	126	0	0.16	#DIV/0!	0	3	0	4	0	5	0	6	2.568197
sp|Q5SY16|NOL9_HUMAN	NOL9 (Nucleolar	Polynucleotide 5~-hydroxyl-KINASE NOL9 OS=Homo sapiens GN=NOL9 PE=1 SV=1	80527	0	1189	0	22	0	0.04	#DIV/0!	0	0	0	1	0	1	0	1	0.693008
sp|P14618|KPYM_HUMAN	PKM	Pyruvate KINASE isozymes M1/M2 OS=Homo sapiens GN=PKM PE=1 SV=4	58470	8	5	1820	3303	5.79	22.89	3.95336788	47	73	58	99	60	100	66	111	-0.16202
sp|O00764|PDXK_HUMAN	PDXK	Pyridoxal kinase OS=Homo sapiens GN=PDXK PE=1 SV=1	35308	566	501	29	85	0.11	0.18	1.63636364	0	1	1	1	1	1	1	1	-0.89229
sp|P60891|PRPS1_HUMAN	PRPS1	Ribose-phosphate pyrophosphokinase 1 OS=Homo sapiens GN=PRPS1 PE=1 SV=2	35325	638	880	22	42	0.14	0.18	1.28571429	0	1	1	1	1	1	2	1	-0.89229
sp|P17858|K6PL_HUMAN	PFKL	6-phosphofructoKINASE, liver type OS=Homo sapiens GN=PFKL PE=1 SV=6	85762	98	269	88	182	0.4	0.21	0.525	1	4	2	5	2	6	2	6	0.486789
tr|E9PK59|E9PK59_HUMAN	SCYL1	N-terminal KINASE-like protein OS=Homo sapiens GN=SCYL1 PE=2 SV=1	86757	0	1058	0	31	0	0.04	#DIV/0!	0	0	0	1	0	1	0	1	0.693008
tr|B3KXE7|B3KXE7_HUMAN	tr|B3KXE7|B3K)	cDNA FLJ45304 fis, clone BRHIP3003984, highly similar to IkappaB KINASE complex-associated protein OS=Hor	112698	0	782	0	50	0	0.03	#DIV/0!	0	1	0	1	0	1	0	1	0.693008
tr|C1PHA2|C1PHA2_HUMAN	KIF5B-ALK	Tyrosine-protein KINASE receptor OS=Homo sapiens GN=KIF5B-ALK PE=2 SV=1	169283	227	197	91	248	0.04	0.17	4.25	2	8	2	8	2	11	3	14	1.310812
tr|B4DNJ6|B4DNJ6_HUMAN	STRAP	Serine-threonine KINASE receptor-associated protein OS=Homo sapiens GN=STRAP PE=2 SV=1	40095	626	676	23	59	0.12	0.17	1.41666667	0	1	1	2	1	1	1	3	-0.89229
tr|Q5JQ10|Q5JQ10_HUMAN	ADK	Adenosine KINASE OS=Homo sapiens GN=ADK PE=2 SV=1	39078	538	989	30	35	0.08	0.08	1	0	0	1	1	1	1	1	2	-0.89229
tr|B2R5T5|B2R5T5_HUMAN	PRKAR1A	Protein KINASE, cAMP-dependent, regulatory, type I, alpha (Tissue specific extinguisher 1), isoform CRA_a OS=	43183	533	679	31	59	0.08	0.08	1	0	1	1	1	1	1	1	2	-0.89229
tr|B0AZV0|B0AZV0_HUMAN	STRAP	Serine-threonine KINASE receptor-associated protein OS=Homo sapiens GN=STRAP PE=2 SV=1	28716	626	628	23	64	0.12	0.12	1	0	1	1	1	1	1	1	1	-0.89229
tr|B4DP56|B4DP56_HUMAN	tr|B4DP56|B4D	cDNA FLJ52237, highly similar to Creatine KINASE B-type (EC 2.7.3.2) OS=Homo sapiens PE=2 SV=1	38840	214	975	97	29	0.18	0.16	0.88888889	2	0	2	1	2	1	2	2	-1.62959
tr|B3KS97|B3KS97_HUMAN	MAP2K2	Mitogen-activated protein KINASE KINASE 2, isoform CRA_d OS=Homo sapiens GN=MAP2K2 PE=2 SV=1	23450	384	621	45	0	0.31	0.16	0.51612903	1	0	2	0	2	0	2	1	-3.21473
tr|G5E9C7|G5E9C7_HUMAN	MAP2K2	Dual-specificity mitogen-activated protein KINASE KINASE 2 OS=Homo sapiens GN=MAP2K2 PE=2 SV=1	34145	384	1205	45	20	0.31	0.07	0.22580645	1	1	2	1	2	1	2	3	-1.62959
tr|B4E1G6|B4E1G6_HUMAN	GALK1	GalactoKINASE OS=Homo sapiens GN=GALK1 PE=2 SV=1	45956	679	0	20	0	0.07	0	0	0	0	1	0	0	0	1	0	-0.89213
tr|A6NIT2|A6NIT2_HUMAN	PHKA1	Phosphorylase b KINASE regulatory subunit alpha, skeletal muscle isoform OS=Homo sapiens GN=PHKA1 PE=2	133462	711	0	17	0	0.02	0	0	0	0	1	0	0	0	1	0	-0.89213
tr|Q8IWY7|Q8IWY7_HUMAN	TTBK2	Tau-tubulin KINASE OS=Homo sapiens GN=TTBK2 PE=2 SV=1	183893	79	0	28	0	0.79	0	0	0	0	1	0	1	0	1	0	-2.47743
tr|B4E2K2|B4E2K2_HUMAN	tr|B4E2K2|B4E2	cDNA FLJ55665, highly similar to A-KINASE anchor protein 2 OS=Homo sapiens PE=2 SV=1	103935	246	0	79	0	0.03	0	0	1	0	1	0	1	0	1	0	-2.47743
sp|Q9UEW8|STK39_HUMAN	STK39	STE20/SPS1-related proline-alanine-rich protein KINASE OS=Homo sapiens GN=STK39 PE=1 SV=3	59950	491	0	35	0	0.05	0	0	0	0	1	0	1	0	1	0	-2.47743
tr|K7EID3|K7EID3_HUMAN	PRKAR1A	cAMP-dependent protein KINASE type I-alpha regulatory subunit (Fragment) OS=Homo sapiens GN=PRKAR1A	19112	533	0	31	0	0.08	0	0	0	0	1	0	1	0	1	0	-2.47743
tr|H0YH33|H0YH33_HUMAN	STRAP	Serine-threonine KINASE receptor-associated protein (Fragment) OS=Homo sapiens GN=STRAP PE=4 SV=1	10763	626	0	23	0	0.12	0	0	0	0	1	0	1	0	1	0	-2.47743
tr|H0Y3C5|H0Y3C5_HUMAN	HCK	Tyrosine-protein KINASE HCK OS=Homo sapiens GN=HCK PE=4 SV=1	59986	628	0	23	0	0.05	0	0	0	0	1	0	1	0	2	0	-2.47743
tr|H0Y3Y5|H0Y3Y5_HUMAN	PPIP5K1	Inositol hexakisphosphate and diphosphoinositol-pentakisphosphate KINASE 1 (Fragment) OS=Homo sapiens (	21913	632	0	22	0	0.15	0	0	0	0	1	0	1	0	2	0	-2.47743
sp|O95747|OXSR1_HUMAN	OXSR1	Serine/threonine-protein KINASE OSR1 OS=Homo sapiens GN=OXSR1 PE=1 SV=1	58271	675	0	20	0	0.06	0	0	0	0	1	0	1	0	2	0	-2.47743
tr|B4DJH7|B4DJH7_HUMAN	tr|B4DJH7|B4D.	cDNA FLJ56916, highly similar to Cell division protein KINASE 8 (EC 2.7.11.22) OS=Homo sapiens PE=2 SV=1	34931	285	0	65	0	0.24	0	0	2	0	2	0	2	0	2	0	-3.21473
tr|H3BRW9|H3BRW9_HUMAN	MAP2K1	Dual-specificity mitogen-activated protein KINASE KINASE 1 OS=Homo sapiens GN=MAP2K1 PE=4 SV=1	24160	384	0	45	0	0.31	0	0	1	0	2	0	2	0	2	0	-3.21473
tr|C9J9S3|C9J9S3_HUMAN	PPP1CB	Serine/threonine-protein phosphatase (Fragment) OS=Homo sapiens GN=PPP1CB PE=2 SV=1	14484	345	700	51	25	0.24	0.22	0.91666667	1	0	1	1	1	1	1	1	-0.89229
tr|H0YDU8|H0YDU8_HUMAN	PPP5C	Serine/threonine-protein phosphatase (Fragment) OS=Homo sapiens GN=PPP5C PE=3 SV=1	55743	596	609	26	67	0.06	0.06	1	0	1	1	1	1	1	1	1	-0.89229
sp|P18031|PTN1_HUMAN	PTPN1	Tyrosine-protein phosphatase non-receptor type 1 OS=Homo sapiens GN=PTPN1 PE=1 SV=1	50505	0	1092	0	29	0	0.07	#DIV/0!	0	0	0	1	0	1	0	1	0.693008
sp|Q8TAP8|PPR35_HUMAN	PPP1R35	Protein phosphatase 1 regulatory subunit 35 OS=Homo sapiens GN=PPP1R35 PE=1 SV=1	28107	0	825	0	47	0	0.12	#DIV/0!	0	1	0	1	0	1	0	1	0.693008
tr|B4DK71|B4DK71_HUMAN	SACM1L	Phosphatidylinositide phosphatase SAC1 OS=Homo sapiens GN=SACM1L PE=2 SV=1	61327	0	653	0	62	0	0.05	#DIV/0!	0	1	0	1	0	1	0	2	0.693008
tr|B4DDF7|B4DDF7_HUMAN	tr|B4DDF7|B4D	cDNA FLJ53296, highly similar to Serine/threonine-protein phosphatase 2A 65 kDa regulatory subunit A alpha	64939	443	263	39	190	0.16	0.56	3.5	1	3	3	9	3	8	3	13	0.388412
tr|J3QT22|J3QT22_HUMAN	PPME1	Protein phosphatase methylesterase 1 OS=Homo sapiens GN=PPME1 PE=3 SV=1	44241	232	884	90	42	0.07	0.24	3.42857143	1	0	1	3	1	3	2	4	0.330464
tr|B4DDZ8|B4DDZ8_HUMAN	tr|B4DDZ8|B4D	cDNA FLJ55048, highly similar to Serine/threonine-protein phosphatase 5 (EC 3.1.3.16) OS=Homo sapiens PE=	55829	596	0	26	0	0.06	0	0	0	0	1	0	1	0	1	0	-2.47743
tr|E1NZA1|E1NZA1_HUMAN	PRIC295	Peroxisome proliferator activated receptor interacting complex protein OS=Homo sapiens GN=PRIC295 PE=2 S	294953	0	167	0	287	0	0.21	#DIV/0!	0	9	0	17	0	17	0	22	4.240211
tr|A8K132|A8K132_HUMAN	tr|A8K132|A8K	cDNA FLJ75476, highly similar to Homo sapiens glutaminase (GLS), mRNA OS=Homo sapiens PE=2 SV=1	74317	0	148	0	315	0	0.54	#DIV/0!	0	7	0	10	0	14	0	16	3.968368
tr|B4DLV7|B4DLV7_HUMAN	tr|B4DLV7|B4D	cDNA FLJ60299, highly similar to Rab GDP dissociation inhibitor beta OS=Homo sapiens PE=2 SV=1	51577	0	108	0	381	0	1.38	#DIV/0!	0	9	0	14	0	14	0	17	3.968368
tr|B4DSL6|B4DSL6_HUMAN	tr|B4DSL6|B4D	cDNA FLJ57190, highly similar to Actin-binding protein anillin OS=Homo sapiens PE=2 SV=1	111977	0	219	0	227	0	0.34	#DIV/0!	0	9	0	11	0	13	0	13	3.865094
sp|O75533|SF3B1_HUMAN	SF3B1	Splicing factor 3B subunit 1 OS=Homo sapiens GN=SF3B1 PE=1 SV=3	146479	0	229	0	217	0	0.25	#DIV/0!	0	6	0	10	0	12	0	13	3.753883
tr|B2RDD7|B2RDD7_HUMAN	tr|B2RDD7|B2R	cDNA, FLJ96564, highly similar to Homo sapiens SKB1 homolog (S. pombe) (SKB1), mRNA OS=Homo sapiens PE	73348	0	271	0	181	0	0.36	#DIV/0!	0	5	0	7	0	11	0	14	3.633408
tr|D6REM6|D6REM6_HUMAN	MATR3	Matrin-3 OS=Homo sapiens GN=MATR3 PE=2 SV=1	88647	0	192	0	228	0	0.38	#DIV/0!	0	7	0	9	0	11	0	12	3.633408
tr|A8K8U1|A8K8U1_HUMAN	tr|A8K8U1|A8K	cDNA FLJ77762, highly similar to Homo sapiens cullin-associated and neddylation-dissociated 1 (CAND1), mRN	137941	0	172	0	284	0	0.32	#DIV/0!	0	10	0	13	0	11	0	15	3.633408
tr|B3KWV6|B3KWV6_HUMAN	tr|B3KWV6|B3K	cDNA FLJ43948 fis, clone TESTI4014924, highly similar to Homo sapiens cytoplasmic FMR1 interacting protein	147082	0	277	0	179	0	0.19	#DIV/0!	0	5	0	8	0	10	0	10	3.501984
sp|O43776|SYNC_HUMAN	NARS	Asparagine--tRNA ligase, cytoplasmic OS=Homo sapiens GN=NARS PE=1 SV=1	63758	0	178	0	270	0	0.57	#DIV/0!	0	6	0	9	0	10	0	11	3.501984
tr|Q6NVW7|Q6NVW7_HUMAN	KPNA2	Importin subunit alpha OS=Homo sapiens GN=KPNA2 PE=2 SV=1	58271	0	169	0	286	0	0.55	#DIV/0!	0	5	0	9	0	10	0	10	3.501984
tr|B3KM58|B3KM58_HUMAN	tr|B3KM58|B3K	cDNA FLJ10358 fis, clone NT2RM2001238, highly similar to Glutaminase kidney isoform, mitochondrial (Fragm	41736	0	96	0	414	0	0.84	#DIV/0!	0	7	0	8	0	10	0	12	3.501984
tr|B4DY30|B4DY30_HUMAN	tr|B4DY30|B4D	cDNA FLJ54078, highly similar to Protein arginine N-methyltransferase 5 (EC 2.1.1.-) OS=Homo sapiens PE=2 S\	68284	0	415	0	106	0	0.26	#DIV/0!	0	3	0	5	0	9	0	11	3.357414
sp|Q08J23|NSUN2_HUMAN	NSUN2	tRNA (cytosine(34)-C(5))-methyltransferase OS=Homo sapiens GN=NSUN2 PE=1 SV=2	87214	0	239	0	210	0	0.29	#DIV/0!	0	5	0	7	0	9	0	12	3.357414
tr|A1KYQ7|A1KYQ7_HUMAN	tr|A1KYQ7|A1K	Cell migration-inducing protein 17 OS=Homo sapiens PE=2 SV=1	106022	0	217	0	231	0	0.31	#DIV/0!	0	8	0	9	0	9	0	9	3.357414
tr|B7Z4B2|B7Z4B2_HUMAN	tr|B7Z4B2|B7Z4	cDNA FLJ56108, highly similar to Puromycin-sensitive aminopeptidase (EC 3.4.11.-) OS=Homo sapiens PE=2 SV	93434	0	184	0	263	0	0.36	#DIV/0!	0	8	0	9	0	9	0	10	3.357414
tr|B4E3J7|B4E3J7_HUMAN	tr|B4E3J7|B4E3	cDNA FLJ51203, highly similar to Thrombospondin-1 OS=Homo sapiens PE=2 SV=1	124032	0	155	0	273	0	0.32	#DIV/0!	0	6	0	10	0	9	0	10	3.357414
tr|B7Z1N6|B7Z1N6_HUMAN	tr|B7Z1N6|B7Z	cDNA FLJ52222, highly similar to Fructose-bisphosphate aldolase C (EC4.1.2.13) OS=Homo sapiens PE=2 SV=1	35800	0	149	0	313	0	0.56	#DIV/0!	0	9	0	9	0	9	0	12	3.357414
tr|B2RDW1|B2RDW1_HUMAN	RPS27A	Ribosomal protein S27a, isoform CRA_c OS=Homo sapiens GN=RPS27A PE=2 SV=1	18296	0	353	0	127	0	0.66	#DIV/0!	0	4	0	6	0	8	0	9	3.196769
tr|B4DZI8|B4DZI8_HUMAN	COPB2	Coatomer protein complex, subunit beta 2 (Beta prime), isoform CRA_b OS=Homo sapiens GN=COPB2 PE=2 S\	99839	0	193	0	251	0	0.34	#DIV/0!	0	6	0	9	0	8	0	10	3.196769
tr|B3KVJ5|B3KVJ5_HUMAN	tr|B3KVJ5|B3K\	cDNA FLJ16648 fis, clone TESTI4035508, highly similar to Structural maintenance of chromosomes 4-like 1 pro	101354	0	437	0	94	0	0.14	#DIV/0!	0	4	0	5	0	7	0	8	3.016016
tr|B3KMV5|B3KMV5_HUMAN	tr|B3KMV5|B3K	cDNA FLJ12728 fis, clone NT2RP2000040, highly similar to Protein FAM62A OS=Homo sapiens PE=2 SV=1	123342	0	302	0	158	0	0.17	#DIV/0!	0	3	0	6	0	7	0	7	3.016016
sp|P49792|RBP2_HUMAN	RANBP2	E3 SUMO-protein ligase RanBP2 OS=Homo sapiens GN=RANBP2 PE=1 SV=2	362365	0	273	0	181	0	0.06	#DIV/0!	0	4	0	6	0	7	0	7	3.016016
tr|G3V198|G3V198_HUMAN	NUP160	Nuclear pore complex protein Nup160 OS=Homo sapiens GN=NUP160 PE=2 SV=1	142365	0	261	0	194	0	0.17	#DIV/0!	0	5	0	7	0	7	0	8	3.016016
tr|B4E1K0|B4E1K0_HUMAN	KIF23	Kinesin-like protein KIF23 OS=Homo sapiens GN=KIF23 PE=2 SV=1	78221	0	255	0	153	0	0.3	#DIV/0!	0	5	0	7	0	7	0	8	3.016016
tr|B0QY89|B0QY89_HUMAN	EIF3EIP	Eukaryotic translation initiation factor 3 subunit L OS=Homo sapiens GN=EIF3EIP PE=4 SV=1	71085	0	228	0	218	0	0.44	#DIV/0!	0	5	0	8	0	7	0	9	3.016016
tr|B4E063|B4E063_HUMAN	tr|B4E063|B4E0	cDNA FLJ54292, highly similar to Kinesin-like protein KIFC1 OS=Homo sapiens PE=2 SV=1	73571	0	222	0	222	0	0.24	#DIV/0!	0	5	0	5	0	7	0	8	3.016016
sp|P46013|KI67_HUMAN	MKI67	Antigen KI-67 OS=Homo sapiens GN=MKI67 PE=1 SV=2	360698	0	732	0	54	0	0.04	#DIV/0!	0	1	0	4	0	6	0	6	2.809386
sp|Q14690|RRP5_HUMAN	PDCD11	Protein RRP5 homolog OS=Homo sapiens GN=PDCD11 PE=1 SV=3	209939	0	579	0	72	0	0.06	#DIV/0!	0	2	0	4	0	6	0	7	2.809386
sp|Q8NI36|WDR36_HUMAN	WDR36	WD repeat-containing protein 36 OS=Homo sapiens GN=WDR36 PE=1 SV=1	106282	0	547	0	77	0	0.16	#DIV/0!	0	1	0	5	0	6	0	6	2.809386
sp|Q5SRE5|NU188_HUMAN	NUP188	Nucleoporin NUP188 homolog OS=Homo sapiens GN=NUP188 PE=1 SV=1	198369	0	432	0	102	0	0.12	#DIV/0!	0	3	0	7	0	6	0	8	2.809386
tr|B4DUF1|B4DUF1_HUMAN	tr|B4DUF1|B4D	cDNA FLJ59760, highly similar to 1,4-alpha-glucan branching enzyme (EC 2.4.1.18) OS=Homo sapiens PE=2 SV=	76254	0	377	0	89	0	0.3	#DIV/0!	0	3	0	6	0	6	0	8	2.809386
tr|B4DZP5|B4DZP5_HUMAN	tr|B4DZP5|B4D	cDNA FLJ51165, highly similar to DNA damage-binding protein 1 OS=Homo sapiens PE=2 SV=1	92726	0	369	0	122	0	0.14	#DIV/0!	0	4	0	5	0	6	0	6	2.809386
sp|P36952|SPB5_HUMAN	SERPINB5	Serpin B5 OS=Homo sapiens GN=SERPINB5 PE=1 SV=2	42530	0	367	0	122	0	0.57	#DIV/0!	0	4	0	6	0	6	0	6	2.809386
tr|Q59H95|Q59H95_HUMAN	tr|Q59H95|Q59	Flightless I homolog variant (Fragment) OS=Homo sapiens PE=2 SV=1	126396	0	350	0	90	0	0.17	#DIV/0!	0	2	0	6	0	6	0	7	2.809386
tr|B4DL79|B4DL79_HUMAN	KIF20A	Kinesin-like protein KIF20A OS=Homo sapiens GN=KIF20A PE=2 SV=1	99066	0	328	0	141	0	0.14	#DIV/0!	0	2	0	4	0	6	0	6	2.809386
tr|K4DI93|K4DI93_HUMAN	CUL4B	Cullin 4B, isoform CRA_e OS=Homo sapiens GN=CUL4B PE=3 SV=1	103318	0	296	0	164	0	0.21	#DIV/0!	0	5	0	6	0	6	0	6	2.809386
tr|Q3SYK8|Q3SYK8_HUMAN	NUMA1	NUMA1 protein (Fragment) OS=Homo sapiens GN=NUMA1 PE=2 SV=1	78666	0	114	0	163	0	0.21	#DIV/0!	0	5	0	6	0	6	0	6	2.809386
tr|G5E9H4|G5E9H4_HUMAN	DCTN1	Dynactin 1 (P150, glued homolog, Drosophila), isoform CRA_a OS=Homo sapiens GN=DCTN1 PE=2 SV=1	127339	0	512	0	82	0	0.14	#DIV/0!	0	1	0	5	0	5	0	5	2.568197
tr|B0YIW5|B0YIW5_HUMAN	ARCN1	Archain 1, isoform CRA_b OS=Homo sapiens GN=ARCN1 PE=2 SV=1	57630	0	511	0	82	0	0.32	#DIV/0!	0	1	0	5	0	5	0	7	2.568197
tr|Q59FA0|Q59FA0_HUMAN	tr|Q59FA0|Q59	DNA polymerase (Fragment) OS=Homo sapiens PE=2 SV=1	113271	0	507	0	83	0	0.15	#DIV/0!	0	3	0	5	0	5	0	6	2.568197
tr|B4DSN3|B4DSN3_HUMAN	tr|B4DSN3|B4D	cDNA FLJ60345, highly similar to Protein transport protein Sec24C OS=Homo sapiens PE=2 SV=1	107974	0	425	0	104	0	0.09	#DIV/0!	0	3	0	3	0	5	0	6	2.568197
sp|Q96I24|FUBP3_HUMAN	FUBP3	Far upstream element-binding protein 3 OS=Homo sapiens GN=FUBP3 PE=1 SV=2	61944	0	416	0	106	0	0.3	#DIV/0!	0	3	0	5	0	5	0	5	2.568197
tr|B3KM90|B3KM90_HUMAN	tr|B3KM90|B3K	cDNA FLJ10529 fis, clone NT2RP2000965, highly similar to Targeting protein for Xklp2 OS=Homo sapiens PE=2	70285	0	407	0	109	0	0.26	#DIV/0!	0	3	0	5	0	5	0	5	2.568197
tr|A8K0D2|A8K0D2_HUMAN	tr|A8K0D2|A8K	cDNA FLJ77740, highly similar to Homo sapiens 7-dehydrocholesterol reductase, mRNA OS=Homo sapiens PE=	55175	0	319	0	147	0	0.26	#DIV/0!	0	3	0	4	0	5	0	5	2.568197
tr|H0Y5W0|H0Y5W0_HUMAN	HUWE1	E3 ubiquitin-protein ligase HUWE1 (Fragment) OS=Homo sapiens GN=HUWE1 PE=4 SV=1	376696	0	309	0	138	0	0.03	#DIV/0!	0	3	0	4	0	5	0	5	2.568197
tr|B4DZC0|B4DZC0_HUMAN	tr|B4DZC0|B4D	cDNA FLJ51771, highly similar to SWI/SNF-related matrix-associatedactin-dependent regulator of chromatin su	117367	0	307	0	153	0	0.09	#DIV/0!	0	3	0	3	0	5	0	6	2.568197
tr|B4DEX7|B4DEX7_HUMAN	tr|B4DEX7|B4D	cDNA FLJ51806, highly similar to Homo sapiens proline-, glutamic acid-, leucine-rich protein 1 (PELP1), mRNA (	97913	0	784	0	50	0	0.1	#DIV/0!	0	1	0	3	0	4	0	4	2.278511
tr|A4D275|A4D275_HUMAN	ARPC1B	Actin related protein 2/3 complex, subunit 1B, 41kDa OS=Homo sapiens GN=ARPC1B PE=2 SV=1	41722	0	691	0	58	0	0.36	#DIV/0!	0	1	0	4	0	4	0	4	2.278511
tr|B4DR36|B4DR36_HUMAN	tr|B4DR36|B4D	cDNA FLJ56414, highly similar to Homo sapiens proline-, glutamic acid-, leucine-rich protein 1 (PELP1), mRNA (	126101	0	610	0	67	0	0.1	#DIV/0!	0	2	0	4	0	4	0	4	2.278511
tr|B4DIT7|B4DIT7_HUMAN	TGM2	Protein-glutamine gamma-glutamyltransferase 2 OS=Homo sapiens GN=TGM2 PE=2 SV=1	69632	0	606	0	67	0	0.2	#DIV/0!	0	1	0	4	0	4	0	7	2.278511
tr|B2RE88|B2RE88_HUMAN	tr|B2RE88|B2RI	cDNA, FLJ96465, highly similar to Homo sapiens solute carrier family 25 (mitochondrial carrier;phosphate carr	40379	0	576	0	37	0	0.42	#DIV/0!	0	0	0	3	0	4	0	5	2.278511
tr|A4D210|A4D210_HUMAN	EIF3S9	Eukaryotic translation initiation factor 3, subunit 9 eta, 116kDa OS=Homo sapiens GN=EIF3S9 PE=4 SV=1	89024	0	517	0	81	0	0.16	#DIV/0!	0	2	0	4	0	4	0	6	2.278511
tr|B7ZMF2|B7ZMF2_HUMAN	FANCI	Fanconi anemia, complementation group I OS=Homo sapiens GN=FANCI PE=2 SV=1	150724	0	505	0	84	0	0.09	#DIV/0!	0	2	0	4	0	4	0	5	2.278511
tr|B4DUT7|B4DUT7_HUMAN	tr|B4DUT7|B4D	cDNA FLJ57604, highly similar to GMP synthase (glutamine-hydrolyzing) (EC 6.3.5.2) OS=Homo sapiens PE=2 S\	71880	0	504	0	76	0	0.23	#DIV/0!	0	2	0	4	0	4	0	5	2.278511
tr|D3DTD2|D3DTD2_HUMAN	TRIO	Triple functional domain (PTPRF interacting), isoform CRA_a OS=Homo sapiens GN=TRIO PE=2 SV=1	270210	0	486	0	90	0	0.05	#DIV/0!	0	3	0	4	0	4	0	6	2.278511
tr|B7Z4B8|B7Z4B8_HUMAN	HNRNPUL1	Heterogeneous nuclear ribonucleoprotein U-like protein 1 OS=Homo sapiens GN=HNRNPUL1 PE=2 SV=1	86639	0	465	0	95	0	0.08	#DIV/0!	0	2	0	2	0	4	0	4	2.278511
tr|B2RDX0|B2RDX0_HUMAN	tr|B2RDX0|B2R	cDNA, FLJ96805, highly similar to Homo sapiens SHC SH2-domain binding protein 1 (SHCBP1), mRNA OS=Homo	76668	0	464	0	95	0	0.18	#DIV/0!	0	3	0	4	0	4	0	4	2.278511
tr|B4DHY1|B4DHY1_HUMAN	HNRNPH3	Heterogeneous nuclear ribonucleoprotein H3 OS=Homo sapiens GN=HNRNPH3 PE=2 SV=1	24925	0	443	0	79	0	0.29	#DIV/0!	0	2	0	2	0	4	0	4	2.278511
sp|Q8WX93|PALLD_HUMAN	PALLD	Palladin OS=Homo sapiens GN=PALLD PE=1 SV=3	151839	0	434	0	101	0	0.09	#DIV/0!	0	3	0	4	0	4	0	5	2.278511
sp|P09960|LKHA4_HUMAN	LTA4H	Leukotriene A-4 hydrolase OS=Homo sapiens GN=LTA4H PE=1 SV=2	69868	0	427	0	104	0	0.22	#DIV/0!	0	3	0	4	0	4	0	4	2.278511
sp|Q27J81|INF2_HUMAN	INF2	Inverted formin-2 OS=Homo sapiens GN=INF2 PE=1 SV=2	136851	0	426	0	104	0	0.1	#DIV/0!	0	3	0	4	0	4	0	4	2.278511
tr|G3V1J5|G3V1J5_HUMAN	DIS3	Exosome complex exonuclease RRP44 OS=Homo sapiens GN=DIS3 PE=2 SV=1	91382	0	400	0	111	0	0.15	#DIV/0!	0	1	0	5	0	4	0	5	2.278511
tr|D3DTY9|D3DTY9_HUMAN	TRIM25	Tripartite motif-containing 25, isoform CRA_a OS=Homo sapiens GN=TRIM25 PE=2 SV=1	48358	0	394	0	91	0	0.19	#DIV/0!	0	2	0	3	0	4	0	5	2.278511
sp|Q92945|FUBP2_HUMAN	KHSRP	Far upstream element-binding protein 2 OS=Homo sapiens GN=KHSRP PE=1 SV=4	73355	0	376	0	118	0	0.19	#DIV/0!	0	3	0	4	0	4	0	5	2.278511
tr|B4DT31|B4DT31_HUMAN	FUBP1	Far upstream element-binding protein 1 OS=Homo sapiens GN=FUBP1 PE=2 SV=1	69993	0	360	0	124	0	0.15	#DIV/0!	0	2	0	4	0	4	0	5	2.278511
tr|H3BLU7|H3BLU7_HUMAN	AKR7A2	Aflatoxin B1 aldehyde reductase member 2 (Fragment) OS=Homo sapiens GN=AKR7A2 PE=4 SV=1	35004	0	348	0	129	0	0.44	#DIV/0!	0	3	0	4	0	4	0	4	2.278511
tr|D3DUW5|D3DUW5_HUMAN	DNM1L	Dynamin 1-like, isoform CRA_c OS=Homo sapiens GN=DNM1L PE=3 SV=1	88442	0	346	0	129	0	0.12	#DIV/0!	0	2	0	3	0	4	0	4	2.278511
tr|A8K5Y7|A8K5Y7_HUMAN	tr|A8K5Y7|A8K	cDNA FLJ78655, highly similar to Homo sapiens exportin 5 (XPO5), mRNA OS=Homo sapiens PE=2 SV=1	138331	0	342	0	135	0	0.1	#DIV/0!	0	3	0	4	0	4	0	5	2.278511
tr|G5E9P1|G5E9P1_HUMAN	ITPR1	Inositol 1,4,5-triphosphate receptor, type 1, isoform CRA_d OS=Homo sapiens GN=ITPR1 PE=2 SV=1	311832	0	333	0	138	0	0.05	#DIV/0!	0	4	0	5	0	4	0	7	2.278511
tr|B3KPA6|B3KPA6_HUMAN	ACADVL	Acyl-Coenzyme A dehydrogenase, very long chain, isoform CRA_e OS=Homo sapiens GN=ACADVL PE=2 SV=1	62941	0	325	0	144	0	0.29	#DIV/0!	0	3	0	5	0	4	0	5	2.278511
tr|B3KY63|B3KY63_HUMAN	tr|B3KY63|B3K	cDNA FLJ16830 fis, clone UTERU3022536, highly similar to Chromodomain helicase-DNA-binding protein 4 (EC	216574	0	312	0	90	0	0.06	#DIV/0!	0	2	0	3	0	4	0	6	2.278511
sp|Q9H3U1|UN45A_HUMAN	UNC45A	Protein unc-45 homolog A OS=Homo sapiens GN=UNC45A PE=1 SV=1	104266	0	308	0	153	0	0.13	#DIV/0!	0	1	0	4	0	4	0	4	2.278511
tr|Q5TZZ9|Q5TZZ9_HUMAN	ANXA1	Annexin OS=Homo sapiens GN=ANXA1 PE=2 SV=1	38918	0	298	0	163	0	0.5	#DIV/0!	0	4	0	5	0	4	0	6	2.278511
tr|A8K4T6|A8K4T6_HUMAN	tr|A8K4T6|A8K	cDNA FLJ76282, highly similar to Homo sapiens proteasome (prosome, macropain) 26S subunit, non-ATPase, 5	56588	0	291	0	166	0	0.25	#DIV/0!	0	3	0	4	0	4	0	4	2.278511
tr|G3XAL0|G3XAL0_HUMAN	MDH2	Malate dehydrogenase OS=Homo sapiens GN=MDH2 PE=2 SV=1	24921	0	221	0	106	0	0.63	#DIV/0!	0	2	0	3	0	4	0	4	2.278511
tr|B4E3M6|B4E3M6_HUMAN	tr|B4E3M6|B4E	cDNA FLJ55446, highly similar to Superkiller viralicidic activity 2-like 2 (EC 3.6.1.-) OS=Homo sapiens PE=2 SV=1	107714	0	1195	0	21	0	0.03	#DIV/0!	0	0	0	3	0	3	0	6	1.915761
sp|Q8NI35|INADL_HUMAN	INADL	InaD-like protein OS=Homo sapiens GN=INADL PE=1 SV=3	197046	0	1158	0	24	0	0.02	#DIV/0!	0	1	0	3	0	3	0	3	1.915761
tr|Q6ZMY0|Q6ZMY0_HUMAN	tr|Q6ZMY0|Q6Z	cDNA FLJ16598 fis, clone TESTI4006473, weakly similar to ATP-dependent RNA helicase A OS=Homo sapiens P	152244	0	1015	0	34	0	0.02	#DIV/0!	0	1	0	3	0	3	0	4	1.915761
sp|Q9Y3Z3|SAMH1_HUMAN	SAMHD1	SAM domain and HD domain-containing protein 1 OS=Homo sapiens GN=SAMHD1 PE=1 SV=2	72896	0	983	0	36	0	0.05	#DIV/0!	0	0	0	1	0	3	0	3	1.915761
tr|Q71UF1|Q71UF1_HUMAN	ACO2	Aconitase OS=Homo sapiens GN=ACO2 PE=2 SV=1	86304	0	937	0	38	0	0.04	#DIV/0!	0	1	0	3	0	3	0	3	1.915761
tr|A2A274|A2A274_HUMAN	ACO2	Aconitase 2, mitochondrial OS=Homo sapiens GN=ACO2 PE=4 SV=1	88563	0	936	0	38	0	0.04	#DIV/0!	0	1	0	3	0	3	0	3	1.915761
sp|Q96CS3|FAF2_HUMAN	FAF2	FAS-associated factor 2 OS=Homo sapiens GN=FAF2 PE=1 SV=2	52933	0	929	0	39	0	0.2	#DIV/0!	0	1	0	3	0	3	0	4	1.915761
tr|B0AZM4|B0AZM4_HUMAN	tr|B0AZM4|B0A	cDNA, FLJ79464, highly similar to Homo sapiens aquarius (Fragment) OS=Homo sapiens PE=2 SV=1	157764	0	899	0	41	0	0.04	#DIV/0!	0	2	0	3	0	3	0	4	1.915761
sp|Q8TCT9|HM13_HUMAN	HM13	Minor histocompatibility antigen H13 OS=Homo sapiens GN=HM13 PE=1 SV=1	41747	0	889	0	41	0	0.26	#DIV/0!	0	1	0	3	0	3	0	4	1.915761
tr|Q5XJ04|Q5XJ04_HUMAN	HMGCS1	3-hydroxy-3-methylglutaryl-Coenzyme A synthase 1 (Soluble) OS=Homo sapiens GN=HMGCS1 PE=2 SV=1	57854	0	888	0	42	0	0.12	#DIV/0!	0	1	0	3	0	3	0	3	1.915761
sp|P12270|TPR_HUMAN	TPR	Nucleoprotein TPR OS=Homo sapiens GN=TPR PE=1 SV=3	267530	0	885	0	42	0	0.02	#DIV/0!	0	2	0	3	0	3	0	3	1.915761
sp|Q9BTC0|DIDO1_HUMAN	DIDO1	Death-inducer obliterator 1 OS=Homo sapiens GN=DIDO1 PE=1 SV=5	245434	0	863	0	44	0	0.04	#DIV/0!	0	0	0	3	0	3	0	3	1.915761
sp|P61221|ABCE1_HUMAN	ABCE1	ATP-binding cassette sub-family E member 1 OS=Homo sapiens GN=ABCE1 PE=1 SV=1	68240	0	860	0	44	0	0.15	#DIV/0!	0	1	0	3	0	3	0	5	1.915761
tr|B4E205|B4E205_HUMAN	tr|B4E205|B4E2	cDNA FLJ61651, highly similar to Protein transport protein Sec24A OS=Homo sapiens PE=2 SV=1	96953	0	852	0	44	0	0.1	#DIV/0!	0	1	0	3	0	3	0	4	1.915761
tr|B2RWN5|B2RWN5_HUMAN	HEATR1	HEAT repeat containing 1 OS=Homo sapiens GN=HEATR1 PE=2 SV=1	244275	0	851	0	44	0	0.03	#DIV/0!	0	1	0	3	0	3	0	4	1.915761
tr|Q4ZG72|Q4ZG72_HUMAN	DDX18	Putative uncharacterized protein DDX18 (Fragment) OS=Homo sapiens GN=DDX18 PE=2 SV=1	61898	0	847	0	44	0	0.14	#DIV/0!	0	1	0	3	0	3	0	4	1.915761
tr|B3KRS5|B3KRS5_HUMAN	HDAC2	Histone deacetylase OS=Homo sapiens GN=HDAC2 PE=2 SV=1	52479	0	843	0	46	0	0.13	#DIV/0!	0	0	0	2	0	3	0	5	1.915761
sp|Q14558|KPRA_HUMAN	PRPSAP1	Phosphoribosyl pyrophosphate synthase-associated protein 1 OS=Homo sapiens GN=PRPSAP1 PE=1 SV=2	39654	0	833	0	46	0	0.22	#DIV/0!	0	1	0	2	0	3	0	3	1.915761
tr|Q0PHS4|Q0PHS4_HUMAN	G6PD	Glucose-6-phosphate dehydrogenase (Fragment) OS=Homo sapiens GN=G6PD PE=2 SV=1	7182	0	830	0	46	0	2.32	#DIV/0!	0	1	0	3	0	3	0	3	1.915761
tr|Q149P0|Q149P0_HUMAN	GBF1	GBF1 protein OS=Homo sapiens GN=GBF1 PE=2 SV=1	207938	0	809	0	47	0	0.02	#DIV/0!	0	1	0	3	0	3	0	3	1.915761
sp|Q5T4S7|UBR4_HUMAN	UBR4	E3 ubiquitin-protein ligase UBR4 OS=Homo sapiens GN=UBR4 PE=1 SV=1	580547	0	773	0	51	0	0.01	#DIV/0!	0	2	0	3	0	3	0	5	1.915761
sp|P54577|SYYC_HUMAN	YARS	Tyrosine--tRNA ligase, cytoplasmic OS=Homo sapiens GN=YARS PE=1 SV=4	59448	0	766	0	51	0	0.11	#DIV/0!	0	1	0	3	0	3	0	4	1.915761
sp|Q92466|DDB2_HUMAN	DDB2	DNA damage-binding protein 2 OS=Homo sapiens GN=DDB2 PE=1 SV=1	48461	0	762	0	51	0	0.07	#DIV/0!	0	1	0	3	0	3	0	3	1.915761
tr|H0Y9V7|H0Y9V7_HUMAN	ATP2C1	Calcium-transporting ATPase type 2C member 1 (Fragment) OS=Homo sapiens GN=ATP2C1 PE=3 SV=1	100184	0	740	0	53	0	0.07	#DIV/0!	0	1	0	2	0	3	0	4	1.915761
tr|B3KMR5|B3KMR5_HUMAN	tr|B3KMR5|B3K	cDNA FLJ12434 fis, clone NT2RM1000037, highly similar to Homo sapiens KIAA0690 protein OS=Homo sapiens	145007	0	737	0	53	0	0.07	#DIV/0!	0	3	0	3	0	3	0	3	1.915761
sp|P49454|CENPF_HUMAN	CENPF	Centromere protein F OS=Homo sapiens GN=CENPF PE=1 SV=2	370844	0	727	0	54	0	0.03	#DIV/0!	0	1	0	3	0	3	0	3	1.915761
sp|P04899|GNAI2_HUMAN	GNAI2	Guanine nucleotide-binding protein G(i) subunit alpha-2 OS=Homo sapiens GN=GNAI2 PE=1 SV=3	40995	0	726	0	55	0	0.26	#DIV/0!	0	3	0	3	0	3	0	4	1.915761
tr|Q96FS1|Q96FS1_HUMAN	CTNND1	CTNND1 protein (Fragment) OS=Homo sapiens GN=CTNND1 PE=2 SV=2	92901	0	711	0	57	0	0.1	#DIV/0!	0	2	0	3	0	3	0	4	1.915761
sp|Q9UHD8|SEPT9_HUMAN	Sep-09	Septin-9 OS=Homo sapiens GN=SEPT9 PE=1 SV=2	65646	0	702	0	57	0	0.05	#DIV/0!	0	1	0	3	0	3	0	4	1.915761
tr|H7C3P6|H7C3P6_HUMAN	NUP98	Nuclear pore complex protein Nup98-Nup96 (Fragment) OS=Homo sapiens GN=NUP98 PE=4 SV=1	86760	0	686	0	58	0	0.12	#DIV/0!	0	2	0	3	0	3	0	3	1.915761

tr|Q6NUR1|Q6NUR1_HUMAN	NCAPG	Non-SMC condensin I complex, subunit G OS=Homo sapiens GN=NCAPG PE=2 SV=1	115205	0	683	0	59	0	0.09	#DIV/0!	0	1	0	3	0	3	0	4	1.915761
tr|G1UI17|G1UI17_HUMAN	AGL	Glycogen debranching enzyme (Fragment) OS=Homo sapiens GN=AGL PE=2 SV=1	145569	0	613	0	66	0	0.07	#DIV/0!	0	2	0	3	0	3	0	3	1.915761
tr|B3KT00|B3KT00_HUMAN	tr|B3KT00|B3KT	cDNA FLJ37368 fis, clone BRAMY2024530, highly similar to Zinc phosphodiesterase ELAC protein 2 (EC 3.1.26.1	91169	0	594	0	69	0	0.11	#DIV/0!	0	2	0	3	0	3	0	6	1.915761
tr|D3DP75|D3DP75_HUMAN	RAB3GAP1	RAB3 GTPase activating protein subunit 1 (Catalytic), isoform CRA_a OS=Homo sapiens GN=RAB3GAP1 PE=2 S	100213	0	592	0	70	0	0.1	#DIV/0!	0	2	0	3	0	3	0	3	1.915761
sp|Q8NI27|THOC2_HUMAN	THOC2	THO complex subunit 2 OS=Homo sapiens GN=THOC2 PE=1 SV=2	184541	0	591	0	70	0	0.1	#DIV/0!	0	3	0	3	0	3	0	3	1.915761
sp|Q9UHX1|PUF60_HUMAN	PUF60	Poly(U)-binding-splicing factor PUF60 OS=Homo sapiens GN=PUF60 PE=1 SV=1	60009	0	589	0	71	0	0.17	#DIV/0!	0	2	0	3	0	3	0	3	1.915761
tr|B0AZQ4|B0AZQ4_HUMAN	tr|B0AZQ4|B0A	cDNA, FLJ79494, highly similar to Structural maintenance of chromosome 3 OS=Homo sapiens PE=2 SV=1	141841	0	562	0	75	0	0.07	#DIV/0!	0	2	0	3	0	3	0	4	1.915761
tr|B2RAM6|B2RAM6_HUMAN	tr|B2RAM6|B2R	cDNA, FLJ95005, highly similar to Homo sapiens kinesin family member 11 (KIF11), mRNA OS=Homo sapiens P	120084	0	561	0	75	0	0.08	#DIV/0!	0	2	0	3	0	3	0	4	1.915761
sp|Q6PKG0|LARP1_HUMAN	LARP1	La-related protein 1 OS=Homo sapiens GN=LARP1 PE=1 SV=2	123833	0	560	0	75	0	0.08	#DIV/0!	0	2	0	3	0	3	0	4	1.915761
sp|Q5K651|SAMD9_HUMAN	SAMD9	Sterile alpha motif domain-containing protein 9 OS=Homo sapiens GN=SAMD9 PE=1 SV=1	185704	0	554	0	76	0	0.04	#DIV/0!	0	1	0	2	0	3	0	3	1.915761
tr|H0Y3P2|H0Y3P2_HUMAN	EIF4G2	Eukaryotic translation initiation factor 4 gamma 2 OS=Homo sapiens GN=EIF4G2 PE=4 SV=1	98569	0	548	0	77	0	0.1	#DIV/0!	0	2	0	3	0	3	0	3	1.915761
tr|Q59GM9|Q59GM9_HUMAN	tr|Q59GM9|Q5	Phosphorylase (Fragment) OS=Homo sapiens PE=2 SV=1	99508	0	542	0	62	0	0.16	#DIV/0!	0	2	0	3	0	3	0	4	1.915761
tr|E3Q0X1|E3Q0X1_HUMAN	HLA-B	MHC class I antigen (Fragment) OS=Homo sapiens GN=HLA-B PE=3 SV=1	31939	0	534	0	79	0	0.22	#DIV/0!	0	2	0	2	0	3	0	3	1.915761
tr|F5H8E5|F5H8E5_HUMAN	USP7	Ubiquitin carboxyl-terminal hydrolase OS=Homo sapiens GN=USP7 PE=2 SV=1	117951	0	526	0	80	0	0.09	#DIV/0!	0	2	0	3	0	3	0	4	1.915761
sp|Q9Y295|DRG1_HUMAN	DRG1	Developmentally-regulated GTP-binding protein 1 OS=Homo sapiens GN=DRG1 PE=1 SV=1	40802	0	513	0	82	0	0.17	#DIV/0!	0	2	0	2	0	3	0	3	1.915761
sp|Q5JVF3|PCID2_HUMAN	PCID2	PCI domain-containing protein 2 OS=Homo sapiens GN=PCID2 PE=1 SV=2	46627	0	509	0	83	0	0.23	#DIV/0!	0	2	0	3	0	3	0	4	1.915761
sp|P49419|AL7A1_HUMAN	ALDH7A1	Alpha-aminoadipic semialdehyde dehydrogenase OS=Homo sapiens GN=ALDH7A1 PE=1 SV=5	59020	0	493	0	87	0	0.11	#DIV/0!	0	2	0	2	0	3	0	3	1.915761
tr|B7ZW05|B7ZW05_HUMAN	AKAP13	AKAP13 protein (Fragment) OS=Homo sapiens GN=AKAP13 PE=2 SV=1	124144	0	492	0	87	0	0.05	#DIV/0!	0	2	0	2	0	3	0	4	1.915761
sp|Q09161|NCBP1_HUMAN	NCBP1	Nuclear cap-binding protein subunit 1 OS=Homo sapiens GN=NCBP1 PE=1 SV=1	92864	0	487	0	89	0	0.11	#DIV/0!	0	3	0	3	0	3	0	3	1.915761
sp|P22059|OSBP1_HUMAN	OSBP	Oxysterol-binding protein 1 OS=Homo sapiens GN=OSBP PE=1 SV=1	90220	0	478	0	92	0	0.11	#DIV/0!	0	2	0	3	0	3	0	3	1.915761
tr|B0QZ18|B0QZ18_HUMAN	CPNE1	Copine I OS=Homo sapiens GN=CPNE1 PE=2 SV=1	60307	0	476	0	92	0	0.17	#DIV/0!	0	2	0	3	0	3	0	4	1.915761
sp|P52907|CAZA1_HUMAN	CAPZA1	F-actin-capping protein subunit alpha-1 OS=Homo sapiens GN=CAPZA1 PE=1 SV=3	33073	0	474	0	93	0	0.33	#DIV/0!	0	2	0	3	0	3	0	3	1.915761
tr|B4DZ87|B4DZ87_HUMAN	tr|B4DZ87|B4D	cDNA FLJ57240, highly similar to Mitochondrial proteins import receptor OS=Homo sapiens PE=2 SV=1	57579	0	473	0	93	0	0.18	#DIV/0!	0	3	0	3	0	3	0	4	1.915761
tr|H3BM93|H3BM93_HUMAN	NUP93	Nuclear pore complex protein Nup93 (Fragment) OS=Homo sapiens GN=NUP93 PE=4 SV=1	19858	0	469	0	69	0	0.27	#DIV/0!	0	1	0	3	0	3	0	3	1.915761
sp|Q9BWF3|RBM4_HUMAN	RBM4	RNA-binding protein 4 OS=Homo sapiens GN=RBM4 PE=1 SV=1	40688	0	449	0	98	0	0.17	#DIV/0!	0	1	0	2	0	3	0	3	1.915761
tr|B4DL39|B4DL39_HUMAN	tr|B4DL39|B4D	cDNA FLJ57316, highly similar to DNA mismatch repair protein Msh2 OS=Homo sapiens PE=2 SV=1	77368	0	448	0	98	0	0.09	#DIV/0!	0	2	0	2	0	3	0	3	1.915761
sp|Q9H0A0|NAT10_HUMAN	NAT10	N-acetyltransferase 10 OS=Homo sapiens GN=NAT10 PE=1 SV=2	116569	0	424	0	104	0	0.16	#DIV/0!	0	2	0	3	0	3	0	3	1.915761
tr|B7ZLC9|B7ZLC9_HUMAN	GEMIN5	GEMIN5 protein OS=Homo sapiens GN=GEMIN5 PE=2 SV=1	170664	0	412	0	108	0	0.06	#DIV/0!	0	2	0	3	0	3	0	4	1.915761
tr|B4DM01|B4DM01_HUMAN	SKIV2L	Helicase SKI2W OS=Homo sapiens GN=SKIV2L PE=2 SV=1	121269	0	408	0	109	0	0.05	#DIV/0!	0	2	0	2	0	3	0	3	1.915761
tr|F5GZ76|F5GZ76_HUMAN	USO1	General vesicular transport factor p115 OS=Homo sapiens GN=USO1 PE=2 SV=1	90003	0	404	0	110	0	0.11	#DIV/0!	0	2	0	3	0	3	0	4	1.915761
tr|Q96F88|Q96F88_HUMAN	POP1	Processing of 1, ribonuclease P/MRP subunit (S. cerevisiae) OS=Homo sapiens GN=POP1 PE=2 SV=1	116318	0	398	0	112	0	0.09	#DIV/0!	0	2	0	3	0	3	0	3	1.915761
tr|H0YL72|H0YL72_HUMAN	IDH3A	Isocitrate dehydrogenase [NAD] subunit alpha, mitochondrial OS=Homo sapiens GN=IDH3A PE=3 SV=1	36162	0	366	0	106	0	0.9	#DIV/0!	0	3	0	3	0	3	0	3	1.915761
sp|O43815|STRN_HUMAN	STRN	Striatin OS=Homo sapiens GN=STRN PE=1 SV=4	86535	0	365	0	123	0	0.08	#DIV/0!	0	1	0	2	0	3	0	3	1.915761
tr|Q6FHX6|Q6FHX6_HUMAN	FEN1	Flap endonuclease 1 OS=Homo sapiens GN=FEN1 PE=2 SV=1	42908	0	364	0	123	0	0.25	#DIV/0!	0	2	0	3	0	3	0	3	1.915761
tr|B3KRR1|B3KRR1_HUMAN	tr|B3KRR1|B3K	cDNA FLJ34725 fis, clone MESAN2005958, highly similar to RNA-binding protein Luc7-like 2 OS=Homo sapiens	40752	0	361	0	95	0	0.31	#DIV/0!	0	2	0	3	0	3	0	3	1.915761
tr|G1UI16|G1UI16_HUMAN	PDS5A	SCC-112 protein, isoform CRA_b OS=Homo sapiens GN=PDS5A PE=2 SV=1	152274	0	343	0	132	0	0.07	#DIV/0!	0	3	0	3	0	3	0	4	1.915761
tr|H0YCK7|H0YCK7_HUMAN	EEF1D	Elongation factor 1-delta (Fragment) OS=Homo sapiens GN=EEF1D PE=4 SV=1	23105	0	259	0	195	0	0.72	#DIV/0!	0	3	0	4	0	3	0	4	1.915761
sp|Q9BW60|ELOV1_HUMAN	ELOVL1	Elongation of very long chain fatty acids protein 1 OS=Homo sapiens GN=ELOVL1 PE=1 SV=1	32755	0	1193	0	21	0	0.1	#DIV/0!	0	0	0	1	0	2	0	2	1.430154
tr|B3KW81|B3KW81_HUMAN	tr|B3KW81|B3K	cDNA FLJ42510 fis, clone BRACE2044473, highly similar to Homo sapiens pericentrin 1 (PCNT1), mRNA OS=Hor	30173	0	1188	0	22	0	0.05	#DIV/0!	0	0	0	1	0	2	0	2	1.430154
sp|Q9UHD1|CHRD1_HUMAN	CHORDC1	Cysteine and histidine-rich domain-containing protein 1 OS=Homo sapiens GN=CHORDC1 PE=1 SV=2	38264	0	1181	0	22	0	0.09	#DIV/0!	0	0	0	1	0	2	0	2	1.430154
sp|Q8N3D4|EH1L1_HUMAN	EHBP1L1	EH domain-binding protein 1-like protein 1 OS=Homo sapiens GN=EHBP1L1 PE=1 SV=2	162554	0	1180	0	22	0	0.02	#DIV/0!	0	0	0	1	0	2	0	2	1.430154
tr|B4DFB6|B4DFB6_HUMAN	DENND5A	DENN domain-containing protein 5A OS=Homo sapiens GN=DENND5A PE=2 SV=1	93173	0	1179	0	22	0	0.04	#DIV/0!	0	0	0	1	0	2	0	3	1.430154
tr|A6NNK5|A6NNK5_HUMAN	TP53BP1	Tumor suppressor p53-binding protein 1 OS=Homo sapiens GN=TP53BP1 PE=2 SV=2	210886	0	1175	0	22	0	0.02	#DIV/0!	0	0	0	1	0	2	0	3	1.430154
sp|P62333|PRS10_HUMAN	PSMC6	26S protease regulatory subunit 10B OS=Homo sapiens GN=PSMC6 PE=1 SV=1	44430	0	1163	0	24	0	0.07	#DIV/0!	0	0	0	1	0	2	0	2	1.430154
tr|A8K564|A8K564_HUMAN	tr|A8K564|A8K	cDNA FLJ76716, highly similar to Homo sapiens WD repeat domain 70 (WDR70), mRNA OS=Homo sapiens PE=	73886	0	1151	0	24	0	0.04	#DIV/0!	0	0	0	1	0	2	0	3	1.430154
tr|B7Z7M6|B7Z7M6_HUMAN	KIF2C	Kinesin-like protein KIF2C OS=Homo sapiens GN=KIF2C PE=2 SV=1	69630	0	1150	0	24	0	0.09	#DIV/0!	0	0	0	2	0	2	0	4	1.430154
sp|Q7Z478|DHX29_HUMAN	DHX29	ATP-dependent RNA helicase DHX29 OS=Homo sapiens GN=DHX29 PE=1 SV=2	156222	0	1136	0	25	0	0.02	#DIV/0!	0	0	0	1	0	2	0	2	1.430154
tr|G3V3B0|G3V3B0_HUMAN	ACIN1	Apoptotic chromatin condensation inducer in the nucleus OS=Homo sapiens GN=ACIN1 PE=2 SV=1	67683	0	1113	0	27	0	0.05	#DIV/0!	0	0	0	1	0	2	0	2	1.430154
tr|Q0VAM0|Q0VAM0_HUMAN	CTNNBL1	Catenin, beta like 1 OS=Homo sapiens GN=CTNNBL1 PE=2 SV=1	65560	0	1112	0	27	0	0.05	#DIV/0!	0	0	0	1	0	2	0	2	1.430154
tr|Q6AHZ7|Q6AHZ7_HUMAN	DKFZp686A111	Putative uncharacterized protein DKFZp686A111 OS=Homo sapiens GN=DKFZp686A111 PE=2 SV=1	240988	0	1109	0	28	0	0.01	#DIV/0!	0	0	0	1	0	2	0	2	1.430154
tr|I3L0S8|I3L0S8_HUMAN	XPO6	Exportin-6 (Fragment) OS=Homo sapiens GN=XPO6 PE=4 SV=1	15327	0	1084	0	29	0	0.22	#DIV/0!	0	0	0	1	0	2	0	2	1.430154
sp|P43034|LIS1_HUMAN	PAFAH1B1	Platelet-activating factor acetylhydrolase IB subunit alpha OS=Homo sapiens GN=PAFAH1B1 PE=1 SV=2	47178	0	1079	0	30	0	0.08	#DIV/0!	0	0	0	1	0	2	0	2	1.430154
tr|B4DL04|B4DL04_HUMAN	tr|B4DL04|B4D	cDNA FLJ51229, highly similar to Ephrin type-A receptor 2 (EC 2.7.10.1) OS=Homo sapiens PE=2 SV=1	73481	0	1052	0	32	0	0.04	#DIV/0!	0	0	0	1	0	2	0	3	1.430154
sp|P05556|ITB1_HUMAN	ITGB1	Integrin beta-1 OS=Homo sapiens GN=ITGB1 PE=1 SV=2	91664	0	1051	0	32	0	0.07	#DIV/0!	0	0	0	2	0	2	0	2	1.430154
tr|B4DJU4|B4DJU4_HUMAN	tr|B4DJU4|B4D	cDNA FLJ53344, highly similar to Splicing factor 1 OS=Homo sapiens PE=2 SV=1	67535	0	1050	0	32	0	0.1	#DIV/0!	0	0	0	2	0	2	0	3	1.430154
tr|B3KNF6|B3KNF6_HUMAN	SEC61A1	Sec61 alpha 1 subunit (S. cerevisiae), isoform CRA_c OS=Homo sapiens GN=SEC61A1 PE=2 SV=1	52687	0	1038	0	33	0	0.06	#DIV/0!	0	1	0	1	0	2	0	3	1.430154
tr|Q2TAM4|Q2TAM4_HUMAN	POLR1B	DNA-directed RNA polymerase OS=Homo sapiens GN=POLR1B PE=2 SV=1	108996	0	1034	0	33	0	0.06	#DIV/0!	0	1	0	2	0	2	0	2	1.430154
tr|G3V1L9|G3V1L9_HUMAN	TJP1	Tight junction protein 1 (Zona occludens 1), isoform CRA_a OS=Homo sapiens GN=TJP1 PE=4 SV=1	197681	0	1030	0	33	0	0.03	#DIV/0!	0	0	0	2	0	2	0	2	1.430154
tr|B3KM68|B3KM68_HUMAN	tr|B3KM68|B3K	cDNA FLJ10398 fis, clone NT2RM4000349, highly similar to Homo sapiens basic leucine zipper and W2 domain	48388	0	1025	0	33	0	0.15	#DIV/0!	0	1	0	2	0	2	0	2	1.430154
sp|Q9NXF1|TEX10_HUMAN	TEX10	Testis-expressed sequence 10 protein OS=Homo sapiens GN=TEX10 PE=1 SV=2	106349	0	1020	0	34	0	0.07	#DIV/0!	0	1	0	1	0	2	0	2	1.430154
tr|H0YEM1|H0YEM1_HUMAN	PUF60	Poly(U)-binding-splicing factor PUF60 (Fragment) OS=Homo sapiens GN=PUF60 PE=4 SV=1	35924	0	1004	0	35	0	0.09	#DIV/0!	0	0	0	1	0	2	0	2	1.430154
tr|B2RB32|B2RB32_HUMAN	tr|B2RB32|B2R	Phosphorylase OS=Homo sapiens PE=2 SV=1	97439	0	998	0	35	0	0.03	#DIV/0!	0	1	0	1	0	2	0	2	1.430154
sp|Q2VIQ3|KIF4B_HUMAN	KIF4B	Chromosome-associated kinesin KIF4B OS=Homo sapiens GN=KIF4B PE=2 SV=2	141829	0	984	0	36	0	0.05	#DIV/0!	0	0	0	2	0	2	0	2	1.430154
tr|B7WPF4|B7WPF4_HUMAN	USP24	Ubiquitin carboxyl-terminal hydrolase OS=Homo sapiens GN=USP24 PE=2 SV=2	280147	0	971	0	37	0	0.01	#DIV/0!	0	1	0	1	0	2	0	2	1.430154
tr|Q6NX51|Q6NX51_HUMAN	EXOC4	Exocyst complex component 4 OS=Homo sapiens GN=EXOC4 PE=2 SV=1	111170	0	962	0	37	0	0.03	#DIV/0!	0	1	0	1	0	2	0	2	1.430154
tr|F5GWE2|F5GWE2_HUMAN	ACOT7	Cytosolic acyl coenzyme A thioester hydrolase OS=Homo sapiens GN=ACOT7 PE=2 SV=1	25090	0	951	0	38	0	0.13	#DIV/0!	0	1	0	1	0	2	0	2	1.430154
sp|P09543|CN37_HUMAN	CNP	2~,3~-cyclic-nucleotide 3~-phosphodiesterase OS=Homo sapiens GN=CNP PE=1 SV=2	47948	0	944	0	38	0	0.14	#DIV/0!	0	1	0	2	0	2	0	3	1.430154
tr|E7ERK9|E7ERK9_HUMAN	EIF2B4	Translation initiation factor eIF-2B subunit delta OS=Homo sapiens GN=EIF2B4 PE=2 SV=1	60248	0	943	0	38	0	0.11	#DIV/0!	0	0	0	2	0	2	0	2	1.430154
sp|P08F94|PKHD1_HUMAN	PKHD1	Fibrocystin OS=Homo sapiens GN=PKHD1 PE=1 SV=1	452120	0	941	0	38	0	0.01	#DIV/0!	0	1	0	1	0	2	0	3	1.430154
sp|Q86X55|CARM1_HUMAN	CARM1	Histone-arginine methyltransferase CARM1 OS=Homo sapiens GN=CARM1 PE=1 SV=3	66382	0	938	0	38	0	0.05	#DIV/0!	0	1	0	1	0	2	0	3	1.430154
tr|Q53G71|Q53G71_HUMAN	tr|Q53G71|Q53	Calreticulin variant (Fragment) OS=Homo sapiens PE=2 SV=1	47061	0	928	0	39	0	0.31	#DIV/0!	0	1	0	3	0	2	0	6	1.430154
tr|A5YM51|A5YM51_HUMAN	MYH7	MYH7 protein (Fragment) OS=Homo sapiens GN=MYH7 PE=2 SV=1	223684	0	921	0	39	0	0.01	#DIV/0!	0	1	0	1	0	2	0	2	1.430154
sp|O75131|CPNE3_HUMAN	CPNE3	Copine-3 OS=Homo sapiens GN=CPNE3 PE=1 SV=1	60947	0	910	0	40	0	0.05	#DIV/0!	0	1	0	1	0	2	0	2	1.430154
tr|Q8WW55|Q8WW55_HUMAN	tr|Q8WW55|Q8	STAT5B protein OS=Homo sapiens PE=2 SV=1	45148	0	891	0	41	0	0.15	#DIV/0!	0	0	0	2	0	2	0	4	1.430154
tr|A0MNN5|A0MNN5_HUMAN	GRWD1	CDW4/GRWD1 OS=Homo sapiens GN=GRWD1 PE=2 SV=1	49787	0	871	0	43	0	0.07	#DIV/0!	0	1	0	1	0	2	0	3	1.430154
tr|B1PBA3|B1PBA3_HUMAN	tr|B1PBA3|B1P	SKNY protein OS=Homo sapiens PE=2 SV=1	98489	0	867	0	43	0	0.03	#DIV/0!	0	1	0	1	0	2	0	3	1.430154
tr|B2R806|B2R806_HUMAN	tr|B2R806|B2R	Eukaryotic translation initiation factor 3 subunit E OS=Homo sapiens PE=2 SV=1	52622	0	866	0	43	0	0.13	#DIV/0!	0	1	0	2	0	2	0	3	1.430154
tr|Q2TU84|Q2TU84_HUMAN	GIG18	Aspartate aminotransferase OS=Homo sapiens GN=GIG18 PE=2 SV=1	46519	0	835	0	46	0	0.15	#DIV/0!	0	1	0	2	0	2	0	2	1.430154
tr|C0KTQ6|C0KTQ6_HUMAN	HLA-Cw	MHC class I antigen (Fragment) OS=Homo sapiens GN=HLA-Cw PE=3 SV=1	34234	0	813	0	47	0	0.1	#DIV/0!	0	1	0	1	0	2	0	2	1.430154
sp|Q14008|CKAP5_HUMAN	CKAP5	Cytoskeleton-associated protein 5 OS=Homo sapiens GN=CKAP5 PE=1 SV=3	227062	0	796	0	49	0	0.03	#DIV/0!	0	1	0	2	0	2	0	4	1.430154
tr|A8K9B9|A8K9B9_HUMAN	tr|A8K9B9|A8K	cDNA FLJ77391, highly similar to Homo sapiens EH-domain containing 4 (EHD4), mRNA OS=Homo sapiens PE=	61346	0	780	0	50	0	0.05	#DIV/0!	0	1	0	1	0	2	0	2	1.430154
tr|F6SBX2|F6SBX2_HUMAN	IARS2	Isoleucine--tRNA ligase, mitochondrial OS=Homo sapiens GN=IARS2 PE=2 SV=1	106884	0	769	0	51	0	0.06	#DIV/0!	0	1	0	2	0	2	0	3	1.430154
tr|B2R6H7|B2R6H7_HUMAN	tr|B2R6H7|B2R	cDNA, FLJ92955, highly similar to Homo sapiens transportin-SR (TRN-SR), mRNA OS=Homo sapiens PE=2 SV=1	105989	0	765	0	51	0	0.06	#DIV/0!	0	2	0	2	0	2	0	3	1.430154
tr|H3BM24|H3BM24_HUMAN	HDAC2	Histone deacetylase 2 (Fragment) OS=Homo sapiens GN=HDAC2 PE=4 SV=1	18917	0	763	0	34	0	0.19	#DIV/0!	0	0	0	1	0	2	0	2	1.430154
tr|F2ZC09|F2ZC09_HUMAN	TRIP6i4	Thyroid hormone receptor interacting protein 6 isoform 4 OS=Homo sapiens GN=TRIP6i4 PE=2 SV=1	17368	0	761	0	52	0	0.43	#DIV/0!	0	1	0	2	0	2	0	2	1.430154
tr|B4DDH8|B4DDH8_HUMAN	tr|B4DDH8|B4D	cDNA FLJ55184, highly similar to Homo sapiens leukocyte receptor cluster (LRC) member 4 (LENG4), mRNA OS	51214	0	750	0	52	0	0.06	#DIV/0!	0	1	0	1	0	2	0	2	1.430154
sp|Q9NRG9|AAAS_HUMAN	AAAS	Aladin OS=Homo sapiens GN=AAAS PE=1 SV=1	60392	0	744	0	52	0	0.11	#DIV/0!	0	1	0	2	0	2	0	2	1.430154
tr|B4E256|B4E256_HUMAN	tr|B4E256|B4E2	cDNA FLJ55348, highly similar to WD repeat protein 6 OS=Homo sapiens PE=2 SV=1	117990	0	743	0	52	0	0.06	#DIV/0!	0	1	0	2	0	2	0	2	1.430154
sp|Q9Y4F1|FARP1_HUMAN	FARP1	FERM, RhoGEF and pleckstrin domain-containing protein 1 OS=Homo sapiens GN=FARP1 PE=1 SV=1	119300	0	741	0	53	0	0.06	#DIV/0!	0	1	0	2	0	2	0	3	1.430154
tr|B5BU08|B5BU08_HUMAN	U2AF1	U2 small nuclear RNA auxillary factor 1 isoform a OS=Homo sapiens GN=U2AF1 PE=2 SV=1	28369	0	736	0	53	0	0.25	#DIV/0!	0	1	0	2	0	2	0	2	1.430154
tr|B2RDI5|B2RDI5_HUMAN	tr|B2RDI5|B2RD	cDNA, FLJ96627, highly similar to Homo sapiens calpain 1, (mu/I) large subunit (CAPN1), mRNA OS=Homo sapi	82529	0	709	0	57	0	0.12	#DIV/0!	0	1	0	3	0	2	0	3	1.430154
sp|O96008|TOM40_HUMAN	TOMM40	Mitochondrial import receptor subunit TOM40 homolog OS=Homo sapiens GN=TOMM40 PE=1 SV=1	38211	0	693	0	57	0	0.4	#DIV/0!	0	2	0	2	0	2	0	2	1.430154
tr|Q7KZJ3|Q7KZJ3_HUMAN	tr|Q7KZJ3|Q7K	Catenin (Cadherin-associated protein), delta 1 OS=Homo sapiens PE=2 SV=1	68531	0	687	0	58	0	0.1	#DIV/0!	0	1	0	2	0	2	0	2	1.430154
tr|G5E988|G5E988_HUMAN	AP3D1	AP-3 complex subunit delta-1 OS=Homo sapiens GN=AP3D1 PE=2 SV=1	126074	0	684	0	55	0	0.14	#DIV/0!	0	1	0	3	0	2	0	3	1.430154
tr|Q6FG59|Q6FG59_HUMAN	CDC37	CDC37 protein OS=Homo sapiens GN=CDC37 PE=2 SV=1	44938	0	682	0	59	0	0.15	#DIV/0!	0	1	0	2	0	2	0	2	1.430154
sp|Q96T76|MMS19_HUMAN	MMS19	MMS19 nucleotide excision repair protein homolog OS=Homo sapiens GN=MMS19 PE=1 SV=2	114928	0	681	0	59	0	0.09	#DIV/0!	0	2	0	3	0	2	0	5	1.430154
sp|Q9NTI5|PDS5B_HUMAN	PDS5B	Sister chromatid cohesion protein PDS5 homolog B OS=Homo sapiens GN=PDS5B PE=1 SV=1	165818	0	680	0	59	0	0.02	#DIV/0!	0	1	0	1	0	2	0	3	1.430154
tr|B3KNV9|B3KNV9_HUMAN	tr|B3KNV9|B3K	cDNA FLJ30554 fis, clone BRAWH2003693 OS=Homo sapiens PE=2 SV=1	112670	0	678	0	59	0	0.06	#DIV/0!	0	1	0	2	0	2	0	2	1.430154
tr|B2R825|B2R825_HUMAN	tr|B2R825|B2R	Phosphorylase OS=Homo sapiens PE=2 SV=1	97405	0	665	0	59	0	0.11	#DIV/0!	0	2	0	2	0	2	0	2	1.430154
sp|O00154|BACH_HUMAN	ACOT7	Cytosolic acyl coenzyme A thioester hydrolase OS=Homo sapiens GN=ACOT7 PE=1 SV=3	42454	0	660	0	61	0	0.16	#DIV/0!	0	2	0	2	0	2	0	3	1.430154
tr|C9J634|C9J634_HUMAN	PDHB	Pyruvate dehydrogenase E1 component subunit beta, mitochondrial OS=Homo sapiens GN=PDHB PE=2 SV=1	39064	0	659	0	61	0	0.25	#DIV/0!	0	1	0	2	0	2	0	3	1.430154
tr|F8W9B5|F8W9B5_HUMAN	PRC1	Protein regulator of cytokinesis 1 OS=Homo sapiens GN=PRC1 PE=2 SV=1	66790	0	651	0	62	0	0.1	#DIV/0!	0	2	0	2	0	2	0	3	1.430154
tr|D3DU41|D3DU41_HUMAN	LUC7L	LUC7-like (S. cerevisiae), isoform CRA_a OS=Homo sapiens GN=LUC7L PE=2 SV=1	34110	0	649	0	63	0	0.2	#DIV/0!	0	1	0	2	0	2	0	2	1.430154
sp|Q6AI08|HEAT6_HUMAN	HEATR6	HEAT repeat-containing protein 6 OS=Homo sapiens GN=HEATR6 PE=1 SV=1	130410	0	646	0	63	0	0.05	#DIV/0!	0	1	0	2	0	2	0	2	1.430154
tr|B4DET0|B4DET0_HUMAN	tr|B4DET0|B4D	cDNA FLJ55900, highly similar to Pre-mRNA-splicing factor ATP-dependent RNA helicase PRP16 (EC 3.6.1.-) OS	140449	0	638	0	63	0	0.05	#DIV/0!	0	1	0	2	0	2	0	2	1.430154
tr|B7Z475|B7Z475_HUMAN	tr|B7Z475|B7Z4	cDNA FLJ55712, highly similar to F-box-like/WD repeat protein TBL1XR1 OS=Homo sapiens PE=2 SV=1	41301	0	619	0	66	0	0.17	#DIV/0!	0	2	0	2	0	2	0	2	1.430154
sp|P42695|CNDD3_HUMAN	NCAPD3	Condensin-2 complex subunit D3 OS=Homo sapiens GN=NCAPD3 PE=1 SV=2	170950	0	604	0	68	0	0.04	#DIV/0!	0	2	0	2	0	2	0	3	1.430154
sp|Q6IA86|ELP2_HUMAN	ELP2	Elongator complex protein 2 OS=Homo sapiens GN=ELP2 PE=1 SV=2	94266	0	598	0	69	0	0.07	#DIV/0!	0	2	0	2	0	2	0	2	1.430154
tr|A8KA19|A8KA19_HUMAN	tr|A8KA19|A8K	cDNA FLJ75831, highly similar to Homo sapiens exportin, tRNA (nuclear export receptor for tRNAs) (XPOT), mR	111104	0	593	0	69	0	0.06	#DIV/0!	0	2	0	2	0	2	0	2	1.430154
tr|H0YDD4|H0YDD4_HUMAN	DLAT	Dihydrolipoyllysine-residue acetyltransferase component of pyruvate dehydrogenase complex, mitochondrial	51511	0	588	0	71	0	0.13	#DIV/0!	0	1	0	2	0	2	0	3	1.430154
sp|Q16222|UAP1_HUMAN	UAP1	UDP-N-acetylhexosamine pyrophosphorylase OS=Homo sapiens GN=UAP1 PE=1 SV=3	59131	0	583	0	72	0	0.11	#DIV/0!	0	1	0	2	0	2	0	2	1.430154
tr|J3KMX1|J3KMX1_HUMAN	NUP88	Nuclear pore complex protein Nup88 (Fragment) OS=Homo sapiens GN=NUP88 PE=4 SV=1	80029	0	581	0	53	0	0.17	#DIV/0!	0	1	0	2	0	2	0	3	1.430154
tr|B4E206|B4E206_HUMAN	tr|B4E206|B4E2	cDNA FLJ55441, highly similar to Nuclear pore complex protein Nup133 OS=Homo sapiens PE=2 SV=1	128349	0	578	0	71	0	0.11	#DIV/0!	0	2	0	3	0	2	0	4	1.430154
tr|A9LSU1|A9LSU1_HUMAN	tr|A9LSU1|A9LS	Type IV collagen alpha 1 (Fragment) OS=Homo sapiens PE=2 SV=1	25998	0	570	0	74	0	0.27	#DIV/0!	0	1	0	2	0	2	0	2	1.430154
tr|Q2KQ72|Q2KQ72_HUMAN	tr|Q2KQ72|Q2K	Chromosome-associated protein E OS=Homo sapiens PE=2 SV=1	86324	0	568	0	74	0	0.07	#DIV/0!	0	2	0	2	0	2	0	3	1.430154
sp|A0AVT1|UBA6_HUMAN	UBA6	Ubiquitin-like modifier-activating enzyme 6 OS=Homo sapiens GN=UBA6 PE=1 SV=1	119207	0	565	0	74	0	0.06	#DIV/0!	0	1	0	2	0	2	0	2	1.430154
tr|B3KWI1|B3KWI1_HUMAN	tr|B3KWI1|B3K	cDNA FLJ43111 fis, clone CTONG2025900, highly similar to Sterile alpha motif domain-containing protein 9 OS	88162	0	555	0	76	0	0.08	#DIV/0!	0	1	0	2	0	2	0	3	1.430154
tr|G0KXF8|G0KXF8_HUMAN	HLA-B	MHC class I antigen (Fragment) OS=Homo sapiens GN=HLA-B PE=3 SV=1	21235	0	538	0	79	0	0.34	#DIV/0!	0	2	0	2	0	2	0	2	1.430154
tr|G1ENK8|G1ENK8_HUMAN	HLA-B	MHC class I antigen (Fragment) OS=Homo sapiens GN=HLA-B PE=3 SV=1	21236	0	537	0	79	0	0.34	#DIV/0!	0	2	0	2	0	2	0	2	1.430154
tr|Q9MYA6|Q9MYA6_HUMAN	HLA-B	MHC class I antigen (Fragment) OS=Homo sapiens GN=HLA-B PE=2 SV=1	21176	0	536	0	79	0	0.34	#DIV/0!	0	2	0	2	0	2	0	3	1.430154
tr|C0KK01|C0KK01_HUMAN	HLA-C	MHC class I antigen (Fragment) OS=Homo sapiens GN=HLA-C PE=3 SV=1	20940	0	535	0	79	0	0.35	#DIV/0!	0	2	0	2	0	2	0	3	1.430154
tr|B3KNC3|B3KNC3_HUMAN	tr|B3KNC3|B3K	cDNA FLJ14222 fis, clone NT2RP3003992, highly similar to Nucleolar complex protein 2 homolog OS=Homo sa	85719	0	524	0	80	0	0.08	#DIV/0!	0	1	0	2	0	2	0	2	1.430154
tr|B7Z6C2|B7Z6C2_HUMAN	tr|B7Z6C2|B7Z6	cDNA FLJ50663, highly similar to Phosphoglucomutase-1 (EC 5.4.2.2) OS=Homo sapiens PE=2 SV=1	64052	0	519	0	81	0	0.11	#DIV/0!	0	2	0	2	0	2	0	4	1.430154
tr|Q9Y4A1|Q9Y4A1_HUMAN	tr|Q9Y4A1|Q9Y	Talin-related protein (Fragment) OS=Homo sapiens PE=2 SV=1	20182	0	500	0	85	0	0.36	#DIV/0!	0	1	0	2	0	2	0	2	1.430154
tr|B4DL07|B4DL07_HUMAN	tr|B4DL07|B4D	cDNA FLJ53353, highly similar to ATP-binding cassette sub-family D member 3 OS=Homo sapiens PE=2 SV=1	78980	0	496	0	86	0	0.08	#DIV/0!	0	2	0	2	0	2	0	3	1.430154
tr|Q59FI0|Q59FI0_HUMAN	tr|Q59FI0|Q59F	Aconitase 1 variant (Fragment) OS=Homo sapiens PE=2 SV=1	86964	0	494	0	64	0	0.16	#DIV/0!	0	1	0	2	0	2	0	2	1.430154
tr|J3KTL8|J3KTL8_HUMAN	SMCHD1	Structural maintenance of chromosomes flexible hinge domain-containing protein 1 (Fragment) OS=Homo sap	156690	0	490	0	88	0	0.04	#DIV/0!	0	2	0	2	0	2	0	2	1.430154
tr|H0YCK3|H0YCK3_HUMAN	ADAR	Double-stranded RNA-specific adenosine deaminase (Fragment) OS=Homo sapiens GN=ADAR PE=4 SV=1	133761	0	480	0	53	0	0.07	#DIV/0!	0	1	0	2	0	2	0	3	1.430154
sp|Q9HD26|GOPC_HUMAN	GOPC	Golgi-associated PDZ and coiled-coil motif-containing protein OS=Homo sapiens GN=GOPC PE=1 SV=1	50888	0	471	0	93	0	0.13	#DIV/0!	0	2	0	2	0	2	0	2	1.430154
tr|H0YH81|H0YH81_HUMAN	ATP5B	ATP synthase subunit beta (Fragment) OS=Homo sapiens GN=ATP5B PE=3 SV=1	38226	0	467	0	94	0	0.18	#DIV/0!	0	2	0	2	0	2	0	2	1.430154
tr|F5GZS0|F5GZS0_HUMAN	DHX36	Probable ATP-dependent RNA helicase DHX36 OS=Homo sapiens GN=DHX36 PE=2 SV=1	113928	0	463	0	95	0	0.06	#DIV/0!	0	1	0	2	0	2	0	4	1.430154
tr|J3KPP4|J3KPP4_HUMAN	LUC7L3	Cisplatin resistance-associated overexpressed protein, isoform CRA_b OS=Homo sapiens GN=LUC7L3 PE=4 SV=	58698	0	458	0	97	0	0.12	#DIV/0!	0	2	0	2	0	2	0	2	1.430154
sp|Q06210|GFPT1_HUMAN	GFPT1	Glutamine--fructose-6-phosphate aminotransferase [isomerizing] 1 OS=Homo sapiens GN=GFPT1 PE=1 SV=3	79555	0	456	0	97	0	0.08	#DIV/0!	0	2	0	2	0	2	0	3	1.430154
sp|O43837|IDH3B_HUMAN	IDH3B	Isocitrate dehydrogenase [NAD] subunit beta, mitochondrial OS=Homo sapiens GN=IDH3B PE=1 SV=2	42442	0	454	0	98	0	0.16	#DIV/0!	0	2	0	2	0	2	0	2	1.430154
tr|B4DFP1|B4DFP1_HUMAN	tr|B4DFP1|B4D	cDNA FLJ51818, highly similar to Phosphoglucomutase-1 (EC 5.4.2.2) OS=Homo sapiens PE=2 SV=1	58987	0	439	0	81	0	0.17	#DIV/0!	0	2	0	2	0	2	0	3	1.430154
tr|Q3LIF4|Q3LIF4_HUMAN	Nbla00271	NOGO-A (Fragment) OS=Homo sapiens GN=Nbla00271 PE=2 SV=1	57162	0	430	0	74	0	0.41	#DIV/0!	0	1	0	2	0	2	0	2	1.430154
tr|Q6NWZ1|Q6NWZ1_HUMAN	CKAP4	CKAP4 protein (Fragment) OS=Homo sapiens GN=CKAP4 PE=2 SV=1	67948	0	429	0	103	0	0.1	#DIV/0!	0	2	0	2	0	2	0	2	1.430154
sp|P30837|AL1B1_HUMAN	ALDH1B1	Aldehyde dehydrogenase X, mitochondrial OS=Homo sapiens GN=ALDH1B1 PE=1 SV=3	57626	0	399	0	112	0	0.12	#DIV/0!	0	2	0	2	0	2	0	3	1.430154
tr|H0Y7C0|H0Y7C0_HUMAN	USO1	General vesicular transport factor p115 (Fragment) OS=Homo sapiens GN=USO1 PE=4 SV=1	72345	0	396	0	112	0	0.09	#DIV/0!	0	2	0	2	0	2	0	3	1.430154
tr|B2RNR6|B2RNR6_HUMAN	ZFR	Zinc finger RNA binding protein OS=Homo sapiens GN=ZFR PE=2 SV=1	118081	0	382	0	116	0	0.06	#DIV/0!	0	2	0	2	0	2	0	2	1.430154
tr|I6L9E8|I6L9E8_HUMAN	FAM98A	Family with sequence similarity 98, member A OS=Homo sapiens GN=FAM98A PE=2 SV=1	55595	0	374	0	119	0	0.12	#DIV/0!	0	2	0	2	0	2	0	2	1.430154
tr|Q4KMR3|Q4KMR3_HUMAN	MYO1E	MYO1E variant protein OS=Homo sapiens GN=MYO1E PE=2 SV=1	127552	0	332	0	140	0	0.05	#DIV/0!	0	2	0	2	0	2	0	2	1.430154
tr|B3KY55|B3KY55_HUMAN	tr|B3KY55|B3KY	cDNA FLJ46894 fis, clone UTERU3019708, highly similar to Regulator of nonsense transcripts 1 OS=Homo sapie	41002	0	244	0	46	0	0.2	#DIV/0!	0	1	0	1	0	2	0	2	1.430154
tr|F5H1X8|F5H1X8_HUMAN	LRBA	Lipopolysaccharide-responsive and beige-like anchor protein OS=Homo sapiens GN=LRBA PE=2 SV=1	320211	0	1227	0	17	0	0.01	#DIV/0!	0	0	0	1	0	1	0	2	0.693008
sp|Q9BVI4|NOC4L_HUMAN	NOC4L	Nucleolar complex protein 4 homolog OS=Homo sapiens GN=NOC4L PE=1 SV=1	58830	0	1212	0	20	0	0.11	#DIV/0!	0	0	0	1	0	1	0	2	0.693008
tr|B4DPP0|B4DPP0_HUMAN	tr|B4DPP0|B4D	cDNA FLJ51032, highly similar to CD9 antigen OS=Homo sapiens PE=2 SV=1	16237	0	1208	0	20	0	0.21	#DIV/0!	0	0	0	1	0	1	0	1	0.693008
sp|Q96JQ0|PCD16_HUMAN	DCHS1	Protocadherin-16 OS=Homo sapiens GN=DCHS1 PE=2 SV=1	346712	0	1207	0	20	0	0.01	#DIV/0!	0	0	0	1	0	1	0	1	0.693008
tr|H3BPA5|H3BPA5_HUMAN	RAB40C	Ras-related protein Rab-40C (Fragment) OS=Homo sapiens GN=RAB40C PE=3 SV=1	26831	0	1206	0	20	0	0.12	#DIV/0!	0	1	0	1	0	1	0	1	0.693008
tr|B4DH88|B4DH88_HUMAN	tr|B4DH88|B4D	cDNA FLJ54092, highly similar to Transmembrane 9 superfamily protein member 4 OS=Homo sapiens PE=2 SV	64520	0	1204	0	20	0	0.05	#DIV/0!	0	0	0	1	0	1	0	1	0.693008
tr|B4DHJ3|B4DHJ3_HUMAN	tr|B4DHJ3|B4D	DNA-directed RNA polymerase OS=Homo sapiens PE=2 SV=1	134367	0	1203	0	21	0	0.02	#DIV/0!	0	0	0	1	0	1	0	1	0.693008
tr|B4DLH3|B4DLH3_HUMAN	tr|B4DLH3|B4D	cDNA FLJ55306, highly similar to Zinc finger protein ZFPM2 OS=Homo sapiens PE=2 SV=1	115311	0	1202	0	21	0	0.03	#DIV/0!	0	0	0	1	0	1	0	1	0.693008
tr|B4DFD7|B4DFD7_HUMAN	tr|B4DFD7|B4D	cDNA FLJ55457, highly similar to ATP-dependent RNA helicase DHX8 (EC 3.6.1.-) OS=Homo sapiens PE=2 SV=1	98244	0	1201	0	21	0	0.02	#DIV/0!	0	0	0	1	0	1	0	1	0.693008
sp|B4DYI2|S31C2_HUMAN	SPATA31C2	Spermatogenesis-associated protein 31C2 OS=Homo sapiens GN=SPATA31C2 PE=2 SV=2	125606	0	1200	0	21	0	0.03	#DIV/0!	0	0	0	1	0	1	0	1	0.693008
tr|E9PCX8|E9PCX8_HUMAN	TNS3	Tensin-3 (Fragment) OS=Homo sapiens GN=TNS3 PE=2 SV=1	135142	0	1199	0	21	0	0.05	#DIV/0!	0	0	0	1	0	1	0	1	0.693008
sp|Q8NEL0|CCD54_HUMAN	CCDC54	Coiled-coil domain-containing protein 54 OS=Homo sapiens GN=CCDC54 PE=1 SV=2	38318	0	1198	0	21	0	0.09	#DIV/0!	0	0	0	1	0	1	0	1	0.693008
sp|Q9H4L5|OSBL3_HUMAN	OSBPL3	Oxysterol-binding protein-related protein 3 OS=Homo sapiens GN=OSBPL3 PE=1 SV=1	102130	0	1196	0	21	0	0.03	#DIV/0!	0	0	0	1	0	1	0	2	0.693008
sp|Q4G0J3|LARP7_HUMAN	LARP7	La-related protein 7 OS=Homo sapiens GN=LARP7 PE=1 SV=1	67143	0	1192	0	22	0	0.05	#DIV/0!	0	0	0	1	0	1	0	1	0.693008
tr|Q59FE8|Q59FE8_HUMAN	tr|Q59FE8|Q59	Epithelial protein lost in neoplasm beta variant (Fragment) OS=Homo sapiens PE=2 SV=1	86510	0	1191	0	22	0	0.06	#DIV/0!	0	0	0	1	0	1	0	1	0.693008
sp|Q15061|WDR43_HUMAN	WDR43	WD repeat-containing protein 43 OS=Homo sapiens GN=WDR43 PE=1 SV=3	75813	0	1190	0	22	0	0.04	#DIV/0!	0	0	0	1	0	1	0	1	0.693008
tr|B1AKJ6|B1AKJ6_HUMAN	OSBPL9	Oxysterol-binding protein OS=Homo sapiens GN=OSBPL9 PE=2 SV=1	84417	0	1187	0	22	0	0.04	#DIV/0!	0	0	0	1	0	1	0	1	0.693008
sp|Q6PJG6|BRAT1_HUMAN	BRAT1	BRCA1-associated ATM activator 1 OS=Homo sapiens GN=BRAT1 PE=1 SV=2	89774	0	1186	0	22	0	0.04	#DIV/0!	0	0	0	1	0	1	0	1	0.693008
tr|H0Y4W2|H0Y4W2_HUMAN	TRRAP	Transformation/transcription domain-associated protein (Fragment) OS=Homo sapiens GN=TRRAP PE=4 SV=1	410041	0	1185	0	22	0	0.01	#DIV/0!	0	0	0	1	0	1	0	3	0.693008
tr|B3KSG9|B3KSG9_HUMAN	tr|B3KSG9|B3K	cDNA FLJ36188 fis, clone TESTI2027179, highly similar to Transmembrane 9 superfamily protein member 2 OS	66299	0	1184	0	22	0	0.05	#DIV/0!	0	0	0	1	0	1	0	1	0.693008
tr|E1P5S2|E1P5S2_HUMAN	RBM39	RNA-binding protein 39 OS=Homo sapiens GN=RBM39 PE=4 SV=1	40744	0	1183	0	22	0	0.09	#DIV/0!	0	0	0	1	0	1	0	1	0.693008
tr|A2IXV5|A2IXV5_HUMAN	MIR	MIR-10 OS=Homo sapiens GN=MIR cl-10 PE=2 SV=1	65648	0	1182	0	22	0	0.05	#DIV/0!	0	0	0	1	0	1	0	1	0.693008
tr|J3KPL1|J3KPL1_HUMAN	PNPLA8	Calcium-independent phospholipase A2-gamma OS=Homo sapiens GN=PNPLA8 PE=4 SV=1	82434	0	1178	0	22	0	0.04	#DIV/0!	0	0	0	1	0	1	0	1	0.693008
tr|D3DVL8|D3DVL8_HUMAN	ADCY1	Adenylate cyclase 1 (Brain), isoform CRA_b OS=Homo sapiens GN=ADCY1 PE=2 SV=1	102287	0	1177	0	22	0	0.03	#DIV/0!	0	0	0	1	0	1	0	1	0.693008
tr|Q5JB52|Q5JB52_HUMAN	HNRPLL	Heterogeneous nuclear ribonucleoprotein L-like OS=Homo sapiens GN=HNRPLL PE=2 SV=1	57115	0	1176	0	22	0	0.06	#DIV/0!	0	0	0	1	0	1	0	1	0.693008
tr|H0YE29|H0YE29_HUMAN	ARHGAP1	Rho GTPase-activating protein 1 (Fragment) OS=Homo sapiens GN=ARHGAP1 PE=4 SV=1	38420	0	1174	0	22	0	0.09	#DIV/0!	0	0	0	1	0	1	0	1	0.693008

tr|H0YMJ0|H0YMJ0_HUMAN	MORF4L1	Mortality factor 4-like protein 1 (Fragment) OS=Homo sapiens GN=MORF4L1 PE=4 SV=1	28360	0	1173	0	23	0	0.12	#DIV/0!	0	0	0	1	0	1	0	1	0.693008
tr|B2RBB2|B2RBB2_HUMAN	tr|B2RBB2|B2R	cDNA, FLJ95416, highly similar to Homo sapiens phosphatidylinositol glycan, class S (PIGS), mRNA OS=Homo sa	61818	0	1172	0	23	0	0.05	#DIV/0!	0	0	0	1	0	1	0	1	0.693008
sp|O95425|SVIL_HUMAN	SVIL	Supervillin OS=Homo sapiens GN=SVIL PE=1 SV=2	249417	0	1171	0	23	0	0.01	#DIV/0!	0	0	0	1	0	1	0	2	0.693008
tr|F5H3I5|F5H3I5_HUMAN	CDK5RAP3	CDK5 regulatory subunit-associated protein 3 OS=Homo sapiens GN=CDK5RAP3 PE=2 SV=1	59894	0	1170	0	23	0	0.05	#DIV/0!	0	0	0	1	0	1	0	2	0.693008
tr|D3DVR8|D3DVR8_HUMAN	HD	Huntingtin (Huntington disease), isoform CRA_a OS=Homo sapiens GN=HD PE=2 SV=1	291559	0	1168	0	23	0	0.01	#DIV/0!	0	0	0	1	0	1	0	1	0.693008
sp|O43149|ZZEF1_HUMAN	ZZEF1	Zinc finger ZZ-type and EF-hand domain-containing protein 1 OS=Homo sapiens GN=ZZEF1 PE=1 SV=6	335767	0	1167	0	23	0	0.01	#DIV/0!	0	1	0	1	0	1	0	1	0.693008
sp|Q8IWB1|IPRI_HUMAN	ITPRIP	Inositol 1,4,5-trisphosphate receptor-interacting protein OS=Homo sapiens GN=ITPRIP PE=1 SV=1	62932	0	1165	0	23	0	0.05	#DIV/0!	0	1	0	1	0	1	0	2	0.693008
tr|H0YJE9|H0YJE9_HUMAN	PSMC6	26S protease regulatory subunit 10B (Fragment) OS=Homo sapiens GN=PSMC6 PE=4 SV=1	8758	0	1164	0	24	0	0.4	#DIV/0!	0	0	0	1	0	1	0	2	0.693008
tr|Q6DCA8|Q6DCA8_HUMAN	BCLAF1	BCLAF1 protein (Fragment) OS=Homo sapiens GN=BCLAF1 PE=2 SV=1	77958	0	1162	0	24	0	0.04	#DIV/0!	0	0	0	1	0	1	0	2	0.693008
sp|O76003|GLRX3_HUMAN	GLRX3	Glutaredoxin-3 OS=Homo sapiens GN=GLRX3 PE=1 SV=2	37693	0	1161	0	24	0	0.09	#DIV/0!	0	0	0	1	0	1	0	1	0.693008
tr|H0YCY6|H0YCY6_HUMAN	DAK	FAD-AMP lyase (cyclizing) (Fragment) OS=Homo sapiens GN=DAK PE=4 SV=1	55026	0	1160	0	24	0	0.06	#DIV/0!	0	0	0	1	0	1	0	1	0.693008
tr|Q9BV00|Q9BV00_HUMAN	tr|Q9BV00|Q9E	LMOD1 protein OS=Homo sapiens PE=2 SV=1	30001	0	1157	0	24	0	0.11	#DIV/0!	0	0	0	1	0	1	0	2	0.693008
tr|Q5T5N3|Q5T5N3_HUMAN	IDE	Insulin-degrading enzyme (Fragment) OS=Homo sapiens GN=IDE PE=4 SV=1	29418	0	1156	0	24	0	0.03	#DIV/0!	0	0	0	1	0	1	0	1	0.693008
sp|O95715|CXL14_HUMAN	CXCL14	C-X-C motif chemokine 14 OS=Homo sapiens GN=CXCL14 PE=1 SV=2	13297	0	1155	0	24	0	0.26	#DIV/0!	0	0	0	1	0	1	0	1	0.693008
tr|A4D0W4|A4D0W4_HUMAN	AASS	Aminoadipate-semialdehyde synthase OS=Homo sapiens GN=AASS PE=2 SV=1	102808	0	1154	0	24	0	0.03	#DIV/0!	0	1	0	1	0	1	0	2	0.693008
tr|Q5SY38|Q5SY38_HUMAN	LGTN	Eukaryotic translation initiation factor 2D (Fragment) OS=Homo sapiens GN=LGTN PE=4 SV=1	29568	0	1153	0	24	0	0.11	#DIV/0!	0	0	0	1	0	1	0	1	0.693008
tr|H0Y7U4|H0Y7U4_HUMAN	THOC2	THO complex subunit 2 (Fragment) OS=Homo sapiens GN=THOC2 PE=4 SV=1	44212	0	1148	0	25	0	0.07	#DIV/0!	0	0	0	1	0	1	0	1	0.693008
tr|A1L172|A1L172_HUMAN	ACOT1	Acyl-CoA thioesterase 1 OS=Homo sapiens GN=ACOT1 PE=2 SV=1	46666	0	1147	0	25	0	0.07	#DIV/0!	0	0	0	1	0	1	0	1	0.693008
tr|B3KRG2|B3KRG2_HUMAN	tr|B3KRG2|B3K	cDNA FLJ34181 fis, clone FCBBF3016667, highly similar to TFIIH basal transcription factor complexhelicase XPB	82758	0	1145	0	25	0	0.04	#DIV/0!	0	0	0	1	0	1	0	1	0.693008
sp|Q8WUX1|S38A5_HUMAN	SLC38A5	Sodium-coupled neutral amino acid transporter 5 OS=Homo sapiens GN=SLC38A5 PE=1 SV=1	52107	0	1144	0	25	0	0.06	#DIV/0!	0	0	0	1	0	1	0	1	0.693008
sp|O00487|PSDE_HUMAN	PSMD14	26S proteasome non-ATPase regulatory subunit 14 OS=Homo sapiens GN=PSMD14 PE=1 SV=1	34726	0	1143	0	25	0	0.2	#DIV/0!	0	0	0	1	0	1	0	1	0.693008
tr|B4DRN6|B4DRN6_HUMAN	tr|B4DRN6|B4D	cDNA FLJ51284, highly similar to Adapter-relatedprotein complex 3 mu-1 subunit OS=Homo sapiens PE=2 SV=	41285	0	1142	0	25	0	0.08	#DIV/0!	0	0	0	1	0	1	0	2	0.693008
sp|O76031|CLPX_HUMAN	CLPX	ATP-dependent Clp protease ATP-binding subunit clpX-like, mitochondrial OS=Homo sapiens GN=CLPX PE=1 S\	69922	0	1141	0	25	0	0.06	#DIV/0!	0	0	0	1	0	1	0	1	0.693008
tr|Q6NSG9|Q6NSG9_HUMAN	AKAP12	AKAP12 protein (Fragment) OS=Homo sapiens GN=AKAP12 PE=2 SV=1	19461	0	1140	0	25	0	0.17	#DIV/0!	0	0	0	1	0	1	0	1	0.693008
tr|B2RE56|B2RE56_HUMAN	tr|B2RE56|B2RI	Peptidyl-prolyl cis-trans isomerase OS=Homo sapiens PE=2 SV=1	18225	0	1139	0	25	0	0.19	#DIV/0!	0	0	0	1	0	1	0	1	0.693008
tr|A8K335|A8K335_HUMAN	tr|A8K335|A8K	cDNA FLJ76254, highly similar to Homo sapiens gamma-glutamyl hydrolase (GGH), mRNA OS=Homo sapiens P	36366	0	1138	0	25	0	0.09	#DIV/0!	0	0	0	1	0	1	0	1	0.693008
sp|Q8N1G4|LRC47_HUMAN	LRRC47	Leucine-rich repeat-containing protein 47 OS=Homo sapiens GN=LRRC47 PE=1 SV=1	64004	0	1135	0	26	0	0.05	#DIV/0!	0	0	0	1	0	1	0	1	0.693008
tr|Q6AW98|Q6AW98_HUMAN	DKFZp686P1317	Putative uncharacterized protein DKFZp686P13170 OS=Homo sapiens GN=DKFZp686P13170 PE=2 SV=1	46511	0	1134	0	26	0	0.07	#DIV/0!	0	0	0	1	0	1	0	1	0.693008
tr|F8W805|F8W805_HUMAN	GRM1	Metabotropic glutamate receptor 1 OS=Homo sapiens GN=GRM1 PE=2 SV=1	103232	0	1133	0	26	0	0.03	#DIV/0!	0	0	0	1	0	1	0	2	0.693008
tr|B2RB95|B2RB95_HUMAN	tr|B2RB95|B2R	cDNA, FLJ95381, Homo sapiens tripartite motif-containing 16 (TRIM16), mRNA OS=Homo sapiens PE=2 SV=1	64884	0	1132	0	26	0	0.07	#DIV/0!	0	0	0	1	0	1	0	2	0.693008
sp|O15230|LAMA5_HUMAN	LAMA5	Laminin subunit alpha-5 OS=Homo sapiens GN=LAMA5 PE=1 SV=8	412023	0	1131	0	26	0	0.01	#DIV/0!	0	0	0	1	0	1	0	2	0.693008
tr|O43245|O43245_HUMAN	p65	Protein p65 OS=Homo sapiens GN=p65 PE=2 SV=2	69659	0	1130	0	26	0	0.05	#DIV/0!	0	0	0	1	0	1	0	1	0.693008
tr|E9PPY3|E9PPY3_HUMAN	RRP8	Ribosomal RNA-processing protein 8 OS=Homo sapiens GN=RRP8 PE=2 SV=1	34579	0	1127	0	26	0	0.1	#DIV/0!	0	0	0	1	0	1	0	1	0.693008
tr|H7C3Q8|H7C3Q8_HUMAN	ACAP2	Arf-GAP with coiled-coil, ANK repeat and PH domain-containing protein 2 (Fragment) OS=Homo sapiens GN=A	29966	0	1126	0	26	0	0.11	#DIV/0!	0	0	0	1	0	1	0	3	0.693008
sp|Q15057|ACAP2_HUMAN	ACAP2	Arf-GAP with coiled-coil, ANK repeat and PH domain-containing protein 2 OS=Homo sapiens GN=ACAP2 PE=1	88943	0	1125	0	26	0	0.04	#DIV/0!	0	0	0	1	0	1	0	2	0.693008
tr|B3KMS9|B3KMS9_HUMAN	tr|B3KMS9|B3K	cDNA FLJ12495 fis, clone NT2RM2001652, highly similar to Brefeldin A-inhibited guanine nucleotide-exchange	78721	0	1124	0	0	0	0.03	#DIV/0!	0	0	0	0	0	1	0	1	0.693008
tr|B4DTS5|B4DTS5_HUMAN	tr|B4DTS5|B4D	cDNA FLJ58882, highly similar to 26S proteasome non-ATPase regulatory subunit 11 OS=Homo sapiens PE=2 S	35374	0	1123	0	26	0	0.16	#DIV/0!	0	0	0	1	0	1	0	1	0.693008
sp|P13667|PDIA4_HUMAN	PDIA4	Protein disulfide-isomerase A4 OS=Homo sapiens GN=PDIA4 PE=1 SV=2	73229	0	1121	0	26	0	0.04	#DIV/0!	0	0	0	1	0	1	0	1	0.693008
tr|F8VY02|F8VY02_HUMAN	ERP29	Endoplasmic reticulum resident protein 29 OS=Homo sapiens GN=ERP29 PE=2 SV=1	18161	0	1120	0	26	0	0.19	#DIV/0!	0	0	0	1	0	1	0	2	0.693008
tr|H0YCZ2|H0YCZ2_HUMAN	CASC5	Protein CASC5 (Fragment) OS=Homo sapiens GN=CASC5 PE=4 SV=1	156753	0	1119	0	27	0	0.02	#DIV/0!	0	0	0	1	0	1	0	1	0.693008
tr|G3V582|G3V582_HUMAN	GPHN	Molybdopterin molybdenumtransferase OS=Homo sapiens GN=GPHN PE=2 SV=1	41786	0	1118	0	27	0	0.08	#DIV/0!	0	0	0	1	0	1	0	1	0.693008
sp|O76024|WFS1_HUMAN	WFS1	Wolframin OS=Homo sapiens GN=WFS1 PE=1 SV=2	101367	0	1117	0	27	0	0.03	#DIV/0!	0	0	0	1	0	1	0	1	0.693008
sp|Q08499|PDE4D_HUMAN	PDE4D	cAMP-specific 3~,5~-cyclic phosphodiesterase 4D OS=Homo sapiens GN=PDE4D PE=1 SV=2	91628	0	1116	0	27	0	0.07	#DIV/0!	0	0	0	1	0	1	0	1	0.693008
tr|F4MH62|F4MH62_HUMAN	UTY	Ubiquitously transcribed tetratricopeptide repeat protein Y-linked transcript variant 54 OS=Homo sapiens GN	154568	0	1115	0	27	0	0.02	#DIV/0!	0	0	0	1	0	1	0	1	0.693008
tr|B2R673|B2R673_HUMAN	tr|B2R673|B2R	cDNA, FLJ92818, highly similar to Homo sapiens pyruvate dehydrogenase complex, component X (PDHX), mRN	54184	0	1114	0	27	0	0.06	#DIV/0!	0	0	0	1	0	1	0	1	0.693008
tr|B4DR60|B4DR60_HUMAN	tr|B4DR60|B4D	cDNA FLJ56882, highly similar to Activating signal cointegrator 1 complex subunit 3 (EC 3.6.1.-) (Fragment) OS	119789	0	1111	0	26	0	0.03	#DIV/0!	0	0	0	1	0	1	0	2	0.693008
sp|Q9NYK5|RM39_HUMAN	MRPL39	39S ribosomal protein L39, mitochondrial OS=Homo sapiens GN=MRPL39 PE=1 SV=3	39200	0	1110	0	28	0	0.08	#DIV/0!	0	0	0	1	0	1	0	1	0.693008
tr|Q6ZWH1|Q6ZWH1_HUMAN	tr|Q6ZWH1|Q6	cDNA FLJ41081 fis, clone ADRGL2009755, highly similar to Homo sapiens brain and reproductive organ-expres	45824	0	1108	0	28	0	0.07	#DIV/0!	0	0	0	1	0	1	0	1	0.693008
tr|B4E395|B4E395_HUMAN	tr|B4E395|B4E	cDNA FLJ54252, highly similar to Argininosuccinate synthase (EC 6.3.4.5) OS=Homo sapiens PE=2 SV=1	32986	0	1101	0	28	0	0.1	#DIV/0!	0	0	0	1	0	1	0	1	0.693008
tr|F5GWY5|F5GWY5_HUMAN	PODXL	Podocalyxin OS=Homo sapiens GN=PODXL PE=2 SV=1	59285	0	1100	0	28	0	0.06	#DIV/0!	0	0	0	1	0	1	0	1	0.693008
tr|A0PJ61|A0PJ61_HUMAN	RSL1D1	RSL1D1 protein (Fragment) OS=Homo sapiens GN=RSL1D1 PE=2 SV=1	49688	0	1099	0	28	0	0.07	#DIV/0!	0	0	0	1	0	1	0	3	0.693008
tr|B3KN37|B3KN37_HUMAN	tr|B3KN37|B3K	cDNA FLJ13414 fis, clone PLACE1001748, highly similar to Homo sapiens pitrilysin metallopeptidase 1 (PITRM1	68281	0	1098	0	28	0	0.07	#DIV/0!	0	0	0	1	0	1	0	2	0.693008
tr|B3KNG5|B3KNG5_HUMAN	tr|B3KNG5|B3K	cDNA FLJ14553 fis, clone NT2RM2001785, highly similar to Homo sapiens fatty acid desaturase 2 (FADS2), mR	19263	0	1097	0	28	0	0.19	#DIV/0!	0	1	0	1	0	1	0	2	0.693008
tr|H0Y564|H0Y564_HUMAN	ANAPC1	Anaphase-promoting complex subunit 1 (Fragment) OS=Homo sapiens GN=ANAPC1 PE=4 SV=1	163055	0	1096	0	28	0	0.02	#DIV/0!	0	0	0	1	0	1	0	1	0.693008
sp|Q9NR50|EI2BG_HUMAN	EIF2B3	Translation initiation factor eIF-2B subunit gamma OS=Homo sapiens GN=EIF2B3 PE=1 SV=1	50949	0	1095	0	28	0	0.06	#DIV/0!	0	0	0	1	0	1	0	1	0.693008
tr|Q6FHF5|Q6FHF5_HUMAN	PCNA	Proliferating cell nuclear antigen (Fragment) OS=Homo sapiens GN=PCNA PE=2 SV=1	29029	0	1094	0	28	0	0.21	#DIV/0!	0	0	0	1	0	1	0	1	0.693008
tr|B1ANE3|B1ANE3_HUMAN	MTR	5-methyltetrahydrofolate-homocysteine methyltransferase (Fragment) OS=Homo sapiens GN=MTR PE=3 SV=1	92463	0	1093	0	29	0	0.03	#DIV/0!	0	0	0	1	0	1	0	1	0.693008
tr|B4E2K4|B4E2K4_HUMAN	tr|B4E2K4|B4E	cDNA FLJ54576, highly similar to Aspartyl/asparaginyl beta-hydroxylase (EC 1.14.11.16) OS=Homo sapiens PE=	83685	0	1090	0	29	0	0.04	#DIV/0!	0	0	0	1	0	1	0	1	0.693008
tr|A8K2I7|A8K2I7_HUMAN	tr|A8K2I7|A8K2	cDNA FLJ76072, highly similar to Homo sapiens GIPC PDZ domain containing family, member 1 (GIPC1), transc	36131	0	1086	0	29	0	0.24	#DIV/0!	0	0	0	1	0	1	0	1	0.693008
sp|Q9Y5Q9|TF3C3_HUMAN	GTF3C3	General transcription factor 3C polypeptide 3 OS=Homo sapiens GN=GTF3C3 PE=1 SV=1	102006	0	1085	0	29	0	0.03	#DIV/0!	0	0	0	1	0	1	0	1	0.693008
sp|Q04941|PLP2_HUMAN	PLP2	Proteolipid protein 2 OS=Homo sapiens GN=PLP2 PE=1 SV=1	17022	0	1083	0	30	0	0.2	#DIV/0!	0	0	0	1	0	1	0	1	0.693008
tr|B4DW54|B4DW54_HUMAN	tr|B4DW54|B4D	cDNA FLJ50888, highly similar to Aldehyde dehydrogenase, mitochondrial (EC 1.2.1.3) OS=Homo sapiens PE=2	51467	0	1082	0	29	0	0.14	#DIV/0!	0	0	0	1	0	1	0	1	0.693008
tr|J3KSW6|J3KSW6_HUMAN	DHX40	Probable ATP-dependent RNA helicase DHX40 (Fragment) OS=Homo sapiens GN=DHX40 PE=4 SV=1	30276	0	1081	0	30	0	0.11	#DIV/0!	0	1	0	1	0	1	0	1	0.693008
sp|Q9ULT8|HECD1_HUMAN	HECTD1	E3 ubiquitin-protein ligase HECTD1 OS=Homo sapiens GN=HECTD1 PE=1 SV=3	292225	0	1078	0	30	0	0.03	#DIV/0!	0	0	0	1	0	1	0	1	0.693008
tr|A1A508|A1A508_HUMAN	PRSS3	PRSS3 protein OS=Homo sapiens GN=PRSS3 PE=2 SV=1	27040	0	1077	0	30	0	0.12	#DIV/0!	0	0	0	1	0	1	0	1	0.693008
tr|B2RD79|B2RD79_HUMAN	tr|B2RD79|B2R	Ubiquitin carboxyl-terminal hydrolase OS=Homo sapiens PE=2 SV=1	56462	0	1076	0	30	0	0.06	#DIV/0!	0	0	0	1	0	1	0	1	0.693008
tr|B4DQL0|B4DQL0_HUMAN	MTMR8	Myotubularin-related protein 8 OS=Homo sapiens GN=MTMR8 PE=2 SV=1	78337	0	1075	0	30	0	0.04	#DIV/0!	0	1	0	1	0	1	0	2	0.693008
tr|E9PDG5|E9PDG5_HUMAN	LARP4	La-related protein 4 OS=Homo sapiens GN=LARP4 PE=2 SV=1	50319	0	1074	0	30	0	0.04	#DIV/0!	0	0	0	1	0	1	0	1	0.693008
tr|K7EIJ8|K7EIJ8_HUMAN	KATNAL2	Katanin p60 ATPase-containing subunit A-like 2 (Fragment) OS=Homo sapiens GN=KATNAL2 PE=4 SV=1	38325	0	1071	0	30	0	0.09	#DIV/0!	0	0	0	1	0	1	0	1	0.693008
tr|D3DSQ7|D3DSQ7_HUMAN	XPO7	Exportin 7, isoform CRA_c OS=Homo sapiens GN=XPO7 PE=2 SV=1	100370	0	1069	0	30	0	0.03	#DIV/0!	0	0	0	1	0	1	0	1	0.693008
sp|Q9NQH7|XPP3_HUMAN	XPNPEP3	Probable Xaa-Pro aminopeptidase 3 OS=Homo sapiens GN=XPNPEP3 PE=1 SV=1	57624	0	1067	0	30	0	0.06	#DIV/0!	0	0	0	1	0	1	0	1	0.693008
sp|Q8WX92|NELFB_HUMAN	COBRA1	Negative elongation factor B OS=Homo sapiens GN=COBRA1 PE=1 SV=1	66283	0	1066	0	31	0	0.05	#DIV/0!	0	1	0	1	0	1	0	1	0.693008
tr|Q5T2B5|Q5T2B5_HUMAN	CUL2	Cullin-2 OS=Homo sapiens GN=CUL2 PE=3 SV=1	82933	0	1065	0	31	0	0.04	#DIV/0!	0	0	0	1	0	1	0	1	0.693008
tr|C9JPG4|C9JPG4_HUMAN	DENND2C	DENN domain-containing protein 2C OS=Homo sapiens GN=DENND2C PE=4 SV=1	95378	0	1063	0	31	0	0.04	#DIV/0!	0	0	0	1	0	1	0	1	0.693008
tr|B2RBM8|B2RBM8_HUMAN	tr|B2RBM8|B2F	cDNA, FLJ95596, highly similar to Homo sapiens activity-dependent neuroprotector (ADNP), mRNA OS=Homo	124850	0	1062	0	31	0	0.03	#DIV/0!	0	0	0	1	0	1	0	2	0.693008
tr|Q562T7|Q562T7_HUMAN	ACT	Actin-like protein (Fragment) OS=Homo sapiens GN=ACT PE=3 SV=1	11541	0	1057	0	31	0	0.3	#DIV/0!	0	0	0	1	0	1	0	1	0.693008
tr|B0AZS5|B0AZS5_HUMAN	tr|B0AZS5|B0A	cDNA, FLJ79515, highly similar to Kinesin-like protein KIF2 OS=Homo sapiens PE=2 SV=1	75666	0	1056	0	31	0	0.04	#DIV/0!	0	0	0	1	0	1	0	1	0.693008
tr|Q05DF2|Q05DF2_HUMAN	SF3A2	SF3A2 protein (Fragment) OS=Homo sapiens GN=SF3A2 PE=2 SV=1	51557	0	1055	0	31	0	0.06	#DIV/0!	0	0	0	1	0	1	0	1	0.693008
tr|Q58EY4|Q58EY4_HUMAN	SMARCC1	SWI/SNF related, matrix associated, actin dependent regulator of chromatin, subfamily c, member 1 OS=Hom	123347	0	1048	0	32	0	0.04	#DIV/0!	0	0	0	1	0	1	0	1	0.693008
tr|H7C561|H7C561_HUMAN	SF1	Splicing factor 1 (Fragment) OS=Homo sapiens GN=SF1 PE=4 SV=1	30425	0	1047	0	32	0	0.11	#DIV/0!	0	0	0	1	0	1	0	1	0.693008
tr|A8K4K5|A8K4K5_HUMAN	tr|A8K4K5|A8K	cDNA FLJ77843, highly similar to Homo sapiens TATA box binding protein (TBP)-associated factor, RNA polyme	52895	0	1045	0	32	0	0.06	#DIV/0!	0	1	0	1	0	1	0	1	0.693008
tr|G1UI38|G1UI38_HUMAN	EPT1	Ethanolaminephosphotransferase 1 (Fragment) OS=Homo sapiens GN=EPT1 PE=2 SV=1	23040	0	1044	0	32	0	0.15	#DIV/0!	0	0	0	1	0	1	0	1	0.693008
sp|Q96JM3|CHAP1_HUMAN	CHAMP1	Chromosome alignment-maintaining phosphoprotein 1 OS=Homo sapiens GN=CHAMP1 PE=1 SV=2	90012	0	1043	0	32	0	0.04	#DIV/0!	0	1	0	1	0	1	0	1	0.693008
tr|G3V438|G3V438_HUMAN	AHSA1	Activator of 90 kDa heat shock protein ATPase homolog 1 (Fragment) OS=Homo sapiens GN=AHSA1 PE=2 SV=	23078	0	1042	0	22	0	0.21	#DIV/0!	0	0	0	1	0	1	0	1	0.693008
tr|B3KTC7|B3KTC7_HUMAN	MRE11A	Double-strand break repair protein MRE11A OS=Homo sapiens GN=MRE11A PE=2 SV=1	81362	0	1041	0	32	0	0.04	#DIV/0!	0	0	0	1	0	1	0	2	0.693008
tr|Q5JSK4|Q5JSK4_HUMAN	RNF128	E3 ubiquitin-protein ligase RNF128 (Fragment) OS=Homo sapiens GN=RNF128 PE=4 SV=1	28037	0	1040	0	33	0	0.12	#DIV/0!	0	1	0	1	0	1	0	1	0.693008
tr|Q2VPA3|Q2VPA3_HUMAN	C15orf39	C15orf39 protein (Fragment) OS=Homo sapiens GN=C15orf39 PE=2 SV=1	53823	0	1039	0	33	0	0.06	#DIV/0!	0	1	0	1	0	1	0	1	0.693008
tr|B7Z6H4|B7Z6H4_HUMAN	tr|B7Z6H4|B7Z	DNA-directed RNA polymerase OS=Homo sapiens PE=2 SV=1	155407	0	1037	0	33	0	0.02	#DIV/0!	0	1	0	1	0	1	0	1	0.693008
sp|Q5TFE4|NT5D1_HUMAN	NT5DC1	5~-nucleotidase domain-containing protein 1 OS=Homo sapiens GN=NT5DC1 PE=1 SV=1	52439	0	1036	0	33	0	0.07	#DIV/0!	0	0	0	1	0	1	0	1	0.693008
tr|I0CMJ8|I0CMJ8_HUMAN	PRIM2	DNA primase large subunit (Fragment) OS=Homo sapiens GN=PRIM2 PE=4 SV=1	5179	0	1035	0	33	0	0.7	#DIV/0!	0	0	0	1	0	1	0	1	0.693008
tr|B4E140|B4E140_HUMAN	tr|B4E140|B4E	Transporter OS=Homo sapiens PE=2 SV=1	41144	0	1033	0	33	0	0.08	#DIV/0!	0	0	0	1	0	1	0	1	0.693008
tr|F5GZX4|F5GZX4_HUMAN	POLR1B	DNA-directed RNA polymerase OS=Homo sapiens GN=POLR1B PE=2 SV=1	133921	0	1029	0	33	0	0.02	#DIV/0!	0	1	0	1	0	1	0	1	0.693008
tr|Q13380|Q13380_HUMAN	RBM	RNA binding motif (Fragment) OS=Homo sapiens GN=RBM PE=4 SV=1	4015	0	1028	0	33	0	0.97	#DIV/0!	0	1	0	1	0	1	0	1	0.693008
tr|H0YKN8|H0YKN8_HUMAN	TLE3	Transducin-like enhancer protein 3 OS=Homo sapiens GN=TLE3 PE=4 SV=1	84740	0	1026	0	33	0	0.07	#DIV/0!	0	0	0	1	0	1	0	1	0.693008
tr|G5E9Z2|G5E9Z2_HUMAN	CLPTM1L	Cisplatin resistance related protein CRR9p, isoform CRA_c OS=Homo sapiens GN=CLPTM1L PE=2 SV=1	43366	0	1023	0	33	0	0.08	#DIV/0!	0	1	0	1	0	1	0	1	0.693008
tr|B4DI28|B4DI28_HUMAN	PTBP2	Polypyrimidine tract-binding protein 2 OS=Homo sapiens GN=PTBP2 PE=2 SV=1	34947	0	1022	0	34	0	0.09	#DIV/0!	0	0	0	1	0	1	0	2	0.693008
tr|E9PJ81|E9PJ81_HUMAN	UBXN1	UBX domain-containing protein 1 (Fragment) OS=Homo sapiens GN=UBXN1 PE=2 SV=1	32649	0	1021	0	34	0	0.15	#DIV/0!	0	0	0	1	0	1	0	1	0.693008
tr|B4DM78|B4DM78_HUMAN	tr|B4DM78|B4D	cDNA FLJ58199, highly similar to Fragile X mental retardation syndrome-related protein 1 OS=Homo sapiens P	63280	0	1019	0	0	0	0.05	#DIV/0!	0	0	0	0	0	1	0	1	0.693008
tr|B2R5Y4|B2R5Y4_HUMAN	tr|B2R5Y4|B2R	cDNA, FLJ92684, highly similar to Homo sapiens IK cytokine, down-regulator of HLA II (IK), mRNA OS=Homo sa	65749	0	1018	0	34	0	0.05	#DIV/0!	0	1	0	1	0	1	0	1	0.693008
tr|B3KU09|B3KU09_HUMAN	tr|B3KU09|B3K	cDNA FLJ39034 fis, clone NT2RP7008085, highly similar to Homo sapiens ring finger protein 123 (RNF123), mR	149906	0	1017	0	34	0	0.02	#DIV/0!	0	1	0	1	0	1	0	1	0.693008
sp|A6NNC1|P12LL_HUMAN	sp|A6NNC1|P1	Putative POM121-like protein 1-like OS=Homo sapiens PE=5 SV=3	94800	0	1013	0	34	0	0.03	#DIV/0!	0	1	0	1	0	1	0	1	0.693008
tr|J3KRI4|J3KRI4_HUMAN	DYNC1LI2	Cytoplasmic dynein 1 light intermediate chain 2 (Fragment) OS=Homo sapiens GN=DYNC1LI2 PE=4 SV=1	17767	0	1012	0	34	0	0.42	#DIV/0!	0	0	0	2	0	1	0	2	0.693008
tr|E9PC74|E9PC74_HUMAN	EIF2B5	Translation initiation factor eIF-2B subunit epsilon OS=Homo sapiens GN=EIF2B5 PE=2 SV=1	79096	0	1010	0	35	0	0.04	#DIV/0!	0	1	0	1	0	1	0	1	0.693008
tr|H7C0M3|H7C0M3_HUMAN	BPHL	Valacyclovir hydrolase (Fragment) OS=Homo sapiens GN=BPHL PE=4 SV=1	3314	0	1009	0	35	0	1.2	#DIV/0!	0	1	0	1	0	1	0	1	0.693008
tr|B2RAH7|B2RAH7_HUMAN	tr|B2RAH7|B2R	cDNA, FLJ94921, highly similar to Homo sapiens prolyl endopeptidase (PREP), mRNA OS=Homo sapiens PE=2 S	81594	0	1007	0	35	0	0.04	#DIV/0!	0	0	0	1	0	1	0	1	0.693008
tr|Q3B768|Q3B768_HUMAN	KIAA0406	KIAA0406 protein (Fragment) OS=Homo sapiens GN=KIAA0406 PE=2 SV=1	91343	0	1006	0	35	0	0.04	#DIV/0!	0	1	0	1	0	1	0	1	0.693008
tr|G3V1S7|G3V1S7_HUMAN	HAS1	Hyaluronan synthase 1 OS=Homo sapiens GN=HAS1 PE=2 SV=1	65517	0	1005	0	35	0	0.05	#DIV/0!	0	1	0	1	0	1	0	1	0.693008
sp|Q14126|DSG2_HUMAN	DSG2	Desmoglein-2 OS=Homo sapiens GN=DSG2 PE=1 SV=2	123016	0	1003	0	35	0	0.03	#DIV/0!	0	0	0	1	0	1	0	1	0.693008
tr|G5EA03|G5EA03_HUMAN	LIMCH1	LIM and calponin homology domains-containing protein 1 OS=Homo sapiens GN=LIMCH1 PE=2 SV=1	165820	0	1001	0	35	0	0.03	#DIV/0!	0	0	0	1	0	1	0	1	0.693008
sp|Q68E01|INT3_HUMAN	INTS3	Integrator complex subunit 3 OS=Homo sapiens GN=INTS3 PE=1 SV=1	119533	0	1000	0	35	0	0.03	#DIV/0!	0	1	0	1	0	1	0	2	0.693008
sp|P23368|MAOM_HUMAN	ME2	NAD-dependent malic enzyme, mitochondrial OS=Homo sapiens GN=ME2 PE=1 SV=1	66029	0	999	0	35	0	0.05	#DIV/0!	0	0	0	1	0	1	0	1	0.693008
sp|Q9H488|OFUT1_HUMAN	POFUT1	GDP-fucose protein O-fucosyltransferase 1 OS=Homo sapiens GN=POFUT1 PE=1 SV=1	44383	0	997	0	35	0	0.07	#DIV/0!	0	0	0	1	0	1	0	1	0.693008
sp|Q9NPI1|BRD7_HUMAN	BRD7	Bromodomain-containing protein 7 OS=Homo sapiens GN=BRD7 PE=1 SV=1	74548	0	996	0	35	0	0.04	#DIV/0!	0	1	0	1	0	1	0	1	0.693008
tr|B3KNJ4|B3KNJ4_HUMAN	SAE1	SUMO-1 activating enzyme subunit 1, isoform CRA_a OS=Homo sapiens GN=SAE1 PE=2 SV=1	33705	0	995	0	35	0	0.1	#DIV/0!	0	0	0	1	0	1	0	2	0.693008
sp|Q92888|ARHG1_HUMAN	ARHGEF1	Rho guanine nucleotide exchange factor 1 OS=Homo sapiens GN=ARHGEF1 PE=1 SV=2	103056	0	994	0	35	0	0.03	#DIV/0!	0	0	0	1	0	1	0	1	0.693008
sp|O75436|VP26A_HUMAN	VPS26A	Vacuolar protein sorting-associated protein 26A OS=Homo sapiens GN=VPS26A PE=1 SV=2	38260	0	993	0	35	0	0.09	#DIV/0!	0	1	0	1	0	1	0	1	0.693008
tr|B3KNS8|B3KNS8_HUMAN	tr|B3KNS8|B3K	cDNA FLJ30322 fis, clone BRACE2006703, highly similar to Surfeit locus protein 6 OS=Homo sapiens PE=2 SV=1	41598	0	992	0	35	0	0.08	#DIV/0!	0	1	0	1	0	1	0	2	0.693008
tr|G3V1K3|G3V1K3_HUMAN	PON2	Paraoxonase 2, isoform CRA_a OS=Homo sapiens GN=PON2 PE=2 SV=1	41672	0	990	0	35	0	0.08	#DIV/0!	0	0	0	1	0	1	0	2	0.693008
tr|Q6FI30|Q6FI30_HUMAN	NFIC	Nuclear factor 1 OS=Homo sapiens GN=NFIC PE=2 SV=1	55019	0	988	0	36	0	0.06	#DIV/0!	0	0	0	1	0	1	0	1	0.693008
sp|A6NJB7|PRR19_HUMAN	PRR19	Proline-rich protein 19 OS=Homo sapiens GN=PRR19 PE=2 SV=1	38920	0	987	0	36	0	0.08	#DIV/0!	0	1	0	1	0	1	0	1	0.693008
tr|A8K5S1|A8K5S1_HUMAN	tr|A8K5S1|A8K	cDNA FLJ78650, highly similar to Homo sapiens mucosa associated lymphoid tissue lymphoma translocation g	93725	0	986	0	36	0	0.03	#DIV/0!	0	1	0	1	0	1	0	2	0.693008
tr|B2R6E5|B2R6E5_HUMAN	tr|B2R6E5|B2R	NADPH--cytochrome P450 reductase OS=Homo sapiens PE=2 SV=1	77429	0	985	0	36	0	0.04	#DIV/0!	0	1	0	1	0	1	0	2	0.693008
tr|B2R6G5|B2R6G5_HUMAN	tr|B2R6G5|B2R	cDNA, FLJ92942, highly similar to Homo sapiens T-box, brain, 1 (TBR1), mRNA OS=Homo sapiens PE=2 SV=1	74530	0	982	0	36	0	0.04	#DIV/0!	0	1	0	1	0	1	0	1	0.693008
tr|B3KN69|B3KN69_HUMAN	tr|B3KN69|B3K	cDNA FLJ13804 fis, clone THYRO1000197, highly similar to Poly(A)-specific ribonuclease PARN (EC 3.1.13.4) OS	40330	0	980	0	36	0	0.08	#DIV/0!	0	0	0	1	0	1	0	1	0.693008
tr|H0YDX7|H0YDX7_HUMAN	HBS1L	HBS1-like protein (Fragment) OS=Homo sapiens GN=HBS1L PE=4 SV=1	61030	0	977	0	36	0	0.05	#DIV/0!	0	0	0	1	0	1	0	1	0.693008
tr|B0V2J7|B0V2J7_HUMAN	TAP2	Antigen peptide transporter 2 OS=Homo sapiens GN=TAP2 PE=4 SV=1	72571	0	976	0	36	0	0.05	#DIV/0!	0	1	0	1	0	1	0	2	0.693008
tr|B4DHJ9|B4DHJ9_HUMAN	tr|B4DHJ9|B4D	cDNA FLJ61262, highly similar to Ubiquitin-protein ligase E3C (EC 6.3.2.-) OS=Homo sapiens PE=2 SV=1	107426	0	974	0	36	0	0.03	#DIV/0!	0	1	0	1	0	1	0	1	0.693008
tr|H7C1M2|H7C1M2_HUMAN	SON	Protein SON (Fragment) OS=Homo sapiens GN=SON PE=4 SV=1	160263	0	973	0	36	0	0.02	#DIV/0!	0	1	0	1	0	1	0	1	0.693008
sp|Q6UWE0|LRSM1_HUMAN	LRSAM1	E3 ubiquitin-protein ligase LRSAM1 OS=Homo sapiens GN=LRSAM1 PE=1 SV=1	84567	0	972	0	36	0	0.04	#DIV/0!	0	0	0	1	0	1	0	1	0.693008
tr|B3KM40|B3KM40_HUMAN	tr|B3KM40|B3K	cDNA FLJ10185 fis, clone HEMBA1004509, highly similar to U4/U6.U5 tri-snRNP-associated protein 2 OS=Hom	56618	0	969	0	37	0	0.06	#DIV/0!	0	1	0	1	0	1	0	1	0.693008
tr|K7EIR0|K7EIR0_HUMAN	WDR18	WD repeat-containing protein 18 OS=Homo sapiens GN=WDR18 PE=4 SV=1	45436	0	968	0	37	0	0.07	#DIV/0!	0	1	0	1	0	1	0	1	0.693008
tr|A4QN18|A4QN18_HUMAN	SEC16A	SEC16A protein (Fragment) OS=Homo sapiens GN=SEC16A PE=2 SV=1	212198	0	967	0	37	0	0.01	#DIV/0!	0	1	0	1	0	1	0	2	0.693008
tr|B4DPV3|B4DPV3_HUMAN	tr|B4DPV3|B4D	cDNA FLJ58109, highly similar to Protein diaphanous homolog 3 (Fragment) OS=Homo sapiens PE=2 SV=1	117640	0	965	0	37	0	0.03	#DIV/0!	0	1	0	1	0	1	0	1	0.693008
tr|Q9UFD5|Q9UFD5_HUMAN	DKFZp434N181	Putative uncharacterized protein DKFZp434N181 (Fragment) OS=Homo sapiens GN=DKFZp434N181 PE=2 SV=	49392	0	963	0	37	0	0.07	#DIV/0!	0	1	0	1	0	1	0	1	0.693008
sp|Q9BXW9|FACD2_HUMAN	FANCD2	Fanconi anemia group D2 protein OS=Homo sapiens GN=FANCD2 PE=1 SV=1	168295	0	959	0	37	0	0.02	#DIV/0!	0	1	0	1	0	1	0	1	0.693008
tr|B3KQH2|B3KQH2_HUMAN	tr|B3KQH2|B3K	cDNA FLJ90454 fis, clone NT2RP3001560, highly similar to Cleft lip and palate transmembrane protein 1 OS=H	76247	0	958	0	37	0	0.04	#DIV/0!	0	1	0	1	0	1	0	1	0.693008
sp|Q9Y4C4|MFHA1_HUMAN	MFHAS1	Malignant fibrous histiocytoma-amplified sequence 1 OS=Homo sapiens GN=MFHAS1 PE=1 SV=2	117960	0	956	0	37	0	0.03	#DIV/0!	0	1	0	1	0	1	0	2	0.693008
sp|Q8IXB1|DJC10_HUMAN	DNAJC10	DnaJ homolog subfamily C member 10 OS=Homo sapiens GN=DNAJC10 PE=1 SV=2	92333	0	955	0	37	0	0.09	#DIV/0!	0	1	0	1	0	1	0	1	0.693008
tr|B4DN44|B4DN44_HUMAN	tr|B4DN44|B4D	cDNA FLJ53581, highly similar to Ras and Rab interactor 1 OS=Homo sapiens PE=2 SV=1	65995	0	952	0	38	0	0.05	#DIV/0!	0	1	0	1	0	1	0	1	0.693008
tr|H0Y2P8|H0Y2P8_HUMAN	SRGAP2	SLIT-ROBO Rho GTPase-activating protein 2 (Fragment) OS=Homo sapiens GN=SRGAP2 PE=4 SV=1	111536	0	949	0	38	0	0.03	#DIV/0!	0	1	0	1	0	1	0	1	0.693008
tr|E5RHC5|E5RHC5_HUMAN	ARFGAP1	ADP-ribosylation factor GTPase-activating protein 1 (Fragment) OS=Homo sapiens GN=ARFGAP1 PE=2 SV=2	29395	0	945	0	38	0	0.11	#DIV/0!	0	1	0	1	0	1	0	1	0.693008
sp|Q5D862|FILA2_HUMAN	FLG2	Filaggrin-2 OS=Homo sapiens GN=FLG2 PE=1 SV=1	249296	0	942	0	38	0	0.01	#DIV/0!	0	1	0	1	0	1	0	1	0.693008
tr|Q8N5A0|Q8N5A0_HUMAN	EIF5B	Eukaryotic translation initiation factor 5B OS=Homo sapiens GN=EIF5B PE=2 SV=1	139172	0	940	0	38	0	0.09	#DIV/0!	0	1	0	2	0	1	0	2	0.693008
tr|B4DPW5|B4DPW5_HUMAN	tr|B4DPW5|B4D	cDNA FLJ59374, highly similar to Homo sapiens caldesmon 1 (CALD1), transcript variant 2, mRNA OS=Homo sa	57436	0	934	0	38	0	0.06	#DIV/0!	0	1	0	1	0	1	0	1	0.693008
tr|Q2TB12|Q2TB12_HUMAN	LIG1	DNA ligase OS=Homo sapiens GN=LIG1 PE=2 SV=1	88975	0	932	0	39	0	0.04	#DIV/0!	0	1	0	1	0	1	0	1	0.693008
tr|Q5LJA0|Q5LJA0_HUMAN	TROVE2	60 kDa SS-A/Ro ribonucleoprotein OS=Homo sapiens GN=TROVE2 PE=4 SV=1	59916	0	931	0	39	0	0.05	#DIV/0!	0	1	0	1	0	1	0	1	0.693008
sp|P02748|CO9_HUMAN	C9	Complement component C9 OS=Homo sapiens GN=C9 PE=1 SV=2	64615	0	930	0	39	0	0.05	#DIV/0!	0	1	0	1	0	1	0	2	0.693008
tr|Q5T8U2|Q5T8U2_HUMAN	RPL7A	60S ribosomal protein L7a OS=Homo sapiens GN=RPL7A PE=4 SV=1	16700	0	927	0	39	0	0.2	#DIV/0!	0	1	0	1	0	1	0	1	0.693008
tr|B4DWV9|B4DWV9_HUMAN	tr|B4DWV9|B4	cDNA FLJ53108, highly similar to Guanine nucleotide-binding protein alpha-13 subunit OS=Homo sapiens PE=2	41291	0	926	0	39	0	0.07	#DIV/0!	0	1	0	1	0	1	0	1	0.693008
tr|J3KQ58|J3KQ58_HUMAN	TTC34	Tetratricopeptide repeat protein 34 OS=Homo sapiens GN=TTC34 PE=4 SV=1	40214	0	925	0	39	0	0.08	#DIV/0!	0	1	0	1	0	1	0	1	0.693008
sp|P11055|MYH3_HUMAN	MYH3	Myosin-3 OS=Homo sapiens GN=MYH3 PE=1 SV=3	224850	0	924	0	39	0	0.01	#DIV/0!	0	1	0	1	0	1	0	2	0.693008
tr|B4DRV3|B4DRV3_HUMAN	tr|B4DRV3|B4D	cDNA FLJ51537, highly similar to Myosin heavy chain, skeletal muscle, adult 2 (Fragment) OS=Homo sapiens P	96376	0	923	0	39	0	0.03	#DIV/0!	0	1	0	1	0	1	0	2	0.693008
sp|Q9Y623|MYH4_HUMAN	MYH4	Myosin-4 OS=Homo sapiens GN=MYH4 PE=1 SV=2	223902	0	922	0	39	0	0.01	#DIV/0!	0	1	0	1	0	1	0	2	0.693008
tr|B4DMA4|B4DMA4_HUMAN	tr|B4DMA4|B4	cDNA FLJ58400, highly similar to Cleavage stimulation factor 77 kDa subunit OS=Homo sapiens PE=2 SV=1	75580	0	920	0	39	0	0.05	#DIV/0!	0	1	0	1	0	1	0	1	0.693008
sp|Q6PD62|CTR9_HUMAN	CTR9	RNA polymerase-associated protein CTR9 homolog OS=Homo sapiens GN=CTR9 PE=1 SV=1	134332	0	919	0	39	0	0.02	#DIV/0!	0	1	0	1	0	1	0	1	0.693008
tr|B4E3I3|B4E3I3_HUMAN	tr|B4E3I3|B4E3	cDNA FLJ59614, highly similar to Ubiquitin fusion degradation protein 1 homolog OS=Homo sapiens PE=2 SV=	35730	0	918	0	39	0	0.09	#DIV/0!	0	1	0	1	0	1	0	2	0.693008
tr|G3V5E1|G3V5E1_HUMAN	CCNK	Cyclin-K OS=Homo sapiens GN=CCNK PE=2 SV=1	43776	0	917	0	39	0	0.08	#DIV/0!	0	1	0	1	0	1	0	1	0.693008
tr|Q8N6B4|Q8N6B4_HUMAN	MLL/CALM	Myeloid/lymphoid or mixed-lineage leukemia/clathrin assembly protein fusion protein (Fragment) OS=Homo	63408	0	916	0	40	0	0.05	#DIV/0!	0	1	0	1	0	1	0	1	0.693008
tr|B3KM97|B3KM97_HUMAN	tr|B3KM97|B3K	cDNA FLJ10554 fis, clone NT2RP2002385, highly similar to Synaptic glycoprotein SC2 OS=Homo sapiens PE=2 S	36429	0	915	0	40	0	0.09	#DIV/0!	0	1	0	1	0	1	0	1	0.693008
sp|Q9BVP2|GNL3_HUMAN	GNL3	Guanine nucleotide-binding protein-like 3 OS=Homo sapiens GN=GNL3 PE=1 SV=2	62468	0	914	0	40	0	0.05	#DIV/0!	0	1	0	1	0	1	0	2	0.693008
tr|A4FVA8|A4FVA8_HUMAN	DFNA5	DFNA5 protein (Fragment) OS=Homo sapiens GN=DFNA5 PE=2 SV=1	48389	0	913	0	40	0	0.07	#DIV/0!	0	1	0	1	0	1	0	1	0.693008
tr|G3XAN1|G3XAN1_HUMAN	INTS9	Integrator complex subunit 9 OS=Homo sapiens GN=INTS9 PE=2 SV=1	59772	0	912	0	40	0	0.06	#DIV/0!	0	1	0	1	0	1	0	1	0.693008
tr|H7C455|H7C455_HUMAN	tr|H7C455|H7C	Uncharacterized protein (Fragment) OS=Homo sapiens PE=4 SV=1	22389	0	911	0	23	0	0.1	#DIV/0!	0	0	0	1	0	1	0	1	0.693008
tr|B2RB57|B2RB57_HUMAN	tr|B2RB57|B2R	cDNA, FLJ95321, highly similar to Homo sapiens ATG7 autophagy related 7 homolog (S. cerevisiae) (ATG7), mR	79076	0	909	0	40	0	0.04	#DIV/0!	0	1	0	1	0	1	0	2	0.693008
tr|J3QQ67|J3QQ67_HUMAN	RPL18	60S ribosomal protein L18 (Fragment) OS=Homo sapiens GN=RPL18 PE=3 SV=1	21940	0	908	0	40	0	0.18	#DIV/0!	0	1	0	1	0	1	0	1	0.693008
tr|H0Y2Y8|H0Y2Y8_HUMAN	ZYX	Zyxin (Fragment) OS=Homo sapiens GN=ZYX PE=4 SV=1	58804	0	907	0	40	0	0.06	#DIV/0!	0	1	0	1	0	1	0	2	0.693008
tr|Q5SRT3|Q5SRT3_HUMAN	CLIC1	Chloride intracellular channel 1, isoform CRA_a OS=Homo sapiens GN=CLIC1 PE=2 SV=2	27248	0	905	0	40	0	0.12	#DIV/0!	0	1	0	1	0	1	0	1	0.693008
tr|K7EQ60|K7EQ60_HUMAN	COASY	Bifunctional coenzyme A synthase (Fragment) OS=Homo sapiens GN=COASY PE=4 SV=1	12950	0	903	0	40	0	0.26	#DIV/0!	0	1	0	1	0	1	0	1	0.693008
tr|Q5TZP7|Q5TZP7_HUMAN	APEX1	APEX nuclease (Multifunctional DNA repair enzyme) 1 OS=Homo sapiens GN=APEX1 PE=2 SV=1	35931	0	902	0	40	0	0.17	#DIV/0!	0	1	0	1	0	1	0	1	0.693008

sp|Q9BS26|ERP44_HUMAN	ERP44	Endoplasmic reticulum resident protein 44 OS=Homo sapiens GN=ERP44 PE=1 SV=1	47341	0	901	0	40	0	0.07	#DIV/0!	0	1	0	1	0	1	0	1	0.693008
tr|K7EPI4|K7EPI4_HUMAN	GFAP	Glial fibrillary acidic protein (Fragment) OS=Homo sapiens GN=GFAP PE=4 SV=1	7120	0	898	0	41	0	0.5	#DIV/0!	0	1	0	1	0	1	0	1	0.693008
tr|B1AK81|B1AK81_HUMAN	PIGK	GPI-anchor transamidase OS=Homo sapiens GN=PIGK PE=4 SV=1	34462	0	895	0	41	0	0.1	#DIV/0!	0	1	0	1	0	1	0	1	0.693008
tr|B4DM41|B4DM41_HUMAN	RFC4	Replication factor C (Activator 1) 4, 37kDa, isoform CRA_b OS=Homo sapiens GN=RFC4 PE=2 SV=1	33996	0	894	0	41	0	0.1	#DIV/0!	0	1	0	1	0	1	0	1	0.693008
sp|Q96FV9|THOC1_HUMAN	THOC1	THO complex subunit 1 OS=Homo sapiens GN=THOC1 PE=1 SV=1	76360	0	892	0	41	0	0.04	#DIV/0!	0	1	0	1	0	1	0	1	0.693008
tr|Q2I0A3|Q2I0A3_HUMAN	CFTR	Cystic fibrosis transmembrane conductance regulator ATP-binding cassette sub-family C member 7 OS=Homo	168854	0	890	0	41	0	0.02	#DIV/0!	0	1	0	1	0	1	0	1	0.693008
sp|P61073|CXCR4_HUMAN	CXCR4	C-X-C chemokine receptor type 4 OS=Homo sapiens GN=CXCR4 PE=1 SV=1	40233	0	886	0	42	0	0.08	#DIV/0!	0	1	0	1	0	1	0	1	0.693008
tr|J3KNI9|J3KNI9_HUMAN	TBC1D15	TBC1 domain family member 15 OS=Homo sapiens GN=TBC1D15 PE=4 SV=1	79491	0	883	0	42	0	0.04	#DIV/0!	0	1	0	1	0	1	0	1	0.693008
tr|G3V1P5|G3V1P5_HUMAN	MED15	Mediator of RNA polymerase II transcription subunit 15 OS=Homo sapiens GN=MED15 PE=4 SV=1	80007	0	882	0	42	0	0.04	#DIV/0!	0	1	0	1	0	1	0	1	0.693008
tr|G3V1B3|G3V1B3_HUMAN	RPL21	60S ribosomal protein L21 OS=Homo sapiens GN=RPL21 PE=2 SV=1	9994	0	881	0	42	0	0.35	#DIV/0!	0	1	0	1	0	1	0	1	0.693008
tr|B4DJV2|B4DJV2_HUMAN	CS	Citrate synthase OS=Homo sapiens GN=CS PE=2 SV=1	50628	0	879	0	42	0	0.07	#DIV/0!	0	1	0	1	0	1	0	2	0.693008
sp|Q9Y3L5|RAP2C_HUMAN	RAP2C	Ras-related protein Rap-2c OS=Homo sapiens GN=RAP2C PE=1 SV=1	20959	0	878	0	42	0	0.16	#DIV/0!	0	1	0	1	0	1	0	1	0.693008
sp|Q8IXI1|MIRO2_HUMAN	RHOT2	Mitochondrial Rho GTPase 2 OS=Homo sapiens GN=RHOT2 PE=1 SV=2	69101	0	877	0	42	0	0.14	#DIV/0!	0	1	0	1	0	1	0	1	0.693008
tr|Q96CP5|Q96CP5_HUMAN	PMPCB	PMPCB protein (Fragment) OS=Homo sapiens GN=PMPCB PE=2 SV=2	54216	0	876	0	42	0	0.06	#DIV/0!	0	1	0	1	0	1	0	1	0.693008
tr|H3BSD9|H3BSD9_HUMAN	MYO7B	Unconventional myosin-VIIb (Fragment) OS=Homo sapiens GN=MYO7B PE=4 SV=1	10022	0	875	0	42	0	0.35	#DIV/0!	0	1	0	1	0	1	0	2	0.693008
tr|B2RDG4|B2RDG4_HUMAN	tr|B2RDG4|B2R	cDNA, FLJ96597 OS=Homo sapiens PE=2 SV=1	57796	0	874	0	43	0	0.06	#DIV/0!	0	1	0	1	0	1	0	2	0.693008
sp|Q9UBG0|MRC2_HUMAN	MRC2	C-type mannose receptor 2 OS=Homo sapiens GN=MRC2 PE=1 SV=2	169761	0	873	0	43	0	0.02	#DIV/0!	0	1	0	1	0	1	0	1	0.693008
tr|B1AMX9|B1AMX9_HUMAN	TRAF2	TNF receptor-associated factor 2 OS=Homo sapiens GN=TRAF2 PE=4 SV=1	47848	0	872	0	43	0	0.07	#DIV/0!	0	1	0	1	0	1	0	1	0.693008
tr|B4DMF5|B4DMF5_HUMAN	tr|B4DMF5|B4D	Glutamate dehydrogenase OS=Homo sapiens PE=2 SV=1	56910	0	870	0	43	0	0.06	#DIV/0!	0	1	0	1	0	1	0	1	0.693008
tr|Q05CN7|Q05CN7_HUMAN	GTF3C4	GTF3C4 protein (Fragment) OS=Homo sapiens GN=GTF3C4 PE=2 SV=1	63439	0	868	0	43	0	0.05	#DIV/0!	0	1	0	1	0	1	0	1	0.693008
tr|B3KMN5|B3KMN5_HUMAN	tr|B3KMN5|B3	cDNA FLJ11713 fis, clone HEMBA1005201, highly similar to Homo sapiens NMD3 homolog (NMD3), mRNA OS=	44787	0	864	0	43	0	0.07	#DIV/0!	0	1	0	1	0	1	0	1	0.693008
tr|B4DFN6|B4DFN6_HUMAN	CNP	2~,3~-cyclic-nucleotide 3~-phosphodiesterase OS=Homo sapiens GN=CNP PE=2 SV=1	20461	0	861	0	44	0	0.16	#DIV/0!	0	1	0	1	0	1	0	2	0.693008
tr|B7Z284|B7Z284_HUMAN	tr|B7Z284|B7Z2	DNA-directed RNA polymerase OS=Homo sapiens PE=2 SV=1	138313	0	859	0	44	0	0.02	#DIV/0!	0	1	0	1	0	1	0	2	0.693008
tr|L0R4Y9|L0R4Y9_HUMAN	IQSEC1	Alternative protein IQSEC1 OS=Homo sapiens GN=IQSEC1 PE=4 SV=1	4703	0	856	0	44	0	0.84	#DIV/0!	0	1	0	1	0	1	0	1	0.693008
tr|B4DYP7|B4DYP7_HUMAN	tr|B4DYP7|B4D	cDNA FLJ55435, highly similar to Gamma-tubulin complex component 3 OS=Homo sapiens PE=2 SV=1	103306	0	855	0	44	0	0.03	#DIV/0!	0	1	0	1	0	1	0	1	0.693008
tr|C0JXB8|C0JXB8_HUMAN	AIP	Aryl hydrocarbon receptor interacting protein OS=Homo sapiens GN=AIP PE=2 SV=1	38041	0	854	0	44	0	0.09	#DIV/0!	0	1	0	1	0	1	0	1	0.693008
tr|Q2TAM5|Q2TAM5_HUMAN	RELA	RELA protein OS=Homo sapiens GN=RELA PE=2 SV=1	43355	0	853	0	44	0	0.08	#DIV/0!	0	1	0	1	0	1	0	1	0.693008
tr|B4DFG0|B4DFG0_HUMAN	DEK	Protein DEK OS=Homo sapiens GN=DEK PE=2 SV=1	39704	0	849	0	44	0	0.08	#DIV/0!	0	1	0	1	0	1	0	1	0.693008
tr|H0YL11|H0YL11_HUMAN	IDH2	Isocitrate dehydrogenase [NADP], mitochondrial OS=Homo sapiens GN=IDH2 PE=3 SV=1	35536	0	848	0	22	0	0.23	#DIV/0!	0	0	0	1	0	1	0	1	0.693008
sp|Q8N766|EMC1_HUMAN	EMC1	ER membrane protein complex subunit 1 OS=Homo sapiens GN=EMC1 PE=1 SV=1	112145	0	846	0	45	0	0.03	#DIV/0!	0	1	0	1	0	1	0	1	0.693008
tr|B4DVM9|B4DVM9_HUMAN	tr|B4DVM9|B4D	cDNA FLJ50247, highly similar to Jumonji/ARID domain-containing protein 1A (Fragment) OS=Homo sapiens PE	121122	0	840	0	46	0	0.03	#DIV/0!	0	1	0	1	0	1	0	1	0.693008
sp|O15091|MRRP3_HUMAN	KIAA0391	Mitochondrial ribonuclease P protein 3 OS=Homo sapiens GN=KIAA0391 PE=1 SV=2	68014	0	839	0	46	0	0.05	#DIV/0!	0	1	0	1	0	1	0	1	0.693008
tr|B3KM89|B3KM89_HUMAN	tr|B3KM89|B3K	cDNA FLJ10528 fis, clone NT2RP2000943, highly similar to Protein transport protein Sec24D OS=Homo sapiens	101723	0	838	0	46	0	0.03	#DIV/0!	0	1	0	1	0	1	0	1	0.693008
tr|Q6IPI1|Q6IPI1_HUMAN	RPL29	Ribosomal protein L29 OS=Homo sapiens GN=RPL29 PE=2 SV=1	17997	0	836	0	46	0	0.19	#DIV/0!	0	1	0	1	0	1	0	1	0.693008
tr|Q5U043|Q5U043_HUMAN	tr|Q5U043|Q5U	Alcohol dehydrogenase 5 (Class III), chi polypeptide OS=Homo sapiens PE=2 SV=1	40520	0	834	0	46	0	0.17	#DIV/0!	0	1	0	2	0	1	0	3	0.693008
tr|J3KQS3|J3KQS3_HUMAN	PNPLA6	Neuropathy target esterase OS=Homo sapiens GN=PNPLA6 PE=4 SV=1	152341	0	832	0	46	0	0.02	#DIV/0!	0	1	0	1	0	1	0	1	0.693008
tr|A8K4W5|A8K4W5_HUMAN	tr|A8K4W5|A8K	cDNA FLJ76813, highly similar to Homo sapiens acetyl-Coenzyme A acetyltransferase 2 (acetoacetyl Coenzyme	41866	0	831	0	46	0	0.07	#DIV/0!	0	1	0	1	0	1	0	1	0.693008
tr|H1UBN2|H1UBN2_HUMAN	CCNB1V	Cyclin B1 OS=Homo sapiens GN=CCNB1V PE=2 SV=1	46024	0	829	0	16	0	0.14	#DIV/0!	0	0	0	1	0	1	0	4	0.693008
tr|A8K594|A8K594_HUMAN	tr|A8K594|A8K	cDNA FLJ77256, highly similar to Homo sapiens SH3-domain binding protein 4 (SH3BP4), mRNA OS=Homo sapi	108400	0	826	0	47	0	0.03	#DIV/0!	0	1	0	1	0	1	0	2	0.693008
sp|P33897|ABCD1_HUMAN	ABCD1	ATP-binding cassette sub-family D member 1 OS=Homo sapiens GN=ABCD1 PE=1 SV=2	83398	0	824	0	47	0	0.04	#DIV/0!	0	1	0	1	0	1	0	1	0.693008
tr|H0Y9X5|H0Y9X5_HUMAN	ERAP1	Endoplasmic reticulum aminopeptidase 1 (Fragment) OS=Homo sapiens GN=ERAP1 PE=4 SV=1	17136	0	823	0	47	0	0.2	#DIV/0!	0	1	0	1	0	1	0	1	0.693008
sp|Q32P28|P3H1_HUMAN	LEPRE1	Prolyl 3-hydroxylase 1 OS=Homo sapiens GN=LEPRE1 PE=1 SV=2	84196	0	821	0	47	0	0.14	#DIV/0!	0	1	0	1	0	1	0	1	0.693008
tr|B1VK50|B1VK50_HUMAN	HLA-C	MHC class I antigen (Fragment) OS=Homo sapiens GN=HLA-C PE=3 SV=1	21287	0	820	0	47	0	0.16	#DIV/0!	0	1	0	1	0	1	0	2	0.693008
tr|H6UWQ4|H6UWQ4_HUMAN	HLA-C	MHC class I antigen (Fragment) OS=Homo sapiens GN=HLA-C PE=3 SV=1	32062	0	818	0	47	0	0.1	#DIV/0!	0	1	0	1	0	1	0	3	0.693008
tr|C6K4M2|C6K4M2_HUMAN	HLA-C	MHC class I antigen (Fragment) OS=Homo sapiens GN=HLA-C PE=3 SV=1	20827	0	817	0	47	0	0.16	#DIV/0!	0	1	0	1	0	1	0	3	0.693008
tr|Q5W1M0|Q5W1M0_HUMAN	HLA-C	MHC class I antigen (Fragment) OS=Homo sapiens GN=HLA-C PE=2 SV=1	10534	0	815	0	0	0	0.08	#DIV/0!	0	0	0	0	0	1	0	1	0.693008
tr|Q5JUA8|Q5JUA8_HUMAN	VPS16	Vacuolar protein sorting-associated protein 16 homolog OS=Homo sapiens GN=VPS16 PE=4 SV=1	59823	0	811	0	47	0	0.05	#DIV/0!	0	1	0	1	0	1	0	1	0.693008
tr|B4DYY5|B4DYY5_HUMAN	tr|B4DYY5|B4D	cDNA FLJ55671, highly similar to Ubiquitin-associated protein 2-like OS=Homo sapiens PE=2 SV=1	113662	0	810	0	47	0	0.03	#DIV/0!	0	1	0	1	0	1	0	1	0.693008
tr|B1AHL2|B1AHL2_HUMAN	FBLN1	Fibulin 1 OS=Homo sapiens GN=FBLN1 PE=4 SV=1	82439	0	806	0	48	0	0.04	#DIV/0!	0	1	0	1	0	1	0	1	0.693008
tr|Q2TSD2|Q2TSD2_HUMAN	tr|Q2TSD2|Q2T	Aging-associated gene 7 protein OS=Homo sapiens PE=2 SV=1	41778	0	805	0	48	0	0.08	#DIV/0!	0	1	0	1	0	1	0	1	0.693008
tr|B4DRK5|B4DRK5_HUMAN	tr|B4DRK5|B4D	cDNA FLJ59584, highly similar to Mitochondrial-processing peptidase alpha subunit, mitochondrial (EC 3.4.24.(	28622	0	804	0	48	0	0.12	#DIV/0!	0	1	0	2	0	1	0	2	0.693008
tr|B7Z653|B7Z653_HUMAN	tr|B7Z653|B7Z6	cDNA FLJ61657, highly similar to Band 4.1-like protein 1 OS=Homo sapiens PE=2 SV=1	90347	0	803	0	48	0	0.04	#DIV/0!	0	1	0	1	0	1	0	1	0.693008
tr|H3BSH7|H3BSH7_HUMAN	CIRH1A	Cirhin (Fragment) OS=Homo sapiens GN=CIRH1A PE=4 SV=1	78578	0	802	0	48	0	0.04	#DIV/0!	0	1	0	1	0	1	0	3	0.693008
tr|B3KPF2|B3KPF2_HUMAN	tr|B3KPF2|B3KP	cDNA FLJ31717 fis, clone NT2RI2006640, highly similar to Dynamin-3 (EC 3.6.5.5) OS=Homo sapiens PE=2 SV=1	63057	0	800	0	48	0	0.05	#DIV/0!	0	1	0	1	0	1	0	1	0.693008
tr|Q0VGA5|Q0VGA5_HUMAN	SARS	SARS protein OS=Homo sapiens GN=SARS PE=2 SV=1	58883	0	797	0	49	0	0.06	#DIV/0!	0	1	0	1	0	1	0	1	0.693008
tr|B4DN25|B4DN25_HUMAN	UGDH	UDP-glucose 6-dehydrogenase OS=Homo sapiens GN=UGDH PE=2 SV=1	44786	0	795	0	37	0	0.12	#DIV/0!	0	1	0	1	0	1	0	1	0.693008
tr|A1A693|A1A693_HUMAN	RBM15	RNA binding motif protein 15 isoform L2 OS=Homo sapiens GN=RBM15 PE=2 SV=1	99689	0	792	0	50	0	0.03	#DIV/0!	0	1	0	1	0	1	0	2	0.693008
tr|B2R8G6|B2R8G6_HUMAN	tr|B2R8G6|B2R	cDNA, FLJ93891, Homo sapiens FK506 binding protein 8, 38kDa (FKBP8), mRNA OS=Homo sapiens PE=2 SV=1	38870	0	789	0	50	0	0.09	#DIV/0!	0	1	0	1	0	1	0	1	0.693008
tr|F1D8N4|F1D8N4_HUMAN	NR3C1	Glucocorticoid nuclear receptor variant 1 OS=Homo sapiens GN=NR3C1 PE=2 SV=1	86745	0	786	0	50	0	0.04	#DIV/0!	0	1	0	1	0	1	0	1	0.693008
sp|Q9Y4X5|ARI1_HUMAN	ARIH1	E3 ubiquitin-protein ligase ARIH1 OS=Homo sapiens GN=ARIH1 PE=1 SV=2	65900	0	785	0	50	0	0.1	#DIV/0!	0	1	0	2	0	1	0	2	0.693008
tr|B3KU23|B3KU23_HUMAN	tr|B3KU23|B3K	cDNA FLJ39097 fis, clone NTONG2000977, highly similar to Neurolysin, mitochondrial (EC 3.4.24.16) OS=Homo	70320	0	783	0	50	0	0.07	#DIV/0!	0	1	0	1	0	1	0	1	0.693008
sp|Q96T51|RUFY1_HUMAN	RUFY1	RUN and FYVE domain-containing protein 1 OS=Homo sapiens GN=RUFY1 PE=1 SV=2	80851	0	781	0	50	0	0.04	#DIV/0!	0	1	0	1	0	1	0	2	0.693008
tr|B7ZKR8|B7ZKR8_HUMAN	USP48	USP48 protein OS=Homo sapiens GN=USP48 PE=2 SV=1	115025	0	779	0	50	0	0.03	#DIV/0!	0	1	0	1	0	1	0	1	0.693008
tr|Q548W1|Q548W1_HUMAN	OGT	HRNT1 OS=Homo sapiens GN=OGT PE=2 SV=1	104029	0	774	0	51	0	0.03	#DIV/0!	0	1	0	1	0	1	0	1	0.693008
sp|Q9UKX7|NUP50_HUMAN	NUP50	Nuclear pore complex protein Nup50 OS=Homo sapiens GN=NUP50 PE=1 SV=2	50512	0	770	0	51	0	0.07	#DIV/0!	0	1	0	1	0	1	0	1	0.693008
tr|E7EVL6|E7EVL6_HUMAN	REPIN1	Replication initiator 1 (Fragment) OS=Homo sapiens GN=REPIN1 PE=2 SV=1	16586	0	764	0	51	0	0.2	#DIV/0!	0	1	0	1	0	1	0	1	0.693008
sp|Q00653|NFKB2_HUMAN	NFKB2	Nuclear factor NF-kappa-B p100 subunit OS=Homo sapiens GN=NFKB2 PE=1 SV=4	97373	0	760	0	52	0	0.03	#DIV/0!	0	1	0	1	0	1	0	1	0.693008
tr|B0V0F7|B0V0F7_HUMAN	DHX16	Putative pre-mRNA-splicing factor ATP-dependent RNA helicase DHX16 (Fragment) OS=Homo sapiens GN=DH)	21454	0	759	0	52	0	0.16	#DIV/0!	0	1	0	1	0	1	0	1	0.693008
tr|A2RRS3|A2RRS3_HUMAN	TCHH	TCHH protein OS=Homo sapiens GN=TCHH PE=2 SV=1	44868	0	758	0	52	0	0.07	#DIV/0!	0	1	0	1	0	1	0	1	0.693008
tr|J3QKW7|J3QKW7_HUMAN	DRG2	Developmentally-regulated GTP-binding protein 2 OS=Homo sapiens GN=DRG2 PE=4 SV=1	26892	0	757	0	52	0	0.12	#DIV/0!	0	1	0	1	0	1	0	1	0.693008
tr|B2R856|B2R856_HUMAN	tr|B2R856|B2R	Acyl-coenzyme A oxidase OS=Homo sapiens PE=2 SV=1	78249	0	755	0	52	0	0.04	#DIV/0!	0	1	0	1	0	1	0	1	0.693008
sp|Q86XP3|DDX42_HUMAN	DDX42	ATP-dependent RNA helicase DDX42 OS=Homo sapiens GN=DDX42 PE=1 SV=1	103197	0	754	0	52	0	0.08	#DIV/0!	0	1	0	1	0	1	0	1	0.693008
sp|O14746|TERT_HUMAN	TERT	Telomerase reverse transcriptase OS=Homo sapiens GN=TERT PE=1 SV=1	128570	0	751	0	52	0	0.03	#DIV/0!	0	1	0	1	0	1	0	1	0.693008
tr|J3QKS9|J3QKS9_HUMAN	NOL11	Nucleolar protein 11 OS=Homo sapiens GN=NOL11 PE=4 SV=1	18816	0	748	0	52	0	0.18	#DIV/0!	0	1	0	1	0	1	0	1	0.693008
sp|Q9UK59|DBR1_HUMAN	DBR1	Lariat debranching enzyme OS=Homo sapiens GN=DBR1 PE=1 SV=2	62144	0	746	0	52	0	0.05	#DIV/0!	0	1	0	1	0	1	0	1	0.693008
tr|H3BNB9|H3BNB9_HUMAN	ARIH1	E3 ubiquitin-protein ligase ARIH1 (Fragment) OS=Homo sapiens GN=ARIH1 PE=4 SV=1	22769	0	745	0	52	0	0.15	#DIV/0!	0	1	0	1	0	1	0	1	0.693008
tr|Q6IBS5|Q6IBS5_HUMAN	DLST	DLST protein OS=Homo sapiens GN=DLST PE=2 SV=1	49131	0	739	0	53	0	0.4	#DIV/0!	0	1	0	1	0	1	0	1	0.693008
tr|B7Z2B4|B7Z2B4_HUMAN	RABGAP1	Rab GTPase-activating protein 1 OS=Homo sapiens GN=RABGAP1 PE=2 SV=1	48660	0	738	0	53	0	0.07	#DIV/0!	0	1	0	1	0	1	0	1	0.693008
tr|B4DLG2|B4DLG2_HUMAN	tr|B4DLG2|B4D	cDNA FLJ58196, highly similar to Zinc finger CCCH domain-containing protein 11A OS=Homo sapiens PE=2 SV=	83177	0	735	0	53	0	0.04	#DIV/0!	0	1	0	1	0	1	0	2	0.693008
tr|Q6FHH6|Q6FHH6_HUMAN	LANCL1	LANCL1 protein (Fragment) OS=Homo sapiens GN=LANCL1 PE=2 SV=1	45979	0	734	0	53	0	0.07	#DIV/0!	0	1	0	1	0	1	0	1	0.693008
sp|Q9BR76|COR1B_HUMAN	CORO1B	Coronin-1B OS=Homo sapiens GN=CORO1B PE=1 SV=1	54885	0	725	0	55	0	0.06	#DIV/0!	0	1	0	1	0	1	0	1	0.693008
tr|G5EA24|G5EA24_HUMAN	MCOLN2	Mucolipin 2, isoform CRA_a OS=Homo sapiens GN=MCOLN2 PE=2 SV=1	29581	0	723	0	55	0	0.12	#DIV/0!	0	1	0	1	0	1	0	2	0.693008
tr|B3KM73|B3KM73_HUMAN	tr|B3KM73|B3K	cDNA FLJ10419 fis, clone NT2RP1000163, highly similar to Homo sapiens transforming growth factor beta regu	55418	0	722	0	36	0	0.1	#DIV/0!	0	1	0	1	0	1	0	2	0.693008
tr|A0JLQ5|A0JLQ5_HUMAN	BXDC2	BXDC2 protein (Fragment) OS=Homo sapiens GN=BXDC2 PE=2 SV=1	39430	0	721	0	0	0	0.08	#DIV/0!	0	0	0	0	0	1	0	1	0.693008
tr|H3BTZ5|H3BTZ5_HUMAN	CNN2	Calponin-2 (Fragment) OS=Homo sapiens GN=CNN2 PE=4 SV=1	21173	0	720	0	55	0	0.1	#DIV/0!	0	1	0	1	0	1	0	1	0.693008
tr|B4DR63|B4DR63_HUMAN	PSMC1	26S protease regulatory subunit 4 OS=Homo sapiens GN=PSMC1 PE=2 SV=1	41255	0	718	0	56	0	0.38	#DIV/0!	0	1	0	1	0	1	0	1	0.693008
sp|Q969N2|PIGT_HUMAN	PIGT	GPI transamidase component PIG-T OS=Homo sapiens GN=PIGT PE=1 SV=1	66228	0	716	0	56	0	0.07	#DIV/0!	0	1	0	1	0	1	0	2	0.693008
sp|Q9HCC0|MCCB_HUMAN	MCCC2	Methylcrotonoyl-CoA carboxylase beta chain, mitochondrial OS=Homo sapiens GN=MCCC2 PE=1 SV=1	61808	0	714	0	56	0	0.05	#DIV/0!	0	1	0	1	0	1	0	1	0.693008
tr|B3KXW5|B3KXW5_HUMAN	AP1G1	AP-1 complex subunit gamma-1 OS=Homo sapiens GN=AP1G1 PE=2 SV=1	94992	0	712	0	56	0	0.03	#DIV/0!	0	1	0	1	0	1	0	1	0.693008
tr|B7Z871|B7Z871_HUMAN	tr|B7Z871|B7Z8	cDNA FLJ50930, highly similar to Lipoma-preferred partner OS=Homo sapiens PE=2 SV=1	49854	0	708	0	57	0	0.07	#DIV/0!	0	1	0	1	0	1	0	1	0.693008
sp|Q0VDF9|HSP7E_HUMAN	HSPA14	Heat shock 70 kDa protein 14 OS=Homo sapiens GN=HSPA14 PE=1 SV=1	55444	0	707	0	57	0	0.06	#DIV/0!	0	1	0	1	0	1	0	1	0.693008
sp|Q63ZY3|KANK2_HUMAN	KANK2	KN motif and ankyrin repeat domain-containing protein 2 OS=Homo sapiens GN=KANK2 PE=1 SV=1	91916	0	706	0	57	0	0.14	#DIV/0!	0	1	0	1	0	1	0	1	0.693008
sp|Q9HC07|TM165_HUMAN	TMEM165	Transmembrane protein 165 OS=Homo sapiens GN=TMEM165 PE=1 SV=1	35055	0	704	0	57	0	0.2	#DIV/0!	0	1	0	1	0	1	0	1	0.693008
sp|Q05048|CSTF1_HUMAN	CSTF1	Cleavage stimulation factor subunit 1 OS=Homo sapiens GN=CSTF1 PE=1 SV=1	49125	0	703	0	57	0	0.22	#DIV/0!	0	1	0	1	0	1	0	1	0.693008
tr|B4DDL6|B4DDL6_HUMAN	tr|B4DDL6|B4D	cDNA FLJ54325, highly similar to Myotubularin-related protein 2 (EC 3.1.3.-) OS=Homo sapiens PE=2 SV=1	36644	0	701	0	57	0	0.09	#DIV/0!	0	1	0	1	0	1	0	1	0.693008
tr|A8K8N3|A8K8N3_HUMAN	tr|A8K8N3|A8K	cDNA FLJ78740, highly similar to Homo sapiens sperm associated antigen 5 (SPAG5), mRNA OS=Homo sapiens	135680	0	699	0	57	0	0.02	#DIV/0!	0	1	0	1	0	1	0	2	0.693008
tr|B0QYK0|B0QYK0_HUMAN	EWSR1	Ewing sarcoma breakpoint region 1 OS=Homo sapiens GN=EWSR1 PE=4 SV=1	65174	0	696	0	57	0	0.05	#DIV/0!	0	1	0	1	0	1	0	1	0.693008
sp|Q92626|PXDN_HUMAN	PXDN	Peroxidasin homolog OS=Homo sapiens GN=PXDN PE=1 SV=2	167793	0	692	0	58	0	0.04	#DIV/0!	0	1	0	1	0	1	0	1	0.693008
tr|Q5IRN4|Q5IRN4_HUMAN	MEF2D/DAZAP1	Myocyte enhancer factor 2D/deleted in azoospermia associated protein 1 fusion protein OS=Homo sapiens GN	50610	0	689	0	23	0	0.29	#DIV/0!	0	0	0	1	0	1	0	1	0.693008
tr|H0Y4J2|H0Y4J2_HUMAN	MRPL37	39S ribosomal protein L37, mitochondrial (Fragment) OS=Homo sapiens GN=MRPL37 PE=4 SV=1	29806	0	673	0	59	0	0.11	#DIV/0!	0	1	0	1	0	1	0	1	0.693008
tr|D2JYH5|D2JYH5_HUMAN	COL3A1	Collagen, type III, alpha 1 OS=Homo sapiens GN=COL3A1 PE=2 SV=1	139733	0	668	0	60	0	0.02	#DIV/0!	0	1	0	1	0	1	0	2	0.693008
tr|B0UZZ8|B0UZZ8_HUMAN	WDR46	Chromosome 6 open reading frame 11 OS=Homo sapiens GN=WDR46 PE=2 SV=1	68455	0	667	0	60	0	0.05	#DIV/0!	0	1	0	1	0	1	0	1	0.693008
tr|B2RB52|B2RB52_HUMAN	tr|B2RB52|B2R	cDNA, FLJ95314, highly similar to Homo sapiens transducin (beta)-like 2 (TBL2), transcript variant 1, mRNA OS=	50423	0	666	0	60	0	0.07	#DIV/0!	0	1	0	1	0	1	0	1	0.693008
tr|B4DJE7|B4DJE7_HUMAN	ACADM	Medium-chain-specific acyl-CoA dehydrogenase, mitochondrial OS=Homo sapiens GN=ACADM PE=2 SV=1	25704	0	664	0	60	0	0.13	#DIV/0!	0	1	0	1	0	1	0	1	0.693008
tr|B2RBM7|B2RBM7_HUMAN	tr|B2RBM7|B2R	cDNA, FLJ95595, highly similar to Homo sapiens proteasome (prosome, macropain) 26S subunit, non-ATPase,	43131	0	662	0	61	0	0.13	#DIV/0!	0	1	0	1	0	1	0	1	0.693008
tr|B4DHS5|B4DHS5_HUMAN	tr|B4DHS5|B4D	cDNA FLJ54678, highly similar to N(2),N(2)-dimethylguanosine tRNA methyltransferase (EC 2.1.1.32) OS=Homo	59256	0	661	0	61	0	0.06	#DIV/0!	0	1	0	1	0	1	0	1	0.693008
tr|B4DH17|B4DH17_HUMAN	tr|B4DH17|B4D	cDNA FLJ59298, highly similar to Eukaryotic translation initiation factor 4 gamma 3 OS=Homo sapiens PE=2 SV	134921	0	658	0	61	0	0.02	#DIV/0!	0	1	0	1	0	1	0	3	0.693008
sp|P63096|GNAI1_HUMAN	GNAI1	Guanine nucleotide-binding protein G(i) subunit alpha-1 OS=Homo sapiens GN=GNAI1 PE=1 SV=2	40905	0	655	0	39	0	0.09	#DIV/0!	0	1	0	1	0	1	0	1	0.693008
tr|B3KN28|B3KN28_HUMAN	tr|B3KN28|B3K	cDNA FLJ13370 fis, clone PLACE1000653, highly similar to Phosphoacetylglucosamine mutase (EC 5.4.2.3) OS=	60284	0	652	0	62	0	0.05	#DIV/0!	0	1	0	1	0	1	0	2	0.693008
tr|F5H6D0|F5H6D0_HUMAN	MOGS	Mannosyl-oligosaccharide glucosidase OS=Homo sapiens GN=MOGS PE=2 SV=1	80824	0	648	0	63	0	0.04	#DIV/0!	0	1	0	1	0	1	0	1	0.693008
sp|Q9Y4C2|F115A_HUMAN	FAM115A	Protein FAM115A OS=Homo sapiens GN=FAM115A PE=1 SV=3	103144	0	647	0	63	0	0.03	#DIV/0!	0	1	0	1	0	1	0	2	0.693008
sp|Q13425|SNTB2_HUMAN	SNTB2	Beta-2-syntrophin OS=Homo sapiens GN=SNTB2 PE=1 SV=1	58369	0	637	0	64	0	0.06	#DIV/0!	0	1	0	1	0	1	0	1	0.693008
tr|B4DGX9|B4DGX9_HUMAN	tr|B4DGX9|B4D	cDNA FLJ60509, highly similar to Probable ubiquitin carboxyl-terminalhydrolase FAF-X (EC 3.1.2.15) OS=Homo	66195	0	636	0	0	0	0.02	#DIV/0!	0	0	0	0	0	1	0	2	0.693008
tr|Q6FHJ5|Q6FHJ5_HUMAN	SCAMP3	SCAMP3 protein (Fragment) OS=Homo sapiens GN=SCAMP3 PE=2 SV=1	38674	0	634	0	64	0	0.09	#DIV/0!	0	1	0	1	0	1	0	1	0.693008
tr|Q56VW8|Q56VW8_HUMAN	tr|Q56VW8|Q5	Lung cancer-related protein 9 OS=Homo sapiens PE=2 SV=1	52186	0	633	0	60	0	0.13	#DIV/0!	0	1	0	1	0	1	0	1	0.693008
tr|H0Y8Z9|H0Y8Z9_HUMAN	ACAD9	Acyl-CoA dehydrogenase family member 9, mitochondrial (Fragment) OS=Homo sapiens GN=ACAD9 PE=4 SV=	43503	0	631	0	64	0	0.05	#DIV/0!	0	1	0	1	0	1	0	1	0.693008
tr|J3KQ32|J3KQ32_HUMAN	OLA1	Obg-like ATPase 1 OS=Homo sapiens GN=OLA1 PE=4 SV=1	47136	0	624	0	65	0	0.07	#DIV/0!	0	1	0	1	0	1	0	1	0.693008
tr|A6NN60|A6NN60_HUMAN	CRYZ	Quinone oxidoreductase OS=Homo sapiens GN=CRYZ PE=2 SV=1	31680	0	620	0	65	0	0.11	#DIV/0!	0	1	0	1	0	1	0	2	0.693008
tr|H3BRG4|H3BRG4_HUMAN	UQCRC2	Cytochrome b-c1 complex subunit 2, mitochondrial OS=Homo sapiens GN=UQCRC2 PE=3 SV=1	44778	0	617	0	66	0	0.07	#DIV/0!	0	1	0	1	0	1	0	2	0.693008
tr|H3BQC4|H3BQC4_HUMAN	TGFB1I1	Transforming growth factor beta-1-induced transcript 1 protein (Fragment) OS=Homo sapiens GN=TGFB1I1 PE	23016	0	615	0	66	0	0.15	#DIV/0!	0	1	0	1	0	1	0	1	0.693008
tr|A7E2Y5|A7E2Y5_HUMAN	DNAJC13	DnaJ (Hsp40) homolog, subfamily C, member 13 OS=Homo sapiens GN=DNAJC13 PE=2 SV=1	256572	0	614	0	66	0	0.01	#DIV/0!	0	1	0	1	0	1	0	3	0.693008
sp|Q9UID3|VPS51_HUMAN	VPS51	Vacuolar protein sorting-associated protein 51 homolog OS=Homo sapiens GN=VPS51 PE=1 SV=2	86901	0	605	0	67	0	0.04	#DIV/0!	0	1	0	1	0	1	0	1	0.693008
tr|B3KM50|B3KM50_HUMAN	tr|B3KM50|B3K	cDNA FLJ10310 fis, clone NT2RM2000322, highly similar to Homo sapiens KIAA0859 protein OS=Homo sapiens	39576	0	602	0	52	0	0.08	#DIV/0!	0	1	0	1	0	1	0	1	0.693008
tr|D3DVA5|D3DVA5_HUMAN	ARHGEF2	Rho/rac guanine nucleotide exchange factor (GEF) 2, isoform CRA_a OS=Homo sapiens GN=ARHGEF2 PE=2 SV	116954	0	600	0	68	0	0.03	#DIV/0!	0	1	0	1	0	1	0	2	0.693008
sp|Q8ND04|SMG8_HUMAN	SMG8	Protein SMG8 OS=Homo sapiens GN=SMG8 PE=1 SV=1	110983	0	586	0	71	0	0.03	#DIV/0!	0	1	0	1	0	1	0	1	0.693008
tr|B4DJ81|B4DJ81_HUMAN	NDUFS1	NADH-ubiquinone oxidoreductase 75 kDa subunit, mitochondrial OS=Homo sapiens GN=NDUFS1 PE=2 SV=1	67563	0	584	0	72	0	0.1	#DIV/0!	0	1	0	2	0	1	0	2	0.693008
sp|Q08379|GOGA2_HUMAN	GOLGA2	Golgin subfamily A member 2 OS=Homo sapiens GN=GOLGA2 PE=1 SV=3	113644	0	582	0	72	0	0.03	#DIV/0!	0	1	0	1	0	1	0	4	0.693008
tr|Q0VAB1|Q0VAB1_HUMAN	TIMM50	Translocase of inner mitochondrial membrane 50 homolog (S. cerevisiae) OS=Homo sapiens GN=TIMM50 PE=	50960	0	580	0	72	0	0.06	#DIV/0!	0	1	0	1	0	1	0	1	0.693008
tr|Q5H907|Q5H907_HUMAN	MAGED2	Melanoma antigen family D, 2, isoform CRA_d OS=Homo sapiens GN=MAGED2 PE=4 SV=1	55932	0	574	0	72	0	0.13	#DIV/0!	0	1	0	1	0	1	0	1	0.693008
sp|Q14739|LBR_HUMAN	LBR	Lamin-B receptor OS=Homo sapiens GN=LBR PE=1 SV=2	71057	0	572	0	73	0	0.05	#DIV/0!	0	1	0	1	0	1	0	2	0.693008
tr|H0YEL7|H0YEL7_HUMAN	ACAT1	Acetyl-CoA acetyltransferase, mitochondrial (Fragment) OS=Homo sapiens GN=ACAT1 PE=3 SV=1	14377	0	569	0	74	0	0.24	#DIV/0!	0	1	0	1	0	1	0	2	0.693008
tr|B3KUI0|B3KUI0_HUMAN	tr|B3KUI0|B3KU	cDNA FLJ39945 fis, clone SPLEN2023977, highly similar to Homo sapiens CD97 antigen (CD97), transcript varia	79803	0	566	0	74	0	0.04	#DIV/0!	0	1	0	1	0	1	0	1	0.693008
tr|B3KNP0|B3KNP0_HUMAN	tr|B3KNP0|B3K	cDNA FLJ30074 fis, clone BGGI11000123, highly similar to Homo sapiens formin binding protein 4 (FNBP4), mR	100252	0	553	0	76	0	0.03	#DIV/0!	0	1	0	1	0	1	0	1	0.693008
sp|Q70J99|UN13D_HUMAN	UNC13D	Protein unc-13 homolog D OS=Homo sapiens GN=UNC13D PE=1 SV=1	124288	0	552	0	76	0	0.03	#DIV/0!	0	1	0	1	0	1	0	1	0.693008
tr|D3DRG7|D3DRG7_HUMAN	DPYSL4	Dihydropyrimidinase-like 4, isoform CRA_a OS=Homo sapiens GN=DPYSL4 PE=2 SV=1	55584	0	549	0	77	0	0.06	#DIV/0!	0	1	0	1	0	1	0	1	0.693008
tr|B4DJ92|B4DJ92_HUMAN	tr|B4DJ92|B4DJ	cDNA FLJ57843, highly similar to Homo sapiens neurochondrin (NCDN), transcript variant 3, mRNA OS=Homo	20030	0	544	0	52	0	0.08	#DIV/0!	0	1	0	1	0	1	0	1	0.693008
tr|A6QKW0|A6QKW0_HUMAN	SHINC3	SHINC3 OS=Homo sapiens GN=SHINC3 PE=2 SV=1	28275	0	539	0	79	0	0.12	#DIV/0!	0	1	0	1	0	1	0	1	0.693008
tr|B4DF80|B4DF80_HUMAN	tr|B4DF80|B4D	cDNA FLJ58527, highly similar to Nucleolar protein 6 OS=Homo sapiens PE=2 SV=1	122714	0	532	0	29	0	0.05	#DIV/0!	0	0	0	1	0	1	0	1	0.693008
sp|Q8TAT6|NPL4_HUMAN	NPLOC4	Nuclear protein localization protein 4 homolog OS=Homo sapiens GN=NPLOC4 PE=1 SV=3	69046	0	529	0	80	0	0.23	#DIV/0!	0	1	0	1	0	1	0	1	0.693008
tr|B4DI61|B4DI61_HUMAN	tr|B4DI61|B4DI	cDNA FLJ58182, highly similar to Protein CYR61 OS=Homo sapiens PE=2 SV=1	41592	0	527	0	80	0	0.08	#DIV/0!	0	1	0	1	0	1	0	1	0.693008
tr|B2R858|B2R858_HUMAN	tr|B2R858|B2R	cDNA, FLJ93750, Homo sapiens DEAD (Asp-Glu-Ala-Asp) box polypeptide 6 (DDX6), mRNA OS=Homo sapiens P	53582	0	523	0	80	0	0.13	#DIV/0!	0	1	0	2	0	1	0	2	0.693008
tr|B5ME97|B5ME97_HUMAN	Sep-10	Septin 10, isoform CRA_c OS=Homo sapiens GN=SEPT10 PE=3 SV=2	63703	0	520	0	81	0	0.05	#DIV/0!	0	1	0	1	0	1	0	1	0.693008
sp|Q9H009|NACA2_HUMAN	NACA2	Nascent polypeptide-associated complex subunit alpha-2 OS=Homo sapiens GN=NACA2 PE=1 SV=1	23209	0	503	0	85	0	0.14	#DIV/0!	0	1	0	1	0	1	0	1	0.693008
tr|H0YHX9|H0YHX9_HUMAN	NACA	Nascent polypeptide-associated complex subunit alpha (Fragment) OS=Homo sapiens GN=NACA PE=4 SV=1	22930	0	502	0	85	0	0.15	#DIV/0!	0	1	0	1	0	1	0	1	0.693008
tr|Q6EHZ3|Q6EHZ3_HUMAN	STRF8	Putative uncharacterized protein STRF8 OS=Homo sapiens GN=STRF8 PE=2 SV=1	41361	0	498	0	86	0	0.08	#DIV/0!	0	1	0	1	0	1	0	1	0.693008
tr|B4DRW3|B4DRW3_HUMAN	tr|B4DRW3|B4I	cDNA FLJ57180, highly similar to TAR DNA-binding protein 43 OS=Homo sapiens PE=2 SV=1	35596	0	479	0	92	0	0.1	#DIV/0!	0	1	0	1	0	1	0	1	0.693008
tr|B3KQ73|B3KQ73_HUMAN	tr|B3KQ73|B3K	cDNA FLJ33002 fis, clone THYMU1000217, highly similar to Homo sapiens ubiquitin associated protein 2 (UBAI	37025	0	475	0	58	0	0.07	#DIV/0!	0	1	0	1	0	1	0	1	0.693008
tr|B3KVA0|B3KVA0_HUMAN	tr|B3KVA0|B3K	Cytosine-specific methyltransferase OS=Homo sapiens PE=2 SV=1	61027	0	472	0	33	0	0.04	#DIV/0!	0	1	0	1	0	1	0	1	0.693008
tr|H7BZF2|H7BZF2_HUMAN	DKC1	H/ACA ribonucleoprotein complex subunit 4 (Fragment) OS=Homo sapiens GN=DKC1 PE=4 SV=1	8803	0	450	0	45	0	0.25	#DIV/0!	0	1	0	1	0	1	0	1	0.693008
tr|B3KNR6|B3KNR6_HUMAN	tr|B3KNR6|B3K	cDNA FLJ30255 fis, clone BRACE2002444, highly similar to LAS1-like protein OS=Homo sapiens PE=2 SV=1	27615	0	413	0	49	0	0.12	#DIV/0!	0	1	0	1	0	1	0	2	0.693008
tr|E7EMC7|E7EMC7_HUMAN	SQSTM1	Sequestosome-1 OS=Homo sapiens GN=SQSTM1 PE=2 SV=1	42070	0	390	0	53	0	0.22	#DIV/0!	0	1	0	1	0	1	0	2	0.693008
tr|G3V1T4|G3V1T4_HUMAN	NUP107	Nuclear pore complex protein Nup107 OS=Homo sapiens GN=NUP107 PE=2 SV=1	11398	0	368	0	111	0	0.06	#DIV/0!	0	1	0	1	0	1	0	1	0.693008
tr|H0YFD2|H0YFD2_HUMAN	KRI1	Protein KRI1 homolog (Fragment) OS=Homo sapiens GN=KRI1 PE=4 SV=1	22298	0	1237	0	16	0	0.15	#DIV/0!	0	0	0	1	0	0	0	1	-0.89213
tr|K7EPQ8|K7EPQ8_HUMAN	GADD45B	Growth arrest and DNA damage-inducible protein GADD45 beta OS=Homo sapiens GN=GADD45B PE=4 SV=1	19253	0	1235	0	16	0	0.18	#DIV/0!	0	0	0	1	0	0	0	1	-0.89213
tr|E9PQD7|E9PQD7_HUMAN	RPS2	40S ribosomal protein S2 OS=Homo sapiens GN=RPS2 PE=2 SV=1	25366	0	1234	0	16	0	0.5	#DIV/0!	0	0	0	1	0	0	0	1	-0.89213
tr|G3V0G3|G3V0G3_HUMAN	DDX56	DEAD (Asp-Glu-Ala-Asp) box polypeptide 56, isoform CRA_a OS=Homo sapiens GN=DDX56 PE=2 SV=1	41969	0	1233	0	17	0	0.08	#DIV/0!	0	0	0	1	0	0	0	1	-0.89213
tr|B2RBE6|B2RBE6_HUMAN	tr|B2RBE6|B2R	cDNA, FLJ95470, highly similar to Homo sapiens homolog of yeast Sec5 (SEC5), mRNA OS=Homo sapiens PE=2	105099	0	1232	0	17	0	0.03	#DIV/0!	0	0	0	1	0	0	0	1	-0.89213
tr|B2RDZ9|B2RDZ9_HUMAN	tr|B2RDZ9|B2R	cDNA, FLJ96850 OS=Homo sapiens PE=2 SV=1	32424	0	1231	0	17	0	0.1	#DIV/0!	0	0	0	1	0	0	0	1	-0.89213
tr|H7C1Q7|H7C1Q7_HUMAN	PTGES3L	Putative protein PTGES3L (Fragment) OS=Homo sapiens GN=PTGES3L PE=4 SV=2	19310	0	1230	0	17	0	0.17	#DIV/0!	0	0	0	1	0	0	0	1	-0.89213
sp|Q9NUL7|DDX28_HUMAN	DDX28	Probable ATP-dependent RNA helicase DDX28 OS=Homo sapiens GN=DDX28 PE=1 SV=2	59773	0	1229	0	17	0	0.06	#DIV/0!	0	0	0	1	0	0	0	1	-0.89213
tr|B4E1L0|B4E1L0_HUMAN	tr|B4E1L0|B4E1	Adenylosuccinate synthetase OS=Homo sapiens PE=2 SV=1	48308	0	1225	0	18	0	0.07	#DIV/0!	0	0	0	1	0	0	0	2	-0.89213
sp|A2RU67|K1467_HUMAN	KIAA1467	Uncharacterized protein KIAA1467 OS=Homo sapiens GN=KIAA1467 PE=1 SV=1	67453	0	1224	0	18	0	0.05	#DIV/0!	0	0	0	1	0	0	0	1	-0.89213
sp|Q86V71|ZN429_HUMAN	ZNF429	Zinc finger protein 429 OS=Homo sapiens GN=ZNF429 PE=2 SV=2	80497	0	1222	0	19	0	0.04	#DIV/0!	0	0	0	1	0	0	0	2	-0.89213
tr|B4DKW2|B4DKW2_HUMAN	tr|B4DKW2|B4I	cDNA FLJ56001, highly similar to Putative ATP-dependent RNA helicase DHX57 (EC 3.6.1.-) (Fragment) OS=Hon	105507	0	1221	0	19	0	0.03	#DIV/0!	0	0	0	1	0	0	0	2	-0.89213
sp|Q9NS40|KCNH7_HUMAN	KCNH7	Potassium voltage-gated channel subfamily H member 7 OS=Homo sapiens GN=KCNH7 PE=2 SV=2	136226	0	1218	0	19	0	0.02	#DIV/0!	0	0	0	1	0	0	0	1	-0.89213
sp|Q9UI26|IPO11_HUMAN	IPO11	Importin-11 OS=Homo sapiens GN=IPO11 PE=1 SV=1	113489	0	1216	0	19	0	0.03	#DIV/0!	0	0	0	1	0	0	0	1	-0.89213
tr|H0Y5B5|H0Y5B5_HUMAN	PBRM1	Protein polybromo-1 (Fragment) OS=Homo sapiens GN=PBRM1 PE=4 SV=1	127011	0	1215	0	19	0	0.03	#DIV/0!	0	0	0	1	0	0	0	1	-0.89213
tr|B3KVR1|B3KVR1_HUMAN	SNRPN	Small nuclear ribonucleoprotein-associated protein OS=Homo sapiens GN=SNRPN PE=2 SV=1	25230	0	1214	0	19	0	0.13	#DIV/0!	0	1	0	1	0	0	0	1	-0.89213
tr|J3QRD1|J3QRD1_HUMAN	ALDH3A2	Fatty aldehyde dehydrogenase OS=Homo sapiens GN=ALDH3A2 PE=3 SV=1	45019	0	1213	0	19	0	0.07	#DIV/0!	0	0	0	1	0	0	0	1	-0.89213

tr|A8K4H7|A8K4H7_HUMAN	PPAT	Amidophosphoribosyltransferase OS=Homo sapiens GN=PPAT PE=2 SV=1	58161	0	1211	0	20	0	0.06	#DIV/0!	0	0	0	1	0	0	0	1	-0.89213
tr|A4UCU0|A4UCU0_HUMAN	tr|A4UCU0|A4U	DEAD box polypeptide 47 isoform 1 variant (Fragment) OS=Homo sapiens PE=2 SV=1	19904	0	1209	0	20	0	0.17	#DIV/0!	0	0	0	1	0	0	0	1	-0.89213
tr|H0UI76|H0UI76_HUMAN	NPEPL1	Aminopeptidase-like 1, isoform CRA_d OS=Homo sapiens GN=NPEPL1 PE=4 SV=1	44188	0	1159	0	0	0	0.06	#DIV/0!	0	0	0	0	0	0	0	1	-0.89213
tr|E9PF58|E9PF58_HUMAN	ARPC1A	Actin-related protein 2/3 complex subunit 1A OS=Homo sapiens GN=ARPC1A PE=2 SV=1	31268	0	677	0	0	0	0.08	#DIV/0!	0	0	0	0	0	0	0	1	-0.89213
tr|B4DTM6|B4DTM6_HUMAN	tr|B4DTM6|B4D	cDNA FLJ61391, highly similar to Protein transport protein Sec24B OS=Homo sapiens PE=2 SV=1	130037	0	625	0	18	0	0.07	#DIV/0!	0	0	0	1	0	0	0	1	-0.89213
tr|B3KPF6|B3KPF6_HUMAN	tr|B3KPF6|B3KP	cDNA FLJ31732 fis, clone NT2RI2006856, highly similar to Striatin-4 OS=Homo sapiens PE=2 SV=1	39506	0	518	0	0	0	0.1	#DIV/0!	0	0	0	0	0	0	0	1	-0.89213
tr|E9PBS1|E9PBS1_HUMAN	PAICS	Phosphoribosylaminoimidazole carboxylase (Fragment) OS=Homo sapiens GN=PAICS PE=2 SV=1	46307	270	104	69	384	0.07	1.72	24.5714286	1	11	1	15	2	15	5	16	1.742167
sp|P11388|TOP2A_HUMAN	TOP2A	DNA topoisomerase 2-alpha OS=Homo sapiens GN=TOP2A PE=1 SV=3	175017	606	137	25	329	0.02	0.34	17	0	7	1	16	1	16	2	18	2.569845
tr|J3KR24|J3KR24_HUMAN	IARS	Isoleucine--tRNA ligase, cytoplasmic OS=Homo sapiens GN=IARS PE=4 SV=1	132820	366	147	47	317	0.02	0.34	17	1	8	1	12	2	14	2	17	1.645772
tr|Q2TU64|Q2TU64_HUMAN	tr|Q2TU64|Q2T	PIG48 OS=Homo sapiens PE=2 SV=1	61110	279	142	68	235	0.05	0.73	14.6	1	6	1	8	3	9	3	9	0.549057
tr|B2R9U2|B2R9U2_HUMAN	tr|B2R9U2|B2R	cDNA, FLJ94557, highly similar to Homo sapiens FK506 binding protein 4, 59kDa (FKBP4), mRNA OS=Homo sap	52087	623	202	24	216	0.06	0.85	14.1666667	0	4	1	8	2	8	2	8	0.874173
sp|Q14204|DYHC1_HUMAN	DYNC1H1	Cytoplasmic dynein 1 heavy chain 1 OS=Homo sapiens GN=DYNC1H1 PE=1 SV=5	534809	168	39	129	945	0.02	0.28	14	3	26	4	41	4	49	6	55	2.574804
tr|E7EVA0|E7EVA0_HUMAN	MAP4	Microtubule-associated protein OS=Homo sapiens GN=MAP4 PE=2 SV=1	246546	429	650	40	62	0.01	0.14	14	1	2	1	2	2	2	4	2	-0.89244
tr|Q4ZG57|Q4ZG57_HUMAN	MCM6	Putative uncharacterized protein MCM6 (Fragment) OS=Homo sapiens GN=MCM6 PE=2 SV=1	89803	444	119	39	363	0.04	0.54	13.5	1	11	1	12	2	12	3	12	1.431287
sp|O14980|XPO1_HUMAN	XPO1	Exportin-1 OS=Homo sapiens GN=XPO1 PE=1 SV=1	124447	305	135	59	335	0.03	0.4	13.3333333	1	9	1	13	1	13	3	16	2.279798
tr|Q68CR9|Q68CR9_HUMAN	DKFZp781B112C	Aspartate--tRNA ligase, cytoplasmic OS=Homo sapiens GN=DKFZp781B11202 PE=2 SV=1	46084	344	208	51	236	0.06	0.74	12.3333333	1	5	1	9	2	10	3	11	1.179388
tr|B2RBA6|B2RBA6_HUMAN	tr|B2RBA6|B2R	cDNA, FLJ95407, highly similar to Homo sapiens MCM7 minichromosome maintenance deficient 7 (S. cerevisia	81856	374	133	46	338	0.04	0.48	12	1	10	1	10	1	10	1	12	1.916687
tr|D3DP78|D3DP78_HUMAN	DARS	Aspartyl-tRNA synthetase, isoform CRA_b OS=Homo sapiens GN=DARS PE=2 SV=1	53829	344	211	51	236	0.06	0.71	11.8333333	1	5	1	9	2	10	3	12	1.179388
tr|Q2Q9B7|Q2Q9B7_HUMAN	G6PD	Glucose-6-phosphate 1-dehydrogenase (Fragment) OS=Homo sapiens GN=G6PD PE=2 SV=1	55221	176	89	118	416	0.18	2.01	11.1666667	2	11	2	18	5	20	6	21	1.007919
tr|B4DYA7|B4DYA7_HUMAN	tr|B4DYA7|B4D	Glucose-6-phosphate 1-dehydrogenase OS=Homo sapiens PE=2 SV=1	58757	176	90	123	407	0.18	2.01	11.1666667	2	11	3	16	6	18	7	19	0.618117
tr|B4DLT2|B4DLT2_HUMAN	tr|B4DLT2|B4D	cDNA FLJ56637, highly similar to Nuclear pore complex protein Nup155 OS=Homo sapiens PE=2 SV=1	152734	489	112	35	344	0.02	0.21	10.5	0	6	1	8	1	9	1	9	1.772117
sp|P45974|UBP5_HUMAN	USP5	Ubiquitin carboxyl-terminal hydrolase 5 OS=Homo sapiens GN=USP5 PE=1 SV=2	96638	622	265	24	186	0.03	0.31	10.3333333	0	6	1	8	2	8	2	10	0.874173
tr|H0UID3|H0UID3_HUMAN	AP2B1	Adaptor-related protein complex 2, beta 1 subunit, isoform CRA_d OS=Homo sapiens GN=AP2B1 PE=4 SV=1	106321	648	316	22	147	0.03	0.3	10	0	4	1	8	3	10	3	11	0.693627
sp|P54886|P5CS_HUMAN	ALDH18A1	Delta-1-pyrroline-5-carboxylate synthase OS=Homo sapiens GN=ALDH18A1 PE=1 SV=2	87989	658	194	21	251	0.04	0.39	9.75	1	9	1	9	1	11	1	11	2.048112
tr|A4FU77|A4FU77_HUMAN	SNRNP200	SNRNP200 protein (Fragment) OS=Homo sapiens GN=SNRNP200 PE=2 SV=1	217585	243	50	83	692	0.05	0.48	9.6	2	19	3	28	7	27	8	35	0.984862
tr|A8K492|A8K492_HUMAN	tr|A8K492|A8K	cDNA FLJ76789, highly similar to Homo sapiens methionine-tRNA synthetase (MARS), mRNA OS=Homo sapien	102277	385	95	45	415	0.07	0.66	9.42857143	1	11	2	16	2	16	2	18	1.832546
tr|B3KSH1|B3KSH1_HUMAN	EIF3F	Eukaryotic translation initiation factor 3 subunit F OS=Homo sapiens GN=EIF3F PE=2 SV=1	39236	652	422	22	105	0.08	0.74	9.25	0	2	1	2	1	2	1	2	-0.15514
sp|P53618|COPB_HUMAN	COPB1	Coatomer subunit beta OS=Homo sapiens GN=COPB1 PE=1 SV=3	108214	303	129	59	341	0.06	0.52	8.66666667	1	10	2	14	2	12	3	16	1.431287
tr|A2VCT2|A2VCT2_HUMAN	OGDH	OGDH protein (Fragment) OS=Homo sapiens GN=OGDH PE=2 SV=1	72973	432	227	40	72	0.05	0.43	8.6	1	2	1	4	1	4	1	4	0.693214
tr|B3KW21|B3KW21_HUMAN	tr|B3KW21|B3K	cDNA FLJ41945 fis, clone PLACE6019676, highly similar to Coatomer subunit gamma OS=Homo sapiens PE=2 S	68674	705	344	18	131	0.04	0.34	8.5	0	2	1	8	0	7	1	10	3.016016
tr|B7Z1R5|B7Z1R5_HUMAN	ATP6V1A	V-type proton ATPase catalytic subunit A OS=Homo sapiens GN=ATP6V1A PE=2 SV=1	65037	428	372	40	120	0.05	0.41	8.2	1	3	1	7	1	7	2	8	1.43072
tr|B2RBE0|B2RBE0_HUMAN	tr|B2RBE0|B2R	cDNA, FLJ95462, highly similar to Homo sapiens fatty-acid-Coenzyme A ligase, long-chain 3 (FACL3),mRNA OS=	81266	462	281	37	175	0.04	0.32	8	0	5	1	7	1	7	1	11	1.43072
sp|Q6P2Q9|PRP8_HUMAN	PRPF8	Pre-mRNA-processing-splicing factor 8 OS=Homo sapiens GN=PRPF8 PE=1 SV=2	274738	130	27	167	1260	0.1	0.78	7.8	7	37	8	50	10	56	10	67	1.542495
tr|H0YJ34|H0YJ34_HUMAN	FERMT2	Fermitin family homolog 2 (Fragment) OS=Homo sapiens GN=FERMT2 PE=4 SV=1	73877	306	74	59	488	0.14	1.09	7.78571429	2	11	3	17	3	16	3	20	1.346785
tr|B1AHB0|B1AHB0_HUMAN	MCM5	MCM5 minichromosome maintenance deficient 5, cell division cycle 46 (S. cerevisiae), isoform CRA_a OS=Hon	83031	562	341	29	135	0.04	0.31	7.75	0	3	1	7	1	8	2	10	1.611472
tr|Q5SU16|Q5SU16_HUMAN	TUBB	Beta 5-tubulin OS=Homo sapiens GN=TUBB PE=2 SV=1	50095	25	8	680	2245	3.07	23.26	7.57654723	26	66	33	81	35	87	39	93	0.413334
tr|Q5VZU9|Q5VZU9_HUMAN	TPP2	Tripeptidyl-peptidase 2 OS=Homo sapiens GN=TPP2 PE=4 SV=1	141160	693	290	19	166	0.02	0.15	7.5	0	3	1	6	0	6	1	6	2.809386
sp|Q9UQ35|SRRM2_HUMAN	SRRM2	Serine/arginine repetitive matrix protein 2 OS=Homo sapiens GN=SRRM2 PE=1 SV=2	300179	247	106	78	382	0.02	0.15	7.5	1	9	2	13	2	12	5	14	1.431287
tr|B2R5V9|B2R5V9_HUMAN	tr|B2R5V9|B2R	cDNA, FLJ92652, highly similar to Homo sapiens high density lipoprotein binding protein (vigilin)(HDLBP), mRN	141919	253	224	76	160	0.02	0.15	7.5	1	4	1	5	2	7	2	9	0.69342
tr|B4DT29|B4DT29_HUMAN	tr|B4DT29|B4D	cDNA FLJ51082, highly similar to Actin-like protein 3 OS=Homo sapiens PE=2 SV=1	40959	548	423	30	71	0.08	0.59	7.375	0	2	1	3	1	5	1	7	0.982901
tr|B4DUC5|B4DUC5_HUMAN	CSE1L	Exportin-2 OS=Homo sapiens GN=CSE1L PE=2 SV=1	86045	433	136	40	306	0.08	0.59	7.375	0	9	2	14	4	14	4	15	0.797107
tr|B4DTV0|B4DTV0_HUMAN	tr|B4DTV0|B4D	cDNA FLJ57801, highly similar to Homo sapiens AHNAK nucleoprotein (desmoyokin) (AHNAK), transcript variar	114179	454	268	38	182	0.03	0.22	7.33333333	1	6	1	7	3	8	3	12	0.388412
tr|A6NKB8|A6NKB8_HUMAN	RNPEP	Aminopeptidase B OS=Homo sapiens GN=RNPEP PE=2 SV=1	68752	316	233	57	210	0.05	0.36	7.2	1	5	2	6	2	7	2	7	0.69342
tr|B2R959|B2R959_HUMAN	tr|B2R959|B2R	cDNA, FLJ94229, highly similar to Homo sapiens heterogeneous nuclear ribonucleoprotein L (HNRPL),mRNA O	60838	182	63	117	464	0.23	1.65	7.17391304	4	12	4	17	6	20	8	20	0.766576
tr|B2R7C5|B2R7C5_HUMAN	tr|B2R7C5|B2R	cDNA, FLJ93378, highly similar to Homo sapiens MCM3 minichromosome maintenance deficient 3 (S. cerevisia	91579	479	288	36	168	0.04	0.28	7	1	3	1	7	1	10	1	11	1.916687
sp|Q09666|AHNK_HUMAN	AHNAK	Neuroblast differentiation-associated protein AHNAK OS=Homo sapiens GN=AHNAK PE=1 SV=2	629213	401	188	43	258	0.01	0.07	7	1	9	1	13	3	16	6	26	1.346785
tr|B2R7B5|B2R7B5_HUMAN	tr|B2R7B5|B2R	cDNA, FLJ93365, highly similar to Homo sapiens KH domain containing, RNA binding, signal transduction assoc	48309	347	336	51	100	0.07	0.49	7	1	2	1	5	2	7	4	8	0.69342
tr|A6NE09|A6NE09_HUMAN	RPSAP58	Protein RPSAP58 OS=Homo sapiens GN=RPSAP58 PE=3 SV=1	33002	521	326	31	89	0.1	0.7	7	0	2	1	3	1	4	1	4	0.693214
sp|Q15645|PCH2_HUMAN	TRIP13	Pachytene checkpoint protein 2 homolog OS=Homo sapiens GN=TRIP13 PE=1 SV=2	48863	507	440	33	100	0.07	0.48	6.85714286	0	3	1	6	2	6	2	6	0.486789
sp|O94906|PRP6_HUMAN	PRPF6	Pre-mRNA-processing factor 6 OS=Homo sapiens GN=PRPF6 PE=1 SV=1	107656	492	287	35	169	0.03	0.2	6.66666667	1	4	1	6	1	6	2	8	1.224089
sp|O95782|AP2A1_HUMAN	AP2A1	AP-2 complex subunit alpha-1 OS=Homo sapiens GN=AP2A1 PE=1 SV=3	108561	500	556	34	76	0.03	0.2	6.66666667	0	1	1	3	1	3	2	4	0.330464
tr|Q8IZ29|Q8IZ29_HUMAN	TUBB2C	Tubulin, beta 2C OS=Homo sapiens GN=TUBB2C PE=2 SV=1	50264	27	10	674	2147	3.04	20.07	6.60197368	26	63	32	75	34	81	38	88	0.351319
tr|B1ANR0|B1ANR0_HUMAN	PABPC4	Poly(A) binding protein, cytoplasmic 4 (Inducible form) OS=Homo sapiens GN=PABPC4 PE=4 SV=1	68156	504	315	33	148	0.05	0.33	6.6	0	4	1	6	1	6	2	8	1.224089
tr|Q60FE2|Q60FE2_HUMAN	MYH9	MYH9 variant protein OS=Homo sapiens GN=MYH9 PE=2 SV=1	227646	17	3	900	4623	0.9	5.92	6.57777778	30	121	46	146	52	160	60	176	0.731659
tr|B4DH39|B4DH39_HUMAN	tr|B4DH39|B4D	cDNA FLJ57028, highly similar to Long-chain-fatty-acid--CoA ligase 4 (EC 6.2.1.3) OS=Homo sapiens PE=2 SV=1	73123	529	310	31	152	0.04	0.25	6.25	0	4	1	5	1	7	3	9	1.43072
tr|B3KRS7|B3KRS7_HUMAN	tr|B3KRS7|B3KI	cDNA FLJ34844 fis, clone NT2NE2011154, highly similar to RuvB-like 1 OS=Homo sapiens PE=2 SV=1	34946	351	284	50	120	0.09	0.56	6.22222222	1	4	1	6	1	6	1	6	1.224089
sp|P11498|PYC_HUMAN	PC	Pyruvate carboxylase, mitochondrial OS=Homo sapiens GN=PC PE=1 SV=2	130293	10	4	1295	4595	1.76	10.91	6.19886364	40	129	52	156	56	173	67	190	0.739124
tr|Q8IWP6|Q8IWP6_HUMAN	tr|Q8IWP6|Q8I	Class IVb beta tubulin OS=Homo sapiens PE=2 SV=1	50177	28	11	654	2079	3.05	18.9	6.19672131	27	62	32	73	34	79	37	86	0.31511
tr|B4DMJ6|B4DMJ6_HUMAN	tr|B4DMJ6|B4D	cDNA FLJ50996, highly similar to 60S ribosomal protein L4 OS=Homo sapiens PE=2 SV=1	46269	163	79	132	445	0.51	3.16	6.19607843	4	11	6	19	8	18	9	20	0.230425
tr|D3DTH7|D3DTH7_HUMAN	MYO1C	Myosin IC, isoform CRA_a OS=Homo sapiens GN=MYO1C PE=2 SV=1	99613	284	118	65	365	0.07	0.43	6.14285714	1	10	3	11	3	14	4	15	1.160011
tr|H0YD14|H0YD14_HUMAN	MYOF	Myoferlin (Fragment) OS=Homo sapiens GN=MYOF PE=4 SV=1	135822	220	77	95	338	0.07	0.43	6.14285714	3	10	3	15	4	16	4	19	0.98388
sp|O75369|FLNB_HUMAN	FLNB	Filamin-B OS=Homo sapiens GN=FLNB PE=1 SV=2	280157	45	14	463	1916	0.22	1.35	6.13636364	14	58	18	79	20	82	28	91	1.124772
tr|B2R5P6|B2R5P6_HUMAN	TXNRD1	Thioredoxin reductase 1, cytoplasmic OS=Homo sapiens GN=TXNRD1 PE=2 SV=1	55253	108	71	204	507	0.26	1.58	6.07692308	3	11	5	17	5	16	6	19	0.694039
tr|F1T0B3|F1T0B3_HUMAN	DDX1	ATP-dependent RNA helicase DDX1 OS=Homo sapiens GN=DDX1 PE=2 SV=1	74838	501	314	33	151	0.09	0.54	6	0	5	2	10	3	11	3	11	0.825051
tr|J3KQL0|J3KQL0_HUMAN	CPS1	Carbamoyl-phosphate synthase [ammonia], mitochondrial OS=Homo sapiens GN=CPS1 PE=3 SV=1	166686	204	42	102	916	0.15	0.9	6	4	27	7	35	10	38	12	46	0.985845
sp|Q7L2E3|DHX30_HUMAN	DHX30	Putative ATP-dependent RNA helicase DHX30 OS=Homo sapiens GN=DHX30 PE=1 SV=1	134938	397	245	43	208	0.05	0.3	6	1	7	2	11	3	13	4	14	1.056737
tr|D3DTW3|D3DTW3_HUMAN	IGF2BP1	Insulin-like growth factor 2 mRNA binding protein 1, isoform CRA_a OS=Homo sapiens GN=IGF2BP1 PE=2 SV=1	48111	556	550	29	77	0.05	0.3	6	0	3	1	4	1	3	1	7	0.330464
sp|P52597|HNRPF_HUMAN	HNRNPF	Heterogeneous nuclear ribonucleoprotein F OS=Homo sapiens GN=HNRNPF PE=1 SV=3	45985	226	209	91	238	0.07	0.41	5.85714286	1	6	1	7	2	7	3	8	0.69342
tr|F8VPD4|F8VPD4_HUMAN	CAD	CAD protein OS=Homo sapiens GN=CAD PE=2 SV=1	238097	317	91	57	431	0.04	0.23	5.75	1	9	3	15	4	17	5	18	1.06895
sp|O76094|SRP72_HUMAN	SRP72	Signal recognition particle 72 kDa protein OS=Homo sapiens GN=SRP72 PE=1 SV=3	75130	455	688	38	58	0.04	0.23	5.75	1	1	1	4	1	3	1	5	0.330464
tr|B5BU25|B5BU25_HUMAN	U2AF2	U2 small nuclear RNA auxiliary factor 2 isoform b OS=Homo sapiens GN=U2AF2 PE=2 SV=1	53445	260	289	74	145	0.13	0.74	5.69230769	1	3	2	5	2	6	2	6	0.486789
tr|Q59FV6|Q59FV6_HUMAN	tr|Q59FV6|Q59	ARP3 actin-related protein 3 homolog variant (Fragment) OS=Homo sapiens PE=2 SV=1	42517	548	462	30	95	0.08	0.45	5.625	0	3	1	5	1	7	1	9	1.43072
sp|O95373|IPO7_HUMAN	IPO7	Importin-7 OS=Homo sapiens GN=IPO7 PE=1 SV=1	120751	162	76	132	508	0.08	0.45	5.625	3	12	3	15	3	15	4	19	1.256407
sp|P49411|EFTU_HUMAN	TUFM	Elongation factor Tu, mitochondrial OS=Homo sapiens GN=TUFM PE=1 SV=2	49852	210	130	99	340	0.29	1.61	5.55172414	3	12	4	15	4	14	5	18	0.797107
tr|B4DFM0|B4DFM0_HUMAN	FARSLB	Phenylalanine-tRNA synthetase-like, beta subunit, isoform CRA_c OS=Homo sapiens GN=FARSLB PE=2 SV=1	55353	360	771	48	47	0.06	0.33	5.5	1	1	1	5	1	6	3	6	1.224089
tr|B4DYL7|B4DYL7_HUMAN	tr|B4DYL7|B4D	cDNA FLJ58490, highly similar to Homo sapiens CCR4-NOT transcription complex, subunit 1 (CNOT1), transcrip	140991	388	393	45	86	0.02	0.11	5.5	1	3	1	7	1	7	2	9	1.43072
tr|F5H1A8|F5H1A8_HUMAN	GSN	Gelsolin OS=Homo sapiens GN=GSN PE=2 SV=1	81776	297	351	61	128	0.04	0.22	5.5	1	3	1	5	1	5	2	6	0.982901
tr|A8K8N7|A8K8N7_HUMAN	PFAS	Phosphoribosylformylglycinamidine synthase (FGAR amidotransferase), isoform CRA_b OS=Homo sapiens GN=	146286	380	124	46	352	0.05	0.27	5.4	0	7	2	11	3	11	5	13	0.825051
tr|H0Y8E6|H0Y8E6_HUMAN	MCM2	DNA replication licensing factor MCM2 (Fragment) OS=Homo sapiens GN=MCM2 PE=3 SV=1	94792	211	183	98	261	0.07	0.37	5.28571429	1	9	2	9	2	9	2	10	1.034818
tr|B4DSI9|B4DSI9_HUMAN	tr|B4DSI9|B4DS	cDNA FLJ56483, highly similar to Eukaryotic translation initiation factor 4 gamma 1 OS=Homo sapiens PE=2 SV	167220	669	226	20	219	0.04	0.21	5.25	0	6	2	10	1	10	3	12	1.916687
sp|Q15155|NOMO1_HUMAN	NOMO1	Nodal modulator 1 OS=Homo sapiens GN=NOMO1 PE=1 SV=5	135209	400	957	43	37	0.04	0.21	5.25	1	1	1	1	1	1	1	1	-0.89229
tr|Q6DC98|Q6DC98_HUMAN	LMNB1	LMNB1 protein (Fragment) OS=Homo sapiens GN=LMNB1 PE=2 SV=1	38289	219	176	95	216	0.18	0.94	5.22222222	2	5	2	6	2	6	4	6	0.486789
tr|Q59GV0|Q59GV0_HUMAN	tr|Q59GV0|Q59	Propionyl Coenzyme A carboxylase, beta polypeptide variant (Fragment) OS=Homo sapiens PE=2 SV=1	60578	331	411	53	109	0.05	0.26	5.2	1	3	1	4	1	4	2	5	0.693214
tr|B2ZZ90|B2ZZ90_HUMAN	ACACA	Acetyl-Coenzyme A carboxylase alpha OS=Homo sapiens GN=ACACA PE=2 SV=1	267095	31	6	628	2748	0.44	2.28	5.18181818	25	80	30	105	38	114	46	132	0.688196
tr|B5MCX3|B5MCX3_HUMAN	Sep-02	Septin-2 OS=Homo sapiens GN=SEPT2 PE=2 SV=1	37145	543	499	30	78	0.09	0.46	5.11111111	1	2	1	4	2	3	3	4	-0.40684
tr|B2RU06|B2RU06_HUMAN	EIF4G1	EIF4G1 protein OS=Homo sapiens GN=EIF4G1 PE=2 SV=1	176807	669	225	20	219	0.04	0.2	5	0	6	2	10	1	10	3	12	1.916687
tr|E7ERW8|E7ERW8_HUMAN	DIAPH1	Protein diaphanous homolog 1 OS=Homo sapiens GN=DIAPH1 PE=2 SV=1	135945	184	635	116	64	0.02	0.1	5	1	3	1	4	1	6	2	6	1.224089
tr|B5LY69|B5LY69_HUMAN	UMPS	Uridine monophosphate synthetase isoform G OS=Homo sapiens GN=UMPS PE=2 SV=1	40514	302	865	59	43	0.1	0.5	5	1	1	1	1	1	1	1	1	-0.89229
tr|Q6IPN6|Q6IPN6_HUMAN	EEF1A1	Elongation factor 1-alpha OS=Homo sapiens GN=EEF1A1 PE=2 SV=1	50433	70	54	295	715	1.14	5.69	4.99122807	11	24	13	41	18	45	21	48	0.408303
sp|P04350|TBB4A_HUMAN	TUBB4A	Tubulin beta-4A chain OS=Homo sapiens GN=TUBB4A PE=1 SV=2	50010	42	18	509	1577	1.96	9.59	4.89285714	21	50	27	59	29	64	32	70	0.238295
tr|F5H6E2|F5H6E2_HUMAN	MYO1C	Unconventional myosin-Ic OS=Homo sapiens GN=MYO1C PE=2 SV=1	119719	284	115	65	372	0.07	0.34	4.85714286	1	10	3	11	3	14	4	15	1.160011
sp|P53621|COPA_HUMAN	COPA	Coatomer subunit alpha OS=Homo sapiens GN=COPA PE=1 SV=2	139797	181	70	118	550	0.18	0.87	4.83333333	5	18	7	27	8	28	11	34	0.855666
sp|P06733|ENOA_HUMAN	ENO1	Alpha-enolase OS=Homo sapiens GN=ENO1 PE=1 SV=2	47481	40	32	520	1097	1.39	6.49	4.66906475	13	30	18	39	19	46	25	51	0.363564
tr|B1AH89|B1AH89_HUMAN	TTLL12	Tubulin tyrosine ligase-like family, member 12 OS=Homo sapiens GN=TTLL12 PE=4 SV=1	74185	104	52	209	736	0.41	1.91	4.65853659	6	18	8	26	10	26	11	31	0.444811
tr|B4DEX8|B4DEX8_HUMAN	MAT2A	S-adenosylmethionine synthase OS=Homo sapiens GN=MAT2A PE=2 SV=1	40027	333	349	52	128	0.08	0.37	4.625	1	3	1	4	1	4	1	5	0.693214
sp|Q00610|CLH1_HUMAN	CLTC	Clathrin heavy chain 1 OS=Homo sapiens GN=CLTC PE=1 SV=5	193260	30	12	629	2014	0.52	2.39	4.59615385	19	46	25	77	24	83	28	92	0.883835
tr|B2R5U1|B2R5U1_HUMAN	tr|B2R5U1|B2R	cDNA, FLJ92620, highly similar to Homo sapiens staphylococcal nuclease domain containing 1 (SND1),mRNA O	100294	150	82	144	486	0.17	0.78	4.58823529	4	11	5	19	10	22	12	22	0.208022
tr|B4DHQ3|B4DHQ3_HUMAN	tr|B4DHQ3|B4D	cDNA FLJ56437, highly similar to Phosphoserine aminotransferase (EC 2.6.1.52) OS=Homo sapiens PE=2 SV=1	45783	552	391	30	114	0.07	0.32	4.57142857	0	3	1	4	1	4	2	4	0.693214
tr|B3KM77|B3KM77_HUMAN	tr|B3KM77|B3K	cDNA FLJ10435 fis, clone NT2RP1000493, highly similar to Splicing factor 3B subunit 3 OS=Homo sapiens PE=2	56736	356	181	49	234	0.06	0.27	4.5	1	7	1	7	1	7	1	7	1.43072
tr|E5KNY5|E5KNY5_HUMAN	LRPPRC	Leucine-rich PPR-motif containing OS=Homo sapiens GN=LRPPRC PE=2 SV=1	159003	329	150	53	312	0.08	0.36	4.5	1	11	4	15	5	16	7	18	0.694039
tr|B4DGQ0|B4DGQ0_HUMAN	LASP1	LIM and SH3 domain protein 1 OS=Homo sapiens GN=LASP1 PE=2 SV=1	23167	568	822	29	37	0.14	0.63	4.5	0	1	1	2	1	2	1	2	-0.15514
tr|B4DXX7|B4DXX7_HUMAN	DDX3Y	ATP-dependent RNA helicase DDX3Y OS=Homo sapiens GN=DDX3Y PE=2 SV=1	73342	217	139	95	210	0.19	0.84	4.42105263	2	8	5	12	6	13	6	15	0.16265
tr|Q60FE5|Q60FE5_HUMAN	FLNA	Filamin A OS=Homo sapiens GN=FLNA PE=2 SV=1	280790	9	2	1306	4630	0.86	3.8	4.41860465	41	123	60	166	75	186	88	205	0.421089
tr|A0JLS5|A0JLS5_HUMAN	TBL3	TBL3 protein (Fragment) OS=Homo sapiens GN=TBL3 PE=2 SV=1	64079	418	389	42	114	0.05	0.22	4.4	1	3	1	4	1	4	1	4	0.693214
tr|Q5HY54|Q5HY54_HUMAN	FLNA	Filamin-A OS=Homo sapiens GN=FLNA PE=2 SV=1	279115	9	1	1304	4654	0.86	3.74	4.34883721	41	123	59	164	74	185	87	203	0.432724
tr|Q53QX9|Q53QX9_HUMAN	HK2	Putative uncharacterized protein HK2 (Fragment) OS=Homo sapiens GN=HK2 PE=2 SV=1	64652	599	543	26	41	0.03	0.13	4.33333333	0	0	1	2	1	2	2	2	-0.15514
tr|B7ZKM0|B7ZKM0_HUMAN	SART3	SART3 protein OS=Homo sapiens GN=SART3 PE=2 SV=1	106372	153	83	139	418	0.16	0.69	4.3125	5	8	5	16	7	18	7	19	0.411332
tr|Q0VGD6|Q0VGD6_HUMAN	HNRPR	HNRPR protein (Fragment) OS=Homo sapiens GN=HNRPR PE=2 SV=1	68218	390	257	44	197	0.1	0.43	4.3	1	6	2	8	2	10	4	11	1.179388
sp|P49327|FAS_HUMAN	FASN	Fatty acid synthase OS=Homo sapiens GN=FASN PE=1 SV=3	275877	55	15	394	1888	0.28	1.2	4.28571429	12	51	21	69	23	79	29	88	0.872744
tr|B2R5M9|B2R5M9_HUMAN	tr|B2R5M9|B2R	cDNA, FLJ92537, highly similar to Homo sapiens procollagen-lysine, 2-oxoglutarate 5-dioxygenase (lysine hydr	84022	251	466	76	75	0.04	0.17	4.25	1	2	1	3	1	3	1	3	0.330464
sp|P41250|SYG_HUMAN	GARS	Glycine--tRNA ligase OS=Homo sapiens GN=GARS PE=1 SV=3	83854	392	446	44	99	0.04	0.17	4.25	1	2	1	4	1	3	2	5	0.330464
tr|Q5TCU6|Q5TCU6_HUMAN	TLN1	Talin-1 OS=Homo sapiens GN=TLN1 PE=4 SV=1	260030	48	13	446	1789	0.27	1.14	4.22222222	12	46	19	59	20	61	25	71	0.697152
tr|A5D906|A5D906_HUMAN	TUBB2A	TUBB2A protein (Fragment) OS=Homo sapiens GN=TUBB2A PE=2 SV=1	28128	25	16	143	774	3.07	12.43	4.04885993	4	22	7	27	8	30	8	34	0.953874
tr|F8W9S7|F8W9S7_HUMAN	GAPVD1	GTPase-activating protein and VPS9 domain-containing protein 1 OS=Homo sapiens GN=GAPVD1 PE=2 SV=1	158790	697	459	18	96	0.02	0.08	4	0	3	1	4	0	4	2	5	2.278511
tr|A6NEM2|A6NEM2_HUMAN	HCFC1	HCF N-terminal chain 5 OS=Homo sapiens GN=HCFC1 PE=2 SV=2	215338	508	483	33	90	0.02	0.08	4	0	2	1	5	1	5	2	5	0.982901
tr|A2BDK6|A2BDK6_HUMAN	MAP1B	Microtubule-associated protein 1B OS=Homo sapiens GN=MAP1B PE=2 SV=1	257692	511	729	32	54	0.01	0.04	4	0	1	1	3	1	2	2	3	-0.15514
sp|P26038|MOES_HUMAN	MSN	Moesin OS=Homo sapiens GN=MSN PE=1 SV=3	67892	119	55	179	700	0.46	1.83	3.97826087	7	19	8	24	11	27	12	32	0.366852
tr|B3KMX0|B3KMX0_HUMAN	tr|B3KMX0|B3K	cDNA FLJ12837 fis, clone NT2RP2003228, highly similar to DNA replication licensing factor MCM4 OS=Homo sa	97114	240	251	85	202	0.1	0.39	3.9	2	7	3	10	4	11	5	12	0.462147
tr|B4DSQ5|B4DSQ5_HUMAN	tr|B4DSQ5|B4D	cDNA FLJ53608, highly similar to Protein transport protein Sec23A OS=Homo sapiens PE=2 SV=1	83787	474	187	37	259	0.08	0.31	3.875	0	6	2	8	2	8	3	9	0.874173
sp|P46063|RECQ1_HUMAN	RECQL	ATP-dependent DNA helicase Q1 OS=Homo sapiens GN=RECQL PE=1 SV=3	74436	439	157	39	298	0.14	0.54	3.85714286	1	6	3	10	3	10	3	11	0.693627
tr|B4DXC4|B4DXC4_HUMAN	tr|B4DXC4|B4D	cDNA FLJ58636, moderately similar to Atlastin OS=Homo sapiens PE=2 SV=1	59162	451	657	38	45	0.06	0.23	3.83333333	1	0	1	3	1	4	1	5	0.693214
sp|O43175|SERA_HUMAN	PHGDH	D-3-phosphoglycerate dehydrogenase OS=Homo sapiens GN=PHGDH PE=1 SV=4	57356	131	107	166	382	0.25	0.95	3.8	3	9	4	13	4	14	4	14	0.797107
tr|A5PHJ9|A5PHJ9_HUMAN	PDHA1/LOC790	PDHA1/LOC79064 protein (Fragment) OS=Homo sapiens GN=PDHA1/LOC79064 PE=2 SV=1	34113	516	442	32	83	0.1	0.38	3.8	0	2	1	2	1	3	1	3	0.330464
tr|B3KS31|B3KS31_HUMAN	TUBB6	Tubulin, beta 6, isoform CRA_a OS=Homo sapiens GN=TUBB6 PE=2 SV=1	42324	73	57	273	589	0.83	3.15	3.79518072	10	20	14	26	17	30	20	35	-0.09096
tr|B5BU01|B5BU01_HUMAN	EIF2S2	Eukaryotic translation initiation factor 2 beta OS=Homo sapiens GN=EIF2S2 PE=2 SV=1	38648	654	1027	22	33	0.09	0.34	3.77777778	0	0	1	1	1	1	1	1	-0.89229
tr|B2RDN4|B2RDN4_HUMAN	tr|B2RDN4|B2R	cDNA, FLJ96693, highly similar to Homo sapiens block of proliferation 1 (BOP1), mRNA OS=Homo sapiens PE=2	84219	505	563	33	54	0.08	0.3	3.75	0	1	2	5	2	5	3	7	0.245601
tr|Q2TSD0|Q2TSD0_HUMAN	tr|Q2TSD0|Q2T	Glyceraldehyde-3-phosphate dehydrogenase OS=Homo sapiens PE=2 SV=1	36197	18	21	891	1425	3.44	12.86	3.73837209	23	34	31	46	31	52	36	58	-0.15617
tr|B4DJ60|B4DJ60_HUMAN	tr|B4DJ60|B4DJ	cDNA FLJ55072, highly similar to Succinate dehydrogenase (ubiquinone) flavoprotein subunit, mitochondrial (I	68233	393	235	44	211	0.1	0.37	3.7	0	5	2	6	3	6	5	11	0.001029
tr|B2RWP9|B2RWP9_HUMAN	MYH10	MYH10 protein OS=Homo sapiens GN=MYH10 PE=2 SV=1	230858	200	102	104	407	0.06	0.22	3.66666667	3	11	4	14	4	22	6	25	1.432421
sp|Q8TCJ2|STT3B_HUMAN	STT3B	Dolichyl-diphosphooligosaccharide--protein glycosyltransferase subunit STT3B OS=Homo sapiens GN=STT3B P	94241	700	645	18	63	0.03	0.11	3.66666667	0	1	1	3	2	3	3	4	-0.40684
tr|J3KPM9|J3KPM9_HUMAN	STAT1	Signal transducer and activator of transcription 1-alpha/beta OS=Homo sapiens GN=STAT1 PE=4 SV=1	83878	364	218	48	207	0.08	0.29	3.625	1	5	2	6	2	6	2	6	0.486789
sp|P26599|PTBP1_HUMAN	PTBP1	Polypyrimidine tract-binding protein 1 OS=Homo sapiens GN=PTBP1 PE=1 SV=1	57357	567	152	29	310	0.18	0.65	3.61111111	0	5	3	9	3	9	3	12	0.549057
sp|Q08211|DHX9_HUMAN	DHX9	ATP-dependent RNA helicase A OS=Homo sapiens GN=DHX9 PE=1 SV=4	142181	96	45	218	903	0.41	1.48	3.6097561	8	30	16	42	18	44	22	49	0.376061
tr|Q8TC62|Q8TC62_HUMAN	Sep-07	Septin 7 OS=Homo sapiens GN=SEPT7 PE=2 SV=3	48913	531	506	31	84	0.07	0.25	3.57142857	1	2	1	3	1	3	2	3	0.330464
tr|B4DR68|B4DR68_HUMAN	tr|B4DR68|B4D	cDNA FLJ58608, highly similar to Heat shock protein 75 kDa, mitochondrial OS=Homo sapiens PE=2 SV=1	74408	169	190	127	257	0.09	0.32	3.55555556	2	6	3	6	3	7	5	7	0.207659
sp|Q14683|SMC1A_HUMAN	SMC1A	Structural maintenance of chromosomes protein 1A OS=Homo sapiens GN=SMC1A PE=1 SV=2	143771	581	623	28	65	0.02	0.07	3.5	0	1	1	3	1	4	1	5	0.693214
tr|I3L1L3|I3L1L3_HUMAN	MYBBP1A	Myb-binding protein 1A (Fragment) OS=Homo sapiens GN=MYBBP1A PE=4 SV=1	141124	698	385	18	115	0.02	0.07	3.5	0	2	1	3	1	4	2	5	0.693214
tr|B4DY09|B4DY09_HUMAN	tr|B4DY09|B4D	cDNA FLJ51660, highly similar to Interleukin enhancer-binding factor 2 OS=Homo sapiens PE=2 SV=1	39057	383	358	45	125	0.08	0.28	3.5	1	2	1	3	1	3	1	3	0.330464
tr|B4DG42|B4DG42_HUMAN	tr|B4DG42|B4D	cDNA FLJ53753, highly similar to Myeloid-associated differentiation marker OS=Homo sapiens PE=2 SV=1	31889	609	297	25	163	0.1	0.35	3.5	0	3	1	3	1	4	1	4	0.693214
tr|B4DMB5|B4DMB5_HUMAN	tr|B4DMB5|B4D	cDNA FLJ60075, highly similar to CTP synthase 1 (EC 6.3.4.2) OS=Homo sapiens PE=2 SV=1	56542	449	170	38	285	0.19	0.66	3.47368421	1	7	3	9	3	9	4	10	0.549057
tr|H0YH88|H0YH88_HUMAN	NAP1L1	Nucleosome assembly protein 1-like 1 (Fragment) OS=Homo sapiens GN=NAP1L1 PE=3 SV=1	21179	417	595	33	69	0.16	0.55	3.4375	0	1	1	3	1	3	1	3	0.330464
tr|B2R8R5|B2R8R5_HUMAN	tr|B2R8R5|B2R	cDNA, FLJ94025, highly similar to Homo sapiens tripartite motif-containing 28 (TRIM28), mRNA OS=Homo sapi	90271	320	247	56	205	0.07	0.24	3.42857143	1	5	3	6	2	8	4	9	0.874173
sp|Q15392|DHC24_HUMAN	DHCR24	Delta(24)-sterol reductase OS=Homo sapiens GN=DHCR24 PE=1 SV=2	60803	442	733	39	53	0.05	0.17	3.4	1	1	1	3	2	4	3	5	-0.04409
sp|O15427|MOT4_HUMAN	SLC16A3	Monocarboxylate transporter 4 OS=Homo sapiens GN=SLC16A3 PE=1 SV=1	50064	362	461	48	95	0.14	0.47	3.35714286	2	2	2	6	2	6	2	6	0.486789
tr|A0PJ92|A0PJ92_HUMAN	NOP56	NOP56 protein (Fragment) OS=Homo sapiens GN=NOP56 PE=2 SV=1	50068	311	165	58	274	0.14	0.47	3.35714286	1	7	2	8	3	7	3	8	0.207659
sp|O00116|ADAS_HUMAN	AGPS	Alkyldihydroxyacetonephosphate synthase, peroxisomal OS=Homo sapiens GN=AGPS PE=1 SV=1	73664	448	383	38	116	0.09	0.3	3.33333333	1	3	2	6	2	6	3	6	0.486789
sp|O00232|PSD12_HUMAN	PSMD12	26S proteasome non-ATPase regulatory subunit 12 OS=Homo sapiens GN=PSMD12 PE=1 SV=3	53270	283	285	66	170	0.06	0.2	3.33333333	1	2	1	3	1	3	1	4	0.330464
tr|B4DKS0|B4DKS0_HUMAN	tr|B4DKS0|B4D	cDNA FLJ53381, highly similar to Monocarboxylate transporter 1 OS=Homo sapiens PE=2 SV=1	52487	598	370	26	102	0.06	0.2	3.33333333	0	1	1	2	1	2	1	2	-0.15514
sp|P07355|ANXA2_HUMAN	ANXA2	Annexin A2 OS=Homo sapiens GN=ANXA2 PE=1 SV=2	38808	100	73	214	544	1.26	4.13	3.27777778	8	11	11	23	12	24	13	27	0.079032
sp|O00425|IF2B3_HUMAN	IGF2BP3	Insulin-like growth factor 2 mRNA-binding protein 3 OS=Homo sapiens GN=IGF2BP3 PE=1 SV=2	64008	556	629	29	64	0.05	0.16	3.2	0	3	1	3	2	3	2	3	-0.40684
tr|G3XAD8|G3XAD8_HUMAN	STIP1	Stress-induced-phosphoprotein 1 OS=Homo sapiens GN=STIP1 PE=2 SV=1	68721	271	203	69	242	0.21	0.67	3.19047619	2	9	4	11	4	11	5	13	0.462147
tr|I3L312|I3L312_HUMAN	P4HB	Protein disulfide-isomerase (Fragment) OS=Homo sapiens GN=P4HB PE=4 SV=1	26348	536	234	30	74	0.27	0.86	3.18518519	1	2	2	4	1	3	2	5	0.330464
tr|F5GX33|F5GX33_HUMAN	SLC25A13	Calcium-binding mitochondrial carrier protein Aralar2 OS=Homo sapiens GN=SLC25A13 PE=2 SV=1	62548	353	387	49	95	0.11	0.35	3.18181818	1	2	2	6	3	6	4	8	0.001029
tr|B4DR45|B4DR45_HUMAN	tr|B4DR45|B4D	cDNA FLJ61339, highly similar to Alanyl-tRNA synthetase (EC 6.1.1.7) OS=Homo sapiens PE=2 SV=1	110017	238	295	86	165	0.06	0.19	3.16666667	1	5	2	6	2	6	2	6	0.486789
tr|Q6IAV5|Q6IAV5_HUMAN	OXCT	Succinyl-CoA:3-ketoacid-coenzyme A transferase OS=Homo sapiens GN=OXCT PE=2 SV=1	56612	209	250	100	188	0.18	0.57	3.16666667	1	5	3	7	5	7	6	10	-0.44509
tr|B7Z6M1|B7Z6M1_HUMAN	PLS3	Plastin-3 OS=Homo sapiens GN=PLS3 PE=2 SV=1	66047	282	153	66	309	0.34	1.07	3.14705882	1	9	6	15	6	15	8	18	0.362319
tr|B2RB23|B2RB23_HUMAN	tr|B2RB23|B2R	cDNA, FLJ95265, highly similar to Homo sapiens acetyl-Coenzyme A acyltransferase 2 (mitochondrial 3-oxoacy	42439	544	380	30	117	0.08	0.25	3.125	0	2	1	3	1	4	1	4	0.693214
tr|B4DFN9|B4DFN9_HUMAN	tr|B4DFN9|B4D	cDNA FLJ54303, highly similar to Heat shock 70 kDa protein 1 OS=Homo sapiens PE=2 SV=1	62699	80	59	252	542	0.58	1.8	3.10344828	7	17	10	21	10	20	10	22	0.07336
tr|B0UZ98|B0UZ98_HUMAN	BAT1	HLA-B associated transcript 1 (Fragment) OS=Homo sapiens GN=BAT1 PE=2 SV=1	28623	394	674	44	33	0.09	0.27	3	1	0	1	1	1	1	1	2	-0.89229
tr|H0Y327|H0Y327_HUMAN	MTHFD1L	Monofunctional C1-tetrahydrofolate synthase, mitochondrial (Fragment) OS=Homo sapiens GN=MTHFD1L PE=	36808	398	447	43	16	0.09	0.27	3	1	0	1	1	1	0	1	1	-2.47743
tr|Q2NLA0|Q2NLA0_HUMAN	COPG2	Coatomer subunit gamma OS=Homo sapiens GN=COPG2 PE=2 SV=1	82400	705	484	18	90	0.04	0.12	3	0	1	1	3	0	3	1	3	1.915761
tr|H0YBW4|H0YBW4_HUMAN	PLAA	Phospholipase A-2-activating protein (Fragment) OS=Homo sapiens GN=PLAA PE=4 SV=1	31645	539	280	30	156	0.11	0.33	3	0	3	1	4	1	4	1	4	0.693214
tr|Q68DB1|Q68DB1_HUMAN	DKFZp686F2281	Metastasis-associated protein MTA2 OS=Homo sapiens GN=DKFZp686F2281 PE=2 SV=1	55515	676	330	20	58	0.06	0.18	3	0	2	1	2	1	3	1	3	0.330464
tr|B1PS43|B1PS43_HUMAN	MYH11	Myosin heavy chain 11 smooth muscle isoform OS=Homo sapiens GN=MYH11 PE=2 SV=1	235004	142	88	155	441	0.06	0.18	3	5	14	5	14	9	17	11	20	-0.01073
sp|Q9UMS4|PRP19_HUMAN	PRPF19	Pre-mRNA-processing factor 19 OS=Homo sapiens GN=PRPF19 PE=1 SV=1	55603	237	201	87	247	0.26	0.78	3	7	8	5	8	6	11	11	15	-0.27582
tr|Q5TB52|Q5TB52_HUMAN	PAPSS2	3''-phosphoadenosine 5''-phosphosulfate synthase 2, isoform CRA_b OS=Homo sapiens GN=PAPSS2 PE=2 SV=1	70027	688	482	19	90	0.05	0.15	3	0	1	1	3	0	4	1	5	2.278511
tr|B2RDF2|B2RDF2_HUMAN	PES1	Pescadillo homolog 1 OS=Homo sapiens GN=PES1 PE=2 SV=1	68359	488	455	35	97	0.05	0.15	3	1	4	1	5	1	5	3	6	0.982901
tr|B4DFL1|B4DFL1_HUMAN	tr|B4DFL1|B4DI	Dihydrolipoyl dehydrogenase OS=Homo sapiens PE=2 SV=1	49381	666	477	21	92	0.07	0.21	3	0	2	1	4	1	3	1	5	0.330464
tr|B2RE46|B2RE46_HUMAN	tr|B2RE46|B2RI	cDNA, FLJ96923, highly similar to Homo sapiens ribophorin II (RPN2), mRNA OS=Homo sapiens PE=2 SV=1	69405	589	457	27	97	0.05	0.15	3	0	2	1	3	1	2	2	4	-0.04409

tr|B4E022|B4E022_HUMAN	TKT	Transketolase OS=Homo sapiens GN=TKT PE=2 SV=1	63410	35	40	578	939	1.36	4.04	2.97058824	17	26	24	39	26	45	32	50	-0.11285
tr|B4DVJ0|B4DVJ0_HUMAN	tr|B4DVJ0|B4DV	Glucose-6-phosphate isomerase OS=Homo sapiens PE=2 SV=1	60181	117	132	182	339	0.3	0.89	2.96666667	4	7	5	12	6	11	6	14	-0.06904
tr|B4DE36|B4DE36_HUMAN	tr|B4DE36|B4D	Glucose-6-phosphate isomerase OS=Homo sapiens PE=2 SV=1	60376	117	131	182	339	0.3	0.89	2.96666667	4	7	5	12	7	11	7	14	-0.27582
tr|J3KPS3|J3KPS3_HUMAN	ALDOA	Fructose-bisphosphate aldolase OS=Homo sapiens GN=ALDOA PE=3 SV=1	40249	63	36	338	979	1.79	5.26	2.93854749	11	26	15	28	17	33	18	39	0.044934
tr|B4DWJ2|B4DWJ2_HUMAN	QARS	Glutamine--tRNA ligase OS=Homo sapiens GN=QARS PE=2 SV=1	87437	277	128	68	341	0.16	0.47	2.9375	3	9	4	10	5	11	5	12	0.172306
tr|Q2NKY5|Q2NKY5_HUMAN	TUBB6	TUBB6 protein OS=Homo sapiens GN=TUBB6 PE=2 SV=1	50514	65	41	317	920	0.88	2.54	2.88636364	12	24	16	30	17	35	19	39	0.128953
tr|B4E0S6|B4E0S6_HUMAN	tr|B4E0S6|B4E0	cDNA FLJ55635, highly similar to pre-mRNA-splicing factorATP-dependent RNA helicase DHX15 (EC 3.6.1.-) OS=	90289	183	159	117	296	0.15	0.43	2.86666667	2	8	4	10	4	12	7	15	0.582621
tr|B2RBR9|B2RBR9_HUMAN	tr|B2RBR9|B2R	cDNA, FLJ95650, highly similar to Homo sapiens karyopherin (importin) beta 1 (KPNB1), mRNA OS=Homo sapie	98434	171	92	126	430	0.22	0.63	2.86363636	4	11	6	15	9	19	9	23	0.145755
sp|Q14764|MVP_HUMAN	MVP	Major vault protein OS=Homo sapiens GN=MVP PE=1 SV=4	99551	304	1128	59	26	0.07	0.2	2.85714286	2	0	2	1	3	1	3	1	-2.11535
sp|P13639|EF2_HUMAN	EEF2	Elongation factor 2 OS=Homo sapiens GN=EEF2 PE=1 SV=4	96246	41	25	511	1339	1.23	3.51	2.85365854	13	40	26	57	27	62	32	71	0.294445
tr|Q6DEN2|Q6DEN2_HUMAN	DPYSL3	DPYSL3 protein OS=Homo sapiens GN=DPYSL3 PE=2 SV=1	74320	490	109	35	380	0.19	0.54	2.84210526	0	8	4	10	3	15	8	16	1.256407
tr|B7Z601|B7Z601_HUMAN	tr|B7Z601|B7Z6	cDNA FLJ57187, highly similar to Glycerol-3-phosphate dehydrogenase, mitochondrial (EC 1.1.99.5) OS=Homo	56047	512	388	32	99	0.06	0.17	2.83333333	0	3	1	3	2	3	2	3	-0.40684
sp|P08238|HS90B_HUMAN	HSP90AB1	Heat shock protein HSP 90-beta OS=Homo sapiens GN=HSP90AB1 PE=1 SV=4	83554	32	22	622	1447	1.24	3.48	2.80645161	18	35	28	43	29	53	35	57	-0.03346
sp|Q9UG63|ABCF2_HUMAN	ABCF2	ATP-binding cassette sub-family F member 2 OS=Homo sapiens GN=ABCF2 PE=1 SV=2	71815	673	841	20	46	0.05	0.14	2.8	0	1	1	3	1	3	2	5	0.330464
tr|Q05D08|Q05D08_HUMAN	PA2G4	PA2G4 protein (Fragment) OS=Homo sapiens GN=PA2G4 PE=2 SV=1	45579	286	431	64	102	0.15	0.42	2.8	1	4	2	5	3	5	3	8	-0.24016
tr|B3KX19|B3KX19_HUMAN	tr|B3KX19|B3KX	cDNA FLJ44500 fis, clone UTERU3000828, highly similar to 116 kDa U5 small nuclear ribonucleoprotein compo	109111	111	99	193	410	0.27	0.75	2.77777778	4	10	8	19	8	19	10	23	0.306554
sp|Q01650|LAT1_HUMAN	SLC7A5	Large neutral amino acids transporter small subunit 1 OS=Homo sapiens GN=SLC7A5 PE=1 SV=2	55659	239	161	85	295	0.12	0.33	2.75	1	7	3	7	4	7	5	7	-0.15524
tr|B2RCM2|B2RCM2_HUMAN	tr|B2RCM2|B2R	cDNA, FLJ96156, highly similar to Homo sapiens leucyl-tRNA synthetase (LARS), mRNA OS=Homo sapiens PE=2	135550	257	143	75	321	0.1	0.27	2.7	2	8	4	10	3	10	5	11	0.693627
sp|P53396|ACLY_HUMAN	ACLY	ATP-citrate synthase OS=Homo sapiens GN=ACLY PE=1 SV=3	121674	20	17	845	1582	1.28	3.42	2.671875	21	43	32	61	34	61	40	70	-0.05853
sp|Q9Y2A7|NCKP1_HUMAN	NCKAP1	Nck-associated protein 1 OS=Homo sapiens GN=NCKAP1 PE=1 SV=1	130018	685	438	19	101	0.03	0.08	2.66666667	0	2	1	3	0	2	1	4	1.430154
sp|Q9Y5B9|SP16H_HUMAN	SUPT16H	FACT complex subunit SPT16 OS=Homo sapiens GN=SUPT16H PE=1 SV=1	120409	506	768	33	51	0.03	0.08	2.66666667	0	2	1	3	1	4	2	4	0.693214
tr|Q1L838|Q1L838_HUMAN	GTBP	GTBP-ALT OS=Homo sapiens GN=GTBP PE=2 SV=1	122267	559	381	29	116	0.03	0.08	2.66666667	0	2	1	3	1	4	1	5	0.693214
tr|B2R6D0|B2R6D0_HUMAN	tr|B2R6D0|B2R	cDNA, FLJ92896, highly similar to Homo sapiens proteasome (prosome, macropain) 26S subunit, non-ATPase,	106797	386	313	45	151	0.06	0.16	2.66666667	0	4	2	5	2	6	4	7	0.486789
tr|B4DQJ8|B4DQJ8_HUMAN	PGD	6-phosphogluconate dehydrogenase, decarboxylating OS=Homo sapiens GN=PGD PE=2 SV=1	52352	293	175	62	277	0.2	0.53	2.65	1	7	3	8	2	9	3	9	1.034818
tr|Q53GN4|Q53GN4_HUMAN	WDR1	WD repeat domain 1, isoform CRA_a (Fragment) OS=Homo sapiens GN=WDR1 PE=2 SV=1	66822	137	65	160	566	0.4	1.05	2.625	4	14	7	18	7	20	9	22	0.559791
tr|A8K2S1|A8K2S1_HUMAN	tr|A8K2S1|A8K2	cDNA FLJ75258, highly similar to Homo sapiens phospholipase A2, group IVA (cytosolic, calcium-dependent) (F	85695	369	335	47	137	0.08	0.21	2.625	1	5	2	5	2	5	5	10	0.245601
tr|A8K7F6|A8K7F6_HUMAN	tr|A8K7F6|A8K7	cDNA FLJ78244, highly similar to Homo sapiens eukaryotic translation initiation factor 4A, isoform 1 (EIF4A1),	46321	52	64	404	594	1.62	4.22	2.60493827	10	17	14	24	15	26	20	31	-0.11874
sp|P17987|TCPA_HUMAN	TCP1	T-complex protein 1 subunit alpha OS=Homo sapiens GN=TCP1 PE=1 SV=1	60819	197	171	106	285	0.3	0.78	2.6	2	7	5	11	8	10	10	11	-0.58815
tr|H0YMR4|H0YMR4_HUMAN	IPO4	Importin-4 (Fragment) OS=Homo sapiens GN=IPO4 PE=4 SV=1	60104	655	371	22	64	0.05	0.13	2.6	0	1	1	1	1	1	1	1	-0.89229
sp|P11021|GRP78_HUMAN	HSPA5	78 kDa glucose-regulated protein OS=Homo sapiens GN=HSPA5 PE=1 SV=2	72402	129	97	170	413	0.19	0.49	2.57894737	3	9	5	10	6	11	6	11	-0.06904
tr|Q6IBR0|Q6IBR0_HUMAN	RPN1	RPN1 protein OS=Homo sapiens GN=RPN1 PE=2 SV=1	68678	170	220	127	226	0.26	0.67	2.57692308	4	8	5	11	8	12	8	13	-0.33625
sp|P22314|UBA1_HUMAN	UBA1	Ubiquitin-like modifier-activating enzyme 1 OS=Homo sapiens GN=UBA1 PE=1 SV=3	118858	114	86	187	464	0.28	0.72	2.57142857	5	12	10	20	10	20	12	23	0.07336
tr|B4DFK9|B4DFK9_HUMAN	tr|B4DFK9|B4D	cDNA FLJ57964, highly similar to Heterogeneous nuclear ribonucleoprotein H~ OS=Homo sapiens PE=2 SV=1	44637	272	195	69	201	0.15	0.38	2.53333333	1	5	2	5	1	5	2	6	0.982901
sp|P68366|TBA4A_HUMAN	TUBA4A	Tubulin alpha-4A chain OS=Homo sapiens GN=TUBA4A PE=1 SV=1	50634	22	26	796	1327	3.01	7.6	2.52491694	20	35	31	49	31	52	36	52	-0.15617
tr|J3KR97|J3KR97_HUMAN	TBCD	Tubulin-specific chaperone D OS=Homo sapiens GN=TBCD PE=4 SV=1	138346	714	808	15	47	0.02	0.05	2.5	0	1	1	2	0	3	1	4	1.915761
tr|B4DKD0|B4DKD0_HUMAN	tr|B4DKD0|B4D	DNA topoisomerase 2 (Fragment) OS=Homo sapiens PE=2 SV=1	135015	606	575	25	72	0.02	0.05	2.5	0	1	1	2	1	3	3	5	0.330464
tr|B4DJ30|B4DJ30_HUMAN	tr|B4DJ30|B4DJ	cDNA FLJ61290, highly similar to Neutral alpha-glucosidase AB OS=Homo sapiens PE=2 SV=1	113539	47	38	446	950	0.62	1.55	2.5	12	24	19	34	17	34	22	42	0.08755
tr|B2R6X5|B2R6X5_HUMAN	tr|B2R6X5|B2R6	cDNA, FLJ93166, highly similar to Homo sapiens heat shock 70kDa protein 6 (HSP70B~) (HSPA6), mRNA OS=Ho	71370	193	199	112	248	0.2	0.5	2.5	3	8	5	9	6	10	6	10	-0.20046
tr|A6NN80|A6NN80_HUMAN	ANXA6	Annexin OS=Homo sapiens GN=ANXA6 PE=3 SV=3	75571	349	337	50	137	0.14	0.35	2.5	1	3	3	7	3	7	3	9	0.207659
tr|B2R4K7|B2R4K7_HUMAN	tr|B2R4K7|B2R4	60S ribosomal protein L6 OS=Homo sapiens PE=2 SV=1	32739	276	231	68	216	0.47	1.17	2.4893617	2	6	4	9	3	10	5	13	0.693627
sp|P23526|SAHH_HUMAN	AHCY	Adenosylhomocysteinase OS=Homo sapiens GN=AHCY PE=1 SV=4	48255	195	110	111	379	0.49	1.21	2.46938776	2	10	6	13	6	13	8	13	0.16265
tr|Q9UJU1|Q9UJU1_HUMAN	VIL2	Cytovillin 2 (Fragment) OS=Homo sapiens GN=VIL2 PE=2 SV=1	16294	236	354	88	127	0.46	1.13	2.45652174	2	4	2	5	2	5	3	5	0.245601
tr|F5H3A1|F5H3A1_HUMAN	ATP1A1	Sodium/potassium-transporting ATPase subunit alpha-1 OS=Homo sapiens GN=ATP1A1 PE=2 SV=1	114239	324	232	55	215	0.09	0.22	2.44444444	2	7	3	8	8	10	12	13	-0.58815
sp|P15121|ALDR_HUMAN	AKR1B1	Aldose reductase OS=Homo sapiens GN=AKR1B1 PE=1 SV=3	36230	185	292	116	166	0.42	1.02	2.42857143	3	5	4	8	5	9	5	10	-0.10369
tr|F2Z2U8|F2Z2U8_HUMAN	MYH14	Myosin-14 OS=Homo sapiens GN=MYH14 PE=2 SV=1	232102	152	213	140	233	0.07	0.17	2.42857143	5	10	5	11	7	12	8	13	-0.15535
tr|J3QSX4|J3QSX4_HUMAN	BUB3	Mitotic checkpoint protein BUB3 OS=Homo sapiens GN=BUB3 PE=4 SV=1	16498	681	401	20	71	0.2	0.48	2.4	0	2	1	2	0	2	1	2	1.430154
tr|H0Y4R1|H0Y4R1_HUMAN	IMPDH2	Inosine-5~-monophosphate dehydrogenase 2 (Fragment) OS=Homo sapiens GN=IMPDH2 PE=3 SV=1	51549	177	105	122	392	0.36	0.86	2.38888889	2	9	5	10	5	11	6	12	0.172306
tr|F5H4R9|F5H4R9_HUMAN	DNM2	Dynamin-2 OS=Homo sapiens GN=DNM2 PE=2 SV=1	98218	713	612	16	66	0.03	0.07	2.33333333	0	2	1	2	0	2	2	4	1.430154
tr|B0V043|B0V043_HUMAN	VARS	Valine--tRNA ligase OS=Homo sapiens GN=VARS PE=3 SV=1	141632	275	100	69	408	0.15	0.35	2.33333333	2	10	6	14	6	16	8	23	0.452697
tr|H7C1U0|H7C1U0_HUMAN	APEH	Acylamino-acid-releasing enzyme (Fragment) OS=Homo sapiens GN=APEH PE=4 SV=1	35132	319	320	56	76	0.09	0.21	2.33333333	1	2	1	2	1	2	1	2	-0.15514
tr|B0QYT3|B0QYT3_HUMAN	RANGAP1	Ran GTPase activating protein 1 (Fragment) OS=Homo sapiens GN=RANGAP1 PE=2 SV=1	27122	574	363	28	37	0.12	0.28	2.33333333	0	1	1	1	1	1	1	1	-0.89229
tr|B4DUQ1|B4DUQ1_HUMAN	tr|B4DUQ1|B4D	cDNA FLJ54552, highly similar to Heterogeneous nuclear ribonucleoprotein K OS=Homo sapiens PE=2 SV=1	48708	147	121	147	360	0.58	1.34	2.31034483	3	11	7	14	7	14	8	16	0.059138
sp|P05165|PCCA_HUMAN	PCCA	Propionyl-CoA carboxylase alpha chain, mitochondrial OS=Homo sapiens GN=PCCA PE=1 SV=4	80635	99	69	216	555	0.49	1.13	2.30612245	6	16	10	19	13	19	13	22	-0.36254
tr|G3V2B8|G3V2B8_HUMAN	MTHFD1	C-1-tetrahydrofolate synthase, cytoplasmic OS=Homo sapiens GN=MTHFD1 PE=2 SV=1	102152	112	111	192	376	0.33	0.76	2.3030303	5	11	9	19	12	19	12	21	-0.25118
tr|B7Z2Z8|B7Z2Z8_HUMAN	tr|B7Z2Z8|B7Z2	T-complex protein 1 subunit delta OS=Homo sapiens PE=2 SV=1	52781	113	293	188	138	0.27	0.62	2.2962963	4	4	5	7	7	7	7	9	-0.89321
tr|B1AMS2|B1AMS2_HUMAN	Sep-06	Septin 6 OS=Homo sapiens GN=SEPT6 PE=3 SV=1	49614	532	611	31	39	0.07	0.16	2.28571429	1	1	1	1	1	1	1	1	-0.89229
tr|H0YDE4|H0YDE4_HUMAN	PRMT1	Protein arginine N-methyltransferase 1 (Fragment) OS=Homo sapiens GN=PRMT1 PE=4 SV=1	27345	437	329	39	134	0.26	0.59	2.26923077	1	3	2	4	2	4	2	4	-0.04409
tr|B4E091|B4E091_HUMAN	tr|B4E091|B4E0	cDNA FLJ55438, highly similar to Splicing factor 3 subunit 1 OS=Homo sapiens PE=2 SV=1	77415	482	896	36	41	0.04	0.09	2.25	1	1	1	2	1	2	1	2	-0.15514
tr|Q3B7A7|Q3B7A7_HUMAN	GART	Phosphoribosylglycinamide formyltransferase, phosphoribosylglycinamide synthetase, phosphoribosylaminoir	108909	341	185	51	248	0.13	0.29	2.23076923	1	6	4	8	3	9	4	13	0.549057
tr|Q59G92|Q59G92_HUMAN	tr|Q59G92|Q59	HLA-B associated transcript 1 variant (Fragment) OS=Homo sapiens PE=2 SV=1	35528	394	1088	44	29	0.09	0.2	2.22222222	1	0	1	2	2	3	2	4	-0.40684
tr|B3GQS7|B3GQS7_HUMAN	HSPD1	Mitochondrial heat shock 60kD protein 1 variant 1 OS=Homo sapiens GN=HSPD1 PE=2 SV=1	60813	16	49	904	714	1.72	3.81	2.21511628	24	22	27	28	26	26	33	33	-0.89616
sp|Q9NZB2|F120A_HUMAN	FAM120A	Constitutive coactivator of PPAR-gamma-like protein 1 OS=Homo sapiens GN=FAM120A PE=1 SV=2	123008	263	405	73	110	0.05	0.11	2.2	2	3	2	4	2	4	2	5	-0.04409
tr|B4DX08|B4DX08_HUMAN	tr|B4DX08|B4D	cDNA FLJ58784, highly similar to T-complex protein 1 subunit epsilon OS=Homo sapiens PE=2 SV=1	57521	90	189	229	185	0.4	0.88	2.2	6	5	6	8	6	7	6	9	-0.68643
tr|H0YF33|H0YF33_HUMAN	NASP	Nuclear autoantigenic sperm protein (Fragment) OS=Homo sapiens GN=NASP PE=4 SV=1	31883	542	1089	30	29	0.1	0.22	2.2	0	0	1	2	2	2	3	2	-0.89244
tr|E7ENU7|E7ENU7_HUMAN	RPL15	Ribosomal protein L15 (Fragment) OS=Homo sapiens GN=RPL15 PE=2 SV=1	20596	534	558	31	76	0.16	0.35	2.1875	0	2	1	2	1	3	1	3	0.330464
tr|E7EQV9|E7EQV9_HUMAN	RPL15	Ribosomal protein L15 (Fragment) OS=Homo sapiens GN=RPL15 PE=2 SV=1	20611	534	557	31	76	0.16	0.35	2.1875	0	2	1	2	1	3	1	3	0.330464
tr|A8K2Y2|A8K2Y2_HUMAN	tr|A8K2Y2|A8K2	cDNA FLJ78120, highly similar to Homo sapiens eukaryotic translation initiation factor 2, subunit 3 gamma, 52	51648	475	433	37	102	0.06	0.13	2.16666667	1	2	1	2	1	3	1	6	0.330464
tr|Q2F839|Q2F839_HUMAN	tr|Q2F839|Q2F	Heat shock 70 kDa protein 9B (Fragment) OS=Homo sapiens PE=2 SV=1	19208	81	338	59	136	0.42	0.91	2.16666667	2	3	2	4	2	4	2	5	-0.04409
tr|B4DJE3|B4DJE3_HUMAN	tr|B4DJE3|B4DJ	cDNA FLJ52929, highly similar to Dolichyl-diphosphooligosaccharide--proteinglycosyltransferase 48 kDa subun	46620	604	1064	26	31	0.07	0.15	2.14285714	0	0	1	2	1	2	2	2	-0.15514
sp|O43286|B4GT5_HUMAN	B4GALT5	Beta-1,4-galactosyltransferase 5 OS=Homo sapiens GN=B4GALT5 PE=2 SV=1	45318	651	979	22	36	0.07	0.15	2.14285714	0	1	1	2	1	2	1	2	-0.15514
tr|B4DUY3|B4DUY3_HUMAN	tr|B4DUY3|B4D	cDNA FLJ56668, highly similar to Nucleolar protein NOP5 OS=Homo sapiens PE=2 SV=1	16345	515	260	32	69	0.21	0.45	2.14285714	0	1	1	2	1	2	1	2	-0.15514
tr|B3KX72|B3KX72_HUMAN	tr|B3KX72|B3KX	cDNA FLJ44920 fis, clone BRAMY3011501, highly similar to Heterogeneous nuclear ribonucleoprotein U OS=Ho	83774	59	51	367	639	0.58	1.24	2.13793103	10	15	14	24	16	23	20	26	-0.38315
sp|Q12792|TWF1_HUMAN	TWF1	Twinfilin-1 OS=Homo sapiens GN=TWF1 PE=1 SV=3	40429	575	452	28	98	0.08	0.17	2.125	0	2	1	2	1	3	1	3	0.330464
tr|B3KT66|B3KT66_HUMAN	PSMD6	Proteasome (Prosome, macropain) 26S subunit, non-ATPase, 6, isoform CRA_c OS=Homo sapiens GN=PSMD6	40600	399	970	43	37	0.08	0.17	2.125	1	0	1	2	1	2	1	2	-0.15514
tr|E9PCI9|E9PCI9_HUMAN	FDPS	Farnesyl pyrophosphate synthase OS=Homo sapiens GN=FDPS PE=2 SV=1	40849	405	828	43	47	0.08	0.17	2.125	1	1	1	2	1	2	1	2	-0.15514
tr|B4DLB8|B4DLB8_HUMAN	tr|B4DLB8|B4D	cDNA FLJ52205, highly similar to Beta-1,4-galactosyltransferase 1 (EC 2.4.1.-) OS=Homo sapiens PE=2 SV=1	40269	650	787	22	50	0.08	0.17	2.125	0	1	1	2	1	2	1	3	-0.15514
tr|Q5TZX1|Q5TZX1_HUMAN	GNAI3	Guanine nucleotide binding protein (G protein), alpha inhibiting activity polypeptide 3 OS=Homo sapiens GN=(	41076	376	698	46	57	0.08	0.17	2.125	1	2	1	2	1	2	1	2	-0.15514
tr|Q5M7Z5|Q5M7Z5_HUMAN	GRHPR	GRHPR protein (Fragment) OS=Homo sapiens GN=GRHPR PE=2 SV=1	37314	659	935	21	38	0.09	0.19	2.11111111	0	0	1	2	1	2	1	2	-0.15514
tr|B2ZZ89|B2ZZ89_HUMAN	SPTBN1	Spectrin beta non-erythrocytic 1 OS=Homo sapiens GN=SPTBN1 PE=2 SV=1	275237	49	30	431	1167	0.22	0.46	2.09090909	11	29	17	33	20	38	24	44	0.01726
sp|Q14247|SRC8_HUMAN	CTTN	Src substrate cortactin OS=Homo sapiens GN=CTTN PE=1 SV=2	61720	378	272	46	181	0.11	0.23	2.09090909	1	3	2	4	3	4	3	4	-0.52985
tr|Q53G58|Q53G58_HUMAN	tr|Q53G58|Q53	Coronin (Fragment) OS=Homo sapiens PE=2 SV=1	53931	242	324	83	145	0.13	0.27	2.07692308	1	3	2	4	2	4	4	4	-0.04409
tr|A8K4D5|A8K4D5_HUMAN	tr|A8K4D5|A8K	Kynureninase OS=Homo sapiens PE=2 SV=1	52819	266	521	71	81	0.13	0.27	2.07692308	2	2	2	4	3	4	3	5	-0.52985
tr|Q6NZ55|Q6NZ55_HUMAN	RPL13	60S ribosomal protein L13 OS=Homo sapiens GN=RPL13 PE=2 SV=1	24308	422	671	41	60	0.14	0.29	2.07142857	1	1	1	2	2	2	2	2	-0.89244
tr|B4DTG2|B4DTG2_HUMAN	EEF1G	Elongation factor 1-gamma OS=Homo sapiens GN=EEF1G PE=2 SV=1	56456	93	80	225	488	0.86	1.77	2.05813953	5	14	15	19	14	21	18	23	-0.32475
tr|D3DPU2|D3DPU2_HUMAN	CAP1	Adenylyl cyclase-associated protein OS=Homo sapiens GN=CAP1 PE=3 SV=1	51926	172	162	125	292	0.36	0.74	2.05555556	4	7	5	9	6	10	7	13	-0.20046
sp|O00410|IPO5_HUMAN	IPO5	Importin-5 OS=Homo sapiens GN=IPO5 PE=1 SV=4	125032	85	78	236	498	0.23	0.47	2.04347826	7	11	8	16	10	17	15	20	-0.15545
tr|I3L2C7|I3L2C7_HUMAN	GEMIN4	Gem-associated protein 4 OS=Homo sapiens GN=GEMIN4 PE=4 SV=1	120456	709	960	17	37	0.03	0.06	2	0	1	1	2	0	4	1	4	2.278511
tr|H7C3A1|H7C3A1_HUMAN	SRRT	Serrate RNA effector molecule homolog (Fragment) OS=Homo sapiens GN=SRRT PE=4 SV=1	57154	701	697	18	57	0.06	0.12	2	0	1	1	2	0	2	1	2	1.430154
tr|B3KNL2|B3KNL2_HUMAN	RUVBL2	RuvB-like 2 (E. coli), isoform CRA_d OS=Homo sapiens GN=RUVBL2 PE=2 SV=1	47603	584	414	28	83	0.14	0.28	2	0	1	2	3	1	5	2	5	0.982901
tr|Q8WUM5|Q8WUM5_HUMAN	GEMIN4	Gem (Nuclear organelle) associated protein 4 OS=Homo sapiens GN=GEMIN4 PE=2 SV=1	121732	709	961	17	29	0.03	0.06	2	0	0	1	1	1	3	2	3	0.330464
sp|P13010|XRCC5_HUMAN	XRCC5	X-ray repair cross-complementing protein 5 OS=Homo sapiens GN=XRCC5 PE=1 SV=3	83222	128	144	172	319	0.36	0.72	2	6	10	8	14	8	18	9	21	0.230425
tr|B4DH52|B4DH52_HUMAN	tr|B4DH52|B4D	cDNA FLJ55916, highly similar to General transcription factor II-I OS=Homo sapiens PE=2 SV=1	112359	554	402	29	111	0.06	0.12	2	0	3	2	4	2	4	3	5	-0.04409
tr|B4E0E1|B4E0E1_HUMAN	tr|B4E0E1|B4E0	cDNA FLJ53442, highly similar to Poly (ADP-ribose) polymerase 1 (EC 2.4.2.30) OS=Homo sapiens PE=2 SV=1	111855	549	323	30	146	0.06	0.12	2	0	4	2	4	2	4	2	6	-0.04409
tr|Q2TB59|Q2TB59_HUMAN	NNT	Nicotinamide nucleotide transhydrogenase OS=Homo sapiens GN=NNT PE=2 SV=1	114552	234	318	88	147	0.06	0.12	2	1	4	2	4	2	4	3	5	-0.04409
tr|E7ET15|E7ET15_HUMAN	U2SURP	U2 snRNP-associated SURP motif-containing protein OS=Homo sapiens GN=U2SURP PE=2 SV=1	118631	647	981	22	36	0.03	0.06	2	0	0	1	2	1	2	2	2	-0.15514
sp|O95831|AIFM1_HUMAN	AIFM1	Apoptosis-inducing factor 1, mitochondrial OS=Homo sapiens GN=AIFM1 PE=1 SV=1	67144	326	844	54	45	0.05	0.1	2	1	1	1	2	1	2	2	2	-0.15514
tr|I3L0N3|I3L0N3_HUMAN	NSF	Vesicle-fusing ATPase OS=Homo sapiens GN=NSF PE=3 SV=1	82553	412	798	42	49	0.04	0.08	2	1	1	1	2	1	2	1	7	-0.15514
tr|F8W930|F8W930_HUMAN	IGF2BP2	Insulin-like growth factor 2 mRNA-binding protein 2 OS=Homo sapiens GN=IGF2BP2 PE=2 SV=1	66859	556	728	29	54	0.05	0.1	2	0	2	1	2	1	2	1	2	-0.15514
tr|J3KN69|J3KN69_HUMAN	NCEH1	Arylacetamide deacetylase-like 1 OS=Homo sapiens GN=NCEH1 PE=4 SV=1	49431	663	675	21	59	0.07	0.14	2	0	1	1	2	1	2	1	2	-0.15514
tr|B3KXU8|B3KXU8_HUMAN	tr|B3KXU8|B3K	cDNA FLJ46087 fis, clone TESTI2009497, highly similar to GPI-anchored protein p137 OS=Homo sapiens PE=2 S	70489	367	599	47	68	0.05	0.1	2	1	2	1	2	1	2	1	2	-0.15514
sp|O14929|HAT1_HUMAN	HAT1	Histone acetyltransferase type B catalytic subunit OS=Homo sapiens GN=HAT1 PE=1 SV=1	49880	343	305	51	154	0.07	0.14	2	1	2	1	2	1	2	1	4	-0.15514
sp|Q14315|FLNC_HUMAN	FLNC	Filamin-C OS=Homo sapiens GN=FLNC PE=1 SV=3	293407	143	198	153	248	0.06	0.12	2	5	8	6	12	10	14	11	18	-0.42729
sp|Q6PGP7|TTC37_HUMAN	TTC37	Tetratricopeptide repeat protein 37 OS=Homo sapiens GN=TTC37 PE=1 SV=1	177485	498	1061	34	31	0.02	0.04	2	1	0	1	2	1	1	1	3	-0.89229
sp|Q9NPQ8|RIC8A_HUMAN	RIC8A	Synembryn-A OS=Homo sapiens GN=RIC8A PE=1 SV=3	60185	496	837	34	46	0.05	0.1	2	0	1	1	1	1	1	1	1	-0.89229
tr|J3KNE5|J3KNE5_HUMAN	HARS	Histidine--tRNA ligase, cytoplasmic OS=Homo sapiens GN=HARS PE=4 SV=1	50581	672	597	20	30	0.07	0.14	2	0	0	1	1	1	1	2	2	-0.89229
tr|B3KUZ8|B3KUZ8_HUMAN	tr|B3KUZ8|B3K	Aspartate aminotransferase OS=Homo sapiens PE=2 SV=1	41699	522	545	31	77	0.08	0.16	2	0	1	1	2	1	1	2	2	-0.89229
tr|H0YLA4|H0YLA4_HUMAN	SORD	Sorbitol dehydrogenase OS=Homo sapiens GN=SORD PE=3 SV=1	36833	473	752	37	52	0.09	0.18	2	0	1	1	1	2	1	2	2	-1.62959
sp|O00629|IMA4_HUMAN	KPNA4	Importin subunit alpha-4 OS=Homo sapiens GN=KPNA4 PE=1 SV=1	58364	472	705	37	57	0.06	0.12	2	0	1	1	2	2	1	2	3	-1.62959
tr|F5H7Y0|F5H7Y0_HUMAN	PDLIM5	PDZ and LIM domain protein 5 OS=Homo sapiens GN=PDLIM5 PE=2 SV=1	52332	175	117	109	325	0.41	0.81	1.97560976	2	8	5	9	5	9	6	11	-0.10369
tr|B4DQX0|B4DQX0_HUMAN	tr|B4DQX0|B4D	cDNA FLJ59219, highly similar to Poly(A)-binding protein 1 OS=Homo sapiens PE=2 SV=1	67239	215	179	96	260	0.33	0.65	1.96969697	3	7	6	10	6	12	10	13	0.051438
tr|J3KN86|J3KN86_HUMAN	RPS3	40S ribosomal protein S3 OS=Homo sapiens GN=RPS3 PE=3 SV=1	28640	480	827	36	47	0.12	0.23	1.91666667	1	1	1	1	1	1	4	1	-0.89229
sp|P07814|SYEP_HUMAN	EPRS	Bifunctional glutamate/proline--tRNA ligase OS=Homo sapiens GN=EPRS PE=1 SV=5	172080	57	60	380	636	0.33	0.63	1.90909091	9	15	15	26	15	30	17	34	0.084797
sp|Q15149|PLEC_HUMAN	PLEC	Plectin OS=Homo sapiens GN=PLEC PE=1 SV=3	533462	74	43	270	909	0.11	0.21	1.90909091	11	22	17	31	18	35	32	43	0.048448
sp|Q96TA1|NIBL1_HUMAN	FAM129B	Niban-like protein 1 OS=Homo sapiens GN=FAM129B PE=1 SV=3	84598	155	386	137	114	0.16	0.3	1.875	4	4	4	7	4	7	4	8	-0.15524
tr|A4QPB0|A4QPB0_HUMAN	IQGAP1	IQ motif containing GTPase activating protein 1 OS=Homo sapiens GN=IQGAP1 PE=2 SV=1	189789	84	37	239	955	0.31	0.58	1.87096774	8	20	17	27	18	34	21	37	0.007044
tr|B4DR70|B4DR70_HUMAN	tr|B4DR70|B4D	cDNA FLJ58049, highly similar to RNA-binding protein FUS OS=Homo sapiens PE=2 SV=1	45013	450	788	38	50	0.07	0.13	1.85714286	1	1	1	2	1	2	1	2	-0.15514
sp|Q3ZCM7|TBB8_HUMAN	TUBB8	Tubulin beta-8 chain OS=Homo sapiens GN=TUBB8 PE=1 SV=2	50257	67	62	306	600	0.77	1.43	1.85714286	13	23	17	25	19	28	21	33	-0.34596
tr|F5H5D3|F5H5D3_HUMAN	TUBA1C	Tubulin alpha-1C chain OS=Homo sapiens GN=TUBA1C PE=2 SV=1	58606	21	24	809	1421	2.92	5.4	1.84931507	24	36	34	52	37	57	41	59	-0.27758
tr|B2R6J2|B2R6J2_HUMAN	tr|B2R6J2|B2R6	cDNA, FLJ92973, highly similar to Homo sapiens villin 2 (ezrin) (VIL2), mRNA OS=Homo sapiens PE=2 SV=1	69472	126	174	173	277	0.32	0.59	1.84375	6	9	6	11	8	13	8	17	-0.22504
tr|Q5U077|Q5U077_HUMAN	LDHB	L-lactate dehydrogenase OS=Homo sapiens GN=LDHB PE=2 SV=1	36900	39	67	530	559	1.81	3.32	1.83425414	13	16	16	21	18	23	20	23	-0.54888
tr|K7EIH8|K7EIH8_HUMAN	DNAJC7	DnaJ homolog subfamily C member 7 (Fragment) OS=Homo sapiens GN=DNAJC7 PE=4 SV=1	30449	527	749	31	52	0.06	0.11	1.83333333	0	1	1	1	1	1	1	1	-0.89229
sp|P00390|GSHR_HUMAN	GSR	Glutathione reductase, mitochondrial OS=Homo sapiens GN=GSR PE=1 SV=2	56791	313	460	58	96	0.18	0.33	1.83333333	1	2	3	5	4	4	4	6	-0.89275
tr|B2R5W2|B2R5W2_HUMAN	tr|B2R5W2|B2R	cDNA, FLJ92657, highly similar to Homo sapiens heterogeneous nuclear ribonucleoprotein C (C1/C2) (HNRPC),	31986	178	379	122	117	0.35	0.64	1.82857143	3	5	3	5	3	7	4	7	0.207659
tr|Q2XPP3|Q2XPP3_HUMAN	tr|Q2XPP3|Q2X	Type II 3a-hydroxysteroid dehydrogenase variant OS=Homo sapiens PE=2 SV=1	37201	203	419	102	105	0.29	0.53	1.82758621	3	2	3	5	3	5	4	5	-0.24016
tr|G3V2A3|G3V2A3_HUMAN	TUBB3	Tubulin beta-3 chain (Fragment) OS=Homo sapiens GN=TUBB3 PE=2 SV=1	20929	25	28	147	543	3.07	5.58	1.81758958	5	14	6	17	7	21	7	26	0.628684
tr|B4DF96|B4DF96_HUMAN	EIF2A	Eukaryotic translation initiation factor 2A OS=Homo sapiens GN=EIF2A PE=2 SV=1	58357	206	294	101	111	0.32	0.58	1.8125	2	3	5	6	4	8	5	9	0.025508
tr|H3BLZ8|H3BLZ8_HUMAN	DDX17	Probable ATP-dependent RNA helicase DDX17 OS=Homo sapiens GN=DDX17 PE=3 SV=1	81073	166	204	130	242	0.37	0.67	1.81081081	3	5	9	14	9	14	10	19	-0.28257
tr|Q59ES3|Q59ES3_HUMAN	tr|Q59ES3|Q59	Solute carrier family 1 (Neutral amino acid transporter), member 5 variant (Fragment) OS=Homo sapiens PE=2	57810	290	778	63	50	0.1	0.18	1.8	1	1	1	3	1	2	1	3	-0.15514
tr|B3KMW5|B3KMW5_HUMAN	tr|B3KMW5|B3	cDNA FLJ12796 fis, clone NT2RP2002058, highly similar to WD repeat protein 3 OS=Homo sapiens PE=2 SV=1	71519	408	694	43	33	0.05	0.09	1.8	1	0	1	2	1	2	1	4	-0.15514
tr|B3KQT2|B3KQT2_HUMAN	tr|B3KQT2|B3K	cDNA PSEC0148 fis, clone PLACE1007202, highly similar to Protein disulfide-isomerase A3 (EC 5.3.4.1) OS=Hon	55268	546	559	30	76	0.19	0.34	1.78947368	0	2	3	5	2	4	8	7	-0.04409
tr|Q59EG8|Q59EG8_HUMAN	tr|Q59EG8|Q59	Proteasome 26S non-ATPase subunit 2 variant (Fragment) OS=Homo sapiens PE=2 SV=1	101322	198	210	105	237	0.14	0.25	1.78571429	1	4	4	7	4	8	5	11	0.025508
sp|P68363|TBA1B_HUMAN	TUBA1B	Tubulin alpha-1B chain OS=Homo sapiens GN=TUBA1B PE=1 SV=1	50804	13	19	1091	1557	5.58	9.89	1.77240143	31	39	43	56	45	60	49	62	-0.48533
sp|Q71U36|TBA1A_HUMAN	TUBA1A	Tubulin alpha-1A chain OS=Homo sapiens GN=TUBA1A PE=1 SV=1	50788	15	23	1022	1428	5.18	9.17	1.77027027	30	36	41	52	43	56	46	58	-0.51922
sp|Q92621|NU205_HUMAN	NUP205	Nuclear pore complex protein Nup205 OS=Homo sapiens GN=NUP205 PE=1 SV=3	230171	415	299	42	163	0.04	0.07	1.75	0	3	3	5	3	5	5	7	-0.24016
tr|G5E9Q2|G5E9Q2_HUMAN	CSDE1	Cold shock domain containing E1, RNA-binding, isoform CRA_d OS=Homo sapiens GN=CSDE1 PE=2 SV=1	94539	312	311	58	152	0.11	0.19	1.72727273	1	3	3	5	3	6	3	6	0.001029
tr|E9PHF7|E9PHF7_HUMAN	MCCC1	Methylcrotonoyl-CoA carboxylase subunit alpha, mitochondrial OS=Homo sapiens GN=MCCC1 PE=2 SV=1	68745	102	58	214	629	0.83	1.4	1.68674699	7	17	13	23	11	25	16	30	0.257557
tr|B2RCT6|B2RCT6_HUMAN	tr|B2RCT6|B2R	cDNA, FLJ96276, highly similar to Homo sapiens G1 to S phase transition 1 (GSPT1), mRNA OS=Homo sapiens F	56320	391	1049	44	30	0.12	0.2	1.66666667	1	1	2	2	2	4	2	4	-0.04409
tr|Q5HYM2|Q5HYM2_HUMAN	DKFZp686O246	Putative uncharacterized protein DKFZp686O2462 (Fragment) OS=Homo sapiens GN=DKFZp686O2462 PE=2 S\	72627	569	276	29	179	0.09	0.15	1.66666667	0	3	2	4	2	4	2	5	-0.04409
tr|Q4J697|Q4J697_HUMAN	PMCA2	Plasma membrane Ca(2+)-ATPase isoform 2 (Fragment) OS=Homo sapiens GN=PMCA2 PE=2 SV=1	122274	528	497	31	46	0.03	0.05	1.66666667	0	1	1	1	1	1	2	1	-0.89229
tr|B4DPZ3|B4DPZ3_HUMAN	tr|B4DPZ3|B4D	cDNA FLJ53290, highly similar to Cytoplasmic dynein 1 intermediate chain 2 OS=Homo sapiens PE=2 SV=1	70869	298	421	60	105	0.09	0.15	1.66666667	2	2	2	3	2	2	2	4	-0.89244
sp|Q1KMD3|HNRL2_HUMAN	HNRNPUL2	Heterogeneous nuclear ribonucleoprotein U-like protein 2 OS=Homo sapiens GN=HNRNPUL2 PE=1 SV=1	85622	249	168	78	287	0.21	0.35	1.66666667	2	6	5	8	6	10	6	11	-0.20046
tr|B7Z4V2|B7Z4V2_HUMAN	tr|B7Z4V2|B7Z4	cDNA FLJ51907, highly similar to Stress-70 protein, mitochondrial OS=Homo sapiens PE=2 SV=1	72641	81	140	251	326	0.42	0.7	1.66666667	7	9	8	12	8	13	8	14	-0.22504
sp|Q92973|TNPO1_HUMAN	TNPO1	Transportin-1 OS=Homo sapiens GN=TNPO1 PE=1 SV=2	103771	139	339	158	136	0.17	0.28	1.64705882	3	4	5	8	8	9	8	9	-0.73272
tr|J3KTA4|J3KTA4_HUMAN	DDX5	Probable ATP-dependent RNA helicase DDX5 OS=Homo sapiens GN=DDX5 PE=3 SV=1	69557	97	180	218	268	0.45	0.74	1.64444444	7	6	9	12	10	14	10	15	-0.42729
tr|B4DHG3|B4DHG3_HUMAN	tr|B4DHG3|B4D	cDNA FLJ55329, highly similar to Procollagen-lysine,2-oxoglutarate5-dioxygenase 2 (EC 1.14.11.4) OS=Homo sa	81716	453	362	38	124	0.08	0.13	1.625	0	3	2	3	2	3	2	3	-0.40684
sp|Q9NR12|PDLI7_HUMAN	PDLIM7	PDZ and LIM domain protein 7 OS=Homo sapiens GN=PDLIM7 PE=1 SV=1	50896	396	663	43	60	0.13	0.21	1.61538462	0	1	2	3	2	4	4	5	-0.04409
tr|Q05BW9|Q05BW9_HUMAN	PAPSS1	PAPSS1 protein (Fragment) OS=Homo sapiens GN=PAPSS1 PE=2 SV=1	65264	553	403	29	110	0.1	0.16	1.6	0	3	2	3	1	3	2	3	0.330464
sp|Q14432|PDE3A_HUMAN	PDE3A	cGMP-inhibited 3~,5~-cyclic phosphodiesterase A OS=Homo sapiens GN=PDE3A PE=1 SV=3	126440	493	485	35	90	0.05	0.08	1.6	1	3	2	3	2	3	2	3	-0.40684
tr|Q96QL0|Q96QL0_HUMAN	RPL3	Ribosomal protein L3 OS=Homo sapiens GN=RPL3 PE=2 SV=1	46376	245	274	82	159	0.32	0.51	1.59375	4	5	4	6	5	7	7	9	-0.44509
sp|P23381|SYWC_HUMAN	WARS	Tryptophan--tRNA ligase, cytoplasmic OS=Homo sapiens GN=WARS PE=1 SV=2	53474	145	253	148	201	0.27	0.43	1.59259259	3	4	4	6	5	6	5	6	-0.65172
tr|K9JA46|K9JA46_HUMAN	EL52	Epididymis luminal secretory protein 52 OS=Homo sapiens GN=EL52 PE=2 SV=1	85006	46	48	462	799	1.05	1.67	1.59047619	15	22	25	28	24	34	29	35	-0.40023
sp|Q9Y3I0|RTCB_HUMAN	C22orf28	tRNA-splicing ligase RtcB homolog OS=Homo sapiens GN=C22orf28 PE=1 SV=1	55688	458	887	38	42	0.12	0.19	1.58333333	0	1	2	3	2	3	2	4	-0.40684
sp|Q9NY65|TBA8_HUMAN	TUBA8	Tubulin alpha-8 chain OS=Homo sapiens GN=TUBA8 PE=1 SV=1	50746	34	47	592	874	1.74	2.75	1.58045977	15	23	23	33	23	34	27	34	-0.33977
tr|B2R7M3|B2R7M3_HUMAN	tr|B2R7M3|B2R	cDNA, FLJ93510, highly similar to Homo sapiens JTV1 gene (JTV1), mRNA OS=Homo sapiens PE=2 SV=1	35767	308	567	58	74	0.19	0.3	1.57894737	2	3	2	3	2	3	2	3	-0.40684
sp|Q9Y305|ACOT9_HUMAN	ACOT9	Acyl-coenzyme A thioesterase 9, mitochondrial OS=Homo sapiens GN=ACOT9 PE=1 SV=2	50269	637	790	22	50	0.07	0.11	1.57142857	0	1	1	1	1	3	2	3	0.330464
tr|B2RAQ8|B2RAQ8_HUMAN	tr|B2RAQ8|B2R	cDNA, FLJ95058, highly similar to Homo sapiens carnitine palmitoyltransferase 1A (liver) (CPT1A),nuclear gene	88967	354	384	49	115	0.07	0.11	1.57142857	1	2	2	3	3	4	3	4	-0.52985
tr|Q53ET2|Q53ET2_HUMAN	tr|Q53ET2|Q53	Dihydropyrimidinase-like 2 variant (Fragment) OS=Homo sapiens PE=2 SV=1	62688	342	207	51	238	0.23	0.36	1.56521739	1	5	4	6	3	7	5	8	0.207659
sp|Q9BYZ2|LDH6B_HUMAN	LDHAL6B	L-lactate dehydrogenase A-like 6B OS=Homo sapiens GN=LDHAL6B PE=1 SV=3	42372	327	317	54	147	0.16	0.25	1.5625	1	4	2	4	2	4	2	4	-0.04409
tr|E9PBU3|E9PBU3_HUMAN	ATIC	Phosphoribosylaminoimidazolecarboxamide formyltransferase OS=Homo sapiens GN=ATIC PE=2 SV=1	64996	72	177	274	273	0.64	0.99	1.546875	8	6	11	14	12	16	13	18	-0.49273
tr|G3V1S6|G3V1S6_HUMAN	GALNT2	Polypeptide N-acetylgalactosaminyltransferase 2 soluble form OS=Homo sapiens GN=GALNT2 PE=2 SV=1	61440	254	546	76	77	0.11	0.17	1.54545455	2	2	2	3	2	4	2	4	-0.04409
tr|H0Y5G9|H0Y5G9_HUMAN	NRD1	Nardilysin (Fragment) OS=Homo sapiens GN=NRD1 PE=3 SV=1	62196	225	279	92	73	0.11	0.17	1.54545455	2	2	2	4	2	4	2	4	-0.04409
tr|B4DII5|B4DII5_HUMAN	tr|B4DII5|B4DII	Importin subunit alpha OS=Homo sapiens PE=2 SV=1	60345	573	435	28	101	0.11	0.17	1.54545455	0	2	2	3	3	3	3	3	-0.8926
tr|Q05CK9|Q05CK9_HUMAN	SYNCRIP	SYNCRIP protein (Fragment) OS=Homo sapiens GN=SYNCRIP PE=2 SV=1	50847	252	453	76	98	0.13	0.2	1.53846154	2	3	2	4	2	5	2	6	0.245601
tr|Q05CU6|Q05CU6_HUMAN	RDX	RDX protein (Fragment) OS=Homo sapiens GN=RDX PE=2 SV=1	40814	126	352	94	127	0.32	0.48	1.5	3	5	3	6	4	7	4	9	-0.15524
tr|Q9BSV4|Q9BSV4_HUMAN	SFPQ	SFPQ protein (Fragment) OS=Homo sapiens GN=SFPQ PE=2 SV=2	68702	228	242	91	209	0.26	0.39	1.5	2	5	5	7	6	8	7	10	-0.50568
sp|O00505|IMA3_HUMAN	KPNA3	Importin subunit alpha-3 OS=Homo sapiens GN=KPNA3 PE=1 SV=2	58288	406	1046	43	32	0.12	0.18	1.5	0	1	2	3	2	2	2	3	-0.89244
sp|Q96P70|IPO9_HUMAN	IPO9	Importin-9 OS=Homo sapiens GN=IPO9 PE=1 SV=3	116858	580	508	28	83	0.06	0.09	1.5	0	2	2	3	2	2	2	5	-0.89244
tr|J3KN66|J3KN66_HUMAN	TOR1AIP1	Torsin-1A-interacting protein 1 OS=Homo sapiens GN=TOR1AIP1 PE=4 SV=1	67952	707	618	17	66	0.1	0.15	1.5	0	1	1	1	0	1	1	1	0.693008
tr|H0YER0|H0YER0_HUMAN	PABPC4	Polyadenylate-binding protein 4 (Fragment) OS=Homo sapiens GN=PABPC4 PE=4 SV=1	7211	215	1011	33	35	0.33	0.49	1.48484848	0	0	1	1	1	2	1	2	-0.15514

tr|I3L492|I3L492_HUMAN	ETF1	Eukaryotic peptide chain release factor subunit 1 (Fragment) OS=Homo sapiens GN=ETF1 PE=4 SV=1	23028	274	528	69	54	0.15	0.22	1.46666667	1	1	1	1	1	1	1	1	-0.89229
tr|B4DRH6|B4DRH6_HUMAN	tr|B4DRH6|B4D	cDNA FLJ54509, highly similar to Trifunctional enzyme subunit alpha, mitochondrial OS=Homo sapiens PE=2 S\	79007	133	264	164	185	0.28	0.41	1.46428571	5	4	6	9	7	8	7	11	-0.71246
tr|H0Y804|H0Y804_HUMAN	AKR1C1	Aldo-keto reductase family 1 member C1 (Fragment) OS=Homo sapiens GN=AKR1C1 PE=4 SV=1	28617	186	214	61	94	0.67	0.98	1.46268657	2	2	4	4	4	3	6	4	-1.2555
tr|Q562P0|Q562P0_HUMAN	ACT	Actin-like protein (Fragment) OS=Homo sapiens GN=ACT PE=2 SV=1	11479	151	134	143	337	1.87	2.73	1.45989305	7	11	8	15	10	16	11	22	-0.24052
tr|K7EPJ3|K7EPJ3_HUMAN	DDX39A	ATP-dependent RNA helicase DDX39A (Fragment) OS=Homo sapiens GN=DDX39A PE=4 SV=1	21690	395	1166	44	23	0.11	0.16	1.45454545	1	0	1	1	1	1	2	4	-0.89229
tr|Q4W4Y1|Q4W4Y1_HUMAN	DRIP4	Dopamine receptor interacting protein 4 OS=Homo sapiens GN=DRIP4 PE=2 SV=1	96646	136	186	160	260	0.18	0.26	1.44444444	2	5	5	7	5	7	6	7	-0.44509
tr|B4DN87|B4DN87_HUMAN	tr|B4DN87|B4D	cDNA FLJ52569, highly similar to Collagen-binding protein 2 OS=Homo sapiens PE=2 SV=1	44290	233	249	89	179	0.43	0.62	1.44186047	4	5	5	6	5	7	5	11	-0.44509
tr|Q7Z5T5|Q7Z5T5_HUMAN	EIF3A	EIF3A protein (Fragment) OS=Homo sapiens GN=EIF3A PE=2 SV=1	97203	192	236	112	211	0.14	0.2	1.42857143	2	6	4	9	5	10	8	12	0.040881
tr|D3DQA6|D3DQA6_HUMAN	DDX46	DEAD (Asp-Glu-Ala-Asp) box polypeptide 46, isoform CRA_a OS=Homo sapiens GN=DDX46 PE=3 SV=1	100958	595	392	26	113	0.07	0.1	1.42857143	0	2	2	3	3	4	3	5	-0.52985
sp|Q9H853|TBA4B_HUMAN	TUBA4B	Putative tubulin-like protein alpha-4B OS=Homo sapiens GN=TUBA4B PE=5 SV=2	27819	146	252	147	202	0.4	0.57	1.425	3	5	4	6	4	6	6	6	-0.36188
tr|Q53GX7|Q53GX7_HUMAN	tr|Q53GX7|Q53	Threonyl-tRNA synthetase variant (Fragment) OS=Homo sapiens PE=2 SV=1	84304	94	127	221	343	0.64	0.91	1.421875	6	9	13	18	17	24	18	26	-0.40807
tr|B1AHC9|B1AHC9_HUMAN	XRCC6	X-ray repair complementing defective repair in Chinese hamster cells 6 (Ku autoantigen, 70kDa) OS=Homo sap	64528	116	173	184	278	0.64	0.91	1.421875	5	5	10	13	11	12	12	19	-0.77336
tr|K7EIG1|K7EIG1_HUMAN	CLUH	Clustered mitochondria protein homolog OS=Homo sapiens GN=CLUH PE=3 SV=1	140903	438	603	39	68	0.05	0.07	1.4	1	1	2	3	2	3	2	3	-0.40684
sp|Q5T9A4|ATD3B_HUMAN	ATAD3B	ATPase family AAA domain-containing protein 3B OS=Homo sapiens GN=ATAD3B PE=1 SV=1	73098	281	541	68	78	0.1	0.14	1.4	2	2	2	3	2	3	2	5	-0.40684
tr|B3KNM0|B3KNM0_HUMAN	tr|B3KNM0|B3K	cDNA FLJ14916 fis, clone PLACE1006958, highly similar to Heat shock 70 kDa protein 4L (Fragment) OS=Homo	64588	191	283	113	168	0.1	0.14	1.4	2	3	2	3	2	3	3	3	-0.40684
tr|B7Z5E2|B7Z5E2_HUMAN	PSMC2	26S protease regulatory subunit 7 OS=Homo sapiens GN=PSMC2 PE=2 SV=1	33594	610	573	25	48	0.1	0.14	1.4	0	1	1	1	1	1	1	1	-0.89229
tr|Q5T081|Q5T081_HUMAN	RCC1	CHC1 protein OS=Homo sapiens GN=RCC1 PE=2 SV=1	45397	269	409	70	109	0.23	0.32	1.39130435	2	3	3	4	2	4	4	4	-0.04409
tr|B4DEM7|B4DEM7_HUMAN	CCT8	T-complex protein 1 subunit theta OS=Homo sapiens GN=CCT8 PE=2 SV=1	58179	268	246	70	206	0.18	0.25	1.38888889	2	4	3	4	5	4	6	4	-1.18259
sp|P55072|TERA_HUMAN	VCP	Transitional endoplasmic reticulum ATPase OS=Homo sapiens GN=VCP PE=1 SV=4	89950	64	56	326	689	0.65	0.9	1.38461538	7	18	14	19	14	20	15	21	-0.39364
tr|Q53SS8|Q53SS8_HUMAN	PCBP1	Poly(RC) binding protein 1 OS=Homo sapiens GN=PCBP1 PE=2 SV=1	37987	110	256	197	197	0.29	0.4	1.37931034	3	4	3	4	3	4	3	5	-0.52985
tr|B4DKZ9|B4DKZ9_HUMAN	tr|B4DKZ9|B4D	cDNA FLJ55705, highly similar to Threonyl-tRNA synthetase, cytoplasmic (EC 6.1.1.3) OS=Homo sapiens PE=2 S	71171	94	166	191	288	0.64	0.88	1.375	5	7	10	15	13	20	14	22	-0.29021
sp|Q96HC4|PDLI5_HUMAN	PDLIM5	PDZ and LIM domain protein 5 OS=Homo sapiens GN=PDLIM5 PE=1 SV=5	65102	175	122	123	357	0.41	0.56	1.36585366	3	9	7	10	7	11	8	14	-0.27582
tr|B2R9K8|B2R9K8_HUMAN	tr|B2R9K8|B2R	cDNA, FLJ94440, highly similar to Homo sapiens chaperonin containing TCP1, subunit 6A (zeta 1)(CCT6A), mRN	58386	118	215	181	233	0.47	0.64	1.36170213	5	5	7	9	9	13	10	13	-0.38584
tr|H0Y8G5|H0Y8G5_HUMAN	HNRNPD	Heterogeneous nuclear ribonucleoprotein D0 (Fragment) OS=Homo sapiens GN=HNRNPD PE=4 SV=1	29877	229	730	90	36	0.37	0.5	1.35135135	3	0	3	2	4	3	4	3	-1.2555
tr|B7Z4F6|B7Z4F6_HUMAN	tr|B7Z4F6|B7Z4	cDNA FLJ54912, highly similar to 60 kDa heat shock protein, mitochondrial OS=Homo sapiens PE=2 SV=1	58705	16	68	699	558	1.72	2.32	1.34883721	18	17	21	22	20	22	27	27	-0.76056
tr|B4DNN4|B4DNN4_HUMAN	tr|B4DNN4|B4D	Ribonucleoside-diphosphate reductase OS=Homo sapiens PE=2 SV=1	87252	156	258	136	197	0.29	0.39	1.34482759	5	6	7	9	8	10	8	13	-0.58815
sp|P16615|AT2A2_HUMAN	ATP2A2	Sarcoplasmic/endoplasmic reticulum calcium ATPase 2 OS=Homo sapiens GN=ATP2A2 PE=1 SV=1	116336	149	103	145	400	0.32	0.43	1.34375	4	9	10	13	12	14	13	17	-0.6795
tr|E9PIM9|E9PIM9_HUMAN	RNH1	Ribonuclease inhibitor (Fragment) OS=Homo sapiens GN=RNH1 PE=2 SV=1	22362	431	300	40	110	0.15	0.2	1.33333333	0	2	1	2	1	2	1	2	-0.15514
tr|B3KNN7|B3KNN7_HUMAN	tr|B3KNN7|B3K	cDNA FLJ30049 fis, clone ADRGL1000033, highly similar to 26S proteasome non-ATPase regulatory subunit 3 C	57311	273	322	69	146	0.18	0.24	1.33333333	3	4	3	4	3	4	3	4	-0.52985
tr|H7BZJ3|H7BZJ3_HUMAN	PDIA3	Thioredoxin (Fragment) OS=Homo sapiens GN=PDIA3 PE=3 SV=1	13739	546	1107	22	28	0.19	0.25	1.31578947	0	0	1	1	1	1	1	2	-0.89229
tr|B4DXW6|B4DXW6_HUMAN	tr|B4DXW6|B4I	cDNA FLJ50285, highly similar to Arginyl-tRNA synthetase (EC 6.1.1.19) OS=Homo sapiens PE=2 SV=1	52771	235	200	88	186	0.35	0.46	1.31428571	3	6	5	6	6	8	7	9	-0.50568
tr|B3KU96|B3KU96_HUMAN	tr|B3KU96|B3K	cDNA FLJ39433 fis, clone PROST2004142, highly similar to GDP-L-fucose synthetase (EC 1.1.1.271) OS=Homo s	17856	371	904	47	37	0.19	0.24	1.26315789	1	1	1	1	1	1	1	1	-0.89229
tr|B0YJC4|B0YJC4_HUMAN	VIM	Vimentin OS=Homo sapiens GN=VIM PE=3 SV=1	49680	66	164	312	267	1.03	1.3	1.26213592	9	10	11	13	12	14	15	15	-0.6795
tr|Q53HF2|Q53HF2_HUMAN	tr|Q53HF2|Q53	Heat shock 70kDa protein 8 isoform 2 variant (Fragment) OS=Homo sapiens PE=2 SV=1	53580	51	61	426	621	1.45	1.83	1.26206897	12	13	17	23	19	25	19	30	-0.50696
tr|B4DH02|B4DH02_HUMAN	tr|B4DH02|B4D	cDNA FLJ50510, highly similar to Heat shock 70 kDa protein 4 OS=Homo sapiens PE=2 SV=1	95157	123	145	175	319	0.23	0.29	1.26086957	4	6	6	7	5	7	6	7	-0.44509
sp|P18206|VINC_HUMAN	VCL	Vinculin OS=Homo sapiens GN=VCL PE=1 SV=4	124292	68	160	299	295	0.37	0.46	1.24324324	8	9	12	13	13	14	13	18	-0.79087
tr|B4DPP6|B4DPP6_HUMAN	tr|B4DPP6|B4D	cDNA FLJ54371, highly similar to Serum albumin OS=Homo sapiens PE=2 SV=1	72370	222	243	93	208	0.25	0.31	1.24	3	6	5	7	6	7	10	9	-0.68643
tr|B4DKT3|B4DKT3_HUMAN	tr|B4DKT3|B4D	cDNA FLJ58390, highly similar to Aldo-keto reductase family 1 member C3homolog (EC 1.-.-.-) OS=Homo sapie	15755	186	238	42	164	0.67	0.82	1.2238806	1	3	1	5	1	5	1	5	0.982901
tr|B3KW67|B3KW67_HUMAN	tr|B3KW67|B3K	cDNA FLJ42347 fis, clone UTERU2003399, highly similar to Actin, gamma-enteric smooth muscle OS=Homo sap	42189	38	44	538	907	2.61	3.19	1.22222222	19	26	27	32	32	41	37	48	-0.54279
sp|P08729|K2C7_HUMAN	KRT7	Keratin, type II cytoskeletal 7 OS=Homo sapiens GN=KRT7 PE=1 SV=5	51411	259	230	74	217	0.45	0.55	1.22222222	2	7	6	7	7	7	9	7	-0.89321
sp|Q15046|SYK_HUMAN	KARS	Lysine--tRNA ligase OS=Homo sapiens GN=KARS PE=1 SV=3	68461	265	262	71	193	0.32	0.39	1.21875	1	5	6	7	6	8	7	9	-0.50568
tr|B4DT47|B4DT47_HUMAN	tr|B4DT47|B4D	cDNA FLJ54507, highly similar to Heat shock 70 kDa protein 4 OS=Homo sapiens PE=2 SV=1	77886	123	191	147	257	0.23	0.28	1.2173913	3	5	5	6	4	7	5	7	-0.15524
tr|Q5I6Y6|Q5I6Y6_HUMAN	LMNA	Lamin A/C transcript variant 1 OS=Homo sapiens GN=LMNA PE=2 SV=1	74322	95	98	221	412	0.75	0.91	1.21333333	7	12	13	15	15	16	15	19	-0.80407
tr|B0QYA5|B0QYA5_HUMAN	EIF3D	Eukaryotic translation initiation factor 3 subunit D (Fragment) OS=Homo sapiens GN=EIF3D PE=4 SV=1	31937	710	445	17	99	0.1	0.12	1.2	0	1	1	2	0	2	2	2	1.430154
sp|P68032|ACTC_HUMAN	ACTC1	Actin, alpha cardiac muscle 1 OS=Homo sapiens GN=ACTC1 PE=1 SV=1	42334	23	33	771	1086	6.63	7.86	1.18552036	25	33	40	46	48	58	55	66	-0.62733
tr|Q2VPA0|Q2VPA0_HUMAN	LONP1	Lon protease homolog (Fragment) OS=Homo sapiens GN=LONP1 PE=2 SV=1	95436	414	564	42	74	0.11	0.13	1.18181818	2	2	3	4	3	4	3	5	-0.52985
tr|B4DUL5|B4DUL5_HUMAN	tr|B4DUL5|B4D	cDNA FLJ51625, highly similar to Ubiquinol-cytochrome-c reductase complex coreprotein I, mitochondrial (EC	40917	357	495	48	86	0.17	0.2	1.17647059	1	3	2	3	2	3	2	3	-0.40684
tr|Q96K98|Q96K98_HUMAN	tr|Q96K98|Q96	cDNA FLJ14414 fis, clone HEMBA1004847, highly similar to SIGNAL RECOGNITION PARTICLE 68 KD PROTEIN O	37365	180	347	119	102	0.18	0.21	1.16666667	2	3	2	3	2	4	4	5	-0.04409
tr|A8K6Q8|A8K6Q8_HUMAN	tr|A8K6Q8|A8K	cDNA FLJ75881, highly similar to Homo sapiens transferrin receptor (p90, CD71) (TFRC), mRNA OS=Homo sapi	85276	122	154	176	303	0.3	0.35	1.16666667	6	7	7	8	7	10	8	11	-0.40725
tr|A6NG51|A6NG51_HUMAN	SPTAN1	Spectrin alpha chain, non-erythrocytic 1 OS=Homo sapiens GN=SPTAN1 PE=2 SV=2	285628	167	301	130	158	0.06	0.07	1.16666667	2	3	5	6	6	7	9	9	-0.68643
sp|Q9NR30|DDX21_HUMAN	DDX21	Nucleolar RNA helicase 2 OS=Homo sapiens GN=DDX21 PE=1 SV=5	87804	148	237	147	211	0.34	0.39	1.14705882	4	6	8	10	8	10	9	12	-0.58815
tr|A8K525|A8K525_HUMAN	tr|A8K525|A8K	cDNA FLJ76817, highly similar to Homo sapiens non-POU domain containing, octamer-binding (NONO), mRNA	54367	75	206	266	241	0.8	0.91	1.1375	10	5	11	11	12	12	14	12	-0.89399
tr|F8SKU2|F8SKU2_HUMAN	HLA-A	MHC class I antigen (Fragment) OS=Homo sapiens GN=HLA-A PE=3 SV=1	21143	639	533	22	0	0.16	0.18	1.125	0	0	1	0	2	0	3	1	-3.21473
sp|P00338|LDHA_HUMAN	LDHA	L-lactate dehydrogenase A chain OS=Homo sapiens GN=LDHA PE=1 SV=2	36950	44	93	481	421	2.33	2.62	1.12446352	10	13	19	16	19	17	24	19	-1.05155
sp|Q9P287|BCCIP_HUMAN	BCCIP	BRCA2 and CDKN1A-interacting protein OS=Homo sapiens GN=BCCIP PE=1 SV=1	36299	503	1105	33	28	0.09	0.1	1.11111111	0	1	1	1	1	1	3	1	-0.89229
tr|O95138|O95138_HUMAN	tr|O95138|O95	Nuclear autoantigenic sperm protein autosomal variant (Fragment) OS=Homo sapiens PE=2 SV=1	34492	542	1070	30	30	0.1	0.11	1.1	0	0	1	1	2	1	3	1	-1.62959
tr|J3QQM1|J3QQM1_HUMAN	PSMC5	26S protease regulatory subunit 8 (Fragment) OS=Homo sapiens GN=PSMC5 PE=4 SV=1	29442	314	1226	57	17	0.11	0.12	1.09090909	1	0	1	1	1	0	2	1	-2.47743
sp|Q04695|K1C17_HUMAN	KRT17	Keratin, type I cytoskeletal 17 OS=Homo sapiens GN=KRT17 PE=1 SV=2	48361	24	81	683	486	2.99	3.26	1.090301	19	16	27	23	32	26	35	30	-1.19262
sp|P43490|NAMPT_HUMAN	NAMPT	Nicotinamide phosphoribosyltransferase OS=Homo sapiens GN=NAMPT PE=1 SV=1	55772	520	410	32	109	0.12	0.13	1.08333333	0	1	1	2	1	2	3	2	-0.15514
tr|H7C4C8|H7C4C8_HUMAN	CCT8	T-complex protein 1 subunit theta (Fragment) OS=Homo sapiens GN=CCT8 PE=3 SV=1	35913	268	304	70	155	0.18	0.19	1.05555556	2	2	3	2	4	2	5	3	-1.74111
tr|Q5T7Q0|Q5T7Q0_HUMAN	DNAJA1	DnaJ (Hsp40) homolog, subfamily A, member 1, isoform CRA_c OS=Homo sapiens GN=DNAJA1 PE=2 SV=1	45581	703	710	18	57	0.07	0.07	1	0	1	1	1	0	3	1	3	1.915761
tr|B0UX83|B0UX83_HUMAN	BAG6	HLA-B associated transcript 3 OS=Homo sapiens GN=BAG6 PE=4 SV=1	119085	704	596	18	69	0.03	0.03	1	0	1	1	1	0	2	1	2	1.430154
tr|Q96CV8|Q96CV8_HUMAN	THOP1	Thimet oligopeptidase 1 OS=Homo sapiens GN=THOP1 PE=2 SV=1	79686	278	397	68	112	0.16	0.16	1	1	3	1	3	1	5	1	5	0.982901
sp|Q8NBJ5|GT251_HUMAN	GLT25D1	Procollagen galactosyltransferase 1 OS=Homo sapiens GN=GLT25D1 PE=1 SV=1	71933	523	1032	31	33	0.09	0.09	1	0	0	2	2	1	3	2	4	0.330464
sp|P38919|IF4A3_HUMAN	EIF4A3	Eukaryotic initiation factor 4A-III OS=Homo sapiens GN=EIF4A3 PE=1 SV=4	47126	262	267	74	184	0.4	0.4	1	1	4	5	5	4	8	6	9	0.025508
tr|A0MNP2|A0MNP2_HUMAN	WDR57	CDW11/WDR57 OS=Homo sapiens GN=WDR57 PE=2 SV=1	39742	413	948	42	38	0.17	0.17	1	1	0	2	2	2	4	3	5	-0.04409
tr|Q0P512|Q0P512_HUMAN	SLC2A1	Solute carrier family 2 (Facilitated glucose transporter), member 1 OS=Homo sapiens GN=SLC2A1 PE=2 SV=1	54361	377	991	46	35	0.06	0.06	1	1	1	1	1	1	2	1	2	-0.15514
tr|B3KPZ0|B3KPZ0_HUMAN	tr|B3KPZ0|B3K	cDNA FLJ32485 fis, clone SKNMC2001596, highly similar to Ankyrin repeat and FYVE domain-containing protei	121468	387	954	45	38	0.03	0.03	1	1	1	1	1	1	2	1	2	-0.15514
tr|H0Y5J4|H0Y5J4_HUMAN	DBNL	Drebrin-like protein (Fragment) OS=Homo sapiens GN=DBNL PE=4 SV=1	40475	466	742	37	52	0.08	0.08	1	1	1	1	1	1	2	1	2	-0.15514
tr|B3KNM6|B3KNM6_HUMAN	tr|B3KNM6|B3K	cDNA FLJ14968 fis, clone THYRO1000288, highly similar to CAAX prenyl protease 1 homolog (EC 3.4.24.84) OS-	27279	547	669	30	60	0.12	0.12	1	1	2	1	2	1	2	1	2	-0.15514
tr|G8JLB6|G8JLB6_HUMAN	HNRNPH1	Heterogeneous nuclear ribonucleoprotein H OS=Homo sapiens GN=HNRNPH1 PE=4 SV=1	51482	207	156	101	298	0.45	0.45	1	2	7	6	8	5	8	6	10	-0.26433
tr|Q13727|Q13727_HUMAN	tr|Q13727|Q13	AHNAK-related protein (Fragment) OS=Homo sapiens PE=2 SV=1	57703	402	893	43	41	0.06	0.06	1	1	1	1	1	2	3	3	7	-0.40684
tr|B4DP08|B4DP08_HUMAN	ALG1	Chitobiosyldiphosphodolichol beta-mannosyltransferase OS=Homo sapiens GN=ALG1 PE=2 SV=1	40938	452	724	38	55	0.17	0.17	1	0	1	2	2	2	3	2	3	-0.40684
tr|H0Y2W2|H0Y2W2_HUMAN	ATAD3A	ATPase family AAA domain-containing protein 3A (Fragment) OS=Homo sapiens GN=ATAD3A PE=4 SV=1	64600	281	630	68	64	0.1	0.1	1	2	2	2	2	2	3	2	5	-0.40684
tr|Q5JP02|Q5JP02_HUMAN	RBBP7	Histone-binding protein RBBP7 (Fragment) OS=Homo sapiens GN=RBBP7 PE=4 SV=1	19626	321	551	37	27	0.22	0.22	1	1	0	1	1	2	3	2	3	-0.40684
tr|D2CFK9|D2CFK9_HUMAN	GTPBP4	Nucleolar GTP-binding protein 1 OS=Homo sapiens GN=GTPBP4 PE=2 SV=1	74317	264	334	72	138	0.14	0.14	1	2	3	3	3	3	4	3	4	-0.52985
tr|G3V0E8|G3V0E8_HUMAN	PCBP2	Poly(RC) binding protein 2, isoform CRA_f OS=Homo sapiens GN=PCBP2 PE=2 SV=1	33817	132	306	166	154	0.32	0.32	1	3	3	3	3	3	4	5	4	-0.52985
tr|B3KPL5|B3KPL5_HUMAN	tr|B3KPL5|B3K	cDNA FLJ31917 fis, clone NT2RP7004925, highly similar to Sprouty-related, EVH1 domain-containing protein 2	48711	657	1194	21	21	0.07	0.07	1	0	0	1	2	16	18	51	33	-0.72919
sp|P58107|EPIPL_HUMAN	EPPK1	Epiplakin OS=Homo sapiens GN=EPPK1 PE=1 SV=2	557674	56	126	390	344	0.07	0.07	1	10	7	12	12	12	13	13	15	-0.78277
tr|B4DN13|B4DN13_HUMAN	tr|B4DN13|B4D	cDNA FLJ59136, highly similar to Transmembrane 9 superfamily protein member 3 OS=Homo sapiens PE=2 SV	29629	624	1197	23	21	0.11	0.11	1	0	0	1	1	1	1	1	1	-0.89229
tr|B3KMZ6|B3KMZ6_HUMAN	tr|B3KMZ6|B3K	cDNA FLJ13058 fis, clone NT2RP3001587, highly similar to Ubiquitin-like 1-activating enzyme E1B OS=Homo sa	71688	590	1122	27	26	0.05	0.05	1	0	1	1	1	1	1	1	1	-0.89229
sp|Q96EK5|KBP_HUMAN	KIAA1279	KIF1-binding protein OS=Homo sapiens GN=KIAA1279 PE=1 SV=1	72453	649	1106	22	28	0.05	0.05	1	0	0	1	1	1	1	1	1	-0.89229
tr|F2Z2J7|F2Z2J7_HUMAN	TTLL7	Tubulin polyglutamylase TTLL7 OS=Homo sapiens GN=TTLL7 PE=2 SV=1	55925	526	1087	31	29	0.06	0.06	1	1	1	1	1	1	1	1	1	-0.89229
tr|B4DM97|B4DM97_HUMAN	tr|B4DM97|B4D	cDNA FLJ55002, highly similar to Alpha-centractin OS=Homo sapiens PE=2 SV=1	38365	561	1080	29	30	0.09	0.09	1	0	0	1	1	1	1	1	1	-0.89229
tr|H0YA24|H0YA24_HUMAN	PLRG1	Pleiotropic regulator 1 (Fragment) OS=Homo sapiens GN=PLRG1 PE=4 SV=1	17588	456	1060	38	31	0.19	0.19	1	1	0	1	1	1	1	2	1	-0.89229
tr|B3KPR5|B3KPR5_HUMAN	tr|B3KPR5|B3K	cDNA FLJ32094 fis, clone OCBBF2000986, highly similar to Homo sapiens elongation factor Tu GTP binding dor	97046	633	978	22	36	0.03	0.03	1	0	0	1	1	1	1	1	2	-0.89229
tr|B2R4C0|B2R4C0_HUMAN	tr|B2R4C0|B2R	60S ribosomal protein L18a OS=Homo sapiens PE=2 SV=1	21024	514	900	32	40	0.16	0.16	1	0	1	1	1	1	1	2	1	-0.89229
tr|Q68E00|Q68E00_HUMAN	DKFZp686G2045	Putative uncharacterized protein DKFZp686G2045 (Fragment) OS=Homo sapiens GN=DKFZp686G2045 PE=2 S\	32787	485	858	36	44	0.1	0.1	1	0	1	1	1	1	1	1	1	-0.89229
tr|H0Y7S3|H0Y7S3_HUMAN	ATP2B2	Plasma membrane calcium-transporting ATPase 2 (Fragment) OS=Homo sapiens GN=ATP2B2 PE=3 SV=1	122272	528	842	31	46	0.03	0.03	1	0	1	1	1	1	1	3	2	-0.89229
tr|Q6UJZ6|Q6UJZ6_HUMAN	HLA-C	MHC class I antigen (Fragment) OS=Homo sapiens GN=HLA-C PE=2 SV=1	21221	639	816	22	47	0.16	0.16	1	0	1	1	1	1	1	1	3	-0.89229
tr|B3KSQ7|B3KSQ7_HUMAN	DBN1	Drebrin 1, isoform CRA_d OS=Homo sapiens GN=DBN1 PE=2 SV=1	66053	478	812	36	47	0.05	0.05	1	0	1	1	1	1	1	1	1	-0.89229
tr|B2RAX6|B2RAX6_HUMAN	tr|B2RAX6|B2R	cDNA, FLJ95176, Homo sapiens CGI-48 protein (CGI-48), mRNA OS=Homo sapiens PE=2 SV=1	58127	605	807	25	47	0.06	0.06	1	0	1	1	1	1	1	1	1	-0.89229
tr|Q2NKW8|Q2NKW8_HUMAN	AHCYL1	Adenosylhomocysteinase (Fragment) OS=Homo sapiens GN=AHCYL1 PE=2 SV=1	57630	509	715	33	56	0.06	0.06	1	0	1	1	1	1	1	1	1	-0.89229
tr|G0ZJH6|G0ZJH6_HUMAN	Ube4B	UFD2A-III/UBE4B-III splice isoform OS=Homo sapiens GN=Ube4B PE=2 SV=1	152890	667	656	21	61	0.02	0.02	1	0	1	1	1	1	1	1	2	-0.89229
tr|E0YTJ7|E0YTJ7_HUMAN	HLA-B	MHC class I antigen (Fragment) OS=Homo sapiens GN=HLA-B PE=3 SV=1	21175	639	644	22	63	0.16	0.16	1	0	1	1	1	1	1	1	1	-0.89229
tr|F6KRT8|F6KRT8_HUMAN	HLA-B	MHC class I antigen (Fragment) OS=Homo sapiens GN=HLA-B PE=3 SV=1	21119	639	643	22	63	0.16	0.16	1	0	1	1	1	1	1	1	2	-0.89229
tr|B3KUJ0|B3KUJ0_HUMAN	tr|B3KUJ0|B3K	cDNA FLJ39996 fis, clone STOMA2002166, highly similar to Splicing factor 3B subunit 4 OS=Homo sapiens PE=2	44512	464	640	37	63	0.07	0.07	1	1	1	1	1	1	1	2	1	-0.89229
tr|J3QQX0|J3QQX0_HUMAN	GPS1	COP9 signalosome complex subunit 1 (Fragment) OS=Homo sapiens GN=GPS1 PE=4 SV=1	8703	636	632	22	64	0.4	0.4	1	0	1	1	1	1	1	1	1	-0.89229
tr|K7EM16|K7EM16_HUMAN	VASP	Vasodilator-stimulated phosphoprotein (Fragment) OS=Homo sapiens GN=VASP PE=4 SV=1	20260	363	622	48	57	0.17	0.17	1	1	1	1	1	1	1	1	2	-0.89229
sp|Q6KB66|K2C80_HUMAN	KRT80	Keratin, type II cytoskeletal 80 OS=Homo sapiens GN=KRT80 PE=1 SV=2	51007	564	608	29	67	0.06	0.06	1	0	1	1	1	1	1	1	1	-0.89229
tr|B4DLD4|B4DLD4_HUMAN	tr|B4DLD4|B4D	cDNA FLJ56738, highly similar to Fibrillarin OS=Homo sapiens PE=2 SV=1	30279	423	601	41	68	0.11	0.11	1	1	1	1	1	1	1	1	1	-0.89229
tr|J3KTE4|J3KTE4_HUMAN	RPL19	Ribosomal protein L19 OS=Homo sapiens GN=RPL19 PE=3 SV=1	23347	336	531	52	79	0.14	0.14	1	1	1	1	1	1	1	1	1	-0.89229
tr|B3KWR2|B3KWR2_HUMAN	tr|B3KWR2|B3K	cDNA FLJ43638 fis, clone SPLEN2039845, highly similar to IMPORTIN ALPHA-1 SUBUNIT OS=Homo sapiens PE=	38087	573	522	22	54	0.11	0.11	1	0	1	1	1	1	1	1	1	-0.89229
tr|B3KV72|B3KV72_HUMAN	tr|B3KV72|B3K	cDNA FLJ16204 fis, clone CTONG2019232, highly similar to Proteasome-associated protein ECM29 homolog (F	78791	579	468	28	22	0.04	0.04	1	0	0	1	1	1	1	1	1	-0.89229
sp|Q68EM7|RHG17_HUMAN	ARHGAP17	Rho GTPase-activating protein 17 OS=Homo sapiens GN=ARHGAP17 PE=1 SV=1	95776	328	1146	54	25	0.03	0.03	1	1	0	1	1	2	2	2	3	-0.89244
sp|Q96SI9|STRBP_HUMAN	STRBP	Spermatid perinuclear RNA-binding protein OS=Homo sapiens GN=STRBP PE=1 SV=1	74290	471	777	37	50	0.09	0.09	1	0	0	2	2	2	2	2	3	-0.89244
tr|Q58FF4|Q58FF4_HUMAN	HSP90Bf	Heat shock protein 90Bf OS=Homo sapiens GN=HSP90Bf PE=1 SV=1	41833	407	756	43	52	0.16	0.16	1	1	1	2	2	2	2	2	3	-0.89244
sp|Q2NL82|TSR1_HUMAN	TSR1	Pre-rRNA-processing protein TSR1 homolog OS=Homo sapiens GN=TSR1 PE=1 SV=1	92151	230	747	90	52	0.07	0.07	1	2	1	2	2	2	2	2	2	-0.89244
tr|L0R5A1|L0R5A1_HUMAN	CSF2RB	Alternative protein CSF2RB OS=Homo sapiens GN=CSF2RB PE=4 SV=1	11752	486	1059	36	31	0.29	0.29	1	1	2	1	3	3	3	5	4	-0.8926
tr|B4DGU4|B4DGU4_HUMAN	CTNNB1	Catenin beta-1 OS=Homo sapiens GN=CTNNB1 PE=2 SV=1	85339	340	654	52	62	0.12	0.12	1	2	2	3	3	3	3	3	3	-0.8926
tr|B3KQQ3|B3KQQ3_HUMAN	tr|B3KQQ3|B3K	cDNA PSEC0016 fis, clone NT2RM1001076, highly similar to Procollagen-lysine,2-oxoglutarate 5-dioxygenase 3	85161	325	428	54	104	0.12	0.12	1	1	3	3	3	3	3	3	3	-0.8926
sp|P42166|LAP2A_HUMAN	TMPO	Lamina-associated polypeptide 2, isoform alpha OS=Homo sapiens GN=TMPO PE=1 SV=2	76016	241	321	84	146	0.24	0.24	1	1	3	5	5	5	5	5	6	-0.8929
tr|J3KPF3|J3KPF3_HUMAN	SLC3A2	4F2 cell-surface antigen heavy chain OS=Homo sapiens GN=SLC3A2 PE=4 SV=1	68230	60	85	366	464	1.33	1.33	1	12	14	20	19	20	19	20	20	-0.96756
sp|P52895|AK1C2_HUMAN	AKR1C2	Aldo-keto reductase family 1 member C2 OS=Homo sapiens GN=AKR1C2 PE=1 SV=3	37111	186	406	116	109	0.67	0.67	1	3	3	6	6	7	5	9	6	-1.34103
tr|A0AV58|A0AV58_HUMAN	STRN3	Striatin, calmodulin binding protein 3 OS=Homo sapiens GN=STRN3 PE=2 SV=1	78069	419	775	42	50	0.09	0.09	1	1	1	2	2	3	2	3	5	-1.3782
tr|A8MW61|A8MW61_HUMAN	PLRG1	Pleiotropic regulator 1 OS=Homo sapiens GN=PLRG1 PE=2 SV=1	57545	476	1103	36	28	0.12	0.12	1	1	0	2	2	2	1	4	2	-1.62959
tr|A8K781|A8K781_HUMAN	tr|A8K781|A8K	cDNA FLJ75299, highly similar to Xenopus laevis proteasome (prosome, macropain) 26S subunit, ATPase 3, mR	47578	603	791	26	50	0.07	0.07	1	0	1	1	1	2	1	2	2	-1.62959
tr|Q0IJ56|Q0IJ56_HUMAN	ST13	ST13 protein (Fragment) OS=Homo sapiens GN=ST13 PE=2 SV=1	35164	382	731	46	54	0.09	0.09	1	1	1	1	1	2	1	3	2	-1.62959
tr|B4DU00|B4DU00_HUMAN	tr|B4DU00|B4D	cDNA FLJ54547, highly similar to Alpha-1 catenin (Cadherin-associated protein) OS=Homo sapiens PE=2 SV=1	89332	389	626	45	41	0.07	0.07	1	0	1	2	1	2	1	4	1	-1.62959
tr|Q53RS3|Q53RS3_HUMAN	DDEF2	Putative uncharacterized protein DDEF2 (Fragment) OS=Homo sapiens GN=DDEF2 PE=2 SV=1	70046	441	966	39	37	0.05	0.05	1	1	1	1	1	4	2	4	2	-1.74111
tr|J3KQE5|J3KQE5_HUMAN	RAN	GTP-binding nuclear protein Ran (Fragment) OS=Homo sapiens GN=RAN PE=4 SV=1	27027	571	1236	29	16	0.12	0.12	1	0	0	1	1	1	0	1	1	-2.47743
tr|F1T0A5|F1T0A5_HUMAN	PRPF31	PRP31 pre-mRNA processing factor 31 homolog (Yeast), isoform CRA_a OS=Homo sapiens GN=PRPF31 PE=2 S\	55649	661	1219	21	19	0.06	0.06	1	0	0	1	1	1	0	2	1	-2.47743
tr|E2FIL8|E2FIL8_HUMAN	HLA-A	MHC class I antigen (Fragment) OS=Homo sapiens GN=HLA-A PE=3 SV=1	38417	639	819	22	0	0.16	0.16	1	0	0	1	0	1	0	2	1	-2.47743
tr|A0N4V7|A0N4V7_HUMAN	Tcr-alpha	HCG2039797 (Fragment) OS=Homo sapiens GN=Tcr-alpha PE=4 SV=1	2269	212	685	98	59	1.81	1.81	1	5	3	15	3	31	4	46	4	-3.70916
sp|Q05639|EF1A2_HUMAN	EEF1A2	Elongation factor 1-alpha 2 OS=Homo sapiens GN=EEF1A2 PE=1 SV=1	50780	70	146	238	319	1.14	1.13	0.99122807	8	9	9	16	12	19	14	21	-0.25118
tr|E0X098|E0X098_HUMAN	TES	Testin variant (Fragment) OS=Homo sapiens GN=TES PE=2 SV=1	22009	430	1031	40	27	0.15	0.14	0.93333333	1	0	1	1	1	1	1	2	-0.89229
tr|D2JYH4|D2JYH4_HUMAN	ACTA2	Actin, alpha 2, smooth muscle, aorta OS=Homo sapiens GN=ACTA2 PE=2 SV=1	42381	23	35	702	1024	6.63	6.04	0.91101056	23	29	35	41	43	51	49	58	-0.6538
sp|Q13867|BLMH_HUMAN	BLMH	Bleomycin hydrolase OS=Homo sapiens GN=BLMH PE=1 SV=1	53155	332	1054	52	31	0.2	0.18	0.9	1	0	3	2	2	3	4	4	-0.40684
sp|Q9NVI7|ATD3A_HUMAN	ATAD3A	ATPase family AAA domain-containing protein 3A OS=Homo sapiens GN=ATAD3A PE=1 SV=2	71610	281	672	68	59	0.1	0.09	0.9	2	1	2	2	2	3	2	5	-0.40684
tr|B1AHE4|B1AHE4_HUMAN	ATXN10	Ataxin 10 (Fragment) OS=Homo sapiens GN=ATXN10 PE=4 SV=1	22299	470	373	37	59	0.38	0.34	0.89473684	0	1	2	1	2	1	2	1	-1.62959
tr|B4DXG8|B4DXG8_HUMAN	tr|B4DXG8|B4D	cDNA FLJ55260, highly similar to Exosome complex exonuclease RRP45 (EC 3.1.13.-) OS=Homo sapiens PE=2 S\	44161	457	571	38	73	0.18	0.16	0.88888889	1	1	1	2	1	2	1	2	-0.15514
tr|Q53GU8|Q53GU8_HUMAN	tr|Q53GU8|Q53	Transforming growth factor, beta-induced, 68kDa variant (Fragment) OS=Homo sapiens PE=2 SV=1	75231	358	1137	48	25	0.09	0.08	0.88888889	1	0	2	1	2	1	2	1	-1.62959
tr|H3BND9|H3BND9_HUMAN	PCBP2	Poly(rC)-binding protein 2 (Fragment) OS=Homo sapiens GN=PCBP2 PE=4 SV=1	12425	132	1149	35	24	0.32	0.28	0.875	1	0	1	1	1	1	2	1	-0.89229
sp|P52272|HNRPM_HUMAN	HNRNPM	Heterogeneous nuclear ribonucleoprotein M OS=Homo sapiens GN=HNRNPM PE=1 SV=3	77749	164	278	132	179	0.39	0.34	0.87179487	7	5	8	8	11	8	12	9	-1.33047
tr|B4DF68|B4DF68_HUMAN	tr|B4DF68|B4D	cDNA FLJ55325, highly similar to Heat-shock protein 105 kDa OS=Homo sapiens PE=2 SV=1	86500	123	345	47	132	0.23	0.2	0.86956522	1	2	1	5	1	5	1	5	0.982901
tr|A6NFQ9|A6NFQ9_HUMAN	Sep-08	Septin-8 OS=Homo sapiens GN=SEPT8 PE=2 SV=1	51181	532	933	31	39	0.07	0.06	0.85714286	1	1	1	1	1	2	1	2	-0.15514
sp|P26232|CTNA2_HUMAN	CTNNA2	Catenin alpha-2 OS=Homo sapiens GN=CTNNA2 PE=1 SV=5	106045	389	627	31	65	0.07	0.06	0.85714286	0	2	1	2	1	2	1	2	-0.15514
tr|H6VRF8|H6VRF8_HUMAN	KRT1	Keratin 1 OS=Homo sapiens GN=KRT1 PE=3 SV=1	66184	6	29	2032	1196	5.01	4.2	0.83832335	55	37	64	41	69	45	76	52	-1.51839
tr|H6VRG2|H6VRG2_HUMAN	KRT1	Keratin 1 OS=Homo sapiens GN=KRT1 PE=3 SV=1	66197	7	31	2027	1115	5.01	4.2	0.83832335	55	35	65	39	70	43	77	50	-1.60455
tr|B3KMV8|B3KMV8_HUMAN	tr|B3KMV8|B3K	cDNA FLJ12766 fis, clone NT2RP2001520, highly similar to Calcium-binding mitochondrial carrier protein Arala	75130	353	516	41	81	0.11	0.09	0.81818182	1	2	1	2	1	3	1	4	0.330464
tr|Q6IBT3|Q6IBT3_HUMAN	CCT7	CCT7 protein OS=Homo sapiens GN=CCT7 PE=2 SV=1	59804	161	331	133	140	0.38	0.31	0.81578947	2	4	6	5	5	5	7	7	-0.8929
tr|Q5HYG8|Q5HYG8_HUMAN	DKFZp686P092C	Putative uncharacterized protein DKFZp686P09201 OS=Homo sapiens GN=DKFZp686P09201 PE=2 SV=1	53831	86	223	235	221	0.61	0.48	0.78688525	4	6	8	7	9	7	10	8	-1.23492
tr|A8K4Z4|A8K4Z4_HUMAN	tr|A8K4Z4|A8K	cDNA FLJ75549, highly similar to Homo sapiens ribosomal protein, large, P0 (RPLP0), transcript variant 1, mRN	34389	109	530	201	79	0.74	0.58	0.78378378	6	2	6	4	6	3	6	4	-1.78668
tr|Q14666|Q14666_HUMAN	tr|Q14666|Q14	Radiated keratinocyte mRNA 266 (keratin-related protein) (Fragment) OS=Homo sapiens PE=2 SV=1	30933	24	66	451	421	2.99	2.33	0.77926421	12	12	17	17	20	18	21	21	-1.04368
sp|P04259|K2C6B_HUMAN	KRT6B	Keratin, type II cytoskeletal 6B OS=Homo sapiens GN=KRT6B PE=1 SV=5	60315	79	241	252	209	0.79	0.61	0.7721519	10	8	13	10	15	10	17	11	-1.45723
tr|D6REX3|D6REX3_HUMAN	SEC31A	Protein transport protein Sec31A OS=Homo sapiens GN=SEC31A PE=2 SV=1	137110	121	470	178	93	0.13	0.1	0.76923077	4	3	5	4	4	4	5	5	-0.89275
tr|B7ZB91|B7ZB91_HUMAN	TNRC6B	Trinucleotide repeat containing 6B (Fragment) OS=Homo sapiens GN=TNRC6B PE=4 SV=1	81825	359	862	48	44	0.13	0.1	0.76923077	1	1	3	1	2	1	3	1	-1.62959
tr|B7Z438|B7Z438_HUMAN	tr|B7Z438|B7Z4	cDNA FLJ56352, highly similar to Succinyl-CoA ligase (GDP-forming) subunit alpha, mitochondrial (EC 6.2.1.4) (	26801	467	1228	37	17	0.12	0.09	0.75	1	0	1	1	1	0	1	1	-2.47743
sp|P16066|ANPRA_HUMAN	NPR1	Atrial natriuretic peptide receptor 1 OS=Homo sapiens GN=NPR1 PE=1 SV=1	119813	620	1223	24	19	0.04	0.03	0.75	0	0	1	1	1	0	1	1	-2.47743
tr|Q0IIN1|Q0IIN1_HUMAN	KRT77	Keratin 77 OS=Homo sapiens GN=KRT77 PE=2 SV=1	62050	76	275	264	180	0.23	0.17	0.73913043	10	6	10	6	10	6	12	6	-1.58627
tr|Q562M3|Q562M3_HUMAN	ACT	Actin-like protein (Fragment) OS=Homo sapiens GN=ACT PE=2 SV=1	11529	103	101	213	408	7.06	5.21	0.73796034	10	14	14	19	17	22	20	30	-0.53129
tr|A0PJ76|A0PJ76_HUMAN	NCAPD2	NCAPD2 protein (Fragment) OS=Homo sapiens GN=NCAPD2 PE=2 SV=1	44957	494	488	34	44	0.15	0.11	0.73333333	0	1	2	2	2	2	2	2	-0.89244
sp|P46087|NOP2_HUMAN	NOP2	Putative ribosomal RNA methyltransferase NOP2 OS=Homo sapiens GN=NOP2 PE=1 SV=2	89589	296	491	61	87	0.15	0.11	0.73333333	2	3	4	3	5	3	6	5	-1.54534
tr|B4DY32|B4DY32_HUMAN	tr|B4DY32|B4D	Asparagine synthetase OS=Homo sapiens PE=2 SV=1	55055	141	540	156	79	0.26	0.19	0.73076923	3	2	5	3	6	4	6	4	-1.42393
tr|B3KTA3|B3KTA3_HUMAN	tr|B3KTA3|B3K	Fascin OS=Homo sapiens PE=2 SV=1	52814	53	141	403	293	1.07	0.78	0.72897196	9	7	14	10	16	10	20	12	-1.54776
tr|A5PLK7|A5PLK7_HUMAN	RCC2	RCC2 protein (Fragment) OS=Homo sapiens GN=RCC2 PE=2 SV=1	50331	165	303	131	129	0.46	0.33	0.7173913	4	4	6	4	6	5	6	6	-1.13425
tr|Q05DV5|Q05DV5_HUMAN	ZC3HAV1	ZC3HAV1 protein (Fragment) OS=Homo sapiens GN=ZC3HAV1 PE=2 SV=1	49964	224	254	92	199	0.14	0.1	0.71428571	1	2	2	3	2	4	2	4	-0.04409
tr|B4E2P4|B4E2P4_HUMAN	tr|B4E2P4|B4E	cDNA FLJ58474, highly similar to Xaa-Pro aminopeptidase 1 (EC 3.4.11.9) OS=Homo sapiens PE=2 SV=1	50020	615	964	24	37	0.07	0.05	0.71428571	0	1	1	1	1	1	1	1	-0.89229
tr|Q53XC0|Q53XC0_HUMAN	EIF2S1	Eukaryotic translation initiation factor 2, subunit 1 alpha, 35kDa, isoform CRA_a OS=Homo sapiens GN=EIF2S1	36374	208	577	100	72	0.42	0.3	0.71428571	4	2	4	3	4	4	4	7	-0.89275
tr|O60747|O60747_HUMAN	tr|O60747|O60	Putative G-binding protein (Fragment) OS=Homo sapiens PE=4 SV=1	65775	264	420	72	105	0.14	0.1	0.71428571	2	2	3	2	3	2	3	3	-1.3782
tr|J3KSN7|J3KSN7_HUMAN	EIF4A2	Eukaryotic initiation factor 4A-II (Fragment) OS=Homo sapiens GN=EIF4A2 PE=4 SV=1	12120	52	120	116	138	1.62	1.15	0.70987654	1	2	3	2	2	2	3	3	-0.89244
tr|H0YGW7|H0YGW7_HUMAN	ABCF1	ATP-binding cassette sub-family F member 1 (Fragment) OS=Homo sapiens GN=ABCF1 PE=3 SV=1	68075	144	282	151	171	0.27	0.19	0.7037037	4	4	5	5	5	6	7	6	-0.65172
sp|Q5T2N8|ATD3C_HUMAN	ATAD3C	ATPase family AAA domain-containing protein 3C OS=Homo sapiens GN=ATAD3C PE=2 SV=2	46635	281	845	68	45	0.1	0.07	0.7	2	1	2	1	2	1	2	3	-1.62959
sp|P02533|K1C14_HUMAN	KRT14	Keratin, type I cytoskeletal 14 OS=Homo sapiens GN=KRT14 PE=1 SV=4	51872	33	138	604	328	2.22	1.52	0.68468468	19	10	25	16	29	18	30	21	-1.5718
sp|P35237|SPB6_HUMAN	SERPINB6	Serpin B6 OS=Homo sapiens GN=SERPINB6 PE=1 SV=3	42936	120	1102	179	28	0.25	0.17	0.68	3	0	3	1	3	1	3	1	-2.11535
tr|B1AKY9|B1AKY9_HUMAN	ATP1A2	ATPase, Na+/K+ transporting, alpha 2 (+) polypeptide OS=Homo sapiens GN=ATP1A2 PE=3 SV=1	112046	324	515	33	81	0.09	0.06	0.66666667	1	2	2	3	6	3	9	7	-1.78668
sp|P53675|CLH2_HUMAN	CLTCL1	Clathrin heavy chain 2 OS=Homo sapiens GN=CLTCL1 PE=1 SV=2	189020	30	94	107	417	0.52	0.34	0.65384615	2	11	2	14	2	14	4	17	1.645772
tr|B4DW52|B4DW52_HUMAN	tr|B4DW52|B4I	cDNA FLJ55253, highly similar to Actin, cytoplasmic 1 OS=Homo sapiens PE=2 SV=1	38950	4	9	3120	2241	27.29	17.82	0.65298644	74	58	108	81	123	92	134	113	-1.33414
sp|Q9NV06|DCA13_HUMAN	DCAF13	DDB1- and CUL4-associated factor 13 OS=Homo sapiens GN=DCAF13 PE=1 SV=2	51996	435	753	39	52	0.2	0.13	0.65	1	1	3	2	3	2	6	2	-1.3782

tr|Q8TE01|Q8TE01_HUMAN	derp12	DERP12 (Dermal papilla derived protein 12) OS=Homo sapiens GN=derp12 PE=2 SV=1	38340	69	240	297	209	0.79	0.51	0.64556962	6	4	7	5	8	5	8	6	-1.52194
sp|P62917|RL8_HUMAN	RPL8	60S ribosomal protein L8 OS=Homo sapiens GN=RPL8 PE=1 SV=2	28235	231	801	90	48	0.28	0.18	0.64285714	2	1	2	1	4	1	5	1	-2.47825
sp|Q09028|RBBP4_HUMAN	RBBP4	Histone-binding protein RBBP4 OS=Homo sapiens GN=RBBP4 PE=1 SV=3	47911	321	850	55	44	0.22	0.14	0.63636364	2	1	3	2	4	5	4	6	-0.60306
tr|B2RCM3|B2RCM3_HUMAN	tr|B2RCM3|B2R	cDNA, FLJ96158, highly similar to Homo sapiens calpain 2, (m/II) large subunit (CAPN2), mRNA OS=Homo sapie	80883	173	489	125	81	0.22	0.14	0.63636364	3	2	7	2	7	2	8	2	-2.47908
tr|F4ZW64|F4ZW64_HUMAN	tr|F4ZW64|F4Z	NF90a OS=Homo sapiens PE=2 SV=1	76369	154	359	137	125	0.46	0.29	0.63043478	3	3	9	6	10	8	11	10	-1.19889
tr|B7XH59|B7XH59_HUMAN	HLA-B	MHC class I antigen (Fragment) OS=Homo sapiens GN=HLA-B PE=3 SV=1	31924	639	642	22	63	0.16	0.1	0.625	0	1	1	1	1	2	1	3	-0.15514
tr|Q1KLZ0|Q1KLZ0_HUMAN	PS1TP5BP1	HCG15971, isoform CRA_a OS=Homo sapiens GN=PS1TP5BP1 PE=2 SV=1	42052	1	7	3367	2431	31.43	19.6	0.62360802	82	65	119	89	130	96	141	118	-1.35428
tr|Q5CAQ5|Q5CAQ5_HUMAN	TRA1	Tumor rejection antigen (Gp96) 1 OS=Homo sapiens GN=TRA1 PE=2 SV=1	92567	140	216	157	233	0.37	0.23	0.62162162	4	5	9	7	10	7	11	11	-1.37964
tr|K7EK77|K7EK77_HUMAN	ATP5A1	ATP synthase subunit alpha, mitochondrial (Fragment) OS=Homo sapiens GN=ATP5A1 PE=3 SV=1	22243	135	116	160	210	1.33	0.8	0.60150376	5	7	6	7	6	7	6	7	-0.68643
tr|B9A041|B9A041_HUMAN	MDH1	Malate dehydrogenase, cytoplasmic OS=Homo sapiens GN=MDH1 PE=2 SV=1	23195	309	670	58	60	0.31	0.18	0.58064516	2	1	2	1	2	1	2	2	-1.62959
sp|P13645|K1C10_HUMAN	KRT10	Keratin, type I cytoskeletal 10 OS=Homo sapiens GN=KRT10 PE=1 SV=6	59020	12	34	1226	1029	4.67	2.67	0.57173448	34	24	42	27	47	32	54	35	-1.44963
tr|B3KM80|B3KM80_HUMAN	NCL	Nucleolin, isoform CRA_c OS=Homo sapiens GN=NCL PE=2 SV=1	58576	71	182	283	265	1.03	0.55	0.53398058	9	5	14	8	15	10	15	10	-1.45723
tr|B4DQG5|B4DQG5_HUMAN	tr|B4DQG5|B4D	cDNA FLJ54122, highly similar to Cytosol aminopeptidase (EC 3.4.11.1) OS=Homo sapiens PE=2 SV=1	39662	368	1068	47	30	0.15	0.08	0.53333333	1	1	1	1	1	1	1	1	-0.89229
tr|B3KTJ9|B3KTJ9_HUMAN	tr|B3KTJ9|B3KT	cDNA FLJ38393 fis, clone FEBRA2007212 OS=Homo sapiens PE=2 SV=1	103493	82	248	251	204	0.32	0.17	0.53125	7	4	9	5	9	7	12	8	-1.23492
sp|Q58FF8|H90B2_HUMAN	HSP90AB2P	Putative heat shock protein HSP 90-beta 2 OS=Homo sapiens GN=HSP90AB2P PE=1 SV=2	44492	32	205	97	242	1.24	0.65	0.52419355	3	5	5	7	5	7	6	9	-0.44509
tr|H0Y9Q7|H0Y9Q7_HUMAN	CANX	Calnexin (Fragment) OS=Homo sapiens GN=CANX PE=3 SV=1	18990	352	340	49	74	0.39	0.2	0.51282051	1	1	2	2	3	3	5	3	-0.8926
tr|F5CL10|F5CL10_HUMAN	HLA-Cw	MHC class I antigen OS=Homo sapiens GN=HLA-Cw PE=3 SV=1	41120	639	814	22	47	0.16	0.08	0.5	0	1	1	1	1	2	1	3	-0.15514
tr|B2RBE5|B2RBE5_HUMAN	tr|B2RBE5|B2R	cDNA, FLJ95468, highly similar to Homo sapiens transcriptional coactivator tubedown-100 (TBDN100),transcri	102490	446	897	38	41	0.06	0.03	0.5	0	1	2	1	2	2	2	4	-0.89244
tr|B4DZ88|B4DZ88_HUMAN	KTN1	KTN1 protein OS=Homo sapiens GN=KTN1 PE=2 SV=1	153179	322	1217	55	19	0.04	0.02	0.5	2	0	2	1	3	2	4	7	-1.3782
sp|P27348|1433T_HUMAN	YWHAQ	14-3-3 protein theta OS=Homo sapiens GN=YWHAQ PE=1 SV=1	28032	258	713	74	56	0.4	0.2	0.5	2	1	3	1	3	2	3	2	-1.3782
sp|Q9UHR4|BI2L1_HUMAN	BAIAP2L1	Brain-specific angiogenesis inhibitor 1-associated protein 2-like protein 1 OS=Homo sapiens GN=BAIAP2L1 PE=	57189	613	717	24	56	0.12	0.06	0.5	0	1	2	1	2	1	4	1	-1.62959
tr|E9PRA5|E9PRA5_HUMAN	ATP1A4	Sodium/potassium-transporting ATPase subunit alpha-4 OS=Homo sapiens GN=ATP1A4 PE=2 SV=1	57835	289	1104	63	28	0.12	0.06	0.5	0	0	6	2	10	2	19	5	-2.96551
sp|P53004|BIEA_HUMAN	BLVRA	Biliverdin reductase A OS=Homo sapiens GN=BLVRA PE=1 SV=2	33692	403	906	43	40	0.21	0.1	0.47619048	1	1	2	1	2	1	3	1	-1.62959
tr|B0AZS6|B0AZS6_HUMAN	YWHAZ	14-3-3 protein zeta/delta OS=Homo sapiens GN=YWHAZ PE=2 SV=1	19174	497	946	34	38	0.38	0.18	0.47368421	0	1	2	1	2	1	2	1	-1.62959
sp|Q562R1|ACTBL_HUMAN	ACTBL2	Beta-actin-like protein 2 OS=Homo sapiens GN=ACTBL2 PE=1 SV=2	42318	61	158	349	297	1.47	0.69	0.46938776	13	7	16	13	24	19	27	27	-1.22606
tr|B2R4P2|B2R4P2_HUMAN	tr|B2R4P2|B2R	cDNA, FLJ92164, highly similar to Homo sapiens peroxiredoxin 1 (PRDX1), mRNA OS=Homo sapiens PE=2 SV=1	22414	481	1091	36	29	0.32	0.15	0.46875	0	0	2	1	2	1	2	1	-1.62959
tr|B2R5M8|B2R5M8_HUMAN	tr|B2R5M8|B2R	Isocitrate dehydrogenase [NADP] OS=Homo sapiens PE=2 SV=1	46905	555	776	29	50	0.15	0.07	0.46666667	0	1	2	1	3	2	3	6	-1.3782
sp|P08779|K1C16_HUMAN	KRT16	Keratin, type I cytoskeletal 16 OS=Homo sapiens GN=KRT16 PE=1 SV=4	51578	62	286	342	169	1.38	0.64	0.46376812	14	6	17	9	21	12	21	14	-1.67941
tr|B3KY95|B3KY95_HUMAN	tr|B3KY95|B3K	cDNA FLJ16143 fis, clone BRAMY2038516, highly similar to Protein disulfide-isomerase A6 (EC 5.3.4.1) OS=Hor	53655	445	587	38	71	0.13	0.06	0.46153846	0	1	2	1	2	1	2	2	-1.62959
tr|A2RUM7|A2RUM7_HUMAN	RPL5	Ribosomal protein L5 OS=Homo sapiens GN=RPL5 PE=2 SV=1	34569	187	444	115	100	0.44	0.2	0.45454545	2	1	4	2	5	2	7	2	-2.03095
tr|B7TY16|B7TY16_HUMAN	ACTN1	Actinin alpha 1 isoform 3 OS=Homo sapiens GN=ACTN1 PE=2 SV=1	107644	50	84	428	467	0.77	0.35	0.45454545	15	9	21	10	22	11	29	12	-1.86581
tr|Q7KZ86|Q7KZ86_HUMAN	tr|Q7KZ86|Q7K	Plakoglobin (Fragment) OS=Homo sapiens PE=2 SV=1	19007	26	163	37	31	1.74	0.79	0.45402299	1	0	2	2	2	2	2	2	-0.89244
tr|H0YIE7|H0YIE7_HUMAN	HSPA1L	Heat shock 70 kDa protein 1-like OS=Homo sapiens GN=HSPA1L PE=3 SV=1	25488	51	113	132	208	1.45	0.65	0.44827586	3	5	4	5	4	5	4	5	-0.60306
tr|E7EN38|E7EN38_HUMAN	RAD50	DNA repair protein RAD50 (Fragment) OS=Homo sapiens GN=RAD50 PE=2 SV=1	77138	563	1024	29	0	0.09	0.04	0.44444444	0	0	2	0	1	2	2	2	-0.15514
sp|P07737|PROF1_HUMAN	PFN1	Profilin-1 OS=Homo sapiens GN=PFN1 PE=1 SV=2	15216	436	869	39	43	0.5	0.22	0.44	1	1	2	1	2	1	2	2	-1.62959
tr|B2R8N0|B2R8N0_HUMAN	tr|B2R8N0|B2R	cDNA, FLJ93975, highly similar to Homo sapiens epoxide hydrolase 1, microsomal (xenobiotic) (EPHX1), mRNA	53129	115	395	184	113	0.62	0.27	0.43548387	6	4	8	4	8	4	8	5	-1.81163
sp|P35908|K22E_HUMAN	KRT2	Keratin, type II cytoskeletal 2 epidermal OS=Homo sapiens GN=KRT2 PE=1 SV=2	65678	11	46	1255	897	4.79	2.07	0.43215031	41	20	48	26	49	28	57	28	-1.70001
tr|H0YAF8|H0YAF8_HUMAN	GNB2L1	Guanine nucleotide-binding protein subunit beta-2-like 1 (Fragment) OS=Homo sapiens GN=GNB2L1 PE=4 SV=	22373	216	1016	96	34	0.52	0.22	0.42307692	2	0	3	1	3	1	4	1	-2.11535
sp|P22626|ROA2_HUMAN	HNRNPA2B1	Heterogeneous nuclear ribonucleoproteins A2/B1 OS=Homo sapiens GN=HNRNPA2B1 PE=1 SV=2	37464	77	375	256	118	0.97	0.4	0.41237113	7	2	8	4	9	4	10	5	-1.97243
tr|F8W8T8|F8W8T8_HUMAN	ACACB	Acetyl-CoA carboxylase 2 OS=Homo sapiens GN=ACACB PE=2 SV=1	269823	31	87	128	449	0.44	0.18	0.40909091	4	13	4	15	6	18	9	20	0.618117
sp|P78371|TCPB_HUMAN	CCT2	T-complex protein 1 subunit beta OS=Homo sapiens GN=CCT2 PE=1 SV=4	57794	37	123	563	355	1.86	0.75	0.40322581	14	7	20	10	20	10	23	11	-1.86226
tr|Q5U0Q1|Q5U0Q1_HUMAN	DKFZp686L1159	Putative uncharacterized protein DKFZp686L1159 OS=Homo sapiens GN=DKFZp686L1159 PE=2 SV=1	52189	294	418	62	105	0.58	0.22	0.37931034	2	2	2	2	2	2	2	2	-0.89244
sp|P35527|K1C9_HUMAN	KRT9	Keratin, type I cytoskeletal 9 OS=Homo sapiens GN=KRT9 PE=1 SV=3	62255	14	125	1044	346	3.01	1.06	0.35215947	25	8	36	14	40	17	44	17	-2.11306
tr|A8K7A4|A8K7A4_HUMAN	tr|A8K7A4|A8K	cDNA FLJ76904, highly similar to Homo sapiens methionine adenosyltransferase II, beta (MAT2B), transcript va	36692	280	950	68	38	0.37	0.13	0.35135135	1	1	1	1	1	2	1	2	-0.15514
tr|F8VTQ5|F8VTQ5_HUMAN	HNRNPA1	Heterogeneous nuclear ribonucleoprotein A1 (Fragment) OS=Homo sapiens GN=HNRNPA1 PE=2 SV=1	16640	101	327	87	0	0.77	0.27	0.35064935	2	0	4	0	5	0	7	1	-4.35324
tr|F5H6T1|F5H6T1_HUMAN	ACTR2	Actin-related protein 2 OS=Homo sapiens GN=ACTR2 PE=2 SV=1	34729	244	525	82	80	0.2	0.07	0.35	2	1	2	1	2	1	4	2	-1.62959
sp|O60518|RNBP6_HUMAN	RANBP6	Ran-binding protein 6 OS=Homo sapiens GN=RANBP6 PE=1 SV=2	126173	85	1002	36	35	0.23	0.08	0.34782609	1	0	1	2	2	2	2	4	-0.89244
tr|B3KY12|B3KY12_HUMAN	tr|B3KY12|B3K	cDNA FLJ46581 fis, clone THYMU3043200, highly similar to Splicing factor 3A subunit 3 OS=Homo sapiens PE=	59082	495	1152	34	24	0.18	0.06	0.33333333	1	0	3	1	2	1	3	1	-1.62959
tr|D6RAT0|D6RAT0_HUMAN	RPS3A	40S ribosomal protein S3a OS=Homo sapiens GN=RPS3A PE=3 SV=1	26099	223	1072	93	30	0.44	0.14	0.31818182	2	0	3	1	4	1	5	1	-2.47825
tr|F5H0C8|F5H0C8_HUMAN	ENO2	Enolase OS=Homo sapiens GN=ENO2 PE=2 SV=1	35026	40	196	112	250	1.39	0.44	0.31654676	2	5	2	5	2	6	4	6	0.486789
sp|P13647|K2C5_HUMAN	KRT5	Keratin, type II cytoskeletal 5 OS=Homo sapiens GN=KRT5 PE=1 SV=3	62568	58	270	378	182	0.95	0.29	0.30526316	13	4	15	5	20	5	24	6	-2.79605
tr|A4ZU86|A4ZU86_HUMAN	NPM1	Truncated nucleolar phosphoprotein B23 OS=Homo sapiens GN=NPM1 PE=2 SV=1	30074	138	590	160	57	0.69	0.21	0.30434783	4	1	5	1	5	1	5	2	-2.76809
sp|O43707|ACTN4_HUMAN	ACTN4	Alpha-actinin-4 OS=Homo sapiens GN=ACTN4 PE=1 SV=2	105245	19	75	849	524	2.11	0.63	0.2985782	26	12	41	16	44	18	52	19	-2.16994
tr|B4DTA2|B4DTA2_HUMAN	tr|B4DTA2|B4D	cDNA FLJ60148, highly similar to Homo sapiens heterogeneous nuclear ribonucleoprotein D-like (HNRPDL), tra	30367	229	1053	39	32	0.37	0.11	0.2972973	1	0	1	1	1	2	1	5	-0.15514
tr|Q5JQZ9|Q5JQZ9_HUMAN	CDC2L6	Cell division cycle 2-like 6 (CDK8-like) (Fragment) OS=Homo sapiens GN=CDC2L6 PE=4 SV=1	29552	285	1210	65	20	0.24	0.07	0.29166667	2	0	2	1	3	0	3	1	-3.70049
sp|Q58FG0|HS905_HUMAN	HSP90AA5P	Putative heat shock protein HSP 90-alpha A5 OS=Homo sapiens GN=HSP90AA5P PE=1 SV=1	38942	46	441	36	100	1.05	0.28	0.26666667	0	2	2	3	2	5	2	6	0.245601
sp|Q9NXW2|DJB12_HUMAN	DNAJB12	DnaJ homolog subfamily B member 12 OS=Homo sapiens GN=DNAJB12 PE=1 SV=4	42021	124	451	174	98	0.57	0.15	0.26315789	5	2	7	2	9	2	9	2	-2.82078
tr|F8VRQ1|F8VRQ1_HUMAN	HNRNPA1	Heterogeneous nuclear ribonucleoprotein A1 OS=Homo sapiens GN=HNRNPA1 PE=2 SV=1	33249	101	378	214	117	0.77	0.2	0.25974026	4	2	6	2	7	2	9	3	-2.47908
sp|Q6IBS0|TWF2_HUMAN	TWF2	Twinfilin-2 OS=Homo sapiens GN=TWF2 PE=1 SV=2	39751	535	616	30	66	0.08	0.02	0.25	0	1	1	1	1	1	1	1	-0.89229
tr|B7Z8J4|B7Z8J4_HUMAN	NMT1	Glycylpeptide N-tetradecanoyltransferase 1 OS=Homo sapiens GN=NMT1 PE=2 SV=1	15265	350	514	50	38	0.49	0.12	0.24489796	1	1	2	1	2	2	2	2	-0.89244
tr|K7ERE3|K7ERE3_HUMAN	KRT13	Keratin, type I cytoskeletal 13 OS=Homo sapiens GN=KRT13 PE=3 SV=1	45574	88	639	234	63	0.63	0.15	0.23809524	9	2	10	3	11	3	12	4	-2.61148
tr|K0J110|K0J110_HUMAN	mcdrh	Malignant cell derived RNA helicase OS=Homo sapiens GN=mcdrh PE=2 SV=1	82499	148	857	61	44	0.34	0.08	0.23529412	2	0	2	2	2	2	2	2	-0.89244
tr|D3DTL4|D3DTL4_HUMAN	ENO3	Enolase OS=Homo sapiens GN=ENO3 PE=2 SV=1	37167	40	151	112	235	1.39	0.31	0.22302158	2	4	2	4	2	4	4	4	-0.04409
tr|A4D1P0|A4D1P0_HUMAN	LOC402299	Uncharacterized protein OS=Homo sapiens GN=LOC402299 PE=4 SV=1	42109	185	772	40	51	0.42	0.08	0.19047619	1	1	1	1	1	2	1	2	-0.15514
tr|F8VU64|F8VU64_HUMAN	KRT8	Keratin, type II cytoskeletal 8 OS=Homo sapiens GN=KRT8 PE=2 SV=1	37274	58	939	61	36	0.95	0.17	0.17894737	3	0	4	1	6	1	7	2	-3.00944
tr|A8K2H9|A8K2H9_HUMAN	tr|A8K2H9|A8K	cDNA FLJ78503, highly similar to Homo sapiens keratin 13 (KRT13), transcript variant 1, mRNA OS=Homo sapie	49828	125	794	174	49	0.47	0.07	0.14893617	5	1	6	1	8	1	10	3	-3.39713
sp|Q58FF6|H90B4_HUMAN	HSP90AB4P	Putative heat shock protein HSP 90-beta 4 OS=Homo sapiens GN=HSP90AB4P PE=5 SV=1	58855	32	481	77	92	1.24	0.18	0.14516129	3	2	5	3	3	5	5	6	-0.24016
sp|O95678|K2C75_HUMAN	KRT75	Keratin, type II cytoskeletal 75 OS=Homo sapiens GN=KRT75 PE=1 SV=2	59809	11	266	94	83	4.79	0.53	0.11064718	2	2	3	3	3	4	5	4	-0.52985
tr|J3KR91|J3KR91_HUMAN	IQGAP2	Ras GTPase-activating-like protein IQGAP2 OS=Homo sapiens GN=IQGAP2 PE=4 SV=1	123072	84	1220	32	19	0.31	0.03	0.09677419	0	0	1	1	1	1	1	3	-0.89229
tr|F8W0F6|F8W0F6_HUMAN	TUBA1A	Tubulin alpha-1A chain OS=Homo sapiens GN=TUBA1A PE=2 SV=1	25114	13	212	100	234	5.58	0.28	0.05017921	2	3	2	3	3	3	3	5	-0.8926
tr|Q562V5|Q562V5_HUMAN	ACT	Actin-like protein (Fragment) OS=Homo sapiens GN=ACT PE=2 SV=1	11481	1	355	136	126	31.43	0.69	0.02195355	6	5	6	5	7	6	8	10	-1.09984
tr|Q562Q1|Q562Q1_HUMAN	ACT	Actin-like protein (Fragment) OS=Homo sapiens GN=ACT PE=3 SV=1	11548	5	0	0	0	0.44	0	0	0	0	0	0	0	0	1	0	-0.89213
tr|E9PQ69|E9PQ69_HUMAN	GANAB	Neutral alpha-glucosidase AB OS=Homo sapiens GN=GANAB PE=2 SV=1	8560	47	0	0	0	0.62	0	0	0	0	0	0	0	0	1	0	-0.89213
tr|F8WBS8|F8WBS8_HUMAN	PSMD2	26S proteasome non-ATPase regulatory subunit 2 OS=Homo sapiens GN=PSMD2 PE=2 SV=1	6579	198	0	0	0	0.14	0	0	0	0	0	0	0	0	1	0	-0.89213
tr|A8K171|A8K171_HUMAN	tr|A8K171|A8K	cDNA FLJ77253, highly similar to Homo sapiens nuclear receptor interacting protein 1 (NRIP1), mRNA OS=Hom	127609	401	0	0	0	0.01	0	0	0	0	0	0	0	0	1	0	-0.89213
tr|H0Y386|H0Y386_HUMAN	PABPC1L	Polyadenylate-binding protein 1-like (Fragment) OS=Homo sapiens GN=PABPC1L PE=4 SV=1	16787	404	0	0	0	0.1	0	0	0	0	0	0	0	0	1	0	-0.89213
tr|Q4VBX7|Q4VBX7_HUMAN	NUP205	NUP205 protein (Fragment) OS=Homo sapiens GN=NUP205 PE=2 SV=1	38620	415	0	0	0	0.04	0	0	0	0	0	0	0	0	1	0	-0.89213
tr|Q9NQS8|Q9NQS8_HUMAN	RPS3	Ribosomal protein S3 (Fragment) OS=Homo sapiens GN=RPS3 PE=2 SV=1	17270	480	0	0	0	0.12	0	0	0	0	0	0	0	0	2	0	-0.89213
tr|H7BYY8|H7BYY8_HUMAN	PES1	Pescadillo homolog OS=Homo sapiens GN=PES1 PE=4 SV=1	1501	488	0	0	0	0.05	0	0	0	0	0	0	0	0	1	0	-0.89213
sp|Q14117|DPYS_HUMAN	DPYS	Dihydropyrimidinase OS=Homo sapiens GN=DPYS PE=1 SV=1	57107	490	0	0	0	0.19	0	0	0	0	0	0	0	0	1	0	-0.89213
tr|B4DM94|B4DM94_HUMAN	tr|B4DM94|B4D	60S ribosomal protein L18a OS=Homo sapiens PE=2 SV=1	17672	514	0	0	0	0.16	0	0	0	0	0	0	0	0	1	0	-0.89213
tr|D6RD96|D6RD96_HUMAN	ACSL4	Long-chain-fatty-acid--CoA ligase 4 (Fragment) OS=Homo sapiens GN=ACSL4 PE=2 SV=1	16399	529	0	0	0	0.04	0	0	0	0	0	0	0	0	1	0	-0.89213
tr|I3L3P5|I3L3P5_HUMAN	P4HB	Protein disulfide-isomerase (Fragment) OS=Homo sapiens GN=P4HB PE=4 SV=1	16923	536	0	19	0	0.27	0	0	0	0	1	0	0	0	1	0	-0.89213
tr|H7C1T1|H7C1T1_HUMAN	Sep-02	Septin-2 (Fragment) OS=Homo sapiens GN=SEPT2 PE=4 SV=1	3094	543	0	0	0	0.09	0	0	0	0	0	0	0	0	1	0	-0.89213
tr|B7Z4W3|B7Z4W3_HUMAN	tr|B7Z4W3|B7Z	cDNA FLJ57543, highly similar to Bifunctional 3~-phosphoadenosine5~-phosphosulfate synthetase 1 OS=Homo	17852	553	0	19	0	0.1	0	0	0	0	1	0	0	0	1	0	-0.89213
tr|J3KN33|J3KN33_HUMAN	CNTLN	Centlein OS=Homo sapiens GN=CNTLN PE=4 SV=1	162069	680	0	20	0	0.02	0	0	0	0	1	0	0	0	2	0	-0.89213
tr|Q3MNE1|Q3MNE1_HUMAN	tr|Q3MNE1|Q3	Adaptor-related protein complex 3 beta 1 subunit (Fragment) OS=Homo sapiens PE=2 SV=1	89828	682	0	20	0	0.04	0	0	0	0	1	0	0	0	1	0	-0.89213
tr|B4DZD5|B4DZD5_HUMAN	tr|B4DZD5|B4D	cDNA FLJ60991, highly similar to FKBP12-rapamycin complex-associated protein OS=Homo sapiens PE=2 SV=1	158783	683	0	20	0	0.02	0	0	0	0	1	0	0	0	2	0	-0.89213
tr|Q9UH66|Q9UH66_HUMAN	tr|Q9UH66|Q9U	7h3 protein (Fragment) OS=Homo sapiens PE=2 SV=1	49963	684	0	19	0	0.07	0	0	0	0	1	0	0	0	2	0	-0.89213
tr|G5E9Q3|G5E9Q3_HUMAN	TSEN2	tRNA splicing endonuclease 2 homolog (S. cerevisiae), isoform CRA_b OS=Homo sapiens GN=TSEN2 PE=2 SV=1	45959	686	0	19	0	0.07	0	0	0	0	1	0	0	0	1	0	-0.89213
tr|E9PQG7|E9PQG7_HUMAN	MLL	MLL cleavage product C180 OS=Homo sapiens GN=MLL PE=2 SV=1	436345	687	0	19	0	0.01	0	0	0	0	1	0	0	0	1	0	-0.89213
tr|G3V448|G3V448_HUMAN	TMX1	Thioredoxin-related transmembrane protein 1 OS=Homo sapiens GN=TMX1 PE=2 SV=1	12018	689	0	19	0	0.29	0	0	0	0	1	0	0	0	1	0	-0.89213
tr|D3DUL0|D3DUL0_HUMAN	ASCIZ	ATM/ATR-Substrate Chk2-Interacting Zn2+-finger protein, isoform CRA_b OS=Homo sapiens GN=ASCIZ PE=2 S	79474	690	0	19	0	0.04	0	0	0	0	1	0	0	0	1	0	-0.89213
tr|H7C4X2|H7C4X2_HUMAN	PCMT1	Protein-L-isoaspartate(D-aspartate) O-methyltransferase (Fragment) OS=Homo sapiens GN=PCMT1 PE=4 SV=1	7342	691	0	19	0	0.48	0	0	0	0	1	0	0	0	2	0	-0.89213
tr|B3KND7|B3KND7_HUMAN	tr|B3KND7|B3K	cDNA FLJ14378 fis, clone HEMBA1001804, highly similar to Zinc finger protein 281 (Fragment) OS=Homo sapie	58221	692	0	19	0	0.06	0	0	0	0	1	0	0	0	1	0	-0.89213
tr|H0YE81|H0YE81_HUMAN	NDUFV1	NADH dehydrogenase [ubiquinone] flavoprotein 1, mitochondrial (Fragment) OS=Homo sapiens GN=NDUFV1	14494	694	0	19	0	0.23	0	0	0	0	1	0	0	0	1	0	-0.89213
tr|H0Y304|H0Y304_HUMAN	DMD	Dystrophin (Fragment) OS=Homo sapiens GN=DMD PE=4 SV=1	160176	695	0	19	0	0.02	0	0	0	0	1	0	0	0	1	0	-0.89213
tr|Q5T097|Q5T097_HUMAN	UTRN	Utrophin OS=Homo sapiens GN=UTRN PE=4 SV=1	114423	696	0	19	0	0.03	0	0	0	0	1	0	0	0	1	0	-0.89213
tr|B4DY47|B4DY47_HUMAN	PHB	Prohibitin OS=Homo sapiens GN=PHB PE=2 SV=1	17064	699	0	18	0	0.2	0	0	0	0	1	0	0	0	1	0	-0.89213
tr|J3KQ96|J3KQ96_HUMAN	TCOF1	Treacle protein (Fragment) OS=Homo sapiens GN=TCOF1 PE=4 SV=1	144268	702	0	18	0	0.02	0	0	0	0	1	0	0	0	2	0	-0.89213
tr|B0UZL4|B0UZL4_HUMAN	BAT3	HLA-B associated transcript 3 (Fragment) OS=Homo sapiens GN=BAT3 PE=2 SV=1	15422	704	0	18	0	0.03	0	0	0	0	1	0	0	0	1	0	-0.89213
tr|J3KMW7|J3KMW7_HUMAN	UBR5	E3 ubiquitin-protein ligase UBR5 OS=Homo sapiens GN=UBR5 PE=4 SV=1	312224	706	0	18	0	0.01	0	0	0	0	1	0	0	0	1	0	-0.89213
tr|H0Y4R4|H0Y4R4_HUMAN	TOR1AIP1	Torsin-1A-interacting protein 1 (Fragment) OS=Homo sapiens GN=TOR1AIP1 PE=4 SV=1	33844	707	0	17	0	0.1	0	0	0	0	1	0	0	0	1	0	-0.89213
sp|P54826|GAS1_HUMAN	GAS1	Growth arrest-specific protein 1 OS=Homo sapiens GN=GAS1 PE=1 SV=2	36867	708	0	17	0	0.09	0	0	0	0	1	0	0	0	1	0	-0.89213
tr|H0YHD9|H0YHD9_HUMAN	KRT72	Keratin, type II cytoskeletal 72 (Fragment) OS=Homo sapiens GN=KRT72 PE=4 SV=1	14802	11	0	43	0	4.79	0	0	1	0	1	0	1	0	2	0	-2.47743
tr|Q6ZPD6|Q6ZPD6_HUMAN	tr|Q6ZPD6|Q6Z	cDNA FLJ25992 fis, clone DMC03508 OS=Homo sapiens PE=2 SV=1	41924	12	0	51	0	4.67	0	0	1	0	1	0	1	0	1	0	-2.47743
tr|B9VP24|B9VP24_HUMAN	HSPD1	60 kDa chaperonin (Fragment) OS=Homo sapiens GN=HSPD1 PE=4 SV=1	19880	16	0	40	0	1.72	0	0	1	0	1	0	1	0	1	0	-2.47743
tr|B7Z2Y4|B7Z2Y4_HUMAN	tr|B7Z2Y4|B7Z2	cDNA FLJ56961, highly similar to Clathrin heavy chain 2 (CLH-22) OS=Homo sapiens PE=2 SV=1	54629	30	0	60	0	0.52	0	0	1	0	1	0	1	0	2	0	-2.47743
tr|F8VS61|F8VS61_HUMAN	KRT77	Keratin, type II cytoskeletal 1b OS=Homo sapiens GN=KRT77 PE=2 SV=1	25704	76	0	43	0	0.23	0	0	1	0	1	0	1	0	3	0	-2.47743
tr|H0YC58|H0YC58_HUMAN	KIAA1967	DBIRD complex subunit KIAA1967 (Fragment) OS=Homo sapiens GN=KIAA1967 PE=4 SV=1	19387	82	0	45	0	0.32	0	0	1	0	1	0	1	0	2	0	-2.47743
tr|A2RRC9|A2RRC9_HUMAN	IQGAP3	IQ motif containing GTPase activating protein 3 OS=Homo sapiens GN=IQGAP3 PE=2 SV=1	185145	84	0	32	0	0.31	0	0	0	0	1	0	1	0	1	0	-2.47743
tr|Q659G2|Q659G2_HUMAN	DKFZp586B2023	Putative uncharacterized protein DKFZp586B2023 (Fragment) OS=Homo sapiens GN=DKFZp586B2023 PE=2 SV	7133	124	0	41	0	0.57	0	0	1	0	1	0	1	0	1	0	-2.47743
tr|F8W1G6|F8W1G6_HUMAN	PCBP2	Poly(rC)-binding protein 2 (Fragment) OS=Homo sapiens GN=PCBP2 PE=2 SV=2	22263	132	0	35	0	0.32	0	0	1	0	1	0	1	0	3	0	-2.47743
tr|Q58FF2|Q58FF2_HUMAN	GRP94c	Heat shock protein 94c OS=Homo sapiens GN=GRP94c PE=2 SV=1	52205	140	0	22	0	0.37	0	0	0	0	1	0	1	0	2	0	-2.47743
tr|G3V423|G3V423_HUMAN	WARS	T1-TrpRS (Fragment) OS=Homo sapiens GN=WARS PE=2 SV=1	15836	145	0	34	0	0.27	0	0	1	0	1	0	1	0	1	0	-2.47743
tr|Q8NI92|Q8NI92_HUMAN	OK/SW-cl.65	DEAD/H(Asp-Glu-Ala-Asp/His)box polypeptide 21 OS=Homo sapiens GN=OK/SW-cl.65 PE=2 SV=1	16499	148	0	21	0	0.34	0	0	0	0	1	0	1	0	1	0	-2.47743
tr|K7EQ75|K7EQ75_HUMAN	ILF3	Interleukin enhancer-binding factor 3 (Fragment) OS=Homo sapiens GN=ILF3 PE=4 SV=1	6139	154	0	29	0	0.46	0	0	0	0	1	0	1	0	1	0	-2.47743
tr|Q6RUP9|Q6RUP9_HUMAN	IMPDH2	Inosine monophosphate dehydrogenase type II (Fragment) OS=Homo sapiens GN=IMPDH2 PE=2 SV=1	7313	177	0	25	0	0.36	0	0	0	0	1	0	1	0	1	0	-2.47743
tr|Q9BUV4|Q9BUV4_HUMAN	RPL5	RPL5 protein OS=Homo sapiens GN=RPL5 PE=2 SV=1	12182	187	0	39	0	0.44	0	0	0	0	1	0	1	0	2	0	-2.47743
tr|B3KV39|B3KV39_HUMAN	tr|B3KV39|B3K	cDNA FLJ16099 fis, clone TESTI2008278, highly similar to Homo sapiens forkhead box K1 (FOXK1), mRNA OS=H	50383	188	0	115	0	0.07	0	0	1	0	1	0	1	0	2	0	-2.47743
tr|D6R9D3|D6R9D3_HUMAN	HNRNPH1	Heterogeneous nuclear ribonucleoprotein H OS=Homo sapiens GN=HNRNPH1 PE=4 SV=1	4404	207	0	23	0	0.45	0	0	0	0	1	0	1	0	1	0	-2.47743
tr|H0Y9U2|H0Y9U2_HUMAN	SFPQ	Splicing factor, proline- and glutamine-rich (Fragment) OS=Homo sapiens GN=SFPQ PE=4 SV=1	9491	228	0	55	0	0.26	0	0	1	0	1	0	1	0	1	0	-2.47743
tr|B4DE40|B4DE40_HUMAN	SMS	Spermine synthase OS=Homo sapiens GN=SMS PE=2 SV=1	31596	248	0	78	0	0.11	0	0	1	0	1	0	1	0	1	0	-2.47743
tr|Q5JXB7|Q5JXB7_HUMAN	PLOD1	Procollagen-lysine,2-oxoglutarate 5-dioxygenase 1 (Fragment) OS=Homo sapiens GN=PLOD1 PE=4 SV=1	31911	251	0	76	0	0.04	0	0	1	0	1	0	1	0	1	0	-2.47743
tr|B4DXK4|B4DXK4_HUMAN	KRT72	Keratin, type II cytoskeletal 72 OS=Homo sapiens GN=KRT72 PE=2 SV=1	36031	259	0	0	0	0.45	0	0	0	0	0	0	1	0	1	0	-2.47743
tr|K7EKB6|K7EKB6_HUMAN	THOP1	Thimet oligopeptidase (Fragment) OS=Homo sapiens GN=THOP1 PE=4 SV=1	20792	278	0	68	0	0.16	0	0	1	0	1	0	1	0	1	0	-2.47743
tr|E5RFS9|E5RFS9_HUMAN	MAT2B	Methionine adenosyltransferase 2 subunit beta OS=Homo sapiens GN=MAT2B PE=2 SV=1	9467	280	0	68	0	0.37	0	0	1	0	1	0	1	0	1	0	-2.47743
tr|H0YC45|H0YC45_HUMAN	ATP6V1B2	V-type proton ATPase subunit B, brain isoform (Fragment) OS=Homo sapiens GN=ATP6V1B2 PE=4 SV=1	5569	287	0	64	0	0.65	0	0	1	0	1	0	1	0	1	0	-2.47743
tr|B4DE27|B4DE27_HUMAN	tr|B4DE27|B4D	cDNA FLJ58765, highly similar to Neutral amino acid transporter B(0) OS=Homo sapiens PE=2 SV=1	33987	290	0	63	0	0.1	0	0	1	0	1	0	1	0	1	0	-2.47743
sp|P81605|DCD_HUMAN	DCD	Dermcidin OS=Homo sapiens GN=DCD PE=1 SV=2	11391	295	0	62	0	0.3	0	0	1	0	1	0	1	0	1	0	-2.47743
tr|A1L3A7|A1L3A7_HUMAN	NUFIP2	Nuclear fragile X mental retardation protein interacting protein 2 OS=Homo sapiens GN=NUFIP2 PE=2 SV=1	76132	301	0	59	0	0.04	0	0	1	0	1	0	1	0	1	0	-2.47743
tr|B5LY64|B5LY64_HUMAN	UMPS	Orotidine 5~-phosphate decarboxylase OS=Homo sapiens GN=UMPS PE=2 SV=1	33258	302	0	59	0	0.1	0	0	1	0	1	0	1	0	1	0	-2.47743
tr|B3KPC1|B3KPC1_HUMAN	tr|B3KPC1|B3K	cDNA FLJ31582 fis, clone NT2RI2002117, highly similar to Protein pelota homolog OS=Homo sapiens PE=2 SV=	43807	307	0	59	0	0.08	0	0	1	0	1	0	1	0	1	0	-2.47743
tr|C9JRL4|C9JRL4_HUMAN	MDH1	Malate dehydrogenase, cytoplasmic (Fragment) OS=Homo sapiens GN=MDH1 PE=2 SV=1	13207	309	0	39	0	0.31	0	0	1	0	1	0	1	0	1	0	-2.47743
sp|Q5TZA2|CROCC_HUMAN	CROCC	Rootletin OS=Homo sapiens GN=CROCC PE=1 SV=1	228787	310	0	58	0	0.01	0	0	1	0	1	0	1	0	2	0	-2.47743
tr|C9JPP3|C9JPP3_HUMAN	RBBP4	Histone-binding protein RBBP4 (Fragment) OS=Homo sapiens GN=RBBP4 PE=2 SV=2	9655	315	0	57	0	0.36	0	0	1	0	1	0	1	0	1	0	-2.47743
tr|Q5RZ99|Q5RZ99_HUMAN	tr|Q5RZ99|Q5R	Apoptosis-inducing factor (Fragment) OS=Homo sapiens PE=2 SV=1	24524	326	0	54	0	0.05	0	0	1	0	1	0	1	0	1	0	-2.47743
tr|I3L4P6|I3L4P6_HUMAN	ARHGAP17	Rho GTPase-activating protein 17 (Fragment) OS=Homo sapiens GN=ARHGAP17 PE=4 SV=1	8676	328	0	54	0	0.03	0	0	1	0	1	0	1	0	1	0	-2.47743
tr|A8K3Q9|A8K3Q9_HUMAN	tr|A8K3Q9|A8K	cDNA FLJ76611, highly similar to Homo sapiens ribosomal protein L14 (RPL14), mRNA OS=Homo sapiens PE=2	23554	330	0	53	0	0.14	0	0	1	0	1	0	1	0	1	0	-2.47743
sp|Q9Y6M5|ZNT1_HUMAN	SLC30A1	Zinc transporter 1 OS=Homo sapiens GN=SLC30A1 PE=1 SV=3	56290	334	0	52	0	0.06	0	0	1	0	1	0	1	0	1	0	-2.47743
tr|A8KA82|A8KA82_HUMAN	DNAJC3	DnaJ (Hsp40) homolog, subfamily C, member 3 OS=Homo sapiens GN=DNAJC3 PE=2 SV=1	58000	337	0	52	0	0.06	0	0	1	0	1	0	1	0	1	0	-2.47743
tr|B2RAR2|B2RAR2_HUMAN	tr|B2RAR2|B2R	cDNA, FLJ95064, highly similar to Homo sapiens nin one binding protein (NOB1P), mRNA OS=Homo sapiens PE	47015	338	0	52	0	0.07	0	0	1	0	1	0	1	0	1	0	-2.47743
tr|A8K517|A8K517_HUMAN	RPS23	Ribosomal protein S23, isoform CRA_a OS=Homo sapiens GN=RPS23 PE=2 SV=1	15969	339	0	52	0	0.21	0	0	1	0	1	0	1	0	1	0	-2.47743
tr|D6RF19|D6RF19_HUMAN	DPYSL3	Dihydropyrimidinase-related protein 3 (Fragment) OS=Homo sapiens GN=DPYSL3 PE=2 SV=1	10214	342	0	20	0	0.23	0	0	0	0	1	0	1	0	1	0	-2.47743
tr|H0YC77|H0YC77_HUMAN	ANXA6	Annexin (Fragment) OS=Homo sapiens GN=ANXA6 PE=3 SV=1	10357	349	0	27	0	0.14	0	0	0	0	1	0	1	0	1	0	-2.47743
tr|K7EN42|K7EN42_HUMAN	NMT1	Glycylpeptide N-tetradecanoyltransferase 1 (Fragment) OS=Homo sapiens GN=NMT1 PE=4 SV=1	9699	350	0	31	0	0.49	0	0	0	0	1	0	1	0	1	0	-2.47743
tr|B7Z8A2|B7Z8A2_HUMAN	tr|B7Z8A2|B7Z8	cDNA FLJ51671, highly similar to Prenylcysteine oxidase (EC 1.8.3.5) OS=Homo sapiens PE=2 SV=1	54918	355	0	49	0	0.06	0	0	1	0	1	0	1	0	1	0	-2.47743
tr|D6RBX4|D6RBX4_HUMAN	TGFBI	Transforming growth factor-beta-induced protein ig-h3 OS=Homo sapiens GN=TGFBI PE=2 SV=1	7268	358	0	31	0	0.09	0	0	0	0	1	0	1	0	1	0	-2.47743
tr|A6NKV8|A6NKV8_HUMAN	PAIP1	Polyadenylate-binding protein-interacting protein 1 OS=Homo sapiens GN=PAIP1 PE=2 SV=1	42379	361	0	48	0	0.08	0	0	1	0	1	0	1	0	1	0	-2.47743
tr|H0Y9Q1|H0Y9Q1_HUMAN	LAP3	Cytosol aminopeptidase (Fragment) OS=Homo sapiens GN=LAP3 PE=4 SV=1	22641	368	0	47	0	0.15	0	0	1	0	1	0	1	0	1	0	-2.47743
tr|E9PP60|E9PP60_HUMAN	TSTA3	GDP-L-fucose synthase OS=Homo sapiens GN=TSTA3 PE=2 SV=1	12325	371	0	47	0	0.19	0	0	1	0	1	0	1	0	1	0	-2.47743
tr|F8WJN3|F8WJN3_HUMAN	CPSF6	Cleavage and polyadenylation-specificity factor subunit 6 OS=Homo sapiens GN=CPSF6 PE=2 SV=1	52409	379	0	46	0	0.06	0	0	1	0	1	0	1	0	1	0	-2.47743
tr|H0YGH1|H0YGH1_HUMAN	PFAS	Phosphoribosylformylglycinamidine synthase (Fragment) OS=Homo sapiens GN=PFAS PE=4 SV=2	41755	380	0	37	0	0.05	0	0	0	0	1	0	1	0	1	0	-2.47743
tr|B4DT09|B4DT09_HUMAN	tr|B4DT09|B4D	cDNA FLJ60145, highly similar to Homo sapiens dendritic cell protein (hfl-B5), mRNA OS=Homo sapiens PE=2 S	13697	381	0	46	0	0.25	0	0	1	0	1	0	1	0	1	0	-2.47743
sp|Q8NFI4|F10A5_HUMAN	ST13P5	Putative protein FAM10A5 OS=Homo sapiens GN=ST13P5 PE=5 SV=1	41579	382	0	0	0	0.09	0	0	0	0	0	0	1	0	2	0	-2.47743
tr|E5RGY7|E5RGY7_HUMAN	CTNNA1	Catenin alpha-1 (Fragment) OS=Homo sapiens GN=CTNNA1 PE=2 SV=1	14895	389	0	35	0	0.07	0	0	0	0	1	0	1	0	1	0	-2.47743
tr|B4DNB2|B4DNB2_HUMAN	tr|B4DNB2|B4D	cDNA FLJ58919, moderately similar to Succinate dehydrogenase (ubiquinone) flavoprotein subunit, mitochond	14636	393	0	0	0	0.1	0	0	0	0	0	0	1	0	2	0	-2.47743
tr|Q1LZN2|Q1LZN2_HUMAN	NOMO3	NOMO3 protein (Fragment) OS=Homo sapiens GN=NOMO3 PE=2 SV=1	90388	400	0	43	0	0.04	0	0	1	0	1	0	1	0	1	0	-2.47743
sp|Q9NSB2|KRT84_HUMAN	KRT84	Keratin, type II cuticular Hb4 OS=Homo sapiens GN=KRT84 PE=2 SV=2	65942	409	0	43	0	0.05	0	0	1	0	1	0	1	0	3	0	-2.47743
tr|B4DRG7|B4DRG7_HUMAN	tr|B4DRG7|B4D	cDNA FLJ60336, highly similar to Condensin complex subunit 2 OS=Homo sapiens PE=2 SV=1	80877	410	0	43	0	0.04	0	0	1	0	1	0	1	0	1	0	-2.47743
sp|Q9NSB4|KRT82_HUMAN	KRT82	Keratin, type II cuticular Hb2 OS=Homo sapiens GN=KRT82 PE=1 SV=3	57985	411	0	43	0	0.06	0	0	1	0	1	0	1	0	2	0	-2.47743
tr|H0YGE9|H0YGE9_HUMAN	M6PR	Cation-dependent mannose-6-phosphate receptor (Fragment) OS=Homo sapiens GN=M6PR PE=4 SV=1	11814	416	0	42	0	0.29	0	0	1	0	1	0	1	0	1	0	-2.47743
tr|B4DPY2|B4DPY2_HUMAN	tr|B4DPY2|B4D	cDNA FLJ59286, highly similar to Pre-mRNA-processing factor 40 homolog A (Fragment) OS=Homo sapiens PE=	93124	425	0	41	0	0.04	0	0	1	0	1	0	1	0	1	0	-2.47743
tr|Q5JR94|Q5JR94_HUMAN	RPS8	40S ribosomal protein S8 OS=Homo sapiens GN=RPS8 PE=2 SV=1	24475	426	0	41	0	0.14	0	0	1	0	1	0	1	0	2	0	-2.47743
tr|A4D1I7|A4D1I7_HUMAN	DNCI1	Dynein, cytoplasmic 1, intermediate chain 1, isoform CRA_e OS=Homo sapiens GN=DNCI1 PE=2 SV=1	73423	427	0	40	0	0.04	0	0	1	0	1	0	1	0	1	0	-2.47743
tr|B4E363|B4E363_HUMAN	FARSA	Phenylalanine--tRNA ligase alpha subunit OS=Homo sapiens GN=FARSA PE=2 SV=1	54181	447	0	38	0	0.06	0	0	1	0	1	0	1	0	1	0	-2.47743
tr|H0Y9L5|H0Y9L5_HUMAN	EXOSC9	Exosome complex component RRP45 (Fragment) OS=Homo sapiens GN=EXOSC9 PE=4 SV=1	18940	457	0	38	0	0.18	0	0	1	0	1	0	1	0	1	0	-2.47743
tr|B3KME2|B3KME2_HUMAN	tr|B3KME2|B3K	cDNA FLJ10772 fis, clone NT2RP4000243, highly similar to Cartilage-associated protein OS=Homo sapiens PE=	47101	459	0	38	0	0.07	0	0	1	0	1	0	1	0	1	0	-2.47743
sp|Q9H9F9|ARP5_HUMAN	ACTR5	Actin-related protein 5 OS=Homo sapiens GN=ACTR5 PE=1 SV=2	68825	460	0	38	0	0.05	0	0	1	0	1	0	1	0	2	0	-2.47743

tr|H7C1M0|H7C1M0_HUMAN	FANCL	E3 ubiquitin-protein ligase FANCL (Fragment) OS=Homo sapiens GN=FANCL PE=4 SV=1	33067	461	0	37	0	0.1	0	0	1	0	1	0	1	0	1	0	-2.47743
tr|Q5SZ64|Q5SZ64_HUMAN	SF3B4	Splicing factor 3B subunit 4 (Fragment) OS=Homo sapiens GN=SF3B4 PE=4 SV=1	20733	464	0	37	0	0.07	0	0	1	0	1	0	1	0	1	0	-2.47743
sp|Q6ZU64|CC108_HUMAN	CCDC108	Coiled-coil domain-containing protein 108 OS=Homo sapiens GN=CCDC108 PE=2 SV=2	220247	465	0	37	0	0.01	0	0	1	0	1	0	1	0	1	0	-2.47743
tr|C9J7L0|C9J7L0_HUMAN	RBBP7	Histone-binding protein RBBP7 (Fragment) OS=Homo sapiens GN=RBBP7 PE=2 SV=1	7740	468	0	0	0	0.07	0	0	0	0	0	0	1	0	1	0	-2.47743
tr|H0YIX9|H0YIX9_HUMAN	R3HDM2	R3H domain-containing protein 2 (Fragment) OS=Homo sapiens GN=R3HDM2 PE=4 SV=1	31776	469	0	37	0	0.1	0	0	1	0	1	0	1	0	1	0	-2.47743
tr|D6RA26|D6RA26_HUMAN	PLRG1	Pleiotropic regulator 1 (Fragment) OS=Homo sapiens GN=PLRG1 PE=2 SV=1	23562	476	0	18	0	0.12	0	0	0	0	1	0	1	0	2	0	-2.47743
tr|B7WPL0|B7WPL0_HUMAN	RIC8B	Synembryn-B OS=Homo sapiens GN=RIC8B PE=2 SV=1	64252	483	0	36	0	0.05	0	0	1	0	1	0	1	0	1	0	-2.47743
tr|C9JNW5|C9JNW5_HUMAN	RPL24	60S ribosomal protein L24 OS=Homo sapiens GN=RPL24 PE=4 SV=1	17646	487	0	35	0	0.19	0	0	0	0	1	0	1	0	2	0	-2.47743
tr|E9PI04|E9PI04_HUMAN	RIC8A	Synembryn-A (Fragment) OS=Homo sapiens GN=RIC8A PE=2 SV=1	16936	496	0	34	0	0.05	0	0	0	0	1	0	1	0	1	0	-2.47743
tr|B4DDC9|B4DDC9_HUMAN	tr|B4DDC9|B4D	cDNA FLJ59448, highly similar to Trifunctional enzyme subunit beta, mitochondrial OS=Homo sapiens PE=2 SV	49937	499	0	34	0	0.07	0	0	0	0	1	0	1	0	1	0	-2.47743
tr|B4DXJ6|B4DXJ6_HUMAN	tr|B4DXJ6|B4D)	cDNA FLJ61453, highly similar to Adapter-relatedprotein complex 2 alpha- 1 subunit OS=Homo sapiens PE=2 S	55511	500	0	34	0	0.03	0	0	0	0	1	0	1	0	1	0	-2.47743
sp|Q8NFZ5|TNIP2_HUMAN	TNIP2	TNFAIP3-interacting protein 2 OS=Homo sapiens GN=TNIP2 PE=1 SV=1	49240	502	0	33	0	0.07	0	0	1	0	1	0	1	0	2	0	-2.47743
sp|Q7Z494|NPHP3_HUMAN	NPHP3	Nephrocystin-3 OS=Homo sapiens GN=NPHP3 PE=1 SV=1	151967	510	0	32	0	0.02	0	0	1	0	1	0	1	0	1	0	-2.47743
tr|Q5H9P1|Q5H9P1_HUMAN	DKFZp686F1345	Putative uncharacterized protein DKFZp686F1345 (Fragment) OS=Homo sapiens GN=DKFZp686F1345 PE=2 SV	193061	511	0	32	0	0.01	0	0	0	0	1	0	1	0	1	0	-2.47743
tr|A0JLS9|A0JLS9_HUMAN	ZEB1	ZEB1 protein (Fragment) OS=Homo sapiens GN=ZEB1 PE=2 SV=1	118576	513	0	32	0	0.03	0	0	1	0	1	0	1	0	1	0	-2.47743
tr|J3KRB3|J3KRB3_HUMAN	RPL17	60S ribosomal protein L17 (Fragment) OS=Homo sapiens GN=RPL17 PE=3 SV=1	12356	517	0	32	0	0.28	0	0	0	0	1	0	1	0	1	0	-2.47743
tr|B4DUH7|B4DUH7_HUMAN	tr|B4DUH7|B4D	cDNA FLJ60162, highly similar to Chloride anion exchanger OS=Homo sapiens PE=2 SV=1	52632	518	0	32	0	0.06	0	0	1	0	1	0	1	0	1	0	-2.47743
tr|E9PNN6|E9PNN6_HUMAN	ALDH3A1	Aldehyde dehydrogenase, dimeric NADP-preferring OS=Homo sapiens GN=ALDH3A1 PE=2 SV=1	44518	519	0	32	0	0.07	0	0	0	0	1	0	1	0	1	0	-2.47743
tr|B7Z8W6|B7Z8W6_HUMAN	tr|B7Z8W6|B7Z	cDNA FLJ55038, highly similar to Nicotinamide phosphoribosyltransferase (EC 2.4.2.12) OS=Homo sapiens PE=	26905	520	0	32	0	0.12	0	0	0	0	1	0	1	0	3	0	-2.47743
tr|L0R4T3|L0R4T3_HUMAN	ABCF2	Alternative protein ABCF2 OS=Homo sapiens GN=ABCF2 PE=4 SV=1	7359	524	0	31	0	0.48	0	0	1	0	1	0	1	0	1	0	-2.47743
tr|B4E0D5|B4E0D5_HUMAN	tr|B4E0D5|B4E(	cDNA FLJ54218, highly similar to Acidic leucine-rich nuclear phosphoprotein 32 family member E OS=Homo sa	19540	525	0	31	0	0.17	0	0	0	0	1	0	1	0	1	0	-2.47743
tr|B7Z9W6|B7Z9W6_HUMAN	tr|B7Z9W6|B7Z	cDNA, FLJ78980, highly similar to DnaJ homolog subfamily C member 7 OS=Homo sapiens PE=2 SV=1	50776	527	0	31	0	0.06	0	0	0	0	1	0	1	0	1	0	-2.47743
tr|A1L3B5|A1L3B5_HUMAN	ATP2B3	ATP2B3 protein OS=Homo sapiens GN=ATP2B3 PE=2 SV=1	96278	528	0	31	0	0.03	0	0	0	0	1	0	1	0	1	0	-2.47743
sp|Q96PE2|ARHGH_HUMAN	ARHGEF17	Rho guanine nucleotide exchange factor 17 OS=Homo sapiens GN=ARHGEF17 PE=1 SV=1	223645	537	0	30	0	0.01	0	0	1	0	1	0	1	0	1	0	-2.47743
tr|K7EKM4|K7EKM4_HUMAN	SRM	Spermidine synthase OS=Homo sapiens GN=SRM PE=4 SV=1	10680	540	0	30	0	0.32	0	0	0	0	1	0	1	0	1	0	-2.47743
tr|H0YK49|H0YK49_HUMAN	ETFA	Electron transfer flavoprotein subunit alpha, mitochondrial OS=Homo sapiens GN=ETFA PE=4 SV=1	24259	545	0	30	0	0.14	0	0	0	0	1	0	1	0	1	0	-2.47743
tr|B6F210|B6F210_HUMAN	GSS	Glutathione synthetase OS=Homo sapiens GN=GSS PE=2 SV=1	40495	551	0	30	0	0.08	0	0	0	0	1	0	1	0	1	0	-2.47743
tr|H3BRB3|H3BRB3_HUMAN	KIF22	Kinesin-like protein KIF22 OS=Homo sapiens GN=KIF22 PE=3 SV=1	67996	557	0	29	0	0.05	0	0	0	0	1	0	1	0	1	0	-2.47743
tr|B4DS04|B4DS04_HUMAN	tr|B4DS04|B4D	cDNA FLJ54142, highly similar to Protein transport protein Sec23B OS=Homo sapiens PE=2 SV=1	41720	558	0	27	0	0.08	0	0	0	0	1	0	1	0	1	0	-2.47743
sp|Q96A33|CCD47_HUMAN	CCDC47	Coiled-coil domain-containing protein 47 OS=Homo sapiens GN=CCDC47 PE=1 SV=1	56123	560	0	29	0	0.06	0	0	0	0	1	0	1	0	1	0	-2.47743
tr|I3L361|I3L361_HUMAN	SGSM2	Small G protein-signaling modulator 2 (Fragment) OS=Homo sapiens GN=SGSM2 PE=4 SV=2	1642	570	0	29	0	2.38	0	0	0	0	1	0	1	0	1	0	-2.47743
tr|G3XAF4|G3XAF4_HUMAN	L1CAM	L1 cell adhesion molecule, isoform CRA_a OS=Homo sapiens GN=L1CAM PE=4 SV=1	139790	572	0	28	0	0.05	0	0	1	0	2	0	1	0	2	0	-2.47743
tr|B4DNG6|B4DNG6_HUMAN	tr|B4DNG6|B4D	Annexin OS=Homo sapiens PE=3 SV=1	12870	577	0	28	0	0.27	0	0	0	0	1	0	1	0	1	0	-2.47743
sp|Q16322|KCA10_HUMAN	KCNA10	Potassium voltage-gated channel subfamily A member 10 OS=Homo sapiens GN=KCNA10 PE=1 SV=2	58147	578	0	28	0	0.06	0	0	1	0	1	0	1	0	1	0	-2.47743
tr|J3KN32|J3KN32_HUMAN	RCOR1	REST corepressor 1 OS=Homo sapiens GN=RCOR1 PE=4 SV=1	53409	583	0	28	0	0.06	0	0	0	0	1	0	1	0	1	0	-2.47743
tr|H0YCU9|H0YCU9_HUMAN	TAGLN	Transgelin (Fragment) OS=Homo sapiens GN=TAGLN PE=4 SV=1	16833	585	0	28	0	0.2	0	0	0	0	1	0	1	0	1	0	-2.47743
tr|A6NE42|A6NE42_HUMAN	TGIF1	Homeobox protein TGIF1 OS=Homo sapiens GN=TGIF1 PE=3 SV=2	27983	586	0	28	0	0.12	0	0	0	0	1	0	1	0	1	0	-2.47743
tr|B3KY60|B3KY60_HUMAN	tr|B3KY60|B3K\	cDNA FLJ16777 fis, clone BRHIP2029567, highly similar to Cell division cycle 5-like protein OS=Homo sapiens P	92448	587	0	27	0	0.04	0	0	0	0	1	0	1	0	1	0	-2.47743
tr|Q9NZ23|Q9NZ23_HUMAN	YA61	Drug-sensitive protein 1 OS=Homo sapiens GN=YA61 PE=2 SV=1	14858	588	0	27	0	0.23	0	0	0	0	1	0	1	0	1	0	-2.47743
tr|B2RDF5|B2RDF5_HUMAN	tr|B2RDF5|B2R	cDNA, FLJ96587, highly similar to Homo sapiens SUMO-1 activating enzyme subunit 2 (UBA2), mRNA OS=Hom	71705	590	0	27	0	0.05	0	0	0	0	1	0	1	0	1	0	-2.47743
tr|H3BS19|H3BS19_HUMAN	ZNF469	Zinc finger protein 469 OS=Homo sapiens GN=ZNF469 PE=4 SV=1	417347	591	0	27	0	0.01	0	0	0	0	1	0	1	0	1	0	-2.47743
tr|H0Y512|H0Y512_HUMAN	APMAP	Adipocyte plasma membrane-associated protein (Fragment) OS=Homo sapiens GN=APMAP PE=4 SV=1	45662	592	0	27	0	0.07	0	0	0	0	1	0	1	0	1	0	-2.47743
tr|B7Z4L9|B7Z4L9_HUMAN	tr|B7Z4L9|B7Z4	cDNA FLJ55342, highly similar to Kelch-like protein 13 OS=Homo sapiens PE=2 SV=1	56884	593	0	26	0	0.06	0	0	0	0	1	0	1	0	1	0	-2.47743
tr|Q5T8C6|Q5T8C6_HUMAN	CDC16	Cell division cycle protein 16 homolog OS=Homo sapiens GN=CDC16 PE=4 SV=1	55239	594	0	26	0	0.06	0	0	0	0	1	0	1	0	1	0	-2.47743
sp|Q0VD83|APOBR_HUMAN	APOBR	Apolipoprotein B receptor OS=Homo sapiens GN=APOBR PE=1 SV=1	115207	597	0	26	0	0.03	0	0	0	0	1	0	1	0	1	0	-2.47743
tr|Q8TC04|Q8TC04_HUMAN	KRT23	Keratin 23 (Histone deacetylase inducible) OS=Homo sapiens GN=KRT23 PE=2 SV=1	48318	600	0	26	0	0.07	0	0	0	0	1	0	1	0	1	0	-2.47743
tr|B4DNK3|B4DNK3_HUMAN	tr|B4DNK3|B4D	cDNA FLJ52127, highly similar to Multisynthetase complex auxiliary component p43 OS=Homo sapiens PE=2 S\	29955	601	0	26	0	0.11	0	0	0	0	1	0	1	0	1	0	-2.47743
tr|B4E2I9|B4E2I9_HUMAN	tr|B4E2I9|B4E2	cDNA FLJ55486, highly similar to Semaphorin-3C OS=Homo sapiens PE=2 SV=1	88481	602	0	26	0	0.04	0	0	0	0	1	0	1	0	2	0	-2.47743
tr|J3KNP0|J3KNP0_HUMAN	RAB11FIP1	Rab11 family-interacting protein 1 OS=Homo sapiens GN=RAB11FIP1 PE=4 SV=1	137482	607	0	25	0	0.02	0	0	0	0	1	0	1	0	2	0	-2.47743
tr|J3KSB9|J3KSB9_HUMAN	tr|J3KSB9|J3KS|	Uncharacterized protein (Fragment) OS=Homo sapiens PE=4 SV=1	8877	608	0	25	0	0.39	0	0	0	0	1	0	1	0	1	0	-2.47743
tr|H3BMW5|H3BMW5_HUMAN	DNAJA2	DnaJ homolog subfamily A member 2 (Fragment) OS=Homo sapiens GN=DNAJA2 PE=4 SV=1	15134	611	0	24	0	0.23	0	0	0	0	1	0	1	0	1	0	-2.47743
tr|B4DVB8|B4DVB8_HUMAN	ELAVL1	ELAV-like protein 1 OS=Homo sapiens GN=ELAVL1 PE=2 SV=1	39200	612	0	24	0	0.08	0	0	0	0	1	0	1	0	1	0	-2.47743
tr|Q9H6Q0|Q9H6Q0_HUMAN	tr|Q9H6Q0|Q9I	cDNA: FLJ22001 fis, clone HEP06629 OS=Homo sapiens PE=2 SV=1	17198	614	0	0	0	0.05	0	0	0	0	0	0	1	0	5	0	-2.47743
tr|Q4VAN1|Q4VAN1_HUMAN	BTN1A1	BTN1A1 protein OS=Homo sapiens GN=BTN1A1 PE=2 SV=1	55878	617	0	24	0	0.06	0	0	0	0	1	0	1	0	1	0	-2.47743
tr|Q2TQ40|Q2TQ40_HUMAN	TNFRSF17	B cell maturation antigen transcript variant 4 OS=Homo sapiens GN=TNFRSF17 PE=2 SV=1	15460	618	0	24	0	0.22	0	0	0	0	1	0	1	0	1	0	-2.47743
tr|B4DU06|B4DU06_HUMAN	tr|B4DU06|B4D	Guanylate cyclase OS=Homo sapiens PE=2 SV=1	91756	620	0	24	0	0.04	0	0	0	0	1	0	1	0	1	0	-2.47743
tr|B0QXZ5|B0QXZ5_HUMAN	DNAL4	Dynein light chain 4, axonemal OS=Homo sapiens GN=DNAL4 PE=4 SV=1	7786	621	0	24	0	0.47	0	0	0	0	1	0	1	0	1	0	-2.47743
tr|F5H120|F5H120_HUMAN	FKBP4	Peptidyl-prolyl cis-trans isomerase FKBP4, N-terminally processed OS=Homo sapiens GN=FKBP4 PE=2 SV=1	5528	623	0	24	0	0.06	0	0	0	0	1	0	1	0	1	0	-2.47743
tr|K7EPB5|K7EPB5_HUMAN	ELAVL3	ELAV-like protein 3 (Fragment) OS=Homo sapiens GN=ELAVL3 PE=4 SV=1	9728	625	0	23	0	0.36	0	0	0	0	1	0	1	0	1	0	-2.47743
tr|B4DFE4|B4DFE4_HUMAN	tr|B4DFE4|B4D	cDNA FLJ59618, highly similar to Exosome component 10 OS=Homo sapiens PE=2 SV=1	61733	627	0	23	0	0.05	0	0	0	0	1	0	1	0	1	0	-2.47743
sp|Q9Y5P6|GMPPB_HUMAN	GMPPB	Mannose-1-phosphate guanyltransferase beta OS=Homo sapiens GN=GMPPB PE=1 SV=2	40379	629	0	23	0	0.08	0	0	0	0	1	0	1	0	1	0	-2.47743
tr|B0QZK4|B0QZK4_HUMAN	HP1BP3	Heterochromatin protein 1-binding protein 3 (Fragment) OS=Homo sapiens GN=HP1BP3 PE=4 SV=1	28725	631	0	23	0	0.25	0	0	0	0	2	0	1	0	3	0	-2.47743
tr|Q5VSQ6|Q5VSQ6_HUMAN	P4HA1	Procollagen-proline, 2-oxoglutarate 4-dioxygenase (Proline 4-hydroxylase), alpha polypeptide I variant (Fragm	61214	634	0	22	0	0.05	0	0	0	0	1	0	1	0	2	0	-2.47743
tr|A8KAK1|A8KAK1_HUMAN	tr|A8KAK1|A8K	cDNA FLJ77398, highly similar to Homo sapiens UDP-glucose ceramide glucosyltransferase-like 1, transcript va	175467	635	0	22	0	0.02	0	0	0	0	1	0	1	0	1	0	-2.47743
tr|B1ALA9|B1ALA9_HUMAN	PRPS1	Phosphoribosyl pyrophosphate synthetase 1 OS=Homo sapiens GN=PRPS1 PE=3 SV=1	24468	638	0	22	0	0.14	0	0	0	0	1	0	1	0	2	0	-2.47743
tr|A0JHM7|A0JHM7_HUMAN	HLA-A	MHC class I antigen (Fragment) OS=Homo sapiens GN=HLA-A PE=3 SV=1	31850	639	0	22	0	0.16	0	0	0	0	1	0	1	0	2	0	-2.47743
tr|G0UE14|G0UE14_HUMAN	HLA-A	MHC class I antigen (Fragment) OS=Homo sapiens GN=HLA-A PE=3 SV=1	31877	641	0	22	0	0.1	0	0	0	0	1	0	1	0	3	0	-2.47743
tr|I1U8N0|I1U8N0_HUMAN	HLA-A	MHC class I antigen (Fragment) OS=Homo sapiens GN=HLA-A PE=3 SV=1	31658	642	0	22	0	0.16	0	0	0	0	1	0	1	0	1	0	-2.47743
tr|F2XI33|F2XI33_HUMAN	HLA-A	MHC class I antigen (Fragment) OS=Homo sapiens GN=HLA-A PE=3 SV=1	31658	643	0	22	0	0.09	0	0	0	0	1	0	1	0	1	0	-2.47743
tr|H7C4V2|H7C4V2_HUMAN	U2SURP	U2 snRNP-associated SURP motif-containing protein (Fragment) OS=Homo sapiens GN=U2SURP PE=4 SV=1	33796	647	0	22	0	0.03	0	0	0	0	1	0	1	0	1	0	-2.47743
tr|B4DQ49|B4DQ49_HUMAN	tr|B4DQ49|B4D	cDNA FLJ54374, highly similar to Homo sapiens keratin 78 (KRT78), mRNA OS=Homo sapiens PE=2 SV=1	31874	653	0	22	0	0.1	0	0	0	0	1	0	1	0	3	0	-2.47743
tr|Q96I16|Q96I16_HUMAN	tr|Q96I16|Q96I	EIF2S2 protein OS=Homo sapiens PE=2 SV=1	24902	654	0	22	0	0.09	0	0	0	0	1	0	1	0	1	0	-2.47743
tr|Q5T946|Q5T946_HUMAN	GRHPR	Glyoxylate reductase/hydroxypyruvate reductase OS=Homo sapiens GN=GRHPR PE=4 SV=1	43965	659	0	21	0	0.09	0	0	0	0	1	0	1	0	1	0	-2.47743
tr|C9J935|C9J935_HUMAN	SETD9	SET domain-containing protein 9 (Fragment) OS=Homo sapiens GN=SETD9 PE=2 SV=1	5515	660	0	21	0	0.65	0	0	0	0	1	0	1	0	1	0	-2.47743
tr|E7EVX8|E7EVX8_HUMAN	PRPF31	U4/U6 small nuclear ribonucleoprotein Prp31 OS=Homo sapiens GN=PRPF31 PE=2 SV=1	54960	661	0	21	0	0.06	0	0	0	0	1	0	1	0	2	0	-2.47743
tr|B3KRG8|B3KRG8_HUMAN	tr|B3KRG8|B3K	Lipase OS=Homo sapiens PE=2 SV=1	45736	662	0	21	0	0.07	0	0	0	0	1	0	1	0	1	0	-2.47743
tr|Q6UWE9|Q6UWE9_HUMAN	UNQ2550	SFVP2550 OS=Homo sapiens GN=UNQ2550 PE=4 SV=1	11890	664	0	21	0	0.29	0	0	1	0	1	0	1	0	1	0	-2.47743
tr|J3QT54|J3QT54_HUMAN	CPSF7	Cleavage and polyadenylation-specificity factor subunit 7 (Fragment) OS=Homo sapiens GN=CPSF7 PE=4 SV=1	22762	665	0	21	0	0.15	0	0	0	0	1	0	1	0	1	0	-2.47743
tr|B4DK80|B4DK80_HUMAN	TOP3A	DNA topoisomerase OS=Homo sapiens GN=TOP3A PE=2 SV=1	103082	668	0	20	0	0.03	0	0	0	0	1	0	1	0	1	0	-2.47743
tr|A0AVG7|A0AVG7_HUMAN	BCAS1	Breast carcinoma amplified sequence 1 OS=Homo sapiens GN=BCAS1 PE=2 SV=1	61929	670	0	20	0	0.05	0	0	0	0	1	0	1	0	1	0	-2.47743
tr|H0YJL6|H0YJL6_HUMAN	EVL	Ena/VASP-like protein (Fragment) OS=Homo sapiens GN=EVL PE=4 SV=1	10347	671	0	20	0	0.34	0	0	0	0	1	0	1	0	1	0	-2.47743
tr|D6RF05|D6RF05_HUMAN	HARS	Histidine--tRNA ligase, cytoplasmic OS=Homo sapiens GN=HARS PE=2 SV=1	6740	672	0	20	0	0.07	0	0	0	0	1	0	1	0	1	0	-2.47743
sp|P55212|CASP6_HUMAN	CASP6	Caspase-6 OS=Homo sapiens GN=CASP6 PE=1 SV=2	33859	674	0	20	0	0.1	0	0	0	0	1	0	1	0	1	0	-2.47743
tr|B4DE48|B4DE48_HUMAN	tr|B4DE48|B4D	cDNA FLJ53824, highly similar to Sorting nexin-17 OS=Homo sapiens PE=2 SV=1	50009	677	0	20	0	0.07	0	0	0	0	1	0	1	0	1	0	-2.47743
sp|O95159|ZFPL1_HUMAN	ZFPL1	Zinc finger protein-like 1 OS=Homo sapiens GN=ZFPL1 PE=1 SV=2	34833	678	0	20	0	0.1	0	0	0	0	1	0	1	0	1	0	-2.47743
tr|B4DS66|B4DS66_HUMAN	tr|B4DS66|B4D	cDNA FLJ54290, highly similar to Mitochondrial inner membrane protein OS=Homo sapiens PE=2 SV=1	73509	712	0	17	0	0.04	0	0	0	0	1	0	1	0	6	0	-2.47743
tr|B7Z4P8|B7Z4P8_HUMAN	ACTN2	Alpha-actinin-2 OS=Homo sapiens GN=ACTN2 PE=2 SV=1	44675	19	0	51	0	2.11	0	0	1	0	2	0	2	0	2	0	-3.21473
tr|K7EMS3|K7EMS3_HUMAN	KRT19	Keratin, type I cytoskeletal 19 (Fragment) OS=Homo sapiens GN=KRT19 PE=4 SV=1	20174	24	0	63	0	2.99	0	0	1	0	1	0	2	0	2	0	-3.21473
tr|G3V2E8|G3V2E8_HUMAN	ACTN1	Alpha-actinin-1 (Fragment) OS=Homo sapiens GN=ACTN1 PE=2 SV=1	9345	50	0	67	0	0.77	0	0	1	0	2	0	2	0	2	0	-3.21473
tr|J3KQA8|J3KQA8_HUMAN	HNRNPA1	Heterogeneous nuclear ribonucleoprotein A1 OS=Homo sapiens GN=HNRNPA1 PE=4 SV=1	11467	101	0	67	0	0.77	0	0	1	0	2	0	2	0	3	0	-3.21473
tr|C9JTG5|C9JTG5_HUMAN	KRT15	Keratin, type I cytoskeletal 15 (Fragment) OS=Homo sapiens GN=KRT15 PE=2 SV=1	25020	106	0	63	0	0.47	0	0	1	0	1	0	2	0	3	0	-3.21473
tr|Q8IXH2|Q8IXH2_HUMAN	tr|Q8IXH2|Q8IX	MSTP057 OS=Homo sapiens PE=2 SV=1	30034	120	0	143	0	0.25	0	0	2	0	2	0	2	0	2	0	-3.21473
tr|C9J057|C9J057_HUMAN	ASNS	Asparagine synthetase [glutamine-hydrolyzing] (Fragment) OS=Homo sapiens GN=ASNS PE=2 SV=1	24946	141	0	32	0	0.26	0	0	0	0	1	0	2	0	2	0	-3.21473
tr|Q2YD88|Q2YD88_HUMAN	FAM129B	FAM129B protein (Fragment) OS=Homo sapiens GN=FAM129B PE=2 SV=1	35264	155	0	86	0	0.16	0	0	2	0	2	0	2	0	2	0	-3.21473
tr|Q5JVP3|Q5JVP3_HUMAN	PTGR1	Leukotriene B4 12-hydroxydehydrogenase, isoform CRA_d OS=Homo sapiens GN=PTGR1 PE=4 SV=1	19441	190	0	113	0	0.38	0	0	2	0	2	0	2	0	2	0	-3.21473
tr|B3KPU1|B3KPU1_HUMAN	GNB2	Guanine nucleotide-binding protein G(I)/G(S)/G(T) subunit beta-2 OS=Homo sapiens GN=GNB2 PE=2 SV=1	26598	194	0	111	0	0.27	0	0	1	0	2	0	2	0	2	0	-3.21473
tr|B2R6K4|B2R6K4_HUMAN	tr|B2R6K4|B2R(	cDNA, FLJ92996, highly similar to Homo sapiens guanine nucleotide binding protein (G protein), beta polypept	38075	199	0	105	0	0.18	0	0	1	0	2	0	2	0	2	0	-3.21473
tr|D6RF23|D6RF23_HUMAN	GNB2L1	Guanine nucleotide-binding protein subunit beta-2-like 1 OS=Homo sapiens GN=GNB2L1 PE=2 SV=1	11411	216	0	59	0	0.52	0	0	1	0	2	0	2	0	3	0	-3.21473
tr|A0MNN4|A0MNN4_HUMAN	SMU1	CDW3/SMU1 OS=Homo sapiens GN=SMU1 PE=2 SV=1	58134	221	0	94	0	0.12	0	0	1	0	2	0	2	0	2	0	-3.21473
tr|B7Z462|B7Z462_HUMAN	tr|B7Z462|B7Z4	cDNA FLJ58016, highly similar to Polypeptide N-acetylgalactosaminyltransferase2 (EC 2.4.1.41) OS=Homo sapi	41208	254	0	76	0	0.11	0	0	2	0	2	0	2	0	2	0	-3.21473
sp|Q6IS14|IF5AL_HUMAN	EIF5AL1	Eukaryotic translation initiation factor 5A-1-like OS=Homo sapiens GN=EIF5AL1 PE=1 SV=2	16990	256	0	75	0	0.44	0	0	1	0	2	0	2	0	2	0	-3.21473
tr|Q53S41|Q53S41_HUMAN	YWHAQ	Putative uncharacterized protein YWHAQ (Fragment) OS=Homo sapiens GN=YWHAQ PE=2 SV=1	16744	258	0	42	0	0.4	0	0	1	0	2	0	2	0	2	0	-3.21473
tr|B4DGT1|B4DGT1_HUMAN	tr|B4DGT1|B4D	cDNA FLJ59116, highly similar to Spectrin alpha chain, brain OS=Homo sapiens PE=2 SV=1	151883	261	0	74	0	0.04	0	0	1	0	2	0	2	0	5	0	-3.21473
tr|Q2QD09|Q2QD09_HUMAN	tr|Q2QD09|Q2(	Triosephosphate isomerase (Fragment) OS=Homo sapiens PE=2 SV=1	27211	267	0	71	0	0.26	0	0	1	0	2	0	2	0	2	0	-3.21473
tr|B1ANK7|B1ANK7_HUMAN	FH	Fumarate hydratase OS=Homo sapiens GN=FH PE=2 SV=1	54773	288	0	64	0	0.12	0	0	1	0	2	0	2	0	5	0	-3.21473
tr|E5RH42|E5RH42_HUMAN	G3BP1	Ras GTPase-activating protein-binding protein 1 (Fragment) OS=Homo sapiens GN=G3BP1 PE=2 SV=1	13338	294	0	62	0	0.58	0	0	2	0	2	0	2	0	2	0	-3.21473
sp|P04040|CATA_HUMAN	CAT	Catalase OS=Homo sapiens GN=CAT PE=1 SV=3	59947	299	0	60	0	0.11	0	0	1	0	2	0	2	0	2	0	-3.21473
tr|B4DMY3|B4DMY3_HUMAN	tr|B4DMY3|B4I	cDNA FLJ60713, highly similar to Homo sapiens heterogeneous nuclear ribonucleoprotein A/B (HNRPAB), tran:	35101	318	0	56	0	0.2	0	0	1	0	2	0	2	0	2	0	-3.21473
tr|B4DMP0|B4DMP0_HUMAN	tr|B4DMP0|B4I	cDNA FLJ52189, highly similar to Procollagen-lysine,2-oxoglutarate5-dioxygenase 3 (EC 1.14.11.4) OS=Homo s	37539	325	0	48	0	0.12	0	0	1	0	2	0	2	0	2	0	-3.21473
tr|G3V180|G3V180_HUMAN	DPP3	Dipeptidyl peptidase 3 OS=Homo sapiens GN=DPP3 PE=2 SV=1	84666	335	0	52	0	0.08	0	0	1	0	2	0	2	0	2	0	-3.21473
tr|E5RHF2|E5RHF2_HUMAN	COPS5	COP9 signalosome complex subunit 5 (Fragment) OS=Homo sapiens GN=COPS5 PE=2 SV=1	16655	346	0	51	0	0.45	0	0	0	0	2	0	2	0	2	0	-3.21473
tr|Q6NZ52|Q6NZ52_HUMAN	RPL27A	Ribosomal protein L27a OS=Homo sapiens GN=RPL27A PE=2 SV=1	16582	348	0	50	0	0.2	0	0	1	0	1	0	2	0	2	0	-3.21473
sp|P01024|CO3_HUMAN	C3	Complement C3 OS=Homo sapiens GN=C3 PE=1 SV=2	188569	370	0	47	0	0.03	0	0	1	0	2	0	2	0	2	0	-3.21473
tr|A5D904|A5D904_HUMAN	RPS9	RPS9 protein (Fragment) OS=Homo sapiens GN=RPS9 PE=2 SV=1	13210	434	0	40	0	0.58	0	0	1	0	2	0	2	0	2	0	-3.21473
tr|Q7Z3K9|Q7Z3K9_HUMAN	DKFZp781L0540	Putative uncharacterized protein DKFZp781L0540 (Fragment) OS=Homo sapiens GN=DKFZp781L0540 PE=2 SV	90081	440	0	39	0	0.04	0	0	1	0	1	0	2	0	2	0	-3.21473
tr|D3DVQ1|D3DVQ1_HUMAN	LETM1	Leucine zipper-EF-hand containing transmembrane protein 1, isoform CRA_a OS=Homo sapiens GN=LETM1 PE	63730	463	0	37	0	0.11	0	0	1	0	2	0	2	0	4	0	-3.21473
tr|H0Y7N2|H0Y7N2_HUMAN	ATXN10	Ataxin-10 (Fragment) OS=Homo sapiens GN=ATXN10 PE=4 SV=1	19107	470	0	37	0	0.38	0	0	0	0	2	0	2	0	2	0	-3.21473
tr|C1KES1|C1KES1_HUMAN	LRRC16B	LRRC16B (Fragment) OS=Homo sapiens GN=LRRC16B PE=4 SV=1	4125	530	0	31	0	0.94	0	0	0	0	1	0	2	0	2	0	-3.21473
tr|B3KNB4|B3KNB4_HUMAN	tr|B3KNB4|B3K	cDNA FLJ14168 fis, clone NT2RP2001440, highly similar to 14-3-3 protein gamma OS=Homo sapiens PE=2 SV=1	28453	541	0	30	0	0.25	0	0	0	0	2	0	2	0	2	0	-3.21473
tr|B2RD27|B2RD27_HUMAN	tr|B2RD27|B2R	cDNA, FLJ96428, highly similar to Homo sapiens proteasome (prosome, macropain) 26S subunit, non-ATPase,	37046	550	0	30	0	0.19	0	0	0	0	2	0	2	0	2	0	-3.21473
sp|O75367|H2AY_HUMAN	H2AFY	Core histone macro-H2A.1 OS=Homo sapiens GN=H2AFY PE=1 SV=4	39764	565	0	29	0	0.08	0	0	0	0	1	0	2	0	2	0	-3.21473
tr|H3BNV2|H3BNV2_HUMAN	NQO1	NAD(P)H dehydrogenase [quinone] 1 OS=Homo sapiens GN=NQO1 PE=4 SV=1	28533	576	0	28	0	0.25	0	0	0	0	2	0	2	0	2	0	-3.21473
tr|B7ZA86|B7ZA86_HUMAN	tr|B7ZA86|B7Z/	cDNA, FLJ79100, highly similar to 14-3-3 protein epsilon (14-3-3E) OS=Homo sapiens PE=2 SV=1	19138	582	0	28	0	0.18	0	0	0	0	1	0	2	0	2	0	-3.21473
tr|D3DSQ0|D3DSQ0_HUMAN	PCM1	Pericentriolar material 1, isoform CRA_a OS=Homo sapiens GN=PCM1 PE=2 SV=1	230041	619	0	24	0	0.01	0	0	0	0	1	0	2	0	3	0	-3.21473
sp|P25490|TYY1_HUMAN	YY1	Transcriptional repressor protein YY1 OS=Homo sapiens GN=YY1 PE=1 SV=2	45141	630	0	23	0	0.07	0	0	0	0	1	0	2	0	2	0	-3.21473
tr|I0BW39|I0BW39_HUMAN	HLA-A	MHC class I antigen (Fragment) OS=Homo sapiens GN=HLA-A PE=4 SV=1	8492	640	0	22	0	0.16	0	0	0	0	1	0	2	0	2	0	-3.21473
tr|H3BUF6|H3BUF6_HUMAN	ATXN2L	Ataxin-2-like protein OS=Homo sapiens GN=ATXN2L PE=4 SV=1	112929	656	0	22	0	0.03	0	0	0	0	1	0	2	0	2	0	-3.21473
tr|Q5STZ7|Q5STZ7_HUMAN	ABCF1	ATP-binding cassette sub-family F member 1 OS=Homo sapiens GN=ABCF1 PE=4 SV=2	27429	144	0	112	0	0.27	0	0	2	0	3	0	3	0	3	0	-3.70049
tr|C9JWY7|C9JWY7_HUMAN	CAPN2	Calpain-2 catalytic subunit (Fragment) OS=Homo sapiens GN=CAPN2 PE=2 SV=1	14668	173	0	48	0	0.22	0	0	0	0	4	0	3	0	4	0	-3.70049
tr|B4DMU0|B4DMU0_HUMAN	PYCR1	Pyrroline-5-carboxylate reductase OS=Homo sapiens GN=PYCR1 PE=2 SV=1	36186	174	0	125	0	0.19	0	0	2	0	2	0	3	0	3	0	-3.70049
tr|H0Y8L7|H0Y8L7_HUMAN	RPS3A	40S ribosomal protein S3a (Fragment) OS=Homo sapiens GN=RPS3A PE=3 SV=1	22294	223	0	74	0	0.44	0	0	1	0	2	0	3	0	4	0	-3.70049
tr|Q0VGL3|Q0VGL3_HUMAN	RPL13A	RPL13A protein OS=Homo sapiens GN=RPL13A PE=2 SV=1	16706	300	0	59	0	0.74	0	0	3	0	3	0	3	0	3	0	-3.70049
tr|B4DDR2|B4DDR2_HUMAN	tr|B4DDR2|B4D	cDNA FLJ54588, highly similar to Major vault protein OS=Homo sapiens PE=2 SV=1	98526	304	0	59	0	0.07	0	0	2	0	2	0	3	0	3	0	-3.70049
sp|Q13151|ROA0_HUMAN	HNRNPA0	Heterogeneous nuclear ribonucleoprotein A0 OS=Homo sapiens GN=HNRNPA0 PE=1 SV=1	30993	323	0	55	0	0.36	0	0	2	0	3	0	3	0	3	0	-3.70049
sp|Q7RTS7|K2C74_HUMAN	KRT74	Keratin, type II cytoskeletal 74 OS=Homo sapiens GN=KRT74 PE=1 SV=2	58229	373	0	47	0	0.12	0	0	2	0	2	0	3	0	5	0	-3.70049
tr|B2R6J4|B2R6J4_HUMAN	tr|B2R6J4|B2R(	cDNA, FLJ92975, highly similar to Homo sapiens nucleosome assembly protein 1-like 4 (NAP1L4), mRNA OS=H	42940	417	0	42	0	0.16	0	0	0	0	2	0	3	0	3	0	-3.70049
tr|B2R659|B2R659_HUMAN	tr|B2R659|B2R(	cDNA, FLJ92803, highly similar to Homo sapiens hydroxysteroid (17-beta) dehydrogenase 4 (HSD17B4), mRNA	80018	484	0	36	0	0.13	0	0	0	0	3	0	3	0	4	0	-3.70049
tr|H7BYX5|H7BYX5_HUMAN	FSCN1	Fascin (Fragment) OS=Homo sapiens GN=FSCN1 PE=4 SV=1	15688	53	0	188	0	1.07	0	0	4	0	5	0	4	0	7	0	-4.0634
tr|E9PKU4|E9PKU4_HUMAN	RPL8	60S ribosomal protein L8 (Fragment) OS=Homo sapiens GN=RPL8 PE=2 SV=1	25806	231	0	90	0	0.28	0	0	2	0	2	0	4	0	5	0	-4.0634
sp|P21796|VDAC1_HUMAN	VDAC1	Voltage-dependent anion-selective channel protein 1 OS=Homo sapiens GN=VDAC1 PE=1 SV=2	30868	250	0	78	0	0.36	0	0	1	0	3	0	4	0	4	0	-4.0634
sp|Q14CN4|K2C72_HUMAN	KRT72	Keratin, type II cytoskeletal 72 OS=Homo sapiens GN=KRT72 PE=1 SV=2	56470	372	0	47	0	0.12	0	0	2	0	2	0	4	0	5	0	-4.0634
sp|A2RTY3|CQ066_HUMAN	C17orf66	Uncharacterized protein C17orf66 OS=Homo sapiens GN=C17orf66 PE=2 SV=2	66437	477	0	36	0	0.05	0	0	0	0	1	0	4	0	8	0	-4.0634
tr|B4DKM5|B4DKM5_HUMAN	VDAC2	Voltage-dependent anion-selective channel protein 2 OS=Homo sapiens GN=VDAC2 PE=2 SV=1	27804	196	0	108	0	0.76	0	0	1	0	5	0	5	0	5	0	-4.35324
tr|B3KVL0|B3KVL0_HUMAN	tr|B3KVL0|B3K\	cDNA FLJ16691 fis, clone TRACH3000692, highly similar to Paxillin OS=Homo sapiens PE=2 SV=1	65600	159	0	133	0	0.28	0	0	3	0	5	0	6	0	6	0	-4.59458
tr|F8VQP2|F8VQP2_HUMAN	ATXN2	Ataxin-2 OS=Homo sapiens GN=ATXN2 PE=2 SV=1	117012	157	0	135	0	0.21	0	0	4	0	7	0	10	0	10	0	-5.28779
sp|Q96RW7|HMCN1_HUMAN	HMCN1	Hemicentin-1 OS=Homo sapiens GN=HMCN1 PE=1 SV=2	623265	291	0	63	0	0.01	0	0	7	0	7	0	11	0	16	0	-5.41937

#18	Hela lysate unsyn MLN-2_2μM																		
#04	Hela live syn NP_2μM																		
Accession	GS	Name	Pro_Mass	04_prot_hit	18_prot_hit	04_prot_sc	18_prot_sc	04_emPAI	18_emPAI	emPAI ratio	04_No>I	18_No>I	04_No>HI	18_No>HI	04_No>20	18_No>20	04_No=All	18_No=All	Rsc
sp|Q96SB4|SRPK1_HUMAN	SRPK1	SRSF protein kinase 1 OS=Homo sapiens GN=SRPK1 PE=1 SV=2	74963	0	610	0	62	0	0.04	#DIV/0!	0	1	0	1	0	3	0	3	1.941386
sp|Q9Y6E0|STK24_HUMAN	STK24	Serine/threonine-protein kinase 24 OS=Homo sapiens GN=STK24 PE=1 SV=1	49562	0	1084	0	28	0	0.08	#DIV/0!	0	1	0	1	0	1	0	1	0.718617
sp|O75116|ROCK2_HUMAN	ROCK2	Rho-associated protein kinase 2 OS=Homo sapiens GN=ROCK2 PE=1 SV=4	161939	375	523	46	74	0.05	0.16	3.2	1	1	2	4	1	7	3	8	1.45637
sp|Q16513|PKN2_HUMAN	PKN2	Serine/threonine-protein kinase N2 OS=Homo sapiens GN=PKN2 PE=1 SV=1	112820	0	963	0	35	0	0.03	#DIV/0!	0	1	0	1	0	1	0	1	0.718617
sp|P51812|KS6A3_HUMAN	RPS6KA3 (	Ribosomal protein S6 kinase alpha-3 OS=Homo sapiens GN=RPS6KA3 PE=1 SV=1	84025	0	1189	0	22	0	0.04	#DIV/0!	0	0	0	1	0	1	0	1	0.718617
sp|Q15418|KS6A1_HUMAN	RPS6KA1	Ribosomal protein S6 kinase alpha-1 OS=Homo sapiens GN=RPS6KA1 PE=1 SV=2	83070	0	881	0	39	0	0.05	#DIV/0!	0	1	0	1	0	1	0	1	0.718617
sp|Q13418|ILK_HUMAN	ILK	Integrin-linked protein kinase OS=Homo sapiens GN=ILK PE=2 SV=1	44776	421	360	41	115	0.07	0.65	9.2857143	1	3	1	7	1	7	1	9	1.45637
sp|P78527|PRKDC_HUMAN	PRKDC	DNA-dependent protein kinase catalytic subunit OS=Homo sapiens GN=PRKDC PE=1 SV=3	473749	83	24	241	1214	0.07	0.44	6.2857143	7	34	10	53	11	65	17	76	1.651839
sp|Q99570|PI3R4_HUMAN	PIK3R4	Phosphoinositide 3-kinase regulatory subunit 4 OS=Homo sapiens GN=PIK3R4 PE=1 SV=3	154318	0	991	0	34	0	0.02	#DIV/0!	0	1	0	1	0	1	0	1	0.718617
sp|P19367|HXK1_HUMAN	HK1	Hexokinase-1 OS=Homo sapiens GN=HK1 PE=1 SV=3	103561	0	308	0	133	0	0.16	#DIV/0!	0	4	0	5	0	5	0	9	2.593839
tr|J3KNX3|J3KNX3_HUMAN	PFKM	6-phosphofructokinase OS=Homo sapiens GN=PFKM PE=3 SV=1	94107	0	499	0	78	0	0.11	#DIV/0!	0	2	0	3	0	3	0	4	1.941386
sp|Q16822|PCKGM_HUMAN	PCK2	Phosphoenolpyruvate carboxykinase [GTP], mitochondrial OS=Homo sapiens GN=PCK2 PE=1 SV=3	71483	0	378	0	91	0	0.19	#DIV/0!	0	2	0	2	0	2	0	2	1.455771
sp|Q9Y223|GLCNE_HUMAN	GNE	Bifunctional UDP-N-acetylglucosamine 2-epimerase/N-acetylmannosamine kinase OS=Homo sapiens GN=GNE PE	80193	0	1187	0	22	0	0.04	#DIV/0!	0	0	0	1	0	1	0	1	0.718617
sp|Q01813|K6PP_HUMAN	PFKP	6-phosphofructokinase type C OS=Homo sapiens GN=PFKP PE=1 SV=2	86454	98	49	217	708	0.4	1.27	3.175	5	19	9	22	9	23	10	27	0.441372
sp|P00558|PGK1_HUMAN	PGK1	Phosphoglycerate kinase 1 OS=Homo sapiens GN=PGK1 PE=1 SV=3	44985	134	86	161	411	0.76	1.7	2.2368421	5	10	8	14	9	17	9	19	0.014933
sp|P40763|STAT3_HUMAN	STAT3	Signal transducer and activator of transcription 3 OS=Homo sapiens GN=STAT3 PE=1 SV=2	88810	0	928	0	36	0	0.04	#DIV/0!	0	1	0	1	0	1	0	2	0.718617
tr|B3KUN1|B3KUN1_HUMAN	PPP2CA	Serine/threonine-protein phosphatase OS=Homo sapiens GN=PPP2CA PE=2 SV=1	36142	0	457	0	88	0	0.39	#DIV/0!	0	2	0	3	0	3	0	3	1.941386
tr|F8VYE8|F8VYE8_HUMAN	PPP1CC	Serine/threonine-protein phosphatase OS=Homo sapiens GN=PPP1CC PE=2 SV=1	35669	0	557	0	68	0	0.35	#DIV/0!	0	1	0	3	0	2	0	4	1.455771
tr|C9J9S3|C9J9S3_HUMAN	PPP1CB	Serine/threonine-protein phosphatase (Fragment) OS=Homo sapiens GN=PPP1CB PE=2 SV=1	14484	345	456	51	88	0.24	1.33	5.5416667	1	2	1	4	1	3	1	5	0.35608
sp|O00743|PPP6_HUMAN	PPP6C	Serine/threonine-protein phosphatase 6 catalytic subunit OS=Homo sapiens GN=PPP6C PE=1 SV=1	35806	0	798	0	44	0	0.09	#DIV/0!	0	1	0	1	0	1	0	1	0.718617
sp|O43684|BUB3_HUMAN	BUB3	Mitotic checkpoint protein BUB3 OS=Homo sapiens GN=BUB3 PE=1 SV=1	37587	681	648	20	51	0.2	0.34	1.7	0	1	1	2	0	2	1	2	1.455771
sp|Q06124|PTN11_HUMAN	PTPN11	Tyrosine-protein phosphatase non-receptor type 11 OS=Homo sapiens GN=PTPN11 PE=1 SV=2	68964	0	805	0	44	0	0.1	#DIV/0!	0	1	0	2	0	2	0	2	1.455771
sp|O15355|PPM1G_HUMAN	PPM1G	Protein phosphatase 1G OS=Homo sapiens GN=PPM1G PE=1 SV=1	59919	0	959	0	35	0	0.11	#DIV/0!	0	0	0	2	0	2	0	2	1.455771
sp|P29966|MARCS_HUMAN	MARCKS	Myristoylated alanine-rich C-kinase substrate OS=Homo sapiens GN=MARCKS PE=1 SV=4	31707	0	564	0	67	0	2.29	#DIV/0!	0	1	0	2	0	2	0	2	1.455771
sp|O75170|PP6R2_HUMAN	PPP6R2	Serine/threonine-protein phosphatase 6 regulatory subunit 2 OS=Homo sapiens GN=PPP6R2 PE=1 SV=2	106358	0	1147	0	24	0	0.03	#DIV/0!	0	0	0	1	0	1	0	1	0.718617
sp|O15357|SHIP2_HUMAN	INPPL1	Phosphatidylinositol 3,4,5-trisphosphate 5-phosphatase 2 OS=Homo sapiens GN=INPPL1 PE=1 SV=2	139311	0	1023	0	32	0	0.23	#DIV/0!	0	0	0	1	0	1	0	1	0.718617
sp|O60271|JIP4_HUMAN	SPAG9	C-Jun-amino-terminal kinase-interacting protein 4 OS=Homo sapiens GN=SPAG9 PE=1 SV=4	146913	0	811	0	43	0	0.04	#DIV/0!	0	1	0	1	0	1	0	1	0.718617
sp|Q8TAP8|PPR35_HUMAN	PPP1R35	Protein phosphatase 1 regulatory subunit 35 OS=Homo sapiens GN=PPP1R35 PE=1 SV=1	28107	0	768	0	46	0	0.12	#DIV/0!	0	1	0	1	0	1	0	1	0.718617
tr|B4DK71|B4DK71_HUMAN	SACM1L	Phosphatidylinositide phosphatase SAC1 OS=Homo sapiens GN=SACM1L PE=2 SV=1	61327	0	687	0	54	0	0.05	#DIV/0!	0	1	0	1	0	1	0	1	0.718617
tr|C1PHA2|C1PHA2_HUMAN	KIF5B-ALK	Tyrosine-protein kinase receptor OS=Homo sapiens GN=KIF5B-ALK PE=2 SV=1	169283	227	233	91	181	0.04	0.16	4	2	2	2	5	2	8	3	11	0.899821
tr|B4DDF7|B4DDF7_HUMAN	tr|B4DDF	cDNA FLJ53296, highly similar to Serine/threonine-protein phosphatase 2A 65 kDa regulatory subunit A alpha iso	64939	443	229	39	185	0.16	0.34	2.125	1	6	3	6	3	6	3	8	0.026651
tr|B0AZV0|B0AZV0_HUMAN	STRAP	Serine-threonine kinase receptor-associated protein OS=Homo sapiens GN=STRAP PE=2 SV=1	28716	626	624	23	57	0.12	0.17	1.4166667	0	1	1	1	1	1	1	1	-0.86669
sp|P52789|HXK2_HUMAN	HK2	Hexokinase-2 OS=Homo sapiens GN=HK2 PE=1 SV=2	103739	599	507	26	76	0.03	0.1	3.3333333	0	2	1	3	1	2	2	4	-0.12954
sp|P14618|KPYM_HUMAN	PKM	Pyruvate kinase isozymes M1/M2 OS=Homo sapiens GN=PKM PE=1 SV=4	58470	8	7	1820	2130	5.79	11.39	1.9671848	47	54	58	64	60	68	66	80	-0.69525
sp|O00764|PDXK_HUMAN	PDXK	Pyridoxal kinase OS=Homo sapiens GN=PDXK PE=1 SV=1	35308	566	1053	29	30	0.11	0.18	1.6363636	0	0	1	1	1	1	2	1	-0.86669
tr|J3QT22|J3QT22_HUMAN	PPME1	Protein phosphatase methylesterase 1 OS=Homo sapiens GN=PPME1 PE=3 SV=1	44241	232	822	90	42	0.07	0.07	1	1	1	1	1	1	2	2	2	-0.12954
tr|H0YDU8|H0YDU8_HUMAN	PPP5C	Serine/threonine-protein phosphatase (Fragment) OS=Homo sapiens GN=PPP5C PE=3 SV=1	55743	596	692	26	52	0.06	0.06	1	0	1	1	1	1	2	1	2	-0.12954
sp|P17858|K6PL_HUMAN	PFKL	6-phosphofructokinase, liver type OS=Homo sapiens GN=PFKL PE=1 SV=6	85762	98	259	88	164	0.4	0.21	0.525	1	3	2	5	2	5	2	6	0.271224
tr|B2R5T5|B2R5T5_HUMAN	PRKAR1A	Protein kinase, cAMP-dependent, regulatory, type I, alpha (Tissue specific extinguisher 1), isoform CRA_a OS=Hor	43183	533	780	31	45	0.08	0.08	1	0	1	1	1	1	1	1	2	-0.86669
sp|O95747|OXSR1_HUMAN	OXSR1	Serine/threonine-protein kinase OSR1 OS=Homo sapiens GN=OXSR1 PE=1 SV=1	58271	675	718	20	51	0.06	0.06	1	0	1	1	1	1	1	2	1	-0.86669
sp|P12277|KCRB_HUMAN	CKB	Creatine kinase B-type OS=Homo sapiens GN=CKB PE=1 SV=1	42902	214	794	97	44	0.18	0.18	1	2	1	2	2	2	2	2	2	-0.86684
tr|A4QPA9|A4QPA9_HUMAN	MAP2K1 (	Mitogen-activated protein kinase kinase 1 OS=Homo sapiens GN=MAP2K1 PE=2 SV=1	43753	384	1047	45	31	0.31	0.08	0.2580645	1	1	2	1	2	1	2	2	-1.604
tr|B4E1G6|B4E1G6_HUMAN	GALK1	Galactokinase OS=Homo sapiens GN=GALK1 PE=2 SV=1	45956	679	0	20	0	0.07	0	0	0	0	1	0	0	0	1	0	-0.86653
tr|A6NIT2|A6NIT2_HUMAN	PHKA1	Phosphorylase b kinase regulatory subunit alpha, skeletal muscle isoform OS=Homo sapiens GN=PHKA1 PE=2 SV=	133462	711	0	17	0	0.02	0	0	0	0	1	0	0	0	1	0	-0.86653
tr|Q8IWY7|Q8IWY7_HUMAN	TTBK2	Tau-tubulin kinase OS=Homo sapiens GN=TTBK2 PE=2 SV=1	183893	79	0	28	0	0.79	0	0	0	0	1	0	1	0	1	0	-2.45184
tr|B4E2K2|B4E2K2_HUMAN	tr|B4E2K2	cDNA FLJ55665, highly similar to A-kinase anchor protein 2 OS=Homo sapiens PE=2 SV=1	103935	246	0	79	0	0.03	0	0	1	0	1	0	1	0	1	0	-2.45184
sp|Q9UEW8|STK39_HUMAN	STK39	STE20/SPS1-related proline-alanine-rich protein kinase OS=Homo sapiens GN=STK39 PE=1 SV=3	59950	491	0	35	0	0.05	0	0	0	0	1	0	1	0	1	0	-2.45184
tr|Q5JQ10|Q5JQ10_HUMAN	ADK	Adenosine kinase OS=Homo sapiens GN=ADK PE=2 SV=1	39078	538	0	30	0	0.08	0	0	0	0	1	0	1	0	1	0	-2.45184
tr|A8MU39|A8MU39_HUMAN	PPP5C	Serine/threonine-protein phosphatase OS=Homo sapiens GN=PPP5C PE=2 SV=1	42823	596	0	26	0	0.06	0	0	0	0	1	0	1	0	1	0	-2.45184
tr|H0YH33|H0YH33_HUMAN	STRAP	Serine-threonine kinase receptor-associated protein (Fragment) OS=Homo sapiens GN=STRAP PE=4 SV=1	10763	626	0	23	0	0.12	0	0	0	0	1	0	1	0	1	0	-2.45184
tr|H0Y3C5|H0Y3C5_HUMAN	HCK	Tyrosine-protein kinase HCK OS=Homo sapiens GN=HCK PE=4 SV=1	59986	628	0	23	0	0.05	0	0	0	0	1	0	1	0	2	0	-2.45184
tr|H0Y3Y5|H0Y3Y5_HUMAN	PPIP5K1	Inositol hexakisphosphate and diphosphoinositol-pentakisphosphate kinase 1 (Fragment) OS=Homo sapiens GN=	21913	632	0	22	0	0.15	0	0	0	0	1	0	1	0	2	0	-2.45184
tr|B3KS97|B3KS97_HUMAN	MAP2K2	Mitogen-activated protein kinase kinase 2, isoform CRA_d OS=Homo sapiens GN=MAP2K2 PE=2 SV=1	23450	384	0	45	0	0.31	0	0	1	0	2	0	2	0	2	0	-3.18915
tr|D6REM6|D6REM6_HUMAN	MATR3	Matrin-3 OS=Homo sapiens GN=MATR3 PE=2 SV=1	88647	0	140	0	230	0	0.72	#DIV/0!	0	9	0	15	0	17	0	20	4.265954
tr|E1NZA1|E1NZA1_HUMAN	PRIC295	Peroxisome proliferator activated receptor interacting complex protein OS=Homo sapiens GN=PRIC295 PE=2 SV=	294953	0	114	0	325	0	0.18	#DIV/0!	0	12	0	15	0	16	0	17	4.180876
tr|A8K8U1|A8K8U1_HUMAN	tr|A8K8U	cDNA FLJ77762, highly similar to Homo sapiens cullin-associated and neddylation-dissociated 1 (CAND1), mRNA C	137941	0	113	0	326	0	0.32	#DIV/0!	0	11	0	13	0	15	0	18	4.09049
tr|B7Z4B2|B7Z4B2_HUMAN	tr|B7Z4B2	cDNA FLJ56108, highly similar to Puromycin-sensitive aminopeptidase (EC 3.4.11.-) OS=Homo sapiens PE=2 SV=1	93434	0	165	0	250	0	0.36	#DIV/0!	0	6	0	9	0	12	0	12	3.779584
sp|Q08J23|NSUN2_HUMAN	NSUN2	tRNA (cytosine(34)-C(5))-methyltransferase OS=Homo sapiens GN=NSUN2 PE=1 SV=2	87214	0	221	0	190	0	0.34	#DIV/0!	0	6	0	9	0	11	0	12	3.659101
sp|O43776|SYNC_HUMAN	NARS	Asparagine--tRNA ligase, cytoplasmic OS=Homo sapiens GN=NARS PE=1 SV=1	63758	0	121	0	315	0	0.74	#DIV/0!	0	9	0	11	0	11	0	11	3.659101
tr|A1KYQ7|A1KYQ7_HUMAN	tr|A1KYQ	Cell migration-inducing protein 17 OS=Homo sapiens PE=2 SV=1	106022	0	210	0	196	0	0.28	#DIV/0!	0	7	0	8	0	10	0	11	3.527668
tr|B4DLV7|B4DLV7_HUMAN	tr|B4DLV	cDNA FLJ60299, highly similar to Rab GDP dissociation inhibitor beta OS=Homo sapiens PE=2 SV=1	51577	0	192	0	208	0	0.54	#DIV/0!	0	7	0	7	0	9	0	11	3.383089
tr|B7Z1N6|B7Z1N6_HUMAN	tr|B7Z1N	cDNA FLJ52222, highly similar to Fructose-bisphosphate aldolase C (EC4.1.2.13) OS=Homo sapiens PE=2 SV=1	35800	0	81	0	441	0	0.56	#DIV/0!	0	8	0	9	0	9	0	11	3.383089
tr|B3KRS5|B3KRS5_HUMAN	HDAC2	Histone deacetylase OS=Homo sapiens GN=HDAC2 PE=2 SV=1	52479	0	345	0	121	0	0.28	#DIV/0!	0	3	0	4	0	8	0	9	3.222436
sp|Q96I24|FUBP3_HUMAN	FUBP3	Far upstream element-binding protein 3 OS=Homo sapiens GN=FUBP3 PE=1 SV=2	61944	0	344	0	121	0	0.3	#DIV/0!	0	4	0	5	0	8	0	8	3.222436
sp|P09960|LKHA4_HUMAN	LTA4H	Leukotriene A-4 hydrolase OS=Homo sapiens GN=LTA4H PE=1 SV=2	69868	0	249	0	169	0	0.49	#DIV/0!	0	5	0	8	0	8	0	8	3.222436
tr|H0YH81|H0YH81_HUMAN	ATP5B	ATP synthase subunit beta (Fragment) OS=Homo sapiens GN=ATP5B PE=3 SV=1	38226	0	196	0	206	0	0.79	#DIV/0!	0	6	0	7	0	8	0	8	3.222436
tr|Q6IT96|Q6IT96_HUMAN	HDAC1	Histone deacetylase OS=Homo sapiens GN=HDAC1 PE=2 SV=1	55638	0	528	0	73	0	0.19	#DIV/0!	0	2	0	3	0	7	0	8	3.041675
sp|O75533|SF3B1_HUMAN	SF3B1	Splicing factor 3B subunit 1 OS=Homo sapiens GN=SF3B1 PE=1 SV=3	146479	0	484	0	81	0	0.12	#DIV/0!	0	3	0	5	0	7	0	8	3.041675
tr|B3KWV6|B3KWV6_HUMAN	tr|B3KWV	cDNA FLJ43948 fis, clone TESTI4014924, highly similar to Homo sapiens cytoplasmic FMR1 interacting protein 1 ((	147082	0	449	0	91	0	0.14	#DIV/0!	0	2	0	6	0	7	0	8	3.041675
sp|P54577|SYYC_HUMAN	YARS	Tyrosine--tRNA ligase, cytoplasmic OS=Homo sapiens GN=YARS PE=1 SV=4	59448	0	410	0	101	0	0.31	#DIV/0!	0	5	0	5	0	7	0	8	3.041675
sp|Q5T4S7|UBR4_HUMAN	UBR4	E3 ubiquitin-protein ligase UBR4 OS=Homo sapiens GN=UBR4 PE=1 SV=1	580547	0	323	0	127	0	0.02	#DIV/0!	0	4	0	4	0	7	0	9	3.041675
tr|B4DT31|B4DT31_HUMAN	FUBP1	Far upstream element-binding protein 1 OS=Homo sapiens GN=FUBP1 PE=2 SV=1	69993	0	231	0	183	0	0.48	#DIV/0!	0	3	0	4	0	7	0	7	3.041675
tr|B7ZMF2|B7ZMF2_HUMAN	FANCI	Fanconi anemia, complementation group I OS=Homo sapiens GN=FANCI PE=2 SV=1	150724	0	583	0	65	0	0.09	#DIV/0!	0	2	0	4	0	6	0	7	2.835036
tr|B4DZC0|B4DZC0_HUMAN	tr|B4DZC	cDNA FLJ51771, highly similar to SWI/SNF-related matrix-associatedactin-dependent regulator of chromatin subf	117367	0	571	0	66	0	0.09	#DIV/0!	0	2	0	3	0	6	0	6	2.835036
tr|A0PJ61|A0PJ61_HUMAN	RSL1D1	RSL1D1 protein (Fragment) OS=Homo sapiens GN=RSL1D1 PE=2 SV=1	49688	0	421	0	99	0	0.57	#DIV/0!	0	3	0	7	0	6	0	9	2.835036
tr|K4DI93|K4DI93_HUMAN	CUL4B	Cullin 4B, isoform CRA_e OS=Homo sapiens GN=CUL4B PE=3 SV=1	103318	0	393	0	105	0	0.13	#DIV/0!	0	4	0	4	0	6	0	7	2.835036
sp|Q9H3U1|UN45A_HUMAN	UNC45A	Protein unc-45 homolog A OS=Homo sapiens GN=UNC45A PE=1 SV=1	104266	0	343	0	121	0	0.13	#DIV/0!	0	3	0	4	0	6	0	6	2.835036
tr|H3BM93|H3BM93_HUMAN	NUP93	Nuclear pore complex protein Nup93 (Fragment) OS=Homo sapiens GN=NUP93 PE=4 SV=1	19858	0	299	0	100	0	0.29	#DIV/0!	0	2	0	6	0	6	0	6	2.835036
tr|B2RDD7|B2RDD7_HUMAN	tr|B2RDD	cDNA, FLJ96564, highly similar to Homo sapiens SKB1 homolog (S. pombe) (SKB1), mRNA OS=Homo sapiens PE=2	73348	0	273	0	151	0	0.3	#DIV/0!	0	4	0	6	0	6	0	6	2.835036
tr|B0QY89|B0QY89_HUMAN	EIF3EIP	Eukaryotic translation initiation factor 3 subunit L OS=Homo sapiens GN=EIF3EIP PE=4 SV=1	71085	0	252	0	168	0	0.31	#DIV/0!	0	6	0	6	0	6	0	7	2.835036
tr|A8K132|A8K132_HUMAN	tr|A8K132	cDNA FLJ75476, highly similar to Homo sapiens glutaminase (GLS), mRNA OS=Homo sapiens PE=2 SV=1	74317	0	225	0	187	0	0.3	#DIV/0!	0	5	0	6	0	6	0	6	2.835036
tr|Q6NVW7|Q6NVW7_HUMAN	KPNA2	Importin subunit alpha OS=Homo sapiens GN=KPNA2 PE=2 SV=1	58271	0	204	0	200	0	0.32	#DIV/0!	0	5	0	5	0	6	0	7	2.835036
tr|B3KNW8|B3KNW8_HUMAN	tr|B3KNW	cDNA FLJ30614 fis, clone CTONG2001139 OS=Homo sapiens PE=2 SV=1	124437	0	756	0	47	0	0.08	#DIV/0!	0	1	0	3	0	5	0	6	2.593839
tr|Q59FA0|Q59FA0_HUMAN	tr|Q59FA	DNA polymerase (Fragment) OS=Homo sapiens PE=2 SV=1	113271	0	727	0	50	0	0.11	#DIV/0!	0	1	0	4	0	5	0	6	2.593839
sp|P46977|STT3A_HUMAN	STT3A	Dolichyl-diphosphooligosaccharide--protein glycosyltransferase subunit STT3A OS=Homo sapiens GN=STT3A PE=:	81104	0	660	0	57	0	0.08	#DIV/0!	0	2	0	2	0	5	0	5	2.593839
tr|H0YCK3|H0YCK3_HUMAN	ADAR	Double-stranded RNA-specific adenosine deaminase (Fragment) OS=Homo sapiens GN=ADAR PE=4 SV=1	133761	0	590	0	64	0	0.08	#DIV/0!	0	1	0	3	0	5	0	8	2.593839
tr|H0Y5W0|H0Y5W0_HUMAN	HUWE1	E3 ubiquitin-protein ligase HUWE1 (Fragment) OS=Homo sapiens GN=HUWE1 PE=4 SV=1	376696	0	480	0	82	0	0.03	#DIV/0!	0	3	0	5	0	5	0	5	2.593839
tr|A8K5Y7|A8K5Y7_HUMAN	tr|A8K5Y	cDNA FLJ78655, highly similar to Homo sapiens exportin 5 (XPO5), mRNA OS=Homo sapiens PE=2 SV=1	138331	0	474	0	83	0	0.15	#DIV/0!	0	2	0	6	0	5	0	7	2.593839
tr|B0UZZ8|B0UZZ8_HUMAN	WDR46	Chromosome 6 open reading frame 11 OS=Homo sapiens GN=WDR46 PE=2 SV=1	68455	0	471	0	84	0	0.15	#DIV/0!	0	1	0	3	0	5	0	6	2.593839
sp|Q9UHD8|SEPT9_HUMAN	Sep-09	Septin-9 OS=Homo sapiens GN=SEPT9 PE=1 SV=2	65646	0	444	0	91	0	0.22	#DIV/0!	0	2	0	4	0	5	0	5	2.593839
tr|B4DZI8|B4DZI8_HUMAN	COPB2	Coatomer protein complex, subunit beta 2 (Beta prime), isoform CRA_b OS=Homo sapiens GN=COPB2 PE=2 SV=1	99839	0	427	0	96	0	0.18	#DIV/0!	0	3	0	5	0	5	0	7	2.593839
tr|Q6NWZ1|Q6NWZ1_HUMAN	CKAP4	CKAP4 protein (Fragment) OS=Homo sapiens GN=CKAP4 PE=2 SV=1	67948	0	383	0	107	0	0.25	#DIV/0!	0	2	0	4	0	5	0	6	2.593839
tr|G5E9H4|G5E9H4_HUMAN	DCTN1	Dynactin 1 (P150, glued homolog, Drosophila), isoform CRA_a OS=Homo sapiens GN=DCTN1 PE=2 SV=1	127339	0	361	0	115	0	0.11	#DIV/0!	0	3	0	4	0	5	0	6	2.593839
tr|B4DZP5|B4DZP5_HUMAN	tr|B4DZP	cDNA FLJ51165, highly similar to DNA damage-binding protein 1 OS=Homo sapiens PE=2 SV=1	92726	0	351	0	119	0	0.11	#DIV/0!	0	2	0	4	0	5	0	5	2.593839
tr|B3KRR1|B3KRR1_HUMAN	tr|B3KRR	cDNA FLJ34725 fis, clone MESAN2005958, highly similar to RNA-binding protein Luc7-like 2 OS=Homo sapiens PE	40752	0	349	0	99	0	0.5	#DIV/0!	0	3	0	5	0	5	0	6	2.593839
sp|Q9BVP2|GNL3_HUMAN	GNL3	Guanine nucleotide-binding protein-like 3 OS=Homo sapiens GN=GNL3 PE=1 SV=2	62468	0	313	0	132	0	0.29	#DIV/0!	0	2	0	5	0	5	0	8	2.593839
tr|H3BLU7|H3BLU7_HUMAN	AKR7A2	Aflatoxin B1 aldehyde reductase member 2 (Fragment) OS=Homo sapiens GN=AKR7A2 PE=4 SV=1	35004	0	274	0	143	0	0.37	#DIV/0!	0	2	0	4	0	5	0	6	2.593839
sp|O43815|STRN_HUMAN	STRN	Striatin OS=Homo sapiens GN=STRN PE=1 SV=4	86535	0	260	0	163	0	0.2	#DIV/0!	0	4	0	5	0	5	0	6	2.593839
sp|Q92945|FUBP2_HUMAN	KHSRP	Far upstream element-binding protein 2 OS=Homo sapiens GN=KHSRP PE=1 SV=4	73355	0	240	0	175	0	0.24	#DIV/0!	0	4	0	5	0	5	0	7	2.593839
tr|Q5TZZ9|Q5TZZ9_HUMAN	ANXA1	Annexin OS=Homo sapiens GN=ANXA1 PE=2 SV=1	38918	0	211	0	195	0	0.5	#DIV/0!	0	4	0	5	0	5	0	6	2.593839
tr|A8K4T6|A8K4T6_HUMAN	tr|A8K4T	cDNA FLJ76282, highly similar to Homo sapiens proteasome (prosome, macropain) 26S subunit, non-ATPase, 5 (P	56588	0	207	0	197	0	0.33	#DIV/0!	0	5	0	5	0	5	0	5	2.593839
tr|G3XAL0|G3XAL0_HUMAN	MDH2	Malate dehydrogenase OS=Homo sapiens GN=MDH2 PE=2 SV=1	24921	0	116	0	223	0	0.98	#DIV/0!	0	5	0	5	0	5	0	5	2.593839
tr|B0AZM4|B0AZM4_HUMAN	tr|B0AZM	cDNA, FLJ79464, highly similar to Homo sapiens aquarius (Fragment) OS=Homo sapiens PE=2 SV=1	157764	0	1041	0	31	0	0.04	#DIV/0!	0	0	0	2	0	4	0	4	2.304144
tr|G3V529|G3V529_HUMAN	DDX24	ATP-dependent RNA helicase DDX24 OS=Homo sapiens GN=DDX24 PE=2 SV=1	91994	0	1021	0	32	0	0.07	#DIV/0!	0	0	0	2	0	4	0	5	2.304144
tr|B4DUF1|B4DUF1_HUMAN	tr|B4DUF	cDNA FLJ59760, highly similar to 1,4-alpha-glucan branching enzyme (EC 2.4.1.18) OS=Homo sapiens PE=2 SV=1	76254	0	921	0	37	0	0.09	#DIV/0!	0	0	0	2	0	4	0	6	2.304144
tr|B4DFN6|B4DFN6_HUMAN	CNP	2~,3~-cyclic-nucleotide 3~-phosphodiesterase OS=Homo sapiens GN=CNP PE=2 SV=1	20461	0	882	0	39	0	0.58	#DIV/0!	0	1	0	3	0	4	0	4	2.304144
tr|B4E3M6|B4E3M6_HUMAN	tr|B4E3M	cDNA FLJ55446, highly similar to Superkiller viralicidic activity 2-like 2 (EC 3.6.1.-) OS=Homo sapiens PE=2 SV=1	107714	0	799	0	44	0	0.06	#DIV/0!	0	1	0	2	0	4	0	5	2.304144
tr|B3KY63|B3KY63_HUMAN	tr|B3KY63	cDNA FLJ16830 fis, clone UTERU3022536, highly similar to Chromodomain helicase-DNA-binding protein 4 (EC 3.(	216574	0	793	0	44	0	0.05	#DIV/0!	0	2	0	3	0	4	0	9	2.304144
sp|Q8NI36|WDR36_HUMAN	WDR36	WD repeat-containing protein 36 OS=Homo sapiens GN=WDR36 PE=1 SV=1	106282	0	771	0	46	0	0.06	#DIV/0!	0	1	0	2	0	4	0	5	2.304144
tr|G3V3B0|G3V3B0_HUMAN	ACIN1	Apoptotic chromatin condensation inducer in the nucleus OS=Homo sapiens GN=ACIN1 PE=2 SV=1	67683	0	759	0	47	0	0.21	#DIV/0!	0	1	0	4	0	4	0	5	2.304144
sp|Q08379|GOGA2_HUMAN	GOLGA2	Golgin subfamily A member 2 OS=Homo sapiens GN=GOLGA2 PE=1 SV=3	113644	0	735	0	49	0	0.03	#DIV/0!	0	1	0	1	0	4	0	4	2.304144
sp|Q14690|RRP5_HUMAN	PDCD11	Protein RRP5 homolog OS=Homo sapiens GN=PDCD11 PE=1 SV=3	209939	0	612	0	62	0	0.03	#DIV/0!	0	1	0	2	0	4	0	5	2.304144
tr|B4DN01|B4DN01_HUMAN	tr|B4DN0	cDNA FLJ57332, highly similar to COP9 signalosome complex subunit 3 OS=Homo sapiens PE=2 SV=1	33950	0	607	0	62	0	0.45	#DIV/0!	0	1	0	4	0	4	0	4	2.304144
tr|B2RE88|B2RE88_HUMAN	tr|B2RE88	cDNA, FLJ96465, highly similar to Homo sapiens solute carrier family 25 (mitochondrial carrier;phosphate carrier)	40379	0	582	0	65	0	0.17	#DIV/0!	0	2	0	2	0	4	0	4	2.304144
tr|F5H8E5|F5H8E5_HUMAN	USP7	Ubiquitin carboxyl-terminal hydrolase OS=Homo sapiens GN=USP7 PE=2 SV=1	117951	0	537	0	71	0	0.09	#DIV/0!	0	2	0	3	0	4	0	7	2.304144
tr|A8KA19|A8KA19_HUMAN	tr|A8KA1	cDNA FLJ75831, highly similar to Homo sapiens exportin, tRNA (nuclear export receptor for tRNAs) (XPOT), mRNA	111104	0	531	0	72	0	0.09	#DIV/0!	0	2	0	3	0	4	0	5	2.304144
tr|F4NBX2|F4NBX2_HUMAN	HLA-B	MHC class I antigen (Fragment) OS=Homo sapiens GN=HLA-B PE=3 SV=1	38566	0	492	0	80	0	0.39	#DIV/0!	0	3	0	4	0	4	0	5	2.304144
tr|H7C1M2|H7C1M2_HUMAN	SON	Protein SON (Fragment) OS=Homo sapiens GN=SON PE=4 SV=1	160263	0	488	0	80	0	0.08	#DIV/0!	0	2	0	4	0	4	0	4	2.304144
tr|B4DSN3|B4DSN3_HUMAN	tr|B4DSN	cDNA FLJ60345, highly similar to Protein transport protein Sec24C OS=Homo sapiens PE=2 SV=1	107974	0	483	0	82	0	0.13	#DIV/0!	0	3	0	4	0	4	0	4	2.304144
tr|B4DL07|B4DL07_HUMAN	tr|B4DL07	cDNA FLJ53353, highly similar to ATP-binding cassette sub-family D member 3 OS=Homo sapiens PE=2 SV=1	78980	0	477	0	83	0	0.13	#DIV/0!	0	2	0	3	0	4	0	4	2.304144
tr|B4DM78|B4DM78_HUMAN	tr|B4DM7	cDNA FLJ58199, highly similar to Fragile X mental retardation syndrome-related protein 1 OS=Homo sapiens PE=2	63280	0	476	0	83	0	0.28	#DIV/0!	0	1	0	4	0	4	0	5	2.304144
tr|H7BZT7|H7BZT7_HUMAN	ESD	S-formylglutathione hydrolase (Fragment) OS=Homo sapiens GN=ESD PE=4 SV=1	25806	0	446	0	62	0	0.64	#DIV/0!	0	2	0	4	0	4	0	4	2.304144
tr|H0YCK7|H0YCK7_HUMAN	EEF1D	Elongation factor 1-delta (Fragment) OS=Homo sapiens GN=EEF1D PE=4 SV=1	23105	0	412	0	98	0	0.14	#DIV/0!	0	2	0	2	0	4	0	4	2.304144
sp|Q9H0A0|NAT10_HUMAN	NAT10	N-acetyltransferase 10 OS=Homo sapiens GN=NAT10 PE=1 SV=2	116569	0	400	0	103	0	0.09	#DIV/0!	0	2	0	3	0	4	0	4	2.304144
tr|B7Z4B8|B7Z4B8_HUMAN	HNRNPUL	Heterogeneous nuclear ribonucleoprotein U-like protein 1 OS=Homo sapiens GN=HNRNPUL1 PE=2 SV=1	86639	0	396	0	104	0	0.12	#DIV/0!	0	2	0	3	0	4	0	5	2.304144
tr|H0YN14|H0YN14_HUMAN	IPO4	Importin-4 OS=Homo sapiens GN=IPO4 PE=4 SV=1	103193	0	382	0	107	0	0.13	#DIV/0!	0	2	0	4	0	4	0	5	2.304144
sp|P49792|RBP2_HUMAN	RANBP2	E3 SUMO-protein ligase RanBP2 OS=Homo sapiens GN=RANBP2 PE=1 SV=2	362365	0	333	0	124	0	0.04	#DIV/0!	0	4	0	4	0	4	0	9	2.304144
tr|G1UI16|G1UI16_HUMAN	PDS5A	SCC-112 protein, isoform CRA_b OS=Homo sapiens GN=PDS5A PE=2 SV=1	152274	0	327	0	126	0	0.09	#DIV/0!	0	4	0	4	0	4	0	5	2.304144
tr|A8K0D2|A8K0D2_HUMAN	tr|A8K0D	cDNA FLJ77740, highly similar to Homo sapiens 7-dehydrocholesterol reductase, mRNA OS=Homo sapiens PE=2 S	55175	0	317	0	130	0	0.19	#DIV/0!	0	2	0	3	0	4	0	4	2.304144
tr|D3DU41|D3DU41_HUMAN	LUC7L	LUC7-like (S. cerevisiae), isoform CRA_a OS=Homo sapiens GN=LUC7L PE=2 SV=1	34110	0	314	0	112	0	0.62	#DIV/0!	0	2	0	4	0	4	0	5	2.304144
sp|P49419|AL7A1_HUMAN	ALDH7A1	Alpha-aminoadipic semialdehyde dehydrogenase OS=Homo sapiens GN=ALDH7A1 PE=1 SV=5	59020	0	300	0	138	0	0.24	#DIV/0!	0	4	0	4	0	4	0	5	2.304144
tr|B4DIT7|B4DIT7_HUMAN	TGM2	Protein-glutamine gamma-glutamyltransferase 2 OS=Homo sapiens GN=TGM2 PE=2 SV=1	69632	0	237	0	177	0	0.2	#DIV/0!	0	4	0	4	0	4	0	5	2.304144
tr|B3KM58|B3KM58_HUMAN	tr|B3KM5	cDNA FLJ10358 fis, clone NT2RM2001238, highly similar to Glutaminase kidney isoform, mitochondrial (Fragment	41736	0	199	0	205	0	0.36	#DIV/0!	0	4	0	4	0	4	0	4	2.304144
tr|Q3SYK8|Q3SYK8_HUMAN	NUMA1	NUMA1 protein (Fragment) OS=Homo sapiens GN=NUMA1 PE=2 SV=1	78666	0	145	0	146	0	0.13	#DIV/0!	0	3	0	4	0	4	0	4	2.304144
sp|Q7Z478|DHX29_HUMAN	DHX29	ATP-dependent RNA helicase DHX29 OS=Homo sapiens GN=DHX29 PE=1 SV=2	156222	0	1203	0	20	0	0.02	#DIV/0!	0	0	0	1	0	3	0	3	1.941386
sp|P57740|NU107_HUMAN	NUP107	Nuclear pore complex protein Nup107 OS=Homo sapiens GN=NUP107 PE=1 SV=1	107048	0	1149	0	24	0	0.05	#DIV/0!	0	0	0	1	0	3	0	3	1.941386
sp|O15020|SPTN2_HUMAN	SPTBN2	Spectrin beta chain, non-erythrocytic 2 OS=Homo sapiens GN=SPTBN2 PE=1 SV=3	272526	0	1144	0	25	0	0.01	#DIV/0!	0	0	0	1	0	3	0	4	1.941386
tr|G3V394|G3V394_HUMAN	MYO5A	Unconventional myosin-Va OS=Homo sapiens GN=MYO5A PE=2 SV=1	217049	0	1141	0	25	0	0.03	#DIV/0!	0	0	0	1	0	3	0	3	1.941386
sp|Q9H9A6|LRC40_HUMAN	LRRC40	Leucine-rich repeat-containing protein 40 OS=Homo sapiens GN=LRRC40 PE=1 SV=1	68720	0	1098	0	27	0	0.05	#DIV/0!	0	0	0	1	0	3	0	3	1.941386
tr|B2RDI5|B2RDI5_HUMAN	tr|B2RDI5	cDNA, FLJ96627, highly similar to Homo sapiens calpain 1, (mu/I) large subunit (CAPN1), mRNA OS=Homo sapiens	82529	0	1040	0	31	0	0.04	#DIV/0!	0	1	0	1	0	3	0	3	1.941386
sp|Q8IWB1|IPRI_HUMAN	ITPRIP	Inositol 1,4,5-trisphosphate receptor-interacting protein OS=Homo sapiens GN=ITPRIP PE=1 SV=1	62932	0	1032	0	31	0	0.05	#DIV/0!	0	1	0	1	0	3	0	3	1.941386
sp|Q08945|SSRP1_HUMAN	SSRP1	FACT complex subunit SSRP1 OS=Homo sapiens GN=SSRP1 PE=1 SV=1	81367	0	1012	0	33	0	0.04	#DIV/0!	0	0	0	1	0	3	0	5	1.941386
sp|P24928|RPB1_HUMAN	POLR2A	DNA-directed RNA polymerase II subunit RPB1 OS=Homo sapiens GN=POLR2A PE=1 SV=2	218408	0	976	0	34	0	0.01	#DIV/0!	0	0	0	1	0	3	0	3	1.941386
tr|B4DH29|B4DH29_HUMAN	tr|B4DH2	DNA-directed RNA polymerase OS=Homo sapiens PE=2 SV=1	126564	0	974	0	34	0	0.05	#DIV/0!	0	0	0	2	0	3	0	4	1.941386
tr|Q6DCA8|Q6DCA8_HUMAN	BCLAF1	BCLAF1 protein (Fragment) OS=Homo sapiens GN=BCLAF1 PE=2 SV=1	77958	0	967	0	35	0	0.13	#DIV/0!	0	0	0	3	0	3	0	3	1.941386
tr|B2RDW1|B2RDW1_HUMAN	RPS27A	Ribosomal protein S27a, isoform CRA_c OS=Homo sapiens GN=RPS27A PE=2 SV=1	18296	0	867	0	39	0	0.18	#DIV/0!	0	0	0	2	0	3	0	4	1.941386
sp|Q8WUX1|S38A5_HUMAN	SLC38A5	Sodium-coupled neutral amino acid transporter 5 OS=Homo sapiens GN=SLC38A5 PE=1 SV=1	52107	0	861	0	40	0	0.2	#DIV/0!	0	0	0	3	0	3	0	3	1.941386
sp|Q8TCT9|HM13_HUMAN	HM13	Minor histocompatibility antigen H13 OS=Homo sapiens GN=HM13 PE=1 SV=1	41747	0	833	0	42	0	0.16	#DIV/0!	0	0	0	2	0	3	0	3	1.941386
tr|Q96F88|Q96F88_HUMAN	POP1	Processing of 1, ribonuclease P/MRP subunit (S. cerevisiae) OS=Homo sapiens GN=POP1 PE=2 SV=1	116318	0	801	0	44	0	0.09	#DIV/0!	0	0	0	3	0	3	0	4	1.941386
sp|Q9UHD1|CHRD1_HUMAN	CHORDC1	Cysteine and histidine-rich domain-containing protein 1 OS=Homo sapiens GN=CHORDC1 PE=1 SV=2	38264	0	782	0	45	0	0.28	#DIV/0!	0	0	0	3	0	3	0	3	1.941386
tr|B4DYY5|B4DYY5_HUMAN	tr|B4DYY	cDNA FLJ55671, highly similar to Ubiquitin-associated protein 2-like OS=Homo sapiens PE=2 SV=1	113662	0	762	0	46	0	0.06	#DIV/0!	0	1	0	2	0	3	0	3	1.941386
sp|Q8WX93|PALLD_HUMAN	PALLD	Palladin OS=Homo sapiens GN=PALLD PE=1 SV=3	151839	0	760	0	46	0	0.05	#DIV/0!	0	1	0	2	0	3	0	3	1.941386
sp|A0AVT1|UBA6_HUMAN	UBA6	Ubiquitin-like modifier-activating enzyme 6 OS=Homo sapiens GN=UBA6 PE=1 SV=1	119207	0	757	0	47	0	0.06	#DIV/0!	0	1	0	2	0	3	0	3	1.941386
tr|H7C3P6|H7C3P6_HUMAN	NUP98	Nuclear pore complex protein Nup98-Nup96 (Fragment) OS=Homo sapiens GN=NUP98 PE=4 SV=1	86760	0	754	0	47	0	0.12	#DIV/0!	0	1	0	3	0	3	0	4	1.941386
tr|G3V3T3|G3V3T3_HUMAN	ACIN1	Apoptotic chromatin condensation inducer in the nucleus (Fragment) OS=Homo sapiens GN=ACIN1 PE=2 SV=1	21170	0	745	0	48	0	0.08	#DIV/0!	0	1	0	3	0	3	0	3	1.941386
tr|B3KT00|B3KT00_HUMAN	tr|B3KT0	cDNA FLJ37368 fis, clone BRAMY2024530, highly similar to Zinc phosphodiesterase ELAC protein 2 (EC 3.1.26.11)	91169	0	741	0	48	0	0.07	#DIV/0!	0	1	0	2	0	3	0	3	1.941386
tr|B0QZ18|B0QZ18_HUMAN	CPNE1	Copine I OS=Homo sapiens GN=CPNE1 PE=2 SV=1	60307	0	726	0	50	0	0.17	#DIV/0!	0	1	0	3	0	3	0	3	1.941386
tr|B4DL39|B4DL39_HUMAN	tr|B4DL3	cDNA FLJ57316, highly similar to DNA mismatch repair protein Msh2 OS=Homo sapiens PE=2 SV=1	77368	0	714	0	51	0	0.13	#DIV/0!	0	1	0	3	0	3	0	3	1.941386
tr|Q56VW8|Q56VW8_HUMAN	tr|Q56VW	Lung cancer-related protein 9 OS=Homo sapiens PE=2 SV=1	52186	0	658	0	57	0	0.2	#DIV/0!	0	0	0	3	0	3	0	3	1.941386
tr|E3Q0X1|E3Q0X1_HUMAN	HLA-B	MHC class I antigen (Fragment) OS=Homo sapiens GN=HLA-B PE=3 SV=1	31939	0	594	0	63	0	0.35	#DIV/0!	0	2	0	3	0	3	0	3	1.941386
tr|J3QQ67|J3QQ67_HUMAN	RPL18	60S ribosomal protein L18 (Fragment) OS=Homo sapiens GN=RPL18 PE=3 SV=1	21940	0	581	0	65	0	0.39	#DIV/0!	0	2	0	2	0	3	0	3	1.941386
tr|H0Y2Y8|H0Y2Y8_HUMAN	ZYX	Zyxin (Fragment) OS=Homo sapiens GN=ZYX PE=4 SV=1	58804	0	574	0	66	0	0.18	#DIV/0!	0	2	0	3	0	3	0	4	1.941386
tr|B3KM89|B3KM89_HUMAN	tr|B3KM8	cDNA FLJ10528 fis, clone NT2RP2000943, highly similar to Protein transport protein Sec24D OS=Homo sapiens PE	101723	0	572	0	66	0	0.1	#DIV/0!	0	1	0	3	0	3	0	3	1.941386
tr|G3V1J5|G3V1J5_HUMAN	DIS3	Exosome complex exonuclease RRP44 OS=Homo sapiens GN=DIS3 PE=2 SV=1	91382	0	563	0	41	0	0.09	#DIV/0!	0	1	0	1	0	3	0	3	1.941386
sp|Q14692|BMS1_HUMAN	BMS1	Ribosome biogenesis protein BMS1 homolog OS=Homo sapiens GN=BMS1 PE=1 SV=1	146571	0	555	0	69	0	0.05	#DIV/0!	0	1	0	2	0	3	0	3	1.941386
tr|B3KNV9|B3KNV9_HUMAN	tr|B3KNV	cDNA FLJ30554 fis, clone BRAWH2003693 OS=Homo sapiens PE=2 SV=1	112670	0	548	0	62	0	0.05	#DIV/0!	0	1	0	2	0	3	0	3	1.941386
sp|Q9UHX1|PUF60_HUMAN	PUF60	Poly(U)-binding-splicing factor PUF60 OS=Homo sapiens GN=PUF60 PE=1 SV=1	60009	0	545	0	70	0	0.17	#DIV/0!	0	2	0	3	0	3	0	3	1.941386
tr|A4D275|A4D275_HUMAN	ARPC1B	Actin related protein 2/3 complex, subunit 1B, 41kDa OS=Homo sapiens GN=ARPC1B PE=2 SV=1	41722	0	518	0	75	0	0.26	#DIV/0!	0	2	0	3	0	3	0	5	1.941386
tr|F5H583|F5H583_HUMAN	CYFIP2	Cytoplasmic FMR1-interacting protein 2 OS=Homo sapiens GN=CYFIP2 PE=2 SV=1	138666	0	517	0	75	0	0.08	#DIV/0!	0	2	0	3	0	3	0	5	1.941386
sp|P55268|LAMB2_HUMAN	LAMB2	Laminin subunit beta-2 OS=Homo sapiens GN=LAMB2 PE=1 SV=2	202982	0	510	0	76	0	0.05	#DIV/0!	0	1	0	3	0	3	0	4	1.941386
tr|C0KK01|C0KK01_HUMAN	HLA-C	MHC class I antigen (Fragment) OS=Homo sapiens GN=HLA-C PE=3 SV=1	20940	0	506	0	76	0	0.56	#DIV/0!	0	3	0	3	0	3	0	4	1.941386
sp|Q06210|GFPT1_HUMAN	GFPT1	Glutamine--fructose-6-phosphate aminotransferase [isomerizing] 1 OS=Homo sapiens GN=GFPT1 PE=1 SV=3	79555	0	472	0	84	0	0.08	#DIV/0!	0	2	0	2	0	3	0	3	1.941386
sp|Q8NI27|THOC2_HUMAN	THOC2	THO complex subunit 2 OS=Homo sapiens GN=THOC2 PE=1 SV=2	184541	0	450	0	90	0	0.1	#DIV/0!	0	3	0	3	0	3	0	4	1.941386
tr|A4D210|A4D210_HUMAN	EIF3S9	Eukaryotic translation initiation factor 3, subunit 9 eta, 116kDa OS=Homo sapiens GN=EIF3S9 PE=4 SV=1	89024	0	448	0	91	0	0.07	#DIV/0!	0	2	0	2	0	3	0	3	1.941386
tr|G3V198|G3V198_HUMAN	NUP160	Nuclear pore complex protein Nup160 OS=Homo sapiens GN=NUP160 PE=2 SV=1	142365	0	437	0	74	0	0.1	#DIV/0!	0	2	0	3	0	3	0	4	1.941386
sp|P22059|OSBP1_HUMAN	OSBP	Oxysterol-binding protein 1 OS=Homo sapiens GN=OSBP PE=1 SV=1	90220	0	394	0	105	0	0.11	#DIV/0!	0	2	0	3	0	3	0	3	1.941386
sp|P04899|GNAI2_HUMAN	GNAI2	Guanine nucleotide-binding protein G(i) subunit alpha-2 OS=Homo sapiens GN=GNAI2 PE=1 SV=3	40995	0	388	0	106	0	0.26	#DIV/0!	0	3	0	3	0	3	0	3	1.941386
tr|G5E988|G5E988_HUMAN	AP3D1	AP-3 complex subunit delta-1 OS=Homo sapiens GN=AP3D1 PE=2 SV=1	126074	0	387	0	107	0	0.08	#DIV/0!	0	3	0	3	0	3	0	3	1.941386

tr|B5BU08|B5BU08_HUMAN	U2AF1	U2 small nuclear RNA auxillary factor 1 isoform a OS=Homo sapiens GN=U2AF1 PE=2 SV=1	28369	0	385	0	107	0	0.39	#DIV/0!	0	2	0	3	0	3	0	3	1.941386
tr|B2RNR6|B2RNR6_HUMAN	ZFR	Zinc finger RNA binding protein OS=Homo sapiens GN=ZFR PE=2 SV=1	118081	0	379	0	109	0	0.06	#DIV/0!	0	2	0	2	0	3	0	3	1.941386
tr|B3KPA6|B3KPA6_HUMAN	ACADVL	Acyl-Coenzyme A dehydrogenase, very long chain, isoform CRA_e OS=Homo sapiens GN=ACADVL PE=2 SV=1	62941	0	377	0	110	0	0.17	#DIV/0!	0	2	0	3	0	3	0	5	1.941386
tr|Q4KMR3|Q4KMR3_HUMAN	MYO1E	MYO1E variant protein OS=Homo sapiens GN=MYO1E PE=2 SV=1	127552	0	373	0	111	0	0.08	#DIV/0!	0	2	0	3	0	3	0	3	1.941386
sp|Q9H2M9|RBGPR_HUMAN	RAB3GAP2	Rab3 GTPase-activating protein non-catalytic subunit OS=Homo sapiens GN=RAB3GAP2 PE=1 SV=1	157482	0	352	0	119	0	0.06	#DIV/0!	0	3	0	3	0	3	0	3	1.941386
sp|P43034|LIS1_HUMAN	PAFAH1B1	Platelet-activating factor acetylhydrolase IB subunit alpha OS=Homo sapiens GN=PAFAH1B1 PE=1 SV=2	47178	0	346	0	120	0	0.27	#DIV/0!	0	3	0	3	0	3	0	4	1.941386
tr|D3DTY9|D3DTY9_HUMAN	TRIM25	Tripartite motif-containing 25, isoform CRA_a OS=Homo sapiens GN=TRIM25 PE=2 SV=1	48358	0	321	0	127	0	0.22	#DIV/0!	0	3	0	3	0	3	0	4	1.941386
tr|Q6FHX6|Q6FHX6_HUMAN	FEN1	Flap endonuclease 1 OS=Homo sapiens GN=FEN1 PE=2 SV=1	42908	0	309	0	133	0	0.16	#DIV/0!	0	2	0	2	0	3	0	3	1.941386
tr|H0YHX9|H0YHX9_HUMAN	NACA	Nascent polypeptide-associated complex subunit alpha (Fragment) OS=Homo sapiens GN=NACA PE=4 SV=1	22930	0	282	0	147	0	0.5	#DIV/0!	0	3	0	3	0	3	0	3	1.941386
tr|B4DJV2|B4DJV2_HUMAN	CS	Citrate synthase OS=Homo sapiens GN=CS PE=2 SV=1	50628	0	1229	0	16	0	0.07	#DIV/0!	0	0	0	1	0	2	0	3	1.455771
tr|Q59ES2|Q59ES2_HUMAN	tr|Q59ES2	Inositol 1,4,5-trisphosphate receptor type 3 variant (Fragment) OS=Homo sapiens PE=2 SV=1	269361	0	1199	0	21	0	0.01	#DIV/0!	0	0	0	1	0	2	0	4	1.455771
tr|B0YIW5|B0YIW5_HUMAN	ARCN1	Archain 1, isoform CRA_b OS=Homo sapiens GN=ARCN1 PE=2 SV=1	57630	0	1172	0	23	0	0.06	#DIV/0!	0	0	0	1	0	2	0	2	1.455771
tr|B1AKJ6|B1AKJ6_HUMAN	OSBPL9	Oxysterol-binding protein OS=Homo sapiens GN=OSBPL9 PE=2 SV=1	84417	0	1163	0	23	0	0.04	#DIV/0!	0	0	0	1	0	2	0	2	1.455771
tr|I3L4X2|I3L4X2_HUMAN	ABCC1	Multidrug resistance-associated protein 1 (Fragment) OS=Homo sapiens GN=ABCC1 PE=3 SV=1	161588	0	1157	0	24	0	0.02	#DIV/0!	0	0	0	1	0	2	0	2	1.455771
tr|B4DF80|B4DF80_HUMAN	tr|B4DF80	cDNA FLJ58527, highly similar to Nucleolar protein 6 OS=Homo sapiens PE=2 SV=1	122714	0	1133	0	25	0	0.03	#DIV/0!	0	0	0	1	0	2	0	2	1.455771
tr|Q96FS1|Q96FS1_HUMAN	CTNND1	CTNND1 protein (Fragment) OS=Homo sapiens GN=CTNND1 PE=2 SV=2	92901	0	1109	0	27	0	0.03	#DIV/0!	0	0	0	1	0	2	0	2	1.455771
tr|B1PBA3|B1PBA3_HUMAN	tr|B1PBA3	SKNY protein OS=Homo sapiens PE=2 SV=1	98489	0	1108	0	27	0	0.03	#DIV/0!	0	0	0	1	0	2	0	3	1.455771
tr|Q4ZG72|Q4ZG72_HUMAN	DDX18	Putative uncharacterized protein DDX18 (Fragment) OS=Homo sapiens GN=DDX18 PE=2 SV=1	61898	0	1105	0	27	0	0.05	#DIV/0!	0	1	0	1	0	2	0	3	1.455771
tr|B0AZQ4|B0AZQ4_HUMAN	tr|B0AZQ4	cDNA, FLJ79494, highly similar to Structural maintenance of chromosome 3 OS=Homo sapiens PE=2 SV=1	141841	0	1101	0	27	0	0.02	#DIV/0!	0	0	0	1	0	2	0	3	1.455771
tr|Q7Z586|Q7Z586_HUMAN	tr|Q7Z586	Putative uncharacterized protein (Fragment) OS=Homo sapiens PE=2 SV=1	105456	0	1077	0	29	0	0.01	#DIV/0!	0	0	0	1	0	2	0	2	1.455771
tr|Q5JB52|Q5JB52_HUMAN	HNRPLL	Heterogeneous nuclear ribonucleoprotein L-like OS=Homo sapiens GN=HNRPLL PE=2 SV=1	57115	0	1066	0	30	0	0.06	#DIV/0!	0	0	0	1	0	2	0	3	1.455771
sp|Q9BR76|COR1B_HUMAN	CORO1B	Coronin-1B OS=Homo sapiens GN=CORO1B PE=1 SV=1	54885	0	1024	0	32	0	0.12	#DIV/0!	0	0	0	2	0	2	0	2	1.455771
sp|Q8IYJ1|CPNE9_HUMAN	CPNE9	Copine-9 OS=Homo sapiens GN=CPNE9 PE=1 SV=3	62281	0	1020	0	32	0	0.11	#DIV/0!	0	0	0	2	0	2	0	2	1.455771
tr|G3XAI2|G3XAI2_HUMAN	LAMB1	Laminin subunit beta-1 OS=Homo sapiens GN=LAMB1 PE=2 SV=1	207703	0	1019	0	32	0	0.03	#DIV/0!	0	0	0	2	0	2	0	4	1.455771
sp|P04181|OAT_HUMAN	OAT	Ornithine aminotransferase, mitochondrial OS=Homo sapiens GN=OAT PE=1 SV=1	48846	0	1016	0	32	0	0.07	#DIV/0!	0	1	0	1	0	2	0	2	1.455771
tr|J3KT10|J3KT10_HUMAN	NUP85	Nuclear pore complex protein Nup85 OS=Homo sapiens GN=NUP85 PE=4 SV=1	70931	0	998	0	34	0	0.05	#DIV/0!	0	0	0	1	0	2	0	2	1.455771
sp|Q9UBL6|CPNE7_HUMAN	CPNE7	Copine-7 OS=Homo sapiens GN=CPNE7 PE=2 SV=1	71390	0	997	0	34	0	0.05	#DIV/0!	0	0	0	1	0	2	0	2	1.455771
tr|B3KMR5|B3KMR5_HUMAN	tr|B3KMR	cDNA FLJ12434 fis, clone NT2RM1000037, highly similar to Homo sapiens KIAA0690 protein OS=Homo sapiens PE	145007	0	986	0	34	0	0.07	#DIV/0!	0	1	0	3	0	2	0	3	1.455771
tr|B2RB90|B2RB90_HUMAN	tr|B2RB90	cDNA, FLJ95375, highly similar to Homo sapiens glutamine-fructose-6-phosphate transaminase 2 (GFPT2), mRNA	77651	0	983	0	34	0	0.04	#DIV/0!	0	1	0	1	0	2	0	2	1.455771
tr|J3KTL8|J3KTL8_HUMAN	SMCHD1	Structural maintenance of chromosomes flexible hinge domain-containing protein 1 (Fragment) OS=Homo sapier	156690	0	964	0	35	0	0.04	#DIV/0!	0	0	0	2	0	2	0	2	1.455771
tr|H0YE88|H0YE88_HUMAN	TEAD1	Transcriptional enhancer factor TEF-1 OS=Homo sapiens GN=TEAD1 PE=4 SV=1	48296	0	962	0	35	0	0.07	#DIV/0!	0	1	0	1	0	2	0	2	1.455771
tr|B4DM01|B4DM01_HUMAN	SKIV2L	Helicase SKI2W OS=Homo sapiens GN=SKIV2L PE=2 SV=1	121269	0	954	0	35	0	0.03	#DIV/0!	0	1	0	1	0	2	0	4	1.455771
tr|Q0PHS4|Q0PHS4_HUMAN	G6PD	Glucose-6-phosphate dehydrogenase (Fragment) OS=Homo sapiens GN=G6PD PE=2 SV=1	7182	0	952	0	35	0	0.49	#DIV/0!	0	1	0	1	0	2	0	2	1.455771
sp|Q16222|UAP1_HUMAN	UAP1	UDP-N-acetylhexosamine pyrophosphorylase OS=Homo sapiens GN=UAP1 PE=1 SV=3	59131	0	941	0	36	0	0.11	#DIV/0!	0	0	0	2	0	2	0	2	1.455771
tr|B4DR36|B4DR36_HUMAN	tr|B4DR36	cDNA FLJ56414, highly similar to Homo sapiens proline-, glutamic acid-, leucine-rich protein 1 (PELP1), mRNA OS=	126101	0	934	0	36	0	0.07	#DIV/0!	0	0	0	2	0	2	0	2	1.455771
sp|Q8NB90|SPAT5_HUMAN	SPATA5	Spermatogenesis-associated protein 5 OS=Homo sapiens GN=SPATA5 PE=1 SV=3	98470	0	929	0	36	0	0.03	#DIV/0!	0	1	0	1	0	2	0	2	1.455771
tr|B2RWN5|B2RWN5_HUMAN	HEATR1	HEAT repeat containing 1 OS=Homo sapiens GN=HEATR1 PE=2 SV=1	244275	0	922	0	37	0	0.04	#DIV/0!	0	1	0	3	0	2	0	5	1.455771
sp|Q6UXN9|WDR82_HUMAN	WDR82	WD repeat-containing protein 82 OS=Homo sapiens GN=WDR82 PE=1 SV=1	35456	0	916	0	37	0	0.09	#DIV/0!	0	1	0	1	0	2	0	4	1.455771
sp|Q9UID3|VPS51_HUMAN	VPS51	Vacuolar protein sorting-associated protein 51 homolog OS=Homo sapiens GN=VPS51 PE=1 SV=2	86901	0	909	0	38	0	0.04	#DIV/0!	0	1	0	1	0	2	0	2	1.455771
tr|B4E263|B4E263_HUMAN	tr|B4E263	cDNA FLJ53502, highly similar to HEAT repeat-containing protein 1 OS=Homo sapiens PE=2 SV=1	128535	0	905	0	38	0	0.05	#DIV/0!	0	1	0	2	0	2	0	3	1.455771
tr|B3KM97|B3KM97_HUMAN	tr|B3KM9	cDNA FLJ10554 fis, clone NT2RP2002385, highly similar to Synaptic glycoprotein SC2 OS=Homo sapiens PE=2 SV=	36429	0	894	0	38	0	0.19	#DIV/0!	0	0	0	2	0	2	0	3	1.455771
tr|B2RE56|B2RE56_HUMAN	tr|B2RE56	Peptidyl-prolyl cis-trans isomerase OS=Homo sapiens PE=2 SV=1	18225	0	886	0	39	0	0.41	#DIV/0!	0	0	0	2	0	2	0	2	1.455771
sp|Q6P1J9|CDC73_HUMAN	CDC73	Parafibromin OS=Homo sapiens GN=CDC73 PE=1 SV=1	60653	0	880	0	39	0	0.09	#DIV/0!	0	1	0	1	0	2	0	2	1.455771
tr|B2RB95|B2RB95_HUMAN	tr|B2RB95	cDNA, FLJ95381, Homo sapiens tripartite motif-containing 16 (TRIM16), mRNA OS=Homo sapiens PE=2 SV=1	64884	0	879	0	39	0	0.03	#DIV/0!	0	1	0	1	0	2	0	2	1.455771
tr|F5GWY5|F5GWY5_HUMAN	PODXL	Podocalyxin OS=Homo sapiens GN=PODXL PE=2 SV=1	59285	0	871	0	39	0	0.06	#DIV/0!	0	1	0	1	0	2	0	2	1.455771
tr|A8K8X0|A8K8X0_HUMAN	tr|A8K8X0	cDNA FLJ75187, highly similar to Homo sapiens nap1 P120 OS=Homo sapiens PE=2 SV=1	113035	0	865	0	39	0	0.06	#DIV/0!	0	0	0	2	0	2	0	3	1.455771
sp|Q5TFE4|NT5D1_HUMAN	NT5DC1	5~-nucleotidase domain-containing protein 1 OS=Homo sapiens GN=NT5DC1 PE=1 SV=1	52439	0	854	0	40	0	0.06	#DIV/0!	0	1	0	1	0	2	0	2	1.455771
tr|A0JLQ5|A0JLQ5_HUMAN	BXDC2	BXDC2 protein (Fragment) OS=Homo sapiens GN=BXDC2 PE=2 SV=1	39430	0	852	0	35	0	0.26	#DIV/0!	0	1	0	2	0	2	0	3	1.455771
tr|B4DMF5|B4DMF5_HUMAN	tr|B4DMF	Glutamate dehydrogenase OS=Homo sapiens PE=2 SV=1	56910	0	846	0	41	0	0.05	#DIV/0!	0	1	0	1	0	2	0	2	1.455771
tr|Q6EHZ3|Q6EHZ3_HUMAN	STRF8	Putative uncharacterized protein STRF8 OS=Homo sapiens GN=STRF8 PE=2 SV=1	41361	0	840	0	41	0	0.08	#DIV/0!	0	1	0	1	0	2	0	2	1.455771
tr|B3KTC7|B3KTC7_HUMAN	MRE11A	Double-strand break repair protein MRE11A OS=Homo sapiens GN=MRE11A PE=2 SV=1	81362	0	832	0	42	0	0.08	#DIV/0!	0	0	0	2	0	2	0	4	1.455771
sp|P05556|ITB1_HUMAN	ITGB1	Integrin beta-1 OS=Homo sapiens GN=ITGB1 PE=1 SV=2	91664	0	828	0	42	0	0.07	#DIV/0!	0	1	0	2	0	2	0	3	1.455771
tr|B3KY04|B3KY04_HUMAN	tr|B3KY04	cDNA FLJ46506 fis, clone THYMU3030752, highly similar to BTB/POZ domain-containing protein KCTD12 OS=Hom	35994	0	826	0	42	0	0.19	#DIV/0!	0	1	0	2	0	2	0	2	1.455771
tr|B2R6H7|B2R6H7_HUMAN	tr|B2R6H7	cDNA, FLJ92955, highly similar to Homo sapiens transportin-SR (TRN-SR), mRNA OS=Homo sapiens PE=2 SV=1	105989	0	821	0	43	0	0.06	#DIV/0!	0	1	0	2	0	2	0	3	1.455771
tr|B4DPW5|B4DPW5_HUMAN	tr|B4DPW	cDNA FLJ59374, highly similar to Homo sapiens caldesmon 1 (CALD1), transcript variant 2, mRNA OS=Homo sapie	57436	0	818	0	43	0	0.06	#DIV/0!	0	1	0	1	0	2	0	2	1.455771
tr|B3KMV5|B3KMV5_HUMAN	tr|B3KMV	cDNA FLJ12728 fis, clone NT2RP2000040, highly similar to Protein FAM62A OS=Homo sapiens PE=2 SV=1	123342	0	813	0	43	0	0.08	#DIV/0!	0	1	0	3	0	2	0	6	1.455771
tr|F6IQC8|F6IQC8_HUMAN	HLA-C	MHC class I antigen (Fragment) OS=Homo sapiens GN=HLA-C PE=3 SV=1	39543	0	808	0	43	0	0.17	#DIV/0!	0	1	0	2	0	2	0	3	1.455771
sp|P27694|RFA1_HUMAN	RPA1	Replication protein A 70 kDa DNA-binding subunit OS=Homo sapiens GN=RPA1 PE=1 SV=2	68723	0	804	0	44	0	0.05	#DIV/0!	0	1	0	1	0	2	0	4	1.455771
tr|B2RDG1|B2RDG1_HUMAN	tr|B2RDG	cDNA, FLJ96593 OS=Homo sapiens PE=2 SV=1	59687	0	803	0	44	0	0.11	#DIV/0!	0	1	0	2	0	2	0	2	1.455771
sp|Q5SRE5|NU188_HUMAN	NUP188	Nucleoporin NUP188 homolog OS=Homo sapiens GN=NUP188 PE=1 SV=1	198369	0	802	0	44	0	0.03	#DIV/0!	0	1	0	2	0	2	0	4	1.455771
tr|H3BRG4|H3BRG4_HUMAN	UQCRC2	Cytochrome b-c1 complex subunit 2, mitochondrial OS=Homo sapiens GN=UQCRC2 PE=3 SV=1	44778	0	783	0	28	0	0.14	#DIV/0!	0	0	0	1	0	2	0	2	1.455771
sp|O15091|MRRP3_HUMAN	KIAA0391	Mitochondrial ribonuclease P protein 3 OS=Homo sapiens GN=KIAA0391 PE=1 SV=2	68014	0	777	0	45	0	0.1	#DIV/0!	0	1	0	2	0	2	0	2	1.455771
tr|Q149P0|Q149P0_HUMAN	GBF1	GBF1 protein OS=Homo sapiens GN=GBF1 PE=2 SV=1	207938	0	766	0	46	0	0.02	#DIV/0!	0	1	0	1	0	2	0	4	1.455771
tr|E7EVL6|E7EVL6_HUMAN	REPIN1	Replication initiator 1 (Fragment) OS=Homo sapiens GN=REPIN1 PE=2 SV=1	16586	0	758	0	47	0	0.1	#DIV/0!	0	1	0	2	0	2	0	2	1.455771
sp|Q96T76|MMS19_HUMAN	MMS19	MMS19 nucleotide excision repair protein homolog OS=Homo sapiens GN=MMS19 PE=1 SV=2	114928	0	753	0	47	0	0.09	#DIV/0!	0	2	0	3	0	2	0	3	1.455771
tr|B4E312|B4E312_HUMAN	tr|B4E312	cDNA FLJ53422, highly similar to TATA-binding protein-associated factor 2N OS=Homo sapiens PE=2 SV=1	40282	0	734	0	49	0	0.08	#DIV/0!	0	1	0	1	0	2	0	2	1.455771
tr|Q9MY63|Q9MY63_HUMAN	HLA-Cw	Leucocyte antigen (Fragment) OS=Homo sapiens GN=HLA-Cw PE=3 SV=1	26578	0	733	0	49	0	0.27	#DIV/0!	0	2	0	2	0	2	0	2	1.455771
tr|A6PVH9|A6PVH9_HUMAN	CPNE1	Copine I OS=Homo sapiens GN=CPNE1 PE=4 SV=1	53641	0	724	0	50	0	0.13	#DIV/0!	0	1	0	2	0	2	0	2	1.455771
sp|O96008|TOM40_HUMAN	TOMM40	Mitochondrial import receptor subunit TOM40 homolog OS=Homo sapiens GN=TOMM40 PE=1 SV=1	38211	0	721	0	51	0	0.4	#DIV/0!	0	1	0	2	0	2	0	2	1.455771
tr|B4DW54|B4DW54_HUMAN	tr|B4DW5	cDNA FLJ50888, highly similar to Aldehyde dehydrogenase, mitochondrial (EC 1.2.1.3) OS=Homo sapiens PE=2 SV	51467	0	694	0	34	0	0.12	#DIV/0!	0	1	0	1	0	2	0	2	1.455771
tr|B4DNM8|B4DNM8_HUMAN	tr|B4DNM	cDNA FLJ53395, highly similar to Prolyl 3-hydroxylase 1 (EC 1.14.11.7) OS=Homo sapiens PE=2 SV=1	69572	0	677	0	49	0	0.12	#DIV/0!	0	1	0	2	0	2	0	2	1.455771
sp|Q5JWF2|GNAS1_HUMAN	GNAS	Guanine nucleotide-binding protein G(s) subunit alpha isoforms XLas OS=Homo sapiens GN=GNAS PE=1 SV=2	111697	0	676	0	55	0	0.06	#DIV/0!	0	1	0	2	0	2	0	2	1.455771
tr|B4DN25|B4DN25_HUMAN	UGDH	UDP-glucose 6-dehydrogenase OS=Homo sapiens GN=UGDH PE=2 SV=1	44786	0	668	0	56	0	0.15	#DIV/0!	0	2	0	2	0	2	0	2	1.455771
tr|J3KNY6|J3KNY6_HUMAN	RGPD4	RanBP2-like and GRIP domain-containing protein 4 OS=Homo sapiens GN=RGPD4 PE=4 SV=1	199555	0	666	0	56	0	0.03	#DIV/0!	0	2	0	2	0	2	0	5	1.455771
sp|Q8IXI1|MIRO2_HUMAN	RHOT2	Mitochondrial Rho GTPase 2 OS=Homo sapiens GN=RHOT2 PE=1 SV=2	69101	0	664	0	56	0	0.05	#DIV/0!	0	1	0	1	0	2	0	2	1.455771
tr|Q6FGM0|Q6FGM0_HUMAN	SH3GL1	SH3 domain GRB2-like 1 OS=Homo sapiens GN=SH3GL1 PE=2 SV=1	41692	0	661	0	57	0	0.16	#DIV/0!	0	1	0	2	0	2	0	2	1.455771
tr|B4DHS5|B4DHS5_HUMAN	tr|B4DHS5	cDNA FLJ54678, highly similar to N(2),N(2)-dimethylguanosine tRNA methyltransferase (EC 2.1.1.32) OS=Homo sa	59256	0	653	0	58	0	0.11	#DIV/0!	0	2	0	2	0	2	0	2	1.455771
sp|P61073|CXCR4_HUMAN	CXCR4	C-X-C chemokine receptor type 4 OS=Homo sapiens GN=CXCR4 PE=1 SV=1	40233	0	649	0	59	0	0.17	#DIV/0!	0	2	0	2	0	2	0	2	1.455771
sp|Q5JVF3|PCID2_HUMAN	PCID2	PCI domain-containing protein 2 OS=Homo sapiens GN=PCID2 PE=1 SV=2	46627	0	642	0	59	0	0.15	#DIV/0!	0	1	0	2	0	2	0	2	1.455771
tr|E0WMV4|E0WMV4_HUMAN	HLA-C	MHC class I antigen (Fragment) OS=Homo sapiens GN=HLA-C PE=3 SV=1	31992	0	641	0	59	0	0.22	#DIV/0!	0	2	0	2	0	2	0	3	1.455771
tr|B1VK50|B1VK50_HUMAN	HLA-C	MHC class I antigen (Fragment) OS=Homo sapiens GN=HLA-C PE=3 SV=1	21287	0	639	0	59	0	0.34	#DIV/0!	0	2	0	2	0	2	0	5	1.455771
tr|G0KXF8|G0KXF8_HUMAN	HLA-B	MHC class I antigen (Fragment) OS=Homo sapiens GN=HLA-B PE=3 SV=1	21235	0	636	0	60	0	0.34	#DIV/0!	0	2	0	2	0	2	0	2	1.455771
tr|G1ENK8|G1ENK8_HUMAN	HLA-B	MHC class I antigen (Fragment) OS=Homo sapiens GN=HLA-B PE=3 SV=1	21236	0	635	0	60	0	0.34	#DIV/0!	0	2	0	2	0	2	0	2	1.455771
tr|A6NN60|A6NN60_HUMAN	CRYZ	Quinone oxidoreductase OS=Homo sapiens GN=CRYZ PE=2 SV=1	31680	0	634	0	60	0	0.11	#DIV/0!	0	1	0	1	0	2	0	3	1.455771
tr|Q29694|Q29694_HUMAN	HLA-B	MHC class I HLA-B antigen (Fragment) OS=Homo sapiens GN=HLA-B PE=2 SV=1	20434	0	633	0	60	0	0.36	#DIV/0!	0	2	0	2	0	2	0	4	1.455771
tr|B4DDH8|B4DDH8_HUMAN	tr|B4DDH	cDNA FLJ55184, highly similar to Homo sapiens leukocyte receptor cluster (LRC) member 4 (LENG4), mRNA OS=H	51214	0	631	0	60	0	0.13	#DIV/0!	0	1	0	2	0	2	0	2	1.455771
tr|E7EMC7|E7EMC7_HUMAN	SQSTM1	Sequestosome-1 OS=Homo sapiens GN=SQSTM1 PE=2 SV=1	42070	0	627	0	61	0	0.22	#DIV/0!	0	1	0	2	0	2	0	2	1.455771
tr|D3DWL9|D3DWL9_HUMAN	CPSF1	Cleavage and polyadenylation specific factor 1, 160kDa, isoform CRA_a OS=Homo sapiens GN=CPSF1 PE=2 SV=1	153087	0	625	0	61	0	0.02	#DIV/0!	0	1	0	1	0	2	0	3	1.455771
tr|H0YJS8|H0YJS8_HUMAN	PSMC6	26S protease regulatory subunit 10B (Fragment) OS=Homo sapiens GN=PSMC6 PE=4 SV=1	22376	0	623	0	61	0	0.15	#DIV/0!	0	1	0	1	0	2	0	2	1.455771
tr|F6SBX2|F6SBX2_HUMAN	IARS2	Isoleucine--tRNA ligase, mitochondrial OS=Homo sapiens GN=IARS2 PE=2 SV=1	106884	0	618	0	61	0	0.03	#DIV/0!	0	1	0	1	0	2	0	6	1.455771
tr|B2RDG4|B2RDG4_HUMAN	tr|B2RDG	cDNA, FLJ96597 OS=Homo sapiens PE=2 SV=1	57796	0	609	0	62	0	0.06	#DIV/0!	0	1	0	1	0	2	0	2	1.455771
sp|P52907|CAZA1_HUMAN	CAPZA1	F-actin-capping protein subunit alpha-1 OS=Homo sapiens GN=CAPZA1 PE=1 SV=3	33073	0	604	0	62	0	0.21	#DIV/0!	0	1	0	2	0	2	0	3	1.455771
tr|H0Y3P2|H0Y3P2_HUMAN	EIF4G2	Eukaryotic translation initiation factor 4 gamma 2 OS=Homo sapiens GN=EIF4G2 PE=4 SV=1	98569	0	597	0	63	0	0.07	#DIV/0!	0	1	0	2	0	2	0	2	1.455771
tr|K0P2X1|K0P2X1_HUMAN	HLA-C	MHC class I antigen OS=Homo sapiens GN=HLA-C PE=3 SV=1	41357	0	593	0	29	0	0.28	#DIV/0!	0	0	0	1	0	2	0	2	1.455771
sp|Q9BW92|SYTM_HUMAN	TARS2	Threonine--tRNA ligase, mitochondrial OS=Homo sapiens GN=TARS2 PE=1 SV=1	81841	0	587	0	64	0	0.08	#DIV/0!	0	1	0	2	0	2	0	2	1.455771
tr|B0QYK0|B0QYK0_HUMAN	EWSR1	Ewing sarcoma breakpoint region 1 OS=Homo sapiens GN=EWSR1 PE=4 SV=1	65174	0	585	0	64	0	0.1	#DIV/0!	0	1	0	2	0	2	0	2	1.455771
tr|B4E3G4|B4E3G4_HUMAN	tr|B4E3G4	cDNA FLJ50383, highly similar to CRSP complex subunit 3 OS=Homo sapiens PE=2 SV=1	116828	0	580	0	65	0	0.06	#DIV/0!	0	2	0	2	0	2	0	2	1.455771
sp|Q96CS3|FAF2_HUMAN	FAF2	FAS-associated factor 2 OS=Homo sapiens GN=FAF2 PE=1 SV=2	52933	0	579	0	65	0	0.13	#DIV/0!	0	1	0	2	0	2	0	3	1.455771
sp|P35251|RFC1_HUMAN	RFC1	Replication factor C subunit 1 OS=Homo sapiens GN=RFC1 PE=1 SV=4	128688	0	577	0	66	0	0.05	#DIV/0!	0	1	0	2	0	2	0	2	1.455771
sp|P61221|ABCE1_HUMAN	ABCE1	ATP-binding cassette sub-family E member 1 OS=Homo sapiens GN=ABCE1 PE=1 SV=1	68240	0	576	0	66	0	0.14	#DIV/0!	0	1	0	2	0	2	0	2	1.455771
tr|G5E9L5|G5E9L5_HUMAN	DDX17	DEAD (Asp-Glu-Ala-Asp) box polypeptide 17, isoform CRA_a OS=Homo sapiens GN=DDX17 PE=4 SV=1	19061	0	570	0	66	0	0.18	#DIV/0!	0	1	0	1	0	2	0	2	1.455771
sp|Q14739|LBR_HUMAN	LBR	Lamin-B receptor OS=Homo sapiens GN=LBR PE=1 SV=2	71057	0	566	0	67	0	0.05	#DIV/0!	0	1	0	1	0	2	0	2	1.455771
sp|Q27J81|INF2_HUMAN	INF2	Inverted formin-2 OS=Homo sapiens GN=INF2 PE=1 SV=2	136851	0	565	0	67	0	0.05	#DIV/0!	0	2	0	2	0	2	0	2	1.455771
tr|J3KQ32|J3KQ32_HUMAN	OLA1	Obg-like ATPase 1 OS=Homo sapiens GN=OLA1 PE=4 SV=1	47136	0	562	0	67	0	0.07	#DIV/0!	0	1	0	1	0	2	0	2	1.455771
sp|O76031|CLPX_HUMAN	CLPX	ATP-dependent Clp protease ATP-binding subunit clpX-like, mitochondrial OS=Homo sapiens GN=CLPX PE=1 SV=2	69922	0	559	0	68	0	0.12	#DIV/0!	0	1	0	2	0	2	0	2	1.455771
tr|E1P5S2|E1P5S2_HUMAN	RBM39	RNA-binding protein 39 OS=Homo sapiens GN=RBM39 PE=4 SV=1	40744	0	544	0	70	0	0.19	#DIV/0!	0	2	0	2	0	2	0	2	1.455771
tr|Q53G71|Q53G71_HUMAN	tr|Q53G7	Calreticulin variant (Fragment) OS=Homo sapiens PE=2 SV=1	47061	0	542	0	70	0	0.29	#DIV/0!	0	2	0	2	0	2	0	2	1.455771
tr|H0YL72|H0YL72_HUMAN	IDH3A	Isocitrate dehydrogenase [NAD] subunit alpha, mitochondrial OS=Homo sapiens GN=IDH3A PE=3 SV=1	36162	0	536	0	72	0	0.38	#DIV/0!	0	2	0	2	0	2	0	2	1.455771
tr|B4DSP0|B4DSP0_HUMAN	hCG_1642	HCG1642748, isoform CRA_b OS=Homo sapiens GN=hCG_1642748 PE=2 SV=1	45559	0	534	0	72	0	0.07	#DIV/0!	0	1	0	1	0	2	0	2	1.455771
tr|Q0VAB1|Q0VAB1_HUMAN	TIMM50	Translocase of inner mitochondrial membrane 50 homolog (S. cerevisiae) OS=Homo sapiens GN=TIMM50 PE=2 S	50960	0	530	0	73	0	0.13	#DIV/0!	0	2	0	2	0	2	0	2	1.455771
sp|Q9NRG9|AAAS_HUMAN	AAAS	Aladin OS=Homo sapiens GN=AAAS PE=1 SV=1	60392	0	525	0	73	0	0.12	#DIV/0!	0	2	0	2	0	2	0	2	1.455771
tr|Q6NUR1|Q6NUR1_HUMAN	NCAPG	Non-SMC condensin I complex, subunit G OS=Homo sapiens GN=NCAPG PE=2 SV=1	115205	0	513	0	76	0	0.06	#DIV/0!	0	1	0	2	0	2	0	2	1.455771
sp|P62333|PRS10_HUMAN	PSMC6	26S protease regulatory subunit 10B OS=Homo sapiens GN=PSMC6 PE=1 SV=1	44430	0	511	0	76	0	0.15	#DIV/0!	0	2	0	2	0	2	0	2	1.455771
sp|P36952|SPB5_HUMAN	SERPINB5	Serpin B5 OS=Homo sapiens GN=SERPINB5 PE=1 SV=2	42530	0	498	0	78	0	0.16	#DIV/0!	0	2	0	2	0	2	0	3	1.455771
tr|J3KMX1|J3KMX1_HUMAN	NUP88	Nuclear pore complex protein Nup88 (Fragment) OS=Homo sapiens GN=NUP88 PE=4 SV=1	80029	0	490	0	68	0	0.17	#DIV/0!	0	2	0	2	0	2	0	2	1.455771
sp|P30837|AL1B1_HUMAN	ALDH1B1	Aldehyde dehydrogenase X, mitochondrial OS=Homo sapiens GN=ALDH1B1 PE=1 SV=3	57626	0	489	0	80	0	0.06	#DIV/0!	0	1	0	1	0	2	0	2	1.455771
tr|B2R858|B2R858_HUMAN	tr|B2R858	cDNA, FLJ93750, Homo sapiens DEAD (Asp-Glu-Ala-Asp) box polypeptide 6 (DDX6), mRNA OS=Homo sapiens PE=	53582	0	487	0	80	0	0.13	#DIV/0!	0	2	0	2	0	2	0	3	1.455771
sp|P40222|TXLNA_HUMAN	TXLNA	Alpha-taxilin OS=Homo sapiens GN=TXLNA PE=1 SV=3	62195	0	440	0	92	0	0.11	#DIV/0!	0	1	0	2	0	2	0	3	1.455771
tr|B4DE85|B4DE85_HUMAN	PTDSS1	Phosphatidylserine synthase 1 OS=Homo sapiens GN=PTDSS1 PE=2 SV=1	35127	0	436	0	84	0	0.19	#DIV/0!	0	2	0	2	0	2	0	2	1.455771
tr|I6L9E8|I6L9E8_HUMAN	FAM98A	Family with sequence similarity 98, member A OS=Homo sapiens GN=FAM98A PE=2 SV=1	55595	0	414	0	100	0	0.12	#DIV/0!	0	2	0	2	0	2	0	2	1.455771
tr|Q2KQ72|Q2KQ72_HUMAN	tr|Q2KQ7	Chromosome-associated protein E OS=Homo sapiens PE=2 SV=1	86324	0	407	0	89	0	0.07	#DIV/0!	0	2	0	2	0	2	0	2	1.455771
tr|A7E2Y5|A7E2Y5_HUMAN	DNAJC13	DnaJ (Hsp40) homolog, subfamily C, member 13 OS=Homo sapiens GN=DNAJC13 PE=2 SV=1	256572	0	404	0	102	0	0.03	#DIV/0!	0	2	0	2	0	2	0	2	1.455771
tr|B3KPX1|B3KPX1_HUMAN	tr|B3KPX1	cDNA FLJ32394 fis, clone SKMUS2000078, highly similar to Very-long-chain specific acyl-CoA dehydrogenase, mit	38899	0	372	0	111	0	0.18	#DIV/0!	0	2	0	2	0	2	0	3	1.455771
tr|Q5VZA6|Q5VZA6_HUMAN	ACO1	Cytoplasmic aconitate hydratase OS=Homo sapiens GN=ACO1 PE=4 SV=1	48888	0	369	0	108	0	0.12	#DIV/0!	0	2	0	2	0	2	0	2	1.455771
tr|B4DRW3|B4DRW3_HUMAN	tr|B4DRW	cDNA FLJ57180, highly similar to TAR DNA-binding protein 43 OS=Homo sapiens PE=2 SV=1	35596	0	358	0	116	0	0.19	#DIV/0!	0	2	0	2	0	2	0	2	1.455771
tr|B4DFP1|B4DFP1_HUMAN	tr|B4DFP1	cDNA FLJ51818, highly similar to Phosphoglucomutase-1 (EC 5.4.2.2) OS=Homo sapiens PE=2 SV=1	58987	0	324	0	93	0	0.17	#DIV/0!	0	2	0	2	0	2	0	2	1.455771
tr|Q6ZVI6|Q6ZVI6_HUMAN	tr|Q6ZVI6	cDNA FLJ42537 fis, clone BRACE3004058, highly similar to NADH-cytochrome B5 reductase (EC 1.6.2.2) OS=Homo	29141	0	306	0	97	0	0.32	#DIV/0!	0	2	0	2	0	2	0	2	1.455771
tr|J3KPP4|J3KPP4_HUMAN	LUC7L3	Cisplatin resistance-associated overexpressed protein, isoform CRA_b OS=Homo sapiens GN=LUC7L3 PE=4 SV=1	58698	0	304	0	136	0	0.12	#DIV/0!	0	2	0	2	0	2	0	2	1.455771
tr|Q5H907|Q5H907_HUMAN	MAGED2	Melanoma antigen family D, 2, isoform CRA_d OS=Homo sapiens GN=MAGED2 PE=4 SV=1	55932	0	243	0	173	0	0.12	#DIV/0!	0	2	0	2	0	2	0	2	1.455771
tr|B3KVT9|B3KVT9_HUMAN	tr|B3KVT9	cDNA FLJ41424 fis, clone BRHIP2003748, highly similar to Tumor suppressor p53-binding protein 1 OS=Homo sap	43438	0	193	0	38	0	0.1	#DIV/0!	0	0	0	2	0	2	0	2	1.455771
tr|B2R806|B2R806_HUMAN	tr|B2R806	Eukaryotic translation initiation factor 3 subunit E OS=Homo sapiens PE=2 SV=1	52622	0	1224	0	17	0	0.06	#DIV/0!	0	0	0	1	0	1	0	2	0.718617
sp|O14647|CHD2_HUMAN	CHD2	Chromodomain-helicase-DNA-binding protein 2 OS=Homo sapiens GN=CHD2 PE=1 SV=2	212183	0	1214	0	19	0	0.03	#DIV/0!	0	0	0	1	0	1	0	2	0.718617
tr|Q59GM9|Q59GM9_HUMAN	tr|Q59GM	Phosphorylase (Fragment) OS=Homo sapiens PE=2 SV=1	99508	0	1212	0	19	0	0.04	#DIV/0!	0	0	0	1	0	1	0	2	0.718617
tr|B4DI19|B4DI19_HUMAN	tr|B4DI19	cDNA FLJ59524, highly similar to Cysteinyl-tRNA synthetase (EC 6.1.1.16) OS=Homo sapiens PE=2 SV=1	76209	0	1206	0	20	0	0.04	#DIV/0!	0	0	0	1	0	1	0	1	0.718617
tr|B3KNR6|B3KNR6_HUMAN	tr|B3KNR6	cDNA FLJ30255 fis, clone BRACE2002444, highly similar to LAS1-like protein OS=Homo sapiens PE=2 SV=1	27615	0	1205	0	20	0	0.04	#DIV/0!	0	0	0	1	0	1	0	1	0.718617
tr|H0Y524|H0Y524_HUMAN	CPNE1	Copine-1 (Fragment) OS=Homo sapiens GN=CPNE1 PE=4 SV=1	21729	0	1204	0	20	0	0.15	#DIV/0!	0	0	0	1	0	1	0	2	0.718617
sp|Q92888|ARHG1_HUMAN	ARHGEF1	Rho guanine nucleotide exchange factor 1 OS=Homo sapiens GN=ARHGEF1 PE=1 SV=2	103056	0	1201	0	21	0	0.03	#DIV/0!	0	0	0	1	0	1	0	1	0.718617
tr|Q5T6L4|Q5T6L4_HUMAN	ASS	Argininosuccinate synthetase, isoform CRA_a OS=Homo sapiens GN=ASS PE=2 SV=1	46786	0	1200	0	21	0	0.07	#DIV/0!	0	0	0	1	0	1	0	1	0.718617
tr|D3DUW5|D3DUW5_HUMAN	DNM1L	Dynamin 1-like, isoform CRA_c OS=Homo sapiens GN=DNM1L PE=3 SV=1	88442	0	1195	0	21	0	0.04	#DIV/0!	0	0	0	1	0	1	0	1	0.718617
sp|Q9Y2H6|FND3A_HUMAN	FNDC3A	Fibronectin type-III domain-containing protein 3A OS=Homo sapiens GN=FNDC3A PE=1 SV=4	133763	0	1194	0	22	0	0.04	#DIV/0!	0	0	0	1	0	1	0	1	0.718617
sp|Q9UK61|F208A_HUMAN	FAM208A	Protein FAM208A OS=Homo sapiens GN=FAM208A PE=1 SV=3	190453	0	1190	0	22	0	0.02	#DIV/0!	0	0	0	1	0	1	0	1	0.718617
tr|A4FVA8|A4FVA8_HUMAN	DFNA5	DFNA5 protein (Fragment) OS=Homo sapiens GN=DFNA5 PE=2 SV=1	48389	0	1188	0	22	0	0.07	#DIV/0!	0	0	0	1	0	1	0	1	0.718617
tr|B3KNM8|B3KNM8_HUMAN	tr|B3KNM	cDNA FLJ14980 fis, clone Y79AA1000181, highly similar to Homo sapiens CGI-01 protein mRNA OS=Homo sapiens	69355	0	1186	0	22	0	0.05	#DIV/0!	0	0	0	1	0	1	0	1	0.718617
tr|D3DVR8|D3DVR8_HUMAN	HD	Huntingtin (Huntington disease), isoform CRA_a OS=Homo sapiens GN=HD PE=2 SV=1	291559	0	1185	0	22	0	0.01	#DIV/0!	0	0	0	1	0	1	0	1	0.718617
tr|A8MV53|A8MV53_HUMAN	PPAN	HCG2033702, isoform CRA_a OS=Homo sapiens GN=PPAN PE=4 SV=1	47344	0	1184	0	22	0	0.07	#DIV/0!	0	0	0	1	0	1	0	1	0.718617
tr|G1UI38|G1UI38_HUMAN	EPT1	Ethanolaminephosphotransferase 1 (Fragment) OS=Homo sapiens GN=EPT1 PE=2 SV=1	23040	0	1183	0	22	0	0.15	#DIV/0!	0	0	0	1	0	1	0	1	0.718617
sp|Q9NRW7|VPS45_HUMAN	VPS45	Vacuolar protein sorting-associated protein 45 OS=Homo sapiens GN=VPS45 PE=1 SV=1	65435	0	1180	0	22	0	0.07	#DIV/0!	0	0	0	1	0	1	0	1	0.718617
tr|G5EA36|G5EA36_HUMAN	CDC27	Cell division cycle 27, isoform CRA_c OS=Homo sapiens GN=CDC27 PE=2 SV=1	92764	0	1178	0	22	0	0.3	#DIV/0!	0	0	0	1	0	1	0	1	0.718617
sp|Q96JM3|CHAP1_HUMAN	CHAMP1	Chromosome alignment-maintaining phosphoprotein 1 OS=Homo sapiens GN=CHAMP1 PE=1 SV=2	90012	0	1176	0	22	0	0.04	#DIV/0!	0	0	0	1	0	1	0	1	0.718617
sp|Q9BTC0|DIDO1_HUMAN	DIDO1	Death-inducer obliterator 1 OS=Homo sapiens GN=DIDO1 PE=1 SV=5	245434	0	1175	0	22	0	0.01	#DIV/0!	0	0	0	1	0	1	0	1	0.718617
tr|Q5T2B5|Q5T2B5_HUMAN	CUL2	Cullin-2 OS=Homo sapiens GN=CUL2 PE=3 SV=1	82933	0	1173	0	23	0	0.04	#DIV/0!	0	0	0	1	0	1	0	2	0.718617
tr|B4DFD7|B4DFD7_HUMAN	tr|B4DFD7	cDNA FLJ55457, highly similar to ATP-dependent RNA helicase DHX8 (EC 3.6.1.-) OS=Homo sapiens PE=2 SV=1	98244	0	1171	0	23	0	0.03	#DIV/0!	0	0	0	1	0	1	0	1	0.718617
tr|Q4G0F4|Q4G0F4_HUMAN	POLRMT	DNA-directed RNA polymerase OS=Homo sapiens GN=POLRMT PE=2 SV=1	140194	0	1169	0	23	0	0.03	#DIV/0!	0	0	0	1	0	1	0	3	0.718617
tr|A4D0W4|A4D0W4_HUMAN	AASS	Aminoadipate-semialdehyde synthase OS=Homo sapiens GN=AASS PE=2 SV=1	102808	0	1168	0	23	0	0.03	#DIV/0!	0	1	0	1	0	1	0	1	0.718617
tr|E9PF58|E9PF58_HUMAN	ARPC1A	Actin-related protein 2/3 complex subunit 1A OS=Homo sapiens GN=ARPC1A PE=2 SV=1	31268	0	1167	0	23	0	0.08	#DIV/0!	0	0	0	1	0	1	0	2	0.718617
tr|D3DTD2|D3DTD2_HUMAN	TRIO	Triple functional domain (PTPRF interacting), isoform CRA_a OS=Homo sapiens GN=TRIO PE=2 SV=1	270210	0	1166	0	23	0	0.01	#DIV/0!	0	0	0	1	0	1	0	1	0.718617
tr|A8K6H9|A8K6H9_HUMAN	tr|A8K6H9	cDNA FLJ75876, highly similar to Homo sapiens solute carrier family 38, member 2 (SLC38A2), mRNA OS=Homo s	56360	0	1165	0	23	0	0.06	#DIV/0!	0	0	0	1	0	1	0	1	0.718617
tr|A8K972|A8K972_HUMAN	tr|A8K972	cDNA FLJ76457, highly similar to Homo sapiens leucine carboxyl methyltransferase 2 (LCMT2), mRNA OS=Homo s	76406	0	1164	0	23	0	0.04	#DIV/0!	0	0	0	1	0	1	0	1	0.718617
tr|H0Y7V3|H0Y7V3_HUMAN	MMS19	MMS19 nucleotide excision repair protein homolog (Fragment) OS=Homo sapiens GN=MMS19 PE=4 SV=1	10029	0	1162	0	23	0	0.35	#DIV/0!	0	1	0	1	0	1	0	2	0.718617
tr|A8K4V2|A8K4V2_HUMAN	tr|A8K4V2	cDNA FLJ75930, highly similar to Homo sapiens NADH dehydrogenase (ubiquinone) 1 alpha subcomplex, 9, 39kDa	42596	0	1161	0	23	0	0.08	#DIV/0!	0	0	0	1	0	1	0	1	0.718617
sp|Q9NUL7|DDX28_HUMAN	DDX28	Probable ATP-dependent RNA helicase DDX28 OS=Homo sapiens GN=DDX28 PE=1 SV=2	59773	0	1156	0	24	0	0.17	#DIV/0!	0	0	0	3	0	1	0	3	0.718617
tr|G5E9P1|G5E9P1_HUMAN	ITPR1	Inositol 1,4,5-triphosphate receptor, type 1, isoform CRA_d OS=Homo sapiens GN=ITPR1 PE=2 SV=1	311832	0	1152	0	24	0	0.02	#DIV/0!	0	0	0	2	0	1	0	4	0.718617
sp|Q9H488|OFUT1_HUMAN	POFUT1	GDP-fucose protein O-fucosyltransferase 1 OS=Homo sapiens GN=POFUT1 PE=1 SV=1	44383	0	1151	0	24	0	0.07	#DIV/0!	0	0	0	1	0	1	0	1	0.718617
sp|O60287|NPA1P_HUMAN	URB1	Nucleolar pre-ribosomal-associated protein 1 OS=Homo sapiens GN=URB1 PE=1 SV=4	256508	0	1150	0	24	0	0.01	#DIV/0!	0	1	0	1	0	1	0	2	0.718617
tr|K7EIR0|K7EIR0_HUMAN	WDR18	WD repeat-containing protein 18 OS=Homo sapiens GN=WDR18 PE=4 SV=1	45436	0	1146	0	24	0	0.07	#DIV/0!	0	0	0	1	0	1	0	1	0.718617
sp|Q7Z4Q2|HEAT3_HUMAN	HEATR3	HEAT repeat-containing protein 3 OS=Homo sapiens GN=HEATR3 PE=1 SV=2	75789	0	1142	0	25	0	0.04	#DIV/0!	0	0	0	1	0	1	0	2	0.718617
tr|B2R6N0|B2R6N0_HUMAN	tr|B2R6N0	cDNA, FLJ93029, highly similar to Homo sapiens COP9 constitutive photomorphogenic homolog subunit 2 (Arabid	51868	0	1138	0	25	0	0.06	#DIV/0!	0	0	0	1	0	1	0	1	0.718617
tr|Q2TAM4|Q2TAM4_HUMAN	POLR1B	DNA-directed RNA polymerase OS=Homo sapiens GN=POLR1B PE=2 SV=1	108996	0	1136	0	25	0	0.03	#DIV/0!	0	0	0	1	0	1	0	2	0.718617
tr|E5KS55|E5KS55_HUMAN	tr|E5KS55	Mitochondrial succinyl-CoA ligase [ADP-forming] subunit beta OS=Homo sapiens PE=3 SV=1	50641	0	1130	0	25	0	0.07	#DIV/0!	0	0	0	1	0	1	0	1	0.718617
sp|Q9H4L5|OSBL3_HUMAN	OSBPL3	Oxysterol-binding protein-related protein 3 OS=Homo sapiens GN=OSBPL3 PE=1 SV=1	102130	0	1129	0	26	0	0.03	#DIV/0!	0	0	0	1	0	1	0	1	0.718617
tr|B1ANM7|B1ANM7_HUMAN	FAF1	FAS-associated factor 1 OS=Homo sapiens GN=FAF1 PE=4 SV=1	56305	0	1125	0	26	0	0.06	#DIV/0!	0	0	0	1	0	1	0	1	0.718617
sp|Q5K651|SAMD9_HUMAN	SAMD9	Sterile alpha motif domain-containing protein 9 OS=Homo sapiens GN=SAMD9 PE=1 SV=1	185704	0	1122	0	26	0	0.02	#DIV/0!	0	0	0	1	0	1	0	1	0.718617
tr|B4DH22|B4DH22_HUMAN	tr|B4DH22	cDNA FLJ59884, highly similar to Secernin-1 OS=Homo sapiens PE=2 SV=1	25067	0	1118	0	26	0	0.13	#DIV/0!	0	0	0	1	0	1	0	1	0.718617
sp|Q9P265|DIP2B_HUMAN	DIP2B	Disco-interacting protein 2 homolog B OS=Homo sapiens GN=DIP2B PE=1 SV=3	173606	0	1117	0	26	0	0.02	#DIV/0!	0	0	0	1	0	1	0	1	0.718617
tr|F5H315|F5H315_HUMAN	XAB2	Pre-mRNA-splicing factor SYF1 OS=Homo sapiens GN=XAB2 PE=2 SV=1	100416	0	1116	0	26	0	0.03	#DIV/0!	0	0	0	1	0	1	0	1	0.718617
tr|H0YEH2|H0YEH2_HUMAN	PUM1	Pumilio homolog 1 (Fragment) OS=Homo sapiens GN=PUM1 PE=4 SV=1	120836	0	1115	0	26	0	0.03	#DIV/0!	0	1	0	1	0	1	0	1	0.718617
tr|E9PC74|E9PC74_HUMAN	EIF2B5	Translation initiation factor eIF-2B subunit epsilon OS=Homo sapiens GN=EIF2B5 PE=2 SV=1	79096	0	1114	0	27	0	0.04	#DIV/0!	0	0	0	1	0	1	0	1	0.718617
tr|B3KVA0|B3KVA0_HUMAN	tr|B3KVA0	Cytosine-specific methyltransferase OS=Homo sapiens PE=2 SV=1	61027	0	1113	0	27	0	0.02	#DIV/0!	0	0	0	1	0	1	0	1	0.718617
tr|Q2TU84|Q2TU84_HUMAN	GIG18	Aspartate aminotransferase OS=Homo sapiens GN=GIG18 PE=2 SV=1	46519	0	1112	0	27	0	0.15	#DIV/0!	0	0	0	2	0	1	0	2	0.718617
tr|H0Y650|H0Y650_HUMAN	HEATR2	HEAT repeat-containing protein 2 (Fragment) OS=Homo sapiens GN=HEATR2 PE=4 SV=1	73507	0	1111	0	27	0	0.04	#DIV/0!	0	0	0	1	0	1	0	1	0.718617
tr|B7Z7E9|B7Z7E9_HUMAN	GOT1	Aspartate aminotransferase OS=Homo sapiens GN=GOT1 PE=2 SV=1	44404	0	1110	0	27	0	0.07	#DIV/0!	0	0	0	1	0	1	0	1	0.718617
tr|E5KND5|E5KND5_HUMAN	tr|E5KND5	Mitochondrial elongation factor G OS=Homo sapiens PE=2 SV=1	84103	0	1107	0	27	0	0.07	#DIV/0!	0	0	0	1	0	1	0	1	0.718617

tr|A1A5C4|A1A5C4_HUMAN	RRBP1	RRBP1 protein OS=Homo sapiens GN=RRBP1 PE=2 SV=1	103162	0	1106	0	27	0	0.09	#DIV/0!	0	0	0	1	0	1	0	1	0.718617
tr|A8K4H7|A8K4H7_HUMAN	PPAT	Amidophosphoribosyltransferase OS=Homo sapiens GN=PPAT PE=2 SV=1	58161	0	1104	0	27	0	0.06	#DIV/0!	0	0	0	1	0	1	0	2	0.718617
tr|Q5XJ04|Q5XJ04_HUMAN	HMGCS1	3-hydroxy-3-methylglutaryl-Coenzyme A synthase 1 (Soluble) OS=Homo sapiens GN=HMGCS1 PE=2 SV=1	57854	0	1102	0	27	0	0.06	#DIV/0!	0	0	0	1	0	1	0	1	0.718617
tr|E7EMP9|E7EMP9_HUMAN	DROSHA	Ribonuclease 3 OS=Homo sapiens GN=DROSHA PE=3 SV=1	156774	0	1100	0	27	0	0.02	#DIV/0!	0	0	0	1	0	1	0	1	0.718617
tr|B2R694|B2R694_HUMAN	tr|B2R694	cDNA, FLJ92849, highly similar to Homo sapiens lanosterol synthase (2,3-oxidosqualene-lanosterol cyclase) (LSS),	84568	0	1099	0	27	0	0.04	#DIV/0!	0	0	0	1	0	1	0	1	0.718617
tr|K7ERT7|K7ERT7_HUMAN	VAT1	Synaptic vesicle membrane protein VAT-1 homolog (Fragment) OS=Homo sapiens GN=VAT1 PE=4 SV=1	15164	0	1097	0	27	0	0.08	#DIV/0!	0	0	0	1	0	1	0	1	0.718617
tr|H0Y8D1|H0Y8D1_HUMAN	PRSS1	Trypsin-1 (Fragment) OS=Homo sapiens GN=PRSS1 PE=3 SV=1	15697	0	1096	0	27	0	0.22	#DIV/0!	0	0	0	1	0	1	0	1	0.718617
sp|Q03169|TNAP2_HUMAN	TNFAIP2	Tumor necrosis factor alpha-induced protein 2 OS=Homo sapiens GN=TNFAIP2 PE=1 SV=2	73015	0	1093	0	27	0	0.08	#DIV/0!	0	1	0	1	0	1	0	1	0.718617
tr|B4DZ55|B4DZ55_HUMAN	tr|B4DZ55	cDNA FLJ52097, weakly similar to Homo sapiens transmembrane and tetratricopeptide repeat containing 1 (TMT	104668	0	1091	0	28	0	0.03	#DIV/0!	0	1	0	1	0	1	0	1	0.718617
tr|F5H667|F5H667_HUMAN	ASPH	Aspartyl/asparaginyl beta-hydroxylase OS=Homo sapiens GN=ASPH PE=2 SV=1	83672	0	1090	0	28	0	0.08	#DIV/0!	0	0	0	2	0	1	0	4	0.718617
sp|Q9NR09|BIRC6_HUMAN	BIRC6	Baculoviral IAP repeat-containing protein 6 OS=Homo sapiens GN=BIRC6 PE=1 SV=2	536192	0	1086	0	28	0	0.01	#DIV/0!	0	0	0	1	0	1	0	1	0.718617
tr|B4DYP7|B4DYP7_HUMAN	tr|B4DYP7	cDNA FLJ55435, highly similar to Gamma-tubulin complex component 3 OS=Homo sapiens PE=2 SV=1	103306	0	1083	0	28	0	0.03	#DIV/0!	0	0	0	1	0	1	0	1	0.718617
tr|Q6AI22|Q6AI22_HUMAN	DKFZp686	Putative uncharacterized protein DKFZp686H16106 (Fragment) OS=Homo sapiens GN=DKFZp686H16106 PE=2 SV	321236	0	1082	0	28	0	0.01	#DIV/0!	0	0	0	1	0	1	0	2	0.718617
sp|Q68E01|INT3_HUMAN	INTS3	Integrator complex subunit 3 OS=Homo sapiens GN=INTS3 PE=1 SV=1	119533	0	1081	0	28	0	0.03	#DIV/0!	0	0	0	1	0	1	0	1	0.718617
tr|B4E2K4|B4E2K4_HUMAN	tr|B4E2K4	cDNA FLJ54576, highly similar to Aspartyl/asparaginyl beta-hydroxylase (EC 1.14.11.16) OS=Homo sapiens PE=2 S	83685	0	1080	0	28	0	0.08	#DIV/0!	0	0	0	2	0	1	0	4	0.718617
tr|B2R5U7|B2R5U7_HUMAN	tr|B2R5U7	cDNA, FLJ92633, highly similar to Homo sapiens CCAAT-box-binding transcription factor (CBF2), mRNA OS=Homo	114755	0	1073	0	29	0	0.03	#DIV/0!	0	0	0	1	0	1	0	1	0.718617
tr|G3V0G3|G3V0G3_HUMAN	DDX56	DEAD (Asp-Glu-Ala-Asp) box polypeptide 56, isoform CRA_a OS=Homo sapiens GN=DDX56 PE=2 SV=1	41969	0	1072	0	29	0	0.07	#DIV/0!	0	0	0	1	0	1	0	1	0.718617
tr|F8W805|F8W805_HUMAN	GRM1	Metabotropic glutamate receptor 1 OS=Homo sapiens GN=GRM1 PE=2 SV=1	103232	0	1071	0	29	0	0.03	#DIV/0!	0	1	0	1	0	1	0	1	0.718617
sp|P61011|SRP54_HUMAN	SRP54	Signal recognition particle 54 kDa protein OS=Homo sapiens GN=SRP54 PE=1 SV=1	55953	0	1068	0	29	0	0.07	#DIV/0!	0	0	0	1	0	1	0	1	0.718617
sp|Q86X55|CARM1_HUMAN	CARM1	Histone-arginine methyltransferase CARM1 OS=Homo sapiens GN=CARM1 PE=1 SV=3	66382	0	1065	0	30	0	0.05	#DIV/0!	0	0	0	1	0	1	0	2	0.718617
tr|B3KU09|B3KU09_HUMAN	tr|B3KU09	cDNA FLJ39034 fis, clone NT2RP7008085, highly similar to Homo sapiens ring finger protein 123 (RNF123), mRNA	149906	0	1064	0	30	0	0.02	#DIV/0!	0	1	0	1	0	1	0	1	0.718617
tr|F5GZS0|F5GZS0_HUMAN	DHX36	Probable ATP-dependent RNA helicase DHX36 OS=Homo sapiens GN=DHX36 PE=2 SV=1	113928	0	1062	0	30	0	0.03	#DIV/0!	0	0	0	1	0	1	0	2	0.718617
tr|Q2TAM5|Q2TAM5_HUMAN	RELA	RELA protein OS=Homo sapiens GN=RELA PE=2 SV=1	43355	0	1061	0	30	0	0.08	#DIV/0!	0	0	0	1	0	1	0	1	0.718617
tr|F8W8W7|F8W8W7_HUMAN	TGOLN2	Trans-Golgi network integral membrane protein 2 OS=Homo sapiens GN=TGOLN2 PE=2 SV=1	29554	0	1058	0	30	0	0.07	#DIV/0!	0	0	0	1	0	1	0	1	0.718617
sp|Q9Y4F1|FARP1_HUMAN	FARP1	FERM, RhoGEF and pleckstrin domain-containing protein 1 OS=Homo sapiens GN=FARP1 PE=1 SV=1	119300	0	1057	0	30	0	0.03	#DIV/0!	0	0	0	1	0	1	0	2	0.718617
sp|Q9UBB6|NCDN_HUMAN	NCDN	Neurochondrin OS=Homo sapiens GN=NCDN PE=1 SV=1	80239	0	1056	0	30	0	0.04	#DIV/0!	0	1	0	1	0	1	0	1	0.718617
tr|F5H6D0|F5H6D0_HUMAN	MOGS	Mannosyl-oligosaccharide glucosidase OS=Homo sapiens GN=MOGS PE=2 SV=1	80824	0	1055	0	30	0	0.04	#DIV/0!	0	0	0	1	0	1	0	2	0.718617
tr|H3BSD9|H3BSD9_HUMAN	MYO7B	Unconventional myosin-VIIb (Fragment) OS=Homo sapiens GN=MYO7B PE=4 SV=1	10022	0	1054	0	30	0	0.35	#DIV/0!	0	1	0	1	0	1	0	1	0.718617
tr|H0YDX7|H0YDX7_HUMAN	HBS1L	HBS1-like protein (Fragment) OS=Homo sapiens GN=HBS1L PE=4 SV=1	61030	0	1044	0	31	0	0.05	#DIV/0!	0	0	0	1	0	1	0	1	0.718617
tr|I3L445|I3L445_HUMAN	PELP1	Proline-, glutamic acid- and leucine-rich protein 1 (Fragment) OS=Homo sapiens GN=PELP1 PE=4 SV=1	32684	0	1037	0	31	0	0.1	#DIV/0!	0	0	0	1	0	1	0	3	0.718617
tr|H0Y7P1|H0Y7P1_HUMAN	MED12	Mediator of RNA polymerase II transcription subunit 12 (Fragment) OS=Homo sapiens GN=MED12 PE=4 SV=1	240034	0	1035	0	31	0	0.03	#DIV/0!	0	1	0	1	0	1	0	1	0.718617
tr|B4DRF4|B4DRF4_HUMAN	PTPLAD1	Very-long-chain (3R)-3-hydroxyacyl-[acyl-carrier protein] dehydratase 3 OS=Homo sapiens GN=PTPLAD1 PE=2 SV	36635	0	1034	0	31	0	0.09	#DIV/0!	0	0	0	1	0	1	0	1	0.718617
tr|B3KU23|B3KU23_HUMAN	tr|B3KU23	cDNA FLJ39097 fis, clone NTONG2000977, highly similar to Neurolysin, mitochondrial (EC 3.4.24.16) OS=Homo sa	70320	0	1031	0	32	0	0.07	#DIV/0!	0	0	0	1	0	1	0	1	0.718617
tr|B4DV73|B4DV73_HUMAN	tr|B4DV73	cDNA FLJ51330, highly similar to Far upstream element-binding protein 2 OS=Homo sapiens PE=2 SV=1	38334	0	1029	0	32	0	0.09	#DIV/0!	0	0	0	1	0	1	0	3	0.718617
sp|Q8N3C0|ASCC3_HUMAN	ASCC3	Activating signal cointegrator 1 complex subunit 3 OS=Homo sapiens GN=ASCC3 PE=1 SV=3	252898	0	1028	0	32	0	0.01	#DIV/0!	0	0	0	1	0	1	0	1	0.718617
tr|B4DEE5|B4DEE5_HUMAN	tr|B4DEE5	cDNA FLJ60230, highly similar to Arsenical pump-driving ATPase (EC 3.6.3.16) OS=Homo sapiens PE=2 SV=1	36162	0	1027	0	32	0	0.09	#DIV/0!	0	0	0	1	0	1	0	1	0.718617
tr|G3V1S7|G3V1S7_HUMAN	HAS1	Hyaluronan synthase 1 OS=Homo sapiens GN=HAS1 PE=2 SV=1	65517	0	1025	0	32	0	0.05	#DIV/0!	0	1	0	1	0	1	0	1	0.718617
tr|G3V161|G3V161_HUMAN	KBTBD3	Kelch repeat and BTB (POZ) domain containing 3, isoform CRA_a OS=Homo sapiens GN=KBTBD3 PE=2 SV=1	61834	0	1017	0	32	0	0.06	#DIV/0!	0	1	0	1	0	1	0	2	0.718617
tr|B4DUT7|B4DUT7_HUMAN	tr|B4DUT	cDNA FLJ57604, highly similar to GMP synthase (glutamine-hydrolyzing) (EC 6.3.5.2) OS=Homo sapiens PE=2 SV=	71880	0	1011	0	33	0	0.05	#DIV/0!	0	1	0	1	0	1	0	2	0.718617
tr|H0YJ30|H0YJ30_HUMAN	GPHN	Molybdopterin molybdenumtransferase (Fragment) OS=Homo sapiens GN=GPHN PE=4 SV=1	30436	0	1010	0	33	0	0.11	#DIV/0!	0	0	0	1	0	1	0	1	0.718617
tr|I7GPQ7|I7GPQ7_HUMAN	tr|I7GPQ7	cDNA FLJ75793 OS=Homo sapiens PE=2 SV=1	67286	0	1008	0	33	0	0.05	#DIV/0!	0	0	0	1	0	1	0	1	0.718617
tr|Q548W1|Q548W1_HUMAN	OGT	HRNT1 OS=Homo sapiens GN=OGT PE=2 SV=1	104029	0	1007	0	33	0	0.03	#DIV/0!	0	0	0	1	0	1	0	2	0.718617
tr|J3KRI4|J3KRI4_HUMAN	DYNC1LI2	Cytoplasmic dynein 1 light intermediate chain 2 (Fragment) OS=Homo sapiens GN=DYNC1LI2 PE=4 SV=1	17767	0	1006	0	33	0	0.19	#DIV/0!	0	0	0	1	0	1	0	1	0.718617
sp|O75131|CPNE3_HUMAN	CPNE3	Copine-3 OS=Homo sapiens GN=CPNE3 PE=1 SV=1	60947	0	996	0	34	0	0.05	#DIV/0!	0	0	0	1	0	1	0	3	0.718617
tr|H7C0M3|H7C0M3_HUMAN	BPHL	Valacyclovir hydrolase (Fragment) OS=Homo sapiens GN=BPHL PE=4 SV=1	3314	0	994	0	34	0	1.2	#DIV/0!	0	1	0	1	0	1	0	1	0.718617
tr|J3QRD1|J3QRD1_HUMAN	ALDH3A2	Fatty aldehyde dehydrogenase OS=Homo sapiens GN=ALDH3A2 PE=3 SV=1	45019	0	993	0	34	0	0.07	#DIV/0!	0	1	0	1	0	1	0	1	0.718617
tr|H0Y564|H0Y564_HUMAN	ANAPC1	Anaphase-promoting complex subunit 1 (Fragment) OS=Homo sapiens GN=ANAPC1 PE=4 SV=1	163055	0	992	0	34	0	0.02	#DIV/0!	0	0	0	1	0	1	0	1	0.718617
tr|B1AK81|B1AK81_HUMAN	PIGK	GPI-anchor transamidase OS=Homo sapiens GN=PIGK PE=4 SV=1	34462	0	990	0	34	0	0.1	#DIV/0!	0	0	0	1	0	1	0	1	0.718617
sp|Q63HN8|RN213_HUMAN	RNF213	E3 ubiquitin-protein ligase RNF213 OS=Homo sapiens GN=RNF213 PE=1 SV=3	598557	0	989	0	34	0	0.01	#DIV/0!	0	1	0	1	0	1	0	2	0.718617
tr|A8K594|A8K594_HUMAN	tr|A8K594	cDNA FLJ77256, highly similar to Homo sapiens SH3-domain binding protein 4 (SH3BP4), mRNA OS=Homo sapien	108400	0	988	0	34	0	0.03	#DIV/0!	0	0	0	1	0	1	0	2	0.718617
sp|P54920|SNAA_HUMAN	NAPA	Alpha-soluble NSF attachment protein OS=Homo sapiens GN=NAPA PE=1 SV=3	33667	0	987	0	34	0	0.1	#DIV/0!	0	0	0	1	0	1	0	1	0.718617
tr|A4QN18|A4QN18_HUMAN	SEC16A	SEC16A protein (Fragment) OS=Homo sapiens GN=SEC16A PE=2 SV=1	212198	0	982	0	34	0	0.01	#DIV/0!	0	1	0	1	0	1	0	2	0.718617
tr|B4DHY1|B4DHY1_HUMAN	HNRNPH3	Heterogeneous nuclear ribonucleoprotein H3 OS=Homo sapiens GN=HNRNPH3 PE=2 SV=1	24925	0	980	0	30	0	0.19	#DIV/0!	0	0	0	1	0	1	0	2	0.718617
tr|Q6ZWH1|Q6ZWH1_HUMAN	tr|Q6ZWH	cDNA FLJ41081 fis, clone ADRGL2009755, highly similar to Homo sapiens brain and reproductive organ-expressed	45824	0	979	0	34	0	0.07	#DIV/0!	0	1	0	1	0	1	0	1	0.718617
tr|B7Z3P1|B7Z3P1_HUMAN	TBCE	Tubulin-specific chaperone E OS=Homo sapiens GN=TBCE PE=2 SV=1	65666	0	978	0	34	0	0.05	#DIV/0!	0	0	0	1	0	1	0	2	0.718617
sp|Q6PKG0|LARP1_HUMAN	LARP1	La-related protein 1 OS=Homo sapiens GN=LARP1 PE=1 SV=2	123833	0	973	0	34	0	0.03	#DIV/0!	0	0	0	1	0	1	0	1	0.718617
tr|D3DUE6|D3DUE6_HUMAN	N-PAC	Cytokine-like nuclear factor n-pac, isoform CRA_c OS=Homo sapiens GN=N-PAC PE=2 SV=1	61517	0	972	0	34	0	0.05	#DIV/0!	0	1	0	1	0	1	0	1	0.718617
tr|B3KQ43|B3KQ43_HUMAN	tr|B3KQ43	cDNA FLJ32791 fis, clone TESTI2002365, highly similar to helicase MOV-10 (EC 3.6.1.-) OS=Homo sapiens PE=2 SV	47208	0	971	0	34	0	0.03	#DIV/0!	0	0	0	1	0	1	0	1	0.718617
tr|B3KMC9|B3KMC9_HUMAN	tr|B3KMC	cDNA FLJ10711 fis, clone NT2RP3000917, highly similar to 5~-3~ exoribonuclease 2 (EC 3.1.11.-) OS=Homo sapier	109456	0	970	0	34	0	0.03	#DIV/0!	0	1	0	1	0	1	0	1	0.718617
tr|B2R960|B2R960_HUMAN	tr|B2R960	cDNA, FLJ94230, highly similar to Homo sapiens thioredoxin-like 1 (TXNL1), mRNA OS=Homo sapiens PE=2 SV=1	32616	0	969	0	35	0	0.1	#DIV/0!	0	1	0	1	0	1	0	1	0.718617
sp|Q9BXW9|FACD2_HUMAN	FANCD2	Fanconi anemia group D2 protein OS=Homo sapiens GN=FANCD2 PE=1 SV=1	168295	0	968	0	35	0	0.02	#DIV/0!	0	0	0	1	0	1	0	1	0.718617
sp|Q52LJ0|FA98B_HUMAN	FAM98B	Protein FAM98B OS=Homo sapiens GN=FAM98B PE=1 SV=1	37566	0	966	0	35	0	0.09	#DIV/0!	0	1	0	1	0	1	0	1	0.718617
tr|B4E3W1|B4E3W1_HUMAN	tr|B4E3W	cDNA FLJ57204, highly similar to Homo sapiens ancient ubiquitous protein 1 (AUP1), transcript variant 2, mRNA (	51668	0	961	0	35	0	0.06	#DIV/0!	0	0	0	1	0	1	0	1	0.718617
tr|G1UI17|G1UI17_HUMAN	AGL	Glycogen debranching enzyme (Fragment) OS=Homo sapiens GN=AGL PE=2 SV=1	145569	0	957	0	35	0	0.02	#DIV/0!	0	0	0	1	0	1	0	1	0.718617
tr|B7Z475|B7Z475_HUMAN	tr|B7Z475	cDNA FLJ55712, highly similar to F-box-like/WD repeat protein TBL1XR1 OS=Homo sapiens PE=2 SV=1	41301	0	956	0	35	0	0.08	#DIV/0!	0	0	0	1	0	1	0	1	0.718617
tr|B4DFG0|B4DFG0_HUMAN	DEK	Protein DEK OS=Homo sapiens GN=DEK PE=2 SV=1	39704	0	955	0	35	0	0.08	#DIV/0!	0	0	0	1	0	1	0	1	0.718617
tr|B4DK35|B4DK35_HUMAN	tr|B4DK35	cDNA FLJ61591, highly similar to Probable global transcription activator SNF2L2 (EC 3.6.1.-) (Fragment) OS=Homo	112636	0	951	0	35	0	0.03	#DIV/0!	0	0	0	1	0	1	0	2	0.718617
tr|G3V1P5|G3V1P5_HUMAN	MED15	Mediator of RNA polymerase II transcription subunit 15 OS=Homo sapiens GN=MED15 PE=4 SV=1	80007	0	950	0	35	0	0.04	#DIV/0!	0	0	0	1	0	1	0	1	0.718617
tr|G3V5E1|G3V5E1_HUMAN	CCNK	Cyclin-K OS=Homo sapiens GN=CCNK PE=2 SV=1	43776	0	949	0	35	0	0.08	#DIV/0!	0	0	0	1	0	1	0	1	0.718617
tr|H0Y3K4|H0Y3K4_HUMAN	RBM33	RNA-binding protein 33 (Fragment) OS=Homo sapiens GN=RBM33 PE=4 SV=1	100636	0	948	0	35	0	0.03	#DIV/0!	0	1	0	1	0	1	0	1	0.718617
tr|B2R6G5|B2R6G5_HUMAN	tr|B2R6G5	cDNA, FLJ92942, highly similar to Homo sapiens T-box, brain, 1 (TBR1), mRNA OS=Homo sapiens PE=2 SV=1	74530	0	939	0	36	0	0.04	#DIV/0!	0	1	0	1	0	1	0	1	0.718617
sp|Q53EP0|FND3B_HUMAN	FNDC3B	Fibronectin type III domain-containing protein 3B OS=Homo sapiens GN=FNDC3B PE=1 SV=2	134970	0	938	0	36	0	0.02	#DIV/0!	0	1	0	1	0	1	0	1	0.718617
sp|Q9BW60|ELOV1_HUMAN	ELOVL1	Elongation of very long chain fatty acids protein 1 OS=Homo sapiens GN=ELOVL1 PE=1 SV=1	32755	0	937	0	36	0	0.1	#DIV/0!	0	1	0	1	0	1	0	1	0.718617
tr|H7C5W5|H7C5W5_HUMAN	PRPH	Peripherin (Fragment) OS=Homo sapiens GN=PRPH PE=3 SV=1	22980	0	936	0	36	0	0.15	#DIV/0!	0	1	0	1	0	1	0	3	0.718617
tr|H3BQC4|H3BQC4_HUMAN	TGFB1I1	Transforming growth factor beta-1-induced transcript 1 protein (Fragment) OS=Homo sapiens GN=TGFB1I1 PE=4	23016	0	935	0	36	0	0.15	#DIV/0!	0	0	0	1	0	1	0	1	0.718617
tr|B4DET0|B4DET0_HUMAN	tr|B4DET0	cDNA FLJ55900, highly similar to Pre-mRNA-splicing factor ATP-dependent RNA helicase PRP16 (EC 3.6.1.-) OS=H	140449	0	933	0	36	0	0.02	#DIV/0!	0	1	0	1	0	1	0	2	0.718617
tr|A8K2M0|A8K2M0_HUMAN	PSMC4	Proteasome (Prosome, macropain) 26S subunit, ATPase, 4, isoform CRA_b OS=Homo sapiens GN=PSMC4 PE=2 S\	47451	0	931	0	36	0	0.07	#DIV/0!	0	1	0	1	0	1	0	2	0.718617
sp|Q92624|APBP2_HUMAN	APPBP2	Amyloid protein-binding protein 2 OS=Homo sapiens GN=APPBP2 PE=1 SV=2	67666	0	930	0	36	0	0.05	#DIV/0!	0	1	0	1	0	1	0	1	0.718617
tr|G3V438|G3V438_HUMAN	AHSA1	Activator of 90 kDa heat shock protein ATPase homolog 1 (Fragment) OS=Homo sapiens GN=AHSA1 PE=2 SV=1	23078	0	926	0	37	0	0.34	#DIV/0!	0	0	0	1	0	1	0	1	0.718617
tr|A8K6V7|A8K6V7_HUMAN	tr|A8K6V7	cDNA FLJ76053, highly similar to Homo sapiens Ras-GTPase activating protein SH3 domain-binding protein 2 (G3	50842	0	924	0	37	0	0.06	#DIV/0!	0	1	0	1	0	1	0	1	0.718617
tr|B3KNF6|B3KNF6_HUMAN	SEC61A1	Sec61 alpha 1 subunit (S. cerevisiae), isoform CRA_c OS=Homo sapiens GN=SEC61A1 PE=2 SV=1	52687	0	923	0	37	0	0.06	#DIV/0!	0	1	0	1	0	1	0	1	0.718617
sp|Q14558|KPRA_HUMAN	PRPSAP1	Phosphoribosyl pyrophosphate synthase-associated protein 1 OS=Homo sapiens GN=PRPSAP1 PE=1 SV=2	39654	0	919	0	37	0	0.17	#DIV/0!	0	1	0	2	0	1	0	2	0.718617
sp|P49750|YLPM1_HUMAN	YLPM1	YLP motif-containing protein 1 OS=Homo sapiens GN=YLPM1 PE=1 SV=3	220077	0	914	0	37	0	0.02	#DIV/0!	0	1	0	1	0	1	0	1	0.718617
tr|B3KPF2|B3KPF2_HUMAN	tr|B3KPF2	cDNA FLJ31717 fis, clone NT2RI2006640, highly similar to Dynamin-3 (EC 3.6.5.5) OS=Homo sapiens PE=2 SV=1	63057	0	913	0	37	0	0.05	#DIV/0!	0	1	0	1	0	1	0	1	0.718617
sp|P80723|BASP1_HUMAN	BASP1	Brain acid soluble protein 1 OS=Homo sapiens GN=BASP1 PE=1 SV=2	22680	0	912	0	37	0	0.15	#DIV/0!	0	1	0	1	0	1	0	3	0.718617
sp|Q14008|CKAP5_HUMAN	CKAP5	Cytoskeleton-associated protein 5 OS=Homo sapiens GN=CKAP5 PE=1 SV=3	227062	0	908	0	38	0	0.01	#DIV/0!	0	0	0	1	0	1	0	2	0.718617
tr|B0V2J7|B0V2J7_HUMAN	TAP2	Antigen peptide transporter 2 OS=Homo sapiens GN=TAP2 PE=4 SV=1	72571	0	906	0	38	0	0.05	#DIV/0!	0	1	0	1	0	1	0	2	0.718617
sp|P09497|CLCB_HUMAN	CLTB	Clathrin light chain B OS=Homo sapiens GN=CLTB PE=1 SV=1	25289	0	903	0	38	0	0.13	#DIV/0!	0	1	0	1	0	1	0	2	0.718617
sp|Q15269|PWP2_HUMAN	PWP2	Periodic tryptophan protein 2 homolog OS=Homo sapiens GN=PWP2 PE=1 SV=2	103357	0	901	0	38	0	0.06	#DIV/0!	0	1	0	2	0	1	0	2	0.718617
tr|K7EIJ8|K7EIJ8_HUMAN	KATNAL2	Katanin p60 ATPase-containing subunit A-like 2 (Fragment) OS=Homo sapiens GN=KATNAL2 PE=4 SV=1	38325	0	900	0	38	0	0.09	#DIV/0!	0	1	0	1	0	1	0	1	0.718617
tr|B2R7V4|B2R7V4_HUMAN	tr|B2R7V4	cDNA, FLJ93619, highly similar to Homo sapiens PRP4 pre-mRNA processing factor 4 homolog (yeast) (PRPF4), m	59097	0	898	0	38	0	0.06	#DIV/0!	0	1	0	1	0	1	0	1	0.718617
tr|B4E206|B4E206_HUMAN	tr|B4E206	cDNA FLJ55441, highly similar to Nuclear pore complex protein Nup133 OS=Homo sapiens PE=2 SV=1	128349	0	896	0	38	0	0.03	#DIV/0!	0	1	0	1	0	1	0	1	0.718617
tr|B3KXW5|B3KXW5_HUMAN	AP1G1	AP-1 complex subunit gamma-1 OS=Homo sapiens GN=AP1G1 PE=2 SV=1	94992	0	895	0	38	0	0.03	#DIV/0!	0	1	0	1	0	1	0	1	0.718617
tr|B4DKE6|B4DKE6_HUMAN	tr|B4DKE6	cDNA FLJ60629, highly similar to Replication factor C subunit 3 OS=Homo sapiens PE=2 SV=1	35551	0	891	0	38	0	0.09	#DIV/0!	0	1	0	1	0	1	0	1	0.718617
sp|Q7Z3U7|MON2_HUMAN	MON2	Protein MON2 homolog OS=Homo sapiens GN=MON2 PE=1 SV=2	192475	0	890	0	38	0	0.02	#DIV/0!	0	1	0	1	0	1	0	2	0.718617
tr|F2YGG7|F2YGG7_HUMAN	tr|F2YGG7	Epidermal growth factor receptor OS=Homo sapiens PE=2 SV=1	132588	0	877	0	39	0	0.02	#DIV/0!	0	1	0	1	0	1	0	1	0.718617
tr|B4DYZ1|B4DYZ1_HUMAN	tr|B4DYZ1	cDNA FLJ59786, highly similar to Probable ATP-dependent RNA helicase DDX31 (EC 3.6.1.-) OS=Homo sapiens PE-	81565	0	876	0	39	0	0.04	#DIV/0!	0	1	0	1	0	1	0	3	0.718617
tr|B7Z2B4|B7Z2B4_HUMAN	RABGAP1	Rab GTPase-activating protein 1 OS=Homo sapiens GN=RABGAP1 PE=2 SV=1	48660	0	875	0	39	0	0.03	#DIV/0!	0	1	0	1	0	1	0	1	0.718617
tr|Q5T8U2|Q5T8U2_HUMAN	RPL7A	60S ribosomal protein L7a OS=Homo sapiens GN=RPL7A PE=4 SV=1	16700	0	873	0	39	0	0.15	#DIV/0!	0	1	0	1	0	1	0	1	0.718617
tr|B2RAH7|B2RAH7_HUMAN	tr|B2RAH7	cDNA, FLJ94921, highly similar to Homo sapiens prolyl endopeptidase (PREP), mRNA OS=Homo sapiens PE=2 SV=	81594	0	869	0	39	0	0.04	#DIV/0!	0	1	0	1	0	1	0	1	0.718617
tr|H7C455|H7C455_HUMAN	tr|H7C455	Uncharacterized protein (Fragment) OS=Homo sapiens PE=4 SV=1	22389	0	866	0	39	0	0.15	#DIV/0!	0	1	0	1	0	1	0	1	0.718617
tr|H0YL11|H0YL11_HUMAN	IDH2	Isocitrate dehydrogenase [NADP], mitochondrial OS=Homo sapiens GN=IDH2 PE=3 SV=1	35536	0	863	0	40	0	0.09	#DIV/0!	0	1	0	1	0	1	0	1	0.718617
tr|Q59EZ3|Q59EZ3_HUMAN	tr|Q59EZ3	Insulin-like growth factor 2 receptor variant (Fragment) OS=Homo sapiens PE=2 SV=1	272672	0	860	0	40	0	0.01	#DIV/0!	0	1	0	1	0	1	0	1	0.718617
sp|Q9H9V9|JMJD4_HUMAN	JMJD4	JmjC domain-containing protein 4 OS=Homo sapiens GN=JMJD4 PE=2 SV=2	52973	0	851	0	40	0	0.06	#DIV/0!	0	1	0	1	0	1	0	1	0.718617
tr|B3KTN5|B3KTN5_HUMAN	tr|B3KTN5	cDNA FLJ38538 fis, clone HCHON2001407, highly similar to LanC-like protein 2 OS=Homo sapiens PE=2 SV=1	51676	0	849	0	40	0	0.06	#DIV/0!	0	1	0	1	0	1	0	2	0.718617
tr|K7EPI4|K7EPI4_HUMAN	GFAP	Glial fibrillary acidic protein (Fragment) OS=Homo sapiens GN=GFAP PE=4 SV=1	7120	0	848	0	41	0	0.5	#DIV/0!	0	1	0	1	0	1	0	1	0.718617
tr|E9PDG5|E9PDG5_HUMAN	LARP4	La-related protein 4 OS=Homo sapiens GN=LARP4 PE=2 SV=1	50319	0	847	0	41	0	0.04	#DIV/0!	0	1	0	1	0	1	0	1	0.718617
sp|Q9UBU9|NXF1_HUMAN	NXF1	Nuclear RNA export factor 1 OS=Homo sapiens GN=NXF1 PE=1 SV=1	70652	0	845	0	41	0	0.05	#DIV/0!	0	1	0	1	0	1	0	1	0.718617
tr|B4E074|B4E074_HUMAN	tr|B4E074	cDNA FLJ58655, highly similar to Notchless homolog 1 OS=Homo sapiens PE=2 SV=1	51211	0	844	0	41	0	0.06	#DIV/0!	0	1	0	1	0	1	0	2	0.718617
tr|H0Y4J2|H0Y4J2_HUMAN	MRPL37	39S ribosomal protein L37, mitochondrial (Fragment) OS=Homo sapiens GN=MRPL37 PE=4 SV=1	29806	0	839	0	41	0	0.11	#DIV/0!	0	1	0	1	0	1	0	1	0.718617
sp|Q15061|WDR43_HUMAN	WDR43	WD repeat-containing protein 43 OS=Homo sapiens GN=WDR43 PE=1 SV=3	75813	0	838	0	41	0	0.04	#DIV/0!	0	1	0	1	0	1	0	2	0.718617
sp|Q8WX92|NELFB_HUMAN	COBRA1	Negative elongation factor B OS=Homo sapiens GN=COBRA1 PE=1 SV=1	66283	0	837	0	41	0	0.05	#DIV/0!	0	1	0	1	0	1	0	1	0.718617
tr|B2RAM6|B2RAM6_HUMAN	tr|B2RAM	cDNA, FLJ95005, highly similar to Homo sapiens kinesin family member 11 (KIF11), mRNA OS=Homo sapiens PE=2	120084	0	834	0	41	0	0.03	#DIV/0!	0	1	0	1	0	1	0	2	0.718617
tr|A4FU51|A4FU51_HUMAN	NARG1L	NMDA receptor regulated 1-like OS=Homo sapiens GN=NARG1L PE=2 SV=1	102424	0	831	0	42	0	0.03	#DIV/0!	0	1	0	1	0	1	0	2	0.718617
sp|O43837|IDH3B_HUMAN	IDH3B	Isocitrate dehydrogenase [NAD] subunit beta, mitochondrial OS=Homo sapiens GN=IDH3B PE=1 SV=2	42442	0	827	0	42	0	0.2	#DIV/0!	0	1	0	1	0	1	0	1	0.718617
tr|C9JNQ3|C9JNQ3_HUMAN	PRPSAP1	Phosphoribosyl pyrophosphate synthase-associated protein 1 (Fragment) OS=Homo sapiens GN=PRPSAP1 PE=2 S	11609	0	823	0	42	0	0.3	#DIV/0!	0	1	0	1	0	1	0	1	0.718617
sp|P78357|CNTP1_HUMAN	CNTNAP1	Contactin-associated protein 1 OS=Homo sapiens GN=CNTNAP1 PE=1 SV=1	158220	0	820	0	43	0	0.02	#DIV/0!	0	1	0	1	0	1	0	1	0.718617
sp|Q13425|SNTB2_HUMAN	SNTB2	Beta-2-syntrophin OS=Homo sapiens GN=SNTB2 PE=1 SV=1	58369	0	817	0	43	0	0.06	#DIV/0!	0	1	0	1	0	1	0	2	0.718617
tr|F5H740|F5H740_HUMAN	VDAC3	Voltage-dependent anion-selective channel protein 3 OS=Homo sapiens GN=VDAC3 PE=2 SV=1	31080	0	816	0	43	0	0.11	#DIV/0!	0	1	0	1	0	1	0	1	0.718617
tr|H0YAY2|H0YAY2_HUMAN	KIAA0196	WASH complex subunit strumpellin (Fragment) OS=Homo sapiens GN=KIAA0196 PE=4 SV=1	82716	0	815	0	43	0	0.04	#DIV/0!	0	1	0	1	0	1	0	1	0.718617
tr|B3KM47|B3KM47_HUMAN	tr|B3KM4	cDNA FLJ10273 fis, clone HEMBB1001137, highly similar to SEC23-interacting protein OS=Homo sapiens PE=2 SV=	111730	0	812	0	43	0	0.08	#DIV/0!	0	1	0	1	0	1	0	2	0.718617
tr|G3V1K3|G3V1K3_HUMAN	PON2	Paraoxonase 2, isoform CRA_a OS=Homo sapiens GN=PON2 PE=2 SV=1	41672	0	806	0	44	0	0.08	#DIV/0!	0	1	0	1	0	1	0	1	0.718617
tr|B3KNP0|B3KNP0_HUMAN	tr|B3KNP0	cDNA FLJ30074 fis, clone BGGI11000123, highly similar to Homo sapiens formin binding protein 4 (FNBP4), mRNA	100252	0	800	0	44	0	0.03	#DIV/0!	0	1	0	1	0	1	0	1	0.718617
tr|B3KM73|B3KM73_HUMAN	tr|B3KM7	cDNA FLJ10419 fis, clone NT2RP1000163, highly similar to Homo sapiens transforming growth factor beta regulat	55418	0	796	0	44	0	0.06	#DIV/0!	0	1	0	1	0	1	0	2	0.718617
tr|B3KNC3|B3KNC3_HUMAN	tr|B3KNC	cDNA FLJ14222 fis, clone NT2RP3003992, highly similar to Nucleolar complex protein 2 homolog OS=Homo sapie	85719	0	791	0	44	0	0.04	#DIV/0!	0	1	0	1	0	1	0	1	0.718617
tr|B4DZ87|B4DZ87_HUMAN	tr|B4DZ87	cDNA FLJ57240, highly similar to Mitochondrial proteins import receptor OS=Homo sapiens PE=2 SV=1	57579	0	787	0	45	0	0.06	#DIV/0!	0	1	0	1	0	1	0	2	0.718617
tr|B2R6E5|B2R6E5_HUMAN	tr|B2R6E5	NADPH--cytochrome P450 reductase OS=Homo sapiens PE=2 SV=1	77429	0	786	0	45	0	0.04	#DIV/0!	0	1	0	1	0	1	0	1	0.718617
tr|Q2TSD2|Q2TSD2_HUMAN	tr|Q2TSD2	Aging-associated gene 7 protein OS=Homo sapiens PE=2 SV=1	41778	0	785	0	45	0	0.08	#DIV/0!	0	1	0	1	0	1	0	1	0.718617
sp|Q8TDD1|DDX54_HUMAN	DDX54	ATP-dependent RNA helicase DDX54 OS=Homo sapiens GN=DDX54 PE=1 SV=2	98819	0	781	0	45	0	0.03	#DIV/0!	0	1	0	1	0	1	0	2	0.718617
sp|P13667|PDIA4_HUMAN	PDIA4	Protein disulfide-isomerase A4 OS=Homo sapiens GN=PDIA4 PE=1 SV=2	73229	0	775	0	46	0	0.04	#DIV/0!	0	1	0	1	0	1	0	1	0.718617
tr|A2A2Q9|A2A2Q9_HUMAN	C20orf4	Chromosome 20 open reading frame 4 OS=Homo sapiens GN=C20orf4 PE=4 SV=1	45575	0	774	0	46	0	0.07	#DIV/0!	0	1	0	1	0	1	0	2	0.718617
tr|Q5DT20|Q5DT20_HUMAN	HRNR	Hornerin OS=Homo sapiens GN=HRNR PE=2 SV=1	283111	0	773	0	46	0	0.01	#DIV/0!	0	1	0	1	0	1	0	1	0.718617
tr|B0V0F7|B0V0F7_HUMAN	DHX16	Putative pre-mRNA-splicing factor ATP-dependent RNA helicase DHX16 (Fragment) OS=Homo sapiens GN=DHX16	21454	0	772	0	46	0	0.16	#DIV/0!	0	1	0	1	0	1	0	1	0.718617
tr|Q6IBU0|Q6IBU0_HUMAN	EIF5	EIF5 protein OS=Homo sapiens GN=EIF5 PE=2 SV=1	49577	0	769	0	46	0	0.15	#DIV/0!	0	1	0	1	0	1	0	1	0.718617
tr|E9PQD7|E9PQD7_HUMAN	RPS2	40S ribosomal protein S2 OS=Homo sapiens GN=RPS2 PE=2 SV=1	25366	0	767	0	46	0	0.5	#DIV/0!	0	1	0	1	0	1	0	1	0.718617
tr|B3KNG5|B3KNG5_HUMAN	tr|B3KNG	cDNA FLJ14553 fis, clone NT2RM2001785, highly similar to Homo sapiens fatty acid desaturase 2 (FADS2), mRNA	19263	0	765	0	34	0	0.14	#DIV/0!	0	1	0	1	0	1	0	2	0.718617
sp|Q9Y295|DRG1_HUMAN	DRG1	Developmentally-regulated GTP-binding protein 1 OS=Homo sapiens GN=DRG1 PE=1 SV=1	40802	0	764	0	46	0	0.08	#DIV/0!	0	1	0	1	0	1	0	1	0.718617
tr|Q0VGA5|Q0VGA5_HUMAN	SARS	SARS protein OS=Homo sapiens GN=SARS PE=2 SV=1	58883	0	755	0	47	0	0.06	#DIV/0!	0	1	0	1	0	1	0	3	0.718617
tr|Q6IPI1|Q6IPI1_HUMAN	RPL29	Ribosomal protein L29 OS=Homo sapiens GN=RPL29 PE=2 SV=1	17997	0	749	0	48	0	0.19	#DIV/0!	0	1	0	1	0	1	0	1	0.718617
sp|Q5D862|FILA2_HUMAN	FLG2	Filaggrin-2 OS=Homo sapiens GN=FLG2 PE=1 SV=1	249296	0	748	0	48	0	0.01	#DIV/0!	0	1	0	1	0	1	0	1	0.718617
tr|G3V1U5|G3V1U5_HUMAN	GOLT1B	Golgi transport 1 homolog B (S. cerevisiae), isoform CRA_c OS=Homo sapiens GN=GOLT1B PE=2 SV=1	8208	0	747	0	48	0	0.43	#DIV/0!	0	1	0	1	0	1	0	1	0.718617
tr|F5GYT3|F5GYT3_HUMAN	LPCAT3	Lysophospholipid acyltransferase 5 OS=Homo sapiens GN=LPCAT3 PE=2 SV=1	22378	0	737	0	49	0	0.06	#DIV/0!	0	1	0	1	0	1	0	1	0.718617
tr|B3KVJ5|B3KVJ5_HUMAN	tr|B3KVJ5	cDNA FLJ16648 fis, clone TESTI4035508, highly similar to Structural maintenance of chromosomes 4-like 1 protei	101354	0	736	0	49	0	0.03	#DIV/0!	0	1	0	1	0	1	0	4	0.718617
sp|Q00653|NFKB2_HUMAN	NFKB2	Nuclear factor NF-kappa-B p100 subunit OS=Homo sapiens GN=NFKB2 PE=1 SV=4	97373	0	731	0	50	0	0.03	#DIV/0!	0	1	0	1	0	1	0	1	0.718617
tr|B3KVR1|B3KVR1_HUMAN	SNRPN	Small nuclear ribonucleoprotein-associated protein OS=Homo sapiens GN=SNRPN PE=2 SV=1	25230	0	730	0	50	0	0.13	#DIV/0!	0	1	0	1	0	1	0	1	0.718617
tr|A2A274|A2A274_HUMAN	ACO2	Aconitase 2, mitochondrial OS=Homo sapiens GN=ACO2 PE=4 SV=1	88563	0	728	0	50	0	0.04	#DIV/0!	0	1	0	1	0	1	0	1	0.718617
tr|B0AZS5|B0AZS5_HUMAN	tr|B0AZS5	cDNA, FLJ79515, highly similar to Kinesin-like protein KIF2 OS=Homo sapiens PE=2 SV=1	75666	0	725	0	50	0	0.04	#DIV/0!	0	1	0	1	0	1	0	1	0.718617
tr|H7C4G8|H7C4G8_HUMAN	NT5DC2	5~-nucleotidase domain-containing protein 2 (Fragment) OS=Homo sapiens GN=NT5DC2 PE=4 SV=1	30207	0	722	0	0	0	0.05	#DIV/0!	0	0	0	0	0	1	0	1	0.718617
tr|O95654|O95654_HUMAN	tr|O95654	P150Glued homolog (Fragment) OS=Homo sapiens PE=2 SV=1	46656	0	720	0	51	0	0.07	#DIV/0!	0	1	0	1	0	1	0	2	0.718617
tr|J3KR44|J3KR44_HUMAN	OTUB1	Ubiquitin thioesterase OTUB1 OS=Homo sapiens GN=OTUB1 PE=4 SV=1	35649	0	711	0	51	0	0.09	#DIV/0!	0	1	0	1	0	1	0	1	0.718617
sp|A9QM74|IMA8_HUMAN	KPNA7	Importin subunit alpha-8 OS=Homo sapiens GN=KPNA7 PE=1 SV=1	57529	0	707	0	52	0	0.06	#DIV/0!	0	1	0	1	0	1	0	1	0.718617
tr|Q59H95|Q59H95_HUMAN	tr|Q59H95	Flightless I homolog variant (Fragment) OS=Homo sapiens PE=2 SV=1	126396	0	706	0	52	0	0.02	#DIV/0!	0	1	0	1	0	1	0	1	0.718617
tr|B2RD79|B2RD79_HUMAN	tr|B2RD79	Ubiquitin carboxyl-terminal hydrolase OS=Homo sapiens PE=2 SV=1	56462	0	704	0	52	0	0.06	#DIV/0!	0	1	0	1	0	1	0	1	0.718617
sp|Q96T51|RUFY1_HUMAN	RUFY1	RUN and FYVE domain-containing protein 1 OS=Homo sapiens GN=RUFY1 PE=1 SV=2	80851	0	703	0	52	0	0.04	#DIV/0!	0	1	0	1	0	1	0	2	0.718617
sp|Q12800|TFCP2_HUMAN	TFCP2	Alpha-globin transcription factor CP2 OS=Homo sapiens GN=TFCP2 PE=1 SV=2	57676	0	699	0	52	0	0.07	#DIV/0!	0	1	0	1	0	1	0	1	0.718617
tr|B4DTM6|B4DTM6_HUMAN	tr|B4DTM	cDNA FLJ61391, highly similar to Protein transport protein Sec24B OS=Homo sapiens PE=2 SV=1	130037	0	698	0	52	0	0.03	#DIV/0!	0	1	0	1	0	1	0	1	0.718617
tr|B4E205|B4E205_HUMAN	tr|B4E205	cDNA FLJ61651, highly similar to Protein transport protein Sec24A OS=Homo sapiens PE=2 SV=1	96953	0	697	0	52	0	0.03	#DIV/0!	0	1	0	1	0	1	0	2	0.718617
sp|Q14669|TRIPC_HUMAN	TRIP12	E3 ubiquitin-protein ligase TRIP12 OS=Homo sapiens GN=TRIP12 PE=1 SV=1	222234	0	693	0	52	0	0.01	#DIV/0!	0	1	0	1	0	1	0	1	0.718617
tr|H0Y4C2|H0Y4C2_HUMAN	HLA-F	HLA class I histocompatibility antigen, alpha chain F (Fragment) OS=Homo sapiens GN=HLA-F PE=3 SV=1	25349	0	686	0	54	0	0.13	#DIV/0!	0	1	0	1	0	1	0	4	0.718617
tr|B4E1L0|B4E1L0_HUMAN	tr|B4E1L0	Adenylosuccinate synthetase OS=Homo sapiens PE=2 SV=1	48308	0	682	0	54	0	0.07	#DIV/0!	0	1	0	1	0	1	0	1	0.718617
tr|B5BUD2|B5BUD2_HUMAN	RFC2	Replication factor C 2 isoform 1 (Fragment) OS=Homo sapiens GN=RFC2 PE=2 SV=1	39616	0	680	0	55	0	0.08	#DIV/0!	0	1	0	1	0	1	0	1	0.718617
sp|Q86XP3|DDX42_HUMAN	DDX42	ATP-dependent RNA helicase DDX42 OS=Homo sapiens GN=DDX42 PE=1 SV=1	103197	0	674	0	56	0	0.08	#DIV/0!	0	1	0	1	0	1	0	3	0.718617
sp|Q7Z5L9|I2BP2_HUMAN	IRF2BP2	Interferon regulatory factor 2-binding protein 2 OS=Homo sapiens GN=IRF2BP2 PE=1 SV=2	61728	0	670	0	56	0	0.05	#DIV/0!	0	1	0	1	0	1	0	1	0.718617
sp|P04792|HSPB1_HUMAN	HSPB1	Heat shock protein beta-1 OS=Homo sapiens GN=HSPB1 PE=1 SV=2	22826	0	665	0	56	0	0.16	#DIV/0!	0	1	0	1	0	1	0	2	0.718617
sp|O00231|PSD11_HUMAN	PSMD11	26S proteasome non-ATPase regulatory subunit 11 OS=Homo sapiens GN=PSMD11 PE=1 SV=3	47719	0	659	0	57	0	0.07	#DIV/0!	0	1	0	1	0	1	0	1	0.718617
tr|B4DWV9|B4DWV9_HUMAN	tr|B4DWV	cDNA FLJ53108, highly similar to Guanine nucleotide-binding protein alpha-13 subunit OS=Homo sapiens PE=2 S\	41291	0	656	0	57	0	0.08	#DIV/0!	0	1	0	1	0	1	0	1	0.718617
tr|J3QKS9|J3QKS9_HUMAN	NOL11	Nucleolar protein 11 OS=Homo sapiens GN=NOL11 PE=4 SV=1	18816	0	655	0	58	0	0.04	#DIV/0!	0	1	0	1	0	1	0	1	0.718617
sp|P37837|TALDO_HUMAN	TALDO1	Transaldolase OS=Homo sapiens GN=TALDO1 PE=1 SV=2	37688	0	654	0	58	0	0.09	#DIV/0!	0	1	0	1	0	1	0	2	0.718617
tr|D3DSQ7|D3DSQ7_HUMAN	XPO7	Exportin 7, isoform CRA_c OS=Homo sapiens GN=XPO7 PE=2 SV=1	100370	0	647	0	55	0	0.05	#DIV/0!	0	1	0	1	0	1	0	1	0.718617
tr|E9PHM2|E9PHM2_HUMAN	LARS2	Probable leucine--tRNA ligase, mitochondrial OS=Homo sapiens GN=LARS2 PE=2 SV=1	97782	0	646	0	59	0	0.03	#DIV/0!	0	1	0	1	0	1	0	2	0.718617
tr|Q5W1M0|Q5W1M0_HUMAN	HLA-C	MHC class I antigen (Fragment) OS=Homo sapiens GN=HLA-C PE=2 SV=1	10534	0	638	0	0	0	0.17	#DIV/0!	0	0	0	0	0	1	0	1	0.718617
tr|B4DR63|B4DR63_HUMAN	PSMC1	26S protease regulatory subunit 4 OS=Homo sapiens GN=PSMC1 PE=2 SV=1	41255	0	628	0	60	0	0.08	#DIV/0!	0	1	0	1	0	1	0	1	0.718617
tr|B4DM41|B4DM41_HUMAN	RFC4	Replication factor C (Activator 1) 4, 37kDa, isoform CRA_b OS=Homo sapiens GN=RFC4 PE=2 SV=1	33996	0	615	0	62	0	0.1	#DIV/0!	0	1	0	1	0	1	0	1	0.718617
sp|Q9GZL7|WDR12_HUMAN	WDR12	Ribosome biogenesis protein WDR12 OS=Homo sapiens GN=WDR12 PE=1 SV=2	48191	0	611	0	62	0	0.07	#DIV/0!	0	1	0	1	0	1	0	2	0.718617
tr|Q5LJA0|Q5LJA0_HUMAN	TROVE2	60 kDa SS-A/Ro ribonucleoprotein OS=Homo sapiens GN=TROVE2 PE=4 SV=1	59916	0	606	0	62	0	0.05	#DIV/0!	0	1	0	1	0	1	0	1	0.718617
tr|B3KN28|B3KN28_HUMAN	tr|B3KN28	cDNA FLJ13370 fis, clone PLACE1000653, highly similar to Phosphoacetylglucosamine mutase (EC 5.4.2.3) OS=Ho	60284	0	602	0	63	0	0.05	#DIV/0!	0	1	0	1	0	1	0	1	0.718617
tr|B1ALA7|B1ALA7_HUMAN	PRPS1	Phosphoribosyl pyrophosphate synthetase 1 OS=Homo sapiens GN=PRPS1 PE=4 SV=1	18730	0	598	0	63	0	0.18	#DIV/0!	0	1	0	1	0	1	0	1	0.718617
tr|B3KY55|B3KY55_HUMAN	tr|B3KY55	cDNA FLJ46894 fis, clone UTERU3019708, highly similar to Regulator of nonsense transcripts 1 OS=Homo sapiens	41002	0	595	0	31	0	0.08	#DIV/0!	0	1	0	1	0	1	0	1	0.718617
tr|B2R8G6|B2R8G6_HUMAN	tr|B2R8G6	cDNA, FLJ93891, Homo sapiens FK506 binding protein 8, 38kDa (FKBP8), mRNA OS=Homo sapiens PE=2 SV=1	38870	0	592	0	63	0	0.09	#DIV/0!	0	1	0	1	0	1	0	1	0.718617
tr|H7C0C0|H7C0C0_HUMAN	GTF3C3	General transcription factor 3C polypeptide 3 (Fragment) OS=Homo sapiens GN=GTF3C3 PE=4 SV=1	16723	0	584	0	41	0	0.1	#DIV/0!	0	1	0	1	0	1	0	1	0.718617
tr|B2RCV1|B2RCV1_HUMAN	tr|B2RCV1	cDNA, FLJ96310, highly similar to Homo sapiens solute carrier family 25 (mitochondrial carrier; adenine nucleotic	33062	0	578	0	65	0	0.1	#DIV/0!	0	1	0	1	0	1	0	2	0.718617
tr|H0YEL7|H0YEL7_HUMAN	ACAT1	Acetyl-CoA acetyltransferase, mitochondrial (Fragment) OS=Homo sapiens GN=ACAT1 PE=3 SV=1	14377	0	573	0	66	0	0.07	#DIV/0!	0	1	0	1	0	1	0	1	0.718617

sp|Q92626|PXDN_HUMAN	PXDN	Peroxidasin homolog OS=Homo sapiens GN=PXDN PE=1 SV=2	167793	0	569	0	66	0	0.04	#DIV/0!	0	1	0	1	0	1	0	1	0.718617
tr|C9J634|C9J634_HUMAN	PDHB	Pyruvate dehydrogenase E1 component subunit beta, mitochondrial OS=Homo sapiens GN=PDHB PE=2 SV=1	39064	0	550	0	63	0	0.17	#DIV/0!	0	1	0	1	0	1	0	1	0.718617
tr|H0Y8Z9|H0Y8Z9_HUMAN	ACAD9	Acyl-CoA dehydrogenase family member 9, mitochondrial (Fragment) OS=Homo sapiens GN=ACAD9 PE=4 SV=1	43503	0	549	0	70	0	0.05	#DIV/0!	0	1	0	1	0	1	0	1	0.718617
tr|B0I1S9|B0I1S9_HUMAN	MYO1B	MYO1B variant protein OS=Homo sapiens GN=MYO1B PE=2 SV=1	132928	0	547	0	70	0	0.02	#DIV/0!	0	1	0	1	0	1	0	2	0.718617
tr|B4DJE7|B4DJE7_HUMAN	ACADM	Medium-chain-specific acyl-CoA dehydrogenase, mitochondrial OS=Homo sapiens GN=ACADM PE=2 SV=1	25704	0	541	0	70	0	0.13	#DIV/0!	0	1	0	1	0	1	0	1	0.718617
tr|B7ZLC9|B7ZLC9_HUMAN	GEMIN5	GEMIN5 protein OS=Homo sapiens GN=GEMIN5 PE=2 SV=1	170664	0	540	0	71	0	0.04	#DIV/0!	0	1	0	1	0	1	0	1	0.718617
tr|B3KUI0|B3KUI0_HUMAN	tr|B3KUI0	cDNA FLJ39945 fis, clone SPLEN2023977, highly similar to Homo sapiens CD97 antigen (CD97), transcript variant	79803	0	539	0	71	0	0.04	#DIV/0!	0	1	0	1	0	1	0	1	0.718617
tr|H0YNQ8|H0YNQ8_HUMAN	MYO1E	Unconventional myosin-Ie (Fragment) OS=Homo sapiens GN=MYO1E PE=4 SV=1	37550	0	538	0	71	0	0.09	#DIV/0!	0	1	0	1	0	1	0	2	0.718617
tr|B2RBM7|B2RBM7_HUMAN	tr|B2RBM	cDNA, FLJ95595, highly similar to Homo sapiens proteasome (prosome, macropain) 26S subunit, non-ATPase, 13	43131	0	535	0	72	0	0.13	#DIV/0!	0	1	0	1	0	1	0	1	0.718617
tr|H7BZF2|H7BZF2_HUMAN	DKC1	H/ACA ribonucleoprotein complex subunit 4 (Fragment) OS=Homo sapiens GN=DKC1 PE=4 SV=1	8803	0	519	0	31	0	0.18	#DIV/0!	0	1	0	1	0	1	0	1	0.718617
tr|Q6FHF5|Q6FHF5_HUMAN	PCNA	Proliferating cell nuclear antigen (Fragment) OS=Homo sapiens GN=PCNA PE=2 SV=1	29029	0	509	0	76	0	0.11	#DIV/0!	0	1	0	1	0	1	0	1	0.718617
sp|Q9BWF3|RBM4_HUMAN	RBM4	RNA-binding protein 4 OS=Homo sapiens GN=RBM4 PE=1 SV=1	40688	0	497	0	79	0	0.08	#DIV/0!	0	1	0	1	0	1	0	2	0.718617
tr|H0Y7C0|H0Y7C0_HUMAN	USO1	General vesicular transport factor p115 (Fragment) OS=Homo sapiens GN=USO1 PE=4 SV=1	72345	0	493	0	80	0	0.05	#DIV/0!	0	1	0	1	0	1	0	1	0.718617
tr|H0Y2P0|H0Y2P0_HUMAN	CD44	CD44 antigen (Fragment) OS=Homo sapiens GN=CD44 PE=4 SV=1	31332	0	485	0	81	0	0.11	#DIV/0!	0	1	0	1	0	1	0	1	0.718617
sp|Q9H009|NACA2_HUMAN	NACA2	Nascent polypeptide-associated complex subunit alpha-2 OS=Homo sapiens GN=NACA2 PE=1 SV=1	23209	0	468	0	84	0	0.14	#DIV/0!	0	1	0	1	0	1	0	1	0.718617
tr|H0Y4Z6|H0Y4Z6_HUMAN	PYGB	Phosphorylase (Fragment) OS=Homo sapiens GN=PYGB PE=3 SV=1	27767	0	462	0	0	0	0.19	#DIV/0!	0	0	0	0	0	1	0	1	0.718617
sp|P33897|ABCD1_HUMAN	ABCD1	ATP-binding cassette sub-family D member 1 OS=Homo sapiens GN=ABCD1 PE=1 SV=2	83398	0	460	0	87	0	0.04	#DIV/0!	0	1	0	1	0	1	0	1	0.718617
tr|B3KPF6|B3KPF6_HUMAN	tr|B3KPF6	cDNA FLJ31732 fis, clone NT2RI2006856, highly similar to Striatin-4 OS=Homo sapiens PE=2 SV=1	39506	0	438	0	28	0	0.2	#DIV/0!	0	0	0	1	0	1	0	1	0.718617
tr|B3KMB7|B3KMB7_HUMAN	PYCR2	Pyrroline-5-carboxylate reductase family, member 2, isoform CRA_a OS=Homo sapiens GN=PYCR2 PE=2 SV=1	22559	0	413	0	61	0	0.2	#DIV/0!	0	1	0	1	0	1	0	1	0.718617
tr|B4DK32|B4DK32_HUMAN	tr|B4DK32	cDNA FLJ61095, highly similar to Ubiquitin conjugation factor E4 A (Fragment) OS=Homo sapiens PE=2 SV=1	120816	0	1232	0	15	0	0.03	#DIV/0!	0	0	0	1	0	0	0	1	-0.86653
tr|H0Y360|H0Y360_HUMAN	AMPD2	AMP deaminase 2 (Fragment) OS=Homo sapiens GN=AMPD2 PE=4 SV=1	99192	0	1231	0	15	0	0.03	#DIV/0!	0	0	0	1	0	0	0	1	-0.86653
sp|Q32MZ4|LRRF1_HUMAN	LRRFIP1	Leucine-rich repeat flightless-interacting protein 1 OS=Homo sapiens GN=LRRFIP1 PE=1 SV=2	89826	0	1230	0	16	0	0.04	#DIV/0!	0	0	0	1	0	0	0	2	-0.86653
sp|O76003|GLRX3_HUMAN	GLRX3	Glutaredoxin-3 OS=Homo sapiens GN=GLRX3 PE=1 SV=2	37693	0	1228	0	16	0	0.09	#DIV/0!	0	0	0	1	0	0	0	1	-0.86653
tr|A4UCU0|A4UCU0_HUMAN	tr|A4UCU	DEAD box polypeptide 47 isoform 1 variant (Fragment) OS=Homo sapiens PE=2 SV=1	19904	0	1226	0	16	0	0.17	#DIV/0!	0	0	0	1	0	0	0	1	-0.86653
tr|E5RHS5|E5RHS5_HUMAN	EIF3E	Eukaryotic translation initiation factor 3 subunit E (Fragment) OS=Homo sapiens GN=EIF3E PE=2 SV=1	26961	0	1223	0	17	0	0.41	#DIV/0!	0	0	0	1	0	0	0	1	-0.86653
tr|B4DKW2|B4DKW2_HUMAN	tr|B4DKW	cDNA FLJ56001, highly similar to Putative ATP-dependent RNA helicase DHX57 (EC 3.6.1.-) (Fragment) OS=Homo	105507	0	1221	0	18	0	0.03	#DIV/0!	0	0	0	1	0	0	0	1	-0.86653
sp|P0C0S8|H2A1_HUMAN	HIST1H2A	Histone H2A type 1 OS=Homo sapiens GN=HIST1H2AG PE=1 SV=2	14083	0	1218	0	19	0	0.24	#DIV/0!	0	0	0	1	0	0	0	1	-0.86653
tr|H0YL71|H0YL71_HUMAN	SMAD3	Mothers against decapentaplegic homolog 3 (Fragment) OS=Homo sapiens GN=SMAD3 PE=4 SV=1	10544	0	1217	0	19	0	0.33	#DIV/0!	0	0	0	1	0	0	0	1	-0.86653
tr|D3DTC2|D3DTC2_HUMAN	AYTL2	Acyltransferase like 2, isoform CRA_a OS=Homo sapiens GN=AYTL2 PE=2 SV=1	54725	0	1216	0	19	0	0.06	#DIV/0!	0	0	0	1	0	0	0	1	-0.86653
tr|B4DIS0|B4DIS0_HUMAN	tr|B4DIS0	cDNA FLJ57840, highly similar to Chromodomain-helicase-DNA-binding protein 7 (EC 3.6.1.-) OS=Homo sapiens P	139212	0	1215	0	0	0	0.01	#DIV/0!	0	0	0	0	0	0	0	2	-0.86653
tr|J3KQS3|J3KQS3_HUMAN	PNPLA6	Neuropathy target esterase OS=Homo sapiens GN=PNPLA6 PE=4 SV=1	152341	0	1209	0	20	0	0.02	#DIV/0!	0	0	0	1	0	0	0	2	-0.86653
tr|B4DDL6|B4DDL6_HUMAN	tr|B4DDL6	cDNA FLJ54325, highly similar to Myotubularin-related protein 2 (EC 3.1.3.-) OS=Homo sapiens PE=2 SV=1	36644	0	1208	0	20	0	0.09	#DIV/0!	0	0	0	1	0	0	0	1	-0.86653
tr|Q3LIF4|Q3LIF4_HUMAN	Nbla0027	NOGO-A (Fragment) OS=Homo sapiens GN=Nbla00271 PE=2 SV=1	57162	0	1191	0	0	0	0.09	#DIV/0!	0	0	0	0	0	0	0	1	-0.86653
tr|Q53G42|Q53G42_HUMAN	tr|Q53G4	mRNA decapping enzyme variant (Fragment) OS=Homo sapiens PE=2 SV=1	38757	0	1127	0	0	0	0.09	#DIV/0!	0	0	0	0	0	0	0	1	-0.86653
sp|O00507|USP9Y_HUMAN	USP9Y	Probable ubiquitin carboxyl-terminal hydrolase FAF-Y OS=Homo sapiens GN=USP9Y PE=1 SV=2	294370	0	1049	0	19	0	0.04	#DIV/0!	0	0	0	1	0	0	0	1	-0.86653
tr|B4DN27|B4DN27_HUMAN	tr|B4DN2	cDNA FLJ57057, highly similar to Small subunit processome component 20 homolog OS=Homo sapiens PE=2 SV=	62656	0	1001	0	0	0	0.01	#DIV/0!	0	0	0	0	0	0	0	1	-0.86653
tr|G1UI18|G1UI18_HUMAN	PSME4	Proteasome activator complex subunit 4 (Fragment) OS=Homo sapiens GN=PSME4 PE=2 SV=1	130054	0	874	0	21	0	0.05	#DIV/0!	0	1	0	2	0	0	0	2	-0.86653
sp|O14980|XPO1_HUMAN	XPO1	Exportin-1 OS=Homo sapiens GN=XPO1 PE=1 SV=1	124447	305	130	59	300	0.03	0.44	14.666667	1	7	1	14	1	16	3	17	2.595571
tr|Q2TU64|Q2TU64_HUMAN	tr|Q2TU6	PIG48 OS=Homo sapiens PE=2 SV=1	61110	279	135	68	213	0.05	0.73	14.6	1	7	1	9	3	11	3	14	0.850716
tr|F5H793|F5H793_HUMAN	LDHB	L-lactate dehydrogenase B chain (Fragment) OS=Homo sapiens GN=LDHB PE=2 SV=1	10857	39	80	391	443	1.81	26.33	14.546961	8	12	10	15	10	15	12	16	-0.30527
sp|Q68EM7|RHG17_HUMAN	ARHGAP1	Rho GTPase-activating protein 17 OS=Homo sapiens GN=ARHGAP17 PE=1 SV=1	95776	328	1059	54	30	0.03	0.4	13.333333	1	0	1	1	2	1	2	1	-1.604
tr|B4DHQ3|B4DHQ3_HUMAN	tr|B4DHQ	cDNA FLJ56437, highly similar to Phosphoserine aminotransferase (EC 2.6.1.52) OS=Homo sapiens PE=2 SV=1	45783	552	203	30	201	0.07	0.87	12.428571	0	7	1	9	1	9	2	9	1.797783
tr|B2RBA6|B2RBA6_HUMAN	tr|B2RBA6	cDNA, FLJ95407, highly similar to Homo sapiens MCM7 minichromosome maintenance deficient 7 (S. cerevisiae)	81856	374	208	46	188	0.04	0.48	12	1	6	1	9	1	9	1	11	1.797783
tr|J3KR24|J3KR24_HUMAN	IARS	Isoleucine--tRNA ligase, cytoplasmic OS=Homo sapiens GN=IARS PE=4 SV=1	132820	366	139	47	267	0.02	0.24	12	1	5	1	9	2	14	2	15	1.671471
tr|Q59GV0|Q59GV0_HUMAN	tr|Q59GV	Propionyl Coenzyme A carboxylase, beta polypeptide variant (Fragment) OS=Homo sapiens PE=2 SV=1	60578	331	143	53	281	0.05	0.59	11.8	1	7	1	9	1	11	2	12	2.073795
sp|Q9UQ35|SRRM2_HUMAN	SRRM2	Serine/arginine repetitive matrix protein 2 OS=Homo sapiens GN=SRRM2 PE=1 SV=2	300179	247	107	78	339	0.02	0.22	11	1	7	2	12	2	14	5	15	1.671471
tr|D3DP78|D3DP78_HUMAN	DARS	Aspartyl-tRNA synthetase, isoform CRA_b OS=Homo sapiens GN=DARS PE=2 SV=1	53829	344	168	51	212	0.06	0.65	10.833333	1	5	1	8	2	9	3	9	1.060474
tr|B4DLT2|B4DLT2_HUMAN	tr|B4DLT2	cDNA FLJ56637, highly similar to Nuclear pore complex protein Nup155 OS=Homo sapiens PE=2 SV=1	152734	489	149	35	254	0.02	0.21	10.5	0	8	1	8	1	8	1	10	1.63713
tr|E9PBS1|E9PBS1_HUMAN	PAICS	Phosphoribosylaminoimidazole carboxylase (Fragment) OS=Homo sapiens GN=PAICS PE=2 SV=1	46307	270	179	69	204	0.07	0.7	10	1	5	1	7	2	7	5	8	0.71906
sp|P54886|P5CS_HUMAN	ALDH18A1	Delta-1-pyrroline-5-carboxylate synthase OS=Homo sapiens GN=ALDH18A1 PE=1 SV=2	87989	658	335	21	123	0.04	0.39	9.75	1	3	1	9	1	8	1	13	1.63713
tr|Q4ZG57|Q4ZG57_HUMAN	MCM6	Putative uncharacterized protein MCM6 (Fragment) OS=Homo sapiens GN=MCM6 PE=2 SV=1	89803	444	247	39	170	0.04	0.38	9.5	1	7	1	9	2	8	3	9	0.899821
sp|Q14204|DYHC1_HUMAN	DYNC1H1	Cytoplasmic dynein 1 heavy chain 1 OS=Homo sapiens GN=DYNC1H1 PE=1 SV=5	534809	168	75	129	480	0.02	0.18	9	3	18	4	27	4	32	6	44	1.990574
sp|P11498|PYC_HUMAN	PC	Pyruvate carboxylase, mitochondrial OS=Homo sapiens GN=PC PE=1 SV=2	130293	10	1	1295	4465	1.76	13.88	7.8863636	40	124	52	160	56	171	67	188	0.748563
tr|A8K781|A8K781_HUMAN	tr|A8K781	cDNA FLJ75299, highly similar to Xenopus laevis proteasome (prosome, macropain) 26S subunit, ATPase 3, mRNA	47578	603	466	26	79	0.07	0.53	7.5714286	0	2	1	5	2	5	2	5	0.271224
tr|B2R9U2|B2R9U2_HUMAN	tr|B2R9U	cDNA, FLJ94557, highly similar to Homo sapiens FK506 binding protein 4, 59kDa (FKBP4), mRNA OS=Homo sapier	52087	623	294	24	142	0.06	0.45	7.5	0	3	1	6	2	7	2	9	0.71906
sp|P49411|EFTU_HUMAN	TUFM	Elongation factor Tu, mitochondrial OS=Homo sapiens GN=TUFM PE=1 SV=2	49852	210	70	99	510	0.29	2.17	7.4827586	3	14	4	18	4	17	5	21	1.094655
sp|Q9UG63|ABCF2_HUMAN	ABCF2	ATP-binding cassette sub-family F member 2 OS=Homo sapiens GN=ABCF2 PE=1 SV=2	71815	673	424	20	97	0.05	0.37	7.4	0	1	1	7	1	7	2	7	1.45637
tr|B4DTV0|B4DTV0_HUMAN	tr|B4DTV0	cDNA FLJ57801, highly similar to Homo sapiens AHNAK nucleoprotein (desmoyokin) (AHNAK), transcript variant 1	114179	454	217	38	194	0.03	0.22	7.3333333	1	5	1	7	3	12	3	13	0.971198
tr|B2R7C5|B2R7C5_HUMAN	tr|B2R7C5	cDNA, FLJ93378, highly similar to Homo sapiens MCM3 minichromosome maintenance deficient 3 (S. cerevisiae)	91579	479	288	36	146	0.04	0.28	7	1	4	1	7	1	10	1	13	1.942362
tr|E7EVA0|E7EVA0_HUMAN	MAP4	Microtubule-associated protein OS=Homo sapiens GN=MAP4 PE=2 SV=1	246546	429	1124	40	26	0.01	0.07	7	1	0	1	1	2	1	4	1	-1.604
sp|O00425|IF2B3_HUMAN	IGF2BP3	Insulin-like growth factor 2 mRNA-binding protein 3 OS=Homo sapiens GN=IGF2BP3 PE=1 SV=2	64008	556	275	29	150	0.05	0.35	7	0	5	1	6	2	7	2	7	0.71906
tr|I3L2F9|I3L2F9_HUMAN	tr|I3L2F9	Uncharacterized protein OS=Homo sapiens PE=3 SV=1	46054	25	6	275	460	3.07	21.45	6.9869707	12	20	15	25	18	29	19	31	-0.19407
tr|B2ZZ90|B2ZZ90_HUMAN	ACACA	Acetyl-Coenzyme A carboxylase alpha OS=Homo sapiens GN=ACACA PE=2 SV=1	267095	31	5	628	3005	0.44	3.04	6.9090909	25	94	30	125	38	135	46	146	0.961372
tr|H0YLA4|H0YLA4_HUMAN	SORD	Sorbitol dehydrogenase OS=Homo sapiens GN=SORD PE=3 SV=1	36833	473	1036	37	25	0.09	0.61	6.7777778	0	0	1	1	2	1	2	1	-1.604
tr|Q68DB1|Q68DB1_HUMAN	DKFZp686	Metastasis-associated protein MTA2 OS=Homo sapiens GN=DKFZp686F2281 PE=2 SV=1	55515	676	200	20	52	0.06	0.4	6.6666667	0	1	1	4	1	5	1	5	1.008534
tr|H0UID3|H0UID3_HUMAN	AP2B1	Adaptor-related protein complex 2, beta 1 subunit, isoform CRA_d OS=Homo sapiens GN=AP2B1 PE=4 SV=1	106321	648	194	22	206	0.03	0.2	6.6666667	0	6	1	6	3	9	3	10	0.574704
tr|B1ANR0|B1ANR0_HUMAN	PABPC4	Poly(A) binding protein, cytoplasmic 4 (Inducible form) OS=Homo sapiens GN=PABPC4 PE=4 SV=1	68156	504	287	33	146	0.05	0.33	6.6	0	4	1	6	1	7	2	7	1.45637
tr|B1AHB0|B1AHB0_HUMAN	MCM5	MCM5 minichromosome maintenance deficient 5, cell division cycle 46 (S. cerevisiae), isoform CRA_a OS=Homo	83031	562	283	29	147	0.04	0.26	6.5	0	4	1	6	1	7	2	10	1.45637
sp|P11021|GRP78_HUMAN	HSPA5	78 kDa glucose-regulated protein OS=Homo sapiens GN=HSPA5 PE=1 SV=2	72402	129	51	170	664	0.19	1.22	6.4210526	3	17	5	20	6	22	6	23	0.926967
tr|B2RE46|B2RE46_HUMAN	tr|B2RE46	cDNA, FLJ96923, highly similar to Homo sapiens ribophorin II (RPN2), mRNA OS=Homo sapiens PE=2 SV=1	69405	589	189	27	210	0.05	0.32	6.4	0	6	1	6	2	7	2	7	0.71906
tr|B4DUQ9|B4DUQ9_HUMAN	tr|B4DUQ	cDNA FLJ50873, highly similar to DNA replication licensing factor MCM3 OS=Homo sapiens PE=2 SV=1	86213	479	301	36	137	0.04	0.25	6.25	1	3	1	6	1	8	1	11	1.63713
tr|Q5SU16|Q5SU16_HUMAN	TUBB	Beta 5-tubulin OS=Homo sapiens GN=TUBB PE=2 SV=1	50095	25	9	680	1983	3.07	19.03	6.1986971	26	67	33	81	35	86	39	90	0.422566
tr|E7ERW8|E7ERW8_HUMAN	DIAPH1	Protein diaphanous homolog 1 OS=Homo sapiens GN=DIAPH1 PE=2 SV=1	135945	184	334	116	124	0.02	0.12	6	1	5	1	5	1	5	2	5	1.008534
tr|B2R5V9|B2R5V9_HUMAN	tr|B2R5V9	cDNA, FLJ92652, highly similar to Homo sapiens high density lipoprotein binding protein (vigilin)(HDLBP), mRNA	141919	253	315	76	131	0.02	0.12	6	1	4	1	5	2	7	2	11	0.71906
tr|Q5T7Q0|Q5T7Q0_HUMAN	DNAJA1	DnaJ (Hsp40) homolog, subfamily A, member 1, isoform CRA_c OS=Homo sapiens GN=DNAJA1 PE=2 SV=1	45581	703	553	18	69	0.07	0.42	6	0	3	1	5	0	7	1	8	3.041675
sp|Q7L2E3|DHX30_HUMAN	DHX30	Putative ATP-dependent RNA helicase DHX30 OS=Homo sapiens GN=DHX30 PE=1 SV=1	134938	397	159	43	256	0.05	0.3	6	1	10	2	11	3	11	4	12	0.850716
tr|B2R4K7|B2R4K7_HUMAN	tr|B2R4K7	60S ribosomal protein L6 OS=Homo sapiens PE=2 SV=1	32739	276	156	68	258	0.47	2.8	5.9574468	2	5	4	9	3	10	5	13	0.719282
tr|B2R7B5|B2R7B5_HUMAN	tr|B2R7B5	cDNA, FLJ93365, highly similar to Homo sapiens KH domain containing, RNA binding, signal transduction associat	48309	347	554	51	57	0.07	0.41	5.8571429	1	3	1	4	2	4	4	5	-0.01847
tr|A4FU77|A4FU77_HUMAN	SNRNP200	SNRNP200 protein (Fragment) OS=Homo sapiens GN=SNRNP200 PE=2 SV=1	217585	243	133	83	294	0.05	0.29	5.8	2	11	3	17	7	20	8	23	0.585493
tr|B4DMJ6|B4DMJ6_HUMAN	tr|B4DMJ	cDNA FLJ50996, highly similar to 60S ribosomal protein L4 OS=Homo sapiens PE=2 SV=1	46269	163	77	132	428	0.51	2.9	5.6862745	4	12	6	18	8	18	9	18	0.256101
sp|P05165|PCCA_HUMAN	PCCA	Propionyl-CoA carboxylase alpha chain, mitochondrial OS=Homo sapiens GN=PCCA PE=1 SV=4	80635	99	37	216	857	0.49	2.73	5.5714286	6	23	10	33	13	35	13	38	0.530461
sp|Q15645|PCH2_HUMAN	TRIP13	Pachytene checkpoint protein 2 homolog OS=Homo sapiens GN=TRIP13 PE=1 SV=2	48863	507	281	33	148	0.07	0.39	5.5714286	0	4	1	5	2	6	2	7	0.512421
tr|Q6NTA2|Q6NTA2_HUMAN	HNRNPL	HNRNPL protein (Fragment) OS=Homo sapiens GN=HNRNPL PE=2 SV=2	62515	182	63	117	563	0.23	1.27	5.5217391	4	12	4	18	6	17	8	21	0.563453
tr|B3KW21|B3KW21_HUMAN	tr|B3KW2	cDNA FLJ41945 fis, clone PLACE6019676, highly similar to Coatomer subunit gamma OS=Homo sapiens PE=2 SV=	68674	705	311	18	112	0.04	0.22	5.5	0	2	1	4	0	3	1	5	1.941386
tr|B2RBE0|B2RBE0_HUMAN	tr|B2RBE0	cDNA, FLJ95462, highly similar to Homo sapiens fatty-acid-Coenzyme A ligase, long-chain 3 (FACL3),mRNA OS=Ho	81266	462	258	37	164	0.04	0.22	5.5	0	5	1	5	1	6	1	6	1.24973
tr|I3L1L3|I3L1L3_HUMAN	MYBBP1A	Myb-binding protein 1A (Fragment) OS=Homo sapiens GN=MYBBP1A PE=4 SV=1	141124	698	362	18	115	0.02	0.11	5.5	0	3	1	5	1	5	2	5	1.008534
tr|B4DFL1|B4DFL1_HUMAN	tr|B4DFL1	Dihydrolipoyl dehydrogenase OS=Homo sapiens PE=2 SV=1	49381	666	340	21	122	0.07	0.38	5.4285714	0	2	1	5	1	5	1	5	1.008534
tr|B1AH89|B1AH89_HUMAN	TTLL12	Tubulin tyrosine ligase-like family, member 12 OS=Homo sapiens GN=TTLL12 PE=4 SV=1	74185	104	46	209	744	0.41	2.22	5.4146341	6	21	8	33	10	34	11	35	0.852653
tr|Q0VGD6|Q0VGD6_HUMAN	HNRPR	HNRPR protein (Fragment) OS=Homo sapiens GN=HNRPR PE=2 SV=1	68218	390	201	44	201	0.1	0.53	5.3	1	6	2	9	2	10	4	11	1.205053
sp|Q6P2Q9|PRP8_HUMAN	PRPF8	Pre-mRNA-processing-splicing factor 8 OS=Homo sapiens GN=PRPF8 PE=1 SV=2	274738	130	50	167	690	0.1	0.53	5.3	7	24	8	36	10	41	10	50	1.120492
sp|O95373|IPO7_HUMAN	IPO7	Importin-7 OS=Homo sapiens GN=IPO7 PE=1 SV=1	120751	162	100	132	376	0.08	0.42	5.25	3	10	3	15	3	15	4	16	1.282105
sp|P41250|SYG_HUMAN	GARS	Glycine--tRNA ligase OS=Homo sapiens GN=GARS PE=1 SV=3	83854	392	429	44	96	0.04	0.21	5.25	1	3	1	5	1	5	2	6	1.008534
tr|A6NKB8|A6NKB8_HUMAN	RNPEP	Aminopeptidase B OS=Homo sapiens GN=RNPEP PE=2 SV=1	68752	316	332	57	124	0.05	0.26	5.2	1	4	2	5	2	6	2	8	0.512421
tr|E7ENU7|E7ENU7_HUMAN	RPL15	Ribosomal protein L15 (Fragment) OS=Homo sapiens GN=RPL15 PE=2 SV=1	20596	534	423	31	83	0.16	0.83	5.1875	0	2	1	4	1	4	1	4	0.718839
tr|B4DMJ2|B4DMJ2_HUMAN	tr|B4DMJ	cDNA FLJ50994, moderately similar to 60S ribosomal protein L4 OS=Homo sapiens PE=2 SV=1	27217	163	163	93	251	0.51	2.57	5.0392157	3	8	3	11	5	12	6	14	0.318434
tr|A2BDK6|A2BDK6_HUMAN	MAP1B	Microtubule-associated protein 1B OS=Homo sapiens GN=MAP1B PE=2 SV=1	257692	511	458	32	88	0.01	0.05	5	0	2	1	4	1	4	2	5	0.718839
tr|B4DT29|B4DT29_HUMAN	tr|B4DT29	cDNA FLJ51082, highly similar to Actin-like protein 3 OS=Homo sapiens PE=2 SV=1	40959	548	371	30	90	0.08	0.4	5	0	3	1	4	1	4	1	5	0.718839
tr|I3L239|I3L239_HUMAN	TRAP1	Heat shock protein 75 kDa, mitochondrial (Fragment) OS=Homo sapiens GN=TRAP1 PE=4 SV=1	24972	179	235	121	179	0.13	0.65	5	2	4	2	5	2	5	4	6	0.271224
tr|H0Y8E6|H0Y8E6_HUMAN	MCM2	DNA replication licensing factor MCM2 (Fragment) OS=Homo sapiens GN=MCM2 PE=3 SV=1	94792	211	216	98	186	0.07	0.35	5	1	8	2	9	2	9	2	10	1.060474
tr|H7BZJ3|H7BZJ3_HUMAN	PDIA3	Thioredoxin (Fragment) OS=Homo sapiens GN=PDIA3 PE=3 SV=1	13739	546	409	22	101	0.19	0.94	4.9473684	0	2	1	3	1	4	1	4	0.718839
sp|P04350|TBB4A_HUMAN	TUBB4A	Tubulin beta-4A chain OS=Homo sapiens GN=TUBB4A PE=1 SV=2	50010	42	13	509	1606	1.96	9.59	4.8928571	21	52	27	63	29	69	32	72	0.372825
tr|B4DH52|B4DH52_HUMAN	tr|B4DH5	cDNA FLJ55916, highly similar to General transcription factor II-I OS=Homo sapiens PE=2 SV=1	112359	554	239	29	176	0.06	0.29	4.8333333	0	6	2	9	2	9	3	10	1.060474
tr|J3KQL0|J3KQL0_HUMAN	CPS1	Carbamoyl-phosphate synthase [ammonia], mitochondrial OS=Homo sapiens GN=CPS1 PE=3 SV=1	166686	204	62	102	570	0.15	0.72	4.8	4	19	7	29	10	31	12	35	0.720841
sp|O75369|FLNB_HUMAN	FLNB	Filamin-B OS=Homo sapiens GN=FLNB PE=1 SV=2	280157	45	17	463	1468	0.22	1.05	4.7727273	14	45	18	64	20	71	28	79	0.942335
tr|B4DVB8|B4DVB8_HUMAN	ELAVL1	ELAV-like protein 1 OS=Homo sapiens GN=ELAVL1 PE=2 SV=1	39200	612	430	24	96	0.08	0.38	4.75	0	1	1	4	1	4	1	4	0.718839
tr|B2R959|B2R959_HUMAN	tr|B2R959	cDNA, FLJ94229, highly similar to Homo sapiens heterogeneous nuclear ribonucleoprotein L (HNRPL),mRNA OS=H	60838	182	96	117	381	0.23	1.09	4.7391304	4	9	4	14	6	14	8	18	0.291584
tr|B4DKS0|B4DKS0_HUMAN	tr|B4DKS0	cDNA FLJ53381, highly similar to Monocarboxylate transporter 1 OS=Homo sapiens PE=2 SV=1	52487	598	322	26	112	0.06	0.28	4.6666667	0	1	1	3	1	3	1	3	0.35608
sp|P45974|UBP5_HUMAN	USP5	Ubiquitin carboxyl-terminal hydrolase 5 OS=Homo sapiens GN=USP5 PE=1 SV=2	96638	622	416	24	100	0.03	0.14	4.6666667	0	2	1	4	2	4	2	5	-0.01847
tr|A5D906|A5D906_HUMAN	TUBB2A	TUBB2A protein (Fragment) OS=Homo sapiens GN=TUBB2A PE=2 SV=1	28128	25	11	143	678	3.07	14.25	4.6416938	4	23	7	28	8	29	8	31	0.931368
sp|P52597|HNRPF_HUMAN	HNRNPF	Heterogeneous nuclear ribonucleoprotein F OS=Homo sapiens GN=HNRNPF PE=1 SV=3	45985	226	395	91	105	0.07	0.32	4.5714286	1	2	1	4	2	4	3	5	-0.01847
tr|B4DFM0|B4DFM0_HUMAN	FARSLB	Phenylalanine-tRNA synthetase-like, beta subunit, isoform CRA_c OS=Homo sapiens GN=FARSLB PE=2 SV=1	55353	360	558	48	63	0.06	0.27	4.5	1	3	1	4	1	6	3	7	1.24973
tr|B2RBR9|B2RBR9_HUMAN	tr|B2RBR9	cDNA, FLJ95650, highly similar to Homo sapiens karyopherin (importin) beta 1 (KPNB1), mRNA OS=Homo sapiens	98434	171	53	126	630	0.22	0.99	4.5	4	15	6	21	9	20	9	21	0.243768
tr|Q2Q9B7|Q2Q9B7_HUMAN	G6PD	Glucose-6-phosphate 1-dehydrogenase (Fragment) OS=Homo sapiens GN=G6PD PE=2 SV=1	55221	176	184	118	195	0.18	0.79	4.3888889	2	6	2	9	5	10	6	11	0.066518
tr|B4DYA7|B4DYA7_HUMAN	tr|B4DYA7	Glucose-6-phosphate 1-dehydrogenase OS=Homo sapiens PE=2 SV=1	58757	176	185	123	210	0.18	0.79	4.3888889	2	6	3	9	6	9	7	10	-0.31941
tr|Q59FV6|Q59FV6_HUMAN	tr|Q59FV6	ARP3 actin-related protein 3 homolog variant (Fragment) OS=Homo sapiens PE=2 SV=1	42517	548	452	30	90	0.08	0.35	4.375	0	3	1	4	1	5	1	6	1.008534
tr|B4DUC5|B4DUC5_HUMAN	CSE1L	Exportin-2 OS=Homo sapiens GN=CSE1L PE=2 SV=1	86045	433	310	40	132	0.08	0.35	4.375	0	3	2	8	4	9	4	11	0.21179
sp|O94906|PRP6_HUMAN	PRPF6	Pre-mRNA-processing factor 6 OS=Homo sapiens GN=PRPF6 PE=1 SV=1	107656	492	298	35	139	0.03	0.13	4.3333333	1	4	1	4	1	4	2	4	0.718839
tr|B0UZ98|B0UZ98_HUMAN	BAT1	HLA-B associated transcript 1 (Fragment) OS=Homo sapiens GN=BAT1 PE=2 SV=1	28623	394	381	44	64	0.09	0.39	4.3333333	1	2	1	2	1	2	1	2	-0.12954
tr|D3DTH7|D3DTH7_HUMAN	MYO1C	Myosin IC, isoform CRA_a OS=Homo sapiens GN=MYO1C PE=2 SV=1	99613	284	218	65	193	0.07	0.3	4.2857143	1	5	3	8	3	8	4	8	0.414051
tr|A8K492|A8K492_HUMAN	tr|A8K492	cDNA FLJ76789, highly similar to Homo sapiens methionine-tRNA synthetase (MARS), mRNA OS=Homo sapiens P	102277	385	228	45	186	0.07	0.29	4.1428571	1	7	2	8	2	8	2	8	0.899821
sp|O14929|HAT1_HUMAN	HAT1	Histone acetyltransferase type B catalytic subunit OS=Homo sapiens GN=HAT1 PE=1 SV=1	49880	343	262	51	162	0.07	0.29	4.1428571	1	3	1	4	1	4	1	4	0.718839
tr|B3KRS7|B3KRS7_HUMAN	tr|B3KRS7	cDNA FLJ34844 fis, clone NT2NE2011154, highly similar to RuvB-like 1 OS=Homo sapiens PE=2 SV=1	34946	351	363	50	103	0.09	0.37	4.1111111	1	3	1	4	1	4	1	4	0.718839
tr|B2RCM2|B2RCM2_HUMAN	tr|B2RCM	cDNA, FLJ96156, highly similar to Homo sapiens leucyl-tRNA synthetase (LARS), mRNA OS=Homo sapiens PE=2 SV	135550	257	102	75	373	0.1	0.4	4	2	10	4	14	3	17	5	21	1.457569
sp|O15427|MOT4_HUMAN	SLC16A3	Monocarboxylate transporter 4 OS=Homo sapiens GN=SLC16A3 PE=1 SV=1	50064	362	406	48	102	0.14	0.56	4	2	3	2	8	2	10	2	11	1.205053
sp|Q09666|AHNK_HUMAN	AHNAK	Neuroblast differentiation-associated protein AHNAK OS=Homo sapiens GN=AHNAK PE=1 SV=2	629213	401	215	43	194	0.01	0.04	4	1	5	1	8	3	14	6	16	1.185701
tr|H0YD14|H0YD14_HUMAN	MYOF	Myoferlin (Fragment) OS=Homo sapiens GN=MYOF PE=4 SV=1	135822	220	117	95	191	0.07	0.28	4	3	7	3	11	4	11	4	11	0.487802
tr|A0PJ92|A0PJ92_HUMAN	NOP56	NOP56 protein (Fragment) OS=Homo sapiens GN=NOP56 PE=2 SV=1	50068	311	222	58	190	0.14	0.56	4	1	5	2	8	3	8	3	8	0.414051
sp|P07355|ANXA2_HUMAN	ANXA2	Annexin A2 OS=Homo sapiens GN=ANXA2 PE=1 SV=2	38808	100	58	214	603	1.26	5.04	4	8	17	11	24	12	29	13	31	0.373598
tr|B5MCX3|B5MCX3_HUMAN	Sep-02	Septin-2 OS=Homo sapiens GN=SEPT2 PE=2 SV=1	37145	543	348	30	89	0.09	0.36	4	1	2	1	3	2	3	3	3	-0.38123
tr|B3KS31|B3KS31_HUMAN	TUBB6	Tubulin, beta 6, isoform CRA_a OS=Homo sapiens GN=TUBB6 PE=2 SV=1	42324	73	44	273	747	0.83	3.31	3.9879518	10	24	14	33	17	39	20	42	0.309478
sp|P26038|MOES_HUMAN	MSN	Moesin OS=Homo sapiens GN=MSN PE=1 SV=3	67892	119	68	179	530	0.46	1.83	3.9782609	7	16	8	24	11	26	12	30	0.338947
tr|B4DDB6|B4DDB6_HUMAN	HNRPA3	Heterogeneous nuclear ribonucleoprotein A3, isoform CRA_a OS=Homo sapiens GN=HNRPA3 PE=2 SV=1	37234	201	291	103	136	0.19	0.75	3.9473684	2	3	2	6	2	7	2	8	0.71906
tr|B7Z6M1|B7Z6M1_HUMAN	PLS3	Plastin-3 OS=Homo sapiens GN=PLS3 PE=2 SV=1	66047	282	93	66	397	0.34	1.34	3.9411765	1	13	6	19	6	22	8	24	0.926967
tr|I3L312|I3L312_HUMAN	P4HB	Protein disulfide-isomerase (Fragment) OS=Homo sapiens GN=P4HB PE=4 SV=1	26348	536	195	30	67	0.27	1.06	3.9259259	1	1	2	7	1	5	2	7	1.008534
tr|Q2F839|Q2F839_HUMAN	tr|Q2F839	Heat shock 70 kDa protein 9B (Fragment) OS=Homo sapiens PE=2 SV=1	19208	81	177	59	234	0.42	1.64	3.9047619	2	4	2	7	2	7	2	8	0.71906
tr|B4DR68|B4DR68_HUMAN	tr|B4DR68	cDNA FLJ58608, highly similar to Heat shock protein 75 kDa, mitochondrial OS=Homo sapiens PE=2 SV=1	74408	169	118	127	319	0.09	0.35	3.8888889	2	7	3	8	3	9	5	9	0.574704
sp|Q09028|RBBP4_HUMAN	RBBP4	Histone-binding protein RBBP4 OS=Homo sapiens GN=RBBP4 PE=1 SV=3	47911	321	738	55	49	0.22	0.85	3.8636364	2	2	3	4	4	4	4	4	-0.86715
tr|B4DR70|B4DR70_HUMAN	tr|B4DR70	cDNA FLJ58049, highly similar to RNA-binding protein FUS OS=Homo sapiens PE=2 SV=1	45013	450	496	38	79	0.07	0.27	3.8571429	1	2	1	4	1	5	1	6	1.008534
tr|A6NG51|A6NG51_HUMAN	SPTAN1	Spectrin alpha chain, non-erythrocytic 1 OS=Homo sapiens GN=SPTAN1 PE=2 SV=2	285628	167	76	130	425	0.06	0.23	3.8333333	2	14	5	17	6	22	9	25	0.926967
sp|P53618|COPB_HUMAN	COPB1	Coatomer subunit beta OS=Homo sapiens GN=COPB1 PE=1 SV=3	108214	303	213	59	195	0.06	0.23	3.8333333	1	5	2	7	2	8	3	11	0.899821
tr|Q60FE2|Q60FE2_HUMAN	MYH9	MYH9 variant protein OS=Homo sapiens GN=MYH9 PE=2 SV=1	227646	17	4	900	3267	0.9	3.45	3.8333333	30	83	46	111	52	127	60	138	0.419725
sp|O43175|SERA_HUMAN	PHGDH	D-3-phosphoglycerate dehydrogenase OS=Homo sapiens GN=PHGDH PE=1 SV=4	57356	131	127	166	308	0.25	0.95	3.8	3	9	4	14	4	12	4	16	0.608285
tr|A6NE09|A6NE09_HUMAN	RPSAP58	Protein RPSAP58 OS=Homo sapiens GN=RPSAP58 PE=3 SV=1	33002	521	277	31	82	0.1	0.38	3.8	0	2	1	2	1	2	1	3	-0.12954
tr|E5KNY5|E5KNY5_HUMAN	LRPPRC	Leucine-rich PPR-motif containing OS=Homo sapiens GN=LRPPRC PE=2 SV=1	159003	329	122	53	314	0.08	0.3	3.75	1	10	4	13	5	14	7	16	0.532936
tr|Q2TSD0|Q2TSD0_HUMAN	tr|Q2TSD0	Glyceraldehyde-3-phosphate dehydrogenase OS=Homo sapiens PE=2 SV=1	36197	18	21	891	1168	3.44	12.86	3.7383721	23	29	31	40	31	43	36	48	-0.40343
tr|Q2NKY5|Q2NKY5_HUMAN	TUBB6	TUBB6 protein OS=Homo sapiens GN=TUBB6 PE=2 SV=1	50514	65	34	317	937	0.88	3.28	3.7272727	12	28	16	35	17	41	19	44	0.381115
sp|P25490|TYY1_HUMAN	YY1	Transcriptional repressor protein YY1 OS=Homo sapiens GN=YY1 PE=1 SV=2	45141	630	1211	23	19	0.07	0.26	3.7142857	0	0	1	1	2	0	2	1	-3.18915
sp|Q9Y5B9|SP16H_HUMAN	SUPT16H	FACT complex subunit SPT16 OS=Homo sapiens GN=SUPT16H PE=1 SV=1	120409	506	516	33	75	0.03	0.11	3.6666667	0	2	1	4	1	5	2	5	1.008534
tr|Q1L838|Q1L838_HUMAN	GTBP	GTBP-ALT OS=Homo sapiens GN=GTBP PE=2 SV=1	122267	559	347	29	120	0.03	0.11	3.6666667	0	3	1	4	1	5	1	6	1.008534
tr|Q2TB59|Q2TB59_HUMAN	NNT	Nicotinamide nucleotide transhydrogenase OS=Homo sapiens GN=NNT PE=2 SV=1	114552	234	307	88	133	0.06	0.22	3.6666667	1	3	2	7	2	7	3	7	0.71906
tr|F5H3A1|F5H3A1_HUMAN	ATP1A1	Sodium/potassium-transporting ATPase subunit alpha-1 OS=Homo sapiens GN=ATP1A1 PE=2 SV=1	114239	324	171	55	242	0.09	0.33	3.6666667	2	6	3	11	8	17	12	23	0.175742
tr|A8K3Q9|A8K3Q9_HUMAN	tr|A8K3Q9	cDNA FLJ76611, highly similar to Homo sapiens ribosomal protein L14 (RPL14), mRNA OS=Homo sapiens PE=2 SV	23554	330	740	53	30	0.14	0.51	3.6428571	1	0	1	1	1	1	1	1	-0.86669
tr|B2ZZ89|B2ZZ89_HUMAN	SPTBN1	Spectrin beta non-erythrocytic 1 OS=Homo sapiens GN=SPTBN1 PE=2 SV=1	275237	49	16	431	1473	0.22	0.8	3.6363636	11	36	17	53	20	59	24	68	0.675002
tr|B4DF96|B4DF96_HUMAN	EIF2A	Eukaryotic translation initiation factor 2A OS=Homo sapiens GN=EIF2A PE=2 SV=1	58357	206	126	101	254	0.32	1.15	3.59375	2	7	5	12	4	16	5	17	1.009577
tr|Q96QL0|Q96QL0_HUMAN	RPL3	Ribosomal protein L3 OS=Homo sapiens GN=RPL3 PE=2 SV=1	46376	245	151	82	230	0.32	1.13	3.53125	4	8	4	10	5	13	7	13	0.429654
tr|Q5VZU9|Q5VZU9_HUMAN	TPP2	Tripeptidyl-peptidase 2 OS=Homo sapiens GN=TPP2 PE=4 SV=1	141160	693	789	19	44	0.02	0.07	3.5	0	0	1	3	0	3	1	6	1.941386
tr|B4DH39|B4DH39_HUMAN	tr|B4DH39	cDNA FLJ57028, highly similar to Long-chain-fatty-acid--CoA ligase 4 (EC 6.2.1.3) OS=Homo sapiens PE=2 SV=1	73123	529	364	31	114	0.04	0.14	3.5	0	2	1	3	1	6	3	7	1.24973
tr|B5BU25|B5BU25_HUMAN	U2AF2	U2 small nuclear RNA auxiliary factor 2 isoform b OS=Homo sapiens GN=U2AF2 PE=2 SV=1	53445	260	420	74	99	0.13	0.45	3.4615385	1	2	2	4	2	4	2	4	-0.01847
tr|Q60FE5|Q60FE5_HUMAN	FLNA	Filamin A OS=Homo sapiens GN=FLNA PE=2 SV=1	280790	9	3	1306	3805	0.86	2.95	3.4302326	41	102	60	147	75	161	88	182	0.23511
sp|P08238|HS90B_HUMAN	HSP90AB1	Heat shock protein HSP 90-beta OS=Homo sapiens GN=HSP90AB1 PE=1 SV=4	83554	32	25	622	1213	1.24	4.25	3.4274194	18	31	28	45	29	50	35	56	-0.09151
tr|B2R5P6|B2R5P6_HUMAN	TXNRD1	Thioredoxin reductase 1, cytoplasmic OS=Homo sapiens GN=TXNRD1 PE=2 SV=1	55253	108	91	204	398	0.26	0.89	3.4230769	3	8	5	11	5	13	6	13	0.429654
sp|Q9NZB2|F120A_HUMAN	FAM120A	Constitutive coactivator of PPAR-gamma-like protein 1 OS=Homo sapiens GN=FAM120A PE=1 SV=2	123008	263	250	73	168	0.05	0.17	3.4	2	5	2	6	2	6	2	7	0.512421
tr|A8K8N7|A8K8N7_HUMAN	PFAS	Phosphoribosylformylglycinamidine synthase (FGAR amidotransferase), isoform CRA_b OS=Homo sapiens GN=PF	146286	380	180	46	229	0.05	0.17	3.4	0	5	2	7	3	8	5	10	0.414051
tr|Q05CK9|Q05CK9_HUMAN	SYNCRIP	SYNCRIP protein (Fragment) OS=Homo sapiens GN=SYNCRIP PE=2 SV=1	50847	252	241	76	175	0.13	0.44	3.3846154	2	4	2	8	2	8	2	8	0.899821
tr|Q5HY54|Q5HY54_HUMAN	FLNA	Filamin-A OS=Homo sapiens GN=FLNA PE=2 SV=1	279115	9	2	1304	3785	0.86	2.9	3.372093	41	101	59	144	74	157	87	177	0.217744
tr|H7C3A1|H7C3A1_HUMAN	SRRT	Serrate RNA effector molecule homolog (Fragment) OS=Homo sapiens GN=SRRT PE=4 SV=1	57154	701	586	18	64	0.06	0.2	3.3333333	0	1	1	1	0	1	1	1	0.718617
tr|A8K2Y2|A8K2Y2_HUMAN	tr|A8K2Y2	cDNA FLJ78120, highly similar to Homo sapiens eukaryotic translation initiation factor 2, subunit 3 gamma, 52kDa	51648	475	551	37	70	0.06	0.2	3.3333333	1	2	1	3	1	3	1	4	0.35608
tr|Q53QX9|Q53QX9_HUMAN	HK2	Putative uncharacterized protein HK2 (Fragment) OS=Homo sapiens GN=HK2 PE=2 SV=1	64652	599	503	26	77	0.03	0.1	3.3333333	0	2	1	2	1	2	2	3	-0.12954
tr|K7EK45|K7EK45_HUMAN	PTBP1	Polypyrimidine tract-binding protein 1 (Fragment) OS=Homo sapiens GN=PTBP1 PE=4 SV=1	27322	567	271	21	153	0.18	0.59	3.2777778	0	3	1	4	1	4	1	5	0.718839
tr|B4DXX7|B4DXX7_HUMAN	DDX3Y	ATP-dependent RNA helicase DDX3Y OS=Homo sapiens GN=DDX3Y PE=2 SV=1	73342	217	162	95	235	0.19	0.62	3.2631579	2	10	5	11	6	12	6	12	0.077082
tr|F8VPD4|F8VPD4_HUMAN	CAD	CAD protein OS=Homo sapiens GN=CAD PE=2 SV=1	238097	317	182	57	223	0.04	0.13	3.25	1	4	3	9	4	13	5	16	0.719505
tr|E9PCI9|E9PCI9_HUMAN	FDPS	Farnesyl pyrophosphate synthase OS=Homo sapiens GN=FDPS PE=2 SV=1	40849	405	603	43	63	0.08	0.26	3.25	1	1	1	3	1	4	1	4	0.718839
sp|Q00610|CLH1_HUMAN	CLTC	Clathrin heavy chain 1 OS=Homo sapiens GN=CLTC PE=1 SV=5	193260	30	12	629	1665	0.52	1.69	3.25	19	43	25	62	24	69	28	76	0.642486
tr|B4E0S6|B4E0S6_HUMAN	tr|B4E0S6	cDNA FLJ55635, highly similar to pre-mRNA-splicing factorATP-dependent RNA helicase DHX15 (EC 3.6.1.-) OS=Ho	90289	183	134	117	293	0.15	0.48	3.2	2	8	4	11	4	12	7	13	0.608285
tr|B7Z1R5|B7Z1R5_HUMAN	ATP6V1A	V-type proton ATPase catalytic subunit A OS=Homo sapiens GN=ATP6V1A PE=2 SV=1	65037	428	354	40	119	0.05	0.16	3.2	1	2	1	3	1	4	2	4	0.718839
tr|B7Z5E2|B7Z5E2_HUMAN	PSMC2	26S protease regulatory subunit 7 OS=Homo sapiens GN=PSMC2 PE=2 SV=1	33594	610	326	25	82	0.1	0.32	3.2	0	2	1	3	1	4	1	4	0.718839
tr|Q6IBR0|Q6IBR0_HUMAN	RPN1	RPN1 protein OS=Homo sapiens GN=RPN1 PE=2 SV=1	68678	170	119	127	318	0.26	0.83	3.1923077	4	9	5	14	8	14	8	17	-0.09613
tr|J3KNE5|J3KNE5_HUMAN	HARS	Histidine--tRNA ligase, cytoplasmic OS=Homo sapiens GN=HARS PE=4 SV=1	50581	672	386	20	55	0.07	0.22	3.1428571	0	1	1	2	1	2	2	2	-0.12954
sp|P06733|ENOA_HUMAN	ENO1	Alpha-enolase OS=Homo sapiens GN=ENO1 PE=1 SV=2	47481	40	38	520	830	1.39	4.36	3.1366906	13	23	18	30	19	40	25	44	0.188928
tr|B4DWJ2|B4DWJ2_HUMAN	QARS	Glutamine--tRNA ligase OS=Homo sapiens GN=QARS PE=2 SV=1	87437	277	188	68	211	0.16	0.5	3.125	3	8	4	11	5	14	5	17	0.532936
tr|B2RB23|B2RB23_HUMAN	tr|B2RB23	cDNA, FLJ95265, highly similar to Homo sapiens acetyl-Coenzyme A acyltransferase 2 (mitochondrial 3-oxoacyl-C	42439	544	356	30	117	0.08	0.25	3.125	0	2	1	3	1	3	1	5	0.35608
tr|B7ZKM0|B7ZKM0_HUMAN	SART3	SART3 protein OS=Homo sapiens GN=SART3 PE=2 SV=1	106372	153	108	139	320	0.16	0.5	3.125	5	12	5	13	7	16	7	16	0.27158

tr|J3KPM9|J3KPM9_HUMAN	STAT1	Signal transducer and activator of transcription 1-alpha/beta OS=Homo sapiens GN=STAT1 PE=4 SV=1	83878	364	264	48	134	0.08	0.25	3.125	1	4	2	5	2	5	2	6	0.271224
tr|B1Q2N1|B1Q2N1_HUMAN	DDX39	ATP-dependent RNA helicase DDX39A OS=Homo sapiens GN=DDX39 PE=2 SV=1	30925	395	357	44	55	0.11	0.34	3.0909091	1	2	1	2	1	2	2	2	-0.12954
sp|P49327|FAS_HUMAN	FASN	Fatty acid synthase OS=Homo sapiens GN=FASN PE=1 SV=3	275877	55	19	394	1339	0.28	0.86	3.0714286	12	43	21	55	23	59	29	67	0.47693
tr|F8SKU2|F8SKU2_HUMAN	HLA-A	MHC class I antigen (Fragment) OS=Homo sapiens GN=HLA-A PE=3 SV=1	21143	639	491	22	54	0.16	0.49	3.0625	0	1	1	1	2	1	3	2	-1.604
sp|P53621|COPA_HUMAN	COPA	Coatomer subunit alpha OS=Homo sapiens GN=COPA PE=1 SV=2	139797	181	105	118	356	0.18	0.55	3.0555556	5	11	7	19	8	20	11	24	0.404577
tr|H7C4C8|H7C4C8_HUMAN	CCT8	T-complex protein 1 subunit theta (Fragment) OS=Homo sapiens GN=CCT8 PE=3 SV=1	35913	268	320	70	128	0.18	0.55	3.0555556	2	4	3	5	4	7	5	8	-0.12962
tr|B2R5M9|B2R5M9_HUMAN	tr|B2R5M	cDNA, FLJ92537, highly similar to Homo sapiens procollagen-lysine, 2-oxoglutarate 5-dioxygenase (lysine hydroxy	84022	251	425	76	97	0.04	0.12	3	1	2	1	3	1	4	1	6	0.718839
tr|A6NEM2|A6NEM2_HUMAN	HCFC1	HCF N-terminal chain 5 OS=Homo sapiens GN=HCFC1 PE=2 SV=2	215338	508	279	33	148	0.02	0.06	3	0	4	1	4	1	4	2	5	0.718839
tr|B2RAX6|B2RAX6_HUMAN	tr|B2RAX6	cDNA, FLJ95176, Homo sapiens CGI-48 protein (CGI-48), mRNA OS=Homo sapiens PE=2 SV=1	58127	605	742	25	48	0.06	0.18	3	0	1	1	3	1	3	1	3	0.35608
sp|O00629|IMA4_HUMAN	KPNA4	Importin subunit alpha-4 OS=Homo sapiens GN=KPNA4 PE=1 SV=1	58364	472	710	37	51	0.06	0.18	3	0	1	1	1	2	1	2	1	-1.604
tr|Q5TB52|Q5TB52_HUMAN	PAPSS2	3~-phosphoadenosine 5~-phosphosulfate synthase 2, isoform CRA_b OS=Homo sapiens GN=PAPSS2 PE=2 SV=1	70027	688	439	19	93	0.05	0.15	3	0	3	1	3	0	4	4	4	2.304144
tr|B2RDF2|B2RDF2_HUMAN	PES1	Pescadillo homolog 1 OS=Homo sapiens GN=PES1 PE=2 SV=1	68359	488	835	35	41	0.05	0.15	3	1	0	1	3	1	5	3	6	1.008534
tr|D3DQA6|D3DQA6_HUMAN	DDX46	DEAD (Asp-Glu-Ala-Asp) box polypeptide 46, isoform CRA_a OS=Homo sapiens GN=DDX46 PE=3 SV=1	100958	595	219	26	192	0.07	0.21	3	0	5	2	9	3	11	3	13	0.850716
tr|Q53HF2|Q53HF2_HUMAN	tr|Q53HF2	Heat shock 70kDa protein 8 isoform 2 variant (Fragment) OS=Homo sapiens PE=2 SV=1	53580	51	48	426	739	1.45	4.31	2.9724138	12	20	17	32	19	34	19	39	-0.04353
tr|B4E022|B4E022_HUMAN	TKT	Transketolase OS=Homo sapiens GN=TKT PE=2 SV=1	63410	35	47	578	743	1.36	4.04	2.9705882	17	21	24	39	26	45	32	50	-0.08712
tr|B4DUY3|B4DUY3_HUMAN	tr|B4DUY	cDNA FLJ56668, highly similar to Nucleolar protein NOP5 OS=Homo sapiens PE=2 SV=1	16345	515	154	32	39	0.21	0.62	2.952381	0	1	1	1	1	1	1	3	-0.86669
tr|J3KPS3|J3KPS3_HUMAN	ALDOA	Fructose-bisphosphate aldolase OS=Homo sapiens GN=ALDOA PE=3 SV=1	40249	63	29	338	982	1.79	5.26	2.9385475	11	24	15	29	17	37	18	39	0.234137
sp|P17987|TCPA_HUMAN	TCP1	T-complex protein 1 subunit alpha OS=Homo sapiens GN=TCP1 PE=1 SV=1	60819	197	112	106	326	0.3	0.88	2.9333333	2	7	5	12	8	12	10	18	-0.31063
tr|F1T0B3|F1T0B3_HUMAN	DDX1	ATP-dependent RNA helicase DDX1 OS=Homo sapiens GN=DDX1 PE=2 SV=1	74838	501	431	33	95	0.09	0.26	2.8888889	0	4	2	6	3	9	3	10	0.574704
tr|Q9BSV4|Q9BSV4_HUMAN	SFPQ	SFPQ protein (Fragment) OS=Homo sapiens GN=SFPQ PE=2 SV=2	68702	228	147	91	279	0.26	0.75	2.8846154	2	5	5	12	6	12	7	13	0.077082
tr|B2R8R5|B2R8R5_HUMAN	tr|B2R8R5	cDNA, FLJ94025, highly similar to Homo sapiens tripartite motif-containing 28 (TRIM28), mRNA OS=Homo sapien	90271	320	261	56	162	0.07	0.2	2.8571429	1	6	3	6	2	8	4	8	0.899821
tr|B4DDR2|B4DDR2_HUMAN	tr|B4DDR	cDNA FLJ54588, highly similar to Major vault protein OS=Homo sapiens PE=2 SV=1	98526	304	850	59	0	0.07	0.2	2.8571429	2	0	2	0	3	0	3	1	-3.67492
tr|Q5TCU6|Q5TCU6_HUMAN	TLN1	Talin-1 OS=Homo sapiens GN=TLN1 PE=4 SV=1	260030	48	20	446	1243	0.27	0.77	2.8518519	12	30	19	44	20	47	25	53	0.347764
tr|B2R6X5|B2R6X5_HUMAN	tr|B2R6X5	cDNA, FLJ93166, highly similar to Homo sapiens heat shock 70kDa protein 6 (HSP70B~) (HSPA6), mRNA OS=Homo	71370	193	155	112	263	0.2	0.57	2.85	3	8	5	12	6	14	6	15	0.291584
tr|H0Y2W2|H0Y2W2_HUMAN	ATAD3A	ATPase family AAA domain-containing protein 3A (Fragment) OS=Homo sapiens GN=ATAD3A PE=4 SV=1	64600	281	702	68	52	0.1	0.28	2.8	2	3	2	5	2	5	2	5	0.271224
tr|H0YMR4|H0YMR4_HUMAN	IPO4	Importin-4 (Fragment) OS=Homo sapiens GN=IPO4 PE=4 SV=1	60104	655	389	22	19	0.05	0.14	2.8	0	1	1	1	1	0	1	1	-2.45184
tr|H0YDE4|H0YDE4_HUMAN	PRMT1	Protein arginine N-methyltransferase 1 (Fragment) OS=Homo sapiens GN=PRMT1 PE=4 SV=1	27345	437	338	39	89	0.26	0.72	2.7692308	1	3	2	4	2	4	2	4	-0.01847
sp|Q01650|LAT1_HUMAN	SLC7A5	Large neutral amino acids transporter small subunit 1 OS=Homo sapiens GN=SLC7A5 PE=1 SV=2	55659	239	110	85	330	0.12	0.33	2.75	1	7	3	7	4	7	5	8	-0.12962
tr|F5GX33|F5GX33_HUMAN	SLC25A13	Calcium-binding mitochondrial carrier protein Aralar2 OS=Homo sapiens GN=SLC25A13 PE=2 SV=1	62548	353	392	49	87	0.11	0.3	2.7272727	1	2	2	5	3	5	4	6	-0.21455
tr|B4DU00|B4DU00_HUMAN	tr|B4DU00	cDNA FLJ54547, highly similar to Alpha-1 catenin (Cadherin-associated protein) OS=Homo sapiens PE=2 SV=1	89332	389	268	45	111	0.07	0.19	2.7142857	0	3	2	3	2	4	4	5	-0.01847
sp|Q9Y2A7|NCKP1_HUMAN	NCKAP1	Nck-associated protein 1 OS=Homo sapiens GN=NCKAP1 PE=1 SV=1	130018	685	533	19	72	0.03	0.08	2.6666667	0	2	1	3	0	3	1	4	1.941386
tr|B0UX83|B0UX83_HUMAN	BAG6	HLA-B associated transcript 3 OS=Homo sapiens GN=BAG6 PE=4 SV=1	119085	704	365	18	113	0.03	0.08	2.6666667	0	3	1	3	0	3	1	3	1.941386
tr|B4DR45|B4DR45_HUMAN	tr|B4DR45	cDNA FLJ61339, highly similar to Alanyl-tRNA synthetase (EC 6.1.1.7) OS=Homo sapiens PE=2 SV=1	110017	238	432	86	95	0.06	0.16	2.6666667	1	2	2	5	2	7	2	8	0.71906
tr|B2R6D0|B2R6D0_HUMAN	tr|B2R6D0	cDNA, FLJ92896, highly similar to Homo sapiens proteasome (prosome, macropain) 26S subunit, non-ATPase, 1 (I	106797	386	359	45	115	0.06	0.16	2.6666667	0	4	2	5	2	6	4	8	0.512421
tr|B7Z4V2|B7Z4V2_HUMAN	tr|B7Z4V2	cDNA FLJ51907, highly similar to Stress-70 protein, mitochondrial OS=Homo sapiens PE=2 SV=1	72641	81	61	251	572	0.42	1.12	2.6666667	7	14	8	18	8	19	8	20	0.332238
tr|B2R5M8|B2R5M8_HUMAN	tr|B2R5M	Isocitrate dehydrogenase [NADP] OS=Homo sapiens PE=2 SV=1	46905	555	329	29	125	0.15	0.4	2.6666667	0	2	2	5	3	6	3	6	0.026651
tr|B1PS43|B1PS43_HUMAN	MYH11	Myosin heavy chain 11 smooth muscle isoform OS=Homo sapiens GN=MYH11 PE=2 SV=1	235004	142	82	155	439	0.06	0.16	2.6666667	5	10	5	13	9	14	11	14	-0.25694
tr|B3KNL2|B3KNL2_HUMAN	RUVBL2	RuvB-like 2 (E. coli), isoform CRA_d OS=Homo sapiens GN=RUVBL2 PE=2 SV=1	47603	584	453	28	89	0.14	0.37	2.6428571	0	3	2	5	1	7	2	7	1.45637
tr|Q59EG8|Q59EG8_HUMAN	tr|Q59EG8	Proteasome 26S non-ATPase subunit 2 variant (Fragment) OS=Homo sapiens PE=2 SV=1	101322	198	212	105	195	0.14	0.37	2.6428571	1	5	4	10	4	11	5	12	0.487802
tr|B4DJ30|B4DJ30_HUMAN	tr|B4DJ30	cDNA FLJ61290, highly similar to Neutral alpha-glucosidase AB OS=Homo sapiens PE=2 SV=1	113539	47	30	446	1016	0.62	1.63	2.6290323	12	23	19	37	17	44	22	48	0.482377
tr|B4DN87|B4DN87_HUMAN	tr|B4DN87	cDNA FLJ52569, highly similar to Collagen-binding protein 2 OS=Homo sapiens PE=2 SV=1	44290	233	176	89	218	0.43	1.13	2.627907	4	6	5	10	5	11	5	12	0.197951
tr|A8K2S1|A8K2S1_HUMAN	tr|A8K2S1	cDNA FLJ75258, highly similar to Homo sapiens phospholipase A2, group IVA (cytosolic, calcium-dependent) (PLA	85695	369	190	47	209	0.08	0.21	2.625	1	6	2	7	2	6	5	13	0.512421
sp|P13639|EF2_HUMAN	EEF2	Elongation factor 2 OS=Homo sapiens GN=EEF2 PE=1 SV=4	96246	41	26	511	1178	1.23	3.22	2.6178862	13	35	26	51	27	56	32	61	0.173569
tr|Q3B7A7|Q3B7A7_HUMAN	GART	Phosphoribosylglycinamide formyltransferase, phosphoribosylglycinamide synthetase, phosphoribosylaminoimic	108909	341	191	51	208	0.13	0.34	2.6153846	1	6	4	10	3	13	4	13	1.082418
tr|B4DJ60|B4DJ60_HUMAN	tr|B4DJ60	cDNA FLJ55072, highly similar to Succinate dehydrogenase (ubiquinone) flavoprotein subunit, mitochondrial (EC1	68233	393	227	44	186	0.1	0.26	2.6	0	4	2	5	3	6	5	6	0.026651
tr|B4DUQ1|B4DUQ1_HUMAN	tr|B4DUQ	cDNA FLJ54552, highly similar to Heterogeneous nuclear ribonucleoprotein K OS=Homo sapiens PE=2 SV=1	48708	147	109	147	330	0.58	1.5	2.5862069	3	11	7	15	7	18	8	18	0.437017
tr|H0YJ34|H0YJ34_HUMAN	FERMT2	Fermitin family homolog 2 (Fragment) OS=Homo sapiens GN=FERMT2 PE=4 SV=1	73877	306	230	59	185	0.14	0.36	2.5714286	2	4	3	7	3	9	3	10	0.574704
tr|B4DFN9|B4DFN9_HUMAN	tr|B4DFN9	cDNA FLJ54303, highly similar to Heat shock 70 kDa protein 1 OS=Homo sapiens PE=2 SV=1	62699	80	64	252	506	0.58	1.49	2.5689655	7	15	10	19	10	22	10	23	0.233713
tr|A0AV58|A0AV58_HUMAN	STRN3	Striatin, calmodulin binding protein 3 OS=Homo sapiens GN=STRN3 PE=2 SV=1	78069	419	391	42	106	0.09	0.23	2.5555556	1	4	2	5	3	5	3	5	-0.21455
tr|J3QQM1|J3QQM1_HUMAN	PSMC5	26S protease regulatory subunit 8 (Fragment) OS=Homo sapiens GN=PSMC5 PE=4 SV=1	29442	314	428	57	52	0.11	0.28	2.5454545	1	1	1	2	1	2	2	2	-0.12954
tr|B3KM77|B3KM77_HUMAN	tr|B3KM7	cDNA FLJ10435 fis, clone NT2RP1000493, highly similar to Splicing factor 3B subunit 3 OS=Homo sapiens PE=2 SV	56736	356	161	49	212	0.06	0.15	2.5	1	4	1	5	1	7	1	7	1.45637
tr|B2RU06|B2RU06_HUMAN	EIF4G1	EIF4G1 protein OS=Homo sapiens GN=EIF4G1 PE=2 SV=1	176807	669	401	20	103	0.04	0.1	2.5	0	3	2	5	1	5	3	8	1.008534
tr|B4DKD0|B4DKD0_HUMAN	tr|B4DKD	DNA topoisomerase 2 (Fragment) OS=Homo sapiens PE=2 SV=1	135015	606	454	25	89	0.02	0.05	2.5	0	1	1	2	1	4	3	5	0.718839
sp|Q9NVI7|ATD3A_HUMAN	ATAD3A	ATPase family AAA domain-containing protein 3A OS=Homo sapiens GN=ATAD3A PE=1 SV=2	71610	281	515	68	75	0.1	0.25	2.5	2	3	2	6	2	6	2	6	0.512421
sp|Q96P70|IPO9_HUMAN	IPO9	Importin-9 OS=Homo sapiens GN=IPO9 PE=1 SV=3	116858	580	380	28	109	0.06	0.15	2.5	0	3	2	5	2	6	2	7	0.512421
tr|B4DGU4|B4DGU4_HUMAN	CTNNB1	Catenin beta-1 OS=Homo sapiens GN=CTNNB1 PE=2 SV=1	85339	340	278	52	150	0.12	0.3	2.5	2	5	3	7	3	8	3	8	0.414051
tr|B3KNN7|B3KNN7_HUMAN	tr|B3KNN	cDNA FLJ30049 fis, clone ADRGL1000033, highly similar to 26S proteasome non-ATPase regulatory subunit 3 OS=	57311	273	224	69	187	0.18	0.45	2.5	3	6	3	7	3	7	3	8	0.23329
tr|B4DS66|B4DS66_HUMAN	tr|B4DS66	cDNA FLJ54290, highly similar to Mitochondrial inner membrane protein OS=Homo sapiens PE=2 SV=1	73509	712	494	17	79	0.04	0.1	2.5	0	2	1	2	1	2	6	4	-0.12954
sp|Q5T2N8|ATD3C_HUMAN	ATAD3C	ATPase family AAA domain-containing protein 3C OS=Homo sapiens GN=ATAD3C PE=2 SV=2	46635	281	495	68	42	0.1	0.25	2.5	2	2	2	2	2	2	2	2	-0.86684
tr|A6NN80|A6NN80_HUMAN	ANXA6	Annexin OS=Homo sapiens GN=ANXA6 PE=3 SV=3	75571	349	350	50	119	0.14	0.35	2.5	1	4	3	7	3	8	3	11	0.414051
sp|P07737|PROF1_HUMAN	PFN1	Profilin-1 OS=Homo sapiens GN=PFN1 PE=1 SV=2	15216	436	366	39	113	0.5	1.24	2.48	1	3	2	4	2	4	2	4	-0.01847
sp|P18206|VINC_HUMAN	VCL	Vinculin OS=Homo sapiens GN=VCL PE=1 SV=4	124292	68	43	299	775	0.37	0.91	2.4594595	8	23	12	26	13	32	13	35	0.402515
sp|O00116|ADAS_HUMAN	AGPS	Alkyldihydroxyacetonephosphate synthase, peroxisomal OS=Homo sapiens GN=AGPS PE=1 SV=1	73664	448	368	38	112	0.09	0.22	2.4444444	1	3	2	4	2	5	3	8	0.271224
tr|B4DF00|B4DF00_HUMAN	tr|B4DF00	cDNA FLJ53308, highly similar to 2-oxoglutarate dehydrogenase E1 component, mitochondrial (EC 1.2.4.2) OS=Ho	111596	432	443	40	92	0.05	0.12	2.4	1	2	1	5	1	6	1	6	1.24973
tr|Q59FI4|Q59FI4_HUMAN	tr|Q59FI4	Importin 4 variant (Fragment) OS=Homo sapiens PE=2 SV=1	139880	655	390	22	106	0.05	0.12	2.4	0	3	1	5	1	4	1	6	0.718839
tr|F8VRQ1|F8VRQ1_HUMAN	HNRNPA1	Heterogeneous nuclear ribonucleoprotein A1 OS=Homo sapiens GN=HNRNPA1 PE=2 SV=1	33249	101	104	214	363	0.77	1.84	2.3896104	4	8	6	12	7	13	9	14	-0.01849
tr|B2RDN4|B2RDN4_HUMAN	tr|B2RDN	cDNA, FLJ96693, highly similar to Homo sapiens block of proliferation 1 (BOP1), mRNA OS=Homo sapiens PE=2 SV	84219	505	434	33	60	0.08	0.19	2.375	0	1	2	3	2	4	3	6	-0.01847
tr|F5H4R9|F5H4R9_HUMAN	DNM2	Dynamin-2 OS=Homo sapiens GN=DNM2 PE=2 SV=1	98218	713	620	16	61	0.03	0.07	2.3333333	0	2	1	2	0	2	2	2	1.455771
tr|B0V043|B0V043_HUMAN	VARS	Valine--tRNA ligase OS=Homo sapiens GN=VARS PE=3 SV=1	141632	275	83	69	431	0.15	0.35	2.3333333	2	9	6	15	6	15	8	21	0.387988
sp|Q8TCJ2|STT3B_HUMAN	STT3B	Dolichyl-diphosphooligosaccharide--protein glycosyltransferase subunit STT3B OS=Homo sapiens GN=STT3B PE=1	94241	700	920	18	37	0.03	0.07	2.3333333	0	0	1	2	2	4	3	5	-0.01847
tr|B4DE36|B4DE36_HUMAN	tr|B4DE36	Glucose-6-phosphate isomerase OS=Homo sapiens PE=2 SV=1	60376	117	120	182	316	0.3	0.7	2.3333333	4	5	5	10	7	11	7	12	-0.2502
tr|B3KMX0|B3KMX0_HUMAN	tr|B3KMX	cDNA FLJ12837 fis, clone NT2RP2003228, highly similar to DNA replication licensing factor MCM4 OS=Homo sapi	97114	240	370	85	111	0.1	0.23	2.3	2	2	3	6	4	7	5	7	-0.12962
tr|Q8TC62|Q8TC62_HUMAN	Sep-07	Septin 7 OS=Homo sapiens GN=SEPT7 PE=2 SV=3	48913	531	752	31	47	0.07	0.16	2.2857143	1	1	1	2	1	2	2	2	-0.12954
sp|Q08211|DHX9_HUMAN	DHX9	ATP-dependent RNA helicase A OS=Homo sapiens GN=DHX9 PE=1 SV=4	142181	96	66	218	551	0.41	0.93	2.2682927	8	20	16	30	18	33	22	37	-0.00986
tr|B2R7M3|B2R7M3_HUMAN	tr|B2R7M	cDNA, FLJ93510, highly similar to Homo sapiens JTV1 gene (JTV1), mRNA OS=Homo sapiens PE=2 SV=1	35767	308	719	58	51	0.19	0.43	2.2631579	2	1	2	4	2	4	2	4	-0.01847
tr|B4DY09|B4DY09_HUMAN	tr|B4DY09	cDNA FLJ51660, highly similar to Interleukin enhancer-binding factor 2 OS=Homo sapiens PE=2 SV=1	39057	383	701	45	52	0.08	0.18	2.25	1	1	1	2	1	3	1	5	0.35608
tr|J3KRF5|J3KRF5_HUMAN	CLTC	Clathrin heavy chain 1 (Fragment) OS=Homo sapiens GN=CLTC PE=4 SV=1	15819	30	632	40	60	0.52	1.17	2.25	1	2	1	4	2	4	2	6	-0.01847
tr|B3KSH1|B3KSH1_HUMAN	EIF3F	Eukaryotic translation initiation factor 3 subunit F OS=Homo sapiens GN=EIF3F PE=2 SV=1	39236	652	589	22	64	0.08	0.18	2.25	0	1	1	2	1	2	1	2	-0.12954
tr|B4E091|B4E091_HUMAN	tr|B4E091	cDNA FLJ55438, highly similar to Splicing factor 3 subunit 1 OS=Homo sapiens PE=2 SV=1	77415	482	556	36	69	0.04	0.09	2.25	1	2	1	2	1	2	1	3	-0.12954
tr|Q59G92|Q59G92_HUMAN	tr|Q59G9	HLA-B associated transcript 1 variant (Fragment) OS=Homo sapiens PE=2 SV=1	35528	394	588	44	64	0.09	0.2	2.2222222	1	2	1	2	2	3	2	3	-0.38123
tr|Q6IAV5|Q6IAV5_HUMAN	OXCT	Succinyl-CoA:3-ketoacid-coenzyme A transferase OS=Homo sapiens GN=OXCT PE=2 SV=1	56612	209	374	100	111	0.18	0.4	2.2222222	1	3	3	6	5	7	6	7	-0.41947
tr|E7EMU4|E7EMU4_HUMAN	DYNC1I2	Cytoplasmic dynein 1 intermediate chain 2 (Fragment) OS=Homo sapiens GN=DYNC1I2 PE=2 SV=1	24071	298	445	48	65	0.09	0.2	2.2222222	1	1	1	1	1	1	1	1	-0.86669
sp|O00410|IPO5_HUMAN	IPO5	Importin-5 OS=Homo sapiens GN=IPO5 PE=1 SV=4	125032	85	88	236	409	0.23	0.51	2.2173913	7	12	8	16	10	18	15	21	-0.04944
tr|E0X098|E0X098_HUMAN	TES	Testin variant (Fragment) OS=Homo sapiens GN=TES PE=2 SV=1	22009	430	616	40	62	0.15	0.33	2.2	1	1	1	2	1	2	1	2	-0.12954
tr|Q05BW9|Q05BW9_HUMAN	PAPSS1	PAPSS1 protein (Fragment) OS=Homo sapiens GN=PAPSS1 PE=2 SV=1	65264	553	297	29	140	0.1	0.22	2.2	0	4	2	4	1	4	2	4	0.718839
tr|A0JLS5|A0JLS5_HUMAN	TBL3	TBL3 protein (Fragment) OS=Homo sapiens GN=TBL3 PE=2 SV=1	64079	418	417	42	99	0.05	0.11	2.2	1	2	1	2	1	2	1	3	-0.12954
sp|Q15392|DHC24_HUMAN	DHCR24	Delta(24)-sterol reductase OS=Homo sapiens GN=DHCR24 PE=1 SV=2	60803	442	868	39	39	0.05	0.11	2.2	1	1	1	2	2	2	3	2	-0.86684
tr|F6IQP2|F6IQP2_HUMAN	HLA-C	MHC class I antigen (Fragment) OS=Homo sapiens GN=HLA-C PE=3 SV=1	39655	639	630	22	60	0.16	0.35	2.1875	0	2	1	3	1	4	1	5	0.718839
sp|Q15149|PLEC_HUMAN	PLEC	Plectin OS=Homo sapiens GN=PLEC PE=1 SV=3	533462	74	33	270	972	0.11	0.24	2.1818182	11	22	17	35	18	50	32	59	0.585477
tr|H0YBW4|H0YBW4_HUMAN	PLAA	Phospholipase A-2-activating protein (Fragment) OS=Homo sapiens GN=PLAA PE=4 SV=1	31645	539	164	30	191	0.11	0.24	2.1818182	0	3	1	3	1	3	1	3	0.35608
tr|K7EM16|K7EM16_HUMAN	VASP	Vasodilator-stimulated phosphoprotein (Fragment) OS=Homo sapiens GN=VASP PE=4 SV=1	20260	363	543	48	54	0.17	0.37	2.1764706	1	1	1	1	1	2	1	2	-0.12954
tr|B3KQT2|B3KQT2_HUMAN	tr|B3KQT2	cDNA PSEC0148 fis, clone PLACE1007202, highly similar to Protein disulfide-isomerase A3 (EC 5.3.4.1) OS=Homo	55268	546	375	30	110	0.19	0.41	2.1578947	0	2	3	6	2	9	8	9	1.060474
tr|B3KU96|B3KU96_HUMAN	tr|B3KU96	cDNA FLJ39433 fis, clone PROST2004142, highly similar to GDP-L-fucose synthetase (EC 1.1.1.271) OS=Homo sapi	17856	371	705	47	52	0.19	0.41	2.1578947	1	2	1	2	1	2	1	3	-0.12954
tr|B4DJE3|B4DJE3_HUMAN	tr|B4DJE3	cDNA FLJ52929, highly similar to Dolichyl-diphosphooligosaccharide--proteinglycosyltransferase 48 kDa subunit (	46620	604	717	26	51	0.07	0.15	2.1428571	0	2	1	2	1	2	2	4	-0.12954
sp|O43286|B4GT5_HUMAN	B4GALT5	Beta-1,4-galactosyltransferase 5 OS=Homo sapiens GN=B4GALT5 PE=1 SV=1	45318	651	1033	22	31	0.07	0.15	2.1428571	0	2	1	2	1	1	1	2	-0.86669
tr|B2R7Y3|B2R7Y3_HUMAN	tr|B2R7Y3	cDNA, FLJ93657, highly similar to Homo sapiens ribosomal protein L3-like (RPL3L), mRNA OS=Homo sapiens PE=2	46581	420	689	42	53	0.07	0.15	2.1428571	1	1	1	2	2	2	3	3	-0.86684
tr|E9PIM9|E9PIM9_HUMAN	RNH1	Ribonuclease inhibitor (Fragment) OS=Homo sapiens GN=RNH1 PE=2 SV=1	22362	431	481	40	82	0.15	0.32	2.1333333	0	2	1	2	1	2	1	2	-0.12954
tr|Q05D08|Q05D08_HUMAN	PA2G4	PA2G4 protein (Fragment) OS=Homo sapiens GN=PA2G4 PE=2 SV=1	45579	286	467	64	85	0.15	0.32	2.1333333	1	4	2	4	3	4	3	4	-0.50424
tr|B4DLB8|B4DLB8_HUMAN	tr|B4DLB8	cDNA FLJ52205, highly similar to Beta-1,4-galactosyltransferase 1 (EC 2.4.1.-) OS=Homo sapiens PE=2 SV=1	40269	650	843	22	41	0.08	0.17	2.125	0	2	1	2	1	2	1	3	-0.12954
tr|G0ZDW9|G0ZDW9_HUMAN	HLA-C	MHC class I antigen (Fragment) OS=Homo sapiens GN=HLA-C PE=3 SV=1	21251	639	640	22	59	0.16	0.34	2.125	0	2	1	2	1	2	1	4	-0.12954
tr|Q5TZX1|Q5TZX1_HUMAN	GNAI3	Guanine nucleotide binding protein (G protein), alpha inhibiting activity polypeptide 3 OS=Homo sapiens GN=GN	41076	376	600	46	63	0.08	0.17	2.125	1	2	1	2	1	2	1	2	-0.12954
tr|E0YTJ7|E0YTJ7_HUMAN	HLA-B	MHC class I antigen (Fragment) OS=Homo sapiens GN=HLA-B PE=3 SV=1	21175	639	561	22	67	0.16	0.34	2.125	0	2	1	2	1	2	1	3	-0.12954
tr|B4DEX8|B4DEX8_HUMAN	MAT2A	S-adenosylmethionine synthase OS=Homo sapiens GN=MAT2A PE=2 SV=1	40027	333	521	52	74	0.08	0.17	2.125	1	2	1	2	1	2	1	2	-0.12954
tr|B4DEM7|B4DEM7_HUMAN	CCT8	T-complex protein 1 subunit theta OS=Homo sapiens GN=CCT8 PE=2 SV=1	58179	268	245	70	170	0.18	0.38	2.1111111	2	5	3	6	5	7	6	9	-0.41947
tr|B4DQX0|B4DQX0_HUMAN	tr|B4DQX	cDNA FLJ59219, highly similar to Poly(A)-binding protein 1 OS=Homo sapiens PE=2 SV=1	67239	215	166	96	250	0.33	0.69	2.0909091	3	9	6	11	6	12	10	12	0.077082
tr|H0Y4R1|H0Y4R1_HUMAN	IMPDH2	Inosine-5~-monophosphate dehydrogenase 2 (Fragment) OS=Homo sapiens GN=IMPDH2 PE=3 SV=1	51549	177	90	122	399	0.36	0.75	2.0833333	2	9	5	9	5	9	6	10	-0.07806
tr|H0Y4A6|H0Y4A6_HUMAN	VARS	Valine--tRNA ligase (Fragment) OS=Homo sapiens GN=VARS PE=3 SV=1	133055	275	98	69	377	0.15	0.31	2.0666667	2	7	6	13	6	13	8	20	0.188302
tr|B2R6J2|B2R6J2_HUMAN	tr|B2R6J2	cDNA, FLJ92973, highly similar to Homo sapiens villin 2 (ezrin) (VIL2), mRNA OS=Homo sapiens PE=2 SV=1	69472	126	158	173	222	0.32	0.66	2.0625	6	9	6	12	8	18	8	20	0.256101
tr|Q6IPN6|Q6IPN6_HUMAN	EEF1A1	Elongation factor 1-alpha OS=Homo sapiens GN=EEF1A1 PE=2 SV=1	50433	70	72	295	508	1.14	2.33	2.0438596	11	15	13	23	18	27	21	32	-0.29573
tr|F8W9S7|F8W9S7_HUMAN	GAPVD1	GTPase-activating protein and VPS9 domain-containing protein 1 OS=Homo sapiens GN=GAPVD1 PE=2 SV=1	158790	697	524	18	74	0.02	0.04	2	0	1	1	2	0	3	2	3	1.941386
tr|Q2NLA0|Q2NLA0_HUMAN	COPG2	Coatomer subunit gamma OS=Homo sapiens GN=COPG2 PE=2 SV=1	82400	705	422	18	99	0.04	0.08	2	0	2	1	2	0	2	1	3	1.455771
sp|P11388|TOP2A_HUMAN	TOP2A	DNA topoisomerase 2-alpha OS=Homo sapiens GN=TOP2A PE=1 SV=3	175017	606	415	25	100	0.02	0.04	2	0	2	1	2	1	3	2	4	0.35608
sp|Q13867|BLMH_HUMAN	BLMH	Bleomycin hydrolase OS=Homo sapiens GN=BLMH PE=1 SV=1	53155	332	469	52	84	0.2	0.4	2	1	2	3	4	2	4	4	4	-0.01847
tr|I3L0N3|I3L0N3_HUMAN	NSF	Vesicle-fusing ATPase OS=Homo sapiens GN=NSF PE=3 SV=1	82553	412	1139	42	25	0.04	0.08	2	1	0	1	2	1	2	1	4	-0.12954
tr|B4DM97|B4DM97_HUMAN	tr|B4DM9	cDNA FLJ55002, highly similar to Alpha-centractin OS=Homo sapiens PE=2 SV=1	38365	561	927	29	37	0.09	0.18	2	0	0	1	2	1	2	1	2	-0.12954
sp|Q6PGP7|TTC37_HUMAN	TTC37	Tetratricopeptide repeat protein 37 OS=Homo sapiens GN=TTC37 PE=1 SV=1	177485	498	858	34	40	0.02	0.04	2	1	1	1	2	1	2	1	3	-0.12954
tr|B3KUZ8|B3KUZ8_HUMAN	tr|B3KUZ8	Aspartate aminotransferase OS=Homo sapiens PE=2 SV=1	41699	522	681	31	55	0.08	0.16	2	0	1	1	2	1	2	2	3	-0.12954
tr|G0ZJH6|G0ZJH6_HUMAN	Ube4B	UFD2A-III/UBE4B-III splice isoform OS=Homo sapiens GN=Ube4B PE=2 SV=1	152890	667	673	21	56	0.02	0.04	2	0	1	1	2	1	2	1	3	-0.12954
sp|Q9Y305|ACOT9_HUMAN	ACOT9	Acyl-coenzyme A thioesterase 9, mitochondrial OS=Homo sapiens GN=ACOT9 PE=1 SV=2	50269	637	667	22	56	0.07	0.14	2	0	2	1	2	1	2	2	2	-0.12954
tr|E7ET15|E7ET15_HUMAN	U2SURP	U2 snRNP-associated SURP motif-containing protein OS=Homo sapiens GN=U2SURP PE=2 SV=1	118631	647	608	22	62	0.03	0.06	2	0	2	1	2	1	2	2	4	-0.12954
tr|F8W930|F8W930_HUMAN	IGF2BP2	Insulin-like growth factor 2 mRNA-binding protein 2 OS=Homo sapiens GN=IGF2BP2 PE=2 SV=1	66859	556	546	29	70	0.05	0.1	2	0	2	1	2	1	2	1	2	-0.12954
tr|F5H1A8|F5H1A8_HUMAN	GSN	Gelsolin OS=Homo sapiens GN=GSN PE=2 SV=1	81776	297	508	61	76	0.04	0.08	2	1	2	1	2	1	2	2	2	-0.12954
tr|B4DYL7|B4DYL7_HUMAN	tr|B4DYL7	cDNA FLJ58490, highly similar to Homo sapiens CCR4-NOT transcription complex, subunit 1 (CNOT1), transcript v	140991	388	502	45	52	0.02	0.04	2	1	1	1	2	1	2	2	2	-0.12954
tr|G3V2A3|G3V2A3_HUMAN	TUBB3	Tubulin beta-3 chain (Fragment) OS=Homo sapiens GN=TUBB3 PE=2 SV=1	20929	25	22	147	493	3.07	6.01	1.9576547	5	15	6	18	7	19	7	22	0.513155
sp|P22314|UBA1_HUMAN	UBA1	Ubiquitin-like modifier-activating enzyme 1 OS=Homo sapiens GN=UBA1 PE=1 SV=3	118858	114	85	187	420	0.28	0.54	1.9285714	5	11	10	16	10	15	12	19	-0.30527
tr|J3KN86|J3KN86_HUMAN	RPS3	40S ribosomal protein S3 OS=Homo sapiens GN=RPS3 PE=3 SV=1	28640	480	1092	36	27	0.12	0.23	1.9166667	1	0	1	1	1	1	4	1	-0.86669
tr|E9PHF7|E9PHF7_HUMAN	MCCC1	Methylcrotonoyl-CoA carboxylase subunit alpha, mitochondrial OS=Homo sapiens GN=MCCC1 PE=2 SV=1	68745	102	56	214	531	0.83	1.59	1.9156627	7	17	13	25	11	29	16	33	0.494237
sp|Q1KMD3|HNRL2_HUMAN	HNRNPUL	Heterogeneous nuclear ribonucleoprotein U-like protein 2 OS=Homo sapiens GN=HNRNPUL2 PE=1 SV=1	85622	249	144	78	281	0.21	0.4	1.9047619	2	7	5	9	6	10	6	11	-0.17483
tr|B1AHC9|B1AHC9_HUMAN	XRCC6	X-ray repair complementing defective repair in Chinese hamster cells 6 (Ku autoantigen, 70kDa) OS=Homo sapier	64528	116	115	184	325	0.64	1.21	1.890625	5	11	10	16	11	17	12	21	-0.26139
sp|Q96TA1|NIBL1_HUMAN	FAM129B	Niban-like protein 1 OS=Homo sapiens GN=FAM129B PE=1 SV=3	84598	155	198	137	205	0.16	0.3	1.875	4	5	4	7	4	9	4	12	0.21179
tr|G3XAD8|G3XAD8_HUMAN	STIP1	Stress-induced-phosphoprotein 1 OS=Homo sapiens GN=STIP1 PE=2 SV=1	68721	271	187	69	214	0.21	0.39	1.8571429	2	4	4	7	4	9	5	11	0.21179
sp|Q3ZCM7|TBB8_HUMAN	TUBB8	Tubulin beta-8 chain OS=Homo sapiens GN=TUBB8 PE=1 SV=2	50257	67	74	306	489	0.77	1.43	1.8571429	13	21	17	26	19	30	21	33	-0.22208
sp|P14923|PLAK_HUMAN	JUP	Junction plakoglobin OS=Homo sapiens GN=JUP PE=1 SV=3	82434	205	206	102	198	0.26	0.48	1.8461538	3	9	6	11	6	10	7	11	-0.17483
tr|Q5U077|Q5U077_HUMAN	LDHB	L-lactate dehydrogenase OS=Homo sapiens GN=LDHB PE=2 SV=1	36900	39	65	530	553	1.81	3.32	1.8342541	13	17	16	21	18	23	20	25	-0.52325
tr|B0QYT3|B0QYT3_HUMAN	RANGAP1	Ran GTPase activating protein 1 (Fragment) OS=Homo sapiens GN=RANGAP1 PE=2 SV=1	27122	574	501	28	37	0.12	0.22	1.8333333	0	1	1	1	1	2	1	2	-0.12954
tr|Q6DC98|Q6DC98_HUMAN	LMNB1	LMNB1 protein (Fragment) OS=Homo sapiens GN=LMNB1 PE=2 SV=1	38289	219	342	95	101	0.18	0.33	1.8333333	2	2	2	3	2	3	4	3	-0.38123
sp|P07814|SYEP_HUMAN	EPRS	Bifunctional glutamate/proline--tRNA ligase OS=Homo sapiens GN=EPRS PE=1 SV=5	172080	57	73	380	494	0.33	0.6	1.8181818	9	15	15	25	15	27	17	35	-0.03944
tr|B3GQS7|B3GQS7_HUMAN	HSPD1	Mitochondrial heat shock 60kD protein 1 variant 1 OS=Homo sapiens GN=HSPD1 PE=2 SV=1	60813	16	45	904	724	1.72	3.11	1.8081395	24	21	27	27	26	31	33	33	-0.62028
tr|B4DQJ8|B4DQJ8_HUMAN	PGD	6-phosphogluconate dehydrogenase, decarboxylating OS=Homo sapiens GN=PGD PE=2 SV=1	52352	293	257	62	165	0.2	0.36	1.8	1	5	3	5	2	8	3	8	0.899821
tr|B4DE27|B4DE27_HUMAN	tr|B4DE27	cDNA FLJ58765, highly similar to Neutral amino acid transporter B(0) OS=Homo sapiens PE=2 SV=1	33987	290	447	63	61	0.1	0.18	1.8	1	1	1	2	1	3	1	3	0.35608
sp|Q9NV06|DCA13_HUMAN	DCAF13	DDB1- and CUL4-associated factor 13 OS=Homo sapiens GN=DCAF13 PE=1 SV=2	51996	435	330	39	124	0.2	0.36	1.8	1	4	3	5	3	6	6	6	0.026651
tr|B4DZR0|B4DZR0_HUMAN	tr|B4DZR0	cDNA FLJ55529, highly similar to Heat shock 70 kDa protein 4L OS=Homo sapiens PE=2 SV=1	98526	191	305	113	136	0.1	0.18	1.8	2	3	2	5	2	4	3	6	-0.01847
sp|Q9NR30|DDX21_HUMAN	DDX21	Nucleolar RNA helicase 2 OS=Homo sapiens GN=DDX21 PE=1 SV=5	87804	148	111	147	328	0.34	0.61	1.7941176	4	12	8	13	8	15	9	20	0.000277
tr|D3DPU2|D3DPU2_HUMAN	CAP1	Adenylyl cyclase-associated protein OS=Homo sapiens GN=CAP1 PE=3 SV=1	51926	172	150	125	274	0.36	0.64	1.7777778	4	7	5	8	6	10	7	10	-0.17483
tr|B4DPP6|B4DPP6_HUMAN	tr|B4DPP6	cDNA FLJ54371, highly similar to Serum albumin OS=Homo sapiens PE=2 SV=1	72370	222	226	93	186	0.25	0.44	1.76	3	7	5	8	6	11	10	14	-0.0434
tr|A0PBY4|A0PBY4_HUMAN	HLA-A	MHC class I antigen (Fragment) OS=Homo sapiens GN=HLA-A PE=3 SV=1	32047	639	505	22	68	0.16	0.28	1.75	0	2	1	2	1	3	1	4	0.35608
tr|B4DHG3|B4DHG3_HUMAN	tr|B4DHG	cDNA FLJ55329, highly similar to Procollagen-lysine,2-oxoglutarate5-dioxygenase 2 (EC 1.14.11.4) OS=Homo sapi	81716	453	679	38	55	0.08	0.14	1.75	0	1	2	2	2	3	2	3	-0.38123
tr|Q53GN4|Q53GN4_HUMAN	WDR1	WD repeat domain 1, isoform CRA_a (Fragment) OS=Homo sapiens GN=WDR1 PE=2 SV=1	66822	137	173	160	242	0.4	0.7	1.75	4	8	7	12	7	15	9	17	0.181194
tr|G5E9Q2|G5E9Q2_HUMAN	CSDE1	Cold shock domain containing E1, RNA-binding, isoform CRA_d OS=Homo sapiens GN=CSDE1 PE=2 SV=1	94539	312	337	58	123	0.11	0.19	1.7272727	1	3	3	5	3	7	3	9	0.23329
sp|Q14247|SRC8_HUMAN	CTTN	Src substrate cortactin OS=Homo sapiens GN=CTTN PE=1 SV=2	61720	378	435	46	94	0.11	0.19	1.7272727	1	1	2	2	3	2	3	2	-1.35261
sp|P55072|TERA_HUMAN	VCP	Transitional endoplasmic reticulum ATPase OS=Homo sapiens GN=VCP PE=1 SV=4	89950	64	57	326	610	0.65	1.12	1.7230769	7	19	14	22	14	25	15	28	-0.05219
tr|D2CFK9|D2CFK9_HUMAN	GTPBP4	Nucleolar GTP-binding protein 1 OS=Homo sapiens GN=GTPBP4 PE=2 SV=1	74317	264	341	72	121	0.14	0.24	1.7142857	2	5	3	5	3	5	3	5	-0.21455
tr|B4DRR0|B4DRR0_HUMAN	tr|B4DRR0	cDNA FLJ53910, highly similar to Keratin, type II cytoskeletal 6A OS=Homo sapiens PE=2 SV=1	58088	92	97	225	379	0.83	1.42	1.7108434	9	14	12	18	15	22	17	24	-0.32989
tr|B2R5U1|B2R5U1_HUMAN	tr|B2R5U1	cDNA, FLJ92620, highly similar to Homo sapiens staphylococcal nuclease domain containing 1 (SND1),mRNA OS=I	100294	150	169	144	246	0.17	0.29	1.7058824	4	5	5	9	10	9	12	11	-1.01267
tr|B4DH02|B4DH02_HUMAN	tr|B4DH02	cDNA FLJ50510, highly similar to Heat shock 70 kDa protein 4 OS=Homo sapiens PE=2 SV=1	95157	123	99	175	376	0.23	0.39	1.6956522	4	8	6	9	5	10	6	10	0.066518
tr|B2R659|B2R659_HUMAN	tr|B2R659	cDNA, FLJ92803, highly similar to Homo sapiens hydroxysteroid (17-beta) dehydrogenase 4 (HSD17B4), mRNA OS	80018	484	284	36	147	0.13	0.22	1.6923077	0	5	3	5	3	5	4	5	-0.21455
tr|J3KPF3|J3KPF3_HUMAN	SLC3A2	4F2 cell-surface antigen heavy chain OS=Homo sapiens GN=SLC3A2 PE=4 SV=1	68230	60	42	366	782	1.33	2.24	1.6842105	12	21	20	27	20	26	20	30	-0.49814
tr|B4DPZ3|B4DPZ3_HUMAN	tr|B4DPZ3	cDNA FLJ53290, highly similar to Cytoplasmic dynein 1 intermediate chain 2 OS=Homo sapiens PE=2 SV=1	70869	298	455	60	89	0.09	0.15	1.6666667	2	2	2	3	2	3	2	3	-0.38123
tr|A2RUM7|A2RUM7_HUMAN	RPL5	Ribosomal protein L5 OS=Homo sapiens GN=RPL5 PE=2 SV=1	34569	187	254	115	167	0.44	0.73	1.6590909	2	4	4	6	5	7	7	7	-0.41947
sp|P15121|ALDR_HUMAN	AKR1B1	Aldose reductase OS=Homo sapiens GN=AKR1B1 PE=1 SV=3	36230	185	512	116	76	0.42	0.69	1.6428571	3	3	4	6	5	7	5	7	-0.41947
tr|Q6NZ55|Q6NZ55_HUMAN	RPL13	60S ribosomal protein L13 OS=Homo sapiens GN=RPL13 PE=2 SV=1	24308	422	1074	41	29	0.14	0.23	1.6428571	1	0	1	1	2	1	2	1	-1.604
sp|P13010|XRCC5_HUMAN	XRCC5	X-ray repair cross-complementing protein 5 OS=Homo sapiens GN=XRCC5 PE=1 SV=3	83222	128	128	172	307	0.36	0.59	1.6388889	6	8	8	12	8	12	9	13	-0.31063
tr|F5CL10|F5CL10_HUMAN	HLA-Cw	MHC class I antigen OS=Homo sapiens GN=HLA-Cw PE=3 SV=1	41120	639	629	22	60	0.16	0.26	1.625	0	2	1	3	1	4	1	5	0.718839
sp|P53396|ACLY_HUMAN	ACLY	ATP-citrate synthase OS=Homo sapiens GN=ACLY PE=1 SV=3	121674	20	18	845	1348	1.28	2.05	1.6015625	21	38	32	48	34	53	40	61	-0.2353
tr|F5H6T1|F5H6T1_HUMAN	ACTR2	Actin-related protein 2 OS=Homo sapiens GN=ACTR2 PE=2 SV=1	34729	244	419	82	99	0.2	0.32	1.6	2	3	2	4	2	4	4	4	-0.01847
tr|B3KNM0|B3KNM0_HUMAN	tr|B3KNM	cDNA FLJ14916 fis, clone PLACE1006958, highly similar to Heat shock 70 kDa protein 4L (Fragment) OS=Homo sa	64588	191	303	113	136	0.1	0.16	1.6	2	3	2	3	2	3	3	4	-0.38123
tr|Q68E00|Q68E00_HUMAN	DKFZp686	Putative uncharacterized protein DKFZp686G2045 (Fragment) OS=Homo sapiens GN=DKFZp686G2045 PE=2 SV=2	32787	485	810	36	41	0.1	0.16	1.6	0	1	1	1	1	1	1	1	-0.86669
tr|A5PHJ9|A5PHJ9_HUMAN	PDHA1/LO	PDHA1/LOC79064 protein (Fragment) OS=Homo sapiens GN=PDHA1/LOC79064 PE=2 SV=1	34113	516	695	32	34	0.1	0.16	1.6	0	1	1	1	1	1	1	1	-0.86669
tr|K9JA46|K9JA46_HUMAN	EL52	Epididymis luminal secretory protein 52 OS=Homo sapiens GN=EL52 PE=2 SV=1	85006	46	41	462	793	1.05	1.67	1.5904762	15	22	25	31	24	34	29	38	-0.37457
tr|B2RCT6|B2RCT6_HUMAN	tr|B2RCT6	cDNA, FLJ96276, highly similar to Homo sapiens G1 to S phase transition 1 (GSPT1), mRNA OS=Homo sapiens PE=	56320	391	878	44	39	0.12	0.19	1.5833333	1	1	2	3	2	3	2	3	-0.38123
tr|Q7Z5T5|Q7Z5T5_HUMAN	EIF3A	EIF3A protein (Fragment) OS=Homo sapiens GN=EIF3A PE=2 SV=1	97203	192	186	112	217	0.14	0.22	1.5714286	2	5	4	10	5	14	8	17	0.532936
sp|P58107|EPIPL_HUMAN	EPPK1	Epiplakin OS=Homo sapiens GN=EPPK1 PE=1 SV=2	557674	56	101	390	376	0.07	0.11	1.5714286	10	11	12	18	12	19	13	21	-0.22553
sp|Q2NL82|TSR1_HUMAN	TSR1	Pre-rRNA-processing protein TSR1 homolog OS=Homo sapiens GN=TSR1 PE=1 SV=1	92151	230	459	90	88	0.07	0.11	1.5714286	2	2	2	3	2	3	2	3	-0.38123
tr|B2RAQ8|B2RAQ8_HUMAN	tr|B2RAQ	cDNA, FLJ95058, highly similar to Homo sapiens carnitine palmitoyltransferase 1A (liver) (CPT1A),nuclear gene er	88967	354	465	49	86	0.07	0.11	1.5714286	1	2	2	3	3	4	3	4	-0.50424
tr|A4QPB0|A4QPB0_HUMAN	IQGAP1	IQ motif containing GTPase activating protein 1 OS=Homo sapiens GN=IQGAP1 PE=2 SV=1	189789	84	60	239	598	0.31	0.48	1.5483871	8	17	17	23	18	23	21	29	-0.52325
tr|G3V2B8|G3V2B8_HUMAN	MTHFD1	C-1-tetrahydrofolate synthase, cytoplasmic OS=Homo sapiens GN=MTHFD1 PE=2 SV=1	102152	112	146	192	280	0.33	0.51	1.5454545	5	11	9	13	12	16	12	17	-0.46711
tr|A8K4D5|A8K4D5_HUMAN	tr|A8K4D5	Kynureninase OS=Homo sapiens PE=2 SV=1	52819	266	532	71	72	0.13	0.2	1.5384615	2	2	2	3	3	3	3	3	-0.867
tr|B4DUL5|B4DUL5_HUMAN	tr|B4DUL5	cDNA FLJ51625, highly similar to Ubiquinol-cytochrome-c reductase complex coreprotein I, mitochondrial (EC 1.1	40917	357	470	48	84	0.17	0.26	1.5294118	1	2	2	3	2	3	2	3	-0.38123
tr|B4DX08|B4DX08_HUMAN	tr|B4DX08	cDNA FLJ58784, highly similar to T-complex protein 1 subunit epsilon OS=Homo sapiens PE=2 SV=1	57521	90	280	229	127	0.4	0.61	1.525	6	2	6	7	6	7	6	9	-0.66083
tr|A8K7F6|A8K7F6_HUMAN	tr|A8K7F6	cDNA FLJ78244, highly similar to Homo sapiens eukaryotic translation initiation factor 4A, isoform 1 (EIF4A1), mR	46321	52	54	404	623	1.62	2.45	1.5123457	10	16	14	18	15	20	20	22	-0.46457
tr|B4DSQ5|B4DSQ5_HUMAN	tr|B4DSQ5	cDNA FLJ53608, highly similar to Protein transport protein Sec23A OS=Homo sapiens PE=2 SV=1	83787	474	522	37	74	0.08	0.12	1.5	0	1	2	3	2	3	3	3	-0.38123
tr|B4E0E1|B4E0E1_HUMAN	tr|B4E0E1	cDNA FLJ53442, highly similar to Poly (ADP-ribose) polymerase 1 (EC 2.4.2.30) OS=Homo sapiens PE=2 SV=1	111855	549	398	30	104	0.06	0.09	1.5	0	2	2	3	2	3	2	6	-0.38123

tr|B3KX72|B3KX72_HUMAN	tr|B3KX72	cDNA FLJ44920 fis, clone BRAMY3011501, highly similar to Heterogeneous nuclear ribonucleoprotein U OS=Hom	83774	59	52	367	534	0.58	0.87	1.5	10	14	14	18	16	21	20	25	-0.48621
sp|Q3SY84|K2C71_HUMAN	KRT71	Keratin, type II cytoskeletal 71 OS=Homo sapiens GN=KRT71 PE=1 SV=3	57769	372	433	47	84	0.12	0.18	1.5	2	3	2	3	3	4	4	4	-0.50424
sp|Q14315|FLNC_HUMAN	FLNC	Filamin-C OS=Homo sapiens GN=FLNC PE=1 SV=3	293407	143	170	153	242	0.06	0.09	1.5	5	10	6	10	10	11	11	14	-0.73665
tr|J3KN66|J3KN66_HUMAN	TOR1AIP1	Torsin-1A-interacting protein 1 OS=Homo sapiens GN=TOR1AIP1 PE=4 SV=1	67952	707	797	17	44	0.1	0.15	1.5	0	1	1	1	0	1	1	1	0.718617
tr|H3BLZ8|H3BLZ8_HUMAN	DDX17	Probable ATP-dependent RNA helicase DDX17 OS=Homo sapiens GN=DDX17 PE=3 SV=1	81073	166	141	130	281	0.37	0.55	1.4864865	3	9	9	12	9	15	10	15	-0.16053
tr|B4DXW6|B4DXW6_HUMAN	tr|B4DXW	cDNA FLJ50285, highly similar to Arginyl-tRNA synthetase (EC 6.1.1.19) OS=Homo sapiens PE=2 SV=1	52771	235	214	88	116	0.35	0.52	1.4857143	3	4	5	5	6	8	7	8	-0.48007
tr|A8K4Z4|A8K4Z4_HUMAN	tr|A8K4Z4	cDNA FLJ75549, highly similar to Homo sapiens ribosomal protein, large, P0 (RPLP0), transcript variant 1, mRNA (	34389	109	246	201	170	0.74	1.09	1.472973	6	7	6	8	6	9	6	11	-0.31941
tr|I3L492|I3L492_HUMAN	ETF1	Eukaryotic peptide chain release factor subunit 1 (Fragment) OS=Homo sapiens GN=ETF1 PE=4 SV=1	23028	274	475	69	64	0.15	0.22	1.4666667	1	1	1	1	1	1	1	1	-0.86669
tr|Q53G58|Q53G58_HUMAN	tr|Q53G5	Coronin (Fragment) OS=Homo sapiens PE=2 SV=1	53931	242	526	83	73	0.13	0.19	1.4615385	1	3	2	3	2	4	4	4	-0.01847
tr|B7ZB91|B7ZB91_HUMAN	TNRC6B	Trinucleotide repeat containing 6B (Fragment) OS=Homo sapiens GN=TNRC6B PE=4 SV=1	81825	359	842	48	34	0.13	0.19	1.4615385	1	1	3	1	2	1	3	1	-1.604
tr|Q5BKZ2|Q5BKZ2_HUMAN	KPNA1	Importin subunit alpha OS=Homo sapiens GN=KPNA1 PE=2 SV=1	60955	573	925	22	37	0.11	0.16	1.4545455	0	1	1	1	1	1	1	2	-0.86669
sp|P26232|CTNA2_HUMAN	CTNNA2	Catenin alpha-2 OS=Homo sapiens GN=CTNNA2 PE=1 SV=5	106045	389	464	31	86	0.07	0.1	1.4285714	0	2	1	3	1	4	1	4	0.718839
sp|P23526|SAHH_HUMAN	AHCY	Adenosylhomocysteinase OS=Homo sapiens GN=AHCY PE=1 SV=4	48255	195	175	111	237	0.49	0.7	1.4285714	2	8	6	8	6	9	8	12	-0.31941
tr|B1ALA9|B1ALA9_HUMAN	PRPS1	Phosphoribosyl pyrophosphate synthetase 1 OS=Homo sapiens GN=PRPS1 PE=3 SV=1	24468	638	621	22	27	0.14	0.2	1.4285714	0	0	1	1	1	1	2	1	-0.86669
sp|P08729|K2C7_HUMAN	KRT7	Keratin, type II cytoskeletal 7 OS=Homo sapiens GN=KRT7 PE=1 SV=5	51411	259	157	74	257	0.45	0.64	1.4222222	2	8	6	8	7	10	9	12	-0.38163
tr|B4DMB5|B4DMB5_HUMAN	tr|B4DMB	cDNA FLJ60075, highly similar to CTP synthase 1 (EC 6.3.4.2) OS=Homo sapiens PE=2 SV=1	56542	449	296	38	133	0.19	0.27	1.4210526	1	4	3	4	3	4	4	5	-0.50424
sp|P00338|LDHA_HUMAN	LDHA	L-lactate dehydrogenase A chain OS=Homo sapiens GN=LDHA PE=1 SV=2	36950	44	67	481	549	2.33	3.3	1.416309	10	16	19	18	19	24	24	24	-0.53924
tr|F8W1U3|F8W1U3_HUMAN	KRT8	Keratin, type II cytoskeletal 8 (Fragment) OS=Homo sapiens GN=KRT8 PE=2 SV=1	32586	58	79	61	54	0.95	1.34	1.4105263	3	2	4	3	6	4	7	4	-1.39836
sp|Q15046|SYK_HUMAN	KARS	Lysine--tRNA ligase OS=Homo sapiens GN=KARS PE=1 SV=3	68461	265	256	71	166	0.32	0.45	1.40625	1	6	6	8	6	8	7	9	-0.48007
tr|B4DFE4|B4DFE4_HUMAN	tr|B4DFE4	cDNA FLJ59618, highly similar to Exosome component 10 OS=Homo sapiens PE=2 SV=1	61733	627	678	23	45	0.05	0.07	1.4	0	1	1	1	1	1	1	1	-0.86669
tr|B4DFK9|B4DFK9_HUMAN	tr|B4DFK9	cDNA FLJ57964, highly similar to Heterogeneous nuclear ribonucleoprotein H~ OS=Homo sapiens PE=2 SV=1	44637	272	244	69	111	0.15	0.21	1.4	1	2	2	2	1	2	2	4	-0.12954
tr|Q53ET2|Q53ET2_HUMAN	tr|Q53ET2	Dihydropyrimidinase-like 2 variant (Fragment) OS=Homo sapiens PE=2 SV=1	62688	342	266	51	137	0.23	0.32	1.3913043	1	4	4	5	3	6	5	6	0.026651
sp|P26599|PTBP1_HUMAN	PTBP1	Polypyrimidine tract-binding protein 1 OS=Homo sapiens GN=PTBP1 PE=1 SV=1	57357	567	270	29	153	0.18	0.25	1.3888889	0	3	3	4	3	5	3	7	-0.21455
sp|P00390|GSHR_HUMAN	GSR	Glutathione reductase, mitochondrial OS=Homo sapiens GN=GSR PE=1 SV=2	56791	313	376	58	110	0.18	0.25	1.3888889	1	3	3	4	4	5	4	5	-0.57746
tr|Q53SS8|Q53SS8_HUMAN	PCBP1	Poly(RC) binding protein 1 OS=Homo sapiens GN=PCBP1 PE=2 SV=1	37987	110	286	197	146	0.29	0.4	1.3793103	3	3	3	4	3	4	3	4	-0.50424
tr|Q5JP02|Q5JP02_HUMAN	RBBP7	Histone-binding protein RBBP7 (Fragment) OS=Homo sapiens GN=RBBP7 PE=4 SV=1	19626	321	601	37	41	0.22	0.3	1.3636364	1	2	1	3	2	3	2	3	-0.38123
tr|B2R9K8|B2R9K8_HUMAN	tr|B2R9K8	cDNA, FLJ94440, highly similar to Homo sapiens chaperonin containing TCP1, subunit 6A (zeta 1)(CCT6A), mRNA (	58386	118	234	181	181	0.47	0.64	1.3617021	5	4	7	9	9	13	10	14	-0.36022
sp|P46063|RECQ1_HUMAN	RECQL	ATP-dependent DNA helicase Q1 OS=Homo sapiens GN=RECQL PE=1 SV=3	74436	439	312	39	132	0.14	0.19	1.3571429	1	3	3	4	3	5	3	7	-0.21455
tr|Q9UJZ2|Q9UJZ2_HUMAN	VIL2	Cytovillin 2 (Fragment) OS=Homo sapiens GN=VIL2 PE=2 SV=1	18464	119	319	88	90	0.46	0.62	1.3478261	2	3	2	3	2	3	2	4	-0.38123
tr|B1AHE4|B1AHE4_HUMAN	ATXN10	Ataxin 10 (Fragment) OS=Homo sapiens GN=ATXN10 PE=4 SV=1	22299	470	289	37	52	0.38	0.51	1.3421053	0	1	2	1	2	2	2	3	-0.86684
tr|A8K6Q8|A8K6Q8_HUMAN	tr|A8K6Q	cDNA FLJ75881, highly similar to Homo sapiens transferrin receptor (p90, CD71) (TFRC), mRNA OS=Homo sapiens	85276	122	131	176	300	0.3	0.4	1.3333333	6	8	7	9	7	10	8	11	-0.38163
tr|H7C1U0|H7C1U0_HUMAN	APEH	Acylamino-acid-releasing enzyme (Fragment) OS=Homo sapiens GN=APEH PE=4 SV=1	35132	319	325	56	49	0.09	0.12	1.3333333	1	1	1	1	1	1	1	1	-0.86669
tr|H6VRF8|H6VRF8_HUMAN	KRT1	Keratin 1 OS=Homo sapiens GN=KRT1 PE=3 SV=1	66184	6	14	2032	1558	5.01	6.66	1.3293413	55	46	64	55	69	61	76	66	-1.05532
tr|B4DY32|B4DY32_HUMAN	tr|B4DY32	Asparagine synthetase OS=Homo sapiens PE=2 SV=1	55055	141	408	156	101	0.26	0.34	1.3076923	3	3	5	5	6	6	6	7	-0.86747
tr|D6RF19|D6RF19_HUMAN	DPYSL3	Dihydropyrimidinase-related protein 3 (Fragment) OS=Homo sapiens GN=DPYSL3 PE=2 SV=1	10214	342	285	20	0	0.23	0.3	1.3043478	0	0	1	0	1	0	1	1	-2.45184
sp|P23381|SYWC_HUMAN	WARS	Tryptophan--tRNA ligase, cytoplasmic OS=Homo sapiens GN=WARS PE=1 SV=2	53474	145	236	148	177	0.27	0.35	1.2962963	3	5	4	5	5	6	5	7	-0.62611
tr|B4DRH6|B4DRH6_HUMAN	tr|B4DRH	cDNA FLJ54509, highly similar to Trifunctional enzyme subunit alpha, mitochondrial OS=Homo sapiens PE=2 SV=1	79007	133	265	164	158	0.28	0.36	1.2857143	5	4	6	7	7	10	7	10	-0.38163
tr|B4DGQ0|B4DGQ0_HUMAN	LASP1	LIM and SH3 domain protein 1 OS=Homo sapiens GN=LASP1 PE=2 SV=1	23167	568	1075	29	29	0.14	0.18	1.2857143	0	0	1	1	1	1	1	3	-0.86669
tr|F5H7Y0|F5H7Y0_HUMAN	PDLIM5	PDZ and LIM domain protein 5 OS=Homo sapiens GN=PDLIM5 PE=2 SV=1	52332	175	251	109	146	0.41	0.52	1.2682927	2	3	5	5	5	5	6	6	-0.86731
tr|H6VRG2|H6VRG2_HUMAN	KRT1	Keratin 1 OS=Homo sapiens GN=KRT1 PE=3 SV=1	66197	7	15	2027	1525	5.01	6.3	1.257485	55	46	65	54	70	59	77	64	-1.12435
sp|O76094|SRP72_HUMAN	SRP72	Signal recognition particle 72 kDa protein OS=Homo sapiens GN=SRP72 PE=1 SV=3	75130	455	552	38	70	0.04	0.05	1.25	1	1	1	1	1	2	1	3	-0.12954
sp|Q12792|TWF1_HUMAN	TWF1	Twinfilin-1 OS=Homo sapiens GN=TWF1 PE=1 SV=3	40429	575	669	28	56	0.08	0.1	1.25	0	1	1	1	1	1	1	1	-0.86669
tr|A5PLK7|A5PLK7_HUMAN	RCC2	RCC2 protein (Fragment) OS=Homo sapiens GN=RCC2 PE=2 SV=1	50331	165	183	131	191	0.46	0.57	1.2391304	4	6	6	7	6	10	6	13	-0.17483
tr|Q53GX7|Q53GX7_HUMAN	tr|Q53GX	Threonyl-tRNA synthetase variant (Fragment) OS=Homo sapiens PE=2 SV=1	84304	94	148	221	276	0.64	0.79	1.234375	6	11	13	19	17	27	18	32	-0.21521
sp|P52895|AK1C2_HUMAN	AKR1C2	Aldo-keto reductase family 1 member C2 OS=Homo sapiens GN=AKR1C2 PE=1 SV=3	37111	186	255	116	167	0.67	0.82	1.2238806	3	4	6	7	7	7	9	8	-0.86762
tr|B3KW67|B3KW67_HUMAN	tr|B3KW6	cDNA FLJ42347 fis, clone UTERU2003399, highly similar to Actin, gamma-enteric smooth muscle OS=Homo sapie	42189	38	36	538	872	2.61	3.19	1.2222222	19	27	27	37	32	46	37	49	-0.35208
sp|P68032|ACTC_HUMAN	ACTC1	Actin, alpha cardiac muscle 1 OS=Homo sapiens GN=ACTC1 PE=1 SV=1	42334	23	27	771	1109	6.63	7.86	1.1855204	25	37	40	52	48	64	55	66	-0.45969
tr|Q2VPA0|Q2VPA0_HUMAN	LONP1	Lon protease homolog (Fragment) OS=Homo sapiens GN=LONP1 PE=2 SV=1	95436	414	857	42	40	0.11	0.13	1.1818182	2	0	3	4	3	4	3	5	-0.50424
tr|B4DP08|B4DP08_HUMAN	ALG1	Chitobiosyldiphosphodolichol beta-mannosyltransferase OS=Homo sapiens GN=ALG1 PE=2 SV=1	40938	452	617	38	56	0.17	0.2	1.1764706	0	1	2	2	2	2	2	3	-0.86684
sp|Q96HC4|PDLI5_HUMAN	PDLIM5	PDZ and LIM domain protein 5 OS=Homo sapiens GN=PDLIM5 PE=1 SV=5	65102	175	253	123	168	0.41	0.48	1.1707317	3	4	7	8	7	8	8	9	-0.68686
sp|Q9BXP5|SRRT_HUMAN	SRRT	Serrate RNA effector molecule homolog OS=Homo sapiens GN=SRRT PE=1 SV=1	101060	701	599	18	63	0.06	0.07	1.1666667	0	1	1	2	0	2	1	2	1.455771
tr|B7Z8A2|B7Z8A2_HUMAN	tr|B7Z8A2	cDNA FLJ51671, highly similar to Prenylcysteine oxidase (EC 1.8.3.5) OS=Homo sapiens PE=2 SV=1	54918	355	947	49	35	0.06	0.07	1.1666667	1	1	1	1	1	1	1	2	-0.86669
tr|B3KTJ9|B3KTJ9_HUMAN	tr|B3KTJ9	cDNA FLJ38393 fis, clone FEBRA2007212 OS=Homo sapiens PE=2 SV=1	103493	82	136	251	288	0.32	0.37	1.15625	7	6	9	10	9	11	12	14	-0.59192
tr|L0R4T3|L0R4T3_HUMAN	ABCF2	Alternative protein ABCF2 OS=Homo sapiens GN=ABCF2 PE=4 SV=1	7359	524	856	31	34	0.48	0.55	1.1458333	1	1	1	1	1	1	1	2	-0.86669
tr|A8K525|A8K525_HUMAN	tr|A8K525	cDNA FLJ76817, highly similar to Homo sapiens non-POU domain containing, octamer-binding (NONO), mRNA OS	54367	75	132	266	294	0.8	0.91	1.1375	10	7	11	11	12	13	14	14	-0.75718
tr|B4DF68|B4DF68_HUMAN	tr|B4DF68	cDNA FLJ55325, highly similar to Heat-shock protein 105 kDa OS=Homo sapiens PE=2 SV=1	86500	123	353	47	116	0.23	0.26	1.1304348	1	3	1	6	1	6	1	6	1.24973
sp|P16615|AT2A2_HUMAN	ATP2A2	Sarcoplasmic/endoplasmic reticulum calcium ATPase 2 OS=Homo sapiens GN=ATP2A2 PE=1 SV=1	116336	149	106	145	352	0.32	0.36	1.125	4	10	10	11	12	16	13	17	-0.46711
sp|Q9P287|BCCIP_HUMAN	BCCIP	BRCA2 and CDKN1A-interacting protein OS=Homo sapiens GN=BCCIP PE=1 SV=1	36299	503	946	33	35	0.09	0.1	1.1111111	0	1	1	1	1	1	3	1	-0.86669
tr|B3KX19|B3KX19_HUMAN	tr|B3KX19	cDNA FLJ44500 fis, clone UTERU3000828, highly similar to 116 kDa U5 small nuclear ribonucleoprotein compone	109111	111	316	193	131	0.27	0.3	1.1111111	4	4	8	10	8	11	10	12	-0.43111
sp|P13647|K2C5_HUMAN	KRT5	Keratin, type II cytoskeletal 5 OS=Homo sapiens GN=KRT5 PE=1 SV=3	62568	58	125	378	310	0.95	1.05	1.1052632	13	13	15	15	20	18	24	19	-1.01812
tr|H0YH88|H0YH88_HUMAN	NAP1L1	Nucleosome assembly protein 1-like 1 (Fragment) OS=Homo sapiens GN=NAP1L1 PE=3 SV=1	21179	417	904	33	38	0.16	0.17	1.0625	0	0	1	2	1	4	1	4	0.718839
tr|B0YJC4|B0YJC4_HUMAN	VIM	Vimentin OS=Homo sapiens GN=VIM PE=3 SV=1	49680	66	172	312	210	1.03	1.04	1.0097087	9	6	11	11	12	12	15	12	-0.8684
tr|J3KMW7|J3KMW7_HUMAN	UBR5	E3 ubiquitin-protein ligase UBR5 OS=Homo sapiens GN=UBR5 PE=4 SV=1	312224	706	1088	18	28	0.01	0.01	1	0	0	1	1	0	1	1	2	0.718617
tr|J3KR97|J3KR97_HUMAN	TBCD	Tubulin-specific chaperone D OS=Homo sapiens GN=TBCD PE=4 SV=1	138346	714	841	15	41	0.02	0.02	1	0	1	1	1	0	1	1	1	0.718617
sp|O95782|AP2A1_HUMAN	AP2A1	AP-2 complex subunit alpha-1 OS=Homo sapiens GN=AP2A1 PE=1 SV=3	108561	500	807	34	43	0.03	0.03	1	0	1	1	1	1	3	2	3	0.35608
tr|F4ZW63|F4ZW63_HUMAN	tr|F4ZW6	NF45 OS=Homo sapiens PE=2 SV=1	43205	383	663	45	56	0.08	0.08	1	1	1	1	1	1	3	1	4	0.35608
tr|Q2XPP3|Q2XPP3_HUMAN	tr|Q2XPP3	Type II 3a-hydroxysteroid dehydrogenase variant OS=Homo sapiens PE=2 SV=1	37201	203	520	102	74	0.29	0.29	1	3	2	3	3	3	6	4	9	0.026651
sp|Q14432|PDE3A_HUMAN	PDE3A	cGMP-inhibited 3~,5~-cyclic phosphodiesterase A OS=Homo sapiens GN=PDE3A PE=1 SV=3	126440	493	463	35	86	0.05	0.05	1	1	2	2	2	2	4	2	6	-0.01847
sp|O95831|AIFM1_HUMAN	AIFM1	Apoptosis-inducing factor 1, mitochondrial OS=Homo sapiens GN=AIFM1 PE=1 SV=1	67144	326	1085	54	28	0.05	0.05	1	1	0	1	1	1	2	2	3	-0.12954
tr|Q8TC04|Q8TC04_HUMAN	KRT23	Keratin 23 (Histone deacetylase inducible) OS=Homo sapiens GN=KRT23 PE=2 SV=1	48318	600	1000	26	34	0.07	0.07	1	0	1	1	1	1	2	1	2	-0.12954
sp|O00232|PSD12_HUMAN	PSMD12	26S proteasome non-ATPase regulatory subunit 12 OS=Homo sapiens GN=PSMD12 PE=1 SV=3	53270	283	907	66	38	0.06	0.06	1	1	1	1	1	1	2	1	2	-0.12954
sp|Q9NSB4|KRT82_HUMAN	KRT82	Keratin, type II cuticular Hb2 OS=Homo sapiens GN=KRT82 PE=1 SV=3	57985	411	792	43	44	0.06	0.06	1	1	1	1	1	1	2	2	3	-0.12954
tr|D3DTW3|D3DTW3_HUMAN	IGF2BP1	Insulin-like growth factor 2 mRNA binding protein 1, isoform CRA_a OS=Homo sapiens GN=IGF2BP1 PE=2 SV=1	48111	556	744	29	48	0.05	0.05	1	0	1	1	1	1	2	1	2	-0.12954
sp|Q6KB66|K2C80_HUMAN	KRT80	Keratin, type II cytoskeletal 80 OS=Homo sapiens GN=KRT80 PE=1 SV=2	51007	564	691	29	53	0.06	0.06	1	0	1	1	1	1	2	1	3	-0.12954
tr|Q562M3|Q562M3_HUMAN	ACT	Actin-like protein (Fragment) OS=Homo sapiens GN=ACT PE=2 SV=1	11529	103	78	213	457	7.06	7.06	1	10	16	14	23	17	26	20	30	-0.26884
tr|Q13727|Q13727_HUMAN	tr|Q13727	AHNAK-related protein (Fragment) OS=Homo sapiens PE=2 SV=1	57703	402	1159	43	23	0.06	0.06	1	1	0	1	1	2	3	3	3	-0.38123
tr|Q5T081|Q5T081_HUMAN	RCC1	CHC1 protein OS=Homo sapiens GN=RCC1 PE=2 SV=1	45397	269	824	70	42	0.23	0.23	1	2	0	3	3	2	3	4	4	-0.38123
tr|L0R5A1|L0R5A1_HUMAN	CSF2RB	Alternative protein CSF2RB OS=Homo sapiens GN=CSF2RB PE=4 SV=1	11752	486	911	36	37	0.29	0.29	1	1	2	1	2	3	4	5	4	-0.50424
tr|J3KTA4|J3KTA4_HUMAN	DDX5	Probable ATP-dependent RNA helicase DDX5 OS=Homo sapiens GN=DDX5 PE=3 SV=1	69557	97	209	218	197	0.45	0.45	1	7	8	9	9	10	13	10	15	-0.50495
tr|H0Y804|H0Y804_HUMAN	AKR1C1	Aldo-keto reductase family 1 member C1 (Fragment) OS=Homo sapiens GN=AKR1C1 PE=4 SV=1	28617	186	223	61	124	0.67	0.67	1	2	2	4	4	4	5	6	7	-0.57746
tr|G8JLB6|G8JLB6_HUMAN	HNRNPH1	Heterogeneous nuclear ribonucleoprotein H OS=Homo sapiens GN=HNRNPH1 PE=4 SV=1	51482	207	197	101	205	0.45	0.45	1	2	4	6	6	5	6	6	8	-0.62611
tr|B3KVL0|B3KVL0_HUMAN	tr|B3KVL0	cDNA FLJ16691 fis, clone TRACH3000692, highly similar to Paxillin OS=Homo sapiens PE=2 SV=1	65600	159	276	133	150	0.28	0.28	1	3	4	5	5	6	7	6	8	-0.66083
tr|H0YK49|H0YK49_HUMAN	ETFA	Electron transfer flavoprotein subunit alpha, mitochondrial OS=Homo sapiens GN=ETFA PE=4 SV=1	24259	545	1196	30	21	0.14	0.14	1	0	0	1	1	1	1	1	2	-0.86669
sp|Q96EK5|KBP_HUMAN	KIAA1279	KIF1-binding protein OS=Homo sapiens GN=KIAA1279 PE=1 SV=1	72453	649	1143	22	25	0.05	0.05	1	0	0	1	1	1	1	1	1	-0.86669
tr|K7EKM4|K7EKM4_HUMAN	SRM	Spermidine synthase OS=Homo sapiens GN=SRM PE=4 SV=1	10680	540	1128	30	26	0.32	0.32	1	0	0	1	1	1	1	1	1	-0.86669
tr|B3KY60|B3KY60_HUMAN	tr|B3KY60	cDNA FLJ16777 fis, clone BRHIP2029567, highly similar to Cell division cycle 5-like protein OS=Homo sapiens PE=2	92448	587	1123	27	26	0.04	0.04	1	0	0	1	1	1	1	1	3	-0.86669
tr|B4DPY2|B4DPY2_HUMAN	tr|B4DPY2	cDNA FLJ59286, highly similar to Pre-mRNA-processing factor 40 homolog A (Fragment) OS=Homo sapiens PE=2 S	93124	425	1087	41	28	0.04	0.04	1	1	0	1	1	1	1	1	2	-0.86669
tr|Q5JR94|Q5JR94_HUMAN	RPS8	40S ribosomal protein S8 OS=Homo sapiens GN=RPS8 PE=2 SV=1	24475	426	1067	41	29	0.14	0.14	1	1	0	1	1	1	1	2	1	-0.86669
tr|B1AMS2|B1AMS2_HUMAN	Sep-06	Septin 6 OS=Homo sapiens GN=SEPT6 PE=3 SV=1	49614	532	1048	31	31	0.07	0.07	1	1	1	1	1	1	1	1	1	-0.86669
sp|Q6IBS0|TWF2_HUMAN	TWF2	Twinfilin-2 OS=Homo sapiens GN=TWF2 PE=1 SV=2	39751	535	1026	30	32	0.08	0.08	1	0	0	1	1	1	1	1	1	-0.86669
tr|F8WJN3|F8WJN3_HUMAN	CPSF6	Cleavage and polyadenylation-specificity factor subunit 6 OS=Homo sapiens GN=CPSF6 PE=2 SV=1	52409	379	1015	46	32	0.06	0.06	1	1	0	1	1	1	1	1	2	-0.86669
tr|B2R4C0|B2R4C0_HUMAN	tr|B2R4C0	60S ribosomal protein L18a OS=Homo sapiens PE=2 SV=1	21024	514	1005	32	33	0.16	0.16	1	0	0	1	1	1	1	2	2	-0.86669
tr|B7Z438|B7Z438_HUMAN	tr|B7Z438	cDNA FLJ56352, highly similar to Succinyl-CoA ligase (GDP-forming) subunit alpha, mitochondrial (EC 6.2.1.4) OS=	26801	467	981	37	34	0.12	0.12	1	1	1	1	1	1	1	1	2	-0.86669
tr|A8KAK1|A8KAK1_HUMAN	tr|A8KAK1	cDNA FLJ77398, highly similar to Homo sapiens UDP-glucose ceramide glucosyltransferase-like 1, transcript varia	175467	635	975	22	34	0.02	0.02	1	0	1	1	1	1	1	1	1	-0.86669
tr|B4DXC4|B4DXC4_HUMAN	tr|B4DXC4	cDNA FLJ58636, moderately similar to Atlastin OS=Homo sapiens PE=2 SV=1	59162	451	932	38	36	0.06	0.06	1	1	1	1	1	1	1	1	1	-0.86669
tr|B2RDF5|B2RDF5_HUMAN	tr|B2RDF5	cDNA, FLJ96587, highly similar to Homo sapiens SUMO-1 activating enzyme subunit 2 (UBA2), mRNA OS=Homo s	71705	590	902	27	38	0.05	0.05	1	0	1	1	1	1	1	1	1	-0.86669
tr|B4DRG7|B4DRG7_HUMAN	tr|B4DRG7	cDNA FLJ60336, highly similar to Condensin complex subunit 2 OS=Homo sapiens PE=2 SV=1	80877	410	859	43	40	0.04	0.04	1	1	1	1	1	1	1	1	1	-0.86669
tr|B3KNM6|B3KNM6_HUMAN	tr|B3KNM	cDNA FLJ14968 fis, clone THYRO1000288, highly similar to CAAX prenyl protease 1 homolog (EC 3.4.24.84) OS=Ho	27279	547	829	30	37	0.12	0.12	1	1	1	1	1	1	1	1	1	-0.86669
sp|Q9H9F9|ARP5_HUMAN	ACTR5	Actin-related protein 5 OS=Homo sapiens GN=ACTR5 PE=1 SV=2	68825	460	795	38	44	0.05	0.05	1	1	1	1	1	1	1	2	1	-0.86669
tr|H0Y9L5|H0Y9L5_HUMAN	EXOSC9	Exosome complex component RRP45 (Fragment) OS=Homo sapiens GN=EXOSC9 PE=4 SV=1	18940	457	790	38	44	0.18	0.18	1	1	1	1	1	1	1	1	1	-0.86669
tr|Q0P512|Q0P512_HUMAN	SLC2A1	Solute carrier family 2 (Facilitated glucose transporter), member 1 OS=Homo sapiens GN=SLC2A1 PE=2 SV=1	54361	377	784	46	45	0.06	0.06	1	1	1	1	1	1	1	1	2	-0.86669
tr|B4DT20|B4DT20_HUMAN	tr|B4DT20	cDNA FLJ59418, highly similar to Mitochondrial inner membrane protein OS=Homo sapiens PE=2 SV=1	73510	712	778	17	45	0.04	0.04	1	0	1	1	1	1	1	6	4	-0.86669
tr|H0Y5J4|H0Y5J4_HUMAN	DBNL	Drebrin-like protein (Fragment) OS=Homo sapiens GN=DBNL PE=4 SV=1	40475	466	761	37	46	0.08	0.08	1	1	1	1	1	1	1	1	1	-0.86669
tr|B4DG42|B4DG42_HUMAN	tr|B4DG42	cDNA FLJ53753, highly similar to Myeloid-associated differentiation marker OS=Homo sapiens PE=2 SV=1	31889	609	732	25	49	0.1	0.1	1	0	1	1	1	1	1	1	1	-0.86669
tr|Q2NKW8|Q2NKW8_HUMAN	AHCYL1	Adenosylhomocysteinase (Fragment) OS=Homo sapiens GN=AHCYL1 PE=2 SV=1	57630	509	716	33	51	0.06	0.06	1	0	1	1	1	1	1	1	1	-0.86669
tr|B3KXU8|B3KXU8_HUMAN	tr|B3KXU8	cDNA FLJ46087 fis, clone TESTI2009497, highly similar to GPI-anchored protein p137 OS=Homo sapiens PE=2 SV=	70489	367	708	47	52	0.05	0.05	1	1	1	1	1	1	1	1	1	-0.86669
tr|B3KUJ0|B3KUJ0_HUMAN	tr|B3KUJ0	cDNA FLJ39996 fis, clone STOMA2002166, highly similar to Splicing factor 3B subunit 4 OS=Homo sapiens PE=2 S\	44512	464	613	37	62	0.07	0.07	1	1	1	1	1	1	1	2	1	-0.86669
tr|B3KSQ7|B3KSQ7_HUMAN	DBN1	Drebrin 1, isoform CRA_d OS=Homo sapiens GN=DBN1 PE=2 SV=1	66053	478	605	36	62	0.05	0.05	1	0	1	1	1	1	1	1	1	-0.86669
tr|B4DLD4|B4DLD4_HUMAN	tr|B4DLD4	cDNA FLJ56738, highly similar to Fibrillarin OS=Homo sapiens PE=2 SV=1	30279	423	575	41	66	0.11	0.11	1	1	1	1	1	1	1	1	1	-0.86669
tr|J3KTE4|J3KTE4_HUMAN	RPL19	Ribosomal protein L19 OS=Homo sapiens GN=RPL19 PE=3 SV=1	23347	336	527	52	73	0.14	0.14	1	1	1	1	1	1	1	1	1	-0.86669
tr|Q58FF4|Q58FF4_HUMAN	HSP90Bf	Heat shock protein 90Bf OS=Homo sapiens GN=HSP90Bf PE=1 SV=1	41833	407	953	43	35	0.16	0.16	1	1	0	2	2	2	2	2	2	-0.86684
sp|Q96SI9|STRBP_HUMAN	STRBP	Spermatid perinuclear RNA-binding protein OS=Homo sapiens GN=STRBP PE=1 SV=1	74290	471	855	37	40	0.09	0.09	1	0	1	2	2	2	2	2	3	-0.86684
tr|B7Z601|B7Z601_HUMAN	tr|B7Z601	cDNA FLJ57187, highly similar to Glycerol-3-phosphate dehydrogenase, mitochondrial (EC 1.1.99.5) OS=Homo sa	56047	512	568	32	66	0.06	0.06	1	0	1	1	1	2	2	2	3	-0.86684
tr|B9A041|B9A041_HUMAN	MDH1	Malate dehydrogenase, cytoplasmic OS=Homo sapiens GN=MDH1 PE=2 SV=1	23195	309	461	58	87	0.31	0.31	1	2	2	2	2	2	2	2	3	-0.86684
tr|Q0IJ56|Q0IJ56_HUMAN	ST13	ST13 protein (Fragment) OS=Homo sapiens GN=ST13 PE=2 SV=1	35164	382	442	46	92	0.09	0.09	1	1	1	1	1	2	2	3	2	-0.86684
tr|B3KY95|B3KY95_HUMAN	tr|B3KY95	cDNA FLJ16143 fis, clone BRAMY2038516, highly similar to Protein disulfide-isomerase A6 (EC 5.3.4.1) OS=Homo	53655	445	441	38	92	0.13	0.13	1	0	1	2	2	2	2	2	3	-0.86684
sp|Q9BYZ2|LDH6B_HUMAN	LDHAL6B	L-lactate dehydrogenase A-like 6B OS=Homo sapiens GN=LDHAL6B PE=1 SV=3	42372	327	318	54	130	0.16	0.16	1	1	2	2	2	2	2	2	3	-0.86684
tr|Q05DV5|Q05DV5_HUMAN	ZC3HAV1	ZC3HAV1 protein (Fragment) OS=Homo sapiens GN=ZC3HAV1 PE=2 SV=1	49964	224	238	92	177	0.14	0.14	1	1	2	2	2	2	2	2	3	-0.86684
sp|Q92621|NU205_HUMAN	NUP205	Nuclear pore complex protein Nup205 OS=Homo sapiens GN=NUP205 PE=1 SV=3	230171	415	729	42	50	0.04	0.04	1	0	2	3	3	3	3	5	4	-0.867
tr|B2R5W2|B2R5W2_HUMAN	tr|B2R5W	cDNA, FLJ92657, highly similar to Homo sapiens heterogeneous nuclear ribonucleoprotein C (C1/C2) (HNRPC), tra	31986	178	529	122	73	0.35	0.35	1	3	3	3	3	3	3	4	6	-0.867
tr|H0YAF8|H0YAF8_HUMAN	GNB2L1	Guanine nucleotide-binding protein subunit beta-2-like 1 (Fragment) OS=Homo sapiens GN=GNB2L1 PE=4 SV=1	22373	216	479	96	79	0.52	0.52	1	2	2	3	3	3	3	4	3	-0.867
tr|G3V0E8|G3V0E8_HUMAN	PCBP2	Poly(RC) binding protein 2, isoform CRA_f OS=Homo sapiens GN=PCBP2 PE=2 SV=1	33817	132	339	166	123	0.32	0.32	1	3	2	3	3	3	3	5	5	-0.867
sp|Q14CN4|K2C72_HUMAN	KRT72	Keratin, type II cytoskeletal 72 OS=Homo sapiens GN=KRT72 PE=1 SV=2	56470	372	473	47	84	0.12	0.12	1	2	3	2	3	4	4	5	5	-0.86715
tr|B4DKM5|B4DKM5_HUMAN	VDAC2	Voltage-dependent anion-selective channel protein 2 OS=Homo sapiens GN=VDAC2 PE=2 SV=1	27804	196	405	108	102	0.76	0.76	1	1	3	5	5	5	5	5	5	-0.86731
sp|Q92973|TNPO1_HUMAN	TNPO1	Transportin-1 OS=Homo sapiens GN=TNPO1 PE=1 SV=2	103771	139	402	158	103	0.17	0.17	1	3	3	5	5	8	8	8	10	-0.86778
sp|P78371|TCPB_HUMAN	CCT2	T-complex protein 1 subunit beta OS=Homo sapiens GN=CCT2 PE=1 SV=4	57794	37	89	563	405	1.86	1.86	1	14	13	20	19	20	20	23	21	-0.86965
tr|Q0IIN1|Q0IIN1_HUMAN	KRT77	Keratin 77 OS=Homo sapiens GN=KRT77 PE=2 SV=1	62050	76	152	264	268	0.23	0.23	1	10	8	10	8	10	9	12	9	-1.01267
tr|B3KPL5|B3KPL5_HUMAN	tr|B3KPL5	cDNA FLJ31917 fis, clone NT2RP7004925, highly similar to Sprouty-related, EVH1 domain-containing protein 2 OS	48711	657	1192	21	22	0.07	0.07	1	0	0	1	1	16	14	51	38	-1.05581
tr|B2RCM3|B2RCM3_HUMAN	tr|B2RCM	cDNA, FLJ96158, highly similar to Homo sapiens calpain 2, (m/II) large subunit (CAPN2), mRNA OS=Homo sapiens	80883	173	426	125	97	0.22	0.22	1	3	2	7	5	7	6	8	7	-1.07426
sp|P46087|NOP2_HUMAN	NOP2	Putative ribosomal RNA methyltransferase NOP2 OS=Homo sapiens GN=NOP2 PE=1 SV=2	89589	296	336	61	123	0.15	0.15	1	2	4	4	4	5	4	6	4	-1.15701
tr|B7Z2Z8|B7Z2Z8_HUMAN	tr|B7Z2Z8	T-complex protein 1 subunit delta OS=Homo sapiens PE=2 SV=1	52781	113	384	188	107	0.27	0.27	1	4	2	5	4	7	5	7	8	-1.31546
sp|Q9NRF8|PYRG2_HUMAN	CTPS2	CTP synthase 2 OS=Homo sapiens GN=CTPS2 PE=1 SV=1	66320	614	746	24	48	0.05	0.05	1	0	1	1	1	2	1	6	4	-1.604
tr|B4DII5|B4DII5_HUMAN	tr|B4DII5	Importin subunit alpha OS=Homo sapiens PE=2 SV=1	60345	573	671	28	56	0.11	0.11	1	0	1	2	2	3	1	3	3	-2.08977
tr|A0N4V7|A0N4V7_HUMAN	Tcr-alpha	HCG2039797 (Fragment) OS=Homo sapiens GN=Tcr-alpha PE=4 SV=1	2269	212	643	98	59	1.81	1.81	1	5	4	15	8	31	13	46	20	-2.09716
sp|Q96RW7|HMCN1_HUMAN	HMCN1	Hemicentin-1 OS=Homo sapiens GN=HMCN1 PE=1 SV=2	623265	291	883	63	39	0.01	0.01	1	7	1	7	1	11	4	16	5	-2.2232
tr|J3KQE5|J3KQE5_HUMAN	RAN	GTP-binding nuclear protein Ran (Fragment) OS=Homo sapiens GN=RAN PE=4 SV=1	27027	571	1222	29	17	0.12	0.12	1	0	0	1	1	1	0	1	1	-2.45184
tr|H0Y7S3|H0Y7S3_HUMAN	ATP2B2	Plasma membrane calcium-transporting ATPase 2 (Fragment) OS=Homo sapiens GN=ATP2B2 PE=3 SV=1	122272	528	779	31	0	0.03	0.03	1	0	0	1	0	1	0	3	1	-2.45184
sp|P68366|TBA4A_HUMAN	TUBA4A	Tubulin alpha-4A chain OS=Homo sapiens GN=TUBA4A PE=1 SV=1	50634	22	39	796	827	3.01	2.93	0.9734219	20	24	31	30	31	37	36	45	-0.61869
tr|Q5HYG8|Q5HYG8_HUMAN	DKFZp686	Putative uncharacterized protein DKFZp686P09201 OS=Homo sapiens GN=DKFZp686P09201 PE=2 SV=1	53831	86	160	235	253	0.61	0.57	0.9344262	4	7	8	8	9	8	10	11	-1.02859
sp|Q6S8J3|POTEE_HUMAN	POTEE	POTE ankyrin domain family member E OS=Homo sapiens GN=POTEE PE=1 SV=3	122882	5	59	2211	602	0.44	0.41	0.9318182	45	19	66	27	70	29	75	31	-2.14221
tr|H0Y5G9|H0Y5G9_HUMAN	NRD1	Nardilysin (Fragment) OS=Homo sapiens GN=NRD1 PE=3 SV=1	62196	225	478	92	41	0.11	0.1	0.9090909	2	1	2	2	2	3	2	4	-0.38123
tr|B3KM80|B3KM80_HUMAN	NCL	Nucleolin, isoform CRA_c OS=Homo sapiens GN=NCL PE=2 SV=1	58576	71	138	283	286	1.03	0.93	0.9029126	9	8	14	12	15	13	15	15	-1.06855
sp|P08779|K1C16_HUMAN	KRT16	Keratin, type I cytoskeletal 16 OS=Homo sapiens GN=KRT16 PE=1 SV=4	51578	62	94	342	386	1.38	1.24	0.8985507	14	12	17	13	21	19	21	21	-1.01104
tr|B4DTG2|B4DTG2_HUMAN	EEF1G	Elongation factor 1-gamma OS=Homo sapiens GN=EEF1G PE=2 SV=1	56456	93	129	225	306	0.86	0.76	0.8837209	5	9	15	13	14	14	18	21	-0.86871
sp|Q05639|EF1A2_HUMAN	EEF1A2	Elongation factor 1-alpha 2 OS=Homo sapiens GN=EEF1A2 PE=1 SV=1	50780	70	142	238	281	1.14	1	0.877193	8	8	9	12	12	15	14	17	-0.55749
tr|E9PBU3|E9PBU3_HUMAN	ATIC	Phosphoribosylaminoimidazolecarboxamide formyltransferase OS=Homo sapiens GN=ATIC PE=2 SV=1	64996	72	178	274	197	0.64	0.56	0.875	8	6	11	8	12	12	13	14	-0.8684
tr|F4ZW64|F4ZW64_HUMAN	tr|F4ZW6	NF90a OS=Homo sapiens PE=2 SV=1	76369	154	181	137	226	0.46	0.4	0.8695652	3	8	9	10	10	13	11	14	-0.50495
tr|Q6I9U3|Q6I9U3_HUMAN	M6PR	M6PR protein OS=Homo sapiens GN=M6PR PE=2 SV=1	31469	416	836	42	41	0.29	0.25	0.862069	1	1	1	1	1	1	1	1	-0.86669
tr|Q1KLZ0|Q1KLZ0_HUMAN	PS1TP5BP	HCG15971, isoform CRA_a OS=Homo sapiens GN=PS1TP5BP1 PE=2 SV=1	42052	1	8	3367	2076	31.43	26.87	0.8549157	82	62	119	83	130	94	141	101	-1.35973
tr|F5H1A7|F5H1A7_HUMAN	RDX	Radixin OS=Homo sapiens GN=RDX PE=2 SV=1	52703	119	269	88	145	0.46	0.39	0.8478261	2	5	2	7	2	8	2	9	0.899821
tr|B4DW52|B4DW52_HUMAN	tr|B4DW5	cDNA FLJ55253, highly similar to Actin, cytoplasmic 1 OS=Homo sapiens PE=2 SV=1	38950	4	10	3120	1785	27.29	23.03	0.8438989	74	56	108	70	123	83	134	91	-1.45835
tr|K7EK77|K7EK77_HUMAN	ATP5A1	ATP synthase subunit alpha, mitochondrial (Fragment) OS=Homo sapiens GN=ATP5A1 PE=3 SV=1	22243	135	103	160	238	1.33	1.12	0.8421053	5	7	6	7	6	7	6	7	-0.66083
tr|B2R8N0|B2R8N0_HUMAN	tr|B2R8N0	cDNA, FLJ93975, highly similar to Homo sapiens epoxide hydrolase 1, microsomal (xenobiotic) (EPHX1), mRNA OS	53129	115	411	184	101	0.62	0.52	0.8387097	6	3	8	7	8	10	8	13	-0.56254
tr|H0Y8G5|H0Y8G5_HUMAN	HNRNPD	Heterogeneous nuclear ribonucleoprotein D0 (Fragment) OS=Homo sapiens GN=HNRNPD PE=4 SV=1	29877	229	709	90	32	0.37	0.31	0.8378378	3	0	3	1	4	1	4	1	-2.45268
sp|P35908|K22E_HUMAN	KRT2	Keratin, type II cytoskeletal 2 epidermal OS=Homo sapiens GN=KRT2 PE=1 SV=2	65678	11	23	1255	1255	4.79	4	0.8350731	41	35	48	39	49	42	57	42	-1.09549
sp|P22626|ROA2_HUMAN	HNRNPA2	Heterogeneous nuclear ribonucleoproteins A2/B1 OS=Homo sapiens GN=HNRNPA2B1 PE=1 SV=2	37464	77	328	256	125	0.97	0.81	0.8350515	7	5	8	7	9	9	10	11	-0.86793
tr|B3KMV8|B3KMV8_HUMAN	tr|B3KMV	cDNA FLJ12766 fis, clone NT2RP2001520, highly similar to Calcium-binding mitochondrial carrier protein Aralar1	75130	353	776	41	45	0.11	0.09	0.8181818	1	1	1	2	1	2	1	3	-0.12954
tr|A5I8L1|A5I8L1_HUMAN	HLA-A	MHC class I antigen (Fragment) OS=Homo sapiens GN=HLA-A PE=3 SV=1	37328	639	684	22	54	0.16	0.13	0.8125	0	1	1	1	1	2	2	4	-0.12954
tr|A8K7A4|A8K7A4_HUMAN	tr|A8K7A4	cDNA FLJ76904, highly similar to Homo sapiens methionine adenosyltransferase II, beta (MAT2B), transcript varia	36692	280	591	68	64	0.37	0.3	0.8108108	1	2	1	3	1	4	1	4	0.718839
sp|Q9NY65|TBA8_HUMAN	TUBA8	Tubulin alpha-8 chain OS=Homo sapiens GN=TUBA8 PE=1 SV=1	50746	34	69	592	517	1.74	1.41	0.8103448	15	14	23	19	23	23	27	30	-0.87012
tr|K7EIG1|K7EIG1_HUMAN	CLUH	Clustered mitochondria protein homolog OS=Homo sapiens GN=CLUH PE=3 SV=1	140903	438	1181	39	22	0.05	0.04	0.8	1	0	2	1	2	2	2	2	-0.86684
tr|F5H5D3|F5H5D3_HUMAN	TUBA1C	Tubulin alpha-1C chain OS=Homo sapiens GN=TUBA1C PE=2 SV=1	58606	21	32	809	999	2.92	2.33	0.7979452	24	26	34	36	37	44	41	52	-0.62407
tr|B7TY16|B7TY16_HUMAN	ACTN1	Actinin alpha 1 isoform 3 OS=Homo sapiens GN=ACTN1 PE=2 SV=1	107644	50	71	428	509	0.77	0.61	0.7922078	15	14	21	16	22	19	29	21	-1.07698
tr|E5RFU4|E5RFU4_HUMAN	DPYSL2	Dihydropyrimidinase-related protein 2 (Fragment) OS=Homo sapiens GN=DPYSL2 PE=2 SV=1	18320	342	1177	20	22	0.23	0.18	0.7826087	0	0	1	1	1	1	1	2	-0.86669
tr|Q4W4Y1|Q4W4Y1_HUMAN	DRIP4	Dopamine receptor interacting protein 4 OS=Homo sapiens GN=DRIP4 PE=2 SV=1	96646	136	355	160	118	0.18	0.14	0.7777778	2	3	5	4	5	4	6	4	-1.15701
sp|P38919|IF4A3_HUMAN	EIF4A3	Eukaryotic initiation factor 4A-III OS=Homo sapiens GN=EIF4A3 PE=1 SV=4	47126	262	293	74	142	0.4	0.31	0.775	1	3	5	4	4	7	6	8	-0.12962
tr|D6REX3|D6REX3_HUMAN	SEC31A	Protein transport protein Sec31A OS=Homo sapiens GN=SEC31A PE=2 SV=1	137110	121	637	178	59	0.13	0.1	0.7692308	4	2	5	4	4	4	5	4	-0.86715
sp|P13645|K1C10_HUMAN	KRT10	Keratin, type I cytoskeletal 10 OS=Homo sapiens GN=KRT10 PE=1 SV=6	59020	12	35	1226	918	4.67	3.56	0.7623126	34	24	42	30	47	35	54	43	-1.29627
sp|Q562R1|ACTBL_HUMAN	ACTBL2	Beta-actin-like protein 2 OS=Homo sapiens GN=ACTBL2 PE=1 SV=2	42318	61	84	349	426	1.47	1.12	0.7619048	13	12	16	18	24	27	27	28	-0.70305
tr|E5RH42|E5RH42_HUMAN	G3BP1	Ras GTPase-activating protein-binding protein 1 (Fragment) OS=Homo sapiens GN=G3BP1 PE=2 SV=1	13338	294	290	62	47	0.58	0.44	0.7586207	2	1	2	3	2	3	2	4	-0.38123
tr|B3KT66|B3KT66_HUMAN	PSMD6	Proteasome (Prosome, macropain) 26S subunit, non-ATPase, 6, isoform CRA_c OS=Homo sapiens GN=PSMD6 PE	40600	399	862	43	40	0.08	0.06	0.75	1	1	1	1	1	1	1	1	-0.86669
sp|Q9NXW2|DJB12_HUMAN	DNAJB12	DnaJ homolog subfamily B member 12 OS=Homo sapiens GN=DNAJB12 PE=1 SV=4	42021	124	263	174	160	0.57	0.42	0.7368421	5	4	7	5	9	5	9	5	-1.65718
sp|Q9UMS4|PRP19_HUMAN	PRPF19	Pre-mRNA-processing factor 19 OS=Homo sapiens GN=PRPF19 PE=1 SV=1	55603	237	567	87	67	0.26	0.19	0.7307692	2	1	5	3	7	3	11	8	-1.96791
sp|P52788|SPSY_HUMAN	SMS	Spermine synthase OS=Homo sapiens GN=SMS PE=1 SV=2	41698	248	1160	78	23	0.11	0.08	0.7272727	1	0	1	1	1	1	1	1	-0.86669
sp|Q58FF8|H90B2_HUMAN	HSP90AB2	Putative heat shock protein HSP 90-beta 2 OS=Homo sapiens GN=HSP90AB2P PE=1 SV=2	44492	32	167	97	249	1.24	0.9	0.7258065	3	5	5	9	5	9	6	10	-0.07806
sp|P52272|HNRPM_HUMAN	HNRNPM	Heterogeneous nuclear ribonucleoprotein M OS=Homo sapiens GN=HNRNPM PE=1 SV=3	77749	164	202	132	201	0.39	0.28	0.7179487	7	5	8	7	11	10	12	10	-0.99968
tr|B4E2P4|B4E2P4_HUMAN	tr|B4E2P4	cDNA FLJ58474, highly similar to Xaa-Pro aminopeptidase 1 (EC 3.4.11.9) OS=Homo sapiens PE=2 SV=1	50020	615	872	24	39	0.07	0.05	0.7142857	0	1	1	1	1	1	1	1	-0.86669
tr|H0YGW7|H0YGW7_HUMAN	ABCF1	ATP-binding cassette sub-family F member 1 (Fragment) OS=Homo sapiens GN=ABCF1 PE=3 SV=1	68075	144	220	151	191	0.27	0.19	0.7037037	4	4	5	5	5	5	7	5	-0.86731
sp|Q04695|K1C17_HUMAN	KRT17	Keratin, type I cytoskeletal 17 OS=Homo sapiens GN=KRT17 PE=1 SV=2	48361	24	123	683	314	2.99	2.07	0.6923077	19	12	27	17	32	25	35	27	-1.22279
tr|G1EPH8|G1EPH8_HUMAN	HLA-A	MHC class I antigen (Fragment) OS=Homo sapiens GN=HLA-A PE=3 SV=1	21132	639	683	22	54	0.16	0.11	0.6875	0	1	1	1	1	1	2	3	-0.86669
sp|P68363|TBA1B_HUMAN	TUBA1B	Tubulin alpha-1B chain OS=Homo sapiens GN=TUBA1B PE=1 SV=1	50804	13	28	1091	1077	5.58	3.8	0.6810036	31	28	43	37	45	45	49	54	-0.87357
sp|Q71U36|TBA1A_HUMAN	TUBA1A	Tubulin alpha-1A chain OS=Homo sapiens GN=TUBA1A PE=1 SV=1	50788	15	31	1022	1001	5.18	3.49	0.6737452	30	26	41	35	43	43	46	50	-0.87325

tr|B4E3L0|B4E3L0_HUMAN	SMU1	WD40 repeat-containing protein SMU1 OS=Homo sapiens GN=SMU1 PE=2 SV=1	39945	221	889	94	38	0.12	0.08	0.6666667	1	1	2	1	2	1	2	2	-1.604
tr|Q6FHM2|Q6FHM2_HUMAN	GNB2	GNB2 protein OS=Homo sapiens GN=GNB2 PE=2 SV=1	38048	194	943	111	35	0.27	0.18	0.6666667	1	1	2	2	2	1	2	3	-1.604
tr|K7ERE3|K7ERE3_HUMAN	KRT13	Keratin, type I cytoskeletal 13 OS=Homo sapiens GN=KRT13 PE=3 SV=1	45574	88	418	234	99	0.63	0.42	0.6666667	9	4	10	5	11	5	12	7	-1.9335
tr|H0Y9Q7|H0Y9Q7_HUMAN	CANX	Calnexin (Fragment) OS=Homo sapiens GN=CANX PE=3 SV=1	18990	352	267	49	99	0.39	0.25	0.6410256	1	2	2	2	3	2	5	3	-1.35261
sp|Q13151|ROA0_HUMAN	HNRNPA0	Heterogeneous nuclear ribonucleoprotein A0 OS=Homo sapiens GN=HNRNPA0 PE=1 SV=1	30993	323	596	55	63	0.36	0.23	0.6388889	2	2	3	2	3	3	3	3	-0.867
sp|P21796|VDAC1_HUMAN	VDAC1	Voltage-dependent anion-selective channel protein 1 OS=Homo sapiens GN=VDAC1 PE=1 SV=2	30868	250	560	78	68	0.36	0.23	0.6388889	1	2	3	2	4	2	4	2	-1.71553
tr|H0YA24|H0YA24_HUMAN	PLRG1	Pleiotropic regulator 1 (Fragment) OS=Homo sapiens GN=PLRG1 PE=4 SV=1	17588	456	514	38	45	0.19	0.12	0.6315789	1	1	1	1	1	2	2	3	-0.12954
tr|Q6IBT3|Q6IBT3_HUMAN	CCT7	CCT7 protein OS=Homo sapiens GN=CCT7 PE=2 SV=1	59804	161	500	133	77	0.38	0.24	0.6315789	2	2	6	4	5	6	7	8	-0.62611
tr|Q75NY8|Q75NY8_HUMAN	HLA-A	MHC class I antigen (Fragment) OS=Homo sapiens GN=HLA-A PE=3 SV=1	32018	639	788	22	44	0.16	0.1	0.625	0	1	1	1	1	2	3	3	-0.12954
tr|D0W033|D0W033_HUMAN	HLA-A	MHC class I antigen (Fragment) OS=Homo sapiens GN=HLA-A PE=3 SV=1	31801	639	685	22	54	0.16	0.1	0.625	0	1	1	1	1	2	1	3	-0.12954
sp|Q9H853|TBA4B_HUMAN	TUBA4B	Putative tubulin-like protein alpha-4B OS=Homo sapiens GN=TUBA4B PE=5 SV=2	27819	146	292	147	142	0.4	0.25	0.625	3	3	4	3	4	3	6	5	-1.22991
tr|H6UV64|H6UV64_HUMAN	HLA-A	MHC class I antigen (Fragment) OS=Homo sapiens GN=HLA-A PE=3 SV=1	21101	639	770	22	0	0.16	0.1	0.625	0	0	1	0	1	0	2	1	-2.45184
tr|B4DTA2|B4DTA2_HUMAN	tr|B4DTA2	cDNA FLJ60148, highly similar to Homo sapiens heterogeneous nuclear ribonucleoprotein D-like (HNRPDL), trans	30367	229	870	39	39	0.37	0.23	0.6216216	1	0	1	2	1	3	1	3	0.35608
tr|A8K2H9|A8K2H9_HUMAN	tr|A8K2H9	cDNA FLJ78503, highly similar to Homo sapiens keratin 13 (KRT13), transcript variant 1, mRNA OS=Homo sapiens	49828	125	482	174	82	0.47	0.29	0.6170213	5	3	6	4	8	3	10	6	-2.14883
tr|B4DKT3|B4DKT3_HUMAN	tr|B4DKT3	cDNA FLJ58390, highly similar to Aldo-keto reductase family 1 member C3homolog (EC 1.-.-.-) OS=Homo sapiens	15755	186	397	42	77	0.67	0.41	0.6119403	1	1	1	3	1	4	1	4	0.718839
tr|B3KMW5|B3KMW5_HUMAN	tr|B3KMW	cDNA FLJ12796 fis, clone NT2RP2002058, highly similar to WD repeat protein 3 OS=Homo sapiens PE=2 SV=1	71519	408	652	43	0	0.05	0.03	0.6	1	0	1	0	1	1	1	1	-0.86669
tr|Q53RS3|Q53RS3_HUMAN	DDEF2	Putative uncharacterized protein DDEF2 (Fragment) OS=Homo sapiens GN=DDEF2 PE=2 SV=1	70046	441	739	39	48	0.05	0.03	0.6	1	1	1	1	4	2	4	3	-1.71553
tr|B3KV96|B3KV96_HUMAN	tr|B3KV96	cDNA FLJ16285 fis, clone OCBBF2004038, highly similar to Dihydropyrimidinase-related protein 1 OS=Homo sapie	61746	490	688	19	53	0.19	0.11	0.5789474	0	1	1	2	0	3	2	3	1.941386
sp|Q5XKE5|K2C79_HUMAN	KRT79	Keratin, type II cytoskeletal 79 OS=Homo sapiens GN=KRT79 PE=1 SV=2	58085	160	331	133	124	0.32	0.18	0.5625	5	4	6	4	6	7	8	7	-0.66083
tr|F8VU64|F8VU64_HUMAN	KRT8	Keratin, type II cytoskeletal 8 OS=Homo sapiens GN=KRT8 PE=2 SV=1	37274	58	403	61	103	0.95	0.53	0.5578947	3	3	4	5	6	6	7	7	-0.86747
sp|Q8NBJ5|GT251_HUMAN	GLT25D1	Procollagen galactosyltransferase 1 OS=Homo sapiens GN=GLT25D1 PE=1 SV=1	71933	523	1174	31	22	0.09	0.05	0.5555556	0	0	2	1	1	2	2	2	-0.12954
tr|B3KMI9|B3KMI9_HUMAN	tr|B3KMI9	cDNA FLJ11140 fis, clone PLACE1006488, highly similar to Signal recognition particle 68 kDa protein OS=Homo sa	41632	180	1050	119	27	0.18	0.1	0.5555556	2	0	2	1	2	1	3	1	-1.604
tr|Q5HYM2|Q5HYM2_HUMAN	DKFZp686	Putative uncharacterized protein DKFZp686O2462 (Fragment) OS=Homo sapiens GN=DKFZp686O2462 PE=2 SV=1	72627	569	750	29	48	0.09	0.05	0.5555556	0	1	2	1	2	1	2	1	-1.604
tr|B4DNN4|B4DNN4_HUMAN	tr|B4DNN	Ribonucleoside-diphosphate reductase OS=Homo sapiens PE=2 SV=1	87252	156	295	136	141	0.29	0.16	0.5517241	5	4	7	5	8	4	8	7	-1.78607
tr|Q5I6Y6|Q5I6Y6_HUMAN	LMNA	Lamin A/C transcript variant 1 OS=Homo sapiens GN=LMNA PE=2 SV=1	74322	95	137	221	286	0.75	0.41	0.5466667	7	6	13	8	15	11	15	13	-1.30026
sp|P04040|CATA_HUMAN	CAT	Catalase OS=Homo sapiens GN=CAT PE=1 SV=3	59947	299	696	60	52	0.11	0.06	0.5454545	1	1	2	1	2	1	2	1	-1.604
tr|F8W8T8|F8W8T8_HUMAN	ACACB	Acetyl-CoA carboxylase 2 OS=Homo sapiens GN=ACACB PE=2 SV=1	269823	31	87	128	410	0.44	0.23	0.5227273	4	12	4	17	6	18	9	22	0.643812
sp|O43707|ACTN4_HUMAN	ACTN4	Alpha-actinin-4 OS=Homo sapiens GN=ACTN4 PE=1 SV=2	105245	19	40	849	797	2.11	1.09	0.5165877	26	20	41	24	44	26	52	33	-1.62463
sp|P35527|K1C9_HUMAN	KRT9	Keratin, type I cytoskeletal 9 OS=Homo sapiens GN=KRT9 PE=1 SV=3	62255	14	55	1044	615	3.01	1.52	0.5049834	25	17	36	19	40	24	44	25	-1.60095
tr|B4DZ88|B4DZ88_HUMAN	KTN1	KTN1 protein OS=Homo sapiens GN=KTN1 PE=2 SV=1	153179	322	651	55	58	0.04	0.02	0.5	2	1	2	1	3	6	4	6	0.026651
tr|Q5QPE2|Q5QPE2_HUMAN	SEC23B	Protein transport protein Sec23B (Fragment) OS=Homo sapiens GN=SEC23B PE=4 SV=1	37575	474	1198	24	21	0.08	0.04	0.5	0	0	1	1	1	1	1	1	-0.86669
tr|J3KN36|J3KN36_HUMAN	NOMO3	Nodal modulator 3 OS=Homo sapiens GN=NOMO3 PE=4 SV=1	140376	400	1063	43	30	0.04	0.02	0.5	1	0	1	1	1	1	1	2	-0.86669
sp|P43490|NAMPT_HUMAN	NAMPT	Nicotinamide phosphoribosyltransferase OS=Homo sapiens GN=NAMPT PE=1 SV=1	55772	520	715	32	51	0.12	0.06	0.5	0	1	1	1	1	1	3	1	-0.86669
tr|J3KN16|J3KN16_HUMAN	KIAA0368	Protein KIAA0368 OS=Homo sapiens GN=KIAA0368 PE=4 SV=1	225491	579	619	28	61	0.04	0.02	0.5	0	1	1	1	1	1	1	2	-0.86669
tr|A0MNN4|A0MNN4_HUMAN	SMU1	CDW3/SMU1 OS=Homo sapiens GN=SMU1 PE=2 SV=1	58134	221	888	94	38	0.12	0.06	0.5	1	1	2	1	2	2	2	2	-0.86684
tr|B2RBE5|B2RBE5_HUMAN	tr|B2RBE5	cDNA, FLJ95468, highly similar to Homo sapiens transcriptional coactivator tubedown-100 (TBDN100),transcript	102490	446	830	38	42	0.06	0.03	0.5	0	1	2	1	2	2	2	4	-0.86684
sp|O00505|IMA3_HUMAN	KPNA3	Importin subunit alpha-3 OS=Homo sapiens GN=KPNA3 PE=1 SV=2	58288	406	1043	43	31	0.12	0.06	0.5	0	1	2	1	2	1	2	2	-1.604
tr|Q96K98|Q96K98_HUMAN	tr|Q96K98	cDNA FLJ14414 fis, clone HEMBA1004847, highly similar to SIGNAL RECOGNITION PARTICLE 68 KD PROTEIN OS=H	37365	180	1042	119	31	0.18	0.09	0.5	2	0	2	1	2	1	4	2	-1.604
sp|Q9Y3I0|RTCB_HUMAN	C22orf28	tRNA-splicing ligase RtcB homolog OS=Homo sapiens GN=C22orf28 PE=1 SV=1	55688	458	984	38	34	0.12	0.06	0.5	0	0	2	1	2	1	2	2	-1.604
tr|Q14666|Q14666_HUMAN	tr|Q14666	Radiated keratinocyte mRNA 266 (keratin-related protein) (Fragment) OS=Homo sapiens PE=2 SV=1	30933	24	95	451	262	2.99	1.49	0.4983278	12	7	17	11	20	16	21	18	-1.18356
tr|B3KTA3|B3KTA3_HUMAN	tr|B3KTA3	Fascin OS=Homo sapiens PE=2 SV=1	52814	53	205	403	199	1.07	0.53	0.4953271	9	7	14	7	16	10	20	10	-1.52223
tr|B3KPU1|B3KPU1_HUMAN	GNB2	Guanine nucleotide-binding protein G(I)/G(S)/G(T) subunit beta-2 OS=Homo sapiens GN=GNB2 PE=2 SV=1	26598	194	892	111	38	0.27	0.13	0.4814815	1	1	2	1	2	1	2	1	-1.604
sp|Q9NR12|PDLI7_HUMAN	PDLIM7	PDZ and LIM domain protein 7 OS=Homo sapiens GN=PDLIM7 PE=1 SV=1	50896	396	809	43	43	0.13	0.06	0.4615385	0	1	2	1	2	2	4	2	-0.86684
sp|P35900|K1C20_HUMAN	KRT20	Keratin, type I cytoskeletal 20 OS=Homo sapiens GN=KRT20 PE=1 SV=1	48514	24	92	78	56	2.99	1.37	0.458194	2	1	2	1	2	2	2	2	-0.86684
tr|D3DVQ1|D3DVQ1_HUMAN	LETM1	Leucine zipper-EF-hand containing transmembrane protein 1, isoform CRA_a OS=Homo sapiens GN=LETM1 PE=2	63730	463	690	37	53	0.11	0.05	0.4545455	1	1	2	1	2	1	4	1	-1.604
sp|O15131|IMA5_HUMAN	KPNA5	Importin subunit alpha-6 OS=Homo sapiens GN=KPNA5 PE=1 SV=2	61052	573	645	28	59	0.11	0.05	0.4545455	0	1	2	1	2	1	2	1	-1.604
tr|B7Z8J4|B7Z8J4_HUMAN	NMT1	Glycylpeptide N-tetradecanoyltransferase 1 OS=Homo sapiens GN=NMT1 PE=2 SV=1	15265	350	723	50	50	0.49	0.22	0.4489796	1	1	2	1	2	1	2	2	-1.604
tr|B2RD24|B2RD24_HUMAN	tr|B2RD24	cDNA, FLJ96424 OS=Homo sapiens PE=2 SV=1	80216	398	614	43	62	0.09	0.04	0.4444444	1	1	1	1	1	2	1	3	-0.12954
tr|Q562U1|Q562U1_HUMAN	ACT	Actin-like protein (Fragment) OS=Homo sapiens GN=ACT PE=2 SV=1	11548	202	399	103	104	2.69	1.19	0.4423792	4	3	7	6	8	7	11	7	-1.04854
tr|Q5SWC8|Q5SWC8_HUMAN	HP1BP3	Heterochromatin protein 1-binding protein 3 (Fragment) OS=Homo sapiens GN=HP1BP3 PE=4 SV=1	31566	631	819	0	43	0.25	0.11	0.44	0	1	0	1	0	1	1	1	0.718617
tr|B0QZK4|B0QZK4_HUMAN	HP1BP3	Heterochromatin protein 1-binding protein 3 (Fragment) OS=Homo sapiens GN=HP1BP3 PE=4 SV=1	28725	631	864	23	40	0.25	0.11	0.44	0	1	2	2	1	1	3	2	-0.86669
tr|G3XAF4|G3XAF4_HUMAN	L1CAM	L1 cell adhesion molecule, isoform CRA_a OS=Homo sapiens GN=L1CAM PE=4 SV=1	139790	572	662	28	57	0.05	0.02	0.4	1	1	2	1	1	1	2	1	-0.86669
tr|H0YF33|H0YF33_HUMAN	NASP	Nuclear autoantigenic sperm protein (Fragment) OS=Homo sapiens GN=NASP PE=4 SV=1	31883	542	1030	30	32	0.1	0.04	0.4	0	0	1	1	2	2	3	2	-0.86684
sp|O60518|RNBP6_HUMAN	RANBP6	Ran-binding protein 6 OS=Homo sapiens GN=RANBP6 PE=1 SV=2	126173	85	486	36	81	0.23	0.08	0.3478261	1	3	1	3	2	4	2	5	-0.01847
tr|H0YIE7|H0YIE7_HUMAN	HSPA1L	Heat shock 70 kDa protein 1-like OS=Homo sapiens GN=HSPA1L PE=3 SV=1	25488	51	124	132	204	1.45	0.5	0.3448276	3	6	4	6	4	7	4	7	-0.12962
tr|B3KQQ3|B3KQQ3_HUMAN	tr|B3KQQ	cDNA PSEC0016 fis, clone NT2RM1001076, highly similar to Procollagen-lysine,2-oxoglutarate 5-dioxygenase 3 (E	85161	325	1154	54	24	0.12	0.04	0.3333333	1	0	3	1	3	2	3	2	-1.35261
tr|B3KY12|B3KY12_HUMAN	tr|B3KY12	cDNA FLJ46581 fis, clone THYMU3043200, highly similar to Splicing factor 3A subunit 3 OS=Homo sapiens PE=2 S	59082	495	1046	34	31	0.18	0.06	0.3333333	1	1	3	1	3	1	3	1	-1.604
sp|P27348|1433T_HUMAN	YWHAQ	14-3-3 protein theta OS=Homo sapiens GN=YWHAQ PE=1 SV=1	28032	258	1013	74	0	0.4	0.12	0.3	2	0	3	0	3	1	3	2	-2.08977
tr|B4DQX3|B4DQX3_HUMAN	tr|B4DQX	cDNA FLJ58927, highly similar to Endoplasmin (Heat shock protein 90 kDa beta member 1) OS=Homo sapiens PE	18712	32	153	32	142	1.24	0.37	0.2983871	0	3	1	3	1	3	1	3	0.35608
tr|J3QQX0|J3QQX0_HUMAN	GPS1	COP9 signalosome complex subunit 1 (Fragment) OS=Homo sapiens GN=GPS1 PE=4 SV=1	8703	636	626	22	48	0.4	0.11	0.275	0	1	1	1	1	1	1	1	-0.86669
tr|E9PRA5|E9PRA5_HUMAN	ATP1A4	Sodium/potassium-transporting ATPase subunit alpha-4 OS=Homo sapiens GN=ATP1A4 PE=2 SV=1	57835	289	700	63	52	0.12	0.03	0.25	0	0	6	7	10	11	19	22	-0.73665
tr|Q59GH5|Q59GH5_HUMAN	tr|Q59GH	COP9 signalosome subunit 5 variant (Fragment) OS=Homo sapiens PE=2 SV=1	31467	346	910	51	38	0.45	0.11	0.2444444	0	1	2	1	2	1	2	1	-1.604
tr|K0J110|K0J110_HUMAN	mcdrh	Malignant cell derived RNA helicase OS=Homo sapiens GN=mcdrh PE=2 SV=1	82499	148	504	61	76	0.34	0.08	0.2352941	2	2	2	2	2	2	2	3	-0.86684
tr|Q8TE01|Q8TE01_HUMAN	derp12	DERP12 (Dermal papilla derived protein 12) OS=Homo sapiens GN=derp12 PE=2 SV=1	38340	69	451	297	90	0.79	0.18	0.2278481	6	2	7	2	8	2	8	3	-2.63444
tr|D3DTL4|D3DTL4_HUMAN	ENO3	Enolase OS=Homo sapiens GN=ENO3 PE=2 SV=1	37167	40	248	112	109	1.39	0.31	0.2230216	2	1	2	2	2	2	4	2	-0.86684
sp|Q92878|RAD50_HUMAN	RAD50	DNA repair protein RAD50 OS=Homo sapiens GN=RAD50 PE=1 SV=1	154823	563	1076	29	29	0.09	0.02	0.2222222	0	0	2	1	1	2	2	2	-0.12954
sp|P42166|LAP2A_HUMAN	TMPO	Lamina-associated polypeptide 2, isoform alpha OS=Homo sapiens GN=TMPO PE=1 SV=2	76016	241	1207	84	20	0.24	0.04	0.1666667	1	0	5	1	5	1	5	2	-2.74253
tr|A4ZU86|A4ZU86_HUMAN	NPM1	Truncated nucleolar phosphoprotein B23 OS=Homo sapiens GN=NPM1 PE=2 SV=1	30074	138	672	160	56	0.69	0.11	0.1594203	4	1	5	1	5	1	5	1	-2.74253
tr|A1L2Z2|A1L2Z2_HUMAN	MYH14	MYH14 protein OS=Homo sapiens GN=MYH14 PE=2 SV=1	73418	17	174	56	32	0.9	0.12	0.1333333	1	0	1	1	1	1	1	2	-0.86669
tr|B7Z4P8|B7Z4P8_HUMAN	ACTN2	Alpha-actinin-2 OS=Homo sapiens GN=ACTN2 PE=2 SV=1	44675	19	242	51	29	2.11	0.24	0.1137441	1	0	2	2	2	2	2	3	-0.86684
tr|E7ERA5|E7ERA5_HUMAN	MYH10	Myosin-10 (Fragment) OS=Homo sapiens GN=MYH10 PE=2 SV=1	21768	17	232	73	73	0.9	0.09	0.1	1	1	1	1	1	2	1	3	-0.12954
sp|O95678|K2C75_HUMAN	KRT75	Keratin, type II cytoskeletal 75 OS=Homo sapiens GN=KRT75 PE=1 SV=2	59809	11	272	94	152	4.79	0.38	0.0793319	2	6	3	7	3	8	5	8	0.414051
tr|G3XAP5|G3XAP5_HUMAN	LDHC	L-lactate dehydrogenase OS=Homo sapiens GN=LDHC PE=2 SV=1	26552	39	622	67	61	1.81	0.13	0.0718232	1	1	1	1	1	2	1	2	-0.12954
sp|A2RTX5|SYTC2_HUMAN	TARSL2	Probable threonine--tRNA ligase 2, cytoplasmic OS=Homo sapiens GN=TARSL2 PE=1 SV=1	93613	94	644	69	59	0.64	0.04	0.0625	2	2	2	2	2	3	2	4	-0.38123
tr|B2R8Y4|B2R8Y4_HUMAN	tr|B2R8Y4	cDNA, FLJ94117, highly similar to Homo sapiens actinin, alpha 3 (ACTN3), mRNA OS=Homo sapiens PE=2 SV=1	103871	19	367	99	113	2.11	0.1	0.0473934	3	3	3	3	4	4	4	4	-0.50424
tr|Q562V5|Q562V5_HUMAN	ACT	Actin-like protein (Fragment) OS=Homo sapiens GN=ACT PE=2 SV=1	11481	1	302	136	137	31.43	1.2	0.0381801	6	5	6	7	7	10	8	11	-0.38163
sp|P12036|NFH_HUMAN	NEFH	Neurofilament heavy polypeptide OS=Homo sapiens GN=NEFH PE=1 SV=4	112639	11	853	0	40	4.79	0.03	0.006263	0	1	0	1	0	2	1	2	1.455771
sp|Q9H254|SPTN4_HUMAN	SPTBN4	Spectrin beta chain, non-erythrocytic 4 OS=Homo sapiens GN=SPTBN4 PE=1 SV=2	290005	19	985	29	34	2.11	0.01	0.0047393	0	1	1	1	2	2	2	3	-0.86684
tr|K7ELP7|K7ELP7_HUMAN	KRT17	Keratin, type I cytoskeletal 17 (Fragment) OS=Homo sapiens GN=KRT17 PE=4 SV=1	5508	24	0	20	0	2.99	0	0	0	0	1	0	0	0	1	0	-0.86653
tr|E9PQ69|E9PQ69_HUMAN	GANAB	Neutral alpha-glucosidase AB OS=Homo sapiens GN=GANAB PE=2 SV=1	8560	47	0	0	0	0.62	0	0	0	0	0	0	0	0	1	0	-0.86653
tr|E9PLJ0|E9PLJ0_HUMAN	IPO7	Importin-7 OS=Homo sapiens GN=IPO7 PE=2 SV=1	7255	162	0	0	0	0.08	0	0	0	0	0	0	0	0	1	0	-0.86653
tr|Q6RUP8|Q6RUP8_HUMAN	IMPDH2	Inosine monophosphate dehydrogenase type II (Fragment) OS=Homo sapiens GN=IMPDH2 PE=2 SV=1	3145	177	0	0	0	0.36	0	0	0	0	0	0	0	0	1	0	-0.86653
tr|I3L204|I3L204_HUMAN	MYO1C	Unconventional myosin-Ic (Fragment) OS=Homo sapiens GN=MYO1C PE=4 SV=1	34968	284	0	0	0	0.07	0	0	0	0	0	0	0	0	1	0	-0.86653
tr|Q1XBU7|Q1XBU7_HUMAN	tr|Q1XBU	Aging-associated protein 14a OS=Homo sapiens PE=2 SV=1	14000	382	0	0	0	0.09	0	0	0	0	0	0	0	0	1	0	-0.86653
tr|B7Z6J5|B7Z6J5_HUMAN	tr|B7Z6J5	cDNA FLJ56858, highly similar to Succinate dehydrogenase (ubiquinone) flavoprotein subunit, mitochondrial (EC	28776	393	0	0	0	0.1	0	0	0	0	0	0	0	0	1	0	-0.86653
tr|H0Y8W6|H0Y8W6_HUMAN	PDLIM7	PDZ and LIM domain protein 7 (Fragment) OS=Homo sapiens GN=PDLIM7 PE=4 SV=1	12898	396	0	0	0	0.13	0	0	0	0	0	0	0	0	1	0	-0.86653
tr|A8K171|A8K171_HUMAN	tr|A8K171	cDNA FLJ77253, highly similar to Homo sapiens nuclear receptor interacting protein 1 (NRIP1), mRNA OS=Homo s	127609	401	0	0	0	0.01	0	0	0	0	0	0	0	0	1	0	-0.86653
tr|H0Y386|H0Y386_HUMAN	PABPC1L	Polyadenylate-binding protein 1-like (Fragment) OS=Homo sapiens GN=PABPC1L PE=4 SV=1	16787	404	0	0	0	0.1	0	0	0	0	0	0	0	0	1	0	-0.86653
tr|Q9NQS8|Q9NQS8_HUMAN	RPS3	Ribosomal protein S3 (Fragment) OS=Homo sapiens GN=RPS3 PE=2 SV=1	17270	480	0	0	0	0.12	0	0	0	0	0	0	0	0	2	0	-0.86653
tr|H7BYY8|H7BYY8_HUMAN	PES1	Pescadillo homolog OS=Homo sapiens GN=PES1 PE=4 SV=1	1501	488	0	0	0	0.05	0	0	0	0	0	0	0	0	1	0	-0.86653
tr|H7C1T1|H7C1T1_HUMAN	Sep-02	Septin-2 (Fragment) OS=Homo sapiens GN=SEPT2 PE=4 SV=1	3094	543	0	0	0	0.09	0	0	0	0	0	0	0	0	1	0	-0.86653
tr|B2R7G6|B2R7G6_HUMAN	tr|B2R7G6	cDNA, FLJ93437, highly similar to Homo sapiens histidyl-tRNA synthetase-like (HARSL), mRNA OS=Homo sapiens	57594	672	0	0	0	0.07	0	0	0	0	0	0	0	0	1	0	-0.86653
tr|J3KN33|J3KN33_HUMAN	CNTLN	Centlein OS=Homo sapiens GN=CNTLN PE=4 SV=1	162069	680	0	20	0	0.02	0	0	0	0	1	0	0	0	2	0	-0.86653
tr|Q3MNE1|Q3MNE1_HUMAN	tr|Q3MNE	Adaptor-related protein complex 3 beta 1 subunit (Fragment) OS=Homo sapiens PE=2 SV=1	89828	682	0	20	0	0.04	0	0	0	0	1	0	0	0	1	0	-0.86653
tr|B4DZD5|B4DZD5_HUMAN	tr|B4DZD5	cDNA FLJ60991, highly similar to FKBP12-rapamycin complex-associated protein OS=Homo sapiens PE=2 SV=1	158783	683	0	20	0	0.02	0	0	0	0	1	0	0	0	2	0	-0.86653
tr|Q9UH66|Q9UH66_HUMAN	tr|Q9UH6	7h3 protein (Fragment) OS=Homo sapiens PE=2 SV=1	49963	684	0	19	0	0.07	0	0	0	0	1	0	0	0	2	0	-0.86653
tr|G5E9Q3|G5E9Q3_HUMAN	TSEN2	tRNA splicing endonuclease 2 homolog (S. cerevisiae), isoform CRA_b OS=Homo sapiens GN=TSEN2 PE=2 SV=1	45959	686	0	19	0	0.07	0	0	0	0	1	0	0	0	1	0	-0.86653
tr|E9PQG7|E9PQG7_HUMAN	MLL	MLL cleavage product C180 OS=Homo sapiens GN=MLL PE=2 SV=1	436345	687	0	19	0	0.01	0	0	0	0	1	0	0	0	1	0	-0.86653
tr|G3V448|G3V448_HUMAN	TMX1	Thioredoxin-related transmembrane protein 1 OS=Homo sapiens GN=TMX1 PE=2 SV=1	12018	689	0	19	0	0.29	0	0	0	0	1	0	0	0	1	0	-0.86653
tr|D3DUL0|D3DUL0_HUMAN	ASCIZ	ATM/ATR-Substrate Chk2-Interacting Zn2+-finger protein, isoform CRA_b OS=Homo sapiens GN=ASCIZ PE=2 SV=	79474	690	0	19	0	0.04	0	0	0	0	1	0	0	0	1	0	-0.86653
tr|H7C4X2|H7C4X2_HUMAN	PCMT1	Protein-L-isoaspartate(D-aspartate) O-methyltransferase (Fragment) OS=Homo sapiens GN=PCMT1 PE=4 SV=1	7342	691	0	19	0	0.48	0	0	0	0	1	0	0	0	2	0	-0.86653
tr|B3KND7|B3KND7_HUMAN	tr|B3KND	cDNA FLJ14378 fis, clone HEMBA1001804, highly similar to Zinc finger protein 281 (Fragment) OS=Homo sapiens	58221	692	0	19	0	0.06	0	0	0	0	1	0	0	0	1	0	-0.86653
tr|H0YE81|H0YE81_HUMAN	NDUFV1	NADH dehydrogenase [ubiquinone] flavoprotein 1, mitochondrial (Fragment) OS=Homo sapiens GN=NDUFV1 PE	14494	694	0	19	0	0.23	0	0	0	0	1	0	0	0	1	0	-0.86653
tr|H0Y304|H0Y304_HUMAN	DMD	Dystrophin (Fragment) OS=Homo sapiens GN=DMD PE=4 SV=1	160176	695	0	19	0	0.02	0	0	0	0	1	0	0	0	1	0	-0.86653
tr|Q5T097|Q5T097_HUMAN	UTRN	Utrophin OS=Homo sapiens GN=UTRN PE=4 SV=1	114423	696	0	19	0	0.03	0	0	0	0	1	0	0	0	1	0	-0.86653
tr|I3L2H8|I3L2H8_HUMAN	MYBBP1A	Myb-binding protein 1A (Fragment) OS=Homo sapiens GN=MYBBP1A PE=4 SV=1	22828	698	0	18	0	0.02	0	0	0	0	1	0	0	0	1	0	-0.86653
tr|B4DY47|B4DY47_HUMAN	PHB	Prohibitin OS=Homo sapiens GN=PHB PE=2 SV=1	17064	699	0	18	0	0.2	0	0	0	0	1	0	0	0	1	0	-0.86653
tr|J3KQ96|J3KQ96_HUMAN	TCOF1	Treacle protein (Fragment) OS=Homo sapiens GN=TCOF1 PE=4 SV=1	144268	702	0	18	0	0.02	0	0	0	0	1	0	0	0	2	0	-0.86653
tr|B0UZL4|B0UZL4_HUMAN	BAT3	HLA-B associated transcript 3 (Fragment) OS=Homo sapiens GN=BAT3 PE=2 SV=1	15422	704	0	18	0	0.03	0	0	0	0	1	0	0	0	1	0	-0.86653
tr|H0Y4R4|H0Y4R4_HUMAN	TOR1AIP1	Torsin-1A-interacting protein 1 (Fragment) OS=Homo sapiens GN=TOR1AIP1 PE=4 SV=1	33844	707	0	17	0	0.1	0	0	0	0	1	0	0	0	1	0	-0.86653
sp|P54826|GAS1_HUMAN	GAS1	Growth arrest-specific protein 1 OS=Homo sapiens GN=GAS1 PE=1 SV=2	36867	708	0	17	0	0.09	0	0	0	0	1	0	0	0	1	0	-0.86653
tr|B0QYA5|B0QYA5_HUMAN	EIF3D	Eukaryotic translation initiation factor 3 subunit D (Fragment) OS=Homo sapiens GN=EIF3D PE=4 SV=1	31937	710	0	17	0	0.1	0	0	0	0	1	0	0	0	2	0	-0.86653
tr|D6RAW4|D6RAW4_HUMAN	IMMT	Mitochondrial inner membrane protein OS=Homo sapiens GN=IMMT PE=2 SV=1	15918	712	0	0	0	0.04	0	0	0	0	0	0	0	0	1	0	-0.86653
tr|B9VP24|B9VP24_HUMAN	HSPD1	60 kDa chaperonin (Fragment) OS=Homo sapiens GN=HSPD1 PE=4 SV=1	19880	16	0	40	0	1.72	0	0	1	0	1	0	1	0	1	0	-2.45184
tr|B7Z2Y4|B7Z2Y4_HUMAN	tr|B7Z2Y4	cDNA FLJ56961, highly similar to Clathrin heavy chain 2 (CLH-22) OS=Homo sapiens PE=2 SV=1	54629	30	0	60	0	0.52	0	0	1	0	1	0	1	0	2	0	-2.45184
tr|Q9BVS9|Q9BVS9_HUMAN	IPO5	IPO5 protein (Fragment) OS=Homo sapiens GN=IPO5 PE=2 SV=1	36033	85	0	71	0	0.23	0	0	1	0	1	0	1	0	1	0	-2.45184
tr|H0Y8V7|H0Y8V7_HUMAN	SEC31A	Protein transport protein Sec31A (Fragment) OS=Homo sapiens GN=SEC31A PE=4 SV=1	32306	121	0	42	0	0.13	0	0	1	0	2	0	1	0	2	0	-2.45184
tr|Q9BZT4|Q9BZT4_HUMAN	tr|Q9BZT4	PNAS-29 OS=Homo sapiens PE=4 SV=1	8036	137	0	59	0	0.4	0	0	1	0	1	0	1	0	1	0	-2.45184
tr|Q9BTI9|Q9BTI9_HUMAN	NPM1	NPM1 protein (Fragment) OS=Homo sapiens GN=NPM1 PE=2 SV=2	25147	138	0	46	0	0.69	0	0	1	0	1	0	1	0	1	0	-2.45184
tr|Q58FF2|Q58FF2_HUMAN	GRP94c	Heat shock protein 94c OS=Homo sapiens GN=GRP94c PE=2 SV=1	52205	140	0	22	0	0.37	0	0	0	0	1	0	1	0	2	0	-2.45184
tr|C9JT45|C9JT45_HUMAN	ASNS	Asparagine synthetase [glutamine-hydrolyzing] (Fragment) OS=Homo sapiens GN=ASNS PE=2 SV=1	18978	141	0	32	0	0.26	0	0	0	0	1	0	1	0	1	0	-2.45184
tr|G3V277|G3V277_HUMAN	WARS	T1-TrpRS (Fragment) OS=Homo sapiens GN=WARS PE=2 SV=1	23928	145	0	34	0	0.27	0	0	1	0	1	0	1	0	1	0	-2.45184
tr|H0YCY7|H0YCY7_HUMAN	RRM1	Ribonucleoside-diphosphate reductase (Fragment) OS=Homo sapiens GN=RRM1 PE=3 SV=1	14342	156	0	22	0	0.29	0	0	0	0	1	0	1	0	1	0	-2.45184
tr|H0Y929|H0Y929_HUMAN	PDLIM5	PDZ and LIM domain protein 5 (Fragment) OS=Homo sapiens GN=PDLIM5 PE=4 SV=1	9116	175	0	25	0	0.41	0	0	0	0	1	0	1	0	1	0	-2.45184
tr|B3KV39|B3KV39_HUMAN	tr|B3KV39	cDNA FLJ16099 fis, clone TESTI2008278, highly similar to Homo sapiens forkhead box K1 (FOXK1), mRNA OS=Hon	50383	188	0	115	0	0.07	0	0	1	0	1	0	1	0	2	0	-2.45184
tr|D6R9D3|D6R9D3_HUMAN	HNRNPH1	Heterogeneous nuclear ribonucleoprotein H OS=Homo sapiens GN=HNRNPH1 PE=4 SV=1	4404	207	0	23	0	0.45	0	0	0	0	1	0	1	0	1	0	-2.45184
tr|H0Y6X6|H0Y6X6_HUMAN	PABPC4	Polyadenylate-binding protein 4 (Fragment) OS=Homo sapiens GN=PABPC4 PE=4 SV=1	20509	215	0	33	0	0.33	0	0	0	0	1	0	1	0	1	0	-2.45184
tr|D6RHJ5|D6RHJ5_HUMAN	GNB2L1	Guanine nucleotide-binding protein subunit beta-2-like 1 OS=Homo sapiens GN=GNB2L1 PE=2 SV=1	9551	216	0	58	0	0.52	0	0	1	0	1	0	1	0	1	0	-2.45184
tr|H0Y960|H0Y960_HUMAN	DDX4	Probable ATP-dependent RNA helicase DDX4 (Fragment) OS=Homo sapiens GN=DDX4 PE=4 SV=1	33292	217	0	43	0	0.19	0	0	1	0	1	0	1	0	1	0	-2.45184
tr|E9PLA6|E9PLA6_HUMAN	SERPINH1	Serpin H1 (Fragment) OS=Homo sapiens GN=SERPINH1 PE=2 SV=1	8925	233	0	45	0	0.43	0	0	1	0	1	0	1	0	1	0	-2.45184
tr|E5RFJ8|E5RFJ8_HUMAN	MCM4	DNA replication licensing factor MCM4 (Fragment) OS=Homo sapiens GN=MCM4 PE=2 SV=1	23630	240	0	65	0	0.1	0	0	1	0	1	0	1	0	2	0	-2.45184
tr|B4DE40|B4DE40_HUMAN	SMS	Spermine synthase OS=Homo sapiens GN=SMS PE=2 SV=1	31596	248	0	78	0	0.11	0	0	1	0	1	0	1	0	1	0	-2.45184
tr|B4DXK4|B4DXK4_HUMAN	KRT72	Keratin, type II cytoskeletal 72 OS=Homo sapiens GN=KRT72 PE=2 SV=1	36031	259	0	0	0	0.45	0	0	0	0	0	0	1	0	1	0	-2.45184
tr|K7EKB6|K7EKB6_HUMAN	THOP1	Thimet oligopeptidase (Fragment) OS=Homo sapiens GN=THOP1 PE=4 SV=1	20792	278	0	68	0	0.16	0	0	1	0	1	0	1	0	1	0	-2.45184
tr|E5RFS9|E5RFS9_HUMAN	MAT2B	Methionine adenosyltransferase 2 subunit beta OS=Homo sapiens GN=MAT2B PE=2 SV=1	9467	280	0	68	0	0.37	0	0	1	0	1	0	1	0	1	0	-2.45184
tr|H0YC45|H0YC45_HUMAN	ATP6V1B2	V-type proton ATPase subunit B, brain isoform (Fragment) OS=Homo sapiens GN=ATP6V1B2 PE=4 SV=1	5569	287	0	64	0	0.65	0	0	1	0	1	0	1	0	1	0	-2.45184
sp|P81605|DCD_HUMAN	DCD	Dermcidin OS=Homo sapiens GN=DCD PE=1 SV=2	11391	295	0	62	0	0.3	0	0	1	0	1	0	1	0	1	0	-2.45184
tr|F5H5X6|F5H5X6_HUMAN	NOP2	Putative ribosomal RNA methyltransferase NOP2 (Fragment) OS=Homo sapiens GN=NOP2 PE=2 SV=1	27408	296	0	28	0	0.15	0	0	1	0	1	0	1	0	2	0	-2.45184
tr|Q5T0I0|Q5T0I0_HUMAN	GSN	Gelsolin (Fragment) OS=Homo sapiens GN=GSN PE=4 SV=1	29107	297	0	61	0	0.04	0	0	1	0	1	0	1	0	2	0	-2.45184
tr|A1L3A7|A1L3A7_HUMAN	NUFIP2	Nuclear fragile X mental retardation protein interacting protein 2 OS=Homo sapiens GN=NUFIP2 PE=2 SV=1	76132	301	0	59	0	0.04	0	0	1	0	1	0	1	0	1	0	-2.45184
tr|B5LY64|B5LY64_HUMAN	UMPS	Orotidine 5'-phosphate decarboxylase OS=Homo sapiens GN=UMPS PE=2 SV=1	33258	302	0	59	0	0.1	0	0	1	0	1	0	1	0	1	0	-2.45184
tr|B3KPC1|B3KPC1_HUMAN	tr|B3KPC1	cDNA FLJ31582 fis, clone NT2RI2002117, highly similar to Protein pelota homolog OS=Homo sapiens PE=2 SV=1	43807	307	0	59	0	0.08	0	0	1	0	1	0	1	0	1	0	-2.45184
sp|Q5TZA2|CROCC_HUMAN	CROCC	Rootletin OS=Homo sapiens GN=CROCC PE=1 SV=1	228787	310	0	58	0	0.01	0	0	1	0	1	0	1	0	2	0	-2.45184
tr|H0YBD4|H0YBD4_HUMAN	GSR	Glutathione reductase, mitochondrial (Fragment) OS=Homo sapiens GN=GSR PE=4 SV=1	32532	313	0	25	0	0.18	0	0	0	0	1	0	1	0	1	0	-2.45184
tr|J3KRP2|J3KRP2_HUMAN	PSMC5	26S protease regulatory subunit 8 (Fragment) OS=Homo sapiens GN=PSMC5 PE=4 SV=1	27292	314	0	57	0	0.11	0	0	1	0	1	0	1	0	1	0	-2.45184
tr|C9JPP3|C9JPP3_HUMAN	RBBP4	Histone-binding protein RBBP4 (Fragment) OS=Homo sapiens GN=RBBP4 PE=2 SV=2	9655	315	0	57	0	0.36	0	0	1	0	1	0	1	0	1	0	-2.45184
tr|C9JIX5|C9JIX5_HUMAN	PLOD3	Procollagen-lysine,2-oxoglutarate 5-dioxygenase 3 (Fragment) OS=Homo sapiens GN=PLOD3 PE=2 SV=1	8424	325	0	41	0	0.12	0	0	1	0	1	0	1	0	1	0	-2.45184
tr|B4DVF3|B4DVF3_HUMAN	tr|B4DVF3	cDNA FLJ57205, highly similar to Homo sapiens Rho GTPase activating protein 17 (ARHGAP17), transcript variant	41146	328	0	0	0	0.03	0	0	0	0	0	0	1	0	1	0	-2.45184
sp|Q9Y6M5|ZNT1_HUMAN	SLC30A1	Zinc transporter 1 OS=Homo sapiens GN=SLC30A1 PE=1 SV=3	56290	334	0	52	0	0.06	0	0	1	0	1	0	1	0	1	0	-2.45184
tr|A8KA82|A8KA82_HUMAN	DNAJC3	DnaJ (Hsp40) homolog, subfamily C, member 3 OS=Homo sapiens GN=DNAJC3 PE=2 SV=1	58000	337	0	52	0	0.06	0	0	1	0	1	0	1	0	1	0	-2.45184
tr|B2RAR2|B2RAR2_HUMAN	tr|B2RAR2	cDNA, FLJ95064, highly similar to Homo sapiens nin one binding protein (NOB1P), mRNA OS=Homo sapiens PE=2	47015	338	0	52	0	0.07	0	0	1	0	1	0	1	0	1	0	-2.45184
tr|A8K517|A8K517_HUMAN	RPS23	Ribosomal protein S23, isoform CRA_a OS=Homo sapiens GN=RPS23 PE=2 SV=1	15969	339	0	52	0	0.21	0	0	1	0	1	0	1	0	1	0	-2.45184
tr|C9JQM9|C9JQM9_HUMAN	DARS	Aspartate--tRNA ligase, cytoplasmic (Fragment) OS=Homo sapiens GN=DARS PE=2 SV=1	15386	344	0	51	0	0.06	0	0	1	0	1	0	1	0	1	0	-2.45184
tr|K7EN42|K7EN42_HUMAN	NMT1	Glycylpeptide N-tetradecanoyltransferase 1 (Fragment) OS=Homo sapiens GN=NMT1 PE=4 SV=1	9699	350	0	31	0	0.49	0	0	0	0	1	0	1	0	1	0	-2.45184
tr|Q53ST0|Q53ST0_HUMAN	PCYOX1	Putative uncharacterized protein PCYOX1 (Fragment) OS=Homo sapiens GN=PCYOX1 PE=2 SV=1	11626	355	0	49	0	0.06	0	0	1	0	1	0	1	0	1	0	-2.45184
tr|A6NKV8|A6NKV8_HUMAN	PAIP1	Polyadenylate-binding protein-interacting protein 1 OS=Homo sapiens GN=PAIP1 PE=2 SV=1	42379	361	0	48	0	0.08	0	0	1	0	1	0	1	0	1	0	-2.45184
tr|H0Y9Q1|H0Y9Q1_HUMAN	LAP3	Cytosol aminopeptidase (Fragment) OS=Homo sapiens GN=LAP3 PE=4 SV=1	22641	368	0	47	0	0.15	0	0	1	0	1	0	1	0	1	0	-2.45184
tr|J3QL39|J3QL39_HUMAN	PFAS	Phosphoribosylformylglycinamidine synthase (Fragment) OS=Homo sapiens GN=PFAS PE=4 SV=1	9951	380	0	0	0	0.05	0	0	0	0	0	0	1	0	1	0	-2.45184
tr|B4DT09|B4DT09_HUMAN	tr|B4DT09	cDNA FLJ60145, highly similar to Homo sapiens dendritic cell protein (hfl-B5), mRNA OS=Homo sapiens PE=2 SV=	13697	381	0	46	0	0.25	0	0	1	0	1	0	1	0	1	0	-2.45184
tr|B3KPZ0|B3KPZ0_HUMAN	tr|B3KPZ0	cDNA FLJ32485 fis, clone SKNMC2001596, highly similar to Ankyrin repeat and FYVE domain-containing protein 1	121468	387	0	45	0	0.03	0	0	1	0	1	0	1	0	1	0	-2.45184
tr|E5RGY7|E5RGY7_HUMAN	CTNNA1	Catenin alpha-1 (Fragment) OS=Homo sapiens GN=CTNNA1 PE=2 SV=1	14895	389	0	35	0	0.07	0	0	0	0	1	0	1	0	1	0	-2.45184
tr|K7EPJ3|K7EPJ3_HUMAN	DDX39A	ATP-dependent RNA helicase DDX39A (Fragment) OS=Homo sapiens GN=DDX39A PE=4 SV=1	21690	395	0	44	0	0.11	0	0	1	0	1	0	1	0	2	0	-2.45184
tr|H0Y327|H0Y327_HUMAN	MTHFD1L	Monofunctional C1-tetrahydrofolate synthase, mitochondrial (Fragment) OS=Homo sapiens GN=MTHFD1L PE=4	36808	398	0	43	0	0.09	0	0	1	0	1	0	1	0	1	0	-2.45184
tr|Q1LZN2|Q1LZN2_HUMAN	NOMO3	NOMO3 protein (Fragment) OS=Homo sapiens GN=NOMO3 PE=2 SV=1	90388	400	0	43	0	0.04	0	0	1	0	1	0	1	0	1	0	-2.45184
tr|F2Z2D0|F2Z2D0_HUMAN	NUP205	Nuclear pore complex protein Nup205 OS=Homo sapiens GN=NUP205 PE=4 SV=1	23590	415	0	21	0	0.04	0	0	0	0	1	0	1	0	1	0	-2.45184
tr|H0YGE9|H0YGE9_HUMAN	M6PR	Cation-dependent mannose-6-phosphate receptor (Fragment) OS=Homo sapiens GN=M6PR PE=4 SV=1	11814	416	0	42	0	0.29	0	0	1	0	1	0	1	0	1	0	-2.45184
tr|O60250|O60250_HUMAN	tr|O60250	Ribosomal protein L13 (Fragment) OS=Homo sapiens PE=2 SV=2	7385	422	0	41	0	0.14	0	0	1	0	1	0	1	0	1	0	-2.45184
tr|A4D1I7|A4D1I7_HUMAN	DNCI1	Dynein, cytoplasmic 1, intermediate chain 1, isoform CRA_e OS=Homo sapiens GN=DNCI1 PE=2 SV=1	73423	427	0	40	0	0.04	0	0	1	0	1	0	1	0	1	0	-2.45184
tr|A2VCT2|A2VCT2_HUMAN	OGDH	OGDH protein (Fragment) OS=Homo sapiens GN=OGDH PE=2 SV=1	72973	432	0	40	0	0.05	0	0	1	0	1	0	1	0	1	0	-2.45184
tr|E9PI83|E9PI83_HUMAN	PRMT1	Protein arginine N-methyltransferase 1 (Fragment) OS=Homo sapiens GN=PRMT1 PE=2 SV=1	12790	437	0	36	0	0.26	0	0	0	0	1	0	1	0	1	0	-2.45184
tr|B7Z943|B7Z943_HUMAN	tr|B7Z943	cDNA FLJ53131, highly similar to 24-dehydrocholesterol reductase (EC 1.3.1.-) OS=Homo sapiens PE=2 SV=1	29093	442	0	39	0	0.05	0	0	1	0	1	0	1	0	2	0	-2.45184
tr|B4E363|B4E363_HUMAN	FARSA	Phenylalanine--tRNA ligase alpha subunit OS=Homo sapiens GN=FARSA PE=2 SV=1	54181	447	0	38	0	0.06	0	0	1	0	1	0	1	0	1	0	-2.45184
tr|B3KME2|B3KME2_HUMAN	tr|B3KME	cDNA FLJ10772 fis, clone NT2RP4000243, highly similar to Cartilage-associated protein OS=Homo sapiens PE=2 S	47101	459	0	38	0	0.07	0	0	1	0	1	0	1	0	1	0	-2.45184
tr|H7C1M0|H7C1M0_HUMAN	FANCL	E3 ubiquitin-protein ligase FANCL (Fragment) OS=Homo sapiens GN=FANCL PE=4 SV=1	33067	461	0	37	0	0.1	0	0	1	0	1	0	1	0	1	0	-2.45184
tr|Q5SZ64|Q5SZ64_HUMAN	SF3B4	Splicing factor 3B subunit 4 (Fragment) OS=Homo sapiens GN=SF3B4 PE=4 SV=1	20733	464	0	37	0	0.07	0	0	1	0	1	0	1	0	1	0	-2.45184
sp|Q6ZU64|CC108_HUMAN	CCDC108	Coiled-coil domain-containing protein 108 OS=Homo sapiens GN=CCDC108 PE=2 SV=2	220247	465	0	37	0	0.01	0	0	1	0	1	0	1	0	1	0	-2.45184
tr|B4DDP6|B4DDP6_HUMAN	DBNL	Drebrin-like protein OS=Homo sapiens GN=DBNL PE=2 SV=1	43242	466	0	37	0	0.08	0	0	1	0	1	0	1	0	1	0	-2.45184
tr|C9J7L0|C9J7L0_HUMAN	RBBP7	Histone-binding protein RBBP7 (Fragment) OS=Homo sapiens GN=RBBP7 PE=2 SV=1	7740	468	0	0	0	0.07	0	0	0	0	0	0	1	0	1	0	-2.45184
tr|H0YIX9|H0YIX9_HUMAN	R3HDM2	R3H domain-containing protein 2 (Fragment) OS=Homo sapiens GN=R3HDM2 PE=4 SV=1	31776	469	0	37	0	0.1	0	0	1	0	1	0	1	0	1	0	-2.45184
tr|B4DKI2|B4DKI2_HUMAN	tr|B4DKI2	cDNA FLJ60282, highly similar to Sorbitol dehydrogenase (EC 1.1.1.14) OS=Homo sapiens PE=2 SV=1	30315	473	0	37	0	0.09	0	0	0	0	1	0	1	0	1	0	-2.45184
tr|B7Z982|B7Z982_HUMAN	PLRG1	Pleiotropic regulator 1 OS=Homo sapiens GN=PLRG1 PE=2 SV=1	14839	476	0	0	0	0.12	0	0	0	0	0	0	1	0	1	0	-2.45184
tr|B7WPL0|B7WPL0_HUMAN	RIC8B	Synembryn-B OS=Homo sapiens GN=RIC8B PE=2 SV=1	64252	483	0	36	0	0.05	0	0	1	0	1	0	1	0	1	0	-2.45184

tr|C9JNW5|C9JNW5_HUMAN	RPL24	60S ribosomal protein L24 OS=Homo sapiens GN=RPL24 PE=4 SV=1	17646	487	0	35	0	0.19	0	0	0	0	1	0	1	0	2	0	-2.45184
sp|Q9NPQ8|RIC8A_HUMAN	RIC8A	Synembryn-A OS=Homo sapiens GN=RIC8A PE=1 SV=3	60185	496	0	34	0	0.05	0	0	0	0	1	0	1	0	1	0	-2.45184
tr|B4DDC9|B4DDC9_HUMAN	tr|B4DDC	cDNA FLJ59448, highly similar to Trifunctional enzyme subunit beta, mitochondrial OS=Homo sapiens PE=2 SV=1	49937	499	0	34	0	0.07	0	0	0	0	1	0	1	0	1	0	-2.45184
tr|B4DXJ6|B4DXJ6_HUMAN	tr|B4DXJ6	cDNA FLJ61453, highly similar to Adapter-relatedprotein complex 2 alpha- 1 subunit OS=Homo sapiens PE=2 SV=	55511	500	0	34	0	0.03	0	0	0	0	1	0	1	0	1	0	-2.45184
sp|Q8NFZ5|TNIP2_HUMAN	TNIP2	TNFAIP3-interacting protein 2 OS=Homo sapiens GN=TNIP2 PE=1 SV=1	49240	502	0	33	0	0.07	0	0	1	0	1	0	1	0	2	0	-2.45184
sp|Q7Z494|NPHP3_HUMAN	NPHP3	Nephrocystin-3 OS=Homo sapiens GN=NPHP3 PE=1 SV=1	151967	510	0	32	0	0.02	0	0	1	0	1	0	1	0	1	0	-2.45184
tr|A0JLS9|A0JLS9_HUMAN	ZEB1	ZEB1 protein (Fragment) OS=Homo sapiens GN=ZEB1 PE=2 SV=1	118576	513	0	32	0	0.03	0	0	1	0	1	0	1	0	1	0	-2.45184
tr|J3KRB3|J3KRB3_HUMAN	RPL17	60S ribosomal protein L17 (Fragment) OS=Homo sapiens GN=RPL17 PE=3 SV=1	12356	517	0	32	0	0.28	0	0	0	0	1	0	1	0	1	0	-2.45184
tr|B4DUH7|B4DUH7_HUMAN	tr|B4DUH	cDNA FLJ60162, highly similar to Chloride anion exchanger OS=Homo sapiens PE=2 SV=1	52632	518	0	32	0	0.06	0	0	1	0	1	0	1	0	1	0	-2.45184
tr|E9PNN6|E9PNN6_HUMAN	ALDH3A1	Aldehyde dehydrogenase, dimeric NADP-preferring OS=Homo sapiens GN=ALDH3A1 PE=2 SV=1	44518	519	0	32	0	0.07	0	0	0	0	1	0	1	0	1	0	-2.45184
tr|B7Z8W6|B7Z8W6_HUMAN	tr|B7Z8W	cDNA FLJ55038, highly similar to Nicotinamide phosphoribosyltransferase (EC 2.4.2.12) OS=Homo sapiens PE=2 S	26905	520	0	32	0	0.12	0	0	0	0	1	0	1	0	3	0	-2.45184
tr|B4E0D5|B4E0D5_HUMAN	tr|B4E0D5	cDNA FLJ54218, highly similar to Acidic leucine-rich nuclear phosphoprotein 32 family member E OS=Homo sapie	19540	525	0	31	0	0.17	0	0	0	0	1	0	1	0	1	0	-2.45184
tr|F2Z2J7|F2Z2J7_HUMAN	TTLL7	Tubulin polyglutamylase TTLL7 OS=Homo sapiens GN=TTLL7 PE=2 SV=1	55925	526	0	31	0	0.06	0	0	1	0	1	0	1	0	1	0	-2.45184
tr|B7Z9W6|B7Z9W6_HUMAN	tr|B7Z9W	cDNA, FLJ78980, highly similar to DnaJ homolog subfamily C member 7 OS=Homo sapiens PE=2 SV=1	50776	527	0	31	0	0.06	0	0	0	0	1	0	1	0	1	0	-2.45184
tr|A1L3B5|A1L3B5_HUMAN	ATP2B3	ATP2B3 protein OS=Homo sapiens GN=ATP2B3 PE=2 SV=1	96278	528	0	31	0	0.03	0	0	0	0	1	0	1	0	1	0	-2.45184
sp|Q96PE2|ARHGH_HUMAN	ARHGEF17	Rho guanine nucleotide exchange factor 17 OS=Homo sapiens GN=ARHGEF17 PE=1 SV=1	223645	537	0	30	0	0.01	0	0	1	0	1	0	1	0	1	0	-2.45184
tr|B6F210|B6F210_HUMAN	GSS	Glutathione synthetase OS=Homo sapiens GN=GSS PE=2 SV=1	40495	551	0	30	0	0.08	0	0	0	0	1	0	1	0	1	0	-2.45184
tr|H3BRB3|H3BRB3_HUMAN	KIF22	Kinesin-like protein KIF22 OS=Homo sapiens GN=KIF22 PE=3 SV=1	67996	557	0	29	0	0.05	0	0	0	0	1	0	1	0	1	0	-2.45184
tr|B4DS04|B4DS04_HUMAN	tr|B4DS04	cDNA FLJ54142, highly similar to Protein transport protein Sec23B OS=Homo sapiens PE=2 SV=1	41720	558	0	27	0	0.08	0	0	0	0	1	0	1	0	1	0	-2.45184
sp|Q96A33|CCD47_HUMAN	CCDC47	Coiled-coil domain-containing protein 47 OS=Homo sapiens GN=CCDC47 PE=1 SV=1	56123	560	0	29	0	0.06	0	0	0	0	1	0	1	0	1	0	-2.45184
tr|E7EN38|E7EN38_HUMAN	RAD50	DNA repair protein RAD50 (Fragment) OS=Homo sapiens GN=RAD50 PE=2 SV=1	77138	563	0	29	0	0.09	0	0	0	0	2	0	1	0	2	0	-2.45184
tr|I3L361|I3L361_HUMAN	SGSM2	Small G protein-signaling modulator 2 (Fragment) OS=Homo sapiens GN=SGSM2 PE=4 SV=2	1642	570	0	29	0	2.38	0	0	0	0	1	0	1	0	1	0	-2.45184
tr|B3KWR2|B3KWR2_HUMAN	tr|B3KWR	cDNA FLJ43638 fis, clone SPLEN2039845, highly similar to IMPORTIN ALPHA-1 SUBUNIT OS=Homo sapiens PE=2 S	38087	573	0	22	0	0.11	0	0	0	0	1	0	1	0	1	0	-2.45184
tr|B4DNG6|B4DNG6_HUMAN	tr|B4DNG	Annexin OS=Homo sapiens PE=3 SV=1	12870	577	0	28	0	0.27	0	0	0	0	1	0	1	0	1	0	-2.45184
sp|Q16322|KCA10_HUMAN	KCNA10	Potassium voltage-gated channel subfamily A member 10 OS=Homo sapiens GN=KCNA10 PE=1 SV=2	58147	578	0	28	0	0.06	0	0	1	0	1	0	1	0	1	0	-2.45184
tr|B3KV72|B3KV72_HUMAN	tr|B3KV72	cDNA FLJ16204 fis, clone CTONG2019232, highly similar to Proteasome-associated protein ECM29 homolog (Frag	78791	579	0	28	0	0.04	0	0	0	0	1	0	1	0	1	0	-2.45184
sp|Q14683|SMC1A_HUMAN	SMC1A	Structural maintenance of chromosomes protein 1A OS=Homo sapiens GN=SMC1A PE=1 SV=2	143771	581	0	28	0	0.02	0	0	0	0	1	0	1	0	1	0	-2.45184
tr|J3KN32|J3KN32_HUMAN	RCOR1	REST corepressor 1 OS=Homo sapiens GN=RCOR1 PE=4 SV=1	53409	583	0	28	0	0.06	0	0	0	0	1	0	1	0	1	0	-2.45184
tr|H0YCU9|H0YCU9_HUMAN	TAGLN	Transgelin (Fragment) OS=Homo sapiens GN=TAGLN PE=4 SV=1	16833	585	0	28	0	0.2	0	0	0	0	1	0	1	0	1	0	-2.45184
tr|A6NE42|A6NE42_HUMAN	TGIF1	Homeobox protein TGIF1 OS=Homo sapiens GN=TGIF1 PE=3 SV=2	27983	586	0	28	0	0.12	0	0	0	0	1	0	1	0	1	0	-2.45184
tr|Q9NZ23|Q9NZ23_HUMAN	YA61	Drug-sensitive protein 1 OS=Homo sapiens GN=YA61 PE=2 SV=1	14858	588	0	27	0	0.23	0	0	0	0	1	0	1	0	1	0	-2.45184
tr|H3BS19|H3BS19_HUMAN	ZNF469	Zinc finger protein 469 OS=Homo sapiens GN=ZNF469 PE=4 SV=1	417347	591	0	27	0	0.01	0	0	0	0	1	0	1	0	1	0	-2.45184
tr|H0Y512|H0Y512_HUMAN	APMAP	Adipocyte plasma membrane-associated protein (Fragment) OS=Homo sapiens GN=APMAP PE=4 SV=1	45662	592	0	27	0	0.07	0	0	0	0	1	0	1	0	1	0	-2.45184
tr|B7Z4L9|B7Z4L9_HUMAN	tr|B7Z4L9	cDNA FLJ55342, highly similar to Kelch-like protein 13 OS=Homo sapiens PE=2 SV=1	56884	593	0	26	0	0.06	0	0	0	0	1	0	1	0	1	0	-2.45184
tr|Q5T8C6|Q5T8C6_HUMAN	CDC16	Cell division cycle protein 16 homolog OS=Homo sapiens GN=CDC16 PE=4 SV=1	55239	594	0	26	0	0.06	0	0	0	0	1	0	1	0	1	0	-2.45184
sp|Q0VD83|APOBR_HUMAN	APOBR	Apolipoprotein B receptor OS=Homo sapiens GN=APOBR PE=1 SV=1	115207	597	0	26	0	0.03	0	0	0	0	1	0	1	0	1	0	-2.45184
tr|B4DNK3|B4DNK3_HUMAN	tr|B4DNK	cDNA FLJ52127, highly similar to Multisynthetase complex auxiliary component p43 OS=Homo sapiens PE=2 SV=	29955	601	0	26	0	0.11	0	0	0	0	1	0	1	0	1	0	-2.45184
tr|B4E2I9|B4E2I9_HUMAN	tr|B4E2I9	cDNA FLJ55486, highly similar to Semaphorin-3C OS=Homo sapiens PE=2 SV=1	88481	602	0	26	0	0.04	0	0	0	0	1	0	1	0	2	0	-2.45184
tr|J3KNP0|J3KNP0_HUMAN	RAB11FIP1	Rab11 family-interacting protein 1 OS=Homo sapiens GN=RAB11FIP1 PE=4 SV=1	137482	607	0	25	0	0.02	0	0	0	0	1	0	1	0	2	0	-2.45184
tr|J3KSB9|J3KSB9_HUMAN	tr|J3KSB9	Uncharacterized protein (Fragment) OS=Homo sapiens PE=4 SV=1	8877	608	0	25	0	0.39	0	0	0	0	1	0	1	0	1	0	-2.45184
tr|H3BMW5|H3BMW5_HUMAN	DNAJA2	DnaJ homolog subfamily A member 2 (Fragment) OS=Homo sapiens GN=DNAJA2 PE=4 SV=1	15134	611	0	24	0	0.23	0	0	0	0	1	0	1	0	1	0	-2.45184
tr|Q4VAN1|Q4VAN1_HUMAN	BTN1A1	BTN1A1 protein OS=Homo sapiens GN=BTN1A1 PE=2 SV=1	55878	617	0	24	0	0.06	0	0	0	0	1	0	1	0	1	0	-2.45184
tr|Q2TQ40|Q2TQ40_HUMAN	TNFRSF17	B cell maturation antigen transcript variant 4 OS=Homo sapiens GN=TNFRSF17 PE=2 SV=1	15460	618	0	24	0	0.22	0	0	0	0	1	0	1	0	1	0	-2.45184
tr|B4DU06|B4DU06_HUMAN	tr|B4DU0	Guanylate cyclase OS=Homo sapiens PE=2 SV=1	91756	620	0	24	0	0.04	0	0	0	0	1	0	1	0	1	0	-2.45184
tr|B0QXZ5|B0QXZ5_HUMAN	DNAL4	Dynein light chain 4, axonemal OS=Homo sapiens GN=DNAL4 PE=4 SV=1	7786	621	0	24	0	0.47	0	0	0	0	1	0	1	0	1	0	-2.45184
tr|F5H120|F5H120_HUMAN	FKBP4	Peptidyl-prolyl cis-trans isomerase FKBP4, N-terminally processed OS=Homo sapiens GN=FKBP4 PE=2 SV=1	5528	623	0	24	0	0.06	0	0	0	0	1	0	1	0	1	0	-2.45184
tr|B4DN13|B4DN13_HUMAN	tr|B4DN1	cDNA FLJ59136, highly similar to Transmembrane 9 superfamily protein member 3 OS=Homo sapiens PE=2 SV=1	29629	624	0	23	0	0.11	0	0	0	0	1	0	1	0	1	0	-2.45184
tr|K7EPB5|K7EPB5_HUMAN	ELAVL3	ELAV-like protein 3 (Fragment) OS=Homo sapiens GN=ELAVL3 PE=4 SV=1	9728	625	0	23	0	0.36	0	0	0	0	1	0	1	0	1	0	-2.45184
tr|K7EJ37|K7EJ37_HUMAN	EXOSC10	Exosome component 10 OS=Homo sapiens GN=EXOSC10 PE=4 SV=1	13869	627	0	23	0	0.05	0	0	0	0	1	0	1	0	1	0	-2.45184
sp|Q9Y5P6|GMPPB_HUMAN	GMPPB	Mannose-1-phosphate guanyltransferase beta OS=Homo sapiens GN=GMPPB PE=1 SV=2	40379	629	0	23	0	0.08	0	0	0	0	1	0	1	0	1	0	-2.45184
tr|B3KPR5|B3KPR5_HUMAN	tr|B3KPR5	cDNA FLJ32094 fis, clone OCBBF2000986, highly similar to Homo sapiens elongation factor Tu GTP binding domai	97046	633	0	22	0	0.03	0	0	0	0	1	0	1	0	1	0	-2.45184
tr|Q5VSQ6|Q5VSQ6_HUMAN	P4HA1	Procollagen-proline, 2-oxoglutarate 4-dioxygenase (Proline 4-hydroxylase), alpha polypeptide I variant (Fragment	61214	634	0	22	0	0.05	0	0	0	0	1	0	1	0	2	0	-2.45184
tr|Q9MYF7|Q9MYF7_HUMAN	HLA-A	MHC class I antigen (Fragment) OS=Homo sapiens GN=HLA-A PE=2 SV=1	21085	639	0	22	0	0.16	0	0	0	0	1	0	1	0	3	0	-2.45184
tr|B5BU01|B5BU01_HUMAN	EIF2S2	Eukaryotic translation initiation factor 2 beta OS=Homo sapiens GN=EIF2S2 PE=2 SV=1	38648	654	0	22	0	0.09	0	0	0	0	1	0	1	0	1	0	-2.45184
tr|Q5M7Z5|Q5M7Z5_HUMAN	GRHPR	GRHPR protein (Fragment) OS=Homo sapiens GN=GRHPR PE=2 SV=1	37314	659	0	21	0	0.09	0	0	0	0	1	0	1	0	1	0	-2.45184
tr|C9J935|C9J935_HUMAN	SETD9	SET domain-containing protein 9 (Fragment) OS=Homo sapiens GN=SETD9 PE=2 SV=1	5515	660	0	21	0	0.65	0	0	0	0	1	0	1	0	1	0	-2.45184
tr|F1T0A5|F1T0A5_HUMAN	PRPF31	PRP31 pre-mRNA processing factor 31 homolog (Yeast), isoform CRA_a OS=Homo sapiens GN=PRPF31 PE=2 SV=	55649	661	0	21	0	0.06	0	0	0	0	1	0	1	0	2	0	-2.45184
tr|B3KRG8|B3KRG8_HUMAN	tr|B3KRG	Lipase OS=Homo sapiens PE=2 SV=1	45736	662	0	21	0	0.07	0	0	0	0	1	0	1	0	1	0	-2.45184
tr|J3KN69|J3KN69_HUMAN	NCEH1	Arylacetamide deacetylase-like 1 OS=Homo sapiens GN=NCEH1 PE=4 SV=1	49431	663	0	21	0	0.07	0	0	0	0	1	0	1	0	1	0	-2.45184
tr|Q6UWE9|Q6UWE9_HUMAN	UNQ2550	SFVP2550 OS=Homo sapiens GN=UNQ2550 PE=4 SV=1	11890	664	0	21	0	0.29	0	0	1	0	1	0	1	0	1	0	-2.45184
tr|J3QT54|J3QT54_HUMAN	CPSF7	Cleavage and polyadenylation-specificity factor subunit 7 (Fragment) OS=Homo sapiens GN=CPSF7 PE=4 SV=1	22762	665	0	21	0	0.15	0	0	0	0	1	0	1	0	1	0	-2.45184
tr|B4DK80|B4DK80_HUMAN	TOP3A	DNA topoisomerase OS=Homo sapiens GN=TOP3A PE=2 SV=1	103082	668	0	20	0	0.03	0	0	0	0	1	0	1	0	1	0	-2.45184
tr|A0AVG7|A0AVG7_HUMAN	BCAS1	Breast carcinoma amplified sequence 1 OS=Homo sapiens GN=BCAS1 PE=2 SV=1	61929	670	0	20	0	0.05	0	0	0	0	1	0	1	0	1	0	-2.45184
tr|H0YJL6|H0YJL6_HUMAN	EVL	Ena/VASP-like protein (Fragment) OS=Homo sapiens GN=EVL PE=4 SV=1	10347	671	0	20	0	0.34	0	0	0	0	1	0	1	0	1	0	-2.45184
sp|P55212|CASP6_HUMAN	CASP6	Caspase-6 OS=Homo sapiens GN=CASP6 PE=1 SV=2	33859	674	0	20	0	0.1	0	0	0	0	1	0	1	0	1	0	-2.45184
tr|B4DE48|B4DE48_HUMAN	tr|B4DE48	cDNA FLJ53824, highly similar to Sorting nexin-17 OS=Homo sapiens PE=2 SV=1	50009	677	0	20	0	0.07	0	0	0	0	1	0	1	0	1	0	-2.45184
sp|O95159|ZFPL1_HUMAN	ZFPL1	Zinc finger protein-like 1 OS=Homo sapiens GN=ZFPL1 PE=1 SV=2	34833	678	0	20	0	0.1	0	0	0	0	1	0	1	0	1	0	-2.45184
tr|Q8WUM5|Q8WUM5_HUMAN	GEMIN4	Gem (Nuclear organelle) associated protein 4 OS=Homo sapiens GN=GEMIN4 PE=2 SV=1	121732	709	0	17	0	0.03	0	0	0	0	1	0	1	0	2	0	-2.45184
tr|Q7KZ86|Q7KZ86_HUMAN	tr|Q7KZ86	Plakoglobin (Fragment) OS=Homo sapiens PE=2 SV=1	19007	26	0	37	0	1.74	0	0	1	0	2	0	2	0	2	0	-3.18915
tr|G3V241|G3V241_HUMAN	SHMT2	Serine hydroxymethyltransferase, mitochondrial (Fragment) OS=Homo sapiens GN=SHMT2 PE=2 SV=1	15985	86	0	71	0	0.61	0	0	1	0	2	0	2	0	3	0	-3.18915
tr|J3QSY6|J3QSY6_HUMAN	ATP2A2	Sarcoplasmic/endoplasmic reticulum calcium ATPase 2 OS=Homo sapiens GN=ATP2A2 PE=4 SV=1	5838	149	0	53	0	0.32	0	0	1	0	2	0	2	0	2	0	-3.18915
tr|B4DNT9|B4DNT9_HUMAN	tr|B4DNT	cDNA FLJ57756, highly similar to Paxillin OS=Homo sapiens PE=2 SV=1	17270	159	0	44	0	0.28	0	0	1	0	2	0	2	0	2	0	-3.18915
tr|Q5JVP3|Q5JVP3_HUMAN	PTGR1	Leukotriene B4 12-hydroxydehydrogenase, isoform CRA_d OS=Homo sapiens GN=PTGR1 PE=4 SV=1	19441	190	0	113	0	0.38	0	0	2	0	2	0	2	0	2	0	-3.18915
tr|B3KVK2|B3KVK2_HUMAN	GNB1	Guanine nucleotide-binding protein G(I)/G(S)/G(T) subunit beta-1 OS=Homo sapiens GN=GNB1 PE=2 SV=1	26659	199	0	105	0	0.18	0	0	1	0	2	0	2	0	2	0	-3.18915
tr|B4E0W4|B4E0W4_HUMAN	tr|B4E0W	cDNA FLJ61020, highly similar to Heterogeneous nuclear ribonucleoprotein D0 OS=Homo sapiens PE=2 SV=1	23062	229	0	75	0	0.37	0	0	2	0	2	0	2	0	2	0	-3.18915
tr|G3V1S6|G3V1S6_HUMAN	GALNT2	Polypeptide N-acetylgalactosaminyltransferase 2 soluble form OS=Homo sapiens GN=GALNT2 PE=2 SV=1	61440	254	0	76	0	0.11	0	0	2	0	2	0	2	0	2	0	-3.18915
sp|Q6IS14|IF5AL_HUMAN	EIF5AL1	Eukaryotic translation initiation factor 5A-1-like OS=Homo sapiens GN=EIF5AL1 PE=1 SV=2	16990	256	0	75	0	0.44	0	0	1	0	2	0	2	0	2	0	-3.18915
tr|Q2QD09|Q2QD09_HUMAN	tr|Q2QD0	Triosephosphate isomerase (Fragment) OS=Homo sapiens PE=2 SV=1	27211	267	0	71	0	0.26	0	0	1	0	2	0	2	0	2	0	-3.18915
tr|B1ANK7|B1ANK7_HUMAN	FH	Fumarate hydratase OS=Homo sapiens GN=FH PE=2 SV=1	54773	288	0	64	0	0.12	0	0	1	0	2	0	2	0	5	0	-3.18915
tr|B4DMY3|B4DMY3_HUMAN	tr|B4DMY	cDNA FLJ60713, highly similar to Homo sapiens heterogeneous nuclear ribonucleoprotein A/B (HNRPAB), transcr	35101	318	0	56	0	0.2	0	0	1	0	2	0	2	0	2	0	-3.18915
tr|G3V180|G3V180_HUMAN	DPP3	Dipeptidyl peptidase 3 OS=Homo sapiens GN=DPP3 PE=2 SV=1	84666	335	0	52	0	0.08	0	0	1	0	2	0	2	0	2	0	-3.18915
tr|E5RHF2|E5RHF2_HUMAN	COPS5	COP9 signalosome complex subunit 5 (Fragment) OS=Homo sapiens GN=COPS5 PE=2 SV=1	16655	346	0	51	0	0.45	0	0	0	0	2	0	2	0	2	0	-3.18915
tr|Q6NZ52|Q6NZ52_HUMAN	RPL27A	Ribosomal protein L27a OS=Homo sapiens GN=RPL27A PE=2 SV=1	16582	348	0	50	0	0.2	0	0	1	0	1	0	2	0	2	0	-3.18915
tr|H0Y9H1|H0Y9H1_HUMAN	CANX	Calnexin (Fragment) OS=Homo sapiens GN=CANX PE=3 SV=1	21748	352	0	49	0	0.39	0	0	1	0	2	0	2	0	3	0	-3.18915
tr|Q53GU8|Q53GU8_HUMAN	tr|Q53GU	Transforming growth factor, beta-induced, 68kDa variant (Fragment) OS=Homo sapiens PE=2 SV=1	75231	358	0	48	0	0.09	0	0	1	0	2	0	2	0	2	0	-3.18915
sp|P01024|CO3_HUMAN	C3	Complement C3 OS=Homo sapiens GN=C3 PE=1 SV=2	188569	370	0	47	0	0.03	0	0	1	0	2	0	2	0	2	0	-3.18915
tr|H7C314|H7C314_HUMAN	CTTN	Src substrate cortactin (Fragment) OS=Homo sapiens GN=CTTN PE=4 SV=1	25136	378	0	46	0	0.11	0	0	1	0	2	0	2	0	2	0	-3.18915
sp|P53004|BIEA_HUMAN	BLVRA	Biliverdin reductase A OS=Homo sapiens GN=BLVRA PE=1 SV=2	33692	403	0	43	0	0.21	0	0	1	0	2	0	2	0	3	0	-3.18915
tr|A0MNP2|A0MNP2_HUMAN	WDR57	CDW11/WDR57 OS=Homo sapiens GN=WDR57 PE=2 SV=1	39742	413	0	42	0	0.17	0	0	1	0	2	0	2	0	3	0	-3.18915
tr|A5D904|A5D904_HUMAN	RPS9	RPS9 protein (Fragment) OS=Homo sapiens GN=RPS9 PE=2 SV=1	13210	434	0	40	0	0.58	0	0	1	0	2	0	2	0	2	0	-3.18915
tr|Q7Z3K9|Q7Z3K9_HUMAN	DKFZp781	Putative uncharacterized protein DKFZp781L0540 (Fragment) OS=Homo sapiens GN=DKFZp781L0540 PE=2 SV=1	90081	440	0	39	0	0.04	0	0	1	0	1	0	2	0	2	0	-3.18915
tr|H0Y7N2|H0Y7N2_HUMAN	ATXN10	Ataxin-10 (Fragment) OS=Homo sapiens GN=ATXN10 PE=4 SV=1	19107	470	0	37	0	0.38	0	0	0	0	2	0	2	0	2	0	-3.18915
tr|B2R4P2|B2R4P2_HUMAN	tr|B2R4P2	cDNA, FLJ92164, highly similar to Homo sapiens peroxiredoxin 1 (PRDX1), mRNA OS=Homo sapiens PE=2 SV=1	22414	481	0	36	0	0.32	0	0	0	0	2	0	2	0	2	0	-3.18915
tr|A0PJ76|A0PJ76_HUMAN	NCAPD2	NCAPD2 protein (Fragment) OS=Homo sapiens GN=NCAPD2 PE=2 SV=1	44957	494	0	34	0	0.15	0	0	0	0	2	0	2	0	2	0	-3.18915
tr|C1KES1|C1KES1_HUMAN	LRRC16B	LRRC16B (Fragment) OS=Homo sapiens GN=LRRC16B PE=4 SV=1	4125	530	0	31	0	0.94	0	0	0	0	1	0	2	0	2	0	-3.18915
tr|B3KNB4|B3KNB4_HUMAN	tr|B3KNB	cDNA FLJ14168 fis, clone NT2RP2001440, highly similar to 14-3-3 protein gamma OS=Homo sapiens PE=2 SV=1	28453	541	0	30	0	0.25	0	0	0	0	2	0	2	0	2	0	-3.18915
tr|B2RD27|B2RD27_HUMAN	tr|B2RD27	cDNA, FLJ96428, highly similar to Homo sapiens proteasome (prosome, macropain) 26S subunit, non-ATPase, 7 (	37046	550	0	30	0	0.19	0	0	0	0	2	0	2	0	2	0	-3.18915
sp|O75367|H2AY_HUMAN	H2AFY	Core histone macro-H2A.1 OS=Homo sapiens GN=H2AFY PE=1 SV=4	39764	565	0	29	0	0.08	0	0	0	0	1	0	2	0	2	0	-3.18915
tr|A6NLN1|A6NLN1_HUMAN	PTBP1	Polypyrimidine tract binding protein 1, isoform CRA_e OS=Homo sapiens GN=PTBP1 PE=4 SV=3	21899	567	0	26	0	0.18	0	0	0	0	2	0	2	0	2	0	-3.18915
tr|H3BNV2|H3BNV2_HUMAN	NQO1	NAD(P)H dehydrogenase [quinone] 1 OS=Homo sapiens GN=NQO1 PE=4 SV=1	28533	576	0	28	0	0.25	0	0	0	0	2	0	2	0	2	0	-3.18915
sp|Q9UHR4|BI2L1_HUMAN	BAIAP2L1	Brain-specific angiogenesis inhibitor 1-associated protein 2-like protein 1 OS=Homo sapiens GN=BAIAP2L1 PE=1 S	57189	613	0	24	0	0.12	0	0	0	0	2	0	2	0	4	0	-3.18915
tr|D3DSQ0|D3DSQ0_HUMAN	PCM1	Pericentriolar material 1, isoform CRA_a OS=Homo sapiens GN=PCM1 PE=2 SV=1	230041	619	0	24	0	0.01	0	0	0	0	1	0	2	0	3	0	-3.18915
tr|I0BW39|I0BW39_HUMAN	HLA-A	MHC class I antigen (Fragment) OS=Homo sapiens GN=HLA-A PE=4 SV=1	8492	640	0	22	0	0.16	0	0	0	0	1	0	2	0	2	0	-3.18915
tr|H3BUF6|H3BUF6_HUMAN	ATXN2L	Ataxin-2-like protein OS=Homo sapiens GN=ATXN2L PE=4 SV=1	112929	656	0	22	0	0.03	0	0	0	0	1	0	2	0	2	0	-3.18915
sp|P35237|SPB6_HUMAN	SERPINB6	Serpin B6 OS=Homo sapiens GN=SERPINB6 PE=1 SV=3	42936	120	0	179	0	0.25	0	0	3	0	3	0	3	0	3	0	-3.67492
tr|Q6ZVC6|Q6ZVC6_HUMAN	tr|Q6ZVC6	cDNA FLJ42761 fis, clone BRAWH3002574, highly similar to Calpain 2, large OS=Homo sapiens PE=2 SV=1	24572	173	0	82	0	0.22	0	0	2	0	2	0	3	0	3	0	-3.67492
tr|B4DMU0|B4DMU0_HUMAN	PYCR1	Pyrroline-5-carboxylate reductase OS=Homo sapiens GN=PYCR1 PE=2 SV=1	36186	174	0	125	0	0.19	0	0	2	0	2	0	3	0	3	0	-3.67492
tr|A2T926|A2T926_HUMAN	TMPO	Thymopentin OS=Homo sapiens GN=TMPO PE=2 SV=1	27428	241	0	41	0	0.24	0	0	0	0	3	0	3	0	3	0	-3.67492
tr|C9JI87|C9JI87_HUMAN	VDAC1	Voltage-dependent anion-selective channel protein 1 (Fragment) OS=Homo sapiens GN=VDAC1 PE=2 SV=1	20418	250	0	78	0	0.36	0	0	1	0	3	0	3	0	3	0	-3.67492
tr|Q5JQZ9|Q5JQZ9_HUMAN	CDC2L6	Cell division cycle 2-like 6 (CDK8-like) (Fragment) OS=Homo sapiens GN=CDC2L6 PE=4 SV=1	29552	285	0	65	0	0.24	0	0	2	0	2	0	3	0	3	0	-3.67492
tr|Q0VGL3|Q0VGL3_HUMAN	RPL13A	RPL13A protein OS=Homo sapiens GN=RPL13A PE=2 SV=1	16706	300	0	59	0	0.74	0	0	3	0	3	0	3	0	3	0	-3.67492
tr|B2R6J4|B2R6J4_HUMAN	tr|B2R6J4	cDNA, FLJ92975, highly similar to Homo sapiens nucleosome assembly protein 1-like 4 (NAP1L4), mRNA OS=Hom	42940	417	0	42	0	0.16	0	0	0	0	2	0	3	0	3	0	-3.67492
tr|Q53XC0|Q53XC0_HUMAN	EIF2S1	Eukaryotic translation initiation factor 2, subunit 1 alpha, 35kDa, isoform CRA_a OS=Homo sapiens GN=EIF2S1 PE	36374	208	0	100	0	0.42	0	0	4	0	4	0	4	0	4	0	-4.03783
tr|D6RAT0|D6RAT0_HUMAN	RPS3A	40S ribosomal protein S3a OS=Homo sapiens GN=RPS3A PE=3 SV=1	26099	223	0	93	0	0.44	0	0	2	0	3	0	4	0	5	0	-4.03783
tr|E9PKU4|E9PKU4_HUMAN	RPL8	60S ribosomal protein L8 (Fragment) OS=Homo sapiens GN=RPL8 PE=2 SV=1	25806	231	0	90	0	0.28	0	0	2	0	2	0	4	0	5	0	-4.03783
sp|A2RTY3|CQ066_HUMAN	C17orf66	Uncharacterized protein C17orf66 OS=Homo sapiens GN=C17orf66 PE=2 SV=2	66437	477	0	36	0	0.05	0	0	0	0	1	0	4	0	8	0	-4.03783
tr|F8VQP2|F8VQP2_HUMAN	ATXN2	Ataxin-2 OS=Homo sapiens GN=ATXN2 PE=2 SV=1	117012	157	0	135	0	0.21	0	0	4	0	7	0	10	0	10	0	-5.26229

#05	SHSY-5Y live unsyn MLN-2_2µM																		
#08	SHSY-5Y live unsyn NP_2µM																		
Accession	GS	Name	Pro_Mass	08_prot	05_pr	08_prot_scor	05_prot	08_emP	05_emPAI	emPAI rati	08_No>I	05_No>I	08_No>HI	05_No>HI	08_No>20	05_No>20	08_No=All	05_No=All	Rsc
sp|Q05655|KPCD_HUMAN	PRKCD (PKC-δ)	Protein kinase C delta type OS=Homo sapiens GN=PRKCD PE=1 SV=2	78652	0	591	0	61	0	0.05	#DIV/0!	0	1	0	1	0	1	0	1	1.991402
sp|Q16512|PKN1_HUMAN	PKN1	Serine/threonine-protein kinase N1 OS=Homo sapiens GN=PKN1 PE=1 SV=2	104666	0	646	0	55	0	0.21	#DIV/0!	0	1	0	1	0	1	0	1	1.991402
sp|O75116|ROCK2_HUMAN	ROCK2	Rho-associated protein kinase 2 OS=Homo sapiens GN=ROCK2 PE=1 SV=4	161939	826	950	41	29	0.04	0.04	1	1	0	1	1	1	1	3	1	0.406244
sp|P28482|MK01_HUMAN	MAPK1 (ERK2)	Mitogen-activated protein kinase 1 OS=Homo sapiens GN=MAPK1 PE=1 SV=3	41762	0	619	0	57	0	0.16	#DIV/0!	0	1	0	2	0	2	0	2	2.728626
sp|P49841|GSK3B_HUMAN	GSK3B	Glycogen synthase kinase-3 beta OS=Homo sapiens GN=GSK3B PE=1 SV=2	47228	0	483	0	76	0	0.07	#DIV/0!	0	1	0	1	0	1	0	1	1.991402
sp|P20794|MAK_HUMAN	MAK	Serine/threonine-protein kinase MAK OS=Homo sapiens GN=MAK PE=1 SV=2	70764	0	974	0	28	0	0.05	#DIV/0!	0	0	0	1	0	1	0	1	1.991402
sp|P11802|CDK4_HUMAN	CDK4	Cyclin-dependent kinase 4 OS=Homo sapiens GN=CDK4 PE=1 SV=2	33936	0	967	0	28	0	0.1	#DIV/0!	0	0	0	1	0	1	0	1	1.991402
sp|P06493|CDK1_HUMAN	CDK1	Cyclin-dependent kinase 1 OS=Homo sapiens GN=CDK1 PE=1 SV=3	34131	918	644	35	48	0.15	0.58	3.866667	1	0	1	3	1	3	1	3	1.629154
sp|Q96KB5|TOPK_HUMAN	PBK	Lymphokine-activated killer T-cell-originated protein kinase OS=Homo sapiens GN=P	36404	0	858	0	36	0	0.18	#DIV/0!	0	0	0	2	0	3	0	3	3.214312
sp|P33981|TTK_HUMAN	TTK	Dual specificity protein kinase TTK OS=Homo sapiens GN=TTK PE=1 SV=2	97980	1102	678	24	52	0.03	0.07	2.333333	0	1	1	2	2	2	2	3	0.406307
sp|Q96SB4|SRPK1_HUMAN	SRPK1	SRSF protein kinase 1 OS=Homo sapiens GN=SRPK1 PE=1 SV=2	74963	407	868	100	35	0.13	0.09	0.692308	2	0	3	2	3	2	4	2	-0.07931
sp|P07205|PGK2_HUMAN	PGK2	Phosphoglycerate kinase 2 OS=Homo sapiens GN=PGK2 PE=1 SV=3	45166	119	115	331	284	1.03	0.98	0.951456	8	6	9	10	12	11	12	13	0.28639
sp|P00558|PGK1_HUMAN	PGK1	Phosphoglycerate kinase 1 OS=Homo sapiens GN=PGK1 PE=1 SV=3	44985	119	116	334	284	1.03	0.89	0.864078	8	6	10	10	13	11	13	13	0.175163
sp|P32189|GLPK_HUMAN	GK	Glycerol kinase OS=Homo sapiens GN=GK PE=1 SV=3	62060	0	1074	0	22	0	0.26	#DIV/0!	0	0	0	1	0	1	0	1	1.991402
sp|P12532|KCRU_HUMAN	CKMT1A	Creatine kinase U-type, mitochondrial OS=Homo sapiens GN=CKMT1A PE=1 SV=1	47406	0	682	0	52	0	0.07	#DIV/0!	0	1	0	1	0	1	0	1	1.991402
sp|P52789|HXK2_HUMAN	HK2	Hexokinase-2 OS=Homo sapiens GN=HK2 PE=1 SV=2	103739	0	662	0	53	0	0.03	#DIV/0!	0	1	0	1	0	1	0	1	1.991402
sp|Q9H999|PANK3_HUMAN	PANK3	Pantothenate kinase 3 OS=Homo sapiens GN=PANK3 PE=1 SV=1	41467	0	1143	0	17	0	0.08	#DIV/0!	0	0	0	1	0	0	0	1	0.40618
sp|P12277|KCRB_HUMAN	CKB	Creatine kinase B-type OS=Homo sapiens GN=CKB PE=1 SV=1	42902	39	43	798	652	1.82	2.52	1.384615	15	13	22	23	27	28	29	32	0.459692
tr|J3KPD9|J3KPD9_HUMAN	NME2	Nucleoside diphosphate kinase B OS=Homo sapiens GN=NME2 PE=3 SV=1	22522	903	398	36	91	0.32	0.33	1.03125	0	2	2	2	2	3	2	6	0.891993
sp|P62714|PP2AB_HUMAN	PPP2CB	Serine/threonine-protein phosphatase 2A catalytic subunit beta isoform OS=Homo s	36123	1038	570	28	50	0.12	0.3	2.5	0	1	1	2	1	1	2	2	0.406244
sp|P67775|PP2AA_HUMAN	PPP2CA	Serine/threonine-protein phosphatase 2A catalytic subunit alpha isoform OS=Homo	36142	1038	570	28	64	0.12	0.3	2.5	0	1	1	3	1	2	2	3	1.143468
sp|P36873|PP1G_HUMAN	PPP1CC	Serine/threonine-protein phosphatase PP1-gamma catalytic subunit OS=Homo sapie	37701	225	286	166	127	0.52	0.71	1.365385	3	3	4	6	5	6	5	8	0.647765
sp|Q9NTJ5|SAC1_HUMAN	SACM1L	Phosphatidylinositide phosphatase SAC1 OS=Homo sapiens GN=SACM1L PE=1 SV=2	67437	0	1085	0	21	0	0.05	#DIV/0!	0	0	0	1	0	1	0	1	1.991402
sp|Q96HS1|PGAM5_HUMAN	PGAM5	Serine/threonine-protein phosphatase PGAM5, mitochondrial OS=Homo sapiens GN	32213	0	736	0	46	0	0.1	#DIV/0!	0	1	0	1	0	1	0	1	1.991402
sp|P36507|MP2K2_HUMAN	MAP2K2 (MEK2)	Dual specificity mitogen-activated protein kinase kinase 2 OS=Homo sapiens GN=MA	44681	1001	1071	31	22	0.14	0.08	0.571429	0	0	1	1	1	1	1	1	0.406244
sp|Q00005|2ABB_HUMAN	PPP2R2B	Serine/threonine-protein phosphatase 2A 55 kDa regulatory subunit B beta isoform	52077	0	1047	0	24	0	0.06	#DIV/0!	0	0	0	1	0	1	0	2	1.991402
tr|E5RIY1|E5RIY1_HUMAN	PPP2R2A	Serine/threonine-protein phosphatase 2A 55 kDa regulatory subunit B alpha isoform	14798	0	819	0	39	0	0.23	#DIV/0!	0	1	0	1	0	1	0	1	1.991402
sp|O15355|PPM1G_HUMAN	PPM1G	Protein phosphatase 1G OS=Homo sapiens GN=PPM1G PE=1 SV=1	59919	858	191	39	181	0.05	0.31	6.2	1	3	1	5	2	5	3	6	1.544588
tr|E5RFR9|E5RFR9_HUMAN	PPP2R2A	Serine/threonine-protein phosphatase 2A 55 kDa regulatory subunit B alpha isoform	23448	871	822	38	24	0.14	0.2	1.428571	1	0	1	1	1	1	1	1	0.406244
tr|F5H0K8|F5H0K8_HUMAN	KSR1	kinase suppressor of Ras 1 OS=Homo sapiens GN=KSR1 PE=2 SV=1	98138	0	459	0	79	0	0.03	#DIV/0!	0	1	0	1	0	1	0	1	1.991402
tr|D6RAR6|D6RAR6_HUMAN	FASTKD3	FAST kinase domain-containing protein 3 OS=Homo sapiens GN=FASTKD3 PE=2 SV=1	70592	0	956	0	29	0	0.05	#DIV/0!	0	1	0	1	0	1	0	1	1.991402
sp|P29966|MARCS_HUMAN	MARCKS	Myristoylated alanine-rich C-kinase substrate OS=Homo sapiens GN=MARCKS PE=1 S	31707	483	1028	80	25	0.22	0.81	3.681818	1	0	2	1	2	1	3	1	-0.33092
tr|B0AZV0|B0AZV0_HUMAN	STRAP	Serine-threonine kinase receptor-associated protein OS=Homo sapiens GN=STRAP P	28716	412	259	100	136	0.55	0.39	0.709091	2	3	4	3	4	3	4	4	0.043605
tr|B4DPM0|B4DPM0_HUMA	tr|B4DPM0|B4DPM	Pyruvate kinase OS=Homo sapiens PE=2 SV=1	52984	8	798	42	41	6.17	0.05	0.008104	1	1	1	1	1	1	1	2	0.406244
sp|Q5H9R7|PP6R3_HUMAN	PPP6R3	Serine/threonine-protein phosphatase 6 regulatory subunit 3 OS=Homo sapiens GN=	98577	663	781	54	42	0.1	0.12	1.2	1	1	2	1	2	1	2	1	-0.33092
tr|B3KS15|B3KS15_HUMAN	tr|B3KS15|B3KS15	6-phosphofructokinase OS=Homo sapiens PE=2 SV=1	86526	237	154	168	170	0.2	0.2	1	4	4	5	5	6	6	6	6	0.406562
tr|B3KRS6|B3KRS6_HUMAN	tr|B3KRS6|B3KRS6	cDNA FLJ34842 fis, clone NT2NE2011033, highly similar to Serine/threonine-protein	39398	479	688	81	51	0.17	0.17	1	2	2	2	2	2	2	2	2	0.406307
tr|A4D1U5|A4D1U5_HUMAN	FLJ10842	Multiple substrate lipid kinase, isoform CRA_a OS=Homo sapiens GN=FLJ10842 PE=2	47564	747	1103	48	21	0.07	0.07	1	1	0	1	1	1	1	2	1	0.406244
tr|H0YJ50|H0YJ50_HUMAN	VRK1	Serine/threonine-protein kinase VRK1 (Fragment) OS=Homo sapiens GN=VRK1 PE=4	26962	830	937	41	30	0.12	0.12	1	1	0	1	1	1	1	1	1	0.406244
tr|H0Y3C5|H0Y3C5_HUMAN	HCK	Tyrosine-protein kinase HCK OS=Homo sapiens GN=HCK PE=4 SV=1	59986	905	882	36	34	0.05	0.05	1	1	1	1	1	1	1	1	1	0.406244
sp|P17858|K6PL_HUMAN	PFKL	6-phosphofructokinase, liver type OS=Homo sapiens GN=PFKL PE=1 SV=6	85762	685	533	53	68	0.04	0.04	1	1	1	1	1	2	1	3	2	-0.33092
tr|B3KXY9|B3KXY9_HUMAN	tr|B3KXY9|B3KXY9	cDNA FLJ46359 fis, clone TESTI4049786, highly similar to Hexokinase-1 (EC 2.7.1.1) C	107381	336	557	119	66	0.16	0.09	0.5625	4	2	5	3	7	3	8	4	-0.69395
sp|P78527|PRKDC_HUMAN	PRKDC	DNA-dependent protein kinase catalytic subunit OS=Homo sapiens GN=PRKDC PE=1	473749	9	23	1588	1049	0.58	0.32	0.551724	43	25	68	42	73	46	83	54	-0.25671
tr|G1UI32|G1UI32_HUMAN	PDXK	Pyridoxal kinase (Fragment) OS=Homo sapiens GN=PDXK PE=2 SV=1	19070	567	534	65	68	0.38	0.18	0.473684	1	1	2	1	2	1	2	1	-0.33092
tr|H0Y630|H0Y630_HUMAN	STK24	Serine/threonine-protein kinase 24 12 kDa subunit (Fragment) OS=Homo sapiens GN	41209	479	891	81	33	0.17	0.08	0.470588	2	1	2	1	2	1	2	2	-0.33092
sp|P14618|KPYM_HUMAN	PKM	Pyruvate kinase isozymes M1/M2 OS=Homo sapiens GN=PKM PE=1 SV=4	58470	8	18	1592	1210	6.17	2.72	0.440843	35	27	53	39	55	41	58	44	-0.01333
tr|B2R6T7|B2R6T7_HUMAN	tr|B2R6T7|B2R6T7	Ribose-phosphate pyrophosphokinase OS=Homo sapiens PE=2 SV=1	35181	374	496	108	74	0.88	0.31	0.352273	3	2	7	3	8	4	8	5	-0.51189
tr|J3KNX3|J3KNX3_HUMAN	PFKM	6-phosphofructokinase OS=Homo sapiens GN=PFKM PE=3 SV=1	94107	549	713	68	48	0.39	0.03	0.076923	1	1	2	1	2	1	2	1	-0.33092
tr|C1PHA0|C1PHA0_HUMAN	EML4-ALK	Tyrosine-protein kinase receptor OS=Homo sapiens GN=EML4-ALK variant 6 PE=2 SV	120561	971	0	0	0	0.03	0	0	0	0	0	0	0	0	2	0	0.40618
tr|H0YK74|H0YK74_HUMAN	PAK6	Serine/threonine-protein kinase PAK 6 (Fragment) OS=Homo sapiens GN=PAK6 PE=4	12466	1186	0	18	0	0.28	0	0	0	0	1	0	0	0	1	0	0.40618
tr|B4DS37|B4DS37_HUMAN	tr|B4DS37|B4DS37	cDNA FLJ58258, highly similar to Cyclin G-associated kinase (EC 2.7.11.1) OS=Homo s	133373	1195	0	18	0	0.02	0	0	0	0	1	0	0	0	1	0	0.40618
tr|F5H7P4|F5H7P4_HUMAN	AKAP3	A-kinase anchor protein 3 (Fragment) OS=Homo sapiens GN=AKAP3 PE=2 SV=1	18443	1196	0	17	0	0.18	0	0	0	0	1	0	0	0	1	0	0.40618
tr|B4DRH8|B4DRH8_HUMAN	tr|B4DRH8|B4DRH	cDNA FLJ55358, highly similar to Mitogen-activated protein kinase kinase kinase7 (E	46041	1203	0	16	0	0.07	0	0	0	0	1	0	0	0	1	0	0.40618
tr|H0YG84|H0YG84_HUMAN	PRKDC	DNA-dependent protein kinase catalytic subunit (Fragment) OS=Homo sapiens GN=F	10177	9	0	32	0	0.58	0	0	0	0	2	0	1	0	3	0	-1.17898
tr|C9JJV0|C9JJV0_HUMAN	STK25	Serine/threonine-protein kinase 25 (Fragment) OS=Homo sapiens GN=STK25 PE=2 S	8717	479	0	61	0	0.17	0	0	1	0	1	0	1	0	1	0	-1.17898
sp|P49137|MAPK2_HUMAN	MAPKAPK2	MAP kinase-activated protein kinase 2 OS=Homo sapiens GN=MAPKAPK2 PE=1 SV=1	45938	528	0	72	0	0.07	0	0	1	0	1	0	1	0	1	0	-1.17898
tr|B4E108|B4E108_HUMAN	tr|B4E108|B4E108	cDNA FLJ61026, highly similar to Homo sapiens phosphofructokinase, liver (PFKL), tr	27257	685	0	53	0	0.04	0	0	1	0	1	0	1	0	1	0	-1.17898
tr|B4DR72|B4DR72_HUMAN	tr|B4DR72|B4DR7	cDNA FLJ59963, highly similar to Homo sapiens multiple substrate lipid kinase (MULI	27090	747	0	48	0	0.07	0	0	1	0	1	0	1	0	1	0	-1.17898
tr|C9JIG9|C9JIG9_HUMAN	OXSR1	Serine/threonine-protein kinase OSR1 OS=Homo sapiens GN=OXSR1 PE=2 SV=1	52109	926	0	35	0	0.06	0	0	1	0	1	0	1	0	1	0	-1.17898
tr|B2R841|B2R841_HUMAN	tr|B2R841|B2R841	cDNA, FLJ93730, highly similar to Homo sapiens polo-like kinase (Drosophila) (PLK), r	68963	967	0	33	0	0.05	0	0	0	0	1	0	1	0	2	0	-1.17898
tr|F5H5M9|F5H5M9_HUMAN	TNIK	TRAF2 and NCK-interacting protein kinase OS=Homo sapiens GN=TNIK PE=2 SV=1	150042	989	0	31	0	0.02	0	0	1	0	1	0	1	0	5	0	-1.17898
tr|B4DIR9|B4DIR9_HUMAN	TAB2	TGF-beta-activated kinase 1 and MAP3K7-binding protein 2 OS=Homo sapiens GN=T	73273	1010	0	30	0	0.04	0	0	0	0	1	0	1	0	1	0	-1.17898
tr|B4DV85|B4DV85_HUMAN	tr|B4DV85|B4DV8	cDNA FLJ53211, highly similar to A-Raf proto-oncogene serine/threonine-protein kin	52561	1044	0	28	0	0.06	0	0	0	0	1	0	1	0	1	0	-1.17898
tr|H0YFG1|H0YFG1_HUMAN	EPS8	Epidermal growth factor receptor kinase substrate 8 (Fragment) OS=Homo sapiens G	7139	1149	0	21	0	0.5	0	0	0	0	1	0	1	0	1	0	-1.17898
sp|Q96GX5|GWL_HUMAN	MASTL	Serine/threonine-protein kinase greatwall OS=Homo sapiens GN=MASTL PE=1 SV=1	98398	1163	0	21	0	0.03	0	0	0	0	1	0	1	0	1	0	-1.17898
tr|E7ETY4|E7ETY4_HUMAN	MARK2	Serine/threonine-protein kinase MARK2 OS=Homo sapiens GN=MARK2 PE=2 SV=1	78803	1164	0	21	0	0.04	0	0	0	0	1	0	1	0	2	0	-1.17898
tr|D3DUP1|D3DUP1_HUMAN	WNK1	WNK lysine deficient protein kinase 1, isoform CRA_b OS=Homo sapiens GN=WNK1	251457	404	0	101	0	0.03	0	0	2	0	2	0	2	0	2	0	-1.91614
tr|F8VP00|F8VP00_HUMAN	PFKM	6-phosphofructokinase, muscle type (Fragment) OS=Homo sapiens GN=PFKM PE=2 S	18709	549	0	68	0	0.39	0	0	1	0	2	0	2	0	2	0	-1.91614
tr|B4E1G6|B4E1G6_HUMAN	GALK1	Galactokinase OS=Homo sapiens GN=GALK1 PE=2 SV=1	45956	597	0	62	0	0.15	0	0	1	0	2	0	2	0	2	0	-1.91614
sp|Q12802|AKP13_HUMAN	AKAP13	A-kinase anchor protein 13 OS=Homo sapiens GN=AKAP13 PE=1 SV=2	310381	842	0	40	0	0.01	0	0	1	0	1	0	2	0	5	0	-1.91614
tr|G3XAA2|G3XAA2_HUMAN	MAP4K4	Mitogen-activated protein kinase kinase kinase kinase 4 OS=Homo sapiens GN=MAP	134173	1026	0	29	0	0.05	0	0	1	0	2	0	2	0	4	0	-1.91614
tr|B4E1D9|B4E1D9_HUMAN	tr|B4E1D9|B4E1D9	cDNA FLJ55318, highly similar to Casein kinase I isoform alpha (EC 2.7.11.1) OS=Hom	26899	1002	0	31	0	0.26	0	0	1	0	2	0	3	0	3	0	-2.40176
tr|B4DZC9|B4DZC9_HUMAN	tr|B4DZC9|B4DZC9	cDNA FLJ61681, highly similar to STE20-like serine/threonine-protein kinase (EC2.7.1	120359	667	0	54	0	0.08	0	0	1	0	3	0	5	0	6	0	-3.05423
tr|A8KAH7|A8KAH7_HUMAN	tr|A8KAH7|A8KAH	cDNA FLJ75444, highly similar to Homo sapiens protein kinase, cAMP-dependent, re	45818	775	0	45	0	0.23	0	0	1	0	3	0	5	0	6	0	-3.05423
tr|B4DDF7|B4DDF7_HUMAN	tr|B4DDF7|B4DDF	cDNA FLJ53296, highly similar to Serine/threonine-protein phosphatase 2A 65 kDa re	64939	140	158	279	214	0.81	0.34	0.419753	7	3	12	6	13	6	17	7	-0.64925
sp|Q8NCN5|PDPR_HUMAN	PDPR	Pyruvate dehydrogenase phosphatase regulatory subunit, mitochondrial OS=Homo s	100156	1200	0	17	0	0.03	0	0	0	0	1	0	0	0	2	0	0.40618
tr|H0YDU8|H0YDU8_HUMAN	PPP5C	Serine/threonine-protein phosphatase (Fragment) OS=Homo sapiens GN=PPP5C PE=	55743	504	0	76	0	0.06	0	0	1	0	1	0	1	0	1	0	-1.17898
tr|A8K6Y2|A8K6Y2_HUMAN	tr|A8K6Y2|A8K6Y2	cDNA FLJ76895, highly similar to Homo sapiens protein phosphatase 2, regulatory su	70276	733	0	49	0	0.05	0	0	1	0	1	0	1	0	2	0	-1.17898
tr|H0YF12|H0YF12_HUMAN	PTPN11	Tyrosine-protein phosphatase non-receptor type 11 (Fragment) OS=Homo sapiens G	12387	812	0	43	0	0.28	0	0	1	0	1	0	1	0	1	0	-1.17898
sp|Q9Y2T4|2ABG_HUMAN	PPP2R2C	Serine/threonine-protein phosphatase 2A 55 kDa regulatory subunit B gamma isofor	51939	871	0	38	0	0.14	0	0	1	0	1	0	1	0	1	0	-1.17898
tr|J3QT22|J3QT22_HUMAN	PPME1	Protein phosphatase methylesterase 1 OS=Homo sapiens GN=PPME1 PE=3 SV=1	44241	955	0	33	0	0.07	0	0	1	0	1	0	1	0	1	0	-1.17898
sp|O75170|PP6R2_HUMAN	PPP6R2	Serine/threonine-protein phosphatase 6 regulatory subunit 2 OS=Homo sapiens GN=	106358	1120	0	23	0	0.03	0	0	1	0	1	0	1	0	1	0	-1.17898
tr|H0YEN2|H0YEN2_HUMAN	PPP6R3	Serine/threonine-protein phosphatase 6 regulatory subunit 3 (Fragment) OS=Homo	64367	663	0	54	0	0.1	0	0	1	0	2	0	2	0	2	0	-1.91614
sp|Q06124|PTN11_HUMAN	PTPN11	Tyrosine-protein phosphatase non-receptor type 11 OS=Homo sapiens GN=PTPN11	68964	852	0	40	0	0.1	0	0	1	0	2	0	2	0	2	0	-1.91614
tr|A8K8B0|A8K8B0_HUMAN	tr|A8K8B0|A8K8B0	cDNA FLJ76434, highly similar to Homo sapiens protein phosphatase 2 (formerly 2A)	74135	140	0	96	0	0.81	0	0	2	0	3	0	3	0	4	0	-2.40176
tr|C9J9S3|C9J9S3_HUMAN	PPP1CB	Serine/threonine-protein phosphatase (Fragment) OS=Homo sapiens GN=PPP1CB PE	14484	225	0	126	0	0.52	0	0	2	0	3	0	4	0	4	0	-2.76453
sp|Q15181|IPYR_HUMAN	PPA1	Inorganic pyrophosphatase OS=Homo sapiens GN=PPA1 PE=1 SV=2	33095	414	0	99	0	0.61	0	0	2	0	5	0	5	0	5	0	-3.05423
tr|Q1WWK5|Q1WWK5_HUM	Sep-09	SEPT9 protein (Fragment) OS=Homo sapiens GN=SEPT9 PE=2 SV=1	36717	0	413	0	87	0	0.54	#DIV/0!	0	2	0	5	0	5	0	5	3.866906
tr|J3KQ96|J3KQ96_HUMAN	TCOF1	Treacle protein (Fragment) OS=Homo sapiens GN=TCOF1 PE=4 SV=1	144268	0	152	0	219	0	0.12	#DIV/0!	0	4	0	5	0	5	0	6	3.866906
tr|Q6FHQ0|Q6FHQ0_HUMAN	RBBP7	RBBP7 protein (Fragment) OS=Homo sapiens GN=RBBP7 PE=2 SV=1	48132	0	731	0	46	0	0.14	#DIV/0!	0	1	0	2	0	4	0	5	3.577141
tr|A4D110|A4D110_HUMAN	LOC401309	Putative uncharacterized protein OS=Homo sapiens GN=LOC401309 PE=4 SV=1	13436	0	149	0	223	0	0.25	#DIV/0!	0	4	0	4	0	4	0	4	3.577141
tr|Q2HJ12|Q2HJ12_HUMAN	CCDC125	CCDC125 protein OS=Homo sapiens GN=CCDC125 PE=4 SV=1	12142	0	947	0	29	0	0.3	#DIV/0!	0	1	0	1	0	3	0	3	3.214312
tr|Q5XJ04|Q5XJ04_HUMAN	HMGCS1	3-hydroxy-3-methylglutaryl-Coenzyme A synthase 1 (Soluble) OS=Homo sapiens GN=	57854	0	846	0	37	0	0.18	#DIV/0!	0	0	0	3	0	3	0	3	3.214312
tr|Q149P0|Q149P0_HUMAN	GBF1	GBF1 protein OS=Homo sapiens GN=GBF1 PE=2 SV=1	207938	0	709	0	48	0	0.03	#DIV/0!	0	1	0	2	0	3	0	3	3.214312
tr|B3KQI5|B3KQI5_HUMAN	tr|B3KQI5|B3KQI5	cDNA FLJ90522 fis, clone NT2RP4000108, highly similar to Neurofilament triplet L pr	61508	0	704	0	49	0	0.17	#DIV/0!	0	1	0	3	0	3	0	3	3.214312
sp|Q09028|RBBP4_HUMAN	RBBP4	Histone-binding protein RBBP4 OS=Homo sapiens GN=RBBP4 PE=1 SV=3	47911	0	650	0	54	0	0.14	#DIV/0!	0	2	0	2	0	3	0	3	3.214312
sp|Q96A33|CCD47_HUMAN	CCDC47	Coiled-coil domain-containing protein 47 OS=Homo sapiens GN=CCDC47 PE=1 SV=1	56123	0	528	0	69	0	0.12	#DIV/0!	0	1	0	2	0	3	0	4	3.214312
tr|Q5VU21|Q5VU21_HUMAN	SERBP1	PAI-1 mRNA-binding protein variant OS=Homo sapiens GN=SERBP1 PE=2 SV=1	42458	0	510	0	71	0	0.25	#DIV/0!	0	2	0	3	0	3	0	4	3.214312
sp|O43815|STRN_HUMAN	STRN	Striatin OS=Homo sapiens GN=STRN PE=1 SV=4	86535	0	453	0	80	0	0.04	#DIV/0!	0	2	0	2	0	3	0	4	3.214312
tr|Q6IBR8|Q6IBR8_HUMAN	EIF2S2	EIF2S2 protein OS=Homo sapiens GN=EIF2S2 PE=2 SV=1	38706	0	422	0	85	0	0.28	#DIV/0!	0	1	0	3	0	3	0	3	3.214312
sp|O60216|RAD21_HUMAN	RAD21	Double-strand-break repair protein rad21 homolog OS=Homo sapiens GN=RAD21 PE	71930	0	366	0	100	0	0.14	#DIV/0!	0	3	0	3	0	3	0	5	3.214312
tr|B3KXC0|B3KXC0_HUMAN	tr|B3KXC0|B3KXC0	cDNA FLJ45113 fis, clone BRAWH3034743, weakly similar to Mus musculus microtub	68137	0	365	0	100	0	0.15	#DIV/0!	0	2	0	3	0	3	0	3	3.214312
tr|Q5JR04|Q5JR04_HUMAN	MOV10	Mov10, Moloney leukemia virus 10, homolog (Mouse) OS=Homo sapiens GN=MOV1	107941	0	360	0	102	0	0.06	#DIV/0!	0	2	0	2	0	3	0	4	3.214312
tr|B0UX83|B0UX83_HUMAN	BAG6	HLA-B associated transcript 3 OS=Homo sapiens GN=BAG6 PE=4 SV=1	119085	0	351	0	103	0	0.08	#DIV/0!	0	2	0	3	0	3	0	3	3.214312
tr|B4DJW8|B4DJW8_HUMAN	tr|B4DJW8|B4DJW	cDNA FLJ55697, highly similar to Protein transport protein Sec23B OS=Homo sapiens	85347	0	326	0	110	0	0.12	#DIV/0!	0	1	0	3	0	3	0	3	3.214312
tr|Q05BL1|Q05BL1_HUMAN	TP53BP2	TP53BP2 protein OS=Homo sapiens GN=TP53BP2 PE=2 SV=1	116431	0	1075	0	22	0	0.03	#DIV/0!	0	0	0	1	0	2	0	2	2.728626
tr|B3KM47|B3KM47_HUMAN	tr|B3KM47|B3KM4	cDNA FLJ10273 fis, clone HEMBB1001137, highly similar to SEC23-interacting protein	111730	0	1016	0	25	0	0.03	#DIV/0!	0	0	0	1	0	2	0	3	2.728626
tr|J3KTL2|J3KTL2_HUMAN	SRSF1	Serine/arginine-rich-splicing factor 1 OS=Homo sapiens GN=SRSF1 PE=4 SV=1	28426	0	1011	0	26	0	0.25	#DIV/0!	0	0	0	2	0	2	0	2	2.728626
tr|B3KTL8|B3KTL8_HUMAN	tr|B3KTL8|B3KTL8	cDNA FLJ38476 fis, clone FEBRA2022504, highly similar to YTH domain protein 1 OS=	61006	0	1006	0	26	0	0.11	#DIV/0!	0	0	0	2	0	2	0	2	2.728626
tr|B7ZKL3|B7ZKL3_HUMAN	EPS8L2	EPS8L2 protein OS=Homo sapiens GN=EPS8L2 PE=2 SV=1	82898	0	1004	0	26	0	0.04	#DIV/0!	0	1	0	1	0	2	0	3	2.728626
tr|B4DG89|B4DG89_HUMAN	NPLOC4	Nuclear protein localization protein 4 homolog OS=Homo sapiens GN=NPLOC4 PE=2	52144	0	960	0	29	0	0.06	#DIV/0!	0	0	0	1	0	2	0	2	2.728626
sp|P14735|IDE_HUMAN	IDE	Insulin-degrading enzyme OS=Homo sapiens GN=IDE PE=1 SV=4	118692	0	959	0	29	0	0.03	#DIV/0!	0	0	0	1	0	2	0	2	2.728626
sp|Q86Z20|CC125_HUMAN	CCDC125	Coiled-coil domain-containing protein 125 OS=Homo sapiens GN=CCDC125 PE=1 SV=	59218	0	949	0	29	0	0.06	#DIV/0!	0	1	0	1	0	2	0	3	2.728626
sp|Q8TDB6|DTX3L_HUMAN	DTX3L	E3 ubiquitin-protein ligase DTX3L OS=Homo sapiens GN=DTX3L PE=1 SV=1	84585	0	935	0	30	0	0.04	#DIV/0!	0	1	0	1	0	2	0	3	2.728626
tr|Q05BW9|Q05BW9_HUMA	PAPSS1	PAPSS1 protein (Fragment) OS=Homo sapiens GN=PAPSS1 PE=2 SV=1	65264	0	878	0	34	0	0.1	#DIV/0!	0	0	0	2	0	2	0	2	2.728626
tr|B3KM58|B3KM58_HUMAN	tr|B3KM58|B3KM5	cDNA FLJ10358 fis, clone NT2RM2001238, highly similar to Glutaminase kidney isofo	41736	0	840	0	37	0	0.16	#DIV/0!	0	0	0	2	0	2	0	2	2.728626
tr|H0Y7L7|H0Y7L7_HUMAN	PITRM1	Presequence protease, mitochondrial (Fragment) OS=Homo sapiens GN=PITRM1 PE=	29816	0	839	0	37	0	0.24	#DIV/0!	0	1	0	2	0	2	0	3	2.728626
tr|B3KMR5|B3KMR5_HUMAN	tr|B3KMR5|B3KMI	cDNA FLJ12434 fis, clone NT2RM1000037, highly similar to Homo sapiens KIAA0690	145007	0	837	0	37	0	0.02	#DIV/0!	0	1	0	1	0	2	0	2	2.728626
tr|A5Z217|A5Z217_HUMAN	tr|A5Z217|A5Z217	Mutant desmin OS=Homo sapiens PE=2 SV=1	53620	0	816	0	39	0	0.13	#DIV/0!	0	1	0	2	0	2	0	3	2.728626
sp|Q5BKZ1|ZN326_HUMAN	ZNF326	DBIRD complex subunit ZNF326 OS=Homo sapiens GN=ZNF326 PE=1 SV=2	65955	0	789	0	41	0	0.1	#DIV/0!	0	1	0	2	0	2	0	2	2.728626
tr|Q6IBU0|Q6IBU0_HUMAN	EIF5	EIF5 protein OS=Homo sapiens GN=EIF5 PE=2 SV=1	49577	0	776	0	43	0	0.14	#DIV/0!	0	1	0	2	0	2	0	3	2.728626
tr|Q7Z3H5|Q7Z3H5_HUMAN	DKFZp686O0215	Putative uncharacterized protein DKFZp686O0215 (Fragment) OS=Homo sapiens GN	37820	0	771	0	43	0	0.18	#DIV/0!	0	1	0	2	0	2	0	2	2.728626
tr|B7Z600|B7Z600_HUMAN	tr|B7Z600|B7Z600	cDNA FLJ53180, highly similar to Cullin-3 OS=Homo sapiens PE=2 SV=1	63726	0	766	0	44	0	0.11	#DIV/0!	0	1	0	2	0	2	0	2	2.728626
tr|B3KSZ4|B3KSZ4_HUMAN	tr|B3KSZ4|B3KSZ4	cDNA FLJ37346 fis, clone BRAMY2021310, highly similar to Transcriptional repressor	65560	0	757	0	45	0	0.1	#DIV/0!	0	1	0	2	0	2	0	2	2.728626
tr|B2R7Y3|B2R7Y3_HUMAN	tr|B2R7Y3|B2R7Y3	cDNA, FLJ93657, highly similar to Homo sapiens ribosomal protein L3-like (RPL3L), m	46581	0	754	0	45	0	0.15	#DIV/0!	0	1	0	2	0	2	0	2	2.728626
tr|B4DL07|B4DL07_HUMAN	tr|B4DL07|B4DL07	cDNA FLJ53353, highly similar to ATP-binding cassette sub-family D member 3 OS=H	78980	0	749	0	45	0	0.08	#DIV/0!	0	0	0	2	0	2	0	2	2.728626
sp|Q86Y56|HEAT2_HUMAN	HEATR2	HEAT repeat-containing protein 2 OS=Homo sapiens GN=HEATR2 PE=1 SV=4	94774	0	740	0	46	0	0.03	#DIV/0!	0	1	0	1	0	2	0	2	2.728626
tr|B3KMI4|B3KMI4_HUMAN	tr|B3KMI4|B3KMI4	cDNA FLJ11102 fis, clone PLACE1005646, highly similar to Homo sapiens DEAD (Asp-	43468	0	734	0	46	0	0.16	#DIV/0!	0	1	0	2	0	2	0	2	2.728626
sp|Q5TFE4|NT5D1_HUMAN	NT5DC1	5~-nucleotidase domain-containing protein 1 OS=Homo sapiens GN=NT5DC1 PE=1 S	52439	0	725	0	47	0	0.2	#DIV/0!	0	0	0	3	0	2	0	3	2.728626
tr|E0X098|E0X098_HUMAN	TES	Testin variant (Fragment) OS=Homo sapiens GN=TES PE=2 SV=1	22009	0	722	0	47	0	0.33	#DIV/0!	0	1	0	2	0	2	0	2	2.728626
tr|B2RD79|B2RD79_HUMAN	tr|B2RD79|B2RD7	Ubiquitin carboxyl-terminal hydrolase OS=Homo sapiens PE=2 SV=1	56462	0	708	0	48	0	0.12	#DIV/0!	0	1	0	2	0	2	0	2	2.728626
sp|P52907|CAZA1_HUMAN	CAPZA1	F-actin-capping protein subunit alpha-1 OS=Homo sapiens GN=CAPZA1 PE=1 SV=3	33073	0	695	0	50	0	0.21	#DIV/0!	0	1	0	2	0	2	0	3	2.728626
sp|Q96AG4|LRC59_HUMAN	LRRC59	Leucine-rich repeat-containing protein 59 OS=Homo sapiens GN=LRRC59 PE=1 SV=1	35308	0	690	0	51	0	0.2	#DIV/0!	0	1	0	2	0	2	0	2	2.728626
tr|B3KPA6|B3KPA6_HUMAN	ACADVL	Acyl-Coenzyme A dehydrogenase, very long chain, isoform CRA_e OS=Homo sapiens	62941	0	679	0	52	0	0.11	#DIV/0!	0	1	0	2	0	2	0	2	2.728626
tr|B4E3W1|B4E3W1_HUMAN	tr|B4E3W1|B4E3W	cDNA FLJ57204, highly similar to Homo sapiens ancient ubiquitous protein 1 (AUP1),	51668	0	668	0	53	0	0.13	#DIV/0!	0	1	0	2	0	2	0	3	2.728626
sp|Q9NU22|MDN1_HUMAN	MDN1	Midasin OS=Homo sapiens GN=MDN1 PE=1 SV=2	638008	0	649	0	54	0	0.01	#DIV/0!	0	1	0	2	0	2	0	3	2.728626
tr|C9JTZ6|C9JTZ6_HUMAN	NT5DC2	5~-nucleotidase domain-containing protein 2 OS=Homo sapiens GN=NT5DC2 PE=2 S	61649	0	645	0	55	0	0.11	#DIV/0!	0	1	0	2	0	2	0	3	2.728626
tr|H0Y580|H0Y580_HUMAN	EIF2B3	Translation initiation factor eIF-2B subunit gamma (Fragment) OS=Homo sapiens GN	25394	0	621	0	57	0	0.45	#DIV/0!	0	0	0	3	0	2	0	3	2.728626
tr|H7BXZ6|H7BXZ6_HUMAN	RHOT1	Mitochondrial Rho GTPase OS=Homo sapiens GN=RHOT1 PE=3 SV=2	69432	0	604	0	59	0	0.1	#DIV/0!	0	2	0	2	0	2	0	3	2.728626
tr|H3BPE7|H3BPE7_HUMAN	FUS	RNA-binding protein FUS OS=Homo sapiens GN=FUS PE=4 SV=1	53693	0	601	0	60	0	0.13	#DIV/0!	0	2	0	2	0	2	0	2	2.728626
tr|Q8IYQ9|Q8IYQ9_HUMAN	KPNA3	Importin subunit alpha OS=Homo sapiens GN=KPNA3 PE=2 SV=1	58378	0	595	0	60	0	0.12	#DIV/0!	0	1	0	2	0	2	0	2	2.728626
tr|I3L3B0|I3L3B0_HUMAN	C1QBP	Complement component 1 Q subcomponent-binding protein, mitochondrial OS=Hor	20179	0	540	0	68	0	0.36	#DIV/0!	0	1	0	2	0	2	0	3	2.728626
tr|Q59F59|Q59F59_HUMAN	tr|Q59F59|Q59F59	Importin 8 variant (Fragment) OS=Homo sapiens PE=2 SV=1	56228	0	535	0	68	0	0.12	#DIV/0!	0	1	0	2	0	2	0	2	2.728626
sp|Q01081|U2AF1_HUMAN	U2AF1	Splicing factor U2AF 35 kDa subunit OS=Homo sapiens GN=U2AF1 PE=1 SV=3	28368	0	530	0	69	0	0.25	#DIV/0!	0	1	0	2	0	2	0	3	2.728626
tr|G3V0G3|G3V0G3_HUMAN	DDX56	DEAD (Asp-Glu-Ala-Asp) box polypeptide 56, isoform CRA_a OS=Homo sapiens GN=D	41969	0	516	0	70	0	0.16	#DIV/0!	0	2	0	2	0	2	0	2	2.728626
sp|Q9UJW0|DCTN4_HUMAN	DCTN4	Dynactin subunit 4 OS=Homo sapiens GN=DCTN4 PE=1 SV=1	53216	0	508	0	72	0	0.13	#DIV/0!	0	2	0	2	0	2	0	3	2.728626

tr|B4E091|B4E091_HUMAN	tr|B4E091|B4E091	cDNA FLJ55438, highly similar to Splicing factor 3 subunit 1 OS=Homo sapiens PE=2 S	77415	0	482	0	76	0	0.09	#DIV/0!	0	2	0	2	0	2	0	3	2.728626
tr|Q4W5K9|Q4W5K9_HUMA	LIM	Putative uncharacterized protein LIM (Fragment) OS=Homo sapiens GN=LIM PE=2 SV	35413	0	467	0	78	0	0.2	#DIV/0!	0	2	0	2	0	2	0	2	2.728626
tr|Q6EHZ3|Q6EHZ3_HUMAN	STRF8	Putative uncharacterized protein STRF8 OS=Homo sapiens GN=STRF8 PE=2 SV=1	41361	0	455	0	80	0	0.17	#DIV/0!	0	2	0	2	0	2	0	2	2.728626
tr|B3KTS5|B3KTS5_HUMAN	tr|B3KTS5|B3KTS5	cDNA FLJ38670 fis, clone HSYRA2000190, highly similar to Voltage-dependent anion-	30839	0	437	0	82	0	0.23	#DIV/0!	0	2	0	2	0	2	0	2	2.728626
tr|B7Z8A2|B7Z8A2_HUMAN	tr|B7Z8A2|B7Z8A2	cDNA FLJ51671, highly similar to Prenylcysteine oxidase (EC 1.8.3.5) OS=Homo sapie	54918	0	423	0	85	0	0.12	#DIV/0!	0	2	0	2	0	2	0	3	2.728626
sp|Q9Y6C9|MTCH2_HUMAN	MTCH2	Mitochondrial carrier homolog 2 OS=Homo sapiens GN=MTCH2 PE=1 SV=1	33936	0	417	0	86	0	0.2	#DIV/0!	0	2	0	2	0	2	0	4	2.728626
sp|O15042|SR140_HUMAN	U2SURP	U2 snRNP-associated SURP motif-containing protein OS=Homo sapiens GN=U2SURP	118675	0	416	0	86	0	0.06	#DIV/0!	0	2	0	2	0	2	0	4	2.728626
tr|I1E4Y6|I1E4Y6_HUMAN	GIGYF2	PERQ amino acid-rich with GYF domain-containing protein 2 OS=Homo sapiens GN=	152891	0	405	0	89	0	0.02	#DIV/0!	0	1	0	1	0	2	0	3	2.728626
sp|P13667|PDIA4_HUMAN	PDIA4	Protein disulfide-isomerase A4 OS=Homo sapiens GN=PDIA4 PE=1 SV=2	73229	0	402	0	90	0	0.09	#DIV/0!	0	2	0	2	0	2	0	3	2.728626
sp|Q96S66|CLCC1_HUMAN	CLCC1	Chloride channel CLIC-like protein 1 OS=Homo sapiens GN=CLCC1 PE=1 SV=1	62667	0	389	0	95	0	0.11	#DIV/0!	0	2	0	2	0	2	0	2	2.728626
tr|G3XAM7|G3XAM7_HUMA	CTNNA1	Catenin (Cadherin-associated protein), alpha 1, 102kDa, isoform CRA_a OS=Homo sa	93576	0	370	0	100	0	0.07	#DIV/0!	0	2	0	2	0	2	0	2	2.728626
tr|B3KSH1|B3KSH1_HUMAN	EIF3F	Eukaryotic translation initiation factor 3 subunit F OS=Homo sapiens GN=EIF3F PE=2	39236	0	295	0	122	0	0.18	#DIV/0!	0	2	0	2	0	2	0	2	2.728626
sp|Q13011|ECH1_HUMAN	ECH1	Delta(3,5)-Delta(2,4)-dienoyl-CoA isomerase, mitochondrial OS=Homo sapiens GN=E	36136	0	1146	0	17	0	0.09	#DIV/0!	0	0	0	1	0	1	0	3	1.991402
sp|Q12955|ANK3_HUMAN	ANK3	Ankyrin-3 OS=Homo sapiens GN=ANK3 PE=1 SV=3	482394	0	1109	0	20	0	0.01	#DIV/0!	0	0	0	1	0	1	0	2	1.991402
tr|B4E1K7|B4E1K7_HUMAN	STOML2	Stomatin-like protein 2 OS=Homo sapiens GN=STOML2 PE=2 SV=1	33317	0	1107	0	20	0	0.1	#DIV/0!	0	0	0	1	0	1	0	1	1.991402
tr|J3KP74|J3KP74_HUMAN	PRG4	Proteoglycan 4 (Fragment) OS=Homo sapiens GN=PRG4 PE=4 SV=1	89944	0	1099	0	21	0	0.04	#DIV/0!	0	0	0	1	0	1	0	1	1.991402
tr|E7EU01|E7EU01_HUMAN	DYNC1I2	Cytoplasmic dynein 1 intermediate chain 2 OS=Homo sapiens GN=DYNC1I2 PE=2 SV=	14610	0	1098	0	21	0	0.23	#DIV/0!	0	0	0	1	0	1	0	1	1.991402
sp|Q99598|TSNAX_HUMAN	TSNAX	Translin-associated protein X OS=Homo sapiens GN=TSNAX PE=1 SV=1	33206	0	1097	0	21	0	0.1	#DIV/0!	0	0	0	1	0	1	0	1	1.991402
tr|I3L142|I3L142_HUMAN	ABHD12	Monoacylglycerol lipase ABHD12 OS=Homo sapiens GN=ABHD12 PE=4 SV=1	15773	0	1096	0	21	0	0.22	#DIV/0!	0	0	0	1	0	1	0	1	1.991402
tr|B4DMW2|B4DMW2_HUM	ZC3H15	Zinc finger CCCH domain-containing protein 15 OS=Homo sapiens GN=ZC3H15 PE=2	18936	0	1095	0	21	0	0.18	#DIV/0!	0	0	0	1	0	1	0	1	1.991402
tr|B2R7R5|B2R7R5_HUMAN	tr|B2R7R5|B2R7R5	cDNA, FLJ93570, highly similar to Homo sapiens phosphoribosyl pyrophosphate synt	41256	0	1094	0	21	0	0.08	#DIV/0!	0	0	0	1	0	1	0	1	1.991402
tr|Q5SZK5|Q5SZK5_HUMAN	WASF1	WAS protein family, member 1 (Fragment) OS=Homo sapiens GN=WASF1 PE=4 SV=1	21322	0	1093	0	21	0	0.16	#DIV/0!	0	0	0	1	0	1	0	1	1.991402
tr|Q05BU7|Q05BU7_HUMAN	WASF2	WASF2 protein (Fragment) OS=Homo sapiens GN=WASF2 PE=2 SV=1	21567	0	1092	0	21	0	0.16	#DIV/0!	0	0	0	1	0	1	0	1	1.991402
sp|Q9H3U1|UN45A_HUMAN	UNC45A	Protein unc-45 homolog A OS=Homo sapiens GN=UNC45A PE=1 SV=1	104266	0	1089	0	21	0	0.03	#DIV/0!	0	0	0	1	0	1	0	1	1.991402
tr|I6LEL9|I6LEL9_HUMAN	KIR3DL1	KIR3DL1 (Fragment) OS=Homo sapiens GN=KIR3DL1 PE=4 SV=1	35273	0	1088	0	21	0	0.09	#DIV/0!	0	0	0	1	0	1	0	1	1.991402
sp|P62269|RS18_HUMAN	RPS18	40S ribosomal protein S18 OS=Homo sapiens GN=RPS18 PE=1 SV=3	17708	0	1084	0	21	0	0.19	#DIV/0!	0	0	0	1	0	1	0	1	1.991402
tr|B4DUM1|B4DUM1_HUMA	tr|B4DUM1|B4DU	cDNA FLJ58983 OS=Homo sapiens PE=2 SV=1	30159	0	1083	0	21	0	0.11	#DIV/0!	0	0	0	1	0	1	0	1	1.991402
tr|J3KPX7|J3KPX7_HUMAN	PHB2	Prohibitin-2 OS=Homo sapiens GN=PHB2 PE=4 SV=1	33382	0	1082	0	21	0	0.1	#DIV/0!	0	0	0	1	0	1	0	1	1.991402
sp|Q9Y232|CDYL1_HUMAN	CDYL	Chromodomain Y-like protein OS=Homo sapiens GN=CDYL PE=1 SV=2	66953	0	1080	0	22	0	0.05	#DIV/0!	0	0	0	1	0	1	0	1	1.991402
sp|Q9GZT9|EGLN1_HUMAN	EGLN1	Egl nine homolog 1 OS=Homo sapiens GN=EGLN1 PE=1 SV=1	46847	0	1079	0	22	0	0.07	#DIV/0!	0	0	0	1	0	1	0	1	1.991402
sp|Q6NUN7|CK063_HUMAN	C11orf63	Uncharacterized protein C11orf63 OS=Homo sapiens GN=C11orf63 PE=2 SV=1	88686	0	1078	0	22	0	0.04	#DIV/0!	0	0	0	1	0	1	0	1	1.991402
tr|B3KXS7|B3KXS7_HUMAN	tr|B3KXS7|B3KXS7	cDNA FLJ45975 fis, clone PLACE7018479, highly similar to 182 kDa tankyrase 1-bindi	113897	0	1077	0	22	0	0.03	#DIV/0!	0	0	0	1	0	1	0	1	1.991402
tr|B3KY04|B3KY04_HUMAN	tr|B3KY04|B3KY04	cDNA FLJ46506 fis, clone THYMU3030752, highly similar to BTB/POZ domain-contair	35994	0	1069	0	22	0	0.19	#DIV/0!	0	0	0	2	0	1	0	2	1.991402
sp|Q68DN1|CB016_HUMAN	C2orf16	Uncharacterized protein C2orf16 OS=Homo sapiens GN=C2orf16 PE=2 SV=3	225780	0	1067	0	22	0	0.01	#DIV/0!	0	0	0	1	0	1	0	1	1.991402
tr|C8YA62|C8YA62_HUMAN	ND6	NADH-ubiquinone oxidoreductase chain 6 OS=Homo sapiens GN=ND6 PE=3 SV=1	18664	0	1064	0	23	0	0.18	#DIV/0!	0	0	0	1	0	1	0	1	1.991402
tr|H0YLA4|H0YLA4_HUMAN	SORD	Sorbitol dehydrogenase OS=Homo sapiens GN=SORD PE=3 SV=1	36833	0	1063	0	23	0	0.09	#DIV/0!	0	0	0	1	0	1	0	1	1.991402
tr|Q5SZG1|Q5SZG1_HUMAN	PRUNE	Protein prune homolog (Fragment) OS=Homo sapiens GN=PRUNE PE=4 SV=1	19953	0	1059	0	23	0	0.17	#DIV/0!	0	0	0	1	0	1	0	1	1.991402
tr|I3L2Z5|I3L2Z5_HUMAN	MAZ	Myc-associated zinc finger protein (Fragment) OS=Homo sapiens GN=MAZ PE=4 SV=	22914	0	1058	0	23	0	0.15	#DIV/0!	0	0	0	1	0	1	0	1	1.991402
tr|G3V4Z4|G3V4Z4_HUMAN	ALDH6A1	Methylmalonate-semialdehyde dehydrogenase [acylating], mitochondrial OS=Homo	27179	0	1056	0	23	0	0.12	#DIV/0!	0	0	0	1	0	1	0	1	1.991402
sp|Q6DKJ4|NXN_HUMAN	NXN	Nucleoredoxin OS=Homo sapiens GN=NXN PE=1 SV=2	48761	0	1055	0	23	0	0.07	#DIV/0!	0	0	0	1	0	1	0	1	1.991402
sp|Q86UA1|PRP39_HUMAN	PRPF39	Pre-mRNA-processing factor 39 OS=Homo sapiens GN=PRPF39 PE=1 SV=3	78723	0	1054	0	23	0	0.04	#DIV/0!	0	0	0	1	0	1	0	1	1.991402
tr|B3KM27|B3KM27_HUMAN	tr|B3KM27|B3KM2	cDNA FLJ10093 fis, clone HEMBA1002363, highly similar to Structural maintenance c	56010	0	1053	0	23	0	0.06	#DIV/0!	0	0	0	1	0	1	0	2	1.991402
tr|B4DYB3|B4DYB3_HUMAN	tr|B4DYB3|B4DYB	cDNA FLJ50293, moderately similar to Oxygen-regulated protein 1 OS=Homo sapiens	69996	0	1051	0	23	0	0.05	#DIV/0!	0	0	0	1	0	1	0	1	1.991402
tr|D6R968|D6R968_HUMAN	N4BP2L2	NEDD4-binding protein 2-like 2 (Fragment) OS=Homo sapiens GN=N4BP2L2 PE=2 SV	83058	0	1050	0	24	0	0.04	#DIV/0!	0	1	0	1	0	1	0	2	1.991402
tr|H0Y5A7|H0Y5A7_HUMAN	TBX19	T-box transcription factor TBX19 (Fragment) OS=Homo sapiens GN=TBX19 PE=4 SV=	34015	0	1046	0	24	0	0.1	#DIV/0!	0	0	0	1	0	1	0	1	1.991402
sp|Q0VF96|CGNL1_HUMAN	CGNL1	Cingulin-like protein 1 OS=Homo sapiens GN=CGNL1 PE=1 SV=2	149559	0	1045	0	24	0	0.02	#DIV/0!	0	0	0	1	0	1	0	2	1.991402
tr|I3L412|I3L412_HUMAN	ARRB2	Beta-arrestin-2 (Fragment) OS=Homo sapiens GN=ARRB2 PE=4 SV=1	22456	0	1044	0	24	0	0.15	#DIV/0!	0	0	0	1	0	1	0	2	1.991402
tr|J3KQN2|J3KQN2_HUMAN	TNNT2	Troponin T, cardiac muscle OS=Homo sapiens GN=TNNT2 PE=4 SV=1	34949	0	1041	0	24	0	0.1	#DIV/0!	0	0	0	1	0	1	0	1	1.991402
tr|G1UI22|G1UI22_HUMAN	MLLT4	Afadin OS=Homo sapiens GN=MLLT4 PE=2 SV=1	202721	0	1040	0	24	0	0.02	#DIV/0!	0	0	0	1	0	1	0	1	1.991402
tr|B4DGQ0|B4DGQ0_HUMAN	LASP1	LIM and SH3 domain protein 1 OS=Homo sapiens GN=LASP1 PE=2 SV=1	23167	0	1038	0	24	0	0.14	#DIV/0!	0	0	0	1	0	1	0	1	1.991402
sp|Q5T653|RM02_HUMAN	MRPL2	39S ribosomal protein L2, mitochondrial OS=Homo sapiens GN=MRPL2 PE=1 SV=2	33565	0	1037	0	24	0	0.1	#DIV/0!	0	0	0	1	0	1	0	1	1.991402
tr|H0YE68|H0YE68_HUMAN	WHSC1L1	Histone-lysine N-methyltransferase NSD3 (Fragment) OS=Homo sapiens GN=WHSC1	8602	0	1036	0	24	0	0.41	#DIV/0!	0	0	0	1	0	1	0	1	1.991402
sp|Q01780|EXOSX_HUMAN	EXOSC10	Exosome component 10 OS=Homo sapiens GN=EXOSC10 PE=1 SV=2	101566	0	1035	0	24	0	0.03	#DIV/0!	0	0	0	1	0	1	0	2	1.991402
tr|E9PK45|E9PK45_HUMAN	FTH1	Ferritin heavy chain OS=Homo sapiens GN=FTH1 PE=2 SV=1	11824	0	1034	0	24	0	0.29	#DIV/0!	0	0	0	1	0	1	0	1	1.991402
sp|Q9HAU5|RENT2_HUMAN	UPF2	Regulator of nonsense transcripts 2 OS=Homo sapiens GN=UPF2 PE=1 SV=1	148972	0	1033	0	24	0	0.02	#DIV/0!	0	0	0	1	0	1	0	1	1.991402
tr|Q53G79|Q53G79_HUMAN	tr|Q53G79|Q53G7	Carnitine O-palmitoyltransferase II, mitochondrial variant (Fragment) OS=Homo sapi	65197	0	1031	0	25	0	0.05	#DIV/0!	0	0	0	1	0	1	0	2	1.991402
tr|B3KS98|B3KS98_HUMAN	EIF3S3	Eukaryotic translation initiation factor 3 subunit H OS=Homo sapiens GN=EIF3S3 PE=	41726	0	1030	0	25	0	0.08	#DIV/0!	0	0	0	1	0	1	0	1	1.991402
sp|A0FGR8|ESYT2_HUMAN	ESYT2	Extended synaptotagmin-2 OS=Homo sapiens GN=ESYT2 PE=1 SV=1	102807	0	1029	0	25	0	0.03	#DIV/0!	0	0	0	1	0	1	0	2	1.991402
tr|B2RDN3|B2RDN3_HUMAN	tr|B2RDN3|B2RDN	cDNA, FLJ96692, highly similar to Homo sapiens nucleotide binding protein 2 (MinD	29264	0	1025	0	25	0	0.11	#DIV/0!	0	1	0	1	0	1	0	1	1.991402
tr|B2C8Z3|B2C8Z3_HUMAN	TGFB1	Transforming growth factor beta 1 (Fragment) OS=Homo sapiens GN=TGFB1 PE=2 SV	12965	0	1024	0	25	0	0.26	#DIV/0!	0	0	0	1	0	1	0	1	1.991402
sp|Q9NYF0|DACT1_HUMAN	DACT1	Dapper homolog 1 OS=Homo sapiens GN=DACT1 PE=1 SV=2	91145	0	1023	0	25	0	0.04	#DIV/0!	0	0	0	1	0	1	0	1	1.991402
tr|E5KSY5|E5KSY5_HUMAN	tr|E5KSY5|E5KSY5	Mitochondrial twinkle protein OS=Homo sapiens PE=2 SV=1	77619	0	1020	0	25	0	0.04	#DIV/0!	0	0	0	1	0	1	0	1	1.991402
tr|Q8TBT6|Q8TBT6_HUMAN	tr|Q8TBT6|Q8TBT	Putative uncharacterized protein (Fragment) OS=Homo sapiens PE=2 SV=1	34810	0	1019	0	25	0	0.1	#DIV/0!	0	0	0	1	0	1	0	1	1.991402
tr|D6REC4|D6REC4_HUMAN	tr|D6REC4|D6REC	Uncharacterized protein OS=Homo sapiens PE=4 SV=1	52489	0	1018	0	25	0	0.06	#DIV/0!	0	0	0	1	0	1	0	1	1.991402
tr|Q59HG1|Q59HG1_HUMAN	tr|Q59HG1|Q59H	Chromosome-associated kinesin KIF4A variant (Fragment) OS=Homo sapiens PE=2 S	141848	0	1017	0	25	0	0.05	#DIV/0!	0	1	0	2	0	1	0	4	1.991402
tr|F5H3F6|F5H3F6_HUMAN	BANF2	Barrier-to-autointegration factor-like protein OS=Homo sapiens GN=BANF2 PE=2 SV	11517	0	1015	0	25	0	0.32	#DIV/0!	0	1	0	1	0	1	0	1	1.991402
tr|J3KRY8|J3KRY8_HUMAN	ASPSCR1	Tether-containing UBX domain for GLUT4 (Fragment) OS=Homo sapiens GN=ASPSCR	41677	0	1014	0	26	0	0.08	#DIV/0!	0	0	0	1	0	1	0	2	1.991402
tr|F8W805|F8W805_HUMAN	GRM1	Metabotropic glutamate receptor 1 OS=Homo sapiens GN=GRM1 PE=2 SV=1	103232	0	1010	0	26	0	0.03	#DIV/0!	0	0	0	1	0	1	0	1	1.991402
tr|D6RJG0|D6RJG0_HUMAN	CDH10	Cadherin-10 OS=Homo sapiens GN=CDH10 PE=2 SV=1	49572	0	1009	0	26	0	0.07	#DIV/0!	0	0	0	1	0	1	0	1	1.991402
tr|B4DF73|B4DF73_HUMAN	PTCD3	Pentatricopeptide repeat domain-containing protein 3, mitochondrial OS=Homo sap	23713	0	1007	0	26	0	0.14	#DIV/0!	0	0	0	1	0	1	0	1	1.991402
tr|B4DV21|B4DV21_HUMAN	tr|B4DV21|B4DV2	cDNA FLJ50878, highly similar to Dimethylglycine dehydrogenase, mitochondrial (EC	65745	0	1003	0	26	0	0.05	#DIV/0!	0	0	0	1	0	1	0	1	1.991402
tr|A0JLU2|A0JLU2_HUMAN	BUB1	BUB1 protein (Fragment) OS=Homo sapiens GN=BUB1 PE=2 SV=1	93169	0	1002	0	26	0	0.04	#DIV/0!	0	0	0	1	0	1	0	1	1.991402
sp|Q9NQR4|NIT2_HUMAN	NIT2	Omega-amidase NIT2 OS=Homo sapiens GN=NIT2 PE=1 SV=1	30988	0	999	0	27	0	0.11	#DIV/0!	0	0	0	1	0	1	0	1	1.991402
tr|B4E2E5|B4E2E5_HUMAN	tr|B4E2E5|B4E2E5	cDNA FLJ56289, highly similar to Homo sapiens sperm specific antigen 2 (SSFA2), mR	120102	0	998	0	27	0	0.03	#DIV/0!	0	0	0	1	0	1	0	1	1.991402
tr|B3KPM8|B3KPM8_HUMAN	tr|B3KPM8|B3KPN	cDNA FLJ31974 fis, clone NT2RP7008167, weakly similar to 85.1 kDa PROTEIN IN GRI	112930	0	997	0	27	0	0.03	#DIV/0!	0	0	0	1	0	1	0	1	1.991402
sp|Q9GZN4|BSSP4_HUMAN	PRSS22	Brain-specific serine protease 4 OS=Homo sapiens GN=PRSS22 PE=1 SV=1	34394	0	996	0	27	0	0.1	#DIV/0!	0	0	0	1	0	1	0	1	1.991402
tr|J3KS31|J3KS31_HUMAN	ZNF207	Zinc finger protein 207 (Fragment) OS=Homo sapiens GN=ZNF207 PE=4 SV=1	35132	0	994	0	27	0	0.09	#DIV/0!	0	0	0	1	0	1	0	1	1.991402
tr|B4DNP0|B4DNP0_HUMAN	tr|B4DNP0|B4DNF	cDNA FLJ59362, highly similar to Signal transducer and activator of transcription 3 O	76912	0	992	0	27	0	0.04	#DIV/0!	0	0	0	1	0	1	0	2	1.991402
tr|G3V180|G3V180_HUMAN	DPP3	Dipeptidyl peptidase 3 OS=Homo sapiens GN=DPP3 PE=2 SV=1	84666	0	990	0	27	0	0.04	#DIV/0!	0	0	0	1	0	1	0	1	1.991402
tr|B7ZMH4|B7ZMH4_HUMAN	ODZ1	ODZ1 protein OS=Homo sapiens GN=ODZ1 PE=2 SV=1	310528	0	989	0	27	0	0.01	#DIV/0!	0	0	0	1	0	1	0	1	1.991402
tr|G0Z349|G0Z349_HUMAN	IRF6	Interferon regulatory factor 6 OS=Homo sapiens GN=IRF6 PE=2 SV=1	53666	0	988	0	27	0	0.06	#DIV/0!	0	0	0	1	0	1	0	1	1.991402
sp|P55107|BMP3B_HUMAN	GDF10	Bone morphogenetic protein 3B OS=Homo sapiens GN=GDF10 PE=2 SV=1	53545	0	987	0	27	0	0.06	#DIV/0!	0	0	0	1	0	1	0	1	1.991402
sp|O94761|RECQ4_HUMAN	RECQL4	ATP-dependent DNA helicase Q4 OS=Homo sapiens GN=RECQL4 PE=1 SV=1	134989	0	986	0	27	0	0.02	#DIV/0!	0	0	0	1	0	1	0	1	1.991402
tr|B4DXJ1|B4DXJ1_HUMAN	SEC13	Protein SEC13 homolog OS=Homo sapiens GN=SEC13 PE=2 SV=1	41405	0	984	0	28	0	0.08	#DIV/0!	0	0	0	1	0	1	0	1	1.991402
tr|B4DM01|B4DM01_HUMA	SKIV2L	Helicase SKI2W OS=Homo sapiens GN=SKIV2L PE=2 SV=1	121269	0	983	0	28	0	0.03	#DIV/0!	0	0	0	1	0	1	0	1	1.991402
tr|B4DP31|B4DP31_HUMAN	PRPSAP1	Phosphoribosyl pyrophosphate synthase-associated protein 1 OS=Homo sapiens GN	31474	0	982	0	28	0	0.11	#DIV/0!	0	0	0	1	0	1	0	1	1.991402
sp|Q5SRE5|NU188_HUMAN	NUP188	Nucleoporin NUP188 homolog OS=Homo sapiens GN=NUP188 PE=1 SV=1	198369	0	981	0	28	0	0.02	#DIV/0!	0	0	0	1	0	1	0	1	1.991402
tr|H7C547|H7C547_HUMAN	ATP1B3	Sodium/potassium-transporting ATPase subunit beta-3 (Fragment) OS=Homo sapien	4914	0	980	0	28	0	0.75	#DIV/0!	0	0	0	1	0	1	0	1	1.991402
tr|B7Z1V5|B7Z1V5_HUMAN	tr|B7Z1V5|B7Z1V5	cDNA FLJ55678, highly similar to Syntaxin-binding protein 1 OS=Homo sapiens PE=2	63779	0	977	0	28	0	0.05	#DIV/0!	0	0	0	1	0	1	0	1	1.991402
tr|B1AM49|B1AM49_HUMAN	ELAVL2	ELAV-like protein 2 OS=Homo sapiens GN=ELAVL2 PE=4 SV=1	42733	0	976	0	28	0	0.08	#DIV/0!	0	0	0	1	0	1	0	1	1.991402
tr|B3KMG0|B3KMG0_HUMA	GLE1	Nucleoporin GLE1 OS=Homo sapiens GN=GLE1 PE=2 SV=1	50703	0	975	0	28	0	0.06	#DIV/0!	0	0	0	1	0	1	0	2	1.991402
sp|O95425|SVIL_HUMAN	SVIL	Supervillin OS=Homo sapiens GN=SVIL PE=1 SV=2	249417	0	973	0	28	0	0.01	#DIV/0!	0	0	0	1	0	1	0	1	1.991402
tr|G3V1C3|G3V1C3_HUMAN	API5	Apoptosis inhibitor 5 OS=Homo sapiens GN=API5 PE=2 SV=1	57867	0	972	0	28	0	0.06	#DIV/0!	0	0	0	1	0	1	0	1	1.991402
tr|B4DQV4|B4DQV4_HUMAN	SNX4	Sorting nexin-4 OS=Homo sapiens GN=SNX4 PE=2 SV=1	35873	0	971	0	28	0	0.09	#DIV/0!	0	0	0	1	0	1	0	1	1.991402
tr|Q5SRN1|Q5SRN1_HUMAN	CDC40	Cell division cycle 40 homolog (S. cerevisiae) OS=Homo sapiens GN=CDC40 PE=4 SV=	61362	0	969	0	28	0	0.05	#DIV/0!	0	0	0	1	0	1	0	1	1.991402
tr|H0YB78|H0YB78_HUMAN	SLC12A7	Solute carrier family 12 member 7 (Fragment) OS=Homo sapiens GN=SLC12A7 PE=4	39776	0	966	0	29	0	0.08	#DIV/0!	0	0	0	1	0	1	0	1	1.991402
tr|B7Z233|B7Z233_HUMAN	ACAT2	Acetyl-CoA acetyltransferase, cytosolic OS=Homo sapiens GN=ACAT2 PE=2 SV=1	45184	0	964	0	29	0	0.07	#DIV/0!	0	0	0	1	0	1	0	1	1.991402
tr|D3DRS9|D3DRS9_HUMAN	SEPHS1	Selenophosphate synthetase 1 major variant OS=Homo sapiens GN=SEPHS1 PE=2 SV	43396	0	962	0	29	0	0.08	#DIV/0!	0	0	0	1	0	1	0	1	1.991402
sp|Q96G25|MED8_HUMAN	MED8	Mediator of RNA polymerase II transcription subunit 8 OS=Homo sapiens GN=MED8	29176	0	958	0	29	0	0.11	#DIV/0!	0	0	0	1	0	1	0	1	1.991402
tr|J3KPP7|J3KPP7_HUMAN	ARRB1	Beta-arrestin-1 OS=Homo sapiens GN=ARRB1 PE=4 SV=1	47626	0	957	0	29	0	0.07	#DIV/0!	0	0	0	1	0	1	0	1	1.991402
tr|Q5JRI7|Q5JRI7_HUMAN	TCEA2	Transcription elongation factor A (SII), 2 (Fragment) OS=Homo sapiens GN=TCEA2 PE	27987	0	952	0	29	0	0.25	#DIV/0!	0	0	0	2	0	1	0	2	1.991402
tr|B3KXM6|B3KXM6_HUMAN	tr|B3KXM6|B3KXN	cDNA FLJ45735 fis, clone JCMLC2000273, highly similar to Integrin alpha-M OS=Hom	62962	0	951	0	29	0	0.05	#DIV/0!	0	0	0	1	0	1	0	1	1.991402
sp|P82673|RT35_HUMAN	MRPS35	28S ribosomal protein S35, mitochondrial OS=Homo sapiens GN=MRPS35 PE=1 SV=1	37106	0	948	0	29	0	0.09	#DIV/0!	0	0	0	1	0	1	0	2	1.991402
sp|Q8TAA9|VANG1_HUMAN	VANGL1	Vang-like protein 1 OS=Homo sapiens GN=VANGL1 PE=1 SV=1	60222	0	946	0	29	0	0.05	#DIV/0!	0	0	0	1	0	1	0	1	1.991402
tr|A0JLP4|A0JLP4_HUMAN	SON	SON protein (Fragment) OS=Homo sapiens GN=SON PE=2 SV=1	14298	0	945	0	29	0	0.24	#DIV/0!	0	0	0	1	0	1	0	1	1.991402
tr|Q05DF2|Q05DF2_HUMAN	SF3A2	SF3A2 protein (Fragment) OS=Homo sapiens GN=SF3A2 PE=2 SV=1	51557	0	943	0	30	0	0.06	#DIV/0!	0	0	0	1	0	1	0	1	1.991402
tr|A0PJK4|A0PJK4_HUMAN	NUP43	NUP43 protein (Fragment) OS=Homo sapiens GN=NUP43 PE=2 SV=1	31994	0	941	0	30	0	0.1	#DIV/0!	0	0	0	1	0	1	0	2	1.991402
tr|B4DI47|B4DI47_HUMAN	tr|B4DI47|B4DI47_	cDNA FLJ51858, highly similar to Integral membrane protein GPR137B OS=Homo sap	30483	0	939	0	30	0	0.11	#DIV/0!	0	0	0	1	0	1	0	1	1.991402
tr|Q3LIE7|Q3LIE7_HUMAN	Nbla03646	Putative uncharacterized protein Nbla03646 OS=Homo sapiens GN=Nbla03646 PE=2	50031	0	934	0	30	0	0.07	#DIV/0!	0	0	0	1	0	1	0	1	1.991402
tr|B2R4W8|B2R4W8_HUMAN	hCG_1994130	HCG1994130, isoform CRA_a OS=Homo sapiens GN=hCG_1994130 PE=2 SV=1	14944	0	932	0	30	0	0.23	#DIV/0!	0	0	0	1	0	1	0	1	1.991402
tr|B0VJZ1|B0VJZ1_HUMAN	LENG9	Leukocyte receptor cluster (LRC) member 9 OS=Homo sapiens GN=LENG9 PE=2 SV=1	51306	0	931	0	30	0	0.06	#DIV/0!	0	0	0	1	0	1	0	1	1.991402
tr|A8K7A1|A8K7A1_HUMAN	tr|A8K7A1|A8K7A1	cDNA FLJ77346, highly similar to Homo sapiens DEAD (Asp-Glu-Ala-Asp) box polypep	54877	0	930	0	31	0	0.06	#DIV/0!	0	0	0	1	0	1	0	1	1.991402
tr|B3KNM1|B3KNM1_HUMA	tr|B3KNM1|B3KNI	cDNA FLJ14918 fis, clone PLACE1007239, highly similar to Transcription elongation fa	31177	0	928	0	31	0	0.11	#DIV/0!	0	0	0	1	0	1	0	1	1.991402
tr|E5KS55|E5KS55_HUMAN	tr|E5KS55|E5KS55	Mitochondrial succinyl-CoA ligase [ADP-forming] subunit beta OS=Homo sapiens PE=	50641	0	927	0	31	0	0.07	#DIV/0!	0	1	0	1	0	1	0	1	1.991402
tr|F8W9K7|F8W9K7_HUMAN	NDUFS8	NADH dehydrogenase [ubiquinone] iron-sulfur protein 8, mitochondrial (Fragment)	14129	0	926	0	31	0	0.24	#DIV/0!	0	0	0	1	0	1	0	1	1.991402
sp|Q6P1J9|CDC73_HUMAN	CDC73	Parafibromin OS=Homo sapiens GN=CDC73 PE=1 SV=1	60653	0	922	0	31	0	0.05	#DIV/0!	0	1	0	1	0	1	0	1	1.991402
tr|B4DJ07|B4DJ07_HUMAN	EXOC7	Exocyst complex component 7 OS=Homo sapiens GN=EXOC7 PE=2 SV=1	74854	0	921	0	31	0	0.04	#DIV/0!	0	0	0	1	0	1	0	1	1.991402
tr|E9PH29|E9PH29_HUMAN	PRDX3	Thioredoxin-dependent peroxide reductase, mitochondrial OS=Homo sapiens GN=PI	26107	0	917	0	31	0	0.13	#DIV/0!	0	0	0	1	0	1	0	1	1.991402
tr|B3KNK5|B3KNK5_HUMAN	tr|B3KNK5|B3KNK	cDNA FLJ14795 fis, clone NT2RP4001219, highly similar to Homo sapiens DnaJ (Hsp4	70004	0	911	0	32	0	0.05	#DIV/0!	0	0	0	1	0	1	0	1	1.991402
tr|A8K8X0|A8K8X0_HUMAN	tr|A8K8X0|A8K8X0	cDNA FLJ75187, highly similar to Homo sapiens nap1 P120 OS=Homo sapiens PE=2 S	113035	0	910	0	32	0	0.03	#DIV/0!	0	0	0	1	0	1	0	2	1.991402
tr|B3KU96|B3KU96_HUMAN	tr|B3KU96|B3KU9	cDNA FLJ39433 fis, clone PROST2004142, highly similar to GDP-L-fucose synthetase (	17856	0	909	0	32	0	0.19	#DIV/0!	0	0	0	1	0	1	0	1	1.991402
tr|E7EN32|E7EN32_HUMAN	MEN1	Menin OS=Homo sapiens GN=MEN1 PE=2 SV=1	61666	0	908	0	32	0	0.05	#DIV/0!	0	0	0	1	0	1	0	1	1.991402
sp|Q9NRN7|ADPPT_HUMAN	AASDHPPT	L-aminoadipate-semialdehyde dehydrogenase-phosphopantetheinyl transferase OS=	35981	0	907	0	32	0	0.09	#DIV/0!	0	0	0	1	0	1	0	1	1.991402
tr|B3KMN5|B3KMN5_HUMA	tr|B3KMN5|B3KM	cDNA FLJ11713 fis, clone HEMBA1005201, highly similar to Homo sapiens NMD3 hor	44787	0	906	0	32	0	0.07	#DIV/0!	0	0	0	1	0	1	0	1	1.991402
tr|A6NJ11|A6NJ11_HUMAN	UFD1L	Ubiquitin fusion degradation protein 1 homolog OS=Homo sapiens GN=UFD1L PE=2	33585	0	902	0	32	0	0.1	#DIV/0!	0	0	0	1	0	1	0	1	1.991402
tr|A7VJC9|A7VJC9_HUMAN	STP	Salt-tolerant protein OS=Homo sapiens GN=STP PE=2 SV=1	62769	0	901	0	32	0	0.05	#DIV/0!	0	0	0	1	0	1	0	2	1.991402
tr|B2RA56|B2RA56_HUMAN	tr|B2RA56|B2RA5	cDNA, FLJ94705, highly similar to Homo sapiens nicalin (LOC56926), mRNA OS=Hom	61891	0	900	0	33	0	0.05	#DIV/0!	0	0	0	1	0	1	0	1	1.991402
sp|Q16352|AINX_HUMAN	INA	Alpha-internexin OS=Homo sapiens GN=INA PE=1 SV=2	55528	0	898	0	33	0	0.06	#DIV/0!	0	0	0	1	0	1	0	2	1.991402
tr|G3V161|G3V161_HUMAN	KBTBD3	Kelch repeat and BTB (POZ) domain containing 3, isoform CRA_a OS=Homo sapiens	61834	0	896	0	33	0	0.06	#DIV/0!	0	1	0	1	0	1	0	1	1.991402
tr|B7Z7X8|B7Z7X8_HUMAN	ATL2	Atlastin-2 OS=Homo sapiens GN=ATL2 PE=2 SV=1	65108	0	895	0	33	0	0.05	#DIV/0!	0	0	0	1	0	1	0	2	1.991402
tr|G3V1N8|G3V1N8_HUMAN	VPS26A	Vacuolar protein sorting 26 homolog A (Yeast), isoform CRA_a OS=Homo sapiens GN	25547	0	894	0	33	0	0.13	#DIV/0!	0	0	0	1	0	1	0	1	1.991402
tr|B3KU09|B3KU09_HUMAN	tr|B3KU09|B3KU0	cDNA FLJ39034 fis, clone NT2RP7008085, highly similar to Homo sapiens ring finger	149906	0	893	0	33	0	0.02	#DIV/0!	0	1	0	1	0	1	0	1	1.991402
tr|A4D112|A4D112_HUMAN	LOC340268	Hypothetical gene supported by AB033109; AY101765; NM_015692 OS=Homo sapie	87612	0	890	0	33	0	0.04	#DIV/0!	0	0	0	1	0	1	0	1	1.991402
tr|A1Z1A7|A1Z1A7_HUMAN	NKRF	NF-kappaB repressing factor (Fragment) OS=Homo sapiens GN=NKRF PE=2 SV=1	12653	0	885	0	34	0	0.27	#DIV/0!	0	0	0	1	0	1	0	1	1.991402
tr|B2RCM3|B2RCM3_HUMAN	tr|B2RCM3|B2RCN	cDNA, FLJ96158, highly similar to Homo sapiens calpain 2, (m/II) large subunit (CAPN	80883	0	884	0	34	0	0.04	#DIV/0!	0	0	0	1	0	1	0	1	1.991402
sp|Q8TCG1|CIP2A_HUMAN	KIAA1524	Protein CIP2A OS=Homo sapiens GN=KIAA1524 PE=1 SV=2	103318	0	879	0	34	0	0.03	#DIV/0!	0	0	0	1	0	1	0	3	1.991402
tr|B7Z553|B7Z553_HUMAN	tr|B7Z553|B7Z553	cDNA FLJ51266, highly similar to Vitronectin OS=Homo sapiens PE=2 SV=1	23761	0	877	0	34	0	0.14	#DIV/0!	0	0	0	1	0	1	0	1	1.991402
tr|G3LGQ6|G3LGQ6_HUMAN	HFE	Hereditary hemochromatosis protein (Fragment) OS=Homo sapiens GN=HFE PE=2 SV	7963	0	876	0	35	0	0.44	#DIV/0!	0	1	0	1	0	1	0	1	1.991402
tr|B4E3G4|B4E3G4_HUMAN	tr|B4E3G4|B4E3G	cDNA FLJ50383, highly similar to CRSP complex subunit 3 OS=Homo sapiens PE=2 SV	116828	0	875	0	35	0	0.03	#DIV/0!	0	0	0	1	0	1	0	2	1.991402
sp|Q15075|EEA1_HUMAN	EEA1	Early endosome antigen 1 OS=Homo sapiens GN=EEA1 PE=1 SV=2	163337	0	873	0	35	0	0.02	#DIV/0!	0	0	0	1	0	1	0	1	1.991402
tr|J3QQW9|J3QQW9_HUMA	SUZ12	Polycomb protein SUZ12 OS=Homo sapiens GN=SUZ12 PE=4 SV=1	81012	0	871	0	35	0	0.04	#DIV/0!	0	0	0	1	0	1	0	1	1.991402
tr|G3V282|G3V282_HUMAN	OSGEP	Probable tRNA threonylcarbamoyladenosine biosynthesis protein OSGEP (Fragment)	19468	0	870	0	35	0	0.17	#DIV/0!	0	0	0	1	0	1	0	1	1.991402
tr|H0YKL9|H0YKL9_HUMAN	ANXA2	Annexin (Fragment) OS=Homo sapiens GN=ANXA2 PE=3 SV=1	12113	0	869	0	35	0	0.28	#DIV/0!	0	0	0	1	0	1	0	1	1.991402
tr|B2RDE1|B2RDE1_HUMAN	tr|B2RDE1|B2RDE	cDNA, FLJ96568, highly similar to Homo sapiens tropomyosin 3 (TPM3), mRNA OS=H	29228	0	866	0	35	0	0.11	#DIV/0!	0	0	0	1	0	1	0	1	1.991402
tr|B4DYB1|B4DYB1_HUMAN	tr|B4DYB1|B4DYB	cDNA FLJ60956, highly similar to DNA-directed RNA polymerase I-associated factor 5	19500	0	865	0	35	0	0.17	#DIV/0!	0	0	0	1	0	1	0	1	1.991402
tr|B4DIW8|B4DIW8_HUMAN	tr|B4DIW8|B4DIW	cDNA FLJ61282, highly similar to Mitochondrial 28S ribosomal protein S5 OS=Homo	32387	0	864	0	35	0	0.1	#DIV/0!	0	0	0	1	0	1	0	1	1.991402
tr|B1AKC7|B1AKC7_HUMAN	RASAL2	RAS protein activator like 2 OS=Homo sapiens GN=RASAL2 PE=4 SV=1	144153	0	861	0	35	0	0.02	#DIV/0!	0	0	0	1	0	1	0	1	1.991402
sp|Q8WX92|NELFB_HUMAN	COBRA1	Negative elongation factor B OS=Homo sapiens GN=COBRA1 PE=1 SV=1	66283	0	859	0	35	0	0.05	#DIV/0!	0	0	0	1	0	1	0	1	1.991402
tr|O60382|O60382_HUMAN	KIAA0324	KIAA0324 (Fragment) OS=Homo sapiens GN=KIAA0324 PE=2 SV=1	191877	0	855	0	36	0	0.03	#DIV/0!	0	0	0	2	0	1	0	3	1.991402
tr|B4DUZ3|B4DUZ3_HUMAN	tr|B4DUZ3|B4DUZ	cDNA FLJ51105, highly similar to Arfaptin-2 OS=Homo sapiens PE=2 SV=1	41783	0	854	0	36	0	0.08	#DIV/0!	0	0	0	1	0	1	0	1	1.991402
tr|Q53TH1|Q53TH1_HUMAN	PSMD14	Putative uncharacterized protein PSMD14 (Fragment) OS=Homo sapiens GN=PSMD1	18165	0	850	0	36	0	0.19	#DIV/0!	0	0	0	1	0	1	0	1	1.991402

tr|F5H1H2|F5H1H2_HUMAN	CCAR1	Cell division cycle and apoptosis regulator protein 1 (Fragment) OS=Homo sapiens G	76191	0	844	0	37	0	0.04	#DIV/0!	0	1	0	1	0	1	0	1	1.991402
tr|I3L2L6|I3L2L6_HUMAN	SRRM2	Serine/arginine repetitive matrix protein 2 OS=Homo sapiens GN=SRRM2 PE=4 SV=1	227649	0	843	0	37	0	0.01	#DIV/0!	0	0	0	1	0	1	0	2	1.991402
tr|D6RCC8|D6RCC8_HUMAN	PPAT	Amidophosphoribosyltransferase OS=Homo sapiens GN=PPAT PE=2 SV=1	21362	0	842	0	37	0	0.16	#DIV/0!	0	0	0	1	0	1	0	1	1.991402
tr|H0Y448|H0Y448_HUMAN	CTTNBP2	Cortactin-binding protein 2 (Fragment) OS=Homo sapiens GN=CTTNBP2 PE=4 SV=1	127487	0	838	0	37	0	0.03	#DIV/0!	0	1	0	1	0	1	0	2	1.991402
tr|B4E0P8|B4E0P8_HUMAN	tr|B4E0P8|B4E0P8	cDNA FLJ53007, highly similar to UBA/UBX 33.3 kDa protein OS=Homo sapiens PE=2	23041	0	835	0	37	0	0.15	#DIV/0!	0	1	0	1	0	1	0	1	1.991402
tr|D3DTC3|D3DTC3_HUMAN	hCG_1742968	HCG1742968, isoform CRA_c OS=Homo sapiens GN=hCG_1742968 PE=4 SV=1	20573	0	834	0	37	0	0.16	#DIV/0!	0	1	0	1	0	1	0	1	1.991402
tr|B4DMI9|B4DMI9_HUMAN	tr|B4DMI9|B4DMI	cDNA FLJ55894, highly similar to Discs large homolog 7 OS=Homo sapiens PE=2 SV=1	89501	0	833	0	37	0	0.04	#DIV/0!	0	0	0	1	0	1	0	1	1.991402
tr|B5MC40|B5MC40_HUMAN	GATAD2A	GATA zinc finger domain containing 2A, isoform CRA_a OS=Homo sapiens GN=GATA	68434	0	830	0	37	0	0.1	#DIV/0!	0	1	0	2	0	1	0	2	1.991402
tr|F5H740|F5H740_HUMAN	VDAC3	Voltage-dependent anion-selective channel protein 3 OS=Homo sapiens GN=VDAC3	31080	0	827	0	38	0	0.11	#DIV/0!	0	1	0	1	0	1	0	1	1.991402
tr|H0YAS9|H0YAS9_HUMAN	EMC2	ER membrane protein complex subunit 2 (Fragment) OS=Homo sapiens GN=EMC2 P	16089	0	826	0	38	0	0.21	#DIV/0!	0	1	0	1	0	1	0	1	1.991402
tr|B3KY95|B3KY95_HUMAN	tr|B3KY95|B3KY95	cDNA FLJ16143 fis, clone BRAMY2038516, highly similar to Protein disulfide-isomera	53655	0	823	0	38	0	0.06	#DIV/0!	0	1	0	1	0	1	0	3	1.991402
tr|B2R657|B2R657_HUMAN	tr|B2R657|B2R657	Annexin OS=Homo sapiens PE=2 SV=1	52892	0	821	0	38	0	0.06	#DIV/0!	0	1	0	1	0	1	0	2	1.991402
tr|G3V448|G3V448_HUMAN	TMX1	Thioredoxin-related transmembrane protein 1 OS=Homo sapiens GN=TMX1 PE=2 SV	12018	0	820	0	38	0	0.29	#DIV/0!	0	1	0	1	0	1	0	1	1.991402
tr|Q5QPL9|Q5QPL9_HUMAN	RALY	RNA binding protein, autoantigenic (HnRNP-associated with lethal yellow homolog (	24707	0	818	0	39	0	0.14	#DIV/0!	0	1	0	1	0	1	0	1	1.991402
sp|Q96JP5|ZFP91_HUMAN	ZFP91	E3 ubiquitin-protein ligase ZFP91 OS=Homo sapiens GN=ZFP91 PE=1 SV=1	64261	0	817	0	39	0	0.05	#DIV/0!	0	1	0	1	0	1	0	1	1.991402
tr|B0LPE5|B0LPE5_HUMAN	AKT1	V-akt murine thymoma viral oncogene homolog 1 OS=Homo sapiens GN=AKT1 PE=2	56050	0	812	0	40	0	0.06	#DIV/0!	0	1	0	1	0	1	0	1	1.991402
tr|G5EA24|G5EA24_HUMAN	MCOLN2	Mucolipin 2, isoform CRA_a OS=Homo sapiens GN=MCOLN2 PE=2 SV=1	29581	0	811	0	40	0	0.11	#DIV/0!	0	1	0	1	0	1	0	1	1.991402
tr|B4DDI7|B4DDI7_HUMAN	tr|B4DDI7|B4DDI7	NADPH:adrenodoxin oxidoreductase, mitochondrial OS=Homo sapiens PE=2 SV=1	57663	0	810	0	40	0	0.06	#DIV/0!	0	1	0	1	0	1	0	1	1.991402
sp|Q9H6W3|NO66_HUMAN	NO66	Bifunctional lysine-specific demethylase and histidyl-hydroxylase NO66 OS=Homo sa	71384	0	809	0	40	0	0.05	#DIV/0!	0	1	0	1	0	1	0	1	1.991402
sp|Q9BTV5|FSD1_HUMAN	FSD1	Fibronectin type III and SPRY domain-containing protein 1 OS=Homo sapiens GN=FSI	56240	0	806	0	40	0	0.06	#DIV/0!	0	1	0	1	0	1	0	1	1.991402
tr|H7C4H2|H7C4H2_HUMAN	SRPRB	Signal recognition particle receptor subunit beta (Fragment) OS=Homo sapiens GN=S	17716	0	805	0	40	0	0.19	#DIV/0!	0	1	0	1	0	1	0	2	1.991402
tr|H0Y6N5|H0Y6N5_HUMAN	SUN1	SUN domain-containing protein 1 (Fragment) OS=Homo sapiens GN=SUN1 PE=4 SV=	71569	0	802	0	40	0	0.05	#DIV/0!	0	1	0	1	0	1	0	1	1.991402
tr|K7EPI4|K7EPI4_HUMAN	GFAP	Glial fibrillary acidic protein (Fragment) OS=Homo sapiens GN=GFAP PE=4 SV=1	7120	0	794	0	41	0	0.5	#DIV/0!	0	1	0	1	0	1	0	1	1.991402
sp|Q6NVY1|HIBCH_HUMAN	HIBCH	3-hydroxyisobutyryl-CoA hydrolase, mitochondrial OS=Homo sapiens GN=HIBCH PE=	43797	0	793	0	41	0	0.08	#DIV/0!	0	1	0	1	0	1	0	1	1.991402
tr|B2R7F8|B2R7F8_HUMAN	tr|B2R7F8|B2R7F8	cDNA, FLJ93426, highly similar to Homo sapiens plasminogen (PLG), mRNA OS=Hom	93217	0	792	0	41	0	0.04	#DIV/0!	0	1	0	1	0	1	0	1	1.991402
tr|B2R9X3|B2R9X3_HUMAN	tr|B2R9X3|B2R9X3	cDNA, FLJ94599, highly similar to Homo sapiens GDP-mannose 4,6-dehydratase (GM	42166	0	787	0	42	0	0.08	#DIV/0!	0	1	0	1	0	1	0	1	1.991402
sp|O60870|KIN17_HUMAN	KIN	DNA/RNA-binding protein KIN17 OS=Homo sapiens GN=KIN PE=1 SV=2	45745	0	785	0	42	0	0.07	#DIV/0!	0	1	0	1	0	1	0	1	1.991402
tr|B3KM34|B3KM34_HUMAN	tr|B3KM34|B3KM3	cDNA FLJ10132 fis, clone HEMBA1003046, highly similar to Mitochondrial-processing	55093	0	783	0	42	0	0.06	#DIV/0!	0	1	0	1	0	1	0	1	1.991402
tr|B2R5S4|B2R5S4_HUMAN	tr|B2R5S4|B2R5S4	cDNA, FLJ92598, highly similar to Homo sapiens polymerase (DNA directed), delta 2,	51914	0	780	0	43	0	0.06	#DIV/0!	0	1	0	1	0	1	0	1	1.991402
tr|B4DPE5|B4DPE5_HUMAN	tr|B4DPE5|B4DPE5	cDNA FLJ58206, highly similar to Tuftelin-interacting protein 11 OS=Homo sapiens P	61633	0	775	0	43	0	0.05	#DIV/0!	0	1	0	1	0	1	0	2	1.991402
tr|B4DHA6|B4DHA6_HUMAN	tr|B4DHA6|B4DHA	cDNA FLJ60526, highly similar to Probable rRNA-processing protein EBP2 OS=Homo	15345	0	774	0	43	0	0.22	#DIV/0!	0	1	0	1	0	1	0	1	1.991402
tr|A4D0P7|A4D0P7_HUMAN	ORC5L	Origin recognition complex, subunit 5-like (Yeast) OS=Homo sapiens GN=ORC5L PE=	50764	0	773	0	43	0	0.06	#DIV/0!	0	1	0	1	0	1	0	1	1.991402
tr|Q5U043|Q5U043_HUMAN	tr|Q5U043|Q5U04	Alcohol dehydrogenase 5 (Class III), chi polypeptide OS=Homo sapiens PE=2 SV=1	40520	0	770	0	43	0	0.08	#DIV/0!	0	1	0	1	0	1	0	1	1.991402
tr|Q59G88|Q59G88_HUMAN	tr|Q59G88|Q59G8	Coronin (Fragment) OS=Homo sapiens PE=3 SV=1	60602	0	769	0	43	0	0.05	#DIV/0!	0	1	0	1	0	1	0	1	1.991402
tr|B7ZKT7|B7ZKT7_HUMAN	FYCO1	FYVE and coiled-coil domain-containing protein 1 OS=Homo sapiens GN=FYCO1 PE=	170608	0	768	0	44	0	0.02	#DIV/0!	0	1	0	1	0	1	0	2	1.991402
tr|B2RD27|B2RD27_HUMAN	tr|B2RD27|B2RD2	cDNA, FLJ96428, highly similar to Homo sapiens proteasome (prosome, macropain)	37046	0	767	0	44	0	0.09	#DIV/0!	0	1	0	1	0	1	0	1	1.991402
tr|H0YA82|H0YA82_HUMAN	LARP7	La-related protein 7 (Fragment) OS=Homo sapiens GN=LARP7 PE=4 SV=1	38466	0	765	0	44	0	0.09	#DIV/0!	0	1	0	1	0	1	0	2	1.991402
tr|H0Y6I6|H0Y6I6_HUMAN	FBXL18	F-box/LRR-repeat protein 18 (Fragment) OS=Homo sapiens GN=FBXL18 PE=4 SV=1	76506	0	763	0	44	0	0.04	#DIV/0!	0	1	0	1	0	1	0	1	1.991402
tr|G5EA30|G5EA30_HUMAN	CELF1	CUG triplet repeat, RNA binding protein 1, isoform CRA_c OS=Homo sapiens GN=CEL	55564	0	760	0	44	0	0.06	#DIV/0!	0	1	0	1	0	1	0	2	1.991402
tr|H0YE88|H0YE88_HUMAN	TEAD1	Transcriptional enhancer factor TEF-1 OS=Homo sapiens GN=TEAD1 PE=4 SV=1	48296	0	759	0	44	0	0.07	#DIV/0!	0	1	0	1	0	1	0	1	1.991402
tr|Q6LES9|Q6LES9_HUMAN	RFC5	RFC5 protein (Fragment) OS=Homo sapiens GN=RFC5 PE=2 SV=1	38658	0	755	0	45	0	0.09	#DIV/0!	0	1	0	1	0	1	0	1	1.991402
sp|Q9HCC0|MCCB_HUMAN	MCCC2	Methylcrotonoyl-CoA carboxylase beta chain, mitochondrial OS=Homo sapiens GN=	61808	0	750	0	45	0	0.05	#DIV/0!	0	1	0	1	0	1	0	1	1.991402
tr|H0YHM6|H0YHM6_HUMA	ALDH7A1	Alpha-aminoadipic semialdehyde dehydrogenase (Fragment) OS=Homo sapiens GN=	26344	0	748	0	45	0	0.13	#DIV/0!	0	1	0	1	0	1	0	1	1.991402
tr|B7Z8W6|B7Z8W6_HUMAN	tr|B7Z8W6|B7Z8W	cDNA FLJ55038, highly similar to Nicotinamide phosphoribosyltransferase (EC 2.4.2.	26905	0	747	0	46	0	0.12	#DIV/0!	0	1	0	1	0	1	0	1	1.991402
tr|B4DRJ1|B4DRJ1_HUMAN	tr|B4DRJ1|B4DRJ1	cDNA FLJ55041, highly similar to 2-hydroxyphytanoyl-CoA lyase (EC 4.1.-.-) OS=Hom	44949	0	746	0	46	0	0.07	#DIV/0!	0	1	0	1	0	1	0	1	1.991402
tr|B4DN85|B4DN85_HUMAN	tr|B4DN85|B4DN8	E3 ubiquitin-protein ligase OS=Homo sapiens PE=2 SV=1	93815	0	744	0	46	0	0.03	#DIV/0!	0	1	0	1	0	1	0	1	1.991402
tr|B4DKZ7|B4DKZ7_HUMAN	GATAD2A	Transcriptional repressor p66-alpha OS=Homo sapiens GN=GATAD2A PE=2 SV=1	47063	0	743	0	46	0	0.07	#DIV/0!	0	1	0	1	0	1	0	1	1.991402
tr|B3KUL1|B3KUL1_HUMAN	tr|B3KUL1|B3KUL1	cDNA FLJ40132 fis, clone TESTI2012155, highly similar to NUCLEOPORIN-LIKE PROTE	59275	0	737	0	46	0	0.06	#DIV/0!	0	1	0	1	0	1	0	1	1.991402
sp|Q9Y4W2|LAS1L_HUMAN	LAS1L	Ribosomal biogenesis protein LAS1L OS=Homo sapiens GN=LAS1L PE=1 SV=2	83982	0	735	0	46	0	0.04	#DIV/0!	0	1	0	1	0	1	0	2	1.991402
tr|Q59GH7|Q59GH7_HUMAN	tr|Q59GH7|Q59GH	Structure specific recognition protein 1 variant (Fragment) OS=Homo sapiens PE=2 S	59907	0	730	0	46	0	0.05	#DIV/0!	0	1	0	1	0	1	0	1	1.991402
tr|Q5HYM3|Q5HYM3_HUMA	DKFZp686C0249	Oxysterol-binding protein OS=Homo sapiens GN=DKFZp686C0249 PE=2 SV=1	101862	0	729	0	46	0	0.03	#DIV/0!	0	1	0	1	0	1	0	1	1.991402
tr|B4DEM9|B4DEM9_HUMA	tr|B4DEM9|B4DEN	cDNA FLJ52257, highly similar to Polymerase delta-interacting protein 2 OS=Homo s	40075	0	724	0	47	0	0.08	#DIV/0!	0	1	0	1	0	1	0	1	1.991402
tr|E5KLB5|E5KLB5_HUMAN	tr|E5KLB5|E5KLB5	DNA ligase OS=Homo sapiens PE=2 SV=1	114317	0	711	0	48	0	0.03	#DIV/0!	0	1	0	1	0	1	0	1	1.991402
tr|E5RK64|E5RK64_HUMAN	VAPB	Vesicle-associated membrane protein-associated protein B/C OS=Homo sapiens GN=	7910	0	706	0	49	0	0.45	#DIV/0!	0	1	0	1	0	1	0	1	1.991402
tr|B2RE34|B2RE34_HUMAN	tr|B2RE34|B2RE34	cDNA, FLJ96901, highly similar to Homo sapiens Rac GTPase activating protein 1 (RA	71608	0	701	0	49	0	0.05	#DIV/0!	0	1	0	1	0	1	0	2	1.991402
tr|Q6NXR6|Q6NXR6_HUMAN	SETD3	Histone-lysine N-methyltransferase setd3 OS=Homo sapiens GN=SETD3 PE=2 SV=1	33536	0	698	0	50	0	0.1	#DIV/0!	0	1	0	1	0	1	0	1	1.991402
tr|Q2TU84|Q2TU84_HUMAN	GIG18	Aspartate aminotransferase OS=Homo sapiens GN=GIG18 PE=2 SV=1	46519	0	696	0	50	0	0.07	#DIV/0!	0	1	0	1	0	1	0	1	1.991402
tr|Q6IBN4|Q6IBN4_HUMAN	PECI	PECI protein OS=Homo sapiens GN=PECI PE=2 SV=1	40510	0	692	0	51	0	0.08	#DIV/0!	0	1	0	1	0	1	0	1	1.991402
tr|B3KTW2|B3KTW2_HUMAN	tr|B3KTW2|B3KTV	cDNA FLJ38853 fis, clone MESAN2010321, highly similar to LIM domain and actin-bir	51596	0	686	0	51	0	0.06	#DIV/0!	0	1	0	1	0	1	0	1	1.991402
tr|B4DF68|B4DF68_HUMAN	tr|B4DF68|B4DF68	cDNA FLJ55325, highly similar to Heat-shock protein 105 kDa OS=Homo sapiens PE=	86500	0	684	0	52	0	0.04	#DIV/0!	0	1	0	1	0	1	0	1	1.991402
tr|B3KN28|B3KN28_HUMAN	tr|B3KN28|B3KN2	cDNA FLJ13370 fis, clone PLACE1000653, highly similar to Phosphoacetylglucosamin	60284	0	681	0	52	0	0.05	#DIV/0!	0	1	0	1	0	1	0	1	1.991402
tr|B4DPT1|B4DPT1_HUMAN	tr|B4DPT1|B4DPT	cDNA FLJ51524, highly similar to Set1/Ash2 histone methyltransferase complex subu	55688	0	677	0	52	0	0.06	#DIV/0!	0	1	0	1	0	1	0	1	1.991402
tr|Q5SQP8|Q5SQP8_HUMAN	CTBP2	C-terminal-binding protein 2 OS=Homo sapiens GN=CTBP2 PE=4 SV=1	56808	0	671	0	53	0	0.06	#DIV/0!	0	1	0	1	0	1	0	1	1.991402
tr|Q5T7C4|Q5T7C4_HUMAN	HMGB1	High mobility group protein B1 OS=Homo sapiens GN=HMGB1 PE=4 SV=1	18470	0	664	0	53	0	0.18	#DIV/0!	0	1	0	1	0	1	0	2	1.991402
tr|Q6ZVI6|Q6ZVI6_HUMAN	tr|Q6ZVI6|Q6ZVI6	cDNA FLJ42537 fis, clone BRACE3004058, highly similar to NADH-cytochrome B5 red	29141	0	653	0	54	0	0.11	#DIV/0!	0	1	0	1	0	1	0	1	1.991402
tr|A8K0D4|A8K0D4_HUMAN	tr|A8K0D4|A8K0D	cDNA FLJ78693, highly similar to Homo sapiens TAF6-like RNA polymerase II, p300/C	68270	0	643	0	55	0	0.05	#DIV/0!	0	1	0	1	0	1	0	1	1.991402
tr|B4DZ70|B4DZ70_HUMAN	ME1	Malic enzyme OS=Homo sapiens GN=ME1 PE=2 SV=1	55701	0	642	0	55	0	0.06	#DIV/0!	0	1	0	1	0	1	0	1	1.991402
sp|Q9H1E5|TMX4_HUMAN	TMX4	Thioredoxin-related transmembrane protein 4 OS=Homo sapiens GN=TMX4 PE=1 SV	39270	0	639	0	55	0	0.08	#DIV/0!	0	1	0	1	0	1	0	1	1.991402
tr|B4DLZ1|B4DLZ1_HUMAN	tr|B4DLZ1|B4DLZ1	cDNA FLJ56574, highly similar to GEM-interacting protein OS=Homo sapiens PE=2 SV	104609	0	638	0	55	0	0.03	#DIV/0!	0	1	0	1	0	1	0	1	1.991402
tr|H0YHS6|H0YHS6_HUMAN	YARS2	Tyrosine--tRNA ligase, mitochondrial (Fragment) OS=Homo sapiens GN=YARS2 PE=3	32279	0	637	0	55	0	0.1	#DIV/0!	0	1	0	1	0	1	0	1	1.991402
tr|B4DLV8|B4DLV8_HUMAN	RPL9	Ribosomal protein L9, isoform CRA_d OS=Homo sapiens GN=RPL9 PE=2 SV=1	19997	0	636	0	55	0	0.17	#DIV/0!	0	1	0	1	0	1	0	1	1.991402
tr|F8VS10|F8VS10_HUMAN	SPATS2	Spermatogenesis-associated serine-rich protein 2 OS=Homo sapiens GN=SPATS2 PE=	32626	0	635	0	55	0	0.1	#DIV/0!	0	1	0	1	0	1	0	1	1.991402
tr|B4E1K0|B4E1K0_HUMAN	KIF23	Kinesin-like protein KIF23 OS=Homo sapiens GN=KIF23 PE=2 SV=1	78221	0	633	0	55	0	0.04	#DIV/0!	0	1	0	1	0	1	0	1	1.991402
tr|H3BPN4|H3BPN4_HUMAN	EDC3	Enhancer of mRNA-decapping protein 3 (Fragment) OS=Homo sapiens GN=EDC3 PE=	23715	0	632	0	56	0	0.14	#DIV/0!	0	1	0	1	0	1	0	1	1.991402
tr|B0QZ36|B0QZ36_HUMAN	RAE1	RAE1 RNA export 1 homolog (S. pombe) (Fragment) OS=Homo sapiens GN=RAE1 PE=	14945	0	629	0	56	0	0.23	#DIV/0!	0	1	0	1	0	1	0	1	1.991402
tr|H0Y360|H0Y360_HUMAN	AMPD2	AMP deaminase 2 (Fragment) OS=Homo sapiens GN=AMPD2 PE=4 SV=1	99192	0	626	0	56	0	0.03	#DIV/0!	0	1	0	1	0	1	0	1	1.991402
tr|B4DJM9|B4DJM9_HUMAN	SARS2	Serine--tRNA ligase, mitochondrial OS=Homo sapiens GN=SARS2 PE=2 SV=1	26696	0	625	0	56	0	0.12	#DIV/0!	0	1	0	1	0	1	0	1	1.991402
tr|B4DYM8|B4DYM8_HUMA	tr|B4DYM8|B4DYN	cDNA FLJ60373, highly similar to Zinc finger CCCH domain-containing protein11A OS	89647	0	612	0	58	0	0.04	#DIV/0!	0	1	0	1	0	1	0	2	1.991402
tr|Q2NL95|Q2NL95_HUMAN	DDX19B	DDX19B protein (Fragment) OS=Homo sapiens GN=DDX19B PE=2 SV=1	51068	0	609	0	59	0	0.06	#DIV/0!	0	1	0	1	0	1	0	1	1.991402
tr|B3KN49|B3KN49_HUMAN	tr|B3KN49|B3KN4	cDNA FLJ13562 fis, clone PLACE1008080, highly similar to Homo sapiens hexamethyl	40868	0	584	0	62	0	0.08	#DIV/0!	0	1	0	1	0	1	0	1	1.991402
tr|Q5JTE4|Q5JTE4_HUMAN	POLR1C	Polymerase (RNA) I polypeptide C, 30kDa OS=Homo sapiens GN=POLR1C PE=2 SV=1	36043	0	583	0	62	0	0.09	#DIV/0!	0	1	0	1	0	1	0	1	1.991402
sp|Q6ZN55|ZN574_HUMAN	ZNF574	Zinc finger protein 574 OS=Homo sapiens GN=ZNF574 PE=1 SV=2	101175	0	579	0	63	0	0.03	#DIV/0!	0	1	0	1	0	1	0	1	1.991402
tr|Q0QD40|Q0QD40_HUMA	RDH11	Retinol dehydrogenase 11 (Fragment) OS=Homo sapiens GN=RDH11 PE=2 SV=1	18929	0	575	0	63	0	0.18	#DIV/0!	0	1	0	1	0	1	0	1	1.991402
tr|B1AQP8|B1AQP8_HUMAN	EPHX1	Epoxide hydrolase 1 (Fragment) OS=Homo sapiens GN=EPHX1 PE=4 SV=1	16430	0	568	0	64	0	0.21	#DIV/0!	0	1	0	1	0	1	0	1	1.991402
tr|B3KMG5|B3KMG5_HUMA	tr|B3KMG5|B3KM	cDNA FLJ10938 fis, clone OVARC1001038, highly similar to Protein ariadne-2 homolo	50060	0	566	0	64	0	0.07	#DIV/0!	0	1	0	1	0	1	0	1	1.991402
tr|B4DN87|B4DN87_HUMAN	tr|B4DN87|B4DN8	cDNA FLJ52569, highly similar to Collagen-binding protein 2 OS=Homo sapiens PE=2	44290	0	559	0	65	0	0.07	#DIV/0!	0	1	0	1	0	1	0	1	1.991402
sp|Q9NVH2|INT7_HUMAN	INTS7	Integrator complex subunit 7 OS=Homo sapiens GN=INTS7 PE=1 SV=1	108021	0	541	0	68	0	0.03	#DIV/0!	0	1	0	1	0	1	0	1	1.991402
tr|Q86WV4|Q86WV4_HUMA	MRPS9	MRPS9 protein (Fragment) OS=Homo sapiens GN=MRPS9 PE=2 SV=1	35297	0	525	0	69	0	0.09	#DIV/0!	0	1	0	1	0	1	0	1	1.991402
tr|H1UBN2|H1UBN2_HUMA	CCNB1V	Cyclin B1 OS=Homo sapiens GN=CCNB1V PE=2 SV=1	46024	0	523	0	70	0	0.07	#DIV/0!	0	1	0	1	0	1	0	2	1.991402
tr|B4DQ08|B4DQ08_HUMAN	tr|B4DQ08|B4DQ0	cDNA FLJ53406, highly similar to U3 small nucleolar RNA-associated protein 14 hom	82068	0	521	0	70	0	0.04	#DIV/0!	0	1	0	1	0	1	0	1	1.991402
tr|Q7Z780|Q7Z780_HUMAN	U2AF1	U2 small nuclear RNA auxiliary factor 1 OS=Homo sapiens GN=U2AF1 PE=2 SV=1	20087	0	512	0	71	0	0.17	#DIV/0!	0	1	0	1	0	1	0	2	1.991402
tr|B3KMQ6|B3KMQ6_HUMA	tr|B3KMQ6|B3KM	cDNA FLJ12172 fis, clone MAMMA1000684, highly similar to Opioid growth factor re	73894	0	505	0	72	0	0.04	#DIV/0!	0	1	0	1	0	1	0	1	1.991402
sp|Q7L2J0|MEPCE_HUMAN	MEPCE	7SK snRNA methylphosphate capping enzyme OS=Homo sapiens GN=MEPCE PE=1 S	74937	0	499	0	73	0	0.04	#DIV/0!	0	1	0	1	0	1	0	1	1.991402
tr|B2RD14|B2RD14_HUMAN	tr|B2RD14|B2RD1	cDNA, FLJ96414, highly similar to Homo sapiens ubiquitin carboxyl-terminal esterase	25167	0	493	0	74	0	0.13	#DIV/0!	0	1	0	1	0	1	0	1	1.991402
tr|H0YCY6|H0YCY6_HUMAN	DAK	FAD-AMP lyase (cyclizing) (Fragment) OS=Homo sapiens GN=DAK PE=4 SV=1	55026	0	491	0	75	0	0.06	#DIV/0!	0	1	0	1	0	1	0	1	1.991402
sp|O15047|SET1A_HUMAN	SETD1A	Histone-lysine N-methyltransferase SETD1A OS=Homo sapiens GN=SETD1A PE=1 SV=	186718	0	490	0	75	0	0.02	#DIV/0!	0	1	0	1	0	1	0	2	1.991402
tr|Q1W6G4|Q1W6G4_HUMA	LUC7L	LUC7-like (S. cerevisiae) OS=Homo sapiens GN=LUC7L PE=2 SV=1	38781	0	472	0	77	0	0.09	#DIV/0!	0	1	0	1	0	1	0	3	1.991402
sp|Q96GS4|CQ059_HUMAN	C17orf59	Uncharacterized protein C17orf59 OS=Homo sapiens GN=C17orf59 PE=1 SV=2	37603	0	457	0	79	0	0.09	#DIV/0!	0	1	0	1	0	1	0	1	1.991402
tr|J3K000|J3K000_HUMAN	PEPD	PEPD protein OS=Homo sapiens GN=PEPD PE=2 SV=1	55345	0	451	0	80	0	0.06	#DIV/0!	0	1	0	1	0	1	0	1	1.991402
tr|Q53G42|Q53G42_HUMAN	tr|Q53G42|Q53G4	mRNA decapping enzyme variant (Fragment) OS=Homo sapiens PE=2 SV=1	38757	0	450	0	80	0	0.09	#DIV/0!	0	1	0	1	0	1	0	1	1.991402
sp|Q96LA8|ANM6_HUMAN	PRMT6	Protein arginine N-methyltransferase 6 OS=Homo sapiens GN=PRMT6 PE=1 SV=1	42253	0	444	0	81	0	0.08	#DIV/0!	0	1	0	1	0	1	0	1	1.991402
sp|Q6P6C2|ALKB5_HUMAN	ALKBH5	RNA demethylase ALKBH5 OS=Homo sapiens GN=ALKBH5 PE=1 SV=2	44571	0	407	0	88	0	0.07	#DIV/0!	0	1	0	1	0	1	0	1	1.991402
sp|O75475|PSIP1_HUMAN	PSIP1	PC4 and SFRS1-interacting protein OS=Homo sapiens GN=PSIP1 PE=1 SV=1	60181	0	400	0	91	0	0.05	#DIV/0!	0	1	0	1	0	1	0	1	1.991402
tr|A8MU44|A8MU44_HUMA	HOOK1	Protein Hook homolog 1 OS=Homo sapiens GN=HOOK1 PE=2 SV=2	80485	0	379	0	97	0	0.04	#DIV/0!	0	1	0	1	0	1	0	1	1.991402
tr|K7ZRV2|K7ZRV2_HUMAN	TPRBK	TPR-containing big gene cloned at Keio OS=Homo sapiens GN=TPRBK PE=2 SV=1	272653	0	328	0	110	0	0.01	#DIV/0!	0	1	0	1	0	1	0	1	1.991402
tr|F8VVL1|F8VVL1_HUMAN	DENR	Density-regulated protein OS=Homo sapiens GN=DENR PE=2 SV=1	18158	0	318	0	113	0	0.19	#DIV/0!	0	1	0	1	0	1	0	1	1.991402
sp|Q68E01|INT3_HUMAN	INTS3	Integrator complex subunit 3 OS=Homo sapiens GN=INTS3 PE=1 SV=1	119533	0	300	0	119	0	0.03	#DIV/0!	0	1	0	1	0	1	0	1	1.991402
tr|K7EKR9|K7EKR9_HUMAN	RAVER1	Ribonucleoprotein PTB-binding 1 (Fragment) OS=Homo sapiens GN=RAVER1 PE=4 S	10993	0	1151	0	15	0	0.31	#DIV/0!	0	0	0	1	0	0	0	1	0.40618
sp|Q8N137|CNTRB_HUMAN	CNTROB	Centrobin OS=Homo sapiens GN=CNTROB PE=1 SV=1	101476	0	1150	0	16	0	0.03	#DIV/0!	0	0	0	1	0	0	0	1	0.40618
tr|B3KP24|B3KP24_HUMAN	tr|B3KP24|B3KP24	cDNA FLJ31012 fis, clone HLUNG2000217, highly similar to Guanine nucleotide-bindi	31909	0	1147	0	17	0	0.1	#DIV/0!	0	0	0	1	0	0	0	1	0.40618
tr|H0YJL6|H0YJL6_HUMAN	EVL	Ena/VASP-like protein (Fragment) OS=Homo sapiens GN=EVL PE=4 SV=1	10347	0	1145	0	17	0	0.34	#DIV/0!	0	0	0	1	0	0	0	1	0.40618
tr|C9JMY0|C9JMY0_HUMAN	LENG8	Leukocyte receptor cluster member 8 OS=Homo sapiens GN=LENG8 PE=2 SV=1	5901	0	1144	0	17	0	0.61	#DIV/0!	0	0	0	1	0	0	0	1	0.40618
sp|Q9P225|DYH2_HUMAN	DNAH2	Dynein heavy chain 2, axonemal OS=Homo sapiens GN=DNAH2 PE=2 SV=3	510796	0	1141	0	17	0	0.01	#DIV/0!	0	0	0	1	0	0	0	1	0.40618
tr|F5H1X8|F5H1X8_HUMAN	LRBA	Lipopolysaccharide-responsive and beige-like anchor protein OS=Homo sapiens GN=	320211	0	1140	0	17	0	0.01	#DIV/0!	0	0	0	1	0	0	0	1	0.40618
tr|B4DYM9|B4DYM9_HUMA	tr|B4DYM9|B4DYN	cDNA FLJ51643, moderately similar to Homo sapiens 1-aminocyclopropane-1-carbox	14826	0	1137	0	17	0	0.23	#DIV/0!	0	0	0	1	0	0	0	1	0.40618
tr|J3KQ16|J3KQ16_HUMAN	KCNMA1	Calcium-activated potassium channel subunit alpha-1 OS=Homo sapiens GN=KCNMA	139880	0	1136	0	18	0	0.02	#DIV/0!	0	0	0	1	0	0	0	2	0.40618
sp|A1L0T0|ILVBL_HUMAN	ILVBL	Acetolactate synthase-like protein OS=Homo sapiens GN=ILVBL PE=1 SV=2	68452	0	1134	0	18	0	0.05	#DIV/0!	0	0	0	1	0	0	0	1	0.40618
tr|H0YHP3|H0YHP3_HUMAN	CNOT2	CCR4-NOT transcription complex subunit 2 (Fragment) OS=Homo sapiens GN=CNOT	9794	0	1133	0	18	0	0.36	#DIV/0!	0	0	0	1	0	0	0	1	0.40618
tr|B4DWA5|B4DWA5_HUMA	tr|B4DWA5|B4DW	cDNA FLJ52464, highly similar to GTPase, IMAP family member 4 OS=Homo sapiens	39223	0	1132	0	18	0	0.08	#DIV/0!	0	0	0	1	0	0	0	1	0.40618
tr|F5H667|F5H667_HUMAN	ASPH	Aspartyl/asparaginyl beta-hydroxylase OS=Homo sapiens GN=ASPH PE=2 SV=1	83672	0	1131	0	18	0	0.04	#DIV/0!	0	0	0	1	0	0	0	1	0.40618
tr|B4E2K4|B4E2K4_HUMAN	tr|B4E2K4|B4E2K4	cDNA FLJ54576, highly similar to Aspartyl/asparaginyl beta-hydroxylase (EC 1.14.11.	83685	0	1130	0	18	0	0.04	#DIV/0!	0	0	0	1	0	0	0	1	0.40618
tr|A1L172|A1L172_HUMAN	ACOT1	Acyl-CoA thioesterase 1 OS=Homo sapiens GN=ACOT1 PE=2 SV=1	46666	0	1128	0	18	0	0.07	#DIV/0!	0	0	0	1	0	0	0	1	0.40618
tr|B3KTF7|B3KTF7_HUMAN	tr|B3KTF7|B3KTF7	cDNA FLJ38176 fis, clone FCBBF1000077, highly similar to Exocyst complex compone	66519	0	1127	0	18	0	0.05	#DIV/0!	0	0	0	1	0	0	0	1	0.40618
sp|Q9H9B4|SFXN1_HUMAN	SFXN1	Sideroflexin-1 OS=Homo sapiens GN=SFXN1 PE=1 SV=4	35881	0	1125	0	18	0	0.09	#DIV/0!	0	0	0	1	0	0	0	1	0.40618
sp|Q9GZL7|WDR12_HUMAN	WDR12	Ribosome biogenesis protein WDR12 OS=Homo sapiens GN=WDR12 PE=1 SV=2	48191	0	1124	0	18	0	0.07	#DIV/0!	0	0	0	1	0	0	0	1	0.40618
tr|B3KNA1|B3KNA1_HUMAN	tr|B3KNA1|B3KNA	cDNA FLJ14021 fis, clone HEMBA1002513, highly similar to Histone deacetylase 6 (F	90775	0	1121	0	19	0	0.04	#DIV/0!	0	0	0	1	0	0	0	1	0.40618
sp|Q96EG3|ZN837_HUMAN	ZNF837	Zinc finger protein 837 OS=Homo sapiens GN=ZNF837 PE=2 SV=2	60208	0	1120	0	19	0	0.05	#DIV/0!	0	0	0	1	0	0	0	1	0.40618
tr|Q9BV95|Q9BV95_HUMAN	tr|Q9BV95|Q9BV9	LIMS2 protein OS=Homo sapiens PE=2 SV=1	4890	0	1119	0	19	0	0.75	#DIV/0!	0	0	0	1	0	0	0	1	0.40618
sp|Q9Y4C5|CHST2_HUMAN	CHST2	Carbohydrate sulfotransferase 2 OS=Homo sapiens GN=CHST2 PE=1 SV=2	58391	0	1118	0	19	0	0.06	#DIV/0!	0	0	0	1	0	0	0	1	0.40618
tr|H0YJ14|H0YJ14_HUMAN	FANCM	Fanconi anemia group M protein (Fragment) OS=Homo sapiens GN=FANCM PE=4 SV	116030	0	1117	0	19	0	0.03	#DIV/0!	0	0	0	1	0	0	0	1	0.40618
tr|H0YHN5|H0YHN5_HUMAN	NCKAP1L	Nck-associated protein 1-like (Fragment) OS=Homo sapiens GN=NCKAP1L PE=4 SV=1	8739	0	1116	0	19	0	0.39	#DIV/0!	0	0	0	1	0	0	0	1	0.40618
tr|A5PKT8|A5PKT8_HUMAN	FAM43B	Family with sequence similarity 43, member B OS=Homo sapiens GN=FAM43B PE=2	37223	0	1115	0	19	0	0.09	#DIV/0!	0	0	0	1	0	0	0	1	0.40618
tr|H7C2X0|H7C2X0_HUMAN	EIF2B5	Translation initiation factor eIF-2B subunit epsilon (Fragment) OS=Homo sapiens GN	9441	0	1113	0	20	0	0.37	#DIV/0!	0	0	0	1	0	0	0	1	0.40618
tr|Q06DW9|Q06DW9_HUMA	WDR13	WDR13 OS=Homo sapiens GN=WDR13 PE=2 SV=1	40260	0	1112	0	20	0	0.08	#DIV/0!	0	1	0	1	0	0	0	1	0.40618
sp|Q96QE3|ATAD5_HUMAN	ATAD5	ATPase family AAA domain-containing protein 5 OS=Homo sapiens GN=ATAD5 PE=1	209494	0	1105	0	21	0	0.03	#DIV/0!	0	0	0	2	0	0	0	2	0.40618
sp|P57740|NU107_HUMAN	NUP107	Nuclear pore complex protein Nup107 OS=Homo sapiens GN=NUP107 PE=1 SV=1	107048	986	431	31	83	0.03	0.3	10	0	1	1	1	1	1	2	1	0.406244
tr|H0YH87|H0YH87_HUMAN	ATXN2	Ataxin-2 (Fragment) OS=Homo sapiens GN=ATXN2 PE=4 SV=1	98626	490	463	78	78	0.03	0.28	9.333333	1	1	1	2	1	2	1	2	1.143468
sp|P00390|GSHR_HUMAN	GSR	Glutathione reductase, mitochondrial OS=Homo sapiens GN=GSR PE=1 SV=2	56791	1139	292	22	123	0.06	0.4	6.666667	0	3	1	6	1	6	1	7	2.523016
tr|E2QRB3|E2QRB3_HUMAN	PYCR1	Pyrroline-5-carboxylate reductase 1, isoform CRA_c OS=Homo sapiens GN=PYCR1 PE	30422	911	140	35	190	0.11	0.69	6.272727	0	5	1	5	1	5	2	5	2.281749
tr|A6NG51|A6NG51_HUMAN	SPTAN1	Spectrin alpha chain, non-erythrocytic 1 OS=Homo sapiens GN=SPTAN1 PE=2 SV=2	285628	409	99	100	273	0.02	0.11	5.5	2	5	2	8	2	9	3	11	2.33412
tr|G3V3Y6|G3V3Y6_HUMAN	APEX1	DNA-(apurinic or apyrimidinic site) lyase (Fragment) OS=Homo sapiens GN=APEX1 P	4488	1043	545	28	67	0.17	0.84	4.941176	0	1	1	1	1	1	1	1	0.406244
tr|B2RDN4|B2RDN4_HUMAN	tr|B2RDN4|B2RDN	cDNA, FLJ96693, highly similar to Homo sapiens block of proliferation 1 (BOP1), mR	84219	1125	361	23	48	0.04	0.19	4.75	0	1	1	3	1	2	1	3	1.143468
sp|Q8N0Y7|PGAM4_HUMAN	PGAM4	Probable phosphoglycerate mutase 4 OS=Homo sapiens GN=PGAM4 PE=2 SV=1	28930	726	392	50	30	0.12	0.55	4.583333	1	0	1	2	1	1	1	3	0.406244
sp|Q9Y2W1|TR150_HUMAN	THRAP3	Thyroid hormone receptor-associated protein 3 OS=Homo sapiens GN=THRAP3 PE=	108658	1080	247	26	142	0.03	0.13	4.333333	0	3	1	4	1	4	1	5	1.991983
tr|H3BP71|H3BP71_HUMAN	RNF40	E3 ubiquitin-protein ligase BRE1B OS=Homo sapiens GN=RNF40 PE=4 SV=1	109696	1067	596	26	60	0.03	0.13	4.333333	0	1	1	1	2	1	2	1	-0.33092
tr|Q6FG59|Q6FG59_HUMAN	CDC37	CDC37 protein OS=Homo sapiens GN=CDC37 PE=2 SV=1	44938	473	571	82	45	0.15	0.63	4.2	2	1	2	1	2	1	3	1	-0.33092
tr|F5H2U1|F5H2U1_HUMAN	CBS	Cystathionine beta-synthase OS=Homo sapiens GN=CBS PE=2 SV=1	51641	1118	296	23	96	0.06	0.25	4.166667	0	3	1	3	1	4	1	4	1.991983
tr|B3KXU8|B3KXU8_HUMAN	tr|B3KXU8|B3KXU	cDNA FLJ46087 fis, clone TESTI2009497, highly similar to GPI-anchored protein p137	70489	1061	275	26	116	0.05	0.2	4	0	3	1	3	1	3	1	3	1.629154
tr|B3KRK4|B3KRK4_HUMAN	tr|B3KRK4|B3KRK4	cDNA FLJ34459 fis, clone HLUNG2002916, highly similar to SRC SUBSTRATE CORTAC	26264	495	235	77	90	0.13	0.52	4	1	2	1	2	1	2	1	2	1.143468
tr|B5MBZ0|B5MBZ0_HUMAN	EML4	Echinoderm microtubule-associated protein-like 4 OS=Homo sapiens GN=EML4 PE=	111192	971	923	32	31	0.03	0.12	4	0	0	1	1	1	1	3	1	0.406244
tr|Q2Q9B7|Q2Q9B7_HUMAN	G6PD	Glucose-6-phosphate 1-dehydrogenase (Fragment) OS=Homo sapiens GN=G6PD PE=	55221	472	244	82	99	0.12	0.45	3.75	2	2	2	3	3	5	6	5	1.058965

tr|D3YTE0|D3YTE0_HUMAN	KRI1	Protein KRI1 homolog OS=Homo sapiens GN=KRI1 PE=2 SV=1	83005	958	705	33	49	0.04	0.15	3.75	0	1	1	1	1	1	1	1	0.406244
tr|Q5H928|Q5H928_HUMAN	HSD17B10	3-hydroxyacyl-CoA dehydrogenase type-2 OS=Homo sapiens GN=HSD17B10 PE=4 SV	17498	782	189	45	183	0.19	0.7	3.684211	1	3	1	3	1	3	1	3	1.629154
tr|J3QL54|J3QL54_HUMAN	NUP85	Nuclear pore complex protein Nup85 OS=Homo sapiens GN=NUP85 PE=4 SV=1	62746	880	685	37	51	0.05	0.17	3.4	1	2	1	3	1	2	1	3	1.143468
tr|B0QZ18|B0QZ18_HUMAN	CPNE1	Copine I OS=Homo sapiens GN=CPNE1 PE=2 SV=1	60307	1008	442	30	81	0.05	0.17	3.4	0	1	1	3	2	3	3	3	0.891993
sp|O00116|ADAS_HUMAN	AGPS	Alkyldihydroxyacetonephosphate synthase, peroxisomal OS=Homo sapiens GN=AGP	73664	648	828	56	38	0.05	0.17	3.4	1	1	1	1	1	1	2	1	0.406244
tr|B4DUL5|B4DUL5_HUMAN	tr|B4DUL5|B4DUL	cDNA FLJ51625, highly similar to Ubiquinol-cytochrome-c reductase complex corepr	40917	870	661	38	51	0.08	0.27	3.375	1	1	1	3	1	3	2	3	1.629154
tr|G3V1A4|G3V1A4_HUMAN	CFL1	Cofilin 1 (Non-muscle), isoform CRA_a OS=Homo sapiens GN=CFL1 PE=4 SV=1	17029	550	255	68	138	0.44	1.48	3.363636	2	3	2	5	3	5	3	6	1.058965
tr|F8WA43|F8WA43_HUMAN	CTNND1	Catenin delta-1 OS=Homo sapiens GN=CTNND1 PE=2 SV=1	106284	1095	309	25	115	0.03	0.1	3.333333	0	3	1	3	1	3	1	3	1.629154
tr|D3DQA6|D3DQA6_HUMAN	DDX46	DEAD (Asp-Glu-Ala-Asp) box polypeptide 46, isoform CRA_a OS=Homo sapiens GN=D	100958	1040	723	28	47	0.03	0.1	3.333333	0	1	1	3	1	2	1	3	1.143468
sp|P49756|RBM25_HUMAN	RBM25	RNA-binding protein 25 OS=Homo sapiens GN=RBM25 PE=1 SV=3	100467	1083	347	25	104	0.03	0.1	3.333333	1	2	1	3	2	3	2	3	0.891993
tr|G1UI18|G1UI18_HUMAN	PSME4	Proteasome activator complex subunit 4 (Fragment) OS=Homo sapiens GN=PSME4 F	130054	1006	914	30	32	0.03	0.1	3.333333	1	2	1	2	1	1	1	2	0.406244
tr|B4DHQ3|B4DHQ3_HUMAN	tr|B4DHQ3|B4DHQ	cDNA FLJ56437, highly similar to Phosphoserine aminotransferase (EC 2.6.1.52) OS=	45783	943	410	34	87	0.07	0.23	3.285714	0	3	1	3	1	3	1	4	1.629154
tr|F5H6D0|F5H6D0_HUMAN	MOGS	Mannosyl-oligosaccharide glucosidase OS=Homo sapiens GN=MOGS PE=2 SV=1	80824	1133	478	22	77	0.04	0.13	3.25	0	1	1	3	1	2	1	4	1.143468
tr|B4DT31|B4DT31_HUMAN	FUBP1	Far upstream element-binding protein 1 OS=Homo sapiens GN=FUBP1 PE=2 SV=1	69993	478	164	81	209	0.1	0.32	3.2	1	4	2	6	2	6	3	7	1.785855
tr|Q14400|Q14400_HUMAN	GLUD1	GLUD1 protein (Fragment) OS=Homo sapiens GN=GLUD1 PE=2 SV=1	28791	664	202	36	175	0.23	0.73	3.173913	0	4	1	5	1	5	2	5	2.281749
tr|A4ZU86|A4ZU86_HUMAN	NPM1	Truncated nucleolar phosphoprotein B23 OS=Homo sapiens GN=NPM1 PE=2 SV=1	30074	271	98	147	275	0.37	1.17	3.162162	3	5	3	9	4	9	4	9	1.485732
tr|H0Y8R1|H0Y8R1_HUMAN	GRSF1	G-rich sequence factor 1 (Fragment) OS=Homo sapiens GN=GRSF1 PE=4 SV=1	47637	586	421	63	85	0.07	0.22	3.142857	1	2	1	3	1	3	1	3	1.629154
sp|Q9Y266|NUDC_HUMAN	NUDC	Nuclear migration protein nudC OS=Homo sapiens GN=NUDC PE=1 SV=1	38276	982	375	32	99	0.09	0.28	3.111111	0	2	1	3	1	3	1	4	1.629154
tr|B4DGU4|B4DGU4_HUMAN	CTNNB1	Catenin beta-1 OS=Homo sapiens GN=CTNNB1 PE=2 SV=1	85339	1052	641	27	55	0.04	0.12	3	1	1	1	3	2	4	2	5	1.254822
tr|Q6AHZ7|Q6AHZ7_HUMAN	DKFZp686A111	Putative uncharacterized protein DKFZp686A111 OS=Homo sapiens GN=DKFZp686A	240988	707	716	51	48	0.01	0.03	3	1	1	1	2	1	2	3	3	1.143468
tr|H0Y7P1|H0Y7P1_HUMAN	MED12	Mediator of RNA polymerase II transcription subunit 12 (Fragment) OS=Homo sapie	240034	1096	961	25	29	0.01	0.03	3	1	0	1	1	1	1	1	1	0.406244
sp|Q5T8P6|RBM26_HUMAN	RBM26	RNA-binding protein 26 OS=Homo sapiens GN=RBM26 PE=1 SV=3	113927	1071	489	26	75	0.03	0.09	3	0	1	1	1	1	1	2	1	0.406244
tr|F8W930|F8W930_HUMAN	IGF2BP2	Insulin-like growth factor 2 mRNA-binding protein 2 OS=Homo sapiens GN=IGF2BP2	66859	1093	471	25	77	0.05	0.15	3	0	2	2	3	1	3	3	3	1.629154
tr|B4DDM6|B4DDM6_HUMA	BUB3	Mitotic checkpoint protein BUB3 OS=Homo sapiens GN=BUB3 PE=2 SV=1	28620	998	758	31	39	0.12	0.34	2.833333	0	1	1	2	3	2	3	3	-0.07931
tr|H0Y2W2|H0Y2W2_HUMA	ATAD3A	ATPase family AAA domain-containing protein 3A (Fragment) OS=Homo sapiens GN=	64600	964	515	33	68	0.05	0.14	2.8	0	2	1	2	2	2	2	2	0.406307
tr|H0Y3Y4|H0Y3Y4_HUMAN	Sep-07	Septin-7 (Fragment) OS=Homo sapiens GN=SEPT7 PE=3 SV=1	43294	576	404	64	90	0.16	0.44	2.75	3	2	3	5	3	5	3	7	1.058965
tr|B3KMZ6|B3KMZ6_HUMAN	tr|B3KMZ6|B3KMZ	cDNA FLJ13058 fis, clone NT2RP3001587, highly similar to Ubiquitin-like 1-activating	71688	325	221	125	154	0.14	0.37	2.642857	2	3	3	5	5	4	6	5	0.116732
tr|B7ZKT9|B7ZKT9_HUMAN	KIAA1033	KIAA1033 protein OS=Homo sapiens GN=KIAA1033 PE=2 SV=1	137456	1003	753	30	45	0.02	0.05	2.5	0	0	1	2	1	3	1	3	1.629154
tr|B4DYY5|B4DYY5_HUMAN	tr|B4DYY5|B4DYY5	cDNA FLJ55671, highly similar to Ubiquitin-associated protein 2-like OS=Homo sapie	113662	646	647	57	55	0.06	0.15	2.5	1	2	2	4	2	4	2	5	1.254822
tr|I3L0L3|I3L0L3_HUMAN	NSF	Vesicle-fusing ATPase (Fragment) OS=Homo sapiens GN=NSF PE=4 SV=1	18140	604	881	28	34	0.08	0.19	2.375	1	0	1	1	1	2	1	2	1.143468
tr|A8MW61|A8MW61_HUM/	PLRG1	Pleiotropic regulator 1 OS=Homo sapiens GN=PLRG1 PE=2 SV=1	57545	898	1027	36	25	0.06	0.14	2.333333	1	0	1	1	1	2	2	2	1.143468
sp|P52948|NUP98_HUMAN	NUP98	Nuclear pore complex protein Nup98-Nup96 OS=Homo sapiens GN=NUP98 PE=1 SV	198597	966	509	33	72	0.03	0.07	2.333333	0	1	2	4	3	5	3	5	1.058965
tr|H0YFF5|H0YFF5_HUMAN	CLPB	Caseinolytic peptidase B protein homolog (Fragment) OS=Homo sapiens GN=CLPB P	37480	642	1066	57	23	0.09	0.21	2.333333	1	0	1	1	1	1	1	1	0.406244
sp|Q00653|NFKB2_HUMAN	NFKB2	Nuclear factor NF-kappa-B p100 subunit OS=Homo sapiens GN=NFKB2 PE=1 SV=4	97373	1101	829	24	38	0.03	0.07	2.333333	0	1	1	1	1	1	2	1	0.406244
tr|D6RG13|D6RG13_HUMAN	RPS3A	40S ribosomal protein S3a (Fragment) OS=Homo sapiens GN=RPS3A PE=2 SV=1	25819	608	439	60	82	0.27	0.63	2.333333	2	1	2	4	3	4	3	5	0.7692
tr|A8K4X6|A8K4X6_HUMAN	tr|A8K4X6|A8K4X6	cDNA FLJ75223, highly similar to Homo sapiens adenylosuccinate lyase (ADSL) OS=H	48997	480	522	80	70	0.13	0.3	2.307692	3	2	3	4	3	3	3	6	0.406371
tr|E7EW20|E7EW20_HUMAN	MYO6	Unconventional myosin-VI OS=Homo sapiens GN=MYO6 PE=2 SV=1	150111	650	147	56	224	0.07	0.16	2.285714	2	6	3	7	3	7	3	7	1.506943
tr|B2R5M8|B2R5M8_HUMAN	tr|B2R5M8|B2R5N	Isocitrate dehydrogenase [NADP] OS=Homo sapiens PE=2 SV=1	46905	1104	779	24	43	0.07	0.16	2.285714	0	1	1	2	2	2	3	2	0.406307
tr|B4DY32|B4DY32_HUMAN	tr|B4DY32|B4DY32	Asparagine synthetase OS=Homo sapiens PE=2 SV=1	55055	351	181	116	194	0.26	0.59	2.269231	3	5	4	10	7	10	7	13	0.89283
tr|F5GX05|F5GX05_HUMAN	EPB41L3	Band 4.1-like protein 3 OS=Homo sapiens GN=EPB41L3 PE=2 SV=1	98856	424	494	37	74	0.12	0.27	2.25	0	1	2	1	2	1	2	1	-0.33092
sp|Q96C36|P5CR2_HUMAN	PYCR2	Pyrroline-5-carboxylate reductase 2 OS=Homo sapiens GN=PYCR2 PE=1 SV=1	33958	425	335	94	107	0.2	0.45	2.25	2	2	2	4	2	4	2	4	1.254822
tr|H0YJA2|H0YJA2_HUMAN	ZC3H14	Zinc finger CCCH domain-containing protein 14 (Fragment) OS=Homo sapiens GN=ZC	74241	809	454	43	80	0.04	0.09	2.25	1	2	1	2	1	2	2	2	1.143468
tr|B3KU62|B3KU62_HUMAN	tr|B3KU62|B3KU6	cDNA FLJ39243 fis, clone OCBBF2008283, highly similar to Protein NDRG1 OS=Homo	38948	795	676	44	52	0.08	0.18	2.25	0	1	2	2	2	2	2	3	0.406307
tr|Q6DCA8|Q6DCA8_HUMAN	BCLAF1	BCLAF1 protein (Fragment) OS=Homo sapiens GN=BCLAF1 PE=2 SV=1	77958	792	386	44	96	0.04	0.09	2.25	1	2	1	2	2	2	3	3	0.406307
tr|G3V359|G3V359_HUMAN	APEX1	DNA-(apurinic or apyrimidinic site) lyase (Fragment) OS=Homo sapiens GN=APEX1 P	19384	1043	569	28	64	0.17	0.38	2.235294	0	1	1	2	1	2	1	2	1.143468
tr|B0LM41|B0LM41_HUMAN	RBM14/RBM4	Protein RBM14-RBM4 OS=Homo sapiens GN=RBM14/RBM4 fusion PE=2 SV=1	37410	247	276	164	130	0.18	0.4	2.222222	2	2	2	4	3	4	3	4	0.7692
tr|A8K4Z4|A8K4Z4_HUMAN	tr|A8K4Z4|A8K4Z4	cDNA FLJ75549, highly similar to Homo sapiens ribosomal protein, large, P0 (RPLP0),	34389	393	153	103	218	0.45	0.98	2.177778	2	7	5	8	6	8	6	8	0.794103
tr|B4DWI8|B4DWI8_HUMAN	tr|B4DWI8|B4DWI	cDNA FLJ57805, highly similar to Homo sapiens paraspeckle component 1 (PSPC1), t	52660	1137	683	22	52	0.06	0.13	2.166667	0	1	1	2	2	3	2	3	0.891993
tr|I6L9E8|I6L9E8_HUMAN	FAM98A	Family with sequence similarity 98, member A OS=Homo sapiens GN=FAM98A PE=2	55595	315	223	127	157	0.19	0.41	2.157895	2	3	3	6	3	6	4	6	1.300233
tr|B5BUB5|B5BUB5_HUMAN	SSB	Autoantigen La (Fragment) OS=Homo sapiens GN=SSB PE=2 SV=1	47009	623	339	59	106	0.07	0.15	2.142857	1	2	1	2	1	2	2	4	1.143468
sp|P51991|ROA3_HUMAN	HNRNPA3	Heterogeneous nuclear ribonucleoprotein A3 OS=Homo sapiens GN=HNRNPA3 PE=1	39799	197	133	202	246	0.49	1.05	2.142857	4	6	5	11	10	11	11	13	0.53832
tr|A0MNP2|A0MNP2_HUMA	WDR57	CDW11/WDR57 OS=Homo sapiens GN=WDR57 PE=2 SV=1	39742	1168	594	20	61	0.08	0.17	2.125	0	1	1	2	2	4	6	6	1.254822
tr|B2RBM7|B2RBM7_HUMA	tr|B2RBM7|B2RBN	cDNA, FLJ95595, highly similar to Homo sapiens proteasome (prosome, macropain)	43131	750	256	48	138	0.16	0.34	2.125	0	2	2	4	2	4	2	5	1.254822
tr|H0Y449|H0Y449_HUMAN	YBX1	Nuclease-sensitive element-binding protein 1 (Fragment) OS=Homo sapiens GN=YBX	42333	439	576	90	63	0.16	0.34	2.125	1	2	2	2	2	2	2	2	0.406307
sp|O76094|SRP72_HUMAN	SRP72	Signal recognition particle 72 kDa protein OS=Homo sapiens GN=SRP72 PE=1 SV=3	75130	661	228	54	151	0.09	0.19	2.111111	2	4	2	4	2	4	2	4	1.254822
tr|Q6FHM2|Q6FHM2_HUMA	GNB2	GNB2 protein OS=Homo sapiens GN=GNB2 PE=2 SV=1	38048	617	425	59	0	0.09	0.19	2.111111	1	0	1	0	1	1	1	1	0.406244
tr|B4DFL1|B4DFL1_HUMAN	tr|B4DFL1|B4DFL1	Dihydrolipoyl dehydrogenase OS=Homo sapiens PE=2 SV=1	49381	525	324	72	110	0.14	0.29	2.071429	2	3	2	4	2	4	3	4	1.254822
tr|F8VQ10|F8VQ10_HUMAN	DDX39B	Spliceosome RNA helicase DDX39B OS=Homo sapiens GN=DDX39B PE=4 SV=1	51226	311	142	128	232	0.55	1.11	2.018182	3	6	7	12	11	11	11	13	0.40688
tr|B4DQ15|B4DQ15_HUMAN	tr|B4DQ15|B4DQ1	cDNA FLJ51525, highly similar to Homo sapiens aldehyde dehydrogenase 16 family,	67899	1205	658	16	53	0.05	0.1	2	0	1	1	2	0	2	1	2	2.728626
sp|P12270|TPR_HUMAN	TPR	Nucleoprotein TPR OS=Homo sapiens GN=TPR PE=1 SV=3	267530	818	979	42	28	0.01	0.02	2	1	0	1	2	1	4	3	6	1.991983
sp|Q96P70|IPO9_HUMAN	IPO9	Importin-9 OS=Homo sapiens GN=IPO9 PE=1 SV=3	116858	773	445	45	81	0.06	0.12	2	1	1	2	4	1	4	3	4	1.991983
tr|D3DWL9|D3DWL9_HUMA	CPSF1	Cleavage and polyadenylation specific factor 1, 160kDa, isoform CRA_a OS=Homo sa	153087	990	395	31	94	0.02	0.04	2	0	3	1	3	1	3	4	3	1.629154
tr|G3V5E1|G3V5E1_HUMAN	CCNK	Cyclin-K OS=Homo sapiens GN=CCNK PE=2 SV=1	43776	877	697	38	50	0.08	0.16	2	1	1	1	2	1	2	1	2	1.143468
sp|O43837|IDH3B_HUMAN	IDH3B	Isocitrate dehydrogenase [NAD] subunit beta, mitochondrial OS=Homo sapiens GN=	42442	622	411	59	87	0.08	0.16	2	1	2	1	2	1	2	1	2	1.143468
tr|B3KSQ7|B3KSQ7_HUMAN	DBN1	Drebrin 1, isoform CRA_d OS=Homo sapiens GN=DBN1 PE=2 SV=1	66053	638	382	57	97	0.05	0.1	2	1	2	1	2	1	2	1	2	1.143468
sp|Q8TCS8|PNPT1_HUMAN	PNPT1	Polyribonucleotide nucleotidyltransferase 1, mitochondrial OS=Homo sapiens GN=P	86524	1056	356	27	102	0.04	0.08	2	0	2	1	2	1	2	3	2	1.143468
sp|P08243|ASNS_HUMAN	ASNS	Asparagine synthetase [glutamine-hydrolyzing] OS=Homo sapiens GN=ASNS PE=1 SV	64899	291	167	136	208	0.28	0.56	2	4	4	5	10	7	10	7	13	0.89283
sp|P46013|KI67_HUMAN	MKI67	Antigen KI-67 OS=Homo sapiens GN=MKI67 PE=1 SV=2	360698	872	918	38	31	0.01	0.02	2	1	0	1	2	2	2	3	4	0.406307
tr|B4DZE5|B4DZE5_HUMAN	tr|B4DZE5|B4DZE5	cDNA FLJ58806, highly similar to DNA-directed RNA polymerase, mitochondrial (EC 2	106234	714	707	51	49	0.03	0.06	2	1	0	1	2	2	2	3	3	0.406307
tr|B0AZM4|B0AZM4_HUMAN	tr|B0AZM4|B0AZN	cDNA, FLJ79464, highly similar to Homo sapiens aquarius (Fragment) OS=Homo sapi	157764	947	607	33	59	0.02	0.04	2	1	1	1	2	2	2	4	3	0.406307
tr|E7EQT4|E7EQT4_HUMAN	ACIN1	Apoptotic chromatin condensation inducer in the nucleus OS=Homo sapiens GN=AC	147674	896	940	36	30	0.02	0.04	2	1	0	1	2	1	1	3	3	0.406244
tr|A5D904|A5D904_HUMAN	RPS9	RPS9 protein (Fragment) OS=Homo sapiens GN=RPS9 PE=2 SV=1	13210	662	469	54	78	0.26	0.51	1.961538	1	3	1	3	2	3	2	3	0.891993
tr|B4DGN5|B4DGN5_HUMAN	GLUD1	Glutamate dehydrogenase OS=Homo sapiens GN=GLUD1 PE=2 SV=1	46945	664	201	54	175	0.23	0.45	1.956522	0	4	3	5	3	5	4	5	1.058965
tr|B4DJ30|B4DJ30_HUMAN	tr|B4DJ30|B4DJ30	cDNA FLJ61290, highly similar to Neutral alpha-glucosidase AB OS=Homo sapiens PE	113539	45	20	707	1107	0.82	1.55	1.890244	15	27	24	41	22	40	30	45	1.260263
tr|E9PKU7|E9PKU7_HUMAN	GANAB	Neutral alpha-glucosidase AB OS=Homo sapiens GN=GANAB PE=2 SV=1	96768	45	33	660	846	0.82	1.54	1.878049	14	20	22	33	20	29	27	34	0.934884
tr|Q5CAQ5|Q5CAQ5_HUMAN	TRA1	Tumor rejection antigen (Gp96) 1 OS=Homo sapiens GN=TRA1 PE=2 SV=1	92567	189	75	211	409	0.28	0.52	1.857143	6	8	7	12	7	13	9	16	1.256177
tr|Q59FA0|Q59FA0_HUMAN	tr|Q59FA0|Q59FA	DNA polymerase (Fragment) OS=Homo sapiens PE=2 SV=1	113271	510	371	75	93	0.06	0.11	1.833333	2	2	2	3	2	4	3	5	1.254822
sp|P12956|XRCC6_HUMAN	XRCC6	X-ray repair cross-complementing protein 6 OS=Homo sapiens GN=XRCC6 PE=1 SV=2	70084	127	71	303	423	0.66	1.21	1.833333	7	10	11	16	17	19	21	20	0.563901
tr|B4DEX8|B4DEX8_HUMAN	MAT2A	S-adenosylmethionine synthase OS=Homo sapiens GN=MAT2A PE=2 SV=1	40027	299	257	132	138	0.27	0.49	1.814815	3	3	4	5	4	5	6	7	0.6962
tr|J3KT10|J3KT10_HUMAN	NUP85	Nuclear pore complex protein Nup85 OS=Homo sapiens GN=NUP85 PE=4 SV=1	70931	880	680	37	52	0.05	0.09	1.8	1	2	1	2	1	2	1	2	1.143468
tr|B7Z611|B7Z611_HUMAN	tr|B7Z611|B7Z611	cDNA FLJ50848, highly similar to Homo sapiens suppressor of var1, 3-like 1 (S. cerevi	71856	659	663	55	53	0.05	0.09	1.8	1	1	1	2	1	2	1	2	1.143468
tr|I6L9B1|I6L9B1_HUMAN	EPB41L2	EPB41L2 protein OS=Homo sapiens GN=EPB41L2 PE=2 SV=1	71998	823	561	41	65	0.05	0.09	1.8	1	1	1	2	1	2	3	4	1.143468
tr|B4DPZ3|B4DPZ3_HUMAN	tr|B4DPZ3|B4DPZ	cDNA FLJ53290, highly similar to Cytoplasmic dynein 1 intermediate chain 2 OS=Hon	70869	552	391	68	95	0.05	0.09	1.8	1	2	1	2	1	2	1	2	1.143468
tr|B2R959|B2R959_HUMAN	tr|B2R959|B2R959	cDNA, FLJ94229, highly similar to Homo sapiens heterogeneous nuclear ribonucleop	60838	94	29	396	937	1.32	2.36	1.787879	14	26	16	32	19	36	24	40	1.316233
tr|G3V3M6|G3V3M6_HUMA	APEX1	DNA-(apurinic or apyrimidinic site) lyase (Fragment) OS=Homo sapiens GN=APEX1 P	29457	1043	588	28	61	0.17	0.3	1.764706	0	1	1	3	1	2	1	3	1.143468
sp|O00148|DX39A_HUMAN	DDX39A	ATP-dependent RNA helicase DDX39A OS=Homo sapiens GN=DDX39A PE=1 SV=2	49611	342	251	118	141	0.38	0.67	1.763158	3	5	5	8	8	9	9	10	0.567413
sp|A6NHL2|TBAL3_HUMAN	TUBAL3	Tubulin alpha chain-like 3 OS=Homo sapiens GN=TUBAL3 PE=1 SV=2	50675	432	468	92	78	0.21	0.37	1.761905	2	4	3	6	3	5	5	6	1.058965
tr|B4DJT7|B4DJT7_HUMAN	tr|B4DJT7|B4DJT7	cDNA FLJ56511, highly similar to Neurofilament triplet M protein OS=Homo sapiens	78895	616	246	60	114	0.08	0.14	1.75	2	2	2	3	2	4	3	5	1.254822
tr|H0YNT2|H0YNT2_HUMAN	TLE3	Transducin-like enhancer protein 3 OS=Homo sapiens GN=TLE3 PE=4 SV=1	77590	1005	963	30	29	0.04	0.07	1.75	0	0	1	1	1	1	1	1	0.406244
tr|B3KQF0|B3KQF0_HUMAN	tr|B3KQF0|B3KQF	cDNA FLJ90354 fis, clone NT2RP2003390, highly similar to Translocation protein SEC	88343	1048	518	27	70	0.04	0.07	1.75	0	1	1	1	1	1	1	2	0.406244
tr|J3KSD8|J3KSD8_HUMAN	BLMH	Bleomycin hydrolase (Fragment) OS=Homo sapiens GN=BLMH PE=4 SV=1	30628	695	539	52	40	0.23	0.4	1.73913	1	0	2	2	2	2	2	2	0.406307
sp|Q15046|SYK_HUMAN	KARS	Lysine--tRNA ligase OS=Homo sapiens GN=KARS PE=1 SV=3	68461	484	242	80	144	0.15	0.26	1.733333	2	3	3	5	4	6	4	6	0.937467
tr|B4DGP8|B4DGP8_HUMAN	CANX	Calnexin OS=Homo sapiens GN=CANX PE=2 SV=1	71971	251	94	161	327	0.25	0.43	1.72	3	6	5	10	5	10	5	11	1.340679
tr|Q6NTA2|Q6NTA2_HUMAN	HNRNPL	HNRNPL protein (Fragment) OS=Homo sapiens GN=HNRNPL PE=2 SV=2	62515	63	19	539	1153	1.52	2.6	1.710526	18	32	20	38	23	39	28	42	1.161001
tr|B2ZZ89|B2ZZ89_HUMAN	SPTBN1	Spectrin beta non-erythrocytic 1 OS=Homo sapiens GN=SPTBN1 PE=2 SV=1	275237	73	25	474	984	0.22	0.37	1.681818	13	21	17	27	17	28	19	31	1.113727
tr|B4DNL5|B4DNL5_HUMAN	tr|B4DNL5|B4DNL	cDNA FLJ59361, highly similar to Protein disulfide-isomerase (EC 5.3.4.1) OS=Homo s	55746	466	487	83	76	0.12	0.2	1.666667	1	2	2	3	3	3	4	3	0.406371
tr|B3KMV5|B3KMV5_HUMA	tr|B3KMV5|B3KM	cDNA FLJ12728 fis, clone NT2RP2000040, highly similar to Protein FAM62A OS=Hom	123342	912	1052	35	23	0.03	0.05	1.666667	0	0	1	2	1	1	2	4	0.406244
sp|P27635|RL10_HUMAN	RPL10	60S ribosomal protein L10 OS=Homo sapiens GN=RPL10 PE=1 SV=4	25044	485	666	80	53	0.28	0.46	1.642857	2	1	2	3	4	3	4	4	0.043605
tr|Q59GH5|Q59GH5_HUMAN	tr|Q59GH5|Q59GH	COP9 signalosome subunit 5 variant (Fragment) OS=Homo sapiens PE=2 SV=1	31467	631	1021	58	15	0.11	0.18	1.636364	1	0	1	1	1	0	1	1	-1.17898
tr|B3KUZ8|B3KUZ8_HUMAN	tr|B3KUZ8|B3KUZ	Aspartate aminotransferase OS=Homo sapiens PE=2 SV=1	41699	438	538	90	68	0.16	0.26	1.625	2	2	2	3	2	2	2	3	0.406307
tr|J3KRX5|J3KRX5_HUMAN	RPL17	60S ribosomal protein L17 (Fragment) OS=Homo sapiens GN=RPL17 PE=3 SV=1	20386	521	581	73	62	0.36	0.58	1.611111	2	1	2	3	2	3	2	3	0.891993
tr|A8K639|A8K639_HUMAN	GMPS	Guanine monphosphate synthetase, isoform CRA_b OS=Homo sapiens GN=GMPS PE	77408	204	194	193	179	0.23	0.37	1.608696	5	5	6	8	7	8	8	8	0.587457
sp|Q9NSD9|SYFB_HUMAN	FARSB	Phenylalanine--tRNA ligase beta subunit OS=Homo sapiens GN=FARSB PE=1 SV=3	66701	541	274	70	131	0.1	0.16	1.6	2	2	2	3	2	5	3	6	1.544588
sp|Q9NZB2|F120A_HUMAN	FAM120A	Constitutive coactivator of PPAR-gamma-like protein 1 OS=Homo sapiens GN=FAM1	123008	569	456	65	79	0.05	0.08	1.6	1	1	2	3	3	4	4	4	0.7692
tr|B7Z1R5|B7Z1R5_HUMAN	ATP6V1A	V-type proton ATPase catalytic subunit A OS=Homo sapiens GN=ATP6V1A PE=2 SV=1	65037	796	297	44	122	0.1	0.16	1.6	1	2	2	3	3	3	3	3	0.406371
tr|B4DFQ5|B4DFQ5_HUMAN	tr|B4DFQ5|B4DFQ	cDNA FLJ58134, highly similar to Cleavage stimulation factor 77 kDa subunit OS=Hor	72538	902	700	36	44	0.05	0.08	1.6	1	1	1	1	1	1	1	1	0.406244
tr|Q96CV8|Q96CV8_HUMAN	THOP1	Thimet oligopeptidase 1 OS=Homo sapiens GN=THOP1 PE=2 SV=1	79686	371	899	110	33	0.1	0.16	1.6	2	0	2	1	2	1	3	1	-0.33092
tr|D3DVQ1|D3DVQ1_HUMA	LETM1	Leucine zipper-EF-hand containing transmembrane protein 1, isoform CRA_a OS=Ho	63730	462	670	84	0	0.05	0.08	1.6	1	0	1	0	1	0	1	1	-1.17898
tr|Q6IAM0|Q6IAM0_HUMAN	EIF3S4	Eukaryotic translation initiation factor 3 subunit G OS=Homo sapiens GN=EIF3S4 PE=	35864	703	381	51	97	0.19	0.3	1.578947	1	3	2	3	2	3	2	3	0.891993
tr|B3KQT9|B3KQT9_HUMAN	tr|B3KQT9|B3KQT	cDNA PSEC0175 fis, clone OVARC1000169, highly similar to Protein disulfide-isomera	54468	515	227	74	152	0.26	0.41	1.576923	2	3	4	6	3	8	5	8	1.687774
tr|Q7Z3K9|Q7Z3K9_HUMAN	DKFZp781L0540	Putative uncharacterized protein DKFZp781L0540 (Fragment) OS=Homo sapiens GN=	90081	671	418	54	86	0.07	0.11	1.571429	2	1	2	3	2	2	2	3	0.406307
sp|Q13151|ROA0_HUMAN	HNRNPA0	Heterogeneous nuclear ribonucleoprotein A0 OS=Homo sapiens GN=HNRNPA0 PE=1	30993	346	408	117	87	0.23	0.36	1.565217	2	2	2	3	4	3	5	4	0.043605
sp|P51116|FXR2_HUMAN	FXR2	Fragile X mental retardation syndrome-related protein 2 OS=Homo sapiens GN=FXR2	74520	437	262	91	135	0.09	0.14	1.555556	2	3	2	3	2	3	4	3	0.891993
tr|B4DR68|B4DR68_HUMAN	tr|B4DR68|B4DR6	cDNA FLJ58608, highly similar to Heat shock protein 75 kDa, mitochondrial OS=Hom	74408	124	125	323	243	0.35	0.54	1.542857	6	8	8	11	8	11	9	14	0.843566
tr|Q2TA84|Q2TA84_HUMAN	FKBP5	FK506 binding protein 5 OS=Homo sapiens GN=FKBP5 PE=2 SV=1	51659	713	363	51	101	0.13	0.2	1.538462	1	3	2	3	2	3	2	3	0.891993
tr|Q5U0Q1|Q5U0Q1_HUMA	DKFZp686L1159	Putative uncharacterized protein DKFZp686L1159 OS=Homo sapiens GN=DKFZp686L	52189	418	341	97	106	0.13	0.2	1.538462	2	3	2	3	2	3	3	4	0.891993
tr|B3KY60|B3KY60_HUMAN	tr|B3KY60|B3KY60	cDNA FLJ16777 fis, clone BRHIP2029567, highly similar to Cell division cycle 5-like pr	92448	344	124	117	266	0.15	0.23	1.533333	2	5	4	6	4	7	6	8	1.144177
tr|B2R8R5|B2R8R5_HUMAN	tr|B2R8R5|B2R8R5	cDNA, FLJ94025, highly similar to Homo sapiens tripartite motif-containing 28 (TRIM	90271	137	109	284	298	0.43	0.65	1.511628	7	7	14	14	17	14	17	16	0.135182
tr|K7EIG7|K7EIG7_HUMAN	MYO1D	Unconventional myosin-Id OS=Homo sapiens GN=MYO1D PE=4 SV=1	106738	993	623	31	56	0.06	0.09	1.5	0	1	2	3	2	4	3	4	1.254822
sp|Q9NX58|LYAR_HUMAN	LYAR	Cell growth-regulating nucleolar protein OS=Homo sapiens GN=LYAR PE=1 SV=2	44044	523	551	72	66	0.16	0.24	1.5	2	1	2	3	2	3	2	4	0.891993
tr|I3L0N3|I3L0N3_HUMAN	NSF	Vesicle-fusing ATPase OS=Homo sapiens GN=NSF PE=3 SV=1	82553	604	883	61	34	0.08	0.12	1.5	2	0	2	3	3	4	3	5	0.7692
tr|F5H8E5|F5H8E5_HUMAN	USP7	Ubiquitin carboxyl-terminal hydrolase OS=Homo sapiens GN=USP7 PE=2 SV=1	117951	677	302	53	117	0.06	0.09	1.5	1	2	2	3	3	3	6	4	0.406371
tr|H7C5W9|H7C5W9_HUMA	ATP2A2	Sarcoplasmic/endoplasmic reticulum calcium ATPase 2 (Fragment) OS=Homo sapien	104121	463	364	83	66	0.06	0.09	1.5	2	2	2	2	2	2	4	3	0.406307
tr|A8K1Z2|A8K1Z2_HUMAN	tr|A8K1Z2|A8K1Z2	cDNA FLJ78475, highly similar to Homo sapiens glycerol-3-phosphate dehydrogenase	81197	578	651	64	54	0.04	0.06	1.5	1	1	1	1	1	1	3	1	0.406244
tr|B3KRR1|B3KRR1_HUMAN	tr|B3KRR1|B3KRR1	cDNA FLJ34725 fis, clone MESAN2005958, highly similar to RNA-binding protein Luc7	40752	595	231	62	97	0.08	0.12	1.5	1	1	1	1	1	1	1	3	0.406244
tr|B4DNN4|B4DNN4_HUMA	tr|B4DNN4|B4DNI	Ribonucleoside-diphosphate reductase OS=Homo sapiens PE=2 SV=1	87252	294	337	134	69	0.12	0.18	1.5	4	1	4	3	5	4	5	4	0.116732
tr|B4DX55|B4DX55_HUMAN	tr|B4DX55|B4DX5	cDNA FLJ58171, moderately similar to Melanoma-associated antigen D2 OS=Homo s	55637	606	134	61	97	0.13	0.19	1.461538	1	1	1	1	1	1	1	1	0.406244
tr|E7ENU4|E7ENU4_HUMAN	ADAR	Double-stranded RNA-specific adenosine deaminase OS=Homo sapiens GN=ADAR PE	142051	319	323	127	111	0.07	0.1	1.428571	2	2	3	4	4	4	4	4	0.406434
sp|O75533|SF3B1_HUMAN	SF3B1	Splicing factor 3B subunit 1 OS=Homo sapiens GN=SF3B1 PE=1 SV=3	146479	145	63	275	466	0.39	0.55	1.410256	8	12	15	20	13	19	19	20	0.939079
tr|B4DLA3|B4DLA3_HUMAN	tr|B4DLA3|B4DLA3	cDNA FLJ58509, highly similar to Cullin-1 OS=Homo sapiens PE=2 SV=1	82091	263	215	152	158	0.22	0.31	1.409091	4	3	5	5	5	5	6	5	0.406498
tr|H0Y7C0|H0Y7C0_HUMAN	USO1	General vesicular transport factor p115 (Fragment) OS=Homo sapiens GN=USO1 PE=	72345	681	331	53	98	0.05	0.07	1.4	1	1	1	1	1	1	1	1	0.406244
tr|J3QT54|J3QT54_HUMAN	CPSF7	Cleavage and polyadenylation-specificity factor subunit 7 (Fragment) OS=Homo sapi	22762	978	587	32	40	0.15	0.21	1.4	1	1	1	2	1	2	1	3	1.143468
tr|Q0VGD6|Q0VGD6_HUMA	HNRPR	HNRPR protein (Fragment) OS=Homo sapiens GN=HNRPR PE=2 SV=1	68218	146	85	274	357	0.6	0.84	1.4	10	10	12	15	11	17	17	18	1.014157
sp|P54886|P5CS_HUMAN	ALDH18A1	Delta-1-pyrroline-5-carboxylate synthase OS=Homo sapiens GN=ALDH18A1 PE=1 SV	87989	195	123	204	269	0.44	0.61	1.386364	7	8	11	13	13	12	14	15	0.295717
sp|P49411|EFTU_HUMAN	TUFM	Elongation factor Tu, mitochondrial OS=Homo sapiens GN=TUFM PE=1 SV=2	49852	179	173	220	204	0.57	0.78	1.368421	5	4	7	9	8	10	9	12	0.712062
sp|P11021|GRP78_HUMAN	HSPA5	78 kDa glucose-regulated protein OS=Homo sapiens GN=HSPA5 PE=1 SV=2	72402	79	53	443	543	0.63	0.86	1.365079	12	13	13	15	13	14	13	15	0.51036
tr|H0YJC0|H0YJC0_HUMAN	PSMC6	26S protease regulatory subunit 10B (Fragment) OS=Homo sapiens GN=PSMC6 PE=4	29919	1012	585	30	62	0.11	0.15	1.363636	0	2	1	2	1	2	1	3	1.143468
tr|Q5HYM2|Q5HYM2_HUMA	DKFZp686O2462	Putative uncharacterized protein DKFZp686O2462 (Fragment) OS=Homo sapiens GN	72627	697	271	52	131	0.14	0.19	1.357143	1	3	3	4	3	4	3	5	0.7692
tr|Q2L6I2|Q2L6I2_HUMAN	ABCF1	ABC50 protein OS=Homo sapiens GN=ABCF1 PE=3 SV=1	92080	187	70	214	373	0.23	0.31	1.347826	4	8	6	9	6	9	7	10	0.954827
tr|H0Y4T7|H0Y4T7_HUMAN	MTA1	Metastasis-associated protein MTA1 (Fragment) OS=Homo sapiens GN=MTA1 PE=4	57747	824	195	41	97	0.06	0.08	1.333333	1	1	1	2	2	2	2	2	0.406307
sp|Q8WWM7|ATX2L_HUMA	ATXN2L	Ataxin-2-like protein OS=Homo sapiens GN=ATXN2L PE=1 SV=2	113589	533	573	71	63	0.09	0.12	1.333333	1	1	3	4	4	6	7	7	0.937467
sp|Q7L2E3|DHX30_HUMAN	DHX30	Putative ATP-dependent RNA helicase DHX30 OS=Homo sapiens GN=DHX30 PE=1 SV	134938	321	369	125	100	0.1	0.13	1.3	3	2	4	5	5	5	6	6	0.406498
sp|P00338|LDHA_HUMAN	LDHA	L-lactate dehydrogenase A chain OS=Homo sapiens GN=LDHA PE=1 SV=2	36950	180	69	219	430	1.8	2.33	1.294444	8	13	13	17	17	18	20	21	0.487693
tr|B4DQR2|B4DQR2_HUMAN	tr|B4DQR2|B4DQR	cDNA FLJ57562, highly similar to Cleavage and polyadenylation specificityfactor 73 k	46241	1143	565	22	64	0.07	0.09	1.285714	0	1	1	2	1	2	1	2	1.143468
tr|B1AH89|B1AH89_HUMAN	TTLL12	Tubulin tyrosine ligase-like family, member 12 OS=Homo sapiens GN=TTLL12 PE=4 S	74185	235	120	168	277	0.24	0.3	1.25	5	6	5	6	5	8	7	9	1.035306
tr|B0AZQ4|B0AZQ4_HUMAN	tr|B0AZQ4|B0AZQ	cDNA, FLJ79494, highly similar to Structural maintenance of chromosome 3 OS=Hom	141841	267	95	150	326	0.12	0.15	1.25	3	5	5	6	7	7	7	7	0.406625
tr|B4DKU3|B4DKU3_HUMAN	tr|B4DKU3|B4DKU	cDNA FLJ53449, highly similar to rRNA methyltransferase 3 (EC 2.1.1.-) OS=Homo sa	82984	430	353	93	100	0.08	0.1	1.25	1	1	2	2	2	2	2	2	0.406307
tr|B4DK79|B4DK79_HUMAN	tr|B4DK79|B4DK79	cDNA FLJ58742, highly similar to Leucine-rich repeat-containing protein 41 OS=Hom	74302	452	912	85	32	0.04	0.05	1.25	1	0	1	1	1	1	1	1	0.406244
sp|P41250|SYG_HUMAN	GARS	Glycine--tRNA ligase OS=Homo sapiens GN=GARS PE=1 SV=3	83854	415	233	98	149	0.17	0.21	1.235294	3	3	4	5	4	6	6	6	0.937467
tr|B4DF96|B4DF96_HUMAN	EIF2A	Eukaryotic translation initiation factor 2A OS=Homo sapiens GN=EIF2A PE=2 SV=1	58357	293	161	135	146	0.47	0.58	1.234043	2	3	7	7	7	11	8	13	1.024333
tr|B3KS36|B3KS36_HUMAN	tr|B3KS36|B3KS36	cDNA FLJ35376 fis, clone SKMUS2004044, highly similar to Homo sapiens ribosomal	40234	113	56	345	524	1.8	2.22	1.233333	12	13	13	18	14	19	14	20	0.83579

tr|Q53SS8|Q53SS8_HUMAN	PCBP1	Poly(RC) binding protein 1 OS=Homo sapiens GN=PCBP1 PE=2 SV=1	37987	175	118	222	280	0.65	0.8	1.230769	4	5	6	8	6	8	7	10	0.794103
tr|B4DGH9|B4DGH9_HUMAN	tr|B4DGH9|B4DGH	cDNA FLJ58772, highly similar to Fragile X mental retardation syndrome-related prot	51203	361	428	114	80	0.13	0.16	1.230769	2	1	2	2	2	2	3	2	0.406307
tr|Q6IBN1|Q6IBN1_HUMAN	HNRPK	HNRPK protein OS=Homo sapiens GN=HNRPK PE=2 SV=1	51281	77	51	459	547	1.85	2.27	1.227027	15	17	21	20	21	22	23	25	0.473366
tr|B4DUQ1|B4DUQ1_HUMAN	tr|B4DUQ1|B4DU(	cDNA FLJ54552, highly similar to Heterogeneous nuclear ribonucleoprotein K OS=Ho	48708	77	54	459	540	1.85	2.25	1.216216	15	16	21	19	21	21	23	25	0.407518
tr|B3KM80|B3KM80_HUMAN	NCL	Nucleolin, isoform CRA_c OS=Homo sapiens GN=NCL PE=2 SV=1	58576	222	260	179	136	0.39	0.47	1.205128	3	3	6	8	7	6	8	9	0.199915
tr|F5H335|F5H335_HUMAN	EIF3A	Eukaryotic translation initiation factor 3 subunit A OS=Homo sapiens GN=EIF3A PE=2	162936	74	86	473	333	0.49	0.59	1.204082	14	6	21	13	25	16	27	18	-0.22259
tr|A4D210|A4D210_HUMAN	EIF3S9	Eukaryotic translation initiation factor 3, subunit 9 eta, 116kDa OS=Homo sapiens GI	89024	200	192	200	180	0.2	0.24	1.2	5	3	5	6	5	6	5	6	0.647765
sp|P53621|COPA_HUMAN	COPA	Coatomer subunit alpha OS=Homo sapiens GN=COPA PE=1 SV=2	139797	396	310	103	115	0.15	0.18	1.2	4	2	6	7	6	7	7	9	0.613272
sp|Q15393|SF3B3_HUMAN	SF3B3	Splicing factor 3B subunit 3 OS=Homo sapiens GN=SF3B3 PE=1 SV=4	136575	120	209	331	167	0.21	0.25	1.190476	10	3	11	8	12	7	14	10	-0.33132
tr|B4DQJ8|B4DQJ8_HUMAN	PGD	6-phosphogluconate dehydrogenase, decarboxylating OS=Homo sapiens GN=PGD PE	52352	193	187	209	185	0.53	0.63	1.188679	4	4	7	8	6	9	8	9	0.954827
tr|Q9BU08|Q9BU08_HUMAN	tr|Q9BU08|Q9BU(	Putative uncharacterized protein (Fragment) OS=Homo sapiens PE=2 SV=1	59887	134	101	287	314	1.22	1.45	1.188525	8	11	15	18	18	20	22	24	0.555944
sp|Q15019|SEPT2_HUMAN	Sep-02	Septin-2 OS=Homo sapiens GN=SEPT2 PE=1 SV=1	41689	553	546	68	67	0.16	0.19	1.1875	1	1	2	2	2	2	2	3	0.406307
tr|B4DZJ7|B4DZJ7_HUMAN	tr|B4DZJ7|B4DZJ7	cDNA FLJ55629, highly similar to Transcription elongation factor SPT5 OS=Homo sap	118511	568	340	65	106	0.06	0.07	1.166667	1	2	2	2	2	2	3	2	0.406307
tr|A6NN80|A6NN80_HUMAN	ANXA6	Annexin OS=Homo sapiens GN=ANXA6 PE=3 SV=3	75571	446	342	88	105	0.24	0.28	1.166667	2	3	5	4	5	4	7	6	0.116732
tr|B4DGB4|B4DGB4_HUMAN	PLS3	Plastin-3 OS=Homo sapiens GN=PLS3 PE=2 SV=1	69820	241	267	166	133	0.38	0.44	1.157895	5	4	7	8	9	7	11	10	0.065197
tr|B2R6D0|B2R6D0_HUMAN	tr|B2R6D0|B2R6D(	cDNA, FLJ92896, highly similar to Homo sapiens proteasome (prosome, macropain)]	106797	302	144	131	231	0.27	0.31	1.148148	2	6	8	9	10	9	10	10	0.262167
tr|H0Y9U2|H0Y9U2_HUMAN	SFPQ	Splicing factor, proline- and glutamine-rich (Fragment) OS=Homo sapiens GN=SFPQ I	9491	122	186	140	185	0.75	0.86	1.146667	3	4	4	4	3	4	4	5	0.7692
tr|B2RDD7|B2RDD7_HUMAN	tr|B2RDD7|B2RDD	cDNA, FLJ96564, highly similar to Homo sapiens SKB1 homolog (S. pombe) (SKB1), m	73348	275	325	145	110	0.3	0.34	1.133333	4	1	6	3	6	3	7	3	-0.4873
tr|E9PK47|E9PK47_HUMAN	PYGL	Phosphorylase OS=Homo sapiens GN=PYGL PE=2 SV=1	94410	388	163	105	195	0.23	0.26	1.130435	3	5	6	6	5	6	7	7	0.647765
sp|P08670|VIME_HUMAN	VIM	Vimentin OS=Homo sapiens GN=VIM PE=1 SV=4	53676	128	96	293	326	1.04	1.17	1.125	9	9	12	14	14	14	15	17	0.407071
tr|B4DP06|B4DP06_HUMAN	tr|B4DP06|B4DP0(	cDNA FLJ57133, highly similar to Bifunctional purine biosynthesis protein PURH OS=I	59138	116	64	343	464	0.81	0.91	1.123457	8	8	11	13	11	12	11	15	0.527433
sp|O43776|SYNC_HUMAN	NARS	Asparagine--tRNA ligase, cytoplasmic OS=Homo sapiens GN=NARS PE=1 SV=1	63758	110	87	349	346	0.74	0.83	1.121622	8	8	11	15	11	14	11	18	0.742077
sp|Q9UHX1|PUF60_HUMAN	PUF60	Poly(U)-binding-splicing factor PUF60 OS=Homo sapiens GN=PUF60 PE=1 SV=1	60009	557	717	67	48	0.17	0.19	1.117647	2	1	3	2	3	2	3	2	-0.07931
tr|B4DQX0|B4DQX0_HUMAN	tr|B4DQX0|B4DQX	cDNA FLJ59219, highly similar to Poly(A)-binding protein 1 OS=Homo sapiens PE=2 S	67239	115	108	344	301	0.69	0.77	1.115942	8	9	11	13	12	13	14	15	0.518234
tr|Q6P151|Q6P151_HUMAN	SNW1	SNW1 protein (Fragment) OS=Homo sapiens GN=SNW1 PE=2 SV=1	36026	975	903	32	32	0.09	0.1	1.111111	0	0	1	1	1	1	2	1	0.406244
tr|B4DHX4|B4DHX4_HUMAN	tr|B4DHX4|B4DHX	cDNA FLJ52902, highly similar to Rab GDP dissociation inhibitor alpha OS=Homo sap	47505	254	138	159	210	0.5	0.55	1.1	5	5	6	6	6	7	7	7	0.613272
sp|P55072|TERA_HUMAN	VCP	Transitional endoplasmic reticulum ATPase OS=Homo sapiens GN=VCP PE=1 SV=4	89950	93	50	396	550	0.54	0.59	1.092593	9	10	12	13	11	13	14	16	0.638724
tr|Q5LJA0|Q5LJA0_HUMAN	TROVE2	60 kDa SS-A/Ro ribonucleoprotein OS=Homo sapiens GN=TROVE2 PE=4 SV=1	59916	360	804	114	40	0.11	0.12	1.090909	2	1	2	1	2	2	2	2	0.406307
tr|B4DUS9|B4DUS9_HUMAN	BPNT1	3~(2~),5~-bisphosphate nucleotidase 1 OS=Homo sapiens GN=BPNT1 PE=2 SV=1	29455	1042	315	28	114	0.11	0.12	1.090909	0	1	1	1	1	1	1	1	0.406244
sp|P46060|RAGP1_HUMAN	RANGAP1	Ran GTPase-activating protein 1 OS=Homo sapiens GN=RANGAP1 PE=1 SV=1	63958	331	659	123	53	0.11	0.12	1.090909	2	1	2	2	5	2	5	4	-0.73178
tr|B4DE59|B4DE59_HUMAN	tr|B4DE59|B4DE5	cDNA FLJ60424, highly similar to Junction plakoglobin OS=Homo sapiens PE=2 SV=1	62862	674	474	54	0	0.11	0.12	1.090909	2	0	2	0	3	1	4	1	-0.81654
tr|Q6NZ55|Q6NZ55_HUMAN	RPL13	60S ribosomal protein L13 OS=Homo sapiens GN=RPL13 PE=2 SV=1	24308	402	519	101	70	0.47	0.51	1.085106	2	1	3	2	4	2	6	2	-0.44208
tr|Q5TCU6|Q5TCU6_HUMAN	TLN1	Talin-1 OS=Homo sapiens GN=TLN1 PE=4 SV=1	260030	186	89	214	341	0.12	0.13	1.083333	6	6	9	10	9	9	11	10	0.406753
sp|Q6P2Q9|PRP8_HUMAN	PRPF8	Pre-mRNA-processing-splicing factor 8 OS=Homo sapiens GN=PRPF8 PE=1 SV=2	274738	46	37	699	724	0.39	0.42	1.076923	23	21	29	31	33	33	40	40	0.408287
tr|B4DJ60|B4DJ60_HUMAN	tr|B4DJ60|B4DJ60	cDNA FLJ55072, highly similar to Succinate dehydrogenase (ubiquinone) flavoprotei	68233	219	105	180	269	0.46	0.48	1.043478	4	6	8	8	8	8	8	8	0.406689
sp|P40925|MDHC_HUMAN	MDH1	Malate dehydrogenase, cytoplasmic OS=Homo sapiens GN=MDH1 PE=1 SV=4	36631	352	280	116	129	0.3	0.31	1.033333	2	2	3	2	3	2	3	3	-0.07931
tr|Q6UWE9|Q6UWE9_HUMA	UNQ2550	SFVP2550 OS=Homo sapiens GN=UNQ2550 PE=4 SV=1	11890	1172	1091	20	21	0.29	0.29	1	1	1	1	1	0	1	1	1	1.991402
tr|H0Y5B5|H0Y5B5_HUMAN	PBRM1	Protein polybromo-1 (Fragment) OS=Homo sapiens GN=PBRM1 PE=4 SV=1	127011	1009	920	30	31	0.03	0.03	1	0	0	1	1	1	3	1	3	1.629154
sp|Q8N1G2|MTR1_HUMAN	FTSJD2	Cap-specific mRNA (nucleoside-2~-O-)-methyltransferase 1 OS=Homo sapiens GN=F1	96172	787	892	44	33	0.03	0.03	1	1	0	1	1	1	3	1	3	1.629154
tr|B2RAU8|B2RAU8_HUMAN	tr|B2RAU8|B2RAU	cDNA, FLJ95131, highly similar to Homo sapiens nucleolar and coiled-body phospho	73561	1110	272	24	131	0.04	0.04	1	0	3	1	3	1	3	1	3	1.629154
sp|Q13733|AT1A4_HUMAN	ATP1A4	Sodium/potassium-transporting ATPase subunit alpha-4 OS=Homo sapiens GN=ATP1	115119	884	778	37	43	0.06	0.06	1	0	0	2	4	2	5	2	8	1.544588
tr|B4DR45|B4DR45_HUMAN	tr|B4DR45|B4DR4	cDNA FLJ61339, highly similar to Alanyl-tRNA synthetase (EC 6.1.1.7) OS=Homo sapie	110017	798	327	44	110	0.12	0.12	1	0	2	4	4	2	4	5	4	1.254822
tr|B4DJE3|B4DJE3_HUMAN	tr|B4DJE3|B4DJE3	cDNA FLJ52929, highly similar to Dolichyl-diphosphooligosaccharide--proteinglycosy	46620	996	1026	31	25	0.07	0.07	1	1	0	1	1	1	2	1	2	1.143468
tr|B2RBM8|B2RBM8_HUMAI	tr|B2RBM8|B2RBN	cDNA, FLJ95596, highly similar to Homo sapiens activity-dependent neuroprotector (	124850	956	968	33	28	0.03	0.03	1	0	0	1	1	1	2	2	2	1.143468
sp|Q52LJ0|FA98B_HUMAN	FAM98B	Protein FAM98B OS=Homo sapiens GN=FAM98B PE=1 SV=1	37566	505	867	76	35	0.09	0.09	1	1	0	1	1	1	2	1	2	1.143468
tr|B4DJV2|B4DJV2_HUMAN	CS	Citrate synthase OS=Homo sapiens GN=CS PE=2 SV=1	50628	868	841	38	37	0.07	0.07	1	1	1	1	1	1	2	3	2	1.143468
sp|Q2NL82|TSR1_HUMAN	TSR1	Pre-rRNA-processing protein TSR1 homolog OS=Homo sapiens GN=TSR1 PE=1 SV=1	92151	1170	832	20	37	0.04	0.04	1	0	1	1	1	1	2	1	2	1.143468
tr|B2R713|B2R713_HUMAN	tr|B2R713|B2R713	cDNA, FLJ93224 OS=Homo sapiens PE=2 SV=1	82921	878	824	38	38	0.04	0.04	1	1	1	1	1	1	2	1	2	1.143468
tr|B5BUD2|B5BUD2_HUMAN	RFC2	Replication factor C 2 isoform 1 (Fragment) OS=Homo sapiens GN=RFC2 PE=2 SV=1	39616	545	814	68	39	0.08	0.08	1	1	1	1	1	1	2	1	2	1.143468
sp|Q7Z5L9|I2BP2_HUMAN	IRF2BP2	Interferon regulatory factor 2-binding protein 2 OS=Homo sapiens GN=IRF2BP2 PE=1	61728	613	733	60	46	0.05	0.05	1	1	1	1	1	1	2	2	2	1.143468
sp|Q9UBB4|ATX10_HUMAN	ATXN10	Ataxin-10 OS=Homo sapiens GN=ATXN10 PE=1 SV=1	54196	769	477	46	77	0.06	0.06	1	1	1	1	1	1	2	1	2	1.143468
tr|H0YHC3|H0YHC3_HUMAN	NAP1L1	Nucleosome assembly protein 1-like 1 (Fragment) OS=Homo sapiens GN=NAP1L1 PE	23517	289	283	136	128	0.49	0.49	1	3	2	4	4	4	6	4	6	0.937467
tr|A8K6U7|A8K6U7_HUMAN	tr|A8K6U7|A8K6U	cDNA FLJ78252, highly similar to Homo sapiens heterogeneous nuclear ribonucleopr	96189	258	210	156	165	0.18	0.18	1	3	3	5	5	5	7	5	8	0.854475
tr|B2R8Z8|B2R8Z8_HUMAN	tr|B2R8Z8|B2R8Z8	cDNA, FLJ94136, highly similar to Homo sapiens synaptotagmin binding, cytoplasmic	69787	185	157	215	215	0.51	0.51	1	6	6	9	9	9	12	12	12	0.803459
sp|P24928|RPB1_HUMAN	POLR2A	DNA-directed RNA polymerase II subunit RPB1 OS=Homo sapiens GN=POLR2A PE=1	218408	348	874	116	35	0.05	0.05	1	2	0	3	3	3	4	3	5	0.7692
sp|Q9NYU2|UGGG1_HUMAN	UGGT1	UDP-glucose:glycoprotein glucosyltransferase 1 OS=Homo sapiens GN=UGGT1 PE=1	177819	669	667	54	53	0.06	0.06	1	1	2	3	3	3	4	6	6	0.7692
tr|B3KPL5|B3KPL5_HUMAN	tr|B3KPL5|B3KPL5	cDNA FLJ31917 fis, clone NT2RP7004925, highly similar to Sprouty-related, EVH1 do	48711	1126	1070	23	22	0.07	0.07	1	0	0	1	2	8	10	15	26	0.712062
sp|P01023|A2MG_HUMAN	A2M	Alpha-2-macroglobulin OS=Homo sapiens GN=A2M PE=1 SV=3	164613	474	622	81	56	0.06	0.06	1	3	1	4	3	4	5	5	5	0.6962
tr|B4DY09|B4DY09_HUMAN	tr|B4DY09|B4DY0	cDNA FLJ51660, highly similar to Interleukin enhancer-binding factor 2 OS=Homo sa	39057	273	350	146	103	0.5	0.5	1	2	3	5	5	5	6	5	6	0.647765
tr|F5H5D3|F5H5D3_HUMAN	TUBA1C	Tubulin alpha-1C chain OS=Homo sapiens GN=TUBA1C PE=2 SV=1	58606	19	15	1165	1315	2.71	2.71	1	31	37	45	50	48	56	54	64	0.631607
tr|Q9BSV4|Q9BSV4_HUMAN	SFPQ	SFPQ protein (Fragment) OS=Homo sapiens GN=SFPQ PE=2 SV=2	68702	122	74	330	418	0.75	0.75	1	10	10	14	14	14	16	17	18	0.594003
tr|G3V1U9|G3V1U9_HUMAN	TUBA1A	Tubulin alpha-1A chain OS=Homo sapiens GN=TUBA1A PE=2 SV=1	46781	24	16	1049	1310	4.14	4.14	1	30	36	44	48	47	53	54	59	0.582365
tr|A8K2X8|A8K2X8_HUMAN	tr|A8K2X8|A8K2X8	cDNA FLJ78433, highly similar to Homo sapiens chaperonin containing TCP1, subunit	60148	134	104	287	308	1.22	1.22	1	8	11	15	17	18	19	22	23	0.483535
sp|Q9P258|RCC2_HUMAN	RCC2	Protein RCC2 OS=Homo sapiens GN=RCC2 PE=1 SV=2	56790	383	183	107	189	0.4	0.4	1	4	5	6	6	8	8	11	12	0.406689
tr|Q562V5|Q562V5_HUMAN	ACT	Actin-like protein (Fragment) OS=Homo sapiens GN=ACT PE=2 SV=1	11481	469	409	82	87	0.3	0.3	1	3	3	3	3	6	6	9	7	0.406562
sp|Q9Y3I0|RTCB_HUMAN	C22orf28	tRNA-splicing ligase RtcB homolog OS=Homo sapiens GN=C22orf28 PE=1 SV=1	55688	305	193	130	179	0.41	0.41	1	5	5	6	6	6	6	7	6	0.406562
sp|P13489|RINI_HUMAN	RNH1	Ribonuclease inhibitor OS=Homo sapiens GN=RNH1 PE=1 SV=2	51766	312	281	128	129	0.28	0.28	1	2	4	4	4	4	4	5	4	0.406434
tr|D3DPU2|D3DPU2_HUMAN	CAP1	Adenylyl cyclase-associated protein OS=Homo sapiens GN=CAP1 PE=3 SV=1	51926	288	252	137	138	0.28	0.28	1	3	4	4	4	4	4	4	4	0.406434
tr|Q0VGA5|Q0VGA5_HUMAN	SARS	SARS protein OS=Homo sapiens GN=SARS PE=2 SV=1	58883	264	239	152	145	0.24	0.24	1	4	3	4	4	4	4	4	5	0.406434
sp|Q8NI27|THOC2_HUMAN	THOC2	THO complex subunit 2 OS=Homo sapiens GN=THOC2 PE=1 SV=2	184541	250	199	162	176	0.07	0.07	1	3	4	4	4	4	4	5	4	0.406434
tr|A0PJ92|A0PJ92_HUMAN	NOP56	NOP56 protein (Fragment) OS=Homo sapiens GN=NOP56 PE=2 SV=1	50068	397	550	102	67	0.21	0.21	1	2	1	3	3	3	3	6	3	0.406371
tr|B7ZB91|B7ZB91_HUMAN	TNRC6B	Trinucleotide repeat containing 6B (Fragment) OS=Homo sapiens GN=TNRC6B PE=4	81825	480	520	80	70	0.13	0.13	1	3	2	3	3	3	3	3	5	0.406371
tr|Q58FF4|Q58FF4_HUMAN	HSP90Bf	Heat shock protein 90Bf OS=Homo sapiens GN=HSP90Bf PE=1 SV=1	41833	841	476	40	77	0.16	0.16	1	1	2	2	3	3	3	3	3	0.406371
tr|B3KXI2|B3KXI2_HUMAN	SEC23A	Protein transport protein Sec23A OS=Homo sapiens GN=SEC23A PE=2 SV=1	63574	555	461	67	79	0.22	0.22	1	1	2	4	4	3	3	5	4	0.406371
tr|B7Z7P8|B7Z7P8_HUMAN	ETF1	Eukaryotic peptide chain release factor subunit 1 OS=Homo sapiens GN=ETF1 PE=2 S	47731	406	368	101	100	0.22	0.22	1	2	2	3	3	3	3	5	4	0.406371
tr|B4DM97|B4DM97_HUMAI	tr|B4DM97|B4DM	cDNA FLJ55002, highly similar to Alpha-centractin OS=Homo sapiens PE=2 SV=1	38365	358	220	114	157	0.28	0.28	1	2	3	3	3	3	3	3	4	0.406371
sp|Q7L0Y3|MRRP1_HUMAN	TRMT10C	Mitochondrial ribonuclease P protein 1 OS=Homo sapiens GN=TRMT10C PE=1 SV=2	47602	991	904	31	32	0.07	0.07	1	0	0	1	1	2	2	2	2	0.406307
tr|Q4ZG72|Q4ZG72_HUMAN	DDX18	Putative uncharacterized protein DDX18 (Fragment) OS=Homo sapiens GN=DDX18 P	61898	799	886	43	34	0.11	0.11	1	1	1	2	2	2	2	3	4	0.406307
sp|P54577|SYYC_HUMAN	YARS	Tyrosine--tRNA ligase, cytoplasmic OS=Homo sapiens GN=YARS PE=1 SV=4	59448	584	669	64	53	0.11	0.11	1	1	2	2	2	2	2	3	4	0.406307
tr|B4DI28|B4DI28_HUMAN	PTBP2	Polypyrimidine tract-binding protein 2 OS=Homo sapiens GN=PTBP2 PE=2 SV=1	34947	758	655	47	54	0.2	0.2	1	1	1	2	2	2	2	2	2	0.406307
tr|Q6FI97|Q6FI97_HUMAN	BAF53A	BAF53A protein OS=Homo sapiens GN=BAF53A PE=2 SV=1	47864	718	556	51	66	0.14	0.14	1	1	1	2	3	2	2	2	3	0.406307
tr|B4DPJ2|B4DPJ2_HUMAN	tr|B4DPJ2|B4DPJ2	Annexin OS=Homo sapiens PE=2 SV=1	46025	486	498	79	73	0.15	0.15	1	1	2	2	2	2	2	2	2	0.406307
tr|B2R5U7|B2R5U7_HUMAN	tr|B2R5U7|B2R5U	cDNA, FLJ92633, highly similar to Homo sapiens CCAAT-box-binding transcription fac	114755	551	424	68	84	0.06	0.06	1	2	2	2	2	2	2	3	2	0.406307
sp|O14929|HAT1_HUMAN	HAT1	Histone acetyltransferase type B catalytic subunit OS=Homo sapiens GN=HAT1 PE=1	49880	278	401	142	91	0.14	0.14	1	2	2	2	2	2	2	2	3	0.406307
tr|B4DSP0|B4DSP0_HUMAN	hCG_1642748	HCG1642748, isoform CRA_b OS=Homo sapiens GN=hCG_1642748 PE=2 SV=1	45559	390	385	104	96	0.15	0.15	1	2	2	2	2	2	2	2	2	0.406307
tr|B4DKR1|B4DKR1_HUMAN	tr|B4DKR1|B4DKR	cDNA FLJ51884, highly similar to Mitochondrial inner membrane protein OS=Homo s	82864	704	355	51	102	0.08	0.08	1	1	2	2	2	2	2	3	5	0.406307
tr|B3KML1|B3KML1_HUMAN	tr|B3KML1|B3KMl	cDNA FLJ11308 fis, clone PLACE1010074, highly similar to Sorting nexin-2 OS=Homo	58550	892	338	36	106	0.12	0.12	1	0	2	2	2	2	2	2	2	0.406307
tr|F8WJN3|F8WJN3_HUMAN	CPSF6	Cleavage and polyadenylation-specificity factor subunit 6 OS=Homo sapiens GN=CPS	52409	403	330	101	109	0.13	0.13	1	2	2	2	2	2	2	2	4	0.406307
tr|Q5JXH7|Q5JXH7_HUMAN	FHL1	Four and a half LIM domains 1 (Fragment) OS=Homo sapiens GN=FHL1 PE=4 SV=1	24735	1100	1102	24	21	0.14	0.14	1	1	0	1	1	1	1	1	2	0.406244
tr|F2Z303|F2Z303_HUMAN	UMPS	Orotate phosphoribosyltransferase OS=Homo sapiens GN=UMPS PE=4 SV=1	7082	751	1087	48	21	0.5	0.5	1	1	0	1	1	1	1	1	1	0.406244
tr|B4DTX6|B4DTX6_HUMAN	tr|B4DTX6|B4DTX(	cDNA FLJ54311, highly similar to Ran-binding protein 6 OS=Homo sapiens PE=2 SV=1	79646	1187	1072	18	22	0.04	0.04	1	0	0	1	1	1	1	3	2	0.406244
tr|Q5JY65|Q5JY65_HUMAN	CRNKL1	Crooked neck-like protein 1 OS=Homo sapiens GN=CRNKL1 PE=4 SV=1	99625	929	1068	34	22	0.03	0.03	1	0	1	1	1	1	1	1	1	0.406244
tr|B0AZR9|B0AZR9_HUMAN	GNAS	GNAS complex locus, isoform CRA_b OS=Homo sapiens GN=GNAS PE=2 SV=1	39373	1085	1061	25	23	0.08	0.08	1	0	1	1	1	1	1	1	3	0.406244
sp|O60884|DNJA2_HUMAN	DNAJA2	DnaJ homolog subfamily A member 2 OS=Homo sapiens GN=DNAJA2 PE=1 SV=1	46344	1136	1057	22	23	0.07	0.07	1	0	0	1	1	1	1	2	1	0.406244
tr|H0YE81|H0YE81_HUMAN	NDUFV1	NADH dehydrogenase [ubiquinone] flavoprotein 1, mitochondrial (Fragment) OS=Ho	14494	1039	1049	28	24	0.23	0.23	1	0	0	1	1	1	1	1	1	0.406244
tr|Q2TAM4|Q2TAM4_HUMA	POLR1B	DNA-directed RNA polymerase OS=Homo sapiens GN=POLR1B PE=2 SV=1	108996	1157	1042	21	24	0.03	0.03	1	0	0	1	1	1	1	1	1	0.406244
sp|P28074|PSB5_HUMAN	PSMB5	Proteasome subunit beta type-5 OS=Homo sapiens GN=PSMB5 PE=1 SV=3	28633	844	1005	40	26	0.12	0.12	1	0	0	1	1	1	1	1	1	0.406244
tr|B3KY12|B3KY12_HUMAN	tr|B3KY12|B3KY12	cDNA FLJ46581 fis, clone THYMU3043200, highly similar to Splicing factor 3A subuni	59082	717	993	51	27	0.06	0.06	1	1	0	1	1	1	1	1	2	0.406244
sp|P51398|RT29_HUMAN	DAP3	28S ribosomal protein S29, mitochondrial OS=Homo sapiens GN=DAP3 PE=1 SV=1	45880	847	978	40	28	0.07	0.07	1	1	0	1	1	1	1	1	1	0.406244
tr|A6NKV8|A6NKV8_HUMAN	PAIP1	Polyadenylate-binding protein-interacting protein 1 OS=Homo sapiens GN=PAIP1 PE	42379	861	954	39	29	0.08	0.08	1	1	0	1	1	1	1	1	1	0.406244
tr|Q6ZWH1|Q6ZWH1_HUMA	tr|Q6ZWH1|Q6ZW	cDNA FLJ41081 fis, clone ADRGL2009755, highly similar to Homo sapiens brain and r	45824	973	953	32	29	0.07	0.07	1	0	0	1	1	1	1	1	1	0.406244
tr|B1ANK7|B1ANK7_HUMAN	FH	Fumarate hydratase OS=Homo sapiens GN=FH PE=2 SV=1	54773	790	938	44	30	0.06	0.06	1	1	0	1	1	1	1	1	2	0.406244
tr|G3V1S7|G3V1S7_HUMAN	HAS1	Hyaluronan synthase 1 OS=Homo sapiens GN=HAS1 PE=2 SV=1	65517	845	936	40	30	0.05	0.05	1	1	1	1	1	1	1	1	1	0.406244
tr|I4AY87|I4AY87_HUMAN	tr|I4AY87|I4AY87_	Macrophage migration inhibitory factor (Fragment) OS=Homo sapiens PE=2 SV=1	12639	603	925	61	31	0.27	0.27	1	1	0	1	1	1	1	1	1	0.406244
sp|Q96IJ6|GMPPA_HUMAN	GMPPA	Mannose-1-phosphate guanyltransferase alpha OS=Homo sapiens GN=GMPPA PE=1	46604	1013	916	30	32	0.07	0.07	1	0	0	1	1	1	1	1	1	0.406244
tr|B3KRZ3|B3KRZ3_HUMAN	IMPDH	Inosine-5~-monophosphate dehydrogenase OS=Homo sapiens GN=IMPDH PE=2 SV=	56729	914	872	35	35	0.06	0.06	1	1	1	1	1	1	1	1	1	0.406244
tr|B4E2W0|B4E2W0_HUMAN	HADHB	3-ketoacyl-CoA thiolase OS=Homo sapiens GN=HADHB PE=2 SV=1	49076	614	862	60	35	0.07	0.07	1	1	1	1	1	1	1	2	1	0.406244
tr|A4D0W4|A4D0W4_HUMA	AASS	Aminoadipate-semialdehyde synthase OS=Homo sapiens GN=AASS PE=2 SV=1	102808	1167	860	20	35	0.03	0.03	1	0	1	1	1	1	1	1	1	0.406244
sp|P0CW22|RS17L_HUMAN	RPS17L	40S ribosomal protein S17-like OS=Homo sapiens GN=RPS17L PE=3 SV=1	15597	819	857	42	36	0.22	0.22	1	1	0	1	1	1	1	1	1	0.406244
tr|B4DL79|B4DL79_HUMAN	KIF20A	Kinesin-like protein KIF20A OS=Homo sapiens GN=KIF20A PE=2 SV=1	99066	895	856	36	36	0.03	0.03	1	1	0	1	1	1	1	1	2	0.406244
tr|E7ESZ7|E7ESZ7_HUMAN	NDUFA10	NADH dehydrogenase [ubiquinone] 1 alpha subcomplex subunit 10, mitochondrial C	45052	1165	851	20	36	0.07	0.07	1	0	0	1	1	1	1	1	1	0.406244
sp|Q32MZ4|LRRF1_HUMAN	LRRFIP1	Leucine-rich repeat flightless-interacting protein 1 OS=Homo sapiens GN=LRRFIP1 PE	89826	1155	848	21	36	0.04	0.04	1	0	1	1	1	1	1	1	1	0.406244
sp|Q9Y295|DRG1_HUMAN	DRG1	Developmentally-regulated GTP-binding protein 1 OS=Homo sapiens GN=DRG1 PE=1	40802	784	836	44	37	0.08	0.08	1	1	1	1	1	1	1	1	1	0.406244
tr|Q9UPE4|Q9UPE4_HUMAN	hTIM44	Putative mitochondrial inner membrane protein import receptor OS=Homo sapiens	47508	981	825	32	38	0.07	0.07	1	0	1	1	1	1	1	2	2	0.406244
tr|J3QKW7|J3QKW7_HUMAN	DRG2	Developmentally-regulated GTP-binding protein 2 OS=Homo sapiens GN=DRG2 PE=4	26892	645	803	57	40	0.12	0.12	1	1	1	1	1	1	1	1	1	0.406244
tr|B3KVR1|B3KVR1_HUMAN	SNRPN	Small nuclear ribonucleoprotein-associated protein OS=Homo sapiens GN=SNRPN PE	25230	749	801	48	40	0.13	0.13	1	1	1	1	1	1	1	1	1	0.406244
sp|Q9BTE3|MCMBP_HUMAN	MCMBP	Mini-chromosome maintenance complex-binding protein OS=Homo sapiens GN=MC	73789	1015	799	30	41	0.04	0.04	1	0	1	1	1	1	1	1	1	0.406244
tr|B5MCP9|B5MCP9_HUMAN	RPS7	40S ribosomal protein S7 OS=Homo sapiens GN=RPS7 PE=2 SV=1	21299	1062	796	26	41	0.16	0.16	1	0	1	1	1	1	1	1	1	0.406244
tr|B3KUP9|B3KUP9_HUMAN	tr|B3KUP9|B3KUP	cDNA FLJ40361 fis, clone TESTI2034407, highly similar to NEDD8-activating enzyme E	60951	723	777	50	43	0.05	0.05	1	1	1	1	1	1	1	1	1	0.406244
tr|A2A2Q9|A2A2Q9_HUMAN	C20orf4	Chromosome 20 open reading frame 4 OS=Homo sapiens GN=C20orf4 PE=4 SV=1	45575	1074	756	26	45	0.07	0.07	1	0	1	1	1	1	1	1	1	0.406244
tr|F2Z2G2|F2Z2G2_HUMAN	EEF1B2	Elongation factor 1-beta OS=Homo sapiens GN=EEF1B2 PE=4 SV=1	7611	544	745	69	46	0.46	0.46	1	1	1	1	1	1	1	1	1	0.406244
tr|B2RBE0|B2RBE0_HUMAN	tr|B2RBE0|B2RBE(	cDNA, FLJ95462, highly similar to Homo sapiens fatty-acid-Coenzyme A ligase, long-c	81266	822	739	42	46	0.04	0.04	1	1	1	1	1	1	1	1	1	0.406244
tr|B4DKI9|B4DKI9_HUMAN	tr|B4DKI9|B4DKI9	cDNA FLJ54799, highly similar to Mitochondrial 28S ribosomal protein S27 OS=Homo	22078	815	726	42	47	0.15	0.15	1	1	1	1	1	1	1	2	1	0.406244
tr|G1UI17|G1UI17_HUMAN	AGL	Glycogen debranching enzyme (Fragment) OS=Homo sapiens GN=AGL PE=2 SV=1	145569	1017	721	29	47	0.02	0.02	1	0	1	1	1	1	1	1	1	0.406244
tr|F6U211|F6U211_HUMAN	RPS10	40S ribosomal protein S10 OS=Homo sapiens GN=RPS10 PE=2 SV=1	19912	670	710	54	48	0.17	0.17	1	1	1	1	1	1	1	1	1	0.406244
tr|G3V3E6|G3V3E6_HUMAN	ERO1L	ERO1-like protein alpha OS=Homo sapiens GN=ERO1L PE=4 SV=1	30351	700	703	52	49	0.11	0.11	1	1	1	1	1	1	1	1	1	0.406244
tr|Q1JQ76|Q1JQ76_HUMAN	RPL10A	Ribosomal protein (Fragment) OS=Homo sapiens GN=RPL10A PE=2 SV=1	23692	732	657	49	54	0.14	0.14	1	1	1	1	1	1	1	1	1	0.406244
tr|F5H4C2|F5H4C2_HUMAN	RANBP3	Ran-binding protein 3 OS=Homo sapiens GN=RANBP3 PE=2 SV=1	47206	1070	630	26	56	0.07	0.07	1	0	1	1	1	1	1	2	1	0.406244
tr|Q5T0F3|Q5T0F3_HUMAN	GNL2	Guanine nucleotide binding protein-like 2 (Nucleolar) OS=Homo sapiens GN=GNL2 P	83831	791	617	44	58	0.04	0.04	1	1	1	1	1	1	1	1	2	0.406244
tr|Q6IAV5|Q6IAV5_HUMAN	OXCT	Succinyl-CoA:3-ketoacid-coenzyme A transferase OS=Homo sapiens GN=OXCT PE=2	56612	542	615	69	58	0.06	0.06	1	1	1	1	1	1	1	1	2	0.406244
tr|B3KTA3|B3KTA3_HUMAN	tr|B3KTA3|B3KTA3	Fascin OS=Homo sapiens PE=2 SV=1	52814	559	614	67	58	0.06	0.06	1	1	1	1	1	1	1	1	1	0.406244
tr|B4DLG2|B4DLG2_HUMAN	tr|B4DLG2|B4DLG	cDNA FLJ58196, highly similar to Zinc finger CCCH domain-containing protein 11A OS	83177	712	611	51	58	0.04	0.04	1	1	1	1	1	1	1	1	2	0.406244
tr|E5RI99|E5RI99_HUMAN	RPL30	60S ribosomal protein L30 (Fragment) OS=Homo sapiens GN=RPL30 PE=2 SV=1	12819	976	574	32	63	0.27	0.27	1	0	1	1	1	1	1	1	1	0.406244
tr|B7Z3P1|B7Z3P1_HUMAN	TBCE	Tubulin-specific chaperone E OS=Homo sapiens GN=TBCE PE=2 SV=1	65666	864	567	39	64	0.05	0.05	1	1	1	1	1	1	1	1	2	0.406244
tr|J3KQ32|J3KQ32_HUMAN	OLA1	Obg-like ATPase 1 OS=Homo sapiens GN=OLA1 PE=4 SV=1	47136	1162	564	21	64	0.07	0.07	1	0	1	1	1	1	1	1	1	0.406244
tr|B4DR63|B4DR63_HUMAN	PSMC1	26S protease regulatory subunit 4 OS=Homo sapiens GN=PSMC1 PE=2 SV=1	41255	889	555	37	66	0.08	0.08	1	0	1	1	1	1	1	2	1	0.406244
sp|O75367|H2AY_HUMAN	H2AFY	Core histone macro-H2A.1 OS=Homo sapiens GN=H2AFY PE=1 SV=4	39764	561	552	66	66	0.08	0.08	1	1	1	1	1	1	1	1	2	0.406244
tr|B4DM84|B4DM84_HUMAI	tr|B4DM84|B4DM	cDNA FLJ60694, highly similar to Deubiquitinating protein VCIP135 (EC 3.4.22.-) OS=I	87230	832	526	41	69	0.04	0.04	1	1	1	1	1	1	1	1	2	0.406244
sp|Q9P2D3|HTR5B_HUMAN	HEATR5B	HEAT repeat-containing protein 5B OS=Homo sapiens GN=HEATR5B PE=1 SV=2	226839	788	517	44	70	0.01	0.01	1	1	1	1	1	1	1	1	2	0.406244
tr|B4DUV6|B4DUV6_HUMAN	TRMT6	cDNA FLJ51319, highly similar to tRNA (adenine-N(1)-)-methyltransferase non-cataly	36701	881	514	37	71	0.09	0.09	1	1	1	1	1	1	1	2	1	0.406244
tr|H7C4F6|H7C4F6_HUMAN	KPNA4	Importin subunit alpha-4 (Fragment) OS=Homo sapiens GN=KPNA4 PE=4 SV=1	19041	985	511	31	71	0.18	0.18	1	0	1	1	1	1	1	1	1	0.406244
tr|C0JXB8|C0JXB8_HUMAN	AIP	Aryl hydrocarbon receptor interacting protein OS=Homo sapiens GN=AIP PE=2 SV=1	38041	1158	504	21	72	0.09	0.09	1	0	1	1	1	1	1	1	1	0.406244
tr|Q5T1D1|Q5T1D1_HUMAN	RP11-632C17__A.1	Ribosomal protein L29 (RPL29) pseudogene OS=Homo sapiens GN=RP11-632C17__A	17471	883	502	37	72	0.19	0.19	1	1	1	1	1	1	1	2	1	0.406244
tr|G8IFA7|G8IFA7_HUMAN	tr|G8IFA7|G8IFA7	p90 OS=Homo sapiens PE=2 SV=1	59481	363	497	114	73	0.06	0.06	1	1	1	1	1	1	1	1	1	0.406244
tr|C9JDR0|C9JDR0_HUMAN	NSDHL	Sterol-4-alpha-carboxylate 3-dehydrogenase, decarboxylating (Fragment) OS=Homo	28356	950	470	33	77	0.12	0.12	1	0	1	1	1	1	1	1	1	0.406244
tr|Q5TGM0|Q5TGM0_HUMA	VTA1	Vacuolar protein sorting-associated protein VTA1 homolog OS=Homo sapiens GN=VT	27485	327	462	125	79	0.12	0.12	1	1	1	1	1	1	1	1	1	0.406244
tr|F8VS81|F8VS81_HUMAN	TWF1	Twinfilin-1 (Fragment) OS=Homo sapiens GN=TWF1 PE=2 SV=1	33615	680	449	53	80	0.1	0.1	1	1	1	1	1	1	1	1	1	0.406244
sp|Q9UHR4|BI2L1_HUMAN	BAIAP2L1	Brain-specific angiogenesis inhibitor 1-associated protein 2-like protein 1 OS=Homo	57189	641	441	57	81	0.06	0.06	1	1	1	1	1	1	1	2	1	0.406244
sp|Q7Z4V5|HDGR2_HUMAN	HDGFRP2	Hepatoma-derived growth factor-related protein 2 OS=Homo sapiens GN=HDGFRP2	74443	699	348	52	104	0.04	0.04	1	1	1	1	1	1	1	1	2	0.406244
tr|Q5U077|Q5U077_HUMAN	LDHB	L-lactate dehydrogenase OS=Homo sapiens GN=LDHB PE=2 SV=1	36900	90	52	408	544	2.96	2.96	1	13	14	18	21	21	20	23	23	0.338546
tr|F1T0B3|F1T0B3_HUMAN	DDX1	ATP-dependent RNA helicase DDX1 OS=Homo sapiens GN=DDX1 PE=2 SV=1	74838	176	92	222	333	0.6	0.6	1	6	8	11	11	12	11	13	13	0.28639

sp|P25705|ATPA_HUMAN	ATP5A1	ATP synthase subunit alpha, mitochondrial OS=Homo sapiens GN=ATP5A1 PE=1 SV=	59828	99	269	369	132	0.62	0.62	1	9	4	10	9	10	9	11	9	0.262167
tr|B2RDX5|B2RDX5_HUMAN	tr|B2RDX5|B2RDX	cDNA, FLJ96812, highly similar to Homo sapiens threonyl-tRNA synthetase (TARS), m	82974	135	113	286	285	0.72	0.72	1	10	9	16	14	20	18	21	20	0.258836
tr|B3KS31|B3KS31_HUMAN	TUBB6	Tubulin, beta 6, isoform CRA_a OS=Homo sapiens GN=TUBB6 PE=2 SV=1	42324	81	68	442	430	1.66	1.66	1	17	16	18	20	23	20	28	25	0.20983
tr|B3KWY7|B3KWY7_HUMAN	tr|B3KWY7|B3KW	cDNA FLJ44270 fis, clone TLIVE2008229, highly similar to SIGNAL RECOGNITION PAR	66524	272	211	146	165	0.34	0.34	1	5	4	6	6	7	6	8	9	0.199915
sp|Q3ZCM7|TBB8_HUMAN	TUBB8	Tubulin beta-8 chain OS=Homo sapiens GN=TUBB8 PE=1 SV=2	50257	61	61	575	476	1.28	1.28	1	26	21	28	24	30	25	34	28	0.148482
tr|E7EQV9|E7EQV9_HUMAN	RPL15	Ribosomal protein L15 (Fragment) OS=Homo sapiens GN=RPL15 PE=2 SV=1	20611	380	464	107	78	0.83	0.83	1	3	2	4	4	5	4	5	4	0.116732
tr|F8VRQ1|F8VRQ1_HUMAN	HNRNPA1	Heterogeneous nuclear ribonucleoprotein A1 OS=Homo sapiens GN=HNRNPA1 PE=2	33249	49	41	660	688	2.13	2.13	1	16	16	21	17	22	17	28	17	0.043714
tr|E9PKZ0|E9PKZ0_HUMAN	RPL8	60S ribosomal protein L8 OS=Homo sapiens GN=RPL8 PE=4 SV=1	22603	320	314	125	114	0.51	0.51	1	3	2	3	3	4	3	5	5	0.043605
tr|B4E0S6|B4E0S6_HUMAN	tr|B4E0S6|B4E0S6	cDNA FLJ55635, highly similar to pre-mRNA-splicing factorATP-dependent RNA helic	90289	218	236	181	148	0.24	0.24	1	5	3	6	6	8	6	11	8	0.019148
tr|A6NEM2|A6NEM2_HUMA	HCFC1	HCF N-terminal chain 5 OS=Homo sapiens GN=HCFC1 PE=2 SV=2	215338	465	590	83	61	0.05	0.05	1	2	1	3	3	3	2	4	4	-0.07931
sp|Q6L8Q7|PDE12_HUMAN	PDE12	2~,5~-phosphodiesterase 12 OS=Homo sapiens GN=PDE12 PE=1 SV=2	68221	752	582	48	62	0.1	0.1	1	1	1	2	2	3	2	3	2	-0.07931
tr|B1AHB0|B1AHB0_HUMAN	MCM5	MCM5 minichromosome maintenance deficient 5, cell division cycle 46 (S. cerevisia	83031	570	577	65	63	0.08	0.08	1	1	1	2	2	3	2	5	3	-0.07931
tr|K4DI93|K4DI93_HUMAN	CUL4B	Cullin 4B, isoform CRA_e OS=Homo sapiens GN=CUL4B PE=3 SV=1	103318	629	486	58	76	0.06	0.06	1	2	1	2	2	3	2	3	2	-0.07931
tr|H0Y9G6|H0Y9G6_HUMAN	MRPL3	39S ribosomal protein L3, mitochondrial (Fragment) OS=Homo sapiens GN=MRPL3 P	40898	828	1060	41	23	0.08	0.08	1	1	0	1	1	2	1	2	2	-0.33092
sp|Q9Y3Z3|SAMH1_HUMAN	SAMHD1	SAM domain and HD domain-containing protein 1 OS=Homo sapiens GN=SAMHD1 P	72896	741	1043	49	24	0.09	0.09	1	1	0	2	2	2	1	4	2	-0.33092
sp|Q8NI36|WDR36_HUMAN	WDR36	WD repeat-containing protein 36 OS=Homo sapiens GN=WDR36 PE=1 SV=1	106282	1166	888	20	34	0.03	0.03	1	0	1	1	1	2	1	2	1	-0.33092
sp|O76003|GLRX3_HUMAN	GLRX3	Glutaredoxin-3 OS=Homo sapiens GN=GLRX3 PE=1 SV=2	37693	838	852	40	36	0.09	0.09	1	1	1	1	1	2	1	2	4	-0.33092
sp|Q9H9A6|LRC40_HUMAN	LRRC40	Leucine-rich repeat-containing protein 40 OS=Homo sapiens GN=LRRC40 PE=1 SV=1	68720	1033	813	28	40	0.05	0.05	1	0	1	1	1	2	1	2	1	-0.33092
tr|B4DZ87|B4DZ87_HUMAN	tr|B4DZ87|B4DZ8	cDNA FLJ57240, highly similar to Mitochondrial proteins import receptor OS=Homo	57579	565	732	66	46	0.12	0.12	1	1	1	2	1	2	1	3	1	-0.33092
tr|H3BLU7|H3BLU7_HUMAN	AKR7A2	Aflatoxin B1 aldehyde reductase member 2 (Fragment) OS=Homo sapiens GN=AKR7	35004	535	628	71	56	0.2	0.2	1	2	1	2	2	2	1	2	3	-0.33092
sp|Q96I24|FUBP3_HUMAN	FUBP3	Far upstream element-binding protein 3 OS=Homo sapiens GN=FUBP3 PE=1 SV=2	61944	927	620	35	57	0.05	0.05	1	0	1	1	1	2	1	4	1	-0.33092
tr|K7EKG4|K7EKG4_HUMAN	TOMM40	Mitochondrial import receptor subunit TOM40 homolog (Fragment) OS=Homo sapie	18328	1078	593	26	61	0.18	0.18	1	0	1	1	1	2	1	2	1	-0.33092
tr|B4DUS0|B4DUS0_HUMAN	tr|B4DUS0|B4DUS	cDNA FLJ60898, highly similar to Homo sapiens BRCA2 and CDKN1A interacting prot	33249	1119	553	23	66	0.1	0.1	1	0	1	1	1	2	1	2	1	-0.33092
tr|A8K670|A8K670_HUMAN	tr|A8K670|A8K67	cDNA FLJ75703, highly similar to Homo sapiens nitric oxide synthase interacting prot	33676	743	507	48	72	0.1	0.1	1	1	1	1	1	2	1	3	2	-0.33092
tr|J3KMW7|J3KMW7_HUMA	UBR5	E3 ubiquitin-protein ligase UBR5 OS=Homo sapiens GN=UBR5 PE=4 SV=1	312224	1049	475	27	77	0.01	0.01	1	0	1	1	1	2	1	2	1	-0.33092
tr|Q05DV5|Q05DV5_HUMAN	ZC3HAV1	ZC3HAV1 protein (Fragment) OS=Homo sapiens GN=ZC3HAV1 PE=2 SV=1	49964	366	289	111	124	0.14	0.14	1	1	1	2	2	2	1	2	2	-0.33092
tr|B2RDW1|B2RDW1_HUMA	RPS27A	Ribosomal protein S27a, isoform CRA_c OS=Homo sapiens GN=RPS27A PE=2 SV=1	18296	213	357	184	102	0.4	0.4	1	5	3	7	4	11	5	11	8	-0.65881
sp|O00507|USP9Y_HUMAN	USP9Y	Probable ubiquitin carboxyl-terminal hydrolase FAF-Y OS=Homo sapiens GN=USP9Y	294370	737	913	49	32	0.01	0.01	1	1	0	1	1	3	1	3	2	-0.81654
tr|B4DH17|B4DH17_HUMAN	tr|B4DH17|B4DH1	cDNA FLJ59298, highly similar to Eukaryotic translation initiation factor 4 gamma 3 C	134921	716	718	51	48	0.02	0.02	1	1	1	1	1	3	1	3	3	-0.81654
tr|B2RAG9|B2RAG9_HUMAN	tr|B2RAG9|B2RAG	cDNA, FLJ94908, highly similar to Homo sapiens PPAR binding protein (PPARBP), mR	167569	977	1135	32	18	0.02	0.02	1	1	1	1	1	1	0	1	4	-1.17898
tr|B4DRK5|B4DRK5_HUMAN	tr|B4DRK5|B4DRK	cDNA FLJ59584, highly similar to Mitochondrial-processing peptidase alpha subunit,	28622	945	1126	33	18	0.12	0.12	1	1	0	1	1	1	0	2	1	-1.17898
tr|A0N4V7|A0N4V7_HUMAN	Tcr-alpha	HCG2039797 (Fragment) OS=Homo sapiens GN=Tcr-alpha PE=4 SV=1	2269	781	853	45	36	1.81	1.81	1	2	1	2	1	4	1	4	3	-1.1793
tr|B4DFG0|B4DFG0_HUMAN	DEK	Protein DEK OS=Homo sapiens GN=DEK PE=2 SV=1	39704	1032	1129	28	18	0.08	0.08	1	0	0	1	1	2	0	2	1	-1.91614
tr|F8VSC4|F8VSC4_HUMAN	METAP2	Methionine aminopeptidase 2 (Fragment) OS=Homo sapiens GN=METAP2 PE=2 SV=	17286	686	188	52	46	0.43	0.41	0.953488	1	1	2	1	2	1	2	1	-0.33092
tr|I3L1L3|I3L1L3_HUMAN	MYBBP1A	Myb-binding protein 1A (Fragment) OS=Homo sapiens GN=MYBBP1A PE=4 SV=1	141124	328	217	124	156	0.17	0.16	0.941176	3	6	7	6	6	8	9	8	0.794103
tr|B4DXL9|B4DXL9_HUMAN	tr|B4DXL9|B4DXL9	cDNA FLJ53677, highly similar to Drebrin-like protein OS=Homo sapiens PE=2 SV=1	42985	503	503	76	48	0.16	0.15	0.9375	1	1	2	1	3	1	4	1	-0.81654
tr|J3KPE3|J3KPE3_HUMAN	GNB2L1	Guanine nucleotide-binding protein subunit beta-2-like 1 OS=Homo sapiens GN=GN	30541	100	58	368	522	1.54	1.44	0.935065	7	12	10	13	11	15	11	16	0.838552
sp|P11142|HSP7C_HUMAN	HSPA8	Heat shock cognate 71 kDa protein OS=Homo sapiens GN=HSPA8 PE=1 SV=1	71082	57	40	602	691	1.7	1.58	0.929412	16	19	27	25	26	28	30	31	0.513328
tr|A8MXP9|A8MXP9_HUMA	MATR3	Matrin-3 OS=Homo sapiens GN=MATR3 PE=4 SV=1	100419	117	88	336	344	0.67	0.62	0.925373	11	9	16	16	19	18	20	18	0.331182
tr|B4DF50|B4DF50_HUMAN	tr|B4DF50|B4DF5	cDNA FLJ60458, highly similar to SWI/SNF-related matrix-associated actin-dependen	28346	413	689	36	51	0.13	0.12	0.923077	0	1	1	1	1	1	1	1	0.406244
sp|P34931|HS71L_HUMAN	HSPA1L	Heat shock 70 kDa protein 1-like OS=Homo sapiens GN=HSPA1L PE=1 SV=2	70730	26	27	981	973	1.16	1.07	0.922414	23	21	33	34	33	35	37	42	0.492465
tr|Q5BJF5|Q5BJF5_HUMAN	SHMT2	Serine hydroxymethyltransferase (Fragment) OS=Homo sapiens GN=SHMT2 PE=2 SV	53561	86	62	416	375	1.17	1.07	0.91453	12	8	15	12	16	14	17	15	0.220267
tr|B3KX11|B3KX11_HUMAN	tr|B3KX11|B3KX11	cDNA FLJ44436 fis, clone UTERU2019706, highly similar to T-complex protein 1 subu	58479	76	84	466	357	1.27	1.15	0.905512	10	11	15	15	18	15	23	16	0.151291
sp|P49327|FAS_HUMAN	FASN	Fatty acid synthase OS=Homo sapiens GN=FASN PE=1 SV=3	275877	3	7	1817	1647	0.93	0.84	0.903226	46	43	59	65	65	65	78	75	0.41035
tr|E9PPU1|E9PPU1_HUMAN	RPS3	40S ribosomal protein S3 OS=Homo sapiens GN=RPS3 PE=2 SV=1	17397	269	197	149	116	1.03	0.93	0.902913	3	3	5	5	5	5	6	7	0.406498
tr|G5E9B2|G5E9B2_HUMAN	CCT8	Chaperonin containing TCP1, subunit 8 (Theta), isoform CRA_a OS=Homo sapiens G	60011	69	66	487	453	1.23	1.11	0.902439	14	13	16	15	19	17	19	18	0.250752
tr|F2Z2C0|F2Z2C0_HUMAN	DIS3	Exosome complex exonuclease RRP44 OS=Homo sapiens GN=DIS3 PE=2 SV=1	64376	666	245	54	116	0.1	0.09	0.9	2	2	2	2	2	3	2	4	0.891993
tr|B4DFK6|B4DFK6_HUMAN	CNN3	Calponin-3 OS=Homo sapiens GN=CNN3 PE=2 SV=1	31889	1011	919	30	31	0.1	0.09	0.9	0	0	1	1	1	1	1	1	0.406244
tr|Q2NKY5|Q2NKY5_HUMAN	TUBB6	TUBB6 protein OS=Homo sapiens GN=TUBB6 PE=2 SV=1	50514	55	45	617	616	1.42	1.27	0.894366	18	17	21	21	26	22	28	29	0.170731
tr|B7Z9L0|B7Z9L0_HUMAN	CCT4	T-complex protein 1 subunit delta OS=Homo sapiens GN=CCT4 PE=2 SV=1	52811	95	166	389	208	1.2	1.07	0.891667	8	6	15	13	18	16	20	18	0.241748
tr|A8K492|A8K492_HUMAN	tr|A8K492|A8K492	cDNA FLJ76789, highly similar to Homo sapiens methionine-tRNA synthetase (MARS	102277	80	177	442	197	0.55	0.49	0.890909	12	4	14	9	14	7	14	10	-0.54583
tr|K9JA46|K9JA46_HUMAN	EL52	Epididymis luminal secretory protein 52 OS=Homo sapiens GN=EL52 PE=2 SV=1	85006	17	22	1239	1052	2	1.78	0.89	31	27	43	46	47	48	50	55	0.439505
tr|H3BP78|H3BP78_HUMAN	FANCI	Fanconi anemia group I protein (Fragment) OS=Homo sapiens GN=FANCI PE=4 SV=1	124065	923	427	35	65	0.08	0.07	0.875	0	1	3	2	4	3	5	3	0.043605
tr|B4DLV7|B4DLV7_HUMAN	tr|B4DLV7|B4DLV	cDNA FLJ60299, highly similar to Rab GDP dissociation inhibitor beta OS=Homo sapi	51577	98	81	374	365	0.86	0.75	0.872093	10	8	10	9	10	11	11	12	0.53832
sp|Q9UMS4|PRP19_HUMAN	PRPF19	Pre-mRNA-processing factor 19 OS=Homo sapiens GN=PRPF19 PE=1 SV=1	55603	139	117	279	284	0.78	0.68	0.871795	9	7	10	9	10	12	14	16	0.658874
sp|Q13885|TBB2A_HUMAN	TUBB2A	Tubulin beta-2A chain OS=Homo sapiens GN=TUBB2A PE=1 SV=1	50274	15	12	1357	1361	8.18	7.13	0.871638	50	42	62	54	69	56	77	65	0.108445
tr|Q53G71|Q53G71_HUMAN	tr|Q53G71|Q53G7	Calreticulin variant (Fragment) OS=Homo sapiens PE=2 SV=1	47061	635	660	57	53	0.23	0.2	0.869565	1	1	3	2	3	2	4	2	-0.07931
sp|Q9BVA1|TBB2B_HUMAN	TUBB2B	Tubulin beta-2B chain OS=Homo sapiens GN=TUBB2B PE=1 SV=1	50377	15	11	1357	1370	8.18	7.09	0.866748	50	43	62	54	69	56	77	65	0.108445
sp|O43175|SERA_HUMAN	PHGDH	D-3-phosphoglycerate dehydrogenase OS=Homo sapiens GN=PHGDH PE=1 SV=4	57356	154	130	261	253	0.75	0.65	0.866667	7	6	11	10	11	10	12	10	0.275376
sp|P04350|TBB4A_HUMAN	TUBB4A	Tubulin beta-4A chain OS=Homo sapiens GN=TUBB4A PE=1 SV=2	50010	16	17	1299	1232	7.74	6.7	0.865633	46	43	61	54	63	55	70	65	0.213859
sp|Q92621|NU205_HUMAN	NUP205	Nuclear pore complex protein Nup205 OS=Homo sapiens GN=NUP205 PE=1 SV=3	230171	324	224	125	155	0.07	0.06	0.857143	4	3	5	4	5	4	8	5	0.116732
tr|B3KPS3|B3KPS3_HUMAN	tr|B3KPS3|B3KPS3	cDNA FLJ32131 fis, clone PEBLM2000267, highly similar to Tubulin alpha-ubiquitous	46725	18	10	1209	1417	5.31	4.51	0.849341	33	40	50	52	52	57	60	65	0.54206
tr|D6R9I9|D6R9I9_HUMAN	ABCE1	ATP-binding cassette sub-family E member 1 OS=Homo sapiens GN=ABCE1 PE=2 SV=	47919	448	387	88	96	0.39	0.33	0.846154	1	3	5	6	4	5	6	6	0.6962
tr|B4DH52|B4DH52_HUMAN	tr|B4DH52|B4DH5	cDNA FLJ55916, highly similar to General transcription factor II-I OS=Homo sapiens F	112359	234	270	169	132	0.26	0.22	0.846154	5	4	8	7	10	9	12	10	0.262167
tr|B2R5V9|B2R5V9_HUMAN	tr|B2R5V9|B2R5V	cDNA, FLJ92652, highly similar to Homo sapiens high density lipoprotein binding pro	141919	353	293	115	123	0.12	0.1	0.833333	2	4	5	4	5	5	6	6	0.406498
tr|Q6IBN0|Q6IBN0_HUMAN	PSMD3	PSMD3 protein OS=Homo sapiens GN=PSMD3 PE=2 SV=1	61022	221	390	179	95	0.3	0.25	0.833333	5	3	5	4	7	5	10	5	-0.04135
sp|Q14008|CKAP5_HUMAN	CKAP5	Cytoskeleton-associated protein 5 OS=Homo sapiens GN=CKAP5 PE=1 SV=3	227062	183	1149	216	16	0.12	0.1	0.833333	5	1	8	1	9	0	10	1	-3.84351
tr|A2RUM7|A2RUM7_HUMA	RPL5	Ribosomal protein L5 OS=Homo sapiens GN=RPL5 PE=2 SV=1	34569	149	143	270	231	1.08	0.9	0.833333	8	7	9	9	10	10	11	11	0.406816
sp|Q9NZI8|IF2B1_HUMAN	IGF2BP1	Insulin-like growth factor 2 mRNA-binding protein 1 OS=Homo sapiens GN=IGF2BP1	63783	190	240	210	144	0.42	0.35	0.833333	5	5	8	6	9	6	12	6	-0.14151
tr|B2R858|B2R858_HUMAN	tr|B2R858|B2R858	cDNA, FLJ93750, Homo sapiens DEAD (Asp-Glu-Ala-Asp) box polypeptide 6 (DDX6), n	53582	191	226	209	152	0.52	0.43	0.826923	6	4	7	6	7	6	8	7	0.199915
sp|Q9BXP5|SRRT_HUMAN	SRRT	Serrate RNA effector molecule homolog OS=Homo sapiens GN=SRRT PE=1 SV=1	101060	377	311	108	115	0.17	0.14	0.823529	2	3	5	4	7	4	8	4	-0.33112
tr|B2R7C5|B2R7C5_HUMAN	tr|B2R7C5|B2R7C	cDNA, FLJ93378, highly similar to Homo sapiens MCM3 minichromosome maintenar	91579	265	212	152	164	0.28	0.23	0.821429	5	5	7	6	9	6	9	6	-0.14151
sp|P04406|G3P_HUMAN	GAPDH	Glyceraldehyde-3-phosphate dehydrogenase OS=Homo sapiens GN=GAPDH PE=1 SV	36201	13	21	1386	1074	6.5	5.3	0.815385	29	24	40	37	42	40	46	48	0.338803
sp|Q15645|PCH2_HUMAN	TRIP13	Pachytene checkpoint protein 2 homolog OS=Homo sapiens GN=TRIP13 PE=1 SV=2	48863	401	258	101	137	0.48	0.39	0.8125	3	3	6	5	7	5	8	5	-0.04135
tr|F5GWQ3|F5GWQ3_HUMA	ENO2	Gamma-enolase OS=Homo sapiens GN=ENO2 PE=2 SV=1	13396	229	547	103	67	0.31	0.25	0.806452	1	1	1	2	3	3	3	3	0.406371
sp|P35527|K1C9_HUMAN	KRT9	Keratin, type I cytoskeletal 9 OS=Homo sapiens GN=KRT9 PE=1 SV=3	62255	214	112	183	287	0.36	0.29	0.805556	4	4	6	5	7	5	9	5	-0.04135
tr|B4E1E0|B4E1E0_HUMAN	tr|B4E1E0|B4E1E0	cDNA FLJ58563, highly similar to CTP synthase 1 (EC 6.3.4.2) OS=Homo sapiens PE=2	50233	338	213	119	160	0.46	0.37	0.804348	3	4	6	5	8	5	8	8	-0.22212
sp|P12268|IMDH2_HUMAN	IMPDH2	Inosine-5~-monophosphate dehydrogenase 2 OS=Homo sapiens GN=IMPDH2 PE=1 S	56226	66	79	513	388	1.22	0.98	0.803279	12	10	14	11	14	10	14	12	-0.05963
tr|A2A274|A2A274_HUMAN	ACO2	Aconitase 2, mitochondrial OS=Homo sapiens GN=ACO2 PE=4 SV=1	88563	182	317	216	113	0.2	0.16	0.8	5	3	5	4	7	4	7	5	-0.33112
tr|B4DVY1|B4DVY1_HUMAN	EIF3D	Eukaryotic translation initiation factor 3 subunit D OS=Homo sapiens GN=EIF3D PE=2	58731	303	254	131	138	0.39	0.31	0.794872	3	4	6	5	7	5	9	5	-0.04135
tr|A8K5I0|A8K5I0_HUMAN	HSPA1A	Heat shock 70kDa protein 1A OS=Homo sapiens GN=HSPA1A PE=2 SV=1	70294	11	9	1514	1571	4.42	3.51	0.794118	37	37	62	61	63	68	68	80	0.520877
tr|B4DRW3|B4DRW3_HUMA	tr|B4DRW3|B4DR	cDNA FLJ57180, highly similar to TAR DNA-binding protein 43 OS=Homo sapiens PE=	35596	308	291	130	92	0.19	0.15	0.789474	2	1	2	1	2	1	2	2	-0.33092
tr|B4E043|B4E043_HUMAN	KHDRBS1	KH domain containing, RNA binding, signal transduction associated 1, isoform CRA_	38772	514	702	74	49	0.28	0.22	0.785714	2	0	3	3	3	3	3	3	0.406371
tr|E5KNY5|E5KNY5_HUMAN	LRPPRC	Leucine-rich PPR-motif containing OS=Homo sapiens GN=LRPPRC PE=2 SV=1	159003	52	44	634	623	0.77	0.6	0.779221	21	17	28	24	29	25	33	31	0.196772
tr|H0YL11|H0YL11_HUMAN	IDH2	Isocitrate dehydrogenase [NADP], mitochondrial OS=Homo sapiens GN=IDH2 PE=3 S	35536	658	656	55	54	0.09	0.07	0.777778	1	1	1	1	1	2	2	3	1.143468
tr|B7Z463|B7Z463_HUMAN	NPEPPS	Puromycin-sensitive aminopeptidase OS=Homo sapiens GN=NPEPPS PE=2 SV=1	95083	511	484	74	76	0.18	0.14	0.777778	2	2	5	4	4	4	6	5	0.406434
tr|A8K2Y2|A8K2Y2_HUMAN	tr|A8K2Y2|A8K2Y2	cDNA FLJ78120, highly similar to Homo sapiens eukaryotic translation initiation fact	51648	356	222	115	157	0.36	0.28	0.777778	2	4	5	4	6	5	7	5	0.165295
tr|B4DQ51|B4DQ51_HUMAN	ACADSB	Short/branched chain-specific acyl-CoA dehydrogenase, mitochondrial OS=Homo sa	36287	719	1138	50	17	0.09	0.07	0.777778	1	0	1	1	1	0	2	1	-1.17898
tr|A8K781|A8K781_HUMAN	tr|A8K781|A8K781	cDNA FLJ75299, highly similar to Xenopus laevis proteasome (prosome, macropain)	47578	548	592	68	61	0.31	0.24	0.774194	2	1	4	2	4	2	6	2	-0.44208
tr|G5E9M5|G5E9M5_HUMA	ILF3	Interleukin enhancer binding factor 3, 90kDa, isoform CRA_b OS=Homo sapiens GN=	96147	62	42	550	687	1.23	0.95	0.772358	19	19	29	23	30	26	31	29	0.204237
tr|B2RBD5|B2RBD5_HUMAN	tr|B2RBD5|B2RBD	cDNA, FLJ95457, highly similar to Homo sapiens tubulin, beta, 4 (TUBB4), mRNA OS=	50828	32	26	913	976	2.98	2.3	0.771812	26	28	35	31	41	35	46	43	0.181945
sp|P13010|XRCC5_HUMAN	XRCC5	X-ray repair cross-complementing protein 5 OS=Homo sapiens GN=XRCC5 PE=1 SV=	83222	108	97	350	325	0.47	0.36	0.765957	8	6	10	9	11	10	14	12	0.275376
tr|B7Z213|B7Z213_HUMAN	tr|B7Z213|B7Z213	cDNA FLJ50130, highly similar to Heterogeneous nuclear ribonucleoprotein Q OS=H	28561	185	412	42	87	0.51	0.39	0.764706	0	2	2	3	2	3	2	3	0.891993
tr|Q5T2B7|Q5T2B7_HUMAN	CUL2	Cullin-2 OS=Homo sapiens GN=CUL2 PE=4 SV=1	80893	334	485	120	76	0.17	0.13	0.764706	3	2	4	3	4	3	4	3	0.043605
tr|B4DTG2|B4DTG2_HUMAN	EEF1G	Elongation factor 1-gamma OS=Homo sapiens GN=EEF1G PE=2 SV=1	56456	48	31	667	865	1.93	1.47	0.761658	18	19	25	23	28	25	33	26	0.246721
tr|B4DVB8|B4DVB8_HUMAN	ELAVL1	ELAV-like protein 1 OS=Homo sapiens GN=ELAVL1 PE=2 SV=1	39200	362	290	114	124	0.5	0.38	0.76	4	3	5	4	5	4	5	4	0.116732
sp|Q12904|AIMP1_HUMAN	AIMP1	Aminoacyl tRNA synthase complex-interacting multifunctional protein 1 OS=Homo s	34616	652	241	56	144	0.58	0.44	0.758621	0	3	5	4	5	4	6	6	0.116732
tr|Q59EG8|Q59EG8_HUMAN	tr|Q59EG8|Q59EG	Proteasome 26S non-ATPase subunit 2 variant (Fragment) OS=Homo sapiens PE=2 S	101322	385	203	106	174	0.33	0.25	0.757576	1	6	9	7	10	9	13	10	0.262167
tr|B2R491|B2R491_HUMAN	RPS4X	Ribosomal protein S4, X-linked, isoform CRA_c OS=Homo sapiens GN=RPS4X PE=2 S	29807	203	531	193	69	0.7	0.53	0.757143	3	2	5	4	5	5	7	5	0.406498
sp|P23526|SAHH_HUMAN	AHCY	Adenosylhomocysteinase OS=Homo sapiens GN=AHCY PE=1 SV=4	48255	142	122	277	270	1.07	0.81	0.757009	7	8	13	10	13	12	17	13	0.295717
tr|B2R6X5|B2R6X5_HUMAN	tr|B2R6X5|B2R6X	cDNA, FLJ93166, highly similar to Homo sapiens heat shock 70kDa protein 6 (HSP70	71370	105	111	356	293	0.57	0.43	0.754386	11	8	15	12	16	12	18	14	0.005623
sp|P04075|ALDOA_HUMAN	ALDOA	Fructose-bisphosphate aldolase A OS=Homo sapiens GN=ALDOA PE=1 SV=2	39851	14	3	1366	1699	6.34	4.78	0.753943	28	32	43	45	47	49	54	57	0.46921
tr|B2RAM6|B2RAM6_HUMA	tr|B2RAM6|B2RAM	cDNA, FLJ95005, highly similar to Homo sapiens kinesin family member 11 (KIF11), n	120084	392	430	104	83	0.08	0.06	0.75	2	2	3	2	3	3	3	3	0.406371
tr|Q3MNE1|Q3MNE1_HUMA	tr|Q3MNE1|Q3MN	Adaptor-related protein complex 3 beta 1 subunit (Fragment) OS=Homo sapiens PE=	89828	1045	1142	28	17	0.04	0.03	0.75	0	0	1	1	1	0	1	2	-1.17898
tr|B7Z1N6|B7Z1N6_HUMAN	tr|B7Z1N6|B7Z1N	cDNA FLJ52222, highly similar to Fructose-bisphosphate aldolase C (EC4.1.2.13) OS=	35800	71	65	482	459	0.56	0.42	0.75	8	6	14	11	16	12	17	14	0.005623
sp|Q9Y230|RUVB2_HUMAN	RUVBL2	RuvB-like 2 OS=Homo sapiens GN=RUVBL2 PE=1 SV=3	51296	174	419	223	86	0.55	0.41	0.745455	6	3	7	5	9	4	9	5	-0.67255
tr|E7EQG2|E7EQG2_HUMAN	EIF4A2	Eukaryotic initiation factor 4A-II OS=Homo sapiens GN=EIF4A2 PE=2 SV=1	41492	64	60	519	486	1.15	0.85	0.73913	11	11	14	15	16	17	17	18	0.492347
tr|K7EIG1|K7EIG1_HUMAN	CLUH	Clustered mitochondria protein homolog OS=Homo sapiens GN=CLUH PE=3 SV=1	140903	178	372	221	99	0.23	0.17	0.73913	5	3	9	7	10	6	12	7	-0.2861
tr|G5E9H4|G5E9H4_HUMAN	DCTN1	Dynactin 1 (P150, glued homolog, Drosophila), isoform CRA_a OS=Homo sapiens GN	127339	350	237	116	148	0.19	0.14	0.736842	4	4	7	5	8	5	9	7	-0.22212
tr|B4DHK9|B4DHK9_HUMAN	tr|B4DHK9|B4DHK	cDNA FLJ51656, highly similar to Actin-like protein 2 OS=Homo sapiens PE=2 SV=1	39175	296	376	133	98	0.38	0.28	0.736842	3	3	4	3	5	3	5	3	-0.2461
sp|Q9NY65|TBA8_HUMAN	TUBA8	Tubulin alpha-8 chain OS=Homo sapiens GN=TUBA8 PE=1 SV=1	50746	42	34	720	820	1.92	1.41	0.734375	20	25	31	34	31	39	34	44	0.736742
tr|H7C441|H7C441_HUMAN	THOC5	THO complex subunit 5 homolog (Fragment) OS=Homo sapiens GN=THOC5 PE=4 SV=	22943	1016	602	29	60	0.15	0.11	0.733333	0	1	1	1	3	1	3	1	-0.81654
tr|H3BLZ8|H3BLZ8_HUMAN	DDX17	Probable ATP-dependent RNA helicase DDX17 OS=Homo sapiens GN=DDX17 PE=3 S	81073	96	165	381	209	0.67	0.49	0.731343	13	7	15	11	16	13	23	14	0.116914
tr|B2RBA6|B2RBA6_HUMAN	tr|B2RBA6|B2RBA	cDNA, FLJ95407, highly similar to Homo sapiens MCM7 minichromosome maintenar	81856	236	362	168	101	0.37	0.27	0.72973	5	2	8	6	10	7	10	7	-0.07939
sp|P54136|SYRC_HUMAN	RARS	Arginine--tRNA ligase, cytoplasmic OS=Homo sapiens GN=RARS PE=1 SV=2	76129	284	184	140	188	0.4	0.29	0.725	6	4	8	7	8	7	10	10	0.225858
tr|B3KX19|B3KX19_HUMAN	tr|B3KX19|B3KX19	cDNA FLJ44500 fis, clone UTERU3000828, highly similar to 116 kDa U5 small nuclear	109111	138	114	280	285	0.47	0.34	0.723404	7	8	13	10	13	13	15	14	0.407007
sp|P26599|PTBP1_HUMAN	PTBP1	Polypyrimidine tract-binding protein 1 OS=Homo sapiens GN=PTBP1 PE=1 SV=1	57357	47	82	672	363	1.18	0.85	0.720339	12	7	15	13	15	13	18	13	0.207307
tr|G8JLB6|G8JLB6_HUMAN	HNRNPH1	Heterogeneous nuclear ribonucleoprotein H OS=Homo sapiens GN=HNRNPH1 PE=4	51482	83	90	426	338	0.75	0.54	0.72	10	9	12	9	13	12	14	17	0.295717
tr|A8K525|A8K525_HUMAN	tr|A8K525|A8K525	cDNA FLJ76817, highly similar to Homo sapiens non-POU domain containing, octame	54367	75	48	467	588	1.42	1.02	0.71831	13	16	19	16	20	20	22	20	0.407454
tr|B1ANR0|B1ANR0_HUMAN	PABPC4	Poly(A) binding protein, cytoplasmic 4 (Inducible form) OS=Homo sapiens GN=PABP	68156	160	205	240	172	0.46	0.33	0.717391	8	4	8	6	9	6	10	7	-0.14151
sp|Q9C0C9|UBE2O_HUMAN	UBE2O	Ubiquitin-conjugating enzyme E2 O OS=Homo sapiens GN=UBE2O PE=1 SV=3	142631	939	492	34	74	0.07	0.05	0.714286	1	1	2	2	2	3	4	3	0.891993
tr|B7ZKR9|B7ZKR9_HUMAN	POLR1A	DNA-directed RNA polymerase OS=Homo sapiens GN=POLR1A PE=2 SV=1	196658	405	393	101	95	0.07	0.05	0.714286	3	3	4	3	4	3	4	4	0.043605
tr|B2RDF2|B2RDF2_HUMAN	PES1	Pescadillo homolog 1 OS=Homo sapiens GN=PES1 PE=2 SV=1	68359	534	263	71	135	0.21	0.15	0.714286	1	3	4	3	4	3	4	3	0.043605
tr|B3KRS5|B3KRS5_HUMAN	HDAC2	Histone deacetylase OS=Homo sapiens GN=HDAC2 PE=2 SV=1	52479	300	460	132	79	0.28	0.2	0.714286	4	3	4	3	5	3	6	4	-0.2461
tr|H0YER0|H0YER0_HUMAN	PABPC4	Polyadenylate-binding protein 4 (Fragment) OS=Homo sapiens GN=PABPC4 PE=4 SV	7211	115	537	51	68	0.69	0.49	0.710145	1	1	1	1	1	2	1	3	1.143468
tr|Q5SU16|Q5SU16_HUMAN	TUBB	Beta 5-tubulin OS=Homo sapiens GN=TUBB PE=2 SV=1	50095	2	4	1882	1696	15.54	11.03	0.709781	64	55	79	66	85	69	97	78	0.108541
tr|Q8IWP6|Q8IWP6_HUMAN	tr|Q8IWP6|Q8IWP	Class IVb beta tubulin OS=Homo sapiens PE=2 SV=1	50177	5	8	1694	1610	14.43	10.22	0.708247	59	52	75	63	80	65	88	74	0.109898
tr|B4DMA2|B4DMA2_HUMA	tr|B4DMA2|B4DM	cDNA FLJ54023, highly similar to Heat shock protein HSP 90-beta OS=Homo sapiens	79487	7	14	1628	1286	4.25	2.99	0.703529	40	33	55	52	61	55	69	63	0.260423
tr|D0PNI1|D0PNI1_HUMAN	YWHAZ	Tyrosine 3-monooxygenase/tryptophan 5-monooxygenase activation protein zeta p	27899	329	243	124	143	0.57	0.4	0.701754	4	2	4	3	5	3	6	3	-0.2461
tr|Q5JR94|Q5JR94_HUMAN	RPS8	40S ribosomal protein S8 OS=Homo sapiens GN=RPS8 PE=2 SV=1	24475	215	751	183	45	0.67	0.47	0.701493	5	1	5	3	6	2	7	4	-0.97299
tr|B2RBS8|B2RBS8_HUMAN	tr|B2RBS8|B2RBS8	cDNA, FLJ95666, highly similar to Homo sapiens albumin (ALB), mRNA OS=Homo sap	71343	292	532	135	69	0.2	0.14	0.7	3	1	4	3	5	4	6	5	0.116732
tr|D3DU83|D3DU83_HUMAN	RPS2	Ribosomal protein S2, isoform CRA_c OS=Homo sapiens GN=RPS2 PE=4 SV=1	19208	698	634	52	44	0.5	0.35	0.7	1	1	1	1	1	1	1	1	0.406244
sp|Q92945|FUBP2_HUMAN	KHSRP	Far upstream element-binding protein 2 OS=Homo sapiens GN=KHSRP PE=1 SV=4	73355	91	83	405	359	0.69	0.48	0.695652	9	8	14	9	16	10	16	11	-0.24643
tr|F8VTY8|F8VTY8_HUMAN	PA2G4	Proliferation-associated protein 2G4 OS=Homo sapiens GN=PA2G4 PE=2 SV=1	42062	159	107	242	302	1.88	1.3	0.691489	8	7	14	11	15	12	18	12	0.096017
tr|B4DJX1|B4DJX1_HUMAN	tr|B4DJX1|B4DJX1	cDNA FLJ50978, highly similar to Dihydrolipoyllysine-residue acetyltransferasecomp	63057	240	346	166	104	0.29	0.2	0.689655	4	2	5	4	6	4	6	4	-0.12447
sp|P22626|ROA2_HUMAN	HNRNPA2B1	Heterogeneous nuclear ribonucleoproteins A2/B1 OS=Homo sapiens GN=HNRNPA2	37464	40	35	746	779	2.56	1.76	0.6875	15	14	18	20	23	20	27	22	0.20983
tr|B4DGB3|B4DGB3_HUMAN	tr|B4DGB3|B4DGB	cDNA FLJ61233, highly similar to Kinesin light chain 1 OS=Homo sapiens PE=2 SV=1	65883	316	648	127	54	0.16	0.11	0.6875	2	1	3	2	3	2	5	2	-0.07931
tr|H7C3T4|H7C3T4_HUMAN	PRDX4	Peroxiredoxin-4 (Fragment) OS=Homo sapiens GN=PRDX4 PE=4 SV=1	18485	246	673	164	52	0.95	0.65	0.684211	4	0	4	3	5	3	5	3	-0.2461
tr|B4DVZ8|B4DVZ8_HUMAN	tr|B4DVZ8|B4DVZ	cDNA FLJ52432, highly similar to Leukotriene A-4 hydrolase (EC 3.3.2.6) OS=Homo sa	64246	268	329	150	75	0.22	0.15	0.681818	3	2	4	2	4	2	5	2	-0.44208
tr|B4DZB1|B4DZB1_HUMAN	tr|B4DZB1|B4DZB	cDNA FLJ59645, highly similar to 116 kDa U5 small nuclear ribonucleoprotein compo	104668	138	121	262	274	0.47	0.32	0.680851	7	8	12	9	12	12	14	13	0.406944
tr|B4DV73|B4DV73_HUMAN	tr|B4DV73|B4DV7	cDNA FLJ51330, highly similar to Far upstream element-binding protein 2 OS=Homo	38334	158	155	243	217	0.94	0.64	0.680851	6	5	8	6	10	7	10	8	-0.07939
tr|B2R9K8|B2R9K8_HUMAN	tr|B2R9K8|B2R9K	cDNA, FLJ94440, highly similar to Homo sapiens chaperonin containing TCP1, subuni	58386	141	156	277	216	0.64	0.43	0.671875	8	5	11	11	16	8	19	12	-0.55181
sp|Q9BWU0|NADAP_HUMAN	SLC4A1AP	Kanadaptin OS=Homo sapiens GN=SLC4A1AP PE=1 SV=1	89500	1183	965	19	29	0.06	0.04	0.666667	0	0	1	1	0	1	1	1	1.991402
tr|Q96L67|Q96L67_HUMAN	NRD1	Nardilysin OS=Homo sapiens GN=NRD1 PE=2 SV=1	109243	513	443	74	0	0.03	0.02	0.666667	1	0	1	0	1	2	2	3	1.143468
tr|J3KN16|J3KN16_HUMAN	KIAA0368	Protein KIAA0368 OS=Homo sapiens GN=KIAA0368 PE=4 SV=1	225491	678	321	53	111	0.06	0.04	0.666667	1	2	4	3	3	3	6	5	0.406371
sp|P55795|HNRH2_HUMAN	HNRNPH2	Heterogeneous nuclear ribonucleoprotein H2 OS=Homo sapiens GN=HNRNPH2 PE=1	49517	112	162	347	211	0.57	0.38	0.666667	7	6	9	7	10	9	11	13	0.262167
sp|Q562R1|ACTBL_HUMAN	ACTBL2	Beta-actin-like protein 2 OS=Homo sapiens GN=ACTBL2 PE=1 SV=2	42318	156	219	260	158	0.69	0.46	0.666667	7	4	12	8	14	12	17	12	0.192428
sp|A6NHR9|SMHD1_HUMAN	SMCHD1	Structural maintenance of chromosomes flexible hinge domain-containing protein 1	227942	467	1111	83	20	0.03	0.02	0.666667	2	0	2	1	2	1	3	3	-0.33092
tr|Q2M1V9|Q2M1V9_HUMA	BTAF1	BTAF1 RNA polymerase II, B-TFIID transcription factor-associated, 170kDa OS=Homo	209037	720	762	50	44	0.03	0.02	0.666667	2	1	2	1	2	1	3	1	-0.33092
sp|Q14651|PLSI_HUMAN	PLS1	Plastin-1 OS=Homo sapiens GN=PLS1 PE=1 SV=2	70608	498	501	77	72	0.15	0.1	0.666667	2	1	3	2	4	2	5	3	-0.44208
tr|A8K5Y7|A8K5Y7_HUMAN	tr|A8K5Y7|A8K5Y7	cDNA FLJ78655, highly similar to Homo sapiens exportin 5 (XPO5), mRNA OS=Homo	138331	202	438	195	82	0.15	0.1	0.666667	4	3	6	4	7	3	9	5	-0.69395

tr|B4DYQ1|B4DYQ1_HUMAN	tr|B4DYQ1|B4DYQ	cDNA FLJ57671, highly similar to SWI/SNF-related matrix-associated actin-dependen	112027	378	782	107	42	0.15	0.1	0.666667	3	0	6	2	6	2	6	2	-0.97299
tr|J3KR91|J3KR91_HUMAN	IQGAP2	Ras GTPase-activating-like protein IQGAP2 OS=Homo sapiens GN=IQGAP2 PE=4 SV=1	123072	1140	995	22	0	0.03	0.02	0.666667	0	0	1	0	1	0	1	1	-1.17898
tr|A6NDY9|A6NDY9_HUMAN	FLNA	Filamin A OS=Homo sapiens GN=FLNA PE=2 SV=4	248165	125	178	321	193	0.18	0.12	0.666667	6	5	13	9	11	9	15	9	0.130727
sp|P07814|SYEP_HUMAN	EPRS	Bifunctional glutamate/proline--tRNA ligase OS=Homo sapiens GN=EPRS PE=1 SV=5	172080	30	57	932	524	0.72	0.48	0.666667	23	16	31	21	34	19	39	23	-0.41867
tr|F6SBX2|F6SBX2_HUMAN	IARS2	Isoleucine--tRNA ligase, mitochondrial OS=Homo sapiens GN=IARS2 PE=2 SV=1	106884	529	436	71	82	0.09	0.06	0.666667	1	2	3	2	4	2	5	2	-0.44208
tr|Q8IZ29|Q8IZ29_HUMAN	TUBB2C	Tubulin, beta 2C OS=Homo sapiens GN=TUBB2C PE=2 SV=1	50264	4	5	1730	1664	15.34	10.17	0.662973	59	52	75	63	80	64	87	73	0.087441
tr|B5BUB1|B5BUB1_HUMAN	RUVBL1	RuvB-like 1 (Fragment) OS=Homo sapiens GN=RUVBL1 PE=2 SV=1	50526	196	624	203	56	0.56	0.37	0.660714	5	2	7	5	8	6	9	7	0.019148
sp|Q02790|FKBP4_HUMAN	FKBP4	Peptidyl-prolyl cis-trans isomerase FKBP4 OS=Homo sapiens GN=FKBP4 PE=1 SV=3	52057	157	179	249	196	0.96	0.63	0.65625	5	5	11	8	11	8	12	10	-0.03
sp|O94906|PRP6_HUMAN	PRPF6	Pre-mRNA-processing factor 6 OS=Homo sapiens GN=PRPF6 PE=1 SV=1	107656	280	563	142	65	0.2	0.13	0.65	3	1	6	4	5	5	7	7	0.406498
tr|E9PHA6|E9PHA6_HUMAN	MSH2	DNA mismatch repair protein Msh2 OS=Homo sapiens GN=MSH2 PE=2 SV=1	103977	188	358	212	102	0.2	0.13	0.65	4	2	6	3	9	3	10	4	-1.03537
tr|K7ENG2|K7ENG2_HUMAN	U2AF2	Splicing factor U2AF 65 kDa subunit OS=Homo sapiens GN=U2AF2 PE=4 SV=1	34222	209	218	186	158	0.91	0.59	0.648352	5	4	7	5	7	5	7	6	-0.04135
tr|B4DMY3|B4DMY3_HUMAN	tr|B4DMY3|B4DM	cDNA FLJ60713, highly similar to Homo sapiens heterogeneous nuclear ribonucleopr	35101	220	225	180	154	0.88	0.57	0.647727	7	5	9	6	10	6	10	8	-0.2861
tr|Q53ET2|Q53ET2_HUMAN	tr|Q53ET2|Q53ET2	Dihydropyrimidinase-like 2 variant (Fragment) OS=Homo sapiens PE=2 SV=1	62688	450	572	86	64	0.17	0.11	0.647059	2	2	3	2	3	2	4	3	-0.07931
tr|Q0IIN1|Q0IIN1_HUMAN	KRT77	Keratin 77 OS=Homo sapiens GN=KRT77 PE=2 SV=1	62050	198	435	202	83	0.17	0.11	0.647059	5	3	6	3	6	4	7	4	-0.12447
tr|Q5BKZ2|Q5BKZ2_HUMAN	KPNA1	Importin subunit alpha OS=Homo sapiens GN=KPNA1 PE=2 SV=1	60955	435	320	91	111	0.17	0.11	0.647059	3	2	3	2	4	2	4	3	-0.44208
tr|F5H0C8|F5H0C8_HUMAN	ENO2	Enolase OS=Homo sapiens GN=ENO2 PE=2 SV=1	35026	229	548	174	67	0.31	0.2	0.645161	3	1	5	3	7	3	7	4	-0.69395
tr|B2RDV7|B2RDV7_HUMAN	tr|B2RDV7|B2RDV	cDNA, FLJ96789, highly similar to Homo sapiens dihydrouridine synthase 3-like (S. ce	73714	440	343	90	104	0.14	0.09	0.642857	2	2	3	2	3	2	3	2	-0.07931
sp|P45974|UBP5_HUMAN	USP5	Ubiquitin carboxyl-terminal hydrolase 5 OS=Homo sapiens GN=USP5 PE=1 SV=2	96638	426	481	94	76	0.11	0.07	0.636364	2	1	3	2	4	2	4	2	-0.44208
tr|B4E1L0|B4E1L0_HUMAN	tr|B4E1L0|B4E1L0	Adenylosuccinate synthetase OS=Homo sapiens PE=2 SV=1	48308	399	394	101	95	0.22	0.14	0.636364	1	1	3	2	4	2	5	3	-0.44208
sp|P20700|LMNB1_HUMAN	LMNB1	Lamin-B1 OS=Homo sapiens GN=LMNB1 PE=1 SV=2	66653	381	131	107	252	0.33	0.21	0.636364	5	4	6	5	6	6	7	8	0.406562
tr|Q53HE2|Q53HE2_HUMAN	tr|Q53HE2|Q53HE	Triosephosphate isomerase (Fragment) OS=Homo sapiens PE=2 SV=1	26981	297	313	133	104	0.79	0.5	0.632911	3	2	5	3	4	4	5	4	0.406434
tr|Q6FHF5|Q6FHF5_HUMAN	PCNA	Proliferating cell nuclear antigen (Fragment) OS=Homo sapiens GN=PCNA PE=2 SV=1	29029	487	578	79	63	0.38	0.24	0.631579	2	2	3	2	3	4	5	4	0.7692
tr|Q6IAX5|Q6IAX5_HUMAN	EIF3S6	Eukaryotic translation initiation factor 3 subunit E OS=Homo sapiens GN=EIF3S6 PE=	52572	210	135	186	242	0.84	0.53	0.630952	6	4	10	7	12	7	13	10	-0.33132
tr|B7ZKM0|B7ZKM0_HUMAN	SART3	SART3 protein OS=Homo sapiens GN=SART3 PE=2 SV=1	106372	171	216	225	158	0.27	0.17	0.62963	8	3	8	3	9	4	11	4	-0.67255
tr|A8K3K1|A8K3K1_HUMAN	tr|A8K3K1|A8K3K1	cDNA FLJ78096, highly similar to Homo sapiens actin, alpha, cardiac muscle (ACTC), r	42362	65	126	516	259	2.32	1.46	0.62931	19	10	27	18	30	23	32	23	0.030126
tr|B5BTY4|B5BTY4_HUMAN	DDX3X	ATP-dependent RNA helicase DDX3X OS=Homo sapiens GN=DDX3X PE=2 SV=1	73525	60	119	582	280	1.1	0.69	0.627273	16	10	19	13	18	15	21	16	0.151291
sp|Q9BYG3|MK67I_HUMAN	MKI67IP	MKI67 FHA domain-interacting nucleolar phosphoprotein OS=Homo sapiens GN=MK	34372	599	610	61	58	0.32	0.2	0.625	1	1	3	2	2	2	3	2	0.406307
sp|P78371|TCPB_HUMAN	CCT2	T-complex protein 1 subunit beta OS=Homo sapiens GN=CCT2 PE=1 SV=4	57794	29	32	936	852	2.77	1.71	0.617329	22	18	29	25	32	26	34	34	0.112214
tr|B2R659|B2R659_HUMAN	tr|B2R659|B2R659	cDNA, FLJ92803, highly similar to Homo sapiens hydroxysteroid (17-beta) dehydroge	80018	395	598	103	60	0.13	0.08	0.615385	2	2	3	2	3	2	8	3	-0.07931
tr|E7EN95|E7EN95_HUMAN	FLNB	Filamin-B OS=Homo sapiens GN=FLNB PE=2 SV=1	258059	192	185	209	185	0.13	0.08	0.615385	5	5	11	6	14	9	17	9	-0.20428
tr|B4DLT2|B4DLT2_HUMAN	tr|B4DLT2|B4DLT2	cDNA FLJ56637, highly similar to Nuclear pore complex protein Nup155 OS=Homo sa	152734	153	168	263	207	0.26	0.16	0.615385	6	6	11	7	11	7	11	8	-0.21083
tr|B2R5W3|B2R5W3_HUMAN	tr|B2R5W3|B2R5V	cDNA, FLJ92658, highly similar to Homo sapiens poly (ADP-ribose) polymerase family	113813	89	129	411	254	0.49	0.3	0.612245	13	8	15	9	16	10	17	12	-0.24643
sp|P16152|CBR1_HUMAN	CBR1	Carbonyl reductase [NADPH] 1 OS=Homo sapiens GN=CBR1 PE=1 SV=3	30641	508	1122	75	19	0.18	0.11	0.611111	1	0	1	1	1	0	1	1	-1.17898
tr|H0Y4Z6|H0Y4Z6_HUMAN	PYGB	Phosphorylase (Fragment) OS=Homo sapiens GN=PYGB PE=3 SV=1	27767	388	560	21	35	0.23	0.14	0.608696	0	0	1	1	1	2	1	2	1.143468
tr|Q6NZ52|Q6NZ52_HUMAN	RPL27A	Ribosomal protein L27a OS=Homo sapiens GN=RPL27A PE=2 SV=1	16582	560	944	67	30	0.75	0.45	0.6	1	0	3	2	3	2	3	3	-0.07931
sp|Q7KZ85|SPT6H_HUMAN	SUPT6H	Transcription elongation factor SPT6 OS=Homo sapiens GN=SUPT6H PE=1 SV=2	200203	630	374	58	99	0.05	0.03	0.6	1	1	3	2	3	2	6	3	-0.07931
sp|P42166|LAP2A_HUMAN	TMPO	Lamina-associated polypeptide 2, isoform alpha OS=Homo sapiens GN=TMPO PE=1 S	76016	233	312	170	115	0.4	0.24	0.6	4	3	9	6	9	6	11	10	-0.14151
tr|G5E988|G5E988_HUMAN	AP3D1	AP-3 complex subunit delta-1 OS=Homo sapiens GN=AP3D1 PE=2 SV=1	126074	684	1062	53	23	0.05	0.03	0.6	1	0	2	1	2	1	3	2	-0.33092
tr|Q0P607|Q0P607_HUMAN	SFRS15	Splicing factor, arginine/serine-rich 15 OS=Homo sapiens GN=SFRS15 PE=2 SV=1	126004	859	942	39	30	0.05	0.03	0.6	0	0	2	1	2	1	2	1	-0.33092
tr|B4DK32|B4DK32_HUMAN	tr|B4DK32|B4DK32	cDNA FLJ61095, highly similar to Ubiquitin conjugation factor E4 A (Fragment) OS=H	120816	702	784	52	42	0.05	0.03	0.6	1	1	2	1	2	1	2	1	-0.33092
tr|K7EP06|K7EP06_HUMAN	RNMT	mRNA cap guanine-N7 methyltransferase OS=Homo sapiens GN=RNMT PE=4 SV=1	35330	930	693	34	51	0.2	0.12	0.6	0	1	2	1	2	1	2	1	-0.33092
tr|B2R6H7|B2R6H7_HUMAN	tr|B2R6H7|B2R6H	cDNA, FLJ92955, highly similar to Homo sapiens transportin-SR (TRN-SR), mRNA OS=	105989	461	791	84	41	0.1	0.06	0.6	1	0	3	2	4	2	5	3	-0.44208
tr|Q59GY2|Q59GY2_HUMAN	tr|Q59GY2|Q59GY	Ribosomal protein L4 variant (Fragment) OS=Homo sapiens PE=2 SV=1	49307	143	106	276	303	1.32	0.79	0.598485	8	8	13	10	15	14	18	16	0.31066
sp|P62424|RL7A_HUMAN	RPL7A	60S ribosomal protein L7a OS=Homo sapiens GN=RPL7A PE=1 SV=2	30148	283	349	140	103	0.87	0.52	0.597701	3	2	6	4	5	4	6	4	0.116732
tr|Q59GS3|Q59GS3_HUMAN	tr|Q59GS3|Q59GS	Proteasome 26S ATPase subunit 5 variant (Fragment) OS=Homo sapiens PE=2 SV=1	38907	309	397	129	85	0.39	0.23	0.589744	3	1	4	2	4	2	4	3	-0.44208
tr|B3KPS9|B3KPS9_HUMAN	tr|B3KPS9|B3KPS9	cDNA FLJ32140 fis, clone PLACE5000058, highly similar to Importin-4 OS=Homo sapi	104593	436	332	91	108	0.17	0.1	0.588235	3	2	5	3	6	3	7	3	-0.4873
tr|J3KTA4|J3KTA4_HUMAN	DDX5	Probable ATP-dependent RNA helicase DDX5 OS=Homo sapiens GN=DDX5 PE=3 SV=1	69557	88	93	412	330	1.4	0.82	0.585714	15	9	21	14	23	16	28	16	-0.10437
sp|O94776|MTA2_HUMAN	MTA2	Metastasis-associated protein MTA2 OS=Homo sapiens GN=MTA2 PE=1 SV=1	75717	216	278	182	130	0.24	0.14	0.583333	4	2	5	3	5	3	5	4	-0.2461
tr|Q2VPA0|Q2VPA0_HUMAN	LONP1	Lon protease homolog (Fragment) OS=Homo sapiens GN=LONP1 PE=2 SV=1	95436	211	640	185	55	0.31	0.18	0.580645	5	2	8	5	8	5	10	7	-0.22212
sp|P15121|ALDR_HUMAN	AKR1B1	Aldose reductase OS=Homo sapiens GN=AKR1B1 PE=1 SV=3	36230	593	603	62	59	0.19	0.11	0.578947	2	1	2	1	2	1	2	1	-0.33092
sp|Q96PK6|RBM14_HUMAN	RBM14	RNA-binding protein 14 OS=Homo sapiens GN=RBM14 PE=1 SV=2	69620	177	169	222	206	0.45	0.26	0.577778	3	4	8	5	9	5	9	6	-0.38278
tr|G3V2B8|G3V2B8_HUMAN	MTHFD1	C-1-tetrahydrofolate synthase, cytoplasmic OS=Homo sapiens GN=MTHFD1 PE=2 SV	102152	107	132	351	250	0.71	0.41	0.577465	13	7	18	11	22	11	24	13	-0.56356
sp|P22234|PUR6_HUMAN	PAICS	Multifunctional protein ADE2 OS=Homo sapiens GN=PAICS PE=1 SV=3	47790	123	305	330	117	1.08	0.62	0.574074	7	5	11	7	13	7	16	10	-0.44254
tr|Q3B7A7|Q3B7A7_HUMAN	GART	Phosphoribosylglycinamide formyltransferase, phosphoribosylglycinamide synthetas	108909	50	49	653	551	0.98	0.56	0.571429	14	8	23	16	24	16	30	20	-0.16468
sp|O00232|PSD12_HUMAN	PSMD12	26S proteasome non-ATPase regulatory subunit 12 OS=Homo sapiens GN=PSMD12 P	53270	307	429	130	84	0.35	0.2	0.571429	3	2	5	3	5	3	7	3	-0.2461
sp|P22059|OSBP1_HUMAN	OSBP	Oxysterol-binding protein 1 OS=Homo sapiens GN=OSBP PE=1 SV=1	90220	850	1104	40	21	0.07	0.04	0.571429	1	0	2	1	1	1	2	2	0.406244
sp|A5YKK6|CNOT1_HUMAN	CNOT1	CCR4-NOT transcription complex subunit 1 OS=Homo sapiens GN=CNOT1 PE=1 SV=2	269106	389	605	105	59	0.07	0.04	0.571429	4	1	6	3	4	3	7	4	0.043605
sp|P53004|BIEA_HUMAN	BLVRA	Biliverdin reductase A OS=Homo sapiens GN=BLVRA PE=1 SV=2	33692	207	307	189	116	1.33	0.76	0.571429	5	5	10	6	10	6	12	8	-0.2861
tr|E9PDG5|E9PDG5_HUMAN	LARP4	La-related protein 4 OS=Homo sapiens GN=LARP4 PE=2 SV=1	50319	802	432	43	77	0.14	0.08	0.571429	1	1	2	1	2	1	2	1	-0.33092
sp|P49792|RBP2_HUMAN	RANBP2	E3 SUMO-protein ligase RanBP2 OS=Homo sapiens GN=RANBP2 PE=1 SV=2	362365	367	294	111	122	0.07	0.04	0.571429	4	2	8	4	8	4	11	5	-0.51189
sp|Q6S8J3|POTEE_HUMAN	POTEE	POTE ankyrin domain family member E OS=Homo sapiens GN=POTEE PE=1 SV=3	122882	43	28	714	956	0.41	0.23	0.560976	17	22	28	41	28	42	31	45	0.988112
sp|P22314|UBA1_HUMAN	UBA1	Ubiquitin-like modifier-activating enzyme 1 OS=Homo sapiens GN=UBA1 PE=1 SV=3	118858	51	59	636	487	0.82	0.46	0.560976	17	10	24	16	23	16	27	21	-0.10437
tr|D3DRX6|D3DRX6_HUMAN	KIF5B	Kinesin family member 5B, isoform CRA_a OS=Homo sapiens GN=KIF5B PE=3 SV=1	110331	168	182	229	192	0.34	0.19	0.558824	7	4	10	6	12	7	14	8	-0.33132
tr|H0Y3P2|H0Y3P2_HUMAN	EIF4G2	Eukaryotic translation initiation factor 4 gamma 2 OS=Homo sapiens GN=EIF4G2 PE=	98569	421	473	96	77	0.18	0.1	0.555556	2	2	5	3	5	4	8	4	0.116732
tr|F5GX68|F5GX68_HUMAN	DNMT1	Cytosine-specific methyltransferase OS=Homo sapiens GN=DNMT1 PE=2 SV=1	185605	382	433	107	83	0.09	0.05	0.555556	3	1	5	3	6	4	9	5	-0.12447
tr|Q6IBT3|Q6IBT3_HUMAN	CCT7	CCT7 protein OS=Homo sapiens GN=CCT7 PE=2 SV=1	59804	34	76	901	408	1.62	0.9	0.555556	18	12	23	13	23	13	24	15	-0.39465
tr|B4DUA2|B4DUA2_HUMAN	tr|B4DUA2|B4DUA	Proliferating cell nuclear antigen OS=Homo sapiens PE=2 SV=1	16275	487	712	46	48	0.38	0.21	0.552632	1	1	2	1	2	1	3	2	-0.33092
tr|Q1L838|Q1L838_HUMAN	GTBP	GTBP-ALT OS=Homo sapiens GN=GTBP PE=2 SV=1	122267	212	303	184	117	0.2	0.11	0.55	5	3	7	4	9	6	10	6	-0.14151
sp|P61158|ARP3_HUMAN	ACTR3	Actin-related protein 3 OS=Homo sapiens GN=ACTR3 PE=1 SV=3	47797	411	488	100	76	0.4	0.22	0.55	2	2	5	3	6	3	6	3	-0.4873
sp|P13929|ENOB_HUMAN	ENO3	Beta-enolase OS=Homo sapiens GN=ENO3 PE=1 SV=5	47299	144	230	275	149	0.4	0.22	0.55	5	2	7	4	8	4	8	5	-0.51189
tr|J3KR24|J3KR24_HUMAN	IARS	Isoleucine--tRNA ligase, cytoplasmic OS=Homo sapiens GN=IARS PE=4 SV=1	132820	151	232	266	149	0.31	0.17	0.548387	6	3	11	7	16	7	18	8	-0.73264
sp|P52272|HNRPM_HUMAN	HNRNPM	Heterogeneous nuclear ribonucleoprotein M OS=Homo sapiens GN=HNRNPM PE=1 S	77749	37	46	862	608	2.18	1.19	0.545872	26	18	34	22	39	25	44	30	-0.22633
tr|K7EIS0|K7EIS0_HUMAN	TUBG1	Tubulin gamma chain (Fragment) OS=Homo sapiens GN=TUBG1 PE=3 SV=1	29797	1129	720	22	0	0.11	0.06	0.545455	0	0	1	0	1	1	1	1	0.406244
sp|Q14204|DYHC1_HUMAN	DYNC1H1	Cytoplasmic dynein 1 heavy chain 1 OS=Homo sapiens GN=DYNC1H1 PE=1 SV=5	534809	44	78	711	396	0.22	0.12	0.545455	23	12	33	19	39	22	48	28	-0.40768
tr|B4DTA2|B4DTA2_HUMAN	tr|B4DTA2|B4DTA	cDNA FLJ60148, highly similar to Homo sapiens heterogeneous nuclear ribonucleopr	30367	276	480	144	76	0.68	0.37	0.544118	4	2	6	3	7	3	7	3	-0.69395
sp|O14654|IRS4_HUMAN	IRS4	Insulin receptor substrate 4 OS=Homo sapiens GN=IRS4 PE=1 SV=1	134711	133	214	287	159	0.24	0.13	0.541667	8	3	9	7	9	9	10	12	0.406753
tr|H0Y8G5|H0Y8G5_HUMAN	HNRNPD	Heterogeneous nuclear ribonucleoprotein D0 (Fragment) OS=Homo sapiens GN=HN	29877	266	446	150	81	0.7	0.37	0.528571	4	2	6	3	7	3	8	3	-0.69395
sp|Q1KMD3|HNRL2_HUMAN	HNRNPUL2	Heterogeneous nuclear ribonucleoprotein U-like protein 2 OS=Homo sapiens GN=HN	85622	228	248	174	142	0.4	0.21	0.525	6	4	9	5	8	6	10	7	0.019148
tr|A8MUD9|A8MUD9_HUMAN	RPL7	60S ribosomal protein L7 OS=Homo sapiens GN=RPL7 PE=3 SV=1	24474	317	345	127	104	0.9	0.47	0.522222	4	3	5	3	7	3	7	5	-0.69395
sp|P13639|EF2_HUMAN	EEF2	Elongation factor 2 OS=Homo sapiens GN=EEF2 PE=1 SV=4	96246	1	6	2167	1655	6.45	3.36	0.52093	54	48	92	65	95	67	111	80	-0.09566
tr|Q562M3|Q562M3_HUMAN	ACT	Actin-like protein (Fragment) OS=Homo sapiens GN=ACT PE=2 SV=1	11529	161	148	239	223	5.21	2.69	0.516315	9	8	16	15	20	18	24	19	0.258836
tr|B7ZAK1|B7ZAK1_HUMAN	tr|B7ZAK1|B7ZAK1	cDNA, FLJ79215, highly similar to Tubulin beta-7 chain OS=Homo sapiens PE=2 SV=1	42054	2	13	1556	1336	15.54	7.96	0.512227	52	42	63	51	66	53	77	60	0.093199
sp|Q00610|CLH1_HUMAN	CLTC	Clathrin heavy chain 1 OS=Homo sapiens GN=CLTC PE=1 SV=5	193260	25	38	1034	714	1.02	0.52	0.509804	24	19	42	26	44	28	50	33	-0.23802
tr|B3KX96|B3KX96_HUMAN	tr|B3KX96|B3KX96	cDNA FLJ45003 fis, clone BRAWH3011623, highly similar to Heterogeneous nuclear r	32368	255	80	158	341	1.18	0.6	0.508475	6	7	8	8	7	8	8	8	0.587457
sp|P38919|IF4A3_HUMAN	EIF4A3	Eukaryotic initiation factor 4A-III OS=Homo sapiens GN=EIF4A3 PE=1 SV=4	47126	152	175	265	199	0.61	0.31	0.508197	7	4	8	5	9	8	9	9	0.246029
sp|Q15366|PCBP2_HUMAN	PCBP2	Poly(rC)-binding protein 2 OS=Homo sapiens GN=PCBP2 PE=1 SV=1	38955	132	102	289	313	1.26	0.64	0.507937	6	6	10	6	10	6	11	7	-0.2861
tr|Q59GF8|Q59GF8_HUMAN	tr|Q59GF8|Q59GF	Heat shock 70kDa protein 4 isoform a variant (Fragment) OS=Homo sapiens PE=2 SV	88805	53	67	626	444	1.3	0.66	0.507692	15	11	23	15	24	16	25	20	-0.16468
tr|B0YIW5|B0YIW5_HUMAN	ARCN1	Archain 1, isoform CRA_b OS=Homo sapiens GN=ARCN1 PE=2 SV=1	57630	825	849	41	36	0.12	0.06	0.5	1	0	2	1	2	2	4	2	0.406307
tr|B7Z4U7|B7Z4U7_HUMAN	SCFD1	Sec1 family domain containing 1, isoform CRA_b OS=Homo sapiens GN=SCFD1 PE=2	65362	516	654	74	54	0.1	0.05	0.5	1	1	2	1	1	1	2	2	0.406244
tr|B2RNR6|B2RNR6_HUMAN	ZFR	Zinc finger RNA binding protein OS=Homo sapiens GN=ZFR PE=2 SV=1	118081	326	206	125	172	0.12	0.06	0.5	2	2	4	2	4	3	4	3	0.043605
tr|B0QY89|B0QY89_HUMAN	EIF3EIP	Eukaryotic translation initiation factor 3 subunit L OS=Homo sapiens GN=EIF3EIP PE=	71085	114	150	345	222	0.5	0.25	0.5	9	4	9	6	11	7	11	7	-0.21083
tr|B4DHG3|B4DHG3_HUMAN	tr|B4DHG3|B4DHG	cDNA FLJ55329, highly similar to Procollagen-lysine,2-oxoglutarate5-dioxygenase 2 (	81716	444	863	89	35	0.08	0.04	0.5	1	0	2	1	2	1	2	1	-0.33092
sp|A0AVT1|UBA6_HUMAN	UBA6	Ubiquitin-like modifier-activating enzyme 6 OS=Homo sapiens GN=UBA6 PE=1 SV=1	119207	721	606	50	59	0.06	0.03	0.5	2	1	2	1	2	1	2	1	-0.33092
tr|A8KA19|A8KA19_HUMAN	tr|A8KA19|A8KA19	cDNA FLJ75831, highly similar to Homo sapiens exportin, tRNA (nuclear export recep	111104	762	586	47	62	0.06	0.03	0.5	1	1	2	1	2	1	4	2	-0.33092
sp|Q9UID3|VPS51_HUMAN	VPS51	Vacuolar protein sorting-associated protein 51 homolog OS=Homo sapiens GN=VPS5	86901	494	529	78	69	0.08	0.04	0.5	1	1	2	1	2	1	2	1	-0.33092
tr|B0QYK0|B0QYK0_HUMAN	EWSR1	Ewing sarcoma breakpoint region 1 OS=Homo sapiens GN=EWSR1 PE=4 SV=1	65174	776	527	45	69	0.1	0.05	0.5	1	1	2	1	2	1	2	2	-0.33092
tr|B2RAH7|B2RAH7_HUMAN	tr|B2RAH7|B2RAH	cDNA, FLJ94921, highly similar to Homo sapiens prolyl endopeptidase (PREP), mRNA	81594	928	383	35	97	0.08	0.04	0.5	0	1	2	1	2	1	2	1	-0.33092
tr|Q4LE64|Q4LE64_HUMAN	NUMA1	NUMA1 variant protein (Fragment) OS=Homo sapiens GN=NUMA1 variant protein P	239798	253	887	160	34	0.06	0.03	0.5	4	0	4	1	4	2	5	3	-0.44208
sp|Q08J23|NSUN2_HUMAN	NSUN2	tRNA (cytosine(34)-C(5))-methyltransferase OS=Homo sapiens GN=NSUN2 PE=1 SV=	87214	290	434	136	83	0.16	0.08	0.5	2	1	4	2	4	2	5	3	-0.44208
tr|Q8N5A0|Q8N5A0_HUMAN	EIF5B	Eukaryotic translation initiation factor 5B OS=Homo sapiens GN=EIF5B PE=2 SV=1	139172	304	250	130	141	0.2	0.1	0.5	4	4	8	4	8	4	8	5	-0.51189
tr|B2RCS5|B2RCS5_HUMAN	ACTN2	Actinin, alpha 2, isoform CRA_b OS=Homo sapiens GN=ACTN2 PE=2 SV=1	104425	298	306	133	117	0.2	0.1	0.5	4	2	6	3	7	3	7	5	-0.69395
sp|Q14684|RRP1B_HUMAN	RRP1B	Ribosomal RNA processing protein 1 homolog B OS=Homo sapiens GN=RRP1B PE=1 S	84774	375	631	108	56	0.16	0.08	0.5	3	1	4	2	5	2	6	2	-0.73178
tr|D3DWB6|D3DWB6_HUMAN	USP9X	Ubiquitin carboxyl-terminal hydrolase OS=Homo sapiens GN=USP9X PE=2 SV=1	274331	554	415	67	87	0.04	0.02	0.5	2	1	3	2	5	2	5	4	-0.73178
tr|B2ZZ90|B2ZZ90_HUMAN	ACACA	Acetyl-Coenzyme A carboxylase alpha OS=Homo sapiens GN=ACACA PE=2 SV=1	267095	817	933	42	30	0.04	0.02	0.5	0	0	3	2	3	1	4	3	-0.81654
sp|Q9UI26|IPO11_HUMAN	IPO11	Importin-11 OS=Homo sapiens GN=IPO11 PE=1 SV=1	113489	899	788	36	42	0.06	0.03	0.5	0	1	2	1	3	1	4	2	-0.81654
sp|Q5T4S7|UBR4_HUMAN	UBR4	E3 ubiquitin-protein ligase UBR4 OS=Homo sapiens GN=UBR4 PE=1 SV=1	580547	619	786	59	42	0.02	0.01	0.5	2	1	4	2	3	1	5	2	-0.81654
tr|A2A3R6|A2A3R6_HUMAN	RPS6	40S ribosomal protein S6 OS=Homo sapiens GN=RPS6 PE=2 SV=1	28834	457	741	84	46	0.24	0.12	0.5	2	1	2	1	3	1	4	2	-0.81654
tr|E9KL44|E9KL44_HUMAN	tr|E9KL44|E9KL44	Epididymis tissue sperm binding protein Li 14m OS=Homo sapiens PE=2 SV=1	83688	167	249	231	142	0.26	0.13	0.5	5	2	6	3	8	3	9	3	-0.87471
sp|P11388|TOP2A_HUMAN	TOP2A	DNA topoisomerase 2-alpha OS=Homo sapiens GN=TOP2A PE=1 SV=3	175017	706	1123	51	18	0.04	0.02	0.5	1	0	2	1	2	0	3	2	-1.91614
tr|Q5JR95|Q5JR95_HUMAN	RPS8	40S ribosomal protein S8 OS=Homo sapiens GN=RPS8 PE=4 SV=1	22094	215	738	142	46	0.67	0.33	0.492537	4	1	4	2	5	2	6	3	-0.73178
sp|O00425|IF2B3_HUMAN	IGF2BP3	Insulin-like growth factor 2 mRNA-binding protein 3 OS=Homo sapiens GN=IGF2BP3	64008	173	190	224	183	0.57	0.28	0.491228	8	5	10	5	9	5	10	5	-0.38278
tr|B2RBR9|B2RBR9_HUMAN	tr|B2RBR9|B2RBR	cDNA, FLJ95650, highly similar to Homo sapiens karyopherin (importin) beta 1 (KPNE	98434	54	77	623	398	0.99	0.48	0.484848	14	9	21	13	24	13	26	16	-0.45497
tr|H0YK49|H0YK49_HUMAN	ETFA	Electron transfer flavoprotein subunit alpha, mitochondrial OS=Homo sapiens GN=ET	24259	748	991	48	27	0.29	0.14	0.482759	1	0	2	1	1	1	2	1	0.406244
tr|B4DKM5|B4DKM5_HUMAN	VDAC2	Voltage-dependent anion-selective channel protein 2 OS=Homo sapiens GN=VDAC2	27804	709	562	51	65	0.25	0.12	0.48	1	1	2	1	3	1	3	1	-0.81654
sp|P40222|TXLNA_HUMAN	TXLNA	Alpha-taxilin OS=Homo sapiens GN=TXLNA PE=1 SV=3	62195	301	273	132	131	0.23	0.11	0.478261	2	2	4	2	4	2	5	2	-0.44208
tr|A6NKB8|A6NKB8_HUMAN	RNPEP	Aminopeptidase B OS=Homo sapiens GN=RNPEP PE=2 SV=1	68752	502	354	76	102	0.21	0.1	0.47619	1	2	4	2	3	3	4	3	0.406371
tr|B2RCV1|B2RCV1_HUMAN	tr|B2RCV1|B2RCV	cDNA, FLJ96310, highly similar to Homo sapiens solute carrier family 25 (mitochondr	33062	632	955	57	29	0.21	0.1	0.47619	1	0	2	1	3	1	3	1	-0.81654
sp|O43707|ACTN4_HUMAN	ACTN4	Alpha-actinin-4 OS=Homo sapiens GN=ACTN4 PE=1 SV=2	105245	41	47	729	594	1.43	0.68	0.475524	19	13	29	17	31	22	34	28	-0.07961
sp|P53396|ACLY_HUMAN	ACLY	ATP-citrate synthase OS=Homo sapiens GN=ACLY PE=1 SV=3	121674	20	39	1097	707	1.53	0.72	0.470588	30	20	44	25	46	25	56	32	-0.46308
tr|Q5T081|Q5T081_HUMAN	RCC1	CHC1 protein OS=Homo sapiens GN=RCC1 PE=2 SV=1	45397	497	403	77	90	0.32	0.15	0.46875	3	2	4	2	4	2	4	2	-0.44208
tr|A8K7N0|A8K7N0_HUMAN	tr|A8K7N0|A8K7N	cDNA FLJ75556, highly similar to Homo sapiens ribosomal protein L14, mRNA OS=Ho	23745	705	764	51	44	0.3	0.14	0.466667	1	1	2	1	2	1	2	1	-0.33092
tr|D2CFK9|D2CFK9_HUMAN	GTPBP4	Nucleolar GTP-binding protein 1 OS=Homo sapiens GN=GTPBP4 PE=2 SV=1	74317	355	388	115	95	0.3	0.14	0.466667	3	2	6	3	6	3	7	3	-0.4873
sp|Q96SI9|STRBP_HUMAN	STRBP	Spermatid perinuclear RNA-binding protein OS=Homo sapiens GN=STRBP PE=1 SV=1	74290	279	447	142	80	0.3	0.14	0.466667	5	3	8	3	8	4	11	5	-0.51189
tr|Q5QTS3|Q5QTS3_HUMAN	tr|Q5QTS3|Q5QTS	FWP004 OS=Homo sapiens PE=2 SV=1	23704	843	1090	40	21	0.3	0.14	0.466667	1	0	2	1	3	1	3	2	-0.81654
tr|B4DLR3|B4DLR3_HUMAN	tr|B4DLR3|B4DLR3	cDNA FLJ54020, highly similar to Heterogeneous nuclear ribonucleoprotein U OS=Ho	87548	12	36	1423	757	2.49	1.16	0.465863	37	23	54	29	56	28	61	34	-0.58482
tr|C9IZE4|C9IZE4_HUMAN	PSMD6	26S proteasome non-ATPase regulatory subunit 6 OS=Homo sapiens GN=PSMD6 PE=	52346	341	772	118	43	0.28	0.13	0.464286	3	1	4	2	3	1	5	4	-0.81654
tr|B2R5P6|B2R5P6_HUMAN	TXNRD1	Thioredoxin reductase 1, cytoplasmic OS=Homo sapiens GN=TXNRD1 PE=2 SV=1	55253	206	264	190	134	0.41	0.19	0.463415	6	2	6	3	6	3	7	3	-0.4873
tr|Q53XC0|Q53XC0_HUMAN	EIF2S1	Eukaryotic translation initiation factor 2, subunit 1 alpha, 35kDa, isoform CRA_a OS=	36374	130	265	292	134	1.19	0.55	0.462185	7	3	9	5	9	7	10	7	0.065197
tr|B4E3M6|B4E3M6_HUMAN	tr|B4E3M6|B4E3M	cDNA FLJ55446, highly similar to Superkiller viralicidic activity 2-like 2 (EC 3.6.1.-) OS	107714	575	970	64	28	0.13	0.06	0.461538	0	0	4	2	5	3	6	4	-0.2461
tr|Q0VAB1|Q0VAB1_HUMAN	TIMM50	Translocase of inner mitochondrial membrane 50 homolog (S. cerevisiae) OS=Homo	50960	369	815	110	39	0.13	0.06	0.461538	2	1	2	1	2	1	2	1	-0.33092
tr|J3KMX2|J3KMX2_HUMAN	SMARCD2	SWI/SNF-related matrix-associated actin-dependent regulator of chromatin subfami	52433	413	414	100	87	0.13	0.06	0.461538	1	1	2	1	2	1	2	1	-0.33092
sp|P02533|K1C14_HUMAN	KRT14	Keratin, type I cytoskeletal 14 OS=Homo sapiens GN=KRT14 PE=1 SV=4	51872	729	795	49	41	0.13	0.06	0.461538	2	1	2	1	3	1	4	1	-0.81654
tr|B3KTJ9|B3KTJ9_HUMAN	tr|B3KTJ9|B3KTJ9	cDNA FLJ38393 fis, clone FEBRA2007212 OS=Homo sapiens PE=2 SV=1	103493	131	238	290	147	0.37	0.17	0.459459	6	3	10	5	10	5	10	7	-0.52737
tr|F8VPD4|F8VPD4_HUMAN	CAD	CAD protein OS=Homo sapiens GN=CAD PE=2 SV=1	238097	82	91	433	334	0.35	0.16	0.457143	11	8	22	12	22	13	27	17	-0.33172
sp|P35998|PRS7_HUMAN	PSMC2	26S protease regulatory subunit 7 OS=Homo sapiens GN=PSMC2 PE=1 SV=3	49002	372	298	109	121	0.22	0.1	0.454545	3	2	4	2	4	3	5	4	0.043605
tr|D0PQI2|D0PQI2_HUMAN	ENAH	Enabled-like protein variant hMenaDv6 OS=Homo sapiens GN=ENAH PE=2 SV=1	60426	808	694	43	50	0.11	0.05	0.454545	1	1	2	1	2	1	3	2	-0.33092
sp|Q69YN2|C19L1_HUMAN	CWF19L1	CWF19-like protein 1 OS=Homo sapiens GN=CWF19L1 PE=1 SV=2	61322	624	549	59	67	0.11	0.05	0.454545	2	1	2	1	2	1	2	1	-0.33092
sp|Q9H3P7|GCP60_HUMAN	ACBD3	Golgi resident protein GCP60 OS=Homo sapiens GN=ACBD3 PE=1 SV=4	60841	417	406	98	88	0.11	0.05	0.454545	2	1	2	1	2	1	2	1	-0.33092
tr|B2RU06|B2RU06_HUMAN	EIF4G1	EIF4G1 protein OS=Homo sapiens GN=EIF4G1 PE=2 SV=1	176807	103	208	357	168	0.22	0.1	0.454545	8	4	11	5	12	5	16	9	-0.77929
tr|Q60FE2|Q60FE2_HUMAN	MYH9	MYH9 variant protein OS=Homo sapiens GN=MYH9 PE=2 SV=1	227646	33	73	911	418	0.53	0.24	0.45283	22	13	31	15	38	17	44	20	-0.73436
tr|H0YCK7|H0YCK7_HUMAN	EEF1D	Elongation factor 1-delta (Fragment) OS=Homo sapiens GN=EEF1D PE=4 SV=1	23105	286	176	138	197	0.31	0.14	0.451613	2	3	2	4	2	4	2	4	1.254822
tr|J3KTE4|J3KTE4_HUMAN	RPL19	Ribosomal protein L19 OS=Homo sapiens GN=RPL19 PE=3 SV=1	23347	349	1001	116	26	0.31	0.14	0.451613	2	0	2	1	2	1	3	1	-0.33092
sp|P14550|AK1A1_HUMAN	AKR1A1	Alcohol dehydrogenase [NADP(+)] OS=Homo sapiens GN=AKR1A1 PE=1 SV=3	36892	816	752	42	45	0.2	0.09	0.45	1	1	1	1	1	1	1	1	0.406244
tr|F2Z393|F2Z393_HUMAN	TALDO1	Transaldolase OS=Homo sapiens GN=TALDO1 PE=2 SV=1	35535	365	479	111	76	0.2	0.09	0.45	2	1	2	1	2	1	3	1	-0.33092
tr|Q6FHX6|Q6FHX6_HUMAN	FEN1	Flap endonuclease 1 OS=Homo sapiens GN=FEN1 PE=2 SV=1	42908	400	304	101	117	0.4	0.18	0.45	2	1	2	2	2	1	2	2	-0.33092
tr|F5H1Y3|F5H1Y3_HUMAN	FEN1	Flap endonuclease 1 (Fragment) OS=Homo sapiens GN=FEN1 PE=2 SV=1	18377	400	299	101	120	0.4	0.18	0.45	2	1	2	1	2	1	2	1	-0.33092
tr|J3KT29|J3KT29_HUMAN	RPL23	60S ribosomal protein L23 OS=Homo sapiens GN=RPL23 PE=3 SV=1	13241	489	426	79	84	0.58	0.26	0.448276	1	1	2	1	2	1	2	1	-0.33092
tr|B0V0F8|B0V0F8_HUMAN	DHX16	Putative pre-mRNA-splicing factor ATP-dependent RNA helicase DHX16 (Fragment) (	77228	772	558	45	65	0.09	0.04	0.444444	1	1	2	1	2	1	3	1	-0.33092
sp|P11177|ODPB_HUMAN	PDHB	Pyruvate dehydrogenase E1 component subunit beta, mitochondrial OS=Homo sapie	39550	573	1032	65	25	0.27	0.12	0.444444	2	0	3	1	3	1	4	1	-0.81654
tr|B3KRE0|B3KRE0_HUMAN	tr|B3KRE0|B3KRE0	cDNA FLJ34086 fis, clone FCBBF3005320, highly similar to Double-stranded RNA-bin	40204	423	790	95	28	0.27	0.12	0.444444	1	0	3	1	3	1	3	1	-0.81654
tr|B4E022|B4E022_HUMAN	TKT	Transketolase OS=Homo sapiens GN=TKT PE=2 SV=1	63410	201	198	197	177	0.66	0.29	0.439394	4	3	11	7	13	7	15	9	-0.44254

sp|Q9H853|TBA4B_HUMAN	TUBA4B	Putative tubulin-like protein alpha-4B OS=Homo sapiens GN=TUBA4B PE=5 SV=2	27819	205	336	191	107	0.57	0.25	0.438596	3	4	7	4	6	7	7	9	0.613272
tr|A8K8U1|A8K8U1_HUMAN	tr|A8K8U1|A8K8U	cDNA FLJ77762, highly similar to Homo sapiens cullin-associated and neddylation-di	137941	67	200	501	175	0.6	0.26	0.433333	15	6	22	10	24	11	25	14	-0.68681
sp|P60842|IF4A1_HUMAN	EIF4A1	Eukaryotic initiation factor 4A-I OS=Homo sapiens GN=EIF4A1 PE=1 SV=1	46353	23	30	1052	923	6.86	2.96	0.431487	30	22	40	30	43	34	46	37	0.072165
tr|B4DSK9|B4DSK9_HUMAN	tr|B4DSK9|B4DSK	cDNA FLJ60860, highly similar to Histone deacetylase 1 OS=Homo sapiens PE=2 SV=1	27596	300	367	45	40	0.28	0.12	0.428571	1	1	1	1	1	1	2	2	0.406244
tr|F6S0T5|F6S0T5_HUMAN	KDM1A	Lysine-specific histone demethylase 1A OS=Homo sapiens GN=KDM1A PE=2 SV=1	95677	724	985	50	27	0.07	0.03	0.428571	2	0	2	1	2	1	2	4	-0.33092
tr|D3DSQ7|D3DSQ7_HUMAN	XPO7	Exportin 7, isoform CRA_c OS=Homo sapiens GN=XPO7 PE=2 SV=1	100370	634	929	57	31	0.07	0.03	0.428571	1	0	2	1	2	1	3	1	-0.33092
tr|H3BQQ2|H3BQQ2_HUMA	ZNF598	Zinc finger protein 598 OS=Homo sapiens GN=ZNF598 PE=4 SV=1	94712	481	333	80	107	0.07	0.03	0.428571	2	1	2	1	2	1	2	1	-0.33092
tr|H6VRF8|H6VRF8_HUMAN	KRT1	Keratin 1 OS=Homo sapiens GN=KRT1 PE=3 SV=1	66184	31	100	917	316	1.64	0.7	0.426829	25	8	27	13	28	14	31	14	-0.57058
sp|Q08211|DHX9_HUMAN	DHX9	ATP-dependent RNA helicase A OS=Homo sapiens GN=DHX9 PE=1 SV=4	142181	27	55	964	533	1.43	0.61	0.426573	25	15	44	22	47	24	54	25	-0.55195
sp|O14980|XPO1_HUMAN	XPO1	Exportin-1 OS=Homo sapiens GN=XPO1 PE=1 SV=1	124447	166	380	234	97	0.33	0.14	0.424242	6	3	11	5	13	9	18	11	-0.10099
tr|Q1KLZ0|Q1KLZ0_HUMAN	PS1TP5BP1	HCG15971, isoform CRA_a OS=Homo sapiens GN=PS1TP5BP1 PE=2 SV=1	42052	6	1	1655	2029	11.13	4.69	0.421384	43	46	67	74	73	79	79	81	0.525654
tr|B4DUC5|B4DUC5_HUMAN	CSE1L	Exportin-2 OS=Homo sapiens GN=CSE1L PE=2 SV=1	86045	129	172	292	131	0.57	0.24	0.421053	4	2	12	5	12	5	14	5	-0.77929
sp|Q92973|TNPO1_HUMAN	TNPO1	Transportin-1 OS=Homo sapiens GN=TNPO1 PE=1 SV=2	103771	314	580	127	62	0.24	0.1	0.416667	3	2	7	3	7	4	9	6	-0.33112
tr|Q6IPS9|Q6IPS9_HUMAN	EEF1A1	Elongation factor 1-alpha OS=Homo sapiens GN=EEF1A1 PE=2 SV=1	50451	59	110	587	297	2.12	0.88	0.415094	18	7	28	10	31	10	39	13	-1.18226
tr|B2R5U1|B2R5U1_HUMAN	tr|B2R5U1|B2R5U	cDNA, FLJ92620, highly similar to Homo sapiens staphylococcal nuclease domain cor	100294	102	170	360	206	0.52	0.21	0.403846	9	6	14	6	14	6	15	10	-0.75254
tr|A0RZB8|A0RZB8_HUMAN	DIAPH1	Diaphanous-1 OS=Homo sapiens GN=DIAPH1 PE=2 SV=1	140767	813	1012	42	26	0.05	0.02	0.4	1	0	2	1	3	2	4	2	-0.07931
tr|Q5VZU9|Q5VZU9_HUMAN	TPP2	Tripeptidyl-peptidase 2 OS=Homo sapiens GN=TPP2 PE=4 SV=1	141160	811	399	43	91	0.05	0.02	0.4	1	1	2	1	2	1	3	2	-0.33092
sp|Q9ULT8|HECD1_HUMAN	HECTD1	E3 ubiquitin-protein ligase HECTD1 OS=Homo sapiens GN=HECTD1 PE=1 SV=3	292225	232	536	170	68	0.05	0.02	0.4	4	1	4	1	5	1	7	1	-1.46901
tr|B4DW52|B4DW52_HUMA	tr|B4DW52|B4DW	cDNA FLJ55253, highly similar to Actin, cytoplasmic 1 OS=Homo sapiens PE=2 SV=1	38950	10	2	1559	1963	12.58	5.01	0.398251	40	46	63	73	68	80	75	83	0.646576
sp|Q9BVP2|GNL3_HUMAN	GNL3	Guanine nucleotide-binding protein-like 3 OS=Homo sapiens GN=GNL3 PE=1 SV=2	62468	333	288	121	126	0.43	0.17	0.395349	3	3	7	3	7	3	8	5	-0.69395
tr|B0V043|B0V043_HUMAN	VARS	Valine--tRNA ligase OS=Homo sapiens GN=VARS PE=3 SV=1	141632	72	141	481	234	0.38	0.15	0.394737	11	5	16	7	16	9	17	12	-0.39108
tr|E1NZA1|E1NZA1_HUMAN	PRIC295	Peroxisome proliferator activated receptor interacting complex protein OS=Homo sa	294953	163	151	236	222	0.23	0.09	0.391304	7	7	19	8	20	10	25	12	-0.56037
sp|Q9NR30|DDX21_HUMAN	DDX21	Nucleolar RNA helicase 2 OS=Homo sapiens GN=DDX21 PE=1 SV=5	87804	58	174	591	203	1.41	0.55	0.390071	18	7	28	12	27	14	34	18	-0.51885
sp|Q96EK5|KBP_HUMAN	KIAA1279	KIF1-binding protein OS=Homo sapiens GN=KIAA1279 PE=1 SV=1	72453	181	396	219	94	0.36	0.14	0.388889	4	1	7	3	6	2	8	3	-0.97299
sp|O95373|IPO7_HUMAN	IPO7	Importin-7 OS=Homo sapiens GN=IPO7 PE=1 SV=1	120751	70	180	485	195	0.62	0.24	0.387097	14	5	18	8	19	8	21	9	-0.7934
tr|J3KPF3|J3KPF3_HUMAN	SLC3A2	4F2 cell-surface antigen heavy chain OS=Homo sapiens GN=SLC3A2 PE=4 SV=1	68230	245	440	164	81	0.26	0.1	0.384615	4	2	5	2	5	3	6	3	-0.2461
tr|B7TY16|B7TY16_HUMAN	ACTN1	Actinin alpha 1 isoform 3 OS=Homo sapiens GN=ACTN1 PE=2 SV=1	107644	109	137	349	240	0.52	0.2	0.384615	11	5	14	6	16	8	18	11	-0.55181
tr|Q6IBR0|Q6IBR0_HUMAN	RPN1	RPN1 protein OS=Homo sapiens GN=RPN1 PE=2 SV=1	68678	429	373	93	99	0.26	0.1	0.384615	3	2	5	2	5	2	7	3	-0.73178
tr|B7ZA86|B7ZA86_HUMAN	tr|B7ZA86|B7ZA8	cDNA, FLJ79100, highly similar to 14-3-3 protein epsilon (14-3-3E) OS=Homo sapiens	19138	227	234	175	148	1.65	0.63	0.381818	6	2	6	5	8	5	8	5	-0.22212
tr|D6RER5|D6RER5_HUMAN	Sep-11	Septin-11 OS=Homo sapiens GN=SEPT11 PE=2 SV=1	50119	441	627	89	56	0.21	0.08	0.380952	3	1	4	1	4	1	4	2	-1.1793
sp|P04181|OAT_HUMAN	OAT	Ornithine aminotransferase, mitochondrial OS=Homo sapiens GN=OAT PE=1 SV=1	48846	162	378	236	98	0.58	0.22	0.37931	7	2	7	3	7	4	7	4	-0.33112
tr|B4DF74|B4DF74_HUMAN	tr|B4DF74|B4DF7	cDNA FLJ61103, highly similar to CLIP-associating protein 2 OS=Homo sapiens PE=2 S	23515	730	308	0	97	0.16	0.06	0.375	0	2	0	2	0	2	1	2	2.728626
tr|B4DH29|B4DH29_HUMAN	tr|B4DH29|B4DH2	DNA-directed RNA polymerase OS=Homo sapiens PE=2 SV=1	126564	499	1101	77	21	0.08	0.03	0.375	2	0	3	1	2	2	5	2	0.406307
tr|F5GWX5|F5GWX5_HUMA	CHD4	Chromodomain-helicase-DNA-binding protein 4 OS=Homo sapiens GN=CHD4 PE=2 S	218449	150	377	268	98	0.16	0.06	0.375	7	2	10	4	9	5	12	7	-0.38278
tr|B4DZC0|B4DZC0_HUMAN	tr|B4DZC0|B4DZC	cDNA FLJ51771, highly similar to SWI/SNF-related matrix-associatedactin-dependent	117367	208	420	189	85	0.32	0.12	0.375	5	3	10	4	11	6	13	7	-0.41754
sp|Q7Z6Z7|HUWE1_HUMAN	HUWE1	E3 ubiquitin-protein ligase HUWE1 OS=Homo sapiens GN=HUWE1 PE=1 SV=3	485523	106	207	353	170	0.08	0.03	0.375	7	2	11	5	12	6	19	6	-0.53803
tr|Q6IAX2|Q6IAX2_HUMAN	RPL21	RPL21 protein OS=Homo sapiens GN=RPL21 PE=2 SV=1	18610	364	597	111	60	0.94	0.35	0.37234	3	1	4	1	4	1	4	1	-1.1793
tr|G3V529|G3V529_HUMAN	DDX24	ATP-dependent RNA helicase DDX24 OS=Homo sapiens GN=DDX24 PE=2 SV=1	91994	1036	831	28	37	0.11	0.04	0.363636	0	1	3	1	2	1	4	1	-0.33092
tr|A1KYQ7|A1KYQ7_HUMAN	tr|A1KYQ7|A1KYQ	Cell migration-inducing protein 17 OS=Homo sapiens PE=2 SV=1	106022	87	103	415	312	0.44	0.16	0.363636	12	6	12	6	14	7	15	7	-0.54583
tr|B3KMS0|B3KMS0_HUMAN	tr|B3KMS0|B3KM	cDNA FLJ12449 fis, clone NT2RM1000314, highly similar to Condensin complex subu	158464	248	301	163	118	0.11	0.04	0.363636	3	2	5	2	6	2	13	2	-0.97299
sp|P10768|ESTD_HUMAN	ESD	S-formylglutathione hydrolase OS=Homo sapiens GN=ESD PE=1 SV=2	31956	1076	880	26	34	0.28	0.1	0.357143	0	0	2	1	1	1	2	1	0.406244
sp|Q9UJS0|CMC2_HUMAN	SLC25A13	Calcium-binding mitochondrial carrier protein Aralar2 OS=Homo sapiens GN=SLC25A	74528	651	1073	56	22	0.14	0.05	0.357143	1	0	3	1	3	1	3	2	-0.81654
sp|Q9BQA1|MEP50_HUMAN	WDR77	Methylosome protein 50 OS=Homo sapiens GN=WDR77 PE=1 SV=1	37442	860	1139	39	17	0.28	0.1	0.357143	1	0	1	1	1	0	1	1	-1.17898
sp|Q58FF6|H90B4_HUMAN	HSP90AB4P	Putative heat shock protein HSP 90-beta 4 OS=Homo sapiens GN=HSP90AB4P PE=5 S	58855	322	847	125	37	0.31	0.11	0.354839	3	0	6	2	6	3	7	5	-0.4873
tr|Q15244|Q15244_HUMAN	tr|Q15244|Q1524	Phosphoribosylpyrophosphate synthetase isoform I (Fragment) OS=Homo sapiens P	4443	374	282	56	35	0.88	0.31	0.352273	2	1	2	1	2	1	2	1	-0.33092
sp|P52597|HNRPF_HUMAN	HNRNPF	Heterogeneous nuclear ribonucleoprotein F OS=Homo sapiens GN=HNRNPF PE=1 SV	45985	111	160	349	213	1.14	0.4	0.350877	9	5	12	5	12	6	14	9	-0.53803
sp|O00410|IPO5_HUMAN	IPO5	Importin-5 OS=Homo sapiens GN=IPO5 PE=1 SV=4	125032	126	204	310	173	0.4	0.14	0.35	8	4	13	5	13	5	16	7	-0.89052
tr|B7Z4V2|B7Z4V2_HUMAN	tr|B7Z4V2|B7Z4V	cDNA FLJ51907, highly similar to Stress-70 protein, mitochondrial OS=Homo sapiens	72641	97	159	378	213	0.86	0.3	0.348837	10	5	17	6	17	6	20	6	-1.02443
sp|P63241|IF5A1_HUMAN	EIF5A	Eukaryotic translation initiation factor 5A-1 OS=Homo sapiens GN=EIF5A PE=1 SV=2	17049	379	359	107	102	3.24	1.13	0.348765	2	3	8	4	7	4	8	4	-0.33112
tr|A8K6Q8|A8K6Q8_HUMAN	tr|A8K6Q8|A8K6Q	cDNA FLJ75881, highly similar to Homo sapiens transferrin receptor (p90, CD71) (TF	85276	184	448	215	80	0.35	0.12	0.342857	6	1	8	3	9	3	9	3	-1.03537
tr|B4DE36|B4DE36_HUMAN	tr|B4DE36|B4DE3	Glucose-6-phosphate isomerase OS=Homo sapiens PE=2 SV=1	60376	92	268	397	133	0.89	0.3	0.337079	12	4	13	5	13	7	13	8	-0.44254
sp|Q14315|FLNC_HUMAN	FLNC	Filamin-C OS=Homo sapiens GN=FLNC PE=1 SV=3	293407	654	800	55	41	0.03	0.01	0.333333	1	1	3	1	2	1	3	2	-0.33092
tr|B4DF22|B4DF22_HUMAN	tr|B4DF22|B4DF2	cDNA FLJ55615, highly similar to SWI/SNF-related matrix-associatedactin-dependent	112428	378	544	52	26	0.15	0.05	0.333333	1	0	2	1	2	1	2	1	-0.33092
tr|D3DUW6|D3DUW6_HUM	DNM1L	Dynamin 1-like, isoform CRA_f OS=Homo sapiens GN=DNM1L PE=2 SV=1	86007	636	672	57	52	0.12	0.04	0.333333	1	1	3	1	3	1	4	1	-0.81654
tr|Q2NLA0|Q2NLA0_HUMAN	COPG2	Coatomer subunit gamma OS=Homo sapiens GN=COPG2 PE=2 SV=1	82400	243	458	165	79	0.12	0.04	0.333333	3	1	3	1	3	1	3	1	-0.81654
sp|P53618|COPB_HUMAN	COPB1	Coatomer subunit beta OS=Homo sapiens GN=COPB1 PE=1 SV=3	108214	165	352	235	103	0.27	0.09	0.333333	8	1	8	3	9	5	10	5	-0.38278
tr|A4FU77|A4FU77_HUMAN	SNRNP200	SNRNP200 protein (Fragment) OS=Homo sapiens GN=SNRNP200 PE=2 SV=1	217585	21	127	1046	258	0.54	0.18	0.333333	27	6	33	12	38	11	41	16	-1.34163
sp|P14868|SYDC_HUMAN	DARS	Aspartate--tRNA ligase, cytoplasmic OS=Homo sapiens GN=DARS PE=1 SV=2	57499	238	608	167	59	0.4	0.13	0.325	4	1	6	2	8	2	8	3	-1.3604
tr|H0YL72|H0YL72_HUMAN	IDH3A	Isocitrate dehydrogenase [NAD] subunit alpha, mitochondrial OS=Homo sapiens GN	36162	710	797	51	41	0.28	0.09	0.321429	1	1	1	1	1	1	1	2	0.406244
tr|B3KXZ4|B3KXZ4_HUMAN	tr|B3KXZ4|B3KXZ4	cDNA FLJ46429 fis, clone THYMU3014372, highly similar to DNA replication licensing	91898	172	322	224	111	0.47	0.15	0.319149	7	3	11	4	10	4	12	5	-0.81713
tr|Q53F48|Q53F48_HUMAN	tr|Q53F48|Q53F4	Heterogeneous nuclear ribonucleoprotein H3 isoform a variant (Fragment) OS=Hom	36959	386	699	105	50	0.41	0.13	0.317073	3	1	4	1	3	1	5	1	-0.81654
tr|B4DG47|B4DG47_HUMAN	tr|B4DG47|B4DG4	cDNA FLJ56967, highly similar to Adapter-relatedprotein complex 1 beta-1 subunit C	54387	445	1148	88	16	0.19	0.06	0.315789	2	0	3	1	2	0	3	1	-1.91614
sp|O75643|U520_HUMAN	SNRNP200	U5 small nuclear ribonucleoprotein 200 kDa helicase OS=Homo sapiens GN=SNRNP2	246006	21	128	1089	258	0.54	0.17	0.314815	28	6	35	13	40	11	44	17	-1.41509
tr|Q0QF37|Q0QF37_HUMAN	MDH2	Malate dehydrogenase (Fragment) OS=Homo sapiens GN=MDH2 PE=2 SV=1	32405	84	266	423	133	2.55	0.8	0.313725	12	5	13	6	13	6	14	7	-0.64925
tr|H0Y4X3|H0Y4X3_HUMAN	RBM39	RNA-binding protein 39 (Fragment) OS=Homo sapiens GN=RBM39 PE=4 SV=1	36959	306	1114	130	19	0.29	0.09	0.310345	3	0	3	1	3	0	3	1	-2.40176
tr|D3DNL3|D3DNL3_HUMAN	GFM1	G elongation factor, mitochondrial 1, isoform CRA_c OS=Homo sapiens GN=GFM1 P	46282	596	808	62	40	0.23	0.07	0.304348	1	1	3	1	2	1	3	1	-0.33092
sp|Q5JVF3|PCID2_HUMAN	PCID2	PCI domain-containing protein 2 OS=Homo sapiens GN=PCID2 PE=1 SV=2	46627	804	665	43	53	0.23	0.07	0.304348	1	1	3	1	3	1	3	1	-0.81654
tr|B2RBE5|B2RBE5_HUMAN	tr|B2RBE5|B2RBE	cDNA, FLJ95468, highly similar to Homo sapiens transcriptional coactivator tubedow	102490	368	452	110	80	0.33	0.1	0.30303	3	3	9	3	9	3	12	3	-1.03537
tr|B7Z7L9|B7Z7L9_HUMAN	tr|B7Z7L9|B7Z7L9	cDNA FLJ56173, highly similar to U4/U6.U5 tri-snRNP-associated protein 2 OS=Hom	56747	546	742	68	46	0.33	0.1	0.30303	2	1	5	1	5	1	5	1	-1.46901
sp|P23381|SYWC_HUMAN	WARS	Tryptophan--tRNA ligase, cytoplasmic OS=Homo sapiens GN=WARS PE=1 SV=2	53474	373	1000	109	27	0.2	0.06	0.3	3	0	3	1	3	1	4	3	-0.81654
sp|Q9H0B6|KLC2_HUMAN	KLC2	Kinesin light chain 2 OS=Homo sapiens GN=KLC2 PE=1 SV=1	69291	384	691	106	51	0.2	0.06	0.3	1	1	4	1	4	1	4	1	-1.1793
tr|B2RWP9|B2RWP9_HUMA	MYH10	MYH10 protein OS=Homo sapiens GN=MYH10 PE=2 SV=1	230858	121	146	331	227	0.2	0.06	0.3	10	4	14	4	16	5	19	8	-1.18062
sp|O75131|CPNE3_HUMAN	CPNE3	Copine-3 OS=Homo sapiens GN=CPNE3 PE=1 SV=1	60947	628	554	58	66	0.17	0.05	0.294118	2	1	3	1	3	1	4	1	-0.81654
tr|A8K8N7|A8K8N7_HUMAN	PFAS	Phosphoribosylformylglycinamidine synthase (FGAR amidotransferase), isoform CRA	146286	323	344	125	104	0.17	0.05	0.294118	3	2	7	2	6	2	7	2	-0.97299
tr|B4E063|B4E063_HUMAN	tr|B4E063|B4E063	cDNA FLJ54292, highly similar to Kinesin-like protein KIFC1 OS=Homo sapiens PE=2 S	73571	626	1013	58	0	0.14	0.04	0.285714	1	0	3	0	2	1	3	2	-0.33092
tr|B3KT66|B3KT66_HUMAN	PSMD6	Proteasome (Prosome, macropain) 26S subunit, non-ATPase, 6, isoform CRA_c OS=H	40600	341	761	83	44	0.28	0.08	0.285714	2	1	3	1	2	1	3	2	-0.33092
tr|G1UI16|G1UI16_HUMAN	PDS5A	SCC-112 protein, isoform CRA_b OS=Homo sapiens GN=PDS5A PE=2 SV=1	152274	434	1100	92	21	0.07	0.02	0.285714	2	0	3	1	4	1	4	2	-1.1793
sp|O94822|LTN1_HUMAN	LTN1	E3 ubiquitin-protein ligase listerin OS=Homo sapiens GN=LTN1 PE=1 SV=6	203103	657	1110	55	20	0.07	0.02	0.285714	1	0	4	1	4	0	5	3	-2.76453
tr|B4DWJ2|B4DWJ2_HUMAN	QARS	Glutamine--tRNA ligase OS=Homo sapiens GN=QARS PE=2 SV=1	87437	217	279	181	129	0.44	0.12	0.272727	4	3	10	3	11	4	16	6	-0.94857
tr|A4QPB0|A4QPB0_HUMAN	IQGAP1	IQ motif containing GTPase activating protein 1 OS=Homo sapiens GN=IQGAP1 PE=2	189789	249	466	162	78	0.11	0.03	0.272727	4	1	6	1	7	1	11	2	-1.91686
sp|Q8WUM4|PDC6I_HUMAN	PDCD6IP	Programmed cell death 6-interacting protein OS=Homo sapiens GN=PDCD6IP PE=1 S	96590	194	284	204	128	0.26	0.07	0.269231	5	2	7	2	7	2	7	3	-1.17963
tr|Q6NVW7|Q6NVW7_HUM	KPNA2	Importin subunit alpha OS=Homo sapiens GN=KPNA2 PE=2 SV=1	58271	118	253	335	138	0.93	0.25	0.268817	8	2	12	4	12	4	12	5	-1.06906
tr|B3KML9|B3KML9_HUMAN	tr|B3KML9|B3KML	cDNA FLJ11352 fis, clone HEMBA1000020, highly similar to Tubulin beta-2C chain OS	44916	4	24	1222	990	15.34	4.1	0.267275	48	37	55	41	60	44	65	49	-0.03728
tr|B4DED6|B4DED6_HUMAN	tr|B4DED6|B4DED	cDNA FLJ56545, highly similar to ATP-dependent RNA helicase DDX50 (EC 3.6.1.-) OS	72683	458	616	84	58	0.19	0.05	0.263158	4	1	5	1	5	3	5	3	-0.2461
sp|Q14258|TRI25_HUMAN	TRIM25	E3 ubiquitin/ISG15 ligase TRIM25 OS=Homo sapiens GN=TRIM25 PE=1 SV=2	72581	394	719	103	47	0.19	0.05	0.263158	3	1	4	1	5	2	5	3	-0.73178
tr|B2RCM2|B2RCM2_HUMA	tr|B2RCM2|B2RCM	cDNA, FLJ96156, highly similar to Homo sapiens leucyl-tRNA synthetase (LARS), mRN	135550	281	613	142	58	0.27	0.07	0.259259	5	2	10	3	10	3	12	5	-1.17996
sp|P54652|HSP72_HUMAN	HSPA2	Heat shock-related 70 kDa protein 2 OS=Homo sapiens GN=HSPA2 PE=1 SV=1	70263	57	145	245	230	1.7	0.44	0.258824	7	6	9	9	9	12	10	12	0.803459
tr|A8K690|A8K690_HUMAN	tr|A8K690|A8K690	cDNA FLJ76863, highly similar to Homo sapiens stress-induced-phosphoprotein 1 (H	63243	78	139	445	237	1.75	0.45	0.257143	13	6	20	9	21	15	28	16	-0.06611
sp|P06733|ENOA_HUMAN	ENO1	Alpha-enolase OS=Homo sapiens GN=ENO1 PE=1 SV=2	47481	22	72	1078	421	5.55	1.39	0.25045	28	11	39	18	43	18	48	23	-0.83107
tr|E7ERK9|E7ERK9_HUMAN	EIF2B4	Translation initiation factor eIF-2B subunit delta OS=Homo sapiens GN=EIF2B4 PE=2	60248	488	543	79	67	0.2	0.05	0.25	2	1	2	1	2	1	2	1	-0.33092
sp|Q9UG63|ABCF2_HUMAN	ABCF2	ATP-binding cassette sub-family F member 2 OS=Homo sapiens GN=ABCF2 PE=1 SV=	71815	538	465	70	78	0.2	0.05	0.25	1	1	4	1	4	2	5	3	-0.44208
sp|Q14690|RRP5_HUMAN	PDCD11	Protein RRP5 homolog OS=Homo sapiens GN=PDCD11 PE=1 SV=3	209939	563	807	66	40	0.08	0.02	0.25	2	1	5	1	5	2	6	6	-0.73178
sp|Q9H0A0|NAT10_HUMAN	NAT10	N-acetyltransferase 10 OS=Homo sapiens GN=NAT10 PE=1 SV=2	116569	518	1039	73	24	0.12	0.03	0.25	1	0	4	1	6	1	6	1	-1.71021
tr|K7EQ02|K7EQ02_HUMAN	DAZAP1	DAZ-associated protein 1 (Fragment) OS=Homo sapiens GN=DAZAP1 PE=4 SV=1	24960	512	905	74	32	0.29	0.07	0.241379	1	0	2	1	2	1	2	1	-0.33092
sp|P17987|TCPA_HUMAN	TCP1	T-complex protein 1 subunit alpha OS=Homo sapiens GN=TCP1 PE=1 SV=1	60819	56	171	617	206	1.58	0.37	0.234177	14	5	18	7	20	9	24	11	-0.70502
tr|B3GQS7|B3GQS7_HUMAN	HSPD1	Mitochondrial heat shock 60kD protein 1 variant 1 OS=Homo sapiens GN=HSPD1 PE	60813	85	136	423	241	1.58	0.37	0.234177	15	6	18	7	19	8	23	10	-0.7934
tr|B2R4K7|B2R4K7_HUMAN	tr|B2R4K7|B2R4K	60S ribosomal protein L6 OS=Homo sapiens PE=2 SV=1	32739	147	542	273	68	1.89	0.44	0.232804	7	2	11	3	12	3	12	4	-1.43189
sp|O75844|FACE1_HUMAN	ZMPSTE24	CAAX prenyl protease 1 homolog OS=Homo sapiens GN=ZMPSTE24 PE=1 SV=2	55063	675	1022	54	25	0.26	0.06	0.230769	1	0	2	1	2	1	2	1	-0.33092
tr|B4DSN3|B4DSN3_HUMAN	tr|B4DSN3|B4DSN	cDNA FLJ60345, highly similar to Protein transport protein Sec24C OS=Homo sapien	107974	410	715	100	48	0.13	0.03	0.230769	3	1	5	1	5	2	5	2	-0.73178
tr|B2RCT6|B2RCT6_HUMAN	tr|B2RCT6|B2RCT	cDNA, FLJ96276, highly similar to Homo sapiens G1 to S phase transition 1 (GSPT1),	56320	540	1065	70	23	0.26	0.06	0.230769	3	1	4	1	4	1	5	2	-1.1793
tr|Q1L6K4|Q1L6K4_HUMAN	PDCD8	Apoptosis-inducing factor 1, mitochondrial OS=Homo sapiens GN=PDCD8 PE=2 SV=1	26131	420	728	96	47	0.44	0.1	0.227273	2	2	3	2	3	2	3	2	-0.07931
sp|Q8N1G4|LRC47_HUMAN	LRRC47	Leucine-rich repeat-containing protein 47 OS=Homo sapiens GN=LRRC47 PE=1 SV=1	64004	313	316	127	114	0.22	0.05	0.227273	4	2	4	2	5	2	6	2	-0.73178
tr|Q68DI0|Q68DI0_HUMAN	DKFZp781K0743	Putative uncharacterized protein DKFZp781K0743 OS=Homo sapiens GN=DKFZp781	106527	136	261	285	136	0.44	0.1	0.227273	8	2	12	3	14	3	16	6	-1.64641
sp|Q9Y678|COPG1_HUMAN	COPG1	Coatomer subunit gamma-1 OS=Homo sapiens GN=COPG1 PE=1 SV=1	98967	261	845	155	37	0.22	0.05	0.227273	3	1	6	1	6	1	7	1	-1.71021
sp|Q10567|AP1B1_HUMAN	AP1B1	AP-1 complex subunit beta-1 OS=Homo sapiens GN=AP1B1 PE=1 SV=2	105482	136	652	208	54	0.44	0.1	0.227273	5	1	7	2	9	1	9	4	-2.25828
tr|G3XAB4|G3XAB4_HUMAN	ACAT1	Acetyl-CoA acetyltransferase, mitochondrial OS=Homo sapiens GN=ACAT1 PE=2 SV=	17449	231	277	172	96	1.03	0.23	0.223301	4	2	4	2	4	2	4	2	-0.44208
tr|B0AZS5|B0AZS5_HUMAN	tr|B0AZS5|B0AZS5	cDNA, FLJ79515, highly similar to Kinesin-like protein KIF2 OS=Homo sapiens PE=2 S	75666	449	506	86	72	0.18	0.04	0.222222	3	1	4	1	4	1	4	1	-1.1793
tr|A7E2E1|A7E2E1_HUMAN	SMARCA4	SWI/SNF related, matrix associated, actin dependent regulator of chromatin, subfam	185100	345	727	117	47	0.09	0.02	0.222222	3	1	5	1	6	1	9	3	-1.71021
tr|B4E205|B4E205_HUMAN	tr|B4E205|B4E205	cDNA FLJ61651, highly similar to Protein transport protein Sec24A OS=Homo sapien	96953	455	1086	85	21	0.14	0.03	0.214286	2	0	4	1	4	1	4	2	-1.1793
tr|F5H4R9|F5H4R9_HUMAN	DNM2	Dynamin-2 OS=Homo sapiens GN=DNM2 PE=2 SV=1	98218	335	1008	120	26	0.14	0.03	0.214286	3	1	4	1	4	1	5	1	-1.1793
tr|B3KMX0|B3KMX0_HUMAN	tr|B3KMX0|B3KM	cDNA FLJ12837 fis, clone NT2RP2003228, highly similar to DNA replication licensing	97114	493	513	78	71	0.14	0.03	0.214286	2	1	4	1	4	1	5	4	-1.1793
sp|P11498|PYC_HUMAN	PC	Pyruvate carboxylase, mitochondrial OS=Homo sapiens GN=PC PE=1 SV=2	130293	101	589	367	61	0.49	0.1	0.204082	11	1	16	4	18	4	20	8	-1.63583
tr|J3KRI6|J3KRI6_HUMAN	CCT6B	T-complex protein 1 subunit zeta-2 (Fragment) OS=Homo sapiens GN=CCT6B PE=4 S	14807	141	687	31	0	0.64	0.13	0.203125	0	0	1	0	1	0	1	1	-1.17898
tr|G5E9Q2|G5E9Q2_HUMAN	CSDE1	Cold shock domain containing E1, RNA-binding, isoform CRA_d OS=Homo sapiens G	94539	257	618	157	57	0.15	0.03	0.2	3	1	4	1	4	1	6	3	-1.1793
sp|Q14683|SMC1A_HUMAN	SMC1A	Structural maintenance of chromosomes protein 1A OS=Homo sapiens GN=SMC1A	143771	155	924	260	31	0.25	0.05	0.2	7	0	10	2	12	3	14	4	-1.43189
tr|B7Z4F6|B7Z4F6_HUMAN	tr|B7Z4F6|B7Z4F6	cDNA FLJ54912, highly similar to 60 kDa heat shock protein, mitochondrial OS=Hom	58705	85	229	309	151	1.58	0.31	0.196203	12	5	15	6	15	7	18	9	-0.64224
sp|Q58FG0|HS905_HUMAN	HSP90AA5P	Putative heat shock protein HSP 90-alpha A5 OS=Homo sapiens GN=HSP90AA5P PE=	38942	17	384	159	96	2	0.39	0.195	3	2	7	5	7	7	7	8	0.406625
sp|Q92900|RENT1_HUMAN	UPF1	Regulator of nonsense transcripts 1 OS=Homo sapiens GN=UPF1 PE=1 SV=2	125578	256	319	157	112	0.26	0.05	0.192308	4	2	9	2	11	3	12	4	-1.3114
tr|A0PJ61|A0PJ61_HUMAN	RSL1D1	RSL1D1 protein (Fragment) OS=Homo sapiens GN=RSL1D1 PE=2 SV=1	49688	468	1081	82	22	0.38	0.07	0.184211	2	0	5	1	6	2	7	2	-0.97299
tr|F5H3A1|F5H3A1_HUMAN	ATP1A1	Sodium/potassium-transporting ATPase subunit alpha-1 OS=Homo sapiens GN=ATP	114239	170	714	227	48	0.33	0.06	0.181818	6	1	10	2	11	2	13	4	-1.79709
sp|P62249|RS16_HUMAN	RPS16	40S ribosomal protein S16 OS=Homo sapiens GN=RPS16 PE=1 SV=2	16549	531	1048	71	24	1.11	0.2	0.18018	4	1	4	1	4	1	4	2	-1.1793
tr|B4DH16|B4DH16_HUMAN	tr|B4DH16|B4DH1	cDNA FLJ54709, highly similar to Methylcrotonoyl-CoA carboxylase subunit alpha, m	47761	259	600	117	60	0.39	0.07	0.179487	3	1	6	1	5	1	7	3	-1.46901
tr|B4DN01|B4DN01_HUMAN	tr|B4DN01|B4DN0	cDNA FLJ57332, highly similar to COP9 signalosome complex subunit 3 OS=Homo sa	33950	343	500	117	73	0.59	0.1	0.169492	3	1	5	1	5	1	5	1	-1.46901
tr|Q4ZG57|Q4ZG57_HUMAN	MCM6	Putative uncharacterized protein MCM6 (Fragment) OS=Homo sapiens GN=MCM6 P	89803	357	897	114	33	0.24	0.04	0.166667	3	1	6	1	7	1	7	3	-1.91686
sp|P13645|K1C10_HUMAN	KRT10	Keratin, type I cytoskeletal 10 OS=Homo sapiens GN=KRT10 PE=1 SV=6	59020	28	196	956	178	2.48	0.38	0.153226	23	6	25	6	27	6	28	6	-1.67847
tr|B3KNB4|B3KNB4_HUMAN	tr|B3KNB4|B3KNB	cDNA FLJ14168 fis, clone NT2RP2001440, highly similar to 14-3-3 protein gamma OS	28453	227	915	57	32	1.65	0.25	0.151515	1	0	1	2	2	2	2	2	0.406307
tr|B4DY04|B4DY04_HUMAN	tr|B4DY04|B4DY0	cDNA FLJ51144, highly similar to 14-3-3 protein theta OS=Homo sapiens PE=2 SV=1	14194	227	287	57	58	1.65	0.25	0.151515	1	1	1	1	2	1	2	1	-0.33092
sp|Q4VXU2|PAP1L_HUMAN	PABPC1L	Polyadenylate-binding protein 1-like OS=Homo sapiens GN=PABPC1L PE=2 SV=1	68976	115	889	57	33	0.69	0.1	0.144928	1	1	2	2	2	2	2	3	0.406307
sp|P46063|RECQ1_HUMAN	RECQL	ATP-dependent DNA helicase Q1 OS=Homo sapiens GN=RECQL PE=1 SV=3	74436	387	1106	105	20	0.3	0.04	0.133333	3	0	6	1	6	1	6	1	-1.71021
tr|E9PHF7|E9PHF7_HUMAN	MCCC1	Methylcrotonoyl-CoA carboxylase subunit alpha, mitochondrial OS=Homo sapiens G	68745	259	599	155	60	0.39	0.05	0.128205	4	1	7	1	6	2	8	3	-0.97299
tr|B4DZI8|B4DZI8_HUMAN	COPB2	Coatomer protein complex, subunit beta 2 (Beta prime), isoform CRA_b OS=Homo s	99839	244	1108	165	20	0.25	0.03	0.12	6	0	7	1	7	1	7	2	-1.91686
tr|Q6NUR1|Q6NUR1_HUMA	NCAPG	Non-SMC condensin I complex, subunit G OS=Homo sapiens GN=NCAPG PE=2 SV=1	115205	239	1076	166	22	0.25	0.03	0.12	3	0	8	1	8	1	8	2	-2.09762
sp|P13647|K2C5_HUMAN	KRT5	Keratin, type II cytoskeletal 5 OS=Homo sapiens GN=KRT5 PE=1 SV=3	62568	224	495	178	0	0.43	0.05	0.116279	5	0	7	0	8	0	9	1	-3.68285
tr|Q6PJT4|Q6PJT4_HUMAN	MSN	MSN protein (Fragment) OS=Homo sapiens GN=MSN PE=2 SV=1	38916	226	674	153	52	0.46	0.05	0.108696	4	1	6	1	6	1	7	1	-1.71021
sp|P26038|MOES_HUMAN	MSN	Moesin OS=Homo sapiens GN=MSN PE=1 SV=3	67892	226	675	176	52	0.46	0.05	0.108696	6	1	8	1	8	1	10	2	-2.09762
tr|H7BZF2|H7BZF2_HUMAN	DKC1	H/ACA ribonucleoprotein complex subunit 4 (Fragment) OS=Homo sapiens GN=DKC	8803	524	524	72	44	1.74	0.12	0.068966	3	1	3	1	3	1	3	1	-0.81654
sp|P35908|K22E_HUMAN	KRT2	Keratin, type II cytoskeletal 2 epidermal OS=Homo sapiens GN=KRT2 PE=1 SV=2	65678	35	334	895	107	2.07	0.1	0.048309	20	2	27	2	30	3	34	3	-2.7223
tr|I3L253|I3L253_HUMAN	TRAP1	Heat shock protein 75 kDa, mitochondrial (Fragment) OS=Homo sapiens GN=TRAP1	6581	124	0	0	0	0.35	0	0	0	0	0	0	0	0	1	0	0.40618
tr|Q3LIE3|Q3LIE3_HUMAN	Nbla04137	Putative uncharacterized protein Nbla04137 (Fragment) OS=Homo sapiens GN=Nbla	33497	168	0	0	0	0.34	0	0	0	0	0	0	0	0	1	0	0.40618
tr|F8WDM3|F8WDM3_HUM	MCM2	DNA replication licensing factor MCM2 OS=Homo sapiens GN=MCM2 PE=2 SV=1	16182	172	0	0	0	0.47	0	0	0	0	0	0	0	0	1	0	0.40618
tr|E2QRM3|E2QRM3_HUMA	XPO5	Exportin-5 OS=Homo sapiens GN=XPO5 PE=2 SV=1	8973	202	0	0	0	0.15	0	0	0	0	0	0	0	0	1	0	0.40618
tr|Q53HI6|Q53HI6_HUMAN	tr|Q53HI6|Q53HI6	Ribosomal protein S4, Y-linked 1 Y isoform variant (Fragment) OS=Homo sapiens PE=	29607	203	0	0	0	0.7	0	0	0	0	0	0	0	0	2	0	0.40618
tr|Q5TBN3|Q5TBN3_HUMAN	LCP1	Plastin-2 (Fragment) OS=Homo sapiens GN=LCP1 PE=4 SV=1	27575	241	0	0	0	0.38	0	0	0	0	0	0	0	0	1	0	0.40618
tr|A0PJ76|A0PJ76_HUMAN	NCAPD2	NCAPD2 protein (Fragment) OS=Homo sapiens GN=NCAPD2 PE=2 SV=1	44957	248	0	0	0	0.11	0	0	0	0	0	0	0	0	1	0	0.40618
tr|H0Y8G2|H0Y8G2_HUMAN	COPG1	Coatomer subunit gamma-1 (Fragment) OS=Homo sapiens GN=COPG1 PE=4 SV=1	8747	261	0	15	0	0.22	0	0	0	0	1	0	0	0	1	0	0.40618
tr|E5RK19|E5RK19_HUMAN	HDAC2	Histone deacetylase 2 (Fragment) OS=Homo sapiens GN=HDAC2 PE=2 SV=1	6474	300	0	0	0	0.28	0	0	0	0	0	0	0	0	1	0	0.40618
tr|B4DXF1|B4DXF1_HUMAN	tr|B4DXF1|B4DXF	cDNA FLJ57223 OS=Homo sapiens PE=2 SV=1	17079	305	0	0	0	0.41	0	0	0	0	0	0	0	0	1	0	0.40618
tr|B4DFH8|B4DFH8_HUMAN	tr|B4DFH8|B4DFH	cDNA FLJ52270, highly similar to Fragile X mental retardation 1 protein OS=Homo sa	28273	361	0	0	0	0.13	0	0	0	0	0	0	0	0	1	0	0.40618
tr|Q5JXT2|Q5JXT2_HUMAN	NOP56	Nucleolar protein 56 (Fragment) OS=Homo sapiens GN=NOP56 PE=4 SV=1	31576	397	0	0	0	0.21	0	0	0	0	0	0	0	0	2	0	0.40618
tr|J3QSH1|J3QSH1_HUMAN	SMCHD1	Structural maintenance of chromosomes flexible hinge domain-containing protein 1	16870	467	0	0	0	0.03	0	0	0	0	0	0	0	0	1	0	0.40618
tr|Q7Z6A1|Q7Z6A1_HUMAN	PCNA	Proliferating cell nuclear antigen (Fragment) OS=Homo sapiens GN=PCNA PE=2 SV=1	8271	487	0	0	0	0.38	0	0	0	0	0	0	0	0	1	0	0.40618

tr|B2WTN6|B2WTN6_HUMA	rpB2	RpB2 (Fragment) OS=Homo sapiens GN=rpB2 PE=2 SV=1	5732	499	0	0	0	0.08	0	0	0	0	0	0	0	0	2	0	0.40618
tr|H3BSQ5|H3BSQ5_HUMAN	ATXN2L	Ataxin-2-like protein (Fragment) OS=Homo sapiens GN=ATXN2L PE=4 SV=1	10164	533	0	0	0	0.09	0	0	0	0	0	0	0	0	1	0	0.40618
tr|H3BSV8|H3BSV8_HUMAN	GSPT1	Eukaryotic peptide chain release factor GTP-binding subunit ERF3A (Fragment) OS=H	22213	540	0	0	0	0.26	0	0	0	0	0	0	0	0	1	0	0.40618
tr|F8WFD7|F8WFD7_HUMAN	MCM5	DNA replication licensing factor MCM5 OS=Homo sapiens GN=MCM5 PE=2 SV=1	6550	570	0	0	0	0.08	0	0	0	0	0	0	0	0	1	0	0.40618
tr|F5GYK7|F5GYK7_HUMAN	GPD2	Glycerol-3-phosphate dehydrogenase, mitochondrial OS=Homo sapiens GN=GPD2 P	41828	578	0	0	0	0.04	0	0	0	0	0	0	0	0	2	0	0.40618
tr|B7Z842|B7Z842_HUMAN	tr|B7Z842|B7Z842	Ubiquitin carboxyl-terminal hydrolase OS=Homo sapiens PE=2 SV=1	59074	677	0	0	0	0.06	0	0	0	0	0	0	0	0	1	0	0.40618
tr|Q59FR7|Q59FR7_HUMAN	tr|Q59FR7|Q59FR	Putative uncharacterized protein (Fragment) OS=Homo sapiens PE=2 SV=1	28771	799	0	0	0	0.11	0	0	0	0	0	0	0	0	1	0	0.40618
tr|H0YA24|H0YA24_HUMAN	PLRG1	Pleiotropic regulator 1 (Fragment) OS=Homo sapiens GN=PLRG1 PE=4 SV=1	17588	898	0	0	0	0.06	0	0	0	0	0	0	0	0	1	0	0.40618
tr|D6RD96|D6RD96_HUMAN	ACSL4	Long-chain-fatty-acid--CoA ligase 4 (Fragment) OS=Homo sapiens GN=ACSL4 PE=2 S\	16399	931	0	0	0	0.09	0	0	0	0	0	0	0	0	1	0	0.40618
tr|Q5SXN0|Q5SXN0_HUMAN	PMPCA	Mitochondrial-processing peptidase subunit alpha OS=Homo sapiens GN=PMPCA PE	15995	945	0	0	0	0.12	0	0	0	0	0	0	0	0	1	0	0.40618
tr|J3QSF3|J3QSF3_HUMAN	ATAD3C	ATPase family AAA domain-containing protein 3C (Fragment) OS=Homo sapiens GN=	14904	965	0	0	0	0.07	0	0	0	0	0	0	0	0	1	0	0.40618
tr|B4DEF4|B4DEF4_HUMAN	tr|B4DEF4|B4DEF4	cDNA FLJ60434, highly similar to Cleavage and polyadenylation specificity factor 160	36825	990	0	0	0	0.02	0	0	0	0	0	0	0	0	1	0	0.40618
tr|C9J4N6|C9J4N6_HUMAN	IDH1	Isocitrate dehydrogenase [NADP] cytoplasmic (Fragment) OS=Homo sapiens GN=IDH	17822	1104	0	0	0	0.07	0	0	0	0	0	0	0	0	1	0	0.40618
sp|Q13224|NMDE2_HUMAN	GRIN2B	Glutamate receptor ionotropic, NMDA 2B OS=Homo sapiens GN=GRIN2B PE=1 SV=3	167972	1174	0	20	0	0.02	0	0	0	0	1	0	0	0	1	0	0.40618
tr|A6QKW0|A6QKW0_HUMA	SHINC3	SHINC3 OS=Homo sapiens GN=SHINC3 PE=2 SV=1	28275	1175	0	20	0	0.12	0	0	0	0	1	0	0	0	1	0	0.40618
tr|B3KM97|B3KM97_HUMAN	tr|B3KM97|B3KM9	cDNA FLJ10554 fis, clone NT2RP2002385, highly similar to Synaptic glycoprotein SC2	36429	1176	0	20	0	0.09	0	0	0	0	1	0	0	0	2	0	0.40618
tr|B3KNB0|B3KNB0_HUMAN	tr|B3KNB0|B3KNB	cDNA FLJ14159 fis, clone NT2RM2001360, highly similar to Homo sapiens clone C40	36422	1177	0	19	0	0.09	0	0	0	0	1	0	0	0	1	0	0.40618
tr|Q05CP4|Q05CP4_HUMAN	EIF2AK2	EIF2AK2 protein (Fragment) OS=Homo sapiens GN=EIF2AK2 PE=2 SV=1	40940	1178	0	19	0	0.08	0	0	0	0	1	0	0	0	1	0	0.40618
tr|B4DH46|B4DH46_HUMAN	tr|B4DH46|B4DH4	cDNA FLJ60519, highly similar to WD repeat protein 68 OS=Homo sapiens PE=2 SV=1	19542	1179	0	19	0	0.17	0	0	0	0	1	0	0	0	1	0	0.40618
tr|B7ZBM3|B7ZBM3_HUMAN	FOXP4	Forkhead box P4 OS=Homo sapiens GN=FOXP4 PE=4 SV=1	72641	1180	0	19	0	0.05	0	0	0	0	1	0	0	0	1	0	0.40618
tr|H0YJU4|H0YJU4_HUMAN	YY1	Transcriptional repressor protein YY1 (Fragment) OS=Homo sapiens GN=YY1 PE=4 S\	13138	1182	0	19	0	0.26	0	0	0	0	1	0	0	0	1	0	0.40618
tr|B4DV74|B4DV74_HUMAN	tr|B4DV74|B4DV7	cDNA FLJ53855, highly similar to Kanadaptin OS=Homo sapiens PE=2 SV=1	56134	1183	0	19	0	0.06	0	0	0	0	1	0	0	0	1	0	0.40618
tr|Q5VSQ6|Q5VSQ6_HUMAN	P4HA1	Procollagen-proline, 2-oxoglutarate 4-dioxygenase (Proline 4-hydroxylase), alpha po	61214	1184	0	19	0	0.05	0	0	0	0	1	0	0	0	1	0	0.40618
tr|B4E2N1|B4E2N1_HUMAN	tr|B4E2N1|B4E2N	cDNA FLJ56560, highly similar to Caspase recruitment domain-containing protein 6 (	55261	1185	0	18	0	0.06	0	0	1	0	1	0	0	0	1	0	0.40618
tr|H0YDT4|H0YDT4_HUMAN	NDUFS8	NADH dehydrogenase [ubiquinone] iron-sulfur protein 8, mitochondrial (Fragment) (	10209	1188	0	18	0	0.34	0	0	0	0	1	0	0	0	1	0	0.40618
tr|A1A508|A1A508_HUMAN	PRSS3	PRSS3 protein OS=Homo sapiens GN=PRSS3 PE=2 SV=1	27040	1189	0	18	0	0.12	0	0	0	0	1	0	0	0	1	0	0.40618
tr|B2RAR3|B2RAR3_HUMAN	tr|B2RAR3|B2RAR	Queuine tRNA-ribosyltransferase OS=Homo sapiens PE=2 SV=1	44645	1191	0	18	0	0.07	0	0	0	0	1	0	0	0	1	0	0.40618
sp|Q7Z4Q2|HEAT3_HUMAN	HEATR3	HEAT repeat-containing protein 3 OS=Homo sapiens GN=HEATR3 PE=1 SV=2	75789	1192	0	18	0	0.04	0	0	0	0	1	0	0	0	1	0	0.40618
tr|B4DJU4|B4DJU4_HUMAN	tr|B4DJU4|B4DJU4	cDNA FLJ53344, highly similar to Splicing factor 1 OS=Homo sapiens PE=2 SV=1	67535	1193	0	18	0	0.05	0	0	0	0	1	0	0	0	2	0	0.40618
sp|Q6ZMV8|ZN730_HUMAN	ZNF730	Putative zinc finger protein 730 OS=Homo sapiens GN=ZNF730 PE=2 SV=1	60769	1194	0	18	0	0.06	0	0	0	0	1	0	0	0	1	0	0.40618
tr|F4MHH8|F4MHH8_HUMA	UTY	Ubiquitously transcribed tetratricopeptide repeat protein Y-linked transcript variant	121442	1198	0	17	0	0.03	0	0	0	0	1	0	0	0	1	0	0.40618
tr|H0YAQ8|H0YAQ8_HUMAN	PPIL6	Peptidyl-prolyl cis-trans isomerase-like 6 (Fragment) OS=Homo sapiens GN=PPIL6 PE	19590	1199	0	17	0	0.17	0	0	0	0	1	0	0	0	1	0	0.40618
sp|Q6UWZ7|F175A_HUMAN	FAM175A	BRCA1-A complex subunit Abraxas OS=Homo sapiens GN=FAM175A PE=1 SV=2	47033	1201	0	17	0	0.07	0	0	0	0	1	0	0	0	1	0	0.40618
tr|H0UI08|H0UI08_HUMAN	IMPG1	Interphotoreceptor matrix proteoglycan 1, isoform CRA_a OS=Homo sapiens GN=IM	64359	1202	0	16	0	0.05	0	0	0	0	1	0	0	0	1	0	0.40618
tr|A4UCS6|A4UCS6_HUMAN	tr|A4UCS6|A4UCS	Peroxiredoxin 6 (Fragment) OS=Homo sapiens PE=2 SV=1	9011	1204	0	16	0	0.39	0	0	0	0	1	0	0	0	1	0	0.40618
sp|Q5C9Z4|NOM1_HUMAN	NOM1	Nucleolar MIF4G domain-containing protein 1 OS=Homo sapiens GN=NOM1 PE=1 S\	96768	1206	0	16	0	0.03	0	0	0	0	1	0	0	0	1	0	0.40618
tr|B4DJQ5|B4DJQ5_HUMAN	tr|B4DJQ5|B4DJQ5	cDNA FLJ59211, highly similar to Glucosidase 2 subunit beta OS=Homo sapiens PE=2	61066	1207	0	15	0	0.05	0	0	0	0	1	0	0	0	1	0	0.40618
sp|O14556|G3PT_HUMAN	GAPDHS	Glyceraldehyde-3-phosphate dehydrogenase, testis-specific OS=Homo sapiens GN=(	44815	13	0	41	0	6.5	0	0	1	0	1	0	1	0	1	0	-1.17898
tr|Q5T7D5|Q5T7D5_HUMAN	ALDOB	Fructose-bisphosphate aldolase B OS=Homo sapiens GN=ALDOB PE=4 SV=1	24512	14	0	39	0	6.34	0	0	1	0	1	0	1	0	1	0	-1.17898
tr|D3DX41|D3DX41_HUMAN	MGC16703	Alpha tubulin-like, isoform CRA_a OS=Homo sapiens GN=MGC16703 PE=3 SV=1	17215	18	0	0	0	5.31	0	0	0	0	0	0	1	0	1	0	-1.17898
tr|F5H5N6|F5H5N6_HUMAN	CLTCL1	Clathrin heavy chain 2 (Fragment) OS=Homo sapiens GN=CLTCL1 PE=2 SV=1	18833	25	0	31	0	1.02	0	0	0	0	1	0	1	0	1	0	-1.17898
tr|A1L2Z2|A1L2Z2_HUMAN	MYH14	MYH14 protein OS=Homo sapiens GN=MYH14 PE=2 SV=1	73418	33	0	61	0	0.53	0	0	1	0	1	0	1	0	1	0	-1.17898
tr|Q92862|Q92862_HUMAN	Dnchc1	Dynein heavy chain (Fragment) OS=Homo sapiens GN=Dnchc1 PE=2 SV=1	8529	44	0	38	0	0.22	0	0	1	0	1	0	1	0	1	0	-1.17898
tr|Q712V1|Q712V1_HUMAN	UBE1	Ubiquitin activating enzyme E1 (Fragment) OS=Homo sapiens GN=UBE1 PE=2 SV=1	4483	51	0	31	0	0.82	0	0	0	0	1	0	1	0	1	0	-1.17898
tr|K7EL29|K7EL29_HUMAN	TUBB6	Tubulin beta-6 chain (Fragment) OS=Homo sapiens GN=TUBB6 PE=4 SV=1	14526	55	0	0	0	1.42	0	0	0	0	0	0	1	0	1	0	-1.17898
tr|H0Y960|H0Y960_HUMAN	DDX4	Probable ATP-dependent RNA helicase DDX4 (Fragment) OS=Homo sapiens GN=DDX	33292	60	0	53	0	1.1	0	0	1	0	1	0	1	0	1	0	-1.17898
tr|K7EQ75|K7EQ75_HUMAN	ILF3	Interleukin enhancer-binding factor 3 (Fragment) OS=Homo sapiens GN=ILF3 PE=4 S	6139	62	0	30	0	1.23	0	0	0	0	1	0	1	0	1	0	-1.17898
tr|F5H6I6|F5H6I6_HUMAN	CAND1	Cullin-associated NEDD8-dissociated protein 1 (Fragment) OS=Homo sapiens GN=CA	6112	67	0	23	0	0.6	0	0	0	0	1	0	1	0	1	0	-1.17898
tr|H0Y3X9|H0Y3X9_HUMAN	VARS	Valine--tRNA ligase (Fragment) OS=Homo sapiens GN=VARS PE=4 SV=1	9798	72	0	0	0	0.38	0	0	0	0	0	0	1	0	1	0	-1.17898
tr|F5H2V6|F5H2V6_HUMAN	MARS	Methionine--tRNA ligase, cytoplasmic OS=Homo sapiens GN=MARS PE=2 SV=2	15629	80	0	74	0	0.55	0	0	1	0	1	0	1	0	1	0	-1.17898
tr|J3KQL0|J3KQL0_HUMAN	CPS1	Carbamoyl-phosphate synthase [ammonia], mitochondrial OS=Homo sapiens GN=CF	166686	82	0	30	0	0.35	0	0	0	0	1	0	1	0	1	0	-1.17898
tr|B9VP24|B9VP24_HUMAN	HSPD1	60 kDa chaperonin (Fragment) OS=Homo sapiens GN=HSPD1 PE=4 SV=1	19880	85	0	36	0	1.58	0	0	1	0	1	0	1	0	1	0	-1.17898
tr|H3BPE3|H3BPE3_HUMAN	EIF3C	Eukaryotic translation initiation factor 3 subunit C (Fragment) OS=Homo sapiens GN	15394	87	0	36	0	0.44	0	0	1	0	1	0	1	0	1	0	-1.17898
tr|C1IK54|C1IK54_HUMAN	DDX5-ETV4	DEAD box polypeptide 5/ets variant protein 4 fusion protein OS=Homo sapiens GN=I	58580	88	0	48	0	1.4	0	0	1	0	1	0	1	0	1	0	-1.17898
tr|H7C0V6|H7C0V6_HUMAN	EIF4G1	Eukaryotic translation initiation factor 4 gamma 1 (Fragment) OS=Homo sapiens GN	20313	103	0	96	0	0.22	0	0	1	0	2	0	1	0	2	0	-1.17898
tr|H0Y7U1|H0Y7U1_HUMAN	HUWE1	E3 ubiquitin-protein ligase HUWE1 (Fragment) OS=Homo sapiens GN=HUWE1 PE=4	24114	106	0	0	0	0.08	0	0	0	0	0	0	1	0	3	0	-1.17898
tr|H7C3A0|H7C3A0_HUMAN	EIF3L	Eukaryotic translation initiation factor 3 subunit L (Fragment) OS=Homo sapiens GN	14793	114	0	40	0	0.5	0	0	1	0	1	0	1	0	1	0	-1.17898
tr|D6R8Z5|D6R8Z5_HUMAN	MATR3	Matrin-3 (Fragment) OS=Homo sapiens GN=MATR3 PE=2 SV=1	10976	117	0	58	0	0.67	0	0	1	0	1	0	1	0	1	0	-1.17898
tr|Q86TQ3|Q86TQ3_HUMAN	tr|Q86TQ3|Q86TC	Similar to filamin C, gamma (Actin binding protein 280) (Fragment) OS=Homo sapien	19163	125	0	37	0	0.18	0	0	0	0	2	0	1	0	2	0	-1.17898
tr|B4DM12|B4DM12_HUMA	tr|B4DM12|B4DM	cDNA FLJ51242, moderately similar to Eukaryotic translation initiation factor 2 subu	15318	130	0	61	0	1.19	0	0	1	0	1	0	1	0	1	0	-1.17898
tr|F8VR69|F8VR69_HUMAN	RPL6	60S ribosomal protein L6 (Fragment) OS=Homo sapiens GN=RPL6 PE=2 SV=1	7285	147	0	47	0	1.89	0	0	1	0	1	0	1	0	1	0	-1.17898
tr|Q96RV6|Q96RV6_HUMAN	tr|Q96RV6|Q96RV	3-phosphoglycerate dehydrogenase (Fragment) OS=Homo sapiens PE=2 SV=1	4275	154	0	38	0	0.75	0	0	1	0	1	0	1	0	1	0	-1.17898
tr|E9PKQ1|E9PKQ1_HUMAN	COPB1	Coatomer subunit beta (Fragment) OS=Homo sapiens GN=COPB1 PE=2 SV=1	15816	165	0	36	0	0.27	0	0	1	0	1	0	1	0	1	0	-1.17898
tr|Q6P4R2|Q6P4R2_HUMAN	PCDHAC2	Protocadherin alpha subfamily C, 2 OS=Homo sapiens GN=PCDHAC2 PE=2 SV=1	110037	178	0	52	0	0.23	0	0	1	0	1	0	1	0	1	0	-1.17898
tr|C9J809|C9J809_HUMAN	MSH2	DNA mismatch repair protein Msh2 (Fragment) OS=Homo sapiens GN=MSH2 PE=2 S	15442	188	0	0	0	0.2	0	0	0	0	0	0	1	0	1	0	-1.17898
tr|F8WDK9|F8WDK9_HUMA	PDCD6IP	Programmed cell death 6-interacting protein OS=Homo sapiens GN=PDCD6IP PE=2 S	10956	194	0	68	0	0.26	0	0	1	0	1	0	1	0	1	0	-1.17898
tr|H7C4I3|H7C4I3_HUMAN	RUVBL1	RuvB-like 1 (Fragment) OS=Homo sapiens GN=RUVBL1 PE=4 SV=1	25136	196	0	37	0	0.56	0	0	1	0	1	0	1	0	1	0	-1.17898
tr|B4DFD7|B4DFD7_HUMAN	tr|B4DFD7|B4DFD	cDNA FLJ55457, highly similar to ATP-dependent RNA helicase DHX8 (EC 3.6.1.-) OS=	98244	218	0	0	0	0.24	0	0	0	0	0	0	1	0	1	0	-1.17898
tr|B4DJB0|B4DJB0_HUMAN	tr|B4DJB0|B4DJB0	cDNA FLJ60092, highly similar to 14-3-3 protein epsilon (14-3-3E) OS=Homo sapiens	16088	227	0	64	0	1.65	0	0	1	0	1	0	1	0	1	0	-1.17898
tr|Q2KQ74|Q2KQ74_HUMAN	tr|Q2KQ74|Q2KQ7	HECTD1 (Fragment) OS=Homo sapiens PE=2 SV=1	136749	232	0	75	0	0.05	0	0	1	0	1	0	1	0	3	0	-1.17898
tr|H7C278|H7C278_HUMAN	DARS	Aspartate--tRNA ligase, cytoplasmic (Fragment) OS=Homo sapiens GN=DARS PE=4 S\	20171	238	0	43	0	0.4	0	0	1	0	1	0	1	0	1	0	-1.17898
tr|Q16791|Q16791_HUMAN	tr|Q16791|Q1679	Mammary dihydrolipoamide acetyltransferase, mature sequence (Fragment) OS=Ho	29551	240	0	36	0	0.29	0	0	1	0	1	0	1	0	1	0	-1.17898
tr|F6X838|F6X838_HUMAN	COPG2	Coatomer subunit gamma-2 OS=Homo sapiens GN=COPG2 PE=4 SV=1	28200	243	0	51	0	0.12	0	0	1	0	1	0	1	0	1	0	-1.17898
tr|F5H0E2|F5H0E2_HUMAN	SLC3A2	4F2 cell-surface antigen heavy chain (Fragment) OS=Homo sapiens GN=SLC3A2 PE=2	15744	245	0	93	0	0.26	0	0	1	0	1	0	1	0	1	0	-1.17898
tr|Q96PA3|Q96PA3_HUMAN	tr|Q96PA3|Q96PA	IQGAP1 protein (Fragment) OS=Homo sapiens PE=2 SV=1	10782	249	0	73	0	0.11	0	0	1	0	1	0	1	0	1	0	-1.17898
tr|D6RFL1|D6RFL1_HUMAN	CANX	Calnexin (Fragment) OS=Homo sapiens GN=CANX PE=2 SV=1	15265	251	0	32	0	0.25	0	0	0	0	1	0	1	0	1	0	-1.17898
tr|H0YE16|H0YE16_HUMAN	RDX	Radixin (Fragment) OS=Homo sapiens GN=RDX PE=4 SV=1	28941	262	0	56	0	0.32	0	0	1	0	1	0	1	0	1	0	-1.17898
tr|Q49AK0|Q49AK0_HUMAN	LTA4H	LTA4H protein OS=Homo sapiens GN=LTA4H PE=2 SV=1	17306	268	0	76	0	0.22	0	0	1	0	1	0	1	0	1	0	-1.17898
tr|Q504R3|Q504R3_HUMAN	CLEC2D	Alternative protein CLEC2D OS=Homo sapiens GN=CLEC2D PE=2 SV=1	23902	271	0	0	0	0.37	0	0	0	0	0	0	1	0	1	0	-1.17898
tr|H0YJD3|H0YJD3_HUMAN	PRMT5	Protein arginine N-methyltransferase 5 (Fragment) OS=Homo sapiens GN=PRMT5 PE	20375	275	0	43	0	0.3	0	0	1	0	1	0	1	0	1	0	-1.17898
tr|B0QYA4|B0QYA4_HUMAN	EIF3D	Eukaryotic translation initiation factor 3 subunit D (Fragment) OS=Homo sapiens GN	19404	303	0	37	0	0.39	0	0	1	0	1	0	1	0	1	0	-1.17898
tr|J3KPU5|J3KPU5_HUMAN	TNPO1	Transportin-1 OS=Homo sapiens GN=TNPO1 PE=4 SV=1	13684	314	0	32	0	0.24	0	0	0	0	1	0	1	0	1	0	-1.17898
tr|G3V2P7|G3V2P7_HUMAN	KLC1	Kinesin light chain 1 (Fragment) OS=Homo sapiens GN=KLC1 PE=2 SV=1	17637	316	0	34	0	0.16	0	0	0	0	1	0	1	0	2	0	-1.17898
tr|F2Z2D0|F2Z2D0_HUMAN	NUP205	Nuclear pore complex protein Nup205 OS=Homo sapiens GN=NUP205 PE=4 SV=1	23590	324	0	55	0	0.07	0	0	1	0	1	0	1	0	1	0	-1.17898
tr|H0Y8W1|H0Y8W1_HUMA	ZFR	Zinc finger RNA-binding protein (Fragment) OS=Homo sapiens GN=ZFR PE=4 SV=1	20174	326	0	51	0	0.12	0	0	1	0	1	0	1	0	1	0	-1.17898
tr|I3L2H8|I3L2H8_HUMAN	MYBBP1A	Myb-binding protein 1A (Fragment) OS=Homo sapiens GN=MYBBP1A PE=4 SV=1	22828	328	0	30	0	0.17	0	0	0	0	1	0	1	0	2	0	-1.17898
tr|C9JLV6|C9JLV6_HUMAN	MDH1	Malate dehydrogenase, cytoplasmic (Fragment) OS=Homo sapiens GN=MDH1 PE=2	12411	352	0	35	0	0.3	0	0	0	0	1	0	1	0	1	0	-1.17898
tr|B3KN94|B3KN94_HUMAN	tr|B3KN94|B3KN9	cDNA FLJ13982 fis, clone Y79AA1001711, highly similar to 60 kDa SS-A/Ro ribonucle	62704	360	0	43	0	0.11	0	0	1	0	1	0	1	0	1	0	-1.17898
tr|F8WBP7|F8WBP7_HUMAN	RANBP2	Putative peptidyl-prolyl cis-trans isomerase OS=Homo sapiens GN=RANBP2 PE=2 SV	6034	367	0	46	0	0.07	0	0	1	0	1	0	1	0	1	0	-1.17898
tr|C9JLS9|C9JLS9_HUMAN	PSMC2	26S protease regulatory subunit 7 (Fragment) OS=Homo sapiens GN=PSMC2 PE=4 S\	14685	372	0	40	0	0.22	0	0	1	0	1	0	1	0	1	0	-1.17898
tr|G3V3H8|G3V3H8_HUMAN	WARS	T1-TrpRS (Fragment) OS=Homo sapiens GN=WARS PE=2 SV=1	25636	373	0	41	0	0.2	0	0	1	0	1	0	1	0	2	0	-1.17898
tr|B4DFR3|B4DFR3_HUMAN	tr|B4DFR3|B4DFR	cDNA FLJ55771, highly similar to Lamin-A/C OS=Homo sapiens PE=2 SV=1	25697	381	0	49	0	0.33	0	0	1	0	1	0	1	0	1	0	-1.17898
tr|K7ENW7|K7ENW7_HUMA	DNMT1	DNA (cytosine-5)-methyltransferase 1 (Fragment) OS=Homo sapiens GN=DNMT1 PE	44102	382	0	22	0	0.09	0	0	0	0	1	0	1	0	1	0	-1.17898
tr|A8MX29|A8MX29_HUMAN	KLC2	Kinesin light chain 2 OS=Homo sapiens GN=KLC2 PE=2 SV=1	36716	384	0	21	0	0.2	0	0	0	0	1	0	1	0	1	0	-1.17898
tr|F8WA66|F8WA66_HUMAN	RECQL	ATP-dependent DNA helicase Q1 (Fragment) OS=Homo sapiens GN=RECQL PE=2 SV=	22447	387	0	36	0	0.3	0	0	1	0	1	0	1	0	1	0	-1.17898
tr|B3KS60|B3KS60_HUMAN	tr|B3KS60|B3KS60	cDNA FLJ35566 fis, clone SPLEN2005474, highly similar to Homo sapiens CCR4-NOT t	36426	389	0	38	0	0.07	0	0	1	0	1	0	1	0	1	0	-1.17898
tr|O60250|O60250_HUMAN	tr|O60250|O6025	Ribosomal protein L13 (Fragment) OS=Homo sapiens PE=2 SV=2	7385	402	0	24	0	0.47	0	0	0	0	1	0	1	0	2	0	-1.17898
tr|D6RCB3|D6RCB3_HUMAN	ETF1	Eukaryotic peptide chain release factor subunit 1 OS=Homo sapiens GN=ETF1 PE=2 S	10476	406	0	30	0	0.22	0	0	0	0	1	0	1	0	1	0	-1.17898
tr|B3KXL9|B3KXL9_HUMAN	tr|B3KXL9|B3KXL9	cDNA FLJ45690 fis, clone FCBBF5000384, highly similar to SWI/SNF-related matrix-as	50398	413	0	36	0	0.13	0	0	0	0	1	0	1	0	1	0	-1.17898
tr|F5H571|F5H571_HUMAN	USP5	Ubiquitin carboxyl-terminal hydrolase 5 (Fragment) OS=Homo sapiens GN=USP5 PE=	15755	426	0	40	0	0.11	0	0	1	0	1	0	1	0	1	0	-1.17898
tr|H0Y9L6|H0Y9L6_HUMAN	PDS5A	Sister chromatid cohesion protein PDS5 homolog A (Fragment) OS=Homo sapiens GI	14071	434	0	24	0	0.07	0	0	0	0	1	0	1	0	1	0	-1.17898
tr|Q05D43|Q05D43_HUMAN	YBX1	YBX1 protein (Fragment) OS=Homo sapiens GN=YBX1 PE=2 SV=1	29487	439	0	83	0	0.16	0	0	1	0	1	0	1	0	1	0	-1.17898
tr|D6W636|D6W636_HUMA	DUS3L	Dihydrouridine synthase 3-like (S. cerevisiae), isoform CRA_a OS=Homo sapiens GN=	36425	440	0	25	0	0.14	0	0	0	0	1	0	1	0	1	0	-1.17898
tr|H0Y9G8|H0Y9G8_HUMAN	Sep-11	Septin-11 (Fragment) OS=Homo sapiens GN=SEPT11 PE=4 SV=1	9813	441	0	65	0	0.21	0	0	1	0	1	0	1	0	1	0	-1.17898
tr|B3KWS3|B3KWS3_HUMAN	PLOD2	Procollagen-lysine,2-oxoglutarate 5-dioxygenase 2 OS=Homo sapiens GN=PLOD2 PE	49567	444	0	20	0	0.08	0	0	0	0	1	0	1	0	1	0	-1.17898
tr|J3KR44|J3KR44_HUMAN	OTUB1	Ubiquitin thioesterase OTUB1 OS=Homo sapiens GN=OTUB1 PE=4 SV=1	35649	456	0	85	0	0.09	0	0	1	0	1	0	1	0	1	0	-1.17898
tr|H3BRG4|H3BRG4_HUMAN	UQCRC2	Cytochrome b-c1 complex subunit 2, mitochondrial OS=Homo sapiens GN=UQCRC2	44778	460	0	84	0	0.07	0	0	1	0	1	0	1	0	2	0	-1.17898
tr|B4DJS0|B4DJS0_HUMAN	tr|B4DJS0|B4DJS0	cDNA FLJ56766, highly similar to Protein disulfide-isomerase (EC5.3.4.1) OS=Homo s	26835	466	0	79	0	0.12	0	0	1	0	1	0	1	0	2	0	-1.17898
tr|K7EKQ2|K7EKQ2_HUMAN	CDC37	Hsp90 co-chaperone Cdc37 (Fragment) OS=Homo sapiens GN=CDC37 PE=4 SV=1	42032	473	0	51	0	0.15	0	0	1	0	1	0	1	0	2	0	-1.17898
tr|B3KQH2|B3KQH2_HUMAN	tr|B3KQH2|B3KQH	cDNA FLJ90454 fis, clone NT2RP3001560, highly similar to Cleft lip and palate transm	76247	482	0	80	0	0.04	0	0	1	0	1	0	1	0	1	0	-1.17898
tr|H3BPV7|H3BPV7_HUMAN	KARS	Lysine--tRNA ligase (Fragment) OS=Homo sapiens GN=KARS PE=4 SV=1	12352	484	0	45	0	0.15	0	0	1	0	1	0	1	0	1	0	-1.17898
tr|Q5T0G9|Q5T0G9_HUMAN	ANXA11	Annexin A11 (Fragment) OS=Homo sapiens GN=ANXA11 PE=4 SV=1	20474	486	0	21	0	0.15	0	0	0	0	1	0	1	0	1	0	-1.17898
tr|B3KT59|B3KT59_HUMAN	tr|B3KT59|B3KT59	cDNA FLJ37698 fis, clone BRHIP2015679, highly similar to Ataxin-2 OS=Homo sapien	45662	490	0	78	0	0.03	0	0	1	0	1	0	1	0	1	0	-1.17898
tr|H0YDX9|H0YDX9_HUMAN	VPS51	Vacuolar protein sorting-associated protein 51 homolog (Fragment) OS=Homo sapie	7976	494	0	25	0	0.08	0	0	0	0	1	0	1	0	1	0	-1.17898
tr|E9PKG3|E9PKG3_HUMAN	CTTN	Src substrate cortactin (Fragment) OS=Homo sapiens GN=CTTN PE=2 SV=1	5789	495	0	77	0	0.13	0	0	1	0	1	0	1	0	1	0	-1.17898
tr|B4DFN6|B4DFN6_HUMAN	CNP	2~,3~-cyclic-nucleotide 3~-phosphodiesterase OS=Homo sapiens GN=CNP PE=2 SV=1	20461	501	0	77	0	0.16	0	0	1	0	1	0	1	0	1	0	-1.17898
tr|B3KMC2|B3KMC2_HUMA	tr|B3KMC2|B3KM	cDNA FLJ10676 fis, clone NT2RP2006464, highly similar to WD repeat and HMG-box	73526	507	0	75	0	0.04	0	0	1	0	1	0	1	0	1	0	-1.17898
tr|B4DFK7|B4DFK7_HUMAN	CBR1	Carbonyl reductase 1, isoform CRA_c OS=Homo sapiens GN=CBR1 PE=2 SV=1	19092	508	0	75	0	0.18	0	0	1	0	1	0	1	0	1	0	-1.17898
tr|Q5IRN4|Q5IRN4_HUMAN	MEF2D/DAZAP1	Myocyte enhancer factor 2D/deleted in azoospermia associated protein 1 fusion pro	50610	512	0	25	0	0.29	0	0	0	0	1	0	1	0	1	0	-1.17898
tr|J3KR78|J3KR78_HUMAN	QTRTD1	Queuine tRNA-ribosyltransferase subunit QTRTD1 OS=Homo sapiens GN=QTRTD1 PE	49085	522	0	72	0	0.07	0	0	1	0	1	0	1	0	1	0	-1.17898
sp|Q13425|SNTB2_HUMAN	SNTB2	Beta-2-syntrophin OS=Homo sapiens GN=SNTB2 PE=1 SV=1	58369	527	0	72	0	0.06	0	0	1	0	1	0	1	0	2	0	-1.17898
tr|H0YHX9|H0YHX9_HUMAN	NACA	Nascent polypeptide-associated complex subunit alpha (Fragment) OS=Homo sapier	22930	537	0	70	0	0.15	0	0	1	0	1	0	1	0	2	0	-1.17898
tr|Q53G25|Q53G25_HUMAN	tr|Q53G25|Q53G2	Ribosomal protein S5 variant (Fragment) OS=Homo sapiens PE=2 SV=1	23121	539	0	70	0	0.15	0	0	1	0	1	0	1	0	1	0	-1.17898
tr|E9PRB8|E9PRB8_HUMAN	PSMC3	26S protease regulatory subunit 6A OS=Homo sapiens GN=PSMC3 PE=2 SV=1	13661	548	0	38	0	0.31	0	0	1	0	1	0	1	0	1	0	-1.17898
tr|F8WB65|F8WB65_HUMAN	Sep-02	Septin-2 OS=Homo sapiens GN=SEPT2 PE=2 SV=1	8930	553	0	30	0	0.16	0	0	0	0	1	0	1	0	1	0	-1.17898
tr|J3QQX0|J3QQX0_HUMAN	GPS1	COP9 signalosome complex subunit 1 (Fragment) OS=Homo sapiens GN=GPS1 PE=4	8703	558	0	67	0	0.4	0	0	1	0	1	0	1	0	1	0	-1.17898
tr|B4E2H2|B4E2H2_HUMAN	tr|B4E2H2|B4E2H	cDNA FLJ56559, highly similar to FKBP12-rapamycin complex-associated protein OS=	196418	562	0	66	0	0.02	0	0	1	0	1	0	1	0	2	0	-1.17898
tr|H7C0T0|H7C0T0_HUMAN	FAM120A	Constitutive coactivator of PPAR-gamma-like protein 1 (Fragment) OS=Homo sapien	54253	569	0	0	0	0.05	0	0	0	0	0	0	1	0	1	0	-1.17898
tr|H3BM93|H3BM93_HUMA	NUP93	Nuclear pore complex protein Nup93 (Fragment) OS=Homo sapiens GN=NUP93 PE=	19858	572	0	65	0	0.17	0	0	1	0	1	0	1	0	1	0	-1.17898
tr|Q59G68|Q59G68_HUMAN	tr|Q59G68|Q59G6	Putative uncharacterized protein (Fragment) OS=Homo sapiens PE=2 SV=1	18527	573	0	16	0	0.27	0	0	0	0	1	0	1	0	2	0	-1.17898
tr|H0Y4J2|H0Y4J2_HUMAN	MRPL37	39S ribosomal protein L37, mitochondrial (Fragment) OS=Homo sapiens GN=MRPL3	29806	583	0	64	0	0.11	0	0	1	0	1	0	1	0	1	0	-1.17898
tr|A4D1P0|A4D1P0_HUMAN	LOC402299	Uncharacterized protein OS=Homo sapiens GN=LOC402299 PE=4 SV=1	42109	593	0	52	0	0.19	0	0	1	0	1	0	1	0	1	0	-1.17898
tr|Q9H9M0|Q9H9M0_HUMA	GFM1	Elongation factor G, mitochondrial OS=Homo sapiens GN=GFM1 PE=2 SV=1	16764	596	0	33	0	0.23	0	0	0	0	2	0	1	0	2	0	-1.17898
tr|I3L2G1|I3L2G1_HUMAN	NSF	Vesicle-fusing ATPase (Fragment) OS=Homo sapiens GN=NSF PE=4 SV=1	15320	604	0	51	0	0.08	0	0	1	0	1	0	1	0	1	0	-1.17898
tr|B4DWM5|B4DWM5_HUM	tr|B4DWM5|B4DV	cDNA FLJ61436, highly similar to Melanoma-associated antigen D2 OS=Homo sapien	26229	606	0	61	0	0.13	0	0	1	0	1	0	1	0	1	0	-1.17898
tr|H0YJF9|H0YJF9_HUMAN	DLST	Dihydrolipoyllysine-residue succinyltransferase component of 2-oxoglutarate dehyd	8710	607	0	60	0	0.4	0	0	1	0	1	0	1	0	2	0	-1.17898
tr|D6RGE0|D6RGE0_HUMAN	RPS3A	40S ribosomal protein S3a (Fragment) OS=Homo sapiens GN=RPS3A PE=2 SV=1	7338	608	0	45	0	0.27	0	0	1	0	1	0	1	0	1	0	-1.17898
tr|Q71US4|Q71US4_HUMAN	tr|Q71US4|Q71US	EIF-2B-delta-like protein OS=Homo sapiens PE=2 SV=1	58031	611	0	60	0	0.06	0	0	1	0	1	0	1	0	2	0	-1.17898
tr|B4DDC9|B4DDC9_HUMAN	tr|B4DDC9|B4DDC	cDNA FLJ59448, highly similar to Trifunctional enzyme subunit beta, mitochondrial C	49937	614	0	60	0	0.07	0	0	1	0	1	0	1	0	1	0	-1.17898
sp|P42695|CNDD3_HUMAN	NCAPD3	Condensin-2 complex subunit D3 OS=Homo sapiens GN=NCAPD3 PE=1 SV=2	170950	625	0	59	0	0.02	0	0	1	0	1	0	1	0	2	0	-1.17898
tr|G3V1U5|G3V1U5_HUMAN	GOLT1B	Golgi transport 1 homolog B (S. cerevisiae), isoform CRA_c OS=Homo sapiens GN=G(	8208	627	0	58	0	0.43	0	0	1	0	1	0	1	0	1	0	-1.17898
tr|H0YB26|H0YB26_HUMAN	CPNE3	Copine-3 (Fragment) OS=Homo sapiens GN=CPNE3 PE=4 SV=1	29941	628	0	38	0	0.17	0	0	1	0	1	0	1	0	2	0	-1.17898
sp|Q9H0C2|ADT4_HUMAN	SLC25A31	ADP/ATP translocase 4 OS=Homo sapiens GN=SLC25A31 PE=2 SV=1	35285	632	0	53	0	0.21	0	0	1	0	1	0	1	0	1	0	-1.17898
tr|H0Y6E7|H0Y6E7_HUMAN	RBMX	RNA-binding motif protein, X chromosome, N-terminally processed (Fragment) OS=H	31837	633	0	57	0	0.1	0	0	1	0	1	0	1	0	1	0	-1.17898
tr|H0YEN0|H0YEN0_HUMAN	RIC8A	Synembryn-A (Fragment) OS=Homo sapiens GN=RIC8A PE=4 SV=1	32826	640	0	57	0	0.1	0	0	1	0	1	0	1	0	1	0	-1.17898
tr|Q7Z777|Q7Z777_HUMAN	CLPB	CLPB protein OS=Homo sapiens GN=CLPB PE=2 SV=1	36436	642	0	57	0	0.09	0	0	1	0	1	0	1	0	1	0	-1.17898
tr|B3KQ12|B3KQ12_HUMAN	tr|B3KQ12|B3KQ1	cDNA FLJ32606 fis, clone STOMA1000186, highly similar to Homo sapiens acyl-CoA t	40814	644	0	57	0	0.08	0	0	1	0	1	0	1	0	2	0	-1.17898
tr|H0Y5H6|H0Y5H6_HUMAN	UBAP2L	Ubiquitin-associated protein 2-like (Fragment) OS=Homo sapiens GN=UBAP2L PE=4	40310	646	0	29	0	0.06	0	0	0	0	1	0	1	0	1	0	-1.17898
tr|D3YTB1|D3YTB1_HUMAN	RPL32	60S ribosomal protein L32 (Fragment) OS=Homo sapiens GN=RPL32 PE=2 SV=1	15721	647	0	56	0	0.22	0	0	1	0	1	0	1	0	1	0	-1.17898
tr|B7Z3Q4|B7Z3Q4_HUMAN	tr|B7Z3Q4|B7Z3Q	cDNA FLJ51873, highly similar to Alkyldihydroxyacetonephosphate synthase, peroxis	64547	648	0	56	0	0.05	0	0	1	0	1	0	1	0	2	0	-1.17898
tr|H7C1W5|H7C1W5_HUMA	MMS19	MMS19 nucleotide excision repair protein homolog (Fragment) OS=Homo sapiens G	16833	649	0	56	0	0.2	0	0	1	0	1	0	1	0	1	0	-1.17898

<table>
<tr><th></th><th></th><th></th><th></th><th></th><th></th><th></th><th></th><th></th><th></th><th></th><th></th><th></th><th></th><th></th><th></th><th></th><th></th><th></th><th></th></tr>
<tr><td>sp|Q6UXN9|WDR82_HUMAN</td><td>WDR82</td><td>WD repeat-containing protein 82 OS=Homo sapiens GN=WDR82 PE=1 SV=1</td><td>35456</td><td>653</td><td>0</td><td>56</td><td>0</td><td>0.09</td><td>0</td><td>0</td><td>1</td><td>0</td><td>1</td><td>0</td><td>1</td><td>0</td><td>5</td><td>0</td><td>-1.17898</td></tr>
<tr><td>tr|G3V3F1|G3V3F1_HUMAN</td><td>NEDD1</td><td>Neural cell expressed, developmentally down-regulated 1, isoform CRA_c OS=Homo</td><td>73292</td><td>655</td><td>0</td><td>55</td><td>0</td><td>0.04</td><td>0</td><td>0</td><td>1</td><td>0</td><td>1</td><td>0</td><td>1</td><td>0</td><td>1</td><td>0</td><td>-1.17898</td></tr>
<tr><td>tr|Q8TEN0|Q8TEN0_HUMAN</td><td>FLJ00163</td><td>FLJ00163 protein (Fragment) OS=Homo sapiens GN=FLJ00163 PE=2 SV=1</td><td>56335</td><td>656</td><td>0</td><td>55</td><td>0</td><td>0.06</td><td>0</td><td>0</td><td>1</td><td>0</td><td>1</td><td>0</td><td>1</td><td>0</td><td>1</td><td>0</td><td>-1.17898</td></tr>
<tr><td>tr|Q1WWM3|Q1WWM3_HU</td><td>EPB41</td><td>EPB41 protein (Fragment) OS=Homo sapiens GN=EPB41 PE=2 SV=1</td><td>83618</td><td>668</td><td>0</td><td>54</td><td>0</td><td>0.04</td><td>0</td><td>0</td><td>1</td><td>0</td><td>1</td><td>0</td><td>1</td><td>0</td><td>1</td><td>0</td><td>-1.17898</td></tr>
<tr><td>tr|H0YEX5|H0YEX5_HUMAN</td><td>SF3B2</td><td>Splicing factor 3B subunit 2 (Fragment) OS=Homo sapiens GN=SF3B2 PE=4 SV=1</td><td>35044</td><td>671</td><td>0</td><td>38</td><td>0</td><td>0.07</td><td>0</td><td>0</td><td>1</td><td>0</td><td>1</td><td>0</td><td>1</td><td>0</td><td>1</td><td>0</td><td>-1.17898</td></tr>
<tr><td>tr|Q6IB71|Q6IB71_HUMAN</td><td>PSMA3</td><td>Proteasome subunit alpha type OS=Homo sapiens GN=PSMA3 PE=2 SV=1</td><td>28625</td><td>672</td><td>0</td><td>54</td><td>0</td><td>0.12</td><td>0</td><td>0</td><td>1</td><td>0</td><td>1</td><td>0</td><td>1</td><td>0</td><td>1</td><td>0</td><td>-1.17898</td></tr>
<tr><td>sp|Q9Y5Q9|TF3C3_HUMAN</td><td>GTF3C3</td><td>General transcription factor 3C polypeptide 3 OS=Homo sapiens GN=GTF3C3 PE=1 S</td><td>102006</td><td>673</td><td>0</td><td>54</td><td>0</td><td>0.03</td><td>0</td><td>0</td><td>1</td><td>0</td><td>1</td><td>0</td><td>1</td><td>0</td><td>2</td><td>0</td><td>-1.17898</td></tr>
<tr><td>tr|H3BNB9|H3BNB9_HUMAN</td><td>ARIH1</td><td>E3 ubiquitin-protein ligase ARIH1 (Fragment) OS=Homo sapiens GN=ARIH1 PE=4 SV=</td><td>22769</td><td>682</td><td>0</td><td>53</td><td>0</td><td>0.15</td><td>0</td><td>0</td><td>1</td><td>0</td><td>1</td><td>0</td><td>1</td><td>0</td><td>1</td><td>0</td><td>-1.17898</td></tr>
<tr><td>tr|G0ZJH6|G0ZJH6_HUMAN</td><td>Ube4B</td><td>UFD2A-III/UBE4B-III splice isoform OS=Homo sapiens GN=Ube4B PE=2 SV=1</td><td>152890</td><td>691</td><td>0</td><td>52</td><td>0</td><td>0.02</td><td>0</td><td>0</td><td>1</td><td>0</td><td>1</td><td>0</td><td>1</td><td>0</td><td>1</td><td>0</td><td>-1.17898</td></tr>
<tr><td>tr|B0I1S9|B0I1S9_HUMAN</td><td>MYO1B</td><td>MYO1B variant protein OS=Homo sapiens GN=MYO1B PE=2 SV=1</td><td>132928</td><td>692</td><td>0</td><td>52</td><td>0</td><td>0.02</td><td>0</td><td>0</td><td>1</td><td>0</td><td>1</td><td>0</td><td>1</td><td>0</td><td>2</td><td>0</td><td>-1.17898</td></tr>
<tr><td>sp|P62851|RS25_HUMAN</td><td>RPS25</td><td>40S ribosomal protein S25 OS=Homo sapiens GN=RPS25 PE=1 SV=1</td><td>13791</td><td>696</td><td>0</td><td>52</td><td>0</td><td>0.25</td><td>0</td><td>0</td><td>1</td><td>0</td><td>1</td><td>0</td><td>1</td><td>0</td><td>1</td><td>0</td><td>-1.17898</td></tr>
<tr><td>tr|H0YE27|H0YE27_HUMAN</td><td>RPS2</td><td>40S ribosomal protein S2 (Fragment) OS=Homo sapiens GN=RPS2 PE=4 SV=1</td><td>7140</td><td>698</td><td>0</td><td>52</td><td>0</td><td>0.5</td><td>0</td><td>0</td><td>1</td><td>0</td><td>1</td><td>0</td><td>1</td><td>0</td><td>1</td><td>0</td><td>-1.17898</td></tr>
<tr><td>tr|B4DZM3|B4DZM3_HUMAN</td><td>tr|B4DZM3|B4DZM</td><td>cDNA FLJ61500, highly similar to NNP-1 protein OS=Homo sapiens PE=2 SV=1</td><td>46521</td><td>701</td><td>0</td><td>52</td><td>0</td><td>0.07</td><td>0</td><td>0</td><td>1</td><td>0</td><td>1</td><td>0</td><td>1</td><td>0</td><td>1</td><td>0</td><td>-1.17898</td></tr>
<tr><td>tr|B7Z7P0|B7Z7P0_HUMAN</td><td>UBE4A</td><td>Ubiquitin conjugation factor E4 A OS=Homo sapiens GN=UBE4A PE=2 SV=1</td><td>62305</td><td>702</td><td>0</td><td>44</td><td>0</td><td>0.05</td><td>0</td><td>0</td><td>1</td><td>0</td><td>1</td><td>0</td><td>1</td><td>0</td><td>1</td><td>0</td><td>-1.17898</td></tr>
<tr><td>tr|A0PJ62|A0PJ62_HUMAN</td><td>RPL14</td><td>RPL14 protein (Fragment) OS=Homo sapiens GN=RPL14 PE=2 SV=1</td><td>14716</td><td>705</td><td>0</td><td>32</td><td>0</td><td>0.3</td><td>0</td><td>0</td><td>0</td><td>0</td><td>1</td><td>0</td><td>1</td><td>0</td><td>1</td><td>0</td><td>-1.17898</td></tr>
<tr><td>tr|Q5JSD1|Q5JSD1_HUMAN</td><td>VDAC2</td><td>Voltage-dependent anion-selective channel protein 2 (Fragment) OS=Homo sapiens</td><td>22544</td><td>709</td><td>0</td><td>26</td><td>0</td><td>0.25</td><td>0</td><td>0</td><td>0</td><td>0</td><td>1</td><td>0</td><td>1</td><td>0</td><td>1</td><td>0</td><td>-1.17898</td></tr>
<tr><td>tr|H0YKD0|H0YKD0_HUMAN</td><td>IDH3A</td><td>Isocitrate dehydrogenase [NAD] subunit alpha, mitochondrial (Fragment) OS=Homo</td><td>12184</td><td>710</td><td>0</td><td>51</td><td>0</td><td>0.28</td><td>0</td><td>0</td><td>1</td><td>0</td><td>1</td><td>0</td><td>1</td><td>0</td><td>1</td><td>0</td><td>-1.17898</td></tr>
<tr><td>sp|A2A3N6|PIPSL_HUMAN</td><td>PIPSL</td><td>Putative PIP5K1A and PSMD4-like protein OS=Homo sapiens GN=PIPSL PE=5 SV=1</td><td>95729</td><td>715</td><td>0</td><td>51</td><td>0</td><td>0.03</td><td>0</td><td>0</td><td>1</td><td>0</td><td>1</td><td>0</td><td>1</td><td>0</td><td>1</td><td>0</td><td>-1.17898</td></tr>
<tr><td>tr|D3DNS0|D3DNS0_HUMAN</td><td>ACTL6A</td><td>Actin-like 6A, isoform CRA_b OS=Homo sapiens GN=ACTL6A PE=2 SV=1</td><td>25781</td><td>718</td><td>0</td><td>31</td><td>0</td><td>0.14</td><td>0</td><td>0</td><td>0</td><td>0</td><td>1</td><td>0</td><td>1</td><td>0</td><td>1</td><td>0</td><td>-1.17898</td></tr>
<tr><td>tr|B4E0W6|B4E0W6_HUMAN</td><td>BTAF1</td><td>TATA-binding protein-associated factor 172 OS=Homo sapiens GN=BTAF1 PE=2 SV=1</td><td>77112</td><td>720</td><td>0</td><td>30</td><td>0</td><td>0.03</td><td>0</td><td>0</td><td>1</td><td>0</td><td>1</td><td>0</td><td>1</td><td>0</td><td>1</td><td>0</td><td>-1.17898</td></tr>
<tr><td>tr|H0Y8S8|H0Y8S8_HUMAN</td><td>UBA6</td><td>Ubiquitin-like modifier-activating enzyme 6 (Fragment) OS=Homo sapiens GN=UBA6</td><td>31255</td><td>721</td><td>0</td><td>35</td><td>0</td><td>0.06</td><td>0</td><td>0</td><td>1</td><td>0</td><td>1</td><td>0</td><td>1</td><td>0</td><td>1</td><td>0</td><td>-1.17898</td></tr>
<tr><td>tr|B7ZLC9|B7ZLC9_HUMAN</td><td>GEMIN5</td><td>GEMIN5 protein OS=Homo sapiens GN=GEMIN5 PE=2 SV=1</td><td>170664</td><td>722</td><td>0</td><td>50</td><td>0</td><td>0.02</td><td>0</td><td>0</td><td>1</td><td>0</td><td>1</td><td>0</td><td>1</td><td>0</td><td>1</td><td>0</td><td>-1.17898</td></tr>
<tr><td>tr|B3KM73|B3KM73_HUMAN</td><td>tr|B3KM73|B3KM7</td><td>cDNA FLJ10419 fis, clone NT2RP1000163, highly similar to Homo sapiens transformi</td><td>55418</td><td>725</td><td>0</td><td>50</td><td>0</td><td>0.06</td><td>0</td><td>0</td><td>1</td><td>0</td><td>1</td><td>0</td><td>1</td><td>0</td><td>1</td><td>0</td><td>-1.17898</td></tr>
<tr><td>tr|A4D2J6|A4D2J6_HUMAN</td><td>PGAM2</td><td>Phosphoglycerate mutase OS=Homo sapiens GN=PGAM2 PE=3 SV=1</td><td>28544</td><td>726</td><td>0</td><td>50</td><td>0</td><td>0.12</td><td>0</td><td>0</td><td>1</td><td>0</td><td>1</td><td>0</td><td>1</td><td>0</td><td>1</td><td>0</td><td>-1.17898</td></tr>
<tr><td>tr|H7C4X8|H7C4X8_HUMAN</td><td>CLASP2</td><td>CLIP-associating protein 2 (Fragment) OS=Homo sapiens GN=CLASP2 PE=4 SV=1</td><td>20981</td><td>730</td><td>0</td><td>49</td><td>0</td><td>0.16</td><td>0</td><td>0</td><td>1</td><td>0</td><td>1</td><td>0</td><td>1</td><td>0</td><td>2</td><td>0</td><td>-1.17898</td></tr>
<tr><td>tr|Q3BDU5|Q3BDU5_HUMAN</td><td>tr|Q3BDU5|Q3BDU</td><td>Rhabdomyosarcoma antigen MU-RMS-40.12 OS=Homo sapiens PE=2 SV=1</td><td>55605</td><td>735</td><td>0</td><td>49</td><td>0</td><td>0.06</td><td>0</td><td>0</td><td>1</td><td>0</td><td>1</td><td>0</td><td>1</td><td>0</td><td>2</td><td>0</td><td>-1.17898</td></tr>
<tr><td>tr|A0JNT2|A0JNT2_HUMAN</td><td>KRT83</td><td>KRT83 protein OS=Homo sapiens GN=KRT83 PE=2 SV=1</td><td>50978</td><td>736</td><td>0</td><td>49</td><td>0</td><td>0.06</td><td>0</td><td>0</td><td>1</td><td>0</td><td>1</td><td>0</td><td>1</td><td>0</td><td>1</td><td>0</td><td>-1.17898</td></tr>
<tr><td>tr|H0Y5T1|H0Y5T1_HUMAN</td><td>CLASP1</td><td>CLIP-associating protein 1 (Fragment) OS=Homo sapiens GN=CLASP1 PE=4 SV=1</td><td>76997</td><td>739</td><td>0</td><td>25</td><td>0</td><td>0.04</td><td>0</td><td>0</td><td>0</td><td>0</td><td>1</td><td>0</td><td>1</td><td>0</td><td>1</td><td>0</td><td>-1.17898</td></tr>
<tr><td>tr|H0YDT5|H0YDT5_HUMAN</td><td>ACADM</td><td>Medium-chain-specific acyl-CoA dehydrogenase, mitochondrial (Fragment) OS=Hom</td><td>7051</td><td>740</td><td>0</td><td>49</td><td>0</td><td>0.5</td><td>0</td><td>0</td><td>1</td><td>0</td><td>1</td><td>0</td><td>1</td><td>0</td><td>1</td><td>0</td><td>-1.17898</td></tr>
<tr><td>tr|A6NDZ3|A6NDZ3_HUMAN</td><td>SAMHD1</td><td>SAM domain and HD domain-containing protein 1 OS=Homo sapiens GN=SAMHD1 P</td><td>20356</td><td>741</td><td>0</td><td>22</td><td>0</td><td>0.09</td><td>0</td><td>0</td><td>0</td><td>0</td><td>1</td><td>0</td><td>1</td><td>0</td><td>1</td><td>0</td><td>-1.17898</td></tr>
<tr><td>tr|Q59ES3|Q59ES3_HUMAN</td><td>tr|Q59ES3|Q59ES3</td><td>Solute carrier family 1 (Neutral amino acid transporter), member 5 variant (Fragmen</td><td>57810</td><td>742</td><td>0</td><td>48</td><td>0</td><td>0.06</td><td>0</td><td>0</td><td>1</td><td>0</td><td>1</td><td>0</td><td>1</td><td>0</td><td>1</td><td>0</td><td>-1.17898</td></tr>
<tr><td>tr|B4DJ92|B4DJ92_HUMAN</td><td>tr|B4DJ92|B4DJ92</td><td>cDNA FLJ57843, highly similar to Homo sapiens neurochondrin (NCDN), transcript va</td><td>20030</td><td>746</td><td>0</td><td>48</td><td>0</td><td>0.17</td><td>0</td><td>0</td><td>1</td><td>0</td><td>1</td><td>0</td><td>1</td><td>0</td><td>1</td><td>0</td><td>-1.17898</td></tr>
<tr><td>tr|B4DVS2|B4DVS2_HUMAN</td><td>tr|B4DVS2|B4DVS</td><td>cDNA FLJ57617 OS=Homo sapiens PE=2 SV=1</td><td>89176</td><td>753</td><td>0</td><td>48</td><td>0</td><td>0.04</td><td>0</td><td>0</td><td>1</td><td>0</td><td>1</td><td>0</td><td>1</td><td>0</td><td>1</td><td>0</td><td>-1.17898</td></tr>
<tr><td>tr|K7EJT5|K7EJT5_HUMAN</td><td>RPL22</td><td>60S ribosomal protein L22 (Fragment) OS=Homo sapiens GN=RPL22 PE=4 SV=1</td><td>5080</td><td>756</td><td>0</td><td>47</td><td>0</td><td>0.72</td><td>0</td><td>0</td><td>1</td><td>0</td><td>1</td><td>0</td><td>1</td><td>0</td><td>1</td><td>0</td><td>-1.17898</td></tr>
<tr><td>tr|Q68E00|Q68E00_HUMAN</td><td>DKFZp686G2045</td><td>Putative uncharacterized protein DKFZp686G2045 (Fragment) OS=Homo sapiens GN</td><td>32787</td><td>759</td><td>0</td><td>47</td><td>0</td><td>0.1</td><td>0</td><td>0</td><td>1</td><td>0</td><td>1</td><td>0</td><td>1</td><td>0</td><td>1</td><td>0</td><td>-1.17898</td></tr>
<tr><td>tr|E9PCI9|E9PCI9_HUMAN</td><td>FDPS</td><td>Farnesyl pyrophosphate synthase OS=Homo sapiens GN=FDPS PE=2 SV=1</td><td>40849</td><td>760</td><td>0</td><td>47</td><td>0</td><td>0.08</td><td>0</td><td>0</td><td>1</td><td>0</td><td>1</td><td>0</td><td>1</td><td>0</td><td>1</td><td>0</td><td>-1.17898</td></tr>
<tr><td>tr|B4DI19|B4DI19_HUMAN</td><td>tr|B4DI19|B4DI19</td><td>cDNA FLJ59524, highly similar to Cysteinyl-tRNA synthetase (EC 6.1.1.16) OS=Homo</td><td>76209</td><td>761</td><td>0</td><td>47</td><td>0</td><td>0.04</td><td>0</td><td>0</td><td>1</td><td>0</td><td>1</td><td>0</td><td>1</td><td>0</td><td>1</td><td>0</td><td>-1.17898</td></tr>
<tr><td>tr|A2AB15|A2AB15_HUMAN</td><td>DHX16</td><td>Putative pre-mRNA-splicing factor ATP-dependent RNA helicase DHX16 (Fragment)</td><td>34860</td><td>763</td><td>0</td><td>47</td><td>0</td><td>0.1</td><td>0</td><td>0</td><td>1</td><td>0</td><td>1</td><td>0</td><td>1</td><td>0</td><td>1</td><td>0</td><td>-1.17898</td></tr>
<tr><td>tr|B5BU72|B5BU72_HUMAN</td><td>PICALM</td><td>Phosphatidylinositol-binding clathrin assembly protein isoform 2 OS=Homo sapiens</td><td>66523</td><td>766</td><td>0</td><td>46</td><td>0</td><td>0.05</td><td>0</td><td>0</td><td>1</td><td>0</td><td>1</td><td>0</td><td>1</td><td>0</td><td>1</td><td>0</td><td>-1.17898</td></tr>
<tr><td>sp|Q8NFW8|NEUA_HUMAN</td><td>CMAS</td><td>N-acylneuraminate cytidylyltransferase OS=Homo sapiens GN=CMAS PE=1 SV=2</td><td>49033</td><td>767</td><td>0</td><td>46</td><td>0</td><td>0.07</td><td>0</td><td>0</td><td>1</td><td>0</td><td>1</td><td>0</td><td>1</td><td>0</td><td>1</td><td>0</td><td>-1.17898</td></tr>
<tr><td>tr|Q7Z524|Q7Z524_HUMAN</td><td>tr|Q7Z524|Q7Z52</td><td>HUMEEP OS=Homo sapiens PE=2 SV=1</td><td>51391</td><td>769</td><td>0</td><td>46</td><td>0</td><td>0.06</td><td>0</td><td>0</td><td>1</td><td>0</td><td>1</td><td>0</td><td>1</td><td>0</td><td>1</td><td>0</td><td>-1.17898</td></tr>
<tr><td>tr|B3KSI3|B3KSI3_HUMAN</td><td>BCAT2</td><td>Branched-chain-amino-acid aminotransferase OS=Homo sapiens GN=BCAT2 PE=2 SV</td><td>40232</td><td>770</td><td>0</td><td>46</td><td>0</td><td>0.08</td><td>0</td><td>0</td><td>1</td><td>0</td><td>1</td><td>0</td><td>1</td><td>0</td><td>1</td><td>0</td><td>-1.17898</td></tr>
<tr><td>sp|A9QM74|IMA8_HUMAN</td><td>KPNA7</td><td>Importin subunit alpha-8 OS=Homo sapiens GN=KPNA7 PE=1 SV=1</td><td>57529</td><td>774</td><td>0</td><td>45</td><td>0</td><td>0.06</td><td>0</td><td>0</td><td>1</td><td>0</td><td>1</td><td>0</td><td>1</td><td>0</td><td>1</td><td>0</td><td>-1.17898</td></tr>
<tr><td>tr|B4DS71|B4DS71_HUMAN</td><td>tr|B4DS71|B4DS71</td><td>cDNA FLJ57081, moderately similar to WD repeat protein 1 OS=Homo sapiens PE=2</td><td>22638</td><td>777</td><td>0</td><td>45</td><td>0</td><td>0.15</td><td>0</td><td>0</td><td>1</td><td>0</td><td>1</td><td>0</td><td>1</td><td>0</td><td>1</td><td>0</td><td>-1.17898</td></tr>
<tr><td>tr|A8MX94|A8MX94_HUMAN</td><td>GSTP1</td><td>Glutathione S-transferase P OS=Homo sapiens GN=GSTP1 PE=2 SV=1</td><td>19696</td><td>779</td><td>0</td><td>45</td><td>0</td><td>0.17</td><td>0</td><td>0</td><td>1</td><td>0</td><td>1</td><td>0</td><td>1</td><td>0</td><td>1</td><td>0</td><td>-1.17898</td></tr>
<tr><td>tr|B2RB52|B2RB52_HUMAN</td><td>tr|B2RB52|B2RB52</td><td>cDNA, FLJ95314, highly similar to Homo sapiens transducin (beta)-like 2 (TBL2), trans</td><td>50423</td><td>785</td><td>0</td><td>44</td><td>0</td><td>0.07</td><td>0</td><td>0</td><td>1</td><td>0</td><td>1</td><td>0</td><td>1</td><td>0</td><td>1</td><td>0</td><td>-1.17898</td></tr>
<tr><td>tr|B4DKE6|B4DKE6_HUMAN</td><td>tr|B4DKE6|B4DKE6</td><td>cDNA FLJ60629, highly similar to Replication factor C subunit 3 OS=Homo sapiens PE</td><td>35551</td><td>786</td><td>0</td><td>44</td><td>0</td><td>0.09</td><td>0</td><td>0</td><td>1</td><td>0</td><td>1</td><td>0</td><td>1</td><td>0</td><td>1</td><td>0</td><td>-1.17898</td></tr>
<tr><td>sp|Q9Y2A7|NCKP1_HUMAN</td><td>NCKAP1</td><td>Nck-associated protein 1 OS=Homo sapiens GN=NCKAP1 PE=1 SV=1</td><td>130018</td><td>797</td><td>0</td><td>44</td><td>0</td><td>0.03</td><td>0</td><td>0</td><td>1</td><td>0</td><td>1</td><td>0</td><td>1</td><td>0</td><td>2</td><td>0</td><td>-1.17898</td></tr>
<tr><td>tr|A1L377|A1L377_HUMAN</td><td>CABC1</td><td>CABC1 protein OS=Homo sapiens GN=CABC1 PE=2 SV=1</td><td>44564</td><td>801</td><td>0</td><td>43</td><td>0</td><td>0.07</td><td>0</td><td>0</td><td>1</td><td>0</td><td>1</td><td>0</td><td>1</td><td>0</td><td>1</td><td>0</td><td>-1.17898</td></tr>
<tr><td>tr|Q6P4E2|Q6P4E2_HUMAN</td><td>LARP4</td><td>LARP4 protein OS=Homo sapiens GN=LARP4 PE=2 SV=1</td><td>42404</td><td>802</td><td>0</td><td>42</td><td>0</td><td>0.14</td><td>0</td><td>0</td><td>1</td><td>0</td><td>1</td><td>0</td><td>1</td><td>0</td><td>1</td><td>0</td><td>-1.17898</td></tr>
<tr><td>tr|B3KPF2|B3KPF2_HUMAN</td><td>tr|B3KPF2|B3KPF2</td><td>cDNA FLJ31717 fis, clone NT2RI2006640, highly similar to Dynamin-3 (EC 3.6.5.5) OS</td><td>63057</td><td>803</td><td>0</td><td>43</td><td>0</td><td>0.05</td><td>0</td><td>0</td><td>1</td><td>0</td><td>1</td><td>0</td><td>1</td><td>0</td><td>2</td><td>0</td><td>-1.17898</td></tr>
<tr><td>tr|B2RN89|B2RN89_HUMAN</td><td>PCDHA8</td><td>Protocadherin alpha 8 OS=Homo sapiens GN=PCDHA8 PE=2 SV=1</td><td>89242</td><td>805</td><td>0</td><td>43</td><td>0</td><td>0.04</td><td>0</td><td>0</td><td>1</td><td>0</td><td>1</td><td>0</td><td>1</td><td>0</td><td>1</td><td>0</td><td>-1.17898</td></tr>
<tr><td>sp|Q5JTZ9|SYAM_HUMAN</td><td>AARS2</td><td>Alanine--tRNA ligase, mitochondrial OS=Homo sapiens GN=AARS2 PE=1 SV=1</td><td>108299</td><td>806</td><td>0</td><td>43</td><td>0</td><td>0.03</td><td>0</td><td>0</td><td>1</td><td>0</td><td>1</td><td>0</td><td>1</td><td>0</td><td>3</td><td>0</td><td>-1.17898</td></tr>
<tr><td>tr|Q5SRT3|Q5SRT3_HUMAN</td><td>CLIC1</td><td>Chloride intracellular channel 1, isoform CRA_a OS=Homo sapiens GN=CLIC1 PE=2 S</td><td>27248</td><td>810</td><td>0</td><td>43</td><td>0</td><td>0.12</td><td>0</td><td>0</td><td>1</td><td>0</td><td>1</td><td>0</td><td>1</td><td>0</td><td>1</td><td>0</td><td>-1.17898</td></tr>
<tr><td>tr|B4DVR3|B4DVR3_HUMAN</td><td>tr|B4DVR3|B4DVR</td><td>cDNA FLJ55595, highly similar to Protein diaphanous homolog 1 OS=Homo sapiens P</td><td>63522</td><td>813</td><td>0</td><td>35</td><td>0</td><td>0.05</td><td>0</td><td>0</td><td>1</td><td>0</td><td>1</td><td>0</td><td>1</td><td>0</td><td>2</td><td>0</td><td>-1.17898</td></tr>
<tr><td>tr|Q5T621|Q5T621_HUMAN</td><td>AKR1A1</td><td>Alcohol dehydrogenase [NADP(+)] (Fragment) OS=Homo sapiens GN=AKR1A1 PE=4 S</td><td>16677</td><td>816</td><td>0</td><td>42</td><td>0</td><td>0.2</td><td>0</td><td>0</td><td>1</td><td>0</td><td>1</td><td>0</td><td>1</td><td>0</td><td>1</td><td>0</td><td>-1.17898</td></tr>
<tr><td>tr|F8W8T8|F8W8T8_HUMAN</td><td>ACACB</td><td>Acetyl-CoA carboxylase 2 OS=Homo sapiens GN=ACACB PE=2 SV=1</td><td>269823</td><td>817</td><td>0</td><td>32</td><td>0</td><td>0.04</td><td>0</td><td>0</td><td>0</td><td>0</td><td>1</td><td>0</td><td>1</td><td>0</td><td>1</td><td>0</td><td>-1.17898</td></tr>
<tr><td>tr|A0PJC9|A0PJC9_HUMAN</td><td>TPR</td><td>TPR protein (Fragment) OS=Homo sapiens GN=TPR PE=2 SV=1</td><td>53894</td><td>818</td><td>0</td><td>42</td><td>0</td><td>0.01</td><td>0</td><td>0</td><td>1</td><td>0</td><td>1</td><td>0</td><td>1</td><td>0</td><td>2</td><td>0</td><td>-1.17898</td></tr>
<tr><td>tr|Q5VVC8|Q5VVC8_HUMAN</td><td>RPL11</td><td>60S ribosomal protein L11 (Fragment) OS=Homo sapiens GN=RPL11 PE=4 SV=1</td><td>20167</td><td>820</td><td>0</td><td>42</td><td>0</td><td>0.17</td><td>0</td><td>0</td><td>1</td><td>0</td><td>1</td><td>0</td><td>1</td><td>0</td><td>1</td><td>0</td><td>-1.17898</td></tr>
<tr><td>tr|B1AMX9|B1AMX9_HUMAN</td><td>TRAF2</td><td>TNF receptor-associated factor 2 OS=Homo sapiens GN=TRAF2 PE=4 SV=1</td><td>47848</td><td>821</td><td>0</td><td>42</td><td>0</td><td>0.07</td><td>0</td><td>0</td><td>1</td><td>0</td><td>1</td><td>0</td><td>1</td><td>0</td><td>1</td><td>0</td><td>-1.17898</td></tr>
<tr><td>tr|H7C260|H7C260_HUMAN</td><td>ACSL3</td><td>Long-chain-fatty-acid--CoA ligase 3 (Fragment) OS=Homo sapiens GN=ACSL3 PE=4 S</td><td>23676</td><td>822</td><td>0</td><td>42</td><td>0</td><td>0.04</td><td>0</td><td>0</td><td>1</td><td>0</td><td>1</td><td>0</td><td>1</td><td>0</td><td>1</td><td>0</td><td>-1.17898</td></tr>
<tr><td>tr|F8W9Y9|F8W9Y9_HUMAN</td><td>MTA1</td><td>Metastasis-associated protein MTA1 OS=Homo sapiens GN=MTA1 PE=2 SV=1</td><td>28772</td><td>824</td><td>0</td><td>0</td><td>0</td><td>0.06</td><td>0</td><td>0</td><td>0</td><td>0</td><td>0</td><td>0</td><td>1</td><td>0</td><td>1</td><td>0</td><td>-1.17898</td></tr>
<tr><td>sp|O60287|NPA1P_HUMAN</td><td>URB1</td><td>Nucleolar pre-ribosomal-associated protein 1 OS=Homo sapiens GN=URB1 PE=1 SV=</td><td>256508</td><td>827</td><td>0</td><td>41</td><td>0</td><td>0.01</td><td>0</td><td>0</td><td>1</td><td>0</td><td>1</td><td>0</td><td>1</td><td>0</td><td>2</td><td>0</td><td>-1.17898</td></tr>
<tr><td>tr|B7Z871|B7Z871_HUMAN</td><td>tr|B7Z871|B7Z871</td><td>cDNA FLJ50930, highly similar to Lipoma-preferred partner OS=Homo sapiens PE=2 S</td><td>49854</td><td>831</td><td>0</td><td>41</td><td>0</td><td>0.07</td><td>0</td><td>0</td><td>1</td><td>0</td><td>1</td><td>0</td><td>1</td><td>0</td><td>1</td><td>0</td><td>-1.17898</td></tr>
<tr><td>tr|B4DHJ9|B4DHJ9_HUMAN</td><td>tr|B4DHJ9|B4DHJ9</td><td>cDNA FLJ61262, highly similar to Ubiquitin-protein ligase E3C (EC 6.3.2.-) OS=Homo s</td><td>107426</td><td>835</td><td>0</td><td>41</td><td>0</td><td>0.03</td><td>0</td><td>0</td><td>1</td><td>0</td><td>1</td><td>0</td><td>1</td><td>0</td><td>1</td><td>0</td><td>-1.17898</td></tr>
<tr><td>tr|B3KNJ4|B3KNJ4_HUMAN</td><td>SAE1</td><td>SUMO-1 activating enzyme subunit 1, isoform CRA_a OS=Homo sapiens GN=SAE1 PE</td><td>33705</td><td>836</td><td>0</td><td>40</td><td>0</td><td>0.21</td><td>0</td><td>0</td><td>1</td><td>0</td><td>2</td><td>0</td><td>1</td><td>0</td><td>2</td><td>0</td><td>-1.17898</td></tr>
<tr><td>sp|Q14739|LBR_HUMAN</td><td>LBR</td><td>Lamin-B receptor OS=Homo sapiens GN=LBR PE=1 SV=2</td><td>71057</td><td>837</td><td>0</td><td>40</td><td>0</td><td>0.05</td><td>0</td><td>0</td><td>1</td><td>0</td><td>1</td><td>0</td><td>1</td><td>0</td><td>1</td><td>0</td><td>-1.17898</td></tr>
<tr><td>tr|A3KFI9|A3KFI9_HUMAN</td><td>CSTF1</td><td>Cleavage stimulation factor subunit 1 (Fragment) OS=Homo sapiens GN=CSTF1 PE=4</td><td>15755</td><td>840</td><td>0</td><td>40</td><td>0</td><td>0.22</td><td>0</td><td>0</td><td>1</td><td>0</td><td>1</td><td>0</td><td>1</td><td>0</td><td>1</td><td>0</td><td>-1.17898</td></tr>
<tr><td>sp|Q16222|UAP1_HUMAN</td><td>UAP1</td><td>UDP-N-acetylhexosamine pyrophosphorylase OS=Homo sapiens GN=UAP1 PE=1 SV=</td><td>59131</td><td>846</td><td>0</td><td>40</td><td>0</td><td>0.06</td><td>0</td><td>0</td><td>1</td><td>0</td><td>1</td><td>0</td><td>1</td><td>0</td><td>2</td><td>0</td><td>-1.17898</td></tr>
<tr><td>tr|B7ZCA0|B7ZCA0_HUMAN</td><td>SYNGAP1</td><td>Ras GTPase-activating protein SynGAP OS=Homo sapiens GN=SYNGAP1 PE=2 SV=1</td><td>143744</td><td>849</td><td>0</td><td>40</td><td>0</td><td>0.02</td><td>0</td><td>0</td><td>1</td><td>0</td><td>1</td><td>0</td><td>1</td><td>0</td><td>2</td><td>0</td><td>-1.17898</td></tr>
<tr><td>tr|B3KPZ0|B3KPZ0_HUMAN</td><td>tr|B3KPZ0|B3KPZ0</td><td>cDNA FLJ32485 fis, clone SKNMC2001596, highly similar to Ankyrin repeat and FYVE</td><td>121468</td><td>853</td><td>0</td><td>39</td><td>0</td><td>0.03</td><td>0</td><td>0</td><td>1</td><td>0</td><td>1</td><td>0</td><td>1</td><td>0</td><td>1</td><td>0</td><td>-1.17898</td></tr>
<tr><td>tr|E5RHH7|E5RHH7_HUMAN</td><td>ARFGAP1</td><td>ADP-ribosylation factor GTPase-activating protein 1 (Fragment) OS=Homo sapiens G</td><td>16483</td><td>856</td><td>0</td><td>39</td><td>0</td><td>0.21</td><td>0</td><td>0</td><td>1</td><td>0</td><td>1</td><td>0</td><td>1</td><td>0</td><td>1</td><td>0</td><td>-1.17898</td></tr>
<tr><td>tr|Q4LE79|Q4LE79_HUMAN</td><td>DSP</td><td>DSP variant protein (Fragment) OS=Homo sapiens GN=DSP variant protein PE=2 SV=</td><td>267367</td><td>857</td><td>0</td><td>39</td><td>0</td><td>0.01</td><td>0</td><td>0</td><td>1</td><td>0</td><td>1</td><td>0</td><td>1</td><td>0</td><td>2</td><td>0</td><td>-1.17898</td></tr>
<tr><td>tr|B4DJP0|B4DJP0_HUMAN</td><td>tr|B4DJP0|B4DJP0</td><td>cDNA FLJ60601, highly similar to Methylosome protein 50 OS=Homo sapiens PE=2 S</td><td>12271</td><td>860</td><td>0</td><td>39</td><td>0</td><td>0.28</td><td>0</td><td>0</td><td>1</td><td>0</td><td>1</td><td>0</td><td>1</td><td>0</td><td>1</td><td>0</td><td>-1.17898</td></tr>
<tr><td>tr|D6RJF2|D6RJF2_HUMAN</td><td>PAIP1</td><td>Polyadenylate-binding protein-interacting protein 1 (Fragment) OS=Homo sapiens G</td><td>22826</td><td>861</td><td>0</td><td>39</td><td>0</td><td>0.08</td><td>0</td><td>0</td><td>1</td><td>0</td><td>1</td><td>0</td><td>1</td><td>0</td><td>1</td><td>0</td><td>-1.17898</td></tr>
<tr><td>tr|Q6FGY5|Q6FGY5_HUMAN</td><td>CKAP1</td><td>CKAP1 protein OS=Homo sapiens GN=CKAP1 PE=2 SV=1</td><td>21959</td><td>862</td><td>0</td><td>39</td><td>0</td><td>0.15</td><td>0</td><td>0</td><td>1</td><td>0</td><td>1</td><td>0</td><td>1</td><td>0</td><td>1</td><td>0</td><td>-1.17898</td></tr>
<tr><td>tr|B0AZM0|B0AZM0_HUMAN</td><td>tr|B0AZM0|B0AZM</td><td>cDNA, FLJ79460, highly similar to Voltage-gated potassium channel subunitbeta-2 O</td><td>39027</td><td>865</td><td>0</td><td>39</td><td>0</td><td>0.08</td><td>0</td><td>0</td><td>1</td><td>0</td><td>1</td><td>0</td><td>1</td><td>0</td><td>1</td><td>0</td><td>-1.17898</td></tr>
<tr><td>tr|D3DVA5|D3DVA5_HUMAN</td><td>ARHGEF2</td><td>Rho/rac guanine nucleotide exchange factor (GEF) 2, isoform CRA_a OS=Homo sapie</td><td>116954</td><td>866</td><td>0</td><td>38</td><td>0</td><td>0.03</td><td>0</td><td>0</td><td>1</td><td>0</td><td>1</td><td>0</td><td>1</td><td>0</td><td>1</td><td>0</td><td>-1.17898</td></tr>
<tr><td>tr|F8VP03|F8VP03_HUMAN</td><td>tr|F8VP03|F8VP03</td><td>Uncharacterized protein (Fragment) OS=Homo sapiens PE=2 SV=1</td><td>11638</td><td>868</td><td>0</td><td>38</td><td>0</td><td>0.07</td><td>0</td><td>0</td><td>1</td><td>0</td><td>1</td><td>0</td><td>1</td><td>0</td><td>2</td><td>0</td><td>-1.17898</td></tr>
<tr><td>tr|H0YH81|H0YH81_HUMAN</td><td>ATP5B</td><td>ATP synthase subunit beta (Fragment) OS=Homo sapiens GN=ATP5B PE=3 SV=1</td><td>38226</td><td>873</td><td>0</td><td>38</td><td>0</td><td>0.09</td><td>0</td><td>0</td><td>1</td><td>0</td><td>1</td><td>0</td><td>1</td><td>0</td><td>1</td><td>0</td><td>-1.17898</td></tr>
<tr><td>sp|Q9UKX7|NUP50_HUMAN</td><td>NUP50</td><td>Nuclear pore complex protein Nup50 OS=Homo sapiens GN=NUP50 PE=1 SV=2</td><td>50512</td><td>874</td><td>0</td><td>38</td><td>0</td><td>0.07</td><td>0</td><td>0</td><td>1</td><td>0</td><td>1</td><td>0</td><td>1</td><td>0</td><td>1</td><td>0</td><td>-1.17898</td></tr>
<tr><td>tr|A8K6K7|A8K6K7_HUMAN</td><td>tr|A8K6K7|A8K6K7</td><td>cDNA FLJ76881, highly similar to Homo sapiens glycogen synthase 1 (muscle) (GYS1)</td><td>84559</td><td>875</td><td>0</td><td>38</td><td>0</td><td>0.04</td><td>0</td><td>0</td><td>1</td><td>0</td><td>1</td><td>0</td><td>1</td><td>0</td><td>1</td><td>0</td><td>-1.17898</td></tr>
<tr><td>tr|F6VDE0|F6VDE0_HUMAN</td><td>SMARCA2</td><td>Probable global transcription activator SNF2L2 (Fragment) OS=Homo sapiens GN=SN</td><td>61946</td><td>879</td><td>0</td><td>0</td><td>0</td><td>0.03</td><td>0</td><td>0</td><td>0</td><td>0</td><td>0</td><td>0</td><td>1</td><td>0</td><td>3</td><td>0</td><td>-1.17898</td></tr>
<tr><td>tr|A2VCT2|A2VCT2_HUMAN</td><td>OGDH</td><td>OGDH protein (Fragment) OS=Homo sapiens GN=OGDH PE=2 SV=1</td><td>72973</td><td>886</td><td>0</td><td>37</td><td>0</td><td>0.05</td><td>0</td><td>0</td><td>1</td><td>0</td><td>1</td><td>0</td><td>1</td><td>0</td><td>1</td><td>0</td><td>-1.17898</td></tr>
<tr><td>sp|Q96JM3|CHAP1_HUMAN</td><td>CHAMP1</td><td>Chromosome alignment-maintaining phosphoprotein 1 OS=Homo sapiens GN=CHAM</td><td>90012</td><td>888</td><td>0</td><td>37</td><td>0</td><td>0.04</td><td>0</td><td>0</td><td>1</td><td>0</td><td>1</td><td>0</td><td>1</td><td>0</td><td>1</td><td>0</td><td>-1.17898</td></tr>
<tr><td>tr|Q6ZPD6|Q6ZPD6_HUMAN</td><td>tr|Q6ZPD6|Q6ZPD</td><td>cDNA FLJ25992 fis, clone DMC03508 OS=Homo sapiens PE=2 SV=1</td><td>41924</td><td>890</td><td>0</td><td>37</td><td>0</td><td>0.08</td><td>0</td><td>0</td><td>1</td><td>0</td><td>1</td><td>0</td><td>1</td><td>0</td><td>2</td><td>0</td><td>-1.17898</td></tr>
<tr><td>sp|Q9P0N8|MARH2_HUMAN</td><td>Mar-02</td><td>E3 ubiquitin-protein ligase MARCH2 OS=Homo sapiens GN=MARCH2 PE=1 SV=1</td><td>27947</td><td>891</td><td>0</td><td>37</td><td>0</td><td>0.12</td><td>0</td><td>0</td><td>1</td><td>0</td><td>1</td><td>0</td><td>1</td><td>0</td><td>1</td><td>0</td><td>-1.17898</td></tr>
<tr><td>tr|B2R4C1|B2R4C1_HUMAN</td><td>tr|B2R4C1|B2R4C1</td><td>cDNA, FLJ92036, highly similar to Homo sapiens ribosomal protein L31 (RPL31), mRN</td><td>14486</td><td>893</td><td>0</td><td>36</td><td>0</td><td>0.23</td><td>0</td><td>0</td><td>1</td><td>0</td><td>1</td><td>0</td><td>1</td><td>0</td><td>1</td><td>0</td><td>-1.17898</td></tr>
<tr><td>tr|B4DQZ7|B4DQZ7_HUMAN</td><td>tr|B4DQZ7|B4DQZ</td><td>cDNA FLJ50838, highly similar to Apoptotic chromatin condensation inducer in thenu</td><td>122827</td><td>896</td><td>0</td><td>36</td><td>0</td><td>0.02</td><td>0</td><td>0</td><td>1</td><td>0</td><td>1</td><td>0</td><td>1</td><td>0</td><td>2</td><td>0</td><td>-1.17898</td></tr>
<tr><td>tr|Q96HI4|Q96HI4_HUMAN</td><td>NMT1</td><td>Glycylpeptide N-tetradecanoyltransferase (Fragment) OS=Homo sapiens GN=NMT1</td><td>56910</td><td>900</td><td>0</td><td>36</td><td>0</td><td>0.06</td><td>0</td><td>0</td><td>0</td><td>0</td><td>1</td><td>0</td><td>1</td><td>0</td><td>2</td><td>0</td><td>-1.17898</td></tr>
<tr><td>tr|H0Y8B6|H0Y8B6_HUMAN</td><td>NVL</td><td>Nuclear valosin-containing protein-like (Fragment) OS=Homo sapiens GN=NVL PE=3</td><td>82802</td><td>901</td><td>0</td><td>36</td><td>0</td><td>0.04</td><td>0</td><td>0</td><td>1</td><td>0</td><td>1</td><td>0</td><td>1</td><td>0</td><td>1</td><td>0</td><td>-1.17898</td></tr>
<tr><td>tr|H0YNU5|H0YNU5_HUMAN</td><td>BLM</td><td>Bloom syndrome protein OS=Homo sapiens GN=BLM PE=4 SV=1</td><td>145978</td><td>904</td><td>0</td><td>36</td><td>0</td><td>0.02</td><td>0</td><td>0</td><td>1</td><td>0</td><td>1</td><td>0</td><td>1</td><td>0</td><td>1</td><td>0</td><td>-1.17898</td></tr>
<tr><td>tr|H3BM14|H3BM14_HUMAN</td><td>NUB1</td><td>NEDD8 ultimate buster 1 OS=Homo sapiens GN=NUB1 PE=4 SV=1</td><td>72732</td><td>906</td><td>0</td><td>36</td><td>0</td><td>0.05</td><td>0</td><td>0</td><td>0</td><td>0</td><td>1</td><td>0</td><td>1</td><td>0</td><td>2</td><td>0</td><td>-1.17898</td></tr>
<tr><td>tr|D3DVR8|D3DVR8_HUMAN</td><td>HD</td><td>Huntingtin (Huntington disease), isoform CRA_a OS=Homo sapiens GN=HD PE=2 SV=</td><td>291559</td><td>907</td><td>0</td><td>35</td><td>0</td><td>0.01</td><td>0</td><td>0</td><td>0</td><td>0</td><td>1</td><td>0</td><td>1</td><td>0</td><td>2</td><td>0</td><td>-1.17898</td></tr>
<tr><td>sp|Q9NYK5|RM39_HUMAN</td><td>MRPL39</td><td>39S ribosomal protein L39, mitochondrial OS=Homo sapiens GN=MRPL39 PE=1 SV=3</td><td>39200</td><td>908</td><td>0</td><td>35</td><td>0</td><td>0.08</td><td>0</td><td>0</td><td>1</td><td>0</td><td>1</td><td>0</td><td>1</td><td>0</td><td>1</td><td>0</td><td>-1.17898</td></tr>
<tr><td>tr|H0YJ30|H0YJ30_HUMAN</td><td>GPHN</td><td>Molybdopterin molybdenumtransferase (Fragment) OS=Homo sapiens GN=GPHN PE</td><td>30436</td><td>909</td><td>0</td><td>35</td><td>0</td><td>0.11</td><td>0</td><td>0</td><td>0</td><td>0</td><td>1</td><td>0</td><td>1</td><td>0</td><td>2</td><td>0</td><td>-1.17898</td></tr>
<tr><td>tr|J3KP72|J3KP72_HUMAN</td><td>PCMT1</td><td>Protein-L-isoaspartate O-methyltransferase OS=Homo sapiens GN=PCMT1 PE=3 SV=</td><td>30524</td><td>913</td><td>0</td><td>35</td><td>0</td><td>0.11</td><td>0</td><td>0</td><td>0</td><td>0</td><td>1</td><td>0</td><td>1</td><td>0</td><td>1</td><td>0</td><td>-1.17898</td></tr>
<tr><td>tr|Q5T7U4|Q5T7U4_HUMAN</td><td>GTF3C5</td><td>General transcription factor 3C polypeptide 5 (Fragment) OS=Homo sapiens GN=GTF</td><td>28666</td><td>915</td><td>0</td><td>35</td><td>0</td><td>0.12</td><td>0</td><td>0</td><td>0</td><td>0</td><td>1</td><td>0</td><td>1</td><td>0</td><td>1</td><td>0</td><td>-1.17898</td></tr>
<tr><td>sp|Q14160|SCRIB_HUMAN</td><td>SCRIB</td><td>Protein scribble homolog OS=Homo sapiens GN=SCRIB PE=1 SV=4</td><td>175748</td><td>920</td><td>0</td><td>35</td><td>0</td><td>0.04</td><td>0</td><td>0</td><td>1</td><td>0</td><td>2</td><td>0</td><td>1</td><td>0</td><td>2</td><td>0</td><td>-1.17898</td></tr>
<tr><td>tr|A0JLS5|A0JLS5_HUMAN</td><td>TBL3</td><td>TBL3 protein (Fragment) OS=Homo sapiens GN=TBL3 PE=2 SV=1</td><td>64079</td><td>922</td><td>0</td><td>35</td><td>0</td><td>0.05</td><td>0</td><td>0</td><td>1</td><td>0</td><td>1</td><td>0</td><td>1</td><td>0</td><td>1</td><td>0</td><td>-1.17898</td></tr>
<tr><td>tr|Q2YD88|Q2YD88_HUMAN</td><td>FAM129B</td><td>FAM129B protein (Fragment) OS=Homo sapiens GN=FAM129B PE=2 SV=1</td><td>35264</td><td>924</td><td>0</td><td>35</td><td>0</td><td>0.09</td><td>0</td><td>0</td><td>0</td><td>0</td><td>1</td><td>0</td><td>1</td><td>0</td><td>1</td><td>0</td><td>-1.17898</td></tr>
<tr><td>tr|Q2TAM5|Q2TAM5_HUMA</td><td>RELA</td><td>RELA protein OS=Homo sapiens GN=RELA PE=2 SV=1</td><td>43355</td><td>925</td><td>0</td><td>35</td><td>0</td><td>0.08</td><td>0</td><td>0</td><td>0</td><td>0</td><td>1</td><td>0</td><td>1</td><td>0</td><td>1</td><td>0</td><td>-1.17898</td></tr>
<tr><td>tr|B7Z8S9|B7Z8S9_HUMAN</td><td>tr|B7Z8S9|B7Z8S9</td><td>cDNA FLJ61036, highly similar to Homo sapiens RNA (guanine-7-) methyltransferase</td><td>15420</td><td>930</td><td>0</td><td>23</td><td>0</td><td>0.2</td><td>0</td><td>0</td><td>0</td><td>0</td><td>1</td><td>0</td><td>1</td><td>0</td><td>1</td><td>0</td><td>-1.17898</td></tr>
<tr><td>sp|P0C0S8|H2A1_HUMAN</td><td>HIST1H2AG</td><td>Histone H2A type 1 OS=Homo sapiens GN=HIST1H2AG PE=1 SV=2</td><td>14083</td><td>932</td><td>0</td><td>34</td><td>0</td><td>0.24</td><td>0</td><td>0</td><td>1</td><td>0</td><td>1</td><td>0</td><td>1</td><td>0</td><td>1</td><td>0</td><td>-1.17898</td></tr>
<tr><td>tr|Q5U0I6|Q5U0I6_HUMAN</td><td>RAB1A</td><td>RAB1A protein OS=Homo sapiens GN=RAB1A PE=2 SV=1</td><td>22891</td><td>934</td><td>0</td><td>34</td><td>0</td><td>0.15</td><td>0</td><td>0</td><td>0</td><td>0</td><td>1</td><td>0</td><td>1</td><td>0</td><td>1</td><td>0</td><td>-1.17898</td></tr>
<tr><td>tr|Q6AWA7|Q6AWA7_HUMA</td><td>DKFZp686J1525</td><td>Putative uncharacterized protein DKFZp686J1525 (Fragment) OS=Homo sapiens GN=</td><td>52127</td><td>935</td><td>0</td><td>34</td><td>0</td><td>0.06</td><td>0</td><td>0</td><td>0</td><td>0</td><td>1</td><td>0</td><td>1</td><td>0</td><td>1</td><td>0</td><td>-1.17898</td></tr>
<tr><td>tr|B3KPX5|B3KPX5_HUMAN</td><td>tr|B3KPX5|B3KPX5</td><td>cDNA FLJ32418 fis, clone SKMUS2000863, weakly similar to PROBABLE ASPARAGINY</td><td>54523</td><td>936</td><td>0</td><td>34</td><td>0</td><td>0.06</td><td>0</td><td>0</td><td>1</td><td>0</td><td>1</td><td>0</td><td>1</td><td>0</td><td>1</td><td>0</td><td>-1.17898</td></tr>
<tr><td>tr|E7ETK0|E7ETK0_HUMAN</td><td>RPS24</td><td>40S ribosomal protein S24 OS=Homo sapiens GN=RPS24 PE=2 SV=1</td><td>15187</td><td>938</td><td>0</td><td>34</td><td>0</td><td>0.22</td><td>0</td><td>0</td><td>0</td><td>0</td><td>1</td><td>0</td><td>1</td><td>0</td><td>1</td><td>0</td><td>-1.17898</td></tr>
<tr><td>sp|P62280|RS11_HUMAN</td><td>RPS11</td><td>40S ribosomal protein S11 OS=Homo sapiens GN=RPS11 PE=1 SV=3</td><td>18590</td><td>940</td><td>0</td><td>34</td><td>0</td><td>0.18</td><td>0</td><td>0</td><td>0</td><td>0</td><td>1</td><td>0</td><td>1</td><td>0</td><td>2</td><td>0</td><td>-1.17898</td></tr>
<tr><td>tr|B5BU44|B5BU44_HUMAN</td><td>CRADD</td><td>CASP2 and RIPK1 domain containing adaptor with death domain OS=Homo sapiens</td><td>22774</td><td>941</td><td>0</td><td>34</td><td>0</td><td>0.15</td><td>0</td><td>0</td><td>0</td><td>0</td><td>1</td><td>0</td><td>1</td><td>0</td><td>1</td><td>0</td><td>-1.17898</td></tr>
<tr><td>sp|A6NJG6|ARGFX_HUMAN</td><td>ARGFX</td><td>Arginine-fifty homeobox OS=Homo sapiens GN=ARGFX PE=2 SV=1</td><td>35766</td><td>946</td><td>0</td><td>33</td><td>0</td><td>0.09</td><td>0</td><td>0</td><td>1</td><td>0</td><td>1</td><td>0</td><td>1</td><td>0</td><td>1</td><td>0</td><td>-1.17898</td></tr>
<tr><td>tr|H0YH15|H0YH15_HUMAN</td><td>AQR</td><td>Aquarius homolog (Mouse), isoform CRA_a OS=Homo sapiens GN=AQR PE=4 SV=1</td><td>76390</td><td>947</td><td>0</td><td>0</td><td>0</td><td>0.02</td><td>0</td><td>0</td><td>0</td><td>0</td><td>0</td><td>0</td><td>1</td><td>0</td><td>2</td><td>0</td><td>-1.17898</td></tr>
<tr><td>tr|K7EN59|K7EN59_HUMAN</td><td>CCDC151</td><td>Coiled-coil domain-containing protein 151 OS=Homo sapiens GN=CCDC151 PE=4 SV=</td><td>62098</td><td>952</td><td>0</td><td>33</td><td>0</td><td>0.06</td><td>0</td><td>0</td><td>1</td><td>0</td><td>1</td><td>0</td><td>1</td><td>0</td><td>2</td><td>0</td><td>-1.17898</td></tr>
<tr><td>tr|Q8TCA8|Q8TCA8_HUMAN</td><td>ANKRD5</td><td>Ankyrin repeat domain 5 OS=Homo sapiens GN=ANKRD5 PE=2 SV=1</td><td>87252</td><td>953</td><td>0</td><td>33</td><td>0</td><td>0.04</td><td>0</td><td>0</td><td>1</td><td>0</td><td>1</td><td>0</td><td>1</td><td>0</td><td>2</td><td>0</td><td>-1.17898</td></tr>
<tr><td>sp|Q13057|COASY_HUMAN</td><td>COASY</td><td>Bifunctional coenzyme A synthase OS=Homo sapiens GN=COASY PE=1 SV=4</td><td>62632</td><td>954</td><td>0</td><td>33</td><td>0</td><td>0.05</td><td>0</td><td>0</td><td>1</td><td>0</td><td>1</td><td>0</td><td>1</td><td>0</td><td>2</td><td>0</td><td>-1.17898</td></tr>
<tr><td>tr|J3KRE2|J3KRE2_HUMAN</td><td>ARHGDIA</td><td>Rho GDP-dissociation inhibitor 1 OS=Homo sapiens GN=ARHGDIA PE=4 SV=1</td><td>14805</td><td>957</td><td>0</td><td>33</td><td>0</td><td>0.23</td><td>0</td><td>0</td><td>0</td><td>0</td><td>1</td><td>0</td><td>1</td><td>0</td><td>1</td><td>0</td><td>-1.17898</td></tr>
<tr><td>tr|Q13380|Q13380_HUMAN</td><td>RBM</td><td>RNA binding motif (Fragment) OS=Homo sapiens GN=RBM PE=4 SV=1</td><td>4015</td><td>959</td><td>0</td><td>33</td><td>0</td><td>0.97</td><td>0</td><td>0</td><td>1</td><td>0</td><td>1</td><td>0</td><td>1</td><td>0</td><td>1</td><td>0</td><td>-1.17898</td></tr>
<tr><td>tr|O00370|O00370_HUMAN</td><td>tr|O00370|O00370</td><td>Putative p150 OS=Homo sapiens PE=2 SV=1</td><td>150000</td><td>960</td><td>0</td><td>33</td><td>0</td><td>0.02</td><td>0</td><td>0</td><td>0</td><td>0</td><td>1</td><td>0</td><td>1</td><td>0</td><td>1</td><td>0</td><td>-1.17898</td></tr>
<tr><td>sp|Q9UK61|F208A_HUMAN</td><td>FAM208A</td><td>Protein FAM208A OS=Homo sapiens GN=FAM208A PE=1 SV=3</td><td>190453</td><td>962</td><td>0</td><td>33</td><td>0</td><td>0.02</td><td>0</td><td>0</td><td>0</td><td>0</td><td>1</td><td>0</td><td>1</td><td>0</td><td>2</td><td>0</td><td>-1.17898</td></tr>
<tr><td>tr|F2Z2Q4|F2Z2Q4_HUMAN</td><td>TRMT11</td><td>tRNA (guanine(10)-N2)-methyltransferase homolog OS=Homo sapiens GN=TRMT11</td><td>39270</td><td>963</td><td>0</td><td>33</td><td>0</td><td>0.08</td><td>0</td><td>0</td><td>0</td><td>0</td><td>1</td><td>0</td><td>1</td><td>0</td><td>2</td><td>0</td><td>-1.17898</td></tr>
<tr><td>tr|Q5SV16|Q5SV16_HUMAN</td><td>ATAD3A</td><td>ATPase family AAA domain-containing protein 3A (Fragment) OS=Homo sapiens GN=</td><td>22896</td><td>964</td><td>0</td><td>33</td><td>0</td><td>0.05</td><td>0</td><td>0</td><td>0</td><td>0</td><td>1</td><td>0</td><td>1</td><td>0</td><td>1</td><td>0</td><td>-1.17898</td></tr>
<tr><td>tr|Q86TI1|Q86TI1_HUMAN</td><td>NUP98/HOXC13</td><td>Putative uncharacterized protein NUP98/HOXC13 (Fragment) OS=Homo sapiens GN=</td><td>37142</td><td>966</td><td>0</td><td>29</td><td>0</td><td>0.03</td><td>0</td><td>0</td><td>0</td><td>0</td><td>1</td><td>0</td><td>1</td><td>0</td><td>1</td><td>0</td><td>-1.17898</td></tr>
<tr><td>sp|Q9H9J2|RM44_HUMAN</td><td>MRPL44</td><td>39S ribosomal protein L44, mitochondrial OS=Homo sapiens GN=MRPL44 PE=1 SV=1</td><td>37854</td><td>968</td><td>0</td><td>33</td><td>0</td><td>0.09</td><td>0</td><td>0</td><td>0</td><td>0</td><td>1</td><td>0</td><td>1</td><td>0</td><td>1</td><td>0</td><td>-1.17898</td></tr>
<tr><td>tr|A1L3A7|A1L3A7_HUMAN</td><td>NUFIP2</td><td>Nuclear fragile X mental retardation protein interacting protein 2 OS=Homo sapiens</td><td>76132</td><td>969</td><td>0</td><td>33</td><td>0</td><td>0.04</td><td>0</td><td>0</td><td>0</td><td>0</td><td>1</td><td>0</td><td>1</td><td>0</td><td>1</td><td>0</td><td>-1.17898</td></tr>
<tr><td>tr|B7ZMI1|B7ZMI1_HUMAN</td><td>MED12L</td><td>MED12L protein OS=Homo sapiens GN=MED12L PE=2 SV=1</td><td>206075</td><td>970</td><td>0</td><td>32</td><td>0</td><td>0.02</td><td>0</td><td>0</td><td>0</td><td>0</td><td>1</td><td>0</td><td>1</td><td>0</td><td>1</td><td>0</td><td>-1.17898</td></tr>
<tr><td>tr|B4DX96|B4DX96_HUMAN</td><td>tr|B4DX96|B4DX96</td><td>cDNA FLJ57646, highly similar to Zinc finger protein 38 OS=Homo sapiens PE=2 SV=1</td><td>51790</td><td>974</td><td>0</td><td>32</td><td>0</td><td>0.06</td><td>0</td><td>0</td><td>0</td><td>0</td><td>1</td><td>0</td><td>1</td><td>0</td><td>1</td><td>0</td><td>-1.17898</td></tr>
<tr><td>tr|E9PC74|E9PC74_HUMAN</td><td>EIF2B5</td><td>Translation initiation factor eIF-2B subunit epsilon OS=Homo sapiens GN=EIF2B5 PE=</td><td>79096</td><td>979</td><td>0</td><td>32</td><td>0</td><td>0.04</td><td>0</td><td>0</td><td>1</td><td>0</td><td>1</td><td>0</td><td>1</td><td>0</td><td>2</td><td>0</td><td>-1.17898</td></tr>
<tr><td>tr|A4FVC0|A4FVC0_HUMAN</td><td>EIF2C2</td><td>EIF2C2 protein (Fragment) OS=Homo sapiens GN=EIF2C2 PE=2 SV=1</td><td>92636</td><td>980</td><td>0</td><td>32</td><td>0</td><td>0.04</td><td>0</td><td>0</td><td>0</td><td>0</td><td>1</td><td>0</td><td>1</td><td>0</td><td>1</td><td>0</td><td>-1.17898</td></tr>
<tr><td>tr|B7Z5S1|B7Z5S1_HUMAN</td><td>tr|B7Z5S1|B7Z5S1</td><td>cDNA FLJ61699, highly similar to Homo sapiens androgen-induced proliferation inhib</td><td>149900</td><td>984</td><td>0</td><td>32</td><td>0</td><td>0.02</td><td>0</td><td>0</td><td>1</td><td>0</td><td>1</td><td>0</td><td>1</td><td>0</td><td>1</td><td>0</td><td>-1.17898</td></tr>
<tr><td>tr|B4DZ67|B4DZ67_HUMAN</td><td>NUP107</td><td>Nuclear pore complex protein Nup107 OS=Homo sapiens GN=NUP107 PE=2 SV=1</td><td>103851</td><td>986</td><td>0</td><td>31</td><td>0</td><td>0.03</td><td>0</td><td>0</td><td>0</td><td>0</td><td>1</td><td>0</td><td>1</td><td>0</td><td>2</td><td>0</td><td>-1.17898</td></tr>
<tr><td>sp|Q8N122|RPTOR_HUMAN</td><td>RPTOR</td><td>Regulatory-associated protein of mTOR OS=Homo sapiens GN=RPTOR PE=1 SV=1</td><td>150653</td><td>987</td><td>0</td><td>31</td><td>0</td><td>0.02</td><td>0</td><td>0</td><td>0</td><td>0</td><td>1</td><td>0</td><td>1</td><td>0</td><td>3</td><td>0</td><td>-1.17898</td></tr>
<tr><td>tr|G3V346|G3V346_HUMAN</td><td>SRP54</td><td>Signal recognition particle 54 kDa protein (Fragment) OS=Homo sapiens GN=SRP54 P</td><td>4785</td><td>988</td><td>0</td><td>31</td><td>0</td><td>0.77</td><td>0</td><td>0</td><td>0</td><td>0</td><td>1</td><td>0</td><td>1</td><td>0</td><td>1</td><td>0</td><td>-1.17898</td></tr>
<tr><td>tr|J3KRL0|J3KRL0_HUMAN</td><td>MYO1D</td><td>Unconventional myosin-Id OS=Homo sapiens GN=MYO1D PE=4 SV=1</td><td>50023</td><td>993</td><td>0</td><td>22</td><td>0</td><td>0.06</td><td>0</td><td>0</td><td>0</td><td>0</td><td>1</td><td>0</td><td>1</td><td>0</td><td>1</td><td>0</td><td>-1.17898</td></tr>
<tr><td>tr|B2R6G5|B2R6G5_HUMAN</td><td>tr|B2R6G5|B2R6G</td><td>cDNA, FLJ92942, highly similar to Homo sapiens T-box, brain, 1 (TBR1), mRNA OS=Ho</td><td>74530</td><td>994</td><td>0</td><td>31</td><td>0</td><td>0.04</td><td>0</td><td>0</td><td>0</td><td>0</td><td>1</td><td>0</td><td>1</td><td>0</td><td>1</td><td>0</td><td>-1.17898</td></tr>
<tr><td>tr|Q05DL3|Q05DL3_HUMAN</td><td>CSNK1A1</td><td>CSNK1A1 protein (Fragment) OS=Homo sapiens GN=CSNK1A1 PE=2 SV=1</td><td>26799</td><td>995</td><td>0</td><td>31</td><td>0</td><td>0.12</td><td>0</td><td>0</td><td>1</td><td>0</td><td>1</td><td>0</td><td>1</td><td>0</td><td>1</td><td>0</td><td>-1.17898</td></tr>
<tr><td>tr|F5GZI5|F5GZI5_HUMAN</td><td>ZAN</td><td>Zonadhesin OS=Homo sapiens GN=ZAN PE=2 SV=1</td><td>299445</td><td>997</td><td>0</td><td>31</td><td>0</td><td>0.01</td><td>0</td><td>0</td><td>0</td><td>0</td><td>1</td><td>0</td><td>1</td><td>0</td><td>3</td><td>0</td><td>-1.17898</td></tr>
<tr><td>sp|Q9NSB4|KRT82_HUMAN</td><td>KRT82</td><td>Keratin, type II cuticular Hb2 OS=Homo sapiens GN=KRT82 PE=1 SV=3</td><td>57985</td><td>999</td><td>0</td><td>31</td><td>0</td><td>0.06</td><td>0</td><td>0</td><td>0</td><td>0</td><td>1</td><td>0</td><td>1</td><td>0</td><td>1</td><td>0</td><td>-1.17898</td></tr>
<tr><td>tr|J3KSL8|J3KSL8_HUMAN</td><td>TRAPPC8</td><td>Trafficking protein particle complex subunit 8 OS=Homo sapiens GN=TRAPPC8 PE=4</td><td>105091</td><td>1000</td><td>0</td><td>31</td><td>0</td><td>0.03</td><td>0</td><td>0</td><td>0</td><td>0</td><td>1</td><td>0</td><td>1</td><td>0</td><td>2</td><td>0</td><td>-1.17898</td></tr>
<tr><td>tr|B4DS83|B4DS83_HUMAN</td><td>tr|B4DS83|B4DS83</td><td>cDNA FLJ53179, highly similar to Nucleolar protein 10 OS=Homo sapiens PE=2 SV=1</td><td>74879</td><td>1004</td><td>0</td><td>30</td><td>0</td><td>0.04</td><td>0</td><td>0</td><td>0</td><td>0</td><td>1</td><td>0</td><td>1</td><td>0</td><td>1</td><td>0</td><td>-1.17898</td></tr>
<tr><td>tr|B4DGY8|B4DGY8_HUMAN</td><td>tr|B4DGY8|B4DGY</td><td>cDNA FLJ53528 OS=Homo sapiens PE=2 SV=1</td><td>22981</td><td>1007</td><td>0</td><td>30</td><td>0</td><td>0.15</td><td>0</td><td>0</td><td>0</td><td>0</td><td>1</td><td>0</td><td>1</td><td>0</td><td>1</td><td>0</td><td>-1.17898</td></tr>
<tr><td>tr|B4DFM1|B4DFM1_HUMAN</td><td>tr|B4DFM1|B4DFM</td><td>cDNA FLJ53034, highly similar to AP-2 complex subunit mu-1 OS=Homo sapiens PE=2</td><td>44226</td><td>1018</td><td>0</td><td>29</td><td>0</td><td>0.07</td><td>0</td><td>0</td><td>0</td><td>0</td><td>1</td><td>0</td><td>1</td><td>0</td><td>1</td><td>0</td><td>-1.17898</td></tr>
<tr><td>tr|Q4G0X8|Q4G0X8_HUMAN</td><td>BRD4</td><td>BRD4 protein OS=Homo sapiens GN=BRD4 PE=2 SV=1</td><td>89032</td><td>1019</td><td>0</td><td>29</td><td>0</td><td>0.04</td><td>0</td><td>0</td><td>0</td><td>0</td><td>1</td><td>0</td><td>1</td><td>0</td><td>2</td><td>0</td><td>-1.17898</td></tr>
<tr><td>tr|B4DEF9|B4DEF9_HUMAN</td><td>tr|B4DEF9|B4DEF9</td><td>cDNA FLJ59241, highly similar to Transducin-like enhancer protein 1 OS=Homo sapie</td><td>82574</td><td>1020</td><td>0</td><td>20</td><td>0</td><td>0.14</td><td>0</td><td>0</td><td>0</td><td>0</td><td>1</td><td>0</td><td>1</td><td>0</td><td>1</td><td>0</td><td>-1.17898</td></tr>
<tr><td>tr|H3BQ23|H3BQ23_HUMAN</td><td>CIAPIN1</td><td>Anamorsin (Fragment) OS=Homo sapiens GN=CIAPIN1 PE=4 SV=1</td><td>13738</td><td>1022</td><td>0</td><td>29</td><td>0</td><td>0.25</td><td>0</td><td>0</td><td>0</td><td>0</td><td>1</td><td>0</td><td>1</td><td>0</td><td>1</td><td>0</td><td>-1.17898</td></tr>
<tr><td>tr|B4DXG8|B4DXG8_HUMAN</td><td>tr|B4DXG8|B4DXG</td><td>cDNA FLJ55260, highly similar to Exosome complex exonuclease RRP45 (EC 3.1.13.-)</td><td>44161</td><td>1023</td><td>0</td><td>29</td><td>0</td><td>0.07</td><td>0</td><td>0</td><td>0</td><td>0</td><td>1</td><td>0</td><td>1</td><td>0</td><td>1</td><td>0</td><td>-1.17898</td></tr>
<tr><td>sp|Q6PKG0|LARP1_HUMAN</td><td>LARP1</td><td>La-related protein 1 OS=Homo sapiens GN=LARP1 PE=1 SV=2</td><td>123833</td><td>1024</td><td>0</td><td>29</td><td>0</td><td>0.03</td><td>0</td><td>0</td><td>0</td><td>0</td><td>1</td><td>0</td><td>1</td><td>0</td><td>1</td><td>0</td><td>-1.17898</td></tr>
<tr><td>tr|B4DME9|B4DME9_HUMAN</td><td>KLC4</td><td>Kinesin light chain 4 OS=Homo sapiens GN=KLC4 PE=2 SV=1</td><td>60003</td><td>1025</td><td>0</td><td>29</td><td>0</td><td>0.05</td><td>0</td><td>0</td><td>0</td><td>0</td><td>1</td><td>0</td><td>1</td><td>0</td><td>1</td><td>0</td><td>-1.17898</td></tr>
<tr><td>tr|B2R4D8|B2R4D8_HUMAN</td><td>tr|B2R4D8|B2R4D</td><td>60S ribosomal protein L27 OS=Homo sapiens PE=2 SV=1</td><td>15760</td><td>1027</td><td>0</td><td>29</td><td>0</td><td>0.22</td><td>0</td><td>0</td><td>0</td><td>0</td><td>1</td><td>0</td><td>1</td><td>0</td><td>3</td><td>0</td><td>-1.17898</td></tr>
<tr><td>tr|H3BS86|H3BS86_HUMAN</td><td>STUB1</td><td>E3 ubiquitin-protein ligase CHIP (Fragment) OS=Homo sapiens GN=STUB1 PE=4 SV=1</td><td>24812</td><td>1028</td><td>0</td><td>29</td><td>0</td><td>0.13</td><td>0</td><td>0</td><td>0</td><td>0</td><td>1</td><td>0</td><td>1</td><td>0</td><td>1</td><td>0</td><td>-1.17898</td></tr>
<tr><td>tr|Q5TBP5|Q5TBP5_HUMAN</td><td>TAF4</td><td>TAF4 RNA polymerase II, TATA box binding protein (TBP)-associated factor, 135kDa (</td><td>97594</td><td>1029</td><td>0</td><td>29</td><td>0</td><td>0.03</td><td>0</td><td>0</td><td>0</td><td>0</td><td>1</td><td>0</td><td>1</td><td>0</td><td>1</td><td>0</td><td>-1.17898</td></tr>
<tr><td>tr|H0Y5B4|H0Y5B4_HUMAN</td><td>RPL36A</td><td>60S ribosomal protein L36a (Fragment) OS=Homo sapiens GN=RPL36A PE=3 SV=1</td><td>15576</td><td>1035</td><td>0</td><td>28</td><td>0</td><td>0.22</td><td>0</td><td>0</td><td>0</td><td>0</td><td>1</td><td>0</td><td>1</td><td>0</td><td>1</td><td>0</td><td>-1.17898</td></tr>
</table>

tr|B1ANM7|B1ANM7_HUMA	FAF1	FAS-associated factor 1 OS=Homo sapiens GN=FAF1 PE=4 SV=1	56305	1037	0	28	0	0.06	0	0	0	0	1	0	1	0	1	0	-1.17898
tr|E9PJL9|E9PJL9_HUMAN	NDUFV1	NADH dehydrogenase [ubiquinone] flavoprotein 1, mitochondrial (Fragment) OS=Ho	10704	1039	0	28	0	0.23	0	0	0	0	1	0	1	0	1	0	-1.17898
tr|D6RJA6|D6RJA6_HUMAN	DDX46	DEAD (Asp-Glu-Ala-Asp) box polypeptide 46, isoform CRA_b OS=Homo sapiens GN=D	54216	1040	0	28	0	0.03	0	0	0	0	1	0	1	0	1	0	-1.17898
sp|Q5D862|FILA2_HUMAN	FLG2	Filaggrin-2 OS=Homo sapiens GN=FLG2 PE=1 SV=1	249296	1041	0	28	0	0.01	0	0	1	0	1	0	1	0	1	0	-1.17898
tr|A6NF51|A6NF51_HUMAN	BPNT1	3~(2~),5~-bisphosphate nucleotidase 1 OS=Homo sapiens GN=BPNT1 PE=2 SV=2	32029	1042	0	28	0	0.11	0	0	0	0	1	0	1	0	1	0	-1.17898
tr|B4DYZ7|B4DYZ7_HUMAN	tr|B4DYZ7|B4DYZ7	cDNA FLJ54316, highly similar to DNA polymerase subunit delta 3 OS=Homo sapiens	51595	1046	0	27	0	0.06	0	0	0	0	1	0	1	0	1	0	-1.17898
tr|J3QS89|J3QS89_HUMAN	FA2H	Fatty acid 2-hydroxylase (Fragment) OS=Homo sapiens GN=FA2H PE=4 SV=1	22573	1047	0	27	0	0.15	0	0	0	0	1	0	1	0	1	0	-1.17898
tr|E5RFK7|E5RFK7_HUMAN	UBR5	E3 ubiquitin-protein ligase UBR5 (Fragment) OS=Homo sapiens GN=UBR5 PE=2 SV=1	19270	1049	0	27	0	0.01	0	0	0	0	1	0	1	0	1	0	-1.17898
tr|B7Z4A9|B7Z4A9_HUMAN	tr|B7Z4A9|B7Z4A9	cDNA FLJ60125, highly similar to Pre-B-cell leukemia transcription factor 3 OS=Homo	46743	1050	0	27	0	0.07	0	0	0	0	1	0	1	0	1	0	-1.17898
tr|B4DYE3|B4DYE3_HUMAN	tr|B4DYE3|B4DYE3	cDNA FLJ58091, highly similar to Chloride channel protein 2 OS=Homo sapiens PE=2	50506	1051	0	27	0	0.07	0	0	0	0	1	0	1	0	1	0	-1.17898
sp|P80217|IN35_HUMAN	IFI35	Interferon-induced 35 kDa protein OS=Homo sapiens GN=IFI35 PE=1 SV=5	31698	1053	0	27	0	0.1	0	0	0	0	1	0	1	0	1	0	-1.17898
tr|B4E074|B4E074_HUMAN	tr|B4E074|B4E074	cDNA FLJ58655, highly similar to Notchless homolog 1 OS=Homo sapiens PE=2 SV=1	51211	1054	0	27	0	0.06	0	0	0	0	1	0	1	0	1	0	-1.17898
tr|B4E0D5|B4E0D5_HUMAN	tr|B4E0D5|B4E0D5	cDNA FLJ54218, highly similar to Acidic leucine-rich nuclear phosphoprotein 32 fami	19540	1055	0	27	0	0.17	0	0	0	0	1	0	1	0	1	0	-1.17898
sp|P19623|SPEE_HUMAN	SRM	Spermidine synthase OS=Homo sapiens GN=SRM PE=1 SV=1	34373	1057	0	27	0	0.2	0	0	0	0	2	0	1	0	2	0	-1.17898
tr|J3KSP2|J3KSP2_HUMAN	RPL38	60S ribosomal protein L38 (Fragment) OS=Homo sapiens GN=RPL38 PE=3 SV=1	2557	1058	0	27	0	1.53	0	0	0	0	1	0	1	0	1	0	-1.17898
tr|H0YF33|H0YF33_HUMAN	NASP	Nuclear autoantigenic sperm protein (Fragment) OS=Homo sapiens GN=NASP PE=4 S	31883	1059	0	27	0	0.1	0	0	0	0	1	0	1	0	2	0	-1.17898
tr|H0YIK6|H0YIK6_HUMAN	SCYL2	SCY1-like protein 2 (Fragment) OS=Homo sapiens GN=SCYL2 PE=4 SV=1	13212	1060	0	27	0	0.26	0	0	0	0	1	0	1	0	1	0	-1.17898
sp|P0DJD0|RGPD1_HUMAN	RGPD1	RANBP2-like and GRIP domain-containing protein 1 OS=Homo sapiens GN=RGPD1 PE	198022	1063	0	26	0	0.03	0	0	0	0	2	0	1	0	3	0	-1.17898
tr|Q0VAM0|Q0VAM0_HUMA	CTNNBL1	Catenin, beta like 1 OS=Homo sapiens GN=CTNNBL1 PE=2 SV=1	65560	1064	0	26	0	0.05	0	0	0	0	1	0	1	0	1	0	-1.17898
sp|P23368|MAOM_HUMAN	ME2	NAD-dependent malic enzyme, mitochondrial OS=Homo sapiens GN=ME2 PE=1 SV=1	66029	1066	0	26	0	0.05	0	0	0	0	1	0	1	0	1	0	-1.17898
tr|E9PR18|E9PR18_HUMAN	FAM111A	Protein FAM111A (Fragment) OS=Homo sapiens GN=FAM111A PE=2 SV=1	21407	1068	0	26	0	0.16	0	0	0	0	1	0	1	0	1	0	-1.17898
tr|B4DW54|B4DW54_HUMA	tr|B4DW54|B4DW	cDNA FLJ50888, highly similar to Aldehyde dehydrogenase, mitochondrial (EC 1.2.1.3	51467	1069	0	26	0	0.06	0	0	0	0	1	0	1	0	1	0	-1.17898
tr|Q53GE1|Q53GE1_HUMAN	RANBP3	RAN binding protein 3 isoform RANBP3-a variant (Fragment) OS=Homo sapiens GN=	59924	1070	0	26	0	0.07	0	0	0	0	1	0	1	0	2	0	-1.17898
tr|B3KY61|B3KY61_HUMAN	tr|B3KY61|B3KY61	cDNA FLJ16787 fis, clone PLACE6013222, weakly similar to Mus musculus RNA bindi	89204	1071	0	26	0	0.03	0	0	0	0	1	0	1	0	1	0	-1.17898
tr|D3DQF6|D3DQF6_HUMAN	POU4F3	POU domain, class 4, transcription factor 3, isoform CRA_a OS=Homo sapiens GN=P	84396	1072	0	26	0	0.04	0	0	0	0	1	0	1	0	2	0	-1.17898
sp|Q86UY8|NT5D3_HUMAN	NT5DC3	5~-nucleotidase domain-containing protein 3 OS=Homo sapiens GN=NT5DC3 PE=2 S	63721	1073	0	26	0	0.05	0	0	0	0	1	0	1	0	1	0	-1.17898
tr|H7BZT7|H7BZT7_HUMAN	ESD	S-formylglutathione hydrolase (Fragment) OS=Homo sapiens GN=ESD PE=4 SV=1	25806	1076	0	26	0	0.28	0	0	0	0	2	0	1	0	2	0	-1.17898
tr|B4DN25|B4DN25_HUMAN	UGDH	UDP-glucose 6-dehydrogenase OS=Homo sapiens GN=UGDH PE=2 SV=1	44786	1077	0	26	0	0.07	0	0	0	0	1	0	1	0	1	0	-1.17898
sp|Q2TAC2|CCD57_HUMAN	CCDC57	Coiled-coil domain-containing protein 57 OS=Homo sapiens GN=CCDC57 PE=2 SV=2	103390	1079	0	26	0	0.03	0	0	0	0	1	0	1	0	1	0	-1.17898
tr|Q05CP8|Q05CP8_HUMAN	CCDC6	CCDC6 protein (Fragment) OS=Homo sapiens GN=CCDC6 PE=2 SV=1	38254	1081	0	25	0	0.09	0	0	0	0	1	0	1	0	1	0	-1.17898
tr|K7EMD6|K7EMD6_HUMA	SGTA	Small glutamine-rich tetratricopeptide repeat-containing protein alpha (Fragment) O	18009	1082	0	25	0	0.19	0	0	0	0	1	0	1	0	1	0	-1.17898
tr|B4DPX9|B4DPX9_HUMAN	tr|B4DPX9|B4DPX9	cDNA FLJ56221, highly similar to YTH domain protein 3 OS=Homo sapiens PE=2 SV=1	64596	1084	0	25	0	0.05	0	0	0	0	1	0	1	0	1	0	-1.17898
tr|H0YH61|H0YH61_HUMAN	MUC19	Mucin-19 (Fragment) OS=Homo sapiens GN=MUC19 PE=4 SV=1	11135	1086	0	25	0	0.31	0	0	0	0	1	0	1	0	1	0	-1.17898
sp|Q4KMP7|TB10B_HUMAN	TBC1D10B	TBC1 domain family member 10B OS=Homo sapiens GN=TBC1D10B PE=1 SV=3	87658	1087	0	25	0	0.04	0	0	1	0	1	0	1	0	1	0	-1.17898
tr|B0QYR0|B0QYR0_HUMAN	BTBD3	BTB/POZ domain-containing protein 3 (Fragment) OS=Homo sapiens GN=BTBD3 PE=	11110	1088	0	25	0	0.31	0	0	0	0	1	0	1	0	1	0	-1.17898
tr|G0XQ39|G0XQ39_HUMAN	STIM1	STIM1L OS=Homo sapiens GN=STIM1 PE=2 SV=1	88933	1089	0	25	0	0.04	0	0	0	0	1	0	1	0	2	0	-1.17898
tr|G3V5V3|G3V5V3_HUMAN	NEMF	Nuclear export mediator factor NEMF (Fragment) OS=Homo sapiens GN=NEMF PE=2	114348	1090	0	25	0	0.03	0	0	0	0	1	0	1	0	1	0	-1.17898
tr|A8MV66|A8MV66_HUMA	GPR123	Probable G-protein-coupled receptor 123 OS=Homo sapiens GN=GPR123 PE=3 SV=1	51296	1091	0	25	0	0.06	0	0	0	0	1	0	1	0	1	0	-1.17898
tr|H0Y792|H0Y792_HUMAN	DOPEY2	Protein dopey-2 (Fragment) OS=Homo sapiens GN=DOPEY2 PE=4 SV=1	5545	1092	0	25	0	0.7	0	0	0	0	1	0	1	0	1	0	-1.17898
tr|Q59H97|Q59H97_HUMAN	tr|Q59H97|Q59H9	Zinc finger protein ZNF-U69274 variant (Fragment) OS=Homo sapiens PE=2 SV=1	81027	1094	0	25	0	0.04	0	0	0	0	1	0	1	0	2	0	-1.17898
tr|E9PRE2|E9PRE2_HUMAN	CTNND1	Catenin delta-1 (Fragment) OS=Homo sapiens GN=CTNND1 PE=2 SV=1	29211	1095	0	25	0	0.03	0	0	0	0	1	0	1	0	1	0	-1.17898
tr|Q7Z2E0|Q7Z2E0_HUMAN	TNRC11	TNRC11 protein (Fragment) OS=Homo sapiens GN=TNRC11 PE=2 SV=1	31092	1096	0	25	0	0.01	0	0	1	0	1	0	1	0	1	0	-1.17898
tr|B2RCJ6|B2RCJ6_HUMAN	tr|B2RCJ6|B2RCJ6	cDNA, FLJ96114, highly similar to Homo sapiens bromodomain and WD repeat doma	138515	1097	0	24	0	0.02	0	0	0	0	1	0	1	0	1	0	-1.17898
tr|J3KR97|J3KR97_HUMAN	TBCD	Tubulin-specific chaperone D OS=Homo sapiens GN=TBCD PE=4 SV=1	138346	1098	0	24	0	0.02	0	0	1	0	1	0	1	0	1	0	-1.17898
tr|H0YKD8|H0YKD8_HUMAN	RPL28	60S ribosomal protein L28 OS=Homo sapiens GN=RPL28 PE=4 SV=1	19174	1099	0	24	0	0.18	0	0	0	0	1	0	1	0	1	0	-1.17898
tr|F5GX80|F5GX80_HUMAN	ZCCHC8	Zinc finger CCHC domain-containing protein 8 (Fragment) OS=Homo sapiens GN=ZC	23636	1103	0	24	0	0.14	0	0	0	0	1	0	1	0	1	0	-1.17898
sp|Q5VSL9|STRP1_HUMAN	STRIP1	Striatin-interacting protein 1 OS=Homo sapiens GN=STRIP1 PE=1 SV=1	96256	1105	0	24	0	0.03	0	0	0	0	1	0	1	0	2	0	-1.17898
tr|B4DHC6|B4DHC6_HUMAN	tr|B4DHC6|B4DHC	cDNA FLJ57928, highly similar to Excitatory amino acid transporter 1 OS=Homo sapie	47096	1106	0	24	0	0.07	0	0	0	0	1	0	1	0	1	0	-1.17898
tr|B3KRC6|B3KRC6_HUMAN	tr|B3KRC6|B3KRC6	cDNA FLJ34004 fis, clone FCBBF1000232, highly similar to Cytochrome P450 51A1 (E	51732	1107	0	24	0	0.06	0	0	0	0	1	0	1	0	1	0	-1.17898
tr|I3L2F4|I3L2F4_HUMAN	EDC4	Enhancer of mRNA-decapping protein 4 (Fragment) OS=Homo sapiens GN=EDC4 PE=	14939	1108	0	24	0	0.23	0	0	0	0	1	0	1	0	2	0	-1.17898
sp|Q9H2M9|RBGPR_HUMAN	RAB3GAP2	Rab3 GTPase-activating protein non-catalytic subunit OS=Homo sapiens GN=RAB3GA	157482	1109	0	24	0	0.02	0	0	0	0	1	0	1	0	1	0	-1.17898
sp|O14686|MLL2_HUMAN	MLL2	Histone-lysine N-methyltransferase MLL2 OS=Homo sapiens GN=MLL2 PE=1 SV=2	599575	1111	0	24	0	0.01	0	0	0	0	1	0	1	0	4	0	-1.17898
tr|B2R9G9|B2R9G9_HUMAN	tr|B2R9G9|B2R9G	cDNA, FLJ94385, highly similar to Homo sapiens cofactor required for Sp1 transcripti	65805	1112	0	24	0	0.05	0	0	0	0	1	0	1	0	1	0	-1.17898
tr|J3KTG2|J3KTG2_HUMAN	DHX38	Pre-mRNA-splicing factor ATP-dependent RNA helicase PRP16 (Fragment) OS=Homo	13784	1114	0	23	0	0.25	0	0	0	0	1	0	1	0	1	0	-1.17898
tr|H0Y955|H0Y955_HUMAN	METAP1	Methionine aminopeptidase 1 (Fragment) OS=Homo sapiens GN=METAP1 PE=4 SV=1	19559	1115	0	23	0	0.17	0	0	0	0	1	0	1	0	1	0	-1.17898
tr|A0AUM2|A0AUM2_HUMA	FOLR2	FOLR2 protein (Fragment) OS=Homo sapiens GN=FOLR2 PE=2 SV=1	27870	1116	0	23	0	0.12	0	0	0	0	1	0	1	0	1	0	-1.17898
tr|H0YI90|H0YI90_HUMAN	DDI2	Protein DDI1 homolog 2 (Fragment) OS=Homo sapiens GN=DDI2 PE=4 SV=1	11954	1117	0	23	0	0.29	0	0	0	0	1	0	1	0	1	0	-1.17898
tr|G3V2H3|G3V2H3_HUMAN	PNP	Purine nucleoside phosphorylase (Fragment) OS=Homo sapiens GN=PNP PE=2 SV=1	10579	1121	0	23	0	0.33	0	0	0	0	1	0	1	0	1	0	-1.17898
tr|B3KY11|B3KY11_HUMAN	tr|B3KY11|B3KY11	cDNA FLJ46571 fis, clone THYMU3041428, highly similar to Probable ATP-dependent	93519	1122	0	23	0	0.04	0	0	0	0	1	0	1	0	1	0	-1.17898
tr|E9PBB1|E9PBB1_HUMAN	CLIP4	CAP-Gly domain-containing linker protein 4 OS=Homo sapiens GN=CLIP4 PE=2 SV=1	11112	1124	0	23	0	0.31	0	0	0	0	1	0	1	0	1	0	-1.17898
tr|H0YAT9|H0YAT9_HUMAN	FGF20	Fibroblast growth factor 20 (Fragment) OS=Homo sapiens GN=FGF20 PE=4 SV=1	12983	1127	0	23	0	0.26	0	0	0	0	1	0	1	0	1	0	-1.17898
tr|B4DJ81|B4DJ81_HUMAN	NDUFS1	NADH-ubiquinone oxidoreductase 75 kDa subunit, mitochondrial OS=Homo sapiens	67563	1128	0	22	0	0.05	0	0	0	0	1	0	1	0	1	0	-1.17898
tr|H3BMZ1|H3BMZ1_HUMA	PTPLAD1	Very-long-chain (3R)-3-hydroxyacyl-[acyl-carrier protein] dehydratase 3 (Fragment)	16267	1130	0	22	0	0.22	0	0	0	0	1	0	1	0	1	0	-1.17898
tr|G5E933|G5E933_HUMAN	SBF1	Myotubularin-related protein 5 OS=Homo sapiens GN=SBF1 PE=2 SV=1	210422	1131	0	22	0	0.02	0	0	0	0	1	0	1	0	1	0	-1.17898
tr|B2WTI3|B2WTI3_HUMAN	JMJD6	Bifunctional arginine demethylase and lysyl-hydroxylase JMJD6 OS=Homo sapiens G	39331	1132	0	22	0	0.08	0	0	0	0	1	0	1	0	1	0	-1.17898
tr|A7BI36|A7BI36_HUMAN	RRBP1	p180/ribosome receptor OS=Homo sapiens GN=RRBP1 PE=2 SV=2	166048	1134	0	22	0	0.02	0	0	0	0	1	0	1	0	2	0	-1.17898
sp|Q6IA86|ELP2_HUMAN	ELP2	Elongator complex protein 2 OS=Homo sapiens GN=ELP2 PE=1 SV=2	94266	1135	0	22	0	0.03	0	0	0	0	1	0	1	0	1	0	-1.17898
tr|H7C2X7|H7C2X7_HUMAN	LSG1	Large subunit GTPase 1 homolog (Fragment) OS=Homo sapiens GN=LSG1 PE=4 SV=1	32482	1141	0	22	0	0.1	0	0	0	0	1	0	1	0	1	0	-1.17898
tr|J3KN33|J3KN33_HUMAN	CNTLN	Centlein OS=Homo sapiens GN=CNTLN PE=4 SV=1	162069	1142	0	22	0	0.02	0	0	0	0	1	0	1	0	2	0	-1.17898
tr|B4E126|B4E126_HUMAN	tr|B4E126|B4E126	cDNA FLJ61199, highly similar to UBX domain-containing protein 7 OS=Homo sapien	39093	1144	0	22	0	0.08	0	0	0	0	1	0	1	0	1	0	-1.17898
tr|H3BTN2|H3BTN2_HUMAN	MGA	MAX gene-associated protein (Fragment) OS=Homo sapiens GN=MGA PE=4 SV=1	9116	1145	0	22	0	0.38	0	0	0	0	1	0	1	0	1	0	-1.17898
tr|H3BST5|H3BST5_HUMAN	RHOT2	Mitochondrial Rho GTPase 2 (Fragment) OS=Homo sapiens GN=RHOT2 PE=4 SV=2	23300	1147	0	21	0	0.14	0	0	0	0	1	0	1	0	1	0	-1.17898
sp|Q9Y3L5|RAP2C_HUMAN	RAP2C	Ras-related protein Rap-2c OS=Homo sapiens GN=RAP2C PE=1 SV=1	20959	1148	0	21	0	0.16	0	0	0	0	1	0	1	0	1	0	-1.17898
tr|Q75MH1|Q75MH1_HUMA	RPS26	HCG1745083 OS=Homo sapiens GN=RPS26 PE=2 SV=1	13319	1150	0	21	0	0.26	0	0	0	0	1	0	1	0	1	0	-1.17898
tr|B4DPV3|B4DPV3_HUMAN	tr|B4DPV3|B4DPV	cDNA FLJ58109, highly similar to Protein diaphanous homolog 3 (Fragment) OS=Hom	117640	1151	0	21	0	0.03	0	0	0	0	1	0	1	0	1	0	-1.17898
tr|K7EP82|K7EP82_HUMAN	RPRD1A	Regulation of nuclear pre-mRNA domain-containing protein 1A (Fragment) OS=Hom	13852	1152	0	21	0	0.25	0	0	0	0	1	0	1	0	1	0	-1.17898
tr|H0Y4P3|H0Y4P3_HUMAN	VPRBP	Protein VPRBP (Fragment) OS=Homo sapiens GN=VPRBP PE=4 SV=1	121870	1153	0	21	0	0.03	0	0	1	0	1	0	1	0	1	0	-1.17898
tr|F1T0J4|F1T0J4_HUMAN	HMX1	Homeobox protein HMX1 OS=Homo sapiens GN=HMX1 PE=2 SV=1	20170	1154	0	21	0	0.17	0	0	0	0	1	0	1	0	1	0	-1.17898
sp|Q8TBP0|TBC16_HUMAN	TBC1D16	TBC1 domain family member 16 OS=Homo sapiens GN=TBC1D16 PE=2 SV=1	87400	1156	0	21	0	0.04	0	0	0	0	1	0	1	0	1	0	-1.17898
tr|F5GZX4|F5GZX4_HUMAN	POLR1B	DNA-directed RNA polymerase OS=Homo sapiens GN=POLR1B PE=2 SV=1	133921	1157	0	21	0	0.03	0	0	0	0	1	0	1	0	1	0	-1.17898
tr|D2CVN5|D2CVN5_HUMAN	AIP	Aryl hydrocarbon receptor-interacting protein AIP722 OS=Homo sapiens GN=AIP PE=	31094	1158	0	21	0	0.09	0	0	0	0	1	0	1	0	1	0	-1.17898
sp|Q9NVV4|PAPD1_HUMAN	MTPAP	Poly(A) RNA polymerase, mitochondrial OS=Homo sapiens GN=MTPAP PE=1 SV=1	66985	1159	0	21	0	0.05	0	0	0	0	1	0	1	0	1	0	-1.17898
tr|D2DGW5|D2DGW5_HUMA	DTNA	Alpha-dystrobrevin (Fragment) OS=Homo sapiens GN=DTNA PE=2 SV=1	28310	1160	0	21	0	0.12	0	0	0	0	1	0	1	0	1	0	-1.17898
tr|Q5XJ20|Q5XJ20_HUMAN	BCR	BCR protein (Fragment) OS=Homo sapiens GN=BCR PE=2 SV=1	6741	1161	0	21	0	0.53	0	0	0	0	1	0	1	0	1	0	-1.17898
tr|Q8N1B9|Q8N1B9_HUMAN	NDUFA10	NADH dehydrogenase (Ubiquinone) 1 alpha subcomplex, 10, 42kDa, isoform CRA_c (	22644	1165	0	20	0	0.07	0	0	0	0	1	0	1	0	1	0	-1.17898
tr|B4DFY7|B4DFY7_HUMAN	tr|B4DFY7|B4DFY7	cDNA FLJ57358, highly similar to Alpha-aminoadipic semialdehyde synthase, mitoch	45400	1167	0	20	0	0.03	0	0	0	0	1	0	1	0	1	0	-1.17898
sp|Q9BVI4|NOC4L_HUMAN	NOC4L	Nucleolar complex protein 4 homolog OS=Homo sapiens GN=NOC4L PE=1 SV=1	58830	1169	0	20	0	0.06	0	0	0	0	1	0	1	0	1	0	-1.17898
tr|Q5JSY8|Q5JSY8_HUMAN	TLE4	Transducin-like enhancer protein 4 (Fragment) OS=Homo sapiens GN=TLE4 PE=4 SV=	22150	1171	0	20	0	0.15	0	0	0	0	1	0	1	0	2	0	-1.17898
tr|B3KPC8|B3KPC8_HUMAN	tr|B3KPC8|B3KPC8	cDNA FLJ31621 fis, clone NT2RI2003205, highly similar to Transportin-2 OS=Homo sa	79954	1173	0	20	0	0.04	0	0	0	0	1	0	1	0	4	0	-1.17898
tr|Q0VF99|Q0VF99_HUMAN	OSBP2	Oxysterol-binding protein OS=Homo sapiens GN=OSBP2 PE=2 SV=1	102085	1190	0	18	0	0.03	0	0	0	0	1	0	1	0	2	0	-1.17898
tr|K7EQQ8|K7EQQ8_HUMAN	TRMT1	tRNA (guanine(26)-N(2))-dimethyltransferase (Fragment) OS=Homo sapiens GN=TRM	32946	1197	0	17	0	0.1	0	0	0	0	1	0	1	0	3	0	-1.17898
tr|K7ERE3|K7ERE3_HUMAN	KRT13	Keratin, type I cytoskeletal 13 OS=Homo sapiens GN=KRT13 PE=3 SV=1	45574	28	0	77	0	2.48	0	0	2	0	2	0	2	0	3	0	-1.91614
tr|J3QRG4|J3QRG4_HUMAN	KPNB1	Importin subunit beta-1 (Fragment) OS=Homo sapiens GN=KPNB1 PE=4 SV=1	6734	54	0	126	0	0.99	0	0	2	0	2	0	2	0	2	0	-1.91614
tr|Q562S2|Q562S2_HUMAN	ACT	Actin-like protein (Fragment) OS=Homo sapiens GN=ACT PE=2 SV=1	11529	65	0	37	0	2.32	0	0	1	0	1	0	2	0	2	0	-1.91614
tr|B3KNG9|B3KNG9_HUMAN	tr|B3KNG9|B3KNG	cDNA FLJ14581 fis, clone NT2RM4001382, highly similar to Importin-7 OS=Homo sap	22930	70	0	67	0	0.62	0	0	2	0	2	0	2	0	2	0	-1.91614
tr|Q59G78|Q59G78_HUMAN	tr|Q59G78|Q59G7	Lactate dehydrogenase B variant (Fragment) OS=Homo sapiens PE=2 SV=1	15109	90	0	68	0	2.96	0	0	1	0	1	0	2	0	2	0	-1.91614
tr|K7ENR8|K7ENR8_HUMAN	GPI	Glucose-6-phosphate isomerase (Fragment) OS=Homo sapiens GN=GPI PE=4 SV=1	13927	92	0	67	0	0.89	0	0	2	0	2	0	2	0	2	0	-1.91614
tr|H0Y8S0|H0Y8S0_HUMAN	HSPA9	Stress-70 protein, mitochondrial (Fragment) OS=Homo sapiens GN=HSPA9 PE=4 SV=	11326	97	0	37	0	0.86	0	0	0	0	3	0	2	0	4	0	-1.91614
tr|K7ENJ4|K7ENJ4_HUMAN	ATP5A1	ATP synthase subunit alpha, mitochondrial (Fragment) OS=Homo sapiens GN=ATP5A	21383	99	0	68	0	0.62	0	0	1	0	2	0	2	0	2	0	-1.91614
tr|C9JZ53|C9JZ53_HUMAN	IPO5	Importin-5 (Fragment) OS=Homo sapiens GN=IPO5 PE=2 SV=1	8736	126	0	62	0	0.4	0	0	1	0	2	0	2	0	2	0	-1.91614
tr|H0YC58|H0YC58_HUMAN	KIAA1967	DBIRD complex subunit KIAA1967 (Fragment) OS=Homo sapiens GN=KIAA1967 PE=4	19387	131	0	75	0	0.37	0	0	1	0	2	0	2	0	2	0	-1.91614
tr|D6RCS6|D6RCS6_HUMAN	TARS	Threonine--tRNA ligase, cytoplasmic OS=Homo sapiens GN=TARS PE=2 SV=1	6912	135	0	0	0	0.72	0	0	0	0	0	0	2	0	2	0	-1.91614
tr|B1AK51|B1AK51_HUMAN	CHD5	Uncharacterized protein OS=Homo sapiens GN=CHD5 PE=4 SV=1	136766	150	0	106	0	0.16	0	0	2	0	2	0	2	0	2	0	-1.91614
tr|H0YFD6|H0YFD6_HUMAN	HADHA	Trifunctional enzyme subunit alpha, mitochondrial (Fragment) OS=Homo sapiens GN	27376	167	0	91	0	0.26	0	0	2	0	2	0	2	0	2	0	-1.91614
tr|B4E3R6|B4E3R6_HUMAN	tr|B4E3R6|B4E3R6	cDNA FLJ51777, highly similar to Squamous cell carcinoma antigen recognized byT-c	82792	171	0	84	0	0.27	0	0	2	0	2	0	2	0	2	0	-1.91614
tr|O95325|O95325_HUMAN	tr|O95325|O9532	Proteasome subunit p58 OS=Homo sapiens PE=2 SV=1	13463	221	0	37	0	0.3	0	0	1	0	1	0	2	0	2	0	-1.91614
tr|B7Z4C7|B7Z4C7_HUMAN	tr|B7Z4C7|B7Z4C7	cDNA FLJ57321, moderately similar to Moesin OS=Homo sapiens PE=2 SV=1	23899	226	0	60	0	0.46	0	0	2	0	2	0	2	0	2	0	-1.91614
tr|A6NJJ0|A6NJJ0_HUMAN	PRDX4	Peroxiredoxin-4 (Fragment) OS=Homo sapiens GN=PRDX4 PE=2 SV=1	21403	246	0	100	0	0.95	0	0	2	0	2	0	2	0	2	0	-1.91614
tr|Q6MZY7|Q6MZY7_HUMA	DKFZp686C17209	Putative uncharacterized protein DKFZp686C17209 OS=Homo sapiens GN=DKFZp686	40730	306	0	115	0	0.29	0	0	2	0	2	0	2	0	2	0	-1.91614
tr|E9PMN0|E9PMN0_HUMA	RNH1	Ribonuclease inhibitor OS=Homo sapiens GN=RNH1 PE=2 SV=1	18835	312	0	64	0	0.28	0	0	1	0	2	0	2	0	2	0	-1.91614
tr|Q9BSK3|Q9BSK3_HUMAN	tr|Q9BSK3|Q9BSK	Putative uncharacterized protein (Fragment) OS=Homo sapiens PE=2 SV=1	23583	331	0	123	0	0.11	0	0	2	0	2	0	2	0	2	0	-1.91614
tr|B4DJ53|B4DJ53_HUMAN	tr|B4DJ53|B4DJ53	cDNA FLJ56447, highly similar to Dynamin-2 (EC 3.6.5.5) OS=Homo sapiens PE=2 SV=	68037	335	0	49	0	0.14	0	0	1	0	2	0	2	0	2	0	-1.91614
tr|A5D6X0|A5D6X0_HUMAN	SMARCA4	SMARCA4 protein (Fragment) OS=Homo sapiens GN=SMARCA4 PE=2 SV=1	63022	345	0	58	0	0.09	0	0	1	0	2	0	2	0	4	0	-1.91614
tr|B4DZR0|B4DZR0_HUMAN	tr|B4DZR0|B4DZR	cDNA FLJ55529, highly similar to Heat shock 70 kDa protein 4L OS=Homo sapiens PE	98526	347	0	116	0	0.07	0	0	2	0	2	0	2	0	6	0	-1.91614
tr|H7BZC3|H7BZC3_HUMAN	HDLBP	Vigilin (Fragment) OS=Homo sapiens GN=HDLBP PE=4 SV=1	30057	353	0	52	0	0.12	0	0	1	0	2	0	2	0	3	0	-1.91614
tr|K7EP46|K7EP46_HUMAN	THOP1	Thimet oligopeptidase OS=Homo sapiens GN=THOP1 PE=3 SV=1	65876	371	0	110	0	0.1	0	0	2	0	2	0	2	0	3	0	-1.91614
tr|H7C1K0|H7C1K0_HUMAN	SRRT	Serrate RNA effector molecule homolog (Fragment) OS=Homo sapiens GN=SRRT PE=	5606	377	0	55	0	0.17	0	0	1	0	1	0	2	0	2	0	-1.91614
tr|B4DRQ4|B4DRQ4_HUMAN	tr|B4DRQ4|B4DRQ	Phosphorylase OS=Homo sapiens PE=2 SV=1	67932	388	0	34	0	0.23	0	0	1	0	2	0	2	0	3	0	-1.91614
tr|F2Z3G4|F2Z3G4_HUMAN	KPNA1	Importin subunit alpha-1 OS=Homo sapiens GN=KPNA1 PE=4 SV=1	20944	435	0	45	0	0.17	0	0	1	0	1	0	2	0	2	0	-1.91614
tr|D6R9M0|D6R9M0_HUMA	KIF2A	Kinesin-like protein KIF2A (Fragment) OS=Homo sapiens GN=KIF2A PE=2 SV=1	32900	449	0	57	0	0.18	0	0	2	0	2	0	2	0	2	0	-1.91614
tr|B4DUP5|B4DUP5_HUMAN	tr|B4DUP5|B4DUP	cDNA FLJ60165, highly similar to Translation initiation factor eIF-2B subunit delta OS	34946	488	0	79	0	0.2	0	0	2	0	2	0	2	0	2	0	-1.91614
tr|A4UCU0|A4UCU0_HUMAN	tr|A4UCU0|A4UCU	DEAD box polypeptide 47 isoform 1 variant (Fragment) OS=Homo sapiens PE=2 SV=1	19904	491	0	78	0	0.37	0	0	2	0	2	0	2	0	2	0	-1.91614
tr|E5RG31|E5RG31_HUMAN	MCM4	DNA replication licensing factor MCM4 (Fragment) OS=Homo sapiens GN=MCM4 PE	31785	493	0	54	0	0.14	0	0	1	0	2	0	2	0	2	0	-1.91614
tr|B2R6X2|B2R6X2_HUMAN	tr|B2R6X2|B2R6X2	cDNA, FLJ93161, highly similar to Homo sapiens glucuronidase, beta (GUSB), mRNA (	75001	496	0	77	0	0.04	0	0	1	0	1	0	2	0	2	0	-1.91614
tr|B2RDE0|B2RDE0_HUMAN	tr|B2RDE0|B2RDE	cDNA, FLJ96567, highly similar to Homo sapiens propionyl Coenzyme A carboxylase,	77992	509	0	75	0	0.09	0	0	1	0	2	0	2	0	3	0	-1.91614
tr|J3KN36|J3KN36_HUMAN	NOMO3	Nodal modulator 3 OS=Homo sapiens GN=NOMO3 PE=4 SV=1	140376	530	0	71	0	0.05	0	0	2	0	2	0	2	0	2	0	-1.91614
sp|Q9BYZ2|LDH6B_HUMAN	LDHAL6B	L-lactate dehydrogenase A-like 6B OS=Homo sapiens GN=LDHAL6B PE=1 SV=3	42372	536	0	71	0	0.16	0	0	2	0	2	0	2	0	3	0	-1.91614
tr|Q6IBS5|Q6IBS5_HUMAN	DLST	DLST protein OS=Homo sapiens GN=DLST PE=2 SV=1	49131	547	0	68	0	0.14	0	0	2	0	2	0	2	0	2	0	-1.91614
tr|B4DR88|B4DR88_HUMAN	tr|B4DR88|B4DR8	cDNA FLJ58252, highly similar to Homo sapiens DEAH (Asp-Glu-Ala-His) box polypep	73228	564	0	66	0	0.09	0	0	2	0	2	0	2	0	2	0	-1.91614
tr|H0YBW4|H0YBW4_HUMA	PLAA	Phospholipase A-2-activating protein (Fragment) OS=Homo sapiens GN=PLAA PE=4 S	31645	566	0	65	0	0.22	0	0	1	0	2	0	2	0	2	0	-1.91614
tr|Q0IJ56|Q0IJ56_HUMAN	ST13	ST13 protein (Fragment) OS=Homo sapiens GN=ST13 PE=2 SV=1	35164	577	0	64	0	0.09	0	0	1	0	1	0	2	0	2	0	-1.91614
sp|Q8TDD1|DDX54_HUMAN	DDX54	ATP-dependent RNA helicase DDX54 OS=Homo sapiens GN=DDX54 PE=1 SV=2	98819	580	0	64	0	0.07	0	0	2	0	2	0	2	0	2	0	-1.91614
tr|B4DSA8|B4DSA8_HUMAN	tr|B4DSA8|B4DSA	cDNA FLJ51067, highly similar to DNA damage-binding protein 1 OS=Homo sapiens P	112974	581	0	64	0	0.06	0	0	1	0	2	0	2	0	2	0	-1.91614
tr|H0YMV8|H0YMV8_HUMA	RPS27L	40S ribosomal protein S27 OS=Homo sapiens GN=RPS27L PE=3 SV=1	11851	587	0	63	0	0.66	0	0	2	0	2	0	2	0	2	0	-1.91614
tr|G3V150|G3V150_HUMAN	B3GAT3	Beta-1,3-glucuronyltransferase 3 (Glucuronosyltransferase I), isoform CRA_a OS=Ho	35170	588	0	0	0	0.2	0	0	0	0	0	0	2	0	2	0	-1.91614
tr|B0QZ65|B0QZ65_HUMAN	GAPVD1	GTPase-activating protein and VPS9 domain-containing protein 1 OS=Homo sapiens	56996	591	0	62	0	0.12	0	0	1	0	2	0	2	0	4	0	-1.91614
tr|B2RCD8|B2RCD8_HUMAN	tr|B2RCD8|B2RCD	cDNA, FLJ96012, highly similar to Homo sapiens interferon regulatory factor 2 bindir	62587	592	0	62	0	0.11	0	0	1	0	2	0	2	0	2	0	-1.91614
tr|Q0VAS5|Q0VAS5_HUMAN	HIST1H4H	Histone H4 OS=Homo sapiens GN=HIST1H4H PE=2 SV=1	11364	594	0	62	0	0.7	0	0	1	0	2	0	2	0	2	0	-1.91614
tr|B0QZK4|B0QZK4_HUMAN	HP1BP3	Heterochromatin protein 1-binding protein 3 (Fragment) OS=Homo sapiens GN=HP1	28725	600	0	61	0	0.25	0	0	2	0	2	0	2	0	2	0	-1.91614
tr|B4E3Q9|B4E3Q9_HUMAN	tr|B4E3Q9|B4E3Q	cDNA FLJ59659, highly similar to Vinculin OS=Homo sapiens PE=2 SV=1	101217	601	0	61	0	0.07	0	0	2	0	2	0	2	0	4	0	-1.91614
tr|Q7Z7R0|Q7Z7R0_HUMAN	tr|Q7Z7R0|Q7Z7R	Smooth muscle myosin heavy chain (Fragment) OS=Homo sapiens PE=2 SV=1	121940	605	0	61	0	0.03	0	0	1	0	1	0	2	0	3	0	-1.91614
tr|I3L2U8|I3L2U8_HUMAN	PAFAH1B1	Platelet-activating factor acetylhydrolase IB subunit alpha OS=Homo sapiens GN=PA	39493	610	0	60	0	0.17	0	0	2	0	2	0	2	0	2	0	-1.91614
sp|Q86UE4|LYRIC_HUMAN	MTDH	Protein LYRIC OS=Homo sapiens GN=MTDH PE=1 SV=2	63856	612	0	60	0	0.11	0	0	1	0	2	0	2	0	3	0	-1.91614
sp|P62263|RS14_HUMAN	RPS14	40S ribosomal protein S14 OS=Homo sapiens GN=RPS14 PE=1 SV=3	16434	615	0	60	0	0.45	0	0	1	0	2	0	2	0	3	0	-1.91614
tr|B2RBV7|B2RBV7_HUMAN	tr|B2RBV7|B2RBV	cDNA, FLJ95721, highly similar to Homo sapiens cullin 4A (CUL4A), mRNA OS=Homo	77212	629	0	41	0	0.06	0	0	1	0	1	0	2	0	2	0	-1.91614
tr|F8VZ52|F8VZ52_HUMAN	DNM1L	Dynamin-1-like protein (Fragment) OS=Homo sapiens GN=DNM1L PE=2 SV=1	28970	636	0	32	0	0.12	0	0	0	0	2	0	2	0	2	0	-1.91614
tr|G1UI19|G1UI19_HUMAN	KPNA6	Importin subunit alpha-7 (Fragment) OS=Homo sapiens GN=KPNA6 PE=2 SV=1	28256	660	0	40	0	0.11	0	0	1	0	1	0	2	0	2	0	-1.91614
tr|Q59FQ4|Q59FQ4_HUMAN	tr|Q59FQ4|Q59FQ	Glutamate dehydrogenase 1 variant (Fragment) OS=Homo sapiens PE=2 SV=1	32587	664	0	40	0	0.23	0	0	0	0	2	0	2	0	2	0	-1.91614
tr|A5PHJ9|A5PHJ9_HUMAN	PDHA1/LOC79064	PDHA1/LOC79064 protein (Fragment) OS=Homo sapiens GN=PDHA1/LOC79064 PE=2	34113	665	0	54	0	0.1	0	0	1	0	1	0	2	0	2	0	-1.91614
tr|B3KNM6|B3KNM6_HUMA	tr|B3KNM6|B3KNI	cDNA FLJ14968 fis, clone THYRO1000288, highly similar to CAAX prenyl protease 1 h	27279	675	0	54	0	0.26	0	0	1	0	2	0	2	0	2	0	-1.91614
sp|Q9UBU9|NXF1_HUMAN	NXF1	Nuclear RNA export factor 1 OS=Homo sapiens GN=NXF1 PE=1 SV=1	70652	683	0	53	0	0.1	0	0	1	0	2	0	2	0	2	0	-1.91614

tr|Q53T70|Q53T70_HUMAN	RAB10	Putative uncharacterized protein RAB10 (Fragment) OS=Homo sapiens GN=RAB10 P	17970	690	0	52	0	0.41	0	0	1	0	2	0	2	0	2	0	-1.91614
tr|B2R643|B2R643_HUMAN	tr|B2R643|B2R643	cDNA, FLJ92777, highly similar to Homo sapiens myosin IA (MYO1A), mRNA OS=Hom	119252	693	0	52	0	0.03	0	0	1	0	1	0	2	0	2	0	-1.91614
tr|B3KN30|B3KN30_HUMAN	tr|B3KN30|B3KN3	cDNA FLJ13372 fis, clone PLACE1000706, highly similar to Transcription intermediary	84930	694	0	52	0	0.04	0	0	1	0	1	0	2	0	2	0	-1.91614
tr|A0AV56|A0AV56_HUMAN	SAFB	SAFB protein OS=Homo sapiens GN=SAFB PE=2 SV=1	103249	708	0	51	0	0.06	0	0	1	0	2	0	2	0	2	0	-1.91614
tr|B4DMU7|B4DMU7_HUMA	tr|B4DMU7|B4DM	cDNA FLJ59351, highly similar to Striatin-4 OS=Homo sapiens PE=2 SV=1	69271	727	0	50	0	0.1	0	0	2	0	2	0	2	0	2	0	-1.91614
tr|J3KQE5|J3KQE5_HUMAN	RAN	GTP-binding nuclear protein Ran (Fragment) OS=Homo sapiens GN=RAN PE=4 SV=1	27027	728	0	50	0	0.26	0	0	0	0	2	0	2	0	3	0	-1.91614
tr|B2RD24|B2RD24_HUMAN	tr|B2RD24|B2RD2	cDNA, FLJ96424 OS=Homo sapiens PE=2 SV=1	80216	738	0	49	0	0.04	0	0	1	0	1	0	2	0	2	0	-1.91614
tr|Q59H95|Q59H95_HUMAN	tr|Q59H95|Q59H9	Flightless I homolog variant (Fragment) OS=Homo sapiens PE=2 SV=1	126396	744	0	48	0	0.05	0	0	1	0	2	0	2	0	3	0	-1.91614
tr|I3L2C7|I3L2C7_HUMAN	GEMIN4	Gem-associated protein 4 OS=Homo sapiens GN=GEMIN4 PE=4 SV=1	120456	754	0	47	0	0.06	0	0	2	0	2	0	2	0	2	0	-1.91614
tr|B2R4C0|B2R4C0_HUMAN	tr|B2R4C0|B2R4C	60S ribosomal protein L18a OS=Homo sapiens PE=2 SV=1	21024	755	0	47	0	0.16	0	0	1	0	1	0	2	0	2	0	-1.91614
tr|H0YN07|H0YN07_HUMAN	IPO4	Importin-4 (Fragment) OS=Homo sapiens GN=IPO4 PE=4 SV=1	33644	764	0	47	0	0.21	0	0	2	0	2	0	2	0	3	0	-1.91614
tr|A0AV58|A0AV58_HUMAN	STRN3	Striatin, calmodulin binding protein 3 OS=Homo sapiens GN=STRN3 PE=2 SV=1	78069	765	0	46	0	0.09	0	0	2	0	2	0	2	0	2	0	-1.91614
tr|B4DM41|B4DM41_HUMA	RFC4	Replication factor C (Activator 1) 4, 37kDa, isoform CRA_b OS=Homo sapiens GN=RF	33996	768	0	46	0	0.1	0	0	1	0	1	0	2	0	2	0	-1.91614
tr|B4E206|B4E206_HUMAN	tr|B4E206|B4E206	cDNA FLJ55441, highly similar to Nuclear pore complex protein Nup133 OS=Homo sa	128349	771	0	46	0	0.05	0	0	1	0	2	0	2	0	2	0	-1.91614
tr|B4DLY2|B4DLY2_HUMAN	tr|B4DLY2|B4DLY2	cDNA FLJ56600, highly similar to Large proline-rich protein BAT2 OS=Homo sapiens	146160	778	0	45	0	0.05	0	0	0	0	2	0	2	0	2	0	-1.91614
sp|Q6PI48|SYDM_HUMAN	DARS2	Aspartate--tRNA ligase, mitochondrial OS=Homo sapiens GN=DARS2 PE=1 SV=1	74086	780	0	45	0	0.04	0	0	1	0	1	0	2	0	2	0	-1.91614
tr|B3KSI7|B3KSI7_HUMAN	tr|B3KSI7|B3KSI7_	cDNA FLJ36374 fis, clone THYMU2008185, highly similar to Xaa-Pro aminopeptidase	62680	783	0	45	0	0.11	0	0	0	0	2	0	2	0	3	0	-1.91614
sp|Q7RTS7|K2C74_HUMAN	KRT74	Keratin, type II cytoskeletal 74 OS=Homo sapiens GN=KRT74 PE=1 SV=2	58229	789	0	44	0	0.12	0	0	1	0	2	0	2	0	4	0	-1.91614
tr|C9JA17|C9JA17_HUMAN	ATP6V1A	V-type proton ATPase catalytic subunit A (Fragment) OS=Homo sapiens GN=ATP6V1	20286	793	0	44	0	0.17	0	0	1	0	1	0	2	0	2	0	-1.91614
sp|Q93034|CUL5_HUMAN	CUL5	Cullin-5 OS=Homo sapiens GN=CUL5 PE=1 SV=4	91468	807	0	43	0	0.04	0	0	1	0	1	0	2	0	3	0	-1.91614
tr|Q6FHJ5|Q6FHJ5_HUMAN	SCAMP3	SCAMP3 protein (Fragment) OS=Homo sapiens GN=SCAMP3 PE=2 SV=1	38674	814	0	42	0	0.18	0	0	1	0	2	0	2	0	2	0	-1.91614
tr|K7ES28|K7ES28_HUMAN	SBNO2	Protein strawberry notch homolog 2 OS=Homo sapiens GN=SBNO2 PE=4 SV=1	150363	834	0	41	0	0.02	0	0	1	0	1	0	2	0	2	0	-1.91614
sp|Q7Z3U7|MON2_HUMAN	MON2	Protein MON2 homolog OS=Homo sapiens GN=MON2 PE=1 SV=2	192475	855	0	39	0	0.03	0	0	1	0	2	0	2	0	3	0	-1.91614
tr|B4DZ88|B4DZ88_HUMAN	KTN1	KTN1 protein OS=Homo sapiens GN=KTN1 PE=2 SV=1	153179	876	0	38	0	0.02	0	0	1	0	1	0	2	0	2	0	-1.91614
tr|Q5VXN0|Q5VXN0_HUMAN	BXDC1	Brix domain containing 1 (Fragment) OS=Homo sapiens GN=BXDC1 PE=4 SV=1	24760	882	0	37	0	0.14	0	0	1	0	1	0	2	0	3	0	-1.91614
tr|B7Z438|B7Z438_HUMAN	tr|B7Z438|B7Z438	cDNA FLJ56352, highly similar to Succinyl-CoA ligase (GDP-forming) subunit alpha, m	26801	894	0	36	0	0.26	0	0	1	0	2	0	2	0	2	0	-1.91614
sp|Q9BPU6|DPYL5_HUMAN	DPYSL5	Dihydropyrimidinase-related protein 5 OS=Homo sapiens GN=DPYSL5 PE=1 SV=1	61952	897	0	36	0	0.05	0	0	0	0	1	0	2	0	3	0	-1.91614
tr|Q5HY91|Q5HY91_HUMAN	DKFZp686E10165	Putative uncharacterized protein DKFZp686E10165 (Fragment) OS=Homo sapiens G	21179	899	0	36	0	0.06	0	0	0	0	2	0	2	0	2	0	-1.91614
tr|B4DZH4|B4DZH4_HUMAN	tr|B4DZH4|B4DZH	cDNA FLJ59078, highly similar to Ran-binding protein 2-like 6 OS=Homo sapiens PE=	127140	910	0	35	0	0.05	0	0	0	0	2	0	2	0	2	0	-1.91614
tr|B4DDH9|B4DDH9_HUMAN	tr|B4DDH9|B4DDH	cDNA FLJ53820, highly similar to U4/U6.U5 tri-snRNP-associated protein 1 OS=Homo	73566	916	0	35	0	0.04	0	0	0	0	1	0	2	0	2	0	-1.91614
tr|D3DS96|D3DS96_HUMAN	BAZ1A	Bromodomain adjacent to zinc finger domain, 1A, isoform CRA_c OS=Homo sapiens	180117	917	0	35	0	0.04	0	0	0	0	2	0	2	0	3	0	-1.91614
tr|B2RE88|B2RE88_HUMAN	tr|B2RE88|B2RE88	cDNA, FLJ96465, highly similar to Homo sapiens solute carrier family 25 (mitochondr	40379	921	0	35	0	0.17	0	0	0	0	2	0	2	0	2	0	-1.91614
sp|Q9NQH7|XPP3_HUMAN	XPNPEP3	Probable Xaa-Pro aminopeptidase 3 OS=Homo sapiens GN=XPNPEP3 PE=1 SV=1	57624	933	0	34	0	0.06	0	0	1	0	2	0	2	0	2	0	-1.91614
tr|F8W1U3|F8W1U3_HUMA	KRT8	Keratin, type II cytoskeletal 8 (Fragment) OS=Homo sapiens GN=KRT8 PE=2 SV=1	32586	937	0	34	0	0.21	0	0	1	0	2	0	2	0	2	0	-1.91614
tr|K7ES11|K7ES11_HUMAN	UBE2O	Ubiquitin-conjugating enzyme E2 O (Fragment) OS=Homo sapiens GN=UBE2O PE=4 S	89434	939	0	34	0	0.07	0	0	1	0	2	0	2	0	4	0	-1.91614
tr|B4DE91|B4DE91_HUMAN	tr|B4DE91|B4DE9	cDNA FLJ55534, highly similar to 4-trimethylaminobutyraldehyde dehydrogenase (E	53648	942	0	34	0	0.06	0	0	0	0	1	0	2	0	3	0	-1.91614
sp|Q86VV8|RTTN_HUMAN	RTTN	Rotatin OS=Homo sapiens GN=RTTN PE=1 SV=3	252290	948	0	33	0	0.01	0	0	1	0	1	0	2	0	2	0	-1.91614
sp|Q06830|PRDX1_HUMAN	PRDX1	Peroxiredoxin-1 OS=Homo sapiens GN=PRDX1 PE=1 SV=1	22324	949	0	33	0	0.15	0	0	1	0	1	0	2	0	3	0	-1.91614
tr|J3QQY1|J3QQY1_HUMAN	CDK5RAP3	CDK5 regulatory subunit-associated protein 3 (Fragment) OS=Homo sapiens GN=CD	32228	951	0	33	0	0.1	0	0	1	0	1	0	2	0	2	0	-1.91614
tr|J3KNE0|J3KNE0_HUMAN	RGPD3	RanBP2-like and GRIP domain-containing protein 3 OS=Homo sapiens GN=RGPD3 PE	199639	961	0	33	0	0.05	0	0	0	0	3	0	2	0	3	0	-1.91614
tr|B3KUU8|B3KUU8_HUMAN	tr|B3KUU8|B3KUU	cDNA FLJ40670 fis, clone THYMU2020959, highly similar to Dynamin-2 OS=Homo sa	67964	972	0	32	0	0.05	0	0	0	0	1	0	2	0	2	0	-1.91614
tr|B4E0H3|B4E0H3_HUMAN	tr|B4E0H3|B4E0H	cDNA FLJ53386, highly similar to Signal recognition particle receptor subunit alpha C	66987	1014	0	30	0	0.1	0	0	0	0	2	0	2	0	2	0	-1.91614
tr|F5GZF3|F5GZF3_HUMAN	THOC5	THO complex subunit 5 homolog OS=Homo sapiens GN=THOC5 PE=2 SV=1	14985	1016	0	0	0	0.15	0	0	0	0	0	0	2	0	2	0	-1.91614
tr|J3KNI9|J3KNI9_HUMAN	TBC1D15	TBC1 domain family member 15 OS=Homo sapiens GN=TBC1D15 PE=4 SV=1	79491	1021	0	29	0	0.08	0	0	0	0	2	0	2	0	2	0	-1.91614
sp|P49750|YLPM1_HUMAN	YLPM1	YLP motif-containing protein 1 OS=Homo sapiens GN=YLPM1 PE=1 SV=3	220077	1031	0	28	0	0.01	0	0	0	0	1	0	2	0	2	0	-1.91614
tr|B4DNW3|B4DNW3_HUMA	tr|B4DNW3|B4DN	cDNA FLJ51914, highly similar to Protein DEK OS=Homo sapiens PE=2 SV=1	40421	1032	0	28	0	0.08	0	0	0	0	1	0	2	0	2	0	-1.91614
tr|Q7Z4W0|Q7Z4W0_HUMA	tr|Q7Z4W0|Q7Z4	ATP-dependent RNA helicase OS=Homo sapiens PE=2 SV=1	53113	1034	0	28	0	0.06	0	0	0	0	1	0	2	0	2	0	-1.91614
sp|Q8NB90|SPAT5_HUMAN	SPATA5	Spermatogenesis-associated protein 5 OS=Homo sapiens GN=SPATA5 PE=1 SV=3	98470	1065	0	26	0	0.03	0	0	0	0	1	0	2	0	2	0	-1.91614
tr|B4DMM1|B4DMM1_HUM	tr|B4DMM1|B4DN	cDNA FLJ58265, highly similar to Ubiquitin-protein ligase BRE1B (EC 6.3.2.-) OS=Hom	79571	1067	0	26	0	0.03	0	0	0	0	1	0	2	0	2	0	-1.91614
tr|Q6IBT1|Q6IBT1_HUMAN	PSMB7	Proteasome subunit beta type OS=Homo sapiens GN=PSMB7 PE=2 SV=1	30260	1075	0	26	0	0.23	0	0	0	0	2	0	2	0	2	0	-1.91614
tr|B4E318|B4E318_HUMAN	BCCIP	BRCA2 and CDKN1A-interacting protein OS=Homo sapiens GN=BCCIP PE=2 SV=1	30064	1119	0	23	0	0.1	0	0	0	0	1	0	2	0	2	0	-1.91614
sp|Q14669|TRIPC_HUMAN	TRIP12	E3 ubiquitin-protein ligase TRIP12 OS=Homo sapiens GN=TRIP12 PE=1 SV=1	222234	1123	0	23	0	0.01	0	0	0	0	1	0	2	0	2	0	-1.91614
tr|B7Z6H4|B7Z6H4_HUMAN	tr|B7Z6H4|B7Z6H	DNA-directed RNA polymerase OS=Homo sapiens PE=2 SV=1	155407	1138	0	22	0	0.02	0	0	0	0	1	0	2	0	2	0	-1.91614
sp|Q15061|WDR43_HUMAN	WDR43	WD repeat-containing protein 43 OS=Homo sapiens GN=WDR43 PE=1 SV=3	75813	1146	0	22	0	0.04	0	0	0	0	1	0	2	0	3	0	-1.91614
sp|Q8NBJ5|GT251_HUMAN	GLT25D1	Procollagen galactosyltransferase 1 OS=Homo sapiens GN=GLT25D1 PE=1 SV=1	71933	1181	0	19	0	0.05	0	0	0	0	1	0	2	0	4	0	-1.91614
tr|G3V5M4|G3V5M4_HUMA	ACTN1	Alpha-actinin-1 (Fragment) OS=Homo sapiens GN=ACTN1 PE=2 SV=1	14086	41	0	74	0	1.43	0	0	3	0	3	0	3	0	3	0	-2.40176
tr|G3V2X9|G3V2X9_HUMAN	ACTN1	Alpha-actinin-1 (Fragment) OS=Homo sapiens GN=ACTN1 PE=2 SV=1	16418	109	0	74	0	0.52	0	0	3	0	3	0	3	0	4	0	-2.40176
tr|H3BSS4|H3BSS4_HUMAN	PCBP2	Poly(rC)-binding protein 2 (Fragment) OS=Homo sapiens GN=PCBP2 PE=4 SV=1	19494	132	0	89	0	1.26	0	0	1	0	3	0	3	0	3	0	-2.40176
tr|Q5TCC5|Q5TCC5_HUMAN	IARS	Isoleucine--tRNA ligase, cytoplasmic (Fragment) OS=Homo sapiens GN=IARS PE=4 SV	19001	151	0	93	0	0.31	0	0	1	0	2	0	3	0	3	0	-2.40176
tr|H0YEU6|H0YEU6_HUMAN	PABPC4	Polyadenylate-binding protein 4 (Fragment) OS=Homo sapiens GN=PABPC4 PE=4 SV	27445	160	0	93	0	0.46	0	0	3	0	3	0	3	0	3	0	-2.40176
tr|B3KNQ8|B3KNQ8_HUMAN	tr|B3KNQ8|B3KNC	cDNA FLJ30174 fis, clone BRACE2000975, highly similar to Sodium/potassium-transp	48576	170	0	64	0	0.33	0	0	1	0	3	0	3	0	3	0	-2.40176
tr|H0Y9Z8|H0Y9Z8_HUMAN	NCAPG	Condensin complex subunit 3 (Fragment) OS=Homo sapiens GN=NCAPG PE=4 SV=1	30654	239	0	93	0	0.25	0	0	1	0	3	0	3	0	3	0	-2.40176
tr|B4E2C9|B4E2C9_HUMAN	tr|B4E2C9|B4E2C9	cDNA FLJ61545, highly similar to Coatomer subunit beta OS=Homo sapiens PE=2 SV=	30965	244	0	77	0	0.25	0	0	3	0	3	0	3	0	3	0	-2.40176
tr|D6RD83|D6RD83_HUMAN	HNRNPD	Heterogeneous nuclear ribonucleoprotein D0 (Fragment) OS=Homo sapiens GN=HN	9992	266	0	73	0	0.7	0	0	2	0	3	0	3	0	4	0	-2.40176
tr|C9JIJ5|C9JIJ5_HUMAN	RPL7	60S ribosomal protein L7 (Fragment) OS=Homo sapiens GN=RPL7 PE=2 SV=1	11885	317	0	29	0	0.9	0	0	2	0	2	0	3	0	3	0	-2.40176
tr|Q0D2M2|Q0D2M2_HUMA	HIST1H2BC	HIST1H2BC protein OS=Homo sapiens GN=HIST1H2BC PE=2 SV=1	13826	337	0	119	0	0.93	0	0	2	0	3	0	3	0	3	0	-2.40176
tr|Q548W1|Q548W1_HUMA	OGT	HRNT1 OS=Homo sapiens GN=OGT PE=2 SV=1	104029	354	0	115	0	0.13	0	0	3	0	4	0	3	0	4	0	-2.40176
tr|B4DT29|B4DT29_HUMAN	tr|B4DT29|B4DT2	cDNA FLJ51082, highly similar to Actin-like protein 3 OS=Homo sapiens PE=2 SV=1	40959	411	0	79	0	0.4	0	0	2	0	3	0	3	0	3	0	-2.40176
tr|B3KY28|B3KY28_HUMAN	tr|B3KY28|B3KY28	cDNA FLJ46689 fis, clone TRACH3012106, highly similar to Band 4.1-like protein 3 OS	85088	424	0	94	0	0.12	0	0	1	0	3	0	3	0	3	0	-2.40176
tr|H0YMR4|H0YMR4_HUMA	IPO4	Importin-4 (Fragment) OS=Homo sapiens GN=IPO4 PE=4 SV=1	60104	436	0	47	0	0.17	0	0	2	0	2	0	3	0	3	0	-2.40176
tr|A8K9E1|A8K9E1_HUMAN	tr|A8K9E1|A8K9E1	cDNA FLJ77957, highly similar to Homo sapiens HBS1-like (S. cerevisiae) (HBS1L), mR	75969	443	0	89	0	0.14	0	0	2	0	3	0	3	0	5	0	-2.40176
tr|E5RJF5|E5RJF5_HUMAN	ANXA6	Annexin (Fragment) OS=Homo sapiens GN=ANXA6 PE=2 SV=1	14825	446	0	34	0	0.24	0	0	0	0	3	0	3	0	3	0	-2.40176
tr|B3KNC3|B3KNC3_HUMAN	tr|B3KNC3|B3KNC	cDNA FLJ14222 fis, clone NT2RP3003992, highly similar to Nucleolar complex protei	85719	451	0	86	0	0.08	0	0	2	0	2	0	3	0	3	0	-2.40176
tr|Q96F88|Q96F88_HUMAN	POP1	Processing of 1, ribonuclease P/MRP subunit (S. cerevisiae) OS=Homo sapiens GN=P	116318	453	0	85	0	0.09	0	0	2	0	3	0	3	0	4	0	-2.40176
tr|B3KSU9|B3KSU9_HUMAN	tr|B3KSU9|B3KSU	cDNA FLJ37014 fis, clone BRACE2010203, highly similar to Zinc phosphodiesterase E	66302	470	0	82	0	0.16	0	0	3	0	3	0	3	0	4	0	-2.40176
tr|Q53G58|Q53G58_HUMAN	tr|Q53G58|Q53G5	Coronin (Fragment) OS=Homo sapiens PE=2 SV=1	53931	471	0	82	0	0.19	0	0	2	0	3	0	3	0	4	0	-2.40176
tr|B7Z1Q4|B7Z1Q4_HUMAN	tr|B7Z1Q4|B7Z1Q	cDNA FLJ57955, highly similar to Adapter-relatedprotein complex 2 alpha- 2 subunit	75682	475	0	81	0	0.14	0	0	2	0	3	0	3	0	3	0	-2.40176
tr|Q8N9K4|Q8N9K4_HUMAN	tr|Q8N9K4|Q8N9K	cDNA FLJ36998 fis, clone BRACE2007295, highly similar to ALPHA-ADAPTIN A OS=Ho	52284	477	0	81	0	0.2	0	0	2	0	3	0	3	0	3	0	-2.40176
tr|B3KXH0|B3KXH0_HUMAN	tr|B3KXH0|B3KXH	cDNA FLJ45395 fis, clone BRHIP3027191, highly similar to 150 kDa oxygen-regulated	110601	492	0	78	0	0.09	0	0	1	0	3	0	3	0	3	0	-2.40176
tr|D3DTH7|D3DTH7_HUMAN	MYO1C	Myosin IC, isoform CRA_a OS=Homo sapiens GN=MYO1C PE=2 SV=1	99613	506	0	75	0	0.14	0	0	1	0	4	0	3	0	4	0	-2.40176
tr|Q05CW7|Q05CW7_HUMA	NAT10	NAT10 protein (Fragment) OS=Homo sapiens GN=NAT10 PE=2 SV=1	63029	518	0	53	0	0.12	0	0	1	0	2	0	3	0	3	0	-2.40176
sp|Q9Y5B9|SP16H_HUMAN	SUPT16H	FACT complex subunit SPT16 OS=Homo sapiens GN=SUPT16H PE=1 SV=1	120409	574	0	65	0	0.06	0	0	2	0	2	0	3	0	4	0	-2.40176
sp|Q9Y6G9|DC1L1_HUMAN	DYNC1LI1	Cytoplasmic dynein 1 light intermediate chain 1 OS=Homo sapiens GN=DYNC1LI1 PE	56829	579	0	64	0	0.12	0	0	1	0	2	0	3	0	3	0	-2.40176
tr|B2R7X3|B2R7X3_HUMAN	tr|B2R7X3|B2R7X	cDNA, FLJ93645, highly similar to Homo sapiens chromatin assembly factor 1, subun	61936	585	0	64	0	0.17	0	0	2	0	3	0	3	0	4	0	-2.40176
tr|B2R8Y6|B2R8Y6_HUMAN	tr|B2R8Y6|B2R8Y6	cDNA, FLJ94121, highly similar to Homo sapiens stromal antigen 2 (STAG2), mRNA O	135116	598	0	62	0	0.05	0	0	1	0	2	0	3	0	3	0	-2.40176
tr|B3KN82|B3KN82_HUMAN	tr|B3KN82|B3KN8	cDNA FLJ13913 fis, clone Y79AA1000231, highly similar to Nucleolar protein NOP5 O	52769	602	0	61	0	0.27	0	0	2	0	4	0	3	0	5	0	-2.40176
tr|Q6IPN0|Q6IPN0_HUMAN	RTN4	RTN4 protein OS=Homo sapiens GN=RTN4 PE=2 SV=1	36952	620	0	59	0	0.29	0	0	1	0	3	0	3	0	3	0	-2.40176
tr|Q59FI9|Q59FI9_HUMAN	tr|Q59FI9|Q59FI9_	Ribosomal protein L12 variant (Fragment) OS=Homo sapiens PE=2 SV=1	21755	621	0	59	0	0.33	0	0	2	0	2	0	3	0	3	0	-2.40176
tr|E7ETD6|E7ETD6_HUMAN	BPTF	Nucleosome-remodeling factor subunit BPTF OS=Homo sapiens GN=BPTF PE=2 SV=3	310562	637	0	57	0	0.04	0	0	1	0	4	0	3	0	4	0	-2.40176
tr|B5BU24|B5BU24_HUMAN	YWHAB	14-3-3 protein beta/alpha OS=Homo sapiens GN=YWHAB PE=2 SV=1	28209	643	0	57	0	0.12	0	0	1	0	1	0	3	0	3	0	-2.40176
tr|F5GWP8|F5GWP8_HUMA	JUP	Junction plakoglobin OS=Homo sapiens GN=JUP PE=2 SV=1	66708	674	0	54	0	0.11	0	0	2	0	2	0	3	0	4	0	-2.40176
tr|B7Z6F7|B7Z6F7_HUMAN	tr|B7Z6F7|B7Z6F7	cDNA FLJ61705, highly similar to Symplekin OS=Homo sapiens PE=2 SV=1	141898	676	0	53	0	0.05	0	0	1	0	2	0	3	0	4	0	-2.40176
tr|J3QST3|J3QST3_HUMAN	KRT3	Keratin, type II cytoskeletal 3 OS=Homo sapiens GN=KRT3 PE=3 SV=1	64636	679	0	53	0	0.16	0	0	2	0	3	0	3	0	4	0	-2.40176
sp|P08779|K1C16_HUMAN	KRT16	Keratin, type I cytoskeletal 16 OS=Homo sapiens GN=KRT16 PE=1 SV=4	51578	729	0	49	0	0.13	0	0	2	0	2	0	3	0	4	0	-2.40176
tr|J3KPM9|J3KPM9_HUMAN	STAT1	Signal transducer and activator of transcription 1-alpha/beta OS=Homo sapiens GN=	83878	734	0	49	0	0.12	0	0	1	0	3	0	3	0	3	0	-2.40176
sp|A3KN83|SBNO1_HUMAN	SBNO1	Protein strawberry notch homolog 1 OS=Homo sapiens GN=SBNO1 PE=1 SV=1	154900	833	0	41	0	0.02	0	0	1	0	1	0	3	0	3	0	-2.40176
sp|P35251|RFC1_HUMAN	RFC1	Replication factor C subunit 1 OS=Homo sapiens GN=RFC1 PE=1 SV=4	128688	839	0	40	0	0.03	0	0	1	0	1	0	3	0	3	0	-2.40176
sp|Q96RW7|HMCN1_HUMA	HMCN1	Hemicentin-1 OS=Homo sapiens GN=HMCN1 PE=1 SV=2	623265	867	0	38	0	0.01	0	0	1	0	1	0	3	0	4	0	-2.40176
tr|B7WPF4|B7WPF4_HUMAN	USP24	Ubiquitin carboxyl-terminal hydrolase OS=Homo sapiens GN=USP24 PE=2 SV=2	280147	1113	0	24	0	0.01	0	0	0	0	1	0	3	0	3	0	-2.40176
tr|Q96EL6|Q96EL6_HUMAN	tr|Q96EL6|Q96EL6	Putative uncharacterized protein (Fragment) OS=Homo sapiens PE=2 SV=1	61329	136	0	125	0	0.44	0	0	3	0	4	0	4	0	6	0	-2.76453
tr|H0Y7K8|H0Y7K8_HUMAN	SMC1A	Structural maintenance of chromosomes protein 1A (Fragment) OS=Homo sapiens G	33648	155	0	107	0	0.25	0	0	2	0	4	0	4	0	6	0	-2.76453
tr|Q2TU79|Q2TU79_HUMAN	PIG44	Cell proliferation-inducing protein 57 OS=Homo sapiens GN=PIG44 PE=2 SV=1	55601	281	0	68	0	0.27	0	0	2	0	4	0	4	0	5	0	-2.76453
tr|B7Z9W6|B7Z9W6_HUMAN	tr|B7Z9W6|B7Z9W	cDNA, FLJ78980, highly similar to DnaJ homolog subfamily C member 7 OS=Homo sa	50776	282	0	141	0	0.37	0	0	3	0	5	0	4	0	5	0	-2.76453
tr|A4FU51|A4FU51_HUMAN	NARG1L	NMDA receptor regulated 1-like OS=Homo sapiens GN=NARG1L PE=2 SV=1	102424	368	0	56	0	0.33	0	0	1	0	2	0	4	0	4	0	-2.76453
tr|F5GZS0|F5GZS0_HUMAN	DHX36	Probable ATP-dependent RNA helicase DHX36 OS=Homo sapiens GN=DHX36 PE=2 S	113928	376	0	108	0	0.09	0	0	3	0	3	0	4	0	4	0	-2.76453
sp|P46087|NOP2_HUMAN	NOP2	Putative ribosomal RNA methyltransferase NOP2 OS=Homo sapiens GN=NOP2 PE=1	89589	419	0	97	0	0.11	0	0	2	0	3	0	4	0	5	0	-2.76453
sp|Q06210|GFPT1_HUMAN	GFPT1	Glutamine--fructose-6-phosphate aminotransferase [isomerizing] 1 OS=Homo sapie	79555	431	0	92	0	0.18	0	0	2	0	4	0	4	0	6	0	-2.76453
tr|B4DFM5|B4DFM5_HUMA	tr|B4DFM5|B4DFN	cDNA FLJ51779, highly similar to Vacuolar ATP synthase subunit B, brain isoform (EC	43446	433	0	92	0	0.34	0	0	2	0	4	0	4	0	4	0	-2.76453
tr|B4DUR9|B4DUR9_HUMAN	AHSA1	Activator of 90 kDa heat shock protein ATPase homolog 1 OS=Homo sapiens GN=AH	32490	442	0	89	0	0.48	0	0	3	0	4	0	4	0	4	0	-2.76453
tr|C9J9K3|C9J9K3_HUMAN	RPSA	40S ribosomal protein SA (Fragment) OS=Homo sapiens GN=RPSA PE=2 SV=1	29601	447	0	88	0	0.53	0	0	1	0	4	0	4	0	4	0	-2.76453
tr|B4DYP7|B4DYP7_HUMAN	tr|B4DYP7|B4DYP	cDNA FLJ55435, highly similar to Gamma-tubulin complex component 3 OS=Homo s	103306	464	0	83	0	0.1	0	0	2	0	3	0	4	0	4	0	-2.76453
tr|I3L3U9|I3L3U9_HUMAN	RSL1D1	Ribosomal L1 domain-containing protein 1 (Fragment) OS=Homo sapiens GN=RSL1D	33993	468	0	64	0	0.38	0	0	2	0	3	0	4	0	5	0	-2.76453
tr|Q6I7N8|Q6I7N8_HUMAN	HELLS	Lymphoid specific helicase variant9 OS=Homo sapiens GN=HELLS PE=2 SV=1	94530	500	0	77	0	0.15	0	0	1	0	4	0	4	0	6	0	-2.76453
tr|B3KWV6|B3KWV6_HUMA	tr|B3KWV6|B3KW	cDNA FLJ43948 fis, clone TESTI4014924, highly similar to Homo sapiens cytoplasmic	147082	543	0	69	0	0.04	0	0	2	0	2	0	4	0	4	0	-2.76453
tr|E9PFG1|E9PFG1_HUMAN	USP9X	Probable ubiquitin carboxyl-terminal hydrolase FAF-X OS=Homo sapiens GN=USP9X	117432	554	0	50	0	0.04	0	0	1	0	2	0	4	0	4	0	-2.76453
tr|G3V198|G3V198_HUMAN	NUP160	Nuclear pore complex protein Nup160 OS=Homo sapiens GN=NUP160 PE=2 SV=1	142365	571	0	65	0	0.07	0	0	2	0	3	0	4	0	4	0	-2.76453
tr|G3V203|G3V203_HUMAN	RPL18	60S ribosomal protein L18 OS=Homo sapiens GN=RPL18 PE=2 SV=1	18859	589	0	63	0	0.64	0	0	2	0	3	0	4	0	6	0	-2.76453
tr|A8K6V7|A8K6V7_HUMAN	tr|A8K6V7|A8K6V	cDNA FLJ76053, highly similar to Homo sapiens Ras-GTPase activating protein SH3 d	50842	618	0	59	0	0.29	0	0	1	0	4	0	4	0	5	0	-2.76453
tr|B2R7M3|B2R7M3_HUMA	tr|B2R7M3|B2R7N	cDNA, FLJ93510, highly similar to Homo sapiens JTV1 gene (JTV1), mRNA OS=Homo	35767	639	0	57	0	0.43	0	0	1	0	4	0	4	0	4	0	-2.76453
tr|B2RE56|B2RE56_HUMAN	tr|B2RE56|B2RE56	Peptidyl-prolyl cis-trans isomerase OS=Homo sapiens PE=2 SV=1	18225	800	0	43	0	0.41	0	0	0	0	2	0	4	0	5	0	-2.76453
tr|B7Z8Z8|B7Z8Z8_HUMAN	tr|B7Z8Z8|B7Z8Z8	cDNA FLJ61702, highly similar to 80 kDa MCM3-associated protein OS=Homo sapien	181264	863	0	39	0	0.02	0	0	1	0	1	0	4	0	5	0	-2.76453
tr|A0MNN4|A0MNN4_HUMA	SMU1	CDW3/SMU1 OS=Homo sapiens GN=SMU1 PE=2 SV=1	58134	869	0	38	0	0.18	0	0	0	0	3	0	4	0	5	0	-2.76453
tr|B4E2E1|B4E2E1_HUMAN	tr|B4E2E1|B4E2E1	cDNA FLJ54322, highly similar to Protein transport protein Sec24B OS=Homo sapiens	97141	919	0	35	0	0.14	0	0	0	0	4	0	4	0	4	0	-2.76453
tr|Q9P172|Q9P172_HUMAN	tr|Q9P172|Q9P17	PRO2281 OS=Homo sapiens PE=2 SV=1	37451	21	0	142	0	0.54	0	0	2	0	4	0	5	0	7	0	-3.05423
sp|Q01546|K22O_HUMAN	KRT76	Keratin, type II cytoskeletal 2 oral OS=Homo sapiens GN=KRT76 PE=1 SV=2	66370	35	0	88	0	2.07	0	0	4	0	5	0	5	0	5	0	-3.05423
tr|F5GZI8|F5GZI8_HUMAN	TCP1	T-complex protein 1 subunit alpha OS=Homo sapiens GN=TCP1 PE=2 SV=1	10411	56	0	226	0	1.58	0	0	4	0	4	0	5	0	5	0	-3.05423
tr|H0YI76|H0YI76_HUMAN	KRT5	Keratin, type II cytoskeletal 5 (Fragment) OS=Homo sapiens GN=KRT5 PE=3 SV=1	23267	224	0	104	0	0.43	0	0	3	0	4	0	5	0	6	0	-3.05423
sp|P07737|PROF1_HUMAN	PFN1	Profilin-1 OS=Homo sapiens GN=PFN1 PE=1 SV=2	15216	230	0	173	0	1.24	0	0	5	0	5	0	5	0	7	0	-3.05423
tr|B4DJ26|B4DJ26_HUMAN	PFAS	Phosphoribosylformylglycinamidine synthase (FGAR amidotransferase), isoform CRA	98876	323	0	118	0	0.17	0	0	3	0	6	0	5	0	6	0	-3.05423
sp|P27694|RFA1_HUMAN	RPA1	Replication protein A 70 kDa DNA-binding subunit OS=Homo sapiens GN=RPA1 PE=1	68723	332	0	122	0	0.21	0	0	3	0	4	0	5	0	5	0	-3.05423
tr|G5E9H0|G5E9H0_HUMAN	USP39	U4/U6.U5 tri-snRNP-associated protein 2 OS=Homo sapiens GN=USP39 PE=2 SV=1	56722	546	0	68	0	0.33	0	0	2	0	5	0	5	0	5	0	-3.05423
tr|Q8IYN5|Q8IYN5_HUMAN	CKAP5	CKAP5 protein OS=Homo sapiens GN=CKAP5 PE=2 SV=1	107753	183	0	169	0	0.12	0	0	4	0	5	0	6	0	7	0	-3.29543
tr|B2RE46|B2RE46_HUMAN	tr|B2RE46|B2RE46	cDNA, FLJ96923, highly similar to Homo sapiens ribophorin II (RPN2), mRNA OS=Hon	69405	340	0	118	0	0.32	0	0	4	0	6	0	6	0	6	0	-3.29543
tr|B4E3C3|B4E3C3_HUMAN	tr|B4E3C3|B4E3C3	cDNA FLJ55610, highly similar to Protein arginine N-methyltransferase 1 (EC 2.1.1.-)	41150	416	0	98	0	0.59	0	0	2	0	6	0	6	0	7	0	-3.29543
sp|O00231|PSD11_HUMAN	PSMD11	26S proteasome non-ATPase regulatory subunit 11 OS=Homo sapiens GN=PSMD11	47719	520	0	73	0	0.49	0	0	2	0	6	0	6	0	8	0	-3.29543
tr|B3KXX5|B3KXX5_HUMAN	tr|B3KXX5|B3KXX5	Structural maintenance of chromosomes protein OS=Homo sapiens PE=2 SV=1	145050	277	0	142	0	0.12	0	0	4	0	5	0	7	0	10	0	-3.50208
sp|Q92878|RAD50_HUMAN	RAD50	DNA repair protein RAD50 OS=Homo sapiens GN=RAD50 PE=1 SV=1	154823	370	0	110	0	0.11	0	0	3	0	5	0	7	0	9	0	-3.50208
sp|Q9UHD1|CHRD1_HUMAN	CHORDC1	Cysteine and histidine-rich domain-containing protein 1 OS=Homo sapiens GN=CHO	38264	422	0	96	0	0.64	0	0	2	0	6	0	7	0	7	0	-3.50208
tr|L0R849|L0R849_HUMAN	EDARADD	Alternative protein EDARADD OS=Homo sapiens GN=EDARADD PE=4 SV=1	42657	22	0	104	0	5.55	0	0	4	0	7	0	8	0	9	0	-3.68285

#02	Hela live syn PU-1 2 uM																		
#04	Hela live syn NP_2µM																		
Accession	GS	Name	Pro_Mas	04_prot_	02_prot_hit_num	04_prot_	02_prot_	04_emPA	02_emPA	emPAi ratio	04_No>l	02_No>l	04_No>Hl	02_No>Hl	04_No>20	02_No>20	04_No=All	02_No=All	Rsc
sp|P06493|CDK1_HU	CDK1	Cyclin-dependent kinase 1 OS=Homo sapiens GN=CDK1 PE=1 SV=3	34131	0	949	0	22	0	0.1	#DIV/0!	0	0	0	1	0	1	0	2	1.542273
sp|P50750|CDK9_HU	CDK9	Cyclin-dependent kinase 9 OS=Homo sapiens GN=CDK9 PE=1 SV=3	43149	0	723	0	37	0	0.08	#DIV/0!	0	1	0	1	0	1	0	1	1.542273
sp|P28482|MK01_HU	MAPK1	Mitogen-activated protein kinase 1 OS=Homo sapiens GN=MAPK1 PE=1 SV=3	41762	0	590	0	49	0	0.16	#DIV/0!	0	1	0	2	0	2	0	2	2.279573
sp|Q9Y6E0|STK24_HU	STK24	Serine/threonine-protein kinase 24 OS=Homo sapiens GN=STK24 PE=1 SV=1	49562	0	961	0	21	0	0.08	#DIV/0!	0	0	0	1	0	1	0	1	1.542273
sp|P78527|PRKDC_HU	PRKDC	DNA-dependent protein kinase catalytic subunit OS=Homo sapiens GN=PRKDC PE=1 SV=3	473749	83	107	241	229	0.07	0.08	1.142857143	7	6	10	11	11	14	17	17	0.292289
sp|O15111|IKKA_HU	CHUK	Inhibitor of nuclear factor kappa-B kinase subunit alpha OS=Homo sapiens GN=CHUK PE=1 SV=2	85725	0	952	0	21	0	0.04	#DIV/0!	0	0	0	1	0	1	0	1	1.542273
sp|P00558|PGK1_HU	PGK1	Phosphoglycerate kinase 1 OS=Homo sapiens GN=PGK1 PE=1 SV=3	44985	134	55	161	361	0.76	1.03	1.355263158	5	8	8	11	9	15	9	17	0.665163
tr|J3KNX3|J3KNX3_H	PFKM	6-phosphofructokinase OS=Homo sapiens GN=PFKM PE=3 SV=1	94107	0	236	0	120	0	0.12	#DIV/0!	0	3	0	3	0	4	0	4	3.128237
sp|P13861|KAP2_HU	PRKAR2A (PK	cAMP-dependent protein kinase type II-alpha regulatory subunit OS=Homo sapiens GN=PRKAR2A PE=1 SV=2	45832	0	600	0	46	0	0.15	#DIV/0!	0	1	0	2	0	2	0	3	2.279573
sp|P48426|PI42A_HU	PIP4K2A (PIP	Phosphatidylinositol 5-phosphate 4-kinase type-2 alpha OS=Homo sapiens GN=PIP4K2A PE=1 SV=2	46424	0	825	0	29	0	0.08	#DIV/0!	0	0	0	1	0	1	0	1	1.542273
sp|P14618|KPYM_HU	PKM	Pyruvate kinase isozymes M1/M2 OS=Homo sapiens GN=PKM PE=1 SV=4	58470	8	5	1820	1705	5.79	5.08	0.877374784	47	45	58	64	60	68	66	76	0.138241
sp|P48426|PI42A_HU	PIP4K2A (PIP	Phosphatidylinositol 5-phosphate 4-kinase type-2 alpha OS=Homo sapiens GN=PIP4K2A PE=1 SV=2	46424	0	825	0	29	0	0.08	#DIV/0!	0	0	0	1	0	1	0	1	1.542273
sp|P40763|STAT3_HU	STAT3	Signal transducer and activator of transcription 3 OS=Homo sapiens GN=STAT3 PE=1 SV=2	88810	0	815	0	30	0	0.09	#DIV/0!	0	0	0	2	0	2	0	2	2.279573
sp|P62136|PP1A_HU	PPP1CA	Serine/threonine-protein phosphatase PP1-alpha catalytic subunit OS=Homo sapiens GN=PPP1CA PE=1 SV=1	38229	0	285	0	105	0	0.52	#DIV/0!	0	2	0	5	0	6	0	6	3.65942
sp|P67775|PP2AA_HU	PPP2CA	Serine/threonine-protein phosphatase 2A catalytic subunit alpha isoform OS=Homo sapiens GN=PPP2CA PE=1 SV=1	36142	0	494	0	62	0	0.09	#DIV/0!	0	1	0	1	0	1	0	1	1.542273
sp|P62140|PP1B_HU	PPP1CB	Serine/threonine-protein phosphatase PP1-beta catalytic subunit OS=Homo sapiens GN=PPP1CB PE=1 SV=3	37961	345	263	51	52	0.24	0.52	2.166666667	1	1	1	2	1	2	1	2	0.694266
sp|O75369|FLNB_HU	FLNB	Filamin-B OS=Homo sapiens GN=FLNB PE=1 SV=2	280157	45	14	463	1029	0.22	0.52	2.363636364	14	26	18	38	20	38	28	38	0.872057
tr|Q60FE5|Q60FE5_H	FLNA	Filamin A OS=Homo sapiens GN=FLNA PE=2 SV=1	280790	9	3	1306	2417	0.86	1.33	1.546511628	41	56	60	83	75	84	88	91	0.12174
tr|B4E2K2|B4E2K2_H	tr|B4E2K2|B	cDNA FLJ55665, highly similar to A-kinase anchor protein 2 OS=Homo sapiens PE=2 SV=1	103935	246	728	79	36	0.03	0.03	1	1	0	1	1	1	1	1	2	-0.04303
tr|B2R5T5|B2R5T5_H	PRKAR1A	Protein kinase, cAMP-dependent, regulatory, type I, alpha (Tissue specific extinguisher 1), isoform CRA_a OS=Homo sapi	43183	533	510	31	60	0.08	0.08	1	0	1	1	1	1	1	1	1	-0.04303
tr|B4DP56|B4DP56_H	tr|B4DP56|B	cDNA FLJ52237, highly similar to Creatine kinase B-type (EC 2.7.3.2) OS=Homo sapiens PE=2 SV=1	38840	214	662	97	41	0.18	0.16	0.888888889	2	0	2	2	2	2	2	2	-0.04304
sp|Q01813|K6PP_HU	PFKP	6-phosphofructokinase type C OS=Homo sapiens GN=PFKP PE=1 SV=2	86454	98	82	217	272	0.4	0.35	0.875	5	7	9	8	9	8	10	9	-0.20391
tr|E9PF63|E9PF63_H	ROCK2	Rho-associated protein kinase 2 OS=Homo sapiens GN=ROCK2 PE=2 SV=1	134266	375	888	46	25	0.05	0.04	0.8	1	0	2	1	1	1	3	1	-0.04303
sp|Q14315|FLNC_HU	FLNC	Filamin-C OS=Homo sapiens GN=FLNC PE=1 SV=3	293407	143	235	153	120	0.06	0.06	1	5	4	6	6	10	5	11	6	-0.97768
tr|H0Y9D8|H0Y9D8_H	PPA2	Inorganic pyrophosphatase 2, mitochondrial (Fragment) OS=Homo sapiens GN=PPA2 PE=4 SV=1	25904	0	973	0	20	0	0.13	#DIV/0!	0	0	0	1	0	1	0	1	1.542273
sp|Q5SWA1|PR15B_H	PPP1R15B	Protein phosphatase 1 regulatory subunit 15B OS=Homo sapiens GN=PPP1R15B PE=1 SV=1	80072	0	925	0	23	0	0.04	#DIV/0!	0	0	0	1	0	1	0	1	1.542273
sp|O15357|SHIP2_HU	INPPL1	Phosphatidylinositol 3,4,5-trisphosphate 5-phosphatase 2 OS=Homo sapiens GN=INPPL1 PE=1 SV=2	139311	0	876	0	26	0	0.02	#DIV/0!	0	0	0	1	0	1	0	1	1.542273
tr|B2RAH5|B2RAH5_H	tr|B2RAH5|B	cDNA, FLJ94919, highly similar to Homo sapiens protein phosphatase 1, regulatory (inhibitor) subunit 12A (PPP1R12A), n	115658	0	867	0	26	0	0.08	#DIV/0!	0	0	0	1	0	1	0	1	1.542273
tr|J3KSW8|J3KSW8_H	MPRIP	Myosin phosphatase Rho-interacting protein (Fragment) OS=Homo sapiens GN=MPRIP PE=4 SV=1	96384	0	638	0	43	0	0.03	#DIV/0!	0	1	0	1	0	1	0	1	1.542273
tr|B4DDF7|B4DDF7_H	tr|B4DDF7|B	cDNA FLJ53296, highly similar to Serine/threonine-protein phosphatase 2A 65 kDa regulatory subunit A alpha isoform O	64939	443	552	39	54	0.16	0.23	1.4375	1	0	3	3	3	5	3	6	0.609691
tr|J3QSX4|J3QSX4_HU	BUB3	Mitotic checkpoint protein BUB3 OS=Homo sapiens GN=BUB3 PE=4 SV=1	16498	681	632	20	44	0.2	0.1	0.5	0	1	1	1	0	1	1	1	1.542273
tr|J3QT22|J3QT22_HU	PPME1	Protein phosphatase methylesterase 1 OS=Homo sapiens GN=PPME1 PE=3 SV=1	44241	232	906	90	24	0.07	0.07	1	1	0	1	1	1	1	2	1	-0.04303
tr|B4DVZ9|B4DVZ9_H	tr|B4DVZ9|B	cDNA FLJ61177, highly similar to Phosphatidylinositol-4-phosphate 5-kinasetype-1 gamma (EC 2.7.1.68) OS=Homo sapie	70427	0	790	0	32	0	0.05	#DIV/0!	0	0	0	1	0	2	0	2	2.279573
sp|P29966|MARCS_H	MARCKS	Myristoylated alanine-rich C-kinase substrate OS=Homo sapiens GN=MARCKS PE=1 SV=4	31707	0	837	0	29	0	0.81	#DIV/0!	0	0	0	1	0	1	0	1	1.542273
tr|D3DQR0|D3DQR0_	PACSIN3	Protein kinase C and casein kinase substrate in neurons 3, isoform CRA_b OS=Homo sapiens GN=PACSIN3 PE=2 SV=1	48901	0	542	0	55	0	0.07	#DIV/0!	0	1	0	1	0	1	0	1	1.542273
tr|B0AZV0|B0AZV0_H	STRAP	Serine-threonine kinase receptor-associated protein OS=Homo sapiens GN=STRAP PE=2 SV=1	28716	626	466	23	66	0.12	0.12	1	0	1	1	1	1	1	1	1	-0.04303
tr|Q5JQZ9|Q5JQZ9_H	CDC2L6	Cell division cycle 2-like 6 (CDK8-like) (Fragment) OS=Homo sapiens GN=CDC2L6 PE=4 SV=1	29552	285	0	65	0	0.24	0	0	2	0	2	0	3	0	3	0	-2.85141
sp|P07205|PGK2_HU	PGK2	Phosphoglycerate kinase 2 OS=Homo sapiens GN=PGK2 PE=1 SV=3	45166	134	229	81	122	0.76	0.24	0.315789474	1	3	3	4	4	6	4	6	0.488119
sp|P17858|K6PL_HU	PFKL	6-phosphofructokinase, liver type OS=Homo sapiens GN=PFKL PE=1 SV=6	85762	98	313	88	97	0.4	0.08	0.2	1	2	2	2	2	3	2	4	0.442717
sp|Q9P287|BCCIP_HU	BCCIP	BRCA2 and CDKN1A-interacting protein OS=Homo sapiens GN=BCCIP PE=1 SV=1	36299	503	0	33	0	0.09	0	0	0	0	1	0	1	0	3	0	-1.62833
tr|H0YDU8|H0YDU8_	PPP5C	Serine/threonine-protein phosphatase (Fragment) OS=Homo sapiens GN=PPP5C PE=3 SV=1	55743	596	0	26	0	0.06	0	0	0	0	1	0	1	0	1	0	-1.62833
tr|A4QPA9|A4QPA9_	MAP2K1 (ME	Mitogen-activated protein kinase kinase 1 OS=Homo sapiens GN=MAP2K1 PE=2 SV=1	43753	384	823	45	30	0.31	0.16	0.516129032	1	1	2	2	2	2	2	2	-0.04304
tr|B3KS97|B3KS97_H	MAP2K2 (ME	Mitogen-activated protein kinase kinase 2, isoform CRA_d OS=Homo sapiens GN=MAP2K2 PE=2 SV=1	23450	384	948	45	0	0.31	0.07	0.225806452	1	0	2	0	2	0	2	1	-2.36564
tr|B4E1G6|B4E1G6_H	GALK1	Galactokinase OS=Homo sapiens GN=GALK1 PE=2 SV=1	45956	679	0	20	0	0.07	0	0	0	0	1	0	0	0	1	0	-0.04302
tr|A6NIT2|A6NIT2_HU	PHKA1	Phosphorylase b kinase regulatory subunit alpha, skeletal muscle isoform OS=Homo sapiens GN=PHKA1 PE=2 SV=1	133462	711	0	17	0	0.02	0	0	0	0	1	0	0	0	1	0	-0.04302
tr|H0Y757|H0Y757_H	PFKP	6-phosphofructokinase type C (Fragment) OS=Homo sapiens GN=PFKP PE=4 SV=1	11746	98	0	31	0	0.4	0	0	0	0	1	0	1	0	1	0	-1.62833
tr|G3V4N7|G3V4N7_	CKB	Creatine kinase B-type (Fragment) OS=Homo sapiens GN=CKB PE=2 SV=1	24272	214	0	51	0	0.18	0	0	1	0	1	0	1	0	1	0	-1.62833
tr|B7Z418|B7Z418_H	ILK	Integrin-linked protein kinase OS=Homo sapiens GN=ILK PE=2 SV=1	44776	421	0	41	0	0.07	0	0	1	0	1	0	1	0	1	0	-1.62833
sp|Q9UEW8|STK39_H	STK39	STE20/SPS1-related proline-alanine-rich protein kinase OS=Homo sapiens GN=STK39 PE=1 SV=3	59950	491	0	35	0	0.05	0	0	0	0	1	0	1	0	1	0	-1.62833
tr|K7EID3|K7EID3_HU	PRKAR1A	cAMP-dependent protein kinase type I-alpha regulatory subunit (Fragment) OS=Homo sapiens GN=PRKAR1A PE=4 SV=1	19112	533	0	31	0	0.08	0	0	0	0	1	0	1	0	1	0	-1.62833
tr|Q5JQ10|Q5JQ10_H	ADK	Adenosine kinase OS=Homo sapiens GN=ADK PE=2 SV=1	39078	538	0	30	0	0.08	0	0	0	0	1	0	1	0	1	0	-1.62833
tr|F2Z2Y4|F2Z2Y4_HU	PDXK	Pyridoxal kinase OS=Homo sapiens GN=PDXK PE=2 SV=1	30790	566	0	29	0	0.11	0	0	0	0	1	0	1	0	1	0	-1.62833
tr|A8K2U2|A8K2U2_H	tr|A8K2U2|A	cDNA FLJ75392, highly similar to Homo sapiens hexokinase II (HKII) mRNA OS=Homo sapiens PE=2 SV=1	103718	599	0	26	0	0.03	0	0	0	0	1	0	1	0	2	0	-1.62833
tr|H0YH33|H0YH33_H	STRAP	Serine-threonine kinase receptor-associated protein (Fragment) OS=Homo sapiens GN=STRAP PE=4 SV=1	10763	626	0	23	0	0.12	0	0	0	0	1	0	1	0	1	0	-1.62833
tr|H0Y3C5|H0Y3C5_H	HCK	Tyrosine-protein kinase HCK OS=Homo sapiens GN=HCK PE=4 SV=1	59986	628	0	23	0	0.05	0	0	0	0	1	0	1	0	2	0	-1.62833
tr|H0Y3Y5|H0Y3Y5_H	PPIP5K1	Inositol hexakisphosphate and diphosphoinositol-pentakisphosphate kinase 1 (Fragment) OS=Homo sapiens GN=PPIP5K	21913	632	0	22	0	0.15	0	0	0	0	1	0	1	0	2	0	-1.62833
sp|O95747|OXSR1_H	OXSR1	Serine/threonine-protein kinase OSR1 OS=Homo sapiens GN=OXSR1 PE=1 SV=1	58271	675	0	20	0	0.06	0	0	0	0	1	0	1	0	2	0	-1.62833
tr|C1PHA2|C1PHA2_H	KIF5B-ALK	Tyrosine-protein kinase receptor OS=Homo sapiens GN=KIF5B-ALK PE=2 SV=1	169283	227	0	91	0	0.04	0	0	2	0	2	0	2	0	3	0	-2.36564
tr|H3BRW9|H3BRW9_	MAP2K1	Dual-specificity mitogen-activated protein kinase kinase 1 OS=Homo sapiens GN=MAP2K1 PE=4 SV=1	24160	384	0	45	0	0.31	0	0	1	0	2	0	2	0	2	0	-2.36564
sp|O75533|SF3B1_HU	SF3B1	Splicing factor 3B subunit 1 OS=Homo sapiens GN=SF3B1 PE=1 SV=3	146479	0	109	0	226	0	0.25	#DIV/0!	0	7	0	10	0	11	0	13	4.484215
sp|O43776|SYNC_HU	NARS	Asparagine--tRNA ligase, cytoplasmic OS=Homo sapiens GN=NARS PE=1 SV=1	63758	0	121	0	206	0	0.5	#DIV/0!	0	4	0	9	0	9	0	10	4.207911
tr|B7Z1N6|B7Z1N6_H	tr|B7Z1N6|B	cDNA FLJ52222, highly similar to Fructose-bisphosphate aldolase C (EC4.1.2.13) OS=Homo sapiens PE=2 SV=1	35800	0	66	0	329	0	0.3	#DIV/0!	0	4	0	7	0	9	0	10	4.207911
tr|B4DLV7|B4DLV7_H	tr|B4DLV7|B	cDNA FLJ60299, highly similar to Rab GDP dissociation inhibitor beta OS=Homo sapiens PE=2 SV=1	51577	0	166	0	163	0	0.45	#DIV/0!	0	3	0	6	0	7	0	7	3.866206
tr|A4D210|A4D210_H	EIF3S9	Eukaryotic translation initiation factor 3, subunit 9 eta, 116kDa OS=Homo sapiens GN=EIF3S9 PE=4 SV=1	89024	0	258	0	113	0	0.16	#DIV/0!	0	3	0	4	0	6	0	6	3.65942
tr|Q14400|Q14400_H	GLUD1	GLUD1 protein (Fragment) OS=Homo sapiens GN=GLUD1 PE=2 SV=1	28791	0	183	0	151	0	0.93	#DIV/0!	0	5	0	6	0	6	0	6	3.65942
tr|B4DMF5|B4DMF5_	tr|B4DMF5|	Glutamate dehydrogenase OS=Homo sapiens PE=2 SV=1	56910	0	175	0	154	0	0.68	#DIV/0!	0	5	0	7	0	6	0	7	3.65942
tr|D6REM6|D6REM6_	MATR3	Matrin-3 OS=Homo sapiens GN=MATR3 PE=2 SV=1	88647	0	157	0	173	0	0.24	#DIV/0!	0	4	0	6	0	6	0	6	3.65942
tr|B7Z4B2|B7Z4B2_H	tr|B7Z4B2|B	cDNA FLJ56108, highly similar to Puromycin-sensitive aminopeptidase (EC 3.4.11.-) OS=Homo sapiens PE=2 SV=1	93434	0	442	0	70	0	0.18	#DIV/0!	0	1	0	5	0	5	0	5	3.418078
sp|Q92626|PXDN_HU	PXDN	Peroxidasin homolog OS=Homo sapiens GN=PXDN PE=1 SV=2	167793	0	249	0	117	0	0.08	#DIV/0!	0	3	0	4	0	5	0	5	3.418078
tr|A2A274|A2A274_H	ACO2	Aconitase 2, mitochondrial OS=Homo sapiens GN=ACO2 PE=4 SV=1	88563	0	218	0	126	0	0.2	#DIV/0!	0	2	0	5	0	5	0	5	3.418078
sp|P13667|PDIA4_HU	PDIA4	Protein disulfide-isomerase A4 OS=Homo sapiens GN=PDIA4 PE=1 SV=2	73229	0	198	0	140	0	0.19	#DIV/0!	0	3	0	4	0	5	0	7	3.418078
tr|Q5H907|Q5H907_	MAGED2	Melanoma antigen family D, 2, isoform CRA_d OS=Homo sapiens GN=MAGED2 PE=4 SV=1	55932	0	150	0	178	0	0.26	#DIV/0!	0	5	0	6	0	5	0	7	3.418078
sp|P09960|LKHA4_HU	LTA4H	Leukotriene A-4 hydrolase OS=Homo sapiens GN=LTA4H PE=1 SV=2	69868	0	108	0	228	0	0.26	#DIV/0!	0	5	0	5	0	5	0	5	3.418078
tr|B4DY28|B4DY28_H	tr|B4DY28|B	cDNA FLJ61189, highly similar to Cysteine and glycine-rich protein 1 OS=Homo sapiens PE=2 SV=1	20884	0	90	0	255	0	1.11	#DIV/0!	0	5	0	5	0	5	0	5	3.418078
sp|Q08J23|NSUN2_H	NSUN2	tRNA (cytosine(34)-C(5))-methyltransferase OS=Homo sapiens GN=NSUN2 PE=1 SV=2	87214	0	819	0	30	0	0.11	#DIV/0!	0	0	0	3	0	4	0	4	3.128237
tr|H7C0A4|H7C0A4_H	HDLBP	Vigilin (Fragment) OS=Homo sapiens GN=HDLBP PE=4 SV=1	37126	0	551	0	54	0	0.29	#DIV/0!	0	1	0	3	0	4	0	5	3.128237
sp|P31150|GDIA_HU	GDI1	Rab GDP dissociation inhibitor alpha OS=Homo sapiens GN=GDI1 PE=1 SV=2	51177	0	517	0	58	0	0.21	#DIV/0!	0	0	0	3	0	4	0	4	3.128237
tr|Q6FHF5|Q6FHF5_H	PCNA	Proliferating cell nuclear antigen (Fragment) OS=Homo sapiens GN=PCNA PE=2 SV=1	29029	0	358	0	85	0	0.38	#DIV/0!	0	2	0	3	0	4	0	4	3.128237
tr|E9PQD7|E9PQD7_	RPS2	40S ribosomal protein S2 OS=Homo sapiens GN=RPS2 PE=2 SV=1	25366	0	328	0	93	0	0.49	#DIV/0!	0	2	0	4	0	4	0	5	3.128237
tr|Q5T8U2|Q5T8U2_	RPL7A	60S ribosomal protein L7a OS=Homo sapiens GN=RPL7A PE=4 SV=1	16700	0	242	0	118	0	1.1	#DIV/0!	0	4	0	4	0	4	0	4	3.128237
tr|B4DN25|B4DN25_	UGDH	UDP-glucose 6-dehydrogenase OS=Homo sapiens GN=UGDH PE=2 SV=1	44786	0	230	0	121	0	0.26	#DIV/0!	0	4	0	4	0	4	0	5	3.128237
tr|B4DT31|B4DT31_H	FUBP1	Far upstream element-binding protein 1 OS=Homo sapiens GN=FUBP1 PE=2 SV=1	69993	0	195	0	141	0	0.2	#DIV/0!	0	3	0	4	0	4	0	5	3.128237
tr|Q96FS1|Q96FS1_H	CTNND1	CTNND1 protein (Fragment) OS=Homo sapiens GN=CTNND1 PE=2 SV=2	92901	0	193	0	142	0	0.06	#DIV/0!	0	2	0	2	0	4	0	4	3.128237
tr|Q59FE8|Q59FE8_H	tr|Q59FE8|Q	Epithelial protein lost in neoplasm beta variant (Fragment) OS=Homo sapiens PE=2 SV=1	86510	0	174	0	155	0	0.2	#DIV/0!	0	3	0	3	0	4	0	5	3.128237
tr|Q5TZZ9|Q5TZZ9_H	ANXA1	Annexin OS=Homo sapiens GN=ANXA1 PE=2 SV=1	38918	0	173	0	156	0	0.39	#DIV/0!	0	3	0	4	0	4	0	5	3.128237
tr|A8K132|A8K132_H	tr|A8K132|A	cDNA FLJ75476, highly similar to Homo sapiens glutaminase (GLS), mRNA OS=Homo sapiens PE=2 SV=1	74317	0	120	0	209	0	0.24	#DIV/0!	0	4	0	6	0	4	0	8	3.128237
tr|B3KM58|B3KM58_	tr|B3KM58|E	cDNA FLJ10358 fis, clone NT2RM2001238, highly similar to Glutaminase kidney isoform, mitochondrial (Fragment) OS=H	41736	0	110	0	225	0	0.36	#DIV/0!	0	4	0	5	0	4	0	6	3.128237
tr|H0Y397|H0Y397_H	ARHGAP40	Rho GTPase-activating protein 40 (Fragment) OS=Homo sapiens GN=ARHGAP40 PE=4 SV=1	17367	0	994	0	19	0	0.2	#DIV/0!	0	0	0	1	0	3	0	5	2.765333
tr|B4DZC0|B4DZC0_H	tr|B4DZC0|B	cDNA FLJ51771, highly similar to SWI/SNF-related matrix-associatedactin-dependent regulator of chromatin subfamily A	117367	0	737	0	36	0	0.06	#DIV/0!	0	1	0	2	0	3	0	3	2.765333
tr|B3KSG9|B3KSG9_H	tr|B3KSG9|B	cDNA FLJ36188 fis, clone TESTI2027179, highly similar to Transmembrane 9 superfamily protein member 2 OS=Homo sa	66299	0	727	0	36	0	0.21	#DIV/0!	0	0	0	4	0	3	0	4	2.765333
tr|D3DUW5|D3DUW5	DNM1L	Dynamin 1-like, isoform CRA_c OS=Homo sapiens GN=DNM1L PE=3 SV=1	88442	0	724	0	37	0	0.16	#DIV/0!	0	0	0	4	0	3	0	4	2.765333
tr|I6L9E8|I6L9E8_HU	FAM98A	Family with sequence similarity 98, member A OS=Homo sapiens GN=FAM98A PE=2 SV=1	55595	0	657	0	41	0	0.12	#DIV/0!	0	1	0	2	0	3	0	4	2.765333
sp|Q9Y266|NUDC_HU	NUDC	Nuclear migration protein nudC OS=Homo sapiens GN=NUDC PE=1 SV=1	38276	0	619	0	45	0	0.28	#DIV/0!	0	0	0	3	0	3	0	3	2.765333
tr|B4E3J7|B4E3J7_HU	tr|B4E3J7|B4	cDNA FLJ51203, highly similar to Thrombospondin-1 OS=Homo sapiens PE=2 SV=1	124032	0	607	0	46	0	0.05	#DIV/0!	0	1	0	2	0	3	0	3	2.765333
sp|P54577|SYYC_HU	YARS	Tyrosine--tRNA ligase, cytoplasmic OS=Homo sapiens GN=YARS PE=1 SV=4	59448	0	467	0	66	0	0.11	#DIV/0!	0	2	0	2	0	3	0	4	2.765333
sp|Q9BVP2|GNL3_HU	GNL3	Guanine nucleotide-binding protein-like 3 OS=Homo sapiens GN=GNL3 PE=1 SV=2	62468	0	452	0	68	0	0.11	#DIV/0!	0	2	0	2	0	3	0	3	2.765333
tr|A8K2I7|A8K2I7_HU	tr|A8K2I7|A8	cDNA FLJ76072, highly similar to Homo sapiens GIPC PDZ domain containing family, member 1 (GIPC1), transcript varian	36131	0	432	0	71	0	0.3	#DIV/0!	0	1	0	3	0	3	0	3	2.765333
sp|Q9UHD8|SEPT9_H	Sep-09	Septin-9 OS=Homo sapiens GN=SEPT9 PE=1 SV=2	65646	0	409	0	76	0	0.3	#DIV/0!	0	2	0	3	0	3	0	3	2.765333
sp|P04899|GNAI2_HU	GNAI2	Guanine nucleotide-binding protein G(i) subunit alpha-2 OS=Homo sapiens GN=GNAI2 PE=1 SV=3	40995	0	404	0	77	0	0.26	#DIV/0!	0	2	0	3	0	3	0	4	2.765333
tr|A2A3R6|A2A3R6_H	RPS6	40S ribosomal protein S6 OS=Homo sapiens GN=RPS6 PE=2 SV=1	28834	0	391	0	80	0	0.39	#DIV/0!	0	2	0	3	0	3	0	3	2.765333
tr|G3XAI2|G3XAI2_HU	LAMB1	Laminin subunit beta-1 OS=Homo sapiens GN=LAMB1 PE=2 SV=1	207703	0	388	0	80	0	0.07	#DIV/0!	0	1	0	2	0	3	0	4	2.765333
tr|B2R491|B2R491_H	RPS4X	Ribosomal protein S4, X-linked, isoform CRA_c OS=Homo sapiens GN=RPS4X PE=2 SV=1	29807	0	378	0	81	0	0.24	#DIV/0!	0	2	0	2	0	3	0	4	2.765333
tr|Q5VU21|Q5VU21_	SERBP1	PAI-1 mRNA-binding protein variant OS=Homo sapiens GN=SERBP1 PE=2 SV=1	42458	0	365	0	85	0	0.25	#DIV/0!	0	2	0	3	0	3	0	3	2.765333
tr|B4DR63|B4DR63_H	PSMC1	26S protease regulatory subunit 4 OS=Homo sapiens GN=PSMC1 PE=2 SV=1	41255	0	327	0	94	0	0.26	#DIV/0!	0	1	0	3	0	3	0	3	2.765333
sp|Q9Y6C9|MTCH2_H	MTCH2	Mitochondrial carrier homolog 2 OS=Homo sapiens GN=MTCH2 PE=1 SV=1	33936	0	324	0	94	0	0.32	#DIV/0!	0	1	0	3	0	3	0	5	2.765333
tr|Q6ZVI6|Q6ZVI6_HU	tr|Q6ZVI6|Q	cDNA FLJ42537 fis, clone BRACE3004058, highly similar to NADH-cytochrome B5 reductase (EC 1.6.2.2) OS=Homo sapien	29141	0	316	0	96	0	0.38	#DIV/0!	0	2	0	3	0	3	0	3	2.765333
sp|Q00653|NFKB2_H	NFKB2	Nuclear factor NF-kappa-B p100 subunit OS=Homo sapiens GN=NFKB2 PE=1 SV=4	97373	0	297	0	102	0	0.1	#DIV/0!	0	2	0	3	0	3	0	3	2.765333
tr|E1NZA1|E1NZA1_H	PRIC295	Peroxisome proliferator activated receptor interacting complex protein OS=Homo sapiens GN=PRIC295 PE=2 SV=1	294953	0	290	0	104	0	0.07	#DIV/0!	0	2	0	6	0	3	0	6	2.765333
sp|Q8N1G4|LRC47_H	LRRC47	Leucine-rich repeat-containing protein 47 OS=Homo sapiens GN=LRRC47 PE=1 SV=1	64004	0	284	0	105	0	0.11	#DIV/0!	0	2	0	3	0	3	0	3	2.765333
tr|Q0VGA5|Q0VGA5_	SARS	SARS protein OS=Homo sapiens GN=SARS PE=2 SV=1	58883	0	268	0	109	0	0.18	#DIV/0!	0	3	0	3	0	3	0	5	2.765333
tr|B2RNR6|B2RNR6_	ZFR	Zinc finger RNA binding protein OS=Homo sapiens GN=ZFR PE=2 SV=1	118081	0	247	0	118	0	0.09	#DIV/0!	0	2	0	3	0	3	0	3	2.765333
tr|B3KRS5|B3KRS5_H	HDAC2	Histone deacetylase OS=Homo sapiens GN=HDAC2 PE=2 SV=1	52479	0	241	0	119	0	0.2	#DIV/0!	0	3	0	3	0	3	0	3	2.765333
tr|B2R858|B2R858_H	tr|B2R858|B	cDNA, FLJ93750, Homo sapiens DEAD (Asp-Glu-Ala-Asp) box polypeptide 6 (DDX6), mRNA OS=Homo sapiens PE=2 SV=1	53582	0	240	0	119	0	0.2	#DIV/0!	0	3	0	3	0	3	0	3	2.765333
tr|G3V1A4|G3V1A4_	CFL1	Cofilin 1 (Non-muscle), isoform CRA_a OS=Homo sapiens GN=CFL1 PE=4 SV=1	17029	0	232	0	121	0	0.72	#DIV/0!	0	2	0	3	0	3	0	3	2.765333
tr|B4DL07|B4DL07_H	tr|B4DL07|B	cDNA FLJ53353, highly similar to ATP-binding cassette sub-family D member 3 OS=Homo sapiens PE=2 SV=1	78980	0	216	0	127	0	0.13	#DIV/0!	0	3	0	3	0	3	0	3	2.765333
tr|Q6FHX6|Q6FHX6_	FEN1	Flap endonuclease 1 OS=Homo sapiens GN=FEN1 PE=2 SV=1	42908	0	215	0	127	0	0.28	#DIV/0!	0	2	0	3	0	3	0	3	2.765333
sp|P36952|SPB5_HU	SERPINB5	Serpin B5 OS=Homo sapiens GN=SERPINB5 PE=1 SV=2	42530	0	214	0	127	0	0.25	#DIV/0!	0	3	0	3	0	3	0	3	2.765333
tr|Q6EHZ3|Q6EHZ3_	STRF8	Putative uncharacterized protein STRF8 OS=Homo sapiens GN=STRF8 PE=2 SV=1	41361	0	211	0	131	0	0.26	#DIV/0!	0	2	0	3	0	3	0	3	2.765333
tr|H0Y7C0|H0Y7C0_H	USO1	General vesicular transport factor p115 (Fragment) OS=Homo sapiens GN=USO1 PE=4 SV=1	72345	0	206	0	108	0	0.09	#DIV/0!	0	1	0	2	0	3	0	3	2.765333
tr|B0AZQ4|B0AZQ4_	tr|B0AZQ4|B	cDNA, FLJ79494, highly similar to Structural maintenance of chromosome 3 OS=Homo sapiens PE=2 SV=1	141841	0	199	0	140	0	0.07	#DIV/0!	0	3	0	3	0	3	0	3	2.765333
tr|A1KYQ7|A1KYQ7_	tr|A1KYQ7|A	Cell migration-inducing protein 17 OS=Homo sapiens PE=2 SV=1	106022	0	194	0	142	0	0.06	#DIV/0!	0	2	0	2	0	3	0	4	2.765333
sp|Q92945|FUBP2_H	KHSRP	Far upstream element-binding protein 2 OS=Homo sapiens GN=KHSRP PE=1 SV=4	73355	0	117	0	213	0	0.14	#DIV/0!	0	3	0	3	0	3	0	4	2.765333
tr|B3KNK9|B3KNK9_	GOLPH2	Golgi phosphoprotein 2, isoform CRA_a OS=Homo sapiens GN=GOLPH2 PE=2 SV=1	45477	0	984	0	20	0	0.07	#DIV/0!	0	0	0	1	0	2	0	4	2.279573
tr|B2R806|B2R806_H	tr|B2R806|B	Eukaryotic translation initiation factor 3 subunit E OS=Homo sapiens PE=2 SV=1	52622	0	974	0	20	0	0.06	#DIV/0!	0	0	0	1	0	2	0	3	2.279573
tr|H0YEP8|H0YEP8_H	SERPINH1	Serpin H1 (Fragment) OS=Homo sapiens GN=SERPINH1 PE=3 SV=1	6605	0	964	0	21	0	0.54	#DIV/0!	0	0	0	1	0	2	0	2	2.279573
tr|F5GX68|F5GX68_H	DNMT1	Cytosine-specific methyltransferase OS=Homo sapiens GN=DNMT1 PE=2 SV=1	185605	0	909	0	24	0	0.02	#DIV/0!	0	1	0	1	0	2	0	2	2.279573
tr|B4DFL2|B4DFL2_H	IDH2	Isocitrate dehydrogenase [NADP] OS=Homo sapiens GN=IDH2 PE=2 SV=1	45550	0	878	0	26	0	0.07	#DIV/0!	0	0	0	1	0	2	0	2	2.279573
tr|B7ZMF2|B7ZMF2_	FANCI	Fanconi anemia, complementation group I OS=Homo sapiens GN=FANCI PE=2 SV=1	150724	0	861	0	27	0	0.02	#DIV/0!	0	0	0	1	0	2	0	3	2.279573
tr|B2RAU8|B2RAU8_	tr|B2RAU8|B	cDNA, FLJ95131, highly similar to Homo sapiens nucleolar and coiled-body phosphoprotein 1 (NOLC1), mRNA OS=Homo	73561	0	852	0	28	0	0.09	#DIV/0!	0	1	0	2	0	2	0	2	2.279573
tr|D3DVA5|D3DVA5_	ARHGEF2	Rho/rac guanine nucleotide exchange factor (GEF) 2, isoform CRA_a OS=Homo sapiens GN=ARHGEF2 PE=2 SV=1	116954	0	847	0	28	0	0.03	#DIV/0!	0	0	0	1	0	2	0	3	2.279573
tr|H0Y8R1|H0Y8R1_H	GRSF1	G-rich sequence factor 1 (Fragment) OS=Homo sapiens GN=GRSF1 PE=4 SV=1	47637	0	821	0	30	0	0.07	#DIV/0!	0	0	0	1	0	2	0	2	2.279573
tr|B3KU23|B3KU23_H	tr|B3KU23|B	cDNA FLJ39097 fis, clone NTONG2000977, highly similar to Neurolysin, mitochondrial (EC 3.4.24.16) OS=Homo sapiens P	70320	0	809	0	31	0	0.1	#DIV/0!	0	0	0	2	0	2	0	2	2.279573
sp|Q96T88|UHRF1_H	UHRF1	E3 ubiquitin-protein ligase UHRF1 OS=Homo sapiens GN=UHRF1 PE=1 SV=1	91297	0	803	0	31	0	0.04	#DIV/0!	0	0	0	1	0	2	0	3	2.279573
tr|E5RFZ5|E5RFZ5_HU	RAD21	Double-strand-break repair protein rad21 homolog OS=Homo sapiens GN=RAD21 PE=2 SV=1	20161	0	795	0	32	0	0.36	#DIV/0!	0	1	0	2	0	2	0	2	2.279573
tr|Q3SYK8|Q3SYK8_H	NUMA1	NUMA1 protein (Fragment) OS=Homo sapiens GN=NUMA1 PE=2 SV=1	78666	0	779	0	32	0	0.01	#DIV/0!	0	0	0	1	0	2	0	2	2.279573
tr|F5GZI3|F5GZI3_HU	PARD3	Partitioning defective 3 homolog OS=Homo sapiens GN=PARD3 PE=2 SV=1	82242	0	764	0	33	0	0.04	#DIV/0!	0	0	0	1	0	2	0	2	2.279573
tr|B4DF80|B4DF80_H	tr|B4DF80|B	cDNA FLJ58527, highly similar to Nucleolar protein 6 OS=Homo sapiens PE=2 SV=1	122714	0	762	0	33	0	0.05	#DIV/0!	0	0	0	2	0	2	0	2	2.279573
sp|O76003|GLRX3_HU	GLRX3	Glutaredoxin-3 OS=Homo sapiens GN=GLRX3 PE=1 SV=2	37693	0	739	0	35	0	0.18	#DIV/0!	0	0	0	2	0	2	0	2	2.279573
tr|D3DWL9|D3DWL9_	CPSF1	Cleavage and polyadenylation specific factor 1, 160kDa, isoform CRA_a OS=Homo sapiens GN=CPSF1 PE=2 SV=1	153087	0	733	0	36	0	0.04	#DIV/0!	0	0	0	3	0	2	0	3	2.279573
sp|Q9Y4G6|TLN2_HU	TLN2	Talin-2 OS=Homo sapiens GN=TLN2 PE=1 SV=4	273781	0	725	0	36	0	0.02	#DIV/0!	0	0	0	1	0	2	0	3	2.279573
tr|B3KNK5|B3KNK5_H	tr|B3KNK5|B	cDNA FLJ14795 fis, clone NT2RP4001219, highly similar to Homo sapiens DnaJ (Hsp40) homolog, subfamily C, member 1(	70004	0	715	0	37	0	0.1	#DIV/0!	0	0	0	2	0	2	0	3	2.279573
sp|Q8NI35|INADL_HU	INADL	InaD-like protein OS=Homo sapiens GN=INADL PE=1 SV=3	197046	0	712	0	38	0	0.02	#DIV/0!	0	1	0	1	0	2	0	2	2.279573
tr|G3V161|G3V161_H	KBTBD3	Kelch repeat and BTB (POZ) domain containing 3, isoform CRA_a OS=Homo sapiens GN=KBTBD3 PE=2 SV=1	61834	0	710	0	38	0	0.06	#DIV/0!	0	1	0	1	0	2	0	2	2.279573
tr|B3KY63|B3KY63_H	tr|B3KY63|B	cDNA FLJ16830 fis, clone UTERU3022536, highly similar to Chromodomain helicase-DNA-binding protein 4 (EC 3.6.1.-) O	216574	0	705	0	38	0	0.03	#DIV/0!	0	2	0	2	0	2	0	4	2.279573
sp|P14550|AK1A1_HU	AKR1A1	Alcohol dehydrogenase [NADP(+)] OS=Homo sapiens GN=AKR1A1 PE=1 SV=3	36892	0	695	0	38	0	0.19	#DIV/0!	0	1	0	2	0	2	0	3	2.279573
sp|P37837|TALDO_HU	TALDO1	Transaldolase OS=Homo sapiens GN=TALDO1 PE=1 SV=2	37688	0	694	0	38	0	0.18	#DIV/0!	0	0	0	2	0	2	0	2	2.279573
tr|B7Z8Z8|B7Z8Z8_HU	tr|B7Z8Z8|B	cDNA FLJ61702, highly similar to 80 kDa MCM3-associated protein OS=Homo sapiens PE=2 SV=1	181264	0	690	0	39	0	0.02	#DIV/0!	0	1	0	1	0	2	0	4	2.279573
tr|Q4KMR3|Q4KMR3_	MYO1E	MYO1E variant protein OS=Homo sapiens GN=MYO1E PE=2 SV=1	127552	0	681	0	39	0	0.05	#DIV/0!	0	1	0	2	0	2	0	2	2.279573
sp|Q92466|DDB2_HU	DDB2	DNA damage-binding protein 2 OS=Homo sapiens GN=DDB2 PE=1 SV=1	48461	0	668	0	40	0	0.07	#DIV/0!	0	1	0	1	0	2	0	3	2.279573
tr|F5H740|F5H740_H	VDAC3	Voltage-dependent anion-selective channel protein 3 OS=Homo sapiens GN=VDAC3 PE=2 SV=1	31080	0	658	0	41	0	0.11	#DIV/0!	0	1	0	1	0	2	0	2	2.279573
sp|Q96IJ6|GMPPA_H	GMPPA	Mannose-1-phosphate guanyltransferase alpha OS=Homo sapiens GN=GMPPA PE=1 SV=1	46604	0	649	0	42	0	0.07	#DIV/0!	0	1	0	1	0	2	0	2	2.279573
tr|B3KST5|B3KST5_H	COPS4	COP9 constitutive photomorphogenic homolog subunit 4 (Arabidopsis), isoform CRA_a OS=Homo sapiens GN=COPS4 PE	40457	0	644	0	43	0	0.07	#DIV/0!	0	1	0	1	0	2	0	2	2.279573
tr|B2R5U7|B2R5U7_H	tr|B2R5U7|B	cDNA, FLJ92633, highly similar to Homo sapiens CCAAT-box-binding transcription factor (CBF2), mRNA OS=Homo sapien	114755	0	639	0	43	0	0.06	#DIV/0!	0	1	0	2	0	2	0	2	2.279573
tr|B4DZ87|B4DZ87_H	tr|B4DZ87|B	cDNA FLJ57240, highly similar to Mitochondrial proteins import receptor OS=Homo sapiens PE=2 SV=1	57579	0	631	0	44	0	0.26	#DIV/0!	0	1	0	2	0	2	0	2	2.279573
tr|B4DH17|B4DH17_H	tr|B4DH17|B	cDNA FLJ59298, highly similar to Eukaryotic translation initiation factor 4 gamma 3 OS=Homo sapiens PE=2 SV=1	134921	0	629	0	44	0	0.02	#DIV/0!	0	1	0	1	0	2	0	3	2.279573
tr|B2RDI5|B2RDI5_HU	tr|B2RDI5|B	cDNA, FLJ96627, highly similar to Homo sapiens calpain 1, (mu/I) large subunit (CAPN1), mRNA OS=Homo sapiens PE=2 S	82529	0	615	0	45	0	0.04	#DIV/0!	0	1	0	1	0	2	0	2	2.279573
tr|Q9BXV5|Q9BXV5_	tr|Q9BXV5|C	PNAS-139 OS=Homo sapiens PE=2 SV=1	22952	0	601	0	46	0	0.31	#DIV/0!	0	1	0	2	0	2	0	2	2.279573
sp|Q8N766|EMC1_HU	EMC1	ER membrane protein complex subunit 1 OS=Homo sapiens GN=EMC1 PE=1 SV=1	112145	0	586	0	49	0	0.03	#DIV/0!	0	1	0	1	0	2	0	2	2.279573
tr|A8K6V7|A8K6V7_H	tr|A8K6V7|A	cDNA FLJ76053, highly similar to Homo sapiens Ras-GTPase activating protein SH3 domain-binding protein 2 (G3BP2), tra	50842	0	579	0	50	0	0.24	#DIV/0!	0	1	0	1	0	2	0	2	2.279573
tr|Q5SRT3|Q5SRT3_H	CLIC1	Chloride intracellular channel 1, isoform CRA_a OS=Homo sapiens GN=CLIC1 PE=2 SV=2	27248	0	577	0	51	0	0.26	#DIV/0!	0	1	0	2	0	2	0	2	2.279573
sp|Q8IVT2|CS021_HU	C19orf21	Uncharacterized protein C19orf21 OS=Homo sapiens GN=C19orf21 PE=1 SV=1	75482	0	576	0	51	0	0.09	#DIV/0!	0	1	0	2	0	2	0	2	2.279573

sp|Q8WX93|PALLD_H	PALLD	Palladin OS=Homo sapiens GN=PALLD PE=1 SV=3	151839	0	569	0	51	0	0.04	#DIV/0!	0	1	0	2	0	2	0	4	2.279573
tr|B7Z213|B7Z213_H	tr|B7Z213|B	cDNA FLJ50130, highly similar to Heterogeneous nuclear ribonucleoprotein Q OS=Homo sapiens PE=2 SV=1	28561	0	562	0	52	0	0.25	#DIV/0!	0	1	0	2	0	2	0	2	2.279573
tr|Q0D2Q6|Q0D2Q6_	PGAM1	Phosphoglycerate mutase 1 (Brain) OS=Homo sapiens GN=PGAM1 PE=2 SV=1	28888	0	559	0	53	0	0.24	#DIV/0!	0	1	0	2	0	2	0	2	2.279573
tr|B4DUT7|B4DUT7_	tr|B4DUT7|B	cDNA FLJ57604, highly similar to GMP synthase (glutamine-hydrolyzing) (EC 6.3.5.2) OS=Homo sapiens PE=2 SV=1	71880	0	557	0	53	0	0.09	#DIV/0!	0	1	0	2	0	2	0	3	2.279573
sp|Q14684|RRP1B_H	RRP1B	Ribosomal RNA processing protein 1 homolog B OS=Homo sapiens GN=RRP1B PE=1 SV=3	84774	0	553	0	53	0	0.08	#DIV/0!	0	1	0	2	0	2	0	2	2.279573
sp|P27635|RL10_HUM	RPL10	60S ribosomal protein L10 OS=Homo sapiens GN=RPL10 PE=1 SV=4	25044	0	541	0	55	0	0.28	#DIV/0!	0	1	0	2	0	2	0	3	2.279573
tr|A0JLQ5|A0JLQ5_H	BXDC2	BXDC2 protein (Fragment) OS=Homo sapiens GN=BXDC2 PE=2 SV=1	39430	0	530	0	57	0	0.17	#DIV/0!	0	1	0	2	0	2	0	2	2.279573
sp|P49792|RBP2_HU	RANBP2	E3 SUMO-protein ligase RanBP2 OS=Homo sapiens GN=RANBP2 PE=1 SV=2	362365	0	518	0	58	0	0.02	#DIV/0!	0	1	0	2	0	2	0	4	2.279573
tr|D0PQI2|D0PQI2_H	ENAH	Enabled-like protein variant hMenaDv6 OS=Homo sapiens GN=ENAH PE=2 SV=1	60426	0	503	0	60	0	0.11	#DIV/0!	0	2	0	2	0	2	0	2	2.279573
sp|Q05048|CSTF1_HU	CSTF1	Cleavage stimulation factor subunit 1 OS=Homo sapiens GN=CSTF1 PE=1 SV=1	49125	0	500	0	61	0	0.14	#DIV/0!	0	1	0	2	0	2	0	2	2.279573
sp|Q70J99|UN13D_H	UNC13D	Protein unc-13 homolog D OS=Homo sapiens GN=UNC13D PE=1 SV=1	124288	0	493	0	62	0	0.05	#DIV/0!	0	1	0	2	0	2	0	2	2.279573
sp|O15031|PLXB2_HU	PLXNB2	Plexin-B2 OS=Homo sapiens GN=PLXNB2 PE=1 SV=3	207734	0	491	0	62	0	0.03	#DIV/0!	0	1	0	1	0	2	0	2	2.279573
sp|Q4G0J3|LARP7_HU	LARP7	La-related protein 7 OS=Homo sapiens GN=LARP7 PE=1 SV=1	67143	0	490	0	62	0	0.05	#DIV/0!	0	1	0	1	0	2	0	2	2.279573
tr|F5H667|F5H667_H	ASPH	Aspartyl/asparaginyl beta-hydroxylase OS=Homo sapiens GN=ASPH PE=2 SV=1	83672	0	489	0	62	0	0.08	#DIV/0!	0	1	0	2	0	2	0	2	2.279573
tr|B2R774|B2R774_H	tr|B2R774|B	cDNA, FLJ93313, highly similar to Homo sapiens lectin, mannose-binding, 1 (LMAN1), mRNA OS=Homo sapiens PE=2 SV=	57768	0	487	0	63	0	0.18	#DIV/0!	0	2	0	3	0	2	0	3	2.279573
tr|B4E2K4|B4E2K4_H	tr|B4E2K4|B	cDNA FLJ54576, highly similar to Aspartyl/asparaginyl beta-hydroxylase (EC 1.14.11.16) OS=Homo sapiens PE=2 SV=1	83685	0	484	0	63	0	0.08	#DIV/0!	0	1	0	2	0	2	0	2	2.279573
sp|Q6PKG0|LARP1_H	LARP1	La-related protein 1 OS=Homo sapiens GN=LARP1 PE=1 SV=2	123833	0	482	0	64	0	0.05	#DIV/0!	0	1	0	2	0	2	0	3	2.279573
tr|A8K0D2|A8K0D2_H	tr|A8K0D2|A	cDNA FLJ77740, highly similar to Homo sapiens 7-dehydrocholesterol reductase, mRNA OS=Homo sapiens PE=2 SV=1	55175	0	469	0	66	0	0.12	#DIV/0!	0	1	0	2	0	2	0	3	2.279573
tr|G5E9H4|G5E9H4_H	DCTN1	Dynactin 1 (P150, glued homolog, Drosophila), isoform CRA_a OS=Homo sapiens GN=DCTN1 PE=2 SV=1	127339	0	461	0	66	0	0.05	#DIV/0!	0	1	0	2	0	2	0	2	2.279573
tr|B2R825|B2R825_H	tr|B2R825|B	Phosphorylase OS=Homo sapiens PE=2 SV=1	97405	0	459	0	67	0	0.07	#DIV/0!	0	2	0	2	0	2	0	2	2.279573
sp|Q07065|CKAP4_H	CKAP4	Cytoskeleton-associated protein 4 OS=Homo sapiens GN=CKAP4 PE=1 SV=2	66097	0	455	0	68	0	0.1	#DIV/0!	0	1	0	2	0	2	0	2	2.279573
tr|A0JLU4|A0JLU4_HU	YBX1	YBX1 protein (Fragment) OS=Homo sapiens GN=YBX1 PE=2 SV=1	21417	0	447	0	52	0	0.16	#DIV/0!	0	2	0	2	0	2	0	2	2.279573
tr|F1D8N4|F1D8N4_H	NR3C1	Glucocorticoid nuclear receptor variant 1 OS=Homo sapiens GN=NR3C1 PE=2 SV=1	86745	0	431	0	72	0	0.04	#DIV/0!	0	1	0	1	0	2	0	2	2.279573
sp|Q08499|PDE4D_H	PDE4D	cAMP-specific 3~,5~-cyclic phosphodiesterase 4D OS=Homo sapiens GN=PDE4D PE=1 SV=2	91628	0	430	0	72	0	0.07	#DIV/0!	0	1	0	2	0	2	0	2	2.279573
tr|B7Z4B8|B7Z4B8_H	HNRNPUL1	Heterogeneous nuclear ribonucleoprotein U-like protein 1 OS=Homo sapiens GN=HNRNPUL1 PE=2 SV=1	86639	0	425	0	73	0	0.12	#DIV/0!	0	1	0	3	0	2	0	3	2.279573
tr|Q5XJ04|Q5XJ04_H	HMGCS1	3-hydroxy-3-methylglutaryl-Coenzyme A synthase 1 (Soluble) OS=Homo sapiens GN=HMGCS1 PE=2 SV=1	57854	0	424	0	73	0	0.12	#DIV/0!	0	1	0	2	0	2	0	4	2.279573
tr|B4DYY5|B4DYY5_H	tr|B4DYY5|B	cDNA FLJ55671, highly similar to Ubiquitin-associated protein 2-like OS=Homo sapiens PE=2 SV=1	113662	0	417	0	75	0	0.06	#DIV/0!	0	2	0	2	0	2	0	4	2.279573
tr|H7C3P6|H7C3P6_H	NUP98	Nuclear pore complex protein Nup98-Nup96 (Fragment) OS=Homo sapiens GN=NUP98 PE=4 SV=1	86760	0	413	0	75	0	0.12	#DIV/0!	0	2	0	3	0	2	0	3	2.279573
sp|Q9NX58|LYAR_HU	LYAR	Cell growth-regulating nucleolar protein OS=Homo sapiens GN=LYAR PE=1 SV=2	44044	0	408	0	76	0	0.16	#DIV/0!	0	2	0	2	0	2	0	2	2.279573
tr|B7Z2D6|B7Z2D6_H	tr|B7Z2D6|B	cDNA FLJ53863, highly similar to Cystathionine beta-synthase (EC 4.2.1.22) OS=Homo sapiens PE=2 SV=1	56409	0	403	0	77	0	0.12	#DIV/0!	0	2	0	2	0	2	0	2	2.279573
tr|Q5JR04|Q5JR04_H	MOV10	Mov10, Moloney leukemia virus 10, homolog (Mouse) OS=Homo sapiens GN=MOV10 PE=4 SV=1	107941	0	376	0	82	0	0.06	#DIV/0!	0	2	0	2	0	2	0	2	2.279573
sp|P52907|CAZA1_HU	CAPZA1	F-actin-capping protein subunit alpha-1 OS=Homo sapiens GN=CAPZA1 PE=1 SV=3	33073	0	375	0	82	0	0.21	#DIV/0!	0	2	0	2	0	2	0	2	2.279573
tr|G3V1J5|G3V1J5_H	DIS3	Exosome complex exonuclease RRP44 OS=Homo sapiens GN=DIS3 PE=2 SV=1	91382	0	374	0	27	0	0.09	#DIV/0!	0	0	0	2	0	2	0	3	2.279573
tr|B4DN01|B4DN01_	tr|B4DN01|B	cDNA FLJ57332, highly similar to COP9 signalosome complex subunit 3 OS=Homo sapiens PE=2 SV=1	33950	0	373	0	83	0	0.17	#DIV/0!	0	2	0	2	0	2	0	2	2.279573
sp|P55268|LAMB2_H	LAMB2	Laminin subunit beta-2 OS=Homo sapiens GN=LAMB2 PE=1 SV=2	202982	0	362	0	85	0	0.03	#DIV/0!	0	2	0	2	0	2	0	2	2.279573
tr|A8K8Z4|A8K8Z4_H	tr|A8K8Z4|A	cDNA FLJ78071, highly similar to Human MHC class III complement component C6 mRNA OS=Homo sapiens PE=2 SV=1	108298	0	357	0	86	0	0.03	#DIV/0!	0	2	0	2	0	2	0	2	2.279573
tr|F5H6D0|F5H6D0_H	MOGS	Mannosyl-oligosaccharide glucosidase OS=Homo sapiens GN=MOGS PE=2 SV=1	80824	0	352	0	87	0	0.07	#DIV/0!	0	1	0	2	0	2	0	2	2.279573
tr|B2R7F8|B2R7F8_H	tr|B2R7F8|B	cDNA, FLJ93426, highly similar to Homo sapiens plasminogen (PLG), mRNA OS=Homo sapiens PE=2 SV=1	93217	0	346	0	90	0	0.07	#DIV/0!	0	1	0	2	0	2	0	2	2.279573
sp|Q9Y2W1|TR150_H	THRAP3	Thyroid hormone receptor-associated protein 3 OS=Homo sapiens GN=THRAP3 PE=1 SV=2	108658	0	338	0	92	0	0.06	#DIV/0!	0	2	0	2	0	2	0	3	2.279573
sp|Q86XP3|DDX42_H	DDX42	ATP-dependent RNA helicase DDX42 OS=Homo sapiens GN=DDX42 PE=1 SV=1	103197	0	336	0	92	0	0.06	#DIV/0!	0	2	0	2	0	2	0	4	2.279573
tr|A4D275|A4D275_H	ARPC1B	Actin related protein 2/3 complex, subunit 1B, 41kDa OS=Homo sapiens GN=ARPC1B PE=2 SV=1	41722	0	321	0	95	0	0.16	#DIV/0!	0	1	0	2	0	2	0	5	2.279573
tr|J3KNQ4|J3KNQ4_H	PARVA	Alpha-parvin OS=Homo sapiens GN=PARVA PE=4 SV=1	46618	0	304	0	101	0	0.15	#DIV/0!	0	2	0	2	0	2	0	2	2.279573
tr|B4DJK4|B4DJK4_H	tr|B4DJK4|B	cDNA FLJ54005, highly similar to Transcription elongation factor SPT5 OS=Homo sapiens PE=2 SV=1	97196	0	302	0	101	0	0.07	#DIV/0!	0	2	0	2	0	2	0	2	2.279573
sp|Q96S66|CLCC1_HU	CLCC1	Chloride channel CLIC-like protein 1 OS=Homo sapiens GN=CLCC1 PE=1 SV=1	62667	0	299	0	102	0	0.11	#DIV/0!	0	2	0	2	0	2	0	2	2.279573
tr|B2RB95|B2RB95_H	tr|B2RB95|B	cDNA, FLJ95381, Homo sapiens tripartite motif-containing 16 (TRIM16), mRNA OS=Homo sapiens PE=2 SV=1	64884	0	293	0	103	0	0.06	#DIV/0!	0	2	0	2	0	2	0	2	2.279573
tr|B4DRW3|B4DRW3	tr|B4DRW3|	cDNA FLJ57180, highly similar to TAR DNA-binding protein 43 OS=Homo sapiens PE=2 SV=1	35596	0	253	0	110	0	0.24	#DIV/0!	0	2	0	2	0	2	0	2	2.279573
tr|A8MWK3|A8MWK	CDH2	Cadherin-2 OS=Homo sapiens GN=CDH2 PE=2 SV=1	97379	0	239	0	119	0	0.07	#DIV/0!	0	2	0	2	0	2	0	2	2.279573
tr|G3XAL0|G3XAL0_H	MDH2	Malate dehydrogenase OS=Homo sapiens GN=MDH2 PE=2 SV=1	24921	0	219	0	112	0	0.34	#DIV/0!	0	2	0	2	0	2	0	3	2.279573
tr|H0YCK7|H0YCK7_H	EEF1D	Elongation factor 1-delta (Fragment) OS=Homo sapiens GN=EEF1D PE=4 SV=1	23105	0	144	0	183	0	0.5	#DIV/0!	0	2	0	3	0	2	0	4	2.279573
tr|B2RAN1|B2RAN1_	tr|B2RAN1|B	cDNA, FLJ95012, highly similar to Homo sapiens UDP-glucose pyrophosphorylase 2 (UGP2), mRNA OS=Homo sapiens PE=	57042	0	1021	0	16	0	0.06	#DIV/0!	0	0	0	1	0	1	0	3	1.542273
tr|A8K4T6|A8K4T6_H	tr|A8K4T6|A	cDNA FLJ76282, highly similar to Homo sapiens proteasome (prosome, macropain) 26S subunit, non-ATPase, 5 (PSMD5),	56588	0	1018	0	17	0	0.06	#DIV/0!	0	0	0	1	0	1	0	2	1.542273
sp|Q8N6G5|CGAT2_H	CSGALNACT2	Chondroitin sulfate N-acetylgalactosaminyltransferase 2 OS=Homo sapiens GN=CSGALNACT2 PE=2 SV=1	62989	0	1014	0	17	0	0.05	#DIV/0!	0	0	0	1	0	1	0	2	1.542273
tr|B3KWV6|B3KWV6_	tr|B3KWV6|	cDNA FLJ43948 fis, clone TESTI4014924, highly similar to Homo sapiens cytoplasmic FMR1 interacting protein 1 (CYFIP1)	147082	0	981	0	20	0	0.02	#DIV/0!	0	0	0	1	0	1	0	3	1.542273
tr|B4DGD5|B4DGD5_	DNAJC11	DnaJ homolog subfamily C member 11 OS=Homo sapiens GN=DNAJC11 PE=2 SV=1	52854	0	980	0	20	0	0.06	#DIV/0!	0	0	0	1	0	1	0	1	1.542273
sp|Q14739|LBR_HUM	LBR	Lamin-B receptor OS=Homo sapiens GN=LBR PE=1 SV=2	71057	0	978	0	20	0	0.05	#DIV/0!	0	0	0	1	0	1	0	1	1.542273
sp|Q8IVF2|AHNK2_H	AHNAK2	Protein AHNAK2 OS=Homo sapiens GN=AHNAK2 PE=1 SV=2	617383	0	977	0	20	0	0.01	#DIV/0!	0	0	0	1	0	1	0	2	1.542273
sp|Q9BXW9|FACD2_	FANCD2	Fanconi anemia group D2 protein OS=Homo sapiens GN=FANCD2 PE=1 SV=1	168295	0	976	0	20	0	0.02	#DIV/0!	0	0	0	1	0	1	0	1	1.542273
sp|P53701|CCHL_HU	HCCS	Cytochrome c-type heme lyase OS=Homo sapiens GN=HCCS PE=1 SV=1	30981	0	975	0	20	0	0.11	#DIV/0!	0	0	0	1	0	1	0	1	1.542273
sp|Q7L0Y3|MRRP1_H	TRMT10C	Mitochondrial ribonuclease P protein 1 OS=Homo sapiens GN=TRMT10C PE=1 SV=2	47602	0	972	0	20	0	0.07	#DIV/0!	0	0	0	1	0	1	0	1	1.542273
tr|K7EJA8|K7EJA8_HU	ST6GALNAC1	Alpha-N-acetylgalactosaminide alpha-2,6-sialyltransferase 1 OS=Homo sapiens GN=ST6GALNAC1 PE=4 SV=1	7383	0	969	0	20	0	0.48	#DIV/0!	0	0	0	1	0	1	0	1	1.542273
tr|B3KVX1|B3KVX1_H	tr|B3KVX1|B	cDNA FLJ41678 fis, clone HCASM2003076, highly similar to Leucine-rich repeat-containing protein 49 OS=Homo sapiens	78289	0	968	0	21	0	0.04	#DIV/0!	0	0	0	1	0	1	0	1	1.542273
sp|P62280|RS11_HUM	RPS11	40S ribosomal protein S11 OS=Homo sapiens GN=RPS11 PE=1 SV=3	18590	0	967	0	21	0	0.18	#DIV/0!	0	0	0	1	0	1	0	1	1.542273
tr|G3V529|G3V529_H	DDX24	ATP-dependent RNA helicase DDX24 OS=Homo sapiens GN=DDX24 PE=2 SV=1	91994	0	966	0	21	0	0.04	#DIV/0!	0	0	0	1	0	1	0	1	1.542273
tr|Q59GI3|Q59GI3_H	tr|Q59GI3|Q	I-kappa-B-related protein variant (Fragment) OS=Homo sapiens PE=2 SV=1	98150	0	965	0	21	0	0.03	#DIV/0!	0	0	0	1	0	1	0	1	1.542273
sp|Q5K651|SAMD9_H	SAMD9	Sterile alpha motif domain-containing protein 9 OS=Homo sapiens GN=SAMD9 PE=1 SV=1	185704	0	963	0	21	0	0.04	#DIV/0!	0	0	0	1	0	1	0	1	1.542273
tr|J3KN41|J3KN41_HU	ITGA6	Integrin alpha-6 heavy chain OS=Homo sapiens GN=ITGA6 PE=3 SV=1	127724	0	962	0	21	0	0.03	#DIV/0!	0	0	0	1	0	1	0	1	1.542273
tr|B4DQG8|B4DQG8_	tr|B4DQG8|	cDNA FLJ54124, highly similar to DNA methyltransferase 1-associated protein 1 OS=Homo sapiens PE=2 SV=1	52000	0	960	0	21	0	0.06	#DIV/0!	0	0	0	1	0	1	0	1	1.542273
tr|B3KY99|B3KY99_H	tr|B3KY99|B	cDNA FLJ16244 fis, clone HCHON2000508, highly similar to TBC1 domain family member 2 OS=Homo sapiens PE=2 SV=1	88971	0	959	0	21	0	0.04	#DIV/0!	0	0	0	1	0	1	0	1	1.542273
tr|B3KPZ2|B3KPZ2_H	tr|B3KPZ2|B	cDNA FLJ32487 fis, clone SKNSH1000002, highly similar to Prostaglandin E synthase 2 (EC 5.3.99.3) OS=Homo sapiens PE	42114	0	958	0	21	0	0.08	#DIV/0!	0	0	0	1	0	1	0	1	1.542273
tr|B4DJE7|B4DJE7_HU	ACADM	Medium-chain-specific acyl-CoA dehydrogenase, mitochondrial OS=Homo sapiens GN=ACADM PE=2 SV=1	25704	0	957	0	21	0	0.13	#DIV/0!	0	0	0	1	0	1	0	1	1.542273
tr|B1ANM7|B1ANM7	FAF1	FAS-associated factor 1 OS=Homo sapiens GN=FAF1 PE=4 SV=1	56305	0	956	0	21	0	0.06	#DIV/0!	0	0	0	1	0	1	0	1	1.542273
tr|H0Y650|H0Y650_H	HEATR2	HEAT repeat-containing protein 2 (Fragment) OS=Homo sapiens GN=HEATR2 PE=4 SV=1	73507	0	955	0	21	0	0.04	#DIV/0!	0	0	0	1	0	1	0	1	1.542273
sp|Q2M389|WASH7_	KIAA1033	WASH complex subunit 7 OS=Homo sapiens GN=KIAA1033 PE=1 SV=2	137343	0	953	0	21	0	0.02	#DIV/0!	0	0	0	1	0	1	0	1	1.542273
tr|B4E2N8|B4E2N8_H	ANKUB1	Protein ANKUB1 OS=Homo sapiens GN=ANKUB1 PE=2 SV=1	57362	0	951	0	21	0	0.06	#DIV/0!	0	0	0	1	0	1	0	1	1.542273
tr|Q96F88|Q96F88_H	POP1	Processing of 1, ribonuclease P/MRP subunit (S. cerevisiae) OS=Homo sapiens GN=POP1 PE=2 SV=1	116318	0	950	0	21	0	0.03	#DIV/0!	0	0	0	1	0	1	0	1	1.542273
tr|B3KY04|B3KY04_H	tr|B3KY04|B	cDNA FLJ46506 fis, clone THYMU3030752, highly similar to BTB/POZ domain-containing protein KCTD12 OS=Homo sapie	35994	0	946	0	22	0	0.09	#DIV/0!	0	0	0	1	0	1	0	1	1.542273
tr|G3XAB8|G3XAB8_	TNRC6C	Trinucleotide repeat containing 6C, isoform CRA_a OS=Homo sapiens GN=TNRC6C PE=4 SV=1	179781	0	945	0	22	0	0.02	#DIV/0!	0	0	0	1	0	1	0	1	1.542273
sp|P01023|A2MG_HU	A2M	Alpha-2-macroglobulin OS=Homo sapiens GN=A2M PE=1 SV=3	164613	0	943	0	22	0	0.02	#DIV/0!	0	0	0	1	0	1	0	2	1.542273
tr|Q6IBU0|Q6IBU0_H	EIF5	EIF5 protein OS=Homo sapiens GN=EIF5 PE=2 SV=1	49577	0	942	0	22	0	0.07	#DIV/0!	0	0	0	1	0	1	0	1	1.542273
tr|D3DUR9|D3DUR9_	ZNF384	Zinc finger protein 384, isoform CRA_a OS=Homo sapiens GN=ZNF384 PE=2 SV=1	64191	0	941	0	22	0	0.05	#DIV/0!	0	0	0	1	0	1	0	1	1.542273
tr|B2R950|B2R950_H	tr|B2R950|B	cDNA, FLJ94213, highly similar to Homo sapiens pregnancy-zone protein (PZP), mRNA OS=Homo sapiens PE=2 SV=1	165250	0	940	0	22	0	0.02	#DIV/0!	0	0	0	1	0	1	0	1	1.542273
tr|K7EJ76|K7EJ76_HU	ASXL3	Putative Polycomb group protein ASXL3 OS=Homo sapiens GN=ASXL3 PE=4 SV=1	35347	0	939	0	22	0	0.09	#DIV/0!	0	0	0	1	0	1	0	1	1.542273
tr|Q6IB29|Q6IB29_HU	EBNA1BP2	EBNA1 binding protein 2 OS=Homo sapiens GN=EBNA1BP2 PE=2 SV=1	34887	0	938	0	22	0	0.1	#DIV/0!	0	0	0	1	0	1	0	1	1.542273
tr|J3KQ32|J3KQ32_H	OLA1	Obg-like ATPase 1 OS=Homo sapiens GN=OLA1 PE=4 SV=1	47136	0	937	0	22	0	0.07	#DIV/0!	0	0	0	1	0	1	0	2	1.542273
tr|B4DYB1|B4DYB1_H	tr|B4DYB1|B	cDNA FLJ60956, highly similar to DNA-directed RNA polymerase I-associated factor 53 kDa subunit (EC 2.7.7.6) OS=Homo	19500	0	936	0	22	0	0.17	#DIV/0!	0	0	0	1	0	1	0	1	1.542273
tr|G3V1W0|G3V1W0_	hCG_203871	HCG2038717 OS=Homo sapiens GN=hCG_2038717 PE=4 SV=1	9582	0	934	0	23	0	0.36	#DIV/0!	0	0	0	1	0	1	0	1	1.542273
sp|Q86YC3|LRC33_HU	LRRC33	Leucine-rich repeat-containing protein 33 OS=Homo sapiens GN=LRRC33 PE=2 SV=1	77457	0	933	0	23	0	0.04	#DIV/0!	0	1	0	1	0	1	0	1	1.542273
tr|B2RE56|B2RE56_H	tr|B2RE56|B	Peptidyl-prolyl cis-trans isomerase OS=Homo sapiens PE=2 SV=1	18225	0	932	0	23	0	0.19	#DIV/0!	0	0	0	1	0	1	0	1	1.542273
tr|H0YKH6|H0YKH6_	CERS2	Ceramide synthase 2 (Fragment) OS=Homo sapiens GN=CERS2 PE=3 SV=1	26788	0	931	0	23	0	0.12	#DIV/0!	0	0	0	1	0	1	0	1	1.542273
sp|P24928|RPB1_HU	POLR2A	DNA-directed RNA polymerase II subunit RPB1 OS=Homo sapiens GN=POLR2A PE=1 SV=2	218408	0	930	0	23	0	0.01	#DIV/0!	0	0	0	1	0	1	0	3	1.542273
tr|I6L9B1|I6L9B1_HU	EPB41L2	EPB41L2 protein OS=Homo sapiens GN=EPB41L2 PE=2 SV=1	71998	0	929	0	0	0	0.03	#DIV/0!	0	0	0	0	0	1	0	2	1.542273
tr|A2A2Q9|A2A2Q9_	C20orf4	Chromosome 20 open reading frame 4 OS=Homo sapiens GN=C20orf4 PE=4 SV=1	45575	0	928	0	23	0	0.07	#DIV/0!	0	0	0	1	0	1	0	1	1.542273
tr|Q59ER2|Q59ER2_H	tr|Q59ER2|C	Cytochrome P450, family 46 variant (Fragment) OS=Homo sapiens PE=2 SV=1	23889	0	927	0	23	0	0.14	#DIV/0!	0	0	0	1	0	1	0	1	1.542273
tr|B4DM78|B4DM78_	tr|B4DM78|	cDNA FLJ58199, highly similar to Fragile X mental retardation syndrome-related protein 1 OS=Homo sapiens PE=2 SV=1	63280	0	926	0	23	0	0.06	#DIV/0!	0	0	0	1	0	1	0	2	1.542273
tr|B3KMN5|B3KMN5	tr|B3KMN5|	cDNA FLJ11713 fis, clone HEMBA1005201, highly similar to Homo sapiens NMD3 homolog (NMD3), mRNA OS=Homo sap	44787	0	924	0	23	0	0.07	#DIV/0!	0	0	0	1	0	1	0	1	1.542273
tr|Q59H44|Q59H44_	tr|Q59H44|C	Lymphocyte antigen 75 variant (Fragment) OS=Homo sapiens PE=2 SV=1	157120	0	920	0	23	0	0.02	#DIV/0!	0	0	0	1	0	1	0	1	1.542273
sp|Q0VAA2|CN16B_H	C14orf166B	Uncharacterized protein C14orf166B OS=Homo sapiens GN=C14orf166B PE=2 SV=2	54899	0	919	0	23	0	0.06	#DIV/0!	0	0	0	1	0	1	0	1	1.542273
tr|K7X9Y0|K7X9Y0_H	ND1	NADH-ubiquinone oxidoreductase chain 1 (Fragment) OS=Homo sapiens GN=ND1 PE=3 SV=1	33139	0	917	0	23	0	0.1	#DIV/0!	0	0	0	1	0	1	0	1	1.542273
sp|Q8TCG1|CIP2A_HU	KIAA1524	Protein CIP2A OS=Homo sapiens GN=KIAA1524 PE=1 SV=2	103318	0	916	0	23	0	0.03	#DIV/0!	0	0	0	1	0	1	0	2	1.542273
sp|Q8TCS8|PNPT1_H	PNPT1	Polyribonucleotide nucleotidyltransferase 1, mitochondrial OS=Homo sapiens GN=PNPT1 PE=1 SV=2	86524	0	915	0	24	0	0.04	#DIV/0!	0	0	0	1	0	1	0	2	1.542273
sp|P22059|OSBP1_HU	OSBP	Oxysterol-binding protein 1 OS=Homo sapiens GN=OSBP PE=1 SV=1	90220	0	914	0	24	0	0.04	#DIV/0!	0	0	0	1	0	1	0	1	1.542273
tr|B7Z507|B7Z507_H	tr|B7Z507|B	cDNA FLJ51036, highly similar to Matrix metalloproteinase-9 (EC3.4.24.35) OS=Homo sapiens PE=2 SV=1	72478	0	912	0	24	0	0.05	#DIV/0!	0	0	0	1	0	1	0	1	1.542273
tr|H0YIY3|H0YIY3_HU	PCNXL4	Pecanex-like protein 4 (Fragment) OS=Homo sapiens GN=PCNXL4 PE=4 SV=1	60728	0	911	0	24	0	0.05	#DIV/0!	0	0	0	1	0	1	0	1	1.542273
tr|B7ZKL3|B7ZKL3_H	EPS8L2	EPS8L2 protein OS=Homo sapiens GN=EPS8L2 PE=2 SV=1	82898	0	910	0	24	0	0.04	#DIV/0!	0	0	0	1	0	1	0	1	1.542273
tr|B2RBM8|B2RBM8_	tr|B2RBM8|	cDNA, FLJ95596, highly similar to Homo sapiens activity-dependent neuroprotector (ADNP), mRNA OS=Homo sapiens PE	124850	0	908	0	24	0	0.03	#DIV/0!	0	0	0	1	0	1	0	2	1.542273
sp|Q9H0A0|NAT10_H	NAT10	N-acetyltransferase 10 OS=Homo sapiens GN=NAT10 PE=1 SV=2	116569	0	907	0	24	0	0.03	#DIV/0!	0	0	0	1	0	1	0	1	1.542273
tr|H0YDU2|H0YDU2_	MUS81	Crossover junction endonuclease MUS81 (Fragment) OS=Homo sapiens GN=MUS81 PE=4 SV=1	45581	0	905	0	24	0	0.07	#DIV/0!	0	0	0	1	0	1	0	2	1.542273
tr|B3KSE4|B3KSE4_H	tr|B3KSE4|B	cDNA FLJ36076 fis, clone TESTI2019760, highly similar to TRANSCRIPTIONAL REPRESSOR CTCF OS=Homo sapiens PE=2 SV	30924	0	901	0	24	0	0.11	#DIV/0!	0	0	0	1	0	1	0	1	1.542273
tr|J3KQF5|J3KQF5_HU	AGBL1	Cytosolic carboxypeptidase 4 OS=Homo sapiens GN=AGBL1 PE=4 SV=1	121440	0	900	0	25	0	0.03	#DIV/0!	0	0	0	1	0	1	0	1	1.542273
tr|B4DK35|B4DK35_H	tr|B4DK35|B	cDNA FLJ61591, highly similar to Probable global transcription activator SNF2L2 (EC 3.6.1.-) (Fragment) OS=Homo sapien	112636	0	898	0	25	0	0.03	#DIV/0!	0	0	0	1	0	1	0	1	1.542273
tr|B7Z2R8|B7Z2R8_H	tr|B7Z2R8|B	cDNA FLJ53324, highly similar to Tight junction protein ZO-2 OS=Homo sapiens PE=2 SV=1	137474	0	896	0	25	0	0.02	#DIV/0!	0	0	0	1	0	1	0	2	1.542273
tr|H0Y5W0|H0Y5W0_	HUWE1	E3 ubiquitin-protein ligase HUWE1 (Fragment) OS=Homo sapiens GN=HUWE1 PE=4 SV=1	376696	0	895	0	25	0	0.01	#DIV/0!	0	0	0	1	0	1	0	1	1.542273
tr|B2RA56|B2RA56_H	tr|B2RA56|B	cDNA, FLJ94705, highly similar to Homo sapiens nicalin (LOC56926), mRNA OS=Homo sapiens PE=2 SV=1	61891	0	894	0	25	0	0.05	#DIV/0!	0	0	0	1	0	1	0	1	1.542273
tr|Q4G0F4|Q4G0F4_	POLRMT	DNA-directed RNA polymerase OS=Homo sapiens GN=POLRMT PE=2 SV=1	140194	0	893	0	25	0	0.02	#DIV/0!	0	0	0	1	0	1	0	2	1.542273
tr|H7C4H2|H7C4H2_	SRPRB	Signal recognition particle receptor subunit beta (Fragment) OS=Homo sapiens GN=SRPRB PE=4 SV=1	17716	0	892	0	25	0	0.19	#DIV/0!	0	0	0	1	0	1	0	1	1.542273
sp|Q8TAT6|NPL4_HU	NPLOC4	Nuclear protein localization protein 4 homolog OS=Homo sapiens GN=NPLOC4 PE=1 SV=3	69046	0	891	0	25	0	0.05	#DIV/0!	0	0	0	1	0	1	0	2	1.542273
sp|P30837|AL1B1_HU	ALDH1B1	Aldehyde dehydrogenase X, mitochondrial OS=Homo sapiens GN=ALDH1B1 PE=1 SV=3	57626	0	890	0	25	0	0.06	#DIV/0!	0	0	0	1	0	1	0	1	1.542273
tr|B3KU92|B3KU92_H	LOC90826	Putative protein arginine N-methyltransferase 10 OS=Homo sapiens GN=LOC90826 PE=2 SV=1	82717	0	889	0	25	0	0.04	#DIV/0!	0	0	0	1	0	1	0	1	1.542273
tr|B3KN37|B3KN37_H	tr|B3KN37|B	cDNA FLJ13414 fis, clone PLACE1001748, highly similar to Homo sapiens pitrilysin metallopeptidase 1 (PITRM1), mRNA C	68281	0	887	0	26	0	0.05	#DIV/0!	0	0	0	1	0	1	0	2	1.542273
sp|Q8WXX0|DYH7_H	DNAH7	Dynein heavy chain 7, axonemal OS=Homo sapiens GN=DNAH7 PE=1 SV=2	464398	0	885	0	26	0	0.01	#DIV/0!	0	0	0	1	0	1	0	3	1.542273
tr|J3QTM1|J3QTM1_	DNAH12	Dynein heavy chain 12, axonemal OS=Homo sapiens GN=DNAH12 PE=4 SV=1	92035	0	884	0	26	0	0.04	#DIV/0!	0	0	0	1	0	1	0	1	1.542273
tr|B4DGD0|B4DGD0_	tr|B4DGD0|	cDNA FLJ50491, highly similar to Amyloid beta A4 protein (APP) (ABPP)(Alzheimer disease amyloid protein) (Cerebral vas	81611	0	883	0	26	0	0.04	#DIV/0!	0	0	0	1	0	1	0	1	1.542273
sp|P21399|ACOC_HU	ACO1	Cytoplasmic aconitate hydratase OS=Homo sapiens GN=ACO1 PE=1 SV=3	98850	0	882	0	26	0	0.04	#DIV/0!	0	0	0	1	0	1	0	1	1.542273
tr|H3BMJ8|H3BMJ8_	TCF25	Transcription factor 25 (Fragment) OS=Homo sapiens GN=TCF25 PE=4 SV=1	23584	0	881	0	26	0	0.14	#DIV/0!	0	0	0	1	0	1	0	1	1.542273
tr|F8W6C2|F8W6C2_	SPATS2L	SPATS2-like protein (Fragment) OS=Homo sapiens GN=SPATS2L PE=2 SV=1	30163	0	879	0	26	0	0.05	#DIV/0!	0	0	0	1	0	1	0	1	1.542273
sp|Q9Y662|HS3SB_HU	HS3ST3B1	Heparan sulfate glucosamine 3-O-sulfotransferase 3B1 OS=Homo sapiens GN=HS3ST3B1 PE=1 SV=1	43639	0	874	0	26	0	0.08	#DIV/0!	0	0	0	1	0	1	0	1	1.542273
tr|F5H1X8|F5H1X8_H	LRBA	Lipopolysaccharide-responsive and beige-like anchor protein OS=Homo sapiens GN=LRBA PE=2 SV=1	320211	0	873	0	26	0	0.01	#DIV/0!	0	0	0	1	0	1	0	2	1.542273
tr|F6S0T5|F6S0T5_HU	KDM1A	Lysine-specific histone demethylase 1A OS=Homo sapiens GN=KDM1A PE=2 SV=1	95677	0	872	0	26	0	0.03	#DIV/0!	0	0	0	1	0	1	0	1	1.542273
tr|F5GZS0|F5GZS0_H	DHX36	Probable ATP-dependent RNA helicase DHX36 OS=Homo sapiens GN=DHX36 PE=2 SV=1	113928	0	871	0	26	0	0.03	#DIV/0!	0	0	0	1	0	1	0	1	1.542273
sp|Q6ZS92|YD022_HU	sp|Q6ZS92|Y	Putative uncharacterized protein FLJ45721 OS=Homo sapiens PE=5 SV=2	18436	0	869	0	26	0	0.18	#DIV/0!	0	0	0	1	0	1	0	1	1.542273
tr|B3KNR6|B3KNR6_H	tr|B3KNR6|B	cDNA FLJ30255 fis, clone BRACE2002444, highly similar to LAS1-like protein OS=Homo sapiens PE=2 SV=1	27615	0	866	0	26	0	0.12	#DIV/0!	0	0	0	1	0	1	0	1	1.542273
tr|H0Y9N5|H0Y9N5_H	TROVE2	60 kDa SS-A/Ro ribonucleoprotein (Fragment) OS=Homo sapiens GN=TROVE2 PE=4 SV=1	22439	0	865	0	26	0	0.15	#DIV/0!	0	0	0	1	0	1	0	1	1.542273
tr|H3BRD4|H3BRD4_	ZFP106	Protein ZFP106 (Fragment) OS=Homo sapiens GN=ZFP106 PE=4 SV=1	25159	0	864	0	27	0	0.13	#DIV/0!	0	0	0	1	0	1	0	1	1.542273
tr|H0Y9V9|H0Y9V9_H	RPL9	60S ribosomal protein L9 (Fragment) OS=Homo sapiens GN=RPL9 PE=4 SV=1	21671	0	863	0	27	0	0.34	#DIV/0!	0	0	0	1	0	1	0	1	1.542273
tr|B3KW81|B3KW81_	tr|B3KW81|B	cDNA FLJ42510 fis, clone BRACE2044473, highly similar to Homo sapiens pericentrin 1 (PCNT1), mRNA OS=Homo sapiens	30173	0	862	0	27	0	0.11	#DIV/0!	0	0	0	1	0	1	0	1	1.542273
tr|Q05DF2|Q05DF2_H	SF3A2	SF3A2 protein (Fragment) OS=Homo sapiens GN=SF3A2 PE=2 SV=1	51557	0	860	0	27	0	0.06	#DIV/0!	0	0	0	1	0	1	0	1	1.542273
sp|Q5SRE5|NU188_H	NUP188	Nucleoporin NUP188 homolog OS=Homo sapiens GN=NUP188 PE=1 SV=1	198369	0	857	0	27	0	0.02	#DIV/0!	0	0	0	1	0	1	0	1	1.542273
tr|Q9UPE4|Q9UPE4_	hTIM44	Putative mitochondrial inner membrane protein import receptor OS=Homo sapiens GN=hTIM44 PE=2 SV=1	47508	0	856	0	27	0	0.07	#DIV/0!	0	0	0	1	0	1	0	1	1.542273
sp|Q9Y3Z3|SAMH1_H	SAMHD1	SAM domain and HD domain-containing protein 1 OS=Homo sapiens GN=SAMHD1 PE=1 SV=2	72896	0	855	0	27	0	0.05	#DIV/0!	0	0	0	1	0	1	0	1	1.542273
tr|F5H4C2|F5H4C2_H	RANBP3	Ran-binding protein 3 OS=Homo sapiens GN=RANBP3 PE=2 SV=1	47206	0	854	0	27	0	0.07	#DIV/0!	0	0	0	1	0	1	0	1	1.542273
tr|Q59EZ3|Q59EZ3_H	tr|Q59EZ3|Q	Insulin-like growth factor 2 receptor variant (Fragment) OS=Homo sapiens PE=2 SV=1	272672	0	853	0	28	0	0.01	#DIV/0!	0	0	0	1	0	1	0	2	1.542273
tr|A4D1T0|A4D1T0_H	ZC3HDC1	Poly (ADP-ribose) polymerase family, member 12, isoform CRA_b OS=Homo sapiens GN=ZC3HDC1 PE=2 SV=1	80496	0	851	0	28	0	0.04	#DIV/0!	0	0	0	1	0	1	0	3	1.542273
tr|B4DHY1|B4DHY1_	HNRNPH3	Heterogeneous nuclear ribonucleoprotein H3 OS=Homo sapiens GN=HNRNPH3 PE=2 SV=1	24925	0	848	0	28	0	0.09	#DIV/0!	0	0	0	1	0	1	0	1	1.542273
tr|D6RGD1|D6RGD1_	MYO10	Unconventional myosin-X (Fragment) OS=Homo sapiens GN=MYO10 PE=2 SV=1	96861	0	846	0	28	0	0.03	#DIV/0!	0	0	0	1	0	1	0	2	1.542273
sp|Q9C0J8|WDR33_H	WDR33	pre-mRNA 3~ end processing protein WDR33 OS=Homo sapiens GN=WDR33 PE=1 SV=2	146198	0	845	0	28	0	0.02	#DIV/0!	0	0	0	1	0	1	0	1	1.542273
sp|P01598|KV106_HU	sp|P01598|K	Ig kappa chain V-I region EU OS=Homo sapiens PE=1 SV=1	11895	0	844	0	28	0	0.29	#DIV/0!	0	0	0	1	0	1	0	1	1.542273
sp|Q8IWV7|UBR1_HU	UBR1	E3 ubiquitin-protein ligase UBR1 OS=Homo sapiens GN=UBR1 PE=1 SV=1	203445	0	843	0	28	0	0.02	#DIV/0!	0	0	0	1	0	1	0	1	1.542273
sp|Q8IWL8|STH_HUM	STH	Saitohin OS=Homo sapiens GN=STH PE=1 SV=1	13986	0	842	0	28	0	0.24	#DIV/0!	0	0	0	1	0	1	0	1	1.542273
tr|B2R894|B2R894_H	MRPL38	Mitochondrial ribosomal protein L38, isoform CRA_b OS=Homo sapiens GN=MRPL38 PE=2 SV=1	41017	0	840	0	28	0	0.09	#DIV/0!	0	1	0	1	0	1	0	1	1.542273
tr|Q5IRN4|Q5IRN4_H	MEF2D/DAZA	Myocyte enhancer factor 2D/deleted in azoospermia associated protein 1 fusion protein OS=Homo sapiens GN=MEF2D/	50610	0	839	0	29	0	0.07	#DIV/0!	0	0	0	1	0	1	0	1	1.542273
tr|B4E3M6|B4E3M6_	tr|B4E3M6|E	cDNA FLJ55446, highly similar to Superkiller viralicidic activity 2-like 2 (EC 3.6.1.-) OS=Homo sapiens PE=2 SV=1	107714	0	838	0	29	0	0.03	#DIV/0!	0	0	0	1	0	1	0	1	1.542273
tr|A4D110|A4D110_H	LOC401309	Putative uncharacterized protein OS=Homo sapiens GN=LOC401309 PE=4 SV=1	13436	0	836	0	29	0	0.24	#DIV/0!	0	0	0	1	0	1	0	1	1.542273
tr|B4DK47|B4DK47_H	TBC1D7	TBC1 domain family member 7 OS=Homo sapiens GN=TBC1D7 PE=2 SV=1	21126	0	835	0	29	0	0.16	#DIV/0!	0	0	0	1	0	1	0	1	1.542273
sp|O95425|SVIL_HUM	SVIL	Supervillin OS=Homo sapiens GN=SVIL PE=1 SV=2	249417	0	833	0	29	0	0.01	#DIV/0!	0	0	0	1	0	1	0	1	1.542273
tr|E9PF58|E9PF58_HU	ARPC1A	Actin-related protein 2/3 complex subunit 1A OS=Homo sapiens GN=ARPC1A PE=2 SV=1	31268	0	831	0	29	0	0.11	#DIV/0!	0	0	0	1	0	1	0	1	1.542273
tr|J3KR44|J3KR44_HU	OTUB1	Ubiquitin thioesterase OTUB1 OS=Homo sapiens GN=OTUB1 PE=4 SV=1	35649	0	830	0	29	0	0.09	#DIV/0!	0	0	0	1	0	1	0	1	1.542273
tr|B4E206|B4E206_H	tr|B4E206|B	cDNA FLJ55441, highly similar to Nuclear pore complex protein Nup133 OS=Homo sapiens PE=2 SV=1	128349	0	829	0	29	0	0.03	#DIV/0!	0	0	0	1	0	1	0	1	1.542273
tr|J3KTD7|J3KTD7_HU	SMG8	Protein SMG8 (Fragment) OS=Homo sapiens GN=SMG8 PE=4 SV=1	30526	0	828	0	29	0	0.11	#DIV/0!	0	0	0	1	0	1	0	1	1.542273
sp|P54296|MYOM2_	MYOM2	Myomesin-2 OS=Homo sapiens GN=MYOM2 PE=1 SV=2	166332	0	827	0	29	0	0.02	#DIV/0!	0	0	0	1	0	1	0	3	1.542273
sp|O60832|DKC1_HU	DKC1	H/ACA ribonucleoprotein complex subunit 4 OS=Homo sapiens GN=DKC1 PE=1 SV=3	58094	0	826	0	29	0	0.06	#DIV/0!	0	0	0	1	0	1	0	1	1.542273
tr|H7C0M3|H7C0M3_	BPHL	Valacyclovir hydrolase (Fragment) OS=Homo sapiens GN=BPHL PE=4 SV=1	3314	0	824	0	29	0	1.2	#DIV/0!	0	1	0	1	0	1	0	1	1.542273
tr|H0YIZ8|H0YIZ8_HU	tr|H0YIZ8|H	Uncharacterized protein (Fragment) OS=Homo sapiens PE=4 SV=1	13542	0	822	0	30	0	0.25	#DIV/0!	0	0	0	1	0	1	0	1	1.542273
sp|Q53EP0|FND3B_H	FNDC3B	Fibronectin type III domain-containing protein 3B OS=Homo sapiens GN=FNDC3B PE=1 SV=2	134970	0	820	0	30	0	0.02	#DIV/0!	0	0	0	1	0	1	0	2	1.542273
tr|C0JXB8|C0JXB8_HU	AIP	Aryl hydrocarbon receptor interacting protein OS=Homo sapiens GN=AIP PE=2 SV=1	38041	0	817	0	30	0	0.09	#DIV/0!	0	0	0	1	0	1	0	1	1.542273
tr|H0YKU5|H0YKU5_	COPS2	COP9 signalosome complex subunit 2 (Fragment) OS=Homo sapiens GN=COPS2 PE=4 SV=1	31061	0	816	0	30	0	0.11	#DIV/0!	0	0	0	1	0	1	0	1	1.542273

tr|B4DRK5|B4DRK5_H	tr|B4DRK5|B	cDNA FLJ59584, highly similar to Mitochondrial-processing peptidase alpha subunit, mitochondrial (EC 3.4.24.64) OS=Ho	28622	0	814	0	30	0	0.12	#DIV/0!	0	0	0	2	0	1	0	2	1.542273
sp|Q9Y295|DRG1_HU	DRG1	Developmentally-regulated GTP-binding protein 1 OS=Homo sapiens GN=DRG1 PE=1 SV=1	40802	0	813	0	30	0	0.08	#DIV/0!	0	0	0	1	0	1	0	1	1.542273
tr|H0YKX1|H0YKX1_H	RTF1	RNA polymerase-associated protein RTF1 homolog (Fragment) OS=Homo sapiens GN=RTF1 PE=4 SV=1	20249	0	812	0	30	0	0.17	#DIV/0!	0	1	0	1	0	1	0	1	1.542273
tr|B4DRU9|B4DRU9_	tr|B4DRU9|B	cDNA FLJ57179, highly similar to Homo sapiens ATP-binding cassette, sub-family F (GCN20), member 3 (ABCF3), mRNA O	75926	0	811	0	31	0	0.04	#DIV/0!	0	0	0	1	0	1	0	1	1.542273
tr|B2RDW1|B2RDW1	RPS27A	Ribosomal protein S27a, isoform CRA_c OS=Homo sapiens GN=RPS27A PE=2 SV=1	18296	0	808	0	31	0	0.18	#DIV/0!	0	0	0	2	0	1	0	3	1.542273
tr|B3KPP7|B3KPP7_H	tr|B3KPP7|B	cDNA FLJ32033 fis, clone NTONG2000265, highly similar to Autophagy-related protein 9A OS=Homo sapiens PE=2 SV=1	95384	0	804	0	31	0	0.03	#DIV/0!	0	1	0	1	0	1	0	1	1.542273
tr|H0Y5F2|H0Y5F2_H	C1orf167	Uncharacterized protein C1orf167 (Fragment) OS=Homo sapiens GN=C1orf167 PE=4 SV=1	69441	0	802	0	31	0	0.05	#DIV/0!	0	0	0	1	0	1	0	1	1.542273
tr|B3KM47|B3KM47_	tr|B3KM47|E	cDNA FLJ10273 fis, clone HEMBB1001137, highly similar to SEC23-interacting protein OS=Homo sapiens PE=2 SV=1	111730	0	799	0	31	0	0.03	#DIV/0!	0	0	0	1	0	1	0	2	1.542273
tr|H0YAS9|H0YAS9_H	EMC2	ER membrane protein complex subunit 2 (Fragment) OS=Homo sapiens GN=EMC2 PE=4 SV=1	16089	0	797	0	31	0	0.21	#DIV/0!	0	0	0	1	0	1	0	1	1.542273
tr|D3DVR8|D3DVR8_	HD	Huntingtin (Huntington disease), isoform CRA_a OS=Homo sapiens GN=HD PE=2 SV=1	291559	0	796	0	31	0	0.01	#DIV/0!	0	0	0	1	0	1	0	1	1.542273
tr|A8MZ87|A8MZ87_	KLC2	Kinesin light chain 2 OS=Homo sapiens GN=KLC2 PE=2 SV=1	57767	0	794	0	32	0	0.12	#DIV/0!	0	0	0	2	0	1	0	2	1.542273
sp|Q9HC07|TM165_H	TMEM165	Transmembrane protein 165 OS=Homo sapiens GN=TMEM165 PE=1 SV=1	35055	0	792	0	32	0	0.43	#DIV/0!	0	0	0	1	0	1	0	1	1.542273
tr|B4DFM1|B4DFM1_	tr|B4DFM1|E	cDNA FLJ53034, highly similar to AP-2 complex subunit mu-1 OS=Homo sapiens PE=2 SV=1	44226	0	791	0	32	0	0.08	#DIV/0!	0	0	0	1	0	1	0	1	1.542273
sp|A2A3N6|PIPSL_HU	PIPSL	Putative PIP5K1A and PSMD4-like protein OS=Homo sapiens GN=PIPSL PE=5 SV=1	95729	0	789	0	0	0	0.05	#DIV/0!	0	0	0	0	0	1	0	1	1.542273
tr|B4DMA4|B4DMA4	tr|B4DMA4|	cDNA FLJ58400, highly similar to Cleavage stimulation factor 77 kDa subunit OS=Homo sapiens PE=2 SV=1	75580	0	788	0	32	0	0.04	#DIV/0!	0	0	0	1	0	1	0	1	1.542273
tr|G0XQ39|G0XQ39_	STIM1	STIM1L OS=Homo sapiens GN=STIM1 PE=2 SV=1	88933	0	787	0	32	0	0.04	#DIV/0!	0	0	0	1	0	1	0	1	1.542273
tr|H0YE29|H0YE29_H	ARHGAP1	Rho GTPase-activating protein 1 (Fragment) OS=Homo sapiens GN=ARHGAP1 PE=4 SV=1	38420	0	786	0	32	0	0.09	#DIV/0!	0	0	0	1	0	1	0	1	1.542273
tr|B4DLY2|B4DLY2_H	tr|B4DLY2|B	cDNA FLJ56600, highly similar to Large proline-rich protein BAT2 OS=Homo sapiens PE=2 SV=1	146160	0	785	0	32	0	0.02	#DIV/0!	0	1	0	1	0	1	0	1	1.542273
tr|A2RU80|A2RU80_	MPP4	MPP4 protein OS=Homo sapiens GN=MPP4 PE=2 SV=1	72737	0	782	0	32	0	0.05	#DIV/0!	0	0	0	1	0	1	0	1	1.542273
sp|Q9UHD1|CHRD1_	CHORDC1	Cysteine and histidine-rich domain-containing protein 1 OS=Homo sapiens GN=CHORDC1 PE=1 SV=2	38264	0	781	0	32	0	0.09	#DIV/0!	0	0	0	1	0	1	0	1	1.542273
tr|B3KN49|B3KN49_H	tr|B3KN49|B	cDNA FLJ13562 fis, clone PLACE1008080, highly similar to Homo sapiens hexamethylene bis-acetamide inducible 1 (HEXI	40868	0	780	0	32	0	0.08	#DIV/0!	0	0	0	1	0	1	0	1	1.542273
tr|G3V2E7|G3V2E7_H	KLC1	Kinesin light chain 1 OS=Homo sapiens GN=KLC1 PE=2 SV=1	63133	0	778	0	33	0	0.05	#DIV/0!	0	0	0	1	0	1	0	1	1.542273
tr|B4DJU4|B4DJU4_H	tr|B4DJU4|B	cDNA FLJ53344, highly similar to Splicing factor 1 OS=Homo sapiens PE=2 SV=1	67535	0	777	0	33	0	0.05	#DIV/0!	0	0	0	1	0	1	0	1	1.542273
tr|B2R9S6|B2R9S6_H	tr|B2R9S6|B	cDNA, FLJ94537, highly similar to Homo sapiens MCF.2 cell line derived transforming sequence (MCF2), mRNA OS=Homo	108533	0	775	0	33	0	0.03	#DIV/0!	0	0	0	1	0	1	0	2	1.542273
tr|B0V109|B0V109_H	FLOT1	Flotillin-1 (Fragment) OS=Homo sapiens GN=FLOT1 PE=4 SV=1	39810	0	774	0	33	0	0.08	#DIV/0!	0	0	0	1	0	1	0	3	1.542273
sp|Q9UKX7|NUP50_H	NUP50	Nuclear pore complex protein Nup50 OS=Homo sapiens GN=NUP50 PE=1 SV=2	50512	0	768	0	33	0	0.07	#DIV/0!	0	0	0	1	0	1	0	1	1.542273
sp|Q7KYR7|BT2A1_H	BTN2A1	Butyrophilin subfamily 2 member A1 OS=Homo sapiens GN=BTN2A1 PE=1 SV=3	60393	0	765	0	33	0	0.05	#DIV/0!	0	0	0	1	0	1	0	1	1.542273
tr|B0QY89|B0QY89_H	EIF3EIP	Eukaryotic translation initiation factor 3 subunit L OS=Homo sapiens GN=EIF3EIP PE=4 SV=1	71085	0	763	0	33	0	0.05	#DIV/0!	0	0	0	1	0	1	0	2	1.542273
sp|P35658|NU214_H	NUP214	Nuclear pore complex protein Nup214 OS=Homo sapiens GN=NUP214 PE=1 SV=2	214230	0	760	0	33	0	0.24	#DIV/0!	0	0	0	1	0	1	0	1	1.542273
tr|H0Y6E7|H0Y6E7_H	RBMX	RNA-binding motif protein, X chromosome, N-terminally processed (Fragment) OS=Homo sapiens GN=RBMX PE=4 SV=2	31837	0	759	0	34	0	0.08	#DIV/0!	0	0	0	1	0	1	0	1	1.542273
sp|Q9NQH7|XPP3_HU	XPNPEP3	Probable Xaa-Pro aminopeptidase 3 OS=Homo sapiens GN=XPNPEP3 PE=1 SV=1	57624	0	758	0	34	0	0.06	#DIV/0!	0	1	0	1	0	1	0	1	1.542273
tr|G5EA24|G5EA24_H	MCOLN2	Mucolipin 2, isoform CRA_a OS=Homo sapiens GN=MCOLN2 PE=2 SV=1	29581	0	757	0	34	0	0.12	#DIV/0!	0	1	0	1	0	1	0	2	1.542273
tr|F5H0D0|F5H0D0_H	EIF2B1	Translation initiation factor eIF-2B subunit alpha OS=Homo sapiens GN=EIF2B1 PE=2 SV=1	22558	0	755	0	34	0	0.15	#DIV/0!	0	0	0	1	0	1	0	1	1.542273
tr|F5H0J3|F5H0J3_HU	NDUFA9	NADH dehydrogenase [ubiquinone] 1 alpha subcomplex subunit 9, mitochondrial OS=Homo sapiens GN=NDUFA9 PE=2 S	15894	0	749	0	34	0	0.21	#DIV/0!	0	0	0	1	0	1	0	1	1.542273
sp|Q7Z5H4|VN1R5_H	VN1R5	Vomeronasal type-1 receptor 5 OS=Homo sapiens GN=VN1R5 PE=2 SV=2	41265	0	748	0	35	0	0.08	#DIV/0!	0	1	0	1	0	1	0	1	1.542273
tr|Q6FHH6|Q6FHH6_	LANCL1	LANCL1 protein (Fragment) OS=Homo sapiens GN=LANCL1 PE=2 SV=1	45979	0	747	0	35	0	0.07	#DIV/0!	0	1	0	1	0	1	0	3	1.542273
tr|Q6FGM0|Q6FGM0	SH3GL1	SH3 domain GRB2-like 1 OS=Homo sapiens GN=SH3GL1 PE=2 SV=1	41692	0	746	0	35	0	0.08	#DIV/0!	0	0	0	1	0	1	0	1	1.542273
sp|Q6DKJ4|NXN_HUN	NXN	Nucleoredoxin OS=Homo sapiens GN=NXN PE=1 SV=2	48761	0	745	0	35	0	0.07	#DIV/0!	0	0	0	1	0	1	0	1	1.542273
sp|Q6NVY1|HIBCH_H	HIBCH	3-hydroxyisobutyryl-CoA hydrolase, mitochondrial OS=Homo sapiens GN=HIBCH PE=1 SV=2	43797	0	744	0	35	0	0.08	#DIV/0!	0	0	0	1	0	1	0	1	1.542273
tr|B3KMK8|B3KMK8_	STBD1	Starch-binding domain-containing protein 1 OS=Homo sapiens GN=STBD1 PE=2 SV=1	23751	0	738	0	36	0	0.14	#DIV/0!	0	0	0	1	0	1	0	1	1.542273
tr|B4DSL6|B4DSL6_H	tr|B4DSL6|B	cDNA FLJ57190, highly similar to Actin-binding protein anillin OS=Homo sapiens PE=2 SV=1	111977	0	735	0	36	0	0.03	#DIV/0!	0	0	0	1	0	1	0	1	1.542273
tr|G5EA03|G5EA03_H	LIMCH1	LIM and calponin homology domains-containing protein 1 OS=Homo sapiens GN=LIMCH1 PE=2 SV=1	165820	0	734	0	36	0	0.03	#DIV/0!	0	0	0	1	0	1	0	1	1.542273
tr|B7Z1V5|B7Z1V5_H	tr|B7Z1V5|B	cDNA FLJ55678, highly similar to Syntaxin-binding protein 1 OS=Homo sapiens PE=2 SV=1	63779	0	731	0	36	0	0.05	#DIV/0!	0	1	0	1	0	1	0	1	1.542273
sp|Q96EN8|MOCOS_	MOCOS	Molybdenum cofactor sulfurase OS=Homo sapiens GN=MOCOS PE=1 SV=2	99084	0	722	0	37	0	0.03	#DIV/0!	0	1	0	1	0	1	0	1	1.542273
tr|Q5U043|Q5U043_	tr|Q5U043|C	Alcohol dehydrogenase 5 (Class III), chi polypeptide OS=Homo sapiens PE=2 SV=1	40520	0	721	0	37	0	0.08	#DIV/0!	0	0	0	1	0	1	0	2	1.542273
tr|B4DDH8|B4DDH8_	tr|B4DDH8|E	cDNA FLJ55184, highly similar to Homo sapiens leukocyte receptor cluster (LRC) member 4 (LENG4), mRNA OS=Homo sa	51214	0	720	0	37	0	0.06	#DIV/0!	0	0	0	1	0	1	0	2	1.542273
tr|Q1JQ76|Q1JQ76_H	RPL10A	Ribosomal protein (Fragment) OS=Homo sapiens GN=RPL10A PE=2 SV=1	23692	0	718	0	37	0	0.14	#DIV/0!	0	1	0	1	0	1	0	1	1.542273
tr|Q59H95|Q59H95_	tr|Q59H95|C	Flightless I homolog variant (Fragment) OS=Homo sapiens PE=2 SV=1	126396	0	716	0	37	0	0.03	#DIV/0!	0	0	0	1	0	1	0	1	1.542273
tr|F8W8W7|F8W8W7	TGOLN2	Trans-Golgi network integral membrane protein 2 OS=Homo sapiens GN=TGOLN2 PE=2 SV=1	29554	0	713	0	37	0	0.07	#DIV/0!	0	1	0	1	0	1	0	1	1.542273
tr|G0KXF8|G0KXF8_H	HLA-B	MHC class I antigen (Fragment) OS=Homo sapiens GN=HLA-B PE=3 SV=1	21235	0	709	0	38	0	0.16	#DIV/0!	0	0	0	1	0	1	0	1	1.542273
tr|G1ENK8|G1ENK8_	HLA-B	MHC class I antigen (Fragment) OS=Homo sapiens GN=HLA-B PE=3 SV=1	21236	0	708	0	38	0	0.16	#DIV/0!	0	0	0	1	0	1	0	1	1.542273
tr|Q58EY4|Q58EY4_H	SMARCC1	SWI/SNF related, matrix associated, actin dependent regulator of chromatin, subfamily c, member 1 OS=Homo sapiens (	123347	0	701	0	38	0	0.08	#DIV/0!	0	1	0	3	0	1	0	3	1.542273
tr|B3KUL1|B3KUL1_H	tr|B3KUL1|B	cDNA FLJ40132 fis, clone TESTI2012155, highly similar to NUCLEOPORIN-LIKE PROTEIN RIP OS=Homo sapiens PE=2 SV=1	59275	0	699	0	38	0	0.06	#DIV/0!	0	1	0	1	0	1	0	1	1.542273
tr|H0Y6I6|H0Y6I6_HU	FBXL18	F-box/LRR-repeat protein 18 (Fragment) OS=Homo sapiens GN=FBXL18 PE=4 SV=1	76506	0	698	0	38	0	0.04	#DIV/0!	0	1	0	1	0	1	0	1	1.542273
tr|Q8TBT6|Q8TBT6_H	tr|Q8TBT6|C	Putative uncharacterized protein (Fragment) OS=Homo sapiens PE=2 SV=1	34810	0	697	0	38	0	0.1	#DIV/0!	0	1	0	1	0	1	0	1	1.542273
tr|B4E0X4|B4E0X4_H	tr|B4E0X4|B	cDNA FLJ51433 OS=Homo sapiens PE=2 SV=1	10805	0	696	0	38	0	0.32	#DIV/0!	0	1	0	1	0	1	0	1	1.542273
sp|P62333|PRS10_HU	PSMC6	26S protease regulatory subunit 10B OS=Homo sapiens GN=PSMC6 PE=1 SV=1	44430	0	693	0	38	0	0.07	#DIV/0!	0	1	0	1	0	1	0	1	1.542273
tr|E9PPY3|E9PPY3_H	RRP8	Ribosomal RNA-processing protein 8 OS=Homo sapiens GN=RRP8 PE=2 SV=1	34579	0	691	0	39	0	0.1	#DIV/0!	0	1	0	1	0	1	0	1	1.542273
tr|Q5T5N3|Q5T5N3_	IDE	Insulin-degrading enzyme (Fragment) OS=Homo sapiens GN=IDE PE=4 SV=1	29418	0	688	0	39	0	0.11	#DIV/0!	0	1	0	1	0	1	0	1	1.542273
tr|B4DYZ1|B4DYZ1_H	tr|B4DYZ1|B	cDNA FLJ59786, highly similar to Probable ATP-dependent RNA helicase DDX31 (EC 3.6.1.-) OS=Homo sapiens PE=2 SV=1	81565	0	687	0	39	0	0.04	#DIV/0!	0	1	0	1	0	1	0	1	1.542273
tr|G3V198|G3V198_H	NUP160	Nuclear pore complex protein Nup160 OS=Homo sapiens GN=NUP160 PE=2 SV=1	142365	0	686	0	39	0	0.02	#DIV/0!	0	1	0	1	0	1	0	2	1.542273
tr|F5H2V5|F5H2V5_H	FADS3	Fatty acid desaturase 3 OS=Homo sapiens GN=FADS3 PE=2 SV=1	43907	0	684	0	39	0	0.08	#DIV/0!	0	1	0	1	0	1	0	1	1.542273
tr|A8K1E4|A8K1E4_H	tr|A8K1E4|A	cDNA FLJ77411, highly similar to Homo sapiens gamma-tubulin complex component GCP5 (GCP5) mRNA OS=Homo sapie	119129	0	682	0	39	0	0.03	#DIV/0!	0	1	0	1	0	1	0	1	1.542273
tr|Q59FI9|Q59FI9_HU	tr|Q59FI9|Q	Ribosomal protein L12 variant (Fragment) OS=Homo sapiens PE=2 SV=1	21755	0	680	0	40	0	0.15	#DIV/0!	0	1	0	1	0	1	0	1	1.542273
tr|Q149P0|Q149P0_H	GBF1	GBF1 protein OS=Homo sapiens GN=GBF1 PE=2 SV=1	207938	0	679	0	40	0	0.02	#DIV/0!	0	1	0	1	0	1	0	1	1.542273
tr|Q5JXH7|Q5JXH7_H	FHL1	Four and a half LIM domains 1 (Fragment) OS=Homo sapiens GN=FHL1 PE=4 SV=1	24735	0	678	0	40	0	0.14	#DIV/0!	0	1	0	1	0	1	0	1	1.542273
tr|H0YLE5|H0YLE5_H	MYO1E	Unconventional myosin-Ie (Fragment) OS=Homo sapiens GN=MYO1E PE=4 SV=1	21833	0	677	0	40	0	0.15	#DIV/0!	0	1	0	1	0	1	0	1	1.542273
sp|Q13136|LIPA1_HU	PPFIA1	Liprin-alpha-1 OS=Homo sapiens GN=PPFIA1 PE=1 SV=1	136265	0	674	0	40	0	0.02	#DIV/0!	0	1	0	1	0	1	0	2	1.542273
tr|J3KT29|J3KT29_HU	RPL23	60S ribosomal protein L23 OS=Homo sapiens GN=RPL23 PE=3 SV=1	13241	0	673	0	40	0	0.26	#DIV/0!	0	1	0	1	0	1	0	1	1.542273
tr|H0YCY6|H0YCY6_H	DAK	FAD-AMP lyase (cyclizing) (Fragment) OS=Homo sapiens GN=DAK PE=4 SV=1	55026	0	669	0	40	0	0.06	#DIV/0!	0	1	0	1	0	1	0	1	1.542273
tr|K4DI93|K4DI93_HU	CUL4B	Cullin 4B, isoform CRA_e OS=Homo sapiens GN=CUL4B PE=3 SV=1	103318	0	667	0	40	0	0.03	#DIV/0!	0	1	0	1	0	1	0	1	1.542273
tr|G3V5E1|G3V5E1_H	CCNK	Cyclin-K OS=Homo sapiens GN=CCNK PE=2 SV=1	43776	0	665	0	41	0	0.08	#DIV/0!	0	1	0	1	0	1	0	3	1.542273
sp|Q969S3|ZN622_HU	ZNF622	Zinc finger protein 622 OS=Homo sapiens GN=ZNF622 PE=1 SV=1	54808	0	664	0	41	0	0.06	#DIV/0!	0	1	0	1	0	1	0	2	1.542273
tr|D3DUJ0|D3DUJ0_H	AFG3L2	AFG3 ATPase family gene 3-like 2 (Yeast), isoform CRA_a (Fragment) OS=Homo sapiens GN=AFG3L2 PE=2 SV=1	84677	0	661	0	41	0	0.04	#DIV/0!	0	1	0	1	0	1	0	1	1.542273
tr|B4DK32|B4DK32_H	tr|B4DK32|B	cDNA FLJ61095, highly similar to Ubiquitin conjugation factor E4 A (Fragment) OS=Homo sapiens PE=2 SV=1	120816	0	660	0	41	0	0.03	#DIV/0!	0	1	0	1	0	1	0	1	1.542273
tr|H0YIQ2|H0YIQ2_H	YLPM1	YLP motif-containing protein 1 (Fragment) OS=Homo sapiens GN=YLPM1 PE=4 SV=1	180345	0	659	0	41	0	0.02	#DIV/0!	0	1	0	1	0	1	0	1	1.542273
sp|Q9GZL7|WDR12_H	WDR12	Ribosome biogenesis protein WDR12 OS=Homo sapiens GN=WDR12 PE=1 SV=2	48191	0	656	0	41	0	0.11	#DIV/0!	0	1	0	1	0	1	0	1	1.542273
tr|Q5D1D5|Q5D1D5_	tr|Q5D1D5|(	Guanylate binding protein 1 OS=Homo sapiens PE=2 SV=1	68387	0	655	0	41	0	0.05	#DIV/0!	0	1	0	1	0	1	0	1	1.542273
tr|A8K8U1|A8K8U1_H	tr|A8K8U1|A	cDNA FLJ77762, highly similar to Homo sapiens cullin-associated and neddylation-dissociated 1 (CAND1), mRNA OS=Hon	137941	0	654	0	41	0	0.07	#DIV/0!	0	1	0	1	0	1	0	1	1.542273
tr|H0Y4J2|H0Y4J2_HU	MRPL37	39S ribosomal protein L37, mitochondrial (Fragment) OS=Homo sapiens GN=MRPL37 PE=4 SV=1	29806	0	652	0	42	0	0.11	#DIV/0!	0	1	0	1	0	1	0	1	1.542273
tr|Q13380|Q13380_H	RBM	RNA binding motif (Fragment) OS=Homo sapiens GN=RBM PE=4 SV=1	4015	0	648	0	42	0	0.97	#DIV/0!	0	1	0	1	0	1	0	1	1.542273
tr|B3KPA6|B3KPA6_H	ACADVL	Acyl-Coenzyme A dehydrogenase, very long chain, isoform CRA_e OS=Homo sapiens GN=ACADVL PE=2 SV=1	62941	0	647	0	42	0	0.05	#DIV/0!	0	1	0	1	0	1	0	1	1.542273
sp|Q6P1L5|F117B_HU	FAM117B	Protein FAM117B OS=Homo sapiens GN=FAM117B PE=1 SV=2	62500	0	642	0	43	0	0.05	#DIV/0!	0	1	0	1	0	1	0	1	1.542273
tr|J3KRI4|J3KRI4_HU	DYNC1LI2	Cytoplasmic dynein 1 light intermediate chain 2 (Fragment) OS=Homo sapiens GN=DYNC1LI2 PE=4 SV=1	17767	0	641	0	43	0	0.19	#DIV/0!	0	1	0	1	0	1	0	1	1.542273
tr|B3KM42|B3KM42_	tr|B3KM42|E	cDNA FLJ10219 fis, clone HEMBA1007018, highly similar to Cytoplasmic dynein 1 light intermediate chain 1 OS=Homo sa	41226	0	637	0	43	0	0.08	#DIV/0!	0	1	0	1	0	1	0	1	1.542273
tr|Q4ZG72|Q4ZG72_	DDX18	Putative uncharacterized protein DDX18 (Fragment) OS=Homo sapiens GN=DDX18 PE=2 SV=1	61898	0	635	0	43	0	0.05	#DIV/0!	0	1	0	1	0	1	0	1	1.542273
tr|B4DKA8|B4DKA8_	USP8	Ubiquitin carboxyl-terminal hydrolase OS=Homo sapiens GN=USP8 PE=2 SV=1	115921	0	630	0	44	0	0.03	#DIV/0!	0	1	0	1	0	1	0	2	1.542273
tr|B4DH43|B4DH43_	tr|B4DH43|B	cDNA FLJ56433, highly similar to Collagen alpha-2(IV) chain OS=Homo sapiens PE=2 SV=1	83428	0	628	0	44	0	0.04	#DIV/0!	0	1	0	1	0	1	0	1	1.542273
tr|Q59FA0|Q59FA0_	tr|Q59FA0|C	DNA polymerase (Fragment) OS=Homo sapiens PE=2 SV=1	113271	0	627	0	44	0	0.03	#DIV/0!	0	1	0	1	0	1	0	1	1.542273
sp|Q8TAA9|VANG1_H	VANGL1	Vang-like protein 1 OS=Homo sapiens GN=VANGL1 PE=1 SV=1	60222	0	626	0	44	0	0.05	#DIV/0!	0	1	0	1	0	1	0	1	1.542273
sp|O96008|TOM40_H	TOMM40	Mitochondrial import receptor subunit TOM40 homolog OS=Homo sapiens GN=TOMM40 PE=1 SV=1	38211	0	623	0	45	0	0.15	#DIV/0!	0	1	0	1	0	1	0	1	1.542273
tr|Q0VAB1|Q0VAB1_	TIMM50	Translocase of inner mitochondrial membrane 50 homolog (S. cerevisiae) OS=Homo sapiens GN=TIMM50 PE=2 SV=1	50960	0	620	0	45	0	0.06	#DIV/0!	0	1	0	1	0	1	0	1	1.542273
sp|Q9UI26|IPO11_HU	IPO11	Importin-11 OS=Homo sapiens GN=IPO11 PE=1 SV=1	113489	0	618	0	45	0	0.16	#DIV/0!	0	1	0	1	0	1	0	1	1.542273
tr|Q53TH1|Q53TH1_	PSMD14	Putative uncharacterized protein PSMD14 (Fragment) OS=Homo sapiens GN=PSMD14 PE=2 SV=1	18165	0	616	0	42	0	0.2	#DIV/0!	0	1	0	1	0	1	0	1	1.542273
sp|Q9H3U1|UN45A_	UNC45A	Protein unc-45 homolog A OS=Homo sapiens GN=UNC45A PE=1 SV=1	104266	0	613	0	45	0	0.03	#DIV/0!	0	1	0	1	0	1	0	1	1.542273
sp|A1L0T0|ILVBL_HU	ILVBL	Acetolactate synthase-like protein OS=Homo sapiens GN=ILVBL PE=1 SV=2	68452	0	612	0	45	0	0.05	#DIV/0!	0	1	0	1	0	1	0	1	1.542273
sp|Q9UHN6|TMEM2_	TMEM2	Transmembrane protein 2 OS=Homo sapiens GN=TMEM2 PE=1 SV=1	155702	0	611	0	45	0	0.04	#DIV/0!	0	1	0	1	0	1	0	1	1.542273
tr|B2R7V4|B2R7V4_H	tr|B2R7V4|B	cDNA, FLJ93619, highly similar to Homo sapiens PRP4 pre-mRNA processing factor 4 homolog (yeast) (PRPF4), mRNA OS	59097	0	608	0	46	0	0.06	#DIV/0!	0	1	0	1	0	1	0	1	1.542273
tr|B4DUF1|B4DUF1_H	tr|B4DUF1|B	cDNA FLJ59760, highly similar to 1,4-alpha-glucan branching enzyme (EC 2.4.1.18) OS=Homo sapiens PE=2 SV=1	76254	0	606	0	46	0	0.06	#DIV/0!	0	1	0	1	0	1	0	1	1.542273
tr|J3KPP4|J3KPP4_HU	LUC7L3	Cisplatin resistance-associated overexpressed protein, isoform CRA_b OS=Homo sapiens GN=LUC7L3 PE=4 SV=1	58698	0	602	0	46	0	0.06	#DIV/0!	0	1	0	1	0	1	0	1	1.542273
tr|B4DL04|B4DL04_H	tr|B4DL04|B	cDNA FLJ51229, highly similar to Ephrin type-A receptor 2 (EC 2.7.10.1) OS=Homo sapiens PE=2 SV=1	73481	0	597	0	47	0	0.04	#DIV/0!	0	1	0	1	0	1	0	1	1.542273
tr|H7C3T4|H7C3T4_H	PRDX4	Peroxiredoxin-4 (Fragment) OS=Homo sapiens GN=PRDX4 PE=4 SV=1	18485	0	594	0	48	0	0.18	#DIV/0!	0	1	0	1	0	1	0	1	1.542273
tr|B4DL39|B4DL39_H	tr|B4DL39|B	cDNA FLJ57316, highly similar to DNA mismatch repair protein Msh2 OS=Homo sapiens PE=2 SV=1	77368	0	593	0	48	0	0.04	#DIV/0!	0	1	0	1	0	1	0	1	1.542273
tr|H3BQT9|H3BQT9_	DHX38	Pre-mRNA-splicing factor ATP-dependent RNA helicase PRP16 (Fragment) OS=Homo sapiens GN=DHX38 PE=4 SV=1	15733	0	592	0	48	0	0.22	#DIV/0!	0	1	0	1	0	1	0	1	1.542273
tr|F5GWY5|F5GWY5_	PODXL	Podocalyxin OS=Homo sapiens GN=PODXL PE=2 SV=1	59285	0	589	0	49	0	0.06	#DIV/0!	0	1	0	1	0	1	0	1	1.542273
tr|A8MUD9|A8MUD9	RPL7	60S ribosomal protein L7 OS=Homo sapiens GN=RPL7 PE=3 SV=1	24474	0	588	0	49	0	0.29	#DIV/0!	0	1	0	2	0	1	0	4	1.542273
sp|P49419|AL7A1_HU	ALDH7A1	Alpha-aminoadipic semialdehyde dehydrogenase OS=Homo sapiens GN=ALDH7A1 PE=1 SV=5	59020	0	587	0	49	0	0.13	#DIV/0!	0	1	0	1	0	1	0	1	1.542273
tr|H7C4P4|H7C4P4_H	U2SURP	U2 snRNP-associated SURP motif-containing protein (Fragment) OS=Homo sapiens GN=U2SURP PE=4 SV=1	10874	0	583	0	50	0	0.32	#DIV/0!	0	1	0	1	0	1	0	1	1.542273
tr|B2R6E2|B2R6E2_H	tr|B2R6E2|B	cDNA, FLJ92910, highly similar to Homo sapiens programmed cell death 4 (neoplastic transformation inhibitor) (PDCD4),	51998	0	580	0	50	0	0.06	#DIV/0!	0	1	0	1	0	1	0	1	1.542273
tr|B4DF50|B4DF50_H	tr|B4DF50|B	cDNA FLJ60458, highly similar to SWI/SNF-related matrix-associated actin-dependent regulator of chromatin subfamily D	28346	0	578	0	51	0	0.12	#DIV/0!	0	1	0	1	0	1	0	1	1.542273
sp|Q8IXI1|MIRO2_HU	RHOT2	Mitochondrial Rho GTPase 2 OS=Homo sapiens GN=RHOT2 PE=1 SV=2	69101	0	575	0	51	0	0.14	#DIV/0!	0	1	0	1	0	1	0	1	1.542273
tr|B3KM89|B3KM89_	tr|B3KM89|E	cDNA FLJ10528 fis, clone NT2RP2000943, highly similar to Protein transport protein Sec24D OS=Homo sapiens PE=2 SV=	101723	0	574	0	51	0	0.03	#DIV/0!	0	1	0	1	0	1	0	2	1.542273
tr|B2C8Z3|B2C8Z3_H	TGFB1	Transforming growth factor beta 1 (Fragment) OS=Homo sapiens GN=TGFB1 PE=2 SV=1	12965	0	573	0	51	0	0.26	#DIV/0!	0	1	0	1	0	1	0	1	1.542273
tr|Q53G71|Q53G71_	tr|Q53G71|C	Calreticulin variant (Fragment) OS=Homo sapiens PE=2 SV=1	47061	0	570	0	51	0	0.14	#DIV/0!	0	1	0	1	0	1	0	1	1.542273
tr|B2RB52|B2RB52_H	tr|B2RB52|B	cDNA, FLJ95314, highly similar to Homo sapiens transducin (beta)-like 2 (TBL2), transcript variant 1, mRNA OS=Homo sap	50423	0	566	0	52	0	0.07	#DIV/0!	0	1	0	1	0	1	0	3	1.542273
sp|Q9NVH2|INT7_HU	INTS7	Integrator complex subunit 7 OS=Homo sapiens GN=INTS7 PE=1 SV=1	108021	0	560	0	53	0	0.03	#DIV/0!	0	1	0	1	0	1	0	1	1.542273
tr|F6SBX2|F6SBX2_HU	IARS2	Isoleucine--tRNA ligase, mitochondrial OS=Homo sapiens GN=IARS2 PE=2 SV=1	106884	0	558	0	53	0	0.03	#DIV/0!	0	1	0	1	0	1	0	1	1.542273
tr|Q3LIF4|Q3LIF4_HU	Nbla00271	NOGO-A (Fragment) OS=Homo sapiens GN=Nbla00271 PE=2 SV=1	57162	0	556	0	53	0	0.06	#DIV/0!	0	1	0	1	0	1	0	1	1.542273
sp|P11047|LAMC1_H	LAMC1	Laminin subunit gamma-1 OS=Homo sapiens GN=LAMC1 PE=1 SV=3	183191	0	555	0	53	0	0.02	#DIV/0!	0	1	0	1	0	1	0	3	1.542273
tr|A2A2L5|A2A2L5_H	C20orf43	Chromosome 20 open reading frame 43 (Fragment) OS=Homo sapiens GN=C20orf43 PE=4 SV=1	31759	0	550	0	54	0	0.1	#DIV/0!	0	1	0	1	0	1	0	1	1.542273
sp|Q96I24|FUBP3_HU	FUBP3	Far upstream element-binding protein 3 OS=Homo sapiens GN=FUBP3 PE=1 SV=2	61944	0	549	0	54	0	0.11	#DIV/0!	0	1	0	2	0	1	0	3	1.542273
tr|B7Z4U7|B7Z4U7_H	SCFD1	Sec1 family domain containing 1, isoform CRA_b OS=Homo sapiens GN=SCFD1 PE=2 SV=1	65362	0	548	0	54	0	0.05	#DIV/0!	0	1	0	1	0	1	0	1	1.542273
tr|B4DTS5|B4DTS5_H	tr|B4DTS5|B	cDNA FLJ58882, highly similar to 26S proteasome non-ATPase regulatory subunit 11 OS=Homo sapiens PE=2 SV=1	35374	0	547	0	35	0	0.14	#DIV/0!	0	0	0	1	0	1	0	1	1.542273
tr|H3BRG4|H3BRG4_	UQCRC2	Cytochrome b-c1 complex subunit 2, mitochondrial OS=Homo sapiens GN=UQCRC2 PE=3 SV=1	44778	0	545	0	54	0	0.15	#DIV/0!	0	1	0	2	0	1	0	2	1.542273
sp|Q969N2|PIGT_HU	PIGT	GPI transamidase component PIG-T OS=Homo sapiens GN=PIGT PE=1 SV=1	66228	0	544	0	55	0	0.07	#DIV/0!	0	1	0	1	0	1	0	2	1.542273
tr|B4E3L4|B4E3L4_HU	tr|B4E3L4|B	cDNA FLJ57039, highly similar to CREB-binding protein (EC 2.3.1.48) (Fragment) OS=Homo sapiens PE=2 SV=1	133738	0	540	0	56	0	0.05	#DIV/0!	0	1	0	2	0	1	0	2	1.542273
tr|B3KXA5|B3KXA5_H	tr|B3KXA5|B	cDNA FLJ45045 fis, clone BRAWH3021580, highly similar to Restin (Cytoplasmic linker protein 170 alpha-2) OS=Homo sa	54802	0	539	0	56	0	0.06	#DIV/0!	0	1	0	1	0	1	0	3	1.542273
sp|Q14677|EPN4_HU	CLINT1	Clathrin interactor 1 OS=Homo sapiens GN=CLINT1 PE=1 SV=1	68273	0	538	0	56	0	0.05	#DIV/0!	0	1	0	1	0	1	0	1	1.542273
tr|Q59GM9|Q59GM9	tr|Q59GM9|	Phosphorylase (Fragment) OS=Homo sapiens PE=2 SV=1	99508	0	537	0	56	0	0.03	#DIV/0!	0	1	0	1	0	1	0	1	1.542273
tr|B7Z2B4|B7Z2B4_H	RABGAP1	Rab GTPase-activating protein 1 OS=Homo sapiens GN=RABGAP1 PE=2 SV=1	48660	0	535	0	56	0	0.07	#DIV/0!	0	1	0	1	0	1	0	1	1.542273
tr|Q6IPI1|Q6IPI1_HU	RPL29	Ribosomal protein L29 OS=Homo sapiens GN=RPL29 PE=2 SV=1	17997	0	532	0	56	0	0.19	#DIV/0!	0	1	0	1	0	1	0	2	1.542273
sp|Q71RC2|LARP4_H	LARP4	La-related protein 4 OS=Homo sapiens GN=LARP4 PE=1 SV=3	81230	0	531	0	56	0	0.04	#DIV/0!	0	1	0	1	0	1	0	1	1.542273
tr|H0Y4T7|H0Y4T7_H	MTA1	Metastasis-associated protein MTA1 (Fragment) OS=Homo sapiens GN=MTA1 PE=4 SV=1	57747	0	529	0	57	0	0.06	#DIV/0!	0	1	0	1	0	1	0	1	1.542273
tr|G5EA30|G5EA30_H	CELF1	CUG triplet repeat, RNA binding protein 1, isoform CRA_c OS=Homo sapiens GN=CELF1 PE=2 SV=1	55564	0	528	0	57	0	0.06	#DIV/0!	0	1	0	1	0	1	0	2	1.542273
tr|E7EMC7|E7EMC7_	SQSTM1	Sequestosome-1 OS=Homo sapiens GN=SQSTM1 PE=2 SV=1	42070	0	524	0	57	0	0.1	#DIV/0!	0	1	0	1	0	1	0	2	1.542273
tr|H3BLU7|H3BLU7_	AKR7A2	Aflatoxin B1 aldehyde reductase member 2 (Fragment) OS=Homo sapiens GN=AKR7A2 PE=4 SV=1	35004	0	522	0	58	0	0.09	#DIV/0!	0	1	0	1	0	1	0	1	1.542273
tr|B4DM84|B4DM84_	tr|B4DM84|	cDNA FLJ60694, highly similar to Deubiquitinating protein VCIP135 (EC 3.4.22.-) OS=Homo sapiens PE=2 SV=1	87230	0	521	0	58	0	0.04	#DIV/0!	0	1	0	1	0	1	0	1	1.542273
tr|F8VS10|F8VS10_H	SPATS2	Spermatogenesis-associated serine-rich protein 2 OS=Homo sapiens GN=SPATS2 PE=2 SV=1	32626	0	519	0	58	0	0.1	#DIV/0!	0	1	0	1	0	1	0	1	1.542273
tr|B3KNE7|B3KNE7_H	tr|B3KNE7|B	cDNA FLJ14468 fis, clone MAMMA1000734, highly similar to Translocation protein SEC63 homolog OS=Homo sapiens PE	48242	0	516	0	58	0	0.07	#DIV/0!	0	1	0	1	0	1	0	1	1.542273
tr|Q2NL95|Q2NL95_H	DDX19B	DDX19B protein (Fragment) OS=Homo sapiens GN=DDX19B PE=2 SV=1	51068	0	515	0	59	0	0.06	#DIV/0!	0	1	0	1	0	1	0	1	1.542273
tr|B3KTN6|B3KTN6_H	tr|B3KTN6|B	cDNA FLJ38544 fis, clone HCHON2001598, highly similar to Integrin alpha-11 OS=Homo sapiens PE=2 SV=1	49190	0	514	0	59	0	0.07	#DIV/0!	0	1	0	1	0	1	0	1	1.542273
tr|B2RBM7|B2RBM7_	tr|B2RBM7|E	cDNA, FLJ95595, highly similar to Homo sapiens proteasome (prosome, macropain) 26S subunit, non-ATPase, 13 (PSMD1	43131	0	512	0	59	0	0.28	#DIV/0!	0	1	0	2	0	1	0	2	1.542273
tr|Q14730|Q14730_H	tr|Q14730|C	La 4.1 protein (Fragment) OS=Homo sapiens PE=2 SV=1	33852	0	511	0	59	0	0.1	#DIV/0!	0	1	0	1	0	1	0	1	1.542273
sp|Q96CS3|FAF2_HU	FAF2	FAS-associated factor 2 OS=Homo sapiens GN=FAF2 PE=1 SV=2	52933	0	509	0	60	0	0.13	#DIV/0!	0	1	0	1	0	1	0	1	1.542273
tr|B4DMR3|B4DMR3	tr|B4DMR3|	cDNA FLJ51896, highly similar to Glia-derived nexin OS=Homo sapiens PE=2 SV=1	37305	0	508	0	60	0	0.09	#DIV/0!	0	1	0	1	0	1	0	1	1.542273
tr|B4DKU3|B4DKU3_	tr|B4DKU3|E	cDNA FLJ53449, highly similar to rRNA methyltransferase 3 (EC 2.1.1.-) OS=Homo sapiens PE=2 SV=1	82984	0	507	0	60	0	0.04	#DIV/0!	0	1	0	1	0	1	0	1	1.542273
tr|B4DQ15|B4DQ15_	tr|B4DQ15|E	cDNA FLJ51525, highly similar to Homo sapiens aldehyde dehydrogenase 16 family, member A1 (ALDH16A1), mRNA OS=	67899	0	501	0	61	0	0.05	#DIV/0!	0	1	0	1	0	1	0	1	1.542273
tr|Q6IBS5|Q6IBS5_HU	DLST	DLST protein OS=Homo sapiens GN=DLST PE=2 SV=1	49131	0	499	0	61	0	0.4	#DIV/0!	0	1	0	1	0	1	0	1	1.542273
tr|B4DQ08|B4DQ08_	tr|B4DQ08|E	cDNA FLJ53406, highly similar to U3 small nucleolar RNA-associated protein 14 homolog A OS=Homo sapiens PE=2 SV=1	82068	0	498	0	61	0	0.04	#DIV/0!	0	1	0	1	0	1	0	2	1.542273
tr|G3V1B3|G3V1B3_H	RPL21	60S ribosomal protein L21 OS=Homo sapiens GN=RPL21 PE=2 SV=1	9994	0	495	0	61	0	0.35	#DIV/0!	0	1	0	1	0	1	0	1	1.542273
tr|H0Y3P2|H0Y3P2_H	EIF4G2	Eukaryotic translation initiation factor 4 gamma 2 OS=Homo sapiens GN=EIF4G2 PE=4 SV=1	98569	0	485	0	63	0	0.03	#DIV/0!	0	1	0	1	0	1	0	2	1.542273
tr|B4DK79|B4DK79_H	tr|B4DK79|B	cDNA FLJ58742, highly similar to Leucine-rich repeat-containing protein 41 OS=Homo sapiens PE=2 SV=1	74302	0	479	0	64	0	0.04	#DIV/0!	0	1	0	1	0	1	0	1	1.542273
tr|B4DFK7|B4DFK7_H	CBR1	Carbonyl reductase 1, isoform CRA_c OS=Homo sapiens GN=CBR1 PE=2 SV=1	19092	0	478	0	64	0	0.18	#DIV/0!	0	1	0	1	0	1	0	1	1.542273
tr|K7EIV0|K7EIV0_HU	CAPNS1	Calpain small subunit 1 OS=Homo sapiens GN=CAPNS1 PE=4 SV=1	21970	0	473	0	65	0	0.15	#DIV/0!	0	1	0	1	0	1	0	1	1.542273
tr|E9PDU6|E9PDU6_	CNN3	Calponin-3 (Fragment) OS=Homo sapiens GN=CNN3 PE=2 SV=1	20309	0	471	0	54	0	0.22	#DIV/0!	0	1	0	1	0	1	0	1	1.542273
sp|Q14669|TRIPC_HU	TRIP12	E3 ubiquitin-protein ligase TRIP12 OS=Homo sapiens GN=TRIP12 PE=1 SV=1	222234	0	468	0	66	0	0.01	#DIV/0!	0	1	0	1	0	1	0	1	1.542273
tr|Q53G42|Q53G42_	tr|Q53G42|C	mRNA decapping enzyme variant (Fragment) OS=Homo sapiens PE=2 SV=1	38757	0	465	0	66	0	0.09	#DIV/0!	0	1	0	1	0	1	0	1	1.542273
sp|Q9UJA5|TRM6_HU	TRMT6	tRNA (adenine(58)-N(1))-methyltransferase non-catalytic subunit TRM6 OS=Homo sapiens GN=TRMT6 PE=1 SV=1	56049	0	464	0	66	0	0.06	#DIV/0!	0	1	0	1	0	1	0	1	1.542273
tr|B4DJS7|B4DJS7_HU	SNX6	Sorting nexin 6, isoform CRA_e OS=Homo sapiens GN=SNX6 PE=2 SV=1	34196	0	457	0	67	0	0.1	#DIV/0!	0	1	0	1	0	1	0	1	1.542273
sp|Q49AR2|CE022_H	C5orf22	UPF0489 protein C5orf22 OS=Homo sapiens GN=C5orf22 PE=1 SV=2	50506	0	453	0	68	0	0.07	#DIV/0!	0	1	0	1	0	1	0	1	1.542273
tr|Q6AWB1|Q6AWB1	DKFZp686E07	Dynactin subunit 1 OS=Homo sapiens GN=DKFZp686E0752 PE=2 SV=1	99198	0	450	0	68	0	0.18	#DIV/0!	0	1	0	1	0	1	0	1	1.542273
tr|B5ME97|B5ME97_	Sep-10	Septin 10, isoform CRA_c OS=Homo sapiens GN=SEPT10 PE=3 SV=2	63703	0	449	0	68	0	0.05	#DIV/0!	0	1	0	1	0	1	0	1	1.542273
sp|Q9NTI5|PDS5B_HU	PDS5B	Sister chromatid cohesion protein PDS5 homolog B OS=Homo sapiens GN=PDS5B PE=1 SV=1	165818	0	446	0	69	0	0.1	#DIV/0!	0	1	0	1	0	1	0	2	1.542273
tr|Q6NWZ1|Q6NWZ1	CKAP4	CKAP4 protein (Fragment) OS=Homo sapiens GN=CKAP4 PE=2 SV=1	67948	0	443	0	69	0	0.05	#DIV/0!	0	1	0	1	0	1	0	1	1.542273
sp|A0AVT1|UBA6_HU	UBA6	Ubiquitin-like modifier-activating enzyme 6 OS=Homo sapiens GN=UBA6 PE=1 SV=1	119207	0	437	0	71	0	0.03	#DIV/0!	0	1	0	1	0	1	0	3	1.542273

sp|O43815|STRN_HU	STRN	Striatin OS=Homo sapiens GN=STRN PE=1 SV=4	86535	0	435	0	71	0	0.04	#DIV/0!	0	1	0	1	0	1	0	2	1.542273
tr|B0AZS5|B0AZS5_H	tr|B0AZS5|B	cDNA, FLJ79515, highly similar to Kinesin-like protein KIF2 OS=Homo sapiens PE=2 SV=1	75666	0	434	0	71	0	0.04	#DIV/0!	0	1	0	1	0	1	0	1	1.542273
tr|J3KMX1|J3KMX1_H	NUP88	Nuclear pore complex protein Nup88 (Fragment) OS=Homo sapiens GN=NUP88 PE=4 SV=1	80029	0	433	0	71	0	0.04	#DIV/0!	0	1	0	1	0	1	0	1	1.542273
tr|H3BTZ5|H3BTZ5_H	CNN2	Calponin-2 (Fragment) OS=Homo sapiens GN=CNN2 PE=4 SV=1	21173	0	429	0	31	0	0.33	#DIV/0!	0	0	0	1	0	1	0	1	1.542273
sp|Q92896|GSLG1_H	GLG1	Golgi apparatus protein 1 OS=Homo sapiens GN=GLG1 PE=1 SV=2	138341	0	422	0	74	0	0.02	#DIV/0!	0	1	0	1	0	1	0	2	1.542273
tr|A4D1M6|A4D1M6_	LOC392793	HCG19809 OS=Homo sapiens GN=LOC392793 PE=3 SV=1	25046	0	420	0	74	0	0.13	#DIV/0!	0	1	0	1	0	1	0	1	1.542273
sp|Q96GS4|CQ059_H	C17orf59	Uncharacterized protein C17orf59 OS=Homo sapiens GN=C17orf59 PE=1 SV=2	37603	0	419	0	74	0	0.09	#DIV/0!	0	1	0	1	0	1	0	1	1.542273
tr|D3DU41|D3DU41_	LUC7L	LUC7-like (S. cerevisiae), isoform CRA_a OS=Homo sapiens GN=LUC7L PE=2 SV=1	34110	0	418	0	75	0	0.09	#DIV/0!	0	1	0	1	0	1	0	1	1.542273
tr|Q16083|Q16083_H	tr|Q16083|C	U1 small nuclear ribonucleoprotein 1SNRP homolog protein (Fragment) OS=Homo sapiens PE=2 SV=1	11203	0	407	0	47	0	0.07	#DIV/0!	0	1	0	1	0	1	0	1	1.542273
tr|B3KRB2|B3KRB2_H	tr|B3KRB2|B	cDNA FLJ33964 fis, clone CTONG2019029, highly similar to Pseudouridylate synthase 7 homolog (EC 5.4.99.-) OS=Homo	75303	0	401	0	77	0	0.04	#DIV/0!	0	1	0	1	0	1	0	1	1.542273
sp|O43837|IDH3B_HU	IDH3B	Isocitrate dehydrogenase [NAD] subunit beta, mitochondrial OS=Homo sapiens GN=IDH3B PE=1 SV=2	42442	0	398	0	78	0	0.08	#DIV/0!	0	1	0	1	0	1	0	1	1.542273
sp|O15397|IPO8_HU	IPO8	Importin-8 OS=Homo sapiens GN=IPO8 PE=1 SV=2	120945	0	390	0	80	0	0.03	#DIV/0!	0	1	0	1	0	1	0	4	1.542273
tr|D3DTY9|D3DTY9_H	TRIM25	Tripartite motif-containing 25, isoform CRA_a OS=Homo sapiens GN=TRIM25 PE=2 SV=1	48358	0	385	0	80	0	0.07	#DIV/0!	0	1	0	1	0	1	0	1	1.542273
tr|B4E1L0|B4E1L0_H	tr|B4E1L0|B	Adenylosuccinate synthetase OS=Homo sapiens PE=2 SV=1	48308	0	383	0	81	0	0.07	#DIV/0!	0	1	0	1	0	1	0	1	1.542273
tr|B4DDT3|B4DDT3_H	tr|B4DDT3|B	cDNA FLJ54622, highly similar to Prothrombin (EC 3.4.21.5) OS=Homo sapiens PE=2 SV=1	54084	0	380	0	81	0	0.06	#DIV/0!	0	1	0	1	0	1	0	1	1.542273
tr|B2R5S4|B2R5S4_H	tr|B2R5S4|B	cDNA, FLJ92598, highly similar to Homo sapiens polymerase (DNA directed), delta 2, regulatory subunit 50kDa (POLD2),	51914	0	370	0	84	0	0.06	#DIV/0!	0	1	0	1	0	1	0	1	1.542273
tr|I1E4Y6|I1E4Y6_HU	GIGYF2	PERQ amino acid-rich with GYF domain-containing protein 2 OS=Homo sapiens GN=GIGYF2 PE=4 SV=1	152891	0	366	0	85	0	0.02	#DIV/0!	0	1	0	1	0	1	0	2	1.542273
tr|Q6NVW7|Q6NVW7	KPNA2	Importin subunit alpha OS=Homo sapiens GN=KPNA2 PE=2 SV=1	58271	0	355	0	86	0	0.18	#DIV/0!	0	1	0	3	0	1	0	3	1.542273
tr|C9JDR0|C9JDR0_H	NSDHL	Sterol-4-alpha-carboxylate 3-dehydrogenase, decarboxylating (Fragment) OS=Homo sapiens GN=NSDHL PE=2 SV=1	28356	0	347	0	90	0	0.12	#DIV/0!	0	1	0	1	0	1	0	1	1.542273
sp|Q9H3P7|GCP60_H	ACBD3	Golgi resident protein GCP60 OS=Homo sapiens GN=ACBD3 PE=1 SV=4	60841	0	345	0	90	0	0.05	#DIV/0!	0	1	0	1	0	1	0	1	1.542273
tr|J3KS65|J3KS65_HU	KPNA2	Importin subunit alpha-2 (Fragment) OS=Homo sapiens GN=KPNA2 PE=4 SV=1	16311	0	341	0	91	0	0.21	#DIV/0!	0	1	0	1	0	1	0	1	1.542273
tr|B3KMQ6|B3KMQ6	tr|B3KMQ6|	cDNA FLJ12172 fis, clone MAMMA1000684, highly similar to Opioid growth factor receptor OS=Homo sapiens PE=2 SV=1	73894	0	332	0	93	0	0.04	#DIV/0!	0	1	0	1	0	1	0	1	1.542273
tr|B3KVT9|B3KVT9_H	tr|B3KVT9|B	cDNA FLJ41424 fis, clone BRHIP2003748, highly similar to Tumor suppressor p53-binding protein 1 OS=Homo sapiens PE	43438	0	320	0	96	0	0.08	#DIV/0!	0	1	0	1	0	1	0	1	1.542273
tr|B3KRR1|B3KRR1_H	tr|B3KRR1|B	cDNA FLJ34725 fis, clone MESAN2005958, highly similar to RNA-binding protein Luc7-like 2 OS=Homo sapiens PE=2 SV=1	40752	0	319	0	76	0	0.12	#DIV/0!	0	1	0	1	0	1	0	1	1.542273
tr|B5BU61|B5BU61_H	HDAC1	Histone deacetylase OS=Homo sapiens GN=HDAC1 PE=2 SV=1	55611	0	317	0	38	0	0.21	#DIV/0!	0	1	0	1	0	1	0	2	1.542273
tr|B4DSM4|B4DSM4_	tr|B4DSM4|E	cDNA FLJ57495, highly similar to MKI67 FHA domain-interacting nucleolar phosphoprotein OS=Homo sapiens PE=2 SV=1	24894	0	305	0	100	0	0.13	#DIV/0!	0	1	0	1	0	1	0	1	1.542273
sp|Q6P6C2|ALKB5_HU	ALKBH5	RNA demethylase ALKBH5 OS=Homo sapiens GN=ALKBH5 PE=1 SV=2	44571	0	295	0	103	0	0.07	#DIV/0!	0	1	0	1	0	1	0	1	1.542273
tr|B4DV73|B4DV73_H	tr|B4DV73|B	cDNA FLJ51330, highly similar to Far upstream element-binding protein 2 OS=Homo sapiens PE=2 SV=1	38334	0	264	0	111	0	0.09	#DIV/0!	0	1	0	1	0	1	0	2	1.542273
tr|J3KMX2|J3KMX2_H	SMARCD2	SWI/SNF-related matrix-associated actin-dependent regulator of chromatin subfamily D member 2 OS=Homo sapiens GN	52433	0	237	0	120	0	0.06	#DIV/0!	0	1	0	1	0	1	0	1	1.542273
tr|B2RDD7|B2RDD7_	tr|B2RDD7|E	cDNA, FLJ96564, highly similar to Homo sapiens SKB1 homolog (S. pombe) (SKB1), mRNA OS=Homo sapiens PE=2 SV=1	73348	0	225	0	123	0	0.1	#DIV/0!	0	1	0	1	0	1	0	1	1.542273
tr|B4DWI8|B4DWI8_	tr|B4DWI8|E	cDNA FLJ57805, highly similar to Homo sapiens paraspeckle component 1 (PSPC1), transcript variant alpha, mRNA OS=H	52660	0	1030	0	16	0	0.06	#DIV/0!	0	0	0	1	0	0	0	1	-0.04302
sp|Q05D60|CCD67_H	CCDC67	Coiled-coil domain-containing protein 67 OS=Homo sapiens GN=CCDC67 PE=2 SV=2	71503	0	1028	0	16	0	0.05	#DIV/0!	0	0	0	1	0	0	0	2	-0.04302
tr|B0QZ18|B0QZ18_H	CPNE1	Copine I OS=Homo sapiens GN=CPNE1 PE=2 SV=1	60307	0	1027	0	16	0	0.05	#DIV/0!	0	0	0	1	0	0	0	1	-0.04302
tr|I3L0M9|I3L0M9_H	TCEB2	Transcription elongation factor B polypeptide 2 (Fragment) OS=Homo sapiens GN=TCEB2 PE=4 SV=1	15763	0	1026	0	16	0	0.22	#DIV/0!	0	0	0	1	0	0	0	1	-0.04302
tr|D3DU01|D3DU01_	TMEM49	Transmembrane protein 49, isoform CRA_b OS=Homo sapiens GN=TMEM49 PE=2 SV=1	47923	0	1025	0	16	0	0.07	#DIV/0!	0	0	0	1	0	0	0	1	-0.04302
tr|F1LJ00|F1LJ00_HU	TBC1D18	TBC1 domain family, member 18 OS=Homo sapiens GN=TBC1D18 PE=2 SV=1	93366	0	1024	0	16	0	0.04	#DIV/0!	0	0	0	1	0	0	0	1	-0.04302
tr|B3KMB7|B3KMB7_	PYCR2	Pyrroline-5-carboxylate reductase family, member 2, isoform CRA_a OS=Homo sapiens GN=PYCR2 PE=2 SV=1	22559	0	1023	0	16	0	0.15	#DIV/0!	0	0	0	1	0	0	0	1	-0.04302
sp|Q5JTW2|CEP78_H	CEP78	Centrosomal protein of 78 kDa OS=Homo sapiens GN=CEP78 PE=1 SV=1	77204	0	1022	0	16	0	0.04	#DIV/0!	0	0	0	1	0	0	0	1	-0.04302
tr|B4DNC6|B4DNC6_	tr|B4DNC6|E	cDNA FLJ58850, highly similar to UBA/UBX 33.3 kDa protein OS=Homo sapiens PE=2 SV=1	20726	0	1020	0	17	0	0.16	#DIV/0!	0	0	0	1	0	0	0	1	-0.04302
sp|Q8WU66|TSEAR_H	TSPEAR	Thrombospondin-type laminin G domain and EAR repeat-containing protein OS=Homo sapiens GN=TSPEAR PE=1 SV=2	75390	0	1019	0	17	0	0.04	#DIV/0!	0	0	0	1	0	0	0	1	-0.04302
tr|H0YJ86|H0YJ86_HU	MBIP	MAP3K12-binding inhibitory protein 1 (Fragment) OS=Homo sapiens GN=MBIP PE=4 SV=1	34487	0	1016	0	17	0	0.1	#DIV/0!	0	0	0	1	0	0	0	1	-0.04302
tr|E9PC74|E9PC74_H	EIF2B5	Translation initiation factor eIF-2B subunit epsilon OS=Homo sapiens GN=EIF2B5 PE=2 SV=1	79096	0	1015	0	17	0	0.04	#DIV/0!	0	0	0	1	0	0	0	1	-0.04302
tr|B2R9P8|B2R9P8_H	tr|B2R9P8|B	cDNA, FLJ94500 OS=Homo sapiens PE=2 SV=1	52717	0	1012	0	17	0	0.06	#DIV/0!	0	0	0	1	0	0	0	1	-0.04302
sp|Q5HYK7|SH319_H	SH3D19	SH3 domain-containing protein 19 OS=Homo sapiens GN=SH3D19 PE=1 SV=2	87042	0	1010	0	18	0	0.04	#DIV/0!	0	1	0	1	0	0	0	1	-0.04302
tr|D6R9N1|D6R9N1_	COX7B2	Cytochrome c oxidase subunit 7B2, mitochondrial (Fragment) OS=Homo sapiens GN=COX7B2 PE=2 SV=1	3615	0	1009	0	18	0	1.03	#DIV/0!	0	0	0	1	0	0	0	1	-0.04302
tr|J3QRQ0|J3QRQ0_H	C18orf63	Uncharacterized protein C18orf63 OS=Homo sapiens GN=C18orf63 PE=4 SV=1	73394	0	1007	0	18	0	0.04	#DIV/0!	0	0	0	1	0	0	0	1	-0.04302
tr|B3KS98|B3KS98_H	EIF3S3	Eukaryotic translation initiation factor 3 subunit H OS=Homo sapiens GN=EIF3S3 PE=2 SV=1	41726	0	1005	0	18	0	0.08	#DIV/0!	0	0	0	1	0	0	0	2	-0.04302
tr|B3KSX3|B3KSX3_H	tr|B3KSX3|B	cDNA FLJ37224 fis, clone BRAMY2000052, moderately similar to RNA-binding protein Raly OS=Homo sapiens PE=2 SV=1	32441	0	1004	0	18	0	0.1	#DIV/0!	0	0	0	1	0	0	0	1	-0.04302
tr|B7Z7J0|B7Z7J0_HU	tr|B7Z7J0|B7	cDNA FLJ52635 OS=Homo sapiens PE=2 SV=1	14828	0	1003	0	18	0	0.23	#DIV/0!	0	0	0	1	0	0	0	1	-0.04302
sp|Q9P266|JCAD_HU	KIAA1462	Junctional protein associated with coronary artery disease OS=Homo sapiens GN=KIAA1462 PE=2 SV=3	149343	0	1002	0	18	0	0.02	#DIV/0!	0	0	0	1	0	0	0	1	-0.04302
tr|B8ZZS4|B8ZZS4_HU	ANKZF1	Ankyrin repeat and zinc finger domain-containing protein 1 OS=Homo sapiens GN=ANKZF1 PE=2 SV=1	57937	0	1001	0	18	0	0.06	#DIV/0!	0	0	0	1	0	0	0	1	-0.04302
tr|Q5T446|Q5T446_H	UROD	Uroporphyrinogen decarboxylase (Fragment) OS=Homo sapiens GN=UROD PE=4 SV=1	21673	0	1000	0	19	0	0.16	#DIV/0!	0	0	0	1	0	0	0	2	-0.04302
tr|B3KMR5|B3KMR5_	tr|B3KMR5|E	cDNA FLJ12434 fis, clone NT2RM1000037, highly similar to Homo sapiens KIAA0690 protein OS=Homo sapiens PE=2 SV=	145007	0	999	0	19	0	0.02	#DIV/0!	0	0	0	1	0	0	0	1	-0.04302
sp|Q09161|NCBP1_H	NCBP1	Nuclear cap-binding protein subunit 1 OS=Homo sapiens GN=NCBP1 PE=1 SV=1	92864	0	998	0	19	0	0.04	#DIV/0!	0	0	0	1	0	0	0	3	-0.04302
sp|Q86SK9|SCD5_HU	SCD5	Stearoyl-CoA desaturase 5 OS=Homo sapiens GN=SCD5 PE=2 SV=2	37814	0	996	0	19	0	0.09	#DIV/0!	0	0	0	1	0	0	0	1	-0.04302
tr|H0YLB8|H0YLB8_H	GMPR2	GMP reductase 2 (Fragment) OS=Homo sapiens GN=GMPR2 PE=4 SV=1	18326	0	995	0	19	0	0.18	#DIV/0!	0	0	0	1	0	0	0	1	-0.04302
tr|H0Y2Y8|H0Y2Y8_H	ZYX	Zyxin (Fragment) OS=Homo sapiens GN=ZYX PE=4 SV=1	58804	0	993	0	19	0	0.11	#DIV/0!	0	0	0	2	0	0	0	2	-0.04302
tr|Q5JTE4|Q5JTE4_HU	POLR1C	Polymerase (RNA) I polypeptide C, 30kDa OS=Homo sapiens GN=POLR1C PE=2 SV=1	36043	0	991	0	19	0	0.09	#DIV/0!	0	0	0	1	0	0	0	1	-0.04302
sp|Q69YN2|C19L1_HU	CWF19L1	CWF19-like protein 1 OS=Homo sapiens GN=CWF19L1 PE=1 SV=2	61322	0	990	0	20	0	0.05	#DIV/0!	0	0	0	1	0	0	0	1	-0.04302
tr|C9JLH9|C9JLH9_HU	NLRC3	Protein NLRC3 OS=Homo sapiens GN=NLRC3 PE=2 SV=1	120711	0	989	0	20	0	0.03	#DIV/0!	0	0	0	1	0	0	0	1	-0.04302
tr|A4VCI1|A4VCI1_HU	DNAJC1	DNAJC1 protein (Fragment) OS=Homo sapiens GN=DNAJC1 PE=2 SV=1	25214	0	987	0	20	0	0.13	#DIV/0!	0	0	0	1	0	0	0	1	-0.04302
tr|Q5TZP7|Q5TZP7_H	APEX1	APEX nuclease (Multifunctional DNA repair enzyme) 1 OS=Homo sapiens GN=APEX1 PE=2 SV=1	35931	0	986	0	20	0	0.09	#DIV/0!	0	0	0	1	0	0	0	3	-0.04302
sp|Q06210|GFPT1_HU	GFPT1	Glutamine--fructose-6-phosphate aminotransferase [isomerizing] 1 OS=Homo sapiens GN=GFPT1 PE=1 SV=3	79555	0	985	0	20	0	0.04	#DIV/0!	0	0	0	1	0	0	0	1	-0.04302
tr|H0YHF4|H0YHF4_H	LETMD1	LETM1 domain-containing protein 1 (Fragment) OS=Homo sapiens GN=LETMD1 PE=4 SV=1	10978	0	983	0	20	0	0.31	#DIV/0!	0	0	0	1	0	0	0	1	-0.04302
tr|Q6NSG9|Q6NSG9_	AKAP12	AKAP12 protein (Fragment) OS=Homo sapiens GN=AKAP12 PE=2 SV=1	19461	0	982	0	20	0	0.17	#DIV/0!	0	0	0	1	0	0	0	1	-0.04302
tr|A8K1Y9|A8K1Y9_H	GNAL	Guanine nucleotide binding protein (G protein), alpha activating activity polypeptide, olfactory type, isoform CRA_b OS=	44794	0	783	0	16	0	0.06	#DIV/0!	0	0	0	1	0	0	0	2	-0.04302
tr|I3L495|I3L495_HU	PAFAH1B1	Platelet-activating factor acetylhydrolase IB subunit alpha (Fragment) OS=Homo sapiens GN=PAFAH1B1 PE=4 SV=1	9626	0	584	0	0	0	0.07	#DIV/0!	0	0	0	0	0	0	0	1	-0.04302
tr|B3KSH1|B3KSH1_H	EIF3F	Eukaryotic translation initiation factor 3 subunit F OS=Homo sapiens GN=EIF3F PE=2 SV=1	39236	652	412	22	76	0.08	0.74	9.25	0	1	1	2	1	2	1	2	0.694266
tr|B4DT29|B4DT29_H	tr|B4DT29|B	cDNA FLJ51082, highly similar to Actin-like protein 3 OS=Homo sapiens PE=2 SV=1	40959	548	119	30	102	0.08	0.7	8.75	0	3	1	5	1	5	1	5	1.832772
tr|Q5T7Q0|Q5T7Q0_	DNAJA1	DnaJ (Hsp40) homolog, subfamily A, member 1, isoform CRA_c OS=Homo sapiens GN=DNAJA1 PE=2 SV=1	45581	703	228	18	122	0.07	0.52	7.428571429	0	3	1	6	0	5	1	7	3.418078
tr|K7EJE9|K7EJE9_HU	DNAJC7	DnaJ homolog subfamily C member 7 (Fragment) OS=Homo sapiens GN=DNAJC7 PE=4 SV=1	8423	527	427	31	72	0.06	0.42	7	0	1	1	1	1	1	1	1	-0.04303
tr|Q0VGD6|Q0VGD6_	HNRPR	HNRPR protein (Fragment) OS=Homo sapiens GN=HNRPR PE=2 SV=1	68218	390	85	44	269	0.1	0.68	6.8	1	7	2	11	2	14	4	16	2.497022
tr|Q9NSU9|Q9NSU9_	DKFZp434G0	Putative uncharacterized protein DKFZp434G0719 (Fragment) OS=Homo sapiens GN=DKFZp434G0719 PE=2 SV=1	41107	56	185	169	150	0.07	0.47	6.714285714	4	3	5	5	5	5	5	6	-0.04307
tr|D6RAN1|D6RAN1_	PDLIM7	PDZ and LIM domain protein 7 (Fragment) OS=Homo sapiens GN=PDLIM7 PE=2 SV=1	9510	396	800	33	31	0.13	0.86	6.615384615	0	0	1	2	1	1	2	2	-0.04303
tr|B2R9U2|B2R9U2_H	tr|B2R9U2|B	cDNA, FLJ94557, highly similar to Homo sapiens FK506 binding protein 4, 59kDa (FKBP4), mRNA OS=Homo sapiens PE=2	52087	623	448	24	69	0.06	0.36	6	0	1	1	5	2	6	2	6	1.336804
tr|B2R5V9|B2R5V9_H	tr|B2R5V9|B	cDNA, FLJ92652, highly similar to Homo sapiens high density lipoprotein binding protein (vigilin)(HDLBP), mRNA OS=Hon	141919	253	277	76	107	0.02	0.12	6	1	2	1	5	2	6	2	6	1.336804
sp|P52597|HNRPF_H	HNRNPF	Heterogeneous nuclear ribonucleoprotein F OS=Homo sapiens GN=HNRNPF PE=1 SV=3	45985	226	367	91	84	0.07	0.4	5.714285714	1	2	1	5	2	4	3	5	0.805621
tr|I3L1L3|I3L1L3_HUN	MYBBP1A	Myb-binding protein 1A (Fragment) OS=Homo sapiens GN=MYBBP1A PE=4 SV=1	141124	698	289	18	104	0.02	0.1	5	0	3	1	4	1	4	2	6	1.542931
sp|Q09666|AHNK_HU	AHNAK	Neuroblast differentiation-associated protein AHNAK OS=Homo sapiens GN=AHNAK PE=1 SV=2	629213	401	99	43	245	0.01	0.05	5	1	5	1	9	3	9	6	14	1.399524
tr|B0QYA5|B0QYA5_	EIF3D	Eukaryotic translation initiation factor 3 subunit D (Fragment) OS=Homo sapiens GN=EIF3D PE=4 SV=1	31937	710	222	17	124	0.1	0.49	4.9	0	3	1	4	0	4	2	4	3.128237
tr|B4DH39|B4DH39_	tr|B4DH39|B	cDNA FLJ57028, highly similar to Long-chain-fatty-acid--CoA ligase 4 (EC 6.2.1.3) OS=Homo sapiens PE=2 SV=1	73123	529	294	31	75	0.04	0.19	4.75	0	2	1	3	1	4	3	4	1.542931
tr|B4DY09|B4DY09_H	tr|B4DY09|B	cDNA FLJ51660, highly similar to Interleukin enhancer-binding factor 2 OS=Homo sapiens PE=2 SV=1	39057	383	325	45	94	0.08	0.38	4.75	1	2	1	4	1	3	1	4	1.180027
tr|B2RBA6|B2RBA6_H	tr|B2RBA6|B	cDNA, FLJ95407, highly similar to Homo sapiens MCM7 minichromosome maintenance deficient 7 (S. cerevisiae) (MCM7	81856	374	252	46	116	0.04	0.19	4.75	1	3	1	4	1	3	1	5	1.180027
tr|B4DUC5|B4DUC5_	CSE1L	Exportin-2 OS=Homo sapiens GN=CSE1L PE=2 SV=1	86045	433	96	40	132	0.08	0.38	4.75	0	3	2	7	4	5	4	9	0.246777
tr|B4DEX8|B4DEX8_H	MAT2A	S-adenosylmethionine synthase OS=Homo sapiens GN=MAT2A PE=2 SV=1	40027	333	363	52	85	0.08	0.37	4.625	1	2	1	4	1	3	1	4	1.180027
tr|B4DJ60|B4DJ60_H	tr|B4DJ60|B	cDNA FLJ55072, highly similar to Succinate dehydrogenase (ubiquinone) flavoprotein subunit, mitochondrial (EC1.3.5.1)	68233	393	93	44	252	0.1	0.46	4.6	0	6	2	8	3	8	5	8	1.238725
tr|Q2TU64|Q2TU64_	tr|Q2TU64|C	PIG48 OS=Homo sapiens PE=2 SV=1	61110	279	234	68	121	0.05	0.23	4.6	1	2	1	4	3	5	3	5	0.609691
tr|B5MCX3|B5MCX3_	Sep-02	Septin-2 OS=Homo sapiens GN=SEPT2 PE=2 SV=1	37145	543	330	30	93	0.09	0.41	4.555555556	1	2	1	4	2	4	3	4	0.805621
tr|Q96QL0|Q96QL0_	RPL3	Ribosomal protein L3 OS=Homo sapiens GN=RPL3 PE=2 SV=1	46376	245	36	82	467	0.32	1.44	4.5	4	12	4	14	5	17	7	19	1.630793
tr|B4DLT2|B4DLT2_H	tr|B4DLT2|B	cDNA FLJ56637, highly similar to Nuclear pore complex protein Nup155 OS=Homo sapiens PE=2 SV=1	152734	489	204	35	134	0.02	0.09	4.5	0	3	1	4	1	4	1	5	1.542931
tr|B2RBE0|B2RBE0_H	tr|B2RBE0|B	cDNA, FLJ95462, highly similar to Homo sapiens fatty-acid-Coenzyme A ligase, long-chain 3 (FACL3),mRNA OS=Homo sap	81266	462	233	37	121	0.04	0.17	4.25	0	3	1	4	1	5	1	5	1.832772
tr|Q59G92|Q59G92_	tr|Q59G92|C	HLA-B associated transcript 1 variant (Fragment) OS=Homo sapiens PE=2 SV=1	35528	394	272	44	44	0.09	0.37	4.111111111	1	1	1	2	2	2	2	2	-0.04304
tr|B2R8R5|B2R8R5_H	tr|B2R8R5|B	cDNA, FLJ94025, highly similar to Homo sapiens tripartite motif-containing 28 (TRIM28), mRNA OS=Homo sapiens PE=2	90271	320	291	56	104	0.07	0.28	4	1	2	3	8	2	7	4	8	1.54359
tr|F5H6T1|F5H6T1_H	ACTR2	Actin-related protein 2 OS=Homo sapiens GN=ACTR2 PE=2 SV=1	34729	244	103	82	226	0.2	0.75	3.75	2	5	2	6	2	6	4	6	1.336804
sp|P45974|UBP5_HU	USP5	Ubiquitin carboxyl-terminal hydrolase 5 OS=Homo sapiens GN=USP5 PE=1 SV=2	96638	622	278	24	107	0.03	0.11	3.666666667	0	2	1	3	2	3	2	3	0.442717
tr|Q8TC62|Q8TC62_H	Sep-07	Septin 7 OS=Homo sapiens GN=SEPT7 PE=2 SV=3	48913	531	350	31	88	0.07	0.25	3.571428571	1	1	1	3	1	4	2	4	1.542931
sp|Q15645|PCH2_HU	TRIP13	Pachytene checkpoint protein 2 homolog OS=Homo sapiens GN=TRIP13 PE=1 SV=2	48863	507	726	33	36	0.07	0.25	3.571428571	0	0	1	2	2	2	2	3	-0.04304
tr|E7ENU7|E7ENU7_	RPL15	Ribosomal protein L15 (Fragment) OS=Homo sapiens GN=RPL15 PE=2 SV=1	20596	534	492	31	62	0.16	0.57	3.5625	0	1	1	4	1	3	1	4	1.180027
sp|Q14204|DYHC1_H	DYNC1H1	Cytoplasmic dynein 1 heavy chain 1 OS=Homo sapiens GN=DYNC1H1 PE=1 SV=5	534809	168	178	129	152	0.02	0.07	3.5	3	4	4	11	4	9	6	15	1.03661
tr|B1Q2N1|B1Q2N1_	DDX39	ATP-dependent RNA helicase DDX39A OS=Homo sapiens GN=DDX39 PE=2 SV=1	30925	395	283	44	45	0.11	0.38	3.454545455	1	1	1	3	1	3	2	3	1.180027
sp|Q6IBS0|TWF2_HU	TWF2	Twinfilin-2 OS=Homo sapiens GN=TWF2 PE=1 SV=2	39751	535	164	30	169	0.08	0.27	3.375	0	3	1	3	1	3	1	3	1.180027
tr|B2ZZ89|B2ZZ89_HU	SPTBN1	Spectrin beta non-erythrocytic 1 OS=Homo sapiens GN=SPTBN1 PE=2 SV=1	275237	49	7	431	1541	0.22	0.74	3.363636364	11	38	17	49	20	50	24	57	1.267528
tr|B4DKS0|B4DKS0_H	tr|B4DKS0|B	cDNA FLJ53381, highly similar to Monocarboxylate transporter 1 OS=Homo sapiens PE=2 SV=1	52487	598	342	26	91	0.06	0.2	3.333333333	0	1	1	3	1	3	1	3	1.180027
tr|A8K2Y2|A8K2Y2_H	tr|A8K2Y2|A	cDNA FLJ78120, highly similar to Homo sapiens eukaryotic translation initiation factor 2, subunit 3 gamma, 52kDa (EIF2S	51648	475	248	37	118	0.06	0.2	3.333333333	1	2	1	3	1	3	1	4	1.180027
sp|Q6P2Q9|PRP8_HU	PRPF8	Pre-mRNA-processing-splicing factor 8 OS=Homo sapiens GN=PRPF8 PE=1 SV=2	274738	130	34	167	543	0.1	0.33	3.3	7	16	8	26	10	27	10	34	1.351616
tr|H0Y8E6|H0Y8E6_H	MCM2	DNA replication licensing factor MCM2 (Fragment) OS=Homo sapiens GN=MCM2 PE=3 SV=1	94792	211	244	98	118	0.07	0.23	3.285714286	1	3	2	6	2	7	2	8	1.54359
tr|Q5TZX1|Q5TZX1_H	GNAI3	Guanine nucleotide binding protein (G protein), alpha inhibiting activity polypeptide 3 OS=Homo sapiens GN=GNAI3 PE=	41076	376	354	46	87	0.08	0.26	3.25	1	2	1	3	1	3	1	4	1.180027
tr|B4E2I9|B4E2I9_HU	tr|B4E2I9|B4	cDNA FLJ55486, highly similar to Semaphorin-3C OS=Homo sapiens PE=2 SV=1	88481	602	1031	26	15	0.04	0.13	3.25	0	0	1	1	1	0	2	1	-1.62833
sp|Q96A33|CCD47_H	CCDC47	Coiled-coil domain-containing protein 47 OS=Homo sapiens GN=CCDC47 PE=1 SV=1	56123	560	335	29	93	0.06	0.19	3.166666667	0	3	1	3	1	3	1	3	1.180027
tr|B4DQJ8|B4DQJ8_H	PGD	6-phosphogluconate dehydrogenase, decarboxylating OS=Homo sapiens GN=PGD PE=2 SV=1	52352	293	160	62	171	0.2	0.63	3.15	1	5	3	8	2	8	3	11	1.724496
tr|B2R7B5|B2R7B5_H	tr|B2R7B5|B	cDNA, FLJ93365, highly similar to Homo sapiens KH domain containing, RNA binding, signal transduction associated 1 (K	48309	347	238	51	120	0.07	0.22	3.142857143	1	2	1	3	2	3	4	3	0.442717
tr|Q6FHQ0|Q6FHQ0_	RBBP7	RBBP7 protein (Fragment) OS=Homo sapiens GN=RBBP7 PE=2 SV=1	48132	468	704	37	38	0.07	0.22	3.142857143	1	0	1	3	3	3	3	4	-0.04305
tr|B2RB23|B2RB23_H	tr|B2RB23|B	cDNA, FLJ95265, highly similar to Homo sapiens acetyl-Coenzyme A acyltransferase 2 (mitochondrial 3-oxoacyl-Coenzym	42439	544	400	30	77	0.08	0.25	3.125	0	2	1	3	1	3	1	4	1.180027
tr|B5BU01|B5BU01_H	EIF2S2	Eukaryotic translation initiation factor 2 beta OS=Homo sapiens GN=EIF2S2 PE=2 SV=1	38648	654	598	22	47	0.09	0.28	3.111111111	0	1	1	3	1	2	1	6	0.694266
tr|B2R5U1|B2R5U1_H	tr|B2R5U1|B	cDNA, FLJ92620, highly similar to Homo sapiens staphylococcal nuclease domain containing 1 (SND1),mRNA OS=Homo s	100294	150	88	144	266	0.17	0.52	3.058823529	4	7	5	14	10	13	12	17	0.32045
sp|O94906|PRP6_HU	PRPF6	Pre-mRNA-processing factor 6 OS=Homo sapiens GN=PRPF6 PE=1 SV=1	107656	492	421	35	74	0.03	0.09	3	1	2	1	3	1	3	2	3	1.180027
tr|D3DP78|D3DP78_H	DARS	Aspartyl-tRNA synthetase, isoform CRA_b OS=Homo sapiens GN=DARS PE=2 SV=1	53829	344	326	51	51	0.06	0.18	3	1	1	1	2	2	3	3	3	0.442717
sp|P54886|P5CS_HU	ALDH18A1	Delta-1-pyrroline-5-carboxylate synthase OS=Homo sapiens GN=ALDH18A1 PE=1 SV=2	87989	658	773	21	33	0.04	0.12	3	1	0	1	3	1	1	1	3	-0.04303
tr|H0UID3|H0UID3_H	AP2B1	Adaptor-related protein complex 2, beta 1 subunit, isoform CRA_d OS=Homo sapiens GN=AP2B1 PE=4 SV=1	106321	648	372	22	83	0.03	0.09	3	0	1	1	3	3	2	3	6	-0.52881
sp|O00629|IMA4_HU	KPNA4	Importin subunit alpha-4 OS=Homo sapiens GN=KPNA4 PE=1 SV=1	58364	472	604	37	46	0.06	0.18	3	0	1	1	1	2	1	2	1	-0.78034
sp|O14929|HAT1_HU	HAT1	Histone acetyltransferase type B catalytic subunit OS=Homo sapiens GN=HAT1 PE=1 SV=1	49880	343	170	51	161	0.07	0.21	3	1	3	1	3	1	3	1	3	1.180027
tr|A6NKB8|A6NKB8_	RNPEP	Aminopeptidase B OS=Homo sapiens GN=RNPEP PE=2 SV=1	68752	316	243	57	118	0.05	0.15	3	1	3	2	3	2	3	2	3	0.442717
tr|H7BZJ3|H7BZJ3_HU	PDIA3	Thioredoxin (Fragment) OS=Homo sapiens GN=PDIA3 PE=3 SV=1	13739	546	481	22	64	0.19	0.56	2.947368421	0	1	1	2	1	3	1	3	1.180027
tr|Q05CK9|Q05CK9_H	SYNCRIP	SYNCRIP protein (Fragment) OS=Homo sapiens GN=SYNCRIP PE=2 SV=1	50847	252	155	76	175	0.13	0.38	2.923076923	2	5	2	7	2	8	2	12	1.724496
tr|B7Z8W6|B7Z8W6_	tr|B7Z8W6|E	cDNA FLJ55038, highly similar to Nicotinamide phosphoribosyltransferase (EC 2.4.2.12) OS=Homo sapiens PE=2 SV=1	26905	520	451	32	66	0.12	0.35	2.916666667	0	2	1	4	1	2	3	4	0.694266
tr|B4DNK3|B4DNK3_	tr|B4DNK3|E	cDNA FLJ52127, highly similar to Multisynthetase complex auxiliary component p43 OS=Homo sapiens PE=2 SV=1	29955	601	410	26	30	0.11	0.32	2.909090909	0	0	1	1	1	1	1	1	-0.04303
tr|B3KM77|B3KM77_	tr|B3KM77|E	cDNA FLJ10435 fis, clone NT2RP1000493, highly similar to Splicing factor 3B subunit 3 OS=Homo sapiens PE=2 SV=1	56736	356	168	49	142	0.06	0.17	2.833333333	1	4	1	4	1	4	1	4	1.542931
tr|A6NG51|A6NG51_	SPTAN1	Spectrin alpha chain, non-erythrocytic 1 OS=Homo sapiens GN=SPTAN1 PE=2 SV=2	285628	167	43	130	376	0.06	0.17	2.833333333	2	9	5	13	6	16	9	17	1.304217
sp|Q9UG63|ABCF2_H	ABCF2	ATP-binding cassette sub-family F member 2 OS=Homo sapiens GN=ABCF2 PE=1 SV=2	71815	673	382	20	81	0.05	0.14	2.8	0	2	1	3	1	4	2	5	1.542931
tr|Q05D08|Q05D08_	PA2G4	PA2G4 protein (Fragment) OS=Homo sapiens GN=PA2G4 PE=2 SV=1	45579	286	276	64	108	0.15	0.42	2.8	1	4	2	5	3	5	3	6	0.609691
tr|B4DDB6|B4DDB6_	HNRPA3	Heterogeneous nuclear ribonucleoprotein A3, isoform CRA_a OS=Homo sapiens GN=HNRPA3 PE=2 SV=1	37234	201	145	103	182	0.19	0.53	2.789473684	2	5	2	5	2	5	2	6	1.095462
tr|B2R7C5|B2R7C5_H	tr|B2R7C5|B	cDNA, FLJ93378, highly similar to Homo sapiens MCM3 minichromosome maintenance deficient 3 (S. cerevisiae) (MCM3	91579	479	506	36	60	0.04	0.11	2.75	1	1	1	3	1	3	1	4	1.180027
tr|Q4ZG57|Q4ZG57_	MCM6	Putative uncharacterized protein MCM6 (Fragment) OS=Homo sapiens GN=MCM6 PE=2 SV=1	89803	444	379	39	81	0.04	0.11	2.75	1	2	1	3	2	3	3	3	0.442717
tr|I3L0N3|I3L0N3_HU	NSF	Vesicle-fusing ATPase OS=Homo sapiens GN=NSF PE=3 SV=1	82553	412	805	42	31	0.04	0.11	2.75	1	0	1	1	1	1	1	1	-0.04303
sp|O14980|XPO1_HU	XPO1	Exportin-1 OS=Homo sapiens GN=XPO1 PE=1 SV=1	124447	305	339	59	92	0.03	0.08	2.666666667	1	2	1	3	1	3	3	5	1.180027
tr|B2R6D0|B2R6D0_H	tr|B2R6D0|B	cDNA, FLJ92896, highly similar to Homo sapiens proteasome (prosome, macropain) 26S subunit, non-ATPase, 1 (PSMD1)	106797	386	151	45	178	0.06	0.16	2.666666667	0	5	2	5	2	5	4	6	1.095462
tr|F1T0B3|F1T0B3_HU	DDX1	ATP-dependent RNA helicase DDX1 OS=Homo sapiens GN=DDX1 PE=2 SV=1	74838	501	220	33	125	0.09	0.24	2.666666667	0	3	2	5	3	5	3	5	0.609691
tr|B4DR68|B4DR68_H	tr|B4DR68|B	cDNA FLJ58608, highly similar to Heat shock protein 75 kDa, mitochondrial OS=Homo sapiens PE=2 SV=1	74408	169	203	127	135	0.09	0.24	2.666666667	2	3	3	5	3	4	5	6	0.319851
sp|O43175|SERA_HU	PHGDH	D-3-phosphoglycerate dehydrogenase OS=Homo sapiens GN=PHGDH PE=1 SV=4	57356	131	113	166	222	0.25	0.65	2.6	3	7	4	9	4	9	4	12	1.03661
sp|O95831|AIFM1_HU	AIFM1	Apoptosis-inducing factor 1, mitochondrial OS=Homo sapiens GN=AIFM1 PE=1 SV=1	67144	326	736	54	36	0.05	0.13	2.6	1	1	1	1	1	1	2	1	-0.04303
tr|B4DMJ6|B4DMJ6_	tr|B4DMJ6|E	cDNA FLJ50996, highly similar to 60S ribosomal protein L4 OS=Homo sapiens PE=2 SV=1	46269	163	44	132	364	0.51	1.32	2.588235294	4	11	6	12	8	14	9	14	0.729422
tr|B2R959|B2R959_H	tr|B2R959|B	cDNA, FLJ94229, highly similar to Homo sapiens heterogeneous nuclear ribonucleoprotein L (HNRPL),mRNA OS=Homo sa	60838	182	58	117	231	0.23	0.59	2.565217391	4	7	4	10	6	11	8	13	0.78171
tr|B3KW21|B3KW21_	tr|B3KW21|E	cDNA FLJ41945 fis, clone PLACE6019676, highly similar to Coatomer subunit gamma OS=Homo sapiens PE=2 SV=1	68674	705	740	18	35	0.04	0.1	2.5	0	0	1	2	0	3	1	4	2.765333
tr|B2RU06|B2RU06_H	EIF4G1	EIF4G1 protein OS=Homo sapiens GN=EIF4G1 PE=2 SV=1	176807	669	260	20	113	0.04	0.1	2.5	0	4	2	5	1	5	3	6	1.832772
tr|B4E0E1|B4E0E1_H	tr|B4E0E1|B	cDNA FLJ53442, highly similar to Poly (ADP-ribose) polymerase 1 (EC 2.4.2.30) OS=Homo sapiens PE=2 SV=1	111855	549	256	30	115	0.06	0.15	2.5	0	4	2	5	2	5	2	6	1.095462
tr|B9ZVX0|B9ZVX0_H	DIAPH1	Diaphanous homolog 1 (Drosophila), isoform CRA_a OS=Homo sapiens GN=DIAPH1 PE=4 SV=1	139473	184	801	116	31	0.02	0.05	2.5	1	1	1	2	1	1	2	3	-0.04303
tr|H0YAF8|H0YAF8_H	GNB2L1	Guanine nucleotide-binding protein subunit beta-2-like 1 (Fragment) OS=Homo sapiens GN=GNB2L1 PE=4 SV=1	22373	216	71	96	278	0.52	1.29	2.480769231	2	5	3	6	3	7	4	7	1.057819
tr|B4DL37|B4DL37_H	AKR1C3	Aldo-keto reductase family 1 member C3 OS=Homo sapiens GN=AKR1C3 PE=2 SV=1	23691	203	212	83	130	0.29	0.7	2.413793103	2	3	2	4	2	4	3	4	0.805621
sp|P26599|PTBP1_HU	PTBP1	Polypyrimidine tract-binding protein 1 OS=Homo sapiens GN=PTBP1 PE=1 SV=1	57357	567	156	29	174	0.18	0.42	2.333333333	0	3	3	5	3	5	3	6	0.609691
tr|B7Z8A2|B7Z8A2_H	tr|B7Z8A2|B	cDNA FLJ51671, highly similar to Prenylcysteine oxidase (EC 1.8.3.5) OS=Homo sapiens PE=2 SV=1	54918	355	904	49	24	0.06	0.14	2.333333333	1	0	1	2	1	1	1	2	-0.04303
tr|H0Y804|H0Y804_H	AKR1C1	Aldo-keto reductase family 1 member C1 (Fragment) OS=Homo sapiens GN=AKR1C1 PE=4 SV=1	28617	186	116	61	93	0.67	1.56	2.328358209	2	3	4	5	4	4	6	5	-0.04306
sp|P42330|AK1C3_HU	AKR1C3	Aldo-keto reductase family 1 member C3 OS=Homo sapiens GN=AKR1C3 PE=1 SV=4	37229	203	136	102	191	0.29	0.67	2.310344828	3	4	3	6	3	6	4	6	0.851034
tr|Q2XPP3|Q2XPP3_H	tr|Q2XPP3|C	Type II 3a-hydroxysteroid dehydrogenase variant OS=Homo sapiens PE=2 SV=1	37201	203	213	102	129	0.29	0.67	2.310344828	3	3	3	6	3	5	4	6	0.609691
sp|P11021|GRP78_HU	HSPA5	78 kDa glucose-regulated protein OS=Homo sapiens GN=HSPA5 PE=1 SV=2	72402	129	105	170	233	0.19	0.43	2.263157895	3	6	5	9	6	9	6	9	0.505407
tr|B2R4K7|B2R4K7_H	tr|B2R4K7|B	60S ribosomal protein L6 OS=Homo sapiens PE=2 SV=1	32739	276	344	68	76	0.47	1.06	2.255319149	2	3	4	5	3	6	5	6	0.851034
tr|B4E091|B4E091_H	tr|B4E091|B	cDNA FLJ55438, highly similar to Splicing factor 3 subunit 1 OS=Homo sapiens PE=2 SV=1	77415	482	340	36	91	0.04	0.09	2.25	1	2	1	2	1	2	1	2	0.694266
tr|B4DVB8|B4DVB8_	ELAVL1	ELAV-like protein 1 OS=Homo sapiens GN=ELAVL1 PE=2 SV=1	39200	612	318	24	96	0.08	0.18	2.25	0	2	1	2	1	2	1	2	0.694266
tr|E5KNY5|E5KNY5_H	LRPPRC	Leucine-rich PPR-motif containing OS=Homo sapiens GN=LRPPRC PE=2 SV=1	159003	329	141	53	187	0.08	0.18	2.25	1	5	4	8	5	8	7	9	0.58596
tr|Q59FC9|Q59FC9_H	tr|Q59FC9|C	Enigma homolog (Fragment) OS=Homo sapiens PE=2 SV=1	50413	175	77	109	239	0.41	0.92	2.243902439	2	5	5	10	5	10	6	14	0.891484
tr|Q5SU16|Q5SU16_	TUBB	Beta 5-tubulin OS=Homo sapiens GN=TUBB PE=2 SV=1	50095	25	9	680	1166	3.07	6.7	2.182410423	26	35	33	49	35	55	39	59	0.608035
sp|P04350|TBB4A_HU	TUBB4A	Tubulin beta-4A chain OS=Homo sapiens GN=TUBB4A PE=1 SV=2	50010	42	17	509	891	1.96	4.25	2.168367347	21	29	27	43	29	49	32	53	0.710131
tr|B7Z9W6|B7Z9W6_	tr|B7Z9W6|E	cDNA, FLJ78980, highly similar to DnaJ homolog subfamily C member 7 OS=Homo sapiens PE=2 SV=1	50776	527	439	31	71	0.06	0.13	2.166666667	0	1	1	2	1	2	1	2	0.694266
sp|Q9Y3I0|RTCB_HUN	C22orf28	tRNA-splicing ligase RtcB homolog OS=Homo sapiens GN=C22orf28 PE=1 SV=1	55688	458	171	38	157	0.12	0.26	2.166666667	0	3	2	4	2	3	2	4	0.442717
sp|Q9NR12|PDLI7_HU	PDLIM7	PDZ and LIM domain protein 7 OS=Homo sapiens GN=PDLIM7 PE=1 SV=1	50896	396	361	43	85	0.13	0.28	2.153846154	0	2	2	4	2	4	4	4	0.805621
tr|B4DQX0|B4DQX0_	tr|B4DQX0|E	cDNA FLJ59219, highly similar to Poly(A)-binding protein 1 OS=Homo sapiens PE=2 SV=1	67239	215	139	96	173	0.33	0.71	2.151515152	3	5	6	10	6	10	10	10	0.650131

sp|O95373|IPO7_HU	IPO7	Importin-7 OS=Homo sapiens GN=IPO7 PE=1 SV=1	120751	162	356	132	86	0.08	0.17	2.125	3	2	3	6	3	6	4	8	0.851034
sp|O75367|H2AY_HU	H2AFY	Core histone macro-H2A.1 OS=Homo sapiens GN=H2AFY PE=1 SV=4	39764	565	310	29	98	0.08	0.17	2.125	0	2	1	2	2	2	2	2	-0.04304
tr|Q5M7Z5|Q5M7Z5_	GRHPR	GRHPR protein (Fragment) OS=Homo sapiens GN=GRHPR PE=2 SV=1	37314	659	850	21	28	0.09	0.19	2.111111111	0	0	1	2	1	2	1	2	0.694266
tr|B4DEM7|B4DEM7_	CCT8	T-complex protein 1 subunit theta OS=Homo sapiens GN=CCT8 PE=2 SV=1	58179	268	128	70	201	0.18	0.38	2.111111111	2	4	3	6	5	6	6	8	0.198269
tr|Q75L23|Q75L23_H	PSMC2	Putative uncharacterized protein PSMC2 (Fragment) OS=Homo sapiens GN=PSMC2 PE=2 SV=1	46202	610	625	25	44	0.1	0.21	2.1	0	1	1	1	1	1	1	1	-0.04303
tr|B4DZ53|B4DZ53_H	tr|B4DZ53|B	cDNA FLJ59643, highly similar to Neutral alpha-glucosidase AB OS=Homo sapiens PE=2 SV=1	96752	47	15	400	920	0.62	1.3	2.096774194	10	21	17	31	15	28	19	38	0.839879
tr|B7Z481|B7Z481_H	tr|B7Z481|B	cDNA FLJ54018, highly similar to PDZ and LIM domain protein 5 OS=Homo sapiens PE=2 SV=1	52359	175	73	109	297	0.41	0.84	2.048780488	2	6	5	10	5	11	6	14	1.023063
tr|K7EPF6|K7EPF6_H	PGD	6-phosphogluconate dehydrogenase, decarboxylating (Fragment) OS=Homo sapiens GN=PGD PE=3 SV=1	28143	293	334	62	93	0.2	0.4	2	1	3	2	3	2	5	2	6	1.095462
sp|Q96P70|IPO9_HU	IPO9	Importin-9 OS=Homo sapiens GN=IPO9 PE=1 SV=3	116858	580	384	28	81	0.06	0.12	2	0	2	2	4	2	4	2	4	0.805621
sp|P41250|SYG_HUM	GARS	Glycine--tRNA ligase OS=Homo sapiens GN=GARS PE=1 SV=3	83854	392	776	44	33	0.04	0.08	2	1	0	1	2	1	2	2	2	0.694266
tr|A8KAK1|A8KAK1_H	tr|A8KAK1|A	cDNA FLJ77398, highly similar to Homo sapiens UDP-glucose ceramide glucosyltransferase-like 1, transcript variant 2, mF	175467	635	622	22	45	0.02	0.04	2	0	1	1	2	1	2	1	3	0.694266
tr|B4DFM0|B4DFM0_	FARSLB	Phenylalanine-tRNA synthetase-like, beta subunit, isoform CRA_c OS=Homo sapiens GN=FARSLB PE=2 SV=1	55353	360	525	48	57	0.06	0.12	2	1	1	1	2	1	2	3	2	0.694266
tr|B4DFL1|B4DFL1_H	tr|B4DFL1|B	Dihydrolipoyl dehydrogenase OS=Homo sapiens PE=2 SV=1	49381	666	315	21	96	0.07	0.14	2	0	2	1	2	1	2	1	2	0.694266
tr|B3KSQ7|B3KSQ7_H	DBN1	Drebrin 1, isoform CRA_d OS=Homo sapiens GN=DBN1 PE=2 SV=1	66053	478	197	36	141	0.05	0.1	2	0	2	1	2	1	2	1	2	0.694266
tr|A6NN80|A6NN80_	ANXA6	Annexin OS=Homo sapiens GN=ANXA6 PE=3 SV=3	75571	349	323	50	94	0.14	0.28	2	1	2	3	4	3	5	3	6	0.609691
tr|A4FU77|A4FU77_H	SNRNP200	SNRNP200 protein (Fragment) OS=Homo sapiens GN=SNRNP200 PE=2 SV=1	217585	243	149	83	180	0.05	0.1	2	2	5	3	8	7	10	8	11	0.443336
tr|B1PS43|B1PS43_H	MYH11	Myosin heavy chain 11 smooth muscle isoform OS=Homo sapiens GN=MYH11 PE=2 SV=1	235004	142	76	155	290	0.06	0.12	2	5	9	5	11	9	12	11	13	0.353818
tr|B4DY16|B4DY16_H	tr|B4DY16|B	cDNA FLJ51385, highly similar to Ran GTPase-activating protein 1 OS=Homo sapiens PE=2 SV=1	58178	574	554	28	53	0.12	0.24	2	0	1	1	1	1	1	1	1	-0.04303
tr|B4DKD0|B4DKD0_	tr|B4DKD0|B	DNA topoisomerase 2 (Fragment) OS=Homo sapiens PE=2 SV=1	135015	606	526	25	33	0.02	0.04	2	0	0	1	1	1	1	3	2	-0.04303
tr|D6RDU5|D6RDU5_	Sep-11	Septin-11 (Fragment) OS=Homo sapiens GN=SEPT11 PE=2 SV=1	43371	532	251	31	48	0.07	0.14	2	1	1	1	1	1	1	1	1	-0.04303
tr|B2RE46|B2RE46_H	tr|B2RE46|B	cDNA, FLJ96923, highly similar to Homo sapiens ribophorin II (RPN2), mRNA OS=Homo sapiens PE=2 SV=1	69405	589	700	27	38	0.05	0.1	2	0	0	1	2	2	2	2	2	-0.04304
tr|B4DJ30|B4DJ30_H	tr|B4DJ30|B	cDNA FLJ61290, highly similar to Neutral alpha-glucosidase AB OS=Homo sapiens PE=2 SV=1	113539	47	13	446	1082	0.62	1.21	1.951612903	12	26	19	37	17	34	22	45	0.941753
sp|Q9UMS4|PRP19_H	PRPF19	Pre-mRNA-processing factor 19 OS=Homo sapiens GN=PRPF19 PE=1 SV=1	55603	237	142	87	187	0.26	0.5	1.923076923	2	5	5	7	7	8	11	10	0.137813
tr|Q2Q9B7|Q2Q9B7_	G6PD	Glucose-6-phosphate 1-dehydrogenase (Fragment) OS=Homo sapiens GN=G6PD PE=2 SV=1	55221	176	209	118	132	0.18	0.34	1.888888889	2	3	2	5	5	6	6	6	0.198269
tr|B4DYA7|B4DYA7_H	tr|B4DYA7|B	Glucose-6-phosphate 1-dehydrogenase OS=Homo sapiens PE=2 SV=1	58757	176	210	123	120	0.18	0.34	1.888888889	2	3	3	4	6	5	7	6	-0.28443
tr|Q8IWP6|Q8IWP6_	tr|Q8IWP6|C	Class IVb beta tubulin OS=Homo sapiens PE=2 SV=1	50177	28	11	654	1135	3.05	5.75	1.885245902	27	35	32	50	34	56	37	61	0.675705
tr|Q8IZ29|Q8IZ29_HU	TUBB2C	Tubulin, beta 2C OS=Homo sapiens GN=TUBB2C PE=2 SV=1	50264	27	10	674	1145	3.04	5.72	1.881578947	26	34	32	50	34	57	38	61	0.701354
tr|G3XAD8|G3XAD8_	STIP1	Stress-induced-phosphoprotein 1 OS=Homo sapiens GN=STIP1 PE=2 SV=1	68721	271	146	69	182	0.21	0.39	1.857142857	2	5	4	7	4	8	5	10	0.875811
tr|B4DR70|B4DR70_H	tr|B4DR70|B	cDNA FLJ58049, highly similar to RNA-binding protein FUS OS=Homo sapiens PE=2 SV=1	45013	450	572	38	51	0.07	0.13	1.857142857	1	1	1	2	1	2	1	3	0.694266
sp|P00390|GSHR_HU	GSR	Glutathione reductase, mitochondrial OS=Homo sapiens GN=GSR PE=1 SV=2	56791	313	296	58	102	0.18	0.33	1.833333333	1	3	3	5	4	5	4	5	0.246777
tr|B4DN87|B4DN87_	tr|B4DN87|B	cDNA FLJ52569, highly similar to Collagen-binding protein 2 OS=Homo sapiens PE=2 SV=1	44290	233	184	89	150	0.43	0.78	1.813953488	4	5	5	8	5	8	5	8	0.58596
tr|B4DWJ2|B4DWJ2_	QARS	Glutamine--tRNA ligase OS=Homo sapiens GN=QARS PE=2 SV=1	87437	277	200	68	137	0.16	0.29	1.8125	3	2	4	7	5	9	5	11	0.746759
tr|H0YDE4|H0YDE4_H	PRMT1	Protein arginine N-methyltransferase 1 (Fragment) OS=Homo sapiens GN=PRMT1 PE=4 SV=1	27345	437	585	39	48	0.26	0.47	1.807692308	1	0	2	4	2	4	2	4	0.805621
sp|P17987|TCPA_HU	TCP1	T-complex protein 1 subunit alpha OS=Homo sapiens GN=TCP1 PE=1 SV=1	60819	197	397	106	78	0.3	0.54	1.8	2	3	5	5	8	4	10	5	-0.96198
tr|A2VCT2|A2VCT2_H	OGDH	OGDH protein (Fragment) OS=Homo sapiens GN=OGDH PE=2 SV=1	72973	432	348	40	29	0.05	0.09	1.8	1	0	1	1	1	1	1	1	-0.04303
tr|Q53SS8|Q53SS8_H	PCBP1	Poly(RC) binding protein 1 OS=Homo sapiens GN=PCBP1 PE=2 SV=1	37987	110	132	197	195	0.29	0.52	1.793103448	3	4	3	5	3	4	3	5	0.319851
tr|B3KQT2|B3KQT2_H	tr|B3KQT2|B	cDNA PSEC0148 fis, clone PLACE1007202, highly similar to Protein disulfide-isomerase A3 (EC 5.3.4.1) OS=Homo sapiens	55268	546	112	30	222	0.19	0.34	1.789473684	0	4	3	5	2	6	8	8	1.336804
tr|Q59EG8|Q59EG8_	tr|Q59EG8|C	Proteasome 26S non-ATPase subunit 2 variant (Fragment) OS=Homo sapiens PE=2 SV=1	101322	198	131	105	195	0.14	0.25	1.785714286	1	5	4	7	4	7	5	8	0.694905
tr|F5GWY2|F5GWY2_	ATIC	Phosphoribosylaminoimidazolecarboxamide formyltransferase OS=Homo sapiens GN=ATIC PE=2 SV=1	59092	72	59	237	352	0.64	1.13	1.765625	7	10	10	15	11	16	12	19	0.479372
sp|Q96HC4|PDLI5_HU	PDLIM5	PDZ and LIM domain protein 5 OS=Homo sapiens GN=PDLIM5 PE=1 SV=5	65102	175	57	123	357	0.41	0.72	1.756097561	3	8	7	11	7	12	8	15	0.695544
sp|P52895|AK1C2_HU	AKR1C2	Aldo-keto reductase family 1 member C2 OS=Homo sapiens GN=AKR1C2 PE=1 SV=3	37111	186	181	116	151	0.67	1.16	1.731343284	3	5	6	9	7	7	9	9	-0.04309
tr|E9PBU3|E9PBU3_H	ATIC	Phosphoribosylaminoimidazolecarboxamide formyltransferase OS=Homo sapiens GN=ATIC PE=2 SV=1	64996	72	60	274	352	0.64	1.1	1.71875	8	10	11	16	12	17	13	21	0.443956
tr|H0YD14|H0YD14_H	MYOF	Myoferlin (Fragment) OS=Homo sapiens GN=MYOF PE=4 SV=1	135822	220	262	95	44	0.07	0.12	1.714285714	3	1	3	3	4	4	4	5	-0.04306
sp|Q9Y2A7|NCKP1_H	NCKAP1	Nck-associated protein 1 OS=Homo sapiens GN=NCKAP1 PE=1 SV=1	130018	685	689	19	39	0.03	0.05	1.666666667	0	1	1	1	0	1	1	1	1.542273
tr|B7Z438|B7Z438_H	tr|B7Z438|B	cDNA FLJ56352, highly similar to Succinyl-CoA ligase (GDP-forming) subunit alpha, mitochondrial (EC 6.2.1.4) OS=Homo s	26801	467	605	37	46	0.12	0.2	1.666666667	1	1	1	2	1	2	1	2	0.694266
sp|P01024|CO3_HUM	C3	Complement C3 OS=Homo sapiens GN=C3 PE=1 SV=2	188569	370	719	47	37	0.03	0.05	1.666666667	1	0	2	3	2	3	2	4	0.442717
sp|P07237|PDIA1_HU	P4HB	Protein disulfide-isomerase OS=Homo sapiens GN=P4HB PE=1 SV=3	57480	536	312	30	97	0.27	0.45	1.666666667	1	4	2	6	1	6	2	6	2.074114
sp|P15121|ALDR_HU	AKR1B1	Aldose reductase OS=Homo sapiens GN=AKR1B1 PE=1 SV=3	36230	185	162	116	171	0.42	0.7	1.666666667	3	4	4	5	5	5	5	7	-0.04307
tr|B7Z2Z8|B7Z2Z8_H	tr|B7Z2Z8|B	T-complex protein 1 subunit delta OS=Homo sapiens PE=2 SV=1	52781	113	571	188	51	0.27	0.45	1.666666667	4	2	5	5	7	4	7	7	-0.78106
sp|P49327|FAS_HUM	FASN	Fatty acid synthase OS=Homo sapiens GN=FASN PE=1 SV=3	275877	55	23	394	740	0.28	0.46	1.642857143	12	18	21	32	23	30	29	41	0.335242
tr|Q6NZ55|Q6NZ55_	RPL13	60S ribosomal protein L13 OS=Homo sapiens GN=RPL13 PE=2 SV=1	24308	422	480	41	64	0.14	0.23	1.642857143	1	1	1	1	2	1	2	1	-0.78034
tr|Q2VPA0|Q2VPA0_	LONP1	Lon protease homolog (Fragment) OS=Homo sapiens GN=LONP1 PE=2 SV=1	95436	414	282	42	107	0.11	0.18	1.636363636	2	2	3	5	3	4	3	5	0.319851
tr|A6NKV8|A6NKV8_	PAIP1	Polyadenylate-binding protein-interacting protein 1 OS=Homo sapiens GN=PAIP1 PE=2 SV=1	42379	361	595	48	45	0.08	0.13	1.625	1	1	1	1	1	2	1	2	0.694266
tr|B7TY16|B7TY16_H	ACTN1	Actinin alpha 1 isoform 3 OS=Homo sapiens GN=ACTN1 PE=2 SV=1	107644	50	24	428	694	0.77	1.25	1.623376623	15	18	21	28	22	30	29	36	0.398324
sp|Q9NZB2|F120A_H	FAM120A	Constitutive coactivator of PPAR-gamma-like protein 1 OS=Homo sapiens GN=FAM120A PE=1 SV=2	123008	263	143	73	184	0.05	0.08	1.6	2	3	2	3	2	4	2	4	0.805621
tr|Q7Z5T5|Q7Z5T5_H	EIF3A	EIF3A protein (Fragment) OS=Homo sapiens GN=EIF3A PE=2 SV=1	97203	192	163	112	169	0.14	0.22	1.571428571	2	3	4	6	5	7	8	7	0.405054
sp|Q9Y305|ACOT9_H	ACOT9	Acyl-coenzyme A thioesterase 9, mitochondrial OS=Homo sapiens GN=ACOT9 PE=1 SV=2	50269	637	1006	22	18	0.07	0.11	1.571428571	0	0	1	1	1	1	2	2	-0.04303
tr|B4DF96|B4DF96_H	EIF2A	Eukaryotic translation initiation factor 2A OS=Homo sapiens GN=EIF2A PE=2 SV=1	58357	206	102	101	157	0.32	0.5	1.5625	2	3	5	6	4	6	5	8	0.488119
sp|Q8NBJ5|GT251_H	GLT25D1	Procollagen galactosyltransferase 1 OS=Homo sapiens GN=GLT25D1 PE=1 SV=1	71933	523	300	31	102	0.09	0.14	1.555555556	0	2	2	3	1	4	2	4	1.542931
tr|Q5HYM2|Q5HYM2	DKFZp686O2	Putative uncharacterized protein DKFZp686O2462 (Fragment) OS=Homo sapiens GN=DKFZp686O2462 PE=2 SV=1	72627	569	349	29	89	0.09	0.14	1.555555556	0	3	2	3	2	4	2	4	0.805621
tr|B1AHC9|B1AHC9_	XRCC6	X-ray repair complementing defective repair in Chinese hamster cells 6 (Ku autoantigen, 70kDa) OS=Homo sapiens GN=X	64528	116	49	184	378	0.64	0.99	1.546875	5	8	10	14	11	13	12	15	0.18886
tr|B5BU25|B5BU25_H	U2AF2	U2 small nuclear RNA auxiliary factor 2 isoform b OS=Homo sapiens GN=U2AF2 PE=2 SV=1	53445	260	333	74	93	0.13	0.2	1.538461538	1	2	2	3	2	4	2	4	0.805621
tr|B3KY95|B3KY95_H	tr|B3KY95|B	cDNA FLJ16143 fis, clone BRAMY2038516, highly similar to Protein disulfide-isomerase A6 (EC 5.3.4.1) OS=Homo sapiens	53655	445	640	38	43	0.13	0.2	1.538461538	0	0	2	3	2	3	2	3	0.442717
tr|A8K4D5|A8K4D5_H	tr|A8K4D5|A	Kynureninase OS=Homo sapiens PE=2 SV=1	52819	266	676	71	40	0.13	0.2	1.538461538	2	0	2	3	3	4	3	5	0.319851
tr|B4DUL5|B4DUL5_H	tr|B4DUL5|B	cDNA FLJ51625, highly similar to Ubiquinol-cytochrome-c reductase complex coreprotein I, mitochondrial (EC 1.10.2.2) (	40917	357	257	48	114	0.17	0.26	1.529411765	1	3	2	3	2	3	2	3	0.442717
tr|A8K7F6|A8K7F6_H	tr|A8K7F6|A	cDNA FLJ78244, highly similar to Homo sapiens eukaryotic translation initiation factor 4A, isoform 1 (EIF4A1), mRNA OS=	46321	52	37	404	495	1.62	2.45	1.512345679	10	13	14	21	15	23	20	23	0.559899
sp|P08238|HS90B_H	HSP90AB1	Heat shock protein HSP 90-beta OS=Homo sapiens GN=HSP90AB1 PE=1 SV=4	83554	32	16	622	896	1.24	1.87	1.508064516	18	23	28	31	29	37	35	39	0.305548
tr|B3KS31|B3KS31_H	TUBB6	Tubulin, beta 6, isoform CRA_a OS=Homo sapiens GN=TUBB6 PE=2 SV=1	42324	73	52	273	365	0.83	1.25	1.506024096	10	11	14	13	17	18	20	19	0.03731
tr|H0Y4R1|H0Y4R1_H	IMPDH2	Inosine-5~-monophosphate dehydrogenase 2 (Fragment) OS=Homo sapiens GN=IMPDH2 PE=3 SV=1	51549	177	91	122	254	0.36	0.54	1.5	2	6	5	7	5	7	6	8	0.405054
tr|A6NEM2|A6NEM2_	HCFC1	HCF N-terminal chain 5 OS=Homo sapiens GN=HCFC1 PE=2 SV=2	215338	508	750	33	34	0.02	0.03	1.5	0	0	1	2	1	2	2	2	0.694266
tr|J3KN86|J3KN86_H	RPS3	40S ribosomal protein S3 OS=Homo sapiens GN=RPS3 PE=3 SV=1	28640	480	634	36	44	0.12	0.18	1.5	1	1	1	1	1	2	4	3	0.694266
tr|B2RWP9|B2RWP9_	MYH10	MYH10 protein OS=Homo sapiens GN=MYH10 PE=2 SV=1	230858	200	123	104	205	0.06	0.09	1.5	3	6	4	6	4	6	6	7	0.488119
tr|B4DR45|B4DR45_H	tr|B4DR45|B	cDNA FLJ61339, highly similar to Alanyl-tRNA synthetase (EC 6.1.1.7) OS=Homo sapiens PE=2 SV=1	110017	238	477	86	64	0.06	0.09	1.5	1	0	2	3	2	3	2	4	0.442717
tr|F5H1A8|F5H1A8_H	GSN	Gelsolin OS=Homo sapiens GN=GSN PE=2 SV=1	81776	297	751	61	34	0.04	0.06	1.5	1	0	1	1	1	1	2	1	-0.04303
tr|B4DYL7|B4DYL7_H	tr|B4DYL7|B	cDNA FLJ58490, highly similar to Homo sapiens CCR4-NOT transcription complex, subunit 1 (CNOT1), transcript variant 1	140991	388	614	45	45	0.02	0.03	1.5	1	1	1	1	1	1	2	1	-0.04303
sp|Q14683|SMC1A_H	SMC1A	Structural maintenance of chromosomes protein 1A OS=Homo sapiens GN=SMC1A PE=1 SV=2	143771	581	568	28	52	0.02	0.03	1.5	0	1	1	1	1	1	1	2	-0.04303
tr|Q6IBR0|Q6IBR0_H	RPN1	RPN1 protein OS=Homo sapiens GN=RPN1 PE=2 SV=1	68678	170	135	127	192	0.26	0.39	1.5	4	4	5	7	8	6	8	8	-0.4308
tr|B4DSQ5|B4DSQ5_	tr|B4DSQ5|B	cDNA FLJ53608, highly similar to Protein transport protein Sec23A OS=Homo sapiens PE=2 SV=1	83787	474	523	37	27	0.08	0.12	1.5	0	0	2	2	2	1	3	3	-0.78034
tr|Q05DV5|Q05DV5_	ZC3HAV1	ZC3HAV1 protein (Fragment) OS=Homo sapiens GN=ZC3HAV1 PE=2 SV=1	49964	224	179	92	152	0.14	0.21	1.5	1	2	2	3	2	3	2	3	0.442717
sp|O15427|MOT4_HU	SLC16A3	Monocarboxylate transporter 4 OS=Homo sapiens GN=SLC16A3 PE=1 SV=1	50064	362	567	48	52	0.14	0.21	1.5	2	1	2	3	2	2	2	3	-0.04304
tr|Q3B7A7|Q3B7A7_	GART	Phosphoribosylglycinamide formyltransferase, phosphoribosylglycinamide synthetase, phosphoribosylaminoimidazole s	108909	341	486	51	63	0.13	0.19	1.461538462	1	1	4	6	3	5	4	7	0.609691
tr|A8K492|A8K492_H	tr|A8K492|A	cDNA FLJ76789, highly similar to Homo sapiens methionine-tRNA synthetase (MARS), mRNA OS=Homo sapiens PE=2 SV=	102277	385	621	45	45	0.07	0.1	1.428571429	1	1	2	3	2	3	2	4	0.442717
tr|B4DU00|B4DU00_	tr|B4DU00|B	cDNA FLJ54547, highly similar to Alpha-1 catenin (Cadherin-associated protein) OS=Homo sapiens PE=2 SV=1	89332	389	472	45	63	0.07	0.1	1.428571429	0	1	2	2	2	3	4	3	0.442717
tr|E9PBS1|E9PBS1_H	PAICS	Phosphoribosylaminoimidazole carboxylase (Fragment) OS=Homo sapiens GN=PAICS PE=2 SV=1	46307	270	683	69	39	0.07	0.1	1.428571429	1	1	1	1	2	2	5	3	-0.04304
tr|E9PKU4|E9PKU4_H	RPL8	60S ribosomal protein L8 (Fragment) OS=Homo sapiens GN=RPL8 PE=2 SV=1	25806	231	476	90	16	0.28	0.4	1.428571429	2	0	2	1	4	0	5	1	-3.21432
tr|G3V0E8|G3V0E8_H	PCBP2	Poly(RC) binding protein 2, isoform CRA_f OS=Homo sapiens GN=PCBP2 PE=2 SV=1	33817	132	129	166	197	0.32	0.45	1.40625	3	4	3	4	3	4	5	4	0.319851
tr|G3XAF4|G3XAF4_H	L1CAM	L1 cell adhesion molecule, isoform CRA_a OS=Homo sapiens GN=L1CAM PE=4 SV=1	139790	572	337	28	92	0.05	0.07	1.4	1	2	2	3	1	3	2	3	1.180027
tr|K7EIG1|K7EIG1_H	CLUH	Clustered mitochondria protein homolog OS=Homo sapiens GN=CLUH PE=3 SV=1	140903	438	207	39	132	0.05	0.07	1.4	1	1	2	3	2	3	2	3	0.442717
tr|H0Y2W2|H0Y2W2_	ATAD3A	ATPase family AAA domain-containing protein 3A (Fragment) OS=Homo sapiens GN=ATAD3A PE=4 SV=1	64600	281	311	68	76	0.1	0.14	1.4	2	2	2	2	2	2	2	2	-0.04304
tr|A8K8N7|A8K8N7_H	PFAS	Phosphoribosylformylglycinamidine synthase (FGAR amidotransferase), isoform CRA_b OS=Homo sapiens GN=PFAS PE=2	146286	380	438	46	71	0.05	0.07	1.4	0	2	2	3	3	3	5	3	-0.04305
tr|J3QT54|J3QT54_H	CPSF7	Cleavage and polyadenylation-specificity factor subunit 7 (Fragment) OS=Homo sapiens GN=CPSF7 PE=4 SV=1	22762	665	454	21	57	0.15	0.21	1.4	0	2	1	2	1	2	1	2	0.694266
tr|B4DFN9|B4DFN9_	tr|B4DFN9|B	cDNA FLJ54303, highly similar to Heat shock 70 kDa protein 1 OS=Homo sapiens PE=2 SV=1	62699	80	89	252	235	0.58	0.81	1.396551724	7	5	10	12	10	12	10	14	0.209084
tr|Q6IAV5|Q6IAV5_H	OXCT	Succinyl-CoA:3-ketoacid-coenzyme A transferase OS=Homo sapiens GN=OXCT PE=2 SV=1	56612	209	246	100	103	0.18	0.25	1.388888889	1	2	3	3	5	3	6	3	-0.69582
tr|Q2QD09|Q2QD09_	tr|Q2QD09|(	Triosephosphate isomerase (Fragment) OS=Homo sapiens PE=2 SV=1	27211	267	392	71	68	0.26	0.36	1.384615385	1	1	2	1	2	2	2	2	-0.04304
tr|B4DUQ1|B4DUQ1_	tr|B4DUQ1|B	cDNA FLJ54552, highly similar to Heterogeneous nuclear ribonucleoprotein K OS=Homo sapiens PE=2 SV=1	48708	147	111	147	223	0.58	0.8	1.379310345	3	6	7	9	7	10	8	11	0.443336
tr|G5E9Q2|G5E9Q2_	CSDE1	Cold shock domain containing E1, RNA-binding, isoform CRA_d OS=Homo sapiens GN=CSDE1 PE=2 SV=1	94539	312	152	58	177	0.11	0.15	1.363636364	1	3	3	4	3	5	3	5	0.609691
tr|H0Y9Q7|H0Y9Q7_	CANX	Calnexin (Fragment) OS=Homo sapiens GN=CANX PE=3 SV=1	18990	352	75	49	109	0.39	0.53	1.358974359	1	2	2	3	3	3	5	3	-0.04305
sp|O43707|ACTN4_H	ACTN4	Alpha-actinin-4 OS=Homo sapiens GN=ACTN4 PE=1 SV=2	105245	19	6	849	1635	2.11	2.85	1.3507109	26	43	41	53	44	55	52	65	0.278924
tr|B2R5P6|B2R5P6_H	TXNRD1	Thioredoxin reductase 1, cytoplasmic OS=Homo sapiens GN=TXNRD1 PE=2 SV=1	55253	108	118	204	198	0.26	0.35	1.346153846	3	3	5	5	5	6	6	8	0.198269
tr|B3KQQ3|B3KQQ3_	tr|B3KQQ3|B	cDNA PSEC0016 fis, clone NT2RM1001076, highly similar to Procollagen-lysine,2-oxoglutarate 5-dioxygenase 3 (EC 1.14.1	85161	325	387	54	54	0.12	0.16	1.333333333	1	1	3	3	3	4	3	5	0.319851
sp|Q68EM7|RHG17_H	ARHGAP17	Rho GTPase-activating protein 17 OS=Homo sapiens GN=ARHGAP17 PE=1 SV=1	95776	328	1032	54	15	0.03	0.04	1.333333333	1	0	1	1	2	0	2	1	-2.36564
tr|A8K1Z2|A8K1Z2_H	tr|A8K1Z2|A	cDNA FLJ78475, highly similar to Homo sapiens glycerol-3-phosphate dehydrogenase 2 (mitochondrial) (GPD2), mRNA O	81197	512	1029	32	16	0.06	0.08	1.333333333	0	0	1	1	2	0	2	1	-2.36564
tr|F5H3A1|F5H3A1_H	ATP1A1	Sodium/potassium-transporting ATPase subunit alpha-1 OS=Homo sapiens GN=ATP1A1 PE=2 SV=1	114239	324	254	55	115	0.09	0.12	1.333333333	2	4	3	4	8	4	12	8	-0.96198
sp|Q96TA1|NIBL1_HU	FAM129B	Niban-like protein 1 OS=Homo sapiens GN=FAM129B PE=1 SV=3	84598	155	274	137	108	0.16	0.21	1.3125	4	3	4	5	4	4	4	6	-0.04306
sp|P49411|EFTU_HU	TUFM	Elongation factor Tu, mitochondrial OS=Homo sapiens GN=TUFM PE=1 SV=2	49852	210	159	99	172	0.29	0.38	1.310344828	3	4	4	5	4	5	5	5	0.246777
tr|Q2NKY5|Q2NKY5_	TUBB6	TUBB6 protein OS=Homo sapiens GN=TUBB6 PE=2 SV=1	50514	65	35	317	522	0.88	1.14	1.295454545	12	13	16	16	17	19	19	21	0.113594
sp|Q15149|PLEC_HU	PLEC	Plectin OS=Homo sapiens GN=PLEC PE=1 SV=3	533462	74	27	270	686	0.11	0.14	1.272727273	11	15	17	22	18	24	32	30	0.364063
tr|B4DTG2|B4DTG2_	EEF1G	Elongation factor 1-gamma OS=Homo sapiens GN=EEF1G PE=2 SV=1	56456	93	32	225	570	0.86	1.09	1.26744186	5	14	15	17	14	19	18	21	0.385931
sp|P13639|EF2_HUM	EEF2	Elongation factor 2 OS=Homo sapiens GN=EEF2 PE=1 SV=4	96246	41	18	511	879	1.23	1.55	1.260162602	13	19	26	35	27	36	32	40	0.368193
sp|Q12792|TWF1_HU	TWF1	Twinfilin-1 OS=Homo sapiens GN=TWF1 PE=1 SV=3	40429	575	793	28	32	0.08	0.1	1.25	0	0	1	1	1	1	1	1	-0.04303
tr|B2R6X5|B2R6X5_H	tr|B2R6X5|B	cDNA, FLJ93166, highly similar to Homo sapiens heat shock 70kDa protein 6 (HSP70B~) (HSPA6), mRNA OS=Homo sapier	71370	193	221	112	124	0.2	0.25	1.25	3	3	5	6	6	6	6	7	-0.04308
tr|B4DE36|B4DE36_H	tr|B4DE36|B	Glucose-6-phosphate isomerase OS=Homo sapiens PE=2 SV=1	60376	117	130	182	195	0.3	0.37	1.233333333	4	4	5	6	7	7	7	10	-0.04309
tr|Q9BSV4|Q9BSV4_H	SFPQ	SFPQ protein (Fragment) OS=Homo sapiens GN=SFPQ PE=2 SV=2	68702	228	104	91	237	0.26	0.32	1.230769231	2	6	5	6	6	7	7	8	0.163701
tr|F8WEJ5|F8WEJ5_H	ASNS	Asparagine synthetase [glutamine-hydrolyzing] OS=Homo sapiens GN=ASNS PE=2 SV=1	46395	141	565	156	52	0.26	0.32	1.230769231	3	0	5	4	6	3	6	4	-0.93717
tr|B4DMU0|B4DMU0	PYCR1	Pyrroline-5-carboxylate reductase OS=Homo sapiens GN=PYCR1 PE=2 SV=1	36186	174	217	125	127	0.19	0.23	1.210526316	2	2	2	2	3	2	3	2	-0.52881
tr|B4DUI5|B4DUI5_H	tr|B4DUI5|B	Triosephosphate isomerase OS=Homo sapiens PE=2 SV=1	23085	267	458	71	67	0.26	0.31	1.192307692	1	1	2	2	2	3	2	4	0.442717
tr|Q60FE2|Q60FE2_H	MYH9	MYH9 variant protein OS=Homo sapiens GN=MYH9 PE=2 SV=1	227646	17	8	900	1472	0.9	1.07	1.188888889	30	40	46	57	52	62	60	69	0.211366
tr|B1AH89|B1AH89_H	TTLL12	Tubulin tyrosine ligase-like family, member 12 OS=Homo sapiens GN=TTLL12 PE=4 SV=1	74185	104	72	209	301	0.41	0.48	1.170731707	6	7	8	10	10	12	11	12	0.209084
tr|A5D906|A5D906_H	TUBB2A	TUBB2A protein (Fragment) OS=Homo sapiens GN=TUBB2A PE=2 SV=1	28128	25	21	143	364	3.07	3.59	1.169381107	4	10	7	15	8	18	8	20	1.082234
sp|P13010|XRCC5_HU	XRCC5	X-ray repair cross-complementing protein 5 OS=Homo sapiens GN=XRCC5 PE=1 SV=3	83222	128	100	172	244	0.36	0.42	1.166666667	6	6	8	10	8	11	9	15	0.393998
tr|Q96K98|Q96K98_H	tr|Q96K98|C	cDNA FLJ14414 fis, clone HEMBA1004847, highly similar to SIGNAL RECOGNITION PARTICLE 68 KD PROTEIN OS=Homo sa	37365	180	292	119	37	0.18	0.21	1.166666667	2	1	2	2	2	1	4	2	-0.78034
sp|Q3ZCM7|TBB8_HU	TUBB8	Tubulin beta-8 chain OS=Homo sapiens GN=TUBB8 PE=1 SV=2	50257	67	42	306	437	0.77	0.89	1.155844156	13	17	17	21	19	24	21	26	0.287769
tr|J3KSZ0|J3KSZ0_HU	EIF4A1	Eukaryotic initiation factor 4A-I (Fragment) OS=Homo sapiens GN=EIF4A1 PE=3 SV=1	26927	52	122	99	206	1.62	1.86	1.148148148	3	5	4	10	5	12	8	13	1.143692
sp|P58107|EPIPL_HU	EPPK1	Epiplakin OS=Homo sapiens GN=EPPK1 PE=1 SV=2	557674	56	40	390	462	0.07	0.08	1.142857143	10	10	12	13	12	13	13	16	0.068221
tr|Q53GX7|Q53GX7_	tr|Q53GX7|C	Threonyl-tRNA synthetase variant (Fragment) OS=Homo sapiens PE=2 SV=1	84304	94	98	221	247	0.64	0.72	1.125	6	8	13	14	17	18	18	20	0.03731
sp|P22314|UBA1_HU	UBA1	Ubiquitin-like modifier-activating enzyme 1 OS=Homo sapiens GN=UBA1 PE=1 SV=3	118858	114	114	187	221	0.28	0.31	1.107142857	5	3	10	10	10	8	12	12	-0.34865
tr|Q05BW9|Q05BW9	PAPSS1	PAPSS1 protein (Fragment) OS=Homo sapiens GN=PAPSS1 PE=2 SV=1	65264	553	1017	29	17	0.1	0.11	1.1	0	0	2	1	1	0	2	1	-1.62833
tr|A5PHJ9|A5PHJ9_H	PDHA1/LOC7	PDHA1/LOC79064 protein (Fragment) OS=Homo sapiens GN=PDHA1/LOC79064 PE=2 SV=1	34113	516	944	32	0	0.1	0.11	1.1	0	0	1	0	1	0	1	1	-1.62833
tr|K9JA46|K9JA46_HU	EL52	Epididymis luminal secretory protein 52 OS=Homo sapiens GN=EL52 PE=2 SV=1	85006	46	39	462	482	1.05	1.13	1.076190476	15	13	25	23	24	23	29	26	-0.10372
tr|B2R6K4|B2R6K4_H	tr|B2R6K4|B	cDNA, FLJ92996, highly similar to Homo sapiens guanine nucleotide binding protein (G protein), beta polypeptide 1 (GNE	38075	199	633	105	44	0.18	0.19	1.055555556	1	1	2	2	2	2	2	2	-0.04304
sp|Q6IS14|IF5AL_HU	EIF5AL1	Eukaryotic translation initiation factor 5A-1-like OS=Homo sapiens GN=EIF5AL1 PE=1 SV=2	16990	256	935	75	21	0.44	0.46	1.045454545	1	0	2	1	2	1	2	1	-0.78034
tr|Q5TCU6|Q5TCU6_	TLN1	Talin-1 OS=Homo sapiens GN=TLN1 PE=4 SV=1	260030	48	29	446	645	0.27	0.28	1.037037037	12	15	19	20	20	20	25	23	-0.04323
tr|B4DX08|B4DX08_H	tr|B4DX08|B	cDNA FLJ58784, highly similar to T-complex protein 1 subunit epsilon OS=Homo sapiens PE=2 SV=1	57521	90	261	229	73	0.4	0.41	1.025	6	1	6	4	6	4	6	5	-0.57427
tr|J3KPS3|J3KPS3_HU	ALDOA	Fructose-bisphosphate aldolase OS=Homo sapiens GN=ALDOA PE=3 SV=1	40249	63	20	338	813	1.79	1.82	1.016759777	11	14	15	20	17	21	18	22	0.255127
tr|J3KR97|J3KR97_HU	TBCD	Tubulin-specific chaperone D OS=Homo sapiens GN=TBCD PE=4 SV=1	138346	714	670	15	40	0.02	0.02	1	0	1	1	1	0	2	1	2	2.279573
tr|G3V448|G3V448_H	TMX1	Thioredoxin-related transmembrane protein 1 OS=Homo sapiens GN=TMX1 PE=2 SV=1	12018	689	859	19	27	0.29	0.29	1	0	0	1	1	0	1	1	1	1.542273
tr|Q5VZU9|Q5VZU9_	TPP2	Tripeptidyl-peptidase 2 OS=Homo sapiens GN=TPP2 PE=4 SV=1	141160	693	582	19	50	0.02	0.02	1	0	1	1	1	0	1	1	1	1.542273
tr|B0UX83|B0UX83_H	BAG6	HLA-B associated transcript 3 OS=Homo sapiens GN=BAG6 PE=4 SV=1	119085	704	561	18	52	0.03	0.03	1	0	1	1	1	0	1	1	2	1.542273
tr|A0MNP2|A0MNP2_	WDR57	CDW11/WDR57 OS=Homo sapiens GN=WDR57 PE=2 SV=1	39742	413	309	42	98	0.17	0.17	1	1	2	2	2	2	4	3	5	0.805621
tr|B1AHB0|B1AHB0_	MCM5	MCM5 minichromosome maintenance deficient 5, cell division cycle 46 (S. cerevisiae), isoform CRA_a OS=Homo sapiens	83031	562	954	29	21	0.04	0.04	1	0	0	1	1	1	2	2	2	0.694266
tr|B4DJE3|B4DJE3_HU	tr|B4DJE3|B	cDNA FLJ52929, highly similar to Dolichyl-diphosphooligosaccharide--proteinglycosyltransferase 48 kDa subunit (EC2.4.1	46620	604	870	26	26	0.07	0.07	1	0	0	1	1	1	2	2	2	0.694266
tr|B3KXU8|B3KXU8_H	tr|B3KXU8|B	cDNA FLJ46087 fis, clone TESTI2009497, highly similar to GPI-anchored protein p137 OS=Homo sapiens PE=2 SV=1	70489	367	596	47	47	0.05	0.05	1	1	1	1	1	1	2	1	2	0.694266
tr|Q2NKW8|Q2NKW8	AHCYL1	Adenosylhomocysteinase (Fragment) OS=Homo sapiens GN=AHCYL1 PE=2 SV=1	57630	509	563	33	52	0.06	0.06	1	0	1	1	1	1	2	1	4	0.694266
sp|Q15046|SYK_HUM	KARS	Lysine--tRNA ligase OS=Homo sapiens GN=KARS PE=1 SV=3	68461	265	147	71	181	0.32	0.32	1	1	6	6	6	6	9	7	9	0.505407
tr|Q9UJU1|Q9UJU1_	VIL2	Cytovillin 2 (Fragment) OS=Homo sapiens GN=VIL2 PE=2 SV=1	16294	236	280	88	107	0.46	0.46	1	2	2	2	2	2	3	3	3	0.442717
tr|B4DMB5|B4DMB5_	tr|B4DMB5|(	cDNA FLJ60075, highly similar to CTP synthase 1 (EC 6.3.4.2) OS=Homo sapiens PE=2 SV=1	56542	449	275	38	108	0.19	0.19	1	1	1	3	3	3	4	4	4	0.319851
tr|Q6DEN2|Q6DEN2_	DPYSL3	DPYSL3 protein OS=Homo sapiens GN=DPYSL3 PE=2 SV=1	74320	490	126	35	202	0.19	0.19	1	0	4	4	4	3	4	8	4	0.319851
tr|Q562M3|Q562M3_	ACT	Actin-like protein (Fragment) OS=Homo sapiens GN=ACT PE=2 SV=1	11529	103	78	213	288	7.06	7.06	1	10	12	14	17	17	21	20	23	0.255127
tr|B4DH02|B4DH02_	tr|B4DH02|B	cDNA FLJ50510, highly similar to Heat shock 70 kDa protein 4 OS=Homo sapiens PE=2 SV=1	95157	123	134	175	193	0.23	0.23	1	4	4	6	6	5	6	6	7	0.198269
tr|Q53GN4|Q53GN4_	WDR1	WD repeat domain 1, isoform CRA_a (Fragment) OS=Homo sapiens GN=WDR1 PE=2 SV=1	66822	137	101	160	243	0.4	0.4	1	4	6	7	7	7	8	9	8	0.137813
tr|B4DHQ3|B4DHQ3_	tr|B4DHQ3|B	cDNA FLJ56437, highly similar to Phosphoserine aminotransferase (EC 2.6.1.52) OS=Homo sapiens PE=2 SV=1	45783	552	988	30	20	0.07	0.07	1	0	0	1	1	1	1	2	3	-0.04303
tr|H0Y4Q3|H0Y4Q3_	RANGAP1	Ran GTPase-activating protein 1 (Fragment) OS=Homo sapiens GN=RANGAP1 PE=4 SV=1	27233	574	947	28	22	0.12	0.12	1	0	0	1	1	1	1	1	1	-0.04303
tr|B3KV72|B3KV72_H	tr|B3KV72|B	cDNA FLJ16204 fis, clone CTONG2019232, highly similar to Proteasome-associated protein ECM29 homolog (Fragment) (	78791	579	918	28	23	0.04	0.04	1	0	0	1	1	1	1	1	1	-0.04303
sp|Q96EK5|KBP_HUM	KIAA1279	KIF1-binding protein OS=Homo sapiens GN=KIAA1279 PE=1 SV=1	72453	649	903	22	24	0.05	0.05	1	0	0	1	1	1	1	1	1	-0.04303
sp|Q9Y5B9|SP16H_H	SUPT16H	FACT complex subunit SPT16 OS=Homo sapiens GN=SUPT16H PE=1 SV=1	120409	506	899	33	25	0.03	0.03	1	0	0	1	1	1	1	2	1	-0.04303
tr|Q0P512|Q0P512_H	SLC2A1	Solute carrier family 2 (Facilitated glucose transporter), member 1 OS=Homo sapiens GN=SLC2A1 PE=2 SV=1	54361	377	841	46	28	0.06	0.06	1	1	0	1	1	1	1	1	1	-0.04303
tr|B4DFE4|B4DFE4_H	tr|B4DFE4|B	cDNA FLJ59618, highly similar to Exosome component 10 OS=Homo sapiens PE=2 SV=1	61733	627	834	23	29	0.05	0.05	1	0	0	1	1	1	1	1	1	-0.04303
tr|B3KY60|B3KY60_H	tr|B3KY60|B	cDNA FLJ16777 fis, clone BRHIP2029567, highly similar to Cell division cycle 5-like protein OS=Homo sapiens PE=2 SV=1	92448	587	772	27	33	0.04	0.04	1	0	1	1	1	1	1	1	1	-0.04303
tr|B3KME2|B3KME2_	tr|B3KME2|B	cDNA FLJ10772 fis, clone NT2RP4000243, highly similar to Cartilage-associated protein OS=Homo sapiens PE=2 SV=1	47101	459	771	38	33	0.07	0.07	1	1	1	1	1	1	1	1	1	-0.04303
tr|F8WJN3|F8WJN3_	CPSF6	Cleavage and polyadenylation-specificity factor subunit 6 OS=Homo sapiens GN=CPSF6 PE=2 SV=1	52409	379	752	46	34	0.06	0.06	1	1	0	1	1	1	1	1	3	-0.04303
tr|J3KQE5|J3KQE5_H	RAN	GTP-binding nuclear protein Ran (Fragment) OS=Homo sapiens GN=RAN PE=4 SV=1	27027	571	730	29	36	0.12	0.12	1	0	0	1	1	1	1	1	1	-0.04303
tr|E7ERW8|E7ERW8_	DIAPH1	Protein diaphanous homolog 1 OS=Homo sapiens GN=DIAPH1 PE=2 SV=1	135945	184	675	116	40	0.02	0.02	1	1	1	1	1	1	1	2	2	-0.04303
sp|O43286|B4GT5_H	B4GALT5	Beta-1,4-galactosyltransferase 5 OS=Homo sapiens GN=B4GALT5 PE=2 SV=1	45318	651	672	22	40	0.07	0.07	1	0	1	1	1	1	1	1	1	-0.04303
tr|B4DLB8|B4DLB8_H	tr|B4DLB8|B	cDNA FLJ52205, highly similar to Beta-1,4-galactosyltransferase 1 (EC 2.4.1.-) OS=Homo sapiens PE=2 SV=1	40269	650	671	22	40	0.08	0.08	1	0	1	1	1	1	1	1	1	-0.04303
tr|B4E2W0|B4E2W0_	HADHB	3-ketoacyl-CoA thiolase OS=Homo sapiens GN=HADHB PE=2 SV=1	49076	499	663	34	41	0.07	0.07	1	0	1	1	1	1	1	1	1	-0.04303

tr|B4DXC4|B4DXC4_H	tr|B4DXC4|B	cDNA FLJ58636, moderately similar to Atlastin OS=Homo sapiens PE=2 SV=1	59162	451	650	38	42	0.06	0.06	1	1	1	1	1	1	1	1	1	-0.04303
tr|H0Y9T9|H0Y9T9_H	SEC31A	Protein transport protein Sec31A (Fragment) OS=Homo sapiens GN=SEC31A PE=4 SV=1	26151	121	645	95	42	0.13	0.13	1	1	1	1	1	1	1	1	1	-0.04303
tr|E9PIM9|E9PIM9_H	RNH1	Ribonuclease inhibitor (Fragment) OS=Homo sapiens GN=RNH1 PE=2 SV=1	22362	431	609	40	45	0.15	0.15	1	0	1	1	1	1	1	1	1	-0.04303
tr|B2R5M9|B2R5M9_	tr|B2R5M9|B	cDNA, FLJ92537, highly similar to Homo sapiens procollagen-lysine, 2-oxoglutarate 5-dioxygenase (lysine hydroxylase, Eh	84022	251	504	76	60	0.04	0.04	1	1	1	1	1	1	1	1	1	-0.04303
sp|O00232|PSD12_HU	PSMD12	26S proteasome non-ATPase regulatory subunit 12 OS=Homo sapiens GN=PSMD12 PE=1 SV=3	53270	283	502	66	61	0.06	0.06	1	1	1	1	1	1	1	1	1	-0.04303
tr|B2RDF2|B2RDF2_H	PES1	Pescadillo homolog 1 OS=Homo sapiens GN=PES1 PE=2 SV=1	68359	488	496	35	61	0.05	0.05	1	1	1	1	1	1	1	3	1	-0.04303
tr|H0YH88|H0YH88_H	NAP1L1	Nucleosome assembly protein 1-like 1 (Fragment) OS=Homo sapiens GN=NAP1L1 PE=3 SV=1	21179	417	441	33	70	0.16	0.16	1	0	1	1	1	1	1	1	1	-0.04303
sp|O76094|SRP72_HU	SRP72	Signal recognition particle 72 kDa protein OS=Homo sapiens GN=SRP72 PE=1 SV=3	75130	455	396	38	79	0.04	0.04	1	1	1	1	1	1	1	1	1	-0.04303
tr|H0YBW4|H0YBW4_	PLAA	Phospholipase A-2-activating protein (Fragment) OS=Homo sapiens GN=PLAA PE=4 SV=1	31645	539	381	30	81	0.11	0.11	1	0	1	1	1	1	1	1	1	-0.04303
sp|P04040|CATA_HU	CAT	Catalase OS=Homo sapiens GN=CAT PE=1 SV=3	59947	299	807	60	31	0.11	0.11	1	1	0	2	2	2	2	2	2	-0.04304
tr|B2RBE5|B2RBE5_H	tr|B2RBE5|B	cDNA, FLJ95468, highly similar to Homo sapiens transcriptional coactivator tubedown-100 (TBDN100),transcript variant	102490	446	784	38	32	0.06	0.06	1	0	0	2	2	2	2	2	2	-0.04304
sp|Q14432|PDE3A_H	PDE3A	cGMP-inhibited 3~,5~-cyclic phosphodiesterase A OS=Homo sapiens GN=PDE3A PE=1 SV=3	126440	493	533	35	56	0.05	0.05	1	1	2	2	2	2	2	2	2	-0.04304
tr|D3DVQ1|D3DVQ1_	LETM1	Leucine zipper-EF-hand containing transmembrane protein 1, isoform CRA_a OS=Homo sapiens GN=LETM1 PE=2 SV=1	63730	463	497	37	61	0.11	0.11	1	1	2	2	2	2	2	4	2	-0.04304
tr|B4DH52|B4DH52_H	tr|B4DH52|B	cDNA FLJ55916, highly similar to General transcription factor II-I OS=Homo sapiens PE=2 SV=1	112359	554	460	29	67	0.06	0.06	1	0	1	2	2	2	2	3	3	-0.04304
tr|B3KNB4|B3KNB4_H	tr|B3KNB4|B	cDNA FLJ14168 fis, clone NT2RP2001440, highly similar to 14-3-3 protein gamma OS=Homo sapiens PE=2 SV=1	28453	541	445	30	69	0.25	0.25	1	0	2	2	2	2	2	2	2	-0.04304
tr|J3KPM9|J3KPM9_H	STAT1	Signal transducer and activator of transcription 1-alpha/beta OS=Homo sapiens GN=STAT1 PE=4 SV=1	83878	364	353	48	87	0.08	0.08	1	1	1	2	2	2	2	2	3	-0.04304
tr|D2CFK9|D2CFK9_H	GTPBP4	Nucleolar GTP-binding protein 1 OS=Homo sapiens GN=GTPBP4 PE=2 SV=1	74317	264	389	72	80	0.14	0.14	1	2	2	3	3	3	3	3	3	-0.04305
sp|Q13151|ROA0_HU	HNRNPA0	Heterogeneous nuclear ribonucleoprotein A0 OS=Homo sapiens GN=HNRNPA0 PE=1 SV=1	30993	323	364	55	85	0.36	0.36	1	2	3	3	3	3	3	3	4	-0.04305
tr|A0PJ92|A0PJ92_HU	NOP56	NOP56 protein (Fragment) OS=Homo sapiens GN=NOP56 PE=2 SV=1	50068	311	359	58	85	0.14	0.14	1	1	2	2	2	3	3	3	3	-0.04305
sp|Q9H853|TBA4B_H	TUBA4B	Putative tubulin-like protein alpha-4B OS=Homo sapiens GN=TUBA4B PE=5 SV=2	27819	146	440	147	70	0.4	0.4	1	3	1	4	4	4	4	6	7	-0.04306
sp|P23526|SAHH_HU	AHCY	Adenosylhomocysteinase OS=Homo sapiens GN=AHCY PE=1 SV=4	48255	195	269	111	109	0.49	0.49	1	2	2	6	6	6	6	8	8	-0.04308
sp|P22626|ROA2_HU	HNRNPA2B1	Heterogeneous nuclear ribonucleoproteins A2/B1 OS=Homo sapiens GN=HNRNPA2B1 PE=1 SV=2	37464	77	62	256	347	0.97	0.97	1	7	7	8	10	9	9	10	11	-0.04311
sp|P53621|COPA_HU	COPA	Coatomer subunit alpha OS=Homo sapiens GN=COPA PE=1 SV=2	139797	181	255	118	115	0.18	0.18	1	5	2	7	7	8	7	11	10	-0.22401
sp|P08729|K2C7_HUI	KRT7	Keratin, type II cytoskeletal 7 OS=Homo sapiens GN=KRT7 PE=1 SV=5	51411	259	201	74	137	0.45	0.45	1	2	4	6	6	7	6	9	6	-0.24988
tr|F8VRQ1|F8VRQ1_H	HNRNPA1	Heterogeneous nuclear ribonucleoprotein A1 OS=Homo sapiens GN=HNRNPA1 PE=2 SV=1	33249	101	87	214	267	0.77	0.77	1	4	6	6	6	7	6	9	8	-0.24988
tr|B0V043|B0V043_H	VARS	Valine--tRNA ligase OS=Homo sapiens GN=VARS PE=3 SV=1	141632	275	148	69	180	0.15	0.15	1	2	4	6	6	6	5	8	6	-0.28443
sp|P07355|ANXA2_H	ANXA2	Annexin A2 OS=Homo sapiens GN=ANXA2 PE=1 SV=2	38808	100	124	214	204	1.26	1.26	1	8	7	11	10	12	10	13	11	-0.29535
tr|A8K525|A8K525_H	tr|A8K525|A	cDNA FLJ76817, highly similar to Homo sapiens non-POU domain containing, octamer-binding (NONO), mRNA OS=Homo	54367	75	92	266	253	0.8	0.8	1	10	6	11	11	12	10	14	12	-0.29535
tr|A2RUM7|A2RUM7	RPL5	Ribosomal protein L5 OS=Homo sapiens GN=RPL5 PE=2 SV=1	34569	187	226	115	123	0.44	0.44	1	2	4	4	4	5	4	7	4	-0.33291
tr|B2RBR9|B2RBR9_H	tr|B2RBR9|B	cDNA, FLJ95650, highly similar to Homo sapiens karyopherin (importin) beta 1 (KPNB1), mRNA OS=Homo sapiens PE=2 S	98434	171	79	126	283	0.22	0.22	1	4	4	6	6	9	7	9	9	-0.38482
tr|F8VPD4|F8VPD4_H	CAD	CAD protein OS=Homo sapiens GN=CAD PE=2 SV=1	238097	317	377	57	81	0.04	0.04	1	1	2	3	3	4	3	5	3	-0.40597
sp|Q08211|DHX9_HU	DHX9	ATP-dependent RNA helicase A OS=Homo sapiens GN=DHX9 PE=1 SV=4	142181	96	51	218	377	0.41	0.41	1	8	8	16	15	18	13	22	16	-0.49945
tr|D3DTH7|D3DTH7_	MYO1C	Myosin IC, isoform CRA_a OS=Homo sapiens GN=MYO1C PE=2 SV=1	99613	284	536	65	56	0.07	0.07	1	1	2	3	2	3	2	4	2	-0.52881
tr|B4DDR2|B4DDR2_	tr|B4DDR2|B	cDNA FLJ54588, highly similar to Major vault protein OS=Homo sapiens PE=2 SV=1	98526	304	368	59	84	0.07	0.07	1	2	2	2	2	3	2	3	4	-0.52881
tr|B4DII5|B4DII5_HUI	tr|B4DII5|B4	Importin subunit alpha OS=Homo sapiens PE=2 SV=1	60345	573	191	28	144	0.11	0.11	1	0	2	2	2	3	2	3	3	-0.52881
tr|B2R6J2|B2R6J2_HU	tr|B2R6J2|B2	cDNA, FLJ92973, highly similar to Homo sapiens villin 2 (ezrin) (VIL2), mRNA OS=Homo sapiens PE=2 SV=1	69472	126	169	173	162	0.32	0.32	1	6	4	6	6	8	5	8	7	-0.67214
tr|B1ANK7|B1ANK7_	FH	Fumarate hydratase OS=Homo sapiens GN=FH PE=2 SV=1	54773	288	979	64	20	0.12	0.12	1	1	0	2	2	2	1	5	2	-0.78034
sp|Q15392|DHC24_H	DHCR24	Delta(24)-sterol reductase OS=Homo sapiens GN=DHCR24 PE=1 SV=2	60803	442	754	39	34	0.05	0.05	1	1	0	1	1	2	1	3	2	-0.78034
tr|Q6NZ52|Q6NZ52_	RPL27A	Ribosomal protein L27a OS=Homo sapiens GN=RPL27A PE=2 SV=1	16582	348	692	50	39	0.2	0.2	1	1	1	1	1	2	1	2	1	-0.78034
tr|Q6DC98|Q6DC98_	LMNB1	LMNB1 protein (Fragment) OS=Homo sapiens GN=LMNB1 PE=2 SV=1	38289	219	534	95	56	0.18	0.18	1	2	1	2	2	2	1	4	2	-0.78034
sp|O00425|IF2B3_HU	IGF2BP3	Insulin-like growth factor 2 mRNA-binding protein 3 OS=Homo sapiens GN=IGF2BP3 PE=1 SV=2	64008	556	527	29	57	0.05	0.05	1	0	1	1	1	2	1	2	1	-0.78034
tr|B4DY32|B4DY32_H	tr|B4DY32|B	Asparagine synthetase OS=Homo sapiens PE=2 SV=1	55055	141	393	156	79	0.26	0.26	1	3	1	5	4	6	3	6	4	-0.93717
sp|Q14247|SRC8_HU	CTTN	Src substrate cortactin OS=Homo sapiens GN=CTTN PE=1 SV=2	61720	378	399	46	78	0.11	0.11	1	1	1	2	1	3	1	3	1	-1.26611
tr|B4DXX7|B4DXX7_	DDX3Y	ATP-dependent RNA helicase DDX3Y OS=Homo sapiens GN=DDX3Y PE=2 SV=1	73342	217	288	95	46	0.19	0.19	1	2	1	5	2	6	2	6	2	-1.42293
tr|H0YF33|H0YF33_H	NASP	Nuclear autoantigenic sperm protein (Fragment) OS=Homo sapiens GN=NASP PE=4 SV=1	31883	542	1011	30	18	0.1	0.1	1	0	0	1	1	2	0	3	2	-2.36564
tr|A0N4V7|A0N4V7_	Tcr-alpha	HCG2039797 (Fragment) OS=Homo sapiens GN=Tcr-alpha PE=4 SV=1	2269	212	470	98	65	1.81	1.81	1	5	3	15	3	31	3	46	7	-3.22265
tr|H0YA24|H0YA24_H	PLRG1	Pleiotropic regulator 1 (Fragment) OS=Homo sapiens GN=PLRG1 PE=4 SV=1	17588	456	331	38	57	0.19	0.18	0.947368421	1	1	1	1	1	1	2	1	-0.04303
tr|E0X098|E0X098_H	TES	Testin variant (Fragment) OS=Homo sapiens GN=TES PE=2 SV=1	22009	430	395	40	24	0.15	0.14	0.933333333	1	0	1	1	1	1	1	1	-0.04303
tr|B7ZB91|B7ZB91_H	TNRC6B	Trinucleotide repeat containing 6B (Fragment) OS=Homo sapiens GN=TNRC6B PE=4 SV=1	81825	359	666	48	36	0.13	0.12	0.923076923	1	1	3	1	2	1	3	1	-0.78034
sp|P07814|SYEP_HUI	EPRS	Bifunctional glutamate/proline--tRNA ligase OS=Homo sapiens GN=EPRS PE=1 SV=5	172080	57	63	380	339	0.33	0.3	0.909090909	9	9	15	14	15	14	17	18	-0.13973
tr|Q53HV2|Q53HV2_	tr|Q53HV2|Q	Chaperonin containing TCP1, subunit 7 (Eta) variant (Fragment) OS=Homo sapiens PE=2 SV=1	59816	161	273	133	108	0.38	0.34	0.894736842	2	3	6	5	5	5	7	6	-0.04307
tr|Q53GU8|Q53GU8_	tr|Q53GU8|Q	Transforming growth factor, beta-induced, 68kDa variant (Fragment) OS=Homo sapiens PE=2 SV=1	75231	358	1008	48	18	0.09	0.08	0.888888889	1	0	2	1	2	0	2	1	-2.36564
tr|Q9UJZ2|Q9UJZ2_H	VIL2	Cytovillin 2 (Fragment) OS=Homo sapiens GN=VIL2 PE=2 SV=1	18464	119	279	88	107	0.46	0.4	0.869565217	2	2	2	2	2	2	2	4	-0.04304
sp|O00410|IPO5_HUI	IPO5	Importin-5 OS=Homo sapiens GN=IPO5 PE=1 SV=4	125032	85	95	236	248	0.23	0.2	0.869565217	7	6	8	7	10	7	15	7	-0.52955
sp|P14923|PLAK_HUI	JUP	Junction plakoglobin OS=Homo sapiens GN=JUP PE=1 SV=3	82434	205	196	102	141	0.26	0.22	0.846153846	3	4	6	5	6	4	7	5	-0.57427
tr|B2R8N0|B2R8N0_H	tr|B2R8N0|B	cDNA, FLJ93975, highly similar to Homo sapiens epoxide hydrolase 1, microsomal (xenobiotic) (EPHX1), mRNA OS=Homo	53129	115	177	184	152	0.62	0.52	0.838709677	6	4	8	8	8	12	8	14	0.514627
tr|B4DNN4|B4DNN4_	tr|B4DNN4|E	Ribonucleoside-diphosphate reductase OS=Homo sapiens PE=2 SV=1	87252	156	188	136	122	0.29	0.24	0.827586207	5	3	7	5	8	5	8	6	-0.67214
tr|B3KTA3|B3KTA3_H	tr|B3KTA3|B	Fascin OS=Homo sapiens PE=2 SV=1	52814	53	50	403	326	1.07	0.88	0.822429907	9	6	14	11	16	10	20	12	-0.69727
tr|B4E022|B4E022_H	TKT	Transketolase OS=Homo sapiens GN=TKT PE=2 SV=1	63410	35	45	578	392	1.36	1.11	0.816176471	17	12	24	18	26	21	32	24	-0.34662
tr|H0YGW7|H0YGW7	ABCF1	ATP-binding cassette sub-family F member 1 (Fragment) OS=Homo sapiens GN=ABCF1 PE=3 SV=1	68075	144	115	151	163	0.27	0.22	0.814814815	4	3	5	4	5	3	7	5	-0.69582
sp|P68366|TBA4A_HU	TUBA4A	Tubulin alpha-4A chain OS=Homo sapiens GN=TUBA4A PE=1 SV=1	50634	22	22	796	843	3.01	2.45	0.813953488	20	23	31	35	31	37	36	44	0.210219
tr|Q5I6Y6|Q5I6Y6_HU	LMNA	Lamin A/C transcript variant 1 OS=Homo sapiens GN=LMNA PE=2 SV=1	74322	95	46	221	389	0.75	0.61	0.813333333	7	7	13	11	15	12	15	14	-0.35452
tr|G8JLB6|G8JLB6_HU	HNRNPH1	Heterogeneous nuclear ribonucleoprotein H OS=Homo sapiens GN=HNRNPH1 PE=4 SV=1	51482	207	137	101	189	0.45	0.36	0.8	2	5	6	7	5	6	6	7	0.198269
tr|Q53HF2|Q53HF2_H	tr|Q53HF2|C	Heat shock 70kDa protein 8 isoform 2 variant (Fragment) OS=Homo sapiens PE=2 SV=1	53580	51	48	426	355	1.45	1.16	0.8	12	10	17	19	19	19	19	20	-0.04322
tr|Q2TSD0|Q2TSD0_H	tr|Q2TSD0|C	Glyceraldehyde-3-phosphate dehydrogenase OS=Homo sapiens PE=2 SV=1	36197	18	25	891	624	3.44	2.72	0.790697674	23	12	31	23	31	22	36	25	-0.53179
tr|B0YJC4|B0YJC4_HU	VIM	Vimentin OS=Homo sapiens GN=VIM PE=3 SV=1	49680	66	47	312	319	1.03	0.81	0.786407767	9	8	11	10	12	11	15	12	-0.16377
sp|P06733|ENOA_HU	ENO1	Alpha-enolase OS=Homo sapiens GN=ENO1 PE=1 SV=2	47481	40	94	520	249	1.39	1.09	0.784172662	13	7	18	14	19	14	25	18	-0.47231
sp|P38919|IF4A3_HU	EIF4A3	Eukaryotic initiation factor 4A-III OS=Homo sapiens GN=EIF4A3 PE=1 SV=4	47126	262	180	74	152	0.4	0.31	0.775	1	4	5	5	4	5	6	8	0.246777
sp|Q00610|CLH1_HU	CLTC	Clathrin heavy chain 1 OS=Homo sapiens GN=CLTC PE=1 SV=5	193260	30	33	629	570	0.52	0.4	0.769230769	19	14	25	20	24	20	28	21	-0.30177
tr|B7Z6M1|B7Z6M1_	PLS3	Plastin-3 OS=Homo sapiens GN=PLS3 PE=2 SV=1	66047	282	227	66	123	0.34	0.26	0.764705882	1	3	6	5	6	5	8	7	-0.28443
sp|Q92973|TNPO1_H	TNPO1	Transportin-1 OS=Homo sapiens GN=TNPO1 PE=1 SV=2	103771	139	711	158	38	0.17	0.13	0.764705882	3	0	5	4	8	4	8	5	-0.96198
tr|G3V2B8|G3V2B8_H	MTHFD1	C-1-tetrahydrofolate synthase, cytoplasmic OS=Homo sapiens GN=MTHFD1 PE=2 SV=1	102152	112	182	192	151	0.33	0.25	0.757575758	5	5	9	7	12	7	12	10	-0.78178
tr|Q5U077|Q5U077_	LDHB	L-lactate dehydrogenase OS=Homo sapiens GN=LDHB PE=2 SV=1	36900	39	54	530	366	1.81	1.36	0.751381215	13	10	16	13	18	14	20	15	-0.39602
tr|D6RB78|D6RB78_H	PDLIM5	PDZ and LIM domain protein 5 OS=Homo sapiens GN=PDLIM5 PE=2 SV=1	68940	189	176	74	154	0.52	0.39	0.75	1	4	6	7	5	7	7	11	0.405054
sp|P42166|LAP2A_HU	TMPO	Lamina-associated polypeptide 2, isoform alpha OS=Homo sapiens GN=TMPO PE=1 SV=2	76016	241	414	84	75	0.24	0.18	0.75	1	2	5	4	5	5	5	5	-0.04307
sp|Q6S8J3|POTEE_HU	POTEE	POTE ankyrin domain family member E OS=Homo sapiens GN=POTEE PE=1 SV=3	122882	5	4	2211	2010	0.44	0.33	0.75	45	49	66	64	70	63	75	70	-0.19698
sp|P23381|SYWC_HU	WARS	Tryptophan--tRNA ligase, cytoplasmic OS=Homo sapiens GN=WARS PE=1 SV=2	53474	145	259	148	113	0.27	0.2	0.740740741	3	2	4	3	5	3	5	3	-0.69582
tr|F4ZW64|F4ZW64_	tr|F4ZW64|F	NF90a OS=Homo sapiens PE=2 SV=1	76369	154	231	137	121	0.46	0.34	0.739130435	3	4	9	7	10	9	11	9	-0.18785
tr|Q0IIN1|Q0IIN1_HU	KRT77	Keratin 77 OS=Homo sapiens GN=KRT77 PE=2 SV=1	62050	76	322	264	95	0.23	0.17	0.739130435	10	3	10	4	10	4	12	4	-1.26752
tr|B4E0S6|B4E0S6_HU	tr|B4E0S6|B	cDNA FLJ55635, highly similar to pre-mRNA-splicing factorATP-dependent RNA helicase DHX15 (EC 3.6.1.-) OS=Homo sap	90289	183	329	117	93	0.15	0.11	0.733333333	2	2	4	3	4	3	7	3	-0.40597
sp|P52272|HNRPM_H	HNRNPM	Heterogeneous nuclear ribonucleoprotein M OS=Homo sapiens GN=HNRNPM PE=1 SV=3	77749	164	161	132	171	0.39	0.28	0.717948718	7	3	8	6	11	6	12	7	-0.86793
tr|A5PLK7|A5PLK7_H	RCC2	RCC2 protein (Fragment) OS=Homo sapiens GN=RCC2 PE=2 SV=1	50331	165	127	131	188	0.46	0.33	0.717391304	4	4	6	4	6	4	6	4	-0.57427
sp|P26038|MOES_HU	MSN	Moesin OS=Homo sapiens GN=MSN PE=1 SV=3	67892	119	106	179	229	0.46	0.33	0.717391304	7	6	8	6	11	7	12	8	-0.66114
tr|Q53XC0|Q53XC0_H	EIF2S1	Eukaryotic translation initiation factor 2, subunit 1 alpha, 35kDa, isoform CRA_a OS=Homo sapiens GN=EIF2S1 PE=2 SV=1	36374	208	360	100	85	0.42	0.3	0.714285714	4	3	4	3	4	3	4	3	-0.40597
tr|B2RCM2|B2RCM2_	tr|B2RCM2|E	cDNA, FLJ96156, highly similar to Homo sapiens leucyl-tRNA synthetase (LARS), mRNA OS=Homo sapiens PE=2 SV=1	135550	257	617	75	45	0.1	0.07	0.7	2	0	4	3	3	3	5	4	-0.04305
tr|A8K6Q8|A8K6Q8_	tr|A8K6Q8|A	cDNA FLJ75881, highly similar to Homo sapiens transferrin receptor (p90, CD71) (TFRC), mRNA OS=Homo sapiens PE=2 S	85276	122	83	176	272	0.3	0.21	0.7	6	5	7	5	7	5	8	6	-0.49122
tr|F2VNS8|F2VNS8_H	HLA-C	MHC class I antigen (Fragment) OS=Homo sapiens GN=HLA-C PE=3 SV=1	21240	639	706	22	38	0.16	0.11	0.6875	0	0	1	1	2	1	2	1	-0.78034
sp|P00338|LDHA_HU	LDHA	L-lactate dehydrogenase A chain OS=Homo sapiens GN=LDHA PE=1 SV=2	36950	44	68	481	320	2.33	1.57	0.673819742	10	10	19	14	19	15	24	17	-0.37576
tr|B3KX72|B3KX72_H	tr|B3KX72|B	cDNA FLJ44920 fis, clone BRAMY3011501, highly similar to Heterogeneous nuclear ribonucleoprotein U OS=Homo sapie	83774	59	86	367	206	0.58	0.39	0.672413793	10	5	14	7	16	8	20	12	-1.00279
tr|Q68DB1|Q68DB1_	DKFZp686F22	Metastasis-associated protein MTA2 OS=Homo sapiens GN=DKFZp686F2281 PE=2 SV=1	55515	676	456	20	0	0.06	0.04	0.666666667	0	0	1	0	1	1	1	1	-0.04303
tr|B4DGU4|B4DGU4_	CTNNB1	Catenin beta-1 OS=Homo sapiens GN=CTNNB1 PE=2 SV=1	85339	340	591	52	49	0.12	0.08	0.666666667	2	1	3	2	3	2	3	3	-0.52881
tr|J3KQL0|J3KQL0_HU	CPS1	Carbamoyl-phosphate synthase [ammonia], mitochondrial OS=Homo sapiens GN=CPS1 PE=3 SV=1	166686	204	165	102	167	0.15	0.1	0.666666667	4	4	7	5	10	6	12	8	-0.73634
tr|B3KNN7|B3KNN7_	tr|B3KNN7|E	cDNA FLJ30049 fis, clone ADRGL1000033, highly similar to 26S proteasome non-ATPase regulatory subunit 3 OS=Homo s	57311	273	599	69	47	0.18	0.12	0.666666667	3	0	3	2	3	2	3	2	-0.52881
tr|B0AZS6|B0AZS6_H	YWHAZ	14-3-3 protein zeta/delta OS=Homo sapiens GN=YWHAZ PE=2 SV=1	19174	497	314	34	30	0.38	0.25	0.657894737	0	0	2	1	2	1	2	1	-0.78034
tr|Q5T081|Q5T081_H	RCC1	CHC1 protein OS=Homo sapiens GN=RCC1 PE=2 SV=1	45397	269	702	70	38	0.23	0.15	0.652173913	2	0	3	2	2	2	4	2	-0.04304
tr|Q53ET2|Q53ET2_H	tr|Q53ET2|Q	Dihydropyrimidinase-like 2 variant (Fragment) OS=Homo sapiens PE=2 SV=1	62688	342	192	51	104	0.23	0.15	0.652173913	1	2	4	2	3	2	5	3	-0.52881
sp|Q9NV06|DCA13_H	DCAF13	DDB1- and CUL4-associated factor 13 OS=Homo sapiens GN=DCAF13 PE=1 SV=2	51996	435	743	39	35	0.2	0.13	0.65	1	0	3	1	3	1	6	1	-1.26611
tr|H0Y8G5|H0Y8G5_H	HNRNPD	Heterogeneous nuclear ribonucleoprotein D0 (Fragment) OS=Homo sapiens GN=HNRNPD PE=4 SV=1	29877	229	406	90	76	0.37	0.24	0.648648649	3	2	3	2	4	3	4	3	-0.40597
tr|B4DRH6|B4DRH6_	tr|B4DRH6|E	cDNA FLJ54509, highly similar to Trifunctional enzyme subunit alpha, mitochondrial OS=Homo sapiens PE=2 SV=1	79007	133	436	164	71	0.28	0.18	0.642857143	5	1	6	4	7	4	7	5	-0.78106
sp|P46063|RECQ1_HU	RECQL	ATP-dependent DNA helicase Q1 OS=Homo sapiens GN=RECQL PE=1 SV=3	74436	439	636	39	43	0.14	0.09	0.642857143	1	1	3	2	3	1	3	4	-1.26611
tr|D6R9L0|D6R9L0_H	GNB2L1	Guanine nucleotide-binding protein subunit beta-2-like 1 (Fragment) OS=Homo sapiens GN=GNB2L1 PE=2 SV=1	33009	216	172	58	157	0.52	0.33	0.634615385	1	2	1	3	1	3	1	4	1.180027
tr|B3GQS7|B3GQS7_	HSPD1	Mitochondrial heat shock 60kD protein 1 variant 1 OS=Homo sapiens GN=HSPD1 PE=2 SV=1	60813	16	26	904	698	1.72	1.09	0.63372093	24	16	27	17	26	17	33	19	-0.64494
tr|Q6IBT3|Q6IBT3_HU	CCT7	CCT7 protein OS=Homo sapiens GN=CCT7 PE=2 SV=1	59804	161	266	133	110	0.38	0.24	0.631578947	2	3	6	4	5	5	7	5	-0.04307
tr|J3KPF3|J3KPF3_HU	SLC3A2	4F2 cell-surface antigen heavy chain OS=Homo sapiens GN=SLC3A2 PE=4 SV=1	68230	60	41	366	446	1.33	0.84	0.631578947	12	9	20	14	20	15	20	16	-0.44826
tr|B4DKM5|B4DKM5_	VDAC2	Voltage-dependent anion-selective channel protein 2 OS=Homo sapiens GN=VDAC2 PE=2 SV=1	27804	196	138	108	158	0.76	0.48	0.631578947	1	3	5	3	5	3	5	3	-0.69582
tr|B4DZB1|B4DZB1_H	tr|B4DZB1|B	cDNA FLJ59645, highly similar to 116 kDa U5 small nuclear ribonucleoprotein component OS=Homo sapiens PE=2 SV=1	104668	111	187	193	148	0.27	0.17	0.62962963	4	4	8	5	8	6	10	8	-0.4308
tr|C5J3Q8|C5J3Q8_H	HLA-B	MHC class I antigen (Fragment) OS=Homo sapiens GN=HLA-B PE=3 SV=1	31979	639	707	22	38	0.16	0.1	0.625	0	0	1	1	1	1	1	2	-0.04303
tr|B4DEN9|B4DEN9_	tr|B4DEN9|E	cDNA FLJ56617, highly similar to Ribosome biogenesis protein BOP1 OS=Homo sapiens PE=2 SV=1	72443	505	766	33	33	0.08	0.05	0.625	0	0	2	1	2	2	3	2	-0.04304
tr|Q96HI1|Q96HI1_H	tr|Q96HI1|Q	Similar to plastin 3 (T isoform) (Fragment) OS=Homo sapiens PE=2 SV=1	46203	282	224	63	123	0.34	0.21	0.617647059	1	3	5	4	5	4	5	4	-0.33291
tr|A4QPB0|A4QPB0_H	IQGAP1	IQ motif containing GTPase activating protein 1 OS=Homo sapiens GN=IQGAP1 PE=2 SV=1	189789	84	84	239	270	0.31	0.19	0.612903226	8	8	17	10	18	10	21	16	-0.86302
tr|A8K4Z4|A8K4Z4_H	tr|A8K4Z4|A	cDNA FLJ75549, highly similar to Homo sapiens ribosomal protein, large, P0 (RPLP0), transcript variant 1, mRNA OS=Hom	34389	109	223	201	123	0.74	0.45	0.608108108	6	3	6	4	6	4	6	4	-0.57427
tr|Q59ES3|Q59ES3_H	tr|Q59ES3|Q	Solute carrier family 1 (Neutral amino acid transporter), member 5 variant (Fragment) OS=Homo sapiens PE=2 SV=1	57810	290	767	63	33	0.1	0.06	0.6	1	1	1	1	1	1	1	1	-0.04303
tr|B3KX19|B3KX19_H	tr|B3KX19|B	cDNA FLJ44500 fis, clone UTERU3000828, highly similar to 116 kDa U5 small nuclear ribonucleoprotein component OS=H	109111	111	186	193	148	0.27	0.16	0.592592593	4	4	8	5	8	6	10	8	-0.4308
sp|Q9NR30|DDX21_H	DDX21	Nucleolar RNA helicase 2 OS=Homo sapiens GN=DDX21 PE=1 SV=5	87804	148	301	147	102	0.34	0.2	0.588235294	4	4	6	5	6	6	9	6	-0.4308
sp|P68363|TBA1B_H	TUBA1B	Tubulin alpha-1B chain OS=Homo sapiens GN=TUBA1B PE=1 SV=1	50804	13	12	1091	1118	5.58	3.24	0.580645161	31	29	43	41	45	43	49	51	-0.10901
tr|Q9UE89|Q9UE89_H	tr|Q9UE89|Q	Beta-actin (Fragment) OS=Homo sapiens PE=2 SV=1	7908	1	19	448	877	31.43	18.24	0.580337257	9	19	13	25	13	25	14	25	0.878406
tr|J3KRX5|J3KRX5_H	RPL17	60S ribosomal protein L17 (Fragment) OS=Homo sapiens GN=RPL17 PE=3 SV=1	20386	517	877	32	26	0.28	0.16	0.571428571	0	0	1	1	1	1	1	1	-0.04303
tr|B2RAQ8|B2RAQ8_H	tr|B2RAQ8|B	cDNA, FLJ95058, highly similar to Homo sapiens carnitine palmitoyltransferase 1A (liver) (CPT1A),nuclear gene encoding	88967	354	405	49	77	0.07	0.04	0.571428571	1	1	2	2	3	2	3	2	-0.52881
sp|Q1KMD3|HNRL2_H	HNRNPUL2	Heterogeneous nuclear ribonucleoprotein U-like protein 2 OS=Homo sapiens GN=HNRNPUL2 PE=1 SV=1	85622	249	581	78	50	0.21	0.12	0.571428571	2	2	5	3	6	4	6	5	-0.57427
sp|P16615|AT2A2_H	ATP2A2	Sarcoplasmic/endoplasmic reticulum calcium ATPase 2 OS=Homo sapiens GN=ATP2A2 PE=1 SV=1	116336	149	127	144	176	0.32	0.18	0.5625	4	4	10	6	12	7	13	8	-0.78178
tr|B4DPZ3|B4DPZ3_H	tr|B4DPZ3|B	cDNA FLJ53290, highly similar to Cytoplasmic dynein 1 intermediate chain 2 OS=Homo sapiens PE=2 SV=1	70869	298	281	60	107	0.09	0.05	0.555555556	2	1	2	1	2	1	2	1	-0.78034
sp|P53396|ACLY_HU	ACLY	ATP-citrate synthase OS=Homo sapiens GN=ACLY PE=1 SV=3	121674	20	31	845	576	1.28	0.7	0.546875	21	15	32	26	34	30	40	32	-0.2225
tr|B4DQG5|B4DQG5_H	tr|B4DQG5|B	cDNA FLJ54122, highly similar to Cytosol aminopeptidase (EC 3.4.11.1) OS=Homo sapiens PE=2 SV=1	39662	368	371	47	84	0.15	0.08	0.533333333	1	1	1	1	1	1	1	1	-0.04303
tr|B2R5M8|B2R5M8_H	tr|B2R5M8|B	Isocitrate dehydrogenase [NADP] OS=Homo sapiens PE=2 SV=1	46905	555	875	29	26	0.15	0.08	0.533333333	0	0	2	1	3	1	3	1	-1.26611
tr|B4DPP6|B4DPP6_H	tr|B4DPP6|B	cDNA FLJ54371, highly similar to Serum albumin OS=Homo sapiens PE=2 SV=1	72370	222	474	93	65	0.25	0.13	0.52	3	1	5	2	6	2	10	3	-1.42293
tr|G3V2A3|G3V2A3_H	TUBB3	Tubulin beta-3 chain (Fragment) OS=Homo sapiens GN=TUBB3 PE=2 SV=1	20929	25	28	147	396	3.07	1.57	0.511400651	5	9	6	10	7	11	7	13	0.574915
sp|P55072|TERA_HU	VCP	Transitional endoplasmic reticulum ATPase OS=Homo sapiens GN=VCP PE=1 SV=4	89950	64	53	326	371	0.65	0.33	0.507692308	7	7	14	8	14	11	15	12	-0.37859
sp|P78371|TCPB_HU	CCT2	T-complex protein 1 subunit beta OS=Homo sapiens GN=CCT2 PE=1 SV=4	57794	37	61	563	348	1.86	0.94	0.505376344	14	7	20	13	20	12	23	15	-0.7596
tr|H7C3A1|H7C3A1_H	SRRT	Serrate RNA effector molecule homolog (Fragment) OS=Homo sapiens GN=SRRT PE=4 SV=1	57154	701	307	18	0	0.06	0.03	0.5	0	0	0	0	0	0	1	2	-0.04302
tr|A4UTT5|A4UTT5_H	HLA-A	MHC class I antigen (Fragment) OS=Homo sapiens GN=HLA-A PE=3 SV=1	40867	639	922	22	23	0.16	0.08	0.5	0	0	1	0	1	1	1	1	-0.04303
tr|B3KNL2|B3KNL2_H	RUVBL2	RuvB-like 2 (E. coli), isoform CRA_d OS=Homo sapiens GN=RUVBL2 PE=2 SV=1	47603	584	810	28	31	0.14	0.07	0.5	0	0	1	1	1	1	2	1	-0.04303
sp|P53618|COPB_HU	COPB1	Coatomer subunit beta OS=Homo sapiens GN=COPB1 PE=1 SV=3	108214	303	546	59	54	0.06	0.03	0.5	1	1	2	1	2	2	3	2	-0.04304
sp|Q01650|LAT1_HU	SLC7A5	Large neutral amino acids transporter small subunit 1 OS=Homo sapiens GN=SLC7A5 PE=1 SV=2	55659	239	286	85	105	0.12	0.06	0.5	1	2	3	3	4	3	5	3	-0.40597
tr|B4DZ88|B4DZ88_H	KTN1	KTN1 protein OS=Homo sapiens GN=KTN1 PE=2 SV=1	153179	322	832	55	29	0.04	0.02	0.5	2	0	2	1	2	2	4	3	-0.52881
tr|G3V180|G3V180_H	DPP3	Dipeptidyl peptidase 3 OS=Homo sapiens GN=DPP3 PE=2 SV=1	84666	335	923	52	23	0.08	0.04	0.5	1	0	1	1	2	1	2	1	-0.78034
tr|A8K2S1|A8K2S1_H	tr|A8K2S1|A	cDNA FLJ75258, highly similar to Homo sapiens phospholipase A2, group IVA (cytosolic, calcium-dependent) (PLA2G4A),	85695	369	921	47	23	0.08	0.04	0.5	1	0	1	1	2	1	5	1	-0.78034
sp|Q9BYZ2|LDH6B_H	LDHAL6B	L-lactate dehydrogenase A-like 6B OS=Homo sapiens GN=LDHAL6B PE=1 SV=3	42372	327	902	54	24	0.16	0.08	0.5	1	0	2	1	2	1	2	1	-0.78034
tr|B2RCT6|B2RCT6_H	tr|B2RCT6|B	cDNA, FLJ96276, highly similar to Homo sapiens G1 to S phase transition 1 (GSPT1), mRNA OS=Homo sapiens PE=2 SV=1	56320	391	897	44	25	0.12	0.06	0.5	1	1	2	1	2	1	2	1	-0.78034
sp|O00505|IMA3_HU	KPNA3	Importin subunit alpha-3 OS=Homo sapiens GN=KPNA3 PE=1 SV=2	58288	406	651	43	42	0.12	0.06	0.5	0	1	2	1	2	1	2	1	-0.78034
tr|Q58FF4|Q58FF4_H	HSP90Bf	Heat shock protein 90Bf OS=Homo sapiens GN=HSP90Bf PE=1 SV=1	41833	407	646	43	42	0.16	0.08	0.5	1	1	2	1	2	1	2	1	-0.78034
tr|B4DHG3|B4DHG3_H	tr|B4DHG3|B	cDNA FLJ55329, highly similar to Procollagen-lysine,2-oxoglutarate5-dioxygenase 2 (EC 1.14.11.4) OS=Homo sapiens PE=	81716	453	402	38	77	0.08	0.04	0.5	0	1	1	2	1	2	1	2	-0.78034
tr|E9PPU0|E9PPU0_H	EPPK1	Epiplakin OS=Homo sapiens GN=EPPK1 PE=4 SV=1	266001	107	140	205	188	0.1	0.05	0.5	6	3	8	4	7	4	9	6	-0.78106
tr|E9PRA5|E9PRA5_H	ATP1A4	Sodium/potassium-transporting ATPase subunit alpha-4 OS=Homo sapiens GN=ATP1A4 PE=2 SV=1	57835	289	818	63	30	0.12	0.06	0.5	0	0	6	2	10	2	19	3	-2.11619
tr|Q8TE01|Q8TE01_H	derp12	DERP12 (Dermal papilla derived protein 12) OS=Homo sapiens GN=derp12 PE=2 SV=1	38340	69	189	297	147	0.79	0.39	0.493670886	6	4	7	4	8	4	8	5	-0.96198
tr|Q5HYG8|Q5HYG8_H	DKFZp686P09	Putative uncharacterized protein DKFZp686P09201 OS=Homo sapiens GN=DKFZp686P09201 PE=2 SV=1	53831	86	426	235	73	0.61	0.3	0.491803279	4	1	8	4	9	3	10	6	-1.48569
tr|Q6IPN6|Q6IPN6_H	EEF1A1	Elongation factor 1-alpha OS=Homo sapiens GN=EEF1A1 PE=2 SV=1	50433	70	167	295	162	1.14	0.56	0.49122807	11	4	13	7	18	10	21	13	-0.86302
tr|B3KPU1|B3KPU1_H	GNB2	Guanine nucleotide-binding protein G(I)/G(S)/G(T) subunit beta-2 OS=Homo sapiens GN=GNB2 PE=2 SV=1	26598	194	761	111	33	0.27	0.13	0.481481481	1	0	2	1	2	1	2	1	-0.78034
sp|P53004|BIEA_HU	BLVRA	Biliverdin reductase A OS=Homo sapiens GN=BLVRA PE=1 SV=2	33692	403	643	43	43	0.21	0.1	0.476190476	1	1	2	1	2	1	2	1	-0.78034
tr|B2R7M3|B2R7M3_H	tr|B2R7M3|B	cDNA, FLJ93510, highly similar to Homo sapiens JTV1 gene (JTV1), mRNA OS=Homo sapiens PE=2 SV=1	35767	308	858	58	27	0.19	0.09	0.473684211	2	0	2	1	2	1	2	1	-0.78034
sp|P50552|VASP_HU	VASP	Vasodilator-stimulated phosphoprotein OS=Homo sapiens GN=VASP PE=1 SV=3	39976	363	685	48	39	0.17	0.08	0.470588235	1	1	1	1	1	3	1	3	1.180027
tr|B4DP08|B4DP08_H	ALG1	Chitobiosyldiphosphodolichol beta-mannosyltransferase OS=Homo sapiens GN=ALG1 PE=2 SV=1	40938	452	729	38	36	0.17	0.08	0.470588235	0	1	2	1	2	1	2	1	-0.78034
sp|Q06830|PRDX1_H	PRDX1	Peroxiredoxin-1 OS=Homo sapiens GN=PRDX1 PE=1 SV=1	22324	481	770	36	33	0.32	0.15	0.46875	0	0	2	1	2	1	2	1	-0.78034
tr|I3L492|I3L492_HU	ETF1	Eukaryotic peptide chain release factor subunit 1 (Fragment) OS=Homo sapiens GN=ETF1 PE=4 SV=1	23028	274	717	69	0	0.15	0.07	0.466666667	1	0	1	0	1	2	1	2	0.694266
tr|Q53G58|Q53G58_H	tr|Q53G58|Q	Coronin (Fragment) OS=Homo sapiens PE=2 SV=1	53931	242	603	83	46	0.13	0.06	0.461538462	1	1	2	1	2	1	2	1	-0.78034
sp|P18206|VINC_HU	VCL	Vinculin OS=Homo sapiens GN=VCL PE=1 SV=4	124292	68	133	299	194	0.37	0.17	0.459459459	8	3	12	5	13	6	13	7	-1.09994
tr|B3KM80|B3KM80_H	NCL	Nucleolin, isoform CRA_c OS=Homo sapiens GN=NCL PE=2 SV=1	58576	71	125	283	203	1.03	0.47	0.45631068	9	5	14	8	15	8	15	8	-0.91225
sp|P0CG38|POTEI_HU	POTEI	POTE ankyrin domain family member I OS=Homo sapiens GN=POTEI PE=1 SV=1	122858	5	81	195	280	0.44	0.2	0.454545455	6	11	11	15	12	17	14	20	0.443956
tr|Q5BKZ2|Q5BKZ2_H	KPNA1	Importin subunit alpha OS=Homo sapiens GN=KPNA1 PE=2 SV=1	60955	573	428	22	72	0.11	0.05	0.454545455	0	1	1	1	1	3	0	3	-0.04303
tr|H0Y5G9|H0Y5G9_H	NRD1	Nardilysin (Fragment) OS=Homo sapiens GN=NRD1 PE=3 SV=1	62196	225	343	92	71	0.11	0.05	0.454545455	1	1	2	1	2	2	2	2	-0.04304
sp|P40925|MDHC_HU	MDH1	Malate dehydrogenase, cytoplasmic OS=Homo sapiens GN=MDH1 PE=1 SV=4	36631	309	806	58	31	0.31	0.14	0.451612903	2	0	2	1	2	1	2	1	-0.78034
tr|K7EM20|K7EM20_H	YWHAE	14-3-3 protein epsilon (Fragment) OS=Homo sapiens GN=YWHAE PE=4 SV=1	12918	258	394	28	79	0.4	0.18	0.45	0	1	1	1	1	1	1	1	-0.04303
tr|B4DMY3|B4DMY3_H	tr|B4DMY3|B	cDNA FLJ60713, highly similar to Homo sapiens heterogeneous nuclear ribonucleoprotein A/B (HNRPAB), transcript varia	35101	318	462	56	66	0.2	0.09	0.45	1	1	2	2	2	2	2	2	-0.04304
tr|H7C393|H7C393_H	APEH	Acylamino-acid-releasing enzyme (Fragment) OS=Homo sapiens GN=APEH PE=4 SV=1	32397	319	868	56	26	0.09	0.04	0.444444444	1	0	1	1	1	1	1	1	-0.04303
tr|J3KTA4|J3KTA4_H	DDX5	Probable ATP-dependent RNA helicase DDX5 OS=Homo sapiens GN=DDX5 PE=3 SV=1	69557	97	250	218	117	0.45	0.2	0.444444444	7	3	9	4	10	6	10	7	-0.73634
sp|Q96SI9|STRBP_H	STRBP	Spermatid perinuclear RNA-binding protein OS=Homo sapiens GN=STRBP PE=1 SV=1	74290	471	880	37	26	0.09	0.04	0.444444444	0	0	2	1	2	1	2	2	-0.78034

<table>
<tr><td>tr|B4DZT3|B4DZT3_H</td><td>tr|B4DZT3|B</td><td>cDNA FLJ50934, highly similar to Lamin-B1 OS=Homo sapiens PE=2 SV=1</td><td>43178</td><td>219</td><td>505</td><td>95</td><td>60</td><td>0.18</td><td>0.08</td><td>0.444444444</td><td>2</td><td>1</td><td>2</td><td>1</td><td>2</td><td>1</td><td>2</td><td>1</td><td>-0.78034</td></tr>
<tr><td>sp|P68032|ACTC_HU</td><td>ACTC1</td><td>Actin, alpha cardiac muscle 1 OS=Homo sapiens GN=ACTC1 PE=1 SV=1</td><td>42334</td><td>23</td><td>30</td><td>771</td><td>634</td><td>6.63</td><td>2.86</td><td>0.431372549</td><td>25</td><td>25</td><td>40</td><td>34</td><td>48</td><td>39</td><td>55</td><td>44</td><td>-0.34268</td></tr>
<tr><td>tr|F8VQP2|F8VQP2_H</td><td>ATXN2</td><td>Ataxin-2 OS=Homo sapiens GN=ATXN2 PE=2 SV=1</td><td>117012</td><td>157</td><td>202</td><td>135</td><td>136</td><td>0.21</td><td>0.09</td><td>0.428571429</td><td>4</td><td>3</td><td>7</td><td>3</td><td>10</td><td>3</td><td>10</td><td>4</td><td>-1.63043</td></tr>
<tr><td>tr|Q9HB74|Q9HB74_</td><td>tr|Q9HB74|Q</td><td>PNAS-102 OS=Homo sapiens PE=2 SV=1</td><td>19284</td><td>90</td><td>798</td><td>127</td><td>31</td><td>0.4</td><td>0.17</td><td>0.425</td><td>3</td><td>0</td><td>3</td><td>1</td><td>3</td><td>1</td><td>3</td><td>2</td><td>-1.26611</td></tr>
<tr><td>tr|F5GWP8|F5GWP8_</td><td>JUP</td><td>Junction plakoglobin OS=Homo sapiens GN=JUP PE=2 SV=1</td><td>66708</td><td>26</td><td>70</td><td>676</td><td>313</td><td>1.74</td><td>0.7</td><td>0.402298851</td><td>20</td><td>12</td><td>27</td><td>13</td><td>31</td><td>14</td><td>34</td><td>14</td><td>-1.16834</td></tr>
<tr><td>sp|Q7L2E3|DHX30_H</td><td>DHX30</td><td>Putative ATP-dependent RNA helicase DHX30 OS=Homo sapiens GN=DHX30 PE=1 SV=1</td><td>134938</td><td>397</td><td>992</td><td>43</td><td>19</td><td>0.05</td><td>0.02</td><td>0.4</td><td>1</td><td>0</td><td>2</td><td>1</td><td>3</td><td>0</td><td>4</td><td>2</td><td>-2.85141</td></tr>
<tr><td>tr|Q9UJZ7|Q9UJZ7_H</td><td>tr|Q9UJZ7|Q</td><td>Ezrin (Fragment) OS=Homo sapiens PE=2 SV=1</td><td>19133</td><td>119</td><td>444</td><td>51</td><td>69</td><td>0.46</td><td>0.18</td><td>0.391304348</td><td>1</td><td>1</td><td>1</td><td>1</td><td>1</td><td>2</td><td>1</td><td>3</td><td>0.694266</td></tr>
<tr><td>tr|Q4W4Y1|Q4W4Y1_</td><td>DRIP4</td><td>Dopamine receptor interacting protein 4 OS=Homo sapiens GN=DRIP4 PE=2 SV=1</td><td>96646</td><td>136</td><td>351</td><td>160</td><td>88</td><td>0.18</td><td>0.07</td><td>0.388888889</td><td>2</td><td>1</td><td>5</td><td>2</td><td>5</td><td>2</td><td>6</td><td>2</td><td>-1.18158</td></tr>
<tr><td>sp|Q562R1|ACTBL_HU</td><td>ACTBL2</td><td>Beta-actin-like protein 2 OS=Homo sapiens GN=ACTBL2 PE=1 SV=2</td><td>42318</td><td>61</td><td>74</td><td>349</td><td>295</td><td>1.47</td><td>0.57</td><td>0.387755102</td><td>13</td><td>8</td><td>16</td><td>14</td><td>24</td><td>14</td><td>27</td><td>22</td><td>-0.80335</td></tr>
<tr><td>tr|D6REX3|D6REX3_H</td><td>SEC31A</td><td>Protein transport protein Sec31A OS=Homo sapiens GN=SEC31A PE=2 SV=1</td><td>137110</td><td>121</td><td>703</td><td>178</td><td>38</td><td>0.13</td><td>0.05</td><td>0.384615385</td><td>4</td><td>1</td><td>5</td><td>2</td><td>4</td><td>2</td><td>5</td><td>2</td><td>-0.89173</td></tr>
<tr><td>tr|H0Y9H1|H0Y9H1_H</td><td>CANX</td><td>Calnexin (Fragment) OS=Homo sapiens GN=CANX PE=3 SV=1</td><td>21748</td><td>352</td><td>564</td><td>49</td><td>52</td><td>0.39</td><td>0.15</td><td>0.384615385</td><td>1</td><td>1</td><td>2</td><td>1</td><td>2</td><td>2</td><td>3</td><td>2</td><td>-0.04304</td></tr>
<tr><td>tr|B2R9K8|B2R9K8_H</td><td>tr|B2R9K8|B</td><td>cDNA, FLJ94440, highly similar to Homo sapiens chaperonin containing TCP1, subunit 6A (zeta 1)(CCT6A), mRNA OS=Hon</td><td>58386</td><td>118</td><td>265</td><td>181</td><td>110</td><td>0.47</td><td>0.18</td><td>0.382978723</td><td>5</td><td>3</td><td>7</td><td>4</td><td>9</td><td>5</td><td>10</td><td>6</td><td>-0.83295</td></tr>
<tr><td>tr|B4DQX3|B4DQX3_</td><td>tr|B4DQX3|B</td><td>cDNA FLJ58927, highly similar to Endoplasmin (Heat shock protein 90 kDa beta member 1) OS=Homo sapiens PE=2 SV=1</td><td>18712</td><td>32</td><td>67</td><td>32</td><td>79</td><td>1.24</td><td>0.47</td><td>0.379032258</td><td>0</td><td>2</td><td>1</td><td>3</td><td>1</td><td>3</td><td>1</td><td>4</td><td>1.180027</td></tr>
<tr><td>tr|B4DXW6|B4DXW6</td><td>tr|B4DXW6|</td><td>cDNA FLJ50285, highly similar to Arginyl-tRNA synthetase (EC 6.1.1.19) OS=Homo sapiens PE=2 SV=1</td><td>52771</td><td>235</td><td>267</td><td>88</td><td>30</td><td>0.35</td><td>0.13</td><td>0.371428571</td><td>3</td><td>1</td><td>5</td><td>1</td><td>6</td><td>2</td><td>7</td><td>2</td><td>-1.42293</td></tr>
<tr><td>tr|Q96HI4|Q96HI4_H</td><td>NMT1</td><td>Glycylpeptide N-tetradecanoyltransferase (Fragment) OS=Homo sapiens GN=NMT1 PE=2 SV=2</td><td>56910</td><td>350</td><td>543</td><td>50</td><td>55</td><td>0.49</td><td>0.18</td><td>0.367346939</td><td>1</td><td>1</td><td>2</td><td>3</td><td>2</td><td>3</td><td>2</td><td>3</td><td>0.442717</td></tr>
<tr><td>tr|K7ERE3|K7ERE3_H</td><td>KRT13</td><td>Keratin, type I cytoskeletal 13 OS=Homo sapiens GN=KRT13 PE=3 SV=1</td><td>45574</td><td>88</td><td>411</td><td>234</td><td>76</td><td>0.63</td><td>0.23</td><td>0.365079365</td><td>9</td><td>2</td><td>10</td><td>3</td><td>11</td><td>3</td><td>12</td><td>3</td><td>-1.76202</td></tr>
<tr><td>tr|D6RAT0|D6RAT0_</td><td>RPS3A</td><td>40S ribosomal protein S3a OS=Homo sapiens GN=RPS3A PE=3 SV=1</td><td>26099</td><td>223</td><td>753</td><td>93</td><td>34</td><td>0.44</td><td>0.16</td><td>0.363636364</td><td>2</td><td>0</td><td>3</td><td>1</td><td>4</td><td>2</td><td>5</td><td>3</td><td>-0.89173</td></tr>
<tr><td>tr|D3DPU2|D3DPU2_</td><td>CAP1</td><td>Adenylyl cyclase-associated protein OS=Homo sapiens GN=CAP1 PE=3 SV=1</td><td>51926</td><td>172</td><td>270</td><td>125</td><td>109</td><td>0.36</td><td>0.13</td><td>0.361111111</td><td>4</td><td>2</td><td>5</td><td>2</td><td>6</td><td>3</td><td>7</td><td>3</td><td>-0.93717</td></tr>
<tr><td>tr|B4DW52|B4DW52</td><td>tr|B4DW52|</td><td>cDNA FLJ55253, highly similar to Actin, cytoplasmic 1 OS=Homo sapiens PE=2 SV=1</td><td>38950</td><td>4</td><td>2</td><td>3120</td><td>3120</td><td>27.29</td><td>9.64</td><td>0.353242946</td><td>74</td><td>78</td><td>108</td><td>108</td><td>123</td><td>117</td><td>134</td><td>128</td><td>-0.11821</td></tr>
<tr><td>tr|H3BLZ8|H3BLZ8_H</td><td>DDX17</td><td>Probable ATP-dependent RNA helicase DDX17 OS=Homo sapiens GN=DDX17 PE=3 SV=1</td><td>81073</td><td>166</td><td>483</td><td>130</td><td>64</td><td>0.37</td><td>0.13</td><td>0.351351351</td><td>3</td><td>2</td><td>9</td><td>4</td><td>9</td><td>5</td><td>10</td><td>7</td><td>-0.83295</td></tr>
<tr><td>sp|P27348|1433T_HU</td><td>YWHAQ</td><td>14-3-3 protein theta OS=Homo sapiens GN=YWHAQ PE=1 SV=1</td><td>28032</td><td>258</td><td>653</td><td>74</td><td>42</td><td>0.4</td><td>0.14</td><td>0.35</td><td>2</td><td>1</td><td>3</td><td>1</td><td>3</td><td>1</td><td>3</td><td>1</td><td>-1.26611</td></tr>
<tr><td>tr|B4DF68|B4DF68_H</td><td>tr|B4DF68|B</td><td>cDNA FLJ55325, highly similar to Heat-shock protein 105 kDa OS=Homo sapiens PE=2 SV=1</td><td>86500</td><td>123</td><td>245</td><td>47</td><td>118</td><td>0.23</td><td>0.08</td><td>0.347826087</td><td>1</td><td>2</td><td>1</td><td>2</td><td>1</td><td>2</td><td>1</td><td>3</td><td>0.694266</td></tr>
<tr><td>sp|Q58FF8|H90B2_H</td><td>HSP90AB2P</td><td>Putative heat shock protein HSP 90-beta 2 OS=Homo sapiens GN=HSP90AB2P PE=1 SV=2</td><td>44492</td><td>32</td><td>190</td><td>97</td><td>146</td><td>1.24</td><td>0.43</td><td>0.346774194</td><td>3</td><td>3</td><td>5</td><td>5</td><td>5</td><td>5</td><td>6</td><td>5</td><td>-0.04307</td></tr>
<tr><td>tr|E5RH42|E5RH42_H</td><td>G3BP1</td><td>Ras GTPase-activating protein-binding protein 1 (Fragment) OS=Homo sapiens GN=G3BP1 PE=2 SV=1</td><td>13338</td><td>294</td><td>756</td><td>62</td><td>24</td><td>0.58</td><td>0.2</td><td>0.344827586</td><td>2</td><td>0</td><td>2</td><td>2</td><td>2</td><td>1</td><td>2</td><td>2</td><td>-0.78034</td></tr>
<tr><td>tr|B1AHE4|B1AHE4_H</td><td>ATXN10</td><td>Ataxin 10 (Fragment) OS=Homo sapiens GN=ATXN10 PE=4 SV=1</td><td>22299</td><td>470</td><td>303</td><td>37</td><td>71</td><td>0.38</td><td>0.13</td><td>0.342105263</td><td>0</td><td>1</td><td>2</td><td>1</td><td>2</td><td>1</td><td>2</td><td>1</td><td>-0.78034</td></tr>
<tr><td>tr|B3KY12|B3KY12_H</td><td>tr|B3KY12|B</td><td>cDNA FLJ46581 fis, clone THYMU3043200, highly similar to Splicing factor 3A subunit 3 OS=Homo sapiens PE=2 SV=1</td><td>59082</td><td>495</td><td>742</td><td>34</td><td>35</td><td>0.18</td><td>0.06</td><td>0.333333333</td><td>1</td><td>0</td><td>3</td><td>1</td><td>2</td><td>1</td><td>3</td><td>1</td><td>-0.78034</td></tr>
<tr><td>tr|Q1KLZ0|Q1KLZ0_H</td><td>PS1TP5BP1</td><td>HCG15971, isoform CRA_a OS=Homo sapiens GN=PS1TP5BP1 PE=2 SV=1</td><td>42052</td><td>1</td><td>1</td><td>3367</td><td>3223</td><td>31.43</td><td>10.25</td><td>0.32612154</td><td>82</td><td>81</td><td>119</td><td>110</td><td>130</td><td>114</td><td>141</td><td>123</td><td>-0.23858</td></tr>
<tr><td>sp|Q09028|RBBP4_H</td><td>RBBP4</td><td>Histone-binding protein RBBP4 OS=Homo sapiens GN=RBBP4 PE=1 SV=3</td><td>47911</td><td>321</td><td>610</td><td>55</td><td>45</td><td>0.22</td><td>0.07</td><td>0.318181818</td><td>2</td><td>1</td><td>3</td><td>1</td><td>4</td><td>4</td><td>4</td><td>5</td><td>-0.04306</td></tr>
<tr><td>tr|Q96CV8|Q96CV8_</td><td>THOP1</td><td>Thimet oligopeptidase 1 OS=Homo sapiens GN=THOP1 PE=2 SV=1</td><td>79686</td><td>278</td><td>732</td><td>68</td><td>36</td><td>0.16</td><td>0.05</td><td>0.3125</td><td>1</td><td>1</td><td>1</td><td>1</td><td>1</td><td>1</td><td>1</td><td>1</td><td>-0.04303</td></tr>
<tr><td>tr|B2R659|B2R659_H</td><td>tr|B2R659|B</td><td>cDNA, FLJ92803, highly similar to Homo sapiens hydroxysteroid (17-beta) dehydrogenase 4 (HSD17B4), mRNA OS=Homo</td><td>80018</td><td>484</td><td>913</td><td>36</td><td>24</td><td>0.13</td><td>0.04</td><td>0.307692308</td><td>0</td><td>0</td><td>3</td><td>1</td><td>3</td><td>2</td><td>4</td><td>2</td><td>-0.52881</td></tr>
<tr><td>sp|O60884|DNJA2_H</td><td>DNAJA2</td><td>DnaJ homolog subfamily A member 2 OS=Homo sapiens GN=DNAJA2 PE=1 SV=1</td><td>46344</td><td>611</td><td>769</td><td>24</td><td>33</td><td>0.23</td><td>0.07</td><td>0.304347826</td><td>0</td><td>0</td><td>1</td><td>1</td><td>1</td><td>1</td><td>1</td><td>2</td><td>-0.04303</td></tr>
<tr><td>sp|P06748|NPM_HUM</td><td>NPM1</td><td>Nucleophosmin OS=Homo sapiens GN=NPM1 PE=1 SV=2</td><td>32726</td><td>138</td><td>416</td><td>160</td><td>75</td><td>0.69</td><td>0.21</td><td>0.304347826</td><td>4</td><td>1</td><td>5</td><td>2</td><td>5</td><td>2</td><td>5</td><td>3</td><td>-1.18158</td></tr>
<tr><td>tr|B3KMX0|B3KMX0_</td><td>tr|B3KMX0|</td><td>cDNA FLJ12837 fis, clone NT2RP2003228, highly similar to DNA replication licensing factor MCM4 OS=Homo sapiens PE=</td><td>97114</td><td>240</td><td>513</td><td>85</td><td>59</td><td>0.1</td><td>0.03</td><td>0.3</td><td>2</td><td>1</td><td>3</td><td>1</td><td>4</td><td>2</td><td>5</td><td>3</td><td>-0.89173</td></tr>
<tr><td>sp|Q14651|PLSI_HUM</td><td>PLS1</td><td>Plastin-1 OS=Homo sapiens GN=PLS1 PE=1 SV=2</td><td>70608</td><td>282</td><td>369</td><td>57</td><td>84</td><td>0.34</td><td>0.1</td><td>0.294117647</td><td>1</td><td>2</td><td>3</td><td>2</td><td>3</td><td>2</td><td>3</td><td>2</td><td>-0.52881</td></tr>
<tr><td>tr|B3KX96|B3KX96_H</td><td>tr|B3KX96|B</td><td>cDNA FLJ45003 fis, clone BRAWH3011623, highly similar to Heterogeneous nuclear ribonucleoproteins C OS=Homo sapie</td><td>32368</td><td>178</td><td>971</td><td>122</td><td>20</td><td>0.35</td><td>0.1</td><td>0.285714286</td><td>3</td><td>1</td><td>3</td><td>1</td><td>3</td><td>1</td><td>4</td><td>1</td><td>-1.26611</td></tr>
<tr><td>tr|B4DUY3|B4DUY3_</td><td>tr|B4DUY3|B</td><td>cDNA FLJ56668, highly similar to Nucleolar protein NOP5 OS=Homo sapiens PE=2 SV=1</td><td>16345</td><td>515</td><td>1013</td><td>32</td><td>17</td><td>0.21</td><td>0.06</td><td>0.285714286</td><td>0</td><td>0</td><td>1</td><td>1</td><td>1</td><td>0</td><td>1</td><td>1</td><td>-1.62833</td></tr>
<tr><td>sp|P46087|NOP2_HU</td><td>NOP2</td><td>Putative ribosomal RNA methyltransferase NOP2 OS=Homo sapiens GN=NOP2 PE=1 SV=2</td><td>89589</td><td>296</td><td>423</td><td>61</td><td>73</td><td>0.15</td><td>0.04</td><td>0.266666667</td><td>2</td><td>1</td><td>4</td><td>1</td><td>5</td><td>2</td><td>6</td><td>2</td><td>-1.18158</td></tr>
<tr><td>sp|Q05639|EF1A2_H</td><td>EEF1A2</td><td>Elongation factor 1-alpha 2 OS=Homo sapiens GN=EEF1A2 PE=1 SV=1</td><td>50780</td><td>70</td><td>308</td><td>238</td><td>98</td><td>1.14</td><td>0.29</td><td>0.254385965</td><td>8</td><td>2</td><td>9</td><td>4</td><td>12</td><td>7</td><td>14</td><td>9</td><td>-0.78178</td></tr>
<tr><td>sp|Q92621|NU205_H</td><td>NUP205</td><td>Nuclear pore complex protein Nup205 OS=Homo sapiens GN=NUP205 PE=1 SV=3</td><td>230171</td><td>415</td><td>624</td><td>42</td><td>44</td><td>0.04</td><td>0.01</td><td>0.25</td><td>0</td><td>1</td><td>3</td><td>1</td><td>3</td><td>1</td><td>5</td><td>3</td><td>-1.26611</td></tr>
<tr><td>tr|B7ZKM0|B7ZKM0_</td><td>SART3</td><td>SART3 protein OS=Homo sapiens GN=SART3 PE=2 SV=1</td><td>106372</td><td>153</td><td>849</td><td>139</td><td>28</td><td>0.16</td><td>0.04</td><td>0.25</td><td>5</td><td>0</td><td>5</td><td>1</td><td>7</td><td>1</td><td>7</td><td>2</td><td>-2.36703</td></tr>
<tr><td>sp|Q7Z3Y7|K1C28_H</td><td>KRT28</td><td>Keratin, type I cytoskeletal 28 OS=Homo sapiens GN=KRT28 PE=1 SV=2</td><td>51163</td><td>12</td><td>65</td><td>131</td><td>45</td><td>4.67</td><td>1.07</td><td>0.229122056</td><td>6</td><td>1</td><td>6</td><td>1</td><td>7</td><td>1</td><td>7</td><td>1</td><td>-2.36703</td></tr>
<tr><td>tr|H0YIE7|H0YIE7_HU</td><td>HSPA1L</td><td>Heat shock 70 kDa protein 1-like OS=Homo sapiens GN=HSPA1L PE=3 SV=1</td><td>25488</td><td>51</td><td>158</td><td>132</td><td>148</td><td>1.45</td><td>0.33</td><td>0.227586207</td><td>3</td><td>4</td><td>4</td><td>6</td><td>4</td><td>6</td><td>4</td><td>7</td><td>0.488119</td></tr>
<tr><td>sp|Q92878|RAD50_H</td><td>RAD50</td><td>DNA repair protein RAD50 OS=Homo sapiens GN=RAD50 PE=1 SV=1</td><td>154823</td><td>563</td><td>475</td><td>29</td><td>65</td><td>0.09</td><td>0.02</td><td>0.222222222</td><td>0</td><td>1</td><td>2</td><td>1</td><td>1</td><td>1</td><td>2</td><td>4</td><td>-0.04303</td></tr>
<tr><td>sp|P11498|PYC_HUM</td><td>PC</td><td>Pyruvate carboxylase, mitochondrial OS=Homo sapiens GN=PC PE=1 SV=2</td><td>130293</td><td>10</td><td>64</td><td>1295</td><td>339</td><td>1.76</td><td>0.38</td><td>0.215909091</td><td>40</td><td>9</td><td>52</td><td>13</td><td>56</td><td>14</td><td>67</td><td>18</td><td>-2.01993</td></tr>
<tr><td>sp|P02533|K1C14_H</td><td>KRT14</td><td>Keratin, type I cytoskeletal 14 OS=Homo sapiens GN=KRT14 PE=1 SV=4</td><td>51872</td><td>33</td><td>97</td><td>604</td><td>247</td><td>2.22</td><td>0.45</td><td>0.202702703</td><td>19</td><td>7</td><td>25</td><td>7</td><td>29</td><td>7</td><td>30</td><td>7</td><td>-2.02644</td></tr>
<tr><td>sp|P35527|K1C9_HUM</td><td>KRT9</td><td>Keratin, type I cytoskeletal 9 OS=Homo sapiens GN=KRT9 PE=1 SV=3</td><td>62255</td><td>14</td><td>80</td><td>1044</td><td>281</td><td>3.01</td><td>0.59</td><td>0.196013289</td><td>25</td><td>5</td><td>36</td><td>9</td><td>40</td><td>10</td><td>44</td><td>12</td><td>-2.00103</td></tr>
<tr><td>tr|H6VRF8|H6VRF8_H</td><td>KRT1</td><td>Keratin 1 OS=Homo sapiens GN=KRT1 PE=3 SV=1</td><td>66184</td><td>6</td><td>38</td><td>2032</td><td>491</td><td>5.01</td><td>0.97</td><td>0.193612774</td><td>55</td><td>11</td><td>64</td><td>15</td><td>69</td><td>16</td><td>76</td><td>16</td><td>-2.13614</td></tr>
<tr><td>tr|B3KTJ9|B3KTJ9_HU</td><td>tr|B3KTJ9|B3</td><td>cDNA FLJ38393 fis, clone FEBRA2007212 OS=Homo sapiens PE=2 SV=1</td><td>103493</td><td>82</td><td>306</td><td>251</td><td>99</td><td>0.32</td><td>0.06</td><td>0.1875</td><td>7</td><td>2</td><td>9</td><td>2</td><td>9</td><td>2</td><td>12</td><td>4</td><td>-1.97145</td></tr>
<tr><td>tr|B2RCM3|B2RCM3_</td><td>tr|B2RCM3|</td><td>cDNA, FLJ96158, highly similar to Homo sapiens calpain 2, (m/II) large subunit (CAPN2), mRNA OS=Homo sapiens PE=2 S</td><td>80883</td><td>173</td><td>886</td><td>125</td><td>26</td><td>0.22</td><td>0.04</td><td>0.181818182</td><td>3</td><td>0</td><td>7</td><td>1</td><td>7</td><td>1</td><td>8</td><td>2</td><td>-2.36703</td></tr>
<tr><td>tr|B2ZZ90|B2ZZ90_H</td><td>ACACA</td><td>Acetyl-Coenzyme A carboxylase alpha OS=Homo sapiens GN=ACACA PE=2 SV=1</td><td>267095</td><td>31</td><td>386</td><td>628</td><td>80</td><td>0.44</td><td>0.08</td><td>0.181818182</td><td>25</td><td>3</td><td>30</td><td>6</td><td>38</td><td>5</td><td>46</td><td>6</td><td>-2.86183</td></tr>
<tr><td>tr|B3KVL0|B3KVL0_H</td><td>tr|B3KVL0|B</td><td>cDNA FLJ16691 fis, clone TRACH3000692, highly similar to Paxillin OS=Homo sapiens PE=2 SV=1</td><td>65600</td><td>159</td><td>741</td><td>133</td><td>35</td><td>0.28</td><td>0.05</td><td>0.178571429</td><td>3</td><td>1</td><td>5</td><td>1</td><td>6</td><td>1</td><td>6</td><td>2</td><td>-2.16023</td></tr>
<tr><td>tr|A4ZU86|A4ZU86_H</td><td>NPM1</td><td>Truncated nucleolar phosphoprotein B23 OS=Homo sapiens GN=NPM1 PE=2 SV=1</td><td>30074</td><td>138</td><td>415</td><td>160</td><td>75</td><td>0.69</td><td>0.11</td><td>0.15942029</td><td>4</td><td>1</td><td>5</td><td>1</td><td>5</td><td>1</td><td>5</td><td>2</td><td>-1.91888</td></tr>
<tr><td>sp|P13645|K1C10_H</td><td>KRT10</td><td>Keratin, type I cytoskeletal 10 OS=Homo sapiens GN=KRT10 PE=1 SV=6</td><td>59020</td><td>12</td><td>69</td><td>1226</td><td>316</td><td>4.67</td><td>0.72</td><td>0.154175589</td><td>34</td><td>7</td><td>42</td><td>10</td><td>47</td><td>11</td><td>54</td><td>11</td><td>-2.10189</td></tr>
<tr><td>sp|P08779|K1C16_H</td><td>KRT16</td><td>Keratin, type I cytoskeletal 16 OS=Homo sapiens GN=KRT16 PE=1 SV=4</td><td>51578</td><td>62</td><td>287</td><td>342</td><td>105</td><td>1.38</td><td>0.2</td><td>0.144927536</td><td>14</td><td>3</td><td>17</td><td>3</td><td>21</td><td>4</td><td>21</td><td>4</td><td>-2.30527</td></tr>
<tr><td>tr|F5H0C8|F5H0C8_H</td><td>ENO2</td><td>Enolase OS=Homo sapiens GN=ENO2 PE=2 SV=1</td><td>35026</td><td>40</td><td>463</td><td>112</td><td>66</td><td>1.39</td><td>0.2</td><td>0.143884892</td><td>2</td><td>1</td><td>2</td><td>2</td><td>2</td><td>3</td><td>4</td><td>4</td><td>0.442717</td></tr>
<tr><td>sp|Q9NXW2|DJB12_H</td><td>DNAJB12</td><td>DnaJ homolog subfamily B member 12 OS=Homo sapiens GN=DNAJB12 PE=1 SV=4</td><td>42021</td><td>124</td><td>970</td><td>174</td><td>20</td><td>0.57</td><td>0.07</td><td>0.122807018</td><td>5</td><td>0</td><td>7</td><td>1</td><td>9</td><td>1</td><td>9</td><td>4</td><td>-2.70875</td></tr>
<tr><td>tr|B7Z4V2|B7Z4V2_H</td><td>tr|B7Z4V2|B</td><td>cDNA FLJ51907, highly similar to Stress-70 protein, mitochondrial OS=Homo sapiens PE=2 SV=1</td><td>72641</td><td>81</td><td>997</td><td>251</td><td>19</td><td>0.42</td><td>0.05</td><td>0.119047619</td><td>7</td><td>0</td><td>8</td><td>1</td><td>8</td><td>1</td><td>8</td><td>3</td><td>-2.54794</td></tr>
<tr><td>tr|B2R8Y4|B2R8Y4_H</td><td>tr|B2R8Y4|B</td><td>cDNA, FLJ94117, highly similar to Homo sapiens actinin, alpha 3 (ACTN3), mRNA OS=Homo sapiens PE=2 SV=1</td><td>103871</td><td>19</td><td>153</td><td>99</td><td>177</td><td>2.11</td><td>0.24</td><td>0.113744076</td><td>3</td><td>5</td><td>3</td><td>8</td><td>3</td><td>8</td><td>4</td><td>10</td><td>1.238725</td></tr>
<tr><td>tr|D3DTL4|D3DTL4_H</td><td>ENO3</td><td>Enolase OS=Homo sapiens GN=ENO3 PE=2 SV=1</td><td>37167</td><td>40</td><td>298</td><td>112</td><td>69</td><td>1.39</td><td>0.14</td><td>0.100719424</td><td>2</td><td>1</td><td>2</td><td>1</td><td>2</td><td>2</td><td>4</td><td>3</td><td>-0.04304</td></tr>
<tr><td>sp|P35908|K22E_HUM</td><td>KRT2</td><td>Keratin, type II cytoskeletal 2 epidermal OS=Homo sapiens GN=KRT2 PE=1 SV=2</td><td>65678</td><td>11</td><td>205</td><td>1255</td><td>134</td><td>4.79</td><td>0.48</td><td>0.100208768</td><td>41</td><td>3</td><td>48</td><td>9</td><td>49</td><td>8</td><td>57</td><td>9</td><td>-2.59919</td></tr>
<tr><td>tr|K7EK77|K7EK77_H</td><td>ATP5A1</td><td>ATP synthase subunit alpha, mitochondrial (Fragment) OS=Homo sapiens GN=ATP5A1 PE=3 SV=1</td><td>22243</td><td>135</td><td>520</td><td>160</td><td>0</td><td>1.33</td><td>0.12</td><td>0.090225564</td><td>5</td><td>0</td><td>6</td><td>0</td><td>6</td><td>1</td><td>6</td><td>1</td><td>-2.16023</td></tr>
<tr><td>tr|A1L2Z2|A1L2Z2_H</td><td>MYH14</td><td>MYH14 protein OS=Homo sapiens GN=MYH14 PE=2 SV=1</td><td>73418</td><td>17</td><td>271</td><td>56</td><td>54</td><td>0.9</td><td>0.06</td><td>0.066666667</td><td>1</td><td>1</td><td>1</td><td>1</td><td>1</td><td>2</td><td>1</td><td>2</td><td>0.694266</td></tr>
<tr><td>sp|P04259|K2C6B_H</td><td>KRT6B</td><td>Keratin, type II cytoskeletal 6B OS=Homo sapiens GN=KRT6B PE=1 SV=5</td><td>60315</td><td>79</td><td>488</td><td>252</td><td>63</td><td>0.79</td><td>0.05</td><td>0.063291139</td><td>10</td><td>1</td><td>13</td><td>1</td><td>15</td><td>2</td><td>17</td><td>3</td><td>-2.67979</td></tr>
<tr><td>tr|Q562V5|Q562V5_H</td><td>ACT</td><td>Actin-like protein (Fragment) OS=Homo sapiens GN=ACT PE=2 SV=1</td><td>11481</td><td>1</td><td>208</td><td>136</td><td>132</td><td>31.43</td><td>1.87</td><td>0.059497296</td><td>6</td><td>6</td><td>6</td><td>7</td><td>7</td><td>10</td><td>8</td><td>11</td><td>0.443336</td></tr>
<tr><td>sp|A6NHL2|TBAL3_H</td><td>TUBAL3</td><td>Tubulin alpha chain-like 3 OS=Homo sapiens GN=TUBAL3 PE=1 SV=2</td><td>50675</td><td>13</td><td>714</td><td>48</td><td>37</td><td>5.58</td><td>0.21</td><td>0.037634409</td><td>2</td><td>1</td><td>2</td><td>3</td><td>3</td><td>4</td><td>3</td><td>6</td><td>0.319851</td></tr>
<tr><td>tr|H7C2E7|H7C2E7_H</td><td>FLNA</td><td>Filamin-A (Fragment) OS=Homo sapiens GN=FLNA PE=4 SV=1</td><td>10032</td><td>9</td><td>0</td><td>0</td><td>0</td><td>0.86</td><td>0</td><td>0</td><td>0</td><td>0</td><td>0</td><td>0</td><td>0</td><td>0</td><td>1</td><td>0</td><td>-0.04302</td></tr>
<tr><td>tr|E9PQ69|E9PQ69_H</td><td>GANAB</td><td>Neutral alpha-glucosidase AB OS=Homo sapiens GN=GANAB PE=2 SV=1</td><td>8560</td><td>47</td><td>0</td><td>0</td><td>0</td><td>0.62</td><td>0</td><td>0</td><td>0</td><td>0</td><td>0</td><td>0</td><td>0</td><td>0</td><td>1</td><td>0</td><td>-0.04302</td></tr>
<tr><td>tr|Q8WY81|Q8WY81_</td><td>tr|Q8WY81|</td><td>PP3895 OS=Homo sapiens PE=2 SV=1</td><td>15132</td><td>114</td><td>0</td><td>15</td><td>0</td><td>0.28</td><td>0</td><td>0</td><td>0</td><td>0</td><td>1</td><td>0</td><td>0</td><td>0</td><td>1</td><td>0</td><td>-0.04302</td></tr>
<tr><td>tr|E9PLJ0|E9PLJ0_HU</td><td>IPO7</td><td>Importin-7 OS=Homo sapiens GN=IPO7 PE=2 SV=1</td><td>7255</td><td>162</td><td>0</td><td>0</td><td>0</td><td>0.08</td><td>0</td><td>0</td><td>0</td><td>0</td><td>0</td><td>0</td><td>0</td><td>0</td><td>1</td><td>0</td><td>-0.04302</td></tr>
<tr><td>tr|Q6RUP8|Q6RUP8_</td><td>IMPDH2</td><td>Inosine monophosphate dehydrogenase type II (Fragment) OS=Homo sapiens GN=IMPDH2 PE=2 SV=1</td><td>3145</td><td>177</td><td>0</td><td>0</td><td>0</td><td>0.36</td><td>0</td><td>0</td><td>0</td><td>0</td><td>0</td><td>0</td><td>0</td><td>0</td><td>1</td><td>0</td><td>-0.04302</td></tr>
<tr><td>tr|F8WAT3|F8WAT3_</td><td>EIF2A</td><td>Eukaryotic translation initiation factor 2A OS=Homo sapiens GN=EIF2A PE=2 SV=1</td><td>7970</td><td>206</td><td>0</td><td>18</td><td>0</td><td>0.32</td><td>0</td><td>0</td><td>0</td><td>0</td><td>1</td><td>0</td><td>0</td><td>0</td><td>1</td><td>0</td><td>-0.04302</td></tr>
<tr><td>tr|F8VR69|F8VR69_H</td><td>RPL6</td><td>60S ribosomal protein L6 (Fragment) OS=Homo sapiens GN=RPL6 PE=2 SV=1</td><td>7285</td><td>276</td><td>0</td><td>0</td><td>0</td><td>0.47</td><td>0</td><td>0</td><td>0</td><td>0</td><td>0</td><td>0</td><td>0</td><td>0</td><td>1</td><td>0</td><td>-0.04302</td></tr>
<tr><td>tr|B7Z6J5|B7Z6J5_HU</td><td>tr|B7Z6J5|B7</td><td>cDNA FLJ56858, highly similar to Succinate dehydrogenase (ubiquinone) flavoprotein subunit, mitochondrial (EC 1.3.5.1)</td><td>28776</td><td>393</td><td>0</td><td>0</td><td>0</td><td>0.1</td><td>0</td><td>0</td><td>0</td><td>0</td><td>0</td><td>0</td><td>0</td><td>0</td><td>1</td><td>0</td><td>-0.04302</td></tr>
<tr><td>tr|H0Y8W6|H0Y8W6_</td><td>PDLIM7</td><td>PDZ and LIM domain protein 7 (Fragment) OS=Homo sapiens GN=PDLIM7 PE=4 SV=1</td><td>12898</td><td>396</td><td>0</td><td>0</td><td>0</td><td>0.13</td><td>0</td><td>0</td><td>0</td><td>0</td><td>0</td><td>0</td><td>0</td><td>0</td><td>1</td><td>0</td><td>-0.04302</td></tr>
<tr><td>tr|A8K171|A8K171_H</td><td>tr|A8K171|A</td><td>cDNA FLJ77253, highly similar to Homo sapiens nuclear receptor interacting protein 1 (NRIP1), mRNA OS=Homo sapiens</td><td>127609</td><td>401</td><td>0</td><td>0</td><td>0</td><td>0.01</td><td>0</td><td>0</td><td>0</td><td>0</td><td>0</td><td>0</td><td>0</td><td>0</td><td>1</td><td>0</td><td>-0.04302</td></tr>
<tr><td>tr|H0Y386|H0Y386_H</td><td>PABPC1L</td><td>Polyadenylate-binding protein 1-like (Fragment) OS=Homo sapiens GN=PABPC1L PE=4 SV=1</td><td>16787</td><td>404</td><td>0</td><td>0</td><td>0</td><td>0.1</td><td>0</td><td>0</td><td>0</td><td>0</td><td>0</td><td>0</td><td>0</td><td>0</td><td>1</td><td>0</td><td>-0.04302</td></tr>
<tr><td>tr|E9PQX2|E9PQX2_H</td><td>RPS3</td><td>40S ribosomal protein S3 OS=Homo sapiens GN=RPS3 PE=2 SV=1</td><td>5066</td><td>480</td><td>0</td><td>0</td><td>0</td><td>0.12</td><td>0</td><td>0</td><td>0</td><td>0</td><td>0</td><td>0</td><td>0</td><td>0</td><td>1</td><td>0</td><td>-0.04302</td></tr>
<tr><td>tr|H7BYY8|H7BYY8_H</td><td>PES1</td><td>Pescadillo homolog OS=Homo sapiens GN=PES1 PE=4 SV=1</td><td>1501</td><td>488</td><td>0</td><td>0</td><td>0</td><td>0.05</td><td>0</td><td>0</td><td>0</td><td>0</td><td>0</td><td>0</td><td>0</td><td>0</td><td>1</td><td>0</td><td>-0.04302</td></tr>
<tr><td>sp|Q14117|DPYS_HU</td><td>DPYS</td><td>Dihydropyrimidinase OS=Homo sapiens GN=DPYS PE=1 SV=1</td><td>57107</td><td>490</td><td>0</td><td>0</td><td>0</td><td>0.19</td><td>0</td><td>0</td><td>0</td><td>0</td><td>0</td><td>0</td><td>0</td><td>0</td><td>1</td><td>0</td><td>-0.04302</td></tr>
<tr><td>tr|D6RD96|D6RD96_</td><td>ACSL4</td><td>Long-chain-fatty-acid--CoA ligase 4 (Fragment) OS=Homo sapiens GN=ACSL4 PE=2 SV=1</td><td>16399</td><td>529</td><td>0</td><td>0</td><td>0</td><td>0.04</td><td>0</td><td>0</td><td>0</td><td>0</td><td>0</td><td>0</td><td>0</td><td>0</td><td>1</td><td>0</td><td>-0.04302</td></tr>
<tr><td>tr|H7C1T1|H7C1T1_H</td><td>Sep-02</td><td>Septin-2 (Fragment) OS=Homo sapiens GN=SEPT2 PE=4 SV=1</td><td>3094</td><td>543</td><td>0</td><td>0</td><td>0</td><td>0.09</td><td>0</td><td>0</td><td>0</td><td>0</td><td>0</td><td>0</td><td>0</td><td>0</td><td>1</td><td>0</td><td>-0.04302</td></tr>
<tr><td>tr|B7Z4W3|B7Z4W3_</td><td>tr|B7Z4W3|E</td><td>cDNA FLJ57543, highly similar to Bifunctional 3~-phosphoadenosine5~-phosphosulfate synthetase 1 OS=Homo sapiens P</td><td>17852</td><td>553</td><td>0</td><td>19</td><td>0</td><td>0.1</td><td>0</td><td>0</td><td>0</td><td>0</td><td>1</td><td>0</td><td>0</td><td>0</td><td>1</td><td>0</td><td>-0.04302</td></tr>
<tr><td>tr|J3KN33|J3KN33_H</td><td>CNTLN</td><td>Centlein OS=Homo sapiens GN=CNTLN PE=4 SV=1</td><td>162069</td><td>680</td><td>0</td><td>20</td><td>0</td><td>0.02</td><td>0</td><td>0</td><td>0</td><td>0</td><td>1</td><td>0</td><td>0</td><td>0</td><td>2</td><td>0</td><td>-0.04302</td></tr>
<tr><td>tr|Q3MNE1|Q3MNE1</td><td>tr|Q3MNE1|</td><td>Adaptor-related protein complex 3 beta 1 subunit (Fragment) OS=Homo sapiens PE=2 SV=1</td><td>89828</td><td>682</td><td>0</td><td>20</td><td>0</td><td>0.04</td><td>0</td><td>0</td><td>0</td><td>0</td><td>1</td><td>0</td><td>0</td><td>0</td><td>1</td><td>0</td><td>-0.04302</td></tr>
<tr><td>tr|B4DZD5|B4DZD5_H</td><td>tr|B4DZD5|B</td><td>cDNA FLJ60991, highly similar to FKBP12-rapamycin complex-associated protein OS=Homo sapiens PE=2 SV=1</td><td>158783</td><td>683</td><td>0</td><td>20</td><td>0</td><td>0.02</td><td>0</td><td>0</td><td>0</td><td>0</td><td>1</td><td>0</td><td>0</td><td>0</td><td>2</td><td>0</td><td>-0.04302</td></tr>
<tr><td>tr|Q9UH66|Q9UH66_</td><td>tr|Q9UH66|(</td><td>7h3 protein (Fragment) OS=Homo sapiens PE=2 SV=1</td><td>49963</td><td>684</td><td>0</td><td>19</td><td>0</td><td>0.07</td><td>0</td><td>0</td><td>0</td><td>0</td><td>1</td><td>0</td><td>0</td><td>0</td><td>2</td><td>0</td><td>-0.04302</td></tr>
<tr><td>tr|G5E9Q3|G5E9Q3_</td><td>TSEN2</td><td>tRNA splicing endonuclease 2 homolog (S. cerevisiae), isoform CRA_b OS=Homo sapiens GN=TSEN2 PE=2 SV=1</td><td>45959</td><td>686</td><td>0</td><td>19</td><td>0</td><td>0.07</td><td>0</td><td>0</td><td>0</td><td>0</td><td>1</td><td>0</td><td>0</td><td>0</td><td>1</td><td>0</td><td>-0.04302</td></tr>
<tr><td>tr|E9PQG7|E9PQG7_</td><td>MLL</td><td>MLL cleavage product C180 OS=Homo sapiens GN=MLL PE=2 SV=1</td><td>436345</td><td>687</td><td>0</td><td>19</td><td>0</td><td>0.01</td><td>0</td><td>0</td><td>0</td><td>0</td><td>1</td><td>0</td><td>0</td><td>0</td><td>1</td><td>0</td><td>-0.04302</td></tr>
<tr><td>tr|Q5TB52|Q5TB52_H</td><td>PAPSS2</td><td>3~-phosphoadenosine 5~-phosphosulfate synthase 2, isoform CRA_b OS=Homo sapiens GN=PAPSS2 PE=2 SV=1</td><td>70027</td><td>688</td><td>0</td><td>19</td><td>0</td><td>0.05</td><td>0</td><td>0</td><td>0</td><td>0</td><td>1</td><td>0</td><td>0</td><td>0</td><td>4</td><td>0</td><td>-0.04302</td></tr>
<tr><td>tr|D3DUL0|D3DUL0_</td><td>ASCIZ</td><td>ATM/ATR-Substrate Chk2-Interacting Zn2+-finger protein, isoform CRA_b OS=Homo sapiens GN=ASCIZ PE=2 SV=1</td><td>79474</td><td>690</td><td>0</td><td>19</td><td>0</td><td>0.04</td><td>0</td><td>0</td><td>0</td><td>0</td><td>1</td><td>0</td><td>0</td><td>0</td><td>1</td><td>0</td><td>-0.04302</td></tr>
<tr><td>tr|H7C4X2|H7C4X2_H</td><td>PCMT1</td><td>Protein-L-isoaspartate(D-aspartate) O-methyltransferase (Fragment) OS=Homo sapiens GN=PCMT1 PE=4 SV=1</td><td>7342</td><td>691</td><td>0</td><td>19</td><td>0</td><td>0.48</td><td>0</td><td>0</td><td>0</td><td>0</td><td>1</td><td>0</td><td>0</td><td>0</td><td>2</td><td>0</td><td>-0.04302</td></tr>
<tr><td>tr|B3KND7|B3KND7_</td><td>tr|B3KND7|E</td><td>cDNA FLJ14378 fis, clone HEMBA1001804, highly similar to Zinc finger protein 281 (Fragment) OS=Homo sapiens PE=2 S\</td><td>58221</td><td>692</td><td>0</td><td>19</td><td>0</td><td>0.06</td><td>0</td><td>0</td><td>0</td><td>0</td><td>1</td><td>0</td><td>0</td><td>0</td><td>1</td><td>0</td><td>-0.04302</td></tr>
<tr><td>tr|H0YE81|H0YE81_H</td><td>NDUFV1</td><td>NADH dehydrogenase [ubiquinone] flavoprotein 1, mitochondrial (Fragment) OS=Homo sapiens GN=NDUFV1 PE=4 SV=1</td><td>14494</td><td>694</td><td>0</td><td>19</td><td>0</td><td>0.23</td><td>0</td><td>0</td><td>0</td><td>0</td><td>1</td><td>0</td><td>0</td><td>0</td><td>1</td><td>0</td><td>-0.04302</td></tr>
<tr><td>tr|H0Y304|H0Y304_H</td><td>DMD</td><td>Dystrophin (Fragment) OS=Homo sapiens GN=DMD PE=4 SV=1</td><td>160176</td><td>695</td><td>0</td><td>19</td><td>0</td><td>0.02</td><td>0</td><td>0</td><td>0</td><td>0</td><td>1</td><td>0</td><td>0</td><td>0</td><td>1</td><td>0</td><td>-0.04302</td></tr>
<tr><td>tr|Q5T097|Q5T097_H</td><td>UTRN</td><td>Utrophin OS=Homo sapiens GN=UTRN PE=4 SV=1</td><td>114423</td><td>696</td><td>0</td><td>19</td><td>0</td><td>0.03</td><td>0</td><td>0</td><td>0</td><td>0</td><td>1</td><td>0</td><td>0</td><td>0</td><td>1</td><td>0</td><td>-0.04302</td></tr>
<tr><td>tr|F8W9S7|F8W9S7_</td><td>GAPVD1</td><td>GTPase-activating protein and VPS9 domain-containing protein 1 OS=Homo sapiens GN=GAPVD1 PE=2 SV=1</td><td>158790</td><td>697</td><td>0</td><td>18</td><td>0</td><td>0.02</td><td>0</td><td>0</td><td>0</td><td>0</td><td>1</td><td>0</td><td>0</td><td>0</td><td>2</td><td>0</td><td>-0.04302</td></tr>
<tr><td>tr|B4DY47|B4DY47_H</td><td>PHB</td><td>Prohibitin OS=Homo sapiens GN=PHB PE=2 SV=1</td><td>17064</td><td>699</td><td>0</td><td>18</td><td>0</td><td>0.2</td><td>0</td><td>0</td><td>0</td><td>0</td><td>1</td><td>0</td><td>0</td><td>0</td><td>1</td><td>0</td><td>-0.04302</td></tr>
<tr><td>tr|J3KQ96|J3KQ96_H</td><td>TCOF1</td><td>Treacle protein (Fragment) OS=Homo sapiens GN=TCOF1 PE=4 SV=1</td><td>144268</td><td>702</td><td>0</td><td>18</td><td>0</td><td>0.02</td><td>0</td><td>0</td><td>0</td><td>0</td><td>1</td><td>0</td><td>0</td><td>0</td><td>2</td><td>0</td><td>-0.04302</td></tr>
<tr><td>tr|Q2NLA0|Q2NLA0_</td><td>COPG2</td><td>Coatomer subunit gamma OS=Homo sapiens GN=COPG2 PE=2 SV=1</td><td>82400</td><td>705</td><td>0</td><td>18</td><td>0</td><td>0.04</td><td>0</td><td>0</td><td>0</td><td>0</td><td>1</td><td>0</td><td>0</td><td>0</td><td>1</td><td>0</td><td>-0.04302</td></tr>
<tr><td>tr|J3KMW7|J3KMW7_</td><td>UBR5</td><td>E3 ubiquitin-protein ligase UBR5 OS=Homo sapiens GN=UBR5 PE=4 SV=1</td><td>312224</td><td>706</td><td>0</td><td>18</td><td>0</td><td>0.01</td><td>0</td><td>0</td><td>0</td><td>0</td><td>1</td><td>0</td><td>0</td><td>0</td><td>1</td><td>0</td><td>-0.04302</td></tr>
<tr><td>tr|H0Y4R4|H0Y4R4_H</td><td>TOR1AIP1</td><td>Torsin-1A-interacting protein 1 (Fragment) OS=Homo sapiens GN=TOR1AIP1 PE=4 SV=1</td><td>33844</td><td>707</td><td>0</td><td>17</td><td>0</td><td>0.1</td><td>0</td><td>0</td><td>0</td><td>0</td><td>1</td><td>0</td><td>0</td><td>0</td><td>1</td><td>0</td><td>-0.04302</td></tr>
<tr><td>sp|P54826|GAS1_HU</td><td>GAS1</td><td>Growth arrest-specific protein 1 OS=Homo sapiens GN=GAS1 PE=1 SV=2</td><td>36867</td><td>708</td><td>0</td><td>17</td><td>0</td><td>0.09</td><td>0</td><td>0</td><td>0</td><td>0</td><td>1</td><td>0</td><td>0</td><td>0</td><td>1</td><td>0</td><td>-0.04302</td></tr>
<tr><td>tr|F5H4R9|F5H4R9_H</td><td>DNM2</td><td>Dynamin-2 OS=Homo sapiens GN=DNM2 PE=2 SV=1</td><td>98218</td><td>713</td><td>0</td><td>16</td><td>0</td><td>0.03</td><td>0</td><td>0</td><td>0</td><td>0</td><td>1</td><td>0</td><td>0</td><td>0</td><td>2</td><td>0</td><td>-0.04302</td></tr>
<tr><td>tr|Q2PS10|Q2PS10_H</td><td>MYH9</td><td>Non-muscle myosin heavy chain (Fragment) OS=Homo sapiens GN=MYH9 PE=2 SV=1</td><td>8156</td><td>17</td><td>0</td><td>48</td><td>0</td><td>0.9</td><td>0</td><td>0</td><td>1</td><td>0</td><td>1</td><td>0</td><td>1</td><td>0</td><td>2</td><td>0</td><td>-1.62833</td></tr>
<tr><td>tr|Q16768|Q16768_H</td><td>GAPD</td><td>Glyceraldehyde 3-phosphate dehydrogenase (Fragment) OS=Homo sapiens GN=GAPD PE=2 SV=1</td><td>4891</td><td>18</td><td>0</td><td>53</td><td>0</td><td>3.44</td><td>0</td><td>0</td><td>1</td><td>0</td><td>1</td><td>0</td><td>1</td><td>0</td><td>2</td><td>0</td><td>-1.62833</td></tr>
<tr><td>sp|Q8N1A0|KT222_H</td><td>KRT222</td><td>Keratin-like protein KRT222 OS=Homo sapiens GN=KRT222 PE=2 SV=1</td><td>34308</td><td>24</td><td>0</td><td>47</td><td>0</td><td>2.99</td><td>0</td><td>0</td><td>1</td><td>0</td><td>1</td><td>0</td><td>1</td><td>0</td><td>1</td><td>0</td><td>-1.62833</td></tr>
<tr><td>tr|K7ENV3|K7ENV3_H</td><td>KRT16</td><td>Keratin, type I cytoskeletal 16 (Fragment) OS=Homo sapiens GN=KRT16 PE=4 SV=1</td><td>4775</td><td>33</td><td>0</td><td>48</td><td>0</td><td>2.22</td><td>0</td><td>0</td><td>1</td><td>0</td><td>1</td><td>0</td><td>1</td><td>0</td><td>1</td><td>0</td><td>-1.62833</td></tr>
<tr><td>tr|F5GZR9|F5GZR9_H</td><td>SLC3A2</td><td>4F2 cell-surface antigen heavy chain (Fragment) OS=Homo sapiens GN=SLC3A2 PE=2 SV=1</td><td>5250</td><td>60</td><td>0</td><td>28</td><td>0</td><td>1.33</td><td>0</td><td>0</td><td>0</td><td>0</td><td>1</td><td>0</td><td>1</td><td>0</td><td>1</td><td>0</td><td>-1.62833</td></tr>
<tr><td>tr|C9IZA5|C9IZA5_HU</td><td>VCP</td><td>Transitional endoplasmic reticulum ATPase (Fragment) OS=Homo sapiens GN=VCP PE=2 SV=1</td><td>18590</td><td>64</td><td>0</td><td>73</td><td>0</td><td>0.65</td><td>0</td><td>0</td><td>1</td><td>0</td><td>1</td><td>0</td><td>1</td><td>0</td><td>1</td><td>0</td><td>-1.62833</td></tr>
<tr><td>tr|F8VS61|F8VS61_HU</td><td>KRT77</td><td>Keratin, type II cytoskeletal 1b OS=Homo sapiens GN=KRT77 PE=2 SV=1</td><td>25704</td><td>76</td><td>0</td><td>43</td><td>0</td><td>0.23</td><td>0</td><td>0</td><td>1</td><td>0</td><td>1</td><td>0</td><td>1</td><td>0</td><td>3</td><td>0</td><td>-1.62833</td></tr>
<tr><td>tr|A2RRC9|A2RRC9_H</td><td>IQGAP3</td><td>IQ motif containing GTPase activating protein 3 OS=Homo sapiens GN=IQGAP3 PE=2 SV=1</td><td>185145</td><td>84</td><td>0</td><td>32</td><td>0</td><td>0.31</td><td>0</td><td>0</td><td>0</td><td>0</td><td>1</td><td>0</td><td>1</td><td>0</td><td>1</td><td>0</td><td>-1.62833</td></tr>
<tr><td>tr|D6RCS6|D6RCS6_H</td><td>TARS</td><td>Threonine--tRNA ligase, cytoplasmic OS=Homo sapiens GN=TARS PE=2 SV=1</td><td>6912</td><td>94</td><td>0</td><td>53</td><td>0</td><td>0.64</td><td>0</td><td>0</td><td>1</td><td>0</td><td>1</td><td>0</td><td>1</td><td>0</td><td>1</td><td>0</td><td>-1.62833</td></tr>
<tr><td>tr|K7EJ74|K7EJ74_HU</td><td>EFTUD2</td><td>116 kDa U5 small nuclear ribonucleoprotein component (Fragment) OS=Homo sapiens GN=EFTUD2 PE=4 SV=1</td><td>15026</td><td>111</td><td>0</td><td>39</td><td>0</td><td>0.27</td><td>0</td><td>0</td><td>1</td><td>0</td><td>1</td><td>0</td><td>1</td><td>0</td><td>2</td><td>0</td><td>-1.62833</td></tr>
<tr><td>tr|H0Y8V7|H0Y8V7_H</td><td>SEC31A</td><td>Protein transport protein Sec31A (Fragment) OS=Homo sapiens GN=SEC31A PE=4 SV=1</td><td>32306</td><td>121</td><td>0</td><td>42</td><td>0</td><td>0.13</td><td>0</td><td>0</td><td>1</td><td>0</td><td>2</td><td>0</td><td>1</td><td>0</td><td>2</td><td>0</td><td>-1.62833</td></tr>
<tr><td>tr|B4DSE8|B4DSE8_H</td><td>tr|B4DSE8|B</td><td>cDNA FLJ50643, highly similar to Heat shock 70 kDa protein 4 OS=Homo sapiens PE=2 SV=1</td><td>38275</td><td>123</td><td>0</td><td>48</td><td>0</td><td>0.23</td><td>0</td><td>0</td><td>1</td><td>0</td><td>1</td><td>0</td><td>1</td><td>0</td><td>1</td><td>0</td><td>-1.62833</td></tr>
<tr><td>tr|Q659G2|Q659G2_</td><td>DKFZp586B20</td><td>Putative uncharacterized protein DKFZp586B2023 (Fragment) OS=Homo sapiens GN=DKFZp586B2023 PE=2 SV=1</td><td>7133</td><td>124</td><td>0</td><td>41</td><td>0</td><td>0.57</td><td>0</td><td>0</td><td>1</td><td>0</td><td>1</td><td>0</td><td>1</td><td>0</td><td>1</td><td>0</td><td>-1.62833</td></tr>
<tr><td>tr|F8WDK9|F8WDK9_</td><td>PDCD6IP</td><td>Programmed cell death 6-interacting protein OS=Homo sapiens GN=PDCD6IP PE=2 SV=1</td><td>10956</td><td>136</td><td>0</td><td>61</td><td>0</td><td>0.18</td><td>0</td><td>0</td><td>1</td><td>0</td><td>1</td><td>0</td><td>1</td><td>0</td><td>1</td><td>0</td><td>-1.62833</td></tr>
<tr><td>tr|Q9BZT4|Q9BZT4_H</td><td>tr|Q9BZT4|Q</td><td>PNAS-29 OS=Homo sapiens PE=4 SV=1</td><td>8036</td><td>137</td><td>0</td><td>59</td><td>0</td><td>0.4</td><td>0</td><td>0</td><td>1</td><td>0</td><td>1</td><td>0</td><td>1</td><td>0</td><td>1</td><td>0</td><td>-1.62833</td></tr>
<tr><td>tr|Q9NX34|Q9NX34_</td><td>tr|Q9NX34|C</td><td>cDNA FLJ20465 fis, clone KAT06236 OS=Homo sapiens PE=2 SV=1</td><td>19417</td><td>138</td><td>0</td><td>46</td><td>0</td><td>0.69</td><td>0</td><td>0</td><td>1</td><td>0</td><td>1</td><td>0</td><td>1</td><td>0</td><td>1</td><td>0</td><td>-1.62833</td></tr>
<tr><td>tr|G3V423|G3V423_H</td><td>WARS</td><td>T1-TrpRS (Fragment) OS=Homo sapiens GN=WARS PE=2 SV=1</td><td>15836</td><td>145</td><td>0</td><td>34</td><td>0</td><td>0.27</td><td>0</td><td>0</td><td>1</td><td>0</td><td>1</td><td>0</td><td>1</td><td>0</td><td>1</td><td>0</td><td>-1.62833</td></tr>
<tr><td>tr|H0YHU8|H0YHU8_</td><td>SART3</td><td>Squamous cell carcinoma antigen recognized by T-cells 3 (Fragment) OS=Homo sapiens GN=SART3 PE=4 SV=1</td><td>16246</td><td>153</td><td>0</td><td>45</td><td>0</td><td>0.16</td><td>0</td><td>0</td><td>1</td><td>0</td><td>1</td><td>0</td><td>1</td><td>0</td><td>1</td><td>0</td><td>-1.62833</td></tr>
<tr><td>tr|K7EQ75|K7EQ75_H</td><td>ILF3</td><td>Interleukin enhancer-binding factor 3 (Fragment) OS=Homo sapiens GN=ILF3 PE=4 SV=1</td><td>6139</td><td>154</td><td>0</td><td>29</td><td>0</td><td>0.46</td><td>0</td><td>0</td><td>0</td><td>0</td><td>1</td><td>0</td><td>1</td><td>0</td><td>1</td><td>0</td><td>-1.62833</td></tr>
<tr><td>tr|H0YCY7|H0YCY7_H</td><td>RRM1</td><td>Ribonucleoside-diphosphate reductase (Fragment) OS=Homo sapiens GN=RRM1 PE=3 SV=1</td><td>14342</td><td>156</td><td>0</td><td>22</td><td>0</td><td>0.29</td><td>0</td><td>0</td><td>0</td><td>0</td><td>1</td><td>0</td><td>1</td><td>0</td><td>1</td><td>0</td><td>-1.62833</td></tr>
<tr><td>tr|Q5T0R9|Q5T0R9_H</td><td>CAP1</td><td>Adenylyl cyclase-associated protein (Fragment) OS=Homo sapiens GN=CAP1 PE=3 SV=1</td><td>28902</td><td>172</td><td>0</td><td>51</td><td>0</td><td>0.36</td><td>0</td><td>0</td><td>1</td><td>0</td><td>1</td><td>0</td><td>1</td><td>0</td><td>1</td><td>0</td><td>-1.62833</td></tr>
<tr><td>tr|J3KSA9|J3KSA9_HU</td><td>PYCR1</td><td>Pyrroline-5-carboxylate reductase 1, mitochondrial (Fragment) OS=Homo sapiens GN=PYCR1 PE=4 SV=1</td><td>11860</td><td>174</td><td>0</td><td>0</td><td>0</td><td>0.19</td><td>0</td><td>0</td><td>0</td><td>0</td><td>0</td><td>0</td><td>1</td><td>0</td><td>1</td><td>0</td><td>-1.62833</td></tr>
<tr><td>tr|Q0PHS6|Q0PHS6_</td><td>G6PD</td><td>Glucose-6-phosphate dehydrogenase (Fragment) OS=Homo sapiens GN=G6PD PE=2 SV=1</td><td>4926</td><td>176</td><td>0</td><td>0</td><td>0</td><td>0.18</td><td>0</td><td>0</td><td>0</td><td>0</td><td>0</td><td>0</td><td>1</td><td>0</td><td>2</td><td>0</td><td>-1.62833</td></tr>
<tr><td>tr|A4D1P0|A4D1P0_H</td><td>LOC402299</td><td>Uncharacterized protein OS=Homo sapiens GN=LOC402299 PE=4 SV=1</td><td>42109</td><td>185</td><td>0</td><td>40</td><td>0</td><td>0.42</td><td>0</td><td>0</td><td>1</td><td>0</td><td>1</td><td>0</td><td>1</td><td>0</td><td>1</td><td>0</td><td>-1.62833</td></tr>
<tr><td>tr|B3KV39|B3KV39_H</td><td>tr|B3KV39|B</td><td>cDNA FLJ16099 fis, clone TESTI2008278, highly similar to Homo sapiens forkhead box K1 (FOXK1), mRNA OS=Homo sapie</td><td>50383</td><td>188</td><td>0</td><td>115</td><td>0</td><td>0.07</td><td>0</td><td>0</td><td>1</td><td>0</td><td>1</td><td>0</td><td>1</td><td>0</td><td>2</td><td>0</td><td>-1.62833</td></tr>
<tr><td>tr|Q05CV6|Q05CV6_</td><td>CPS1</td><td>CPS1 protein (Fragment) OS=Homo sapiens GN=CPS1 PE=2 SV=1</td><td>22462</td><td>204</td><td>0</td><td>32</td><td>0</td><td>0.15</td><td>0</td><td>0</td><td>0</td><td>0</td><td>1</td><td>0</td><td>1</td><td>0</td><td>1</td><td>0</td><td>-1.62833</td></tr>
<tr><td>tr|D6R9D3|D6R9D3_</td><td>HNRNPH1</td><td>Heterogeneous nuclear ribonucleoprotein H OS=Homo sapiens GN=HNRNPH1 PE=4 SV=1</td><td>4404</td><td>207</td><td>0</td><td>23</td><td>0</td><td>0.45</td><td>0</td><td>0</td><td>0</td><td>0</td><td>1</td><td>0</td><td>1</td><td>0</td><td>1</td><td>0</td><td>-1.62833</td></tr>
<tr><td>sp|Q5JQF8|PAP1M_H</td><td>PABPC1L2A</td><td>Polyadenylate-binding protein 1-like 2 OS=Homo sapiens GN=PABPC1L2A PE=2 SV=1</td><td>22956</td><td>215</td><td>0</td><td>43</td><td>0</td><td>0.33</td><td>0</td><td>0</td><td>1</td><td>0</td><td>1</td><td>0</td><td>1</td><td>0</td><td>1</td><td>0</td><td>-1.62833</td></tr>
<tr><td>tr|H0Y960|H0Y960_H</td><td>DDX4</td><td>Probable ATP-dependent RNA helicase DDX4 (Fragment) OS=Homo sapiens GN=DDX4 PE=4 SV=1</td><td>33292</td><td>217</td><td>0</td><td>43</td><td>0</td><td>0.19</td><td>0</td><td>0</td><td>1</td><td>0</td><td>1</td><td>0</td><td>1</td><td>0</td><td>1</td><td>0</td><td>-1.62833</td></tr>
<tr><td>tr|H7C013|H7C013_H</td><td>ALB</td><td>Serum albumin (Fragment) OS=Homo sapiens GN=ALB PE=4 SV=1</td><td>23414</td><td>222</td><td>0</td><td>24</td><td>0</td><td>0.25</td><td>0</td><td>0</td><td>0</td><td>0</td><td>1</td><td>0</td><td>1</td><td>0</td><td>2</td><td>0</td><td>-1.62833</td></tr>
<tr><td>tr|D6RI02|D6RI02_HU</td><td>RPS3A</td><td>40S ribosomal protein S3a OS=Homo sapiens GN=RPS3A PE=2 SV=1</td><td>10609</td><td>223</td><td>0</td><td>72</td><td>0</td><td>0.44</td><td>0</td><td>0</td><td>1</td><td>0</td><td>1</td><td>0</td><td>1</td><td>0</td><td>1</td><td>0</td><td>-1.62833</td></tr>
<tr><td>tr|D6RD83|D6RD83_</td><td>HNRNPD</td><td>Heterogeneous nuclear ribonucleoprotein D0 (Fragment) OS=Homo sapiens GN=HNRNPD PE=2 SV=1</td><td>9992</td><td>229</td><td>0</td><td>0</td><td>0</td><td>0.37</td><td>0</td><td>0</td><td>0</td><td>0</td><td>0</td><td>0</td><td>1</td><td>0</td><td>1</td><td>0</td><td>-1.62833</td></tr>
<tr><td>tr|H0YGZ5|H0YGZ5_H</td><td>PRPF19</td><td>Pre-mRNA-processing factor 19 (Fragment) OS=Homo sapiens GN=PRPF19 PE=4 SV=1</td><td>13367</td><td>237</td><td>0</td><td>62</td><td>0</td><td>0.26</td><td>0</td><td>0</td><td>1</td><td>0</td><td>1</td><td>0</td><td>1</td><td>0</td><td>1</td><td>0</td><td>-1.62833</td></tr>
<tr><td>tr|Q96QB2|Q96QB2_</td><td>lat1</td><td>Sodium-independent neutral amino acid transporter LAT1 OS=Homo sapiens GN=lat1 PE=2 SV=1</td><td>55772</td><td>239</td><td>0</td><td>35</td><td>0</td><td>0.12</td><td>0</td><td>0</td><td>0</td><td>0</td><td>1</td><td>0</td><td>1</td><td>0</td><td>1</td><td>0</td><td>-1.62833</td></tr>
<tr><td>tr|E5RFJ8|E5RFJ8_HU</td><td>MCM4</td><td>DNA replication licensing factor MCM4 (Fragment) OS=Homo sapiens GN=MCM4 PE=2 SV=1</td><td>23630</td><td>240</td><td>0</td><td>65</td><td>0</td><td>0.1</td><td>0</td><td>0</td><td>1</td><td>0</td><td>1</td><td>0</td><td>1</td><td>0</td><td>2</td><td>0</td><td>-1.62833</td></tr>
<tr><td>tr|B4DE40|B4DE40_H</td><td>SMS</td><td>Spermine synthase OS=Homo sapiens GN=SMS PE=2 SV=1</td><td>31596</td><td>248</td><td>0</td><td>78</td><td>0</td><td>0.11</td><td>0</td><td>0</td><td>1</td><td>0</td><td>1</td><td>0</td><td>1</td><td>0</td><td>1</td><td>0</td><td>-1.62833</td></tr>
<tr><td>tr|Q5JXB7|Q5JXB7_H</td><td>PLOD1</td><td>Procollagen-lysine,2-oxoglutarate 5-dioxygenase 1 (Fragment) OS=Homo sapiens GN=PLOD1 PE=4 SV=1</td><td>31911</td><td>251</td><td>0</td><td>76</td><td>0</td><td>0.04</td><td>0</td><td>0</td><td>1</td><td>0</td><td>1</td><td>0</td><td>1</td><td>0</td><td>1</td><td>0</td><td>-1.62833</td></tr>
<tr><td>tr|Q53RR5|Q53RR5_</td><td>YWHAQ</td><td>Putative uncharacterized protein YWHAQ (Fragment) OS=Homo sapiens GN=YWHAQ PE=2 SV=1</td><td>11306</td><td>258</td><td>0</td><td>57</td><td>0</td><td>0.4</td><td>0</td><td>0</td><td>1</td><td>0</td><td>1</td><td>0</td><td>1</td><td>0</td><td>1</td><td>0</td><td>-1.62833</td></tr>
<tr><td>tr|Q3B834|Q3B834_H</td><td>KRT7</td><td>KRT7 protein (Fragment) OS=Homo sapiens GN=KRT7 PE=2 SV=1</td><td>12922</td><td>259</td><td>0</td><td>0</td><td>0</td><td>0.45</td><td>0</td><td>0</td><td>0</td><td>0</td><td>0</td><td>0</td><td>1</td><td>0</td><td>1</td><td>0</td><td>-1.62833</td></tr>
<tr><td>tr|H7C2U0|H7C2U0_</td><td>CCT8</td><td>T-complex protein 1 subunit theta (Fragment) OS=Homo sapiens GN=CCT8 PE=4 SV=1</td><td>11522</td><td>268</td><td>0</td><td>0</td><td>0</td><td>0.18</td><td>0</td><td>0</td><td>0</td><td>0</td><td>0</td><td>0</td><td>1</td><td>0</td><td>1</td><td>0</td><td>-1.62833</td></tr>
<tr><td>tr|K7EKB6|K7EKB6_H</td><td>THOP1</td><td>Thimet oligopeptidase (Fragment) OS=Homo sapiens GN=THOP1 PE=4 SV=1</td><td>20792</td><td>278</td><td>0</td><td>68</td><td>0</td><td>0.16</td><td>0</td><td>0</td><td>1</td><td>0</td><td>1</td><td>0</td><td>1</td><td>0</td><td>1</td><td>0</td><td>-1.62833</td></tr>
<tr><td>tr|Q5SZX8|Q5SZX8_H</td><td>CCT3</td><td>Chaperonin containing TCP1, subunit 3 (Gamma) OS=Homo sapiens GN=CCT3 PE=2 SV=1</td><td>18150</td><td>279</td><td>0</td><td>0</td><td>0</td><td>0.05</td><td>0</td><td>0</td><td>0</td><td>0</td><td>0</td><td>0</td><td>1</td><td>0</td><td>1</td><td>0</td><td>-1.62833</td></tr>
<tr><td>tr|E5RFS9|E5RFS9_HU</td><td>MAT2B</td><td>Methionine adenosyltransferase 2 subunit beta OS=Homo sapiens GN=MAT2B PE=2 SV=1</td><td>9467</td><td>280</td><td>0</td><td>68</td><td>0</td><td>0.37</td><td>0</td><td>0</td><td>1</td><td>0</td><td>1</td><td>0</td><td>1</td><td>0</td><td>1</td><td>0</td><td>-1.62833</td></tr>
<tr><td>tr|H0YC45|H0YC45_H</td><td>ATP6V1B2</td><td>V-type proton ATPase subunit B, brain isoform (Fragment) OS=Homo sapiens GN=ATP6V1B2 PE=4 SV=1</td><td>5569</td><td>287</td><td>0</td><td>64</td><td>0</td><td>0.65</td><td>0</td><td>0</td><td>1</td><td>0</td><td>1</td><td>0</td><td>1</td><td>0</td><td>1</td><td>0</td><td>-1.62833</td></tr>
<tr><td>tr|B4DE27|B4DE27_H</td><td>tr|B4DE27|B</td><td>cDNA FLJ58765, highly similar to Neutral amino acid transporter B(0) OS=Homo sapiens PE=2 SV=1</td><td>33987</td><td>290</td><td>0</td><td>63</td><td>0</td><td>0.1</td><td>0</td><td>0</td><td>1</td><td>0</td><td>1</td><td>0</td><td>1</td><td>0</td><td>1</td><td>0</td><td>-1.62833</td></tr>
<tr><td>sp|P81605|DCD_HUM</td><td>DCD</td><td>Dermcidin OS=Homo sapiens GN=DCD PE=1 SV=2</td><td>11391</td><td>295</td><td>0</td><td>62</td><td>0</td><td>0.3</td><td>0</td><td>0</td><td>1</td><td>0</td><td>1</td><td>0</td><td>1</td><td>0</td><td>1</td><td>0</td><td>-1.62833</td></tr>
<tr><td>tr|Q5T0I0|Q5T0I0_HU</td><td>GSN</td><td>Gelsolin (Fragment) OS=Homo sapiens GN=GSN PE=4 SV=1</td><td>29107</td><td>297</td><td>0</td><td>61</td><td>0</td><td>0.04</td><td>0</td><td>0</td><td>1</td><td>0</td><td>1</td><td>0</td><td>1</td><td>0</td><td>2</td><td>0</td><td>-1.62833</td></tr>
<tr><td>tr|B4DX93|B4DX93_H</td><td>tr|B4DX93|B</td><td>cDNA FLJ55471, highly similar to Cytoplasmic dynein 1 intermediate chain 2 OS=Homo sapiens PE=2 SV=1</td><td>40089</td><td>298</td><td>0</td><td>40</td><td>0</td><td>0.09</td><td>0</td><td>0</td><td>1</td><td>0</td><td>1</td><td>0</td><td>1</td><td>0</td><td>1</td><td>0</td><td>-1.62833</td></tr>
<tr><td>tr|Q8TAK2|Q8TAK2_H</td><td>tr|Q8TAK2|C</td><td>Similar to catalase (Fragment) OS=Homo sapiens PE=2 SV=1</td><td>26093</td><td>299</td><td>0</td><td>49</td><td>0</td><td>0.11</td><td>0</td><td>0</td><td>1</td><td>0</td><td>1</td><td>0</td><td>1</td><td>0</td><td>1</td><td>0</td><td>-1.62833</td></tr>
<tr><td>tr|A1L3A7|A1L3A7_H</td><td>NUFIP2</td><td>Nuclear fragile X mental retardation protein interacting protein 2 OS=Homo sapiens GN=NUFIP2 PE=2 SV=1</td><td>76132</td><td>301</td><td>0</td><td>59</td><td>0</td><td>0.04</td><td>0</td><td>0</td><td>1</td><td>0</td><td>1</td><td>0</td><td>1</td><td>0</td><td>1</td><td>0</td><td>-1.62833</td></tr>
<tr><td>tr|B5LY64|B5LY64_HU</td><td>UMPS</td><td>Orotidine 5~-phosphate decarboxylase OS=Homo sapiens GN=UMPS PE=2 SV=1</td><td>33258</td><td>302</td><td>0</td><td>59</td><td>0</td><td>0.1</td><td>0</td><td>0</td><td>1</td><td>0</td><td>1</td><td>0</td><td>1</td><td>0</td><td>1</td><td>0</td><td>-1.62833</td></tr>
<tr><td>tr|H3BNF6|H3BNF6_</td><td>MVP</td><td>Major vault protein (Fragment) OS=Homo sapiens GN=MVP PE=4 SV=1</td><td>16538</td><td>304</td><td>0</td><td>38</td><td>0</td><td>0.07</td><td>0</td><td>0</td><td>1</td><td>0</td><td>1</td><td>0</td><td>1</td><td>0</td><td>1</td><td>0</td><td>-1.62833</td></tr>
<tr><td>tr|B3KPC1|B3KPC1_H</td><td>tr|B3KPC1|B</td><td>cDNA FLJ31582 fis, clone NT2RI2002117, highly similar to Protein pelota homolog OS=Homo sapiens PE=2 SV=1</td><td>43807</td><td>307</td><td>0</td><td>59</td><td>0</td><td>0.08</td><td>0</td><td>0</td><td>1</td><td>0</td><td>1</td><td>0</td><td>1</td><td>0</td><td>1</td><td>0</td><td>-1.62833</td></tr>
<tr><td>sp|Q5TZA2|CROCC_H</td><td>CROCC</td><td>Rootletin OS=Homo sapiens GN=CROCC PE=1 SV=1</td><td>228787</td><td>310</td><td>0</td><td>58</td><td>0</td><td>0.01</td><td>0</td><td>0</td><td>1</td><td>0</td><td>1</td><td>0</td><td>1</td><td>0</td><td>2</td><td>0</td><td>-1.62833</td></tr>
<tr><td>tr|J3QQM1|J3QQM1_</td><td>PSMC5</td><td>26S protease regulatory subunit 8 (Fragment) OS=Homo sapiens GN=PSMC5 PE=4 SV=1</td><td>29442</td><td>314</td><td>0</td><td>57</td><td>0</td><td>0.11</td><td>0</td><td>0</td><td>1</td><td>0</td><td>1</td><td>0</td><td>1</td><td>0</td><td>2</td><td>0</td><td>-1.62833</td></tr>
<tr><td>tr|C9JPP3|C9JPP3_HU</td><td>RBBP4</td><td>Histone-binding protein RBBP4 (Fragment) OS=Homo sapiens GN=RBBP4 PE=2 SV=2</td><td>9655</td><td>315</td><td>0</td><td>57</td><td>0</td><td>0.36</td><td>0</td><td>0</td><td>1</td><td>0</td><td>1</td><td>0</td><td>1</td><td>0</td><td>1</td><td>0</td><td>-1.62833</td></tr>
<tr><td>tr|H7C2E4|H7C2E4_H</td><td>CAD</td><td>CAD protein (Fragment) OS=Homo sapiens GN=CAD PE=3 SV=1</td><td>32547</td><td>317</td><td>0</td><td>40</td><td>0</td><td>0.04</td><td>0</td><td>0</td><td>1</td><td>0</td><td>1</td><td>0</td><td>1</td><td>0</td><td>1</td><td>0</td><td>-1.62833</td></tr>
<tr><td>tr|H7C1U0|H7C1U0_</td><td>APEH</td><td>Acylamino-acid-releasing enzyme (Fragment) OS=Homo sapiens GN=APEH PE=4 SV=1</td><td>35132</td><td>319</td><td>0</td><td>56</td><td>0</td><td>0.09</td><td>0</td><td>0</td><td>1</td><td>0</td><td>1</td><td>0</td><td>1</td><td>0</td><td>1</td><td>0</td><td>-1.62833</td></tr>
<tr><td>tr|C9JIX5|C9JIX5_HUM</td><td>PLOD3</td><td>Procollagen-lysine,2-oxoglutarate 5-dioxygenase 3 (Fragment) OS=Homo sapiens GN=PLOD3 PE=2 SV=1</td><td>8424</td><td>325</td><td>0</td><td>41</td><td>0</td><td>0.12</td><td>0</td><td>0</td><td>1</td><td>0</td><td>1</td><td>0</td><td>1</td><td>0</td><td>1</td><td>0</td><td>-1.62833</td></tr>
<tr><td>tr|Q5RZ99|Q5RZ99_H</td><td>tr|Q5RZ99|C</td><td>Apoptosis-inducing factor (Fragment) OS=Homo sapiens PE=2 SV=1</td><td>24524</td><td>326</td><td>0</td><td>54</td><td>0</td><td>0.05</td><td>0</td><td>0</td><td>1</td><td>0</td><td>1</td><td>0</td><td>1</td><td>0</td><td>1</td><td>0</td><td>-1.62833</td></tr>
<tr><td>tr|I3L4P6|I3L4P6_HU</td><td>ARHGAP17</td><td>Rho GTPase-activating protein 17 (Fragment) OS=Homo sapiens GN=ARHGAP17 PE=4 SV=1</td><td>8676</td><td>328</td><td>0</td><td>54</td><td>0</td><td>0.03</td><td>0</td><td>0</td><td>1</td><td>0</td><td>1</td><td>0</td><td>1</td><td>0</td><td>1</td><td>0</td><td>-1.62833</td></tr>
<tr><td>tr|A8K3Q9|A8K3Q9_</td><td>tr|A8K3Q9|A</td><td>cDNA FLJ76611, highly similar to Homo sapiens ribosomal protein L14 (RPL14), mRNA OS=Homo sapiens PE=2 SV=1</td><td>23554</td><td>330</td><td>0</td><td>53</td><td>0</td><td>0.14</td><td>0</td><td>0</td><td>1</td><td>0</td><td>1</td><td>0</td><td>1</td><td>0</td><td>1</td><td>0</td><td>-1.62833</td></tr>
</table>

tr|Q59GV0|Q59GV0_	tr|Q59GV0|(	Propionyl Coenzyme A carboxylase, beta polypeptide variant (Fragment) OS=Homo sapiens PE=2 SV=1	60578	331	0	53	0	0.05	0	0	1	0	1	0	1	0	2	0	-1.62833
sp|Q9Y6M5|ZNT1_HU	SLC30A1	Zinc transporter 1 OS=Homo sapiens GN=SLC30A1 PE=1 SV=3	56290	334	0	52	0	0.06	0	0	1	0	1	0	1	0	1	0	-1.62833
tr|J3KTE4|J3KTE4_HU	RPL19	Ribosomal protein L19 OS=Homo sapiens GN=RPL19 PE=3 SV=1	23347	336	0	52	0	0.14	0	0	1	0	1	0	1	0	1	0	-1.62833
tr|A8KA82|A8KA82_H	DNAJC3	DnaJ (Hsp40) homolog, subfamily C, member 3 OS=Homo sapiens GN=DNAJC3 PE=2 SV=1	58000	337	0	52	0	0.06	0	0	1	0	1	0	1	0	1	0	-1.62833
tr|B2RAR2|B2RAR2_H	tr|B2RAR2|B	cDNA, FLJ95064, highly similar to Homo sapiens nin one binding protein (NOB1P), mRNA OS=Homo sapiens PE=2 SV=1	47015	338	0	52	0	0.07	0	0	1	0	1	0	1	0	1	0	-1.62833
tr|A8K517|A8K517_H	RPS23	Ribosomal protein S23, isoform CRA_a OS=Homo sapiens GN=RPS23 PE=2 SV=1	15969	339	0	52	0	0.21	0	0	1	0	1	0	1	0	1	0	-1.62833
tr|H7C5I9|H7C5I9_HU	GART	Phosphoribosylformylglycinamidine cyclo-ligase (Fragment) OS=Homo sapiens GN=GART PE=4 SV=1	2640	341	0	23	0	0.13	0	0	0	0	1	0	1	0	1	0	-1.62833
tr|E5RFU4|E5RFU4_H	DPYSL2	Dihydropyrimidinase-related protein 2 (Fragment) OS=Homo sapiens GN=DPYSL2 PE=2 SV=1	18320	342	0	20	0	0.23	0	0	0	0	1	0	1	0	1	0	-1.62833
tr|C9JQM9|C9JQM9_	DARS	Aspartate--tRNA ligase, cytoplasmic (Fragment) OS=Homo sapiens GN=DARS PE=2 SV=1	15386	344	0	51	0	0.06	0	0	1	0	1	0	1	0	1	0	-1.62833
tr|E9PLX7|E9PLX7_HU	RPL27A	60S ribosomal protein L27a OS=Homo sapiens GN=RPL27A PE=2 SV=1	12526	348	0	0	0	0.2	0	0	0	0	0	0	1	0	1	0	-1.62833
tr|E5RJF5|E5RJF5_HU	ANXA6	Annexin (Fragment) OS=Homo sapiens GN=ANXA6 PE=2 SV=1	14825	349	0	40	0	0.14	0	0	1	0	1	0	1	0	1	0	-1.62833
tr|B3KRS7|B3KRS7_H	tr|B3KRS7|B	cDNA FLJ34844 fis, clone NT2NE2011154, highly similar to RuvB-like 1 OS=Homo sapiens PE=2 SV=1	34946	351	0	50	0	0.09	0	0	1	0	1	0	1	0	1	0	-1.62833
tr|Q53ST0|Q53ST0_H	PCYOX1	Putative uncharacterized protein PCYOX1 (Fragment) OS=Homo sapiens GN=PCYOX1 PE=2 SV=1	11626	355	0	49	0	0.06	0	0	1	0	1	0	1	0	1	0	-1.62833
tr|D6RBX4|D6RBX4_H	TGFBI	Transforming growth factor-beta-induced protein ig-h3 OS=Homo sapiens GN=TGFBI PE=2 SV=1	7268	358	0	31	0	0.09	0	0	0	0	1	0	1	0	1	0	-1.62833
tr|J3KTI8|J3KTI8_HUN	SLC16A3	Monocarboxylate transporter 4 (Fragment) OS=Homo sapiens GN=SLC16A3 PE=4 SV=1	32793	362	0	37	0	0.14	0	0	1	0	1	0	1	0	1	0	-1.62833
tr|K7EM16|K7EM16_	VASP	Vasodilator-stimulated phosphoprotein (Fragment) OS=Homo sapiens GN=VASP PE=4 SV=1	20260	363	0	48	0	0.17	0	0	1	0	1	0	1	0	1	0	-1.62833
tr|H0Y9Q1|H0Y9Q1_	LAP3	Cytosol aminopeptidase (Fragment) OS=Homo sapiens GN=LAP3 PE=4 SV=1	22641	368	0	47	0	0.15	0	0	1	0	1	0	1	0	1	0	-1.62833
tr|B3KU96|B3KU96_H	tr|B3KU96|B	cDNA FLJ39433 fis, clone PROST2004142, highly similar to GDP-L-fucose synthetase (EC 1.1.1.271) OS=Homo sapiens PE=	17856	371	0	47	0	0.19	0	0	1	0	1	0	1	0	1	0	-1.62833
tr|B4DDR6|B4DDR6_	tr|B4DDR6|E	cDNA FLJ60414, highly similar to Solute carrier family 2, facilitated glucose transporter member 1 OS=Homo sapiens PE=	50229	377	0	46	0	0.06	0	0	1	0	1	0	1	0	1	0	-1.62833
tr|J3QL39|J3QL39_HU	PFAS	Phosphoribosylformylglycinamidine synthase (Fragment) OS=Homo sapiens GN=PFAS PE=4 SV=1	9951	380	0	0	0	0.05	0	0	0	0	0	0	1	0	1	0	-1.62833
tr|B4DT09|B4DT09_H	tr|B4DT09|B	cDNA FLJ60145, highly similar to Homo sapiens dendritic cell protein (hfl-B5), mRNA OS=Homo sapiens PE=2 SV=1	13697	381	0	46	0	0.25	0	0	1	0	1	0	1	0	1	0	-1.62833
tr|H0YHV5|H0YHV5_	MARS	Methionine--tRNA ligase, cytoplasmic (Fragment) OS=Homo sapiens GN=MARS PE=4 SV=1	27023	385	0	30	0	0.07	0	0	0	0	1	0	1	0	1	0	-1.62833
tr|B3KPZ0|B3KPZ0_H	tr|B3KPZ0|B	cDNA FLJ32485 fis, clone SKNMC2001596, highly similar to Ankyrin repeat and FYVE domain-containing protein 1 (Fragm	121468	387	0	45	0	0.03	0	0	1	0	1	0	1	0	1	0	-1.62833
tr|E5RGY7|E5RGY7_H	CTNNA1	Catenin alpha-1 (Fragment) OS=Homo sapiens GN=CTNNA1 PE=2 SV=1	14895	389	0	35	0	0.07	0	0	0	0	1	0	1	0	1	0	-1.62833
tr|Q6W6M7|Q6W6M	tr|Q6W6M7	Antigen MLAA-43 (Fragment) OS=Homo sapiens PE=2 SV=1	23572	397	0	0	0	0.05	0	0	0	0	0	0	1	0	1	0	-1.62833
tr|H0Y327|H0Y327_H	MTHFD1L	Monofunctional C1-tetrahydrofolate synthase, mitochondrial (Fragment) OS=Homo sapiens GN=MTHFD1L PE=4 SV=1	36808	398	0	43	0	0.09	0	0	1	0	1	0	1	0	1	0	-1.62833
tr|B3KT66|B3KT66_H	PSMD6	Proteasome (Prosome, macropain) 26S subunit, non-ATPase, 6, isoform CRA_c OS=Homo sapiens GN=PSMD6 PE=2 SV=1	40600	399	0	43	0	0.08	0	0	1	0	1	0	1	0	1	0	-1.62833
tr|Q1LZN2|Q1LZN2_H	NOMO3	NOMO3 protein (Fragment) OS=Homo sapiens GN=NOMO3 PE=2 SV=1	90388	400	0	43	0	0.04	0	0	1	0	1	0	1	0	1	0	-1.62833
tr|E9PCI9|E9PCI9_HU	FDPS	Farnesyl pyrophosphate synthase OS=Homo sapiens GN=FDPS PE=2 SV=1	40849	405	0	43	0	0.08	0	0	1	0	1	0	1	0	1	0	-1.62833
tr|B3KMW5|B3KMW5	tr|B3KMW5|	cDNA FLJ12796 fis, clone NT2RP2002058, highly similar to WD repeat protein 3 OS=Homo sapiens PE=2 SV=1	71519	408	0	43	0	0.05	0	0	1	0	1	0	1	0	1	0	-1.62833
sp|Q9NSB2|KRT84_H	KRT84	Keratin, type II cuticular Hb4 OS=Homo sapiens GN=KRT84 PE=2 SV=2	65942	409	0	43	0	0.05	0	0	1	0	1	0	1	0	3	0	-1.62833
tr|B4DRG7|B4DRG7_	tr|B4DRG7|E	cDNA FLJ60336, highly similar to Condensin complex subunit 2 OS=Homo sapiens PE=2 SV=1	80877	410	0	43	0	0.04	0	0	1	0	1	0	1	0	1	0	-1.62833
sp|Q9NSB4|KRT82_H	KRT82	Keratin, type II cuticular Hb2 OS=Homo sapiens GN=KRT82 PE=1 SV=3	57985	411	0	43	0	0.06	0	0	1	0	1	0	1	0	2	0	-1.62833
tr|H0YGE9|H0YGE9_H	M6PR	Cation-dependent mannose-6-phosphate receptor (Fragment) OS=Homo sapiens GN=M6PR PE=4 SV=1	11814	416	0	42	0	0.29	0	0	1	0	1	0	1	0	1	0	-1.62833
tr|A0JLS5|A0JLS5_HU	TBL3	TBL3 protein (Fragment) OS=Homo sapiens GN=TBL3 PE=2 SV=1	64079	418	0	42	0	0.05	0	0	1	0	1	0	1	0	1	0	-1.62833
tr|O60250|O60250_H	tr|O60250|O	Ribosomal protein L13 (Fragment) OS=Homo sapiens PE=2 SV=2	7385	422	0	41	0	0.14	0	0	1	0	1	0	1	0	1	0	-1.62833
tr|B4DLD4|B4DLD4_H	tr|B4DLD4|B	cDNA FLJ56738, highly similar to Fibrillarin OS=Homo sapiens PE=2 SV=1	30279	423	0	41	0	0.11	0	0	1	0	1	0	1	0	1	0	-1.62833
tr|B4DPY2|B4DPY2_H	tr|B4DPY2|B	cDNA FLJ59286, highly similar to Pre-mRNA-processing factor 40 homolog A (Fragment) OS=Homo sapiens PE=2 SV=1	93124	425	0	41	0	0.04	0	0	1	0	1	0	1	0	1	0	-1.62833
tr|Q5JR94|Q5JR94_H	RPS8	40S ribosomal protein S8 OS=Homo sapiens GN=RPS8 PE=2 SV=1	24475	426	0	41	0	0.14	0	0	1	0	1	0	1	0	2	0	-1.62833
tr|A4D1I7|A4D1I7_HU	DNCI1	Dynein, cytoplasmic 1, intermediate chain 1, isoform CRA_e OS=Homo sapiens GN=DNCI1 PE=2 SV=1	73423	427	0	40	0	0.04	0	0	1	0	1	0	1	0	1	0	-1.62833
tr|B7Z1R5|B7Z1R5_H	ATP6V1A	V-type proton ATPase catalytic subunit A OS=Homo sapiens GN=ATP6V1A PE=2 SV=1	65037	428	0	40	0	0.05	0	0	1	0	1	0	1	0	2	0	-1.62833
tr|B7Z5L5|B7Z5L5_HU	tr|B7Z5L5|B7	cDNA FLJ59160, highly similar to Testin OS=Homo sapiens PE=2 SV=1	17178	430	0	40	0	0.15	0	0	1	0	1	0	1	0	1	0	-1.62833
tr|B4E363|B4E363_H	FARSA	Phenylalanine--tRNA ligase alpha subunit OS=Homo sapiens GN=FARSA PE=2 SV=1	54181	447	0	38	0	0.06	0	0	1	0	1	0	1	0	1	0	-1.62833
tr|K7EID2|K7EID2_HU	ALG1	Chitobiosyldiphosphodolichol beta-mannosyltransferase (Fragment) OS=Homo sapiens GN=ALG1 PE=4 SV=1	19593	452	0	31	0	0.17	0	0	0	0	1	0	1	0	1	0	-1.62833
tr|B3KWS3|B3KWS3_	PLOD2	Procollagen-lysine,2-oxoglutarate 5-dioxygenase 2 OS=Homo sapiens GN=PLOD2 PE=2 SV=1	49567	453	0	29	0	0.08	0	0	0	0	1	0	1	0	1	0	-1.62833
tr|H0Y9L5|H0Y9L5_H	EXOSC9	Exosome complex component RRP45 (Fragment) OS=Homo sapiens GN=EXOSC9 PE=4 SV=1	18940	457	0	38	0	0.18	0	0	1	0	1	0	1	0	1	0	-1.62833
sp|Q9H9F9|ARP5_HU	ACTR5	Actin-related protein 5 OS=Homo sapiens GN=ACTR5 PE=1 SV=2	68825	460	0	38	0	0.05	0	0	1	0	1	0	1	0	2	0	-1.62833
tr|H7C1M0|H7C1M0_	FANCL	E3 ubiquitin-protein ligase FANCL (Fragment) OS=Homo sapiens GN=FANCL PE=4 SV=1	33067	461	0	37	0	0.1	0	0	1	0	1	0	1	0	1	0	-1.62833
tr|B3KUJ0|B3KUJ0_H	tr|B3KUJ0|B	cDNA FLJ39996 fis, clone STOMA2002166, highly similar to Splicing factor 3B subunit 4 OS=Homo sapiens PE=2 SV=1	44512	464	0	37	0	0.07	0	0	1	0	1	0	1	0	2	0	-1.62833
sp|Q6ZU64|CC108_H	CCDC108	Coiled-coil domain-containing protein 108 OS=Homo sapiens GN=CCDC108 PE=2 SV=2	220247	465	0	37	0	0.01	0	0	1	0	1	0	1	0	1	0	-1.62833
tr|H0Y5J4|H0Y5J4_HU	DBNL	Drebrin-like protein (Fragment) OS=Homo sapiens GN=DBNL PE=4 SV=1	40475	466	0	37	0	0.08	0	0	1	0	1	0	1	0	1	0	-1.62833
tr|C9J7L0|C9J7L0_HU	RBBP7	Histone-binding protein RBBP7 (Fragment) OS=Homo sapiens GN=RBBP7 PE=2 SV=1	7740	468	0	0	0	0.07	0	0	0	0	0	0	1	0	1	0	-1.62833
tr|H0YIX9|H0YIX9_HU	R3HDM2	R3H domain-containing protein 2 (Fragment) OS=Homo sapiens GN=R3HDM2 PE=4 SV=1	31776	469	0	37	0	0.1	0	0	1	0	1	0	1	0	1	0	-1.62833
tr|B7Z982|B7Z982_H	PLRG1	Pleiotropic regulator 1 OS=Homo sapiens GN=PLRG1 PE=2 SV=1	14839	476	0	0	0	0.12	0	0	0	0	0	0	1	0	1	0	-1.62833
tr|B7WPL0|B7WPL0_	RIC8B	Synembryn-B OS=Homo sapiens GN=RIC8B PE=2 SV=1	64252	483	0	36	0	0.05	0	0	1	0	1	0	1	0	1	0	-1.62833
tr|Q68E00|Q68E00_H	DKFZp686G2	Putative uncharacterized protein DKFZp686G2045 (Fragment) OS=Homo sapiens GN=DKFZp686G2045 PE=2 SV=2	32787	485	0	36	0	0.1	0	0	0	0	1	0	1	0	1	0	-1.62833
tr|C9JNW5|C9JNW5_	RPL24	60S ribosomal protein L24 OS=Homo sapiens GN=RPL24 PE=4 SV=1	17646	487	0	35	0	0.19	0	0	0	0	1	0	1	0	2	0	-1.62833
sp|Q9NPQ8|RIC8A_H	RIC8A	Synembryn-A OS=Homo sapiens GN=RIC8A PE=1 SV=3	60185	496	0	34	0	0.05	0	0	0	0	1	0	1	0	1	0	-1.62833
sp|Q6PGP7|TTC37_H	TTC37	Tetratricopeptide repeat protein 37 OS=Homo sapiens GN=TTC37 PE=1 SV=1	177485	498	0	34	0	0.02	0	0	1	0	1	0	1	0	1	0	-1.62833
tr|B4DDC9|B4DDC9_	tr|B4DDC9|E	cDNA FLJ59448, highly similar to Trifunctional enzyme subunit beta, mitochondrial OS=Homo sapiens PE=2 SV=1	49937	499	0	34	0	0.07	0	0	0	0	1	0	1	0	1	0	-1.62833
sp|O95782|AP2A1_H	AP2A1	AP-2 complex subunit alpha-1 OS=Homo sapiens GN=AP2A1 PE=1 SV=3	108561	500	0	34	0	0.03	0	0	0	0	1	0	1	0	2	0	-1.62833
sp|Q8NFZ5|TNIP2_HU	TNIP2	TNFAIP3-interacting protein 2 OS=Homo sapiens GN=TNIP2 PE=1 SV=1	49240	502	0	33	0	0.07	0	0	1	0	1	0	1	0	2	0	-1.62833
sp|Q7Z494|NPHP3_H	NPHP3	Nephrocystin-3 OS=Homo sapiens GN=NPHP3 PE=1 SV=1	151967	510	0	32	0	0.02	0	0	1	0	1	0	1	0	1	0	-1.62833
tr|A2BDK6|A2BDK6_H	MAP1B	Microtubule-associated protein 1B OS=Homo sapiens GN=MAP1B PE=2 SV=1	257692	511	0	32	0	0.01	0	0	0	0	1	0	1	0	2	0	-1.62833
tr|A0JLS9|A0JLS9_HU	ZEB1	ZEB1 protein (Fragment) OS=Homo sapiens GN=ZEB1 PE=2 SV=1	118576	513	0	32	0	0.03	0	0	1	0	1	0	1	0	1	0	-1.62833
tr|B2R4C0|B2R4C0_H	tr|B2R4C0|B	60S ribosomal protein L18a OS=Homo sapiens PE=2 SV=1	21024	514	0	32	0	0.16	0	0	0	0	1	0	1	0	2	0	-1.62833
sp|P29803|ODPAT_H	PDHA2	Pyruvate dehydrogenase E1 component subunit alpha, testis-specific form, mitochondrial OS=Homo sapiens GN=PDHA2	43647	516	0	32	0	0.1	0	0	0	0	1	0	1	0	1	0	-1.62833
tr|J3KRB3|J3KRB3_HU	RPL17	60S ribosomal protein L17 (Fragment) OS=Homo sapiens GN=RPL17 PE=3 SV=1	12356	517	0	32	0	0.28	0	0	0	0	1	0	1	0	1	0	-1.62833
tr|B4DUH7|B4DUH7_	tr|B4DUH7|E	cDNA FLJ60162, highly similar to Chloride anion exchanger OS=Homo sapiens PE=2 SV=1	52632	518	0	32	0	0.06	0	0	1	0	1	0	1	0	1	0	-1.62833
tr|E9PNN6|E9PNN6_	ALDH3A1	Aldehyde dehydrogenase, dimeric NADP-preferring OS=Homo sapiens GN=ALDH3A1 PE=2 SV=1	44518	519	0	32	0	0.07	0	0	0	0	1	0	1	0	1	0	-1.62833
tr|A6NE09|A6NE09_H	RPSAP58	Protein RPSAP58 OS=Homo sapiens GN=RPSAP58 PE=3 SV=1	33002	521	0	31	0	0.1	0	0	0	0	1	0	1	0	1	0	-1.62833
tr|B3KUZ8|B3KUZ8_H	tr|B3KUZ8|B	Aspartate aminotransferase OS=Homo sapiens PE=2 SV=1	41699	522	0	31	0	0.08	0	0	0	0	1	0	1	0	2	0	-1.62833
tr|L0R4T3|L0R4T3_HU	ABCF2	Alternative protein ABCF2 OS=Homo sapiens GN=ABCF2 PE=4 SV=1	7359	524	0	31	0	0.48	0	0	1	0	1	0	1	0	1	0	-1.62833
tr|B4E0D5|B4E0D5_H	tr|B4E0D5|B	cDNA FLJ54218, highly similar to Acidic leucine-rich nuclear phosphoprotein 32 family member E OS=Homo sapiens PE=2	19540	525	0	31	0	0.17	0	0	0	0	1	0	1	0	1	0	-1.62833
tr|F2Z2J7|F2Z2J7_HU	TTLL7	Tubulin polyglutamylase TTLL7 OS=Homo sapiens GN=TTLL7 PE=2 SV=1	55925	526	0	31	0	0.06	0	0	1	0	1	0	1	0	1	0	-1.62833
tr|H0Y7S3|H0Y7S3_H	ATP2B2	Plasma membrane calcium-transporting ATPase 2 (Fragment) OS=Homo sapiens GN=ATP2B2 PE=3 SV=1	122272	528	0	31	0	0.03	0	0	0	0	1	0	1	0	3	0	-1.62833
sp|Q6ZU15|SEP14_HU	Sep-14	Septin-14 OS=Homo sapiens GN=SEPT14 PE=1 SV=2	50449	531	0	31	0	0.07	0	0	1	0	1	0	1	0	1	0	-1.62833
tr|B1AMS2|B1AMS2_	Sep-06	Septin 6 OS=Homo sapiens GN=SEPT6 PE=3 SV=1	49614	532	0	31	0	0.07	0	0	1	0	1	0	1	0	1	0	-1.62833
tr|I3L312|I3L312_HU	P4HB	Protein disulfide-isomerase (Fragment) OS=Homo sapiens GN=P4HB PE=4 SV=1	26348	536	0	30	0	0.27	0	0	1	0	2	0	1	0	2	0	-1.62833
sp|Q96PE2|ARHGH_H	ARHGEF17	Rho guanine nucleotide exchange factor 17 OS=Homo sapiens GN=ARHGEF17 PE=1 SV=1	223645	537	0	30	0	0.01	0	0	1	0	1	0	1	0	1	0	-1.62833
tr|K7EKM4|K7EKM4_	SRM	Spermidine synthase OS=Homo sapiens GN=SRM PE=4 SV=1	10680	540	0	30	0	0.32	0	0	0	0	1	0	1	0	1	0	-1.62833
tr|H0YK49|H0YK49_H	ETFA	Electron transfer flavoprotein subunit alpha, mitochondrial OS=Homo sapiens GN=ETFA PE=4 SV=1	24259	545	0	30	0	0.14	0	0	0	0	1	0	1	0	1	0	-1.62833
tr|B3KNM6|B3KNM6	tr|B3KNM6|	cDNA FLJ14968 fis, clone THYRO1000288, highly similar to CAAX prenyl protease 1 homolog (EC 3.4.24.84) OS=Homo sap	27279	547	0	30	0	0.12	0	0	1	0	1	0	1	0	1	0	-1.62833
tr|B6F210|B6F210_H	GSS	Glutathione synthetase OS=Homo sapiens GN=GSS PE=2 SV=1	40495	551	0	30	0	0.08	0	0	0	0	1	0	1	0	1	0	-1.62833
tr|D3DTW3|D3DTW3	IGF2BP1	Insulin-like growth factor 2 mRNA binding protein 1, isoform CRA_a OS=Homo sapiens GN=IGF2BP1 PE=2 SV=1	48111	556	0	29	0	0.05	0	0	0	0	1	0	1	0	1	0	-1.62833
tr|H3BRB3|H3BRB3_	KIF22	Kinesin-like protein KIF22 OS=Homo sapiens GN=KIF22 PE=3 SV=1	67996	557	0	29	0	0.05	0	0	0	0	1	0	1	0	1	0	-1.62833
tr|B4DS04|B4DS04_H	tr|B4DS04|B	cDNA FLJ54142, highly similar to Protein transport protein Sec23B OS=Homo sapiens PE=2 SV=1	41720	558	0	27	0	0.08	0	0	0	0	1	0	1	0	1	0	-1.62833
tr|Q1L838|Q1L838_H	GTBP	GTBP-ALT OS=Homo sapiens GN=GTBP PE=2 SV=1	122267	559	0	29	0	0.03	0	0	0	0	1	0	1	0	1	0	-1.62833
tr|B4DM97|B4DM97_	tr|B4DM97|	cDNA FLJ55002, highly similar to Alpha-centractin OS=Homo sapiens PE=2 SV=1	38365	561	0	29	0	0.09	0	0	0	0	1	0	1	0	1	0	-1.62833
tr|E7EN38|E7EN38_H	RAD50	DNA repair protein RAD50 (Fragment) OS=Homo sapiens GN=RAD50 PE=2 SV=1	77138	563	0	29	0	0.09	0	0	0	0	2	0	1	0	2	0	-1.62833
sp|Q6KB66|K2C80_H	KRT80	Keratin, type II cytoskeletal 80 OS=Homo sapiens GN=KRT80 PE=1 SV=2	51007	564	0	29	0	0.06	0	0	0	0	1	0	1	0	1	0	-1.62833
tr|B4DJC3|B4DJC3_H	H2AFY	Histone H2A OS=Homo sapiens GN=H2AFY PE=2 SV=1	21706	565	0	0	0	0.08	0	0	0	0	0	0	1	0	1	0	-1.62833
tr|B4DGQ0|B4DGQ0_	LASP1	LIM and SH3 domain protein 1 OS=Homo sapiens GN=LASP1 PE=2 SV=1	23167	568	0	29	0	0.14	0	0	0	0	1	0	1	0	1	0	-1.62833
tr|I3L361|I3L361_HU	SGSM2	Small G protein-signaling modulator 2 (Fragment) OS=Homo sapiens GN=SGSM2 PE=4 SV=2	1642	570	0	29	0	2.38	0	0	0	0	1	0	1	0	1	0	-1.62833
tr|B0QYT3|B0QYT3_H	RANGAP1	Ran GTPase activating protein 1 (Fragment) OS=Homo sapiens GN=RANGAP1 PE=2 SV=1	27122	574	0	28	0	0.12	0	0	0	0	1	0	1	0	1	0	-1.62833
tr|B4DNG6|B4DNG6_	tr|B4DNG6|E	Annexin OS=Homo sapiens PE=3 SV=1	12870	577	0	28	0	0.27	0	0	0	0	1	0	1	0	1	0	-1.62833
sp|Q16322|KCA10_HU	KCNA10	Potassium voltage-gated channel subfamily A member 10 OS=Homo sapiens GN=KCNA10 PE=1 SV=2	58147	578	0	28	0	0.06	0	0	1	0	1	0	1	0	1	0	-1.62833
tr|J3KN32|J3KN32_HU	RCOR1	REST corepressor 1 OS=Homo sapiens GN=RCOR1 PE=4 SV=1	53409	583	0	28	0	0.06	0	0	0	0	1	0	1	0	1	0	-1.62833
tr|B4DW30|B4DW30_	tr|B4DW30|	cDNA FLJ56743, highly similar to RuvB-like 2 (EC 3.6.1.-) OS=Homo sapiens PE=2 SV=1	27273	584	0	24	0	0.14	0	0	0	0	1	0	1	0	1	0	-1.62833
tr|H0YCU9|H0YCU9_	TAGLN	Transgelin (Fragment) OS=Homo sapiens GN=TAGLN PE=4 SV=1	16833	585	0	28	0	0.2	0	0	0	0	1	0	1	0	1	0	-1.62833
tr|A6NE42|A6NE42_H	TGIF1	Homeobox protein TGIF1 OS=Homo sapiens GN=TGIF1 PE=3 SV=2	27983	586	0	28	0	0.12	0	0	0	0	1	0	1	0	1	0	-1.62833
tr|Q9NZ23|Q9NZ23_H	YA61	Drug-sensitive protein 1 OS=Homo sapiens GN=YA61 PE=2 SV=1	14858	588	0	27	0	0.23	0	0	0	0	1	0	1	0	1	0	-1.62833
tr|B2RDF5|B2RDF5_H	tr|B2RDF5|B	cDNA, FLJ96587, highly similar to Homo sapiens SUMO-1 activating enzyme subunit 2 (UBA2), mRNA OS=Homo sapiens I	71705	590	0	27	0	0.05	0	0	0	0	1	0	1	0	1	0	-1.62833
tr|H3BS19|H3BS19_H	ZNF469	Zinc finger protein 469 OS=Homo sapiens GN=ZNF469 PE=4 SV=1	417347	591	0	27	0	0.01	0	0	0	0	1	0	1	0	1	0	-1.62833
tr|H0Y512|H0Y512_H	APMAP	Adipocyte plasma membrane-associated protein (Fragment) OS=Homo sapiens GN=APMAP PE=4 SV=1	45662	592	0	27	0	0.07	0	0	0	0	1	0	1	0	1	0	-1.62833
tr|B7Z4L9|B7Z4L9_HU	tr|B7Z4L9|B7	cDNA FLJ55342, highly similar to Kelch-like protein 13 OS=Homo sapiens PE=2 SV=1	56884	593	0	26	0	0.06	0	0	0	0	1	0	1	0	1	0	-1.62833
tr|Q5T8C6|Q5T8C6_H	CDC16	Cell division cycle protein 16 homolog OS=Homo sapiens GN=CDC16 PE=4 SV=1	55239	594	0	26	0	0.06	0	0	0	0	1	0	1	0	1	0	-1.62833
sp|Q0VD83|APOBR_H	APOBR	Apolipoprotein B receptor OS=Homo sapiens GN=APOBR PE=1 SV=1	115207	597	0	26	0	0.03	0	0	0	0	1	0	1	0	1	0	-1.62833
tr|Q8TC04|Q8TC04_H	KRT23	Keratin 23 (Histone deacetylase inducible) OS=Homo sapiens GN=KRT23 PE=2 SV=1	48318	600	0	26	0	0.07	0	0	0	0	1	0	1	0	1	0	-1.62833
tr|D6R937|D6R937_H	AIMP1	Endothelial monocyte-activating polypeptide 2 (Fragment) OS=Homo sapiens GN=AIMP1 PE=2 SV=1	16568	601	0	26	0	0.11	0	0	0	0	1	0	1	0	1	0	-1.62833
tr|B2RAX6|B2RAX6_H	tr|B2RAX6|B	cDNA, FLJ95176, Homo sapiens CGI-48 protein (CGI-48), mRNA OS=Homo sapiens PE=2 SV=1	58127	605	0	25	0	0.06	0	0	0	0	1	0	1	0	1	0	-1.62833
tr|J3KNP0|J3KNP0_HU	RAB11FIP1	Rab11 family-interacting protein 1 OS=Homo sapiens GN=RAB11FIP1 PE=4 SV=1	137482	607	0	25	0	0.02	0	0	0	0	1	0	1	0	2	0	-1.62833
tr|J3KSB9|J3KSB9_HU	tr|J3KSB9|J3	Uncharacterized protein (Fragment) OS=Homo sapiens PE=4 SV=1	8877	608	0	25	0	0.39	0	0	0	0	1	0	1	0	1	0	-1.62833
tr|B4DG42|B4DG42_	tr|B4DG42|B	cDNA FLJ53753, highly similar to Myeloid-associated differentiation marker OS=Homo sapiens PE=2 SV=1	31889	609	0	25	0	0.1	0	0	0	0	1	0	1	0	1	0	-1.62833
tr|B7Z5E2|B7Z5E2_H	PSMC2	26S protease regulatory subunit 7 OS=Homo sapiens GN=PSMC2 PE=2 SV=1	33594	610	0	25	0	0.1	0	0	0	0	1	0	1	0	1	0	-1.62833
tr|H3BMW5|H3BMW	DNAJA2	DnaJ homolog subfamily A member 2 (Fragment) OS=Homo sapiens GN=DNAJA2 PE=4 SV=1	15134	611	0	24	0	0.23	0	0	0	0	1	0	1	0	1	0	-1.62833
tr|B4E2P4|B4E2P4_H	tr|B4E2P4|B	cDNA FLJ58474, highly similar to Xaa-Pro aminopeptidase 1 (EC 3.4.11.9) OS=Homo sapiens PE=2 SV=1	50020	615	0	24	0	0.07	0	0	0	0	1	0	1	0	1	0	-1.62833
tr|Q4VAN1|Q4VAN1_	BTN1A1	BTN1A1 protein OS=Homo sapiens GN=BTN1A1 PE=2 SV=1	55878	617	0	24	0	0.06	0	0	0	0	1	0	1	0	1	0	-1.62833
tr|Q2TQ40|Q2TQ40_	TNFRSF17	B cell maturation antigen transcript variant 4 OS=Homo sapiens GN=TNFRSF17 PE=2 SV=1	15460	618	0	24	0	0.22	0	0	0	0	1	0	1	0	1	0	-1.62833
tr|B4DU06|B4DU06_	tr|B4DU06|E	Guanylate cyclase OS=Homo sapiens PE=2 SV=1	91756	620	0	24	0	0.04	0	0	0	0	1	0	1	0	1	0	-1.62833
tr|B0QXZ5|B0QXZ5_H	DNAL4	Dynein light chain 4, axonemal OS=Homo sapiens GN=DNAL4 PE=4 SV=1	7786	621	0	24	0	0.47	0	0	0	0	1	0	1	0	1	0	-1.62833
tr|F5H120|F5H120_H	FKBP4	Peptidyl-prolyl cis-trans isomerase FKBP4, N-terminally processed OS=Homo sapiens GN=FKBP4 PE=2 SV=1	5528	623	0	24	0	0.06	0	0	0	0	1	0	1	0	1	0	-1.62833
tr|B4DN13|B4DN13_	tr|B4DN13|B	cDNA FLJ59136, highly similar to Transmembrane 9 superfamily protein member 3 OS=Homo sapiens PE=2 SV=1	29629	624	0	23	0	0.11	0	0	0	0	1	0	1	0	1	0	-1.62833
tr|K7EPB5|K7EPB5_H	ELAVL3	ELAV-like protein 3 (Fragment) OS=Homo sapiens GN=ELAVL3 PE=4 SV=1	9728	625	0	23	0	0.36	0	0	0	0	1	0	1	0	1	0	-1.62833
sp|Q9Y5P6|GMPPB_H	GMPPB	Mannose-1-phosphate guanyltransferase beta OS=Homo sapiens GN=GMPPB PE=1 SV=2	40379	629	0	23	0	0.08	0	0	0	0	1	0	1	0	1	0	-1.62833
tr|B0QZK4|B0QZK4_H	HP1BP3	Heterochromatin protein 1-binding protein 3 (Fragment) OS=Homo sapiens GN=HP1BP3 PE=4 SV=1	28725	631	0	23	0	0.25	0	0	0	0	2	0	1	0	3	0	-1.62833
tr|B3KPR5|B3KPR5_H	tr|B3KPR5|B	cDNA FLJ32094 fis, clone OCBBF2000986, highly similar to Homo sapiens elongation factor Tu GTP binding domain conta	97046	633	0	22	0	0.03	0	0	0	0	1	0	1	0	1	0	-1.62833
tr|Q5VSQ6|Q5VSQ6_	P4HA1	Procollagen-proline, 2-oxoglutarate 4-dioxygenase (Proline 4-hydroxylase), alpha polypeptide I variant (Fragment) OS=H	61214	634	0	22	0	0.05	0	0	0	0	1	0	1	0	2	0	-1.62833
tr|J3QQX0|J3QQX0_H	GPS1	COP9 signalosome complex subunit 1 (Fragment) OS=Homo sapiens GN=GPS1 PE=4 SV=1	8703	636	0	22	0	0.4	0	0	0	0	1	0	1	0	1	0	-1.62833
tr|B1ALA9|B1ALA9_H	PRPS1	Phosphoribosyl pyrophosphate synthetase 1 OS=Homo sapiens GN=PRPS1 PE=3 SV=1	24468	638	0	22	0	0.14	0	0	0	0	1	0	1	0	2	0	-1.62833
tr|G0UE14|G0UE14_	HLA-A	MHC class I antigen (Fragment) OS=Homo sapiens GN=HLA-A PE=3 SV=1	31877	641	0	22	0	0.1	0	0	0	0	1	0	1	0	3	0	-1.62833
tr|F2X5X4|F2X5X4_HU	HLA-A	MHC class I antigen (Fragment) OS=Homo sapiens GN=HLA-A PE=3 SV=1	21229	642	0	22	0	0.16	0	0	0	0	1	0	1	0	2	0	-1.62833
tr|F6IR35|F6IR35_HU	HLA-A	MHC class I antigen (Fragment) OS=Homo sapiens GN=HLA-A PE=2 SV=1	38417	643	0	22	0	0.09	0	0	0	0	1	0	1	0	2	0	-1.62833
tr|E0YTJ8|E0YTJ8_HU	HLA-B	MHC class I antigen (Fragment) OS=Homo sapiens GN=HLA-B PE=3 SV=1	21237	644	0	22	0	0.16	0	0	0	0	1	0	1	0	2	0	-1.62833
tr|D5FZU2|D5FZU2_H	HLA-B	MHC class I antigen (Fragment) OS=Homo sapiens GN=HLA-B PE=3 SV=1	21233	645	0	22	0	0.16	0	0	0	0	1	0	1	0	2	0	-1.62833
tr|H6SSQ4|H6SSQ4_	HLA-C	MHC class I antigen (Fragment) OS=Homo sapiens GN=HLA-C PE=3 SV=1	31856	646	0	22	0	0.1	0	0	0	0	1	0	1	0	2	0	-1.62833
tr|E7ET15|E7ET15_HU	U2SURP	U2 snRNP-associated SURP motif-containing protein OS=Homo sapiens GN=U2SURP PE=2 SV=1	118631	647	0	22	0	0.03	0	0	0	0	1	0	1	0	2	0	-1.62833
tr|B4DQ49|B4DQ49_	tr|B4DQ49|E	cDNA FLJ54374, highly similar to Homo sapiens keratin 78 (KRT78), mRNA OS=Homo sapiens PE=2 SV=1	31874	653	0	22	0	0.1	0	0	0	0	1	0	1	0	3	0	-1.62833
tr|H0YMR4|H0YMR4_	IPO4	Importin-4 (Fragment) OS=Homo sapiens GN=IPO4 PE=4 SV=1	60104	655	0	22	0	0.05	0	0	0	0	1	0	1	0	1	0	-1.62833
tr|C9J935|C9J935_HU	SETD9	SET domain-containing protein 9 (Fragment) OS=Homo sapiens GN=SETD9 PE=2 SV=1	5515	660	0	21	0	0.65	0	0	0	0	1	0	1	0	1	0	-1.62833
tr|F1T0A5|F1T0A5_H	PRPF31	PRP31 pre-mRNA processing factor 31 homolog (Yeast), isoform CRA_a OS=Homo sapiens GN=PRPF31 PE=2 SV=1	55649	661	0	21	0	0.06	0	0	0	0	1	0	1	0	2	0	-1.62833
tr|B3KRG8|B3KRG8_H	tr|B3KRG8|B	Lipase OS=Homo sapiens PE=2 SV=1	45736	662	0	21	0	0.07	0	0	0	0	1	0	1	0	1	0	-1.62833
tr|J3KN69|J3KN69_HU	NCEH1	Arylacetamide deacetylase-like 1 OS=Homo sapiens GN=NCEH1 PE=4 SV=1	49431	663	0	21	0	0.07	0	0	0	0	1	0	1	0	1	0	-1.62833
tr|Q6UWE9|Q6UWE9	UNQ2550	SFVP2550 OS=Homo sapiens GN=UNQ2550 PE=4 SV=1	11890	664	0	21	0	0.29	0	0	1	0	1	0	1	0	1	0	-1.62833
tr|G0ZJH6|G0ZJH6_H	Ube4B	UFD2A-III/UBE4B-III splice isoform OS=Homo sapiens GN=Ube4B PE=2 SV=1	152890	667	0	21	0	0.02	0	0	0	0	1	0	1	0	1	0	-1.62833
tr|B4DK80|B4DK80_H	TOP3A	DNA topoisomerase OS=Homo sapiens GN=TOP3A PE=2 SV=1	103082	668	0	20	0	0.03	0	0	0	0	1	0	1	0	1	0	-1.62833
tr|A0AVG7|A0AVG7_	BCAS1	Breast carcinoma amplified sequence 1 OS=Homo sapiens GN=BCAS1 PE=2 SV=1	61929	670	0	20	0	0.05	0	0	0	0	1	0	1	0	1	0	-1.62833
tr|H0YJL6|H0YJL6_HU	EVL	Ena/VASP-like protein (Fragment) OS=Homo sapiens GN=EVL PE=4 SV=1	10347	671	0	20	0	0.34	0	0	0	0	1	0	1	0	1	0	-1.62833
tr|J3KNE5|J3KNE5_HU	HARS	Histidine--tRNA ligase, cytoplasmic OS=Homo sapiens GN=HARS PE=4 SV=1	50581	672	0	20	0	0.07	0	0	0	0	1	0	1	0	2	0	-1.62833
sp|P55212|CASP6_HU	CASP6	Caspase-6 OS=Homo sapiens GN=CASP6 PE=1 SV=2	33859	674	0	20	0	0.1	0	0	0	0	1	0	1	0	1	0	-1.62833
tr|B4DE48|B4DE48_H	tr|B4DE48|B	cDNA FLJ53824, highly similar to Sorting nexin-17 OS=Homo sapiens PE=2 SV=1	50009	677	0	20	0	0.07	0	0	0	0	1	0	1	0	1	0	-1.62833
sp|O95159|ZFPL1_HU	ZFPL1	Zinc finger protein-like 1 OS=Homo sapiens GN=ZFPL1 PE=1 SV=2	34833	678	0	20	0	0.1	0	0	0	0	1	0	1	0	1	0	-1.62833
tr|Q8WUM5|Q8WUM	GEMIN4	Gem (Nuclear organelle) associated protein 4 OS=Homo sapiens GN=GEMIN4 PE=2 SV=1	121732	709	0	17	0	0.03	0	0	0	0	1	0	1	0	2	0	-1.62833
tr|B4DS66|B4DS66_H	tr|B4DS66|B	cDNA FLJ54290, highly similar to Mitochondrial inner membrane protein OS=Homo sapiens PE=2 SV=1	73509	712	0	17	0	0.04	0	0	0	0	1	0	1	0	6	0	-1.62833
sp|Q7Z3Y8|K1C27_HU	KRT27	Keratin, type I cytoskeletal 27 OS=Homo sapiens GN=KRT27 PE=1 SV=2	50419	12	0	72	0	4.67	0	0	2	0	2	0	2	0	2	0	-2.36564
tr|B7Z4P8|B7Z4P8_H	ACTN2	Alpha-actinin-2 OS=Homo sapiens GN=ACTN2 PE=2 SV=1	44675	19	0	51	0	2.11	0	0	1	0	2	0	2	0	2	0	-2.36564
tr|Q7KZ86|Q7KZ86_H	tr|Q7KZ86|Q	Plakoglobin (Fragment) OS=Homo sapiens PE=2 SV=1	19007	26	0	37	0	1.74	0	0	1	0	2	0	2	0	2	0	-2.36564
sp|P53675|CLH2_HU	CLTCL1	Clathrin heavy chain 2 OS=Homo sapiens GN=CLTCL1 PE=1 SV=2	189020	30	0	107	0	0.52	0	0	2	0	2	0	2	0	4	0	-2.36564
tr|Q2F839|Q2F839_H	tr|Q2F839|Q	Heat shock 70 kDa protein 9B (Fragment) OS=Homo sapiens PE=2 SV=1	19208	81	0	59	0	0.42	0	0	2	0	2	0	2	0	2	0	-2.36564
tr|E5RHJ4|E5RHJ4_HU	KIAA1967	DBIRD complex subunit KIAA1967 (Fragment) OS=Homo sapiens GN=KIAA1967 PE=2 SV=1	31413	82	0	137	0	0.32	0	0	2	0	3	0	2	0	3	0	-2.36564
tr|J3KQA8|J3KQA8_H	HNRNPA1	Heterogeneous nuclear ribonucleoprotein A1 OS=Homo sapiens GN=HNRNPA1 PE=4 SV=1	11467	101	0	67	0	0.77	0	0	1	0	1	0	2	0	3	0	-2.36564
tr|B4DKJ0|B4DKJ0_HU	tr|B4DKJ0|B	cDNA FLJ58539, highly similar to Keratin, type II cytoskeletal 4 OS=Homo sapiens PE=2 SV=1	55657	105	0	61	0	0.7	0	0	2	0	2	0	2	0	4	0	-2.36564
tr|E9PQI3|E9PQI3_HU	TXNRD1	Thioredoxin reductase 1, cytoplasmic (Fragment) OS=Homo sapiens GN=TXNRD1 PE=2 SV=1	5610	108	0	145	0	0.26	0	0	1	0	2	0	2	0	2	0	-2.36564
tr|Q59F85|Q59F85_H	tr|Q59F85|Q	Glucose phosphate isomerase variant (Fragment) OS=Homo sapiens PE=2 SV=1	56563	117	0	0	0	0.3	0	0	0	0	0	0	2	0	2	0	-2.36564
tr|B4DX20|B4DX20_H	CCT6B	T-complex protein 1 subunit zeta-2 OS=Homo sapiens GN=CCT6B PE=2 SV=1	54066	118	0	46	0	0.47	0	0	1	0	1	0	2	0	3	0	-2.36564

tr|H3BSS4|H3BSS4_H	PCBP2	Poly(rC)-binding protein 2 (Fragment) OS=Homo sapiens GN=PCBP2 PE=4 SV=1	19494	132	0	154	0	0.32	0	0	2	0	2	0	2	0	2	0	-2.36564
sp|Q58FF3|ENPLL_HU	HSP90B2P	Putative endoplasmin-like protein OS=Homo sapiens GN=HSP90B2P PE=5 SV=1	46343	140	0	41	0	0.37	0	0	1	0	2	0	2	0	2	0	-2.36564
tr|B3KS35|B3KS35_HU	tr|B3KS35|B3	cDNA FLJ35372 fis, clone SKMUS2001742, highly similar to Sarcoplasmic/endoplasmic reticulum calcium ATPase 1 (EC 3.	32698	149	0	55	0	0.32	0	0	1	0	2	0	2	0	2	0	-2.36564
tr|B3KWH4|B3KWH4_	tr|B3KWH4|I	cDNA FLJ43092 fis, clone COLON2002520, highly similar to Myosin-14 (Fragment) OS=Homo sapiens PE=2 SV=1	73003	152	0	70	0	0.07	0	0	2	0	2	0	2	0	2	0	-2.36564
tr|Q2YD88|Q2YD88_H	FAM129B	FAM129B protein (Fragment) OS=Homo sapiens GN=FAM129B PE=2 SV=1	35264	155	0	86	0	0.16	0	0	2	0	2	0	2	0	2	0	-2.36564
tr|B4DNT9|B4DNT9_H	tr|B4DNT9|B	cDNA FLJ57756, highly similar to Paxillin OS=Homo sapiens PE=2 SV=1	17270	159	0	44	0	0.28	0	0	1	0	2	0	2	0	2	0	-2.36564
tr|J3QR48|J3QR48_HU	KPNB1	Importin subunit beta-1 (Fragment) OS=Homo sapiens GN=KPNB1 PE=4 SV=1	16589	171	0	0	0	0.22	0	0	0	0	0	0	2	0	2	0	-2.36564
tr|B4DPK8|B4DPK8_H	tr|B4DPK8|B	cDNA FLJ55127, highly similar to Heterogeneous nuclear ribonucleoprotein L OS=Homo sapiens PE=2 SV=1	26147	182	0	0	0	0.23	0	0	0	0	0	0	2	0	2	0	-2.36564
tr|Q5JVP3|Q5JVP3_H	PTGR1	Leukotriene B4 12-hydroxydehydrogenase, isoform CRA_d OS=Homo sapiens GN=PTGR1 PE=4 SV=1	19441	190	0	113	0	0.38	0	0	2	0	2	0	2	0	2	0	-2.36564
tr|D6RJ96|D6RJ96_HU	HSPA4L	Heat shock 70 kDa protein 4L (Fragment) OS=Homo sapiens GN=HSPA4L PE=2 SV=1	59957	191	0	113	0	0.1	0	0	2	0	2	0	2	0	3	0	-2.36564
tr|A0MNN4|A0MNN4	SMU1	CDW3/SMU1 OS=Homo sapiens GN=SMU1 PE=2 SV=1	58134	221	0	94	0	0.12	0	0	1	0	2	0	2	0	2	0	-2.36564
sp|Q2NL82|TSR1_HU	TSR1	Pre-rRNA-processing protein TSR1 homolog OS=Homo sapiens GN=TSR1 PE=1 SV=1	92151	230	0	90	0	0.07	0	0	2	0	2	0	2	0	2	0	-2.36564
tr|E9PP36|E9PP36_HU	RPL8	60S ribosomal protein L8 OS=Homo sapiens GN=RPL8 PE=2 SV=1	16227	231	0	33	0	0.28	0	0	1	0	1	0	2	0	2	0	-2.36564
tr|E9PQ34|E9PQ34_H	SERPINH1	Serpin H1 (Fragment) OS=Homo sapiens GN=SERPINH1 PE=2 SV=1	15406	233	0	40	0	0.43	0	0	1	0	2	0	2	0	2	0	-2.36564
tr|Q2TB59|Q2TB59_H	NNT	Nicotinamide nucleotide transhydrogenase OS=Homo sapiens GN=NNT PE=2 SV=1	114552	234	0	88	0	0.06	0	0	1	0	2	0	2	0	3	0	-2.36564
sp|Q9UQ35|SRRM2_H	SRRM2	Serine/arginine repetitive matrix protein 2 OS=Homo sapiens GN=SRRM2 PE=1 SV=2	300179	247	0	78	0	0.02	0	0	1	0	2	0	2	0	5	0	-2.36564
tr|G3V1S6|G3V1S6_H	GALNT2	Polypeptide N-acetylgalactosaminyltransferase 2 soluble form OS=Homo sapiens GN=GALNT2 PE=2 SV=1	61440	254	0	76	0	0.11	0	0	2	0	2	0	2	0	2	0	-2.36564
tr|B3KXA9|B3KXA9_H	tr|B3KXA9|B	cDNA FLJ45065 fis, clone BRAWH3024506, highly similar to Leucyl-tRNA synthetase, cytoplasmic (EC 6.1.1.4) OS=Homo s	38658	257	0	49	0	0.1	0	0	1	0	2	0	2	0	2	0	-2.36564
tr|D6RF62|D6RF62_H	PAICS	Phosphoribosylaminoimidazole carboxylase OS=Homo sapiens GN=PAICS PE=4 SV=1	37658	270	0	69	0	0.07	0	0	1	0	1	0	2	0	4	0	-2.36564
tr|C9JAM8|C9JAM8_H	PLS1	Plastin-1 (Fragment) OS=Homo sapiens GN=PLS1 PE=2 SV=1	29616	282	0	48	0	0.34	0	0	1	0	2	0	2	0	2	0	-2.36564
tr|F5GYR3|F5GYR3_H	NOP2	Putative ribosomal RNA methyltransferase NOP2 (Fragment) OS=Homo sapiens GN=NOP2 PE=2 SV=1	30665	296	0	28	0	0.15	0	0	1	0	1	0	2	0	3	0	-2.36564
tr|B9A041|B9A041_H	MDH1	Malate dehydrogenase, cytoplasmic OS=Homo sapiens GN=MDH1 PE=2 SV=1	23195	309	0	58	0	0.31	0	0	2	0	2	0	2	0	2	0	-2.36564
sp|Q13867|BLMH_HU	BLMH	Bleomycin hydrolase OS=Homo sapiens GN=BLMH PE=1 SV=1	53155	332	0	52	0	0.2	0	0	1	0	3	0	2	0	4	0	-2.36564
tr|E5RHF2|E5RHF2_H	COPS5	COP9 signalosome complex subunit 5 (Fragment) OS=Homo sapiens GN=COPS5 PE=2 SV=1	16655	346	0	51	0	0.45	0	0	0	0	2	0	2	0	2	0	-2.36564
tr|B7Z8J4|B7Z8J4_HU	NMT1	Glycylpeptide N-tetradecanoyltransferase 1 OS=Homo sapiens GN=NMT1 PE=2 SV=1	15265	350	0	50	0	0.49	0	0	1	0	2	0	2	0	2	0	-2.36564
tr|J3KR24|J3KR24_HU	IARS	Isoleucine--tRNA ligase, cytoplasmic OS=Homo sapiens GN=IARS PE=4 SV=1	132820	366	0	47	0	0.02	0	0	1	0	1	0	2	0	2	0	-2.36564
tr|H7C314|H7C314_H	CTTN	Src substrate cortactin (Fragment) OS=Homo sapiens GN=CTTN PE=4 SV=1	25136	378	0	46	0	0.11	0	0	1	0	2	0	2	0	2	0	-2.36564
tr|Q0IJ56|Q0IJ56_HU	ST13	ST13 protein (Fragment) OS=Homo sapiens GN=ST13 PE=2 SV=1	35164	382	0	46	0	0.09	0	0	1	0	1	0	2	0	3	0	-2.36564
tr|E7EVA0|E7EVA0_H	MAP4	Microtubule-associated protein OS=Homo sapiens GN=MAP4 PE=2 SV=1	246546	429	0	40	0	0.01	0	0	1	0	1	0	2	0	4	0	-2.36564
tr|A5D904|A5D904_H	RPS9	RPS9 protein (Fragment) OS=Homo sapiens GN=RPS9 PE=2 SV=1	13210	434	0	40	0	0.58	0	0	1	0	2	0	2	0	2	0	-2.36564
sp|P07737|PROF1_HU	PFN1	Profilin-1 OS=Homo sapiens GN=PFN1 PE=1 SV=2	15216	436	0	39	0	0.5	0	0	1	0	2	0	2	0	2	0	-2.36564
tr|Q7Z3K9|Q7Z3K9_H	DKFZp781L05	Putative uncharacterized protein DKFZp781L0540 (Fragment) OS=Homo sapiens GN=DKFZp781L0540 PE=2 SV=1	90081	440	0	39	0	0.04	0	0	1	0	1	0	2	0	2	0	-2.36564
tr|F5H0H1|F5H0H1_H	DHCR24	Delta(24)-sterol reductase OS=Homo sapiens GN=DHCR24 PE=2 SV=1	35100	442	0	39	0	0.05	0	0	1	0	1	0	2	0	2	0	-2.36564
sp|O00116|ADAS_HU	AGPS	Alkyldihydroxyacetonephosphate synthase, peroxisomal OS=Homo sapiens GN=AGPS PE=1 SV=1	73664	448	0	38	0	0.09	0	0	1	0	2	0	2	0	3	0	-2.36564
tr|H0Y7N2|H0Y7N2_H	ATXN10	Ataxin-10 (Fragment) OS=Homo sapiens GN=ATXN10 PE=4 SV=1	19107	470	0	37	0	0.38	0	0	0	0	2	0	2	0	2	0	-2.36564
tr|H0YLA4|H0YLA4_H	SORD	Sorbitol dehydrogenase OS=Homo sapiens GN=SORD PE=3 SV=1	36833	473	0	37	0	0.09	0	0	0	0	1	0	2	0	2	0	-2.36564
tr|B2R4P2|B2R4P2_H	tr|B2R4P2|B	cDNA, FLJ92164, highly similar to Homo sapiens peroxiredoxin 1 (PRDX1), mRNA OS=Homo sapiens PE=2 SV=1	22414	481	0	36	0	0.32	0	0	0	0	2	0	2	0	2	0	-2.36564
tr|E7EPL9|E7EPL9_HU	HSD17B4	(3R)-hydroxyacyl-CoA dehydrogenase OS=Homo sapiens GN=HSD17B4 PE=2 SV=1	51239	484	0	26	0	0.13	0	0	0	0	2	0	2	0	3	0	-2.36564
tr|A0PJ76|A0PJ76_HU	NCAPD2	NCAPD2 protein (Fragment) OS=Homo sapiens GN=NCAPD2 PE=2 SV=1	44957	494	0	34	0	0.15	0	0	0	0	2	0	2	0	2	0	-2.36564
tr|B4DW90|B4DW90_	tr|B4DW90|I	cDNA FLJ58737, highly similar to Splicing factor 3A subunit 3 OS=Homo sapiens PE=2 SV=1	52737	495	0	34	0	0.18	0	0	1	0	3	0	2	0	3	0	-2.36564
tr|B2RDN4|B2RDN4_	tr|B2RDN4|E	cDNA, FLJ96693, highly similar to Homo sapiens block of proliferation 1 (BOP1), mRNA OS=Homo sapiens PE=2 SV=1	84219	505	0	33	0	0.08	0	0	0	0	2	0	2	0	3	0	-2.36564
tr|B7Z601|B7Z601_HU	tr|B7Z601|B7	cDNA FLJ57187, highly similar to Glycerol-3-phosphate dehydrogenase, mitochondrial (EC 1.1.99.5) OS=Homo sapiens PE	56047	512	0	32	0	0.06	0	0	0	0	1	0	2	0	2	0	-2.36564
tr|C1KES1|C1KES1_HU	LRRC16B	LRRC16B (Fragment) OS=Homo sapiens GN=LRRC16B PE=4 SV=1	4125	530	0	31	0	0.94	0	0	0	0	1	0	2	0	2	0	-2.36564
tr|B2RD27|B2RD27_H	tr|B2RD27|B	cDNA, FLJ96428, highly similar to Homo sapiens proteasome (prosome, macropain) 26S subunit, non-ATPase, 7 (Mov34	37046	550	0	30	0	0.19	0	0	0	0	2	0	2	0	2	0	-2.36564
tr|A6NLN1|A6NLN1_H	PTBP1	Polypyrimidine tract binding protein 1, isoform CRA_e OS=Homo sapiens GN=PTBP1 PE=4 SV=3	21899	567	0	26	0	0.18	0	0	0	0	2	0	2	0	2	0	-2.36564
tr|Q5TFJ7|Q5TFJ7_HU	KPNA6	Importin subunit alpha-7 (Fragment) OS=Homo sapiens GN=KPNA6 PE=4 SV=1	36005	573	0	28	0	0.11	0	0	0	0	2	0	2	0	2	0	-2.36564
tr|H3BNV2|H3BNV2_	NQO1	NAD(P)H dehydrogenase [quinone] 1 OS=Homo sapiens GN=NQO1 PE=4 SV=1	28533	576	0	28	0	0.25	0	0	0	0	2	0	2	0	2	0	-2.36564
tr|Q5JYR7|Q5JYR7_HU	RPN2	Dolichyl-diphosphooligosaccharide--protein glycosyltransferase subunit 2 (Fragment) OS=Homo sapiens GN=RPN2 PE=4	37257	589	0	27	0	0.05	0	0	0	0	1	0	2	0	2	0	-2.36564
tr|A8K781|A8K781_H	tr|A8K781|A	cDNA FLJ75299, highly similar to Xenopus laevis proteasome (prosome, macropain) 26S subunit, ATPase 3, mRNA OS=Ho	47578	603	0	26	0	0.07	0	0	0	0	1	0	2	0	2	0	-2.36564
sp|Q9UHR4|BI2L1_HU	BAIAP2L1	Brain-specific angiogenesis inhibitor 1-associated protein 2-like protein 1 OS=Homo sapiens GN=BAIAP2L1 PE=1 SV=2	57189	613	0	24	0	0.12	0	0	0	0	2	0	2	0	4	0	-2.36564
sp|Q9NRF8|PYRG2_H	CTPS2	CTP synthase 2 OS=Homo sapiens GN=CTPS2 PE=1 SV=1	66320	614	0	24	0	0.05	0	0	0	0	1	0	2	0	6	0	-2.36564
sp|Q99456|K1C12_HU	KRT12	Keratin, type I cytoskeletal 12 OS=Homo sapiens GN=KRT12 PE=1 SV=1	53592	616	0	24	0	0.06	0	0	0	0	1	0	2	0	3	0	-2.36564
tr|D3DSQ0|D3DSQ0_	PCM1	Pericentriolar material 1, isoform CRA_a OS=Homo sapiens GN=PCM1 PE=2 SV=1	230041	619	0	24	0	0.01	0	0	0	0	1	0	2	0	3	0	-2.36564
sp|P25490|TYY1_HUM	YY1	Transcriptional repressor protein YY1 OS=Homo sapiens GN=YY1 PE=1 SV=2	45141	630	0	23	0	0.07	0	0	0	0	1	0	2	0	2	0	-2.36564
tr|F8SKU2|F8SKU2_H	HLA-A	MHC class I antigen (Fragment) OS=Homo sapiens GN=HLA-A PE=3 SV=1	21143	639	0	22	0	0.16	0	0	0	0	1	0	2	0	3	0	-2.36564
tr|D5FHQ7|D5FHQ7_	HLA-A	MHC class I antigen (Fragment) OS=Homo sapiens GN=HLA-A PE=3 SV=1	21188	640	0	22	0	0.16	0	0	0	0	1	0	2	0	3	0	-2.36564
tr|H3BUF6|H3BUF6_H	ATXN2L	Ataxin-2-like protein OS=Homo sapiens GN=ATXN2L PE=4 SV=1	112929	656	0	22	0	0.03	0	0	0	0	1	0	2	0	2	0	-2.36564
sp|Q8TCJ2|STT3B_HU	STT3B	Dolichyl-diphosphooligosaccharide--protein glycosyltransferase subunit STT3B OS=Homo sapiens GN=STT3B PE=1 SV=1	94241	700	0	18	0	0.03	0	0	0	0	1	0	2	0	3	0	-2.36564
sp|O95678|K2C75_HU	KRT75	Keratin, type II cytoskeletal 75 OS=Homo sapiens GN=KRT75 PE=1 SV=2	59809	11	0	94	0	4.79	0	0	2	0	3	0	3	0	5	0	-2.85141
sp|P35237|SPB6_HUM	SERPINB6	Serpin B6 OS=Homo sapiens GN=SERPINB6 PE=1 SV=3	42936	120	0	179	0	0.25	0	0	3	0	3	0	3	0	3	0	-2.85141
tr|K7ENJ4|K7ENJ4_HU	ATP5A1	ATP synthase subunit alpha, mitochondrial (Fragment) OS=Homo sapiens GN=ATP5A1 PE=4 SV=1	21383	135	0	74	0	1.33	0	0	3	0	3	0	3	0	3	0	-2.85141
tr|Q5STZ7|Q5STZ7_H	ABCF1	ATP-binding cassette sub-family F member 1 OS=Homo sapiens GN=ABCF1 PE=4 SV=2	27429	144	0	112	0	0.27	0	0	2	0	3	0	3	0	3	0	-2.85141
tr|B2R5W2|B2R5W2_	tr|B2R5W2|E	cDNA, FLJ92657, highly similar to Homo sapiens heterogeneous nuclear ribonucleoprotein C (C1/C2) (HNRPC), transcript	31986	178	0	122	0	0.35	0	0	3	0	3	0	3	0	4	0	-2.85141
tr|Q5T7N0|Q5T7N0_H	RPL5	60S ribosomal protein L5 OS=Homo sapiens GN=RPL5 PE=4 SV=1	13870	187	0	90	0	0.44	0	0	1	0	2	0	3	0	5	0	-2.85141
tr|J3QST3|J3QST3_HU	KRT3	Keratin, type II cytoskeletal 3 OS=Homo sapiens GN=KRT3 PE=3 SV=1	64636	255	0	76	0	0.16	0	0	3	0	3	0	3	0	6	0	-2.85141
tr|Q0VGL3|Q0VGL3_H	RPL13A	RPL13A protein OS=Homo sapiens GN=RPL13A PE=2 SV=1	16706	300	0	59	0	0.74	0	0	3	0	3	0	3	0	3	0	-2.85141
tr|H0YJ34|H0YJ34_HU	FERMT2	Fermitin family homolog 2 (Fragment) OS=Homo sapiens GN=FERMT2 PE=4 SV=1	73877	306	0	59	0	0.14	0	0	2	0	3	0	3	0	3	0	-2.85141
tr|F5GX33|F5GX33_H	SLC25A13	Calcium-binding mitochondrial carrier protein Aralar2 OS=Homo sapiens GN=SLC25A13 PE=2 SV=1	62548	353	0	49	0	0.11	0	0	1	0	2	0	3	0	4	0	-2.85141
sp|Q7RTS7|K2C74_HU	KRT74	Keratin, type II cytoskeletal 74 OS=Homo sapiens GN=KRT74 PE=1 SV=2	58229	373	0	47	0	0.12	0	0	2	0	2	0	3	0	5	0	-2.85141
tr|B2R6J4|B2R6J4_HU	tr|B2R6J4|B2	cDNA, FLJ92975, highly similar to Homo sapiens nucleosome assembly protein 1-like 4 (NAP1L4), mRNA OS=Homo sapie	42940	417	0	42	0	0.16	0	0	0	0	2	0	3	0	3	0	-2.85141
tr|A0AV58|A0AV58_H	STRN3	Striatin, calmodulin binding protein 3 OS=Homo sapiens GN=STRN3 PE=2 SV=1	78069	419	0	42	0	0.09	0	0	1	0	2	0	3	0	3	0	-2.85141
tr|L0R5A1|L0R5A1_H	CSF2RB	Alternative protein CSF2RB OS=Homo sapiens GN=CSF2RB PE=4 SV=1	11752	486	0	36	0	0.29	0	0	1	0	1	0	3	0	5	0	-2.85141
tr|Q6FI37|Q6FI37_HU	IDH1	Isocitrate dehydrogenase [NADP] OS=Homo sapiens GN=IDH1 PE=2 SV=1	46933	555	0	29	0	0.15	0	0	0	0	2	0	3	0	3	0	-2.85141
tr|D3DQA6|D3DQA6_	DDX46	DEAD (Asp-Glu-Ala-Asp) box polypeptide 46, isoform CRA_a OS=Homo sapiens GN=DDX46 PE=3 SV=1	100958	595	0	26	0	0.07	0	0	0	0	2	0	3	0	3	0	-2.85141
tr|J3QSF1|J3QSF1_HU	DDX5	Probable ATP-dependent RNA helicase DDX5 (Fragment) OS=Homo sapiens GN=DDX5 PE=4 SV=1	18277	97	0	63	0	0.45	0	0	1	0	3	0	4	0	4	0	-3.21432
tr|F5GZI8|F5GZI8_HU	TCP1	T-complex protein 1 subunit alpha OS=Homo sapiens GN=TCP1 PE=2 SV=1	10411	197	0	64	0	0.3	0	0	1	0	2	0	4	0	4	0	-3.21432
sp|P21796|VDAC1_HU	VDAC1	Voltage-dependent anion-selective channel protein 1 OS=Homo sapiens GN=VDAC1 PE=1 SV=2	30868	250	0	78	0	0.36	0	0	1	0	3	0	4	0	4	0	-3.21432
sp|Q14CN4|K2C72_H	KRT72	Keratin, type II cytoskeletal 72 OS=Homo sapiens GN=KRT72 PE=1 SV=2	56470	372	0	47	0	0.12	0	0	2	0	2	0	4	0	5	0	-3.21432
tr|Q53RS3|Q53RS3_H	DDEF2	Putative uncharacterized protein DDEF2 (Fragment) OS=Homo sapiens GN=DDEF2 PE=2 SV=1	70046	441	0	39	0	0.05	0	0	1	0	1	0	4	0	4	0	-3.21432
sp|A2RTY3|CQ066_HU	C17orf66	Uncharacterized protein C17orf66 OS=Homo sapiens GN=C17orf66 PE=2 SV=2	66437	477	0	36	0	0.05	0	0	0	0	1	0	4	0	8	0	-3.21432
tr|F8VP67|F8VP67_H	KRT8	Keratin, type II cytoskeletal 8 (Fragment) OS=Homo sapiens GN=KRT8 PE=2 SV=1	13580	6	0	50	0	5.01	0	0	2	0	3	0	5	0	5	0	-3.50418
tr|K7EIE7|K7EIE7_HU	ACLY	ATP-citrate synthase (Fragment) OS=Homo sapiens GN=ACLY PE=4 SV=1	14168	20	0	220	0	1.28	0	0	3	0	4	0	5	0	6	0	-3.50418
tr|K7ENW6|K7ENW6_	KRT16	Keratin, type I cytoskeletal 16 (Fragment) OS=Homo sapiens GN=KRT16 PE=4 SV=1	18735	62	0	109	0	1.38	0	0	3	0	4	0	5	0	5	0	-3.50418
sp|Q01546|K22O_HU	KRT76	Keratin, type II cytoskeletal 2 oral OS=Homo sapiens GN=KRT76 PE=1 SV=2	66370	213	0	97	0	0.21	0	0	4	0	4	0	5	0	9	0	-3.50418
tr|B3KT59|B3KT59_H	tr|B3KT59|B	cDNA FLJ37698 fis, clone BRHIP2015679, highly similar to Ataxin-2 OS=Homo sapiens PE=2 SV=1	45662	157	0	91	0	0.21	0	0	3	0	4	0	6	0	6	0	-3.74553
sp|Q5XKE5|K2C79_HU	KRT79	Keratin, type II cytoskeletal 79 OS=Homo sapiens GN=KRT79 PE=1 SV=2	58085	160	0	133	0	0.32	0	0	5	0	6	0	6	0	8	0	-3.74553
tr|B9VP19|B9VP19_H	HSPD1	60 kDa chaperonin (Fragment) OS=Homo sapiens GN=HSPD1 PE=2 SV=1	19897	16	0	268	0	1.72	0	0	7	0	8	0	7	0	8	0	-3.95232
tr|B4DIR2|B4DIR2_HU	tr|B4DIR2|B4	cDNA FLJ52592, highly similar to Acetyl-CoA carboxylase 1 (EC 6.4.1.2) OS=Homo sapiens PE=2 SV=1	54395	31	0	95	0	0.44	0	0	4	0	4	0	7	0	7	0	-3.95232
sp|P05787|K2C8_HUM	KRT8	Keratin, type II cytoskeletal 8 OS=Homo sapiens GN=KRT8 PE=1 SV=7	53671	79	0	62	0	0.79	0	0	3	0	5	0	7	0	8	0	-3.95232
tr|B4DN77|B4DN77_H	tr|B4DN77|B	cDNA FLJ58517, highly similar to Calpain-2 catalytic subunit (EC 3.4.22.53) OS=Homo sapiens PE=2 SV=1	72392	173	0	125	0	0.22	0	0	3	0	7	0	7	0	8	0	-3.95232
tr|F5GWW2|F5GWW	LDHA	L-lactate dehydrogenase A chain OS=Homo sapiens GN=LDHA PE=4 SV=1	4934	44	0	189	0	2.33	0	0	4	0	7	0	8	0	9	0	-4.13324
tr|E9PHF7|E9PHF7_H	MCCC1	Methylcrotonoyl-CoA carboxylase subunit alpha, mitochondrial OS=Homo sapiens GN=MCCC1 PE=2 SV=1	68745	102	0	214	0	0.83	0	0	7	0	13	0	11	0	16	0	-4.57037
sp|Q96RW7|HMCN1_	HMCN1	Hemicentin-1 OS=Homo sapiens GN=HMCN1 PE=1 SV=2	623265	291	0	63	0	0.01	0	0	7	0	7	0	11	0	16	0	-4.57037
sp|P05165|PCCA_HU	PCCA	Propionyl-CoA carboxylase alpha chain, mitochondrial OS=Homo sapiens GN=PCCA PE=1 SV=4	80635	99	0	216	0	0.49	0	0	6	0	10	0	13	0	13	0	-4.80239
tr|B3KPL5|B3KPL5_HU	tr|B3KPL5|B3	cDNA FLJ31917 fis, clone NT2RP7004925, highly similar to Sprouty-related, EVH1 domain-containing protein 2 OS=Homo	48711	657	0	21	0	0.07	0	0	0	0	1	0	16	0	51	0	-5.09293
sp|P13647|K2C5_HUM	KRT5	Keratin, type II cytoskeletal 5 OS=Homo sapiens GN=KRT5 PE=1 SV=3	62568	58	0	378	0	0.95	0	0	13	0	15	0	20	0	24	0	-5.40747

#13	Hela live syn PU-1_0.2μM																		
#04	Hela live syn NP_2μM																		
Accession	GS	Name	Pro_Mass	04_prot_hit_n	13_prot_hit_n	04_prot_score	13_prot_score	04_emPAI	13_emPAI	emPAI ratio	04_No>I	13_No>I	04_No>HI	13_No>HI	04_No>20	13_No>20	04_No=All	13_No=All	Rsc
sp|Q16513|PKN2_HUMAN	PKN2	Serine/threonine-protein kinase N2 OS=Homo sapiens GN=PKN2 PE=1 SV=1	112820	0	863	0	44	0	0.03	#DIV/0!	0	1	0	1	0	1	0	1	0.665884
sp|P41743|KPCI_HUMAN	PRKCI	Protein kinase C iota type OS=Homo sapiens GN=PRKCI PE=1 SV=2	69188	0	1152	0	26	0	0.05	#DIV/0!	0	0	0	1	0	1	0	1	0.665884
sp|O75116|ROCK2_HUMAN	ROCK2	Rho-associated protein kinase 2 OS=Homo sapiens GN=ROCK2 PE=1 SV=4	161939	375	770	46	51	0.05	0.06	1.2	1	1	2	3	1	4	3	4	0.666081
sp|P48736|PK3CG_HUMAN	PIK3CG	Phosphatidylinositol 4,5-bisphosphate 3-kinase catalytic subunit gamma isoform OS=Homo sapiens GN=PI	127571	0	1207	0	23	0	0.03	#DIV/0!	0	0	0	1	0	1	0	2	0.665884
sp|P78527|PRKDC_HUMAN	PRKDC	DNA-dependent protein kinase catalytic subunit OS=Homo sapiens GN=PRKDC PE=1 SV=3	473749	83	11	241	1701	0.07	0.51	7.285714286	7	44	10	59	11	69	17	81	1.684732
tr|D3DN97|D3DN97_HUMAN	MYLK	Myosin, light polypeptide kinase, isoform CRA_d OS=Homo sapiens GN=MYLK PE=2 SV=1	205484	0	969	0	37	0	0.02	#DIV/0!	0	1	0	1	0	1	0	1	0.665884
sp|Q9H1R3|MYLK2_HUMAN	MYLK2	Myosin light chain kinase 2, skeletal/cardiac muscle OS=Homo sapiens GN=MYLK2 PE=1 SV=3	65214	0	970	0	37	0	0.05	#DIV/0!	0	1	0	1	0	1	0	1	0.665884
sp|Q13131|AAPK1_HUMAN	PRKAA1	5'-AMP-activated protein kinase catalytic subunit alpha-1 OS=Homo sapiens GN=PRKAA1 PE=1 SV=4	64596	0	926	0	39	0	0.05	#DIV/0!	0	1	0	1	0	1	0	1	0.665884
sp|P50750|CDK9_HUMAN	CDK9	Cyclin-dependent kinase 9 OS=Homo sapiens GN=CDK9 PE=1 SV=3	43149	0	1083	0	30	0	0.08	#DIV/0!	0	0	0	1	0	1	0	2	0.665884
sp|Q16539|MK14_HUMAN	MAPK14	Mitogen-activated protein kinase 14 OS=Homo sapiens GN=MAPK14 PE=1 SV=3	41495	0	968	0	37	0	0.09	#DIV/0!	0	1	0	1	0	1	0	1	0.665884
sp|Q13523|PRP4B_HUMAN	PRPF4B	Serine/threonine-protein kinase PRP4 homolog OS=Homo sapiens GN=PRPF4B PE=1 SV=3	117258	0	852	0	44	0	0.26	#DIV/0!	0	1	0	1	0	1	0	1	0.665884
sp|Q96SB4|SRPK1_HUMAN	SRPK1	SRSF protein kinase 1 OS=Homo sapiens GN=SRPK1 PE=1 SV=2	74963	0	485	0	93	0	0.13	#DIV/0!	0	2	0	3	0	5	0	5	2.541064
sp|Q9H4A3|WNK1_HUMAN	WNK1	Serine/threonine-protein kinase WNK1 OS=Homo sapiens GN=WNK1 PE=1 SV=2	251552	0	767	0	51	0	0.01	#DIV/0!	0	1	0	1	0	1	0	2	0.665884
sp|Q8N4C8|MINK1_HUMAN	MINK1	Misshapen-like kinase 1 (Fragment) OS=Homo sapiens GN=MINK1 PE=4 SV=1	45115	0	911	0	0	0	0.02	#DIV/0!	0	0	0	0	0	1	0	3	0.665884
sp|Q13153|PAK1_HUMAN	PAK1	Serine/threonine-protein kinase PAK 1 OS=Homo sapiens GN=PAK1 PE=1 SV=2	60894	0	1175	0	25	0	0.06	#DIV/0!	0	0	0	1	0	1	0	1	0.665884
sp|Q9H2G2|SLK_HUMAN	SLK	STE20-like serine/threonine-protein kinase OS=Homo sapiens GN=SLK PE=1 SV=1	143234	0	995	0	35	0	0.03	#DIV/0!	0	0	0	1	0	1	0	1	0.665884
sp|Q9Y6E0|STK24_HUMAN	STK24	Serine/threonine-protein kinase 24 OS=Homo sapiens GN=STK24 PE=1 SV=1	49562	0	898	0	42	0	0.08	#DIV/0!	0	1	0	1	0	1	0	1	0.665884
sp|P43405|KSYK_HUMAN	SYK	Tyrosine-protein kinase SYK OS=Homo sapiens GN=SYK PE=1 SV=1	72533	0	1054	0	32	0	0.12	#DIV/0!	0	1	0	1	0	1	0	1	0.665884
sp|Q13418|ILK_HUMAN	ILK	Integrin-linked protein kinase OS=Homo sapiens GN=ILK PE=1 SV=2	51899	421	587	41	75	0.07	0.24	3.428571429	1	1	1	3	1	4	1	4	0.666081
sp|Q9UIG0|BAZ1B_HUMAN	BAZ1B	Tyrosine-protein kinase BAZ1B OS=Homo sapiens GN=BAZ1B PE=1 SV=2	172392	0	786	0	49	0	0.02	#DIV/0!	0	1	0	1	0	1	0	1	0.665884
tr|B4E1G6|B4E1G6_HUMAN	GALK1	Galactokinase OS=Homo sapiens GN=GALK1 PE=2 SV=1	45956	679	1176	20	25	0.07	0.2	2.857142857	0	0	1	1	0	1	1	1	0.665884
sp|P19367|HXK1_HUMAN	HK1	Hexokinase-1 OS=Homo sapiens GN=HK1 PE=1 SV=3	103561	0	321	0	155	0	0.2	#DIV/0!	0	4	0	6	0	6	0	8	2.78225
sp|P52789|HXK2_HUMAN	HK2	Hexokinase-2 OS=Homo sapiens GN=HK2 PE=1 SV=2	103739	599	872	26	43	0.03	0.1	3.333333333	0	1	1	3	1	3	2	3	0.303333
sp|P29966|MARCS_HUMAN	MARCKS	Myristoylated alanine-rich C-kinase substrate OS=Homo sapiens GN=MARCKS PE=1 SV=4	31707	0	676	0	62	0	0.81	#DIV/0!	0	1	0	1	0	1	0	1	0.665884
sp|Q16822|PCKGM_HUMAN	PCK2	Phosphoenolpyruvate carboxykinase [GTP], mitochondrial OS=Homo sapiens GN=PCK2 PE=1 SV=3	71483	0	658	0	66	0	0.06	#DIV/0!	0	1	0	1	0	1	0	2	0.665884
tr|J3KNX3|J3KNX3_HUMAN	PFKM	6-phosphofructokinase OS=Homo sapiens GN=PFKM PE=3 SV=1	94107	0	364	0	129	0	0.15	#DIV/0!	0	2	0	4	0	4	0	4	2.25138
tr|J3KNX3|J3KNX3_HUMAN	PFKM	6-phosphofructokinase, muscle type (Fragment) OS=Homo sapiens GN=PFKM PE=4 SV=1	4671	0	899	0	42	0	0.79	#DIV/0!	0	1	0	1	0	1	0	1	0.665884
sp|P42356|PI4KA_HUMAN	PI4KA	Phosphatidylinositol 4-kinase alpha OS=Homo sapiens GN=PI4KA PE=4 SV=1	239244	0	1167	0	25	0	0.01	#DIV/0!	0	0	0	1	0	1	0	3	0.665884
sp|P67775|PP2AA_HUMAN	PPP2CA	Serine/threonine-protein phosphatase 2A catalytic subunit alpha isoform OS=Homo sapiens GN=PPP2CA P	36142	0	494	0	91	0	0.25	#DIV/0!	0	2	0	2	0	3	0	3	1.888632
sp|O00743|PPP6_HUMAN	PPP6C	Serine/threonine-protein phosphatase 6 catalytic subunit OS=Homo sapiens GN=PPP6C PE=1 SV=1	35806	0	903	0	41	0	0.09	#DIV/0!	0	1	0	1	0	1	0	1	0.665884
sp|P40763|STAT3_HUMAN	STAT3	Signal transducer and activator of transcription 3 OS=Homo sapiens GN=STAT3 PE=1 SV=2	88810	0	1130	0	27	0	0.08	#DIV/0!	0	0	0	2	0	2	0	4	1.403027
sp|Q06124|PTN11_HUMAN	PTPN11	Tyrosine-protein phosphatase non-receptor type 11 OS=Homo sapiens GN=PTPN11 PE=1 SV=2	68964	0	749	0	53	0	0.1	#DIV/0!	0	2	0	2	0	2	0	2	1.403027
tr|C1PHA2|C1PHA2_HUMAN	KIF5B-ALK	Tyrosine-protein kinase receptor OS=Homo sapiens GN=KIF5B-ALK PE=2 SV=1	169283	227	232	91	223	0.04	0.17	4.25	2	6	2	8	2	9	3	10	1.007671
sp|O15355|PPM1G_HUMAN	PPM1G	Protein phosphatase 1G OS=Homo sapiens GN=PPM1G PE=1 SV=1	59919	0	534	0	83	0	0.17	#DIV/0!	0	1	0	3	0	3	0	3	1.888632
sp|O15357|SHIP2_HUMAN	INPPL1	Phosphatidylinositol 3,4,5-trisphosphate 5-phosphatase 2 OS=Homo sapiens GN=INPPL1 PE=1 SV=2	139311	0	1064	0	32	0	0.02	#DIV/0!	0	0	0	1	0	3	0	3	1.888632
tr|B3KPZ7|B3KPZ7_HUMAN	tr|B3KPZ7	cDNA FLJ32517 fis, clone SMINT1000117, highly similar to Pyruvate dehydrogenase (lipoamide)-phosphata	61329	0	1137	0	27	0	0.05	#DIV/0!	0	0	0	1	0	1	0	1	0.665884
tr|H0Y8C4|H0Y8C4_HUMAN	PPP2R5D	Serine/threonine-protein phosphatase 2A 56 kDa regulatory subunit delta isoform (Fragment) OS=Homo s	59637	0	1135	0	27	0	0.06	#DIV/0!	0	0	0	1	0	1	0	1	0.665884
sp|Q8TAP8|PPR35_HUMAN	PPP1R35	Protein phosphatase 1 regulatory subunit 35 OS=Homo sapiens GN=PPP1R35 PE=1 SV=1	28107	0	812	0	47	0	0.12	#DIV/0!	0	1	0	1	0	1	0	1	0.665884
tr|B4DK71|B4DK71_HUMAN	SACM1L	Phosphatidylinositide phosphatase SAC1 OS=Homo sapiens GN=SACM1L PE=2 SV=1	61327	0	659	0	66	0	0.05	#DIV/0!	0	1	0	1	0	1	0	1	0.665884
tr|B4DDF7|B4DDF7_HUMAN	tr|B4DDF7	cDNA FLJ53296, highly similar to Serine/threonine-protein phosphatase 2A 65 kDa regulatory subunit A alp	64939	443	210	39	247	0.16	0.64	4	1	7	3	10	3	9	3	11	0.521908
tr|G3V105|G3V105_HUMAN	IKBKB	Inhibitor of kappa light polypeptide gene enhancer in B-cells, kinase beta, isoform CRA_b OS=Homo sapien	64497	0	1284	0	16	0	0.05	#DIV/0!	0	0	0	1	0	0	0	1	-0.91926
tr|B3KX60|B3KX60_HUMAN	tr|B3KX60	cDNA FLJ44825 fis, clone BRACE3046609, highly similar to Homo sapiens inhibitor of Bruton agammaglobu	33742	0	1257	0	19	0	0.1	#DIV/0!	0	0	0	1	0	0	0	1	-0.91926
tr|B7Z2K7|B7Z2K7_HUMAN	tr|B7Z2K7	cDNA FLJ53289, highly similar to Ribosomal protein S6 kinase alpha-1 (EC 2.7.11.1) OS=Homo sapiens PE=2	67793	0	1024	0	0	0	0.04	#DIV/0!	0	0	0	0	0	0	0	2	-0.91926
tr|F2Z2Y4|F2Z2Y4_HUMAN	PDXK	Pyridoxal kinase OS=Homo sapiens GN=PDXK PE=2 SV=1	30790	566	631	29	69	0.11	0.38	3.454545455	0	1	1	2	1	2	1	2	-0.18227
sp|P14618|KPYM_HUMAN	PKM	Pyruvate kinase isozymes M1/M2 OS=Homo sapiens GN=PKM PE=1 SV=4	58470	8	4	1820	2788	5.79	17.17	2.965457686	47	68	58	89	60	93	66	101	-0.29491
sp|P00558|PGK1_HUMAN	PGK1	Phosphoglycerate kinase 1 OS=Homo sapiens GN=PGK1 PE=1 SV=3	44985	134	98	161	422	0.76	1.51	1.986842105	5	11	8	13	9	16	9	17	-0.12297
sp|Q01813|K6PP_HUMAN	PFKP	6-phosphofructokinase type C OS=Homo sapiens GN=PFKP PE=1 SV=2	86454	98	69	217	593	0.4	0.68	1.7	5	14	9	15	9	16	10	18	-0.12297
tr|B0AZV0|B0AZV0_HUMAN	STRAP	Serine-threonine kinase receptor-associated protein OS=Homo sapiens GN=STRAP PE=2 SV=1	28716	626	608	23	63	0.12	0.17	1.416666667	0	1	1	1	1	1	1	1	-0.91941
tr|B4DP56|B4DP56_HUMAN	tr|B4DP56	cDNA FLJ52237, highly similar to Creatine kinase B-type (EC 2.7.3.2) OS=Homo sapiens PE=2 SV=1	38840	214	339	97	91	0.18	0.25	1.388888889	2	1	2	2	2	2	2	3	-0.91957
tr|B4E2K2|B4E2K2_HUMAN	tr|B4E2K2	cDNA FLJ55665, highly similar to A-kinase anchor protein 2 OS=Homo sapiens PE=2 SV=1	103935	246	1165	79	25	0.03	0.03	1	1	0	1	1	1	1	1	1	-0.91941
sp|O95747|OXSR1_HUMAN	OXSR1	Serine/threonine-protein kinase OSR1 OS=Homo sapiens GN=OXSR1 PE=1 SV=1	58271	675	876	20	43	0.06	0.06	1	0	1	1	1	1	1	2	1	-0.91941
tr|B3KS97|B3KS97_HUMAN	MAP2K2	Mitogen-activated protein kinase kinase 2, isoform CRA_d OS=Homo sapiens GN=MAP2K2 PE=2 SV=1	23450	384	615	45	38	0.31	0.15	0.483870968	1	0	2	1	2	1	2	1	-1.65672
sp|P17858|K6PL_HUMAN	PFKL	6-phosphofructokinase, liver type OS=Homo sapiens GN=PFKL PE=1 SV=6	85762	98	263	88	197	0.4	0.16	0.4	1	4	2	4	2	4	2	4	-0.07122
tr|C8CHJ6|C8CHJ6_HUMAN	GALK	Galactokinase (Fragment) OS=Homo sapiens GN=GALK PE=2 SV=1	6801	679	0	20	0	0.07	0	0	0	0	1	0	0	0	1	0	-0.91926
tr|A6NIT2|A6NIT2_HUMAN	PHKA1	Phosphorylase b kinase regulatory subunit alpha, skeletal muscle isoform OS=Homo sapiens GN=PHKA1 PE	133462	711	0	17	0	0.02	0	0	0	0	1	0	0	0	1	0	-0.91926
tr|Q8IWY7|Q8IWY7_HUMAN	TTBK2	Tau-tubulin kinase OS=Homo sapiens GN=TTBK2 PE=2 SV=1	183893	79	0	28	0	0.79	0	0	0	0	1	0	1	0	1	0	-2.50455
tr|C9JVY5|C9JVY5_HUMAN	AKAP2	A-kinase anchor protein 2 (Fragment) OS=Homo sapiens GN=AKAP2 PE=2 SV=1	85573	246	0	79	0	0.03	0	0	1	0	1	0	1	0	1	0	-2.50455
sp|Q9UEW8|STK39_HUMAN	STK39	STE20/SPS1-related proline-alanine-rich protein kinase OS=Homo sapiens GN=STK39 PE=1 SV=3	59950	491	0	35	0	0.05	0	0	0	0	1	0	1	0	1	0	-2.50455
tr|B2R5T5|B2R5T5_HUMAN	PRKAR1A	Protein kinase, cAMP-dependent, regulatory, type I, alpha (Tissue specific extinguisher 1), isoform CRA_a C	43183	533	0	31	0	0.08	0	0	0	0	1	0	1	0	1	0	-2.50455
tr|Q5JQ10|Q5JQ10_HUMAN	ADK	Adenosine kinase OS=Homo sapiens GN=ADK PE=2 SV=1	39078	538	0	30	0	0.08	0	0	0	0	1	0	1	0	1	0	-2.50455
tr|H0YH33|H0YH33_HUMAN	STRAP	Serine-threonine kinase receptor-associated protein (Fragment) OS=Homo sapiens GN=STRAP PE=4 SV=1	10763	626	0	23	0	0.12	0	0	0	0	1	0	1	0	1	0	-2.50455
tr|H0Y3C5|H0Y3C5_HUMAN	HCK	Tyrosine-protein kinase HCK OS=Homo sapiens GN=HCK PE=4 SV=1	59986	628	0	23	0	0.05	0	0	0	0	1	0	1	0	2	0	-2.50455
tr|H0Y3Y5|H0Y3Y5_HUMAN	PPIP5K1	Inositol hexakisphosphate and diphosphoinositol-pentakisphosphate kinase 1 (Fragment) OS=Homo sapier	21913	632	0	22	0	0.15	0	0	0	0	1	0	1	0	2	0	-2.50455
tr|H3BRW9|H3BRW9_HUMAN	MAP2K1	Dual-specificity mitogen-activated protein kinase kinase 1 OS=Homo sapiens GN=MAP2K1 PE=4 SV=1	24160	384	0	45	0	0.31	0	0	1	0	2	0	2	0	2	0	-3.24186
tr|C9J9S3|C9J9S3_HUMAN	PPP1CB	Serine/threonine-protein phosphatase (Fragment) OS=Homo sapiens GN=PPP1CB PE=2 SV=1	14484	345	354	51	78	0.24	0.49	2.041666667	1	1	1	2	1	1	1	2	-0.91941
tr|J3QT22|J3QT22_HUMAN	PPME1	Protein phosphatase methylesterase 1 OS=Homo sapiens GN=PPME1 PE=3 SV=1	44241	232	809	90	47	0.07	0.07	1	1	1	1	1	1	2	2	3	-0.18227
tr|H0YDU8|H0YDU8_HUMAN	PPP5C	Serine/threonine-protein phosphatase (Fragment) OS=Homo sapiens GN=PPP5C PE=3 SV=1	55743	596	0	26	0	0.06	0	0	0	0	1	0	1	0	1	0	-2.50455
tr|E1NZA1|E1NZA1_HUMAN	PRIC295	Peroxisome proliferator activated receptor interacting complex protein OS=Homo sapiens GN=PRIC295 PE	294953	0	135	0	349	0	0.22	#DIV/0!	0	8	0	18	0	20	0	22	4.441853
tr|D6REM6|D6REM6_HUMAN	MATR3	Matrin-3 OS=Homo sapiens GN=MATR3 PE=2 SV=1	88647	0	97	0	374	0	0.96	#DIV/0!	0	9	0	21	0	20	0	22	4.441853
tr|A8K8U1|A8K8U1_HUMAN	tr|A8K8U1	cDNA FLJ77762, highly similar to Homo sapiens cullin-associated and neddylation-dissociated 1 (CAND1), n	137941	0	110	0	389	0	0.42	#DIV/0!	0	11	0	15	0	18	0	19	4.293398
tr|B4DLV7|B4DLV7_HUMAN	tr|B4DLV7	cDNA FLJ60299, highly similar to Rab GDP dissociation inhibitor beta OS=Homo sapiens PE=2 SV=1	51577	0	127	0	360	0	1.1	#DIV/0!	0	11	0	12	0	17	0	18	4.21305
sp|O75533|SF3B1_HUMAN	SF3B1	Splicing factor 3B subunit 1 OS=Homo sapiens GN=SF3B1 PE=1 SV=3	146479	0	150	0	324	0	0.39	#DIV/0!	0	11	0	15	0	16	0	19	4.127983
tr|B3KPA6|B3KPA6_HUMAN	ACADVL	Acyl-Coenzyme A dehydrogenase, very long chain, isoform CRA_e OS=Homo sapiens GN=ACADVL PE=2 SV	62941	0	242	0	217	0	0.75	#DIV/0!	0	7	0	11	0	13	0	14	3.837943
sp|Q08J23|NSUN2_HUMAN	NSUN2	tRNA (cytosine(34)-C(5))-methyltransferase OS=Homo sapiens GN=NSUN2 PE=1 SV=2	87214	0	223	0	228	0	0.5	#DIV/0!	0	5	0	11	0	13	0	18	3.837943
sp|O43776|SYNC_HUMAN	NARS	Asparagine--tRNA ligase, cytoplasmic OS=Homo sapiens GN=NARS PE=1 SV=1	63758	0	124	0	366	0	0.92	#DIV/0!	0	10	0	13	0	13	0	13	3.837943
tr|B7Z1N6|B7Z1N6_HUMAN	tr|B7Z1N6	cDNA FLJ52222, highly similar to Fructose-bisphosphate aldolase C (EC4.1.2.13) OS=Homo sapiens PE=2 SV	35800	0	92	0	463	0	0.56	#DIV/0!	0	8	0	9	0	13	0	16	3.837943
tr|A1KYQ7|A1KYQ7_HUMAN	tr|A1KYQ7	Cell migration-inducing protein 17 OS=Homo sapiens PE=2 SV=1	106022	0	144	0	333	0	0.36	#DIV/0!	0	9	0	10	0	12	0	12	3.726733
tr|B4DIT7|B4DIT7_HUMAN	TGM2	Protein-glutamine gamma-glutamyltransferase 2 OS=Homo sapiens GN=TGM2 PE=2 SV=1	69632	0	148	0	330	0	0.59	#DIV/0!	0	9	0	10	0	11	0	14	3.606261
tr|B2RDW1|B2RDW1_HUMAN	RPS27A	Ribosomal protein S27a, isoform CRA_c OS=Homo sapiens GN=RPS27A PE=2 SV=1	18296	0	360	0	132	0	0.66	#DIV/0!	0	4	0	7	0	9	0	9	3.330271
tr|B7Z4B2|B7Z4B2_HUMAN	tr|B7Z4B2	cDNA FLJ56108, highly similar to Puromycin-sensitive aminopeptidase (EC 3.4.11.-) OS=Homo sapiens PE=2	93434	0	308	0	164	0	0.32	#DIV/0!	0	5	0	8	0	9	0	10	3.330271
tr|B3KY63|B3KY63_HUMAN	tr|B3KY63	cDNA FLJ16830 fis, clone UTERU3022536, highly similar to Chromodomain helicase-DNA-binding protein 4	216574	0	449	0	103	0	0.08	#DIV/0!	0	4	0	5	0	8	0	10	3.169629
tr|B4DZC0|B4DZC0_HUMAN	tr|B4DZC0	cDNA FLJ51771, highly similar to SWI/SNF-related matrix-associatedactin-dependent regulator of chromati	117367	0	347	0	137	0	0.18	#DIV/0!	0	3	0	6	0	8	0	11	3.169629
sp|Q9UHD8|SEPT9_HUMAN	Sep-09	Septin-9 OS=Homo sapiens GN=SEPT9 PE=1 SV=2	65646	0	316	0	158	0	0.34	#DIV/0!	0	4	0	6	0	8	0	9	3.169629
tr|Q5TZZ9|Q5TZZ9_HUMAN	ANXA1	Annexin OS=Homo sapiens GN=ANXA1 PE=2 SV=1	38918	0	217	0	235	0	0.92	#DIV/0!	0	7	0	8	0	8	0	8	3.169629
sp|P31150|GDIA_HUMAN	GDI1	Rab GDP dissociation inhibitor alpha OS=Homo sapiens GN=GDI1 PE=1 SV=2	51177	0	552	0	81	0	0.31	#DIV/0!	0	3	0	4	0	7	0	8	2.988879
sp|Q9Y2W1|TR150_HUMAN	THRAP3	Thyroid hormone receptor-associated protein 3 OS=Homo sapiens GN=THRAP3 PE=1 SV=2	108658	0	307	0	167	0	0.23	#DIV/0!	0	5	0	7	0	7	0	8	2.988879
tr|B3KRS5|B3KRS5_HUMAN	HDAC2	Histone deacetylase OS=Homo sapiens GN=HDAC2 PE=2 SV=1	52479	0	300	0	170	0	0.44	#DIV/0!	0	5	0	6	0	7	0	9	2.988879
tr|Q5T3N1|Q5T3N1_HUMAN	ANXA1	Annexin A1 (Fragment) OS=Homo sapiens GN=ANXA1 PE=4 SV=1	22741	0	285	0	177	0	1.28	#DIV/0!	0	5	0	6	0	7	0	7	2.988879
sp|O43815|STRN_HUMAN	STRN	Striatin OS=Homo sapiens GN=STRN PE=1 SV=4	86535	0	255	0	207	0	0.3	#DIV/0!	0	5	0	7	0	7	0	8	2.988879
tr|H7C3P6|H7C3P6_HUMAN	NUP98	Nuclear pore complex protein Nup98-Nup96 (Fragment) OS=Homo sapiens GN=NUP98 PE=4 SV=1	86760	0	582	0	76	0	0.07	#DIV/0!	0	1	0	4	0	6	0	6	2.78225
sp|Q96I24|FUBP3_HUMAN	FUBP3	Far upstream element-binding protein 3 OS=Homo sapiens GN=FUBP3 PE=1 SV=2	61944	0	413	0	112	0	0.3	#DIV/0!	0	5	0	5	0	6	0	6	2.78225
tr|Q59EC0|Q59EC0_HUMAN	tr|Q59EC0	Adenosine deaminase, RNA-specific isoform ADAR-a variant (Fragment) OS=Homo sapiens PE=2 SV=1	139001	0	398	0	119	0	0.1	#DIV/0!	0	3	0	4	0	6	0	10	2.78225
sp|P80723|BASP1_HUMAN	BASP1	Brain acid soluble protein 1 OS=Homo sapiens GN=BASP1 PE=1 SV=2	22680	0	395	0	120	0	0.99	#DIV/0!	0	4	0	5	0	6	0	7	2.78225
sp|A0AVT1|UBA6_HUMAN	UBA6	Ubiquitin-like modifier-activating enzyme 6 OS=Homo sapiens GN=UBA6 PE=1 SV=1	119207	0	392	0	120	0	0.14	#DIV/0!	0	3	0	5	0	6	0	6	2.78225
tr|Q59FA0|Q59FA0_HUMAN	tr|Q59FA0	DNA polymerase (Fragment) OS=Homo sapiens PE=2 SV=1	113271	0	365	0	129	0	0.22	#DIV/0!	0	2	0	7	0	6	0	8	2.78225
tr|B0AZQ4|B0AZQ4_HUMAN	tr|B0AZQ4	cDNA, FLJ79494, highly similar to Structural maintenance of chromosome 3 OS=Homo sapiens PE=2 SV=1	141841	0	296	0	173	0	0.15	#DIV/0!	0	6	0	6	0	6	0	7	2.78225
sp|P09960|LKHA4_HUMAN	LTA4H	Leukotriene A-4 hydrolase OS=Homo sapiens GN=LTA4H PE=1 SV=2	69868	0	249	0	212	0	0.32	#DIV/0!	0	6	0	6	0	6	0	6	2.78225
tr|B0QY89|B0QY89_HUMAN	EIF3EIP	Eukaryotic translation initiation factor 3 subunit L OS=Homo sapiens GN=EIF3EIP PE=4 SV=1	71085	0	220	0	230	0	0.31	#DIV/0!	0	5	0	6	0	6	0	6	2.78225
tr|A4D210|A4D210_HUMAN	EIF3S9	Eukaryotic translation initiation factor 3, subunit 9 eta, 116kDa OS=Homo sapiens GN=EIF3S9 PE=4 SV=1	89024	0	720	0	56	0	0.16	#DIV/0!	0	1	0	4	0	5	0	6	2.541064
tr|G3V198|G3V198_HUMAN	NUP160	Nuclear pore complex protein Nup160 OS=Homo sapiens GN=NUP160 PE=2 SV=1	142365	0	578	0	77	0	0.12	#DIV/0!	0	1	0	5	0	5	0	6	2.541064
tr|B3KTC7|B3KTC7_HUMAN	MRE11A	Double-strand break repair protein MRE11A OS=Homo sapiens GN=MRE11A PE=2 SV=1	81362	0	527	0	83	0	0.17	#DIV/0!	0	2	0	4	0	5	0	7	2.541064
tr|Q5TZP7|Q5TZP7_HUMAN	APEX1	APEX nuclease (Multifunctional DNA repair enzyme) 1 OS=Homo sapiens GN=APEX1 PE=2 SV=1	35931	0	512	0	87	0	0.42	#DIV/0!	0	2	0	4	0	5	0	5	2.541064
tr|B3KWV6|B3KWV6_HUMAN	tr|B3KWV	cDNA FLJ43948 fis, clone TESTI4014924, highly similar to Homo sapiens cytoplasmic FMR1 interacting prot	147082	0	437	0	107	0	0.09	#DIV/0!	0	3	0	4	0	5	0	6	2.541064
sp|P35658|NU214_HUMAN	NUP214	Nuclear pore complex protein Nup214 OS=Homo sapiens GN=NUP214 PE=1 SV=2	214230	0	396	0	119	0	0.11	#DIV/0!	0	3	0	5	0	5	0	6	2.541064
sp|Q9NUL7|DDX28_HUMAN	DDX28	Probable ATP-dependent RNA helicase DDX28 OS=Homo sapiens GN=DDX28 PE=1 SV=2	59773	0	383	0	122	0	0.31	#DIV/0!	0	3	0	5	0	5	0	6	2.541064
tr|A2A274|A2A274_HUMAN	ACO2	Aconitase 2, mitochondrial OS=Homo sapiens GN=ACO2 PE=4 SV=1	88563	0	368	0	128	0	0.24	#DIV/0!	0	4	0	6	0	5	0	7	2.541064
tr|Q58EY4|Q58EY4_HUMAN	SMARCC1	SWI/SNF related, matrix associated, actin dependent regulator of chromatin, subfamily c, member 1 OS=H	123347	0	366	0	128	0	0.19	#DIV/0!	0	2	0	5	0	5	0	6	2.541064
tr|D3DUW5|D3DUW5_HUMAN	DNM1L	Dynamin 1-like, isoform CRA_c OS=Homo sapiens GN=DNM1L PE=3 SV=1	88442	0	352	0	134	0	0.16	#DIV/0!	0	4	0	4	0	5	0	5	2.541064
tr|H0YH81|H0YH81_HUMAN	ATP5B	ATP synthase subunit beta (Fragment) OS=Homo sapiens GN=ATP5B PE=3 SV=1	38226	0	323	0	154	0	0.39	#DIV/0!	0	4	0	5	0	5	0	5	2.541064
tr|B5BU61|B5BU61_HUMAN	HDAC1	Histone deacetylase OS=Homo sapiens GN=HDAC1 PE=2 SV=1	55611	0	315	0	66	0	0.33	#DIV/0!	0	2	0	3	0	5	0	7	2.541064
tr|Q6DCA8|Q6DCA8_HUMAN	BCLAF1	BCLAF1 protein (Fragment) OS=Homo sapiens GN=BCLAF1 PE=2 SV=1	77958	0	312	0	162	0	0.18	#DIV/0!	0	3	0	4	0	5	0	6	2.541064
tr|Q4KMR3|Q4KMR3_HUMAN	MYO1E	MYO1E variant protein OS=Homo sapiens GN=MYO1E PE=2 SV=1	127552	0	290	0	175	0	0.11	#DIV/0!	0	4	0	4	0	5	0	5	2.541064
tr|Q6NVW7|Q6NVW7_HUMAN	KPNA2	Importin subunit alpha OS=Homo sapiens GN=KPNA2 PE=2 SV=1	58271	0	289	0	176	0	0.32	#DIV/0!	0	3	0	5	0	5	0	6	2.541064
sp|Q96PK6|RBM14_HUMAN	RBM14	RNA-binding protein 14 OS=Homo sapiens GN=RBM14 PE=1 SV=2	69620	0	278	0	180	0	0.26	#DIV/0!	0	2	0	5	0	5	0	5	2.541064
tr|B2RNR6|B2RNR6_HUMAN	ZFR	Zinc finger RNA binding protein OS=Homo sapiens GN=ZFR PE=2 SV=1	118081	0	234	0	220	0	0.15	#DIV/0!	0	5	0	5	0	5	0	6	2.541064
tr|G3XAL0|G3XAL0_HUMAN	MDH2	Malate dehydrogenase OS=Homo sapiens GN=MDH2 PE=2 SV=1	24921	0	174	0	141	0	1.18	#DIV/0!	0	3	0	4	0	5	0	6	2.541064
tr|Q5VZA6|Q5VZA6_HUMAN	ACO1	Cytoplasmic aconitate hydratase OS=Homo sapiens GN=ACO1 PE=4 SV=1	48888	0	169	0	91	0	0.38	#DIV/0!	0	2	0	4	0	5	0	5	2.541064
tr|B7Z8Z8|B7Z8Z8_HUMAN	tr|B7Z8Z8	cDNA FLJ61702, highly similar to 80 kDa MCM3-associated protein OS=Homo sapiens PE=2 SV=1	181264	0	943	0	38	0	0.02	#DIV/0!	0	2	0	2	0	4	0	5	2.25138
tr|B4E3M6|B4E3M6_HUMAN	tr|B4E3M6	cDNA FLJ55446, highly similar to Superkiller viralicidic activity 2-like 2 (EC 3.6.1.-) OS=Homo sapiens PE=2 S	107714	0	902	0	41	0	0.09	#DIV/0!	0	0	0	3	0	4	0	5	2.25138
tr|B2R6H7|B2R6H7_HUMAN	tr|B2R6H7	cDNA, FLJ92955, highly similar to Homo sapiens transportin-SR (TRN-SR), mRNA OS=Homo sapiens PE=2 SV	105989	0	883	0	43	0	0.13	#DIV/0!	0	2	0	4	0	4	0	4	2.25138
sp|P01023|A2MG_HUMAN	A2M	Alpha-2-macroglobulin OS=Homo sapiens GN=A2M PE=1 SV=3	164613	0	851	0	44	0	0.06	#DIV/0!	0	1	0	3	0	4	0	4	2.25138
tr|Q149P0|Q149P0_HUMAN	GBF1	GBF1 protein OS=Homo sapiens GN=GBF1 PE=2 SV=1	207938	0	807	0	47	0	0.05	#DIV/0!	0	1	0	3	0	4	0	6	2.25138
tr|B4E2K4|B4E2K4_HUMAN	tr|B4E2K4	cDNA FLJ54576, highly similar to Aspartyl/asparaginyl beta-hydroxylase (EC 1.14.11.16) OS=Homo sapiens	83685	0	754	0	52	0	0.08	#DIV/0!	0	1	0	2	0	4	0	4	2.25138
tr|A0MNN5|A0MNN5_HUMAN	GRWD1	CDW4/GRWD1 OS=Homo sapiens GN=GRWD1 PE=2 SV=1	49787	0	737	0	54	0	0.07	#DIV/0!	0	1	0	1	0	4	0	5	2.25138
tr|B3KNV9|B3KNV9_HUMAN	tr|B3KNV9	cDNA FLJ30554 fis, clone BRAWH2003693 OS=Homo sapiens PE=2 SV=1	112670	0	668	0	52	0	0.08	#DIV/0!	0	2	0	4	0	4	0	4	2.25138
sp|Q13619|CUL4A_HUMAN	CUL4A	Cullin-4A OS=Homo sapiens GN=CUL4A PE=1 SV=3	88138	0	636	0	68	0	0.13	#DIV/0!	0	2	0	3	0	4	0	5	2.25138
tr|Q5H907|Q5H907_HUMAN	MAGED2	Melanoma antigen family D, 2, isoform CRA_d OS=Homo sapiens GN=MAGED2 PE=4 SV=1	55932	0	618	0	71	0	0.19	#DIV/0!	0	1	0	3	0	4	0	4	2.25138
tr|B3KT00|B3KT00_HUMAN	tr|B3KT00	cDNA FLJ37368 fis, clone BRAMY2024530, highly similar to Zinc phosphodiesterase ELAC protein 2 (EC 3.1.	91169	0	614	0	71	0	0.11	#DIV/0!	0	1	0	3	0	4	0	5	2.25138
tr|B4DJV2|B4DJV2_HUMAN	CS	Citrate synthase OS=Homo sapiens GN=CS PE=2 SV=1	50628	0	583	0	76	0	0.21	#DIV/0!	0	3	0	3	0	4	0	5	2.25138
tr|B2RDI5|B2RDI5_HUMAN	tr|B2RDI5	cDNA, FLJ96627, highly similar to Homo sapiens calpain 1, (mu/I) large subunit (CAPN1), mRNA OS=Homo s	82529	0	569	0	75	0	0.22	#DIV/0!	0	2	0	4	0	4	0	6	2.25138
sp|Q9H0A0|NAT10_HUMAN	NAT10	N-acetyltransferase 10 OS=Homo sapiens GN=NAT10 PE=1 SV=2	116569	0	547	0	82	0	0.12	#DIV/0!	0	1	0	4	0	4	0	5	2.25138
tr|Q53G71|Q53G71_HUMAN	tr|Q53G71	Calreticulin variant (Fragment) OS=Homo sapiens PE=2 SV=1	47061	0	500	0	91	0	0.39	#DIV/0!	0	2	0	3	0	4	0	4	2.25138
tr|K4DI93|K4DI93_HUMAN	CUL4B	Cullin 4B, isoform CRA_e OS=Homo sapiens GN=CUL4B PE=3 SV=1	103318	0	479	0	95	0	0.1	#DIV/0!	0	3	0	3	0	4	0	5	2.25138
tr|G3V1L9|G3V1L9_HUMAN	TJP1	Tight junction protein 1 (Zona occludens 1), isoform CRA_a OS=Homo sapiens GN=TJP1 PE=4 SV=1	197681	0	430	0	108	0	0.09	#DIV/0!	0	2	0	5	0	4	0	5	2.25138
sp|P36952|SPB5_HUMAN	SERPINB5	Serpin B5 OS=Homo sapiens GN=SERPINB5 PE=1 SV=2	42530	0	425	0	110	0	0.35	#DIV/0!	0	3	0	4	0	4	0	4	2.25138
sp|Q9Y6C9|MTCH2_HUMAN	MTCH2	Mitochondrial carrier homolog 2 OS=Homo sapiens GN=MTCH2 PE=1 SV=1	33936	0	405	0	116	0	0.32	#DIV/0!	0	4	0	4	0	4	0	4	2.25138
tr|G3V3B0|G3V3B0_HUMAN	ACIN1	Apoptotic chromatin condensation inducer in the nucleus OS=Homo sapiens GN=ACIN1 PE=2 SV=1	67683	0	399	0	79	0	0.12	#DIV/0!	0	2	0	4	0	4	0	5	2.25138
tr|H0YCK3|H0YCK3_HUMAN	ADAR	Double-stranded RNA-specific adenosine deaminase (Fragment) OS=Homo sapiens GN=ADAR PE=4 SV=1	133761	0	377	0	84	0	0.12	#DIV/0!	0	2	0	4	0	4	0	8	2.25138
tr|B3KM89|B3KM89_HUMAN	tr|B3KM8	cDNA FLJ10528 fis, clone NT2RP2000943, highly similar to Protein transport protein Sec24D OS=Homo sap	101723	0	373	0	126	0	0.1	#DIV/0!	0	2	0	3	0	4	0	5	2.25138
tr|B2RDD7|B2RDD7_HUMAN	tr|B2RDD7	cDNA, FLJ96564, highly similar to Homo sapiens SKB1 homolog (S. pombe) (SKB1), mRNA OS=Homo sapien	73348	0	336	0	144	0	0.19	#DIV/0!	0	3	0	4	0	4	0	5	2.25138
tr|B7Z4B8|B7Z4B8_HUMAN	HNRNPUL	Heterogeneous nuclear ribonucleoprotein U-like protein 1 OS=Homo sapiens GN=HNRNPUL1 PE=2 SV=1	86639	0	327	0	149	0	0.14	#DIV/0!	0	2	0	4	0	4	0	6	2.25138
tr|H3BLU7|H3BLU7_HUMAN	AKR7A2	Aflatoxin B1 aldehyde reductase member 2 (Fragment) OS=Homo sapiens GN=AKR7A2 PE=4 SV=1	35004	0	264	0	196	0	0.44	#DIV/0!	0	4	0	4	0	4	0	4	2.25138
tr|Q6NWZ1|Q6NWZ1_HUMAN	CKAP4	CKAP4 protein (Fragment) OS=Homo sapiens GN=CKAP4 PE=2 SV=1	67948	0	213	0	183	0	0.27	#DIV/0!	0	4	0	4	0	4	0	4	2.25138
tr|J3QTQ0|J3QTQ0_HUMAN	DST	Dystonin OS=Homo sapiens GN=DST PE=4 SV=1	826674	0	1206	0	23	0	0	#DIV/0!	0	0	0	1	0	3	0	7	1.888632
tr|B2R950|B2R950_HUMAN	tr|B2R950	cDNA, FLJ94213, highly similar to Homo sapiens pregnancy-zone protein (PZP), mRNA OS=Homo sapiens P	165250	0	1077	0	31	0	0.04	#DIV/0!	0	0	0	2	0	3	0	3	1.888632
sp|A7E2Y1|MYH7B_HUMAN	MYH7B	Myosin-7B OS=Homo sapiens GN=MYH7B PE=2 SV=3	222392	0	1065	0	31	0	0.01	#DIV/0!	0	0	0	1	0	3	0	5	1.888632
sp|Q9H9A6|LRC40_HUMAN	LRRC40	Leucine-rich repeat-containing protein 40 OS=Homo sapiens GN=LRRC40 PE=1 SV=1	68720	0	997	0	35	0	0.1	#DIV/0!	0	1	0	2	0	3	0	4	1.888632
tr|Q59GM9|Q59GM9_HUMAN	tr|Q59GM	Phosphorylase (Fragment) OS=Homo sapiens PE=2 SV=1	99508	0	963	0	37	0	0.12	#DIV/0!	0	0	0	3	0	3	0	5	1.888632
tr|A4D275|A4D275_HUMAN	ARPC1B	Actin related protein 2/3 complex, subunit 1B, 41kDa OS=Homo sapiens GN=ARPC1B PE=2 SV=1	41722	0	956	0	37	0	0.26	#DIV/0!	0	1	0	3	0	3	0	3	1.888632
sp|Q5T8P6|RBM26_HUMAN	RBM26	RNA-binding protein 26 OS=Homo sapiens GN=RBM26 PE=1 SV=3	113927	0	934	0	39	0	0.06	#DIV/0!	0	1	0	2	0	3	0	3	1.888632
tr|J3QRR3|J3QRR3_HUMAN	PSMC5	26S protease regulatory subunit 8 (Fragment) OS=Homo sapiens GN=PSMC5 PE=4 SV=1	13944	0	904	0	41	0	0.24	#DIV/0!	0	1	0	1	0	3	0	4	1.888632
sp|Q6UXN9|WDR82_HUMAN	WDR82	WD repeat-containing protein 82 OS=Homo sapiens GN=WDR82 PE=1 SV=1	35456	0	868	0	44	0	0.09	#DIV/0!	0	1	0	1	0	3	0	3	1.888632
tr|J3KTL8|J3KTL8_HUMAN	SMCHD1	Structural maintenance of chromosomes flexible hinge domain-containing protein 1 (Fragment) OS=Homo	156690	0	866	0	44	0	0.04	#DIV/0!	0	1	0	2	0	3	0	5	1.888632
tr|B4DJU4|B4DJU4_HUMAN	tr|B4DJU4	cDNA FLJ53344, highly similar to Splicing factor 1 OS=Homo sapiens PE=2 SV=1	67535	0	857	0	44	0	0.15	#DIV/0!	0	0	0	3	0	3	0	3	1.888632
tr|H0YDD4|H0YDD4_HUMAN	DLAT	Dihydrolipoyllysine-residue acetyltransferase component of pyruvate dehydrogenase complex, mitochond	51511	0	853	0	44	0	0.13	#DIV/0!	0	1	0	2	0	3	0	3	1.888632
tr|Q5T2B5|Q5T2B5_HUMAN	CUL2	Cullin-2 OS=Homo sapiens GN=CUL2 PE=3 SV=1	82933	0	844	0	45	0	0.08	#DIV/0!	0	1	0	2	0	3	0	3	1.888632
tr|B2RMN7|B2RMN7_HUMAN	SPTB	Spectrin, beta, erythrocytic OS=Homo sapiens GN=SPTB PE=2 SV=1	247271	0	840	0	45	0	0.01	#DIV/0!	0	1	0	1	0	3	0	7	1.888632
tr|B3KMV5|B3KMV5_HUMAN	tr|B3KMV	cDNA FLJ12728 fis, clone NT2RP2000040, highly similar to Protein FAM62A OS=Homo sapiens PE=2 SV=1	123342	0	835	0	45	0	0.08	#DIV/0!	0	2	0	3	0	3	0	4	1.888632
sp|P62333|PRS10_HUMAN	PSMC6	26S protease regulatory subunit 10B OS=Homo sapiens GN=PSMC6 PE=1 SV=1	44430	0	832	0	46	0	0.24	#DIV/0!	0	1	0	3	0	3	0	4	1.888632
sp|O96008|TOM40_HUMAN	TOMM40	Mitochondrial import receptor subunit TOM40 homolog OS=Homo sapiens GN=TOMM40 PE=1 SV=1	38211	0	830	0	46	0	0.28	#DIV/0!	0	1	0	3	0	3	0	4	1.888632
tr|B3KY56|B3KY56_HUMAN	tr|B3KY56	cDNA FLJ46898 fis, clone UTERU3022168, highly similar to Protein FAM62A OS=Homo sapiens PE=2 SV=1	117833	0	813	0	47	0	0.06	#DIV/0!	0	2	0	2	0	3	0	3	1.888632
sp|Q96T76|MMS19_HUMAN	MMS19	MMS19 nucleotide excision repair protein homolog OS=Homo sapiens GN=MMS19 PE=1 SV=2	114928	0	808	0	47	0	0.09	#DIV/0!	0	1	0	3	0	3	0	3	1.888632
tr|Q59H95|Q59H95_HUMAN	tr|Q59H95	Flightless I homolog variant (Fragment) OS=Homo sapiens PE=2 SV=1	126396	0	782	0	50	0	0.07	#DIV/0!	0	3	0	3	0	3	0	3	1.888632
tr|B7Z2R8|B7Z2R8_HUMAN	tr|B7Z2R8	cDNA FLJ53324, highly similar to Tight junction protein ZO-2 OS=Homo sapiens PE=2 SV=1	137474	0	759	0	52	0	0.02	#DIV/0!	0	1	0	1	0	3	0	4	1.888632
tr|A0JLQ5|A0JLQ5_HUMAN	BXDC2	BXDC2 protein (Fragment) OS=Homo sapiens GN=BXDC2 PE=2 SV=1	39430	0	745	0	53	0	0.27	#DIV/0!	0	2	0	3	0	3	0	3	1.888632
sp|Q9NTI5|PDS5B_HUMAN	PDS5B	Sister chromatid cohesion protein PDS5 homolog B OS=Homo sapiens GN=PDS5B PE=1 SV=1	165818	0	732	0	55	0	0.04	#DIV/0!	0	1	0	2	0	3	0	4	1.888632
tr|B4DZP5|B4DZP5_HUMAN	tr|B4DZP5	cDNA FLJ51165, highly similar to DNA damage-binding protein 1 OS=Homo sapiens PE=2 SV=1	92726	0	730	0	55	0	0.11	#DIV/0!	0	1	0	3	0	3	0	4	1.888632
tr|B0QYK0|B0QYK0_HUMAN	EWSR1	Ewing sarcoma breakpoint region 1 OS=Homo sapiens GN=EWSR1 PE=4 SV=1	65174	0	713	0	57	0	0.1	#DIV/0!	0	2	0	2	0	3	0	3	1.888632
tr|A4FU51|A4FU51_HUMAN	NARG1L	NMDA receptor regulated 1-like OS=Homo sapiens GN=NARG1L PE=2 SV=1	102424	0	689	0	61	0	0.03	#DIV/0!	0	1	0	1	0	3	0	4	1.888632
tr|B3KVJ5|B3KVJ5_HUMAN	tr|B3KVJ5	cDNA FLJ16648 fis, clone TESTI4035508, highly similar to Structural maintenance of chromosomes 4-like 1	101354	0	686	0	61	0	0.07	#DIV/0!	0	1	0	2	0	3	0	3	1.888632
tr|H3BM93|H3BM93_HUMAN	NUP93	Nuclear pore complex protein Nup93 (Fragment) OS=Homo sapiens GN=NUP93 PE=4 SV=1	19858	0	679	0	53	0	0.17	#DIV/0!	0	1	0	3	0	3	0	4	1.888632
tr|F6S0T5|F6S0T5_HUMAN	KDM1A	Lysine-specific histone demethylase 1A OS=Homo sapiens GN=KDM1A PE=2 SV=1	95677	0	672	0	63	0	0.07	#DIV/0!	0	2	0	2	0	3	0	3	1.888632
tr|B7ZMF2|B7ZMF2_HUMAN	FANCI	Fanconi anemia, complementation group I OS=Homo sapiens GN=FANCI PE=2 SV=1	150724	0	671	0	63	0	0.08	#DIV/0!	0	2	0	3	0	3	0	4	1.888632
tr|B4DM01|B4DM01_HUMAN	SKIV2L	Helicase SKI2W OS=Homo sapiens GN=SKIV2L PE=2 SV=1	121269	0	667	0	64	0	0.08	#DIV/0!	0	1	0	3	0	3	0	3	1.888632
tr|I1E4Y6|I1E4Y6_HUMAN	GIGYF2	PERQ amino acid-rich with GYF domain-containing protein 2 OS=Homo sapiens GN=GIGYF2 PE=4 SV=1	152891	0	660	0	66	0	0.04	#DIV/0!	0	2	0	2	0	3	0	4	1.888632
tr|B4DL39|B4DL39_HUMAN	tr|B4DL39	cDNA FLJ57316, highly similar to DNA mismatch repair protein Msh2 OS=Homo sapiens PE=2 SV=1	77368	0	657	0	66	0	0.06	#DIV/0!	0	1	0	2	0	3	0	3	1.888632
tr|A0PJ61|A0PJ61_HUMAN	RSL1D1	RSL1D1 protein (Fragment) OS=Homo sapiens GN=RSL1D1 PE=2 SV=1	49688	0	649	0	68	0	0.57	#DIV/0!	0	2	0	5	0	3	0	5	1.888632
sp|P13667|PDIA4_HUMAN	PDIA4	Protein disulfide-isomerase A4 OS=Homo sapiens GN=PDIA4 PE=1 SV=2	73229	0	640	0	68	0	0.14	#DIV/0!	0	2	0	3	0	3	0	3	1.888632

sp|Q00653|NFKB2_HUMAN	NFKB2	Nuclear factor NF-kappa-B p100 subunit OS=Homo sapiens GN=NFKB2 PE=1 SV=4	97373	0	638	0	68	0	0.07	#DIV/0!	0	2	0	2	0	3	0	5	1.888632
sp|Q9Y4G6|TLN2_HUMAN	TLN2	Talin-2 OS=Homo sapiens GN=TLN2 PE=1 SV=4	273781	0	635	0	69	0	0.04	#DIV/0!	0	2	0	3	0	3	0	5	1.888632
tr|A8K4H7|A8K4H7_HUMAN	PPAT	Amidophosphoribosyltransferase OS=Homo sapiens GN=PPAT PE=2 SV=1	58161	0	634	0	69	0	0.55	#DIV/0!	0	1	0	3	0	3	0	3	1.888632
tr|B1AK85|B1AK85_HUMAN	CAPZB	F-actin-capping protein subunit beta OS=Homo sapiens GN=CAPZB PE=2 SV=1	29703	0	607	0	72	0	0.37	#DIV/0!	0	2	0	3	0	3	0	3	1.888632
sp|Q8NI27|THOC2_HUMAN	THOC2	THO complex subunit 2 OS=Homo sapiens GN=THOC2 PE=1 SV=2	184541	0	602	0	72	0	0.1	#DIV/0!	0	3	0	3	0	3	0	4	1.888632
tr|B4DYY5|B4DYY5_HUMAN	tr|B4DYY5	cDNA FLJ55671, highly similar to Ubiquitin-associated protein 2-like OS=Homo sapiens PE=2 SV=1	113662	0	595	0	74	0	0.09	#DIV/0!	0	2	0	3	0	3	0	4	1.888632
sp|P54577|SYYC_HUMAN	YARS	Tyrosine--tRNA ligase, cytoplasmic OS=Homo sapiens GN=YARS PE=1 SV=4	59448	0	588	0	75	0	0.11	#DIV/0!	0	1	0	2	0	3	0	4	1.888632
tr|B4DH17|B4DH17_HUMAN	tr|B4DH17	cDNA FLJ59298, highly similar to Eukaryotic translation initiation factor 4 gamma 3 OS=Homo sapiens PE=2	134921	0	580	0	77	0	0.07	#DIV/0!	0	3	0	3	0	3	0	3	1.888632
sp|Q9BR76|COR1B_HUMAN	CORO1B	Coronin-1B OS=Homo sapiens GN=CORO1B PE=1 SV=1	54885	0	574	0	78	0	0.12	#DIV/0!	0	1	0	2	0	3	0	3	1.888632
sp|P49792|RBP2_HUMAN	RANBP2	E3 SUMO-protein ligase RanBP2 OS=Homo sapiens GN=RANBP2 PE=1 SV=2	362365	0	573	0	78	0	0.03	#DIV/0!	0	2	0	3	0	3	0	4	1.888632
tr|J3KR44|J3KR44_HUMAN	OTUB1	Ubiquitin thioesterase OTUB1 OS=Homo sapiens GN=OTUB1 PE=4 SV=1	35649	0	553	0	81	0	0.31	#DIV/0!	0	2	0	3	0	3	0	3	1.888632
sp|Q6PKG0|LARP1_HUMAN	LARP1	La-related protein 1 OS=Homo sapiens GN=LARP1 PE=1 SV=2	123833	0	536	0	83	0	0.05	#DIV/0!	0	2	0	2	0	3	0	5	1.888632
tr|B4E1L0|B4E1L0_HUMAN	tr|B4E1L0	Adenylosuccinate synthetase OS=Homo sapiens PE=2 SV=1	48308	0	526	0	84	0	0.14	#DIV/0!	0	2	0	2	0	3	0	4	1.888632
tr|B3KY04|B3KY04_HUMAN	tr|B3KY04	cDNA FLJ46506 fis, clone THYMU3030752, highly similar to BTB/POZ domain-containing protein KCTD12 O	35994	0	523	0	85	0	0.19	#DIV/0!	0	2	0	2	0	3	0	3	1.888632
sp|Q92466|DDB2_HUMAN	DDB2	DNA damage-binding protein 2 OS=Homo sapiens GN=DDB2 PE=1 SV=1	48461	0	518	0	86	0	0.07	#DIV/0!	0	1	0	1	0	3	0	4	1.888632
sp|P51116|FXR2_HUMAN	FXR2	Fragile X mental retardation syndrome-related protein 2 OS=Homo sapiens GN=FXR2 PE=1 SV=2	74520	0	516	0	86	0	0.14	#DIV/0!	0	1	0	3	0	3	0	4	1.888632
sp|P22059|OSBP1_HUMAN	OSBP	Oxysterol-binding protein 1 OS=Homo sapiens GN=OSBP PE=1 SV=1	90220	0	507	0	89	0	0.07	#DIV/0!	0	1	0	2	0	3	0	5	1.888632
tr|B4DFP1|B4DFP1_HUMAN	tr|B4DFP1	cDNA FLJ51818, highly similar to Phosphoglucomutase-1 (EC 5.4.2.2) OS=Homo sapiens PE=2 SV=1	58987	0	496	0	91	0	0.18	#DIV/0!	0	2	0	3	0	3	0	4	1.888632
sp|Q92626|PXDN_HUMAN	PXDN	Peroxidasin homolog OS=Homo sapiens GN=PXDN PE=1 SV=2	167793	0	495	0	91	0	0.06	#DIV/0!	0	2	0	3	0	3	0	3	1.888632
sp|Q9H3U1|UN45A_HUMAN	UNC45A	Protein unc-45 homolog A OS=Homo sapiens GN=UNC45A PE=1 SV=1	104266	0	490	0	93	0	0.06	#DIV/0!	0	2	0	2	0	3	0	3	1.888632
tr|B2R774|B2R774_HUMAN	tr|B2R774	cDNA, FLJ93313, highly similar to Homo sapiens lectin, mannose-binding, 1 (LMAN1), mRNA OS=Homo sap	57768	0	482	0	94	0	0.18	#DIV/0!	0	3	0	3	0	3	0	3	1.888632
tr|H7BZT7|H7BZT7_HUMAN	ESD	S-formylglutathione hydrolase (Fragment) OS=Homo sapiens GN=ESD PE=4 SV=1	25806	0	472	0	74	0	0.49	#DIV/0!	0	2	0	3	0	3	0	3	1.888632
tr|B4DUF1|B4DUF1_HUMAN	tr|B4DUF1	cDNA FLJ59760, highly similar to 1,4-alpha-glucan branching enzyme (EC 2.4.1.18) OS=Homo sapiens PE=2	76254	0	464	0	74	0	0.2	#DIV/0!	0	2	0	3	0	3	0	3	1.888632
sp|P04899|GNAI2_HUMAN	GNAI2	Guanine nucleotide-binding protein G(i) subunit alpha-2 OS=Homo sapiens GN=GNAI2 PE=1 SV=3	40995	0	459	0	100	0	0.26	#DIV/0!	0	2	0	3	0	3	0	3	1.888632
sp|Q92945|FUBP2_HUMAN	KHSRP	Far upstream element-binding protein 2 OS=Homo sapiens GN=KHSRP PE=1 SV=4	73355	0	452	0	102	0	0.14	#DIV/0!	0	3	0	3	0	3	0	5	1.888632
sp|P30837|AL1B1_HUMAN	ALDH1B1	Aldehyde dehydrogenase X, mitochondrial OS=Homo sapiens GN=ALDH1B1 PE=1 SV=3	57626	0	448	0	104	0	0.18	#DIV/0!	0	3	0	3	0	3	0	4	1.888632
tr|Q0VGA5|Q0VGA5_HUMAN	SARS	SARS protein OS=Homo sapiens GN=SARS PE=2 SV=1	58883	0	442	0	106	0	0.18	#DIV/0!	0	3	0	3	0	3	0	5	1.888632
tr|B3KM58|B3KM58_HUMAN	tr|B3KM5	cDNA FLJ10358 fis, clone NT2RM2001238, highly similar to Glutaminase kidney isoform, mitochondrial (Fra	41736	0	429	0	108	0	0.26	#DIV/0!	0	2	0	3	0	3	0	4	1.888632
tr|B4DT31|B4DT31_HUMAN	FUBP1	Far upstream element-binding protein 1 OS=Homo sapiens GN=FUBP1 PE=2 SV=1	69993	0	426	0	110	0	0.22	#DIV/0!	0	2	0	2	0	3	0	3	1.888632
tr|B4DPW5|B4DPW5_HUMAN	tr|B4DPW	cDNA FLJ59374, highly similar to Homo sapiens caldesmon 1 (CALD1), transcript variant 2, mRNA OS=Hom	57436	0	412	0	112	0	0.18	#DIV/0!	0	3	0	3	0	3	0	4	1.888632
tr|G5E9H4|G5E9H4_HUMAN	DCTN1	Dynactin 1 (P150, glued homolog, Drosophila), isoform CRA_a OS=Homo sapiens GN=DCTN1 PE=2 SV=1	127339	0	388	0	121	0	0.08	#DIV/0!	0	2	0	3	0	3	0	5	1.888632
tr|B4DTS5|B4DTS5_HUMAN	tr|B4DTS5	cDNA FLJ58882, highly similar to 26S proteasome non-ATPase regulatory subunit 11 OS=Homo sapiens PE=	35374	0	378	0	93	0	0.31	#DIV/0!	0	3	0	3	0	3	0	3	1.888632
tr|H0YHX9|H0YHX9_HUMAN	NACA	Nascent polypeptide-associated complex subunit alpha (Fragment) OS=Homo sapiens GN=NACA PE=4 SV=	22930	0	332	0	146	0	0.5	#DIV/0!	0	3	0	3	0	3	0	3	1.888632
tr|H0YCK7|H0YCK7_HUMAN	EEF1D	Elongation factor 1-delta (Fragment) OS=Homo sapiens GN=EEF1D PE=4 SV=1	23105	0	284	0	178	0	0.2	#DIV/0!	0	3	0	4	0	3	0	4	1.888632
tr|E7ERK9|E7ERK9_HUMAN	EIF2B4	Translation initiation factor eIF-2B subunit delta OS=Homo sapiens GN=EIF2B4 PE=2 SV=1	60248	0	1241	0	20	0	0.05	#DIV/0!	0	0	0	1	0	2	0	2	1.403027
tr|Q8N5A0|Q8N5A0_HUMAN	EIF5B	Eukaryotic translation initiation factor 5B OS=Homo sapiens GN=EIF5B PE=2 SV=1	139172	0	1224	0	22	0	0.02	#DIV/0!	0	0	0	1	0	2	0	5	1.403027
sp|Q969N2|PIGT_HUMAN	PIGT	GPI transamidase component PIG-T OS=Homo sapiens GN=PIGT PE=1 SV=1	66228	0	1222	0	22	0	0.07	#DIV/0!	0	0	0	1	0	2	0	2	1.403027
sp|Q9BTC0|DIDO1_HUMAN	DIDO1	Death-inducer obliterator 1 OS=Homo sapiens GN=DIDO1 PE=1 SV=5	245434	0	1215	0	22	0	0.03	#DIV/0!	0	0	0	2	0	2	0	5	1.403027
tr|B4DM73|B4DM73_HUMAN	tr|B4DM7	cDNA FLJ60078, highly similar to CLIP-associating protein 2 (Fragment) OS=Homo sapiens PE=2 SV=1	116274	0	1184	0	24	0	0.02	#DIV/0!	0	0	0	1	0	2	0	4	1.403027
tr|D3DVA5|D3DVA5_HUMAN	ARHGEF2	Rho/rac guanine nucleotide exchange factor (GEF) 2, isoform CRA_a OS=Homo sapiens GN=ARHGEF2 PE=2	116954	0	1183	0	24	0	0.03	#DIV/0!	0	0	0	1	0	2	0	2	1.403027
sp|Q9UBG0|MRC2_HUMAN	MRC2	C-type mannose receptor 2 OS=Homo sapiens GN=MRC2 PE=1 SV=2	169761	0	1178	0	25	0	0.02	#DIV/0!	0	0	0	1	0	2	0	2	1.403027
tr|B7Z7F0|B7Z7F0_HUMAN	tr|B7Z7F0	cDNA FLJ56420, highly similar to Aspartyl aminopeptidase (EC 3.4.11.21) OS=Homo sapiens PE=2 SV=1	55086	0	1161	0	26	0	0.13	#DIV/0!	0	0	0	2	0	2	0	2	1.403027
tr|G5EA36|G5EA36_HUMAN	CDC27	Cell division cycle 27, isoform CRA_c OS=Homo sapiens GN=CDC27 PE=2 SV=1	92764	0	1151	0	26	0	0.04	#DIV/0!	0	0	0	1	0	2	0	2	1.403027
sp|Q68E01|INT3_HUMAN	INTS3	Integrator complex subunit 3 OS=Homo sapiens GN=INTS3 PE=1 SV=1	119533	0	1142	0	27	0	0.06	#DIV/0!	0	0	0	2	0	2	0	3	1.403027
tr|H3BSD9|H3BSD9_HUMAN	MYO7B	Unconventional myosin-VIIb (Fragment) OS=Homo sapiens GN=MYO7B PE=4 SV=1	10022	0	1120	0	28	0	0.35	#DIV/0!	0	1	0	1	0	2	0	2	1.403027
tr|H7C2T8|H7C2T8_HUMAN	UBAP2L	Ubiquitin-associated protein 2-like (Fragment) OS=Homo sapiens GN=UBAP2L PE=4 SV=1	41506	0	1118	0	28	0	0.08	#DIV/0!	0	0	0	1	0	2	0	2	1.403027
tr|B3KVA0|B3KVA0_HUMAN	tr|B3KVA0	Cytosine-specific methyltransferase OS=Homo sapiens PE=2 SV=1	61027	0	1114	0	29	0	0.05	#DIV/0!	0	0	0	1	0	2	0	2	1.403027
tr|Q5JB52|Q5JB52_HUMAN	HNRPLL	Heterogeneous nuclear ribonucleoprotein L-like OS=Homo sapiens GN=HNRPLL PE=2 SV=1	57115	0	1110	0	29	0	0.12	#DIV/0!	0	0	0	2	0	2	0	2	1.403027
sp|Q9Y5K6|CD2AP_HUMAN	CD2AP	CD2-associated protein OS=Homo sapiens GN=CD2AP PE=1 SV=1	71635	0	1107	0	29	0	0.09	#DIV/0!	0	1	0	2	0	2	0	2	1.403027
tr|B4DL07|B4DL07_HUMAN	tr|B4DL07	cDNA FLJ53353, highly similar to ATP-binding cassette sub-family D member 3 OS=Homo sapiens PE=2 SV=	78980	0	1104	0	30	0	0.04	#DIV/0!	0	0	0	1	0	2	0	4	1.403027
sp|Q9H3P7|GCP60_HUMAN	ACBD3	Golgi resident protein GCP60 OS=Homo sapiens GN=ACBD3 PE=1 SV=4	60841	0	1094	0	30	0	0.05	#DIV/0!	0	0	0	1	0	2	0	2	1.403027
tr|E5KLJ5|E5KLJ5_HUMAN	OPA1	Dynamin-like 120 kDa protein, mitochondrial OS=Homo sapiens GN=OPA1 PE=3 SV=1	118240	0	1089	0	30	0	0.03	#DIV/0!	0	0	0	1	0	2	0	2	1.403027
tr|B0QYV7|B0QYV7_HUMAN	ARFGAP3	ADP-ribosylation factor GTPase activating protein 3 (Fragment) OS=Homo sapiens GN=ARFGAP3 PE=2 SV=1	30052	0	1071	0	31	0	0.23	#DIV/0!	0	0	0	2	0	2	0	2	1.403027
tr|F6SBX2|F6SBX2_HUMAN	IARS2	Isoleucine--tRNA ligase, mitochondrial OS=Homo sapiens GN=IARS2 PE=2 SV=1	106884	0	1058	0	32	0	0.03	#DIV/0!	0	0	0	1	0	2	0	3	1.403027
tr|B3KM68|B3KM68_HUMAN	tr|B3KM6	cDNA FLJ10398 fis, clone NT2RM4000349, highly similar to Homo sapiens basic leucine zipper and W2 dom	48388	0	1050	0	32	0	0.07	#DIV/0!	0	1	0	1	0	2	0	4	1.403027
sp|Q14558|KPRA_HUMAN	PRPSAP1	Phosphoribosyl pyrophosphate synthase-associated protein 1 OS=Homo sapiens GN=PRPSAP1 PE=1 SV=2	39654	0	1043	0	33	0	0.22	#DIV/0!	0	0	0	2	0	2	0	2	1.403027
tr|B3KSJ6|B3KSJ6_HUMAN	tr|B3KSJ6	Aldehyde dehydrogenase OS=Homo sapiens PE=2 SV=1	44674	0	1041	0	33	0	0.15	#DIV/0!	0	0	0	2	0	2	0	2	1.403027
tr|H0Y360|H0Y360_HUMAN	AMPD2	AMP deaminase 2 (Fragment) OS=Homo sapiens GN=AMPD2 PE=4 SV=1	99192	0	1028	0	34	0	0.03	#DIV/0!	0	0	0	1	0	2	0	3	1.403027
tr|Q4G0F4|Q4G0F4_HUMAN	POLRMT	DNA-directed RNA polymerase OS=Homo sapiens GN=POLRMT PE=2 SV=1	140194	0	1025	0	34	0	0.03	#DIV/0!	0	0	0	1	0	2	0	3	1.403027
tr|A0PJ47|A0PJ47_HUMAN	SAFB2	SAFB2 protein (Fragment) OS=Homo sapiens GN=SAFB2 PE=2 SV=1	57340	0	1021	0	34	0	0.12	#DIV/0!	0	0	0	2	0	2	0	2	1.403027
tr|A3KPE3|A3KPE3_HUMAN	FOXC1	FOXC1 protein OS=Homo sapiens GN=FOXC1 PE=2 SV=1	24051	0	1020	0	34	0	0.14	#DIV/0!	0	0	0	1	0	2	0	2	1.403027
tr|B2RB32|B2RB32_HUMAN	tr|B2RB32	Phosphorylase OS=Homo sapiens PE=2 SV=1	97439	0	1018	0	34	0	0.07	#DIV/0!	0	0	0	2	0	2	0	4	1.403027
sp|Q9UKX7|NUP50_HUMAN	NUP50	Nuclear pore complex protein Nup50 OS=Homo sapiens GN=NUP50 PE=1 SV=2	50512	0	1007	0	35	0	0.07	#DIV/0!	0	0	0	1	0	2	0	2	1.403027
tr|B2RE88|B2RE88_HUMAN	tr|B2RE88	cDNA, FLJ96465, highly similar to Homo sapiens solute carrier family 25 (mitochondrial carrier;phosphate c	40379	0	1001	0	35	0	0.08	#DIV/0!	0	0	0	1	0	2	0	2	1.403027
tr|B4DHS5|B4DHS5_HUMAN	tr|B4DHS5	cDNA FLJ54678, highly similar to N(2),N(2)-dimethylguanosine tRNA methyltransferase (EC 2.1.1.32) OS=H	59256	0	996	0	35	0	0.11	#DIV/0!	0	1	0	2	0	2	0	3	1.403027
tr|J3QRS9|J3QRS9_HUMAN	ZNF207	Zinc finger protein 207 OS=Homo sapiens GN=ZNF207 PE=4 SV=1	52849	0	979	0	36	0	0.06	#DIV/0!	0	0	0	1	0	2	0	2	1.403027
sp|Q16222|UAP1_HUMAN	UAP1	UDP-N-acetylhexosamine pyrophosphorylase OS=Homo sapiens GN=UAP1 PE=1 SV=3	59131	0	972	0	36	0	0.06	#DIV/0!	0	0	0	1	0	2	0	3	1.403027
tr|B7Z475|B7Z475_HUMAN	tr|B7Z475	cDNA FLJ55712, highly similar to F-box-like/WD repeat protein TBL1XR1 OS=Homo sapiens PE=2 SV=1	41301	0	966	0	37	0	0.17	#DIV/0!	0	0	0	2	0	2	0	2	1.403027
tr|Q4ZG72|Q4ZG72_HUMAN	DDX18	Putative uncharacterized protein DDX18 (Fragment) OS=Homo sapiens GN=DDX18 PE=2 SV=1	61898	0	962	0	37	0	0.11	#DIV/0!	0	1	0	2	0	2	0	2	1.403027
tr|H0YJS8|H0YJS8_HUMAN	PSMC6	26S protease regulatory subunit 10B (Fragment) OS=Homo sapiens GN=PSMC6 PE=4 SV=1	22376	0	957	0	37	0	0.32	#DIV/0!	0	1	0	2	0	2	0	3	1.403027
tr|Q6MZP3|Q6MZP3_HUMAN	DKFZp686	Putative uncharacterized protein DKFZp686I05169 OS=Homo sapiens GN=DKFZp686I05169 PE=2 SV=1	146779	0	954	0	37	0	0.02	#DIV/0!	0	0	0	1	0	2	0	2	1.403027
tr|A1A693|A1A693_HUMAN	RBM15	RNA binding motif protein 15 isoform L2 OS=Homo sapiens GN=RBM15 PE=2 SV=1	99689	0	932	0	39	0	0.07	#DIV/0!	0	1	0	2	0	2	0	2	1.403027
tr|G3V0J0|G3V0J0_HUMAN	FMR1	Fragile X mental retardation 1, isoform CRA_e OS=Homo sapiens GN=FMR1 PE=2 SV=1	67030	0	931	0	39	0	0.1	#DIV/0!	0	1	0	2	0	2	0	4	1.403027
sp|P46013|KI67_HUMAN	MKI67	Antigen KI-67 OS=Homo sapiens GN=MKI67 PE=1 SV=2	360698	0	915	0	40	0	0.01	#DIV/0!	0	1	0	1	0	2	0	6	1.403027
tr|I3L1M4|I3L1M4_HUMAN	ALDH3A2	Fatty aldehyde dehydrogenase (Fragment) OS=Homo sapiens GN=ALDH3A2 PE=3 SV=1	18870	0	913	0	31	0	0.24	#DIV/0!	0	0	0	2	0	2	0	2	1.403027
tr|B2RB06|B2RB06_HUMAN	tr|B2RB06	cDNA, FLJ95242, highly similar to Homo sapiens L-3-hydroxyacyl-Coenzyme A dehydrogenase, short chain (	34297	0	912	0	40	0	0.1	#DIV/0!	0	1	0	1	0	2	0	3	1.403027
tr|Q53S54|Q53S54_HUMAN	CUL3	Putative uncharacterized protein CUL3 (Fragment) OS=Homo sapiens GN=CUL3 PE=2 SV=1	86981	0	910	0	40	0	0.05	#DIV/0!	0	1	0	1	0	2	0	2	1.403027
sp|Q32MZ4|LRRF1_HUMAN	LRRFIP1	Leucine-rich repeat flightless-interacting protein 1 OS=Homo sapiens GN=LRRFIP1 PE=1 SV=2	89826	0	909	0	40	0	0.04	#DIV/0!	0	1	0	1	0	2	0	2	1.403027
tr|Q6IBS5|Q6IBS5_HUMAN	DLST	DLST protein OS=Homo sapiens GN=DLST PE=2 SV=1	49131	0	906	0	41	0	0.14	#DIV/0!	0	1	0	2	0	2	0	2	1.403027
tr|Q2NL95|Q2NL95_HUMAN	DDX19B	DDX19B protein (Fragment) OS=Homo sapiens GN=DDX19B PE=2 SV=1	51068	0	897	0	42	0	0.06	#DIV/0!	0	1	0	1	0	2	0	2	1.403027
sp|Q5JVF3|PCID2_HUMAN	PCID2	PCI domain-containing protein 2 OS=Homo sapiens GN=PCID2 PE=1 SV=2	46627	0	894	0	42	0	0.15	#DIV/0!	0	1	0	2	0	2	0	2	1.403027
tr|B4DW54|B4DW54_HUMAN	tr|B4DW5	cDNA FLJ50888, highly similar to Aldehyde dehydrogenase, mitochondrial (EC 1.2.1.3) OS=Homo sapiens P	51467	0	893	0	42	0	0.06	#DIV/0!	0	1	0	1	0	2	0	3	1.403027
tr|G3V161|G3V161_HUMAN	KBTBD3	Kelch repeat and BTB (POZ) domain containing 3, isoform CRA_a OS=Homo sapiens GN=KBTBD3 PE=2 SV=1	61834	0	882	0	43	0	0.06	#DIV/0!	0	1	0	1	0	2	0	3	1.403027
tr|F2YGG7|F2YGG7_HUMAN	tr|F2YGG7	Epidermal growth factor receptor OS=Homo sapiens PE=2 SV=1	132588	0	881	0	43	0	0.05	#DIV/0!	0	1	0	2	0	2	0	2	1.403027
sp|Q63ZY3|KANK2_HUMAN	KANK2	KN motif and ankyrin repeat domain-containing protein 2 OS=Homo sapiens GN=KANK2 PE=1 SV=1	91916	0	880	0	43	0	0.04	#DIV/0!	0	1	0	1	0	2	0	2	1.403027
tr|H0YE88|H0YE88_HUMAN	TEAD1	Transcriptional enhancer factor TEF-1 OS=Homo sapiens GN=TEAD1 PE=4 SV=1	48296	0	878	0	43	0	0.07	#DIV/0!	0	1	0	1	0	2	0	2	1.403027
sp|Q96AG4|LRC59_HUMAN	LRRC59	Leucine-rich repeat-containing protein 59 OS=Homo sapiens GN=LRRC59 PE=1 SV=1	35308	0	860	0	44	0	0.09	#DIV/0!	0	1	0	1	0	2	0	2	1.403027
tr|H0YEH2|H0YEH2_HUMAN	PUM1	Pumilio homolog 1 (Fragment) OS=Homo sapiens GN=PUM1 PE=4 SV=1	120836	0	854	0	44	0	0.03	#DIV/0!	0	1	0	1	0	2	0	2	1.403027
tr|G5E9D3|G5E9D3_HUMAN	CAST	Calpastatin OS=Homo sapiens GN=CAST PE=2 SV=1	80615	0	850	0	44	0	0.08	#DIV/0!	0	0	0	2	0	2	0	3	1.403027
tr|Q6FG59|Q6FG59_HUMAN	CDC37	CDC37 protein OS=Homo sapiens GN=CDC37 PE=2 SV=1	44938	0	849	0	45	0	0.07	#DIV/0!	0	1	0	1	0	2	0	3	1.403027
tr|B4DSN3|B4DSN3_HUMAN	tr|B4DSN3	cDNA FLJ60345, highly similar to Protein transport protein Sec24C OS=Homo sapiens PE=2 SV=1	107974	0	842	0	45	0	0.09	#DIV/0!	0	1	0	3	0	2	0	4	1.403027
tr|D3DTC2|D3DTC2_HUMAN	AYTL2	Acyltransferase like 2, isoform CRA_a OS=Homo sapiens GN=AYTL2 PE=2 SV=1	54725	0	839	0	45	0	0.12	#DIV/0!	0	0	0	2	0	2	0	2	1.403027
tr|Q2KQ72|Q2KQ72_HUMAN	tr|Q2KQ72	Chromosome-associated protein E OS=Homo sapiens PE=2 SV=1	86324	0	834	0	46	0	0.05	#DIV/0!	0	1	0	2	0	2	0	3	1.403027
sp|P09497|CLCB_HUMAN	CLTB	Clathrin light chain B OS=Homo sapiens GN=CLTB PE=1 SV=1	25289	0	831	0	46	0	0.13	#DIV/0!	0	1	0	1	0	2	0	3	1.403027
sp|Q9Y2H6|FND3A_HUMAN	FNDC3A	Fibronectin type-III domain-containing protein 3A OS=Homo sapiens GN=FNDC3A PE=1 SV=4	133763	0	827	0	46	0	0.05	#DIV/0!	0	0	0	2	0	2	0	2	1.403027
tr|I3L445|I3L445_HUMAN	PELP1	Proline-, glutamic acid- and leucine-rich protein 1 (Fragment) OS=Homo sapiens GN=PELP1 PE=4 SV=1	32684	0	816	0	47	0	0.1	#DIV/0!	0	1	0	1	0	2	0	2	1.403027
tr|B4DR36|B4DR36_HUMAN	tr|B4DR36	cDNA FLJ56414, highly similar to Homo sapiens proline-, glutamic acid-, leucine-rich protein 1 (PELP1), mRI	126101	0	815	0	47	0	0.03	#DIV/0!	0	1	0	1	0	2	0	3	1.403027
tr|B4DEX7|B4DEX7_HUMAN	tr|B4DEX7	cDNA FLJ51806, highly similar to Homo sapiens proline-, glutamic acid-, leucine-rich protein 1 (PELP1), mRI	97913	0	814	0	47	0	0.31	#DIV/0!	0	1	0	1	0	2	0	3	1.403027
tr|B4DXJ1|B4DXJ1_HUMAN	SEC13	Protein SEC13 homolog OS=Homo sapiens GN=SEC13 PE=2 SV=1	41405	0	805	0	48	0	0.17	#DIV/0!	0	0	0	2	0	2	0	2	1.403027
tr|J3KPX7|J3KPX7_HUMAN	PHB2	Prohibitin-2 OS=Homo sapiens GN=PHB2 PE=4 SV=1	33382	0	804	0	48	0	0.1	#DIV/0!	0	1	0	1	0	2	0	2	1.403027
tr|B4DH43|B4DH43_HUMAN	tr|B4DH43	cDNA FLJ56433, highly similar to Collagen alpha-2(IV) chain OS=Homo sapiens PE=2 SV=1	83428	0	800	0	48	0	0.04	#DIV/0!	0	1	0	1	0	2	0	2	1.403027
tr|B4DK35|B4DK35_HUMAN	tr|B4DK35	cDNA FLJ61591, highly similar to Probable global transcription activator SNF2L2 (EC 3.6.1.-) (Fragment) OS	112636	0	794	0	49	0	0.04	#DIV/0!	0	1	0	2	0	2	0	3	1.403027
tr|Q7Z586|Q7Z586_HUMAN	tr|Q7Z586	Putative uncharacterized protein (Fragment) OS=Homo sapiens PE=2 SV=1	105456	0	791	0	49	0	0.03	#DIV/0!	0	1	0	1	0	2	0	3	1.403027
tr|B3KXW5|B3KXW5_HUMAN	AP1G1	AP-1 complex subunit gamma-1 OS=Homo sapiens GN=AP1G1 PE=2 SV=1	94992	0	790	0	49	0	0.07	#DIV/0!	0	1	0	2	0	2	0	2	1.403027
tr|E9PG89|E9PG89_HUMAN	CHD3	Chromodomain-helicase-DNA-binding protein 3 OS=Homo sapiens GN=CHD3 PE=2 SV=1	234431	0	783	0	50	0	0.01	#DIV/0!	0	1	0	1	0	2	0	4	1.403027
sp|P52907|CAZA1_HUMAN	CAPZA1	F-actin-capping protein subunit alpha-1 OS=Homo sapiens GN=CAPZA1 PE=1 SV=3	33073	0	779	0	50	0	0.21	#DIV/0!	0	1	0	2	0	2	0	2	1.403027
tr|F5H8E5|F5H8E5_HUMAN	USP7	Ubiquitin carboxyl-terminal hydrolase OS=Homo sapiens GN=USP7 PE=2 SV=1	117951	0	774	0	50	0	0.06	#DIV/0!	0	1	0	2	0	2	0	3	1.403027
tr|B3KNK5|B3KNK5_HUMAN	tr|B3KNK5	cDNA FLJ14795 fis, clone NT2RP4001219, highly similar to Homo sapiens DnaJ (Hsp40) homolog, subfamily	70004	0	773	0	50	0	0.07	#DIV/0!	0	1	0	2	0	2	0	3	1.403027
sp|Q9Y4F1|FARP1_HUMAN	FARP1	FERM, RhoGEF and pleckstrin domain-containing protein 1 OS=Homo sapiens GN=FARP1 PE=1 SV=1	119300	0	766	0	51	0	0.06	#DIV/0!	0	1	0	2	0	2	0	2	1.403027
sp|Q09161|NCBP1_HUMAN	NCBP1	Nuclear cap-binding protein subunit 1 OS=Homo sapiens GN=NCBP1 PE=1 SV=1	92864	0	763	0	51	0	0.07	#DIV/0!	0	1	0	2	0	2	0	2	1.403027
tr|B4E205|B4E205_HUMAN	tr|B4E205	cDNA FLJ61651, highly similar to Protein transport protein Sec24A OS=Homo sapiens PE=2 SV=1	96953	0	757	0	52	0	0.1	#DIV/0!	0	1	0	3	0	2	0	4	1.403027
tr|B4DQ15|B4DQ15_HUMAN	tr|B4DQ15	cDNA FLJ51525, highly similar to Homo sapiens aldehyde dehydrogenase 16 family, member A1 (ALDH16A	67899	0	755	0	52	0	0.1	#DIV/0!	0	2	0	2	0	2	0	2	1.403027
tr|A7E2E1|A7E2E1_HUMAN	SMARCA4	SWI/SNF related, matrix associated, actin dependent regulator of chromatin, subfamily a, member 4 OS=H	185100	0	742	0	54	0	0.04	#DIV/0!	0	2	0	2	0	2	0	4	1.403027
tr|B4DHY1|B4DHY1_HUMAN	HNRNPH3	Heterogeneous nuclear ribonucleoprotein H3 OS=Homo sapiens GN=HNRNPH3 PE=2 SV=1	24925	0	741	0	54	0	0.29	#DIV/0!	0	1	0	2	0	2	0	2	1.403027
sp|P98160|PGBM_HUMAN	HSPG2	Basement membrane-specific heparan sulfate proteoglycan core protein OS=Homo sapiens GN=HSPG2 PE	479253	0	728	0	56	0	0.01	#DIV/0!	0	1	0	2	0	2	0	2	1.403027
tr|B4DZI8|B4DZI8_HUMAN	COPB2	Coatomer protein complex, subunit beta 2 (Beta prime), isoform CRA_b OS=Homo sapiens GN=COPB2 PE=	99839	0	726	0	56	0	0.07	#DIV/0!	0	1	0	2	0	2	0	2	1.403027
tr|B4E206|B4E206_HUMAN	tr|B4E206	cDNA FLJ55441, highly similar to Nuclear pore complex protein Nup133 OS=Homo sapiens PE=2 SV=1	128349	0	721	0	56	0	0.05	#DIV/0!	0	1	0	2	0	2	0	2	1.403027
tr|B4DJ81|B4DJ81_HUMAN	NDUFS1	NADH-ubiquinone oxidoreductase 75 kDa subunit, mitochondrial OS=Homo sapiens GN=NDUFS1 PE=2 SV=	67563	0	716	0	57	0	0.1	#DIV/0!	0	1	0	2	0	2	0	3	1.403027
tr|B4DN25|B4DN25_HUMAN	UGDH	UDP-glucose 6-dehydrogenase OS=Homo sapiens GN=UGDH PE=2 SV=1	44786	0	711	0	57	0	0.15	#DIV/0!	0	2	0	2	0	2	0	3	1.403027
tr|B0QZ18|B0QZ18_HUMAN	CPNE1	Copine I OS=Homo sapiens GN=CPNE1 PE=2 SV=1	60307	0	701	0	59	0	0.11	#DIV/0!	0	1	0	2	0	2	0	3	1.403027
sp|Q92896|GSLG1_HUMAN	GLG1	Golgi apparatus protein 1 OS=Homo sapiens GN=GLG1 PE=1 SV=2	138341	0	696	0	59	0	0.02	#DIV/0!	0	1	0	1	0	2	0	6	1.403027
tr|E9PDG5|E9PDG5_HUMAN	LARP4	La-related protein 4 OS=Homo sapiens GN=LARP4 PE=2 SV=1	50319	0	693	0	60	0	0.14	#DIV/0!	0	2	0	2	0	2	0	2	1.403027
tr|A8K6V7|A8K6V7_HUMAN	tr|A8K6V7	cDNA FLJ76053, highly similar to Homo sapiens Ras-GTPase activating protein SH3 domain-binding protein	50842	0	688	0	61	0	0.13	#DIV/0!	0	1	0	2	0	2	0	2	1.403027
tr|H0Y8X1|H0Y8X1_HUMAN	SDHA	Succinate dehydrogenase [ubiquinone] flavoprotein subunit, mitochondrial (Fragment) OS=Homo sapiens	13856	0	682	0	61	0	0.55	#DIV/0!	0	1	0	2	0	2	0	3	1.403027
tr|B3KRB2|B3KRB2_HUMAN	tr|B3KRB2	cDNA FLJ33964 fis, clone CTONG2019029, highly similar to Pseudouridylate synthase 7 homolog (EC 5.4.99	75303	0	677	0	62	0	0.09	#DIV/0!	0	2	0	2	0	2	0	4	1.403027
tr|A1A5C4|A1A5C4_HUMAN	RRBP1	RRBP1 protein OS=Homo sapiens GN=RRBP1 PE=2 SV=1	103162	0	674	0	62	0	0.04	#DIV/0!	0	1	0	2	0	2	0	3	1.403027
tr|B4DNM8|B4DNM8_HUMAN	tr|B4DNM	cDNA FLJ53395, highly similar to Prolyl 3-hydroxylase 1 (EC 1.14.11.7) OS=Homo sapiens PE=2 SV=1	69572	0	663	0	65	0	0.1	#DIV/0!	0	1	0	2	0	2	0	2	1.403027
tr|B9EG90|B9EG90_HUMAN	TOP1	Topoisomerase (DNA) I OS=Homo sapiens GN=TOP1 PE=2 SV=1	91099	0	653	0	67	0	0.07	#DIV/0!	0	2	0	2	0	2	0	2	1.403027
tr|B4DZ87|B4DZ87_HUMAN	tr|B4DZ87	cDNA FLJ57240, highly similar to Mitochondrial proteins import receptor OS=Homo sapiens PE=2 SV=1	57579	0	652	0	67	0	0.12	#DIV/0!	0	2	0	2	0	2	0	2	1.403027
sp|P27694|RFA1_HUMAN	RPA1	Replication protein A 70 kDa DNA-binding subunit OS=Homo sapiens GN=RPA1 PE=1 SV=2	68723	0	651	0	67	0	0.1	#DIV/0!	0	2	0	2	0	2	0	4	1.403027
tr|H0Y532|H0Y532_HUMAN	SELENBP1	Selenium-binding protein 1 (Fragment) OS=Homo sapiens GN=SELENBP1 PE=4 SV=1	34415	0	645	0	68	0	0.08	#DIV/0!	0	1	0	1	0	2	0	2	1.403027
tr|Q14400|Q14400_HUMAN	GLUD1	GLUD1 protein (Fragment) OS=Homo sapiens GN=GLUD1 PE=2 SV=1	28791	0	632	0	69	0	0.25	#DIV/0!	0	2	0	2	0	2	0	3	1.403027
tr|Q6EHZ3|Q6EHZ3_HUMAN	STRF8	Putative uncharacterized protein STRF8 OS=Homo sapiens GN=STRF8 PE=2 SV=1	41361	0	630	0	69	0	0.17	#DIV/0!	0	2	0	2	0	2	0	2	1.403027
tr|B3KRR1|B3KRR1_HUMAN	tr|B3KRR1	cDNA FLJ34725 fis, clone MESAN2005958, highly similar to RNA-binding protein Luc7-like 2 OS=Homo sapi	40752	0	625	0	69	0	0.08	#DIV/0!	0	1	0	1	0	2	0	4	1.403027
tr|B4DMF5|B4DMF5_HUMAN	tr|B4DMF	Glutamate dehydrogenase OS=Homo sapiens PE=2 SV=1	56910	0	621	0	70	0	0.12	#DIV/0!	0	1	0	2	0	2	0	3	1.403027
sp|Q96CS3|FAF2_HUMAN	FAF2	FAS-associated factor 2 OS=Homo sapiens GN=FAF2 PE=1 SV=2	52933	0	619	0	71	0	0.13	#DIV/0!	0	2	0	2	0	2	0	2	1.403027
tr|A9LSU1|A9LSU1_HUMAN	tr|A9LSU1	Type IV collagen alpha 1 (Fragment) OS=Homo sapiens PE=2 SV=1	25998	0	613	0	71	0	0.27	#DIV/0!	0	1	0	2	0	2	0	2	1.403027
tr|Q0VF99|Q0VF99_HUMAN	OSBP2	Oxysterol-binding protein OS=Homo sapiens GN=OSBP2 PE=2 SV=1	102085	0	611	0	71	0	0.03	#DIV/0!	0	1	0	1	0	2	0	3	1.403027
tr|B3KNC3|B3KNC3_HUMAN	tr|B3KNC3	cDNA FLJ14222 fis, clone NT2RP3003992, highly similar to Nucleolar complex protein 2 homolog OS=Homo	85719	0	601	0	72	0	0.08	#DIV/0!	0	1	0	2	0	2	0	2	1.403027
tr|Q6IPN0|Q6IPN0_HUMAN	RTN4	RTN4 protein OS=Homo sapiens GN=RTN4 PE=2 SV=1	36952	0	594	0	74	0	0.19	#DIV/0!	0	2	0	2	0	2	0	2	1.403027
sp|P49419|AL7A1_HUMAN	ALDH7A1	Alpha-aminoadipic semialdehyde dehydrogenase OS=Homo sapiens GN=ALDH7A1 PE=1 SV=5	59020	0	590	0	74	0	0.18	#DIV/0!	0	1	0	3	0	2	0	4	1.403027
sp|Q8N1G4|LRC47_HUMAN	LRRC47	Leucine-rich repeat-containing protein 47 OS=Homo sapiens GN=LRRC47 PE=1 SV=1	64004	0	584	0	76	0	0.16	#DIV/0!	0	1	0	3	0	2	0	3	1.403027
sp|O00507|USP9Y_HUMAN	USP9Y	Probable ubiquitin carboxyl-terminal hydrolase FAF-Y OS=Homo sapiens GN=USP9Y PE=1 SV=2	294370	0	572	0	78	0	0.01	#DIV/0!	0	1	0	1	0	2	0	5	1.403027
tr|B4DGX9|B4DGX9_HUMAN	tr|B4DGX9	cDNA FLJ60509, highly similar to Probable ubiquitin carboxyl-terminalhydrolase FAF-X (EC 3.1.2.15) OS=Ho	66195	0	571	0	78	0	0.01	#DIV/0!	0	1	0	1	0	2	0	3	1.403027
sp|P61221|ABCE1_HUMAN	ABCE1	ATP-binding cassette sub-family E member 1 OS=Homo sapiens GN=ABCE1 PE=1 SV=1	68240	0	570	0	79	0	0.14	#DIV/0!	0	1	0	2	0	2	0	2	1.403027
sp|Q9Y295|DRG1_HUMAN	DRG1	Developmentally-regulated GTP-binding protein 1 OS=Homo sapiens GN=DRG1 PE=1 SV=1	40802	0	567	0	79	0	0.17	#DIV/0!	0	2	0	2	0	2	0	3	1.403027
sp|Q96S66|CLCC1_HUMAN	CLCC1	Chloride channel CLIC-like protein 1 OS=Homo sapiens GN=CLCC1 PE=1 SV=1	62667	0	565	0	80	0	0.11	#DIV/0!	0	2	0	2	0	2	0	2	1.403027
tr|B0AZS5|B0AZS5_HUMAN	tr|B0AZS5	cDNA, FLJ79515, highly similar to Kinesin-like protein KIF2 OS=Homo sapiens PE=2 SV=1	75666	0	563	0	80	0	0.09	#DIV/0!	0	2	0	2	0	2	0	3	1.403027
tr|B7Z7U0|B7Z7U0_HUMAN	tr|B7Z7U0	cDNA FLJ61739, highly similar to Serine/arginine repetitive matrix protein 1 OS=Homo sapiens PE=2 SV=1	103792	0	561	0	80	0	0.06	#DIV/0!	0	2	0	2	0	2	0	2	1.403027
tr|H0Y5W0|H0Y5W0_HUMAN	HUWE1	E3 ubiquitin-protein ligase HUWE1 (Fragment) OS=Homo sapiens GN=HUWE1 PE=4 SV=1	376696	0	556	0	80	0	0.01	#DIV/0!	0	1	0	2	0	2	0	2	1.403027
tr|B4DM78|B4DM78_HUMAN	tr|B4DM7	cDNA FLJ58199, highly similar to Fragile X mental retardation syndrome-related protein 1 OS=Homo sapier	63280	0	554	0	81	0	0.13	#DIV/0!	0	2	0	2	0	2	0	2	1.403027
tr|F5GZS0|F5GZS0_HUMAN	DHX36	Probable ATP-dependent RNA helicase DHX36 OS=Homo sapiens GN=DHX36 PE=2 SV=1	113928	0	541	0	82	0	0.07	#DIV/0!	0	1	0	2	0	2	0	2	1.403027
tr|Q0VAB1|Q0VAB1_HUMAN	TIMM50	Translocase of inner mitochondrial membrane 50 homolog (S. cerevisiae) OS=Homo sapiens GN=TIMM50 I	50960	0	537	0	83	0	0.13	#DIV/0!	0	2	0	2	0	2	0	2	1.403027
tr|B4DUT7|B4DUT7_HUMAN	tr|B4DUT7	cDNA FLJ57604, highly similar to GMP synthase (glutamine-hydrolyzing) (EC 6.3.5.2) OS=Homo sapiens PE=	71880	0	535	0	76	0	0.13	#DIV/0!	0	2	0	2	0	2	0	5	1.403027
tr|H3BRG4|H3BRG4_HUMAN	UQCRC2	Cytochrome b-c1 complex subunit 2, mitochondrial OS=Homo sapiens GN=UQCRC2 PE=3 SV=1	44778	0	520	0	66	0	0.22	#DIV/0!	0	1	0	2	0	2	0	2	1.403027
sp|Q08379|GOGA2_HUMAN	GOLGA2	Golgin subfamily A member 2 OS=Homo sapiens GN=GOLGA2 PE=1 SV=3	113644	0	519	0	85	0	0.06	#DIV/0!	0	2	0	2	0	2	0	2	1.403027
tr|D3DUE6|D3DUE6_HUMAN	N-PAC	Cytokine-like nuclear factor n-pac, isoform CRA_c OS=Homo sapiens GN=N-PAC PE=2 SV=1	61517	0	511	0	88	0	0.11	#DIV/0!	0	2	0	2	0	2	0	2	1.403027
tr|E7EMC7|E7EMC7_HUMAN	SQSTM1	Sequestosome-1 OS=Homo sapiens GN=SQSTM1 PE=2 SV=1	42070	0	509	0	53	0	0.35	#DIV/0!	0	1	0	2	0	2	0	3	1.403027
tr|B2R858|B2R858_HUMAN	tr|B2R858	cDNA, FLJ93750, Homo sapiens DEAD (Asp-Glu-Ala-Asp) box polypeptide 6 (DDX6), mRNA OS=Homo sapier	53582	0	504	0	67	0	0.19	#DIV/0!	0	1	0	2	0	2	0	3	1.403027
tr|I6L9E8|I6L9E8_HUMAN	FAM98A	Family with sequence similarity 98, member A OS=Homo sapiens GN=FAM98A PE=2 SV=1	55595	0	499	0	91	0	0.12	#DIV/0!	0	1	0	2	0	2	0	2	1.403027
tr|B7ZLC9|B7ZLC9_HUMAN	GEMIN5	GEMIN5 protein OS=Homo sapiens GN=GEMIN5 PE=2 SV=1	170664	0	493	0	92	0	0.06	#DIV/0!	0	2	0	3	0	2	0	3	1.403027
tr|H0YEL7|H0YEL7_HUMAN	ACAT1	Acetyl-CoA acetyltransferase, mitochondrial (Fragment) OS=Homo sapiens GN=ACAT1 PE=3 SV=1	14377	0	491	0	93	0	0.15	#DIV/0!	0	1	0	2	0	2	0	2	1.403027
tr|B3KPF6|B3KPF6_HUMAN	tr|B3KPF6	cDNA FLJ31732 fis, clone NT2RI2006856, highly similar to Striatin-4 OS=Homo sapiens PE=2 SV=1	39506	0	480	0	63	0	0.26	#DIV/0!	0	2	0	2	0	2	0	2	1.403027
tr|J3KPP4|J3KPP4_HUMAN	LUC7L3	Cisplatin resistance-associated overexpressed protein, isoform CRA_b OS=Homo sapiens GN=LUC7L3 PE=4	58698	0	465	0	99	0	0.12	#DIV/0!	0	2	0	2	0	2	0	2	1.403027
sp|Q7Z5L9|I2BP2_HUMAN	IRF2BP2	Interferon regulatory factor 2-binding protein 2 OS=Homo sapiens GN=IRF2BP2 PE=1 SV=2	61728	0	463	0	99	0	0.11	#DIV/0!	0	2	0	2	0	2	0	2	1.403027
tr|C9J634|C9J634_HUMAN	PDHB	Pyruvate dehydrogenase E1 component subunit beta, mitochondrial OS=Homo sapiens GN=PDHB PE=2 SV	39064	0	447	0	76	0	0.38	#DIV/0!	0	2	0	2	0	2	0	3	1.403027
tr|Q6IBU0|Q6IBU0_HUMAN	EIF5	EIF5 protein OS=Homo sapiens GN=EIF5 PE=2 SV=1	49577	0	444	0	106	0	0.14	#DIV/0!	0	1	0	2	0	2	0	2	1.403027
tr|A8K4T6|A8K4T6_HUMAN	tr|A8K4T6	cDNA FLJ76282, highly similar to Homo sapiens proteasome (prosome, macropain) 26S subunit, non-ATPas	56588	0	439	0	107	0	0.12	#DIV/0!	0	1	0	2	0	2	0	3	1.403027
tr|E1P5S2|E1P5S2_HUMAN	RBM39	RNA-binding protein 39 OS=Homo sapiens GN=RBM39 PE=4 SV=1	40744	0	438	0	107	0	0.26	#DIV/0!	0	2	0	3	0	2	0	3	1.403027
tr|Q6MZY7|Q6MZY7_HUMAN	DKFZp686	Putative uncharacterized protein DKFZp686C17209 OS=Homo sapiens GN=DKFZp686C17209 PE=2 SV=1	40730	0	428	0	109	0	0.17	#DIV/0!	0	2	0	2	0	2	0	2	1.403027
tr|B4DR63|B4DR63_HUMAN	PSMC1	26S protease regulatory subunit 4 OS=Homo sapiens GN=PSMC1 PE=2 SV=1	41255	0	423	0	110	0	0.17	#DIV/0!	0	2	0	2	0	2	0	2	1.403027
tr|B2R8G6|B2R8G6_HUMAN	tr|B2R8G6	cDNA, FLJ93891, Homo sapiens FK506 binding protein 8, 38kDa (FKBP8), mRNA OS=Homo sapiens PE=2 SV	38870	0	415	0	112	0	0.18	#DIV/0!	0	2	0	2	0	2	0	2	1.403027
tr|G5E988|G5E988_HUMAN	AP3D1	AP-3 complex subunit delta-1 OS=Homo sapiens GN=AP3D1 PE=2 SV=1	126074	0	414	0	105	0	0.1	#DIV/0!	0	2	0	2	0	2	0	5	1.403027
tr|Q6FHX6|Q6FHX6_HUMAN	FEN1	Flap endonuclease 1 OS=Homo sapiens GN=FEN1 PE=2 SV=1	42908	0	409	0	113	0	0.18	#DIV/0!	0	1	0	2	0	2	0	3	1.403027
tr|Q6ZVI6|Q6ZVI6_HUMAN	tr|Q6ZVI6	cDNA FLJ42537 fis, clone BRACE3004058, highly similar to NADH-cytochrome B5 reductase (EC 1.6.2.2) OS	29141	0	406	0	63	0	0.32	#DIV/0!	0	1	0	2	0	2	0	2	1.403027
tr|B3KQ43|B3KQ43_HUMAN	tr|B3KQ43	cDNA FLJ32791 fis, clone TESTI2002365, highly similar to helicase MOV-10 (EC 3.6.1.-) OS=Homo sapiens P	47208	0	394	0	110	0	0.09	#DIV/0!	0	2	0	2	0	2	0	3	1.403027
tr|B0I1S9|B0I1S9_HUMAN	MYO1B	MYO1B variant protein OS=Homo sapiens GN=MYO1B PE=2 SV=1	132928	0	391	0	120	0	0.05	#DIV/0!	0	2	0	2	0	2	0	3	1.403027
tr|B2R7F8|B2R7F8_HUMAN	tr|B2R7F8	cDNA, FLJ93426, highly similar to Homo sapiens plasminogen (PLG), mRNA OS=Homo sapiens PE=2 SV=1	93217	0	367	0	128	0	0.07	#DIV/0!	0	2	0	2	0	2	0	2	1.403027
sp|P40222|TXLNA_HUMAN	TXLNA	Alpha-taxilin OS=Homo sapiens GN=TXLNA PE=1 SV=3	62195	0	361	0	131	0	0.11	#DIV/0!	0	2	0	2	0	2	0	2	1.403027
sp|P11047|LAMC1_HUMAN	LAMC1	Laminin subunit gamma-1 OS=Homo sapiens GN=LAMC1 PE=1 SV=3	183191	0	353	0	133	0	0.04	#DIV/0!	0	2	0	2	0	2	0	2	1.403027
tr|D3DTY9|D3DTY9_HUMAN	TRIM25	Tripartite motif-containing 25, isoform CRA_a OS=Homo sapiens GN=TRIM25 PE=2 SV=1	48358	0	345	0	115	0	0.19	#DIV/0!	0	2	0	2	0	2	0	3	1.403027
sp|Q9BWF3|RBM4_HUMAN	RBM4	RNA-binding protein 4 OS=Homo sapiens GN=RBM4 PE=1 SV=1	40688	0	341	0	104	0	0.29	#DIV/0!	0	1	0	2	0	2	0	2	1.403027

tr|Q3SYK8|Q3SYK8_HUMAN	NUMA1	NUMA1 protein (Fragment) OS=Homo sapiens GN=NUMA1 PE=2 SV=1	78666	0	237	0	94	0	0.08	#DIV/0!	0	2	0	2	0	2	0	3	1.403027
tr|A8K2M0|A8K2M0_HUMAN	PSMC4	Proteasome (Prosome, macropain) 26S subunit, ATPase, 4, isoform CRA_b OS=Homo sapiens GN=PSMC4 P	47451	0	1276	0	17	0	0.07	#DIV/0!	0	0	0	1	0	1	0	2	0.665884
tr|D3DP12|D3DP12_HUMAN	DCHS2	Dachsous 2 (Drosophila), isoform CRA_b OS=Homo sapiens GN=DCHS2 PE=4 SV=1	280417	0	1268	0	18	0	0.01	#DIV/0!	0	0	0	1	0	1	0	3	0.665884
sp|Q9UID3|VPS51_HUMAN	VPS51	Vacuolar protein sorting-associated protein 51 homolog OS=Homo sapiens GN=VPS51 PE=1 SV=2	86901	0	1260	0	19	0	0.15	#DIV/0!	0	0	0	1	0	1	0	2	0.665884
sp|Q5SRE5|NU188_HUMAN	NUP188	Nucleoporin NUP188 homolog OS=Homo sapiens GN=NUP188 PE=1 SV=1	198369	0	1252	0	20	0	0.03	#DIV/0!	0	0	0	2	0	1	0	5	0.665884
tr|Q96CP5|Q96CP5_HUMAN	PMPCB	PMPCB protein (Fragment) OS=Homo sapiens GN=PMPCB PE=2 SV=2	54216	0	1244	0	20	0	0.06	#DIV/0!	0	0	0	1	0	1	0	2	0.665884
tr|G3V2J6|G3V2J6_HUMAN	TDP1	Tyrosyl-DNA phosphodiesterase 1 (Fragment) OS=Homo sapiens GN=TDP1 PE=2 SV=1	25066	0	1243	0	20	0	0.13	#DIV/0!	0	0	0	1	0	1	0	1	0.665884
tr|H3BNI0|H3BNI0_HUMAN	ZNF19	Zinc finger protein 19 OS=Homo sapiens GN=ZNF19 PE=4 SV=1	48887	0	1240	0	20	0	0.07	#DIV/0!	0	0	0	1	0	1	0	1	0.665884
sp|Q5R3I4|TTC38_HUMAN	TTC38	Tetratricopeptide repeat protein 38 OS=Homo sapiens GN=TTC38 PE=1 SV=1	53267	0	1239	0	20	0	0.06	#DIV/0!	0	0	0	1	0	1	0	1	0.665884
tr|A8K972|A8K972_HUMAN	tr|A8K972	cDNA FLJ76457, highly similar to Homo sapiens leucine carboxyl methyltransferase 2 (LCMT2), mRNA OS=H	76406	0	1238	0	20	0	0.04	#DIV/0!	0	0	0	1	0	1	0	1	0.665884
tr|H7C455|H7C455_HUMAN	tr|H7C455	Uncharacterized protein (Fragment) OS=Homo sapiens PE=4 SV=1	22389	0	1236	0	21	0	0.15	#DIV/0!	0	0	0	1	0	1	0	1	0.665884
tr|Q8TDG7|Q8TDG7_HUMAN	tr|Q8TDG	CLL-associated antigen KW-6 (Fragment) OS=Homo sapiens PE=2 SV=1	25926	0	1235	0	21	0	0.13	#DIV/0!	0	0	0	1	0	1	0	1	0.665884
sp|Q9P253|VPS18_HUMAN	VPS18	Vacuolar protein sorting-associated protein 18 homolog OS=Homo sapiens GN=VPS18 PE=1 SV=2	111484	0	1234	0	21	0	0.03	#DIV/0!	0	0	0	1	0	1	0	1	0.665884
tr|I7GPQ7|I7GPQ7_HUMAN	tr|I7GPQ7	cDNA FLJ75793 OS=Homo sapiens PE=2 SV=1	67286	0	1233	0	21	0	0.05	#DIV/0!	0	0	0	1	0	1	0	2	0.665884
tr|B2R7R5|B2R7R5_HUMAN	tr|B2R7R5	cDNA, FLJ93570, highly similar to Homo sapiens phosphoribosyl pyrophosphate synthetase-associated pro	41256	0	1232	0	21	0	0.08	#DIV/0!	0	0	0	1	0	1	0	1	0.665884
tr|G5E933|G5E933_HUMAN	SBF1	Myotubularin-related protein 5 OS=Homo sapiens GN=SBF1 PE=2 SV=1	210422	0	1231	0	21	0	0.02	#DIV/0!	0	0	0	1	0	1	0	1	0.665884
tr|B4DH22|B4DH22_HUMAN	tr|B4DH22	cDNA FLJ59884, highly similar to Secernin-1 OS=Homo sapiens PE=2 SV=1	25067	0	1230	0	21	0	0.13	#DIV/0!	0	0	0	1	0	1	0	1	0.665884
sp|Q8IWB1|IPRI_HUMAN	ITPRIP	Inositol 1,4,5-trisphosphate receptor-interacting protein OS=Homo sapiens GN=ITPRIP PE=1 SV=1	62932	0	1229	0	21	0	0.05	#DIV/0!	0	1	0	1	0	1	0	2	0.665884
tr|Q8NE02|Q8NE02_HUMAN	AKAP8	AKAP8 protein OS=Homo sapiens GN=AKAP8 PE=2 SV=1	76689	0	1228	0	21	0	0.04	#DIV/0!	0	0	0	1	0	1	0	2	0.665884
sp|P61011|SRP54_HUMAN	SRP54	Signal recognition particle 54 kDa protein OS=Homo sapiens GN=SRP54 PE=1 SV=1	55953	0	1227	0	21	0	0.07	#DIV/0!	0	0	0	1	0	1	0	1	0.665884
tr|Q2TAM4|Q2TAM4_HUMAN	POLR1B	DNA-directed RNA polymerase OS=Homo sapiens GN=POLR1B PE=2 SV=1	108996	0	1226	0	21	0	0.03	#DIV/0!	0	0	0	1	0	1	0	1	0.665884
tr|B4DIJ4|B4DIJ4_HUMAN	tr|B4DIJ4	cDNA FLJ51207, highly similar to VPS10 domain-containing receptor SorCS2 OS=Homo sapiens PE=2 SV=1	113194	0	1225	0	22	0	0.03	#DIV/0!	0	0	0	1	0	1	0	1	0.665884
tr|B3KRC6|B3KRC6_HUMAN	tr|B3KRC6	cDNA FLJ34004 fis, clone FCBBF1000232, highly similar to Cytochrome P450 51A1 (EC 1.14.13.70) OS=Hom	51732	0	1223	0	22	0	0.06	#DIV/0!	0	0	0	1	0	1	0	2	0.665884
tr|B3KN69|B3KN69_HUMAN	tr|B3KN69	cDNA FLJ13804 fis, clone THYRO1000197, highly similar to Poly(A)-specific ribonuclease PARN (EC 3.1.13.4)	40330	0	1221	0	22	0	0.08	#DIV/0!	0	0	0	1	0	1	0	1	0.665884
tr|B2RTX8|B2RTX8_HUMAN	WAPAL	WAPAL protein OS=Homo sapiens GN=WAPAL PE=2 SV=1	133797	0	1219	0	22	0	0.02	#DIV/0!	0	0	0	1	0	1	0	2	0.665884
tr|B4DE19|B4DE19_HUMAN	ELP3	Elongator complex protein 3 OS=Homo sapiens GN=ELP3 PE=2 SV=1	49772	0	1218	0	22	0	0.07	#DIV/0!	0	0	0	1	0	1	0	1	0.665884
tr|A7Y9J9|A7Y9J9_HUMAN	MUC5AC	Mucin 5AC, oligomeric mucus/gel-forming OS=Homo sapiens GN=MUC5AC PE=2 SV=1	666520	0	1217	0	22	0	0	#DIV/0!	0	0	0	1	0	1	0	2	0.665884
tr|Q8N9H7|Q8N9H7_HUMAN	tr|Q8N9H	cDNA FLJ37128 fis, clone BRACE2022928 OS=Homo sapiens PE=2 SV=1	16502	0	1216	0	22	0	0.2	#DIV/0!	0	0	0	1	0	1	0	1	0.665884
tr|B3KU05|B3KU05_HUMAN	tr|B3KU05	cDNA FLJ39026 fis, clone NT2RP7005675 OS=Homo sapiens PE=2 SV=1	130427	0	1214	0	22	0	0.03	#DIV/0!	0	0	0	1	0	1	0	1	0.665884
tr|J3KMX1|J3KMX1_HUMAN	NUP88	Nuclear pore complex protein Nup88 (Fragment) OS=Homo sapiens GN=NUP88 PE=4 SV=1	80029	0	1213	0	22	0	0.04	#DIV/0!	0	0	0	1	0	1	0	1	0.665884
tr|A0AVG2|A0AVG2_HUMAN	TRPM8	TRPM8 protein OS=Homo sapiens GN=TRPM8 PE=2 SV=1	80015	0	1212	0	22	0	0.04	#DIV/0!	0	0	0	1	0	1	0	1	0.665884
tr|Q16380|Q16380_HUMAN	metastasis	Metastasis-associated gene protein (Fragment) OS=Homo sapiens GN=metastasis-associated gene PE=2 SV	11817	0	1211	0	22	0	0.29	#DIV/0!	0	0	0	1	0	1	0	2	0.665884
tr|Q0VAM0|Q0VAM0_HUMAN	CTNNBL1	Catenin, beta like 1 OS=Homo sapiens GN=CTNNBL1 PE=2 SV=1	65560	0	1210	0	22	0	0.05	#DIV/0!	0	0	0	1	0	1	0	1	0.665884
tr|B2R6H3|B2R6H3_HUMAN	tr|B2R6H3	cDNA, FLJ92951, highly similar to Homo sapiens kinesin family member 18A (KIF18A), mRNA OS=Homo sap	103326	0	1209	0	23	0	0.03	#DIV/0!	0	0	0	1	0	1	0	1	0.665884
tr|B4DRW3|B4DRW3_HUMAN	tr|B4DRW	cDNA FLJ57180, highly similar to TAR DNA-binding protein 43 OS=Homo sapiens PE=2 SV=1	35596	0	1208	0	23	0	0.1	#DIV/0!	0	0	0	1	0	1	0	1	0.665884
tr|J3KSW6|J3KSW6_HUMAN	DHX40	Probable ATP-dependent RNA helicase DHX40 (Fragment) OS=Homo sapiens GN=DHX40 PE=4 SV=1	30276	0	1205	0	23	0	0.11	#DIV/0!	0	0	0	1	0	1	0	1	0.665884
tr|B2R7T6|B2R7T6_HUMAN	tr|B2R7T6	cDNA, FLJ93596, highly similar to Homo sapiens sulfide quinone reductase-like (yeast) (SQRDL), mRNA OS=	50199	0	1204	0	23	0	0.07	#DIV/0!	0	0	0	1	0	1	0	1	0.665884
tr|B2R5U7|B2R5U7_HUMAN	tr|B2R5U7	cDNA, FLJ92633, highly similar to Homo sapiens CCAAT-box-binding transcription factor (CBF2), mRNA OS=	114755	0	1203	0	23	0	0.03	#DIV/0!	0	0	0	1	0	1	0	1	0.665884
tr|B3KM90|B3KM90_HUMAN	tr|B3KM9	cDNA FLJ10529 fis, clone NT2RP2000965, highly similar to Targeting protein for Xklp2 OS=Homo sapiens PE	70285	0	1199	0	23	0	0.05	#DIV/0!	0	0	0	1	0	1	0	2	0.665884
tr|B4DH45|B4DH45_HUMAN	tr|B4DH45	cDNA FLJ53322, highly similar to Homo sapiens sorbin and SH3 domain containing 1 (SORBS1), transcript v	49097	0	1198	0	23	0	0.07	#DIV/0!	0	0	0	1	0	1	0	1	0.665884
tr|B4DDL6|B4DDL6_HUMAN	tr|B4DDL6	cDNA FLJ54325, highly similar to Myotubularin-related protein 2 (EC 3.1.3.-) OS=Homo sapiens PE=2 SV=1	36644	0	1197	0	24	0	0.09	#DIV/0!	0	0	0	1	0	1	0	1	0.665884
sp|P78357|CNTP1_HUMAN	CNTNAP1	Contactin-associated protein 1 OS=Homo sapiens GN=CNTNAP1 PE=1 SV=1	158220	0	1196	0	24	0	0.02	#DIV/0!	0	0	0	1	0	1	0	1	0.665884
sp|P37837|TALDO_HUMAN	TALDO1	Transaldolase OS=Homo sapiens GN=TALDO1 PE=1 SV=2	37688	0	1195	0	24	0	0.09	#DIV/0!	0	0	0	1	0	1	0	1	0.665884
tr|H0Y4S9|H0Y4S9_HUMAN	KPNA3	Importin subunit alpha-3 (Fragment) OS=Homo sapiens GN=KPNA3 PE=4 SV=1	10174	0	1194	0	24	0	0.34	#DIV/0!	0	0	0	1	0	1	0	2	0.665884
tr|H0YJ87|H0YJ87_HUMAN	ZC3H14	Zinc finger CCCH domain-containing protein 14 (Fragment) OS=Homo sapiens GN=ZC3H14 PE=4 SV=1	8686	0	1193	0	24	0	0.4	#DIV/0!	0	0	0	1	0	1	0	1	0.665884
sp|Q96T51|RUFY1_HUMAN	RUFY1	RUN and FYVE domain-containing protein 1 OS=Homo sapiens GN=RUFY1 PE=1 SV=2	80851	0	1192	0	24	0	0.04	#DIV/0!	0	0	0	1	0	1	0	2	0.665884
tr|Q5T5N3|Q5T5N3_HUMAN	IDE	Insulin-degrading enzyme (Fragment) OS=Homo sapiens GN=IDE PE=4 SV=1	29418	0	1191	0	23	0	0.06	#DIV/0!	0	0	0	1	0	1	0	1	0.665884
tr|H0YB78|H0YB78_HUMAN	SLC12A7	Solute carrier family 12 member 7 (Fragment) OS=Homo sapiens GN=SLC12A7 PE=4 SV=1	39776	0	1189	0	24	0	0.08	#DIV/0!	0	0	0	1	0	1	0	1	0.665884
tr|K7ES11|K7ES11_HUMAN	UBE2O	Ubiquitin-conjugating enzyme E2 O (Fragment) OS=Homo sapiens GN=UBE2O PE=4 SV=1	89434	0	1188	0	24	0	0.04	#DIV/0!	0	0	0	1	0	1	0	1	0.665884
tr|A8MV53|A8MV53_HUMAN	PPAN	HCG2033702, isoform CRA_a OS=Homo sapiens GN=PPAN PE=4 SV=1	47344	0	1187	0	24	0	0.07	#DIV/0!	0	0	0	1	0	1	0	1	0.665884
tr|H3BS29|H3BS29_HUMAN	Sep-01	Septin-1 (Fragment) OS=Homo sapiens GN=SEPT1 PE=3 SV=1	35905	0	1186	0	24	0	0.1	#DIV/0!	0	1	0	1	0	1	0	1	0.665884
sp|P11182|ODB2_HUMAN	DBT	Lipoamide acyltransferase component of branched-chain alpha-keto acid dehydrogenase complex, mitoch	53852	0	1185	0	24	0	0.1	#DIV/0!	0	0	0	1	0	1	0	1	0.665884
tr|B7Z7K9|B7Z7K9_HUMAN	tr|B7Z7K9	cDNA FLJ51382 OS=Homo sapiens PE=2 SV=1	48343	0	1182	0	24	0	0.07	#DIV/0!	0	0	0	1	0	1	0	1	0.665884
tr|B3KM73|B3KM73_HUMAN	tr|B3KM7	cDNA FLJ10419 fis, clone NT2RP1000163, highly similar to Homo sapiens transforming growth factor beta	55418	0	1181	0	24	0	0.19	#DIV/0!	0	0	0	3	0	1	0	3	0.665884
tr|F2Z2T0|F2Z2T0_HUMAN	RABL6	Rab-like protein 6 OS=Homo sapiens GN=RABL6 PE=2 SV=1	54224	0	1180	0	25	0	0.06	#DIV/0!	0	0	0	1	0	1	0	1	0.665884
tr|B3KV00|B3KV00_HUMAN	tr|B3KV00	cDNA FLJ41005 fis, clone UTERU2017613, highly similar to Homo sapiens zinc finger protein 289, ID1 regul	42367	0	1177	0	25	0	0.08	#DIV/0!	0	0	0	1	0	1	0	1	0.665884
tr|H0Y2V6|H0Y2V6_HUMAN	CEP170	Centrosomal protein of 170 kDa (Fragment) OS=Homo sapiens GN=CEP170 PE=4 SV=1	172379	0	1174	0	0	0	0.03	#DIV/0!	0	0	0	0	0	1	0	2	0.665884
sp|Q9P2P5|HECW2_HUMAN	HECW2	E3 ubiquitin-protein ligase HECW2 OS=Homo sapiens GN=HECW2 PE=1 SV=2	176630	0	1173	0	25	0	0.02	#DIV/0!	0	0	0	1	0	1	0	1	0.665884
tr|B4DEM6|B4DEM6_HUMAN	TOE1	Target of EGR1 protein 1 OS=Homo sapiens GN=TOE1 PE=2 SV=1	48544	0	1172	0	25	0	0.07	#DIV/0!	0	0	0	1	0	1	0	1	0.665884
sp|Q9Y4W2|LAS1L_HUMAN	LAS1L	Ribosomal biogenesis protein LAS1L OS=Homo sapiens GN=LAS1L PE=1 SV=2	83982	0	1171	0	25	0	0.15	#DIV/0!	0	0	0	1	0	1	0	2	0.665884
tr|B2R694|B2R694_HUMAN	tr|B2R694	cDNA, FLJ92849, highly similar to Homo sapiens lanosterol synthase (2,3-oxidosqualene-lanosterol cyclase)	84568	0	1170	0	25	0	0.04	#DIV/0!	0	0	0	1	0	1	0	2	0.665884
tr|A8KAQ5|A8KAQ5_HUMAN	tr|A8KAQ5	cDNA FLJ77404, highly similar to Homo sapiens small nuclear ribonucleoprotein 70kDa polypeptide (RNP a	51522	0	1168	0	25	0	0.06	#DIV/0!	0	0	0	1	0	1	0	1	0.665884
tr|B3KPB8|B3KPB8_HUMAN	tr|B3KPB8	cDNA FLJ31563 fis, clone NT2RI2001449, highly similar to Homo sapiens stimulated by retinoic acid gene 6	74268	0	1163	0	25	0	0.04	#DIV/0!	0	0	0	1	0	1	0	1	0.665884
tr|B3KS26|B3KS26_HUMAN	tr|B3KS26	cDNA FLJ16813 fis, clone THYMU3030231, highly similar to Ribonucleoside-diphosphate reductase M2 sub	34818	0	1162	0	25	0	0.1	#DIV/0!	0	0	0	1	0	1	0	1	0.665884
tr|B2RWN5|B2RWN5_HUMAN	HEATR1	HEAT repeat containing 1 OS=Homo sapiens GN=HEATR1 PE=2 SV=1	244275	0	1160	0	26	0	0.02	#DIV/0!	0	1	0	1	0	1	0	1	0.665884
tr|H0YDX7|H0YDX7_HUMAN	HBS1L	HBS1-like protein (Fragment) OS=Homo sapiens GN=HBS1L PE=4 SV=1	61030	0	1158	0	26	0	0.05	#DIV/0!	0	0	0	1	0	1	0	1	0.665884
tr|Q1RMZ3|Q1RMZ3_HUMAN	NCOA6	NCOA6 protein (Fragment) OS=Homo sapiens GN=NCOA6 PE=2 SV=1	39584	0	1157	0	26	0	0.08	#DIV/0!	0	0	0	1	0	1	0	1	0.665884
tr|B4DN85|B4DN85_HUMAN	tr|B4DN85	E3 ubiquitin-protein ligase OS=Homo sapiens PE=2 SV=1	93815	0	1156	0	26	0	0.12	#DIV/0!	0	0	0	1	0	1	0	1	0.665884
sp|Q14677|EPN4_HUMAN	CLINT1	Clathrin interactor 1 OS=Homo sapiens GN=CLINT1 PE=1 SV=1	68273	0	1153	0	26	0	0.05	#DIV/0!	0	0	0	1	0	1	0	1	0.665884
sp|O15031|PLXB2_HUMAN	PLXNB2	Plexin-B2 OS=Homo sapiens GN=PLXNB2 PE=1 SV=3	207734	0	1150	0	26	0	0.02	#DIV/0!	0	0	0	1	0	1	0	2	0.665884
tr|A6NNK5|A6NNK5_HUMAN	TP53BP1	Tumor suppressor p53-binding protein 1 OS=Homo sapiens GN=TP53BP1 PE=2 SV=2	210886	0	1149	0	26	0	0.02	#DIV/0!	0	0	0	1	0	1	0	1	0.665884
tr|B4DUZ3|B4DUZ3_HUMAN	tr|B4DUZ3	cDNA FLJ51105, highly similar to Arfaptin-2 OS=Homo sapiens PE=2 SV=1	41783	0	1148	0	26	0	0.08	#DIV/0!	0	0	0	1	0	1	0	1	0.665884
tr|D6R968|D6R968_HUMAN	N4BP2L2	NEDD4-binding protein 2-like 2 (Fragment) OS=Homo sapiens GN=N4BP2L2 PE=2 SV=1	83058	0	1147	0	26	0	0.04	#DIV/0!	0	1	0	1	0	1	0	2	0.665884
sp|O60287|NPA1P_HUMAN	URB1	Nucleolar pre-ribosomal-associated protein 1 OS=Homo sapiens GN=URB1 PE=1 SV=4	256508	0	1146	0	26	0	0.01	#DIV/0!	0	1	0	1	0	1	0	2	0.665884
sp|P08F94|PKHD1_HUMAN	PKHD1	Fibrocystin OS=Homo sapiens GN=PKHD1 PE=1 SV=1	452120	0	1145	0	26	0	0.01	#DIV/0!	0	1	0	1	0	1	0	2	0.665884
tr|D6RAZ8|D6RAZ8_HUMAN	ANXA3	Annexin (Fragment) OS=Homo sapiens GN=ANXA3 PE=2 SV=1	11594	0	1144	0	26	0	0.3	#DIV/0!	0	0	0	1	0	1	0	2	0.665884
tr|B4DJ92|B4DJ92_HUMAN	tr|B4DJ92	cDNA FLJ57843, highly similar to Homo sapiens neurochondrin (NCDN), transcript variant 3, mRNA OS=Hor	20030	0	1143	0	26	0	0.17	#DIV/0!	0	0	0	1	0	1	0	1	0.665884
tr|H0Y9H2|H0Y9H2_HUMAN	ADD1	Alpha-adducin (Fragment) OS=Homo sapiens GN=ADD1 PE=4 SV=1	48831	0	1138	0	27	0	0.04	#DIV/0!	0	0	0	1	0	1	0	3	0.665884
sp|Q9NP92|RT30_HUMAN	MRPS30	28S ribosomal protein S30, mitochondrial OS=Homo sapiens GN=MRPS30 PE=1 SV=2	50960	0	1132	0	27	0	0.06	#DIV/0!	0	0	0	1	0	1	0	2	0.665884
tr|B7Z3P1|B7Z3P1_HUMAN	TBCE	Tubulin-specific chaperone E OS=Homo sapiens GN=TBCE PE=2 SV=1	65666	0	1131	0	27	0	0.05	#DIV/0!	0	0	0	1	0	1	0	1	0.665884
tr|H0Y9V7|H0Y9V7_HUMAN	ATP2C1	Calcium-transporting ATPase type 2C member 1 (Fragment) OS=Homo sapiens GN=ATP2C1 PE=3 SV=1	100184	0	1129	0	27	0	0.03	#DIV/0!	0	0	0	1	0	1	0	2	0.665884
tr|Q05CN7|Q05CN7_HUMAN	GTF3C4	GTF3C4 protein (Fragment) OS=Homo sapiens GN=GTF3C4 PE=2 SV=1	63439	0	1128	0	27	0	0.05	#DIV/0!	0	0	0	1	0	1	0	1	0.665884
tr|B7ZW05|B7ZW05_HUMAN	AKAP13	AKAP13 protein (Fragment) OS=Homo sapiens GN=AKAP13 PE=2 SV=1	124144	0	1127	0	27	0	0.01	#DIV/0!	0	0	0	1	0	1	0	2	0.665884
tr|H0Y2P0|H0Y2P0_HUMAN	CD44	CD44 antigen (Fragment) OS=Homo sapiens GN=CD44 PE=4 SV=1	31332	0	1125	0	28	0	0.11	#DIV/0!	0	0	0	1	0	1	0	1	0.665884
tr|F5H6D0|F5H6D0_HUMAN	MOGS	Mannosyl-oligosaccharide glucosidase OS=Homo sapiens GN=MOGS PE=2 SV=1	80824	0	1124	0	28	0	0.04	#DIV/0!	0	0	0	1	0	1	0	1	0.665884
tr|J3KSL7|J3KSL7_HUMAN	RECQL5	ATP-dependent DNA helicase Q5 (Fragment) OS=Homo sapiens GN=RECQL5 PE=4 SV=1	22648	0	1123	0	28	0	0.15	#DIV/0!	0	0	0	1	0	1	0	3	0.665884
tr|D6RAL1|D6RAL1_HUMAN	GFM2	Ribosome-releasing factor 2, mitochondrial (Fragment) OS=Homo sapiens GN=GFM2 PE=2 SV=1	32208	0	1122	0	28	0	0.1	#DIV/0!	0	0	0	1	0	1	0	1	0.665884
tr|C9JDR0|C9JDR0_HUMAN	NSDHL	Sterol-4-alpha-carboxylate 3-dehydrogenase, decarboxylating (Fragment) OS=Homo sapiens GN=NSDHL PE	28356	0	1121	0	28	0	0.12	#DIV/0!	0	0	0	1	0	1	0	1	0.665884
sp|Q9BVP2|GNL3_HUMAN	GNL3	Guanine nucleotide-binding protein-like 3 OS=Homo sapiens GN=GNL3 PE=1 SV=2	62468	0	1119	0	28	0	0.05	#DIV/0!	0	0	0	1	0	1	0	3	0.665884
tr|Q9BXV5|Q9BXV5_HUMAN	tr|Q9BXV5	PNAS-139 OS=Homo sapiens PE=2 SV=1	22952	0	1117	0	28	0	0.15	#DIV/0!	0	0	0	1	0	1	0	2	0.665884
tr|B2R806|B2R806_HUMAN	tr|B2R806	Eukaryotic translation initiation factor 3 subunit E OS=Homo sapiens PE=2 SV=1	52622	0	1115	0	28	0	0.13	#DIV/0!	0	1	0	2	0	1	0	2	0.665884
sp|Q14126|DSG2_HUMAN	DSG2	Desmoglein-2 OS=Homo sapiens GN=DSG2 PE=1 SV=2	123016	0	1112	0	29	0	0.03	#DIV/0!	0	0	0	1	0	1	0	2	0.665884
tr|G3V438|G3V438_HUMAN	AHSA1	Activator of 90 kDa heat shock protein ATPase homolog 1 (Fragment) OS=Homo sapiens GN=AHSA1 PE=2 S	23078	0	1109	0	29	0	0.14	#DIV/0!	0	0	0	1	0	1	0	1	0.665884
tr|E9PHM2|E9PHM2_HUMAN	LARS2	Probable leucine--tRNA ligase, mitochondrial OS=Homo sapiens GN=LARS2 PE=2 SV=1	97782	0	1103	0	30	0	0.03	#DIV/0!	0	0	0	1	0	1	0	2	0.665884
tr|H0Y843|H0Y843_HUMAN	EFCAB5	EF-hand calcium-binding domain-containing protein 5 (Fragment) OS=Homo sapiens GN=EFCAB5 PE=4 SV=	137592	0	1101	0	30	0	0.05	#DIV/0!	0	1	0	2	0	1	0	2	0.665884
tr|F8W805|F8W805_HUMAN	GRM1	Metabotropic glutamate receptor 1 OS=Homo sapiens GN=GRM1 PE=2 SV=1	103232	0	1100	0	30	0	0.03	#DIV/0!	0	1	0	1	0	1	0	1	0.665884
tr|B4DWV9|B4DWV9_HUMAN	tr|B4DWV	cDNA FLJ53108, highly similar to Guanine nucleotide-binding protein alpha-13 subunit OS=Homo sapiens P	41291	0	1099	0	30	0	0.07	#DIV/0!	0	0	0	1	0	1	0	1	0.665884
sp|P01137|TGFB1_HUMAN	TGFB1	Transforming growth factor beta-1 OS=Homo sapiens GN=TGFB1 PE=1 SV=2	44997	0	1097	0	30	0	0.07	#DIV/0!	0	0	0	1	0	1	0	1	0.665884
tr|G3V195|G3V195_HUMAN	EML3	Echinoderm microtubule associated protein like 3, isoform CRA_c OS=Homo sapiens GN=EML3 PE=2 SV=1	74262	0	1096	0	30	0	0.04	#DIV/0!	0	0	0	1	0	1	0	1	0.665884
tr|B4DGG1|B4DGG1_HUMAN	ITFG3	Protein ITFG3 OS=Homo sapiens GN=ITFG3 PE=2 SV=1	58252	0	1095	0	30	0	0.06	#DIV/0!	0	0	0	1	0	1	0	1	0.665884
tr|Q6FHF5|Q6FHF5_HUMAN	PCNA	Proliferating cell nuclear antigen (Fragment) OS=Homo sapiens GN=PCNA PE=2 SV=1	29029	0	1092	0	30	0	0.11	#DIV/0!	0	0	0	1	0	1	0	1	0.665884
tr|B4DGP9|B4DGP9_HUMAN	tr|B4DGP9	cDNA FLJ54102, highly similar to Beta-soluble NSF attachment protein OS=Homo sapiens PE=2 SV=1	29061	0	1091	0	30	0	0.11	#DIV/0!	0	0	0	1	0	1	0	1	0.665884
tr|Q6IPI1|Q6IPI1_HUMAN	RPL29	Ribosomal protein L29 OS=Homo sapiens GN=RPL29 PE=2 SV=1	17997	0	1090	0	30	0	0.19	#DIV/0!	0	0	0	1	0	1	0	1	0.665884
sp|Q9UNE7|CHIP_HUMAN	STUB1	E3 ubiquitin-protein ligase CHIP OS=Homo sapiens GN=STUB1 PE=1 SV=2	35290	0	1088	0	30	0	0.09	#DIV/0!	0	0	0	1	0	1	0	2	0.665884
tr|A1L172|A1L172_HUMAN	ACOT1	Acyl-CoA thioesterase 1 OS=Homo sapiens GN=ACOT1 PE=2 SV=1	46666	0	1087	0	30	0	0.07	#DIV/0!	0	0	0	1	0	1	0	1	0.665884
tr|A2A2L5|A2A2L5_HUMAN	C20orf43	Chromosome 20 open reading frame 43 (Fragment) OS=Homo sapiens GN=C20orf43 PE=4 SV=1	31759	0	1086	0	30	0	0.1	#DIV/0!	0	0	0	1	0	1	0	1	0.665884
tr|Q5DT20|Q5DT20_HUMAN	HRNR	Hornerin OS=Homo sapiens GN=HRNR PE=2 SV=1	283111	0	1085	0	30	0	0.01	#DIV/0!	0	0	0	1	0	1	0	1	0.665884
sp|Q6PD62|CTR9_HUMAN	CTR9	RNA polymerase-associated protein CTR9 homolog OS=Homo sapiens GN=CTR9 PE=1 SV=1	134332	0	1084	0	30	0	0.02	#DIV/0!	0	1	0	1	0	1	0	1	0.665884
tr|B4DEA3|B4DEA3_HUMAN	tr|B4DEA3	cDNA FLJ56531, highly similar to UV excision repair protein RAD23 homolog B OS=Homo sapiens PE=2 SV=	42340	0	1082	0	31	0	0.08	#DIV/0!	0	1	0	1	0	1	0	1	0.665884
tr|B7Z1V5|B7Z1V5_HUMAN	tr|B7Z1V5	cDNA FLJ55678, highly similar to Syntaxin-binding protein 1 OS=Homo sapiens PE=2 SV=1	63779	0	1081	0	31	0	0.05	#DIV/0!	0	0	0	1	0	1	0	2	0.665884
tr|B4DWS5|B4DWS5_HUMAN	tr|B4DWS	cDNA FLJ57639, highly similar to Brix domain-containing protein 1 OS=Homo sapiens PE=2 SV=1	29109	0	1080	0	31	0	0.11	#DIV/0!	0	0	0	1	0	1	0	1	0.665884
sp|O75131|CPNE3_HUMAN	CPNE3	Copine-3 OS=Homo sapiens GN=CPNE3 PE=1 SV=1	60947	0	1078	0	31	0	0.05	#DIV/0!	0	0	0	1	0	1	0	1	0.665884
tr|Q2TAM5|Q2TAM5_HUMAN	RELA	RELA protein OS=Homo sapiens GN=RELA PE=2 SV=1	43355	0	1075	0	31	0	0.08	#DIV/0!	0	0	0	1	0	1	0	1	0.665884
tr|B4DG47|B4DG47_HUMAN	tr|B4DG47	cDNA FLJ56967, highly similar to Adapter-relatedprotein complex 1 beta-1 subunit OS=Homo sapiens PE=2	54387	0	1074	0	31	0	0.06	#DIV/0!	0	0	0	1	0	1	0	2	0.665884
tr|B0QYA3|B0QYA3_HUMAN	EIF3D	Eukaryotic translation initiation factor 3 subunit D (Fragment) OS=Homo sapiens GN=EIF3D PE=4 SV=1	20111	0	1070	0	31	0	0.17	#DIV/0!	0	0	0	1	0	1	0	2	0.665884
tr|B4DRV3|B4DRV3_HUMAN	tr|B4DRV3	cDNA FLJ51537, highly similar to Myosin heavy chain, skeletal muscle, adult 2 (Fragment) OS=Homo sapier	96376	0	1069	0	31	0	0.03	#DIV/0!	0	0	0	1	0	1	0	2	0.665884
sp|Q9Y623|MYH4_HUMAN	MYH4	Myosin-4 OS=Homo sapiens GN=MYH4 PE=1 SV=2	223902	0	1068	0	31	0	0.01	#DIV/0!	0	0	0	1	0	1	0	2	0.665884
tr|B4DFX9|B4DFX9_HUMAN	tr|B4DFX9	cDNA FLJ51822, highly similar to Myosin-13 OS=Homo sapiens PE=2 SV=1	77256	0	1067	0	31	0	0.04	#DIV/0!	0	0	0	1	0	1	0	2	0.665884
tr|A5YM51|A5YM51_HUMAN	MYH7	MYH7 protein (Fragment) OS=Homo sapiens GN=MYH7 PE=2 SV=1	223684	0	1066	0	31	0	0.01	#DIV/0!	0	0	0	1	0	1	0	2	0.665884
tr|H7C0C0|H7C0C0_HUMAN	GTF3C3	General transcription factor 3C polypeptide 3 (Fragment) OS=Homo sapiens GN=GTF3C3 PE=4 SV=1	16723	0	1063	0	32	0	0.2	#DIV/0!	0	1	0	1	0	1	0	1	0.665884
tr|E9PPY3|E9PPY3_HUMAN	RRP8	Ribosomal RNA-processing protein 8 OS=Homo sapiens GN=RRP8 PE=2 SV=1	34579	0	1062	0	32	0	0.1	#DIV/0!	0	0	0	1	0	1	0	1	0.665884
tr|B4DKA8|B4DKA8_HUMAN	USP8	Ubiquitin carboxyl-terminal hydrolase OS=Homo sapiens GN=USP8 PE=2 SV=1	115921	0	1060	0	32	0	0.03	#DIV/0!	0	0	0	1	0	1	0	3	0.665884
sp|Q6NVY1|HIBCH_HUMAN	HIBCH	3-hydroxyisobutyryl-CoA hydrolase, mitochondrial OS=Homo sapiens GN=HIBCH PE=1 SV=2	43797	0	1059	0	32	0	0.08	#DIV/0!	0	0	0	1	0	1	0	1	0.665884
tr|J3KN41|J3KN41_HUMAN	ITGA6	Integrin alpha-6 heavy chain OS=Homo sapiens GN=ITGA6 PE=3 SV=1	127724	0	1057	0	32	0	0.03	#DIV/0!	0	0	0	1	0	1	0	1	0.665884
tr|B4DEM8|B4DEM8_HUMAN	tr|B4DEM	cDNA FLJ50118, highly similar to Splicing factor, arginine/serine-rich 4 OS=Homo sapiens PE=2 SV=1	18388	0	1056	0	32	0	0.18	#DIV/0!	0	0	0	1	0	1	0	1	0.665884
tr|B4DLU2|B4DLU2_HUMAN	tr|B4DLU2	cDNA FLJ56496, highly similar to Integrin alpha-IIb OS=Homo sapiens PE=2 SV=1	56966	0	1055	0	32	0	0.06	#DIV/0!	0	0	0	1	0	1	0	1	0.665884
tr|A0JLU4|A0JLU4_HUMAN	YBX1	YBX1 protein (Fragment) OS=Homo sapiens GN=YBX1 PE=2 SV=1	21417	0	1053	0	32	0	0.16	#DIV/0!	0	1	0	1	0	1	0	1	0.665884
tr|B4DEP6|B4DEP6_HUMAN	tr|B4DEP6	cDNA FLJ59206, highly similar to Eukaryotic translation initiation factor 4B OS=Homo sapiens PE=2 SV=1	66146	0	1052	0	32	0	0.05	#DIV/0!	0	0	0	1	0	1	0	1	0.665884
sp|Q6YHK3|CD109_HUMAN	CD109	CD109 antigen OS=Homo sapiens GN=CD109 PE=1 SV=2	162500	0	1051	0	32	0	0.02	#DIV/0!	0	1	0	1	0	1	0	1	0.665884
tr|B0AZM4|B0AZM4_HUMAN	tr|B0AZM	cDNA, FLJ79464, highly similar to Homo sapiens aquarius (Fragment) OS=Homo sapiens PE=2 SV=1	157764	0	1049	0	32	0	0.02	#DIV/0!	0	0	0	1	0	1	0	1	0.665884
tr|D3DVR8|D3DVR8_HUMAN	HD	Huntingtin (Huntington disease), isoform CRA_a OS=Homo sapiens GN=HD PE=2 SV=1	291559	0	1048	0	32	0	0.01	#DIV/0!	0	0	0	1	0	1	0	2	0.665884
tr|B4DI28|B4DI28_HUMAN	PTBP2	Polypyrimidine tract-binding protein 2 OS=Homo sapiens GN=PTBP2 PE=2 SV=1	34947	0	1047	0	32	0	0.09	#DIV/0!	0	0	0	1	0	1	0	1	0.665884
tr|H0YCY6|H0YCY6_HUMAN	DAK	FAD-AMP lyase (cyclizing) (Fragment) OS=Homo sapiens GN=DAK PE=4 SV=1	55026	0	1046	0	32	0	0.06	#DIV/0!	0	0	0	1	0	1	0	1	0.665884
tr|F5H315|F5H315_HUMAN	XAB2	Pre-mRNA-splicing factor SYF1 OS=Homo sapiens GN=XAB2 PE=2 SV=1	100416	0	1045	0	32	0	0.03	#DIV/0!	0	1	0	1	0	1	0	1	0.665884
sp|P05556|ITB1_HUMAN	ITGB1	Integrin beta-1 OS=Homo sapiens GN=ITGB1 PE=1 SV=2	91664	0	1044	0	33	0	0.22	#DIV/0!	0	1	0	1	0	1	0	1	0.665884
tr|E9PC74|E9PC74_HUMAN	EIF2B5	Translation initiation factor eIF-2B subunit epsilon OS=Homo sapiens GN=EIF2B5 PE=2 SV=1	79096	0	1040	0	33	0	0.04	#DIV/0!	0	1	0	1	0	1	0	1	0.665884
tr|J3KQB0|J3KQB0_HUMAN	THUMPD1	THUMP domain-containing protein 1 OS=Homo sapiens GN=THUMPD1 PE=4 SV=1	49032	0	1038	0	33	0	0.07	#DIV/0!	0	0	0	1	0	1	0	1	0.665884
tr|B2R6G5|B2R6G5_HUMAN	tr|B2R6G5	cDNA, FLJ92942, highly similar to Homo sapiens T-box, brain, 1 (TBR1), mRNA OS=Homo sapiens PE=2 SV=1	74530	0	1037	0	33	0	0.04	#DIV/0!	0	0	0	1	0	1	0	1	0.665884
sp|Q9Y266|NUDC_HUMAN	NUDC	Nuclear migration protein nudC OS=Homo sapiens GN=NUDC PE=1 SV=1	38276	0	1036	0	33	0	0.09	#DIV/0!	0	0	0	1	0	1	0	1	0.665884
tr|B2R6E2|B2R6E2_HUMAN	tr|B2R6E2	cDNA, FLJ92910, highly similar to Homo sapiens programmed cell death 4 (neoplastic transformation inhib	51998	0	1035	0	33	0	0.06	#DIV/0!	0	0	0	1	0	1	0	1	0.665884
tr|B4DN01|B4DN01_HUMAN	tr|B4DN01	cDNA FLJ57332, highly similar to COP9 signalosome complex subunit 3 OS=Homo sapiens PE=2 SV=1	33950	0	1034	0	33	0	0.1	#DIV/0!	0	0	0	1	0	1	0	1	0.665884
tr|Q13380|Q13380_HUMAN	RBM	RNA binding motif (Fragment) OS=Homo sapiens GN=RBM PE=4 SV=1	4015	0	1033	0	33	0	0.97	#DIV/0!	0	1	0	1	0	1	0	1	0.665884
tr|I0CMJ8|I0CMJ8_HUMAN	PRIM2	DNA primase large subunit (Fragment) OS=Homo sapiens GN=PRIM2 PE=4 SV=1	5179	0	1032	0	33	0	0.7	#DIV/0!	0	0	0	1	0	1	0	1	0.665884
tr|H0YL71|H0YL71_HUMAN	SMAD3	Mothers against decapentaplegic homolog 3 (Fragment) OS=Homo sapiens GN=SMAD3 PE=4 SV=1	10544	0	1031	0	33	0	0.33	#DIV/0!	0	1	0	1	0	1	0	1	0.665884
tr|B4DKE6|B4DKE6_HUMAN	tr|B4DKE6	cDNA FLJ60629, highly similar to Replication factor C subunit 3 OS=Homo sapiens PE=2 SV=1	35551	0	1029	0	34	0	0.09	#DIV/0!	0	0	0	1	0	1	0	1	0.665884
tr|B3KPF2|B3KPF2_HUMAN	tr|B3KPF2	cDNA FLJ31717 fis, clone NT2RI2006640, highly similar to Dynamin-3 (EC 3.6.5.5) OS=Homo sapiens PE=2 S	63057	0	1026	0	34	0	0.05	#DIV/0!	0	0	0	1	0	1	0	1	0.665884
tr|Q7Z4G3|Q7Z4G3_HUMAN	tr|Q7Z4G3	IL1RAPL1-dystrophin fusion protein (Fragment) OS=Homo sapiens PE=2 SV=1	16074	0	1023	0	34	0	0.23	#DIV/0!	0	1	0	1	0	1	0	1	0.665884
tr|L0R5D0|L0R5D0_HUMAN	SRGAP1	Alternative protein SRGAP1 OS=Homo sapiens GN=SRGAP1 PE=4 SV=1	9510	0	1022	0	34	0	0.36	#DIV/0!	0	1	0	1	0	1	0	1	0.665884
tr|B3KW56|B3KW56_HUMAN	EIF3S6	Eukaryotic translation initiation factor 3 subunit E OS=Homo sapiens GN=EIF3S6 PE=2 SV=1	46815	0	1019	0	34	0	0.19	#DIV/0!	0	1	0	1	0	1	0	1	0.665884
tr|A0AV56|A0AV56_HUMAN	SAFB	SAFB protein OS=Homo sapiens GN=SAFB PE=2 SV=1	103249	0	1017	0	34	0	0.03	#DIV/0!	0	0	0	1	0	1	0	1	0.665884
tr|B2RAH7|B2RAH7_HUMAN	tr|B2RAH7	cDNA, FLJ94921, highly similar to Homo sapiens prolyl endopeptidase (PREP), mRNA OS=Homo sapiens PE=	81594	0	1015	0	34	0	0.04	#DIV/0!	0	1	0	1	0	1	0	2	0.665884
tr|Q6MZF7|Q6MZF7_HUMAN	DKFZp686	Putative uncharacterized protein DKFZp686E11248 (Fragment) OS=Homo sapiens GN=DKFZp686E11248 PE	29013	0	1014	0	34	0	0.11	#DIV/0!	0	1	0	1	0	1	0	1	0.665884
tr|H0Y8H0|H0Y8H0_HUMAN	DDX60L	Probable ATP-dependent RNA helicase DDX60-like (Fragment) OS=Homo sapiens GN=DDX60L PE=4 SV=1	102233	0	1013	0	34	0	0.03	#DIV/0!	0	1	0	1	0	1	0	2	0.665884
tr|Q59FE8|Q59FE8_HUMAN	tr|Q59FE8	Epithelial protein lost in neoplasm beta variant (Fragment) OS=Homo sapiens PE=2 SV=1	86510	0	1012	0	34	0	0.06	#DIV/0!	0	0	0	1	0	1	0	2	0.665884
sp|O60343|TBCD4_HUMAN	TBC1D4	TBC1 domain family member 4 OS=Homo sapiens GN=TBC1D4 PE=1 SV=2	148068	0	1010	0	35	0	0.02	#DIV/0!	0	0	0	1	0	1	0	2	0.665884
tr|B2RDG1|B2RDG1_HUMAN	tr|B2RDG1	cDNA, FLJ96593 OS=Homo sapiens PE=2 SV=1	59687	0	1009	0	35	0	0.06	#DIV/0!	0	0	0	1	0	1	0	1	0.665884
tr|B4DQF3|B4DQF3_HUMAN	TARS2	Threonine--tRNA ligase, mitochondrial OS=Homo sapiens GN=TARS2 PE=2 SV=1	18740	0	1008	0	32	0	0.08	#DIV/0!	0	0	0	1	0	1	0	1	0.665884
tr|H0Y9G6|H0Y9G6_HUMAN	MRPL3	39S ribosomal protein L3, mitochondrial (Fragment) OS=Homo sapiens GN=MRPL3 PE=3 SV=1	40898	0	1006	0	35	0	0.08	#DIV/0!	0	1	0	1	0	1	0	1	0.665884
tr|K7ERT7|K7ERT7_HUMAN	VAT1	Synaptic vesicle membrane protein VAT-1 homolog (Fragment) OS=Homo sapiens GN=VAT1 PE=4 SV=1	15164	0	1005	0	35	0	0.22	#DIV/0!	0	0	0	1	0	1	0	1	0.665884
tr|H7C0M3|H7C0M3_HUMAN	BPHL	Valacyclovir hydrolase (Fragment) OS=Homo sapiens GN=BPHL PE=4 SV=1	3314	0	1004	0	35	0	1.2	#DIV/0!	0	1	0	1	0	1	0	1	0.665884
tr|G3V529|G3V529_HUMAN	DDX24	ATP-dependent RNA helicase DDX24 OS=Homo sapiens GN=DDX24 PE=2 SV=1	91994	0	1003	0	35	0	0.04	#DIV/0!	0	0	0	1	0	1	0	1	0.665884
tr|A8JZZ8|A8JZZ8_HUMAN	tr|A8JZZ8	cDNA FLJ77826, highly similar to Homo sapiens BCS1-like (yeast), mRNA OS=Homo sapiens PE=2 SV=1	47692	0	1000	0	35	0	0.07	#DIV/0!	0	1	0	1	0	1	0	1	0.665884
tr|B4E2S2|B4E2S2_HUMAN	tr|B4E2S2	cDNA FLJ61556, highly similar to Poly (ADP-ribose) polymerase 4 (EC 2.4.2.30) OS=Homo sapiens PE=2 SV=	113804	0	999	0	35	0	0.03	#DIV/0!	0	0	0	1	0	1	0	1	0.665884
sp|Q0VDF9|HSP7E_HUMAN	HSPA14	Heat shock 70 kDa protein 14 OS=Homo sapiens GN=HSPA14 PE=1 SV=1	55444	0	998	0	35	0	0.06	#DIV/0!	0	0	0	1	0	1	0	1	0.665884
sp|P49756|RBM25_HUMAN	RBM25	RNA-binding protein 25 OS=Homo sapiens GN=RBM25 PE=1 SV=3	100467	0	994	0	35	0	0.03	#DIV/0!	0	0	0	1	0	1	0	2	0.665884
sp|Q9NQH7|XPP3_HUMAN	XPNPEP3	Probable Xaa-Pro aminopeptidase 3 OS=Homo sapiens GN=XPNPEP3 PE=1 SV=1	57624	0	993	0	35	0	0.06	#DIV/0!	0	1	0	1	0	1	0	1	0.665884
tr|A8K9B9|A8K9B9_HUMAN	tr|A8K9B9	cDNA FLJ77391, highly similar to Homo sapiens EH-domain containing 4 (EHD4), mRNA OS=Homo sapiens	61346	0	992	0	35	0	0.05	#DIV/0!	0	0	0	1	0	1	0	2	0.665884
tr|G5EA03|G5EA03_HUMAN	LIMCH1	LIM and calponin homology domains-containing protein 1 OS=Homo sapiens GN=LIMCH1 PE=2 SV=1	165820	0	991	0	24	0	0.06	#DIV/0!	0	0	0	1	0	1	0	1	0.665884
tr|A6NN60|A6NN60_HUMAN	CRYZ	Quinone oxidoreductase OS=Homo sapiens GN=CRYZ PE=2 SV=1	31680	0	990	0	36	0	0.11	#DIV/0!	0	0	0	1	0	1	0	1	0.665884
tr|G3V1C3|G3V1C3_HUMAN	API5	Apoptosis inhibitor 5 OS=Homo sapiens GN=API5 PE=2 SV=1	57867	0	983	0	36	0	0.06	#DIV/0!	0	0	0	1	0	1	0	1	0.665884
sp|Q8WX92|NELFB_HUMAN	COBRA1	Negative elongation factor B OS=Homo sapiens GN=COBRA1 PE=1 SV=1	66283	0	982	0	36	0	0.05	#DIV/0!	0	1	0	1	0	1	0	1	0.665884
tr|G1UI17|G1UI17_HUMAN	AGL	Glycogen debranching enzyme (Fragment) OS=Homo sapiens GN=AGL PE=2 SV=1	145569	0	980	0	36	0	0.02	#DIV/0!	0	1	0	1	0	1	0	1	0.665884
sp|Q9NXF1|TEX10_HUMAN	TEX10	Testis-expressed sequence 10 protein OS=Homo sapiens GN=TEX10 PE=1 SV=2	106349	0	978	0	36	0	0.2	#DIV/0!	0	1	0	1	0	1	0	1	0.665884
tr|B2MV13|B2MV13_HUMAN	LTF	Lactoferrin OS=Homo sapiens GN=LTF PE=2 SV=1	80228	0	975	0	36	0	0.04	#DIV/0!	0	1	0	1	0	1	0	1	0.665884
sp|P16152|CBR1_HUMAN	CBR1	Carbonyl reductase [NADPH] 1 OS=Homo sapiens GN=CBR1 PE=1 SV=3	30641	0	973	0	36	0	0.11	#DIV/0!	0	1	0	1	0	1	0	2	0.665884
tr|I6L9B1|I6L9B1_HUMAN	EPB41L2	EPB41L2 protein OS=Homo sapiens GN=EPB41L2 PE=2 SV=1	71998	0	965	0	37	0	0.05	#DIV/0!	0	0	0	1	0	1	0	1	0.665884
tr|B4DVN5|B4DVN5_HUMAN	FHOD1	FH1/FH2 domain-containing protein 1 OS=Homo sapiens GN=FHOD1 PE=2 SV=1	81910	0	964	0	37	0	0.04	#DIV/0!	0	1	0	1	0	1	0	1	0.665884
tr|B3KR24|B3KR24_HUMAN	tr|B3KR24	cDNA FLJ33515 fis, clone BRAMY2005662, highly similar to Methylcrotonoyl-CoA carboxylase beta chain, n	25101	0	961	0	37	0	0.13	#DIV/0!	0	1	0	1	0	1	0	1	0.665884
tr|H7C2R4|H7C2R4_HUMAN	DIEXF	Digestive organ expansion factor homolog (Fragment) OS=Homo sapiens GN=DIEXF PE=4 SV=1	28503	0	959	0	37	0	0.04	#DIV/0!	0	1	0	1	0	1	0	1	0.665884
tr|B4DYZ7|B4DYZ7_HUMAN	tr|B4DYZ7	cDNA FLJ54316, highly similar to DNA polymerase subunit delta 3 OS=Homo sapiens PE=2 SV=1	51595	0	958	0	37	0	0.06	#DIV/0!	0	1	0	1	0	1	0	1	0.665884
tr|B3KY11|B3KY11_HUMAN	tr|B3KY11	cDNA FLJ46571 fis, clone THYMU3041428, highly similar to Probable ATP-dependent RNA helicase DDX23 (	93519	0	955	0	37	0	0.04	#DIV/0!	0	1	0	1	0	1	0	2	0.665884
sp|O14776|TCRG1_HUMAN	TCERG1	Transcription elongation regulator 1 OS=Homo sapiens GN=TCERG1 PE=1 SV=2	124051	0	952	0	37	0	0.03	#DIV/0!	0	1	0	1	0	1	0	2	0.665884

tr|A4D1I0|A4D1I0_HUMAN	PPP1R9A	HCG1741805, isoform CRA_c OS=Homo sapiens GN=PPP1R9A PE=2 SV=1	148567	0	951	0	38	0	0.02	#DIV/0!	0	1	0	1	0	1	0	2	0.665884
tr|G3V1S7|G3V1S7_HUMAN	HAS1	Hyaluronan synthase 1 OS=Homo sapiens GN=HAS1 PE=2 SV=1	65517	0	947	0	38	0	0.05	#DIV/0!	0	1	0	1	0	1	0	2	0.665884
tr|K7EKI4|K7EKI4_HUMAN	MRPL4	39S ribosomal protein L4, mitochondrial OS=Homo sapiens GN=MRPL4 PE=4 SV=1	14161	0	946	0	38	0	0.24	#DIV/0!	0	1	0	1	0	1	0	1	0.665884
tr|B3KUI0|B3KUI0_HUMAN	tr|B3KUI0	cDNA FLJ39945 fis, clone SPLEN2023977, highly similar to Homo sapiens CD97 antigen (CD97), transcript va	79803	0	945	0	38	0	0.04	#DIV/0!	0	1	0	1	0	1	0	1	0.665884
tr|K7EPI4|K7EPI4_HUMAN	GFAP	Glial fibrillary acidic protein (Fragment) OS=Homo sapiens GN=GFAP PE=4 SV=1	7120	0	944	0	38	0	0.5	#DIV/0!	0	1	0	1	0	1	0	1	0.665884
tr|B4DQ08|B4DQ08_HUMAN	tr|B4DQ0	cDNA FLJ53406, highly similar to U3 small nucleolar RNA-associated protein 14 homolog A OS=Homo sapie	82068	0	942	0	38	0	0.04	#DIV/0!	0	1	0	1	0	1	0	1	0.665884
tr|B3KUG0|B3KUG0_HUMAN	tr|B3KUG	cDNA FLJ39778 fis, clone SPLEN2001710, highly similar to Kelch domain-containing protein 4 OS=Homo sa	27277	0	941	0	38	0	0.12	#DIV/0!	0	1	0	1	0	1	0	1	0.665884
tr|B2RA56|B2RA56_HUMAN	tr|B2RA56	cDNA, FLJ94705, highly similar to Homo sapiens nicalin (LOC56926), mRNA OS=Homo sapiens PE=2 SV=1	61891	0	940	0	38	0	0.05	#DIV/0!	0	1	0	1	0	1	0	2	0.665884
tr|Q6IAM0|Q6IAM0_HUMAN	EIF3S4	Eukaryotic translation initiation factor 3 subunit G OS=Homo sapiens GN=EIF3S4 PE=2 SV=1	35864	0	939	0	38	0	0.09	#DIV/0!	0	1	0	1	0	1	0	1	0.665884
tr|A8K8Z4|A8K8Z4_HUMAN	tr|A8K8Z4	cDNA FLJ78071, highly similar to Human MHC class III complement component C6 mRNA OS=Homo sapien	108298	0	937	0	38	0	0.03	#DIV/0!	0	1	0	1	0	1	0	1	0.665884
tr|A8KA19|A8KA19_HUMAN	tr|A8KA19	cDNA FLJ75831, highly similar to Homo sapiens exportin, tRNA (nuclear export receptor for tRNAs) (XPOT),	111104	0	936	0	38	0	0.03	#DIV/0!	0	1	0	1	0	1	0	1	0.665884
tr|Q5W7F7|Q5W7F7_HUMAN	UBE3A	Ubiquitin ligase E3A isoform 1 OS=Homo sapiens GN=UBE3A PE=2 SV=1	98751	0	935	0	38	0	0.03	#DIV/0!	0	1	0	1	0	1	0	2	0.665884
tr|B4E0N6|B4E0N6_HUMAN	tr|B4E0N6	cDNA FLJ56280, highly similar to Endoplasmic reticulum-Golgi intermediate compartment protein 1 OS=Ho	26584	0	929	0	39	0	0.18	#DIV/0!	0	1	0	1	0	1	0	1	0.665884
tr|F5H740|F5H740_HUMAN	VDAC3	Voltage-dependent anion-selective channel protein 3 OS=Homo sapiens GN=VDAC3 PE=2 SV=1	31080	0	928	0	39	0	0.11	#DIV/0!	0	1	0	1	0	1	0	1	0.665884
tr|J3KRI4|J3KRI4_HUMAN	DYNC1LI2	Cytoplasmic dynein 1 light intermediate chain 2 (Fragment) OS=Homo sapiens GN=DYNC1LI2 PE=4 SV=1	17767	0	925	0	39	0	0.19	#DIV/0!	0	1	0	1	0	1	0	1	0.665884
tr|B4DL14|B4DL14_HUMAN	ATP5C1	ATP synthase gamma chain OS=Homo sapiens GN=ATP5C1 PE=2 SV=1	27552	0	924	0	39	0	0.26	#DIV/0!	0	1	0	2	0	1	0	2	0.665884
sp|Q92917|GPKOW_HUMAN	GPKOW	G patch domain and KOW motifs-containing protein OS=Homo sapiens GN=GPKOW PE=1 SV=2	52425	0	923	0	39	0	0.06	#DIV/0!	0	1	0	1	0	1	0	1	0.665884
tr|B3KM40|B3KM40_HUMAN	tr|B3KM4	cDNA FLJ10185 fis, clone HEMBA1004509, highly similar to U4/U6.U5 tri-snRNP-associated protein 2 OS=H	56618	0	922	0	39	0	0.06	#DIV/0!	0	1	0	1	0	1	0	1	0.665884
tr|E9PM46|E9PM46_HUMAN	USP47	Ubiquitin carboxyl-terminal hydrolase OS=Homo sapiens GN=USP47 PE=2 SV=1	155912	0	921	0	40	0	0.02	#DIV/0!	0	1	0	1	0	1	0	1	0.665884
tr|A8K8X0|A8K8X0_HUMAN	tr|A8K8X0	cDNA FLJ75187, highly similar to Homo sapiens nap1 P120 OS=Homo sapiens PE=2 SV=1	113035	0	920	0	40	0	0.03	#DIV/0!	0	1	0	1	0	1	0	1	0.665884
tr|H7C4G8|H7C4G8_HUMAN	NT5DC2	5~-nucleotidase domain-containing protein 2 (Fragment) OS=Homo sapiens GN=NT5DC2 PE=4 SV=1	30207	0	919	0	40	0	0.05	#DIV/0!	0	1	0	1	0	1	0	1	0.665884
sp|Q9UHX1|PUF60_HUMAN	PUF60	Poly(U)-binding-splicing factor PUF60 OS=Homo sapiens GN=PUF60 PE=1 SV=1	60009	0	908	0	40	0	0.05	#DIV/0!	0	1	0	1	0	1	0	1	0.665884
tr|Q6ZNZ8|Q6ZNZ8_HUMAN	tr|Q6ZNZ8	cDNA FLJ26816 fis, clone PRS06437 OS=Homo sapiens PE=2 SV=1	54459	0	907	0	41	0	0.06	#DIV/0!	0	1	0	1	0	1	0	1	0.665884
tr|E9PJ81|E9PJ81_HUMAN	UBXN1	UBX domain-containing protein 1 (Fragment) OS=Homo sapiens GN=UBXN1 PE=2 SV=1	32649	0	905	0	41	0	0.15	#DIV/0!	0	1	0	1	0	1	0	1	0.665884
tr|B2R6E5|B2R6E5_HUMAN	tr|B2R6E5	NADPH--cytochrome P450 reductase OS=Homo sapiens PE=2 SV=1	77429	0	900	0	42	0	0.04	#DIV/0!	0	1	0	1	0	1	0	2	0.665884
sp|Q9GZL7|WDR12_HUMAN	WDR12	Ribosome biogenesis protein WDR12 OS=Homo sapiens GN=WDR12 PE=1 SV=2	48191	0	896	0	42	0	0.07	#DIV/0!	0	1	0	1	0	1	0	1	0.665884
tr|Q96LK9|Q96LK9_HUMAN	tr|Q96LK9	cDNA FLJ25413 fis, clone TST03402, highly similar to 3-OXO-5-BETA-STEROID 4-DEHYDROGENASE (EC 1.3.9	16911	0	895	0	42	0	0.2	#DIV/0!	0	1	0	1	0	1	0	1	0.665884
tr|B4E3J7|B4E3J7_HUMAN	tr|B4E3J7	cDNA FLJ51203, highly similar to Thrombospondin-1 OS=Homo sapiens PE=2 SV=1	124032	0	892	0	42	0	0.03	#DIV/0!	0	1	0	1	0	1	0	1	0.665884
tr|D6RGG3|D6RGG3_HUMAN	COL12A1	Collagen alpha-1(XII) chain OS=Homo sapiens GN=COL12A1 PE=2 SV=1	334193	0	891	0	42	0	0.01	#DIV/0!	0	1	0	1	0	1	0	2	0.665884
tr|Q2TU84|Q2TU84_HUMAN	GIG18	Aspartate aminotransferase OS=Homo sapiens GN=GIG18 PE=2 SV=1	46519	0	889	0	42	0	0.07	#DIV/0!	0	1	0	1	0	1	0	2	0.665884
sp|Q86XP3|DDX42_HUMAN	DDX42	ATP-dependent RNA helicase DDX42 OS=Homo sapiens GN=DDX42 PE=1 SV=1	103197	0	888	0	42	0	0.05	#DIV/0!	0	1	0	1	0	1	0	2	0.665884
tr|J3KR12|J3KR12_HUMAN	tr|J3KR12	Uncharacterized protein OS=Homo sapiens PE=4 SV=1	36981	0	887	0	42	0	0.09	#DIV/0!	0	1	0	1	0	1	0	1	0.665884
tr|G3V2E7|G3V2E7_HUMAN	KLC1	Kinesin light chain 1 OS=Homo sapiens GN=KLC1 PE=2 SV=1	63133	0	886	0	42	0	0.11	#DIV/0!	0	1	0	1	0	1	0	1	0.665884
sp|Q96HU8|DIRA2_HUMAN	DIRAS2	GTP-binding protein Di-Ras2 OS=Homo sapiens GN=DIRAS2 PE=1 SV=1	22813	0	884	0	43	0	0.15	#DIV/0!	0	1	0	1	0	1	0	1	0.665884
tr|B3GQE6|B3GQE6_HUMAN	DDX27	DEAD box polypeptide 27 OS=Homo sapiens GN=DDX27 PE=2 SV=1	86980	0	879	0	43	0	0.04	#DIV/0!	0	1	0	1	0	1	0	1	0.665884
tr|A4QN18|A4QN18_HUMAN	SEC16A	SEC16A protein (Fragment) OS=Homo sapiens GN=SEC16A PE=2 SV=1	212198	0	877	0	43	0	0.01	#DIV/0!	0	1	0	1	0	1	0	1	0.665884
sp|Q03169|TNAP2_HUMAN	TNFAIP2	Tumor necrosis factor alpha-induced protein 2 OS=Homo sapiens GN=TNFAIP2 PE=1 SV=2	73015	0	869	0	44	0	0.04	#DIV/0!	0	1	0	1	0	1	0	1	0.665884
tr|F5H1X8|F5H1X8_HUMAN	LRBA	Lipopolysaccharide-responsive and beige-like anchor protein OS=Homo sapiens GN=LRBA PE=2 SV=1	320211	0	867	0	44	0	0.22	#DIV/0!	0	1	0	1	0	1	0	1	0.665884
sp|P23368|MAOM_HUMAN	ME2	NAD-dependent malic enzyme, mitochondrial OS=Homo sapiens GN=ME2 PE=1 SV=1	66029	0	865	0	44	0	0.05	#DIV/0!	0	1	0	1	0	1	0	1	0.665884
sp|Q7Z3S7|CA2D4_HUMAN	CACNA2D4	Voltage-dependent calcium channel subunit alpha-2/delta-4 OS=Homo sapiens GN=CACNA2D4 PE=1 SV=2	129168	0	864	0	44	0	0.03	#DIV/0!	0	1	0	1	0	1	0	1	0.665884
sp|Q9BTE3|MCMBP_HUMAN	MCMBP	Mini-chromosome maintenance complex-binding protein OS=Homo sapiens GN=MCMBP PE=1 SV=2	73789	0	862	0	44	0	0.04	#DIV/0!	0	1	0	1	0	1	0	1	0.665884
tr|G3V1P5|G3V1P5_HUMAN	MED15	Mediator of RNA polymerase II transcription subunit 15 OS=Homo sapiens GN=MED15 PE=4 SV=1	80007	0	861	0	44	0	0.04	#DIV/0!	0	1	0	1	0	1	0	1	0.665884
tr|Q8N6B4|Q8N6B4_HUMAN	MLL/CALM	Myeloid/lymphoid or mixed-lineage leukemia/clathrin assembly protein fusion protein (Fragment) OS=Hor	63408	0	859	0	44	0	0.05	#DIV/0!	0	1	0	1	0	1	0	1	0.665884
sp|Q9NUQ9|FA49B_HUMAN	FAM49B	Protein FAM49B OS=Homo sapiens GN=FAM49B PE=1 SV=1	37010	0	858	0	44	0	0.09	#DIV/0!	0	1	0	1	0	1	0	2	0.665884
tr|B4DFG0|B4DFG0_HUMAN	DEK	Protein DEK OS=Homo sapiens GN=DEK PE=2 SV=1	39704	0	856	0	44	0	0.08	#DIV/0!	0	1	0	1	0	1	0	1	0.665884
sp|P04181|OAT_HUMAN	OAT	Ornithine aminotransferase, mitochondrial OS=Homo sapiens GN=OAT PE=1 SV=1	48846	0	855	0	44	0	0.07	#DIV/0!	0	1	0	1	0	1	0	3	0.665884
tr|Q6ZME7|Q6ZME7_HUMAN	tr|Q6ZME	cDNA FLJ23972 fis, clone HEP18230, highly similar to 65 kDa FK506-binding protein (EC 5.2.1.8) OS=Homo	60053	0	848	0	45	0	0.05	#DIV/0!	0	1	0	1	0	1	0	1	0.665884
tr|E5RHC5|E5RHC5_HUMAN	ARFGAP1	ADP-ribosylation factor GTPase-activating protein 1 (Fragment) OS=Homo sapiens GN=ARFGAP1 PE=2 SV=	29395	0	847	0	45	0	0.11	#DIV/0!	0	1	0	1	0	1	0	1	0.665884
tr|A0JNT2|A0JNT2_HUMAN	KRT83	KRT83 protein OS=Homo sapiens GN=KRT83 PE=2 SV=1	50978	0	846	0	45	0	0.06	#DIV/0!	0	1	0	1	0	1	0	1	0.665884
tr|E7EVL6|E7EVL6_HUMAN	REPIN1	Replication initiator 1 (Fragment) OS=Homo sapiens GN=REPIN1 PE=2 SV=1	16586	0	837	0	45	0	0.2	#DIV/0!	0	1	0	1	0	1	0	1	0.665884
sp|Q96JM3|CHAP1_HUMAN	CHAMP1	Chromosome alignment-maintaining phosphoprotein 1 OS=Homo sapiens GN=CHAMP1 PE=1 SV=2	90012	0	836	0	45	0	0.04	#DIV/0!	0	1	0	1	0	1	0	1	0.665884
tr|B7Z556|B7Z556_HUMAN	tr|B7Z556	cDNA FLJ56822, highly similar to Alpha-2-HS-glycoprotein OS=Homo sapiens PE=2 SV=1	27850	0	833	0	46	0	0.12	#DIV/0!	0	1	0	1	0	1	0	1	0.665884
tr|B4DLG2|B4DLG2_HUMAN	tr|B4DLG2	cDNA FLJ58196, highly similar to Zinc finger CCCH domain-containing protein 11A OS=Homo sapiens PE=2	83177	0	829	0	46	0	0.04	#DIV/0!	0	1	0	1	0	1	0	1	0.665884
tr|B2R704|B2R704_HUMAN	tr|B2R704	cDNA, FLJ93207, highly similar to Homo sapiens microtubule-associated protein 7 (MAP7), mRNA OS=Hom	84046	0	826	0	46	0	0.04	#DIV/0!	0	1	0	1	0	1	0	2	0.665884
tr|Q5LJA0|Q5LJA0_HUMAN	TROVE2	60 kDa SS-A/Ro ribonucleoprotein OS=Homo sapiens GN=TROVE2 PE=4 SV=1	59916	0	825	0	46	0	0.05	#DIV/0!	0	1	0	1	0	1	0	1	0.665884
tr|J3KQ32|J3KQ32_HUMAN	OLA1	Obg-like ATPase 1 OS=Homo sapiens GN=OLA1 PE=4 SV=1	47136	0	823	0	46	0	0.07	#DIV/0!	0	1	0	1	0	1	0	2	0.665884
tr|H0YL72|H0YL72_HUMAN	IDH3A	Isocitrate dehydrogenase [NAD] subunit alpha, mitochondrial OS=Homo sapiens GN=IDH3A PE=3 SV=1	36162	0	822	0	46	0	0.09	#DIV/0!	0	1	0	1	0	1	0	1	0.665884
sp|Q5TFE4|NT5D1_HUMAN	NT5DC1	5~-nucleotidase domain-containing protein 1 OS=Homo sapiens GN=NT5DC1 PE=1 SV=1	52439	0	821	0	46	0	0.07	#DIV/0!	0	1	0	1	0	1	0	2	0.665884
tr|B5BUD2|B5BUD2_HUMAN	RFC2	Replication factor C 2 isoform 1 (Fragment) OS=Homo sapiens GN=RFC2 PE=2 SV=1	39616	0	820	0	46	0	0.08	#DIV/0!	0	1	0	1	0	1	0	1	0.665884
tr|B3KXH0|B3KXH0_HUMAN	tr|B3KXH	cDNA FLJ45395 fis, clone BRHIP3027191, highly similar to 150 kDa oxygen-regulated protein (Orp150) OS=	110601	0	818	0	47	0	0.03	#DIV/0!	0	1	0	1	0	1	0	1	0.665884
tr|A8K5Y7|A8K5Y7_HUMAN	tr|A8K5Y7	cDNA FLJ78655, highly similar to Homo sapiens exportin 5 (XPO5), mRNA OS=Homo sapiens PE=2 SV=1	138331	0	811	0	47	0	0.28	#DIV/0!	0	1	0	1	0	1	0	1	0.665884
tr|B4DET0|B4DET0_HUMAN	tr|B4DET0	cDNA FLJ55900, highly similar to Pre-mRNA-splicing factor ATP-dependent RNA helicase PRP16 (EC 3.6.1.-)	140449	0	810	0	47	0	0.02	#DIV/0!	0	1	0	1	0	1	0	1	0.665884
tr|Q14730|Q14730_HUMAN	tr|Q14730	La 4.1 protein (Fragment) OS=Homo sapiens PE=2 SV=1	33852	0	802	0	48	0	0.1	#DIV/0!	0	1	0	1	0	1	0	1	0.665884
tr|C9J164|C9J164_HUMAN	RAPH1	Ras-associated and pleckstrin homology domains-containing protein 1 OS=Homo sapiens GN=RAPH1 PE=2	67592	0	801	0	48	0	0.02	#DIV/0!	0	1	0	1	0	1	0	2	0.665884
tr|H0Y3Y9|H0Y3Y9_HUMAN	PSMD4	26S proteasome non-ATPase regulatory subunit 4 (Fragment) OS=Homo sapiens GN=PSMD4 PE=4 SV=1	21105	0	799	0	39	0	0.17	#DIV/0!	0	1	0	1	0	1	0	1	0.665884
tr|B2RCV1|B2RCV1_HUMAN	tr|B2RCV1	cDNA, FLJ96310, highly similar to Homo sapiens solute carrier family 25 (mitochondrial carrier; adenine nu	33062	0	795	0	49	0	0.1	#DIV/0!	0	1	0	1	0	1	0	1	0.665884
tr|Q6ZWH1|Q6ZWH1_HUMAN	tr|Q6ZWH	cDNA FLJ41081 fis, clone ADRGL2009755, highly similar to Homo sapiens brain and reproductive organ-exp	45824	0	793	0	49	0	0.07	#DIV/0!	0	1	0	1	0	1	0	1	0.665884
tr|B2RDW9|B2RDW9_HUMAN	tr|B2RDW	cDNA, FLJ96803, highly similar to Homo sapiens GTP binding protein 1 (GTPBP1), mRNA OS=Homo sapiens	64008	0	792	0	49	0	0.05	#DIV/0!	0	1	0	1	0	1	0	1	0.665884
tr|G3V1J5|G3V1J5_HUMAN	DIS3	Exosome complex exonuclease RRP44 OS=Homo sapiens GN=DIS3 PE=2 SV=1	91382	0	789	0	0	0	0.05	#DIV/0!	0	0	0	0	0	1	0	1	0.665884
sp|Q14684|RRP1B_HUMAN	RRP1B	Ribosomal RNA processing protein 1 homolog B OS=Homo sapiens GN=RRP1B PE=1 SV=3	84774	0	784	0	49	0	0.04	#DIV/0!	0	1	0	1	0	1	0	2	0.665884
tr|H7C166|H7C166_HUMAN	DLG1	Disks large homolog 1 (Fragment) OS=Homo sapiens GN=DLG1 PE=4 SV=1	30646	0	781	0	50	0	0.11	#DIV/0!	0	1	0	1	0	1	0	1	0.665884
sp|Q6IA86|ELP2_HUMAN	ELP2	Elongator complex protein 2 OS=Homo sapiens GN=ELP2 PE=1 SV=2	94266	0	776	0	50	0	0.03	#DIV/0!	0	1	0	1	0	1	0	1	0.665884
tr|G3V5E1|G3V5E1_HUMAN	CCNK	Cyclin-K OS=Homo sapiens GN=CCNK PE=2 SV=1	43776	0	771	0	51	0	0.08	#DIV/0!	0	1	0	1	0	1	0	2	0.665884
tr|B2RBM7|B2RBM7_HUMAN	tr|B2RBM	cDNA, FLJ95595, highly similar to Homo sapiens proteasome (prosome, macropain) 26S subunit, non-ATPa	43131	0	765	0	51	0	0.08	#DIV/0!	0	1	0	1	0	1	0	2	0.665884
tr|Q59EZ3|Q59EZ3_HUMAN	tr|Q59EZ3	Insulin-like growth factor 2 receptor variant (Fragment) OS=Homo sapiens PE=2 SV=1	272672	0	764	0	51	0	0.01	#DIV/0!	0	1	0	1	0	1	0	3	0.665884
tr|H0Y9X5|H0Y9X5_HUMAN	ERAP1	Endoplasmic reticulum aminopeptidase 1 (Fragment) OS=Homo sapiens GN=ERAP1 PE=4 SV=1	17136	0	761	0	51	0	0.2	#DIV/0!	0	1	0	1	0	1	0	1	0.665884
sp|Q05048|CSTF1_HUMAN	CSTF1	Cleavage stimulation factor subunit 1 OS=Homo sapiens GN=CSTF1 PE=1 SV=1	49125	0	756	0	52	0	0.22	#DIV/0!	0	1	0	1	0	1	0	1	0.665884
tr|B7Z871|B7Z871_HUMAN	tr|B7Z871	cDNA FLJ50930, highly similar to Lipoma-preferred partner OS=Homo sapiens PE=2 SV=1	49854	0	752	0	52	0	0.07	#DIV/0!	0	1	0	1	0	1	0	1	0.665884
tr|E9PQD7|E9PQD7_HUMAN	RPS2	40S ribosomal protein S2 OS=Homo sapiens GN=RPS2 PE=2 SV=1	25366	0	751	0	53	0	0.5	#DIV/0!	0	1	0	1	0	1	0	1	0.665884
tr|H0YK42|H0YK42_HUMAN	SNX1	Sorting nexin-1 OS=Homo sapiens GN=SNX1 PE=4 SV=1	35628	0	750	0	53	0	0.09	#DIV/0!	0	1	0	1	0	1	0	1	0.665884
tr|B4DM41|B4DM41_HUMAN	RFC4	Replication factor C (Activator 1) 4, 37kDa, isoform CRA_b OS=Homo sapiens GN=RFC4 PE=2 SV=1	33996	0	748	0	53	0	0.1	#DIV/0!	0	1	0	1	0	1	0	1	0.665884
tr|B2RBB2|B2RBB2_HUMAN	tr|B2RBB2	cDNA, FLJ95416, highly similar to Homo sapiens phosphatidylinositol glycan, class S (PIGS), mRNA OS=Hom	61818	0	744	0	53	0	0.05	#DIV/0!	0	1	0	1	0	1	0	1	0.665884
tr|H0Y8Z9|H0Y8Z9_HUMAN	ACAD9	Acyl-CoA dehydrogenase family member 9, mitochondrial (Fragment) OS=Homo sapiens GN=ACAD9 PE=4	43503	0	739	0	54	0	0.08	#DIV/0!	0	1	0	1	0	1	0	1	0.665884
tr|D3DRG7|D3DRG7_HUMAN	DPYSL4	Dihydropyrimidinase-like 4, isoform CRA_a OS=Homo sapiens GN=DPYSL4 PE=2 SV=1	55584	0	738	0	54	0	0.06	#DIV/0!	0	1	0	1	0	1	0	1	0.665884
tr|B4DV74|B4DV74_HUMAN	tr|B4DV74	cDNA FLJ53855, highly similar to Kanadaptin OS=Homo sapiens PE=2 SV=1	56134	0	736	0	54	0	0.06	#DIV/0!	0	1	0	1	0	1	0	2	0.665884
sp|Q7Z4V5|HDGR2_HUMAN	HDGFRP2	Hepatoma-derived growth factor-related protein 2 OS=Homo sapiens GN=HDGFRP2 PE=1 SV=1	74443	0	729	0	56	0	0.04	#DIV/0!	0	1	0	1	0	1	0	1	0.665884
tr|B4DJK4|B4DJK4_HUMAN	tr|B4DJK4	cDNA FLJ54005, highly similar to Transcription elongation factor SPT5 OS=Homo sapiens PE=2 SV=1	97196	0	724	0	56	0	0.03	#DIV/0!	0	1	0	1	0	1	0	1	0.665884
tr|I3L495|I3L495_HUMAN	PAFAH1B1	Platelet-activating factor acetylhydrolase IB subunit alpha (Fragment) OS=Homo sapiens GN=PAFAH1B1 PE	9626	0	723	0	27	0	0.22	#DIV/0!	0	0	0	1	0	1	0	1	0.665884
tr|B1ANM7|B1ANM7_HUMAN	FAF1	FAS-associated factor 1 OS=Homo sapiens GN=FAF1 PE=4 SV=1	56305	0	719	0	56	0	0.06	#DIV/0!	0	1	0	1	0	1	0	1	0.665884
tr|Q56VW8|Q56VW8_HUMAN	tr|Q56VW	Lung cancer-related protein 9 OS=Homo sapiens PE=2 SV=1	52186	0	712	0	57	0	0.06	#DIV/0!	0	1	0	1	0	1	0	1	0.665884
sp|Q14008|CKAP5_HUMAN	CKAP5	Cytoskeleton-associated protein 5 OS=Homo sapiens GN=CKAP5 PE=1 SV=3	227062	0	710	0	57	0	0.18	#DIV/0!	0	1	0	1	0	1	0	1	0.665884
sp|Q14669|TRIPC_HUMAN	TRIP12	E3 ubiquitin-protein ligase TRIP12 OS=Homo sapiens GN=TRIP12 PE=1 SV=1	222234	0	709	0	57	0	0.01	#DIV/0!	0	1	0	1	0	1	0	2	0.665884
sp|Q9H4F8|SMOC1_HUMAN	SMOC1	SPARC-related modular calcium-binding protein 1 OS=Homo sapiens GN=SMOC1 PE=1 SV=1	49443	0	707	0	58	0	0.07	#DIV/0!	0	1	0	1	0	1	0	1	0.665884
tr|B3KUZ7|B3KUZ7_HUMAN	tr|B3KUZ7	cDNA FLJ40986 fis, clone UTERU2014898, highly similar to Vacuolar ATP synthase subunit H (EC 3.6.3.14) (	56445	0	698	0	59	0	0.06	#DIV/0!	0	1	0	1	0	1	0	1	0.665884
sp|Q9BS26|ERP44_HUMAN	ERP44	Endoplasmic reticulum resident protein 44 OS=Homo sapiens GN=ERP44 PE=1 SV=1	47341	0	697	0	59	0	0.07	#DIV/0!	0	1	0	1	0	1	0	1	0.665884
tr|Q05CP4|Q05CP4_HUMAN	EIF2AK2	EIF2AK2 protein (Fragment) OS=Homo sapiens GN=EIF2AK2 PE=2 SV=1	40940	0	694	0	60	0	0.08	#DIV/0!	0	1	0	1	0	1	0	1	0.665884
sp|A9QM74|IMA8_HUMAN	KPNA7	Importin subunit alpha-8 OS=Homo sapiens GN=KPNA7 PE=1 SV=1	57529	0	680	0	62	0	0.06	#DIV/0!	0	1	0	1	0	1	0	1	0.665884
sp|Q96HE7|ERO1A_HUMAN	ERO1L	ERO1-like protein alpha OS=Homo sapiens GN=ERO1L PE=1 SV=2	55213	0	665	0	65	0	0.06	#DIV/0!	0	1	0	1	0	1	0	2	0.665884
tr|Q5XJ04|Q5XJ04_HUMAN	HMGCS1	3-hydroxy-3-methylglutaryl-Coenzyme A synthase 1 (Soluble) OS=Homo sapiens GN=HMGCS1 PE=2 SV=1	57854	0	664	0	65	0	0.12	#DIV/0!	0	1	0	2	0	1	0	3	0.665884
tr|H0Y4J2|H0Y4J2_HUMAN	MRPL37	39S ribosomal protein L37, mitochondrial (Fragment) OS=Homo sapiens GN=MRPL37 PE=4 SV=1	29806	0	655	0	67	0	0.11	#DIV/0!	0	1	0	1	0	1	0	1	0.665884
tr|B2RAU8|B2RAU8_HUMAN	tr|B2RAU	cDNA, FLJ95131, highly similar to Homo sapiens nucleolar and coiled-body phosphoprotein 1 (NOLC1), mR	73561	0	654	0	67	0	0.04	#DIV/0!	0	1	0	1	0	1	0	2	0.665884
sp|Q27J81|INF2_HUMAN	INF2	Inverted formin-2 OS=Homo sapiens GN=INF2 PE=1 SV=2	136851	0	647	0	68	0	0.02	#DIV/0!	0	1	0	1	0	1	0	1	0.665884
tr|H0YL11|H0YL11_HUMAN	IDH2	Isocitrate dehydrogenase [NADP], mitochondrial OS=Homo sapiens GN=IDH2 PE=3 SV=1	35536	0	639	0	58	0	0.15	#DIV/0!	0	1	0	1	0	1	0	1	0.665884
tr|B4DSP0|B4DSP0_HUMAN	hCG_1642	HCG1642748, isoform CRA_b OS=Homo sapiens GN=hCG_1642748 PE=2 SV=1	45559	0	628	0	69	0	0.07	#DIV/0!	0	1	0	1	0	1	0	1	0.665884
tr|A8K0D2|A8K0D2_HUMAN	tr|A8K0D2	cDNA FLJ77740, highly similar to Homo sapiens 7-dehydrocholesterol reductase, mRNA OS=Homo sapiens	55175	0	623	0	70	0	0.06	#DIV/0!	0	1	0	1	0	1	0	1	0.665884
tr|B7Z2B4|B7Z2B4_HUMAN	RABGAP1	Rab GTPase-activating protein 1 OS=Homo sapiens GN=RABGAP1 PE=2 SV=1	48660	0	622	0	70	0	0.03	#DIV/0!	0	1	0	1	0	1	0	1	0.665884
tr|B3KQ73|B3KQ73_HUMAN	tr|B3KQ73	cDNA FLJ33002 fis, clone THYMU1000217, highly similar to Homo sapiens ubiquitin associated protein 2 (U	37025	0	616	0	42	0	0.07	#DIV/0!	0	1	0	1	0	1	0	1	0.665884
tr|B4DR25|B4DR25_HUMAN	tr|B4DR25	Oxysterol-binding protein OS=Homo sapiens PE=2 SV=1	51440	0	612	0	71	0	0.06	#DIV/0!	0	1	0	1	0	1	0	3	0.665884
tr|B3KMC2|B3KMC2_HUMAN	tr|B3KMC	cDNA FLJ10676 fis, clone NT2RP2006464, highly similar to WD repeat and HMG-box DNA-binding protein 1	73526	0	604	0	72	0	0.09	#DIV/0!	0	1	0	2	0	1	0	2	0.665884
tr|H0Y424|H0Y424_HUMAN	LMO7	LIM domain only protein 7 (Fragment) OS=Homo sapiens GN=LMO7 PE=4 SV=1	121750	0	603	0	72	0	0.03	#DIV/0!	0	1	0	1	0	1	0	2	0.665884
tr|J3KMX2|J3KMX2_HUMAN	SMARCD2	SWI/SNF-related matrix-associated actin-dependent regulator of chromatin subfamily D member 2 OS=Ho	52433	0	598	0	73	0	0.06	#DIV/0!	0	1	0	1	0	1	0	1	0.665884
sp|Q9Y3Z3|SAMH1_HUMAN	SAMHD1	SAM domain and HD domain-containing protein 1 OS=Homo sapiens GN=SAMHD1 PE=1 SV=2	72896	0	596	0	74	0	0.05	#DIV/0!	0	1	0	1	0	1	0	1	0.665884
tr|B3KN28|B3KN28_HUMAN	tr|B3KN28	cDNA FLJ13370 fis, clone PLACE1000653, highly similar to Phosphoacetylglucosamine mutase (EC 5.4.2.3) (	60284	0	593	0	74	0	0.05	#DIV/0!	0	1	0	1	0	1	0	2	0.665884
sp|Q8IXI1|MIRO2_HUMAN	RHOT2	Mitochondrial Rho GTPase 2 OS=Homo sapiens GN=RHOT2 PE=1 SV=2	69101	0	591	0	74	0	0.05	#DIV/0!	0	1	0	1	0	1	0	1	0.665884
sp|Q9H009|NACA2_HUMAN	NACA2	Nascent polypeptide-associated complex subunit alpha-2 OS=Homo sapiens GN=NACA2 PE=1 SV=1	23209	0	586	0	75	0	0.14	#DIV/0!	0	1	0	1	0	1	0	1	0.665884
tr|H0Y2Y8|H0Y2Y8_HUMAN	ZYX	Zyxin (Fragment) OS=Homo sapiens GN=ZYX PE=4 SV=1	58804	0	581	0	76	0	0.06	#DIV/0!	0	1	0	1	0	1	0	3	0.665884
tr|H0Y7C0|H0Y7C0_HUMAN	USO1	General vesicular transport factor p115 (Fragment) OS=Homo sapiens GN=USO1 PE=4 SV=1	72345	0	559	0	80	0	0.05	#DIV/0!	0	1	0	1	0	1	0	1	0.665884
tr|B4DRK5|B4DRK5_HUMAN	tr|B4DRK5	cDNA FLJ59584, highly similar to Mitochondrial-processing peptidase alpha subunit, mitochondrial (EC 3.4.	28622	0	557	0	50	0	0.12	#DIV/0!	0	1	0	1	0	1	0	1	0.665884
tr|B7Z653|B7Z653_HUMAN	tr|B7Z653	cDNA FLJ61657, highly similar to Band 4.1-like protein 1 OS=Homo sapiens PE=2 SV=1	90347	0	555	0	81	0	0.03	#DIV/0!	0	1	0	1	0	1	0	1	0.665884
tr|B4DTM6|B4DTM6_HUMAN	tr|B4DTM	cDNA FLJ61391, highly similar to Protein transport protein Sec24B OS=Homo sapiens PE=2 SV=1	130037	0	551	0	44	0	0.07	#DIV/0!	0	1	0	1	0	1	0	1	0.665884
sp|Q13425|SNTB2_HUMAN	SNTB2	Beta-2-syntrophin OS=Homo sapiens GN=SNTB2 PE=1 SV=1	58369	0	548	0	82	0	0.06	#DIV/0!	0	1	0	1	0	1	0	1	0.665884
sp|Q06210|GFPT1_HUMAN	GFPT1	Glutamine--fructose-6-phosphate aminotransferase [isomerizing] 1 OS=Homo sapiens GN=GFPT1 PE=1 SV	79555	0	531	0	83	0	0.08	#DIV/0!	0	1	0	2	0	1	0	3	0.665884
tr|B2RB90|B2RB90_HUMAN	tr|B2RB90	cDNA, FLJ95375, highly similar to Homo sapiens glutamine-fructose-6-phosphate transaminase 2 (GFPT2),	77651	0	522	0	85	0	0.04	#DIV/0!	0	1	0	1	0	1	0	1	0.665884
sp|Q5F1R6|DJC21_HUMAN	DNAJC21	DnaJ homolog subfamily C member 21 OS=Homo sapiens GN=DNAJC21 PE=1 SV=2	62445	0	515	0	87	0	0.05	#DIV/0!	0	1	0	1	0	1	0	1	0.665884
tr|D6RD16|D6RD16_HUMAN	CANX	Calnexin OS=Homo sapiens GN=CANX PE=2 SV=1	10341	0	505	0	90	0	0.34	#DIV/0!	0	1	0	1	0	1	0	2	0.665884
tr|Q14756|Q14756_HUMAN	LLDBP	LLDBP (Fragment) OS=Homo sapiens GN=LLDBP PE=2 SV=1	58557	0	492	0	77	0	0.05	#DIV/0!	0	1	0	1	0	1	0	1	0.665884
tr|G3V1T4|G3V1T4_HUMAN	NUP107	Nuclear pore complex protein Nup107 OS=Homo sapiens GN=NUP107 PE=2 SV=1	11398	0	484	0	73	0	0.09	#DIV/0!	0	1	0	1	0	1	0	1	0.665884
tr|A0PJC9|A0PJC9_HUMAN	TPR	TPR protein (Fragment) OS=Homo sapiens GN=TPR PE=2 SV=1	53894	0	424	0	31	0	0.06	#DIV/0!	0	0	0	1	0	1	0	1	0.665884
tr|K7EQ60|K7EQ60_HUMAN	COASY	Bifunctional coenzyme A synthase (Fragment) OS=Homo sapiens GN=COASY PE=4 SV=1	12950	0	417	0	55	0	0.17	#DIV/0!	0	1	0	1	0	1	0	1	0.665884
tr|H7BZD1|H7BZD1_HUMAN	GLS	Glutaminase kidney isoform, mitochondrial (Fragment) OS=Homo sapiens GN=GLS PE=4 SV=1	12786	0	403	0	58	0	0.24	#DIV/0!	0	1	0	1	0	1	0	2	0.665884
tr|B3KMN2|B3KMN2_HUMAN	tr|B3KMN	cDNA FLJ11695 fis, clone HEMBA1005019, highly similar to Homo sapiens SCC-112 protein (SCC-112), mRN	36724	0	328	0	67	0	0.09	#DIV/0!	0	1	0	1	0	1	0	1	0.665884
tr|H0Y4Z6|H0Y4Z6_HUMAN	PYGB	Phosphorylase (Fragment) OS=Homo sapiens GN=PYGB PE=3 SV=1	27767	0	325	0	31	0	0.3	#DIV/0!	0	0	0	1	0	1	0	1	0.665884
tr|B3KY55|B3KY55_HUMAN	tr|B3KY55	cDNA FLJ46894 fis, clone UTERU3019708, highly similar to Regulator of nonsense transcripts 1 OS=Homo s	41002	0	286	0	51	0	0.2	#DIV/0!	0	1	0	1	0	1	0	1	0.665884
sp|Q4G0F5|VP26B_HUMAN	VPS26B	Vacuolar protein sorting-associated protein 26B OS=Homo sapiens GN=VPS26B PE=1 SV=2	39244	0	1286	0	15	0	0.08	#DIV/0!	0	0	0	1	0	0	0	1	-0.91926
tr|B4DF80|B4DF80_HUMAN	tr|B4DF80	cDNA FLJ58527, highly similar to Nucleolar protein 6 OS=Homo sapiens PE=2 SV=1	122714	0	1285	0	15	0	0.03	#DIV/0!	0	0	0	1	0	0	0	2	-0.91926
tr|A2A3F3|A2A3F3_HUMAN	TRPM3	Transient receptor potential cation channel subfamily M member 3 OS=Homo sapiens GN=TRPM3 PE=4 SV	179906	0	1283	0	16	0	0.02	#DIV/0!	0	0	0	1	0	0	0	1	-0.91926
sp|Q9NUQ3|TXLNG_HUMAN	TXLNG	Gamma-taxilin OS=Homo sapiens GN=TXLNG PE=1 SV=2	61061	0	1282	0	16	0	0.05	#DIV/0!	0	0	0	1	0	0	0	2	-0.91926
tr|E9PIQ6|E9PIQ6_HUMAN	POLA2	DNA polymerase alpha subunit B (Fragment) OS=Homo sapiens GN=POLA2 PE=2 SV=1	15999	0	1281	0	16	0	0.21	#DIV/0!	0	0	0	1	0	0	0	1	-0.91926
sp|A5D8W1|CG063_HUMAN	C7orf63	Uncharacterized protein C7orf63 OS=Homo sapiens GN=C7orf63 PE=2 SV=3	107070	0	1280	0	17	0	0.03	#DIV/0!	0	0	0	1	0	0	0	1	-0.91926
tr|A0PJK8|A0PJK8_HUMAN	SCRIB	SCRIB protein (Fragment) OS=Homo sapiens GN=SCRIB PE=2 SV=1	71166	0	1278	0	17	0	0.05	#DIV/0!	0	0	0	1	0	0	0	1	-0.91926
tr|C1K3L2|C1K3L2_HUMAN	tr|C1K3L2	CMP-N-acetylneuraminic acid hydroxylase OS=Homo sapiens PE=2 SV=1	58714	0	1277	0	17	0	0.06	#DIV/0!	0	0	0	1	0	0	0	1	-0.91926
sp|Q96EN8|MOCOS_HUMAN	MOCOS	Molybdenum cofactor sulfurase OS=Homo sapiens GN=MOCOS PE=1 SV=2	99084	0	1275	0	17	0	0.03	#DIV/0!	0	0	0	1	0	0	0	1	-0.91926
tr|H7C1N0|H7C1N0_HUMAN	C7orf10	CaiB/baiF CoA-transferase family protein C7orf10 (Fragment) OS=Homo sapiens GN=C7orf10 PE=4 SV=1	5431	0	1274	0	17	0	0.67	#DIV/0!	0	0	0	1	0	0	0	1	-0.91926
tr|Q53T85|Q53T85_HUMAN	FLJ14126	Putative uncharacterized protein FLJ14126 OS=Homo sapiens GN=FLJ14126 PE=2 SV=1	17207	0	1273	0	17	0	0.2	#DIV/0!	0	0	0	1	0	0	0	1	-0.91926
tr|Q86TD5|Q86TD5_HUMAN	DKFZp451	Putative uncharacterized protein DKFZp451F083 OS=Homo sapiens GN=DKFZp451F083 PE=2 SV=1	60173	0	1272	0	17	0	0.05	#DIV/0!	0	0	0	1	0	0	0	1	-0.91926
tr|J3KS45|J3KS45_HUMAN	TMCO1	Transmembrane and coiled-coil domain-containing protein 1 (Fragment) OS=Homo sapiens GN=TMCO1 PE	16741	0	1271	0	17	0	0.2	#DIV/0!	0	0	0	1	0	0	0	1	-0.91926
tr|B1AKJ6|B1AKJ6_HUMAN	OSBPL9	Oxysterol-binding protein OS=Homo sapiens GN=OSBPL9 PE=2 SV=1	84417	0	1270	0	17	0	0.04	#DIV/0!	0	0	0	1	0	0	0	1	-0.91926
tr|Q504W7|Q504W7_HUMAN	OCRL	Oculocerebrorenal syndrome of Lowe OS=Homo sapiens GN=OCRL PE=2 SV=1	104312	0	1269	0	17	0	0.03	#DIV/0!	0	0	0	1	0	0	0	1	-0.91926
sp|Q9BVI4|NOC4L_HUMAN	NOC4L	Nucleolar complex protein 4 homolog OS=Homo sapiens GN=NOC4L PE=1 SV=1	58830	0	1267	0	18	0	0.11	#DIV/0!	0	0	0	1	0	0	0	1	-0.91926
tr|E5RK48|E5RK48_HUMAN	RDH10	Retinol dehydrogenase 10 OS=Homo sapiens GN=RDH10 PE=2 SV=1	20236	0	1266	0	19	0	0.17	#DIV/0!	0	0	0	1	0	0	0	1	-0.91926
sp|Q8TE73|DYH5_HUMAN	DNAH5	Dynein heavy chain 5, axonemal OS=Homo sapiens GN=DNAH5 PE=1 SV=3	532504	0	1265	0	19	0	0.01	#DIV/0!	0	0	0	1	0	0	0	4	-0.91926
tr|B4DVV7|B4DVV7_HUMAN	tr|B4DVV7	cDNA FLJ51339, highly similar to Methionyl-tRNA synthetase, mitochondrial (EC 6.1.1.10) OS=Homo sapier	59183	0	1263	0	19	0	0.06	#DIV/0!	0	0	0	1	0	0	0	2	-0.91926
sp|Q15061|WDR43_HUMAN	WDR43	WD repeat-containing protein 43 OS=Homo sapiens GN=WDR43 PE=1 SV=3	75813	0	1262	0	19	0	0.04	#DIV/0!	0	0	0	1	0	0	0	2	-0.91926
tr|B4E1K7|B4E1K7_HUMAN	STOML2	Stomatin-like protein 2 OS=Homo sapiens GN=STOML2 PE=2 SV=1	33317	0	1261	0	19	0	0.1	#DIV/0!	0	0	0	1	0	0	0	1	-0.91926
tr|H0Y7P1|H0Y7P1_HUMAN	MED12	Mediator of RNA polymerase II transcription subunit 12 (Fragment) OS=Homo sapiens GN=MED12 PE=4 SV	240034	0	1259	0	19	0	0.01	#DIV/0!	0	1	0	1	0	0	0	1	-0.91926
tr|B2RE56|B2RE56_HUMAN	tr|B2RE56	Peptidyl-prolyl cis-trans isomerase OS=Homo sapiens PE=2 SV=1	18225	0	1256	0	19	0	0.19	#DIV/0!	0	0	0	1	0	0	0	1	-0.91926
tr|H0YGY9|H0YGY9_HUMAN	TRPT1	tRNA 2~-phosphotransferase 1 (Fragment) OS=Homo sapiens GN=TRPT1 PE=4 SV=1	12598	0	1255	0	19	0	0.27	#DIV/0!	0	0	0	1	0	0	0	1	-0.91926
tr|G3V1K3|G3V1K3_HUMAN	PON2	Paraoxonase 2, isoform CRA_a OS=Homo sapiens GN=PON2 PE=2 SV=1	41672	0	1254	0	19	0	0.08	#DIV/0!	0	0	0	1	0	0	0	1	-0.91926
tr|I3L3B0|I3L3B0_HUMAN	C1QBP	Complement component 1 Q subcomponent-binding protein, mitochondrial OS=Homo sapiens GN=C1QBP	20179	0	1253	0	19	0	0.17	#DIV/0!	0	0	0	1	0	0	0	1	-0.91926
tr|B1ANE3|B1ANE3_HUMAN	MTR	5-methyltetrahydrofolate-homocysteine methyltransferase (Fragment) OS=Homo sapiens GN=MTR PE=3 S	92463	0	1251	0	20	0	0.04	#DIV/0!	0	0	0	1	0	0	0	1	-0.91926
tr|B4DFN6|B4DFN6_HUMAN	CNP	2~,3~-cyclic-nucleotide 3~-phosphodiesterase OS=Homo sapiens GN=CNP PE=2 SV=1	20461	0	1250	0	0	0	0.07	#DIV/0!	0	0	0	0	0	0	0	1	-0.91926
tr|B4DSA4|B4DSA4_HUMAN	tr|B4DSA4	cDNA FLJ56930, highly similar to Ferrochelatase, mitochondrial (EC 4.99.1.1) OS=Homo sapiens PE=2 SV=1	32775	0	1249	0	20	0	0.1	#DIV/0!	0	0	0	1	0	0	0	1	-0.91926
tr|H3BMM6|H3BMM6_HUMAN	GGA2	ADP-ribosylation factor-binding protein GGA2 OS=Homo sapiens GN=GGA2 PE=4 SV=1	24917	0	1248	0	20	0	0.13	#DIV/0!	0	0	0	1	0	0	0	1	-0.91926
tr|F8VS10|F8VS10_HUMAN	SPATS2	Spermatogenesis-associated serine-rich protein 2 OS=Homo sapiens GN=SPATS2 PE=2 SV=1	32626	0	1247	0	20	0	0.1	#DIV/0!	0	0	0	1	0	0	0	1	-0.91926
tr|B3KVR1|B3KVR1_HUMAN	SNRPN	Small nuclear ribonucleoprotein-associated protein OS=Homo sapiens GN=SNRPN PE=2 SV=1	25230	0	1246	0	20	0	0.13	#DIV/0!	0	1	0	1	0	0	0	1	-0.91926
sp|Q5VTL8|PR38B_HUMAN	PRPF38B	Pre-mRNA-splicing factor 38B OS=Homo sapiens GN=PRPF38B PE=1 SV=1	64657	0	1245	0	20	0	0.05	#DIV/0!	0	0	0	1	0	0	0	2	-0.91926
tr|Q53G42|Q53G42_HUMAN	tr|Q53G42	mRNA decapping enzyme variant (Fragment) OS=Homo sapiens PE=2 SV=1	38757	0	1126	0	19	0	0.18	#DIV/0!	0	0	0	1	0	0	0	1	-0.91926
tr|B3KW81|B3KW81_HUMAN	tr|B3KW8	cDNA FLJ42510 fis, clone BRACE2044473, highly similar to Homo sapiens pericentrin 1 (PCNT1), mRNA OS=	30173	0	1011	0	0	0	0.05	#DIV/0!	0	0	0	0	0	0	0	1	-0.91926
tr|Q8N274|Q8N274_HUMAN	tr|Q8N27	cDNA FLJ33834 fis, clone CTONG2004264, moderately similar to NEUROBLAST DIFFERENTIATION ASSOCIA	86111	401	371	43	127	0.01	0.25	25	1	5	1	6	2	8	4	10	0.847028
tr|Q2TU64|Q2TU64_HUMAN	tr|Q2TU6	PIG48 OS=Homo sapiens PE=2 SV=1	61110	279	137	68	285	0.05	0.93	18.6	1	7	1	10	3	12	3	12	0.91837
tr|E9PBS1|E9PBS1_HUMAN	PAICS	Phosphoribosylaminoimidazole carboxylase (Fragment) OS=Homo sapiens GN=PAICS PE=2 SV=1	46307	270	131	69	329	0.07	1.23	17.57142857	1	8	1	11	2	13	5	17	1.515342
tr|B2RBA6|B2RBA6_HUMAN	tr|B2RBA	cDNA, FLJ95407, highly similar to Homo sapiens MCM7 minichromosome maintenance deficient 7 (S. cere	81856	374	215	46	237	0.04	0.67	16.75	1	8	1	12	1	12	1	14	2.141435
tr|B2R7B5|B2R7B5_HUMAN	tr|B2R7B5	cDNA, FLJ93365, highly similar to Homo sapiens KH domain containing, RNA binding, signal transduction as	48309	347	238	51	205	0.07	1.13	16.14285714	1	4	1	9	2	9	4	10	1.007671
tr|H0UID3|H0UID3_HUMAN	AP2B1	Adaptor-related protein complex 2, beta 1 subunit, isoform CRA_d OS=Homo sapiens GN=AP2B1 PE=4 SV=	106321	648	268	22	194	0.03	0.4	13.33333333	0	7	1	11	3	11	3	13	0.797898
tr|B2R9U2|B2R9U2_HUMAN	tr|B2R9U2	cDNA, FLJ94557, highly similar to Homo sapiens FK506 binding protein 4, 59kDa (FKBP4), mRNA OS=Homo	52087	623	189	24	232	0.06	0.74	12.33333333	0	7	1	8	2	9	2	10	1.007671
tr|B4DTV0|B4DTV0_HUMAN	tr|B4DTV	cDNA FLJ57801, highly similar to Homo sapiens AHNAK nucleoprotein (desmoyokin) (AHNAK), transcript va	114179	454	182	38	271	0.03	0.36	12	1	7	1	11	3	13	3	16	1.02958
tr|J3KR24|J3KR24_HUMAN	IARS	Isoleucine--tRNA ligase, cytoplasmic OS=Homo sapiens GN=IARS PE=4 SV=1	132820	366	251	47	192	0.02	0.22	11	1	4	1	6	2	12	2	15	1.404133
tr|E7EVA0|E7EVA0_HUMAN	MAP4	Microtubule-associated protein OS=Homo sapiens GN=MAP4 PE=2 SV=1	246546	429	441	40	106	0.01	0.11	11	1	3	1	4	2	4	4	5	-0.07122
tr|Q9NSU9|Q9NSU9_HUMAN	DKFZp434	Putative uncharacterized protein DKFZp434G0719 (Fragment) OS=Homo sapiens GN=DKFZp434G0719 PE=	41107	56	248	169	214	0.07	0.72	10.28571429	4	5	5	7	5	7	5	7	-0.47223
sp|O14980|XPO1_HUMAN	XPO1	Exportin-1 OS=Homo sapiens GN=XPO1 PE=1 SV=1	124447	305	221	59	228	0.03	0.3	10	1	7	1	10	1	13	3	15	2.252644
sp|P11388|TOP2A_HUMAN	TOP2A	DNA topoisomerase 2-alpha OS=Homo sapiens GN=TOP2A PE=1 SV=3	175017	606	173	25	282	0.02	0.2	10	0	8	1	10	1	10	2	14	1.88954
sp|Q09666|AHNK_HUMAN	AHNAK	Neuroblast differentiation-associated protein AHNAK OS=Homo sapiens GN=AHNAK PE=1 SV=2	629213	401	115	43	376	0.01	0.1	10	1	11	1	19	3	23	6	30	1.831061

tr|I3L1L3|I3L1L3_HUMAN	MYBBP1A	Myb-binding protein 1A (Fragment) OS=Homo sapiens GN=MYBBP1A PE=4 SV=1	141124	698	291	18	175	0.02	0.2	10	0	3	1	8	1	8	2	9	1.58433
tr|B2R5V9|B2R5V9_HUMAN	tr|B2R5V9	cDNA, FLJ92652, highly similar to Homo sapiens high density lipoprotein binding protein (vigilin)(HDLBP), m	141919	253	208	76	251	0.02	0.2	10	1	6	1	8	2	11	2	11	1.283661
tr|Q6NTA2|Q6NTA2_HUMAN	HNRNPL	HNRNPL protein (Fragment) OS=Homo sapiens GN=HNRNPL PE=2 SV=2	62515	182	49	117	815	0.23	2.25	9.782608696	4	21	4	25	6	27	8	28	1.164451
tr|B4DH39|B4DH39_HUMAN	tr|B4DH39	cDNA FLJ57028, highly similar to Long-chain-fatty-acid--CoA ligase 4 (EC 6.2.1.3) OS=Homo sapiens PE=2 SV	73123	529	254	31	208	0.04	0.36	9	0	4	1	7	1	9	3	9	1.744973
tr|B2R959|B2R959_HUMAN	tr|B2R959	cDNA, FLJ94229, highly similar to Homo sapiens heterogeneous nuclear ribonucleoprotein L (HNRPL),mRNA	60838	182	60	117	661	0.23	2.02	8.782608696	4	17	4	21	6	24	8	25	0.997267
tr|Q05D08|Q05D08_HUMAN	PA2G4	PA2G4 protein (Fragment) OS=Homo sapiens GN=PA2G4 PE=2 SV=1	45579	286	297	64	162	0.15	1.3	8.666666667	1	6	2	10	3	11	3	12	0.797898
tr|Q2Q9B7|Q2Q9B7_HUMAN	G6PD	Glucose-6-phosphate 1-dehydrogenase (Fragment) OS=Homo sapiens GN=G6PD PE=2 SV=1	55221	176	100	118	383	0.18	1.53	8.5	2	10	2	15	5	17	6	20	0.751938
tr|B4DYA7|B4DYA7_HUMAN	tr|B4DYA7	Glucose-6-phosphate 1-dehydrogenase OS=Homo sapiens PE=2 SV=1	58757	176	101	123	409	0.18	1.53	8.5	2	10	3	15	6	17	7	20	0.510593
tr|B2R7C5|B2R7C5_HUMAN	tr|B2R7C5	cDNA, FLJ93378, highly similar to Homo sapiens MCM3 minichromosome maintenance deficient 3 (S. cerev	91579	479	199	36	257	0.04	0.32	8	1	6	1	9	1	12	1	15	2.141435
sp|O95831|AIFM1_HUMAN	AIFM1	Apoptosis-inducing factor 1, mitochondrial OS=Homo sapiens GN=AIFM1 PE=1 SV=1	67144	326	545	54	82	0.05	0.4	8	1	4	1	7	1	7	2	7	1.40358
tr|B4DLT2|B4DLT2_HUMAN	tr|B4DLT2	cDNA FLJ56637, highly similar to Nuclear pore complex protein Nup155 OS=Homo sapiens PE=2 SV=1	152734	489	197	35	257	0.02	0.16	8	0	6	1	8	1	7	1	9	1.40358
tr|D3DP78|D3DP78_HUMAN	DARS	Aspartyl-tRNA synthetase, isoform CRA_b OS=Homo sapiens GN=DARS PE=2 SV=1	53829	344	292	51	118	0.06	0.48	8	1	4	1	6	2	7	3	7	0.666278
tr|Q2F839|Q2F839_HUMAN	tr|Q2F839	Heat shock 70 kDa protein 9B (Fragment) OS=Homo sapiens PE=2 SV=1	19208	81	165	59	297	0.42	3.28	7.80952381	2	6	2	9	2	9	2	10	1.007671
tr|A2VCT2|A2VCT2_HUMAN	OGDH	OGDH protein (Fragment) OS=Homo sapiens GN=OGDH PE=2 SV=1	72973	432	245	40	129	0.05	0.39	7.8	1	4	1	7	1	6	1	7	1.196951
tr|B4DS46|B4DS46_HUMAN	tr|B4DS46	cDNA FLJ55751, highly similar to DNA replication licensing factor MCM3 OS=Homo sapiens PE=2 SV=1	84347	479	198	36	257	0.04	0.31	7.75	1	6	1	8	1	11	1	13	2.020963
sp|O94906|PRP6_HUMAN	PRPF6	Pre-mRNA-processing factor 6 OS=Homo sapiens GN=PRPF6 PE=1 SV=1	107656	492	319	35	156	0.03	0.23	7.666666667	1	5	1	7	1	7	2	7	1.40358
tr|B4DJ60|B4DJ60_HUMAN	tr|B4DJ60	cDNA FLJ55072, highly similar to Succinate dehydrogenase (ubiquinone) flavoprotein subunit, mitochondri	68233	393	149	44	328	0.1	0.76	7.6	0	9	2	12	3	13	5	14	1.02958
sp|Q6P2Q9|PRP8_HUMAN	PRPF8	Pre-mRNA-processing-splicing factor 8 OS=Homo sapiens GN=PRPF8 PE=1 SV=2	274738	130	43	167	910	0.1	0.74	7.4	7	34	8	47	10	50	10	60	1.352184
tr|B4DT29|B4DT29_HUMAN	tr|B4DT29	cDNA FLJ51082, highly similar to Actin-like protein 3 OS=Homo sapiens PE=2 SV=1	40959	548	318	30	80	0.08	0.59	7.375	0	1	1	4	1	5	1	5	0.955766
tr|Q4ZG57|Q4ZG57_HUMAN	MCM6	Putative uncharacterized protein MCM6 (Fragment) OS=Homo sapiens GN=MCM6 PE=2 SV=1	89803	444	178	39	277	0.04	0.29	7.25	1	7	1	7	2	8	3	9	0.847028
tr|B4DR68|B4DR68_HUMAN	tr|B4DR68	cDNA FLJ58608, highly similar to Heat shock protein 75 kDa, mitochondrial OS=Homo sapiens PE=2 SV=1	74408	169	164	127	299	0.09	0.65	7.222222222	2	7	3	10	3	10	5	11	0.666476
tr|A6NKB8|A6NKB8_HUMAN	RNPEP	Aminopeptidase B OS=Homo sapiens GN=RNPEP PE=2 SV=1	68752	316	257	57	190	0.05	0.36	7.2	1	5	2	6	2	7	2	9	0.666278
sp|Q14204|DYHC1_HUMAN	DYNC1H1	Cytoplasmic dynein 1 heavy chain 1 OS=Homo sapiens GN=DYNC1H1 PE=1 SV=5	534809	168	96	129	448	0.02	0.14	7	3	9	4	22	4	26	6	33	1.642021
tr|B2R4K7|B2R4K7_HUMAN	tr|B2R4K7	60S ribosomal protein L6 OS=Homo sapiens PE=2 SV=1	32739	276	119	68	369	0.47	3.29	7	2	12	4	14	3	15	5	15	1.229244
sp|Q14683|SMC1A_HUMAN	SMC1A	Structural maintenance of chromosomes protein 1A OS=Homo sapiens GN=SMC1A PE=1 SV=2	143771	581	579	28	77	0.02	0.14	7	0	3	1	6	1	6	1	11	1.196951
tr|Q6ZQN2|Q6ZQN2_HUMAN	tr|Q6ZQN	cDNA FLJ46846 fis, clone UTERU3004635, moderately similar to Neuroblast differentiation associated prot	180689	401	643	43	68	0.01	0.07	7	1	3	1	4	2	5	3	7	0.218464
tr|B4E0E1|B4E0E1_HUMAN	tr|B4E0E1	cDNA FLJ53442, highly similar to Poly (ADP-ribose) polymerase 1 (EC 2.4.2.30) OS=Homo sapiens PE=2 SV=	111855	549	128	30	358	0.06	0.41	6.833333333	0	7	2	12	2	12	2	19	1.404133
tr|Q0VGD6|Q0VGD6_HUMAN	HNRPR	HNRPR protein (Fragment) OS=Homo sapiens GN=HNRPR PE=2 SV=1	68218	390	171	44	284	0.1	0.68	6.8	1	8	2	12	2	12	4	12	1.404133
tr|B7Z1R5|B7Z1R5_HUMAN	ATP6V1A	V-type proton ATPase catalytic subunit A OS=Homo sapiens GN=ATP6V1A PE=2 SV=1	65037	428	326	40	149	0.05	0.34	6.8	1	4	1	6	1	7	2	8	1.40358
sp|P26038|MOES_HUMAN	MSN	Moesin OS=Homo sapiens GN=MSN PE=1 SV=3	67892	119	40	179	948	0.46	3.12	6.782608696	7	28	8	33	11	39	12	43	0.864207
tr|H0YLA4|H0YLA4_HUMAN	SORD	Sorbitol dehydrogenase OS=Homo sapiens GN=SORD PE=3 SV=1	36833	473	585	37	52	0.09	0.61	6.777777778	0	1	1	1	2	1	2	2	-1.65672
tr|B2RBE0|B2RBE0_HUMAN	tr|B2RBE0	cDNA, FLJ95462, highly similar to Homo sapiens fatty-acid-Coenzyme A ligase, long-chain 3 (FACL3),mRNA	81266	462	276	37	181	0.04	0.27	6.75	0	4	1	6	1	6	1	6	1.196951
tr|J3KPS3|J3KPS3_HUMAN	ALDOA	Fructose-bisphosphate aldolase OS=Homo sapiens GN=ALDOA PE=3 SV=1	40249	63	24	338	1201	1.79	11.83	6.608938547	11	29	15	36	17	44	18	46	0.429309
tr|B1ANR0|B1ANR0_HUMAN	PABPC4	Poly(A) binding protein, cytoplasmic 4 (Inducible form) OS=Homo sapiens GN=PABPC4 PE=4 SV=1	68156	504	287	33	177	0.05	0.33	6.6	0	6	1	6	1	7	2	8	1.40358
tr|A8K8N7|A8K8N7_HUMAN	PFAS	Phosphoribosylformylglycinamidine synthase (FGAR amidotransferase), isoform CRA_b OS=Homo sapiens	146286	380	125	46	365	0.05	0.33	6.6	0	9	2	13	3	14	5	15	1.132851
tr|B2R5M9|B2R5M9_HUMAN	tr|B2R5M	cDNA, FLJ92537, highly similar to Homo sapiens procollagen-lysine, 2-oxoglutarate 5-dioxygenase (lysine h	84022	251	216	76	237	0.04	0.26	6.5	1	6	1	7	1	9	1	10	1.744973
sp|P41250|SYG_HUMAN	GARS	Glycine--tRNA ligase OS=Homo sapiens GN=GARS PE=1 SV=3	83854	392	455	44	101	0.04	0.26	6.5	1	4	1	6	1	7	2	8	1.40358
tr|B5MCX3|B5MCX3_HUMAN	Sep-02	Septin-2 OS=Homo sapiens GN=SEPT2 PE=2 SV=1	37145	543	356	30	109	0.09	0.58	6.444444444	1	3	1	5	2	5	3	5	0.218464
tr|A4FU77|A4FU77_HUMAN	SNRNP200	SNRNP200 protein (Fragment) OS=Homo sapiens GN=SNRNP200 PE=2 SV=1	217585	243	78	83	446	0.05	0.32	6.4	2	12	3	19	7	22	8	24	0.667266
tr|Q96QL0|Q96QL0_HUMAN	RPL3	Ribosomal protein L3 OS=Homo sapiens GN=RPL3 PE=2 SV=1	46376	245	103	82	385	0.32	2.03	6.34375	4	10	4	13	5	13	7	13	0.37683
sp|P06733|ENOA_HUMAN	ENO1	Alpha-enolase OS=Homo sapiens GN=ENO1 PE=1 SV=2	47481	40	34	520	1059	1.39	8.8	6.330935252	13	30	18	42	19	48	25	57	0.39741
tr|J3KQL0|J3KQL0_HUMAN	CPS1	Carbamoyl-phosphate synthase [ammonia], mitochondrial OS=Homo sapiens GN=CPS1 PE=3 SV=1	166686	204	42	102	915	0.15	0.94	6.266666667	4	26	7	36	10	38	12	43	0.958616
sp|P54886|P5CS_HUMAN	ALDH18A1	Delta-1-pyrroline-5-carboxylate synthase OS=Homo sapiens GN=ALDH18A1 PE=1 SV=2	87989	658	380	21	124	0.04	0.25	6.25	1	3	1	6	1	5	1	8	0.955766
tr|Q5SU16|Q5SU16_HUMAN	TUBB	Beta 5-tubulin OS=Homo sapiens GN=TUBB PE=2 SV=1	50095	25	7	680	1966	3.07	19.03	6.198697068	26	62	33	75	35	83	39	92	0.317715
tr|Q8IZ29|Q8IZ29_HUMAN	TUBB2C	Tubulin, beta 2C OS=Homo sapiens GN=TUBB2C PE=2 SV=1	50264	27	8	674	1928	3.04	18.77	6.174342105	26	64	32	75	34	82	38	88	0.341715
tr|A8KAK1|A8KAK1_HUMAN	tr|A8KAK1	cDNA FLJ77398, highly similar to Homo sapiens UDP-glucose ceramide glucosyltransferase-like 1, transcrip	175467	635	477	22	97	0.02	0.12	6	0	1	1	6	1	8	1	9	1.58433
tr|B4DKR8|B4DKR8_HUMAN	tr|B4DKR8	cDNA FLJ55700, moderately similar to Vigilin OS=Homo sapiens PE=2 SV=1	84696	253	610	0	72	0.02	0.12	6	0	2	0	3	1	5	1	6	0.955766
sp|P45974|UBP5_HUMAN	USP5	Ubiquitin carboxyl-terminal hydrolase 5 OS=Homo sapiens GN=USP5 PE=1 SV=2	96638	622	306	24	167	0.03	0.18	6	0	3	1	5	2	5	2	7	0.218464
tr|Q8IWP6|Q8IWP6_HUMAN	tr|Q8IWP6	Class IVb beta tubulin OS=Homo sapiens PE=2 SV=1	50177	28	10	654	1850	3.05	17.67	5.793442623	27	61	32	72	34	78	37	84	0.269296
tr|B4DWJ2|B4DWJ2_HUMAN	QARS	Glutamine--tRNA ligase OS=Homo sapiens GN=QARS PE=2 SV=1	87437	277	138	68	309	0.16	0.92	5.75	3	8	4	16	5	16	5	18	0.666871
sp|O76094|SRP72_HUMAN	SRP72	Signal recognition particle 72 kDa protein OS=Homo sapiens GN=SRP72 PE=1 SV=3	75130	455	540	38	82	0.04	0.23	5.75	1	1	1	4	1	4	1	6	0.666081
sp|P52597|HNRPF_HUMAN	HNRNPF	Heterogeneous nuclear ribonucleoprotein F OS=Homo sapiens GN=HNRNPF PE=1 SV=3	45985	226	379	91	125	0.07	0.4	5.714285714	1	4	1	5	2	5	3	6	0.218464
tr|B1AH89|B1AH89_HUMAN	TTLL12	Tubulin tyrosine ligase-like family, member 12 OS=Homo sapiens GN=TTLL12 PE=4 SV=1	74185	104	45	209	890	0.41	2.31	5.634146341	6	21	8	32	10	35	11	41	0.841041
tr|Q59FV6|Q59FV6_HUMAN	tr|Q59FV6	ARP3 actin-related protein 3 homolog variant (Fragment) OS=Homo sapiens PE=2 SV=1	42517	548	436	30	107	0.08	0.45	5.625	0	2	1	5	1	6	1	8	1.196951
sp|O00425|IF2B3_HUMAN	IGF2BP3	Insulin-like growth factor 2 mRNA-binding protein 3 OS=Homo sapiens GN=IGF2BP3 PE=1 SV=2	64008	556	331	29	147	0.05	0.28	5.6	0	4	1	5	2	5	2	6	0.218464
sp|Q15645|PCH2_HUMAN	TRIP13	Pachytene checkpoint protein 2 homolog OS=Homo sapiens GN=TRIP13 PE=1 SV=2	48863	507	508	33	88	0.07	0.39	5.571428571	0	2	1	5	2	4	2	9	-0.07122
tr|A8K4D5|A8K4D5_HUMAN	tr|A8K4D5	Kynureninase OS=Homo sapiens PE=2 SV=1	52819	266	275	71	151	0.13	0.72	5.538461538	2	5	2	8	3	9	3	9	0.521908
sp|Q14247|SRC8_HUMAN	CTTN	Src substrate cortactin OS=Homo sapiens GN=CTTN PE=1 SV=2	61720	378	233	46	222	0.11	0.6	5.454545455	1	8	2	9	3	9	3	9	0.521908
tr|B4DFL1|B4DFL1_HUMAN	tr|B4DFL1	Dihydrolipoyl dehydrogenase OS=Homo sapiens PE=2 SV=1	49381	666	357	21	133	0.07	0.38	5.428571429	0	4	1	5	1	5	1	6	0.955766
tr|A6NE09|A6NE09_HUMAN	RPSAP58	Protein RPSAP58 OS=Homo sapiens GN=RPSAP58 PE=3 SV=1	33002	521	404	31	67	0.1	0.53	5.3	0	2	1	2	1	2	1	3	-0.18227
tr|B4DSI9|B4DSI9_HUMAN	tr|B4DSI9	cDNA FLJ56483, highly similar to Eukaryotic translation initiation factor 4 gamma 1 OS=Homo sapiens PE=2	167220	669	202	20	255	0.04	0.21	5.25	0	8	2	10	1	13	3	15	2.252644
tr|B1AHB0|B1AHB0_HUMAN	MCM5	MCM5 minichromosome maintenance deficient 5, cell division cycle 46 (S. cerevisiae), isoform CRA_a OS=H	83031	562	382	29	123	0.04	0.21	5.25	0	3	1	5	1	6	2	9	1.196951
sp|P04350|TBB4A_HUMAN	TUBB4A	Tubulin beta-4A chain OS=Homo sapiens GN=TUBB4A PE=1 SV=2	50010	42	13	509	1589	1.96	10.28	5.244897959	21	53	27	63	29	68	32	73	0.298488
tr|B4DMJ6|B4DMJ6_HUMAN	tr|B4DMJ6	cDNA FLJ50996, highly similar to 60S ribosomal protein L4 OS=Homo sapiens PE=2 SV=1	46269	163	83	132	432	0.51	2.65	5.196078431	4	11	6	16	8	17	9	17	0.122897
sp|P08238|HS90B_HUMAN	HSP90AB1	Heat shock protein HSP 90-beta OS=Homo sapiens GN=HSP90AB1 PE=1 SV=4	83554	32	12	622	1643	1.24	6.39	5.153225806	18	46	28	58	29	61	35	66	0.141717
tr|E5KNY5|E5KNY5_HUMAN	LRPPRC	Leucine-rich PPR-motif containing OS=Homo sapiens GN=LRPPRC PE=2 SV=1	159003	329	85	53	491	0.08	0.41	5.125	1	14	4	17	5	19	7	21	0.908413
tr|J3KR97|J3KR97_HUMAN	TBCD	Tubulin-specific chaperone D OS=Homo sapiens GN=TBCD PE=4 SV=1	138346	714	385	15	121	0.02	0.1	5	0	2	1	4	0	6	1	8	2.78225
tr|B2RU06|B2RU06_HUMAN	EIF4G1	EIF4G1 protein OS=Homo sapiens GN=EIF4G1 PE=2 SV=1	176807	669	201	20	255	0.04	0.2	5	0	8	2	10	1	13	3	15	2.252644
tr|B2ZZ89|B2ZZ89_HUMAN	SPTBN1	Spectrin beta non-erythrocytic 1 OS=Homo sapiens GN=SPTBN1 PE=2 SV=1	275237	49	9	431	1890	0.22	1.1	5	11	47	17	64	20	67	24	75	0.805224
sp|P49411|EFTU_HUMAN	TUFM	Elongation factor Tu, mitochondrial OS=Homo sapiens GN=TUFM PE=1 SV=2	49852	210	102	99	419	0.29	1.45	5	3	12	4	14	4	14	5	14	0.769945
sp|P11021|GRP78_HUMAN	HSPA5	78 kDa glucose-regulated protein OS=Homo sapiens GN=HSPA5 PE=1 SV=2	72402	129	56	170	704	0.19	0.95	5	3	16	5	17	6	18	6	20	0.590942
tr|I3L239|I3L239_HUMAN	TRAP1	Heat shock protein 75 kDa, mitochondrial (Fragment) OS=Homo sapiens GN=TRAP1 PE=4 SV=1	24972	179	346	121	139	0.13	0.65	5	2	4	2	5	2	5	4	7	0.218464
tr|H0Y8E6|H0Y8E6_HUMAN	MCM2	DNA replication licensing factor MCM2 (Fragment) OS=Homo sapiens GN=MCM2 PE=3 SV=1	94792	211	187	98	267	0.07	0.35	5	1	7	2	10	2	9	2	10	1.007671
tr|H0YD14|H0YD14_HUMAN	MYOF	Myoferlin (Fragment) OS=Homo sapiens GN=MYOF PE=4 SV=1	135822	220	82	95	280	0.07	0.35	5	3	7	3	12	4	12	4	15	0.555464
tr|D3DTH7|D3DTH7_HUMAN	MYO1C	Myosin IC, isoform CRA_a OS=Homo sapiens GN=MYO1C PE=2 SV=1	99613	284	160	65	305	0.07	0.34	4.857142857	1	7	3	9	3	9	4	11	0.521908
tr|B3KQT2|B3KQT2_HUMAN	tr|B3KQT2	cDNA PSEC0148 fis, clone PLACE1007202, highly similar to Protein disulfide-isomerase A3 (EC 5.3.4.1) OS=H	55268	546	363	30	98	0.19	0.91	4.789473684	0	4	3	10	2	9	8	11	1.007671
tr|H0YAF8|H0YAF8_HUMAN	GNB2L1	Guanine nucleotide-binding protein subunit beta-2-like 1 (Fragment) OS=Homo sapiens GN=GNB2L1 PE=4	22373	216	95	96	376	0.52	2.49	4.788461538	2	8	3	10	3	10	4	10	0.666476
tr|B4DVB8|B4DVB8_HUMAN	ELAVL1	ELAV-like protein 1 OS=Homo sapiens GN=ELAVL1 PE=2 SV=1	39200	612	419	24	111	0.08	0.38	4.75	0	4	1	4	1	5	1	5	0.955766
tr|B4DY09|B4DY09_HUMAN	tr|B4DY09	cDNA FLJ51660, highly similar to Interleukin enhancer-binding factor 2 OS=Homo sapiens PE=2 SV=1	39057	383	702	45	58	0.08	0.38	4.75	1	2	1	4	1	3	1	4	0.303333
tr|A8K492|A8K492_HUMAN	tr|A8K492	cDNA FLJ76789, highly similar to Homo sapiens methionine-tRNA synthetase (MARS), mRNA OS=Homo sap	102277	385	265	45	196	0.07	0.33	4.714285714	1	6	2	9	2	12	2	12	1.404133
tr|A8K781|A8K781_HUMAN	tr|A8K781	cDNA FLJ75299, highly similar to Xenopus laevis proteasome (prosome, macropain) 26S subunit, ATPase 3,	47578	603	488	26	84	0.07	0.33	4.714285714	0	2	1	3	2	3	2	3	-0.43397
tr|B2R5P6|B2R5P6_HUMAN	TXNRD1	Thioredoxin reductase 1, cytoplasmic OS=Homo sapiens GN=TXNRD1 PE=2 SV=1	55253	108	87	204	446	0.26	1.21	4.653846154	3	10	5	14	5	14	6	14	0.480102
tr|Q5T7Q0|Q5T7Q0_HUMAN	DNAJA1	DnaJ (Hsp40) homolog, subfamily A, member 1, isoform CRA_c OS=Homo sapiens GN=DNAJA1 PE=2 SV=1	45581	703	560	18	80	0.07	0.32	4.571428571	0	2	1	4	0	6	1	10	2.78225
tr|B4DHQ3|B4DHQ3_HUMAN	tr|B4DHQ	cDNA FLJ56437, highly similar to Phosphoserine aminotransferase (EC 2.6.1.52) OS=Homo sapiens PE=2 SV	45783	552	606	30	72	0.07	0.32	4.571428571	0	3	1	4	1	4	2	4	0.666081
tr|B5BU25|B5BU25_HUMAN	U2AF2	U2 small nuclear RNA auxiliary factor 2 isoform b OS=Homo sapiens GN=U2AF2 PE=2 SV=1	53445	260	338	74	131	0.13	0.59	4.538461538	1	4	2	4	2	4	2	5	-0.07122
tr|Q2TSD0|Q2TSD0_HUMAN	tr|Q2TSD0	Glyceraldehyde-3-phosphate dehydrogenase OS=Homo sapiens PE=2 SV=1	36197	18	17	891	1261	3.44	15.51	4.50872093	23	32	31	43	31	49	36	54	-0.2689
tr|B4DFM0|B4DFM0_HUMAN	FARSLB	Phenylalanine-tRNA synthetase-like, beta subunit, isoform CRA_c OS=Homo sapiens GN=FARSLB PE=2 SV=	55353	360	372	48	101	0.06	0.27	4.5	1	3	1	4	1	5	3	6	0.955766
tr|A6NEM2|A6NEM2_HUMAN	HCFC1	HCF N-terminal chain 5 OS=Homo sapiens GN=HCFC1 PE=2 SV=2	215338	508	387	33	121	0.02	0.09	4.5	0	3	1	6	1	8	2	8	1.58433
sp|Q9UQ35|SRRM2_HUMAN	SRRM2	Serine/arginine repetitive matrix protein 2 OS=Homo sapiens GN=SRRM2 PE=1 SV=2	300179	247	219	78	233	0.02	0.09	4.5	1	5	2	8	2	10	5	10	1.152239
tr|B4DEM7|B4DEM7_HUMAN	CCT8	T-complex protein 1 subunit theta OS=Homo sapiens GN=CCT8 PE=2 SV=1	58179	268	106	70	401	0.18	0.8	4.444444444	2	9	3	10	5	10	6	14	0.013727
tr|F5H6E2|F5H6E2_HUMAN	MYO1C	Unconventional myosin-Ic OS=Homo sapiens GN=MYO1C PE=2 SV=1	119719	284	162	65	301	0.07	0.31	4.428571429	1	8	3	10	3	10	4	12	0.666476
tr|B3KMX0|B3KMX0_HUMAN	tr|B3KMX	cDNA FLJ12837 fis, clone NT2RP2003228, highly similar to DNA replication licensing factor MCM4 OS=Hom	97114	240	207	85	251	0.1	0.44	4.4	2	11	3	11	4	12	5	12	0.555464
tr|D3DTW3|D3DTW3_HUMAN	IGF2BP1	Insulin-like growth factor 2 mRNA binding protein 1, isoform CRA_a OS=Homo sapiens GN=IGF2BP1 PE=2 S	48111	556	476	29	71	0.05	0.22	4.4	0	1	1	3	1	3	1	3	0.303333
tr|B2RB23|B2RB23_HUMAN	tr|B2RB23	cDNA, FLJ95265, highly similar to Homo sapiens acetyl-Coenzyme A acyltransferase 2 (mitochondrial 3-oxo	42439	544	454	30	102	0.08	0.35	4.375	0	2	1	4	1	5	1	6	0.955766
tr|B4DJ30|B4DJ30_HUMAN	tr|B4DJ30	cDNA FLJ61290, highly similar to Neutral alpha-glucosidase AB OS=Homo sapiens PE=2 SV=1	113539	47	25	446	1286	0.62	2.69	4.338709677	12	32	19	49	17	50	22	56	0.61286
tr|B7ZKM0|B7ZKM0_HUMAN	SART3	SART3 protein OS=Homo sapiens GN=SART3 PE=2 SV=1	106372	153	63	139	571	0.16	0.69	4.3125	5	14	5	17	7	19	7	23	0.460281
tr|Q60FE2|Q60FE2_HUMAN	MYH9	MYH9 variant protein OS=Homo sapiens GN=MYH9 PE=2 SV=1	227646	17	1	900	3752	0.9	3.85	4.277777778	30	95	46	116	52	123	60	137	0.319307
sp|O95373|IPO7_HUMAN	IPO7	Importin-7 OS=Homo sapiens GN=IPO7 PE=1 SV=1	120751	162	145	132	331	0.08	0.34	4.25	3	8	3	12	3	12	4	14	0.91837
tr|I3L0N3|I3L0N3_HUMAN	NSF	Vesicle-fusing ATPase OS=Homo sapiens GN=NSF PE=3 SV=1	82553	412	550	42	81	0.04	0.17	4.25	1	2	1	4	1	4	1	6	0.666081
tr|Q05CK9|Q05CK9_HUMAN	SYNCRIP	SYNCRIP protein (Fragment) OS=Homo sapiens GN=SYNCRIP PE=2 SV=1	50847	252	240	76	218	0.13	0.55	4.230769231	2	6	2	7	2	8	2	8	0.847028
sp|Q7L2E3|DHX30_HUMAN	DHX30	Putative ATP-dependent RNA helicase DHX30 OS=Homo sapiens GN=DHX30 PE=1 SV=1	134938	397	343	43	140	0.05	0.21	4.2	1	5	2	8	3	8	4	10	0.361266
tr|B3KRS7|B3KRS7_HUMAN	tr|B3KRS7	cDNA FLJ34844 fis, clone NT2NE2011154, highly similar to RuvB-like 1 OS=Homo sapiens PE=2 SV=1	34946	351	446	50	91	0.09	0.37	4.111111111	1	3	1	4	1	4	1	5	0.666081
tr|B2R8R5|B2R8R5_HUMAN	tr|B2R8R5	cDNA, FLJ94025, highly similar to Homo sapiens tripartite motif-containing 28 (TRIM28), mRNA OS=Homo s	90271	320	305	56	168	0.07	0.28	4	1	5	3	7	2	8	4	8	0.847028
sp|Q9NZB2|F120A_HUMAN	FAM120A	Constitutive coactivator of PPAR-gamma-like protein 1 OS=Homo sapiens GN=FAM120A PE=1 SV=2	123008	263	186	73	269	0.05	0.2	4	2	6	2	7	2	8	2	13	0.847028
tr|Q6DEN2|Q6DEN2_HUMAN	DPYSL3	DPYSL3 protein OS=Homo sapiens GN=DPYSL3 PE=2 SV=1	74320	490	154	35	318	0.19	0.75	3.947368421	0	9	4	13	3	14	8	15	1.132851
tr|B4DDB6|B4DDB6_HUMAN	HNRPA3	Heterogeneous nuclear ribonucleoprotein A3, isoform CRA_a OS=Homo sapiens GN=HNRPA3 PE=2 SV=1	37234	201	295	103	163	0.19	0.75	3.947368421	2	4	2	6	2	7	2	10	0.666278
tr|F5GX33|F5GX33_HUMAN	SLC25A13	Calcium-binding mitochondrial carrier protein Aralar2 OS=Homo sapiens GN=SLC25A13 PE=2 SV=1	62548	353	298	49	172	0.11	0.43	3.909090909	1	6	2	7	3	8	4	8	0.361266
tr|Q6IBR0|Q6IBR0_HUMAN	RPN1	RPN1 protein OS=Homo sapiens GN=RPN1 PE=2 SV=1	68678	170	108	127	392	0.26	1.01	3.884615385	4	12	5	15	8	16	8	16	0.03783
tr|B4DSQ5|B4DSQ5_HUMAN	tr|B4DSQ5	cDNA FLJ53608, highly similar to Protein transport protein Sec23A OS=Homo sapiens PE=2 SV=1	83787	474	304	37	126	0.08	0.31	3.875	0	3	2	6	2	7	3	7	0.666278
tr|H0Y4R1|H0Y4R1_HUMAN	IMPDH2	Inosine-5~-monophosphate dehydrogenase 2 (Fragment) OS=Homo sapiens GN=IMPDH2 PE=3 SV=1	51549	177	81	122	513	0.36	1.38	3.833333333	2	13	5	15	5	15	6	16	0.576495
sp|O43175|SERA_HUMAN	PHGDH	D-3-phosphoglycerate dehydrogenase OS=Homo sapiens GN=PHGDH PE=1 SV=4	57356	131	129	166	356	0.25	0.95	3.8	3	9	4	12	4	12	4	13	0.555464
tr|A6NN80|A6NN80_HUMAN	ANXA6	Annexin OS=Homo sapiens GN=ANXA6 PE=3 SV=3	75571	349	212	50	245	0.14	0.53	3.785714286	1	9	3	10	3	12	3	14	0.91837
tr|F5H6T1|F5H6T1_HUMAN	ACTR2	Actin-related protein 2 OS=Homo sapiens GN=ACTR2 PE=2 SV=1	34729	244	184	82	269	0.2	0.75	3.75	2	7	2	8	2	9	4	9	1.007671
tr|A8K2S1|A8K2S1_HUMAN	tr|A8K2S1	cDNA FLJ75258, highly similar to Homo sapiens phospholipase A2, group IVA (cytosolic, calcium-dependen	85695	369	270	47	191	0.08	0.3	3.75	1	5	2	7	2	6	5	12	0.45965
sp|P13010|XRCC5_HUMAN	XRCC5	X-ray repair cross-complementing protein 5 OS=Homo sapiens GN=XRCC5 PE=1 SV=3	83222	128	112	172	387	0.36	1.34	3.722222222	6	9	8	23	8	25	9	27	0.667464
sp|P25490|TYY1_HUMAN	YY1	Transcriptional repressor protein YY1 OS=Homo sapiens GN=YY1 PE=1 SV=2	45141	630	1169	23	25	0.07	0.26	3.714285714	0	0	1	1	2	1	2	1	-1.65672
tr|Q1L838|Q1L838_HUMAN	GTBP	GTBP-ALT OS=Homo sapiens GN=GTBP PE=2 SV=1	122267	559	475	29	97	0.03	0.11	3.666666667	0	3	1	4	1	5	1	7	0.955766
tr|Q6IAV5|Q6IAV5_HUMAN	OXCT	Succinyl-CoA:3-ketoacid-coenzyme A transferase OS=Homo sapiens GN=OXCT PE=2 SV=1	56612	209	193	100	237	0.18	0.66	3.666666667	1	7	3	8	5	9	6	10	-0.13084
sp|O75369|FLNB_HUMAN	FLNB	Filamin-B OS=Homo sapiens GN=FLNB PE=1 SV=2	280157	45	28	463	1179	0.22	0.8	3.636363636	14	33	18	51	20	61	28	67	0.669849
tr|A4QPB0|A4QPB0_HUMAN	IQGAP1	IQ motif containing GTPase activating protein 1 OS=Homo sapiens GN=IQGAP1 PE=2 SV=1	189789	84	22	239	1332	0.31	1.12	3.612903226	8	28	17	45	18	49	21	52	0.503318
sp|P26599|PTBP1_HUMAN	PTBP1	Polypyrimidine tract-binding protein 1 OS=Homo sapiens GN=PTBP1 PE=1 SV=1	57357	567	185	29	269	0.18	0.65	3.611111111	0	3	3	9	3	9	3	12	0.521908
tr|G3XAD8|G3XAD8_HUMAN	STIP1	Stress-induced-phosphoprotein 1 OS=Homo sapiens GN=STIP1 PE=2 SV=1	68721	271	156	69	317	0.21	0.75	3.571428571	2	7	4	12	4	14	5	15	0.769945
tr|Q8TC62|Q8TC62_HUMAN	Sep-07	Septin 7 OS=Homo sapiens GN=SEPT7 PE=2 SV=3	48913	531	538	31	83	0.07	0.25	3.571428571	1	2	1	3	1	3	2	3	0.303333
tr|A5D906|A5D906_HUMAN	TUBB2A	TUBB2A protein (Fragment) OS=Homo sapiens GN=TUBB2A PE=2 SV=1	28128	25	16	143	603	3.07	10.83	3.527687296	4	19	7	22	8	26	8	30	0.723138
tr|B3KW21|B3KW21_HUMAN	tr|B3KW2	cDNA FLJ41945 fis, clone PLACE6019676, highly similar to Coatomer subunit gamma OS=Homo sapiens PE=	68674	705	458	18	96	0.04	0.14	3.5	0	2	1	3	0	5	1	5	2.541064
tr|Q5VZU9|Q5VZU9_HUMAN	TPP2	Tripeptidyl-peptidase 2 OS=Homo sapiens GN=TPP2 PE=4 SV=1	141160	693	704	19	58	0.02	0.07	3.5	0	2	1	3	0	2	1	4	1.403027
tr|E7ERW8|E7ERW8_HUMAN	DIAPH1	Protein diaphanous homolog 1 OS=Homo sapiens GN=DIAPH1 PE=2 SV=1	135945	184	605	116	72	0.02	0.07	3.5	1	3	1	3	1	4	2	5	0.666081
tr|B2RBE5|B2RBE5_HUMAN	tr|B2RBE5	cDNA, FLJ95468, highly similar to Homo sapiens transcriptional coactivator tubedown-100 (TBDN100),tran	102490	446	558	38	80	0.06	0.21	3.5	0	2	2	6	2	6	2	9	0.45965
tr|Q6DC98|Q6DC98_HUMAN	LMNB1	LMNB1 protein (Fragment) OS=Homo sapiens GN=LMNB1 PE=2 SV=1	38289	219	136	95	206	0.18	0.62	3.444444444	2	6	2	6	2	6	4	6	0.45965
tr|B2RAQ8|B2RAQ8_HUMAN	tr|B2RAQ8	cDNA, FLJ95058, highly similar to Homo sapiens carnitine palmitoyltransferase 1A (liver) (CPT1A),nuclear g	88967	354	358	49	133	0.07	0.24	3.428571429	1	4	2	6	3	5	3	7	-0.2673
tr|H0Y5F5|H0Y5F5_HUMAN	PABPC4	Polyadenylate-binding protein 4 (Fragment) OS=Homo sapiens GN=PABPC4 PE=4 SV=1	60492	504	699	33	59	0.05	0.17	3.4	0	3	1	3	1	5	2	6	0.955766
tr|B4DSF8|B4DSF8_HUMAN	tr|B4DSF8	cDNA FLJ57278 OS=Homo sapiens PE=2 SV=1	40477	367	620	47	70	0.05	0.17	3.4	1	1	1	2	1	2	1	2	-0.18227
sp|P49327|FAS_HUMAN	FASN	Fatty acid synthase OS=Homo sapiens GN=FASN PE=1 SV=3	275877	55	20	394	1388	0.28	0.95	3.392857143	12	41	21	58	23	63	29	68	0.518327
tr|D3DPU2|D3DPU2_HUMAN	CAP1	Adenylyl cyclase-associated protein OS=Homo sapiens GN=CAP1 PE=3 SV=1	51926	172	141	125	343	0.36	1.22	3.388888889	4	5	5	14	6	16	7	19	0.425526
tr|B4DHG3|B4DHG3_HUMAN	tr|B4DHG	cDNA FLJ55329, highly similar to Procollagen-lysine,2-oxoglutarate5-dioxygenase 2 (EC 1.14.11.4) OS=Hom	81716	453	283	38	178	0.08	0.27	3.375	0	5	2	6	2	7	2	7	0.666278
sp|Q6IBS0|TWF2_HUMAN	TWF2	Twinfilin-2 OS=Homo sapiens GN=TWF2 PE=1 SV=2	39751	535	397	30	119	0.08	0.27	3.375	0	2	1	3	1	4	1	4	0.666081
tr|B4DLB8|B4DLB8_HUMAN	tr|B4DLB8	cDNA FLJ52205, highly similar to Beta-1,4-galactosyltransferase 1 (EC 2.4.1.-) OS=Homo sapiens PE=2 SV=1	40269	650	715	22	57	0.08	0.27	3.375	0	1	1	3	1	3	1	3	0.303333
tr|B4DEX8|B4DEX8_HUMAN	MAT2A	S-adenosylmethionine synthase OS=Homo sapiens GN=MAT2A PE=2 SV=1	40027	333	349	52	136	0.08	0.27	3.375	1	3	1	3	1	3	1	4	0.303333
tr|G3V0E8|G3V0E8_HUMAN	PCBP2	Poly(RC) binding protein 2, isoform CRA_f OS=Homo sapiens GN=PCBP2 PE=2 SV=1	33817	132	209	166	200	0.32	1.08	3.375	3	4	3	7	3	7	5	7	0.180515
tr|B3KS31|B3KS31_HUMAN	TUBB6	Tubulin, beta 6, isoform CRA_a OS=Homo sapiens GN=TUBB6 PE=2 SV=1	42324	73	62	273	608	0.83	2.79	3.361445783	10	21	14	29	17	34	20	37	0.060315
tr|B2R5U1|B2R5U1_HUMAN	tr|B2R5U1	cDNA, FLJ92620, highly similar to Homo sapiens staphylococcal nuclease domain containing 1 (SND1),mRN	100294	150	118	144	374	0.17	0.57	3.352941176	4	10	5	15	10	20	12	24	0.046172
sp|Q9Y2A7|NCKP1_HUMAN	NCKAP1	Nck-associated protein 1 OS=Homo sapiens GN=NCKAP1 PE=1 SV=1	130018	685	637	19	68	0.03	0.1	3.333333333	0	1	1	4	0	6	1	6	2.78225
tr|B2R6D0|B2R6D0_HUMAN	tr|B2R6D0	cDNA, FLJ92896, highly similar to Homo sapiens proteasome (prosome, macropain) 26S subunit, non-ATPa	106797	386	272	45	188	0.06	0.2	3.333333333	0	5	2	6	2	7	4	9	0.666278
tr|H7C3A1|H7C3A1_HUMAN	SRRT	Serrate RNA effector molecule homolog (Fragment) OS=Homo sapiens GN=SRRT PE=4 SV=1	57154	701	734	18	54	0.06	0.2	3.333333333	0	1	1	1	0	1	1	1	0.665884
sp|P53618|COPB_HUMAN	COPB1	Coatomer subunit beta OS=Homo sapiens GN=COPB1 PE=1 SV=3	108214	303	389	59	121	0.06	0.2	3.333333333	1	4	2	6	2	6	3	9	0.45965
tr|A8K2Y2|A8K2Y2_HUMAN	tr|A8K2Y2	cDNA FLJ78120, highly similar to Homo sapiens eukaryotic translation initiation factor 2, subunit 3 gamma,	51648	475	501	37	90	0.06	0.2	3.333333333	1	3	1	3	1	3	1	5	0.303333
tr|A8K3Q7|A8K3Q7_HUMAN	tr|A8K3Q7	Annexin OS=Homo sapiens PE=2 SV=1	76140	349	211	43	246	0.14	0.46	3.285714286	1	9	2	9	2	12	2	13	1.404133
tr|Q59EG8|Q59EG8_HUMAN	tr|Q59EG8	Proteasome 26S non-ATPase subunit 2 variant (Fragment) OS=Homo sapiens PE=2 SV=1	101322	198	147	105	330	0.14	0.46	3.285714286	1	12	4	12	4	12	5	12	0.555464
sp|Q9Y305|ACOT9_HUMAN	ACOT9	Acyl-coenzyme A thioesterase 9, mitochondrial OS=Homo sapiens GN=ACOT9 PE=1 SV=2	50269	637	1061	22	32	0.07	0.23	3.285714286	0	0	1	2	1	2	2	2	-0.18227
tr|Q53SS8|Q53SS8_HUMAN	PCBP1	Poly(RC) binding protein 1 OS=Homo sapiens GN=PCBP1 PE=2 SV=1	37987	110	179	197	277	0.29	0.95	3.275862069	3	6	3	8	3	8	3	9	0.361266
tr|Q5TCU6|Q5TCU6_HUMAN	TLN1	Talin-1 OS=Homo sapiens GN=TLN1 PE=4 SV=1	260030	48	14	446	1485	0.27	0.88	3.259259259	12	40	19	49	20	50	25	58	0.383578
tr|F5H1A8|F5H1A8_HUMAN	GSN	Gelsolin OS=Homo sapiens GN=GSN PE=2 SV=1	81776	297	513	61	87	0.04	0.13	3.25	1	2	1	3	1	3	2	7	0.303333
tr|B3GQS7|B3GQS7_HUMAN	HSPD1	Mitochondrial heat shock 60kD protein 1 variant 1 OS=Homo sapiens GN=HSPD1 PE=2 SV=1	60813	16	37	904	951	1.72	5.59	3.25	24	23	27	36	26	38	33	42	-0.38238
tr|H0YK49|H0YK49_HUMAN	ETFA	Electron transfer flavoprotein subunit alpha, mitochondrial OS=Homo sapiens GN=ETFA PE=4 SV=1	24259	545	673	30	52	0.14	0.45	3.214285714	0	1	1	2	1	2	1	2	-0.18227
tr|B4E022|B4E022_HUMAN	TKT	Transketolase OS=Homo sapiens GN=TKT PE=2 SV=1	63410	35	27	578	1235	1.36	4.3	3.161764706	17	36	24	43	26	51	32	57	0.039651
tr|Q2NKY5|Q2NKY5_HUMAN	TUBB6	TUBB6 protein OS=Homo sapiens GN=TUBB6 PE=2 SV=1	50514	65	52	317	745	0.88	2.77	3.147727273	12	23	16	32	17	38	19	42	0.219291
tr|Q6IPN6|Q6IPN6_HUMAN	EEF1A1	Elongation factor 1-alpha OS=Homo sapiens GN=EEF1A1 PE=2 SV=1	50433	70	80	295	515	1.14	3.58	3.140350877	11	18	13	30	18	34	21	37	-0.02019
tr|B4DFK9|B4DFK9_HUMAN	tr|B4DFK9	cDNA FLJ57964, highly similar to Heterogeneous nuclear ribonucleoprotein H~ OS=Homo sapiens PE=2 SV=	44637	272	196	69	208	0.15	0.47	3.133333333	1	6	2	6	1	7	2	8	1.40358
tr|B0UZ98|B0UZ98_HUMAN	BAT1	HLA-B associated transcript 1 (Fragment) OS=Homo sapiens GN=BAT1 PE=2 SV=1	28623	394	589	44	53	0.09	0.28	3.111111111	1	1	1	2	1	2	1	3	-0.18227
sp|P13639|EF2_HUMAN	EEF2	Elongation factor 2 OS=Homo sapiens GN=EEF2 PE=1 SV=4	96246	41	15	511	1522	1.23	3.82	3.105691057	13	45	26	62	27	68	32	79	0.400448
tr|F8W9S7|F8W9S7_HUMAN	GAPVD1	GTPase-activating protein and VPS9 domain-containing protein 1 OS=Homo sapiens GN=GAPVD1 PE=2 SV=	158790	697	460	18	100	0.02	0.06	3	0	3	1	3	0	4	2	4	2.25138
tr|B4DYL7|B4DYL7_HUMAN	tr|B4DYL7	cDNA FLJ58490, highly similar to Homo sapiens CCR4-NOT transcription complex, subunit 1 (CNOT1), trans	140991	388	478	45	59	0.02	0.06	3	1	3	1	3	1	5	2	5	0.955766
sp|O95782|AP2A1_HUMAN	AP2A1	AP-2 complex subunit alpha-1 OS=Homo sapiens GN=AP2A1 PE=1 SV=3	108561	500	661	34	65	0.03	0.09	3	0	1	1	3	1	3	2	4	0.303333
tr|Q13727|Q13727_HUMAN	tr|Q13727	AHNAK-related protein (Fragment) OS=Homo sapiens PE=2 SV=1	57703	402	627	43	69	0.06	0.18	3	1	3	1	3	2	4	3	5	-0.07122
sp|Q6DD88|ATLA3_HUMAN	ATL3	Atlastin-3 OS=Homo sapiens GN=ATL3 PE=1 SV=1	60960	451	1076	38	31	0.06	0.18	3	1	0	1	1	1	1	1	1	-0.91941
sp|O00629|IMA4_HUMAN	KPNA4	Importin subunit alpha-4 OS=Homo sapiens GN=KPNA4 PE=1 SV=1	58364	472	817	37	47	0.06	0.18	3	0	1	1	1	2	1	2	1	-1.65672
tr|Q5TB52|Q5TB52_HUMAN	PAPSS2	3~-phosphoadenosine 5~-phosphosulfate synthase 2, isoform CRA_b OS=Homo sapiens GN=PAPSS2 PE=2	70027	688	342	19	141	0.05	0.15	3	0	3	1	3	0	3	4	3	1.888632
tr|B3KXU8|B3KXU8_HUMAN	tr|B3KXU8	cDNA FLJ46087 fis, clone TESTI2009497, highly similar to GPI-anchored protein p137 OS=Homo sapiens PE	70489	367	633	47	69	0.05	0.15	3	1	1	1	3	1	3	1	3	0.303333
tr|B1AMS2|B1AMS2_HUMAN	Sep-06	Septin 6 OS=Homo sapiens GN=SEPT6 PE=3 SV=1	49614	532	411	31	38	0.07	0.21	3	1	1	1	1	1	1	1	1	-0.91941
tr|B4DE36|B4DE36_HUMAN	tr|B4DE36	Glucose-6-phosphate isomerase OS=Homo sapiens PE=2 SV=1	60376	117	146	182	331	0.3	0.89	2.966666667	4	8	5	12	7	14	7	16	0.03197
tr|H7BZJ3|H7BZJ3_HUMAN	PDIA3	Thioredoxin (Fragment) OS=Homo sapiens GN=PDIA3 PE=3 SV=1	13739	546	666	22	64	0.19	0.56	2.947368421	0	1	1	2	1	2	1	3	-0.18227
tr|Q60FE5|Q60FE5_HUMAN	FLNA	Filamin A OS=Homo sapiens GN=FLNA PE=2 SV=1	280790	9	2	1306	3397	0.86	2.52	2.930232558	41	90	60	128	75	138	88	154	-0.04461
tr|Q3B7A7|Q3B7A7_HUMAN	GART	Phosphoribosylglycinamide formyltransferase, phosphoribosylglycinamide synthetase, phosphoribosylami	108909	341	163	51	300	0.13	0.38	2.923076923	1	6	4	11	3	16	4	18	1.31962
tr|B4DGU4|B4DGU4_HUMAN	CTNNB1	Catenin beta-1 OS=Homo sapiens GN=CTNNB1 PE=2 SV=1	85339	340	310	52	163	0.12	0.35	2.916666667	2	6	3	8	3	8	3	9	0.361266
sp|Q15149|PLEC_HUMAN	PLEC	Plectin OS=Homo sapiens GN=PLEC PE=1 SV=3	533462	74	21	270	1339	0.11	0.32	2.909090909	11	37	17	46	18	61	32	69	0.818622
tr|B7Z4V2|B7Z4V2_HUMAN	tr|B7Z4V2	cDNA FLJ51907, highly similar to Stress-70 protein, mitochondrial OS=Homo sapiens PE=2 SV=1	72641	81	67	251	611	0.42	1.22	2.904761905	7	15	8	22	8	24	8	26	0.609571
sp|Q96TA1|NIBL1_HUMAN	FAM129B	Niban-like protein 1 OS=Homo sapiens GN=FAM129B PE=1 SV=3	84598	155	256	137	206	0.16	0.46	2.875	4	7	4	10	4	10	4	13	0.303569
tr|Q7Z5T5|Q7Z5T5_HUMAN	EIF3A	EIF3A protein (Fragment) OS=Homo sapiens GN=EIF3A PE=2 SV=1	97203	192	139	112	341	0.14	0.4	2.857142857	2	13	4	16	5	16	8	20	0.666871
tr|B7Z6M1|B7Z6M1_HUMAN	PLS3	Plastin-3 OS=Homo sapiens GN=PLS3 PE=2 SV=1	66047	282	109	66	382	0.34	0.97	2.852941176	1	10	6	14	6	16	8	17	0.425526
tr|Q5HY54|Q5HY54_HUMAN	FLNA	Filamin-A OS=Homo sapiens GN=FLNA PE=2 SV=1	279115	9	3	1304	3357	0.86	2.44	2.837209302	41	89	59	125	74	136	87	151	-0.04638
tr|B3KM77|B3KM77_HUMAN	tr|B3KM7	cDNA FLJ10435 fis, clone NT2RP1000493, highly similar to Splicing factor 3B subunit 3 OS=Homo sapiens P	56736	356	262	49	148	0.06	0.17	2.833333333	1	3	1	3	1	4	1	4	0.666081
tr|Q59FC9|Q59FC9_HUMAN	tr|Q59FC9	Enigma homolog (Fragment) OS=Homo sapiens PE=2 SV=1	50413	175	104	109	403	0.41	1.16	2.829268293	2	10	5	12	5	12	6	13	0.265621

tr|F8VPD4|F8VPD4_HUMAN	CAD	CAD protein OS=Homo sapiens GN=CAD PE=2 SV=1	238097	317	302	57	169	0.04	0.11	2.75	1	5	3	8	4	7	5	8	-0.18239
tr|B0V043|B0V043_HUMAN	VARS	Valine--tRNA ligase OS=Homo sapiens GN=VARS PE=3 SV=1	141632	275	151	69	324	0.15	0.41	2.733333333	2	8	6	17	6	16	8	21	0.425526
tr|B4DR45|B4DR45_HUMAN	tr|B4DR45	cDNA FLJ61339, highly similar to Alanyl-tRNA synthetase (EC 6.1.1.7) OS=Homo sapiens PE=2 SV=1	110017	238	525	86	84	0.06	0.16	2.666666667	1	1	2	5	2	6	2	7	0.45965
tr|H0Y4A6|H0Y4A6_HUMAN	VARS	Valine--tRNA ligase (Fragment) OS=Homo sapiens GN=VARS PE=3 SV=1	133055	275	180	69	276	0.15	0.4	2.666666667	2	7	6	16	6	16	8	21	0.425526
tr|A6NG51|A6NG51_HUMAN	SPTAN1	Spectrin alpha chain, non-erythrocytic 1 OS=Homo sapiens GN=SPTAN1 PE=2 SV=2	285628	167	114	130	327	0.06	0.16	2.666666667	2	6	5	12	6	14	9	18	0.238757
sp|P07355|ANXA2_HUMAN	ANXA2	Annexin A2 OS=Homo sapiens GN=ANXA2 PE=1 SV=2	38808	100	66	214	612	1.26	3.36	2.666666667	8	18	11	23	12	25	13	31	0.109724
sp|Q96HC4|PDLI5_HUMAN	PDLIM5	PDZ and LIM domain protein 5 OS=Homo sapiens GN=PDLIM5 PE=1 SV=5	65102	175	89	123	477	0.41	1.09	2.658536585	3	11	7	16	7	15	8	17	0.128363
sp|Q09028|RBBP4_HUMAN	RBBP4	Histone-binding protein RBBP4 OS=Homo sapiens GN=RBBP4 PE=1 SV=3	47911	321	703	55	58	0.22	0.58	2.636363636	2	1	3	3	4	4	4	4	-0.91989
tr|B7Z481|B7Z481_HUMAN	tr|B7Z481	cDNA FLJ54018, highly similar to PDZ and LIM domain protein 5 OS=Homo sapiens PE=2 SV=1	52359	175	93	109	455	0.41	1.08	2.634146341	2	11	5	13	5	13	6	13	0.37683
tr|B4DN87|B4DN87_HUMAN	tr|B4DN87	cDNA FLJ52569, highly similar to Collagen-binding protein 2 OS=Homo sapiens PE=2 SV=1	44290	233	239	89	192	0.43	1.13	2.627906977	4	7	5	10	5	10	5	11	0.013727
tr|B0YJC4|B0YJC4_HUMAN	VIM	Vimentin OS=Homo sapiens GN=VIM PE=3 SV=1	49680	66	74	312	483	1.03	2.7	2.621359223	9	17	11	22	12	23	15	26	-0.00847
sp|Q08211|DHX9_HUMAN	DHX9	ATP-dependent RNA helicase A OS=Homo sapiens GN=DHX9 PE=1 SV=4	142181	96	53	218	733	0.41	1.07	2.609756098	8	19	16	33	18	37	22	42	0.100637
tr|B3KSQ7|B3KSQ7_HUMAN	DBN1	Drebrin 1, isoform CRA_d OS=Homo sapiens GN=DBN1 PE=2 SV=1	66053	478	280	36	136	0.05	0.13	2.6	0	2	1	2	1	2	1	3	-0.18227
tr|E9PNN6|E9PNN6_HUMAN	ALDH3A1	Aldehyde dehydrogenase, dimeric NADP-preferring OS=Homo sapiens GN=ALDH3A1 PE=2 SV=1	44518	519	1042	32	33	0.07	0.18	2.571428571	0	0	1	3	1	2	1	3	-0.18227
sp|O00410|IPO5_HUMAN	IPO5	Importin-5 OS=Homo sapiens GN=IPO5 PE=1 SV=4	125032	85	76	236	533	0.23	0.59	2.565217391	7	16	8	18	10	21	15	23	0.115063
tr|B2R6J2|B2R6J2_HUMAN	tr|B2R6J2	cDNA, FLJ92973, highly similar to Homo sapiens villin 2 (ezrin) (VIL2), mRNA OS=Homo sapiens PE=2 SV=1	69472	126	116	173	375	0.32	0.82	2.5625	6	13	6	16	8	21	8	22	0.420591
tr|A0AV58|A0AV58_HUMAN	STRN3	Striatin, calmodulin binding protein 3 OS=Homo sapiens GN=STRN3 PE=2 SV=1	78069	419	271	42	188	0.09	0.23	2.555555556	1	5	2	5	3	5	3	5	-0.2673
tr|B2RWP9|B2RWP9_HUMAN	MYH10	MYH10 protein OS=Homo sapiens GN=MYH10 PE=2 SV=1	230858	200	261	104	201	0.06	0.15	2.5	3	5	4	10	4	10	6	14	0.303569
tr|J3KPM9|J3KPM9_HUMAN	STAT1	Signal transducer and activator of transcription 1-alpha/beta OS=Homo sapiens GN=STAT1 PE=4 SV=1	83878	364	420	48	93	0.08	0.2	2.5	1	3	2	4	2	4	2	4	-0.07122
tr|B4DKD0|B4DKD0_HUMAN	tr|B4DKD0	DNA topoisomerase 2 (Fragment) OS=Homo sapiens PE=2 SV=1	135015	606	467	25	99	0.02	0.05	2.5	0	2	1	2	1	2	3	5	-0.18227
tr|B1PS43|B1PS43_HUMAN	MYH11	Myosin heavy chain 11 smooth muscle isoform OS=Homo sapiens GN=MYH11 PE=2 SV=1	235004	142	111	155	387	0.06	0.15	2.5	5	11	5	12	9	15	11	16	-0.21335
tr|B1Q2N1|B1Q2N1_HUMAN	DDX39	ATP-dependent RNA helicase DDX39A OS=Homo sapiens GN=DDX39 PE=2 SV=1	30925	395	646	44	44	0.11	0.27	2.454545455	1	1	1	2	1	3	2	4	0.303333
tr|D3DQA6|D3DQA6_HUMAN	DDX46	DEAD (Asp-Glu-Ala-Asp) box polypeptide 46, isoform CRA_a OS=Homo sapiens GN=DDX46 PE=3 SV=1	100958	595	451	26	102	0.07	0.17	2.428571429	0	2	2	5	3	5	3	6	-0.2673
tr|B2RCM2|B2RCM2_HUMAN	tr|B2RCM	cDNA, FLJ96156, highly similar to Homo sapiens leucyl-tRNA synthetase (LARS), mRNA OS=Homo sapiens P	135550	257	273	75	185	0.1	0.24	2.4	2	6	4	9	3	13	5	16	1.02958
tr|K7EIG1|K7EIG1_HUMAN	CLUH	Clustered mitochondria protein homolog OS=Homo sapiens GN=CLUH PE=3 SV=1	140903	438	351	39	134	0.05	0.12	2.4	1	3	2	5	2	4	2	5	-0.07122
tr|A5PHJ9|A5PHJ9_HUMAN	PDHA1/LC	PDHA1/LOC79064 protein (Fragment) OS=Homo sapiens GN=PDHA1/LOC79064 PE=2 SV=1	34113	516	775	32	50	0.1	0.24	2.4	0	2	1	2	1	2	1	2	-0.18227
tr|F8VRQ1|F8VRQ1_HUMAN	HNRNPA1	Heterogeneous nuclear ribonucleoprotein A1 OS=Homo sapiens GN=HNRNPA1 PE=2 SV=1	33249	101	72	214	572	0.77	1.84	2.38961039	4	11	6	13	7	15	9	17	0.128363
tr|B2RCM3|B2RCM3_HUMAN	tr|B2RCM	cDNA, FLJ96158, highly similar to Homo sapiens calpain 2, (m/II) large subunit (CAPN2), mRNA OS=Homo s	80883	173	143	125	315	0.22	0.52	2.363636364	3	8	7	10	7	12	8	13	-0.18251
tr|B4DH02|B4DH02_HUMAN	tr|B4DH02	cDNA FLJ50510, highly similar to Heat shock 70 kDa protein 4 OS=Homo sapiens PE=2 SV=1	95157	123	91	175	463	0.23	0.54	2.347826087	4	10	6	12	5	12	6	14	0.265621
tr|B4DF96|B4DF96_HUMAN	EIF2A	Eukaryotic translation initiation factor 2A OS=Homo sapiens GN=EIF2A PE=2 SV=1	58357	206	191	101	252	0.32	0.75	2.34375	2	7	5	10	4	13	5	14	0.666673
sp|Q9P287|BCCIP_HUMAN	BCCIP	BRCA2 and CDKN1A-interacting protein OS=Homo sapiens GN=BCCIP PE=1 SV=1	36299	503	901	33	42	0.09	0.21	2.333333333	0	2	1	2	1	2	3	2	-0.18227
tr|H7C4C8|H7C4C8_HUMAN	CCT8	T-complex protein 1 subunit theta (Fragment) OS=Homo sapiens GN=CCT8 PE=3 SV=1	35913	268	374	70	126	0.18	0.42	2.333333333	2	3	3	4	4	5	5	7	-0.63021
tr|Q2XPP3|Q2XPP3_HUMAN	tr|Q2XPP3	Type II 3a-hydroxysteroid dehydrogenase variant OS=Homo sapiens PE=2 SV=1	37201	203	362	102	130	0.29	0.67	2.310344828	3	4	3	6	3	6	4	7	-0.02611
sp|P07814|SYEP_HUMAN	EPRS	Bifunctional glutamate/proline--tRNA ligase OS=Homo sapiens GN=EPRS PE=1 SV=5	172080	57	57	380	702	0.33	0.76	2.303030303	9	18	15	31	15	33	17	37	0.193463
tr|B7Z5E2|B7Z5E2_HUMAN	PSMC2	26S protease regulatory subunit 7 OS=Homo sapiens GN=PSMC2 PE=2 SV=1	33594	610	544	25	59	0.1	0.23	2.3	0	1	1	2	1	2	1	2	-0.18227
tr|B7Z2Z8|B7Z2Z8_HUMAN	tr|B7Z2Z8	T-complex protein 1 subunit delta OS=Homo sapiens PE=2 SV=1	52781	113	194	188	262	0.27	0.62	2.296296296	4	8	5	8	7	8	7	10	-0.73962
tr|B3KX72|B3KX72_HUMAN	tr|B3KX72	cDNA FLJ44920 fis, clone BRAMY3011501, highly similar to Heterogeneous nuclear ribonucleoprotein U OS	83774	59	47	367	690	0.58	1.33	2.293103448	10	15	14	22	16	29	20	33	-0.08123
tr|Q5U077|Q5U077_HUMAN	LDHB	L-lactate dehydrogenase OS=Homo sapiens GN=LDHB PE=2 SV=1	36900	39	58	530	680	1.81	4.13	2.281767956	13	22	16	27	18	27	20	27	-0.34861
tr|B2R7M3|B2R7M3_HUMAN	tr|B2R7M	cDNA, FLJ93510, highly similar to Homo sapiens JTV1 gene (JTV1), mRNA OS=Homo sapiens PE=2 SV=1	35767	308	641	58	68	0.19	0.43	2.263157895	2	2	2	4	2	4	2	6	-0.07122
sp|P23381|SYWC_HUMAN	WARS	Tryptophan--tRNA ligase, cytoplasmic OS=Homo sapiens GN=WARS PE=1 SV=2	53474	145	214	148	240	0.27	0.61	2.259259259	3	5	4	8	5	9	5	11	-0.13084
tr|B4E091|B4E091_HUMAN	tr|B4E091	cDNA FLJ55438, highly similar to Splicing factor 3 subunit 1 OS=Homo sapiens PE=2 SV=1	77415	482	568	36	79	0.04	0.09	2.25	1	1	1	2	1	2	1	2	-0.18227
tr|B4DPY2|B4DPY2_HUMAN	tr|B4DPY2	cDNA FLJ59286, highly similar to Pre-mRNA-processing factor 40 homolog A (Fragment) OS=Homo sapiens	93124	425	988	41	36	0.04	0.09	2.25	1	1	1	1	1	1	1	1	-0.91941
tr|E9PBU3|E9PBU3_HUMAN	ATIC	Phosphoribosylaminoimidazolecarboxamide formyltransferase OS=Homo sapiens GN=ATIC PE=2 SV=1	64996	72	107	274	363	0.64	1.43	2.234375	8	12	11	17	12	18	13	20	-0.35449
sp|P55072|TERA_HUMAN	VCP	Transitional endoplasmic reticulum ATPase OS=Homo sapiens GN=VCP PE=1 SV=4	89950	64	46	326	872	0.65	1.45	2.230769231	7	21	14	28	14	28	15	35	0.055925
tr|Q59G92|Q59G92_HUMAN	tr|Q59G92	HLA-B associated transcript 1 variant (Fragment) OS=Homo sapiens PE=2 SV=1	35528	394	747	44	53	0.09	0.2	2.222222222	1	1	1	2	2	4	2	5	-0.07122
tr|Q9NZ23|Q9NZ23_HUMAN	YA61	Drug-sensitive protein 1 OS=Homo sapiens GN=YA61 PE=2 SV=1	14858	588	796	27	49	0.23	0.51	2.217391304	0	2	1	2	1	3	1	3	0.303333
tr|B1ALA9|B1ALA9_HUMAN	PRPS1	Phosphoribosyl pyrophosphate synthetase 1 OS=Homo sapiens GN=PRPS1 PE=3 SV=1	24468	638	421	22	70	0.14	0.31	2.214285714	0	1	1	2	1	2	2	3	-0.18227
tr|B4DXX7|B4DXX7_HUMAN	DDX3Y	ATP-dependent RNA helicase DDX3Y OS=Homo sapiens GN=DDX3Y PE=2 SV=1	73342	217	279	95	180	0.19	0.42	2.210526316	2	6	5	9	6	10	6	12	-0.22762
sp|P52272|HNRPM_HUMAN	HNRNPM	Heterogeneous nuclear ribonucleoprotein M OS=Homo sapiens GN=HNRNPM PE=1 SV=3	77749	164	90	132	472	0.39	0.86	2.205128205	7	11	8	15	11	15	12	19	-0.48965
tr|B0QZK4|B0QZK4_HUMAN	HP1BP3	Heterochromatin protein 1-binding protein 3 (Fragment) OS=Homo sapiens GN=HP1BP3 PE=4 SV=1	28725	631	542	23	82	0.25	0.55	2.2	0	3	2	4	1	5	3	6	0.955766
tr|B4DMY3|B4DMY3_HUMAN	tr|B4DMY	cDNA FLJ60713, highly similar to Homo sapiens heterogeneous nuclear ribonucleoprotein A/B (HNRPAB), t	35101	318	533	56	83	0.2	0.44	2.2	1	4	2	5	2	5	2	5	0.218464
sp|Q15392|DHC24_HUMAN	DHCR24	Delta(24)-sterol reductase OS=Homo sapiens GN=DHCR24 PE=1 SV=2	60803	442	692	39	60	0.05	0.11	2.2	1	2	1	2	2	4	3	5	-0.07122
sp|Q1KMD3|HNRL2_HUMAN	HNRNPUL	Heterogeneous nuclear ribonucleoprotein U-like protein 2 OS=Homo sapiens GN=HNRNPUL2 PE=1 SV=1	85622	249	130	78	355	0.21	0.46	2.19047619	2	8	5	10	6	13	6	13	0.135486
tr|H3BLZ8|H3BLZ8_HUMAN	DDX17	Probable ATP-dependent RNA helicase DDX17 OS=Homo sapiens GN=DDX17 PE=3 SV=1	81073	166	84	130	492	0.37	0.81	2.189189189	3	14	9	17	9	17	10	18	-0.0379
sp|P00338|LDHA_HUMAN	LDHA	L-lactate dehydrogenase A chain OS=Homo sapiens GN=LDHA PE=1 SV=2	36950	44	61	481	651	2.33	5.06	2.17167382	10	19	19	23	19	26	24	33	-0.47851
sp|O00232|PSD12_HUMAN	PSMD12	26S proteasome non-ATPase regulatory subunit 12 OS=Homo sapiens GN=PSMD12 PE=1 SV=3	53270	283	981	66	36	0.06	0.13	2.166666667	1	1	1	2	1	4	1	4	0.666081
tr|B4E363|B4E363_HUMAN	FARSA	Phenylalanine--tRNA ligase alpha subunit OS=Homo sapiens GN=FARSA PE=2 SV=1	54181	447	1136	38	27	0.06	0.13	2.166666667	1	0	1	2	1	2	1	4	-0.18227
tr|B4DKS0|B4DKS0_HUMAN	tr|B4DKS0	cDNA FLJ53381, highly similar to Monocarboxylate transporter 1 OS=Homo sapiens PE=2 SV=1	52487	598	683	26	23	0.06	0.13	2.166666667	0	0	1	1	1	1	1	1	-0.91941
tr|B7Z8W6|B7Z8W6_HUMAN	tr|B7Z8W	cDNA FLJ55038, highly similar to Nicotinamide phosphoribosyltransferase (EC 2.4.2.12) OS=Homo sapiens	26905	520	393	32	41	0.12	0.26	2.166666667	0	1	1	1	1	1	3	2	-0.91941
tr|B7Z601|B7Z601_HUMAN	tr|B7Z601	cDNA FLJ57187, highly similar to Glycerol-3-phosphate dehydrogenase, mitochondrial (EC 1.1.99.5) OS=Ho	56047	512	445	32	95	0.06	0.13	2.166666667	0	2	1	2	2	2	2	3	-0.91957
tr|G3V2B8|G3V2B8_HUMAN	MTHFD1	C-1-tetrahydrofolate synthase, cytoplasmic OS=Homo sapiens GN=MTHFD1 PE=2 SV=1	102152	112	94	192	451	0.33	0.71	2.151515152	5	15	9	17	12	22	12	24	-0.07138
sp|O43286|B4GT5_HUMAN	B4GALT5	Beta-1,4-galactosyltransferase 5 OS=Homo sapiens GN=B4GALT5 PE=2 SV=1	45318	651	967	22	37	0.07	0.15	2.142857143	0	2	1	2	1	2	1	2	-0.18227
tr|B4DX08|B4DX08_HUMAN	tr|B4DX08	cDNA FLJ58784, highly similar to T-complex protein 1 subunit epsilon OS=Homo sapiens PE=2 SV=1	57521	90	176	229	264	0.4	0.85	2.125	6	9	6	10	6	11	6	12	-0.0962
tr|H0YH88|H0YH88_HUMAN	NAP1L1	Nucleosome assembly protein 1-like 1 (Fragment) OS=Homo sapiens GN=NAP1L1 PE=3 SV=1	21179	417	971	33	37	0.16	0.34	2.125	0	1	1	2	1	2	1	2	-0.18227
tr|H0Y5J4|H0Y5J4_HUMAN	DBNL	Drebrin-like protein (Fragment) OS=Homo sapiens GN=DBNL PE=4 SV=1	40475	466	506	37	89	0.08	0.17	2.125	1	2	1	2	1	2	1	2	-0.18227
tr|Q5M7Z5|Q5M7Z5_HUMAN	GRHPR	GRHPR protein (Fragment) OS=Homo sapiens GN=GRHPR PE=2 SV=1	37314	659	690	21	61	0.09	0.19	2.111111111	0	2	1	2	1	3	1	3	0.303333
tr|B4DMB5|B4DMB5_HUMAN	tr|B4DMB	cDNA FLJ60075, highly similar to CTP synthase 1 (EC 6.3.4.2) OS=Homo sapiens PE=2 SV=1	56542	449	293	38	169	0.19	0.4	2.105263158	1	6	3	6	3	7	4	8	0.180515
tr|H0Y5G9|H0Y5G9_HUMAN	NRD1	Nardilysin (Fragment) OS=Homo sapiens GN=NRD1 PE=3 SV=1	62196	225	303	92	45	0.11	0.23	2.090909091	2	3	2	4	2	3	2	5	-0.43397
tr|I3L492|I3L492_HUMAN	ETF1	Eukaryotic peptide chain release factor subunit 1 (Fragment) OS=Homo sapiens GN=ETF1 PE=4 SV=1	23028	274	381	69	40	0.15	0.31	2.066666667	1	1	1	1	1	1	1	1	-0.91941
sp|Q3ZCM7|TBB8_HUMAN	TUBB8	Tubulin beta-8 chain OS=Homo sapiens GN=TUBB8 PE=1 SV=2	50257	67	73	306	561	0.77	1.59	2.064935065	13	24	17	28	19	32	21	35	-0.18299
tr|H0YAS6|H0YAS6_HUMAN	PABPC1	Polyadenylate-binding protein 1 (Fragment) OS=Homo sapiens GN=PABPC1 PE=4 SV=1	17943	215	375	48	126	0.33	0.68	2.060606061	1	3	2	3	2	4	2	5	-0.07122
tr|B4DFN9|B4DFN9_HUMAN	tr|B4DFN9	cDNA FLJ54303, highly similar to Heat shock 70 kDa protein 1 OS=Homo sapiens PE=2 SV=1	62699	80	79	252	450	0.58	1.19	2.051724138	7	16	10	21	10	22	10	23	0.18083
tr|A2RUM7|A2RUM7_HUMAN	RPL5	Ribosomal protein L5 OS=Homo sapiens GN=RPL5 PE=2 SV=1	34569	187	229	115	224	0.44	0.9	2.045454545	2	6	4	9	5	10	7	11	0.013727
tr|B4DNG6|B4DNG6_HUMAN	tr|B4DNG	Annexin OS=Homo sapiens PE=3 SV=1	12870	577	340	28	107	0.27	0.55	2.037037037	0	2	1	2	1	2	1	2	-0.18227
sp|P17987|TCPA_HUMAN	TCP1	T-complex protein 1 subunit alpha OS=Homo sapiens GN=TCP1 PE=1 SV=1	60819	197	246	106	214	0.3	0.61	2.033333333	2	4	5	9	8	12	10	14	-0.36342
sp|P15121|ALDR_HUMAN	AKR1B1	Aldose reductase OS=Homo sapiens GN=AKR1B1 PE=1 SV=3	36230	185	350	116	136	0.42	0.85	2.023809524	3	5	4	7	5	9	5	14	-0.13084
sp|P52895|AK1C2_HUMAN	AKR1C2	Aldo-keto reductase family 1 member C2 OS=Homo sapiens GN=AKR1C2 PE=1 SV=3	37111	186	158	116	165	0.67	1.35	2.014925373	3	5	6	6	7	6	9	7	-1.12699
tr|Q2NLA0|Q2NLA0_HUMAN	COPG2	Coatomer subunit gamma OS=Homo sapiens GN=COPG2 PE=2 SV=1	82400	705	432	18	108	0.04	0.08	2	0	2	1	2	0	2	1	2	1.403027
sp|Q9Y5B9|SP16H_HUMAN	SUPT16H	FACT complex subunit SPT16 OS=Homo sapiens GN=SUPT16H PE=1 SV=1	120409	506	1016	33	34	0.03	0.06	2	0	0	1	2	1	4	2	4	0.666081
tr|Q5QPE2|Q5QPE2_HUMAN	SEC23B	Protein transport protein Sec23B (Fragment) OS=Homo sapiens GN=SEC23B PE=4 SV=1	37575	474	644	24	65	0.08	0.16	2	0	1	1	3	1	3	1	3	0.303333
sp|O14929|HAT1_HUMAN	HAT1	Histone acetyltransferase type B catalytic subunit OS=Homo sapiens GN=HAT1 PE=1 SV=1	49880	343	481	51	95	0.07	0.14	2	1	2	1	2	1	3	1	4	0.303333
tr|H3BUF6|H3BUF6_HUMAN	ATXN2L	Ataxin-2-like protein OS=Homo sapiens GN=ATXN2L PE=4 SV=1	112929	656	843	22	45	0.03	0.06	2	0	0	1	2	2	4	2	5	-0.07122
sp|Q9NRF8|PYRG2_HUMAN	CTPS2	CTP synthase 2 OS=Homo sapiens GN=CTPS2 PE=1 SV=1	66320	614	714	24	57	0.05	0.1	2	0	2	1	2	2	4	6	5	-0.07122
tr|H0Y2W2|H0Y2W2_HUMAN	ATAD3A	ATPase family AAA domain-containing protein 3A (Fragment) OS=Homo sapiens GN=ATAD3A PE=4 SV=1	64600	281	626	68	34	0.1	0.2	2	2	1	2	2	2	4	2	4	-0.07122
tr|B5BU01|B5BU01_HUMAN	EIF2S2	Eukaryotic translation initiation factor 2 beta OS=Homo sapiens GN=EIF2S2 PE=2 SV=1	38648	654	777	22	50	0.09	0.18	2	0	1	1	2	1	2	1	2	-0.18227
tr|G0ZJH6|G0ZJH6_HUMAN	Ube4B	UFD2A-III/UBE4B-III splice isoform OS=Homo sapiens GN=Ube4B PE=2 SV=1	152890	667	733	21	54	0.02	0.04	2	0	1	1	2	1	2	1	2	-0.18227
tr|Q2VPA0|Q2VPA0_HUMAN	LONP1	Lon protease homolog (Fragment) OS=Homo sapiens GN=LONP1 PE=2 SV=1	95436	414	453	42	102	0.11	0.22	2	2	4	3	6	3	5	3	7	-0.2673
sp|Q9NPQ8|RIC8A_HUMAN	RIC8A	Synembryn-A OS=Homo sapiens GN=RIC8A PE=1 SV=3	60185	496	953	34	37	0.05	0.1	2	0	1	1	1	1	1	1	1	-0.91941
tr|J3KNE5|J3KNE5_HUMAN	HARS	Histidine--tRNA ligase, cytoplasmic OS=Homo sapiens GN=HARS PE=4 SV=1	50581	672	819	20	34	0.07	0.14	2	0	0	1	1	1	1	2	1	-0.91941
tr|B4DDC9|B4DDC9_HUMAN	tr|B4DDC9	cDNA FLJ59448, highly similar to Trifunctional enzyme subunit beta, mitochondrial OS=Homo sapiens PE=2	49937	499	662	34	56	0.07	0.14	2	0	1	1	1	1	1	1	1	-0.91941
tr|H0YMR4|H0YMR4_HUMAN	IPO4	Importin-4 (Fragment) OS=Homo sapiens GN=IPO4 PE=4 SV=1	60104	655	489	22	35	0.05	0.1	2	0	1	1	1	1	1	1	1	-0.91941
tr|B2RE46|B2RE46_HUMAN	tr|B2RE46	cDNA, FLJ96923, highly similar to Homo sapiens ribophorin II (RPN2), mRNA OS=Homo sapiens PE=2 SV=1	69405	589	642	27	68	0.05	0.1	2	0	1	1	2	2	2	2	2	-0.91957
tr|B4DQX0|B4DQX0_HUMAN	tr|B4DQX0	cDNA FLJ59219, highly similar to Poly(A)-binding protein 1 OS=Homo sapiens PE=2 SV=1	67239	215	170	96	278	0.33	0.65	1.96969697	3	8	6	10	6	13	10	13	0.135486
tr|G3V2A3|G3V2A3_HUMAN	TUBB3	Tubulin beta-3 chain (Fragment) OS=Homo sapiens GN=TUBB3 PE=2 SV=1	20929	25	36	147	450	3.07	6.01	1.957654723	5	13	6	14	7	18	7	22	0.384154
tr|B2RBR9|B2RBR9_HUMAN	tr|B2RBR9	cDNA, FLJ95650, highly similar to Homo sapiens karyopherin (importin) beta 1 (KPNB1), mRNA OS=Homo s	98434	171	121	126	367	0.22	0.43	1.954545455	4	9	6	11	9	13	9	15	-0.41301
tr|Q53GN4|Q53GN4_HUMAN	WDR1	WD repeat domain 1, isoform CRA_a (Fragment) OS=Homo sapiens GN=WDR1 PE=2 SV=1	66822	137	99	160	419	0.4	0.78	1.95	4	12	7	15	7	15	9	17	0.128363
tr|H0Y8G5|H0Y8G5_HUMAN	HNRNPD	Heterogeneous nuclear ribonucleoprotein D0 (Fragment) OS=Homo sapiens GN=HNRNPD PE=4 SV=1	29877	229	521	90	84	0.37	0.72	1.945945946	3	3	3	4	4	4	4	4	-0.91989
sp|P22314|UBA1_HUMAN	UBA1	Ubiquitin-like modifier-activating enzyme 1 OS=Homo sapiens GN=UBA1 PE=1 SV=3	118858	114	86	187	488	0.28	0.54	1.928571429	5	12	10	17	10	17	12	18	-0.18263
sp|P23526|SAHH_HUMAN	AHCY	Adenosylhomocysteinase OS=Homo sapiens GN=AHCY PE=1 SV=4	48255	195	205	111	252	0.49	0.94	1.918367347	2	7	6	10	6	11	8	11	-0.0962
tr|A8K6Q8|A8K6Q8_HUMAN	tr|A8K6Q8	cDNA FLJ75881, highly similar to Homo sapiens transferrin receptor (p90, CD71) (TFRC), mRNA OS=Homo s	85276	122	120	176	369	0.3	0.57	1.9	6	9	7	12	7	12	8	13	-0.18251
tr|B4DRH6|B4DRH6_HUMAN	tr|B4DRH6	cDNA FLJ54509, highly similar to Trifunctional enzyme subunit alpha, mitochondrial OS=Homo sapiens PE=	79007	133	269	164	183	0.28	0.53	1.892857143	5	5	6	10	7	10	7	10	-0.43441
tr|Q53GU8|Q53GU8_HUMAN	tr|Q53GU	Transforming growth factor, beta-induced, 68kDa variant (Fragment) OS=Homo sapiens PE=2 SV=1	75231	358	938	48	38	0.09	0.17	1.888888889	1	1	2	2	2	2	2	3	-0.91957
tr|Q6IBT3|Q6IBT3_HUMAN	CCT7	CCT7 protein OS=Homo sapiens GN=CCT7 PE=2 SV=1	59804	161	175	133	280	0.38	0.71	1.868421053	2	6	6	10	5	12	7	13	0.265621
tr|B4DR70|B4DR70_HUMAN	tr|B4DR70	cDNA FLJ58049, highly similar to RNA-binding protein FUS OS=Homo sapiens PE=2 SV=1	45013	450	1072	38	31	0.07	0.13	1.857142857	1	0	1	2	1	3	1	5	0.303333
tr|F2Z2U8|F2Z2U8_HUMAN	MYH14	Myosin-14 OS=Homo sapiens GN=MYH14 PE=2 SV=1	232102	152	203	140	254	0.07	0.13	1.857142857	5	8	5	10	7	11	8	12	-0.30298
tr|B2R6X5|B2R6X5_HUMAN	tr|B2R6X5	cDNA, FLJ93166, highly similar to Homo sapiens heat shock 70kDa protein 6 (HSP70B~) (HSPA6), mRNA OS	71370	193	224	112	228	0.2	0.37	1.85	3	8	5	9	6	11	6	11	-0.0962
sp|P18206|VINC_HUMAN	VCL	Vinculin OS=Homo sapiens GN=VCL PE=1 SV=4	124292	68	71	299	585	0.37	0.68	1.837837838	8	16	12	21	13	20	13	22	-0.31741
tr|Q2NKW8|Q2NKW8_HUMAN	AHCYL1	Adenosylhomocysteinase (Fragment) OS=Homo sapiens GN=AHCYL1 PE=2 SV=1	57630	509	984	33	36	0.06	0.11	1.833333333	0	1	1	2	1	2	1	2	-0.18227
tr|K7EIH8|K7EIH8_HUMAN	DNAJC7	DnaJ homolog subfamily C member 7 (Fragment) OS=Homo sapiens GN=DNAJC7 PE=4 SV=1	30449	527	949	31	38	0.06	0.11	1.833333333	0	1	1	1	1	1	1	1	-0.91941
tr|B4DUQ1|B4DUQ1_HUMAN	tr|B4DUQ	cDNA FLJ54552, highly similar to Heterogeneous nuclear ribonucleoprotein K OS=Homo sapiens PE=2 SV=1	48708	147	188	147	267	0.58	1.06	1.827586207	3	8	7	13	7	17	8	19	0.303806
tr|B0QYA5|B0QYA5_HUMAN	EIF3D	Eukaryotic translation initiation factor 3 subunit D (Fragment) OS=Homo sapiens GN=EIF3D PE=4 SV=1	31937	710	443	17	91	0.1	0.18	1.8	0	2	1	2	0	3	2	3	1.888632
tr|B4DQJ8|B4DQJ8_HUMAN	PGD	6-phosphogluconate dehydrogenase, decarboxylating OS=Homo sapiens GN=PGD PE=2 SV=1	52352	293	244	62	215	0.2	0.36	1.8	1	5	3	5	2	5	3	5	0.218464
sp|Q9NR30|DDX21_HUMAN	DDX21	Nucleolar RNA helicase 2 OS=Homo sapiens GN=DDX21 PE=1 SV=5	87804	148	122	147	367	0.34	0.61	1.794117647	4	12	8	13	8	16	9	16	0.03783
sp|O00116|ADAS_HUMAN	AGPS	Alkyldihydroxyacetonephosphate synthase, peroxisomal OS=Homo sapiens GN=AGPS PE=1 SV=1	73664	448	524	38	84	0.09	0.16	1.777777778	1	1	2	3	2	3	3	4	-0.43397
tr|B3KNN7|B3KNN7_HUMAN	tr|B3KNN7	cDNA FLJ30049 fis, clone ADRGL1000033, highly similar to 26S proteasome non-ATPase regulatory subunit	57311	273	401	69	110	0.18	0.32	1.777777778	3	2	3	4	3	4	3	4	-0.55698
tr|I3L312|I3L312_HUMAN	P4HB	Protein disulfide-isomerase (Fragment) OS=Homo sapiens GN=P4HB PE=4 SV=1	26348	536	294	30	50	0.27	0.48	1.777777778	1	2	2	2	1	4	2	5	0.666081
tr|B2R9K8|B2R9K8_HUMAN	tr|B2R9K8	cDNA, FLJ94440, highly similar to Homo sapiens chaperonin containing TCP1, subunit 6A (zeta 1)(CCT6A), r	58386	118	266	181	195	0.47	0.83	1.765957447	5	7	7	11	9	14	10	17	-0.30974
sp|Q92621|NU205_HUMAN	NUP205	Nuclear pore complex protein Nup205 OS=Homo sapiens GN=NUP205 PE=1 SV=3	230171	415	705	42	58	0.04	0.07	1.75	0	4	3	5	3	5	5	7	-0.2673
tr|B4DZ88|B4DZ88_HUMAN	KTN1	KTN1 protein OS=Homo sapiens GN=KTN1 PE=2 SV=1	153179	322	543	55	82	0.04	0.07	1.75	2	3	2	3	3	5	4	6	-0.2673
tr|Q7Z3K9|Q7Z3K9_HUMAN	DKFZp781	Putative uncharacterized protein DKFZp781L0540 (Fragment) OS=Homo sapiens GN=DKFZp781L0540 PE=2	90081	440	974	39	36	0.04	0.07	1.75	1	0	1	2	2	3	2	5	-0.43397
tr|B2RDN4|B2RDN4_HUMAN	tr|B2RDN4	cDNA, FLJ96693, highly similar to Homo sapiens block of proliferation 1 (BOP1), mRNA OS=Homo sapiens P	84219	505	735	33	54	0.08	0.14	1.75	0	1	2	3	2	3	3	3	-0.43397
tr|B3KY60|B3KY60_HUMAN	tr|B3KY60	cDNA FLJ16777 fis, clone BRHIP2029567, highly similar to Cell division cycle 5-like protein OS=Homo sapier	92448	587	1164	27	25	0.04	0.07	1.75	0	1	1	2	1	1	1	2	-0.91941
tr|B3KMV8|B3KMV8_HUMAN	tr|B3KMV	cDNA FLJ12766 fis, clone NT2RP2001520, highly similar to Calcium-binding mitochondrial carrier protein A	75130	353	408	41	114	0.11	0.19	1.727272727	1	4	1	4	1	5	1	5	0.955766
tr|G5E9Q2|G5E9Q2_HUMAN	CSDE1	Cold shock domain containing E1, RNA-binding, isoform CRA_d OS=Homo sapiens GN=CSDE1 PE=2 SV=1	94539	312	324	58	153	0.11	0.19	1.727272727	1	4	3	5	3	5	3	6	-0.2673
tr|B4DNN4|B4DNN4_HUMAN	tr|B4DNN	Ribonucleoside-diphosphate reductase OS=Homo sapiens PE=2 SV=1	87252	156	166	136	294	0.29	0.5	1.724137931	5	6	7	12	8	11	8	15	-0.48389
tr|D2CFK9|D2CFK9_HUMAN	GTPBP4	Nucleolar GTP-binding protein 1 OS=Homo sapiens GN=GTPBP4 PE=2 SV=1	74317	264	369	72	127	0.14	0.24	1.714285714	2	3	3	5	3	6	3	6	-0.02611
tr|B4DU00|B4DU00_HUMAN	tr|B4DU00	cDNA FLJ54547, highly similar to Alpha-1 catenin (Cadherin-associated protein) OS=Homo sapiens PE=2 SV	89332	389	566	45	79	0.07	0.12	1.714285714	0	2	2	3	2	4	4	5	-0.07122
tr|H0YJ34|H0YJ34_HUMAN	FERMT2	Fermitin family homolog 2 (Fragment) OS=Homo sapiens GN=FERMT2 PE=4 SV=1	73877	306	329	59	148	0.14	0.24	1.714285714	2	3	3	5	3	5	3	5	-0.2673
tr|B4DT47|B4DT47_HUMAN	tr|B4DT47	cDNA FLJ54507, highly similar to Heat shock 70 kDa protein 4 OS=Homo sapiens PE=2 SV=1	77886	123	167	147	294	0.23	0.39	1.695652174	3	7	5	8	4	8	5	11	-0.00164
tr|K9JA46|K9JA46_HUMAN	EL52	Epididymis luminal secretory protein 52 OS=Homo sapiens GN=EL52 PE=2 SV=1	85006	46	48	462	833	1.05	1.78	1.695238095	15	26	25	32	24	37	29	41	-0.30664
tr|B4DXW6|B4DXW6_HUMAN	tr|B4DXW	cDNA FLJ50285, highly similar to Arginyl-tRNA synthetase (EC 6.1.1.19) OS=Homo sapiens PE=2 SV=1	52771	235	168	88	162	0.35	0.59	1.685714286	3	6	5	7	6	8	7	9	-0.53283
tr|G5E9M5|G5E9M5_HUMAN	ILF3	Interleukin enhancer binding factor 3, 90kDa, isoform CRA_b OS=Homo sapiens GN=ILF3 PE=2 SV=1	96147	154	134	137	351	0.46	0.77	1.673913043	3	12	9	17	10	17	11	19	-0.18263
tr|F4ZW64|F4ZW64_HUMAN	tr|F4ZW64	NF90a OS=Homo sapiens PE=2 SV=1	76369	154	113	137	324	0.46	0.77	1.673913043	3	10	9	15	10	15	11	17	-0.35807
tr|F5H4R9|F5H4R9_HUMAN	DNM2	Dynamin-2 OS=Homo sapiens GN=DNM2 PE=2 SV=1	98218	713	1027	16	34	0.03	0.05	1.666666667	0	0	1	1	0	1	2	1	0.665884
tr|A8K7F6|A8K7F6_HUMAN	tr|A8K7F6	cDNA FLJ78244, highly similar to Homo sapiens eukaryotic translation initiation factor 4A, isoform 1 (EIF4A	46321	52	68	404	611	1.62	2.7	1.666666667	10	17	14	20	15	22	20	24	-0.38273
tr|F5H3A1|F5H3A1_HUMAN	ATP1A1	Sodium/potassium-transporting ATPase subunit alpha-1 OS=Homo sapiens GN=ATP1A1 PE=2 SV=1	114239	324	599	55	73	0.09	0.15	1.666666667	2	2	3	5	8	6	12	9	-1.3079
tr|G8JLB6|G8JLB6_HUMAN	HNRNPH1	Heterogeneous nuclear ribonucleoprotein H OS=Homo sapiens GN=HNRNPH1 PE=4 SV=1	51482	207	155	101	318	0.45	0.75	1.666666667	2	8	6	11	5	12	6	13	0.265621
sp|Q00610|CLH1_HUMAN	CLTC	Clathrin heavy chain 1 OS=Homo sapiens GN=CLTC PE=1 SV=5	193260	30	39	629	952	0.52	0.86	1.653846154	19	25	25	37	24	40	28	45	-0.19508
tr|J3QQM1|J3QQM1_HUMAN	PSMC5	26S protease regulatory subunit 8 (Fragment) OS=Homo sapiens GN=PSMC5 PE=4 SV=1	29442	314	678	57	62	0.11	0.18	1.636363636	1	2	1	2	1	5	2	5	0.955766
tr|B3KT66|B3KT66_HUMAN	PSMD6	Proteasome (Prosome, macropain) 26S subunit, non-ATPase, 6, isoform CRA_c OS=Homo sapiens GN=PSM	40600	399	687	43	33	0.08	0.13	1.625	1	0	1	1	1	1	1	1	-0.91941
tr|B9A041|B9A041_HUMAN	MDH1	Malate dehydrogenase, cytoplasmic OS=Homo sapiens GN=MDH1 PE=2 SV=1	23195	309	359	58	132	0.31	0.5	1.612903226	2	2	2	3	2	3	2	4	-0.43397
sp|P53621|COPA_HUMAN	COPA	Coatomer subunit alpha OS=Homo sapiens GN=COPA PE=1 SV=2	139797	181	195	118	260	0.18	0.29	1.611111111	5	9	7	11	8	12	11	14	-0.36342
tr|B2R6K4|B2R6K4_HUMAN	tr|B2R6K4	cDNA, FLJ92996, highly similar to Homo sapiens guanine nucleotide binding protein (G protein), beta polyp	38075	199	528	105	83	0.18	0.29	1.611111111	1	3	2	3	2	3	2	3	-0.43397
tr|B4E0S6|B4E0S6_HUMAN	tr|B4E0S6	cDNA FLJ55635, highly similar to pre-mRNA-splicing factorATP-dependent RNA helicase DHX15 (EC 3.6.1.-)	90289	183	247	117	214	0.15	0.24	1.6	2	5	4	6	4	7	7	7	-0.18239
sp|P46087|NOP2_HUMAN	NOP2	Putative ribosomal RNA methyltransferase NOP2 OS=Homo sapiens GN=NOP2 PE=1 SV=2	89589	296	267	61	195	0.15	0.24	1.6	2	6	4	6	5	8	6	9	-0.29148
tr|I3L2B0|I3L2B0_HUMAN	CLUH	Clustered mitochondria protein homolog OS=Homo sapiens GN=CLUH PE=3 SV=1	86269	438	431	21	108	0.05	0.08	1.6	0	1	1	2	1	3	1	3	0.303333
tr|Q05BW9|Q05BW9_HUMAN	PAPSS1	PAPSS1 protein (Fragment) OS=Homo sapiens GN=PAPSS1 PE=2 SV=1	65264	553	330	29	147	0.1	0.16	1.6	0	3	2	3	1	3	2	3	0.303333
sp|Q14432|PDE3A_HUMAN	PDE3A	cGMP-inhibited 3~,5~-cyclic phosphodiesterase A OS=Homo sapiens GN=PDE3A PE=1 SV=3	126440	493	407	35	114	0.05	0.08	1.6	1	3	2	3	2	5	2	5	0.218464
sp|Q01650|LAT1_HUMAN	SLC7A5	Large neutral amino acids transporter small subunit 1 OS=Homo sapiens GN=SLC7A5 PE=1 SV=2	55659	239	288	85	177	0.12	0.19	1.583333333	1	4	3	4	4	4	5	6	-0.91989
sp|P58107|EPIPL_HUMAN	EPPK1	Epiplakin OS=Homo sapiens GN=EPPK1 PE=1 SV=2	557674	56	75	390	536	0.07	0.11	1.571428571	10	13	12	18	12	19	13	25	-0.27837
sp|Q9BYZ2|LDH6B_HUMAN	LDHAL6B	L-lactate dehydrogenase A-like 6B OS=Homo sapiens GN=LDHAL6B PE=1 SV=3	42372	327	333	54	146	0.16	0.25	1.5625	1	4	2	4	2	4	2	5	-0.07122
tr|H0YBG6|H0YBG6_HUMAN	HSPA9	Stress-70 protein, mitochondrial (Fragment) OS=Homo sapiens GN=HSPA9 PE=4 SV=1	18491	81	530	38	83	0.42	0.65	1.547619048	1	1	1	3	1	3	1	4	0.303333
tr|B4DII5|B4DII5_HUMAN	tr|B4DII5	Importin subunit alpha OS=Homo sapiens PE=2 SV=1	60345	573	469	28	98	0.11	0.17	1.545454545	0	2	2	3	3	3	3	3	-0.91973
tr|B3KY95|B3KY95_HUMAN	tr|B3KY95	cDNA FLJ16143 fis, clone BRAMY2038516, highly similar to Protein disulfide-isomerase A6 (EC 5.3.4.1) OS=	53655	445	461	38	100	0.13	0.2	1.538461538	0	2	2	3	2	3	2	4	-0.43397
tr|B4DP08|B4DP08_HUMAN	ALG1	Chitobiosyldiphosphodolichol beta-mannosyltransferase OS=Homo sapiens GN=ALG1 PE=2 SV=1	40938	452	503	38	90	0.17	0.26	1.529411765	0	3	2	3	2	3	2	4	-0.43397
tr|J3KN86|J3KN86_HUMAN	RPS3	40S ribosomal protein S3 OS=Homo sapiens GN=RPS3 PE=3 SV=1	28640	480	609	36	72	0.12	0.18	1.5	1	1	1	1	1	2	4	3	-0.18227
sp|O94776|MTA2_HUMAN	MTA2	Metastasis-associated protein MTA2 OS=Homo sapiens GN=MTA2 PE=1 SV=1	75717	676	462	20	100	0.06	0.09	1.5	0	2	1	2	1	2	1	2	-0.18227
tr|B4DH52|B4DH52_HUMAN	tr|B4DH52	cDNA FLJ55916, highly similar to General transcription factor II-I OS=Homo sapiens PE=2 SV=1	112359	554	576	29	78	0.06	0.09	1.5	0	2	2	3	2	3	3	4	-0.43397
tr|B3KNL2|B3KNL2_HUMAN	RUVBL2	RuvB-like 2 (E. coli), isoform CRA_d OS=Homo sapiens GN=RUVBL2 PE=2 SV=1	47603	584	440	28	73	0.14	0.21	1.5	0	2	2	2	1	2	2	2	-0.18227
tr|H0YER0|H0YER0_HUMAN	PABPC4	Polyadenylate-binding protein 4 (Fragment) OS=Homo sapiens GN=PABPC4 PE=4 SV=1	7211	215	948	33	38	0.33	0.49	1.484848485	0	1	1	1	1	1	1	2	-0.91941
sp|P68366|TBA4A_HUMAN	TUBA4A	Tubulin alpha-4A chain OS=Homo sapiens GN=TUBA4A PE=1 SV=1	50634	22	29	796	1171	3.01	4.45	1.478405316	20	32	31	37	31	44	36	48	-0.42342
sp|P53396|ACLY_HUMAN	ACLY	ATP-citrate synthase OS=Homo sapiens GN=ACLY PE=1 SV=3	121674	20	23	845	1324	1.28	1.89	1.4765625	21	33	32	47	34	53	40	54	-0.28834
tr|B4DUY3|B4DUY3_HUMAN	tr|B4DUY3	cDNA FLJ56668, highly similar to Nucleolar protein NOP5 OS=Homo sapiens PE=2 SV=1	16345	515	562	32	29	0.21	0.31	1.476190476	0	0	1	2	1	2	1	3	-0.18227
tr|B4DKT3|B4DKT3_HUMAN	tr|B4DKT3	cDNA FLJ58390, highly similar to Aldo-keto reductase family 1 member C3homolog (EC 1.-.-.-) OS=Homo sa	15755	186	225	42	172	0.67	0.98	1.462686567	1	4	1	5	1	4	1	5	0.666081
tr|Q53G58|Q53G58_HUMAN	tr|Q53G58	Coronin (Fragment) OS=Homo sapiens PE=2 SV=1	53931	242	497	83	91	0.13	0.19	1.461538462	1	3	2	3	2	4	4	4	-0.07122
tr|H0YBW4|H0YBW4_HUMAN	PLAA	Phospholipase A-2-activating protein (Fragment) OS=Homo sapiens GN=PLAA PE=4 SV=1	31645	539	355	30	118	0.11	0.16	1.454545455	0	2	1	2	1	3	1	3	0.303333
tr|K7EK45|K7EK45_HUMAN	PTBP1	Polypyrimidine tract-binding protein 1 (Fragment) OS=Homo sapiens GN=PTBP1 PE=4 SV=1	27322	567	400	21	118	0.18	0.26	1.444444444	0	1	1	2	1	2	1	4	-0.18227
tr|Q96I16|Q96I16_HUMAN	tr|Q96I16	EIF2S2 protein OS=Homo sapiens PE=2 SV=1	24902	654	769	22	51	0.09	0.13	1.444444444	0	1	1	1	1	1	1	1	-0.91941
tr|Q53HF2|Q53HF2_HUMAN	tr|Q53HF2	Heat shock 70kDa protein 8 isoform 2 variant (Fragment) OS=Homo sapiens PE=2 SV=1	53580	51	54	426	711	1.45	2.09	1.44137931	12	23	17	30	19	31	19	32	-0.22826
tr|Q5I6Y6|Q5I6Y6_HUMAN	LMNA	Lamin A/C transcript variant 1 OS=Homo sapiens GN=LMNA PE=2 SV=1	74322	95	77	221	530	0.75	1.08	1.44	7	14	13	17	15	20	15	22	-0.51739
tr|Q53GX7|Q53GX7_HUMAN	tr|Q53GX7	Threonyl-tRNA synthetase variant (Fragment) OS=Homo sapiens PE=2 SV=1	84304	94	157	221	317	0.64	0.91	1.421875	6	10	13	20	17	27	18	30	-0.2681
sp|P21796|VDAC1_HUMAN	VDAC1	Voltage-dependent anion-selective channel protein 1 OS=Homo sapiens GN=VDAC1 PE=1 SV=2	30868	250	281	78	179	0.36	0.51	1.416666667	1	4	3	4	4	4	4	6	-0.91989
tr|B4DTG2|B4DTG2_HUMAN	EEF1G	Elongation factor 1-gamma OS=Homo sapiens GN=EEF1G PE=2 SV=1	56456	93	70	225	592	0.86	1.21	1.406976744	5	14	15	18	14	22	18	24	-0.28618
tr|B1AHC9|B1AHC9_HUMAN	XRCC6	X-ray repair complementing defective repair in Chinese hamster cells 6 (Ku autoantigen, 70kDa) OS=Homo	64528	116	105	184	402	0.64	0.9	1.40625	5	11	10	14	11	15	12	22	-0.48965
tr|B4DPP6|B4DPP6_HUMAN	tr|B4DPP6	cDNA FLJ54371, highly similar to Serum albumin OS=Homo sapiens PE=2 SV=1	72370	222	282	93	178	0.25	0.35	1.4	3	5	5	7	6	7	10	13	-0.71358
tr|Q53ET2|Q53ET2_HUMAN	tr|Q53ET2	Dihydropyrimidinase-like 2 variant (Fragment) OS=Homo sapiens PE=2 SV=1	62688	342	192	51	237	0.23	0.32	1.391304348	1	5	4	5	3	6	5	6	-0.02611
sp|Q13151|ROA0_HUMAN	HNRNPA0	Heterogeneous nuclear ribonucleoprotein A0 OS=Homo sapiens GN=HNRNPA0 PE=1 SV=1	30993	323	301	55	170	0.36	0.5	1.388888889	2	4	3	4	3	5	3	5	-0.2673
sp|P05165|PCCA_HUMAN	PCCA	Propionyl-CoA carboxylase alpha chain, mitochondrial OS=Homo sapiens GN=PCCA PE=1 SV=4	80635	99	132	216	351	0.49	0.68	1.387755102	6	10	10	13	13	16	13	18	-0.63128
tr|H0YDE4|H0YDE4_HUMAN	PRMT1	Protein arginine N-methyltransferase 1 (Fragment) OS=Homo sapiens GN=PRMT1 PE=4 SV=1	27345	437	532	39	83	0.26	0.36	1.384615385	1	2	2	4	2	4	2	4	-0.07122
tr|B4DTA2|B4DTA2_HUMAN	tr|B4DTA2	cDNA FLJ60148, highly similar to Homo sapiens heterogeneous nuclear ribonucleoprotein D-like (HNRPDL)	30367	229	529	39	83	0.37	0.51	1.378378378	1	2	1	5	1	5	1	5	0.955766
tr|E0YTG9|E0YTG9_HUMAN	HLA-A	MHC class I antigen (Fragment) OS=Homo sapiens GN=HLA-A PE=3 SV=1	21255	639	772	22	51	0.16	0.22	1.375	0	1	1	2	1	4	3	4	0.666081
tr|H0YGW7|H0YGW7_HUMAN	ABCF1	ATP-binding cassette sub-family F member 1 (Fragment) OS=Homo sapiens GN=ABCF1 PE=3 SV=1	68075	144	204	151	254	0.27	0.37	1.37037037	4	6	5	9	5	9	7	9	-0.13084

sp|P46063|RECQ1_HUMAN	RECQL	ATP-dependent DNA helicase Q1 OS=Homo sapiens GN=RECQL PE=1 SV=3	74436	439	410	39	112	0.14	0.19	1.357142857	1	3	3	4	3	4	3	4	-0.55698
sp|Q14315|FLNC_HUMAN	FLNC	Filamin-C OS=Homo sapiens GN=FLNC PE=1 SV=3	293407	143	230	153	224	0.06	0.08	1.333333333	5	7	6	8	10	11	11	13	-0.78942
tr|F1T0B3|F1T0B3_HUMAN	DDX1	ATP-dependent RNA helicase DDX1 OS=Homo sapiens GN=DDX1 PE=2 SV=1	74838	501	457	33	101	0.09	0.12	1.333333333	0	3	2	3	3	4	3	5	-0.55698
tr|B2R659|B2R659_HUMAN	tr|B2R659	cDNA, FLJ92803, highly similar to Homo sapiens hydroxysteroid (17-beta) dehydrogenase 4 (HSD17B4), mR	80018	484	471	36	98	0.13	0.17	1.307692308	0	2	3	4	3	4	4	5	-0.55698
tr|B4DY32|B4DY32_HUMAN	tr|B4DY32	Asparagine synthetase OS=Homo sapiens PE=2 SV=1	55055	141	402	156	117	0.26	0.34	1.307692308	3	3	5	5	6	4	6	5	-1.45108
sp|O43707|ACTN4_HUMAN	ACTN4	Alpha-actinin-4 OS=Homo sapiens GN=ACTN4 PE=1 SV=2	105245	19	18	849	1448	2.11	2.73	1.293838863	26	38	41	48	44	56	52	58	-0.57969
tr|B4DGQ0|B4DGQ0_HUMAN	LASP1	LIM and SH3 domain protein 1 OS=Homo sapiens GN=LASP1 PE=2 SV=1	23167	568	1113	29	29	0.14	0.18	1.285714286	0	0	1	1	1	2	1	2	-0.18227
tr|B7TY16|B7TY16_HUMAN	ACTN1	Actinin alpha 1 isoform 3 OS=Homo sapiens GN=ACTN1 PE=2 SV=1	107644	50	44	428	863	0.77	0.98	1.272727273	15	20	21	24	22	28	29	29	-0.58065
sp|Q562R1|ACTBL_HUMAN	ACTBL2	Beta-actin-like protein 2 OS=Homo sapiens GN=ACTBL2 PE=1 SV=2	42318	61	88	349	481	1.47	1.87	1.272108844	13	15	16	21	24	25	27	32	-0.86518
sp|Q9UMS4|PRP19_HUMAN	PRPF19	Pre-mRNA-processing factor 19 OS=Homo sapiens GN=PRPF19 PE=1 SV=1	55603	237	450	87	103	0.26	0.33	1.269230769	2	3	5	5	7	7	11	9	-0.92037
tr|J3KPF3|J3KPF3_HUMAN	SLC3A2	4F2 cell-surface antigen heavy chain OS=Homo sapiens GN=SLC3A2 PE=4 SV=1	68230	60	55	366	707	1.33	1.68	1.263157895	12	17	20	24	20	26	20	28	-0.551
tr|B3KX19|B3KX19_HUMAN	tr|B3KX19	cDNA FLJ44500 fis, clone UTERU3000828, highly similar to 116 kDa U5 small nuclear ribonucleoprotein cor	109111	111	159	193	312	0.27	0.34	1.259259259	4	6	8	10	8	12	10	14	-0.36342
sp|Q12792|TWF1_HUMAN	TWF1	Twinfilin-1 OS=Homo sapiens GN=TWF1 PE=1 SV=3	40429	575	474	28	97	0.08	0.1	1.25	0	1	1	1	1	1	1	1	-0.91941
tr|A5PLK7|A5PLK7_HUMAN	RCC2	RCC2 protein (Fragment) OS=Homo sapiens GN=RCC2 PE=2 SV=1	50331	165	259	131	188	0.46	0.57	1.239130435	4	4	6	6	6	8	6	9	-0.53283
tr|B3KM80|B3KM80_HUMAN	NCL	Nucleolin, isoform CRA_c OS=Homo sapiens GN=NCL PE=2 SV=1	58576	71	64	283	617	1.03	1.27	1.233009709	9	13	14	16	15	19	15	20	-0.58972
tr|Q59ES3|Q59ES3_HUMAN	tr|Q59ES3	Solute carrier family 1 (Neutral amino acid transporter), member 5 variant (Fragment) OS=Homo sapiens P	57810	290	592	63	74	0.1	0.12	1.2	1	1	1	2	1	3	1	3	0.303333
tr|B4DW52|B4DW52_HUMAN	tr|B4DW5	cDNA FLJ55253, highly similar to Actin, cytoplasmic 1 OS=Homo sapiens PE=2 SV=1	38950	4	6	3120	2036	27.29	32.3	1.18358373	74	58	108	80	123	98	134	113	-1.26998
sp|Q6S8J3|POTEE_HUMAN	POTEE	POTE ankyrin domain family member E OS=Homo sapiens GN=POTEE PE=1 SV=3	122882	5	33	2211	1083	0.44	0.52	1.181818182	45	27	66	43	70	45	75	54	-1.56671
sp|Q92973|TNPO1_HUMAN	TNPO1	Transportin-1 OS=Homo sapiens GN=TNPO1 PE=1 SV=2	103771	139	390	158	121	0.17	0.2	1.176470588	3	4	5	6	8	7	8	9	-1.10127
sp|P22626|ROA2_HUMAN	HNRNPA2	Heterogeneous nuclear ribonucleoproteins A2/B1 OS=Homo sapiens GN=HNRNPA2B1 PE=1 SV=2	37464	77	140	256	345	0.97	1.14	1.175257732	7	8	8	11	9	14	10	18	-0.30974
tr|Q1KLZ0|Q1KLZ0_HUMAN	PS1TP5BP	HCG15971, isoform CRA_a OS=Homo sapiens GN=PS1TP5BP1 PE=2 SV=1	42052	1	5	3367	2391	31.43	36.72	1.168310531	82	68	119	93	130	105	141	121	-1.25164
tr|B7Z8A2|B7Z8A2_HUMAN	tr|B7Z8A2	cDNA FLJ51671, highly similar to Prenylcysteine oxidase (EC 1.8.3.5) OS=Homo sapiens PE=2 SV=1	54918	355	1105	49	29	0.06	0.07	1.166666667	1	0	1	1	1	1	1	1	-0.91941
tr|B4DQG5|B4DQG5_HUMAN	tr|B4DQG	cDNA FLJ54122, highly similar to Cytosol aminopeptidase (EC 3.4.11.1) OS=Homo sapiens PE=2 SV=1	39662	368	871	47	43	0.15	0.17	1.133333333	1	2	1	2	1	2	1	2	-0.18227
tr|Q9BSV4|Q9BSV4_HUMAN	SFPQ	SFPQ protein (Fragment) OS=Homo sapiens GN=SFPQ PE=2 SV=2	68702	228	226	91	226	0.26	0.29	1.115384615	2	5	5	7	6	9	7	11	-0.37219
tr|J3QSX4|J3QSX4_HUMAN	BUB3	Mitotic checkpoint protein BUB3 OS=Homo sapiens GN=BUB3 PE=4 SV=1	16498	681	798	20	44	0.2	0.22	1.1	0	1	1	1	0	1	1	2	0.665884
sp|P68032|ACTC_HUMAN	ACTC1	Actin, alpha cardiac muscle 1 OS=Homo sapiens GN=ACTC1 PE=1 SV=1	42334	23	38	771	1004	6.63	7.22	1.088989442	25	38	40	48	48	58	55	65	-0.65468
sp|P11498|PYC_HUMAN	PC	Pyruvate carboxylase, mitochondrial OS=Homo sapiens GN=PC PE=1 SV=2	130293	10	19	1295	1399	1.76	1.9	1.079545455	40	33	52	45	56	49	67	54	-1.12028
tr|F5H5D3|F5H5D3_HUMAN	TUBA1C	Tubulin alpha-1C chain OS=Homo sapiens GN=TUBA1C PE=2 SV=1	58606	21	32	809	1086	2.92	3.14	1.075342466	24	32	34	39	37	46	41	52	-0.6132
sp|P68363|TBA1B_HUMAN	TUBA1B	Tubulin alpha-1B chain OS=Homo sapiens GN=TUBA1B PE=1 SV=1	50804	13	26	1091	1279	5.58	5.76	1.032258065	31	36	43	42	45	49	49	54	-0.80416
tr|J3KQ96|J3KQ96_HUMAN	TCOF1	Treacle protein (Fragment) OS=Homo sapiens GN=TCOF1 PE=4 SV=1	144268	702	617	18	71	0.02	0.02	1	0	1	1	1	0	2	2	2	1.403027
tr|B0UX83|B0UX83_HUMAN	BAG6	HLA-B associated transcript 3 OS=Homo sapiens GN=BAG6 PE=4 SV=1	119085	704	1190	18	24	0.03	0.03	1	0	0	1	1	0	1	1	1	0.665884
tr|B4DZD5|B4DZD5_HUMAN	tr|B4DZD5	cDNA FLJ60991, highly similar to FKBP12-rapamycin complex-associated protein OS=Homo sapiens PE=2 S\	158783	683	985	20	36	0.02	0.02	1	0	0	1	1	0	1	2	1	0.665884
tr|E3SWF7|E3SWF7_HUMAN	HLA-A	MHC class I antigen (Fragment) OS=Homo sapiens GN=HLA-A PE=3 SV=1	21021	639	1220	22	22	0.16	0.16	1	0	0	1	1	1	3	1	3	0.303333
tr|E7ET15|E7ET15_HUMAN	U2SURP	U2 snRNP-associated SURP motif-containing protein OS=Homo sapiens GN=U2SURP PE=2 SV=1	118631	647	1179	22	25	0.03	0.03	1	0	0	1	1	1	2	2	3	-0.18227
sp|Q9UG63|ABCF2_HUMAN	ABCF2	ATP-binding cassette sub-family F member 2 OS=Homo sapiens GN=ABCF2 PE=1 SV=2	71815	673	1111	20	29	0.05	0.05	1	0	0	1	1	1	2	2	4	-0.18227
tr|Q6UWE9|Q6UWE9_HUMAN	UNQ2550	SFVP2550 OS=Homo sapiens GN=UNQ2550 PE=4 SV=1	11890	664	1093	21	30	0.29	0.29	1	1	2	1	2	1	2	1	2	-0.18227
tr|E9PCI9|E9PCI9_HUMAN	FDPS	Farnesyl pyrophosphate synthase OS=Homo sapiens GN=FDPS PE=2 SV=1	40849	405	874	43	43	0.08	0.08	1	1	1	1	1	1	2	1	4	-0.18227
tr|J3QT54|J3QT54_HUMAN	CPSF7	Cleavage and polyadenylation-specificity factor subunit 7 (Fragment) OS=Homo sapiens GN=CPSF7 PE=4 S\	22762	665	873	21	43	0.15	0.15	1	0	1	1	1	1	2	1	2	-0.18227
tr|B4DDP6|B4DDP6_HUMAN	DBNL	Drebrin-like protein OS=Homo sapiens GN=DBNL PE=2 SV=1	43242	466	670	37	63	0.08	0.08	1	1	1	1	1	1	2	1	2	-0.18227
tr|D6REX3|D6REX3_HUMAN	SEC31A	Protein transport protein Sec31A OS=Homo sapiens GN=SEC31A PE=2 SV=1	137110	121	348	178	137	0.13	0.13	1	4	4	5	5	4	7	5	9	-0.18239
sp|P01024|CO3_HUMAN	C3	Complement C3 OS=Homo sapiens GN=C3 PE=1 SV=2	188569	370	740	47	54	0.03	0.03	1	1	1	2	2	2	3	2	3	-0.43397
sp|O15427|MOT4_HUMAN	SLC16A3	Monocarboxylate transporter 4 OS=Homo sapiens GN=SLC16A3 PE=1 SV=1	50064	362	650	48	67	0.14	0.14	1	2	2	2	2	2	3	2	5	-0.43397
sp|Q96P70|IPO9_HUMAN	IPO9	Importin-9 OS=Homo sapiens GN=IPO9 PE=1 SV=3	116858	580	648	28	68	0.06	0.06	1	0	2	2	2	2	3	2	3	-0.43397
tr|Q58FF4|Q58FF4_HUMAN	HSP90Bf	Heat shock protein 90Bf OS=Homo sapiens GN=HSP90Bf PE=1 SV=1	41833	407	624	43	70	0.16	0.16	1	1	2	2	2	2	3	2	3	-0.43397
sp|Q96SI9|STRBP_HUMAN	STRBP	Spermatid perinuclear RNA-binding protein OS=Homo sapiens GN=STRBP PE=1 SV=1	74290	471	600	37	73	0.09	0.09	1	0	2	2	2	2	3	2	4	-0.43397
tr|Q5JP02|Q5JP02_HUMAN	RBBP7	Histone-binding protein RBBP7 (Fragment) OS=Homo sapiens GN=RBBP7 PE=4 SV=1	19626	321	546	37	47	0.22	0.22	1	1	1	2	2	2	3	2	3	-0.43397
sp|Q15046|SYK_HUMAN	KARS	Lysine--tRNA ligase OS=Homo sapiens GN=KARS PE=1 SV=3	68461	265	309	71	164	0.32	0.32	1	1	5	6	6	6	8	7	9	-0.53283
tr|B3KPL5|B3KPL5_HUMAN	tr|B3KPL5	cDNA FLJ31917 fis, clone NT2RP7004925, highly similar to Sprouty-related, EVH1 domain-containing prote	48711	657	1140	21	27	0.07	0.07	1	0	0	1	3	16	21	51	42	-0.53904
tr|B2R5W2|B2R5W2_HUMAN	tr|B2R5W	cDNA, FLJ92657, highly similar to Homo sapiens heterogeneous nuclear ribonucleoprotein C (C1/C2) (HNRI	31986	178	433	122	108	0.35	0.35	1	3	3	3	3	3	4	4	4	-0.55698
sp|Q9H853|TBA4B_HUMAN	TUBA4B	Putative tubulin-like protein alpha-4B OS=Homo sapiens GN=TUBA4B PE=5 SV=2	27819	146	299	147	171	0.4	0.4	1	3	4	4	5	4	5	6	5	-0.63021
tr|J3KTA4|J3KTA4_HUMAN	DDX5	Probable ATP-dependent RNA helicase DDX5 OS=Homo sapiens GN=DDX5 PE=3 SV=1	69557	97	181	218	275	0.45	0.45	1	7	7	9	10	10	11	10	13	-0.78942
tr|B3KUJ0|B3KUJ0_HUMAN	tr|B3KUJ0	cDNA FLJ39996 fis, clone STOMA2002166, highly similar to Splicing factor 3B subunit 4 OS=Homo sapiens F	44512	464	1159	37	26	0.07	0.07	1	1	0	1	1	1	1	2	1	-0.91941
tr|B7Z438|B7Z438_HUMAN	tr|B7Z438	cDNA FLJ56352, highly similar to Succinyl-CoA ligase (GDP-forming) subunit alpha, mitochondrial (EC 6.2.1.	26801	467	1154	37	26	0.12	0.12	1	1	0	1	1	1	1	1	1	-0.91941
sp|Q6PGP7|TTC37_HUMAN	TTC37	Tetratricopeptide repeat protein 37 OS=Homo sapiens GN=TTC37 PE=1 SV=1	177485	498	1141	34	27	0.02	0.02	1	1	0	1	1	1	1	1	2	-0.91941
sp|Q16322|KCA10_HUMAN	KCNA10	Potassium voltage-gated channel subfamily A member 10 OS=Homo sapiens GN=KCNA10 PE=1 SV=2	58147	578	1133	28	27	0.06	0.06	1	1	1	1	1	1	1	1	1	-0.91941
tr|B2RAX6|B2RAX6_HUMAN	tr|B2RAX6	cDNA, FLJ95176, Homo sapiens CGI-48 protein (CGI-48), mRNA OS=Homo sapiens PE=2 SV=1	58127	605	1102	25	30	0.06	0.06	1	0	0	1	1	1	1	1	1	-0.91941
tr|Q5TZX1|Q5TZX1_HUMAN	GNAI3	Guanine nucleotide binding protein (G protein), alpha inhibiting activity polypeptide 3 OS=Homo sapiens G	41076	376	1098	46	30	0.08	0.08	1	1	0	1	1	1	1	1	2	-0.91941
tr|E0X098|E0X098_HUMAN	TES	Testin variant (Fragment) OS=Homo sapiens GN=TES PE=2 SV=1	22009	430	1039	40	33	0.15	0.15	1	1	0	1	1	1	1	1	1	-0.91941
tr|A0JLS5|A0JLS5_HUMAN	TBL3	TBL3 protein (Fragment) OS=Homo sapiens GN=TBL3 PE=2 SV=1	64079	418	1002	42	35	0.05	0.05	1	1	0	1	1	1	1	1	1	-0.91941
tr|B4DJE3|B4DJE3_HUMAN	tr|B4DJE3	cDNA FLJ52929, highly similar to Dolichyl-diphosphooligosaccharide--proteinglycosyltransferase 48 kDa su	46620	604	933	26	39	0.07	0.07	1	0	1	1	1	1	1	2	1	-0.91941
tr|B3KPR5|B3KPR5_HUMAN	tr|B3KPR5	cDNA FLJ32094 fis, clone OCBBF2000986, highly similar to Homo sapiens elongation factor Tu GTP binding	97046	633	918	22	40	0.03	0.03	1	0	1	1	1	1	1	1	1	-0.91941
tr|B7WPL0|B7WPL0_HUMAN	RIC8B	Synembryn-B OS=Homo sapiens GN=RIC8B PE=2 SV=1	64252	483	916	36	40	0.05	0.05	1	1	1	1	1	1	1	1	1	-0.91941
tr|F8WJN3|F8WJN3_HUMAN	CPSF6	Cleavage and polyadenylation-specificity factor subunit 6 OS=Homo sapiens GN=CPSF6 PE=2 SV=1	52409	379	838	46	45	0.06	0.06	1	1	1	1	1	1	1	1	1	-0.91941
tr|B2RDF2|B2RDF2_HUMAN	PES1	Pescadillo homolog 1 OS=Homo sapiens GN=PES1 PE=2 SV=1	68359	488	828	35	46	0.05	0.05	1	1	1	1	1	1	1	3	3	-0.91941
tr|B2R4C0|B2R4C0_HUMAN	tr|B2R4C0	60S ribosomal protein L18a OS=Homo sapiens PE=2 SV=1	21024	514	824	32	46	0.16	0.16	1	0	1	1	1	1	1	2	1	-0.91941
tr|B2RDF5|B2RDF5_HUMAN	tr|B2RDF5	cDNA, FLJ96587, highly similar to Homo sapiens SUMO-1 activating enzyme subunit 2 (UBA2), mRNA OS=H	71705	590	778	27	50	0.05	0.05	1	0	1	1	1	1	1	1	3	-0.91941
tr|E7ENU7|E7ENU7_HUMAN	RPL15	Ribosomal protein L15 (Fragment) OS=Homo sapiens GN=RPL15 PE=2 SV=1	20596	534	768	31	51	0.16	0.16	1	0	1	1	1	1	1	1	2	-0.91941
tr|F8W930|F8W930_HUMAN	IGF2BP2	Insulin-like growth factor 2 mRNA-binding protein 2 OS=Homo sapiens GN=IGF2BP2 PE=2 SV=1	66859	556	762	29	51	0.05	0.05	1	0	1	1	1	1	1	1	2	-0.91941
tr|Q68E00|Q68E00_HUMAN	DKFZp686	Putative uncharacterized protein DKFZp686G2045 (Fragment) OS=Homo sapiens GN=DKFZp686G2045 PE=	32787	485	758	36	52	0.1	0.1	1	0	1	1	1	1	1	1	1	-0.91941
tr|E5RFS9|E5RFS9_HUMAN	MAT2B	Methionine adenosyltransferase 2 subunit beta OS=Homo sapiens GN=MAT2B PE=2 SV=1	9467	280	743	68	53	0.37	0.37	1	1	1	1	1	1	1	1	1	-0.91941
tr|B4DLD4|B4DLD4_HUMAN	tr|B4DLD4	cDNA FLJ56738, highly similar to Fibrillarin OS=Homo sapiens PE=2 SV=1	30279	423	708	41	58	0.11	0.11	1	1	1	1	1	1	1	1	2	-0.91941
tr|A2BDK6|A2BDK6_HUMAN	MAP1B	Microtubule-associated protein 1B OS=Homo sapiens GN=MAP1B PE=2 SV=1	257692	511	700	32	59	0.01	0.01	1	0	1	1	1	1	1	2	2	-0.91941
tr|B3KUZ8|B3KUZ8_HUMAN	tr|B3KUZ8	Aspartate aminotransferase OS=Homo sapiens PE=2 SV=1	41699	522	685	31	61	0.08	0.08	1	0	1	1	1	1	1	2	1	-0.91941
sp|O00505|IMA3_HUMAN	KPNA3	Importin subunit alpha-3 OS=Homo sapiens GN=KPNA3 PE=1 SV=2	58288	406	977	43	36	0.12	0.12	1	0	1	2	2	2	2	2	2	-0.91957
tr|F8SKU2|F8SKU2_HUMAN	HLA-A	MHC class I antigen (Fragment) OS=Homo sapiens GN=HLA-A PE=3 SV=1	21143	639	731	22	0	0.16	0.16	1	0	0	1	0	2	2	3	2	-0.91957
tr|Q5HYM2|Q5HYM2_HUMAN	DKFZp686	Putative uncharacterized protein DKFZp686O2462 (Fragment) OS=Homo sapiens GN=DKFZp686O2462 PE=	72627	569	691	29	61	0.09	0.09	1	0	2	2	2	2	2	2	4	-0.91957
tr|B4DPZ3|B4DPZ3_HUMAN	tr|B4DPZ3	cDNA FLJ53290, highly similar to Cytoplasmic dynein 1 intermediate chain 2 OS=Homo sapiens PE=2 SV=1	70869	298	498	60	91	0.09	0.09	1	2	1	2	2	2	2	2	2	-0.91957
sp|Q9Y3I0|RTCB_HUMAN	C22orf28	tRNA-splicing ligase RtcB homolog OS=Homo sapiens GN=C22orf28 PE=1 SV=1	55688	458	466	38	99	0.12	0.12	1	0	2	2	2	2	2	2	3	-0.91957
tr|L0R5A1|L0R5A1_HUMAN	CSF2RB	Alternative protein CSF2RB OS=Homo sapiens GN=CSF2RB PE=4 SV=1	11752	486	1079	36	31	0.29	0.29	1	1	1	1	1	3	3	5	4	-0.91973
tr|Q53XC0|Q53XC0_HUMAN	EIF2S1	Eukaryotic translation initiation factor 2, subunit 1 alpha, 35kDa, isoform CRA_a OS=Homo sapiens GN=EIF	36374	208	517	100	86	0.42	0.42	1	4	3	4	4	4	4	4	4	-0.91989
sp|P42166|LAP2A_HUMAN	TMPO	Lamina-associated polypeptide 2, isoform alpha OS=Homo sapiens GN=TMPO PE=1 SV=2	76016	241	334	84	146	0.24	0.24	1	1	3	5	5	5	5	5	5	-0.92005
tr|Q4W4Y1|Q4W4Y1_HUMAN	DRIP4	Dopamine receptor interacting protein 4 OS=Homo sapiens GN=DRIP4 PE=2 SV=1	96646	136	258	160	204	0.18	0.18	1	2	4	5	5	5	5	6	6	-0.92005
tr|Q562U1|Q562U1_HUMAN	ACT	Actin-like protein (Fragment) OS=Homo sapiens GN=ACT PE=2 SV=1	11548	202	470	103	98	2.69	2.69	1	4	2	7	8	8	7	11	11	-1.10127
sp|P00390|GSHR_HUMAN	GSR	Glutathione reductase, mitochondrial OS=Homo sapiens GN=GSR PE=1 SV=2	56791	313	718	58	57	0.18	0.18	1	1	1	3	3	4	3	4	4	-1.28264
sp|Q68EM7|RHG17_HUMAN	ARHGAP17	Rho GTPase-activating protein 17 OS=Homo sapiens GN=ARHGAP17 PE=1 SV=1	95776	328	976	54	36	0.03	0.03	1	1	1	1	2	1	2	4	-1.65672
tr|Q0IJ56|Q0IJ56_HUMAN	ST13	ST13 protein (Fragment) OS=Homo sapiens GN=ST13 PE=2 SV=1	35164	382	656	46	66	0.09	0.09	1	1	1	1	1	2	1	3	1	-1.65672
sp|O75367|H2AY_HUMAN	H2AFY	Core histone macro-H2A.1 OS=Homo sapiens GN=H2AFY PE=1 SV=4	39764	565	502	29	90	0.08	0.08	1	0	1	1	1	2	1	2	2	-1.65672
sp|Q96RW7|HMCN1_HUMAN	HMCN1	Hemicentin-1 OS=Homo sapiens GN=HMCN1 PE=1 SV=2	623265	291	930	63	39	0.01	0.01	1	7	1	7	1	11	5	16	5	-1.9862
tr|B4DDR2|B4DDR2_HUMAN	tr|B4DDR2	cDNA FLJ54588, highly similar to Major vault protein OS=Homo sapiens PE=2 SV=1	98526	304	487	59	60	0.07	0.07	1	2	1	2	1	3	1	3	2	-2.14248
tr|B5LY64|B5LY64_HUMAN	UMPS	Orotidine 5'-phosphate decarboxylase OS=Homo sapiens GN=UMPS PE=2 SV=1	33258	302	1264	59	19	0.1	0.1	1	1	0	1	1	1	0	1	1	-2.50455
tr|B2RAR2|B2RAR2_HUMAN	tr|B2RAR2	cDNA, FLJ95064, highly similar to Homo sapiens nin one binding protein (NOB1P), mRNA OS=Homo sapien	47015	338	1258	52	19	0.07	0.07	1	1	0	1	1	1	0	1	1	-2.50455
tr|B4DS66|B4DS66_HUMAN	tr|B4DS66	cDNA FLJ54290, highly similar to Mitochondrial inner membrane protein OS=Homo sapiens PE=2 SV=1	73509	712	1073	17	0	0.04	0.04	1	0	0	1	0	1	0	6	1	-2.50455
tr|A0N4V7|A0N4V7_HUMAN	Tcr-alpha	HCG2039797 (Fragment) OS=Homo sapiens GN=Tcr-alpha PE=4 SV=1	2269	212	841	98	45	1.81	1.81	1	5	3	15	4	31	6	46	7	-3.20548
sp|Q05639|EF1A2_HUMAN	EEF1A2	Elongation factor 1-alpha 2 OS=Homo sapiens GN=EEF1A2 PE=1 SV=1	50780	70	252	238	211	1.14	1.13	0.99122807	8	6	9	13	12	17	14	17	-0.43484
tr|F5H1A7|F5H1A7_HUMAN	RDX	Radixin OS=Homo sapiens GN=RDX PE=2 SV=1	52703	119	231	88	211	0.46	0.45	0.97826087	2	8	2	9	2	11	2	12	1.283661
tr|H0Y9Q7|H0Y9Q7_HUMAN	CANX	Calnexin (Fragment) OS=Homo sapiens GN=CANX PE=3 SV=1	18990	352	260	49	115	0.39	0.37	0.948717949	1	2	2	2	3	2	5	2	-1.40534
sp|Q71U36|TBA1A_HUMAN	TUBA1A	Tubulin alpha-1A chain OS=Homo sapiens GN=TUBA1A PE=1 SV=1	50788	15	30	1022	1151	5.18	4.89	0.944015444	30	33	41	38	43	45	46	50	-0.86091
tr|Q9BS14|Q9BS14_HUMAN	GANAB	GANAB protein (Fragment) OS=Homo sapiens GN=GANAB PE=2 SV=1	34786	47	384	50	122	0.62	0.58	0.935483871	1	5	1	5	1	6	1	7	1.196951
tr|B7ZB91|B7ZB91_HUMAN	TNRC6B	Trinucleotide repeat containing 6B (Fragment) OS=Homo sapiens GN=TNRC6B PE=4 SV=1	81825	359	695	48	37	0.13	0.12	0.923076923	1	1	3	1	2	1	3	1	-1.65672
tr|B0QYT3|B0QYT3_HUMAN	RANGAP1	Ran GTPase activating protein 1 (Fragment) OS=Homo sapiens GN=RANGAP1 PE=2 SV=1	27122	574	549	28	36	0.12	0.11	0.916666667	0	0	1	1	1	1	1	1	-0.91941
tr|H0YJS4|H0YJS4_HUMAN	EIF2S1	Eukaryotic translation initiation factor 2 subunit 1 (Fragment) OS=Homo sapiens GN=EIF2S1 PE=4 SV=1	29243	208	514	85	87	0.42	0.38	0.904761905	3	3	3	3	3	3	3	3	-0.91973
sp|Q9NY65|TBA8_HUMAN	TUBA8	Tubulin alpha-8 chain OS=Homo sapiens GN=TUBA8 PE=1 SV=1	50746	34	59	592	677	1.74	1.57	0.902298851	15	18	23	22	23	27	27	30	-0.69543
sp|Q13867|BLMH_HUMAN	BLMH	Bleomycin hydrolase OS=Homo sapiens GN=BLMH PE=1 SV=1	53155	332	725	52	56	0.2	0.18	0.9	1	1	3	2	2	2	4	4	-0.91957
tr|H7C393|H7C393_HUMAN	APEH	Acylamino-acid-releasing enzyme (Fragment) OS=Homo sapiens GN=APEH PE=4 SV=1	32397	319	435	56	61	0.09	0.08	0.888888889	1	1	1	1	1	1	1	1	-0.91941
tr|Q96HI4|Q96HI4_HUMAN	NMT1	Glycylpeptide N-tetradecanoyltransferase (Fragment) OS=Homo sapiens GN=NMT1 PE=2 SV=2	56910	350	875	50	43	0.49	0.43	0.87755102	1	1	2	1	2	1	2	1	-1.65672
tr|E9PIM9|E9PIM9_HUMAN	RNH1	Ribonuclease inhibitor (Fragment) OS=Homo sapiens GN=RNH1 PE=2 SV=1	22362	431	468	40	57	0.15	0.13	0.866666667	0	1	1	1	1	1	1	1	-0.91941
tr|Q6I9U3|Q6I9U3_HUMAN	M6PR	M6PR protein OS=Homo sapiens GN=M6PR PE=2 SV=1	31469	416	914	42	40	0.29	0.25	0.862068966	1	1	1	1	1	1	1	1	-0.91941
tr|D2JYH4|D2JYH4_HUMAN	ACTA2	Actin, alpha 2, smooth muscle, aorta OS=Homo sapiens GN=ACTA2 PE=2 SV=1	42381	23	41	702	945	6.63	5.53	0.834087481	23	35	35	44	43	52	49	58	-0.65321
tr|B3KTA3|B3KTA3_HUMAN	tr|B3KTA3	Fascin OS=Homo sapiens PE=2 SV=1	52814	53	190	403	229	1.07	0.88	0.822429907	9	8	14	11	16	11	20	15	-1.44352
tr|Q8TE01|Q8TE01_HUMAN	derp12	DERP12 (Dermal papilla derived protein 12) OS=Homo sapiens GN=derp12 PE=2 SV=1	38340	69	235	297	220	0.79	0.64	0.810126582	6	6	7	6	8	6	8	6	-1.3079
sp|P08729|K2C7_HUMAN	KRT7	Keratin, type II cytoskeletal 7 OS=Homo sapiens GN=KRT7 PE=1 SV=5	51411	259	274	74	183	0.45	0.36	0.8	2	5	6	5	7	8	9	9	-0.73962
tr|H6VRF8|H6VRF8_HUMAN	KRT1	Keratin 1 OS=Homo sapiens GN=KRT1 PE=3 SV=1	66184	6	31	2032	1138	5.01	3.95	0.788423154	55	31	64	39	69	45	76	48	-1.54575
tr|H6VRG2|H6VRG2_HUMAN	KRT1	Keratin 1 OS=Homo sapiens GN=KRT1 PE=3 SV=1	66197	7	35	2027	1039	5.01	3.95	0.788423154	55	29	65	37	70	43	77	46	-1.63191
sp|P38919|IF4A3_HUMAN	EIF4A3	Eukaryotic initiation factor 4A-III OS=Homo sapiens GN=EIF4A3 PE=1 SV=4	47126	262	241	74	218	0.4	0.31	0.775	1	4	5	5	4	8	6	9	-0.00164
sp|Q01546|K22O_HUMAN	KRT76	Keratin, type II cytoskeletal 2 oral OS=Homo sapiens GN=KRT76 PE=1 SV=2	66370	213	706	97	58	0.21	0.16	0.761904762	4	2	4	3	5	3	9	5	-1.57248
tr|A4ZU86|A4ZU86_HUMAN	NPM1	Truncated nucleolar phosphoprotein B23 OS=Homo sapiens GN=NPM1 PE=2 SV=1	30074	138	337	160	143	0.69	0.52	0.753623188	4	4	5	4	5	5	5	6	-0.92005
tr|B4DUC5|B4DUC5_HUMAN	CSE1L	Exportin-2 OS=Homo sapiens GN=CSE1L PE=2 SV=1	86045	433	669	40	64	0.08	0.06	0.75	0	1	2	2	4	2	4	3	-1.76824
tr|B4DKM5|B4DKM5_HUMAN	VDAC2	Voltage-dependent anion-selective channel protein 2 OS=Homo sapiens GN=VDAC2 PE=2 SV=1	27804	196	311	108	162	0.76	0.57	0.75	1	5	5	5	5	5	5	5	-0.92005
tr|A8K525|A8K525_HUMAN	tr|A8K525	cDNA FLJ76817, highly similar to Homo sapiens non-POU domain containing, octamer-binding (NONO), mR	54367	75	250	266	212	0.8	0.6	0.75	10	5	11	8	12	9	14	9	-1.31762
tr|Q0IIN1|Q0IIN1_HUMAN	KRT77	Keratin 77 OS=Homo sapiens GN=KRT77 PE=2 SV=1	62050	76	317	264	157	0.23	0.17	0.739130435	10	4	10	5	10	5	12	6	-1.85462
tr|Q562M3|Q562M3_HUMAN	ACT	Actin-like protein (Fragment) OS=Homo sapiens GN=ACT PE=2 SV=1	11529	103	117	213	375	7.06	5.21	0.73796034	10	13	14	18	17	23	20	30	-0.49558
tr|Q05DV5|Q05DV5_HUMAN	ZC3HAV1	ZC3HAV1 protein (Fragment) OS=Homo sapiens GN=ZC3HAV1 PE=2 SV=1	49964	224	322	92	151	0.14	0.1	0.714285714	1	2	2	2	2	3	2	3	-0.43397
tr|B4E2P4|B4E2P4_HUMAN	tr|B4E2P4	cDNA FLJ58474, highly similar to Xaa-Pro aminopeptidase 1 (EC 3.4.11.9) OS=Homo sapiens PE=2 SV=1	50020	615	1139	24	27	0.07	0.05	0.714285714	0	0	1	1	1	1	1	1	-0.91941
tr|A0PJ92|A0PJ92_HUMAN	NOP56	NOP56 protein (Fragment) OS=Homo sapiens GN=NOP56 PE=2 SV=1	50068	311	376	58	126	0.14	0.1	0.714285714	1	2	2	2	3	2	3	2	-1.40534
tr|E7ES43|E7ES43_HUMAN	HSPA4L	Heat shock 70 kDa protein 4L OS=Homo sapiens GN=HSPA4L PE=2 SV=2	98623	191	629	113	69	0.1	0.07	0.7	2	1	2	2	2	2	3	3	-0.91957
tr|B4DF68|B4DF68_HUMAN	tr|B4DF68	cDNA FLJ55325, highly similar to Heat-shock protein 105 kDa OS=Homo sapiens PE=2 SV=1	86500	123	483	47	94	0.23	0.16	0.695652174	1	2	1	4	1	5	1	6	0.955766
tr|B2R8N0|B2R8N0_HUMAN	tr|B2R8N0	cDNA, FLJ93975, highly similar to Homo sapiens epoxide hydrolase 1, microsomal (xenobiotic) (EPHX1), mR	53129	115	335	184	145	0.62	0.43	0.693548387	6	5	8	6	8	7	8	9	-1.10127
sp|P35237|SPB6_HUMAN	SERPINB6	Serpin B6 OS=Homo sapiens GN=SERPINB6 PE=1 SV=3	42936	120	1134	179	27	0.25	0.17	0.68	3	0	3	1	3	2	3	2	-1.40534
tr|K7EK77|K7EK77_HUMAN	ATP5A1	ATP synthase subunit alpha, mitochondrial (Fragment) OS=Homo sapiens GN=ATP5A1 PE=3 SV=1	22243	135	200	160	132	1.33	0.9	0.676691729	5	5	6	7	6	7	6	7	-0.71358
tr|B3KPU1|B3KPU1_HUMAN	GNB2	Guanine nucleotide-binding protein G(I)/G(S)/G(T) subunit beta-2 OS=Homo sapiens GN=GNB2 PE=2 SV=1	26598	194	473	111	0	0.27	0.18	0.666666667	1	0	2	0	2	0	2	1	-3.24186
tr|E9PHF7|E9PHF7_HUMAN	MCCC1	Methylcrotonoyl-CoA carboxylase subunit alpha, mitochondrial OS=Homo sapiens GN=MCCC1 PE=2 SV=1	68745	102	177	214	278	0.83	0.55	0.662650602	7	6	13	11	11	11	16	13	-0.921
tr|B4DRR0|B4DRR0_HUMAN	tr|B4DRR0	cDNA FLJ53910, highly similar to Keratin, type II cytoskeletal 6A OS=Homo sapiens PE=2 SV=1	58088	92	344	225	139	0.83	0.55	0.662650602	9	5	12	8	15	9	17	10	-1.62897
tr|H0YC69|H0YC69_HUMAN	KIAA1967	DBIRD complex subunit KIAA1967 (Fragment) OS=Homo sapiens GN=KIAA1967 PE=4 SV=1	16355	82	1166	45	25	0.32	0.21	0.65625	1	0	1	1	1	1	2	2	-0.91941
tr|B3KTJ9|B3KTJ9_HUMAN	tr|B3KTJ9	cDNA FLJ38393 fis, clone FEBRA2007212 OS=Homo sapiens PE=2 SV=1	103493	82	218	251	235	0.32	0.21	0.65625	7	4	9	6	9	6	12	7	-1.4687
tr|Q5T081|Q5T081_HUMAN	RCC1	CHC1 protein OS=Homo sapiens GN=RCC1 PE=2 SV=1	45397	269	787	70	49	0.23	0.15	0.652173913	2	2	3	2	2	3	4	3	-0.43397
tr|Q5HYG8|Q5HYG8_HUMAN	DKFZp686	Putative uncharacterized protein DKFZp686P09201 OS=Homo sapiens GN=DKFZp686P09201 PE=2 SV=1	53831	86	253	235	211	0.61	0.39	0.639344262	4	4	8	5	9	5	10	6	-1.70989
sp|Q04695|K1C17_HUMAN	KRT17	Keratin, type I cytoskeletal 17 OS=Homo sapiens GN=KRT17 PE=1 SV=2	48361	24	142	683	338	2.99	1.87	0.62541806	19	13	27	16	32	19	35	20	-1.66363
tr|Q96CV8|Q96CV8_HUMAN	THOP1	Thimet oligopeptidase 1 OS=Homo sapiens GN=THOP1 PE=2 SV=1	79686	278	427	68	109	0.16	0.1	0.625	1	2	1	2	1	4	1	5	0.666081
tr|K7EKM4|K7EKM4_HUMAN	SRM	Spermidine synthase OS=Homo sapiens GN=SRM PE=4 SV=1	10680	540	722	30	44	0.32	0.2	0.625	0	1	1	1	1	1	1	1	-0.91941
sp|Q58FF8|H90B2_HUMAN	HSP90AB2	Putative heat shock protein HSP 90-beta 2 OS=Homo sapiens GN=HSP90AB2P PE=1 SV=2	44492	32	228	97	225	1.24	0.77	0.620967742	3	6	5	8	5	9	6	10	-0.13084
tr|B2Z290|B2Z290_HUMAN	ACACA	Acetyl-Coenzyme A carboxylase alpha OS=Homo sapiens GN=ACACA PE=2 SV=1	267095	31	152	628	320	0.44	0.27	0.613636364	25	10	30	20	38	20	46	28	-1.83776
sp|Q01780|EXOSX_HUMAN	EXOSC10	Exosome component 10 OS=Homo sapiens GN=EXOSC10 PE=1 SV=2	101566	627	788	23	49	0.05	0.03	0.6	0	1	1	1	1	1	1	1	-0.91941
sp|Q2NL82|TSR1_HUMAN	TSR1	Pre-rRNA-processing protein TSR1 homolog OS=Homo sapiens GN=TSR1 PE=1 SV=1	92151	230	986	90	36	0.07	0.04	0.571428571	2	0	2	1	2	1	2	4	-1.65672
sp|P78371|TCPB_HUMAN	CCT2	T-complex protein 1 subunit beta OS=Homo sapiens GN=CCT2 PE=1 SV=4	57794	37	161	563	301	1.86	1.05	0.564516129	14	7	20	14	20	13	23	15	-1.52634
sp|Q5XKE5|K2C79_HUMAN	KRT79	Keratin, type II cytoskeletal 79 OS=Homo sapiens GN=KRT79 PE=1 SV=2	58085	160	456	133	101	0.32	0.18	0.5625	5	3	6	3	6	3	8	4	-1.81382
sp|Q8NBJ5|GT251_HUMAN	GLT25D1	Procollagen galactosyltransferase 1 OS=Homo sapiens GN=GLT25D1 PE=1 SV=1	71933	523	1116	31	28	0.09	0.05	0.555555556	0	0	2	1	1	3	2	4	0.303333
sp|P53675|CLH2_HUMAN	CLTCL1	Clathrin heavy chain 2 OS=Homo sapiens GN=CLTCL1 PE=1 SV=2	189020	30	206	107	251	0.52	0.28	0.538461538	2	6	2	11	2	10	4	13	1.152239
sp|P13647|K2C5_HUMAN	KRT5	Keratin, type II cytoskeletal 5 OS=Homo sapiens GN=KRT5 PE=1 SV=3	62568	58	222	378	228	0.95	0.51	0.536842105	13	4	15	8	20	9	24	12	-2.03402
sp|Q7L2H7|EIF3M_HUMAN	EIF3M	Eukaryotic translation initiation factor 3 subunit M OS=Homo sapiens GN=EIF3M PE=1 SV=1	42932	381	1242	46	20	0.25	0.13	0.52	1	0	1	1	1	1	1	2	-0.91941
tr|A8K7A4|A8K7A4_HUMAN	tr|A8K7A4	cDNA FLJ76904, highly similar to Homo sapiens methionine adenosyltransferase II, beta (MAT2B), transcrip	36692	280	797	68	49	0.37	0.19	0.513513514	1	1	1	2	1	1	1	2	-0.91941
tr|H0YIE7|H0YIE7_HUMAN	HSPA1L	Heat shock 70 kDa protein 1-like OS=Homo sapiens GN=HSPA1L PE=3 SV=1	25488	51	123	132	205	1.45	0.73	0.503448276	3	5	4	6	4	6	4	6	-0.38902
tr|J3KN36|J3KN36_HUMAN	NOMO3	Nodal modulator 3 OS=Homo sapiens GN=NOMO3 PE=4 SV=1	140376	400	1237	43	0	0.04	0.02	0.5	1	0	1	0	1	1	1	1	-0.91941
tr|B3KV72|B3KV72_HUMAN	tr|B3KV72	cDNA FLJ16204 fis, clone CTONG2019232, highly similar to Proteasome-associated protein ECM29 homolo	78791	579	803	28	0	0.04	0.02	0.5	0	0	1	0	1	1	1	1	-0.91941
sp|Q9UHR4|BI2L1_HUMAN	BAIAP2L1	Brain-specific angiogenesis inhibitor 1-associated protein 2-like protein 1 OS=Homo sapiens GN=BAIAP2L1	57189	613	564	24	80	0.12	0.06	0.5	0	1	2	1	2	2	4	3	-0.91957
tr|Q2TB59|Q2TB59_HUMAN	NNT	Nicotinamide nucleotide transhydrogenase OS=Homo sapiens GN=NNT PE=2 SV=1	114552	234	785	88	49	0.06	0.03	0.5	1	1	2	1	2	1	3	1	-1.65672
tr|B1ANK7|B1ANK7_HUMAN	FH	Fumarate hydratase OS=Homo sapiens GN=FH PE=2 SV=1	54773	288	575	64	78	0.12	0.06	0.5	1	1	2	1	2	1	5	1	-1.65672
tr|B3KNM6|B3KNM6_HUMAN	tr|B3KNM	cDNA FLJ14968 fis, clone THYRO1000288, highly similar to CAAX prenyl protease 1 homolog (EC 3.4.24.84)	27279	547	1279	30	0	0.12	0.06	0.5	1	0	1	0	1	0	1	1	-2.50455
sp|Q14CN4|K2C72_HUMAN	KRT72	Keratin, type II cytoskeletal 72 OS=Homo sapiens GN=KRT72 PE=1 SV=2	56470	372	1200	47	0	0.12	0.06	0.5	2	0	2	0	4	1	5	3	-2.50539
tr|E9PRA5|E9PRA5_HUMAN	ATP1A4	Sodium/potassium-transporting ATPase subunit alpha-4 OS=Homo sapiens GN=ATP1A4 PE=2 SV=1	57835	289	1155	63	26	0.12	0.06	0.5	0	0	6	2	10	3	19	5	-2.50705
sp|P02533|K1C14_HUMAN	KRT14	Keratin, type I cytoskeletal 14 OS=Homo sapiens GN=KRT14 PE=1 SV=4	51872	33	153	604	319	2.22	1.09	0.490990991	19	10	25	12	29	15	30	17	-1.85481
tr|H3BNV2|H3BNV2_HUMAN	NQO1	NAD(P)H dehydrogenase [quinone] 1 OS=Homo sapiens GN=NQO1 PE=4 SV=1	28533	576	717	28	57	0.25	0.12	0.48	0	1	2	1	2	1	2	1	-1.65672
sp|P53004|BIEA_HUMAN	BLVRA	Biliverdin reductase A OS=Homo sapiens GN=BLVRA PE=1 SV=2	33692	403	989	43	36	0.21	0.1	0.476190476	1	0	2	1	2	1	3	1	-1.65672
sp|P16615|AT2A2_HUMAN	ATP2A2	Sarcoplasmic/endoplasmic reticulum calcium ATPase 2 OS=Homo sapiens GN=ATP2A2 PE=1 SV=1	116336	149	434	145	108	0.32	0.15	0.46875	4	3	10	5	12	6	13	7	-1.86564
tr|B2R5M8|B2R5M8_HUMAN	tr|B2R5M	Isocitrate dehydrogenase [NADP] OS=Homo sapiens PE=2 SV=1	46905	555	927	29	39	0.15	0.07	0.466666667	0	1	2	1	3	3	3	3	-0.91973
sp|P08779|K1C16_HUMAN	KRT16	Keratin, type I cytoskeletal 16 OS=Homo sapiens GN=KRT16 PE=1 SV=4	51578	62	183	342	270	1.38	0.64	0.463768116	14	8	17	8	21	12	21	13	-1.7066
sp|Q6IS14|IF5AL_HUMAN	EIF5AL1	Eukaryotic translation initiation factor 5A-1-like OS=Homo sapiens GN=EIF5AL1 PE=1 SV=2	16990	256	1202	75	23	0.44	0.2	0.454545455	1	0	2	1	2	1	2	1	-1.65672
sp|P27348|1433T_HUMAN	YWHAQ	14-3-3 protein theta OS=Homo sapiens GN=YWHAQ PE=1 SV=1	28032	258	960	74	37	0.4	0.18	0.45	2	1	3	1	3	1	3	1	-2.14248
tr|B2RD24|B2RD24_HUMAN	tr|B2RD24	cDNA, FLJ96424 OS=Homo sapiens PE=2 SV=1	80216	398	760	43	51	0.09	0.04	0.444444444	1	1	1	1	1	2	1	3	-0.18227
tr|Q96K98|Q96K98_HUMAN	tr|Q96K98	cDNA FLJ14414 fis, clone HEMBA1004847, highly similar to SIGNAL RECOGNITION PARTICLE 68 KD PROTEIN	37365	180	727	119	0	0.18	0.08	0.444444444	2	0	2	0	2	0	4	1	-3.24186
sp|Q14651|PLSI_HUMAN	PLS1	Plastin-1 OS=Homo sapiens GN=PLS1 PE=1 SV=2	70608	282	416	57	111	0.34	0.15	0.441176471	1	3	3	3	3	3	3	4	-0.91973
tr|A8K4Z4|A8K4Z4_HUMAN	tr|A8K4Z4	cDNA FLJ75549, highly similar to Homo sapiens ribosomal protein, large, P0 (RPLP0), transcript variant 1, m	34389	109	577	201	77	0.74	0.32	0.432432432	6	2	6	3	6	4	6	4	-1.45108
sp|P35527|K1C9_HUMAN	KRT9	Keratin, type I cytoskeletal 9 OS=Homo sapiens GN=KRT9 PE=1 SV=3	62255	14	65	1044	612	3.01	1.28	0.425249169	25	14	36	18	40	20	44	22	-1.9115
tr|J3QQX0|J3QQX0_HUMAN	GPS1	COP9 signalosome complex subunit 1 (Fragment) OS=Homo sapiens GN=GPS1 PE=4 SV=1	8703	636	753	22	44	0.4	0.17	0.425	0	1	1	2	1	1	1	2	-0.91941
tr|Q14666|Q14666_HUMAN	tr|Q14666	Radiated keratinocyte mRNA 266 (keratin-related protein) (Fragment) OS=Homo sapiens PE=2 SV=1	30933	24	126	451	285	2.99	1.26	0.421404682	12	10	17	12	20	13	21	14	-1.52634
tr|B2RCT6|B2RCT6_HUMAN	tr|B2RCT6	cDNA, FLJ96276, highly similar to Homo sapiens G1 to S phase transition 1 (GSPT1), mRNA OS=Homo sapie	56320	391	950	44	0	0.12	0.05	0.416666667	1	0	2	0	2	1	2	2	-1.65672
tr|G3XAF4|G3XAF4_HUMAN	L1CAM	L1 cell adhesion molecule, isoform CRA_a OS=Homo sapiens GN=L1CAM PE=4 SV=1	139790	572	597	28	73	0.05	0.02	0.4	1	1	2	1	1	1	2	2	-0.91941
tr|H0YF33|H0YF33_HUMAN	NASP	Nuclear autoantigenic sperm protein (Fragment) OS=Homo sapiens GN=NASP PE=4 SV=1	31883	542	870	30	44	0.1	0.04	0.4	0	1	1	1	2	2	3	2	-0.91957
tr|H0Y9L5|H0Y9L5_HUMAN	EXOSC9	Exosome complex component RRP45 (Fragment) OS=Homo sapiens GN=EXOSC9 PE=4 SV=1	18940	457	1106	38	0	0.18	0.07	0.388888889	1	0	1	0	1	1	1	1	-0.91941
tr|B2RA03|B2RA03_HUMAN	tr|B2RA03	cDNA, FLJ94640, highly similar to Homo sapiens keratin 18 (KRT18), mRNA OS=Homo sapiens PE=2 SV=1	48000	127	681	88	62	0.36	0.14	0.388888889	4	2	4	2	5	2	6	3	-2.05808
sp|P13645|K1C10_HUMAN	KRT10	Keratin, type I cytoskeletal 10 OS=Homo sapiens GN=KRT10 PE=1 SV=6	59020	12	51	1226	771	4.67	1.8	0.385438972	34	17	42	20	47	20	54	21	-2.14388
sp|P35908|K22E_HUMAN	KRT2	Keratin, type II cytoskeletal 2 epidermal OS=Homo sapiens GN=KRT2 PE=1 SV=2	65678	11	50	1255	777	4.79	1.79	0.373695198	41	18	48	23	49	25	57	29	-1.8883
sp|Q58FG0|HS905_HUMAN	HSP90AA5	Putative heat shock protein HSP 90-alpha A5 OS=Homo sapiens GN=HSP90AA5P PE=1 SV=1	38942	46	320	36	156	1.05	0.39	0.371428571	0	3	2	5	2	7	2	9	0.666278

tr|B7Z4V1|B7Z4V1_HUMAN	tr|B7Z4V1	cDNA FLJ55401, highly similar to Alpha-actinin-2 OS=Homo sapiens PE=2 SV=1	32962	50	227	103	126	0.77	0.28	0.363636364	3	3	4	4	4	6	4	6	-0.38902
tr|B4DUL5|B4DUL5_HUMAN	tr|B4DUL5	cDNA FLJ51625, highly similar to Ubiquinol-cytochrome-c reductase complex coreprotein I, mitochondrial	40917	357	780	48	50	0.17	0.06	0.352941176	1	1	2	1	2	1	2	1	-1.65672
tr|E5RIZ6|E5RIZ6_HUMAN	G3BP1	Ras GTPase-activating protein-binding protein 1 (Fragment) OS=Homo sapiens GN=G3BP1 PE=2 SV=1	17032	294	539	62	22	0.58	0.2	0.344827586	2	0	2	1	2	1	2	1	-1.65672
tr|Q7KZ86|Q7KZ86_HUMAN	tr|Q7KZ86	Plakoglobin (Fragment) OS=Homo sapiens PE=2 SV=1	19007	26	172	37	71	1.74	0.6	0.344827586	1	1	2	3	2	3	2	3	-0.43397
tr|H0Y7N2|H0Y7N2_HUMAN	ATXN10	Ataxin-10 (Fragment) OS=Homo sapiens GN=ATXN10 PE=4 SV=1	19107	470	675	37	0	0.38	0.13	0.342105263	0	0	2	0	2	0	2	1	-3.24186
tr|B4DQX3|B4DQX3_HUMAN	tr|B4DQX3	cDNA FLJ58927, highly similar to Endoplasmin (Heat shock protein 90 kDa beta member 1) OS=Homo sapie	18712	32	133	32	80	1.24	0.42	0.338709677	0	1	1	2	1	2	1	2	-0.18227
tr|B3KQQ3|B3KQQ3_HUMAN	tr|B3KQQ	cDNA PSEC0016 fis, clone NT2RM1001076, highly similar to Procollagen-lysine,2-oxoglutarate 5-dioxygena	85161	325	486	54	93	0.12	0.04	0.333333333	1	2	3	2	3	2	3	2	-1.40534
tr|D6RAT0|D6RAT0_HUMAN	RPS3A	40S ribosomal protein S3a OS=Homo sapiens GN=RPS3A PE=3 SV=1	26099	223	890	93	42	0.44	0.14	0.318181818	2	1	3	1	4	1	5	1	-2.50539
sp|Q9NV06|DCA13_HUMAN	DCAF13	DDB1- and CUL4-associated factor 13 OS=Homo sapiens GN=DCAF13 PE=1 SV=2	51996	435	746	39	53	0.2	0.06	0.3	1	1	3	1	3	1	6	1	-2.14248
tr|Q16195|Q16195_HUMAN	keratin	Keratin (Fragment) OS=Homo sapiens GN=keratin PE=2 SV=1	27757	62	422	109	110	1.38	0.41	0.297101449	3	3	4	3	5	5	5	5	-0.92005
tr|F8VQP2|F8VQP2_HUMAN	ATXN2	Ataxin-2 OS=Homo sapiens GN=ATXN2 PE=2 SV=1	117012	157	510	135	88	0.21	0.06	0.285714286	4	2	7	2	10	2	10	2	-2.99265
tr|B4DX20|B4DX20_HUMAN	CCT6B	T-complex protein 1 subunit zeta-2 OS=Homo sapiens GN=CCT6B PE=2 SV=1	54066	118	885	46	42	0.47	0.13	0.276595745	1	2	1	2	2	2	3	3	-0.91957
sp|Q58FF6|H90B4_HUMAN	HSP90AB4	Putative heat shock protein HSP 90-beta 4 OS=Homo sapiens GN=HSP90AB4P PE=5 SV=1	58855	32	386	77	121	1.24	0.31	0.25	3	4	5	5	3	5	5	6	-0.2673
tr|F5H0C8|F5H0C8_HUMAN	ENO2	Enolase OS=Homo sapiens GN=ENO2 PE=2 SV=1	35026	40	313	112	162	1.39	0.31	0.223021583	2	3	2	3	2	4	4	5	-0.07122
tr|E7EN38|E7EN38_HUMAN	RAD50	DNA repair protein RAD50 (Fragment) OS=Homo sapiens GN=RAD50 PE=2 SV=1	77138	563	806	29	0	0.09	0.02	0.222222222	0	0	2	0	1	0	2	2	-2.50455
sp|O60518|RNBP6_HUMAN	RANBP6	Ran-binding protein 6 OS=Homo sapiens GN=RANBP6 PE=1 SV=2	126173	85	1108	36	29	0.23	0.04	0.173913043	1	0	1	1	2	1	2	2	-1.65672
tr|D3DTL4|D3DTL4_HUMAN	ENO3	Enolase OS=Homo sapiens GN=ENO3 PE=2 SV=1	37167	40	236	112	149	1.39	0.22	0.158273381	2	2	2	2	2	3	4	6	-0.43397
tr|B3KMS0|B3KMS0_HUMAN	tr|B3KMS0	cDNA FLJ12449 fis, clone NT2RM1000314, highly similar to Condensin complex subunit 1 OS=Homo sapien	158464	494	1030	34	34	0.15	0.02	0.133333333	0	0	2	1	2	3	2	3	-0.43397
tr|C1IK54|C1IK54_HUMAN	DDX5-ETV	DEAD box polypeptide 5/ets variant protein 4 fusion protein OS=Homo sapiens GN=DDX5-ETV4 fusion PE=	58580	97	917	51	40	0.45	0.06	0.133333333	1	1	1	1	1	1	1	2	-0.91941
tr|J3KR91|J3KR91_HUMAN	IQGAP2	Ras GTPase-activating-like protein IQGAP2 OS=Homo sapiens GN=IQGAP2 PE=4 SV=1	123072	84	370	32	127	0.31	0.04	0.129032258	0	2	1	2	1	3	1	5	0.303333
sp|O95678|K2C75_HUMAN	KRT75	Keratin, type II cytoskeletal 75 OS=Homo sapiens GN=KRT75 PE=1 SV=2	59809	11	314	94	79	4.79	0.61	0.127348643	2	2	3	3	3	3	5	4	-0.91973
tr|B2R8Y4|B2R8Y4_HUMAN	tr|B2R8Y4	cDNA, FLJ94117, highly similar to Homo sapiens actinin, alpha 3 (ACTN3), mRNA OS=Homo sapiens PE=2 SV	103871	19	277	99	180	2.11	0.24	0.113744076	3	5	3	7	3	8	4	9	0.361266
tr|F8VP67|F8VP67_HUMAN	KRT8	Keratin, type II cytoskeletal 8 (Fragment) OS=Homo sapiens GN=KRT8 PE=2 SV=1	13580	6	243	50	82	5.01	0.43	0.085828343	2	1	3	3	5	3	5	5	-1.57248
tr|A2RRC9|A2RRC9_HUMAN	IQGAP3	IQ motif containing GTPase activating protein 3 OS=Homo sapiens GN=IQGAP3 PE=2 SV=1	185145	84	987	32	36	0.31	0.02	0.064516129	0	0	1	1	1	1	1	4	-0.91941
tr|Q562V5|Q562V5_HUMAN	ACT	Actin-like protein (Fragment) OS=Homo sapiens GN=ACT PE=2 SV=1	11481	1	418	136	111	31.43	1.2	0.038180083	6	4	6	5	7	8	8	10	-0.73962
sp|P35900|K1C20_HUMAN	KRT20	Keratin, type I cytoskeletal 20 OS=Homo sapiens GN=KRT20 PE=1 SV=1	48514	24	845	78	45	2.99	0.07	0.023411371	2	1	2	1	2	1	2	2	-1.65672
sp|P12036|NFH_HUMAN	NEFH	Neurofilament heavy polypeptide OS=Homo sapiens GN=NEFH PE=1 SV=4	112639	11	1201	0	23	4.79	0.03	0.006263048	0	0	0	1	0	1	1	2	0.665884
sp|Q9H254|SPTN4_HUMAN	SPTBN4	Spectrin beta chain, non-erythrocytic 4 OS=Homo sapiens GN=SPTBN4 PE=1 SV=2	290005	19	684	29	61	2.11	0.01	0.004739336	0	1	1	1	2	2	2	5	-0.91957
tr|K7ELP7|K7ELP7_HUMAN	KRT17	Keratin, type I cytoskeletal 17 (Fragment) OS=Homo sapiens GN=KRT17 PE=4 SV=1	5508	24	0	20	0	2.99	0	0	0	0	1	0	0	0	1	0	-0.91926
tr|E9PQ69|E9PQ69_HUMAN	GANAB	Neutral alpha-glucosidase AB OS=Homo sapiens GN=GANAB PE=2 SV=1	8560	47	0	0	0	0.62	0	0	0	0	0	0	0	0	1	0	-0.91926
tr|Q8WY81|Q8WY81_HUMAN	tr|Q8WY8	PP3895 OS=Homo sapiens PE=2 SV=1	15132	114	0	15	0	0.28	0	0	0	0	1	0	0	0	1	0	-0.91926
tr|E9PLJ0|E9PLJ0_HUMAN	IPO7	Importin-7 OS=Homo sapiens GN=IPO7 PE=2 SV=1	7255	162	0	0	0	0.08	0	0	0	0	0	0	0	0	1	0	-0.91926
tr|B7Z6J5|B7Z6J5_HUMAN	tr|B7Z6J5	cDNA FLJ56858, highly similar to Succinate dehydrogenase (ubiquinone) flavoprotein subunit, mitochondri	28776	393	0	0	0	0.1	0	0	0	0	0	0	0	0	1	0	-0.91926
tr|A8K171|A8K171_HUMAN	tr|A8K171	cDNA FLJ77253, highly similar to Homo sapiens nuclear receptor interacting protein 1 (NRIP1), mRNA OS=H	127609	401	0	0	0	0.01	0	0	0	0	0	0	0	0	1	0	-0.91926
tr|H0Y386|H0Y386_HUMAN	PABPC1L	Polyadenylate-binding protein 1-like (Fragment) OS=Homo sapiens GN=PABPC1L PE=4 SV=1	16787	404	0	0	0	0.1	0	0	0	0	0	0	0	0	1	0	-0.91926
tr|Q4VBX7|Q4VBX7_HUMAN	NUP205	NUP205 protein (Fragment) OS=Homo sapiens GN=NUP205 PE=2 SV=1	38620	415	0	0	0	0.04	0	0	0	0	0	0	0	0	1	0	-0.91926
tr|Q53SG6|Q53SG6_HUMAN	AGPS	Putative uncharacterized protein AGPS (Fragment) OS=Homo sapiens GN=AGPS PE=2 SV=1	36640	448	0	17	0	0.09	0	0	0	0	1	0	0	0	1	0	-0.91926
tr|E9PQX2|E9PQX2_HUMAN	RPS3	40S ribosomal protein S3 OS=Homo sapiens GN=RPS3 PE=2 SV=1	5066	480	0	0	0	0.12	0	0	0	0	0	0	0	0	1	0	-0.91926
tr|H7BYY8|H7BYY8_HUMAN	PES1	Pescadillo homolog OS=Homo sapiens GN=PES1 PE=4 SV=1	1501	488	0	0	0	0.05	0	0	0	0	0	0	0	0	1	0	-0.91926
sp|Q14117|DPYS_HUMAN	DPYS	Dihydropyrimidinase OS=Homo sapiens GN=DPYS PE=1 SV=1	57107	490	0	0	0	0.19	0	0	0	0	0	0	0	0	1	0	-0.91926
tr|B4DM94|B4DM94_HUMAN	tr|B4DM9	60S ribosomal protein L18a OS=Homo sapiens PE=2 SV=1	17672	514	0	0	0	0.16	0	0	0	0	0	0	0	0	1	0	-0.91926
tr|A8K482|A8K482_HUMAN	tr|A8K482	Aspartate aminotransferase OS=Homo sapiens PE=2 SV=1	47885	522	0	0	0	0.08	0	0	0	0	0	0	0	0	1	0	-0.91926
tr|D6RD96|D6RD96_HUMAN	ACSL4	Long-chain-fatty-acid--CoA ligase 4 (Fragment) OS=Homo sapiens GN=ACSL4 PE=2 SV=1	16399	529	0	0	0	0.04	0	0	0	0	0	0	0	0	1	0	-0.91926
tr|H7C1T1|H7C1T1_HUMAN	Sep-02	Septin-2 (Fragment) OS=Homo sapiens GN=SEPT2 PE=4 SV=1	3094	543	0	0	0	0.09	0	0	0	0	0	0	0	0	1	0	-0.91926
tr|J3KTB7|J3KTB7_HUMAN	TOP2A	DNA topoisomerase 2-alpha (Fragment) OS=Homo sapiens GN=TOP2A PE=4 SV=1	17149	606	0	0	0	0.02	0	0	0	0	0	0	0	0	1	0	-0.91926
tr|J3KN33|J3KN33_HUMAN	CNTLN	Centlein OS=Homo sapiens GN=CNTLN PE=4 SV=1	162069	680	0	20	0	0.02	0	0	0	0	1	0	0	0	2	0	-0.91926
tr|Q3MNE1|Q3MNE1_HUMAN	tr|Q3MNE	Adaptor-related protein complex 3 beta 1 subunit (Fragment) OS=Homo sapiens PE=2 SV=1	89828	682	0	20	0	0.04	0	0	0	0	1	0	0	0	1	0	-0.91926
tr|Q9UH66|Q9UH66_HUMAN	tr|Q9UH6	7h3 protein (Fragment) OS=Homo sapiens PE=2 SV=1	49963	684	0	19	0	0.07	0	0	0	0	1	0	0	0	2	0	-0.91926
tr|G5E9Q3|G5E9Q3_HUMAN	TSEN2	tRNA splicing endonuclease 2 homolog (S. cerevisiae), isoform CRA_b OS=Homo sapiens GN=TSEN2 PE=2 S	45959	686	0	19	0	0.07	0	0	0	0	1	0	0	0	1	0	-0.91926
tr|E9PQG7|E9PQG7_HUMAN	MLL	MLL cleavage product C180 OS=Homo sapiens GN=MLL PE=2 SV=1	436345	687	0	19	0	0.01	0	0	0	0	1	0	0	0	1	0	-0.91926
tr|G3V448|G3V448_HUMAN	TMX1	Thioredoxin-related transmembrane protein 1 OS=Homo sapiens GN=TMX1 PE=2 SV=1	12018	689	0	19	0	0.29	0	0	0	0	1	0	0	0	1	0	-0.91926
tr|D3DUL0|D3DUL0_HUMAN	ASCIZ	ATM/ATR-Substrate Chk2-Interacting Zn2+-finger protein, isoform CRA_b OS=Homo sapiens GN=ASCIZ PE=	79474	690	0	19	0	0.04	0	0	0	0	1	0	0	0	1	0	-0.91926
tr|H7C4X2|H7C4X2_HUMAN	PCMT1	Protein-L-isoaspartate(D-aspartate) O-methyltransferase (Fragment) OS=Homo sapiens GN=PCMT1 PE=4 S	7342	691	0	19	0	0.48	0	0	0	0	1	0	0	0	2	0	-0.91926
tr|B3KND7|B3KND7_HUMAN	tr|B3KND	cDNA FLJ14378 fis, clone HEMBA1001804, highly similar to Zinc finger protein 281 (Fragment) OS=Homo sa	58221	692	0	19	0	0.06	0	0	0	0	1	0	0	0	1	0	-0.91926
tr|H0YE81|H0YE81_HUMAN	NDUFV1	NADH dehydrogenase [ubiquinone] flavoprotein 1, mitochondrial (Fragment) OS=Homo sapiens GN=NDUF	14494	694	0	19	0	0.23	0	0	0	0	1	0	0	0	1	0	-0.91926
tr|H0Y304|H0Y304_HUMAN	DMD	Dystrophin (Fragment) OS=Homo sapiens GN=DMD PE=4 SV=1	160176	695	0	19	0	0.02	0	0	0	0	1	0	0	0	1	0	-0.91926
tr|Q5T097|Q5T097_HUMAN	UTRN	Utrophin OS=Homo sapiens GN=UTRN PE=4 SV=1	114423	696	0	19	0	0.03	0	0	0	0	1	0	0	0	1	0	-0.91926
tr|B4DY47|B4DY47_HUMAN	PHB	Prohibitin OS=Homo sapiens GN=PHB PE=2 SV=1	17064	699	0	18	0	0.2	0	0	0	0	1	0	0	0	1	0	-0.91926
tr|B0UZL4|B0UZL4_HUMAN	BAT3	HLA-B associated transcript 3 (Fragment) OS=Homo sapiens GN=BAT3 PE=2 SV=1	15422	704	0	18	0	0.03	0	0	0	0	1	0	0	0	1	0	-0.91926
tr|J3KMW7|J3KMW7_HUMAN	UBR5	E3 ubiquitin-protein ligase UBR5 OS=Homo sapiens GN=UBR5 PE=4 SV=1	312224	706	0	18	0	0.01	0	0	0	0	1	0	0	0	1	0	-0.91926
tr|H0Y4R4|H0Y4R4_HUMAN	TOR1AIP1	Torsin-1A-interacting protein 1 (Fragment) OS=Homo sapiens GN=TOR1AIP1 PE=4 SV=1	33844	707	0	17	0	0.1	0	0	0	0	1	0	0	0	1	0	-0.91926
sp|P54826|GAS1_HUMAN	GAS1	Growth arrest-specific protein 1 OS=Homo sapiens GN=GAS1 PE=1 SV=2	36867	708	0	17	0	0.09	0	0	0	0	1	0	0	0	1	0	-0.91926
tr|H0YHD9|H0YHD9_HUMAN	KRT72	Keratin, type II cytoskeletal 72 (Fragment) OS=Homo sapiens GN=KRT72 PE=4 SV=1	14802	11	0	43	0	4.79	0	0	1	0	1	0	1	0	2	0	-2.50455
tr|B9VP24|B9VP24_HUMAN	HSPD1	60 kDa chaperonin (Fragment) OS=Homo sapiens GN=HSPD1 PE=4 SV=1	19880	16	0	40	0	1.72	0	0	1	0	1	0	1	0	1	0	-2.50455
tr|A1L2Z2|A1L2Z2_HUMAN	MYH14	MYH14 protein OS=Homo sapiens GN=MYH14 PE=2 SV=1	73418	17	0	56	0	0.9	0	0	1	0	1	0	1	0	1	0	-2.50455
tr|B7Z2Y4|B7Z2Y4_HUMAN	tr|B7Z2Y4	cDNA FLJ56961, highly similar to Clathrin heavy chain 2 (CLH-22) OS=Homo sapiens PE=2 SV=1	54629	30	0	60	0	0.52	0	0	1	0	1	0	1	0	2	0	-2.50455
tr|F8VS61|F8VS61_HUMAN	KRT77	Keratin, type II cytoskeletal 1b OS=Homo sapiens GN=KRT77 PE=2 SV=1	25704	76	0	43	0	0.23	0	0	1	0	1	0	1	0	3	0	-2.50455
tr|K7ESA0|K7ESA0_HUMAN	ATP5A1	ATP synthase subunit alpha, mitochondrial OS=Homo sapiens GN=ATP5A1 PE=4 SV=1	8259	135	0	54	0	1.33	0	0	1	0	1	0	1	0	1	0	-2.50455
tr|Q9BZT4|Q9BZT4_HUMAN	tr|Q9BZT4	PNAS-29 OS=Homo sapiens PE=4 SV=1	8036	137	0	59	0	0.4	0	0	1	0	1	0	1	0	1	0	-2.50455
tr|B4DSC0|B4DSC0_HUMAN	TNPO1	Transportin-1 OS=Homo sapiens GN=TNPO1 PE=2 SV=1	14552	139	0	67	0	0.17	0	0	1	0	1	0	1	0	1	0	-2.50455
tr|Q58FF2|Q58FF2_HUMAN	GRP94c	Heat shock protein 94c OS=Homo sapiens GN=GRP94c PE=2 SV=1	52205	140	0	22	0	0.37	0	0	0	0	1	0	1	0	2	0	-2.50455
tr|Q6RUP9|Q6RUP9_HUMAN	IMPDH2	Inosine monophosphate dehydrogenase type II (Fragment) OS=Homo sapiens GN=IMPDH2 PE=2 SV=1	7313	177	0	25	0	0.36	0	0	0	0	1	0	1	0	1	0	-2.50455
tr|A4D1P0|A4D1P0_HUMAN	LOC40229	Uncharacterized protein OS=Homo sapiens GN=LOC402299 PE=4 SV=1	42109	185	0	40	0	0.42	0	0	1	0	1	0	1	0	1	0	-2.50455
tr|B3KV39|B3KV39_HUMAN	tr|B3KV39	cDNA FLJ16099 fis, clone TESTI2008278, highly similar to Homo sapiens forkhead box K1 (FOXK1), mRNA O	50383	188	0	115	0	0.07	0	0	1	0	1	0	1	0	2	0	-2.50455
tr|F5H7Y1|F5H7Y1_HUMAN	TCP1	T-complex protein 1 subunit alpha (Fragment) OS=Homo sapiens GN=TCP1 PE=2 SV=1	6597	197	0	0	0	0.3	0	0	0	0	0	0	1	0	1	0	-2.50455
tr|D6R9D3|D6R9D3_HUMAN	HNRNPH1	Heterogeneous nuclear ribonucleoprotein H OS=Homo sapiens GN=HNRNPH1 PE=4 SV=1	4404	207	0	23	0	0.45	0	0	0	0	1	0	1	0	1	0	-2.50455
tr|H0Y960|H0Y960_HUMAN	DDX4	Probable ATP-dependent RNA helicase DDX4 (Fragment) OS=Homo sapiens GN=DDX4 PE=4 SV=1	33292	217	0	43	0	0.19	0	0	1	0	1	0	1	0	1	0	-2.50455
tr|E9PLA6|E9PLA6_HUMAN	SERPINH1	Serpin H1 (Fragment) OS=Homo sapiens GN=SERPINH1 PE=2 SV=1	8925	233	0	45	0	0.43	0	0	1	0	1	0	1	0	1	0	-2.50455
tr|E5RFJ8|E5RFJ8_HUMAN	MCM4	DNA replication licensing factor MCM4 (Fragment) OS=Homo sapiens GN=MCM4 PE=2 SV=1	23630	240	0	65	0	0.1	0	0	1	0	1	0	1	0	2	0	-2.50455
tr|B4DE40|B4DE40_HUMAN	SMS	Spermine synthase OS=Homo sapiens GN=SMS PE=2 SV=1	31596	248	0	78	0	0.11	0	0	1	0	1	0	1	0	1	0	-2.50455
tr|B4DXK4|B4DXK4_HUMAN	KRT72	Keratin, type II cytoskeletal 72 OS=Homo sapiens GN=KRT72 PE=2 SV=1	36031	259	0	0	0	0.45	0	0	0	0	0	0	1	0	1	0	-2.50455
tr|K7EKB6|K7EKB6_HUMAN	THOP1	Thimet oligopeptidase (Fragment) OS=Homo sapiens GN=THOP1 PE=4 SV=1	20792	278	0	68	0	0.16	0	0	1	0	1	0	1	0	1	0	-2.50455
tr|H0YC45|H0YC45_HUMAN	ATP6V1B2	V-type proton ATPase subunit B, brain isoform (Fragment) OS=Homo sapiens GN=ATP6V1B2 PE=4 SV=1	5569	287	0	64	0	0.65	0	0	1	0	1	0	1	0	1	0	-2.50455
tr|B4DE27|B4DE27_HUMAN	tr|B4DE27	cDNA FLJ58765, highly similar to Neutral amino acid transporter B(0) OS=Homo sapiens PE=2 SV=1	33987	290	0	63	0	0.1	0	0	1	0	1	0	1	0	1	0	-2.50455
sp|P81605|DCD_HUMAN	DCD	Dermcidin OS=Homo sapiens GN=DCD PE=1 SV=2	11391	295	0	62	0	0.3	0	0	1	0	1	0	1	0	1	0	-2.50455
tr|B4DX93|B4DX93_HUMAN	tr|B4DX93	cDNA FLJ55471, highly similar to Cytoplasmic dynein 1 intermediate chain 2 OS=Homo sapiens PE=2 SV=1	40089	298	0	40	0	0.09	0	0	1	0	1	0	1	0	1	0	-2.50455
tr|A1L3A7|A1L3A7_HUMAN	NUFIP2	Nuclear fragile X mental retardation protein interacting protein 2 OS=Homo sapiens GN=NUFIP2 PE=2 SV=1	76132	301	0	59	0	0.04	0	0	1	0	1	0	1	0	1	0	-2.50455
tr|B5LY69|B5LY69_HUMAN	UMPS	Uridine monophosphate synthetase isoform G OS=Homo sapiens GN=UMPS PE=2 SV=1	40514	302	0	59	0	0.1	0	0	1	0	1	0	1	0	1	0	-2.50455
tr|H3BP76|H3BP76_HUMAN	MVP	Major vault protein (Fragment) OS=Homo sapiens GN=MVP PE=4 SV=1	10921	304	0	38	0	0.07	0	0	1	0	1	0	1	0	1	0	-2.50455
tr|B3KPC1|B3KPC1_HUMAN	tr|B3KPC1	cDNA FLJ31582 fis, clone NT2RI2002117, highly similar to Protein pelota homolog OS=Homo sapiens PE=2	43807	307	0	59	0	0.08	0	0	1	0	1	0	1	0	1	0	-2.50455
sp|Q5TZA2|CROCC_HUMAN	CROCC	Rootletin OS=Homo sapiens GN=CROCC PE=1 SV=1	228787	310	0	58	0	0.01	0	0	1	0	1	0	1	0	2	0	-2.50455
tr|Q5JXT2|Q5JXT2_HUMAN	NOP56	Nucleolar protein 56 (Fragment) OS=Homo sapiens GN=NOP56 PE=4 SV=1	31576	311	0	0	0	0.14	0	0	0	0	0	0	1	0	1	0	-2.50455
tr|C9JPP3|C9JPP3_HUMAN	RBBP4	Histone-binding protein RBBP4 (Fragment) OS=Homo sapiens GN=RBBP4 PE=2 SV=2	9655	315	0	57	0	0.36	0	0	1	0	1	0	1	0	1	0	-2.50455
tr|H7C1U0|H7C1U0_HUMAN	APEH	Acylamino-acid-releasing enzyme (Fragment) OS=Homo sapiens GN=APEH PE=4 SV=1	35132	319	0	56	0	0.09	0	0	1	0	1	0	1	0	1	0	-2.50455
tr|C9JIX5|C9JIX5_HUMAN	PLOD3	Procollagen-lysine,2-oxoglutarate 5-dioxygenase 3 (Fragment) OS=Homo sapiens GN=PLOD3 PE=2 SV=1	8424	325	0	41	0	0.12	0	0	1	0	1	0	1	0	1	0	-2.50455
tr|I3L4P6|I3L4P6_HUMAN	ARHGAP17	Rho GTPase-activating protein 17 (Fragment) OS=Homo sapiens GN=ARHGAP17 PE=4 SV=1	8676	328	0	54	0	0.03	0	0	1	0	1	0	1	0	1	0	-2.50455
tr|A8K3Q9|A8K3Q9_HUMAN	tr|A8K3Q9	cDNA FLJ76611, highly similar to Homo sapiens ribosomal protein L14 (RPL14), mRNA OS=Homo sapiens P	23554	330	0	53	0	0.14	0	0	1	0	1	0	1	0	1	0	-2.50455
tr|Q59GV0|Q59GV0_HUMAN	tr|Q59GV	Propionyl Coenzyme A carboxylase, beta polypeptide variant (Fragment) OS=Homo sapiens PE=2 SV=1	60578	331	0	53	0	0.05	0	0	1	0	1	0	1	0	2	0	-2.50455
sp|Q9Y6M5|ZNT1_HUMAN	SLC30A1	Zinc transporter 1 OS=Homo sapiens GN=SLC30A1 PE=1 SV=3	56290	334	0	52	0	0.06	0	0	1	0	1	0	1	0	1	0	-2.50455
tr|J3KTE4|J3KTE4_HUMAN	RPL19	Ribosomal protein L19 OS=Homo sapiens GN=RPL19 PE=3 SV=1	23347	336	0	52	0	0.14	0	0	1	0	1	0	1	0	1	0	-2.50455
tr|A8KA82|A8KA82_HUMAN	DNAJC3	DnaJ (Hsp40) homolog, subfamily C, member 3 OS=Homo sapiens GN=DNAJC3 PE=2 SV=1	58000	337	0	52	0	0.06	0	0	1	0	1	0	1	0	1	0	-2.50455
tr|A8K517|A8K517_HUMAN	RPS23	Ribosomal protein S23, isoform CRA_a OS=Homo sapiens GN=RPS23 PE=2 SV=1	15969	339	0	52	0	0.21	0	0	1	0	1	0	1	0	1	0	-2.50455
tr|H7C5I9|H7C5I9_HUMAN	GART	Phosphoribosylformylglycinamidine cyclo-ligase (Fragment) OS=Homo sapiens GN=GART PE=4 SV=1	2640	341	0	23	0	0.13	0	0	0	0	1	0	1	0	1	0	-2.50455
tr|D6RF19|D6RF19_HUMAN	DPYSL3	Dihydropyrimidinase-related protein 3 (Fragment) OS=Homo sapiens GN=DPYSL3 PE=2 SV=1	10214	342	0	20	0	0.23	0	0	0	0	1	0	1	0	1	0	-2.50455
tr|Q53ST0|Q53ST0_HUMAN	PCYOX1	Putative uncharacterized protein PCYOX1 (Fragment) OS=Homo sapiens GN=PCYOX1 PE=2 SV=1	11626	355	0	49	0	0.06	0	0	1	0	1	0	1	0	1	0	-2.50455
tr|D6RBX4|D6RBX4_HUMAN	TGFBI	Transforming growth factor-beta-induced protein ig-h3 OS=Homo sapiens GN=TGFBI PE=2 SV=1	7268	358	0	31	0	0.09	0	0	0	0	1	0	1	0	1	0	-2.50455
tr|A6NKV8|A6NKV8_HUMAN	PAIP1	Polyadenylate-binding protein-interacting protein 1 OS=Homo sapiens GN=PAIP1 PE=2 SV=1	42379	361	0	48	0	0.08	0	0	1	0	1	0	1	0	1	0	-2.50455
tr|K7EM16|K7EM16_HUMAN	VASP	Vasodilator-stimulated phosphoprotein (Fragment) OS=Homo sapiens GN=VASP PE=4 SV=1	20260	363	0	48	0	0.17	0	0	1	0	1	0	1	0	1	0	-2.50455
tr|D2KFR9|D2KFR9_HUMAN	STAT1	Signal transducer and activator of transcription 1-alpha/beta OS=Homo sapiens GN=STAT1 PE=2 SV=1	23360	364	0	42	0	0.08	0	0	1	0	1	0	1	0	1	0	-2.50455
tr|H0Y9Q1|H0Y9Q1_HUMAN	LAP3	Cytosol aminopeptidase (Fragment) OS=Homo sapiens GN=LAP3 PE=4 SV=1	22641	368	0	47	0	0.15	0	0	1	0	1	0	1	0	1	0	-2.50455
tr|B3KU96|B3KU96_HUMAN	tr|B3KU96	cDNA FLJ39433 fis, clone PROST2004142, highly similar to GDP-L-fucose synthetase (EC 1.1.1.271) OS=Hom	17856	371	0	47	0	0.19	0	0	1	0	1	0	1	0	1	0	-2.50455
tr|Q0P512|Q0P512_HUMAN	SLC2A1	Solute carrier family 2 (Facilitated glucose transporter), member 1 OS=Homo sapiens GN=SLC2A1 PE=2 SV=	54361	377	0	46	0	0.06	0	0	1	0	1	0	1	0	1	0	-2.50455
tr|B4DT09|B4DT09_HUMAN	tr|B4DT09	cDNA FLJ60145, highly similar to Homo sapiens dendritic cell protein (hfl-B5), mRNA OS=Homo sapiens PE=	13697	381	0	46	0	0.25	0	0	1	0	1	0	1	0	1	0	-2.50455
sp|Q8NFI4|F10A5_HUMAN	ST13P5	Putative protein FAM10A5 OS=Homo sapiens GN=ST13P5 PE=5 SV=1	41579	382	0	0	0	0.09	0	0	0	0	0	0	1	0	2	0	-2.50455
tr|B3KPZ0|B3KPZ0_HUMAN	tr|B3KPZ0	cDNA FLJ32485 fis, clone SKNMC2001596, highly similar to Ankyrin repeat and FYVE domain-containing pr	121468	387	0	45	0	0.03	0	0	1	0	1	0	1	0	1	0	-2.50455
tr|A6NKP0|A6NKP0_HUMAN	CTNNA3	Catenin alpha-3 OS=Homo sapiens GN=CTNNA3 PE=2 SV=1	26635	389	0	35	0	0.07	0	0	0	0	1	0	1	0	1	0	-2.50455
tr|H0Y327|H0Y327_HUMAN	MTHFD1L	Monofunctional C1-tetrahydrofolate synthase, mitochondrial (Fragment) OS=Homo sapiens GN=MTHFD1L	36808	398	0	43	0	0.09	0	0	1	0	1	0	1	0	1	0	-2.50455
tr|Q1LZN2|Q1LZN2_HUMAN	NOMO3	NOMO3 protein (Fragment) OS=Homo sapiens GN=NOMO3 PE=2 SV=1	90388	400	0	43	0	0.04	0	0	1	0	1	0	1	0	1	0	-2.50455
tr|B3KMW5|B3KMW5_HUMAN	tr|B3KMW	cDNA FLJ12796 fis, clone NT2RP2002058, highly similar to WD repeat protein 3 OS=Homo sapiens PE=2 SV	71519	408	0	43	0	0.05	0	0	1	0	1	0	1	0	1	0	-2.50455
tr|B4DRG7|B4DRG7_HUMAN	tr|B4DRG	cDNA FLJ60336, highly similar to Condensin complex subunit 2 OS=Homo sapiens PE=2 SV=1	80877	410	0	43	0	0.04	0	0	1	0	1	0	1	0	1	0	-2.50455
sp|Q9NSB4|KRT82_HUMAN	KRT82	Keratin, type II cuticular Hb2 OS=Homo sapiens GN=KRT82 PE=1 SV=3	57985	411	0	43	0	0.06	0	0	1	0	1	0	1	0	2	0	-2.50455
tr|H0YGE9|H0YGE9_HUMAN	M6PR	Cation-dependent mannose-6-phosphate receptor (Fragment) OS=Homo sapiens GN=M6PR PE=4 SV=1	11814	416	0	42	0	0.29	0	0	1	0	1	0	1	0	1	0	-2.50455
tr|Q5JR94|Q5JR94_HUMAN	RPS8	40S ribosomal protein S8 OS=Homo sapiens GN=RPS8 PE=2 SV=1	24475	426	0	41	0	0.14	0	0	1	0	1	0	1	0	2	0	-2.50455
tr|A4D1I7|A4D1I7_HUMAN	DNCI1	Dynein, cytoplasmic 1, intermediate chain 1, isoform CRA_e OS=Homo sapiens GN=DNCI1 PE=2 SV=1	73423	427	0	40	0	0.04	0	0	1	0	1	0	1	0	1	0	-2.50455
tr|B3KME9|B3KME9_HUMAN	tr|B3KME	cDNA FLJ10831 fis, clone NT2RP4001148, highly similar to Homo sapiens WD repeats and SOF1 domain co	25732	435	0	28	0	0.2	0	0	0	0	2	0	1	0	4	0	-2.50455
tr|K7EK06|K7EK06_HUMAN	FARSA	Phenylalanine--tRNA ligase alpha subunit (Fragment) OS=Homo sapiens GN=FARSA PE=4 SV=1	18547	447	0	38	0	0.06	0	0	1	0	1	0	1	0	1	0	-2.50455
tr|B4DXC4|B4DXC4_HUMAN	tr|B4DXC4	cDNA FLJ58636, moderately similar to Atlastin OS=Homo sapiens PE=2 SV=1	59162	451	0	38	0	0.06	0	0	1	0	1	0	1	0	1	0	-2.50455
tr|H0YA24|H0YA24_HUMAN	PLRG1	Pleiotropic regulator 1 (Fragment) OS=Homo sapiens GN=PLRG1 PE=4 SV=1	17588	456	0	38	0	0.19	0	0	1	0	1	0	1	0	2	0	-2.50455
tr|B3KME2|B3KME2_HUMAN	tr|B3KME	cDNA FLJ10772 fis, clone NT2RP4000243, highly similar to Cartilage-associated protein OS=Homo sapiens F	47101	459	0	38	0	0.07	0	0	1	0	1	0	1	0	1	0	-2.50455
sp|Q9H9F9|ARP5_HUMAN	ACTR5	Actin-related protein 5 OS=Homo sapiens GN=ACTR5 PE=1 SV=2	68825	460	0	38	0	0.05	0	0	1	0	1	0	1	0	2	0	-2.50455
tr|H7C1M0|H7C1M0_HUMAN	FANCL	E3 ubiquitin-protein ligase FANCL (Fragment) OS=Homo sapiens GN=FANCL PE=4 SV=1	33067	461	0	37	0	0.1	0	0	1	0	1	0	1	0	1	0	-2.50455
tr|Q5SZ64|Q5SZ64_HUMAN	SF3B4	Splicing factor 3B subunit 4 (Fragment) OS=Homo sapiens GN=SF3B4 PE=4 SV=1	20733	464	0	37	0	0.07	0	0	1	0	1	0	1	0	1	0	-2.50455
sp|Q6ZU64|CC108_HUMAN	CCDC108	Coiled-coil domain-containing protein 108 OS=Homo sapiens GN=CCDC108 PE=2 SV=2	220247	465	0	37	0	0.01	0	0	1	0	1	0	1	0	1	0	-2.50455
tr|C9J7L0|C9J7L0_HUMAN	RBBP7	Histone-binding protein RBBP7 (Fragment) OS=Homo sapiens GN=RBBP7 PE=2 SV=1	7740	468	0	0	0	0.07	0	0	0	0	0	0	1	0	1	0	-2.50455
tr|H0YIX9|H0YIX9_HUMAN	R3HDM2	R3H domain-containing protein 2 (Fragment) OS=Homo sapiens GN=R3HDM2 PE=4 SV=1	31776	469	0	37	0	0.1	0	0	1	0	1	0	1	0	1	0	-2.50455
tr|B4DKI2|B4DKI2_HUMAN	tr|B4DKI2	cDNA FLJ60282, highly similar to Sorbitol dehydrogenase (EC 1.1.1.14) OS=Homo sapiens PE=2 SV=1	30315	473	0	37	0	0.09	0	0	0	0	1	0	1	0	1	0	-2.50455
tr|C9JNW5|C9JNW5_HUMAN	RPL24	60S ribosomal protein L24 OS=Homo sapiens GN=RPL24 PE=4 SV=1	17646	487	0	35	0	0.19	0	0	0	0	1	0	1	0	2	0	-2.50455
tr|E9PI04|E9PI04_HUMAN	RIC8A	Synembryn-A (Fragment) OS=Homo sapiens GN=RIC8A PE=2 SV=1	16936	496	0	34	0	0.05	0	0	0	0	1	0	1	0	1	0	-2.50455
sp|Q8NFZ5|TNIP2_HUMAN	TNIP2	TNFAIP3-interacting protein 2 OS=Homo sapiens GN=TNIP2 PE=1 SV=1	49240	502	0	33	0	0.07	0	0	1	0	1	0	1	0	2	0	-2.50455
sp|Q7Z494|NPHP3_HUMAN	NPHP3	Nephrocystin-3 OS=Homo sapiens GN=NPHP3 PE=1 SV=1	151967	510	0	32	0	0.02	0	0	1	0	1	0	1	0	1	0	-2.50455
tr|A0JLS9|A0JLS9_HUMAN	ZEB1	ZEB1 protein (Fragment) OS=Homo sapiens GN=ZEB1 PE=2 SV=1	118576	513	0	32	0	0.03	0	0	1	0	1	0	1	0	1	0	-2.50455
tr|J3KRB3|J3KRB3_HUMAN	RPL17	60S ribosomal protein L17 (Fragment) OS=Homo sapiens GN=RPL17 PE=3 SV=1	12356	517	0	32	0	0.28	0	0	0	0	1	0	1	0	1	0	-2.50455
tr|B4DUH7|B4DUH7_HUMAN	tr|B4DUH	cDNA FLJ60162, highly similar to Chloride anion exchanger OS=Homo sapiens PE=2 SV=1	52632	518	0	32	0	0.06	0	0	1	0	1	0	1	0	1	0	-2.50455
tr|L0R4T3|L0R4T3_HUMAN	ABCF2	Alternative protein ABCF2 OS=Homo sapiens GN=ABCF2 PE=4 SV=1	7359	524	0	31	0	0.48	0	0	1	0	1	0	1	0	1	0	-2.50455
tr|B4E0D5|B4E0D5_HUMAN	tr|B4E0D5	cDNA FLJ54218, highly similar to Acidic leucine-rich nuclear phosphoprotein 32 family member E OS=Hom	19540	525	0	31	0	0.17	0	0	0	0	1	0	1	0	1	0	-2.50455
tr|F2Z2J7|F2Z2J7_HUMAN	TTLL7	Tubulin polyglutamylase TTLL7 OS=Homo sapiens GN=TTLL7 PE=2 SV=1	55925	526	0	31	0	0.06	0	0	1	0	1	0	1	0	1	0	-2.50455
tr|B7Z9W6|B7Z9W6_HUMAN	tr|B7Z9W	cDNA, FLJ78980, highly similar to DnaJ homolog subfamily C member 7 OS=Homo sapiens PE=2 SV=1	50776	527	0	31	0	0.06	0	0	0	0	1	0	1	0	1	0	-2.50455
tr|H0Y7S3|H0Y7S3_HUMAN	ATP2B2	Plasma membrane calcium-transporting ATPase 2 (Fragment) OS=Homo sapiens GN=ATP2B2 PE=3 SV=1	122272	528	0	31	0	0.03	0	0	0	0	1	0	1	0	3	0	-2.50455
sp|Q96PE2|ARHGH_HUMAN	ARHGEF17	Rho guanine nucleotide exchange factor 17 OS=Homo sapiens GN=ARHGEF17 PE=1 SV=1	223645	537	0	30	0	0.01	0	0	1	0	1	0	1	0	1	0	-2.50455
tr|B6F210|B6F210_HUMAN	GSS	Glutathione synthetase OS=Homo sapiens GN=GSS PE=2 SV=1	40495	551	0	30	0	0.08	0	0	0	0	1	0	1	0	1	0	-2.50455
tr|B4DXS4|B4DXS4_HUMAN	tr|B4DXS4	cDNA FLJ57227, highly similar to Isocitrate dehydrogenase OS=Homo sapiens PE=2 SV=1	26824	555	0	0	0	0.15	0	0	0	0	0	0	1	0	1	0	-2.50455
tr|H3BRB3|H3BRB3_HUMAN	KIF22	Kinesin-like protein KIF22 OS=Homo sapiens GN=KIF22 PE=3 SV=1	67996	557	0	29	0	0.05	0	0	0	0	1	0	1	0	1	0	-2.50455
sp|Q96A33|CCD47_HUMAN	CCDC47	Coiled-coil domain-containing protein 47 OS=Homo sapiens GN=CCDC47 PE=1 SV=1	56123	560	0	29	0	0.06	0	0	0	0	1	0	1	0	1	0	-2.50455
tr|B4DM97|B4DM97_HUMAN	tr|B4DM9	cDNA FLJ55002, highly similar to Alpha-centractin OS=Homo sapiens PE=2 SV=1	38365	561	0	29	0	0.09	0	0	0	0	1	0	1	0	1	0	-2.50455
sp|Q6KB66|K2C80_HUMAN	KRT80	Keratin, type II cytoskeletal 80 OS=Homo sapiens GN=KRT80 PE=1 SV=2	51007	564	0	29	0	0.06	0	0	0	0	1	0	1	0	1	0	-2.50455
tr|B4DJC3|B4DJC3_HUMAN	H2AFY	Histone H2A OS=Homo sapiens GN=H2AFY PE=2 SV=1	21706	565	0	0	0	0.08	0	0	0	0	0	0	1	0	1	0	-2.50455
tr|I3L361|I3L361_HUMAN	SGSM2	Small G protein-signaling modulator 2 (Fragment) OS=Homo sapiens GN=SGSM2 PE=4 SV=2	1642	570	0	29	0	2.38	0	0	0	0	1	0	1	0	1	0	-2.50455
tr|J3KQE5|J3KQE5_HUMAN	RAN	GTP-binding nuclear protein Ran (Fragment) OS=Homo sapiens GN=RAN PE=4 SV=1	27027	571	0	29	0	0.12	0	0	0	0	1	0	1	0	1	0	-2.50455
tr|A4ZYW4|A4ZYW4_HUMAN	L1CAM	Non-neural L1CAM OS=Homo sapiens GN=L1CAM PE=2 SV=1	139633	572	0	28	0	0.05	0	0	1	0	2	0	1	0	2	0	-2.50455
tr|C9JYI4|C9JYI4_HUMAN	KPNA1	Importin subunit alpha-1 (Fragment) OS=Homo sapiens GN=KPNA1 PE=2 SV=1	37206	573	0	22	0	0.11	0	0	0	0	1	0	1	0	1	0	-2.50455
tr|Q53G81|Q53G81_HUMAN	tr|Q53G8	NAD(P)H menadione oxidoreductase 1, dioxin-inducible variant (Fragment) OS=Homo sapiens PE=2 SV=1	30857	576	0	22	0	0.25	0	0	0	0	1	0	1	0	1	0	-2.50455
tr|J3KN32|J3KN32_HUMAN	RCOR1	REST corepressor 1 OS=Homo sapiens GN=RCOR1 PE=4 SV=1	53409	583	0	28	0	0.06	0	0	0	0	1	0	1	0	1	0	-2.50455
tr|H0YCU9|H0YCU9_HUMAN	TAGLN	Transgelin (Fragment) OS=Homo sapiens GN=TAGLN PE=4 SV=1	16833	585	0	28	0	0.2	0	0	0	0	1	0	1	0	1	0	-2.50455
tr|A6NE42|A6NE42_HUMAN	TGIF1	Homeobox protein TGIF1 OS=Homo sapiens GN=TGIF1 PE=3 SV=2	27983	586	0	28	0	0.12	0	0	0	0	1	0	1	0	1	0	-2.50455
tr|H3BS19|H3BS19_HUMAN	ZNF469	Zinc finger protein 469 OS=Homo sapiens GN=ZNF469 PE=4 SV=1	417347	591	0	27	0	0.01	0	0	0	0	1	0	1	0	1	0	-2.50455
tr|H0Y512|H0Y512_HUMAN	APMAP	Adipocyte plasma membrane-associated protein (Fragment) OS=Homo sapiens GN=APMAP PE=4 SV=1	45662	592	0	27	0	0.07	0	0	0	0	1	0	1	0	1	0	-2.50455
tr|B7Z4L9|B7Z4L9_HUMAN	tr|B7Z4L9	cDNA FLJ55342, highly similar to Kelch-like protein 13 OS=Homo sapiens PE=2 SV=1	56884	593	0	26	0	0.06	0	0	0	0	1	0	1	0	1	0	-2.50455
tr|Q5T8C6|Q5T8C6_HUMAN	CDC16	Cell division cycle protein 16 homolog OS=Homo sapiens GN=CDC16 PE=4 SV=1	55239	594	0	26	0	0.06	0	0	0	0	1	0	1	0	1	0	-2.50455
sp|Q0VD83|APOBR_HUMAN	APOBR	Apolipoprotein B receptor OS=Homo sapiens GN=APOBR PE=1 SV=1	115207	597	0	26	0	0.03	0	0	0	0	1	0	1	0	1	0	-2.50455
tr|Q8TC04|Q8TC04_HUMAN	KRT23	Keratin 23 (Histone deacetylase inducible) OS=Homo sapiens GN=KRT23 PE=2 SV=1	48318	600	0	26	0	0.07	0	0	0	0	1	0	1	0	1	0	-2.50455
tr|B4DNK3|B4DNK3_HUMAN	tr|B4DNK	cDNA FLJ52127, highly similar to Multisynthetase complex auxiliary component p43 OS=Homo sapiens PE=	29955	601	0	26	0	0.11	0	0	0	0	1	0	1	0	1	0	-2.50455
tr|B4E2I9|B4E2I9_HUMAN	tr|B4E2I9	cDNA FLJ55486, highly similar to Semaphorin-3C OS=Homo sapiens PE=2 SV=1	88481	602	0	26	0	0.04	0	0	0	0	1	0	1	0	2	0	-2.50455
tr|J3KNP0|J3KNP0_HUMAN	RAB11FIP1	Rab11 family-interacting protein 1 OS=Homo sapiens GN=RAB11FIP1 PE=4 SV=1	137482	607	0	25	0	0.02	0	0	0	0	1	0	1	0	2	0	-2.50455
tr|J3KSB9|J3KSB9_HUMAN	tr|J3KSB9	Uncharacterized protein (Fragment) OS=Homo sapiens PE=4 SV=1	8877	608	0	25	0	0.39	0	0	0	0	1	0	1	0	1	0	-2.50455
tr|B4DG42|B4DG42_HUMAN	tr|B4DG42	cDNA FLJ53753, highly similar to Myeloid-associated differentiation marker OS=Homo sapiens PE=2 SV=1	31889	609	0	25	0	0.1	0	0	0	0	1	0	1	0	1	0	-2.50455
tr|H3BMW5|H3BMW5_HUMAN	DNAJA2	DnaJ homolog subfamily A member 2 (Fragment) OS=Homo sapiens GN=DNAJA2 PE=4 SV=1	15134	611	0	24	0	0.23	0	0	0	0	1	0	1	0	1	0	-2.50455
tr|Q4VAN1|Q4VAN1_HUMAN	BTN1A1	BTN1A1 protein OS=Homo sapiens GN=BTN1A1 PE=2 SV=1	55878	617	0	24	0	0.06	0	0	0	0	1	0	1	0	1	0	-2.50455
tr|Q2TQ40|Q2TQ40_HUMAN	TNFRSF17	B cell maturation antigen transcript variant 4 OS=Homo sapiens GN=TNFRSF17 PE=2 SV=1	15460	618	0	24	0	0.22	0	0	0	0	1	0	1	0	1	0	-2.50455
tr|B4DU06|B4DU06_HUMAN	tr|B4DU0	Guanylate cyclase OS=Homo sapiens PE=2 SV=1	91756	620	0	24	0	0.04	0	0	0	0	1	0	1	0	1	0	-2.50455
tr|B0QXZ5|B0QXZ5_HUMAN	DNAL4	Dynein light chain 4, axonemal OS=Homo sapiens GN=DNAL4 PE=4 SV=1	7786	621	0	24	0	0.47	0	0	0	0	1	0	1	0	1	0	-2.50455
tr|B4DN13|B4DN13_HUMAN	tr|B4DN1	cDNA FLJ59136, highly similar to Transmembrane 9 superfamily protein member 3 OS=Homo sapiens PE=2	29629	624	0	23	0	0.11	0	0	0	0	1	0	1	0	1	0	-2.50455
tr|K7EPB5|K7EPB5_HUMAN	ELAVL3	ELAV-like protein 3 (Fragment) OS=Homo sapiens GN=ELAVL3 PE=4 SV=1	9728	625	0	23	0	0.36	0	0	0	0	1	0	1	0	1	0	-2.50455

tr|B4DFE4|B4DFE4_HUMAN	tr|B4DFE4	cDNA FLJ59618, highly similar to Exosome component 10 OS=Homo sapiens PE=2 SV=1	61733	627	0	23	0	0.05	0	0	0	0	1	0	1	0	1	0	-2.50455
sp|Q9Y5P6|GMPPB_HUMAN	GMPPB	Mannose-1-phosphate guanyltransferase beta OS=Homo sapiens GN=GMPPB PE=1 SV=2	40379	629	0	23	0	0.08	0	0	0	0	1	0	1	0	1	0	-2.50455
tr|Q5VSQ6|Q5VSQ6_HUMAN	P4HA1	Procollagen-proline, 2-oxoglutarate 4-dioxygenase (Proline 4-hydroxylase), alpha polypeptide I variant (Fra	61214	634	0	22	0	0.05	0	0	0	0	1	0	1	0	2	0	-2.50455
tr|E0X9P6|E0X9P6_HUMAN	HLA-A	MHC class I antigen (Fragment) OS=Homo sapiens GN=HLA-A PE=3 SV=1	21057	639	0	22	0	0.16	0	0	0	0	1	0	1	0	3	0	-2.50455
tr|F2VNK8|F2VNK8_HUMAN	HLA-B	MHC class I antigen (Fragment) OS=Homo sapiens GN=HLA-B PE=3 SV=1	21213	643	0	22	0	0.09	0	0	0	0	1	0	1	0	1	0	-2.50455
tr|E1Y6P9|E1Y6P9_HUMAN	HLA-B	MHC class I antigen (Fragment) OS=Homo sapiens GN=HLA-B PE=3 SV=1	31912	644	0	22	0	0.16	0	0	0	0	1	0	1	0	2	0	-2.50455
tr|H7C4V2|H7C4V2_HUMAN	U2SURP	U2 snRNP-associated SURP motif-containing protein (Fragment) OS=Homo sapiens GN=U2SURP PE=4 SV=1	33796	647	0	22	0	0.03	0	0	0	0	1	0	1	0	1	0	-2.50455
sp|Q96EK5|KBP_HUMAN	KIAA1279	KIF1-binding protein OS=Homo sapiens GN=KIAA1279 PE=1 SV=1	72453	649	0	22	0	0.05	0	0	0	0	1	0	1	0	1	0	-2.50455
tr|B3KSH1|B3KSH1_HUMAN	EIF3F	Eukaryotic translation initiation factor 3 subunit F OS=Homo sapiens GN=EIF3F PE=2 SV=1	39236	652	0	22	0	0.08	0	0	0	0	1	0	1	0	1	0	-2.50455
tr|C9J935|C9J935_HUMAN	SETD9	SET domain-containing protein 9 (Fragment) OS=Homo sapiens GN=SETD9 PE=2 SV=1	5515	660	0	21	0	0.65	0	0	0	0	1	0	1	0	1	0	-2.50455
tr|F1T0A5|F1T0A5_HUMAN	PRPF31	PRP31 pre-mRNA processing factor 31 homolog (Yeast), isoform CRA_a OS=Homo sapiens GN=PRPF31 PE=	55649	661	0	21	0	0.06	0	0	0	0	1	0	1	0	2	0	-2.50455
tr|B3KRG8|B3KRG8_HUMAN	tr|B3KRG8	Lipase OS=Homo sapiens PE=2 SV=1	45736	662	0	21	0	0.07	0	0	0	0	1	0	1	0	1	0	-2.50455
tr|J3KN69|J3KN69_HUMAN	NCEH1	Arylacetamide deacetylase-like 1 OS=Homo sapiens GN=NCEH1 PE=4 SV=1	49431	663	0	21	0	0.07	0	0	0	0	1	0	1	0	1	0	-2.50455
tr|B4E2Y2|B4E2Y2_HUMAN	tr|B4E2Y2	cDNA FLJ50370, highly similar to Ubiquitin conjugation factor E4 B OS=Homo sapiens PE=2 SV=1	122548	667	0	21	0	0.02	0	0	0	0	1	0	1	0	1	0	-2.50455
tr|B4DK80|B4DK80_HUMAN	TOP3A	DNA topoisomerase OS=Homo sapiens GN=TOP3A PE=2 SV=1	103082	668	0	20	0	0.03	0	0	0	0	1	0	1	0	1	0	-2.50455
tr|A0AVG7|A0AVG7_HUMAN	BCAS1	Breast carcinoma amplified sequence 1 OS=Homo sapiens GN=BCAS1 PE=2 SV=1	61929	670	0	20	0	0.05	0	0	0	0	1	0	1	0	1	0	-2.50455
tr|H0YJL6|H0YJL6_HUMAN	EVL	Ena/VASP-like protein (Fragment) OS=Homo sapiens GN=EVL PE=4 SV=1	10347	671	0	20	0	0.34	0	0	0	0	1	0	1	0	1	0	-2.50455
tr|D6RF05|D6RF05_HUMAN	HARS	Histidine--tRNA ligase, cytoplasmic OS=Homo sapiens GN=HARS PE=2 SV=1	6740	672	0	20	0	0.07	0	0	0	0	1	0	1	0	1	0	-2.50455
sp|P55212|CASP6_HUMAN	CASP6	Caspase-6 OS=Homo sapiens GN=CASP6 PE=1 SV=2	33859	674	0	20	0	0.1	0	0	0	0	1	0	1	0	1	0	-2.50455
tr|Q68DB1|Q68DB1_HUMAN	DKFZp686	Metastasis-associated protein MTA2 OS=Homo sapiens GN=DKFZp686F2281 PE=2 SV=1	55515	676	0	20	0	0.06	0	0	0	0	1	0	1	0	1	0	-2.50455
tr|B4DE48|B4DE48_HUMAN	tr|B4DE48	cDNA FLJ53824, highly similar to Sorting nexin-17 OS=Homo sapiens PE=2 SV=1	50009	677	0	20	0	0.07	0	0	0	0	1	0	1	0	1	0	-2.50455
sp|O95159|ZFPL1_HUMAN	ZFPL1	Zinc finger protein-like 1 OS=Homo sapiens GN=ZFPL1 PE=1 SV=2	34833	678	0	20	0	0.1	0	0	0	0	1	0	1	0	1	0	-2.50455
tr|Q8WUM5|Q8WUM5_HUMAN	GEMIN4	Gem (Nuclear organelle) associated protein 4 OS=Homo sapiens GN=GEMIN4 PE=2 SV=1	121732	709	0	17	0	0.03	0	0	0	0	1	0	1	0	2	0	-2.50455
tr|H7C463|H7C463_HUMAN	IMMT	Mitochondrial inner membrane protein (Fragment) OS=Homo sapiens GN=IMMT PE=4 SV=1	68305	712	0	17	0	0.04	0	0	0	0	1	0	1	0	6	0	-2.50455
tr|B7Z4P8|B7Z4P8_HUMAN	ACTN2	Alpha-actinin-2 OS=Homo sapiens GN=ACTN2 PE=2 SV=1	44675	19	0	51	0	2.11	0	0	1	0	2	0	2	0	2	0	-3.24186
tr|B4DYM0|B4DYM0_HUMAN	tr|B4DYM	cDNA FLJ60371, highly similar to Acetyl-CoA carboxylase 2 (EC 6.4.1.2) OS=Homo sapiens PE=2 SV=1	128678	31	0	53	0	0.44	0	0	1	0	1	0	2	0	3	0	-3.24186
tr|C9J057|C9J057_HUMAN	ASNS	Asparagine synthetase [glutamine-hydrolyzing] (Fragment) OS=Homo sapiens GN=ASNS PE=2 SV=1	24946	141	0	32	0	0.26	0	0	0	0	1	0	2	0	2	0	-3.24186
tr|B3KS35|B3KS35_HUMAN	tr|B3KS35	cDNA FLJ35372 fis, clone SKMUS2001742, highly similar to Sarcoplasmic/endoplasmic reticulum calcium A	32698	149	0	55	0	0.32	0	0	1	0	2	0	2	0	2	0	-3.24186
tr|Q5JVP3|Q5JVP3_HUMAN	PTGR1	Leukotriene B4 12-hydroxydehydrogenase, isoform CRA_d OS=Homo sapiens GN=PTGR1 PE=4 SV=1	19441	190	0	113	0	0.38	0	0	2	0	2	0	2	0	2	0	-3.24186
tr|Q71UM7|Q71UM7_HUMAN	tr|Q71UM	Beta-subunit signal transducing proteins GS/GI (Fragment) OS=Homo sapiens PE=2 SV=1	14203	199	0	105	0	0.18	0	0	1	0	2	0	2	0	2	0	-3.24186
tr|A0MNN4|A0MNN4_HUMAN	SMU1	CDW3/SMU1 OS=Homo sapiens GN=SMU1 PE=2 SV=1	58134	221	0	94	0	0.12	0	0	1	0	2	0	2	0	2	0	-3.24186
tr|G3V1S6|G3V1S6_HUMAN	GALNT2	Polypeptide N-acetylgalactosaminyltransferase 2 soluble form OS=Homo sapiens GN=GALNT2 PE=2 SV=1	61440	254	0	76	0	0.11	0	0	2	0	2	0	2	0	2	0	-3.24186
tr|E9PG15|E9PG15_HUMAN	YWHAQ	14-3-3 protein theta (Fragment) OS=Homo sapiens GN=YWHAQ PE=2 SV=1	17209	258	0	73	0	0.4	0	0	2	0	2	0	2	0	2	0	-3.24186
tr|Q2QD09|Q2QD09_HUMAN	tr|Q2QD0	Triosephosphate isomerase (Fragment) OS=Homo sapiens PE=2 SV=1	27211	267	0	71	0	0.26	0	0	1	0	2	0	2	0	2	0	-3.24186
tr|E5RH42|E5RH42_HUMAN	G3BP1	Ras GTPase-activating protein-binding protein 1 (Fragment) OS=Homo sapiens GN=G3BP1 PE=2 SV=1	13338	294	0	62	0	0.58	0	0	2	0	2	0	2	0	2	0	-3.24186
sp|P04040|CATA_HUMAN	CAT	Catalase OS=Homo sapiens GN=CAT PE=1 SV=3	59947	299	0	60	0	0.11	0	0	1	0	2	0	2	0	2	0	-3.24186
tr|G3V180|G3V180_HUMAN	DPP3	Dipeptidyl peptidase 3 OS=Homo sapiens GN=DPP3 PE=2 SV=1	84666	335	0	52	0	0.08	0	0	1	0	2	0	2	0	2	0	-3.24186
tr|E5RHF2|E5RHF2_HUMAN	COPS5	COP9 signalosome complex subunit 5 (Fragment) OS=Homo sapiens GN=COPS5 PE=2 SV=1	16655	346	0	51	0	0.45	0	0	0	0	2	0	2	0	2	0	-3.24186
tr|Q6NZ52|Q6NZ52_HUMAN	RPL27A	Ribosomal protein L27a OS=Homo sapiens GN=RPL27A PE=2 SV=1	16582	348	0	50	0	0.2	0	0	1	0	1	0	2	0	2	0	-3.24186
tr|B7Z8J4|B7Z8J4_HUMAN	NMT1	Glycylpeptide N-tetradecanoyltransferase 1 OS=Homo sapiens GN=NMT1 PE=2 SV=1	15265	350	0	50	0	0.49	0	0	1	0	2	0	2	0	2	0	-3.24186
sp|Q9NR12|PDLI7_HUMAN	PDLIM7	PDZ and LIM domain protein 7 OS=Homo sapiens GN=PDLIM7 PE=1 SV=1	50896	396	0	43	0	0.13	0	0	0	0	2	0	2	0	4	0	-3.24186
tr|A0MNP2|A0MNP2_HUMAN	WDR57	CDW11/WDR57 OS=Homo sapiens GN=WDR57 PE=2 SV=1	39742	413	0	42	0	0.17	0	0	1	0	2	0	2	0	3	0	-3.24186
tr|B2R7Y3|B2R7Y3_HUMAN	tr|B2R7Y3	cDNA, FLJ93657, highly similar to Homo sapiens ribosomal protein L3-like (RPL3L), mRNA OS=Homo sapien	46581	420	0	42	0	0.07	0	0	1	0	1	0	2	0	3	0	-3.24186
tr|Q6NZ55|Q6NZ55_HUMAN	RPL13	60S ribosomal protein L13 OS=Homo sapiens GN=RPL13 PE=2 SV=1	24308	422	0	41	0	0.14	0	0	1	0	1	0	2	0	2	0	-3.24186
tr|A5D904|A5D904_HUMAN	RPS9	RPS9 protein (Fragment) OS=Homo sapiens GN=RPS9 PE=2 SV=1	13210	434	0	40	0	0.58	0	0	1	0	2	0	2	0	2	0	-3.24186
sp|P07737|PROF1_HUMAN	PFN1	Profilin-1 OS=Homo sapiens GN=PFN1 PE=1 SV=2	15216	436	0	39	0	0.5	0	0	1	0	2	0	2	0	2	0	-3.24186
tr|D3DVQ1|D3DVQ1_HUMAN	LETM1	Leucine zipper-EF-hand containing transmembrane protein 1, isoform CRA_a OS=Homo sapiens GN=LETM1	63730	463	0	37	0	0.11	0	0	1	0	2	0	2	0	4	0	-3.24186
tr|A8MW61|A8MW61_HUMAN	PLRG1	Pleiotropic regulator 1 OS=Homo sapiens GN=PLRG1 PE=2 SV=1	57545	476	0	36	0	0.12	0	0	1	0	2	0	2	0	4	0	-3.24186
tr|B2R4P2|B2R4P2_HUMAN	tr|B2R4P2	cDNA, FLJ92164, highly similar to Homo sapiens peroxiredoxin 1 (PRDX1), mRNA OS=Homo sapiens PE=2 S	22414	481	0	36	0	0.32	0	0	0	0	2	0	2	0	2	0	-3.24186
tr|A0PJ76|A0PJ76_HUMAN	NCAPD2	NCAPD2 protein (Fragment) OS=Homo sapiens GN=NCAPD2 PE=2 SV=1	44957	494	0	34	0	0.15	0	0	0	0	2	0	2	0	2	0	-3.24186
tr|B3KY12|B3KY12_HUMAN	tr|B3KY12	cDNA FLJ46581 fis, clone THYMU3043200, highly similar to Splicing factor 3A subunit 3 OS=Homo sapiens F	59082	495	0	34	0	0.18	0	0	1	0	3	0	2	0	3	0	-3.24186
tr|C1KES1|C1KES1_HUMAN	LRRC16B	LRRC16B (Fragment) OS=Homo sapiens GN=LRRC16B PE=4 SV=1	4125	530	0	31	0	0.94	0	0	0	0	1	0	2	0	2	0	-3.24186
tr|B2RD27|B2RD27_HUMAN	tr|B2RD27	cDNA, FLJ96428, highly similar to Homo sapiens proteasome (prosome, macropain) 26S subunit, non-ATPa	37046	550	0	30	0	0.19	0	0	0	0	2	0	2	0	2	0	-3.24186
sp|Q99456|K1C12_HUMAN	KRT12	Keratin, type I cytoskeletal 12 OS=Homo sapiens GN=KRT12 PE=1 SV=1	53592	616	0	24	0	0.06	0	0	0	0	1	0	2	0	3	0	-3.24186
tr|D3DSQ0|D3DSQ0_HUMAN	PCM1	Pericentriolar material 1, isoform CRA_a OS=Homo sapiens GN=PCM1 PE=2 SV=1	230041	619	0	24	0	0.01	0	0	0	0	1	0	2	0	3	0	-3.24186
tr|I0BW39|I0BW39_HUMAN	HLA-A	MHC class I antigen (Fragment) OS=Homo sapiens GN=HLA-A PE=4 SV=1	8492	640	0	22	0	0.16	0	0	0	0	1	0	2	0	2	0	-3.24186
sp|Q8TCJ2|STT3B_HUMAN	STT3B	Dolichyl-diphosphooligosaccharide--protein glycosyltransferase subunit STT3B OS=Homo sapiens GN=STT3	94241	700	0	18	0	0.03	0	0	0	0	1	0	2	0	3	0	-3.24186
tr|F8VRK6|F8VRK6_HUMAN	ATXN2	Ataxin-2 (Fragment) OS=Homo sapiens GN=ATXN2 PE=2 SV=1	12635	157	0	61	0	0.21	0	0	2	0	3	0	3	0	3	0	-3.72762
tr|B4DMU0|B4DMU0_HUMAN	PYCR1	Pyrroline-5-carboxylate reductase OS=Homo sapiens GN=PYCR1 PE=2 SV=1	36186	174	0	125	0	0.19	0	0	2	0	2	0	3	0	3	0	-3.72762
tr|H0Y8L7|H0Y8L7_HUMAN	RPS3A	40S ribosomal protein S3a (Fragment) OS=Homo sapiens GN=RPS3A PE=3 SV=1	22294	223	0	74	0	0.44	0	0	1	0	2	0	3	0	4	0	-3.72762
tr|Q5JQZ9|Q5JQZ9_HUMAN	CDC2L6	Cell division cycle 2-like 6 (CDK8-like) (Fragment) OS=Homo sapiens GN=CDC2L6 PE=4 SV=1	29552	285	0	65	0	0.24	0	0	2	0	2	0	3	0	3	0	-3.72762
tr|Q0VGL3|Q0VGL3_HUMAN	RPL13A	RPL13A protein OS=Homo sapiens GN=RPL13A PE=2 SV=1	16706	300	0	59	0	0.74	0	0	3	0	3	0	3	0	3	0	-3.72762
tr|D6RFL1|D6RFL1_HUMAN	CANX	Calnexin (Fragment) OS=Homo sapiens GN=CANX PE=2 SV=1	15265	352	0	49	0	0.39	0	0	1	0	2	0	3	0	4	0	-3.72762
tr|B2R6J4|B2R6J4_HUMAN	tr|B2R6J4	cDNA, FLJ92975, highly similar to Homo sapiens nucleosome assembly protein 1-like 4 (NAP1L4), mRNA OS	42940	417	0	42	0	0.16	0	0	0	0	2	0	3	0	3	0	-3.72762
tr|E9PKU4|E9PKU4_HUMAN	RPL8	60S ribosomal protein L8 (Fragment) OS=Homo sapiens GN=RPL8 PE=2 SV=1	25806	231	0	90	0	0.28	0	0	2	0	2	0	4	0	5	0	-4.09053
tr|Q53RS3|Q53RS3_HUMAN	DDEF2	Putative uncharacterized protein DDEF2 (Fragment) OS=Homo sapiens GN=DDEF2 PE=2 SV=1	70046	441	0	39	0	0.05	0	0	1	0	1	0	4	0	4	0	-4.09053
sp|A2RTY3|CQ066_HUMAN	C17orf66	Uncharacterized protein C17orf66 OS=Homo sapiens GN=C17orf66 PE=2 SV=2	66437	477	0	36	0	0.05	0	0	0	0	1	0	4	0	8	0	-4.09053
tr|B3KVL0|B3KVL0_HUMAN	tr|B3KVL0	cDNA FLJ16691 fis, clone TRACH3000692, highly similar to Paxillin OS=Homo sapiens PE=2 SV=1	65600	159	0	133	0	0.28	0	0	3	0	5	0	6	0	6	0	-4.62171
sp|Q9NXW2|DJB12_HUMAN	DNAJB12	DnaJ homolog subfamily B member 12 OS=Homo sapiens GN=DNAJB12 PE=1 SV=4	42021	124	0	174	0	0.57	0	0	5	0	7	0	9	0	9	0	-5.17021

**#10**	**Hela live unsyn PU-1_2μM**																		
#04	Hela live syn NP_2μM																		
Accession	GS	Name	Pro_Mass	04_prot_hi	10_prot_hi	04_prot_sc	10_prot_score	04_emPAI	10_emPAI	emPAI ratio	04_No>I	10_No>I	04_No>HI	10_No>HI	04_No>20	10_No>20	04_No=All	10_No=All	Rsc
sp|Q8IWU2|LMTK2_HUMAN	LMTK2	Serine/threonine-protein kinase LMTK2 OS=Homo sapiens GN=LMTK2 PE=1 SV=2		0	1154	0	26	0	0.02	#DIV/0!	0	0	0	1	0	1	0	2	0.696063
sp|Q96SB4|SRPK1_HUMAN	SRPK1	SRSF protein kinase 1 OS=Homo sapiens GN=SRPK1 PE=1 SV=2	74963	0	353	0	143	0	0.18	#DIV/0!	0	2	0	4	0	4	0	6	2.281563
sp|Q9H4A3|WNK1_HUMAN	WNK1	Serine/threonine-protein kinase WNK1 OS=Homo sapiens GN=WNK1 PE=1 SV=2	251552	0	404	0	119	0	0.04	#DIV/0!	0	3	0	3	0	3	0	5	1.918814
sp|P28482|MK01_HUMAN	MAPK1	Mitogen-activated protein kinase 1 OS=Homo sapiens GN=MAPK1 PE=1 SV=3	41762	0	1121	0	28	0	0.06	#DIV/0!	0	0	0	1	0	1	0	1	0.696063
sp|Q8TD08|MK15_HUMAN	MAPK15	Mitogen-activated protein kinase 15 OS=Homo sapiens GN=MAPK15 PE=1 SV=1	60251	0	1120	0	28	0	0.05	#DIV/0!	0	0	0	1	0	1	0	2	0.696063
sp|Q13464|ROCK1_HUMAN	ROCK1	Rho-associated protein kinase 1 OS=Homo sapiens GN=ROCK1 PE=1 SV=1	159102	0	964	0	39	0	0.02	#DIV/0!	0	1	0	1	0	1	0	4	0.696063
sp|P51812|KS6A3_HUMAN	RPS6KA3	Ribosomal protein S6 kinase alpha-3 OS=Homo sapiens GN=RPS6KA3 PE=1 SV=1	84025	0	522	0	91	0	0.27	#DIV/0!	0	2	0	6	0	5	0	7	2.571248
sp|Q15418|KS6A1_HUMAN	RPS6KA1	Ribosomal protein S6 kinase alpha-1 OS=Homo sapiens GN=RPS6KA1 PE=1 SV=2	83070	0	461	0	106	0	0.27	#DIV/0!	0	2	0	5	0	5	0	6	2.571248
sp|Q13523|PRP4B_HUMAN	PRPF4B	Serine/threonine-protein kinase PRP4 homolog OS=Homo sapiens GN=PRPF4B PE=1 SV=3	117258	0	1124	0	28	0	0.05	#DIV/0!	0	0	0	1	0	1	0	1	0.696063
sp|P19784|CSK22_HUMAN	CSNK2A2	Casein kinase II subunit alpha~ OS=Homo sapiens GN=CSNK2A2 PE=1 SV=1	41358	0	744	0	57	0	0.12	#DIV/0!	0	1	0	1	0	1	0	2	0.696063
sp|P78527|PRKDC_HUMAN	PRKDC	DNA-dependent protein kinase catalytic subunit OS=Homo sapiens GN=PRKDC PE=1 SV=3	473749	83	71	241	573	0.07	0.2	2.8571429	7	13	10	27	11	31	17	39	0.566547
sp|Q96Q15|SMG1_HUMAN	SMG1	Serine/threonine-protein kinase SMG1 OS=Homo sapiens GN=SMG1 PE=1 SV=3	414347	0	1207	0	22	0	0.01	#DIV/0!	0	1	0	1	0	1	0	2	0.696063
sp|Q9UIG0|BAZ1B_HUMAN	BAZ1B	Tyrosine-protein kinase BAZ1B OS=Homo sapiens GN=BAZ1B PE=1 SV=2	172392	0	1022	0	35	0	0.02	#DIV/0!	0	0	0	1	0	1	0	1	0.696063
tr|J3KNX3|J3KNX3_HUMAN	PFKM	6-phosphofructokinase OS=Homo sapiens GN=PFKM PE=3 SV=1	94107	0	224	0	231	0	0.19	#DIV/0!	0	4	0	5	0	5	0	6	2.571248
sp|P19367|HXK1_HUMAN	HK1	Hexokinase-1 OS=Homo sapiens GN=HK1 PE=1 SV=3	103561	0	994	0	36	0	0.06	#DIV/0!	0	0	0	2	0	3	0	5	1.918814
sp|Q8TBX8|PI42C_HUMAN	PIP4K2C	Phosphatidylinositol 5-phosphate 4-kinase type-2 gamma OS=Homo sapiens GN=PIP4K2C PE=1 SV=3	47441	0	1195	0	23	0	0.08	#DIV/0!	0	0	0	1	0	1	0	1	0.696063
sp|Q9BRR6|ADPGK_HUMAN	ADPGK	ADP-dependent glucokinase OS=Homo sapiens GN=ADPGK PE=1 SV=1	54397	0	821	0	49	0	0.07	#DIV/0!	0	1	0	1	0	1	0	1	0.696063
sp|P13861|KAP2_HUMAN	PRKAR2A	cAMP-dependent protein kinase type II-alpha regulatory subunit OS=Homo sapiens GN=PRKAR2A PE=1 SV=2	45832	0	580	0	78	0	0.07	#DIV/0!	0	1	0	1	0	1	0	1	0.696063
sp|P12277|KCRB_HUMAN	CKB	Creatine kinase B-type OS=Homo sapiens GN=CKB PE=1 SV=1	42902	214	230	97	227	0.18	0.77	4.2777778	2	7	2	7	2	7	2	7	0.696475
sp|P42345|MTOR_HUMAN	MTOR	Serine/threonine-protein kinase mTOR OS=Homo sapiens GN=MTOR PE=1 SV=1	290759	683	476	20	21	0.02	0.06	3	0	1	3	0	2	2	5	1.433208
sp|P40763|STAT3_HUMAN	STAT3	Signal transducer and activator of transcription 3 OS=Homo sapiens GN=STAT3 PE=1 SV=2	88810	0	386	0	127	0	0.24	#DIV/0!	0	3	0	6	0	6	0	7	2.812435
sp|P62140|PP1B_HUMAN	PPP1CB	Serine/threonine-protein phosphatase PP1-beta catalytic subunit OS=Homo sapiens GN=PPP1CB PE=1 SV=3	37961	345	498	51	59	0.24	0.35	1.4583333	1	1	1	2	1	2	1	2	-0.15209
sp|P36507|MP2K2_HUMAN	MAP2K2	Dual specificity mitogen-activated protein kinase kinase 2 OS=Homo sapiens GN=MAP2K2 PE=1 SV=1	44681	384	681	45	21	0.31	0.33	1.0645161	1	0	2	2	2	0	2	2	-3.21167
sp|P08631|HCK_HUMAN	HCK	Tyrosine-protein kinase HCK OS=Homo sapiens GN=HCK PE=1 SV=5	60075	628	1064	23	32	0.05	0.05	1	0	0	1	1	1	1	2	1	-0.88923
sp|P55263|ADK_HUMAN	ADK	Adenosine kinase OS=Homo sapiens GN=ADK PE=1 SV=2	40919	538	1272	30	17	0.08	0.08	1	0	0	1	1	1	1	1	2	-0.88923
sp|P10644|KAP0_HUMAN	PRKAR1A	cAMP-dependent protein kinase type I-alpha regulatory subunit OS=Homo sapiens GN=PRKAR1A PE=1 SV=1	43183	533	901	31	42	0.08	0.08	1	0	1	1	1	1	1	1	1	-0.88923
sp|Q02750|MP2K1_HUMAN	MAP2K1	Dual specificity mitogen-activated protein kinase kinase 1 OS=Homo sapiens GN=MAP2K1 PE=1 SV=2	43753	384	623	45	35	0.31	0.24	0.7741935	1	0	2	2	2	1	2	2	-1.62653
sp|P60891|PRPS1_HUMAN	PRPS1	Ribose-phosphate pyrophosphokinase 1 OS=Homo sapiens GN=PRPS1 PE=1 SV=2	35325	638	971	22	38	0.14	0.09	0.6428571	0	1	1	1	1	1	2	1	-0.88923
sp|Q13153|PAK1_HUMAN	PAK1	Serine/threonine-protein kinase PAK 1 OS=Homo sapiens GN=PAK1 PE=1 SV=2	60894	0	1281	0	15	0	0.12	#DIV/0!	0	0	0	1	0	0	0	1	-0.88908
sp|Q8N4C8|MINK1_HUMAN	MINK1	Misshapen-like kinase 1 OS=Homo sapiens GN=MINK1 PE=1 SV=2	150413	0	949	0	0	0	0.02	#DIV/0!	0	0	0	0	0	0	0	3	-0.88908
sp|Q13418|ILK_HUMAN	ILK	Integrin-linked protein kinase OS=Homo sapiens GN=ILK PE=1 SV=2	51899	421	497	41	94	0.07	0.2	2.8571429	1	2	1	2	1	2	1	4	-0.15209
sp|O75116|ROCK2_HUMAN	ROCK2	Rho-associated protein kinase 2 OS=Homo sapiens GN=ROCK2 PE=1 SV=4	161939	375	932	46	40	0.05	0.08	1.6	1	1	2	2	1	2	3	3	-0.15209
sp|P14618|KPYM_HUMAN	PKM	Pyruvate kinase isozymes M1/M2 OS=Homo sapiens GN=PKM PE=1 SV=4	58470	8	3	1820	3536	5.79	22.89	3.9533679	47	82	58	107	60	111	66	124	-0.00706
sp|P00558|PGK1_HUMAN	PGK1	Phosphoglycerate kinase 1 OS=Homo sapiens GN=PGK1 PE=1 SV=3	44985	134	163	161	305	0.76	1.51	1.9868421	5	9	8	14	9	14	9	15	-0.2795
sp|O00764|PDXK_HUMAN	PDXK	Pyridoxal kinase OS=Homo sapiens GN=PDXK PE=1 SV=1	35308	566	442	29	111	0.11	0.18	1.6363636	0	1	1	1	1	1	1	1	-0.88923
sp|Q01813|K6PP_HUMAN	PFKP	6-phosphofructokinase type C OS=Homo sapiens GN=PFKP PE=1 SV=2	86454	98	100	217	459	0.4	0.56	1.4	5	12	9	13	9	15	10	18	-0.18311
sp|P29966|MARCS_HUMAN	MARCKS	Myristoylated alanine-rich C-kinase substrate OS=Homo sapiens GN=MARCKS PE=1 SV=4	31707	0	690	0	64	0	0.35	#DIV/0!	0	1	0	3	0	3	0	4	1.918814
sp|Q8NB16|MLKL_HUMAN	MLKL	Mixed lineage kinase domain-like protein OS=Homo sapiens GN=MLKL PE=1 SV=1	55015	0	1091	0	30	0	0.17	#DIV/0!	0	0	0	1	0	1	0	1	0.696063
sp|O60271|JIP4_HUMAN	SPAG9	C-Jun-amino-terminal kinase-interacting protein 4 OS=Homo sapiens GN=SPAG9 PE=1 SV=4	146913	0	846	0	47	0	0.03	#DIV/0!	0	1	0	1	0	1	0	1	0.696063
sp|Q9UNF0|PACN2_HUMAN	PACSIN2	Protein kinase C and casein kinase substrate in neurons protein 2 OS=Homo sapiens GN=PACSIN2 PE=1 SV=2	56046	0	791	0	52	0	0.19	#DIV/0!	0	1	0	1	0	1	0	1	0.696063
sp|Q9UKS6|PACN3_HUMAN	PACSIN3	Protein kinase C and casein kinase substrate in neurons protein 3 OS=Homo sapiens GN=PACSIN3 PE=1 SV=2	48799	0	774	0	53	0	0.07	#DIV/0!	0	1	0	1	0	1	0	1	0.696063
sp|Q9NYJ8|TAB2_HUMAN	TAB2	TGF-beta-activated kinase 1 and MAP3K7-binding protein 2 OS=Homo sapiens GN=TAB2 PE=1 SV=1	77017	0	748	0	57	0	0.04	#DIV/0!	0	1	0	1	0	1	0	1	0.696063
sp|Q9Y3F4|STRAP_HUMAN	STRAP	Serine-threonine kinase receptor-associated protein OS=Homo sapiens GN=STRAP PE=1 SV=1	38756	626	316	23	158	0.12	0.55	4.5833333	0	4	1	4	1	4	1	4	0.696269
sp|O15357|SHIP2_HUMAN	INPPL1	Phosphatidylinositol 3,4,5-trisphosphate 5-phosphatase 2 OS=Homo sapiens GN=INPPL1 PE=1 SV=2	139311	0	905	0	42	0	0.05	#DIV/0!	0	1	0	2	0	4	0	5	2.281563
tr|B2R665|B2R665_HUMAN	tr|B2R665|B2	cDNA, FLJ92810, highly similar to Homo sapiens protein phosphatase 1G (formerly 2C), magnesium-dependent, gamma isoform (PPM1G), mRN/	59889	0	711	0	61	0	0.17	#DIV/0!	0	1	0	3	0	3	0	3	1.918814
sp|Q13362|2A5G_HUMAN	PPP2R5C	Serine/threonine-protein phosphatase 2A 56 kDa regulatory subunit gamma isoform OS=Homo sapiens GN=PPP2R5C PE=1 SV=3	61421	0	1139	0	27	0	0.05	#DIV/0!	0	0	0	1	0	2	0	3	1.433208
tr|B3KPZ7|B3KPZ7_HUMAN	tr|B3KPZ7|B3	cDNA FLJ32517 fis, clone SMINT1000117, highly similar to Pyruvate dehydrogenase (lipoamide)-phosphatase 1 (EC 3.1.3.43) OS=Homo sapiens I	61329	0	1198	0	23	0	0.05	#DIV/0!	0	0	0	1	0	1	0	1	0.696063
tr|H0Y8C4|H0Y8C4_HUMAN	PPP2R5D	Serine/threonine-protein phosphatase 2A 56 kDa regulatory subunit delta isoform (Fragment) OS=Homo sapiens GN=PPP2R5D PE=4 SV=1	59637	0	1143	0	27	0	0.11	#DIV/0!	0	0	0	2	0	1	0	2	0.696063
tr|H0YJ75|H0YJ75_HUMAN	PPP2R5C	Serine/threonine-protein phosphatase 2A 56 kDa regulatory subunit gamma isoform (Fragment) OS=Homo sapiens GN=PPP2R5C PE=4 SV=1	64365	0	1138	0	27	0	0.05	#DIV/0!	0	0	0	1	0	1	0	2	0.696063
sp|Q06124|PTN11_HUMAN	PTPN11	Tyrosine-protein phosphatase non-receptor type 11 OS=Homo sapiens GN=PTPN11 PE=1 SV=2	68964	0	1116	0	29	0	0.29	#DIV/0!	0	0	0	1	0	1	0	1	0.696063
tr|H0Y380|H0Y380_HUMAN	PTPRF	Receptor-type tyrosine-protein phosphatase F (Fragment) OS=Homo sapiens GN=PTPRF PE=4 SV=1	111922	0	1084	0	31	0	0.03	#DIV/0!	0	0	0	1	0	1	0	1	0.696063
tr|B2RAH5|B2RAH5_HUMAN	tr|B2RAH5|B2	cDNA, FLJ94919, highly similar to Homo sapiens protein phosphatase 1, regulatory (inhibitor) subunit 12A (PPP1R12A), mRNA OS=Homo sapiens	115658	0	1054	0	33	0	0.06	#DIV/0!	0	0	0	2	0	1	0	2	0.696063
tr|H0YHI8|H0YHI8_HUMAN	PPP1R12A	Protein phosphatase 1 regulatory subunit 12A (Fragment) OS=Homo sapiens GN=PPP1R12A PE=4 SV=1	40591	0	1047	0	33	0	0.08	#DIV/0!	0	0	0	1	0	1	0	1	0.696063
tr|K7EJB8|K7EJB8_HUMAN	PPP1R14A	Protein phosphatase 1 regulatory subunit 14A OS=Homo sapiens GN=PPP1R14A PE=4 SV=1	13934	0	1038	0	34	0	0.25	#DIV/0!	0	1	0	1	0	1	0	1	0.696063
sp|Q8TAP8|PPR35_HUMAN	PPP1R35	Protein phosphatase 1 regulatory subunit 35 OS=Homo sapiens GN=PPP1R35 PE=1 SV=1	28107	0	788	0	52	0	0.12	#DIV/0!	0	1	0	1	0	1	0	1	0.696063
sp|Q15345|LRC41_HUMAN	LRRC41	Leucine-rich repeat-containing protein 41 OS=Homo sapiens GN=LRRC41 PE=1 SV=3	89734	0	1086	0	31	0	0.09	#DIV/0!	0	0	0	2	0	2	0	2	1.433208
tr|H7C1Y1|H7C1Y1_HUMAN	PPP1R21	Protein phosphatase 1 regulatory subunit 21 (Fragment) OS=Homo sapiens GN=PPP1R21 PE=4 SV=1	13903	0	1274	0	16	0	0.25	#DIV/0!	0	0	0	1	0	0	0	1	-0.88908
tr|H0Y9D8|H0Y9D8_HUMAN	PPA2	Inorganic pyrophosphatase 2, mitochondrial (Fragment) OS=Homo sapiens GN=PPA2 PE=4 SV=1	25904	0	1271	0	17	0	0.13	#DIV/0!	0	0	0	1	0	0	0	1	-0.88908
sp|Q5H9R7|PP6R3_HUMAN	PPP6R3	Serine/threonine-protein phosphatase 6 regulatory subunit 3 OS=Homo sapiens GN=PPP6R3 PE=1 SV=2	98577	0	1255	0	19	0	0.12	#DIV/0!	0	0	0	1	0	0	0	1	-0.88908
tr|H7C234|H7C234_HUMAN	DUSP28	Dual-specificity phosphatase 28 (Fragment) OS=Homo sapiens GN=DUSP28 PE=4 SV=1	15330	0	1252	0	19	0	0.22	#DIV/0!	0	0	0	1	0	0	0	1	-0.88908
sp|Q96C86|DCPS_HUMAN	DCPS	m7GpppX diphosphatase OS=Homo sapiens GN=DCPS PE=1 SV=2	38756	0	1240	0	20	0	0.09	#DIV/0!	0	0	0	1	0	0	0	1	-0.88908
tr|B4DDF7|B4DDF7_HUMAN	tr|B4DDF7|B4	cDNA FLJ53296, highly similar to Serine/threonine-protein phosphatase 2A 65 kDa regulatory subunit A alpha isoform OS=Homo sapiens PE=2 S	64939	443	260	39	197	0.16	0.28	1.75	1	4	3	6	3	5	3	11	-0.2371
sp|P53041|PPP5_HUMAN	PPP5C	Serine/threonine-protein phosphatase 5 OS=Homo sapiens GN=PPP5C PE=1 SV=1	57412	596	577	26	79	0.06	0.06	1	0	1	1	1	1	1	1	1	-0.88923
tr|B4DDZ8|B4DDZ8_HUMAN	tr|B4DDZ8|B4	cDNA FLJ55048, highly similar to Serine/threonine-protein phosphatase 5 (EC 3.1.3.16) OS=Homo sapiens PE=2 SV=1	55829	596	0	26	0	0.06	0	0	0	0	1	0	1	0	1	0	-2.47437
tr|J3QT22|J3QT22_HUMAN	PPME1	Protein phosphatase methylesterase 1 OS=Homo sapiens GN=PPME1 PE=3 SV=1	44241	232	0	90	0	0.07	0	0	1	0	1	0	1	0	2	0	-2.47437
tr|C1PHA2|C1PHA2_HUMAN	KIF5B-ALK	Tyrosine-protein kinase receptor OS=Homo sapiens GN=KIF5B-ALK PE=2 SV=1	169283	227	417	91	116	0.04	0.08	2	2	1	2	4	2	4	3	4	-0.04103
sp|Q12929|EPS8_HUMAN	EPS8	Epidermal growth factor receptor kinase substrate 8 OS=Homo sapiens GN=EPS8 PE=1 SV=1	92167	0	1083	0	31	0	0.5	#DIV/0!	0	0	0	1	0	1	0	1	0.696063
sp|Q02952|AKA12_HUMAN	AKAP12	A-kinase anchor protein 12 OS=Homo sapiens GN=AKAP12 PE=1 SV=4	191937	0	967	0	38	0	0.03	#DIV/0!	0	1	0	1	0	1	0	1	0.696063
sp|P17858|K6PL_HUMAN	PFKL	6-phosphofructokinase, liver type OS=Homo sapiens GN=PFKL PE=1 SV=6	85762	98	438	88	111	0.4	0.08	0.2	1	2	2	2	2	2	2	3	-0.88938
tr|B1AKP8|B1AKP8_HUMAN	MTOR	Serine/threonine-protein kinase mTOR OS=Homo sapiens GN=MTOR PE=4 SV=1	86359	0	448	0	71	0	0.06	#DIV/0!	0	1	0	1	0	1	0	1	0.696063
tr|A6NIT2|A6NIT2_HUMAN	PHKA1	Phosphorylase b kinase regulatory subunit alpha, skeletal muscle isoform OS=Homo sapiens GN=PHKA1 PE=2 SV=1	133462	711	0	17	0	0.02	0	0	0	0	1	0	0	0	1	0	-0.88908
tr|B4E1G6|B4E1G6_HUMAN	GALK1	Galactokinase OS=Homo sapiens GN=GALK1 PE=2 SV=1	45956	679	0	20	0	0.07	0	0	0	0	1	0	0	0	1	0	-0.88908
sp|O95747|OXSR1_HUMAN	OXSR1	Serine/threonine-protein kinase OSR1 OS=Homo sapiens GN=OXSR1 PE=1 SV=1	58271	675	0	20	0	0.06	0	0	0	0	1	0	1	0	2	0	-2.47437
tr|H0Y3Y5|H0Y3Y5_HUMAN	PPIP5K1	Inositol hexakisphosphate and diphosphoinositol-pentakisphosphate kinase 1 (Fragment) OS=Homo sapiens GN=PPIP5K1 PE=4 SV=1	21913	632	0	22	0	0.15	0	0	0	0	1	0	1	0	2	0	-2.47437
tr|A8K2U2|A8K2U2_HUMAN	tr|A8K2U2|A8	cDNA FLJ75392, highly similar to Homo sapiens hexokinase II (HKII) mRNA OS=Homo sapiens PE=2 SV=1	103718	599	0	26	0	0.03	0	0	0	0	1	0	1	0	2	0	-2.47437
tr|K7EID3|K7EID3_HUMAN	PRKAR1A	cAMP-dependent protein kinase type I-alpha regulatory subunit (Fragment) OS=Homo sapiens GN=PRKAR1A PE=4 SV=1	19112	533	0	31	0	0.08	0	0	0	0	1	0	1	0	1	0	-2.47437
sp|Q9UEW8|STK39_HUMAN	STK39	STE20/SPS1-related proline-alanine-rich protein kinase OS=Homo sapiens GN=STK39 PE=1 SV=3	59950	491	0	35	0	0.05	0	0	0	0	1	0	1	0	1	0	-2.47437
tr|B4E2K2|B4E2K2_HUMAN	tr|B4E2K2|B4	cDNA FLJ55665, highly similar to A-kinase anchor protein 2 OS=Homo sapiens PE=2 SV=1	103935	246	0	79	0	0.03	0	0	1	0	1	0	1	0	1	0	-2.47437
tr|H0Y3Y3|H0Y3Y3_HUMAN	PFKP	6-phosphofructokinase type C (Fragment) OS=Homo sapiens GN=PFKP PE=4 SV=1	17771	98	0	85	0	0.4	0	0	1	0	1	0	1	0	1	0	-2.47437
tr|Q8IWY7|Q8IWY7_HUMAN	TTBK2	Tau-tubulin kinase OS=Homo sapiens GN=TTBK2 PE=2 SV=1	183893	79	0	28	0	0.79	0	0	0	0	1	0	1	0	1	0	-2.47437
sp|O75533|SF3B1_HUMAN	SF3B1	Splicing factor 3B subunit 1 OS=Homo sapiens GN=SF3B1 PE=1 SV=3	146479	0	89	0	489	0	0.45	#DIV/0!	0	13	0	17	0	19	0	22	4.399727
tr|E1NZA1|E1NZA1_HUMAN	PRIC295	Peroxisome proliferator activated receptor interacting complex protein OS=Homo sapiens GN=PRIC295 PE=2 SV=1	294953	0	147	0	328	0	0.18	#DIV/0!	0	10	0	15	0	15	0	18	4.067804
sp|O43776|SYNC_HUMAN	NARS	Asparagine--tRNA ligase, cytoplasmic OS=Homo sapiens GN=NARS PE=1 SV=1	63758	0	127	0	376	0	0.92	#DIV/0!	0	11	0	14	0	14	0	14	3.97141
tr|A8K8U1|A8K8U1_HUMAN	tr|A8K8U1|A8	cDNA FLJ77762, highly similar to Homo sapiens cullin-associated and neddylation-dissociated 1 (CAND1), mRNA OS=Homo sapiens PE=2 SV=1	137941	0	198	0	257	0	0.29	#DIV/0!	0	8	0	11	0	13	0	13	3.868137
tr|B7Z1N6|B7Z1N6_HUMAN	tr|B7Z1N6|B7	cDNA FLJ52222, highly similar to Fructose-bisphosphate aldolase C (EC4.1.2.13) OS=Homo sapiens PE=2 SV=1	35800	0	107	0	432	0	0.56	#DIV/0!	0	8	0	10	0	12	0	18	3.756927
sp|Q08J23|NSUN2_HUMAN	NSUN2	tRNA (cytosine(34)-C(5))-methyltransferase OS=Homo sapiens GN=NSUN2 PE=1 SV=2	87214	0	274	0	185	0	0.29	#DIV/0!	0	5	0	10	0	11	0	14	3.636453
sp|Q92626|PXDN_HUMAN	PXDN	Peroxidasin homolog OS=Homo sapiens GN=PXDN PE=1 SV=2	167793	0	183	0	275	0	0.26	#DIV/0!	0	6	0	12	0	11	0	17	3.636453
tr|B3KMV5|B3KMV5_HUMAN	tr|B3KMV5|B	cDNA FLJ12728 fis, clone NT2RP2000040, highly similar to Protein FAM62A OS=Homo sapiens PE=2 SV=1	123342	0	306	0	164	0	0.23	#DIV/0!	0	5	0	8	0	10	0	10	3.505029
tr|D6REM6|D6REM6_HUMAN	MATR3	Matrin-3 OS=Homo sapiens GN=MATR3 PE=2 SV=1	88647	0	234	0	179	0	0.42	#DIV/0!	0	6	0	10	0	10	0	11	3.505029
tr|B2RDW1|B2RDW1_HUMAN	RPS27A	Ribosomal protein S27a, isoform CRA_c OS=Homo sapiens GN=RPS27A PE=2 SV=1	18296	0	226	0	230	0	0.66	#DIV/0!	0	8	0	10	0	10	0	12	3.505029
tr|B4DMF5|B4DMF5_HUMAN	tr|B4DMF5|B	Glutamate dehydrogenase OS=Homo sapiens PE=2 SV=1	56910	0	152	0	320	0	0.84	#DIV/0!	0	9	0	9	0	10	0	11	3.505029
tr|B0QY89|B0QY89_HUMAN	EIF3EIP	Eukaryotic translation initiation factor 3 subunit L OS=Homo sapiens GN=EIF3EIP PE=4 SV=1	71085	0	189	0	270	0	0.44	#DIV/0!	0	6	0	8	0	9	0	9	3.36046
tr|Q6NWZ1|Q6NWZ1_HUMAN	CKAP4	CKAP4 protein (Fragment) OS=Homo sapiens GN=CKAP4 PE=2 SV=1	67948	0	140	0	347	0	0.53	#DIV/0!	0	7	0	9	0	9	0	9	3.36046
tr|B7Z4B2|B7Z4B2_HUMAN	tr|B7Z4B2|B7	cDNA FLJ56108, highly similar to Puromycin-sensitive aminopeptidase (EC 3.4.11.-) OS=Homo sapiens PE=2 SV=1	93434	0	361	0	140	0	0.24	#DIV/0!	0	3	0	6	0	8	0	9	3.199817
tr|A2A274|A2A274_HUMAN	ACO2	Aconitase 2, mitochondrial OS=Homo sapiens GN=ACO2 PE=4 SV=1	88563	0	236	0	220	0	0.34	#DIV/0!	0	5	0	8	0	8	0	9	3.199817
tr|A1KYQ7|A1KYQ7_HUMAN	tr|A1KYQ7|A1	Cell migration-inducing protein 17 OS=Homo sapiens PE=2 SV=1	106022	0	217	0	236	0	0.28	#DIV/0!	0	6	0	8	0	8	0	9	3.199817
sp|Q9UHD8|SEPT9_HUMAN	Sep-09	Septin-9 OS=Homo sapiens GN=SEPT9 PE=1 SV=2	65646	0	205	0	252	0	0.34	#DIV/0!	0	4	0	6	0	8	0	9	3.199817
tr|B4DLV7|B4DLV7_HUMAN	tr|B4DLV7|B4	cDNA FLJ60299, highly similar to Rab GDP dissociation inhibitor beta OS=Homo sapiens PE=2 SV=1	51577	0	178	0	281	0	0.64	#DIV/0!	0	7	0	8	0	8	0	12	3.199817
sp|Q969N2|PIGT_HUMAN	PIGT	GPI transamidase component PIG-T OS=Homo sapiens GN=PIGT PE=1 SV=1	66228	0	454	0	107	0	0.27	#DIV/0!	0	3	0	5	0	7	0	8	3.019065
tr|A8K4T6|A8K4T6_HUMAN	tr|A8K4T6|A8	cDNA FLJ76282, highly similar to Homo sapiens proteasome (prosome, macropain) 26S subunit, non-ATPase, 5 (PSMD5), mRNA OS=Homo sapie	56588	0	395	0	123	0	0.4	#DIV/0!	0	4	0	6	0	7	0	7	3.019065
tr|B3KPA6|B3KPA6_HUMAN	ACADVL	Acyl-Coenzyme A dehydrogenase, very long chain, isoform CRA_e OS=Homo sapiens GN=ACADVL PE=2 SV=1	62941	0	392	0	123	0	0.43	#DIV/0!	0	4	0	7	0	7	0	7	3.019065
tr|Q0VGA5|Q0VGA5_HUMAN	SARS	SARS protein OS=Homo sapiens GN=SARS PE=2 SV=1	58883	0	290	0	173	0	0.39	#DIV/0!	0	5	0	6	0	7	0	8	3.019065
tr|G3XAI2|G3XAI2_HUMAN	LAMB1	Laminin subunit beta-1 OS=Homo sapiens GN=LAMB1 PE=2 SV=1	207703	0	277	0	183	0	0.12	#DIV/0!	0	4	0	7	0	7	0	8	3.019065
tr|Q53G71|Q53G71_HUMAN	tr|Q53G71|Q	Calreticulin variant (Fragment) OS=Homo sapiens PE=2 SV=1	47061	0	410	0	117	0	0.31	#DIV/0!	0	3	0	4	0	6	0	8	2.812435
tr|A4D210|A4D210_HUMAN	EIF3S9	Eukaryotic translation initiation factor 3, subunit 9 eta, 116kDa OS=Homo sapiens GN=EIF3S9 PE=4 SV=1	89024	0	408	0	118	0	0.24	#DIV/0!	0	3	0	6	0	6	0	9	2.812435
tr|Q59FE8|Q59FE8_HUMAN	tr|Q59FE8|Q5	Epithelial protein lost in neoplasm beta variant (Fragment) OS=Homo sapiens PE=2 SV=1	86510	0	366	0	138	0	0.36	#DIV/0!	0	3	0	5	0	6	0	6	2.812435
tr|B4DUF1|B4DUF1_HUMAN	tr|B4DUF1|B4	cDNA FLJ59760, highly similar to 1,4-alpha-glucan branching enzyme (EC 2.4.1.18) OS=Homo sapiens PE=2 SV=1	76254	0	344	0	145	0	0.2	#DIV/0!	0	3	0	5	0	6	0	7	2.812435
tr|B4DYY5|B4DYY5_HUMAN	tr|B4DYY5|B4	cDNA FLJ55671, highly similar to Ubiquitin-associated protein 2-like OS=Homo sapiens PE=2 SV=1	113662	0	328	0	153	0	0.22	#DIV/0!	0	5	0	7	0	6	0	7	2.812435
tr|A8K132|A8K132_HUMAN	tr|A8K132|A8	cDNA FLJ75476, highly similar to Homo sapiens glutaminase (GLS), mRNA OS=Homo sapiens PE=2 SV=1	74317	0	326	0	153	0	0.24	#DIV/0!	0	3	0	5	0	6	0	6	2.812435
sp|Q92945|FUBP2_HUMAN	KHSRP	Far upstream element-binding protein 2 OS=Homo sapiens GN=KHSRP PE=1 SV=4	73355	0	251	0	203	0	0.36	#DIV/0!	0	4	0	7	0	6	0	7	2.812435
tr|Q14400|Q14400_HUMAN	GLUD1	GLUD1 protein (Fragment) OS=Homo sapiens GN=GLUD1 PE=2 SV=1	28791	0	199	0	257	0	0.93	#DIV/0!	0	6	0	6	0	6	0	7	2.812435
tr|Q4ZG72|Q4ZG72_HUMAN	DDX18	Putative uncharacterized protein DDX18 (Fragment) OS=Homo sapiens GN=DDX18 PE=2 SV=1	61898	0	963	0	39	0	0.17	#DIV/0!	0	1	0	3	0	5	0	5	2.571248
tr|B4DMR3|B4DMR3_HUMAN	tr|B4DMR3|B	cDNA FLJ51896, highly similar to Glia-derived nexin OS=Homo sapiens PE=2 SV=1	37305	0	725	0	60	0	0.41	#DIV/0!	0	2	0	4	0	5	0	6	2.571248
tr|D9UAZ3|D9UAZ3_HUMAN	HLA-C	MHC class I antigen (Fragment) OS=Homo sapiens GN=HLA-C PE=3 SV=1	34263	0	723	0	60	0	0.32	#DIV/0!	0	2	0	3	0	5	0	5	2.571248
tr|Q58EY4|Q58EY4_HUMAN	SMARCC1	SWI/SNF related, matrix associated, actin dependent regulator of chromatin, subfamily c, member 1 OS=Homo sapiens GN=SMARCC1 PE=2 SV=	123347	0	695	0	64	0	0.14	#DIV/0!	0	2	0	5	0	5	0	6	2.571248
tr|A8K639|A8K639_HUMAN	GMPS	Guanine monphosphate synthetase, isoform CRA_b OS=Homo sapiens GN=GMPS PE=2 SV=1	77408	0	684	0	65	0	0.23	#DIV/0!	0	2	0	5	0	5	0	6	2.571248
sp|P61221|ABCE1_HUMAN	ABCE1	ATP-binding cassette sub-family E member 1 OS=Homo sapiens GN=ABCE1 PE=1 SV=1	68240	0	605	0	75	0	0.21	#DIV/0!	0	1	0	4	0	5	0	5	2.571248
tr|E5RJ24|E5RJ24_HUMAN	tr|E5RJ24|E5I	Uncharacterized protein (Fragment) OS=Homo sapiens PE=2 SV=1	53738	0	541	0	85	0	0.2	#DIV/0!	0	2	0	3	0	5	0	5	2.571248
tr|Q5TZP7|Q5TZP7_HUMAN	APEX1	APEX nuclease (Multifunctional DNA repair enzyme) 1 OS=Homo sapiens GN=APEX1 PE=2 SV=1	35931	0	490	0	99	0	0.55	#DIV/0!	0	2	0	5	0	5	0	6	2.571248
sp|P54577|SYYC_HUMAN	YARS	Tyrosine--tRNA ligase, cytoplasmic OS=Homo sapiens GN=YARS PE=1 SV=4	59448	0	477	0	103	0	0.24	#DIV/0!	0	2	0	4	0	5	0	8	2.571248
tr|G3V1J5|G3V1J5_HUMAN	DIS3	Exosome complex exonuclease RRP44 OS=Homo sapiens GN=DIS3 PE=2 SV=1	91382	0	445	0	103	0	0.19	#DIV/0!	0	2	0	5	0	5	0	7	2.571248
tr|A8K5Y7|A8K5Y7_HUMAN	tr|A8K5Y7|A8	cDNA FLJ78655, highly similar to Homo sapiens exportin 5 (XPO5), mRNA OS=Homo sapiens PE=2 SV=1	138331	0	428	0	114	0	0.18	#DIV/0!	0	4	0	5	0	5	0	5	2.571248
tr|B2RBM7|B2RBM7_HUMAN	tr|B2RBM7|B	cDNA, FLJ95595, highly similar to Homo sapiens proteasome (prosome, macropain) 26S subunit, non-ATPase, 13 (PSMD13), mRNA OS=Homo sa	43131	0	425	0	114	0	0.34	#DIV/0!	0	2	0	4	0	5	0	5	2.571248
tr|B4DZ87|B4DZ87_HUMAN	tr|B4DZ87|B4	cDNA FLJ57240, highly similar to Mitochondrial proteins import receptor OS=Homo sapiens PE=2 SV=1	57579	0	396	0	122	0	0.25	#DIV/0!	0	4	0	4	0	5	0	6	2.571248
sp|P30837|AL1B1_HUMAN	ALDH1B1	Aldehyde dehydrogenase X, mitochondrial OS=Homo sapiens GN=ALDH1B1 PE=1 SV=3	57626	0	380	0	130	0	0.32	#DIV/0!	0	3	0	5	0	5	0	5	2.571248
tr|B7Z213|B7Z213_HUMAN	tr|B7Z213|B7	cDNA FLJ50130, highly similar to Heterogeneous nuclear ribonucleoprotein Q OS=Homo sapiens PE=2 SV=1	28561	0	378	0	130	0	0.74	#DIV/0!	0	3	0	5	0	5	0	5	2.571248
tr|B2R774|B2R774_HUMAN	tr|B2R774|B2	cDNA, FLJ93313, highly similar to Homo sapiens lectin, mannose-binding, 1 (LMAN1), mRNA OS=Homo sapiens PE=2 SV=1	57768	0	369	0	137	0	0.25	#DIV/0!	0	3	0	4	0	5	0	5	2.571248
tr|B2R858|B2R858_HUMAN	tr|B2R858|B2	cDNA, FLJ93750, Homo sapiens DEAD (Asp-Glu-Ala-Asp) box polypeptide 6 (DDX6), mRNA OS=Homo sapiens PE=2 SV=1	53582	0	364	0	91	0	0.34	#DIV/0!	0	3	0	4	0	5	0	5	2.571248
tr|Q5H907|Q5H907_HUMAN	MAGED2	Melanoma antigen family D, 2, isoform CRA_d OS=Homo sapiens GN=MAGED2 PE=4 SV=1	55932	0	341	0	146	0	0.19	#DIV/0!	0	4	0	5	0	5	0	6	2.571248
tr|B4DN25|B4DN25_HUMAN	UGDH	UDP-glucose 6-dehydrogenase OS=Homo sapiens GN=UGDH PE=2 SV=1	44786	0	338	0	147	0	0.43	#DIV/0!	0	3	0	5	0	5	0	7	2.571248
tr|Q6FHX6|Q6FHX6_HUMAN	FEN1	Flap endonuclease 1 OS=Homo sapiens GN=FEN1 PE=2 SV=1	42908	0	322	0	154	0	0.35	#DIV/0!	0	4	0	5	0	5	0	7	2.571248
tr|B3KRS5|B3KRS5_HUMAN	HDAC2	Histone deacetylase OS=Homo sapiens GN=HDAC2 PE=2 SV=1	52479	0	317	0	157	0	0.36	#DIV/0!	0	3	0	5	0	5	0	6	2.571248
tr|B0AZQ4|B0AZQ4_HUMAN	tr|B0AZQ4|B(	cDNA, FLJ79494, highly similar to Structural maintenance of chromosome 3 OS=Homo sapiens PE=2 SV=1	141841	0	286	0	175	0	0.12	#DIV/0!	0	4	0	5	0	5	0	5	2.571248
sp|P31150|GDIA_HUMAN	GDI1	Rab GDP dissociation inhibitor alpha OS=Homo sapiens GN=GDI1 PE=1 SV=2	51177	0	266	0	190	0	0.37	#DIV/0!	0	4	0	5	0	5	0	7	2.571248
tr|Q6NVW7|Q6NVW7_HUMAN	KPNA2	Importin subunit alpha OS=Homo sapiens GN=KPNA2 PE=2 SV=1	58271	0	256	0	199	0	0.32	#DIV/0!	0	3	0	5	0	5	0	7	2.571248
sp|Q6PKG0|LARP1_HUMAN	LARP1	La-related protein 1 OS=Homo sapiens GN=LARP1 PE=1 SV=2	123833	0	957	0	39	0	0.05	#DIV/0!	0	0	0	2	0	4	0	5	2.281563
tr|Q5D1D5|Q5D1D5_HUMAN	tr|Q5D1D5|Q	Guanylate binding protein 1 OS=Homo sapiens PE=2 SV=1	68387	0	900	0	43	0	0.15	#DIV/0!	0	1	0	3	0	4	0	4	2.281563
tr|B4DUT7|B4DUT7_HUMAN	tr|B4DUT7|B4	cDNA FLJ57604, highly similar to GMP synthase (glutamine-hydrolyzing) (EC 6.3.5.2) OS=Homo sapiens PE=2 SV=1	71880	0	683	0	65	0	0.2	#DIV/0!	0	2	0	4	0	4	0	5	2.281563
tr|Q59GM9|Q59GM9_HUMAN	tr|Q59GM9|C	Phosphorylase (Fragment) OS=Homo sapiens PE=2 SV=1	99508	0	676	0	66	0	0.14	#DIV/0!	0	1	0	4	0	4	0	4	2.281563
sp|Q9BVP2|GNL3_HUMAN	GNL3	Guanine nucleotide-binding protein-like 3 OS=Homo sapiens GN=GNL3 PE=1 SV=2	62468	0	606	0	75	0	0.17	#DIV/0!	0	2	0	3	0	4	0	4	2.281563
tr|J3K000|J3K000_HUMAN	PEPD	PEPD protein OS=Homo sapiens GN=PEPD PE=2 SV=1	55345	0	604	0	75	0	0.19	#DIV/0!	0	1	0	3	0	4	0	4	2.281563
sp|Q9NTI5|PDS5B_HUMAN	PDS5B	Sister chromatid cohesion protein PDS5 homolog B OS=Homo sapiens GN=PDS5B PE=1 SV=1	165818	0	593	0	77	0	0.04	#DIV/0!	0	1	0	2	0	4	0	5	2.281563
tr|C0KK01|C0KK01_HUMAN	HLA-C	MHC class I antigen (Fragment) OS=Homo sapiens GN=HLA-C PE=3 SV=1	20940	0	579	0	79	0	0.56	#DIV/0!	0	2	0	3	0	4	0	4	2.281563
tr|F4NBX2|F4NBX2_HUMAN	HLA-B	MHC class I antigen (Fragment) OS=Homo sapiens GN=HLA-B PE=3 SV=1	38566	0	572	0	79	0	0.18	#DIV/0!	0	2	0	2	0	4	0	4	2.281563
tr|Q59FA0|Q59FA0_HUMAN	tr|Q59FA0|Q!	DNA polymerase (Fragment) OS=Homo sapiens PE=2 SV=1	113271	0	552	0	83	0	0.09	#DIV/0!	0	2	0	3	0	4	0	4	2.281563
tr|H0YCK3|H0YCK3_HUMAN	ADAR	Double-stranded RNA-specific adenosine deaminase (Fragment) OS=Homo sapiens GN=ADAR PE=4 SV=1	133761	0	549	0	84	0	0.1	#DIV/0!	0	2	0	4	0	4	0	4	2.281563
tr|B4DSN3|B4DSN3_HUMAN	tr|B4DSN3|B4	cDNA FLJ60345, highly similar to Protein transport protein Sec24C OS=Homo sapiens PE=2 SV=1	107974	0	540	0	86	0	0.13	#DIV/0!	0	3	0	4	0	4	0	4	2.281563
tr|B4DDM6|B4DDM6_HUMAN	BUB3	Mitotic checkpoint protein BUB3 OS=Homo sapiens GN=BUB3 PE=2 SV=1	28620	0	529	0	88	0	0.39	#DIV/0!	0	3	0	3	0	4	0	5	2.281563
tr|B4DZC0|B4DZC0_HUMAN	tr|B4DZC0|B4	cDNA FLJ51771, highly similar to SWI/SNF-related matrix-associatedactin-dependent regulator of chromatin subfamily A member5 (EC 3.6.1.-) C	117367	0	520	0	92	0	0.09	#DIV/0!	0	2	0	3	0	4	0	5	2.281563
sp|P52907|CAZA1_HUMAN	CAPZA1	F-actin-capping protein subunit alpha-1 OS=Homo sapiens GN=CAPZA1 PE=1 SV=3	33073	0	510	0	94	0	0.33	#DIV/0!	0	1	0	3	0	4	0	4	2.281563
tr|B7Z4B8|B7Z4B8_HUMAN	HNRNPUL1	Heterogeneous nuclear ribonucleoprotein U-like protein 1 OS=Homo sapiens GN=HNRNPUL1 PE=2 SV=1	86639	0	495	0	99	0	0.08	#DIV/0!	0	2	0	2	0	4	0	5	2.281563
sp|Q9BWF3|RBM4_HUMAN	RBM4	RNA-binding protein 4 OS=Homo sapiens GN=RBM4 PE=1 SV=1	40688	0	486	0	100	0	0.37	#DIV/0!	0	3	0	4	0	4	0	4	2.281563
sp|Q09161|NCBP1_HUMAN	NCBP1	Nuclear cap-binding protein subunit 1 OS=Homo sapiens GN=NCBP1 PE=1 SV=1	92864	0	483	0	101	0	0.15	#DIV/0!	0	4	0	4	0	4	0	4	2.281563
tr|Q6FG59|Q6FG59_HUMAN	CDC37	CDC37 protein OS=Homo sapiens GN=CDC37 PE=2 SV=1	44938	0	481	0	101	0	0.24	#DIV/0!	0	3	0	3	0	4	0	4	2.281563
tr|G3V1L9|G3V1L9_HUMAN	TJP1	Tight junction protein 1 (Zona occludens 1), isoform CRA_a OS=Homo sapiens GN=TJP1 PE=4 SV=1	197681	0	473	0	104	0	0.05	#DIV/0!	0	2	0	3	0	4	0	5	2.281563
tr|K4DI93|K4DI93_HUMAN	CUL4B	Cullin 4B, isoform CRA_e OS=Homo sapiens GN=CUL4B PE=3 SV=1	103318	0	462	0	106	0	0.13	#DIV/0!	0	3	0	4	0	4	0	4	2.281563
tr|A0PJ61|A0PJ61_HUMAN	RSL1D1	RSL1D1 protein (Fragment) OS=Homo sapiens GN=RSL1D1 PE=2 SV=1	49688	0	446	0	109	0	0.21	#DIV/0!	0	2	0	3	0	4	0	5	2.281563
sp|Q86XP3|DDX42_HUMAN	DDX42	ATP-dependent RNA helicase DDX42 OS=Homo sapiens GN=DDX42 PE=1 SV=1	103197	0	429	0	114	0	0.13	#DIV/0!	0	3	0	4	0	4	0	4	2.281563
tr|Q96F88|Q96F88_HUMAN	POP1	Processing of 1, ribonuclease P/MRP subunit (S. cerevisiae) OS=Homo sapiens GN=POP1 PE=2 SV=1	116318	0	423	0	115	0	0.09	#DIV/0!	0	3	0	3	0	4	0	4	2.281563
tr|B0YIW5|B0YIW5_HUMAN	ARCN1	Archain 1, isoform CRA_b OS=Homo sapiens GN=ARCN1 PE=2 SV=1	57630	0	394	0	123	0	0.25	#DIV/0!	0	3	0	4	0	4	0	4	2.281563
tr|B0LM41|B0LM41_HUMAN	RBM14/RBM4	Protein RBM14-RBM4 OS=Homo sapiens GN=RBM14/RBM4 fusion PE=2 SV=1	37410	0	387	0	126	0	0.4	#DIV/0!	0	4	0	4	0	4	0	4	2.281563
tr|B4DN01|B4DN01_HUMAN	tr|B4DN01|B4	cDNA FLJ57332, highly similar to COP9 signalosome complex subunit 3 OS=Homo sapiens PE=2 SV=1	33950	0	367	0	138	0	0.45	#DIV/0!	0	4	0	4	0	4	0	4	2.281563
tr|B4DT31|B4DT31_HUMAN	FUBP1	Far upstream element-binding protein 1 OS=Homo sapiens GN=FUBP1 PE=2 SV=1	69993	0	337	0	148	0	0.15	#DIV/0!	0	2	0	3	0	4	0	6	2.281563
tr|B4DWZ4|B4DWZ4_HUMAN	FEN1	Flap endonuclease 1 OS=Homo sapiens GN=FEN1 PE=2 SV=1	39109	0	332	0	151	0	0.28	#DIV/0!	0	4	0	4	0	4	0	7	2.281563
sp|Q9Y2W1|TR150_HUMAN	THRAP3	Thyroid hormone receptor-associated protein 3 OS=Homo sapiens GN=THRAP3 PE=1 SV=2	108658	0	327	0	153	0	0.16	#DIV/0!	0	4	0	5	0	4	0	5	2.281563
tr|B4DM78|B4DM78_HUMAN	tr|B4DM78|B	cDNA FLJ58199, highly similar to Fragile X mental retardation syndrome-related protein 1 OS=Homo sapiens PE=2 SV=1	63280	0	311	0	162	0	0.28	#DIV/0!	0	3	0	4	0	4	0	5	2.281563
sp|Q96PK6|RBM14_HUMAN	RBM14	RNA-binding protein 14 OS=Homo sapiens GN=RBM14 PE=1 SV=2	69620	0	308	0	162	0	0.2	#DIV/0!	0	4	0	4	0	4	0	4	2.281563
tr|G5E9H4|G5E9H4_HUMAN	DCTN1	Dynactin 1 (P150, glued homolog, Drosophila), isoform CRA_a OS=Homo sapiens GN=DCTN1 PE=2 SV=1	127339	0	299	0	169	0	0.11	#DIV/0!	0	4	0	4	0	4	0	5	2.281563
tr|B2RNR6|B2RNR6_HUMAN	ZFR	Zinc finger RNA binding protein OS=Homo sapiens GN=ZFR PE=2 SV=1	118081	0	298	0	170	0	0.12	#DIV/0!	0	4	0	4	0	4	0	4	2.281563
tr|B3KM58|B3KM58_HUMAN	tr|B3KM58|B	cDNA FLJ10358 fis, clone NT2RM2001238, highly similar to Glutaminase kidney isoform, mitochondrial (Fragment) OS=Homo sapiens PE=2 SV=1	41736	0	269	0	189	0	0.26	#DIV/0!	0	3	0	3	0	4	0	4	2.281563
tr|B4DR63|B4DR63_HUMAN	PSMC1	26S protease regulatory subunit 4 OS=Homo sapiens GN=PSMC1 PE=2 SV=1	41255	0	243	0	182	0	0.29	#DIV/0!	0	4	0	4	0	4	0	5	2.281563
tr|Q9MY63|Q9MY63_HUMAN	HLA-Cw	Leucocyte antigen (Fragment) OS=Homo sapiens GN=HLA-Cw PE=3 SV=1	26578	0	1069	0	32	0	0.13	#DIV/0!	0	0	0	1	0	3	0	3	1.918814
tr|G3V529|G3V529_HUMAN	DDX24	ATP-dependent RNA helicase DDX24 OS=Homo sapiens GN=DDX24 PE=2 SV=1	91994	0	1053	0	33	0	0.07	#DIV/0!	0	0	0	2	0	3	0	5	1.918814
tr|B7ZMF2|B7ZMF2_HUMAN	FANCI	Fanconi anemia, complementation group I OS=Homo sapiens GN=FANCI PE=2 SV=1	150724	0	1023	0	35	0	0.04	#DIV/0!	0	0	0	2	0	3	0	4	1.918814
tr|B4E3M6|B4E3M6_HUMAN	tr|B4E3M6|B4	cDNA FLJ55446, highly similar to Superkiller viralicidic activity 2-like 2 (EC 3.6.1.-) OS=Homo sapiens PE=2 SV=1	107714	0	997	0	36	0	0.09	#DIV/0!	0	1	0	3	0	3	0	3	1.918814
tr|B3KY11|B3KY11_HUMAN	tr|B3KY11|B3	cDNA FLJ46571 fis, clone THYMU3041428, highly similar to Probable ATP-dependent RNA helicase DDX23 (EC 3.6.1.-) OS=Homo sapiens PE=2 SV	93519	0	960	0	39	0	0.07	#DIV/0!	0	1	0	2	0	3	0	5	1.918814
tr|H7C3P6|H7C3P6_HUMAN	NUP98	Nuclear pore complex protein Nup98-Nup96 (Fragment) OS=Homo sapiens GN=NUP98 PE=4 SV=1	86760	0	950	0	39	0	0.12	#DIV/0!	0	0	0	3	0	3	0	4	1.918814
tr|B4DJV2|B4DJV2_HUMAN	CS	Citrate synthase OS=Homo sapiens GN=CS PE=2 SV=1	50628	0	942	0	40	0	0.21	#DIV/0!	0	1	0	3	0	3	0	4	1.918814

sp|Q14677|EPN4_HUMAN	CLINT1	Clathrin interactor 1 OS=Homo sapiens GN=CLINT1 PE=1 SV=1	68273	0	936	0	40	0	0.15	#DIV/0!	0	0	0	3	0	3	0	3	1.918814
tr|B3KY63|B3KY63_HUMAN	tr|B3KY63|B3	cDNA FLJ16830 fis, clone UTERU3022536, highly similar to Chromodomain helicase-DNA-binding protein 4 (EC 3.6.1.-) OS=Homo sapiens PE=2 S	216574	0	929	0	40	0	0.03	#DIV/0!	0	2	0	2	0	3	0	3	1.918814
tr|E7EPN9|E7EPN9_HUMAN	PRRC2C	Protein PRRC2C OS=Homo sapiens GN=PRRC2C PE=2 SV=1	309159	0	908	0	42	0	0.02	#DIV/0!	0	1	0	2	0	3	0	3	1.918814
tr|E0WMV4|E0WMV4_HUMAN	HLA-C	MHC class I antigen (Fragment) OS=Homo sapiens GN=HLA-C PE=3 SV=1	31992	0	895	0	43	0	0.22	#DIV/0!	0	1	0	2	0	3	0	3	1.918814
tr|B4DTS5|B4DTS5_HUMAN	tr|B4DTS5|B4	cDNA FLJ58882, highly similar to 26S proteasome non-ATPase regulatory subunit 11 OS=Homo sapiens PE=2 SV=1	35374	0	881	0	44	0	0.2	#DIV/0!	0	1	0	2	0	3	0	3	1.918814
tr|E7ESC6|E7ESC6_HUMAN	XPO7	Exportin-7 OS=Homo sapiens GN=XPO7 PE=2 SV=1	125356	0	865	0	46	0	0.11	#DIV/0!	0	2	0	4	0	3	0	4	1.918814
tr|H0Y4C2|H0Y4C2_HUMAN	HLA-F	HLA class I histocompatibility antigen, alpha chain F (Fragment) OS=Homo sapiens GN=HLA-F PE=3 SV=1	25349	0	853	0	47	0	0.13	#DIV/0!	0	1	0	1	0	3	0	4	1.918814
tr|B3KMC9|B3KMC9_HUMAN	tr|B3KMC9|B	cDNA FLJ10711 fis, clone NT2RP3000917, highly similar to 5~-3~ exoribonuclease 2 (EC 3.1.11.-) OS=Homo sapiens PE=2 SV=1	109456	0	782	0	53	0	0.09	#DIV/0!	0	1	0	3	0	3	0	4	1.918814
tr|Q05DF2|Q05DF2_HUMAN	SF3A2	SF3A2 protein (Fragment) OS=Homo sapiens GN=SF3A2 PE=2 SV=1	51557	0	771	0	54	0	0.2	#DIV/0!	0	1	0	3	0	3	0	3	1.918814
tr|B4DJU4|B4DJU4_HUMAN	tr|B4DJU4|B4	cDNA FLJ53344, highly similar to Splicing factor 1 OS=Homo sapiens PE=2 SV=1	67535	0	769	0	54	0	0.15	#DIV/0!	0	1	0	3	0	3	0	3	1.918814
tr|A0MNN5|A0MNN5_HUMAN	GRWD1	CDW4/GRWD1 OS=Homo sapiens GN=GRWD1 PE=2 SV=1	49787	0	755	0	55	0	0.07	#DIV/0!	0	1	0	1	0	3	0	3	1.918814
sp|Q27J81|INF2_HUMAN	INF2	Inverted formin-2 OS=Homo sapiens GN=INF2 PE=1 SV=2	136851	0	754	0	55	0	0.05	#DIV/0!	0	1	0	2	0	3	0	5	1.918814
tr|A8KA19|A8KA19_HUMAN	tr|A8KA19|A8	cDNA FLJ75831, highly similar to Homo sapiens exportin, tRNA (nuclear export receptor for tRNAs) (XPOT), mRNA OS=Homo sapiens PE=2 SV=1	111104	0	753	0	55	0	0.06	#DIV/0!	0	1	0	2	0	3	0	4	1.918814
tr|B4E2K4|B4E2K4_HUMAN	tr|B4E2K4|B4	cDNA FLJ54576, highly similar to Aspartyl/asparaginyl beta-hydroxylase (EC 1.14.11.16) OS=Homo sapiens PE=2 SV=1	83685	0	714	0	61	0	0.12	#DIV/0!	0	1	0	3	0	3	0	3	1.918814
tr|B4DSD8|B4DSD8_HUMAN	tr|B4DSD8|B4	Phosphorylase OS=Homo sapiens PE=2 SV=1	86417	0	674	0	66	0	0.12	#DIV/0!	0	1	0	3	0	3	0	3	1.918814
tr|B4DY28|B4DY28_HUMAN	tr|B4DY28|B4	cDNA FLJ61189, highly similar to Cysteine and glycine-rich protein 1 OS=Homo sapiens PE=2 SV=1	20884	0	672	0	66	0	0.35	#DIV/0!	0	2	0	2	0	3	0	3	1.918814
tr|A1A693|A1A693_HUMAN	RBM15	RNA binding motif protein 15 isoform L2 OS=Homo sapiens GN=RBM15 PE=2 SV=1	99689	0	650	0	69	0	0.1	#DIV/0!	0	2	0	3	0	3	0	3	1.918814
sp|P11047|LAMC1_HUMAN	LAMC1	Laminin subunit gamma-1 OS=Homo sapiens GN=LAMC1 PE=1 SV=3	183191	0	646	0	70	0	0.05	#DIV/0!	0	1	0	3	0	3	0	4	1.918814
sp|Q9UN86|G3BP2_HUMAN	G3BP2	Ras GTPase-activating protein-binding protein 2 OS=Homo sapiens GN=G3BP2 PE=1 SV=2	54145	0	629	0	72	0	0.19	#DIV/0!	0	1	0	3	0	3	0	4	1.918814
tr|B3KRC6|B3KRC6_HUMAN	tr|B3KRC6|B3	cDNA FLJ34004 fis, clone FCBBF1000232, highly similar to Cytochrome P450 51A1 (EC 1.14.13.70) OS=Homo sapiens PE=2 SV=1	51732	0	626	0	72	0	0.13	#DIV/0!	0	2	0	2	0	3	0	4	1.918814
tr|B3GQE6|B3GQE6_HUMAN	DDX27	DEAD box polypeptide 27 OS=Homo sapiens GN=DDX27 PE=2 SV=1	86980	0	616	0	74	0	0.08	#DIV/0!	0	1	0	2	0	3	0	3	1.918814
tr|H0Y3P2|H0Y3P2_HUMAN	EIF4G2	Eukaryotic translation initiation factor 4 gamma 2 OS=Homo sapiens GN=EIF4G2 PE=4 SV=1	98569	0	601	0	75	0	0.03	#DIV/0!	0	1	0	1	0	3	0	4	1.918814
tr|Q6EHZ3|Q6EHZ3_HUMAN	STRF8	Putative uncharacterized protein STRF8 OS=Homo sapiens GN=STRF8 PE=2 SV=1	41361	0	599	0	76	0	0.17	#DIV/0!	0	1	0	2	0	3	0	3	1.918814
tr|B2RMN7|B2RMN7_HUMAN	SPTB	Spectrin, beta, erythrocytic OS=Homo sapiens GN=SPTB PE=2 SV=1	247271	0	588	0	78	0	0.03	#DIV/0!	0	2	0	2	0	3	0	4	1.918814
tr|Q6IAM0|Q6IAM0_HUMAN	EIF3S4	Eukaryotic translation initiation factor 3 subunit G OS=Homo sapiens GN=EIF3S4 PE=2 SV=1	35864	0	586	0	78	0	0.3	#DIV/0!	0	2	0	3	0	3	0	3	1.918814
tr|J3KQ32|J3KQ32_HUMAN	OLA1	Obg-like ATPase 1 OS=Homo sapiens GN=OLA1 PE=4 SV=1	47136	0	585	0	78	0	0.14	#DIV/0!	0	2	0	2	0	3	0	3	1.918814
tr|B2ZRW0|B2ZRW0_HUMAN	HLA-B	MHC class I antigen (Fragment) OS=Homo sapiens GN=HLA-B PE=3 SV=1	21200	0	573	0	79	0	0.34	#DIV/0!	0	2	0	2	0	3	0	4	1.918814
sp|P35658|NU214_HUMAN	NUP214	Nuclear pore complex protein Nup214 OS=Homo sapiens GN=NUP214 PE=1 SV=2	214230	0	568	0	79	0	0.03	#DIV/0!	0	1	0	2	0	3	0	3	1.918814
tr|H0Y2Y8|H0Y2Y8_HUMAN	ZYX	Zyxin (Fragment) OS=Homo sapiens GN=ZYX PE=4 SV=1	58804	0	567	0	79	0	0.11	#DIV/0!	0	1	0	2	0	3	0	3	1.918814
sp|Q9NUL7|DDX28_HUMAN	DDX28	Probable ATP-dependent RNA helicase DDX28 OS=Homo sapiens GN=DDX28 PE=1 SV=2	59773	0	558	0	82	0	0.11	#DIV/0!	0	2	0	2	0	3	0	3	1.918814
sp|Q08945|SSRP1_HUMAN	SSRP1	FACT complex subunit SSRP1 OS=Homo sapiens GN=SSRP1 PE=1 SV=1	81367	0	554	0	83	0	0.08	#DIV/0!	0	2	0	2	0	3	0	6	1.918814
tr|A8K0D2|A8K0D2_HUMAN	tr|A8K0D2|A8	cDNA FLJ77740, highly similar to Homo sapiens 7-dehydrocholesterol reductase, mRNA OS=Homo sapiens PE=2 SV=1	55175	0	539	0	86	0	0.12	#DIV/0!	0	2	0	2	0	3	0	3	1.918814
tr|F6SBX2|F6SBX2_HUMAN	IARS2	Isoleucine--tRNA ligase, mitochondrial OS=Homo sapiens GN=IARS2 PE=2 SV=1	106884	0	504	0	95	0	0.09	#DIV/0!	0	3	0	3	0	3	0	4	1.918814
sp|P36952|SPB5_HUMAN	SERPINB5	Serpin B5 OS=Homo sapiens GN=SERPINB5 PE=1 SV=2	42530	0	494	0	99	0	0.16	#DIV/0!	0	1	0	2	0	3	0	3	1.918814
tr|Q6DCA8|Q6DCA8_HUMAN	BCLAF1	BCLAF1 protein (Fragment) OS=Homo sapiens GN=BCLAF1 PE=2 SV=1	77958	0	484	0	101	0	0.09	#DIV/0!	0	2	0	2	0	3	0	3	1.918814
tr|B3KRR1|B3KRR1_HUMAN	tr|B3KRR1|B3	cDNA FLJ34725 fis, clone MESAN2005958, highly similar to RNA-binding protein Luc7-like 2 OS=Homo sapiens PE=2 SV=1	40752	0	482	0	101	0	0.19	#DIV/0!	0	2	0	3	0	3	0	3	1.918814
tr|F5GZS0|F5GZS0_HUMAN	DHX36	Probable ATP-dependent RNA helicase DHX36 OS=Homo sapiens GN=DHX36 PE=2 SV=1	113928	0	460	0	106	0	0.09	#DIV/0!	0	3	0	3	0	3	0	3	1.918814
sp|Q96I24|FUBP3_HUMAN	FUBP3	Far upstream element-binding protein 3 OS=Homo sapiens GN=FUBP3 PE=1 SV=2	61944	0	451	0	107	0	0.17	#DIV/0!	0	3	0	3	0	3	0	3	1.918814
tr|Q5VZA6|Q5VZA6_HUMAN	ACO1	Cytoplasmic aconitate hydratase OS=Homo sapiens GN=ACO1 PE=4 SV=1	48888	0	449	0	108	0	0.1	#DIV/0!	0	2	0	3	0	3	0	3	1.918814
tr|D3DUW5|D3DUW5_HUMAN	DNM1L	Dynamin 1-like, isoform CRA_c OS=Homo sapiens GN=DNM1L PE=3 SV=1	88442	0	440	0	111	0	0.12	#DIV/0!	0	2	0	3	0	3	0	4	1.918814
tr|Q96FS1|Q96FS1_HUMAN	CTNND1	CTNND1 protein (Fragment) OS=Homo sapiens GN=CTNND1 PE=2 SV=2	92901	0	393	0	123	0	0.1	#DIV/0!	0	2	0	3	0	3	0	3	1.918814
tr|B3KM89|B3KM89_HUMAN	tr|B3KM89|B	cDNA FLJ10528 fis, clone NT2RP2000943, highly similar to Protein transport protein Sec24D OS=Homo sapiens PE=2 SV=1	101723	0	391	0	124	0	0.1	#DIV/0!	0	3	0	3	0	3	0	3	1.918814
tr|B3KM73|B3KM73_HUMAN	tr|B3KM73|B	cDNA FLJ10419 fis, clone NT2RP1000163, highly similar to Homo sapiens transforming growth factor beta regulator 4 (TBRG4), transcript varian	55418	0	382	0	112	0	0.28	#DIV/0!	0	2	0	4	0	3	0	5	1.918814
tr|H0Y5W0|H0Y5W0_HUMAN	HUWE1	E3 ubiquitin-protein ligase HUWE1 (Fragment) OS=Homo sapiens GN=HUWE1 PE=4 SV=1	376696	0	375	0	127	0	0.03	#DIV/0!	0	2	0	3	0	3	0	4	1.918814
tr|B2R6E5|B2R6E5_HUMAN	tr|B2R6E5|B2	NADPH--cytochrome P450 reductase OS=Homo sapiens PE=2 SV=1	77429	0	371	0	134	0	0.13	#DIV/0!	0	3	0	3	0	3	0	3	1.918814
tr|H3BRG4|H3BRG4_HUMAN	UQCRC2	Cytochrome b-c1 complex subunit 2, mitochondrial OS=Homo sapiens GN=UQCRC2 PE=3 SV=1	44778	0	359	0	141	0	0.24	#DIV/0!	0	2	0	3	0	3	0	4	1.918814
tr|B3KMN2|B3KMN2_HUMAN	tr|B3KMN2|B	cDNA FLJ11695 fis, clone HEMBA1005019, highly similar to Homo sapiens SCC-112 protein (SCC-112), mRNA OS=Homo sapiens PE=2 SV=1	36724	0	356	0	111	0	0.11	#DIV/0!	0	2	0	3	0	3	0	3	1.918814
tr|B2RAH7|B2RAH7_HUMAN	tr|B2RAH7|B2	cDNA, FLJ94921, highly similar to Homo sapiens prolyl endopeptidase (PREP), mRNA OS=Homo sapiens PE=2 SV=1	81594	0	350	0	144	0	0.13	#DIV/0!	0	3	0	3	0	3	0	4	1.918814
tr|B4E1L0|B4E1L0_HUMAN	tr|B4E1L0|B4	Adenylosuccinate synthetase OS=Homo sapiens PE=2 SV=1	48308	0	313	0	160	0	0.22	#DIV/0!	0	3	0	3	0	3	0	4	1.918814
tr|Q4LE64|Q4LE64_HUMAN	NUMA1	NUMA1 variant protein (Fragment) OS=Homo sapiens GN=NUMA1 variant protein PE=2 SV=1	239798	0	307	0	162	0	0.09	#DIV/0!	0	3	0	3	0	3	0	4	1.918814
tr|I6L9B1|I6L9B1_HUMAN	EPB41L2	EPB41L2 protein OS=Homo sapiens GN=EPB41L2 PE=2 SV=1	71998	0	1234	0	20	0	0.05	#DIV/0!	0	0	0	1	0	2	0	3	1.433208
tr|J3KNQ4|J3KNQ4_HUMAN	PARVA	Alpha-parvin OS=Homo sapiens GN=PARVA PE=4 SV=1	46618	0	1227	0	21	0	0.07	#DIV/0!	0	0	0	1	0	2	0	2	1.433208
tr|B0YJ32|B0YJ32_HUMAN	LAMA3	Laminin alpha-3 chain variant 1 OS=Homo sapiens GN=LAMA3 PE=2 SV=1	375952	0	1211	0	22	0	0.01	#DIV/0!	0	0	0	1	0	2	0	2	1.433208
tr|J3KTL8|J3KTL8_HUMAN	SMCHD1	Structural maintenance of chromosomes flexible hinge domain-containing protein 1 (Fragment) OS=Homo sapiens GN=SMCHD1 PE=4 SV=1	156690	0	1196	0	23	0	0.01	#DIV/0!	0	0	0	1	0	2	0	3	1.433208
tr|F5H8E5|F5H8E5_HUMAN	USP7	Ubiquitin carboxyl-terminal hydrolase OS=Homo sapiens GN=USP7 PE=2 SV=1	117951	0	1171	0	25	0	0.06	#DIV/0!	0	0	0	2	0	2	0	3	1.433208
tr|Q6MZP3|Q6MZP3_HUMAN	DKFZp686I051	Putative uncharacterized protein DKFZp686I05169 OS=Homo sapiens GN=DKFZp686I05169 PE=2 SV=1	146779	0	1159	0	26	0	0.05	#DIV/0!	0	1	0	2	0	2	0	4	1.433208
tr|J3KQA9|J3KQA9_HUMAN	MTUS2	Microtubule-associated tumor suppressor candidate 2 OS=Homo sapiens GN=MTUS2 PE=4 SV=1	151875	0	1153	0	26	0	0.02	#DIV/0!	0	0	0	1	0	2	0	2	1.433208
tr|B3KQ39|B3KQ39_HUMAN	tr|B3KQ39|B3	cDNA FLJ32740 fis, clone TESTI2001339, highly similar to UDP-GLUCOSE 4-EPIMERASE (EC 5.1.3.2) OS=Homo sapiens PE=2 SV=1	30550	0	1145	0	27	0	0.11	#DIV/0!	0	0	0	1	0	2	0	4	1.433208
sp|Q9BVI4|NOC4L_HUMAN	NOC4L	Nucleolar complex protein 4 homolog OS=Homo sapiens GN=NOC4L PE=1 SV=1	58830	0	1134	0	27	0	0.11	#DIV/0!	0	0	0	2	0	2	0	4	1.433208
sp|P27635|RL10_HUMAN	RPL10	60S ribosomal protein L10 OS=Homo sapiens GN=RPL10 PE=1 SV=4	25044	0	1108	0	29	0	0.13	#DIV/0!	0	0	0	1	0	2	0	2	1.433208
sp|Q8IVF2|AHNK2_HUMAN	AHNAK2	Protein AHNAK2 OS=Homo sapiens GN=AHNAK2 PE=1 SV=2	617383	0	1101	0	29	0	0.01	#DIV/0!	0	0	0	1	0	2	0	2	1.433208
sp|Q9Y5L0|TNPO3_HUMAN	TNPO3	Transportin-3 OS=Homo sapiens GN=TNPO3 PE=1 SV=3	105961	0	1078	0	31	0	0.03	#DIV/0!	0	0	0	1	0	2	0	3	1.433208
sp|O43432|IF4G3_HUMAN	EIF4G3	Eukaryotic translation initiation factor 4 gamma 3 OS=Homo sapiens GN=EIF4G3 PE=1 SV=2	177682	0	1072	0	32	0	0.02	#DIV/0!	0	0	0	1	0	2	0	2	1.433208
sp|Q96RP9|EFGM_HUMAN	GFM1	Elongation factor G, mitochondrial OS=Homo sapiens GN=GFM1 PE=1 SV=2	84103	0	1070	0	32	0	0.08	#DIV/0!	0	0	0	2	0	2	0	3	1.433208
tr|B7Z611|B7Z611_HUMAN	tr|B7Z611|B7	cDNA FLJ50848, highly similar to Homo sapiens suppressor of var1, 3-like 1 (S. cerevisiae) (SUPV3L1), mRNA (Fragment) OS=Homo sapiens PE=2	71856	0	1065	0	32	0	0.09	#DIV/0!	0	0	0	2	0	2	0	2	1.433208
sp|P28288|ABCD3_HUMAN	ABCD3	ATP-binding cassette sub-family D member 3 OS=Homo sapiens GN=ABCD3 PE=1 SV=1	75941	0	1062	0	32	0	0.04	#DIV/0!	0	0	0	1	0	2	0	3	1.433208
sp|P24928|RPB1_HUMAN	POLR2A	DNA-directed RNA polymerase II subunit RPB1 OS=Homo sapiens GN=POLR2A PE=1 SV=2	218408	0	1055	0	33	0	0.03	#DIV/0!	0	0	0	2	0	2	0	2	1.433208
sp|P98160|PGBM_HUMAN	HSPG2	Basement membrane-specific heparan sulfate proteoglycan core protein OS=Homo sapiens GN=HSPG2 PE=1 SV=4	479253	0	1036	0	34	0	0.01	#DIV/0!	0	0	0	2	0	2	0	3	1.433208
sp|Q14126|DSG2_HUMAN	DSG2	Desmoglein-2 OS=Homo sapiens GN=DSG2 PE=1 SV=2	123016	0	1035	0	34	0	0.05	#DIV/0!	0	1	0	2	0	2	0	3	1.433208
sp|P40222|TXLNA_HUMAN	TXLNA	Alpha-taxilin OS=Homo sapiens GN=TXLNA PE=1 SV=3	62195	0	1033	0	34	0	0.05	#DIV/0!	0	0	0	1	0	2	0	2	1.433208
sp|Q8NI35|INADL_HUMAN	INADL	InaD-like protein OS=Homo sapiens GN=INADL PE=1 SV=3	197046	0	1020	0	35	0	0.03	#DIV/0!	0	1	0	1	0	2	0	4	1.433208
sp|P14550|AK1A1_HUMAN	AKR1A1	Alcohol dehydrogenase [NADP(+)] OS=Homo sapiens GN=AKR1A1 PE=1 SV=3	36892	0	1011	0	35	0	0.09	#DIV/0!	0	1	0	1	0	2	0	2	1.433208
tr|Q53GY1|Q53GY1_HUMAN	tr|Q53GY1|Q	BCL2-associated athanogene 3 variant (Fragment) OS=Homo sapiens PE=2 SV=1	61680	0	999	0	36	0	0.05	#DIV/0!	0	0	0	1	0	2	0	2	1.433208
tr|H3BPE1|H3BPE1_HUMAN	MACF1	Microtubule-actin cross-linking factor 1, isoforms 1/2/3/5 OS=Homo sapiens GN=MACF1 PE=4 SV=1	861649	0	998	0	36	0	0	#DIV/0!	0	1	0	1	0	2	0	2	1.433208
sp|A1L0T0|ILVBL_HUMAN	ILVBL	Acetolactate synthase-like protein OS=Homo sapiens GN=ILVBL PE=1 SV=2	68452	0	990	0	37	0	0.15	#DIV/0!	0	0	0	3	0	2	0	3	1.433208
sp|Q8NB90|SPAT5_HUMAN	SPATA5	Spermatogenesis-associated protein 5 OS=Homo sapiens GN=SPATA5 PE=1 SV=3	98470	0	986	0	37	0	0.03	#DIV/0!	0	1	0	1	0	2	0	2	1.433208
tr|B3KNE7|B3KNE7_HUMAN	tr|B3KNE7|B3	cDNA FLJ14468 fis, clone MAMMA1000734, highly similar to Translocation protein SEC63 homolog OS=Homo sapiens PE=2 SV=1	48242	0	985	0	37	0	0.04	#DIV/0!	0	0	0	1	0	2	0	2	1.433208
tr|A2A3N8|A2A3N8_HUMAN	ADD1	Adducin 1 (Alpha) OS=Homo sapiens GN=ADD1 PE=4 SV=1	73914	0	972	0	38	0	0.09	#DIV/0!	0	1	0	2	0	2	0	2	1.433208
tr|B3KMS9|B3KMS9_HUMAN	tr|B3KMS9|B	cDNA FLJ12495 fis, clone NT2RM2001652, highly similar to Brefeldin A-inhibited guanine nucleotide-exchange protein 1 OS=Homo sapiens PE=2	78721	0	966	0	38	0	0.08	#DIV/0!	0	0	0	2	0	2	0	2	1.433208
tr|Q59EZ3|Q59EZ3_HUMAN	tr|Q59EZ3|Q5	Insulin-like growth factor 2 receptor variant (Fragment) OS=Homo sapiens PE=2 SV=1	272672	0	965	0	39	0	0.04	#DIV/0!	0	0	0	3	0	2	0	5	1.433208
sp|Q969S3|ZN622_HUMAN	ZNF622	Zinc finger protein 622 OS=Homo sapiens GN=ZNF622 PE=1 SV=1	54808	0	952	0	39	0	0.12	#DIV/0!	0	1	0	2	0	2	0	2	1.433208
tr|E5KLB5|E5KLB5_HUMAN	tr|E5KLB5|E5	DNA ligase OS=Homo sapiens PE=2 SV=1	114317	0	948	0	39	0	0.03	#DIV/0!	0	1	0	1	0	2	0	2	1.433208
tr|B2RDV7|B2RDV7_HUMAN	tr|B2RDV7|B2	cDNA, FLJ96789, highly similar to Homo sapiens dihydrouridine synthase 3-like (S. cerevisiae) (DUS3L), mRNA OS=Homo sapiens PE=2 SV=1	73714	0	946	0	39	0	0.09	#DIV/0!	0	0	0	2	0	2	0	2	1.433208
tr|D3DU41|D3DU41_HUMAN	LUC7L	LUC7-like (S. cerevisiae), isoform CRA_a OS=Homo sapiens GN=LUC7L PE=2 SV=1	34110	0	945	0	39	0	0.21	#DIV/0!	0	1	0	2	0	2	0	2	1.433208
sp|P09497|CLCB_HUMAN	CLTB	Clathrin light chain B OS=Homo sapiens GN=CLTB PE=1 SV=1	25289	0	940	0	40	0	0.13	#DIV/0!	0	1	0	1	0	2	0	2	1.433208
tr|Q6NUR1|Q6NUR1_HUMAN	NCAPG	Non-SMC condensin I complex, subunit G OS=Homo sapiens GN=NCAPG PE=2 SV=1	115205	0	939	0	40	0	0.06	#DIV/0!	0	0	0	2	0	2	0	2	1.433208
sp|Q86TU7|SETD3_HUMAN	SETD3	Histone-lysine N-methyltransferase setd3 OS=Homo sapiens GN=SETD3 PE=1 SV=1	67557	0	926	0	41	0	0.05	#DIV/0!	0	1	0	1	0	2	0	2	1.433208
sp|Q9NP92|RT30_HUMAN	MRPS30	28S ribosomal protein S30, mitochondrial OS=Homo sapiens GN=MRPS30 PE=1 SV=2	50960	0	921	0	41	0	0.13	#DIV/0!	0	1	0	2	0	2	0	3	1.433208
tr|Q8N5A0|Q8N5A0_HUMAN	EIF5B	Eukaryotic translation initiation factor 5B OS=Homo sapiens GN=EIF5B PE=2 SV=1	139172	0	918	0	41	0	0.02	#DIV/0!	0	1	0	1	0	2	0	2	1.433208
tr|C0JSV2|C0JSV2_HUMAN	HLA-G	MHC class I antigen (Fragment) OS=Homo sapiens GN=HLA-G PE=2 SV=1	31871	0	916	0	42	0	0.1	#DIV/0!	0	1	0	1	0	2	0	2	1.433208
tr|B4DMA4|B4DMA4_HUMAN	tr|B4DMA4|B	cDNA FLJ58400, highly similar to Cleavage stimulation factor 77 kDa subunit OS=Homo sapiens PE=2 SV=1	75580	0	904	0	42	0	0.09	#DIV/0!	0	0	0	2	0	2	0	2	1.433208
tr|B7Z1V5|B7Z1V5_HUMAN	tr|B7Z1V5|B7	cDNA FLJ55678, highly similar to Syntaxin-binding protein 1 OS=Homo sapiens PE=2 SV=1	63779	0	890	0	44	0	0.05	#DIV/0!	0	1	0	1	0	2	0	2	1.433208
sp|Q8WX93|PALLD_HUMAN	PALLD	Palladin OS=Homo sapiens GN=PALLD PE=1 SV=3	151839	0	886	0	44	0	0.02	#DIV/0!	0	1	0	1	0	2	0	2	1.433208
tr|H0YL11|H0YL11_HUMAN	IDH2	Isocitrate dehydrogenase [NADP], mitochondrial OS=Homo sapiens GN=IDH2 PE=3 SV=1	35536	0	882	0	26	0	0.23	#DIV/0!	0	0	0	1	0	2	0	3	1.433208
tr|F5H1X8|F5H1X8_HUMAN	LRBA	Lipopolysaccharide-responsive and beige-like anchor protein OS=Homo sapiens GN=LRBA PE=2 SV=1	320211	0	878	0	45	0	0.01	#DIV/0!	0	1	0	1	0	2	0	3	1.433208
tr|B3KMQ6|B3KMQ6_HUMAN	tr|B3KMQ6|B	cDNA FLJ12172 fis, clone MAMMA1000684, highly similar to Opioid growth factor receptor OS=Homo sapiens PE=2 SV=1	73894	0	875	0	45	0	0.09	#DIV/0!	0	1	0	2	0	2	0	2	1.433208
tr|B7Z4U7|B7Z4U7_HUMAN	SCFD1	Sec1 family domain containing 1, isoform CRA_b OS=Homo sapiens GN=SCFD1 PE=2 SV=1	65362	0	873	0	45	0	0.1	#DIV/0!	0	1	0	2	0	2	0	2	1.433208
tr|B4DKU3|B4DKU3_HUMAN	tr|B4DKU3|B	cDNA FLJ53449, highly similar to rRNA methyltransferase 3 (EC 2.1.1.-) OS=Homo sapiens PE=2 SV=1	82984	0	872	0	45	0	0.04	#DIV/0!	0	1	0	1	0	2	0	2	1.433208
sp|P63096|GNAI1_HUMAN	GNAI1	Guanine nucleotide-binding protein G(i) subunit alpha-1 OS=Homo sapiens GN=GNAI1 PE=1 SV=2	40905	0	870	0	45	0	2.14	#DIV/0!	0	2	0	2	0	2	0	2	1.433208
tr|B4DNM8|B4DNM8_HUMAN	tr|B4DNM8|B	cDNA FLJ53395, highly similar to Prolyl 3-hydroxylase 1 (EC 1.14.11.7) OS=Homo sapiens PE=2 SV=1	69572	0	863	0	46	0	0.1	#DIV/0!	0	1	0	2	0	2	0	2	1.433208
tr|G3V198|G3V198_HUMAN	NUP160	Nuclear pore complex protein Nup160 OS=Homo sapiens GN=NUP160 PE=2 SV=1	142365	0	859	0	46	0	0.05	#DIV/0!	0	1	0	2	0	2	0	2	1.433208
tr|B4DG89|B4DG89_HUMAN	NPLOC4	Nuclear protein localization protein 4 homolog OS=Homo sapiens GN=NPLOC4 PE=2 SV=1	52144	0	854	0	0	0	0.05	#DIV/0!	0	0	0	0	0	2	0	2	1.433208
tr|B4DZ54|B4DZ54_HUMAN	tr|B4DZ54|B4	cDNA FLJ61492, highly similar to Homo sapiens ubiquitin associated protein 2 (UBAP2), transcript variant 1, mRNA OS=Homo sapiens PE=2 SV=1	108020	0	852	0	47	0	0.06	#DIV/0!	0	0	0	2	0	2	0	3	1.433208
tr|B4E1K7|B4E1K7_HUMAN	STOML2	Stomatin-like protein 2 OS=Homo sapiens GN=STOML2 PE=2 SV=1	33317	0	850	0	47	0	0.21	#DIV/0!	0	1	0	2	0	2	0	2	1.433208
sp|Q96T76|MMS19_HUMAN	MMS19	MMS19 nucleotide excision repair protein homolog OS=Homo sapiens GN=MMS19 PE=1 SV=2	114928	0	849	0	47	0	0.06	#DIV/0!	0	1	0	2	0	2	0	2	1.433208
tr|D3DSQ7|D3DSQ7_HUMAN	XPO7	Exportin 7, isoform CRA_c OS=Homo sapiens GN=XPO7 PE=2 SV=1	100370	0	841	0	47	0	0.07	#DIV/0!	0	2	0	2	0	2	0	2	1.433208
tr|B3KRB2|B3KRB2_HUMAN	tr|B3KRB2|B3	cDNA FLJ33964 fis, clone CTONG2019029, highly similar to Pseudouridylate synthase 7 homolog (EC 5.4.99.-) OS=Homo sapiens PE=2 SV=1	75303	0	827	0	28	0	0.21	#DIV/0!	0	0	0	2	0	2	0	2	1.433208
sp|Q9BR76|COR1B_HUMAN	CORO1B	Coronin-1B OS=Homo sapiens GN=CORO1B PE=1 SV=1	54885	0	822	0	49	0	0.12	#DIV/0!	0	1	0	2	0	2	0	3	1.433208
sp|Q2M389|WASH7_HUMAN	KIAA1033	WASH complex subunit 7 OS=Homo sapiens GN=KIAA1033 PE=1 SV=2	137343	0	819	0	50	0	0.05	#DIV/0!	0	1	0	2	0	2	0	2	1.433208
tr|A8K4W5|A8K4W5_HUMAN	tr|A8K4W5|A	cDNA FLJ76813, highly similar to Homo sapiens acetyl-Coenzyme A acetyltransferase 2 (acetoacetyl Coenzyme A thiolase), mRNA OS=Homo sap	41866	0	808	0	51	0	0.15	#DIV/0!	0	1	0	2	0	2	0	2	1.433208
tr|H3BM93|H3BM93_HUMAN	NUP93	Nuclear pore complex protein Nup93 (Fragment) OS=Homo sapiens GN=NUP93 PE=4 SV=1	19858	0	803	0	51	0	0.6	#DIV/0!	0	2	0	3	0	2	0	3	1.433208
tr|B4DQ15|B4DQ15_HUMAN	tr|B4DQ15|B	cDNA FLJ51525, highly similar to Homo sapiens aldehyde dehydrogenase 16 family, member A1 (ALDH16A1), mRNA OS=Homo sapiens PE=2 SV	67899	0	798	0	52	0	0.1	#DIV/0!	0	1	0	2	0	2	0	3	1.433208
sp|Q8N1G4|LRC47_HUMAN	LRRC47	Leucine-rich repeat-containing protein 47 OS=Homo sapiens GN=LRRC47 PE=1 SV=1	64004	0	797	0	52	0	0.11	#DIV/0!	0	1	0	2	0	2	0	2	1.433208
tr|H3BRI8|H3BRI8_HUMAN	NUP93	Nuclear pore complex protein Nup93 (Fragment) OS=Homo sapiens GN=NUP93 PE=4 SV=1	17024	0	790	0	52	0	0.44	#DIV/0!	0	2	0	2	0	2	0	2	1.433208
tr|B2R5S4|B2R5S4_HUMAN	tr|B2R5S4|B2	cDNA, FLJ92598, highly similar to Homo sapiens polymerase (DNA directed), delta 2, regulatory subunit 50kDa (POLD2), mRNA OS=Homo sapien	51914	0	777	0	53	0	0.06	#DIV/0!	0	1	0	1	0	2	0	2	1.433208
tr|A8K2M0|A8K2M0_HUMAN	PSMC4	Proteasome (Prosome, macropain) 26S subunit, ATPase, 4, isoform CRA_b OS=Homo sapiens GN=PSMC4 PE=2 SV=1	47451	0	775	0	53	0	0.14	#DIV/0!	0	2	0	2	0	2	0	2	1.433208
tr|B0QZ18|B0QZ18_HUMAN	CPNE1	Copine I OS=Homo sapiens GN=CPNE1 PE=2 SV=1	60307	0	772	0	54	0	0.11	#DIV/0!	0	1	0	2	0	2	0	2	1.433208
tr|B4DH22|B4DH22_HUMAN	tr|B4DH22|B	cDNA FLJ59884, highly similar to Secernin-1 OS=Homo sapiens PE=2 SV=1	25067	0	763	0	54	0	0.28	#DIV/0!	0	1	0	2	0	2	0	2	1.433208
tr|G5E988|G5E988_HUMAN	AP3D1	AP-3 complex subunit delta-1 OS=Homo sapiens GN=AP3D1 PE=2 SV=1	126074	0	762	0	54	0	0.05	#DIV/0!	0	1	0	2	0	2	0	2	1.433208
sp|P51398|RT29_HUMAN	DAP3	28S ribosomal protein S29, mitochondrial OS=Homo sapiens GN=DAP3 PE=1 SV=1	45880	0	756	0	55	0	0.15	#DIV/0!	0	1	0	2	0	2	0	2	1.433208
tr|B4DRK5|B4DRK5_HUMAN	tr|B4DRK5|B4	cDNA FLJ59584, highly similar to Mitochondrial-processing peptidase alpha subunit, mitochondrial (EC 3.4.24.64) OS=Homo sapiens PE=2 SV=1	28622	0	742	0	58	0	0.25	#DIV/0!	0	1	0	2	0	2	0	2	1.433208
tr|Q6YPB0|Q6YPB0_HUMAN	tr|Q6YPB0|Q	Endoplasmic reticulum DnaJ/PDI fusion protein 3 OS=Homo sapiens PE=4 SV=1	38170	0	741	0	58	0	0.18	#DIV/0!	0	1	0	2	0	2	0	2	1.433208
sp|Q9UHX1|PUF60_HUMAN	PUF60	Poly(U)-binding-splicing factor PUF60 OS=Homo sapiens GN=PUF60 PE=1 SV=1	60009	0	738	0	59	0	0.11	#DIV/0!	0	2	0	2	0	2	0	2	1.433208
tr|Q5W1M0|Q5W1M0_HUMAN	HLA-C	MHC class I antigen (Fragment) OS=Homo sapiens GN=HLA-C PE=2 SV=1	10534	0	735	0	52	0	0.26	#DIV/0!	0	1	0	1	0	2	0	2	1.433208
tr|G3V0G3|G3V0G3_HUMAN	DDX56	DEAD (Asp-Glu-Ala-Asp) box polypeptide 56, isoform CRA_a OS=Homo sapiens GN=DDX56 PE=2 SV=1	41969	0	726	0	60	0	0.08	#DIV/0!	0	1	0	1	0	2	0	2	1.433208
tr|B4E0H3|B4E0H3_HUMAN	tr|B4E0H3|B4	cDNA FLJ53386, highly similar to Signal recognition particle receptor subunit alpha OS=Homo sapiens PE=2 SV=1	66987	0	722	0	60	0	0.1	#DIV/0!	0	1	0	2	0	2	0	2	1.433208
tr|H3BUQ2|H3BUQ2_HUMAN	OGFOD1	2-oxoglutarate and iron-dependent oxygenase domain-containing protein 1 OS=Homo sapiens GN=OGFOD1 PE=4 SV=1	58798	0	713	0	61	0	0.11	#DIV/0!	0	2	0	2	0	2	0	2	1.433208
tr|B7Z5X0|B7Z5X0_HUMAN	tr|B7Z5X0|B7	cDNA FLJ57183, highly similar to Ubiquitin-like 1-activating enzyme E1B OS=Homo sapiens PE=2 SV=1	33044	0	712	0	61	0	0.21	#DIV/0!	0	2	0	2	0	2	0	3	1.433208
tr|Q05CN7|Q05CN7_HUMAN	GTF3C4	GTF3C4 protein (Fragment) OS=Homo sapiens GN=GTF3C4 PE=2 SV=1	63439	0	707	0	62	0	0.11	#DIV/0!	0	1	0	2	0	2	0	2	1.433208
tr|B7Z475|B7Z475_HUMAN	tr|B7Z475|B7	cDNA FLJ55712, highly similar to F-box-like/WD repeat protein TBL1XR1 OS=Homo sapiens PE=2 SV=1	41301	0	694	0	64	0	0.17	#DIV/0!	0	1	0	2	0	2	0	2	1.433208
tr|A8K9B9|A8K9B9_HUMAN	tr|A8K9B9|A8	cDNA FLJ77391, highly similar to Homo sapiens EH-domain containing 4 (EHD4), mRNA OS=Homo sapiens PE=2 SV=1	61346	0	687	0	65	0	0.11	#DIV/0!	0	2	0	2	0	2	0	2	1.433208
tr|H3BLU7|H3BLU7_HUMAN	AKR7A2	Aflatoxin B1 aldehyde reductase member 2 (Fragment) OS=Homo sapiens GN=AKR7A2 PE=4 SV=1	35004	0	685	0	65	0	0.2	#DIV/0!	0	1	0	2	0	2	0	3	1.433208
tr|E3Q0X1|E3Q0X1_HUMAN	HLA-B	MHC class I antigen (Fragment) OS=Homo sapiens GN=HLA-B PE=3 SV=1	31939	0	661	0	68	0	0.1	#DIV/0!	0	1	0	1	0	2	0	2	1.433208
tr|F4NBU7|F4NBU7_HUMAN	HLA-B	MHC class I antigen (Fragment) OS=Homo sapiens GN=HLA-B PE=3 SV=1	38476	0	660	0	68	0	0.09	#DIV/0!	0	1	0	1	0	2	0	3	1.433208
sp|Q9H3U1|UN45A_HUMAN	UNC45A	Protein unc-45 homolog A OS=Homo sapiens GN=UNC45A PE=1 SV=1	104266	0	659	0	68	0	0.06	#DIV/0!	0	2	0	2	0	2	0	2	1.433208
tr|Q0VAM0|Q0VAM0_HUMAN	CTNNBL1	Catenin, beta like 1 OS=Homo sapiens GN=CTNNBL1 PE=2 SV=1	65560	0	656	0	68	0	0.1	#DIV/0!	0	2	0	2	0	2	0	2	1.433208
tr|F5H6D0|F5H6D0_HUMAN	MOGS	Mannosyl-oligosaccharide glucosidase OS=Homo sapiens GN=MOGS PE=2 SV=1	80824	0	655	0	50	0	0.07	#DIV/0!	0	1	0	1	0	2	0	3	1.433208
tr|B2R9S4|B2R9S4_HUMAN	tr|B2R9S4|B2	cDNA, FLJ94534, highly similar to Homo sapiens capping protein (actin filament), gelsolin-like(CAPG), mRNA OS=Homo sapiens PE=2 SV=1	38759	0	643	0	70	0	0.18	#DIV/0!	0	1	0	2	0	2	0	3	1.433208
tr|Q6IBU0|Q6IBU0_HUMAN	EIF5	EIF5 protein OS=Homo sapiens GN=EIF5 PE=2 SV=1	49577	0	642	0	70	0	0.14	#DIV/0!	0	1	0	2	0	2	0	2	1.433208
tr|B2RD79|B2RD79_HUMAN	tr|B2RD79|B2	Ubiquitin carboxyl-terminal hydrolase OS=Homo sapiens PE=2 SV=1	56462	0	641	0	71	0	0.12	#DIV/0!	0	1	0	2	0	2	0	2	1.433208
tr|Q7Z586|Q7Z586_HUMAN	tr|Q7Z586|Q	Putative uncharacterized protein (Fragment) OS=Homo sapiens PE=2 SV=1	105456	0	633	0	71	0	0.07	#DIV/0!	0	1	0	2	0	2	0	2	1.433208
tr|A8K6V7|A8K6V7_HUMAN	tr|A8K6V7|A8	cDNA FLJ76053, highly similar to Homo sapiens Ras-GTPase activating protein SH3 domain-binding protein 2 (G3BP2), transcript variant 3, mRN	50842	0	625	0	73	0	0.13	#DIV/0!	0	1	0	2	0	2	0	3	1.433208
tr|E9PDU6|E9PDU6_HUMAN	CNN3	Calponin-3 (Fragment) OS=Homo sapiens GN=CNN3 PE=2 SV=1	20309	0	621	0	57	0	0.35	#DIV/0!	0	2	0	2	0	2	0	2	1.433208
tr|G3V3B0|G3V3B0_HUMAN	ACIN1	Apoptotic chromatin condensation inducer in the nucleus OS=Homo sapiens GN=ACIN1 PE=2 SV=1	67683	0	614	0	74	0	0.05	#DIV/0!	0	2	0	2	0	2	0	2	1.433208
tr|Q548W1|Q548W1_HUMAN	OGT	HRNT1 OS=Homo sapiens GN=OGT PE=2 SV=1	104029	0	610	0	75	0	0.06	#DIV/0!	0	1	0	2	0	2	0	2	1.433208
tr|H0Y4J2|H0Y4J2_HUMAN	MRPL37	39S ribosomal protein L37, mitochondrial (Fragment) OS=Homo sapiens GN=MRPL37 PE=4 SV=1	29806	0	602	0	58	0	0.22	#DIV/0!	0	1	0	2	0	2	0	2	1.433208
tr|A8K4H7|A8K4H7_HUMAN	PPAT	Amidophosphoribosyltransferase OS=Homo sapiens GN=PPAT PE=2 SV=1	58161	0	600	0	76	0	0.12	#DIV/0!	0	2	0	2	0	2	0	2	1.433208
tr|Q5VU21|Q5VU21_HUMAN	SERBP1	PAI-1 mRNA-binding protein variant OS=Homo sapiens GN=SERBP1 PE=2 SV=1	42458	0	596	0	77	0	0.14	#DIV/0!	0	1	0	2	0	2	0	3	1.433208
tr|Q6IBS5|Q6IBS5_HUMAN	DLST	DLST protein OS=Homo sapiens GN=DLST PE=2 SV=1	49131	0	591	0	77	0	0.14	#DIV/0!	0	1	0	2	0	2	0	3	1.433208
tr|Q6ZVI6|Q6ZVI6_HUMAN	tr|Q6ZVI6|Q6	cDNA FLJ42537 fis, clone BRACE3004058, highly similar to NADH-cytochrome B5 reductase (EC 1.6.2.2) OS=Homo sapiens PE=2 SV=1	29141	0	589	0	78	0	0.24	#DIV/0!	0	1	0	2	0	2	0	2	1.433208
tr|B4DIT7|B4DIT7_HUMAN	TGM2	Protein-glutamine gamma-glutamyltransferase 2 OS=Homo sapiens GN=TGM2 PE=2 SV=1	69632	0	583	0	78	0	0.1	#DIV/0!	0	2	0	2	0	2	0	2	1.433208
tr|C7FDR3|C7FDR3_HUMAN	HLA-B	MHC class I antigen (Fragment) OS=Homo sapiens GN=HLA-B PE=3 SV=1	21132	0	576	0	79	0	0.34	#DIV/0!	0	2	0	2	0	2	0	2	1.433208
tr|B4E206|B4E206_HUMAN	tr|B4E206|B4	cDNA FLJ55441, highly similar to Nuclear pore complex protein Nup133 OS=Homo sapiens PE=2 SV=1	128349	0	569	0	79	0	0.05	#DIV/0!	0	2	0	2	0	2	0	2	1.433208
sp|Q5T8P6|RBM26_HUMAN	RBM26	RNA-binding protein 26 OS=Homo sapiens GN=RBM26 PE=1 SV=3	113927	0	564	0	80	0	0.06	#DIV/0!	0	1	0	2	0	2	0	2	1.433208
sp|Q13619|CUL4A_HUMAN	CUL4A	Cullin-4A OS=Homo sapiens GN=CUL4A PE=1 SV=3	88138	0	560	0	82	0	0.09	#DIV/0!	0	2	0	2	0	2	0	2	1.433208
sp|Q9UPT8|ZC3H4_HUMAN	ZC3H4	Zinc finger CCCH domain-containing protein 4 OS=Homo sapiens GN=ZC3H4 PE=1 SV=3	140797	0	559	0	82	0	0.05	#DIV/0!	0	2	0	2	0	2	0	2	1.433208
tr|B0I1S9|B0I1S9_HUMAN	MYO1B	MYO1B variant protein OS=Homo sapiens GN=MYO1B PE=2 SV=1	132928	0	556	0	83	0	0.05	#DIV/0!	0	1	0	2	0	2	0	2	1.433208
tr|B4DWI8|B4DWI8_HUMAN	tr|B4DWI8|B4	cDNA FLJ57805, highly similar to Homo sapiens paraspeckle component 1 (PSPC1), transcript variant alpha, mRNA OS=Homo sapiens PE=2 SV=1	52660	0	553	0	83	0	0.13	#DIV/0!	0	2	0	2	0	2	0	2	1.433208
sp|P55268|LAMB2_HUMAN	LAMB2	Laminin subunit beta-2 OS=Homo sapiens GN=LAMB2 PE=1 SV=2	202982	0	551	0	83	0	0.03	#DIV/0!	0	1	0	2	0	2	0	3	1.433208
tr|B4DFP1|B4DFP1_HUMAN	tr|B4DFP1|B4	cDNA FLJ51818, highly similar to Phosphoglucomutase-1 (EC 5.4.2.2) OS=Homo sapiens PE=2 SV=1	58987	0	545	0	53	0	0.17	#DIV/0!	0	2	0	2	0	2	0	3	1.433208
sp|Q92888|ARHG1_HUMAN	ARHGEF1	Rho guanine nucleotide exchange factor 1 OS=Homo sapiens GN=ARHGEF1 PE=1 SV=2	103056	0	543	0	85	0	0.06	#DIV/0!	0	2	0	2	0	2	0	2	1.433208
tr|Q56VW8|Q56VW8_HUMAN	tr|Q56VW8|Q	Lung cancer-related protein 9 OS=Homo sapiens PE=2 SV=1	52186	0	533	0	74	0	0.28	#DIV/0!	0	1	0	3	0	2	0	3	1.433208
sp|P49792|RBP2_HUMAN	RANBP2	E3 SUMO-protein ligase RanBP2 OS=Homo sapiens GN=RANBP2 PE=1 SV=2	362365	0	528	0	88	0	0.02	#DIV/0!	0	2	0	2	0	2	0	2	1.433208
sp|P09960|LKHA4_HUMAN	LTA4H	Leukotriene A-4 hydrolase OS=Homo sapiens GN=LTA4H PE=1 SV=2	69868	0	527	0	89	0	0.1	#DIV/0!	0	2	0	2	0	2	0	3	1.433208
tr|B3KQ43|B3KQ43_HUMAN	tr|B3KQ43|B3	cDNA FLJ32791 fis, clone TESTI2002365, highly similar to helicase MOV-10 (EC 3.6.1.-) OS=Homo sapiens PE=2 SV=1	47208	0	516	0	73	0	0.06	#DIV/0!	0	1	0	1	0	2	0	2	1.433208
tr|B2R806|B2R806_HUMAN	tr|B2R806|B2	Eukaryotic translation initiation factor 3 subunit E OS=Homo sapiens PE=2 SV=1	52622	0	514	0	25	0	0.13	#DIV/0!	0	0	0	1	0	2	0	3	1.433208
tr|I6L9E8|I6L9E8_HUMAN	FAM98A	Family with sequence similarity 98, member A OS=Homo sapiens GN=FAM98A PE=2 SV=1	55595	0	511	0	94	0	0.2	#DIV/0!	0	1	0	2	0	2	0	2	1.433208
tr|Q5T0F3|Q5T0F3_HUMAN	GNL2	Guanine nucleotide binding protein-like 2 (Nucleolar) OS=Homo sapiens GN=GNL2 PE=2 SV=1	83831	0	502	0	97	0	0.08	#DIV/0!	0	2	0	2	0	2	0	2	1.433208
tr|H0YCK7|H0YCK7_HUMAN	EEF1D	Elongation factor 1-delta (Fragment) OS=Homo sapiens GN=EEF1D PE=4 SV=1	23105	0	480	0	102	0	0.09	#DIV/0!	0	2	0	2	0	2	0	2	1.433208
sp|Q7Z5L9|I2BP2_HUMAN	IRF2BP2	Interferon regulatory factor 2-binding protein 2 OS=Homo sapiens GN=IRF2BP2 PE=1 SV=2	61728	0	475	0	103	0	0.11	#DIV/0!	0	1	0	2	0	2	0	3	1.433208
sp|O15230|LAMA5_HUMAN	LAMA5	Laminin subunit alpha-5 OS=Homo sapiens GN=LAMA5 PE=1 SV=8	412023	0	459	0	106	0	0.02	#DIV/0!	0	1	0	2	0	2	0	2	1.433208
tr|A0JLQ5|A0JLQ5_HUMAN	BXDC2	BXDC2 protein (Fragment) OS=Homo sapiens GN=BXDC2 PE=2 SV=1	39430	0	450	0	55	0	0.36	#DIV/0!	0	2	0	2	0	2	0	2	1.433208
tr|H0YEL7|H0YEL7_HUMAN	ACAT1	Acetyl-CoA acetyltransferase, mitochondrial (Fragment) OS=Homo sapiens GN=ACAT1 PE=3 SV=1	14377	0	444	0	98	0	0.23	#DIV/0!	0	1	0	2	0	2	0	2	1.433208
tr|B2RBB2|B2RBB2_HUMAN	tr|B2RBB2|B2	cDNA, FLJ95416, highly similar to Homo sapiens phosphatidylinositol glycan, class S (PIGS), mRNA OS=Homo sapiens PE=2 SV=1	61818	0	439	0	111	0	0.11	#DIV/0!	0	1	0	2	0	2	0	2	1.433208
tr|E9PDG5|E9PDG5_HUMAN	LARP4	La-related protein 4 OS=Homo sapiens GN=LARP4 PE=2 SV=1	50319	0	433	0	113	0	0.14	#DIV/0!	0	2	0	2	0	2	0	2	1.433208
tr|B4DX99|B4DX99_HUMAN	tr|B4DX99|B4	Malic enzyme OS=Homo sapiens PE=2 SV=1	45378	0	430	0	60	0	0.22	#DIV/0!	0	1	0	2	0	2	0	2	1.433208
sp|P04899|GNAI2_HUMAN	GNAI2	Guanine nucleotide-binding protein G(i) subunit alpha-2 OS=Homo sapiens GN=GNAI2 PE=1 SV=3	40995	0	416	0	117	0	0.18	#DIV/0!	0	2	0	2	0	2	0	2	1.433208
sp|Q9H3P7|GCP60_HUMAN	ACBD3	Golgi resident protein GCP60 OS=Homo sapiens GN=ACBD3 PE=1 SV=4	60841	0	411	0	117	0	0.11	#DIV/0!	0	2	0	2	0	2	0	2	1.433208
sp|Q96S66|CLCC1_HUMAN	CLCC1	Chloride channel CLIC-like protein 1 OS=Homo sapiens GN=CLCC1 PE=1 SV=1	62667	0	409	0	117	0	0.11	#DIV/0!	0	2	0	2	0	2	0	2	1.433208
sp|P51116|FXR2_HUMAN	FXR2	Fragile X mental retardation syndrome-related protein 2 OS=Homo sapiens GN=FXR2 PE=1 SV=2	74520	0	372	0	134	0	0.09	#DIV/0!	0	2	0	2	0	2	0	4	1.433208
tr|F5H583|F5H583_HUMAN	CYFIP2	Cytoplasmic FMR1-interacting protein 2 OS=Homo sapiens GN=CYFIP2 PE=2 SV=1	138666	0	1273	0	16	0	0.02	#DIV/0!	0	0	0	1	0	1	0	2	0.696063
tr|D3DWB6|D3DWB6_HUMAN	USP9X	Ubiquitin carboxyl-terminal hydrolase OS=Homo sapiens GN=USP9X PE=2 SV=1	274331	0	1265	0	18	0	0.01	#DIV/0!	0	0	0	1	0	1	0	3	0.696063

tr|Q0VF97|Q0VF97_HUMAN	ITGB4	Integrin beta OS=Homo sapiens GN=ITGB4 PE=2 SV=1	198538	0	1243	0	20	0	0.02	#DIV/0!	0	0	0	1	0	1	0	2	0.696063
tr|B4DF22|B4DF22_HUMAN	tr|B4DF22|B4	cDNA FLJ55615, highly similar to SWI/SNF-related matrix-associatedactin-dependent regulator of chromatin subfamily C member 2 OS=Homo sa	112428	0	1238	0	20	0	0.03	#DIV/0!	0	0	0	1	0	1	0	3	0.696063
tr|B2RE56|B2RE56_HUMAN	tr|B2RE56|B2	Peptidyl-prolyl cis-trans isomerase OS=Homo sapiens PE=2 SV=1	18225	0	1235	0	20	0	0.19	#DIV/0!	0	0	0	1	0	1	0	2	0.696063
tr|D3DXC9|D3DXC9_HUMAN	SHMT1	Serine hydroxymethyltransferase OS=Homo sapiens GN=SHMT1 PE=3 SV=1	49601	0	1233	0	20	0	0.07	#DIV/0!	0	0	0	1	0	1	0	1	0.696063
sp|Q96J94|PIWL1_HUMAN	PIWIL1	Piwi-like protein 1 OS=Homo sapiens GN=PIWIL1 PE=1 SV=1	99453	0	1232	0	21	0	0.03	#DIV/0!	0	0	0	1	0	1	0	1	0.696063
sp|B2RTY4|MYO9A_HUMAN	MYO9A	Unconventional myosin-IXa OS=Homo sapiens GN=MYO9A PE=1 SV=2	294917	0	1231	0	21	0	0.01	#DIV/0!	0	0	0	1	0	1	0	1	0.696063
tr|B3KTK4|B3KTK4_HUMAN	tr|B3KTK4|B3	cDNA FLJ38410 fis, clone FEBRA2009289, highly similar to Contactin-4 OS=Homo sapiens PE=2 SV=1	78377	0	1230	0	21	0	0.04	#DIV/0!	0	0	0	1	0	1	0	1	0.696063
tr|B0AZU2|B0AZU2_HUMAN	KIAA0930	Uncharacterized protein KIAA0930 OS=Homo sapiens GN=KIAA0930 PE=2 SV=1	43985	0	1229	0	21	0	0.08	#DIV/0!	0	0	0	1	0	1	0	1	0.696063
tr|H0YHA8|H0YHA8_HUMAN	NAV3	Neuron navigator 3 (Fragment) OS=Homo sapiens GN=NAV3 PE=4 SV=1	136766	0	1228	0	21	0	0.01	#DIV/0!	0	0	0	1	0	1	0	1	0.696063
tr|B3KNV9|B3KNV9_HUMAN	tr|B3KNV9|B3	cDNA FLJ30554 fis, clone BRAWH2003693 OS=Homo sapiens PE=2 SV=1	112670	0	1226	0	21	0	0.03	#DIV/0!	0	0	0	1	0	1	0	1	0.696063
sp|Q14684|RRP1B_HUMAN	RRP1B	Ribosomal RNA processing protein 1 homolog B OS=Homo sapiens GN=RRP1B PE=1 SV=3	84774	0	1225	0	21	0	0.04	#DIV/0!	0	0	0	1	0	1	0	1	0.696063
sp|Q9UBG0|MRC2_HUMAN	MRC2	C-type mannose receptor 2 OS=Homo sapiens GN=MRC2 PE=1 SV=2	169761	0	1224	0	21	0	0.02	#DIV/0!	0	0	0	1	0	1	0	1	0.696063
tr|H0YL72|H0YL72_HUMAN	IDH3A	Isocitrate dehydrogenase [NAD] subunit alpha, mitochondrial OS=Homo sapiens GN=IDH3A PE=3 SV=1	36162	0	1223	0	21	0	0.09	#DIV/0!	0	0	0	1	0	1	0	1	0.696063
sp|A4D1P6|WDR91_HUMAN	WDR91	WD repeat-containing protein 91 OS=Homo sapiens GN=WDR91 PE=1 SV=2	84204	0	1221	0	21	0	0.04	#DIV/0!	0	0	0	1	0	1	0	1	0.696063
tr|B2RBM8|B2RBM8_HUMAN	tr|B2RBM8|B	cDNA, FLJ95596, highly similar to Homo sapiens activity-dependent neuroprotector (ADNP), mRNA OS=Homo sapiens PE=2 SV=1	124850	0	1220	0	21	0	0.03	#DIV/0!	0	0	0	1	0	1	0	1	0.696063
tr|B3KU23|B3KU23_HUMAN	tr|B3KU23|B3	cDNA FLJ39097 fis, clone NTONG2000977, highly similar to Neurolysin, mitochondrial (EC 3.4.24.16) OS=Homo sapiens PE=2 SV=1	70320	0	1219	0	21	0	0.05	#DIV/0!	0	0	0	1	0	1	0	1	0.696063
tr|E5RG87|E5RG87_HUMAN	SLC30A8	Zinc transporter 8 (Fragment) OS=Homo sapiens GN=SLC30A8 PE=2 SV=1	9658	0	1218	0	22	0	0.36	#DIV/0!	0	0	0	1	0	1	0	1	0.696063
tr|A8K5Y1|A8K5Y1_HUMAN	tr|A8K5Y1|A8	Microtubule-associated protein OS=Homo sapiens PE=2 SV=1	49596	0	1217	0	22	0	0.07	#DIV/0!	0	0	0	1	0	1	0	1	0.696063
sp|Q9UBE0|SAE1_HUMAN	SAE1	SUMO-activating enzyme subunit 1 OS=Homo sapiens GN=SAE1 PE=1 SV=1	38882	0	1216	0	22	0	0.18	#DIV/0!	0	1	0	2	0	1	0	2	0.696063
tr|A7VJC9|A7VJC9_HUMAN	STP	Salt-tolerant protein OS=Homo sapiens GN=STP PE=2 SV=1	62769	0	1215	0	22	0	0.05	#DIV/0!	0	0	0	1	0	1	0	1	0.696063
sp|Q6P1J9|CDC73_HUMAN	CDC73	Parafibromin OS=Homo sapiens GN=CDC73 PE=1 SV=1	60653	0	1213	0	22	0	0.09	#DIV/0!	0	0	0	1	0	1	0	1	0.696063
tr|B4E2N1|B4E2N1_HUMAN	tr|B4E2N1|B4	cDNA FLJ56560, highly similar to Caspase recruitment domain-containing protein 6 OS=Homo sapiens PE=2 SV=1	55261	0	1212	0	22	0	0.06	#DIV/0!	0	1	0	1	0	1	0	1	0.696063
tr|Q9NS29|Q9NS29_HUMAN	tr|Q9NS29|Q	HDCMC04P OS=Homo sapiens PE=2 SV=1	52679	0	1210	0	22	0	0.07	#DIV/0!	0	1	0	1	0	1	0	1	0.696063
sp|Q8IWB1|IPRI_HUMAN	ITPRIP	Inositol 1,4,5-trisphosphate receptor-interacting protein OS=Homo sapiens GN=ITPRIP PE=1 SV=1	62932	0	1209	0	22	0	0.05	#DIV/0!	0	1	0	1	0	1	0	1	0.696063
tr|Q5TDG2|Q5TDG2_HUMAN	HSD3B1	3 beta-hydroxysteroid dehydrogenase/Delta 5-->4-isomerase type 1 OS=Homo sapiens GN=HSD3B1 PE=2 SV=1	42712	0	1208	0	22	0	0.08	#DIV/0!	0	1	0	1	0	1	0	1	0.696063
sp|O95237|LRAT_HUMAN	LRAT	Lecithin retinol acyltransferase OS=Homo sapiens GN=LRAT PE=1 SV=2	25915	0	1206	0	22	0	0.13	#DIV/0!	0	1	0	1	0	1	0	1	0.696063
tr|Q9HBR1|Q9HBR1_HUMAN	tr|Q9HBR1|Q	Putative uncharacterized protein OS=Homo sapiens PE=2 SV=1	51123	0	1205	0	22	0	0.06	#DIV/0!	0	0	0	1	0	1	0	1	0.696063
tr|B4DR60|B4DR60_HUMAN	tr|B4DR60|B4	cDNA FLJ56882, highly similar to Activating signal cointegrator 1 complex subunit 3 (EC 3.6.1.-) (Fragment) OS=Homo sapiens PE=2 SV=1	119789	0	1203	0	23	0	0.03	#DIV/0!	0	0	0	1	0	1	0	1	0.696063
tr|B4DJ81|B4DJ81_HUMAN	NDUFS1	NADH-ubiquinone oxidoreductase 75 kDa subunit, mitochondrial OS=Homo sapiens GN=NDUFS1 PE=2 SV=1	67563	0	1202	0	23	0	0.1	#DIV/0!	0	0	0	2	0	1	0	2	0.696063
tr|B1AMX9|B1AMX9_HUMAN	TRAF2	TNF receptor-associated factor 2 OS=Homo sapiens GN=TRAF2 PE=4 SV=1	47848	0	1201	0	23	0	0.07	#DIV/0!	0	0	0	1	0	1	0	1	0.696063
tr|H3BS29|H3BS29_HUMAN	Sep-01	Septin-1 (Fragment) OS=Homo sapiens GN=SEPT1 PE=3 SV=1	35905	0	1197	0	23	0	0.09	#DIV/0!	0	1	0	1	0	1	0	1	0.696063
sp|O76031|CLPX_HUMAN	CLPX	ATP-dependent Clp protease ATP-binding subunit clpX-like, mitochondrial OS=Homo sapiens GN=CLPX PE=1 SV=2	69922	0	1194	0	24	0	0.06	#DIV/0!	0	0	0	1	0	1	0	1	0.696063
tr|H0YE29|H0YE29_HUMAN	ARHGAP1	Rho GTPase-activating protein 1 (Fragment) OS=Homo sapiens GN=ARHGAP1 PE=4 SV=1	38420	0	1193	0	24	0	0.15	#DIV/0!	0	0	0	1	0	1	0	2	0.696063
sp|Q5D862|FILA2_HUMAN	FLG2	Filaggrin-2 OS=Homo sapiens GN=FLG2 PE=1 SV=1	249296	0	1190	0	24	0	0.01	#DIV/0!	0	1	0	1	0	1	0	1	0.696063
tr|Q9Y656|Q9Y656_HUMAN	tr|Q9Y656|Q	WSB-1 isoform OS=Homo sapiens PE=1 SV=1	25050	0	1189	0	24	0	0.14	#DIV/0!	0	1	0	1	0	1	0	1	0.696063
tr|B7Z871|B7Z871_HUMAN	tr|B7Z871|B7	cDNA FLJ50930, highly similar to Lipoma-preferred partner OS=Homo sapiens PE=2 SV=1	49854	0	1188	0	24	0	0.07	#DIV/0!	0	0	0	1	0	1	0	1	0.696063
tr|F5GYC3|F5GYC3_HUMAN	VPS45	Vacuolar protein sorting-associated protein 45 OS=Homo sapiens GN=VPS45 PE=2 SV=1	50247	0	1187	0	24	0	0.07	#DIV/0!	0	0	0	1	0	1	0	2	0.696063
tr|B7ZLH8|B7ZLH8_HUMAN	EVPL	EVPL protein OS=Homo sapiens GN=EVPL PE=2 SV=1	235047	0	1186	0	24	0	0.01	#DIV/0!	0	0	0	1	0	1	0	1	0.696063
tr|D6R970|D6R970_HUMAN	SUB1	Activated RNA polymerase II transcriptional coactivator p15 OS=Homo sapiens GN=SUB1 PE=2 SV=1	9100	0	1184	0	24	0	0.38	#DIV/0!	0	0	0	1	0	1	0	1	0.696063
tr|B0AZM4|B0AZM4_HUMAN	tr|B0AZM4|B	cDNA, FLJ79464, highly similar to Homo sapiens aquarius (Fragment) OS=Homo sapiens PE=2 SV=1	157764	0	1183	0	24	0	0.02	#DIV/0!	0	0	0	1	0	1	0	1	0.696063
sp|O76003|GLRX3_HUMAN	GLRX3	Glutaredoxin-3 OS=Homo sapiens GN=GLRX3 PE=1 SV=2	37693	0	1182	0	24	0	0.09	#DIV/0!	0	0	0	1	0	1	0	1	0.696063
sp|Q96Q11|TRNT1_HUMAN	TRNT1	CCA tRNA nucleotidyltransferase 1, mitochondrial OS=Homo sapiens GN=TRNT1 PE=1 SV=2	50324	0	1181	0	25	0	0.07	#DIV/0!	0	0	0	1	0	1	0	1	0.696063
tr|Q6FHH6|Q6FHH6_HUMAN	LANCL1	LANCL1 protein (Fragment) OS=Homo sapiens GN=LANCL1 PE=2 SV=1	45979	0	1180	0	25	0	0.07	#DIV/0!	0	0	0	1	0	1	0	1	0.696063
tr|B4DTM6|B4DTM6_HUMAN	tr|B4DTM6|B	cDNA FLJ61391, highly similar to Protein transport protein Sec24B OS=Homo sapiens PE=2 SV=1	130037	0	1179	0	25	0	0.03	#DIV/0!	0	1	0	1	0	1	0	1	0.696063
tr|B3KNJ4|B3KNJ4_HUMAN	SAE1	SUMO-1 activating enzyme subunit 1, isoform CRA_a OS=Homo sapiens GN=SAE1 PE=2 SV=1	33705	0	1178	0	25	0	0.1	#DIV/0!	0	1	0	1	0	1	0	1	0.696063
sp|Q5TFE4|NT5D1_HUMAN	NT5DC1	5'-nucleotidase domain-containing protein 1 OS=Homo sapiens GN=NT5DC1 PE=1 SV=1	52439	0	1177	0	25	0	0.06	#DIV/0!	0	0	0	1	0	1	0	1	0.696063
tr|I3L2E3|I3L2E3_HUMAN	LCMT1	Leucine carboxyl methyltransferase 1 (Fragment) OS=Homo sapiens GN=LCMT1 PE=4 SV=1	10759	0	1176	0	25	0	0.32	#DIV/0!	0	0	0	1	0	1	0	1	0.696063
tr|Q9UQ39|Q9UQ39_HUMAN	tr|Q9UQ39|Q	RNA binding protein (Fragment) OS=Homo sapiens PE=2 SV=1	106629	0	1175	0	25	0	0.03	#DIV/0!	0	0	0	1	0	1	0	2	0.696063
tr|H0Y3Y0|H0Y3Y0_HUMAN	GTF3C5	General transcription factor 3C polypeptide 5 (Fragment) OS=Homo sapiens GN=GTF3C5 PE=4 SV=1	22648	0	1173	0	25	0	0.15	#DIV/0!	0	0	0	1	0	1	0	1	0.696063
tr|Q5SRT3|Q5SRT3_HUMAN	CLIC1	Chloride intracellular channel 1, isoform CRA_a OS=Homo sapiens GN=CLIC1 PE=2 SV=2	27248	0	1172	0	25	0	0.12	#DIV/0!	0	0	0	1	0	1	0	1	0.696063
sp|Q14558|KPRA_HUMAN	PRPSAP1	Phosphoribosyl pyrophosphate synthase-associated protein 1 OS=Homo sapiens GN=PRPSAP1 PE=1 SV=2	39654	0	1170	0	25	0	0.11	#DIV/0!	0	0	0	1	0	1	0	1	0.696063
tr|B2R6E2|B2R6E2_HUMAN	tr|B2R6E2|B2	cDNA, FLJ92910, highly similar to Homo sapiens programmed cell death 4 (neoplastic transformation inhibitor) (PDCD4), transcript variant 1, mF	51998	0	1169	0	25	0	0.06	#DIV/0!	0	0	0	1	0	1	0	1	0.696063
tr|A1A528|A1A528_HUMAN	ZW10	Centromere/kinetochore protein zw10 homolog OS=Homo sapiens GN=ZW10 PE=2 SV=1	77556	0	1168	0	25	0	0.04	#DIV/0!	0	0	0	1	0	1	0	1	0.696063
sp|Q9H1B7|I2BPL_HUMAN	IRF2BPL	Interferon regulatory factor 2-binding protein-like OS=Homo sapiens GN=IRF2BPL PE=1 SV=1	83576	0	1167	0	25	0	0.04	#DIV/0!	0	0	0	1	0	1	0	1	0.696063
sp|Q7Z5H4|VN1R5_HUMAN	VN1R5	Vomeronasal type-1 receptor 5 OS=Homo sapiens GN=VN1R5 PE=2 SV=2	41265	0	1166	0	25	0	0.08	#DIV/0!	0	0	0	1	0	1	0	1	0.696063
sp|Q03169|TNAP2_HUMAN	TNFAIP2	Tumor necrosis factor alpha-induced protein 2 OS=Homo sapiens GN=TNFAIP2 PE=1 SV=2	73015	0	1165	0	25	0	0.34	#DIV/0!	0	0	0	1	0	1	0	1	0.696063
tr|H0YB22|H0YB22_HUMAN	RPS14	40S ribosomal protein S14 (Fragment) OS=Homo sapiens GN=RPS14 PE=4 SV=1	13220	0	1164	0	25	0	0.26	#DIV/0!	0	0	0	1	0	1	0	1	0.696063
tr|Q6ZRP2|Q6ZRP2_HUMAN	tr|Q6ZRP2|Q	cDNA FLJ46208 fis, clone TESTI4010979 OS=Homo sapiens PE=2 SV=1	15703	0	1163	0	26	0	0.22	#DIV/0!	0	0	0	1	0	1	0	1	0.696063
tr|B4DN88|B4DN88_HUMAN	RBMS1	RNA-binding motif, single-stranded-interacting protein 1 OS=Homo sapiens GN=RBMS1 PE=2 SV=1	46168	0	1162	0	26	0	0.17	#DIV/0!	0	0	0	1	0	1	0	1	0.696063
tr|F8VXS0|F8VXS0_HUMAN	tr|F8VXS0|F8	Uncharacterized protein (Fragment) OS=Homo sapiens PE=4 SV=1	25472	0	1161	0	26	0	0.13	#DIV/0!	0	0	0	1	0	1	0	1	0.696063
tr|H3BND4|H3BND4_HUMAN	PDXDC1	Pyridoxal-dependent decarboxylase domain-containing protein 1 OS=Homo sapiens GN=PDXDC1 PE=3 SV=1	89733	0	1160	0	26	0	0.04	#DIV/0!	0	0	0	1	0	1	0	1	0.696063
tr|Q2KQ72|Q2KQ72_HUMAN	tr|Q2KQ72|Q	Chromosome-associated protein E OS=Homo sapiens PE=2 SV=1	86324	0	1157	0	26	0	0.04	#DIV/0!	0	0	0	1	0	1	0	2	0.696063
sp|P83111|LACTB_HUMAN	LACTB	Serine beta-lactamase-like protein LACTB, mitochondrial OS=Homo sapiens GN=LACTB PE=1 SV=2	61111	0	1156	0	26	0	0.05	#DIV/0!	0	0	0	1	0	1	0	2	0.696063
tr|F5H3F6|F5H3F6_HUMAN	BANF2	Barrier-to-autointegration factor-like protein OS=Homo sapiens GN=BANF2 PE=2 SV=1	11517	0	1155	0	26	0	0.3	#DIV/0!	0	1	0	1	0	1	0	1	0.696063
tr|H0Y642|H0Y642_HUMAN	OPN1MW2	Medium-wave-sensitive opsin 1 (Fragment) OS=Homo sapiens GN=OPN1MW2 PE=3 SV=1	18501	0	1151	0	26	0	0.18	#DIV/0!	0	0	0	1	0	1	0	1	0.696063
tr|H0YLV5|H0YLV5_HUMAN	GMPR2	GMP reductase 2 OS=Homo sapiens GN=GMPR2 PE=3 SV=1	36342	0	1150	0	26	0	0.56	#DIV/0!	0	0	0	1	0	1	0	1	0.696063
tr|C0JXB8|C0JXB8_HUMAN	AIP	Aryl hydrocarbon receptor interacting protein OS=Homo sapiens GN=AIP PE=2 SV=1	38041	0	1148	0	27	0	0.09	#DIV/0!	0	0	0	1	0	1	0	1	0.696063
tr|Q5XJ04|Q5XJ04_HUMAN	HMGCS1	3-hydroxy-3-methylglutaryl-Coenzyme A synthase 1 (Soluble) OS=Homo sapiens GN=HMGCS1 PE=2 SV=1	57854	0	1147	0	27	0	0.06	#DIV/0!	0	0	0	1	0	1	0	1	0.696063
tr|B3KMR5|B3KMR5_HUMAN	tr|B3KMR5|B	cDNA FLJ12434 fis, clone NT2RM1000037, highly similar to Homo sapiens KIAA0690 protein OS=Homo sapiens PE=2 SV=1	145007	0	1146	0	27	0	0.05	#DIV/0!	0	1	0	2	0	1	0	3	0.696063
sp|Q9UHN6|TMEM2_HUMAN	TMEM2	Transmembrane protein 2 OS=Homo sapiens GN=TMEM2 PE=1 SV=1	155702	0	1144	0	27	0	0.02	#DIV/0!	0	0	0	1	0	1	0	1	0.696063
tr|F2YGG7|F2YGG7_HUMAN	tr|F2YGG7|F2	Epidermal growth factor receptor OS=Homo sapiens PE=2 SV=1	132588	0	1142	0	27	0	0.02	#DIV/0!	0	0	0	1	0	1	0	1	0.696063
tr|B4DR36|B4DR36_HUMAN	tr|B4DR36|B4	cDNA FLJ56414, highly similar to Homo sapiens proline-, glutamic acid-, leucine-rich protein 1 (PELP1), mRNA OS=Homo sapiens PE=2 SV=1	126101	0	1141	0	27	0	0.31	#DIV/0!	0	0	0	1	0	1	0	1	0.696063
tr|B4DP44|B4DP44_HUMAN	tr|B4DP44|B4	cDNA FLJ60751, highly similar to Protein arginine N-methyltransferase 3 (EC 2.1.1.-) OS=Homo sapiens PE=2 SV=1	52013	0	1140	0	27	0	0.06	#DIV/0!	0	1	0	1	0	1	0	1	0.696063
tr|D3DVR8|D3DVR8_HUMAN	HD	Huntingtin (Huntington disease), isoform CRA_a OS=Homo sapiens GN=HD PE=2 SV=1	291559	0	1135	0	27	0	0.01	#DIV/0!	0	0	0	1	0	1	0	1	0.696063
tr|A6NLD2|A6NLD2_HUMAN	tr|A6NLD2|A6	cDNA FLJ11885 fis, clone HEMBA1007203, highly similar to Transmembrane GTPase MFN2 (EC 3.6.5.-) OS=Homo sapiens PE=2 SV=4	53523	0	1133	0	27	0	0.06	#DIV/0!	0	0	0	1	0	1	0	1	0.696063
tr|E5RH83|E5RH83_HUMAN	TTI2	TELO2-interacting protein 2 OS=Homo sapiens GN=TTI2 PE=2 SV=1	38123	0	1132	0	28	0	0.09	#DIV/0!	0	0	0	1	0	1	0	1	0.696063
tr|B2RDI5|B2RDI5_HUMAN	tr|B2RDI5|B2	cDNA, FLJ96627, highly similar to Homo sapiens calpain 1, (mu/I) large subunit (CAPN1), mRNA OS=Homo sapiens PE=2 SV=1	82529	0	1131	0	26	0	0.12	#DIV/0!	0	0	0	2	0	1	0	2	0.696063
tr|B1AZV3|B1AZV3_HUMAN	TAP1	Antigen peptide transporter 1 OS=Homo sapiens GN=TAP1 PE=4 SV=1	87691	0	1130	0	28	0	0.04	#DIV/0!	0	0	0	1	0	1	0	1	0.696063
tr|Q2TU84|Q2TU84_HUMAN	GIG18	Aspartate aminotransferase OS=Homo sapiens GN=GIG18 PE=2 SV=1	46519	0	1129	0	28	0	0.07	#DIV/0!	0	0	0	1	0	1	0	1	0.696063
tr|B4DSH9|B4DSH9_HUMAN	tr|B4DSH9|B4	cDNA FLJ60857, highly similar to Polymerase delta-interacting protein 3 OS=Homo sapiens PE=2 SV=1	20618	0	1128	0	28	0	0.16	#DIV/0!	0	0	0	1	0	1	0	1	0.696063
sp|P21283|VATC1_HUMAN	ATP6V1C1	V-type proton ATPase subunit C 1 OS=Homo sapiens GN=ATP6V1C1 PE=1 SV=4	44085	0	1127	0	28	0	0.07	#DIV/0!	0	0	0	1	0	1	0	1	0.696063
sp|Q16222|UAP1_HUMAN	UAP1	UDP-N-acetylhexosamine pyrophosphorylase OS=Homo sapiens GN=UAP1 PE=1 SV=3	59131	0	1126	0	28	0	0.06	#DIV/0!	0	0	0	1	0	1	0	1	0.696063
tr|H7C4F5|H7C4F5_HUMAN	LYST	Lysosomal-trafficking regulator (Fragment) OS=Homo sapiens GN=LYST PE=4 SV=1	20104	0	1125	0	28	0	0.17	#DIV/0!	0	0	0	1	0	1	0	1	0.696063
tr|J3QRD1|J3QRD1_HUMAN	ALDH3A2	Fatty aldehyde dehydrogenase OS=Homo sapiens GN=ALDH3A2 PE=3 SV=1	45019	0	1119	0	28	0	0.15	#DIV/0!	0	0	0	2	0	1	0	2	0.696063
sp|Q06210|GFPT1_HUMAN	GFPT1	Glutamine--fructose-6-phosphate aminotransferase [isomerizing] 1 OS=Homo sapiens GN=GFPT1 PE=1 SV=3	79555	0	1118	0	29	0	0.04	#DIV/0!	0	0	0	1	0	1	0	2	0.696063
sp|P46013|KI67_HUMAN	MKI67	Antigen KI-67 OS=Homo sapiens GN=MKI67 PE=1 SV=2	360698	0	1117	0	29	0	0.01	#DIV/0!	0	0	0	1	0	1	0	1	0.696063
tr|B2RCY5|B2RCY5_HUMAN	tr|B2RCY5|B2	cDNA, FLJ96373, highly similar to Homo sapiens X-ray repair complementing defective repair in Chinese hamster cells 1 (XRCC1), mRNA OS=Hon	69836	0	1115	0	29	0	0.05	#DIV/0!	0	0	0	1	0	1	0	1	0.696063
sp|Q6ZTR5|CX022_HUMAN	CXorf22	Uncharacterized protein CXorf22 OS=Homo sapiens GN=CXorf22 PE=2 SV=3	111537	0	1113	0	29	0	0.03	#DIV/0!	0	0	0	1	0	1	0	1	0.696063
tr|B7Z2D6|B7Z2D6_HUMAN	tr|B7Z2D6|B7	cDNA FLJ53863, highly similar to Cystathionine beta-synthase (EC 4.2.1.22) OS=Homo sapiens PE=2 SV=1	56409	0	1111	0	29	0	0.13	#DIV/0!	0	0	0	1	0	1	0	1	0.696063
tr|B4DR97|B4DR97_HUMAN	tr|B4DR97|B4	cDNA FLJ61343, highly similar to SECIS-binding protein 2 OS=Homo sapiens PE=2 SV=1	88575	0	1109	0	29	0	0.04	#DIV/0!	0	0	0	1	0	1	0	1	0.696063
tr|Q6ZW36|Q6ZW36_HUMAN	tr|Q6ZW36|Q	cDNA FLJ41667 fis, clone FEBRA2028366 OS=Homo sapiens PE=2 SV=1	36158	0	1107	0	29	0	0.09	#DIV/0!	0	0	0	1	0	1	0	1	0.696063
tr|E9PL37|E9PL37_HUMAN	CAPN1	Calpain-1 catalytic subunit (Fragment) OS=Homo sapiens GN=CAPN1 PE=2 SV=1	21231	0	1106	0	29	0	0.16	#DIV/0!	0	0	0	1	0	1	0	1	0.696063
tr|Q14730|Q14730_HUMAN	tr|Q14730|Q1	La 4.1 protein (Fragment) OS=Homo sapiens PE=2 SV=1	33852	0	1105	0	29	0	0.1	#DIV/0!	0	1	0	1	0	1	0	2	0.696063
tr|B5BUB5|B5BUB5_HUMAN	SSB	Autoantigen La (Fragment) OS=Homo sapiens GN=SSB PE=2 SV=1	47009	0	1104	0	29	0	0.07	#DIV/0!	0	1	0	1	0	1	0	2	0.696063
tr|B4DH53|B4DH53_HUMAN	MAP1S	MAP1S light chain OS=Homo sapiens GN=MAP1S PE=2 SV=1	110689	0	1103	0	29	0	0.03	#DIV/0!	0	0	0	1	0	1	0	1	0.696063
tr|B2RB95|B2RB95_HUMAN	tr|B2RB95|B2	cDNA, FLJ95381, Homo sapiens tripartite motif-containing 16 (TRIM16), mRNA OS=Homo sapiens PE=2 SV=1	64884	0	1102	0	29	0	0.03	#DIV/0!	0	0	0	1	0	1	0	1	0.696063
sp|Q9UBK8|MTRR_HUMAN	MTRR	Methionine synthase reductase OS=Homo sapiens GN=MTRR PE=1 SV=3	81386	0	1100	0	29	0	0.04	#DIV/0!	0	0	0	1	0	1	0	1	0.696063
tr|Q8TBJ6|Q8TBJ6_HUMAN	ST6GALNAC1	ST6GALNAC1 protein OS=Homo sapiens GN=ST6GALNAC1 PE=2 SV=1	52413	0	1099	0	29	0	0.06	#DIV/0!	0	0	0	1	0	1	0	1	0.696063
sp|Q8IXI1|MIRO2_HUMAN	RHOT2	Mitochondrial Rho GTPase 2 OS=Homo sapiens GN=RHOT2 PE=1 SV=2	69101	0	1097	0	29	0	0.14	#DIV/0!	0	0	0	1	0	1	0	1	0.696063
tr|B4DV28|B4DV28_HUMAN	tr|B4DV28|B4	cDNA FLJ54170, highly similar to Cytosolic nonspecific dipeptidase OS=Homo sapiens PE=2 SV=1	51811	0	1095	0	30	0	0.08	#DIV/0!	0	0	0	1	0	1	0	1	0.696063
tr|B2R894|B2R894_HUMAN	MRPL38	Mitochondrial ribosomal protein L38, isoform CRA_b OS=Homo sapiens GN=MRPL38 PE=2 SV=1	41017	0	1094	0	30	0	0.09	#DIV/0!	0	1	0	1	0	1	0	1	0.696063
sp|Q9H2M9|RBGPR_HUMAN	RAB3GAP2	Rab3 GTPase-activating protein non-catalytic subunit OS=Homo sapiens GN=RAB3GAP2 PE=1 SV=1	157482	0	1093	0	30	0	0.05	#DIV/0!	0	0	0	1	0	1	0	1	0.696063
tr|H0Y360|H0Y360_HUMAN	AMPD2	AMP deaminase 2 (Fragment) OS=Homo sapiens GN=AMPD2 PE=4 SV=1	99192	0	1092	0	30	0	0.03	#DIV/0!	0	0	0	1	0	1	0	1	0.696063
tr|B7Z2B7|B7Z2B7_HUMAN	tr|B7Z2B7|B7	cDNA FLJ60493 OS=Homo sapiens PE=2 SV=1	22350	0	1090	0	30	0	0.15	#DIV/0!	0	0	0	1	0	1	0	1	0.696063
tr|B3KM40|B3KM40_HUMAN	tr|B3KM40|B	cDNA FLJ10185 fis, clone HEMBA1004509, highly similar to U4/U6.U5 tri-snRNP-associated protein 2 OS=Homo sapiens PE=2 SV=1	56618	0	1088	0	30	0	0.06	#DIV/0!	0	0	0	1	0	1	0	1	0.696063
tr|B4DTA7|B4DTA7_HUMAN	tr|B4DTA7|B4	cDNA FLJ50207, highly similar to Pappalysin-1 (EC 3.4.24.79) OS=Homo sapiens PE=2 SV=1	64336	0	1087	0	30	0	0.05	#DIV/0!	0	0	0	1	0	1	0	1	0.696063
tr|H7BZF2|H7BZF2_HUMAN	DKC1	H/ACA ribonucleoprotein complex subunit 4 (Fragment) OS=Homo sapiens GN=DKC1 PE=4 SV=1	8803	0	1082	0	31	0	0.4	#DIV/0!	0	1	0	1	0	1	0	1	0.696063
tr|B3KPQ7|B3KPQ7_HUMAN	tr|B3KPQ7|B3	cDNA FLJ32066 fis, clone OCBBF1000091, highly similar to Homo sapiens sorbin and SH3 domain containing 2 (SORBS2), transcript variant 2, mR	135617	0	1081	0	31	0	0.02	#DIV/0!	0	0	0	1	0	1	0	1	0.696063
tr|Q4LE48|Q4LE48_HUMAN	STAG1	STAG1 variant protein (Fragment) OS=Homo sapiens GN=STAG1 variant protein PE=2 SV=1	147272	0	1079	0	31	0	0.05	#DIV/0!	0	1	0	1	0	1	0	1	0.696063
tr|F1D8N4|F1D8N4_HUMAN	NR3C1	Glucocorticoid nuclear receptor variant 1 OS=Homo sapiens GN=NR3C1 PE=2 SV=1	86745	0	1077	0	31	0	0.04	#DIV/0!	0	0	0	1	0	1	0	1	0.696063
sp|Q9BS26|ERP44_HUMAN	ERP44	Endoplasmic reticulum resident protein 44 OS=Homo sapiens GN=ERP44 PE=1 SV=1	47341	0	1076	0	31	0	0.07	#DIV/0!	0	0	0	1	0	1	0	1	0.696063
tr|Q5SQP8|Q5SQP8_HUMAN	CTBP2	C-terminal-binding protein 2 OS=Homo sapiens GN=CTBP2 PE=4 SV=1	56808	0	1075	0	31	0	0.06	#DIV/0!	0	0	0	1	0	1	0	1	0.696063
sp|Q9NTX7|RN146_HUMAN	RNF146	E3 ubiquitin-protein ligase RNF146 OS=Homo sapiens GN=RNF146 PE=1 SV=1	39610	0	1074	0	31	0	0.08	#DIV/0!	0	1	0	1	0	1	0	1	0.696063
sp|Q9UKV8|AGO2_HUMAN	EIF2C2	Protein argonaute-2 OS=Homo sapiens GN=EIF2C2 PE=1 SV=3	98400	0	1073	0	32	0	0.04	#DIV/0!	0	0	0	1	0	1	0	1	0.696063
tr|F6U211|F6U211_HUMAN	RPS10	40S ribosomal protein S10 OS=Homo sapiens GN=RPS10 PE=2 SV=1	19912	0	1071	0	32	0	0.17	#DIV/0!	0	1	0	1	0	1	0	1	0.696063
sp|Q92995|UBP13_HUMAN	USP13	Ubiquitin carboxyl-terminal hydrolase 13 OS=Homo sapiens GN=USP13 PE=1 SV=2	98006	0	1068	0	32	0	0.03	#DIV/0!	0	0	0	1	0	1	0	1	0.696063
tr|I3L1M4|I3L1M4_HUMAN	ALDH3A2	Fatty aldehyde dehydrogenase (Fragment) OS=Homo sapiens GN=ALDH3A2 PE=3 SV=1	18870	0	1066	0	32	0	0.18	#DIV/0!	0	0	0	1	0	1	0	1	0.696063
tr|B0V0F7|B0V0F7_HUMAN	DHX16	Putative pre-mRNA-splicing factor ATP-dependent RNA helicase DHX16 (Fragment) OS=Homo sapiens GN=DHX16 PE=4 SV=1	21454	0	1061	0	32	0	0.16	#DIV/0!	0	0	0	1	0	1	0	1	0.696063
tr|B2RAG9|B2RAG9_HUMAN	tr|B2RAG9|B2	cDNA, FLJ94908, highly similar to Homo sapiens PPAR binding protein (PPARBP), mRNA OS=Homo sapiens PE=2 SV=1	167569	0	1059	0	32	0	0.02	#DIV/0!	0	1	0	1	0	1	0	1	0.696063
tr|E9PC74|E9PC74_HUMAN	EIF2B5	Translation initiation factor eIF-2B subunit epsilon OS=Homo sapiens GN=EIF2B5 PE=2 SV=1	79096	0	1056	0	32	0	0.04	#DIV/0!	0	1	0	1	0	1	0	1	0.696063
tr|H3BNQ7|H3BNQ7_HUMAN	ABAT	4-aminobutyrate aminotransferase, mitochondrial OS=Homo sapiens GN=ABAT PE=3 SV=1	58741	0	1052	0	33	0	0.06	#DIV/0!	0	1	0	1	0	1	0	1	0.696063
sp|Q13136|LIPA1_HUMAN	PPFIA1	Liprin-alpha-1 OS=Homo sapiens GN=PPFIA1 PE=1 SV=1	136265	0	1051	0	33	0	0.13	#DIV/0!	0	1	0	1	0	1	0	1	0.696063
tr|J3QTJ6|J3QTJ6_HUMAN	FSIP2	Fibrous sheath-interacting protein 2 OS=Homo sapiens GN=FSIP2 PE=4 SV=1	795150	0	1050	0	33	0	0	#DIV/0!	0	1	0	1	0	1	0	2	0.696063
tr|B4DSE9|B4DSE9_HUMAN	tr|B4DSE9|B4	cDNA FLJ58278, highly similar to Homo sapiens WD repeat domain 44 (WDR44), mRNA OS=Homo sapiens PE=2 SV=1	91610	0	1049	0	33	0	0.04	#DIV/0!	0	0	0	1	0	1	0	1	0.696063
tr|H0Y650|H0Y650_HUMAN	HEATR2	HEAT repeat-containing protein 2 (Fragment) OS=Homo sapiens GN=HEATR2 PE=4 SV=1	73507	0	1048	0	33	0	0.04	#DIV/0!	0	0	0	1	0	1	0	1	0.696063
tr|B4DEP6|B4DEP6_HUMAN	tr|B4DEP6|B4	cDNA FLJ59206, highly similar to Eukaryotic translation initiation factor 4B OS=Homo sapiens PE=2 SV=1	66146	0	1045	0	33	0	0.05	#DIV/0!	0	0	0	1	0	1	0	2	0.696063
tr|B4DJE7|B4DJE7_HUMAN	ACADM	Medium-chain-specific acyl-CoA dehydrogenase, mitochondrial OS=Homo sapiens GN=ACADM PE=2 SV=1	25704	0	1044	0	33	0	0.13	#DIV/0!	0	0	0	1	0	1	0	1	0.696063
tr|Q6IB29|Q6IB29_HUMAN	EBNA1BP2	EBNA1 binding protein 2 OS=Homo sapiens GN=EBNA1BP2 PE=2 SV=1	34887	0	1042	0	33	0	0.1	#DIV/0!	0	0	0	1	0	1	0	1	0.696063
tr|B2R642|B2R642_HUMAN	tr|B2R642|B2	cDNA, FLJ92775, highly similar to Homo sapiens melanoma cell adhesion molecule (MCAM), mRNA OS=Homo sapiens PE=2 SV=1	72560	0	1041	0	33	0	0.05	#DIV/0!	0	0	0	1	0	1	0	1	0.696063
tr|H0Y3K4|H0Y3K4_HUMAN	RBM33	RNA-binding protein 33 (Fragment) OS=Homo sapiens GN=RBM33 PE=4 SV=1	100636	0	1037	0	34	0	0.07	#DIV/0!	0	1	0	2	0	1	0	3	0.696063
tr|G3V161|G3V161_HUMAN	KBTBD3	Kelch repeat and BTB (POZ) domain containing 3, isoform CRA_a OS=Homo sapiens GN=KBTBD3 PE=2 SV=1	61834	0	1034	0	34	0	0.06	#DIV/0!	0	1	0	1	0	1	0	2	0.696063
tr|B3KMK2|B3KMK2_HUMAN	tr|B3KMK2|B	cDNA FLJ11229 fis, clone PLACE1008356, highly similar to Homo sapiens meningioma expressed antigen 5 ((MGEA5), mRNA) OS=Homo sapiens	54835	0	1032	0	34	0	0.06	#DIV/0!	0	0	0	1	0	1	0	1	0.696063
sp|Q6UWE0|LRSM1_HUMAN	LRSAM1	E3 ubiquitin-protein ligase LRSAM1 OS=Homo sapiens GN=LRSAM1 PE=1 SV=1	84567	0	1029	0	34	0	0.04	#DIV/0!	0	0	0	1	0	1	0	1	0.696063
tr|H0Y8Z9|H0Y8Z9_HUMAN	ACAD9	Acyl-CoA dehydrogenase family member 9, mitochondrial (Fragment) OS=Homo sapiens GN=ACAD9 PE=4 SV=1	43503	0	1028	0	35	0	0.08	#DIV/0!	0	1	0	1	0	1	0	1	0.696063
tr|B2RA91|B2RA91_HUMAN	tr|B2RA91|B2	cDNA, FLJ94773, highly similar to Homo sapiens splicing factor, arginine/serine-rich 2, interacting protein (SFRS2IP), mRNA OS=Homo sapiens PE	129817	0	1026	0	35	0	0.03	#DIV/0!	0	0	0	1	0	1	0	1	0.696063
tr|B4DH43|B4DH43_HUMAN	tr|B4DH43|B4	cDNA FLJ56433, highly similar to Collagen alpha-2(IV) chain OS=Homo sapiens PE=2 SV=1	83428	0	1025	0	35	0	0.04	#DIV/0!	0	1	0	1	0	1	0	2	0.696063
tr|A0JLU4|A0JLU4_HUMAN	YBX1	YBX1 protein (Fragment) OS=Homo sapiens GN=YBX1 PE=2 SV=1	21417	0	1024	0	35	0	0.16	#DIV/0!	0	1	0	1	0	1	0	1	0.696063
sp|P62333|PRS10_HUMAN	PSMC6	26S protease regulatory subunit 10B OS=Homo sapiens GN=PSMC6 PE=1 SV=1	44430	0	1021	0	35	0	0.23	#DIV/0!	0	1	0	2	0	1	0	2	0.696063
sp|Q70J99|UN13D_HUMAN	UNC13D	Protein unc-13 homolog D OS=Homo sapiens GN=UNC13D PE=1 SV=1	124288	0	1019	0	35	0	0.21	#DIV/0!	0	0	0	1	0	1	0	1	0.696063
tr|B3KT00|B3KT00_HUMAN	tr|B3KT00|B3	cDNA FLJ37368 fis, clone BRAMY2024530, highly similar to Zinc phosphodiesterase ELAC protein 2 (EC 3.1.26.11) OS=Homo sapiens PE=2 SV=1	91169	0	1018	0	35	0	0.04	#DIV/0!	0	1	0	1	0	1	0	1	0.696063
tr|Q86U96|Q86U96_HUMAN	tr|Q86U96|Q	TUBGCP2 protein OS=Homo sapiens PE=4 SV=1	8795	0	1017	0	35	0	0.4	#DIV/0!	0	0	0	1	0	1	0	1	0.696063
sp|Q5JSP0|FGD3_HUMAN	FGD3	FYVE, RhoGEF and PH domain-containing protein 3 OS=Homo sapiens GN=FGD3 PE=1 SV=1	80206	0	1016	0	35	0	0.04	#DIV/0!	0	1	0	1	0	1	0	1	0.696063
tr|B4DGI6|B4DGI6_HUMAN	tr|B4DGI6|B4	cDNA FLJ50498, highly similar to ATP-dependent RNA helicase DDX24 (EC 3.6.1.-) OS=Homo sapiens PE=2 SV=1	56048	0	1015	0	35	0	0.06	#DIV/0!	0	0	0	1	0	1	0	2	0.696063
tr|B4DYZ1|B4DYZ1_HUMAN	tr|B4DYZ1|B4	cDNA FLJ59786, highly similar to Probable ATP-dependent RNA helicase DDX31 (EC 3.6.1.-) OS=Homo sapiens PE=2 SV=1	81565	0	1014	0	35	0	0.04	#DIV/0!	0	1	0	1	0	1	0	1	0.696063
tr|B7ZKR8|B7ZKR8_HUMAN	USP48	USP48 protein OS=Homo sapiens GN=USP48 PE=2 SV=1	115025	0	1013	0	35	0	0.03	#DIV/0!	0	0	0	1	0	1	0	2	0.696063
tr|G3V1C1|G3V1C1_HUMAN	AKR1E2	1,5-anhydro-D-fructose reductase OS=Homo sapiens GN=AKR1E2 PE=2 SV=1	23420	0	1012	0	35	0	0.14	#DIV/0!	0	1	0	1	0	1	0	1	0.696063
sp|Q9H0I3|CC113_HUMAN	CCDC113	Coiled-coil domain-containing protein 113 OS=Homo sapiens GN=CCDC113 PE=2 SV=1	44249	0	1009	0	35	0	0.07	#DIV/0!	0	1	0	1	0	1	0	1	0.696063
tr|B4E140|B4E140_HUMAN	tr|B4E140|B4	Transporter OS=Homo sapiens PE=2 SV=1	41144	0	1004	0	36	0	0.08	#DIV/0!	0	0	0	1	0	1	0	1	0.696063
tr|F8VQQ3|F8VQQ3_HUMAN	C12orf10	UPF0160 protein MYG1, mitochondrial OS=Homo sapiens GN=C12orf10 PE=2 SV=1	34140	0	1003	0	36	0	0.1	#DIV/0!	0	0	0	1	0	1	0	1	0.696063
tr|B0V0L0|B0V0L0_HUMAN	GNL1	Guanine nucleotide-binding protein-like 1 (Fragment) OS=Homo sapiens GN=GNL1 PE=4 SV=1	45273	0	1002	0	36	0	0.07	#DIV/0!	0	1	0	1	0	1	0	1	0.696063
sp|P78386|KRT85_HUMAN	KRT85	Keratin, type II cuticular Hb5 OS=Homo sapiens GN=KRT85 PE=1 SV=1	57306	0	1001	0	36	0	0.06	#DIV/0!	0	1	0	1	0	1	0	1	0.696063
sp|Q9Y3Z3|SAMH1_HUMAN	SAMHD1	SAM domain and HD domain-containing protein 1 OS=Homo sapiens GN=SAMHD1 PE=1 SV=2	72896	0	1000	0	36	0	0.05	#DIV/0!	0	1	0	1	0	1	0	1	0.696063
sp|Q9H0A0|NAT10_HUMAN	NAT10	N-acetyltransferase 10 OS=Homo sapiens GN=NAT10 PE=1 SV=2	116569	0	996	0	36	0	0.06	#DIV/0!	0	1	0	2	0	1	0	2	0.696063
tr|A0AV56|A0AV56_HUMAN	SAFB	SAFB protein OS=Homo sapiens GN=SAFB PE=2 SV=1	103249	0	995	0	36	0	0.03	#DIV/0!	0	0	0	1	0	1	0	1	0.696063
tr|H0YAV4|H0YAV4_HUMAN	TRPS1	Zinc finger transcription factor Trps1 (Fragment) OS=Homo sapiens GN=TRPS1 PE=4 SV=1	30847	0	992	0	37	0	0.11	#DIV/0!	0	1	0	1	0	1	0	1	0.696063
tr|B4DKE6|B4DKE6_HUMAN	tr|B4DKE6|B4	cDNA FLJ60629, highly similar to Replication factor C subunit 3 OS=Homo sapiens PE=2 SV=1	35551	0	989	0	37	0	0.09	#DIV/0!	0	0	0	1	0	1	0	1	0.696063
tr|B2R5U7|B2R5U7_HUMAN	tr|B2R5U7|B2	cDNA, FLJ92633, highly similar to Homo sapiens CCAAT-box-binding transcription factor (CBF2), mRNA OS=Homo sapiens PE=2 SV=1	114755	0	988	0	37	0	0.03	#DIV/0!	0	0	0	1	0	1	0	1	0.696063
tr|H0YBD0|H0YBD0_HUMAN	AP3B1	AP-3 complex subunit beta-1 (Fragment) OS=Homo sapiens GN=AP3B1 PE=4 SV=1	17084	0	987	0	37	0	0.2	#DIV/0!	0	1	0	1	0	1	0	1	0.696063
tr|H0YJ87|H0YJ87_HUMAN	ZC3H14	Zinc finger CCCH domain-containing protein 14 (Fragment) OS=Homo sapiens GN=ZC3H14 PE=4 SV=1	8686	0	982	0	37	0	0.04	#DIV/0!	0	1	0	1	0	1	0	1	0.696063
tr|B3KSE4|B3KSE4_HUMAN	tr|B3KSE4|B3	cDNA FLJ36076 fis, clone TESTI2019760, highly similar to TRANSCRIPTIONAL REPRESSOR CTCF OS=Homo sapiens PE=2 SV=1	30924	0	981	0	37	0	0.11	#DIV/0!	0	1	0	1	0	1	0	1	0.696063
tr|A4FUT8|A4FUT8_HUMAN	JMJD1B	JMJD1B protein (Fragment) OS=Homo sapiens GN=JMJD1B PE=2 SV=1	170603	0	980	0	37	0	0.04	#DIV/0!	0	1	0	2	0	1	0	2	0.696063
sp|Q7L0Y3|MRRP1_HUMAN	TRMT10C	Mitochondrial ribonuclease P protein 1 OS=Homo sapiens GN=TRMT10C PE=1 SV=2	47602	0	978	0	37	0	0.07	#DIV/0!	0	0	0	1	0	1	0	2	0.696063
tr|B7Z7U0|B7Z7U0_HUMAN	tr|B7Z7U0|B7	cDNA FLJ61739, highly similar to Serine/arginine repetitive matrix protein 1 OS=Homo sapiens PE=2 SV=1	103792	0	977	0	37	0	0.03	#DIV/0!	0	1	0	1	0	1	0	2	0.696063
tr|Q7Z2Y2|Q7Z2Y2_HUMAN	DKFZp686P10	Putative uncharacterized protein DKFZp686P10119 (Fragment) OS=Homo sapiens GN=DKFZp686P10119 PE=2 SV=1	9616	0	976	0	38	0	0.36	#DIV/0!	0	1	0	1	0	1	0	2	0.696063
tr|B1AK66|B1AK66_HUMAN	SNIP1	Smad nuclear interacting protein 1 OS=Homo sapiens GN=SNIP1 PE=2 SV=1	45807	0	975	0	38	0	0.07	#DIV/0!	0	1	0	1	0	1	0	1	0.696063
tr|B4DLG2|B4DLG2_HUMAN	tr|B4DLG2|B4	cDNA FLJ58196, highly similar to Zinc finger CCCH domain-containing protein 11A OS=Homo sapiens PE=2 SV=1	83177	0	973	0	38	0	0.04	#DIV/0!	0	1	0	1	0	1	0	2	0.696063
tr|B2R6X2|B2R6X2_HUMAN	tr|B2R6X2|B2	cDNA, FLJ93161, highly similar to Homo sapiens glucuronidase, beta (GUSB), mRNA OS=Homo sapiens PE=2 SV=1	75001	0	970	0	38	0	0.04	#DIV/0!	0	0	0	1	0	1	0	1	0.696063
sp|Q9UBV2|SE1L1_HUMAN	SEL1L	Protein sel-1 homolog 1 OS=Homo sapiens GN=SEL1L PE=1 SV=3	89212	0	969	0	38	0	0.04	#DIV/0!	0	1	0	1	0	1	0	2	0.696063
sp|Q6NVY1|HIBCH_HUMAN	HIBCH	3-hydroxyisobutyryl-CoA hydrolase, mitochondrial OS=Homo sapiens GN=HIBCH PE=1 SV=2	43797	0	962	0	39	0	0.08	#DIV/0!	0	1	0	1	0	1	0	1	0.696063
tr|Q6ZWH1|Q6ZWH1_HUMAN	tr|Q6ZWH1|Q	cDNA FLJ41081 fis, clone ADRGL2009755, highly similar to Homo sapiens brain and reproductive organ-expressed protein (BRE) mRNA OS=Hom	45824	0	961	0	39	0	0.07	#DIV/0!	0	1	0	1	0	1	0	1	0.696063
tr|B4DYP7|B4DYP7_HUMAN	tr|B4DYP7|B4	cDNA FLJ55435, highly similar to Gamma-tubulin complex component 3 OS=Homo sapiens PE=2 SV=1	103306	0	959	0	39	0	0.03	#DIV/0!	0	1	0	1	0	1	0	1	0.696063
sp|Q7Z6I6|RHG30_HUMAN	ARHGAP30	Rho GTPase-activating protein 30 OS=Homo sapiens GN=ARHGAP30 PE=1 SV=3	119649	0	954	0	39	0	0.03	#DIV/0!	0	1	0	1	0	1	0	1	0.696063
tr|E9PJ81|E9PJ81_HUMAN	UBXN1	UBX domain-containing protein 1 (Fragment) OS=Homo sapiens GN=UBXN1 PE=2 SV=1	32649	0	953	0	39	0	0.15	#DIV/0!	0	1	0	1	0	1	0	1	0.696063
sp|Q6PD62|CTR9_HUMAN	CTR9	RNA polymerase-associated protein CTR9 homolog OS=Homo sapiens GN=CTR9 PE=1 SV=1	134332	0	951	0	39	0	0.02	#DIV/0!	0	1	0	1	0	1	0	1	0.696063
sp|Q00653|NFKB2_HUMAN	NFKB2	Nuclear factor NF-kappa-B p100 subunit OS=Homo sapiens GN=NFKB2 PE=1 SV=4	97373	0	944	0	40	0	0.07	#DIV/0!	0	1	0	1	0	1	0	1	0.696063
tr|B1AK81|B1AK81_HUMAN	PIGK	GPI-anchor transamidase OS=Homo sapiens GN=PIGK PE=4 SV=1	34462	0	943	0	40	0	0.1	#DIV/0!	0	1	0	1	0	1	0	1	0.696063
tr|H7C0M3|H7C0M3_HUMAN	BPHL	Valacyclovir hydrolase (Fragment) OS=Homo sapiens GN=BPHL PE=4 SV=1	3314	0	941	0	40	0	1.2	#DIV/0!	0	1	0	1	0	1	0	1	0.696063
tr|E9PL10|E9PL10_HUMAN	BTF3L4	Transcription factor BTF3 homolog 4 OS=Homo sapiens GN=BTF3L4 PE=2 SV=1	15769	0	938	0	40	0	0.21	#DIV/0!	0	1	0	1	0	1	0	1	0.696063
tr|Q2VIM1|Q2VIM1_HUMAN	tr|Q2VIM1|Q	RcCDC20 (Fragment) OS=Homo sapiens PE=2 SV=1	50517	0	935	0	40	0	0.07	#DIV/0!	0	1	0	1	0	1	0	1	0.696063
sp|Q9Y295|DRG1_HUMAN	DRG1	Developmentally-regulated GTP-binding protein 1 OS=Homo sapiens GN=DRG1 PE=1 SV=1	40802	0	934	0	40	0	0.08	#DIV/0!	0	1	0	1	0	1	0	1	0.696063
tr|H7C4G8|H7C4G8_HUMAN	NT5DC2	5'-nucleotidase domain-containing protein 2 (Fragment) OS=Homo sapiens GN=NT5DC2 PE=4 SV=1	30207	0	933	0	30	0	0.11	#DIV/0!	0	0	0	1	0	1	0	1	0.696063
tr|G0XQ39|G0XQ39_HUMAN	STIM1	STIM1L OS=Homo sapiens GN=STIM1 PE=2 SV=1	88933	0	931	0	40	0	0.04	#DIV/0!	0	1	0	1	0	1	0	1	0.696063

sp|Q86UE4|LYRIC_HUMAN	MTDH	Protein LYRIC OS=Homo sapiens GN=MTDH PE=1 SV=2	63856	0	930	0	40	0	0.05	#DIV/0!	0	1	0	1	0	1	0	1	0.696063
tr|G3V2E7|G3V2E7_HUMAN	KLC1	Kinesin light chain 1 OS=Homo sapiens GN=KLC1 PE=2 SV=1	63133	0	927	0	41	0	0.11	#DIV/0!	0	1	0	1	0	1	0	1	0.696063
tr|H0YG10|H0YG10_HUMAN	GNL2	Nucleolar GTP-binding protein 2 (Fragment) OS=Homo sapiens GN=GNL2 PE=4 SV=1	26716	0	925	0	41	0	0.12	#DIV/0!	0	1	0	1	0	1	0	2	0.696063
sp|O15031|PLXB2_HUMAN	PLXNB2	Plexin-B2 OS=Homo sapiens GN=PLXNB2 PE=1 SV=3	207734	0	922	0	41	0	0.03	#DIV/0!	0	1	0	1	0	1	0	2	0.696063
tr|Q2TB12|Q2TB12_HUMAN	LIG1	DNA ligase OS=Homo sapiens GN=LIG1 PE=2 SV=1	88975	0	920	0	41	0	0.04	#DIV/0!	0	1	0	1	0	1	0	2	0.696063
tr|H0YGH7|H0YGH7_HUMAN	PLCZ1	1-phosphatidylinositol 4,5-bisphosphate phosphodiesterase zeta-1 (Fragment) OS=Homo sapiens GN=PLCZ1 PE=4 SV=1	47240	0	919	0	41	0	0.07	#DIV/0!	0	1	0	1	0	1	0	1	0.696063
sp|Q8NI27|THOC2_HUMAN	THOC2	THO complex subunit 2 OS=Homo sapiens GN=THOC2 PE=1 SV=2	184541	0	917	0	42	0	0.03	#DIV/0!	0	1	0	1	0	1	0	1	0.696063
tr|D6R968|D6R968_HUMAN	N4BP2L2	NEDD4-binding protein 2-like 2 (Fragment) OS=Homo sapiens GN=N4BP2L2 PE=2 SV=1	83058	0	915	0	42	0	0.04	#DIV/0!	0	1	0	1	0	1	0	2	0.696063
sp|O60287|NPA1P_HUMAN	URB1	Nucleolar pre-ribosomal-associated protein 1 OS=Homo sapiens GN=URB1 PE=1 SV=4	256508	0	914	0	42	0	0.01	#DIV/0!	0	1	0	1	0	1	0	2	0.696063
sp|Q08499|PDE4D_HUMAN	PDE4D	cAMP-specific 3~,5~-cyclic phosphodiesterase 4D OS=Homo sapiens GN=PDE4D PE=1 SV=2	91628	0	913	0	42	0	0.04	#DIV/0!	0	1	0	1	0	1	0	2	0.696063
tr|A9LSU1|A9LSU1_HUMAN	tr|A9LSU1|A9	Type IV collagen alpha 1 (Fragment) OS=Homo sapiens PE=2 SV=1	25998	0	912	0	42	0	0.13	#DIV/0!	0	1	0	1	0	1	0	1	0.696063
tr|B7ZLC9|B7ZLC9_HUMAN	GEMIN5	GEMIN5 protein OS=Homo sapiens GN=GEMIN5 PE=2 SV=1	170664	0	909	0	42	0	0.04	#DIV/0!	0	1	0	2	0	1	0	2	0.696063
tr|B3KN37|B3KN37_HUMAN	tr|B3KN37|B3	cDNA FLJ13414 fis, clone PLACE1001748, highly similar to Homo sapiens pitrilysin metallopeptidase 1 (PITRM1), mRNA OS=Homo sapiens PE=2 S	68281	0	906	0	42	0	0.03	#DIV/0!	0	1	0	1	0	1	0	2	0.696063
tr|B3KST5|B3KST5_HUMAN	COPS4	COP9 constitutive photomorphogenic homolog subunit 4 (Arabidopsis), isoform CRA_a OS=Homo sapiens GN=COPS4 PE=2 SV=1	40457	0	903	0	40	0	0.15	#DIV/0!	0	1	0	1	0	1	0	1	0.696063
tr|B4DF50|B4DF50_HUMAN	tr|B4DF50|B4	cDNA FLJ60458, highly similar to SWI/SNF-related matrix-associated actin-dependent regulator of chromatin subfamily D member 1 OS=Homo s	28346	0	902	0	42	0	0.12	#DIV/0!	0	1	0	1	0	1	0	1	0.696063
tr|Q2TSD2|Q2TSD2_HUMAN	tr|Q2TSD2|Q	Aging-associated gene 7 protein OS=Homo sapiens PE=2 SV=1	41778	0	899	0	43	0	0.08	#DIV/0!	0	1	0	1	0	1	0	1	0.696063
tr|B2R7M1|B2R7M1_HUMAN	tr|B2R7M1|B	cDNA, FLJ93507, highly similar to Homo sapiens ATPase, H+ transporting, lysosomal 38kDa, V0 subunit d isoform 1 (ATP6V0D1), mRNA OS=Hom	40747	0	898	0	43	0	0.08	#DIV/0!	0	1	0	1	0	1	0	1	0.696063
tr|E7EVL6|E7EVL6_HUMAN	REPIN1	Replication initiator 1 (Fragment) OS=Homo sapiens GN=REPIN1 PE=2 SV=1	16586	0	897	0	43	0	0.2	#DIV/0!	0	1	0	1	0	1	0	1	0.696063
sp|P49756|RBM25_HUMAN	RBM25	RNA-binding protein 25 OS=Homo sapiens GN=RBM25 PE=1 SV=3	100467	0	893	0	43	0	0.12	#DIV/0!	0	1	0	1	0	1	0	1	0.696063
tr|G5E9V1|G5E9V1_HUMAN	TFG	Protein TFG OS=Homo sapiens GN=TFG PE=4 SV=1	43051	0	891	0	43	0	0.08	#DIV/0!	0	1	0	1	0	1	0	2	0.696063
tr|D3DTD2|D3DTD2_HUMAN	TRIO	Triple functional domain (PTPRF interacting), isoform CRA_a OS=Homo sapiens GN=TRIO PE=2 SV=1	270210	0	889	0	44	0	0.01	#DIV/0!	0	1	0	1	0	1	0	1	0.696063
tr|H0YCE3|H0YCE3_HUMAN	TCIRG1	V-type proton ATPase 116 kDa subunit a isoform 3 (Fragment) OS=Homo sapiens GN=TCIRG1 PE=4 SV=1	33055	0	888	0	44	0	0.1	#DIV/0!	0	1	0	1	0	1	0	1	0.696063
tr|G3V1K3|G3V1K3_HUMAN	PON2	Paraoxonase 2, isoform CRA_a OS=Homo sapiens GN=PON2 PE=2 SV=1	41672	0	887	0	44	0	0.08	#DIV/0!	0	1	0	1	0	1	0	1	0.696063
tr|H0Y539|H0Y539_HUMAN	SHC1	SHC-transforming protein 1 (Fragment) OS=Homo sapiens GN=SHC1 PE=4 SV=1	32712	0	884	0	44	0	0.1	#DIV/0!	0	1	0	1	0	1	0	1	0.696063
tr|F8VS10|F8VS10_HUMAN	SPATS2	Spermatogenesis-associated serine-rich protein 2 OS=Homo sapiens GN=SPATS2 PE=2 SV=1	32626	0	883	0	44	0	0.1	#DIV/0!	0	1	0	1	0	1	0	1	0.696063
tr|G3V438|G3V438_HUMAN	AHSA1	Activator of 90 kDa heat shock protein ATPase homolog 1 (Fragment) OS=Homo sapiens GN=AHSA1 PE=2 SV=1	23078	0	880	0	44	0	0.14	#DIV/0!	0	1	0	1	0	1	0	2	0.696063
tr|G1UI18|G1UI18_HUMAN	PSME4	Proteasome activator complex subunit 4 (Fragment) OS=Homo sapiens GN=PSME4 PE=2 SV=1	130054	0	879	0	45	0	0.02	#DIV/0!	0	1	0	1	0	1	0	1	0.696063
tr|B4DWH7|B4DWH7_HUMAN	tr|B4DWH7|B	cDNA FLJ57632, highly similar to Vacuolar ATP synthase subunit B, kidney isoform (EC 3.6.3.14) OS=Homo sapiens PE=2 SV=1	54602	0	877	0	45	0	0.06	#DIV/0!	0	1	0	1	0	1	0	2	0.696063
tr|A8MUD9|A8MUD9_HUMAN	RPL7	60S ribosomal protein L7 OS=Homo sapiens GN=RPL7 PE=3 SV=1	24474	0	876	0	45	0	0.29	#DIV/0!	0	1	0	2	0	1	0	2	0.696063
tr|B2RB52|B2RB52_HUMAN	tr|B2RB52|B2	cDNA, FLJ95314, highly similar to Homo sapiens transducin (beta)-like 2 (TBL2), transcript variant 1, mRNA OS=Homo sapiens PE=2 SV=1	50423	0	869	0	46	0	0.07	#DIV/0!	0	1	0	1	0	1	0	1	0.696063
tr|G3V1P5|G3V1P5_HUMAN	MED15	Mediator of RNA polymerase II transcription subunit 15 OS=Homo sapiens GN=MED15 PE=4 SV=1	80007	0	868	0	46	0	0.04	#DIV/0!	0	1	0	1	0	1	0	1	0.696063
tr|H0YK42|H0YK42_HUMAN	SNX1	Sorting nexin-1 OS=Homo sapiens GN=SNX1 PE=4 SV=1	35628	0	867	0	46	0	0.09	#DIV/0!	0	1	0	1	0	1	0	1	0.696063
tr|F6XEV2|F6XEV2_HUMAN	MICU1	Calcium uptake protein 1, mitochondrial OS=Homo sapiens GN=MICU1 PE=2 SV=1	54915	0	866	0	46	0	0.06	#DIV/0!	0	1	0	1	0	1	0	1	0.696063
tr|J3QSY3|J3QSY3_HUMAN	GDA	Guanine deaminase OS=Homo sapiens GN=GDA PE=4 SV=1	46321	0	864	0	46	0	0.07	#DIV/0!	0	1	0	1	0	1	0	1	0.696063
tr|B2R7V4|B2R7V4_HUMAN	tr|B2R7V4|B2	cDNA, FLJ93619, highly similar to Homo sapiens PRP4 pre-mRNA processing factor 4 homolog (yeast) (PRPF4), mRNA OS=Homo sapiens PE=2 SV	59097	0	862	0	46	0	0.06	#DIV/0!	0	1	0	1	0	1	0	1	0.696063
tr|Q3LIF4|Q3LIF4_HUMAN	Nbla00271	NOGO-A (Fragment) OS=Homo sapiens GN=Nbla00271 PE=2 SV=1	57162	0	861	0	46	0	0.06	#DIV/0!	0	1	0	1	0	1	0	1	0.696063
sp|P49750|YLPM1_HUMAN	YLPM1	YLP motif-containing protein 1 OS=Homo sapiens GN=YLPM1 PE=1 SV=3	220077	0	858	0	46	0	0.02	#DIV/0!	0	1	0	1	0	1	0	1	0.696063
tr|B3KY55|B3KY55_HUMAN	tr|B3KY55|B3	cDNA FLJ46894 fis, clone UTERU3019708, highly similar to Regulator of nonsense transcripts 1 OS=Homo sapiens PE=2 SV=1	41002	0	856	0	32	0	0.08	#DIV/0!	0	0	0	1	0	1	0	1	0.696063
sp|Q8IVT2|CS021_HUMAN	C19orf21	Uncharacterized protein C19orf21 OS=Homo sapiens GN=C19orf21 PE=1 SV=1	75482	0	847	0	47	0	0.04	#DIV/0!	0	1	0	1	0	1	0	1	0.696063
tr|B3KUZ7|B3KUZ7_HUMAN	tr|B3KUZ7|B3	cDNA FLJ40986 fis, clone UTERU2014898, highly similar to Vacuolar ATP synthase subunit H (EC 3.6.3.14) OS=Homo sapiens PE=2 SV=1	56445	0	845	0	47	0	0.06	#DIV/0!	0	1	0	1	0	1	0	1	0.696063
tr|D3DTC3|D3DTC3_HUMAN	hCG_1742968	HCG1742968, isoform CRA_c OS=Homo sapiens GN=hCG_1742968 PE=4 SV=1	20573	0	842	0	47	0	0.16	#DIV/0!	0	1	0	1	0	1	0	1	0.696063
sp|O96008|TOM40_HUMAN	TOMM40	Mitochondrial import receptor subunit TOM40 homolog OS=Homo sapiens GN=TOMM40 PE=1 SV=1	38211	0	840	0	47	0	0.09	#DIV/0!	0	1	0	1	0	1	0	2	0.696063
tr|B4DSP0|B4DSP0_HUMAN	hCG_1642748	HCG1642748, isoform CRA_b OS=Homo sapiens GN=hCG_1642748 PE=2 SV=1	45559	0	839	0	48	0	0.07	#DIV/0!	0	1	0	1	0	1	0	1	0.696063
tr|A4UCU0|A4UCU0_HUMAN	tr|A4UCU0|A	DEAD box polypeptide 47 isoform 1 variant (Fragment) OS=Homo sapiens PE=2 SV=1	19904	0	838	0	48	0	0.06	#DIV/0!	0	1	0	1	0	1	0	1	0.696063
sp|Q8IX12|CCAR1_HUMAN	CCAR1	Cell division cycle and apoptosis regulator protein 1 OS=Homo sapiens GN=CCAR1 PE=1 SV=2	133423	0	830	0	48	0	0.04	#DIV/0!	0	1	0	1	0	1	0	2	0.696063
sp|Q96HE7|ERO1A_HUMAN	ERO1L	ERO1-like protein alpha OS=Homo sapiens GN=ERO1L PE=1 SV=2	55213	0	828	0	49	0	0.11	#DIV/0!	0	1	0	1	0	1	0	1	0.696063
sp|P02748|CO9_HUMAN	C9	Complement component C9 OS=Homo sapiens GN=C9 PE=1 SV=2	64615	0	826	0	49	0	0.05	#DIV/0!	0	1	0	1	0	1	0	1	0.696063
tr|Q5LJ97|Q5LJ97_HUMAN	TROVE2	TROVE domain family, member 2 (Fragment) OS=Homo sapiens GN=TROVE2 PE=2 SV=1	28360	0	825	0	30	0	0.11	#DIV/0!	0	1	0	1	0	1	0	1	0.696063
sp|Q9H814|PHAX_HUMAN	PHAX	Phosphorylated adapter RNA export protein OS=Homo sapiens GN=PHAX PE=1 SV=1	44546	0	824	0	49	0	0.07	#DIV/0!	0	1	0	1	0	1	0	1	0.696063
tr|H7C1L6|H7C1L6_HUMAN	CUL3	Cullin-3 (Fragment) OS=Homo sapiens GN=CUL3 PE=4 SV=1	22753	0	818	0	0	0	0.04	#DIV/0!	0	0	0	0	0	1	0	1	0.696063
tr|B4DHS5|B4DHS5_HUMAN	tr|B4DHS5|B4	cDNA FLJ54678, highly similar to N(2),N(2)-dimethylguanosine tRNA methyltransferase (EC 2.1.1.32) OS=Homo sapiens PE=2 SV=1	59256	0	816	0	50	0	0.06	#DIV/0!	0	1	0	1	0	1	0	1	0.696063
tr|G3V1T4|G3V1T4_HUMAN	NUP107	Nuclear pore complex protein Nup107 OS=Homo sapiens GN=NUP107 PE=2 SV=1	11398	0	814	0	42	0	0.06	#DIV/0!	0	1	0	1	0	1	0	1	0.696063
tr|B4DUS9|B4DUS9_HUMAN	BPNT1	3~(2~),5~-bisphosphate nucleotidase 1 OS=Homo sapiens GN=BPNT1 PE=2 SV=1	29455	0	812	0	50	0	0.11	#DIV/0!	0	1	0	1	0	1	0	2	0.696063
tr|Q05BU7|Q05BU7_HUMAN	WASF2	WASF2 protein (Fragment) OS=Homo sapiens GN=WASF2 PE=2 SV=1	21567	0	809	0	50	0	0.16	#DIV/0!	0	1	0	1	0	1	0	1	0.696063
tr|I0CMJ8|I0CMJ8_HUMAN	PRIM2	DNA primase large subunit (Fragment) OS=Homo sapiens GN=PRIM2 PE=4 SV=1	5179	0	806	0	51	0	0.7	#DIV/0!	0	1	0	1	0	1	0	1	0.696063
tr|A6NN60|A6NN60_HUMAN	CRYZ	Quinone oxidoreductase OS=Homo sapiens GN=CRYZ PE=2 SV=1	31680	0	805	0	51	0	0.11	#DIV/0!	0	1	0	1	0	1	0	2	0.696063
tr|B3KVA0|B3KVA0_HUMAN	tr|B3KVA0|B3	Cytosine-specific methyltransferase OS=Homo sapiens PE=2 SV=1	61027	0	804	0	0	0	0.02	#DIV/0!	0	0	0	0	0	1	0	1	0.696063
tr|Q6IPI1|Q6IPI1_HUMAN	RPL29	Ribosomal protein L29 OS=Homo sapiens GN=RPL29 PE=2 SV=1	17997	0	802	0	51	0	0.19	#DIV/0!	0	1	0	1	0	1	0	1	0.696063
sp|Q8TDD1|DDX54_HUMAN	DDX54	ATP-dependent RNA helicase DDX54 OS=Homo sapiens GN=DDX54 PE=1 SV=2	98819	0	800	0	51	0	0.03	#DIV/0!	0	1	0	1	0	1	0	3	0.696063
tr|B3KML1|B3KML1_HUMAN	tr|B3KML1|B3	cDNA FLJ11308 fis, clone PLACE1010074, highly similar to Sorting nexin-2 OS=Homo sapiens PE=2 SV=1	58550	0	799	0	52	0	0.06	#DIV/0!	0	1	0	1	0	1	0	1	0.696063
sp|Q5BKZ1|ZN326_HUMAN	ZNF326	DBIRD complex subunit ZNF326 OS=Homo sapiens GN=ZNF326 PE=1 SV=2	65955	0	795	0	52	0	0.05	#DIV/0!	0	1	0	1	0	1	0	1	0.696063
sp|Q9HCC0|MCCB_HUMAN	MCCC2	Methylcrotonoyl-CoA carboxylase beta chain, mitochondrial OS=Homo sapiens GN=MCCC2 PE=1 SV=1	61808	0	793	0	52	0	0.05	#DIV/0!	0	1	0	1	0	1	0	1	0.696063
tr|H3BP71|H3BP71_HUMAN	RNF40	E3 ubiquitin-protein ligase BRE1B OS=Homo sapiens GN=RNF40 PE=4 SV=1	109696	0	792	0	52	0	0.03	#DIV/0!	0	1	0	1	0	1	0	2	0.696063
sp|Q07954|LRP1_HUMAN	LRP1	Prolow-density lipoprotein receptor-related protein 1 OS=Homo sapiens GN=LRP1 PE=1 SV=2	523150	0	789	0	52	0	0.01	#DIV/0!	0	1	0	1	0	1	0	2	0.696063
tr|B4DX52|B4DX52_HUMAN	DNAJB1	DnaJ homolog subfamily B member 1 OS=Homo sapiens GN=DNAJB1 PE=2 SV=1	27170	0	785	0	52	0	0.12	#DIV/0!	0	1	0	1	0	1	0	1	0.696063
tr|A2A2L5|A2A2L5_HUMAN	C20orf43	Chromosome 20 open reading frame 43 (Fragment) OS=Homo sapiens GN=C20orf43 PE=4 SV=1	31759	0	784	0	52	0	0.2	#DIV/0!	0	1	0	1	0	1	0	1	0.696063
tr|Q59H95|Q59H95_HUMAN	tr|Q59H95|Q	Flightless I homolog variant (Fragment) OS=Homo sapiens PE=2 SV=1	126396	0	780	0	53	0	0.02	#DIV/0!	0	1	0	1	0	1	0	2	0.696063
tr|B7Z592|B7Z592_HUMAN	tr|B7Z592|B7	cDNA FLJ61635, highly similar to Homo sapiens likely ortholog of mouse immediate early response, erythropoietin 4 (LEREPO4), mRNA OS=Hom	45262	0	779	0	53	0	0.07	#DIV/0!	0	1	0	1	0	1	0	1	0.696063
tr|H0YFV6|H0YFV6_HUMAN	METTL3	N6-adenosine-methyltransferase 70 kDa subunit (Fragment) OS=Homo sapiens GN=METTL3 PE=4 SV=1	36576	0	776	0	53	0	0.07	#DIV/0!	0	1	0	1	0	1	0	1	0.696063
sp|Q9UKX7|NUP50_HUMAN	NUP50	Nuclear pore complex protein Nup50 OS=Homo sapiens GN=NUP50 PE=1 SV=2	50512	0	770	0	54	0	0.07	#DIV/0!	0	1	0	1	0	1	0	1	0.696063
sp|Q96IJ6|GMPPA_HUMAN	GMPPA	Mannose-1-phosphate guanyltransferase alpha OS=Homo sapiens GN=GMPPA PE=1 SV=1	46604	0	759	0	55	0	0.07	#DIV/0!	0	1	0	1	0	1	0	3	0.696063
sp|Q6PI48|SYDM_HUMAN	DARS2	Aspartate--tRNA ligase, mitochondrial OS=Homo sapiens GN=DARS2 PE=1 SV=1	74086	0	751	0	56	0	0.04	#DIV/0!	0	1	0	1	0	1	0	1	0.696063
tr|H7BY14|H7BY14_HUMAN	SAMD11	Sterile alpha motif domain-containing protein 11 (Fragment) OS=Homo sapiens GN=SAMD11 PE=4 SV=1	63302	0	750	0	56	0	0.06	#DIV/0!	0	1	0	1	0	1	0	1	0.696063
sp|Q6DKJ4|NXN_HUMAN	NXN	Nucleoredoxin OS=Homo sapiens GN=NXN PE=1 SV=2	48761	0	749	0	56	0	0.07	#DIV/0!	0	1	0	1	0	1	0	1	0.696063
tr|B7Z2B4|B7Z2B4_HUMAN	RABGAP1	Rab GTPase-activating protein 1 OS=Homo sapiens GN=RABGAP1 PE=2 SV=1	48660	0	747	0	57	0	0.07	#DIV/0!	0	1	0	1	0	1	0	3	0.696063
tr|D3DNS0|D3DNS0_HUMAN	ACTL6A	Actin-like 6A, isoform CRA_b OS=Homo sapiens GN=ACTL6A PE=2 SV=1	25781	0	745	0	57	0	0.13	#DIV/0!	0	1	0	1	0	1	0	1	0.696063
tr|E9PN86|E9PN86_HUMAN	EIF3M	Eukaryotic translation initiation factor 3 subunit M (Fragment) OS=Homo sapiens GN=EIF3M PE=2 SV=1	11896	0	743	0	57	0	0.29	#DIV/0!	0	1	0	1	0	1	0	1	0.696063
sp|Q9GZL7|WDR12_HUMAN	WDR12	Ribosome biogenesis protein WDR12 OS=Homo sapiens GN=WDR12 PE=1 SV=2	48191	0	740	0	58	0	0.07	#DIV/0!	0	1	0	1	0	1	0	1	0.696063
tr|Q149P0|Q149P0_HUMAN	GBF1	GBF1 protein OS=Homo sapiens GN=GBF1 PE=2 SV=1	207938	0	739	0	59	0	0.03	#DIV/0!	0	1	0	2	0	1	0	4	0.696063
sp|O15269|SPTC1_HUMAN	SPTLC1	Serine palmitoyltransferase 1 OS=Homo sapiens GN=SPTLC1 PE=1 SV=1	53281	0	731	0	59	0	0.06	#DIV/0!	0	1	0	1	0	1	0	1	0.696063
sp|Q6KC79|NIPBL_HUMAN	NIPBL	Nipped-B-like protein OS=Homo sapiens GN=NIPBL PE=1 SV=2	317907	0	730	0	59	0	0.01	#DIV/0!	0	1	0	1	0	1	0	2	0.696063
tr|B7Z7P2|B7Z7P2_HUMAN	tr|B7Z7P2|B7	cDNA FLJ61633, highly similar to Homo sapiens ubiquitin associated protein 2 (UBAP2), transcript variant 1, mRNA OS=Homo sapiens PE=2 SV=1	73550	0	729	0	33	0	0.09	#DIV/0!	0	0	0	1	0	1	0	2	0.696063
tr|Q9Y4A1|Q9Y4A1_HUMAN	tr|Q9Y4A1|Q	Talin-related protein (Fragment) OS=Homo sapiens PE=2 SV=1	20182	0	728	0	59	0	0.17	#DIV/0!	0	1	0	1	0	1	0	1	0.696063
sp|Q8N0X7|SPG20_HUMAN	SPG20	Spartin OS=Homo sapiens GN=SPG20 PE=1 SV=1	73358	0	721	0	60	0	0.07	#DIV/0!	0	1	0	1	0	1	0	1	0.696063
sp|Q9UI26|IPO11_HUMAN	IPO11	Importin-11 OS=Homo sapiens GN=IPO11 PE=1 SV=1	113489	0	717	0	60	0	0.06	#DIV/0!	0	1	0	1	0	1	0	1	0.696063
tr|Q0VAB1|Q0VAB1_HUMAN	TIMM50	Translocase of inner mitochondrial membrane 50 homolog (S. cerevisiae) OS=Homo sapiens GN=TIMM50 PE=2 SV=1	50960	0	715	0	61	0	0.13	#DIV/0!	0	1	0	2	0	1	0	2	0.696063
tr|Q5T5N3|Q5T5N3_HUMAN	IDE	Insulin-degrading enzyme (Fragment) OS=Homo sapiens GN=IDE PE=4 SV=1	29418	0	706	0	42	0	0.08	#DIV/0!	0	1	0	1	0	1	0	1	0.696063
tr|B4DF80|B4DF80_HUMAN	tr|B4DF80|B4	cDNA FLJ58527, highly similar to Nucleolar protein 6 OS=Homo sapiens PE=2 SV=1	122714	0	705	0	29	0	0.05	#DIV/0!	0	0	0	1	0	1	0	1	0.696063
tr|B2C8Z3|B2C8Z3_HUMAN	TGFB1	Transforming growth factor beta 1 (Fragment) OS=Homo sapiens GN=TGFB1 PE=2 SV=1	12965	0	703	0	44	0	0.24	#DIV/0!	0	1	0	1	0	1	0	1	0.696063
sp|P49419|AL7A1_HUMAN	ALDH7A1	Alpha-aminoadipic semialdehyde dehydrogenase OS=Homo sapiens GN=ALDH7A1 PE=1 SV=5	59020	0	702	0	63	0	0.13	#DIV/0!	0	1	0	1	0	1	0	1	0.696063
tr|B4DU69|B4DU69_HUMAN	tr|B4DU69|B4	cDNA FLJ56583, highly similar to Argininosuccinate lyase (EC 4.3.2.1) OS=Homo sapiens PE=2 SV=1	24664	0	700	0	63	0	0.14	#DIV/0!	0	1	0	1	0	1	0	1	0.696063
tr|C9J634|C9J634_HUMAN	PDHB	Pyruvate dehydrogenase E1 component subunit beta, mitochondrial OS=Homo sapiens GN=PDHB PE=2 SV=1	39064	0	699	0	63	0	0.12	#DIV/0!	0	1	0	1	0	1	0	1	0.696063
tr|Q6FI30|Q6FI30_HUMAN	NFIC	Nuclear factor 1 OS=Homo sapiens GN=NFIC PE=2 SV=1	55019	0	697	0	64	0	0.06	#DIV/0!	0	1	0	1	0	1	0	1	0.696063
tr|Q8N6B4|Q8N6B4_HUMAN	MLL/CALM	Myeloid/lymphoid or mixed-lineage leukemia/clathrin assembly protein fusion protein (Fragment) OS=Homo sapiens GN=MLL/CALM fusion PE=	63408	0	692	0	64	0	0.05	#DIV/0!	0	1	0	1	0	1	0	1	0.696063
sp|Q96CS3|FAF2_HUMAN	FAF2	FAS-associated factor 2 OS=Homo sapiens GN=FAF2 PE=1 SV=2	52933	0	689	0	64	0	0.13	#DIV/0!	0	1	0	1	0	1	0	1	0.696063
tr|B3KTW0|B3KTW0_HUMAN	tr|B3KTW0|B	cDNA FLJ38844 fis, clone MESAN2003662, highly similar to Cullin-1 OS=Homo sapiens PE=2 SV=1	70469	0	682	0	65	0	0.05	#DIV/0!	0	1	0	1	0	1	0	1	0.696063
sp|Q9UJA5|TRM6_HUMAN	TRMT6	tRNA (adenine(58)-N(1))-methyltransferase non-catalytic subunit TRM6 OS=Homo sapiens GN=TRMT6 PE=1 SV=1	56049	0	679	0	66	0	0.09	#DIV/0!	0	1	0	1	0	1	0	1	0.696063
tr|J3KMX1|J3KMX1_HUMAN	NUP88	Nuclear pore complex protein Nup88 (Fragment) OS=Homo sapiens GN=NUP88 PE=4 SV=1	80029	0	673	0	64	0	0.11	#DIV/0!	0	1	0	1	0	1	0	1	0.696063
tr|I1E4Y6|I1E4Y6_HUMAN	GIGYF2	PERQ amino acid-rich with GYF domain-containing protein 2 OS=Homo sapiens GN=GIGYF2 PE=4 SV=1	152891	0	669	0	67	0	0.02	#DIV/0!	0	1	0	1	0	1	0	2	0.696063
tr|H0Y5T1|H0Y5T1_HUMAN	CLASP1	CLIP-associating protein 1 (Fragment) OS=Homo sapiens GN=CLASP1 PE=4 SV=1	76997	0	667	0	67	0	0.02	#DIV/0!	0	1	0	1	0	1	0	1	0.696063
tr|B3KXH0|B3KXH0_HUMAN	tr|B3KXH0|B3	cDNA FLJ45395 fis, clone BRHIP3027191, highly similar to 150 kDa oxygen-regulated protein (Orp150) OS=Homo sapiens PE=2 SV=1	110601	0	663	0	68	0	0.03	#DIV/0!	0	1	0	1	0	1	0	3	0.696063
tr|H0Y4L0|H0Y4L0_HUMAN	HLA-B	HLA class I histocompatibility antigen, B-35 alpha chain (Fragment) OS=Homo sapiens GN=HLA-B PE=3 SV=1	26083	0	662	0	68	0	0.13	#DIV/0!	0	1	0	1	0	1	0	2	0.696063
sp|Q8TAA9|VANG1_HUMAN	VANGL1	Vang-like protein 1 OS=Homo sapiens GN=VANGL1 PE=1 SV=1	60222	0	658	0	68	0	0.05	#DIV/0!	0	1	0	1	0	1	0	1	0.696063
tr|A8MZF9|A8MZF9_HUMAN	DRG2	Developmentally-regulated GTP-binding protein 2 OS=Homo sapiens GN=DRG2 PE=2 SV=1	38504	0	657	0	68	0	0.09	#DIV/0!	0	1	0	1	0	1	0	1	0.696063
tr|J3KPP4|J3KPP4_HUMAN	LUC7L3	Cisplatin resistance-associated overexpressed protein, isoform CRA_b OS=Homo sapiens GN=LUC7L3 PE=4 SV=1	58698	0	654	0	68	0	0.06	#DIV/0!	0	1	0	1	0	1	0	1	0.696063
tr|B3KN28|B3KN28_HUMAN	tr|B3KN28|B3	cDNA FLJ13370 fis, clone PLACE1000653, highly similar to Phosphoacetylglucosamine mutase (EC 5.4.2.3) OS=Homo sapiens PE=2 SV=1	60284	0	651	0	69	0	0.05	#DIV/0!	0	1	0	1	0	1	0	1	0.696063
sp|P51531|SMCA2_HUMAN	SMARCA2	Probable global transcription activator SNF2L2 OS=Homo sapiens GN=SMARCA2 PE=1 SV=2	181794	0	647	0	70	0	0.04	#DIV/0!	0	1	0	1	0	1	0	3	0.696063
tr|B3KQ73|B3KQ73_HUMAN	tr|B3KQ73|B3	cDNA FLJ33002 fis, clone THYMU1000217, highly similar to Homo sapiens ubiquitin associated protein 2 (UBAP2), transcript variant 1, mRNA OS	37025	0	640	0	31	0	0.09	#DIV/0!	0	0	0	1	0	1	0	1	0.696063
tr|H0YJF9|H0YJF9_HUMAN	DLST	Dihydrolipoyllysine-residue succinyltransferase component of 2-oxoglutarate dehydrogenase complex, mitochondrial (Fragment) OS=Homo sap	8710	0	639	0	71	0	0.4	#DIV/0!	0	1	0	1	0	1	0	2	0.696063
tr|B7Z2R8|B7Z2R8_HUMAN	tr|B7Z2R8|B7	cDNA FLJ53324, highly similar to Tight junction protein ZO-2 OS=Homo sapiens PE=2 SV=1	137474	0	637	0	71	0	0.02	#DIV/0!	0	1	0	1	0	1	0	1	0.696063
tr|B2R791|B2R791_HUMAN	tr|B2R791|B2	cDNA, FLJ93335, highly similar to Homo sapiens PRP3 pre-mRNA processing factor 3 homolog (yeast) (PRPF3), mRNA OS=Homo sapiens PE=2 SV	77666	0	632	0	71	0	0.04	#DIV/0!	0	1	0	1	0	1	0	1	0.696063
tr|B3KTG4|B3KTG4_HUMAN	tr|B3KTG4|B3	cDNA FLJ38197 fis, clone FCBBF1000367, highly similar to Ubiquitin carboxyl-terminal hydrolase 14 (EC 3.1.2.15) OS=Homo sapiens PE=2 SV=1	43086	0	622	0	73	0	0.08	#DIV/0!	0	1	0	1	0	1	0	1	0.696063
tr|G3V5E1|G3V5E1_HUMAN	CCNK	Cyclin-K OS=Homo sapiens GN=CCNK PE=2 SV=1	43776	0	618	0	73	0	0.08	#DIV/0!	0	1	0	1	0	1	0	1	0.696063
tr|B4DM73|B4DM73_HUMAN	tr|B4DM73|B	cDNA FLJ60078, highly similar to CLIP-associating protein 2 (Fragment) OS=Homo sapiens PE=2 SV=1	116274	0	615	0	74	0	0.02	#DIV/0!	0	1	0	1	0	1	0	2	0.696063
tr|B7ZW05|B7ZW05_HUMAN	AKAP13	AKAP13 protein (Fragment) OS=Homo sapiens GN=AKAP13 PE=2 SV=1	124144	0	607	0	75	0	0.03	#DIV/0!	0	1	0	1	0	1	0	1	0.696063
tr|B4DG70|B4DG70_HUMAN	tr|B4DG70|B4	cDNA FLJ52624, highly similar to 2-hydroxyacylsphingosine1-beta-galactosyltransferase (EC 2.4.1.45) OS=Homo sapiens PE=2 SV=1	33947	0	597	0	76	0	0.1	#DIV/0!	0	1	0	1	0	1	0	2	0.696063
sp|Q49AR2|CE022_HUMAN	C5orf22	UPF0489 protein C5orf22 OS=Homo sapiens GN=C5orf22 PE=1 SV=2	50506	0	594	0	77	0	0.07	#DIV/0!	0	1	0	1	0	1	0	2	0.696063
tr|B4DK86|B4DK86_HUMAN	tr|B4DK86|B4	cDNA FLJ54466, moderately similar to Rattus norvegicus ubiquitin specific protease 19 (Usp19), mRNA OS=Homo sapiens PE=2 SV=1	56274	0	592	0	77	0	0.02	#DIV/0!	0	1	0	1	0	1	0	2	0.696063
tr|B3KNK5|B3KNK5_HUMAN	tr|B3KNK5|B3	cDNA FLJ14795 fis, clone NT2RP4001219, highly similar to Homo sapiens DnaJ (Hsp40) homolog, subfamily C, member 10 (DNAJC10), mRNA OS-	70004	0	590	0	39	0	0.11	#DIV/0!	0	1	0	1	0	1	0	1	0.696063
sp|A0AVT1|UBA6_HUMAN	UBA6	Ubiquitin-like modifier-activating enzyme 6 OS=Homo sapiens GN=UBA6 PE=1 SV=1	119207	0	587	0	78	0	0.03	#DIV/0!	0	1	0	1	0	1	0	1	0.696063
tr|B4DK35|B4DK35_HUMAN	tr|B4DK35|B4	cDNA FLJ61591, highly similar to Probable global transcription activator SNF2L2 (EC 3.6.1.-) (Fragment) OS=Homo sapiens PE=2 SV=1	112636	0	563	0	70	0	0.07	#DIV/0!	0	1	0	1	0	1	0	2	0.696063
tr|D3DTY9|D3DTY9_HUMAN	TRIM25	Tripartite motif-containing 25, isoform CRA_a OS=Homo sapiens GN=TRIM25 PE=2 SV=1	48358	0	561	0	81	0	0.07	#DIV/0!	0	1	0	1	0	1	0	1	0.696063
tr|Q4KMR3|Q4KMR3_HUMAN	MYO1E	MYO1E variant protein OS=Homo sapiens GN=MYO1E PE=2 SV=1	127552	0	548	0	84	0	0.03	#DIV/0!	0	1	0	1	0	1	0	2	0.696063
tr|B4YAH7|B4YAH7_HUMAN	ALDH2	ALDH2 (Fragment) OS=Homo sapiens GN=ALDH2 PE=2 SV=1	26844	0	537	0	86	0	0.12	#DIV/0!	0	1	0	1	0	1	0	1	0.696063
tr|Q5IRN4|Q5IRN4_HUMAN	MEF2D/DAZA	Myocyte enhancer factor 2D/deleted in azoospermia associated protein 1 fusion protein OS=Homo sapiens GN=MEF2D/DAZAP1 fusion PE=2 SV	50610	0	535	0	38	0	0.29	#DIV/0!	0	1	0	1	0	1	0	1	0.696063
sp|Q5F1R6|DJC21_HUMAN	DNAJC21	DnaJ homolog subfamily C member 21 OS=Homo sapiens GN=DNAJC21 PE=1 SV=2	62445	0	524	0	90	0	0.05	#DIV/0!	0	1	0	1	0	1	0	1	0.696063
sp|Q13057|COASY_HUMAN	COASY	Bifunctional coenzyme A synthase OS=Homo sapiens GN=COASY PE=1 SV=4	62632	0	523	0	90	0	0.11	#DIV/0!	0	1	0	2	0	1	0	4	0.696063
tr|H0Y827|H0Y827_HUMAN	FKBP10	Peptidyl-prolyl cis-trans isomerase FKBP10 (Fragment) OS=Homo sapiens GN=FKBP10 PE=4 SV=1	42521	0	521	0	91	0	0.08	#DIV/0!	0	1	0	1	0	1	0	1	0.696063
tr|K7EQ60|K7EQ60_HUMAN	COASY	Bifunctional coenzyme A synthase (Fragment) OS=Homo sapiens GN=COASY PE=4 SV=1	12950	0	515	0	92	0	0.26	#DIV/0!	0	1	0	1	0	1	0	2	0.696063
tr|K7EIS0|K7EIS0_HUMAN	TUBG1	Tubulin gamma chain (Fragment) OS=Homo sapiens GN=TUBG1 PE=3 SV=1	29797	0	512	0	72	0	0.13	#DIV/0!	0	1	0	1	0	1	0	1	0.696063
tr|H0Y7C0|H0Y7C0_HUMAN	USO1	General vesicular transport factor p115 (Fragment) OS=Homo sapiens GN=USO1 PE=4 SV=1	72345	0	488	0	95	0	0.07	#DIV/0!	0	1	0	1	0	1	0	1	0.696063
tr|H0Y4Z6|H0Y4Z6_HUMAN	PYGB	Phosphorylase (Fragment) OS=Homo sapiens GN=PYGB PE=3 SV=1	27767	0	478	0	21	0	0.22	#DIV/0!	0	0	0	1	0	1	0	1	0.696063
tr|B3KMC2|B3KMC2_HUMAN	tr|B3KMC2|B	cDNA FLJ10676 fis, clone NT2RP2006464, highly similar to WD repeat and HMG-box DNA-binding protein 1 OS=Homo sapiens PE=2 SV=1	73526	0	470	0	104	0	0.04	#DIV/0!	0	1	0	1	0	1	0	1	0.696063
tr|G5EA03|G5EA03_HUMAN	LIMCH1	LIM and calponin homology domains-containing protein 1 OS=Homo sapiens GN=LIMCH1 PE=2 SV=1	165820	0	467	0	46	0	0.06	#DIV/0!	0	1	0	1	0	1	0	1	0.696063
tr|H0YAM1|H0YAM1_HUMAN	GRSF1	G-rich sequence factor 1 (Fragment) OS=Homo sapiens GN=GRSF1 PE=4 SV=1	13410	0	452	0	71	0	0.14	#DIV/0!	0	1	0	1	0	1	0	1	0.696063
tr|A0PJC9|A0PJC9_HUMAN	TPR	TPR protein (Fragment) OS=Homo sapiens GN=TPR PE=2 SV=1	53894	0	424	0	34	0	0.05	#DIV/0!	0	0	0	1	0	1	0	1	0.696063
tr|H3BM24|H3BM24_HUMAN	HDAC2	Histone deacetylase 2 (Fragment) OS=Homo sapiens GN=HDAC2 PE=4 SV=1	18917	0	292	0	43	0	0.41	#DIV/0!	0	1	0	1	0	1	0	1	0.696063
tr|J3KT29|J3KT29_HUMAN	RPL23	60S ribosomal protein L23 OS=Homo sapiens GN=RPL23 PE=3 SV=1	13241	0	1280	0	15	0	0.26	#DIV/0!	0	0	0	1	0	0	0	1	-0.88908
sp|Q8TCS8|PNPT1_HUMAN	PNPT1	Polyribonucleotide nucleotidyltransferase 1, mitochondrial OS=Homo sapiens GN=PNPT1 PE=1 SV=2	86524	0	1279	0	15	0	0.04	#DIV/0!	0	1	0	1	0	0	0	1	-0.88908
tr|B3KUR4|B3KUR4_HUMAN	tr|B3KUR4|B3	cDNA FLJ40462 fis, clone TESTI2042062, highly similar to Transcription initiation factor TFIID subunit 6 OS=Homo sapiens PE=2 SV=1	64691	0	1277	0	16	0	0.05	#DIV/0!	0	0	0	1	0	0	0	1	-0.88908
tr|B4DMI9|B4DMI9_HUMAN	tr|B4DMI9|B4	cDNA FLJ55894, highly similar to Discs large homolog 7 OS=Homo sapiens PE=2 SV=1	89501	0	1276	0	16	0	0.04	#DIV/0!	0	0	0	1	0	0	0	1	-0.88908
sp|Q9UHD1|CHRD1_HUMAN	CHORDC1	Cysteine and histidine-rich domain-containing protein 1 OS=Homo sapiens GN=CHORDC1 PE=1 SV=2	38264	0	1275	0	16	0	0.09	#DIV/0!	0	0	0	1	0	0	0	1	-0.88908
tr|B1AW85|B1AW85_HUMAN	SLC30A7	Solute carrier family 30 (Zinc transporter), member 7 (Fragment) OS=Homo sapiens GN=SLC30A7 PE=2 SV=1	10416	0	1270	0	17	0	0.33	#DIV/0!	0	0	0	1	0	0	0	1	-0.88908
tr|B4DQ08|B4DQ08_HUMAN	tr|B4DQ08|B4	cDNA FLJ53406, highly similar to U3 small nucleolar RNA-associated protein 14 homolog A OS=Homo sapiens PE=2 SV=1	82068	0	1269	0	17	0	0.04	#DIV/0!	0	0	0	1	0	0	0	1	-0.88908
tr|Q5JPD9|Q5JPD9_HUMAN	DKFZp313I242	Coiled-coil domain-containing protein 132 OS=Homo sapiens GN=DKFZp313I2429 PE=2 SV=1	68615	0	1268	0	17	0	0.05	#DIV/0!	0	0	0	1	0	0	0	1	-0.88908
sp|P27694|RFA1_HUMAN	RPA1	Replication protein A 70 kDa DNA-binding subunit OS=Homo sapiens GN=RPA1 PE=1 SV=2	68723	0	1267	0	18	0	0.09	#DIV/0!	0	0	0	1	0	0	0	2	-0.88908
sp|O00507|USP9Y_HUMAN	USP9Y	Probable ubiquitin carboxyl-terminal hydrolase FAF-Y OS=Homo sapiens GN=USP9Y PE=1 SV=2	294370	0	1266	0	18	0	0.01	#DIV/0!	0	0	0	1	0	0	0	3	-0.88908
sp|Q7Z4Q2|HEAT3_HUMAN	HEATR3	HEAT repeat-containing protein 3 OS=Homo sapiens GN=HEATR3 PE=1 SV=2	75789	0	1264	0	18	0	0.04	#DIV/0!	0	0	0	1	0	0	0	1	-0.88908
tr|A1L172|A1L172_HUMAN	ACOT1	Acyl-CoA thioesterase 1 OS=Homo sapiens GN=ACOT1 PE=2 SV=1	46666	0	1263	0	18	0	0.07	#DIV/0!	0	0	0	1	0	0	0	1	-0.88908
sp|Q5R3I4|TTC38_HUMAN	TTC38	Tetratricopeptide repeat protein 38 OS=Homo sapiens GN=TTC38 PE=1 SV=1	53267	0	1262	0	18	0	0.06	#DIV/0!	0	0	0	1	0	0	0	1	-0.88908
tr|B3KNK9|B3KNK9_HUMAN	GOLPH2	Golgi phosphoprotein 2, isoform CRA_a OS=Homo sapiens GN=GOLPH2 PE=2 SV=1	45477	0	1261	0	18	0	0.07	#DIV/0!	0	0	0	1	0	0	0	1	-0.88908
sp|A6BM72|MEG11_HUMAN	MEGF11	Multiple epidermal growth factor-like domains protein 11 OS=Homo sapiens GN=MEGF11 PE=2 SV=3	119091	0	1260	0	18	0	0.03	#DIV/0!	0	0	0	1	0	0	0	1	-0.88908
tr|B4DPX9|B4DPX9_HUMAN	tr|B4DPX9|B4	cDNA FLJ56221, highly similar to YTH domain protein 3 OS=Homo sapiens PE=2 SV=1	64596	0	1259	0	18	0	0.05	#DIV/0!	0	0	0	1	0	0	0	1	-0.88908
sp|Q9Y512|SAM50_HUMAN	SAMM50	Sorting and assembly machinery component 50 homolog OS=Homo sapiens GN=SAMM50 PE=1 SV=3	52342	0	1258	0	19	0	0.06	#DIV/0!	0	0	0	1	0	0	0	1	-0.88908
tr|H0Y4V9|H0Y4V9_HUMAN	LARP4B	La-related protein 4B (Fragment) OS=Homo sapiens GN=LARP4B PE=4 SV=1	36998	0	1257	0	19	0	0.09	#DIV/0!	0	0	0	1	0	0	0	1	-0.88908
sp|Q53EP0|FND3B_HUMAN	FNDC3B	Fibronectin type III domain-containing protein 3B OS=Homo sapiens GN=FNDC3B PE=1 SV=2	134970	0	1256	0	19	0	0.02	#DIV/0!	0	0	0	1	0	0	0	1	-0.88908
tr|D0PQI2|D0PQI2_HUMAN	ENAH	Enabled-like protein variant hMenaDv6 OS=Homo sapiens GN=ENAH PE=2 SV=1	60426	0	1254	0	19	0	0.05	#DIV/0!	0	0	0	1	0	0	0	1	-0.88908
sp|Q9H9A5|CNOTA_HUMAN	CNOT10	CCR4-NOT transcription complex subunit 10 OS=Homo sapiens GN=CNOT10 PE=1 SV=1	83398	0	1253	0	19	0	0.04	#DIV/0!	0	0	0	1	0	0	0	1	-0.88908
tr|Q4LE61|Q4LE61_HUMAN	SYMPK	SYMPK variant protein (Fragment) OS=Homo sapiens GN=SYMPK variant protein PE=2 SV=1	119459	0	1251	0	19	0	0.03	#DIV/0!	0	0	0	1	0	0	0	1	-0.88908
tr|D3VVH4|D3VVH4_HUMAN	ATXN3	Ataxin 3 variant ref (Fragment) OS=Homo sapiens GN=ATXN3 PE=2 SV=1	45518	0	1250	0	19	0	0.07	#DIV/0!	0	0	0	1	0	0	0	1	-0.88908
tr|B4DQV4|B4DQV4_HUMAN	SNX4	Sorting nexin-4 OS=Homo sapiens GN=SNX4 PE=2 SV=1	35873	0	1249	0	19	0	0.09	#DIV/0!	0	0	0	1	0	0	0	2	-0.88908
tr|Q6IPX4|Q6IPX4_HUMAN	RPS16	40S ribosomal protein S16 OS=Homo sapiens GN=RPS16 PE=2 SV=1	17267	0	1248	0	19	0	0.2	#DIV/0!	0	0	0	1	0	0	0	1	-0.88908
tr|I3L1C9|I3L1C9_HUMAN	SIRT7	NAD-dependent protein deacetylase sirtuin-7 OS=Homo sapiens GN=SIRT7 PE=4 SV=1	36251	0	1247	0	19	0	0.09	#DIV/0!	0	0	0	1	0	0	0	2	-0.88908
tr|Q1RMC9|Q1RMC9_HUMAN	ERBB2IP	ERBB2IP protein OS=Homo sapiens GN=ERBB2IP PE=2 SV=1	154105	0	1246	0	19	0	0.02	#DIV/0!	0	0	0	1	0	0	0	2	-0.88908
tr|A1L1C6|A1L1C6_HUMAN	LRRC7	LRRC7 protein OS=Homo sapiens GN=LRRC7 PE=2 SV=1	91836	0	1245	0	20	0	0.04	#DIV/0!	0	0	0	1	0	0	0	1	-0.88908
sp|Q96G03|PGM2_HUMAN	PGM2	Phosphoglucomutase-2 OS=Homo sapiens GN=PGM2 PE=1 SV=4	68754	0	1244	0	20	0	0.06	#DIV/0!	0	0	0	1	0	0	0	2	-0.88908
tr|B3KSZ8|B3KSZ8_HUMAN	tr|B3KSZ8|B3	cDNA FLJ37362 fis, clone BRAMY2024004, highly similar to Splicing factor, arginine/serine-rich 16 OS=Homo sapiens PE=2 SV=1	74889	0	1242	0	20	0	0.04	#DIV/0!	0	0	0	1	0	0	0	1	-0.88908
tr|I3L174|I3L174_HUMAN	HCFC1R1	Host cell factor C1 regulator 1 (Fragment) OS=Homo sapiens GN=HCFC1R1 PE=4 SV=1	13542	0	1241	0	20	0	0.25	#DIV/0!	0	0	0	1	0	0	0	1	-0.88908
tr|B3KWV6|B3KWV6_HUMAN	tr|B3KWV6|B	cDNA FLJ43948 fis, clone TESTI4014924, highly similar to Homo sapiens cytoplasmic FMR1 interacting protein 1 (CYFIP1), transcript variant 1, m	147082	0	1239	0	20	0	0.13	#DIV/0!	0	0	0	2	0	0	0	2	-0.88908
tr|A8K1Y9|A8K1Y9_HUMAN	GNAL	Guanine nucleotide binding protein (G protein), alpha activating activity polypeptide, olfactory type, isoform CRA_b OS=Homo sapiens GN=GNA	44794	0	1149	0	0	0	0.08	#DIV/0!	0	0	0	0	0	0	0	1	-0.88908
tr|B4DQR0|B4DQR0_HUMAN	tr|B4DQR0|B4	cDNA FLJ54377 OS=Homo sapiens PE=2 SV=1	91332	0	923	0	0	0	0.03	#DIV/0!	0	0	0	0	0	0	0	1	-0.88908
tr|H0Y424|H0Y424_HUMAN	LMO7	LIM domain only protein 7 (Fragment) OS=Homo sapiens GN=LMO7 PE=4 SV=1	121750	0	811	0	0	0	0.02	#DIV/0!	0	0	0	0	0	0	0	1	-0.88908
tr|B4DZ17|B4DZ17_HUMAN	tr|B4DZ17|B4	cDNA FLJ59789, highly similar to Fragile X mental retardation 1 protein OS=Homo sapiens PE=2 SV=1	28910	0	645	0	0	0	0.05	#DIV/0!	0	0	0	0	0	0	0	2	-0.88908
tr|Q8N274|Q8N274_HUMAN	tr|Q8N274|Q	cDNA FLJ33834 fis, clone CTONG2004264, moderately similar to NEUROBLAST DIFFERENTIATION ASSOCIATED PROTEIN AHNAK OS=Homo sapie	86111	401	373	43	133	0.01	0.3	30	1	4	1	7	2	9	4	11	1.03787
tr|Q2TU64|Q2TU64_HUMAN	tr|Q2TU64|Q	PIG48 OS=Homo sapiens PE=2 SV=1	61110	279	126	68	286	0.05	1.15	23	1	7	1	12	3	13	3	14	1.059788
tr|B2R5M9|B2R5M9_HUMAN	tr|B2R5M9|B	cDNA, FLJ92537, highly similar to Homo sapiens procollagen-lysine, 2-oxoglutarate 5-dioxygenase (lysine hydroxylase, Ehlers-Danlos syndrome t	84022	251	109	76	404	0.04	0.76	19	1	6	1	13	1	13	1	14	2.282843
tr|B4DTV0|B4DTV0_HUMAN	tr|B4DTV0|B4	cDNA FLJ57801, highly similar to Homo sapiens AHNAK nucleoprotein (desmoyokin) (AHNAK), transcript variant 1, mRNA (Fragment) OS=Homo	114179	454	204	38	253	0.03	0.48	16	1	7	1	14	3	17	3	19	1.4349
tr|B2R9U2|B2R9U2_HUMAN	tr|B2R9U2|B2	cDNA, FLJ94557, highly similar to Homo sapiens FK506 binding protein 4, 59kDa (FKBP4), mRNA OS=Homo sapiens PE=2 SV=1	52087	623	179	24	240	0.06	0.85	14.166667	0	5	1	9	2	10	2	12	1.182439
tr|B2R7B5|B2R7B5_HUMAN	tr|B2R7B5|B2	cDNA, FLJ93365, highly similar to Homo sapiens KH domain containing, RNA binding, signal transduction associated 1 (KHDRBS1), mRNA OS=Ho	48309	347	193	51	231	0.07	0.98	14	1	6	1	9	2	10	4	10	1.182439
tr|B1ANR0|B1ANR0_HUMAN	PABPC4	Poly(A) binding protein, cytoplasmic 4 (Inducible form) OS=Homo sapiens GN=PABPC4 PE=4 SV=1	68156	504	285	33	175	0.05	0.68	13.6	0	6	1	11	1	11	2	15	2.051159
tr|B4DY09|B4DY09_HUMAN	tr|B4DY09|B4	cDNA FLJ51660, highly similar to Interleukin enhancer-binding factor 2 OS=Homo sapiens PE=2 SV=1	39057	383	184	45	275	0.08	1.08	13.5	1	6	1	9	1	8	1	10	1.614523
sp|O14980|XPO1_HUMAN	XPO1	Exportin-1 OS=Homo sapiens GN=XPO1 PE=1 SV=1	124447	305	162	59	305	0.03	0.4	13.333333	1	8	1	13	1	13	3	15	2.282843

sp|Q09666|AHNK_HUMAN	AHNAK	Neuroblast differentiation-associated protein AHNAK OS=Homo sapiens GN=AHNAK PE=1 SV=2	629213	401	137	43	351	0.01	0.13	13	1	10	1	23	3	28	6	35	2.140481
tr|E7EVA0|E7EVA0_HUMAN	MAP4	Microtubule-associated protein OS=Homo sapiens GN=MAP4 PE=2 SV=1	246546	429	570	40	79	0.01	0.12	12	1	2	1	3	2	3	4	3	-0.40378
tr|Q05CK9|Q05CK9_HUMAN	SYNCRIP	SYNCRIP protein (Fragment) OS=Homo sapiens GN=SYNCRIP PE=2 SV=1	50847	252	88	76	440	0.13	1.51	11.615385	2	14	2	20	2	21	2	21	2.218358
tr|Q8TC62|Q8TC62_HUMAN	Sep-07	Septin 7 OS=Homo sapiens GN=SEPT7 PE=2 SV=3	48913	531	406	31	118	0.07	0.8	11.428571	1	4	1	8	1	7	2	9	1.433772
tr|Q0VGD6|Q0VGD6_HUMAN	HNRPR	HNRPR protein (Fragment) OS=Homo sapiens GN=HNRPR PE=2 SV=1	68218	390	117	44	402	0.1	1.12	11.2	1	13	2	20	2	20	4	21	2.149465
sp|P45974|UBP5_HUMAN	USP5	Ubiquitin carboxyl-terminal hydrolase 5 OS=Homo sapiens GN=USP5 PE=1 SV=2	96638	622	149	24	323	0.03	0.31	10.333333	0	6	1	9	2	8	2	11	0.877226
tr|B4DT29|B4DT29_HUMAN	tr|B4DT29|B4	cDNA FLJ51082, highly similar to Actin-like protein 3 OS=Homo sapiens PE=2 SV=1	40959	548	142	30	145	0.08	0.82	10.25	0	4	1	6	1	9	1	9	1.775167
tr|Q2Q9B7|Q2Q9B7_HUMAN	G6PD	Glucose-6-phosphate 1-dehydrogenase (Fragment) OS=Homo sapiens GN=G6PD PE=2 SV=1	55221	176	95	118	466	0.18	1.84	10.222222	2	13	2	17	5	21	6	21	1.079859
tr|A8KAK1|A8KAK1_HUMAN	tr|A8KAK1|A8	cDNA FLJ77398, highly similar to Homo sapiens UDP-glucose ceramide glucosyltransferase-like 1, transcript variant 2, mRNA OS=Homo sapiens I	175467	635	190	22	269	0.02	0.2	10	0	9	1	10	1	11	1	11	2.051159
tr|B4DYA7|B4DYA7_HUMAN	tr|B4DYA7|B4	Glucose-6-phosphate 1-dehydrogenase OS=Homo sapiens PE=2 SV=1	58757	176	93	123	464	0.18	1.77	9.8333333	2	12	3	18	6	21	7	22	0.838519
tr|B4DVJ0|B4DVJ0_HUMAN	tr|B4DVJ0|B4	Glucose-6-phosphate isomerase OS=Homo sapiens PE=2 SV=1	60181	117	41	182	935	0.3	2.78	9.2666667	4	21	5	28	6	32	6	33	1.436597
tr|Q96QL0|Q96QL0_HUMAN	RPL3	Ribosomal protein L3 OS=Homo sapiens GN=RPL3 PE=2 SV=1	46376	245	58	82	645	0.32	2.95	9.21875	4	17	4	23	5	24	7	27	1.268843
tr|A8K2Y2|A8K2Y2_HUMAN	tr|A8K2Y2|A8	cDNA FLJ78120, highly similar to Homo sapiens eukaryotic translation initiation factor 2, subunit 3 gamma, 52kDa (EIF2S3), mRNA OS=Homo sap	51648	475	296	37	170	0.06	0.54	9	1	4	1	7	1	7	1	8	1.433772
tr|Q6ZQN2|Q6ZQN2_HUMAN	tr|Q6ZQN2|Q	cDNA FLJ46846 fis, clone UTERU3004635, moderately similar to Neuroblast differentiation associated protein AHNAK (Fragment) OS=Homo sap	180689	401	509	43	94	0.01	0.09	9	1	3	1	5	2	9	3	12	1.03787
tr|Q59FV6|Q59FV6_HUMAN	tr|Q59FV6|Q	ARP3 actin-related protein 3 homolog variant (Fragment) OS=Homo sapiens PE=2 SV=1	42517	548	173	30	294	0.08	0.69	8.625	0	7	1	9	1	12	1	12	2.171633
tr|B4DE36|B4DE36_HUMAN	tr|B4DE36|B4	Glucose-6-phosphate isomerase OS=Homo sapiens PE=2 SV=1	60376	117	40	182	918	0.3	2.54	8.4666667	4	21	5	26	7	31	7	32	1.184547
tr|B7Z1R5|B7Z1R5_HUMAN	ATP6V1A	V-type proton ATPase catalytic subunit A OS=Homo sapiens GN=ATP6V1A PE=2 SV=1	65037	428	239	40	217	0.05	0.41	8.2	1	5	1	7	1	7	2	8	1.433772
sp|O14929|HAT1_HUMAN	HAT1	Histone acetyltransferase type B catalytic subunit OS=Homo sapiens GN=HAT1 PE=1 SV=1	49880	343	221	51	233	0.07	0.57	8.1428571	1	5	1	7	1	8	1	9	1.614523
tr|B2R7C5|B2R7C5_HUMAN	tr|B2R7C5|B2	cDNA, FLJ93378, highly similar to Homo sapiens MCM3 minichromosome maintenance deficient 3 (S. cerevisiae) (MCM3), mRNA OS=Homo sapi	91579	479	279	36	179	0.04	0.32	8	1	4	1	8	1	9	1	10	1.775167
tr|I3L1L3|I3L1L3_HUMAN	MYBBP1A	Myb-binding protein 1A (Fragment) OS=Homo sapiens GN=MYBBP1A PE=4 SV=1	141124	698	210	18	235	0.02	0.16	8	0	6	1	6	1	6	2	8	1.227142
tr|J3KR24|J3KR24_HUMAN	IARS	Isoleucine--tRNA ligase, cytoplasmic OS=Homo sapiens GN=IARS PE=4 SV=1	132820	366	321	47	154	0.02	0.16	8	1	3	1	6	2	8	2	9	0.877226
sp|P41250|SYG_HUMAN	GARS	Glycine--tRNA ligase OS=Homo sapiens GN=GARS PE=1 SV=3	83854	392	453	44	107	0.04	0.31	7.75	1	2	1	7	1	7	2	9	1.433772
tr|B2R8R5|B2R8R5_HUMAN	tr|B2R8R5|B2	cDNA, FLJ94025, highly similar to Homo sapiens tripartite motif-containing 28 (TRIM28), mRNA OS=Homo sapiens PE=2 SV=1	90271	320	203	56	254	0.07	0.53	7.5714286	1	6	3	13	2	14	4	15	1.648819
tr|F8WJN3|F8WJN3_HUMAN	CPSF6	Cleavage and polyadenylation-specificity factor subunit 6 OS=Homo sapiens GN=CPSF6 PE=2 SV=1	52409	379	261	46	196	0.06	0.44	7.3333333	1	4	1	6	1	6	1	6	1.227142
tr|E9PBS1|E9PBS1_HUMAN	PAICS	Phosphoribosylaminoimidazole carboxylase (Fragment) OS=Homo sapiens GN=PAICS PE=2 SV=1	46307	270	259	69	198	0.07	0.51	7.2857143	1	4	1	6	2	6	5	8	0.489845
tr|B4DJ30|B4DJ30_HUMAN	tr|B4DJ30|B4	cDNA FLJ61290, highly similar to Neutral alpha-glucosidase AB OS=Homo sapiens PE=2 SV=1	113539	47	12	446	2139	0.62	4.5	7.2580645	12	54	19	77	17	89	22	96	1.475822
tr|J3KPS3|J3KPS3_HUMAN	ALDOA	Fructose-bisphosphate aldolase OS=Homo sapiens GN=ALDOA PE=3 SV=1	40249	63	22	338	1495	1.79	12.9	7.2067039	11	35	15	43	17	50	18	55	0.64318
tr|A6NKB8|A6NKB8_HUMAN	RNPEP	Aminopeptidase B OS=Homo sapiens GN=RNPEP PE=2 SV=1	68752	316	263	57	195	0.05	0.36	7.2	1	4	2	7	2	8	2	10	0.877226
tr|B4DZ53|B4DZ53_HUMAN	tr|B4DZ53|B4	cDNA FLJ59643, highly similar to Neutral alpha-glucosidase AB OS=Homo sapiens PE=2 SV=1	96752	47	16	400	1816	0.62	4.46	7.1935484	10	46	17	63	15	71	19	79	1.324377
sp|O95373|IPO7_HUMAN	IPO7	Importin-7 OS=Homo sapiens GN=IPO7 PE=1 SV=1	120751	162	84	132	493	0.08	0.57	7.125	3	13	3	18	3	18	4	21	1.51525
tr|B4DLT2|B4DLT2_HUMAN	tr|B4DLT2|B4	cDNA FLJ56637, highly similar to Nuclear pore complex protein Nup155 OS=Homo sapiens PE=2 SV=1	152734	489	325	35	153	0.02	0.14	7	0	4	1	6	1	7	1	7	1.433772
tr|B4E091|B4E091_HUMAN	tr|B4E091|B4	cDNA FLJ55438, highly similar to Splicing factor 3 subunit 1 OS=Homo sapiens PE=2 SV=1	77415	482	388	36	126	0.04	0.28	7	1	4	1	6	1	5	1	6	0.985955
tr|A6NG51|A6NG51_HUMAN	SPTAN1	Spectrin alpha chain, non-erythrocytic 1 OS=Homo sapiens GN=SPTAN1 PE=2 SV=2	285628	167	36	130	996	0.06	0.41	6.8333333	2	23	5	29	6	30	9	39	1.344607
tr|B4DQJ8|B4DQJ8_HUMAN	PGD	6-phosphogluconate dehydrogenase, decarboxylating OS=Homo sapiens GN=PGD PE=2 SV=1	52352	293	106	62	432	0.2	1.35	6.75	1	13	3	15	2	16	3	17	1.83559
tr|B2RBE0|B2RBE0_HUMAN	tr|B2RBE0|B2	cDNA, FLJ95462, highly similar to Homo sapiens fatty-acid-Coenzyme A ligase, long-chain 3 (FACL3),mRNA OS=Homo sapiens PE=2 SV=1	81266	462	310	37	159	0.04	0.27	6.75	0	4	1	5	1	5	1	5	0.985955
tr|B3KQT2|B3KQT2_HUMAN	tr|B3KQT2|B3	cDNA PSEC0148 fis, clone PLACE1007202, highly similar to Protein disulfide-isomerase A3 (EC 5.3.4.1) OS=Homo sapiens PE=2 SV=1	55268	546	114	30	375	0.19	1.27	6.6842105	0	9	3	14	2	13	8	18	1.545546
tr|Q5SU16|Q5SU16_HUMAN	TUBB	Beta 5-tubulin OS=Homo sapiens GN=TUBB PE=2 SV=1	50095	25	10	680	2182	3.07	20.35	6.6286645	26	67	33	82	35	86	39	95	0.399628
sp|P04040|CATA_HUMAN	CAT	Catalase OS=Homo sapiens GN=CAT PE=1 SV=3	59947	299	170	60	296	0.11	0.71	6.4545455	1	7	2	10	2	10	2	11	1.182439
sp|O00629|IMA4_HUMAN	KPNA4	Importin subunit alpha-4 OS=Homo sapiens GN=KPNA4 PE=1 SV=1	58364	472	500	37	97	0.06	0.38	6.3333333	0	2	1	2	2	2	2	2	-0.88938
sp|P08238|HS90B_HUMAN	HSP90AB1	Heat shock protein HSP 90-beta OS=Homo sapiens GN=HSP90AB1 PE=1 SV=4	83554	32	11	622	2176	1.24	7.62	6.1451613	18	51	28	72	29	75	35	85	0.470523
tr|B2R5U1|B2R5U1_HUMAN	tr|B2R5U1|B2	cDNA, FLJ92620, highly similar to Homo sapiens staphylococcal nuclease domain containing 1 (SND1),mRNA OS=Homo sapiens PE=2 SV=1	100294	150	53	144	716	0.17	1.03	6.0588235	4	17	5	22	10	27	12	30	0.501486
tr|H7C2Y0|H7C2Y0_HUMAN	Sep-02	Septin-2 (Fragment) OS=Homo sapiens GN=SEPT2 PE=4 SV=1	21750	543	465	30	105	0.09	0.54	6	1	3	1	3	1	3	2	4	0.33352
sp|P11388|TOP2A_HUMAN	TOP2A	DNA topoisomerase 2-alpha OS=Homo sapiens GN=TOP2A PE=1 SV=3	175017	606	398	25	122	0.02	0.12	6	0	3	1	6	1	6	2	10	1.227142
tr|B2R5V9|B2R5V9_HUMAN	tr|B2R5V9|B2	cDNA, FLJ92652, highly similar to Homo sapiens high density lipoprotein binding protein (vigilin)(HDLBP), mRNA OS=Homo sapiens PE=2 SV=1	141919	253	384	76	128	0.02	0.12	6	1	2	1	5	2	5	2	6	0.248657
tr|Q2HJC8|Q2HJC8_HUMAN	HDLBP	HDLBP protein (Fragment) OS=Homo sapiens GN=HDLBP PE=2 SV=1	65655	253	331	0	57	0.02	0.12	6	0	1	0	1	1	1	1	1	-0.88923
tr|B2R6D0|B2R6D0_HUMAN	tr|B2R6D0|B2	cDNA, FLJ92896, highly similar to Homo sapiens proteasome (prosome, macropain) 26S subunit, non-ATPase, 1 (PSMD1), mRNA OS=Homo sapie	106797	386	159	45	309	0.06	0.35	5.8333333	0	8	2	10	2	12	4	14	1.434336
sp|O00232|PSD12_HUMAN	PSMD12	26S proteasome non-ATPase regulatory subunit 12 OS=Homo sapiens GN=PSMD12 PE=1 SV=3	53270	283	222	66	232	0.06	0.35	5.8333333	1	3	1	5	1	5	1	6	0.985955
sp|Q6P2Q9|PRP8_HUMAN	PRPF8	Pre-mRNA-processing-splicing factor 8 OS=Homo sapiens GN=PRPF8 PE=1 SV=2	274738	130	37	167	1012	0.1	0.58	5.8	7	30	8	39	10	39	10	46	1.026062
tr|A6NN80|A6NN80_HUMAN	ANXA6	Annexin OS=Homo sapiens GN=ANXA6 PE=3 SV=3	75571	349	131	50	366	0.14	0.81	5.7857143	1	12	3	14	3	17	3	21	1.4349
tr|B2RB23|B2RB23_HUMAN	tr|B2RB23|B2	cDNA, FLJ95265, highly similar to Homo sapiens acetyl-Coenzyme A acyltransferase 2 (mitochondrial 3-oxoacyl-Coenzyme A thiolase) (ACAA2), r	42439	544	275	30	185	0.08	0.46	5.75	0	4	1	5	1	5	1	5	0.985955
tr|H0Y8E6|H0Y8E6_HUMAN	MCM2	DNA replication licensing factor MCM2 (Fragment) OS=Homo sapiens GN=MCM2 PE=3 SV=1	94792	211	169	98	300	0.07	0.4	5.7142857	1	9	2	10	2	11	2	11	1.313862
sp|P52597|HNRPF_HUMAN	HNRNPF	Heterogeneous nuclear ribonucleoprotein F OS=Homo sapiens GN=HNRNPF PE=1 SV=3	45985	226	235	91	221	0.07	0.4	5.7142857	1	5	1	6	2	7	3	7	0.696475
tr|B2ZZ89|B2ZZ89_HUMAN	SPTBN1	Spectrin beta non-erythrocytic 1 OS=Homo sapiens GN=SPTBN1 PE=2 SV=1	275237	49	8	431	2622	0.22	1.25	5.6818182	11	61	17	72	20	78	24	85	1.055371
tr|A2VCT2|A2VCT2_HUMAN	OGDH	OGDH protein (Fragment) OS=Homo sapiens GN=OGDH PE=2 SV=1	72973	432	187	40	151	0.05	0.28	5.6	1	3	1	4	1	4	1	5	0.696269
tr|B4DJ60|B4DJ60_HUMAN	tr|B4DJ60|B4	cDNA FLJ55072, highly similar to Succinate dehydrogenase (ubiquinone) flavoprotein subunit, mitochondrial (EC1.3.5.1) OS=Homo sapiens PE=2	68233	393	141	44	307	0.1	0.56	5.6	0	5	2	8	3	9	5	10	0.552111
tr|B2R5P6|B2R5P6_HUMAN	TXNRD1	Thioredoxin reductase 1, cytoplasmic OS=Homo sapiens GN=TXNRD1 PE=2 SV=1	55253	108	75	204	488	0.26	1.45	5.5769231	3	12	5	16	5	17	6	19	0.78216
tr|B0YJC4|B0YJC4_HUMAN	VIM	Vimentin OS=Homo sapiens GN=VIM PE=3 SV=1	49680	66	42	312	831	1.03	5.71	5.5436893	9	21	11	32	12	34	15	38	0.577704
tr|Q6NTA2|Q6NTA2_HUMAN	HNRNPL	HNRNPL protein (Fragment) OS=Homo sapiens GN=HNRNPL PE=2 SV=2	62515	182	85	117	493	0.23	1.27	5.5217391	4	13	4	18	6	23	8	25	0.967202
tr|Q8IZ29|Q8IZ29_HUMAN	TUBB2C	Tubulin, beta 2C OS=Homo sapiens GN=TUBB2C PE=2 SV=1	50264	27	13	674	2125	3.04	16.41	5.3980263	26	65	32	77	34	84	38	91	0.407074
sp|P13010|XRCC5_HUMAN	XRCC5	X-ray repair cross-complementing protein 5 OS=Homo sapiens GN=XRCC5 PE=1 SV=3	83222	128	57	172	680	0.36	1.84	5.1111111	6	19	8	28	8	31	9	37	1.003643
tr|B5MCX3|B5MCX3_HUMAN	Sep-02	Septin-2 OS=Homo sapiens GN=SEPT2 PE=2 SV=1	37145	543	253	30	201	0.09	0.46	5.1111111	1	5	1	5	2	5	3	6	0.248657
tr|H3BUF6|H3BUF6_HUMAN	ATXN2L	Ataxin-2-like protein OS=Homo sapiens GN=ATXN2L PE=4 SV=1	112929	656	304	22	166	0.03	0.15	5	0	3	1	5	2	6	2	7	0.489845
tr|D5FHQ7|D5FHQ7_HUMAN	HLA-A	MHC class I antigen (Fragment) OS=Homo sapiens GN=HLA-A PE=3 SV=1	21188	640	432	22	113	0.16	0.8	5	0	3	1	4	2	6	3	6	0.489845
tr|A8K8N7|A8K8N7_HUMAN	PFAS	Phosphoribosylformylglycinamidine synthase (FGAR amidotransferase), isoform CRA_b OS=Homo sapiens GN=PFAS PE=2 SV=1	146286	380	201	46	255	0.05	0.25	5	0	4	2	10	3	7	5	11	0.210716
tr|B5LY69|B5LY69_HUMAN	UMPS	Uridine monophosphate synthetase isoform G OS=Homo sapiens GN=UMPS PE=2 SV=1	40514	302	1067	59	32	0.1	0.5	5	1	0	1	1	1	1	1	1	-0.88923
tr|H7BZJ3|H7BZJ3_HUMAN	PDIA3	Thioredoxin (Fragment) OS=Homo sapiens GN=PDIA3 PE=3 SV=1	13739	546	760	22	55	0.19	0.94	4.9473684	0	1	1	4	1	3	1	4	0.33352
sp|Q6IBS0|TWF2_HUMAN	TWF2	Twinfilin-2 OS=Homo sapiens GN=TWF2 PE=1 SV=2	39751	535	265	30	191	0.08	0.38	4.75	0	4	1	4	1	4	1	4	0.696269
tr|B4DMJ6|B4DMJ6_HUMAN	tr|B4DMJ6|B	cDNA FLJ50996, highly similar to 60S ribosomal protein L4 OS=Homo sapiens PE=2 SV=1	46269	163	86	132	444	0.51	2.42	4.745098	4	12	6	18	8	18	9	22	0.233483
tr|Q8IWP6|Q8IWP6_HUMAN	tr|Q8IWP6|Q	Class IVb beta tubulin OS=Homo sapiens PE=2 SV=1	50177	28	14	654	2013	3.05	14.43	4.7311475	27	62	32	74	34	83	37	89	0.389718
sp|O43175|SERA_HUMAN	PHGDH	D-3-phosphoglycerate dehydrogenase OS=Homo sapiens GN=PHGDH PE=1 SV=4	57356	131	92	166	477	0.25	1.18	4.72	3	12	4	17	4	19	4	21	1.228476
tr|Q59EG8|Q59EG8_HUMAN	tr|Q59EG8|Q	Proteasome 26S non-ATPase subunit 2 variant (Fragment) OS=Homo sapiens PE=2 SV=1	101322	198	124	105	387	0.14	0.66	4.7142857	1	10	4	16	4	16	5	21	0.98693
tr|F5GX33|F5GX33_HUMAN	SLC25A13	Calcium-binding mitochondrial carrier protein Aralar2 OS=Homo sapiens GN=SLC25A13 PE=2 SV=1	62548	353	255	49	200	0.11	0.51	4.6363636	1	5	2	8	3	7	4	8	0.210716
sp|Q9NZB2|F120A_HUMAN	FAM120A	Constitutive coactivator of PPAR-gamma-like protein 1 OS=Homo sapiens GN=FAM120A PE=1 SV=2	123008	263	164	73	304	0.05	0.23	4.6	2	7	2	8	2	8	2	8	0.877226
tr|B4DU00|B4DU00_HUMAN	tr|B4DU00|B	cDNA FLJ54547, highly similar to Alpha-1 catenin (Cadherin-associated protein) OS=Homo sapiens PE=2 SV=1	89332	389	237	45	134	0.07	0.32	4.5714286	0	4	2	6	2	6	4	8	0.489845
tr|B4DHQ3|B4DHQ3_HUMAN	tr|B4DHQ3|B	cDNA FLJ56437, highly similar to Phosphoserine aminotransferase (EC 2.6.1.52) OS=Homo sapiens PE=2 SV=1	45783	552	492	30	99	0.07	0.32	4.5714286	0	3	1	4	1	3	2	5	0.33352
sp|P04350|TBB4A_HUMAN	TUBB4A	Tubulin beta-4A chain OS=Homo sapiens GN=TUBB4A PE=1 SV=2	50010	42	21	509	1611	1.96	8.93	4.5561224	21	53	27	62	29	70	32	75	0.370767
tr|B4DSQ5|B4DSQ5_HUMAN	tr|B4DSQ5|B4	cDNA FLJ53608, highly similar to Protein transport protein Sec23A OS=Homo sapiens PE=2 SV=1	83787	474	246	37	172	0.08	0.36	4.5	0	6	2	7	2	7	3	7	0.696475
tr|A8K781|A8K781_HUMAN	tr|A8K781|A8	cDNA FLJ75299, highly similar to Xenopus laevis proteasome (prosome, macropain) 26S subunit, ATPase 3, mRNA OS=Homo sapiens PE=2 SV=1	47578	603	612	26	74	0.07	0.31	4.4285714	0	2	1	4	2	4	2	5	-0.04103
sp|Q96TA1|NIBL1_HUMAN	FAM129B	Niban-like protein 1 OS=Homo sapiens GN=FAM129B PE=1 SV=3	84598	155	123	137	387	0.16	0.7	4.375	4	10	4	14	4	14	4	14	0.800159
tr|Q9BSV4|Q9BSV4_HUMAN	SFPQ	SFPQ protein (Fragment) OS=Homo sapiens GN=SFPQ PE=2 SV=2	68702	228	74	91	527	0.26	1.13	4.3461538	2	15	5	18	6	22	7	23	0.904287
tr|H0YLA4|H0YLA4_HUMAN	SORD	Sorbitol dehydrogenase OS=Homo sapiens GN=SORD PE=3 SV=1	36833	473	421	37	106	0.09	0.39	4.3333333	0	1	1	3	2	3	2	3	-0.40378
tr|C9J1E7|C9J1E7_HUMAN	AP1B1	AP-1 complex subunit beta-1 (Fragment) OS=Homo sapiens GN=AP1B1 PE=2 SV=1	66014	648	595	22	69	0.03	0.13	4.3333333	0	1	1	3	3	3	3	3	-0.88954
tr|A8K492|A8K492_HUMAN	tr|A8K492|A8	cDNA FLJ76789, highly similar to Homo sapiens methionine-tRNA synthetase (MARS), mRNA OS=Homo sapiens PE=2 SV=1	102277	385	300	45	168	0.07	0.3	4.2857143	1	3	2	6	2	6	2	6	0.489845
sp|Q15645|PCH2_HUMAN	TRIP13	Pachytene checkpoint protein 2 homolog OS=Homo sapiens GN=TRIP13 PE=1 SV=2	48863	507	503	33	96	0.07	0.3	4.2857143	0	2	1	4	2	5	2	7	0.248657
tr|Q6FHQ0|Q6FHQ0_HUMAN	RBBP7	RBBP7 protein (Fragment) OS=Homo sapiens GN=RBBP7 PE=2 SV=1	48132	468	582	37	78	0.07	0.3	4.2857143	1	2	1	4	3	6	3	6	0.004086
tr|B2R959|B2R959_HUMAN	tr|B2R959|B2	cDNA, FLJ94229, highly similar to Homo sapiens heterogeneous nuclear ribonucleoprotein L (HNRPL),mRNA OS=Homo sapiens PE=2 SV=1	60838	182	166	117	302	0.23	0.98	4.2608696	4	9	4	13	6	18	8	19	0.62117
tr|B4DSI9|B4DSI9_HUMAN	tr|B4DSI9|B4	cDNA FLJ56483, highly similar to Eukaryotic translation initiation factor 4 gamma 1 OS=Homo sapiens PE=2 SV=1	167220	669	241	20	216	0.04	0.17	4.25	0	4	2	8	1	11	3	11	2.051159
tr|B2RBA6|B2RBA6_HUMAN	tr|B2RBA6|B2	cDNA, FLJ95407, highly similar to Homo sapiens MCM7 minichromosome maintenance deficient 7 (S. cerevisiae) (MCM7), mRNA OS=Homo sapi	81856	374	335	46	149	0.04	0.17	4.25	1	3	1	4	1	5	1	7	0.985955
tr|Q59G92|Q59G92_HUMAN	tr|Q59G92|Q	HLA-B associated transcript 1 variant (Fragment) OS=Homo sapiens PE=2 SV=1	35528	394	288	44	47	0.09	0.38	4.2222222	1	1	1	1	2	1	2	2	-1.62653
sp|P26599|PTBP1_HUMAN	PTBP1	Polypyrimidine tract-binding protein 1 OS=Homo sapiens GN=PTBP1 PE=1 SV=1	57357	567	110	29	421	0.18	0.75	4.1666667	0	9	3	12	3	11	3	14	0.828104
tr|B4DWJ2|B4DWJ2_HUMAN	QARS	Glutamine--tRNA ligase OS=Homo sapiens GN=QARS PE=2 SV=1	87437	277	119	68	381	0.16	0.66	4.125	3	9	4	13	5	14	5	17	0.510321
tr|E5KNY5|E5KNY5_HUMAN	LRPPRC	Leucine-rich PPR-motif containing OS=Homo sapiens GN=LRPPRC PE=2 SV=1	159003	329	132	53	365	0.08	0.33	4.125	1	11	4	14	5	13	7	17	0.407048
tr|B7Z5E2|B7Z5E2_HUMAN	PSMC2	26S protease regulatory subunit 7 OS=Homo sapiens GN=PSMC2 PE=2 SV=1	33594	610	319	25	120	0.1	0.41	4.1	0	4	1	4	1	4	1	6	0.696269
tr|B2R8N0|B2R8N0_HUMAN	tr|B2R8N0|B2	cDNA, FLJ93975, highly similar to Homo sapiens epoxide hydrolase 1, microsomal (xenobiotic) (EPHX1), mRNA OS=Homo sapiens PE=2 SV=1	53129	115	64	184	596	0.62	2.53	4.0806452	6	16	8	22	8	25	8	27	0.697711
tr|B2RU06|B2RU06_HUMAN	EIF4G1	EIF4G1 protein OS=Homo sapiens GN=EIF4G1 PE=2 SV=1	176807	669	238	20	217	0.04	0.16	4	0	4	2	8	1	11	3	11	2.051159
sp|Q14204|DYHC1_HUMAN	DYNC1H1	Cytoplasmic dynein 1 heavy chain 1 OS=Homo sapiens GN=DYNC1H1 PE=1 SV=5	534809	168	168	129	300	0.02	0.08	4	3	7	4	13	4	15	6	20	0.896553
tr|A2BDK6|A2BDK6_HUMAN	MAP1B	Microtubule-associated protein 1B OS=Homo sapiens GN=MAP1B PE=2 SV=1	257692	511	544	32	85	0.01	0.04	4	0	3	1	3	1	4	2	4	0.696269
sp|Q14247|SRC8_HUMAN	CTTN	Src substrate cortactin OS=Homo sapiens GN=CTTN PE=1 SV=2	61720	378	216	46	237	0.11	0.44	4	1	6	2	7	3	7	3	7	0.210716
tr|D3DRX6|D3DRX6_HUMAN	KIF5B	Kinesin family member 5B, isoform CRA_a OS=Homo sapiens GN=KIF5B PE=3 SV=1	110331	227	422	91	115	0.04	0.16	4	2	1	2	5	2	4	3	5	-0.04103
sp|P13639|EF2_HUMAN	EEF2	Elongation factor 2 OS=Homo sapiens GN=EEF2 PE=1 SV=4	96246	41	15	511	1964	1.23	4.89	3.9756098	13	53	26	75	27	79	32	94	0.647674
sp|O43707|ACTN4_HUMAN	ACTN4	Alpha-actinin-4 OS=Homo sapiens GN=ACTN4 PE=1 SV=2	105245	19	9	849	2604	2.11	8.35	3.957346	26	69	41	92	44	105	52	113	0.360543
sp|O75369|FLNB_HUMAN	FLNB	Filamin-B OS=Homo sapiens GN=FLNB PE=1 SV=2	280157	45	19	463	1698	0.22	0.87	3.9545455	14	45	18	58	20	61	28	66	0.700197
tr|J3KQL0|J3KQL0_HUMAN	CPS1	Carbamoyl-phosphate synthase [ammonia], mitochondrial OS=Homo sapiens GN=CPS1 PE=3 SV=1	166686	204	61	102	629	0.15	0.59	3.9333333	4	20	7	24	10	25	12	28	0.392192
tr|B4DUQ1|B4DUQ1_HUMAN	tr|B4DUQ1|B	cDNA FLJ54552, highly similar to Heterogeneous nuclear ribonucleoprotein K OS=Homo sapiens PE=2 SV=1	48708	147	62	147	606	0.58	2.27	3.9137931	3	18	7	21	7	21	8	24	0.631737
tr|B2RBR9|B2RBR9_HUMAN	tr|B2RBR9|B2	cDNA, FLJ95650, highly similar to Homo sapiens karyopherin (importin) beta 1 (KPNB1), mRNA OS=Homo sapiens PE=2 SV=1	98434	171	68	126	584	0.22	0.86	3.9090909	4	13	6	20	9	22	9	25	0.355804
tr|F1T0B3|F1T0B3_HUMAN	DDX1	ATP-dependent RNA helicase DDX1 OS=Homo sapiens GN=DDX1 PE=2 SV=1	74838	501	291	33	173	0.09	0.35	3.8888889	0	5	2	8	3	9	3	10	0.552111
tr|B4DR68|B4DR68_HUMAN	tr|B4DR68|B4	cDNA FLJ58608, highly similar to Heat shock protein 75 kDa, mitochondrial OS=Homo sapiens PE=2 SV=1	74408	169	121	127	389	0.09	0.35	3.8888889	2	8	3	8	3	9	5	10	0.552111
tr|B0V043|B0V043_HUMAN	VARS	Valine--tRNA ligase OS=Homo sapiens GN=VARS PE=3 SV=1	141632	275	99	69	462	0.15	0.58	3.8666667	2	12	6	20	6	19	8	24	0.697298
tr|F5H6T1|F5H6T1_HUMAN	ACTR2	Actin-related protein 2 OS=Homo sapiens GN=ACTR2 PE=2 SV=1	34729	244	233	82	210	0.2	0.76	3.8	2	6	2	7	2	6	4	8	0.489845
tr|A8K2S1|A8K2S1_HUMAN	tr|A8K2S1|A8	cDNA FLJ75258, highly similar to Homo sapiens phospholipase A2, group IVA (cytosolic, calcium-dependent) (PLA2G4A), mRNA OS=Homo sapier	85695	369	303	47	166	0.08	0.3	3.75	1	4	2	7	2	7	5	9	0.696475
tr|B4DS66|B4DS66_HUMAN	tr|B4DS66|B4	cDNA FLJ54290, highly similar to Mitochondrial inner membrane protein OS=Homo sapiens PE=2 SV=1	73509	712	385	17	128	0.04	0.15	3.75	0	2	1	3	1	3	6	5	0.33352
tr|Q4ZG57|Q4ZG57_HUMAN	MCM6	Putative uncharacterized protein MCM6 (Fragment) OS=Homo sapiens GN=MCM6 PE=2 SV=1	89803	444	323	39	154	0.04	0.15	3.75	1	3	1	4	2	4	3	5	-0.04103
tr|Q7Z3K9|Q7Z3K9_HUMAN	DKFZp781L05	Putative uncharacterized protein DKFZp781L0540 (Fragment) OS=Homo sapiens GN=DKFZp781L0540 PE=2 SV=1	90081	440	357	39	141	0.04	0.15	3.75	1	3	1	4	2	4	2	4	-0.04103
tr|Q9NSU9|Q9NSU9_HUMAN	DKFZp434G07	Putative uncharacterized protein DKFZp434G0719 (Fragment) OS=Homo sapiens GN=DKFZp434G0719 PE=2 SV=1	41107	56	390	169	125	0.07	0.26	3.7142857	4	3	5	3	5	3	5	3	-1.54228
tr|B4E0E1|B4E0E1_HUMAN	tr|B4E0E1|B4	cDNA FLJ53442, highly similar to Poly (ADP-ribose) polymerase 1 (EC 2.4.2.30) OS=Homo sapiens PE=2 SV=1	111855	549	293	30	172	0.06	0.22	3.6666667	0	3	2	8	2	7	2	11	0.696475
tr|J3KPM9|J3KPM9_HUMAN	STAT1	Signal transducer and activator of transcription 1-alpha/beta OS=Homo sapiens GN=STAT1 PE=4 SV=1	83878	364	457	48	106	0.08	0.29	3.625	1	2	2	6	2	6	2	8	0.489845
tr|F8SKU2|F8SKU2_HUMAN	HLA-A	MHC class I antigen (Fragment) OS=Homo sapiens GN=HLA-A PE=3 SV=1	21143	639	377	22	43	0.16	0.58	3.625	0	1	1	2	2	4	3	4	-0.04103
tr|G3XAF4|G3XAF4_HUMAN	L1CAM	L1 cell adhesion molecule, isoform CRA_a OS=Homo sapiens GN=L1CAM PE=4 SV=1	139790	572	244	28	210	0.05	0.18	3.6	1	6	2	7	1	7	2	9	1.433772
tr|B4DMY3|B4DMY3_HUMAN	tr|B4DMY3|B	cDNA FLJ60713, highly similar to Homo sapiens heterogeneous nuclear ribonucleoprotein A/B (HNRPAB), transcript variant 1, mRNA OS=Homo s	35101	318	262	56	196	0.2	0.72	3.6	1	7	2	7	2	7	2	7	0.696475
tr|B3KXU8|B3KXU8_HUMAN	tr|B3KXU8|B3	cDNA FLJ46087 fis, clone TESTI2009497, highly similar to GPI-anchored protein p137 OS=Homo sapiens PE=2 SV=1	70489	367	526	47	63	0.05	0.18	3.6	1	1	1	2	1	2	1	3	-0.15209
tr|E7ENU7|E7ENU7_HUMAN	RPL15	Ribosomal protein L15 (Fragment) OS=Homo sapiens GN=RPL15 PE=2 SV=1	20596	534	746	31	57	0.16	0.57	3.5625	0	1	1	3	1	3	1	4	0.33352
tr|E9PBU3|E9PBU3_HUMAN	ATIC	Phosphoribosylaminoimidazolecarboxamide formyltransferase OS=Homo sapiens GN=ATIC PE=2 SV=1	64996	72	48	274	688	0.64	2.26	3.53125	8	17	11	23	12	24	13	28	0.082094
tr|B7Z2Z8|B7Z2Z8_HUMAN	tr|B7Z2Z8|B7	T-complex protein 1 subunit delta OS=Homo sapiens PE=2 SV=1	52781	113	186	188	224	0.27	0.95	3.5185185	4	6	5	10	7	12	7	13	-0.15229
tr|F5H7Y0|F5H7Y0_HUMAN	PDLIM5	PDZ and LIM domain protein 5 OS=Homo sapiens GN=PDLIM5 PE=2 SV=1	52332	175	128	109	345	0.41	1.44	3.5121951	2	9	5	13	5	13	6	15	0.407048
tr|J3KR97|J3KR97_HUMAN	TBCD	Tubulin-specific chaperone D OS=Homo sapiens GN=TBCD PE=4 SV=1	138346	714	688	15	64	0.02	0.07	3.5	0	2	1	3	0	3	1	3	1.918814
tr|B4DH39|B4DH39_HUMAN	tr|B4DH39|B4	cDNA FLJ57028, highly similar to Long-chain-fatty-acid--CoA ligase 4 (EC 6.2.1.3) OS=Homo sapiens PE=2 SV=1	73123	529	412	31	117	0.04	0.14	3.5	0	2	1	3	1	5	3	5	0.985955
tr|E7ERW8|E7ERW8_HUMAN	DIAPH1	Protein diaphanous homolog 1 OS=Homo sapiens GN=DIAPH1 PE=2 SV=1	135945	184	634	116	71	0.02	0.07	3.5	1	2	1	3	1	5	2	5	0.985955
tr|B4DFM0|B4DFM0_HUMAN	FARSLB	Phenylalanine-tRNA synthetase-like, beta subunit, isoform CRA_c OS=Homo sapiens GN=FARSLB PE=2 SV=1	55353	360	405	48	88	0.06	0.21	3.5	1	1	1	3	1	5	3	6	0.985955
tr|B4DKZ9|B4DKZ9_HUMAN	tr|B4DKZ9|B4	cDNA FLJ55705, highly similar to Threonyl-tRNA synthetase, cytoplasmic (EC 6.1.1.3) OS=Homo sapiens PE=2 SV=1	71171	94	66	191	586	0.64	2.23	3.484375	5	21	10	28	13	33	14	35	0.423726
tr|Q05D08|Q05D08_HUMAN	PA2G4	PA2G4 protein (Fragment) OS=Homo sapiens GN=PA2G4 PE=2 SV=1	45579	286	342	64	145	0.15	0.52	3.4666667	1	5	2	6	3	6	3	7	0.004086
tr|B3KX72|B3KX72_HUMAN	tr|B3KX72|B3	cDNA FLJ44920 fis, clone BRAMY3011501, highly similar to Heterogeneous nuclear ribonucleoprotein U OS=Homo sapiens PE=2 SV=1	83774	59	38	367	820	0.58	2.01	3.4655172	10	23	14	32	16	35	20	37	0.217239
tr|B4DEM7|B4DEM7_HUMAN	CCT8	T-complex protein 1 subunit theta OS=Homo sapiens GN=CCT8 PE=2 SV=1	58179	268	156	70	310	0.18	0.62	3.4444444	2	8	3	9	5	9	6	10	-0.10063
sp|Q9Y3I0|RTCB_HUMAN	C22orf28	tRNA-splicing ligase RtcB homolog OS=Homo sapiens GN=C22orf28 PE=1 SV=1	55688	458	271	38	186	0.12	0.41	3.4166667	0	4	2	6	2	6	2	6	0.489845
tr|B3KNM6|B3KNM6_HUMAN	tr|B3KNM6|B	cDNA FLJ14968 fis, clone THYRO1000288, highly similar to CAAX prenyl protease 1 homolog (EC 3.4.24.84) OS=Homo sapiens PE=2 SV=1	27279	547	491	30	99	0.12	0.41	3.4166667	1	3	1	3	1	3	1	3	0.33352
tr|B7Z8W6|B7Z8W6_HUMAN	tr|B7Z8W6|B	cDNA FLJ55038, highly similar to Nicotinamide phosphoribosyltransferase (EC 2.4.2.12) OS=Homo sapiens PE=2 SV=1	26905	520	250	32	51	0.12	0.41	3.4166667	0	1	1	2	1	2	3	3	-0.15209
tr|A8K4D5|A8K4D5_HUMAN	tr|A8K4D5|A8	Kynureninase OS=Homo sapiens PE=2 SV=1	52819	266	280	71	104	0.13	0.44	3.3846154	2	3	2	5	3	7	3	7	0.210716
tr|B4DLB8|B4DLB8_HUMAN	tr|B4DLB8|B4	cDNA FLJ52205, highly similar to Beta-1,4-galactosyltransferase 1 (EC 2.4.1.-) OS=Homo sapiens PE=2 SV=1	40269	650	727	22	60	0.08	0.27	3.375	0	2	1	3	1	3	1	4	0.33352
sp|O75367|H2AY_HUMAN	H2AFY	Core histone macro-H2A.1 OS=Homo sapiens GN=H2AFY PE=1 SV=4	39764	565	455	29	107	0.08	0.27	3.375	0	2	1	3	2	4	2	5	-0.04103
tr|B4DN87|B4DN87_HUMAN	tr|B4DN87|B4	cDNA FLJ52569, highly similar to Collagen-binding protein 2 OS=Homo sapiens PE=2 SV=1	44290	233	76	89	483	0.43	1.44	3.3488372	4	12	5	12	5	12	5	13	0.295837
tr|Q53GX7|Q53GX7_HUMAN	tr|Q53GX7|Q	Threonyl-tRNA synthetase variant (Fragment) OS=Homo sapiens PE=2 SV=1	84304	94	52	221	721	0.64	2.14	3.34375	6	25	13	32	17	37	18	38	0.211449
tr|F5H4R9|F5H4R9_HUMAN	DNM2	Dynamin-2 OS=Homo sapiens GN=DNM2 PE=2 SV=1	98218	713	691	16	64	0.03	0.1	3.3333333	0	1	1	3	0	2	2	3	1.433208
tr|B4DKS0|B4DKS0_HUMAN	tr|B4DKS0|B4	cDNA FLJ53381, highly similar to Monocarboxylate transporter 1 OS=Homo sapiens PE=2 SV=1	52487	598	718	26	41	0.06	0.2	3.3333333	0	1	1	2	1	2	1	2	-0.15209
tr|H0UID3|H0UID3_HUMAN	AP2B1	Adaptor-related protein complex 2, beta 1 subunit, isoform CRA_d OS=Homo sapiens GN=AP2B1 PE=4 SV=1	106321	648	630	22	72	0.03	0.1	3.3333333	0	1	1	3	3	4	3	4	-0.52679
tr|B5BU25|B5BU25_HUMAN	U2AF2	U2 small nuclear RNA auxiliary factor 2 isoform b OS=Homo sapiens GN=U2AF2 PE=2 SV=1	53445	260	232	74	224	0.13	0.43	3.3076923	1	6	2	6	2	6	2	6	0.489845
tr|Q5T7Q0|Q5T7Q0_HUMAN	DNAJA1	DnaJ (Hsp40) homolog, subfamily A, member 1, isoform CRA_c OS=Homo sapiens GN=DNAJA1 PE=2 SV=1	45581	703	334	18	150	0.07	0.23	3.2857143	0	3	1	3	0	5	1	6	2.571248
tr|B4E2I9|B4E2I9_HUMAN	tr|B4E2I9|B4E	cDNA FLJ55486, highly similar to Semaphorin-3C OS=Homo sapiens PE=2 SV=1	88481	602	1063	26	32	0.04	0.13	3.25	0	0	1	1	1	2	2	2	-0.15209
tr|B3KT93|B3KT93_HUMAN	tr|B3KT93|B3	cDNA FLJ37875 fis, clone BRSSN2018771, highly similar to Poly(A)-binding protein 1 OS=Homo sapiens PE=2 SV=1	70799	215	144	96	335	0.33	1.06	3.2121212	3	10	6	16	5	18	9	21	0.86251
tr|B4DQX0|B4DQX0_HUMAN	tr|B4DQX0|B	cDNA FLJ59219, highly similar to Poly(A)-binding protein 1 OS=Homo sapiens PE=2 SV=1	67239	215	143	96	299	0.33	1.06	3.2121212	3	9	6	14	6	18	10	21	0.62117
tr|A5PHJ9|A5PHJ9_HUMAN	PDHA1/LOC79	PDHA1/LOC79064 protein (Fragment) OS=Homo sapiens GN=PDHA1/LOC79064 PE=2 SV=1	34113	516	710	32	61	0.1	0.32	3.2	0	1	1	3	1	4	1	4	0.696269
tr|D3DTW3|D3DTW3_HUMAN	IGF2BP1	Insulin-like growth factor 2 mRNA binding protein 1, isoform CRA_a OS=Homo sapiens GN=IGF2BP1 PE=2 SV=1	48111	556	581	29	78	0.05	0.16	3.2	0	3	1	3	1	3	1	5	0.33352
sp|O00425|IF2B3_HUMAN	IGF2BP3	Insulin-like growth factor 2 mRNA-binding protein 3 OS=Homo sapiens GN=IGF2BP3 PE=1 SV=2	64008	556	624	29	73	0.05	0.16	3.2	0	2	1	3	2	3	2	4	-0.40378
sp|Q96A33|CCD47_HUMAN	CCDC47	Coiled-coil domain-containing protein 47 OS=Homo sapiens GN=CCDC47 PE=1 SV=1	56123	560	675	29	66	0.06	0.19	3.1666667	0	2	1	3	1	3	1	3	0.33352
tr|B1AH89|B1AH89_HUMAN	TTLL12	Tubulin tyrosine ligase-like family, member 12 OS=Homo sapiens GN=TTLL12 PE=4 SV=1	74185	104	72	209	559	0.41	1.28	3.1219512	6	14	8	19	10	17	11	21	-0.15238
sp|P49411|EFTU_HUMAN	TUFM	Elongation factor Tu, mitochondrial OS=Homo sapiens GN=TUFM PE=1 SV=2	49852	210	171	99	295	0.29	0.9	3.1034483	3	7	4	11	4	13	5	15	0.696886
tr|B0QYA5|B0QYA5_HUMAN	EIF3D	Eukaryotic translation initiation factor 3 subunit D (Fragment) OS=Homo sapiens GN=EIF3D PE=4 SV=1	31937	710	383	17	104	0.1	0.31	3.1	0	2	1	3	0	3	2	3	1.918814
tr|B3KS31|B3KS31_HUMAN	TUBB6	Tubulin, beta 6, isoform CRA_a OS=Homo sapiens GN=TUBB6 PE=2 SV=1	42324	73	70	273	556	0.83	2.56	3.0843373	10	18	14	23	17	29	20	32	-0.13616
tr|B7TY16|B7TY16_HUMAN	ACTN1	Actinin alpha 1 isoform 3 OS=Homo sapiens GN=ACTN1 PE=2 SV=1	107644	50	30	428	1157	0.77	2.37	3.0779221	15	30	21	40	22	47	29	50	0.19005
tr|B4DSX0|B4DSX0_HUMAN	tr|B4DSX0|B4	cDNA FLJ61380, highly similar to Alpha-actinin-4 OS=Homo sapiens PE=2 SV=1	47819	19	32	369	1166	2.11	6.39	3.028436	10	32	15	39	15	43	18	46	0.602404
tr|I3ZN76|I3ZN76_HUMAN	HLA-A	MHC class I antigen OS=Homo sapiens GN=HLA-A PE=3 SV=1	41296	639	513	22	93	0.16	0.48	3	0	3	1	4	1	6	2	6	1.227142
sp|P54886|P5CS_HUMAN	ALDH18A1	Delta-1-pyrroline-5-carboxylate synthase OS=Homo sapiens GN=ALDH18A1 PE=1 SV=2	87989	658	351	21	143	0.04	0.12	3	1	3	1	3	1	5	1	6	0.985955
tr|Q13727|Q13727_HUMAN	tr|Q13727|Q	AHNAK-related protein (Fragment) OS=Homo sapiens PE=2 SV=1	57703	402	666	43	67	0.06	0.18	3	1	2	1	3	2	7	3	8	0.696475
tr|F8W9S7|F8W9S7_HUMAN	GAPVD1	GTPase-activating protein and VPS9 domain-containing protein 1 OS=Homo sapiens GN=GAPVD1 PE=2 SV=1	158790	697	911	18	42	0.02	0.06	3	0	1	1	1	0	1	2	1	0.696063
sp|O94906|PRP6_HUMAN	PRPF6	Pre-mRNA-processing factor 6 OS=Homo sapiens GN=PRPF6 PE=1 SV=1	107656	492	466	35	105	0.03	0.09	3	1	3	1	3	1	3	2	3	0.33352
tr|I3L0N3|I3L0N3_HUMAN	NSF	Vesicle-fusing ATPase OS=Homo sapiens GN=NSF PE=3 SV=1	82553	412	843	42	47	0.04	0.12	3	1	1	1	3	1	3	1	5	0.33352
sp|Q9UMS4|PRP19_HUMAN	PRPF19	Pre-mRNA-processing factor 19 OS=Homo sapiens GN=PRPF19 PE=1 SV=1	55603	237	242	87	215	0.26	0.78	3	2	8	5	10	7	12	11	12	-0.15229
tr|Q5TB52|Q5TB52_HUMAN	PAPSS2	3~-phosphoadenosine 5~-phosphosulfate synthase 2, isoform CRA_b OS=Homo sapiens GN=PAPSS2 PE=2 SV=1	70027	688	531	19	87	0.05	0.15	3	0	1	1	3	0	3	4	3	1.918814
tr|B4DFL1|B4DFL1_HUMAN	tr|B4DFL1|B4	Dihydrolipoyl dehydrogenase OS=Homo sapiens PE=2 SV=1	49381	666	648	21	70	0.07	0.21	3	0	1	1	4	1	3	1	4	0.33352
tr|B2RDF2|B2RDF2_HUMAN	PES1	Pescadillo homolog 1 OS=Homo sapiens GN=PES1 PE=2 SV=1	68359	488	489	35	100	0.05	0.15	3	1	3	1	3	1	3	3	4	0.33352
tr|B4DLI2|B4DLI2_HUMAN	tr|B4DLI2|B4	cDNA FLJ55714, highly similar to Dolichyl-diphosphooligosaccharide--protein glycosyltransferase 48 kDa subunit (EC 2.4.1.119) OS=Homo sapier	49120	604	813	26	50	0.07	0.21	3	0	1	1	3	1	2	2	4	-0.15209
tr|B4DDR2|B4DDR2_HUMAN	tr|B4DDR2|B4	cDNA FLJ54588, highly similar to Major vault protein OS=Homo sapiens PE=2 SV=1	98526	304	195	59	227	0.07	0.21	3	2	5	2	5	3	5	3	6	-0.2371
tr|B1AMS2|B1AMS2_HUMAN	Sep-06	Septin 6 OS=Homo sapiens GN=SEPT6 PE=3 SV=1	49614	532	352	31	39	0.07	0.21	3	1	1	1	1	1	1	1	1	-0.88923
sp|P11021|GRP78_HUMAN	HSPA5	78 kDa glucose-regulated protein OS=Homo sapiens GN=HSPA5 PE=1 SV=2	72402	129	104	170	437	0.19	0.56	2.9473684	3	10	5	11	6	11	6	11	-0.06598
tr|H0YA24|H0YA24_HUMAN	PLRG1	Pleiotropic regulator 1 (Fragment) OS=Homo sapiens GN=PLRG1 PE=4 SV=1	17588	456	278	38	72	0.19	0.56	2.9473684	1	1	1	2	1	2	2	2	-0.15209
sp|P22314|UBA1_HUMAN	UBA1	Ubiquitin-like modifier-activating enzyme 1 OS=Homo sapiens GN=UBA1 PE=1 SV=3	118858	114	51	187	726	0.28	0.82	2.9285714	5	18	10	25	10	26	12	28	0.447867
tr|Q3B7A7|Q3B7A7_HUMAN	GART	Phosphoribosylglycinamide formyltransferase, phosphoribosylglycinamide synthetase, phosphoribosylaminoimidazole synthetase OS=Homo sap	108909	341	154	51	312	0.13	0.38	2.9230769	1	8	4	11	3	11	4	15	0.828104
tr|B3KQQ3|B3KQQ3_HUMAN	tr|B3KQQ3|B	cDNA PSEC0016 fis, clone NT2RM1001076, highly similar to Procollagen-lysine,2-oxoglutarate 5-dioxygenase 3 (EC 1.14.11.4) OS=Homo sapiens	85161	325	202	54	254	0.12	0.35	2.9166667	1	6	3	8	3	7	3	9	0.210716
tr|J3QQM1|J3QQM1_HUMAN	PSMC5	26S protease regulatory subunit 8 (Fragment) OS=Homo sapiens GN=PSMC5 PE=4 SV=1	29442	314	518	57	58	0.11	0.32	2.9090909	1	1	1	2	1	2	2	2	-0.15209
tr|B4DXX7|B4DXX7_HUMAN	DDX3Y	ATP-dependent RNA helicase DDX3Y OS=Homo sapiens GN=DDX3Y PE=2 SV=1	73342	217	175	95	155	0.19	0.55	2.8947368	2	4	5	7	6	8	6	10	-0.50261
tr|A5D906|A5D906_HUMAN	TUBB2A	TUBB2A protein (Fragment) OS=Homo sapiens GN=TUBB2A PE=2 SV=1	28128	25	23	143	569	3.07	8.79	2.8631922	4	16	7	22	8	25	8	30	0.697711
sp|P53004|BIEA_HUMAN	BLVRA	Biliverdin reductase A OS=Homo sapiens GN=BLVRA PE=1 SV=2	33692	403	874	43	45	0.21	0.6	2.8571429	1	0	2	5	2	5	3	5	0.248657
tr|B2RAQ8|B2RAQ8_HUMAN	tr|B2RAQ8|B	cDNA, FLJ95058, highly similar to Homo sapiens carnitine palmitoyltransferase 1A (liver) (CPT1A),nuclear gene encoding mitochondrial protein,	88967	354	469	49	104	0.07	0.2	2.8571429	1	3	2	5	3	5	3	5	-0.2371
tr|B3KSM6|B3KSM6_HUMAN	tr|B3KSM6|B	cDNA FLJ36606 fis, clone TRACH2015654, highly similar to HEAT SHOCK 70 kDa PROTEIN 6 OS=Homo sapiens PE=2 SV=1	71384	193	214	112	238	0.2	0.57	2.85	3	8	5	12	6	12	6	13	0.054498

tr|A8K7F6|A8K7F6_HUMAN	tr|A8K7F6|A8	cDNA FLJ78244, highly similar to Homo sapiens eukaryotic translation initiation factor 4A, isoform 1 (EIF4A1), mRNA OS=Homo sapiens PE=2 SV	46321	52	46	404	847	1.62	4.59	2.8333333	10	23	14	29	15	31	20	35	0.134584
tr|Q2XPP3|Q2XPP3_HUMAN	tr|Q2XPP3|Q	Type II 3a-hydroxysteroid dehydrogenase variant OS=Homo sapiens PE=2 SV=1	37201	203	315	102	160	0.29	0.82	2.8275862	3	5	3	7	3	8	4	8	0.391467
tr|Q2VPA0|Q2VPA0_HUMAN	LONP1	Lon protease homolog (Fragment) OS=Homo sapiens GN=LONP1 PE=2 SV=1	95436	414	270	42	188	0.11	0.31	2.8181818	2	7	3	8	3	10	3	10	0.69668
tr|Q2TSD0|Q2TSD0_HUMAN	tr|Q2TSD0|Q	Glyceraldehyde-3-phosphate dehydrogenase OS=Homo sapiens PE=2 SV=1	36197	18	25	891	1238	3.44	9.65	2.8052326	23	31	31	40	31	43	36	49	-0.42601
sp|Q96EK5|KBP_HUMAN	KIAA1279	KIF1-binding protein OS=Homo sapiens GN=KIAA1279 PE=1 SV=1	72453	649	447	22	109	0.05	0.14	2.8	0	3	1	3	1	4	1	4	0.696269
sp|Q9UG63|ABCF2_HUMAN	ABCF2	ATP-binding cassette sub-family F member 2 OS=Homo sapiens GN=ABCF2 PE=1 SV=2	71815	673	363	20	140	0.05	0.14	2.8	0	3	1	3	1	3	2	3	0.33352
tr|B2RDF5|B2RDF5_HUMAN	tr|B2RDF5|B2	cDNA, FLJ96587, highly similar to Homo sapiens SUMO-1 activating enzyme subunit 2 (UBA2), mRNA OS=Homo sapiens PE=2 SV=1	71705	590	719	27	39	0.05	0.14	2.8	0	1	1	2	1	1	1	3	-0.88923
sp|Q8NBJ5|GT251_HUMAN	GLT25D1	Procollagen galactosyltransferase 1 OS=Homo sapiens GN=GLT25D1 PE=1 SV=1	71933	523	436	31	112	0.09	0.25	2.7777778	0	3	2	5	1	8	2	10	1.614523
tr|I3L312|I3L312_HUMAN	P4HB	Protein disulfide-isomerase (Fragment) OS=Homo sapiens GN=P4HB PE=4 SV=1	26348	536	330	30	95	0.27	0.75	2.7777778	1	2	2	4	1	4	2	4	0.696269
tr|K9JA46|K9JA46_HUMAN	EL52	Epididymis luminal secretory protein 52 OS=Homo sapiens GN=EL52 PE=2 SV=1	85006	46	26	462	1397	1.05	2.9	2.7619048	15	33	25	47	24	52	29	61	0.211817
tr|Q53SS8|Q53SS8_HUMAN	PCBP1	Poly(RC) binding protein 1 OS=Homo sapiens GN=PCBP1 PE=2 SV=1	37987	110	245	197	209	0.29	0.8	2.7586207	3	5	3	9	3	9	3	13	0.552111
tr|B4DHG3|B4DHG3_HUMAN	tr|B4DHG3|B	cDNA FLJ55329, highly similar to Procollagen-lysine,2-oxoglutarate5-dioxygenase 2 (EC 1.14.11.4) OS=Homo sapiens PE=2 SV=1	81716	453	287	38	175	0.08	0.22	2.75	0	4	2	5	2	4	2	5	-0.04103
tr|B4DZB1|B4DZB1_HUMAN	tr|B4DZB1|B4	cDNA FLJ59645, highly similar to 116 kDa U5 small nuclear ribonucleoprotein component OS=Homo sapiens PE=2 SV=1	104668	111	98	193	465	0.27	0.74	2.7407407	4	13	8	18	8	19	10	20	0.309611
tr|Q60FE5|Q60FE5_HUMAN	FLNA	Filamin A OS=Homo sapiens GN=FLNA PE=2 SV=1	280790	9	4	1306	3526	0.86	2.32	2.6976744	41	91	60	129	75	134	88	149	-0.05691
sp|P26038|MOES_HUMAN	MSN	Moesin OS=Homo sapiens GN=MSN PE=1 SV=3	67892	119	79	179	503	0.46	1.23	2.673913	7	12	8	17	11	23	12	24	0.14242
tr|B4DR45|B4DR45_HUMAN	tr|B4DR45|B4	cDNA FLJ61339, highly similar to Alanyl-tRNA synthetase (EC 6.1.1.7) OS=Homo sapiens PE=2 SV=1	110017	238	370	86	134	0.06	0.16	2.6666667	1	4	2	5	2	6	2	7	0.489845
tr|B2R5M8|B2R5M8_HUMAN	tr|B2R5M8|B	Isocitrate dehydrogenase [NADP] OS=Homo sapiens PE=2 SV=1	46905	555	340	29	146	0.15	0.4	2.6666667	0	4	2	5	3	6	3	8	0.004086
sp|P53618|COPB_HUMAN	COPB1	Coatomer subunit beta OS=Homo sapiens GN=COPB1 PE=1 SV=3	108214	303	397	59	122	0.06	0.16	2.6666667	1	2	2	5	2	4	3	5	-0.04103
tr|Q5HY54|Q5HY54_HUMAN	FLNA	Filamin-A OS=Homo sapiens GN=FLNA PE=2 SV=1	279115	9	5	1304	3465	0.86	2.29	2.6627907	41	90	59	127	74	132	87	147	-0.05932
sp|Q96HC4|PDLI5_HUMAN	PDLIM5	PDZ and LIM domain protein 5 OS=Homo sapiens GN=PDLIM5 PE=1 SV=5	65102	175	134	123	360	0.41	1.09	2.6585366	3	9	7	15	7	14	8	17	0.062198
tr|Q2NKY5|Q2NKY5_HUMAN	TUBB6	TUBB6 protein OS=Homo sapiens GN=TUBB6 PE=2 SV=1	50514	65	50	317	734	0.88	2.32	2.6363636	12	22	16	27	17	31	19	36	-0.04117
sp|P23381|SYWC_HUMAN	WARS	Tryptophan--tRNA ligase, cytoplasmic OS=Homo sapiens GN=WARS PE=1 SV=2	53474	145	151	148	322	0.27	0.71	2.6296296	3	6	4	9	5	10	5	10	0.04394
sp|Q15046|SYK_HUMAN	KARS	Lysine--tRNA ligase OS=Homo sapiens GN=KARS PE=1 SV=3	68461	265	138	71	348	0.32	0.84	2.625	1	8	6	13	6	14	7	14	0.268981
tr|B4DH02|B4DH02_HUMAN	tr|B4DH02|B4	cDNA FLJ50510, highly similar to Heat shock 70 kDa protein 4 OS=Homo sapiens PE=2 SV=1	95157	123	105	175	434	0.23	0.6	2.6086957	4	11	6	13	5	16	6	17	0.697092
tr|B3KMX0|B3KMX0_HUMAN	tr|B3KMX0|B	cDNA FLJ12837 fis, clone NT2RP2003228, highly similar to DNA replication licensing factor MCM4 OS=Homo sapiens PE=2 SV=1	97114	240	399	85	121	0.1	0.26	2.6	2	5	3	7	4	8	5	9	0.028566
tr|B3KX19|B3KX19_HUMAN	tr|B3KX19|B3	cDNA FLJ44500 fis, clone UTERU3000828, highly similar to 116 kDa U5 small nuclear ribonucleoprotein component OS=Homo sapiens PE=2 SV=	109111	111	97	193	465	0.27	0.7	2.5925926	4	13	8	18	8	19	10	20	0.309611
tr|B7Z6M1|B7Z6M1_HUMAN	PLS3	Plastin-3 OS=Homo sapiens GN=PLS3 PE=2 SV=1	66047	282	146	66	332	0.34	0.88	2.5882353	1	10	6	13	6	15	8	16	0.365375
tr|B4DDB6|B4DDB6_HUMAN	HNRPA3	Heterogeneous nuclear ribonucleoprotein A3, isoform CRA_a OS=Homo sapiens GN=HNRPA3 PE=2 SV=1	37234	201	254	103	184	0.19	0.49	2.5789474	2	2	2	4	2	5	2	5	0.248657
tr|D3DTH7|D3DTH7_HUMAN	MYO1C	Myosin IC, isoform CRA_a OS=Homo sapiens GN=MYO1C PE=2 SV=1	99613	284	297	65	151	0.07	0.18	2.5714286	1	3	3	5	3	5	4	7	-0.2371
tr|Q5CAQ5|Q5CAQ5_HUMAN	TRA1	Tumor rejection antigen (Gp96) 1 OS=Homo sapiens GN=TRA1 PE=2 SV=1	92567	140	115	157	408	0.37	0.94	2.5405405	4	8	9	20	10	20	11	23	0.076422
tr|B4DFK9|B4DFK9_HUMAN	tr|B4DFK9|B4	cDNA FLJ57964, highly similar to Heterogeneous nuclear ribonucleoprotein H~ OS=Homo sapiens PE=2 SV=1	44637	272	197	69	186	0.15	0.38	2.5333333	1	5	2	6	1	7	2	9	1.433772
tr|B4E0S6|B4E0S6_HUMAN	tr|B4E0S6|B4	cDNA FLJ55635, highly similar to pre-mRNA-splicing factorATP-dependent RNA helicase DHX15 (EC 3.6.1.-) OS=Homo sapiens PE=2 SV=1	90289	183	252	117	202	0.15	0.38	2.5333333	2	5	4	9	4	9	7	11	0.18921
tr|Q53ET2|Q53ET2_HUMAN	tr|Q53ET2|Q5	Dihydropyrimidinase-like 2 variant (Fragment) OS=Homo sapiens PE=2 SV=1	62688	342	145	51	333	0.23	0.58	2.5217391	1	9	4	9	3	9	5	9	0.552111
tr|Q2NLA0|Q2NLA0_HUMAN	COPG2	Coatomer subunit gamma OS=Homo sapiens GN=COPG2 PE=2 SV=1	82400	705	458	18	71	0.04	0.1	2.5	0	1	1	1	0	1	1	1	0.696063
tr|J3KQ96|J3KQ96_HUMAN	TCOF1	Treacle protein (Fragment) OS=Homo sapiens GN=TCOF1 PE=4 SV=1	144268	702	947	18	39	0.02	0.05	2.5	0	1	1	2	0	1	2	3	0.696063
sp|Q96P70|IPO9_HUMAN	IPO9	Importin-9 OS=Homo sapiens GN=IPO9 PE=1 SV=3	116858	580	414	28	117	0.06	0.15	2.5	0	2	2	5	2	5	2	7	0.248657
tr|B4DGU4|B4DGU4_HUMAN	CTNNB1	Catenin beta-1 OS=Homo sapiens GN=CTNNB1 PE=2 SV=1	85339	340	249	52	205	0.12	0.3	2.5	2	6	3	7	3	7	3	7	0.210716
tr|B2R6X5|B2R6X5_HUMAN	tr|B2R6X5|B2	cDNA, FLJ93166, highly similar to Homo sapiens heat shock 70kDa protein 6 (HSP70B~) (HSPA6), mRNA OS=Homo sapiens PE=2 SV=1	71370	193	213	112	238	0.2	0.5	2.5	3	8	5	11	6	12	6	12	0.054498
sp|Q5T2N8|ATD3C_HUMAN	ATAD3C	ATPase family AAA domain-containing protein 3C OS=Homo sapiens GN=ATAD3C PE=2 SV=2	46635	281	294	68	79	0.1	0.25	2.5	2	1	2	2	2	2	2	3	-0.88938
tr|D2CFK9|D2CFK9_HUMAN	GTPBP4	Nucleolar GTP-binding protein 1 OS=Homo sapiens GN=GTPBP4 PE=2 SV=1	74317	264	219	72	235	0.14	0.35	2.5	2	6	3	7	3	7	3	7	0.210716
tr|B2R4K7|B2R4K7_HUMAN	tr|B2R4K7|B2	60S ribosomal protein L6 OS=Homo sapiens PE=2 SV=1	32739	276	365	68	139	0.47	1.17	2.4893617	2	5	4	8	3	6	5	8	0.004086
tr|B4DMB5|B4DMB5_HUMAN	tr|B4DMB5|B	cDNA FLJ60075, highly similar to CTP synthase 1 (EC 6.3.4.2) OS=Homo sapiens PE=2 SV=1	56542	449	220	38	234	0.19	0.46	2.4210526	1	5	3	6	3	6	4	7	0.004086
sp|Q92973|TNPO1_HUMAN	TNPO1	Transportin-1 OS=Homo sapiens GN=TNPO1 PE=1 SV=2	103771	139	174	158	290	0.17	0.41	2.4117647	3	8	5	11	8	11	8	15	-0.45366
tr|Q5TCU6|Q5TCU6_HUMAN	TLN1	Talin-1 OS=Homo sapiens GN=TLN1 PE=4 SV=1	260030	48	24	446	1418	0.27	0.65	2.4074074	12	31	19	41	20	46	25	47	0.29414
tr|G3V0E8|G3V0E8_HUMAN	PCBP2	Poly(RC) binding protein 2, isoform CRA_f OS=Homo sapiens GN=PCBP2 PE=2 SV=1	33817	132	229	166	205	0.32	0.77	2.40625	3	6	3	7	3	7	5	8	0.210716
tr|J3QSX4|J3QSX4_HUMAN	BUB3	Mitotic checkpoint protein BUB3 OS=Homo sapiens GN=BUB3 PE=4 SV=1	16498	681	530	20	57	0.2	0.48	2.4	0	1	1	2	0	1	1	2	0.696063
tr|B2RCM2|B2RCM2_HUMAN	tr|B2RCM2|B	cDNA, FLJ96156, highly similar to Homo sapiens leucyl-tRNA synthetase (LARS), mRNA OS=Homo sapiens PE=2 SV=1	135550	257	209	75	241	0.1	0.24	2.4	2	4	4	9	3	9	5	13	0.552111
tr|K7EIG1|K7EIG1_HUMAN	CLUH	Clustered mitochondria protein homolog OS=Homo sapiens GN=CLUH PE=3 SV=1	140903	438	302	39	167	0.05	0.12	2.4	1	3	2	5	2	6	2	10	0.489845
tr|A6NE09|A6NE09_HUMAN	RPSAP58	Protein RPSAP58 OS=Homo sapiens GN=RPSAP58 PE=3 SV=1	33002	521	613	31	46	0.1	0.24	2.4	0	1	1	1	1	1	1	1	-0.88923
sp|O95831|AIFM1_HUMAN	AIFM1	Apoptosis-inducing factor 1, mitochondrial OS=Homo sapiens GN=AIFM1 PE=1 SV=1	67144	326	955	54	39	0.05	0.12	2.4	1	1	1	1	1	1	2	2	-0.88923
tr|B4DUM2|B4DUM2_HUMAN	tr|B4DUM2|B	cDNA FLJ53891, highly similar to Adenylosuccinate lyase (EC 4.3.2.2) OS=Homo sapiens PE=2 SV=1	34875	359	837	27	48	0.13	0.31	2.3846154	0	1	1	3	2	4	2	4	-0.04103
tr|H7C3A1|H7C3A1_HUMAN	SRRT	Serrate RNA effector molecule homolog (Fragment) OS=Homo sapiens GN=SRRT PE=4 SV=1	57154	701	471	18	76	0.06	0.14	2.3333333	0	2	1	3	0	3	1	3	1.918814
tr|B7Z8A2|B7Z8A2_HUMAN	tr|B7Z8A2|B7	cDNA FLJ51671, highly similar to Prenylcysteine oxidase (EC 1.8.3.5) OS=Homo sapiens PE=2 SV=1	54918	355	767	49	54	0.06	0.14	2.3333333	1	1	1	2	1	2	1	2	-0.15209
sp|Q8TCJ2|STT3B_HUMAN	STT3B	Dolichyl-diphosphooligosaccharide--protein glycosyltransferase subunit STT3B OS=Homo sapiens GN=STT3B PE=1 SV=1	94241	700	885	18	44	0.03	0.07	2.3333333	0	1	1	2	2	3	3	4	-0.40378
tr|Q68DB1|Q68DB1_HUMAN	DKFZp686F22	Metastasis-associated protein MTA2 OS=Homo sapiens GN=DKFZp686F2281 PE=2 SV=1	55515	676	349	20	35	0.06	0.14	2.3333333	0	0	1	1	1	1	1	1	-0.88923
tr|Q6IBR0|Q6IBR0_HUMAN	RPN1	RPN1 protein OS=Homo sapiens GN=RPN1 PE=2 SV=1	68678	170	125	127	384	0.26	0.6	2.3076923	4	9	5	10	8	14	8	14	-0.11871
sp|P49327|FAS_HUMAN	FASN	Fatty acid synthase OS=Homo sapiens GN=FASN PE=1 SV=3	275877	55	29	394	1300	0.28	0.64	2.2857143	12	34	21	43	23	47	29	54	0.126981
tr|Q5T0B1|Q5T0B1_HUMAN	RCC1	CHC1 protein OS=Homo sapiens GN=RCC1 PE=2 SV=1	45397	269	343	70	145	0.23	0.52	2.2608696	2	2	3	6	2	6	4	7	0.489845
tr|B4E022|B4E022_HUMAN	TKT	Transketolase OS=Homo sapiens GN=TKT PE=2 SV=1	63410	35	31	578	1161	1.36	3.07	2.2573529	17	33	24	42	26	47	32	56	-0.04735
tr|C5IWY8|C5IWY8_HUMAN	HLA-A	MHC class I antigen OS=Homo sapiens GN=HLA-A PE=3 SV=1	41236	639	505	22	95	0.16	0.36	2.25	0	3	1	4	1	7	3	7	1.433772
tr|B3KSH1|B3KSH1_HUMAN	EIF3F	Eukaryotic translation initiation factor 3 subunit F OS=Homo sapiens GN=EIF3F PE=2 SV=1	39236	652	611	22	75	0.08	0.18	2.25	0	2	1	2	1	2	1	2	-0.15209
tr|B1AHB0|B1AHB0_HUMAN	MCM5	MCM5 minichromosome maintenance deficient 5, cell division cycle 46 (S. cerevisiae), isoform CRA_a OS=Homo sapiens GN=MCM5 PE=2 SV=1	83031	562	1008	29	36	0.04	0.09	2.25	0	0	1	2	1	2	2	4	-0.15209
tr|B3KNN7|B3KNN7_HUMAN	tr|B3KNN7|B	cDNA FLJ30049 fis, clone ADRGL1000033, highly similar to 26S proteasome non-ATPase regulatory subunit 3 OS=Homo sapiens PE=2 SV=1	57311	273	284	69	176	0.18	0.4	2.2222222	3	5	3	6	3	7	3	9	0.210716
tr|Q6DC98|Q6DC98_HUMAN	LMNB1	LMNB1 protein (Fragment) OS=Homo sapiens GN=LMNB1 PE=2 SV=1	38289	219	215	95	163	0.18	0.4	2.2222222	2	3	2	4	2	4	4	5	-0.04103
sp|P00390|GSHR_HUMAN	GSR	Glutathione reductase, mitochondrial OS=Homo sapiens GN=GSR PE=1 SV=2	56791	313	295	58	171	0.18	0.4	2.2222222	1	5	3	6	4	7	4	9	-0.15219
tr|Q9NZ23|Q9NZ23_HUMAN	YA61	Drug-sensitive protein 1 OS=Homo sapiens GN=YA61 PE=2 SV=1	14858	588	701	27	63	0.23	0.51	2.2173913	0	2	1	2	1	3	1	4	0.33352
tr|F4ZW64|F4ZW64_HUMAN	tr|F4ZW64|F4	NF90a OS=Homo sapiens PE=2 SV=1	76369	154	82	137	408	0.46	1.02	2.2173913	3	12	9	21	10	20	11	26	0.076422
tr|B4DFN9|B4DFN9_HUMAN	tr|B4DFN9|B4	cDNA FLJ54303, highly similar to Heat shock 70 kDa protein 1 OS=Homo sapiens PE=2 SV=1	62699	80	101	252	433	0.58	1.28	2.2068966	7	11	10	19	10	20	10	22	0.076422
tr|Q05BW9|Q05BW9_HUMAN	PAPSS1	PAPSS1 protein (Fragment) OS=Homo sapiens GN=PAPSS1 PE=2 SV=1	65264	553	362	29	140	0.1	0.22	2.2	0	3	2	4	1	5	2	6	0.985955
sp|Q5T9A4|ATD3B_HUMAN	ATAD3B	ATPase family AAA domain-containing protein 3B OS=Homo sapiens GN=ATAD3B PE=1 SV=1	73098	281	345	68	144	0.1	0.22	2.2	2	2	2	6	2	6	2	6	0.489845
tr|H0Y2W2|H0Y2W2_HUMAN	ATAD3A	ATPase family AAA domain-containing protein 3A (Fragment) OS=Homo sapiens GN=ATAD3A PE=4 SV=1	64600	281	374	68	132	0.1	0.22	2.2	2	3	2	4	2	4	2	6	-0.04103
tr|A4FU77|A4FU77_HUMAN	SNRNP200	SNRNP200 protein (Fragment) OS=Homo sapiens GN=SNRNP200 PE=2 SV=1	217585	243	211	83	205	0.05	0.11	2.2	2	6	3	7	7	8	8	13	-0.70939
tr|Q0GC70|Q0GC70_HUMAN	HLA-A	MHC class I antigen (Fragment) OS=Homo sapiens GN=HLA-A PE=3 SV=1	31863	639	733	22	59	0.16	0.35	2.1875	0	2	1	3	1	6	2	6	1.227142
tr|H2AM05|H2AM05_HUMAN	HLA-A	MHC class I antigen (Fragment) OS=Homo sapiens GN=HLA-A PE=3 SV=1	31675	639	737	22	59	0.16	0.35	2.1875	0	2	1	3	1	5	1	5	0.985955
tr|K4TZQ2|K4TZQ2_HUMAN	HLA-A	MHC class I antigen (Fragment) OS=Homo sapiens GN=HLA-A PE=3 SV=1	31799	639	736	22	59	0.16	0.35	2.1875	0	2	1	3	1	5	1	5	0.985955
tr|D0W033|D0W033_HUMAN	HLA-A	MHC class I antigen (Fragment) OS=Homo sapiens GN=HLA-A PE=3 SV=1	31801	639	734	22	59	0.16	0.35	2.1875	0	2	1	3	1	5	1	5	0.985955
tr|A0MNW2|A0MNW2_HUMAN	HLA-B	MHC class I antigen (Fragment) OS=Homo sapiens GN=HLA-B PE=3 SV=1	31749	639	578	22	79	0.16	0.35	2.1875	0	2	1	3	1	5	1	5	0.985955
tr|B7XH59|B7XH59_HUMAN	HLA-B	MHC class I antigen (Fragment) OS=Homo sapiens GN=HLA-B PE=3 SV=1	31924	639	565	22	80	0.16	0.35	2.1875	0	2	1	3	1	5	1	5	0.985955
sp|Q15149|PLEC_HUMAN	PLEC	Plectin OS=Homo sapiens GN=PLEC PE=1 SV=3	533462	74	35	270	1042	0.11	0.24	2.1818182	11	28	17	37	18	38	32	48	0.169093
tr|H0Y9Q7|H0Y9Q7_HUMAN	CANX	Calnexin (Fragment) OS=Homo sapiens GN=CANX PE=3 SV=1	18990	352	167	49	149	0.39	0.85	2.1794872	1	3	2	5	3	5	5	6	-0.2371
tr|Q2F839|Q2F839_HUMAN	tr|Q2F839|Q2	Heat shock 70 kDa protein 9B (Fragment) OS=Homo sapiens PE=2 SV=1	19208	81	257	59	199	0.42	0.91	2.1666667	2	4	2	4	2	4	2	5	-0.04103
tr|G3V2B8|G3V2B8_HUMAN	MTHFD1	C-1-tetrahydrofolate synthase, cytoplasmic OS=Homo sapiens GN=MTHFD1 PE=2 SV=1	102152	112	133	192	361	0.33	0.71	2.1515152	5	10	9	17	12	19	12	22	-0.24811
tr|G3XAD8|G3XAD8_HUMAN	STIP1	Stress-induced-phosphoprotein 1 OS=Homo sapiens GN=STIP1 PE=2 SV=1	68721	271	212	69	240	0.21	0.45	2.1428571	2	7	4	8	4	8	5	10	0.028566
sp|O43286|B4GT5_HUMAN	B4GALT5	Beta-1,4-galactosyltransferase 5 OS=Homo sapiens GN=B4GALT5 PE=2 SV=1	45318	651	993	22	37	0.07	0.15	2.1428571	0	2	1	2	1	2	1	2	-0.15209
tr|B4DJE3|B4DJE3_HUMAN	tr|B4DJE3|B4	cDNA FLJ52929, highly similar to Dolichyl-diphosphooligosaccharide--proteinglycosyltransferase 48 kDa subunit (EC2.4.1.119) OS=Homo sapiens	46620	604	796	26	52	0.07	0.15	2.1428571	0	1	1	2	1	2	2	3	-0.15209
tr|H0YD14|H0YD14_HUMAN	MYOF	Myoferlin (Fragment) OS=Homo sapiens GN=MYOF PE=4 SV=1	135822	220	264	95	67	0.07	0.15	2.1428571	3	1	3	2	4	2	4	4	-1.73804
tr|D9ZNB2|D9ZNB2_HUMAN	HLA-A	MHC class I antigen (Fragment) OS=Homo sapiens GN=HLA-A PE=3 SV=1	21389	639	894	22	43	0.16	0.34	2.125	0	1	1	2	1	3	2	4	0.33352
tr|E0YTJ7|E0YTJ7_HUMAN	HLA-B	MHC class I antigen (Fragment) OS=Homo sapiens GN=HLA-B PE=3 SV=1	21175	639	575	22	79	0.16	0.34	2.125	0	2	1	2	1	3	1	3	0.33352
tr|F2X601|F2X601_HUMAN	HLA-A	MHC class I antigen (Fragment) OS=Homo sapiens GN=HLA-A PE=3 SV=1	21322	639	574	22	79	0.16	0.34	2.125	0	2	1	2	1	3	2	4	0.33352
tr|B4DEX8|B4DEX8_HUMAN	MAT2A	S-adenosylmethionine synthase OS=Homo sapiens GN=MAT2A PE=2 SV=1	40027	333	519	52	92	0.08	0.17	2.125	1	2	1	2	1	3	1	4	0.33352
tr|Q5HYM2|Q5HYM2_HUMAN	DKFZp686O24	Putative uncharacterized protein DKFZp686O2462 (Fragment) OS=Homo sapiens GN=DKFZp686O2462 PE=2 SV=1	72627	569	305	29	144	0.09	0.19	2.1111111	0	4	2	4	2	4	2	4	-0.04103
sp|O00116|ADAS_HUMAN	AGPS	Alkyldihydroxyacetonephosphate synthase, peroxisomal OS=Homo sapiens GN=AGPS PE=1 SV=1	73664	448	346	38	144	0.09	0.19	2.1111111	1	3	2	4	2	4	3	5	-0.04103
tr|Q5M7Z5|Q5M7Z5_HUMAN	GRHPR	GRHPR protein (Fragment) OS=Homo sapiens GN=GRHPR PE=2 SV=1	37314	659	1200	21	23	0.09	0.19	2.1111111	0	0	1	2	1	2	1	2	-0.15209
tr|F5H5D3|F5H5D3_HUMAN	TUBA1C	Tubulin alpha-1C chain OS=Homo sapiens GN=TUBA1C PE=2 SV=1	58606	21	18	809	1781	2.92	6.14	2.1027397	24	42	34	63	37	65	41	77	-0.08511
tr|B3GQS7|B3GQS7_HUMAN	HSPD1	Mitochondrial heat shock 60kD protein 1 variant 1 OS=Homo sapiens GN=HSPD1 PE=2 SV=1	60813	16	27	904	1278	1.72	3.57	2.0755814	24	28	27	33	26	33	33	39	-0.55363
tr|Q7Z5T5|Q7Z5T5_HUMAN	EIF3A	EIF3A protein (Fragment) OS=Homo sapiens GN=EIF3A PE=2 SV=1	97203	192	129	112	368	0.14	0.29	2.0714286	2	9	4	13	5	15	8	20	0.606715
tr|Q6FI37|Q6FI37_HUMAN	IDH1	Isocitrate dehydrogenase [NADP] OS=Homo sapiens GN=IDH1 PE=2 SV=1	46933	555	339	29	147	0.15	0.31	2.0666667	0	4	2	4	3	5	3	7	-0.2371
tr|B4DTG2|B4DTG2_HUMAN	EEF1G	Elongation factor 1-gamma OS=Homo sapiens GN=EEF1G PE=2 SV=1	56456	93	56	225	698	0.86	1.77	2.0581395	5	17	15	23	14	23	18	25	-0.193
sp|Q71U36|TBA1A_HUMAN	TUBA1A	Tubulin alpha-1A chain OS=Homo sapiens GN=TUBA1A PE=1 SV=1	50788	15	20	1022	1624	5.18	10.66	2.0579151	30	38	41	58	43	61	46	72	-0.39287
sp|P68363|TBA1B_HUMAN	TUBA1B	Tubulin alpha-1B chain OS=Homo sapiens GN=TUBA1B PE=1 SV=1	50804	13	17	1091	1845	5.58	11.48	2.0573477	31	43	43	63	45	66	49	78	-0.34472
sp|O00410|IPO5_HUMAN	IPO5	Importin-5 OS=Homo sapiens GN=IPO5 PE=1 SV=4	125032	85	150	236	322	0.23	0.47	2.0434783	7	11	8	16	10	17	15	20	-0.15238
tr|J3KTA4|J3KTA4_HUMAN	DDX5	Probable ATP-dependent RNA helicase DDX5 OS=Homo sapiens GN=DDX5 PE=3 SV=1	69557	97	116	218	406	0.45	0.91	2.0222222	7	11	9	14	10	16	10	16	-0.23745
tr|Q60FE2|Q60FE2_HUMAN	MYH9	MYH9 variant protein OS=Homo sapiens GN=MYH9 PE=2 SV=1	227646	17	7	900	2640	0.9	1.82	2.0222222	30	64	46	77	52	82	60	87	-0.23959
sp|P68366|TBA4A_HUMAN	TUBA4A	Tubulin alpha-4A chain OS=Homo sapiens GN=TUBA4A PE=1 SV=1	50634	22	28	796	1332	3.01	6.07	2.0166113	20	32	31	49	31	52	36	61	-0.15309
tr|H0Y804|H0Y804_HUMAN	AKR1C1	Aldo-keto reductase family 1 member C1 (Fragment) OS=Homo sapiens GN=AKR1C1 PE=4 SV=1	28617	186	180	61	89	0.67	1.35	2.0149254	2	3	4	5	4	7	6	7	-0.15219
tr|B5A9M9|B5A9M9_HUMAN	HLA-A	MHC class I antigen (Fragment) OS=Homo sapiens GN=HLA-A PE=3 SV=1	34234	639	506	22	95	0.16	0.32	2	0	3	1	3	1	5	3	5	0.985955
tr|B4DM97|B4DM97_HUMAN	tr|B4DM97|B	cDNA FLJ55002, highly similar to Alpha-centractin OS=Homo sapiens PE=2 SV=1	38365	561	485	29	100	0.09	0.18	2	0	2	1	3	1	4	1	4	0.696269
tr|E7ET15|E7ET15_HUMAN	U2SURP	U2 snRNP-associated SURP motif-containing protein OS=Homo sapiens GN=U2SURP PE=2 SV=1	118631	647	403	22	119	0.03	0.06	2	0	2	1	2	1	3	2	3	0.33352
sp|O95782|AP2A1_HUMAN	AP2A1	AP-2 complex subunit alpha-1 OS=Homo sapiens GN=AP2A1 PE=1 SV=3	108561	500	907	34	42	0.03	0.06	2	0	0	1	2	1	3	2	3	0.33352
tr|B1AHC9|B1AHC9_HUMAN	XRCC6	X-ray repair complementing defective repair in Chinese hamster cells 6 (Ku autoantigen, 70kDa) OS=Homo sapiens GN=XRCC6 PE=4 SV=1	64528	116	81	184	470	0.64	1.28	2	5	8	10	17	11	22	12	26	0.079505
tr|B3KSQ7|B3KSQ7_HUMAN	DBN1	Drebrin 1, isoform CRA_d OS=Homo sapiens GN=DBN1 PE=2 SV=1	66053	478	431	36	113	0.05	0.1	2	0	2	1	2	1	2	1	2	-0.15209
tr|F5H1A8|F5H1A8_HUMAN	GSN	Gelsolin OS=Homo sapiens GN=GSN PE=2 SV=1	81776	297	407	61	118	0.04	0.08	2	1	2	1	2	1	2	2	2	-0.15209
tr|B4DNN4|B4DNN4_HUMAN	tr|B4DNN4|B	Ribonucleoside-diphosphate reductase OS=Homo sapiens PE=2 SV=1	87252	156	118	136	341	0.29	0.58	2	5	9	7	13	8	13	8	16	-0.22198
sp|Q13867|BLMH_HUMAN	BLMH	Bleomycin hydrolase OS=Homo sapiens GN=BLMH PE=1 SV=1	53155	332	668	52	67	0.2	0.4	2	1	1	3	4	2	3	4	4	-0.40378
tr|H0YMR4|H0YMR4_HUMAN	IPO4	Importin-4 (Fragment) OS=Homo sapiens GN=IPO4 PE=4 SV=1	60104	655	716	22	44	0.05	0.1	2	0	1	1	1	1	1	1	2	-0.88923
tr|B4DDC9|B4DDC9_HUMAN	tr|B4DDC9|B4	cDNA FLJ59448, highly similar to Trifunctional enzyme subunit beta, mitochondrial OS=Homo sapiens PE=2 SV=1	49937	499	773	34	43	0.07	0.14	2	0	1	1	1	1	1	1	1	-0.88923
sp|Q9NPQ8|RIC8A_HUMAN	RIC8A	Synembryn-A OS=Homo sapiens GN=RIC8A PE=1 SV=3	60185	496	831	34	48	0.05	0.1	2	0	1	1	1	1	1	1	1	-0.88923
tr|B4DYL7|B4DYL7_HUMAN	tr|B4DYL7|B4	cDNA FLJ58490, highly similar to Homo sapiens CCR4-NOT transcription complex, subunit 1 (CNOT1), transcript variant 1, mRNA OS=Homo sapie	140991	388	983	45	26	0.02	0.04	2	1	1	1	2	1	1	2	2	-0.88923
sp|P55072|TERA_HUMAN	VCP	Transitional endoplasmic reticulum ATPase OS=Homo sapiens GN=VCP PE=1 SV=4	89950	64	54	326	701	0.65	1.28	1.9692308	7	15	14	25	14	23	15	27	-0.193
tr|A4QPB0|A4QPB0_HUMAN	IQGAP1	IQ motif containing GTPase activating protein 1 OS=Homo sapiens GN=IQGAP1 PE=2 SV=1	189789	84	45	239	869	0.31	0.61	1.9677419	8	19	17	28	18	33	21	42	-0.0325
tr|B4DW52|B4DW52_HUMAN	tr|B4DW52|B	cDNA FLJ55253, highly similar to Actin, cytoplasmic 1 OS=Homo sapiens PE=2 SV=1	38950	4	2	3120	4725	27.29	53.31	1.9534628	74	98	108	146	123	160	134	184	-0.52344
sp|Q1KMD3|HNRL2_HUMAN	HNRNPUL2	Heterogeneous nuclear ribonucleoprotein U-like protein 2 OS=Homo sapiens GN=HNRNPUL2 PE=1 SV=1	85622	249	281	78	178	0.21	0.4	1.9047619	2	5	5	10	6	10	6	12	-0.1974
tr|B3KTJ9|B3KTJ9_HUMAN	tr|B3KTJ9|B3	cDNA FLJ38393 fis, clone FEBRA2007212 OS=Homo sapiens PE=2 SV=1	103493	82	111	251	419	0.32	0.6	1.875	7	11	9	15	9	18	12	18	0.072687
tr|H0Y5J4|H0Y5J4_HUMAN	DBNL	Drebrin-like protein (Fragment) OS=Homo sapiens GN=DBNL PE=4 SV=1	40475	466	778	37	53	0.08	0.15	1.875	1	1	1	2	1	2	1	2	-0.15209
tr|E9PKT8|E9PKT8_HUMAN	NAP1L4	Nucleosome assembly protein 1-like 4 (Fragment) OS=Homo sapiens GN=NAP1L4 PE=2 SV=1	19202	417	381	42	0	0.16	0.3	1.875	0	0	2	0	3	0	3	1	-3.69743
tr|Q6IBT3|Q6IBT3_HUMAN	CCT7	CCT7 protein OS=Homo sapiens GN=CCT7 PE=2 SV=1	59804	161	157	133	309	0.38	0.71	1.8684211	2	7	6	10	5	10	7	10	0.04394
tr|H0Y4R1|H0Y4R1_HUMAN	IMPDH2	Inosine-5~-monophosphate dehydrogenase 2 (Fragment) OS=Homo sapiens GN=IMPDH2 PE=3 SV=1	51549	177	182	122	262	0.36	0.67	1.8611111	2	6	5	8	5	9	6	10	-0.10063
sp|P26232|CTNA2_HUMAN	CTNNA2	Catenin alpha-2 OS=Homo sapiens GN=CTNNA2 PE=1 SV=5	106045	389	301	31	167	0.07	0.13	1.8571429	0	4	1	4	1	4	1	4	0.696269
sp|P06733|ENOA_HUMAN	ENO1	Alpha-enolase OS=Homo sapiens GN=ENO1 PE=1 SV=2	47481	40	59	520	642	1.39	2.58	1.8561151	13	15	18	26	19	32	25	39	-0.15269
tr|D6REX3|D6REX3_HUMAN	SEC31A	Protein transport protein Sec31A OS=Homo sapiens GN=SEC31A PE=2 SV=1	137110	121	227	178	229	0.13	0.24	1.8461538	4	5	5	9	4	9	5	10	0.18921
tr|Q6IAV5|Q6IAV5_HUMAN	OXCT	Succinyl-CoA:3-ketoacid-coenzyme A transferase OS=Homo sapiens GN=OXCT PE=2 SV=1	56612	209	268	100	177	0.18	0.33	1.8333333	1	4	3	4	5	5	6	6	-0.88984
tr|B4DNK3|B4DNK3_HUMAN	tr|B4DNK3|B4	cDNA FLJ52127, highly similar to Multisynthetase complex auxiliary component p43 OS=Homo sapiens PE=2 SV=1	29955	601	833	26	39	0.11	0.2	1.8181818	0	1	1	1	1	1	1	1	-0.88923
sp|P22626|ROA2_HUMAN	HNRNPA2B1	Heterogeneous nuclear ribonucleoproteins A2/B1 OS=Homo sapiens GN=HNRNPA2B1 PE=1 SV=2	37464	77	69	256	579	0.97	1.76	1.814433	7	12	8	13	9	15	10	18	-0.18311
sp|P38919|IF4A3_HUMAN	EIF4A3	Eukaryotic initiation factor 4A-III OS=Homo sapiens GN=EIF4A3 PE=1 SV=4	47126	262	148	74	327	0.4	0.72	1.8	1	6	5	9	4	10	6	10	0.333779
tr|Q53HF2|Q53HF2_HUMAN	tr|Q53HF2|Q5	Heat shock 70kDa protein 8 isoform 2 variant (Fragment) OS=Homo sapiens PE=2 SV=1	53580	51	44	426	859	1.45	2.54	1.7517241	12	23	17	31	19	31	19	34	-0.19795
tr|E2FIL8|E2FIL8_HUMAN	HLA-A	MHC class I antigen (Fragment) OS=Homo sapiens GN=HLA-A PE=3 SV=1	38417	639	724	22	60	0.16	0.28	1.75	0	2	1	3	1	4	2	4	0.696269
tr|B3KY60|B3KY60_HUMAN	tr|B3KY60|B3	cDNA FLJ16777 fis, clone BRHIP2029567, highly similar to Cell division cycle 5-like protein OS=Homo sapiens PE=2 SV=1	92448	587	468	27	105	0.04	0.07	1.75	0	1	1	2	1	2	1	3	-0.15209
tr|B3KV72|B3KV72_HUMAN	tr|B3KV72|B3	cDNA FLJ16204 fis, clone CTONG2019232, highly similar to Proteasome-associated protein ECM29 homolog (Fragment) OS=Homo sapiens PE=2	78791	579	415	28	63	0.04	0.07	1.75	0	1	1	1	1	1	1	1	-0.88923
tr|Q53GN4|Q53GN4_HUMAN	WDR1	WD repeat domain 1, isoform CRA_a (Fragment) OS=Homo sapiens GN=WDR1 PE=2 SV=1	66822	137	130	160	366	0.4	0.7	1.75	4	8	7	12	7	12	9	13	-0.15229
sp|P17516|AK1C4_HUMAN	AKR1C4	Aldo-keto reductase family 1 member C4 OS=Homo sapiens GN=AKR1C4 PE=1 SV=3	37442	186	158	42	63	0.67	1.16	1.7313433	1	2	1	3	1	3	2	3	0.33352
tr|G5E9Q2|G5E9Q2_HUMAN	CSDE1	Cold shock domain containing E1, RNA-binding, isoform CRA_d OS=Homo sapiens GN=CSDE1 PE=2 SV=1	94539	312	329	58	153	0.11	0.19	1.7272727	1	4	3	5	3	6	3	7	0.004086
tr|B4DRT0|B4DRT0_HUMAN	RBBP4	Histone-binding protein RBBP4 OS=Homo sapiens GN=RBBP4 PE=2 SV=1	19586	321	463	37	105	0.22	0.38	1.7272727	1	2	1	2	2	3	2	3	-0.40378
sp|P07814|SYEP_HUMAN	EPRS	Bifunctional glutamate/proline--tRNA ligase OS=Homo sapiens GN=EPRS PE=1 SV=5	172080	57	60	380	639	0.33	0.57	1.7272727	9	18	15	24	15	26	17	34	-0.11567
tr|Q1KLZ0|Q1KLZ0_HUMAN	PS1TP5BP1	HCG15971, isoform CRA_a OS=Homo sapiens GN=PS1TP5BP1 PE=2 SV=1	42052	1	1	3367	4899	31.43	54.06	1.7200127	82	102	119	152	130	156	141	180	-0.64251
tr|Q5HYG8|Q5HYG8_HUMAN	DKFZp686P09	Putative uncharacterized protein DKFZp686P09201 OS=Homo sapiens GN=DKFZp686P09201 PE=2 SV=1	53831	86	102	235	442	0.61	1.04	1.704918	4	10	8	13	9	15	10	18	-0.18311
sp|P07355|ANXA2_HUMAN	ANXA2	Annexin A2 OS=Homo sapiens GN=ANXA2 PE=1 SV=2	38808	100	83	214	494	1.26	2.14	1.6984127	8	13	11	17	12	20	13	22	-0.17578
tr|B7ZB91|B7ZB91_HUMAN	TNRC6B	Trinucleotide repeat containing 6B (Fragment) OS=Homo sapiens GN=TNRC6B PE=4 SV=1	81825	359	493	48	99	0.13	0.22	1.6923077	1	2	3	5	2	5	3	5	0.248657
tr|K7EKB6|K7EKB6_HUMAN	THOP1	Thimet oligopeptidase (Fragment) OS=Homo sapiens GN=THOP1 PE=4 SV=1	20792	278	420	68	30	0.16	0.27	1.6875	1	0	1	1	1	1	1	1	-0.88923
tr|B2RBE5|B2RBE5_HUMAN	tr|B2RBE5|B2	cDNA, FLJ95468, highly similar to Homo sapiens transcriptional coactivator tubedown-100 (TBDN100),transcript variant 1, mRNA OS=Homo sapi	102490	446	696	38	64	0.06	0.1	1.6666667	0	2	2	3	2	5	2	7	0.248657
sp|Q3ZCM7|TBB8_HUMAN	TUBB8	Tubulin beta-8 chain OS=Homo sapiens GN=TUBB8 PE=1 SV=2	50257	67	63	306	623	0.77	1.28	1.6623377	13	23	17	28	19	34	21	37	-0.06617
sp|P23526|SAHH_HUMAN	AHCY	Adenosylhomocysteinase OS=Homo sapiens GN=AHCY PE=1 SV=4	48255	195	347	111	144	0.49	0.81	1.6530612	2	5	6	9	6	12	8	12	0.054498
tr|Q58FF4|Q58FF4_HUMAN	HSP90Bf	Heat shock protein 90Bf OS=Homo sapiens GN=HSP90Bf PE=1 SV=1	41833	407	598	43	76	0.16	0.26	1.625	1	2	2	3	2	4	2	4	-0.04103
tr|B3KT66|B3KT66_HUMAN	PSMD6	Proteasome (Prosome, macropain) 26S subunit, non-ATPase, 6, isoform CRA_c OS=Homo sapiens GN=PSMD6 PE=2 SV=1	40600	399	678	43	66	0.08	0.13	1.625	1	2	1	2	1	2	1	3	-0.15209
tr|B4DUC5|B4DUC5_HUMAN	CSE1L	Exportin-2 OS=Homo sapiens GN=CSE1L PE=2 SV=1	86045	433	437	40	69	0.08	0.13	1.625	0	1	2	3	4	3	4	3	-1.25244
sp|P14923|PLAK_HUMAN	JUP	Junction plakoglobin OS=Homo sapiens GN=JUP PE=1 SV=3	82434	205	172	102	294	0.26	0.42	1.6153846	3	7	6	10	6	10	7	10	-0.1974
tr|Q9UJZ2|Q9UJZ2_HUMAN	VIL2	Cytovillin 2 (Fragment) OS=Homo sapiens GN=VIL2 PE=2 SV=1	18464	119	176	88	141	0.46	0.74	1.6086957	2	2	2	4	2	5	2	6	0.248657
sp|Q14432|PDE3A_HUMAN	PDE3A	cGMP-inhibited 3~,5~-cyclic phosphodiesterase A OS=Homo sapiens GN=PDE3A PE=1 SV=3	126440	493	419	35	116	0.05	0.08	1.6	1	2	2	3	2	4	2	4	-0.04103
sp|Q9Y305|ACOT9_HUMAN	ACOT9	Acyl-coenzyme A thioesterase 9, mitochondrial OS=Homo sapiens GN=ACOT9 PE=1 SV=2	50269	637	1060	22	32	0.07	0.11	1.5714286	0	0	1	1	1	1	2	1	-0.88923
sp|Q6S8J3|POTEE_HUMAN	POTEE	POTE ankyrin domain family member E OS=Homo sapiens GN=POTEE PE=1 SV=3	122882	5	6	2211	2789	0.44	0.69	1.5681818	45	52	66	79	70	79	75	90	-0.72492
tr|B0QZK4|B0QZK4_HUMAN	HP1BP3	Heterochromatin protein 1-binding protein 3 (Fragment) OS=Homo sapiens GN=HP1BP3 PE=4 SV=1	28725	631	834	23	48	0.25	0.39	1.56	0	1	2	3	1	3	3	3	0.33352
tr|B4DUL5|B4DUL5_HUMAN	tr|B4DUL5|B4	cDNA FLJ51625, highly similar to Ubiquinol-cytochrome-c reductase complex coreprotein I, mitochondrial (EC 1.10.2.2) OS=Homo sapiens PE=2	40917	357	671	48	66	0.17	0.26	1.5294118	1	2	2	3	2	3	2	4	-0.40378
tr|J3KRF5|J3KRF5_HUMAN	CLTC	Clathrin heavy chain 1 (Fragment) OS=Homo sapiens GN=CLTC PE=4 SV=1	15819	30	609	40	75	0.52	0.79	1.5192308	1	1	1	3	2	3	2	3	-0.40378
sp|Q9UQ35|SRRM2_HUMAN	SRRM2	Serine/arginine repetitive matrix protein 2 OS=Homo sapiens GN=SRRM2 PE=1 SV=2	300179	247	815	78	50	0.02	0.03	1.5	1	1	2	3	2	4	5	5	-0.04103
tr|A6NEM2|A6NEM2_HUMAN	HCFC1	HCF N-terminal chain 5 OS=Homo sapiens GN=HCFC1 PE=2 SV=2	215338	508	507	33	95	0.02	0.03	1.5	0	2	1	2	1	2	2	2	-0.15209
tr|B2RWP9|B2RWP9_HUMAN	MYH10	MYH10 protein OS=Homo sapiens GN=MYH10 PE=2 SV=1	230858	200	358	104	141	0.06	0.09	1.5	3	3	4	6	4	7	6	9	-0.15219
tr|B4DH52|B4DH52_HUMAN	tr|B4DH52|B4	cDNA FLJ55916, highly similar to General transcription factor II-I OS=Homo sapiens PE=2 SV=1	112359	554	464	29	105	0.06	0.09	1.5	0	3	2	3	2	3	3	3	-0.40378
tr|B2RDN4|B2RDN4_HUMAN	tr|B2RDN4|B2	cDNA, FLJ96693, highly similar to Homo sapiens block of proliferation 1 (BOP1), mRNA OS=Homo sapiens PE=2 SV=1	84219	505	314	33	57	0.08	0.12	1.5	0	1	2	2	2	3	3	4	-0.40378
tr|Q2TB59|Q2TB59_HUMAN	NNT	Nicotinamide nucleotide transhydrogenase OS=Homo sapiens GN=NNT PE=2 SV=1	114552	234	360	88	141	0.06	0.09	1.5	1	3	2	3	2	3	3	3	-0.40378
tr|B4DPY2|B4DPY2_HUMAN	tr|B4DPY2|B4	cDNA FLJ59286, highly similar to Pre-mRNA-processing factor 40 homolog A (Fragment) OS=Homo sapiens PE=2 SV=1	93124	425	680	41	42	0.04	0.06	1.5	1	1	1	1	1	1	1	1	-0.88923
sp|O15427|MOT4_HUMAN	SLC16A3	Monocarboxylate transporter 4 OS=Homo sapiens GN=SLC16A3 PE=1 SV=1	50064	362	851	48	47	0.14	0.21	1.5	2	1	2	3	2	3	2	3	-0.40378
tr|Q05DV5|Q05DV5_HUMAN	ZC3HAV1	ZC3HAV1 protein (Fragment) OS=Homo sapiens GN=ZC3HAV1 PE=2 SV=1	49964	224	501	92	97	0.14	0.21	1.5	1	1	2	3	2	3	2	3	-0.40378
tr|B4DF68|B4DF68_HUMAN	tr|B4DF68|B4	cDNA FLJ55325, highly similar to Heat-shock protein 105 kDa OS=Homo sapiens PE=2 SV=1	86500	123	192	47	202	0.23	0.34	1.4782609	1	5	1	6	1	8	1	9	1.614523
tr|J3KPF3|J3KPF3_HUMAN	SLC3A2	4F2 cell-surface antigen heavy chain OS=Homo sapiens GN=SLC3A2 PE=4 SV=1	68230	60	47	366	814	1.33	1.95	1.4661654	12	18	20	24	20	24	20	27	-0.63426
sp|P52895|AK1C2_HUMAN	AKR1C2	Aldo-keto reductase family 1 member C2 OS=Homo sapiens GN=AKR1C2 PE=1 SV=3	37111	186	312	116	161	0.67	0.98	1.4626866	3	6	6	8	7	9	9	9	-0.54875
tr|B4DX08|B4DX08_HUMAN	tr|B4DX08|B4	cDNA FLJ58784, highly similar to T-complex protein 1 subunit epsilon OS=Homo sapiens PE=2 SV=1	57521	90	208	229	206	0.4	0.58	1.45	6	6	6	7	6	10	6	11	-0.1974
sp|Q9NY65|TBA8_HUMAN	TUBA8	Tubulin alpha-8 chain OS=Homo sapiens GN=TUBA8 PE=1 SV=1	50746	34	39	592	955	1.74	2.52	1.4482759	15	25	23	36	23	38	27	45	-0.17771
tr|B3KRS7|B3KRS7_HUMAN	tr|B3KRS7|B3	cDNA FLJ34844 fis, clone NT2NE2011154, highly similar to RuvB-like 1 OS=Homo sapiens PE=2 SV=1	34946	351	1039	50	29	0.09	0.13	1.4444444	1	1	1	1	1	1	1	1	-0.88923
tr|A0AV58|A0AV58_HUMAN	STRN3	Striatin, calmodulin binding protein 3 OS=Homo sapiens GN=STRN3 PE=2 SV=1	78069	419	732	42	59	0.09	0.13	1.4444444	1	1	2	3	3	3	3	4	-0.88954
tr|A8K525|A8K525_HUMAN	tr|A8K525|A8	cDNA FLJ76817, highly similar to Homo sapiens non-POU domain containing, octamer-binding (NONO), mRNA OS=Homo sapiens PE=2 SV=1	54367	75	77	266	514	0.8	1.15	1.4375	10	10	11	14	12	15	14	17	-0.58003
tr|Q9BS14|Q9BS14_HUMAN	GANAB	GANAB protein (Fragment) OS=Homo sapiens GN=GANAB PE=2 SV=1	34786	47	188	50	270	0.62	0.89	1.4354839	1	8	1	9	1	13	1	14	2.282843
tr|H0Y8G5|H0Y8G5_HUMAN	HNRNPD	Heterogeneous nuclear ribonucleoprotein D0 (Fragment) OS=Homo sapiens GN=HNRNPD PE=4 SV=1	29877	229	368	90	138	0.37	0.53	1.4324324	3	6	3	6	4	8	4	8	0.028566
sp|P58107|EPIPL_HUMAN	EPPK1	Epiplakin OS=Homo sapiens GN=EPPK1 PE=1 SV=2	557674	56	67	390	585	0.07	0.1	1.4285714	10	15	12	17	12	17	13	18	-0.40459

tr|E9PKU4|E9PKU4_HUMAN	RPL8	60S ribosomal protein L8 (Fragment) OS=Homo sapiens GN=RPL8 PE=2 SV=1	25806	231	652	90	0	0.28	0.4	1.4285714	2	0	2	0	4	0	5	1	-4.06033
sp|Q15392|DHC24_HUMAN	DHCR24	Delta(24)-sterol reductase OS=Homo sapiens GN=DHCR24 PE=1 SV=2	60803	442	1174	39	25	0.05	0.07	1.4	1	0	1	1	2	1	3	1	-1.62653
tr|E3Q1J2|E3Q1J2_HUMAN	HLA-B	MHC class I antigen (Fragment) OS=Homo sapiens GN=HLA-B PE=3 SV=1	31790	639	571	22	79	0.16	0.22	1.375	0	2	1	2	1	4	1	5	0.696269
tr|B3KPZ8|B3KPZ8_HUMAN	tr|B3KPZ8|B3	cDNA FLJ32530 fis, clone SMINT2000185, highly similar to TRANSKETOLASE (EC 2.2.1.1) OS=Homo sapiens PE=2 SV=1	39205	35	65	475	590	1.36	1.87	1.375	15	17	19	19	22	23	26	29	-0.82953
tr|G3V2A3|G3V2A3_HUMAN	TUBB3	Tubulin beta-3 chain (Fragment) OS=Homo sapiens GN=TUBB3 PE=2 SV=1	20929	25	34	147	447	3.07	4.12	1.3420195	5	12	6	16	7	21	7	25	0.631737
tr|Q562M3|Q562M3_HUMAN	ACT	Actin-like protein (Fragment) OS=Homo sapiens GN=ACT PE=2 SV=1	11529	103	103	213	441	7.06	9.47	1.3413598	10	14	14	25	17	28	20	35	-0.1861
tr|A8K6Q8|A8K6Q8_HUMAN	tr|A8K6Q8|A8	cDNA FLJ75881, highly similar to Homo sapiens transferrin receptor (p90, CD71) (TFRC), mRNA OS=Homo sapiens PE=2 SV=1	85276	122	136	176	355	0.3	0.4	1.3333333	6	8	7	9	7	9	8	12	-0.54875
sp|P46087|NOP2_HUMAN	NOP2	Putative ribosomal RNA methyltransferase NOP2 OS=Homo sapiens GN=NOP2 PE=1 SV=2	89589	296	401	61	120	0.15	0.2	1.3333333	2	2	4	5	5	5	6	6	-0.88984
sp|Q14315|FLNC_HUMAN	FLNC	Filamin-C OS=Homo sapiens GN=FLNC PE=1 SV=3	293407	143	223	153	232	0.06	0.08	1.3333333	5	7	6	9	10	10	11	11	-0.8906
tr|B7Z601|B7Z601_HUMAN	tr|B7Z601|B7	cDNA FLJ57187, highly similar to Glycerol-3-phosphate dehydrogenase, mitochondrial (EC 1.1.99.5) OS=Homo sapiens PE=2 SV=1	56047	512	635	32	39	0.06	0.08	1.3333333	0	1	1	1	2	1	2	1	-1.62653
tr|H7C1U0|H7C1U0_HUMAN	APEH	Acylamino-acid-releasing enzyme (Fragment) OS=Homo sapiens GN=APEH PE=4 SV=1	35132	319	619	56	63	0.09	0.12	1.3333333	1	1	1	2	1	2	1	2	-0.15209
sp|P15121|ALDR_HUMAN	AKR1B1	Aldose reductase OS=Homo sapiens GN=AKR1B1 PE=1 SV=3	36230	185	282	116	177	0.42	0.55	1.3095238	3	4	4	5	5	6	5	8	-0.64865
sp|P52272|HNRPM_HUMAN	HNRNPM	Heterogeneous nuclear ribonucleoprotein M OS=Homo sapiens GN=HNRNPM PE=1 SV=3	77749	164	120	132	397	0.39	0.51	1.3076923	7	7	8	10	11	14	12	16	-0.5558
tr|B3KV39|B3KV39_HUMAN	tr|B3KV39|B3	cDNA FLJ16099 fis, clone TESTI2008278, highly similar to Homo sapiens forkhead box K1 (FOXK1), mRNA OS=Homo sapiens PE=2 SV=1	50383	188	1096	115	20	0.07	0.09	1.2857143	1	0	1	1	1	2	2	3	-0.15209
tr|B4DGQ0|B4DGQ0_HUMAN	LASP1	LIM and SH3 domain protein 1 OS=Homo sapiens GN=LASP1 PE=2 SV=1	23167	568	761	29	54	0.14	0.18	1.2857143	0	1	1	1	1	1	1	1	-0.88923
tr|Q6DEN2|Q6DEN2_HUMAN	DPYSL3	DPYSL3 protein OS=Homo sapiens GN=DPYSL3 PE=2 SV=1	74320	490	181	35	276	0.19	0.24	1.2631579	0	5	4	5	3	5	8	8	-0.2371
tr|A0JHM7|A0JHM7_HUMAN	HLA-A	MHC class I antigen (Fragment) OS=Homo sapiens GN=HLA-A PE=3 SV=1	31850	639	720	22	52	0.16	0.2	1.25	0	1	1	1	1	3	2	3	0.33352
sp|Q12792|TWF1_HUMAN	TWF1	Twinfilin-1 OS=Homo sapiens GN=TWF1 PE=1 SV=3	40429	575	653	28	68	0.08	0.1	1.25	0	1	1	1	1	1	1	1	-0.88923
sp|O76094|SRP72_HUMAN	SRP72	Signal recognition particle 72 kDa protein OS=Homo sapiens GN=SRP72 PE=1 SV=3	75130	455	1030	38	34	0.04	0.05	1.25	1	0	1	1	1	1	1	1	-0.88923
sp|P17987|TCPA_HUMAN	TCP1	T-complex protein 1 subunit alpha OS=Homo sapiens GN=TCP1 PE=1 SV=1	60819	197	320	106	154	0.3	0.37	1.2333333	2	4	5	6	8	7	10	7	-1.07105
tr|B3KM80|B3KM80_HUMAN	NCL	Nucleolin, isoform CRA_c OS=Homo sapiens GN=NCL PE=2 SV=1	58576	71	96	283	466	1.03	1.27	1.2330097	9	13	14	17	15	18	15	20	-0.63558
tr|Q5I6Y6|Q5I6Y6_HUMAN	LMNA	Lamin A/C transcript variant 1 OS=Homo sapiens GN=LMNA PE=2 SV=1	74322	95	80	221	502	0.75	0.91	1.2133333	7	12	13	15	15	17	15	19	-0.71593
tr|B4DZY9|B4DZY9_HUMAN	tr|B4DZY9|B4	cDNA FLJ59103, highly similar to T-complex protein 1 subunit epsilon OS=Homo sapiens PE=2 SV=1	32544	90	354	126	142	0.4	0.48	1.2	3	4	3	4	3	7	3	7	0.210716
sp|P53396|ACLY_HUMAN	ACLY	ATP-citrate synthase OS=Homo sapiens GN=ACLY PE=1 SV=3	121674	20	33	845	1131	1.28	1.53	1.1953125	21	30	32	41	34	41	40	46	-0.62652
tr|H0YIV0|H0YIV0_HUMAN	HSP90B1	Endoplasmin (Fragment) OS=Homo sapiens GN=HSP90B1 PE=4 SV=1	17033	140	820	37	49	0.37	0.44	1.1891892	0	1	2	2	3	3	3	4	-0.88954
sp|P68032|ACTC_HUMAN	ACTC1	Actin, alpha cardiac muscle 1 OS=Homo sapiens GN=ACTC1 PE=1 SV=1	42334	23	43	771	911	6.63	7.86	1.1855204	25	33	40	58	48	63	55	76	-0.50498
tr|B4DP08|B4DP08_HUMAN	ALG1	Chitobiosyldiphosphodolichol beta-mannosyltransferase OS=Homo sapiens GN=ALG1 PE=2 SV=1	40938	452	318	38	89	0.17	0.2	1.1764706	0	1	2	1	2	1	2	1	-1.62653
tr|B3KM77|B3KM77_HUMAN	tr|B3KM77|B	cDNA FLJ10435 fis, clone NT2RP1000493, highly similar to Splicing factor 3B subunit 3 OS=Homo sapiens PE=2 SV=1	56736	356	474	49	55	0.06	0.07	1.1666667	1	1	1	1	1	1	1	2	-0.88923
tr|B1PS43|B1PS43_HUMAN	MYH11	Myosin heavy chain 11 smooth muscle isoform OS=Homo sapiens GN=MYH11 PE=2 SV=1	235004	142	231	155	226	0.06	0.07	1.1666667	5	6	5	6	9	6	11	8	-1.43848
sp|Q9NR30|DDX21_HUMAN	DDX21	Nucleolar RNA helicase 2 OS=Homo sapiens GN=DDX21 PE=1 SV=5	87804	148	348	147	144	0.34	0.39	1.1470588	4	6	8	9	8	8	9	9	-0.8903
sp|Q08211|DHX9_HUMAN	DHX9	ATP-dependent RNA helicase A OS=Homo sapiens GN=DHX9 PE=1 SV=4	142181	96	90	218	480	0.41	0.47	1.1463415	8	12	16	17	18	22	22	26	-0.60871
tr|A4ZU86|A4ZU86_HUMAN	NPM1	Truncated nucleolar phosphoprotein B23 OS=Homo sapiens GN=NPM1 PE=2 SV=1	30074	138	276	160	172	0.69	0.79	1.1449275	4	4	5	4	5	6	5	8	-0.64865
tr|B4DF96|B4DF96_HUMAN	EIF2A	Eukaryotic translation initiation factor 2A OS=Homo sapiens GN=EIF2A PE=2 SV=1	58357	206	479	101	94	0.32	0.36	1.125	2	3	5	5	4	7	5	9	-0.15219
sp|Q00610|CLH1_HUMAN	CLTC	Clathrin heavy chain 1 OS=Homo sapiens GN=CLTC PE=1 SV=5	193260	30	55	629	699	0.52	0.57	1.0961538	19	19	25	28	24	29	28	30	-0.62392
tr|B4DII5|B4DII5_HUMAN	tr|B4DII5|B4D	Importin subunit alpha OS=Homo sapiens PE=2 SV=1	60345	573	1204	28	22	0.11	0.12	1.0909091	0	0	2	1	3	2	3	2	-1.37514
tr|B2R4C0|B2R4C0_HUMAN	tr|B2R4C0|B2	60S ribosomal protein L18a OS=Homo sapiens PE=2 SV=1	21024	514	1027	32	35	0.16	0.17	1.0625	0	0	1	1	1	1	2	1	-0.88923
tr|B7ZKM0|B7ZKM0_HUMAN	SART3	SART3 protein OS=Homo sapiens GN=SART3 PE=2 SV=1	106372	153	532	139	87	0.16	0.17	1.0625	5	2	5	3	7	3	7	3	-1.9904
tr|B4DTA2|B4DTA2_HUMAN	tr|B4DTA2|B4	cDNA FLJ60148, highly similar to Homo sapiens heterogeneous nuclear ribonucleoprotein D-like (HNRPDL), transcript variant 2, mRNA OS=Hom	30367	229	546	39	84	0.37	0.37	1	1	3	1	4	1	5	1	5	0.985955
tr|J3QT27|J3QT27_HUMAN	PCBP3	Poly(rC)-binding protein 3 (Fragment) OS=Homo sapiens GN=PCBP3 PE=4 SV=1	34649	132	333	35	150	0.32	0.32	1	1	4	1	5	1	5	1	7	0.985955
tr|B0UX83|B0UX83_HUMAN	BAG6	HLA-B associated transcript 3 OS=Homo sapiens GN=BAG6 PE=4 SV=1	119085	704	603	18	75	0.03	0.03	1	0	1	1	1	0	1	1	3	0.696063
tr|Q3MNE1|Q3MNE1_HUMAN	tr|Q3MNE1|Q	Adaptor-related protein complex 3 beta 1 subunit (Fragment) OS=Homo sapiens PE=2 SV=1	89828	682	892	20	43	0.04	0.04	1	0	1	1	1	0	1	1	1	0.696063
sp|Q14683|SMC1A_HUMAN	SMC1A	Structural maintenance of chromosomes protein 1A OS=Homo sapiens GN=SMC1A PE=1 SV=2	143771	581	968	28	38	0.02	0.02	1	0	1	1	1	1	3	1	4	0.33352
tr|B4DDP6|B4DDP6_HUMAN	DBNL	Drebrin-like protein OS=Homo sapiens GN=DBNL PE=2 SV=1	43242	466	752	37	56	0.08	0.08	1	1	1	1	1	1	2	1	2	-0.15209
sp|P50552|VASP_HUMAN	VASP	Vasodilator-stimulated phosphoprotein OS=Homo sapiens GN=VASP PE=1 SV=3	39976	363	855	48	46	0.17	0.17	1	1	1	1	2	1	2	1	2	-0.15209
tr|B2R659|B2R659_HUMAN	tr|B2R659|B2	cDNA, FLJ92803, highly similar to Homo sapiens hydroxysteroid (17-beta) dehydrogenase 4 (HSD17B4), mRNA OS=Homo sapiens PE=2 SV=1	80018	484	542	36	85	0.13	0.13	1	0	2	3	3	3	5	4	6	-0.2371
sp|Q9H853|TBA4B_HUMAN	TUBA4B	Putative tubulin-like protein alpha-4B OS=Homo sapiens GN=TUBA4B PE=5 SV=2	27819	146	248	147	207	0.4	0.4	1	3	5	4	7	4	6	6	9	-0.35882
tr|B3KNM0|B3KNM0_HUMAN	tr|B3KNM0|B	cDNA FLJ14916 fis, clone PLACE1006958, highly similar to Heat shock 70 kDa protein 4L (Fragment) OS=Homo sapiens PE=2 SV=1	64588	191	536	113	86	0.1	0.1	1	2	2	2	3	2	3	3	4	-0.40378
tr|A5PLK7|A5PLK7_HUMAN	RCC2	RCC2 protein (Fragment) OS=Homo sapiens GN=RCC2 PE=2 SV=1	50331	165	228	131	229	0.46	0.46	1	4	4	6	6	6	8	6	10	-0.50261
sp|P18206|VINC_HUMAN	VCL	Vinculin OS=Homo sapiens GN=VCL PE=1 SV=4	124292	68	139	299	348	0.37	0.37	1	8	7	12	12	13	15	13	18	-0.6914
tr|H3BLZ8|H3BLZ8_HUMAN	DDX17	Probable ATP-dependent RNA helicase DDX17 OS=Homo sapiens GN=DDX17 PE=3 SV=1	81073	166	185	130	274	0.37	0.37	1	3	6	9	8	9	10	10	10	-0.74588
tr|Q6UWE9|Q6UWE9_HUMAN	UNQ2550	SFVP2550 OS=Homo sapiens GN=UNQ2550 PE=4 SV=1	11890	664	1237	21	20	0.29	0.29	1	1	1	1	1	1	1	1	1	-0.88923
tr|B5BU01|B5BU01_HUMAN	EIF2S2	Eukaryotic translation initiation factor 2 beta OS=Homo sapiens GN=EIF2S2 PE=2 SV=1	38648	654	781	22	53	0.09	0.09	1	0	1	1	1	1	1	1	3	-0.88923
tr|B4DKD0|B4DKD0_HUMAN	tr|B4DKD0|B4	DNA topoisomerase 2 (Fragment) OS=Homo sapiens PE=2 SV=1	135015	606	1043	25	33	0.02	0.02	1	0	0	1	1	1	1	3	4	-0.88923
sp|Q6KB66|K2C80_HUMAN	KRT80	Keratin, type II cytoskeletal 80 OS=Homo sapiens GN=KRT80 PE=1 SV=2	51007	564	1058	29	32	0.06	0.06	1	0	0	1	1	1	1	1	2	-0.88923
tr|Q1L838|Q1L838_HUMAN	GTBP	GTBP-ALT OS=Homo sapiens GN=GTBP PE=2 SV=1	122267	559	765	29	54	0.03	0.03	1	0	1	1	1	1	1	1	2	-0.88923
tr|F8W930|F8W930_HUMAN	IGF2BP2	Insulin-like growth factor 2 mRNA-binding protein 2 OS=Homo sapiens GN=IGF2BP2 PE=2 SV=1	66859	556	910	29	42	0.05	0.05	1	0	1	1	1	1	1	1	1	-0.88923
tr|H0YBW4|H0YBW4_HUMAN	PLAA	Phospholipase A-2-activating protein (Fragment) OS=Homo sapiens GN=PLAA PE=4 SV=1	31645	539	665	30	67	0.11	0.11	1	0	1	1	1	1	1	1	1	-0.88923
tr|B7Z9W6|B7Z9W6_HUMAN	tr|B7Z9W6|B	cDNA, FLJ78980, highly similar to DnaJ homolog subfamily C member 7 OS=Homo sapiens PE=2 SV=1	50776	527	801	31	51	0.06	0.06	1	0	1	1	1	1	1	1	1	-0.88923
tr|B3KUZ8|B3KUZ8_HUMAN	tr|B3KUZ8|B3	Aspartate aminotransferase OS=Homo sapiens PE=2 SV=1	41699	522	534	31	87	0.08	0.08	1	0	1	1	1	1	1	2	1	-0.88923
tr|Q2NKW8|Q2NKW8_HUMAN	AHCYL1	Adenosylhomocysteinase (Fragment) OS=Homo sapiens GN=AHCYL1 PE=2 SV=1	57630	509	979	33	37	0.06	0.06	1	0	1	1	1	1	1	1	3	-0.88923
sp|Q6PGP7|TTC37_HUMAN	TTC37	Tetratricopeptide repeat protein 37 OS=Homo sapiens GN=TTC37 PE=1 SV=1	177485	498	766	34	54	0.02	0.02	1	1	1	1	1	1	1	1	1	-0.88923
tr|B3KUJ0|B3KUJ0_HUMAN	tr|B3KUJ0|B3	cDNA FLJ39996 fis, clone STOMA2002166, highly similar to Splicing factor 3B subunit 4 OS=Homo sapiens PE=2 SV=1	44512	464	836	37	48	0.07	0.07	1	1	1	1	1	1	1	2	1	-0.88923
tr|B3KME2|B3KME2_HUMAN	tr|B3KME2|B	cDNA FLJ10772 fis, clone NT2RP4000243, highly similar to Cartilage-associated protein OS=Homo sapiens PE=2 SV=1	47101	459	1010	38	35	0.07	0.07	1	1	1	1	1	1	1	1	1	-0.88923
tr|E0X098|E0X098_HUMAN	TES	Testin variant (Fragment) OS=Homo sapiens GN=TES PE=2 SV=1	22009	430	991	40	37	0.15	0.15	1	1	1	1	1	1	1	1	2	-0.88923
tr|Q5JR94|Q5JR94_HUMAN	RPS8	40S ribosomal protein S8 OS=Homo sapiens GN=RPS8 PE=2 SV=1	24475	426	1031	41	34	0.14	0.14	1	1	0	1	1	1	1	2	1	-0.88923
tr|Q5TZX1|Q5TZX1_HUMAN	GNAI3	Guanine nucleotide binding protein (G protein), alpha inhibiting activity polypeptide 3 OS=Homo sapiens GN=GNAI3 PE=2 SV=1	41076	376	974	46	38	0.08	0.08	1	1	1	1	1	1	1	1	1	-0.88923
tr|B3KU96|B3KU96_HUMAN	tr|B3KU96|B3	cDNA FLJ39433 fis, clone PROST2004142, highly similar to GDP-L-fucose synthetase (EC 1.1.1.271) OS=Homo sapiens PE=2 SV=1	17856	371	764	47	50	0.19	0.19	1	1	1	1	1	1	1	1	1	-0.88923
tr|A6NKV8|A6NKV8_HUMAN	PAIP1	Polyadenylate-binding protein-interacting protein 1 OS=Homo sapiens GN=PAIP1 PE=2 SV=1	42379	361	1123	48	28	0.08	0.08	1	1	0	1	1	1	1	1	1	-0.88923
tr|B2RAR2|B2RAR2_HUMAN	tr|B2RAR2|B2	cDNA, FLJ95064, highly similar to Homo sapiens nin one binding protein (NOB1P), mRNA OS=Homo sapiens PE=2 SV=1	47015	338	1185	52	24	0.07	0.07	1	1	0	1	1	1	1	1	1	-0.88923
tr|A8KA82|A8KA82_HUMAN	DNAJC3	DnaJ (Hsp40) homolog, subfamily C, member 3 OS=Homo sapiens GN=DNAJC3 PE=2 SV=1	58000	337	1089	52	30	0.06	0.06	1	1	0	1	1	1	1	1	1	-0.88923
sp|Q9Y6M5|ZNT1_HUMAN	SLC30A1	Zinc transporter 1 OS=Homo sapiens GN=SLC30A1 PE=1 SV=3	56290	334	562	52	80	0.06	0.06	1	1	1	1	1	1	1	1	1	-0.88923
tr|A1L3A7|A1L3A7_HUMAN	NUFIP2	Nuclear fragile X mental retardation protein interacting protein 2 OS=Homo sapiens GN=NUFIP2 PE=2 SV=1	76132	301	627	59	72	0.04	0.04	1	1	1	1	1	1	1	1	1	-0.88923
sp|Q68EM7|RHG17_HUMAN	ARHGAP17	Rho GTPase-activating protein 17 OS=Homo sapiens GN=ARHGAP17 PE=1 SV=1	95776	328	860	54	46	0.03	0.03	1	1	1	1	1	2	2	2	2	-0.88938
sp|Q9BYZ2|LDH6B_HUMAN	LDHAL6B	L-lactate dehydrogenase A-like 6B OS=Homo sapiens GN=LDHAL6B PE=1 SV=3	42372	327	525	54	89	0.16	0.16	1	1	2	2	2	2	2	2	2	-0.88938
tr|Q2QD09|Q2QD09_HUMAN	tr|Q2QD09|Q	Triosephosphate isomerase (Fragment) OS=Homo sapiens PE=2 SV=1	27211	267	807	71	51	0.26	0.26	1	1	1	2	2	2	2	2	2	-0.88938
tr|Q53G58|Q53G58_HUMAN	tr|Q53G58|Q	Coronin (Fragment) OS=Homo sapiens PE=2 SV=1	53931	242	664	83	67	0.13	0.13	1	1	2	2	2	2	2	4	3	-0.88938
sp|Q2NL82|TSR1_HUMAN	TSR1	Pre-rRNA-processing protein TSR1 homolog OS=Homo sapiens GN=TSR1 PE=1 SV=1	92151	230	547	90	84	0.07	0.07	1	2	2	2	2	2	2	2	2	-0.88938
sp|Q7L2E3|DHX30_HUMAN	DHX30	Putative ATP-dependent RNA helicase DHX30 OS=Homo sapiens GN=DHX30 PE=1 SV=1	134938	397	1005	43	36	0.05	0.05	1	1	0	2	2	3	3	4	5	-0.88954
sp|Q09028|RBBP4_HUMAN	RBBP4	Histone-binding protein RBBP4 OS=Homo sapiens GN=RBBP4 PE=1 SV=3	47911	321	472	55	104	0.22	0.22	1	2	2	3	3	4	4	4	5	-0.88969
sp|Q01650|LAT1_HUMAN	SLC7A5	Large neutral amino acids transporter small subunit 1 OS=Homo sapiens GN=SLC7A5 PE=1 SV=2	55659	239	456	85	107	0.12	0.12	1	2	3	2	4	4	5	4	4	-0.88969
tr|B7Z4V2|B7Z4V2_HUMAN	tr|B7Z4V2|B7	cDNA FLJ51907, highly similar to Stress-70 protein, mitochondrial OS=Homo sapiens PE=2 SV=1	72641	81	153	251	320	0.42	0.42	1	7	7	8	8	8	8	8	9	-0.8903
tr|Q6IPN6|Q6IPN6_HUMAN	EEF1A1	Elongation factor 1-alpha OS=Homo sapiens GN=EEF1A1 PE=2 SV=1	50433	70	165	295	304	1.14	1.14	1	11	10	13	16	18	18	21	18	-0.89183
tr|B4DPP6|B4DPP6_HUMAN	tr|B4DPP6|B4	cDNA FLJ54371, highly similar to Serum albumin OS=Homo sapiens PE=2 SV=1	72370	222	434	93	113	0.25	0.25	1	3	3	5	6	6	4	10	6	-1.42087
sp|Q96RW7|HMCN1_HUMAN	HMCN1	Hemicentin-1 OS=Homo sapiens GN=HMCN1 PE=1 SV=2	623265	291	896	63	43	0.01	0.01	1	7	1	7	1	11	2	16	4	-3.094
tr|A0N4V7|A0N4V7_HUMAN	Tcr-alpha	HCG2039797 (Fragment) OS=Homo sapiens GN=Tcr-alpha PE=4 SV=1	2269	212	848	98	47	1.81	1.81	1	5	2	15	2	31	3	46	4	-4.06877
tr|F8VRQ1|F8VRQ1_HUMAN	HNRNPA1	Heterogeneous nuclear ribonucleoprotein A1 OS=Homo sapiens GN=HNRNPA1 PE=2 SV=1	33249	101	191	214	268	0.77	0.74	0.961039	4	6	6	6	7	7	9	8	-0.89015
tr|B2RCM3|B2RCM3_HUMAN	tr|B2RCM3|B	cDNA, FLJ96158, highly similar to Homo sapiens calpain 2, (m/II) large subunit (CAPN2), mRNA OS=Homo sapiens PE=2 SV=1	80883	173	309	125	162	0.22	0.21	0.9545455	3	4	7	5	7	7	8	8	-0.89015
tr|Q9HB74|Q9HB74_HUMAN	tr|Q9HB74|Q	PNAS-102 OS=Homo sapiens PE=2 SV=1	19284	90	787	127	52	0.4	0.38	0.95	3	1	3	2	3	5	3	6	-0.2371
tr|Q96K98|Q96K98_HUMAN	tr|Q96K98|Q	cDNA FLJ14414 fis, clone HEMBA1004847, highly similar to SIGNAL RECOGNITION PARTICLE 68 KD PROTEIN OS=Homo sapiens PE=2 SV=1	37365	180	786	119	34	0.18	0.17	0.9444444	2	0	2	1	2	1	4	1	-1.62653
tr|I3L492|I3L492_HUMAN	ETF1	Eukaryotic peptide chain release factor subunit 1 (Fragment) OS=Homo sapiens GN=ETF1 PE=4 SV=1	23028	274	496	69	0	0.15	0.14	0.9333333	1	0	1	0	1	0	1	1	-2.47437
tr|H0YAF8|H0YAF8_HUMAN	GNB2L1	Guanine nucleotide-binding protein subunit beta-2-like 1 (Fragment) OS=Homo sapiens GN=GNB2L1 PE=4 SV=1	22373	216	379	96	130	0.52	0.48	0.9230769	2	2	3	2	3	2	4	2	-1.37514
tr|G3V1S6|G3V1S6_HUMAN	GALNT2	Polypeptide N-acetylgalactosaminyltransferase 2 soluble form OS=Homo sapiens GN=GALNT2 PE=2 SV=1	61440	254	555	76	83	0.11	0.1	0.9090909	2	1	2	2	2	2	2	3	-0.88938
tr|H0Y7N2|H0Y7N2_HUMAN	ATXN10	Ataxin-10 (Fragment) OS=Homo sapiens GN=ATXN10 PE=4 SV=1	19107	470	336	37	45	0.38	0.34	0.8947368	0	1	2	1	2	2	2	2	-0.88938
tr|Q5T946|Q5T946_HUMAN	GRHPR	Glyoxylate reductase/hydroxypyruvate reductase OS=Homo sapiens GN=GRHPR PE=4 SV=1	43965	659	1199	21	23	0.09	0.08	0.8888889	0	0	1	1	1	1	1	1	-0.88923
tr|B6F210|B6F210_HUMAN	GSS	Glutathione synthetase OS=Homo sapiens GN=GSS PE=2 SV=1	40495	551	677	30	0	0.08	0.07	0.875	0	0	1	0	1	1	1	1	-0.88923
tr|F2Z2U8|F2Z2U8_HUMAN	MYH14	Myosin-14 OS=Homo sapiens GN=MYH14 PE=2 SV=1	232102	152	355	140	142	0.07	0.06	0.8571429	5	4	5	4	7	4	8	5	-1.62765
tr|F8WEJ5|F8WEJ5_HUMAN	ASNS	Asparagine synthetase [glutamine-hydrolyzing] OS=Homo sapiens GN=ASNS PE=2 SV=1	46395	141	376	156	114	0.26	0.22	0.8461538	3	2	5	3	6	4	6	4	-1.42087
tr|B2R9K8|B2R9K8_HUMAN	tr|B2R9K8|B2	cDNA, FLJ94440, highly similar to Homo sapiens chaperonin containing TCP1, subunit 6A (zeta 1)(CCT6A), mRNA OS=Homo sapiens PE=2 SV=1	58386	118	194	181	258	0.47	0.39	0.8297872	5	6	7	7	9	7	10	8	-1.23185
tr|B3KMV8|B3KMV8_HUMAN	tr|B3KMV8|B	cDNA FLJ12766 fis, clone NT2RP2001520, highly similar to Calcium-binding mitochondrial carrier protein Aralar1 OS=Homo sapiens PE=2 SV=1	75130	353	686	41	65	0.11	0.09	0.8181818	1	2	1	2	1	2	1	3	-0.15209
tr|H0YGW7|H0YGW7_HUMAN	ABCF1	ATP-binding cassette sub-family F member 1 (Fragment) OS=Homo sapiens GN=ABCF1 PE=3 SV=1	68075	144	225	151	211	0.27	0.22	0.8148148	4	5	5	5	5	5	7	6	-0.88984
tr|G8JLB6|G8JLB6_HUMAN	HNRNPH1	Heterogeneous nuclear ribonucleoprotein H OS=Homo sapiens GN=HNRNPH1 PE=4 SV=1	51482	207	196	101	258	0.45	0.36	0.8	2	7	6	9	5	10	6	14	0.04394
tr|A8K4Z4|A8K4Z4_HUMAN	tr|A8K4Z4|A8	cDNA FLJ75549, highly similar to Homo sapiens ribosomal protein, large, P0 (RPLP0), transcript variant 1, mRNA OS=Homo sapiens PE=2 SV=1	34389	109	413	201	117	0.74	0.58	0.7837838	6	4	6	5	6	6	6	7	-0.88999
tr|Q4W4Y1|Q4W4Y1_HUMAN	DRIP4	Dopamine receptor interacting protein 4 OS=Homo sapiens GN=DRIP4 PE=2 SV=1	96646	136	289	160	173	0.18	0.14	0.7777778	2	4	5	4	5	4	6	4	-1.17953
tr|B7Z4V1|B7Z4V1_HUMAN	tr|B7Z4V1|B7	cDNA FLJ55401, highly similar to Alpha-actinin-2 OS=Homo sapiens PE=2 SV=1	32962	50	113	103	234	0.77	0.59	0.7662338	3	6	4	7	4	7	4	8	-0.15219
sp|Q562R1|ACTBL_HUMAN	ACTBL2	Beta-actin-like protein 2 OS=Homo sapiens GN=ACTBL2 PE=1 SV=2	42318	61	112	349	413	1.47	1.12	0.7619048	13	15	16	21	24	23	27	30	-0.95305
tr|B7Z438|B7Z438_HUMAN	tr|B7Z438|B7	cDNA FLJ56352, highly similar to Succinyl-CoA ligase (GDP-forming) subunit alpha, mitochondrial (EC 6.2.1.4) OS=Homo sapiens PE=2 SV=1	26801	467	1214	37	22	0.12	0.09	0.75	1	0	1	1	1	1	1	1	-0.88923
sp|Q92621|NU205_HUMAN	NUP205	Nuclear pore complex protein Nup205 OS=Homo sapiens GN=NUP205 PE=1 SV=3	230171	415	708	42	62	0.04	0.03	0.75	0	1	3	2	3	2	5	2	-1.37514
tr|B4DRH6|B4DRH6_HUMAN	tr|B4DRH6|B	cDNA FLJ54509, highly similar to Trifunctional enzyme subunit alpha, mitochondrial OS=Homo sapiens PE=2 SV=1	79007	133	426	164	99	0.28	0.21	0.75	5	2	6	4	7	4	7	4	-1.62765
tr|A0PJ76|A0PJ76_HUMAN	NCAPD2	NCAPD2 protein (Fragment) OS=Homo sapiens GN=NCAPD2 PE=2 SV=1	44957	494	258	34	56	0.15	0.11	0.7333333	0	1	2	1	2	1	2	1	-1.62653
tr|B4DY32|B4DY32_HUMAN	tr|B4DY32|B4	Asparagine synthetase OS=Homo sapiens PE=2 SV=1	55055	141	538	156	86	0.26	0.19	0.7307692	3	2	5	3	6	5	6	6	-1.13118
sp|Q58FF8|H90B2_HUMAN	HSP90AB2P	Putative heat shock protein HSP 90-beta 2 OS=Homo sapiens GN=HSP90AB2P PE=1 SV=2	44492	32	135	97	355	1.24	0.9	0.7258065	3	9	5	11	5	12	6	15	0.295837
tr|G8JLB2|G8JLB2_HUMAN	XPNPEP1	Xaa-Pro aminopeptidase 1 OS=Homo sapiens GN=XPNPEP1 PE=3 SV=1	72746	615	757	24	55	0.07	0.05	0.7142857	0	1	1	1	1	1	1	1	-0.88923
tr|A0PJ92|A0PJ92_HUMAN	NOP56	NOP56 protein (Fragment) OS=Homo sapiens GN=NOP56 PE=2 SV=1	50068	311	1006	58	31	0.14	0.1	0.7142857	1	0	2	1	3	1	3	2	-2.11229
tr|J3KSN7|J3KSN7_HUMAN	EIF4A2	Eukaryotic initiation factor 4A-II (Fragment) OS=Homo sapiens GN=EIF4A2 PE=4 SV=1	12120	52	122	116	229	1.62	1.15	0.7098765	1	3	3	4	2	5	3	5	0.248657
tr|H0YF33|H0YF33_HUMAN	NASP	Nuclear autoantigenic sperm protein (Fragment) OS=Homo sapiens GN=NASP PE=4 SV=1	31883	542	1278	30	0	0.1	0.07	0.7	0	0	1	0	2	1	3	1	-1.62653
sp|P35237|SPB6_HUMAN	SERPINB6	Serpin B6 OS=Homo sapiens GN=SERPINB6 PE=1 SV=3	42936	120	693	179	64	0.25	0.17	0.68	3	1	3	1	3	2	3	2	-1.37514
sp|P13489|RINI_HUMAN	RNH1	Ribonuclease inhibitor OS=Homo sapiens GN=RNH1 PE=1 SV=2	51766	431	628	40	72	0.15	0.1	0.6666667	0	1	1	1	1	1	1	1	-0.88923
tr|K7EM20|K7EM20_HUMAN	YWHAE	14-3-3 protein epsilon (Fragment) OS=Homo sapiens GN=YWHAE PE=4 SV=1	12918	258	1158	28	26	0.4	0.26	0.65	0	0	1	1	1	1	1	1	-0.88923
tr|A1L196|A1L196_HUMAN	tr|A1L196|A1	Putative uncharacterized protein (Fragment) OS=Homo sapiens PE=2 SV=1	16876	25	200	64	256	3.07	1.99	0.6482085	2	7	3	10	4	12	4	16	0.585676
tr|H0YJ34|H0YJ34_HUMAN	FERMT2	Fermitin family homolog 2 (Fragment) OS=Homo sapiens GN=FERMT2 PE=4 SV=1	73877	306	550	59	83	0.14	0.09	0.6428571	2	2	3	2	3	2	3	2	-1.37514
sp|Q7L2H7|EIF3M_HUMAN	EIF3M	Eukaryotic translation initiation factor 3 subunit M OS=Homo sapiens GN=EIF3M PE=1 SV=1	42932	381	768	46	54	0.25	0.16	0.64	1	1	1	2	1	3	1	3	0.33352
tr|H0Y5G9|H0Y5G9_HUMAN	NRD1	Nardilysin (Fragment) OS=Homo sapiens GN=NRD1 PE=3 SV=1	62196	225	608	92	0	0.11	0.07	0.6363636	2	0	2	0	2	1	2	1	-1.62653
tr|B2R5W2|B2R5W2_HUMAN	tr|B2R5W2|B	cDNA, FLJ92657, highly similar to Homo sapiens heterogeneous nuclear ribonucleoprotein C (C1/C2) (HNRPC), transcript variant 2, mRNA OS=Ho	31986	178	698	122	63	0.35	0.22	0.6285714	3	1	3	2	3	2	4	4	-1.37514
sp|Q04695|K1C17_HUMAN	KRT17	Keratin, type I cytoskeletal 17 OS=Homo sapiens GN=KRT17 PE=1 SV=2	48361	24	108	683	424	2.99	1.87	0.6254181	19	13	27	18	32	19	35	21	-1.63328
sp|Q9Y2X3|NOP58_HUMAN	NOP58	Nucleolar protein 58 OS=Homo sapiens GN=NOP58 PE=1 SV=1	60054	515	584	32	78	0.21	0.13	0.6190476	0	2	1	2	1	2	1	2	-0.15209
sp|Q01780|EXOSX_HUMAN	EXOSC10	Exosome component 10 OS=Homo sapiens GN=EXOSC10 PE=1 SV=2	101566	627	956	23	39	0.05	0.03	0.6	0	1	1	1	1	1	1	1	-0.88923
sp|P42166|LAP2A_HUMAN	TMPO	Lamina-associated polypeptide 2, isoform alpha OS=Homo sapiens GN=TMPO PE=1 SV=2	76016	241	499	84	98	0.24	0.14	0.5833333	1	2	5	3	5	3	5	3	-1.54228
tr|J3KRX5|J3KRX5_HUMAN	RPL17	60S ribosomal protein L17 (Fragment) OS=Homo sapiens GN=RPL17 PE=3 SV=1	20386	517	1152	32	26	0.28	0.16	0.5714286	0	0	1	1	1	1	1	1	-0.88923
tr|Q53XC0|Q53XC0_HUMAN	EIF2S1	Eukaryotic translation initiation factor 2, subunit 1 alpha, 35kDa, isoform CRA_a OS=Homo sapiens GN=EIF2S1 PE=2 SV=1	36374	208	817	100	50	0.42	0.24	0.5714286	4	0	4	2	4	3	4	3	-1.25244
sp|P05787|K2C8_HUMAN	KRT8	Keratin, type II cytoskeletal 8 OS=Homo sapiens GN=KRT8 PE=1 SV=7	53671	79	273	62	32	0.79	0.45	0.5696203	3	0	5	1	7	2	8	3	-2.476
sp|P78371|TCPB_HUMAN	CCT2	T-complex protein 1 subunit beta OS=Homo sapiens GN=CCT2 PE=1 SV=4	57794	37	73	563	545	1.86	1.05	0.5645161	14	8	20	14	20	15	23	19	-1.29639
tr|E7EN38|E7EN38_HUMAN	RAD50	DNA repair protein RAD50 (Fragment) OS=Homo sapiens GN=RAD50 PE=2 SV=1	77138	563	937	29	40	0.09	0.05	0.5555556	0	1	2	1	1	2	2	5	-0.15209
sp|P53621|COPA_HUMAN	COPA	Coatomer subunit alpha OS=Homo sapiens GN=COPA PE=1 SV=2	139797	181	566	118	79	0.18	0.1	0.5555556	5	2	7	4	8	5	11	5	-1.51887
tr|B4DPZ3|B4DPZ3_HUMAN	tr|B4DPZ3|B4	cDNA FLJ53290, highly similar to Cytoplasmic dynein 1 intermediate chain 2 OS=Homo sapiens PE=2 SV=1	70869	298	1046	60	33	0.09	0.05	0.5555556	2	0	2	1	2	1	2	2	-1.62653
tr|B3KTA3|B3KTA3_HUMAN	tr|B3KTA3|B3	Fascin OS=Homo sapiens PE=2 SV=1	52814	53	206	403	251	1.07	0.58	0.5420561	9	5	14	9	16	9	20	10	-1.68924
tr|B4DKM5|B4DKM5_HUMAN	VDAC2	Voltage-dependent anion-selective channel protein 2 OS=Homo sapiens GN=VDAC2 PE=2 SV=1	27804	196	670	108	67	0.76	0.41	0.5394737	1	1	5	3	5	2	5	3	-2.02788
tr|K7EK77|K7EK77_HUMAN	ATP5A1	ATP synthase subunit alpha, mitochondrial (Fragment) OS=Homo sapiens GN=ATP5A1 PE=3 SV=1	22243	135	155	160	167	1.33	0.71	0.5338346	5	5	6	5	6	5	6	5	-1.13118
tr|B4DQG5|B4DQG5_HUMAN	tr|B4DQG5|B	cDNA FLJ54122, highly similar to Cytosol aminopeptidase (EC 3.4.11.1) OS=Homo sapiens PE=2 SV=1	39662	368	984	47	37	0.15	0.08	0.5333333	1	1	1	1	1	1	1	2	-0.88923
tr|B4DXW6|B4DXW6_HUMAN	tr|B4DXW6|B	cDNA FLJ50285, highly similar to Arginyl-tRNA synthetase (EC 6.1.1.19) OS=Homo sapiens PE=2 SV=1	52771	235	517	88	61	0.35	0.18	0.5142857	3	2	5	2	6	3	7	4	-1.78362
tr|B3KNL2|B3KNL2_HUMAN	RUVBL2	RuvB-like 2 (E. coli), isoform CRA_d OS=Homo sapiens GN=RUVBL2 PE=2 SV=1	47603	584	1007	28	36	0.14	0.07	0.5	0	0	2	1	1	2	2	2	-0.15209
tr|B4DY16|B4DY16_HUMAN	tr|B4DY16|B4	cDNA FLJ51385, highly similar to Ran GTPase-activating protein 1 OS=Homo sapiens PE=2 SV=1	58178	574	924	28	41	0.12	0.06	0.5	0	1	1	1	1	1	1	1	-0.88923
tr|B4DZ88|B4DZ88_HUMAN	KTN1	KTN1 protein OS=Homo sapiens GN=KTN1 PE=2 SV=1	153179	322	557	55	83	0.04	0.02	0.5	2	1	2	1	3	2	4	5	-1.37514
sp|Q9UHR4|BI2L1_HUMAN	BAIAP2L1	Brain-specific angiogenesis inhibitor 1-associated protein 2-like protein 1 OS=Homo sapiens GN=BAIAP2L1 PE=1 SV=2	57189	613	709	24	61	0.12	0.06	0.5	0	1	2	1	2	1	4	2	-1.62653
sp|O00505|IMA3_HUMAN	KPNA3	Importin subunit alpha-3 OS=Homo sapiens GN=KPNA3 PE=1 SV=2	58288	406	1137	43	27	0.12	0.06	0.5	0	1	2	1	2	1	2	1	-1.62653
tr|A0MNN4|A0MNN4_HUMAN	SMU1	CDW3/SMU1 OS=Homo sapiens GN=SMU1 PE=2 SV=1	58134	221	1122	94	28	0.12	0.06	0.5	1	0	2	1	2	1	2	1	-1.62653
tr|B2R6J4|B2R6J4_HUMAN	tr|B2R6J4|B2	cDNA, FLJ92975, highly similar to Homo sapiens nucleosome assembly protein 1-like 4 (NAP1L4), mRNA OS=Homo sapiens PE=2 SV=1	42940	417	1112	42	29	0.16	0.08	0.5	0	0	2	1	3	1	3	4	-2.11229
sp|P27348|1433T_HUMAN	YWHAQ	14-3-3 protein theta OS=Homo sapiens GN=YWHAQ PE=1 SV=1	28032	258	487	74	100	0.4	0.2	0.5	2	1	3	1	3	1	3	1	-2.11229
tr|G5E972|G5E972_HUMAN	TMPO	Thymopentin OS=Homo sapiens GN=TMPO PE=2 SV=1	46335	241	832	41	48	0.24	0.12	0.5	0	1	3	1	3	1	3	1	-2.11229
tr|E9PRA5|E9PRA5_HUMAN	ATP1A4	Sodium/potassium-transporting ATPase subunit alpha-4 OS=Homo sapiens GN=ATP1A4 PE=2 SV=1	57835	289	1080	63	31	0.12	0.06	0.5	0	0	6	2	10	2	19	2	-2.96243
tr|Q8TE01|Q8TE01_HUMAN	derp12	DERP12 (Dermal papilla derived protein 12) OS=Homo sapiens GN=derp12 PE=2 SV=1	38340	69	207	297	248	0.79	0.39	0.4936709	6	4	7	4	8	4	8	4	-1.80855
tr|E5RH42|E5RH42_HUMAN	G3BP1	Ras GTPase-activating protein-binding protein 1 (Fragment) OS=Homo sapiens GN=G3BP1 PE=2 SV=1	13338	294	402	62	67	0.58	0.28	0.4827586	2	1	2	2	2	2	2	3	-0.88938
tr|H3BNV2|H3BNV2_HUMAN	NQO1	NAD(P)H dehydrogenase [quinone] 1 OS=Homo sapiens GN=NQO1 PE=4 SV=1	28533	576	1057	28	32	0.25	0.12	0.48	0	0	2	1	2	1	2	1	-1.62653
tr|Q0IIN1|Q0IIN1_HUMAN	KRT77	Keratin 77 OS=Homo sapiens GN=KRT77 PE=2 SV=1	62050	76	418	264	116	0.23	0.11	0.4782609	10	3	10	3	10	4	12	5	-2.11407
sp|P00338|LDHA_HUMAN	LDHA	L-lactate dehydrogenase A chain OS=Homo sapiens GN=LDHA PE=1 SV=2	36950	44	272	481	186	2.33	1.11	0.4763948	10	7	19	8	19	9	24	10	-1.93125
tr|F8VQP2|F8VQP2_HUMAN	ATXN2	Ataxin-2 OS=Homo sapiens GN=ATXN2 PE=2 SV=1	117012	157	283	135	177	0.21	0.1	0.4761905	4	3	7	3	10	3	10	3	-2.47682
tr|B2R7M3|B2R7M3_HUMAN	tr|B2R7M3|B	cDNA, FLJ93510, highly similar to Homo sapiens JTV1 gene (JTV1), mRNA OS=Homo sapiens PE=2 SV=1	35767	308	1098	58	29	0.19	0.09	0.4736842	2	0	2	1	2	1	2	1	-1.62653
sp|P16615|AT2A2_HUMAN	ATP2A2	Sarcoplasmic/endoplasmic reticulum calcium ATPase 2 OS=Homo sapiens GN=ATP2A2 PE=1 SV=1	116336	149	427	145	114	0.32	0.15	0.46875	4	4	10	5	12	4	13	5	-2.36627
sp|Q9NR12|PDLI7_HUMAN	PDLIM7	PDZ and LIM domain protein 7 OS=Homo sapiens GN=PDLIM7 PE=1 SV=1	50896	396	1191	43	24	0.13	0.06	0.4615385	0	0	2	1	2	4	4	5	-0.04103
tr|B3KY95|B3KY95_HUMAN	tr|B3KY95|B3	cDNA FLJ16143 fis, clone BRAMY2038516, highly similar to Protein disulfide-isomerase A6 (EC 5.3.4.1) OS=Homo sapiens PE=2 SV=1	53655	445	857	38	46	0.13	0.06	0.4615385	0	1	2	1	2	1	2	1	-1.62653
tr|Q5U077|Q5U077_HUMAN	LDHB	L-lactate dehydrogenase OS=Homo sapiens GN=LDHB PE=2 SV=1	36900	39	240	530	217	1.81	0.83	0.4585635	13	5	16	7	18	8	20	8	-2.01561
tr|A5D904|A5D904_HUMAN	RPS9	RPS9 protein (Fragment) OS=Homo sapiens GN=RPS9 PE=2 SV=1	13210	434	1040	40	34	0.58	0.26	0.4482759	1	0	2	1	2	2	2	2	-0.88938
sp|P11498|PYC_HUMAN	PC	Pyruvate carboxylase, mitochondrial OS=Homo sapiens GN=PC PE=1 SV=2	130293	10	49	1295	738	1.76	0.77	0.4375	40	17	52	23	56	24	67	27	-2.10892
tr|Q5QTS3|Q5QTS3_HUMAN	tr|Q5QTS3|Q	FWP004 OS=Homo sapiens PE=2 SV=1	23704	300	1110	59	29	0.74	0.3	0.4054054	3	1	3	2	3	2	3	3	-1.37514
tr|J3QT54|J3QT54_HUMAN	CPSF7	Cleavage and polyadenylation-specificity factor subunit 7 (Fragment) OS=Homo sapiens GN=CPSF7 PE=4 SV=1	22762	665	958	21	0	0.15	0.06	0.4	0	0	1	0	1	0	1	1	-2.47437
tr|D3DPU2|D3DPU2_HUMAN	CAP1	Adenylyl cyclase-associated protein OS=Homo sapiens GN=CAP1 PE=3 SV=1	51926	172	389	125	125	0.36	0.13	0.3611111	4	2	5	2	6	2	7	3	-2.26922
tr|B3KVL0|B3KVL0_HUMAN	tr|B3KVL0|B3	cDNA FLJ16691 fis, clone TRACH3000692, highly similar to Paxillin OS=Homo sapiens PE=2 SV=1	65600	159	649	133	69	0.28	0.1	0.3571429	3	2	5	2	6	2	6	2	-2.26922
tr|A8K7A4|A8K7A4_HUMAN	tr|A8K7A4|A8	cDNA FLJ76904, highly similar to Homo sapiens methionine adenosyltransferase II, beta (MAT2B), transcript variant 2, mRNA OS=Homo sapiens	36692	280	1192	68	24	0.37	0.13	0.3513514	1	0	1	1	1	1	1	1	-0.88923
sp|O60518|RNBP6_HUMAN	RANBP6	Ran-binding protein 6 OS=Homo sapiens GN=RANBP6 PE=1 SV=2	126173	85	636	36	71	0.23	0.08	0.3478261	1	2	1	2	2	2	2	2	-0.88938
tr|F5H3A1|F5H3A1_HUMAN	ATP1A1	Sodium/potassium-transporting ATPase subunit alpha-1 OS=Homo sapiens GN=ATP1A1 PE=2 SV=1	114239	324	928	55	41	0.09	0.03	0.3333333	2	1	3	1	8	3	12	4	-2.1713
sp|Q2M2I5|K1C24_HUMAN	KRT24	Keratin, type I cytoskeletal 24 OS=Homo sapiens GN=KRT24 PE=1 SV=1	55567	12	78	147	27	4.67	1.49	0.3190578	6	0	6	1	6	1	6	1	-3.00637
tr|B3KT07|B3KT07_HUMAN	CRMP1	Collapsin response mediator protein 1, isoform CRA_d OS=Homo sapiens GN=CRMP1 PE=2 SV=1	55596	490	704	19	62	0.19	0.06	0.3157895	0	1	1	1	0	1	2	2	0.696063
tr|D0PNI1|D0PNI1_HUMAN	YWHAZ	Tyrosine 3-monooxygenase/tryptophan 5-monooxygenase activation protein zeta polypeptide OS=Homo sapiens GN=YWHAZ PE=2 SV=1	27899	497	844	34	47	0.38	0.12	0.3157895	0	1	2	1	2	1	2	1	-1.62653
sp|Q13151|ROA0_HUMAN	HNRNPA0	Heterogeneous nuclear ribonucleoprotein A0 OS=Homo sapiens GN=HNRNPA0 PE=1 SV=1	30993	323	644	55	70	0.36	0.11	0.3055556	2	1	3	1	3	2	3	2	-1.37514
sp|O60884|DNJA2_HUMAN	DNAJA2	DnaJ homolog subfamily A member 2 OS=Homo sapiens GN=DNAJA2 PE=1 SV=1	46344	611	1222	24	21	0.23	0.07	0.3043478	0	0	1	1	1	1	1	1	-0.88923
tr|H0YIE7|H0YIE7_HUMAN	HSPA1L	Heat shock 70 kDa protein 1-like OS=Homo sapiens GN=HSPA1L PE=3 SV=1	25488	51	218	132	188	1.45	0.44	0.3034483	3	4	4	6	4	7	4	7	-0.15219
tr|D3DTL4|D3DTL4_HUMAN	ENO3	Enolase OS=Homo sapiens GN=ENO3 PE=2 SV=1	37167	40	247	112	151	1.39	0.4	0.2877698	2	2	2	5	2	4	4	5	-0.04103
tr|A4D1Z4|A4D1Z4_HUMAN	KIAA0415	KIAA0415 gene product OS=Homo sapiens GN=KIAA0415 PE=2 SV=1	166512	188	1236	115	20	0.07	0.02	0.2857143	1	0	1	1	1	2	2	4	-0.15209
sp|P46063|RECQ1_HUMAN	RECQL	ATP-dependent DNA helicase Q1 OS=Homo sapiens GN=RECQL PE=1 SV=3	74436	439	829	39	49	0.14	0.04	0.2857143	1	1	3	1	3	1	3	1	-2.11229
tr|J3QQX0|J3QQX0_HUMAN	GPS1	COP9 signalosome complex subunit 1 (Fragment) OS=Homo sapiens GN=GPS1 PE=4 SV=1	8703	636	617	22	73	0.4	0.11	0.275	0	1	1	2	1	2	1	2	-0.15209
sp|Q9NXW2|DJB12_HUMAN	DNAJB12	DnaJ homolog subfamily B member 12 OS=Homo sapiens GN=DNAJB12 PE=1 SV=4	42021	124	441	174	111	0.57	0.15	0.2631579	5	2	7	3	9	3	9	3	-2.3321
tr|B4DUI0|B4DUI0_HUMAN	tr|B4DUI0|B4	cDNA FLJ61414, highly similar to Desmin OS=Homo sapiens PE=2 SV=1	27396	66	638	50	71	1.03	0.26	0.2524272	1	3	1	3	1	3	2	5	0.33352
tr|B4DFM5|B4DFM5_HUMAN	tr|B4DFM5|B	cDNA FLJ51779, highly similar to Vacuolar ATP synthase subunit B, brain isoform (EC 3.6.3.14) OS=Homo sapiens PE=2 SV=1	43446	287	783	64	53	0.65	0.16	0.2461538	1	1	1	2	1	2	1	4	-0.15209

tr|Q96HI4|Q96HI4_HUMAN	NMT1	Glycylpeptide N-tetradecanoyltransferase (Fragment) OS=Homo sapiens GN=NMT1 PE=2 SV=2	56910	350	508	50	95	0.49	0.12	0.244898	1	2	2	2	2	2	2	3	-0.88938
tr|A2RUM7|A2RUM7_HUMAN	RPL5	Ribosomal protein L5 OS=Homo sapiens GN=RPL5 PE=2 SV=1	34569	187	823	115	49	0.44	0.1	0.2272727	2	1	4	1	5	1	7	1	-2.76503
sp|P04259|K2C6B_HUMAN	KRT6B	Keratin, type II cytoskeletal 6B OS=Homo sapiens GN=KRT6B PE=1 SV=5	60315	79	443	252	110	0.79	0.17	0.2151899	10	3	13	3	15	4	17	5	-2.67761
sp|P02533|K1C14_HUMAN	KRT14	Keratin, type I cytoskeletal 14 OS=Homo sapiens GN=KRT14 PE=1 SV=4	51872	33	267	604	190	2.22	0.45	0.2027027	19	5	25	8	29	9	30	9	-2.53182
tr|E5RHH5|E5RHH5_HUMAN	COPS5	COP9 signalosome complex subunit 5 OS=Homo sapiens GN=COPS5 PE=2 SV=1	22265	346	1114	51	29	0.45	0.09	0.2	0	0	2	1	2	1	2	1	-1.62653
tr|H6VRF8|H6VRF8_HUMAN	KRT1	Keratin 1 OS=Homo sapiens GN=KRT1 PE=3 SV=1	66184	6	94	2032	471	5.01	0.97	0.1936128	55	12	64	16	69	19	76	20	-2.74226
tr|H6VRG2|H6VRG2_HUMAN	KRT1	Keratin 1 OS=Homo sapiens GN=KRT1 PE=3 SV=1	66197	7	91	2027	479	5.01	0.97	0.1936128	55	12	65	16	70	19	77	20	-2.7632
sp|Q58FF6|H90B4_HUMAN	HSP90AB4P	Putative heat shock protein HSP 90-beta 4 OS=Homo sapiens GN=HSP90AB4P PE=5 SV=1	58855	32	435	77	113	1.24	0.24	0.1935484	3	3	5	4	3	5	5	7	-0.2371
tr|B7ZAW3|B7ZAW3_HUMAN	tr|B7ZAW3|B	cDNA, FLJ79327, highly similar to Methylcrotonoyl-CoA carboxylase subunit alpha, mitochondrial (EC 6.4.1.4) OS=Homo sapiens PE=2 SV=1	22540	102	794	139	52	0.83	0.15	0.1807229	3	1	4	1	4	1	5	2	-2.47519
sp|P13645|K1C10_HUMAN	KRT10	Keratin, type I cytoskeletal 10 OS=Homo sapiens GN=KRT10 PE=1 SV=6	59020	12	87	1226	491	4.67	0.82	0.1755889	34	10	42	11	47	12	54	15	-2.82859
tr|B4DKJ0|B4DKJ0_HUMAN	tr|B4DKJ0|B4	cDNA FLJ58539, highly similar to Keratin, type II cytoskeletal 4 OS=Homo sapiens PE=2 SV=1	55657	105	758	61	55	0.7	0.12	0.1714286	2	1	2	2	2	3	4	3	-0.40378
tr|B2R8Y4|B2R8Y4_HUMAN	tr|B2R8Y4|B2	cDNA, FLJ94117, highly similar to Homo sapiens actinin, alpha 3 (ACTN3), mRNA OS=Homo sapiens PE=2 SV=1	103871	19	160	99	308	2.11	0.36	0.1706161	3	8	3	10	3	13	4	14	1.059788
tr|E9PHF7|E9PHF7_HUMAN	MCCC1	Methylcrotonoyl-CoA carboxylase subunit alpha, mitochondrial OS=Homo sapiens GN=MCCC1 PE=2 SV=1	68745	102	835	214	48	0.83	0.14	0.1686747	7	1	13	2	11	2	16	3	-3.094
sp|P08779|K1C16_HUMAN	KRT16	Keratin, type I cytoskeletal 16 OS=Homo sapiens GN=KRT16 PE=1 SV=4	51578	62	631	342	72	1.38	0.2	0.1449275	14	2	17	3	21	4	21	5	-3.15168
sp|P35527|K1C9_HUMAN	KRT9	Keratin, type I cytoskeletal 9 OS=Homo sapiens GN=KRT9 PE=1 SV=3	62255	14	177	1044	282	3.01	0.43	0.1428571	25	6	36	7	40	9	44	9	-2.9927
sp|Q58FG1|HS904_HUMAN	HSP90AA4P	Putative heat shock protein HSP 90-alpha A4 OS=Homo sapiens GN=HSP90AA4P PE=5 SV=1	47796	46	324	103	154	1.05	0.14	0.1333333	3	3	4	3	3	4	5	4	-0.52679
tr|J3KR91|J3KR91_HUMAN	IQGAP2	Ras GTPase-activating-like protein IQGAP2 OS=Homo sapiens GN=IQGAP2 PE=4 SV=1	123072	84	810	32	50	0.31	0.03	0.0967742	0	1	1	1	1	1	1	1	-0.88923
sp|P35908|K22E_HUMAN	KRT2	Keratin, type II cytoskeletal 2 epidermal OS=Homo sapiens GN=KRT2 PE=1 SV=2	65678	11	161	1255	306	4.79	0.34	0.0709812	41	7	48	7	49	9	57	14	-3.28522
sp|P13647|K2C5_HUMAN	KRT5	Keratin, type II cytoskeletal 5 OS=Homo sapiens GN=KRT5 PE=1 SV=3	62568	58	620	378	73	0.95	0.05	0.0526316	13	1	15	1	20	3	24	4	-3.44538
sp|Q6ZMR3|LDH6A_HUMAN	LDHAL6A	L-lactate dehydrogenase A-like 6A OS=Homo sapiens GN=LDHAL6A PE=2 SV=1	36826	39	871	67	45	1.81	0.09	0.0497238	1	1	1	1	1	1	1	3	-0.88923
tr|Q562V5|Q562V5_HUMAN	ACT	Actin-like protein (Fragment) OS=Homo sapiens GN=ACT PE=2 SV=1	11481	1	400	136	120	31.43	1.2	0.0381801	6	6	6	7	7	8	8	9	-0.70939
tr|B2ZZ90|B2ZZ90_HUMAN	ACACA	Acetyl-Coenzyme A carboxylase alpha OS=Homo sapiens GN=ACACA PE=2 SV=1	267095	31	1085	628	31	0.44	0.01	0.0227273	25	0	30	1	38	1	46	1	-5.58337
tr|C4AM86|C4AM86_HUMAN	KRT35	Keratin, type I cuticular Ha5 OS=Homo sapiens GN=KRT35 PE=2 SV=1	48816	12	1136	88	27	4.67	0.07	0.0149893	4	0	4	1	4	1	4	2	-2.47519
tr|B4DHH5|B4DHH5_HUMAN	tr|B4DHH5|B	cDNA FLJ53472, highly similar to Dynamin-1 (EC 3.6.5.5) OS=Homo sapiens PE=2 SV=1	89920	713	0	16	0	0.03	0	0	0	0	1	0	0	0	1	0	-0.88908
tr|D6RAW4|D6RAW4_HUMAN	IMMT	Mitochondrial inner membrane protein OS=Homo sapiens GN=IMMT PE=2 SV=1	15918	712	0	0	0	0.04	0	0	0	0	0	0	0	0	1	0	-0.88908
sp|P54826|GAS1_HUMAN	GAS1	Growth arrest-specific protein 1 OS=Homo sapiens GN=GAS1 PE=1 SV=2	36867	708	0	17	0	0.09	0	0	0	0	1	0	0	0	1	0	-0.88908
tr|H0Y4R4|H0Y4R4_HUMAN	TOR1AIP1	Torsin-1A-interacting protein 1 (Fragment) OS=Homo sapiens GN=TOR1AIP1 PE=4 SV=1	33844	707	0	17	0	0.1	0	0	0	0	1	0	0	0	1	0	-0.88908
tr|J3KMW7|J3KMW7_HUMAN	UBR5	E3 ubiquitin-protein ligase UBR5 OS=Homo sapiens GN=UBR5 PE=4 SV=1	312224	706	0	18	0	0.01	0	0	0	0	1	0	0	0	1	0	-0.88908
tr|B0UZL4|B0UZL4_HUMAN	BAT3	HLA-B associated transcript 3 (Fragment) OS=Homo sapiens GN=BAT3 PE=2 SV=1	15422	704	0	18	0	0.03	0	0	0	0	1	0	0	0	1	0	-0.88908
tr|B4DY47|B4DY47_HUMAN	PHB	Prohibitin OS=Homo sapiens GN=PHB PE=2 SV=1	17064	699	0	18	0	0.2	0	0	0	0	1	0	0	0	1	0	-0.88908
tr|B3KMM3|B3KMM3_HUMAN	tr|B3KMM3|B	cDNA FLJ11471 fis, clone HEMBA1001675, highly similar to Mus musculus GTPase activating protein and VPS9 domains 1 (Gapvd1), mRNA OS=H	19935	697	0	0	0	0.02	0	0	0	0	0	0	0	0	1	0	-0.88908
tr|Q5T097|Q5T097_HUMAN	UTRN	Utrophin OS=Homo sapiens GN=UTRN PE=4 SV=1	114423	696	0	19	0	0.03	0	0	0	0	1	0	0	0	1	0	-0.88908
tr|H0Y304|H0Y304_HUMAN	DMD	Dystrophin (Fragment) OS=Homo sapiens GN=DMD PE=4 SV=1	160176	695	0	19	0	0.02	0	0	0	0	1	0	0	0	1	0	-0.88908
tr|H0YE81|H0YE81_HUMAN	NDUFV1	NADH dehydrogenase [ubiquinone] flavoprotein 1, mitochondrial (Fragment) OS=Homo sapiens GN=NDUFV1 PE=4 SV=1	14494	694	0	19	0	0.23	0	0	0	0	1	0	0	0	1	0	-0.88908
tr|Q5VZU9|Q5VZU9_HUMAN	TPP2	Tripeptidyl-peptidase 2 OS=Homo sapiens GN=TPP2 PE=4 SV=1	141160	693	0	19	0	0.02	0	0	0	0	1	0	0	0	1	0	-0.88908
tr|B3KND7|B3KND7_HUMAN	tr|B3KND7|B	cDNA FLJ14378 fis, clone HEMBA1001804, highly similar to Zinc finger protein 281 (Fragment) OS=Homo sapiens PE=2 SV=1	58221	692	0	19	0	0.06	0	0	0	0	1	0	0	0	1	0	-0.88908
tr|H7C4X2|H7C4X2_HUMAN	PCMT1	Protein-L-isoaspartate(D-aspartate) O-methyltransferase (Fragment) OS=Homo sapiens GN=PCMT1 PE=4 SV=1	7342	691	0	19	0	0.48	0	0	0	0	1	0	0	0	2	0	-0.88908
tr|D3DUL0|D3DUL0_HUMAN	ASCIZ	ATM/ATR-Substrate Chk2-Interacting Zn2+-finger protein, isoform CRA_b OS=Homo sapiens GN=ASCIZ PE=2 SV=1	79474	690	0	19	0	0.04	0	0	0	0	1	0	0	0	1	0	-0.88908
tr|G3V448|G3V448_HUMAN	TMX1	Thioredoxin-related transmembrane protein 1 OS=Homo sapiens GN=TMX1 PE=2 SV=1	12018	689	0	19	0	0.29	0	0	0	0	1	0	0	0	1	0	-0.88908
tr|E9PQG7|E9PQG7_HUMAN	MLL	MLL cleavage product C180 OS=Homo sapiens GN=MLL PE=2 SV=1	436345	687	0	19	0	0.01	0	0	0	0	1	0	0	0	1	0	-0.88908
tr|G5E9Q3|G5E9Q3_HUMAN	TSEN2	tRNA splicing endonuclease 2 homolog (S. cerevisiae), isoform CRA_b OS=Homo sapiens GN=TSEN2 PE=2 SV=1	45959	686	0	19	0	0.07	0	0	0	0	1	0	0	0	1	0	-0.88908
sp|Q9Y2A7|NCKP1_HUMAN	NCKAP1	Nck-associated protein 1 OS=Homo sapiens GN=NCKAP1 PE=1 SV=1	130018	685	0	19	0	0.03	0	0	0	0	1	0	0	0	1	0	-0.88908
tr|Q9UH66|Q9UH66_HUMAN	tr|Q9UH66|Q	7h3 protein (Fragment) OS=Homo sapiens PE=2 SV=1	49963	684	0	19	0	0.07	0	0	0	0	1	0	0	0	2	0	-0.88908
tr|J3KN33|J3KN33_HUMAN	CNTLN	Centlein OS=Homo sapiens GN=CNTLN PE=4 SV=1	162069	680	0	20	0	0.02	0	0	0	0	1	0	0	0	2	0	-0.88908
tr|J3KTB7|J3KTB7_HUMAN	TOP2A	DNA topoisomerase 2-alpha (Fragment) OS=Homo sapiens GN=TOP2A PE=4 SV=1	17149	606	0	0	0	0.02	0	0	0	0	0	0	0	0	1	0	-0.88908
tr|H7C1T1|H7C1T1_HUMAN	Sep-02	Septin-2 (Fragment) OS=Homo sapiens GN=SEPT2 PE=4 SV=1	3094	543	0	0	0	0.09	0	0	0	0	0	0	0	0	1	0	-0.88908
tr|D6RD96|D6RD96_HUMAN	ACSL4	Long-chain-fatty-acid--CoA ligase 4 (Fragment) OS=Homo sapiens GN=ACSL4 PE=2 SV=1	16399	529	0	0	0	0.04	0	0	0	0	0	0	0	0	1	0	-0.88908
tr|A8K482|A8K482_HUMAN	tr|A8K482|A8	Aspartate aminotransferase OS=Homo sapiens PE=2 SV=1	47885	522	0	0	0	0.08	0	0	0	0	0	0	0	0	1	0	-0.88908
sp|Q14117|DPYS_HUMAN	DPYS	Dihydropyrimidinase OS=Homo sapiens GN=DPYS PE=1 SV=1	57107	490	0	0	0	0.19	0	0	0	0	0	0	0	0	1	0	-0.88908
tr|H7BYY8|H7BYY8_HUMAN	PES1	Pescadillo homolog OS=Homo sapiens GN=PES1 PE=4 SV=1	1501	488	0	0	0	0.05	0	0	0	0	0	0	0	0	1	0	-0.88908
tr|H0Y386|H0Y386_HUMAN	PABPC1L	Polyadenylate-binding protein 1-like (Fragment) OS=Homo sapiens GN=PABPC1L PE=4 SV=1	16787	404	0	0	0	0.1	0	0	0	0	0	0	0	0	1	0	-0.88908
tr|A8K171|A8K171_HUMAN	tr|A8K171|A8	cDNA FLJ77253, highly similar to Homo sapiens nuclear receptor interacting protein 1 (NRIP1), mRNA OS=Homo sapiens PE=2 SV=1	127609	401	0	0	0	0.01	0	0	0	0	0	0	0	0	1	0	-0.88908
tr|H0Y8W6|H0Y8W6_HUMAN	PDLIM7	PDZ and LIM domain protein 7 (Fragment) OS=Homo sapiens GN=PDLIM7 PE=4 SV=1	12898	396	0	0	0	0.13	0	0	0	0	0	0	0	0	1	0	-0.88908
tr|F8WAT3|F8WAT3_HUMAN	EIF2A	Eukaryotic translation initiation factor 2A OS=Homo sapiens GN=EIF2A PE=2 SV=1	7970	206	0	18	0	0.32	0	0	0	0	1	0	0	0	1	0	-0.88908
tr|Q9BWG0|Q9BWG0_HUMAN	MYH10	MYH10 protein (Fragment) OS=Homo sapiens GN=MYH10 PE=2 SV=1	54557	200	0	0	0	0.06	0	0	0	0	0	0	0	0	1	0	-0.88908
tr|H0YJ21|H0YJ21_HUMAN	DYNC1H1	Cytoplasmic dynein 1 heavy chain 1 (Fragment) OS=Homo sapiens GN=DYNC1H1 PE=4 SV=1	21181	168	0	0	0	0.02	0	0	0	0	0	0	0	0	1	0	-0.88908
tr|F8VU69|F8VU69_HUMAN	KRT5	Keratin, type II cytoskeletal 5 (Fragment) OS=Homo sapiens GN=KRT5 PE=2 SV=1	14387	58	0	0	0	0.95	0	0	0	0	0	0	0	0	2	0	-0.88908
tr|E9PQ69|E9PQ69_HUMAN	GANAB	Neutral alpha-glucosidase AB OS=Homo sapiens GN=GANAB PE=2 SV=1	8560	47	0	0	0	0.62	0	0	0	0	0	0	0	0	1	0	-0.88908
sp|P12036|NFH_HUMAN	NEFH	Neurofilament heavy polypeptide OS=Homo sapiens GN=NEFH PE=1 SV=4	112639	11	0	0	0	4.79	0	0	0	0	0	0	0	0	1	0	-0.88908
tr|Q8WUM5|Q8WUM5_HUMAN	GEMIN4	Gem (Nuclear organelle) associated protein 4 OS=Homo sapiens GN=GEMIN4 PE=2 SV=1	121732	709	0	17	0	0.03	0	0	0	0	1	0	1	0	2	0	-2.47437
sp|O95159|ZFPL1_HUMAN	ZFPL1	Zinc finger protein-like 1 OS=Homo sapiens GN=ZFPL1 PE=1 SV=2	34833	678	0	20	0	0.1	0	0	0	0	1	0	1	0	1	0	-2.47437
tr|B4DE48|B4DE48_HUMAN	tr|B4DE48|B4	cDNA FLJ53824, highly similar to Sorting nexin-17 OS=Homo sapiens PE=2 SV=1	50009	677	0	20	0	0.07	0	0	0	0	1	0	1	0	1	0	-2.47437
sp|P55212|CASP6_HUMAN	CASP6	Caspase-6 OS=Homo sapiens GN=CASP6 PE=1 SV=2	33859	674	0	20	0	0.1	0	0	0	0	1	0	1	0	1	0	-2.47437
tr|J3KNE5|J3KNE5_HUMAN	HARS	Histidine--tRNA ligase, cytoplasmic OS=Homo sapiens GN=HARS PE=4 SV=1	50581	672	0	20	0	0.07	0	0	0	0	1	0	1	0	2	0	-2.47437
tr|H0YJL6|H0YJL6_HUMAN	EVL	Ena/VASP-like protein (Fragment) OS=Homo sapiens GN=EVL PE=4 SV=1	10347	671	0	20	0	0.34	0	0	0	0	1	0	1	0	1	0	-2.47437
tr|A0AVG7|A0AVG7_HUMAN	BCAS1	Breast carcinoma amplified sequence 1 OS=Homo sapiens GN=BCAS1 PE=2 SV=1	61929	670	0	20	0	0.05	0	0	0	0	1	0	1	0	1	0	-2.47437
tr|B4DK80|B4DK80_HUMAN	TOP3A	DNA topoisomerase OS=Homo sapiens GN=TOP3A PE=2 SV=1	103082	668	0	20	0	0.03	0	0	0	0	1	0	1	0	1	0	-2.47437
tr|G0ZJH6|G0ZJH6_HUMAN	Ube4B	UFD2A-III/UBE4B-III splice isoform OS=Homo sapiens GN=Ube4B PE=2 SV=1	152890	667	0	21	0	0.02	0	0	0	0	1	0	1	0	1	0	-2.47437
tr|J3KN69|J3KN69_HUMAN	NCEH1	Arylacetamide deacetylase-like 1 OS=Homo sapiens GN=NCEH1 PE=4 SV=1	49431	663	0	21	0	0.07	0	0	0	0	1	0	1	0	1	0	-2.47437
tr|B3KRG8|B3KRG8_HUMAN	tr|B3KRG8|B3	Lipase OS=Homo sapiens PE=2 SV=1	45736	662	0	21	0	0.07	0	0	0	0	1	0	1	0	1	0	-2.47437
tr|F1T0A5|F1T0A5_HUMAN	PRPF31	PRP31 pre-mRNA processing factor 31 homolog (Yeast), isoform CRA_a OS=Homo sapiens GN=PRPF31 PE=2 SV=1	55649	661	0	21	0	0.06	0	0	0	0	1	0	1	0	2	0	-2.47437
tr|C9J935|C9J935_HUMAN	SETD9	SET domain-containing protein 9 (Fragment) OS=Homo sapiens GN=SETD9 PE=2 SV=1	5515	660	0	21	0	0.65	0	0	0	0	1	0	1	0	1	0	-2.47437
tr|B4DQ49|B4DQ49_HUMAN	tr|B4DQ49|B4	cDNA FLJ54374, highly similar to Homo sapiens keratin 78 (KRT78), mRNA OS=Homo sapiens PE=2 SV=1	31874	653	0	22	0	0.1	0	0	0	0	1	0	1	0	3	0	-2.47437
tr|H7C4V2|H7C4V2_HUMAN	U2SURP	U2 snRNP-associated SURP motif-containing protein (Fragment) OS=Homo sapiens GN=U2SURP PE=4 SV=1	33796	647	0	22	0	0.03	0	0	0	0	1	0	1	0	1	0	-2.47437
tr|I0BW36|I0BW36_HUMAN	HLA-A	MHC class I antigen (Fragment) OS=Homo sapiens GN=HLA-A PE=4 SV=1	8588	639	0	22	0	0.16	0	0	0	0	1	0	1	0	2	0	-2.47437
tr|B1ALA9|B1ALA9_HUMAN	PRPS1	Phosphoribosyl pyrophosphate synthetase 1 OS=Homo sapiens GN=PRPS1 PE=3 SV=1	24468	638	0	22	0	0.14	0	0	0	0	1	0	1	0	2	0	-2.47437
tr|Q5VSQ6|Q5VSQ6_HUMAN	P4HA1	Procollagen-proline, 2-oxoglutarate 4-dioxygenase (Proline 4-hydroxylase), alpha polypeptide I variant (Fragment) OS=Homo sapiens GN=P4HA1	61214	634	0	22	0	0.05	0	0	0	0	1	0	1	0	2	0	-2.47437
tr|B3KPR5|B3KPR5_HUMAN	tr|B3KPR5|B3	cDNA FLJ32094 fis, clone OCBBF2000986, highly similar to Homo sapiens elongation factor Tu GTP binding domain containing 1, transcript varia	97046	633	0	22	0	0.03	0	0	0	0	1	0	1	0	1	0	-2.47437
sp|Q9Y5P6|GMPPB_HUMAN	GMPPB	Mannose-1-phosphate guanyltransferase beta OS=Homo sapiens GN=GMPPB PE=1 SV=2	40379	629	0	23	0	0.08	0	0	0	0	1	0	1	0	1	0	-2.47437
tr|B4DFE4|B4DFE4_HUMAN	tr|B4DFE4|B4	cDNA FLJ59618, highly similar to Exosome component 10 OS=Homo sapiens PE=2 SV=1	61733	627	0	23	0	0.05	0	0	0	0	1	0	1	0	1	0	-2.47437
tr|K7EPB5|K7EPB5_HUMAN	ELAVL3	ELAV-like protein 3 (Fragment) OS=Homo sapiens GN=ELAVL3 PE=4 SV=1	9728	625	0	23	0	0.36	0	0	0	0	1	0	1	0	1	0	-2.47437
tr|B4DN13|B4DN13_HUMAN	tr|B4DN13|B4	cDNA FLJ59136, highly similar to Transmembrane 9 superfamily protein member 3 OS=Homo sapiens PE=2 SV=1	29629	624	0	23	0	0.11	0	0	0	0	1	0	1	0	1	0	-2.47437
tr|B0QXZ5|B0QXZ5_HUMAN	DNAL4	Dynein light chain 4, axonemal OS=Homo sapiens GN=DNAL4 PE=4 SV=1	7786	621	0	24	0	0.47	0	0	0	0	1	0	1	0	1	0	-2.47437
tr|B4DU06|B4DU06_HUMAN	tr|B4DU06|B4	Guanylate cyclase OS=Homo sapiens PE=2 SV=1	91756	620	0	24	0	0.04	0	0	0	0	1	0	1	0	1	0	-2.47437
tr|Q2TQ40|Q2TQ40_HUMAN	TNFRSF17	B cell maturation antigen transcript variant 4 OS=Homo sapiens GN=TNFRSF17 PE=2 SV=1	15460	618	0	24	0	0.22	0	0	0	0	1	0	1	0	1	0	-2.47437
tr|Q4VAN1|Q4VAN1_HUMAN	BTN1A1	BTN1A1 protein OS=Homo sapiens GN=BTN1A1 PE=2 SV=1	55878	617	0	24	0	0.06	0	0	0	0	1	0	1	0	1	0	-2.47437
tr|B4E2P4|B4E2P4_HUMAN	tr|B4E2P4|B4	cDNA FLJ58474, highly similar to Xaa-Pro aminopeptidase 1 (EC 3.4.11.9) OS=Homo sapiens PE=2 SV=1	50020	615	0	24	0	0.07	0	0	0	0	1	0	1	0	1	0	-2.47437
tr|B4DVB8|B4DVB8_HUMAN	ELAVL1	ELAV-like protein 1 OS=Homo sapiens GN=ELAVL1 PE=2 SV=1	39200	612	0	24	0	0.08	0	0	0	0	1	0	1	0	1	0	-2.47437
tr|H3BMW5|H3BMW5_HUMAN	DNAJA2	DnaJ homolog subfamily A member 2 (Fragment) OS=Homo sapiens GN=DNAJA2 PE=4 SV=1	15134	611	0	24	0	0.23	0	0	0	0	1	0	1	0	1	0	-2.47437
tr|B4DG42|B4DG42_HUMAN	tr|B4DG42|B4	cDNA FLJ53753, highly similar to Myeloid-associated differentiation marker OS=Homo sapiens PE=2 SV=1	31889	609	0	25	0	0.1	0	0	0	0	1	0	1	0	1	0	-2.47437
tr|J3KSB9|J3KSB9_HUMAN	tr|J3KSB9|J3K	Uncharacterized protein (Fragment) OS=Homo sapiens PE=4 SV=1	8877	608	0	25	0	0.39	0	0	0	0	1	0	1	0	1	0	-2.47437
tr|J3KNP0|J3KNP0_HUMAN	RAB11FIP1	Rab11 family-interacting protein 1 OS=Homo sapiens GN=RAB11FIP1 PE=4 SV=1	137482	607	0	25	0	0.02	0	0	0	0	1	0	1	0	2	0	-2.47437
tr|B2RAX6|B2RAX6_HUMAN	tr|B2RAX6|B2	cDNA, FLJ95176, Homo sapiens CGI-48 protein (CGI-48), mRNA OS=Homo sapiens PE=2 SV=1	58127	605	0	25	0	0.06	0	0	0	0	1	0	1	0	1	0	-2.47437
tr|Q8TC04|Q8TC04_HUMAN	KRT23	Keratin 23 (Histone deacetylase inducible) OS=Homo sapiens GN=KRT23 PE=2 SV=1	48318	600	0	26	0	0.07	0	0	0	0	1	0	1	0	1	0	-2.47437
sp|Q0VD83|APOBR_HUMAN	APOBR	Apolipoprotein B receptor OS=Homo sapiens GN=APOBR PE=1 SV=1	115207	597	0	26	0	0.03	0	0	0	0	1	0	1	0	1	0	-2.47437
tr|Q5T8C6|Q5T8C6_HUMAN	CDC16	Cell division cycle protein 16 homolog OS=Homo sapiens GN=CDC16 PE=4 SV=1	55239	594	0	26	0	0.06	0	0	0	0	1	0	1	0	1	0	-2.47437
tr|B7Z4L9|B7Z4L9_HUMAN	tr|B7Z4L9|B7	cDNA FLJ55342, highly similar to Kelch-like protein 13 OS=Homo sapiens PE=2 SV=1	56884	593	0	26	0	0.06	0	0	0	0	1	0	1	0	1	0	-2.47437
tr|H0Y512|H0Y512_HUMAN	APMAP	Adipocyte plasma membrane-associated protein (Fragment) OS=Homo sapiens GN=APMAP PE=4 SV=1	45662	592	0	27	0	0.07	0	0	0	0	1	0	1	0	1	0	-2.47437
tr|H3BS19|H3BS19_HUMAN	ZNF469	Zinc finger protein 469 OS=Homo sapiens GN=ZNF469 PE=4 SV=1	417347	591	0	27	0	0.01	0	0	0	0	1	0	1	0	1	0	-2.47437
tr|A6NE42|A6NE42_HUMAN	TGIF1	Homeobox protein TGIF1 OS=Homo sapiens GN=TGIF1 PE=3 SV=2	27983	586	0	28	0	0.12	0	0	0	0	1	0	1	0	1	0	-2.47437
tr|H0YCU9|H0YCU9_HUMAN	TAGLN	Transgelin (Fragment) OS=Homo sapiens GN=TAGLN PE=4 SV=1	16833	585	0	28	0	0.2	0	0	0	0	1	0	1	0	1	0	-2.47437
tr|J3KN32|J3KN32_HUMAN	RCOR1	REST corepressor 1 OS=Homo sapiens GN=RCOR1 PE=4 SV=1	53409	583	0	28	0	0.06	0	0	0	0	1	0	1	0	1	0	-2.47437
sp|Q16322|KCA10_HUMAN	KCNA10	Potassium voltage-gated channel subfamily A member 10 OS=Homo sapiens GN=KCNA10 PE=1 SV=2	58147	578	0	28	0	0.06	0	0	1	0	1	0	1	0	1	0	-2.47437
tr|B4DNG6|B4DNG6_HUMAN	tr|B4DNG6|B	Annexin OS=Homo sapiens PE=3 SV=1	12870	577	0	28	0	0.27	0	0	0	0	1	0	1	0	1	0	-2.47437
tr|Q53G81|Q53G81_HUMAN	tr|Q53G81|Q	NAD(P)H menadione oxidoreductase 1, dioxin-inducible variant (Fragment) OS=Homo sapiens PE=2 SV=1	30857	576	0	22	0	0.25	0	0	0	0	1	0	1	0	1	0	-2.47437
tr|B0QYT3|B0QYT3_HUMAN	RANGAP1	Ran GTPase activating protein 1 (Fragment) OS=Homo sapiens GN=RANGAP1 PE=2 SV=1	27122	574	0	28	0	0.12	0	0	0	0	1	0	1	0	1	0	-2.47437
tr|J3KQE5|J3KQE5_HUMAN	RAN	GTP-binding nuclear protein Ran (Fragment) OS=Homo sapiens GN=RAN PE=4 SV=1	27027	571	0	29	0	0.12	0	0	0	0	1	0	1	0	1	0	-2.47437
tr|I3L361|I3L361_HUMAN	SGSM2	Small G protein-signaling modulator 2 (Fragment) OS=Homo sapiens GN=SGSM2 PE=4 SV=2	1642	570	0	29	0	2.38	0	0	0	0	1	0	1	0	1	0	-2.47437
tr|B4DS04|B4DS04_HUMAN	tr|B4DS04|B4	cDNA FLJ54142, highly similar to Protein transport protein Sec23B OS=Homo sapiens PE=2 SV=1	41720	558	0	27	0	0.08	0	0	0	0	1	0	1	0	1	0	-2.47437
tr|H3BRB3|H3BRB3_HUMAN	KIF22	Kinesin-like protein KIF22 OS=Homo sapiens GN=KIF22 PE=3 SV=1	67996	557	0	29	0	0.05	0	0	0	0	1	0	1	0	1	0	-2.47437
tr|B4DKT5|B4DKT5_HUMAN	tr|B4DKT5|B4	cDNA FLJ53054, highly similar to Insulin-like growth factor 2 mRNA-binding protein 2 OS=Homo sapiens PE=2 SV=1	33360	556	0	29	0	0.05	0	0	0	0	1	0	1	0	1	0	-2.47437
tr|H0YK49|H0YK49_HUMAN	ETFA	Electron transfer flavoprotein subunit alpha, mitochondrial OS=Homo sapiens GN=ETFA PE=4 SV=1	24259	545	0	30	0	0.14	0	0	0	0	1	0	1	0	1	0	-2.47437
tr|K7EKM4|K7EKM4_HUMAN	SRM	Spermidine synthase OS=Homo sapiens GN=SRM PE=4 SV=1	10680	540	0	30	0	0.32	0	0	0	0	1	0	1	0	1	0	-2.47437
sp|Q96PE2|ARHGH_HUMAN	ARHGEF17	Rho guanine nucleotide exchange factor 17 OS=Homo sapiens GN=ARHGEF17 PE=1 SV=1	223645	537	0	30	0	0.01	0	0	1	0	1	0	1	0	1	0	-2.47437
tr|H0Y7S3|H0Y7S3_HUMAN	ATP2B2	Plasma membrane calcium-transporting ATPase 2 (Fragment) OS=Homo sapiens GN=ATP2B2 PE=3 SV=1	122272	528	0	31	0	0.03	0	0	0	0	1	0	1	0	3	0	-2.47437
tr|F2Z2J7|F2Z2J7_HUMAN	TTLL7	Tubulin polyglutamylase TTLL7 OS=Homo sapiens GN=TTLL7 PE=2 SV=1	55925	526	0	31	0	0.06	0	0	1	0	1	0	1	0	1	0	-2.47437
tr|B4E0D5|B4E0D5_HUMAN	tr|B4E0D5|B4	cDNA FLJ54218, highly similar to Acidic leucine-rich nuclear phosphoprotein 32 family member E OS=Homo sapiens PE=2 SV=1	19540	525	0	31	0	0.17	0	0	0	0	1	0	1	0	1	0	-2.47437
tr|L0R4T3|L0R4T3_HUMAN	ABCF2	Alternative protein ABCF2 OS=Homo sapiens GN=ABCF2 PE=4 SV=1	7359	524	0	31	0	0.48	0	0	1	0	1	0	1	0	1	0	-2.47437
tr|E9PNN6|E9PNN6_HUMAN	ALDH3A1	Aldehyde dehydrogenase, dimeric NADP-preferring OS=Homo sapiens GN=ALDH3A1 PE=2 SV=1	44518	519	0	32	0	0.07	0	0	0	0	1	0	1	0	1	0	-2.47437
tr|B4DUH7|B4DUH7_HUMAN	tr|B4DUH7|B	cDNA FLJ60162, highly similar to Chloride anion exchanger OS=Homo sapiens PE=2 SV=1	52632	518	0	32	0	0.06	0	0	1	0	1	0	1	0	1	0	-2.47437
tr|J3KRB3|J3KRB3_HUMAN	RPL17	60S ribosomal protein L17 (Fragment) OS=Homo sapiens GN=RPL17 PE=3 SV=1	12356	517	0	32	0	0.28	0	0	0	0	1	0	1	0	1	0	-2.47437
tr|B4DUY3|B4DUY3_HUMAN	tr|B4DUY3|B4	cDNA FLJ56668, highly similar to Nucleolar protein NOP5 OS=Homo sapiens PE=2 SV=1	16345	515	0	32	0	0.21	0	0	0	0	1	0	1	0	1	0	-2.47437
tr|A0JLS9|A0JLS9_HUMAN	ZEB1	ZEB1 protein (Fragment) OS=Homo sapiens GN=ZEB1 PE=2 SV=1	118576	513	0	32	0	0.03	0	0	1	0	1	0	1	0	1	0	-2.47437
sp|Q7Z494|NPHP3_HUMAN	NPHP3	Nephrocystin-3 OS=Homo sapiens GN=NPHP3 PE=1 SV=1	151967	510	0	32	0	0.02	0	0	1	0	1	0	1	0	1	0	-2.47437
sp|Q9Y5B9|SP16H_HUMAN	SUPT16H	FACT complex subunit SPT16 OS=Homo sapiens GN=SUPT16H PE=1 SV=1	120409	506	0	33	0	0.03	0	0	0	0	1	0	1	0	2	0	-2.47437
sp|Q9P287|BCCIP_HUMAN	BCCIP	BRCA2 and CDKN1A-interacting protein OS=Homo sapiens GN=BCCIP PE=1 SV=1	36299	503	0	33	0	0.09	0	0	0	0	1	0	1	0	3	0	-2.47437
sp|Q8NFZ5|TNIP2_HUMAN	TNIP2	TNFAIP3-interacting protein 2 OS=Homo sapiens GN=TNIP2 PE=1 SV=1	49240	502	0	33	0	0.07	0	0	1	0	1	0	1	0	2	0	-2.47437
tr|E9PI04|E9PI04_HUMAN	RIC8A	Synembryn-A (Fragment) OS=Homo sapiens GN=RIC8A PE=2 SV=1	16936	496	0	34	0	0.05	0	0	0	0	1	0	1	0	1	0	-2.47437
tr|C9JNW5|C9JNW5_HUMAN	RPL24	60S ribosomal protein L24 OS=Homo sapiens GN=RPL24 PE=4 SV=1	17646	487	0	35	0	0.19	0	0	0	0	1	0	1	0	2	0	-2.47437
tr|Q68E00|Q68E00_HUMAN	DKFZp686G20	Putative uncharacterized protein DKFZp686G2045 (Fragment) OS=Homo sapiens GN=DKFZp686G2045 PE=2 SV=2	32787	485	0	36	0	0.1	0	0	0	0	1	0	1	0	1	0	-2.47437
tr|B7WPL0|B7WPL0_HUMAN	RIC8B	Synembryn-B OS=Homo sapiens GN=RIC8B PE=2 SV=1	64252	483	0	36	0	0.05	0	0	1	0	1	0	1	0	1	0	-2.47437
tr|J3KN86|J3KN86_HUMAN	RPS3	40S ribosomal protein S3 OS=Homo sapiens GN=RPS3 PE=3 SV=1	28640	480	0	36	0	0.12	0	0	1	0	1	0	1	0	4	0	-2.47437
tr|H0YIX9|H0YIX9_HUMAN	R3HDM2	R3H domain-containing protein 2 (Fragment) OS=Homo sapiens GN=R3HDM2 PE=4 SV=1	31776	469	0	37	0	0.1	0	0	1	0	1	0	1	0	1	0	-2.47437
sp|Q6ZU64|CC108_HUMAN	CCDC108	Coiled-coil domain-containing protein 108 OS=Homo sapiens GN=CCDC108 PE=2 SV=2	220247	465	0	37	0	0.01	0	0	1	0	1	0	1	0	1	0	-2.47437
tr|Q5SZ64|Q5SZ64_HUMAN	SF3B4	Splicing factor 3B subunit 4 (Fragment) OS=Homo sapiens GN=SF3B4 PE=4 SV=1	20733	464	0	37	0	0.07	0	0	1	0	1	0	1	0	1	0	-2.47437
tr|H7C1M0|H7C1M0_HUMAN	FANCL	E3 ubiquitin-protein ligase FANCL (Fragment) OS=Homo sapiens GN=FANCL PE=4 SV=1	33067	461	0	37	0	0.1	0	0	1	0	1	0	1	0	1	0	-2.47437
sp|Q9H9F9|ARP5_HUMAN	ACTR5	Actin-related protein 5 OS=Homo sapiens GN=ACTR5 PE=1 SV=2	68825	460	0	38	0	0.05	0	0	1	0	1	0	1	0	2	0	-2.47437
tr|H0Y9L5|H0Y9L5_HUMAN	EXOSC9	Exosome complex component RRP45 (Fragment) OS=Homo sapiens GN=EXOSC9 PE=4 SV=1	18940	457	0	38	0	0.18	0	0	1	0	1	0	1	0	1	0	-2.47437
tr|K7EID2|K7EID2_HUMAN	ALG1	Chitobiosyldiphosphodolichol beta-mannosyltransferase (Fragment) OS=Homo sapiens GN=ALG1 PE=4 SV=1	19593	452	0	31	0	0.17	0	0	0	0	1	0	1	0	1	0	-2.47437
tr|B4DXC4|B4DXC4_HUMAN	tr|B4DXC4|B4	cDNA FLJ58636, moderately similar to Atlastin OS=Homo sapiens PE=2 SV=1	59162	451	0	38	0	0.06	0	0	1	0	1	0	1	0	1	0	-2.47437
tr|B4DR70|B4DR70_HUMAN	tr|B4DR70|B4	cDNA FLJ58049, highly similar to RNA-binding protein FUS OS=Homo sapiens PE=2 SV=1	45013	450	0	38	0	0.07	0	0	1	0	1	0	1	0	1	0	-2.47437
tr|B4E363|B4E363_HUMAN	FARSA	Phenylalanine--tRNA ligase alpha subunit OS=Homo sapiens GN=FARSA PE=2 SV=1	54181	447	0	38	0	0.06	0	0	1	0	1	0	1	0	1	0	-2.47437
tr|E9PIM9|E9PIM9_HUMAN	RNH1	Ribonuclease inhibitor (Fragment) OS=Homo sapiens GN=RNH1 PE=2 SV=1	22362	431	0	40	0	0.15	0	0	0	0	1	0	1	0	1	0	-2.47437
tr|B7Z5L5|B7Z5L5_HUMAN	tr|B7Z5L5|B7	cDNA FLJ59160, highly similar to Testin OS=Homo sapiens PE=2 SV=1	17178	430	0	40	0	0.15	0	0	1	0	1	0	1	0	1	0	-2.47437
tr|A4D1I7|A4D1I7_HUMAN	DNCI1	Dynein, cytoplasmic 1, intermediate chain 1, isoform CRA_e OS=Homo sapiens GN=DNCI1 PE=2 SV=1	73423	427	0	40	0	0.04	0	0	1	0	1	0	1	0	1	0	-2.47437
tr|F5H578|F5H578_HUMAN	PRPF40A	Pre-mRNA-processing factor 40 homolog A (Fragment) OS=Homo sapiens GN=PRPF40A PE=2 SV=1	48302	425	0	41	0	0.04	0	0	1	0	1	0	1	0	1	0	-2.47437
tr|B4DLD4|B4DLD4_HUMAN	tr|B4DLD4|B4	cDNA FLJ56738, highly similar to Fibrillarin OS=Homo sapiens PE=2 SV=1	30279	423	0	41	0	0.11	0	0	1	0	1	0	1	0	1	0	-2.47437
tr|A0JLS5|A0JLS5_HUMAN	TBL3	TBL3 protein (Fragment) OS=Homo sapiens GN=TBL3 PE=2 SV=1	64079	418	0	42	0	0.05	0	0	1	0	1	0	1	0	1	0	-2.47437
tr|H0YGE9|H0YGE9_HUMAN	M6PR	Cation-dependent mannose-6-phosphate receptor (Fragment) OS=Homo sapiens GN=M6PR PE=4 SV=1	11814	416	0	42	0	0.29	0	0	1	0	1	0	1	0	1	0	-2.47437
sp|Q9NSB4|KRT82_HUMAN	KRT82	Keratin, type II cuticular Hb2 OS=Homo sapiens GN=KRT82 PE=1 SV=3	57985	411	0	43	0	0.06	0	0	1	0	1	0	1	0	2	0	-2.47437
tr|B4DRG7|B4DRG7_HUMAN	tr|B4DRG7|B4	cDNA FLJ60336, highly similar to Condensin complex subunit 2 OS=Homo sapiens PE=2 SV=1	80877	410	0	43	0	0.04	0	0	1	0	1	0	1	0	1	0	-2.47437
tr|B3KMW5|B3KMW5_HUMAN	tr|B3KMW5|B	cDNA FLJ12796 fis, clone NT2RP2002058, highly similar to WD repeat protein 3 OS=Homo sapiens PE=2 SV=1	71519	408	0	43	0	0.05	0	0	1	0	1	0	1	0	1	0	-2.47437
tr|E9PCI9|E9PCI9_HUMAN	FDPS	Farnesyl pyrophosphate synthase OS=Homo sapiens GN=FDPS PE=2 SV=1	40849	405	0	43	0	0.08	0	0	1	0	1	0	1	0	1	0	-2.47437
tr|Q1LZN2|Q1LZN2_HUMAN	NOMO3	NOMO3 protein (Fragment) OS=Homo sapiens GN=NOMO3 PE=2 SV=1	90388	400	0	43	0	0.04	0	0	1	0	1	0	1	0	1	0	-2.47437
tr|H0Y327|H0Y327_HUMAN	MTHFD1L	Monofunctional C1-tetrahydrofolate synthase, mitochondrial (Fragment) OS=Homo sapiens GN=MTHFD1L PE=4 SV=1	36808	398	0	43	0	0.09	0	0	1	0	1	0	1	0	1	0	-2.47437
tr|Q6W6M7|Q6W6M7_HUMAN	tr|Q6W6M7|(	Antigen MLAA-43 (Fragment) OS=Homo sapiens PE=2 SV=1	23572	397	0	0	0	0.05	0	0	0	0	0	0	1	0	1	0	-2.47437
tr|B4DNB2|B4DNB2_HUMAN	tr|B4DNB2|B4	cDNA FLJ58919, moderately similar to Succinate dehydrogenase (ubiquinone) flavoprotein subunit, mitochondrial (EC1.3.5.1) OS=Homo sapiens	14636	393	0	0	0	0.1	0	0	0	0	0	0	1	0	2	0	-2.47437
tr|E5RGY7|E5RGY7_HUMAN	CTNNA1	Catenin alpha-1 (Fragment) OS=Homo sapiens GN=CTNNA1 PE=2 SV=1	14895	389	0	35	0	0.07	0	0	0	0	1	0	1	0	1	0	-2.47437
tr|B3KPZ0|B3KPZ0_HUMAN	tr|B3KPZ0|B3	cDNA FLJ32485 fis, clone SKNMC2001596, highly similar to Ankyrin repeat and FYVE domain-containing protein 1 (Fragment) OS=Homo sapiens	121468	387	0	45	0	0.03	0	0	1	0	1	0	1	0	1	0	-2.47437
tr|H0YHV5|H0YHV5_HUMAN	MARS	Methionine--tRNA ligase, cytoplasmic (Fragment) OS=Homo sapiens GN=MARS PE=4 SV=1	27023	385	0	30	0	0.07	0	0	0	0	1	0	1	0	1	0	-2.47437
tr|B4DT09|B4DT09_HUMAN	tr|B4DT09|B4	cDNA FLJ60145, highly similar to Homo sapiens dendritic cell protein (hfl-B5), mRNA OS=Homo sapiens PE=2 SV=1	13697	381	0	46	0	0.25	0	0	1	0	1	0	1	0	1	0	-2.47437
tr|J3QL39|J3QL39_HUMAN	PFAS	Phosphoribosylformylglycinamidine synthase (Fragment) OS=Homo sapiens GN=PFAS PE=4 SV=1	9951	380	0	0	0	0.05	0	0	0	0	0	0	1	0	1	0	-2.47437
tr|Q0P512|Q0P512_HUMAN	SLC2A1	Solute carrier family 2 (Facilitated glucose transporter), member 1 OS=Homo sapiens GN=SLC2A1 PE=2 SV=1	54361	377	0	46	0	0.06	0	0	1	0	1	0	1	0	1	0	-2.47437
tr|E9PP60|E9PP60_HUMAN	TSTA3	GDP-L-fucose synthase OS=Homo sapiens GN=TSTA3 PE=2 SV=1	12325	371	0	47	0	0.19	0	0	1	0	1	0	1	0	1	0	-2.47437
tr|H0Y9Q1|H0Y9Q1_HUMAN	LAP3	Cytosol aminopeptidase (Fragment) OS=Homo sapiens GN=LAP3 PE=4 SV=1	22641	368	0	47	0	0.15	0	0	1	0	1	0	1	0	1	0	-2.47437
tr|K7EM16|K7EM16_HUMAN	VASP	Vasodilator-stimulated phosphoprotein (Fragment) OS=Homo sapiens GN=VASP PE=4 SV=1	20260	363	0	48	0	0.17	0	0	1	0	1	0	1	0	1	0	-2.47437
tr|D6RJF2|D6RJF2_HUMAN	PAIP1	Polyadenylate-binding protein-interacting protein 1 (Fragment) OS=Homo sapiens GN=PAIP1 PE=2 SV=1	22826	361	0	48	0	0.08	0	0	1	0	1	0	1	0	1	0	-2.47437
tr|Q53ST0|Q53ST0_HUMAN	PCYOX1	Putative uncharacterized protein PCYOX1 (Fragment) OS=Homo sapiens GN=PCYOX1 PE=2 SV=1	11626	355	0	49	0	0.06	0	0	1	0	1	0	1	0	1	0	-2.47437
tr|D6RF19|D6RF19_HUMAN	DPYSL3	Dihydropyrimidinase-related protein 3 (Fragment) OS=Homo sapiens GN=DPYSL3 PE=2 SV=1	10214	342	0	20	0	0.23	0	0	0	0	1	0	1	0	1	0	-2.47437
tr|H7C5I9|H7C5I9_HUMAN	GART	Phosphoribosylformylglycinamidine cyclo-ligase (Fragment) OS=Homo sapiens GN=GART PE=4 SV=1	2640	341	0	23	0	0.13	0	0	0	0	1	0	1	0	1	0	-2.47437
tr|A8K517|A8K517_HUMAN	RPS23	Ribosomal protein S23, isoform CRA_a OS=Homo sapiens GN=RPS23 PE=2 SV=1	15969	339	0	52	0	0.21	0	0	1	0	1	0	1	0	1	0	-2.47437
tr|J3KTE4|J3KTE4_HUMAN	RPL19	Ribosomal protein L19 OS=Homo sapiens GN=RPL19 PE=3 SV=1	23347	336	0	52	0	0.14	0	0	1	0	1	0	1	0	1	0	-2.47437
tr|Q59GV0|Q59GV0_HUMAN	tr|Q59GV0|Q	Propionyl Coenzyme A carboxylase, beta polypeptide variant (Fragment) OS=Homo sapiens PE=2 SV=1	60578	331	0	53	0	0.05	0	0	1	0	1	0	1	0	2	0	-2.47437
tr|A8K3Q9|A8K3Q9_HUMAN	tr|A8K3Q9|A8	cDNA FLJ76611, highly similar to Homo sapiens ribosomal protein L14 (RPL14), mRNA OS=Homo sapiens PE=2 SV=1	23554	330	0	53	0	0.14	0	0	1	0	1	0	1	0	1	0	-2.47437
tr|I3L4P6|I3L4P6_HUMAN	ARHGAP17	Rho GTPase-activating protein 17 (Fragment) OS=Homo sapiens GN=ARHGAP17 PE=4 SV=1	8676	328	0	54	0	0.03	0	0	1	0	1	0	1	0	1	0	-2.47437
tr|Q1L6K4|Q1L6K4_HUMAN	PDCD8	Apoptosis-inducing factor 1, mitochondrial OS=Homo sapiens GN=PDCD8 PE=2 SV=1	26131	326	0	54	0	0.05	0	0	1	0	1	0	1	0	1	0	-2.47437
tr|C9JIX5|C9JIX5_HUMAN	PLOD3	Procollagen-lysine,2-oxoglutarate 5-dioxygenase 3 (Fragment) OS=Homo sapiens GN=PLOD3 PE=2 SV=1	8424	325	0	41	0	0.12	0	0	1	0	1	0	1	0	1	0	-2.47437
tr|C9JPP3|C9JPP3_HUMAN	RBBP4	Histone-binding protein RBBP4 (Fragment) OS=Homo sapiens GN=RBBP4 PE=2 SV=2	9655	315	0	57	0	0.36	0	0	1	0	1	0	1	0	1	0	-2.47437
sp|Q5TZA2|CROCC_HUMAN	CROCC	Rootletin OS=Homo sapiens GN=CROCC PE=1 SV=1	228787	310	0	58	0	0.01	0	0	1	0	1	0	1	0	2	0	-2.47437
tr|B3KPC1|B3KPC1_HUMAN	tr|B3KPC1|B3	cDNA FLJ31582 fis, clone NT2RI2002117, highly similar to Protein pelota homolog OS=Homo sapiens PE=2 SV=1	43807	307	0	59	0	0.08	0	0	1	0	1	0	1	0	1	0	-2.47437
tr|B2RAX1|B2RAX1_HUMAN	tr|B2RAX1|B2	cDNA, FLJ95169 OS=Homo sapiens PE=2 SV=1	77979	306	0	41	0	0.14	0	0	1	0	1	0	1	0	1	0	-2.47437
tr|B5LY64|B5LY64_HUMAN	UMPS	Orotidine 5'-phosphate decarboxylase OS=Homo sapiens GN=UMPS PE=2 SV=1	33258	302	0	59	0	0.1	0	0	1	0	1	0	1	0	1	0	-2.47437

tr|B4DX93|B4DX93_HUMAN	tr|B4DX93|B4	cDNA FLJ55471, highly similar to Cytoplasmic dynein 1 intermediate chain 2 OS=Homo sapiens PE=2 SV=1	40089	298	0	40	0	0.09	0	0	1	0	1	0	1	0	1	0	-2.47437
tr|Q5T0I0|Q5T0I0_HUMAN	GSN	Gelsolin (Fragment) OS=Homo sapiens GN=GSN PE=4 SV=1	29107	297	0	61	0	0.04	0	0	1	0	1	0	1	0	2	0	-2.47437
sp|P81605|DCD_HUMAN	DCD	Dermcidin OS=Homo sapiens GN=DCD PE=1 SV=2	11391	295	0	62	0	0.3	0	0	1	0	1	0	1	0	1	0	-2.47437
tr|B4DE27|B4DE27_HUMAN	tr|B4DE27|B4	cDNA FLJ58765, highly similar to Neutral amino acid transporter B(0) OS=Homo sapiens PE=2 SV=1	33987	290	0	63	0	0.1	0	0	1	0	1	0	1	0	1	0	-2.47437
tr|H0YC45|H0YC45_HUMAN	ATP6V1B2	V-type proton ATPase subunit B, brain isoform (Fragment) OS=Homo sapiens GN=ATP6V1B2 PE=4 SV=1	5569	287	0	64	0	0.65	0	0	1	0	1	0	1	0	1	0	-2.47437
tr|E5RFS9|E5RFS9_HUMAN	MAT2B	Methionine adenosyltransferase 2 subunit beta OS=Homo sapiens GN=MAT2B PE=2 SV=1	9467	280	0	68	0	0.37	0	0	1	0	1	0	1	0	1	0	-2.47437
tr|Q9NVP8|Q9NVP8_HUMAN	tr|Q9NVP8|Q	cDNA FLJ10595 fis, clone NT2RP2004791, moderately similar to PUTATIVE LEUCYL-TRNA SYNTHETASE, CYTOPLASMIC (EC 6.1.1.4) OS=Homo sap	13706	257	0	50	0	0.1	0	0	1	0	1	0	1	0	1	0	-2.47437
tr|B4DE40|B4DE40_HUMAN	SMS	Spermine synthase OS=Homo sapiens GN=SMS PE=2 SV=1	31596	248	0	78	0	0.11	0	0	1	0	1	0	1	0	1	0	-2.47437
tr|E5RFJ8|E5RFJ8_HUMAN	MCM4	DNA replication licensing factor MCM4 (Fragment) OS=Homo sapiens GN=MCM4 PE=2 SV=1	23630	240	0	65	0	0.1	0	0	1	0	1	0	1	0	2	0	-2.47437
tr|A1L179|A1L179_HUMAN	KIF5C	KIF5C protein (Fragment) OS=Homo sapiens GN=KIF5C PE=2 SV=1	99437	227	0	46	0	0.04	0	0	1	0	1	0	1	0	2	0	-2.47437
tr|H0Y960|H0Y960_HUMAN	DDX4	Probable ATP-dependent RNA helicase DDX4 (Fragment) OS=Homo sapiens GN=DDX4 PE=4 SV=1	33292	217	0	43	0	0.19	0	0	1	0	1	0	1	0	1	0	-2.47437
tr|D6RFZ9|D6RFZ9_HUMAN	GNB2L1	Guanine nucleotide-binding protein subunit beta-2-like 1 (Fragment) OS=Homo sapiens GN=GNB2L1 PE=2 SV=1	22150	216	0	58	0	0.52	0	0	1	0	1	0	1	0	1	0	-2.47437
tr|F5GYL4|F5GYL4_HUMAN	TCP1	T-complex protein 1 subunit alpha OS=Homo sapiens GN=TCP1 PE=2 SV=1	7059	197	0	22	0	0.3	0	0	0	0	1	0	1	0	1	0	-2.47437
tr|Q9BUV4|Q9BUV4_HUMAN	RPL5	RPL5 protein OS=Homo sapiens GN=RPL5 PE=2 SV=1	12182	187	0	39	0	0.44	0	0	0	0	1	0	1	0	2	0	-2.47437
tr|G3V2H6|G3V2H6_HUMAN	HNRNPC	Heterogeneous nuclear ribonucleoproteins C1/C2 (Fragment) OS=Homo sapiens GN=HNRNPC PE=2 SV=1	5961	178	0	37	0	0.35	0	0	1	0	1	0	1	0	1	0	-2.47437
tr|Q6RUP9|Q6RUP9_HUMAN	IMPDH2	Inosine monophosphate dehydrogenase type II (Fragment) OS=Homo sapiens GN=IMPDH2 PE=2 SV=1	7313	177	0	25	0	0.36	0	0	0	0	1	0	1	0	1	0	-2.47437
tr|F8VZQ7|F8VZQ7_HUMAN	ATXN2	Ataxin-2 (Fragment) OS=Homo sapiens GN=ATXN2 PE=2 SV=1	16188	157	0	31	0	0.21	0	0	0	0	1	0	1	0	1	0	-2.47437
tr|K7EQ75|K7EQ75_HUMAN	ILF3	Interleukin enhancer-binding factor 3 (Fragment) OS=Homo sapiens GN=ILF3 PE=4 SV=1	6139	154	0	29	0	0.46	0	0	0	0	1	0	1	0	1	0	-2.47437
tr|H0YHU8|H0YHU8_HUMAN	SART3	Squamous cell carcinoma antigen recognized by T-cells 3 (Fragment) OS=Homo sapiens GN=SART3 PE=4 SV=1	16246	153	0	45	0	0.16	0	0	1	0	1	0	1	0	1	0	-2.47437
tr|Q659G2|Q659G2_HUMAN	DKFZp586B20	Putative uncharacterized protein DKFZp586B2023 (Fragment) OS=Homo sapiens GN=DKFZp586B2023 PE=2 SV=1	7133	124	0	41	0	0.57	0	0	1	0	1	0	1	0	1	0	-2.47437
tr|Q9BR23|Q9BR23_HUMAN	tr|Q9BR23|Q	Putative uncharacterized protein (Fragment) OS=Homo sapiens PE=2 SV=1	23788	104	0	44	0	0.41	0	0	1	0	1	0	1	0	1	0	-2.47437
tr|D6RCS6|D6RCS6_HUMAN	TARS	Threonine--tRNA ligase, cytoplasmic OS=Homo sapiens GN=TARS PE=2 SV=1	6912	94	0	53	0	0.64	0	0	1	0	1	0	1	0	1	0	-2.47437
tr|A2RRC9|A2RRC9_HUMAN	IQGAP3	IQ motif containing GTPase activating protein 3 OS=Homo sapiens GN=IQGAP3 PE=2 SV=1	185145	84	0	32	0	0.31	0	0	0	0	1	0	1	0	1	0	-2.47437
tr|F5GZR9|F5GZR9_HUMAN	SLC3A2	4F2 cell-surface antigen heavy chain (Fragment) OS=Homo sapiens GN=SLC3A2 PE=2 SV=1	5250	60	0	28	0	1.33	0	0	0	0	1	0	1	0	1	0	-2.47437
tr|K7ENV3|K7ENV3_HUMAN	KRT16	Keratin, type I cytoskeletal 16 (Fragment) OS=Homo sapiens GN=KRT16 PE=4 SV=1	4775	33	0	48	0	2.22	0	0	1	0	1	0	1	0	1	0	-2.47437
tr|B7Z2Y4|B7Z2Y4_HUMAN	tr|B7Z2Y4|B7	cDNA FLJ56961, highly similar to Clathrin heavy chain 2 (CLH-22) OS=Homo sapiens PE=2 SV=1	54629	30	0	60	0	0.52	0	0	1	0	1	0	1	0	2	0	-2.47437
sp|Q8N1A0|KT222_HUMAN	KRT222	Keratin-like protein KRT222 OS=Homo sapiens GN=KRT222 PE=2 SV=1	34308	24	0	47	0	2.99	0	0	1	0	1	0	1	0	1	0	-2.47437
tr|A1L2Z2|A1L2Z2_HUMAN	MYH14	MYH14 protein OS=Homo sapiens GN=MYH14 PE=2 SV=1	73418	17	0	56	0	0.9	0	0	1	0	1	0	1	0	1	0	-2.47437
tr|B9VP24|B9VP24_HUMAN	HSPD1	60 kDa chaperonin (Fragment) OS=Homo sapiens GN=HSPD1 PE=4 SV=1	19880	16	0	40	0	1.72	0	0	1	0	1	0	1	0	1	0	-2.47437
tr|I0BW39|I0BW39_HUMAN	HLA-A	MHC class I antigen (Fragment) OS=Homo sapiens GN=HLA-A PE=4 SV=1	8492	640	0	22	0	0.16	0	0	0	0	1	0	2	0	2	0	-3.21167
sp|P25490|TYY1_HUMAN	YY1	Transcriptional repressor protein YY1 OS=Homo sapiens GN=YY1 PE=1 SV=2	45141	630	0	23	0	0.07	0	0	0	0	1	0	2	0	2	0	-3.21167
tr|D3DSQ0|D3DSQ0_HUMAN	PCM1	Pericentriolar material 1, isoform CRA_a OS=Homo sapiens GN=PCM1 PE=2 SV=1	230041	619	0	24	0	0.01	0	0	0	0	1	0	2	0	3	0	-3.21167
sp|Q9NRF8|PYRG2_HUMAN	CTPS2	CTP synthase 2 OS=Homo sapiens GN=CTPS2 PE=1 SV=1	66320	614	0	24	0	0.05	0	0	0	0	1	0	2	0	6	0	-3.21167
tr|B2RE46|B2RE46_HUMAN	tr|B2RE46|B2	cDNA, FLJ96923, highly similar to Homo sapiens ribophorin II (RPN2), mRNA OS=Homo sapiens PE=2 SV=1	69405	589	0	27	0	0.05	0	0	0	0	1	0	2	0	2	0	-3.21167
sp|O15131|IMA5_HUMAN	KPNA5	Importin subunit alpha-6 OS=Homo sapiens GN=KPNA5 PE=1 SV=2	61052	573	0	28	0	0.11	0	0	0	0	2	0	2	0	2	0	-3.21167
tr|B2RD27|B2RD27_HUMAN	tr|B2RD27|B2	cDNA, FLJ96428, highly similar to Homo sapiens proteasome (prosome, macropain) 26S subunit, non-ATPase, 7 (Mov34 homolog) (PSMD7), mRI	37046	550	0	30	0	0.19	0	0	0	0	2	0	2	0	2	0	-3.21167
tr|B3KNB4|B3KNB4_HUMAN	tr|B3KNB4|B3	cDNA FLJ14168 fis, clone NT2RP2001440, highly similar to 14-3-3 protein gamma OS=Homo sapiens PE=2 SV=1	28453	541	0	30	0	0.25	0	0	0	0	2	0	2	0	2	0	-3.21167
tr|C1KES1|C1KES1_HUMAN	LRRC16B	LRRC16B (Fragment) OS=Homo sapiens GN=LRRC16B PE=4 SV=1	4125	530	0	31	0	0.94	0	0	0	0	1	0	2	0	2	0	-3.21167
tr|B0AZS6|B0AZS6_HUMAN	YWHAZ	14-3-3 protein zeta/delta OS=Homo sapiens GN=YWHAZ PE=2 SV=1	19174	497	0	34	0	0.38	0	0	0	0	2	0	2	0	2	0	-3.21167
tr|B3KY12|B3KY12_HUMAN	tr|B3KY12|B3	cDNA FLJ46581 fis, clone THYMU3043200, highly similar to Splicing factor 3A subunit 3 OS=Homo sapiens PE=2 SV=1	59082	495	0	34	0	0.18	0	0	1	0	3	0	2	0	3	0	-3.21167
tr|B2R4P2|B2R4P2_HUMAN	tr|B2R4P2|B2	cDNA, FLJ92164, highly similar to Homo sapiens peroxiredoxin 1 (PRDX1), mRNA OS=Homo sapiens PE=2 SV=1	22414	481	0	36	0	0.32	0	0	0	0	2	0	2	0	2	0	-3.21167
tr|D3DVQ1|D3DVQ1_HUMAN	LETM1	Leucine zipper-EF-hand containing transmembrane protein 1, isoform CRA_a OS=Homo sapiens GN=LETM1 PE=2 SV=1	63730	463	0	37	0	0.11	0	0	1	0	2	0	2	0	4	0	-3.21167
tr|H0YDE4|H0YDE4_HUMAN	PRMT1	Protein arginine N-methyltransferase 1 (Fragment) OS=Homo sapiens GN=PRMT1 PE=4 SV=1	27345	437	0	39	0	0.26	0	0	1	0	2	0	2	0	2	0	-3.21167
sp|P07737|PROF1_HUMAN	PFN1	Profilin-1 OS=Homo sapiens GN=PFN1 PE=1 SV=2	15216	436	0	39	0	0.5	0	0	1	0	2	0	2	0	2	0	-3.21167
tr|Q6NZ55|Q6NZ55_HUMAN	RPL13	60S ribosomal protein L13 OS=Homo sapiens GN=RPL13 PE=2 SV=1	24308	422	0	41	0	0.14	0	0	1	0	1	0	2	0	2	0	-3.21167
tr|C9J1B1|C9J1B1_HUMAN	NAP1L4	Nucleosome assembly protein 1-like 4 (Fragment) OS=Homo sapiens GN=NAP1L4 PE=2 SV=2	11455	417	0	31	0	0.16	0	0	0	0	1	0	2	0	2	0	-3.21167
tr|B4DE72|B4DE72_HUMAN	tr|B4DE72|B4	cDNA FLJ50078, highly similar to Nuclear pore complex protein Nup205 OS=Homo sapiens PE=2 SV=1	106663	415	0	41	0	0.04	0	0	0	0	2	0	2	0	4	0	-3.21167
tr|A0MNP2|A0MNP2_HUMAN	WDR57	CDW11/WDR57 OS=Homo sapiens GN=WDR57 PE=2 SV=1	39742	413	0	42	0	0.17	0	0	1	0	2	0	2	0	3	0	-3.21167
tr|B2RCT6|B2RCT6_HUMAN	tr|B2RCT6|B2	cDNA, FLJ96276, highly similar to Homo sapiens G1 to S phase transition 1 (GSPT1), mRNA OS=Homo sapiens PE=2 SV=1	56320	391	0	44	0	0.12	0	0	1	0	2	0	2	0	2	0	-3.21167
tr|Q0IJ56|Q0IJ56_HUMAN	ST13	ST13 protein (Fragment) OS=Homo sapiens GN=ST13 PE=2 SV=1	35164	382	0	46	0	0.09	0	0	1	0	1	0	2	0	3	0	-3.21167
tr|H0YIG3|H0YIG3_HUMAN	KRT72	Keratin, type II cytoskeletal 72 (Fragment) OS=Homo sapiens GN=KRT72 PE=4 SV=1	21922	372	0	39	0	0.12	0	0	1	0	1	0	2	0	2	0	-3.21167
sp|P01024|CO3_HUMAN	C3	Complement C3 OS=Homo sapiens GN=C3 PE=1 SV=2	188569	370	0	47	0	0.03	0	0	1	0	2	0	2	0	2	0	-3.21167
tr|Q53GU8|Q53GU8_HUMAN	tr|Q53GU8|Q	Transforming growth factor, beta-induced, 68kDa variant (Fragment) OS=Homo sapiens PE=2 SV=1	75231	358	0	48	0	0.09	0	0	1	0	2	0	2	0	2	0	-3.21167
tr|B7Z8J4|B7Z8J4_HUMAN	NMT1	Glycylpeptide N-tetradecanoyltransferase 1 OS=Homo sapiens GN=NMT1 PE=2 SV=1	15265	350	0	50	0	0.49	0	0	1	0	2	0	2	0	2	0	-3.21167
tr|Q6NZ52|Q6NZ52_HUMAN	RPL27A	Ribosomal protein L27a OS=Homo sapiens GN=RPL27A PE=2 SV=1	16582	348	0	50	0	0.2	0	0	1	0	1	0	2	0	2	0	-3.21167
tr|E5RHF2|E5RHF2_HUMAN	COPS5	COP9 signalosome complex subunit 5 (Fragment) OS=Homo sapiens GN=COPS5 PE=2 SV=1	16655	346	0	51	0	0.45	0	0	0	0	2	0	2	0	2	0	-3.21167
tr|D3DP78|D3DP78_HUMAN	DARS	Aspartyl-tRNA synthetase, isoform CRA_b OS=Homo sapiens GN=DARS PE=2 SV=1	53829	344	0	51	0	0.06	0	0	1	0	1	0	2	0	3	0	-3.21167
tr|G3V180|G3V180_HUMAN	DPP3	Dipeptidyl peptidase 3 OS=Homo sapiens GN=DPP3 PE=2 SV=1	84666	335	0	52	0	0.08	0	0	1	0	2	0	2	0	2	0	-3.21167
tr|B9A041|B9A041_HUMAN	MDH1	Malate dehydrogenase, cytoplasmic OS=Homo sapiens GN=MDH1 PE=2 SV=1	23195	309	0	58	0	0.31	0	0	2	0	2	0	2	0	2	0	-3.21167
tr|F5GYR3|F5GYR3_HUMAN	NOP2	Putative ribosomal RNA methyltransferase NOP2 (Fragment) OS=Homo sapiens GN=NOP2 PE=2 SV=1	30665	296	0	28	0	0.15	0	0	1	0	1	0	2	0	3	0	-3.21167
tr|B1ANK7|B1ANK7_HUMAN	FH	Fumarate hydratase OS=Homo sapiens GN=FH PE=2 SV=1	54773	288	0	64	0	0.12	0	0	1	0	2	0	2	0	5	0	-3.21167
tr|Q3KNV1|Q3KNV1_HUMAN	KRT7	KRT7 protein OS=Homo sapiens GN=KRT7 PE=2 SV=1	15081	259	0	21	0	0.45	0	0	0	0	1	0	2	0	2	0	-3.21167
tr|B4DMT8|B4DMT8_HUMAN	tr|B4DMT8|B	cDNA FLJ52118, highly similar to 14-3-3 protein theta OS=Homo sapiens PE=2 SV=1	24050	258	0	42	0	0.4	0	0	1	0	2	0	2	0	2	0	-3.21167
sp|Q6IS14|IF5AL_HUMAN	EIF5AL1	Eukaryotic translation initiation factor 5A-1-like OS=Homo sapiens GN=EIF5AL1 PE=1 SV=2	16990	256	0	75	0	0.44	0	0	1	0	2	0	2	0	2	0	-3.21167
tr|E9PP36|E9PP36_HUMAN	RPL8	60S ribosomal protein L8 OS=Homo sapiens GN=RPL8 PE=2 SV=1	16227	231	0	33	0	0.28	0	0	1	0	1	0	2	0	2	0	-3.21167
tr|B2R6K4|B2R6K4_HUMAN	tr|B2R6K4|B2	cDNA, FLJ92996, highly similar to Homo sapiens guanine nucleotide binding protein (G protein), beta polypeptide 1 (GNB1), mRNA OS=Homo sa	38075	199	0	105	0	0.18	0	0	1	0	2	0	2	0	2	0	-3.21167
tr|B3KPU1|B3KPU1_HUMAN	GNB2	Guanine nucleotide-binding protein G(I)/G(S)/G(T) subunit beta-2 OS=Homo sapiens GN=GNB2 PE=2 SV=1	26598	194	0	111	0	0.27	0	0	1	0	2	0	2	0	2	0	-3.21167
tr|Q5JVP3|Q5JVP3_HUMAN	PTGR1	Leukotriene B4 12-hydroxydehydrogenase, isoform CRA_d OS=Homo sapiens GN=PTGR1 PE=4 SV=1	19441	190	0	113	0	0.38	0	0	2	0	2	0	2	0	2	0	-3.21167
tr|B4DRY6|B4DRY6_HUMAN	tr|B4DRY6|B4	cDNA FLJ57182, highly similar to Paxillin OS=Homo sapiens PE=2 SV=1	36791	159	0	73	0	0.28	0	0	1	0	2	0	2	0	2	0	-3.21167
tr|B3KWH4|B3KWH4_HUMAN	tr|B3KWH4|B	cDNA FLJ43092 fis, clone COLON2002520, highly similar to Myosin-14 (Fragment) OS=Homo sapiens PE=2 SV=1	73003	152	0	70	0	0.07	0	0	2	0	2	0	2	0	2	0	-3.21167
tr|B4DX20|B4DX20_HUMAN	CCT6B	T-complex protein 1 subunit zeta-2 OS=Homo sapiens GN=CCT6B PE=2 SV=1	54066	118	0	46	0	0.47	0	0	1	0	1	0	2	0	3	0	-3.21167
tr|Q5JRS1|Q5JRS1_HUMAN	UBA1	Ubiquitin-like modifier-activating enzyme 1 (Fragment) OS=Homo sapiens GN=UBA1 PE=4 SV=1	18707	114	0	51	0	0.28	0	0	1	0	2	0	2	0	2	0	-3.21167
tr|Q7KZ86|Q7KZ86_HUMAN	tr|Q7KZ86|Q7	Plakoglobin (Fragment) OS=Homo sapiens PE=2 SV=1	19007	26	0	37	0	1.74	0	0	1	0	2	0	2	0	2	0	-3.21167
tr|B7Z4P8|B7Z4P8_HUMAN	ACTN2	Alpha-actinin-2 OS=Homo sapiens GN=ACTN2 PE=2 SV=1	44675	19	0	51	0	2.11	0	0	1	0	2	0	2	0	2	0	-3.21167
sp|Q7Z3Y8|K1C27_HUMAN	KRT27	Keratin, type I cytoskeletal 27 OS=Homo sapiens GN=KRT27 PE=1 SV=2	50419	12	0	72	0	4.67	0	0	2	0	2	0	2	0	2	0	-3.21167
tr|D3DQA6|D3DQA6_HUMAN	DDX46	DEAD (Asp-Glu-Ala-Asp) box polypeptide 46, isoform CRA_a OS=Homo sapiens GN=DDX46 PE=3 SV=1	100958	595	0	26	0	0.07	0	0	0	0	2	0	3	0	3	0	-3.69743
tr|L0R5A1|L0R5A1_HUMAN	CSF2RB	Alternative protein CSF2RB OS=Homo sapiens GN=CSF2RB PE=4 SV=1	11752	486	0	36	0	0.29	0	0	1	0	1	0	3	0	5	0	-3.69743
sp|Q9NV06|DCA13_HUMAN	DCAF13	DDB1- and CUL4-associated factor 13 OS=Homo sapiens GN=DCAF13 PE=1 SV=2	51996	435	0	39	0	0.2	0	0	1	0	3	0	3	0	6	0	-3.69743
tr|B4DS32|B4DS32_HUMAN	tr|B4DS32|B4	cDNA FLJ56236, highly similar to Exportin-2 OS=Homo sapiens PE=2 SV=1	64855	433	0	28	0	0.08	0	0	0	0	1	0	3	0	3	0	-3.69743
tr|Q0VGL3|Q0VGL3_HUMAN	RPL13A	RPL13A protein OS=Homo sapiens GN=RPL13A PE=2 SV=1	16706	300	0	59	0	0.74	0	0	3	0	3	0	3	0	3	0	-3.69743
tr|Q5JQZ9|Q5JQZ9_HUMAN	CDC2L6	Cell division cycle 2-like 6 (CDK8-like) (Fragment) OS=Homo sapiens GN=CDC2L6 PE=4 SV=1	29552	285	0	65	0	0.24	0	0	2	0	2	0	3	0	3	0	-3.69743
tr|J3QST3|J3QST3_HUMAN	KRT3	Keratin, type II cytoskeletal 3 OS=Homo sapiens GN=KRT3 PE=3 SV=1	64636	255	0	76	0	0.16	0	0	3	0	3	0	3	0	6	0	-3.69743
tr|A2T926|A2T926_HUMAN	TMPO	Thymopentin OS=Homo sapiens GN=TMPO PE=2 SV=1	27428	241	0	41	0	0.24	0	0	0	0	3	0	3	0	3	0	-3.69743
tr|Q5JSD1|Q5JSD1_HUMAN	VDAC2	Voltage-dependent anion-selective channel protein 2 (Fragment) OS=Homo sapiens GN=VDAC2 PE=4 SV=1	22544	196	0	97	0	0.76	0	0	1	0	3	0	3	0	3	0	-3.69743
tr|B4DMU0|B4DMU0_HUMAN	PYCR1	Pyrroline-5-carboxylate reductase OS=Homo sapiens GN=PYCR1 PE=2 SV=1	36186	174	0	125	0	0.19	0	0	2	0	2	0	3	0	3	0	-3.69743
tr|C9JWY7|C9JWY7_HUMAN	CAPN2	Calpain-2 catalytic subunit (Fragment) OS=Homo sapiens GN=CAPN2 PE=2 SV=1	14668	173	0	48	0	0.22	0	0	0	0	4	0	3	0	4	0	-3.69743
tr|Q5STZ7|Q5STZ7_HUMAN	ABCF1	ATP-binding cassette sub-family F member 1 OS=Homo sapiens GN=ABCF1 PE=4 SV=2	27429	144	0	112	0	0.27	0	0	2	0	3	0	3	0	3	0	-3.69743
sp|A2RTY3|CQ066_HUMAN	C17orf66	Uncharacterized protein C17orf66 OS=Homo sapiens GN=C17orf66 PE=2 SV=2	66437	477	0	36	0	0.05	0	0	0	0	1	0	4	0	8	0	-4.06033
tr|Q53RS3|Q53RS3_HUMAN	DDEF2	Putative uncharacterized protein DDEF2 (Fragment) OS=Homo sapiens GN=DDEF2 PE=2 SV=1	70046	441	0	39	0	0.05	0	0	1	0	1	0	4	0	4	0	-4.06033
tr|F8VPD4|F8VPD4_HUMAN	CAD	CAD protein OS=Homo sapiens GN=CAD PE=2 SV=1	238097	317	0	57	0	0.04	0	0	1	0	3	0	4	0	5	0	-4.06033
sp|P21796|VDAC1_HUMAN	VDAC1	Voltage-dependent anion-selective channel protein 1 OS=Homo sapiens GN=VDAC1 PE=1 SV=2	30868	250	0	78	0	0.36	0	0	1	0	3	0	4	0	4	0	-4.06033
tr|D6RAT0|D6RAT0_HUMAN	RPS3A	40S ribosomal protein S3a OS=Homo sapiens GN=RPS3A PE=3 SV=1	26099	223	0	93	0	0.44	0	0	2	0	3	0	4	0	5	0	-4.06033
tr|B3KY17|B3KY17_HUMAN	ATP2A1	Sarcoplasmic/endoplasmic reticulum calcium ATPase 1 OS=Homo sapiens GN=ATP2A1 PE=2 SV=1	96392	149	0	86	0	0.32	0	0	2	0	4	0	5	0	5	0	-4.35017
tr|K7ENW6|K7ENW6_HUMAN	KRT16	Keratin, type I cytoskeletal 16 (Fragment) OS=Homo sapiens GN=KRT16 PE=4 SV=1	18735	62	0	109	0	1.38	0	0	3	0	4	0	5	0	5	0	-4.35017
tr|F8VP67|F8VP67_HUMAN	KRT8	Keratin, type II cytoskeletal 8 (Fragment) OS=Homo sapiens GN=KRT8 PE=2 SV=1	13580	6	0	50	0	5.01	0	0	2	0	3	0	5	0	5	0	-4.35017
sp|P05165|PCCA_HUMAN	PCCA	Propionyl-CoA carboxylase alpha chain, mitochondrial OS=Homo sapiens GN=PCCA PE=1 SV=4	80635	99	0	216	0	0.49	0	0	6	0	10	0	13	0	13	0	-5.64828
tr|Q59FY4|Q59FY4_HUMAN	ACACA	Acetyl-CoA carboxylase 1 (Fragment) OS=Homo sapiens GN=ACACA PE=2 SV=1	115059	31	0	225	0	0.44	0	0	10	0	12	0	15	0	18	0	-5.84825
tr|B3KPL5|B3KPL5_HUMAN	tr|B3KPL5|B3	cDNA FLJ31917 fis, clone NT2RP7004925, highly similar to Sprouty-related, EVH1 domain-containing protein 2 OS=Homo sapiens PE=2 SV=1	48711	657	0	21	0	0.07	0	0	0	0	1	0	16	0	51	0	-5.93878

#16	Hela lysate syn PU-1_2μM																		
#14	Hela live syn NP_2μM																		
Accession	GS	Name	Pro_Mass	04_prot_hit_num	16_prot_h	04_prot_scor	16_prot_scor	04_emPAI	16_emPAI	emPAI ratio	04_No>I	16_No>I	04_No>HI	16_No>HI	04_No>20	16_No>20	04_No=All	16_No=All	Rsc
sp|P17612|KAPCA_HUMAN	PRKACA	cAMP-dependent protein kinase catalytic subunit alpha OS=Homo sapiens GN=PRKACA PE=1 SV	40678	0	869	0	47	0	0.08	#DIV/0!	0	1	0	1	0	3	0	3	1.910843
sp|P41743|KPCI_HUMAN	PRKCI	Protein KINASE C iota type OS=Homo sapiens GN=PRKCI PE=1 SV=2	69188	0	1297	0	22	0	0.05	#DIV/0!	0	0	0	1	0	1	0	1	0.68809
sp|O75116|ROCK2_HUMAN	ROCK2	Rho-associated protein kinase 2 OS=Homo sapiens GN=ROCK2 PE=1 SV=4	161939	375	270	46	170	0.05	0.16	3.2	1	5	2	6	1	6	3	10	1.21917
sp|P78527|PRKDC_HUMAN	PRKDC	DNA-dependent protein kinase catalytic subunit OS=Homo sapiens GN=PRKDC PE=1 SV=3	473749	83	17	241	1402	0.07	0.5	7.14285714	7	41	10	56	11	67	17	77	1.664625
sp|Q13555|KCC2G_HUMAN	CAMK2G	Calcium/calmodulin-dependent protein kinase type II subunit gamma OS=Homo sapiens GN=CA	63311	0	983	0	39	0	0.15	#DIV/0!	0	1	0	1	0	1	0	1	0.68809
sp|P48729|KC1A_HUMAN	CSNK1A1	Casein kinase I isoform alpha OS=Homo sapiens GN=CSNK1A1 PE=1 SV=2	39118	0	752	0	57	0	0.12	#DIV/0!	0	1	0	1	0	1	0	1	0.68809
sp|P49761|CLK3_HUMAN	CLK3	Dual specificity protein kinase CLK3 OS=Homo sapiens GN=CLK3 PE=1 SV=3	74267	0	1174	0	29	0	0.04	#DIV/0!	0	0	0	1	0	1	0	1	0.68809
sp|Q96SB4|SRPK1_HUMAN	SRPK1	SRSF protein kinase 1 OS=Homo sapiens GN=SRPK1 PE=1 SV=2	74963	0	750	0	57	0	0.04	#DIV/0!	0	1	0	1	0	1	0	1	0.68809
sp|O14965|AURKA_HUMAN	AURKA	Aurora kinase A OS=Homo sapiens GN=AURKA PE=1 SV=2	46180	0	502	0	89	0	0.32	#DIV/0!	0	3	0	4	0	3	0	4	1.910843
sp|O60566|BUB1B_HUMAN	BUB1B	Mitotic checkpoint serine/threonine-protein KINASE BUB1 beta OS=Homo sapiens GN=BUB1B P	120781	0	1221	0	26	0	0.05	#DIV/0!	0	0	0	2	0	2	0	4	1.425236
sp|Q99570|PI3R4_HUMAN	PIK3R4	Phosphoinositide 3-kinase regulatory subunit 4 OS=Homo sapiens GN=PIK3R4 PE=1 SV=3	154318	0	984	0	39	0	0.04	#DIV/0!	0	1	0	2	0	2	0	2	1.425236
sp|P53350|PLK1_HUMAN	PLK1	Serine/threonine-protein kinase PLK1 OS=Homo sapiens GN=PLK1 PE=1 SV=1	68953	0	441	0	104	0	0.15	#DIV/0!	0	3	0	3	0	3	0	3	1.910843
sp|P33981|TTK_HUMAN	TTK	Dual specificity protein KINASE TTK OS=Homo sapiens GN=TTK PE=1 SV=2	97980	0	1037	0	36	0	0.03	#DIV/0!	0	1	0	1	0	2	0	4	1.425236
sp|Q9H4A3|WNK1_HUMAN	WNK1	Serine/threonine-protein kinase WNK1 OS=Homo sapiens GN=WNK1 PE=1 SV=2	251552	0	862	0	48	0	0.01	#DIV/0!	0	1	0	1	0	1	0	2	0.68809
sp|Q8N4C8|MINK1_HUMAN	MINK1	Misshapen-like kinase 1 OS=Homo sapiens GN=MINK1 PE=1 SV=2	150413	0	1073	0	0	0	0.02	#DIV/0!	0	0	0	0	0	1	0	2	0.68809
sp|Q9NYL2|MLTK_HUMAN	MLTK	Mitogen-activated protein kinase kinase kinase MLT OS=Homo sapiens GN=MLTK PE=1 SV=3	91725	0	1049	0	35	0	0.06	#DIV/0!	0	1	0	1	0	2	0	2	1.425236
sp|Q96KB5|TOPK_HUMAN	PBK	Lymphokine-activated killer T-cell-originated protein kinase OS=Homo sapiens GN=PBK PE=1 SV	36404	0	866	0	48	0	0.09	#DIV/0!	0	1	0	1	0	1	0	1	0.68809
sp|Q13418|ILK_HUMAN	ILK	Integrin-linked protein kinase OS=Homo sapiens GN=ILK PE=1 SV=2	51899	421	366	41	126	0.07	0.65	9.28571429	1	3	1	7	1	7	1	7	1.425801
sp|Q53H12|AGK_HUMAN	AGK	Acylglycerol kinase, mitochondrial OS=Homo sapiens GN=AGK PE=1 SV=2	47564	0	1191	0	28	0	0.07	#DIV/0!	0	0	0	1	0	1	0	1	0.68809
sp|Q9UIG0|BAZ1B_HUMAN	BAZ1B	Tyrosine-protein KINASE BAZ1B OS=Homo sapiens GN=BAZ1B PE=1 SV=2	172392	0	1203	0	27	0	0.04	#DIV/0!	0	0	0	2	0	2	0	3	1.425236
sp|Q9Y223|GLCNE_HUMAN	GNE	Bifunctional UDP-N-acetylglucosamine 2-epimerase/N-acetylmannosamine kinase OS=Homo sa	80193	0	1290	0	22	0	0.04	#DIV/0!	0	0	0	1	0	1	0	1	0.68809
sp|P19367|HXK1_HUMAN	HK1	Hexokinase-1 OS=Homo sapiens GN=HK1 PE=1 SV=3	103561	0	530	0	84	0	0.09	#DIV/0!	0	3	0	3	0	4	0	4	2.273593
sp|Q16822|PCKGM_HUMAN	PCK2	Phosphoenolpyruvate carboxykinase [GTP], mitochondrial OS=Homo sapiens GN=PCK2 PE=1 SV	71483	0	363	0	93	0	0.2	#DIV/0!	0	2	0	3	0	4	0	5	2.273593
sp|P17858|K6PL_HUMAN	PFKL	6-phosphofructoKINASE, liver type OS=Homo sapiens GN=PFKL PE=1 SV=6	85762	98	263	88	173	0.4	0.16	0.4	1	4	2	4	2	5	2	5	0.240681
tr|J3KNX3|J3KNX3_HUMAN	PFKM	6-phosphofructoKINASE OS=Homo sapiens GN=PFKM PE=3 SV=1	94107	0	194	0	245	0	0.31	#DIV/0!	0	7	0	8	0	9	0	9	3.352497
sp|Q01813|K6PP_HUMAN	PFKP	6-phosphofructoKINASE type C OS=Homo sapiens GN=PFKP PE=1 SV=2	86454	98	59	217	636	0.4	1.35	3.375	5	17	9	23	9	25	10	25	0.528933
sp|P42356|PI4KA_HUMAN	PI4KA	Phosphatidylinositol 4-kinase alpha OS=Homo sapiens GN=PI4KA PE=1 SV=3	233622	0	1253	0	24	0	0.02	#DIV/0!	0	0	0	1	0	2	0	2	1.425236
sp|P67775|PP2AA_HUMAN	PPP2CA	Serine/threonine-protein phosphatase 2A catalytic subunit alpha isoform OS=Homo sapiens GN	36142	0	573	0	77	0	0.25	#DIV/0!	0	2	0	2	0	2	0	3	1.425236
sp|P36873|PP1G_HUMAN	PPP1CC	Serine/threonine-protein phosphatase PP1-gamma catalytic subunit OS=Homo sapiens GN=PPP	37701	0	689	0	62	0	0.35	#DIV/0!	0	2	0	3	0	3	0	4	1.910843
sp|P62136|PP1A_HUMAN	PPP1CA	Serine/threonine-protein phosphatase PP1-alpha catalytic subunit OS=Homo sapiens GN=PPP1C	38229	0	688	0	62	0	0.23	#DIV/0!	0	2	0	3	0	4	0	4	2.273593
sp|P40763|STAT3_HUMAN	STAT3	Signal transducer and activator of transcription 3 OS=Homo sapiens GN=STAT3 PE=1 SV=2	88810	0	537	0	83	0	0.12	#DIV/0!	0	2	0	3	0	7	0	8	3.011099
sp|P62140|PP1B_HUMAN	PPP1CB	Serine/threonine-protein phosphatase PP1-beta catalytic subunit OS=Homo sapiens GN=PPP1C	37961	345	572	51	77	0.24	1.33	5.54166667	1	3	1	4	1	4	1	5	0.688295
tr|C1PHA2|C1PHA2_HUMAN	KIF5B-ALK	Tyrosine-protein KINASE receptor OS=Homo sapiens GN=KIF5B-ALK PE=2 SV=1	169283	227	164	91	285	0.04	0.23	5.75	2	7	2	11	2	14	3	16	1.640853
tr|A4D1C5|A4D1C5_HUMAN	PTPN12	Protein tyrosine phosphatase, non-receptor type 12 OS=Homo sapiens GN=PTPN12 PE=2 SV=1	88907	0	921	0	43	0	0.04	#DIV/0!	0	1	0	1	0	1	0	1	0.68809
tr|B4DSN5|B4DSN5_HUMAN	PTPN1	Tyrosine-protein phosphatase non-receptor type OS=Homo sapiens GN=PTPN1 PE=2 SV=1	41747	0	830	0	50	0	0.08	#DIV/0!	0	1	0	1	0	1	0	1	0.68809
tr|H0Y8C4|H0Y8C4_HUMAN	PPP2R5D	Serine/threonine-protein phosphatase 2A 56 kDa regulatory subunit delta isoform (Fragment) O	59637	0	1214	0	27	0	0.11	#DIV/0!	0	0	0	2	0	2	0	2	1.425236
tr|B2R665|B2R665_HUMAN	tr|B2R665|B2	cDNA, FLJ92810, highly similar to Homo sapiens protein phosphatase 1G (formerly 2C), magnesi	59889	0	944	0	42	0	0.11	#DIV/0!	0	1	0	2	0	2	0	2	1.425236
sp|Q5H9R7|PP6R3_HUMAN	PPP6R3	Serine/threonine-protein phosphatase 6 regulatory subunit 3 OS=Homo sapiens GN=PPP6R3 PE	98577	0	804	0	52	0	0.07	#DIV/0!	0	2	0	2	0	2	0	3	1.425236
tr|H0Y380|H0Y380_HUMAN	PTPRF	Receptor-type tyrosine-protein phosphatase F (Fragment) OS=Homo sapiens GN=PTPRF PE=4 SV	111922	0	1284	0	23	0	0.03	#DIV/0!	0	1	0	1	0	1	0	1	0.68809
sp|O15357|SHIP2_HUMAN	INPPL1	Phosphatidylinositol 3,4,5-trisphosphate 5-phosphatase 2 OS=Homo sapiens GN=INPPL1 PE=1 S	139311	0	1158	0	29	0	0.23	#DIV/0!	0	0	0	1	0	1	0	1	0.68809
tr|H0YJ75|H0YJ75_HUMAN	PPP2R5C	Serine/threonine-protein phosphatase 2A 56 kDa regulatory subunit gamma isoform (Fragment	64365	0	1153	0	30	0	0.42	#DIV/0!	0	0	0	1	0	1	0	1	0.68809
sp|Q8TAP8|PPR35_HUMAN	PPP1R35	Protein phosphatase 1 regulatory subunit 35 OS=Homo sapiens GN=PPP1R35 PE=1 SV=1	28107	0	877	0	46	0	0.12	#DIV/0!	0	1	0	1	0	1	0	1	0.68809
tr|B4DK71|B4DK71_HUMAN	SACM1L	Phosphatidylinositide phosphatase SAC1 OS=Homo sapiens GN=SACM1L PE=2 SV=1	61327	0	598	0	72	0	0.05	#DIV/0!	0	1	0	1	0	1	0	1	0.68809
tr|B4DDF7|B4DDF7_HUMAN	tr|B4DDF7|B	cDNA FLJ53296, highly similar to Serine/threonine-protein phosphatase 2A 65 kDa regulatory su	64939	443	436	39	105	0.16	0.34	2.125	1	3	3	6	3	7	3	9	0.202738
sp|Q12802|AKP13_HUMAN	AKAP13	A-KINASE anchor protein 13 OS=Homo sapiens GN=AKAP13 PE=1 SV=2	310381	0	1354	0	15	0	0.08	#DIV/0!	0	0	0	1	0	0	0	1	-0.89705
sp|P00558|PGK1_HUMAN	PGK1	Phosphoglycerate KINASE 1 OS=Homo sapiens GN=PGK1 PE=1 SV=3	44985	134	83	161	489	0.76	1.9	2.5	5	12	8	15	9	17	9	19	-0.01565
sp|P14618|KPYM_HUMAN	PKM	Pyruvate KINASE isozymes M1/M2 OS=Homo sapiens GN=PKM PE=1 SV=4	58470	8	6	1820	2276	5.79	11.39	1.9671848	47	52	58	69	60	74	66	81	-0.60362
tr|B4DP56|B4DP56_HUMAN	tr|B4DP56|B	cDNA FLJ52237, highly similar to Creatine KINASE B-type (EC 2.7.3.2) OS=Homo sapiens PE=2 SV	38840	214	591	97	73	0.18	0.28	1.55555556	2	1	2	3	2	3	2	3	-0.41176
tr|A8K2U2|A8K2U2_HUMAN	tr|A8K2U2|A	cDNA FLJ75392, highly similar to Homo sapiens hexoKINASE II (HKII) mRNA OS=Homo sapiens PE	103718	599	943	26	42	0.03	0.03	1	0	1	1	1	1	1	2	4	-0.89721
tr|B2R5T5|B2R5T5_HUMAN	PRKAR1A	Protein KINASE, cAMP-dependent, regulatory, type I, alpha (Tissue specific extinguisher 1), isofo	43183	533	907	31	44	0.08	0.08	1	0	1	1	1	1	1	1	1	-0.89721
tr|F2Z2Y4|F2Z2Y4_HUMAN	PDXK	Pyridoxal KINASE OS=Homo sapiens GN=PDXK PE=2 SV=1	30790	566	801	29	52	0.11	0.11	1	0	1	1	1	1	1	1	3	-0.89721
tr|B0AZV0|B0AZV0_HUMAN	STRAP	Serine-threonine KINASE receptor-associated protein OS=Homo sapiens GN=STRAP PE=2 SV=1	28716	626	627	23	68	0.12	0.08	0.66666667	0	1	1	1	1	1	1	1	-0.89721
tr|G5E9C7|G5E9C7_HUMAN	MAP2K2	Dual-specificity mitogen-activated protein KINASE KINASE 2 OS=Homo sapiens GN=MAP2K2 PE=	34145	384	916	45	17	0.31	0.15	0.48387097	1	0	2	1	2	0	2	1	-3.21965
tr|A4QPA9|A4QPA9_HUMAN	MAP2K1	Mitogen-activated protein KINASE KINASE 1 OS=Homo sapiens GN=MAP2K1 PE=2 SV=1	43753	384	746	45	57	0.31	0.08	0.25806452	1	1	2	1	2	1	2	1	-1.63451
tr|B4DPM0|B4DPM0_HUMAN	tr|B4DPM0|E	Pyruvate KINASE OS=Homo sapiens PE=2 SV=1	52984	8	806	40	52	5.79	0.06	0.01036269	2	1	2	1	2	1	2	2	-1.63451
tr|B4E1G6|B4E1G6_HUMAN	GALK1	GalactoKINASE OS=Homo sapiens GN=GALK1 PE=2 SV=1	45956	679	0	20	0	0.07	0	0	0	0	1	0	0	0	1	0	-0.89705
tr|A6NIT2|A6NIT2_HUMAN	PHKA1	Phosphorylase b KINASE regulatory subunit alpha, skeletal muscle isoform OS=Homo sapiens GN	133462	711	0	17	0	0.02	0	0	0	0	1	0	0	0	1	0	-0.89705
tr|Q8IWY7|Q8IWY7_HUMAN	TTBK2	Tau-tubulin KINASE OS=Homo sapiens GN=TTBK2 PE=2 SV=1	183893	79	0	28	0	0.79	0	0	0	0	1	0	1	0	1	0	-2.48235
tr|B4E2K2|B4E2K2_HUMAN	tr|B4E2K2|B4	cDNA FLJ55665, highly similar to A-KINASE anchor protein 2 OS=Homo sapiens PE=2 SV=1	103935	246	0	79	0	0.03	0	0	1	0	1	0	1	0	1	0	-2.48235
tr|H0YDW9|H0YDW9_HUMAN	CDK19	Cyclin-dependent KINASE 19 (Fragment) OS=Homo sapiens GN=CDK19 PE=4 SV=1	14459	285	0	0	0	0.24	0	0	0	0	0	0	1	0	1	0	-2.48235
sp|Q9UEW8|STK39_HUMAN	STK39	STE20/SPS1-related proline-alanine-rich protein KINASE OS=Homo sapiens GN=STK39 PE=1 SV=	59950	491	0	35	0	0.05	0	0	0	0	1	0	1	0	1	0	-2.48235
tr|K7EID3|K7EID3_HUMAN	PRKAR1A	cAMP-dependent protein KINASE type I-alpha regulatory subunit (Fragment) OS=Homo sapiens	19112	533	0	31	0	0.08	0	0	0	0	1	0	1	0	1	0	-2.48235
tr|Q5JQ10|Q5JQ10_HUMAN	ADK	Adenosine KINASE OS=Homo sapiens GN=ADK PE=2 SV=1	39078	538	0	30	0	0.08	0	0	0	0	1	0	1	0	1	0	-2.48235
tr|H0YH33|H0YH33_HUMAN	STRAP	Serine-threonine KINASE receptor-associated protein (Fragment) OS=Homo sapiens GN=STRAP	10763	626	0	23	0	0.12	0	0	0	0	1	0	1	0	1	0	-2.48235
tr|H0Y3C5|H0Y3C5_HUMAN	HCK	Tyrosine-protein KINASE HCK OS=Homo sapiens GN=HCK PE=4 SV=1	59986	628	0	23	0	0.05	0	0	0	0	1	0	1	0	2	0	-2.48235
tr|H0Y3Y5|H0Y3Y5_HUMAN	PPIP5K1	Inositol hexakisphosphate and diphosphoinositol-pentakisphosphate KINASE 1 (Fragment) OS=H	21913	632	0	22	0	0.15	0	0	0	0	1	0	1	0	2	0	-2.48235
sp|O95747|OXSR1_HUMAN	OXSR1	Serine/threonine-protein KINASE OSR1 OS=Homo sapiens GN=OXSR1 PE=1 SV=1	58271	675	0	20	0	0.06	0	0	0	0	1	0	1	0	2	0	-2.48235
tr|B3KS97|B3KS97_HUMAN	MAP2K2	Mitogen-activated protein KINASE KINASE 2, isoform CRA_d OS=Homo sapiens GN=MAP2K2 PE=	23450	384	0	45	0	0.31	0	0	1	0	2	0	2	0	2	0	-3.21965
tr|H0YDU8|H0YDU8_HUMAN	PPP5C	Serine/threonine-protein phosphatase (Fragment) OS=Homo sapiens GN=PPP5C PE=3 SV=1	55743	596	817	26	51	0.06	0.06	1	0	1	1	1	1	1	1	1	-0.89721
tr|J3QT22|J3QT22_HUMAN	PPME1	Protein phosphatase methylesterase 1 OS=Homo sapiens GN=PPME1 PE=3 SV=1	44241	232	0	90	0	0.07	0	0	1	0	1	0	1	0	2	0	-2.48235
tr|B4DDZ8|B4DDZ8_HUMAN	tr|B4DDZ8|B	cDNA FLJ55048, highly similar to Serine/threonine-protein phosphatase 5 (EC 3.1.3.16) OS=Hom	55829	596	0	26	0	0.06	0	0	0	0	1	0	1	0	1	0	-2.48235
tr|E1NZA1|E1NZA1_HUMAN	PRIC295	Peroxisome proliferator activated receptor interacting complex protein OS=Homo sapiens GN=F	294953	0	81	0	496	0	0.32	#DIV/0!	0	15	0	25	0	26	0	29	4.835558
tr|D6REM6|D6REM6_HUMAN	MATR3	Matrin-3 OS=Homo sapiens GN=MATR3 PE=2 SV=1	88647	0	175	0	238	0	0.52	#DIV/0!	0	10	0	13	0	18	0	20	4.315646
tr|A8K8U1|A8K8U1_HUMAN	tr|A8K8U1|A	cDNA FLJ77762, highly similar to Homo sapiens cullin-associated and neddylation-dissociated 1	137941	0	104	0	391	0	0.36	#DIV/0!	0	11	0	14	0	16	0	18	4.150226
sp|Q08J23|NSUN2_HUMAN	NSUN2	tRNA (cytosine(34)-C(5))-methyltransferase OS=Homo sapiens GN=NSUN2 PE=1 SV=2	87214	0	181	0	261	0	0.39	#DIV/0!	0	7	0	10	0	13	0	15	3.860178
sp|O75533|SF3B1_HUMAN	SF3B1	Splicing factor 3B subunit 1 OS=Homo sapiens GN=SF3B1 PE=1 SV=3	146479	0	141	0	315	0	0.33	#DIV/0!	0	9	0	13	0	13	0	14	3.860178
tr|B7Z4B2|B7Z4B2_HUMAN	tr|B7Z4B2|B7	cDNA FLJ56108, highly similar to Puromycin-sensitive aminopeptidase (EC 3.4.11.-) OS=Homo sa	93434	0	132	0	331	0	0.51	#DIV/0!	0	10	0	12	0	13	0	14	3.860178
tr|B4E3J7|B4E3J7_HUMAN	tr|B4E3J7|B4	cDNA FLJ51203, highly similar to Thrombospondin-1 OS=Homo sapiens PE=2 SV=1	124032	0	126	0	342	0	0.26	#DIV/0!	0	8	0	10	0	11	0	11	3.628492
tr|A8K132|A8K132_HUMAN	tr|A8K132|A	cDNA FLJ75476, highly similar to Homo sapiens glutaminase (GLS), mRNA OS=Homo sapiens PE=	74317	0	114	0	362	0	0.47	#DIV/0!	0	8	0	10	0	11	0	11	3.628492
tr|B4DLV7|B4DLV7_HUMAN	tr|B4DLV7|B	cDNA FLJ60299, highly similar to Rab GDP dissociation inhibitor beta OS=Homo sapiens PE=2 SV	51577	0	201	0	236	0	0.54	#DIV/0!	0	5	0	7	0	10	0	11	3.497067
sp|O43776|SYNC_HUMAN	NARS	Asparagine--tRNA ligase, cytoplasmic OS=Homo sapiens GN=NARS PE=1 SV=1	63758	0	182	0	259	0	0.57	#DIV/0!	0	7	0	9	0	10	0	12	3.497067
tr|Q5TZZ9|Q5TZZ9_HUMAN	ANXA1	Annexin OS=Homo sapiens GN=ANXA1 PE=2 SV=1	38918	0	178	0	266	0	0.92	#DIV/0!	0	6	0	8	0	10	0	10	3.497067
tr|B3KM58|B3KM58_HUMAN	tr|B3KM58|B	cDNA FLJ10358 fis, clone NT2RM2001238, highly similar to Glutaminase kidney isoform, mitoch	41736	0	89	0	442	0	0.99	#DIV/0!	0	8	0	9	0	10	0	10	3.497067
sp|Q8NI36|WDR36_HUMAN	WDR36	WD repeat-containing protein 36 OS=Homo sapiens GN=WDR36 PE=1 SV=1	106282	0	501	0	90	0	0.16	#DIV/0!	0	2	0	5	0	9	0	9	3.352497
tr|B2RDW1|B2RDW1_HUMAN	RPS27A	Ribosomal protein S27a, isoform CRA_c OS=Homo sapiens GN=RPS27A PE=2 SV=1	18296	0	348	0	131	0	0.4	#DIV/0!	0	5	0	7	0	9	0	11	3.352497
tr|B3KWV6|B3KWV6_HUMAN	tr|B3KWV6|E	cDNA FLJ43948 fis, clone TESTI4014924, highly similar to Homo sapiens cytoplasmic FMR1 intera	147082	0	307	0	145	0	0.14	#DIV/0!	0	5	0	6	0	9	0	12	3.352497
tr|G3V198|G3V198_HUMAN	NUP160	Nuclear pore complex protein Nup160 OS=Homo sapiens GN=NUP160 PE=2 SV=1	142365	0	244	0	180	0	0.22	#DIV/0!	0	7	0	9	0	9	0	12	3.352497
tr|H0YH81|H0YH81_HUMAN	ATP5B	ATP synthase subunit beta (Fragment) OS=Homo sapiens GN=ATP5B PE=3 SV=1	38226	0	220	0	217	0	0.79	#DIV/0!	0	6	0	7	0	9	0	10	3.352497
tr|Q6NVW7|Q6NVW7_HUMAN	KPNA2	Importin subunit alpha OS=Homo sapiens GN=KPNA2 PE=2 SV=1	58271	0	172	0	272	0	0.55	#DIV/0!	0	7	0	9	0	9	0	10	3.352497
tr|B4DZI8|B4DZI8_HUMAN	COPB2	Coatomer protein complex, subunit beta 2 (Beta prime), isoform CRA_b OS=Homo sapiens GN=	99839	0	279	0	164	0	0.29	#DIV/0!	0	4	0	8	0	8	0	10	3.191852
sp|P49792|RBP2_HUMAN	RANBP2	E3 SUMO-protein ligase RanBP2 OS=Homo sapiens GN=RANBP2 PE=1 SV=2	362365	0	272	0	167	0	0.07	#DIV/0!	0	4	0	8	0	8	0	10	3.191852
tr|B0QY89|B0QY89_HUMAN	EIF3EIP	Eukaryotic translation initiation factor 3 subunit L OS=Homo sapiens GN=EIF3EIP PE=4 SV=1	71085	0	224	0	210	0	0.37	#DIV/0!	0	5	0	7	0	8	0	9	3.191852
tr|B7Z1N6|B7Z1N6_HUMAN	tr|B7Z1N6|B	cDNA FLJ52222, highly similar to Fructose-bisphosphate aldolase C (EC4.1.2.13) OS=Homo sapie	35800	0	176	0	267	0	0.19	#DIV/0!	0	6	0	6	0	8	0	8	3.191852
sp|P46977|STT3A_HUMAN	STT3A	Dolichyl-diphosphooligosaccharide--protein glycosyltransferase subunit STT3A OS=Homo sapien	81104	0	1083	0	33	0	0.13	#DIV/0!	0	0	0	3	0	7	0	7	3.011099
tr|Q6IT96|Q6IT96_HUMAN	HDAC1	Histone deacetylase OS=Homo sapiens GN=HDAC1 PE=2 SV=1	55638	0	628	0	68	0	0.33	#DIV/0!	0	2	0	5	0	7	0	9	3.011099
tr|F5H8E5|F5H8E5_HUMAN	USP7	Ubiquitin carboxyl-terminal hydrolase OS=Homo sapiens GN=USP7 PE=2 SV=1	117951	0	454	0	100	0	0.18	#DIV/0!	0	3	0	6	0	7	0	7	3.011099
tr|B3KRS5|B3KRS5_HUMAN	HDAC2	Histone deacetylase OS=Homo sapiens GN=HDAC2 PE=2 SV=1	52479	0	448	0	102	0	0.36	#DIV/0!	0	3	0	5	0	7	0	9	3.011099
tr|B4DSL6|B4DSL6_HUMAN	tr|B4DSL6|B4	cDNA FLJ57190, highly similar to Actin-binding protein anillin OS=Homo sapiens PE=2 SV=1	111977	0	382	0	122	0	0.11	#DIV/0!	0	3	0	4	0	7	0	10	3.011099
tr|B3KM90|B3KM90_HUMAN	tr|B3KM90|B	cDNA FLJ10529 fis, clone NT2RP2000965, highly similar to Targeting protein for Xklp2 OS=Homo	70285	0	299	0	152	0	0.32	#DIV/0!	0	5	0	6	0	7	0	7	3.011099
tr|B2RAM6|B2RAM6_HUMAN	tr|B2RAM6|E	cDNA, FLJ95005, highly similar to Homo sapiens kinesin family member 11 (KIF11), mRNA OS=H	120084	0	292	0	159	0	0.21	#DIV/0!	0	5	0	7	0	7	0	8	3.011099
sp|Q8WX93|PALLD_HUMAN	PALLD	Palladin OS=Homo sapiens GN=PALLD PE=1 SV=3	151839	0	260	0	175	0	0.17	#DIV/0!	0	4	0	6	0	7	0	8	3.011099
tr|A1KYQ7|A1KYQ7_HUMAN	tr|A1KYQ7|A	Cell migration-inducing protein 17 OS=Homo sapiens PE=2 SV=1	106022	0	250	0	185	0	0.16	#DIV/0!	0	5	0	5	0	7	0	7	3.011099
tr|B2RWN5|B2RWN5_HUMAN	HEATR1	HEAT repeat containing 1 OS=Homo sapiens GN=HEATR1 PE=2 SV=1	244275	0	655	0	65	0	0.1	#DIV/0!	0	2	0	7	0	6	0	9	2.804468
sp|Q14558|KPRA_HUMAN	PRPSAP1	Phosphoribosyl pyrophosphate synthase-associated protein 1 OS=Homo sapiens GN=PRPSAP1 F	39654	0	452	0	101	0	0.62	#DIV/0!	0	2	0	6	0	6	0	6	2.804468
tr|B0UZZ8|B0UZZ8_HUMAN	WDR46	Chromosome 6 open reading frame 11 OS=Homo sapiens GN=WDR46 PE=2 SV=1	68455	0	426	0	108	0	0.26	#DIV/0!	0	4	0	5	0	6	0	7	2.804468
sp|Q09161|NCBP1_HUMAN	NCBP1	Nuclear cap-binding protein subunit 1 OS=Homo sapiens GN=NCBP1 PE=1 SV=1	92864	0	399	0	115	0	0.19	#DIV/0!	0	4	0	5	0	6	0	6	2.804468
tr|H3BM93|H3BM93_HUMAN	NUP93	Nuclear pore complex protein Nup93 (Fragment) OS=Homo sapiens GN=NUP93 PE=4 SV=1	19858	0	396	0	50	0	0.32	#DIV/0!	0	0	0	3	0	6	0	6	2.804468
tr|B3KNV9|B3KNV9_HUMAN	tr|B3KNV9|B	cDNA FLJ30554 fis, clone BRAWH2003693 OS=Homo sapiens PE=2 SV=1	112670	0	355	0	99	0	0.17	#DIV/0!	0	3	0	6	0	6	0	7	2.804468
tr|H0Y3Z3|H0Y3Z3_HUMAN	P4HB	Protein disulfide-isomerase (Fragment) OS=Homo sapiens GN=P4HB PE=3 SV=1	31752	0	336	0	134	0	0.64	#DIV/0!	0	3	0	5	0	6	0	7	2.804468
tr|G5E9P1|G5E9P1_HUMAN	ITPR1	Inositol 1,4,5-triphosphate receptor, type 1, isoform CRA_d OS=Homo sapiens GN=ITPR1 PE=2 S	311832	0	323	0	140	0	0.06	#DIV/0!	0	4	0	6	0	6	0	8	2.804468
tr|G5E9H4|G5E9H4_HUMAN	DCTN1	Dynactin 1 (P150, glued homolog, Drosophila), isoform CRA_a OS=Homo sapiens GN=DCTN1 PE	127339	0	289	0	160	0	0.14	#DIV/0!	0	4	0	5	0	6	0	7	2.804468
tr|B2RDD7|B2RDD7_HUMAN	tr|B2RDD7|B	cDNA, FLJ96564, highly similar to Homo sapiens SKB1 homolog (S. pombe) (SKB1), mRNA OS=Ho	73348	0	288	0	160	0	0.24	#DIV/0!	0	4	0	5	0	6	0	6	2.804468
tr|Q6NUR1|Q6NUR1_HUMAN	NCAPG	Non-SMC condensin I complex, subunit G OS=Homo sapiens GN=NCAPG PE=2 SV=1	115205	0	261	0	154	0	0.18	#DIV/0!	0	3	0	5	0	6	0	6	2.804468
tr|Q3SYK8|Q3SYK8_HUMAN	NUMA1	NUMA1 protein (Fragment) OS=Homo sapiens GN=NUMA1 PE=2 SV=1	78666	0	70	0	200	0	0.26	#DIV/0!	0	4	0	5	0	6	0	9	2.804468
tr|H7C3P6|H7C3P6_HUMAN	NUP98	Nuclear pore complex protein Nup98-Nup96 (Fragment) OS=Homo sapiens GN=NUP98 PE=4 SV	86760	0	721	0	59	0	0.2	#DIV/0!	0	1	0	5	0	5	0	6	2.56328
tr|Q149P0|Q149P0_HUMAN	GBF1	GBF1 protein OS=Homo sapiens GN=GBF1 PE=2 SV=1	207938	0	647	0	66	0	0.06	#DIV/0!	0	3	0	4	0	5	0	6	2.56328
tr|Q96FS1|Q96FS1_HUMAN	CTNND1	CTNND1 protein (Fragment) OS=Homo sapiens GN=CTNND1 PE=2 SV=2	92901	0	593	0	73	0	0.1	#DIV/0!	0	1	0	3	0	5	0	6	2.56328
sp|Q5T4S7|UBR4_HUMAN	UBR4	E3 ubiquitin-protein ligase UBR4 OS=Homo sapiens GN=UBR4 PE=1 SV=1	580547	0	527	0	84	0	0.02	#DIV/0!	0	3	0	3	0	5	0	8	2.56328
tr|H0YCK3|H0YCK3_HUMAN	ADAR	Double-stranded RNA-specific adenosine deaminase (Fragment) OS=Homo sapiens GN=ADAR PE	133761	0	481	0	78	0	0.1	#DIV/0!	0	1	0	3	0	5	0	5	2.56328
tr|B4DZC0|B4DZC0_HUMAN	tr|B4DZC0|B	cDNA FLJ51771, highly similar to SWI/SNF-related matrix-associatedactin-dependent regulator c	117367	0	473	0	96	0	0.15	#DIV/0!	0	2	0	5	0	5	0	5	2.56328
sp|A0AVT1|UBA6_HUMAN	UBA6	Ubiquitin-like modifier-activating enzyme 6 OS=Homo sapiens GN=UBA6 PE=1 SV=1	119207	0	471	0	96	0	0.14	#DIV/0!	0	3	0	5	0	5	0	6	2.56328
tr|B2RE88|B2RE88_HUMAN	tr|B2RE88|B2	cDNA, FLJ96465, highly similar to Homo sapiens solute carrier family 25 (mitochondrial carrier;p	40379	0	443	0	102	0	0.37	#DIV/0!	0	2	0	4	0	5	0	5	2.56328
tr|B4DSN3|B4DSN3_HUMAN	tr|B4DSN3|B	cDNA FLJ60345, highly similar to Protein transport protein Sec24C OS=Homo sapiens PE=2 SV=1	107974	0	433	0	107	0	0.16	#DIV/0!	0	3	0	5	0	5	0	6	2.56328
tr|B4DUT7|B4DUT7_HUMAN	tr|B4DUT7|B	cDNA FLJ57604, highly similar to GMP synthase (glutamine-hydrolyzing) (EC 6.3.5.2) OS=Homo s	71880	0	411	0	102	0	0.28	#DIV/0!	0	2	0	5	0	5	0	5	2.56328
sp|Q5SRE5|NU188_HUMAN	NUP188	Nucleoporin NUP188 homolog OS=Homo sapiens GN=NUP188 PE=1 SV=1	198369	0	392	0	117	0	0.1	#DIV/0!	0	3	0	6	0	5	0	8	2.56328
tr|B3KVJ5|B3KVJ5_HUMAN	tr|B3KVJ5|B3	cDNA FLJ16648 fis, clone TESTI4035508, highly similar to Structural maintenance of chromosom	101354	0	371	0	107	0	0.19	#DIV/0!	0	4	0	5	0	5	0	6	2.56328
tr|B4DUF1|B4DUF1_HUMAN	tr|B4DUF1|B	cDNA FLJ59760, highly similar to 1,4-alpha-glucan branching enzyme (EC 2.4.1.18) OS=Homo sap	76254	0	358	0	85	0	0.16	#DIV/0!	0	2	0	3	0	5	0	5	2.56328
sp|P36952|SPB5_HUMAN	SERPINB5	Serpin B5 OS=Homo sapiens GN=SERPINB5 PE=1 SV=2	42530	0	320	0	140	0	0.35	#DIV/0!	0	4	0	4	0	5	0	6	2.56328
tr|Q59FA0|Q59FA0_HUMAN	tr|Q59FA0|Q	DNA polymerase (Fragment) OS=Homo sapiens PE=2 SV=1	113271	0	317	0	142	0	0.11	#DIV/0!	0	3	0	4	0	5	0	6	2.56328
tr|B3KPA6|B3KPA6_HUMAN	ACADVL	Acyl-Coenzyme A dehydrogenase, very long chain, isoform CRA_e OS=Homo sapiens GN=ACADV	62941	0	291	0	159	0	0.29	#DIV/0!	0	4	0	5	0	5	0	6	2.56328
tr|Q6NWZ1|Q6NWZ1_HUMAN	CKAP4	CKAP4 protein (Fragment) OS=Homo sapiens GN=CKAP4 PE=2 SV=1	67948	0	274	0	167	0	0.21	#DIV/0!	0	2	0	4	0	5	0	5	2.56328
sp|Q9H3U1|UN45A_HUMAN	UNC45A	Protein unc-45 homolog A OS=Homo sapiens GN=UNC45A PE=1 SV=1	104266	0	258	0	177	0	0.17	#DIV/0!	0	4	0	5	0	5	0	6	2.56328
sp|Q92945|FUBP2_HUMAN	KHSRP	Far upstream element-binding protein 2 OS=Homo sapiens GN=KHSRP PE=1 SV=4	73355	0	254	0	182	0	0.24	#DIV/0!	0	5	0	5	0	5	0	5	2.56328
tr|A8K4T6|A8K4T6_HUMAN	tr|A8K4T6|A	cDNA FLJ76282, highly similar to Homo sapiens proteasome (prosome, macropain) 26S subunit,	56588	0	248	0	185	0	0.33	#DIV/0!	0	4	0	5	0	5	0	6	2.56328
tr|B0AZM4|B0AZM4_HUMAN	tr|B0AZM4|B	cDNA, FLJ79464, highly similar to Homo sapiens aquarius (Fragment) OS=Homo sapiens PE=2 SV	157764	0	996	0	38	0	0.02	#DIV/0!	0	1	0	1	0	4	0	7	2.273593
sp|Q7L1Q6|BZW1_HUMAN	BZW1	Basic leucine zipper and W2 domain-containing protein 1 OS=Homo sapiens GN=BZW1 PE=1 SV	48184	0	985	0	39	0	0.22	#DIV/0!	0	2	0	3	0	4	0	5	2.273593
sp|Q8TCT9|HM13_HUMAN	HM13	Minor histocompatibility antigen H13 OS=Homo sapiens GN=HM13 PE=1 SV=1	41747	0	845	0	49	0	0.16	#DIV/0!	0	1	0	2	0	4	0	4	2.273593
tr|B4DEX7|B4DEX7_HUMAN	tr|B4DEX7|B	cDNA FLJ51806, highly similar to Homo sapiens proline-, glutamic acid-, leucine-rich protein 1 (P	97913	0	782	0	54	0	0.07	#DIV/0!	0	1	0	2	0	4	0	4	2.273593
tr|B4DR36|B4DR36_HUMAN	tr|B4DR36|B	cDNA FLJ56414, highly similar to Homo sapiens proline-, glutamic acid-, leucine-rich protein 1 (P	126101	0	711	0	60	0	0.08	#DIV/0!	0	1	0	3	0	4	0	4	2.273593
tr|B0QZ18|B0QZ18_HUMAN	CPNE1	Copine I OS=Homo sapiens GN=CPNE1 PE=2 SV=1	60307	0	693	0	62	0	0.17	#DIV/0!	0	2	0	3	0	4	0	4	2.273593
tr|B4DHY1|B4DHY1_HUMAN	HNRNPH3	Heterogeneous nuclear ribonucleoprotein H3 OS=Homo sapiens GN=HNRNPH3 PE=2 SV=1	24925	0	687	0	62	0	0.65	#DIV/0!	0	1	0	4	0	4	0	4	2.273593
tr|B0AZQ4|B0AZQ4_HUMAN	tr|B0AZQ4|B	cDNA, FLJ79494, highly similar to Structural maintenance of chromosome 3 OS=Homo sapiens P	141841	0	671	0	63	0	0.07	#DIV/0!	0	2	0	3	0	4	0	4	2.273593
tr|B4DZ87|B4DZ87_HUMAN	tr|B4DZ87|B	cDNA FLJ57240, highly similar to Mitochondrial proteins import receptor OS=Homo sapiens PE=	57579	0	664	0	64	0	0.25	#DIV/0!	0	2	0	4	0	4	0	4	2.273593
tr|F5H583|F5H583_HUMAN	CYFIP2	Cytoplasmic FMR1-interacting protein 2 OS=Homo sapiens GN=CYFIP2 PE=2 SV=1	138666	0	636	0	67	0	0.07	#DIV/0!	0	2	0	3	0	4	0	7	2.273593
tr|Q4KMR3|Q4KMR3_HUMAN	MYO1E	MYO1E variant protein OS=Homo sapiens GN=MYO1E PE=2 SV=1	127552	0	588	0	74	0	0.08	#DIV/0!	0	2	0	3	0	4	0	4	2.273593
tr|J3KTL8|J3KTL8_HUMAN	SMCHD1	Structural maintenance of chromosomes flexible hinge domain-containing protein 1 (Fragment)	156690	0	560	0	79	0	0.06	#DIV/0!	0	3	0	4	0	4	0	5	2.273593
tr|B4E206|B4E206_HUMAN	tr|B4E206|B4	cDNA FLJ55441, highly similar to Nuclear pore complex protein Nup133 OS=Homo sapiens PE=2	128349	0	535	0	83	0	0.11	#DIV/0!	0	2	0	4	0	4	0	6	2.273593
tr|B3KT00|B3KT00_HUMAN	tr|B3KT00|B3	cDNA FLJ37368 fis, clone BRAMY2024530, highly similar to Zinc phosphodiesterase ELAC protein	91169	0	531	0	84	0	0.15	#DIV/0!	0	4	0	4	0	4	0	7	2.273593
tr|J3KMX1|J3KMX1_HUMAN	NUP88	Nuclear pore complex protein Nup88 (Fragment) OS=Homo sapiens GN=NUP88 PE=4 SV=1	80029	0	486	0	94	0	0.12	#DIV/0!	0	2	0	3	0	4	0	6	2.273593
tr|A8KA19|A8KA19_HUMAN	tr|A8KA19|A	cDNA FLJ75831, highly similar to Homo sapiens exportin, tRNA (nuclear export receptor for tRN	111104	0	466	0	97	0	0.12	#DIV/0!	0	2	0	4	0	4	0	4	2.273593
tr|D3DUW5|D3DUW5_HUMAN	DNM1L	Dynamin 1-like, isoform CRA_c OS=Homo sapiens GN=DNM1L PE=3 SV=1	88442	0	462	0	98	0	0.12	#DIV/0!	0	2	0	3	0	4	0	4	2.273593
tr|H0YL11|H0YL11_HUMAN	IDH2	Isocitrate dehydrogenase [NADP], mitochondrial OS=Homo sapiens GN=IDH2 PE=3 SV=1	35536	0	458	0	95	0	0.23	#DIV/0!	0	2	0	2	0	4	0	5	2.273593
tr|B4DZP5|B4DZP5_HUMAN	tr|B4DZP5|B	cDNA FLJ51165, highly similar to DNA damage-binding protein 1 OS=Homo sapiens PE=2 SV=1	92726	0	444	0	96	0	0.11	#DIV/0!	0	2	0	3	0	4	0	5	2.273593
tr|Q59H95|Q59H95_HUMAN	tr|Q59H95|Q	Flightless I homolog variant (Fragment) OS=Homo sapiens PE=2 SV=1	126396	0	435	0	105	0	0.09	#DIV/0!	0	3	0	4	0	4	0	5	2.273593
tr|D3DTY9|D3DTY9_HUMAN	TRIM25	Tripartite motif-containing 25, isoform CRA_a OS=Homo sapiens GN=TRIM25 PE=2 SV=1	48358	0	417	0	110	0	0.39	#DIV/0!	0	4	0	5	0	4	0	5	2.273593
tr|B2RNR6|B2RNR6_HUMAN	ZFR	Zinc finger RNA binding protein OS=Homo sapiens GN=ZFR PE=2 SV=1	118081	0	386	0	121	0	0.12	#DIV/0!	0	3	0	4	0	4	0	4	2.273593
tr|G3V1J5|G3V1J5_HUMAN	DIS3	Exosome complex exonuclease RRP44 OS=Homo sapiens GN=DIS3 PE=2 SV=1	91382	0	385	0	122	0	0.09	#DIV/0!	0	3	0	3	0	4	0	4	2.273593
tr|A8K5Y7|A8K5Y7_HUMAN	tr|A8K5Y7|A	cDNA FLJ78655, highly similar to Homo sapiens exportin 5 (XPO5), mRNA OS=Homo sapiens PE=	138331	0	383	0	122	0	0.1	#DIV/0!	0	4	0	4	0	4	0	4	2.273593
tr|D2JYH5|D2JYH5_HUMAN	COL3A1	Collagen, type III, alpha 1 OS=Homo sapiens GN=COL3A1 PE=2 SV=1	139733	0	379	0	123	0	0.1	#DIV/0!	0	3	0	4	0	4	0	4	2.273593
tr|B4E063|B4E063_HUMAN	tr|B4E063|B4	cDNA FLJ54292, highly similar to Kinesin-like protein KIFC1 OS=Homo sapiens PE=2 SV=1	73571	0	356	0	128	0	0.19	#DIV/0!	0	4	0	4	0	4	0	6	2.273593
tr|B4DT31|B4DT31_HUMAN	FUBP1	Far upstream element-binding protein 1 OS=Homo sapiens GN=FUBP1 PE=2 SV=1	69993	0	352	0	129	0	0.15	#DIV/0!	0	2	0	3	0	4	0	5	2.273593
tr|F4NBX2|F4NBX2_HUMAN	HLA-B	MHC class I antigen (Fragment) OS=Homo sapiens GN=HLA-B PE=3 SV=1	38566	0	347	0	131	0	0.39	#DIV/0!	0	3	0	4	0	4	0	5	2.273593
sp|O43815|STRN_HUMAN	STRN	Striatin OS=Homo sapiens GN=STRN PE=1 SV=4	86535	0	342	0	132	0	0.12	#DIV/0!	0	2	0	3	0	4	0	4	2.273593
tr|B4DL79|B4DL79_HUMAN	KIF20A	Kinesin-like protein KIF20A OS=Homo sapiens GN=KIF20A PE=2 SV=1	99066	0	313	0	144	0	0.14	#DIV/0!	0	4	0	4	0	4	0	4	2.273593
tr|H0Y3P2|H0Y3P2_HUMAN	EIF4G2	Eukaryotic translation initiation factor 4 gamma 2 OS=Homo sapiens GN=EIF4G2 PE=4 SV=1	98569	0	303	0	149	0	0.14	#DIV/0!	0	3	0	4	0	4	0	5	2.273593
tr|G1UI16|G1UI16_HUMAN	PDS5A	SCC-112 protein, isoform CRA_b OS=Homo sapiens GN=PDS5A PE=2 SV=1	152274	0	282	0	163	0	0.07	#DIV/0!	0	3	0	3	0	4	0	5	2.273593
tr|B3KY63|B3KY63_HUMAN	tr|B3KY63|B3	cDNA FLJ16830 fis, clone UTERU3022536, highly similar to Chromodomain helicase-DNA-binding	216574	0	281	0	77	0	0.06	#DIV/0!	0	1	0	3	0	4	0	11	2.273593
tr|H3BLU7|H3BLU7_HUMAN	AKR7A2	Aflatoxin B1 aldehyde reductase member 2 (Fragment) OS=Homo sapiens GN=AKR7A2 PE=4 SV	35004	0	267	0	148	0	0.37	#DIV/0!	0	3	0	3	0	4	0	4	2.273593
tr|B4E1K0|B4E1K0_HUMAN	KIF23	Kinesin-like protein KIF23 OS=Homo sapiens GN=KIF23 PE=2 SV=1	78221	0	241	0	193	0	0.18	#DIV/0!	0	4	0	4	0	4	0	5	2.273593
tr|H0Y5W0|H0Y5W0_HUMAN	HUWE1	E3 ubiquitin-protein ligase HUWE1 (Fragment) OS=Homo sapiens GN=HUWE1 PE=4 SV=1	376696	0	218	0	199	0	0.03	#DIV/0!	0	4	0	4	0	4	0	7	2.273593
tr|B3KPF2|B3KPF2_HUMAN	tr|B3KPF2|B3	cDNA FLJ31717 fis, clone NT2RI2006640, highly similar to Dynamin-3 (EC 3.6.5.5) OS=Homo sapi	63057	0	1204	0	27	0	0.11	#DIV/0!	0	0	0	2	0	3	0	4	1.910843
tr|F6SBX2|F6SBX2_HUMAN	IARS2	Isoleucine--tRNA ligase, mitochondrial OS=Homo sapiens GN=IARS2 PE=2 SV=1	106884	0	1135	0	31	0	0.03	#DIV/0!	0	0	0	1	0	3	0	4	1.910843
tr|H0Y564|H0Y564_HUMAN	ANAPC1	Anaphase-promoting complex subunit 1 (Fragment) OS=Homo sapiens GN=ANAPC1 PE=4 SV=1	163055	0	1110	0	32	0	0.04	#DIV/0!	0	1	0	2	0	3	0	3	1.910843
sp|Q8IWB1|IPRI_HUMAN	ITPRIP	Inositol 1,4,5-trisphosphate receptor-interacting protein OS=Homo sapiens GN=ITPRIP PE=1 SV	62932	0	1033	0	36	0	0.05	#DIV/0!	0	1	0	1	0	3	0	4	1.910843
tr|Q8N7L7|Q8N7L7_HUMAN	tr|Q8N7L7|Q	cDNA FLJ40893 fis, clone UTERU2001607 OS=Homo sapiens PE=2 SV=1	102422	0	1024	0	37	0	0.1	#DIV/0!	0	1	0	3	0	3	0	4	1.910843

tr|B7Z964|B7Z964_HUMAN	SLMAP	Sarcolemmal membrane-associated protein OS=Homo sapiens GN=SLMAP PE=2 SV=1	42296	0	987	0	38	0	0.08	#DIV/0!	0	1	0	1	0	3	0	3	1.910843
tr|B4DFN6|B4DFN6_HUMAN	CNP	2~,3~-cyclic-nucleotide 3~-phosphodiesterase OS=Homo sapiens GN=CNP PE=2 SV=1	20461	0	933	0	43	0	0.16	#DIV/0!	0	1	0	1	0	3	0	4	1.910843
tr|Q6DCA8|Q6DCA8_HUMAN	BCLAF1	BCLAF1 protein (Fragment) OS=Homo sapiens GN=BCLAF1 PE=2 SV=1	77958	0	898	0	45	0	0.13	#DIV/0!	0	0	0	3	0	3	0	6	1.910843
tr|B3KMR5|B3KMR5_HUMAN	tr|B3KMR5|B	cDNA FLJ12434 fis, clone NT2RM1000037, highly similar to Homo sapiens KIAA0690 protein OS=	145007	0	855	0	48	0	0.07	#DIV/0!	0	3	0	3	0	3	0	3	1.910843
tr|B4E3M6|B4E3M6_HUMAN	tr|B4E3M6|B	cDNA FLJ55446, highly similar to Superkiller viralicidic activity 2-like 2 (EC 3.6.1.-) OS=Homo sapi	107714	0	853	0	48	0	0.09	#DIV/0!	0	1	0	3	0	3	0	3	1.910843
tr|Q05CN7|Q05CN7_HUMAN	GTF3C4	GTF3C4 protein (Fragment) OS=Homo sapiens GN=GTF3C4 PE=2 SV=1	63439	0	844	0	49	0	0.11	#DIV/0!	0	1	0	2	0	3	0	3	1.910843
tr|B4DN25|B4DN25_HUMAN	UGDH	UDP-glucose 6-dehydrogenase OS=Homo sapiens GN=UGDH PE=2 SV=1	44786	0	822	0	51	0	0.24	#DIV/0!	0	2	0	3	0	3	0	4	1.910843
tr|Q0VGA5|Q0VGA5_HUMAN	SARS	SARS protein OS=Homo sapiens GN=SARS PE=2 SV=1	58883	0	814	0	51	0	0.11	#DIV/0!	0	2	0	2	0	3	0	3	1.910843
tr|J3KNY6|J3KNY6_HUMAN	RGPD4	RanBP2-like and GRIP domain-containing protein 4 OS=Homo sapiens GN=RGPD4 PE=4 SV=1	199555	0	798	0	52	0	0.05	#DIV/0!	0	1	0	3	0	3	0	3	1.910843
tr|B3KM97|B3KM97_HUMAN	tr|B3KM97|B	cDNA FLJ10554 fis, clone NT2RP2002385, highly similar to Synaptic glycoprotein SC2 OS=Homo s	36429	0	795	0	53	0	0.09	#DIV/0!	0	1	0	1	0	3	0	4	1.910843
sp|Q14690|RRP5_HUMAN	PDCD11	Protein RRP5 homolog OS=Homo sapiens GN=PDCD11 PE=1 SV=3	209939	0	748	0	57	0	0.05	#DIV/0!	0	1	0	3	0	3	0	4	1.910843
tr|G3V3B0|G3V3B0_HUMAN	ACIN1	Apoptotic chromatin condensation inducer in the nucleus OS=Homo sapiens GN=ACIN1 PE=2 SV	67683	0	732	0	58	0	0.21	#DIV/0!	0	1	0	4	0	3	0	4	1.910843
tr|B4DIC4|B4DIC4_HUMAN	tr|B4DIC4|B4	cDNA FLJ52195, highly similar to LIM and SH3 domain protein 1 OS=Homo sapiens PE=2 SV=1	14987	0	731	0	58	0	0.51	#DIV/0!	0	1	0	2	0	3	0	3	1.910843
tr|K4DI93|K4DI93_HUMAN	CUL4B	Cullin 4B, isoform CRA_e OS=Homo sapiens GN=CUL4B PE=3 SV=1	103318	0	724	0	59	0	0.12	#DIV/0!	0	2	0	3	0	3	0	4	1.910843
tr|Q53G71|Q53G71_HUMAN	tr|Q53G71|Q	Calreticulin variant (Fragment) OS=Homo sapiens PE=2 SV=1	47061	0	719	0	59	0	0.23	#DIV/0!	0	1	0	3	0	3	0	4	1.910843
tr|B3KVA0|B3KVA0_HUMAN	tr|B3KVA0|B	Cytosine-specific methyltransferase OS=Homo sapiens PE=2 SV=1	61027	0	717	0	59	0	0.11	#DIV/0!	0	1	0	2	0	3	0	3	1.910843
sp|P35658|NU214_HUMAN	NUP214	Nuclear pore complex protein Nup214 OS=Homo sapiens GN=NUP214 PE=1 SV=2	214230	0	714	0	60	0	0.07	#DIV/0!	0	1	0	3	0	3	0	3	1.910843
tr|B4DL39|B4DL39_HUMAN	tr|B4DL39|B4	cDNA FLJ57316, highly similar to DNA mismatch repair protein Msh2 OS=Homo sapiens PE=2 SV	77368	0	709	0	60	0	0.09	#DIV/0!	0	1	0	2	0	3	0	3	1.910843
sp|Q2VIQ3|KIF4B_HUMAN	KIF4B	Chromosome-associated kinesin KIF4B OS=Homo sapiens GN=KIF4B PE=2 SV=2	141829	0	707	0	31	0	0.1	#DIV/0!	0	0	0	2	0	3	0	3	1.910843
tr|Q71UF1|Q71UF1_HUMAN	ACO2	Aconitase OS=Homo sapiens GN=ACO2 PE=2 SV=1	86304	0	699	0	61	0	0.12	#DIV/0!	0	1	0	3	0	3	0	4	1.910843
tr|A2A274|A2A274_HUMAN	ACO2	Aconitase 2, mitochondrial OS=Homo sapiens GN=ACO2 PE=4 SV=1	88563	0	698	0	61	0	0.12	#DIV/0!	0	1	0	3	0	3	0	4	1.910843
tr|Q2KQ72|Q2KQ72_HUMAN	tr|Q2KQ72|C	Chromosome-associated protein E OS=Homo sapiens PE=2 SV=1	86324	0	670	0	63	0	0.12	#DIV/0!	0	2	0	3	0	3	0	3	1.910843
tr|B4DL07|B4DL07_HUMAN	tr|B4DL07|B4	cDNA FLJ53353, highly similar to ATP-binding cassette sub-family D member 3 OS=Homo sapien	78980	0	661	0	64	0	0.13	#DIV/0!	0	1	0	3	0	3	0	5	1.910843
sp|O96008|TOM40_HUMAN	TOMM40	Mitochondrial import receptor subunit TOM40 homolog OS=Homo sapiens GN=TOMM40 PE=1	38211	0	659	0	64	0	0.18	#DIV/0!	0	2	0	2	0	3	0	3	1.910843
tr|G1UI17|G1UI17_HUMAN	AGL	Glycogen debranching enzyme (Fragment) OS=Homo sapiens GN=AGL PE=2 SV=1	145569	0	656	0	65	0	0.05	#DIV/0!	0	1	0	2	0	3	0	4	1.910843
sp|P52907|CAZA1_HUMAN	CAPZA1	F-actin-capping protein subunit alpha-1 OS=Homo sapiens GN=CAPZA1 PE=1 SV=3	33073	0	642	0	66	0	0.33	#DIV/0!	0	1	0	3	0	3	0	3	1.910843
tr|B7ZMF2|B7ZMF2_HUMAN	FANCI	Fanconi anemia, complementation group I OS=Homo sapiens GN=FANCI PE=2 SV=1	150724	0	630	0	68	0	0.08	#DIV/0!	0	3	0	3	0	3	0	4	1.910843
sp|Q96I24|FUBP3_HUMAN	FUBP3	Far upstream element-binding protein 3 OS=Homo sapiens GN=FUBP3 PE=1 SV=2	61944	0	625	0	69	0	0.17	#DIV/0!	0	3	0	3	0	3	0	3	1.910843
tr|D3DTD2|D3DTD2_HUMAN	TRIO	Triple functional domain (PTPRF interacting), isoform CRA_a OS=Homo sapiens GN=TRIO PE=2 S	270210	0	624	0	69	0	0.04	#DIV/0!	0	2	0	3	0	3	0	4	1.910843
tr|A8K6V7|A8K6V7_HUMAN	tr|A8K6V7|A	cDNA FLJ76053, highly similar to Homo sapiens Ras-GTPase activating protein SH3 domain-bindi	50842	0	623	0	69	0	0.21	#DIV/0!	0	1	0	3	0	3	0	4	1.910843
tr|B7ZLC9|B7ZLC9_HUMAN	GEMIN5	GEMIN5 protein OS=Homo sapiens GN=GEMIN5 PE=2 SV=1	170664	0	622	0	69	0	0.02	#DIV/0!	0	1	0	1	0	3	0	5	1.910843
tr|B2R6H7|B2R6H7_HUMAN	tr|B2R6H7|B	cDNA, FLJ92955, highly similar to Homo sapiens transportin-SR (TRN-SR), mRNA OS=Homo sapie	105989	0	582	0	74	0	0.06	#DIV/0!	0	2	0	2	0	3	0	5	1.910843
tr|B2RCV1|B2RCV1_HUMAN	tr|B2RCV1|B	cDNA, FLJ96310, highly similar to Homo sapiens solute carrier family 25 (mitochondrial carrier; a	33062	0	559	0	79	0	0.43	#DIV/0!	0	2	0	4	0	3	0	4	1.910843
tr|B3KQB4|B3KQB4_HUMAN	tr|B3KQB4|B	cDNA FLJ90131 fis, clone HEMBB1000447, highly similar to PRA1 family protein 3 OS=Homo sap	21540	0	536	0	83	0	0.34	#DIV/0!	0	1	0	2	0	3	0	3	1.910843
tr|H0Y9V7|H0Y9V7_HUMAN	ATP2C1	Calcium-transporting ATPase type 2C member 1 (Fragment) OS=Homo sapiens GN=ATP2C1 PE=	100184	0	508	0	87	0	0.1	#DIV/0!	0	2	0	3	0	3	0	3	1.910843
tr|B4DET0|B4DET0_HUMAN	tr|B4DET0|B4	cDNA FLJ55900, highly similar to Pre-mRNA-splicing factor ATP-dependent RNA helicase PRP16 (	140449	0	505	0	88	0	0.07	#DIV/0!	0	1	0	3	0	3	0	4	1.910843
sp|P42695|CNDD3_HUMAN	NCAPD3	Condensin-2 complex subunit D3 OS=Homo sapiens GN=NCAPD3 PE=1 SV=2	170950	0	494	0	91	0	0.08	#DIV/0!	0	2	0	4	0	3	0	4	1.910843
sp|Q96T76|MMS19_HUMAN	MMS19	MMS19 nucleotide excision repair protein homolog OS=Homo sapiens GN=MMS19 PE=1 SV=2	114928	0	493	0	91	0	0.09	#DIV/0!	0	2	0	3	0	3	0	5	1.910843
tr|Q56VW8|Q56VW8_HUMAN	tr|Q56VW8|(	Lung cancer-related protein 9 OS=Homo sapiens PE=2 SV=1	52186	0	490	0	93	0	0.2	#DIV/0!	0	1	0	3	0	3	0	3	1.910843
tr|Q0VAB1|Q0VAB1_HUMAN	TIMM50	Translocase of inner mitochondrial membrane 50 homolog (S. cerevisiae) OS=Homo sapiens GN	50960	0	437	0	105	0	0.21	#DIV/0!	0	3	0	3	0	3	0	3	1.910843
tr|B7Z871|B7Z871_HUMAN	tr|B7Z871|B7	cDNA FLJ50930, highly similar to Lipoma-preferred partner OS=Homo sapiens PE=2 SV=1	49854	0	431	0	101	0	0.21	#DIV/0!	0	3	0	3	0	3	0	3	1.910843
sp|P04899|GNAI2_HUMAN	GNAI2	Guanine nucleotide-binding protein G(i) subunit alpha-2 OS=Homo sapiens GN=GNAI2 PE=1 SV=	40995	0	427	0	108	0	0.26	#DIV/0!	0	3	0	3	0	3	0	3	1.910843
tr|E2GJL3|E2GJL3_HUMAN	HLA-B	MHC class I antigen (Fragment) OS=Homo sapiens GN=HLA-B PE=3 SV=1	21222	0	419	0	109	0	0.34	#DIV/0!	0	2	0	2	0	3	0	3	1.910843
tr|H0Y4L0|H0Y4L0_HUMAN	HLA-B	HLA class I histocompatibility antigen, B-35 alpha chain (Fragment) OS=Homo sapiens GN=HLA-E	26083	0	409	0	112	0	0.44	#DIV/0!	0	2	0	3	0	3	0	3	1.910843
tr|E3Q0X1|E3Q0X1_HUMAN	HLA-B	MHC class I antigen (Fragment) OS=Homo sapiens GN=HLA-B PE=3 SV=1	31939	0	406	0	112	0	0.35	#DIV/0!	0	2	0	3	0	3	0	4	1.910843
tr|H0YCK7|H0YCK7_HUMAN	EEF1D	Elongation factor 1-delta (Fragment) OS=Homo sapiens GN=EEF1D PE=4 SV=1	23105	0	401	0	115	0	0.72	#DIV/0!	0	3	0	4	0	3	0	4	1.910843
tr|G3XAL0|G3XAL0_HUMAN	MDH2	Malate dehydrogenase OS=Homo sapiens GN=MDH2 PE=2 SV=1	24921	0	400	0	61	0	0.48	#DIV/0!	0	2	0	2	0	3	0	3	1.910843
sp|P22059|OSBP1_HUMAN	OSBP	Oxysterol-binding protein 1 OS=Homo sapiens GN=OSBP PE=1 SV=1	90220	0	394	0	117	0	0.11	#DIV/0!	0	2	0	3	0	3	0	3	1.910843
sp|Q9H0A0|NAT10_HUMAN	NAT10	N-acetyltransferase 10 OS=Homo sapiens GN=NAT10 PE=1 SV=2	116569	0	387	0	102	0	0.16	#DIV/0!	0	2	0	2	0	3	0	4	1.910843
tr|B4DRW3|B4DRW3_HUMAN	tr|B4DRW3|E	cDNA FLJ57180, highly similar to TAR DNA-binding protein 43 OS=Homo sapiens PE=2 SV=1	35596	0	380	0	122	0	0.31	#DIV/0!	0	2	0	3	0	3	0	3	1.910843
tr|C0KK01|C0KK01_HUMAN	HLA-C	MHC class I antigen (Fragment) OS=Homo sapiens GN=HLA-C PE=3 SV=1	20940	0	357	0	128	0	0.56	#DIV/0!	0	3	0	3	0	3	0	3	1.910843
tr|H3BRG4|H3BRG4_HUMAN	UQCRC2	Cytochrome b-c1 complex subunit 2, mitochondrial OS=Homo sapiens GN=UQCRC2 PE=3 SV=1	44778	0	349	0	130	0	0.22	#DIV/0!	0	2	0	3	0	3	0	5	1.910843
tr|Q5VZA6|Q5VZA6_HUMAN	ACO1	Cytoplasmic aconitate hydratase OS=Homo sapiens GN=ACO1 PE=4 SV=1	48888	0	330	0	91	0	0.18	#DIV/0!	0	2	0	3	0	3	0	3	1.910843
tr|E9PG89|E9PG89_HUMAN	CHD3	Chromodomain-helicase-DNA-binding protein 3 OS=Homo sapiens GN=CHD3 PE=2 SV=1	234431	0	296	0	157	0	0.04	#DIV/0!	0	2	0	3	0	3	0	5	1.910843
tr|H3BQC4|H3BQC4_HUMAN	TGFB1I1	Transforming growth factor beta-1-induced transcript 1 protein (Fragment) OS=Homo sapiens G	23016	0	259	0	161	0	0.28	#DIV/0!	0	3	0	3	0	3	0	3	1.910843
tr|B4DRN6|B4DRN6_HUMAN	tr|B4DRN6|B	cDNA FLJ51284, highly similar to Adapter-relatedprotein complex 3 mu-1 subunit OS=Homo sap	41285	0	1299	0	22	0	0.08	#DIV/0!	0	0	0	1	0	2	0	2	1.425236
tr|B4E256|B4E256_HUMAN	tr|B4E256|B4	cDNA FLJ55348, highly similar to WD repeat protein 6 OS=Homo sapiens PE=2 SV=1	117990	0	1293	0	22	0	0.08	#DIV/0!	0	0	0	1	0	2	0	2	1.425236
tr|B3KN49|B3KN49_HUMAN	tr|B3KN49|B	cDNA FLJ13562 fis, clone PLACE1008080, highly similar to Homo sapiens hexamethylene bis-ace	40868	0	1255	0	24	0	0.08	#DIV/0!	0	0	0	1	0	2	0	2	1.425236
tr|Q2HJ12|Q2HJ12_HUMAN	CCDC125	CCDC125 protein OS=Homo sapiens GN=CCDC125 PE=4 SV=1	12142	0	1251	0	24	0	0.3	#DIV/0!	0	1	0	1	0	2	0	2	1.425236
tr|A8K8T0|A8K8T0_HUMAN	tr|A8K8T0|A	cDNA FLJ78760, highly similar to Homo sapiens integrin, alpha 11 (ITGA11), transcript variant 1,	134468	0	1236	0	25	0	0.02	#DIV/0!	0	0	0	1	0	2	0	4	1.425236
tr|J3KQM2|J3KQM2_HUMAN	ITGA11	Integrin alpha-11 OS=Homo sapiens GN=ITGA11 PE=3 SV=1	134626	0	1235	0	25	0	0.02	#DIV/0!	0	0	0	1	0	2	0	4	1.425236
tr|Q6ZMY0|Q6ZMY0_HUMAN	tr|Q6ZMY0|C	cDNA FLJ16598 fis, clone TESTI4006473, weakly similar to ATP-dependent RNA helicase A OS=H	152244	0	1232	0	25	0	0.02	#DIV/0!	0	0	0	1	0	2	0	2	1.425236
sp|Q99543|DNJC2_HUMAN	DNAJC2	DnaJ homolog subfamily C member 2 OS=Homo sapiens GN=DNAJC2 PE=1 SV=4	72465	0	1226	0	26	0	0.14	#DIV/0!	0	0	0	1	0	2	0	2	1.425236
sp|Q9NTI5|PDS5B_HUMAN	PDS5B	Sister chromatid cohesion protein PDS5 homolog B OS=Homo sapiens GN=PDS5B PE=1 SV=1	165818	0	1205	0	27	0	0.02	#DIV/0!	0	0	0	1	0	2	0	5	1.425236
sp|O76003|GLRX3_HUMAN	GLRX3	Glutaredoxin-3 OS=Homo sapiens GN=GLRX3 PE=1 SV=2	37693	0	1199	0	27	0	0.09	#DIV/0!	0	0	0	1	0	2	0	2	1.425236
sp|Q92466|DDB2_HUMAN	DDB2	DNA damage-binding protein 2 OS=Homo sapiens GN=DDB2 PE=1 SV=1	48461	0	1196	0	27	0	0.07	#DIV/0!	0	0	0	1	0	2	0	3	1.425236
tr|B4DKW2|B4DKW2_HUMAN	tr|B4DKW2|E	cDNA FLJ56001, highly similar to Putative ATP-dependent RNA helicase DHX57 (EC 3.6.1.-) (Frag	105507	0	1193	0	28	0	0.02	#DIV/0!	0	0	0	1	0	2	0	2	1.425236
sp|Q6P1J9|CDC73_HUMAN	CDC73	Parafibromin OS=Homo sapiens GN=CDC73 PE=1 SV=1	60653	0	1170	0	29	0	0.05	#DIV/0!	0	1	0	1	0	2	0	2	1.425236
sp|Q08499|PDE4D_HUMAN	PDE4D	cAMP-specific 3~,5~-cyclic phosphodiesterase 4D OS=Homo sapiens GN=PDE4D PE=1 SV=2	91628	0	1169	0	29	0	0.07	#DIV/0!	0	0	0	1	0	2	0	2	1.425236
tr|Q5T2B5|Q5T2B5_HUMAN	CUL2	Cullin-2 OS=Homo sapiens GN=CUL2 PE=3 SV=1	82933	0	1157	0	29	0	0.04	#DIV/0!	0	1	0	1	0	2	0	2	1.425236
sp|Q8N1G4|LRC47_HUMAN	LRRC47	Leucine-rich repeat-containing protein 47 OS=Homo sapiens GN=LRRC47 PE=1 SV=1	64004	0	1142	0	30	0	0.11	#DIV/0!	0	0	0	2	0	2	0	2	1.425236
sp|O60287|NPA1P_HUMAN	URB1	Nucleolar pre-ribosomal-associated protein 1 OS=Homo sapiens GN=URB1 PE=1 SV=4	256508	0	1120	0	31	0	0.01	#DIV/0!	0	1	0	1	0	2	0	3	1.425236
tr|B2RDI5|B2RDI5_HUMAN	tr|B2RDI5|B2	cDNA, FLJ96627, highly similar to Homo sapiens calpain 1, (mu/l) large subunit (CAPN1), mRNA (	82529	0	1117	0	31	0	0.04	#DIV/0!	0	0	0	1	0	2	0	2	1.425236
tr|G3XAI2|G3XAI2_HUMAN	LAMB1	Laminin subunit beta-1 OS=Homo sapiens GN=LAMB1 PE=2 SV=1	207703	0	1105	0	32	0	0.03	#DIV/0!	0	0	0	2	0	2	0	5	1.425236
tr|B3KT69|B3KT69_HUMAN	tr|B3KT69|B3	cDNA FLJ37759 fis, clone BRHIP2023888 OS=Homo sapiens PE=2 SV=1	100124	0	1095	0	33	0	0.03	#DIV/0!	0	0	0	1	0	2	0	2	1.425236
tr|E5KS55|E5KS55_HUMAN	tr|E5KS55|E5	Mitochondrial succinyl-CoA ligase [ADP-forming] subunit beta OS=Homo sapiens PE=3 SV=1	50641	0	1094	0	33	0	0.07	#DIV/0!	0	1	0	1	0	2	0	3	1.425236
tr|G5E9Z2|G5E9Z2_HUMAN	CLPTM1L	Cisplatin resistance related protein CRR9p, isoform CRA_c OS=Homo sapiens GN=CLPTM1L PE=2	43366	0	1091	0	33	0	0.16	#DIV/0!	0	0	0	2	0	2	0	2	1.425236
tr|A1A5C4|A1A5C4_HUMAN	RRBP1	RRBP1 protein OS=Homo sapiens GN=RRBP1 PE=2 SV=1	103162	0	1058	0	35	0	0.04	#DIV/0!	0	0	0	2	0	2	0	2	1.425236
tr|B3KM68|B3KM68_HUMAN	tr|B3KM68|B	cDNA FLJ10398 fis, clone NT2RM4000349, highly similar to Homo sapiens basic leucine zipper ar	48388	0	1056	0	35	0	0.15	#DIV/0!	0	2	0	2	0	2	0	3	1.425236
tr|B2R960|B2R960_HUMAN	tr|B2R960|B2	cDNA, FLJ94230, highly similar to Homo sapiens thioredoxin-like 1 (TXNL1), mRNA OS=Homo sap	32616	0	1051	0	35	0	0.21	#DIV/0!	0	0	0	2	0	2	0	3	1.425236
sp|P04792|HSPB1_HUMAN	HSPB1	Heat shock protein beta-1 OS=Homo sapiens GN=HSPB1 PE=1 SV=2	22826	0	1050	0	35	0	0.31	#DIV/0!	0	0	0	2	0	2	0	2	1.425236
sp|Q9BR76|COR1B_HUMAN	CORO1B	Coronin-1B OS=Homo sapiens GN=CORO1B PE=1 SV=1	54885	0	1047	0	36	0	0.19	#DIV/0!	0	1	0	3	0	2	0	3	1.425236
sp|Q9BTC0|DIDO1_HUMAN	DIDO1	Death-inducer obliterator 1 OS=Homo sapiens GN=DIDO1 PE=1 SV=5	245434	0	1043	0	36	0	0.03	#DIV/0!	0	0	0	2	0	2	0	3	1.425236
sp|Q5JTZ9|SYAM_HUMAN	AARS2	Alanine--tRNA ligase, mitochondrial OS=Homo sapiens GN=AARS2 PE=1 SV=1	108299	0	1039	0	36	0	0.03	#DIV/0!	0	1	0	1	0	2	0	3	1.425236
tr|Q59GM9|Q59GM9_HUMAN	tr|Q59GM9|(	Phosphorylase (Fragment) OS=Homo sapiens PE=2 SV=1	99508	0	1027	0	37	0	0.12	#DIV/0!	0	1	0	3	0	2	0	4	1.425236
tr|F5GWF8|F5GWF8_HUMAN	ATL3	Atlastin-3 (Fragment) OS=Homo sapiens GN=ATL3 PE=2 SV=1	18563	0	1025	0	37	0	0.18	#DIV/0!	0	1	0	1	0	2	0	2	1.425236
sp|P30837|AL1B1_HUMAN	ALDH1B1	Aldehyde dehydrogenase X, mitochondrial OS=Homo sapiens GN=ALDH1B1 PE=1 SV=3	57626	0	1022	0	37	0	0.29	#DIV/0!	0	0	0	2	0	2	0	2	1.425236
tr|Q58FF9|Q58FF9_HUMAN	HSP90Af	Heat shock protein 90Af OS=Homo sapiens GN=HSP90Af PE=4 SV=1	11851	0	1015	0	37	0	0.31	#DIV/0!	0	1	0	1	0	2	0	2	1.425236
sp|Q5TFE4|NT5D1_HUMAN	NT5DC1	5~-nucleotidase domain-containing protein 1 OS=Homo sapiens GN=NT5DC1 PE=1 SV=1	52439	0	1004	0	38	0	0.06	#DIV/0!	0	1	0	1	0	2	0	2	1.425236
sp|P61221|ABCE1_HUMAN	ABCE1	ATP-binding cassette sub-family E member 1 OS=Homo sapiens GN=ABCE1 PE=1 SV=1	68240	0	997	0	38	0	0.05	#DIV/0!	0	1	0	1	0	2	0	3	1.425236
tr|B3KQE8|B3KQE8_HUMAN	tr|B3KQE8|B	cDNA FLJ90350 fis, clone NT2RP2003210, highly similar to Long-chain fatty acid transport protei	43223	0	982	0	39	0	0.08	#DIV/0!	0	1	0	1	0	2	0	2	1.425236
sp|Q06210|GFPT1_HUMAN	GFPT1	Glutamine--fructose-6-phosphate aminotransferase [isomerizing] 1 OS=Homo sapiens GN=GFPT	79555	0	973	0	40	0	0.04	#DIV/0!	0	1	0	1	0	2	0	2	1.425236
sp|O75131|CPNE3_HUMAN	CPNE3	Copine-3 OS=Homo sapiens GN=CPNE3 PE=1 SV=1	60947	0	971	0	40	0	0.05	#DIV/0!	0	1	0	1	0	2	0	2	1.425236
tr|Q3LIF4|Q3LIF4_HUMAN	Nbla00271	NOGO-A (Fragment) OS=Homo sapiens GN=Nbla00271 PE=2 SV=1	57162	0	966	0	20	0	0.29	#DIV/0!	0	0	0	1	0	2	0	2	1.425236
tr|Q53R94|Q53R94_HUMAN	RTN4	Putative uncharacterized protein RTN4 (Fragment) OS=Homo sapiens GN=RTN4 PE=2 SV=1	19288	0	960	0	40	0	0.38	#DIV/0!	0	0	0	2	0	2	0	2	1.425236
tr|F5H619|F5H619_HUMAN	HEATR5A	HEAT repeat-containing protein 5A OS=Homo sapiens GN=HEATR5A PE=2 SV=1	225926	0	954	0	41	0	0.03	#DIV/0!	0	1	0	2	0	2	0	2	1.425236
sp|Q9UKX7|NUP50_HUMAN	NUP50	Nuclear pore complex protein Nup50 OS=Homo sapiens GN=NUP50 PE=1 SV=2	50512	0	940	0	42	0	0.13	#DIV/0!	0	0	0	2	0	2	0	4	1.425236
tr|J3QSB4|J3QSB4_HUMAN	RPL13	60S ribosomal protein L13 (Fragment) OS=Homo sapiens GN=RPL13 PE=3 SV=1	14740	0	939	0	42	0	0.23	#DIV/0!	0	1	0	1	0	2	0	2	1.425236
tr|Q5W7F7|Q5W7F7_HUMAN	UBE3A	Ubiquitin ligase E3A isoform 1 OS=Homo sapiens GN=UBE3A PE=2 SV=1	98751	0	929	0	43	0	0.03	#DIV/0!	0	1	0	1	0	2	0	3	1.425236
tr|G3V1K3|G3V1K3_HUMAN	PON2	Paraoxonase 2, isoform CRA_a OS=Homo sapiens GN=PON2 PE=2 SV=1	41672	0	925	0	43	0	0.08	#DIV/0!	0	1	0	1	0	2	0	2	1.425236
tr|Q59ES2|Q59ES2_HUMAN	tr|Q59ES2|Q	Inositol 1,4,5-trisphosphate receptor type 3 variant (Fragment) OS=Homo sapiens PE=2 SV=1	269361	0	884	0	46	0	0.04	#DIV/0!	0	1	0	3	0	2	0	6	1.425236
sp|Q9NRG9|AAAS_HUMAN	AAAS	Aladin OS=Homo sapiens GN=AAAS PE=1 SV=1	60392	0	880	0	46	0	0.11	#DIV/0!	0	1	0	2	0	2	0	2	1.425236
tr|Q6ZMG7|Q6ZMG7_HUMAN	ATP13A1	cDNA FLJ23939 fis, clone HEP01940, highly similar to Cation-transporting ATPase 2 (EC 3.6.3.-) C	109818	0	875	0	47	0	0.03	#DIV/0!	0	1	0	1	0	2	0	2	1.425236
tr|B2R5Y4|B2R5Y4_HUMAN	tr|B2R5Y4|B2	cDNA, FLJ92684, highly similar to Homo sapiens IK cytokine, down-regulator of HLA II (IK), mRNA	65749	0	867	0	47	0	0.1	#DIV/0!	0	1	0	2	0	2	0	2	1.425236
tr|B4DYY5|B4DYY5_HUMAN	tr|B4DYY5|B4	cDNA FLJ55671, highly similar to Ubiquitin-associated protein 2-like OS=Homo sapiens PE=2 SV=	113662	0	860	0	48	0	0.06	#DIV/0!	0	1	0	2	0	2	0	2	1.425236
sp|Q5K651|SAMD9_HUMAN	SAMD9	Sterile alpha motif domain-containing protein 9 OS=Homo sapiens GN=SAMD9 PE=1 SV=1	185704	0	856	0	48	0	0.04	#DIV/0!	0	1	0	2	0	2	0	3	1.425236
sp|Q9P253|VPS18_HUMAN	VPS18	Vacuolar protein sorting-associated protein 18 homolog OS=Homo sapiens GN=VPS18 PE=1 SV=	111484	0	838	0	49	0	0.06	#DIV/0!	0	1	0	2	0	2	0	2	1.425236
sp|Q9H9A6|LRC40_HUMAN	LRRC40	Leucine-rich repeat-containing protein 40 OS=Homo sapiens GN=LRRC40 PE=1 SV=1	68720	0	835	0	50	0	0.1	#DIV/0!	0	1	0	2	0	2	0	2	1.425236
tr|J9JID7|J9JID7_HUMAN	LMNB2	Lamin B2, isoform CRA_a OS=Homo sapiens GN=LMNB2 PE=3 SV=1	70020	0	824	0	51	0	0.05	#DIV/0!	0	1	0	1	0	2	0	2	1.425236
tr|B0AZP8|B0AZP8_HUMAN	tr|B0AZP8|B(	cDNA, FLJ79488, highly similar to Homo sapiens solute carrier family 43, member 3 (SLC43A3), r	55033	0	823	0	51	0	0.12	#DIV/0!	0	1	0	2	0	2	0	2	1.425236
tr|B2R825|B2R825_HUMAN	tr|B2R825|B2	Phosphorylase OS=Homo sapiens PE=2 SV=1	97405	0	818	0	51	0	0.07	#DIV/0!	0	1	0	2	0	2	0	4	1.425236
tr|B1AZV3|B1AZV3_HUMAN	TAP1	Antigen peptide transporter 1 OS=Homo sapiens GN=TAP1 PE=4 SV=1	87691	0	815	0	51	0	0.04	#DIV/0!	0	1	0	1	0	2	0	2	1.425236
sp|Q9UI26|IPO11_HUMAN	IPO11	Importin-11 OS=Homo sapiens GN=IPO11 PE=1 SV=1	113489	0	810	0	52	0	0.03	#DIV/0!	0	1	0	1	0	2	0	3	1.425236
tr|E7ERK9|E7ERK9_HUMAN	EIF2B4	Translation initiation factor eIF-2B subunit delta OS=Homo sapiens GN=EIF2B4 PE=2 SV=1	60248	0	808	0	52	0	0.11	#DIV/0!	0	1	0	2	0	2	0	2	1.425236
sp|Q9BXW9|FACD2_HUMAN	FANCD2	Fanconi anemia group D2 protein OS=Homo sapiens GN=FANCD2 PE=1 SV=1	168295	0	803	0	52	0	0.04	#DIV/0!	0	2	0	2	0	2	0	2	1.425236
sp|Q6PKG0|LARP1_HUMAN	LARP1	La-related protein 1 OS=Homo sapiens GN=LARP1 PE=1 SV=2	123833	0	797	0	52	0	0.05	#DIV/0!	0	1	0	2	0	2	0	3	1.425236
tr|B4DM41|B4DM41_HUMAN	RFC4	Replication factor C (Activator 1) 4, 37kDa, isoform CRA_b OS=Homo sapiens GN=RFC4 PE=2 SV	33996	0	790	0	53	0	0.2	#DIV/0!	0	1	0	2	0	2	0	2	1.425236
sp|Q8IXI1|MIRO2_HUMAN	RHOT2	Mitochondrial Rho GTPase 2 OS=Homo sapiens GN=RHOT2 PE=1 SV=2	69101	0	789	0	53	0	0.31	#DIV/0!	0	2	0	2	0	2	0	2	1.425236
tr|A8MUD9|A8MUD9_HUMAN	RPL7	60S ribosomal protein L7 OS=Homo sapiens GN=RPL7 PE=3 SV=1	24474	0	786	0	53	0	0.14	#DIV/0!	0	1	0	1	0	2	0	3	1.425236
tr|B2RB95|B2RB95_HUMAN	tr|B2RB95|B2	cDNA, FLJ95381, Homo sapiens tripartite motif-containing 16 (TRIM16), mRNA OS=Homo sapien	64884	0	781	0	54	0	0.1	#DIV/0!	0	1	0	2	0	2	0	3	1.425236
tr|B4E3W1|B4E3W1_HUMAN	tr|B4E3W1|B	cDNA FLJ57204, highly similar to Homo sapiens ancient ubiquitous protein 1 (AUP1), transcript v	51668	0	772	0	55	0	0.13	#DIV/0!	0	1	0	2	0	2	0	2	1.425236
tr|B7Z2B4|B7Z2B4_HUMAN	RABGAP1	Rab GTPase-activating protein 1 OS=Homo sapiens GN=RABGAP1 PE=2 SV=1	48660	0	771	0	55	0	0.14	#DIV/0!	0	2	0	2	0	2	0	2	1.425236
sp|Q9HD26|GOPC_HUMAN	GOPC	Golgi-associated PDZ and coiled-coil motif-containing protein OS=Homo sapiens GN=GOPC PE=1	50888	0	757	0	56	0	0.13	#DIV/0!	0	1	0	2	0	2	0	3	1.425236
sp|Q14008|CKAP5_HUMAN	CKAP5	Cytoskeleton-associated protein 5 OS=Homo sapiens GN=CKAP5 PE=1 SV=3	227062	0	737	0	58	0	0.03	#DIV/0!	0	1	0	1	0	2	0	2	1.425236
tr|A0MNN5|A0MNN5_HUMAN	GRWD1	CDW4/GRWD1 OS=Homo sapiens GN=GRWD1 PE=2 SV=1	49787	0	733	0	58	0	0.07	#DIV/0!	0	1	0	1	0	2	0	3	1.425236
tr|E9PHM2|E9PHM2_HUMAN	LARS2	Probable leucine--tRNA ligase, mitochondrial OS=Homo sapiens GN=LARS2 PE=2 SV=1	97782	0	728	0	58	0	0.07	#DIV/0!	0	1	0	2	0	2	0	3	1.425236
tr|Q7Z586|Q7Z586_HUMAN	tr|Q7Z586|Q	Putative uncharacterized protein (Fragment) OS=Homo sapiens PE=2 SV=1	105456	0	725	0	58	0	0.03	#DIV/0!	0	1	0	1	0	2	0	2	1.425236
tr|A8K8N3|A8K8N3_HUMAN	tr|A8K8N3|A	cDNA FLJ78740, highly similar to Homo sapiens sperm associated antigen 5 (SPAG5), mRNA OS=	135680	0	723	0	59	0	0.05	#DIV/0!	0	2	0	2	0	2	0	2	1.425236
sp|Q27J81|INF2_HUMAN	INF2	Inverted formin-2 OS=Homo sapiens GN=INF2 PE=1 SV=2	136851	0	722	0	59	0	0.05	#DIV/0!	0	2	0	2	0	2	0	2	1.425236
tr|Q96F88|Q96F88_HUMAN	POP1	Processing of 1, ribonuclease P/MRP subunit (S. cerevisiae) OS=Homo sapiens GN=POP1 PE=2 S\	116318	0	716	0	59	0	0.06	#DIV/0!	0	1	0	2	0	2	0	3	1.425236
tr|B4DJV2|B4DJV2_HUMAN	CS	Citrate synthase OS=Homo sapiens GN=CS PE=2 SV=1	50628	0	715	0	60	0	0.13	#DIV/0!	0	1	0	2	0	2	0	3	1.425236
tr|B4DG47|B4DG47_HUMAN	tr|B4DG47|B	cDNA FLJ56967, highly similar to Adapter-relatedprotein complex 1 beta-1 subunit OS=Homo sa	54387	0	710	0	60	0	0.12	#DIV/0!	0	2	0	2	0	2	0	2	1.425236
tr|B4DTM6|B4DTM6_HUMAN	tr|B4DTM6|E	cDNA FLJ61391, highly similar to Protein transport protein Sec24B OS=Homo sapiens PE=2 SV=1	130037	0	708	0	44	0	0.1	#DIV/0!	0	0	0	2	0	2	0	2	1.425236
tr|B4DE85|B4DE85_HUMAN	PTDSS1	Phosphatidylserine synthase 1 OS=Homo sapiens GN=PTDSS1 PE=2 SV=1	35127	0	697	0	61	0	0.2	#DIV/0!	0	2	0	2	0	2	0	2	1.425236
tr|Q6FG59|Q6FG59_HUMAN	CDC37	CDC37 protein OS=Homo sapiens GN=CDC37 PE=2 SV=1	44938	0	696	0	62	0	0.63	#DIV/0!	0	1	0	2	0	2	0	2	1.425236
tr|B2RB90|B2RB90_HUMAN	tr|B2RB90|B2	cDNA, FLJ95375, highly similar to Homo sapiens glutamine-fructose-6-phosphate transaminase	77651	0	695	0	62	0	0.09	#DIV/0!	0	2	0	2	0	2	0	2	1.425236
sp|Q00653|NFKB2_HUMAN	NFKB2	Nuclear factor NF-kappa-B p100 subunit OS=Homo sapiens GN=NFKB2 PE=1 SV=4	97373	0	694	0	62	0	0.03	#DIV/0!	0	1	0	1	0	2	0	2	1.425236
tr|A0JLQ5|A0JLQ5_HUMAN	BXDC2	BXDC2 protein (Fragment) OS=Homo sapiens GN=BXDC2 PE=2 SV=1	39430	0	691	0	0	0	0.08	#DIV/0!	0	0	0	0	0	2	0	2	1.425236
sp|Q8ND04|SMG8_HUMAN	SMG8	Protein SMG8 OS=Homo sapiens GN=SMG8 PE=1 SV=1	110983	0	685	0	62	0	0.06	#DIV/0!	0	1	0	2	0	2	0	2	1.425236
tr|K7EIR0|K7EIR0_HUMAN	WDR18	WD repeat-containing protein 18 OS=Homo sapiens GN=WDR18 PE=4 SV=1	45436	0	684	0	62	0	0.15	#DIV/0!	0	2	0	2	0	2	0	2	1.425236
sp|P78357|CNTP1_HUMAN	CNTNAP1	Contactin-associated protein 1 OS=Homo sapiens GN=CNTNAP1 PE=1 SV=1	158220	0	683	0	62	0	0.02	#DIV/0!	0	1	0	1	0	2	0	2	1.425236
sp|P49419|AL7A1_HUMAN	ALDH7A1	Alpha-aminoadipic semialdehyde dehydrogenase OS=Homo sapiens GN=ALDH7A1 PE=1 SV=5	59020	0	679	0	63	0	0.11	#DIV/0!	0	1	0	2	0	2	0	2	1.425236
tr|Q6AHZ7|Q6AHZ7_HUMAN	DKFZp686A11	Putative uncharacterized protein DKFZp686A111 OS=Homo sapiens GN=DKFZp686A111 PE=2 SV	240988	0	667	0	64	0	0.03	#DIV/0!	0	1	0	2	0	2	0	3	1.425236
sp|Q9BW60|ELOV1_HUMAN	ELOVL1	Elongation of very long chain fatty acids protein 1 OS=Homo sapiens GN=ELOVL1 PE=1 SV=1	32755	0	662	0	64	0	0.21	#DIV/0!	0	2	0	2	0	2	0	2	1.425236
tr|B4DDH8|B4DDH8_HUMAN	tr|B4DDH8|B	cDNA FLJ55184, highly similar to Homo sapiens leukocyte receptor cluster (LRC) member 4 (LEN	51214	0	660	0	64	0	0.13	#DIV/0!	0	1	0	2	0	2	0	2	1.425236
tr|B2RB52|B2RB52_HUMAN	tr|B2RB52|B2	cDNA, FLJ95314, highly similar to Homo sapiens transducin (beta)-like 2 (TBL2), transcript variar	50423	0	654	0	65	0	0.07	#DIV/0!	0	1	0	1	0	2	0	2	1.425236
tr|B3KQH2|B3KQH2_HUMAN	tr|B3KQH2|B	cDNA FLJ90454 fis, clone NT2RP3001560, highly similar to Cleft lip and palate transmembrane p	76247	0	652	0	65	0	0.09	#DIV/0!	0	1	0	2	0	2	0	2	1.425236
tr|D3DU41|D3DU41_HUMAN	LUC7L	LUC7-like (S. cerevisiae), isoform CRA_a OS=Homo sapiens GN=LUC7L PE=2 SV=1	34110	0	649	0	65	0	0.32	#DIV/0!	0	2	0	3	0	2	0	4	1.425236
sp|Q8NI27|THOC2_HUMAN	THOC2	THO complex subunit 2 OS=Homo sapiens GN=THOC2 PE=1 SV=2	184541	0	645	0	66	0	0.07	#DIV/0!	0	2	0	2	0	2	0	2	1.425236
tr|B4DPV3|B4DPV3_HUMAN	tr|B4DPV3|B	cDNA FLJ58109, highly similar to Protein diaphanous homolog 3 (Fragment) OS=Homo sapiens F	117640	0	644	0	66	0	0.06	#DIV/0!	0	1	0	2	0	2	0	3	1.425236
tr|B4DRK5|B4DRK5_HUMAN	tr|B4DRK5|B	cDNA FLJ59584, highly similar to Mitochondrial-processing peptidase alpha subunit, mitochondr	28622	0	643	0	66	0	0.12	#DIV/0!	0	2	0	2	0	2	0	2	1.425236
tr|A4D210|A4D210_HUMAN	EIF3S9	Eukaryotic translation initiation factor 3, subunit 9 eta, 116kDa OS=Homo sapiens GN=EIF3S9 PE	89024	0	640	0	67	0	0.07	#DIV/0!	0	1	0	2	0	2	0	3	1.425236
tr|B3KMV5|B3KMV5_HUMAN	tr|B3KMV5|E	cDNA FLJ12728 fis, clone NT2RP2000040, highly similar to Protein FAM62A OS=Homo sapiens P	123342	0	638	0	67	0	0.05	#DIV/0!	0	1	0	2	0	2	0	4	1.425236
tr|B8ZZ09|B8ZZ09_HUMAN	LUC7L	LUC7-like (S. cerevisiae) (Fragment) OS=Homo sapiens GN=LUC7L PE=4 SV=1	9530	0	631	0	68	0	0.86	#DIV/0!	0	2	0	2	0	2	0	2	1.425236
tr|F5H6D0|F5H6D0_HUMAN	MOGS	Mannosyl-oligosaccharide glucosidase OS=Homo sapiens GN=MOGS PE=2 SV=1	80824	0	620	0	69	0	0.07	#DIV/0!	0	1	0	2	0	2	0	3	1.425236
tr|B4DMU7|B4DMU7_HUMAN	tr|B4DMU7|I	cDNA FLJ59351, highly similar to Striatin-4 OS=Homo sapiens PE=2 SV=1	69271	0	619	0	69	0	0.1	#DIV/0!	0	2	0	2	0	2	0	2	1.425236
sp|Q9Y295|DRG1_HUMAN	DRG1	Developmentally-regulated GTP-binding protein 1 OS=Homo sapiens GN=DRG1 PE=1 SV=1	40802	0	617	0	69	0	0.17	#DIV/0!	0	2	0	2	0	2	0	2	1.425236
sp|Q9H2M9|RBGPR_HUMAN	RAB3GAP2	Rab3 GTPase-activating protein non-catalytic subunit OS=Homo sapiens GN=RAB3GAP2 PE=1 SV	157482	0	614	0	70	0	0.09	#DIV/0!	0	1	0	2	0	2	0	2	1.425236
sp|O00154|BACH_HUMAN	ACOT7	Cytosolic acyl coenzyme A thioester hydrolase OS=Homo sapiens GN=ACOT7 PE=1 SV=3	42454	0	612	0	70	0	0.22	#DIV/0!	0	1	0	2	0	2	0	2	1.425236
sp|P49454|CENPF_HUMAN	CENPF	Centromere protein F OS=Homo sapiens GN=CENPF PE=1 SV=2	370844	0	607	0	71	0	0.02	#DIV/0!	0	1	0	2	0	2	0	4	1.425236
tr|A6QKW0|A6QKW0_HUMAN	SHINC3	SHINC3 OS=Homo sapiens GN=SHINC3 PE=2 SV=1	28275	0	606	0	71	0	0.12	#DIV/0!	0	1	0	1	0	2	0	2	1.425236
tr|G5EA03|G5EA03_HUMAN	LIMCH1	LIM and calponin homology domains-containing protein 1 OS=Homo sapiens GN=LIMCH1 PE=2	165820	0	602	0	72	0	0.15	#DIV/0!	0	2	0	2	0	2	0	3	1.425236
sp|Q16222|UAP1_HUMAN	UAP1	UDP-N-acetylhexosamine pyrophosphorylase OS=Homo sapiens GN=UAP1 PE=1 SV=3	59131	0	597	0	72	0	0.06	#DIV/0!	0	1	0	1	0	2	0	2	1.425236
tr|B4E3G4|B4E3G4_HUMAN	tr|B4E3G4|B	cDNA FLJ50383, highly similar to CRSP complex subunit 3 OS=Homo sapiens PE=2 SV=1	116828	0	583	0	74	0	0.06	#DIV/0!	0	2	0	2	0	2	0	3	1.425236
tr|F8W9B5|F8W9B5_HUMAN	PRC1	Protein regulator of cytokinesis 1 OS=Homo sapiens GN=PRC1 PE=2 SV=1	66790	0	579	0	75	0	0.1	#DIV/0!	0	2	0	2	0	2	0	6	1.425236
tr|Q2TAM5|Q2TAM5_HUMAN	RELA	RELA protein OS=Homo sapiens GN=RELA PE=2 SV=1	43355	0	571	0	77	0	0.16	#DIV/0!	0	1	0	2	0	2	0	2	1.425236
tr|B3KRR1|B3KRR1_HUMAN	tr|B3KRR1|B	cDNA FLJ34725 fis, clone MESAN2005958, highly similar to RNA-binding protein Luc7-like 2 OS=	40752	0	565	0	58	0	0.31	#DIV/0!	0	2	0	3	0	2	0	4	1.425236
tr|J3KQS3|J3KQS3_HUMAN	PNPLA6	Neuropathy target esterase OS=Homo sapiens GN=PNPLA6 PE=4 SV=1	152341	0	562	0	79	0	0.04	#DIV/0!	0	2	0	2	0	2	0	2	1.425236
tr|A0PJC9|A0PJC9_HUMAN	TPR	TPR protein (Fragment) OS=Homo sapiens GN=TPR PE=2 SV=1	53894	0	561	0	43	0	0.06	#DIV/0!	0	1	0	2	0	2	0	3	1.425236
tr|A6NNK5|A6NNK5_HUMAN	TP53BP1	Tumor suppressor p53-binding protein 1 OS=Homo sapiens GN=TP53BP1 PE=2 SV=2	210886	0	554	0	79	0	0.02	#DIV/0!	0	1	0	1	0	2	0	2	1.425236
tr|A0PJ61|A0PJ61_HUMAN	RSL1D1	RSL1D1 protein (Fragment) OS=Homo sapiens GN=RSL1D1 PE=2 SV=1	49688	0	547	0	81	0	0.07	#DIV/0!	0	1	0	1	0	2	0	3	1.425236
tr|B7Z4B8|B7Z4B8_HUMAN	HNRNPUL1	Heterogeneous nuclear ribonucleoprotein U-like protein 1 OS=Homo sapiens GN=HNRNPUL1 PE	86639	0	543	0	82	0	0.07	#DIV/0!	0	2	0	2	0	2	0	3	1.425236
tr|B4DHS5|B4DHS5_HUMAN	tr|B4DHS5|B	cDNA FLJ54678, highly similar to N(2),N(2)-dimethylguanosine tRNA methyltransferase (EC 2.1.1	59256	0	540	0	82	0	0.11	#DIV/0!	0	2	0	2	0	2	0	2	1.425236
sp|Q08379|GOGA2_HUMAN	GOLGA2	Golgin subfamily A member 2 OS=Homo sapiens GN=GOLGA2 PE=1 SV=3	113644	0	533	0	84	0	0.06	#DIV/0!	0	2	0	2	0	2	0	3	1.425236
tr|B2R858|B2R858_HUMAN	tr|B2R858|B2	cDNA, FLJ93750, Homo sapiens DEAD (Asp-Glu-Ala-Asp) box polypeptide 6 (DDX6), mRNA OS=H	53582	0	523	0	85	0	0.13	#DIV/0!	0	2	0	2	0	2	0	2	1.425236
tr|Q9MY63|Q9MY63_HUMAN	HLA-Cw	Leucocyte antigen (Fragment) OS=Homo sapiens GN=HLA-Cw PE=3 SV=1	26578	0	522	0	85	0	0.27	#DIV/0!	0	2	0	2	0	2	0	2	1.425236
tr|B4DIT7|B4DIT7_HUMAN	TGM2	Protein-glutamine gamma-glutamyltransferase 2 OS=Homo sapiens GN=TGM2 PE=2 SV=1	69632	0	521	0	85	0	0.1	#DIV/0!	0	2	0	2	0	2	0	3	1.425236
sp|Q92626|PXDN_HUMAN	PXDN	Peroxidasin homolog OS=Homo sapiens GN=PXDN PE=1 SV=2	167793	0	519	0	86	0	0.04	#DIV/0!	0	2	0	2	0	2	0	2	1.425236
tr|B4E1L0|B4E1L0_HUMAN	tr|B4E1L0|B4	Adenylosuccinate synthetase OS=Homo sapiens PE=2 SV=1	48308	0	515	0	86	0	0.14	#DIV/0!	0	2	0	2	0	2	0	2	1.425236

sp|Q5JVF3|PCID2_HUMAN	PCID2	PCI domain-containing protein 2 OS=Homo sapiens GN=PCID2 PE=1 SV=2	46627	0	509	0	87	0	0.15	#DIV/0!	0	2	0	2	0	2	0	2	1.425236
sp|Q9BWF3|RBM4_HUMAN	RBM4	RNA-binding protein 4 OS=Homo sapiens GN=RBM4 PE=1 SV=1	40688	0	507	0	87	0	0.18	#DIV/0!	0	1	0	2	0	2	0	2	1.425236
tr|Q6FHJ5|Q6FHJ5_HUMAN	SCAMP3	SCAMP3 protein (Fragment) OS=Homo sapiens GN=SCAMP3 PE=2 SV=1	38674	0	496	0	90	0	0.18	#DIV/0!	0	2	0	2	0	2	0	2	1.425236
tr|H0YN14|H0YN14_HUMAN	IPO4	Importin-4 OS=Homo sapiens GN=IPO4 PE=4 SV=1	103193	0	483	0	94	0	0.06	#DIV/0!	0	2	0	2	0	2	0	2	1.425236
sp|P40222|TXLNA_HUMAN	TXLNA	Alpha-taxilin OS=Homo sapiens GN=TXLNA PE=1 SV=3	62195	0	476	0	95	0	0.11	#DIV/0!	0	1	0	2	0	2	0	2	1.425236
tr|H0YEL7|H0YEL7_HUMAN	ACAT1	Acetyl-CoA acetyltransferase, mitochondrial (Fragment) OS=Homo sapiens GN=ACAT1 PE=3 SV=	14377	0	465	0	63	0	0.43	#DIV/0!	0	1	0	1	0	2	0	2	1.425236
tr|A8K0D2|A8K0D2_HUMAN	tr|A8K0D2|A	cDNA FLJ77740, highly similar to Homo sapiens 7-dehydrocholesterol reductase, mRNA OS=Hom	55175	0	463	0	98	0	0.12	#DIV/0!	0	2	0	2	0	2	0	2	1.425236
sp|P09960|LKHA4_HUMAN	LTA4H	Leukotriene A-4 hydrolase OS=Homo sapiens GN=LTA4H PE=1 SV=2	69868	0	460	0	99	0	0.16	#DIV/0!	0	2	0	3	0	2	0	3	1.425236
tr|E0WMV4|E0WMV4_HUMA	HLA-C	MHC class I antigen (Fragment) OS=Homo sapiens GN=HLA-C PE=3 SV=1	31992	0	451	0	101	0	0.22	#DIV/0!	0	2	0	2	0	2	0	2	1.425236
tr|Q6FHX6|Q6FHX6_HUMAN	FEN1	Flap endonuclease 1 OS=Homo sapiens GN=FEN1 PE=2 SV=1	42908	0	430	0	107	0	0.4	#DIV/0!	0	2	0	2	0	2	0	2	1.425236
tr|B4DM01|B4DM01_HUMAN	SKIV2L	Helicase SKI2W OS=Homo sapiens GN=SKIV2L PE=2 SV=1	121269	0	425	0	108	0	0.05	#DIV/0!	0	2	0	2	0	2	0	4	1.425236
tr|G1ENK8|G1ENK8_HUMAN	HLA-B	MHC class I antigen (Fragment) OS=Homo sapiens GN=HLA-B PE=3 SV=1	21236	0	421	0	109	0	0.34	#DIV/0!	0	2	0	2	0	2	0	2	1.425236
tr|G0KXF8|G0KXF8_HUMAN	HLA-B	MHC class I antigen (Fragment) OS=Homo sapiens GN=HLA-B PE=3 SV=1	21235	0	420	0	109	0	0.34	#DIV/0!	0	2	0	2	0	2	0	2	1.425236
tr|Q548W1|Q548W1_HUMAN	OGT	HRNT1 OS=Homo sapiens GN=OGT PE=2 SV=1	104029	0	412	0	111	0	0.06	#DIV/0!	0	2	0	2	0	2	0	4	1.425236
tr|H0YHX9|H0YHX9_HUMAN	NACA	Nascent polypeptide-associated complex subunit alpha (Fragment) OS=Homo sapiens GN=NACA	22930	0	408	0	112	0	0.31	#DIV/0!	0	2	0	2	0	2	0	2	1.425236
tr|B1AK51|B1AK51_HUMAN	CHD5	Uncharacterized protein OS=Homo sapiens GN=CHD5 PE=4 SV=1	136766	0	398	0	115	0	0.05	#DIV/0!	0	1	0	2	0	2	0	4	1.425236
tr|E7EMC7|E7EMC7_HUMAN	SQSTM1	Sequestosome-1 OS=Homo sapiens GN=SQSTM1 PE=2 SV=1	42070	0	397	0	51	0	0.35	#DIV/0!	0	1	0	1	0	2	0	2	1.425236
sp|Q9HC07|TM165_HUMAN	TMEM165	Transmembrane protein 165 OS=Homo sapiens GN=TMEM165 PE=1 SV=1	35055	0	390	0	119	0	0.45	#DIV/0!	0	2	0	2	0	2	0	2	1.425236
tr|B4DSP0|B4DSP0_HUMAN	hCG_1642748	HCG1642748, isoform CRA_b OS=Homo sapiens GN=hCG_1642748 PE=2 SV=1	45559	0	359	0	128	0	0.15	#DIV/0!	0	2	0	2	0	2	0	2	1.425236
tr|A7E2Y5|A7E2Y5_HUMAN	DNAJC13	DnaJ (Hsp40) homolog, subfamily C, member 13 OS=Homo sapiens GN=DNAJC13 PE=2 SV=1	256572	0	328	0	137	0	0.03	#DIV/0!	0	2	0	2	0	2	0	3	1.425236
sp|O43837|IDH3B_HUMAN	IDH3B	Isocitrate dehydrogenase [NAD] subunit beta, mitochondrial OS=Homo sapiens GN=IDH3B PE=1	42442	0	324	0	139	0	0.16	#DIV/0!	0	2	0	2	0	2	0	2	1.425236
sp|Q7RTS9|DYM_HUMAN	DYM	Dymeclin OS=Homo sapiens GN=DYM PE=1 SV=1	76571	0	1353	0	16	0	0.04	#DIV/0!	0	0	0	1	0	1	0	2	0.68809
tr|B4E3I3|B4E3I3_HUMAN	tr|B4E3I3|B4	cDNA FLJ59614, highly similar to Ubiquitin fusion degradation protein 1 homolog OS=Homo sap	35730	0	1334	0	19	0	0.09	#DIV/0!	0	0	0	1	0	1	0	2	0.68809
tr|H0Y8D1|H0Y8D1_HUMAN	PRSS1	Trypsin-1 (Fragment) OS=Homo sapiens GN=PRSS1 PE=3 SV=1	15697	0	1332	0	0	0	0.12	#DIV/0!	0	0	0	0	0	1	0	1	0.68809
tr|B4DYF0|B4DYF0_HUMAN	tr|B4DYF0|B4	cDNA FLJ61469, highly similar to Zinc finger protein 694 OS=Homo sapiens PE=2 SV=1	89134	0	1320	0	20	0	0.04	#DIV/0!	0	0	0	1	0	1	0	1	0.68809
tr|Q5IRN4|Q5IRN4_HUMAN	MEF2D/DAZA	Myocyte enhancer factor 2D/deleted in azoospermia associated protein 1 fusion protein OS=Ho	50610	0	1316	0	21	0	0.07	#DIV/0!	0	0	0	1	0	1	0	1	0.68809
sp|Q9H488|OFUT1_HUMAN	POFUT1	GDP-fucose protein O-fucosyltransferase 1 OS=Homo sapiens GN=POFUT1 PE=1 SV=1	44383	0	1311	0	21	0	0.07	#DIV/0!	0	0	0	1	0	1	0	1	0.68809
tr|A4D0W4|A4D0W4_HUMAN	AASS	Aminoadipate-semialdehyde synthase OS=Homo sapiens GN=AASS PE=2 SV=1	102808	0	1310	0	21	0	0.03	#DIV/0!	0	0	0	1	0	1	0	1	0.68809
tr|Q2TAM4|Q2TAM4_HUMAN	POLR1B	DNA-directed RNA polymerase OS=Homo sapiens GN=POLR1B PE=2 SV=1	108996	0	1309	0	21	0	0.03	#DIV/0!	0	0	0	1	0	1	0	1	0.68809
tr|B4DJK4|B4DJK4_HUMAN	tr|B4DJK4|B4	cDNA FLJ54005, highly similar to Transcription elongation factor SPT5 OS=Homo sapiens PE=2 S	97196	0	1308	0	21	0	0.03	#DIV/0!	0	0	0	1	0	1	0	1	0.68809
sp|Q8NB90|SPAT5_HUMAN	SPATA5	Spermatogenesis-associated protein 5 OS=Homo sapiens GN=SPATA5 PE=1 SV=3	98470	0	1306	0	21	0	0.03	#DIV/0!	0	0	0	1	0	1	0	2	0.68809
sp|Q9BVI4|NOC4L_HUMAN	NOC4L	Nucleolar complex protein 4 homolog OS=Homo sapiens GN=NOC4L PE=1 SV=1	58830	0	1305	0	21	0	0.22	#DIV/0!	0	0	0	2	0	1	0	2	0.68809
sp|Q86XP3|DDX42_HUMAN	DDX42	ATP-dependent RNA helicase DDX42 OS=Homo sapiens GN=DDX42 PE=1 SV=1	103197	0	1303	0	21	0	0.05	#DIV/0!	0	0	0	1	0	1	0	1	0.68809
tr|B4DTX5|B4DTX5_HUMAN	tr|B4DTX5|B4	cDNA FLJ60072, highly similar to Homo sapiens sorbin and SH3 domain containing 1 (SORBS1), t	84033	0	1302	0	21	0	0.12	#DIV/0!	0	0	0	1	0	1	0	1	0.68809
tr|Q5TZP7|Q5TZP7_HUMAN	APEX1	APEX nuclease (Multifunctional DNA repair enzyme) 1 OS=Homo sapiens GN=APEX1 PE=2 SV=1	35931	0	1301	0	21	0	0.17	#DIV/0!	0	0	0	1	0	1	0	2	0.68809
tr|Q2TU84|Q2TU84_HUMAN	GIG18	Aspartate aminotransferase OS=Homo sapiens GN=GIG18 PE=2 SV=1	46519	0	1296	0	22	0	0.07	#DIV/0!	0	0	0	1	0	1	0	1	0.68809
sp|Q6IA86|ELP2_HUMAN	ELP2	Elongator complex protein 2 OS=Homo sapiens GN=ELP2 PE=1 SV=2	94266	0	1295	0	22	0	0.03	#DIV/0!	0	0	0	1	0	1	0	1	0.68809
tr|Q3B768|Q3B768_HUMAN	KIAA0406	KIAA0406 protein (Fragment) OS=Homo sapiens GN=KIAA0406 PE=2 SV=1	91343	0	1294	0	22	0	0.04	#DIV/0!	0	0	0	1	0	1	0	1	0.68809
tr|B4DI19|B4DI19_HUMAN	tr|B4DI19|B4	cDNA FLJ59524, highly similar to Cysteinyl-tRNA synthetase (EC 6.1.1.16) OS=Homo sapiens PE=	76209	0	1289	0	22	0	0.09	#DIV/0!	0	0	0	2	0	1	0	2	0.68809
sp|P41214|EIF2D_HUMAN	EIF2D	Eukaryotic translation initiation factor 2D OS=Homo sapiens GN=EIF2D PE=1 SV=3	65293	0	1285	0	23	0	0.07	#DIV/0!	0	0	0	1	0	1	0	1	0.68809
tr|Q9H003|Q9H003_HUMAN	ABC-transpor	Putative ATP-binding cassette protein (Fragment) OS=Homo sapiens GN=ABC-transporter PE=2	12105	0	1278	0	23	0	0.28	#DIV/0!	0	0	0	1	0	1	0	1	0.68809
tr|B4DH29|B4DH29_HUMAN	tr|B4DH29|B	DNA-directed RNA polymerase OS=Homo sapiens PE=2 SV=1	126564	0	1276	0	23	0	0.03	#DIV/0!	0	0	0	1	0	1	0	1	0.68809
tr|B2RB57|B2RB57_HUMAN	tr|B2RB57|B	cDNA, FLJ95321, highly similar to Homo sapiens ATG7 autophagy related 7 homolog (S. cerevisia	79076	0	1275	0	23	0	0.04	#DIV/0!	0	0	0	1	0	1	0	1	0.68809
tr|H0YIK5|H0YIK5_HUMAN	RACGAP1	Rac GTPase-activating protein 1 (Fragment) OS=Homo sapiens GN=RACGAP1 PE=4 SV=1	31466	0	1274	0	23	0	0.11	#DIV/0!	0	0	0	1	0	1	0	1	0.68809
tr|B2R806|B2R806_HUMAN	tr|B2R806|B	Eukaryotic translation initiation factor 3 subunit E OS=Homo sapiens PE=2 SV=1	52622	0	1273	0	23	0	0.2	#DIV/0!	0	1	0	3	0	1	0	3	0.68809
tr|Q53HG6|Q53HG6_HUMAN	tr|Q53HG6|Q	Cysteine-tRNA ligase isoform a variant (Fragment) OS=Homo sapiens PE=2 SV=1	83493	0	1269	0	23	0	0.04	#DIV/0!	0	0	0	1	0	1	0	1	0.68809
sp|P55268|LAMB2_HUMAN	LAMB2	Laminin subunit beta-2 OS=Homo sapiens GN=LAMB2 PE=1 SV=2	202982	0	1267	0	24	0	0.02	#DIV/0!	0	0	0	1	0	1	0	2	0.68809
tr|B2R7R5|B2R7R5_HUMAN	tr|B2R7R5|B	cDNA, FLJ93570, highly similar to Homo sapiens phosphoribosyl pyrophosphate synthetase-asso	41256	0	1266	0	24	0	0.12	#DIV/0!	0	0	0	1	0	1	0	1	0.68809
sp|Q9H4L5|OSBL3_HUMAN	OSBPL3	Oxysterol-binding protein-related protein 3 OS=Homo sapiens GN=OSBPL3 PE=1 SV=1	102130	0	1265	0	24	0	0.03	#DIV/0!	0	0	0	1	0	1	0	1	0.68809
tr|B4DV28|B4DV28_HUMAN	tr|B4DV28|B	cDNA FLJ54170, highly similar to Cytosolic nonspecific dipeptidase OS=Homo sapiens PE=2 SV=1	51811	0	1264	0	24	0	0.08	#DIV/0!	0	0	0	1	0	1	0	1	0.68809
tr|G0XQ39|G0XQ39_HUMAN	STIM1	STIM1L OS=Homo sapiens GN=STIM1 PE=2 SV=1	88933	0	1263	0	24	0	0.04	#DIV/0!	0	0	0	1	0	1	0	2	0.68809
sp|Q9UBG0|MRC2_HUMAN	MRC2	C-type mannose receptor 2 OS=Homo sapiens GN=MRC2 PE=1 SV=2	169761	0	1262	0	24	0	0.02	#DIV/0!	0	0	0	1	0	1	0	1	0.68809
sp|Q96ST3|SIN3A_HUMAN	SIN3A	Paired amphipathic helix protein Sin3a OS=Homo sapiens GN=SIN3A PE=1 SV=2	145883	0	1260	0	24	0	0.02	#DIV/0!	0	0	0	1	0	1	0	1	0.68809
tr|G3V0G3|G3V0G3_HUMAN	DDX56	DEAD (Asp-Glu-Ala-Asp) box polypeptide 56, isoform CRA_a OS=Homo sapiens GN=DDX56 PE=2	41969	0	1259	0	24	0	0.08	#DIV/0!	0	0	0	1	0	1	0	1	0.68809
tr|B3KW56|B3KW56_HUMAN	EIF3S6	Eukaryotic translation initiation factor 3 subunit E OS=Homo sapiens GN=EIF3S6 PE=2 SV=1	46815	0	1258	0	24	0	0.15	#DIV/0!	0	1	0	2	0	1	0	2	0.68809
tr|Q53G42|Q53G42_HUMAN	tr|Q53G42|Q	mRNA decapping enzyme variant (Fragment) OS=Homo sapiens PE=2 SV=1	38757	0	1257	0	0	0	0.09	#DIV/0!	0	0	0	0	0	1	0	1	0.68809
sp|B4DYI2|S31C2_HUMAN	SPATA31C2	Spermatogenesis-associated protein 31C2 OS=Homo sapiens GN=SPATA31C2 PE=2 SV=2	125606	0	1250	0	24	0	0.03	#DIV/0!	0	0	0	1	0	1	0	1	0.68809
tr|C9JDR0|C9JDR0_HUMAN	NSDHL	Sterol-4-alpha-carboxylate 3-dehydrogenase, decarboxylating (Fragment) OS=Homo sapiens GN	28356	0	1249	0	24	0	0.12	#DIV/0!	0	0	0	1	0	1	0	2	0.68809
tr|B0AZU2|B0AZU2_HUMAN	KIAA0930	Uncharacterized protein KIAA0930 OS=Homo sapiens GN=KIAA0930 PE=2 SV=1	43985	0	1248	0	24	0	0.08	#DIV/0!	0	0	0	1	0	1	0	1	0.68809
tr|B2RDV7|B2RDV7_HUMAN	tr|B2RDV7|B	cDNA, FLJ96789, highly similar to Homo sapiens dihydrouridine synthase 3-like (S. cerevisiae) (D	73714	0	1246	0	25	0	0.04	#DIV/0!	0	0	0	1	0	1	0	1	0.68809
tr|B2RBM7|B2RBM7_HUMAN	tr|B2RBM7|B	cDNA, FLJ95595, highly similar to Homo sapiens proteasome (prosome, macropain) 26S subunit	43131	0	1245	0	25	0	0.08	#DIV/0!	0	0	0	1	0	1	0	1	0.68809
sp|Q04941|PLP2_HUMAN	PLP2	Proteolipid protein 2 OS=Homo sapiens GN=PLP2 PE=1 SV=1	17022	0	1242	0	25	0	0.2	#DIV/0!	0	0	0	1	0	1	0	1	0.68809
tr|B4E2K4|B4E2K4_HUMAN	tr|B4E2K4|B4	cDNA FLJ54576, highly similar to Aspartyl/asparaginyl beta-hydroxylase (EC 1.14.11.16) OS=Hom	83685	0	1241	0	25	0	0.08	#DIV/0!	0	0	0	2	0	1	0	2	0.68809
tr|A8K4W5|A8K4W5_HUMAN	tr|A8K4W5|A	cDNA FLJ76813, highly similar to Homo sapiens acetyl-Coenzyme A acetyltransferase 2 (acetoac	41866	0	1240	0	25	0	0.07	#DIV/0!	0	0	0	1	0	1	0	1	0.68809
tr|B3KNK9|B3KNK9_HUMAN	GOLPH2	Golgi phosphoprotein 2, isoform CRA_a OS=Homo sapiens GN=GOLPH2 PE=2 SV=1	45477	0	1238	0	0	0	0.07	#DIV/0!	0	0	0	0	0	1	0	1	0.68809
tr|B3KUP9|B3KUP9_HUMAN	tr|B3KUP9|B	cDNA FLJ40361 fis, clone TESTI2034407, highly similar to NEDD8-activating enzyme E1 regulator	60951	0	1234	0	25	0	0.05	#DIV/0!	0	0	0	1	0	1	0	1	0.68809
tr|G3V529|G3V529_HUMAN	DDX24	ATP-dependent RNA helicase DDX24 OS=Homo sapiens GN=DDX24 PE=2 SV=1	91994	0	1233	0	25	0	0.04	#DIV/0!	0	1	0	1	0	1	0	1	0.68809
tr|G5E988|G5E988_HUMAN	AP3D1	AP-3 complex subunit delta-1 OS=Homo sapiens GN=AP3D1 PE=2 SV=1	126074	0	1231	0	0	0	0.03	#DIV/0!	0	0	0	0	0	1	0	1	0.68809
tr|B7Z475|B7Z475_HUMAN	tr|B7Z475|B7	cDNA FLJ55712, highly similar to F-box-like/WD repeat protein TBL1XR1 OS=Homo sapiens PE=2	41301	0	1230	0	26	0	0.08	#DIV/0!	0	0	0	1	0	1	0	1	0.68809
tr|B4DJQ5|B4DJQ5_HUMAN	tr|B4DJQ5|B4	cDNA FLJ59211, highly similar to Glucosidase 2 subunit beta OS=Homo sapiens PE=2 SV=1	61066	0	1227	0	26	0	0.16	#DIV/0!	0	0	0	1	0	1	0	1	0.68809
sp|P35555|FBN1_HUMAN	FBN1	Fibrillin-1 OS=Homo sapiens GN=FBN1 PE=1 SV=3	332664	0	1222	0	26	0	0.01	#DIV/0!	0	0	0	1	0	1	0	1	0.68809
tr|Q6FGM0|Q6FGM0_HUMAN	SH3GL1	SH3 domain GRB2-like 1 OS=Homo sapiens GN=SH3GL1 PE=2 SV=1	41692	0	1220	0	26	0	0.09	#DIV/0!	0	0	0	1	0	1	0	1	0.68809
tr|E5RHK2|E5RHK2_HUMAN	ASPH	Aspartyl/asparaginyl beta-hydroxylase (Fragment) OS=Homo sapiens GN=ASPH PE=2 SV=1	15282	0	1217	0	26	0	0.22	#DIV/0!	0	0	0	1	0	1	0	1	0.68809
tr|G5EA36|G5EA36_HUMAN	CDC27	Cell division cycle 27, isoform CRA_c OS=Homo sapiens GN=CDC27 PE=2 SV=1	92764	0	1216	0	26	0	0.3	#DIV/0!	0	0	0	1	0	1	0	1	0.68809
tr|B3KM47|B3KM47_HUMAN	tr|B3KM47|B	cDNA FLJ10273 fis, clone HEMBB1001137, highly similar to SEC23-interacting protein OS=Homo	111730	0	1215	0	26	0	0.03	#DIV/0!	0	0	0	1	0	1	0	1	0.68809
tr|Q5T8U5|Q5T8U5_HUMAN	SURF4	Surfeit 4 OS=Homo sapiens GN=SURF4 PE=4 SV=1	21342	0	1213	0	27	0	0.16	#DIV/0!	0	0	0	1	0	1	0	1	0.68809
tr|A4D275|A4D275_HUMAN	ARPC1B	Actin related protein 2/3 complex, subunit 1B, 41kDa OS=Homo sapiens GN=ARPC1B PE=2 SV=1	41722	0	1212	0	27	0	0.08	#DIV/0!	0	0	0	1	0	1	0	1	0.68809
tr|Q2VIM1|Q2VIM1_HUMAN	tr|Q2VIM1|Q	RcCDC20 (Fragment) OS=Homo sapiens PE=2 SV=1	50517	0	1211	0	27	0	0.07	#DIV/0!	0	0	0	1	0	1	0	1	0.68809
sp|Q9NUL7|DDX28_HUMAN	DDX28	Probable ATP-dependent RNA helicase DDX28 OS=Homo sapiens GN=DDX28 PE=1 SV=2	59773	0	1209	0	27	0	0.06	#DIV/0!	0	0	0	1	0	1	0	1	0.68809
tr|B4DN44|B4DN44_HUMAN	tr|B4DN44|B	cDNA FLJ53581, highly similar to Ras and Rab interactor 1 OS=Homo sapiens PE=2 SV=1	65995	0	1206	0	27	0	0.05	#DIV/0!	0	1	0	1	0	1	0	1	0.68809
tr|A0JLN5|A0JLN5_HUMAN	SEC62	SEC62 protein (Fragment) OS=Homo sapiens GN=SEC62 PE=2 SV=1	20031	0	1198	0	27	0	0.17	#DIV/0!	0	0	0	1	0	1	0	1	0.68809
tr|G3V1L9|G3V1L9_HUMAN	TJP1	Tight junction protein 1 (Zona occludens 1), isoform CRA_a OS=Homo sapiens GN=TJP1 PE=4 SV	197681	0	1192	0	28	0	0.02	#DIV/0!	0	0	0	1	0	1	0	2	0.68809
tr|J3KSW6|J3KSW6_HUMAN	DHX40	Probable ATP-dependent RNA helicase DHX40 (Fragment) OS=Homo sapiens GN=DHX40 PE=4 S	30276	0	1186	0	28	0	0.11	#DIV/0!	0	1	0	1	0	1	0	1	0.68809
tr|Q4ZG72|Q4ZG72_HUMAN	DDX18	Putative uncharacterized protein DDX18 (Fragment) OS=Homo sapiens GN=DDX18 PE=2 SV=1	61898	0	1185	0	28	0	0.05	#DIV/0!	0	0	0	1	0	1	0	2	0.68809
sp|A1L0T0|ILVBL_HUMAN	ILVBL	Acetolactate synthase-like protein OS=Homo sapiens GN=ILVBL PE=1 SV=2	68452	0	1184	0	28	0	0.05	#DIV/0!	0	0	0	1	0	1	0	1	0.68809
tr|B2RAK1|B2RAK1_HUMAN	tr|B2RAK1|B	cDNA, FLJ94965, highly similar to Homo sapiens leucyl/cystinyl aminopeptidase (LNPEP), mRNA	117783	0	1181	0	28	0	0.03	#DIV/0!	0	0	0	1	0	1	0	2	0.68809
sp|Q13057|COASY_HUMAN	COASY	Bifunctional coenzyme A synthase OS=Homo sapiens GN=COASY PE=1 SV=4	62632	0	1177	0	28	0	0.11	#DIV/0!	0	1	0	2	0	1	0	2	0.68809
tr|B2RBM8|B2RBM8_HUMAN	tr|B2RBM8|B	cDNA, FLJ95596, highly similar to Homo sapiens activity-dependent neuroprotector (ADNP), mR	124850	0	1173	0	29	0	0.03	#DIV/0!	0	0	0	1	0	1	0	2	0.68809
tr|B4DYP7|B4DYP7_HUMAN	tr|B4DYP7|B4	cDNA FLJ55435, highly similar to Gamma-tubulin complex component 3 OS=Homo sapiens PE=2	103306	0	1172	0	29	0	0.03	#DIV/0!	0	0	0	1	0	1	0	2	0.68809
tr|B3KU09|B3KU09_HUMAN	tr|B3KU09|B	cDNA FLJ39034 fis, clone NT2RP7008085, highly similar to Homo sapiens ring finger protein 123	149906	0	1171	0	29	0	0.02	#DIV/0!	0	1	0	1	0	1	0	1	0.68809
tr|J3KRI4|J3KRI4_HUMAN	DYNC1LI2	Cytoplasmic dynein 1 light intermediate chain 2 (Fragment) OS=Homo sapiens GN=DYNC1LI2 PE	17767	0	1168	0	29	0	0.19	#DIV/0!	0	0	0	1	0	1	0	1	0.68809
tr|A8K2I7|A8K2I7_HUMAN	tr|A8K2I7|A8	cDNA FLJ76072, highly similar to Homo sapiens GIPC PDZ domain containing family, member 1 (	36131	0	1166	0	29	0	0.24	#DIV/0!	0	0	0	1	0	1	0	1	0.68809
tr|J3QRS9|J3QRS9_HUMAN	ZNF207	Zinc finger protein 207 OS=Homo sapiens GN=ZNF207 PE=4 SV=1	52849	0	1165	0	29	0	0.16	#DIV/0!	0	0	0	1	0	1	0	1	0.68809
tr|A0JLU4|A0JLU4_HUMAN	YBX1	YBX1 protein (Fragment) OS=Homo sapiens GN=YBX1 PE=2 SV=1	21417	0	1164	0	29	0	0.34	#DIV/0!	0	0	0	2	0	1	0	2	0.68809
tr|B3KU23|B3KU23_HUMAN	tr|B3KU23|B	cDNA FLJ39097 fis, clone NTONG2000977, highly similar to Neurolysin, mitochondrial (EC 3.4.24	70320	0	1163	0	29	0	0.07	#DIV/0!	0	0	0	1	0	1	0	1	0.68809
sp|Q96IJ6|GMPPA_HUMAN	GMPPA	Mannose-1-phosphate guanyltransferase alpha OS=Homo sapiens GN=GMPPA PE=1 SV=1	46604	0	1162	0	29	0	0.07	#DIV/0!	0	0	0	1	0	1	0	1	0.68809
tr|Q2M1V9|Q2M1V9_HUMAN	BTAF1	BTAF1 RNA polymerase II, B-TFIID transcription factor-associated, 170kDa OS=Homo sapiens GN	209037	0	1161	0	29	0	0.02	#DIV/0!	0	0	0	1	0	1	0	2	0.68809
tr|A4FVA8|A4FVA8_HUMAN	DFNA5	DFNA5 protein (Fragment) OS=Homo sapiens GN=DFNA5 PE=2 SV=1	48389	0	1159	0	29	0	0.07	#DIV/0!	0	0	0	1	0	1	0	1	0.68809
tr|B4DEM6|B4DEM6_HUMAN	TOE1	Target of EGR1 protein 1 OS=Homo sapiens GN=TOE1 PE=2 SV=1	48544	0	1156	0	30	0	0.07	#DIV/0!	0	1	0	1	0	1	0	1	0.68809
sp|Q9NQH7|XPP3_HUMAN	XPNPEP3	Probable Xaa-Pro aminopeptidase 3 OS=Homo sapiens GN=XPNPEP3 PE=1 SV=1	57624	0	1152	0	30	0	0.06	#DIV/0!	0	0	0	1	0	1	0	1	0.68809
sp|O76024|WFS1_HUMAN	WFS1	Wolframin OS=Homo sapiens GN=WFS1 PE=1 SV=2	101367	0	1151	0	30	0	0.03	#DIV/0!	0	0	0	1	0	1	0	1	0.68809
tr|B4DFD7|B4DFD7_HUMAN	tr|B4DFD7|B	cDNA FLJ55457, highly similar to ATP-dependent RNA helicase DHX8 (EC 3.6.1.-) OS=Homo sapie	98244	0	1149	0	30	0	0.03	#DIV/0!	0	0	0	1	0	1	0	3	0.68809
tr|B4DN01|B4DN01_HUMAN	tr|B4DN01|B	cDNA FLJ57332, highly similar to COP9 signalosome complex subunit 3 OS=Homo sapiens PE=2 S	33950	0	1148	0	30	0	0.1	#DIV/0!	0	1	0	1	0	1	0	1	0.68809
tr|F5GZS0|F5GZS0_HUMAN	DHX36	Probable ATP-dependent RNA helicase DHX36 OS=Homo sapiens GN=DHX36 PE=2 SV=1	113928	0	1146	0	30	0	0.03	#DIV/0!	0	0	0	1	0	1	0	2	0.68809
tr|B7Z6H4|B7Z6H4_HUMAN	tr|B7Z6H4|B7	DNA-directed RNA polymerase OS=Homo sapiens PE=2 SV=1	155407	0	1145	0	30	0	0.02	#DIV/0!	0	0	0	1	0	1	0	2	0.68809
tr|B4DFM1|B4DFM1_HUMAN	tr|B4DFM1|B	cDNA FLJ53034, highly similar to AP-2 complex subunit mu-1 OS=Homo sapiens PE=2 SV=1	44226	0	1141	0	0	0	0.11	#DIV/0!	0	0	0	0	0	1	0	1	0.68809
sp|Q5F1R6|DJC21_HUMAN	DNAJC21	DnaJ homolog subfamily C member 21 OS=Homo sapiens GN=DNAJC21 PE=1 SV=2	62445	0	1140	0	30	0	0.05	#DIV/0!	0	0	0	1	0	1	0	1	0.68809
sp|Q8NFQ8|TOIP2_HUMAN	TOR1AIP2	Torsin-1A-interacting protein 2 OS=Homo sapiens GN=TOR1AIP2 PE=1 SV=1	51460	0	1138	0	31	0	0.06	#DIV/0!	0	0	0	1	0	1	0	1	0.68809
tr|B3KU05|B3KU05_HUMAN	tr|B3KU05|B	cDNA FLJ39026 fis, clone NT2RP7005675 OS=Homo sapiens PE=2 SV=1	130427	0	1133	0	31	0	0.04	#DIV/0!	0	0	0	1	0	1	0	1	0.68809
tr|H0Y360|H0Y360_HUMAN	AMPD2	AMP deaminase 2 (Fragment) OS=Homo sapiens GN=AMPD2 PE=4 SV=1	99192	0	1131	0	31	0	0.03	#DIV/0!	0	0	0	1	0	1	0	1	0.68809
tr|Q05D43|Q05D43_HUMAN	YBX1	YBX1 protein (Fragment) OS=Homo sapiens GN=YBX1 PE=2 SV=1	29487	0	1130	0	31	0	0.11	#DIV/0!	0	0	0	1	0	1	0	1	0.68809
tr|Q0PHS4|Q0PHS4_HUMAN	G6PD	Glucose-6-phosphate dehydrogenase (Fragment) OS=Homo sapiens GN=G6PD PE=2 SV=1	7182	0	1129	0	31	0	0.49	#DIV/0!	0	1	0	1	0	1	0	1	0.68809
sp|Q9UKV8|AGO2_HUMAN	EIF2C2	Protein argonaute-2 OS=Homo sapiens GN=EIF2C2 PE=1 SV=3	98400	0	1127	0	31	0	0.03	#DIV/0!	0	0	0	1	0	1	0	1	0.68809
tr|B7Z284|B7Z284_HUMAN	tr|B7Z284|B7	DNA-directed RNA polymerase OS=Homo sapiens PE=2 SV=1	138313	0	1122	0	31	0	0.02	#DIV/0!	0	0	0	1	0	1	0	2	0.68809
tr|D6R968|D6R968_HUMAN	N4BP2L2	NEDD4-binding protein 2-like 2 (Fragment) OS=Homo sapiens GN=N4BP2L2 PE=2 SV=1	83058	0	1121	0	31	0	0.04	#DIV/0!	0	1	0	1	0	1	0	2	0.68809
sp|Q99797|MIPEP_HUMAN	MIPEP	Mitochondrial intermediate peptidase OS=Homo sapiens GN=MIPEP PE=1 SV=2	81387	0	1119	0	31	0	0.04	#DIV/0!	0	0	0	1	0	1	0	2	0.68809
tr|B3KUR4|B3KUR4_HUMAN	tr|B3KUR4|B	cDNA FLJ40462 fis, clone TESTI2042062, highly similar to Transcription initiation factor TFIID sub	64691	0	1118	0	31	0	0.05	#DIV/0!	0	0	0	1	0	1	0	1	0.68809
tr|E5KLJ5|E5KLJ5_HUMAN	OPA1	Dynamin-like 120 kDa protein, mitochondrial OS=Homo sapiens GN=OPA1 PE=3 SV=1	118240	0	1116	0	31	0	0.03	#DIV/0!	0	0	0	1	0	1	0	1	0.68809
tr|Q8TEP9|Q8TEP9_HUMAN	FLJ00144	FLJ00144 protein (Fragment) OS=Homo sapiens GN=FLJ00144 PE=2 SV=1	28437	0	1113	0	32	0	0.07	#DIV/0!	0	0	0	1	0	1	0	2	0.68809
tr|Q13380|Q13380_HUMAN	RBM	RNA binding motif (Fragment) OS=Homo sapiens GN=RBM PE=4 SV=1	4015	0	1111	0	32	0	0.97	#DIV/0!	0	1	0	1	0	1	0	1	0.68809
tr|B2R5U7|B2R5U7_HUMAN	tr|B2R5U7|B	cDNA, FLJ92633, highly similar to Homo sapiens CCAAT-box-binding transcription factor (CBF2),	114755	0	1108	0	32	0	0.03	#DIV/0!	0	0	0	1	0	1	0	3	0.68809
sp|Q8IWT6|LRC8A_HUMAN	LRRC8A	Leucine-rich repeat-containing protein 8A OS=Homo sapiens GN=LRRC8A PE=1 SV=1	95051	0	1104	0	32	0	0.03	#DIV/0!	0	0	0	1	0	1	0	1	0.68809
tr|A2A2Q9|A2A2Q9_HUMAN	C20orf4	Chromosome 20 open reading frame 4 OS=Homo sapiens GN=C20orf4 PE=4 SV=1	45575	0	1103	0	32	0	0.07	#DIV/0!	0	0	0	1	0	1	0	1	0.68809
tr|B4E205|B4E205_HUMAN	tr|B4E205|B4	cDNA FLJ61651, highly similar to Protein transport protein Sec24A OS=Homo sapiens PE=2 SV=1	96953	0	1101	0	32	0	0.03	#DIV/0!	0	0	0	1	0	1	0	1	0.68809
tr|H1UBN2|H1UBN2_HUMAN	CCNB1V	Cyclin B1 OS=Homo sapiens GN=CCNB1V PE=2 SV=1	46024	0	1100	0	32	0	0.16	#DIV/0!	0	0	0	1	0	1	0	1	0.68809
tr|A8K5S1|A8K5S1_HUMAN	tr|A8K5S1|A	cDNA FLJ78650, highly similar to Homo sapiens mucosa associated lymphoid tissue lymphoma t	93725	0	1096	0	33	0	0.03	#DIV/0!	0	0	0	1	0	1	0	1	0.68809
tr|B4DJ81|B4DJ81_HUMAN	NDUFS1	NADH-ubiquinone oxidoreductase 75 kDa subunit, mitochondrial OS=Homo sapiens GN=NDUFS	67563	0	1093	0	33	0	0.05	#DIV/0!	0	0	0	1	0	1	0	1	0.68809
tr|B0YIW5|B0YIW5_HUMAN	ARCN1	Archain 1, isoform CRA_b OS=Homo sapiens GN=ARCN1 PE=2 SV=1	57630	0	1092	0	33	0	0.06	#DIV/0!	0	1	0	1	0	1	0	3	0.68809
sp|Q9UK59|DBR1_HUMAN	DBR1	Lariat debranching enzyme OS=Homo sapiens GN=DBR1 PE=1 SV=2	62144	0	1090	0	33	0	0.05	#DIV/0!	0	1	0	1	0	1	0	1	0.68809
tr|A1L172|A1L172_HUMAN	ACOT1	Acyl-CoA thioesterase 1 OS=Homo sapiens GN=ACOT1 PE=2 SV=1	46666	0	1089	0	33	0	0.07	#DIV/0!	0	0	0	1	0	1	0	1	0.68809
sp|Q9BVQ7|SPA5L_HUMAN	SPATA5L1	Spermatogenesis-associated protein 5-like protein 1 OS=Homo sapiens GN=SPATA5L1 PE=1 SV=	81515	0	1088	0	33	0	0.04	#DIV/0!	0	1	0	1	0	1	0	1	0.68809
tr|Q8TAS6|Q8TAS6_HUMAN	LAMB1	LAMB1 protein (Fragment) OS=Homo sapiens GN=LAMB1 PE=2 SV=2	123283	0	1087	0	33	0	0.03	#DIV/0!	0	0	0	1	0	1	0	3	0.68809
tr|D3DP75|D3DP75_HUMAN	RAB3GAP1	RAB3 GTPase activating protein subunit 1 (Catalytic), isoform CRA_a OS=Homo sapiens GN=RAB	100213	0	1085	0	33	0	0.03	#DIV/0!	0	0	0	1	0	1	0	2	0.68809
tr|C0JXB8|C0JXB8_HUMAN	AIP	Aryl hydrocarbon receptor interacting protein OS=Homo sapiens GN=AIP PE=2 SV=1	38041	0	1082	0	33	0	0.09	#DIV/0!	0	1	0	1	0	1	0	1	0.68809
tr|H0YGP2|H0YGP2_HUMAN	GPR126	G-protein-coupled receptor 126 (Fragment) OS=Homo sapiens GN=GPR126 PE=4 SV=1	15676	0	1081	0	33	0	0.23	#DIV/0!	0	1	0	1	0	1	0	1	0.68809
tr|B4DHD5|B4DHD5_HUMAN	tr|B4DHD5|B	cDNA FLJ57979, highly similar to FERM domain-containing protein 4B OS=Homo sapiens PE=2 S	100328	0	1080	0	33	0	0.03	#DIV/0!	0	1	0	1	0	1	0	2	0.68809
sp|Q8WUX1|S38A5_HUMAN	SLC38A5	Sodium-coupled neutral amino acid transporter 5 OS=Homo sapiens GN=SLC38A5 PE=1 SV=1	52107	0	1079	0	34	0	0.06	#DIV/0!	0	0	0	1	0	1	0	1	0.68809
tr|B2RE56|B2RE56_HUMAN	tr|B2RE56|B	Peptidyl-prolyl cis-trans isomerase OS=Homo sapiens PE=2 SV=1	18225	0	1077	0	34	0	0.19	#DIV/0!	0	0	0	1	0	1	0	1	0.68809
tr|Q6ZWH1|Q6ZWH1_HUMAN	tr|Q6ZWH1|(	cDNA FLJ41081 fis, clone ADRGL2009755, highly similar to Homo sapiens brain and reproductive	45824	0	1071	0	34	0	0.07	#DIV/0!	0	1	0	1	0	1	0	2	0.68809
sp|Q52LJ0|FA98B_HUMAN	FAM98B	Protein FAM98B OS=Homo sapiens GN=FAM98B PE=1 SV=1	37566	0	1070	0	34	0	0.09	#DIV/0!	0	0	0	1	0	1	0	1	0.68809
tr|B1PBA3|B1PBA3_HUMAN	tr|B1PBA3|B	SKNY protein OS=Homo sapiens PE=2 SV=1	98489	0	1067	0	34	0	0.03	#DIV/0!	0	1	0	1	0	1	0	2	0.68809
tr|G3XAN1|G3XAN1_HUMAN	INTS9	Integrator complex subunit 9 OS=Homo sapiens GN=INTS9 PE=2 SV=1	59772	0	1066	0	34	0	0.06	#DIV/0!	0	0	0	1	0	1	0	2	0.68809
tr|H7C0C0|H7C0C0_HUMAN	GTF3C3	General transcription factor 3C polypeptide 3 (Fragment) OS=Homo sapiens GN=GTF3C3 PE=4 S	16723	0	1065	0	28	0	0.07	#DIV/0!	0	0	0	1	0	1	0	1	0.68809
tr|H7C0M3|H7C0M3_HUMAN	BPHL	Valacyclovir hydrolase (Fragment) OS=Homo sapiens GN=BPHL PE=4 SV=1	3314	0	1064	0	34	0	1.2	#DIV/0!	0	1	0	1	0	1	0	1	0.68809
tr|H0Y7P1|H0Y7P1_HUMAN	MED12	Mediator of RNA polymerase II transcription subunit 12 (Fragment) OS=Homo sapiens GN=MED	240034	0	1061	0	35	0	0.03	#DIV/0!	0	1	0	1	0	1	0	1	0.68809
sp|Q86X55|CARM1_HUMAN	CARM1	Histone-arginine methyltransferase CARM1 OS=Homo sapiens GN=CARM1 PE=1 SV=3	66382	0	1060	0	35	0	0.08	#DIV/0!	0	0	0	1	0	1	0	1	0.68809
tr|F6S0T5|F6S0T5_HUMAN	KDM1A	Lysine-specific histone demethylase 1A OS=Homo sapiens GN=KDM1A PE=2 SV=1	95677	0	1059	0	35	0	0.03	#DIV/0!	0	0	0	1	0	1	0	2	0.68809
sp|O00231|PSD11_HUMAN	PSMD11	26S proteasome non-ATPase regulatory subunit 11 OS=Homo sapiens GN=PSMD11 PE=1 SV=3	47719	0	1057	0	35	0	0.07	#DIV/0!	0	0	0	1	0	1	0	1	0.68809
tr|B4DQR0|B4DQR0_HUMAN	tr|B4DQR0|B	cDNA FLJ54377 OS=Homo sapiens PE=2 SV=1	91332	0	1055	0	35	0	0.04	#DIV/0!	0	0	0	1	0	1	0	1	0.68809
sp|Q9UPT8|ZC3H4_HUMAN	ZC3H4	Zinc finger CCCH domain-containing protein 4 OS=Homo sapiens GN=ZC3H4 PE=1 SV=3	140797	0	1054	0	35	0	0.02	#DIV/0!	0	1	0	1	0	1	0	1	0.68809
tr|H3BRE0|H3BRE0_HUMAN	FBXO22	F-box only protein 22 OS=Homo sapiens GN=FBXO22 PE=4 SV=1	10696	0	1053	0	35	0	0.32	#DIV/0!	0	0	0	1	0	1	0	1	0.68809
tr|F5H3P6|F5H3P6_HUMAN	FADS1	Fatty acid desaturase 1 OS=Homo sapiens GN=FADS1 PE=2 SV=1	17375	0	1052	0	35	0	0.19	#DIV/0!	0	1	0	1	0	1	0	1	0.68809
sp|Q9NPI1|BRD7_HUMAN	BRD7	Bromodomain-containing protein 7 OS=Homo sapiens GN=BRD7 PE=1 SV=1	74548	0	1048	0	35	0	0.05	#DIV/0!	0	1	0	1	0	1	0	2	0.68809
tr|H7C4G8|H7C4G8_HUMAN	NT5DC2	5~-nucleotidase domain-containing protein 2 (Fragment) OS=Homo sapiens GN=NT5DC2 PE=4 S	30207	0	1046	0	36	0	0.11	#DIV/0!	0	0	0	1	0	1	0	1	0.68809
sp|Q2KHR3|QSER1_HUMAN	QSER1	Glutamine and serine-rich protein 1 OS=Homo sapiens GN=QSER1 PE=1 SV=3	190597	0	1044	0	36	0	0.02	#DIV/0!	0	1	0	1	0	1	0	1	0.68809
tr|B3KN52|B3KN52_HUMAN	tr|B3KN52|B	cDNA FLJ13606 fis, clone PLACE1010579, highly similar to Homo sapiens GULP, engulfment adap	34895	0	1042	0	36	0	0.1	#DIV/0!	0	0	0	1	0	1	0	2	0.68809
tr|B4DK86|B4DK86_HUMAN	tr|B4DK86|B	cDNA FLJ54466, moderately similar to Rattus norvegicus ubiquitin specific protease 19 (Usp19),	56274	0	1041	0	36	0	0.06	#DIV/0!	0	1	0	1	0	1	0	1	0.68809
tr|B4DZG6|B4DZG6_HUMAN	TMEM48	Nucleoporin NDC1 OS=Homo sapiens GN=TMEM48 PE=2 SV=1	38018	0	1038	0	36	0	0.09	#DIV/0!	0	1	0	1	0	1	0	1	0.68809
tr|G3V1S7|G3V1S7_HUMAN	HAS1	Hyaluronan synthase 1 OS=Homo sapiens GN=HAS1 PE=2 SV=1	65517	0	1036	0	36	0	0.05	#DIV/0!	0	1	0	1	0	1	0	2	0.68809
tr|I3L1M4|I3L1M4_HUMAN	ALDH3A2	Fatty aldehyde dehydrogenase (Fragment) OS=Homo sapiens GN=ALDH3A2 PE=3 SV=1	18870	0	1035	0	36	0	0.18	#DIV/0!	0	1	0	1	0	1	0	1	0.68809
sp|Q9C0C2|TB182_HUMAN	TNKS1BP1	182 kDa tankyrase-1-binding protein OS=Homo sapiens GN=TNKS1BP1 PE=1 SV=4	182711	0	1034	0	36	0	0.02	#DIV/0!	0	1	0	1	0	1	0	1	0.68809
sp|Q9BV73|CP250_HUMAN	CEP250	Centrosome-associated protein CEP250 OS=Homo sapiens GN=CEP250 PE=1 SV=2	281880	0	1031	0	37	0	0.01	#DIV/0!	0	1	0	1	0	1	0	2	0.68809
tr|B3KSG1|B3KSG1_HUMAN	tr|B3KSG1|B	cDNA FLJ36146 fis, clone TESTI2025117, highly similar to Ribosome-binding protein 1 OS=Homo	38833	0	1029	0	37	0	0.09	#DIV/0!	0	0	0	1	0	1	0	1	0.68809
tr|A8K594|A8K594_HUMAN	tr|A8K594|A	cDNA FLJ77256, highly similar to Homo sapiens SH3-domain binding protein 4 (SH3BP4), mRNA	108400	0	1028	0	37	0	0.03	#DIV/0!	0	0	0	1	0	1	0	2	0.68809
tr|B4E074|B4E074_HUMAN	tr|B4E074|B4	cDNA FLJ58655, highly similar to Notchless homolog 1 OS=Homo sapiens PE=2 SV=1	51211	0	1023	0	37	0	0.06	#DIV/0!	0	0	0	1	0	1	0	2	0.68809
tr|Q5JB52|Q5JB52_HUMAN	HNRPLL	Heterogeneous nuclear ribonucleoprotein L-like OS=Homo sapiens GN=HNRPLL PE=2 SV=1	57115	0	1021	0	37	0	0.12	#DIV/0!	0	1	0	2	0	1	0	2	0.68809
tr|H7BZJ7|H7BZJ7_HUMAN	FANCD2	Fanconi anemia group D2 protein (Fragment) OS=Homo sapiens GN=FANCD2 PE=4 SV=1	85193	0	1019	0	37	0	0.04	#DIV/0!	0	1	0	1	0	1	0	2	0.68809
sp|Q86TI2|DPP9_HUMAN	DPP9	Dipeptidyl peptidase 9 OS=Homo sapiens GN=DPP9 PE=1 SV=3	98999	0	1018	0	37	0	0.03	#DIV/0!	0	0	0	1	0	1	0	1	0.68809
tr|Q5VWU8|Q5VWU8_HUMA	PARD3	Partitioning defective 3 homolog OS=Homo sapiens GN=PARD3 PE=4 SV=1	110388	0	1017	0	37	0	0.02	#DIV/0!	0	1	0	1	0	1	0	2	0.68809
tr|A8K6H9|A8K6H9_HUMAN	tr|A8K6H9|A	cDNA FLJ75876, highly similar to Homo sapiens solute carrier family 38, member 2 (SLC38A2), m	56360	0	1014	0	37	0	0.06	#DIV/0!	0	1	0	1	0	1	0	1	0.68809
tr|B4DLG2|B4DLG2_HUMAN	tr|B4DLG2|B	cDNA FLJ58196, highly similar to Zinc finger CCCH domain-containing protein 11A OS=Homo sap	83177	0	1013	0	37	0	0.04	#DIV/0!	0	1	0	1	0	1	0	1	0.68809
sp|Q03169|TNAP2_HUMAN	TNFAIP2	Tumor necrosis factor alpha-induced protein 2 OS=Homo sapiens GN=TNFAIP2 PE=1 SV=2	73015	0	1012	0	37	0	0.08	#DIV/0!	0	1	0	1	0	1	0	1	0.68809
sp|P01137|TGFB1_HUMAN	TGFB1	Transforming growth factor beta-1 OS=Homo sapiens GN=TGFB1 PE=1 SV=2	44997	0	1010	0	37	0	0.07	#DIV/0!	0	1	0	1	0	1	0	1	0.68809
sp|Q96FV9|THOC1_HUMAN	THOC1	THO complex subunit 1 OS=Homo sapiens GN=THOC1 PE=1 SV=1	76360	0	1009	0	37	0	0.04	#DIV/0!	0	1	0	1	0	1	0	1	0.68809
tr|Q7Z3D7|Q7Z3D7_HUMAN	DKFZp686E24	Putative uncharacterized protein DKFZp686E2459 OS=Homo sapiens GN=DKFZp686E2459 PE=2	110613	0	1008	0	38	0	0.03	#DIV/0!	0	1	0	1	0	1	0	2	0.68809
tr|K7EPI4|K7EPI4_HUMAN	GFAP	Glial fibrillary acidic protein (Fragment) OS=Homo sapiens GN=GFAP PE=4 SV=1	7120	0	1007	0	38	0	0.5	#DIV/0!	0	1	0	1	0	1	0	1	0.68809
tr|Q969H1|Q969H1_HUMAN	hCG_2036967	HCG2036967 OS=Homo sapiens GN=hCG_2036967 PE=2 SV=1	14112	0	1006	0	38	0	0.25	#DIV/0!	0	1	0	1	0	1	0	1	0.68809
tr|B3KVY3|B3KVY3_HUMAN	tr|B3KVY3|B	cDNA FLJ41723 fis, clone HLUNG2013851 OS=Homo sapiens PE=2 SV=1	20460	0	1005	0	38	0	0.16	#DIV/0!	0	1	0	1	0	1	0	1	0.68809
sp|Q63ZY3|KANK2_HUMAN	KANK2	KN motif and ankyrin repeat domain-containing protein 2 OS=Homo sapiens GN=KANK2 PE=1 SV	91916	0	1001	0	38	0	0.14	#DIV/0!	0	1	0	1	0	1	0	1	0.68809

tr|B3KM73|B3KM73_HUMAN	tr|B3KM73|B	cDNA FLJ10419 fis, clone NT2RP1000163, highly similar to Homo sapiens transforming growth fa	55418	0	1000	0	38	0	0.06	#DIV/0!	0	1	0	1	0	1	0	1	0.68809
tr|Q6ZNZ8|Q6ZNZ8_HUMAN	tr|Q6ZNZ8|Q	cDNA FLJ26816 fis, clone PRS06437 OS=Homo sapiens PE=2 SV=1	54459	0	998	0	38	0	0.06	#DIV/0!	0	1	0	1	0	1	0	1	0.68809
tr|B4DFG0|B4DFG0_HUMAN	DEK	Protein DEK OS=Homo sapiens GN=DEK PE=2 SV=1	39704	0	995	0	38	0	0.08	#DIV/0!	0	1	0	1	0	1	0	1	0.68809
sp|P27635|RL10_HUMAN	RPL10	60S ribosomal protein L10 OS=Homo sapiens GN=RPL10 PE=1 SV=4	25044	0	994	0	38	0	0.34	#DIV/0!	0	1	0	1	0	1	0	1	0.68809
tr|B4DFH9|B4DFH9_HUMAN	tr|B4DFH9|B	cDNA FLJ60472, highly similar to Homo sapiens IQ motif containing with AAA domain (IQCA), mR	90664	0	993	0	38	0	0.04	#DIV/0!	0	1	0	1	0	1	0	1	0.68809
tr|H0Y9X5|H0Y9X5_HUMAN	ERAP1	Endoplasmic reticulum aminopeptidase 1 (Fragment) OS=Homo sapiens GN=ERAP1 PE=4 SV=1	17136	0	992	0	38	0	0.2	#DIV/0!	0	1	0	1	0	1	0	1	0.68809
tr|B2RDG4|B2RDG4_HUMAN	tr|B2RDG4|B	cDNA, FLJ96597 OS=Homo sapiens PE=2 SV=1	57796	0	989	0	38	0	0.06	#DIV/0!	0	1	0	1	0	1	0	2	0.68809
tr|B3KM95|B3KM95_HUMAN	tr|B3KM95|B	Phosphatidate cytidylyltransferase OS=Homo sapiens PE=2 SV=1	51896	0	988	0	38	0	0.06	#DIV/0!	0	1	0	1	0	1	0	1	0.68809
tr|J3KMX2|J3KMX2_HUMAN	SMARCD2	SWI/SNF-related matrix-associated actin-dependent regulator of chromatin subfamily D membe	52433	0	986	0	39	0	0.06	#DIV/0!	0	1	0	1	0	1	0	1	0.68809
tr|G3V1P5|G3V1P5_HUMAN	MED15	Mediator of RNA polymerase II transcription subunit 15 OS=Homo sapiens GN=MED15 PE=4 SV=	80007	0	980	0	39	0	0.04	#DIV/0!	0	1	0	1	0	1	0	1	0.68809
tr|F5GYT3|F5GYT3_HUMAN	LPCAT3	Lysophospholipid acyltransferase 5 OS=Homo sapiens GN=LPCAT3 PE=2 SV=1	22378	0	979	0	39	0	0.15	#DIV/0!	0	1	0	1	0	1	0	1	0.68809
tr|B2R6G5|B2R6G5_HUMAN	tr|B2R6G5|B	cDNA, FLJ92942, highly similar to Homo sapiens T-box, brain, 1 (TBR1), mRNA OS=Homo sapiens	74530	0	976	0	40	0	0.04	#DIV/0!	0	1	0	1	0	1	0	2	0.68809
tr|I3VM53|I3VM53_HUMAN	KDM2A	F-box and leucine-rich repeat protein 11, isoform CRA_a OS=Homo sapiens GN=KDM2A PE=2 SV	135159	0	974	0	40	0	0.02	#DIV/0!	0	1	0	1	0	1	0	1	0.68809
tr|E7ERH3|E7ERH3_HUMAN	TNS3	Tensin-3 OS=Homo sapiens GN=TNS3 PE=2 SV=1	130127	0	970	0	40	0	0.02	#DIV/0!	0	1	0	1	0	1	0	1	0.68809
tr|F5H1X8|F5H1X8_HUMAN	LRBA	Lipopolysaccharide-responsive and beige-like anchor protein OS=Homo sapiens GN=LRBA PE=2	320211	0	968	0	40	0	0.02	#DIV/0!	0	1	0	1	0	1	0	2	0.68809
tr|B3KQX0|B3KQX0_HUMAN	tr|B3KQX0|B	cDNA FLJ33228 fis, clone ASTRO2001122, highly similar to Zinc finger protein 64, isoforms 1 and	59209	0	965	0	40	0	0.06	#DIV/0!	0	1	0	1	0	1	0	1	0.68809
sp|O95140|MFN2_HUMAN	MFN2	Mitofusin-2 OS=Homo sapiens GN=MFN2 PE=1 SV=3	87088	0	964	0	40	0	0.31	#DIV/0!	0	1	0	1	0	1	0	1	0.68809
tr|Q6AI22|Q6AI22_HUMAN	DKFZp686H16	Putative uncharacterized protein DKFZp686H16106 (Fragment) OS=Homo sapiens GN=DKFZp68	321236	0	962	0	25	0	0.01	#DIV/0!	0	0	0	1	0	1	0	3	0.68809
tr|Q2NNQ8|Q2NNQ8_HUMAN	FER1L4	FER-1-like protein 4 OS=Homo sapiens GN=FER1L4 PE=2 SV=1	231472	0	959	0	40	0	0.01	#DIV/0!	0	1	0	1	0	1	0	1	0.68809
tr|B4DUZ3|B4DUZ3_HUMAN	tr|B4DUZ3|B	cDNA FLJ51105, highly similar to Arfaptin-2 OS=Homo sapiens PE=2 SV=1	41783	0	958	0	41	0	0.08	#DIV/0!	0	1	0	1	0	1	0	1	0.68809
tr|B4DS74|B4DS74_HUMAN	tr|B4DS74|B4	Nuclear factor 1 OS=Homo sapiens PE=2 SV=1	50737	0	957	0	41	0	0.06	#DIV/0!	0	1	0	1	0	1	0	1	0.68809
tr|B3KVR1|B3KVR1_HUMAN	SNRPN	Small nuclear ribonucleoprotein-associated protein OS=Homo sapiens GN=SNRPN PE=2 SV=1	25230	0	955	0	41	0	0.13	#DIV/0!	0	1	0	1	0	1	0	1	0.68809
tr|I0CMJ8|I0CMJ8_HUMAN	PRIM2	DNA primase large subunit (Fragment) OS=Homo sapiens GN=PRIM2 PE=4 SV=1	5179	0	953	0	41	0	0.7	#DIV/0!	0	1	0	1	0	1	0	1	0.68809
sp|Q9Y3L5|RAP2C_HUMAN	RAP2C	Ras-related protein Rap-2c OS=Homo sapiens GN=RAP2C PE=1 SV=1	20959	0	952	0	41	0	0.16	#DIV/0!	0	1	0	1	0	1	0	1	0.68809
tr|Q6ZVI6|Q6ZVI6_HUMAN	tr|Q6ZVI6|Q6	cDNA FLJ42537 fis, clone BRACE3004058, highly similar to NADH-cytochrome B5 reductase (EC 1	29141	0	951	0	41	0	0.11	#DIV/0!	0	1	0	1	0	1	0	1	0.68809
sp|Q8IZV5|RDH10_HUMAN	RDH10	Retinol dehydrogenase 10 OS=Homo sapiens GN=RDH10 PE=1 SV=1	38747	0	950	0	41	0	0.09	#DIV/0!	0	1	0	1	0	1	0	1	0.68809
tr|I3L4X2|I3L4X2_HUMAN	ABCC1	Multidrug resistance-associated protein 1 (Fragment) OS=Homo sapiens GN=ABCC1 PE=3 SV=1	161588	0	948	0	41	0	0.02	#DIV/0!	0	1	0	1	0	1	0	1	0.68809
tr|A0PJ47|A0PJ47_HUMAN	SAFB2	SAFB2 protein (Fragment) OS=Homo sapiens GN=SAFB2 PE=2 SV=1	57340	0	942	0	42	0	0.12	#DIV/0!	0	1	0	2	0	1	0	2	0.68809
sp|P46013|KI67_HUMAN	MKI67	Antigen KI-67 OS=Homo sapiens GN=MKI67 PE=1 SV=2	360698	0	941	0	42	0	0.02	#DIV/0!	0	1	0	2	0	1	0	5	0.68809
tr|B0V2J7|B0V2J7_HUMAN	TAP2	Antigen peptide transporter 2 OS=Homo sapiens GN=TAP2 PE=4 SV=1	72571	0	938	0	42	0	0.05	#DIV/0!	0	1	0	1	0	1	0	1	0.68809
tr|B3KY11|B3KY11_HUMAN	tr|B3KY11|B3	cDNA FLJ46571 fis, clone THYMU3041428, highly similar to Probable ATP-dependent RNA helica	93519	0	937	0	42	0	0.04	#DIV/0!	0	1	0	1	0	1	0	3	0.68809
tr|H3BRE8|H3BRE8_HUMAN	RPAP1	RNA polymerase II-associated protein 1 OS=Homo sapiens GN=RPAP1 PE=4 SV=1	124587	0	936	0	43	0	0.03	#DIV/0!	0	1	0	1	0	1	0	2	0.68809
sp|P13667|PDIA4_HUMAN	PDIA4	Protein disulfide-isomerase A4 OS=Homo sapiens GN=PDIA4 PE=1 SV=2	73229	0	935	0	43	0	0.04	#DIV/0!	0	1	0	1	0	1	0	1	0.68809
tr|A2A306|A2A306_HUMAN	UBAP2	Ubiquitin-associated protein 2 OS=Homo sapiens GN=UBAP2 PE=4 SV=1	34828	0	934	0	43	0	0.1	#DIV/0!	0	1	0	1	0	1	0	2	0.68809
tr|B0QYK0|B0QYK0_HUMAN	EWSR1	Ewing sarcoma breakpoint region 1 OS=Homo sapiens GN=EWSR1 PE=4 SV=1	65174	0	931	0	43	0	0.05	#DIV/0!	0	1	0	1	0	1	0	1	0.68809
tr|H0Y650|H0Y650_HUMAN	HEATR2	HEAT repeat-containing protein 2 (Fragment) OS=Homo sapiens GN=HEATR2 PE=4 SV=1	73507	0	930	0	43	0	0.04	#DIV/0!	0	1	0	1	0	1	0	1	0.68809
tr|B3KM50|B3KM50_HUMAN	tr|B3KM50|B	cDNA FLJ10310 fis, clone NT2RM2000322, highly similar to Homo sapiens KIAA0859 protein OS=	39576	0	928	0	43	0	0.04	#DIV/0!	0	1	0	1	0	1	0	1	0.68809
tr|B3KM40|B3KM40_HUMAN	tr|B3KM40|B	cDNA FLJ10185 fis, clone HEMBA1004509, highly similar to U4/U6.U5 tri-snRNP-associated prot	56618	0	927	0	43	0	0.06	#DIV/0!	0	1	0	1	0	1	0	1	0.68809
tr|Q5XJ04|Q5XJ04_HUMAN	HMGCS1	3-hydroxy-3-methylglutaryl-Coenzyme A synthase 1 (Soluble) OS=Homo sapiens GN=HMGCS1 P	57854	0	924	0	43	0	0.12	#DIV/0!	0	1	0	2	0	1	0	2	0.68809
sp|P04181|OAT_HUMAN	OAT	Ornithine aminotransferase, mitochondrial OS=Homo sapiens GN=OAT PE=1 SV=1	48846	0	923	0	43	0	0.07	#DIV/0!	0	1	0	1	0	1	0	1	0.68809
tr|I1E4Y6|I1E4Y6_HUMAN	GIGYF2	PERQ amino acid-rich with GYF domain-containing protein 2 OS=Homo sapiens GN=GIGYF2 PE=4	152891	0	920	0	43	0	0.02	#DIV/0!	0	1	0	1	0	1	0	2	0.68809
tr|F8VX04|F8VX04_HUMAN	SLC38A1	Sodium-coupled neutral amino acid transporter 1 OS=Homo sapiens GN=SLC38A1 PE=2 SV=1	56900	0	918	0	43	0	0.06	#DIV/0!	0	1	0	1	0	1	0	1	0.68809
tr|Q6IPI1|Q6IPI1_HUMAN	RPL29	Ribosomal protein L29 OS=Homo sapiens GN=RPL29 PE=2 SV=1	17997	0	917	0	44	0	0.19	#DIV/0!	0	1	0	1	0	1	0	1	0.68809
tr|A0AV56|A0AV56_HUMAN	SAFB	SAFB protein OS=Homo sapiens GN=SAFB PE=2 SV=1	103249	0	915	0	44	0	0.03	#DIV/0!	0	1	0	1	0	1	0	1	0.68809
sp|Q9BTE3|MCMBP_HUMAN	MCMBP	Mini-chromosome maintenance complex-binding protein OS=Homo sapiens GN=MCMBP PE=1 S	73789	0	913	0	44	0	0.04	#DIV/0!	0	1	0	1	0	1	0	1	0.68809
tr|B4E2S2|B4E2S2_HUMAN	tr|B4E2S2|B4	cDNA FLJ61556, highly similar to Poly (ADP-ribose) polymerase 4 (EC 2.4.2.30) OS=Homo sapien	113804	0	912	0	44	0	0.03	#DIV/0!	0	1	0	1	0	1	0	1	0.68809
sp|O75475|PSIP1_HUMAN	PSIP1	PC4 and SFRS1-interacting protein OS=Homo sapiens GN=PSIP1 PE=1 SV=1	60181	0	911	0	44	0	0.05	#DIV/0!	0	1	0	1	0	1	0	1	0.68809
sp|P61073|CXCR4_HUMAN	CXCR4	C-X-C chemokine receptor type 4 OS=Homo sapiens GN=CXCR4 PE=1 SV=1	40233	0	910	0	44	0	0.08	#DIV/0!	0	1	0	1	0	1	0	1	0.68809
sp|O60343|TBCD4_HUMAN	TBC1D4	TBC1 domain family member 4 OS=Homo sapiens GN=TBC1D4 PE=1 SV=2	148068	0	909	0	44	0	0.02	#DIV/0!	0	1	0	1	0	1	0	2	0.68809
sp|Q96JM3|CHAP1_HUMAN	CHAMP1	Chromosome alignment-maintaining phosphoprotein 1 OS=Homo sapiens GN=CHAMP1 PE=1 SV	90012	0	905	0	44	0	0.04	#DIV/0!	0	1	0	1	0	1	0	1	0.68809
sp|Q9ULI0|ATD2B_HUMAN	ATAD2B	ATPase family AAA domain-containing protein 2B OS=Homo sapiens GN=ATAD2B PE=1 SV=3	166351	0	903	0	44	0	0.02	#DIV/0!	0	1	0	1	0	1	0	1	0.68809
tr|B1AHL2|B1AHL2_HUMAN	FBLN1	Fibulin 1 OS=Homo sapiens GN=FBLN1 PE=4 SV=1	82439	0	902	0	44	0	0.04	#DIV/0!	0	1	0	1	0	1	0	2	0.68809
tr|Q70L73|Q70L73_HUMAN	MKI67	KI67 Antigen (Fragment) OS=Homo sapiens GN=MKI67 PE=2 SV=1	46560	0	900	0	45	0	0.07	#DIV/0!	0	1	0	1	0	1	0	1	0.68809
tr|J3KNI9|J3KNI9_HUMAN	TBC1D15	TBC1 domain family member 15 OS=Homo sapiens GN=TBC1D15 PE=4 SV=1	79491	0	899	0	45	0	0.04	#DIV/0!	0	1	0	1	0	1	0	1	0.68809
tr|B3KM89|B3KM89_HUMAN	tr|B3KM89|B	cDNA FLJ10528 fis, clone NT2RP2000943, highly similar to Protein transport protein Sec24D OS=	101723	0	897	0	45	0	0.07	#DIV/0!	0	1	0	1	0	1	0	1	0.68809
tr|B1AK81|B1AK81_HUMAN	PIGK	GPI-anchor transamidase OS=Homo sapiens GN=PIGK PE=4 SV=1	34462	0	894	0	45	0	0.1	#DIV/0!	0	1	0	1	0	1	0	1	0.68809
tr|J3KQZ1|J3KQZ1_HUMAN	VWA7	von Willebrand factor A domain-containing protein 7 OS=Homo sapiens GN=VWA7 PE=4 SV=1	96313	0	892	0	45	0	0.03	#DIV/0!	0	1	0	1	0	1	0	1	0.68809
tr|Q5H907|Q5H907_HUMAN	MAGED2	Melanoma antigen family D, 2, isoform CRA_d OS=Homo sapiens GN=MAGED2 PE=4 SV=1	55932	0	891	0	46	0	0.13	#DIV/0!	0	1	0	1	0	1	0	1	0.68809
tr|Q2TSD2|Q2TSD2_HUMAN	tr|Q2TSD2|Q	Aging-associated gene 7 protein OS=Homo sapiens PE=2 SV=1	41778	0	890	0	46	0	0.08	#DIV/0!	0	1	0	1	0	1	0	2	0.68809
tr|B0I1S9|B0I1S9_HUMAN	MYO1B	MYO1B variant protein OS=Homo sapiens GN=MYO1B PE=2 SV=1	132928	0	888	0	46	0	0.1	#DIV/0!	0	1	0	1	0	1	0	1	0.68809
tr|Q6IAM0|Q6IAM0_HUMAN	EIF3S4	Eukaryotic translation initiation factor 3 subunit G OS=Homo sapiens GN=EIF3S4 PE=2 SV=1	35864	0	887	0	46	0	0.09	#DIV/0!	0	1	0	1	0	1	0	1	0.68809
sp|P62333|PRS10_HUMAN	PSMC6	26S protease regulatory subunit 10B OS=Homo sapiens GN=PSMC6 PE=1 SV=1	44430	0	886	0	46	0	0.07	#DIV/0!	0	1	0	1	0	1	0	2	0.68809
tr|H0YJC0|H0YJC0_HUMAN	PSMC6	26S protease regulatory subunit 10B (Fragment) OS=Homo sapiens GN=PSMC6 PE=4 SV=1	29919	0	885	0	46	0	0.15	#DIV/0!	0	1	0	1	0	1	0	1	0.68809
tr|B7Z3P1|B7Z3P1_HUMAN	TBCE	Tubulin-specific chaperone E OS=Homo sapiens GN=TBCE PE=2 SV=1	65666	0	883	0	46	0	0.05	#DIV/0!	0	1	0	1	0	1	0	1	0.68809
sp|Q9NR09|BIRC6_HUMAN	BIRC6	Baculoviral IAP repeat-containing protein 6 OS=Homo sapiens GN=BIRC6 PE=1 SV=2	536192	0	881	0	46	0	0.01	#DIV/0!	0	1	0	1	0	1	0	1	0.68809
sp|P09497|CLCB_HUMAN	CLTB	Clathrin light chain B OS=Homo sapiens GN=CLTB PE=1 SV=1	25289	0	879	0	46	0	0.13	#DIV/0!	0	1	0	1	0	1	0	1	0.68809
tr|B2RDX0|B2RDX0_HUMAN	tr|B2RDX0|B	cDNA, FLJ96805, highly similar to Homo sapiens SHC SH2-domain binding protein 1 (SHCBP1), m	76668	0	878	0	46	0	0.04	#DIV/0!	0	1	0	1	0	1	0	1	0.68809
tr|B3KXW5|B3KXW5_HUMAN	AP1G1	AP-1 complex subunit gamma-1 OS=Homo sapiens GN=AP1G1 PE=2 SV=1	94992	0	876	0	47	0	0.03	#DIV/0!	0	1	0	1	0	1	0	1	0.68809
sp|P49750|YLPM1_HUMAN	YLPM1	YLP motif-containing protein 1 OS=Homo sapiens GN=YLPM1 PE=1 SV=3	220077	0	873	0	47	0	0.02	#DIV/0!	0	1	0	1	0	1	0	1	0.68809
tr|B3KNG5|B3KNG5_HUMAN	tr|B3KNG5|B	cDNA FLJ14553 fis, clone NT2RM2001785, highly similar to Homo sapiens fatty acid desaturase 2	19263	0	872	0	29	0	0.22	#DIV/0!	0	1	0	1	0	1	0	2	0.68809
tr|Q5SRT3|Q5SRT3_HUMAN	CLIC1	Chloride intracellular channel 1, isoform CRA_a OS=Homo sapiens GN=CLIC1 PE=2 SV=2	27248	0	865	0	48	0	0.12	#DIV/0!	0	1	0	1	0	1	0	1	0.68809
tr|H0Y2Y8|H0Y2Y8_HUMAN	ZYX	Zyxin (Fragment) OS=Homo sapiens GN=ZYX PE=4 SV=1	58804	0	863	0	48	0	0.06	#DIV/0!	0	1	0	1	0	1	0	2	0.68809
tr|B3KTN5|B3KTN5_HUMAN	tr|B3KTN5|B	cDNA FLJ38538 fis, clone HCHON2001407, highly similar to LanC-like protein 2 OS=Homo sapien	51676	0	861	0	48	0	0.06	#DIV/0!	0	1	0	1	0	1	0	2	0.68809
tr|B3KN37|B3KN37_HUMAN	tr|B3KN37|B	cDNA FLJ13414 fis, clone PLACE1001748, highly similar to Homo sapiens pitrilysin metallopeptid	68281	0	858	0	48	0	0.11	#DIV/0!	0	1	0	1	0	1	0	1	0.68809
tr|A4QN18|A4QN18_HUMAN	SEC16A	SEC16A protein (Fragment) OS=Homo sapiens GN=SEC16A PE=2 SV=1	212198	0	857	0	0	0	0.02	#DIV/0!	0	0	0	0	0	1	0	2	0.68809
tr|B5BUD2|B5BUD2_HUMAN	RFC2	Replication factor C 2 isoform 1 (Fragment) OS=Homo sapiens GN=RFC2 PE=2 SV=1	39616	0	852	0	48	0	0.08	#DIV/0!	0	1	0	1	0	1	0	1	0.68809
sp|Q9UKJ3|GPTC8_HUMAN	GPATCH8	G patch domain-containing protein 8 OS=Homo sapiens GN=GPATCH8 PE=1 SV=2	165010	0	851	0	48	0	0.02	#DIV/0!	0	1	0	1	0	1	0	1	0.68809
tr|Q58EY4|Q58EY4_HUMAN	SMARCC1	SWI/SNF related, matrix associated, actin dependent regulator of chromatin, subfamily c, memb	123347	0	849	0	48	0	0.04	#DIV/0!	0	1	0	1	0	1	0	1	0.68809
tr|B4DRF4|B4DRF4_HUMAN	PTPLAD1	Very-long-chain (3R)-3-hydroxyacyl-[acyl-carrier protein] dehydratase 3 OS=Homo sapiens GN=P	36635	0	848	0	48	0	0.09	#DIV/0!	0	1	0	1	0	1	0	1	0.68809
tr|B7Z4U7|B7Z4U7_HUMAN	SCFD1	Sec1 family domain containing 1, isoform CRA_b OS=Homo sapiens GN=SCFD1 PE=2 SV=1	65362	0	847	0	49	0	0.05	#DIV/0!	0	1	0	1	0	1	0	1	0.68809
tr|B4DWI8|B4DWI8_HUMAN	tr|B4DWI8|B	cDNA FLJ57805, highly similar to Homo sapiens paraspeckle component 1 (PSPC1), transcript va	52660	0	843	0	49	0	0.06	#DIV/0!	0	1	0	1	0	1	0	1	0.68809
sp|Q92995|UBP13_HUMAN	USP13	Ubiquitin carboxyl-terminal hydrolase 13 OS=Homo sapiens GN=USP13 PE=1 SV=2	98006	0	842	0	49	0	0.03	#DIV/0!	0	1	0	1	0	1	0	2	0.68809
tr|Q05DA4|Q05DA4_HUMAN	P4HA2	P4HA2 protein OS=Homo sapiens GN=P4HA2 PE=2 SV=1	57684	0	841	0	49	0	0.06	#DIV/0!	0	1	0	1	0	1	0	1	0.68809
tr|H3BNB9|H3BNB9_HUMAN	ARIH1	E3 ubiquitin-protein ligase ARIH1 (Fragment) OS=Homo sapiens GN=ARIH1 PE=4 SV=1	22769	0	839	0	49	0	0.15	#DIV/0!	0	1	0	1	0	1	0	1	0.68809
sp|Q8NBX0|SCPDL_HUMAN	SCCPDH	Saccharopine dehydrogenase-like oxidoreductase OS=Homo sapiens GN=SCCPDH PE=1 SV=1	47464	0	837	0	49	0	0.07	#DIV/0!	0	1	0	1	0	1	0	1	0.68809
sp|Q5QP74|CJ115_HUMAN	C10orf115	Putative uncharacterized protein C10orf115 OS=Homo sapiens GN=C10orf115 PE=4 SV=2	10394	0	836	0	49	0	0.33	#DIV/0!	0	1	0	1	0	1	0	1	0.68809
tr|B4DNM8|B4DNM8_HUMAN	tr|B4DNM8|E	cDNA FLJ53395, highly similar to Prolyl 3-hydroxylase 1 (EC 1.14.11.7) OS=Homo sapiens PE=2 S	69572	0	834	0	39	0	0.08	#DIV/0!	0	1	0	1	0	1	0	1	0.68809
tr|B4DKE6|B4DKE6_HUMAN	tr|B4DKE6|B4	cDNA FLJ60629, highly similar to Replication factor C subunit 3 OS=Homo sapiens PE=2 SV=1	35551	0	833	0	50	0	0.09	#DIV/0!	0	1	0	1	0	1	0	1	0.68809
tr|B3KMN5|B3KMN5_HUMAN	tr|B3KMN5|E	cDNA FLJ11713 fis, clone HEMBA1005201, highly similar to Homo sapiens NMD3 homolog (NMD	44787	0	828	0	50	0	0.07	#DIV/0!	0	1	0	1	0	1	0	1	0.68809
tr|E5RHC5|E5RHC5_HUMAN	ARFGAP1	ADP-ribosylation factor GTPase-activating protein 1 (Fragment) OS=Homo sapiens GN=ARFGAP1	29395	0	827	0	50	0	0.11	#DIV/0!	0	1	0	1	0	1	0	1	0.68809
tr|H0Y2P8|H0Y2P8_HUMAN	SRGAP2	SLIT-ROBO Rho GTPase-activating protein 2 (Fragment) OS=Homo sapiens GN=SRGAP2 PE=4 SV=	111536	0	826	0	50	0	0.03	#DIV/0!	0	1	0	1	0	1	0	1	0.68809
tr|A0JNT2|A0JNT2_HUMAN	KRT83	KRT83 protein OS=Homo sapiens GN=KRT83 PE=2 SV=1	50978	0	825	0	51	0	0.06	#DIV/0!	0	1	0	1	0	1	0	1	0.68809
tr|H0Y4J2|H0Y4J2_HUMAN	MRPL37	39S ribosomal protein L37, mitochondrial (Fragment) OS=Homo sapiens GN=MRPL37 PE=4 SV=1	29806	0	821	0	51	0	0.11	#DIV/0!	0	1	0	1	0	1	0	1	0.68809
sp|Q96T51|RUFY1_HUMAN	RUFY1	RUN and FYVE domain-containing protein 1 OS=Homo sapiens GN=RUFY1 PE=1 SV=2	80851	0	820	0	51	0	0.04	#DIV/0!	0	1	0	1	0	1	0	2	0.68809
tr|E7EMP9|E7EMP9_HUMAN	DROSHA	Ribonuclease 3 OS=Homo sapiens GN=DROSHA PE=3 SV=1	156774	0	819	0	51	0	0.02	#DIV/0!	0	1	0	1	0	1	0	1	0.68809
sp|Q13425|SNTB2_HUMAN	SNTB2	Beta-2-syntrophin OS=Homo sapiens GN=SNTB2 PE=1 SV=1	58369	0	816	0	51	0	0.06	#DIV/0!	0	1	0	1	0	1	0	1	0.68809
sp|Q9Y266|NUDC_HUMAN	NUDC	Nuclear migration protein nudC OS=Homo sapiens GN=NUDC PE=1 SV=1	38276	0	813	0	51	0	0.09	#DIV/0!	0	1	0	1	0	1	0	1	0.68809
tr|Q6MZM7|Q6MZM7_HUMA	DKFZp686O12	Putative uncharacterized protein DKFZp686O12165 (Fragment) OS=Homo sapiens GN=DKFZp68	242944	0	807	0	52	0	0.01	#DIV/0!	0	1	0	1	0	1	0	1	0.68809
sp|O14746|TERT_HUMAN	TERT	Telomerase reverse transcriptase OS=Homo sapiens GN=TERT PE=1 SV=1	128570	0	805	0	52	0	0.03	#DIV/0!	0	1	0	1	0	1	0	2	0.68809
tr|B7Z653|B7Z653_HUMAN	tr|B7Z653|B7	cDNA FLJ61657, highly similar to Band 4.1-like protein 1 OS=Homo sapiens PE=2 SV=1	90347	0	800	0	52	0	0.04	#DIV/0!	0	1	0	1	0	1	0	1	0.68809
sp|Q96CS3|FAF2_HUMAN	FAF2	FAS-associated factor 2 OS=Homo sapiens GN=FAF2 PE=1 SV=2	52933	0	799	0	52	0	0.06	#DIV/0!	0	1	0	1	0	1	0	1	0.68809
tr|Q59FE8|Q59FE8_HUMAN	tr|Q59FE8|Q	Epithelial protein lost in neoplasm beta variant (Fragment) OS=Homo sapiens PE=2 SV=1	86510	0	796	0	52	0	0.09	#DIV/0!	0	1	0	2	0	1	0	2	0.68809
sp|Q14669|TRIPC_HUMAN	TRIP12	E3 ubiquitin-protein ligase TRIP12 OS=Homo sapiens GN=TRIP12 PE=1 SV=1	222234	0	794	0	53	0	0.01	#DIV/0!	0	1	0	1	0	1	0	1	0.68809
tr|J3KQ32|J3KQ32_HUMAN	OLA1	Obg-like ATPase 1 OS=Homo sapiens GN=OLA1 PE=4 SV=1	47136	0	793	0	53	0	0.07	#DIV/0!	0	1	0	1	0	1	0	2	0.68809
tr|C9J634|C9J634_HUMAN	PDHB	Pyruvate dehydrogenase E1 component subunit beta, mitochondrial OS=Homo sapiens GN=PDH	39064	0	791	0	27	0	0.27	#DIV/0!	0	0	0	1	0	1	0	2	0.68809
sp|Q9NYL9|TMOD3_HUMAN	TMOD3	Tropomodulin-3 OS=Homo sapiens GN=TMOD3 PE=1 SV=1	39741	0	788	0	53	0	0.08	#DIV/0!	0	1	0	1	0	1	0	2	0.68809
tr|H7BZF2|H7BZF2_HUMAN	DKC1	H/ACA ribonucleoprotein complex subunit 4 (Fragment) OS=Homo sapiens GN=DKC1 PE=4 SV=1	8803	0	784	0	46	0	0.12	#DIV/0!	0	1	0	1	0	1	0	1	0.68809
tr|Q6FI30|Q6FI30_HUMAN	NFIC	Nuclear factor 1 OS=Homo sapiens GN=NFIC PE=2 SV=1	55019	0	783	0	54	0	0.06	#DIV/0!	0	1	0	1	0	1	0	1	0.68809
tr|G1UI18|G1UI18_HUMAN	PSME4	Proteasome activator complex subunit 4 (Fragment) OS=Homo sapiens GN=PSME4 PE=2 SV=1	130054	0	778	0	54	0	0.05	#DIV/0!	0	1	0	1	0	1	0	3	0.68809
tr|B3KQ95|B3KQ95_HUMAN	FDFT1	Squalene synthase OS=Homo sapiens GN=FDFT1 PE=2 SV=1	35421	0	777	0	54	0	0.09	#DIV/0!	0	1	0	1	0	1	0	1	0.68809
tr|C9JKF1|C9JKF1_HUMAN	SAMD9	Sterile alpha motif domain-containing protein 9 (Fragment) OS=Homo sapiens GN=SAMD9 PE=2	149867	0	774	0	55	0	0.02	#DIV/0!	0	1	0	1	0	1	0	2	0.68809
sp|P27694|RFA1_HUMAN	RPA1	Replication protein A 70 kDa DNA-binding subunit OS=Homo sapiens GN=RPA1 PE=1 SV=2	68723	0	773	0	55	0	0.09	#DIV/0!	0	1	0	1	0	1	0	2	0.68809
sp|P82933|RT09_HUMAN	MRPS9	28S ribosomal protein S9, mitochondrial OS=Homo sapiens GN=MRPS9 PE=1 SV=2	46034	0	769	0	55	0	0.09	#DIV/0!	0	1	0	1	0	1	0	1	0.68809
tr|B4DWV9|B4DWV9_HUMAN	tr|B4DWV9|E	cDNA FLJ53108, highly similar to Guanine nucleotide-binding protein alpha-13 subunit OS=Hom	41291	0	767	0	55	0	0.08	#DIV/0!	0	1	0	1	0	1	0	1	0.68809
sp|Q5JWF2|GNAS1_HUMAN	GNAS	Guanine nucleotide-binding protein G(s) subunit alpha isoforms XLas OS=Homo sapiens GN=GN	111697	0	766	0	55	0	0.03	#DIV/0!	0	1	0	1	0	1	0	2	0.68809
tr|F8VRN8|F8VRN8_HUMAN	LIMA1	LIM domain and actin-binding protein 1 OS=Homo sapiens GN=LIMA1 PE=2 SV=1	43278	0	765	0	55	0	0.08	#DIV/0!	0	1	0	1	0	1	0	1	0.68809
tr|B4DJE7|B4DJE7_HUMAN	ACADM	Medium-chain-specific acyl-CoA dehydrogenase, mitochondrial OS=Homo sapiens GN=ACADM I	25704	0	764	0	55	0	0.13	#DIV/0!	0	1	0	1	0	1	0	1	0.68809
tr|B4DPW5|B4DPW5_HUMAN	tr|B4DPW5|E	cDNA FLJ59374, highly similar to Homo sapiens caldesmon 1 (CALD1), transcript variant 2, mRNA	57436	0	763	0	55	0	0.06	#DIV/0!	0	1	0	1	0	1	0	1	0.68809
sp|Q9UID3|VPS51_HUMAN	VPS51	Vacuolar protein sorting-associated protein 51 homolog OS=Homo sapiens GN=VPS51 PE=1 SV=	86901	0	761	0	55	0	0.04	#DIV/0!	0	1	0	1	0	1	0	2	0.68809
tr|G3V5E1|G3V5E1_HUMAN	CCNK	Cyclin-K OS=Homo sapiens GN=CCNK PE=2 SV=1	43776	0	760	0	55	0	0.08	#DIV/0!	0	1	0	1	0	1	0	1	0.68809
tr|B4DJ92|B4DJ92_HUMAN	tr|B4DJ92|B4	cDNA FLJ57843, highly similar to Homo sapiens neurochondrin (NCDN), transcript variant 3, mR	20030	0	758	0	55	0	0.08	#DIV/0!	0	1	0	1	0	1	0	1	0.68809
tr|B0V0F7|B0V0F7_HUMAN	DHX16	Putative pre-mRNA-splicing factor ATP-dependent RNA helicase DHX16 (Fragment) OS=Homo sa	21454	0	756	0	50	0	0.09	#DIV/0!	0	1	0	1	0	1	0	1	0.68809
sp|P37837|TALDO_HUMAN	TALDO1	Transaldolase OS=Homo sapiens GN=TALDO1 PE=1 SV=2	37688	0	749	0	57	0	0.09	#DIV/0!	0	1	0	1	0	1	0	2	0.68809
tr|H0YAS8|H0YAS8_HUMAN	CLU	Clusterin beta chain (Fragment) OS=Homo sapiens GN=CLU PE=4 SV=1	16224	0	747	0	57	0	0.21	#DIV/0!	0	1	0	1	0	1	0	1	0.68809
tr|H0Y4C2|H0Y4C2_HUMAN	HLA-F	HLA class I histocompatibility antigen, alpha chain F (Fragment) OS=Homo sapiens GN=HLA-F PE	25349	0	743	0	57	0	0.13	#DIV/0!	0	1	0	1	0	1	0	3	0.68809
sp|Q53EP0|FND3B_HUMAN	FNDC3B	Fibronectin type III domain-containing protein 3B OS=Homo sapiens GN=FNDC3B PE=1 SV=2	134970	0	739	0	57	0	0.02	#DIV/0!	0	1	0	1	0	1	0	1	0.68809
sp|O15091|MRRP3_HUMAN	KIAA0391	Mitochondrial ribonuclease P protein 3 OS=Homo sapiens GN=KIAA0391 PE=1 SV=2	68014	0	735	0	58	0	0.05	#DIV/0!	0	1	0	1	0	1	0	2	0.68809
sp|P54577|SYYC_HUMAN	YARS	Tyrosine--tRNA ligase, cytoplasmic OS=Homo sapiens GN=YARS PE=1 SV=4	59448	0	734	0	58	0	0.06	#DIV/0!	0	1	0	1	0	1	0	1	0.68809
tr|B3KNC3|B3KNC3_HUMAN	tr|B3KNC3|B	cDNA FLJ14222 fis, clone NT2RP3003992, highly similar to Nucleolar complex protein 2 homolog	85719	0	730	0	58	0	0.04	#DIV/0!	0	1	0	1	0	1	0	2	0.68809
tr|A6NN60|A6NN60_HUMAN	CRYZ	Quinone oxidoreductase OS=Homo sapiens GN=CRYZ PE=2 SV=1	31680	0	727	0	58	0	0.11	#DIV/0!	0	1	0	1	0	1	0	1	0.68809
tr|H0YNQ8|H0YNQ8_HUMAN	MYO1E	Unconventional myosin-Ie (Fragment) OS=Homo sapiens GN=MYO1E PE=4 SV=1	37550	0	718	0	59	0	0.09	#DIV/0!	0	1	0	1	0	1	0	2	0.68809
tr|Q6FHH6|Q6FHH6_HUMAN	LANCL1	LANCL1 protein (Fragment) OS=Homo sapiens GN=LANCL1 PE=2 SV=1	45979	0	713	0	60	0	0.07	#DIV/0!	0	1	0	1	0	1	0	1	0.68809
tr|B3KY55|B3KY55_HUMAN	tr|B3KY55|B3	cDNA FLJ46894 fis, clone UTERU3019708, highly similar to Regulator of nonsense transcripts 1 C	41002	0	712	0	0	0	0.05	#DIV/0!	0	0	0	0	0	1	0	1	0.68809
tr|B3KW81|B3KW81_HUMAN	tr|B3KW81|E	cDNA FLJ42510 fis, clone BRACE2044473, highly similar to Homo sapiens pericentrin 1 (PCNT1),	30173	0	705	0	0	0	0.09	#DIV/0!	0	0	0	0	0	1	0	1	0.68809
tr|B4DM84|B4DM84_HUMAN	tr|B4DM84|E	cDNA FLJ60694, highly similar to Deubiquitinating protein VCIP135 (EC 3.4.22.-) OS=Homo sapie	87230	0	701	0	61	0	0.04	#DIV/0!	0	1	0	1	0	1	0	1	0.68809
sp|Q8NAF0|ZN579_HUMAN	ZNF579	Zinc finger protein 579 OS=Homo sapiens GN=ZNF579 PE=1 SV=2	61726	0	682	0	63	0	0.05	#DIV/0!	0	1	0	1	0	1	0	1	0.68809
tr|B3KN28|B3KN28_HUMAN	tr|B3KN28|B	cDNA FLJ13370 fis, clone PLACE1000653, highly similar to Phosphoacetylglucosamine mutase (E	60284	0	675	0	63	0	0.05	#DIV/0!	0	1	0	1	0	1	0	2	0.68809
tr|Q6NX51|Q6NX51_HUMAN	EXOC4	Exocyst complex component 4 OS=Homo sapiens GN=EXOC4 PE=2 SV=1	111170	0	674	0	63	0	0.05	#DIV/0!	0	1	0	1	0	1	0	2	0.68809
tr|H0YJU2|H0YJU2_HUMAN	AHSA1	Activator of 90 kDa heat shock protein ATPase homolog 1 (Fragment) OS=Homo sapiens GN=AH	15052	0	669	0	23	0	0.48	#DIV/0!	0	0	0	1	0	1	0	1	0.68809
sp|Q05048|CSTF1_HUMAN	CSTF1	Cleavage stimulation factor subunit 1 OS=Homo sapiens GN=CSTF1 PE=1 SV=1	49125	0	668	0	64	0	0.07	#DIV/0!	0	1	0	1	0	1	0	2	0.68809
tr|B4DF80|B4DF80_HUMAN	tr|B4DF80|B4	cDNA FLJ58527, highly similar to Nucleolar protein 6 OS=Homo sapiens PE=2 SV=1	122714	0	663	0	25	0	0.05	#DIV/0!	0	0	0	1	0	1	0	1	0.68809
tr|B4DFP1|B4DFP1_HUMAN	tr|B4DFP1|B4	cDNA FLJ51818, highly similar to Phosphoglucomutase-1 (EC 5.4.2.2) OS=Homo sapiens PE=2 SV	58987	0	658	0	38	0	0.17	#DIV/0!	0	1	0	2	0	1	0	2	0.68809
tr|E9PDG5|E9PDG5_HUMAN	LARP4	La-related protein 4 OS=Homo sapiens GN=LARP4 PE=2 SV=1	50319	0	657	0	64	0	0.04	#DIV/0!	0	1	0	1	0	1	0	1	0.68809
tr|D3DSQ7|D3DSQ7_HUMAN	XPO7	Exportin 7, isoform CRA_c OS=Homo sapiens GN=XPO7 PE=2 SV=1	100370	0	641	0	66	0	0.03	#DIV/0!	0	1	0	1	0	1	0	1	0.68809
tr|I6L9E8|I6L9E8_HUMAN	FAM98A	Family with sequence similarity 98, member A OS=Homo sapiens GN=FAM98A PE=2 SV=1	55595	0	639	0	67	0	0.1	#DIV/0!	0	1	0	1	0	1	0	1	0.68809
tr|B3KY04|B3KY04_HUMAN	tr|B3KY04|B3	cDNA FLJ46506 fis, clone THYMU3030752, highly similar to BTB/POZ domain-containing protein	35994	0	626	0	68	0	0.09	#DIV/0!	0	1	0	1	0	1	0	1	0.68809
tr|G3V1U5|G3V1U5_HUMAN	GOLT1B	Golgi transport 1 homolog B (S. cerevisiae), isoform CRA_c OS=Homo sapiens GN=GOLT1B PE=2	8208	0	616	0	70	0	0.43	#DIV/0!	0	1	0	1	0	1	0	1	0.68809
sp|Q14126|DSG2_HUMAN	DSG2	Desmoglein-2 OS=Homo sapiens GN=DSG2 PE=1 SV=2	123016	0	610	0	70	0	0.03	#DIV/0!	0	1	0	1	0	1	0	1	0.68809
tr|B3KXF2|B3KXF2_HUMAN	tr|B3KXF2|B3	cDNA FLJ45314 fis, clone BRHIP3005142, highly similar to Proteasome-associated protein ECM2	146819	0	601	0	72	0	0.02	#DIV/0!	0	1	0	1	0	1	0	1	0.68809
tr|A8K1E4|A8K1E4_HUMAN	tr|A8K1E4|A8	cDNA FLJ77411, highly similar to Homo sapiens gamma-tubulin complex component GCP5 (GCP	119129	0	594	0	73	0	0.03	#DIV/0!	0	1	0	1	0	1	0	1	0.68809
sp|P51116|FXR2_HUMAN	FXR2	Fragile X mental retardation syndrome-related protein 2 OS=Homo sapiens GN=FXR2 PE=1 SV=2	74520	0	585	0	74	0	0.04	#DIV/0!	0	1	0	1	0	1	0	1	0.68809
tr|B3KMT7|B3KMT7_HUMAN	tr|B3KMT7|B	cDNA FLJ12561 fis, clone NT2RM4000798, highly similar to Brefeldin A-inhibited guanine nucleo	87689	0	580	0	58	0	0.05	#DIV/0!	0	1	0	1	0	1	0	2	0.68809
tr|B2RBB2|B2RBB2_HUMAN	tr|B2RBB2|B	cDNA, FLJ95416, highly similar to Homo sapiens phosphatidylinositol glycan, class S (PIGS), mRN	61818	0	576	0	76	0	0.05	#DIV/0!	0	1	0	1	0	1	0	1	0.68809
tr|Q6IBS5|Q6IBS5_HUMAN	DLST	DLST protein OS=Homo sapiens GN=DLST PE=2 SV=1	49131	0	574	0	77	0	0.4	#DIV/0!	0	1	0	1	0	1	0	1	0.68809
tr|B3KUI0|B3KUI0_HUMAN	tr|B3KUI0|B3	cDNA FLJ39945 fis, clone SPLEN2023977, highly similar to Homo sapiens CD97 antigen (CD97), t	79803	0	568	0	78	0	0.04	#DIV/0!	0	1	0	1	0	1	0	1	0.68809
tr|Q2NL95|Q2NL95_HUMAN	DDX19B	DDX19B protein (Fragment) OS=Homo sapiens GN=DDX19B PE=2 SV=1	51068	0	557	0	79	0	0.06	#DIV/0!	0	1	0	1	0	1	0	1	0.68809
tr|E7EVL6|E7EVL6_HUMAN	REPIN1	Replication initiator 1 (Fragment) OS=Homo sapiens GN=REPIN1 PE=2 SV=1	16586	0	551	0	80	0	0.2	#DIV/0!	0	1	0	1	0	1	0	1	0.68809
tr|B3KQ73|B3KQ73_HUMAN	tr|B3KQ73|B	cDNA FLJ33002 fis, clone THYMU1000217, highly similar to Homo sapiens ubiquitin associated p	37025	0	549	0	56	0	0.07	#DIV/0!	0	1	0	1	0	1	0	1	0.68809
tr|Q6IBU0|Q6IBU0_HUMAN	EIF5	EIF5 protein OS=Homo sapiens GN=EIF5 PE=2 SV=1	49577	0	544	0	81	0	0.15	#DIV/0!	0	1	0	1	0	1	0	1	0.68809
tr|B4DR63|B4DR63_HUMAN	PSMC1	26S protease regulatory subunit 4 OS=Homo sapiens GN=PSMC1 PE=2 SV=1	41255	0	532	0	84	0	0.08	#DIV/0!	0	1	0	1	0	1	0	1	0.68809
tr|B4DM73|B4DM73_HUMAN	tr|B4DM73|E	cDNA FLJ60078, highly similar to CLIP-associating protein 2 (Fragment) OS=Homo sapiens PE=2 S	116274	0	514	0	86	0	0.02	#DIV/0!	0	1	0	1	0	1	0	1	0.68809
tr|F1D8N4|F1D8N4_HUMAN	NR3C1	Glucocorticoid nuclear receptor variant 1 OS=Homo sapiens GN=NR3C1 PE=2 SV=1	86745	0	506	0	88	0	0.04	#DIV/0!	0	1	0	1	0	1	0	1	0.68809
tr|G3V1T4|G3V1T4_HUMAN	NUP107	Nuclear pore complex protein Nup107 OS=Homo sapiens GN=NUP107 PE=2 SV=1	11398	0	489	0	68	0	0.09	#DIV/0!	0	1	0	1	0	1	0	1	0.68809
sp|Q9H009|NACA2_HUMAN	NACA2	Nascent polypeptide-associated complex subunit alpha-2 OS=Homo sapiens GN=NACA2 PE=1 SV	23209	0	484	0	94	0	0.14	#DIV/0!	0	1	0	1	0	1	0	1	0.68809
tr|H0Y7C0|H0Y7C0_HUMAN	USO1	General vesicular transport factor p115 (Fragment) OS=Homo sapiens GN=USO1 PE=4 SV=1	72345	0	479	0	95	0	0.05	#DIV/0!	0	1	0	1	0	1	0	2	0.68809
sp|Q14739|LBR_HUMAN	LBR	Lamin-B receptor OS=Homo sapiens GN=LBR PE=1 SV=2	71057	0	470	0	97	0	0.05	#DIV/0!	0	1	0	1	0	1	0	2	0.68809
tr|B3KW93|B3KW93_HUMAN	tr|B3KW93|E	cDNA FLJ42590 fis, clone BRACE3009708, highly similar to Sodium/potassium-transporting ATPa	102434	0	461	0	99	0	0.03	#DIV/0!	0	1	0	1	0	1	0	2	0.68809
tr|J3KPP4|J3KPP4_HUMAN	LUC7L3	Cisplatin resistance-associated overexpressed protein, isoform CRA_b OS=Homo sapiens GN=LU	58698	0	456	0	99	0	0.06	#DIV/0!	0	1	0	1	0	1	0	2	0.68809
sp|O00507|USP9Y_HUMAN	USP9Y	Probable ubiquitin carboxyl-terminal hydrolase FAF-Y OS=Homo sapiens GN=USP9Y PE=1 SV=2	294370	0	416	0	0	0	0.05	#DIV/0!	0	0	0	0	0	1	0	1	0.68809
tr|B2R8G6|B2R8G6_HUMAN	tr|B2R8G6|B	cDNA, FLJ93891, Homo sapiens FK506 binding protein 8, 38kDa (FKBP8), mRNA OS=Homo sapie	38870	0	407	0	112	0	0.09	#DIV/0!	0	1	0	1	0	1	0	1	0.68809
tr|B4DJU4|B4DJU4_HUMAN	tr|B4DJU4|B4	cDNA FLJ53344, highly similar to Splicing factor 1 OS=Homo sapiens PE=2 SV=1	67535	0	1356	0	15	0	0.05	#DIV/0!	0	0	0	1	0	0	0	3	-0.89705
tr|B1AKJ6|B1AKJ6_HUMAN	OSBPL9	Oxysterol-binding protein OS=Homo sapiens GN=OSBPL9 PE=2 SV=1	84417	0	1355	0	15	0	0.04	#DIV/0!	0	0	0	1	0	0	0	1	-0.89705
tr|Q9UFD5|Q9UFD5_HUMAN	DKFZp434N18	Putative uncharacterized protein DKFZp434N181 (Fragment) OS=Homo sapiens GN=DKFZp434N	49392	0	1352	0	16	0	0.07	#DIV/0!	0	0	0	1	0	0	0	1	-0.89705
tr|Q5DT20|Q5DT20_HUMAN	HRNR	Hornerin OS=Homo sapiens GN=HRNR PE=2 SV=1	283111	0	1351	0	16	0	0.01	#DIV/0!	0	0	0	1	0	0	0	1	-0.89705
tr|Q4G0F4|Q4G0F4_HUMAN	POLRMT	DNA-directed RNA polymerase OS=Homo sapiens GN=POLRMT PE=2 SV=1	140194	0	1347	0	17	0	0.03	#DIV/0!	0	0	0	1	0	0	0	1	-0.89705
sp|Q12800|TFCP2_HUMAN	TFCP2	Alpha-globin transcription factor CP2 OS=Homo sapiens GN=TFCP2 PE=1 SV=2	57676	0	1345	0	17	0	0.07	#DIV/0!	0	0	0	1	0	0	0	1	-0.89705
sp|Q2M389|WASH7_HUMAN	KIAA1033	WASH complex subunit 7 OS=Homo sapiens GN=KIAA1033 PE=1 SV=2	137343	0	1342	0	17	0	0.02	#DIV/0!	0	0	0	1	0	0	0	2	-0.89705
tr|Q9UPE4|Q9UPE4_HUMAN	hTIM44	Putative mitochondrial inner membrane protein import receptor OS=Homo sapiens GN=hTIM44	47508	0	1341	0	17	0	0.07	#DIV/0!	0	0	0	1	0	0	0	3	-0.89705
tr|A8K5G2|A8K5G2_HUMAN	tr|A8K5G2|A	cDNA FLJ76292, highly similar to Homo sapiens myotubularin related protein 2 (MTMR2), transc	73877	0	1339	0	18	0	0.04	#DIV/0!	0	0	0	1	0	0	0	1	-0.89705

tr|B3KMC9|B3KMC9_HUMAN	tr|B3KMC9|B	cDNA FLJ10711 fis, clone NT2RP3000917, highly similar to 5~-3~ exoribonuclease 2 (EC 3.1.11.-)	109456	0	1337	0	18	0	0.03	#DIV/0!	0	0	0	1	0	0	0	1	-0.89705
tr|H7C1M2|H7C1M2_HUMAN	SON	Protein SON (Fragment) OS=Homo sapiens GN=SON PE=4 SV=1	160263	0	1333	0	19	0	0.05	#DIV/0!	0	0	0	1	0	0	0	1	-0.89705
tr|B4DR60|B4DR60_HUMAN	tr|B4DR60|B	cDNA FLJ56882, highly similar to Activating signal cointegrator 1 complex subunit 3 (EC 3.6.1.-) (	119789	0	1330	0	0	0	0.01	#DIV/0!	0	0	0	0	0	0	0	1	-0.89705
tr|B4E1K7|B4E1K7_HUMAN	STOML2	Stomatin-like protein 2 OS=Homo sapiens GN=STOML2 PE=2 SV=1	33317	0	1329	0	19	0	0.1	#DIV/0!	0	0	0	1	0	0	0	1	-0.89705
tr|B4DKA8|B4DKA8_HUMAN	USP8	Ubiquitin carboxyl-terminal hydrolase OS=Homo sapiens GN=USP8 PE=2 SV=1	115921	0	1328	0	19	0	0.03	#DIV/0!	0	0	0	1	0	0	0	2	-0.89705
tr|E9PDQ8|E9PDQ8_HUMAN	SUCLG2	Succinyl-CoA ligase [GDP-forming] subunit beta, mitochondrial OS=Homo sapiens GN=SUCLG2 P	41641	0	1106	0	0	0	0.08	#DIV/0!	0	0	0	0	0	0	0	1	-0.89705
tr|B3KNJ4|B3KNJ4_HUMAN	SAE1	SUMO-1 activating enzyme subunit 1, isoform CRA_a OS=Homo sapiens GN=SAE1 PE=2 SV=1	33705	0	1074	0	0	0	0.09	#DIV/0!	0	0	0	0	0	0	0	2	-0.89705
tr|H0Y3Y9|H0Y3Y9_HUMAN	PSMD4	26S proteasome non-ATPase regulatory subunit 4 (Fragment) OS=Homo sapiens GN=PSMD4 PE	21105	0	1026	0	0	0	0.08	#DIV/0!	0	0	0	0	0	0	0	1	-0.89705
tr|B1ANE3|B1ANE3_HUMAN	MTR	5-methyltetrahydrofolate-homocysteine methyltransferase (Fragment) OS=Homo sapiens GN=N	92463	0	991	0	0	0	0.03	#DIV/0!	0	0	0	0	0	0	0	1	-0.89705
tr|Q14756|Q14756_HUMAN	LLDBP	LLDBP (Fragment) OS=Homo sapiens GN=LLDBP PE=2 SV=1	58557	0	949	0	0	0	0.03	#DIV/0!	0	0	0	0	0	0	0	1	-0.89705
tr|B3KNR6|B3KNR6_HUMAN	tr|B3KNR6|B	cDNA FLJ30255 fis, clone BRACE2002444, highly similar to LAS1-like protein OS=Homo sapiens P	27615	0	812	0	0	0	0.04	#DIV/0!	0	0	0	0	0	0	0	1	-0.89705
tr|B4E227|B4E227_HUMAN	tr|B4E227|B	cDNA FLJ61053, highly similar to Nuclear RNA export factor 1 OS=Homo sapiens PE=2 SV=1	24906	0	755	0	0	0	0.05	#DIV/0!	0	0	0	0	0	0	0	1	-0.89705
tr|A0PJK8|A0PJK8_HUMAN	SCRIB	SCRIB protein (Fragment) OS=Homo sapiens GN=SCRIB PE=2 SV=1	71166	0	686	0	0	0	0.02	#DIV/0!	0	0	0	0	0	0	0	1	-0.89705
tr|H0Y7L9|H0Y7L9_HUMAN	ABCD1	ATP-binding cassette sub-family D member 1 (Fragment) OS=Homo sapiens GN=ABCD1 PE=4 SV	24597	0	563	0	0	0	0.04	#DIV/0!	0	0	0	0	0	0	0	1	-0.89705
tr|J3KR24|J3KR24_HUMAN	IARS	Isoleucine--tRNA ligase, cytoplasmic OS=Homo sapiens GN=IARS PE=4 SV=1	132820	366	112	47	364	0.02	0.44	22	1	9	1	15	2	16	2	18	1.827627
tr|B2RBA6|B2RBA6_HUMAN	tr|B2RBA6|B	cDNA, FLJ95407, highly similar to Homo sapiens MCM7 minichromosome maintenance deficient	81856	374	82	46	491	0.04	0.8	20	1	13	1	14	1	14	1	16	2.378154
sp|O14980|XPO1_HUMAN	XPO1	Exportin-1 OS=Homo sapiens GN=XPO1 PE=1 SV=1	124447	305	95	59	428	0.03	0.51	17	1	10	1	16	1	19	3	22	2.806478
sp|Q14204|DYHC1_HUMAN	DYNC1H1	Cytoplasmic dynein 1 heavy chain 1 OS=Homo sapiens GN=DYNC1H1 PE=1 SV=5	534809	168	41	129	887	0.02	0.34	17	3	28	4	48	4	50	6	59	2.598925
sp|P45974|UBP5_HUMAN	USP5	Ubiquitin carboxyl-terminal hydrolase 5 OS=Homo sapiens GN=USP5 PE=1 SV=2	96638	622	180	24	262	0.03	0.49	16.3333333	0	8	1	12	2	12	2	13	1.426368
sp|P11388|TOP2A_HUMAN	TOP2A	DNA topoisomerase 2-alpha OS=Homo sapiens GN=TOP2A PE=1 SV=3	175017	606	125	25	343	0.02	0.27	13.5	0	9	1	13	1	14	2	16	2.378154
tr|H0UID3|H0UID3_HUMAN	AP2B1	Adaptor-related protein complex 2, beta 1 subunit, isoform CRA_d OS=Homo sapiens GN=AP2B	106321	648	183	22	258	0.03	0.4	13.3333333	0	9	1	12	3	13	3	13	1.051817
tr|Q2TU64|Q2TU64_HUMAN	tr|Q2TU64|Q	PIG48 OS=Homo sapiens PE=2 SV=1	61110	279	171	68	179	0.05	0.64	12.8	1	5	1	7	3	10	3	11	0.688706
tr|Q9NSU9|Q9NSU9_HUMAN	DKFZp434G07	Putative uncharacterized protein DKFZp434G0719 (Fragment) OS=Homo sapiens GN=DKFZp434	41107	56	174	169	270	0.07	0.86	12.2857143	4	7	5	8	5	8	5	8	-0.26926
tr|E9PBS1|E9PBS1_HUMAN	PAICS	Phosphoribosylaminoimidazole carboxylase (Fragment) OS=Homo sapiens GN=PAICS PE=2 SV=1	46307	270	163	69	247	0.07	0.82	11.7142857	1	7	1	8	2	8	5	10	0.869253
tr|A8K0G3|A8K0G3_HUMAN	tr|A8K0G3|A	cDNA FLJ78733, highly similar to Homo sapiens adaptor-related protein complex 2, beta 1 subur	106477	648	190	22	252	0.03	0.35	11.6666667	0	9	1	11	3	12	3	13	0.940605
tr|B4DJL0|B4DJL0_HUMAN	tr|B4DJL0|B	cDNA FLJ60278, highly similar to Dolichyl-diphosphooligosaccharide--proteinglycosyltransferase	31401	589	422	27	109	0.05	0.5	10	0	2	1	4	1	4	1	5	0.688295
sp|Q15645|PCH2_HUMAN	TRIP13	Pachytene checkpoint protein 2 homolog OS=Homo sapiens GN=TRIP13 PE=1 SV=2	48863	507	438	33	105	0.07	0.69	9.85714286	0	4	1	8	2	7	2	9	0.6885
tr|B4DLT2|B4DLT2_HUMAN	tr|B4DLT2|B	cDNA FLJ56637, highly similar to Nuclear pore complex protein Nup155 OS=Homo sapiens PE=2	152734	489	122	35	325	0.02	0.19	9.5	0	5	1	8	1	9	1	12	1.767199
sp|Q7L2E3|DHX30_HUMAN	DHX30	Putative ATP-dependent RNA helicase DHX30 OS=Homo sapiens GN=DHX30 PE=1 SV=1	134938	397	179	43	264	0.05	0.47	9.4	1	10	2	16	3	18	4	20	1.507285
tr|E7ENU7|E7ENU7_HUMAN	RPL15	Ribosomal protein L15 (Fragment) OS=Homo sapiens GN=RPL15 PE=2 SV=1	20596	534	345	31	119	0.16	1.47	9.1875	0	4	1	5	1	5	1	5	0.977982
sp|Q6P2Q9|PRP8_HUMAN	PRPF8	Pre-mRNA-processing-splicing factor 8 OS=Homo sapiens GN=PRPF8 PE=1 SV=2	274738	130	26	167	1314	0.1	0.91	9.1	7	39	8	55	10	67	10	77	1.796205
tr|B2RE46|B2RE46_HUMAN	tr|B2RE46|B	cDNA, FLJ96923, highly similar to Homo sapiens ribophorin II (RPN2), mRNA OS=Homo sapiens F	69405	589	271	27	170	0.05	0.45	9	0	5	1	8	2	7	2	8	0.6885
tr|A4FU77|A4FU77_HUMAN	SNRNP200	SNRNP200 protein (Fragment) OS=Homo sapiens GN=SNRNP200 PE=2 SV=1	217585	243	54	83	645	0.05	0.43	8.6	2	20	3	26	7	28	8	32	1.031649
sp|P54886|P5CS_HUMAN	ALDH18A1	Delta-1-pyrroline-5-carboxylate synthase OS=Homo sapiens GN=ALDH18A1 PE=1 SV=2	87989	658	221	21	214	0.04	0.34	8.5	1	6	1	8	1	11	1	14	2.043194
tr|D3DRX6|D3DRX6_HUMAN	KIF5B	Kinesin family member 5B, isoform CRA_a OS=Homo sapiens GN=KIF5B PE=3 SV=1	110331	227	168	91	279	0.04	0.34	8.5	2	7	2	10	2	13	3	16	1.537579
tr|E7ERW8|E7ERW8_HUMAN	DIAPH1	Protein diaphanous homolog 1 OS=Homo sapiens GN=DIAPH1 PE=2 SV=1	135945	184	377	116	123	0.02	0.17	8.5	1	5	1	7	1	7	2	10	1.425801
tr|B2R5V9|B2R5V9_HUMAN	tr|B2R5V9|B	cDNA, FLJ92652, highly similar to Homo sapiens high density lipoprotein binding protein (vigilin)	141919	253	160	76	222	0.02	0.17	8.5	1	5	1	6	2	8	2	10	0.869253
sp|P25490|TYY1_HUMAN	YY1	Transcriptional repressor protein YY1 OS=Homo sapiens GN=YY1 PE=1 SV=2	45141	630	787	23	53	0.07	0.59	8.42857143	0	1	1	2	2	2	2	2	-0.89736
sp|P11498|PYC_HUMAN	PC	Pyruvate carboxylase, mitochondrial OS=Homo sapiens GN=PC PE=1 SV=2	130293	10	1	1295	5174	1.76	14.64	8.31818182	40	138	52	180	56	198	67	214	0.932966
tr|Q4ZG57|Q4ZG57_HUMAN	MCM6	Putative uncharacterized protein MCM6 (Fragment) OS=Homo sapiens GN=MCM6 PE=2 SV=1	89803	444	225	39	210	0.04	0.33	8.25	1	7	1	8	2	9	3	10	1.029898
tr|B7Z9W6|B7Z9W6_HUMAN	tr|B7Z9W6|B	cDNA, FLJ78980, highly similar to DnaJ homolog subfamily C member 7 OS=Homo sapiens PE=2	50776	527	651	31	42	0.06	0.48	8	0	1	1	1	1	2	1	2	-0.16006
tr|B2R8R5|B2R8R5_HUMAN	tr|B2R8R5|B	cDNA, FLJ94025, highly similar to Homo sapiens tripartite motif-containing 28 (TRIM28), mRNA	90271	320	129	56	333	0.07	0.53	7.57142857	1	13	3	14	2	15	4	15	1.737249
sp|Q9UQ35|SRRM2_HUMAN	SRRM2	Serine/arginine repetitive matrix protein 2 OS=Homo sapiens GN=SRRM2 PE=1 SV=2	300179	247	97	78	419	0.02	0.15	7.5	1	11	2	13	2	13	5	14	1.537579
tr|D3DTH7|D3DTH7_HUMAN	MYO1C	Myosin IC, isoform CRA_a OS=Homo sapiens GN=MYO1C PE=2 SV=1	99613	284	148	65	304	0.07	0.52	7.42857143	1	7	3	13	3	13	4	15	1.051817
sp|P53618|COPB_HUMAN	COPB1	Coatomer subunit beta OS=Homo sapiens GN=COPB1 PE=1 SV=3	108214	303	161	59	288	0.06	0.43	7.16666667	1	7	2	12	2	10	3	14	1.174468
tr|F8VPD4|F8VPD4_HUMAN	CAD	CAD protein OS=Homo sapiens GN=CAD PE=2 SV=1	238097	317	87	57	445	0.04	0.28	7	1	12	3	18	4	21	5	27	1.361732
tr|H0YD14|H0YD14_HUMAN	MYOF	Myoferlin (Fragment) OS=Homo sapiens GN=MYOF PE=4 SV=1	135822	220	56	95	391	0.07	0.49	7	3	12	3	16	4	18	4	20	1.144379
tr|B2RBE0|B2RBE0_HUMAN	tr|B2RBE0|B	cDNA, FLJ95462, highly similar to Homo sapiens fatty-acid-Coenzyme A ligase, long-chain 3 (FAC	81266	462	265	37	172	0.04	0.27	6.75	0	6	1	6	1	6	1	7	1.21917
tr|D3DP78|D3DP78_HUMAN	DARS	Aspartyl-tRNA synthetase, isoform CRA_b OS=Homo sapiens GN=DARS PE=2 SV=1	53829	344	233	51	172	0.06	0.4	6.66666667	1	5	1	5	2	5	3	5	0.240681
tr|C9J1E7|C9J1E7_HUMAN	AP1B1	AP-1 complex subunit beta-1 (Fragment) OS=Homo sapiens GN=AP1B1 PE=2 SV=1	66014	648	304	22	138	0.03	0.2	6.66666667	0	4	1	5	3	4	3	5	-0.53477
tr|H0Y8E6|H0Y8E6_HUMAN	MCM2	DNA replication licensing factor MCM2 (Fragment) OS=Homo sapiens GN=MCM2 PE=3 SV=1	94792	211	153	98	273	0.07	0.46	6.57142857	1	8	2	11	2	11	2	11	1.305893
tr|B1AHB0|B1AHB0_HUMAN	MCM5	MCM5 minichromosome maintenance deficient 5, cell division cycle 46 (S. cerevisiae), isoform C	83031	562	340	29	132	0.04	0.26	6.5	0	3	1	6	1	7	2	12	1.425801
tr|Q2NLA0|Q2NLA0_HUMAN	COPG2	Coatomer subunit gamma OS=Homo sapiens GN=COPG2 PE=2 SV=1	82400	705	344	18	71	0.04	0.26	6.5	0	1	1	1	0	1	1	1	0.68809
tr|B4DH39|B4DH39_HUMAN	tr|B4DH39|B	cDNA FLJ57028, highly similar to Long-chain-fatty-acid--CoA ligase 4 (EC 6.2.1.3) OS=Homo sapie	73123	529	314	31	144	0.04	0.25	6.25	0	3	1	5	1	5	3	5	0.977982
tr|B4DUC5|B4DUC5_HUMAN	CSE1L	Exportin-2 OS=Homo sapiens GN=CSE1L PE=2 SV=1	86045	433	156	40	221	0.08	0.5	6.25	0	7	2	10	4	12	4	14	0.577699
tr|B2ZZ90|B2ZZ90_HUMAN	ACACA	Acetyl-Coenzyme A carboxylase alpha OS=Homo sapiens GN=ACACA PE=2 SV=1	267095	31	5	628	3310	0.44	2.71	6.15909091	25	94	30	125	38	136	46	147	0.940821
sp|Q09666|AHNK_HUMAN	AHNAK	Neuroblast differentiation-associated protein AHNAK OS=Homo sapiens GN=AHNAK PE=1 SV=2	629213	401	208	43	229	0.01	0.06	6	1	6	1	12	3	16	6	19	1.341865
tr|A2BDK6|A2BDK6_HUMAN	MAP1B	Microtubule-associated protein 1B OS=Homo sapiens GN=MAP1B PE=2 SV=1	257692	511	264	32	169	0.01	0.06	6	0	3	1	4	1	5	2	6	0.977982
tr|B4DKS0|B4DKS0_HUMAN	tr|B4DKS0|B	cDNA FLJ53381, highly similar to Monocarboxylate transporter 1 OS=Homo sapiens PE=2 SV=1	52487	598	373	26	109	0.06	0.36	6	0	2	1	4	1	4	1	4	0.688295
tr|I3L1L3|I3L1L3_HUMAN	MYBBP1A	Myb-binding protein 1A (Fragment) OS=Homo sapiens GN=MYBBP1A PE=4 SV=1	141124	698	298	18	153	0.02	0.12	6	0	3	1	5	1	4	2	6	0.688295
tr|A6NKB8|A6NKB8_HUMAN	RNPEP	Aminopeptidase B OS=Homo sapiens GN=RNPEP PE=2 SV=1	68752	316	300	57	151	0.05	0.3	6	1	5	2	6	2	5	2	7	0.240681
tr|B2R4K7|B2R4K7_HUMAN	tr|B2R4K7|B	60S ribosomal protein L6 OS=Homo sapiens PE=2 SV=1	32739	276	92	68	434	0.47	2.8	5.95744681	2	11	4	12	3	12	5	14	0.940605
sp|O75369|FLNB_HUMAN	FLNB	Filamin-B OS=Homo sapiens GN=FLNB PE=1 SV=2	280157	45	11	463	1829	0.22	1.3	5.90909091	14	52	18	76	20	80	28	92	1.084069
tr|F1T0B3|F1T0B3_HUMAN	DDX1	ATP-dependent RNA helicase DDX1 OS=Homo sapiens GN=DDX1 PE=2 SV=1	74838	501	309	33	144	0.09	0.53	5.88888889	0	6	2	10	3	11	3	12	0.820131
sp|P52597|HNRPF_HUMAN	HNRNPF	Heterogeneous nuclear ribonucleoprotein F OS=Homo sapiens GN=HNRNPF PE=1 SV=3	45985	226	243	91	189	0.07	0.41	5.85714286	1	4	1	6	2	6	3	9	0.481869
tr|D3DTW3|D3DTW3_HUMAN	IGF2BP1	Insulin-like growth factor 2 mRNA binding protein 1, isoform CRA_a OS=Homo sapiens GN=IGF2	48111	556	512	29	60	0.05	0.29	5.8	0	3	1	4	1	6	1	8	1.21917
tr|B2RB23|B2RB23_HUMAN	tr|B2RB23|B	cDNA, FLJ95265, highly similar to Homo sapiens acetyl-Coenzyme A acyltransferase 2 (mitochon	42439	544	239	30	194	0.08	0.46	5.75	0	3	1	5	1	5	1	5	0.977982
tr|Q1L838|Q1L838_HUMAN	GTBP	GTBP-ALT OS=Homo sapiens GN=GTBP PE=2 SV=1	122267	559	381	29	122	0.03	0.17	5.66666667	0	4	1	6	1	6	1	8	1.21917
tr|B0UZ98|B0UZ98_HUMAN	BAT1	HLA-B associated transcript 1 (Fragment) OS=Homo sapiens GN=BAT1 PE=2 SV=1	28623	394	650	44	41	0.09	0.51	5.66666667	1	1	1	2	1	2	1	2	-0.16006
tr|B4DYL7|B4DYL7_HUMAN	tr|B4DYL7|B	cDNA FLJ58490, highly similar to Homo sapiens CCR4-NOT transcription complex, subunit 1 (CN	140991	388	362	45	81	0.02	0.11	5.5	1	3	1	6	1	5	2	6	0.977982
tr|J3KQL0|J3KQL0_HUMAN	CPS1	Carbamoyl-phosphate synthase [ammonia], mitochondrial OS=Homo sapiens GN=CPS1 PE=3 SV	166686	204	42	102	871	0.15	0.82	5.46666667	4	22	7	33	10	39	12	42	1.018094
sp|P11021|GRP78_HUMAN	HSPA5	78 kDa glucose-regulated protein OS=Homo sapiens GN=HSPA5 PE=1 SV=2	72402	129	64	170	609	0.19	1.03	5.42105263	3	12	5	17	6	19	6	20	0.689322
sp|O94906|PRP6_HUMAN	PRPF6	Pre-mRNA-processing factor 6 OS=Homo sapiens GN=PRPF6 PE=1 SV=1	107656	492	415	35	110	0.03	0.16	5.33333333	1	3	1	5	1	8	2	8	1.606554
tr|A8K492|A8K492_HUMAN	tr|A8K492|A	cDNA FLJ76789, highly similar to Homo sapiens methionine-tRNA synthetase (MARS), mRNA OS	102277	385	157	45	292	0.07	0.37	5.28571429	1	6	2	10	2	9	2	10	1.029898
sp|P41250|SYG_HUMAN	GARS	Glycine--tRNA ligase OS=Homo sapiens GN=GARS PE=1 SV=3	83854	392	393	44	117	0.04	0.21	5.25	1	4	1	5	1	5	2	8	0.977982
tr|B4DS66|B4DS66_HUMAN	tr|B4DS66|B	cDNA FLJ54290, highly similar to Mitochondrial inner membrane protein OS=Homo sapiens PE=	73509	712	442	17	103	0.04	0.21	5.25	0	2	1	4	1	4	6	7	0.688295
sp|Q15155|NOMO1_HUMAN	NOMO1	Nodal modulator 1 OS=Homo sapiens GN=NOMO1 PE=1 SV=5	135209	400	977	43	39	0.04	0.21	5.25	1	1	1	1	1	1	1	1	-0.89721
tr|B3KMX0|B3KMX0_HUMAN	tr|B3KMX0|B	cDNA FLJ12837 fis, clone NT2RP2003228, highly similar to DNA replication licensing factor MCM	97114	240	211	85	225	0.1	0.51	5.1	2	9	3	12	4	13	5	13	0.688911
tr|B3KS31|B3KS31_HUMAN	TUBB6	Tubulin, beta 6, isoform CRA_a OS=Homo sapiens GN=TUBB6 PE=2 SV=1	42324	73	44	273	741	0.83	4.21	5.07228916	10	25	14	33	17	36	20	40	0.164274
sp|P06733|ENOA_HUMAN	ENO1	Alpha-enolase OS=Homo sapiens GN=ENO1 PE=1 SV=2	47481	40	31	520	1154	1.39	7.01	5.04316547	13	32	18	39	19	46	25	53	0.358626
tr|Q68DH9|Q68DH9_HUMAN	DKFZp686M0	Putative uncharacterized protein DKFZp686M088 OS=Homo sapiens GN=DKFZp686M088 PE=2 S	135044	528	360	31	127	0.03	0.15	5	0	2	1	6	1	6	1	7	1.21917
tr|A2VCT2|A2VCT2_HUMAN	OGDH	OGDH protein (Fragment) OS=Homo sapiens GN=OGDH PE=2 SV=1	72973	432	372	40	35	0.05	0.25	5	1	1	1	2	1	2	1	3	-0.16006
tr|H0Y7S3|H0Y7S3_HUMAN	ATP2B2	Plasma membrane calcium-transporting ATPase 2 (Fragment) OS=Homo sapiens GN=ATP2B2 PE	122272	528	283	31	100	0.03	0.15	5	0	2	1	2	1	2	3	2	-0.16006
sp|P05165|PCCA_HUMAN	PCCA	Propionyl-CoA carboxylase alpha chain, mitochondrial OS=Homo sapiens GN=PCCA PE=1 SV=4	80635	99	39	216	919	0.49	2.44	4.97959184	6	26	10	32	13	34	13	38	0.458354
tr|Q2TSD0|Q2TSD0_HUMAN	tr|Q2TSD0|Q	Glyceraldehyde-3-phosphate dehydrogenase OS=Homo sapiens PE=2 SV=1	36197	18	27	891	1100	3.44	17.02	4.94767442	23	28	31	40	31	46	36	52	-0.3373
tr|B3KRS7|B3KRS7_HUMAN	tr|B3KRS7|B	cDNA FLJ34844 fis, clone NT2NE2011154, highly similar to RuvB-like 1 OS=Homo sapiens PE=2 S	34946	351	646	50	66	0.09	0.44	4.88888889	1	3	1	4	1	4	1	4	0.688295
tr|Q60FE2|Q60FE2_HUMAN	MYH9	MYH9 variant protein OS=Homo sapiens GN=MYH9 PE=2 SV=1	227646	17	3	900	4100	0.9	4.36	4.84444444	30	101	46	130	52	147	60	157	0.602447
tr|Q68DB1|Q68DB1_HUMAN	DKFZp686F22	Metastasis-associated protein MTA2 OS=Homo sapiens GN=DKFZp686F2281 PE=2 SV=1	55515	676	223	20	54	0.06	0.29	4.83333333	0	1	1	3	1	3	1	3	0.325545
tr|B4DE27|B4DE27_HUMAN	tr|B4DE27|B	cDNA FLJ58765, highly similar to Neutral amino acid transporter B(0) OS=Homo sapiens PE=2 SV	33987	290	234	63	111	0.1	0.48	4.8	1	3	1	4	1	4	1	4	0.688295
tr|Q59G92|Q59G92_HUMAN	tr|Q59G92|Q	HLA-B associated transcript 1 variant (Fragment) OS=Homo sapiens PE=2 SV=1	35528	394	1072	44	34	0.09	0.43	4.77777778	1	1	1	4	2	5	2	5	0.240681
sp|O95373|IPO7_HUMAN	IPO7	Importin-7 OS=Homo sapiens GN=IPO7 PE=1 SV=1	120751	162	124	132	347	0.08	0.38	4.75	3	9	3	14	3	15	4	17	1.251486
tr|B2R7C5|B2R7C5_HUMAN	tr|B2R7C5|B	cDNA, FLJ93378, highly similar to Homo sapiens MCM3 minichromosome maintenance deficient	91579	479	539	36	82	0.04	0.19	4.75	1	4	1	5	1	6	1	7	1.21917
sp|O76094|SRP72_HUMAN	SRP72	Signal recognition particle 72 kDa protein OS=Homo sapiens GN=SRP72 PE=1 SV=3	75130	455	368	38	125	0.04	0.19	4.75	1	3	1	4	1	4	1	7	0.688295
tr|Q2NKY5|Q2NKY5_HUMAN	TUBB6	TUBB6 protein OS=Homo sapiens GN=TUBB6 PE=2 SV=1	50514	65	38	317	961	0.88	4.17	4.73863636	12	29	16	40	17	42	19	48	0.384927
tr|I3L2F9|I3L2F9_HUMAN	tr|I3L2F9|I3L	Uncharacterized protein OS=Homo sapiens PE=3 SV=1	46054	25	8	275	354	3.07	14.34	4.67100977	12	15	15	18	18	21	19	24	-0.6825
tr|B2R9U2|B2R9U2_HUMAN	tr|B2R9U2|B	cDNA, FLJ94557, highly similar to Homo sapiens FK506 binding protein 4, 59kDa (FKBP4), mRNA	52087	623	429	24	107	0.06	0.28	4.66666667	0	2	1	4	2	4	2	5	-0.04901
sp|Q68EM7|RHG17_HUMAN	ARHGAP17	Rho GTPase-activating protein 17 OS=Homo sapiens GN=ARHGAP17 PE=1 SV=1	95776	328	809	54	52	0.03	0.14	4.66666667	1	1	1	1	2	2	2	3	-0.89736
tr|B2R7B5|B2R7B5_HUMAN	tr|B2R7B5|B	cDNA, FLJ93365, highly similar to Homo sapiens KH domain containing, RNA binding, signal tran	48309	347	294	51	124	0.07	0.32	4.57142857	1	3	1	3	2	5	4	6	0.240681
tr|B5BU25|B5BU25_HUMAN	U2AF2	U2 small nuclear RNA auxiliary factor 2 isoform b OS=Homo sapiens GN=U2AF2 PE=2 SV=1	53445	260	332	74	104	0.13	0.59	4.53846154	1	3	2	4	2	4	2	4	-0.04901
tr|B3KM77|B3KM77_HUMAN	tr|B3KM77|B	cDNA FLJ10435 fis, clone NT2RP1000493, highly similar to Splicing factor 3B subunit 3 OS=Homo	56736	356	110	49	264	0.06	0.27	4.5	1	6	1	7	1	8	1	8	1.606554
tr|A6NEM2|A6NEM2_HUMAN	HCFC1	HCF N-terminal chain 5 OS=Homo sapiens GN=HCFC1 PE=2 SV=2	215338	508	310	33	145	0.02	0.09	4.5	0	4	1	6	1	7	2	8	1.425801
tr|G0ZJH6|G0ZJH6_HUMAN	Ube4B	UFD2A-III/UBE4B-III splice isoform OS=Homo sapiens GN=Ube4B PE=2 SV=1	152890	667	528	21	84	0.02	0.09	4.5	0	1	1	4	1	4	1	5	0.688295
tr|B4DGQ0|B4DGQ0_HUMAN	LASP1	LIM and SH3 domain protein 1 OS=Homo sapiens GN=LASP1 PE=2 SV=1	23167	568	759	29	35	0.14	0.63	4.5	0	0	1	2	1	3	1	4	0.325545
tr|Q5SU16|Q5SU16_HUMAN	TUBB	Beta 5-tubulin OS=Homo sapiens GN=TUBB PE=2 SV=1	50095	25	9	680	1843	3.07	13.56	4.41693811	26	58	33	70	35	75	39	79	0.19339
sp|Q9Y2A7|NCKP1_HUMAN	NCKAP1	Nck-associated protein 1 OS=Homo sapiens GN=NCKAP1 PE=1 SV=1	130018	685	326	19	138	0.03	0.13	4.33333333	0	5	1	5	0	7	1	8	3.011099
tr|A7E2D8|A7E2D8_HUMAN	ATP2B4	ATP2B4 protein OS=Homo sapiens GN=ATP2B4 PE=2 SV=1	129890	528	354	31	129	0.03	0.13	4.33333333	0	2	1	5	1	6	1	6	1.21917
tr|B7ZKM0|B7ZKM0_HUMAN	SART3	SART3 protein OS=Homo sapiens GN=SART3 PE=2 SV=1	106372	153	66	139	548	0.16	0.69	4.3125	5	14	5	17	7	19	7	24	0.482535
tr|I3L0N3|I3L0N3_HUMAN	NSF	Vesicle-fusing ATPase OS=Homo sapiens GN=NSF PE=3 SV=1	82553	412	802	42	52	0.04	0.17	4.25	1	0	1	4	1	5	1	6	0.977982
tr|B2R5M9|B2R5M9_HUMAN	tr|B2R5M9|B	cDNA, FLJ92537, highly similar to Homo sapiens procollagen-lysine, 2-oxoglutarate 5-dioxygena	84022	251	369	76	125	0.04	0.17	4.25	1	4	1	4	1	5	1	6	0.977982
tr|Q05D08|Q05D08_HUMAN	PA2G4	PA2G4 protein (Fragment) OS=Homo sapiens GN=PA2G4 PE=2 SV=1	45579	286	339	64	133	0.15	0.63	4.2	1	5	2	7	3	7	3	7	0.202738
sp|O95831|AIFM1_HUMAN	AIFM1	Apoptosis-inducing factor 1, mitochondrial OS=Homo sapiens GN=AIFM1 PE=1 SV=1	67144	326	566	54	78	0.05	0.21	4.2	1	2	1	4	1	4	2	7	0.688295
sp|Q00610|CLH1_HUMAN	CLTC	Clathrin heavy chain 1 OS=Homo sapiens GN=CLTC PE=1 SV=5	193260	30	7	629	1912	0.52	2.17	4.17307692	19	51	25	74	24	82	28	97	0.861332
tr|B1AH89|B1AH89_HUMAN	TTLL12	Tubulin tyrosine ligase-like family, member 12 OS=Homo sapiens GN=TTLL12 PE=4 SV=1	74185	104	47	209	778	0.41	1.71	4.17073171	6	18	8	27	10	28	11	32	0.545214
sp|Q01650|LAT1_HUMAN	SLC7A5	Large neutral amino acids transporter small subunit 1 OS=Homo sapiens GN=SLC7A5 PE=1 SV=2	55659	239	142	85	315	0.12	0.5	4.16666667	1	8	3	9	4	9	5	11	0.18123
sp|Q9Y305|ACOT9_HUMAN	ACOT9	Acyl-coenzyme A thioesterase 9, mitochondrial OS=Homo sapiens GN=ACOT9 PE=1 SV=2	50269	637	751	22	57	0.07	0.29	4.14285714	0	2	1	4	1	6	2	8	1.21917
tr|Q8IWP6|Q8IWP6_HUMAN	tr|Q8IWP6|Q	Class IVb beta tubulin OS=Homo sapiens PE=2 SV=1	50177	28	12	654	1815	3.05	12.59	4.12786885	27	57	32	68	34	73	37	77	0.195854
tr|E5KNY5|E5KNY5_HUMAN	LRPPRC	Leucine-rich PPR-motif containing OS=Homo sapiens GN=LRPPRC PE=2 SV=1	159003	329	140	53	319	0.08	0.33	4.125	1	10	4	14	5	15	7	17	0.598738
tr|B7Z6M1|B7Z6M1_HUMAN	PLS3	Plastin-3 OS=Homo sapiens GN=PLS3 PE=2 SV=1	66047	282	99	66	411	0.34	1.4	4.11764706	1	12	6	18	6	18	8	20	0.613193
tr|H0YJ34|H0YJ34_HUMAN	FERMT2	Fermitin family homolog 2 (Fragment) OS=Homo sapiens GN=FERMT2 PE=4 SV=1	73877	306	105	59	352	0.14	0.57	4.07142857	2	8	3	11	3	12	3	14	0.940605
tr|F8W9S7|F8W9S7_HUMAN	GAPVD1	GTPase-activating protein and VPS9 domain-containing protein 1 OS=Homo sapiens GN=GAPVD	158790	697	482	18	94	0.02	0.08	4	0	3	1	4	0	4	2	4	2.273593
sp|Q9NZB2|F120A_HUMAN	FAM120A	Constitutive coactivator of PPAR-gamma-like protein 1 OS=Homo sapiens GN=FAM120A PE=1 S	123008	263	242	73	191	0.05	0.2	4	2	4	2	7	2	7	2	7	0.6885
tr|B3KSH1|B3KSH1_HUMAN	EIF3F	Eukaryotic translation initiation factor 3 subunit F OS=Homo sapiens GN=EIF3F PE=2 SV=1	39236	652	570	22	77	0.08	0.32	4	0	1	1	1	1	1	1	1	-0.89721
sp|P26038|MOES_HUMAN	MSN	Moesin OS=Homo sapiens GN=MSN PE=1 SV=3	67892	119	63	179	614	0.46	1.83	3.97826087	7	20	8	25	11	26	12	33	0.308302
tr|B2R5U1|B2R5U1_HUMAN	tr|B2R5U1|B	cDNA, FLJ92620, highly similar to Homo sapiens staphylococcal nuclease domain containing 1 (S	100294	150	90	144	440	0.17	0.67	3.94117647	4	11	5	17	10	21	12	21	0.137324
tr|Q0VGD6|Q0VGD6_HUMAN	HNRPR	HNRPR protein (Fragment) OS=Homo sapiens GN=HNRPR PE=2 SV=1	68218	390	212	44	224	0.1	0.39	3.9	1	6	2	7	2	8	4	9	0.869253
tr|B2R5P6|B2R5P6_HUMAN	TXNRD1	Thioredoxin reductase 1, cytoplasmic OS=Homo sapiens GN=TXNRD1 PE=2 SV=1	55253	108	74	204	495	0.26	0.99	3.80769231	3	10	5	12	5	14	6	15	0.502343
tr|A6NE09|A6NE09_HUMAN	RPSAP58	Protein RPSAP58 OS=Homo sapiens GN=RPSAP58 PE=3 SV=1	33002	521	327	31	72	0.1	0.38	3.8	0	2	1	2	1	2	1	3	-0.16006
tr|Q2Q9B7|Q2Q9B7_HUMAN	G6PD	Glucose-6-phosphate 1-dehydrogenase (Fragment) OS=Homo sapiens GN=G6PD PE=2 SV=1	55221	176	237	118	195	0.18	0.68	3.77777778	2	5	2	9	5	11	6	13	0.167382
tr|B4DR68|B4DR68_HUMAN	tr|B4DR68|B	cDNA FLJ58608, highly similar to Heat shock protein 75 kDa, mitochondrial OS=Homo sapiens PE	74408	169	193	127	218	0.09	0.34	3.77777778	2	5	3	6	3	6	5	7	-0.00389
tr|B4DYA7|B4DYA7_HUMAN	tr|B4DYA7|B	Glucose-6-phosphate 1-dehydrogenase OS=Homo sapiens PE=2 SV=1	58757	176	238	123	179	0.18	0.68	3.77777778	2	4	3	8	6	10	7	12	-0.20539
tr|Q3B7A3|Q3B7A3_HUMAN	Sep-07	SEPT7 protein (Fragment) OS=Homo sapiens GN=SEPT7 PE=2 SV=1	26971	531	653	31	65	0.07	0.26	3.71428571	1	2	1	2	1	3	1	3	0.325545
tr|Q5TCU6|Q5TCU6_HUMAN	TLN1	Talin-1 OS=Homo sapiens GN=TLN1 PE=4 SV=1	260030	48	10	446	1807	0.27	1	3.7037037	12	46	19	60	20	64	25	73	0.761472
tr|Q60FE5|Q60FE5_HUMAN	FLNA	Filamin A OS=Homo sapiens GN=FLNA PE=2 SV=1	280790	9	4	1306	4079	0.86	3.18	3.69767442	41	113	60	156	75	163	88	182	0.221972
tr|Q2TB59|Q2TB59_HUMAN	NNT	Nicotinamide nucleotide transhydrogenase OS=Homo sapiens GN=NNT PE=2 SV=1	114552	234	170	88	274	0.06	0.22	3.66666667	1	5	2	7	2	7	3	7	0.6885
tr|F5H3A1|F5H3A1_HUMAN	ATP1A1	Sodium/potassium-transporting ATPase subunit alpha-1 OS=Homo sapiens GN=ATP1A1 PE=2 SV	114239	324	146	55	308	0.09	0.33	3.66666667	2	6	3	10	8	14	12	18	-0.1267
tr|Q5HY54|Q5HY54_HUMAN	FLNA	Filamin-A OS=Homo sapiens GN=FLNA PE=2 SV=1	279115	9	2	1304	4103	0.86	3.13	3.63953488	41	111	59	153	74	160	87	179	0.214281
tr|B4DF96|B4DF96_HUMAN	EIF2A	Eukaryotic translation initiation factor 2A OS=Homo sapiens GN=EIF2A PE=2 SV=1	58357	206	98	101	275	0.32	1.15	3.59375	2	6	5	11	4	12	5	12	0.577699
tr|Q8TC62|Q8TC62_HUMAN	Sep-07	Septin 7 OS=Homo sapiens GN=SEPT7 PE=2 SV=3	48913	531	513	31	86	0.07	0.25	3.57142857	1	3	1	3	1	3	2	3	0.325545
tr|B4DMJ6|B4DMJ6_HUMAN	tr|B4DMJ6|B	cDNA FLJ50996, highly similar to 60S ribosomal protein L4 OS=Homo sapiens PE=2 SV=1	46269	163	118	132	321	0.51	1.82	3.56862745	4	6	6	11	8	11	9	14	-0.46166
tr|E0X098|E0X098_HUMAN	TES	Testin variant (Fragment) OS=Homo sapiens GN=TES PE=2 SV=1	22009	430	472	40	96	0.15	0.53	3.53333333	1	2	1	3	1	3	1	3	0.325545
tr|Q96QL0|Q96QL0_HUMAN	RPL3	Ribosomal protein L3 OS=Homo sapiens GN=RPL3 PE=2 SV=1	46376	245	147	82	273	0.32	1.13	3.53125	4	6	4	10	5	10	7	12	0.035958
tr|A5D906|A5D906_HUMAN	TUBB2A	TUBB2A protein (Fragment) OS=Homo sapiens GN=TUBB2A PE=2 SV=1	28128	25	16	143	626	3.07	10.83	3.5276873	4	20	7	24	8	25	8	28	0.689734
tr|J3KR97|J3KR97_HUMAN	TBCD	Tubulin-specific chaperone D OS=Homo sapiens GN=TBCD PE=4 SV=1	138346	714	780	15	54	0.02	0.07	3.5	0	1	1	3	0	4	1	4	2.273593
tr|Q5VZU9|Q5VZU9_HUMAN	TPP2	Tripeptidyl-peptidase 2 OS=Homo sapiens GN=TPP2 PE=4 SV=1	141160	693	500	19	90	0.02	0.07	3.5	0	3	1	3	0	4	1	6	2.273593
tr|B2RU06|B2RU06_HUMAN	EIF4G1	EIF4G1 protein OS=Homo sapiens GN=EIF4G1 PE=2 SV=1	176807	669	338	20	133	0.04	0.14	3.5	0	6	2	7	1	7	3	7	1.425801
sp|Q14683|SMC1A_HUMAN	SMC1A	Structural maintenance of chromosomes protein 1A OS=Homo sapiens GN=SMC1A PE=1 SV=2	143771	581	726	28	58	0.02	0.07	3.5	0	1	1	3	1	4	1	6	0.688295
tr|Q6DC98|Q6DC98_HUMAN	LMNB1	LMNB1 protein (Fragment) OS=Homo sapiens GN=LMNB1 PE=2 SV=1	38289	219	162	95	187	0.18	0.62	3.44444444	2	5	2	6	2	7	4	7	0.6885
tr|D5FHQ7|D5FHQ7_HUMAN	HLA-A	MHC class I antigen (Fragment) OS=Homo sapiens GN=HLA-A PE=3 SV=1	21188	640	499	22	90	0.16	0.55	3.4375	0	3	1	3	2	4	3	6	-0.04901
sp|P04350|TBB4A_HUMAN	TUBB4A	Tubulin beta-4A chain OS=Homo sapiens GN=TUBB4A PE=1 SV=2	50010	42	25	509	1335	1.96	6.7	3.41836735	21	44	27	54	29	58	32	63	0.091394
tr|B2RCM2|B2RCM2_HUMAN	tr|B2RCM2|B	cDNA, FLJ96156, highly similar to Homo sapiens leucyl-tRNA synthetase (LARS), mRNA OS=Homo	135550	257	111	75	365	0.1	0.34	3.4	2	10	4	12	3	13	5	15	1.051817
tr|Q59GV0|Q59GV0_HUMAN	tr|Q59GV0|Q	Propionyl Coenzyme A carboxylase, beta polypeptide variant (Fragment) OS=Homo sapiens PE=	60578	331	534	53	83	0.05	0.17	3.4	1	2	1	3	1	4	2	6	0.688295
tr|A8K8N7|A8K8N7_HUMAN	PFAS	Phosphoribosylformylglycinamidine synthase (FGAR amidotransferase), isoform CRA_b OS=Hom	146286	380	215	46	221	0.05	0.17	3.4	0	4	2	7	3	6	5	8	-0.00389
tr|A5PHJ9|A5PHJ9_HUMAN	PDHA1/LOC7	PDHA1/LOC79064 protein (Fragment) OS=Homo sapiens GN=PDHA1/LOC79064 PE=2 SV=1	34113	516	564	32	47	0.1	0.34	3.4	0	1	1	2	1	2	1	3	-0.16006
tr|B4DEX8|B4DEX8_HUMAN	MAT2A	S-adenosylmethionine synthase OS=Homo sapiens GN=MAT2A PE=2 SV=1	40027	333	384	52	122	0.08	0.27	3.375	1	2	1	3	1	4	1	4	0.688295
tr|E9PCI9|E9PCI9_HUMAN	FDPS	Farnesyl pyrophosphate synthase OS=Homo sapiens GN=FDPS PE=2 SV=1	40849	405	1194	43	27	0.08	0.27	3.375	1	0	1	1	1	2	1	2	-0.16006
tr|B2RBR9|B2RBR9_HUMAN	tr|B2RBR9|B	cDNA, FLJ95650, highly similar to Homo sapiens karyopherin (importin) beta 1 (KPNB1), mRNA C	98434	171	69	126	580	0.22	0.74	3.36363636	4	14	6	19	9	18	9	21	0.064697
tr|F5H4R9|F5H4R9_HUMAN	DNM2	Dynamin-2 OS=Homo sapiens GN=DNM2 PE=2 SV=1	98218	713	621	16	69	0.03	0.1	3.33333333	0	1	1	3	0	4	2	4	2.273593
tr|B2R6D0|B2R6D0_HUMAN	tr|B2R6D0|B	cDNA, FLJ92896, highly similar to Homo sapiens proteasome (prosome, macropain) 26S subunit	106797	386	311	45	144	0.06	0.2	3.33333333	0	4	2	6	2	7	4	8	0.6885
tr|H7C3A1|H7C3A1_HUMAN	SRRT	Serrate RNA effector molecule homolog (Fragment) OS=Homo sapiens GN=SRRT PE=4 SV=1	57154	701	870	18	47	0.06	0.2	3.33333333	0	1	1	1	0	1	1	1	0.68809
tr|B4DJ60|B4DJ60_HUMAN	tr|B4DJ60|B	cDNA FLJ55072, highly similar to Succinate dehydrogenase (ubiquinone) flavoprotein subunit, m	68233	393	256	44	181	0.1	0.33	3.3	0	3	2	6	3	7	5	7	0.202738
tr|Q5T7Q0|Q5T7Q0_HUMAN	DNAJA1	DnaJ (Hsp40) homolog, subfamily A, member 1, isoform CRA_c OS=Homo sapiens GN=DNAJA1 F	45581	703	1123	18	31	0.07	0.23	3.28571429	0	0	1	3	0	3	1	4	1.910843
tr|Q59EG8|Q59EG8_HUMAN	tr|Q59EG8|Q	Proteasome 26S non-ATPase subunit 2 variant (Fragment) OS=Homo sapiens PE=2 SV=1	101322	198	135	105	324	0.14	0.46	3.28571429	1	9	4	12	4	12	5	13	0.577699
sp|Q92621|NU205_HUMAN	NUP205	Nuclear pore complex protein Nup205 OS=Homo sapiens GN=NUP205 PE=1 SV=3	230171	415	217	42	218	0.04	0.13	3.25	0	8	3	9	3	10	5	12	0.688706
tr|B4E0S6|B4E0S6_HUMAN	tr|B4E0S6|B	cDNA FLJ55635, highly similar to pre-mRNA-splicing factorATP-dependent RNA helicase DHX15 (	90289	183	189	117	252	0.15	0.48	3.2	2	7	4	11	4	13	7	15	0.688911
tr|J3QSX4|J3QSX4_HUMAN	BUB3	Mitotic checkpoint protein BUB3 OS=Homo sapiens GN=BUB3 PE=4 SV=1	16498	681	518	20	46	0.2	0.64	3.2	0	0	1	2	0	2	1	2	1.425236
sp|O00425|IF2B3_HUMAN	IGF2BP3	Insulin-like growth factor 2 mRNA-binding protein 3 OS=Homo sapiens GN=IGF2BP3 PE=1 SV=2	64008	556	706	29	61	0.05	0.16	3.2	0	3	1	3	2	3	2	3	-0.41176
tr|I3L3I2|I3L3I2_HUMAN	P4HB	Protein disulfide-isomerase (Fragment) OS=Homo sapiens GN=P4HB PE=4 SV=1	26348	536	197	30	58	0.27	0.86	3.18518519	1	1	2	4	1	5	2	6	0.977982
tr|Q2VPA0|Q2VPA0_HUMAN	LONP1	Lon protease homolog (Fragment) OS=Homo sapiens GN=LONP1 PE=2 SV=1	95436	414	273	42	167	0.11	0.35	3.18181818	2	5	3	9	3	9	3	10	0.544135
tr|A6NG51|A6NG51_HUMAN	SPTAN1	Spectrin alpha chain, non-erythrocytic 1 OS=Homo sapiens GN=SPTAN1 PE=2 SV=2	285628	167	101	130	363	0.06	0.19	3.16666667	2	10	5	14	6	16	9	20	0.447773
tr|B4DR45|B4DR45_HUMAN	tr|B4DR45|B	cDNA FLJ61339, highly similar to Alanyl-tRNA synthetase (EC 6.1.1.7) OS=Homo sapiens PE=2 SV	110017	238	262	86	174	0.06	0.19	3.16666667	1	3	2	6	2	5	2	7	0.240681
tr|B4E0E1|B4E0E1_HUMAN	tr|B4E0E1|B	cDNA FLJ53442, highly similar to Poly (ADP-ribose) polymerase 1 (EC 2.4.2.30) OS=Homo sapien	111855	549	253	30	183	0.06	0.19	3.16666667	0	4	2	6	2	5	2	6	0.240681
tr|A4QPB0|A4QPB0_HUMAN	IQGAP1	IQ motif containing GTPase activating protein 1 OS=Homo sapiens GN=IQGAP1 PE=2 SV=1	189789	84	35	239	1057	0.31	0.98	3.16129032	8	29	17	40	18	45	21	49	0.403366
tr|B3KQT2|B3KQT2_HUMAN	tr|B3KQT2|B	cDNA PSEC0148 fis, clone PLACE1007202, highly similar to Protein disulfide-isomerase A3 (EC 5.	55268	546	231	30	179	0.19	0.6	3.15789474	0	5	3	7	2	9	8	10	1.029898
tr|B2R6J2|B2R6J2_HUMAN	tr|B2R6J2|B	cDNA, FLJ92973, highly similar to Homo sapiens villin 2 (ezrin) (VIL2), mRNA OS=Homo sapiens F	69472	126	139	173	311	0.32	1	3.125	6	11	6	15	8	18	8	21	0.225498
tr|B4DT29|B4DT29_HUMAN	tr|B4DT29|B	cDNA FLJ51082, highly similar to Actin-like protein 3 OS=Homo sapiens PE=2 SV=1	40959	548	553	30	69	0.08	0.25	3.125	0	2	1	2	1	2	1	2	-0.16006
tr|A0AV58|A0AV58_HUMAN	STRN3	Striatin, calmodulin binding protein 3 OS=Homo sapiens GN=STRN3 PE=2 SV=1	78069	419	375	42	123	0.09	0.28	3.11111111	1	4	2	6	3	6	3	7	-0.00389
sp|P49411|EFTU_HUMAN	TUFM	Elongation factor Tu, mitochondrial OS=Homo sapiens GN=TUFM PE=1 SV=2	49852	210	134	99	326	0.29	0.9	3.10344828	3	8	4	11	4	11	5	14	0.457225
sp|P08238|HS90B_HUMAN	HSP90AB1	Heat shock protein HSP 90-beta OS=Homo sapiens GN=HSP90AB1 PE=1 SV=4	83554	32	30	622	1241	1.24	3.84	3.09677419	18	34	28	47	29	54	35	58	-0.01151
tr|B2ZZ89|B2ZZ89_HUMAN	SPTBN1	Spectrin beta non-erythrocytic 1 OS=Homo sapiens GN=SPTBN1 PE=2 SV=1	275237	49	19	431	1424	0.22	0.68	3.09090909	11	35	17	44	20	49	24	60	0.376881

tr|H0YLA4|H0YLA4_HUMAN	SORD	Sorbitol dehydrogenase OS=Homo sapiens GN=SORD PE=3 SV=1	36833	473	840	37	49	0.09	0.27	3	0	1	1	1	2	1	2	1	-1.63451
tr|B4DJ30|B4DJ30_HUMAN	tr|B4DJ30|B4	cDNA FLJ61290, highly similar to Neutral alpha-glucosidase AB OS=Homo sapiens PE=2 SV=1	113539	47	34	446	1061	0.62	1.86	3	12	28	19	40	17	44	22	51	0.451631
tr|B2RAX6|B2RAX6_HUMAN	tr|B2RAX6|B	cDNA, FLJ95176, Homo sapiens CGI-48 protein (CGI-48), mRNA OS=Homo sapiens PE=2 SV=1	58127	605	634	25	67	0.06	0.18	3	0	1	1	3	1	3	1	3	0.325545
tr|B1ANR0|B1ANR0_HUMAN	PABPC4	Poly(A) binding protein, cytoplasmic 4 (Inducible form) OS=Homo sapiens GN=PABPC4 PE=4 SV=	68156	504	337	33	133	0.05	0.15	3	0	2	1	3	1	7	2	7	1.425801
tr|B2RDF2|B2RDF2_HUMAN	PES1	Pescadillo homolog 1 OS=Homo sapiens GN=PES1 PE=2 SV=1	68359	488	584	35	74	0.05	0.15	3	1	2	1	3	1	4	3	5	0.688295
sp|P49327|FAS_HUMAN	FASN	Fatty acid synthase OS=Homo sapiens GN=FASN PE=1 SV=3	275877	55	23	394	1361	0.28	0.84	3	12	43	21	54	23	60	29	66	0.470334
tr|B3KXU8|B3KXU8_HUMAN	tr|B3KXU8|B	cDNA FLJ46087 fis, clone TESTI2009497, highly similar to GPI-anchored protein p137 OS=Homo	70489	367	577	47	75	0.05	0.15	3	1	3	1	3	1	3	1	3	0.325545
tr|B4DFL1|B4DFL1_HUMAN	tr|B4DFL1|B4	Dihydrolipoyl dehydrogenase OS=Homo sapiens PE=2 SV=1	49381	666	453	21	100	0.07	0.21	3	0	3	1	4	1	3	1	4	0.325545
tr|D3DQA6|D3DQA6_HUMAN	DDX46	DEAD (Asp-Glu-Ala-Asp) box polypeptide 46, isoform CRA_a OS=Homo sapiens GN=DDX46 PE=3	100958	595	295	26	157	0.07	0.21	3	0	5	2	6	3	7	3	8	0.202738
tr|A8K781|A8K781_HUMAN	tr|A8K781|A	cDNA FLJ75299, highly similar to Xenopus laevis proteasome (prosome, macropain) 26S subunit	47578	603	859	26	31	0.07	0.21	3	0	0	1	1	2	1	2	1	-1.63451
tr|Q8NAN9|Q8NAN9_HUMAN	tr|Q8NAN9|C	cDNA FLJ35058 fis, clone OCBBF2018707, highly similar to DIHYDROPYRIMIDINASE RELATED PR	21515	342	109	19	60	0.23	0.68	2.95652174	0	1	1	2	1	2	2	2	-0.16006
tr|Q2F839|Q2F839_HUMAN	tr|Q2F839|Q	Heat shock 70 kDa protein 9B (Fragment) OS=Homo sapiens PE=2 SV=1	19208	81	213	59	223	0.42	1.24	2.95238095	2	5	2	5	2	5	2	8	0.240681
tr|H7BZJ3|H7BZJ3_HUMAN	PDIA3	Thioredoxin (Fragment) OS=Homo sapiens GN=PDIA3 PE=3 SV=1	13739	546	497	22	90	0.19	0.56	2.94736842	0	2	1	2	1	2	1	3	-0.16006
tr|B0V043|B0V043_HUMAN	VARS	Valine--tRNA ligase OS=Homo sapiens GN=VARS PE=3 SV=1	141632	275	85	69	463	0.15	0.44	2.93333333	2	12	6	19	6	21	8	25	0.830546
tr|E9PHF7|E9PHF7_HUMAN	MCCC1	Methylcrotonoyl-CoA carboxylase subunit alpha, mitochondrial OS=Homo sapiens GN=MCCC1 P	68745	102	48	214	688	0.83	2.43	2.92771084	7	27	13	35	11	40	16	44	0.922764
tr|B2R959|B2R959_HUMAN	tr|B2R959|B2	cDNA, FLJ94229, highly similar to Homo sapiens heterogeneous nuclear ribonucleoprotein L (HN	60838	182	108	117	245	0.23	0.67	2.91304348	4	8	4	9	6	13	8	13	0.157725
tr|B5MCX3|B5MCX3_HUMAN	Sep-02	Septin-2 OS=Homo sapiens GN=SEPT2 PE=2 SV=1	37145	543	319	30	104	0.09	0.26	2.88888889	1	2	1	2	2	2	3	2	-0.89736
tr|B4DR70|B4DR70_HUMAN	tr|B4DR70|B	cDNA FLJ58049, highly similar to RNA-binding protein FUS OS=Homo sapiens PE=2 SV=1	45013	450	558	38	79	0.07	0.2	2.85714286	1	2	1	3	1	4	1	4	0.688295
tr|A6NN80|A6NN80_HUMAN	ANXA6	Annexin OS=Homo sapiens GN=ANXA6 PE=3 SV=3	75571	349	219	50	178	0.14	0.4	2.85714286	1	5	3	7	3	9	3	11	0.544135
tr|B4DMB5|B4DMB5_HUMAN	tr|B4DMB5|B	cDNA FLJ60075, highly similar to CTP synthase 1 (EC 6.3.4.2) OS=Homo sapiens PE=2 SV=1	56542	449	207	38	210	0.19	0.53	2.78947368	1	5	3	7	3	6	4	7	-0.00389
sp|Q15149|PLEC_HUMAN	PLEC	Plectin OS=Homo sapiens GN=PLEC PE=1 SV=3	533462	74	32	270	1144	0.11	0.3	2.72727273	11	34	17	43	18	49	32	57	0.525653
tr|F5GX33|F5GX33_HUMAN	SLC25A13	Calcium-binding mitochondrial carrier protein Aralar2 OS=Homo sapiens GN=SLC25A13 PE=2 SV	62548	353	228	49	168	0.11	0.3	2.72727273	1	3	2	5	3	7	4	7	0.202738
sp|O15427|MOT4_HUMAN	SLC16A3	Monocarboxylate transporter 4 OS=Homo sapiens GN=SLC16A3 PE=1 SV=1	50064	362	477	48	95	0.14	0.38	2.71428571	2	3	2	5	2	6	2	8	0.481869
tr|B4DU00|B4DU00_HUMAN	tr|B4DU00|B	cDNA FLJ54547, highly similar to Alpha-1 catenin (Cadherin-associated protein) OS=Homo sapie	89332	389	367	45	126	0.07	0.19	2.71428571	0	4	2	5	2	6	4	8	0.481869
tr|Q9UJZ2|Q9UJZ2_HUMAN	VIL2	Cytovillin 2 (Fragment) OS=Homo sapiens GN=VIL2 PE=2 SV=1	18464	119	434	88	81	0.46	1.23	2.67391304	2	3	2	3	2	3	2	4	-0.41176
tr|B0UX83|B0UX83_HUMAN	BAG6	HLA-B associated transcript 3 OS=Homo sapiens GN=BAG6 PE=4 SV=1	119085	704	389	18	120	0.03	0.08	2.66666667	0	2	1	3	0	3	1	5	1.910843
sp|P53621|COPA_HUMAN	COPA	Coatomer subunit alpha OS=Homo sapiens GN=COPA PE=1 SV=2	139797	181	107	118	387	0.18	0.48	2.66666667	5	11	7	18	8	20	11	23	0.373958
sp|P26599|PTBP1_HUMAN	PTBP1	Polypyrimidine tract-binding protein 1 OS=Homo sapiens GN=PTBP1 PE=1 SV=1	57357	567	136	29	321	0.18	0.48	2.66666667	0	4	3	7	3	7	3	8	0.202738
tr|A8K2S1|A8K2S1_HUMAN	tr|A8K2S1|A	cDNA FLJ75258, highly similar to Homo sapiens phospholipase A2, group IVA (cytosolic, calcium-	85695	369	302	47	150	0.08	0.21	2.625	1	3	2	6	2	8	5	9	0.869253
tr|B4E022|B4E022_HUMAN	TKT	Transketolase OS=Homo sapiens GN=TKT PE=2 SV=1	63410	35	45	578	809	1.36	3.56	2.61764706	17	29	24	33	26	39	32	46	-0.3229
sp|O43175|SERA_HUMAN	PHGDH	D-3-phosphoglycerate dehydrogenase OS=Homo sapiens GN=PHGDH PE=1 SV=4	57356	131	209	166	228	0.25	0.65	2.6	3	6	4	9	4	9	4	10	0.18123
tr|A0JLS5|A0JLS5_HUMAN	TBL3	TBL3 protein (Fragment) OS=Homo sapiens GN=TBL3 PE=2 SV=1	64079	418	511	42	83	0.05	0.13	2.6	1	2	1	2	1	2	1	2	-0.16006
tr|Q6IBR0|Q6IBR0_HUMAN	RPN1	RPN1 protein OS=Homo sapiens GN=RPN1 PE=2 SV=1	68678	170	150	127	298	0.26	0.67	2.57692308	4	9	5	11	8	14	8	17	-0.1267
sp|P22314|UBA1_HUMAN	UBA1	Ubiquitin-like modifier-activating enzyme 1 OS=Homo sapiens GN=UBA1 PE=1 SV=3	118858	114	76	187	516	0.28	0.72	2.57142857	5	15	10	21	10	21	12	22	0.137324
tr|Q53HF2|Q53HF2_HUMAN	tr|Q53HF2|Q	Heat shock 70kDa protein 8 isoform 2 variant (Fragment) OS=Homo sapiens PE=2 SV=1	53580	51	73	426	548	1.45	3.71	2.55862069	12	15	17	28	19	30	19	34	-0.25269
tr|B4DXX7|B4DXX7_HUMAN	DDX3Y	ATP-dependent RNA helicase DDX3Y OS=Homo sapiens GN=DDX3Y PE=2 SV=1	73342	217	199	95	208	0.19	0.48	2.52631579	2	6	5	9	6	9	6	10	-0.34996
tr|B4DH52|B4DH52_HUMAN	tr|B4DH52|B	cDNA FLJ55916, highly similar to General transcription factor II-I OS=Homo sapiens PE=2 SV=1	112359	554	604	29	71	0.06	0.15	2.5	0	2	2	5	2	5	3	5	0.240681
tr|J3KPM9|J3KPM9_HUMAN	STAT1	Signal transducer and activator of transcription 1-alpha/beta OS=Homo sapiens GN=STAT1 PE=4	83878	364	251	48	185	0.08	0.2	2.5	1	4	2	5	2	5	2	7	0.240681
tr|B1PS43|B1PS43_HUMAN	MYH11	Myosin heavy chain 11 smooth muscle isoform OS=Homo sapiens GN=MYH11 PE=2 SV=1	235004	142	86	155	457	0.06	0.15	2.5	5	12	5	13	9	14	11	14	-0.2875
sp|O00116|ADAS_HUMAN	AGPS	Alkyldihydroxyacetonephosphate synthase, peroxisomal OS=Homo sapiens GN=AGPS PE=1 SV=	73664	448	474	38	96	0.09	0.22	2.44444444	1	3	2	4	2	5	3	5	0.240681
tr|K7EIG1|K7EIG1_HUMAN	CLUH	Clustered mitochondria protein homolog OS=Homo sapiens GN=CLUH PE=3 SV=1	140903	438	567	39	78	0.05	0.12	2.4	1	2	2	5	2	5	2	6	0.240681
sp|Q08211|DHX9_HUMAN	DHX9	ATP-dependent RNA helicase A OS=Homo sapiens GN=DHX9 PE=1 SV=4	142181	96	52	218	705	0.41	0.98	2.3902439	8	21	16	31	18	37	22	38	0.122942
tr|H0Y4R1|H0Y4R1_HUMAN	IMPDH2	Inosine-5~-monophosphate dehydrogenase 2 (Fragment) OS=Homo sapiens GN=IMPDH2 PE=3	51549	177	106	122	388	0.36	0.86	2.38888889	2	8	5	10	5	10	6	13	0.035958
sp|P07355|ANXA2_HUMAN	ANXA2	Annexin A2 OS=Homo sapiens GN=ANXA2 PE=1 SV=2	38808	100	80	214	503	1.26	3.01	2.38888889	8	14	11	18	12	21	13	25	-0.11489
tr|Q3B7A7|Q3B7A7_HUMAN	GART	Phosphoribosylglycinamide formyltransferase, phosphoribosylglycinamide synthetase, phospho	108909	341	200	51	237	0.13	0.31	2.38461538	1	6	4	9	3	9	4	12	0.544135
tr|D3DPU2|D3DPU2_HUMAN	CAP1	Adenylyl cyclase-associated protein OS=Homo sapiens GN=CAP1 PE=3 SV=1	51926	172	127	125	342	0.36	0.85	2.36111111	4	8	5	10	6	12	7	12	0.046513
tr|B3KW67|B3KW67_HUMAN	tr|B3KW67|B	cDNA FLJ42347 fis, clone UTERU2003399, highly similar to Actin, gamma-enteric smooth muscle	42189	38	29	538	1264	2.61	6.11	2.34099617	19	41	27	47	32	50	37	52	-0.26295
tr|A1L3B5|A1L3B5_HUMAN	ATP2B3	ATP2B3 protein OS=Homo sapiens GN=ATP2B3 PE=2 SV=1	96278	528	586	31	74	0.03	0.07	2.33333333	0	1	1	2	1	3	1	3	0.325545
sp|Q8TCJ2|STT3B_HUMAN	STT3B	Dolichyl-diphosphooligosaccharide--protein glycosyltransferase subunit STT3B OS=Homo sapien	94241	700	495	18	90	0.03	0.07	2.33333333	0	2	1	2	2	4	3	4	-0.04901
sp|Q9P287|BCCIP_HUMAN	BCCIP	BRCA2 and CDKN1A-interacting protein OS=Homo sapiens GN=BCCIP PE=1 SV=1	36299	503	906	33	44	0.09	0.21	2.33333333	0	1	1	2	1	2	3	2	-0.16006
tr|B3KPR5|B3KPR5_HUMAN	tr|B3KPR5|B	cDNA FLJ32094 fis, clone OCBBF2000986, highly similar to Homo sapiens elongation factor Tu G	97046	633	590	22	73	0.03	0.07	2.33333333	0	1	1	2	1	2	1	2	-0.16006
sp|Q14315|FLNC_HUMAN	FLNC	Filamin-C OS=Homo sapiens GN=FLNC PE=1 SV=3	293407	143	158	153	290	0.06	0.14	2.33333333	5	10	6	13	10	12	11	16	-0.64671
tr|F2VNS8|F2VNS8_HUMAN	HLA-C	MHC class I antigen (Fragment) OS=Homo sapiens GN=HLA-C PE=3 SV=1	21240	639	346	22	101	0.16	0.37	2.3125	0	2	1	2	2	2	2	2	-0.89736
tr|B2RAQ8|B2RAQ8_HUMAN	tr|B2RAQ8|B	cDNA, FLJ95058, highly similar to Homo sapiens carnitine palmitoyltransferase 1A (liver) (CPT1A	88967	354	376	49	123	0.07	0.16	2.28571429	1	3	2	4	3	5	3	5	-0.24508
tr|B4DII5|B4DII5_HUMAN	tr|B4DII5|B4	Importin subunit alpha OS=Homo sapiens PE=2 SV=1	60345	573	599	28	72	0.11	0.25	2.27272727	0	2	2	2	3	3	3	3	-0.89752
tr|B4E091|B4E091_HUMAN	tr|B4E091|B4	cDNA FLJ55438, highly similar to Splicing factor 3 subunit 1 OS=Homo sapiens PE=2 SV=1	77415	482	603	36	71	0.04	0.09	2.25	1	2	1	2	1	2	1	2	-0.16006
tr|B4DY09|B4DY09_HUMAN	tr|B4DY09|B	cDNA FLJ51660, highly similar to Interleukin enhancer-binding factor 2 OS=Homo sapiens PE=2	39057	383	517	45	86	0.08	0.18	2.25	1	2	1	2	1	2	1	2	-0.16006
tr|B7Z481|B7Z481_HUMAN	tr|B7Z481|B7	cDNA FLJ54018, highly similar to PDZ and LIM domain protein 5 OS=Homo sapiens PE=2 SV=1	52359	175	120	109	346	0.41	0.92	2.24390244	2	8	5	10	5	10	6	12	0.035958
tr|Q6IPN6|Q6IPN6_HUMAN	EEF1A1	Elongation factor 1-alpha OS=Homo sapiens GN=EEF1A1 PE=2 SV=1	50433	70	113	295	363	1.14	2.55	2.23684211	11	14	13	24	18	23	21	28	-0.55382
tr|B4DQJ8|B4DQJ8_HUMAN	PGD	6-phosphogluconate dehydrogenase, decarboxylating OS=Homo sapiens GN=PGD PE=2 SV=1	52352	293	245	62	186	0.2	0.44	2.2	1	5	3	7	2	8	3	8	0.869253
sp|Q9NPQ8|RIC8A_HUMAN	RIC8A	Synembryn-A OS=Homo sapiens GN=RIC8A PE=1 SV=3	60185	496	702	34	61	0.05	0.11	2.2	0	1	1	2	1	3	1	3	0.325545
tr|B4DG42|B4DG42_HUMAN	tr|B4DG42|B	cDNA FLJ53753, highly similar to Myeloid-associated differentiation marker OS=Homo sapiens P	31889	609	510	25	87	0.1	0.22	2.2	0	2	1	2	1	3	1	3	0.325545
tr|Q05BW9|Q05BW9_HUMAN	PAPSS1	PAPSS1 protein (Fragment) OS=Homo sapiens GN=PAPSS1 PE=2 SV=1	65264	553	457	29	99	0.1	0.22	2.2	0	3	2	4	1	3	2	4	0.325545
tr|B7Z5E2|B7Z5E2_HUMAN	PSMC2	26S protease regulatory subunit 7 OS=Homo sapiens GN=PSMC2 PE=2 SV=1	33594	610	364	25	111	0.1	0.22	2.2	0	3	1	3	1	3	1	3	0.325545
sp|Q14432|PDE3A_HUMAN	PDE3A	cGMP-inhibited 3~,5~-cyclic phosphodiesterase A OS=Homo sapiens GN=PDE3A PE=1 SV=3	126440	493	440	35	104	0.05	0.11	2.2	1	3	2	4	2	5	2	6	0.240681
sp|Q15392|DHC24_HUMAN	DHCR24	Delta(24)-sterol reductase OS=Homo sapiens GN=DHCR24 PE=1 SV=2	60803	442	478	39	95	0.05	0.11	2.2	1	3	1	3	2	4	3	4	-0.04901
sp|P23526|SAHH_HUMAN	AHCY	Adenosylhomocysteinase OS=Homo sapiens GN=AHCY PE=1 SV=4	48255	195	159	111	290	0.49	1.07	2.18367347	2	9	6	12	6	11	8	15	-0.07396
sp|O00232|PSD12_HUMAN	PSMD12	26S proteasome non-ATPase regulatory subunit 12 OS=Homo sapiens GN=PSMD12 PE=1 SV=3	53270	283	611	66	70	0.06	0.13	2.16666667	1	1	1	2	1	2	1	2	-0.16006
tr|G3V2B8|G3V2B8_HUMAN	MTHFD1	C-1-tetrahydrofolate synthase, cytoplasmic OS=Homo sapiens GN=MTHFD1 PE=2 SV=1	102152	112	94	192	429	0.33	0.71	2.15151515	5	12	9	17	12	17	12	20	-0.41259
tr|G3XAD8|G3XAD8_HUMAN	STIP1	Stress-induced-phosphoprotein 1 OS=Homo sapiens GN=STIP1 PE=2 SV=1	68721	271	277	69	165	0.21	0.45	2.14285714	2	5	4	8	4	13	5	14	0.688911
tr|B4DJE3|B4DJE3_HUMAN	tr|B4DJE3|B4	cDNA FLJ52929, highly similar to Dolichyl-diphosphooligosaccharide--proteinglycosyltransferase	46620	604	1003	26	38	0.07	0.15	2.14285714	0	1	1	2	1	4	2	5	0.688295
tr|B4DHQ3|B4DHQ3_HUMAN	tr|B4DHQ3|B	cDNA FLJ56437, highly similar to Phosphoserine aminotransferase (EC 2.6.1.52) OS=Homo sapie	45783	552	608	30	71	0.07	0.15	2.14285714	0	2	1	2	1	2	2	2	-0.16006
tr|B4DWJ2|B4DWJ2_HUMAN	QARS	Glutamine--tRNA ligase OS=Homo sapiens GN=QARS PE=2 SV=1	87437	277	206	68	233	0.16	0.34	2.125	3	5	4	8	5	12	5	14	0.287857
tr|B4DHG3|B4DHG3_HUMAN	tr|B4DHG3|B	cDNA FLJ55329, highly similar to Procollagen-lysine,2-oxoglutarate5-dioxygenase 2 (EC 1.14.11.	81716	453	370	38	125	0.08	0.17	2.125	0	3	2	4	2	4	2	4	-0.04901
tr|H0YH88|H0YH88_HUMAN	NAP1L1	Nucleosome assembly protein 1-like 1 (Fragment) OS=Homo sapiens GN=NAP1L1 PE=3 SV=1	21179	417	922	33	43	0.16	0.34	2.125	0	0	1	2	1	2	1	2	-0.16006
tr|B4DLB8|B4DLB8_HUMAN	tr|B4DLB8|B4	cDNA FLJ52205, highly similar to Beta-1,4-galactosyltransferase 1 (EC 2.4.1.-) OS=Homo sapiens	40269	650	895	22	45	0.08	0.17	2.125	0	1	1	2	1	2	1	2	-0.16006
tr|H0Y5J4|H0Y5J4_HUMAN	DBNL	Drebrin-like protein (Fragment) OS=Homo sapiens GN=DBNL PE=4 SV=1	40475	466	762	37	55	0.08	0.17	2.125	1	1	1	2	1	2	1	2	-0.16006
tr|Q5TZX1|Q5TZX1_HUMAN	GNAI3	Guanine nucleotide binding protein (G protein), alpha inhibiting activity polypeptide 3 OS=Hom	41076	376	542	46	82	0.08	0.17	2.125	1	2	1	2	1	2	1	2	-0.16006
tr|E0YTJ7|E0YTJ7_HUMAN	HLA-B	MHC class I antigen (Fragment) OS=Homo sapiens GN=HLA-B PE=3 SV=1	21175	639	541	22	82	0.16	0.34	2.125	0	2	1	2	1	2	1	2	-0.16006
tr|Q2I7H5|Q2I7H5_HUMAN	HLA-Cw	MHC class I antigen (Fragment) OS=Homo sapiens GN=HLA-Cw PE=2 SV=1	21095	639	450	22	101	0.16	0.34	2.125	0	2	1	2	1	2	1	3	-0.16006
tr|F8SKU2|F8SKU2_HUMAN	HLA-A	MHC class I antigen (Fragment) OS=Homo sapiens GN=HLA-A PE=3 SV=1	21143	639	449	22	57	0.16	0.34	2.125	0	1	1	1	2	2	3	4	-0.89736
tr|A8K6Q8|A8K6Q8_HUMAN	tr|A8K6Q8|A	cDNA FLJ75881, highly similar to Homo sapiens transferrin receptor (p90, CD71) (TFRC), mRNA (	85276	122	100	176	392	0.3	0.63	2.1	6	9	7	12	7	13	8	15	-0.04906
tr|H0Y5G9|H0Y5G9_HUMAN	NRD1	Nardilysin (Fragment) OS=Homo sapiens GN=NRD1 PE=3 SV=1	62196	225	595	92	73	0.11	0.23	2.09090909	2	2	2	4	2	5	2	6	0.240681
tr|G5E9Q2|G5E9Q2_HUMAN	CSDE1	Cold shock domain containing E1, RNA-binding, isoform CRA_d OS=Homo sapiens GN=CSDE1 PE	94539	312	255	58	181	0.11	0.23	2.09090909	1	5	3	6	3	7	3	7	0.202738
sp|Q14247|SRC8_HUMAN	CTTN	Src substrate cortactin OS=Homo sapiens GN=CTTN PE=1 SV=2	61720	378	297	46	154	0.11	0.23	2.09090909	1	3	2	4	3	4	3	4	-0.53477
tr|Q6NZ55|Q6NZ55_HUMAN	RPL13	60S ribosomal protein L13 OS=Homo sapiens GN=RPL13 PE=2 SV=1	24308	422	946	41	41	0.14	0.29	2.07142857	1	1	1	2	2	3	2	3	-0.41176
sp|O00410|IPO5_HUMAN	IPO5	Importin-5 OS=Homo sapiens GN=IPO5 PE=1 SV=4	125032	85	78	236	508	0.23	0.47	2.04347826	7	14	8	15	10	16	15	17	-0.24545
tr|B4DE36|B4DE36_HUMAN	tr|B4DE36|B	Glucose-6-phosphate isomerase OS=Homo sapiens PE=2 SV=1	60376	117	133	182	330	0.3	0.61	2.03333333	4	6	5	9	7	11	7	13	-0.28075
sp|P17987|TCPA_HUMAN	TCP1	T-complex protein 1 subunit alpha OS=Homo sapiens GN=TCP1 PE=1 SV=1	60819	197	137	106	321	0.3	0.61	2.03333333	2	8	5	9	8	9	10	9	-0.73765
sp|P53396|ACLY_HUMAN	ACLY	ATP-citrate synthase OS=Homo sapiens GN=ACLY PE=1 SV=3	121674	20	14	845	1605	1.28	2.57	2.0078125	21	46	32	55	34	63	40	70	-0.01695
sp|O14929|HAT1_HUMAN	HAT1	Histone acetyltransferase type B catalytic subunit OS=Homo sapiens GN=HAT1 PE=1 SV=1	49880	343	404	51	114	0.07	0.14	2	1	2	1	2	1	4	1	4	0.688295
tr|H0Y386|H0Y386_HUMAN	PABPC1L	Polyadenylate-binding protein 1-like (Fragment) OS=Homo sapiens GN=PABPC1L PE=4 SV=1	16787	404	914	0	44	0.1	0.2	2	0	1	0	1	0	1	1	2	0.68809
tr|B7Z1R5|B7Z1R5_HUMAN	ATP6V1A	V-type proton ATPase catalytic subunit A OS=Homo sapiens GN=ATP6V1A PE=2 SV=1	65037	428	468	40	97	0.05	0.1	2	1	2	1	2	1	3	2	4	0.325545
sp|O95782|AP2A1_HUMAN	AP2A1	AP-2 complex subunit alpha-1 OS=Homo sapiens GN=AP2A1 PE=1 SV=3	108561	500	447	34	102	0.03	0.06	2	0	2	1	2	1	3	2	6	0.325545
sp|Q96P70|IPO9_HUMAN	IPO9	Importin-9 OS=Homo sapiens GN=IPO9 PE=1 SV=3	116858	580	545	28	81	0.06	0.12	2	0	1	2	4	2	4	2	4	-0.04901
tr|Q9BSV4|Q9BSV4_HUMAN	SFPQ	SFPQ protein (Fragment) OS=Homo sapiens GN=SFPQ PE=2 SV=2	68702	228	196	91	241	0.26	0.52	2	2	5	5	9	6	11	7	13	-0.07396
sp|Q6PGP7|TTC37_HUMAN	TTC37	Tetratricopeptide repeat protein 37 OS=Homo sapiens GN=TTC37 PE=1 SV=1	177485	498	871	34	47	0.02	0.04	2	1	1	1	2	1	2	1	2	-0.16006
tr|F8W930|F8W930_HUMAN	IGF2BP2	Insulin-like growth factor 2 mRNA-binding protein 2 OS=Homo sapiens GN=IGF2BP2 PE=2 SV=1	66859	556	745	29	57	0.05	0.1	2	0	2	1	2	1	2	1	2	-0.16006
tr|B3KUZ8|B3KUZ8_HUMAN	tr|B3KUZ8|B	Aspartate aminotransferase OS=Homo sapiens PE=2 SV=1	41699	522	681	31	63	0.08	0.16	2	0	1	1	2	1	2	2	2	-0.16006
tr|F5H1A8|F5H1A8_HUMAN	GSN	Gelsolin OS=Homo sapiens GN=GSN PE=2 SV=1	81776	297	424	61	108	0.04	0.08	2	1	2	1	2	1	2	2	3	-0.16006
tr|B4DFE4|B4DFE4_HUMAN	tr|B4DFE4|B	cDNA FLJ59618, highly similar to Exosome component 10 OS=Homo sapiens PE=2 SV=1	61733	627	402	23	84	0.05	0.1	2	0	2	1	2	1	2	1	2	-0.16006
tr|B4DKD0|B4DKD0_HUMAN	tr|B4DKD0|B	DNA topoisomerase 2 (Fragment) OS=Homo sapiens PE=2 SV=1	135015	606	374	25	124	0.02	0.04	2	0	2	1	2	1	2	3	6	-0.16006
tr|B4DDB6|B4DDB6_HUMAN	HNRPA3	Heterogeneous nuclear ribonucleoprotein A3, isoform CRA_a OS=Homo sapiens GN=HNRPA3 PE	37234	201	331	103	124	0.19	0.38	2	2	1	2	2	2	3	2	4	-0.41176
tr|H0YN07|H0YN07_HUMAN	IPO4	Importin-4 (Fragment) OS=Homo sapiens GN=IPO4 PE=4 SV=1	33644	655	1344	22	17	0.05	0.1	2	0	0	1	1	1	1	1	3	-0.89721
tr|Q0P512|Q0P512_HUMAN	SLC2A1	Solute carrier family 2 (Facilitated glucose transporter), member 1 OS=Homo sapiens GN=SLC2A	54361	377	904	46	44	0.06	0.12	2	1	1	1	2	1	1	1	3	-0.89721
tr|D6RDU5|D6RDU5_HUMAN	Sep-11	Septin-11 (Fragment) OS=Homo sapiens GN=SEPT11 PE=2 SV=1	43371	532	829	31	29	0.07	0.14	2	1	0	1	1	1	1	1	1	-0.89721
tr|J3KNE5|J3KNE5_HUMAN	HARS	Histidine--tRNA ligase, cytoplasmic OS=Homo sapiens GN=HARS PE=4 SV=1	50581	672	673	20	36	0.07	0.14	2	0	0	1	1	1	1	2	1	-0.89721
tr|H0YMR4|H0YMR4_HUMAN	IPO4	Importin-4 (Fragment) OS=Homo sapiens GN=IPO4 PE=4 SV=1	60104	655	485	22	17	0.05	0.1	2	0	0	1	1	1	1	1	2	-0.89721
sp|P13639|EF2_HUMAN	EEF2	Elongation factor 2 OS=Homo sapiens GN=EEF2 PE=1 SV=4	96246	41	40	511	893	1.23	2.45	1.99186992	13	29	26	45	27	47	32	54	-0.10915
tr|B4DN87|B4DN87_HUMAN	tr|B4DN87|B	cDNA FLJ52569, highly similar to Collagen-binding protein 2 OS=Homo sapiens PE=2 SV=1	44290	233	236	89	172	0.43	0.85	1.97674419	4	6	5	8	5	8	5	8	-0.26926
sp|P18206|VINC_HUMAN	VCL	Vinculin OS=Homo sapiens GN=VCL PE=1 SV=4	124292	68	50	299	710	0.37	0.72	1.94594595	8	15	12	21	13	24	13	25	-0.03727
tr|H0Y8G5|H0Y8G5_HUMAN	HNRNPD	Heterogeneous nuclear ribonucleoprotein D0 (Fragment) OS=Homo sapiens GN=HNRNPD PE=4	29877	229	524	90	72	0.37	0.72	1.94594595	3	2	3	3	4	3	4	3	-1.26042
tr|Q7Z5T5|Q7Z5T5_HUMAN	EIF3A	EIF3A protein (Fragment) OS=Homo sapiens GN=EIF3A PE=2 SV=1	97203	192	192	112	248	0.14	0.27	1.92857143	2	10	4	12	5	14	8	15	0.502343
tr|H0YBW4|H0YBW4_HUMAN	PLAA	Phospholipase A-2-activating protein (Fragment) OS=Homo sapiens GN=PLAA PE=4 SV=1	31645	539	257	30	180	0.11	0.21	1.90909091	0	3	1	4	1	4	1	4	0.688295
tr|Q53GU8|Q53GU8_HUMAN	tr|Q53GU8|C	Transforming growth factor, beta-induced, 68kDa variant (Fragment) OS=Homo sapiens PE=2 SV	75231	358	945	48	42	0.09	0.17	1.88888889	1	1	2	2	2	2	2	2	-0.89736
tr|H3BLZ8|H3BLZ8_HUMAN	DDX17	Probable ATP-dependent RNA helicase DDX17 OS=Homo sapiens GN=DDX17 PE=3 SV=1	81073	166	117	130	354	0.37	0.69	1.86486486	3	9	9	13	9	13	10	15	-0.39077
tr|B7Z4V2|B7Z4V2_HUMAN	tr|B7Z4V2|B7	cDNA FLJ51907, highly similar to Stress-70 protein, mitochondrial OS=Homo sapiens PE=2 SV=1	72641	81	88	251	443	0.42	0.78	1.85714286	7	11	8	13	8	13	8	17	-0.22997
sp|P58107|EPIPL_HUMAN	EPPK1	Epiplakin OS=Homo sapiens GN=EPPK1 PE=1 SV=2	557674	56	58	390	640	0.07	0.13	1.85714286	10	17	12	21	12	23	13	24	0.013798
tr|B4DX08|B4DX08_HUMAN	tr|B4DX08|B	cDNA FLJ58784, highly similar to T-complex protein 1 subunit epsilon OS=Homo sapiens PE=2 SV	57521	90	353	229	123	0.4	0.74	1.85	6	4	6	8	6	9	6	10	-0.34996
tr|B4DXC4|B4DXC4_HUMAN	tr|B4DXC4|B	cDNA FLJ58636, moderately similar to Atlastin OS=Homo sapiens PE=2 SV=1	59162	451	1137	38	31	0.06	0.11	1.83333333	1	1	1	2	1	2	1	3	-0.16006
sp|P00390|GSHR_HUMAN	GSR	Glutathione reductase, mitochondrial OS=Homo sapiens GN=GSR PE=1 SV=2	56791	313	246	58	186	0.18	0.33	1.83333333	1	3	3	5	4	5	4	7	-0.60799
tr|A8K7F6|A8K7F6_HUMAN	tr|A8K7F6|A	cDNA FLJ78244, highly similar to Homo sapiens eukaryotic translation initiation factor 4A, isofor	46321	52	60	404	633	1.62	2.96	1.82716049	10	20	14	23	15	28	20	29	-0.01835
tr|Q5I6Y6|Q5I6Y6_HUMAN	LMNA	Lamin A/C transcript variant 1 OS=Homo sapiens GN=LMNA PE=2 SV=1	74322	95	71	221	557	0.75	1.37	1.82666667	7	16	13	20	15	21	15	23	-0.42624
tr|B4DUY3|B4DUY3_HUMAN	tr|B4DUY3|B	cDNA FLJ56668, highly similar to Nucleolar protein NOP5 OS=Homo sapiens PE=2 SV=1	16345	515	226	32	46	0.21	0.38	1.80952381	0	1	1	1	1	1	1	1	-0.89721
sp|P13010|XRCC5_HUMAN	XRCC5	X-ray repair cross-complementing protein 5 OS=Homo sapiens GN=XRCC5 PE=1 SV=3	83222	128	169	172	275	0.36	0.65	1.80555556	6	8	8	13	8	16	9	17	0.060078
sp|Q9NR30|DDX21_HUMAN	DDX21	Nucleolar RNA helicase 2 OS=Homo sapiens GN=DDX21 PE=1 SV=5	87804	148	138	147	319	0.34	0.61	1.79411765	4	10	8	13	8	12	9	14	-0.34118
tr|Q53SS8|Q53SS8_HUMAN	PCBP1	Poly(RC) binding protein 1 OS=Homo sapiens GN=PCBP1 PE=2 SV=1	37987	110	188	197	252	0.29	0.52	1.79310345	3	5	3	5	3	6	3	8	-0.00389
tr|H0Y327|H0Y327_HUMAN	MTHFD1L	Monofunctional C1-tetrahydrofolate synthase, mitochondrial (Fragment) OS=Homo sapiens GN-	36808	398	459	43	25	0.09	0.16	1.77777778	1	0	1	2	1	2	1	3	-0.16006
tr|H0YDE4|H0YDE4_HUMAN	PRMT1	Protein arginine N-methyltransferase 1 (Fragment) OS=Homo sapiens GN=PRMT1 PE=4 SV=1	27345	437	690	39	59	0.26	0.46	1.76923077	1	2	2	3	2	3	2	4	-0.41176
tr|J3KPS3|J3KPS3_HUMAN	ALDOA	Fructose-bisphosphate aldolase OS=Homo sapiens GN=ALDOA PE=3 SV=1	40249	63	49	338	724	1.79	3.14	1.75418994	11	16	15	23	17	33	18	35	0.039996
tr|Q7Z3K9|Q7Z3K9_HUMAN	DKFZp781L05	Putative uncharacterized protein DKFZp781L0540 (Fragment) OS=Homo sapiens GN=DKFZp781L	90081	440	678	39	63	0.04	0.07	1.75	1	2	1	2	2	3	2	3	-0.41176
tr|B2RDN4|B2RDN4_HUMAN	tr|B2RDN4|B	cDNA, FLJ96693, highly similar to Homo sapiens block of proliferation 1 (BOP1), mRNA OS=Hom	84219	505	316	33	57	0.08	0.14	1.75	0	2	2	2	2	2	3	5	-0.89736
tr|B4DGU4|B4DGU4_HUMAN	CTNNB1	Catenin beta-1 OS=Homo sapiens GN=CTNNB1 PE=2 SV=1	85339	340	391	52	119	0.12	0.21	1.75	2	5	3	5	3	7	3	8	0.202738
tr|Q53GN4|Q53GN4_HUMAN	WDR1	WD repeat domain 1, isoform CRA_a (Fragment) OS=Homo sapiens GN=WDR1 PE=2 SV=1	66822	137	121	160	350	0.4	0.7	1.75	4	11	7	13	7	15	9	16	0.150608
tr|B4DFN9|B4DFN9_HUMAN	tr|B4DFN9|B	cDNA FLJ54303, highly similar to Heat shock 70 kDa protein 1 OS=Homo sapiens PE=2 SV=1	62699	80	91	252	353	0.58	1.01	1.74137931	7	8	10	15	10	16	10	17	-0.24545
tr|B3KX72|B3KX72_HUMAN	tr|B3KX72|B	cDNA FLJ44920 fis, clone BRAMY3011501, highly similar to Heterogeneous nuclear ribonucleopr	83774	59	57	367	565	0.58	1.01	1.74137931	10	16	14	20	16	23	20	27	-0.38809
tr|B3KX19|B3KX19_HUMAN	tr|B3KX19|B	cDNA FLJ44500 fis, clone UTERU3000828, highly similar to 116 kDa U5 small nuclear ribonucleo	109111	111	165	193	282	0.27	0.47	1.74074074	4	5	8	13	8	14	10	17	-0.1267
tr|C9JZ53|C9JZ53_HUMAN	IPO5	Importin-5 (Fragment) OS=Homo sapiens GN=IPO5 PE=2 SV=1	8736	85	492	26	91	0.23	0.4	1.73913043	0	1	1	1	1	1	1	2	-0.89721
tr|B3KMV8|B3KMV8_HUMAN	tr|B3KMV8|B	cDNA FLJ12766 fis, clone NT2RP2001520, highly similar to Calcium-binding mitochondrial carrie	75130	353	677	41	63	0.11	0.19	1.72727273	1	1	1	4	1	3	1	4	0.325545
sp|P55072|TERA_HUMAN	VCP	Transitional endoplasmic reticulum ATPase OS=Homo sapiens GN=VCP PE=1 SV=4	89950	64	67	326	598	0.65	1.12	1.72307692	7	16	14	24	14	24	15	28	-0.1407
tr|B3KNN7|B3KNN7_HUMAN	tr|B3KNN7|B	cDNA FLJ30049 fis, clone ADRGL1000033, highly similar to 26S proteasome non-ATPase regulato	57311	273	322	69	140	0.18	0.31	1.72222222	3	4	3	5	3	5	3	5	-0.24508
sp|Q9NXW2|DJB12_HUMAN	DNAJB12	DnaJ homolog subfamily B member 12 OS=Homo sapiens GN=DNAJB12 PE=1 SV=4	42021	124	154	174	293	0.57	0.98	1.71929825	5	7	7	10	9	10	9	11	-0.75388
tr|B3KTJ9|B3KTJ9_HUMAN	tr|B3KTJ9|B3	cDNA FLJ38393 fis, clone FEBRA2007212 OS=Homo sapiens PE=2 SV=1	103493	82	143	251	314	0.32	0.55	1.71875	7	8	9	14	9	13	12	15	-0.39077
tr|D2CFK9|D2CFK9_HUMAN	GTPBP4	Nucleolar GTP-binding protein 1 OS=Homo sapiens GN=GTPBP4 PE=2 SV=1	74317	264	305	72	147	0.14	0.24	1.71428571	2	5	3	5	3	6	3	7	-0.00389
tr|G3V2A3|G3V2A3_HUMAN	TUBB3	Tubulin beta-3 chain (Fragment) OS=Homo sapiens GN=TUBB3 PE=2 SV=1	20929	25	28	147	531	3.07	5.18	1.68729642	5	15	6	17	7	21	7	25	0.623759
tr|B2RWP9|B2RWP9_HUMAN	MYH10	MYH10 protein OS=Homo sapiens GN=MYH10 PE=2 SV=1	230858	200	229	104	202	0.06	0.1	1.66666667	3	6	4	7	4	14	6	18	0.792185
tr|F8WJN3|F8WJN3_HUMAN	CPSF6	Cleavage and polyadenylation-specificity factor subunit 6 OS=Homo sapiens GN=CPSF6 PE=2 SV	52409	379	1317	46	20	0.06	0.1	1.66666667	1	0	1	1	1	1	1	1	-0.89721
sp|Q09028|RBBP4_HUMAN	RBBP4	Histone-binding protein RBBP4 OS=Homo sapiens GN=RBBP4 PE=1 SV=3	47911	321	1011	55	37	0.22	0.36	1.63636364	2	0	3	2	4	3	4	3	-1.26042
tr|A0EVJ9|A0EVJ9_HUMAN	HLA-A	MHC class I antigen (Fragment) OS=Homo sapiens GN=HLA-A PE=3 SV=1	21189	639	498	22	90	0.16	0.26	1.625	0	3	1	3	1	4	3	6	0.688295
tr|F5CL10|F5CL10_HUMAN	HLA-Cw	MHC class I antigen OS=Homo sapiens GN=HLA-Cw PE=3 SV=1	41120	639	445	22	102	0.16	0.26	1.625	0	2	1	3	1	3	1	4	0.325545
sp|P46087|NOP2_HUMAN	NOP2	Putative ribosomal RNA methyltransferase NOP2 OS=Homo sapiens GN=NOP2 PE=1 SV=2	89589	296	306	61	146	0.15	0.24	1.6	2	3	4	6	5	6	6	6	-0.65664
tr|K9JA46|K9JA46_HUMAN	EL52	Epididymis luminal secretory protein 52 OS=Homo sapiens GN=EL52 PE=2 SV=1	85006	46	43	462	828	1.05	1.67	1.59047619	15	21	25	33	24	35	29	38	-0.36377
tr|B3KNM6|B3KNM6_HUMAN	tr|B3KNM6|E	cDNA FLJ14968 fis, clone THYRO1000288, highly similar to CAAX prenyl protease 1 homolog (EC	27279	547	932	30	32	0.12	0.19	1.58333333	1	1	1	2	1	2	1	3	-0.16006
sp|Q2NL82|TSR1_HUMAN	TSR1	Pre-rRNA-processing protein TSR1 homolog OS=Homo sapiens GN=TSR1 PE=1 SV=1	92151	230	613	90	70	0.07	0.11	1.57142857	2	3	2	3	2	4	2	4	-0.04901
tr|B4DI54|B4DI54_HUMAN	tr|B4DI54|B4	cDNA FLJ56386, highly similar to Heat shock 70 kDa protein 1L OS=Homo sapiens PE=2 SV=1	77912	91	198	226	205	0.28	0.44	1.57142857	5	4	7	7	7	8	7	8	-0.71739
tr|B4DH02|B4DH02_HUMAN	tr|B4DH02|B	cDNA FLJ50510, highly similar to Heat shock 70 kDa protein 4 OS=Homo sapiens PE=2 SV=1	95157	123	145	175	311	0.23	0.36	1.56521739	4	6	6	9	5	10	6	12	0.035958
tr|K7EK77|K7EK77_HUMAN	ATP5A1	ATP synthase subunit alpha, mitochondrial (Fragment) OS=Homo sapiens GN=ATP5A1 PE=3 SV=	22243	135	151	160	297	1.33	2.08	1.56390977	5	8	6	10	6	10	6	13	-0.20539
sp|Q96HC4|PDLI5_HUMAN	PDLIM5	PDZ and LIM domain protein 5 OS=Homo sapiens GN=PDLIM5 PE=1 SV=5	65102	175	116	123	355	0.41	0.64	1.56097561	3	7	7	11	7	11	8	13	-0.28075
tr|B4DTG2|B4DTG2_HUMAN	EEF1G	Elongation factor 1-gamma OS=Homo sapiens GN=EEF1G PE=2 SV=1	56456	93	191	225	249	0.86	1.34	1.55813953	5	6	15	15	14	15	18	20	-0.80284
tr|B2R6X5|B2R6X5_HUMAN	tr|B2R6X5|B	cDNA, FLJ93166, highly similar to Homo sapiens heat shock 70kDa protein 6 (HSP70B~) (HSPA6)	71370	193	335	112	134	0.2	0.31	1.55	3	4	5	6	6	6	6	7	-0.89799
tr|F5GY56|F5GY56_HUMAN	PRPF19	Pre-mRNA-processing factor 19 (Fragment) OS=Homo sapiens GN=PRPF19 PE=2 SV=1	27929	237	266	0	172	0.26	0.4	1.53846154	0	3	0	3	0	3	1	4	1.910843
tr|A8K4D5|A8K4D5_HUMAN	tr|A8K4D5|A	Kynureninase OS=Homo sapiens PE=2 SV=1	52819	266	467	71	97	0.13	0.2	1.53846154	2	2	2	3	3	4	3	4	-0.53477
sp|P68032|ACTC_HUMAN	ACTC1	Actin, alpha cardiac muscle 1 OS=Homo sapiens GN=ACTC1 PE=1 SV=1	42334	23	22	771	1384	6.63	10.11	1.52488688	25	45	40	54	48	59	55	64	-0.60767
sp|P53004|BIEA_HUMAN	BLVRA	Biliverdin reductase A OS=Homo sapiens GN=BLVRA PE=1 SV=2	33692	403	874	43	47	0.21	0.32	1.52380952	1	1	2	3	2	2	3	3	-0.89736
tr|J3KRF5|J3KRF5_HUMAN	CLTC	Clathrin heavy chain 1 (Fragment) OS=Homo sapiens GN=CLTC PE=4 SV=1	15819	30	775	40	54	0.52	0.79	1.51923077	1	2	1	3	2	3	2	3	-0.41176
tr|H0YIV4|H0YIV4_HUMAN	NAP1L1	Nucleosome assembly protein 1-like 1 (Fragment) OS=Homo sapiens GN=NAP1L1 PE=3 SV=1	44972	417	926	33	43	0.16	0.24	1.5	0	0	1	3	1	2	1	3	-0.16006
tr|G3V0E8|G3V0E8_HUMAN	PCBP2	Poly(RC) binding protein 2, isoform CRA_f OS=Homo sapiens GN=PCBP2 PE=2 SV=1	33817	132	252	166	144	0.32	0.48	1.5	3	3	3	3	3	3	5	5	-0.89752
tr|J3KN66|J3KN66_HUMAN	TOR1AIP1	Torsin-1A-interacting protein 1 OS=Homo sapiens GN=TOR1AIP1 PE=4 SV=1	67952	707	692	17	62	0.1	0.15	1.5	0	1	1	1	0	1	1	1	0.68809
sp|P52272|HNRPM_HUMAN	HNRNPM	Heterogeneous nuclear ribonucleoprotein M OS=Homo sapiens GN=HNRNPM PE=1 SV=3	77749	164	123	132	347	0.39	0.58	1.48717949	7	9	8	11	11	14	12	18	-0.5638
tr|I3L492|I3L492_HUMAN	ETF1	Eukaryotic peptide chain release factor subunit 1 (Fragment) OS=Homo sapiens GN=ETF1 PE=4	23028	274	648	69	50	0.15	0.22	1.46666667	1	1	1	1	1	1	1	1	-0.89721
tr|Q53G58|Q53G58_HUMAN	tr|Q53G58|C	Coronin (Fragment) OS=Homo sapiens PE=2 SV=1	53931	242	395	83	116	0.13	0.19	1.46153846	1	3	2	4	2	4	4	4	-0.04901
tr|B3GQS7|B3GQS7_HUMAN	HSPD1	Mitochondrial heat shock 60kD protein 1 variant 1 OS=Homo sapiens GN=HSPD1 PE=2 SV=1	60813	16	61	904	603	1.72	2.51	1.45930233	24	21	27	25	26	29	33	34	-0.74584
tr|Q5BKZ2|Q5BKZ2_HUMAN	KPNA1	Importin subunit alpha OS=Homo sapiens GN=KPNA1 PE=2 SV=1	60955	573	1002	22	38	0.11	0.16	1.45454545	0	1	1	1	1	2	1	2	-0.16006
sp|P07814|SYEP_HUMAN	EPRS	Bifunctional glutamate/proline--tRNA ligase OS=Homo sapiens GN=EPRS PE=1 SV=5	172080	57	77	380	511	0.33	0.48	1.45454545	9	16	15	21	15	26	17	31	-0.12368
sp|P50991|TCPD_HUMAN	CCT4	T-complex protein 1 subunit delta OS=Homo sapiens GN=CCT4 PE=1 SV=4	58401	113	249	188	185	0.27	0.39	1.44444444	4	5	5	6	7	6	7	9	-1.10477
tr|F2Z2U8|F2Z2U8_HUMAN	MYH14	Myosin-14 OS=Homo sapiens GN=MYH14 PE=2 SV=1	232102	152	185	140	253	0.07	0.1	1.42857143	5	8	5	8	7	9	8	10	-0.55674
sp|Q92973|TNPO1_HUMAN	TNPO1	Transportin-1 OS=Homo sapiens GN=TNPO1 PE=1 SV=2	103771	139	423	158	109	0.17	0.24	1.41176471	3	1	5	7	8	7	8	10	-1.07905
tr|B4DFK9|B4DFK9_HUMAN	tr|B4DFK9|B	cDNA FLJ57964, highly similar to Heterogeneous nuclear ribonucleoprotein H~ OS=Homo sapier	44637	272	230	69	146	0.15	0.21	1.4	1	3	2	3	1	3	2	3	0.325545
tr|D2JYH4|D2JYH4_HUMAN	ACTA2	Actin, alpha 2, smooth muscle, aorta OS=Homo sapiens GN=ACTA2 PE=2 SV=1	42381	23	24	702	1349	6.63	9.24	1.39366516	23	43	35	53	43	55	49	60	-0.55013

tr|Q5T081|Q5T081_HUMAN	RCC1	CHC1 protein OS=Homo sapiens GN=RCC1 PE=2 SV=1	45397	269	312	70	144	0.23	0.32	1.39130435	2	3	3	4	2	5	4	5	0.240681
tr|B4DUQ1|B4DUQ1_HUMAN	tr|B4DUQ1|B	cDNA FLJ54552, highly similar to Heterogeneous nuclear ribonucleoprotein K OS=Homo sapiens	48708	147	203	147	235	0.58	0.8	1.37931034	3	9	7	11	7	11	8	15	-0.28075
tr|B4DSQ5|B4DSQ5_HUMAN	tr|B4DSQ5|B	cDNA FLJ53608, highly similar to Protein transport protein Sec23A OS=Homo sapiens PE=2 SV=1	83787	474	315	37	143	0.08	0.11	1.375	0	3	2	4	2	6	3	6	0.481869
tr|D2KZ54|D2KZ54_HUMAN	HLA-A	MHC class I antigen (Fragment) OS=Homo sapiens GN=HLA-A PE=3 SV=1	31862	639	704	22	61	0.16	0.22	1.375	0	2	1	2	1	3	2	3	0.325545
sp|P68366|TBA4A_HUMAN	TUBA4A	Tubulin alpha-4A chain OS=Homo sapiens GN=TUBA4A PE=1 SV=1	50634	22	33	796	1100	3.01	4.1	1.36212625	20	32	31	38	31	42	36	49	-0.46779
sp|P46063|RECQ1_HUMAN	RECQL	ATP-dependent DNA helicase Q1 OS=Homo sapiens GN=RECQL PE=1 SV=3	74436	439	405	39	114	0.14	0.19	1.35714286	1	3	3	4	3	5	3	6	-0.24508
tr|B0QYT3|B0QYT3_HUMAN	RANGAP1	Ran GTPase activating protein 1 (Fragment) OS=Homo sapiens GN=RANGAP1 PE=2 SV=1	27122	574	503	28	47	0.12	0.16	1.33333333	0	1	1	1	1	2	1	2	-0.16006
tr|E9PIM9|E9PIM9_HUMAN	RNH1	Ribonuclease inhibitor (Fragment) OS=Homo sapiens GN=RNH1 PE=2 SV=1	22362	431	269	40	119	0.15	0.2	1.33333333	0	2	1	2	1	2	1	2	-0.16006
tr|Q96K98|Q96K98_HUMAN	tr|Q96K98|Q	cDNA FLJ14414 fis, clone HEMBA1004847, highly similar to SIGNAL RECOGNITION PARTICLE 68 K	37365	180	410	119	95	0.18	0.24	1.33333333	2	3	2	3	2	4	4	5	-0.04901
tr|A8K2X8|A8K2X8_HUMAN	tr|A8K2X8|A	cDNA FLJ78433, highly similar to Homo sapiens chaperonin containing TCP1, subunit 5 (epsilon)	60148	90	351	229	129	0.4	0.53	1.325	6	4	6	8	6	9	6	11	-0.34996
tr|B4DXW6|B4DXW6_HUMAN	tr|B4DXW6|B	cDNA FLJ50285, highly similar to Arginyl-tRNA synthetase (EC 6.1.1.19) OS=Homo sapiens PE=2 S	52771	235	232	88	133	0.35	0.46	1.31428571	3	6	5	7	6	11	7	11	-0.07396
tr|J3KTA4|J3KTA4_HUMAN	DDX5	Probable ATP-dependent RNA helicase DDX5 OS=Homo sapiens GN=DDX5 PE=3 SV=1	69557	97	166	218	281	0.45	0.59	1.31111111	7	8	9	11	10	12	10	17	-0.64671
sp|Q71U36|TBA1A_HUMAN	TUBA1A	Tubulin alpha-1A chain OS=Homo sapiens GN=TUBA1A PE=1 SV=1	50788	15	20	1022	1415	5.18	6.74	1.3011583	30	38	41	46	43	50	46	58	-0.68725
tr|B7Z2Z8|B7Z2Z8_HUMAN	tr|B7Z2Z8|B7	T-complex protein 1 subunit delta OS=Homo sapiens PE=2 SV=1	52781	113	247	188	156	0.27	0.35	1.2962963	4	4	5	4	7	5	7	6	-1.34596
tr|B3KU96|B3KU96_HUMAN	tr|B3KU96|B	cDNA FLJ39433 fis, clone PROST2004142, highly similar to GDP-L-fucose synthetase (EC 1.1.1.27	17856	371	1075	47	27	0.19	0.24	1.26315789	1	0	1	1	1	1	1	1	-0.89721
sp|P15121|ALDR_HUMAN	AKR1B1	Aldose reductase OS=Homo sapiens GN=AKR1B1 PE=1 SV=3	36230	185	596	116	72	0.42	0.53	1.26190476	3	2	4	4	5	5	5	5	-0.89783
sp|Q12792|TWF1_HUMAN	TWF1	Twinfilin-1 OS=Homo sapiens GN=TWF1 PE=1 SV=3	40429	575	754	28	57	0.08	0.1	1.25	0	1	1	1	1	1	1	1	-0.89721
tr|H0YAS6|H0YAS6_HUMAN	PABPC1	Polyadenylate-binding protein 1 (Fragment) OS=Homo sapiens GN=PABPC1 PE=4 SV=1	17943	215	439	48	104	0.33	0.41	1.24242424	1	2	2	2	2	2	2	4	-0.89736
tr|F5H5D3|F5H5D3_HUMAN	TUBA1C	Tubulin alpha-1C chain OS=Homo sapiens GN=TUBA1C PE=2 SV=1	58606	21	21	809	1386	2.92	3.61	1.23630137	24	38	34	46	37	50	41	59	-0.47109
tr|Q5HYM2|Q5HYM2_HUMAN	DKFZp686O24	Putative uncharacterized protein DKFZp686O2462 (Fragment) OS=Homo sapiens GN=DKFZp686	72627	569	556	29	79	0.09	0.11	1.22222222	0	1	2	3	2	3	2	3	-0.41176
tr|Q4W4Y1|Q4W4Y1_HUMAN	DRIP4	Dopamine receptor interacting protein 4 OS=Homo sapiens GN=DRIP4 PE=2 SV=1	96646	136	276	160	161	0.18	0.22	1.22222222	2	5	5	5	5	6	6	7	-0.65664
sp|Q9NY65|TBA8_HUMAN	TUBA8	Tubulin alpha-8 chain OS=Homo sapiens GN=TUBA8 PE=1 SV=1	50746	34	46	592	786	1.74	2.11	1.21264368	15	22	23	26	23	29	27	36	-0.5715
sp|P42166|LAP2A_HUMAN	TMPO	Lamina-associated polypeptide 2, isoform alpha OS=Homo sapiens GN=TMPO PE=1 SV=2	76016	241	365	84	127	0.24	0.29	1.20833333	1	5	5	6	5	7	5	8	-0.45001
sp|P68363|TBA1B_HUMAN	TUBA1B	Tubulin alpha-1B chain OS=Homo sapiens GN=TUBA1B PE=1 SV=1	50804	13	18	1091	1448	5.58	6.74	1.2078853	31	39	43	48	45	52	49	60	-0.69638
tr|B0QYA5|B0QYA5_HUMAN	EIF3D	Eukaryotic translation initiation factor 3 subunit D (Fragment) OS=Homo sapiens GN=EIF3D PE=4	31937	710	896	17	45	0.1	0.12	1.2	0	0	1	2	0	2	2	3	1.425236
tr|B3KMW5|B3KMW5_HUMA	tr|B3KMW5|	cDNA FLJ12796 fis, clone NT2RP2002058, highly similar to WD repeat protein 3 OS=Homo sapie	71519	408	587	43	41	0.05	0.06	1.2	1	1	1	1	1	1	1	1	-0.89721
tr|F5H6T1|F5H6T1_HUMAN	ACTR2	Actin-related protein 2 OS=Homo sapiens GN=ACTR2 PE=2 SV=1	34729	244	680	82	54	0.2	0.24	1.2	2	1	2	2	2	2	4	3	-0.89736
sp|Q1KMD3|HNRL2_HUMAN	HNRNPUL2	Heterogeneous nuclear ribonucleoprotein U-like protein 2 OS=Homo sapiens GN=HNRNPUL2 PE	85622	249	240	78	194	0.21	0.25	1.19047619	2	6	5	6	6	6	6	7	-0.89799
tr|B4DP08|B4DP08_HUMAN	ALG1	Chitobiosyldiphosphodolichol beta-mannosyltransferase OS=Homo sapiens GN=ALG1 PE=2 SV=1	40938	452	736	38	30	0.17	0.2	1.17647059	0	0	2	2	2	3	2	4	-0.41176
tr|J3KPF3|J3KPF3_HUMAN	SLC3A2	4F2 cell-surface antigen heavy chain OS=Homo sapiens GN=SLC3A2 PE=4 SV=1	68230	60	62	366	621	1.33	1.56	1.17293233	12	19	20	23	20	24	20	24	-0.64229
tr|B2R9K8|B2R9K8_HUMAN	tr|B2R9K8|B	cDNA, FLJ94440, highly similar to Homo sapiens chaperonin containing TCP1, subunit 6A (zeta 1	58386	118	378	181	123	0.47	0.55	1.17021277	5	4	7	8	9	8	10	9	-1.0591
tr|A8MW61|A8MW61_HUMA	PLRG1	Pleiotropic regulator 1 OS=Homo sapiens GN=PLRG1 PE=2 SV=1	57545	476	666	36	64	0.12	0.14	1.16666667	1	1	2	1	2	1	4	1	-1.63451
tr|Q5HYG8|Q5HYG8_HUMAN	DKFZp686P09	Putative uncharacterized protein DKFZp686P09201 OS=Homo sapiens GN=DKFZp686P09201 PE	53831	86	214	235	221	0.61	0.7	1.14754098	4	4	8	7	9	9	10	10	-0.89845
tr|A0PJ92|A0PJ92_HUMAN	NOP56	NOP56 protein (Fragment) OS=Homo sapiens GN=NOP56 PE=2 SV=1	50068	311	516	58	86	0.14	0.16	1.14285714	1	1	2	3	3	5	3	7	-0.24508
tr|B1AHC9|B1AHC9_HUMAN	XRCC6	X-ray repair complementing defective repair in Chinese hamster cells 6 (Ku autoantigen, 70kDa)	64528	116	149	184	299	0.64	0.73	1.140625	5	8	10	11	11	13	12	17	-0.66708
tr|A8K525|A8K525_HUMAN	tr|A8K525|A	cDNA FLJ76817, highly similar to Homo sapiens non-POU domain containing, octamer-binding (I	54367	75	128	266	334	0.8	0.91	1.1375	10	7	11	12	12	13	14	16	-0.78771
tr|B0YJC4|B0YJC4_HUMAN	VIM	Vimentin OS=Homo sapiens GN=VIM PE=3 SV=1	49680	66	155	312	228	1.03	1.17	1.13592233	9	9	11	11	12	13	15	13	-0.78771
sp|Q6ZMR3|LDH6A_HUMAN	LDHAL6A	L-lactate dehydrogenase A-like 6A OS=Homo sapiens GN=LDHAL6A PE=2 SV=1	36826	39	84	67	68	1.81	2.05	1.13259669	1	1	1	1	1	1	1	1	-0.89721
tr|F4ZW64|F4ZW64_HUMAN	tr|F4ZW64|F	NF90a OS=Homo sapiens PE=2 SV=1	76369	154	227	137	204	0.46	0.52	1.13043478	3	6	9	10	10	10	11	11	-0.89861
tr|B4DKZ9|B4DKZ9_HUMAN	tr|B4DKZ9|B	cDNA FLJ55705, highly similar to Threonyl-tRNA synthetase, cytoplasmic (EC 6.1.1.3) OS=Homo	71171	94	184	191	254	0.64	0.72	1.125	5	9	10	12	13	15	14	16	-0.69941
sp|P16615|AT2A2_HUMAN	ATP2A2	Sarcoplasmic/endoplasmic reticulum calcium ATPase 2 OS=Homo sapiens GN=ATP2A2 PE=1 SV=	116336	149	131	145	332	0.32	0.36	1.125	4	11	10	11	12	13	13	15	-0.78771
tr|E9PBU3|E9PBU3_HUMAN	ATIC	Phosphoribosylaminoimidazolecarboxamide formyltransferase OS=Homo sapiens GN=ATIC PE=2	64996	72	177	274	235	0.64	0.72	1.125	8	6	11	10	12	11	13	13	-1.01939
tr|B4DQX0|B4DQX0_HUMAN	tr|B4DQX0|B	cDNA FLJ59219, highly similar to Poly(A)-binding protein 1 OS=Homo sapiens PE=2 SV=1	67239	215	195	96	218	0.33	0.37	1.12121212	3	5	6	6	6	9	10	9	-0.34996
tr|E5RFS9|E5RFS9_HUMAN	MAT2B	Methionine adenosyltransferase 2 subunit beta OS=Homo sapiens GN=MAT2B PE=2 SV=1	9467	280	333	68	78	0.37	0.41	1.10810811	1	1	1	1	1	1	1	1	-0.89721
tr|B1AKJ5|B1AKJ5_HUMAN	NRD1	Nardilysin OS=Homo sapiens GN=NRD1 PE=3 SV=1	140352	225	546	92	81	0.11	0.12	1.09090909	2	2	2	5	2	9	2	10	1.029898
tr|J3QQM1|J3QQM1_HUMAN	PSMC5	26S protease regulatory subunit 8 (Fragment) OS=Homo sapiens GN=PSMC5 PE=4 SV=1	29442	314	1180	57	28	0.11	0.12	1.09090909	1	0	1	1	2	2	2	2	-0.16006
tr|D6REX3|D6REX3_HUMAN	SEC31A	Protein transport protein Sec31A OS=Homo sapiens GN=SEC31A PE=2 SV=1	137110	121	414	178	110	0.13	0.14	1.07692308	4	3	5	4	4	4	5	8	-0.89767
tr|B2R5M8|B2R5M8_HUMAN	tr|B2R5M8|B	Isocitrate dehydrogenase [NADP] OS=Homo sapiens PE=2 SV=1	46905	555	637	29	67	0.15	0.16	1.06666667	0	1	2	2	3	1	3	3	-2.12027
tr|H0YGW7|H0YGW7_HUMAN	ABCF1	ATP-binding cassette sub-family F member 1 (Fragment) OS=Homo sapiens GN=ABCF1 PE=3 SV=	68075	144	186	151	253	0.27	0.28	1.03703704	4	5	5	7	5	7	7	7	-0.45001
tr|Q5TB52|Q5TB52_HUMAN	PAPSS2	3~-phosphoadenosine 5~-phosphosulfate synthase 2, isoform CRA_b OS=Homo sapiens GN=PAI	70027	688	893	19	45	0.05	0.05	1	0	1	1	1	0	2	4	2	1.425236
tr|J3KN33|J3KN33_HUMAN	CNTLN	Centlein OS=Homo sapiens GN=CNTLN PE=4 SV=1	162069	680	1229	20	26	0.02	0.02	1	0	0	1	1	0	1	2	2	0.68809
tr|Q8WUM5|Q8WUM5_HUM	GEMIN4	Gem (Nuclear organelle) associated protein 4 OS=Homo sapiens GN=GEMIN4 PE=2 SV=1	121732	709	1086	17	33	0.03	0.03	1	0	1	1	1	1	3	2	4	0.325545
tr|A8K2Y2|A8K2Y2_HUMAN	tr|A8K2Y2|A	cDNA FLJ78120, highly similar to Homo sapiens eukaryotic translation initiation factor 2, subunit	51648	475	908	37	44	0.06	0.06	1	1	1	1	1	1	3	1	3	0.325545
tr|B4DFM0|B4DFM0_HUMAN	FARSLB	Phenylalanine-tRNA synthetase-like, beta subunit, isoform CRA_c OS=Homo sapiens GN=FARSLE	55353	360	832	48	50	0.06	0.06	1	1	1	1	1	1	3	3	3	0.325545
tr|G0KXI8|G0KXI8_HUMAN	HLA-A	MHC class I antigen (Fragment) OS=Homo sapiens GN=HLA-A PE=3 SV=1	21246	639	740	22	57	0.16	0.16	1	1	0	1	1	1	3	2	3	0.325545
sp|Q9Y3I0|RTCB_HUMAN	C22orf28	tRNA-splicing ligase RtcB homolog OS=Homo sapiens GN=C22orf28 PE=1 SV=1	55688	458	1062	38	35	0.12	0.12	1	0	0	2	2	2	4	2	4	-0.04901
tr|H0Y2W2|H0Y2W2_HUMAN	ATAD3A	ATPase family AAA domain-containing protein 3A (Fragment) OS=Homo sapiens GN=ATAD3A PE	64600	281	753	68	57	0.1	0.1	1	2	1	2	2	2	4	2	7	-0.04901
tr|J3QT54|J3QT54_HUMAN	CPSF7	Cleavage and polyadenylation-specificity factor subunit 7 (Fragment) OS=Homo sapiens GN=CPS	22762	665	1178	21	28	0.15	0.15	1	0	1	1	1	1	2	1	2	-0.16006
sp|Q9Y5B9|SP16H_HUMAN	SUPT16H	FACT complex subunit SPT16 OS=Homo sapiens GN=SUPT16H PE=1 SV=1	120409	506	1032	33	36	0.03	0.03	1	0	1	1	1	1	2	2	2	-0.16006
tr|G3XAF4|G3XAF4_HUMAN	L1CAM	L1 cell adhesion molecule, isoform CRA_a OS=Homo sapiens GN=L1CAM PE=4 SV=1	139790	572	975	28	40	0.05	0.05	1	1	1	2	2	1	2	2	2	-0.16006
tr|E7ET15|E7ET15_HUMAN	U2SURP	U2 snRNP-associated SURP motif-containing protein OS=Homo sapiens GN=U2SURP PE=2 SV=1	118631	647	776	22	54	0.03	0.03	1	0	1	1	1	1	2	2	2	-0.16006
tr|B7Z601|B7Z601_HUMAN	tr|B7Z601|B7	cDNA FLJ57187, highly similar to Glycerol-3-phosphate dehydrogenase, mitochondrial (EC 1.1.9	56047	512	1069	32	34	0.06	0.06	1	0	1	1	1	2	3	2	3	-0.41176
tr|Q58FF4|Q58FF4_HUMAN	HSP90Bf	Heat shock protein 90Bf OS=Homo sapiens GN=HSP90Bf PE=1 SV=1	41833	407	967	43	40	0.16	0.16	1	1	1	2	2	2	3	2	3	-0.41176
sp|Q9BYZ2|LDH6B_HUMAN	LDHAL6B	L-lactate dehydrogenase A-like 6B OS=Homo sapiens GN=LDHAL6B PE=1 SV=3	42372	327	361	54	127	0.16	0.16	1	1	3	2	3	2	3	2	4	-0.41176
tr|Q6IBT3|Q6IBT3_HUMAN	CCT7	CCT7 protein OS=Homo sapiens GN=CCT7 PE=2 SV=1	59804	161	428	133	107	0.38	0.38	1	2	5	6	6	5	7	7	7	-0.45001
sp|Q15046|SYK_HUMAN	KARS	Lysine--tRNA ligase OS=Homo sapiens GN=KARS PE=1 SV=3	68461	265	275	71	167	0.32	0.32	1	1	6	6	6	6	8	7	11	-0.5106
sp|Q9NV06|DCA13_HUMAN	DCAF13	DDB1- and CUL4-associated factor 13 OS=Homo sapiens GN=DCAF13 PE=1 SV=2	51996	435	779	39	54	0.2	0.2	1	1	0	3	3	3	4	6	6	-0.53477
sp|Q96TA1|NIBL1_HUMAN	FAM129B	Niban-like protein 1 OS=Homo sapiens GN=FAM129B PE=1 SV=3	84598	155	286	137	160	0.16	0.16	1	4	4	4	4	4	5	4	6	-0.60799
sp|P08729|K2C7_HUMAN	KRT7	Keratin, type II cytoskeletal 7 OS=Homo sapiens GN=KRT7 PE=1 SV=5	51411	259	235	74	198	0.45	0.45	1	2	6	6	6	7	8	9	9	-0.71739
tr|A1L3A7|A1L3A7_HUMAN	NUFIP2	Nuclear fragile X mental retardation protein interacting protein 2 OS=Homo sapiens GN=NUFIP2	76132	301	1315	59	21	0.04	0.04	1	1	0	1	1	1	1	1	1	-0.89721
tr|B7Z438|B7Z438_HUMAN	tr|B7Z438|B7	cDNA FLJ56352, highly similar to Succinyl-CoA ligase (GDP-forming) subunit alpha, mitochondria	26801	467	1239	37	25	0.12	0.12	1	1	0	1	1	1	1	1	1	-0.89721
tr|B4DM97|B4DM97_HUMAN	tr|B4DM97|B	cDNA FLJ55002, highly similar to Alpha-centractin OS=Homo sapiens PE=2 SV=1	38365	561	1219	29	26	0.09	0.09	1	0	0	1	1	1	1	1	1	-0.89721
tr|B4DK80|B4DK80_HUMAN	TOP3A	DNA topoisomerase OS=Homo sapiens GN=TOP3A PE=2 SV=1	103082	668	1218	20	26	0.03	0.03	1	0	0	1	1	1	1	1	1	-0.89721
tr|J3KN69|J3KN69_HUMAN	NCEH1	Arylacetamide deacetylase-like 1 OS=Homo sapiens GN=NCEH1 PE=4 SV=1	49431	663	1197	21	27	0.07	0.07	1	0	0	1	1	1	1	1	2	-0.89721
tr|F2Z2J7|F2Z2J7_HUMAN	TTLL7	Tubulin polyglutamylase TTLL7 OS=Homo sapiens GN=TTLL7 PE=2 SV=1	55925	526	1188	31	28	0.06	0.06	1	1	1	1	1	1	1	1	1	-0.89721
tr|B3KPC1|B3KPC1_HUMAN	tr|B3KPC1|B	cDNA FLJ31582 fis, clone NT2RI2002117, highly similar to Protein pelota homolog OS=Homo sap	43807	307	1182	59	28	0.08	0.08	1	1	0	1	1	1	1	1	1	-0.89721
sp|Q9UG63|ABCF2_HUMAN	ABCF2	ATP-binding cassette sub-family F member 2 OS=Homo sapiens GN=ABCF2 PE=1 SV=2	71815	673	1126	20	31	0.05	0.05	1	0	0	1	1	1	1	2	1	-0.89721
tr|B4DE48|B4DE48_HUMAN	tr|B4DE48|B	cDNA FLJ53824, highly similar to Sorting nexin-17 OS=Homo sapiens PE=2 SV=1	50009	677	1125	20	31	0.07	0.07	1	0	0	1	1	1	1	1	1	-0.89721
tr|B5BU01|B5BU01_HUMAN	EIF2S2	Eukaryotic translation initiation factor 2 beta OS=Homo sapiens GN=EIF2S2 PE=2 SV=1	38648	654	1107	22	32	0.09	0.09	1	0	0	1	1	1	1	1	2	-0.89721
sp|O43286|B4GT5_HUMAN	B4GALT5	Beta-1,4-galactosyltransferase 5 OS=Homo sapiens GN=B4GALT5 PE=2 SV=1	45318	651	1078	22	34	0.07	0.07	1	0	1	1	1	1	1	1	1	-0.89721
tr|B3KY60|B3KY60_HUMAN	tr|B3KY60|B	cDNA FLJ16777 fis, clone BRHIP2029567, highly similar to Cell division cycle 5-like protein OS=H	92448	587	1068	27	34	0.04	0.04	1	0	1	1	1	1	1	1	1	-0.89721
tr|Q8TC04|Q8TC04_HUMAN	KRT23	Keratin 23 (Histone deacetylase inducible) OS=Homo sapiens GN=KRT23 PE=2 SV=1	48318	600	1045	26	36	0.07	0.07	1	0	1	1	1	1	1	1	1	-0.89721
sp|Q6KB66|K2C80_HUMAN	KRT80	Keratin, type II cytoskeletal 80 OS=Homo sapiens GN=KRT80 PE=1 SV=2	51007	564	1030	29	37	0.06	0.06	1	0	1	1	1	1	1	1	1	-0.89721
tr|J3QQX0|J3QQX0_HUMAN	GPS1	COP9 signalosome complex subunit 1 (Fragment) OS=Homo sapiens GN=GPS1 PE=4 SV=1	8703	636	868	22	47	0.4	0.4	1	0	1	1	1	1	1	1	1	-0.89721
tr|E9PNN6|E9PNN6_HUMAN	ALDH3A1	Aldehyde dehydrogenase, dimeric NADP-preferring OS=Homo sapiens GN=ALDH3A1 PE=2 SV=1	44518	519	854	32	48	0.07	0.07	1	0	1	1	1	1	1	1	1	-0.89721
tr|B2RDF5|B2RDF5_HUMAN	tr|B2RDF5|B	cDNA, FLJ96587, highly similar to Homo sapiens SUMO-1 activating enzyme subunit 2 (UBA2), m	71705	590	792	27	53	0.05	0.05	1	0	1	1	1	1	1	1	1	-0.89721
tr|B3KUJ0|B3KUJ0_HUMAN	tr|B3KUJ0|B	cDNA FLJ39996 fis, clone STOMA2002166, highly similar to Splicing factor 3B subunit 4 OS=Hom	44512	464	785	37	53	0.07	0.07	1	1	1	1	1	1	2	1	1	-0.89721
tr|A0EVK1|A0EVK1_HUMAN	HLA-A	MHC class I antigen (Fragment) OS=Homo sapiens GN=HLA-A PE=3 SV=1	21022	639	744	22	57	0.16	0.16	1	0	1	1	1	1	1	1	2	-0.89721
tr|C5IZP5|C5IZP5_HUMAN	HLA-A	MHC class I antigen (Fragment) OS=Homo sapiens GN=HLA-A PE=3 SV=1	21150	639	741	22	57	0.16	0.16	1	0	1	1	1	1	1	1	2	-0.89721
tr|B3KSQ7|B3KSQ7_HUMAN	DBN1	Drebrin 1, isoform CRA_d OS=Homo sapiens GN=DBN1 PE=2 SV=1	66053	478	676	36	63	0.05	0.05	1	0	1	1	1	1	1	1	1	-0.89721
tr|B4DLD4|B4DLD4_HUMAN	tr|B4DLD4|B	cDNA FLJ56738, highly similar to Fibrillarin OS=Homo sapiens PE=2 SV=1	30279	423	615	41	70	0.11	0.11	1	1	1	1	1	1	1	1	1	-0.89721
tr|J3KTE4|J3KTE4_HUMAN	RPL19	Ribosomal protein L19 OS=Homo sapiens GN=RPL19 PE=3 SV=1	23347	336	550	52	80	0.14	0.14	1	1	1	1	1	1	1	1	1	-0.89721
sp|O00629|IMA4_HUMAN	KPNA4	Importin subunit alpha-4 OS=Homo sapiens GN=KPNA4 PE=1 SV=1	58364	472	947	37	41	0.06	0.06	1	0	1	1	1	2	2	2	2	-0.89736
sp|P07737|PROF1_HUMAN	PFN1	Profilin-1 OS=Homo sapiens GN=PFN1 PE=1 SV=2	15216	436	919	39	43	0.5	0.5	1	1	1	2	2	2	2	2	2	-0.89736
tr|B4DUL5|B4DUL5_HUMAN	tr|B4DUL5|B	cDNA FLJ51625, highly similar to Ubiquinol-cytochrome-c reductase complex coreprotein I, mito	40917	357	864	48	48	0.17	0.17	1	1	2	2	2	2	2	2	2	-0.89736
tr|B3KY95|B3KY95_HUMAN	tr|B3KY95|B	cDNA FLJ16143 fis, clone BRAMY2038516, highly similar to Protein disulfide-isomerase A6 (EC 5.	53655	445	491	38	92	0.13	0.13	1	0	1	2	2	2	2	2	2	-0.89736
tr|B4DPZ3|B4DPZ3_HUMAN	tr|B4DPZ3|B	cDNA FLJ53290, highly similar to Cytoplasmic dynein 1 intermediate chain 2 OS=Homo sapiens F	70869	298	432	60	107	0.09	0.09	1	2	2	2	2	2	2	2	2	-0.89736
tr|Q05DV5|Q05DV5_HUMAN	ZC3HAV1	ZC3HAV1 protein (Fragment) OS=Homo sapiens GN=ZC3HAV1 PE=2 SV=1	49964	224	284	92	161	0.14	0.14	1	1	1	2	2	2	2	2	2	-0.89736
tr|L0R5A1|L0R5A1_HUMAN	CSF2RB	Alternative protein CSF2RB OS=Homo sapiens GN=CSF2RB PE=4 SV=1	11752	486	901	36	45	0.29	0.29	1	1	2	1	3	3	3	5	5	-0.89752
tr|Q53XC0|Q53XC0_HUMAN	EIF2S1	Eukaryotic translation initiation factor 2, subunit 1 alpha, 35kDa, isoform CRA_a OS=Homo sapie	36374	208	318	100	141	0.42	0.42	1	4	2	4	4	4	4	4	5	-0.89767
tr|B4DRH6|B4DRH6_HUMAN	tr|B4DRH6|B	cDNA FLJ54509, highly similar to Trifunctional enzyme subunit alpha, mitochondrial OS=Homo s	79007	133	280	164	164	0.28	0.28	1	5	4	6	6	7	7	7	7	-0.89814
tr|Q5U077|Q5U077_HUMAN	LDHB	L-lactate dehydrogenase OS=Homo sapiens GN=LDHB PE=2 SV=1	36900	39	96	530	423	1.81	1.81	1	13	13	16	14	18	18	20	21	-0.89986
tr|Q53GX7|Q53GX7_HUMAN	tr|Q53GX7|Q	Threonyl-tRNA synthetase variant (Fragment) OS=Homo sapiens PE=2 SV=1	84304	94	173	221	270	0.64	0.64	1	6	10	13	13	17	15	18	17	-1.07515
tr|B2RCM3|B2RCM3_HUMAN	tr|B2RCM3|B	cDNA, FLJ96158, highly similar to Homo sapiens calpain 2, (m/II) large subunit (CAPN2), mRNA C	80883	173	278	125	165	0.22	0.22	1	3	3	7	5	7	6	8	7	-1.10477
sp|Q9UMS4|PRP19_HUMAN	PRPF19	Pre-mRNA-processing factor 19 OS=Homo sapiens GN=PRPF19 PE=1 SV=1	55603	237	222	87	212	0.26	0.26	1	2	4	5	4	7	6	11	8	-1.10477
tr|A8K4Z4|A8K4Z4_HUMAN	tr|A8K4Z4|A	cDNA FLJ75549, highly similar to Homo sapiens ribosomal protein, large, P0 (RPLP0), transcript v	34389	109	635	201	67	0.74	0.74	1	6	2	6	6	6	5	6	7	-1.13917
sp|P23381|SYWC_HUMAN	WARS	Tryptophan--tRNA ligase, cytoplasmic OS=Homo sapiens GN=WARS PE=1 SV=2	53474	145	329	148	136	0.27	0.27	1	3	3	4	4	5	4	5	6	-1.18752
tr|H0Y804|H0Y804_HUMAN	AKR1C1	Aldo-keto reductase family 1 member C1 (Fragment) OS=Homo sapiens GN=AKR1C1 PE=4 SV=1	28617	186	290	61	83	0.67	0.67	1	2	2	4	3	4	3	6	4	-1.26042
sp|Q9NR12|PDLI7_HUMAN	PDLIM7	PDZ and LIM domain protein 7 OS=Homo sapiens GN=PDLIM7 PE=1 SV=1	50896	396	1261	43	24	0.13	0.13	1	0	0	2	2	2	1	4	3	-1.63451
sp|O00505|IMA3_HUMAN	KPNA3	Importin subunit alpha-3 OS=Homo sapiens GN=KPNA3 PE=1 SV=2	58288	406	1128	43	31	0.12	0.12	1	0	1	2	2	2	1	2	2	-1.63451
sp|Q9NRF8|PYRG2_HUMAN	CTPS2	CTP synthase 2 OS=Homo sapiens GN=CTPS2 PE=1 SV=1	66320	614	811	24	51	0.05	0.05	1	0	1	1	1	2	1	6	2	-1.63451
tr|Q0IJ56|Q0IJ56_HUMAN	ST13	ST13 protein (Fragment) OS=Homo sapiens GN=ST13 PE=2 SV=1	35164	382	581	46	75	0.09	0.09	1	1	1	1	1	2	1	3	2	-1.63451
tr|Q6IAV5|Q6IAV5_HUMAN	OXCT	Succinyl-CoA:3-ketoacid-coenzyme A transferase OS=Homo sapiens GN=OXCT PE=2 SV=1	56612	209	487	100	91	0.18	0.18	1	1	1	3	2	5	2	6	3	-2.03587
sp|Q6S8J3|POTEE_HUMAN	POTEE	POTE ankyrin domain family member E OS=Homo sapiens GN=POTEE PE=1 SV=3	122882	5	55	2211	673	0.44	0.44	1	45	21	66	28	70	31	75	34	-2.0774
tr|J3KQE5|J3KQE5_HUMAN	RAN	GTP-binding nuclear protein Ran (Fragment) OS=Homo sapiens GN=RAN PE=4 SV=1	27027	571	1322	29	20	0.12	0.12	1	0	0	1	1	1	0	1	1	-2.48235
tr|A0N4V7|A0N4V7_HUMAN	Tcr-alpha	HCG2039797 (Fragment) OS=Homo sapiens GN=Tcr-alpha PE=4 SV=1	2269	212	665	98	64	1.81	1.81	1	5	4	15	5	31	5	46	5	-3.42443
sp|Q13867|BLMH_HUMAN	BLMH	Bleomycin hydrolase OS=Homo sapiens GN=BLMH PE=1 SV=1	53155	332	850	52	48	0.2	0.18	0.9	1	1	3	2	2	2	4	2	-0.89736
tr|H0Y9L5|H0Y9L5_HUMAN	EXOSC9	Exosome complex component RRP45 (Fragment) OS=Homo sapiens GN=EXOSC9 PE=4 SV=1	18940	457	488	38	68	0.18	0.16	0.88888889	1	1	1	1	1	1	1	1	-0.89721
tr|H7C393|H7C393_HUMAN	APEH	Acylamino-acid-releasing enzyme (Fragment) OS=Homo sapiens GN=APEH PE=4 SV=1	32397	319	418	56	57	0.09	0.08	0.88888889	1	1	1	1	1	1	1	1	-0.89721
sp|Q05639|EF1A2_HUMAN	EEF1A2	Elongation factor 1-alpha 2 OS=Homo sapiens GN=EEF1A2 PE=1 SV=1	50780	70	202	238	235	1.14	1	0.87719298	8	8	9	14	12	15	14	17	-0.58804
tr|Q5CAQ5|Q5CAQ5_HUMAN	TRA1	Tumor rejection antigen (Gp96) 1 OS=Homo sapiens GN=TRA1 PE=2 SV=1	92567	140	187	157	253	0.37	0.32	0.86486486	4	6	9	8	10	10	11	10	-0.89861
tr|Q6I9U3|Q6I9U3_HUMAN	M6PR	M6PR protein OS=Homo sapiens GN=M6PR PE=2 SV=1	31469	416	956	42	41	0.29	0.25	0.86206897	1	1	1	1	1	1	1	1	-0.89721
sp|P25705|ATPA_HUMAN	ATP5A1	ATP synthase subunit alpha, mitochondrial OS=Homo sapiens GN=ATP5A1 PE=1 SV=1	59828	135	93	160	433	1.33	1.12	0.84210526	5	12	6	16	6	17	6	19	0.532842
tr|G8JLB6|G8JLB6_HUMAN	HNRNPH1	Heterogeneous nuclear ribonucleoprotein H OS=Homo sapiens GN=HNRNPH1 PE=4 SV=1	51482	207	210	101	228	0.45	0.36	0.8	2	4	6	6	5	7	6	9	-0.45001
sp|P38919|IF4A3_HUMAN	EIF4A3	Eukaryotic initiation factor 4A-III OS=Homo sapiens GN=EIF4A3 PE=1 SV=4	47126	262	293	74	158	0.4	0.31	0.775	1	3	5	4	4	7	6	8	-0.16017
tr|Q05CK9|Q05CK9_HUMAN	SYNCRIP	SYNCRIP protein (Fragment) OS=Homo sapiens GN=SYNCRIP PE=2 SV=1	50847	252	889	76	46	0.13	0.1	0.76923077	2	1	2	2	2	3	2	4	-0.41176
tr|B7ZB91|B7ZB91_HUMAN	TNRC6B	Trinucleotide repeat containing 6B (Fragment) OS=Homo sapiens GN=TNRC6B PE=4 SV=1	81825	359	1195	48	27	0.13	0.1	0.76923077	1	0	3	1	2	1	3	1	-1.63451
sp|Q562R1|ACTBL_HUMAN	ACTBL2	Beta-actin-like protein 2 OS=Homo sapiens GN=ACTBL2 PE=1 SV=2	42318	61	115	349	359	1.47	1.12	0.76190476	13	14	16	17	24	17	27	24	-1.38749
tr|B3KV72|B3KV72_HUMAN	tr|B3KV72|B	cDNA FLJ16204 fis, clone CTONG2019232, highly similar to Proteasome-associated protein ECM	78791	579	609	28	23	0.04	0.03	0.75	0	0	1	1	1	1	1	1	-0.89721
tr|H6VRF8|H6VRF8_HUMAN	KRT1	Keratin 1 OS=Homo sapiens GN=KRT1 PE=3 SV=1	66184	6	36	2032	1056	5.01	3.72	0.74251497	55	30	64	38	69	45	76	49	-1.5234
tr|H6VRG2|H6VRG2_HUMAN	KRT1	Keratin 1 OS=Homo sapiens GN=KRT1 PE=3 SV=1	66197	7	37	2027	964	5.01	3.72	0.74251497	55	29	65	37	70	44	77	48	-1.57659
tr|Q53ET2|Q53ET2_HUMAN	tr|Q53ET2|Q	Dihydropyrimidinase-like 2 variant (Fragment) OS=Homo sapiens PE=2 SV=1	62688	342	525	51	85	0.23	0.17	0.73913043	1	2	4	3	3	4	5	4	-0.53477
tr|Q562M3|Q562M3_HUMAN	ACT	Actin-like protein (Fragment) OS=Homo sapiens GN=ACT PE=2 SV=1	11529	103	102	213	401	7.06	5.21	0.73796034	10	14	14	18	17	22	20	25	-0.53623
tr|B4DY32|B4DY32_HUMAN	tr|B4DY32|B	Asparagine synthetase OS=Homo sapiens PE=2 SV=1	55055	141	475	156	95	0.26	0.19	0.73076923	3	3	5	3	6	3	6	4	-1.79161
tr|A2RUM7|A2RUM7_HUMAN	RPL5	Ribosomal protein L5 OS=Homo sapiens GN=RPL5 PE=2 SV=1	34569	187	738	115	58	0.44	0.32	0.72727273	2	1	4	3	5	3	7	4	-1.55027
sp|Q58FF8|H90B2_HUMAN	HSP90AB2P	Putative heat shock protein HSP 90-beta 2 OS=Homo sapiens GN=HSP90AB2P PE=1 SV=2	44492	32	205	97	234	1.24	0.9	0.72580645	3	6	5	9	5	11	6	13	0.167382
tr|B1AHE4|B1AHE4_HUMAN	ATXN10	Ataxin 10 (Fragment) OS=Homo sapiens GN=ATXN10 PE=4 SV=1	22299	470	413	37	60	0.38	0.27	0.71052632	0	1	2	1	2	1	2	2	-1.63451
sp|Q04695|K1C17_HUMAN	KRT17	Keratin, type I cytoskeletal 17 OS=Homo sapiens GN=KRT17 PE=1 SV=2	48361	24	79	683	507	2.99	2.07	0.69230769	19	17	27	19	32	24	35	30	-1.31115
tr|B4DNN4|B4DNN4_HUMAN	tr|B4DNN4|B	Ribonucleoside-diphosphate reductase OS=Homo sapiens PE=2 SV=1	87252	156	216	136	218	0.29	0.2	0.68965517	5	6	7	6	8	7	8	7	-1.07905
tr|H2AM05|H2AM05_HUMAN	HLA-A	MHC class I antigen (Fragment) OS=Homo sapiens GN=HLA-A PE=3 SV=1	31675	639	742	22	57	0.16	0.11	0.6875	0	1	1	1	1	2	1	2	-0.16006
tr|A0MNN4|A0MNN4_HUMA	SMU1	CDW3/SMU1 OS=Homo sapiens GN=SMU1 PE=2 SV=1	58134	221	981	94	39	0.12	0.08	0.66666667	1	1	2	1	2	1	2	1	-1.63451
tr|Q5JQZ9|Q5JQZ9_HUMAN	CDC2L6	Cell division cycle 2-like 6 (CDK8-like) (Fragment) OS=Homo sapiens GN=CDC2L6 PE=4 SV=1	29552	285	729	65	51	0.24	0.16	0.66666667	2	1	2	1	3	1	3	1	-2.12027
tr|B4DEM7|B4DEM7_HUMAN	CCT8	T-complex protein 1 subunit theta OS=Homo sapiens GN=CCT8 PE=2 SV=1	58179	268	321	70	140	0.18	0.12	0.66666667	2	2	3	2	5	2	6	2	-2.03587
tr|D0PNI1|D0PNI1_HUMAN	YWHAZ	Tyrosine 3-monooxygenase/tryptophan 5-monooxygenase activation protein zeta polypeptide (	27899	497	605	34	71	0.38	0.25	0.65789474	0	2	2	2	2	3	2	3	-0.41176
tr|Q8TE01|Q8TE01_HUMAN	derp12	DERP12 (Dermal papilla derived protein 12) OS=Homo sapiens GN=derp12 PE=2 SV=1	38340	69	403	297	114	0.79	0.51	0.64556962	6	3	7	5	8	6	8	6	-1.28568
tr|B1ALA9|B1ALA9_HUMAN	PRPS1	Phosphoribosyl pyrophosphate synthetase 1 OS=Homo sapiens GN=PRPS1 PE=3 SV=1	24468	638	978	22	0	0.14	0.09	0.64285714	0	0	1	0	1	0	2	1	-2.48235
tr|H0Y9Q7|H0Y9Q7_HUMAN	CANX	Calnexin (Fragment) OS=Homo sapiens GN=CANX PE=3 SV=1	18990	352	287	49	111	0.39	0.25	0.64102564	1	2	2	3	3	4	5	4	-0.53477
tr|Q5JP02|Q5JP02_HUMAN	RBBP7	Histone-binding protein RBBP7 (Fragment) OS=Homo sapiens GN=RBBP7 PE=4 SV=1	19626	321	703	37	28	0.22	0.14	0.63636364	1	0	1	1	2	2	2	2	-0.89736
sp|P27348|1433T_HUMAN	YWHAQ	14-3-3 protein theta OS=Homo sapiens GN=YWHAQ PE=1 SV=1	28032	258	529	74	84	0.4	0.25	0.625	2	2	3	2	3	3	3	3	-0.89752
sp|Q9H853|TBA4B_HUMAN	TUBA4B	Putative tubulin-like protein alpha-4B OS=Homo sapiens GN=TUBA4B PE=5 SV=2	27819	146	350	147	130	0.4	0.25	0.625	3	3	4	4	4	4	6	6	-0.89767
tr|B4DTA2|B4DTA2_HUMAN	tr|B4DTA2|B	cDNA FLJ60148, highly similar to Homo sapiens heterogeneous nuclear ribonucleoprotein D-like	30367	229	831	39	50	0.37	0.23	0.62162162	1	2	1	2	1	2	1	2	-0.16006
tr|B2R659|B2R659_HUMAN	tr|B2R659|B	cDNA, FLJ92803, highly similar to Homo sapiens hydroxysteroid (17-beta) dehydrogenase 4 (HSD	80018	484	1016	36	37	0.13	0.08	0.61538462	0	0	3	2	3	3	4	3	-0.89752
sp|P52895|AK1C2_HUMAN	AKR1C2	Aldo-keto reductase family 1 member C2 OS=Homo sapiens GN=AKR1C2 PE=1 SV=3	37111	186	504	116	89	0.67	0.41	0.6119403	3	3	6	4	7	4	9	5	-1.63565
tr|B3KM80|B3KM80_HUMAN	NCL	Nucleolin, isoform CRA_c OS=Homo sapiens GN=NCL PE=2 SV=1	58576	71	144	283	311	1.03	0.63	0.61165049	9	6	14	9	15	11	15	11	-1.33074
tr|B4DW52|B4DW52_HUMAN	tr|B4DW52|B	cDNA FLJ55253, highly similar to Actin, cytoplasmic 1 OS=Homo sapiens PE=2 SV=1	38950	4	15	3120	1529	27.29	16.35	0.59912056	74	45	108	62	123	72	134	81	-1.69434
tr|Q1KLZ0|Q1KLZ0_HUMAN	PS1TP5BP1	HCG15971, isoform CRA_a OS=Homo sapiens GN=PS1TP5BP1 PE=2 SV=1	42052	1	13	3367	1811	31.43	18.1	0.57588291	82	57	119	74	130	81	141	89	-1.60584
tr|B2R8N0|B2R8N0_HUMAN	tr|B2R8N0|B	cDNA, FLJ93975, highly similar to Homo sapiens epoxide hydrolase 1, microsomal (xenobiotic) (E	53129	115	341	184	132	0.62	0.35	0.56451613	6	3	8	5	8	7	8	9	-1.07905
sp|Q5XKE5|K2C79_HUMAN	KRT79	Keratin, type II cytoskeletal 79 OS=Homo sapiens GN=KRT79 PE=1 SV=2	58085	160	578	133	75	0.32	0.18	0.5625	5	3	6	3	6	4	8	5	-1.42886
tr|B4DPP6|B4DPP6_HUMAN	tr|B4DPP6|B	cDNA FLJ54371, highly similar to Serum albumin OS=Homo sapiens PE=2 SV=1	72370	222	464	93	97	0.25	0.14	0.56	3	3	5	3	6	3	10	4	-1.79161
sp|Q8NBJ5|GT251_HUMAN	GLT25D1	Procollagen galactosyltransferase 1 OS=Homo sapiens GN=GLT25D1 PE=1 SV=1	71933	523	1340	31	18	0.09	0.05	0.55555556	0	0	2	1	1	0	2	3	-2.48235
sp|P35527|K1C9_HUMAN	KRT9	Keratin, type I cytoskeletal 9 OS=Homo sapiens GN=KRT9 PE=1 SV=3	62255	14	51	1044	709	3.01	1.66	0.55149502	25	16	36	20	40	21	44	22	-1.82033
tr|A0PJ76|A0PJ76_HUMAN	NCAPD2	NCAPD2 protein (Fragment) OS=Homo sapiens GN=NCAPD2 PE=2 SV=1	44957	494	334	34	71	0.15	0.08	0.53333333	0	2	2	2	2	2	2	2	-0.89736
tr|F8W8T8|F8W8T8_HUMAN	ACACB	Acetyl-CoA carboxylase 2 OS=Homo sapiens GN=ACACB PE=2 SV=1	269823	31	75	128	526	0.44	0.23	0.52272727	4	15	4	20	6	20	9	22	0.761653
tr|L0R4T3|L0R4T3_HUMAN	ABCF2	Alternative protein ABCF2 OS=Homo sapiens GN=ABCF2 PE=4 SV=1	7359	524	1270	31	23	0.48	0.25	0.52083333	1	0	1	1	1	1	1	1	-0.89721
sp|Q9NVI7|ATD3A_HUMAN	ATAD3A	ATPase family AAA domain-containing protein 3A OS=Homo sapiens GN=ATAD3A PE=1 SV=2	71610	281	1160	68	29	0.1	0.05	0.5	2	0	2	1	2	4	2	7	-0.04901
tr|B3KNL2|B3KNL2_HUMAN	RUVBL2	RuvB-like 2 (E. coli), isoform CRA_d OS=Homo sapiens GN=RUVBL2 PE=2 SV=1	47603	584	1243	28	25	0.14	0.07	0.5	0	0	2	1	1	2	2	2	-0.16006
tr|B4DZ88|B4DZ88_HUMAN	KTN1	KTN1 protein OS=Homo sapiens GN=KTN1 PE=2 SV=1	153179	322	1020	55	37	0.04	0.02	0.5	2	1	2	1	3	4	4	5	-0.53477
tr|Q5QPE2|Q5QPE2_HUMAN	SEC23B	Protein transport protein Sec23B (Fragment) OS=Homo sapiens GN=SEC23B PE=4 SV=1	37575	474	1112	24	32	0.08	0.04	0.5	0	0	1	1	1	1	1	1	-0.89721
sp|P43490|NAMPT_HUMAN	NAMPT	Nicotinamide phosphoribosyltransferase OS=Homo sapiens GN=NAMPT PE=1 SV=1	55772	520	455	32	99	0.12	0.06	0.5	0	1	1	1	1	1	3	1	-0.89721
tr|B2RCT6|B2RCT6_HUMAN	tr|B2RCT6|B	cDNA, FLJ96276, highly similar to Homo sapiens G1 to S phase transition 1 (GSPT1), mRNA OS=H	56320	391	1200	44	27	0.12	0.06	0.5	1	0	2	1	2	2	2	2	-0.89736
tr|B2RBE5|B2RBE5_HUMAN	tr|B2RBE5|B	cDNA, FLJ95468, highly similar to Homo sapiens transcriptional coactivator tubedown-100 (TBD	102490	446	1040	38	36	0.06	0.03	0.5	0	1	2	1	2	2	2	4	-0.89736
tr|E9PRA5|E9PRA5_HUMAN	ATP1A4	Sodium/potassium-transporting ATPase subunit alpha-4 OS=Homo sapiens GN=ATP1A4 PE=2 SV	57835	289	969	63	40	0.12	0.06	0.5	0	0	6	3	10	7	19	8	-1.38458
sp|Q9UHR4|BI2L1_HUMAN	BAIAP2L1	Brain-specific angiogenesis inhibitor 1-associated protein 2-like protein 1 OS=Homo sapiens GN=	57189	613	770	24	55	0.12	0.06	0.5	0	1	2	1	2	1	4	2	-1.63451
tr|B2R6J4|B2R6J4_HUMAN	tr|B2R6J4|B2	cDNA, FLJ92975, highly similar to Homo sapiens nucleosome assembly protein 1-like 4 (NAP1L4)	42940	417	1349	42	16	0.16	0.08	0.5	0	0	2	1	3	1	3	2	-2.12027
sp|O60518|RNBP6_HUMAN	RANBP6	Ran-binding protein 6 OS=Homo sapiens GN=RANBP6 PE=1 SV=2	126173	85	285	36	161	0.23	0.11	0.47826087	1	4	1	4	2	4	2	5	-0.04901
tr|Q0IIN1|Q0IIN1_HUMAN	KRT77	Keratin 77 OS=Homo sapiens GN=KRT77 PE=2 SV=1	62050	76	268	264	172	0.23	0.11	0.47826087	10	4	10	4	10	5	12	5	-1.8324
sp|P0CG39|POTEJ_HUMAN	POTEJ	POTE ankyrin domain family member J OS=Homo sapiens GN=POTEJ PE=3 SV=1	118740	5	204	186	234	0.44	0.21	0.47727273	6	8	10	11	11	14	13	18	-0.5638
tr|B2R7M3|B2R7M3_HUMAN	tr|B2R7M3|B	cDNA, FLJ93510, highly similar to Homo sapiens JTV1 gene (JTV1), mRNA OS=Homo sapiens PE=	35767	308	1084	58	33	0.19	0.09	0.47368421	2	0	2	1	2	1	2	2	-1.63451
sp|P50552|VASP_HUMAN	VASP	Vasodilator-stimulated phosphoprotein OS=Homo sapiens GN=VASP PE=1 SV=3	39976	363	961	48	40	0.17	0.08	0.47058824	1	1	1	1	1	1	1	2	-0.89721

tr|Q2QD09|Q2QD09_HUMAN	tr|Q2QD09|Q	Triosephosphate isomerase (Fragment) OS=Homo sapiens PE=2 SV=1	27211	267	1076	71	34	0.26	0.12	0.46153846	1	0	2	1	2	1	2	2	-1.63451
tr|A5PLK7|A5PLK7_HUMAN	RCC2	RCC2 protein (Fragment) OS=Homo sapiens GN=RCC2 PE=2 SV=1	50331	165	469	131	97	0.46	0.21	0.45652174	4	3	6	3	6	4	6	5	-1.42886
tr|D3DVQ1|D3DVQ1_HUMAN	LETM1	Leucine zipper-EF-hand containing transmembrane protein 1, isoform CRA_a OS=Homo sapiens	63730	463	629	37	68	0.11	0.05	0.45454545	1	1	2	1	2	1	4	1	-1.63451
tr|B7TY16|B7TY16_HUMAN	ACTN1	Actinin alpha 1 isoform 3 OS=Homo sapiens GN=ACTN1 PE=2 SV=1	107644	50	119	428	352	0.77	0.35	0.45454545	15	10	21	11	22	13	29	13	-1.63907
tr|Q562U1|Q562U1_HUMAN	ACT	Actin-like protein (Fragment) OS=Homo sapiens GN=ACT PE=2 SV=1	11548	202	555	103	79	2.69	1.19	0.44237918	4	3	7	4	8	5	11	7	-1.52687
tr|Q7KZ86|Q7KZ86_HUMAN	tr|Q7KZ86|Q	Plakoglobin (Fragment) OS=Homo sapiens PE=2 SV=1	19007	26	152	37	54	1.74	0.73	0.41954023	1	1	2	1	2	1	2	2	-1.63451
tr|F8VRQ1|F8VRQ1_HUMAN	HNRNPA1	Heterogeneous nuclear ribonucleoprotein A1 OS=Homo sapiens GN=HNRNPA1 PE=2 SV=1	33249	101	308	214	145	0.77	0.32	0.41558442	4	2	6	3	7	4	9	4	-1.63565
sp|P78371|TCPB_HUMAN	CCT2	T-complex protein 1 subunit beta OS=Homo sapiens GN=CCT2 PE=1 SV=4	57794	37	103	563	393	1.86	0.75	0.40322581	14	9	20	10	20	10	23	14	-1.86721
sp|Q5T9A4|ATD3B_HUMAN	ATAD3B	ATPase family AAA domain-containing protein 3B OS=Homo sapiens GN=ATAD3B PE=1 SV=1	73098	281	768	68	55	0.1	0.04	0.4	2	1	2	1	2	4	2	6	-0.04901
tr|B2RA03|B2RA03_HUMAN	tr|B2RA03|B	cDNA, FLJ94640, highly similar to Homo sapiens keratin 18 (KRT18), mRNA OS=Homo sapiens PE	48000	127	672	88	63	0.36	0.14	0.38888889	4	2	4	3	5	4	6	4	-1.18752
sp|P02533|K1C14_HUMAN	KRT14	Keratin, type I cytoskeletal 14 OS=Homo sapiens GN=KRT14 PE=1 SV=4	51872	33	130	604	332	2.22	0.85	0.38288288	19	11	25	12	29	13	30	18	-2.03222
sp|Q2M2I5|K1C24_HUMAN	KRT24	Keratin, type I cytoskeletal 24 OS=Homo sapiens GN=KRT24 PE=1 SV=1	55567	12	65	147	63	4.67	1.74	0.37259101	6	2	6	2	6	2	6	2	-2.27722
tr|B7Z8J4|B7Z8J4_HUMAN	NMT1	Glycylpeptide N-tetradecanoyltransferase 1 OS=Homo sapiens GN=NMT1 PE=2 SV=1	15265	350	520	50	52	0.49	0.18	0.36734694	1	1	2	2	2	2	2	2	-0.89736
tr|D3DTL4|D3DTL4_HUMAN	ENO3	Enolase OS=Homo sapiens GN=ENO3 PE=2 SV=1	37167	40	167	112	142	1.39	0.5	0.35971223	2	3	2	3	2	3	4	3	-0.41176
tr|B4DF68|B4DF68_HUMAN	tr|B4DF68|B	cDNA FLJ55325, highly similar to Heat-shock protein 105 kDa OS=Homo sapiens PE=2 SV=1	86500	123	633	47	68	0.23	0.08	0.34782609	1	1	1	2	1	3	1	3	0.325545
tr|E5RH42|E5RH42_HUMAN	G3BP1	Ras GTPase-activating protein-binding protein 1 (Fragment) OS=Homo sapiens GN=G3BP1 PE=2	13338	294	343	62	29	0.58	0.2	0.34482759	2	0	2	1	2	2	2	3	-0.89736
tr|B3KQQ3|B3KQQ3_HUMAN	tr|B3KQQ3|B	cDNA PSEC0016 fis, clone NT2RM1001076, highly similar to Procollagen-lysine,2-oxoglutarate 5-	85161	325	846	54	49	0.12	0.04	0.33333333	1	1	3	1	3	1	3	2	-2.12027
tr|B3KY12|B3KY12_HUMAN	tr|B3KY12|B	cDNA FLJ46581 fis, clone THYMU3043200, highly similar to Splicing factor 3A subunit 3 OS=Hom	59082	495	1281	34	23	0.18	0.06	0.33333333	1	0	3	1	2	1	3	2	-1.63451
tr|K7ERE3|K7ERE3_HUMAN	KRT13	Keratin, type I cytoskeletal 13 OS=Homo sapiens GN=KRT13 PE=3 SV=1	45574	88	388	234	63	0.63	0.21	0.33333333	9	2	10	2	11	2	12	4	-3.10202
tr|B4DKM5|B4DKM5_HUMAN	VDAC2	Voltage-dependent anion-selective channel protein 2 OS=Homo sapiens GN=VDAC2 PE=2 SV=1	27804	196	589	108	73	0.76	0.25	0.32894737	1	2	5	2	5	2	5	3	-2.03587
tr|B3KTA3|B3KTA3_HUMAN	tr|B3KTA3|B	Fascin OS=Homo sapiens PE=2 SV=1	52814	53	325	403	139	1.07	0.35	0.3271028	9	4	14	5	16	6	20	7	-2.2453
sp|P13645|K1C10_HUMAN	KRT10	Keratin, type I cytoskeletal 10 OS=Homo sapiens GN=KRT10 PE=1 SV=6	59020	12	68	1226	586	4.67	1.51	0.32334047	34	19	42	20	47	22	54	24	-1.98694
sp|Q13151|ROA0_HUMAN	HNRNPA0	Heterogeneous nuclear ribonucleoprotein A0 OS=Homo sapiens GN=HNRNPA0 PE=1 SV=1	30993	323	575	55	77	0.36	0.11	0.30555556	2	1	3	1	3	2	3	2	-1.38313
sp|P06748|NPM_HUMAN	NPM1	Nucleophosmin OS=Homo sapiens GN=NPM1 PE=1 SV=2	32726	138	569	160	77	0.69	0.21	0.30434783	4	1	5	2	5	2	5	3	-2.03587
sp|P04259|K2C6B_HUMAN	KRT6B	Keratin, type II cytoskeletal 6B OS=Homo sapiens GN=KRT6B PE=1 SV=5	60315	79	446	252	102	0.79	0.24	0.30379747	10	4	13	4	15	6	17	6	-2.15477
sp|P22626|ROA2_HUMAN	HNRNPA2B1	Heterogeneous nuclear ribonucleoproteins A2/B1 OS=Homo sapiens GN=HNRNPA2B1 PE=1 SV=	37464	77	552	256	80	0.97	0.29	0.29896907	7	2	8	3	9	4	10	4	-1.97736
sp|P35908|K22E_HUMAN	KRT2	Keratin, type II cytoskeletal 2 epidermal OS=Homo sapiens GN=KRT2 PE=1 SV=2	65678	11	53	1255	683	4.79	1.41	0.29436326	41	17	48	20	49	22	57	25	-2.04712
tr|B2R5W2|B2R5W2_HUMAN	tr|B2R5W2|B	cDNA, FLJ92657, highly similar to Homo sapiens heterogeneous nuclear ribonucleoprotein C (C1	31986	178	592	122	73	0.35	0.1	0.28571429	3	1	3	1	3	1	4	1	-2.12027
sp|O43707|ACTN4_HUMAN	ACTN4	Alpha-actinin-4 OS=Homo sapiens GN=ACTN4 PE=1 SV=2	105245	19	72	849	549	2.11	0.58	0.27488152	26	14	41	16	44	18	52	19	-2.17492
tr|Q59GH5|Q59GH5_HUMAN	tr|Q59GH5|Q	COP9 signalosome subunit 5 variant (Fragment) OS=Homo sapiens PE=2 SV=1	31467	346	972	51	40	0.45	0.11	0.24444444	0	1	2	1	2	1	2	1	-1.63451
tr|E7EN38|E7EN38_HUMAN	RAD50	DNA repair protein RAD50 (Fragment) OS=Homo sapiens GN=RAD50 PE=2 SV=1	77138	563	990	29	38	0.09	0.02	0.22222222	0	1	2	1	1	1	2	2	-0.89721
tr|B4DRR0|B4DRR0_HUMAN	tr|B4DRR0|B	cDNA FLJ53910, highly similar to Keratin, type II cytoskeletal 6A OS=Homo sapiens PE=2 SV=1	58088	92	600	225	72	0.83	0.17	0.20481928	9	3	12	3	15	6	17	6	-2.15477
tr|B0QZK4|B0QZK4_HUMAN	HP1BP3	Heterochromatin protein 1-binding protein 3 (Fragment) OS=Homo sapiens GN=HP1BP3 PE=4 S	28725	631	999	23	38	0.25	0.05	0.2	0	1	2	1	1	1	3	1	-0.89721
sp|P13647|K2C5_HUMAN	KRT5	Keratin, type II cytoskeletal 5 OS=Homo sapiens GN=KRT5 PE=1 SV=3	62568	58	526	378	85	0.95	0.18	0.18947368	13	3	15	3	20	6	24	6	-2.55981
sp|Q58FF3|ENPLL_HUMAN	HSP90B2P	Putative endoplasmin-like protein OS=Homo sapiens GN=HSP90B2P PE=5 SV=1	46343	140	963	41	40	0.37	0.07	0.18918919	1	1	2	1	2	2	2	3	-0.89736
tr|B3KVL0|B3KVL0_HUMAN	tr|B3KVL0|B	cDNA FLJ16691 fis, clone TRACH3000692, highly similar to Paxillin OS=Homo sapiens PE=2 SV=1	65600	159	1063	133	34	0.28	0.05	0.17857143	3	0	5	1	6	1	6	1	-3.01436
sp|A2RTX5|SYTC2_HUMAN	TARSL2	Probable threonine--tRNA ligase 2, cytoplasmic OS=Homo sapiens GN=TARSL2 PE=1 SV=1	93613	94	882	69	46	0.64	0.11	0.171875	2	2	2	3	2	3	2	4	-0.41176
tr|A4ZU86|A4ZU86_HUMAN	NPM1	Truncated nucleolar phosphoprotein B23 OS=Homo sapiens GN=NPM1 PE=2 SV=1	30074	138	548	160	81	0.69	0.11	0.15942029	4	1	5	1	5	1	5	2	-2.77302
tr|Q4G170|Q4G170_HUMAN	ACACB	ACACB protein OS=Homo sapiens GN=ACACB PE=2 SV=1	97778	31	538	49	82	0.44	0.07	0.15909091	1	2	1	2	1	2	2	4	-0.16006
tr|Q53FI1|Q53FI1_HUMAN	tr|Q53FI1|Q	L-plastin variant (Fragment) OS=Homo sapiens PE=2 SV=1	70785	282	1175	41	29	0.34	0.05	0.14705882	1	0	1	1	1	3	1	4	0.325545
tr|F8VP67|F8VP67_HUMAN	KRT8	Keratin, type II cytoskeletal 8 (Fragment) OS=Homo sapiens GN=KRT8 PE=2 SV=1	13580	6	301	50	61	5.01	0.43	0.08582834	2	2	3	3	5	4	5	5	-1.18752
tr|G3XAP5|G3XAP5_HUMAN	LDHC	L-lactate dehydrogenase OS=Homo sapiens GN=LDHC PE=2 SV=1	26552	39	632	67	68	1.81	0.13	0.0718232	1	1	1	1	1	2	1	4	-0.16006
tr|A2RRC9|A2RRC9_HUMAN	IQGAP3	IQ motif containing GTPase activating protein 3 OS=Homo sapiens GN=IQGAP3 PE=2 SV=1	185145	84	1189	32	28	0.31	0.02	0.06451613	0	0	1	1	1	2	1	3	-0.16006
tr|Q562V5|Q562V5_HUMAN	ACT	Actin-like protein (Fragment) OS=Homo sapiens GN=ACT PE=2 SV=1	11481	1	480	136	95	31.43	1.2	0.03818008	6	5	6	6	7	7	8	9	-0.89814
sp|A6NHL2|TBAL3_HUMAN	TUBAL3	Tubulin alpha chain-like 3 OS=Homo sapiens GN=TUBAL3 PE=1 SV=2	50675	13	618	48	69	5.58	0.21	0.03763441	2	2	2	3	3	6	3	7	-0.00389
sp|O95678|K2C75_HUMAN	KRT75	Keratin, type II cytoskeletal 75 OS=Homo sapiens GN=KRT75 PE=1 SV=2	59809	11	700	94	61	4.79	0.11	0.02296451	2	2	3	2	3	3	5	3	-0.89752
sp|P12036|NFH_HUMAN	NEFH	Neurofilament heavy polypeptide OS=Homo sapiens GN=NEFH PE=1 SV=4	112639	11	0	0	0	4.79	0	0	0	0	0	0	0	0	1	0	-0.89705
tr|E9PQ69|E9PQ69_HUMAN	GANAB	Neutral alpha-glucosidase AB OS=Homo sapiens GN=GANAB PE=2 SV=1	8560	47	0	0	0	0.62	0	0	0	0	0	0	0	0	1	0	-0.89705
tr|Q6RUP8|Q6RUP8_HUMAN	IMPDH2	Inosine monophosphate dehydrogenase type II (Fragment) OS=Homo sapiens GN=IMPDH2 PE=2	3145	177	0	0	0	0.36	0	0	0	0	0	0	0	0	1	0	-0.89705
tr|Q1XBU7|Q1XBU7_HUMAN	tr|Q1XBU7|Q	Aging-associated protein 14a OS=Homo sapiens PE=2 SV=1	14000	382	0	0	0	0.09	0	0	0	0	0	0	0	0	1	0	-0.89705
tr|B7Z6J5|B7Z6J5_HUMAN	tr|B7Z6J5|B7	cDNA FLJ56858, highly similar to Succinate dehydrogenase (ubiquinone) flavoprotein subunit, m	28776	393	0	0	0	0.1	0	0	0	0	0	0	0	0	1	0	-0.89705
tr|A8K171|A8K171_HUMAN	tr|A8K171|A	cDNA FLJ77253, highly similar to Homo sapiens nuclear receptor interacting protein 1 (NRIP1), r	127609	401	0	0	0	0.01	0	0	0	0	0	0	0	0	1	0	-0.89705
tr|F2Z2Z0|F2Z2Z0_HUMAN	PABPC1L	Polyadenylate-binding protein 1-like OS=Homo sapiens GN=PABPC1L PE=2 SV=1	19642	404	0	0	0	0.1	0	0	0	0	0	0	0	0	1	0	-0.89705
tr|H7BYY8|H7BYY8_HUMAN	PES1	Pescadillo homolog OS=Homo sapiens GN=PES1 PE=4 SV=1	1501	488	0	0	0	0.05	0	0	0	0	0	0	0	0	1	0	-0.89705
sp|Q14117|DPYS_HUMAN	DPYS	Dihydropyrimidinase OS=Homo sapiens GN=DPYS PE=1 SV=1	57107	490	0	0	0	0.19	0	0	0	0	0	0	0	0	1	0	-0.89705
tr|D6RD96|D6RD96_HUMAN	ACSL4	Long-chain-fatty-acid--CoA ligase 4 (Fragment) OS=Homo sapiens GN=ACSL4 PE=2 SV=1	16399	529	0	0	0	0.04	0	0	0	0	0	0	0	0	1	0	-0.89705
tr|I3L3P5|I3L3P5_HUMAN	P4HB	Protein disulfide-isomerase (Fragment) OS=Homo sapiens GN=P4HB PE=4 SV=1	16923	536	0	19	0	0.27	0	0	0	0	1	0	0	0	1	0	-0.89705
tr|H7C1T1|H7C1T1_HUMAN	Sep-02	Septin-2 (Fragment) OS=Homo sapiens GN=SEPT2 PE=4 SV=1	3094	543	0	0	0	0.09	0	0	0	0	0	0	0	0	1	0	-0.89705
tr|B7Z4W3|B7Z4W3_HUMAN	tr|B7Z4W3|B	cDNA FLJ57543, highly similar to Bifunctional 3~-phosphoadenosine5~-phosphosulfate syntheta	17852	553	0	19	0	0.1	0	0	0	0	1	0	0	0	1	0	-0.89705
tr|Q3MNE1|Q3MNE1_HUMAN	tr|Q3MNE1|Q	Adaptor-related protein complex 3 beta 1 subunit (Fragment) OS=Homo sapiens PE=2 SV=1	89828	682	0	20	0	0.04	0	0	0	0	1	0	0	0	1	0	-0.89705
tr|B4DZD5|B4DZD5_HUMAN	tr|B4DZD5|B	cDNA FLJ60991, highly similar to FKBP12-rapamycin complex-associated protein OS=Homo sapie	158783	683	0	20	0	0.02	0	0	0	0	1	0	0	0	2	0	-0.89705
tr|Q9UH66|Q9UH66_HUMAN	tr|Q9UH66|Q	7h3 protein (Fragment) OS=Homo sapiens PE=2 SV=1	49963	684	0	19	0	0.07	0	0	0	0	1	0	0	0	2	0	-0.89705
tr|G5E9Q3|G5E9Q3_HUMAN	TSEN2	tRNA splicing endonuclease 2 homolog (S. cerevisiae), isoform CRA_b OS=Homo sapiens GN=TSI	45959	686	0	19	0	0.07	0	0	0	0	1	0	0	0	1	0	-0.89705
tr|E9PQG7|E9PQG7_HUMAN	MLL	MLL cleavage product C180 OS=Homo sapiens GN=MLL PE=2 SV=1	436345	687	0	19	0	0.01	0	0	0	0	1	0	0	0	1	0	-0.89705
tr|G3V448|G3V448_HUMAN	TMX1	Thioredoxin-related transmembrane protein 1 OS=Homo sapiens GN=TMX1 PE=2 SV=1	12018	689	0	19	0	0.29	0	0	0	0	1	0	0	0	1	0	-0.89705
tr|D3DUL0|D3DUL0_HUMAN	ASCIZ	ATM/ATR-Substrate Chk2-Interacting Zn2+-finger protein, isoform CRA_b OS=Homo sapiens GN	79474	690	0	19	0	0.04	0	0	0	0	1	0	0	0	1	0	-0.89705
tr|H7C4X2|H7C4X2_HUMAN	PCMT1	Protein-L-isoaspartate(D-aspartate) O-methyltransferase (Fragment) OS=Homo sapiens GN=PCN	7342	691	0	19	0	0.48	0	0	0	0	1	0	0	0	2	0	-0.89705
tr|B3KND7|B3KND7_HUMAN	tr|B3KND7|B	cDNA FLJ14378 fis, clone HEMBA1001804, highly similar to Zinc finger protein 281 (Fragment) O	58221	692	0	19	0	0.06	0	0	0	0	1	0	0	0	1	0	-0.89705
tr|H0YE81|H0YE81_HUMAN	NDUFV1	NADH dehydrogenase [ubiquinone] flavoprotein 1, mitochondrial (Fragment) OS=Homo sapiens	14494	694	0	19	0	0.23	0	0	0	0	1	0	0	0	1	0	-0.89705
tr|H0Y304|H0Y304_HUMAN	DMD	Dystrophin (Fragment) OS=Homo sapiens GN=DMD PE=4 SV=1	160176	695	0	19	0	0.02	0	0	0	0	1	0	0	0	1	0	-0.89705
tr|Q5T097|Q5T097_HUMAN	UTRN	Utrophin OS=Homo sapiens GN=UTRN PE=4 SV=1	114423	696	0	19	0	0.03	0	0	0	0	1	0	0	0	1	0	-0.89705
tr|I3L2H8|I3L2H8_HUMAN	MYBBP1A	Myb-binding protein 1A (Fragment) OS=Homo sapiens GN=MYBBP1A PE=4 SV=1	22828	698	0	18	0	0.02	0	0	0	0	1	0	0	0	1	0	-0.89705
tr|B4DY47|B4DY47_HUMAN	PHB	Prohibitin OS=Homo sapiens GN=PHB PE=2 SV=1	17064	699	0	18	0	0.2	0	0	0	0	1	0	0	0	1	0	-0.89705
tr|J3KQ96|J3KQ96_HUMAN	TCOF1	Treacle protein (Fragment) OS=Homo sapiens GN=TCOF1 PE=4 SV=1	144268	702	0	18	0	0.02	0	0	0	0	1	0	0	0	2	0	-0.89705
tr|J3KMW7|J3KMW7_HUMAN	UBR5	E3 ubiquitin-protein ligase UBR5 OS=Homo sapiens GN=UBR5 PE=4 SV=1	312224	706	0	18	0	0.01	0	0	0	0	1	0	0	0	1	0	-0.89705
tr|H0Y4R4|H0Y4R4_HUMAN	TOR1AIP1	Torsin-1A-interacting protein 1 (Fragment) OS=Homo sapiens GN=TOR1AIP1 PE=4 SV=1	33844	707	0	17	0	0.1	0	0	0	0	1	0	0	0	1	0	-0.89705
sp|P54826|GAS1_HUMAN	GAS1	Growth arrest-specific protein 1 OS=Homo sapiens GN=GAS1 PE=1 SV=2	36867	708	0	17	0	0.09	0	0	0	0	1	0	0	0	1	0	-0.89705
tr|I3L2C7|I3L2C7_HUMAN	GEMIN4	Gem-associated protein 4 OS=Homo sapiens GN=GEMIN4 PE=4 SV=1	120456	709	0	17	0	0.03	0	0	0	0	1	0	0	0	1	0	-0.89705
tr|D6RAW4|D6RAW4_HUMAN	IMMT	Mitochondrial inner membrane protein OS=Homo sapiens GN=IMMT PE=2 SV=1	15918	712	0	0	0	0.04	0	0	0	0	0	0	0	0	1	0	-0.89705
tr|Q8WXT3|Q8WXT3_HUMAN	FLNB	Filamin B variant 2 (Fragment) OS=Homo sapiens GN=FLNB PE=2 SV=1	7949	45	0	0	0	0.22	0	0	0	0	0	0	1	0	2	0	-2.48235
tr|J3KR91|J3KR91_HUMAN	IQGAP2	Ras GTPase-activating-like protein IQGAP2 OS=Homo sapiens GN=IQGAP2 PE=4 SV=1	123072	84	0	32	0	0.31	0	0	0	0	1	0	1	0	1	0	-2.48235
tr|D6RCS6|D6RCS6_HUMAN	TARS	Threonine--tRNA ligase, cytoplasmic OS=Homo sapiens GN=TARS PE=2 SV=1	6912	94	0	53	0	0.64	0	0	1	0	1	0	1	0	1	0	-2.48235
tr|H0YLE2|H0YLE2_HUMAN	ANXA2	Annexin A2 (Fragment) OS=Homo sapiens GN=ANXA2 PE=4 SV=1	5333	100	0	57	0	1.26	0	0	1	0	1	0	1	0	1	0	-2.48235
tr|H0Y8V7|H0Y8V7_HUMAN	SEC31A	Protein transport protein Sec31A (Fragment) OS=Homo sapiens GN=SEC31A PE=4 SV=1	32306	121	0	42	0	0.13	0	0	1	0	2	0	1	0	2	0	-2.48235
tr|H3BND9|H3BND9_HUMAN	PCBP2	Poly(rC)-binding protein 2 (Fragment) OS=Homo sapiens GN=PCBP2 PE=4 SV=1	12425	132	0	35	0	0.32	0	0	1	0	1	0	1	0	2	0	-2.48235
tr|Q9BZT4|Q9BZT4_HUMAN	tr|Q9BZT4|Q	PNAS-29 OS=Homo sapiens PE=4 SV=1	8036	137	0	59	0	0.4	0	0	1	0	1	0	1	0	1	0	-2.48235
tr|Q58FF2|Q58FF2_HUMAN	GRP94c	Heat shock protein 94c OS=Homo sapiens GN=GRP94c PE=2 SV=1	52205	140	0	22	0	0.37	0	0	0	0	1	0	1	0	2	0	-2.48235
tr|K7EQ75|K7EQ75_HUMAN	ILF3	Interleukin enhancer-binding factor 3 (Fragment) OS=Homo sapiens GN=ILF3 PE=4 SV=1	6139	154	0	29	0	0.46	0	0	0	0	1	0	1	0	1	0	-2.48235
tr|H0YCY7|H0YCY7_HUMAN	RRM1	Ribonucleoside-diphosphate reductase (Fragment) OS=Homo sapiens GN=RRM1 PE=3 SV=1	14342	156	0	22	0	0.29	0	0	0	0	1	0	1	0	1	0	-2.48235
tr|A4D1P0|A4D1P0_HUMAN	LOC402299	Uncharacterized protein OS=Homo sapiens GN=LOC402299 PE=4 SV=1	42109	185	0	40	0	0.42	0	0	1	0	1	0	1	0	1	0	-2.48235
tr|Q9BUV4|Q9BUV4_HUMAN	RPL5	RPL5 protein OS=Homo sapiens GN=RPL5 PE=2 SV=1	12182	187	0	39	0	0.44	0	0	0	0	1	0	1	0	2	0	-2.48235
tr|B3KV39|B3KV39_HUMAN	tr|B3KV39|B	cDNA FLJ16099 fis, clone TESTI2008278, highly similar to Homo sapiens forkhead box K1 (FOXK1	50383	188	0	115	0	0.07	0	0	1	0	1	0	1	0	2	0	-2.48235
tr|D6R9D3|D6R9D3_HUMAN	HNRNPH1	Heterogeneous nuclear ribonucleoprotein H OS=Homo sapiens GN=HNRNPH1 PE=4 SV=1	4404	207	0	23	0	0.45	0	0	0	0	1	0	1	0	1	0	-2.48235
tr|B7Z528|B7Z528_HUMAN	OXCT1	Succinyl-CoA:3-ketoacid coenzyme A transferase 1, mitochondrial OS=Homo sapiens GN=OXCT1	13520	209	0	0	0	0.18	0	0	0	0	0	0	1	0	1	0	-2.48235
tr|H0YB86|H0YB86_HUMAN	PABPC1	Polyadenylate-binding protein 1 (Fragment) OS=Homo sapiens GN=PABPC1 PE=4 SV=1	18427	215	0	34	0	0.33	0	0	0	0	1	0	1	0	2	0	-2.48235
tr|H0Y960|H0Y960_HUMAN	DDX4	Probable ATP-dependent RNA helicase DDX4 (Fragment) OS=Homo sapiens GN=DDX4 PE=4 SV=	33292	217	0	43	0	0.19	0	0	1	0	1	0	1	0	1	0	-2.48235
tr|H7C013|H7C013_HUMAN	ALB	Serum albumin (Fragment) OS=Homo sapiens GN=ALB PE=4 SV=1	23414	222	0	24	0	0.25	0	0	0	0	1	0	1	0	2	0	-2.48235
tr|B0I1S7|B0I1S7_HUMAN	tr|B0I1S7|B0	KIF5A variant protein OS=Homo sapiens PE=2 SV=1	118189	227	0	46	0	0.04	0	0	1	0	1	0	1	0	1	0	-2.48235
tr|H0Y9U2|H0Y9U2_HUMAN	SFPQ	Splicing factor, proline- and glutamine-rich (Fragment) OS=Homo sapiens GN=SFPQ PE=4 SV=1	9491	228	0	55	0	0.26	0	0	1	0	1	0	1	0	1	0	-2.48235
tr|E9PLA6|E9PLA6_HUMAN	SERPINH1	Serpin H1 (Fragment) OS=Homo sapiens GN=SERPINH1 PE=2 SV=1	8925	233	0	45	0	0.43	0	0	1	0	1	0	1	0	1	0	-2.48235
tr|E5RFJ8|E5RFJ8_HUMAN	MCM4	DNA replication licensing factor MCM4 (Fragment) OS=Homo sapiens GN=MCM4 PE=2 SV=1	23630	240	0	65	0	0.1	0	0	1	0	1	0	1	0	2	0	-2.48235
tr|B4DE40|B4DE40_HUMAN	SMS	Spermine synthase OS=Homo sapiens GN=SMS PE=2 SV=1	31596	248	0	78	0	0.11	0	0	1	0	1	0	1	0	1	0	-2.48235
tr|B4DXK4|B4DXK4_HUMAN	KRT72	Keratin, type II cytoskeletal 72 OS=Homo sapiens GN=KRT72 PE=2 SV=1	36031	259	0	0	0	0.45	0	0	0	0	0	0	1	0	1	0	-2.48235
tr|K7EKB6|K7EKB6_HUMAN	THOP1	Thimet oligopeptidase (Fragment) OS=Homo sapiens GN=THOP1 PE=4 SV=1	20792	278	0	68	0	0.16	0	0	1	0	1	0	1	0	1	0	-2.48235
tr|H0YC45|H0YC45_HUMAN	ATP6V1B2	V-type proton ATPase subunit B, brain isoform (Fragment) OS=Homo sapiens GN=ATP6V1B2 PE-	5569	287	0	64	0	0.65	0	0	1	0	1	0	1	0	1	0	-2.48235
sp|P81605|DCD_HUMAN	DCD	Dermcidin OS=Homo sapiens GN=DCD PE=1 SV=2	11391	295	0	62	0	0.3	0	0	1	0	1	0	1	0	1	0	-2.48235
tr|Q5T0I0|Q5T0I0_HUMAN	GSN	Gelsolin (Fragment) OS=Homo sapiens GN=GSN PE=4 SV=1	29107	297	0	61	0	0.04	0	0	1	0	1	0	1	0	2	0	-2.48235
tr|B4DX93|B4DX93_HUMAN	tr|B4DX93|B	cDNA FLJ55471, highly similar to Cytoplasmic dynein 1 intermediate chain 2 OS=Homo sapiens F	40089	298	0	40	0	0.09	0	0	1	0	1	0	1	0	1	0	-2.48235
tr|B5LY64|B5LY64_HUMAN	UMPS	Orotidine 5~-phosphate decarboxylase OS=Homo sapiens GN=UMPS PE=2 SV=1	33258	302	0	59	0	0.1	0	0	1	0	1	0	1	0	1	0	-2.48235
sp|Q5TZA2|CROCC_HUMAN	CROCC	Rootletin OS=Homo sapiens GN=CROCC PE=1 SV=1	228787	310	0	58	0	0.01	0	0	1	0	1	0	1	0	2	0	-2.48235
tr|H0YBD4|H0YBD4_HUMAN	GSR	Glutathione reductase, mitochondrial (Fragment) OS=Homo sapiens GN=GSR PE=4 SV=1	32532	313	0	25	0	0.18	0	0	0	0	1	0	1	0	1	0	-2.48235
tr|C9JPP3|C9JPP3_HUMAN	RBBP4	Histone-binding protein RBBP4 (Fragment) OS=Homo sapiens GN=RBBP4 PE=2 SV=2	9655	315	0	57	0	0.36	0	0	1	0	1	0	1	0	1	0	-2.48235
tr|H7C1U0|H7C1U0_HUMAN	APEH	Acylamino-acid-releasing enzyme (Fragment) OS=Homo sapiens GN=APEH PE=4 SV=1	35132	319	0	56	0	0.09	0	0	1	0	1	0	1	0	1	0	-2.48235
tr|I3L4P6|I3L4P6_HUMAN	ARHGAP17	Rho GTPase-activating protein 17 (Fragment) OS=Homo sapiens GN=ARHGAP17 PE=4 SV=1	8676	328	0	54	0	0.03	0	0	1	0	1	0	1	0	1	0	-2.48235
tr|A8K3Q9|A8K3Q9_HUMAN	tr|A8K3Q9|A	cDNA FLJ76611, highly similar to Homo sapiens ribosomal protein L14 (RPL14), mRNA OS=Homo	23554	330	0	53	0	0.14	0	0	1	0	1	0	1	0	1	0	-2.48235
sp|Q9Y6M5|ZNT1_HUMAN	SLC30A1	Zinc transporter 1 OS=Homo sapiens GN=SLC30A1 PE=1 SV=3	56290	334	0	52	0	0.06	0	0	1	0	1	0	1	0	1	0	-2.48235
tr|A8KA82|A8KA82_HUMAN	DNAJC3	DnaJ (Hsp40) homolog, subfamily C, member 3 OS=Homo sapiens GN=DNAJC3 PE=2 SV=1	58000	337	0	52	0	0.06	0	0	1	0	1	0	1	0	1	0	-2.48235
tr|B2RAR2|B2RAR2_HUMAN	tr|B2RAR2|B	cDNA, FLJ95064, highly similar to Homo sapiens nin one binding protein (NOB1P), mRNA OS=Ho	47015	338	0	52	0	0.07	0	0	1	0	1	0	1	0	1	0	-2.48235
tr|A8K517|A8K517_HUMAN	RPS23	Ribosomal protein S23, isoform CRA_a OS=Homo sapiens GN=RPS23 PE=2 SV=1	15969	339	0	52	0	0.21	0	0	1	0	1	0	1	0	1	0	-2.48235
tr|H7C5I9|H7C5I9_HUMAN	GART	Phosphoribosylformylglycinamidine cyclo-ligase (Fragment) OS=Homo sapiens GN=GART PE=4 S	2640	341	0	23	0	0.13	0	0	0	0	1	0	1	0	1	0	-2.48235
tr|E5RFU4|E5RFU4_HUMAN	DPYSL2	Dihydropyrimidinase-related protein 2 (Fragment) OS=Homo sapiens GN=DPYSL2 PE=2 SV=1	18320	342	0	20	0	0.23	0	0	0	0	1	0	1	0	1	0	-2.48235
tr|C9JQM9|C9JQM9_HUMAN	DARS	Aspartate--tRNA ligase, cytoplasmic (Fragment) OS=Homo sapiens GN=DARS PE=2 SV=1	15386	344	0	51	0	0.06	0	0	1	0	1	0	1	0	1	0	-2.48235
tr|H0YC77|H0YC77_HUMAN	ANXA6	Annexin (Fragment) OS=Homo sapiens GN=ANXA6 PE=3 SV=1	10357	349	0	27	0	0.14	0	0	0	0	1	0	1	0	1	0	-2.48235
tr|K7EN42|K7EN42_HUMAN	NMT1	Glycylpeptide N-tetradecanoyltransferase 1 (Fragment) OS=Homo sapiens GN=NMT1 PE=4 SV=1	9699	350	0	31	0	0.49	0	0	0	0	1	0	1	0	1	0	-2.48235
tr|B7Z8A2|B7Z8A2_HUMAN	tr|B7Z8A2|B7	cDNA FLJ51671, highly similar to Prenylcysteine oxidase (EC 1.8.3.5) OS=Homo sapiens PE=2 SV=	54918	355	0	49	0	0.06	0	0	1	0	1	0	1	0	1	0	-2.48235
tr|D6RBX4|D6RBX4_HUMAN	TGFBI	Transforming growth factor-beta-induced protein ig-h3 OS=Homo sapiens GN=TGFBI PE=2 SV=1	7268	358	0	31	0	0.09	0	0	0	0	1	0	1	0	1	0	-2.48235
tr|A6NKV8|A6NKV8_HUMAN	PAIP1	Polyadenylate-binding protein-interacting protein 1 OS=Homo sapiens GN=PAIP1 PE=2 SV=1	42379	361	0	48	0	0.08	0	0	1	0	1	0	1	0	1	0	-2.48235
tr|K7EM16|K7EM16_HUMAN	VASP	Vasodilator-stimulated phosphoprotein (Fragment) OS=Homo sapiens GN=VASP PE=4 SV=1	20260	363	0	48	0	0.17	0	0	1	0	1	0	1	0	1	0	-2.48235
tr|H0Y9Q1|H0Y9Q1_HUMAN	LAP3	Cytosol aminopeptidase (Fragment) OS=Homo sapiens GN=LAP3 PE=4 SV=1	22641	368	0	47	0	0.15	0	0	1	0	1	0	1	0	1	0	-2.48235
tr|H0YGH1|H0YGH1_HUMAN	PFAS	Phosphoribosylformylglycinamidine synthase (Fragment) OS=Homo sapiens GN=PFAS PE=4 SV=	41755	380	0	37	0	0.05	0	0	0	0	1	0	1	0	1	0	-2.48235
tr|B4DT09|B4DT09_HUMAN	tr|B4DT09|B	cDNA FLJ60145, highly similar to Homo sapiens dendritic cell protein (hfl-B5), mRNA OS=Homo s	13697	381	0	46	0	0.25	0	0	1	0	1	0	1	0	1	0	-2.48235
tr|B3KPZ0|B3KPZ0_HUMAN	tr|B3KPZ0|B	cDNA FLJ32485 fis, clone SKNMC2001596, highly similar to Ankyrin repeat and FYVE domain-con	121468	387	0	45	0	0.03	0	0	1	0	1	0	1	0	1	0	-2.48235
tr|E5RGY7|E5RGY7_HUMAN	CTNNA1	Catenin alpha-1 (Fragment) OS=Homo sapiens GN=CTNNA1 PE=2 SV=1	14895	389	0	35	0	0.07	0	0	0	0	1	0	1	0	1	0	-2.48235
tr|D6RAN1|D6RAN1_HUMAN	PDLIM7	PDZ and LIM domain protein 7 (Fragment) OS=Homo sapiens GN=PDLIM7 PE=2 SV=1	9510	396	0	33	0	0.13	0	0	0	0	1	0	1	0	2	0	-2.48235
tr|B3KT66|B3KT66_HUMAN	PSMD6	Proteasome (Prosome, macropain) 26S subunit, non-ATPase, 6, isoform CRA_c OS=Homo sapier	40600	399	0	43	0	0.08	0	0	1	0	1	0	1	0	1	0	-2.48235
tr|Q1LZN2|Q1LZN2_HUMAN	NOMO3	NOMO3 protein (Fragment) OS=Homo sapiens GN=NOMO3 PE=2 SV=1	90388	400	0	43	0	0.04	0	0	1	0	1	0	1	0	1	0	-2.48235
tr|B4DRG7|B4DRG7_HUMAN	tr|B4DRG7|B	cDNA FLJ60336, highly similar to Condensin complex subunit 2 OS=Homo sapiens PE=2 SV=1	80877	410	0	43	0	0.04	0	0	1	0	1	0	1	0	1	0	-2.48235
sp|Q9NSB4|KRT82_HUMAN	KRT82	Keratin, type II cuticular Hb2 OS=Homo sapiens GN=KRT82 PE=1 SV=3	57985	411	0	43	0	0.06	0	0	1	0	1	0	1	0	2	0	-2.48235
tr|H0YGE9|H0YGE9_HUMAN	M6PR	Cation-dependent mannose-6-phosphate receptor (Fragment) OS=Homo sapiens GN=M6PR PE=	11814	416	0	42	0	0.29	0	0	1	0	1	0	1	0	1	0	-2.48235
tr|B4DPY2|B4DPY2_HUMAN	tr|B4DPY2|B	cDNA FLJ59286, highly similar to Pre-mRNA-processing factor 40 homolog A (Fragment) OS=Hor	93124	425	0	41	0	0.04	0	0	1	0	1	0	1	0	1	0	-2.48235
tr|Q5JR94|Q5JR94_HUMAN	RPS8	40S ribosomal protein S8 OS=Homo sapiens GN=RPS8 PE=2 SV=1	24475	426	0	41	0	0.14	0	0	1	0	1	0	1	0	2	0	-2.48235
tr|A4D1I7|A4D1I7_HUMAN	DNCI1	Dynein, cytoplasmic 1, intermediate chain 1, isoform CRA_e OS=Homo sapiens GN=DNCI1 PE=2	73423	427	0	40	0	0.04	0	0	1	0	1	0	1	0	1	0	-2.48235
tr|E9PI83|E9PI83_HUMAN	PRMT1	Protein arginine N-methyltransferase 1 (Fragment) OS=Homo sapiens GN=PRMT1 PE=2 SV=1	12790	437	0	36	0	0.26	0	0	0	0	1	0	1	0	1	0	-2.48235
tr|B7Z943|B7Z943_HUMAN	tr|B7Z943|B7	cDNA FLJ53131, highly similar to 24-dehydrocholesterol reductase (EC 1.3.1.-) OS=Homo sapien	29093	442	0	39	0	0.05	0	0	1	0	1	0	1	0	2	0	-2.48235
tr|B4E363|B4E363_HUMAN	FARSA	Phenylalanine--tRNA ligase alpha subunit OS=Homo sapiens GN=FARSA PE=2 SV=1	54181	447	0	38	0	0.06	0	0	1	0	1	0	1	0	1	0	-2.48235
tr|H0YA24|H0YA24_HUMAN	PLRG1	Pleiotropic regulator 1 (Fragment) OS=Homo sapiens GN=PLRG1 PE=4 SV=1	17588	456	0	38	0	0.19	0	0	1	0	1	0	1	0	2	0	-2.48235
tr|B3KME2|B3KME2_HUMAN	tr|B3KME2|B	cDNA FLJ10772 fis, clone NT2RP4000243, highly similar to Cartilage-associated protein OS=Hom	47101	459	0	38	0	0.07	0	0	1	0	1	0	1	0	1	0	-2.48235
sp|Q9H9F9|ARP5_HUMAN	ACTR5	Actin-related protein 5 OS=Homo sapiens GN=ACTR5 PE=1 SV=2	68825	460	0	38	0	0.05	0	0	1	0	1	0	1	0	2	0	-2.48235
tr|H7C1M0|H7C1M0_HUMAN	FANCL	E3 ubiquitin-protein ligase FANCL (Fragment) OS=Homo sapiens GN=FANCL PE=4 SV=1	33067	461	0	37	0	0.1	0	0	1	0	1	0	1	0	1	0	-2.48235
tr|Q5SZ64|Q5SZ64_HUMAN	SF3B4	Splicing factor 3B subunit 4 (Fragment) OS=Homo sapiens GN=SF3B4 PE=4 SV=1	20733	464	0	37	0	0.07	0	0	1	0	1	0	1	0	1	0	-2.48235
sp|Q6ZU64|CC108_HUMAN	CCDC108	Coiled-coil domain-containing protein 108 OS=Homo sapiens GN=CCDC108 PE=2 SV=2	220247	465	0	37	0	0.01	0	0	1	0	1	0	1	0	1	0	-2.48235
tr|C9J7L0|C9J7L0_HUMAN	RBBP7	Histone-binding protein RBBP7 (Fragment) OS=Homo sapiens GN=RBBP7 PE=2 SV=1	7740	468	0	0	0	0.07	0	0	0	0	0	0	1	0	1	0	-2.48235
tr|H0YIX9|H0YIX9_HUMAN	R3HDM2	R3H domain-containing protein 2 (Fragment) OS=Homo sapiens GN=R3HDM2 PE=4 SV=1	31776	469	0	37	0	0.1	0	0	1	0	1	0	1	0	1	0	-2.48235
tr|B4DKI2|B4DKI2_HUMAN	tr|B4DKI2|B4	cDNA FLJ60282, highly similar to Sorbitol dehydrogenase (EC 1.1.1.14) OS=Homo sapiens PE=2 S	30315	473	0	37	0	0.09	0	0	0	0	1	0	1	0	1	0	-2.48235
tr|G3V1W4|G3V1W4_HUMAN	SEC23A	Protein transport protein Sec23A OS=Homo sapiens GN=SEC23A PE=2 SV=1	12904	474	0	24	0	0.08	0	0	0	0	1	0	1	0	1	0	-2.48235
tr|B7Z982|B7Z982_HUMAN	PLRG1	Pleiotropic regulator 1 OS=Homo sapiens GN=PLRG1 PE=2 SV=1	14839	476	0	0	0	0.12	0	0	0	0	0	0	1	0	1	0	-2.48235
tr|J3KN86|J3KN86_HUMAN	RPS3	40S ribosomal protein S3 OS=Homo sapiens GN=RPS3 PE=3 SV=1	28640	480	0	36	0	0.12	0	0	1	0	1	0	1	0	4	0	-2.48235
tr|B7WPL0|B7WPL0_HUMAN	RIC8B	Synembryn-B OS=Homo sapiens GN=RIC8B PE=2 SV=1	64252	483	0	36	0	0.05	0	0	1	0	1	0	1	0	1	0	-2.48235
tr|Q68E00|Q68E00_HUMAN	DKFZp686G2	Putative uncharacterized protein DKFZp686G2045 (Fragment) OS=Homo sapiens GN=DKFZp686	32787	485	0	36	0	0.1	0	0	0	0	1	0	1	0	1	0	-2.48235
tr|C9JNW5|C9JNW5_HUMAN	RPL24	60S ribosomal protein L24 OS=Homo sapiens GN=RPL24 PE=4 SV=1	17646	487	0	35	0	0.19	0	0	0	0	1	0	1	0	2	0	-2.48235
tr|E9PI04|E9PI04_HUMAN	RIC8A	Synembryn-A (Fragment) OS=Homo sapiens GN=RIC8A PE=2 SV=1	16936	496	0	34	0	0.05	0	0	0	0	1	0	1	0	1	0	-2.48235
tr|B4DDC9|B4DDC9_HUMAN	tr|B4DDC9|B	cDNA FLJ59448, highly similar to Trifunctional enzyme subunit beta, mitochondrial OS=Homo sa	49937	499	0	34	0	0.07	0	0	0	0	1	0	1	0	1	0	-2.48235
sp|Q8NFZ5|TNIP2_HUMAN	TNIP2	TNFAIP3-interacting protein 2 OS=Homo sapiens GN=TNIP2 PE=1 SV=1	49240	502	0	33	0	0.07	0	0	1	0	1	0	1	0	2	0	-2.48235
tr|Q2NKW8|Q2NKW8_HUMAN	AHCYL1	Adenosylhomocysteinase (Fragment) OS=Homo sapiens GN=AHCYL1 PE=2 SV=1	57630	509	0	33	0	0.06	0	0	0	0	1	0	1	0	1	0	-2.48235
sp|Q7Z494|NPHP3_HUMAN	NPHP3	Nephrocystin-3 OS=Homo sapiens GN=NPHP3 PE=1 SV=1	151967	510	0	32	0	0.02	0	0	1	0	1	0	1	0	1	0	-2.48235
tr|A0JLS9|A0JLS9_HUMAN	ZEB1	ZEB1 protein (Fragment) OS=Homo sapiens GN=ZEB1 PE=2 SV=1	118576	513	0	32	0	0.03	0	0	1	0	1	0	1	0	1	0	-2.48235
tr|B2R4C0|B2R4C0_HUMAN	tr|B2R4C0|B	60S ribosomal protein L18a OS=Homo sapiens PE=2 SV=1	21024	514	0	32	0	0.16	0	0	0	0	1	0	1	0	2	0	-2.48235
tr|J3KRB3|J3KRB3_HUMAN	RPL17	60S ribosomal protein L17 (Fragment) OS=Homo sapiens GN=RPL17 PE=3 SV=1	12356	517	0	32	0	0.28	0	0	0	0	1	0	1	0	1	0	-2.48235
tr|B4DUH7|B4DUH7_HUMAN	tr|B4DUH7|B	cDNA FLJ60162, highly similar to Chloride anion exchanger OS=Homo sapiens PE=2 SV=1	52632	518	0	32	0	0.06	0	0	1	0	1	0	1	0	1	0	-2.48235
tr|B7Z8W6|B7Z8W6_HUMAN	tr|B7Z8W6|B	cDNA FLJ55038, highly similar to Nicotinamide phosphoribosyltransferase (EC 2.4.2.12) OS=Hom	26905	520	0	32	0	0.12	0	0	0	0	1	0	1	0	3	0	-2.48235
tr|B4E0D5|B4E0D5_HUMAN	tr|B4E0D5|B	cDNA FLJ54218, highly similar to Acidic leucine-rich nuclear phosphoprotein 32 family member	19540	525	0	31	0	0.17	0	0	0	0	1	0	1	0	1	0	-2.48235
sp|Q6ZU15|SEP14_HUMAN	Sep-14	Septin-14 OS=Homo sapiens GN=SEPT14 PE=1 SV=2	50449	531	0	31	0	0.07	0	0	1	0	1	0	1	0	1	0	-2.48235
tr|B1AMS2|B1AMS2_HUMAN	Sep-06	Septin 6 OS=Homo sapiens GN=SEPT6 PE=3 SV=1	49614	532	0	31	0	0.07	0	0	1	0	1	0	1	0	1	0	-2.48235
sp|Q6IBS0|TWF2_HUMAN	TWF2	Twinfilin-2 OS=Homo sapiens GN=TWF2 PE=1 SV=2	39751	535	0	30	0	0.08	0	0	0	0	1	0	1	0	1	0	-2.48235
sp|Q96PE2|ARHGH_HUMAN	ARHGEF17	Rho guanine nucleotide exchange factor 17 OS=Homo sapiens GN=ARHGEF17 PE=1 SV=1	223645	537	0	30	0	0.01	0	0	1	0	1	0	1	0	1	0	-2.48235
tr|K7EKM4|K7EKM4_HUMAN	SRM	Spermidine synthase OS=Homo sapiens GN=SRM PE=4 SV=1	10680	540	0	30	0	0.32	0	0	0	0	1	0	1	0	1	0	-2.48235
tr|H0YK49|H0YK49_HUMAN	ETFA	Electron transfer flavoprotein subunit alpha, mitochondrial OS=Homo sapiens GN=ETFA PE=4 S\	24259	545	0	30	0	0.14	0	0	0	0	1	0	1	0	1	0	-2.48235
tr|B6F210|B6F210_HUMAN	GSS	Glutathione synthetase OS=Homo sapiens GN=GSS PE=2 SV=1	40495	551	0	30	0	0.08	0	0	0	0	1	0	1	0	1	0	-2.48235
tr|C9JJE5|C9JJE5_HUMAN	IDH1	Isocitrate dehydrogenase [NADP] cytoplasmic (Fragment) OS=Homo sapiens GN=IDH1 PE=2 SV=	7068	555	0	23	0	0.15	0	0	0	0	1	0	1	0	1	0	-2.48235

tr|H3BRB3|H3BRB3_HUMAN	KIF22	Kinesin-like protein KIF22 OS=Homo sapiens GN=KIF22 PE=3 SV=1	67996	557	0	29	0	0.05	0	0	0	0	1	0	1	0	1	0	-2.48235
tr|B4DS04|B4DS04_HUMAN	tr|B4DS04|B4	cDNA FLJ54142, highly similar to Protein transport protein Sec23B OS=Homo sapiens PE=2 SV=1	41720	558	0	27	0	0.08	0	0	0	0	1	0	1	0	1	0	-2.48235
sp|Q96A33|CCD47_HUMAN	CCDC47	Coiled-coil domain-containing protein 47 OS=Homo sapiens GN=CCDC47 PE=1 SV=1	56123	560	0	29	0	0.06	0	0	0	0	1	0	1	0	1	0	-2.48235
tr|I3L361|I3L361_HUMAN	SGSM2	Small G protein-signaling modulator 2 (Fragment) OS=Homo sapiens GN=SGSM2 PE=4 SV=2	1642	570	0	29	0	2.38	0	0	0	0	1	0	1	0	1	0	-2.48235
tr|A4ZYW4|A4ZYW4_HUMAN	L1CAM	Non-neural L1CAM OS=Homo sapiens GN=L1CAM PE=2 SV=1	139633	572	0	28	0	0.05	0	0	1	0	2	0	1	0	2	0	-2.48235
tr|B4DNG6|B4DNG6_HUMAN	tr|B4DNG6|B	Annexin OS=Homo sapiens PE=3 SV=1	12870	577	0	28	0	0.27	0	0	0	0	1	0	1	0	1	0	-2.48235
sp|Q16322|KCA10_HUMAN	KCNA10	Potassium voltage-gated channel subfamily A member 10 OS=Homo sapiens GN=KCNA10 PE=1 S	58147	578	0	28	0	0.06	0	0	1	0	1	0	1	0	1	0	-2.48235
tr|J3KN32|J3KN32_HUMAN	RCOR1	REST corepressor 1 OS=Homo sapiens GN=RCOR1 PE=4 SV=1	53409	583	0	28	0	0.06	0	0	0	0	1	0	1	0	1	0	-2.48235
tr|H0YCU9|H0YCU9_HUMAN	TAGLN	Transgelin (Fragment) OS=Homo sapiens GN=TAGLN PE=4 SV=1	16833	585	0	28	0	0.2	0	0	0	0	1	0	1	0	1	0	-2.48235
tr|A6NE42|A6NE42_HUMAN	TGIF1	Homeobox protein TGIF1 OS=Homo sapiens GN=TGIF1 PE=3 SV=2	27983	586	0	28	0	0.12	0	0	0	0	1	0	1	0	1	0	-2.48235
tr|Q9NZ23|Q9NZ23_HUMAN	YA61	Drug-sensitive protein 1 OS=Homo sapiens GN=YA61 PE=2 SV=1	14858	588	0	27	0	0.23	0	0	0	0	1	0	1	0	1	0	-2.48235
tr|H3BS19|H3BS19_HUMAN	ZNF469	Zinc finger protein 469 OS=Homo sapiens GN=ZNF469 PE=4 SV=1	417347	591	0	27	0	0.01	0	0	0	0	1	0	1	0	1	0	-2.48235
tr|H0Y512|H0Y512_HUMAN	APMAP	Adipocyte plasma membrane-associated protein (Fragment) OS=Homo sapiens GN=APMAP PE=	45662	592	0	27	0	0.07	0	0	0	0	1	0	1	0	1	0	-2.48235
tr|B7Z4L9|B7Z4L9_HUMAN	tr|B7Z4L9|B7	cDNA FLJ55342, highly similar to Kelch-like protein 13 OS=Homo sapiens PE=2 SV=1	56884	593	0	26	0	0.06	0	0	0	0	1	0	1	0	1	0	-2.48235
tr|Q5T8C6|Q5T8C6_HUMAN	CDC16	Cell division cycle protein 16 homolog OS=Homo sapiens GN=CDC16 PE=4 SV=1	55239	594	0	26	0	0.06	0	0	0	0	1	0	1	0	1	0	-2.48235
sp|Q0VD83|APOBR_HUMAN	APOBR	Apolipoprotein B receptor OS=Homo sapiens GN=APOBR PE=1 SV=1	115207	597	0	26	0	0.03	0	0	0	0	1	0	1	0	1	0	-2.48235
tr|B4DNK3|B4DNK3_HUMAN	tr|B4DNK3|B	cDNA FLJ52127, highly similar to Multisynthetase complex auxiliary component p43 OS=Homo s	29955	601	0	26	0	0.11	0	0	0	0	1	0	1	0	1	0	-2.48235
tr|B4E2I9|B4E2I9_HUMAN	tr|B4E2I9|B4	cDNA FLJ55486, highly similar to Semaphorin-3C OS=Homo sapiens PE=2 SV=1	88481	602	0	26	0	0.04	0	0	0	0	1	0	1	0	2	0	-2.48235
tr|J3KNP0|J3KNP0_HUMAN	RAB11FIP1	Rab11 family-interacting protein 1 OS=Homo sapiens GN=RAB11FIP1 PE=4 SV=1	137482	607	0	25	0	0.02	0	0	0	0	1	0	1	0	2	0	-2.48235
tr|J3KSB9|J3KSB9_HUMAN	tr|J3KSB9|J3K	Uncharacterized protein (Fragment) OS=Homo sapiens PE=4 SV=1	8877	608	0	25	0	0.39	0	0	0	0	1	0	1	0	1	0	-2.48235
tr|H3BMW5|H3BMW5_HUMA	DNAJA2	DnaJ homolog subfamily A member 2 (Fragment) OS=Homo sapiens GN=DNAJA2 PE=4 SV=1	15134	611	0	24	0	0.23	0	0	0	0	1	0	1	0	1	0	-2.48235
tr|B4DVB8|B4DVB8_HUMAN	ELAVL1	ELAV-like protein 1 OS=Homo sapiens GN=ELAVL1 PE=2 SV=1	39200	612	0	24	0	0.08	0	0	0	0	1	0	1	0	1	0	-2.48235
tr|B4E2P4|B4E2P4_HUMAN	tr|B4E2P4|B4	cDNA FLJ58474, highly similar to Xaa-Pro aminopeptidase 1 (EC 3.4.11.9) OS=Homo sapiens PE=	50020	615	0	24	0	0.07	0	0	0	0	1	0	1	0	1	0	-2.48235
tr|Q4VAN1|Q4VAN1_HUMAN	BTN1A1	BTN1A1 protein OS=Homo sapiens GN=BTN1A1 PE=2 SV=1	55878	617	0	24	0	0.06	0	0	0	0	1	0	1	0	1	0	-2.48235
tr|Q2TQ40|Q2TQ40_HUMAN	TNFRSF17	B cell maturation antigen transcript variant 4 OS=Homo sapiens GN=TNFRSF17 PE=2 SV=1	15460	618	0	24	0	0.22	0	0	0	0	1	0	1	0	1	0	-2.48235
tr|B4DU06|B4DU06_HUMAN	tr|B4DU06|B	Guanylate cyclase OS=Homo sapiens PE=2 SV=1	91756	620	0	24	0	0.04	0	0	0	0	1	0	1	0	1	0	-2.48235
tr|B0QXZ5|B0QXZ5_HUMAN	DNAL4	Dynein light chain 4, axonemal OS=Homo sapiens GN=DNAL4 PE=4 SV=1	7786	621	0	24	0	0.47	0	0	0	0	1	0	1	0	1	0	-2.48235
tr|F5H120|F5H120_HUMAN	FKBP4	Peptidyl-prolyl cis-trans isomerase FKBP4, N-terminally processed OS=Homo sapiens GN=FKBP4	5528	623	0	24	0	0.06	0	0	0	0	1	0	1	0	1	0	-2.48235
tr|B4DN13|B4DN13_HUMAN	tr|B4DN13|B	cDNA FLJ59136, highly similar to Transmembrane 9 superfamily protein member 3 OS=Homo sa	29629	624	0	23	0	0.11	0	0	0	0	1	0	1	0	1	0	-2.48235
tr|K7EPB5|K7EPB5_HUMAN	ELAVL3	ELAV-like protein 3 (Fragment) OS=Homo sapiens GN=ELAVL3 PE=4 SV=1	9728	625	0	23	0	0.36	0	0	0	0	1	0	1	0	1	0	-2.48235
tr|K7EJ37|K7EJ37_HUMAN	EXOSC10	Exosome component 10 OS=Homo sapiens GN=EXOSC10 PE=4 SV=1	13869	627	0	23	0	0.05	0	0	0	0	1	0	1	0	1	0	-2.48235
sp|Q9Y5P6|GMPPB_HUMAN	GMPPB	Mannose-1-phosphate guanyltransferase beta OS=Homo sapiens GN=GMPPB PE=1 SV=2	40379	629	0	23	0	0.08	0	0	0	0	1	0	1	0	1	0	-2.48235
tr|Q5VSQ6|Q5VSQ6_HUMAN	P4HA1	Procollagen-proline, 2-oxoglutarate 4-dioxygenase (Proline 4-hydroxylase), alpha polypeptide I v	61214	634	0	22	0	0.05	0	0	0	0	1	0	1	0	2	0	-2.48235
tr|A8KAK1|A8KAK1_HUMAN	tr|A8KAK1|A	cDNA FLJ77398, highly similar to Homo sapiens UDP-glucose ceramide glucosyltransferase-like 1	175467	635	0	22	0	0.02	0	0	0	0	1	0	1	0	1	0	-2.48235
tr|C0JFR3|C0JFR3_HUMAN	HLA-A	Truncated MHC class I antigen (Fragment) OS=Homo sapiens GN=HLA-A PE=3 SV=1	17701	639	0	22	0	0.16	0	0	0	0	1	0	1	0	1	0	-2.48235
tr|H7C4V2|H7C4V2_HUMAN	U2SURP	U2 snRNP-associated SURP motif-containing protein (Fragment) OS=Homo sapiens GN=U2SURP	33796	647	0	22	0	0.03	0	0	0	0	1	0	1	0	1	0	-2.48235
sp|Q96EK5|KBP_HUMAN	KIAA1279	KIF1-binding protein OS=Homo sapiens GN=KIAA1279 PE=1 SV=1	72453	649	0	22	0	0.05	0	0	0	0	1	0	1	0	1	0	-2.48235
tr|B4DQ49|B4DQ49_HUMAN	tr|B4DQ49|B	cDNA FLJ54374, highly similar to Homo sapiens keratin 78 (KRT78), mRNA OS=Homo sapiens PE	31874	653	0	22	0	0.1	0	0	0	0	1	0	1	0	3	0	-2.48235
tr|Q96I16|Q96I16_HUMAN	tr|Q96I16|Q9	EIF2S2 protein OS=Homo sapiens PE=2 SV=1	24902	654	0	22	0	0.09	0	0	0	0	1	0	1	0	1	0	-2.48235
tr|Q5M7Z5|Q5M7Z5_HUMAN	GRHPR	GRHPR protein (Fragment) OS=Homo sapiens GN=GRHPR PE=2 SV=1	37314	659	0	21	0	0.09	0	0	0	0	1	0	1	0	1	0	-2.48235
tr|C9J935|C9J935_HUMAN	SETD9	SET domain-containing protein 9 (Fragment) OS=Homo sapiens GN=SETD9 PE=2 SV=1	5515	660	0	21	0	0.65	0	0	0	0	1	0	1	0	1	0	-2.48235
tr|F1T0A5|F1T0A5_HUMAN	PRPF31	PRP31 pre-mRNA processing factor 31 homolog (Yeast), isoform CRA_a OS=Homo sapiens GN=P	55649	661	0	21	0	0.06	0	0	0	0	1	0	1	0	2	0	-2.48235
tr|B3KRG8|B3KRG8_HUMAN	tr|B3KRG8|B	Lipase OS=Homo sapiens PE=2 SV=1	45736	662	0	21	0	0.07	0	0	0	0	1	0	1	0	1	0	-2.48235
tr|H7C046|H7C046_HUMAN	NCEH1	Neutral cholesterol ester hydrolase 1 (Fragment) OS=Homo sapiens GN=NCEH1 PE=4 SV=1	34262	663	0	21	0	0.07	0	0	0	0	1	0	1	0	1	0	-2.48235
tr|Q6UWE9|Q6UWE9_HUMAN	UNQ2550	SFVP2550 OS=Homo sapiens GN=UNQ2550 PE=4 SV=1	11890	664	0	21	0	0.29	0	0	1	0	1	0	1	0	1	0	-2.48235
tr|A0AVG7|A0AVG7_HUMAN	BCAS1	Breast carcinoma amplified sequence 1 OS=Homo sapiens GN=BCAS1 PE=2 SV=1	61929	670	0	20	0	0.05	0	0	0	0	1	0	1	0	1	0	-2.48235
tr|H0YJL6|H0YJL6_HUMAN	EVL	Ena/VASP-like protein (Fragment) OS=Homo sapiens GN=EVL PE=4 SV=1	10347	671	0	20	0	0.34	0	0	0	0	1	0	1	0	1	0	-2.48235
tr|D6RF05|D6RF05_HUMAN	HARS	Histidine--tRNA ligase, cytoplasmic OS=Homo sapiens GN=HARS PE=2 SV=1	6740	672	0	20	0	0.07	0	0	0	0	1	0	1	0	1	0	-2.48235
tr|C9JHK9|C9JHK9_HUMAN	ABCF2	ATP-binding cassette sub-family F member 2 (Fragment) OS=Homo sapiens GN=ABCF2 PE=2 SV=	27027	673	0	20	0	0.05	0	0	0	0	1	0	1	0	2	0	-2.48235
sp|P55212|CASP6_HUMAN	CASP6	Caspase-6 OS=Homo sapiens GN=CASP6 PE=1 SV=2	33859	674	0	20	0	0.1	0	0	0	0	1	0	1	0	1	0	-2.48235
sp|O95159|ZFPL1_HUMAN	ZFPL1	Zinc finger protein-like 1 OS=Homo sapiens GN=ZFPL1 PE=1 SV=2	34833	678	0	20	0	0.1	0	0	0	0	1	0	1	0	1	0	-2.48235
tr|B7Z4P8|B7Z4P8_HUMAN	ACTN2	Alpha-actinin-2 OS=Homo sapiens GN=ACTN2 PE=2 SV=1	44675	19	0	51	0	2.11	0	0	1	0	2	0	2	0	2	0	-3.21965
sp|P35900|K1C20_HUMAN	KRT20	Keratin, type I cytoskeletal 20 OS=Homo sapiens GN=KRT20 PE=1 SV=1	48514	24	0	78	0	2.99	0	0	2	0	2	0	2	0	2	0	-3.21965
tr|F8VV57|F8VV57_HUMAN	KRT5	Keratin, type II cytoskeletal 5 (Fragment) OS=Homo sapiens GN=KRT5 PE=2 SV=1	12207	58	0	136	0	0.95	0	0	2	0	2	0	2	0	4	0	-3.21965
tr|G3V241|G3V241_HUMAN	SHMT2	Serine hydroxymethyltransferase, mitochondrial (Fragment) OS=Homo sapiens GN=SHMT2 PE=2	15985	86	0	71	0	0.61	0	0	1	0	2	0	2	0	3	0	-3.21965
tr|B4DX20|B4DX20_HUMAN	CCT6B	T-complex protein 1 subunit zeta-2 OS=Homo sapiens GN=CCT6B PE=2 SV=1	54066	118	0	46	0	0.47	0	0	1	0	1	0	2	0	3	0	-3.21965
tr|C9J057|C9J057_HUMAN	ASNS	Asparagine synthetase [glutamine-hydrolyzing] (Fragment) OS=Homo sapiens GN=ASNS PE=2 SV	24946	141	0	32	0	0.26	0	0	0	0	1	0	2	0	2	0	-3.21965
tr|B4DRY6|B4DRY6_HUMAN	tr|B4DRY6|B	cDNA FLJ57182, highly similar to Paxillin OS=Homo sapiens PE=2 SV=1	36791	159	0	73	0	0.28	0	0	1	0	2	0	2	0	2	0	-3.21965
tr|D3DPI2|D3DPI2_HUMAN	hCG_164122	HCG1641229, isoform CRA_a OS=Homo sapiens GN=hCG_1641229 PE=2 SV=1	32409	178	0	76	0	0.35	0	0	2	0	2	0	2	0	2	0	-3.21965
tr|B4DPK8|B4DPK8_HUMAN	tr|B4DPK8|B	cDNA FLJ55127, highly similar to Heterogeneous nuclear ribonucleoprotein L OS=Homo sapiens	26147	182	0	0	0	0.23	0	0	0	0	0	0	2	0	2	0	-3.21965
tr|Q5JVP3|Q5JVP3_HUMAN	PTGR1	Leukotriene B4 12-hydroxydehydrogenase, isoform CRA_d OS=Homo sapiens GN=PTGR1 PE=4 S	19441	190	0	113	0	0.38	0	0	2	0	2	0	2	0	2	0	-3.21965
tr|B3KNM0|B3KNM0_HUMAN	tr|B3KNM0|E	cDNA FLJ14916 fis, clone PLACE1006958, highly similar to Heat shock 70 kDa protein 4L (Fragme	64588	191	0	113	0	0.1	0	0	2	0	2	0	2	0	3	0	-3.21965
tr|B3KPU1|B3KPU1_HUMAN	GNB2	Guanine nucleotide-binding protein G(I)/G(S)/G(T) subunit beta-2 OS=Homo sapiens GN=GNB2	26598	194	0	111	0	0.27	0	0	1	0	2	0	2	0	2	0	-3.21965
tr|B2R6K4|B2R6K4_HUMAN	tr|B2R6K4|B	cDNA, FLJ92996, highly similar to Homo sapiens guanine nucleotide binding protein (G protein),	38075	199	0	105	0	0.18	0	0	1	0	2	0	2	0	2	0	-3.21965
tr|Q5TCG1|Q5TCG1_HUMAN	SYNCRIP	Synaptotagmin binding, cytoplasmic RNA interacting protein (Fragment) OS=Homo sapiens GN=	20301	252	0	76	0	0.13	0	0	2	0	2	0	2	0	2	0	-3.21965
tr|G3V1S6|G3V1S6_HUMAN	GALNT2	Polypeptide N-acetylgalactosaminyltransferase 2 soluble form OS=Homo sapiens GN=GALNT2 P	61440	254	0	76	0	0.11	0	0	2	0	2	0	2	0	2	0	-3.21965
sp|Q6IS14|IF5AL_HUMAN	EIF5AL1	Eukaryotic translation initiation factor 5A-1-like OS=Homo sapiens GN=EIF5AL1 PE=1 SV=2	16990	256	0	75	0	0.44	0	0	1	0	2	0	2	0	2	0	-3.21965
tr|B1ANK7|B1ANK7_HUMAN	FH	Fumarate hydratase OS=Homo sapiens GN=FH PE=2 SV=1	54773	288	0	64	0	0.12	0	0	1	0	2	0	2	0	5	0	-3.21965
sp|P04040|CATA_HUMAN	CAT	Catalase OS=Homo sapiens GN=CAT PE=1 SV=3	59947	299	0	60	0	0.11	0	0	1	0	2	0	2	0	2	0	-3.21965
tr|B9A041|B9A041_HUMAN	MDH1	Malate dehydrogenase, cytoplasmic OS=Homo sapiens GN=MDH1 PE=2 SV=1	23195	309	0	58	0	0.31	0	0	2	0	2	0	2	0	2	0	-3.21965
tr|B4DMY3|B4DMY3_HUMAN	tr|B4DMY3|E	cDNA FLJ60713, highly similar to Homo sapiens heterogeneous nuclear ribonucleoprotein A/B (I	35101	318	0	56	0	0.2	0	0	1	0	2	0	2	0	2	0	-3.21965
tr|B4DMP0|B4DMP0_HUMAN	tr|B4DMP0|E	cDNA FLJ52189, highly similar to Procollagen-lysine,2-oxoglutarate5-dioxygenase 3 (EC 1.14.11.	37539	325	0	48	0	0.12	0	0	1	0	2	0	2	0	2	0	-3.21965
tr|G3V180|G3V180_HUMAN	DPP3	Dipeptidyl peptidase 3 OS=Homo sapiens GN=DPP3 PE=2 SV=1	84666	335	0	52	0	0.08	0	0	1	0	2	0	2	0	2	0	-3.21965
tr|E5RHF2|E5RHF2_HUMAN	COPS5	COP9 signalosome complex subunit 5 (Fragment) OS=Homo sapiens GN=COPS5 PE=2 SV=1	16655	346	0	51	0	0.45	0	0	0	0	2	0	2	0	2	0	-3.21965
tr|Q6NZ52|Q6NZ52_HUMAN	RPL27A	Ribosomal protein L27a OS=Homo sapiens GN=RPL27A PE=2 SV=1	16582	348	0	50	0	0.2	0	0	1	0	1	0	2	0	2	0	-3.21965
tr|H0Y9H1|H0Y9H1_HUMAN	CANX	Calnexin (Fragment) OS=Homo sapiens GN=CANX PE=3 SV=1	21748	352	0	49	0	0.39	0	0	1	0	2	0	2	0	3	0	-3.21965
sp|P01024|CO3_HUMAN	C3	Complement C3 OS=Homo sapiens GN=C3 PE=1 SV=2	188569	370	0	47	0	0.03	0	0	1	0	2	0	2	0	2	0	-3.21965
tr|H0YIG3|H0YIG3_HUMAN	KRT72	Keratin, type II cytoskeletal 72 (Fragment) OS=Homo sapiens GN=KRT72 PE=4 SV=1	21922	372	0	39	0	0.12	0	0	1	0	1	0	2	0	2	0	-3.21965
tr|A0MNP2|A0MNP2_HUMAN	WDR57	CDW11/WDR57 OS=Homo sapiens GN=WDR57 PE=2 SV=1	39742	413	0	42	0	0.17	0	0	1	0	2	0	2	0	3	0	-3.21965
tr|B2R7Y3|B2R7Y3_HUMAN	tr|B2R7Y3|B	cDNA, FLJ93657, highly similar to Homo sapiens ribosomal protein L3-like (RPL3L), mRNA OS=Ho	46581	420	0	42	0	0.07	0	0	1	0	1	0	2	0	3	0	-3.21965
tr|E7EVA0|E7EVA0_HUMAN	MAP4	Microtubule-associated protein OS=Homo sapiens GN=MAP4 PE=2 SV=1	246546	429	0	40	0	0.01	0	0	1	0	1	0	2	0	4	0	-3.21965
tr|A5D904|A5D904_HUMAN	RPS9	RPS9 protein (Fragment) OS=Homo sapiens GN=RPS9 PE=2 SV=1	13210	434	0	40	0	0.58	0	0	1	0	2	0	2	0	2	0	-3.21965
tr|H0Y7N2|H0Y7N2_HUMAN	ATXN10	Ataxin-10 (Fragment) OS=Homo sapiens GN=ATXN10 PE=4 SV=1	19107	470	0	37	0	0.38	0	0	0	0	2	0	2	0	2	0	-3.21965
tr|B2R4P2|B2R4P2_HUMAN	tr|B2R4P2|B	cDNA, FLJ92164, highly similar to Homo sapiens peroxiredoxin 1 (PRDX1), mRNA OS=Homo sapi	22414	481	0	36	0	0.32	0	0	0	0	2	0	2	0	2	0	-3.21965
tr|C1KES1|C1KES1_HUMAN	LRRC16B	LRRC16B (Fragment) OS=Homo sapiens GN=LRRC16B PE=4 SV=1	4125	530	0	31	0	0.94	0	0	0	0	1	0	2	0	2	0	-3.21965
tr|H0YF33|H0YF33_HUMAN	NASP	Nuclear autoantigenic sperm protein (Fragment) OS=Homo sapiens GN=NASP PE=4 SV=1	31883	542	0	30	0	0.1	0	0	0	0	1	0	2	0	3	0	-3.21965
tr|B2RD27|B2RD27_HUMAN	tr|B2RD27|B	cDNA, FLJ96428, highly similar to Homo sapiens proteasome (prosome, macropain) 26S subunit	37046	550	0	30	0	0.19	0	0	0	0	2	0	2	0	2	0	-3.21965
sp|O75367|H2AY_HUMAN	H2AFY	Core histone macro-H2A.1 OS=Homo sapiens GN=H2AFY PE=1 SV=4	39764	565	0	29	0	0.08	0	0	0	0	1	0	2	0	2	0	-3.21965
tr|A6NLN1|A6NLN1_HUMAN	PTBP1	Polypyrimidine tract binding protein 1, isoform CRA_e OS=Homo sapiens GN=PTBP1 PE=4 SV=3	21899	567	0	26	0	0.18	0	0	0	0	2	0	2	0	2	0	-3.21965
sp|O15131|IMA5_HUMAN	KPNA5	Importin subunit alpha-6 OS=Homo sapiens GN=KPNA5 PE=1 SV=2	61052	573	0	28	0	0.11	0	0	0	0	2	0	2	0	2	0	-3.21965
tr|H3BNV2|H3BNV2_HUMAN	NQO1	NAD(P)H dehydrogenase [quinone] 1 OS=Homo sapiens GN=NQO1 PE=4 SV=1	28533	576	0	28	0	0.25	0	0	0	0	2	0	2	0	2	0	-3.21965
tr|D3DSQ0|D3DSQ0_HUMAN	PCM1	Pericentriolar material 1, isoform CRA_a OS=Homo sapiens GN=PCM1 PE=2 SV=1	230041	619	0	24	0	0.01	0	0	0	0	1	0	2	0	3	0	-3.21965
tr|H3BUF6|H3BUF6_HUMAN	ATXN2L	Ataxin-2-like protein OS=Homo sapiens GN=ATXN2L PE=4 SV=1	112929	656	0	22	0	0.03	0	0	0	0	1	0	2	0	2	0	-3.21965
sp|P35237|SPB6_HUMAN	SERPINB6	Serpin B6 OS=Homo sapiens GN=SERPINB6 PE=1 SV=3	42936	120	0	179	0	0.25	0	0	3	0	3	0	3	0	3	0	-3.70541
tr|C9JWY7|C9JWY7_HUMAN	CAPN2	Calpain-2 catalytic subunit (Fragment) OS=Homo sapiens GN=CAPN2 PE=2 SV=1	14668	173	0	48	0	0.22	0	0	0	0	4	0	3	0	4	0	-3.70541
tr|B4DMU0|B4DMU0_HUMAN	PYCR1	Pyrroline-5-carboxylate reductase OS=Homo sapiens GN=PYCR1 PE=2 SV=1	36186	174	0	125	0	0.19	0	0	2	0	2	0	3	0	3	0	-3.70541
tr|H0YAF8|H0YAF8_HUMAN	GNB2L1	Guanine nucleotide-binding protein subunit beta-2-like 1 (Fragment) OS=Homo sapiens GN=GN	22373	216	0	96	0	0.52	0	0	2	0	3	0	3	0	4	0	-3.70541
tr|Q0VGL3|Q0VGL3_HUMAN	RPL13A	RPL13A protein OS=Homo sapiens GN=RPL13A PE=2 SV=1	16706	300	0	59	0	0.74	0	0	3	0	3	0	3	0	3	0	-3.70541
tr|B4DDR2|B4DDR2_HUMAN	tr|B4DDR2|B	cDNA FLJ54588, highly similar to Major vault protein OS=Homo sapiens PE=2 SV=1	98526	304	0	59	0	0.07	0	0	2	0	2	0	3	0	3	0	-3.70541
sp|Q86Y46|K2C73_HUMAN	KRT73	Keratin, type II cytoskeletal 73 OS=Homo sapiens GN=KRT73 PE=1 SV=1	59457	373	0	47	0	0.12	0	0	2	0	2	0	3	0	5	0	-3.70541
tr|B7Z4V1|B7Z4V1_HUMAN	tr|B7Z4V1|B	cDNA FLJ55401, highly similar to Alpha-actinin-2 OS=Homo sapiens PE=2 SV=1	32962	50	0	103	0	0.77	0	0	3	0	4	0	4	0	4	0	-4.06832
tr|D6RAT0|D6RAT0_HUMAN	RPS3A	40S ribosomal protein S3a OS=Homo sapiens GN=RPS3A PE=3 SV=1	26099	223	0	93	0	0.44	0	0	2	0	3	0	4	0	5	0	-4.06832
tr|E9PKU4|E9PKU4_HUMAN	RPL8	60S ribosomal protein L8 (Fragment) OS=Homo sapiens GN=RPL8 PE=2 SV=1	25806	231	0	90	0	0.28	0	0	2	0	2	0	4	0	5	0	-4.06832
sp|P21796|VDAC1_HUMAN	VDAC1	Voltage-dependent anion-selective channel protein 1 OS=Homo sapiens GN=VDAC1 PE=1 SV=2	30868	250	0	78	0	0.36	0	0	1	0	3	0	4	0	4	0	-4.06832
tr|Q53RS3|Q53RS3_HUMAN	DDEF2	Putative uncharacterized protein DDEF2 (Fragment) OS=Homo sapiens GN=DDEF2 PE=2 SV=1	70046	441	0	39	0	0.05	0	0	1	0	1	0	4	0	4	0	-4.06832
sp|A2RTY3|CQ066_HUMAN	C17orf66	Uncharacterized protein C17orf66 OS=Homo sapiens GN=C17orf66 PE=2 SV=2	66437	477	0	36	0	0.05	0	0	0	0	1	0	4	0	8	0	-4.06832
tr|K7ENW6|K7ENW6_HUMAN	KRT16	Keratin, type I cytoskeletal 16 (Fragment) OS=Homo sapiens GN=KRT16 PE=4 SV=1	18735	62	0	109	0	1.38	0	0	3	0	4	0	5	0	5	0	-4.35816
tr|F8VQP2|F8VQP2_HUMAN	ATXN2	Ataxin-2 OS=Homo sapiens GN=ATXN2 PE=2 SV=1	117012	157	0	135	0	0.21	0	0	4	0	7	0	10	0	10	0	-5.29273
sp|Q96RW7|HMCN1_HUMAN	HMCN1	Hemicentin-1 OS=Homo sapiens GN=HMCN1 PE=1 SV=2	623265	291	0	63	0	0.01	0	0	7	0	7	0	11	0	16	0	-5.42431
tr|B2RCS5|B2RCS5_HUMAN	ACTN2	Actinin, alpha 2, isoform CRA_b OS=Homo sapiens GN=ACTN2 PE=2 SV=1	104425	89	0	229	0	0.45	0	0	9	0	12	0	12	0	13	0	-5.54494
tr|B3KPL5|B3KPL5_HUMAN	tr|B3KPL5|B3	cDNA FLJ31917 fis, clone NT2RP7004925, highly similar to Sprouty-related, EVH1 domain-contai	48711	657	0	21	0	0.07	0	0	0	0	1	0	16	0	51	0	-5.94682

#19	Hela lysate unsyn PU-1 2 uM																		
#04	Hela live syn NP_2μM																		
Accession	GS	Name	Pro_Mass	04_prot_hit_n	19_prot_hit_n	04_prot_score	19_prot_score	04_emPAI	19_emPAI	emPAI ratio	04_No>I	19_No>I	04_No>HI	19_No>HI	04_No>20	19_No>20	04_No=All	19_No=All	Rsc
sp|P22694|KAPCB_HUMAN	PRKACB	cAMP-dependent protein kinase catalytic subunit beta OS=Homo sapiens GN=PRKACB PE=1 SV=2	40711	0	1100	0	26	0	0.16	#DIV/0!	0	0	0	1	0	1	0	1	0.795451
sp|O75116|ROCK2_HUMAN	ROCK2	Rho-associated protein kinase 2 OS=Homo sapiens GN=ROCK2 PE=1 SV=4	161939	375	410	46	88	0.05	0.2	4	1	2	2	5	1	5	3	5	1.085403
sp|Q15418|KS6A1_HUMAN	RPS6KA1	Ribosomal protein S6 kinase alpha-1 OS=Homo sapiens GN=RPS6KA1 PE=1 SV=2	83070	0	807	0	22	0	0.08	#DIV/0!	0	0	0	1	0	1	0	1	0.795451
sp|P78527|PRKDC_HUMAN	PRKDC	DNA-dependent protein kinase catalytic subunit OS=Homo sapiens GN=PRKDC PE=1 SV=3	473749	83	25	241	1120	0.07	0.43	6.142857143	7	33	10	51	11	57	17	72	1.539701
sp|Q13555|KCC2G_HUMAN	CAMK2G	Calcium/calmodulin-dependent protein kinase type II subunit gamma OS=Homo sapiens GN=CAMK2G PE=1 SV=3	63311	0	894	0	37	0	0.15	#DIV/0!	0	1	0	1	0	1	0	1	0.795451
sp|P49761|CLK3_HUMAN	CLK3	Dual specificity protein kinase CLK3 OS=Homo sapiens GN=CLK3 PE=1 SV=3	74267	0	1001	0	31	0	0.04	#DIV/0!	0	0	0	1	0	1	0	1	0.795451
sp|Q96SB4|SRPK1_HUMAN	SRPK1	SRSF protein kinase 1 OS=Homo sapiens GN=SRPK1 PE=1 SV=2	74963	0	363	0	102	0	0.09	#DIV/0!	0	2	0	2	0	2	0	3	1.532612
sp|Q9Y6E0|STK24_HUMAN	STK24	Serine/threonine-protein kinase 24 OS=Homo sapiens GN=STK24 PE=1 SV=1	49562	0	952	0	34	0	0.17	#DIV/0!	0	0	0	2	0	2	0	2	1.532612
sp|Q13418|ILK_HUMAN	ILKTKL	Integrin-linked protein kinase OS=Homo sapiens GN=ILK PE=1 SV=2	51899	421	369	41	100	0.07	0.53	7.571428571	1	1	1	6	1	6	1	7	1.326607
sp|Q9UKS6|PACN3_HUMAN	PACSIN3	Protein kinase C and casein kinase substrate in neurons protein 3 OS=Homo sapiens GN=PACSIN3 PE=1 SV=2	48799	0	902	0	36	0	0.07	#DIV/0!	0	1	0	1	0	1	0	1	0.795451
sp|Q16822|PCKGM_HUMAN	PCK2	Phosphoenolpyruvate carboxykinase [GTP], mitochondrial OS=Homo sapiens GN=PCK2 PE=1 SV=3	71483	0	413	0	59	0	0.14	#DIV/0!	0	1	0	2	0	2	0	2	1.532612
sp|P17858|K6PL_HUMAN	PFKL	6-phosphofructokinase, liver type OS=Homo sapiens GN=PFKL PE=1 SV=6	85762	98	208	88	188	0.4	0.21	0.525	1	4	2	5	2	6	2	8	0.589303
tr|J3KNX3|J3KNX3_HUMAN	PFKM	6-phosphofructokinase OS=Homo sapiens GN=PFKM PE=3 SV=1	94107	0	378	0	96	0	0.15	#DIV/0!	0	4	0	4	0	5	0	6	2.670704
sp|Q01813|K6PP_HUMAN	PFKP	6-phosphofructokinase type C OS=Homo sapiens GN=PFKP PE=1 SV=2	86454	98	57	217	568	0.4	0.95	2.375	5	15	9	18	9	20	10	22	0.320789
sp|P00558|PGK1_HUMAN	PGK1	Phosphoglycerate kinase 1 OS=Homo sapiens GN=PGK1 PE=1 SV=3	44985	134	76	161	453	0.76	1.9	2.5	5	10	8	15	9	19	9	21	0.248443
tr|B3KUN1|B3KUN1_HUMAN	PPP2CA	Serine/threonine-protein phosphatase OS=Homo sapiens GN=PPP2CA PE=2 SV=1	36142	0	711	0	49	0	0.12	#DIV/0!	0	1	0	1	0	1	0	2	0.795451
sp|P40763|STAT3_HUMAN	STAT3	Signal transducer and activator of transcription 3 OS=Homo sapiens GN=STAT3 PE=1 SV=2	88810	0	406	0	89	0	0.08	#DIV/0!	0	2	0	2	0	2	0	3	1.532612
tr|C9J9S3|C9J9S3_HUMAN	PPP1CB	Serine/threonine-protein phosphatase (Fragment) OS=Homo sapiens GN=PPP1CB PE=2 SV=1	14484	345	388	51	93	0.24	0.89	3.708333333	1	3	1	3	1	3	1	3	0.432934
tr|B3KXE7|B3KXE7_HUMAN	tr|B3KXE7	cDNA FLJ45304 fis, clone BRHIP3003984, highly similar to IkappaB kinase complex-associated protein OS=Homo sapiens PE=2 SV=1	112698	0	607	0	59	0	0.03	#DIV/0!	0	1	0	1	0	1	0	1	0.795451
tr|C1PHA2|C1PHA2_HUMAN	KIF5B-ALK	Tyrosine-protein kinase receptor OS=Homo sapiens GN=KIF5B-ALK PE=2 SV=1	169283	227	242	91	159	0.04	0.37	9.25	2	4	2	10	2	10	3	16	1.281965
sp|Q9NTJ5|SAC1_HUMAN	SACM1L	Phosphatidylinositide phosphatase SAC1 OS=Homo sapiens GN=SACM1L PE=1 SV=2	67437	0	534	0	67	0	0.17	#DIV/0!	0	1	0	1	0	1	0	1	0.795451
tr|B4DK71|B4DK71_HUMAN	SACM1L	Phosphatidylinositide phosphatase SAC1 OS=Homo sapiens GN=SACM1L PE=2 SV=1	61327	0	577	0	62	0	0.11	#DIV/0!	0	1	0	2	0	2	0	2	1.532612
sp|Q96C86|DCPS_HUMAN	DCPS	m7GpppX diphosphatase OS=Homo sapiens GN=DCPS PE=1 SV=2	38756	0	1126	0	24	0	0.09	#DIV/0!	0	0	0	1	0	1	0	1	0.795451
tr|B2R665|B2R665_HUMAN	tr|B2R665	cDNA, FLJ92810, highly similar to Homo sapiens protein phosphatase 1G (formerly 2C), magnesium-dependent, gamma isoform (PP	59889	0	1004	0	31	0	0.05	#DIV/0!	0	0	0	1	0	1	0	1	0.795451
sp|Q8TAP8|PPR35_HUMAN	PPP1R35	Protein phosphatase 1 regulatory subunit 35 OS=Homo sapiens GN=PPP1R35 PE=1 SV=1	28107	0	751	0	46	0	0.12	#DIV/0!	0	1	0	1	0	1	0	1	0.795451
tr|B1AL79|B1AL79_HUMAN	PKN2	Protein kinase N2 OS=Homo sapiens GN=PKN2 PE=4 SV=1	68653	0	1034	0	0	0	0.03	#DIV/0!	0	0	0	0	0	0	0	1	-0.78971
tr|A8K2U2|A8K2U2_HUMAN	tr|A8K2U2	cDNA FLJ75392, highly similar to Homo sapiens hexokinase II (HKII) mRNA OS=Homo sapiens PE=2 SV=1	103718	599	599	26	47	0.03	0.11	3.666666667	0	1	1	1	1	1	2	1	-0.78985
sp|P14618|KPYM_HUMAN	PKM	Pyruvate kinase isozymes M1/M2 OS=Homo sapiens GN=PKM PE=1 SV=4	58470	8	6	1820	2285	5.79	13.6	2.348877375	47	52	58	70	60	72	66	81	-0.53495
tr|F2Z2Y4|F2Z2Y4_HUMAN	PDXK	Pyridoxal kinase OS=Homo sapiens GN=PDXK PE=2 SV=1	30790	566	481	29	75	0.11	0.18	1.636363636	0	1	1	1	1	1	1	1	-0.78985
tr|B4DP56|B4DP56_HUMAN	tr|B4DP56	cDNA FLJ52237, highly similar to Creatine kinase B-type (EC 2.7.3.2) OS=Homo sapiens PE=2 SV=1	38840	214	346	97	111	0.18	0.18	1	2	2	2	2	2	3	2	3	-0.30437
tr|B0AZV0|B0AZV0_HUMAN	STRAP	Serine-threonine kinase receptor-associated protein OS=Homo sapiens GN=STRAP PE=2 SV=1	28716	626	616	23	58	0.12	0.12	1	0	1	1	1	1	1	1	1	-0.78985
tr|A4QPA9|A4QPA9_HUMAN	MAP2K1	Mitogen-activated protein kinase kinase 1 OS=Homo sapiens GN=MAP2K1 PE=2 SV=1	43753	384	1117	45	25	0.31	0.07	0.225806452	1	1	2	1	2	1	2	1	-1.52715
tr|B4E1G6|B4E1G6_HUMAN	GALK1	Galactokinase OS=Homo sapiens GN=GALK1 PE=2 SV=1	45956	679	0	20	0	0.07	0	0	0	0	1	0	0	0	1	0	-0.78971
tr|A6NIT2|A6NIT2_HUMAN	PHKA1	Phosphorylase b kinase regulatory subunit alpha, skeletal muscle isoform OS=Homo sapiens GN=PHKA1 PE=2 SV=1	133462	711	0	17	0	0.02	0	0	0	0	1	0	0	0	1	0	-0.78971
tr|Q8IWY7|Q8IWY7_HUMAN	TTBK2	Tau-tubulin kinase OS=Homo sapiens GN=TTBK2 PE=2 SV=1	183893	79	0	28	0	0.79	0	0	0	0	1	0	1	0	1	0	-2.37501
tr|B4E2K2|B4E2K2_HUMAN	tr|B4E2K2	cDNA FLJ55665, highly similar to A-kinase anchor protein 2 OS=Homo sapiens PE=2 SV=1	103935	246	0	79	0	0.03	0	0	1	0	1	0	1	0	1	0	-2.37501
sp|Q9UEW8|STK39_HUMAN	STK39	STE20/SPS1-related proline-alanine-rich protein kinase OS=Homo sapiens GN=STK39 PE=1 SV=3	59950	491	0	35	0	0.05	0	0	0	0	1	0	1	0	1	0	-2.37501
tr|B2R5T5|B2R5T5_HUMAN	PRKAR1A	Protein kinase, cAMP-dependent, regulatory, type I, alpha (Tissue specific extinguisher 1), isoform CRA_a OS=Homo sapiens GN=PRK	43183	533	0	31	0	0.08	0	0	0	0	1	0	1	0	1	0	-2.37501
tr|Q5JQ10|Q5JQ10_HUMAN	ADK	Adenosine kinase OS=Homo sapiens GN=ADK PE=2 SV=1	39078	538	0	30	0	0.08	0	0	0	0	1	0	1	0	1	0	-2.37501
tr|H0YH33|H0YH33_HUMAN	STRAP	Serine-threonine kinase receptor-associated protein (Fragment) OS=Homo sapiens GN=STRAP PE=4 SV=1	10763	626	0	23	0	0.12	0	0	0	0	1	0	1	0	1	0	-2.37501
tr|H0Y3C5|H0Y3C5_HUMAN	HCK	Tyrosine-protein kinase HCK OS=Homo sapiens GN=HCK PE=4 SV=1	59986	628	0	23	0	0.05	0	0	0	0	1	0	1	0	2	0	-2.37501
tr|H0Y3Y5|H0Y3Y5_HUMAN	PPIP5K1	Inositol hexakisphosphate and diphosphoinositol-pentakisphosphate kinase 1 (Fragment) OS=Homo sapiens GN=PPIP5K1 PE=4 SV=1	21913	632	0	22	0	0.15	0	0	0	0	1	0	1	0	2	0	-2.37501
tr|B2R860|B2R860_HUMAN	tr|B2R860	Ribose-phosphate pyrophosphokinase OS=Homo sapiens PE=2 SV=1	35047	638	0	22	0	0.14	0	0	0	0	1	0	1	0	1	0	-2.37501
sp|O95747|OXSR1_HUMAN	OXSR1	Serine/threonine-protein kinase OSR1 OS=Homo sapiens GN=OXSR1 PE=1 SV=1	58271	675	0	20	0	0.06	0	0	0	0	1	0	1	0	2	0	-2.37501
tr|B3KS97|B3KS97_HUMAN	MAP2K2	Mitogen-activated protein kinase kinase 2, isoform CRA_d OS=Homo sapiens GN=MAP2K2 PE=2 SV=1	23450	384	0	45	0	0.31	0	0	1	0	2	0	2	0	2	0	-3.11231
tr|Q6ZP32|Q6ZP32_HUMAN	tr|Q6ZP32	cDNA FLJ26613 fis, clone MPB05565, highly similar to Serine/threonine protein phosphatase 2A, 55 kDa regulatory subunit B, alpha	53547	0	1172	0	19	0	0.06	#DIV/0!	0	0	0	1	0	0	0	2	-0.78971
tr|B4DDF7|B4DDF7_HUMAN	tr|B4DDF7	cDNA FLJ53296, highly similar to Serine/threonine-protein phosphatase 2A 65 kDa regulatory subunit A alpha isoform OS=Homo sap	64939	443	284	39	138	0.16	0.34	2.125	1	4	3	6	3	5	3	7	-0.13767
tr|H0YDU8|H0YDU8_HUMAN	PPP5C	Serine/threonine-protein phosphatase (Fragment) OS=Homo sapiens GN=PPP5C PE=3 SV=1	55743	596	786	26	44	0.06	0.06	1	0	1	1	1	1	1	1	1	-0.78985
tr|J3QT22|J3QT22_HUMAN	PPME1	Protein phosphatase methylesterase 1 OS=Homo sapiens GN=PPME1 PE=3 SV=1	44241	232	0	90	0	0.07	0	0	1	0	1	0	1	0	2	0	-2.37501
tr|E1NZA1|E1NZA1_HUMAN	PRIC295	Peroxisome proliferator activated receptor interacting complex protein OS=Homo sapiens GN=PRIC295 PE=2 SV=1	294953	0	99	0	354	0	0.19	#DIV/0!	0	11	0	16	0	19	0	22	4.499422
tr|A8K8U1|A8K8U1_HUMAN	tr|A8K8U1	cDNA FLJ77762, highly similar to Homo sapiens cullin-associated and neddylation-dissociated 1 (CAND1), mRNA OS=Homo sapiens P	137941	0	130	0	287	0	0.32	#DIV/0!	0	9	0	12	0	15	0	15	4.167431
tr|D6REM6|D6REM6_HUMAN	MATR3	Matrin-3 OS=Homo sapiens GN=MATR3 PE=2 SV=1	88647	0	133	0	253	0	0.42	#DIV/0!	0	6	0	11	0	14	0	22	4.071019
tr|A1KYQ7|A1KYQ7_HUMAN	tr|A1KYQ7	Cell migration-inducing protein 17 OS=Homo sapiens PE=2 SV=1	106022	0	146	0	256	0	0.28	#DIV/0!	0	8	0	8	0	11	0	11	3.736011
sp|O43776|SYNC_HUMAN	NARS	Asparagine--tRNA ligase, cytoplasmic OS=Homo sapiens GN=NARS PE=1 SV=1	63758	0	148	0	252	0	0.65	#DIV/0!	0	6	0	10	0	10	0	11	3.60457
tr|B3KWV6|B3KWV6_HUMAN	tr|B3KWV6	cDNA FLJ43948 fis, clone TESTI4014924, highly similar to Homo sapiens cytoplasmic FMR1 interacting protein 1 (CYFIP1), transcript	147082	0	322	0	117	0	0.17	#DIV/0!	0	5	0	7	0	9	0	12	3.459984
tr|B0QY89|B0QY89_HUMAN	EIF3EIP	Eukaryotic translation initiation factor 3 subunit L OS=Homo sapiens GN=EIF3EIP PE=4 SV=1	71085	0	153	0	238	0	0.44	#DIV/0!	0	6	0	8	0	9	0	9	3.459984
tr|B4DLV7|B4DLV7_HUMAN	tr|B4DLV7	cDNA FLJ60299, highly similar to Rab GDP dissociation inhibitor beta OS=Homo sapiens PE=2 SV=1	51577	0	201	0	192	0	0.64	#DIV/0!	0	5	0	8	0	8	0	10	3.299324
tr|B7Z4B2|B7Z4B2_HUMAN	tr|B7Z4B2	cDNA FLJ56108, highly similar to Puromycin-sensitive aminopeptidase (EC 3.4.11.-) OS=Homo sapiens PE=2 SV=1	93434	0	192	0	200	0	0.36	#DIV/0!	0	6	0	9	0	8	0	9	3.299324
tr|Q5TZZ9|Q5TZZ9_HUMAN	ANXA1	Annexin OS=Homo sapiens GN=ANXA1 PE=2 SV=1	38918	0	147	0	256	0	0.92	#DIV/0!	0	6	0	8	0	8	0	9	3.299324
tr|H0YH81|H0YH81_HUMAN	ATP5B	ATP synthase subunit beta (Fragment) OS=Homo sapiens GN=ATP5B PE=3 SV=1	38226	0	391	0	91	0	0.39	#DIV/0!	0	2	0	4	0	7	0	7	3.118555
sp|Q08J23|NSUN2_HUMAN	NSUN2	tRNA (cytosine(34)-C(5))-methyltransferase OS=Homo sapiens GN=NSUN2 PE=1 SV=2	87214	0	223	0	171	0	0.29	#DIV/0!	0	4	0	9	0	7	0	11	3.118555
tr|B7Z1N6|B7Z1N6_HUMAN	tr|B7Z1N6	cDNA FLJ52222, highly similar to Fructose-bisphosphate aldolase C (EC4.1.2.13) OS=Homo sapiens PE=2 SV=1	35800	0	114	0	314	0	0.38	#DIV/0!	0	5	0	5	0	7	0	7	3.118555
tr|G3V529|G3V529_HUMAN	DDX24	ATP-dependent RNA helicase DDX24 OS=Homo sapiens GN=DDX24 PE=2 SV=1	91994	0	761	0	46	0	0.11	#DIV/0!	0	1	0	3	0	6	0	6	2.911908
tr|B3KY63|B3KY63_HUMAN	tr|B3KY63	cDNA FLJ16830 fis, clone UTERU3022536, highly similar to Chromodomain helicase-DNA-binding protein 4 (EC 3.6.1.-) OS=Homo sap	216574	0	705	0	50	0	0.08	#DIV/0!	0	2	0	5	0	6	0	8	2.911908
sp|Q5T4S7|UBR4_HUMAN	UBR4	E3 ubiquitin-protein ligase UBR4 OS=Homo sapiens GN=UBR4 PE=1 SV=1	580547	0	490	0	74	0	0.03	#DIV/0!	0	1	0	5	0	6	0	6	2.911908
tr|B3KRS5|B3KRS5_HUMAN	HDAC2	Histone deacetylase OS=Homo sapiens GN=HDAC2 PE=2 SV=1	52479	0	474	0	77	0	0.2	#DIV/0!	0	2	0	3	0	6	0	8	2.911908
sp|Q09161|NCBP1_HUMAN	NCBP1	Nuclear cap-binding protein subunit 1 OS=Homo sapiens GN=NCBP1 PE=1 SV=1	92864	0	450	0	82	0	0.15	#DIV/0!	0	3	0	4	0	6	0	6	2.911908
sp|Q92945|FUBP2_HUMAN	KHSRP	Far upstream element-binding protein 2 OS=Homo sapiens GN=KHSRP PE=1 SV=4	73355	0	402	0	90	0	0.19	#DIV/0!	0	3	0	4	0	6	0	6	2.911908
tr|B4DY30|B4DY30_HUMAN	tr|B4DY30	cDNA FLJ54078, highly similar to Protein arginine N-methyltransferase 5 (EC 2.1.1.-) OS=Homo sapiens PE=2 SV=1	68284	0	336	0	112	0	0.26	#DIV/0!	0	3	0	5	0	6	0	7	2.911908
tr|Q6NVW7|Q6NVW7_HUMAN	KPNA2	Importin subunit alpha OS=Homo sapiens GN=KPNA2 PE=2 SV=1	58271	0	316	0	120	0	0.32	#DIV/0!	0	2	0	5	0	6	0	6	2.911908
sp|Q9H0A0|NAT10_HUMAN	NAT10	N-acetyltransferase 10 OS=Homo sapiens GN=NAT10 PE=1 SV=2	116569	0	314	0	121	0	0.15	#DIV/0!	0	3	0	5	0	6	0	9	2.911908
tr|B2RDD7|B2RDD7_HUMAN	tr|B2RDD7	cDNA, FLJ96564, highly similar to Homo sapiens SKB1 homolog (S. pombe) (SKB1), mRNA OS=Homo sapiens PE=2 SV=1	73348	0	303	0	127	0	0.24	#DIV/0!	0	3	0	5	0	6	0	7	2.911908
tr|B3KRR1|B3KRR1_HUMAN	tr|B3KRR1	cDNA FLJ34725 fis, clone MESAN2005958, highly similar to RNA-binding protein Luc7-like 2 OS=Homo sapiens PE=2 SV=1	40752	0	252	0	125	0	0.5	#DIV/0!	0	5	0	5	0	6	0	7	2.911908
sp|P09960|LKHA4_HUMAN	LTA4H	Leukotriene A-4 hydrolase OS=Homo sapiens GN=LTA4H PE=1 SV=2	69868	0	195	0	196	0	0.35	#DIV/0!	0	5	0	6	0	6	0	6	2.911908
sp|Q8NI36|WDR36_HUMAN	WDR36	WD repeat-containing protein 36 OS=Homo sapiens GN=WDR36 PE=1 SV=1	106282	0	716	0	49	0	0.1	#DIV/0!	0	2	0	3	0	5	0	6	2.670704
sp|O75533|SF3B1_HUMAN	SF3B1	Splicing factor 3B subunit 1 OS=Homo sapiens GN=SF3B1 PE=1 SV=3	146479	0	663	0	52	0	0.09	#DIV/0!	0	2	0	4	0	5	0	7	2.670704
tr|Q6I9V5|Q6I9V5_HUMAN	SLC25A6	SLC25A6 protein OS=Homo sapiens GN=SLC25A6 PE=2 SV=1	33073	0	497	0	73	0	0.33	#DIV/0!	0	2	0	3	0	5	0	6	2.670704
tr|F5H583|F5H583_HUMAN	CYFIP2	Cytoplasmic FMR1-interacting protein 2 OS=Homo sapiens GN=CYFIP2 PE=2 SV=1	138666	0	494	0	74	0	0.1	#DIV/0!	0	2	0	4	0	5	0	8	2.670704
tr|B4DUF1|B4DUF1_HUMAN	tr|B4DUF1	cDNA FLJ59760, highly similar to 1,4-alpha-glucan branching enzyme (EC 2.4.1.18) OS=Homo sapiens PE=2 SV=1	76254	0	430	0	85	0	0.18	#DIV/0!	0	1	0	4	0	5	0	5	2.670704
tr|B4DZI8|B4DZI8_HUMAN	COPB2	Coatomer protein complex, subunit beta 2 (Beta prime), isoform CRA_b OS=Homo sapiens GN=COPB2 PE=2 SV=1	99839	0	429	0	85	0	0.14	#DIV/0!	0	2	0	4	0	5	0	5	2.670704
tr|Q59FA0|Q59FA0_HUMAN	tr|Q59FA0	DNA polymerase (Fragment) OS=Homo sapiens PE=2 SV=1	113271	0	427	0	85	0	0.12	#DIV/0!	0	3	0	4	0	5	0	5	2.670704
tr|A0PJ61|A0PJ61_HUMAN	RSL1D1	RSL1D1 protein (Fragment) OS=Homo sapiens GN=RSL1D1 PE=2 SV=1	49688	0	414	0	88	0	0.47	#DIV/0!	0	2	0	6	0	5	0	8	2.670704
tr|D3DU41|D3DU41_HUMAN	LUC7L	LUC7-like (S. cerevisiae), isoform CRA_a OS=Homo sapiens GN=LUC7L PE=2 SV=1	34110	0	313	0	122	0	0.45	#DIV/0!	0	4	0	4	0	5	0	6	2.670704
tr|B4DIT7|B4DIT7_HUMAN	TGM2	Protein-glutamine gamma-glutamyltransferase 2 OS=Homo sapiens GN=TGM2 PE=2 SV=1	69632	0	267	0	147	0	0.26	#DIV/0!	0	2	0	5	0	5	0	5	2.670704
sp|Q9H3U1|UN45A_HUMAN	UNC45A	Protein unc-45 homolog A OS=Homo sapiens GN=UNC45A PE=1 SV=1	104266	0	255	0	153	0	0.13	#DIV/0!	0	4	0	4	0	5	0	5	2.670704
tr|B2RNR6|B2RNR6_HUMAN	ZFR	Zinc finger RNA binding protein OS=Homo sapiens GN=ZFR PE=2 SV=1	118081	0	246	0	156	0	0.15	#DIV/0!	0	5	0	5	0	5	0	5	2.670704
sp|P04899|GNAI2_HUMAN	GNAI2	Guanine nucleotide-binding protein G(i) subunit alpha-2 OS=Homo sapiens GN=GNAI2 PE=1 SV=3	40995	0	221	0	172	0	0.47	#DIV/0!	0	5	0	5	0	5	0	5	2.670704
tr|B3KM58|B3KM58_HUMAN	tr|B3KM58	cDNA FLJ10358 fis, clone NT2RM2001238, highly similar to Glutaminase kidney isoform, mitochondrial (Fragment) OS=Homo sapien	41736	0	218	0	176	0	0.36	#DIV/0!	0	3	0	4	0	5	0	5	2.670704
tr|G3XAL0|G3XAL0_HUMAN	MDH2	Malate dehydrogenase OS=Homo sapiens GN=MDH2 PE=2 SV=1	24921	0	162	0	123	0	0.8	#DIV/0!	0	4	0	4	0	5	0	6	2.670704
tr|H0Y659|H0Y659_HUMAN	HUWE1	E3 ubiquitin-protein ligase HUWE1 (Fragment) OS=Homo sapiens GN=HUWE1 PE=4 SV=1	134352	0	954	0	33	0	0.05	#DIV/0!	0	1	0	2	0	4	0	5	2.381001
tr|Q149P0|Q149P0_HUMAN	GBF1	GBF1 protein OS=Homo sapiens GN=GBF1 PE=2 SV=1	207938	0	882	0	38	0	0.05	#DIV/0!	0	1	0	3	0	4	0	4	2.381001
sp|Q14690|RRP5_HUMAN	PDCD11	Protein RRP5 homolog OS=Homo sapiens GN=PDCD11 PE=1 SV=3	209939	0	864	0	39	0	0.03	#DIV/0!	0	1	0	2	0	4	0	4	2.381001
tr|H0Y5W0|H0Y5W0_HUMAN	HUWE1	E3 ubiquitin-protein ligase HUWE1 (Fragment) OS=Homo sapiens GN=HUWE1 PE=4 SV=1	376696	0	821	0	41	0	0.02	#DIV/0!	0	1	0	3	0	4	0	6	2.381001
sp|P27694|RFA1_HUMAN	RPA1	Replication protein A 70 kDa DNA-binding subunit OS=Homo sapiens GN=RPA1 PE=1 SV=2	68723	0	772	0	45	0	0.15	#DIV/0!	0	1	0	3	0	4	0	5	2.381001
tr|B0QZ18|B0QZ18_HUMAN	CPNE1	Copine I OS=Homo sapiens GN=CPNE1 PE=2 SV=1	60307	0	764	0	45	0	0.24	#DIV/0!	0	1	0	4	0	4	0	4	2.381001
tr|B2RCV1|B2RCV1_HUMAN	tr|B2RCV1	cDNA, FLJ96310, highly similar to Homo sapiens solute carrier family 25 (mitochondrial carrier; adenine nucleotide translocator), me	33062	0	717	0	49	0	0.21	#DIV/0!	0	1	0	2	0	4	0	6	2.381001
tr|B2RDW1|B2RDW1_HUMAN	RPS27A	Ribosomal protein S27a, isoform CRA_c OS=Homo sapiens GN=RPS27A PE=2 SV=1	18296	0	706	0	50	0	0.4	#DIV/0!	0	2	0	3	0	4	0	4	2.381001
tr|I3L4X2|I3L4X2_HUMAN	ABCC1	Multidrug resistance-associated protein 1 (Fragment) OS=Homo sapiens GN=ABCC1 PE=3 SV=1	161588	0	686	0	51	0	0.06	#DIV/0!	0	1	0	3	0	4	0	5	2.381001
sp|P54577|SYYC_HUMAN	YARS	Tyrosine--tRNA ligase, cytoplasmic OS=Homo sapiens GN=YARS PE=1 SV=4	59448	0	638	0	55	0	0.18	#DIV/0!	0	2	0	3	0	4	0	6	2.381001
tr|F4NBW3|F4NBW3_HUMAN	HLA-B	MHC class I antigen (Fragment) OS=Homo sapiens GN=HLA-B PE=3 SV=1	38308	0	631	0	56	0	0.18	#DIV/0!	0	1	0	2	0	4	0	4	2.381001
tr|F4NBX2|F4NBX2_HUMAN	HLA-B	MHC class I antigen (Fragment) OS=Homo sapiens GN=HLA-B PE=3 SV=1	38566	0	629	0	56	0	0.18	#DIV/0!	0	1	0	2	0	4	0	5	2.381001
sp|P46977|STT3A_HUMAN	STT3A	Dolichyl-diphosphooligosaccharide--protein glycosyltransferase subunit STT3A OS=Homo sapiens GN=STT3A PE=1 SV=2	81104	0	608	0	59	0	0.17	#DIV/0!	0	1	0	4	0	4	0	8	2.381001
tr|F6SBX2|F6SBX2_HUMAN	IARS2	Isoleucine--tRNA ligase, mitochondrial OS=Homo sapiens GN=IARS2 PE=2 SV=1	106884	0	604	0	59	0	0.06	#DIV/0!	0	1	0	2	0	4	0	5	2.381001
sp|Q08945|SSRP1_HUMAN	SSRP1	FACT complex subunit SSRP1 OS=Homo sapiens GN=SSRP1 PE=1 SV=1	81367	0	584	0	61	0	0.08	#DIV/0!	0	1	0	2	0	4	0	5	2.381001
tr|B2RDI5|B2RDI5_HUMAN	tr|B2RDI5	cDNA, FLJ96627, highly similar to Homo sapiens calpain 1, (mu/l) large subunit (CAPN1), mRNA OS=Homo sapiens PE=2 SV=1	82529	0	556	0	64	0	0.12	#DIV/0!	0	2	0	3	0	4	0	4	2.381001
tr|K4DI93|K4DI93_HUMAN	CUL4B	Cullin 4B, isoform CRA_e OS=Homo sapiens GN=CUL4B PE=3 SV=1	103318	0	519	0	58	0	0.2	#DIV/0!	0	3	0	3	0	4	0	4	2.381001
sp|Q9Y3Z3|SAMH1_HUMAN	SAMHD1	SAM domain and HD domain-containing protein 1 OS=Homo sapiens GN=SAMHD1 PE=1 SV=2	72896	0	517	0	71	0	0.14	#DIV/0!	0	2	0	3	0	4	0	4	2.381001
sp|Q8TDN6|BRX1_HUMAN	BRIX1	Ribosome biogenesis protein BRX1 homolog OS=Homo sapiens GN=BRIX1 PE=1 SV=2	41660	0	492	0	74	0	0.17	#DIV/0!	0	2	0	2	0	4	0	4	2.381001
tr|D3DTY9|D3DTY9_HUMAN	TRIM25	Tripartite motif-containing 25, isoform CRA_a OS=Homo sapiens GN=TRIM25 PE=2 SV=1	48358	0	432	0	84	0	0.3	#DIV/0!	0	2	0	4	0	4	0	5	2.381001
tr|B2R858|B2R858_HUMAN	tr|B2R858	cDNA, FLJ93750, Homo sapiens DEAD (Asp-Glu-Ala-Asp) box polypeptide 6 (DDX6), mRNA OS=Homo sapiens PE=2 SV=1	53582	0	407	0	89	0	0.27	#DIV/0!	0	2	0	4	0	4	0	5	2.381001
tr|B4DM78|B4DM78_HUMAN	tr|B4DM78	cDNA FLJ58199, highly similar to Fragile X mental retardation syndrome-related protein 1 OS=Homo sapiens PE=2 SV=1	63280	0	375	0	97	0	0.21	#DIV/0!	0	2	0	3	0	4	0	5	2.381001
sp|P49792|RBP2_HUMAN	RANBP2	E3 SUMO-protein ligase RanBP2 OS=Homo sapiens GN=RANBP2 PE=1 SV=2	362365	0	368	0	101	0	0.04	#DIV/0!	0	2	0	4	0	4	0	5	2.381001
tr|B4DZP5|B4DZP5_HUMAN	tr|B4DZP5	cDNA FLJ51165, highly similar to DNA damage-binding protein 1 OS=Homo sapiens PE=2 SV=1	92726	0	366	0	101	0	0.11	#DIV/0!	0	3	0	3	0	4	0	6	2.381001
tr|A8KA19|A8KA19_HUMAN	tr|A8KA19	cDNA FLJ75831, highly similar to Homo sapiens exportin, tRNA (nuclear export receptor for tRNAs) (XPOT), mRNA OS=Homo sapiens	111104	0	353	0	105	0	0.12	#DIV/0!	0	3	0	4	0	4	0	4	2.381001
sp|Q9H2M9|RBGPR_HUMAN	RAB3GAP2	Rab3 GTPase-activating protein non-catalytic subunit OS=Homo sapiens GN=RAB3GAP2 PE=1 SV=1	157482	0	351	0	108	0	0.09	#DIV/0!	0	3	0	4	0	4	0	4	2.381001
tr|H0YCK7|H0YCK7_HUMAN	EEF1D	Elongation factor 1-delta (Fragment) OS=Homo sapiens GN=EEF1D PE=4 SV=1	23105	0	334	0	91	0	0.25	#DIV/0!	0	3	0	4	0	4	0	4	2.381001
tr|Q6NWZ1|Q6NWZ1_HUMAN	CKAP4	CKAP4 protein (Fragment) OS=Homo sapiens GN=CKAP4 PE=2 SV=1	67948	0	298	0	128	0	0.18	#DIV/0!	0	2	0	3	0	4	0	5	2.381001
tr|B4DT31|B4DT31_HUMAN	FUBP1	Far upstream element-binding protein 1 OS=Homo sapiens GN=FUBP1 PE=2 SV=1	69993	0	268	0	146	0	0.15	#DIV/0!	0	3	0	3	0	4	0	4	2.381001
tr|A8K4T6|A8K4T6_HUMAN	tr|A8K4T6	cDNA FLJ76282, highly similar to Homo sapiens proteasome (prosome, macropain) 26S subunit, non-ATPase, 5 (PSMD5), mRNA OS=	56588	0	214	0	177	0	0.25	#DIV/0!	0	3	0	4	0	4	0	5	2.381001
tr|B4DH29|B4DH29_HUMAN	tr|B4DH29	DNA-directed RNA polymerase OS=Homo sapiens PE=2 SV=1	126564	0	1080	0	27	0	0.05	#DIV/0!	0	0	0	2	0	3	0	4	2.018235
tr|B4DI19|B4DI19_HUMAN	tr|B4DI19	cDNA FLJ59524, highly similar to Cysteinyl-tRNA synthetase (EC 6.1.1.16) OS=Homo sapiens PE=2 SV=1	76209	0	1067	0	27	0	0.04	#DIV/0!	0	0	0	1	0	3	0	3	2.018235
tr|A0JLQ5|A0JLQ5_HUMAN	BXDC2	BXDC2 protein (Fragment) OS=Homo sapiens GN=BXDC2 PE=2 SV=1	39430	0	1062	0	28	0	0.08	#DIV/0!	0	1	0	1	0	3	0	4	2.018235
tr|B2R806|B2R806_HUMAN	tr|B2R806	Eukaryotic translation initiation factor 3 subunit E OS=Homo sapiens PE=2 SV=1	52622	0	1047	0	29	0	0.06	#DIV/0!	0	0	0	1	0	3	0	5	2.018235
sp|P31150|GDIA_HUMAN	GDI1	Rab GDP dissociation inhibitor alpha OS=Homo sapiens GN=GDI1 PE=1 SV=2	51177	0	1009	0	30	0	0.14	#DIV/0!	0	0	0	2	0	3	0	4	2.018235
sp|O60287|NPA1P_HUMAN	URB1	Nucleolar pre-ribosomal-associated protein 1 OS=Homo sapiens GN=URB1 PE=1 SV=4	256508	0	973	0	32	0	0.01	#DIV/0!	0	1	0	1	0	3	0	3	2.018235
tr|B4DMF5|B4DMF5_HUMAN	tr|B4DMF5	Glutamate dehydrogenase OS=Homo sapiens PE=2 SV=1	56910	0	960	0	33	0	0.06	#DIV/0!	0	0	0	1	0	3	0	3	2.018235
tr|E9PG89|E9PG89_HUMAN	CHD3	Chromodomain-helicase-DNA-binding protein 3 OS=Homo sapiens GN=CHD3 PE=2 SV=1	234431	0	942	0	34	0	0.03	#DIV/0!	0	0	0	2	0	3	0	5	2.018235
tr|B0UZZ8|B0UZZ8_HUMAN	WDR46	Chromosome 6 open reading frame 11 OS=Homo sapiens GN=WDR46 PE=2 SV=1	68455	0	936	0	34	0	0.05	#DIV/0!	0	0	0	1	0	3	0	3	2.018235
tr|Q5T2B5|Q5T2B5_HUMAN	CUL2	Cullin-2 OS=Homo sapiens GN=CUL2 PE=3 SV=1	82933	0	927	0	35	0	0.08	#DIV/0!	0	1	0	2	0	3	0	3	2.018235
sp|Q9NUL7|DDX28_HUMAN	DDX28	Probable ATP-dependent RNA helicase DDX28 OS=Homo sapiens GN=DDX28 PE=1 SV=2	59773	0	910	0	36	0	0.11	#DIV/0!	0	0	0	2	0	3	0	3	2.018235
tr|B0AZQ4|B0AZQ4_HUMAN	tr|B0AZQ4	cDNA, FLJ79494, highly similar to Structural maintenance of chromosome 3 OS=Homo sapiens PE=2 SV=1	141841	0	876	0	38	0	0.05	#DIV/0!	0	1	0	2	0	3	0	3	2.018235
tr|B5BU08|B5BU08_HUMAN	U2AF1	U2 small nuclear RNA auxillary factor 1 isoform a OS=Homo sapiens GN=U2AF1 PE=2 SV=1	28369	0	872	0	38	0	0.25	#DIV/0!	0	1	0	2	0	3	0	3	2.018235
tr|B3KM97|B3KM97_HUMAN	tr|B3KM97	cDNA FLJ10554 fis, clone NT2RP2002385, highly similar to Synaptic glycoprotein SC2 OS=Homo sapiens PE=2 SV=1	36429	0	856	0	39	0	0.19	#DIV/0!	0	0	0	2	0	3	0	3	2.018235
tr|Q96F88|Q96F88_HUMAN	POP1	Processing of 1, ribonuclease P/MRP subunit (S. cerevisiae) OS=Homo sapiens GN=POP1 PE=2 SV=1	116318	0	828	0	41	0	0.06	#DIV/0!	0	0	0	2	0	3	0	3	2.018235
tr|B7Z475|B7Z475_HUMAN	tr|B7Z475	cDNA FLJ55712, highly similar to F-box-like/WD repeat protein TBL1XR1 OS=Homo sapiens PE=2 SV=1	41301	0	815	0	42	0	0.26	#DIV/0!	0	0	0	3	0	3	0	3	2.018235
tr|E7ERK9|E7ERK9_HUMAN	EIF2B4	Translation initiation factor eIF-2B subunit delta OS=Homo sapiens GN=EIF2B4 PE=2 SV=1	60248	0	781	0	44	0	0.11	#DIV/0!	0	0	0	2	0	3	0	3	2.018235
sp|Q63HN8|RN213_HUMAN	RNF213	E3 ubiquitin-protein ligase RNF213 OS=Homo sapiens GN=RNF213 PE=1 SV=3	598557	0	766	0	45	0	0.01	#DIV/0!	0	1	0	1	0	3	0	6	2.018235
tr|A6PVH9|A6PVH9_HUMAN	CPNE1	Copine I OS=Homo sapiens GN=CPNE1 PE=4 SV=1	53641	0	756	0	46	0	0.2	#DIV/0!	0	1	0	3	0	3	0	3	2.018235
tr|B3KMV5|B3KMV5_HUMAN	tr|B3KMV5	cDNA FLJ12728 fis, clone NT2RP2000040, highly similar to Protein FAM62A OS=Homo sapiens PE=2 SV=1	123342	0	753	0	46	0	0.14	#DIV/0!	0	2	0	5	0	3	0	6	2.018235
tr|B4E3M6|B4E3M6_HUMAN	tr|B4E3M6	cDNA FLJ55446, highly similar to Superkiller viralicidic activity 2-like 2 (EC 3.6.1.-) OS=Homo sapiens PE=2 SV=1	107714	0	718	0	49	0	0.03	#DIV/0!	0	1	0	1	0	3	0	3	2.018235
sp|Q9BVP2|GNL3_HUMAN	GNL3	Guanine nucleotide-binding protein-like 3 OS=Homo sapiens GN=GNL3 PE=1 SV=2	62468	0	712	0	49	0	0.17	#DIV/0!	0	1	0	3	0	3	0	5	2.018235
tr|H0Y9V7|H0Y9V7_HUMAN	ATP2C1	Calcium-transporting ATPase type 2C member 1 (Fragment) OS=Homo sapiens GN=ATP2C1 PE=3 SV=1	100184	0	688	0	50	0	0.1	#DIV/0!	0	1	0	3	0	3	0	3	2.018235
sp|P61221|ABCE1_HUMAN	ABCE1	ATP-binding cassette sub-family E member 1 OS=Homo sapiens GN=ABCE1 PE=1 SV=1	68240	0	669	0	52	0	0.05	#DIV/0!	0	1	0	1	0	3	0	4	2.018235
sp|Q14571|ITPR2_HUMAN	ITPR2	Inositol 1,4,5-trisphosphate receptor type 2 OS=Homo sapiens GN=ITPR2 PE=1 SV=2	311060	0	665	0	52	0	0.01	#DIV/0!	0	1	0	2	0	3	0	4	2.018235
tr|Q59ES2|Q59ES2_HUMAN	tr|Q59ES2	Inositol 1,4,5-trisphosphate receptor type 3 variant (Fragment) OS=Homo sapiens PE=2 SV=1	269361	0	664	0	52	0	0.01	#DIV/0!	0	1	0	2	0	3	0	4	2.018235
sp|P13667|PDIA4_HUMAN	PDIA4	Protein disulfide-isomerase A4 OS=Homo sapiens GN=PDIA4 PE=1 SV=2	73229	0	646	0	54	0	0.14	#DIV/0!	0	1	0	3	0	3	0	3	2.018235
tr|Q59H95|Q59H95_HUMAN	tr|Q59H95	Flightless I homolog variant (Fragment) OS=Homo sapiens PE=2 SV=1	126396	0	644	0	55	0	0.08	#DIV/0!	0	1	0	3	0	3	0	5	2.018235
tr|E3Q0X1|E3Q0X1_HUMAN	HLA-B	MHC class I antigen (Fragment) OS=Homo sapiens GN=HLA-B PE=3 SV=1	31939	0	632	0	56	0	0.22	#DIV/0!	0	1	0	2	0	3	0	4	2.018235
tr|B9WPQ8|B9WPQ8_HUMAN	HLA-B	MHC class I antigen (Fragment) OS=Homo sapiens GN=HLA-B PE=3 SV=1	35888	0	630	0	56	0	0.19	#DIV/0!	0	1	0	2	0	3	0	5	2.018235
tr|A6NN60|A6NN60_HUMAN	CRYZ	Quinone oxidoreductase OS=Homo sapiens GN=CRYZ PE=2 SV=1	31680	0	625	0	56	0	0.22	#DIV/0!	0	1	0	2	0	3	0	3	2.018235
tr|B4DZC0|B4DZC0_HUMAN	tr|B4DZC0	cDNA FLJ51771, highly similar to SWI/SNF-related matrix-associatedactin-dependent regulator of chromatin subfamily A member5 (	117367	0	624	0	56	0	0.06	#DIV/0!	0	1	0	2	0	3	0	5	2.018235
sp|Q96T76|MMS19_HUMAN	MMS19	MMS19 nucleotide excision repair protein homolog OS=Homo sapiens GN=MMS19 PE=1 SV=2	114928	0	623	0	56	0	0.09	#DIV/0!	0	2	0	3	0	3	0	3	2.018235
sp|Q9UHD8|SEPT9_HUMAN	Sep-09	Septin-9 OS=Homo sapiens GN=SEPT9 PE=1 SV=2	65646	0	622	0	56	0	0.68	#DIV/0!	0	1	0	3	0	3	0	3	2.018235
tr|B3KT00|B3KT00_HUMAN	tr|B3KT00	cDNA FLJ37368 fis, clone BRAMY2024530, highly similar to Zinc phosphodiesterase ELAC protein 2 (EC 3.1.26.11) OS=Homo sapiens	91169	0	603	0	59	0	0.11	#DIV/0!	0	1	0	3	0	3	0	3	2.018235
tr|J3KMX1|J3KMX1_HUMAN	NUP88	Nuclear pore complex protein Nup88 (Fragment) OS=Homo sapiens GN=NUP88 PE=4 SV=1	80029	0	589	0	61	0	0.08	#DIV/0!	0	1	0	2	0	3	0	3	2.018235
tr|G3XAI2|G3XAI2_HUMAN	LAMB1	Laminin subunit beta-1 OS=Homo sapiens GN=LAMB1 PE=2 SV=1	207703	0	583	0	61	0	0.05	#DIV/0!	0	1	0	3	0	3	0	3	2.018235
tr|B4DJE7|B4DJE7_HUMAN	ACADM	Medium-chain-specific acyl-CoA dehydrogenase, mitochondrial OS=Homo sapiens GN=ACADM PE=2 SV=1	25704	0	580	0	61	0	0.28	#DIV/0!	0	1	0	2	0	3	0	3	2.018235
tr|B4DR63|B4DR63_HUMAN	PSMC1	26S protease regulatory subunit 4 OS=Homo sapiens GN=PSMC1 PE=2 SV=1	41255	0	558	0	64	0	0.17	#DIV/0!	0	1	0	2	0	3	0	4	2.018235
tr|A8K5Y7|A8K5Y7_HUMAN	tr|A8K5Y7	cDNA FLJ78655, highly similar to Homo sapiens exportin 5 (XPO5), mRNA OS=Homo sapiens PE=2 SV=1	138331	0	552	0	64	0	0.07	#DIV/0!	0	2	0	3	0	3	0	3	2.018235
sp|Q8TCT9|HM13_HUMAN	HM13	Minor histocompatibility antigen H13 OS=Homo sapiens GN=HM13 PE=1 SV=1	41747	0	545	0	65	0	0.26	#DIV/0!	0	3	0	3	0	3	0	3	2.018235
tr|B7Z4B8|B7Z4B8_HUMAN	HNRNPUL1	Heterogeneous nuclear ribonucleoprotein U-like protein 1 OS=Homo sapiens GN=HNRNPUL1 PE=2 SV=1	86639	0	535	0	67	0	0.08	#DIV/0!	0	2	0	2	0	3	0	4	2.018235
tr|B7ZLC9|B7ZLC9_HUMAN	GEMIN5	GEMIN5 protein OS=Homo sapiens GN=GEMIN5 PE=2 SV=1	170664	0	533	0	67	0	0.04	#DIV/0!	0	1	0	2	0	3	0	3	2.018235
sp|Q8WX93|PALLD_HUMAN	PALLD	Palladin OS=Homo sapiens GN=PALLD PE=1 SV=3	151839	0	531	0	68	0	0.05	#DIV/0!	0	2	0	2	0	3	0	3	2.018235
tr|G3V198|G3V198_HUMAN	NUP160	Nuclear pore complex protein Nup160 OS=Homo sapiens GN=NUP160 PE=2 SV=1	142365	0	521	0	66	0	0.06	#DIV/0!	0	2	0	2	0	3	0	4	2.018235
tr|B4DL07|B4DL07_HUMAN	tr|B4DL07	cDNA FLJ53353, highly similar to ATP-binding cassette sub-family D member 3 OS=Homo sapiens PE=2 SV=1	78980	0	515	0	71	0	0.13	#DIV/0!	0	2	0	3	0	3	0	3	2.018235
tr|B4DJV2|B4DJV2_HUMAN	CS	Citrate synthase OS=Homo sapiens GN=CS PE=2 SV=1	50628	0	509	0	72	0	0.21	#DIV/0!	0	2	0	3	0	3	0	3	2.018235
tr|D3DUW5|D3DUW5_HUMAN	DNM1L	Dynamin 1-like, isoform CRA_c OS=Homo sapiens GN=DNM1L PE=3 SV=1	88442	0	486	0	75	0	0.12	#DIV/0!	0	1	0	3	0	3	0	5	2.018235
tr|B2RE88|B2RE88_HUMAN	tr|B2RE88	cDNA, FLJ96465, highly similar to Homo sapiens solute carrier family 25 (mitochondrial carrier;phosphate carrier), member 3 (SLC25	40379	0	477	0	76	0	0.17	#DIV/0!	0	2	0	2	0	3	0	3	2.018235
tr|B4DSN3|B4DSN3_HUMAN	tr|B4DSN3	cDNA FLJ60345, highly similar to Protein transport protein Sec24C OS=Homo sapiens PE=2 SV=1	107974	0	476	0	77	0	0.09	#DIV/0!	0	2	0	3	0	3	0	3	2.018235
tr|B4DN25|B4DN25_HUMAN	UGDH	UDP-glucose 6-dehydrogenase OS=Homo sapiens GN=UGDH PE=2 SV=1	44786	0	473	0	73	0	0.26	#DIV/0!	0	2	0	3	0	3	0	3	2.018235
sp|Q27J81|INF2_HUMAN	INF2	Inverted formin-2 OS=Homo sapiens GN=INF2 PE=1 SV=2	136851	0	465	0	79	0	0.07	#DIV/0!	0	3	0	3	0	3	0	3	2.018235
tr|H3BM93|H3BM93_HUMAN	NUP93	Nuclear pore complex protein Nup93 (Fragment) OS=Homo sapiens GN=NUP93 PE=4 SV=1	19858	0	449	0	62	0	0.25	#DIV/0!	0	2	0	3	0	3	0	4	2.018235
tr|B4DDH8|B4DDH8_HUMAN	tr|B4DDH8	cDNA FLJ55184, highly similar to Homo sapiens leukocyte receptor cluster (LRC) member 4 (LENG4), mRNA OS=Homo sapiens PE=2	51214	0	444	0	83	0	0.21	#DIV/0!	0	2	0	3	0	3	0	3	2.018235
sp|P52907|CAZA1_HUMAN	CAPZA1	F-actin-capping protein subunit alpha-1 OS=Homo sapiens GN=CAPZA1 PE=1 SV=3	33073	0	437	0	84	0	0.33	#DIV/0!	0	2	0	3	0	3	0	3	2.018235
tr|B3KP09|B3KP09_HUMAN	tr|B3KP09	cDNA FLJ30894 fis, clone FEBRA2005416, highly similar to Homo sapiens NOL1/NOP2/Sun domain family, member 2 (NSUN2), mRN	59902	0	428	0	85	0	0.24	#DIV/0!	0	2	0	4	0	3	0	5	2.018235
tr|B4DRW3|B4DRW3_HUMAN	tr|B4DRW3	cDNA FLJ57180, highly similar to TAR DNA-binding protein 43 OS=Homo sapiens PE=2 SV=1	35596	0	412	0	88	0	0.19	#DIV/0!	0	2	0	2	0	3	0	3	2.018235
sp|P30837|AL1B1_HUMAN	ALDH1B1	Aldehyde dehydrogenase X, mitochondrial OS=Homo sapiens GN=ALDH1B1 PE=1 SV=3	57626	0	403	0	90	0	0.12	#DIV/0!	0	2	0	2	0	3	0	3	2.018235
tr|B4DL39|B4DL39_HUMAN	tr|B4DL39	cDNA FLJ57316, highly similar to DNA mismatch repair protein Msh2 OS=Homo sapiens PE=2 SV=1	77368	0	398	0	91	0	0.1	#DIV/0!	0	2	0	3	0	3	0	5	2.018235
tr|A7E2Y5|A7E2Y5_HUMAN	DNAJC13	DnaJ (Hsp40) homolog, subfamily C, member 13 OS=Homo sapiens GN=DNAJC13 PE=2 SV=1	256572	0	394	0	91	0	0.03	#DIV/0!	0	2	0	2	0	3	0	4	2.018235
sp|P36952|SPB5_HUMAN	SERPINB5	Serpin B5 OS=Homo sapiens GN=SERPINB5 PE=1 SV=2	42530	0	387	0	93	0	0.16	#DIV/0!	0	2	0	2	0	3	0	3	2.018235
tr|B4DN49|B4DN49_HUMAN	tr|B4DN49	cDNA FLJ50998, highly similar to DNA mismatch repair protein Msh2 OS=Homo sapiens PE=2 SV=1	97491	0	383	0	94	0	0.07	#DIV/0!	0	2	0	2	0	3	0	4	2.018235
sp|Q96I24|FUBP3_HUMAN	FUBP3	Far upstream element-binding protein 3 OS=Homo sapiens GN=FUBP3 PE=1 SV=2	61944	0	381	0	95	0	0.17	#DIV/0!	0	3	0	3	0	3	0	3	2.018235
tr|A4D275|A4D275_HUMAN	ARPC1B	Actin related protein 2/3 complex, subunit 1B, 41kDa OS=Homo sapiens GN=ARPC1B PE=2 SV=1	41722	0	365	0	101	0	0.16	#DIV/0!	0	1	0	2	0	3	0	3	2.018235
tr|H7C1M2|H7C1M2_HUMAN	SON	Protein SON (Fragment) OS=Homo sapiens GN=SON PE=4 SV=1	160263	0	344	0	111	0	0.06	#DIV/0!	0	3	0	3	0	3	0	3	2.018235
tr|G1UI16|G1UI16_HUMAN	PDS5A	SCC-112 protein, isoform CRA_b OS=Homo sapiens GN=PDS5A PE=2 SV=1	152274	0	337	0	112	0	0.07	#DIV/0!	0	3	0	3	0	3	0	4	2.018235
tr|H3BLU7|H3BLU7_HUMAN	AKR7A2	Aflatoxin B1 aldehyde reductase member 2 (Fragment) OS=Homo sapiens GN=AKR7A2 PE=4 SV=1	35004	0	333	0	107	0	0.27	#DIV/0!	0	2	0	2	0	3	0	4	2.018235
tr|B3KPA6|B3KPA6_HUMAN	ACADVL	Acyl-Coenzyme A dehydrogenase, very long chain, isoform CRA_e OS=Homo sapiens GN=ACADVL PE=2 SV=1	62941	0	296	0	129	0	0.17	#DIV/0!	0	3	0	3	0	3	0	3	2.018235
tr|Q6NUR1|Q6NUR1_HUMAN	NCAPG	Non-SMC condensin I complex, subunit G OS=Homo sapiens GN=NCAPG PE=2 SV=1	115205	0	294	0	132	0	0.09	#DIV/0!	0	2	0	3	0	3	0	5	2.018235
sp|O43815|STRN_HUMAN	STRN	Striatin OS=Homo sapiens GN=STRN PE=1 SV=4	86535	0	286	0	136	0	0.12	#DIV/0!	0	2	0	3	0	3	0	6	2.018235
tr|A8K0D2|A8K0D2_HUMAN	tr|A8K0D2	cDNA FLJ77740, highly similar to Homo sapiens 7-dehydrocholesterol reductase, mRNA OS=Homo sapiens PE=2 SV=1	55175	0	251	0	155	0	0.19	#DIV/0!	0	3	0	3	0	3	0	3	2.018235

tr|B3KVT9|B3KVT9_HUMAN	tr|B3KVT9	cDNA FLJ41424 fis, clone BRHIP2003748, highly similar to Tumor suppressor p53-binding protein 1 OS=Homo sapiens PE=2 SV=1	43438	0	216	0	85	0	0.08	#DIV/0!	0	2	0	3	0	3	0	3	2.018235
sp|Q9NTI5|PDS5B_HUMAN	PDS5B	Sister chromatid cohesion protein PDS5 homolog B OS=Homo sapiens GN=PDS5B PE=1 SV=1	165818	0	1191	0	17	0	0.04	#DIV/0!	0	0	0	2	0	2	0	4	1.532612
tr|H0Y7P1|H0Y7P1_HUMAN	MED12	Mediator of RNA polymerase II transcription subunit 12 (Fragment) OS=Homo sapiens GN=MED12 PE=4 SV=1	240034	0	1147	0	22	0	0.01	#DIV/0!	0	1	0	1	0	2	0	3	1.532612
tr|B4DFN6|B4DFN6_HUMAN	CNP	2~,3~-cyclic-nucleotide 3~-phosphodiesterase OS=Homo sapiens GN=CNP PE=2 SV=1	20461	0	1142	0	23	0	0.16	#DIV/0!	0	0	0	1	0	2	0	3	1.532612
sp|Q9UKX7|NUP50_HUMAN	NUP50	Nuclear pore complex protein Nup50 OS=Homo sapiens GN=NUP50 PE=1 SV=2	50512	0	1128	0	24	0	0.07	#DIV/0!	0	0	0	1	0	2	0	2	1.532612
tr|B2RWN5|B2RWN5_HUMAN	HEATR1	HEAT repeat containing 1 OS=Homo sapiens GN=HEATR1 PE=2 SV=1	244275	0	1096	0	26	0	0.03	#DIV/0!	0	1	0	2	0	2	0	4	1.532612
tr|B3KM68|B3KM68_HUMAN	tr|B3KM6	cDNA FLJ10398 fis, clone NT2RM4000349, highly similar to Homo sapiens basic leucine zipper and W2 domains 2 (BZW2), mRNA OS	48388	0	1072	0	27	0	0.07	#DIV/0!	0	1	0	1	0	2	0	2	1.532612
tr|Q0PHS4|Q0PHS4_HUMAN	G6PD	Glucose-6-phosphate dehydrogenase (Fragment) OS=Homo sapiens GN=G6PD PE=2 SV=1	7182	0	1039	0	29	0	0.49	#DIV/0!	0	1	0	1	0	2	0	2	1.532612
tr|B0YIW5|B0YIW5_HUMAN	ARCN1	Archain 1, isoform CRA_b OS=Homo sapiens GN=ARCN1 PE=2 SV=1	57630	0	1036	0	29	0	0.06	#DIV/0!	0	0	0	1	0	2	0	4	1.532612
tr|B4DN85|B4DN85_HUMAN	tr|B4DN8	E3 ubiquitin-protein ligase OS=Homo sapiens PE=2 SV=1	93815	0	1030	0	29	0	0.03	#DIV/0!	0	0	0	1	0	2	0	2	1.532612
tr|F6IQC8|F6IQC8_HUMAN	HLA-C	MHC class I antigen (Fragment) OS=Homo sapiens GN=HLA-C PE=3 SV=1	39543	0	1024	0	30	0	0.08	#DIV/0!	0	0	0	1	0	2	0	4	1.532612
tr|B7Z2D6|B7Z2D6_HUMAN	tr|B7Z2D6	cDNA FLJ53863, highly similar to Cystathionine beta-synthase (EC 4.2.1.22) OS=Homo sapiens PE=2 SV=1	56409	0	1015	0	30	0	0.06	#DIV/0!	0	0	0	1	0	2	0	3	1.532612
sp|Q9ULT8|HECD1_HUMAN	HECTD1	E3 ubiquitin-protein ligase HECTD1 OS=Homo sapiens GN=HECTD1 PE=1 SV=3	292225	0	1012	0	30	0	0.01	#DIV/0!	0	0	0	1	0	2	0	2	1.532612
tr|B2R7T6|B2R7T6_HUMAN	tr|B2R7T6	cDNA, FLJ93596, highly similar to Homo sapiens sulfide quinone reductase-like (yeast) (SQRDL), mRNA OS=Homo sapiens PE=2 SV=1	50199	0	1006	0	31	0	0.14	#DIV/0!	0	0	0	2	0	2	0	2	1.532612
tr|B2R6E5|B2R6E5_HUMAN	tr|B2R6E5	NADPH--cytochrome P450 reductase OS=Homo sapiens PE=2 SV=1	77429	0	1000	0	31	0	0.04	#DIV/0!	0	0	0	1	0	2	0	3	1.532612
tr|B4DPV3|B4DPV3_HUMAN	tr|B4DPV	cDNA FLJ58109, highly similar to Protein diaphanous homolog 3 (Fragment) OS=Homo sapiens PE=2 SV=1	117640	0	999	0	31	0	0.03	#DIV/0!	0	0	0	1	0	2	0	2	1.532612
tr|F5H8E5|F5H8E5_HUMAN	USP7	Ubiquitin carboxyl-terminal hydrolase OS=Homo sapiens GN=USP7 PE=2 SV=1	117951	0	996	0	31	0	0.03	#DIV/0!	0	0	0	1	0	2	0	4	1.532612
tr|G1UI18|G1UI18_HUMAN	PSME4	Proteasome activator complex subunit 4 (Fragment) OS=Homo sapiens GN=PSME4 PE=2 SV=1	130054	0	981	0	32	0	0.03	#DIV/0!	0	1	0	2	0	2	0	2	1.532612
tr|H7C5W5|H7C5W5_HUMAN	PRPH	Peripherin (Fragment) OS=Homo sapiens GN=PRPH PE=3 SV=1	22980	0	978	0	32	0	0.15	#DIV/0!	0	0	0	1	0	2	0	3	1.532612
tr|B3KMR5|B3KMR5_HUMAN	tr|B3KMR	cDNA FLJ12434 fis, clone NT2RM1000037, highly similar to Homo sapiens KIAA0690 protein OS=Homo sapiens PE=2 SV=1	145007	0	976	0	32	0	0.05	#DIV/0!	0	1	0	2	0	2	0	2	1.532612
tr|G1UI17|G1UI17_HUMAN	AGL	Glycogen debranching enzyme (Fragment) OS=Homo sapiens GN=AGL PE=2 SV=1	145569	0	945	0	34	0	0.07	#DIV/0!	0	0	0	3	0	2	0	3	1.532612
sp|P51116|FXR2_HUMAN	FXR2	Fragile X mental retardation syndrome-related protein 2 OS=Homo sapiens GN=FXR2 PE=1 SV=2	74520	0	938	0	34	0	0.04	#DIV/0!	0	0	0	1	0	2	0	4	1.532612
sp|A0AVT1|UBA6_HUMAN	UBA6	Ubiquitin-like modifier-activating enzyme 6 OS=Homo sapiens GN=UBA6 PE=1 SV=1	119207	0	926	0	35	0	0.06	#DIV/0!	0	0	0	2	0	2	0	2	1.532612
tr|E7ESC6|E7ESC6_HUMAN	XPO7	Exportin-7 OS=Homo sapiens GN=XPO7 PE=2 SV=1	125356	0	922	0	35	0	0.33	#DIV/0!	0	0	0	2	0	2	0	2	1.532612
tr|B3KM47|B3KM47_HUMAN	tr|B3KM4	cDNA FLJ10273 fis, clone HEMBB1001137, highly similar to SEC23-interacting protein OS=Homo sapiens PE=2 SV=1	111730	0	915	0	36	0	0.03	#DIV/0!	0	0	0	1	0	2	0	2	1.532612
sp|Q5JWF2|GNAS1_HUMAN	GNAS	Guanine nucleotide-binding protein G(s) subunit alpha isoforms XLas OS=Homo sapiens GN=GNAS PE=1 SV=2	111697	0	906	0	36	0	0.06	#DIV/0!	0	1	0	2	0	2	0	3	1.532612
tr|Q6IBU0|Q6IBU0_HUMAN	EIF5	EIF5 protein OS=Homo sapiens GN=EIF5 PE=2 SV=1	49577	0	890	0	37	0	0.07	#DIV/0!	0	1	0	1	0	2	0	2	1.532612
tr|H7C3P6|H7C3P6_HUMAN	NUP98	Nuclear pore complex protein Nup98-Nup96 (Fragment) OS=Homo sapiens GN=NUP98 PE=4 SV=1	86760	0	873	0	38	0	0.08	#DIV/0!	0	0	0	2	0	2	0	4	1.532612
tr|I6L9B1|I6L9B1_HUMAN	EPB41L2	EPB41L2 protein OS=Homo sapiens GN=EPB41L2 PE=2 SV=1	71998	0	850	0	39	0	0.05	#DIV/0!	0	1	0	1	0	2	0	2	1.532612
tr|B7Z709|B7Z709_HUMAN	tr|B7Z709	cDNA FLJ50250, highly similar to Structural maintenance of chromosome 1-like 1 protein OS=Homo sapiens PE=2 SV=1	59338	0	848	0	40	0	0.06	#DIV/0!	0	1	0	1	0	2	0	3	1.532612
sp|Q08499|PDE4D_HUMAN	PDE4D	cAMP-specific 3~,5~-cyclic phosphodiesterase 4D OS=Homo sapiens GN=PDE4D PE=1 SV=2	91628	0	846	0	40	0	0.07	#DIV/0!	0	1	0	1	0	2	0	2	1.532612
tr|B3KVJ5|B3KVJ5_HUMAN	tr|B3KVJ5	cDNA FLJ16648 fis, clone TESTI4035508, highly similar to Structural maintenance of chromosomes 4-like 1 protein OS=Homo sapiens	101354	0	840	0	38	0	0.05	#DIV/0!	0	1	0	1	0	2	0	3	1.532612
tr|H0Y4T7|H0Y4T7_HUMAN	MTA1	Metastasis-associated protein MTA1 (Fragment) OS=Homo sapiens GN=MTA1 PE=4 SV=1	57747	0	839	0	29	0	0.13	#DIV/0!	0	0	0	2	0	2	0	2	1.532612
tr|B2RDG1|B2RDG1_HUMAN	tr|B2RDG	cDNA, FLJ96593 OS=Homo sapiens PE=2 SV=1	59687	0	838	0	40	0	0.11	#DIV/0!	0	1	0	2	0	2	0	2	1.532612
tr|F6IQM8|F6IQM8_HUMAN	HLA-C	MHC class I antigen (Fragment) OS=Homo sapiens GN=HLA-C PE=3 SV=1	39497	0	834	0	41	0	0.08	#DIV/0!	0	1	0	1	0	2	0	3	1.532612
tr|Q5W1M0|Q5W1M0_HUMA	HLA-C	MHC class I antigen (Fragment) OS=Homo sapiens GN=HLA-C PE=2 SV=1	10534	0	832	0	41	0	0.08	#DIV/0!	0	1	0	1	0	2	0	2	1.532612
sp|Q6PKG0|LARP1_HUMAN	LARP1	La-related protein 1 OS=Homo sapiens GN=LARP1 PE=1 SV=2	123833	0	829	0	41	0	0.03	#DIV/0!	0	1	0	1	0	2	0	2	1.532612
tr|B3KY04|B3KY04_HUMAN	tr|B3KY04	cDNA FLJ46506 fis, clone THYMU3030752, highly similar to BTB/POZ domain-containing protein KCTD12 OS=Homo sapiens PE=2 SV	35994	0	825	0	41	0	0.19	#DIV/0!	0	1	0	2	0	2	0	2	1.532612
tr|B4DN01|B4DN01_HUMAN	tr|B4DN0	cDNA FLJ57332, highly similar to COP9 signalosome complex subunit 3 OS=Homo sapiens PE=2 SV=1	33950	0	822	0	41	0	0.1	#DIV/0!	0	1	0	1	0	2	0	2	1.532612
tr|B4E206|B4E206_HUMAN	tr|B4E206	cDNA FLJ55441, highly similar to Nuclear pore complex protein Nup133 OS=Homo sapiens PE=2 SV=1	128349	0	819	0	42	0	0.03	#DIV/0!	0	1	0	1	0	2	0	2	1.532612
tr|B1AKJ6|B1AKJ6_HUMAN	OSBPL9	Oxysterol-binding protein OS=Homo sapiens GN=OSBPL9 PE=2 SV=1	84417	0	811	0	42	0	0.08	#DIV/0!	0	1	0	2	0	2	0	2	1.532612
tr|B4DYY5|B4DYY5_HUMAN	tr|B4DYY5	cDNA FLJ55671, highly similar to Ubiquitin-associated protein 2-like OS=Homo sapiens PE=2 SV=1	113662	0	802	0	42	0	0.06	#DIV/0!	0	1	0	2	0	2	0	3	1.532612
sp|Q9UID3|VPS51_HUMAN	VPS51	Vacuolar protein sorting-associated protein 51 homolog OS=Homo sapiens GN=VPS51 PE=1 SV=2	86901	0	795	0	43	0	0.08	#DIV/0!	0	2	0	2	0	2	0	4	1.532612
tr|J3KTL8|J3KTL8_HUMAN	SMCHD1	Structural maintenance of chromosomes flexible hinge domain-containing protein 1 (Fragment) OS=Homo sapiens GN=SMCHD1 PE	156690	0	785	0	44	0	0.04	#DIV/0!	0	1	0	2	0	2	0	3	1.532612
tr|B3KVA0|B3KVA0_HUMAN	tr|B3KVA0	Cytosine-specific methyltransferase OS=Homo sapiens PE=2 SV=1	61027	0	773	0	45	0	0.11	#DIV/0!	0	1	0	2	0	2	0	2	1.532612
tr|B2R6H7|B2R6H7_HUMAN	tr|B2R6H7	cDNA, FLJ92955, highly similar to Homo sapiens transportin-SR (TRN-SR), mRNA OS=Homo sapiens PE=2 SV=1	105989	0	769	0	45	0	0.06	#DIV/0!	0	1	0	2	0	2	0	3	1.532612
sp|Q5T8P6|RBM26_HUMAN	RBM26	RNA-binding protein 26 OS=Homo sapiens GN=RBM26 PE=1 SV=3	113927	0	767	0	45	0	0.03	#DIV/0!	0	1	0	1	0	2	0	2	1.532612
tr|B4DPJ2|B4DPJ2_HUMAN	tr|B4DPJ2	Annexin OS=Homo sapiens PE=2 SV=1	46025	0	765	0	45	0	0.15	#DIV/0!	0	0	0	2	0	2	0	2	1.532612
tr|Q6FHH6|Q6FHH6_HUMAN	LANCL1	LANCL1 protein (Fragment) OS=Homo sapiens GN=LANCL1 PE=2 SV=1	45979	0	748	0	46	0	0.07	#DIV/0!	0	1	0	1	0	2	0	3	1.532612
sp|P37837|TALDO_HUMAN	TALDO1	Transaldolase OS=Homo sapiens GN=TALDO1 PE=1 SV=2	37688	0	735	0	47	0	0.09	#DIV/0!	0	1	0	1	0	2	0	3	1.532612
tr|Q53G71|Q53G71_HUMAN	tr|Q53G7	Calreticulin variant (Fragment) OS=Homo sapiens PE=2 SV=1	47061	0	725	0	48	0	0.29	#DIV/0!	0	1	0	2	0	2	0	2	1.532612
sp|P61073|CXCR4_HUMAN	CXCR4	C-X-C chemokine receptor type 4 OS=Homo sapiens GN=CXCR4 PE=1 SV=1	40233	0	724	0	48	0	0.17	#DIV/0!	0	1	0	2	0	2	0	2	1.532612
sp|P04181|OAT_HUMAN	OAT	Ornithine aminotransferase, mitochondrial OS=Homo sapiens GN=OAT PE=1 SV=1	48846	0	714	0	49	0	0.14	#DIV/0!	0	2	0	2	0	2	0	2	1.532612
tr|H0Y3P2|H0Y3P2_HUMAN	EIF4G2	Eukaryotic translation initiation factor 4 gamma 2 OS=Homo sapiens GN=EIF4G2 PE=4 SV=1	98569	0	707	0	50	0	0.03	#DIV/0!	0	1	0	1	0	2	0	3	1.532612
tr|G3V3B0|G3V3B0_HUMAN	ACIN1	Apoptotic chromatin condensation inducer in the nucleus OS=Homo sapiens GN=ACIN1 PE=2 SV=1	67683	0	703	0	50	0	0.05	#DIV/0!	0	1	0	1	0	2	0	3	1.532612
tr|C0KK01|C0KK01_HUMAN	HLA-C	MHC class I antigen (Fragment) OS=Homo sapiens GN=HLA-C PE=3 SV=1	20940	0	699	0	50	0	0.16	#DIV/0!	0	1	0	1	0	2	0	3	1.532612
tr|Q6FGM0|Q6FGM0_HUMAN	SH3GL1	SH3 domain GRB2-like 1 OS=Homo sapiens GN=SH3GL1 PE=2 SV=1	41692	0	693	0	50	0	0.16	#DIV/0!	0	1	0	2	0	2	0	2	1.532612
tr|B4DET0|B4DET0_HUMAN	tr|B4DET0	cDNA FLJ55900, highly similar to Pre-mRNA-splicing factor ATP-dependent RNA helicase PRP16 (EC 3.6.1.-) OS=Homo sapiens PE=2 S	140449	0	683	0	51	0	0.05	#DIV/0!	0	2	0	2	0	2	0	2	1.532612
tr|B1AJV0|B1AJV0_HUMAN	SMARCA1	SWI/SNF related, matrix associated, actin dependent regulator of chromatin, subfamily a, member 1 (Fragment) OS=Homo sapiens (	117875	0	681	0	51	0	0.03	#DIV/0!	0	1	0	1	0	2	0	4	1.532612
sp|P04792|HSPB1_HUMAN	HSPB1	Heat shock protein beta-1 OS=Homo sapiens GN=HSPB1 PE=1 SV=2	22826	0	674	0	52	0	0.31	#DIV/0!	0	1	0	2	0	2	0	2	1.532612
sp|Q9GZL7|WDR12_HUMAN	WDR12	Ribosome biogenesis protein WDR12 OS=Homo sapiens GN=WDR12 PE=1 SV=2	48191	0	672	0	52	0	0.14	#DIV/0!	0	1	0	2	0	2	0	2	1.532612
tr|B4DUT7|B4DUT7_HUMAN	tr|B4DUT	cDNA FLJ57604, highly similar to GMP synthase (glutamine-hydrolyzing) (EC 6.3.5.2) OS=Homo sapiens PE=2 SV=1	71880	0	671	0	37	0	0.13	#DIV/0!	0	1	0	2	0	2	0	2	1.532612
tr|B7ZMF2|B7ZMF2_HUMAN	FANCI	Fanconi anemia, complementation group I OS=Homo sapiens GN=FANCI PE=2 SV=1	150724	0	668	0	52	0	0.04	#DIV/0!	0	2	0	2	0	2	0	2	1.532612
sp|P05556|ITB1_HUMAN	ITGB1	Integrin beta-1 OS=Homo sapiens GN=ITGB1 PE=1 SV=2	91664	0	661	0	52	0	0.07	#DIV/0!	0	1	0	2	0	2	0	3	1.532612
sp|Q9Y6D5|BIG2_HUMAN	ARFGEF2	Brefeldin A-inhibited guanine nucleotide-exchange protein 2 OS=Homo sapiens GN=ARFGEF2 PE=1 SV=3	204418	0	658	0	53	0	0.03	#DIV/0!	0	1	0	2	0	2	0	3	1.532612
tr|G5E9P1|G5E9P1_HUMAN	ITPR1	Inositol 1,4,5-triphosphate receptor, type 1, isoform CRA_d OS=Homo sapiens GN=ITPR1 PE=2 SV=1	311832	0	656	0	53	0	0.02	#DIV/0!	0	1	0	3	0	2	0	3	1.532612
sp|Q06210|GFPT1_HUMAN	GFPT1	Glutamine--fructose-6-phosphate aminotransferase [isomerizing] 1 OS=Homo sapiens GN=GFPT1 PE=1 SV=3	79555	0	650	0	54	0	0.08	#DIV/0!	0	1	0	2	0	2	0	3	1.532612
tr|H0YHH6|H0YHH6_HUMAN	ATP2B1	Plasma membrane calcium-transporting ATPase 1 (Fragment) OS=Homo sapiens GN=ATP2B1 PE=4 SV=1	18678	0	643	0	55	0	0.18	#DIV/0!	0	1	0	1	0	2	0	3	1.532612
sp|O00154|BACH_HUMAN	ACOT7	Cytosolic acyl coenzyme A thioester hydrolase OS=Homo sapiens GN=ACOT7 PE=1 SV=3	42454	0	634	0	56	0	0.22	#DIV/0!	0	2	0	2	0	2	0	2	1.532612
sp|Q05048|CSTF1_HUMAN	CSTF1	Cleavage stimulation factor subunit 1 OS=Homo sapiens GN=CSTF1 PE=1 SV=1	49125	0	633	0	56	0	0.07	#DIV/0!	0	1	0	1	0	2	0	2	1.532612
tr|H3BQP1|H3BQP1_HUMAN	USP10	Ubiquitin carboxyl-terminal hydrolase 10 (Fragment) OS=Homo sapiens GN=USP10 PE=4 SV=1	12544	0	620	0	57	0	0.27	#DIV/0!	0	1	0	1	0	2	0	3	1.532612
tr|H7BZF2|H7BZF2_HUMAN	DKC1	H/ACA ribonucleoprotein complex subunit 4 (Fragment) OS=Homo sapiens GN=DKC1 PE=4 SV=1	8803	0	606	0	53	0	0.27	#DIV/0!	0	1	0	1	0	2	0	2	1.532612
sp|O75131|CPNE3_HUMAN	CPNE3	Copine-3 OS=Homo sapiens GN=CPNE3 PE=1 SV=1	60947	0	593	0	60	0	0.11	#DIV/0!	0	2	0	2	0	2	0	2	1.532612
sp|Q14558|KPRA_HUMAN	PRPSAP1	Phosphoribosyl pyrophosphate synthase-associated protein 1 OS=Homo sapiens GN=PRPSAP1 PE=1 SV=2	39654	0	587	0	61	0	0.17	#DIV/0!	0	2	0	2	0	2	0	3	1.532612
sp|Q14692|BMS1_HUMAN	BMS1	Ribosome biogenesis protein BMS1 homolog OS=Homo sapiens GN=BMS1 PE=1 SV=1	146571	0	581	0	61	0	0.07	#DIV/0!	0	2	0	3	0	2	0	4	1.532612
tr|Q0VAB1|Q0VAB1_HUMAN	TIMM50	Translocase of inner mitochondrial membrane 50 homolog (S. cerevisiae) OS=Homo sapiens GN=TIMM50 PE=2 SV=1	50960	0	575	0	62	0	0.13	#DIV/0!	0	2	0	2	0	2	0	2	1.532612
tr|B3KM89|B3KM89_HUMAN	tr|B3KM8	cDNA FLJ10528 fis, clone NT2RP2000943, highly similar to Protein transport protein Sec24D OS=Homo sapiens PE=2 SV=1	101723	0	572	0	62	0	0.03	#DIV/0!	0	1	0	1	0	2	0	2	1.532612
tr|Q6FHJ5|Q6FHJ5_HUMAN	SCAMP3	SCAMP3 protein (Fragment) OS=Homo sapiens GN=SCAMP3 PE=2 SV=1	38674	0	554	0	64	0	0.18	#DIV/0!	0	2	0	2	0	2	0	2	1.532612
tr|B4DMU7|B4DMU7_HUMAN	tr|B4DMU	cDNA FLJ59351, highly similar to Striatin-4 OS=Homo sapiens PE=2 SV=1	69271	0	540	0	66	0	0.1	#DIV/0!	0	2	0	2	0	2	0	3	1.532612
tr|Q5H907|Q5H907_HUMAN	MAGED2	Melanoma antigen family D, 2, isoform CRA_d OS=Homo sapiens GN=MAGED2 PE=4 SV=1	55932	0	532	0	67	0	0.27	#DIV/0!	0	1	0	2	0	2	0	2	1.532612
tr|G5E9H4|G5E9H4_HUMAN	DCTN1	Dynactin 1 (P150, glued homolog, Drosophila), isoform CRA_a OS=Homo sapiens GN=DCTN1 PE=2 SV=1	127339	0	526	0	68	0	0.05	#DIV/0!	0	2	0	2	0	2	0	2	1.532612
tr|Q3LIF4|Q3LIF4_HUMAN	Nbla0027	NOGO-A (Fragment) OS=Homo sapiens GN=Nbla00271 PE=2 SV=1	57162	0	518	0	42	0	0.29	#DIV/0!	0	1	0	2	0	2	0	2	1.532612
sp|Q8NI27|THOC2_HUMAN	THOC2	THO complex subunit 2 OS=Homo sapiens GN=THOC2 PE=1 SV=2	184541	0	511	0	71	0	0.04	#DIV/0!	0	2	0	2	0	2	0	3	1.532612
tr|Q6ZVI6|Q6ZVI6_HUMAN	tr|Q6ZVI6	cDNA FLJ42537 fis, clone BRACE3004058, highly similar to NADH-cytochrome B5 reductase (EC 1.6.2.2) OS=Homo sapiens PE=2 SV=1	29141	0	506	0	72	0	0.24	#DIV/0!	0	2	0	2	0	2	0	2	1.532612
sp|Q8IXI1|MIRO2_HUMAN	RHOT2	Mitochondrial Rho GTPase 2 OS=Homo sapiens GN=RHOT2 PE=1 SV=2	69101	0	501	0	73	0	0.1	#DIV/0!	0	2	0	2	0	2	0	2	1.532612
tr|B4DE85|B4DE85_HUMAN	PTDSS1	Phosphatidylserine synthase 1 OS=Homo sapiens GN=PTDSS1 PE=2 SV=1	35127	0	498	0	51	0	0.26	#DIV/0!	0	1	0	2	0	2	0	2	1.532612
tr|B0I1S9|B0I1S9_HUMAN	MYO1B	MYO1B variant protein OS=Homo sapiens GN=MYO1B PE=2 SV=1	132928	0	493	0	74	0	0.02	#DIV/0!	0	1	0	1	0	2	0	2	1.532612
tr|B4DHY1|B4DHY1_HUMAN	HNRNPH3	Heterogeneous nuclear ribonucleoprotein H3 OS=Homo sapiens GN=HNRNPH3 PE=2 SV=1	24925	0	488	0	74	0	0.29	#DIV/0!	0	2	0	2	0	2	0	2	1.532612
tr|Q2TAM5|Q2TAM5_HUMAN	RELA	RELA protein OS=Homo sapiens GN=RELA PE=2 SV=1	43355	0	467	0	78	0	0.16	#DIV/0!	0	2	0	2	0	2	0	2	1.532612
tr|I3L495|I3L495_HUMAN	PAFAH1B1	Platelet-activating factor acetylhydrolase IB subunit alpha (Fragment) OS=Homo sapiens GN=PAFAH1B1 PE=4 SV=1	9626	0	466	0	34	0	0.31	#DIV/0!	0	0	0	2	0	2	0	2	1.532612
sp|P55268|LAMB2_HUMAN	LAMB2	Laminin subunit beta-2 OS=Homo sapiens GN=LAMB2 PE=1 SV=2	202982	0	463	0	79	0	0.03	#DIV/0!	0	1	0	2	0	2	0	4	1.532612
tr|Q6FHX6|Q6FHX6_HUMAN	FEN1	Flap endonuclease 1 OS=Homo sapiens GN=FEN1 PE=2 SV=1	42908	0	454	0	81	0	0.18	#DIV/0!	0	1	0	2	0	2	0	2	1.532612
sp|Q08379|GOGA2_HUMAN	GOLGA2	Golgin subfamily A member 2 OS=Homo sapiens GN=GOLGA2 PE=1 SV=3	113644	0	451	0	81	0	0.06	#DIV/0!	0	2	0	2	0	2	0	3	1.532612
sp|P62333|PRS10_HUMAN	PSMC6	26S protease regulatory subunit 10B OS=Homo sapiens GN=PSMC6 PE=1 SV=1	44430	0	442	0	83	0	0.15	#DIV/0!	0	2	0	2	0	2	0	2	1.532612
sp|Q9BWF3|RBM4_HUMAN	RBM4	RNA-binding protein 4 OS=Homo sapiens GN=RBM4 PE=1 SV=1	40688	0	440	0	83	0	0.08	#DIV/0!	0	1	0	1	0	2	0	2	1.532612
tr|J3KPP4|J3KPP4_HUMAN	LUC7L3	Cisplatin resistance-associated overexpressed protein, isoform CRA_b OS=Homo sapiens GN=LUC7L3 PE=4 SV=1	58698	0	415	0	88	0	0.12	#DIV/0!	0	1	0	2	0	2	0	2	1.532612
tr|I6L9E8|I6L9E8_HUMAN	FAM98A	Family with sequence similarity 98, member A OS=Homo sapiens GN=FAM98A PE=2 SV=1	55595	0	405	0	89	0	0.12	#DIV/0!	0	1	0	2	0	2	0	2	1.532612
sp|P40222|TXLNA_HUMAN	TXLNA	Alpha-taxilin OS=Homo sapiens GN=TXLNA PE=1 SV=3	62195	0	404	0	89	0	0.11	#DIV/0!	0	1	0	2	0	2	0	2	1.532612
tr|J3QQ67|J3QQ67_HUMAN	RPL18	60S ribosomal protein L18 (Fragment) OS=Homo sapiens GN=RPL18 PE=3 SV=1	21940	0	371	0	100	0	0.38	#DIV/0!	0	2	0	2	0	2	0	2	1.532612
tr|Q4KMR3|Q4KMR3_HUMAN	MYO1E	MYO1E variant protein OS=Homo sapiens GN=MYO1E PE=2 SV=1	127552	0	370	0	100	0	0.05	#DIV/0!	0	2	0	2	0	2	0	2	1.532612
tr|H0YCK3|H0YCK3_HUMAN	ADAR	Double-stranded RNA-specific adenosine deaminase (Fragment) OS=Homo sapiens GN=ADAR PE=4 SV=1	133761	0	361	0	73	0	0.09	#DIV/0!	0	2	0	2	0	2	0	5	1.532612
sp|O43837|IDH3B_HUMAN	IDH3B	Isocitrate dehydrogenase [NAD] subunit beta, mitochondrial OS=Homo sapiens GN=IDH3B PE=1 SV=2	42442	0	331	0	113	0	0.16	#DIV/0!	0	2	0	2	0	2	0	3	1.532612
tr|E7EMC7|E7EMC7_HUMAN	SQSTM1	Sequestosome-1 OS=Homo sapiens GN=SQSTM1 PE=2 SV=1	42070	0	320	0	56	0	0.35	#DIV/0!	0	1	0	2	0	2	0	2	1.532612
tr|Q4ZG72|Q4ZG72_HUMAN	DDX18	Putative uncharacterized protein DDX18 (Fragment) OS=Homo sapiens GN=DDX18 PE=2 SV=1	61898	0	1175	0	19	0	0.05	#DIV/0!	0	0	0	1	0	1	0	2	0.795451
tr|B4E0N6|B4E0N6_HUMAN	tr|B4E0N6	cDNA FLJ56280, highly similar to Endoplasmic reticulum-Golgi intermediate compartment protein 1 OS=Homo sapiens PE=2 SV=1	26584	0	1162	0	20	0	0.13	#DIV/0!	0	0	0	1	0	1	0	1	0.795451
sp|P24928|RPB1_HUMAN	POLR2A	DNA-directed RNA polymerase II subunit RPB1 OS=Homo sapiens GN=POLR2A PE=1 SV=2	218408	0	1157	0	21	0	0.04	#DIV/0!	0	0	0	1	0	1	0	1	0.795451
tr|B7Z6H4|B7Z6H4_HUMAN	tr|B7Z6H4	DNA-directed RNA polymerase OS=Homo sapiens PE=2 SV=1	155407	0	1154	0	21	0	0.02	#DIV/0!	0	0	0	1	0	1	0	1	0.795451
sp|Q04941|PLP2_HUMAN	PLP2	Proteolipid protein 2 OS=Homo sapiens GN=PLP2 PE=1 SV=1	17022	0	1152	0	22	0	0.2	#DIV/0!	0	0	0	1	0	1	0	1	0.795451
tr|H0Y6I6|H0Y6I6_HUMAN	FBXL18	F-box/LRR-repeat protein 18 (Fragment) OS=Homo sapiens GN=FBXL18 PE=4 SV=1	76506	0	1151	0	22	0	0.04	#DIV/0!	0	0	0	1	0	1	0	1	0.795451
sp|P14735|IDE_HUMAN	IDE	Insulin-degrading enzyme OS=Homo sapiens GN=IDE PE=1 SV=4	118692	0	1150	0	22	0	0.03	#DIV/0!	0	0	0	1	0	1	0	1	0.795451
tr|B4DJB4|B4DJB4_HUMAN	tr|B4DJB4	cDNA FLJ55931, highly similar to Isocitrate dehydrogenase OS=Homo sapiens PE=2 SV=1	34940	0	1144	0	22	0	0.28	#DIV/0!	0	0	0	1	0	1	0	1	0.795451
tr|B4DWN1|B4DWN1_HUMAN	tr|B4DWN	cDNA FLJ52285, highly similar to Vesicular integral-membrane protein VIP36 OS=Homo sapiens PE=2 SV=1	32842	0	1138	0	23	0	0.1	#DIV/0!	0	0	0	1	0	1	0	1	0.795451
tr|B8QFA1|B8QFA1_HUMAN	PKP2	Plakophilin-2 transcript variant 2b OS=Homo sapiens GN=PKP2 PE=2 SV=1	97841	0	1133	0	24	0	0.03	#DIV/0!	0	1	0	1	0	1	0	2	0.795451
tr|B4DDL6|B4DDL6_HUMAN	tr|B4DDL	cDNA FLJ54325, highly similar to Myotubularin-related protein 2 (EC 3.1.3.-) OS=Homo sapiens PE=2 SV=1	36644	0	1130	0	24	0	0.04	#DIV/0!	0	0	0	1	0	1	0	1	0.795451
tr|B4E2K4|B4E2K4_HUMAN	tr|B4E2K4	cDNA FLJ54576, highly similar to Aspartyl/asparaginyl beta-hydroxylase (EC 1.14.11.16) OS=Homo sapiens PE=2 SV=1	83685	0	1129	0	24	0	0.04	#DIV/0!	0	0	0	1	0	1	0	1	0.795451
tr|H3BNQ7|H3BNQ7_HUMAN	ABAT	4-aminobutyrate aminotransferase, mitochondrial OS=Homo sapiens GN=ABAT PE=3 SV=1	58741	0	1125	0	24	0	0.06	#DIV/0!	0	1	0	1	0	1	0	1	0.795451
sp|Q9H488|OFUT1_HUMAN	POFUT1	GDP-fucose protein O-fucosyltransferase 1 OS=Homo sapiens GN=POFUT1 PE=1 SV=1	44383	0	1124	0	24	0	0.07	#DIV/0!	0	0	0	1	0	1	0	1	0.795451
tr|H0YDD4|H0YDD4_HUMAN	DLAT	Dihydrolipoyllysine-residue acetyltransferase component of pyruvate dehydrogenase complex, mitochondrial (Fragment) OS=Homo	51511	0	1122	0	24	0	0.06	#DIV/0!	0	0	0	1	0	1	0	2	0.795451
tr|Q0VAM0|Q0VAM0_HUMAN	CTNNBL1	Catenin, beta like 1 OS=Homo sapiens GN=CTNNBL1 PE=2 SV=1	65560	0	1120	0	25	0	0.05	#DIV/0!	0	0	0	1	0	1	0	1	0.795451
tr|Q6MZP3|Q6MZP3_HUMAN	DKFZp686	Putative uncharacterized protein DKFZp686I05169 OS=Homo sapiens GN=DKFZp686I05169 PE=2 SV=1	146779	0	1118	0	25	0	0.02	#DIV/0!	0	0	0	1	0	1	0	1	0.795451
tr|Q5XJ04|Q5XJ04_HUMAN	HMGCS1	3-hydroxy-3-methylglutaryl-Coenzyme A synthase 1 (Soluble) OS=Homo sapiens GN=HMGCS1 PE=2 SV=1	57854	0	1115	0	25	0	0.12	#DIV/0!	0	0	0	2	0	1	0	2	0.795451
tr|D3DTC2|D3DTC2_HUMAN	AYTL2	Acyltransferase like 2, isoform CRA_a OS=Homo sapiens GN=AYTL2 PE=2 SV=1	54725	0	1113	0	25	0	0.06	#DIV/0!	0	0	0	1	0	1	0	1	0.795451
tr|K7EKI4|K7EKI4_HUMAN	MRPL4	39S ribosomal protein L4, mitochondrial OS=Homo sapiens GN=MRPL4 PE=4 SV=1	14161	0	1108	0	25	0	0.24	#DIV/0!	0	0	0	1	0	1	0	1	0.795451
sp|Q68E01|INT3_HUMAN	INTS3	Integrator complex subunit 3 OS=Homo sapiens GN=INTS3 PE=1 SV=1	119533	0	1107	0	26	0	0.06	#DIV/0!	0	0	0	2	0	1	0	3	0.795451
tr|F2ZC09|F2ZC09_HUMAN	TRIP6i4	Thyroid hormone receptor interacting protein 6 isoform 4 OS=Homo sapiens GN=TRIP6i4 PE=2 SV=1	17368	0	1106	0	26	0	0.07	#DIV/0!	0	0	0	1	0	1	0	1	0.795451
tr|Q5DT20|Q5DT20_HUMAN	HRNR	Hornerin OS=Homo sapiens GN=HRNR PE=2 SV=1	283111	0	1104	0	26	0	0.01	#DIV/0!	0	0	0	1	0	1	0	2	0.795451
sp|Q9Y2H6|FND3A_HUMAN	FNDC3A	Fibronectin type-III domain-containing protein 3A OS=Homo sapiens GN=FNDC3A PE=1 SV=4	133763	0	1101	0	26	0	0.02	#DIV/0!	0	0	0	1	0	1	0	2	0.795451
tr|Q5TG85|Q5TG85_HUMAN	CHD5	Chromodomain-helicase-DNA-binding protein 5 OS=Homo sapiens GN=CHD5 PE=4 SV=1	92084	0	1099	0	26	0	0.04	#DIV/0!	0	0	0	1	0	1	0	2	0.795451
tr|B9A6K1|B9A6K1_HUMAN	TBC1D5	TBC1 domain family, member 5 OS=Homo sapiens GN=TBC1D5 PE=2 SV=1	89519	0	1098	0	26	0	0.04	#DIV/0!	0	0	0	1	0	1	0	1	0.795451
sp|A9QM74|IMA8_HUMAN	KPNA7	Importin subunit alpha-8 OS=Homo sapiens GN=KPNA7 PE=1 SV=1	57529	0	1097	0	26	0	0.06	#DIV/0!	0	0	0	1	0	1	0	1	0.795451
tr|Q05CN7|Q05CN7_HUMAN	GTF3C4	GTF3C4 protein (Fragment) OS=Homo sapiens GN=GTF3C4 PE=2 SV=1	63439	0	1094	0	26	0	0.05	#DIV/0!	0	0	0	1	0	1	0	2	0.795451
sp|P61011|SRP54_HUMAN	SRP54	Signal recognition particle 54 kDa protein OS=Homo sapiens GN=SRP54 PE=1 SV=1	55953	0	1089	0	27	0	0.07	#DIV/0!	0	0	0	1	0	1	0	1	0.795451
tr|A8MV53|A8MV53_HUMAN	PPAN	HCG2033702, isoform CRA_a OS=Homo sapiens GN=PPAN PE=4 SV=1	47344	0	1081	0	27	0	0.07	#DIV/0!	0	0	0	1	0	1	0	1	0.795451
tr|B4DVV7|B4DVV7_HUMAN	tr|B4DVV	cDNA FLJ51339, highly similar to Methionyl-tRNA synthetase, mitochondrial (EC 6.1.1.10) OS=Homo sapiens PE=2 SV=1	59183	0	1077	0	27	0	0.06	#DIV/0!	0	0	0	1	0	1	0	1	0.795451
tr|E5KND5|E5KND5_HUMAN	tr|E5KND	Mitochondrial elongation factor G OS=Homo sapiens PE=2 SV=1	84103	0	1076	0	27	0	0.07	#DIV/0!	0	0	0	1	0	1	0	1	0.795451
tr|C9JDR0|C9JDR0_HUMAN	NSDHL	Sterol-4-alpha-carboxylate 3-dehydrogenase, decarboxylating (Fragment) OS=Homo sapiens GN=NSDHL PE=2 SV=1	28356	0	1075	0	27	0	0.12	#DIV/0!	0	0	0	1	0	1	0	1	0.795451
tr|B2RAG9|B2RAG9_HUMAN	tr|B2RAG	cDNA, FLJ94908, highly similar to Homo sapiens PPAR binding protein (PPARBP), mRNA OS=Homo sapiens PE=2 SV=1	167569	0	1073	0	27	0	0.02	#DIV/0!	0	0	0	1	0	1	0	1	0.795451
tr|G5E988|G5E988_HUMAN	AP3D1	AP-3 complex subunit delta-1 OS=Homo sapiens GN=AP3D1 PE=2 SV=1	126074	0	1069	0	27	0	0.02	#DIV/0!	0	0	0	1	0	1	0	1	0.795451
sp|Q9NR09|BIRC6_HUMAN	BIRC6	Baculoviral IAP repeat-containing protein 6 OS=Homo sapiens GN=BIRC6 PE=1 SV=2	536192	0	1066	0	28	0	0.01	#DIV/0!	0	0	0	1	0	1	0	1	0.795451
tr|B7Z6C2|B7Z6C2_HUMAN	tr|B7Z6C2	cDNA FLJ50663, highly similar to Phosphoglucomutase-1 (EC 5.4.2.2) OS=Homo sapiens PE=2 SV=1	64052	0	1065	0	28	0	0.05	#DIV/0!	0	0	0	1	0	1	0	2	0.795451
tr|H3BSD9|H3BSD9_HUMAN	MYO7B	Unconventional myosin-VIIb (Fragment) OS=Homo sapiens GN=MYO7B PE=4 SV=1	10022	0	1061	0	28	0	0.35	#DIV/0!	0	1	0	1	0	1	0	2	0.795451
sp|O76031|CLPX_HUMAN	CLPX	ATP-dependent Clp protease ATP-binding subunit clpX-like, mitochondrial OS=Homo sapiens GN=CLPX PE=1 SV=2	69922	0	1060	0	28	0	0.06	#DIV/0!	0	0	0	1	0	1	0	3	0.795451
sp|Q5JTZ9|SYAM_HUMAN	AARS2	Alanine--tRNA ligase, mitochondrial OS=Homo sapiens GN=AARS2 PE=1 SV=1	108299	0	1059	0	28	0	0.03	#DIV/0!	0	0	0	1	0	1	0	3	0.795451
tr|B3KTC7|B3KTC7_HUMAN	MRE11A	Double-strand break repair protein MRE11A OS=Homo sapiens GN=MRE11A PE=2 SV=1	81362	0	1058	0	28	0	0.04	#DIV/0!	0	0	0	1	0	1	0	2	0.795451
sp|Q86X55|CARM1_HUMAN	CARM1	Histone-arginine methyltransferase CARM1 OS=Homo sapiens GN=CARM1 PE=1 SV=3	66382	0	1057	0	28	0	0.05	#DIV/0!	0	0	0	1	0	1	0	2	0.795451
tr|E9PQD7|E9PQD7_HUMAN	RPS2	40S ribosomal protein S2 OS=Homo sapiens GN=RPS2 PE=2 SV=1	25366	0	1055	0	28	0	0.5	#DIV/0!	0	0	0	1	0	1	0	1	0.795451
tr|Q5JUA8|Q5JUA8_HUMAN	VPS16	Vacuolar protein sorting-associated protein 16 homolog OS=Homo sapiens GN=VPS16 PE=4 SV=1	59823	0	1054	0	28	0	0.11	#DIV/0!	0	0	0	1	0	1	0	1	0.795451
tr|B4DI28|B4DI28_HUMAN	PTBP2	Polypyrimidine tract-binding protein 2 OS=Homo sapiens GN=PTBP2 PE=2 SV=1	34947	0	1051	0	29	0	0.09	#DIV/0!	0	0	0	1	0	1	0	1	0.795451
tr|A8K2M0|A8K2M0_HUMAN	PSMC4	Proteasome (Prosome, macropain) 26S subunit, ATPase, 4, isoform CRA_b OS=Homo sapiens GN=PSMC4 PE=2 SV=1	47451	0	1044	0	29	0	0.07	#DIV/0!	0	0	0	1	0	1	0	2	0.795451
tr|B1AK81|B1AK81_HUMAN	PIGK	GPI-anchor transamidase OS=Homo sapiens GN=PIGK PE=4 SV=1	34462	0	1040	0	29	0	0.1	#DIV/0!	0	0	0	1	0	1	0	1	0.795451
sp|P29590|PML_HUMAN	PML	Protein PML OS=Homo sapiens GN=PML PE=1 SV=3	99143	0	1037	0	29	0	0.12	#DIV/0!	0	0	0	1	0	1	0	1	0.795451
tr|B2RB95|B2RB95_HUMAN	tr|B2RB95	cDNA, FLJ95381, Homo sapiens tripartite motif-containing 16 (TRIM16), mRNA OS=Homo sapiens PE=2 SV=1	64884	0	1035	0	29	0	0.07	#DIV/0!	0	0	0	1	0	1	0	3	0.795451
tr|H0YEH2|H0YEH2_HUMAN	PUM1	Pumilio homolog 1 (Fragment) OS=Homo sapiens GN=PUM1 PE=4 SV=1	120836	0	1025	0	30	0	0.03	#DIV/0!	0	0	0	1	0	1	0	1	0.795451
sp|Q9H4L5|OSBL3_HUMAN	OSBPL3	Oxysterol-binding protein-related protein 3 OS=Homo sapiens GN=OSBPL3 PE=1 SV=1	102130	0	1023	0	30	0	0.03	#DIV/0!	0	0	0	1	0	1	0	2	0.795451
sp|Q14126|DSG2_HUMAN	DSG2	Desmoglein-2 OS=Homo sapiens GN=DSG2 PE=1 SV=2	123016	0	1022	0	30	0	0.03	#DIV/0!	0	0	0	1	0	1	0	2	0.795451
tr|B2R774|B2R774_HUMAN	tr|B2R774	cDNA, FLJ93313, highly similar to Homo sapiens lectin, mannose-binding, 1 (LMAN1), mRNA OS=Homo sapiens PE=2 SV=1	57768	0	1021	0	30	0	0.09	#DIV/0!	0	0	0	1	0	1	0	1	0.795451
tr|B3KU23|B3KU23_HUMAN	tr|B3KU2	cDNA FLJ39097 fis, clone NTONG2000977, highly similar to Neurolysin, mitochondrial (EC 3.4.24.16) OS=Homo sapiens PE=2 SV=1	70320	0	1020	0	30	0	0.05	#DIV/0!	0	0	0	1	0	1	0	2	0.795451
tr|G3V1A4|G3V1A4_HUMAN	CFL1	Cofilin 1 (Non-muscle), isoform CRA_a OS=Homo sapiens GN=CFL1 PE=4 SV=1	17029	0	1019	0	30	0	0.2	#DIV/0!	0	0	0	1	0	1	0	1	0.795451
sp|Q14008|CKAP5_HUMAN	CKAP5	Cytoskeleton-associated protein 5 OS=Homo sapiens GN=CKAP5 PE=1 SV=3	227062	0	1018	0	30	0	0.03	#DIV/0!	0	0	0	1	0	1	0	1	0.795451
tr|A8K972|A8K972_HUMAN	tr|A8K972	cDNA FLJ76457, highly similar to Homo sapiens leucine carboxyl methyltransferase 2 (LCMT2), mRNA OS=Homo sapiens PE=2 SV=1	76406	0	1014	0	30	0	0.04	#DIV/0!	0	0	0	1	0	1	0	1	0.795451
sp|Q5QP74|CJ115_HUMAN	C10orf115	Putative uncharacterized protein C10orf115 OS=Homo sapiens GN=C10orf115 PE=4 SV=2	10394	0	1011	0	30	0	0.33	#DIV/0!	0	1	0	1	0	1	0	1	0.795451
tr|F5H1X8|F5H1X8_HUMAN	LRBA	Lipopolysaccharide-responsive and beige-like anchor protein OS=Homo sapiens GN=LRBA PE=2 SV=1	320211	0	1010	0	30	0	0.01	#DIV/0!	0	0	0	1	0	1	0	2	0.795451
tr|Q96FS1|Q96FS1_HUMAN	CTNND1	CTNND1 protein (Fragment) OS=Homo sapiens GN=CTNND1 PE=2 SV=2	92901	0	1008	0	30	0	0.05	#DIV/0!	0	0	0	1	0	1	0	1	0.795451
tr|B4DV74|B4DV74_HUMAN	tr|B4DV7	cDNA FLJ53855, highly similar to Kanadaptin OS=Homo sapiens PE=2 SV=1	56134	0	1007	0	31	0	0.06	#DIV/0!	0	1	0	1	0	1	0	2	0.795451
tr|B4DM41|B4DM41_HUMAN	RFC4	Replication factor C (Activator 1) 4, 37kDa, isoform CRA_b OS=Homo sapiens GN=RFC4 PE=2 SV=1	33996	0	1005	0	31	0	0.1	#DIV/0!	0	0	0	1	0	1	0	2	0.795451
tr|Q59FE8|Q59FE8_HUMAN	tr|Q59FE8	Epithelial protein lost in neoplasm beta variant (Fragment) OS=Homo sapiens PE=2 SV=1	86510	0	1002	0	31	0	0.04	#DIV/0!	0	0	0	1	0	1	0	1	0.795451
sp|Q9HD67|MYO10_HUMAN	MYO10	Unconventional myosin-X OS=Homo sapiens GN=MYO10 PE=1 SV=3	239194	0	998	0	31	0	0.03	#DIV/0!	0	0	0	1	0	1	0	1	0.795451
tr|B2R856|B2R856_HUMAN	tr|B2R856	Acyl-coenzyme A oxidase OS=Homo sapiens PE=2 SV=1	78249	0	997	0	31	0	0.04	#DIV/0!	0	0	0	1	0	1	0	1	0.795451
tr|A8K6V7|A8K6V7_HUMAN	tr|A8K6V7	cDNA FLJ76053, highly similar to Homo sapiens Ras-GTPase activating protein SH3 domain-binding protein 2 (G3BP2), transcript vari	50842	0	994	0	32	0	0.06	#DIV/0!	0	0	0	1	0	1	0	1	0.795451
tr|Q53TH1|Q53TH1_HUMAN	PSMD14	Putative uncharacterized protein PSMD14 (Fragment) OS=Homo sapiens GN=PSMD14 PE=2 SV=1	18165	0	992	0	32	0	0.19	#DIV/0!	0	0	0	1	0	1	0	1	0.795451
sp|O00507|USP9Y_HUMAN	USP9Y	Probable ubiquitin carboxyl-terminal hydrolase FAF-Y OS=Homo sapiens GN=USP9Y PE=1 SV=2	294370	0	990	0	32	0	0.01	#DIV/0!	0	0	0	1	0	1	0	2	0.795451
tr|Q59EM6|Q59EM6_HUMAN	tr|Q59EM	Internexin neuronal intermediate filament protein, alpha variant (Fragment) OS=Homo sapiens PE=2 SV=1	37319	0	989	0	32	0	0.09	#DIV/0!	0	0	0	1	0	1	0	3	0.795451
sp|A1L0T0|ILVBL_HUMAN	ILVBL	Acetolactate synthase-like protein OS=Homo sapiens GN=ILVBL PE=1 SV=2	68452	0	988	0	32	0	0.05	#DIV/0!	0	0	0	1	0	1	0	1	0.795451
tr|Q05DA4|Q05DA4_HUMAN	P4HA2	P4HA2 protein OS=Homo sapiens GN=P4HA2 PE=2 SV=1	57684	0	986	0	32	0	0.06	#DIV/0!	0	0	0	1	0	1	0	1	0.795451
tr|C0JXB8|C0JXB8_HUMAN	AIP	Aryl hydrocarbon receptor interacting protein OS=Homo sapiens GN=AIP PE=2 SV=1	38041	0	985	0	32	0	0.09	#DIV/0!	0	1	0	1	0	1	0	1	0.795451
tr|G3V261|G3V261_HUMAN	ATP10A	Probable phospholipid-transporting ATPase VA OS=Homo sapiens GN=ATP10A PE=2 SV=1	68071	0	984	0	32	0	0.05	#DIV/0!	0	1	0	1	0	1	0	1	0.795451
tr|K7EM59|K7EM59_HUMAN	MRI1	Methylthioribose-1-phosphate isomerase (Fragment) OS=Homo sapiens GN=MRI1 PE=3 SV=1	26783	0	983	0	32	0	0.12	#DIV/0!	0	1	0	1	0	1	0	1	0.795451
tr|B4DYZ1|B4DYZ1_HUMAN	tr|B4DYZ1	cDNA FLJ59786, highly similar to Probable ATP-dependent RNA helicase DDX31 (EC 3.6.1.-) OS=Homo sapiens PE=2 SV=1	81565	0	982	0	32	0	0.04	#DIV/0!	0	1	0	1	0	1	0	1	0.795451
sp|Q9BXW9|FACD2_HUMAN	FANCD2	Fanconi anemia group D2 protein OS=Homo sapiens GN=FANCD2 PE=1 SV=1	168295	0	980	0	32	0	0.02	#DIV/0!	0	0	0	1	0	1	0	2	0.795451
sp|Q86XP3|DDX42_HUMAN	DDX42	ATP-dependent RNA helicase DDX42 OS=Homo sapiens GN=DDX42 PE=1 SV=1	103197	0	979	0	32	0	0.08	#DIV/0!	0	0	0	1	0	1	0	1	0.795451
tr|B4E1K0|B4E1K0_HUMAN	KIF23	Kinesin-like protein KIF23 OS=Homo sapiens GN=KIF23 PE=2 SV=1	78221	0	975	0	32	0	0.04	#DIV/0!	0	0	0	1	0	1	0	1	0.795451
tr|H7C455|H7C455_HUMAN	tr|H7C455	Uncharacterized protein (Fragment) OS=Homo sapiens PE=4 SV=1	22389	0	971	0	33	0	0.15	#DIV/0!	0	1	0	1	0	1	0	1	0.795451
sp|Q9BW60|ELOV1_HUMAN	ELOVL1	Elongation of very long chain fatty acids protein 1 OS=Homo sapiens GN=ELOVL1 PE=1 SV=1	32755	0	970	0	33	0	0.1	#DIV/0!	0	0	0	1	0	1	0	1	0.795451
tr|B3KNV9|B3KNV9_HUMAN	tr|B3KNV	cDNA FLJ30554 fis, clone BRAWH2003693 OS=Homo sapiens PE=2 SV=1	112670	0	969	0	31	0	0.05	#DIV/0!	0	1	0	2	0	1	0	5	0.795451
tr|B1PBA3|B1PBA3_HUMAN	tr|B1PBA	SKNY protein OS=Homo sapiens PE=2 SV=1	98489	0	965	0	33	0	0.03	#DIV/0!	0	1	0	1	0	1	0	2	0.795451
tr|J3QRD1|J3QRD1_HUMAN	ALDH3A2	Fatty aldehyde dehydrogenase OS=Homo sapiens GN=ALDH3A2 PE=3 SV=1	45019	0	963	0	33	0	0.07	#DIV/0!	0	1	0	1	0	1	0	1	0.795451
sp|Q99543|DNJC2_HUMAN	DNAJC2	DnaJ homolog subfamily C member 2 OS=Homo sapiens GN=DNAJC2 PE=1 SV=4	72465	0	958	0	33	0	0.14	#DIV/0!	0	0	0	1	0	1	0	1	0.795451
tr|H7C0M3|H7C0M3_HUMAN	BPHL	Valacyclovir hydrolase (Fragment) OS=Homo sapiens GN=BPHL PE=4 SV=1	3314	0	946	0	34	0	1.2	#DIV/0!	0	1	0	1	0	1	0	1	0.795451
tr|H0Y3K4|H0Y3K4_HUMAN	RBM33	RNA-binding protein 33 (Fragment) OS=Homo sapiens GN=RBM33 PE=4 SV=1	100636	0	944	0	34	0	0.03	#DIV/0!	0	1	0	1	0	1	0	1	0.795451
sp|Q96CS3|FAF2_HUMAN	FAF2	FAS-associated factor 2 OS=Homo sapiens GN=FAF2 PE=1 SV=2	52933	0	943	0	34	0	0.06	#DIV/0!	0	0	0	1	0	1	0	1	0.795451
tr|J3KRI4|J3KRI4_HUMAN	DYNC1LI2	Cytoplasmic dynein 1 light intermediate chain 2 (Fragment) OS=Homo sapiens GN=DYNC1LI2 PE=4 SV=1	17767	0	941	0	34	0	0.19	#DIV/0!	0	0	0	1	0	1	0	1	0.795451
tr|J3KNI9|J3KNI9_HUMAN	TBC1D15	TBC1 domain family member 15 OS=Homo sapiens GN=TBC1D15 PE=4 SV=1	79491	0	939	0	34	0	0.04	#DIV/0!	0	1	0	1	0	1	0	1	0.795451
sp|Q9H9V9|JMJD4_HUMAN	JMJD4	JmjC domain-containing protein 4 OS=Homo sapiens GN=JMJD4 PE=2 SV=2	52973	0	935	0	35	0	0.06	#DIV/0!	0	1	0	1	0	1	0	1	0.795451
tr|A1A5C4|A1A5C4_HUMAN	RRBP1	RRBP1 protein OS=Homo sapiens GN=RRBP1 PE=2 SV=1	103162	0	934	0	35	0	0.02	#DIV/0!	0	0	0	1	0	1	0	1	0.795451

tr|B3KQH2|B3KQH2_HUMAN	tr|B3KQH	cDNA FLJ90454 fis, clone NT2RP3001560, highly similar to Cleft lip and palate transmembrane protein 1 OS=Homo sapiens PE=2 SV=	76247	0	931	0	35	0	0.04	#DIV/0!	0	0	0	1	0	1	0	1	0.795451
tr|Q2TSD2|Q2TSD2_HUMAN	tr|Q2TSD	Aging-associated gene 7 protein OS=Homo sapiens PE=2 SV=1	41778	0	929	0	35	0	0.08	#DIV/0!	0	0	0	1	0	1	0	1	0.795451
tr|B4DK35|B4DK35_HUMAN	tr|B4DK3	cDNA FLJ61591, highly similar to Probable global transcription activator SNF2L2 (EC 3.6.1.-) (Fragment) OS=Homo sapiens PE=2 SV=1	112636	0	925	0	35	0	0.04	#DIV/0!	0	0	0	1	0	1	0	1	0.795451
tr|H0Y8Z9|H0Y8Z9_HUMAN	ACAD9	Acyl-CoA dehydrogenase family member 9, mitochondrial (Fragment) OS=Homo sapiens GN=ACAD9 PE=4 SV=1	43503	0	924	0	35	0	0.05	#DIV/0!	0	0	0	1	0	1	0	1	0.795451
tr|B3KVR1|B3KVR1_HUMAN	SNRPN	Small nuclear ribonucleoprotein-associated protein OS=Homo sapiens GN=SNRPN PE=2 SV=1	25230	0	916	0	36	0	0.13	#DIV/0!	0	1	0	1	0	1	0	1	0.795451
tr|H0YKN8|H0YKN8_HUMAN	TLE3	Transducin-like enhancer protein 3 OS=Homo sapiens GN=TLE3 PE=4 SV=1	84740	0	913	0	36	0	0.07	#DIV/0!	0	0	0	1	0	1	0	1	0.795451
tr|B3KY55|B3KY55_HUMAN	tr|B3KY5	cDNA FLJ46894 fis, clone UTERU3019708, highly similar to Regulator of nonsense transcripts 1 OS=Homo sapiens PE=2 SV=1	41002	0	912	0	36	0	0.03	#DIV/0!	0	1	0	1	0	1	0	1	0.795451
sp|O15091|MRRP3_HUMAN	KIAA0391	Mitochondrial ribonuclease P protein 3 OS=Homo sapiens GN=KIAA0391 PE=1 SV=2	68014	0	911	0	36	0	0.05	#DIV/0!	0	1	0	1	0	1	0	1	0.795451
sp|Q92624|APBP2_HUMAN	APPBP2	Amyloid protein-binding protein 2 OS=Homo sapiens GN=APPBP2 PE=1 SV=2	67666	0	907	0	36	0	0.05	#DIV/0!	0	1	0	1	0	1	0	1	0.795451
sp|Q96S66|CLCC1_HUMAN	CLCC1	Chloride channel CLIC-like protein 1 OS=Homo sapiens GN=CLCC1 PE=1 SV=1	62667	0	900	0	36	0	0.05	#DIV/0!	0	1	0	1	0	1	0	1	0.795451
sp|Q9BVQ7|SPA5L_HUMAN	SPATA5L1	Spermatogenesis-associated protein 5-like protein 1 OS=Homo sapiens GN=SPATA5L1 PE=1 SV=2	81515	0	898	0	36	0	0.04	#DIV/0!	0	1	0	1	0	1	0	1	0.795451
tr|J3KQ58|J3KQ58_HUMAN	TTC34	Tetratricopeptide repeat protein 34 OS=Homo sapiens GN=TTC34 PE=4 SV=1	40214	0	896	0	37	0	0.08	#DIV/0!	0	1	0	1	0	1	0	1	0.795451
sp|Q03169|TNAP2_HUMAN	TNFAIP2	Tumor necrosis factor alpha-induced protein 2 OS=Homo sapiens GN=TNFAIP2 PE=1 SV=2	73015	0	895	0	37	0	0.08	#DIV/0!	0	1	0	1	0	1	0	1	0.795451
tr|Q6IAM0|Q6IAM0_HUMAN	EIF3S4	Eukaryotic translation initiation factor 3 subunit G OS=Homo sapiens GN=EIF3S4 PE=2 SV=1	35864	0	893	0	37	0	0.09	#DIV/0!	0	1	0	1	0	1	0	1	0.795451
tr|B4DP44|B4DP44_HUMAN	tr|B4DP4	cDNA FLJ60751, highly similar to Protein arginine N-methyltransferase 3 (EC 2.1.1.-) OS=Homo sapiens PE=2 SV=1	52013	0	891	0	37	0	0.06	#DIV/0!	0	1	0	1	0	1	0	1	0.795451
tr|Q6ZWH1|Q6ZWH1_HUMAN	tr|Q6ZWH	cDNA FLJ41081 fis, clone ADRGL2009755, highly similar to Homo sapiens brain and reproductive organ-expressed protein (BRE) mRN	45824	0	889	0	37	0	0.07	#DIV/0!	0	1	0	1	0	1	0	1	0.795451
tr|D3DVA5|D3DVA5_HUMAN	ARHGEF2	Rho/rac guanine nucleotide exchange factor (GEF) 2, isoform CRA_a OS=Homo sapiens GN=ARHGEF2 PE=2 SV=1	116954	0	888	0	37	0	0.06	#DIV/0!	0	0	0	2	0	1	0	2	0.795451
sp|Q13057|COASY_HUMAN	COASY	Bifunctional coenzyme A synthase OS=Homo sapiens GN=COASY PE=1 SV=4	62632	0	887	0	37	0	0.18	#DIV/0!	0	1	0	1	0	1	0	1	0.795451
tr|K7EPI4|K7EPI4_HUMAN	GFAP	Glial fibrillary acidic protein (Fragment) OS=Homo sapiens GN=GFAP PE=4 SV=1	7120	0	884	0	37	0	0.5	#DIV/0!	0	1	0	1	0	1	0	1	0.795451
sp|Q9HCC0|MCCB_HUMAN	MCCC2	Methylcrotonoyl-CoA carboxylase beta chain, mitochondrial OS=Homo sapiens GN=MCCC2 PE=1 SV=1	61808	0	883	0	38	0	0.05	#DIV/0!	0	1	0	1	0	1	0	1	0.795451
tr|B4DWV9|B4DWV9_HUMAN	tr|B4DW	cDNA FLJ53108, highly similar to Guanine nucleotide-binding protein alpha-13 subunit OS=Homo sapiens PE=2 SV=1	41291	0	881	0	38	0	0.08	#DIV/0!	0	1	0	1	0	1	0	1	0.795451
sp|Q13425|SNTB2_HUMAN	SNTB2	Beta-2-syntrophin OS=Homo sapiens GN=SNTB2 PE=1 SV=1	58369	0	878	0	38	0	0.06	#DIV/0!	0	1	0	1	0	1	0	1	0.795451
tr|B4DKE6|B4DKE6_HUMAN	tr|B4DKE	cDNA FLJ60629, highly similar to Replication factor C subunit 3 OS=Homo sapiens PE=2 SV=1	35551	0	877	0	38	0	0.09	#DIV/0!	0	1	0	1	0	1	0	1	0.795451
sp|Q96QR8|PURB_HUMAN	PURB	Transcriptional activator protein Pur-beta OS=Homo sapiens GN=PURB PE=1 SV=3	33392	0	871	0	38	0	0.1	#DIV/0!	0	1	0	1	0	1	0	1	0.795451
tr|B4DTN4|B4DTN4_HUMAN	METTL3	N6-adenosine-methyltransferase 70 kDa subunit OS=Homo sapiens GN=METTL3 PE=2 SV=1	27699	0	870	0	38	0	0.12	#DIV/0!	0	1	0	1	0	1	0	1	0.795451
sp|Q9UBU9|NXF1_HUMAN	NXF1	Nuclear RNA export factor 1 OS=Homo sapiens GN=NXF1 PE=1 SV=1	70652	0	869	0	38	0	0.05	#DIV/0!	0	1	0	1	0	1	0	1	0.795451
tr|H0Y8D1|H0Y8D1_HUMAN	PRSS1	Trypsin-1 (Fragment) OS=Homo sapiens GN=PRSS1 PE=3 SV=1	15697	0	867	0	38	0	0.22	#DIV/0!	0	1	0	1	0	1	0	1	0.795451
tr|F5GYT3|F5GYT3_HUMAN	LPCAT3	Lysophospholipid acyltransferase 5 OS=Homo sapiens GN=LPCAT3 PE=2 SV=1	22378	0	866	0	39	0	0.15	#DIV/0!	0	1	0	1	0	1	0	1	0.795451
tr|B4DLG2|B4DLG2_HUMAN	tr|B4DLG	cDNA FLJ58196, highly similar to Zinc finger CCCH domain-containing protein 11A OS=Homo sapiens PE=2 SV=1	83177	0	865	0	39	0	0.04	#DIV/0!	0	1	0	1	0	1	0	1	0.795451
tr|H0YJU2|H0YJU2_HUMAN	AHSA1	Activator of 90 kDa heat shock protein ATPase homolog 1 (Fragment) OS=Homo sapiens GN=AHSA1 PE=4 SV=1	15052	0	862	0	27	0	0.3	#DIV/0!	0	0	0	1	0	1	0	1	0.795451
tr|F5GWY5|F5GWY5_HUMAN	PODXL	Podocalyxin OS=Homo sapiens GN=PODXL PE=2 SV=1	59285	0	859	0	39	0	0.06	#DIV/0!	0	1	0	1	0	1	0	1	0.795451
sp|P49419|AL7A1_HUMAN	ALDH7A1	Alpha-aminoadipic semialdehyde dehydrogenase OS=Homo sapiens GN=ALDH7A1 PE=1 SV=5	59020	0	855	0	39	0	0.13	#DIV/0!	0	1	0	1	0	1	0	1	0.795451
sp|Q96T51|RUFY1_HUMAN	RUFY1	RUN and FYVE domain-containing protein 1 OS=Homo sapiens GN=RUFY1 PE=1 SV=2	80851	0	852	0	39	0	0.04	#DIV/0!	0	1	0	1	0	1	0	2	0.795451
tr|B4E1K7|B4E1K7_HUMAN	STOML2	Stomatin-like protein 2 OS=Homo sapiens GN=STOML2 PE=2 SV=1	33317	0	851	0	39	0	0.1	#DIV/0!	0	1	0	1	0	1	0	1	0.795451
tr|B4E1L0|B4E1L0_HUMAN	tr|B4E1L0	Adenylosuccinate synthetase OS=Homo sapiens PE=2 SV=1	48308	0	847	0	40	0	0.07	#DIV/0!	0	1	0	1	0	1	0	1	0.795451
tr|A4D1I0|A4D1I0_HUMAN	PPP1R9A	HCG1741805, isoform CRA_c OS=Homo sapiens GN=PPP1R9A PE=2 SV=1	148567	0	845	0	40	0	0.02	#DIV/0!	0	1	0	1	0	1	0	2	0.795451
tr|B2RDG4|B2RDG4_HUMAN	tr|B2RDG	cDNA, FLJ96597 OS=Homo sapiens PE=2 SV=1	57796	0	844	0	40	0	0.06	#DIV/0!	0	1	0	1	0	1	0	1	0.795451
sp|Q9UBL6|CPNE7_HUMAN	CPNE7	Copine-7 OS=Homo sapiens GN=CPNE7 PE=2 SV=1	71390	0	842	0	40	0	0.05	#DIV/0!	0	1	0	1	0	1	0	2	0.795451
tr|A2A274|A2A274_HUMAN	ACO2	Aconitase 2, mitochondrial OS=Homo sapiens GN=ACO2 PE=4 SV=1	88563	0	824	0	41	0	0.04	#DIV/0!	0	1	0	1	0	1	0	1	0.795451
tr|Q6AI22|Q6AI22_HUMAN	DKFZp686	Putative uncharacterized protein DKFZp686H16106 (Fragment) OS=Homo sapiens GN=DKFZp686H16106 PE=2 SV=1	321236	0	823	0	30	0	0.01	#DIV/0!	0	1	0	1	0	1	0	1	0.795451
tr|Q6NX51|Q6NX51_HUMAN	EXOC4	Exocyst complex component 4 OS=Homo sapiens GN=EXOC4 PE=2 SV=1	111170	0	818	0	42	0	0.05	#DIV/0!	0	1	0	1	0	1	0	1	0.795451
tr|J3QKW7|J3QKW7_HUMAN	DRG2	Developmentally-regulated GTP-binding protein 2 OS=Homo sapiens GN=DRG2 PE=4 SV=1	26892	0	817	0	42	0	0.2	#DIV/0!	0	1	0	1	0	1	0	1	0.795451
tr|G3V1U5|G3V1U5_HUMAN	GOLT1B	Golgi transport 1 homolog B (S. cerevisiae), isoform CRA_c OS=Homo sapiens GN=GOLT1B PE=2 SV=1	8208	0	816	0	42	0	0.43	#DIV/0!	0	1	0	1	0	1	0	1	0.795451
tr|H0YN14|H0YN14_HUMAN	IPO4	Importin-4 OS=Homo sapiens GN=IPO4 PE=4 SV=1	103193	0	812	0	42	0	0.03	#DIV/0!	0	1	0	1	0	1	0	1	0.795451
sp|Q5SRE5|NU188_HUMAN	NUP188	Nucleoporin NUP188 homolog OS=Homo sapiens GN=NUP188 PE=1 SV=1	198369	0	808	0	42	0	0.02	#DIV/0!	0	1	0	1	0	1	0	2	0.795451
tr|Q59EZ3|Q59EZ3_HUMAN	tr|Q59EZ	Insulin-like growth factor 2 receptor variant (Fragment) OS=Homo sapiens PE=2 SV=1	272672	0	806	0	42	0	0.01	#DIV/0!	0	1	0	1	0	1	0	1	0.795451
sp|P35658|NU214_HUMAN	NUP214	Nuclear pore complex protein Nup214 OS=Homo sapiens GN=NUP214 PE=1 SV=2	214230	0	805	0	42	0	0.03	#DIV/0!	0	1	0	1	0	1	0	1	0.795451
tr|B4E074|B4E074_HUMAN	tr|B4E074	cDNA FLJ58655, highly similar to Notchless homolog 1 OS=Homo sapiens PE=2 SV=1	51211	0	804	0	42	0	0.06	#DIV/0!	0	1	0	1	0	1	0	2	0.795451
tr|B3KNJ4|B3KNJ4_HUMAN	SAE1	SUMO-1 activating enzyme subunit 1, isoform CRA_a OS=Homo sapiens GN=SAE1 PE=2 SV=1	33705	0	801	0	42	0	0.17	#DIV/0!	0	1	0	1	0	1	0	1	0.795451
sp|Q5TFE4|NT5D1_HUMAN	NT5DC1	5'~-nucleotidase domain-containing protein 1 OS=Homo sapiens GN=NT5DC1 PE=1 SV=1	52439	0	799	0	43	0	0.06	#DIV/0!	0	1	0	1	0	1	0	2	0.795451
tr|A4D210|A4D210_HUMAN	EIF3S9	Eukaryotic translation initiation factor 3, subunit 9 eta, 116kDa OS=Homo sapiens GN=EIF3S9 PE=4 SV=1	89024	0	798	0	43	0	0.09	#DIV/0!	0	1	0	2	0	1	0	2	0.795451
tr|Q5U043|Q5U043_HUMAN	tr|Q5U04	Alcohol dehydrogenase 5 (Class III), chi polypeptide OS=Homo sapiens PE=2 SV=1	40520	0	797	0	43	0	0.08	#DIV/0!	0	1	0	1	0	1	0	1	0.795451
tr|E1P5S2|E1P5S2_HUMAN	RBM39	RNA-binding protein 39 OS=Homo sapiens GN=RBM39 PE=4 SV=1	40744	0	796	0	43	0	0.09	#DIV/0!	0	1	0	1	0	1	0	2	0.795451
sp|Q5D862|FILA2_HUMAN	FLG2	Filaggrin-2 OS=Homo sapiens GN=FLG2 PE=1 SV=1	249296	0	794	0	43	0	0.01	#DIV/0!	0	1	0	1	0	1	0	1	0.795451
tr|H0Y2P8|H0Y2P8_HUMAN	SRGAP2	SLIT-ROBO Rho GTPase-activating protein 2 (Fragment) OS=Homo sapiens GN=SRGAP2 PE=4 SV=1	111536	0	793	0	43	0	0.03	#DIV/0!	0	1	0	1	0	1	0	1	0.795451
tr|Q4G0F4|Q4G0F4_HUMAN	POLRMT	DNA-directed RNA polymerase OS=Homo sapiens GN=POLRMT PE=2 SV=1	140194	0	792	0	43	0	0.02	#DIV/0!	0	1	0	1	0	1	0	2	0.795451
tr|B4E0X4|B4E0X4_HUMAN	tr|B4E0X4	cDNA FLJ51433 OS=Homo sapiens PE=2 SV=1	10805	0	787	0	44	0	0.32	#DIV/0!	0	1	0	1	0	1	0	1	0.795451
sp|Q9Y295|DRG1_HUMAN	DRG1	Developmentally-regulated GTP-binding protein 1 OS=Homo sapiens GN=DRG1 PE=1 SV=1	40802	0	783	0	44	0	0.08	#DIV/0!	0	1	0	1	0	1	0	1	0.795451
sp|P09497|CLCB_HUMAN	CLTB	Clathrin light chain B OS=Homo sapiens GN=CLTB PE=1 SV=1	25289	0	782	0	44	0	0.13	#DIV/0!	0	1	0	1	0	1	0	1	0.795451
tr|O95654|O95654_HUMAN	tr|O9565	P150Glued homolog (Fragment) OS=Homo sapiens PE=2 SV=1	46656	0	780	0	44	0	0.07	#DIV/0!	0	1	0	1	0	1	0	2	0.795451
tr|H0YL71|H0YL71_HUMAN	SMAD3	Mothers against decapentaplegic homolog 3 (Fragment) OS=Homo sapiens GN=SMAD3 PE=4 SV=1	10544	0	778	0	44	0	0.33	#DIV/0!	0	1	0	1	0	1	0	1	0.795451
sp|Q8TDD1|DDX54_HUMAN	DDX54	ATP-dependent RNA helicase DDX54 OS=Homo sapiens GN=DDX54 PE=1 SV=2	98819	0	777	0	44	0	0.03	#DIV/0!	0	1	0	1	0	1	0	1	0.795451
tr|Q59FS8|Q59FS8_HUMAN	tr|Q59FS	Eukaryotic translation initiation factor 3, subunit 9 eta, 116kDa isoform b variant (Fragment) OS=Homo sapiens PE=2 SV=1	19913	0	775	0	44	0	0.17	#DIV/0!	0	1	0	1	0	1	0	1	0.795451
tr|Q5SQP8|Q5SQP8_HUMAN	CTBP2	C-terminal-binding protein 2 OS=Homo sapiens GN=CTBP2 PE=4 SV=1	56808	0	771	0	45	0	0.07	#DIV/0!	0	1	0	1	0	1	0	1	0.795451
tr|B4E312|B4E312_HUMAN	tr|B4E312	cDNA FLJ53422, highly similar to TATA-binding protein-associated factor 2N OS=Homo sapiens PE=2 SV=1	40282	0	770	0	45	0	0.08	#DIV/0!	0	1	0	1	0	1	0	2	0.795451
tr|G3V1K3|G3V1K3_HUMAN	PON2	Paraoxonase 2, isoform CRA_a OS=Homo sapiens GN=PON2 PE=2 SV=1	41672	0	768	0	45	0	0.08	#DIV/0!	0	1	0	1	0	1	0	1	0.795451
sp|Q14669|TRIPC_HUMAN	TRIP12	E3 ubiquitin-protein ligase TRIP12 OS=Homo sapiens GN=TRIP12 PE=1 SV=1	222234	0	762	0	45	0	0.01	#DIV/0!	0	1	0	1	0	1	0	1	0.795451
tr|Q8N6B4|Q8N6B4_HUMAN	MLL/CALM	Myeloid/lymphoid or mixed-lineage leukemia/clathrin assembly protein fusion protein (Fragment) OS=Homo sapiens GN=MLL/CALM	63408	0	760	0	28	0	0.1	#DIV/0!	0	0	0	1	0	1	0	1	0.795451
tr|B3KUP9|B3KUP9_HUMAN	tr|B3KUP	cDNA FLJ40361 fis, clone TESTI2034407, highly similar to NEDD8-activating enzyme E1 regulatory subunit OS=Homo sapiens PE=2 SV	60951	0	759	0	46	0	0.05	#DIV/0!	0	1	0	1	0	1	0	1	0.795451
tr|A4UCU0|A4UCU0_HUMAN	tr|A4UCU	DEAD box polypeptide 47 isoform 1 variant (Fragment) OS=Homo sapiens PE=2 SV=1	19904	0	757	0	46	0	0.06	#DIV/0!	0	1	0	1	0	1	0	1	0.795451
tr|Q6DCA8|Q6DCA8_HUMAN	BCLAF1	BCLAF1 protein (Fragment) OS=Homo sapiens GN=BCLAF1 PE=2 SV=1	77958	0	755	0	46	0	0.04	#DIV/0!	0	1	0	1	0	1	0	1	0.795451
tr|J3KQ32|J3KQ32_HUMAN	OLA1	Obg-like ATPase 1 OS=Homo sapiens GN=OLA1 PE=4 SV=1	47136	0	754	0	46	0	0.07	#DIV/0!	0	1	0	1	0	1	0	1	0.795451
sp|Q9BW92|SYTM_HUMAN	TARS2	Threonine--tRNA ligase, mitochondrial OS=Homo sapiens GN=TARS2 PE=1 SV=1	81841	0	752	0	46	0	0.04	#DIV/0!	0	1	0	1	0	1	0	1	0.795451
sp|P80723|BASP1_HUMAN	BASP1	Brain acid soluble protein 1 OS=Homo sapiens GN=BASP1 PE=1 SV=2	22680	0	746	0	46	0	0.15	#DIV/0!	0	1	0	1	0	1	0	1	0.795451
tr|B2R6E2|B2R6E2_HUMAN	tr|B2R6E2	cDNA, FLJ92910, highly similar to Homo sapiens programmed cell death 4 (neoplastic transformation inhibitor) (PDCD4), transcript v	51998	0	744	0	47	0	0.06	#DIV/0!	0	1	0	1	0	1	0	2	0.795451
tr|B0QYK0|B0QYK0_HUMAN	EWSR1	Ewing sarcoma breakpoint region 1 OS=Homo sapiens GN=EWSR1 PE=4 SV=1	65174	0	742	0	47	0	0.05	#DIV/0!	0	1	0	1	0	1	0	2	0.795451
sp|Q32P28|P3H1_HUMAN	LEPRE1	Prolyl 3-hydroxylase 1 OS=Homo sapiens GN=LEPRE1 PE=1 SV=2	84196	0	740	0	47	0	0.14	#DIV/0!	0	1	0	1	0	1	0	1	0.795451
tr|B0V0F7|B0V0F7_HUMAN	DHX16	Putative pre-mRNA-splicing factor ATP-dependent RNA helicase DHX16 (Fragment) OS=Homo sapiens GN=DHX16 PE=4 SV=1	21454	0	739	0	47	0	0.16	#DIV/0!	0	1	0	1	0	1	0	1	0.795451
tr|B0AZS5|B0AZS5_HUMAN	tr|B0AZS5	cDNA, FLJ79515, highly similar to Kinesin-like protein KIF2 OS=Homo sapiens PE=2 SV=1	75666	0	736	0	47	0	0.04	#DIV/0!	0	1	0	1	0	1	0	1	0.795451
sp|P49750|YLPM1_HUMAN	YLPM1	YLP motif-containing protein 1 OS=Homo sapiens GN=YLPM1 PE=1 SV=3	220077	0	733	0	47	0	0.02	#DIV/0!	0	1	0	1	0	1	0	1	0.795451
tr|A4FU51|A4FU51_HUMAN	NARG1L	NMDA receptor regulated 1-like OS=Homo sapiens GN=NARG1L PE=2 SV=1	102424	0	730	0	48	0	0.03	#DIV/0!	0	1	0	1	0	1	0	1	0.795451
tr|B4DHS5|B4DHS5_HUMAN	tr|B4DHS	cDNA FLJ54678, highly similar to N(2),N(2)-dimethylguanosine tRNA methyltransferase (EC 2.1.1.32) OS=Homo sapiens PE=2 SV=1	59256	0	729	0	48	0	0.06	#DIV/0!	0	1	0	1	0	1	0	1	0.795451
sp|Q9UKJ3|GPTC8_HUMAN	GPATCH8	G patch domain-containing protein 8 OS=Homo sapiens GN=GPATCH8 PE=1 SV=2	165010	0	720	0	48	0	0.02	#DIV/0!	0	1	0	1	0	1	0	1	0.795451
tr|B7Z2B4|B7Z2B4_HUMAN	RABGAP1	Rab GTPase-activating protein 1 OS=Homo sapiens GN=RABGAP1 PE=2 SV=1	48660	0	715	0	49	0	0.07	#DIV/0!	0	1	0	1	0	1	0	2	0.795451
tr|B2RB52|B2RB52_HUMAN	tr|B2RB5	cDNA, FLJ95314, highly similar to Homo sapiens transducin (beta)-like 2 (TBL2), transcript variant 1, mRNA OS=Homo sapiens PE=2 S	50423	0	713	0	49	0	0.07	#DIV/0!	0	1	0	1	0	1	0	2	0.795451
tr|E7ERH3|E7ERH3_HUMAN	TNS3	Tensin-3 OS=Homo sapiens GN=TNS3 PE=2 SV=1	130127	0	710	0	49	0	0.03	#DIV/0!	0	1	0	1	0	1	0	1	0.795451
sp|Q7Z5L9|I2BP2_HUMAN	IRF2BP2	Interferon regulatory factor 2-binding protein 2 OS=Homo sapiens GN=IRF2BP2 PE=1 SV=2	61728	0	709	0	49	0	0.05	#DIV/0!	0	1	0	1	0	1	0	1	0.795451
tr|G0KXF8|G0KXF8_HUMAN	HLA-B	MHC class I antigen (Fragment) OS=Homo sapiens GN=HLA-B PE=3 SV=1	21235	0	702	0	50	0	0.16	#DIV/0!	0	1	0	1	0	1	0	2	0.795451
tr|G1ENK8|G1ENK8_HUMAN	HLA-B	MHC class I antigen (Fragment) OS=Homo sapiens GN=HLA-B PE=3 SV=1	21236	0	701	0	50	0	0.16	#DIV/0!	0	1	0	1	0	1	0	2	0.795451
tr|C9WCV1|C9WCV1_HUMAN	HLA-B	MHC class I antigen (Fragment) OS=Homo sapiens GN=HLA-B PE=3 SV=1	21235	0	700	0	50	0	0.16	#DIV/0!	0	1	0	1	0	1	0	3	0.795451
tr|B4E3G4|B4E3G4_HUMAN	tr|B4E3G	cDNA FLJ50383, highly similar to CRSP complex subunit 3 OS=Homo sapiens PE=2 SV=1	116828	0	692	0	47	0	0.07	#DIV/0!	0	1	0	1	0	1	0	1	0.795451
sp|Q5JVF3|PCID2_HUMAN	PCID2	PCI domain-containing protein 2 OS=Homo sapiens GN=PCID2 PE=1 SV=2	46627	0	691	0	50	0	0.07	#DIV/0!	0	1	0	1	0	1	0	2	0.795451
tr|B2R5Y4|B2R5Y4_HUMAN	tr|B2R5Y	cDNA, FLJ92684, highly similar to Homo sapiens IK cytokine, down-regulator of HLA II (IK), mRNA OS=Homo sapiens PE=2 SV=1	65749	0	690	0	50	0	0.05	#DIV/0!	0	1	0	1	0	1	0	1	0.795451
tr|B3KXW5|B3KXW5_HUMAN	AP1G1	AP-1 complex subunit gamma-1 OS=Homo sapiens GN=AP1G1 PE=2 SV=1	94992	0	684	0	51	0	0.03	#DIV/0!	0	1	0	1	0	1	0	1	0.795451
sp|Q9ULD0|OGDHL_HUMAN	OGDHL	2-oxoglutarate dehydrogenase-like, mitochondrial OS=Homo sapiens GN=OGDHL PE=1 SV=3	115264	0	682	0	51	0	0.06	#DIV/0!	0	1	0	2	0	1	0	4	0.795451
tr|G5EA24|G5EA24_HUMAN	MCOLN2	Mucolipin 2, isoform CRA_a OS=Homo sapiens GN=MCOLN2 PE=2 SV=1	29581	0	676	0	52	0	0.11	#DIV/0!	0	1	0	1	0	1	0	1	0.795451
tr|Q6IPI1|Q6IPI1_HUMAN	RPL29	Ribosomal protein L29 OS=Homo sapiens GN=RPL29 PE=2 SV=1	17997	0	673	0	52	0	0.19	#DIV/0!	0	1	0	1	0	1	0	1	0.795451
tr|E7EVL6|E7EVL6_HUMAN	REPIN1	Replication initiator 1 (Fragment) OS=Homo sapiens GN=REPIN1 PE=2 SV=1	16586	0	667	0	52	0	0.2	#DIV/0!	0	1	0	1	0	1	0	1	0.795451
tr|H0Y2P0|H0Y2P0_HUMAN	CD44	CD44 antigen (Fragment) OS=Homo sapiens GN=CD44 PE=4 SV=1	31332	0	662	0	52	0	0.11	#DIV/0!	0	1	0	1	0	1	0	1	0.795451
tr|Q5D1D5|Q5D1D5_HUMAN	tr|Q5D1D	Guanylate binding protein 1 OS=Homo sapiens PE=2 SV=1	68387	0	659	0	53	0	0.08	#DIV/0!	0	1	0	1	0	1	0	1	0.795451
sp|P98160|PGBM_HUMAN	HSPG2	Basement membrane-specific heparan sulfate proteoglycan core protein OS=Homo sapiens GN=HSPG2 PE=1 SV=4	479253	0	655	0	53	0	0.01	#DIV/0!	0	1	0	1	0	1	0	1	0.795451
tr|H0Y4J2|H0Y4J2_HUMAN	MRPL37	39S ribosomal protein L37, mitochondrial (Fragment) OS=Homo sapiens GN=MRPL37 PE=4 SV=1	29806	0	653	0	54	0	0.11	#DIV/0!	0	1	0	1	0	1	0	2	0.795451
tr|H0YAY2|H0YAY2_HUMAN	KIAA0196	WASH complex subunit strumpellin (Fragment) OS=Homo sapiens GN=KIAA0196 PE=4 SV=1	82716	0	652	0	54	0	0.04	#DIV/0!	0	1	0	1	0	1	0	2	0.795451
tr|Q5W7F7|Q5W7F7_HUMAN	UBE3A	Ubiquitin ligase E3A isoform 3 OS=Homo sapiens GN=UBE3A PE=2 SV=1	98751	0	651	0	54	0	0.03	#DIV/0!	0	1	0	1	0	1	0	1	0.795451
tr|B4DFP1|B4DFP1_HUMAN	tr|B4DFP	cDNA FLJ51818, highly similar to Phosphoglucomutase-1 (EC 5.4.2.2) OS=Homo sapiens PE=2 SV=1	58987	0	645	0	28	0	0.11	#DIV/0!	0	0	0	1	0	1	0	1	0.795451
tr|H0YJ30|H0YJ30_HUMAN	GPHN	Molybdopterin molybdenumtransferase (Fragment) OS=Homo sapiens GN=GPHN PE=4 SV=1	30436	0	641	0	48	0	0.16	#DIV/0!	0	1	0	1	0	1	0	1	0.795451
tr|E5KLJ5|E5KLJ5_HUMAN	OPA1	Dynamin-like 120 kDa protein, mitochondrial OS=Homo sapiens GN=OPA1 PE=3 SV=1	118240	0	640	0	55	0	0.03	#DIV/0!	0	1	0	1	0	1	0	1	0.795451
tr|B3KN28|B3KN28_HUMAN	tr|B3KN2	cDNA FLJ13370 fis, clone PLACE1000653, highly similar to Phosphoacetylglucosamine mutase (EC 5.4.2.3) OS=Homo sapiens PE=2 SV	60284	0	626	0	56	0	0.05	#DIV/0!	0	1	0	1	0	1	0	1	0.795451
tr|J3QKS9|J3QKS9_HUMAN	NOL11	Nucleolar protein 11 OS=Homo sapiens GN=NOL11 PE=4 SV=1	18816	0	617	0	58	0	0.18	#DIV/0!	0	1	0	1	0	1	0	1	0.795451
sp|P35251|RFC1_HUMAN	RFC1	Replication factor C subunit 1 OS=Homo sapiens GN=RFC1 PE=1 SV=4	128688	0	605	0	59	0	0.03	#DIV/0!	0	1	0	1	0	1	0	2	0.795451
tr|H0YJS8|H0YJS8_HUMAN	PSMC6	26S protease regulatory subunit 10B (Fragment) OS=Homo sapiens GN=PSMC6 PE=4 SV=1	22376	0	601	0	60	0	0.15	#DIV/0!	0	1	0	1	0	1	0	2	0.795451
tr|E9PHM2|E9PHM2_HUMAN	LARS2	Probable leucine--tRNA ligase, mitochondrial OS=Homo sapiens GN=LARS2 PE=2 SV=1	97782	0	600	0	60	0	0.03	#DIV/0!	0	1	0	1	0	1	0	1	0.795451
sp|Q92626|PXDN_HUMAN	PXDN	Peroxidasin homolog OS=Homo sapiens GN=PXDN PE=1 SV=2	167793	0	586	0	61	0	0.04	#DIV/0!	0	1	0	1	0	1	0	1	0.795451
tr|H0YL11|H0YL11_HUMAN	IDH2	Isocitrate dehydrogenase [NADP], mitochondrial OS=Homo sapiens GN=IDH2 PE=3 SV=1	35536	0	579	0	58	0	0.15	#DIV/0!	0	1	0	1	0	1	0	1	0.795451
tr|B2R8G6|B2R8G6_HUMAN	tr|B2R8G	cDNA, FLJ93891, Homo sapiens FK506 binding protein 8, 38kDa (FKBP8), mRNA OS=Homo sapiens PE=2 SV=1	38870	0	571	0	62	0	0.09	#DIV/0!	0	1	0	1	0	1	0	1	0.795451
sp|P22059|OSBP1_HUMAN	OSBP	Oxysterol-binding protein 1 OS=Homo sapiens GN=OSBP PE=1 SV=1	90220	0	567	0	62	0	0.07	#DIV/0!	0	1	0	2	0	1	0	2	0.795451
sp|Q14739|LBR_HUMAN	LBR	Lamin-B receptor OS=Homo sapiens GN=LBR PE=1 SV=2	71057	0	565	0	63	0	0.05	#DIV/0!	0	1	0	1	0	1	0	1	0.795451
tr|B3KUI0|B3KUI0_HUMAN	tr|B3KUI	cDNA FLJ39945 fis, clone SPLEN2023977, highly similar to Homo sapiens CD97 antigen (CD97), transcript variant 2, mRNA OS=Homo	79803	0	564	0	63	0	0.04	#DIV/0!	0	1	0	1	0	1	0	1	0.795451
tr|B3KNR6|B3KNR6_HUMAN	tr|B3KNR	cDNA FLJ30255 fis, clone BRACE2002444, highly similar to LAS1-like protein OS=Homo sapiens PE=2 SV=1	27615	0	560	0	34	0	0.12	#DIV/0!	0	0	0	1	0	1	0	1	0.795451
tr|F5GZS0|F5GZS0_HUMAN	DHX36	Probable ATP-dependent RNA helicase DHX36 OS=Homo sapiens GN=DHX36 PE=2 SV=1	113928	0	555	0	64	0	0.03	#DIV/0!	0	1	0	1	0	1	0	2	0.795451
tr|D3DWL9|D3DWL9_HUMAN	CPSF1	Cleavage and polyadenylation specific factor 1, 160kDa, isoform CRA_a OS=Homo sapiens GN=CPSF1 PE=2 SV=1	153087	0	553	0	64	0	0.02	#DIV/0!	0	1	0	1	0	1	0	2	0.795451
tr|B4DSP0|B4DSP0_HUMAN	hCG_1642	HCG1642748, isoform CRA_b OS=Homo sapiens GN=hCG_1642748 PE=2 SV=1	45559	0	549	0	65	0	0.07	#DIV/0!	0	1	0	1	0	1	0	1	0.795451
tr|H0Y4Z6|H0Y4Z6_HUMAN	PYGB	Phosphorylase (Fragment) OS=Homo sapiens GN=PYGB PE=3 SV=1	27767	0	546	0	0	0	0.14	#DIV/0!	0	0	0	0	0	1	0	1	0.795451
tr|Q2KQ72|Q2KQ72_HUMAN	tr|Q2KQ7	Chromosome-associated protein E OS=Homo sapiens PE=2 SV=1	86324	0	544	0	65	0	0.02	#DIV/0!	0	1	0	1	0	1	0	1	0.795451
tr|H0Y2Y8|H0Y2Y8_HUMAN	ZYX	Zyxin (Fragment) OS=Homo sapiens GN=ZYX PE=4 SV=1	58804	0	538	0	66	0	0.06	#DIV/0!	0	1	0	1	0	1	0	1	0.795451
tr|B5BUD2|B5BUD2_HUMAN	RFC2	Replication factor C 2 isoform 1 (Fragment) OS=Homo sapiens GN=RFC2 PE=2 SV=1	39616	0	536	0	66	0	0.08	#DIV/0!	0	1	0	1	0	1	0	1	0.795451
tr|B4DGX9|B4DGX9_HUMAN	tr|B4DGX	cDNA FLJ60509, highly similar to Probable ubiquitin carboxyl-terminalhydrolase FAF-X (EC 3.1.2.15) OS=Homo sapiens PE=2 SV=1	66195	0	527	0	32	0	0.03	#DIV/0!	0	0	0	1	0	1	0	2	0.795451
tr|B1ALA7|B1ALA7_HUMAN	PRPS1	Phosphoribosyl pyrophosphate synthetase 1 OS=Homo sapiens GN=PRPS1 PE=4 SV=1	18730	0	523	0	70	0	0.18	#DIV/0!	0	1	0	1	0	1	0	1	0.795451
tr|B0V109|B0V109_HUMAN	FLOT1	Flotillin-1 (Fragment) OS=Homo sapiens GN=FLOT1 PE=4 SV=1	39810	0	514	0	71	0	0.16	#DIV/0!	0	1	0	1	0	1	0	1	0.795451
sp|Q8IWZ3|ANKH1_HUMAN	ANKHD1	Ankyrin repeat and KH domain-containing protein 1 OS=Homo sapiens GN=ANKHD1 PE=1 SV=1	271286	0	508	0	72	0	0.01	#DIV/0!	0	1	0	1	0	1	0	2	0.795451
tr|Q7Z586|Q7Z586_HUMAN	tr|Q7Z58	Putative uncharacterized protein (Fragment) OS=Homo sapiens PE=2 SV=1	105456	0	507	0	72	0	0.04	#DIV/0!	0	1	0	1	0	1	0	1	0.795451
tr|G3V1J5|G3V1J5_HUMAN	DIS3	Exosome complex exonuclease RRP44 OS=Homo sapiens GN=DIS3 PE=2 SV=1	91382	0	483	0	30	0	0.09	#DIV/0!	0	0	0	1	0	1	0	1	0.795451
sp|P33897|ABCD1_HUMAN	ABCD1	ATP-binding cassette sub-family D member 1 OS=Homo sapiens GN=ABCD1 PE=1 SV=2	83398	0	482	0	75	0	0.04	#DIV/0!	0	1	0	1	0	1	0	1	0.795451
sp|Q9H009|NACA2_HUMAN	NACA2	Nascent polypeptide-associated complex subunit alpha-2 OS=Homo sapiens GN=NACA2 PE=1 SV=1	23209	0	469	0	78	0	0.14	#DIV/0!	0	1	0	1	0	1	0	1	0.795451
tr|H0YHX9|H0YHX9_HUMAN	NACA	Nascent polypeptide-associated complex subunit alpha (Fragment) OS=Homo sapiens GN=NACA PE=4 SV=1	22930	0	468	0	78	0	0.15	#DIV/0!	0	1	0	1	0	1	0	1	0.795451
tr|F5H1Y3|F5H1Y3_HUMAN	FEN1	Flap endonuclease 1 (Fragment) OS=Homo sapiens GN=FEN1 PE=2 SV=1	18377	0	443	0	83	0	0.18	#DIV/0!	0	1	0	1	0	1	0	1	0.795451
tr|Q5LJ97|Q5LJ97_HUMAN	TROVE2	TROVE domain family, member 2 (Fragment) OS=Homo sapiens GN=TROVE2 PE=2 SV=1	28360	0	433	0	56	0	0.11	#DIV/0!	0	1	0	1	0	1	0	1	0.795451
tr|B4DTS5|B4DTS5_HUMAN	tr|B4DTS	cDNA FLJ58882, highly similar to 26S proteasome non-ATPase regulatory subunit 11 OS=Homo sapiens PE=2 SV=1	35374	0	424	0	37	0	0.22	#DIV/0!	0	1	0	1	0	1	0	1	0.795451
tr|Q5VZA6|Q5VZA6_HUMAN	ACO1	Cytoplasmic aconitate hydratase OS=Homo sapiens GN=ACO1 PE=4 SV=1	48888	0	421	0	54	0	0.12	#DIV/0!	0	1	0	1	0	1	0	2	0.795451
tr|H0Y7C0|H0Y7C0_HUMAN	USO1	General vesicular transport factor p115 (Fragment) OS=Homo sapiens GN=USO1 PE=4 SV=1	72345	0	409	0	88	0	0.05	#DIV/0!	0	1	0	1	0	1	0	1	0.795451
tr|Q3SYK8|Q3SYK8_HUMAN	NUMA1	NUMA1 protein (Fragment) OS=Homo sapiens GN=NUMA1 PE=2 SV=1	78666	0	309	0	41	0	0.07	#DIV/0!	0	1	0	1	0	1	0	2	0.795451
tr|H7BZD1|H7BZD1_HUMAN	GLS	Glutaminase kidney isoform, mitochondrial (Fragment) OS=Homo sapiens GN=GLS PE=4 SV=1	12786	0	213	0	0	0	0.24	#DIV/0!	0	0	0	0	0	1	0	1	0.795451
tr|F5H6D0|F5H6D0_HUMAN	MOGS	Mannosyl-oligosaccharide glucosidase OS=Homo sapiens GN=MOGS PE=2 SV=1	80824	0	1196	0	15	0	0.04	#DIV/0!	0	0	0	1	0	0	0	1	-0.78971
tr|B4DYF0|B4DYF0_HUMAN	tr|B4DYF	cDNA FLJ61469, highly similar to Zinc finger protein 694 OS=Homo sapiens PE=2 SV=1	89134	0	1195	0	15	0	0.04	#DIV/0!	0	0	0	1	0	0	0	1	-0.78971
sp|Q6P1J9|CDC73_HUMAN	CDC73	Parafibromin OS=Homo sapiens GN=CDC73 PE=1 SV=1	60653	0	1186	0	18	0	0.05	#DIV/0!	0	0	0	1	0	0	0	2	-0.78971
tr|B4DV28|B4DV28_HUMAN	tr|B4DV2	cDNA FLJ54170, highly similar to Cytosolic nonspecific dipeptidase OS=Homo sapiens PE=2 SV=1	51811	0	1185	0	18	0	0.08	#DIV/0!	0	0	0	1	0	0	0	1	-0.78971
tr|B4E205|B4E205_HUMAN	tr|B4E205	cDNA FLJ61651, highly similar to Protein transport protein Sec24A OS=Homo sapiens PE=2 SV=1	96953	0	1182	0	18	0	0.03	#DIV/0!	0	0	0	1	0	0	0	1	-0.78971
tr|B4DTM6|B4DTM6_HUMAN	tr|B4DTM	cDNA FLJ61391, highly similar to Protein transport protein Sec24B OS=Homo sapiens PE=2 SV=1	130037	0	1181	0	18	0	0.03	#DIV/0!	0	0	0	1	0	0	0	1	-0.78971
tr|B4DF80|B4DF80_HUMAN	tr|B4DF8	cDNA FLJ58527, highly similar to Nucleolar protein 6 OS=Homo sapiens PE=2 SV=1	122714	0	1180	0	18	0	0.03	#DIV/0!	0	0	0	1	0	0	0	1	-0.78971
sp|Q99698|LYST_HUMAN	LYST	Lysosomal-trafficking regulator OS=Homo sapiens GN=LYST PE=1 SV=3	434169	0	1179	0	18	0	0.02	#DIV/0!	0	0	0	1	0	0	0	1	-0.78971
tr|F6U211|F6U211_HUMAN	RPS10	40S ribosomal protein S10 OS=Homo sapiens GN=RPS10 PE=2 SV=1	19912	0	1178	0	18	0	0.17	#DIV/0!	0	0	0	1	0	0	0	1	-0.78971
tr|Q6IBS5|Q6IBS5_HUMAN	DLST	DLST protein OS=Homo sapiens GN=DLST PE=2 SV=1	49131	0	1169	0	19	0	0.07	#DIV/0!	0	0	0	1	0	0	0	2	-0.78971
tr|E9PF58|E9PF58_HUMAN	ARPC1A	Actin-related protein 2/3 complex subunit 1A OS=Homo sapiens GN=ARPC1A PE=2 SV=1	31268	0	1168	0	20	0	0.08	#DIV/0!	0	0	0	1	0	0	0	1	-0.78971
tr|B3KNI0|B3KNI0_HUMAN	tr|B3KNI	cDNA FLJ14646 fis, clone NT2RP2001883, highly similar to Homo sapiens CGI-01 protein mRNA OS=Homo sapiens PE=2 SV=1	79495	0	1166	0	20	0	0.05	#DIV/0!	0	0	0	1	0	0	0	1	-0.78971
tr|B2RBB2|B2RBB2_HUMAN	tr|B2RBB	cDNA, FLJ95416, highly similar to Homo sapiens phosphatidylinositol glycan, class S (PIGS), mRNA OS=Homo sapiens PE=2 SV=1	61818	0	1165	0	20	0	0.05	#DIV/0!	0	0	0	1	0	0	0	1	-0.78971
tr|A0PJK8|A0PJK8_HUMAN	SCRIB	SCRIB protein (Fragment) OS=Homo sapiens GN=SCRIB PE=2 SV=1	71166	0	1134	0	0	0	0.02	#DIV/0!	0	0	0	0	0	0	0	1	-0.78971
tr|H3BRG4|H3BRG4_HUMAN	UQCRC2	Cytochrome b-c1 complex subunit 2, mitochondrial OS=Homo sapiens GN=UQCRC2 PE=3 SV=1	44778	0	1116	0	0	0	0.07	#DIV/0!	0	0	0	0	0	0	0	1	-0.78971
tr|B4DJ92|B4DJ92_HUMAN	tr|B4DJ92	cDNA FLJ57843, highly similar to Homo sapiens neurochondrin (NCDN), transcript variant 3, mRNA OS=Homo sapiens PE=2 SV=1	20030	0	1095	0	0	0	0.04	#DIV/0!	0	0	0	0	0	0	0	1	-0.78971
tr|Q56VW8|Q56VW8_HUMAN	tr|Q56VW	Lung cancer-related protein 9 OS=Homo sapiens PE=2 SV=1	52186	0	1086	0	19	0	0.13	#DIV/0!	0	0	0	1	0	0	0	1	-0.78971
tr|B4DFM1|B4DFM1_HUMAN	tr|B4DFM	cDNA FLJ53034, highly similar to AP-2 complex subunit mu-1 OS=Homo sapiens PE=2 SV=1	44226	0	953	0	0	0	0.06	#DIV/0!	0	0	0	0	0	0	0	3	-0.78971
tr|A0PJC9|A0PJC9_HUMAN	TPR	TPR protein (Fragment) OS=Homo sapiens GN=TPR PE=2 SV=1	53894	0	950	0	0	0	0.02	#DIV/0!	0	0	0	0	0	0	0	1	-0.78971
tr|B4DQR0|B4DQR0_HUMAN	tr|B4DQR	cDNA FLJ54377 OS=Homo sapiens PE=2 SV=1	91332	0	738	0	0	0	0.07	#DIV/0!	0	0	0	0	0	0	0	1	-0.78971
tr|C9J634|C9J634_HUMAN	PDHB	Pyruvate dehydrogenase E1 component subunit beta, mitochondrial OS=Homo sapiens GN=PDHB PE=2 SV=1	39064	0	660	0	0	0	0.27	#DIV/0!	0	0	0	0	0	0	0	1	-0.78971
tr|Q2TU64|Q2TU64_HUMAN	tr|Q2TU6	PIG48 OS=Homo sapiens PE=2 SV=1	61110	279	105	68	302	0.05	0.93	18.6	1	8	1	10	3	10	3	12	0.796199
tr|Q59GV0|Q59GV0_HUMAN	tr|Q59GV	Propionyl Coenzyme A carboxylase, beta polypeptide variant (Fragment) OS=Homo sapiens PE=2 SV=1	60578	331	198	53	195	0.05	0.87	17.4	1	5	1	11	1	9	2	12	1.874683
tr|B2RBA6|B2RBA6_HUMAN	tr|B2RBA	cDNA, FLJ95407, highly similar to Homo sapiens MCM7 minichromosome maintenance deficient 7 (S. cerevisiae) (MCM7), mRNA OS	81856	374	166	46	224	0.04	0.6	15	1	6	1	11	1	14	1	15	2.485718
tr|Q9NSU9|Q9NSU9_HUMAN	DKFZp434	Putative uncharacterized protein DKFZp434G0719 (Fragment) OS=Homo sapiens GN=DKFZp434G0719 PE=2 SV=1	41107	56	161	169	232	0.07	0.86	12.28571429	4	7	5	8	5	9	5	9	-0.00114
tr|Q4ZG57|Q4ZG57_HUMAN	MCM6	Putative uncharacterized protein MCM6 (Fragment) OS=Homo sapiens GN=MCM6 PE=2 SV=1	89803	444	145	39	259	0.04	0.48	12	1	9	1	11	2	12	3	14	1.533896
tr|J3KR24|J3KR24_HUMAN	IARS	Isoleucine--tRNA ligase, cytoplasmic OS=Homo sapiens GN=IARS PE=4 SV=1	132820	366	117	47	301	0.02	0.24	12	1	6	1	9	2	10	2	14	1.281965
tr|E9PBS1|E9PBS1_HUMAN	PAICS	Phosphoribosylaminoimidazole carboxylase (Fragment) OS=Homo sapiens GN=PAICS PE=2 SV=1	46307	270	132	69	259	0.07	0.82	11.71428571	1	6	1	8	2	8	5	9	0.976718
sp|O14980|XPO1_HUMAN	XPO1	Exportin-1 OS=Homo sapiens GN=XPO1 PE=1 SV=1	124447	305	152	59	240	0.03	0.33	11	1	7	1	11	1	12	3	17	2.2712
tr|F5H120|F5H120_HUMAN	FKBP4	Peptidyl-prolyl cis-trans isomerase FKBP4, N-terminally processed OS=Homo sapiens GN=FKBP4 PE=2 SV=1	5528	623	570	24	62	0.06	0.65	10.83333333	0	1	1	1	1	1	1	2	-0.78985
tr|B7ZAW0|B7ZAW0_HUMAN	tr|B7ZAW	cDNA, FLJ79324, highly similar to Carbamoyl-phosphate synthase (ammonia), mitochondrial (EC 6.3.4.16) OS=Homo sapiens PE=2 SV	20368	204	125	44	293	0.15	1.51	10.06666667	1	7	1	7	2	8	2	8	0.976718
sp|Q14204|DYHC1_HUMAN	DYNC1H1	Cytoplasmic dynein 1 heavy chain 1 OS=Homo sapiens GN=DYNC1H1 PE=1 SV=5	534809	168	52	129	620	0.02	0.2	10	3	21	4	30	4	34	6	43	2.154213
tr|D3DP78|D3DP78_HUMAN	DARS	Aspartyl-tRNA synthetase, isoform CRA_b OS=Homo sapiens GN=DARS PE=2 SV=1	53829	344	240	51	132	0.06	0.56	9.333333333	1	4	1	7	2	9	3	9	1.137379
sp|Q9UQ35|SRRM2_HUMAN	SRRM2	Serine/arginine repetitive matrix protein 2 OS=Homo sapiens GN=SRRM2 PE=1 SV=2	300179	247	188	78	204	0.02	0.18	9	1	7	2	10	2	10	5	11	1.281965
tr|B2RE46|B2RE46_HUMAN	tr|B2RE4	cDNA, FLJ96923, highly similar to Homo sapiens ribophorin II (RPN2), mRNA OS=Homo sapiens PE=2 SV=1	69405	589	187	27	205	0.05	0.45	9	0	7	1	8	2	8	2	9	0.976718
tr|A8K781|A8K781_HUMAN	tr|A8K78	cDNA FLJ75299, highly similar to Xenopus laevis proteasome (prosome, macropain) 26S subunit, ATPase 3, mRNA OS=Homo sapiens	47578	603	356	26	100	0.07	0.62	8.857142857	0	3	1	4	2	4	2	6	0.058396
tr|H0UID3|H0UID3_HUMAN	AP2B1	Adaptor-related protein complex 2, beta 1 subunit, isoform CRA_d OS=Homo sapiens GN=AP2B1 PE=4 SV=1	106321	648	272	22	145	0.03	0.26	8.666666667	0	5	1	7	3	9	3	11	0.651613
tr|E7ERW8|E7ERW8_HUMAN	DIAPH1	Protein diaphanous homolog 1 OS=Homo sapiens GN=DIAPH1 PE=2 SV=1	135945	184	343	116	112	0.02	0.17	8.5	1	4	1	7	1	9	2	10	1.874683
sp|P11498|PYC_HUMAN	PC	Pyruvate carboxylase, mitochondrial OS=Homo sapiens GN=PC PE=1 SV=2	130293	10	1	1295	5065	1.76	14.64	8.318181818	40	135	52	172	56	191	67	211	0.990137
sp|P52597|HNRPF_HUMAN	HNRNPF	Heterogeneous nuclear ribonucleoprotein F OS=Homo sapiens GN=HNRNPF PE=1 SV=3	45985	226	199	91	195	0.07	0.53	7.571428571	1	4	1	6	2	6	3	8	0.589303
tr|Q5T7Q0|Q5T7Q0_HUMAN	DNAJA1	DnaJ (Hsp40) homolog, subfamily A, member 1, isoform CRA_c OS=Homo sapiens GN=DNAJA1 PE=2 SV=1	45581	703	347	18	110	0.07	0.52	7.428571429	0	4	1	6	0	7	1	7	3.118555
tr|B2ZZ90|B2ZZ90_HUMAN	ACACA	Acetyl-Coenzyme A carboxylase alpha OS=Homo sapiens GN=ACACA PE=2 SV=1	267095	31	3	628	3635	0.44	3.24	7.363636364	25	105	30	140	38	157	46	169	1.260878
sp|P45974|UBP5_HUMAN	USP5	Ubiquitin carboxyl-terminal hydrolase 5 OS=Homo sapiens GN=USP5 PE=1 SV=2	96638	622	236	24	161	0.03	0.22	7.333333333	0	3	1	6	2	8	2	9	0.976718
sp|P54886|P5CS_HUMAN	ALDH18A1	Delta-1-pyrroline-5-carboxylate synthase OS=Homo sapiens GN=ALDH18A1 PE=1 SV=2	87989	658	275	21	143	0.04	0.29	7.25	1	4	1	7	1	7	1	11	1.533254
tr|E7ENU7|E7ENU7_HUMAN	RPL15	Ribosomal protein L15 (Fragment) OS=Homo sapiens GN=RPL15 PE=2 SV=1	20596	534	399	31	76	0.16	1.13	7.0625	0	2	1	4	1	6	1	7	1.326607
tr|E7EVA0|E7EVA0_HUMAN	MAP4	Microtubule-associated protein OS=Homo sapiens GN=MAP4 PE=2 SV=1	246546	429	1085	40	27	0.01	0.07	7	1	0	1	1	2	1	4	1	-1.52715
tr|Q05CK9|Q05CK9_HUMAN	SYNCRIP	SYNCRIP protein (Fragment) OS=Homo sapiens GN=SYNCRIP PE=2 SV=1	50847	252	176	76	216	0.13	0.87	6.692307692	2	5	2	10	2	10	2	10	1.281965
sp|O95782|AP2A1_HUMAN	AP2A1	AP-2 complex subunit alpha-1 OS=Homo sapiens GN=AP2A1 PE=1 SV=3	108561	500	253	34	154	0.03	0.2	6.666666667	0	5	1	6	1	8	2	9	1.714022
tr|H0Y8E6|H0Y8E6_HUMAN	MCM2	DNA replication licensing factor MCM2 (Fragment) OS=Homo sapiens GN=MCM2 PE=3 SV=1	94792	211	203	98	190	0.07	0.45	6.428571429	1	8	2	11	2	11	2	12	1.413406
tr|A6NKB8|A6NKB8_HUMAN	RNPEP	Aminopeptidase B OS=Homo sapiens GN=RNPEP PE=2 SV=1	68752	316	355	57	104	0.05	0.32	6.4	1	3	2	6	2	6	2	9	0.589303
tr|B2R5V9|B2R5V9_HUMAN	tr|B2R5V	cDNA, FLJ92652, highly similar to Homo sapiens high density lipoprotein binding protein (vigilin)(HDLBP), mRNA OS=Homo sapiens P	141919	253	244	76	157	0.02	0.12	6	1	5	1	5	2	5	2	8	0.348098
sp|Q7L2E3|DHX30_HUMAN	DHX30	Putative ATP-dependent RNA helicase DHX30 OS=Homo sapiens GN=DHX30 PE=1 SV=1	134938	397	204	43	189	0.05	0.3	6	1	7	2	11	3	13	4	16	1.159358

tr|B2R7B5|B2R7B5_HUMAN	tr|B2R7B5	cDNA, FLJ93365, highly similar to Homo sapiens KH domain containing, RNA binding, signal transduction associated 1 (KHDRBS1), m	48309	347	380	51	95	0.07	0.41	5.857142857	1	3	1	5	2	5	4	5	0.348098
tr|B2R5P6|B2R5P6_HUMAN	TXNRD1	Thioredoxin reductase 1, cytoplasmic OS=Homo sapiens GN=TXNRD1 PE=2 SV=1	55253	108	95	204	358	0.26	1.52	5.846153846	3	10	5	16	5	16	6	17	0.796699
tr|Q68DB1|Q68DB1_HUMAN	DKFZp686	Metastasis-associated protein MTA2 OS=Homo sapiens GN=DKFZp686F2281 PE=2 SV=1	55515	676	250	20	40	0.06	0.35	5.833333333	0	0	1	3	1	4	1	5	0.7957
tr|Q1L838|Q1L838_HUMAN	GTBP	GTBP-ALT OS=Homo sapiens GN=GTBP PE=2 SV=1	122267	559	273	29	145	0.03	0.17	5.666666667	0	3	1	6	1	7	1	8	1.533254
tr|B0UZ98|B0UZ98_HUMAN	BAT1	HLA-B associated transcript 1 (Fragment) OS=Homo sapiens GN=BAT1 PE=2 SV=1	28623	394	339	44	34	0.09	0.51	5.666666667	1	0	1	2	1	2	1	2	-0.05269
sp|O00425|IF2B3_HUMAN	IGF2BP3	Insulin-like growth factor 2 mRNA-binding protein 3 OS=Homo sapiens GN=IGF2BP3 PE=1 SV=2	64008	556	299	29	128	0.05	0.28	5.6	0	5	1	5	2	6	2	6	0.589303
tr|Q2Q9B7|Q2Q9B7_HUMAN	G6PD	Glucose-6-phosphate 1-dehydrogenase (Fragment) OS=Homo sapiens GN=G6PD PE=2 SV=1	55221	176	174	118	211	0.18	1	5.555555556	2	6	2	11	5	15	6	16	0.706304
tr|B4DYA7|B4DYA7_HUMAN	tr|B4DYA	Glucose-6-phosphate 1-dehydrogenase OS=Homo sapiens PE=2 SV=1	58757	176	175	123	217	0.18	1	5.555555556	2	5	3	11	6	14	7	15	0.368546
sp|P49411|EFTU_HUMAN	TUFM	Elongation factor Tu, mitochondrial OS=Homo sapiens GN=TUFM PE=1 SV=2	49852	210	77	99	446	0.29	1.61	5.551724138	3	11	4	15	4	18	5	21	1.251996
tr|B4DLT2|B4DLT2_HUMAN	tr|B4DLT2	cDNA FLJ56637, highly similar to Nuclear pore complex protein Nup155 OS=Homo sapiens PE=2 SV=1	152734	489	212	35	178	0.02	0.11	5.5	0	4	1	5	1	6	1	7	1.326607
tr|I3L1L3|I3L1L3_HUMAN	MYBBP1A	Myb-binding protein 1A (Fragment) OS=Homo sapiens GN=MYBBP1A PE=4 SV=1	141124	698	374	18	91	0.02	0.11	5.5	0	2	1	4	1	4	2	6	0.7957
tr|Q2NLA0|Q2NLA0_HUMAN	COPG2	Coatomer subunit gamma OS=Homo sapiens GN=COPG2 PE=2 SV=1	82400	705	311	18	63	0.04	0.22	5.5	0	1	1	1	0	1	1	1	0.795451
tr|B4DFL1|B4DFL1_HUMAN	tr|B4DFL1	Dihydrolipoyl dehydrogenase OS=Homo sapiens PE=2 SV=1	49381	666	254	21	153	0.07	0.38	5.428571429	0	4	1	5	1	5	1	5	1.085403
tr|Q5SU16|Q5SU16_HUMAN	TUBB	Beta 5-tubulin OS=Homo sapiens GN=TUBB PE=2 SV=1	50095	25	9	680	2013	3.07	16.63	5.416938111	26	63	33	75	35	82	39	88	0.43112
tr|I3L2F9|I3L2F9_HUMAN	tr|I3L2F9	Uncharacterized protein OS=Homo sapiens PE=3 SV=1	46054	25	8	275	498	3.07	16.41	5.345276873	12	21	15	26	18	30	19	34	-0.06864
tr|J3KNE5|J3KNE5_HUMAN	HARS	Histidine--tRNA ligase, cytoplasmic OS=Homo sapiens GN=HARS PE=4 SV=1	50581	672	330	20	101	0.07	0.37	5.285714286	0	2	1	4	1	4	2	4	0.7957
sp|P41250|SYG_HUMAN	GARS	Glycine--tRNA ligase OS=Homo sapiens GN=GARS PE=1 SV=3	83854	392	408	44	89	0.04	0.21	5.25	1	1	1	5	1	7	2	7	1.533254
tr|B1AHB0|B1AHB0_HUMAN	MCM5	MCM5 minichromosome maintenance deficient 5, cell division cycle 46 (S. cerevisiae), isoform CRA_a OS=Homo sapiens GN=MCM5	83031	562	324	29	116	0.04	0.21	5.25	0	4	1	5	1	6	2	6	1.326607
tr|B4DMJ6|B4DMJ6_HUMAN	tr|B4DMJ	cDNA FLJ50996, highly similar to 60S ribosomal protein L4 OS=Homo sapiens PE=2 SV=1	46269	163	97	132	308	0.51	2.65	5.196078431	4	7	6	14	8	15	9	18	0.077256
tr|Q8IWP6|Q8IWP6_HUMAN	tr|Q8IWP	Class IVb beta tubulin OS=Homo sapiens PE=2 SV=1	50177	28	10	654	1975	3.05	15.44	5.062295082	27	63	32	74	34	80	37	86	0.436883
tr|Q5VZU9|Q5VZU9_HUMAN	TPP2	Tripeptidyl-peptidase 2 OS=Homo sapiens GN=TPP2 PE=4 SV=1	141160	693	732	19	47	0.02	0.1	5	0	1	1	4	0	5	1	6	2.670704
tr|Q05D08|Q05D08_HUMAN	PA2G4	PA2G4 protein (Fragment) OS=Homo sapiens GN=PA2G4 PE=2 SV=1	45579	286	362	64	103	0.15	0.75	5	1	4	2	8	3	9	3	9	0.651613
tr|A4FU77|A4FU77_HUMAN	SNRNP200	SNRNP200 protein (Fragment) OS=Homo sapiens GN=SNRNP200 PE=2 SV=1	217585	243	159	83	233	0.05	0.25	5	2	8	3	15	7	14	8	20	0.161756
tr|F8WJN3|F8WJN3_HUMAN	CPSF6	Cleavage and polyadenylation-specificity factor subunit 6 OS=Homo sapiens GN=CPSF6 PE=2 SV=1	52409	379	1084	46	27	0.06	0.3	5	1	0	1	1	1	1	1	1	-0.78985
tr|B5LY69|B5LY69_HUMAN	UMPS	Uridine monophosphate synthetase isoform G OS=Homo sapiens GN=UMPS PE=2 SV=1	40514	302	619	59	57	0.1	0.5	5	1	1	1	1	1	1	1	1	-0.78985
sp|P05165|PCCA_HUMAN	PCCA	Propionyl-CoA carboxylase alpha chain, mitochondrial OS=Homo sapiens GN=PCCA PE=1 SV=4	80635	99	44	216	797	0.49	2.44	4.979591837	6	22	10	31	13	32	13	35	0.479646
tr|D3DTH7|D3DTH7_HUMAN	MYO1C	Myosin IC, isoform CRA_a OS=Homo sapiens GN=MYO1C PE=2 SV=1	99613	284	220	65	173	0.07	0.34	4.857142857	1	6	3	9	3	10	4	11	0.796199
tr|B2R7C5|B2R7C5_HUMAN	tr|B2R7C5	cDNA, FLJ93378, highly similar to Homo sapiens MCM3 minichromosome maintenance deficient 3 (S. cerevisiae) (MCM3), mRNA OS	91579	479	461	36	79	0.04	0.19	4.75	1	2	1	5	1	6	1	11	1.326607
tr|B4DY09|B4DY09_HUMAN	tr|B4DY09	cDNA FLJ51660, highly similar to Interleukin enhancer-binding factor 2 OS=Homo sapiens PE=2 SV=1	39057	383	282	45	140	0.08	0.38	4.75	1	2	1	4	1	5	1	5	1.085403
tr|F8VPD4|F8VPD4_HUMAN	CAD	CAD protein OS=Homo sapiens GN=CAD PE=2 SV=1	238097	317	165	57	229	0.04	0.19	4.75	1	7	3	13	4	15	5	16	0.99615
tr|E5KNY5|E5KNY5_HUMAN	LRPPRC	Leucine-rich PPR-motif containing OS=Homo sapiens GN=LRPPRC PE=2 SV=1	159003	329	91	53	363	0.08	0.38	4.75	1	15	4	16	5	18	7	21	0.962151
tr|A8K2Y2|A8K2Y2_HUMAN	tr|A8K2Y2	cDNA FLJ78120, highly similar to Homo sapiens eukaryotic translation initiation factor 2, subunit 3 gamma, 52kDa (EIF2S3), mRNA O	51648	475	470	37	78	0.06	0.28	4.666666667	1	2	1	4	1	5	1	5	1.085403
tr|B4DKS0|B4DKS0_HUMAN	tr|B4DKS0	cDNA FLJ53381, highly similar to Monocarboxylate transporter 1 OS=Homo sapiens PE=2 SV=1	52487	598	217	26	148	0.06	0.28	4.666666667	0	2	1	3	1	3	1	3	0.432934
tr|B2R9U2|B2R9U2_HUMAN	tr|B2R9U	cDNA, FLJ94557, highly similar to Homo sapiens FK506 binding protein 4, 59kDa (FKBP4), mRNA OS=Homo sapiens PE=2 SV=1	52087	623	238	24	160	0.06	0.28	4.666666667	0	4	1	4	2	5	2	5	0.348098
tr|Q0VGD6|Q0VGD6_HUMAN	HNRPR	HNRPR protein (Fragment) OS=Homo sapiens GN=HNRPR PE=2 SV=1	68218	390	184	44	207	0.1	0.46	4.6	1	5	2	8	2	9	4	10	1.137379
tr|B7Z5E2|B7Z5E2_HUMAN	PSMC2	26S protease regulatory subunit 7 OS=Homo sapiens GN=PSMC2 PE=2 SV=1	33594	610	349	25	109	0.1	0.46	4.6	0	2	1	4	1	5	1	6	1.085403
tr|J3KQL0|J3KQL0_HUMAN	CPS1	Carbamoyl-phosphate synthase [ammonia], mitochondrial OS=Homo sapiens GN=CPS1 PE=3 SV=1	166686	204	50	102	668	0.15	0.69	4.6	4	21	7	28	10	27	12	29	0.601243
tr|B4DHQ3|B4DHQ3_HUMAN	tr|B4DHQ	cDNA FLJ56437, highly similar to Phosphoserine aminotransferase (EC 2.6.1.52) OS=Homo sapiens PE=2 SV=1	45783	552	484	30	75	0.07	0.32	4.571428571	0	2	1	4	1	4	2	5	0.7957
sp|P04350|TBB4A_HUMAN	TUBB4A	Tubulin beta-4A chain OS=Homo sapiens GN=TUBB4A PE=1 SV=2	50010	42	12	509	1572	1.96	8.93	4.556122449	21	52	27	63	29	69	32	74	0.450317
sp|P53618|COPB_HUMAN	COPB1	Coatomer subunit beta OS=Homo sapiens GN=COPB1 PE=1 SV=3	108214	303	183	59	208	0.06	0.27	4.5	1	5	2	8	2	12	3	14	1.533896
tr|B4DFM0|B4DFM0_HUMAN	FARSLB	Phenylalanine-tRNA synthetase-like, beta subunit, isoform CRA_c OS=Homo sapiens GN=FARSLB PE=2 SV=1	55353	360	537	48	57	0.06	0.27	4.5	1	2	1	4	1	6	3	7	1.326607
tr|B2RB23|B2RB23_HUMAN	tr|B2RB2	cDNA, FLJ95265, highly similar to Homo sapiens acetyl-Coenzyme A acyltransferase 2 (mitochondrial 3-oxoacyl-Coenzyme A thiolase	42439	544	239	30	160	0.08	0.35	4.375	0	3	1	4	1	5	1	5	1.085403
sp|O75369|FLNB_HUMAN	FLNB	Filamin-B OS=Homo sapiens GN=FLNB PE=1 SV=2	280157	45	18	463	1284	0.22	0.96	4.363636364	14	40	18	61	20	66	28	80	0.914222
sp|Q15645|PCH2_HUMAN	TRIP13	Pachytene checkpoint protein 2 homolog OS=Homo sapiens GN=TRIP13 PE=1 SV=2	48863	507	491	33	74	0.07	0.3	4.285714286	0	2	1	4	2	8	2	9	0.976718
tr|B2R5M9|B2R5M9_HUMAN	tr|B2R5M	cDNA, FLJ92537, highly similar to Homo sapiens procollagen-lysine, 2-oxoglutarate 5-dioxygenase (lysine hydroxylase, Ehlers-Danlos	84022	251	292	76	133	0.04	0.17	4.25	1	3	1	4	1	4	1	4	0.7957
tr|Q60FE2|Q60FE2_HUMAN	MYH9	MYH9 variant protein OS=Homo sapiens GN=MYH9 PE=2 SV=1	227646	17	2	900	3670	0.9	3.78	4.2	30	92	46	117	52	133	60	150	0.565318
tr|Q6NTA2|Q6NTA2_HUMAN	HNRNPL	HNRNPL protein (Fragment) OS=Homo sapiens GN=HNRNPL PE=2 SV=2	62515	182	81	117	408	0.23	0.95	4.130434783	4	12	4	14	6	16	8	17	0.555351
tr|B3KRS7|B3KRS7_HUMAN	tr|B3KRS7	cDNA FLJ34844 fis, clone NT2NE2011154, highly similar to RuvB-like 1 OS=Homo sapiens PE=2 SV=1	34946	351	359	50	90	0.09	0.37	4.111111111	1	2	1	4	1	4	1	4	0.7957
tr|A2BDK6|A2BDK6_HUMAN	MAP1B	Microtubule-associated protein 1B OS=Homo sapiens GN=MAP1B PE=2 SV=1	257692	511	335	32	113	0.01	0.04	4	0	3	1	3	1	3	2	4	0.432934
sp|Q6P2Q9|PRP8_HUMAN	PRPF8	Pre-mRNA-processing-splicing factor 8 OS=Homo sapiens GN=PRPF8 PE=1 SV=2	274738	130	78	167	445	0.1	0.39	3.9	7	15	8	28	10	33	10	41	0.887153
tr|B4DR68|B4DR68_HUMAN	tr|B4DR6	cDNA FLJ58608, highly similar to Heat shock protein 75 kDa, mitochondrial OS=Homo sapiens PE=2 SV=1	74408	169	108	127	336	0.09	0.35	3.888888889	2	8	3	8	3	8	5	10	0.490952
tr|B1AH89|B1AH89_HUMAN	TTLL12	Tubulin tyrosine ligase-like family, member 12 OS=Homo sapiens GN=TTLL12 PE=4 SV=1	74185	104	51	209	654	0.41	1.59	3.87804878	6	20	8	24	10	28	11	31	0.65297
tr|B4DT29|B4DT29_HUMAN	tr|B4DT2	cDNA FLJ51082, highly similar to Actin-like protein 3 OS=Homo sapiens PE=2 SV=1	40959	548	425	30	85	0.08	0.31	3.875	0	2	1	4	1	4	1	4	0.7957
tr|B4DR70|B4DR70_HUMAN	tr|B4DR7	cDNA FLJ58049, highly similar to RNA-binding protein FUS OS=Homo sapiens PE=2 SV=1	45013	450	504	38	72	0.07	0.27	3.857142857	1	1	1	4	1	4	1	4	0.7957
tr|A8K8N7|A8K8N7_HUMAN	PFAS	Phosphoribosylformylglycinamidine synthase (FGAR amidotransferase), isoform CRA_b OS=Homo sapiens GN=PFAS PE=2 SV=1	146286	380	137	46	274	0.05	0.19	3.8	0	5	2	8	3	8	5	9	0.490952
tr|A5PHJ9|A5PHJ9_HUMAN	PDHA1/LC	PDHA1/LOC79064 protein (Fragment) OS=Homo sapiens GN=PDHA1/LOC79064 PE=2 SV=1	34113	516	530	32	68	0.1	0.38	3.8	0	2	1	3	1	2	1	3	-0.05269
tr|H0YD14|H0YD14_HUMAN	MYOF	Myoferlin (Fragment) OS=Homo sapiens GN=MYOF PE=4 SV=1	135822	220	90	95	238	0.07	0.26	3.714285714	3	7	3	10	4	11	4	14	0.564731
sp|Q9Y5B9|SP16H_HUMAN	SUPT16H	FACT complex subunit SPT16 OS=Homo sapiens GN=SUPT16H PE=1 SV=1	120409	506	423	33	87	0.03	0.11	3.666666667	0	2	1	4	1	4	2	4	0.7957
sp|P53621|COPA_HUMAN	COPA	Coatomer subunit alpha OS=Homo sapiens GN=COPA PE=1 SV=2	139797	181	84	118	396	0.18	0.66	3.666666667	5	14	7	22	8	22	11	24	0.616287
tr|B4DH52|B4DH52_HUMAN	tr|B4DH5	cDNA FLJ55916, highly similar to General transcription factor II-I OS=Homo sapiens PE=2 SV=1	112359	554	237	29	160	0.06	0.22	3.666666667	0	4	2	7	2	6	3	8	0.589303
tr|B2RBR9|B2RBR9_HUMAN	tr|B2RBR	cDNA, FLJ95650, highly similar to Homo sapiens karyopherin (importin) beta 1 (KPNB1), mRNA OS=Homo sapiens PE=2 SV=1	98434	171	49	126	682	0.22	0.8	3.636363636	4	17	6	18	9	19	9	20	0.248443
sp|P07355|ANXA2_HUMAN	ANXA2	Annexin A2 OS=Homo sapiens GN=ANXA2 PE=1 SV=2	38808	100	48	214	686	1.26	4.57	3.626984127	8	18	11	26	12	27	13	29	0.349026
tr|B4DLD4|B4DLD4_HUMAN	tr|B4DLD	cDNA FLJ56738, highly similar to Fibrillarin OS=Homo sapiens PE=2 SV=1	30279	423	500	41	68	0.11	0.39	3.545454545	1	1	1	1	1	1	1	1	-0.78985
tr|B4E0S6|B4E0S6_HUMAN	tr|B4E0S6	cDNA FLJ55635, highly similar to pre-mRNA-splicing factorATP-dependent RNA helicase DHX15 (EC 3.6.1.-) OS=Homo sapiens PE=2 S	90289	183	87	117	384	0.15	0.53	3.533333333	2	11	4	12	4	14	7	15	0.899739
tr|B4DH39|B4DH39_HUMAN	tr|B4DH3	cDNA FLJ57028, highly similar to Long-chain-fatty-acid--CoA ligase 4 (EC 6.2.1.3) OS=Homo sapiens PE=2 SV=1	73123	529	345	31	111	0.04	0.14	3.5	0	2	1	3	1	5	3	5	1.085403
tr|B4DYL7|B4DYL7_HUMAN	tr|B4DYL7	cDNA FLJ58490, highly similar to Homo sapiens CCR4-NOT transcription complex, subunit 1 (CNOT1), transcript variant 1, mRNA OS=	140991	388	372	45	61	0.02	0.07	3.5	1	3	1	4	1	4	2	4	0.7957
sp|Q14683|SMC1A_HUMAN	SMC1A	Structural maintenance of chromosomes protein 1A OS=Homo sapiens GN=SMC1A PE=1 SV=2	143771	581	857	28	39	0.02	0.07	3.5	0	1	1	2	1	3	1	4	0.432934
tr|B3KM77|B3KM77_HUMAN	tr|B3KM7	cDNA FLJ10435 fis, clone NT2RP1000493, highly similar to Splicing factor 3B subunit 3 OS=Homo sapiens PE=2 SV=1	56736	356	138	49	185	0.06	0.21	3.5	1	4	1	5	1	6	1	6	1.326607
tr|B3KNL2|B3KNL2_HUMAN	RUVBL2	RuvB-like 2 (E. coli), isoform CRA_d OS=Homo sapiens GN=RUVBL2 PE=2 SV=1	47603	584	312	28	123	0.14	0.49	3.5	0	4	2	6	1	8	2	8	1.714022
tr|H0Y2W2|H0Y2W2_HUMAN	ATAD3A	ATPase family AAA domain-containing protein 3A (Fragment) OS=Homo sapiens GN=ATAD3A PE=4 SV=1	64600	281	489	68	74	0.1	0.35	3.5	2	2	2	6	2	6	2	6	0.589303
tr|B4DEM7|B4DEM7_HUMAN	CCT8	T-complex protein 1 subunit theta OS=Homo sapiens GN=CCT8 PE=2 SV=1	58179	268	202	70	188	0.18	0.62	3.444444444	2	6	3	8	5	9	6	10	-0.00114
tr|B7Z6M1|B7Z6M1_HUMAN	PLS3	Plastin-3 OS=Homo sapiens GN=PLS3 PE=2 SV=1	66047	282	110	66	321	0.34	1.17	3.441176471	1	15	6	18	6	19	8	20	0.796949
tr|H0YH88|H0YH88_HUMAN	NAP1L1	Nucleosome assembly protein 1-like 1 (Fragment) OS=Homo sapiens GN=NAP1L1 PE=3 SV=1	21179	417	462	33	79	0.16	0.55	3.4375	0	2	1	3	1	3	1	4	0.432934
tr|B4DWJ2|B4DWJ2_HUMAN	QARS	Glutamine--tRNA ligase OS=Homo sapiens GN=QARS PE=2 SV=1	87437	277	189	68	202	0.16	0.55	3.4375	3	8	4	12	5	12	5	13	0.395376
tr|Q96QL0|Q96QL0_HUMAN	RPL3	Ribosomal protein L3 OS=Homo sapiens GN=RPL3 PE=2 SV=1	46376	245	158	82	182	0.32	1.1	3.4375	4	4	4	9	5	12	7	13	0.395376
tr|B2R8R5|B2R8R5_HUMAN	tr|B2R8R	cDNA, FLJ94025, highly similar to Homo sapiens tripartite motif-containing 28 (TRIM28), mRNA OS=Homo sapiens PE=2 SV=1	90271	320	260	56	150	0.07	0.24	3.428571429	1	5	3	7	2	8	4	9	0.976718
sp|P06733|ENOA_HUMAN	ENO1	Alpha-enolase OS=Homo sapiens GN=ENO1 PE=1 SV=2	47481	40	41	520	858	1.39	4.73	3.402877698	13	24	18	31	19	41	25	46	0.301535
tr|B2R959|B2R959_HUMAN	tr|B2R959	cDNA, FLJ94229, highly similar to Homo sapiens heterogeneous nuclear ribonucleoprotein L (HNRPL),mRNA OS=Homo sapiens PE=2	60838	182	129	117	288	0.23	0.78	3.391304348	4	9	4	11	6	13	8	14	0.265256
tr|B4DEX8|B4DEX8_HUMAN	MAT2A	S-adenosylmethionine synthase OS=Homo sapiens GN=MAT2A PE=2 SV=1	40027	333	386	52	93	0.08	0.27	3.375	1	3	1	3	1	3	1	4	0.432934
tr|E9PCI9|E9PCI9_HUMAN	FDPS	Farnesyl pyrophosphate synthase OS=Homo sapiens GN=FDPS PE=2 SV=1	40849	405	886	43	37	0.08	0.27	3.375	1	1	1	1	1	1	1	1	-0.78985
tr|B3KS31|B3KS31_HUMAN	TUBB6	Tubulin, beta 6, isoform CRA_a OS=Homo sapiens GN=TUBB6 PE=2 SV=1	42324	73	42	273	791	0.83	2.79	3.361445783	10	28	14	31	17	40	20	43	0.422948
tr|A0PJ92|A0PJ92_HUMAN	NOP56	NOP56 protein (Fragment) OS=Homo sapiens GN=NOP56 PE=2 SV=1	50068	311	283	58	140	0.14	0.47	3.357142857	1	2	2	6	3	6	3	6	0.103537
sp|Q9Y2A7|NCKP1_HUMAN	NCKAP1	Nck-associated protein 1 OS=Homo sapiens GN=NCKAP1 PE=1 SV=1	130018	685	384	19	94	0.03	0.1	3.333333333	0	3	1	4	0	3	1	5	2.018235
tr|B2R6D0|B2R6D0_HUMAN	tr|B2R6D	cDNA, FLJ92896, highly similar to Homo sapiens proteasome (prosome, macropain) 26S subunit, non-ATPase, 1 (PSMD1), mRNA OS=	106797	386	367	45	101	0.06	0.2	3.333333333	0	4	2	6	2	6	4	7	0.589303
tr|C9J1E7|C9J1E7_HUMAN	AP1B1	AP-1 complex subunit beta-1 (Fragment) OS=Homo sapiens GN=AP1B1 PE=2 SV=1	66014	648	396	22	91	0.03	0.1	3.333333333	0	2	1	3	3	2	3	3	-1.27576
tr|B2RCM2|B2RCM2_HUMAN	tr|B2RCM	cDNA, FLJ96156, highly similar to Homo sapiens leucyl-tRNA synthetase (LARS), mRNA OS=Homo sapiens PE=2 SV=1	135550	257	100	75	353	0.1	0.33	3.3	2	9	4	12	3	14	5	15	1.262648
tr|A6NE09|A6NE09_HUMAN	RPSAP58	Protein RPSAP58 OS=Homo sapiens GN=RPSAP58 PE=3 SV=1	33002	521	352	31	106	0.1	0.33	3.3	0	3	1	3	1	3	1	3	0.432934
tr|B4DU00|B4DU00_HUMAN	tr|B4DU0	cDNA FLJ54547, highly similar to Alpha-1 catenin (Cadherin-associated protein) OS=Homo sapiens PE=2 SV=1	89332	389	342	45	88	0.07	0.23	3.285714286	0	2	2	5	2	6	4	6	0.589303
sp|P08238|HS90B_HUMAN	HSP90AB1	Heat shock protein HSP 90-beta OS=Homo sapiens GN=HSP90AB1 PE=1 SV=4	83554	32	29	622	1052	1.24	4.04	3.258064516	18	31	28	43	29	47	35	56	-0.10311
tr|B2RBE0|B2RBE0_HUMAN	tr|B2RBE	cDNA, FLJ95462, highly similar to Homo sapiens fatty-acid-Coenzyme A ligase, long-chain 3 (FACL3),mRNA OS=Homo sapiens PE=2 S	81266	462	328	37	114	0.04	0.13	3.25	0	3	1	3	1	5	1	5	1.085403
tr|B3KUZ8|B3KUZ8_HUMAN	tr|B3KUZ	Aspartate aminotransferase OS=Homo sapiens PE=2 SV=1	41699	522	592	31	60	0.08	0.26	3.25	0	1	1	3	1	3	2	3	0.432934
tr|G5E9G0|G5E9G0_HUMAN	RPL3	60S ribosomal protein L3 OS=Homo sapiens GN=RPL3 PE=2 SV=1	40527	245	157	67	234	0.32	1.03	3.21875	3	5	3	9	4	12	6	13	0.685221
tr|B7Z1R5|B7Z1R5_HUMAN	ATP6V1A	V-type proton ATPase catalytic subunit A OS=Homo sapiens GN=ATP6V1A PE=2 SV=1	65037	428	350	40	108	0.05	0.16	3.2	1	3	1	3	1	5	2	5	1.085403
tr|B4DE27|B4DE27_HUMAN	tr|B4DE2	cDNA FLJ58765, highly similar to Neutral amino acid transporter B(0) OS=Homo sapiens PE=2 SV=1	33987	290	229	63	99	0.1	0.32	3.2	1	2	1	2	1	2	1	2	-0.05269
tr|A2VCT2|A2VCT2_HUMAN	OGDH	OGDH protein (Fragment) OS=Homo sapiens GN=OGDH PE=2 SV=1	72973	432	395	40	52	0.05	0.16	3.2	1	1	1	1	1	1	1	1	-0.78985
tr|A8K2S1|A8K2S1_HUMAN	tr|A8K2S1	cDNA FLJ75258, highly similar to Homo sapiens phospholipase A2, group IVA (cytosolic, calcium-dependent) (PLA2G4A), mRNA OS=H	85695	369	194	47	197	0.08	0.25	3.125	1	5	2	8	2	9	5	12	1.137379
tr|B7ZKM0|B7ZKM0_HUMAN	SART3	SART3 protein OS=Homo sapiens GN=SART3 PE=2 SV=1	106372	153	128	139	283	0.16	0.5	3.125	5	8	5	13	7	13	7	14	0.058466
tr|B3KQT2|B3KQT2_HUMAN	tr|B3KQT	cDNA PSEC0148 fis, clone PLACE1007202, highly similar to Protein disulfide-isomerase A3 (EC 5.3.4.1) OS=Homo sapiens PE=2 SV=1	55268	546	308	30	126	0.19	0.59	3.105263158	0	6	3	8	2	8	8	10	0.976718
sp|Q15149|PLEC_HUMAN	PLEC	Plectin OS=Homo sapiens GN=PLEC PE=1 SV=3	533462	74	22	270	1197	0.11	0.34	3.090909091	11	35	17	48	18	60	32	72	0.925396
tr|A5D906|A5D906_HUMAN	TUBB2A	TUBB2A protein (Fragment) OS=Homo sapiens GN=TUBB2A PE=2 SV=1	28128	25	13	143	544	3.07	9.43	3.071661238	4	18	7	19	8	24	8	26	0.739537
tr|H0Y4R1|H0Y4R1_HUMAN	IMPDH2	Inosine-5~-monophosphate dehydrogenase 2 (Fragment) OS=Homo sapiens GN=IMPDH2 PE=3 SV=1	51549	177	82	122	407	0.36	1.1	3.055555556	2	9	5	12	5	13	6	14	0.506603
tr|H7C4C8|H7C4C8_HUMAN	CCT8	T-complex protein 1 subunit theta (Fragment) OS=Homo sapiens GN=CCT8 PE=3 SV=1	35913	268	475	70	77	0.18	0.55	3.055555556	2	3	3	5	4	6	5	6	-0.25937
tr|F8W9S7|F8W9S7_HUMAN	GAPVD1	GTPase-activating protein and VPS9 domain-containing protein 1 OS=Homo sapiens GN=GAPVD1 PE=2 SV=1	158790	697	390	18	92	0.02	0.06	3	0	2	1	3	0	3	2	3	2.018235
sp|O94906|PRP6_HUMAN	PRPF6	Pre-mRNA-processing factor 6 OS=Homo sapiens GN=PRPF6 PE=1 SV=1	107656	492	321	35	118	0.03	0.09	3	1	3	1	3	1	4	2	5	0.7957
tr|B4DJ30|B4DJ30_HUMAN	tr|B4DJ30	cDNA FLJ61290, highly similar to Neutral alpha-glucosidase AB OS=Homo sapiens PE=2 SV=1	113539	47	37	446	922	0.62	1.86	3	12	24	19	39	17	44	22	47	0.55962
sp|Q09666|AHNK_HUMAN	AHNAK	Neuroblast differentiation-associated protein AHNAK OS=Homo sapiens GN=AHNAK PE=1 SV=2	629213	401	326	43	114	0.01	0.03	3	1	3	1	5	3	7	6	10	0.310184
tr|A6NEM2|A6NEM2_HUMAN	HCFC1	HCF N-terminal chain 5 OS=Homo sapiens GN=HCFC1 PE=2 SV=2	215338	508	614	33	58	0.02	0.06	3	0	1	1	1	1	1	2	1	-0.78985
sp|O00629|IMA4_HUMAN	KPNA4	Importin subunit alpha-4 OS=Homo sapiens GN=KPNA4 PE=1 SV=1	58364	472	747	37	46	0.06	0.18	3	0	1	1	1	2	1	2	1	-1.52715
tr|B2RDF2|B2RDF2_HUMAN	PES1	Pescadillo homolog 1 OS=Homo sapiens GN=PES1 PE=2 SV=1	68359	488	434	35	84	0.05	0.15	3	1	2	1	3	1	4	3	5	0.7957
tr|D3DQA6|D3DQA6_HUMAN	DDX46	DEAD (Asp-Glu-Ala-Asp) box polypeptide 46, isoform CRA_a OS=Homo sapiens GN=DDX46 PE=3 SV=1	100958	595	150	26	219	0.07	0.21	3	0	6	2	8	3	9	3	11	0.651613
tr|A8K492|A8K492_HUMAN	tr|A8K492	cDNA FLJ76789, highly similar to Homo sapiens methionine-tRNA synthetase (MARS), mRNA OS=Homo sapiens PE=2 SV=1	102277	385	269	45	146	0.07	0.21	3	1	4	2	6	2	6	2	6	0.589303
tr|B1ANR0|B1ANR0_HUMAN	PABPC4	Poly(A) binding protein, cytoplasmic 4 (Inducible form) OS=Homo sapiens GN=PABPC4 PE=4 SV=1	68156	504	278	33	142	0.05	0.15	3	0	3	1	3	1	3	2	5	0.432934
tr|Q60FE5|Q60FE5_HUMAN	FLNA	Filamin A OS=Homo sapiens GN=FLNA PE=2 SV=1	280790	9	4	1306	3619	0.86	2.56	2.976744186	41	95	60	134	75	147	88	173	0.179922
sp|P11021|GRP78_HUMAN	HSPA5	78 kDa glucose-regulated protein OS=Homo sapiens GN=HSPA5 PE=1 SV=2	72402	129	94	170	358	0.19	0.56	2.947368421	3	9	5	11	6	11	6	13	0.033538
tr|H7BZJ3|H7BZJ3_HUMAN	PDIA3	Thioredoxin (Fragment) OS=Homo sapiens GN=PDIA3 PE=3 SV=1	13739	546	480	22	76	0.19	0.56	2.947368421	0	2	1	2	1	2	1	2	-0.05269
tr|Q5TCU6|Q5TCU6_HUMAN	TLN1	Talin-1 OS=Homo sapiens GN=TLN1 PE=4 SV=1	260030	48	17	446	1302	0.27	0.79	2.925925926	12	34	19	46	20	48	25	53	0.455298
tr|B2ZZ89|B2ZZ89_HUMAN	SPTBN1	Spectrin beta non-erythrocytic 1 OS=Homo sapiens GN=SPTBN1 PE=2 SV=1	275237	49	21	431	1251	0.22	0.64	2.909090909	11	35	17	42	20	48	24	57	0.455298
tr|Q2NKY5|Q2NKY5_HUMAN	TUBB6	TUBB6 protein OS=Homo sapiens GN=TUBB6 PE=2 SV=1	50514	65	33	317	966	0.88	2.54	2.886363636	12	31	16	34	17	44	19	48	0.55962
tr|Q6IBR0|Q6IBR0_HUMAN	RPN1	RPN1 protein OS=Homo sapiens GN=RPN1 PE=2 SV=1	68678	170	118	127	309	0.26	0.75	2.884615385	4	9	5	12	8	14	8	16	-0.01916
tr|Q5HY54|Q5HY54_HUMAN	FLNA	Filamin-A OS=Homo sapiens GN=FLNA PE=2 SV=1	279115	9	5	1304	3526	0.86	2.48	2.88372093	41	92	59	131	74	143	87	167	0.15904
tr|A6NN80|A6NN80_HUMAN	ANXA6	Annexin OS=Homo sapiens GN=ANXA6 PE=3 SV=3	75571	349	247	50	155	0.14	0.4	2.857142857	1	6	3	8	3	10	3	10	0.796199
sp|Q14764|MVP_HUMAN	MVP	Major vault protein OS=Homo sapiens GN=MVP PE=1 SV=4	99551	304	1013	59	30	0.07	0.2	2.857142857	2	0	2	1	3	1	3	1	-2.01292
sp|Q9UG63|ABCF2_HUMAN	ABCF2	ATP-binding cassette sub-family F member 2 OS=Homo sapiens GN=ABCF2 PE=1 SV=2	71815	673	602	20	59	0.05	0.14	2.8	0	2	1	3	1	4	2	4	0.7957
tr|D3DTW3|D3DTW3_HUMAN	IGF2BP1	Insulin-like growth factor 2 mRNA binding protein 1, isoform CRA_a OS=Homo sapiens GN=IGF2BP1 PE=2 SV=1	48111	556	557	29	64	0.05	0.14	2.8	0	2	1	2	1	3	1	4	0.432934
sp|Q9NZB2|F120A_HUMAN	FAM120A	Constitutive coactivator of PPAR-gamma-like protein 1 OS=Homo sapiens GN=FAM120A PE=1 SV=2	123008	263	297	73	128	0.05	0.14	2.8	2	3	2	5	2	5	2	5	0.348098
tr|B4DFN9|B4DFN9_HUMAN	tr|B4DFN	cDNA FLJ54303, highly similar to Heat shock 70 kDa protein 1 OS=Homo sapiens PE=2 SV=1	62699	80	89	252	318	0.58	1.61	2.775862069	7	11	10	19	10	20	10	20	0.17606
sp|Q01650|LAT1_HUMAN	SLC7A5	Large neutral amino acids transporter small subunit 1 OS=Homo sapiens GN=SLC7A5 PE=1 SV=2	55659	239	113	85	315	0.12	0.33	2.75	1	7	3	7	4	7	5	7	-0.05273
tr|B3KS36|B3KS36_HUMAN	tr|B3KS36	cDNA FLJ35376 fis, clone SKMUS2004044, highly similar to Homo sapiens ribosomal protein L3 (RPL3), transcript variant 2, mRNA OS	40234	245	173	64	220	0.32	0.88	2.75	3	5	3	8	4	11	6	13	0.564731
tr|I3L0N3|I3L0N3_HUMAN	NSF	Vesicle-fusing ATPase OS=Homo sapiens GN=NSF PE=3 SV=1	82553	412	932	42	35	0.04	0.11	2.75	1	0	1	1	1	1	1	1	-0.78985
tr|F5GX33|F5GX33_HUMAN	SLC25A13	Calcium-binding mitochondrial carrier protein Aralar2 OS=Homo sapiens GN=SLC25A13 PE=2 SV=1	62548	353	259	49	137	0.11	0.3	2.727272727	1	4	2	5	3	6	4	6	0.103537
sp|O15427|MOT4_HUMAN	SLC16A3	Monocarboxylate transporter 4 OS=Homo sapiens GN=SLC16A3 PE=1 SV=1	50064	362	455	48	81	0.14	0.38	2.714285714	2	2	2	6	2	7	2	7	0.795949
sp|P49327|FAS_HUMAN	FASN	Fatty acid synthase OS=Homo sapiens GN=FASN PE=1 SV=3	275877	55	27	394	1125	0.28	0.76	2.714285714	12	35	21	51	23	55	29	65	0.453125
sp|Q00610|CLH1_HUMAN	CLTC	Clathrin heavy chain 1 OS=Homo sapiens GN=CLTC PE=1 SV=5	193260	30	14	629	1512	0.52	1.39	2.673076923	19	43	25	56	24	64	28	74	0.611252
tr|B1PS43|B1PS43_HUMAN	MYH11	Myosin heavy chain 11 smooth muscle isoform OS=Homo sapiens GN=MYH11 PE=2 SV=1	235004	142	62	155	551	0.06	0.16	2.666666667	5	11	5	14	9	14	11	15	-0.17996
tr|B2R5M8|B2R5M8_HUMAN	tr|B2R5M	Isocitrate dehydrogenase [NADP] OS=Homo sapiens PE=2 SV=1	46905	555	266	29	147	0.15	0.4	2.666666667	0	3	2	5	3	4	3	6	-0.42737
tr|B4DUC5|B4DUC5_HUMAN	CSE1L	Exportin-2 OS=Homo sapiens GN=CSE1L PE=2 SV=1	86045	433	327	40	114	0.08	0.21	2.625	0	4	2	5	4	7	4	7	-0.05273
tr|B4E022|B4E022_HUMAN	TKT	Transketolase OS=Homo sapiens GN=TKT PE=2 SV=1	63410	35	40	578	874	1.36	3.56	2.617647059	17	25	24	39	26	42	32	47	-0.10882
tr|Q3B7A7|Q3B7A7_HUMAN	GART	Phosphoribosylglycinamide formyltransferase, phosphoribosylglycinamide synthetase, phosphoribosylaminoimidazole synthetase O	108909	341	191	51	200	0.13	0.34	2.615384615	1	4	4	10	3	10	4	14	0.796199
tr|B3KSQ7|B3KSQ7_HUMAN	DBN1	Drebrin 1, isoform CRA_d OS=Homo sapiens GN=DBN1 PE=2 SV=1	66053	478	287	36	112	0.05	0.13	2.6	0	2	1	2	1	2	1	2	-0.05269
tr|B4DUQ1|B4DUQ1_HUMAN	tr|B4DUQ	cDNA FLJ54552, highly similar to Heterogeneous nuclear ribonucleoprotein K OS=Homo sapiens PE=2 SV=1	48708	147	88	147	376	0.58	1.5	2.586206897	3	12	7	16	7	17	8	19	0.433647
tr|B4DMB5|B4DMB5_HUMAN	tr|B4DMB	cDNA FLJ60075, highly similar to CTP synthase 1 (EC 6.3.4.2) OS=Homo sapiens PE=2 SV=1	56542	449	168	38	224	0.19	0.49	2.578947368	1	6	3	7	3	7	4	7	0.310184
tr|B4DDB6|B4DDB6_HUMAN	HNRPA3	Heterogeneous nuclear ribonucleoprotein A3, isoform CRA_a OS=Homo sapiens GN=HNRPA3 PE=2 SV=1	37234	201	271	103	133	0.19	0.49	2.578947368	2	2	2	3	2	4	2	5	0.058396
tr|Q9BSV4|Q9BSV4_HUMAN	SFPQ	SFPQ protein (Fragment) OS=Homo sapiens GN=SFPQ PE=2 SV=2	68702	228	151	91	245	0.26	0.67	2.576923077	2	7	5	11	6	13	7	13	0.265256
tr|H0YJ34|H0YJ34_HUMAN	FERMT2	Fermitin family homolog 2 (Fragment) OS=Homo sapiens GN=FERMT2 PE=4 SV=1	73877	306	169	59	224	0.14	0.36	2.571428571	2	4	3	7	3	9	3	9	0.651613
tr|J3QQM1|J3QQM1_HUMAN	PSMC5	26S protease regulatory subunit 8 (Fragment) OS=Homo sapiens GN=PSMC5 PE=4 SV=1	29442	314	615	57	53	0.11	0.28	2.545454545	1	1	2	1	2	2	2	2	-0.05269
tr|B4DFK9|B4DFK9_HUMAN	tr|B4DFK	cDNA FLJ57964, highly similar to Heterogeneous nuclear ribonucleoprotein H~ OS=Homo sapiens PE=2 SV=1	44637	272	211	69	129	0.15	0.38	2.533333333	1	2	2	4	1	4	2	4	0.7957
tr|B0V043|B0V043_HUMAN	VARS	Valine--tRNA ligase OS=Homo sapiens GN=VARS PE=3 SV=1	141632	275	86	69	388	0.15	0.38	2.533333333	2	13	6	15	6	17	8	19	0.640437
tr|B2RU06|B2RU06_HUMAN	EIF4G1	EIF4G1 protein OS=Homo sapiens GN=EIF4G1 PE=2 SV=1	176807	669	569	20	62	0.04	0.1	2.5	0	3	2	5	1	6	3	7	1.326607
tr|B4DKD0|B4DKD0_HUMAN	tr|B4DKD	DNA topoisomerase 2 (Fragment) OS=Homo sapiens PE=2 SV=1	135015	606	400	25	90	0.02	0.05	2.5	0	2	1	2	1	4	3	4	0.7957
sp|Q96P70|IPO9_HUMAN	IPO9	Importin-9 OS=Homo sapiens GN=IPO9 PE=1 SV=3	116858	580	502	28	73	0.06	0.15	2.5	0	2	2	5	2	5	2	5	0.348098
tr|B4E0E1|B4E0E1_HUMAN	tr|B4E0E1	cDNA FLJ53442, highly similar to Poly (ADP-ribose) polymerase 1 (EC 2.4.2.30) OS=Homo sapiens PE=2 SV=1	111855	549	338	30	112	0.06	0.15	2.5	0	2	2	5	2	4	2	6	0.058396
tr|J3KPM9|J3KPM9_HUMAN	STAT1	Signal transducer and activator of transcription 1-alpha/beta OS=Homo sapiens GN=STAT1 PE=4 SV=1	83878	364	231	48	137	0.08	0.2	2.5	1	3	2	4	2	4	2	5	0.058396
tr|B2R4K7|B2R4K7_HUMAN	tr|B2R4K	60S ribosomal protein L6 OS=Homo sapiens PE=2 SV=1	32739	276	131	68	287	0.47	1.17	2.489361702	2	7	4	9	3	11	5	12	0.92764
sp|O00116|ADAS_HUMAN	AGPS	Alkyldihydroxyacetonephosphate synthase, peroxisomal OS=Homo sapiens GN=AGPS PE=1 SV=1	73664	448	379	38	95	0.09	0.22	2.444444444	1	4	2	4	2	4	3	5	0.058396
tr|F5H3A1|F5H3A1_HUMAN	ATP1A1	Sodium/potassium-transporting ATPase subunit alpha-1 OS=Homo sapiens GN=ATP1A1 PE=2 SV=1	114239	324	160	55	233	0.09	0.22	2.444444444	2	6	3	9	8	12	12	19	-0.23367
tr|J3KPS3|J3KPS3_HUMAN	ALDOA	Fructose-bisphosphate aldolase OS=Homo sapiens GN=ALDOA PE=3 SV=1	40249	63	39	338	875	1.79	4.24	2.368715084	11	22	15	28	17	33	18	36	0.147809
tr|B4DE36|B4DE36_HUMAN	tr|B4DE36	Glucose-6-phosphate isomerase OS=Homo sapiens PE=2 SV=1	60376	117	106	182	338	0.3	0.7	2.333333333	4	7	5	10	7	10	7	13	-0.30469
sp|Q8TCJ2|STT3B_HUMAN	STT3B	Dolichyl-diphosphooligosaccharide--protein glycosyltransferase subunit STT3B OS=Homo sapiens GN=STT3B PE=1 SV=1	94241	700	727	18	48	0.03	0.07	2.333333333	0	1	1	2	2	2	3	4	-0.78999
tr|B2RAQ8|B2RAQ8_HUMAN	tr|B2RAQ	cDNA, FLJ95058, highly similar to Homo sapiens carnitine palmitoyltransferase 1A (liver) (CPT1A),nuclear gene encoding mitochondr	88967	354	382	49	95	0.07	0.16	2.285714286	1	3	2	4	3	4	3	4	-0.42737
tr|H0Y4A6|H0Y4A6_HUMAN	VARS	Valine--tRNA ligase (Fragment) OS=Homo sapiens GN=VARS PE=3 SV=1	133055	275	102	69	351	0.15	0.34	2.266666667	2	11	6	13	6	15	8	18	0.464957
sp|O76094|SRP72_HUMAN	SRP72	Signal recognition particle 72 kDa protein OS=Homo sapiens GN=SRP72 PE=1 SV=3	75130	455	499	38	73	0.04	0.09	2.25	1	2	1	2	1	3	1	4	0.432934
tr|B3KSH1|B3KSH1_HUMAN	EIF3F	Eukaryotic translation initiation factor 3 subunit F OS=Homo sapiens GN=EIF3F PE=2 SV=1	39236	652	479	22	76	0.08	0.18	2.25	0	1	1	2	1	3	1	3	0.432934
tr|B4E091|B4E091_HUMAN	tr|B4E091	cDNA FLJ55438, highly similar to Splicing factor 3 subunit 1 OS=Homo sapiens PE=2 SV=1	77415	482	550	36	65	0.04	0.09	2.25	1	2	1	2	1	2	1	2	-0.05269
tr|B4DZ88|B4DZ88_HUMAN	KTN1	KTN1 protein OS=Homo sapiens GN=KTN1 PE=2 SV=1	153179	322	397	55	91	0.04	0.09	2.25	2	3	2	4	3	4	4	6	-0.42737
sp|O43175|SERA_HUMAN	PHGDH	D-3-phosphoglycerate dehydrogenase OS=Homo sapiens GN=PHGDH PE=1 SV=4	57356	131	143	166	260	0.25	0.56	2.24	3	7	4	8	4	12	4	14	0.685221
sp|P26038|MOES_HUMAN	MSN	Moesin OS=Homo sapiens GN=MSN PE=1 SV=3	67892	119	80	179	416	0.46	1.03	2.239130435	7	14	8	16	11	23	12	26	0.242102
tr|B2R5U1|B2R5U1_HUMAN	tr|B2R5U	cDNA, FLJ92620, highly similar to Homo sapiens staphylococcal nuclease domain containing 1 (SND1),mRNA OS=Homo sapiens PE=2	100294	150	139	144	265	0.17	0.38	2.235294118	4	6	5	10	10	11	12	14	-0.6597
tr|Q59G92|Q59G92_HUMAN	tr|Q59G9	HLA-B associated transcript 1 variant (Fragment) OS=Homo sapiens PE=2 SV=1	35528	394	956	44	33	0.09	0.2	2.222222222	1	0	1	2	2	3	2	3	-0.30437
tr|Q0IJ56|Q0IJ56_HUMAN	ST13	ST13 protein (Fragment) OS=Homo sapiens GN=ST13 PE=2 SV=1	35164	382	445	46	83	0.09	0.2	2.222222222	1	1	1	2	2	2	3	3	-0.78999
tr|D6W507|D6W507_HUMAN	hCG_199	HCG1990625, isoform CRA_a OS=Homo sapiens GN=hCG_1990625 PE=2 SV=1	16708	382	411	46	88	0.09	0.2	2.222222222	1	1	1	1	2	2	2	2	-0.78999
tr|B4DFE4|B4DFE4_HUMAN	tr|B4DFE4	cDNA FLJ59618, highly similar to Exosome component 10 OS=Homo sapiens PE=2 SV=1	61733	627	745	23	46	0.05	0.11	2.2	0	1	1	2	1	2	1	3	-0.05269
sp|Q9NPQ8|RIC8A_HUMAN	RIC8A	Synembryn-A OS=Homo sapiens GN=RIC8A PE=1 SV=3	60185	496	726	34	48	0.05	0.11	2.2	0	1	1	2	1	2	1	2	-0.05269
tr|B4DII5|B4DII5_HUMAN	tr|B4DII5	Importin subunit alpha OS=Homo sapiens PE=2 SV=1	60345	573	306	28	126	0.11	0.24	2.181818182	0	3	2	4	3	4	3	4	-0.42737
tr|B3KNM6|B3KNM6_HUMAN	tr|B3KNM	cDNA FLJ14968 fis, clone THYRO1000288, highly similar to CAAX prenyl protease 1 homolog (EC 3.4.24.84) OS=Homo sapiens PE=2 S	27279	547	779	30	42	0.12	0.26	2.166666667	1	1	1	3	1	3	1	3	0.432934
tr|Q2F839|Q2F839_HUMAN	tr|Q2F83	Heat shock 70 kDa protein 9B (Fragment) OS=Homo sapiens PE=2 SV=1	19208	81	243	59	157	0.42	0.91	2.166666667	2	4	2	4	2	5	2	7	0.348098
tr|A6NG51|A6NG51_HUMAN	SPTAN1	Spectrin alpha chain, non-erythrocytic 1 OS=Homo sapiens GN=SPTAN1 PE=2 SV=2	285628	167	104	130	348	0.06	0.13	2.166666667	2	9	5	11	6	12	9	17	0.154028
tr|B7Z9W6|B7Z9W6_HUMAN	tr|B7Z9W	cDNA, FLJ78980, highly similar to DnaJ homolog subfamily C member 7 OS=Homo sapiens PE=2 SV=1	50776	527	704	31	50	0.06	0.13	2.166666667	0	1	1	2	1	2	1	2	-0.05269
sp|Q99456|K1C12_HUMAN	KRT12	Keratin, type I cytoskeletal 12 OS=Homo sapiens GN=KRT12 PE=1 SV=1	53592	616	582	24	61	0.06	0.13	2.166666667	0	2	2	2	2	3	3	3	-0.30437
tr|G3XAD8|G3XAD8_HUMAN	STIP1	Stress-induced-phosphoprotein 1 OS=Homo sapiens GN=STIP1 PE=2 SV=1	68721	271	233	69	161	0.21	0.45	2.142857143	2	5	4	8	4	9	5	9	0.288704
tr|B4DUY3|B4DUY3_HUMAN	tr|B4DUY	cDNA FLJ56668, highly similar to Nucleolar protein NOP5 OS=Homo sapiens PE=2 SV=1	16345	515	232	32	27	0.21	0.45	2.142857143	0	0	1	2	1	2	1	2	-0.05269
tr|E9PIM9|E9PIM9_HUMAN	RNH1	Ribonuclease inhibitor (Fragment) OS=Homo sapiens GN=RNH1 PE=2 SV=1	22362	431	354	40	105	0.15	0.32	2.133333333	0	2	1	2	1	2	1	2	-0.05269
sp|O95373|IPO7_HUMAN	IPO7	Importin-7 OS=Homo sapiens GN=IPO7 PE=1 SV=1	120751	162	178	132	214	0.08	0.17	2.125	3	5	3	7	3	9	4	10	0.651613
tr|Q5TZX1|Q5TZX1_HUMAN	GNAI3	Guanine nucleotide binding protein (G protein), alpha inhibiting activity polypeptide 3 OS=Homo sapiens GN=GNAI3 PE=2 SV=1	41076	376	689	46	50	0.08	0.17	2.125	1	2	1	2	1	2	1	2	-0.05269
tr|E7EP94|E7EP94_HUMAN	HSPA1A	Heat shock 70 kDa protein 1A/1B OS=Homo sapiens GN=HSPA1A PE=3 SV=2	60090	80	177	147	214	0.58	1.23	2.120689655	4	6	6	15	6	16	6	17	0.555351
tr|B3KNN7|B3KNN7_HUMAN	tr|B3KNN	cDNA FLJ30049 fis, clone ADRGL1000033, highly similar to 26S proteasome non-ATPase regulatory subunit 3 OS=Homo sapiens PE=2	57311	273	274	69	143	0.18	0.38	2.111111111	3	5	3	6	3	7	3	8	0.310184
tr|F1T0B3|F1T0B3_HUMAN	DDX1	ATP-dependent RNA helicase DDX1 OS=Homo sapiens GN=DDX1 PE=2 SV=1	74838	501	670	33	52	0.09	0.19	2.111111111	0	2	2	4	3	5	3	7	-0.13767
tr|B4DJ60|B4DJ60_HUMAN	tr|B4DJ60	cDNA FLJ55072, highly similar to Succinate dehydrogenase (ubiquinone) flavoprotein subunit, mitochondrial (EC1.3.5.1) OS=Homo s	68233	393	456	44	81	0.1	0.21	2.1	0	1	2	4	3	4	5	4	-0.42737
tr|B4DGU4|B4DGU4_HUMAN	CTNNB1	Catenin beta-1 OS=Homo sapiens GN=CTNNB1 PE=2 SV=1	85339	340	205	52	188	0.12	0.25	2.083333333	2	6	3	6	3	7	3	8	0.310184
tr|Q53G58|Q53G58_HUMAN	tr|Q53G5	Coronin (Fragment) OS=Homo sapiens PE=2 SV=1	53931	242	305	83	126	0.13	0.27	2.076923077	1	3	2	5	2	5	4	5	0.348098
tr|Q6FIA4|Q6FIA4_HUMAN	IDH1	Isocitrate dehydrogenase [NADP] OS=Homo sapiens GN=IDH1 PE=2 SV=1	46824	555	263	29	149	0.15	0.31	2.066666667	0	3	2	4	3	4	3	5	-0.42737
tr|A4QPB0|A4QPB0_HUMAN	IQGAP1	IQ motif containing GTPase activating protein 1 OS=Homo sapiens GN=IQGAP1 PE=2 SV=1	189789	84	47	239	725	0.31	0.64	2.064516129	8	22	17	29	18	32	21	37	0.023381
tr|B4DP08|B4DP08_HUMAN	ALG1	Chitobiosyldiphosphodolichol beta-mannosyltransferase OS=Homo sapiens GN=ALG1 PE=2 SV=1	40938	452	438	38	53	0.17	0.35	2.058823529	0	1	2	3	2	3	2	5	-0.30437
tr|E9PHF7|E9PHF7_HUMAN	MCCC1	Methylcrotonoyl-CoA carboxylase subunit alpha, mitochondrial OS=Homo sapiens GN=MCCC1 PE=2 SV=1	68745	102	56	214	502	0.83	1.7	2.048192771	7	15	13	26	11	30	16	35	0.619627
tr|J3QSX4|J3QSX4_HUMAN	BUB3	Mitotic checkpoint protein BUB3 OS=Homo sapiens GN=BUB3 PE=4 SV=1	16498	681	472	20	65	0.2	0.4	2	0	1	1	3	0	3	1	3	2.018235

tr|H7C3A1|H7C3A1_HUMAN	SRRT	Serrate RNA effector molecule homolog (Fragment) OS=Homo sapiens GN=SRRT PE=4 SV=1	57154	701	547	18	65	0.06	0.12	2	0	2	1	2	0	2	1	2	1.532612
tr|B4DS66|B4DS66_HUMAN	tr|B4DS66	cDNA FLJ54290, highly similar to Mitochondrial inner membrane protein OS=Homo sapiens PE=2 SV=1	73509	712	561	17	63	0.04	0.08	2	0	2	1	2	1	4	6	5	0.7957
sp|O95831|AIFM1_HUMAN	AIFM1	Apoptosis-inducing factor 1, mitochondrial OS=Homo sapiens GN=AIFM1 PE=1 SV=1	67144	326	1056	54	28	0.05	0.1	2	1	0	1	2	1	3	2	3	0.432934
tr|Q8TC04|Q8TC04_HUMAN	KRT23	Keratin 23 (Histone deacetylase inducible) OS=Homo sapiens GN=KRT23 PE=2 SV=1	48318	600	680	26	51	0.07	0.14	2	0	2	1	2	1	3	1	4	0.432934
tr|B2RDN4|B2RDN4_HUMAN	tr|B2RDN	cDNA, FLJ96693, highly similar to Homo sapiens block of proliferation 1 (BOP1), mRNA OS=Homo sapiens PE=2 SV=1	84219	505	853	33	39	0.08	0.16	2	0	1	2	4	2	4	3	5	0.058396
sp|Q13867|BLMH_HUMAN	BLMH	Bleomycin hydrolase OS=Homo sapiens GN=BLMH PE=1 SV=1	53155	332	447	52	82	0.2	0.4	2	1	3	3	4	2	4	4	5	0.058396
tr|Q2TB59|Q2TB59_HUMAN	NNT	Nicotinamide nucleotide transhydrogenase OS=Homo sapiens GN=NNT PE=2 SV=1	114552	234	245	88	157	0.06	0.12	2	1	3	2	4	2	4	3	5	0.058396
tr|B2RAX6|B2RAX6_HUMAN	tr|B2RAX	cDNA, FLJ95176, Homo sapiens CGI-48 protein (CGI-48), mRNA OS=Homo sapiens PE=2 SV=1	58127	605	909	25	36	0.06	0.12	2	0	0	1	2	1	2	1	2	-0.05269
tr|B3KXU8|B3KXU8_HUMAN	tr|B3KXU	cDNA FLJ46087 fis, clone TESTI2009497, highly similar to GPI-anchored protein p137 OS=Homo sapiens PE=2 SV=1	70489	367	734	47	47	0.05	0.1	2	1	1	1	2	1	2	1	2	-0.05269
tr|F8W930|F8W930_HUMAN	IGF2BP2	Insulin-like growth factor 2 mRNA-binding protein 2 OS=Homo sapiens GN=IGF2BP2 PE=2 SV=1	66859	556	609	29	58	0.05	0.1	2	0	2	1	2	1	2	1	2	-0.05269
tr|Q0P512|Q0P512_HUMAN	SLC2A1	Solute carrier family 2 (Facilitated glucose transporter), member 1 OS=Homo sapiens GN=SLC2A1 PE=2 SV=1	54361	377	594	46	60	0.06	0.12	2	1	2	1	2	1	2	1	2	-0.05269
sp|O14929|HAT1_HUMAN	HAT1	Histone acetyltransferase type B catalytic subunit OS=Homo sapiens GN=HAT1 PE=1 SV=1	49880	343	270	51	146	0.07	0.14	2	1	2	1	2	1	2	1	3	-0.05269
tr|A0AV58|A0AV58_HUMAN	STRN3	Striatin, calmodulin binding protein 3 OS=Homo sapiens GN=STRN3 PE=2 SV=1	78069	419	422	42	87	0.09	0.18	2	1	2	2	4	3	5	3	5	-0.13767
tr|G3V2B8|G3V2B8_HUMAN	MTHFD1	C-1-tetrahydrofolate synthase, cytoplasmic OS=Homo sapiens GN=MTHFD1 PE=2 SV=1	102152	112	93	192	360	0.33	0.66	2	5	12	9	16	12	18	12	19	-0.22465
tr|B7ZB91|B7ZB91_HUMAN	TNRC6B	Trinucleotide repeat containing 6B (Fragment) OS=Homo sapiens GN=TNRC6B PE=4 SV=1	81825	359	464	48	70	0.13	0.26	2	1	3	3	3	2	3	3	3	-0.30437
sp|Q14315|FLNC_HUMAN	FLNC	Filamin-C OS=Homo sapiens GN=FLNC PE=1 SV=3	293407	143	186	153	205	0.06	0.12	2	5	8	6	11	10	12	11	15	-0.53921
tr|D3DSQ0|D3DSQ0_HUMAN	PCM1	Pericentriolar material 1, isoform CRA_a OS=Homo sapiens GN=PCM1 PE=2 SV=1	230041	619	892	24	37	0.01	0.02	2	0	1	1	1	2	2	3	2	-0.78999
tr|B7Z601|B7Z601_HUMAN	tr|B7Z601	cDNA FLJ57187, highly similar to Glycerol-3-phosphate dehydrogenase, mitochondrial (EC 1.1.99.5) OS=Homo sapiens PE=2 SV=1	56047	512	737	32	47	0.06	0.12	2	0	1	1	2	2	2	2	2	-0.78999
sp|Q9NRF8|PYRG2_HUMAN	CTPS2	CTP synthase 2 OS=Homo sapiens GN=CTPS2 PE=1 SV=1	66320	614	539	24	66	0.05	0.1	2	0	2	1	2	2	2	6	5	-0.78999
tr|B4DN87|B4DN87_HUMAN	tr|B4DN8	cDNA FLJ52569, highly similar to Collagen-binding protein 2 OS=Homo sapiens PE=2 SV=1	44290	233	172	89	186	0.43	0.85	1.976744186	4	5	5	8	5	8	5	10	-0.1618
sp|P22314|UBA1_HUMAN	UBA1	Ubiquitin-like modifier-activating enzyme 1 OS=Homo sapiens GN=UBA1 PE=1 SV=3	118858	114	79	187	431	0.28	0.54	1.928571429	5	13	10	17	10	18	12	22	0.027568
sp|P23526|SAHH_HUMAN	AHCY	Adenosylhomocysteinase OS=Homo sapiens GN=AHCY PE=1 SV=4	48255	195	119	111	308	0.49	0.94	1.918367347	2	9	6	11	6	12	8	13	0.154028
tr|H0YBW4|H0YBW4_HUMAN	PLAA	Phospholipase A-2-activating protein (Fragment) OS=Homo sapiens GN=PLAA PE=4 SV=1	31645	539	291	30	115	0.11	0.21	1.909090909	0	2	1	3	1	3	1	3	0.432934
tr|Q8IXW6|Q8IXW6_HUMAN	DPYSL3	Dihydropyrimidinase-like 3 OS=Homo sapiens GN=DPYSL3 PE=2 SV=1	62339	490	319	28	118	0.19	0.36	1.894736842	0	3	3	6	2	6	6	7	0.589303
tr|Q2TSD0|Q2TSD0_HUMAN	tr|Q2TSD	Glyceraldehyde-3-phosphate dehydrogenase OS=Homo sapiens PE=2 SV=1	36197	18	31	891	945	3.44	6.5	1.889534884	23	25	31	32	31	38	36	47	-0.50327
tr|H7C1U0|H7C1U0_HUMAN	APEH	Acylamino-acid-releasing enzyme (Fragment) OS=Homo sapiens GN=APEH PE=4 SV=1	35132	319	235	56	78	0.09	0.17	1.888888889	1	2	1	2	1	2	1	2	-0.05269
sp|Q3ZCM7|TBB8_HUMAN	TUBB8	Tubulin beta-8 chain OS=Homo sapiens GN=TUBB8 PE=1 SV=2	50257	67	59	306	561	0.77	1.43	1.857142857	13	23	17	29	19	32	21	37	-0.05291
tr|F2Z2U8|F2Z2U8_HUMAN	MYH14	Myosin-14 OS=Homo sapiens GN=MYH14 PE=2 SV=1	232102	152	116	140	311	0.07	0.13	1.857142857	5	8	5	10	7	10	8	14	-0.30469
sp|Q08211|DHX9_HUMAN	DHX9	ATP-dependent RNA helicase A OS=Homo sapiens GN=DHX9 PE=1 SV=4	142181	96	66	218	541	0.41	0.76	1.853658537	8	20	16	27	18	28	22	34	-0.16688
tr|B2R6X5|B2R6X5_HUMAN	tr|B2R6X	cDNA, FLJ93166, highly similar to Homo sapiens heat shock 70kDa protein 6 (HSP70B~) (HSPA6), mRNA OS=Homo sapiens PE=2 SV=	71370	193	307	112	126	0.2	0.37	1.85	3	6	5	9	6	9	6	9	-0.24249
sp|P14923|PLAK_HUMAN	JUP	Junction plakoglobin OS=Homo sapiens GN=JUP PE=1 SV=3	82434	205	280	102	141	0.26	0.48	1.846153846	3	6	6	10	6	10	7	11	-0.0979
tr|B2R5W2|B2R5W2_HUMAN	tr|B2R5W	cDNA, FLJ92657, highly similar to Homo sapiens heterogeneous nuclear ribonucleoprotein C (C1/C2) (HNRPC), transcript variant 2, m	31986	178	419	122	88	0.35	0.64	1.828571429	3	4	3	5	3	5	4	5	-0.13767
tr|B4DQJ8|B4DQJ8_HUMAN	PGD	6-phosphogluconate dehydrogenase, decarboxylating OS=Homo sapiens GN=PGD PE=2 SV=1	52352	293	315	62	120	0.2	0.36	1.8	1	4	3	5	2	7	3	8	0.795949
tr|B3KMX0|B3KMX0_HUMAN	tr|B3KMX	cDNA FLJ12837 fis, clone NT2RP2003228, highly similar to DNA replication licensing factor MCM4 OS=Homo sapiens PE=2 SV=1	97114	240	304	85	127	0.1	0.18	1.8	2	3	3	5	4	7	5	9	-0.05273
sp|Q9NV06|DCA13_HUMAN	DCAF13	DDB1- and CUL4-associated factor 13 OS=Homo sapiens GN=DCAF13 PE=1 SV=2	51996	435	302	39	127	0.2	0.36	1.8	1	5	3	5	3	5	6	5	-0.13767
sp|P53396|ACLY_HUMAN	ACLY	ATP-citrate synthase OS=Homo sapiens GN=ACLY PE=1 SV=3	121674	20	15	845	1384	1.28	2.3	1.796875	21	36	32	51	34	53	40	59	-0.15791
sp|P26599|PTBP1_HUMAN	PTBP1	Polypyrimidine tract-binding protein 1 OS=Homo sapiens GN=PTBP1 PE=1 SV=1	57357	567	225	29	169	0.18	0.32	1.777777778	0	3	3	5	3	5	3	7	-0.13767
tr|D3DPU2|D3DPU2_HUMAN	CAP1	Adenylyl cyclase-associated protein OS=Homo sapiens GN=CAP1 PE=3 SV=1	51926	172	122	125	302	0.36	0.64	1.777777778	4	8	5	8	6	9	7	11	-0.24249
tr|B5MCX3|B5MCX3_HUMAN	Sep-02	Septin-2 OS=Homo sapiens GN=SEPT2 PE=2 SV=1	37145	543	340	30	112	0.09	0.16	1.777777778	1	2	1	2	2	3	3	3	-0.30437
sp|Q92621|NU205_HUMAN	NUP205	Nuclear pore complex protein Nup205 OS=Homo sapiens GN=NUP205 PE=1 SV=3	230171	415	373	42	98	0.04	0.07	1.75	0	3	3	5	3	7	5	8	0.310184
tr|E0X9P6|E0X9P6_HUMAN	HLA-A	MHC class I antigen (Fragment) OS=Homo sapiens GN=HLA-A PE=3 SV=1	21057	639	551	22	50	0.16	0.28	1.75	0	1	1	1	1	1	3	1	-0.78985
sp|P17987|TCPA_HUMAN	TCP1	T-complex protein 1 subunit alpha OS=Homo sapiens GN=TCP1 PE=1 SV=1	60819	197	120	106	304	0.3	0.52	1.733333333	2	6	5	8	8	12	10	14	-0.23367
tr|B4DQX0|B4DQX0_HUMAN	tr|B4DQX	cDNA FLJ59219, highly similar to Poly(A)-binding protein 1 OS=Homo sapiens PE=2 SV=1	67239	215	141	96	241	0.33	0.57	1.727272727	3	7	6	9	6	10	10	12	-0.0979
tr|B4DF96|B4DF96_HUMAN	EIF2A	Eukaryotic translation initiation factor 2A OS=Homo sapiens GN=EIF2A PE=2 SV=1	58357	206	181	101	144	0.32	0.55	1.71875	2	4	5	7	4	10	5	14	0.43329
tr|Q6IPN6|Q6IPN6_HUMAN	EEF1A1	Elongation factor 1-alpha OS=Homo sapiens GN=EEF1A1 PE=2 SV=1	50433	70	85	295	392	1.14	1.94	1.701754386	11	16	13	20	18	24	21	29	-0.38585
tr|G3V2A3|G3V2A3_HUMAN	TUBB3	Tubulin beta-3 chain (Fragment) OS=Homo sapiens GN=TUBB3 PE=2 SV=1	20929	25	20	147	488	3.07	5.18	1.687296417	5	15	6	17	7	23	7	25	0.860131
tr|B4DPZ3|B4DPZ3_HUMAN	tr|B4DPZ	cDNA FLJ53290, highly similar to Cytoplasmic dynein 1 intermediate chain 2 OS=Homo sapiens PE=2 SV=1	70869	298	332	60	113	0.09	0.15	1.666666667	2	2	2	3	2	3	2	3	-0.30437
tr|Q59H63|Q59H63_HUMAN	tr|Q59H6	Plasma membrane calcium ATPase 1 isoform 1a variant (Fragment) OS=Homo sapiens PE=2 SV=1	94309	528	590	31	0	0.03	0.05	1.666666667	0	0	1	0	1	0	1	1	-2.37501
tr|B7Z4V2|B7Z4V2_HUMAN	tr|B7Z4V	cDNA FLJ51907, highly similar to Stress-70 protein, mitochondrial OS=Homo sapiens PE=2 SV=1	72641	81	73	251	495	0.42	0.7	1.666666667	7	11	8	13	8	13	8	16	-0.12245
tr|A8K7F6|A8K7F6_HUMAN	tr|A8K7F	cDNA FLJ78244, highly similar to Homo sapiens eukaryotic translation initiation factor 4A, isoform 1 (EIF4A1), mRNA OS=Homo sapie	46321	52	64	404	517	1.62	2.69	1.660493827	10	15	14	19	15	20	20	22	-0.38752
sp|P07737|PROF1_HUMAN	PFN1	Profilin-1 OS=Homo sapiens GN=PFN1 PE=1 SV=2	15216	436	416	39	88	0.5	0.83	1.66	1	3	2	3	2	3	2	3	-0.30437
sp|Q09028|RBBP4_HUMAN	RBBP4	Histone-binding protein RBBP4 OS=Homo sapiens GN=RBBP4 PE=1 SV=3	47911	321	897	55	37	0.22	0.36	1.636363636	2	1	3	2	4	2	4	3	-1.63867
tr|B7Z2Z8|B7Z2Z8_HUMAN	tr|B7Z2Z8	T-complex protein 1 subunit delta OS=Homo sapiens PE=2 SV=1	52781	113	248	188	138	0.27	0.44	1.62962963	4	2	5	5	7	6	7	9	-0.99736
sp|Q96TA1|NIBL1_HUMAN	FAM129B	Niban-like protein 1 OS=Homo sapiens GN=FAM129B PE=1 SV=3	84598	155	348	137	110	0.16	0.26	1.625	4	4	4	6	4	8	4	8	0.128043
tr|B2R6J2|B2R6J2_HUMAN	tr|B2R6J2	cDNA, FLJ92973, highly similar to Homo sapiens villin 2 (ezrin) (VIL2), mRNA OS=Homo sapiens PE=2 SV=1	69472	126	227	173	168	0.32	0.52	1.625	6	6	6	10	8	13	8	18	-0.12245
sp|P18206|VINC_HUMAN	VCL	Vinculin OS=Homo sapiens GN=VCL PE=1 SV=4	124292	68	70	299	521	0.37	0.6	1.621621622	8	13	12	19	13	24	13	26	0.070415
tr|Q5HYG8|Q5HYG8_HUMAN	DKFZp686	Putative uncharacterized protein DKFZp686P09201 OS=Homo sapiens GN=DKFZp686P09201 PE=2 SV=1	53831	86	200	235	193	0.61	0.98	1.606557377	4	6	8	9	9	10	10	10	-0.64641
tr|Q05BW9|Q05BW9_HUMAN	PAPSS1	PAPSS1 protein (Fragment) OS=Homo sapiens GN=PAPSS1 PE=2 SV=1	65264	553	513	29	71	0.1	0.16	1.6	0	2	2	3	1	3	2	3	0.432934
tr|F5H6T1|F5H6T1_HUMAN	ACTR2	Actin-related protein 2 OS=Homo sapiens GN=ACTR2 PE=2 SV=1	34729	244	323	82	116	0.2	0.32	1.6	2	4	2	4	2	5	4	5	0.348098
tr|A8K525|A8K525_HUMAN	tr|A8K525	cDNA FLJ76817, highly similar to Homo sapiens non-POU domain containing, octamer-binding (NONO), mRNA OS=Homo sapiens PE	54367	75	126	266	292	0.8	1.28	1.6	10	8	11	14	12	17	14	20	-0.30502
tr|K9JA46|K9JA46_HUMAN	EL52	Epididymis luminal secretory protein 52 OS=Homo sapiens GN=EL52 PE=2 SV=1	85006	46	45	462	762	1.05	1.67	1.59047619	15	25	25	29	24	32	29	39	-0.38392
tr|K7EM16|K7EM16_HUMAN	VASP	Vasodilator-stimulated phosphoprotein (Fragment) OS=Homo sapiens GN=VASP PE=4 SV=1	20260	363	435	48	78	0.17	0.27	1.588235294	1	2	1	2	1	2	1	2	-0.05269
tr|B2R7M3|B2R7M3_HUMAN	tr|B2R7M	cDNA, FLJ93510, highly similar to Homo sapiens JTV1 gene (JTV1), mRNA OS=Homo sapiens PE=2 SV=1	35767	308	694	58	50	0.19	0.3	1.578947368	2	1	2	3	2	3	2	3	-0.30437
tr|B4DXX7|B4DXX7_HUMAN	DDX3Y	ATP-dependent RNA helicase DDX3Y OS=Homo sapiens GN=DDX3Y PE=2 SV=1	73342	217	258	95	152	0.19	0.3	1.578947368	2	6	5	7	6	7	6	9	-0.58392
sp|P58107|EPIPL_HUMAN	EPPK1	Epiplakin OS=Homo sapiens GN=EPPK1 PE=1 SV=2	557674	56	71	390	511	0.07	0.11	1.571428571	10	13	12	18	12	18	13	21	-0.22465
sp|Q2NL82|TSR1_HUMAN	TSR1	Pre-rRNA-processing protein TSR1 homolog OS=Homo sapiens GN=TSR1 PE=1 SV=1	92151	230	471	90	78	0.07	0.11	1.571428571	2	2	2	3	2	3	2	3	-0.30437
tr|B4E2P4|B4E2P4_HUMAN	tr|B4E2P4	cDNA FLJ58474, highly similar to Xaa-Pro aminopeptidase 1 (EC 3.4.11.9) OS=Homo sapiens PE=2 SV=1	50020	615	635	24	39	0.07	0.11	1.571428571	0	1	1	1	1	1	1	1	-0.78985
tr|B4DH02|B4DH02_HUMAN	tr|B4DH0	cDNA FLJ50510, highly similar to Heat shock 70 kDa protein 4 OS=Homo sapiens PE=2 SV=1	95157	123	92	175	363	0.23	0.36	1.565217391	4	9	6	9	5	9	6	10	-0.00114
tr|J3KRF5|J3KRF5_HUMAN	CLTC	Clathrin heavy chain 1 (Fragment) OS=Homo sapiens GN=CLTC PE=4 SV=1	15819	30	563	40	63	0.52	0.79	1.519230769	1	2	1	3	2	3	2	3	-0.30437
tr|B4DR45|B4DR45_HUMAN	tr|B4DR4	cDNA FLJ61339, highly similar to Alanyl-tRNA synthetase (EC 6.1.1.7) OS=Homo sapiens PE=2 SV=1	110017	238	262	86	149	0.06	0.09	1.5	1	3	2	3	2	6	2	7	0.589303
tr|I3L312|I3L312_HUMAN	P4HB	Protein disulfide-isomerase (Fragment) OS=Homo sapiens GN=P4HB PE=4 SV=1	26348	536	325	30	43	0.27	0.4	1.481481481	1	2	2	3	1	5	2	5	1.085403
sp|P68366|TBA4A_HUMAN	TUBA4A	Tubulin alpha-4A chain OS=Homo sapiens GN=TUBA4A PE=1 SV=1	50634	22	36	796	927	3.01	4.45	1.478405316	20	23	31	35	31	42	36	45	-0.35988
tr|A8K4Z4|A8K4Z4_HUMAN	tr|A8K4Z4	cDNA FLJ75549, highly similar to Homo sapiens ribosomal protein, large, P0 (RPLP0), transcript variant 1, mRNA OS=Homo sapiens P	34389	109	265	201	148	0.74	1.09	1.472972973	6	7	6	8	6	10	6	10	-0.0979
sp|P13010|XRCC5_HUMAN	XRCC5	X-ray repair cross-complementing protein 5 OS=Homo sapiens GN=XRCC5 PE=1 SV=3	83222	128	226	172	168	0.36	0.53	1.472222222	6	6	8	11	8	12	9	14	-0.23367
tr|B4DRH6|B4DRH6_HUMAN	tr|B4DRH	cDNA FLJ54509, highly similar to Trifunctional enzyme subunit alpha, mitochondrial OS=Homo sapiens PE=2 SV=1	79007	133	185	164	199	0.28	0.41	1.464285714	5	6	6	7	7	9	7	9	-0.44928
tr|H0Y327|H0Y327_HUMAN	MTHFD1L	Monofunctional C1-tetrahydrofolate synthase, mitochondrial (Fragment) OS=Homo sapiens GN=MTHFD1L PE=4 SV=1	36808	398	524	43	19	0.09	0.13	1.444444444	1	0	1	1	1	0	1	1	-2.37501
tr|Q53HF2|Q53HF2_HUMAN	tr|Q53HF	Heat shock 70kDa protein 8 isoform 2 variant (Fragment) OS=Homo sapiens PE=2 SV=1	53580	51	58	426	529	1.45	2.09	1.44137931	12	20	17	28	19	26	19	32	-0.34854
sp|O00410|IPO5_HUMAN	IPO5	Importin-5 OS=Homo sapiens GN=IPO5 PE=1 SV=4	125032	85	135	236	278	0.23	0.33	1.434782609	7	9	8	12	10	15	15	15	-0.22828
sp|P26232|CTNA2_HUMAN	CTNNA2	Catenin alpha-2 OS=Homo sapiens GN=CTNNA2 PE=1 SV=5	106045	389	576	31	62	0.07	0.1	1.428571429	0	1	1	3	1	3	1	3	0.432934
tr|Q7Z5T5|Q7Z5T5_HUMAN	EIF3A	EIF3A protein (Fragment) OS=Homo sapiens GN=EIF3A PE=2 SV=1	97203	192	230	112	163	0.14	0.2	1.428571429	2	6	4	9	5	12	8	17	0.395376
sp|Q1KMD3|HNRL2_HUMAN	HNRNPUL	Heterogeneous nuclear ribonucleoprotein U-like protein 2 OS=Homo sapiens GN=HNRNPUL2 PE=1 SV=1	85622	249	170	78	224	0.21	0.3	1.428571429	2	6	5	7	6	8	6	9	-0.40315
tr|B1ALA9|B1ALA9_HUMAN	PRPS1	Phosphoribosyl pyrophosphate synthetase 1 OS=Homo sapiens GN=PRPS1 PE=3 SV=1	24468	638	543	22	26	0.14	0.2	1.428571429	0	0	1	1	1	1	2	1	-0.78985
sp|Q9H853|TBA4B_HUMAN	TUBA4B	Putative tubulin-like protein alpha-4B OS=Homo sapiens GN=TUBA4B PE=5 SV=2	27819	146	228	147	163	0.4	0.57	1.425	3	3	4	5	4	4	6	5	-0.79028
tr|B1AHC9|B1AHC9_HUMAN	XRCC6	X-ray repair complementing defective repair in Chinese hamster cells 6 (Ku autoantigen, 70kDa) OS=Homo sapiens GN=XRCC6 PE=4	64528	116	96	184	357	0.64	0.91	1.421875	5	10	10	13	11	15	12	19	-0.35986
tr|B3KX19|B3KX19_HUMAN	tr|B3KX1	cDNA FLJ44500 fis, clone UTERU3000828, highly similar to 116 kDa U5 small nuclear ribonucleoprotein component OS=Homo sapier	109111	111	124	193	293	0.27	0.38	1.407407407	4	6	8	11	8	13	10	15	-0.12245
sp|P13639|EF2_HUMAN	EEF2	Elongation factor 2 OS=Homo sapiens GN=EEF2 PE=1 SV=4	96246	41	38	511	914	1.23	1.73	1.406504065	13	32	26	36	27	48	32	55	0.029107
tr|E9PBU3|E9PBU3_HUMAN	ATIC	Phosphoribosylaminoimidazolecarboxamide formyltransferase OS=Homo sapiens GN=ATIC PE=2 SV=1	64996	72	140	274	243	0.64	0.9	1.40625	8	7	11	12	12	12	13	15	-0.79142
tr|B3KNM0|B3KNM0_HUMAN	tr|B3KNM	cDNA FLJ14916 fis, clone PLACE1006958, highly similar to Heat shock 70 kDa protein 4L (Fragment) OS=Homo sapiens PE=2 SV=1	64588	191	261	113	150	0.1	0.14	1.4	2	3	2	4	2	4	3	4	0.058396
tr|K7EIG1|K7EIG1_HUMAN	CLUH	Clustered mitochondria protein homolog OS=Homo sapiens GN=CLUH PE=3 SV=1	140903	438	562	39	63	0.05	0.07	1.4	1	2	2	3	2	3	2	3	-0.30437
tr|B4DPP6|B4DPP6_HUMAN	tr|B4DPP	cDNA FLJ54371, highly similar to Serum albumin OS=Homo sapiens PE=2 SV=1	72370	222	209	93	187	0.25	0.35	1.4	3	5	5	7	6	8	10	10	-0.40315
tr|B3KX72|B3KX72_HUMAN	tr|B3KX7	cDNA FLJ44920 fis, clone BRAMY3011501, highly similar to Heterogeneous nuclear ribonucleoprotein U OS=Homo sapiens PE=2 SV=	83774	59	74	367	466	0.58	0.8	1.379310345	10	14	14	18	16	21	20	23	-0.40915
tr|Q53SS8|Q53SS8_HUMAN	PCBP1	Poly(RC) binding protein 1 OS=Homo sapiens GN=PCBP1 PE=2 SV=1	37987	110	522	197	70	0.29	0.4	1.379310345	3	2	3	4	3	4	3	5	-0.42737
sp|Q05639|EF1A2_HUMAN	EEF1A2	Elongation factor 1-alpha 2 OS=Homo sapiens GN=EEF1A2 PE=1 SV=1	50780	70	112	238	316	1.14	1.57	1.377192982	8	12	9	17	12	19	14	21	-0.1485
tr|F6IQP2|F6IQP2_HUMAN	HLA-C	MHC class I antigen (Fragment) OS=Homo sapiens GN=HLA-C PE=3 SV=1	39655	639	827	22	41	0.16	0.22	1.375	0	1	1	2	1	4	1	5	0.7957
tr|A1KWR0|A1KWR0_HUMAN	HLA-B	MHC class I antigen (Fragment) OS=Homo sapiens GN=HLA-B PE=3 SV=1	31888	639	628	22	56	0.16	0.22	1.375	0	1	1	2	1	4	1	5	0.7957
tr|A0MNW2|A0MNW2_HUMA	HLA-B	MHC class I antigen (Fragment) OS=Homo sapiens GN=HLA-B PE=3 SV=1	31749	639	627	22	56	0.16	0.22	1.375	0	1	1	2	1	4	1	4	0.7957
tr|Q75NY8|Q75NY8_HUMAN	HLA-A	MHC class I antigen (Fragment) OS=Homo sapiens GN=HLA-A PE=3 SV=1	32018	639	598	22	60	0.16	0.22	1.375	0	2	1	2	1	2	3	3	-0.05269
tr|A0JCS1|A0JCS1_HUMAN	HLA-B	MHC class I antigen (Fragment) OS=Homo sapiens GN=HLA-B PE=3 SV=1	31890	639	597	22	50	0.16	0.22	1.375	0	1	1	1	1	2	1	2	-0.05269
tr|B4DSQ5|B4DSQ5_HUMAN	tr|B4DSQ	cDNA FLJ53608, highly similar to Protein transport protein Sec23A OS=Homo sapiens PE=2 SV=1	83787	474	591	37	60	0.08	0.11	1.375	0	2	2	2	2	2	3	2	-0.78999
tr|H0Y5G9|H0Y5G9_HUMAN	NRD1	Nardilysin (Fragment) OS=Homo sapiens GN=NRD1 PE=3 SV=1	62196	225	510	92	55	0.11	0.15	1.363636364	2	2	2	4	2	4	2	4	0.058396
tr|B3GQS7|B3GQS7_HUMAN	HSPD1	Mitochondrial heat shock 60kD protein 1 variant 1 OS=Homo sapiens GN=HSPD1 PE=2 SV=1	60813	16	53	904	590	1.72	2.34	1.360465116	24	15	27	23	26	26	33	27	-0.79344
sp|P46063|RECQ1_HUMAN	RECQL	ATP-dependent DNA helicase Q1 OS=Homo sapiens GN=RECQL PE=1 SV=3	74436	439	393	39	91	0.14	0.19	1.357142857	1	2	3	4	3	5	3	5	-0.13767
tr|B3KW67|B3KW67_HUMAN	tr|B3KW6	cDNA FLJ42347 fis, clone UTERU2003399, highly similar to Actin, gamma-enteric smooth muscle OS=Homo sapiens PE=2 SV=1	42189	38	34	538	952	2.61	3.52	1.348659004	19	27	27	38	32	43	37	48	-0.37156
tr|Q9UJZ2|Q9UJZ2_HUMAN	VIL2	Cytovillin 2 (Fragment) OS=Homo sapiens GN=VIL2 PE=2 SV=1	18464	119	295	88	112	0.46	0.62	1.347826087	2	3	2	3	2	3	2	5	-0.30437
tr|B4DHH5|B4DHH5_HUMAN	tr|B4DHH	cDNA FLJ53472, highly similar to Dynamin-1 (EC 3.6.5.5) OS=Homo sapiens PE=2 SV=1	89920	713	1141	16	23	0.03	0.04	1.333333333	0	0	1	1	0	2	1	2	1.532612
sp|Q68EM7|RHG17_HUMAN	ARHGAP1	Rho GTPase-activating protein 17 OS=Homo sapiens GN=ARHGAP17 PE=1 SV=1	95776	328	814	54	42	0.03	0.04	1.333333333	1	1	1	1	2	1	2	1	-1.52715
tr|Q5I6Y6|Q5I6Y6_HUMAN	LMNA	Lamin A/C transcript variant 1 OS=Homo sapiens GN=LMNA PE=2 SV=1	74322	95	75	221	481	0.75	1	1.333333333	7	14	13	16	15	16	15	16	-0.70146
tr|D6RF19|D6RF19_HUMAN	DPYSL3	Dihydropyrimidinase-related protein 3 (Fragment) OS=Homo sapiens GN=DPYSL3 PE=2 SV=1	10214	342	318	20	0	0.23	0.3	1.304347826	0	0	1	0	1	0	1	1	-2.37501
sp|P23381|SYWC_HUMAN	WARS	Tryptophan--tRNA ligase, cytoplasmic OS=Homo sapiens GN=WARS PE=1 SV=2	53474	145	224	148	169	0.27	0.35	1.296296296	3	4	4	5	5	5	5	6	-0.79042
tr|F4ZW64|F4ZW64_HUMAN	tr|F4ZW6	NF90a OS=Homo sapiens PE=2 SV=1	76369	154	142	137	262	0.46	0.59	1.282608696	3	7	9	11	10	14	11	14	-0.32469
sp|Q9UMS4|PRP19_HUMAN	PRPF19	Pre-mRNA-processing factor 19 OS=Homo sapiens GN=PRPF19 PE=1 SV=1	55603	237	276	87	142	0.26	0.33	1.269230769	2	3	5	5	7	6	11	8	-0.99736
tr|H6VRF8|H6VRF8_HUMAN	KRT1	Keratin 1 OS=Homo sapiens GN=KRT1 PE=3 SV=1	66184	6	16	2032	1384	5.01	6.3	1.25748503	55	39	64	53	69	60	76	65	-1.00154
tr|F8SKU2|F8SKU2_HUMAN	HLA-A	MHC class I antigen (Fragment) OS=Homo sapiens GN=HLA-A PE=3 SV=1	21143	639	595	22	0	0.16	0.2	1.25	0	0	1	0	2	2	3	2	-0.78999
sp|Q6IBS0|TWF2_HUMAN	TWF2	Twinfilin-2 OS=Homo sapiens GN=TWF2 PE=1 SV=2	39751	535	721	30	0	0.08	0.1	1.25	0	0	1	0	1	0	1	1	-2.37501
tr|H0YAS6|H0YAS6_HUMAN	PABPC1	Polyadenylate-binding protein 1 (Fragment) OS=Homo sapiens GN=PABPC1 PE=4 SV=1	17943	215	458	48	79	0.33	0.41	1.242424242	1	2	2	2	2	2	2	4	-0.78999
tr|Q53GX7|Q53GX7_HUMAN	tr|Q53GX	Threonyl-tRNA synthetase variant (Fragment) OS=Homo sapiens PE=2 SV=1	84304	94	101	221	352	0.64	0.79	1.234375	6	11	13	19	17	20	18	25	-0.56328
tr|H0Y804|H0Y804_HUMAN	AKR1C1	Aldo-keto reductase family 1 member C1 (Fragment) OS=Homo sapiens GN=AKR1C1 PE=4 SV=1	28617	186	285	61	98	0.67	0.82	1.223880597	2	3	4	4	4	5	6	6	-0.50058
sp|Q15046|SYK_HUMAN	KARS	Lysine--tRNA ligase OS=Homo sapiens GN=KARS PE=1 SV=3	68461	265	222	71	171	0.32	0.39	1.21875	1	5	6	7	6	10	7	11	-0.0979
tr|Q59EG8|Q59EG8_HUMAN	tr|Q59EG	Proteasome 26S non-ATPase subunit 2 variant (Fragment) OS=Homo sapiens PE=2 SV=1	101322	198	264	105	149	0.14	0.17	1.214285714	1	4	4	5	4	8	5	8	0.128043
tr|G8JLB6|G8JLB6_HUMAN	HNRNPH1	Heterogeneous nuclear ribonucleoprotein H OS=Homo sapiens GN=HNRNPH1 PE=4 SV=1	51482	207	171	101	223	0.45	0.54	1.2	2	4	6	7	5	6	6	7	-0.54922
tr|H0YMR4|H0YMR4_HUMAN	IPO4	Importin-4 (Fragment) OS=Homo sapiens GN=IPO4 PE=4 SV=1	60104	655	860	22	15	0.05	0.06	1.2	0	0	1	1	1	0	1	1	-2.37501
tr|H6VRG2|H6VRG2_HUMAN	KRT1	Keratin 1 OS=Homo sapiens GN=KRT1 PE=3 SV=1	66197	7	19	2027	1283	5.01	5.95	1.18762475	55	37	65	51	70	59	77	64	-1.04674
tr|F8VRQ1|F8VRQ1_HUMAN	HNRNPA1	Heterogeneous nuclear ribonucleoprotein A1 OS=Homo sapiens GN=HNRNPA1 PE=2 SV=1	33249	101	180	214	211	0.77	0.91	1.181818182	4	5	6	7	7	6	9	9	-0.99736
tr|J3KPF3|J3KPF3_HUMAN	SLC3A2	4F2 cell-surface antigen heavy chain OS=Homo sapiens GN=SLC3A2 PE=4 SV=1	68230	60	55	366	600	1.33	1.56	1.172932331	12	16	20	21	20	23	20	27	-0.59495
tr|A8K6Q8|A8K6Q8_HUMAN	tr|A8K6Q	cDNA FLJ75881, highly similar to Homo sapiens transferrin receptor (p90, CD71) (TFRC), mRNA OS=Homo sapiens PE=2 SV=1	85276	122	144	176	260	0.3	0.35	1.166666667	6	6	7	8	7	12	8	12	-0.05276
tr|Q6DC98|Q6DC98_HUMAN	LMNB1	LMNB1 protein (Fragment) OS=Homo sapiens GN=LMNB1 PE=2 SV=1	38289	219	452	95	75	0.18	0.21	1.166666667	2	2	2	3	2	3	4	3	-0.30437
tr|A8MW61|A8MW61_HUMA	PLRG1	Pleiotropic regulator 1 OS=Homo sapiens GN=PLRG1 PE=2 SV=1	57545	476	820	36	42	0.12	0.14	1.166666667	1	1	2	1	2	1	4	1	-1.52715
sp|P52272|HNRPM_HUMAN	HNRNPM	Heterogeneous nuclear ribonucleoprotein M OS=Homo sapiens GN=HNRNPM PE=1 SV=3	77749	164	164	132	229	0.39	0.45	1.153846154	7	8	8	10	11	11	12	13	-0.79128
sp|P07814|SYEP_HUMAN	EPRS	Bifunctional glutamate/proline--tRNA ligase OS=Homo sapiens GN=EPRS PE=1 SV=5	172080	57	83	380	401	0.33	0.38	1.151515152	9	12	15	17	15	22	17	27	-0.25282
tr|B7Z4F6|B7Z4F6_HUMAN	tr|B7Z4F6	cDNA FLJ54912, highly similar to 60 kDa heat shock protein, mitochondrial OS=Homo sapiens PE=2 SV=1	58705	16	67	699	525	1.72	1.98	1.151162791	18	13	21	20	20	23	27	24	-0.59495
sp|Q9NR30|DDX21_HUMAN	DDX21	Nucleolar RNA helicase 2 OS=Homo sapiens GN=DDX21 PE=1 SV=5	87804	148	182	147	209	0.34	0.39	1.147058824	4	9	8	9	8	9	9	10	-0.63019
tr|B4DXW6|B4DXW6_HUMAN	tr|B4DXW	cDNA FLJ50285, highly similar to Arginyl-tRNA synthetase (EC 6.1.1.19) OS=Homo sapiens PE=2 SV=1	52771	235	277	88	98	0.35	0.4	1.142857143	3	3	5	5	6	4	7	6	-1.32147
tr|B4DKZ9|B4DKZ9_HUMAN	tr|B4DKZ	cDNA FLJ55705, highly similar to Threonyl-tRNA synthetase, cytoplasmic (EC 6.1.1.3) OS=Homo sapiens PE=2 SV=1	71171	94	111	191	316	0.64	0.72	1.125	5	10	10	15	13	15	14	21	-0.59187
tr|F2VNS8|F2VNS8_HUMAN	HLA-C	MHC class I antigen (Fragment) OS=Homo sapiens GN=HLA-C PE=3 SV=1	21240	639	596	22	0	0.16	0.18	1.125	0	0	1	0	2	1	2	1	-1.52715
sp|Q9P287|BCCIP_HUMAN	BCCIP	BRCA2 and CDKN1A-interacting protein OS=Homo sapiens GN=BCCIP PE=1 SV=1	36299	503	1145	33	22	0.09	0.1	1.111111111	0	0	1	1	1	1	3	1	-0.78985
sp|Q04695|K1C17_HUMAN	KRT17	Keratin, type I cytoskeletal 17 OS=Homo sapiens GN=KRT17 PE=1 SV=2	48361	24	68	683	524	2.99	3.26	1.090301003	19	17	27	25	32	33	35	37	-0.75039
tr|E5RFS9|E5RFS9_HUMAN	MAT2B	Methionine adenosyltransferase 2 subunit beta OS=Homo sapiens GN=MAT2B PE=2 SV=1	9467	280	441	68	69	0.37	0.4	1.081081081	1	1	1	1	1	1	1	1	-0.78985
tr|F5CL10|F5CL10_HUMAN	HLA-Cw	MHC class I antigen OS=Homo sapiens GN=HLA-Cw PE=3 SV=1	41120	639	826	22	41	0.16	0.17	1.0625	0	1	1	2	1	4	1	5	0.7957
tr|B2R4C0|B2R4C0_HUMAN	tr|B2R4C	60S ribosomal protein L18a OS=Homo sapiens PE=2 SV=1	21024	514	1088	32	27	0.16	0.17	1.0625	0	0	1	1	1	1	2	1	-0.78985
tr|B7Z481|B7Z481_HUMAN	tr|B7Z481	cDNA FLJ54018, highly similar to PDZ and LIM domain protein 5 OS=Homo sapiens PE=2 SV=1	52359	175	281	109	140	0.41	0.43	1.048780488	2	3	5	6	5	6	6	6	-0.54922
tr|Q6FHM2|Q6FHM2_HUMAN	GNB2	GNB2 protein OS=Homo sapiens GN=GNB2 PE=2 SV=1	38048	194	1177	111	19	0.27	0.28	1.037037037	1	0	2	1	2	1	2	2	-1.52715
sp|P68363|TBA1B_HUMAN	TUBA1B	Tubulin alpha-1B chain OS=Homo sapiens GN=TUBA1B PE=1 SV=1	50804	13	24	1091	1160	5.58	5.76	1.032258065	31	30	43	44	45	52	49	56	-0.58836
tr|F5H4R9|F5H4R9_HUMAN	DNM2	Dynamin-2 OS=Homo sapiens GN=DNM2 PE=2 SV=1	98218	713	679	16	51	0.03	0.03	1	1	0	1	1	0	2	2	2	1.532612
tr|B0QYA5|B0QYA5_HUMAN	EIF3D	Eukaryotic translation initiation factor 3 subunit D (Fragment) OS=Homo sapiens GN=EIF3D PE=4 SV=1	31937	710	1109	17	25	0.1	0.1	1	0	0	1	1	0	1	2	2	0.795451
tr|I3L2C7|I3L2C7_HUMAN	GEMIN4	Gem-associated protein 4 OS=Homo sapiens GN=GEMIN4 PE=4 SV=1	120456	709	1048	17	29	0.03	0.03	1	0	1	1	1	0	1	1	3	0.795451
tr|B0UX83|B0UX83_HUMAN	BAG6	HLA-B associated transcript 3 OS=Homo sapiens GN=BAG6 PE=4 SV=1	119085	704	385	18	94	0.03	0.03	1	0	1	1	1	0	1	1	1	0.795451
sp|P11388|TOP2A_HUMAN	TOP2A	DNA topoisomerase 2-alpha OS=Homo sapiens GN=TOP2A PE=1 SV=3	175017	606	503	25	73	0.02	0.02	1	0	1	1	1	1	3	2	6	0.432934
sp|Q6KB66|K2C80_HUMAN	KRT80	Keratin, type II cytoskeletal 80 OS=Homo sapiens GN=KRT80 PE=1 SV=2	51007	564	1016	29	30	0.06	0.06	1	0	0	1	1	1	2	1	3	-0.05269
tr|L0R4T3|L0R4T3_HUMAN	ABCF2	Alternative protein ABCF2 OS=Homo sapiens GN=ABCF2 PE=4 SV=1	7359	524	995	31	31	0.48	0.48	1	1	1	1	1	1	2	1	2	-0.05269
tr|B4DXC4|B4DXC4_HUMAN	tr|B4DXC	cDNA FLJ58636, moderately similar to Atlastin OS=Homo sapiens PE=2 SV=1	59162	451	885	38	37	0.06	0.06	1	1	1	1	1	1	2	1	2	-0.05269
tr|B4DJE3|B4DJE3_HUMAN	tr|B4DJE3	cDNA FLJ52929, highly similar to Dolichyl-diphosphooligosaccharide--proteinglycosyltransferase 48 kDa subunit (EC2.4.1.119) OS=Ho	46620	604	861	26	39	0.07	0.07	1	0	1	1	1	1	2	1	2	-0.05269
tr|H0Y5F5|H0Y5F5_HUMAN	PABPC4	Polyadenylate-binding protein 4 (Fragment) OS=Homo sapiens GN=PABPC4 PE=4 SV=1	60492	504	813	33	42	0.05	0.05	1	0	1	1	1	1	2	2	3	-0.05269
tr|E0YTJ7|E0YTJ7_HUMAN	HLA-B	MHC class I antigen (Fragment) OS=Homo sapiens GN=HLA-B PE=3 SV=1	21175	639	723	22	48	0.16	0.16	1	0	1	1	1	1	2	1	2	-0.05269
tr|J3KN16|J3KN16_HUMAN	KIAA0368	Protein KIAA0368 OS=Homo sapiens GN=KIAA0368 PE=4 SV=1	225491	579	611	28	58	0.04	0.04	1	0	1	1	2	1	2	1	2	-0.05269
tr|Q5BKZ2|Q5BKZ2_HUMAN	KPNA1	Importin subunit alpha OS=Homo sapiens GN=KPNA1 PE=2 SV=1	60955	573	548	22	65	0.11	0.11	1	0	2	1	2	1	2	1	2	-0.05269
tr|F5H1A8|F5H1A8_HUMAN	GSN	Gelsolin OS=Homo sapiens GN=GSN PE=2 SV=1	81776	297	512	61	71	0.04	0.04	1	1	1	1	1	1	2	1	2	-0.05269
tr|Q2VPA0|Q2VPA0_HUMAN	LONP1	Lon protease homolog (Fragment) OS=Homo sapiens GN=LONP1 PE=2 SV=1	95436	414	588	42	61	0.11	0.11	1	2	2	3	3	3	5	3	6	-0.13767
tr|D5FHQ7|D5FHQ7_HUMAN	HLA-A	MHC class I antigen (Fragment) OS=Homo sapiens GN=HLA-A PE=3 SV=1	21188	640	698	22	50	0.16	0.16	1	0	1	1	1	2	3	3	3	-0.30437
sp|P53004|BIEA_HUMAN	BLVRA	Biliverdin reductase A OS=Homo sapiens GN=BLVRA PE=1 SV=2	33692	403	585	43	61	0.21	0.21	1	1	1	2	2	2	3	3	3	-0.30437
tr|B3KY95|B3KY95_HUMAN	tr|B3KY9	cDNA FLJ16143 fis, clone BRAMY2038516, highly similar to Protein disulfide-isomerase A6 (EC 5.3.4.1) OS=Homo sapiens PE=2 SV=1	53655	445	436	38	84	0.13	0.13	1	0	1	2	2	2	3	2	3	-0.30437
sp|P55072|TERA_HUMAN	VCP	Transitional endoplasmic reticulum ATPase OS=Homo sapiens GN=VCP PE=1 SV=4	89950	64	65	326	542	0.65	0.65	1	7	12	14	16	14	19	15	23	-0.36331
tr|F5H5D3|F5H5D3_HUMAN	TUBA1C	Tubulin alpha-1C chain OS=Homo sapiens GN=TUBA1C PE=2 SV=1	58606	21	28	809	1084	2.92	2.92	1	24	28	34	41	37	49	41	53	-0.39212
tr|L0R5A1|L0R5A1_HUMAN	CSF2RB	Alternative protein CSF2RB OS=Homo sapiens GN=CSF2RB PE=4 SV=1	11752	486	914	36	36	0.29	0.29	1	1	2	1	2	3	4	5	5	-0.42737
tr|G5E9Q2|G5E9Q2_HUMAN	CSDE1	Cold shock domain containing E1, RNA-binding, isoform CRA_d OS=Homo sapiens GN=CSDE1 PE=2 SV=1	94539	312	431	58	85	0.11	0.11	1	1	2	3	3	3	4	3	5	-0.42737
tr|Q53GN4|Q53GN4_HUMAN	WDR1	WD repeat domain 1, isoform CRA_a (Fragment) OS=Homo sapiens GN=WDR1 PE=2 SV=1	66822	137	196	160	196	0.4	0.4	1	4	5	7	8	7	9	9	10	-0.44928
sp|P68032|ACTC_HUMAN	ACTC1	Actin, alpha cardiac muscle 1 OS=Homo sapiens GN=ACTC1 PE=1 SV=1	42334	23	23	771	1181	6.63	6.63	1	25	35	40	49	48	57	55	63	-0.54927
tr|K7EK77|K7EK77_HUMAN	ATP5A1	ATP synthase subunit alpha, mitochondrial (Fragment) OS=Homo sapiens GN=ATP5A1 PE=3 SV=1	22243	135	257	160	153	1.33	1.33	1	5	4	6	6	6	7	6	8	-0.58392
tr|B4DTG2|B4DTG2_HUMAN	EEF1G	Elongation factor 1-gamma OS=Homo sapiens GN=EEF1G PE=2 SV=1	56456	93	121	225	303	0.86	0.86	1	5	10	15	14	14	16	18	18	-0.60491
tr|B3KTJ9|B3KTJ9_HUMAN	tr|B3KTJ9	cDNA FLJ38393 fis, clone FEBRA2007212 OS=Homo sapiens PE=2 SV=1	103493	82	98	251	355	0.32	0.32	1	7	7	9	9	9	10	12	12	-0.64641
tr|J3KTA4|J3KTA4_HUMAN	DDX5	Probable ATP-dependent RNA helicase DDX5 OS=Homo sapiens GN=DDX5 PE=3 SV=1	69557	97	167	218	224	0.45	0.45	1	7	8	9	9	10	11	10	12	-0.6597
tr|Q8WUM5|Q8WUM5_HUM	GEMIN4	Gem (Nuclear organelle) associated protein 4 OS=Homo sapiens GN=GEMIN4 PE=2 SV=1	121732	709	1049	17	29	0.03	0.03	1	0	1	1	1	1	1	2	3	-0.78985
sp|O00232|PSD12_HUMAN	PSMD12	26S proteasome non-ATPase regulatory subunit 12 OS=Homo sapiens GN=PSMD12 PE=1 SV=3	53270	283	1046	66	29	0.06	0.06	1	1	0	1	1	1	1	1	1	-0.78985
sp|O43286|B4GT5_HUMAN	B4GALT5	Beta-1,4-galactosyltransferase 5 OS=Homo sapiens GN=B4GALT5 PE=2 SV=1	45318	651	1043	22	29	0.07	0.07	1	0	0	1	1	1	1	1	2	-0.78985
tr|B4DLB8|B4DLB8_HUMAN	tr|B4DLB	cDNA FLJ52205, highly similar to Beta-1,4-galactosyltransferase 1 (EC 2.4.1.-) OS=Homo sapiens PE=2 SV=1	40269	650	1042	22	29	0.08	0.08	1	0	0	1	1	1	1	1	3	-0.78985
tr|B4DN13|B4DN13_HUMAN	tr|B4DN1	cDNA FLJ59136, highly similar to Transmembrane 9 superfamily protein member 3 OS=Homo sapiens PE=2 SV=1	29629	624	1041	23	29	0.11	0.11	1	0	1	1	1	1	1	1	2	-0.78985
sp|Q9Y305|ACOT9_HUMAN	ACOT9	Acyl-coenzyme A thioesterase 9, mitochondrial OS=Homo sapiens GN=ACOT9 PE=1 SV=2	50269	637	987	22	32	0.07	0.07	1	0	0	1	1	1	1	2	2	-0.78985
tr|Q9NZ23|Q9NZ23_HUMAN	YA61	Drug-sensitive protein 1 OS=Homo sapiens GN=YA61 PE=2 SV=1	14858	588	964	27	33	0.23	0.23	1	0	1	1	1	1	1	1	1	-0.78985
sp|Q6PGP7|TTC37_HUMAN	TTC37	Tetratricopeptide repeat protein 37 OS=Homo sapiens GN=TTC37 PE=1 SV=1	177485	498	947	34	34	0.02	0.02	1	1	0	1	1	1	1	1	2	-0.78985
tr|H0Y5J4|H0Y5J4_HUMAN	DBNL	Drebrin-like protein (Fragment) OS=Homo sapiens GN=DBNL PE=4 SV=1	40475	466	940	37	34	0.08	0.08	1	1	0	1	1	1	1	1	1	-0.78985
tr|E0X098|E0X098_HUMAN	TES	Testin variant (Fragment) OS=Homo sapiens GN=TES PE=2 SV=1	22009	430	937	40	34	0.15	0.15	1	1	1	1	1	1	1	1	1	-0.78985
tr|E7ET15|E7ET15_HUMAN	U2SURP	U2 snRNP-associated SURP motif-containing protein OS=Homo sapiens GN=U2SURP PE=2 SV=1	118631	647	930	22	35	0.03	0.03	1	0	0	1	1	1	1	2	3	-0.78985
tr|A8KAK1|A8KAK1_HUMAN	tr|A8KAK	cDNA FLJ77398, highly similar to Homo sapiens UDP-glucose ceramide glucosyltransferase-like 1, transcript variant 2, mRNA OS=Hor	175467	635	904	22	36	0.02	0.02	1	0	0	1	1	1	1	1	2	-0.78985
tr|B4DRG7|B4DRG7_HUMAN	tr|B4DRG	cDNA FLJ60336, highly similar to Condensin complex subunit 2 OS=Homo sapiens PE=2 SV=1	80877	410	899	43	36	0.04	0.04	1	1	1	1	1	1	1	1	1	-0.78985
tr|B2RDF5|B2RDF5_HUMAN	tr|B2RDF	cDNA, FLJ96587, highly similar to Homo sapiens SUMO-1 activating enzyme subunit 2 (UBA2), mRNA OS=Homo sapiens PE=2 SV=1	71705	590	863	27	39	0.05	0.05	1	0	1	1	1	1	1	1	1	-0.78985
tr|A6NKV8|A6NKV8_HUMAN	PAIP1	Polyadenylate-binding protein-interacting protein 1 OS=Homo sapiens GN=PAIP1 PE=2 SV=1	42379	361	843	48	40	0.08	0.08	1	1	1	1	1	1	1	1	1	-0.78985
tr|Q2NKW8|Q2NKW8_HUMA	AHCYL1	Adenosylhomocysteinase (Fragment) OS=Homo sapiens GN=AHCYL1 PE=2 SV=1	57630	509	836	33	41	0.06	0.06	1	0	1	1	1	1	1	1	1	-0.78985
sp|Q9Y6M5|ZNT1_HUMAN	SLC30A1	Zinc transporter 1 OS=Homo sapiens GN=SLC30A1 PE=1 SV=3	56290	334	830	52	41	0.06	0.06	1	1	1	1	1	1	1	1	1	-0.78985
tr|H3BMW5|H3BMW5_HUMA	DNAJA2	DnaJ homolog subfamily A member 2 (Fragment) OS=Homo sapiens GN=DNAJA2 PE=4 SV=1	15134	611	809	24	42	0.23	0.23	1	0	1	1	1	1	1	1	1	-0.78985
tr|B3KY60|B3KY60_HUMAN	tr|B3KY6	cDNA FLJ16777 fis, clone BRHIP2029567, highly similar to Cell division cycle 5-like protein OS=Homo sapiens PE=2 SV=1	92448	587	790	27	43	0.04	0.04	1	0	1	1	1	1	1	1	1	-0.78985
tr|B4E363|B4E363_HUMAN	FARSA	Phenylalanine--tRNA ligase alpha subunit OS=Homo sapiens GN=FARSA PE=2 SV=1	54181	447	789	38	43	0.06	0.06	1	1	1	1	1	1	1	1	2	-0.78985
tr|A1L3A7|A1L3A7_HUMAN	NUFIP2	Nuclear fragile X mental retardation protein interacting protein 2 OS=Homo sapiens GN=NUFIP2 PE=2 SV=1	76132	301	776	59	44	0.04	0.04	1	1	1	1	1	1	1	1	1	-0.78985
tr|Q68E00|Q68E00_HUMAN	DKFZp686	Putative uncharacterized protein DKFZp686G2045 (Fragment) OS=Homo sapiens GN=DKFZp686G2045 PE=2 SV=2	32787	485	749	36	46	0.1	0.1	1	0	1	1	1	1	1	1	1	-0.78985
tr|B3KUJ0|B3KUJ0_HUMAN	tr|B3KUJ	cDNA FLJ39996 fis, clone STOMA2002166, highly similar to Splicing factor 3B subunit 4 OS=Homo sapiens PE=2 SV=1	44512	464	677	37	52	0.07	0.07	1	1	1	1	1	1	1	2	1	-0.78985
tr|G0ZJH6|G0ZJH6_HUMAN	Ube4B	UFD2A-III/UBE4B-III splice isoform OS=Homo sapiens GN=Ube4B PE=2 SV=1	152890	667	648	21	54	0.02	0.02	1	1	0	1	1	1	1	1	1	-0.78985

tr|H0Y7S3|H0Y7S3_HUMAN	ATP2B2	Plasma membrane calcium-transporting ATPase 2 (Fragment) OS=Homo sapiens GN=ATP2B2 PE=3 SV=1	122272	528	642	31	55	0.03	0.03	1	0	1	1	1	1	1	3	4	-0.78985
tr|B4DVB8|B4DVB8_HUMAN	ELAVL1	ELAV-like protein 1 OS=Homo sapiens GN=ELAVL1 PE=2 SV=1	39200	612	542	24	65	0.08	0.08	1	0	1	1	1	1	1	1	2	-0.78985
tr|J3KTE4|J3KTE4_HUMAN	RPL19	Ribosomal protein L19 OS=Homo sapiens GN=RPL19 PE=3 SV=1	23347	336	448	52	82	0.14	0.14	1	1	1	1	1	1	1	1	1	-0.78985
tr|Q7Z3K9|Q7Z3K9_HUMAN	DKFZp781	Putative uncharacterized protein DKFZp781L0540 (Fragment) OS=Homo sapiens GN=DKFZp781L0540 PE=2 SV=1	90081	440	972	39	33	0.04	0.04	1	1	0	1	1	2	2	2	2	-0.78999
tr|B2RBE5|B2RBE5_HUMAN	tr|B2RBE5	cDNA, FLJ95468, highly similar to Homo sapiens transcriptional coactivator tubedown-100 (TBDN100),transcript variant 1, mRNA OS	102490	446	741	38	47	0.06	0.06	1	0	1	2	2	2	2	2	6	-0.78999
sp|Q15392|DHC24_HUMAN	DHCR24	Delta(24)-sterol reductase OS=Homo sapiens GN=DHCR24 PE=1 SV=2	60803	442	719	39	48	0.05	0.05	1	1	1	1	1	2	2	3	3	-0.78999
sp|Q9Y3I0|RTCB_HUMAN	C22orf28	tRNA-splicing ligase RtcB homolog OS=Homo sapiens GN=C22orf28 PE=1 SV=1	55688	458	687	38	50	0.12	0.12	1	0	2	2	2	2	2	2	5	-0.78999
sp|Q96SI9|STRBP_HUMAN	STRBP	Spermatid perinuclear RNA-binding protein OS=Homo sapiens GN=STRBP PE=1 SV=1	74290	471	666	37	52	0.09	0.09	1	0	1	2	2	2	2	2	2	-0.78999
tr|B5BU25|B5BU25_HUMAN	U2AF2	U2 small nuclear RNA auxiliary factor 2 isoform b OS=Homo sapiens GN=U2AF2 PE=2 SV=1	53445	260	649	74	54	0.13	0.13	1	1	2	2	2	2	2	2	3	-0.78999
tr|Q58FF4|Q58FF4_HUMAN	HSP90Bf	Heat shock protein 90Bf OS=Homo sapiens GN=HSP90Bf PE=1 SV=1	41833	407	636	43	55	0.16	0.16	1	1	1	2	2	2	2	2	2	-0.78999
tr|H0YDE4|H0YDE4_HUMAN	PRMT1	Protein arginine N-methyltransferase 1 (Fragment) OS=Homo sapiens GN=PRMT1 PE=4 SV=1	27345	437	516	39	62	0.26	0.26	1	1	1	2	2	2	2	2	3	-0.78999
sp|Q9BYZ2|LDH6B_HUMAN	LDHAL6B	L-lactate dehydrogenase A-like 6B OS=Homo sapiens GN=LDHAL6B PE=1 SV=3	42372	327	487	54	75	0.16	0.16	1	1	2	2	2	2	2	2	2	-0.78999
sp|Q14432|PDE3A_HUMAN	PDE3A	cGMP-inhibited 3~,5~-cyclic phosphodiesterase A OS=Homo sapiens GN=PDE3A PE=1 SV=3	126440	493	457	35	80	0.05	0.05	1	1	1	2	2	2	2	2	3	-0.78999
tr|B4DUL5|B4DUL5_HUMAN	tr|B4DUL5	cDNA FLJ51625, highly similar to Ubiquinol-cytochrome-c reductase complex coreprotein I, mitochondrial (EC 1.10.2.2) OS=Homo sa	40917	357	360	48	103	0.17	0.17	1	1	2	2	2	2	2	2	2	-0.78999
tr|B4DHG3|B4DHG3_HUMAN	tr|B4DHG	cDNA FLJ55329, highly similar to Procollagen-lysine,2-oxoglutarate5-dioxygenase 2 (EC 1.14.11.4) OS=Homo sapiens PE=2 SV=1	81716	453	357	38	104	0.08	0.08	1	0	1	2	2	2	2	2	2	-0.78999
tr|Q05DV5|Q05DV5_HUMAN	ZC3HAV1	ZC3HAV1 protein (Fragment) OS=Homo sapiens GN=ZC3HAV1 PE=2 SV=1	49964	224	210	92	182	0.14	0.14	1	1	2	2	2	2	2	2	2	-0.78999
tr|D2CFK9|D2CFK9_HUMAN	GTPBP4	Nucleolar GTP-binding protein 1 OS=Homo sapiens GN=GTPBP4 PE=2 SV=1	74317	264	446	72	83	0.14	0.14	1	2	3	3	3	3	3	3	3	-0.79014
sp|P00390|GSHR_HUMAN	GSR	Glutathione reductase, mitochondrial OS=Homo sapiens GN=GSR PE=1 SV=2	56791	313	568	58	62	0.18	0.18	1	1	2	3	3	4	4	4	4	-0.79028
sp|P38919|IF4A3_HUMAN	EIF4A3	Eukaryotic initiation factor 4A-III OS=Homo sapiens GN=EIF4A3 PE=1 SV=4	47126	262	288	74	135	0.4	0.4	1	1	2	5	5	4	4	6	7	-0.79028
sp|P15121|ALDR_HUMAN	AKR1B1	Aldose reductase OS=Homo sapiens GN=AKR1B1 PE=1 SV=3	36230	185	460	116	79	0.42	0.42	1	3	3	4	4	5	5	5	6	-0.79042
sp|P16615|AT2A2_HUMAN	ATP2A2	Sarcoplasmic/endoplasmic reticulum calcium ATPase 2 OS=Homo sapiens GN=ATP2A2 PE=1 SV=1	116336	149	127	145	291	0.32	0.32	1	4	7	10	10	12	11	13	16	-0.91192
tr|B2R9K8|B2R9K8_HUMAN	tr|B2R9K8	cDNA, FLJ94440, highly similar to Homo sapiens chaperonin containing TCP1, subunit 6A (zeta 1)(CCT6A), mRNA OS=Homo sapiens P	58386	118	301	181	127	0.47	0.47	1	5	4	7	7	9	8	10	10	-0.95166
sp|P52895|AK1C2_HUMAN	AKR1C2	Aldo-keto reductase family 1 member C2 OS=Homo sapiens GN=AKR1C2 PE=1 SV=3	37111	186	341	116	112	0.67	0.67	1	3	3	6	6	7	6	9	9	-0.99736
tr|Q4W4Y1|Q4W4Y1_HUMAN	DRIP4	Dopamine receptor interacting protein 4 OS=Homo sapiens GN=DRIP4 PE=2 SV=1	96646	136	317	160	120	0.18	0.18	1	2	4	5	5	5	4	6	5	-1.08012
tr|E9PRA5|E9PRA5_HUMAN	ATP1A4	Sodium/potassium-transporting ATPase subunit alpha-4 OS=Homo sapiens GN=ATP1A4 PE=2 SV=1	57835	289	678	63	52	0.12	0.12	1	0	0	6	7	10	8	19	19	-1.09638
tr|Q6FHQ0|Q6FHQ0_HUMAN	RBBP7	RBBP7 protein (Fragment) OS=Homo sapiens GN=RBBP7 PE=2 SV=1	48132	468	901	37	36	0.07	0.07	1	1	1	1	1	3	2	3	3	-1.27576
tr|A8K4D5|A8K4D5_HUMAN	tr|A8K4D5	Kynureninase OS=Homo sapiens PE=2 SV=1	52819	266	418	71	88	0.13	0.13	1	2	2	2	2	3	2	3	3	-1.27576
tr|Q6IAV5|Q6IAV5_HUMAN	OXCT	Succinyl-CoA:3-ketoacid-coenzyme A transferase OS=Homo sapiens GN=OXCT PE=2 SV=1	56612	209	389	100	92	0.18	0.18	1	1	2	3	3	5	3	6	4	-1.44289
tr|H3BUF6|H3BUF6_HUMAN	ATXN2L	Ataxin-2-like protein OS=Homo sapiens GN=ATXN2L PE=4 SV=1	112929	656	1064	22	28	0.03	0.03	1	0	0	1	1	2	1	2	3	-1.52715
tr|H0YF33|H0YF33_HUMAN	NASP	Nuclear autoantigenic sperm protein (Fragment) OS=Homo sapiens GN=NASP PE=4 SV=1	31883	542	858	30	39	0.1	0.1	1	0	1	1	1	2	1	3	1	-1.52715
tr|A0N4V7|A0N4V7_HUMAN	Tcr-alpha	HCG2039797 (Fragment) OS=Homo sapiens GN=Tcr-alpha PE=4 SV=1	2269	212	639	98	55	1.81	1.81	1	5	4	15	5	31	15	46	25	-1.82039
tr|J3KQE5|J3KQE5_HUMAN	RAN	GTP-binding nuclear protein Ran (Fragment) OS=Homo sapiens GN=RAN PE=4 SV=1	27027	571	1170	29	19	0.12	0.12	1	0	0	1	1	1	0	1	1	-2.37501
tr|Q6NZ55|Q6NZ55_HUMAN	RPL13	60S ribosomal protein L13 OS=Homo sapiens GN=RPL13 PE=2 SV=1	24308	422	1167	41	20	0.14	0.14	1	1	0	1	1	2	0	2	1	-3.11231
tr|H3BLZ8|H3BLZ8_HUMAN	DDX17	Probable ATP-dependent RNA helicase DDX17 OS=Homo sapiens GN=DDX17 PE=3 SV=1	81073	166	179	130	213	0.37	0.36	0.972972973	3	8	9	8	9	10	10	10	-0.64641
sp|Q71U36|TBA1A_HUMAN	TUBA1A	Tubulin alpha-1A chain OS=Homo sapiens GN=TUBA1A PE=1 SV=1	50788	15	30	1022	1028	5.18	4.89	0.944015444	30	27	41	41	43	49	46	53	-0.6083
tr|B7Z6I1|B7Z6I1_HUMAN	tr|B7Z6I1	cDNA FLJ52755, highly similar to Actin, aortic smooth muscle OS=Homo sapiens PE=2 SV=1	17222	23	60	354	559	6.63	6.2	0.935143288	13	17	19	24	21	29	23	32	-0.33477
tr|I3L492|I3L492_HUMAN	ETF1	Eukaryotic peptide chain release factor subunit 1 (Fragment) OS=Homo sapiens GN=ETF1 PE=4 SV=1	23028	274	647	69	46	0.15	0.14	0.933333333	1	1	1	1	1	1	1	2	-0.78985
tr|B0QYT3|B0QYT3_HUMAN	RANGAP1	Ran GTPase activating protein 1 (Fragment) OS=Homo sapiens GN=RANGAP1 PE=2 SV=1	27122	574	478	28	41	0.12	0.11	0.916666667	0	1	1	1	1	2	1	2	-0.05269
tr|Q53ET2|Q53ET2_HUMAN	tr|Q53ET2	Dihydropyrimidinase-like 2 variant (Fragment) OS=Homo sapiens PE=2 SV=1	62688	342	439	51	84	0.23	0.21	0.913043478	1	3	4	4	3	6	5	6	0.103537
tr|D2JYH4|D2JYH4_HUMAN	ACTA2	Actin, alpha 2, smooth muscle, aorta OS=Homo sapiens GN=ACTA2 PE=2 SV=1	42381	23	26	702	1125	6.63	6.04	0.911010558	23	33	35	45	43	51	49	57	-0.55076
tr|B4DX08|B4DX08_HUMAN	tr|B4DX08	cDNA FLJ58784, highly similar to T-complex protein 1 subunit epsilon OS=Homo sapiens PE=2 SV=1	57521	90	219	229	175	0.4	0.36	0.9	6	5	6	6	6	7	6	12	-0.58392
tr|H0Y7N2|H0Y7N2_HUMAN	ATXN10	Ataxin-10 (Fragment) OS=Homo sapiens GN=ATXN10 PE=4 SV=1	19107	470	420	37	0	0.38	0.34	0.894736842	0	0	2	0	2	1	2	2	-1.52715
tr|H0YLA4|H0YLA4_HUMAN	SORD	Sorbitol dehydrogenase OS=Homo sapiens GN=SORD PE=3 SV=1	36833	473	868	37	0	0.09	0.08	0.888888889	0	0	1	0	2	2	2	3	-0.78999
tr|Q5U077|Q5U077_HUMAN	LDHB	L-lactate dehydrogenase OS=Homo sapiens GN=LDHB PE=2 SV=1	36900	39	155	530	238	1.81	1.58	0.872928177	13	9	16	11	18	10	20	11	-1.61099
tr|A5PLK7|A5PLK7_HUMAN	RCC2	RCC2 protein (Fragment) OS=Homo sapiens GN=RCC2 PE=2 SV=1	50331	165	290	131	113	0.46	0.4	0.869565217	4	3	6	5	6	5	6	7	-1.03177
tr|F8WAM2|F8WAM2_HUMAN	CCT7	T-complex protein 1 subunit eta (Fragment) OS=Homo sapiens GN=CCT7 PE=2 SV=1	10488	161	750	47	46	0.38	0.33	0.868421053	1	1	1	1	1	1	1	2	-0.78985
tr|B7TY16|B7TY16_HUMAN	ACTN1	Actinin alpha 1 isoform 3 OS=Homo sapiens GN=ACTN1 PE=2 SV=1	107644	50	69	428	522	0.77	0.66	0.857142857	15	14	21	18	22	20	29	20	-0.92756
tr|H0YGW7|H0YGW7_HUMAN	ABCF1	ATP-binding cassette sub-family F member 1 (Fragment) OS=Homo sapiens GN=ABCF1 PE=3 SV=1	68075	144	207	151	188	0.27	0.23	0.851851852	4	5	5	6	5	8	7	9	-0.1618
tr|F8WEJ5|F8WEJ5_HUMAN	ASNS	Asparagine synthetase [glutamine-hydrolyzing] OS=Homo sapiens GN=ASNS PE=2 SV=1	46395	141	496	156	16	0.26	0.22	0.846153846	3	0	5	1	6	0	6	1	-4.49218
tr|D6RAF8|D6RAF8_HUMAN	HNRNPD	Heterogeneous nuclear ribonucleoprotein D0 (Fragment) OS=Homo sapiens GN=HNRNPD PE=2 SV=1	23119	229	993	90	32	0.37	0.31	0.837837838	3	0	3	2	4	2	4	2	-1.63867
sp|Q96HC4|PDLI5_HUMAN	PDLIM5	PDZ and LIM domain protein 5 OS=Homo sapiens GN=PDLIM5 PE=1 SV=5	65102	175	249	123	155	0.41	0.34	0.829268293	3	4	7	6	7	7	8	7	-0.79071
tr|B3KMV8|B3KMV8_HUMAN	tr|B3KMV	cDNA FLJ12766 fis, clone NT2RP2001520, highly similar to Calcium-binding mitochondrial carrier protein Aralar1 OS=Homo sapiens P	75130	353	618	41	57	0.11	0.09	0.818181818	1	2	1	2	1	2	1	2	-0.05269
sp|P35527|K1C9_HUMAN	KRT9	Keratin, type I cytoskeletal 9 OS=Homo sapiens GN=KRT9 PE=1 SV=3	62255	14	35	1044	936	3.01	2.44	0.810631229	25	20	36	28	40	29	44	31	-1.25483
tr|A0JLS5|A0JLS5_HUMAN	TBL3	TBL3 protein (Fragment) OS=Homo sapiens GN=TBL3 PE=2 SV=1	64079	418	529	42	68	0.05	0.04	0.8	1	1	1	1	1	1	1	1	-0.78985
tr|H0Y9Q7|H0Y9Q7_HUMAN	CANX	Calnexin (Fragment) OS=Homo sapiens GN=CANX PE=3 SV=1	18990	352	279	49	84	0.39	0.31	0.794871795	1	2	2	2	3	3	5	4	-0.79014
tr|Q9H552|Q9H552_HUMAN	KRT8P11	Protein KRT8P11 OS=Homo sapiens GN=KRT8P11 PE=3 SV=1	55459	58	149	46	0	0.95	0.75	0.789473684	2	0	2	0	2	2	3	2	-0.78999
tr|B4DRR0|B4DRR0_HUMAN	tr|B4DRR	cDNA FLJ53910, highly similar to Keratin, type II cytoskeletal 6A OS=Homo sapiens PE=2 SV=1	58088	92	293	225	133	0.83	0.64	0.771084337	9	6	12	10	15	12	17	14	-1.10278
tr|D6REX3|D6REX3_HUMAN	SEC31A	Protein transport protein Sec31A OS=Homo sapiens GN=SEC31A PE=2 SV=1	137110	121	541	178	65	0.13	0.1	0.769230769	4	2	5	4	4	5	5	6	-0.50058
sp|Q92973|TNPO1_HUMAN	TNPO1	Transportin-1 OS=Homo sapiens GN=TNPO1 PE=1 SV=2	103771	139	505	158	72	0.17	0.13	0.764705882	3	3	5	4	8	5	8	6	-1.41947
sp|Q562R1|ACTBL_HUMAN	ACTBL2	Beta-actin-like protein 2 OS=Homo sapiens GN=ACTBL2 PE=1 SV=2	42318	61	72	349	498	1.47	1.12	0.761904762	13	12	16	16	24	24	27	27	-0.79315
tr|B7Z438|B7Z438_HUMAN	tr|B7Z438	cDNA FLJ56352, highly similar to Succinyl-CoA ligase (GDP-forming) subunit alpha, mitochondrial (EC 6.2.1.4) OS=Homo sapiens PE=2	26801	467	874	37	38	0.12	0.09	0.75	1	1	1	1	1	1	1	1	-0.78985
tr|A1L179|A1L179_HUMAN	KIF5C	KIF5C protein (Fragment) OS=Homo sapiens GN=KIF5C PE=2 SV=1	99437	227	854	46	39	0.04	0.03	0.75	1	1	1	1	1	1	2	2	-0.78985
tr|Q0IIN1|Q0IIN1_HUMAN	KRT77	Keratin 77 OS=Homo sapiens GN=KRT77 PE=2 SV=1	62050	76	190	264	201	0.23	0.17	0.739130435	10	5	10	7	10	9	12	10	-0.93572
tr|Q562M3|Q562M3_HUMAN	ACT	Actin-like protein (Fragment) OS=Homo sapiens GN=ACT PE=2 SV=1	11529	103	61	213	553	7.06	5.21	0.73796034	10	18	14	24	17	29	20	33	-0.03642
sp|P46087|NOP2_HUMAN	NOP2	Putative ribosomal RNA methyltransferase NOP2 OS=Homo sapiens GN=NOP2 PE=1 SV=2	89589	296	329	61	114	0.15	0.11	0.733333333	2	3	4	3	5	4	6	4	-1.08012
tr|B4DY32|B4DY32_HUMAN	tr|B4DY32	Asparagine synthetase OS=Homo sapiens PE=2 SV=1	55055	141	495	156	74	0.26	0.19	0.730769231	3	2	5	3	6	3	6	4	-1.68424
sp|P52788|SPSY_HUMAN	SMS	Spermine synthase OS=Homo sapiens GN=SMS PE=1 SV=2	41698	248	1137	78	23	0.11	0.08	0.727272727	1	0	1	1	1	1	1	1	-0.78985
tr|A2RUM7|A2RUM7_HUMAN	RPL5	Ribosomal protein L5 OS=Homo sapiens GN=RPL5 PE=2 SV=1	34569	187	358	115	104	0.44	0.32	0.727272727	2	2	4	3	5	4	7	5	-1.08012
tr|Q9BS14|Q9BS14_HUMAN	GANAB	GANAB protein (Fragment) OS=Homo sapiens GN=GANAB PE=2 SV=1	34786	47	300	50	128	0.62	0.44	0.709677419	1	4	1	4	1	5	1	6	1.085403
tr|B2R8N0|B2R8N0_HUMAN	tr|B2R8N	cDNA, FLJ93975, highly similar to Homo sapiens epoxide hydrolase 1, microsomal (xenobiotic) (EPHX1), mRNA OS=Homo sapiens PE	53129	115	310	184	124	0.62	0.43	0.693548387	6	4	8	6	8	9	8	12	-0.63019
sp|P78371|TCPB_HUMAN	CCT2	T-complex protein 1 subunit beta OS=Homo sapiens GN=CCT2 PE=1 SV=4	57794	37	103	563	349	1.86	1.29	0.693548387	14	9	20	15	20	13	23	16	-1.39661
sp|P25705|ATPA_HUMAN	ATP5A1	ATP synthase subunit alpha, mitochondrial OS=Homo sapiens GN=ATP5A1 PE=1 SV=1	59828	135	154	160	238	1.33	0.9	0.676691729	5	7	6	12	6	12	6	15	0.154028
tr|Q14666|Q14666_HUMAN	tr|Q14666	Radiated keratinocyte mRNA 266 (keratin-related protein) (Fragment) OS=Homo sapiens PE=2 SV=1	30933	24	63	451	414	2.99	2.02	0.675585284	12	11	17	17	20	22	21	26	-0.65788
tr|Q1KLZ0|Q1KLZ0_HUMAN	PS1TP5BP	HCG15971, isoform CRA_a OS=Homo sapiens GN=PS1TP5BP1 PE=2 SV=1	42052	1	7	3367	2254	31.43	21.22	0.675151129	82	61	119	81	130	89	141	99	-1.36097
tr|B3KQQ3|B3KQQ3_HUMAN	tr|B3KQQ	cDNA PSEC0016 fis, clone NT2RM1001076, highly similar to Procollagen-lysine,2-oxoglutarate 5-dioxygenase 3 (EC 1.14.11.4) OS=Ho	85161	325	376	54	97	0.12	0.08	0.666666667	1	2	3	2	3	2	3	2	-1.27576
tr|G3V0E8|G3V0E8_HUMAN	PCBP2	Poly(RC) binding protein 2, isoform CRA_f OS=Homo sapiens GN=PCBP2 PE=2 SV=1	33817	132	637	166	55	0.32	0.21	0.65625	3	1	3	2	3	2	5	3	-1.27576
tr|G3XAP5|G3XAP5_HUMAN	LDHC	L-lactate dehydrogenase OS=Homo sapiens GN=LDHC PE=2 SV=1	26552	39	134	67	53	1.81	1.17	0.64640884	1	1	1	1	1	1	1	1	-0.78985
sp|O43707|ACTN4_HUMAN	ACTN4	Alpha-actinin-4 OS=Homo sapiens GN=ACTN4 PE=1 SV=2	105245	19	43	849	799	2.11	1.36	0.644549763	26	25	41	31	44	33	52	36	-1.20785
tr|D0W033|D0W033_HUMAN	HLA-A	MHC class I antigen (Fragment) OS=Homo sapiens GN=HLA-A PE=3 SV=1	31801	639	835	22	41	0.16	0.1	0.625	0	1	1	1	1	2	1	2	-0.05269
tr|D0F1S2|D0F1S2_HUMAN	HLA-Cw	MHC class I antigen (Fragment) OS=Homo sapiens GN=HLA-Cw PE=3 SV=1	38632	639	697	22	0	0.16	0.1	0.625	0	0	1	0	1	2	1	3	-0.05269
sp|P27348|1433T_HUMAN	YWHAQ	14-3-3 protein theta OS=Homo sapiens GN=YWHAQ PE=1 SV=1	28032	258	758	74	0	0.4	0.25	0.625	2	0	3	0	3	1	3	1	-2.01292
tr|E0X609|E0X609_HUMAN	HLA-C	MHC class I antigen (Fragment) OS=Homo sapiens GN=HLA-C PE=3 SV=1	21236	639	833	22	0	0.16	0.1	0.625	0	0	1	0	1	0	1	2	-2.37501
sp|P08779|K1C16_HUMAN	KRT16	Keratin, type I cytoskeletal 16 OS=Homo sapiens GN=KRT16 PE=1 SV=4	51578	62	156	342	236	1.38	0.86	0.623188406	14	10	17	11	21	14	21	16	-1.36238
tr|H0Y8G5|H0Y8G5_HUMAN	HNRNPD	Heterogeneous nuclear ribonucleoprotein D0 (Fragment) OS=Homo sapiens GN=HNRNPD PE=4 SV=1	29877	229	921	90	30	0.37	0.23	0.621621622	3	0	3	1	4	1	4	1	-2.37583
sp|P13647|K2C5_HUMAN	KRT5	Keratin, type II cytoskeletal 5 OS=Homo sapiens GN=KRT5 PE=1 SV=3	62568	58	206	378	188	0.95	0.59	0.621052632	13	7	15	10	20	13	24	14	-1.39661
sp|Q58FF8|H90B2_HUMAN	HSP90AB2	Putative heat shock protein HSP 90-beta 2 OS=Homo sapiens GN=HSP90AB2P PE=1 SV=2	44492	32	197	97	195	1.24	0.77	0.620967742	3	5	5	8	5	9	6	11	-0.00114
tr|B4DKT3|B4DKT3_HUMAN	tr|B4DKT3	cDNA FLJ58390, highly similar to Aldo-keto reductase family 1 member C3homolog (EC 1.-.-.-) OS=Homo sapiens PE=2 SV=1	15755	186	459	42	69	0.67	0.41	0.611940299	1	2	1	3	1	3	1	5	0.432934
sp|P35908|K22E_HUMAN	KRT2	Keratin, type II cytoskeletal 2 epidermal OS=Homo sapiens GN=KRT2 PE=1 SV=2	65678	11	32	1255	968	4.79	2.92	0.60960334	41	24	48	33	49	38	57	42	-1.16151
tr|B4DF68|B4DF68_HUMAN	tr|B4DF68	cDNA FLJ55325, highly similar to Heat-shock protein 105 kDa OS=Homo sapiens PE=2 SV=1	86500	123	364	47	102	0.23	0.14	0.608695652	1	2	1	4	1	4	1	4	0.7957
tr|B3KMW5|B3KMW5_HUMA	tr|B3KMW	cDNA FLJ12796 fis, clone NT2RP2002058, highly similar to WD repeat protein 3 OS=Homo sapiens PE=2 SV=1	71519	408	1052	43	29	0.05	0.03	0.6	1	0	1	1	1	1	1	2	-0.78985
tr|Q53RS3|Q53RS3_HUMAN	DDEF2	Putative uncharacterized protein DDEF2 (Fragment) OS=Homo sapiens GN=DDEF2 PE=2 SV=1	70046	441	722	39	48	0.05	0.03	0.6	1	1	1	1	4	1	4	2	-2.37583
tr|B4DW52|B4DW52_HUMAN	tr|B4DW5	cDNA FLJ55253, highly similar to Actin, cytoplasmic 1 OS=Homo sapiens PE=2 SV=1	38950	4	11	3120	1906	27.29	16.35	0.599120557	74	53	108	68	123	80	134	93	-1.43367
tr|B2RCM3|B2RCM3_HUMAN	tr|B2RCM	cDNA, FLJ96158, highly similar to Homo sapiens calpain 2, (m/II) large subunit (CAPN2), mRNA OS=Homo sapiens PE=2 SV=1	80883	173	401	125	90	0.22	0.13	0.590909091	3	3	7	3	7	4	8	7	-1.52826
tr|B3KU96|B3KU96_HUMAN	tr|B3KU9	cDNA FLJ39433 fis, clone PROST2004142, highly similar to GDP-L-fucose synthetase (EC 1.1.1.271) OS=Homo sapiens PE=2 SV=1	17856	371	923	47	0	0.19	0.11	0.578947368	1	0	1	0	1	1	1	1	-0.78985
sp|Q8NBJ5|GT251_HUMAN	GLT25D1	Procollagen galactosyltransferase 1 OS=Homo sapiens GN=GLT25D1 PE=1 SV=1	71933	523	962	31	33	0.09	0.05	0.555555556	0	0	2	1	1	1	2	2	-0.78985
sp|P02533|K1C14_HUMAN	KRT14	Keratin, type I cytoskeletal 14 OS=Homo sapiens GN=KRT14 PE=1 SV=4	51872	33	107	604	338	2.22	1.23	0.554054054	19	11	25	15	29	20	30	23	-1.32073
sp|P04040|CATA_HUMAN	CAT	Catalase OS=Homo sapiens GN=CAT PE=1 SV=3	59947	299	743	60	47	0.11	0.06	0.545454545	1	1	2	1	2	1	2	1	-1.52715
tr|B3KM80|B3KM80_HUMAN	NCL	Nucleolin, isoform CRA_c OS=Homo sapiens GN=NCL PE=2 SV=1	58576	71	241	283	159	1.03	0.55	0.533980583	9	4	14	8	15	11	15	12	-1.22327
tr|K7ERE3|K7ERE3_HUMAN	KRT13	Keratin, type I cytoskeletal 13 OS=Homo sapiens GN=KRT13 PE=3 SV=1	45574	88	417	234	88	0.63	0.32	0.507936508	9	4	10	5	11	5	12	7	-1.85659
tr|E0WMN0|E0WMN0_HUMA	HLA-C	MHC class I antigen (Fragment) OS=Homo sapiens GN=HLA-C PE=3 SV=1	31801	639	831	22	41	0.16	0.08	0.5	0	1	1	1	1	3	1	3	0.432934
tr|J3KN36|J3KN36_HUMAN	NOMO3	Nodal modulator 3 OS=Homo sapiens GN=NOMO3 PE=4 SV=1	140376	400	1017	43	30	0.04	0.02	0.5	1	0	1	1	1	1	1	1	-0.78985
tr|B1ANK7|B1ANK7_HUMAN	FH	Fumarate hydratase OS=Homo sapiens GN=FH PE=2 SV=1	54773	288	1033	64	29	0.12	0.06	0.5	1	0	2	1	2	1	5	1	-1.52715
sp|O00505|IMA3_HUMAN	KPNA3	Importin subunit alpha-3 OS=Homo sapiens GN=KPNA3 PE=1 SV=2	58288	406	791	43	43	0.12	0.06	0.5	0	1	2	1	2	1	2	1	-1.52715
tr|A0MNN4|A0MNN4_HUMAN	SMU1	CDW3/SMU1 OS=Homo sapiens GN=SMU1 PE=2 SV=1	58134	221	612	94	58	0.12	0.06	0.5	1	1	2	1	2	1	2	2	-1.52715
tr|B7Z8W6|B7Z8W6_HUMAN	tr|B7Z8W	cDNA FLJ55038, highly similar to Nicotinamide phosphoribosyltransferase (EC 2.4.2.12) OS=Homo sapiens PE=2 SV=1	26905	520	525	32	0	0.12	0.06	0.5	0	0	1	0	1	0	3	1	-2.37501
sp|P13645|K1C10_HUMAN	KRT10	Keratin, type I cytoskeletal 10 OS=Homo sapiens GN=KRT10 PE=1 SV=6	59020	12	46	1226	732	4.67	2.3	0.492505353	34	22	42	27	47	32	54	38	-1.34692
tr|B0QZK4|B0QZK4_HUMAN	HP1BP3	Heterochromatin protein 1-binding protein 3 (Fragment) OS=Homo sapiens GN=HP1BP3 PE=4 SV=1	28725	631	800	23	42	0.25	0.12	0.48	0	1	2	1	1	1	3	2	-0.78985
tr|B3KNB4|B3KNB4_HUMAN	tr|B3KNB	cDNA FLJ14168 fis, clone NT2RP2001440, highly similar to 14-3-3 protein gamma OS=Homo sapiens PE=2 SV=1	28453	541	1029	30	29	0.25	0.12	0.48	0	0	2	1	2	2	2	2	-0.78999
tr|B2RD27|B2RD27_HUMAN	tr|B2RD2	cDNA, FLJ96428, highly similar to Homo sapiens proteasome (prosome, macropain) 26S subunit, non-ATPase, 7 (Mov34 homolog) (P	37046	550	917	30	36	0.19	0.09	0.473684211	0	1	2	1	2	1	2	1	-1.52715
tr|B2R4P2|B2R4P2_HUMAN	tr|B2R4P	cDNA, FLJ92164, highly similar to Homo sapiens peroxiredoxin 1 (PRDX1), mRNA OS=Homo sapiens PE=2 SV=1	22414	481	1074	36	27	0.32	0.15	0.46875	0	0	2	1	2	1	2	1	-1.52715
sp|Q9NR12|PDLI7_HUMAN	PDLIM7	PDZ and LIM domain protein 7 OS=Homo sapiens GN=PDLIM7 PE=1 SV=1	50896	396	1103	43	26	0.13	0.06	0.461538462	0	0	2	1	2	1	4	2	-1.52715
sp|P0CG38|POTEI_HUMAN	POTEI	POTE ankyrin domain family member I OS=Homo sapiens GN=POTEI PE=2 SV=1	122858	5	109	195	331	0.44	0.2	0.454545455	6	11	11	16	12	17	14	20	-0.30502
sp|Q14247|SRC8_HUMAN	CTTN	Src substrate cortactin OS=Homo sapiens GN=CTTN PE=1 SV=2	61720	378	1173	46	19	0.11	0.05	0.454545455	1	0	2	1	3	0	3	2	-3.59808
tr|B9A041|B9A041_HUMAN	MDH1	Malate dehydrogenase, cytoplasmic OS=Homo sapiens GN=MDH1 PE=2 SV=1	23195	309	810	58	42	0.31	0.14	0.451612903	2	1	2	1	2	1	2	1	-1.52715
tr|B7Z8J4|B7Z8J4_HUMAN	NMT1	Glycylpeptide N-tetradecanoyltransferase 1 OS=Homo sapiens GN=NMT1 PE=2 SV=1	15265	350	903	50	36	0.49	0.22	0.448979592	1	1	2	1	2	1	2	1	-1.52715
tr|E7EN38|E7EN38_HUMAN	RAD50	DNA repair protein RAD50 (Fragment) OS=Homo sapiens GN=RAD50 PE=2 SV=1	77138	563	841	29	22	0.09	0.04	0.444444444	0	0	2	1	1	1	2	1	-0.78985
tr|Q5HYM2|Q5HYM2_HUMAN	DKFZp686	Putative uncharacterized protein DKFZp686O2462 (Fragment) OS=Homo sapiens GN=DKFZp686O2462 PE=2 SV=1	72627	569	1092	29	26	0.09	0.04	0.444444444	0	0	2	1	2	1	2	1	-1.52715
tr|E5RHF2|E5RHF2_HUMAN	COPS5	COP9 signalosome complex subunit 5 (Fragment) OS=Homo sapiens GN=COPS5 PE=2 SV=1	16655	346	803	51	42	0.45	0.2	0.444444444	0	1	2	1	2	1	2	1	-1.52715
sp|Q14651|PLSI_HUMAN	PLS1	Plastin-1 OS=Homo sapiens GN=PLS1 PE=1 SV=2	70608	282	392	57	91	0.34	0.15	0.441176471	1	4	3	4	3	5	3	8	-0.13767
tr|B4DNN4|B4DNN4_HUMAN	tr|B4DNN	Ribonucleoside-diphosphate reductase OS=Homo sapiens PE=2 SV=1	87252	156	256	136	153	0.29	0.12	0.413793103	5	4	7	4	8	4	8	4	-1.70917
tr|F8W8T8|F8W8T8_HUMAN	ACACB	Acetyl-CoA carboxylase 2 OS=Homo sapiens GN=ACACB PE=2 SV=1	269823	31	54	128	607	0.44	0.18	0.409090909	4	15	4	20	6	24	9	24	1.127239
tr|G3XAF4|G3XAF4_HUMAN	L1CAM	L1 cell adhesion molecule, isoform CRA_a OS=Homo sapiens GN=L1CAM PE=4 SV=1	139790	572	610	28	58	0.05	0.02	0.4	1	1	2	1	1	1	2	1	-0.78985
sp|Q5XKE5|K2C79_HUMAN	KRT79	Keratin, type II cytoskeletal 79 OS=Homo sapiens GN=KRT79 PE=1 SV=2	58085	160	485	133	75	0.32	0.12	0.375	5	2	6	2	6	5	8	6	-1.03177
sp|P42166|LAP2A_HUMAN	TMPO	Lamina-associated polypeptide 2, isoform alpha OS=Homo sapiens GN=TMPO PE=1 SV=2	76016	241	453	84	81	0.24	0.09	0.375	1	1	5	2	5	2	5	5	-1.92851
tr|H0YIE7|H0YIE7_HUMAN	HSPA1L	Heat shock 70 kDa protein 1-like OS=Homo sapiens GN=HSPA1L PE=3 SV=1	25488	51	163	132	141	1.45	0.5	0.344827586	3	5	4	5	4	5	4	5	-0.50058
tr|B4DKM5|B4DKM5_HUMAN	VDAC2	Voltage-dependent anion-selective channel protein 2 OS=Homo sapiens GN=VDAC2 PE=2 SV=1	27804	196	728	108	48	0.76	0.25	0.328947368	1	1	5	2	5	3	5	3	-1.44289
tr|B3KTA3|B3KTA3_HUMAN	tr|B3KTA3	Fascin OS=Homo sapiens PE=2 SV=1	52814	53	234	403	161	1.07	0.35	0.327102804	9	5	14	5	16	6	20	7	-2.13792
tr|B2R659|B2R659_HUMAN	tr|B2R659	cDNA, FLJ92803, highly similar to Homo sapiens hydroxysteroid (17-beta) dehydrogenase 4 (HSD17B4), mRNA OS=Homo sapiens PE	80018	484	566	36	62	0.13	0.04	0.307692308	0	1	3	1	3	2	4	3	-1.27576
tr|Q5T081|Q5T081_HUMAN	RCC1	CHC1 protein OS=Homo sapiens GN=RCC1 PE=2 SV=1	45397	269	695	70	50	0.23	0.07	0.304347826	2	1	3	1	2	4	4	4	0.058396
sp|P12036|NFH_HUMAN	NEFH	Neurofilament heavy polypeptide OS=Homo sapiens GN=NEFH PE=1 SV=4	112639	11	123	0	32	4.79	1.44	0.300626305	0	0	0	1	0	1	1	1	0.795451
sp|P22626|ROA2_HUMAN	HNRNPA2	Heterogeneous nuclear ribonucleoproteins A2/B1 OS=Homo sapiens GN=HNRNPA2B1 PE=1 SV=2	37464	77	574	256	62	0.97	0.29	0.298969072	7	2	8	3	9	3	10	4	-2.23274
tr|Q6IBT3|Q6IBT3_HUMAN	CCT7	CCT7 protein OS=Homo sapiens GN=CCT7 PE=2 SV=1	59804	161	837	133	40	0.38	0.11	0.289473684	2	1	6	2	5	3	7	5	-1.44289
tr|K7ENW6|K7ENW6_HUMAN	KRT16	Keratin, type I cytoskeletal 16 (Fragment) OS=Homo sapiens GN=KRT16 PE=4 SV=1	18735	62	426	109	85	1.38	0.39	0.282608696	3	2	4	2	5	3	5	5	-1.44289
tr|Q96K98|Q96K98_HUMAN	tr|Q96K9	cDNA FLJ14414 fis, clone HEMBA1004847, highly similar to SIGNAL RECOGNITION PARTICLE 68 KD PROTEIN OS=Homo sapiens PE=2	37365	180	1027	119	29	0.18	0.05	0.277777778	2	0	2	1	2	1	4	2	-1.52715
tr|J3QQX0|J3QQX0_HUMAN	GPS1	COP9 signalosome complex subunit 1 (Fragment) OS=Homo sapiens GN=GPS1 PE=4 SV=1	8703	636	613	22	47	0.4	0.11	0.275	0	1	1	1	1	1	1	1	-0.78985
tr|Q53XC0|Q53XC0_HUMAN	EIF2S1	Eukaryotic translation initiation factor 2, subunit 1 alpha, 35kDa, isoform CRA_a OS=Homo sapiens GN=EIF2S1 PE=2 SV=1	36374	208	1136	100	24	0.42	0.11	0.261904762	4	0	4	1	4	1	4	1	-2.37583
tr|B4DQX3|B4DQX3_HUMAN	tr|B4DQX	cDNA FLJ58927, highly similar to Endoplasmin (Heat shock protein 90 kDa beta member 1) OS=Homo sapiens PE=2 SV=1	18712	32	136	32	97	1.24	0.28	0.225806452	0	2	1	2	1	2	1	3	-0.05269
tr|E5RH42|E5RH42_HUMAN	G3BP1	Ras GTPase-activating protein-binding protein 1 (Fragment) OS=Homo sapiens GN=G3BP1 PE=2 SV=1	13338	294	528	62	0	0.58	0.13	0.224137931	2	0	2	0	2	0	2	1	-3.11231
tr|F8VU64|F8VU64_HUMAN	KRT8	Keratin, type II cytoskeletal 8 OS=Homo sapiens GN=KRT8 PE=2 SV=1	37274	58	377	61	96	0.95	0.2	0.210526316	3	2	4	4	6	5	7	6	-1.03177
tr|B3KVL0|B3KVL0_HUMAN	tr|B3KVL0	cDNA FLJ16691 fis, clone TRACH3000692, highly similar to Paxillin OS=Homo sapiens PE=2 SV=1	65600	159	520	133	70	0.28	0.05	0.178571429	3	1	5	1	6	1	6	2	-2.90702
tr|E7ERA5|E7ERA5_HUMAN	MYH10	Myosin-10 (Fragment) OS=Homo sapiens GN=MYH10 PE=2 SV=1	21768	17	115	73	56	0.9	0.15	0.166666667	1	1	1	1	1	1	1	2	-0.78985
tr|A4ZU86|A4ZU86_HUMAN	NPM1	Truncated nucleolar phosphoprotein B23 OS=Homo sapiens GN=NPM1 PE=2 SV=1	30074	138	657	160	53	0.69	0.11	0.15942029	4	1	5	1	5	1	5	1	-2.66568
tr|D3DTL4|D3DTL4_HUMAN	ENO3	Enolase OS=Homo sapiens GN=ENO3 PE=2 SV=1	37167	40	193	112	92	1.39	0.22	0.158273381	2	1	2	1	2	1	4	2	-1.52715
tr|B3KMS0|B3KMS0_HUMAN	tr|B3KMS	cDNA FLJ12449 fis, clone NT2RM1000314, highly similar to Condensin complex subunit 1 OS=Homo sapiens PE=2 SV=1	158464	494	957	34	33	0.15	0.02	0.133333333	0	1	2	1	2	3	2	3	-0.30437
tr|J3KR91|J3KR91_HUMAN	IQGAP2	Ras GTPase-activating-like protein IQGAP2 OS=Homo sapiens GN=IQGAP2 PE=4 SV=1	123072	84	966	32	33	0.31	0.04	0.129032258	0	0	1	2	1	3	1	3	0.432934
tr|B4DX20|B4DX20_HUMAN	CCT6B	T-complex protein 1 subunit zeta-2 OS=Homo sapiens GN=CCT6B PE=2 SV=1	54066	118	1156	46	21	0.47	0.06	0.127659574	1	0	1	1	2	1	3	3	-1.52715
sp|O95678|K2C75_HUMAN	KRT75	Keratin, type II cytoskeletal 75 OS=Homo sapiens GN=KRT75 PE=1 SV=2	59809	11	215	94	66	4.79	0.61	0.127348643	2	2	3	2	3	4	5	6	-0.42737
sp|Q9NXW2|DJB12_HUMAN	DNAJB12	DnaJ homolog subfamily B member 12 OS=Homo sapiens GN=DNAJB12 PE=1 SV=4	42021	124	654	174	53	0.57	0.07	0.122807018	5	1	7	1	9	1	9	1	-3.45553
tr|Q8TE01|Q8TE01_HUMAN	derp12	DERP12 (Dermal papilla derived protein 12) OS=Homo sapiens GN=derp12 PE=2 SV=1	38340	69	573	297	62	0.79	0.09	0.113924051	6	1	7	1	8	1	8	1	-3.29472
tr|B7Z7U6|B7Z7U6_HUMAN	tr|B7Z7U	cDNA FLJ51769, highly similar to Ras GTPase-activating-like protein IQGAP2 (Fragment) OS=Homo sapiens PE=2 SV=1	160665	84	955	32	33	0.31	0.02	0.064516129	0	0	1	1	1	2	1	3	-0.05269
sp|Q7Z3Y8|K1C27_HUMAN	KRT27	Keratin, type I cytoskeletal 27 OS=Homo sapiens GN=KRT27 PE=1 SV=2	50419	12	763	72	45	4.67	0.07	0.014989293	2	1	2	1	2	1	2	2	-1.52715
tr|C4AM86|C4AM86_HUMAN	KRT35	Keratin, type I cuticular Ha5 OS=Homo sapiens GN=KRT35 PE=2 SV=1	48816	12	879	88	38	4.67	0.07	0.014989293	4	1	4	2	4	2	4	3	-1.63867
tr|Q562V5|Q562V5_HUMAN	ACT	Actin-like protein (Fragment) OS=Homo sapiens GN=ACT PE=2 SV=1	11481	1	289	136	135	31.43	0.3	0.009545021	6	4	6	4	7	10	8	12	-0.30469
tr|Q562Q1|Q562Q1_HUMAN	ACT	Actin-like protein (Fragment) OS=Homo sapiens GN=ACT PE=3 SV=1	11548	5	0	0	0	0.44	0	0	0	0	0	0	0	0	1	0	-0.78971
tr|H0YIC5|H0YIC5_HUMAN	KRT73	Keratin, type II cytoskeletal 73 (Fragment) OS=Homo sapiens GN=KRT73 PE=3 SV=1	31556	11	0	0	0	4.79	0	0	0	0	0	0	0	0	1	0	-0.78971
tr|K7ELP7|K7ELP7_HUMAN	KRT17	Keratin, type I cytoskeletal 17 (Fragment) OS=Homo sapiens GN=KRT17 PE=4 SV=1	5508	24	0	20	0	2.99	0	0	0	0	1	0	0	0	1	0	-0.78971
tr|E9PQ69|E9PQ69_HUMAN	GANAB	Neutral alpha-glucosidase AB OS=Homo sapiens GN=GANAB PE=2 SV=1	8560	47	0	0	0	0.62	0	0	0	0	0	0	0	0	1	0	-0.78971
tr|F8VU69|F8VU69_HUMAN	KRT5	Keratin, type II cytoskeletal 5 (Fragment) OS=Homo sapiens GN=KRT5 PE=2 SV=1	14387	58	0	0	0	0.95	0	0	0	0	0	0	0	0	2	0	-0.78971
tr|Q8WY81|Q8WY81_HUMAN	tr|Q8WY8	PP3895 OS=Homo sapiens PE=2 SV=1	15132	114	0	15	0	0.28	0	0	0	0	1	0	0	0	1	0	-0.78971
tr|E9PLJ0|E9PLJ0_HUMAN	IPO7	Importin-7 OS=Homo sapiens GN=IPO7 PE=2 SV=1	7255	162	0	0	0	0.08	0	0	0	0	0	0	0	0	1	0	-0.78971
tr|I3L204|I3L204_HUMAN	MYO1C	Unconventional myosin-Ic (Fragment) OS=Homo sapiens GN=MYO1C PE=4 SV=1	34968	284	0	0	0	0.07	0	0	0	0	0	0	0	0	1	0	-0.78971
tr|Q1XBU7|Q1XBU7_HUMAN	tr|Q1XBU	Aging-associated protein 14a OS=Homo sapiens PE=2 SV=1	14000	382	0	0	0	0.09	0	0	0	0	0	0	0	0	1	0	-0.78971
tr|B7Z6J5|B7Z6J5_HUMAN	tr|B7Z6J5	cDNA FLJ56858, highly similar to Succinate dehydrogenase (ubiquinone) flavoprotein subunit, mitochondrial (EC 1.3.5.1) OS=Homo s	28776	393	0	0	0	0.1	0	0	0	0	0	0	0	0	1	0	-0.78971
tr|H0Y386|H0Y386_HUMAN	PABPC1L	Polyadenylate-binding protein 1-like (Fragment) OS=Homo sapiens GN=PABPC1L PE=4 SV=1	16787	404	0	0	0	0.1	0	0	0	0	0	0	0	0	1	0	-0.78971
tr|H7BYY8|H7BYY8_HUMAN	PES1	Pescadillo homolog OS=Homo sapiens GN=PES1 PE=4 SV=1	1501	488	0	0	0	0.05	0	0	0	0	0	0	0	0	1	0	-0.78971
sp|Q14117|DPYS_HUMAN	DPYS	Dihydropyrimidinase OS=Homo sapiens GN=DPYS PE=1 SV=1	57107	490	0	0	0	0.19	0	0	0	0	0	0	0	0	1	0	-0.78971
tr|D6RD96|D6RD96_HUMAN	ACSL4	Long-chain-fatty-acid--CoA ligase 4 (Fragment) OS=Homo sapiens GN=ACSL4 PE=2 SV=1	16399	529	0	0	0	0.04	0	0	0	0	0	0	0	0	1	0	-0.78971
tr|H0YDS9|H0YDS9_HUMAN	NASP	Nuclear autoantigenic sperm protein (Fragment) OS=Homo sapiens GN=NASP PE=4 SV=1	18974	542	0	0	0	0.1	0	0	0	0	0	0	0	0	1	0	-0.78971
tr|H7C1T1|H7C1T1_HUMAN	Sep-02	Septin-2 (Fragment) OS=Homo sapiens GN=SEPT2 PE=4 SV=1	3094	543	0	0	0	0.09	0	0	0	0	0	0	0	0	1	0	-0.78971
tr|B7Z4W3|B7Z4W3_HUMAN	tr|B7Z4W	cDNA FLJ57543, highly similar to Bifunctional 3~-phosphoadenosine5~-phosphosulfate synthetase 1 OS=Homo sapiens PE=2 SV=1	17852	553	0	19	0	0.1	0	0	0	0	1	0	0	0	1	0	-0.78971
tr|J3KN33|J3KN33_HUMAN	CNTLN	Centlein OS=Homo sapiens GN=CNTLN PE=4 SV=1	162069	680	0	20	0	0.02	0	0	0	0	1	0	0	0	2	0	-0.78971
tr|Q3MNE1|Q3MNE1_HUMAN	tr|Q3MNE	Adaptor-related protein complex 3 beta 1 subunit (Fragment) OS=Homo sapiens PE=2 SV=1	89828	682	0	20	0	0.04	0	0	0	0	1	0	0	0	1	0	-0.78971
tr|B4DZD5|B4DZD5_HUMAN	tr|B4DZD	cDNA FLJ60991, highly similar to FKBP12-rapamycin complex-associated protein OS=Homo sapiens PE=2 SV=1	158783	683	0	20	0	0.02	0	0	0	0	1	0	0	0	2	0	-0.78971
tr|Q9UH66|Q9UH66_HUMAN	tr|Q9UH6	7h3 protein (Fragment) OS=Homo sapiens PE=2 SV=1	49963	684	0	19	0	0.07	0	0	0	0	1	0	0	0	2	0	-0.78971
tr|G5E9Q3|G5E9Q3_HUMAN	TSEN2	tRNA splicing endonuclease 2 homolog (S. cerevisiae), isoform CRA_b OS=Homo sapiens GN=TSEN2 PE=2 SV=1	45959	686	0	19	0	0.07	0	0	0	0	1	0	0	0	1	0	-0.78971
tr|E9PQG7|E9PQG7_HUMAN	MLL	MLL cleavage product C180 OS=Homo sapiens GN=MLL PE=2 SV=1	436345	687	0	19	0	0.01	0	0	0	0	1	0	0	0	1	0	-0.78971
tr|Q5TB52|Q5TB52_HUMAN	PAPSS2	3~-phosphoadenosine 5~-phosphosulfate synthase 2, isoform CRA_b OS=Homo sapiens GN=PAPSS2 PE=2 SV=1	70027	688	0	19	0	0.05	0	0	0	0	1	0	0	0	4	0	-0.78971
tr|G3V448|G3V448_HUMAN	TMX1	Thioredoxin-related transmembrane protein 1 OS=Homo sapiens GN=TMX1 PE=2 SV=1	12018	689	0	19	0	0.29	0	0	0	0	1	0	0	0	1	0	-0.78971
tr|D3DUL0|D3DUL0_HUMAN	ASCIZ	ATM/ATR-Substrate Chk2-Interacting Zn2+-finger protein, isoform CRA_b OS=Homo sapiens GN=ASCIZ PE=2 SV=1	79474	690	0	19	0	0.04	0	0	0	0	1	0	0	0	1	0	-0.78971
tr|H7C4X2|H7C4X2_HUMAN	PCMT1	Protein-L-isoaspartate(D-aspartate) O-methyltransferase (Fragment) OS=Homo sapiens GN=PCMT1 PE=4 SV=1	7342	691	0	19	0	0.48	0	0	0	0	1	0	0	0	2	0	-0.78971
tr|B3KND7|B3KND7_HUMAN	tr|B3KND	cDNA FLJ14378 fis, clone HEMBA1001804, highly similar to Zinc finger protein 281 (Fragment) OS=Homo sapiens PE=2 SV=1	58221	692	0	19	0	0.06	0	0	0	0	1	0	0	0	1	0	-0.78971
tr|H0YE81|H0YE81_HUMAN	NDUFV1	NADH dehydrogenase [ubiquinone] flavoprotein 1, mitochondrial (Fragment) OS=Homo sapiens GN=NDUFV1 PE=4 SV=1	14494	694	0	19	0	0.23	0	0	0	0	1	0	0	0	1	0	-0.78971
tr|H0Y304|H0Y304_HUMAN	DMD	Dystrophin (Fragment) OS=Homo sapiens GN=DMD PE=4 SV=1	160176	695	0	19	0	0.01	0	0	0	0	1	0	0	0	1	0	-0.78971
tr|Q5T097|Q5T097_HUMAN	UTRN	Utrophin OS=Homo sapiens GN=UTRN PE=4 SV=1	114423	696	0	19	0	0.01	0	0	0	0	1	0	0	0	1	0	-0.78971
tr|I3L2H8|I3L2H8_HUMAN	MYBBP1A	Myb-binding protein 1A (Fragment) OS=Homo sapiens GN=MYBBP1A PE=4 SV=1	22828	698	0	18	0	0.02	0	0	0	0	1	0	0	0	1	0	-0.78971
tr|B4DY47|B4DY47_HUMAN	PHB	Prohibitin OS=Homo sapiens GN=PHB PE=2 SV=1	17064	699	0	18	0	0.2	0	0	0	0	1	0	0	0	1	0	-0.78971
tr|J3KQ96|J3KQ96_HUMAN	TCOF1	Treacle protein (Fragment) OS=Homo sapiens GN=TCOF1 PE=4 SV=1	144268	702	0	18	0	0.03	0	0	0	0	1	0	0	0	1	0	-0.78971
tr|B0UZL4|B0UZL4_HUMAN	BAT3	HLA-B associated transcript 3 (Fragment) OS=Homo sapiens GN=BAT3 PE=2 SV=1	15422	704	0	18	0	0.03	0	0	0	0	1	0	0	0	1	0	-0.78971
tr|J3KMW7|J3KMW7_HUMAN	UBR5	E3 ubiquitin-protein ligase UBR5 OS=Homo sapiens GN=UBR5 PE=4 SV=1	312224	706	0	18	0	0.01	0	0	0	0	1	0	0	0	1	0	-0.78971
tr|H0Y4R4|H0Y4R4_HUMAN	TOR1AIP1	Torsin-1A-interacting protein 1 (Fragment) OS=Homo sapiens GN=TOR1AIP1 PE=4 SV=1	33844	707	0	17	0	0.1	0	0	0	0	1	0	0	0	1	0	-0.78971
sp|P54826|GAS1_HUMAN	GAS1	Growth arrest-specific protein 1 OS=Homo sapiens GN=GAS1 PE=1 SV=2	36867	708	0	17	0	0.09	0	0	0	0	1	0	0	0	1	0	-0.78971
tr|D6RAW4|D6RAW4_HUMAN	IMMT	Mitochondrial inner membrane protein OS=Homo sapiens GN=IMMT PE=2 SV=1	15918	712	0	0	0	0.04	0	0	0	0	0	0	0	0	1	0	-0.78971
tr|J3KR97|J3KR97_HUMAN	TBCD	Tubulin-specific chaperone D OS=Homo sapiens GN=TBCD PE=4 SV=1	138346	714	0	15	0	0.02	0	0	0	0	1	0	0	0	1	0	-0.78971
tr|Q6ZPD6|Q6ZPD6_HUMAN	tr|Q6ZPD	cDNA FLJ25992 fis, clone DMC03508 OS=Homo sapiens PE=2 SV=1	41924	12	0	51	0	4.67	0	0	1	0	0	0	1	0	0	[illegible]	-2.37501

tr|B9VP24|B9VP24_HUMAN	HSPD1	60 kDa chaperonin (Fragment) OS=Homo sapiens GN=HSPD1 PE=4 SV=1	19880	16	0	40	0	1.72	0	0	1	0	1	0	1	0	1	0	-2.37501
tr|A1L2Z2|A1L2Z2_HUMAN	MYH14	MYH14 protein OS=Homo sapiens GN=MYH14 PE=2 SV=1	73418	17	0	56	0	0.9	0	0	1	0	1	0	1	0	1	0	-2.37501
tr|B7Z2Y4|B7Z2Y4_HUMAN	tr|B7Z2Y4	cDNA FLJ56961, highly similar to Clathrin heavy chain 2 (CLH-22) OS=Homo sapiens PE=2 SV=1	54629	30	0	60	0	0.52	0	0	1	0	1	0	1	0	2	0	-2.37501
tr|Q9BVS9|Q9BVS9_HUMAN	IPO5	IPO5 protein (Fragment) OS=Homo sapiens GN=IPO5 PE=2 SV=1	36033	85	0	71	0	0.23	0	0	1	0	1	0	1	0	1	0	-2.37501
tr|Q659G2|Q659G2_HUMAN	DKFZp586	Putative uncharacterized protein DKFZp586B2023 (Fragment) OS=Homo sapiens GN=DKFZp586B2023 PE=2 SV=1	7133	124	0	41	0	0.57	0	0	1	0	1	0	1	0	1	0	-2.37501
tr|K7ESA0|K7ESA0_HUMAN	ATP5A1	ATP synthase subunit alpha, mitochondrial OS=Homo sapiens GN=ATP5A1 PE=4 SV=1	8259	135	0	54	0	1.33	0	0	1	0	1	0	1	0	1	0	-2.37501
tr|Q9BZT4|Q9BZT4_HUMAN	tr|Q9BZT4	PNAS-29 OS=Homo sapiens PE=4 SV=1	8036	137	0	59	0	0.4	0	0	1	0	1	0	1	0	1	0	-2.37501
tr|Q9BTI9|Q9BTI9_HUMAN	NPM1	NPM1 protein (Fragment) OS=Homo sapiens GN=NPM1 PE=2 SV=2	25147	138	0	46	0	0.69	0	0	1	0	1	0	1	0	1	0	-2.37501
tr|Q8NI92|Q8NI92_HUMAN	OK/SW-cl	DEAD/H(Asp-Glu-Ala-Asp/His)box polypeptide 21 OS=Homo sapiens GN=OK/SW-cl.65 PE=2 SV=1	16499	148	0	21	0	0.34	0	0	0	0	1	0	1	0	1	0	-2.37501
tr|K7EQ75|K7EQ75_HUMAN	ILF3	Interleukin enhancer-binding factor 3 (Fragment) OS=Homo sapiens GN=ILF3 PE=4 SV=1	6139	154	0	29	0	0.46	0	0	0	0	1	0	1	0	1	0	-2.37501
tr|H0YCY7|H0YCY7_HUMAN	RRM1	Ribonucleoside-diphosphate reductase (Fragment) OS=Homo sapiens GN=RRM1 PE=3 SV=1	14342	156	0	22	0	0.29	0	0	0	0	1	0	1	0	1	0	-2.37501
tr|H0Y929|H0Y929_HUMAN	PDLIM5	PDZ and LIM domain protein 5 (Fragment) OS=Homo sapiens GN=PDLIM5 PE=4 SV=1	9116	175	0	25	0	0.41	0	0	0	0	1	0	1	0	1	0	-2.37501
tr|Q9BUV4|Q9BUV4_HUMAN	RPL5	RPL5 protein OS=Homo sapiens GN=RPL5 PE=2 SV=1	12182	187	0	39	0	0.44	0	0	0	0	1	0	1	0	2	0	-2.37501
tr|B3KV39|B3KV39_HUMAN	tr|B3KV39	cDNA FLJ16099 fis, clone TESTI2008278, highly similar to Homo sapiens forkhead box K1 (FOXK1), mRNA OS=Homo sapiens PE=2 SV	50383	188	0	115	0	0.07	0	0	1	0	1	0	1	0	2	0	-2.37501
tr|D6R9D3|D6R9D3_HUMAN	HNRNPH1	Heterogeneous nuclear ribonucleoprotein H OS=Homo sapiens GN=HNRNPH1 PE=4 SV=1	4404	207	0	23	0	0.45	0	0	0	0	1	0	1	0	1	0	-2.37501
tr|A1E286|A1E286_HUMAN	OXCT1	Succinyl-CoA:3-ketoacid CoA transferase (Fragment) OS=Homo sapiens GN=OXCT1 PE=2 SV=1	16361	209	0	32	0	0.18	0	0	0	0	1	0	1	0	2	0	-2.37501
tr|H0Y6X6|H0Y6X6_HUMAN	PABPC4	Polyadenylate-binding protein 4 (Fragment) OS=Homo sapiens GN=PABPC4 PE=4 SV=1	20509	215	0	33	0	0.33	0	0	0	0	1	0	1	0	1	0	-2.37501
tr|H0Y960|H0Y960_HUMAN	DDX4	Probable ATP-dependent RNA helicase DDX4 (Fragment) OS=Homo sapiens GN=DDX4 PE=4 SV=1	33292	217	0	43	0	0.19	0	0	1	0	1	0	1	0	1	0	-2.37501
tr|B4DE40|B4DE40_HUMAN	SMS	Spermine synthase OS=Homo sapiens GN=SMS PE=2 SV=1	31596	248	0	78	0	0.11	0	0	1	0	1	0	1	0	1	0	-2.37501
tr|Q5JXB7|Q5JXB7_HUMAN	PLOD1	Procollagen-lysine,2-oxoglutarate 5-dioxygenase 1 (Fragment) OS=Homo sapiens GN=PLOD1 PE=4 SV=1	31911	251	0	76	0	0.04	0	0	1	0	1	0	1	0	1	0	-2.37501
tr|B4DXK4|B4DXK4_HUMAN	KRT72	Keratin, type II cytoskeletal 72 OS=Homo sapiens GN=KRT72 PE=2 SV=1	36031	259	0	0	0	0.45	0	0	0	0	0	0	1	0	1	0	-2.37501
tr|K7EKB6|K7EKB6_HUMAN	THOP1	Thimet oligopeptidase (Fragment) OS=Homo sapiens GN=THOP1 PE=4 SV=1	20792	278	0	68	0	0.16	0	0	1	0	1	0	1	0	1	0	-2.37501
tr|H0YC45|H0YC45_HUMAN	ATP6V1B2	V-type proton ATPase subunit B, brain isoform (Fragment) OS=Homo sapiens GN=ATP6V1B2 PE=4 SV=1	5569	287	0	64	0	0.65	0	0	1	0	1	0	1	0	1	0	-2.37501
sp|P81605|DCD_HUMAN	DCD	Dermcidin OS=Homo sapiens GN=DCD PE=1 SV=2	11391	295	0	62	0	0.3	0	0	1	0	1	0	1	0	1	0	-2.37501
tr|Q5T0I1|Q5T0I1_HUMAN	GSN	Gelsolin (Fragment) OS=Homo sapiens GN=GSN PE=4 SV=1	26126	297	0	61	0	0.04	0	0	1	0	1	0	1	0	2	0	-2.37501
tr|B5LY64|B5LY64_HUMAN	UMPS	Orotidine 5'-phosphate decarboxylase OS=Homo sapiens GN=UMPS PE=2 SV=1	33258	302	0	59	0	0.1	0	0	1	0	1	0	1	0	1	0	-2.37501
tr|B3KPC1|B3KPC1_HUMAN	tr|B3KPC1	cDNA FLJ31582 fis, clone NT2RI2002117, highly similar to Protein pelota homolog OS=Homo sapiens PE=2 SV=1	43807	307	0	59	0	0.08	0	0	1	0	1	0	1	0	1	0	-2.37501
tr|B8ZZ51|B8ZZ51_HUMAN	MDH1	Malate dehydrogenase, cytoplasmic OS=Homo sapiens GN=MDH1 PE=2 SV=1	18735	309	0	45	0	0.31	0	0	1	0	1	0	1	0	1	0	-2.37501
sp|Q5TZA2|CROCC_HUMAN	CROCC	Rootletin OS=Homo sapiens GN=CROCC PE=1 SV=1	228787	310	0	58	0	0.01	0	0	1	0	1	0	1	0	2	0	-2.37501
tr|J3KRP2|J3KRP2_HUMAN	PSMC5	26S protease regulatory subunit 8 (Fragment) OS=Homo sapiens GN=PSMC5 PE=4 SV=1	27292	314	0	57	0	0.11	0	0	1	0	1	0	1	0	1	0	-2.37501
tr|C9JPP3|C9JPP3_HUMAN	RBBP4	Histone-binding protein RBBP4 (Fragment) OS=Homo sapiens GN=RBBP4 PE=2 SV=2	9655	315	0	57	0	0.36	0	0	1	0	1	0	1	0	1	0	-2.37501
tr|C9JIX5|C9JIX5_HUMAN	PLOD3	Procollagen-lysine,2-oxoglutarate 5-dioxygenase 3 (Fragment) OS=Homo sapiens GN=PLOD3 PE=2 SV=1	8424	325	0	41	0	0.12	0	0	1	0	1	0	1	0	1	0	-2.37501
tr|I3L4P6|I3L4P6_HUMAN	ARHGAP1	Rho GTPase-activating protein 17 (Fragment) OS=Homo sapiens GN=ARHGAP17 PE=4 SV=1	8676	328	0	54	0	0.03	0	0	1	0	1	0	1	0	1	0	-2.37501
tr|A8K3Q9|A8K3Q9_HUMAN	tr|A8K3Q	cDNA FLJ76611, highly similar to Homo sapiens ribosomal protein L14 (RPL14), mRNA OS=Homo sapiens PE=2 SV=1	23554	330	0	53	0	0.14	0	0	1	0	1	0	1	0	1	0	-2.37501
tr|A8KA82|A8KA82_HUMAN	DNAJC3	DnaJ (Hsp40) homolog, subfamily C, member 3 OS=Homo sapiens GN=DNAJC3 PE=2 SV=1	58000	337	0	52	0	0.06	0	0	1	0	1	0	1	0	1	0	-2.37501
tr|B2RAR2|B2RAR2_HUMAN	tr|B2RAR	cDNA, FLJ95064, highly similar to Homo sapiens nin one binding protein (NOB1P), mRNA OS=Homo sapiens PE=2 SV=1	47015	338	0	52	0	0.07	0	0	1	0	1	0	1	0	1	0	-2.37501
tr|A8K517|A8K517_HUMAN	RPS23	Ribosomal protein S23, isoform CRA_a OS=Homo sapiens GN=RPS23 PE=2 SV=1	15969	339	0	52	0	0.21	0	0	1	0	1	0	1	0	1	0	-2.37501
tr|K7EN42|K7EN42_HUMAN	NMT1	Glycylpeptide N-tetradecanoyltransferase 1 (Fragment) OS=Homo sapiens GN=NMT1 PE=4 SV=1	9699	350	0	31	0	0.49	0	0	0	0	1	0	1	0	1	0	-2.37501
tr|B7Z8A2|B7Z8A2_HUMAN	tr|B7Z8A2	cDNA FLJ51671, highly similar to Prenylcysteine oxidase (EC 1.8.3.5) OS=Homo sapiens PE=2 SV=1	54918	355	0	49	0	0.06	0	0	1	0	1	0	1	0	1	0	-2.37501
tr|D6RJF2|D6RJF2_HUMAN	PAIP1	Polyadenylate-binding protein-interacting protein 1 (Fragment) OS=Homo sapiens GN=PAIP1 PE=2 SV=1	22826	361	0	48	0	0.08	0	0	1	0	1	0	1	0	1	0	-2.37501
tr|H0Y9Q1|H0Y9Q1_HUMAN	LAP3	Cytosol aminopeptidase (Fragment) OS=Homo sapiens GN=LAP3 PE=4 SV=1	22641	368	0	47	0	0.15	0	0	1	0	1	0	1	0	1	0	-2.37501
tr|H0YCD9|H0YCD9_HUMAN	CTTN	Src substrate cortactin (Fragment) OS=Homo sapiens GN=CTTN PE=4 SV=1	29938	378	0	0	0	0.11	0	0	0	0	0	0	1	0	1	0	-2.37501
tr|J3KTL4|J3KTL4_HUMAN	PFAS	Phosphoribosylformylglycinamidine synthase (Fragment) OS=Homo sapiens GN=PFAS PE=4 SV=1	11552	380	0	0	0	0.05	0	0	0	0	0	0	1	0	1	0	-2.37501
tr|B4DT09|B4DT09_HUMAN	tr|B4DT09	cDNA FLJ60145, highly similar to Homo sapiens dendritic cell protein (hfl-B5), mRNA OS=Homo sapiens PE=2 SV=1	13697	381	0	46	0	0.25	0	0	1	0	1	0	1	0	1	0	-2.37501
tr|B3KPZ0|B3KPZ0_HUMAN	tr|B3KPZ0	cDNA FLJ32485 fis, clone SKNMC2001596, highly similar to Ankyrin repeat and FYVE domain-containing protein 1 (Fragment) OS=Ho	121468	387	0	45	0	0.03	0	0	1	0	1	0	1	0	1	0	-2.37501
tr|E5RGY7|E5RGY7_HUMAN	CTNNA1	Catenin alpha-1 (Fragment) OS=Homo sapiens GN=CTNNA1 PE=2 SV=1	14895	389	0	35	0	0.07	0	0	0	0	1	0	1	0	1	0	-2.37501
tr|B3KT66|B3KT66_HUMAN	PSMD6	Proteasome (Prosome, macropain) 26S subunit, non-ATPase, 6, isoform CRA_c OS=Homo sapiens GN=PSMD6 PE=2 SV=1	40600	399	0	43	0	0.08	0	0	1	0	1	0	1	0	1	0	-2.37501
tr|Q1LZN2|Q1LZN2_HUMAN	NOMO3	NOMO3 protein (Fragment) OS=Homo sapiens GN=NOMO3 PE=2 SV=1	90388	400	0	43	0	0.04	0	0	1	0	1	0	1	0	1	0	-2.37501
sp|Q9NSB4|KRT82_HUMAN	KRT82	Keratin, type II cuticular Hb2 OS=Homo sapiens GN=KRT82 PE=1 SV=3	57985	411	0	43	0	0.06	0	0	1	0	1	0	1	0	2	0	-2.37501
tr|F2Z2D0|F2Z2D0_HUMAN	NUP205	Nuclear pore complex protein Nup205 OS=Homo sapiens GN=NUP205 PE=4 SV=1	23590	415	0	21	0	0.04	0	0	0	0	1	0	1	0	1	0	-2.37501
tr|H0YGE9|H0YGE9_HUMAN	M6PR	Cation-dependent mannose-6-phosphate receptor (Fragment) OS=Homo sapiens GN=M6PR PE=4 SV=1	11814	416	0	42	0	0.29	0	0	1	0	1	0	1	0	1	0	-2.37501
tr|B4DPY2|B4DPY2_HUMAN	tr|B4DPY2	cDNA FLJ59286, highly similar to Pre-mRNA-processing factor 40 homolog A (Fragment) OS=Homo sapiens PE=2 SV=1	93124	425	0	41	0	0.04	0	0	1	0	1	0	1	0	1	0	-2.37501
tr|Q5JR94|Q5JR94_HUMAN	RPS8	40S ribosomal protein S8 OS=Homo sapiens GN=RPS8 PE=2 SV=1	24475	426	0	41	0	0.14	0	0	1	0	1	0	1	0	2	0	-2.37501
tr|A4D1I7|A4D1I7_HUMAN	DNCI1	Dynein, cytoplasmic 1, intermediate chain 1, isoform CRA_e OS=Homo sapiens GN=DNCI1 PE=2 SV=1	73423	427	0	40	0	0.04	0	0	1	0	1	0	1	0	1	0	-2.37501
tr|E9PI83|E9PI83_HUMAN	PRMT1	Protein arginine N-methyltransferase 1 (Fragment) OS=Homo sapiens GN=PRMT1 PE=2 SV=1	12790	437	0	36	0	0.26	0	0	0	0	1	0	1	0	1	0	-2.37501
tr|B7Z943|B7Z943_HUMAN	tr|B7Z943	cDNA FLJ53131, highly similar to 24-dehydrocholesterol reductase (EC 1.3.1.-) OS=Homo sapiens PE=2 SV=1	29093	442	0	39	0	0.05	0	0	1	0	1	0	1	0	2	0	-2.37501
tr|K7EK06|K7EK06_HUMAN	FARSA	Phenylalanine--tRNA ligase alpha subunit (Fragment) OS=Homo sapiens GN=FARSA PE=4 SV=1	18547	447	0	38	0	0.06	0	0	1	0	1	0	1	0	1	0	-2.37501
tr|B3KWS3|B3KWS3_HUMAN	PLOD2	Procollagen-lysine,2-oxoglutarate 5-dioxygenase 2 OS=Homo sapiens GN=PLOD2 PE=2 SV=1	49567	453	0	29	0	0.08	0	0	0	0	1	0	1	0	1	0	-2.37501
tr|H0YA24|H0YA24_HUMAN	PLRG1	Pleiotropic regulator 1 (Fragment) OS=Homo sapiens GN=PLRG1 PE=4 SV=1	17588	456	0	38	0	0.19	0	0	1	0	1	0	1	0	2	0	-2.37501
tr|H0Y9L5|H0Y9L5_HUMAN	EXOSC9	Exosome complex component RRP45 (Fragment) OS=Homo sapiens GN=EXOSC9 PE=4 SV=1	18940	457	0	38	0	0.18	0	0	1	0	1	0	1	0	1	0	-2.37501
tr|B3KME2|B3KME2_HUMAN	tr|B3KME	cDNA FLJ10772 fis, clone NT2RP4000243, highly similar to Cartilage-associated protein OS=Homo sapiens PE=2 SV=1	47101	459	0	38	0	0.07	0	0	1	0	1	0	1	0	1	0	-2.37501
sp|Q9H9F9|ARP5_HUMAN	ACTR5	Actin-related protein 5 OS=Homo sapiens GN=ACTR5 PE=1 SV=2	68825	460	0	38	0	0.05	0	0	1	0	1	0	1	0	2	0	-2.37501
tr|H7C1M0|H7C1M0_HUMAN	FANCL	E3 ubiquitin-protein ligase FANCL (Fragment) OS=Homo sapiens GN=FANCL PE=4 SV=1	33067	461	0	37	0	0.1	0	0	1	0	1	0	1	0	1	0	-2.37501
tr|Q5SZ64|Q5SZ64_HUMAN	SF3B4	Splicing factor 3B subunit 4 (Fragment) OS=Homo sapiens GN=SF3B4 PE=4 SV=1	20733	464	0	37	0	0.07	0	0	1	0	1	0	1	0	1	0	-2.37501
sp|Q6ZU64|CC108_HUMAN	CCDC108	Coiled-coil domain-containing protein 108 OS=Homo sapiens GN=CCDC108 PE=2 SV=2	220247	465	0	37	0	0.01	0	0	1	0	1	0	1	0	1	0	-2.37501
tr|B4DDP6|B4DDP6_HUMAN	DBNL	Drebrin-like protein OS=Homo sapiens GN=DBNL PE=2 SV=1	43242	466	0	37	0	0.08	0	0	1	0	1	0	1	0	1	0	-2.37501
tr|C9J7L0|C9J7L0_HUMAN	RBBP7	Histone-binding protein RBBP7 (Fragment) OS=Homo sapiens GN=RBBP7 PE=2 SV=1	7740	468	0	0	0	0.07	0	0	0	0	0	0	1	0	1	0	-2.37501
tr|H0YIX9|H0YIX9_HUMAN	R3HDM2	R3H domain-containing protein 2 (Fragment) OS=Homo sapiens GN=R3HDM2 PE=4 SV=1	31776	469	0	37	0	0.1	0	0	1	0	1	0	1	0	1	0	-2.37501
tr|G3V1W4|G3V1W4_HUMAN	SEC23A	Protein transport protein Sec23A OS=Homo sapiens GN=SEC23A PE=2 SV=1	12904	474	0	24	0	0.08	0	0	0	0	1	0	1	0	1	0	-2.37501
tr|B7Z982|B7Z982_HUMAN	PLRG1	Pleiotropic regulator 1 OS=Homo sapiens GN=PLRG1 PE=2 SV=1	14839	476	0	0	0	0.12	0	0	0	0	0	0	1	0	1	0	-2.37501
tr|J3KN86|J3KN86_HUMAN	RPS3	40S ribosomal protein S3 OS=Homo sapiens GN=RPS3 PE=3 SV=1	28640	480	0	36	0	0.12	0	0	1	0	1	0	1	0	4	0	-2.37501
tr|B7WPL0|B7WPL0_HUMAN	RIC8B	Synembryn-B OS=Homo sapiens GN=RIC8B PE=2 SV=1	64252	483	0	36	0	0.05	0	0	1	0	1	0	1	0	1	0	-2.37501
tr|C9JNW5|C9JNW5_HUMAN	RPL24	60S ribosomal protein L24 OS=Homo sapiens GN=RPL24 PE=4 SV=1	17646	487	0	35	0	0.19	0	0	0	0	1	0	1	0	2	0	-2.37501
tr|E9PI04|E9PI04_HUMAN	RIC8A	Synembryn-A (Fragment) OS=Homo sapiens GN=RIC8A PE=2 SV=1	16936	496	0	34	0	0.05	0	0	0	0	1	0	1	0	1	0	-2.37501
tr|B4DDC9|B4DDC9_HUMAN	tr|B4DDC	cDNA FLJ59448, highly similar to Trifunctional enzyme subunit beta, mitochondrial OS=Homo sapiens PE=2 SV=1	49937	499	0	34	0	0.07	0	0	0	0	1	0	1	0	1	0	-2.37501
sp|Q8NFZ5|TNIP2_HUMAN	TNIP2	TNFAIP3-interacting protein 2 OS=Homo sapiens GN=TNIP2 PE=1 SV=1	49240	502	0	33	0	0.07	0	0	1	0	1	0	1	0	2	0	-2.37501
sp|Q7Z494|NPHP3_HUMAN	NPHP3	Nephrocystin-3 OS=Homo sapiens GN=NPHP3 PE=1 SV=1	151967	510	0	32	0	0.02	0	0	1	0	1	0	1	0	1	0	-2.37501
tr|A0JLS9|A0JLS9_HUMAN	ZEB1	ZEB1 protein (Fragment) OS=Homo sapiens GN=ZEB1 PE=2 SV=1	118576	513	0	32	0	0.03	0	0	1	0	1	0	1	0	1	0	-2.37501
tr|J3KRB3|J3KRB3_HUMAN	RPL17	60S ribosomal protein L17 (Fragment) OS=Homo sapiens GN=RPL17 PE=3 SV=1	12356	517	0	32	0	0.28	0	0	0	0	1	0	1	0	1	0	-2.37501
tr|B4DUH7|B4DUH7_HUMAN	tr|B4DUH	cDNA FLJ60162, highly similar to Chloride anion exchanger OS=Homo sapiens PE=2 SV=1	52632	518	0	32	0	0.06	0	0	1	0	1	0	1	0	1	0	-2.37501
tr|E9PNN6|E9PNN6_HUMAN	ALDH3A1	Aldehyde dehydrogenase, dimeric NADP-preferring OS=Homo sapiens GN=ALDH3A1 PE=2 SV=1	44518	519	0	32	0	0.07	0	0	0	0	1	0	1	0	1	0	-2.37501
tr|B4E0D5|B4E0D5_HUMAN	tr|B4E0D5	cDNA FLJ54218, highly similar to Acidic leucine-rich nuclear phosphoprotein 32 family member E OS=Homo sapiens PE=2 SV=1	19540	525	0	31	0	0.17	0	0	0	0	1	0	1	0	1	0	-2.37501
tr|F2Z2J7|F2Z2J7_HUMAN	TTLL7	Tubulin polyglutamylase TTLL7 OS=Homo sapiens GN=TTLL7 PE=2 SV=1	55925	526	0	31	0	0.06	0	0	1	0	1	0	1	0	1	0	-2.37501
tr|A1L3B5|A1L3B5_HUMAN	ATP2B3	ATP2B3 protein OS=Homo sapiens GN=ATP2B3 PE=2 SV=1	96278	528	0	31	0	0.03	0	0	0	0	1	0	1	0	1	0	-2.37501
tr|Q8TC62|Q8TC62_HUMAN	Sep-07	Septin 7 OS=Homo sapiens GN=SEPT7 PE=2 SV=3	48913	531	0	31	0	0.07	0	0	1	0	1	0	1	0	2	0	-2.37501
tr|B1AMS2|B1AMS2_HUMAN	Sep-06	Septin 6 OS=Homo sapiens GN=SEPT6 PE=3 SV=1	49614	532	0	31	0	0.07	0	0	1	0	1	0	1	0	1	0	-2.37501
sp|Q96PE2|ARHGH_HUMAN	ARHGEF17	Rho guanine nucleotide exchange factor 17 OS=Homo sapiens GN=ARHGEF17 PE=1 SV=1	223645	537	0	30	0	0.01	0	0	1	0	1	0	1	0	1	0	-2.37501
tr|K7EKM4|K7EKM4_HUMAN	SRM	Spermidine synthase OS=Homo sapiens GN=SRM PE=4 SV=1	10680	540	0	30	0	0.32	0	0	0	0	1	0	1	0	1	0	-2.37501
tr|H0YK49|H0YK49_HUMAN	ETFA	Electron transfer flavoprotein subunit alpha, mitochondrial OS=Homo sapiens GN=ETFA PE=4 SV=1	24259	545	0	30	0	0.14	0	0	0	0	1	0	1	0	1	0	-2.37501
tr|B6F210|B6F210_HUMAN	GSS	Glutathione synthetase OS=Homo sapiens GN=GSS PE=2 SV=1	40495	551	0	30	0	0.08	0	0	0	0	1	0	1	0	1	0	-2.37501
tr|H3BRB3|H3BRB3_HUMAN	KIF22	Kinesin-like protein KIF22 OS=Homo sapiens GN=KIF22 PE=3 SV=1	67996	557	0	29	0	0.05	0	0	0	0	1	0	1	0	1	0	-2.37501
sp|Q96A33|CCD47_HUMAN	CCDC47	Coiled-coil domain-containing protein 47 OS=Homo sapiens GN=CCDC47 PE=1 SV=1	56123	560	0	29	0	0.06	0	0	0	0	1	0	1	0	1	0	-2.37501
tr|B4DM97|B4DM97_HUMAN	tr|B4DM9	cDNA FLJ55002, highly similar to Alpha-centractin OS=Homo sapiens PE=2 SV=1	38365	561	0	29	0	0.09	0	0	0	0	1	0	1	0	1	0	-2.37501
tr|B4DGQ0|B4DGQ0_HUMAN	LASP1	LIM and SH3 domain protein 1 OS=Homo sapiens GN=LASP1 PE=2 SV=1	23167	568	0	29	0	0.14	0	0	0	0	1	0	1	0	1	0	-2.37501
tr|I3L361|I3L361_HUMAN	SGSM2	Small G protein-signaling modulator 2 (Fragment) OS=Homo sapiens GN=SGSM2 PE=4 SV=2	1642	570	0	29	0	2.38	0	0	0	0	1	0	1	0	1	0	-2.37501
tr|A4ZYW4|A4ZYW4_HUMAN	L1CAM	Non-neural L1CAM OS=Homo sapiens GN=L1CAM PE=2 SV=1	139633	572	0	28	0	0.05	0	0	1	0	2	0	1	0	2	0	-2.37501
tr|B4DNG6|B4DNG6_HUMAN	tr|B4DNG	Annexin OS=Homo sapiens PE=3 SV=1	12870	577	0	28	0	0.27	0	0	0	0	1	0	1	0	1	0	-2.37501
sp|Q16322|KCA10_HUMAN	KCNA10	Potassium voltage-gated channel subfamily A member 10 OS=Homo sapiens GN=KCNA10 PE=1 SV=2	58147	578	0	28	0	0.06	0	0	1	0	1	0	1	0	1	0	-2.37501
tr|B3KV72|B3KV72_HUMAN	tr|B3KV72	cDNA FLJ16204 fis, clone CTONG2019232, highly similar to Proteasome-associated protein ECM29 homolog (Fragment) OS=Homo sa	78791	579	0	28	0	0.04	0	0	0	0	1	0	1	0	1	0	-2.37501
tr|J3KN32|J3KN32_HUMAN	RCOR1	REST corepressor 1 OS=Homo sapiens GN=RCOR1 PE=4 SV=1	53409	583	0	28	0	0.06	0	0	0	0	1	0	1	0	1	0	-2.37501
tr|H0YCU9|H0YCU9_HUMAN	TAGLN	Transgelin (Fragment) OS=Homo sapiens GN=TAGLN PE=4 SV=1	16833	585	0	28	0	0.2	0	0	0	0	1	0	1	0	1	0	-2.37501
tr|A6NE42|A6NE42_HUMAN	TGIF1	Homeobox protein TGIF1 OS=Homo sapiens GN=TGIF1 PE=3 SV=2	27983	586	0	28	0	0.12	0	0	0	0	1	0	1	0	1	0	-2.37501
tr|H3BS19|H3BS19_HUMAN	ZNF469	Zinc finger protein 469 OS=Homo sapiens GN=ZNF469 PE=4 SV=1	417347	591	0	27	0	0.01	0	0	0	0	1	0	1	0	1	0	-2.37501
tr|H0Y512|H0Y512_HUMAN	APMAP	Adipocyte plasma membrane-associated protein (Fragment) OS=Homo sapiens GN=APMAP PE=4 SV=1	45662	592	0	27	0	0.07	0	0	0	0	1	0	1	0	1	0	-2.37501
tr|B7Z4L9|B7Z4L9_HUMAN	tr|B7Z4L9	cDNA FLJ55342, highly similar to Kelch-like protein 13 OS=Homo sapiens PE=2 SV=1	56884	593	0	26	0	0.06	0	0	0	0	1	0	1	0	1	0	-2.37501
tr|Q5T8C6|Q5T8C6_HUMAN	CDC16	Cell division cycle protein 16 homolog OS=Homo sapiens GN=CDC16 PE=4 SV=1	55239	594	0	26	0	0.06	0	0	0	0	1	0	1	0	1	0	-2.37501
sp|Q0VD83|APOBR_HUMAN	APOBR	Apolipoprotein B receptor OS=Homo sapiens GN=APOBR PE=1 SV=1	115207	597	0	26	0	0.03	0	0	0	0	1	0	1	0	1	0	-2.37501
tr|B4DNK3|B4DNK3_HUMAN	tr|B4DNK	cDNA FLJ52127, highly similar to Multisynthetase complex auxiliary component p43 OS=Homo sapiens PE=2 SV=1	29955	601	0	26	0	0.11	0	0	0	0	1	0	1	0	1	0	-2.37501
tr|B4E2I9|B4E2I9_HUMAN	tr|B4E2I9	cDNA FLJ55486, highly similar to Semaphorin-3C OS=Homo sapiens PE=2 SV=1	88481	602	0	26	0	0.04	0	0	0	0	1	0	1	0	2	0	-2.37501
tr|J3KNP0|J3KNP0_HUMAN	RAB11FIP	Rab11 family-interacting protein 1 OS=Homo sapiens GN=RAB11FIP1 PE=4 SV=1	137482	607	0	25	0	0.02	0	0	0	0	1	0	1	0	2	0	-2.37501
tr|J3KSB9|J3KSB9_HUMAN	tr|J3KSB9	Uncharacterized protein (Fragment) OS=Homo sapiens PE=4 SV=1	8877	608	0	25	0	0.39	0	0	0	0	1	0	1	0	1	0	-2.37501
tr|B4DG42|B4DG42_HUMAN	tr|B4DG4	cDNA FLJ53753, highly similar to Myeloid-associated differentiation marker OS=Homo sapiens PE=2 SV=1	31889	609	0	25	0	0.1	0	0	0	0	1	0	1	0	1	0	-2.37501
tr|Q4VAN1|Q4VAN1_HUMAN	BTN1A1	BTN1A1 protein OS=Homo sapiens GN=BTN1A1 PE=2 SV=1	55878	617	0	24	0	0.06	0	0	0	0	1	0	1	0	1	0	-2.37501
tr|Q2TQ40|Q2TQ40_HUMAN	TNFRSF17	B cell maturation antigen transcript variant 4 OS=Homo sapiens GN=TNFRSF17 PE=2 SV=1	15460	618	0	24	0	0.22	0	0	0	0	1	0	1	0	1	0	-2.37501
tr|H0YBA1|H0YBA1_HUMAN	PCM1	Pericentriolar material 1 protein (Fragment) OS=Homo sapiens GN=PCM1 PE=4 SV=1	86629	619	0	24	0	0.01	0	0	0	0	1	0	1	0	1	0	-2.37501
tr|B4DU06|B4DU06_HUMAN	tr|B4DU0	Guanylate cyclase OS=Homo sapiens PE=2 SV=1	91756	620	0	24	0	0.04	0	0	0	0	1	0	1	0	1	0	-2.37501
tr|B0QXZ5|B0QXZ5_HUMAN	DNAL4	Dynein light chain 4, axonemal OS=Homo sapiens GN=DNAL4 PE=4 SV=1	7786	621	0	24	0	0.47	0	0	0	0	1	0	1	0	1	0	-2.37501
tr|K7EPB5|K7EPB5_HUMAN	ELAVL3	ELAV-like protein 3 (Fragment) OS=Homo sapiens GN=ELAVL3 PE=4 SV=1	9728	625	0	23	0	0.36	0	0	0	0	1	0	1	0	1	0	-2.37501
tr|K7EJ37|K7EJ37_HUMAN	EXOSC10	Exosome component 10 OS=Homo sapiens GN=EXOSC10 PE=4 SV=1	13869	627	0	23	0	0.05	0	0	0	0	1	0	1	0	1	0	-2.37501
sp|Q9Y5P6|GMPPB_HUMAN	GMPPB	Mannose-1-phosphate guanyltransferase beta OS=Homo sapiens GN=GMPPB PE=1 SV=2	40379	629	0	23	0	0.08	0	0	0	0	1	0	1	0	1	0	-2.37501
tr|B3KPR5|B3KPR5_HUMAN	tr|B3KPR5	cDNA FLJ32094 fis, clone OCBBF2000986, highly similar to Homo sapiens elongation factor Tu GTP binding domain containing 1, trar	97046	633	0	22	0	0.03	0	0	0	0	1	0	1	0	1	0	-2.37501
tr|Q5VSQ6|Q5VSQ6_HUMAN	P4HA1	Procollagen-proline, 2-oxoglutarate 4-dioxygenase (Proline 4-hydroxylase), alpha polypeptide I variant (Fragment) OS=Homo sapien	61214	634	0	22	0	0.05	0	0	0	0	1	0	1	0	2	0	-2.37501
tr|Q9MYF7|Q9MYF7_HUMAN	HLA-A	MHC class I antigen (Fragment) OS=Homo sapiens GN=HLA-A PE=2 SV=1	21085	639	0	22	0	0.16	0	0	0	0	1	0	1	0	3	0	-2.37501
tr|H7C4V2|H7C4V2_HUMAN	U2SURP	U2 snRNP-associated SURP motif-containing protein (Fragment) OS=Homo sapiens GN=U2SURP PE=4 SV=1	33796	647	0	22	0	0.03	0	0	0	0	1	0	1	0	1	0	-2.37501
sp|Q96EK5|KBP_HUMAN	KIAA1279	KIF1-binding protein OS=Homo sapiens GN=KIAA1279 PE=1 SV=1	72453	649	0	22	0	0.05	0	0	0	0	1	0	1	0	1	0	-2.37501
tr|B4DQ49|B4DQ49_HUMAN	tr|B4DQ4	cDNA FLJ54374, highly similar to Homo sapiens keratin 78 (KRT78), mRNA OS=Homo sapiens PE=2 SV=1	31874	653	0	22	0	0.1	0	0	0	0	1	0	1	0	3	0	-2.37501
tr|B5BU01|B5BU01_HUMAN	EIF2S2	Eukaryotic translation initiation factor 2 beta OS=Homo sapiens GN=EIF2S2 PE=2 SV=1	38648	654	0	22	0	0.09	0	0	0	0	1	0	1	0	1	0	-2.37501
tr|Q5M7Z5|Q5M7Z5_HUMAN	GRHPR	GRHPR protein (Fragment) OS=Homo sapiens GN=GRHPR PE=2 SV=1	37314	659	0	21	0	0.09	0	0	0	0	1	0	1	0	1	0	-2.37501
tr|C9J935|C9J935_HUMAN	SETD9	SET domain-containing protein 9 (Fragment) OS=Homo sapiens GN=SETD9 PE=2 SV=1	5515	660	0	21	0	0.65	0	0	0	0	1	0	1	0	1	0	-2.37501
tr|F1T0A5|F1T0A5_HUMAN	PRPF31	PRP31 pre-mRNA processing factor 31 homolog (Yeast), isoform CRA_a OS=Homo sapiens GN=PRPF31 PE=2 SV=1	55649	661	0	21	0	0.06	0	0	0	0	1	0	1	0	2	0	-2.37501
tr|B3KRG8|B3KRG8_HUMAN	tr|B3KRG	Lipase OS=Homo sapiens PE=2 SV=1	45736	662	0	21	0	0.07	0	0	0	0	1	0	1	0	1	0	-2.37501
tr|J3KN69|J3KN69_HUMAN	NCEH1	Arylacetamide deacetylase-like 1 OS=Homo sapiens GN=NCEH1 PE=4 SV=1	49431	663	0	21	0	0.07	0	0	0	0	1	0	1	0	1	0	-2.37501
tr|Q6UWE9|Q6UWE9_HUMAN	UNQ2550	SFVP2550 OS=Homo sapiens GN=UNQ2550 PE=4 SV=1	11890	664	0	21	0	0.29	0	0	1	0	1	0	1	0	1	0	-2.37501
tr|J3QT54|J3QT54_HUMAN	CPSF7	Cleavage and polyadenylation-specificity factor subunit 7 (Fragment) OS=Homo sapiens GN=CPSF7 PE=4 SV=1	22762	665	0	21	0	0.15	0	0	0	0	1	0	1	0	1	0	-2.37501
tr|B4E2Y2|B4E2Y2_HUMAN	tr|B4E2Y2	cDNA FLJ50370, highly similar to Ubiquitin conjugation factor E4 B OS=Homo sapiens PE=2 SV=1	122548	667	0	21	0	0.02	0	0	0	0	1	0	1	0	1	0	-2.37501
tr|B4DK80|B4DK80_HUMAN	TOP3A	DNA topoisomerase OS=Homo sapiens GN=TOP3A PE=2 SV=1	103082	668	0	20	0	0.03	0	0	0	0	1	0	1	0	1	0	-2.37501
tr|A0AVG7|A0AVG7_HUMAN	BCAS1	Breast carcinoma amplified sequence 1 OS=Homo sapiens GN=BCAS1 PE=2 SV=1	61929	670	0	20	0	0.05	0	0	0	0	1	0	1	0	1	0	-2.37501
tr|H0YJL6|H0YJL6_HUMAN	EVL	Ena/VASP-like protein (Fragment) OS=Homo sapiens GN=EVL PE=4 SV=1	10347	671	0	20	0	0.34	0	0	0	0	1	0	1	0	1	0	-2.37501
tr|D6RF05|D6RF05_HUMAN	HARS	Histidine--tRNA ligase, cytoplasmic OS=Homo sapiens GN=HARS PE=2 SV=1	6740	672	0	20	0	0.07	0	0	0	0	1	0	1	0	1	0	-2.37501
sp|P55212|CASP6_HUMAN	CASP6	Caspase-6 OS=Homo sapiens GN=CASP6 PE=1 SV=2	33859	674	0	20	0	0.1	0	0	0	0	1	0	1	0	1	0	-2.37501
tr|B4DE48|B4DE48_HUMAN	tr|B4DE48	cDNA FLJ53824, highly similar to Sorting nexin-17 OS=Homo sapiens PE=2 SV=1	50009	677	0	20	0	0.07	0	0	0	0	1	0	1	0	1	0	-2.37501
sp|O95159|ZFPL1_HUMAN	ZFPL1	Zinc finger protein-like 1 OS=Homo sapiens GN=ZFPL1 PE=1 SV=2	34833	678	0	20	0	0.1	0	0	0	0	1	0	1	0	1	0	-2.37501
sp|Q9H254|SPTN4_HUMAN	SPTBN4	Spectrin beta chain, non-erythrocytic 4 OS=Homo sapiens GN=SPTBN4 PE=1 SV=2	290005	19	0	29	0	2.11	0	0	0	0	1	0	2	0	2	0	-3.11231
tr|Q7KZ86|Q7KZ86_HUMAN	tr|Q7KZ8	Plakoglobin (Fragment) OS=Homo sapiens PE=2 SV=1	19007	26	0	37	0	1.74	0	0	1	0	2	0	2	0	2	0	-3.11231
tr|G3V2E8|G3V2E8_HUMAN	ACTN1	Alpha-actinin-1 (Fragment) OS=Homo sapiens GN=ACTN1 PE=2 SV=1	9345	50	0	67	0	0.77	0	0	1	0	2	0	2	0	2	0	-3.11231
tr|G3V241|G3V241_HUMAN	SHMT2	Serine hydroxymethyltransferase, mitochondrial (Fragment) OS=Homo sapiens GN=SHMT2 PE=2 SV=1	15985	86	0	71	0	0.61	0	0	1	0	2	0	2	0	3	0	-3.11231
tr|H3BSS4|H3BSS4_HUMAN	PCBP2	Poly(rC)-binding protein 2 (Fragment) OS=Homo sapiens GN=PCBP2 PE=4 SV=1	19494	132	0	154	0	0.32	0	0	2	0	2	0	2	0	2	0	-3.11231
tr|J3QSY6|J3QSY6_HUMAN	ATP2A2	Sarcoplasmic/endoplasmic reticulum calcium ATPase 2 OS=Homo sapiens GN=ATP2A2 PE=4 SV=1	5838	149	0	53	0	0.32	0	0	1	0	2	0	2	0	2	0	-3.11231
tr|Q6ZQU7|Q6ZQU7_HUMAN	tr|Q6ZQU	cDNA FLJ46879 fis, clone UTERU3015011, highly similar to Paxillin OS=Homo sapiens PE=2 SV=1	46149	159	0	73	0	0.28	0	0	1	0	2	0	2	0	2	0	-3.11231
tr|Q5JVP3|Q5JVP3_HUMAN	PTGR1	Leukotriene B4 12-hydroxydehydrogenase, isoform CRA_d OS=Homo sapiens GN=PTGR1 PE=4 SV=1	19441	190	0	113	0	0.38	0	0	2	0	2	0	2	0	2	0	-3.11231
tr|B3KPU1|B3KPU1_HUMAN	GNB2	Guanine nucleotide-binding protein G(I)/G(S)/G(T) subunit beta-2 OS=Homo sapiens GN=GNB2 PE=2 SV=1	26598	194	0	111	0	0.27	0	0	1	0	2	0	2	0	2	0	-3.11231
tr|F5GZ03|F5GZ03_HUMAN	TCP1	T-complex protein 1 subunit alpha (Fragment) OS=Homo sapiens GN=TCP1 PE=2 SV=1	24428	197	0	52	0	0.3	0	0	1	0	2	0	2	0	3	0	-3.11231
tr|B3KVK2|B3KVK2_HUMAN	GNB1	Guanine nucleotide-binding protein G(I)/G(S)/G(T) subunit beta-1 OS=Homo sapiens GN=GNB1 PE=2 SV=1	26659	199	0	105	0	0.18	0	0	1	0	2	0	2	0	2	0	-3.11231
tr|B4E0W4|B4E0W4_HUMAN	tr|B4E0W	cDNA FLJ61020, highly similar to Heterogeneous nuclear ribonucleoprotein D0 OS=Homo sapiens PE=2 SV=1	23062	229	0	75	0	0.37	0	0	2	0	2	0	2	0	2	0	-3.11231
tr|H0YJH7|H0YJH7_HUMAN	TMPO	Thymopentin (Fragment) OS=Homo sapiens GN=TMPO PE=4 SV=1	26907	241	0	38	0	0.24	0	0	0	0	2	0	2	0	2	0	-3.11231
tr|G3V1S6|G3V1S6_HUMAN	GALNT2	Polypeptide N-acetylgalactosaminyltransferase 2 soluble form OS=Homo sapiens GN=GALNT2 PE=2 SV=1	61440	254	0	76	0	0.11	0	0	2	0	2	0	2	0	2	0	-3.11231
sp|Q6IS14|IF5AL_HUMAN	EIF5AL1	Eukaryotic translation initiation factor 5A-1-like OS=Homo sapiens GN=EIF5AL1 PE=1 SV=2	16990	256	0	75	0	0.44	0	0	1	0	2	0	2	0	2	0	-3.11231
tr|E9PG15|E9PG15_HUMAN	YWHAQ	14-3-3 protein theta (Fragment) OS=Homo sapiens GN=YWHAQ PE=2 SV=1	17209	258	0	73	0	0.4	0	0	2	0	2	0	2	0	2	0	-3.11231
tr|Q2QD09|Q2QD09_HUMAN	tr|Q2QD0	Triosephosphate isomerase (Fragment) OS=Homo sapiens PE=2 SV=1	27211	267	0	71	0	0.26	0	0	1	0	2	0	2	0	2	0	-3.11231
tr|E5RIZ6|E5RIZ6_HUMAN	G3BP1	Ras GTPase-activating protein-binding protein 1 (Fragment) OS=Homo sapiens GN=G3BP1 PE=2 SV=1	17032	294	0	62	0	0.58	0	0	2	0	2	0	2	0	2	0	-3.11231
tr|B4DMY3|B4DMY3_HUMAN	tr|B4DMY	cDNA FLJ60713, highly similar to Homo sapiens heterogeneous nuclear ribonucleoprotein A/B (HNRPAB), transcript variant 1, mRNA	35101	318	0	56	0	0.2	0	0	1	0	2	0	2	0	2	0	-3.11231
tr|G3V180|G3V180_HUMAN	DPP3	Dipeptidyl peptidase 3 OS=Homo sapiens GN=DPP3 PE=2 SV=1	84666	335	0	52	0	0.08	0	0	1	0	2	0	2	0	2	0	-3.11231
tr|Q6NZ52|Q6NZ52_HUMAN	RPL27A	Ribosomal protein L27a OS=Homo sapiens GN=RPL27A PE=2 SV=1	16582	348	0	50	0	0.2	0	0	1	0	1	0	2	0	2	0	-3.11231
tr|Q53GU8|Q53GU8_HUMAN	tr|Q53GU	Transforming growth factor, beta-induced, 68kDa variant (Fragment) OS=Homo sapiens PE=2 SV=1	75231	358	0	48	0	0.09	0	0	1	0	2	0	2	0	2	0	-3.11231
sp|P01024|CO3_HUMAN	C3	Complement C3 OS=Homo sapiens GN=C3 PE=1 SV=2	188569	370	0	47	0	0.03	0	0	1	0	2	0	2	0	2	0	-3.11231
tr|B2RCT6|B2RCT6_HUMAN	tr|B2RCT6	cDNA, FLJ96276, highly similar to Homo sapiens G1 to S phase transition 1 (GSPT1), mRNA OS=Homo sapiens PE=2 SV=1	56320	391	0	44	0	0.12	0	0	1	0	2	0	2	0	2	0	-3.11231
tr|Q6ZQN2|Q6ZQN2_HUMAN	tr|Q6ZQN	cDNA FLJ46846 fis, clone UTERU3004635, moderately similar to Neuroblast differentiation associated protein AHNAK (Fragment) OS	180689	401	0	43	0	0.01	0	0	1	0	1	0	2	0	3	0	-3.11231
tr|Q13727|Q13727_HUMAN	tr|Q13727	AHNAK-related protein (Fragment) OS=Homo sapiens PE=2 SV=1	57703	402	0	43	0	0.06	0	0	1	0	1	0	2	0	3	0	-3.11231
tr|A0MNP2|A0MNP2_HUMAN	WDR57	CDW11/WDR57 OS=Homo sapiens GN=WDR57 PE=2 SV=1	39742	413	0	42	0	0.17	0	0	1	0	2	0	2	0	3	0	-3.11231
tr|A5D904|A5D904_HUMAN	RPS9	RPS9 protein (Fragment) OS=Homo sapiens GN=RPS9 PE=2 SV=1	13210	434	0	40	0	0.58	0	0	1	0	2	0	2	0	2	0	-3.11231
tr|D3DVQ1|D3DVQ1_HUMAN	LETM1	Leucine zipper-EF-hand containing transmembrane protein 1, isoform CRA_a OS=Homo sapiens GN=LETM1 PE=2 SV=1	63730	463	0	37	0	0.11	0	0	1	0	2	0	2	0	4	0	-3.11231
tr|A0PJ76|A0PJ76_HUMAN	NCAPD2	NCAPD2 protein (Fragment) OS=Homo sapiens GN=NCAPD2 PE=2 SV=1	44957	494	0	34	0	0.15	0	0	0	0	2	0	2	0	2	0	-3.11231
tr|B3KY12|B3KY12_HUMAN	tr|B3KY12	cDNA FLJ46581 fis, clone THYMU3043200, highly similar to Splicing factor 3A subunit 3 OS=Homo sapiens PE=2 SV=1	59082	495	0	34	0	0.18	0	0	1	0	3	0	2	0	3	0	-3.11231
tr|C1KES1|C1KES1_HUMAN	LRRC16B	LRRC16B (Fragment) OS=Homo sapiens GN=LRRC16B PE=4 SV=1	4125	530	0	31	0	0.94	0	0	0	0	1	0	2	0	2	0	-3.11231
tr|B4DXI8|B4DXI8_HUMAN	PSMD7	26S proteasome non-ATPase regulatory subunit 7 OS=Homo sapiens GN=PSMD7 PE=2 SV=1	28337	550	0	30	0	0.19	0	0	0	0	2	0	2	0	2	0	-3.11231
tr|B4DJW8|B4DJW8_HUMAN	tr|B4DJW	cDNA FLJ55697, highly similar to Protein transport protein Sec23B OS=Homo sapiens PE=2 SV=1	85347	558	0	29	0	0.08	0	0	0	0	2	0	2	0	3	0	-3.11231
sp|O75367|H2AY_HUMAN	H2AFY	Core histone macro-H2A.1 OS=Homo sapiens GN=H2AFY PE=1 SV=4	39764	565	0	29	0	0.08	0	0	0	0	1	0	2	0	2	0	-3.11231
tr|A6NLN1|A6NLN1_HUMAN	PTBP1	Polypyrimidine tract binding protein 1, isoform CRA_e OS=Homo sapiens GN=PTBP1 PE=4 SV=3	21899	567	0	26	0	0.18	0	0	0	0	2	0	2	0	2	0	-3.11231
tr|H3BNV2|H3BNV2_HUMAN	NQO1	NAD(P)H dehydrogenase [quinone] 1 OS=Homo sapiens GN=NQO1 PE=4 SV=1	28533	576	0	28	0	0.25	0	0	0	0	2	0	2	0	2	0	-3.11231
sp|Q9UHR4|BI2L1_HUMAN	BAIAP2L1	Brain-specific angiogenesis inhibitor 1-associated protein 2-like protein 1 OS=Homo sapiens GN=BAIAP2L1 PE=1 SV=2	57189	613	0	24	0	0.12	0	0	0	0	2	0	2	0	4	0	-3.11231
sp|P25490|TYY1_HUMAN	YY1	Transcriptional repressor protein YY1 OS=Homo sapiens GN=YY1 PE=1 SV=2	45141	630	0	23	0	0.07	0	0	0	0	1	0	2	0	2	0	-3.11231
tr|I0BW39|I0BW39_HUMAN	HLA-A	MHC class I antigen (Fragment) OS=Homo sapiens GN=HLA-A PE=4 SV=1	8492	640	0	22	0	0.16	0	0	0	0	1	0	2	0	2	0	-3.11231
sp|P35237|SPB6_HUMAN	SERPINB6	Serpin B6 OS=Homo sapiens GN=SERPINB6 PE=1 SV=3	42936	120	0	179	0	0.25	0	0	3	0	3	0	3	0	3	0	-3.59808
tr|C9JWY7|C9JWY7_HUMAN	CAPN2	Calpain-2 catalytic subunit (Fragment) OS=Homo sapiens GN=CAPN2 PE=2 SV=1	14668	173	0	48	0	0.22	0	0	0	0	4	0	3	0	4	0	-3.59808
tr|B4DMU0|B4DMU0_HUMAN	PYCR1	Pyrroline-5-carboxylate reductase OS=Homo sapiens GN=PYCR1 PE=2 SV=1	36186	174	0	125	0	0.19	0	0	2	0	2	0	3	0	3	0	-3.59808
tr|H0YAF8|H0YAF8_HUMAN	GNB2L1	Guanine nucleotide-binding protein subunit beta-2-like 1 (Fragment) OS=Homo sapiens GN=GNB2L1 PE=4 SV=1	22373	216	0	96	0	0.52	0	0	2	0	3	0	3	0	4	0	-3.59808
tr|Q5JQZ9|Q5JQZ9_HUMAN	CDC2L6	Cell division cycle 2-like 6 (CDK8-like) (Fragment) OS=Homo sapiens GN=CDC2L6 PE=4 SV=1	29552	285	0	65	0	0.24	0	0	2	0	2	0	3	0	3	0	-3.59808
tr|Q0VGL3|Q0VGL3_HUMAN	RPL13A	RPL13A protein OS=Homo sapiens GN=RPL13A PE=2 SV=1	16706	300	0	59	0	0.74	0	0	3	0	3	0	3	0	3	0	-3.59808
tr|B4DDR2|B4DDR2_HUMAN	tr|B4DDR	cDNA FLJ54588, highly similar to Major vault protein OS=Homo sapiens PE=2 SV=1	98526	304	0	59	0	0.07	0	0	2	0	2	0	3	0	3	0	-3.59808
sp|Q13151|ROA0_HUMAN	HNRNPA0	Heterogeneous nuclear ribonucleoprotein A0 OS=Homo sapiens GN=HNRNPA0 PE=1 SV=1	30993	323	0	55	0	0.36	0	0	2	0	3	0	3	0	3	0	-3.59808
sp|Q7RTS7|K2C74_HUMAN	KRT74	Keratin, type II cytoskeletal 74 OS=Homo sapiens GN=KRT74 PE=1 SV=2	58229	373	0	47	0	0.12	0	0	2	0	3	0	3	0	5	0	-3.59808
tr|B2R6J4|B2R6J4_HUMAN	tr|B2R6J4	cDNA, FLJ92975, highly similar to Homo sapiens nucleosome assembly protein 1-like 4 (NAP1L4), mRNA OS=Homo sapiens PE=2 SV=	42940	417	0	42	0	0.16	0	0	0	0	2	0	3	0	3	0	-3.59808
tr|B3KTM9|B3KTM9_HUMAN	tr|B3KTM	cDNA FLJ38511 fis, clone HCHON2000417, highly similar to FASCIN OS=Homo sapiens PE=2 SV=1	24132	53	0	188	0	1.07	0	0	4	0	5	0	4	0	7	0	-3.96099
tr|D6RAT0|D6RAT0_HUMAN	RPS3A	40S ribosomal protein S3a OS=Homo sapiens GN=RPS3A PE=3 SV=1	26099	223	0	93	0	0.44	0	0	2	0	3	0	4	0	5	0	-3.96099

tr|E9PKU4|E9PKU4_HUMAN	RPL8	60S ribosomal protein L8 (Fragment) OS=Homo sapiens GN=RPL8 PE=2 SV=1	25806	231	0	90	0	0.28	0	0	2	0	2	0	4	0	5	0	-3.96099
sp|P21796|VDAC1_HUMAN	VDAC1	Voltage-dependent anion-selective channel protein 1 OS=Homo sapiens GN=VDAC1 PE=1 SV=2	30868	250	0	78	0	0.36	0	0	1	0	3	0	4	0	4	0	-3.96099
sp|Q14CN4|K2C72_HUMAN	KRT72	Keratin, type II cytoskeletal 72 OS=Homo sapiens GN=KRT72 PE=1 SV=2	56470	372	0	47	0	0.12	0	0	2	0	2	0	4	0	5	0	-3.96099
sp|A2RTY3|CQ066_HUMAN	C17orf66	Uncharacterized protein C17orf66 OS=Homo sapiens GN=C17orf66 PE=2 SV=2	66437	477	0	36	0	0.05	0	0	0	0	1	0	4	0	8	0	-3.96099
tr|F8VQP2|F8VQP2_HUMAN	ATXN2	Ataxin-2 OS=Homo sapiens GN=ATXN2 PE=2 SV=1	117012	157	0	135	0	0.21	0	0	4	0	7	0	10	0	10	0	-5.18542
sp|Q96RW7|HMCN1_HUMAN	HMCN1	Hemicentin-1 OS=Homo sapiens GN=HMCN1 PE=1 SV=2	623265	291	0	63	0	0.01	0	0	7	0	7	0	11	0	16	0	-5.317
tr|B3KPL5|B3KPL5_HUMAN	tr|B3KPL5	cDNA FLJ31917 fis, clone NT2RP7004925, highly similar to Sprouty-related, EVH1 domain-containing protein 2 OS=Homo sapiens PE	48711	657	0	21	0	0.07	0	0	0	0	1	0	16	0	51	0	-5.83953

#06	SHSY-5Y live unsyn PU-1_2µM																		
#08	SHSY-5Y live unsyn NP_2µM																		
Accession	GS	Name	Pro_Mass	08_prot_hi	06_prot_hi	08_prot_sc	06_prot_sc	08_emPAI	06_emPAI	emPAI rati	08_No>I	06_No>I	08_No>HI	06_No>HI	08_No>20	06_No>20	08_No=All	06_No=All	Rsc
sp|Q09013|DMPK_HUMAN	DMPK	Myotonin-protein kinase OS=Homo sapiens GN=DMPK PE=1 SV=3	69854	0	769	0	33	0	0.05	#DIV/0!	0	0	0	1	0	1	0	1	2.244499
sp|Q16512|PKN1_HUMAN	PKN1	Serine/threonine-protein kinase N1 OS=Homo sapiens GN=PKN1 PE=1 SV=2	104666	0	610	0	46	0	0.03	#DIV/0!	0	1	0	1	0	1	0	2	2.244499
sp|P17612|KAPCA_HUMAN	PRKACA	cAMP-dependent protein kinase catalytic subunit alpha OS=Homo sapiens GN=PRKACA PE=1 SV=2	40678	0	710	0	37	0	0.08	#DIV/0!	0	1	0	1	0	2	0	2	2.981786
sp|Q05655|KPCD_HUMAN	PRKCD	Protein kinase C delta type OS=Homo sapiens GN=PRKCD PE=1 SV=2	78652	0	535	0	53	0	0.05	#DIV/0!	0	1	0	1	0	1	0	1	2.244499
sp|P51812|KS6A3_HUMAN	RPS6KA3	Ribosomal protein S6 kinase alpha-3 OS=Homo sapiens GN=RPS6KA3 PE=1 SV=1	84025	0	401	0	71	0	0.04	#DIV/0!	0	1	0	1	0	1	0	1	2.244499
sp|P27361|MK03_HUMAN	MAPK3	Mitogen-activated protein kinase 3 OS=Homo sapiens GN=MAPK3 PE=1 SV=4	43450	0	531	0	53	0	0.18	#DIV/0!	0	1	0	2	0	2	0	2	2.981786
sp|P06493|CDK1_HUMAN	CDK1	Cyclin-dependent kinase 1 OS=Homo sapiens GN=CDK1 PE=1 SV=3	34131	918	586	35	30	0.15	0.2	1.333333	1	0	1	1	1	1	1	1	0.659333
sp|P21127|CD11B_HUMAN	CDK11B	Cyclin-dependent kinase 11B OS=Homo sapiens GN=CDK11B PE=1 SV=3	92821	0	742	0	35	0	0.04	#DIV/0!	0	1	0	1	0	1	0	1	2.244499
sp|P28482|MK01_HUMAN	MAPK1	Mitogen-activated protein kinase 1 OS=Homo sapiens GN=MAPK1 PE=1 SV=3	41762	0	533	0	53	0	0.16	#DIV/0!	0	1	0	2	0	2	0	2	2.981786
sp|Q96KB5|TOPK_HUMAN	PBK	Lymphokine-activated killer T-cell-originated protein kinase OS=Homo sapiens GN=PBK PE=1 SV=3	36404	0	974	0	20	0	0.09	#DIV/0!	0	0	0	1	0	1	0	1	2.244499
sp|P55263|ADK_HUMAN	ADK	Adenosine kinase OS=Homo sapiens GN=ADK PE=1 SV=2	40919	0	972	0	20	0	0.08	#DIV/0!	0	0	0	1	0	1	0	1	2.244499
sp|Q53H12|AGK_HUMAN	AGK	Acylglycerol kinase, mitochondrial OS=Homo sapiens GN=AGK PE=1 SV=2	47564	747	700	48	38	0.07	0.07	1	1	1	1	1	1	2	2	2	1.39662
sp|Q96M32|KAD7_HUMAN	AK7	Adenylate kinase 7 OS=Homo sapiens GN=AK7 PE=1 SV=3	83120	0	948	0	22	0	0.04	#DIV/0!	0	0	0	1	0	2	0	2	2.981786
sp|O75969|AKAP3_HUMAN	AKAP3	A-kinase anchor protein 3 OS=Homo sapiens GN=AKAP3 PE=1 SV=2	95433	1196	0	17	0	0.18	0	0	0	0	1	0	0	0	1	0	0.659215
sp|P12277|KCRB_HUMAN	CKB	Creatine kinase B-type OS=Homo sapiens GN=CKB PE=1 SV=1	42902	39	27	798	715	1.82	2.04	1.120879	15	15	22	28	27	28	29	32	0.714266
sp|P52789|HXK2_HUMAN	HK2	Hexokinase-2 OS=Homo sapiens GN=HK2 PE=1 SV=2	103739	0	657	0	42	0	0.06	#DIV/0!	0	1	0	2	0	2	0	2	2.981786
sp|P17858|K6PL_HUMAN	PFKL	6-phosphofructokinase, liver type OS=Homo sapiens GN=PFKL PE=1 SV=6	85762	685	385	53	76	0.04	0.12	3	1	2	1	3	2	3	3	3	1.145199
sp|P08237|K6PF_HUMAN	PFKM	6-phosphofructokinase, muscle type OS=Homo sapiens GN=PFKM PE=1 SV=2	85984	549	811	68	31	0.39	0.03	0.076923	1	1	2	1	2	2	2	2	0.65945
sp|P00558|PGK1_HUMAN	PGK1	Phosphoglycerate kinase 1 OS=Homo sapiens GN=PGK1 PE=1 SV=3	44985	119	111	334	250	1.03	0.84	0.815534	8	6	10	9	13	9	13	10	0.152502
sp|P14618|KPYM_HUMAN	PKM	Pyruvate kinase isozymes M1/M2 OS=Homo sapiens GN=PKM PE=1 SV=4	58470	8	17	1592	1023	6.17	3.15	0.510535	35	24	53	44	55	41	58	47	0.241838
sp|P67775|PP2AA_HUMAN	PPP2CA	Serine/threonine-protein phosphatase 2A catalytic subunit alpha isoform OS=Homo sapiens GN=PPP2CA PE=1 SV=1	36142	1038	834	28	29	0.12	0.25	2.083333	0	0	1	2	1	2	2	2	1.39662
sp|P49137|MAPK2_HUMAN	MAPKAPK2	MAP kinase-activated protein kinase 2 OS=Homo sapiens GN=MAPKAPK2 PE=1 SV=1	45938	528	725	72	36	0.07	0.07	1	1	1	1	1	1	1	1	1	0.659333
sp|Q99986|VRK1_HUMAN	VRK1	Serine/threonine-protein kinase VRK1 OS=Homo sapiens GN=VRK1 PE=1 SV=1	45790	830	819	41	30	0.12	0.12	1	1	0	1	1	1	1	1	1	0.659333
sp|Q96SB4|SRPK1_HUMAN	SRPK1	SRSF protein kinase 1 OS=Homo sapiens GN=SRPK1 PE=1 SV=2	74963	407	659	100	42	0.13	0.09	0.692308	2	2	3	2	3	3	4	3	0.659568
sp|O14976|GAK_HUMAN	GAK	Cyclin-G-associated kinase OS=Homo sapiens GN=GAK PE=1 SV=2	144583	1195	0	18	0	0.02	0	0	0	0	1	0	0	0	1	0	0.659215
sp|P33981|TTK_HUMAN	TTK	Dual specificity protein kinase TTK OS=Homo sapiens GN=TTK PE=1 SV=2	97980	1102	726	24	36	0.03	0.03	1	0	0	1	1	2	2	2	2	0.65945
sp|O43318|M3K7_HUMAN	MAP3K7	Mitogen-activated protein kinase kinase kinase 7 OS=Homo sapiens GN=MAP3K7 PE=1 SV=1	67895	1203	0	16	0	0.07	0	0	0	0	1	0	0	0	1	0	0.659215
sp|Q7Z2Y5|NRK_HUMAN	NRK	Nik-related protein kinase OS=Homo sapiens GN=NRK PE=1 SV=2	179509	989	0	0	0	0.02	0	0	0	0	0	0	0	0	1	0	0.659215
sp|Q9NQU5|PAK6_HUMAN	PAK6	Serine/threonine-protein kinase PAK 6 OS=Homo sapiens GN=PAK6 PE=1 SV=1	75335	1186	0	18	0	0.28	0	0	0	0	1	0	0	0	1	0	0.659215
tr|F8TLS5|F8TLS5_HUMAN	RET	Ret proto-oncogene tyrosine-protein kinase receptor isoform a OS=Homo sapiens GN=RET PE=3 SV=1	126195	0	781	0	33	0	0.03	#DIV/0!	0	1	0	1	0	1	0	2	2.244499
tr|B3KXR2|B3KXR2_HUMAN	tr|B3KXR2	cDNA FLJ45906 fis, clone OCBBF3026979, highly similar to Protein kinase C-binding protein NELL1 OS=Homo sapiens PE=2 SV=1	97850	0	552	0	51	0	0.03	#DIV/0!	0	1	0	1	0	1	0	1	2.244499
sp|P29966|MARCS_HUMAN	MARCKS	Myristoylated alanine-rich C-kinase substrate OS=Homo sapiens GN=MARCKS PE=1 SV=4	31707	483	405	80	71	0.22	0.51	2.318182	1	2	2	2	2	3	3	3	1.145199
tr|C1PHA0|C1PHA0_HUMAN	EML4-ALK	Tyrosine-protein kinase receptor OS=Homo sapiens GN=EML4-ALK variant 6 PE=2 SV=1	120561	971	0	0	0	0.03	0	0	0	0	0	0	0	0	2	0	0.659215
sp|Q8WTR2|DUS19_HUMAN	DUSP19	Dual specificity protein phosphatase 19 OS=Homo sapiens GN=DUSP19 PE=1 SV=1	24521	0	987	0	19	0	0.14	#DIV/0!	0	0	0	1	0	0	0	1	0.659215
tr|H0YHI8|H0YHI8_HUMAN	PPP1R12A	Protein phosphatase 1 regulatory subunit 12A (Fragment) OS=Homo sapiens GN=PPP1R12A PE=4 SV=1	40591	0	879	0	26	0	0.08	#DIV/0!	0	0	0	1	0	1	0	1	2.244499
sp|A6NDG6|PGP_HUMAN	PGP	Phosphoglycolate phosphatase OS=Homo sapiens GN=PGP PE=1 SV=1	34441	0	807	0	31	0	0.1	#DIV/0!	0	0	0	1	0	1	0	1	2.244499
tr|B2R665|B2R665_HUMAN	tr|B2R665	cDNA, FLJ92810, highly similar to Homo sapiens protein phosphatase 1G (formerly 2C), magnesium-dependent, gamma isoform (PPM1G), r	59889	858	178	39	176	0.05	0.17	3.4	1	3	1	3	2	3	3	4	1.145199
sp|Q06124|PTN11_HUMAN	PTPN11	Tyrosine-protein phosphatase non-receptor type 11 OS=Homo sapiens GN=PTPN11 PE=1 SV=2	68964	852	443	40	66	0.1	0.1	1	1	1	2	2	2	3	2	3	1.145199
sp|Q15181|IPYR_HUMAN	PPA1	Inorganic pyrophosphatase OS=Homo sapiens GN=PPA1 PE=1 SV=2	33095	414	315	99	94	0.61	0.33	0.540984	2	2	5	3	5	4	5	5	0.369976
sp|Q8NCN5|PDPR_HUMAN	PDPR	Pyruvate dehydrogenase phosphatase regulatory subunit, mitochondrial OS=Homo sapiens GN=PDPR PE=1 SV=2	100156	1200	0	17	0	0.03	0	0	0	0	1	0	0	0	2	0	0.659215
tr|F5H5M9|F5H5M9_HUMAN	TNIK	TRAF2 and NCK-interacting protein kinase OS=Homo sapiens GN=TNIK PE=2 SV=1	150042	989	751	31	0	0.02	0.02	1	1	0	1	0	1	0	5	2	-0.92595
sp|Q9Y6E0|STK24_HUMAN	STK24	Serine/threonine-protein kinase 24 OS=Homo sapiens GN=STK24 PE=1 SV=1	49562	479	400	81	45	0.17	0.14	0.823529	2	1	2	1	2	1	2	1	-0.07784
tr|B3KS15|B3KS15_HUMAN	tr|B3KS15	6-phosphofructokinase OS=Homo sapiens PE=2 SV=1	86526	237	208	168	104	0.2	0.16	0.8	4	2	5	3	6	3	6	3	-0.23413
tr|B4DZC9|B4DZC9_HUMAN	tr|B4DZC9	cDNA FLJ61681, highly similar to STE20-like serine/threonine-protein kinase (EC2.7.11.1) (Fragment) OS=Homo sapiens PE=2 SV=1	120359	667	415	54	36	0.08	0.05	0.625	1	0	3	1	5	1	6	3	-1.21595
tr|J3KPD9|J3KPD9_HUMAN	NME2	Nucleoside diphosphate kinase B OS=Homo sapiens GN=NME2 PE=3 SV=1	22522	903	583	36	49	0.32	0.19	0.59375	0	1	2	2	2	1	2	2	-0.07784
sp|P78527|PRKDC_HUMAN	PRKDC	DNA-dependent protein kinase catalytic subunit OS=Homo sapiens GN=PRKDC PE=1 SV=3	473749	9	23	1588	742	0.58	0.28	0.482759	43	22	68	37	73	40	83	48	-0.20263
tr|B0AZV0|B0AZV0_HUMAN	STRAP	Serine-threonine kinase receptor-associated protein OS=Homo sapiens GN=STRAP PE=2 SV=1	28716	412	420	100	69	0.55	0.25	0.454545	2	2	4	2	4	2	4	2	-0.18895
tr|B2R6T7|B2R6T7_HUMAN	tr|B2R6T7	Ribose-phosphate pyrophosphokinase OS=Homo sapiens PE=2 SV=1	35181	374	627	108	44	0.88	0.2	0.227273	3	1	7	2	8	2	8	3	-1.10731
tr|B3KXY9|B3KXY9_HUMAN	tr|B3KXY9	cDNA FLJ46359 fis, clone TESTI4049786, highly similar to Hexokinase-1 (EC 2.7.1.1) OS=Homo sapiens PE=2 SV=1	107381	336	607	119	46	0.16	0.03	0.1875	4	1	5	1	7	2	8	3	-0.92653
tr|C1PHA2|C1PHA2_HUMAN	KIF5B-ALK	Tyrosine-protein kinase receptor OS=Homo sapiens GN=KIF5B-ALK PE=2 SV=1	169283	168	748	211	34	0.34	0.04	0.117647	6	1	9	2	11	1	13	3	-2.2813
tr|Q16444|Q16444_HUMAN	tr|Q16444	Phosphoglycerate kinase (Fragment) OS=Homo sapiens PE=2 SV=1	5221	119	0	0	0	1.03	0	0	0	0	0	0	1	0	1	0	-0.92595
tr|O14943|O14943_HUMAN	PFK-P	Phosphofructokinase-P (Fragment) OS=Homo sapiens GN=PFK-P PE=2 SV=1	45974	237	0	97	0	0.2	0	0	3	0	4	0	5	0	5	0	-2.80123
tr|B1AR63|B1AR63_HUMAN	HK1	Hexokinase-1 (Fragment) OS=Homo sapiens GN=HK1 PE=4 SV=1	33098	336	0	49	0	0.16	0	0	1	0	1	0	2	0	2	0	-1.66312
sp|Q9H4A3|WNK1_HUMAN	WNK1	Serine/threonine-protein kinase WNK1 OS=Homo sapiens GN=WNK1 PE=1 SV=2	251552	404	0	101	0	0.03	0	0	2	0	2	0	2	0	2	0	-1.66312
tr|D6RBF8|D6RBF8_HUMAN	SRPK1	SRSF protein kinase 1 (Fragment) OS=Homo sapiens GN=SRPK1 PE=2 SV=1	16752	407	0	93	0	0.13	0	0	2	0	2	0	2	0	2	0	-1.66312
tr|H0YH33|H0YH33_HUMAN	STRAP	Serine-threonine kinase receptor-associated protein (Fragment) OS=Homo sapiens GN=STRAP PE=4 SV=1	10763	412	0	53	0	0.55	0	0	1	0	2	0	2	0	2	0	-1.66312
tr|Q5JV98|Q5JV98_HUMAN	STK24	Serine/threonine-protein kinase 24 12 kDa subunit (Fragment) OS=Homo sapiens GN=STK24 PE=4 SV=1	23284	479	0	42	0	0.17	0	0	1	0	1	0	1	0	1	0	-0.92595
tr|D3YTJ7|D3YTJ7_HUMAN	PRPS2	Ribose-phosphate pyrophosphokinase 2 OS=Homo sapiens GN=PRPS2 PE=2 SV=1	22419	532	0	48	0	0.43	0	0	2	0	3	0	3	0	3	0	-2.14875
tr|F8VP00|F8VP00_HUMAN	PFKM	6-phosphofructokinase, muscle type (Fragment) OS=Homo sapiens GN=PFKM PE=2 SV=1	18709	549	0	68	0	0.39	0	0	1	0	2	0	2	0	2	0	-1.66312
tr|G1UI32|G1UI32_HUMAN	PDXK	Pyridoxal kinase (Fragment) OS=Homo sapiens GN=PDXK PE=2 SV=1	19070	567	0	65	0	0.38	0	0	1	0	2	0	2	0	2	0	-1.66312
tr|B4E1G6|B4E1G6_HUMAN	GALK1	Galactokinase OS=Homo sapiens GN=GALK1 PE=2 SV=1	45956	597	0	62	0	0.15	0	0	1	0	2	0	2	0	2	0	-1.66312
tr|B4E108|B4E108_HUMAN	tr|B4E108	cDNA FLJ61026, highly similar to Homo sapiens phosphofructokinase, liver (PFKL), transcript variant 1, mRNA OS=Homo sapiens PE=2 SV=1	27257	685	0	53	0	0.04	0	0	1	0	1	0	1	0	1	0	-0.92595
tr|B4DR72|B4DR72_HUMAN	tr|B4DR72	cDNA FLJ59963, highly similar to Homo sapiens multiple substrate lipid kinase (MULK), mRNA OS=Homo sapiens PE=2 SV=1	27090	747	0	48	0	0.07	0	0	1	0	1	0	1	0	1	0	-0.92595
tr|A8KAH7|A8KAH7_HUMAN	tr|A8KAH7	cDNA FLJ75444, highly similar to Homo sapiens protein kinase, cAMP-dependent, regulatory, type II, alpha (PRKAR2A), mRNA OS=Homo sap	45818	775	0	45	0	0.23	0	0	1	0	3	0	5	0	6	0	-2.80123
sp|Q12802|AKP13_HUMAN	AKAP13	A-kinase anchor protein 13 OS=Homo sapiens GN=AKAP13 PE=1 SV=2	310381	842	0	40	0	0.01	0	0	1	0	1	0	2	0	5	0	-1.66312
tr|H0Y3C5|H0Y3C5_HUMAN	HCK	Tyrosine-protein kinase HCK OS=Homo sapiens GN=HCK PE=4 SV=1	59986	905	0	36	0	0.05	0	0	1	0	1	0	1	0	1	0	-0.92595
tr|C9JIG9|C9JIG9_HUMAN	OXSR1	Serine/threonine-protein kinase OSR1 OS=Homo sapiens GN=OXSR1 PE=2 SV=1	52109	926	0	35	0	0.06	0	0	1	0	1	0	1	0	1	0	-0.92595
tr|B2R841|B2R841_HUMAN	tr|B2R841	cDNA, FLJ93730, highly similar to Homo sapiens polo-like kinase (Drosophila) (PLK), mRNA OS=Homo sapiens PE=2 SV=1	68963	967	0	33	0	0.05	0	0	0	0	1	0	1	0	2	0	-0.92595
tr|B3KS97|B3KS97_HUMAN	MAP2K2	Mitogen-activated protein kinase kinase 2, isoform CRA_d OS=Homo sapiens GN=MAP2K2 PE=2 SV=1	23450	1001	0	31	0	0.14	0	0	0	0	1	0	1	0	1	0	-0.92595
tr|B4E1D9|B4E1D9_HUMAN	tr|B4E1D9	cDNA FLJ55318, highly similar to Casein kinase I isoform alpha (EC 2.7.11.1) OS=Homo sapiens PE=2 SV=1	26899	1002	0	31	0	0.26	0	0	1	0	2	0	3	0	3	0	-2.14875
tr|B4DIR9|B4DIR9_HUMAN	TAB2	TGF-beta-activated kinase 1 and MAP3K7-binding protein 2 OS=Homo sapiens GN=TAB2 PE=2 SV=1	73273	1010	0	30	0	0.04	0	0	0	0	1	0	1	0	1	0	-0.92595
tr|Q5MD60|Q5MD60_HUMAI	MAP4K4	Mitogen-activated protein kinase kinase kinase kinase 4 isoform OS=Homo sapiens GN=MAP4K4 PE=2 SV=1	119845	1026	0	22	0	0.05	0	0	0	0	1	0	1	0	2	0	-0.92595
tr|B4DV85|B4DV85_HUMAN	tr|B4DV85	cDNA FLJ53211, highly similar to A-Raf proto-oncogene serine/threonine-protein kinase (EC 2.7.11.1) OS=Homo sapiens PE=2 SV=1	52561	1044	0	28	0	0.06	0	0	0	0	1	0	1	0	1	0	-0.92595
tr|H0YFG1|H0YFG1_HUMAN	EPS8	Epidermal growth factor receptor kinase substrate 8 (Fragment) OS=Homo sapiens GN=EPS8 PE=4 SV=1	7139	1149	0	21	0	0.5	0	0	0	0	1	0	1	0	1	0	-0.92595
sp|Q96GX5|GWL_HUMAN	MASTL	Serine/threonine-protein kinase greatwall OS=Homo sapiens GN=MASTL PE=1 SV=1	98398	1163	0	21	0	0.03	0	0	0	0	1	0	1	0	1	0	-0.92595
tr|E7ETY4|E7ETY4_HUMAN	MARK2	Serine/threonine-protein kinase MARK2 OS=Homo sapiens GN=MARK2 PE=2 SV=1	78803	1164	0	21	0	0.04	0	0	0	0	1	0	1	0	2	0	-0.92595
tr|B4DDF7|B4DDF7_HUMAN	tr|B4DDF7	cDNA FLJ53296, highly similar to Serine/threonine-protein phosphatase 2A 65 kDa regulatory subunit A alpha isoform OS=Homo sapiens PE	64939	140	308	279	98	0.81	0.34	0.419753	7	2	12	6	13	4	17	8	-0.92711
sp|P62140|PP1B_HUMAN	PPP1CB	Serine/threonine-protein phosphatase PP1-beta catalytic subunit OS=Homo sapiens GN=PPP1CB PE=1 SV=3	37961	225	901	176	25	0.52	0.07	0.134615	3	0	5	1	6	1	6	2	-1.45716
tr|H0Y3Y6|H0Y3Y6_HUMAN	PPP1CB	Serine/threonine-protein phosphatase PP1-beta catalytic subunit (Fragment) OS=Homo sapiens GN=PPP1CB PE=4 SV=1	10086	225	0	68	0	0.52	0	0	1	0	1	0	1	0	1	0	-0.92595
tr|H0YDU8|H0YDU8_HUMAN	PPP5C	Serine/threonine-protein phosphatase (Fragment) OS=Homo sapiens GN=PPP5C PE=3 SV=1	55743	504	0	76	0	0.06	0	0	1	0	1	0	1	0	1	0	-0.92595
tr|H0YEN2|H0YEN2_HUMAN	PPP6R3	Serine/threonine-protein phosphatase 6 regulatory subunit 3 (Fragment) OS=Homo sapiens GN=PPP6R3 PE=4 SV=1	64367	663	0	54	0	0.1	0	0	1	0	2	0	2	0	2	0	-1.66312
tr|A8K6Y2|A8K6Y2_HUMAN	tr|A8K6Y2	cDNA FLJ76895, highly similar to Homo sapiens protein phosphatase 2, regulatory subunit B (B56), delta isoform (PPP2R5D), transcript varia	70276	733	0	49	0	0.05	0	0	1	0	1	0	1	0	2	0	-0.92595
tr|H0YF12|H0YF12_HUMAN	PTPN11	Tyrosine-protein phosphatase non-receptor type 11 (Fragment) OS=Homo sapiens GN=PTPN11 PE=4 SV=1	12387	812	0	43	0	0.28	0	0	1	0	1	0	1	0	1	0	-0.92595
tr|Q59GB2|Q59GB2_HUMAN	tr|Q59GB2	Protein phosphatase 1G variant (Fragment) OS=Homo sapiens PE=2 SV=1	38757	858	0	0	0	0.05	0	0	0	0	0	0	1	0	2	0	-0.92595
tr|E5RFR9|E5RFR9_HUMAN	PPP2R2A	Serine/threonine-protein phosphatase 2A 55 kDa regulatory subunit B alpha isoform (Fragment) OS=Homo sapiens GN=PPP2R2A PE=2 SV=	23448	871	0	38	0	0.14	0	0	1	0	1	0	1	0	1	0	-0.92595
tr|J3QT22|J3QT22_HUMAN	PPME1	Protein phosphatase methylesterase 1 OS=Homo sapiens GN=PPME1 PE=3 SV=1	44241	955	0	33	0	0.07	0	0	1	0	1	0	1	0	1	0	-0.92595
tr|H0YBN9|H0YBN9_HUMAN	PPP2CB	Serine/threonine-protein phosphatase 2A catalytic subunit beta isoform (Fragment) OS=Homo sapiens GN=PPP2CB PE=4 SV=1	6207	1038	0	28	0	0.12	0	0	0	0	1	0	1	0	1	0	-0.92595
sp|O75170|PP6R2_HUMAN	PPP6R2	Serine/threonine-protein phosphatase 6 regulatory subunit 2 OS=Homo sapiens GN=PPP6R2 PE=1 SV=2	106358	1120	0	23	0	0.03	0	0	1	0	1	0	1	0	1	0	-0.92595
tr|Q8HWM6|Q8HWM6_HUM	HLA-B	MHC class I antigen (Fragment) OS=Homo sapiens GN=HLA-B PE=3 SV=1	18457	0	1021	0	16	0	0.18	#DIV/0!	0	0	0	1	0	0	0	1	0.659215
tr|H3BRB3|H3BRB3_HUMAN	KIF22	Kinesin-like protein KIF22 OS=Homo sapiens GN=KIF22 PE=3 SV=1	67996	0	1020	0	16	0	0.05	#DIV/0!	0	0	0	1	0	0	0	1	0.659215
tr|Q8N2F8|Q8N2F8_HUMAN	tr|Q8N2F8	cDNA PSEC0195 fis, clone HEMBA1001322, highly similar to ALPHA-ADAPTIN C OS=Homo sapiens PE=2 SV=1	52100	0	1019	0	16	0	0.06	#DIV/0!	0	0	0	1	0	0	0	2	0.659215
tr|J3QKM0|J3QKM0_HUMAN	ARHGAP28	Rho GTPase-activating protein 28 (Fragment) OS=Homo sapiens GN=ARHGAP28 PE=4 SV=1	23255	0	1018	0	16	0	0.14	#DIV/0!	0	0	0	1	0	0	0	2	0.659215
sp|Q92743|HTRA1_HUMAN	HTRA1	Serine protease HTRA1 OS=Homo sapiens GN=HTRA1 PE=1 SV=1	52167	0	1013	0	17	0	0.06	#DIV/0!	0	0	0	1	0	0	0	1	0.659215
sp|Q6P1J9|CDC73_HUMAN	CDC73	Parafibromin OS=Homo sapiens GN=CDC73 PE=1 SV=1	60653	0	1012	0	17	0	0.05	#DIV/0!	0	0	0	1	0	0	0	1	0.659215
tr|G3V4Z4|G3V4Z4_HUMAN	ALDH6A1	Methylmalonate-semialdehyde dehydrogenase [acylating], mitochondrial OS=Homo sapiens GN=ALDH6A1 PE=2 SV=1	27179	0	1010	0	0	0	0.06	#DIV/0!	0	0	0	0	0	0	0	1	0.659215
sp|P31994|FCG2B_HUMAN	FCGR2B	Low affinity immunoglobulin gamma Fc region receptor II-b OS=Homo sapiens GN=FCGR2B PE=1 SV=2	34421	0	1008	0	17	0	0.1	#DIV/0!	0	0	0	1	0	0	0	1	0.659215
tr|B4DZ70|B4DZ70_HUMAN	ME1	Malic enzyme OS=Homo sapiens GN=ME1 PE=2 SV=1	55701	0	1006	0	17	0	0.07	#DIV/0!	0	0	0	1	0	1	0	2	2.244499
tr|B2RAR2|B2RAR2_HUMAN	tr|B2RAR2	cDNA, FLJ95064, highly similar to Homo sapiens nin one binding protein (NOB1P), mRNA OS=Homo sapiens PE=2 SV=1	47015	0	1005	0	17	0	0.07	#DIV/0!	0	0	0	1	0	0	0	1	0.659215
tr|A8K2P6|A8K2P6_HUMAN	tr|A8K2P6	cDNA FLJ76083, highly similar to Homo sapiens zinc finger protein 503 (ZNF503), mRNA OS=Homo sapiens PE=2 SV=1	63269	0	1004	0	17	0	0.05	#DIV/0!	0	0	0	1	0	0	0	1	0.659215
tr|B3KME2|B3KME2_HUMAN	tr|B3KME2	cDNA FLJ10772 fis, clone NT2RP4000243, highly similar to Cartilage-associated protein OS=Homo sapiens PE=2 SV=1	47101	0	1003	0	17	0	0.07	#DIV/0!	0	0	0	1	0	0	0	1	0.659215
tr|B4DRJ1|B4DRJ1_HUMAN	tr|B4DRJ1	cDNA FLJ55041, highly similar to 2-hydroxyphytanoyl-CoA lyase (EC 4.1.-.-) OS=Homo sapiens PE=2 SV=1	44949	0	1001	0	18	0	0.07	#DIV/0!	0	0	0	1	0	0	0	1	0.659215
sp|Q2WGJ9|FR1L6_HUMAN	FER1L6	Fer-1-like protein 6 OS=Homo sapiens GN=FER1L6 PE=2 SV=2	210431	0	1000	0	18	0	0.02	#DIV/0!	0	0	0	1	0	1	0	2	2.244499
sp|P54920|SNAA_HUMAN	NAPA	Alpha-soluble NSF attachment protein OS=Homo sapiens GN=NAPA PE=1 SV=3	33667	0	999	0	18	0	0.1	#DIV/0!	0	0	0	1	0	0	0	1	0.659215
tr|B2RE34|B2RE34_HUMAN	tr|B2RE34	cDNA, FLJ96901, highly similar to Homo sapiens Rac GTPase activating protein 1 (RACGAP1), mRNA OS=Homo sapiens PE=2 SV=1	71608	0	998	0	18	0	0.11	#DIV/0!	0	0	0	1	0	0	0	1	0.659215
tr|J3KPZ3|J3KPZ3_HUMAN	MYCBP2	Probable E3 ubiquitin-protein ligase MYCBP2 OS=Homo sapiens GN=MYCBP2 PE=4 SV=1	521463	0	997	0	18	0	0.01	#DIV/0!	0	0	0	1	0	0	0	2	0.659215
tr|H7BXF3|H7BXF3_HUMAN	TRA2B	Transformer-2 protein homolog beta (Fragment) OS=Homo sapiens GN=TRA2B PE=4 SV=1	15221	0	995	0	18	0	0.22	#DIV/0!	0	0	0	1	0	0	0	1	0.659215
tr|H0YET1|H0YET1_HUMAN	PPFIBP2	Liprin-beta-2 (Fragment) OS=Homo sapiens GN=PPFIBP2 PE=4 SV=1	16976	0	994	0	18	0	0.2	#DIV/0!	0	0	0	1	0	0	0	1	0.659215
sp|Q6IBS0|TWF2_HUMAN	TWF2	Twinfilin-2 OS=Homo sapiens GN=TWF2 PE=1 SV=2	39751	0	993	0	19	0	0.08	#DIV/0!	0	0	0	1	0	0	0	1	0.659215
tr|J3KTE2|J3KTE2_HUMAN	DNHD1	Dynein heavy chain domain-containing protein 1 (Fragment) OS=Homo sapiens GN=DNHD1 PE=4 SV=1	22213	0	990	0	19	0	0.15	#DIV/0!	0	0	0	1	0	0	0	1	0.659215
tr|G3V470|G3V470_HUMAN	TEP1	Telomerase protein component 1 OS=Homo sapiens GN=TEP1 PE=2 SV=1	168206	0	989	0	19	0	0.02	#DIV/0!	0	0	0	1	0	0	0	1	0.659215
tr|E5RI52|E5RI52_HUMAN	C5orf45	UPF0544 protein C5orf45 OS=Homo sapiens GN=C5orf45 PE=2 SV=1	21939	0	986	0	19	0	0.15	#DIV/0!	0	0	0	1	0	0	0	1	0.659215
tr|B3KRT1|B3KRT1_HUMAN	tr|B3KRT1	cDNA FLJ34860 fis, clone NT2NE2012790, highly similar to Homo sapiens myo-inositol 1-phosphate synthase A1 (ISYNA1), mRNA OS=Homo	55633	0	983	0	19	0	0.06	#DIV/0!	0	0	0	1	0	0	0	2	0.659215
tr|B3KU09|B3KU09_HUMAN	tr|B3KU09	cDNA FLJ39034 fis, clone NT2RP7008085, highly similar to Homo sapiens ring finger protein 123 (RNF123), mRNA OS=Homo sapiens PE=2 S	149906	0	981	0	20	0	0.02	#DIV/0!	0	0	0	1	0	0	0	1	0.659215
sp|Q3SY69|AL1L2_HUMAN	ALDH1L2	Mitochondrial 10-formyltetrahydrofolate dehydrogenase OS=Homo sapiens GN=ALDH1L2 PE=1 SV=2	102365	0	979	0	20	0	0.03	#DIV/0!	0	0	0	1	0	1	0	1	2.244499
sp|Q14674|ESPL1_HUMAN	ESPL1	Separin OS=Homo sapiens GN=ESPL1 PE=1 SV=3	236564	0	977	0	20	0	0.01	#DIV/0!	0	0	0	1	0	1	0	1	2.244499
tr|B3KPA6|B3KPA6_HUMAN	ACADVL	Acyl-Coenzyme A dehydrogenase, very long chain, isoform CRA_e OS=Homo sapiens GN=ACADVL PE=2 SV=1	62941	0	976	0	20	0	0.05	#DIV/0!	0	0	0	1	0	1	0	1	2.244499
tr|Q2PH61|Q2PH61_HUMAN	HAL	Histidase (Fragment) OS=Homo sapiens GN=HAL PE=2 SV=1	5144	0	975	0	20	0	0.72	#DIV/0!	0	0	0	1	0	1	0	1	2.244499
tr|A4D0Y0|A4D0Y0_HUMAN	LOC402588	Similar to hyaluronoglucosaminidase 1 isoform 1; hyaluronidase 1; tumor suppressor LUCA-1; plasma hyaluronidase OS=Homo sapiens GN=	26365	0	973	0	20	0	0.13	#DIV/0!	0	0	0	1	0	1	0	1	2.244499
tr|B2R657|B2R657_HUMAN	tr|B2R657	Annexin OS=Homo sapiens PE=2 SV=1	52892	0	971	0	20	0	0.06	#DIV/0!	0	0	0	1	0	1	0	1	2.244499
sp|Q9NQ60|EQTN_HUMAN	EQTN	Equatorin OS=Homo sapiens GN=EQTN PE=2 SV=2	32933	0	970	0	21	0	0.1	#DIV/0!	0	0	0	1	0	1	0	1	2.244499
sp|Q8IWL8|STH_HUMAN	STH	Saitohin OS=Homo sapiens GN=STH PE=1 SV=1	13986	0	966	0	21	0	0.24	#DIV/0!	0	0	0	1	0	1	0	1	2.244499
tr|B4DV04|B4DV04_HUMAN	tr|B4DV04	cDNA FLJ56552, highly similar to Signal transducer and activator oftranscription 4 OS=Homo sapiens PE=2 SV=1	79154	0	965	0	21	0	0.04	#DIV/0!	0	0	0	1	0	1	0	2	2.244499
tr|A8K0D4|A8K0D4_HUMAN	tr|A8K0D4	cDNA FLJ78693, highly similar to Homo sapiens TAF6-like RNA polymerase II, p300/CBP-associated factor (PCAF)-associated factor, 65kDa (T	68270	0	964	0	21	0	0.05	#DIV/0!	0	0	0	1	0	1	0	1	2.244499
tr|H0Y2W3|H0Y2W3_HUMAN	FAM35A	Protein FAM35A (Fragment) OS=Homo sapiens GN=FAM35A PE=4 SV=1	63955	0	962	0	21	0	0.05	#DIV/0!	0	0	0	1	0	1	0	1	2.244499
tr|C6JWH3|C6JWH3_HUMAN	CYTB	Cytochrome b (Fragment) OS=Homo sapiens GN=CYTB PE=4 SV=1	2862	0	961	0	21	0	1.32	#DIV/0!	0	0	0	1	0	1	0	1	2.244499
tr|C0JYZ1|C0JYZ1_HUMAN	DNAH11	Dynein, axonemal, heavy chain 11 OS=Homo sapiens GN=DNAH11 PE=2 SV=1	525182	0	959	0	21	0	0.01	#DIV/0!	0	0	0	1	0	1	0	1	2.244499
tr|D2KTB4|D2KTB4_HUMAN	tr|D2KTB4	Helicase (Fragment) OS=Homo sapiens PE=2 SV=1	82709	0	957	0	22	0	0.04	#DIV/0!	0	0	0	1	0	1	0	2	2.244499
sp|Q9UHV7|MED13_HUMAN	MED13	Mediator of RNA polymerase II transcription subunit 13 OS=Homo sapiens GN=MED13 PE=1 SV=3	242680	0	955	0	22	0	0.01	#DIV/0!	0	0	0	1	0	1	0	1	2.244499
tr|D8VER1|D8VER1_HUMAN	GPR33	G protein-coupled receptor 33 OS=Homo sapiens GN=GPR33 PE=2 SV=1	38493	0	953	0	22	0	0.09	#DIV/0!	0	0	0	1	0	1	0	1	2.244499
tr|A1L4L9|A1L4L9_HUMAN	POM121C	POM121C protein OS=Homo sapiens GN=POM121C PE=4 SV=1	8398	0	952	0	22	0	0.42	#DIV/0!	0	0	0	1	0	1	0	1	2.244499
tr|A7E2D7|A7E2D7_HUMAN	EP400	E1A-binding protein p400 (Fragment) OS=Homo sapiens GN=EP400 PE=2 SV=1	189065	0	951	0	22	0	0.02	#DIV/0!	0	0	0	1	0	1	0	1	2.244499
tr|B7Z592|B7Z592_HUMAN	tr|B7Z592	cDNA FLJ61635, highly similar to Homo sapiens likely ortholog of mouse immediate early response, erythropoietin 4 (LEREPO4), mRNA OS=	45262	0	950	0	22	0	0.07	#DIV/0!	0	0	0	1	0	1	0	1	2.244499
tr|B4DEK7|B4DEK7_HUMAN	RAB8A	Ras-related protein Rab-8A OS=Homo sapiens GN=RAB8A PE=2 SV=1	23720	0	947	0	22	0	0.14	#DIV/0!	0	0	0	1	0	1	0	1	2.244499
tr|D6RGC4|D6RGC4_HUMAN	Mar-01	E3 ubiquitin-protein ligase MARCH1 OS=Homo sapiens GN=MARCH1 PE=2 SV=1	62915	0	946	0	22	0	0.05	#DIV/0!	0	0	0	1	0	1	0	2	2.244499
sp|Q01167|FOXK2_HUMAN	FOXK2	Forkhead box protein K2 OS=Homo sapiens GN=FOXK2 PE=1 SV=3	69248	0	945	0	22	0	0.05	#DIV/0!	0	0	0	1	0	1	0	1	2.244499
sp|Q86YC3|LRC33_HUMAN	LRRC33	Leucine-rich repeat-containing protein 33 OS=Homo sapiens GN=LRRC33 PE=2 SV=1	77457	0	942	0	23	0	0.04	#DIV/0!	0	1	0	1	0	1	0	1	2.244499
tr|A8K8Z4|A8K8Z4_HUMAN	tr|A8K8Z4	cDNA FLJ78071, highly similar to Human MHC class III complement component C6 mRNA OS=Homo sapiens PE=2 SV=1	108298	0	939	0	23	0	0.03	#DIV/0!	0	1	0	1	0	1	0	1	2.244499
tr|J3KMX9|J3KMX9_HUMAN	EIF2B1	Translation initiation factor eIF-2B subunit alpha OS=Homo sapiens GN=EIF2B1 PE=3 SV=1	33802	0	938	0	23	0	0.1	#DIV/0!	0	0	0	1	0	1	0	1	2.244499
sp|Q96HA7|TONSL_HUMAN	TONSL	Tonsoku-like protein OS=Homo sapiens GN=TONSL PE=1 SV=2	153001	0	937	0	23	0	0.02	#DIV/0!	0	0	0	1	0	2	0	4	2.981786
tr|A0PJ50|A0PJ50_HUMAN	PARP6	PARP6 protein (Fragment) OS=Homo sapiens GN=PARP6 PE=2 SV=1	64418	0	935	0	23	0	0.05	#DIV/0!	0	0	0	1	0	1	0	1	2.244499
tr|F2Z390|F2Z390_HUMAN	NFKBIL1	NF-kappa-B inhibitor-like protein 1 OS=Homo sapiens GN=NFKBIL1 PE=4 SV=1	14007	0	933	0	23	0	0.24	#DIV/0!	0	0	0	1	0	1	0	2	2.244499
tr|B4E3L4|B4E3L4_HUMAN	tr|B4E3L4	cDNA FLJ57039, highly similar to CREB-binding protein (EC 2.3.1.48) (Fragment) OS=Homo sapiens PE=2 SV=1	133738	0	932	0	23	0	0.02	#DIV/0!	0	0	0	1	0	1	0	1	2.244499
tr|E9PB38|E9PB38_HUMAN	PACS2	Phosphofurin acidic cluster sorting protein 2 OS=Homo sapiens GN=PACS2 PE=2 SV=1	98908	0	931	0	23	0	0.03	#DIV/0!	0	0	0	1	0	1	0	1	2.244499
tr|B4DEM9|B4DEM9_HUMAN	tr|B4DEM9	cDNA FLJ52257, highly similar to Polymerase delta-interacting protein 2 OS=Homo sapiens PE=2 SV=1	40075	0	928	0	23	0	0.08	#DIV/0!	0	0	0	1	0	1	0	1	2.244499
sp|Q13011|ECH1_HUMAN	ECH1	Delta(3,5)-Delta(2,4)-dienoyl-CoA isomerase, mitochondrial OS=Homo sapiens GN=ECH1 PE=1 SV=2	36136	0	927	0	23	0	0.16	#DIV/0!	0	0	0	1	0	1	0	1	2.244499
tr|I3L2E0|I3L2E0_HUMAN	RMI2	RecQ-mediated genome instability protein 2 OS=Homo sapiens GN=RMI2 PE=4 SV=1	2699	0	925	0	23	0	1.46	#DIV/0!	0	0	0	1	0	1	0	1	2.244499
sp|Q6ZT83|YR005_HUMAN	sp|Q6ZT83	Uncharacterized protein FLJ44881 OS=Homo sapiens PE=2 SV=1	14573	0	924	0	24	0	0.23	#DIV/0!	0	0	0	1	0	1	0	1	2.244499
tr|G3V2K5|G3V2K5_HUMAN	EVL	Ena/VASP-like protein (Fragment) OS=Homo sapiens GN=EVL PE=2 SV=1	13024	0	923	0	24	0	0.26	#DIV/0!	0	0	0	1	0	1	0	1	2.244499
tr|Q2TB12|Q2TB12_HUMAN	LIG1	DNA ligase OS=Homo sapiens GN=LIG1 PE=2 SV=1	88975	0	922	0	24	0	0.04	#DIV/0!	0	0	0	1	0	1	0	2	2.244499
tr|A8K8X0|A8K8X0_HUMAN	tr|A8K8X0	cDNA FLJ75187, highly similar to Homo sapiens nap1 P120 OS=Homo sapiens PE=2 SV=1	113035	0	921	0	24	0	0.03	#DIV/0!	0	0	0	1	0	1	0	1	2.244499
tr|I3L2Z5|I3L2Z5_HUMAN	MAZ	Myc-associated zinc finger protein (Fragment) OS=Homo sapiens GN=MAZ PE=4 SV=1	22914	0	919	0	24	0	0.15	#DIV/0!	0	0	0	1	0	1	0	1	2.244499
tr|Q8IUV6|Q8IUV6_HUMAN	CORT	CORT protein (Fragment) OS=Homo sapiens GN=CORT PE=2 SV=1	13475	0	918	0	24	0	0.25	#DIV/0!	0	0	0	1	0	1	0	1	2.244499
tr|Q8N8W3|Q8N8W3_HUMA	tr|Q8N8W	cDNA FLJ38763 fis, clone KIDNE2014119 OS=Homo sapiens PE=2 SV=1	28252	0	915	0	24	0	0.12	#DIV/0!	0	0	0	1	0	1	0	1	2.244499
sp|Q0VD83|APOBR_HUMAN	APOBR	Apolipoprotein B receptor OS=Homo sapiens GN=APOBR PE=1 SV=1	115207	0	913	0	24	0	0.03	#DIV/0!	0	0	0	1	0	1	0	1	2.244499
sp|Q9GZL7|WDR12_HUMAN	WDR12	Ribosome biogenesis protein WDR12 OS=Homo sapiens GN=WDR12 PE=1 SV=2	48191	0	911	0	25	0	0.07	#DIV/0!	0	0	0	1	0	1	0	1	2.244499
tr|A7VJC9|A7VJC9_HUMAN	STP	Salt-tolerant protein OS=Homo sapiens GN=STP PE=2 SV=1	62769	0	909	0	25	0	0.05	#DIV/0!	0	0	0	1	0	1	0	1	2.244499
sp|Q9Y4W2|LAS1L_HUMAN	LAS1L	Ribosomal biogenesis protein LAS1L OS=Homo sapiens GN=LAS1L PE=1 SV=2	83982	0	908	0	25	0	0.12	#DIV/0!	0	0	0	1	0	1	0	1	2.244499
tr|B4DUM1|B4DUM1_HUMA	tr|B4DUM	cDNA FLJ58983 OS=Homo sapiens PE=2 SV=1	30159	0	903	0	25	0	0.11	#DIV/0!	0	0	0	1	0	1	0	1	2.244499
tr|B4DMI9|B4DMI9_HUMAN	tr|B4DMI9	cDNA FLJ55894, highly similar to Discs large homolog 7 OS=Homo sapiens PE=2 SV=1	89501	0	902	0	25	0	0.04	#DIV/0!	0	0	0	1	0	1	0	1	2.244499
tr|B4E172|B4E172_HUMAN	tr|B4E172	cDNA FLJ61526, highly similar to Transcription factor COE1 OS=Homo sapiens PE=2 SV=1	50236	0	899	0	25	0	0.07	#DIV/0!	0	0	0	1	0	1	0	1	2.244499
tr|B4DKF6|B4DKF6_HUMAN	tr|B4DKF6	cDNA FLJ54635, highly similar to Syntaxin-binding protein 5 OS=Homo sapiens PE=2 SV=1	130624	0	898	0	25	0	0.03	#DIV/0!	0	0	0	1	0	1	0	1	2.244499

sp|Q2TBE0|C19L2_HUMAN	CWF19L2	CWF19-like protein 2 OS=Homo sapiens GN=CWF19L2 PE=1 SV=4	104292	0	896	0	25	0	0.03	#DIV/0!	0	0	0	1	0	1	0	2	2.244499
tr|Q6IBU0|Q6IBU0_HUMAN	EIF5	EIF5 protein OS=Homo sapiens GN=EIF5 PE=2 SV=1	49577	0	895	0	25	0	0.07	#DIV/0!	0	0	0	1	0	1	0	1	2.244499
sp|Q9Y6R7|FCGBP_HUMAN	FCGBP	IgGFc-binding protein OS=Homo sapiens GN=FCGBP PE=1 SV=3	596443	0	890	0	26	0	0.01	#DIV/0!	0	0	0	1	0	1	0	1	2.244499
tr|Q5DT20|Q5DT20_HUMAN	HRNR	Hornerin OS=Homo sapiens GN=HRNR PE=2 SV=1	283111	0	887	0	26	0	0.01	#DIV/0!	0	1	0	1	0	1	0	1	2.244499
sp|Q86U70|LDB1_HUMAN	LDB1	LIM domain-binding protein 1 OS=Homo sapiens GN=LDB1 PE=1 SV=2	47130	0	885	0	26	0	0.07	#DIV/0!	0	1	0	1	0	1	0	1	2.244499
sp|P15374|UCHL3_HUMAN	UCHL3	Ubiquitin carboxyl-terminal hydrolase isozyme L3 OS=Homo sapiens GN=UCHL3 PE=1 SV=1	26337	0	883	0	26	0	0.13	#DIV/0!	0	0	0	1	0	1	0	1	2.244499
tr|Q2TU84|Q2TU84_HUMAN	GIG18	Aspartate aminotransferase OS=Homo sapiens GN=GIG18 PE=2 SV=1	46519	0	882	0	26	0	0.15	#DIV/0!	0	0	0	2	0	1	0	3	2.244499
tr|I3L3S6|I3L3S6_HUMAN	SPATA22	Spermatogenesis-associated protein 22 (Fragment) OS=Homo sapiens GN=SPATA22 PE=4 SV=1	5736	0	880	0	26	0	0.63	#DIV/0!	0	0	0	1	0	1	0	1	2.244499
tr|H0Y507|H0Y507_HUMAN	SH3PXD2A	SH3 and PX domain-containing protein 2A (Fragment) OS=Homo sapiens GN=SH3PXD2A PE=4 SV=1	117035	0	878	0	26	0	0.03	#DIV/0!	0	0	0	1	0	1	0	1	2.244499
sp|Q9NVH2|INT7_HUMAN	INTS7	Integrator complex subunit 7 OS=Homo sapiens GN=INTS7 PE=1 SV=1	108021	0	877	0	26	0	0.03	#DIV/0!	0	0	0	1	0	1	0	1	2.244499
tr|B3KQ17|B3KQ17_HUMAN	tr|B3KQ17	cDNA FLJ32627 fis, clone SYNOV1000049, highly similar to NADPH oxidase 4 (EC 1.6.3.-) OS=Homo sapiens PE=2 SV=1	64982	0	875	0	27	0	0.05	#DIV/0!	0	0	0	1	0	1	0	2	2.244499
tr|E9PGF9|E9PGF9_HUMAN	MGEA5	Bifunctional protein NCOAT OS=Homo sapiens GN=MGEA5 PE=2 SV=1	97952	0	874	0	27	0	0.03	#DIV/0!	0	0	0	1	0	2	0	2	2.981786
tr|A4L733|A4L733_HUMAN	PDIA2	Protein disulfide isomerase family A, member 2 variant OS=Homo sapiens GN=PDIA2 PE=2 SV=1	61474	0	873	0	27	0	0.05	#DIV/0!	0	0	0	1	0	1	0	1	2.244499
tr|B4E1N9|B4E1N9_HUMAN	tr|B4E1N9	cDNA FLJ53430, highly similar to Homo sapiens FERM and PDZ domain containing 2 (FRMPD2), transcript variant 3, mRNA (Fragment) OS=H	94370	0	872	0	27	0	0.03	#DIV/0!	0	0	0	1	0	1	0	1	2.244499
tr|B3KM47|B3KM47_HUMAN	tr|B3KM47	cDNA FLJ10273 fis, clone HEMBB1001137, highly similar to SEC23-interacting protein OS=Homo sapiens PE=2 SV=1	111730	0	869	0	27	0	0.03	#DIV/0!	0	0	0	1	0	1	0	1	2.244499
sp|Q96L92|SNX27_HUMAN	SNX27	Sorting nexin-27 OS=Homo sapiens GN=SNX27 PE=1 SV=2	61854	0	867	0	27	0	0.05	#DIV/0!	0	0	0	1	0	1	0	1	2.244499
tr|B3KRK2|B3KRK2_HUMAN	DDX41	DEAD (Asp-Glu-Ala-Asp) box polypeptide 41, isoform CRA_a OS=Homo sapiens GN=DDX41 PE=2 SV=1	56058	0	865	0	27	0	0.06	#DIV/0!	0	0	0	1	0	1	0	1	2.244499
sp|Q58A45|PAN3_HUMAN	PAN3	PAB-dependent poly(A)-specific ribonuclease subunit 3 OS=Homo sapiens GN=PAN3 PE=1 SV=3	96293	0	864	0	27	0	0.03	#DIV/0!	0	0	0	1	0	1	0	1	2.244499
tr|I3L234|I3L234_HUMAN	RSL1D1	Ribosomal L1 domain-containing protein 1 (Fragment) OS=Homo sapiens GN=RSL1D1 PE=4 SV=1	11678	0	862	0	27	0	0.3	#DIV/0!	0	0	0	1	0	1	0	1	2.244499
tr|Q5JTE4|Q5JTE4_HUMAN	POLR1C	Polymerase (RNA) I polypeptide C, 30kDa OS=Homo sapiens GN=POLR1C PE=2 SV=1	36043	0	859	0	27	0	0.09	#DIV/0!	0	0	0	1	0	1	0	1	2.244499
tr|B2RAX5|B2RAX5_HUMAN	tr|B2RAX5	cDNA, FLJ95175, highly similar to Homo sapiens cerebral protein 11 (HUCEP11), mRNA OS=Homo sapiens PE=2 SV=1	53601	0	856	0	28	0	0.06	#DIV/0!	0	0	0	1	0	1	0	1	2.244499
tr|Q3LIC8|Q3LIC8_HUMAN	Nbla10300	Putative uncharacterized protein Nbla10300 (Fragment) OS=Homo sapiens GN=Nbla10300 PE=2 SV=1	53517	0	855	0	28	0	0.06	#DIV/0!	0	0	0	1	0	1	0	2	2.244499
tr|B2RA91|B2RA91_HUMAN	tr|B2RA91	cDNA, FLJ94773, highly similar to Homo sapiens splicing factor, arginine/serine-rich 2, interacting protein (SFRS2IP), mRNA OS=Homo sapier	129817	0	853	0	28	0	0.03	#DIV/0!	0	0	0	1	0	1	0	1	2.244499
tr|A8KAQ5|A8KAQ5_HUMAN	tr|A8KAQ5	cDNA FLJ77404, highly similar to Homo sapiens small nuclear ribonucleoprotein 70kDa polypeptide (RNP antigen) (SNRP70), transcript varia	51522	0	851	0	28	0	0.06	#DIV/0!	0	0	0	1	0	1	0	2	2.244499
tr|B4DDI7|B4DDI7_HUMAN	tr|B4DDI7	NADPH:adrenodoxin oxidoreductase, mitochondrial OS=Homo sapiens PE=2 SV=1	57663	0	850	0	28	0	0.06	#DIV/0!	0	0	0	1	0	1	0	1	2.244499
sp|Q96AG4|LRC59_HUMAN	LRRC59	Leucine-rich repeat-containing protein 59 OS=Homo sapiens GN=LRRC59 PE=1 SV=1	35308	0	849	0	28	0	0.09	#DIV/0!	0	0	0	1	0	1	0	1	2.244499
tr|B2R6D4|B2R6D4_HUMAN	tr|B2R6D4	cDNA, FLJ92901, highly similar to Homo sapiens phosphomannomutase 2 (PMM2), mRNA OS=Homo sapiens PE=2 SV=1	28378	0	844	0	28	0	0.12	#DIV/0!	0	0	0	1	0	1	0	1	2.244499
tr|J3QQW9|J3QQW9_HUMAN	SUZ12	Polycomb protein SUZ12 OS=Homo sapiens GN=SUZ12 PE=4 SV=1	81012	0	833	0	29	0	0.08	#DIV/0!	0	0	0	2	0	2	0	3	2.981786
tr|B4DKZ7|B4DKZ7_HUMAN	GATAD2A	Transcriptional repressor p66-alpha OS=Homo sapiens GN=GATAD2A PE=2 SV=1	47063	0	832	0	0	0	0.05	#DIV/0!	0	0	0	0	0	1	0	1	2.244499
tr|B3KNK5|B3KNK5_HUMAN	tr|B3KNK5	cDNA FLJ14795 fis, clone NT2RP4001219, highly similar to Homo sapiens DnaJ (Hsp40) homolog, subfamily C, member 10 (DNAJC10), mRNA	70004	0	831	0	29	0	0.05	#DIV/0!	0	0	0	1	0	1	0	1	2.244499
tr|B3KQ25|B3KQ25_HUMAN	PSME3	Proteasome activator complex subunit 3 OS=Homo sapiens GN=PSME3 PE=2 SV=1	22555	0	824	0	30	0	0.15	#DIV/0!	0	0	0	1	0	1	0	1	2.244499
tr|Q5U043|Q5U043_HUMAN	tr|Q5U043	Alcohol dehydrogenase 5 (Class III), chi polypeptide OS=Homo sapiens PE=2 SV=1	40520	0	823	0	30	0	0.08	#DIV/0!	0	0	0	1	0	1	0	1	2.244499
tr|H0YNF5|H0YNF5_HUMAN	IDH3A	Isocitrate dehydrogenase [NAD] subunit alpha, mitochondrial OS=Homo sapiens GN=IDH3A PE=3 SV=1	9483	0	820	0	30	0	0.37	#DIV/0!	0	0	0	1	0	1	0	1	2.244499
tr|B6F210|B6F210_HUMAN	GSS	Glutathione synthetase OS=Homo sapiens GN=GSS PE=2 SV=1	40495	0	818	0	31	0	0.08	#DIV/0!	0	1	0	1	0	1	0	1	2.244499
tr|B4DEY3|B4DEY3_HUMAN	tr|B4DEY3	cDNA FLJ56678, highly similar to Homo sapiens CTF18, chromosome transmission fidelity factor 18 homolog, mRNA OS=Homo sapiens PE=2	28192	0	816	0	31	0	0.12	#DIV/0!	0	0	0	1	0	1	0	1	2.244499
tr|B7Z233|B7Z233_HUMAN	ACAT2	Acetyl-CoA acetyltransferase, cytosolic OS=Homo sapiens GN=ACAT2 PE=2 SV=1	45184	0	813	0	27	0	0.16	#DIV/0!	0	1	0	1	0	1	0	1	2.244499
tr|B4DSA4|B4DSA4_HUMAN	tr|B4DSA4	cDNA FLJ56930, highly similar to Ferrochelatase, mitochondrial (EC 4.99.1.1) OS=Homo sapiens PE=2 SV=1	32775	0	810	0	31	0	0.1	#DIV/0!	0	0	0	1	0	1	0	1	2.244499
sp|Q96A33|CCD47_HUMAN	CCDC47	Coiled-coil domain-containing protein 47 OS=Homo sapiens GN=CCDC47 PE=1 SV=1	56123	0	809	0	31	0	0.06	#DIV/0!	0	0	0	1	0	1	0	3	2.244499
tr|G5E9V1|G5E9V1_HUMAN	TFG	Protein TFG OS=Homo sapiens GN=TFG PE=4 SV=1	43051	0	806	0	31	0	0.08	#DIV/0!	0	0	0	1	0	1	0	1	2.244499
tr|B2C8Z3|B2C8Z3_HUMAN	TGFB1	Transforming growth factor beta 1 (Fragment) OS=Homo sapiens GN=TGFB1 PE=2 SV=1	12965	0	805	0	31	0	0.26	#DIV/0!	0	0	0	1	0	1	0	1	2.244499
tr|F1T0A4|F1T0A4_HUMAN	PRPF31	U4/U6 small nuclear ribonucleoprotein Prp31 OS=Homo sapiens GN=PRPF31 PE=2 SV=1	55217	0	804	0	31	0	0.06	#DIV/0!	0	0	0	1	0	1	0	1	2.244499
tr|B4DEV8|B4DEV8_HUMAN	PSMA1	Proteasome subunit alpha type OS=Homo sapiens GN=PSMA1 PE=2 SV=1	18674	0	802	0	32	0	0.18	#DIV/0!	0	1	0	1	0	1	0	1	2.244499
sp|Q6P1N0|C2D1A_HUMAN	CC2D1A	Coiled-coil and C2 domain-containing protein 1A OS=Homo sapiens GN=CC2D1A PE=1 SV=1	104397	0	800	0	32	0	0.03	#DIV/0!	0	1	0	1	0	1	0	2	2.244499
tr|B7Z8W6|B7Z8W6_HUMAN	tr|B7Z8W6	cDNA FLJ55038, highly similar to Nicotinamide phosphoribosyltransferase (EC 2.4.2.12) OS=Homo sapiens PE=2 SV=1	26905	0	799	0	32	0	0.12	#DIV/0!	0	0	0	1	0	1	0	1	2.244499
tr|B4DPT1|B4DPT1_HUMAN	tr|B4DPT1	cDNA FLJ51524, highly similar to Set1/Ash2 histone methyltransferase complex subunit ASH2 OS=Homo sapiens PE=2 SV=1	55688	0	798	0	32	0	0.06	#DIV/0!	0	0	0	1	0	1	0	1	2.244499
sp|Q9H7N4|SFR19_HUMAN	SCAF1	Splicing factor, arginine/serine-rich 19 OS=Homo sapiens GN=SCAF1 PE=1 SV=3	139814	0	794	0	32	0	0.02	#DIV/0!	0	0	0	1	0	1	0	1	2.244499
sp|P62306|RUXF_HUMAN	SNRPF	Small nuclear ribonucleoprotein F OS=Homo sapiens GN=SNRPF PE=1 SV=1	9776	0	792	0	32	0	0.36	#DIV/0!	0	1	0	1	0	1	0	1	2.244499
sp|O95425|SVIL_HUMAN	SVIL	Supervillin OS=Homo sapiens GN=SVIL PE=1 SV=2	249417	0	790	0	32	0	0.01	#DIV/0!	0	0	0	1	0	1	0	1	2.244499
sp|P46109|CRKL_HUMAN	CRKL	Crk-like protein OS=Homo sapiens GN=CRKL PE=1 SV=1	33870	0	787	0	32	0	0.1	#DIV/0!	0	0	0	1	0	1	0	1	2.244499
sp|Q5TFE4|NT5D1_HUMAN	NT5DC1	5~-nucleotidase domain-containing protein 1 OS=Homo sapiens GN=NT5DC1 PE=1 SV=1	52439	0	786	0	32	0	0.15	#DIV/0!	0	0	0	2	0	1	0	2	2.244499
tr|A8K7A4|A8K7A4_HUMAN	tr|A8K7A4	cDNA FLJ76904, highly similar to Homo sapiens methionine adenosyltransferase II, beta (MAT2B), transcript variant 2, mRNA OS=Homo sap	36692	0	785	0	32	0	0.09	#DIV/0!	0	1	0	1	0	2	0	3	2.981786
tr|E5RIP1|E5RIP1_HUMAN	RPS20	40S ribosomal protein S20 OS=Homo sapiens GN=RPS20 PE=2 SV=1	5426	0	784	0	32	0	0.67	#DIV/0!	0	0	0	1	0	1	0	1	2.244499
tr|B2RDG1|B2RDG1_HUMAN	tr|B2RDG1	cDNA, FLJ96593 OS=Homo sapiens PE=2 SV=1	59687	0	783	0	33	0	0.06	#DIV/0!	0	0	0	1	0	1	0	1	2.244499
sp|P00918|CAH2_HUMAN	CA2	Carbonic anhydrase 2 OS=Homo sapiens GN=CA2 PE=1 SV=2	29285	0	778	0	33	0	0.24	#DIV/0!	0	0	0	2	0	2	0	3	2.981786
tr|K9M2P7|K9M2P7_HUMAN	IFNL4	Uncharacterized protein OS=Homo sapiens GN=IFNL4 PE=2 SV=1	15105	0	777	0	33	0	0.23	#DIV/0!	0	0	0	1	0	1	0	1	2.244499
tr|H0Y6C1|H0Y6C1_HUMAN	NT5DC1	5~-nucleotidase domain-containing protein 1 (Fragment) OS=Homo sapiens GN=NT5DC1 PE=4 SV=1	12979	0	774	0	33	0	0.26	#DIV/0!	0	0	0	1	0	1	0	1	2.244499
sp|Q6NVY1|HIBCH_HUMAN	HIBCH	3-hydroxyisobutyryl-CoA hydrolase, mitochondrial OS=Homo sapiens GN=HIBCH PE=1 SV=2	43797	0	770	0	33	0	0.08	#DIV/0!	0	0	0	1	0	1	0	1	2.244499
tr|B3KS98|B3KS98_HUMAN	EIF3S3	Eukaryotic translation initiation factor 3 subunit H OS=Homo sapiens GN=EIF3S3 PE=2 SV=1	41726	0	768	0	33	0	0.08	#DIV/0!	0	0	0	1	0	1	0	2	2.244499
tr|Q8IYQ9|Q8IYQ9_HUMAN	KPNA3	Importin subunit alpha OS=Homo sapiens GN=KPNA3 PE=2 SV=1	58378	0	766	0	33	0	0.06	#DIV/0!	0	0	0	1	0	1	0	1	2.244499
tr|G3XAI2|G3XAI2_HUMAN	LAMB1	Laminin subunit beta-1 OS=Homo sapiens GN=LAMB1 PE=2 SV=1	207703	0	763	0	33	0	0.02	#DIV/0!	0	0	0	1	0	1	0	1	2.244499
tr|G3V161|G3V161_HUMAN	KBTBD3	Kelch repeat and BTB (POZ) domain containing 3, isoform CRA_a OS=Homo sapiens GN=KBTBD3 PE=2 SV=1	61834	0	762	0	33	0	0.06	#DIV/0!	0	2	0	2	0	2	0	2	2.981786
tr|B4DP31|B4DP31_HUMAN	PRPSAP1	Phosphoribosyl pyrophosphate synthase-associated protein 1 OS=Homo sapiens GN=PRPSAP1 PE=2 SV=1	31474	0	759	0	33	0	0.22	#DIV/0!	0	0	0	2	0	2	0	2	2.981786
tr|H0Y8P4|H0Y8P4_HUMAN	UTP15	U3 small nucleolar RNA-associated protein 15 homolog (Fragment) OS=Homo sapiens GN=UTP15 PE=4 SV=1	62020	0	757	0	34	0	0.05	#DIV/0!	0	1	0	1	0	1	0	1	2.244499
tr|A2A2M0|A2A2M0_HUMAN	RPRD1B	Regulation of nuclear pre-mRNA domain containing 1B (Fragment) OS=Homo sapiens GN=RPRD1B PE=4 SV=1	22171	0	756	0	34	0	0.15	#DIV/0!	0	0	0	1	0	1	0	1	2.244499
sp|Q9Y4B5|SOGA2_HUMAN	SOGA2	Protein SOGA2 OS=Homo sapiens GN=SOGA2 PE=1 SV=5	210652	0	755	0	34	0	0.02	#DIV/0!	0	0	0	1	0	1	0	1	2.244499
tr|Q8NBV9|Q8NBV9_HUMAN	tr|Q8NBV9	cDNA FLJ90717 fis, clone PLACE1008657, highly similar to Bovine adseverin OS=Homo sapiens PE=2 SV=1	44919	0	754	0	34	0	0.07	#DIV/0!	0	0	0	1	0	1	0	1	2.244499
tr|J3KMX1|J3KMX1_HUMAN	NUP88	Nuclear pore complex protein Nup88 (Fragment) OS=Homo sapiens GN=NUP88 PE=4 SV=1	80029	0	750	0	34	0	0.04	#DIV/0!	0	0	0	1	0	1	0	1	2.244499
tr|F8W805|F8W805_HUMAN	GRM1	Metabotropic glutamate receptor 1 OS=Homo sapiens GN=GRM1 PE=2 SV=1	103232	0	749	0	34	0	0.03	#DIV/0!	0	1	0	1	0	1	0	1	2.244499
tr|L0R5A1|L0R5A1_HUMAN	CSF2RB	Alternative protein CSF2RB OS=Homo sapiens GN=CSF2RB PE=4 SV=1	11752	0	746	0	34	0	0.29	#DIV/0!	0	1	0	1	0	2	0	2	2.981786
tr|B2R894|B2R894_HUMAN	MRPL38	Mitochondrial ribosomal protein L38, isoform CRA_b OS=Homo sapiens GN=MRPL38 PE=2 SV=1	41017	0	744	0	35	0	0.09	#DIV/0!	0	1	0	1	0	1	0	1	2.244499
tr|H0YD72|H0YD72_HUMAN	PPFIA1	Liprin-alpha-1 (Fragment) OS=Homo sapiens GN=PPFIA1 PE=4 SV=1	26134	0	743	0	35	0	0.13	#DIV/0!	0	1	0	1	0	1	0	1	2.244499
tr|A8K517|A8K517_HUMAN	RPS23	Ribosomal protein S23, isoform CRA_a OS=Homo sapiens GN=RPS23 PE=2 SV=1	15969	0	740	0	35	0	0.21	#DIV/0!	0	1	0	1	0	1	0	1	2.244499
tr|Q5VU21|Q5VU21_HUMAN	SERBP1	PAI-1 mRNA-binding protein variant OS=Homo sapiens GN=SERBP1 PE=2 SV=1	42458	0	739	0	35	0	0.08	#DIV/0!	0	0	0	1	0	2	0	2	2.981786
sp|Q15075|EEA1_HUMAN	EEA1	Early endosome antigen 1 OS=Homo sapiens GN=EEA1 PE=1 SV=2	163337	0	736	0	35	0	0.02	#DIV/0!	0	1	0	1	0	2	0	3	2.981786
tr|B3KMN5|B3KMN5_HUMAN	tr|B3KMN5	cDNA FLJ11713 fis, clone HEMBA1005201, highly similar to Homo sapiens NMD3 homolog (NMD3), mRNA OS=Homo sapiens PE=2 SV=1	44787	0	733	0	35	0	0.07	#DIV/0!	0	0	0	1	0	1	0	1	2.244499
tr|A4VCI1|A4VCI1_HUMAN	DNAJC1	DNAJC1 protein (Fragment) OS=Homo sapiens GN=DNAJC1 PE=2 SV=1	25214	0	731	0	35	0	0.13	#DIV/0!	0	1	0	1	0	1	0	1	2.244499
tr|G3V264|G3V264_HUMAN	SERPINA5	Plasma serine protease inhibitor (Fragment) OS=Homo sapiens GN=SERPINA5 PE=2 SV=1	19059	0	730	0	35	0	0.18	#DIV/0!	0	0	0	1	0	1	0	1	2.244499
tr|Q1WWK5|Q1WWK5_HUM/	Sep-09	SEPT9 protein (Fragment) OS=Homo sapiens GN=SEPT9 PE=2 SV=1	36717	0	729	0	36	0	0.19	#DIV/0!	0	0	0	2	0	2	0	3	2.981786
sp|O75475|PSIP1_HUMAN	PSIP1	PC4 and SFRS1-interacting protein OS=Homo sapiens GN=PSIP1 PE=1 SV=1	60181	0	724	0	36	0	0.11	#DIV/0!	0	1	0	2	0	1	0	2	2.244499
sp|Q5VW32|BROX_HUMAN	BROX	BRO1 domain-containing protein BROX OS=Homo sapiens GN=BROX PE=1 SV=1	46960	0	719	0	36	0	0.07	#DIV/0!	0	0	0	1	0	1	0	1	2.244499
tr|Q5T7C4|Q5T7C4_HUMAN	HMGB1	High mobility group protein B1 OS=Homo sapiens GN=HMGB1 PE=4 SV=1	18470	0	714	0	37	0	0.3	#DIV/0!	0	1	0	1	0	1	0	1	2.244499
tr|B2R6J4|B2R6J4_HUMAN	tr|B2R6J4	cDNA, FLJ92975, highly similar to Homo sapiens nucleosome assembly protein 1-like 4 (NAP1L4), mRNA OS=Homo sapiens PE=2 SV=1	42940	0	713	0	37	0	0.16	#DIV/0!	0	0	0	2	0	2	0	2	2.981786
tr|D6RCC8|D6RCC8_HUMAN	PPAT	Amidophosphoribosyltransferase OS=Homo sapiens GN=PPAT PE=2 SV=1	21362	0	712	0	37	0	0.16	#DIV/0!	0	0	0	1	0	1	0	1	2.244499
tr|F5GZY1|F5GZY1_HUMAN	C6orf211	UPF0364 protein C6orf211 OS=Homo sapiens GN=C6orf211 PE=2 SV=1	37672	0	711	0	37	0	0.09	#DIV/0!	0	0	0	1	0	1	0	1	2.244499
tr|B7Z600|B7Z600_HUMAN	tr|B7Z600	cDNA FLJ53180, highly similar to Cullin-3 OS=Homo sapiens PE=2 SV=1	63726	0	709	0	37	0	0.05	#DIV/0!	0	1	0	1	0	1	0	2	2.244499
sp|O60870|KIN17_HUMAN	KIN	DNA/RNA-binding protein KIN17 OS=Homo sapiens GN=KIN PE=1 SV=2	45745	0	705	0	37	0	0.07	#DIV/0!	0	1	0	1	0	1	0	1	2.244499
sp|Q09028|RBBP4_HUMAN	RBBP4	Histone-binding protein RBBP4 OS=Homo sapiens GN=RBBP4 PE=1 SV=3	47911	0	699	0	38	0	0.22	#DIV/0!	0	1	0	3	0	4	0	7	3.830425
sp|Q68E01|INT3_HUMAN	INTS3	Integrator complex subunit 3 OS=Homo sapiens GN=INTS3 PE=1 SV=1	119533	0	697	0	38	0	0.03	#DIV/0!	0	1	0	1	0	1	0	1	2.244499
tr|K7ELJ2|K7ELJ2_HUMAN	GIPC1	PDZ domain-containing protein GIPC1 (Fragment) OS=Homo sapiens GN=GIPC1 PE=4 SV=1	3892	0	694	0	38	0	0.97	#DIV/0!	0	1	0	1	0	1	0	1	2.244499
tr|Q6IBR8|Q6IBR8_HUMAN	EIF2S2	EIF2S2 protein OS=Homo sapiens GN=EIF2S2 PE=2 SV=1	38706	0	688	0	39	0	0.09	#DIV/0!	0	1	0	1	0	1	0	2	2.244499
tr|B2R7V4|B2R7V4_HUMAN	tr|B2R7V4	cDNA, FLJ93619, highly similar to Homo sapiens PRP4 pre-mRNA processing factor 4 homolog (yeast) (PRPF4), mRNA OS=Homo sapiens PE=	59097	0	687	0	39	0	0.06	#DIV/0!	0	1	0	1	0	1	0	1	2.244499
tr|A1A528|A1A528_HUMAN	ZW10	Centromere/kinetochore protein zw10 homolog OS=Homo sapiens GN=ZW10 PE=2 SV=1	77556	0	685	0	40	0	0.04	#DIV/0!	0	1	0	1	0	1	0	1	2.244499
sp|Q86Y56|HEAT2_HUMAN	HEATR2	HEAT repeat-containing protein 2 OS=Homo sapiens GN=HEATR2 PE=1 SV=4	94774	0	684	0	40	0	0.07	#DIV/0!	0	1	0	2	0	2	0	2	2.981786
sp|Q9UJW0|DCTN4_HUMAN	DCTN4	Dynactin subunit 4 OS=Homo sapiens GN=DCTN4 PE=1 SV=1	53216	0	683	0	40	0	0.06	#DIV/0!	0	1	0	2	0	2	0	2	2.981786
tr|Q7Z780|Q7Z780_HUMAN	U2AF1	U2 small nuclear RNA auxiliary factor 1 OS=Homo sapiens GN=U2AF1 PE=2 SV=1	20087	0	682	0	40	0	0.12	#DIV/0!	0	1	0	1	0	1	0	1	2.244499
tr|B2R9X3|B2R9X3_HUMAN	tr|B2R9X3	cDNA, FLJ94599, highly similar to Homo sapiens GDP-mannose 4,6-dehydratase (GMDS), mRNA OS=Homo sapiens PE=2 SV=1	42166	0	678	0	40	0	0.08	#DIV/0!	0	1	0	1	0	1	0	1	2.244499
tr|A6NJ11|A6NJ11_HUMAN	UFD1L	Ubiquitin fusion degradation protein 1 homolog OS=Homo sapiens GN=UFD1L PE=2 SV=1	33585	0	677	0	40	0	0.1	#DIV/0!	0	1	0	1	0	2	0	2	2.981786
tr|B4DHA6|B4DHA6_HUMAN	tr|B4DHA6	cDNA FLJ60526, highly similar to Probable rRNA-processing protein EBP2 OS=Homo sapiens PE=2 SV=1	15345	0	675	0	41	0	0.22	#DIV/0!	0	1	0	1	0	1	0	1	2.244499
tr|Q53F88|Q53F88_HUMAN	tr|Q53F88	General transcription factor IIE, polypeptide 1 (Alpha subunit, 56kD) variant (Fragment) OS=Homo sapiens PE=2 SV=1	40435	0	674	0	41	0	0.08	#DIV/0!	0	1	0	1	0	1	0	1	2.244499
tr|Q2NKQ6|Q2NKQ6_HUMAN	PPIG	PPIG protein OS=Homo sapiens GN=PPIG PE=2 SV=1	87564	0	672	0	41	0	0.04	#DIV/0!	0	1	0	1	0	1	0	1	2.244499
tr|B2RD14|B2RD14_HUMAN	tr|B2RD14	cDNA, FLJ96414, highly similar to Homo sapiens ubiquitin carboxyl-terminal esterase L1 (ubiquitin thiolesterase) (UCHL1), mRNA OS=Homo	25167	0	670	0	41	0	0.21	#DIV/0!	0	1	0	1	0	1	0	1	2.244499
tr|H0Y4V9|H0Y4V9_HUMAN	LARP4B	La-related protein 4B (Fragment) OS=Homo sapiens GN=LARP4B PE=4 SV=1	36998	0	669	0	41	0	0.09	#DIV/0!	0	1	0	1	0	1	0	1	2.244499
sp|Q6DKJ4|NXN_HUMAN	NXN	Nucleoredoxin OS=Homo sapiens GN=NXN PE=1 SV=2	48761	0	665	0	41	0	0.07	#DIV/0!	0	1	0	1	0	1	0	1	2.244499
tr|B7WP27|B7WP27_HUMAN	CWC22	Pre-mRNA-splicing factor CWC22 homolog OS=Homo sapiens GN=CWC22 PE=2 SV=1	106030	0	663	0	41	0	0.03	#DIV/0!	0	1	0	1	0	1	0	1	2.244499
tr|B1AQP8|B1AQP8_HUMAN	EPHX1	Epoxide hydrolase 1 (Fragment) OS=Homo sapiens GN=EPHX1 PE=4 SV=1	16430	0	662	0	41	0	0.06	#DIV/0!	0	1	0	1	0	1	0	1	2.244499
tr|I3L3B0|I3L3B0_HUMAN	C1QBP	Complement component 1 Q subcomponent-binding protein, mitochondrial OS=Homo sapiens GN=C1QBP PE=4 SV=1	20179	0	656	0	42	0	0.36	#DIV/0!	0	1	0	2	0	1	0	2	2.244499
tr|B4DJS7|B4DJS7_HUMAN	SNX6	Sorting nexin 6, isoform CRA_e OS=Homo sapiens GN=SNX6 PE=2 SV=1	34196	0	650	0	42	0	0.1	#DIV/0!	0	1	0	1	0	1	0	1	2.244499
tr|B2R7F8|B2R7F8_HUMAN	tr|B2R7F8	cDNA, FLJ93426, highly similar to Homo sapiens plasminogen (PLG), mRNA OS=Homo sapiens PE=2 SV=1	93217	0	646	0	42	0	0.04	#DIV/0!	0	1	0	1	0	1	0	1	2.244499
tr|Q6NX52|Q6NX52_HUMAN	ARHGEF1	ARHGEF1 protein OS=Homo sapiens GN=ARHGEF1 PE=2 SV=1	106643	0	645	0	42	0	0.03	#DIV/0!	0	1	0	1	0	1	0	2	2.244499
tr|Q2TSD2|Q2TSD2_HUMAN	tr|Q2TSD2	Aging-associated gene 7 protein OS=Homo sapiens PE=2 SV=1	41778	0	643	0	43	0	0.08	#DIV/0!	0	1	0	1	0	1	0	1	2.244499
tr|Q6EHZ3|Q6EHZ3_HUMAN	STRF8	Putative uncharacterized protein STRF8 OS=Homo sapiens GN=STRF8 PE=2 SV=1	41361	0	642	0	43	0	0.08	#DIV/0!	0	1	0	1	0	1	0	1	2.244499
tr|G5EA30|G5EA30_HUMAN	CELF1	CUG triplet repeat, RNA binding protein 1, isoform CRA_c OS=Homo sapiens GN=CELF1 PE=2 SV=1	55564	0	640	0	43	0	0.06	#DIV/0!	0	1	0	1	0	2	0	2	2.981786
tr|B7ZKT7|B7ZKT7_HUMAN	FYCO1	FYVE and coiled-coil domain-containing protein 1 OS=Homo sapiens GN=FYCO1 PE=2 SV=1	170608	0	635	0	44	0	0.02	#DIV/0!	0	1	0	1	0	2	0	3	2.981786
sp|P13667|PDIA4_HUMAN	PDIA4	Protein disulfide-isomerase A4 OS=Homo sapiens GN=PDIA4 PE=1 SV=2	73229	0	632	0	44	0	0.14	#DIV/0!	0	0	0	3	0	4	0	8	3.830425
tr|J3KSI8|J3KSI8_HUMAN	MRPS7	28S ribosomal protein S7, mitochondrial (Fragment) OS=Homo sapiens GN=MRPS7 PE=4 SV=1	18488	0	631	0	44	0	0.18	#DIV/0!	0	1	0	1	0	1	0	1	2.244499
tr|Q86WV4|Q86WV4_HUMAI	MRPS9	MRPS9 protein (Fragment) OS=Homo sapiens GN=MRPS9 PE=2 SV=1	35297	0	628	0	44	0	0.09	#DIV/0!	0	1	0	1	0	1	0	1	2.244499
tr|H0Y8B3|H0Y8B3_HUMAN	AHCYL2	Adenosylhomocysteinase (Fragment) OS=Homo sapiens GN=AHCYL2 PE=3 SV=1	59219	0	621	0	45	0	0.06	#DIV/0!	0	1	0	1	0	1	0	1	2.244499
tr|B4DQG8|B4DQG8_HUMAN	tr|B4DQG8	cDNA FLJ54124, highly similar to DNA methyltransferase 1-associated protein 1 OS=Homo sapiens PE=2 SV=1	52000	0	614	0	45	0	0.06	#DIV/0!	0	1	0	1	0	1	0	1	2.244499
tr|B3KNP0|B3KNP0_HUMAN	tr|B3KNP0	cDNA FLJ30074 fis, clone BGGI11000123, highly similar to Homo sapiens formin binding protein 4 (FNBP4), mRNA (Fragment) OS=Homo sap	100252	0	613	0	46	0	0.03	#DIV/0!	0	1	0	1	0	1	0	1	2.244499
tr|F5H740|F5H740_HUMAN	VDAC3	Voltage-dependent anion-selective channel protein 3 OS=Homo sapiens GN=VDAC3 PE=2 SV=1	31080	0	608	0	46	0	0.11	#DIV/0!	0	1	0	1	0	1	0	1	2.244499
tr|J3KS54|J3KS54_HUMAN	FLII	Protein flightless-1 homolog OS=Homo sapiens GN=FLII PE=4 SV=1	78779	0	603	0	47	0	0.04	#DIV/0!	0	1	0	1	0	1	0	1	2.244499
tr|C9JTZ6|C9JTZ6_HUMAN	NT5DC2	5~-nucleotidase domain-containing protein 2 OS=Homo sapiens GN=NT5DC2 PE=2 SV=1	61649	0	601	0	47	0	0.23	#DIV/0!	0	0	0	2	0	2	0	2	2.981786
tr|B3KUL1|B3KUL1_HUMAN	tr|B3KUL1	cDNA FLJ40132 fis, clone TESTI2012155, highly similar to NUCLEOPORIN-LIKE PROTEIN RIP OS=Homo sapiens PE=2 SV=1	59275	0	596	0	48	0	0.06	#DIV/0!	0	1	0	1	0	1	0	1	2.244499
tr|G3V0J0|G3V0J0_HUMAN	FMR1	Fragile X mental retardation 1, isoform CRA_e OS=Homo sapiens GN=FMR1 PE=2 SV=1	67030	0	592	0	48	0	0.05	#DIV/0!	0	1	0	1	0	1	0	2	2.244499
tr|B3KSH1|B3KSH1_HUMAN	EIF3F	Eukaryotic translation initiation factor 3 subunit F OS=Homo sapiens GN=EIF3F PE=2 SV=1	39236	0	588	0	48	0	0.18	#DIV/0!	0	1	0	2	0	2	0	2	2.981786
tr|Q6ZPD0|Q6ZPD0_HUMAN	tr|Q6ZPD0	cDNA FLJ26027 fis, clone PNC04328, highly similar to Homo sapiens translocase of outer mitochondrial membrane 34 (TOMM34) OS=Homo	20621	0	580	0	49	0	0.16	#DIV/0!	0	1	0	1	0	1	0	1	2.244499
tr|B3KY95|B3KY95_HUMAN	tr|B3KY95	cDNA FLJ16143 fis, clone BRAMY2038516, highly similar to Protein disulfide-isomerase A6 (EC 5.3.4.1) OS=Homo sapiens PE=2 SV=1	53655	0	577	0	49	0	0.2	#DIV/0!	0	1	0	3	0	3	0	4	3.467534
tr|Q6LES9|Q6LES9_HUMAN	RFC5	RFC5 protein (Fragment) OS=Homo sapiens GN=RFC5 PE=2 SV=1	38658	0	569	0	50	0	0.09	#DIV/0!	0	1	0	1	0	1	0	1	2.244499
tr|H3BPE7|H3BPE7_HUMAN	FUS	RNA-binding protein FUS OS=Homo sapiens GN=FUS PE=4 SV=1	53693	0	561	0	50	0	0.2	#DIV/0!	0	1	0	3	0	2	0	3	2.981786
tr|B2RDW9|B2RDW9_HUMAN	tr|B2RDW9	cDNA, FLJ96803, highly similar to Homo sapiens GTP binding protein 1 (GTPBP1), mRNA OS=Homo sapiens PE=2 SV=1	64008	0	558	0	50	0	0.05	#DIV/0!	0	1	0	1	0	1	0	2	2.244499
tr|A2A2L5|A2A2L5_HUMAN	C20orf43	Chromosome 20 open reading frame 43 (Fragment) OS=Homo sapiens GN=C20orf43 PE=4 SV=1	31759	0	548	0	52	0	0.1	#DIV/0!	0	1	0	1	0	1	0	1	2.244499
tr|G3XL83|G3XL83_HUMAN	HEXA	Hexosaminidase A (Fragment) OS=Homo sapiens GN=HEXA PE=2 SV=1	4956	0	546	0	52	0	0.73	#DIV/0!	0	1	0	1	0	1	0	1	2.244499
tr|Q59F59|Q59F59_HUMAN	tr|Q59F59	Importin 8 variant (Fragment) OS=Homo sapiens PE=2 SV=1	56228	0	544	0	52	0	0.06	#DIV/0!	0	1	0	1	0	1	0	1	2.244499
sp|Q7L2J0|MEPCE_HUMAN	MEPCE	7SK snRNA methylphosphate capping enzyme OS=Homo sapiens GN=MEPCE PE=1 SV=1	74937	0	536	0	53	0	0.04	#DIV/0!	0	1	0	1	0	1	0	1	2.244499
tr|B7Z8A2|B7Z8A2_HUMAN	tr|B7Z8A2	cDNA FLJ51671, highly similar to Prenylcysteine oxidase (EC 1.8.3.5) OS=Homo sapiens PE=2 SV=1	54918	0	534	0	53	0	0.07	#DIV/0!	0	1	0	1	0	1	0	1	2.244499
tr|H0Y855|H0Y855_HUMAN	RRM2	Ribonucleoside-diphosphate reductase subunit M2 (Fragment) OS=Homo sapiens GN=RRM2 PE=4 SV=1	12086	0	525	0	54	0	0.28	#DIV/0!	0	1	0	1	0	1	0	1	2.244499
tr|B5BU99|B5BU99_HUMAN	YTHDF2	High glucose-regulated protein 8 OS=Homo sapiens GN=YTHDF2 PE=2 SV=1	61510	0	521	0	55	0	0.05	#DIV/0!	0	1	0	1	0	1	0	1	2.244499
sp|Q9NP92|RT30_HUMAN	MRPS30	28S ribosomal protein S30, mitochondrial OS=Homo sapiens GN=MRPS30 PE=1 SV=2	50960	0	520	0	55	0	0.06	#DIV/0!	0	1	0	1	0	1	0	1	2.244499
sp|Q96GS4|CQ059_HUMAN	C17orf59	Uncharacterized protein C17orf59 OS=Homo sapiens GN=C17orf59 PE=1 SV=2	37603	0	515	0	55	0	0.09	#DIV/0!	0	1	0	1	0	1	0	1	2.244499
tr|B3KQI5|B3KQI5_HUMAN	tr|B3KQI5	cDNA FLJ90522 fis, clone NT2RP4000108, highly similar to Neurofilament triplet L protein OS=Homo sapiens PE=2 SV=1	61508	0	507	0	56	0	0.05	#DIV/0!	0	1	0	1	0	2	0	3	2.981786
tr|A6NNK5|A6NNK5_HUMAN	TP53BP1	Tumor suppressor p53-binding protein 1 OS=Homo sapiens GN=TP53BP1 PE=2 SV=2	210886	0	505	0	57	0	0.02	#DIV/0!	0	1	0	1	0	1	0	2	2.244499
sp|Q9Y6C9|MTCH2_HUMAN	MTCH2	Mitochondrial carrier homolog 2 OS=Homo sapiens GN=MTCH2 PE=1 SV=1	33936	0	504	0	58	0	0.2	#DIV/0!	0	1	0	2	0	2	0	2	2.981786
tr|Q5JR04|Q5JR04_HUMAN	MOV10	Mov10, Moloney leukemia virus 10, homolog (Mouse) OS=Homo sapiens GN=MOV10 PE=4 SV=1	107941	0	503	0	58	0	0.06	#DIV/0!	0	1	0	2	0	2	0	2	2.981786
sp|O60216|RAD21_HUMAN	RAD21	Double-strand-break repair protein rad21 homolog OS=Homo sapiens GN=RAD21 PE=1 SV=2	71930	0	502	0	58	0	0.59	#DIV/0!	0	3	0	3	0	3	0	3	3.467534
sp|Q8TAT6|NPL4_HUMAN	NPLOC4	Nuclear protein localization protein 4 homolog OS=Homo sapiens GN=NPLOC4 PE=1 SV=3	69046	0	500	0	58	0	0.23	#DIV/0!	0	1	0	1	0	1	0	1	2.244499
tr|B3KVX6|B3KVX6_HUMAN	tr|B3KVX6	cDNA FLJ41699 fis, clone HCHON2004776, highly similar to Homo sapiens cytoskeleton-associated protein 4 (CKAP4), mRNA OS=Homo sapi	58173	0	489	0	60	0	0.06	#DIV/0!	0	1	0	1	0	1	0	1	2.244499
tr|B2R5M9|B2R5M9_HUMAN	tr|B2R5M9	cDNA, FLJ92537, highly similar to Homo sapiens procollagen-lysine, 2-oxoglutarate 5-dioxygenase (lysine hydroxylase, Ehlers-Danlos syndro	84022	0	486	0	60	0	0.04	#DIV/0!	0	1	0	1	0	1	0	1	2.244499
sp|O15047|SET1A_HUMAN	SETD1A	Histone-lysine N-methyltransferase SETD1A OS=Homo sapiens GN=SETD1A PE=1 SV=3	186718	0	483	0	60	0	0.12	#DIV/0!	0	1	0	1	0	1	0	1	2.244499
tr|F5H1H2|F5H1H2_HUMAN	CCAR1	Cell division cycle and apoptosis regulator protein 1 (Fragment) OS=Homo sapiens GN=CCAR1 PE=2 SV=1	76191	0	479	0	61	0	0.04	#DIV/0!	0	1	0	1	0	1	0	1	2.244499
tr|H0YCY6|H0YCY6_HUMAN	DAK	FAD-AMP lyase (cyclizing) (Fragment) OS=Homo sapiens GN=DAK PE=4 SV=1	55026	0	475	0	61	0	0.06	#DIV/0!	0	1	0	1	0	1	0	1	2.244499
tr|H0Y360|H0Y360_HUMAN	AMPD2	AMP deaminase 2 (Fragment) OS=Homo sapiens GN=AMPD2 PE=4 SV=1	99192	0	468	0	63	0	0.03	#DIV/0!	0	1	0	1	0	1	0	1	2.244499
sp|Q9HCC0|MCCB_HUMAN	MCCC2	Methylcrotonoyl-CoA carboxylase beta chain, mitochondrial OS=Homo sapiens GN=MCCC2 PE=1 SV=1	61808	0	467	0	63	0	0.05	#DIV/0!	0	1	0	1	0	1	0	1	2.244499
tr|B4DWK8|B4DWK8_HUMAN	tr|B4DWK8	Catalase OS=Homo sapiens PE=2 SV=1	53482	0	462	0	64	0	0.06	#DIV/0!	0	1	0	1	0	2	0	2	2.981786
tr|J3KNH7|J3KNH7_HUMAN	SENP3	Sentrin-specific protease 3 OS=Homo sapiens GN=SENP3 PE=4 SV=1	65468	0	458	0	64	0	0.05	#DIV/0!	0	1	0	1	0	1	0	1	2.244499
sp|O15042|SR140_HUMAN	U2SURP	U2 snRNP-associated SURP motif-containing protein OS=Homo sapiens GN=U2SURP PE=1 SV=2	118675	0	454	0	65	0	0.08	#DIV/0!	0	2	0	3	0	3	0	4	3.467534
tr|C9JB80|C9JB80_HUMAN	U2SURP	U2 snRNP-associated SURP motif-containing protein (Fragment) OS=Homo sapiens GN=U2SURP PE=2 SV=1	57313	0	449	0	65	0	0.12	#DIV/0!	0	2	0	2	0	3	0	3	3.467534
tr|H8Y0Y3|H8Y0Y3_HUMAN	DCTN1/ALK	DCTN1/ALK fusion protein (Fragment) OS=Homo sapiens GN=DCTN1/ALK fusion PE=2 SV=1	18758	0	446	0	66	0	0.18	#DIV/0!	0	1	0	1	0	1	0	2	2.244499
tr|H0YKL9|H0YKL9_HUMAN	ANXA2	Annexin (Fragment) OS=Homo sapiens GN=ANXA2 PE=3 SV=1	12113	0	440	0	54	0	0.37	#DIV/0!	0	1	0	1	0	1	0	1	2.244499
tr|Q6ZMY0|Q6ZMY0_HUMAN	tr|Q6ZMY0	cDNA FLJ16598 fis, clone TESTI4006473, weakly similar to ATP-dependent RNA helicase A OS=Homo sapiens PE=2 SV=1	152244	0	427	0	67	0	0.02	#DIV/0!	0	1	0	1	0	1	0	1	2.244499
tr|H0Y580|H0Y580_HUMAN	EIF2B3	Translation initiation factor eIF-2B subunit gamma (Fragment) OS=Homo sapiens GN=EIF2B3 PE=4 SV=1	25394	0	422	0	68	0	0.28	#DIV/0!	0	1	0	2	0	2	0	2	2.981786
tr|H3BLS9|H3BLS9_HUMAN	ANKHD1	Ankyrin repeat and KH domain-containing protein 1 (Fragment) OS=Homo sapiens GN=ANKHD1 PE=4 SV=1	73999	0	413	0	70	0	0.09	#DIV/0!	0	1	0	2	0	2	0	3	2.981786
tr|Q6ZVI6|Q6ZVI6_HUMAN	tr|Q6ZVI6	cDNA FLJ42537 fis, clone BRACE3004058, highly similar to NADH-cytochrome B5 reductase (EC 1.6.2.2) OS=Homo sapiens PE=2 SV=1	29141	0	412	0	70	0	0.11	#DIV/0!	0	1	0	1	0	1	0	1	2.244499
tr|F5H170|F5H170_HUMAN	DNAJA4	DnaJ homolog subfamily A member 4 OS=Homo sapiens GN=DNAJA4 PE=2 SV=1	24818	0	411	0	70	0	0.13	#DIV/0!	0	1	0	1	0	2	0	2	2.981786
tr|B4E1K7|B4E1K7_HUMAN	STOML2	Stomatin-like protein 2 OS=Homo sapiens GN=STOML2 PE=2 SV=1	33317	0	402	0	71	0	0.1	#DIV/0!	0	1	0	1	0	1	0	1	2.244499
tr|Q96BS4|Q96BS4_HUMAN	FBL	FBL protein (Fragment) OS=Homo sapiens GN=FBL PE=2 SV=2	28546	0	398	0	72	0	0.25	#DIV/0!	0	1	0	2	0	2	0	2	2.981786

sp|Q96S66|CLCC1_HUMAN	CLCC1	Chloride channel CLIC-like protein 1 OS=Homo sapiens GN=CLCC1 PE=1 SV=1	62667	0	394	0	72	0	0.05	#DIV/0!	0	1	0	1	0	1	0	1	2.244499
tr|B4E091|B4E091_HUMAN	tr|B4E091	cDNA FLJ55438, highly similar to Splicing factor 3 subunit 1 OS=Homo sapiens PE=2 SV=1	77415	0	389	0	74	0	0.13	#DIV/0!	0	1	0	3	0	3	0	4	3.467534
tr|H0YCT5|H0YCT5_HUMAN	RBBP4	Histone-binding protein RBBP4 (Fragment) OS=Homo sapiens GN=RBBP4 PE=4 SV=1	20356	0	379	0	37	0	0.3	#DIV/0!	0	1	0	1	0	2	0	3	2.981786
tr|Q05DF2|Q05DF2_HUMAN	SF3A2	SF3A2 protein (Fragment) OS=Homo sapiens GN=SF3A2 PE=2 SV=1	51557	0	373	0	79	0	0.2	#DIV/0!	0	2	0	3	0	2	0	3	2.981786
tr|J3KPP4|J3KPP4_HUMAN	LUC7L3	Cisplatin resistance-associated overexpressed protein, isoform CRA_b OS=Homo sapiens GN=LUC7L3 PE=4 SV=1	58698	0	367	0	80	0	0.06	#DIV/0!	0	1	0	1	0	1	0	1	2.244499
tr|Q5XJ04|Q5XJ04_HUMAN	HMGCS1	3-hydroxy-3-methylglutaryl-Coenzyme A synthase 1 (Soluble) OS=Homo sapiens GN=HMGCS1 PE=2 SV=1	57854	0	358	0	82	0	0.18	#DIV/0!	0	1	0	4	0	5	0	5	4.120254
tr|G3XAM7|G3XAM7_HUMAN	CTNNA1	Catenin (Cadherin-associated protein), alpha 1, 102kDa, isoform CRA_a OS=Homo sapiens GN=CTNNA1 PE=2 SV=1	93576	0	353	0	83	0	0.11	#DIV/0!	0	2	0	3	0	3	0	5	3.467534
tr|H7C4P4|H7C4P4_HUMAN	U2SURP	U2 snRNP-associated SURP motif-containing protein (Fragment) OS=Homo sapiens GN=U2SURP PE=4 SV=1	10874	0	350	0	83	0	0.32	#DIV/0!	0	1	0	1	0	1	0	1	2.244499
tr|B4DF68|B4DF68_HUMAN	tr|B4DF68	cDNA FLJ55325, highly similar to Heat-shock protein 105 kDa OS=Homo sapiens PE=2 SV=1	86500	0	349	0	83	0	0.08	#DIV/0!	0	1	0	2	0	2	0	2	2.981786
tr|Q1W6G4|Q1W6G4_HUMA	LUC7L	LUC7-like (S. cerevisiae) OS=Homo sapiens GN=LUC7L PE=2 SV=1	38781	0	348	0	84	0	0.09	#DIV/0!	0	1	0	1	0	1	0	1	2.244499
tr|B2R774|B2R774_HUMAN	tr|B2R774	cDNA, FLJ93313, highly similar to Homo sapiens lectin, mannose-binding, 1 (LMAN1), mRNA OS=Homo sapiens PE=2 SV=1	57768	0	342	0	86	0	0.18	#DIV/0!	0	2	0	3	0	3	0	3	3.467534
sp|Q6P6C2|ALKB5_HUMAN	ALKBH5	RNA demethylase ALKBH5 OS=Homo sapiens GN=ALKBH5 PE=1 SV=2	44571	0	334	0	87	0	0.07	#DIV/0!	0	1	0	1	0	1	0	1	2.244499
tr|Q2NL95|Q2NL95_HUMAN	DDX19B	DDX19B protein (Fragment) OS=Homo sapiens GN=DDX19B PE=2 SV=1	51068	0	332	0	88	0	0.06	#DIV/0!	0	1	0	1	0	1	0	1	2.244499
tr|B3KM27|B3KM27_HUMAN	tr|B3KM27	cDNA FLJ10093 fis, clone HEMBA1002363, highly similar to Structural maintenance of chromosome 2-like 1 protein (Fragment) OS=Homo sa	56010	0	331	0	67	0	0.05	#DIV/0!	0	1	0	1	0	1	0	2	2.244499
tr|H0YHM6|H0YHM6_HUMAN	ALDH7A1	Alpha-aminoadipic semialdehyde dehydrogenase (Fragment) OS=Homo sapiens GN=ALDH7A1 PE=4 SV=1	26344	0	323	0	68	0	0.2	#DIV/0!	0	1	0	2	0	1	0	2	2.244499
tr|B0UX83|B0UX83_HUMAN	BAG6	HLA-B associated transcript 3 OS=Homo sapiens GN=BAG6 PE=4 SV=1	119085	0	309	0	97	0	0.08	#DIV/0!	0	2	0	3	0	3	0	3	3.467534
tr|F8VVL1|F8VVL1_HUMAN	DENR	Density-regulated protein OS=Homo sapiens GN=DENR PE=2 SV=1	18158	0	306	0	98	0	0.19	#DIV/0!	0	1	0	1	0	1	0	1	2.244499
tr|Q5T7Q0|Q5T7Q0_HUMAN	DNAJA1	DnaJ (Hsp40) homolog, subfamily A, member 1, isoform CRA_c OS=Homo sapiens GN=DNAJA1 PE=2 SV=1	45581	0	301	0	100	0	0.15	#DIV/0!	0	2	0	2	0	2	0	3	2.981786
tr|I1E4Y6|I1E4Y6_HUMAN	GIGYF2	PERQ amino acid-rich with GYF domain-containing protein 2 OS=Homo sapiens GN=GIGYF2 PE=4 SV=1	152891	0	299	0	100	0	0.02	#DIV/0!	0	1	0	1	0	1	0	1	2.244499
sp|O43815|STRN_HUMAN	STRN	Striatin OS=Homo sapiens GN=STRN PE=1 SV=4	86535	0	271	0	110	0	0.04	#DIV/0!	0	1	0	1	0	1	0	1	2.244499
tr|B2R5S4|B2R5S4_HUMAN	tr|B2R5S4	cDNA, FLJ92598, highly similar to Homo sapiens polymerase (DNA directed), delta 2, regulatory subunit 50kDa (POLD2), mRNA OS=Homo sa	51914	0	263	0	112	0	0.2	#DIV/0!	0	2	0	3	0	3	0	3	3.467534
tr|B3KTS5|B3KTS5_HUMAN	tr|B3KTS5	cDNA FLJ38670 fis, clone HSYRA2000190, highly similar to Voltage-dependent anion-selective channel protein 1 OS=Homo sapiens PE=2 SV	30839	0	248	0	121	0	0.23	#DIV/0!	0	2	0	2	0	2	0	2	2.981786
tr|A4D110|A4D110_HUMAN	LOC401309	Putative uncharacterized protein OS=Homo sapiens GN=LOC401309 PE=4 SV=1	13436	0	202	0	151	0	0.57	#DIV/0!	0	5	0	5	0	5	0	5	4.120254
tr|J3KQ96|J3KQ96_HUMAN	TCOF1	Treacle protein (Fragment) OS=Homo sapiens GN=TCOF1 PE=4 SV=1	144268	0	150	0	203	0	0.14	#DIV/0!	0	4	0	6	0	6	0	6	4.361583
tr|H0YJ87|H0YJ87_HUMAN	ZC3H14	Zinc finger CCCH domain-containing protein 14 (Fragment) OS=Homo sapiens GN=ZC3H14 PE=4 SV=1	8686	809	539	43	53	0.04	0.4	10	1	1	1	1	1	1	1	1	0.659333
sp|P00390|GSHR_HUMAN	GSR	Glutathione reductase, mitochondrial OS=Homo sapiens GN=GSR PE=1 SV=2	56791	1139	219	22	140	0.06	0.4	6.666667	0	4	1	6	1	7	1	8	2.98319
tr|A6NG51|A6NG51_HUMAN	SPTAN1	Spectrin alpha chain, non-erythrocytic 1 OS=Homo sapiens GN=SPTAN1 PE=2 SV=2	285628	409	65	100	309	0.02	0.13	6.5	2	6	2	10	2	12	3	15	2.984596
tr|J3KR97|J3KR97_HUMAN	TBCD	Tubulin-specific chaperone D OS=Homo sapiens GN=TBCD PE=4 SV=1	138346	1098	481	24	61	0.02	0.11	5.5	1	1	1	1	1	1	1	1	0.659333
tr|B4DHQ3|B4DHQ3_HUMAN	tr|B4DHQ3	cDNA FLJ56437, highly similar to Phosphoserine aminotransferase (EC 2.6.1.52) OS=Homo sapiens PE=2 SV=1	45783	943	629	34	44	0.07	0.32	4.571429	0	0	1	4	1	5	1	6	2.535088
tr|B4DR63|B4DR63_HUMAN	PSMC1	26S protease regulatory subunit 4 OS=Homo sapiens GN=PSMC1 PE=2 SV=1	41255	889	242	37	124	0.08	0.36	4.5	0	2	1	4	1	4	2	4	2.245259
tr|G3V1A4|G3V1A4_HUMAN	CFL1	Cofilin 1 (Non-muscle), isoform CRA_a OS=Homo sapiens GN=CFL1 PE=4 SV=1	17029	550	138	68	211	0.44	1.97	4.477273	2	4	2	6	3	6	3	6	1.553618
tr|Q59FQ4|Q59FQ4_HUMAN	tr|Q59FQ4	Glutamate dehydrogenase 1 variant (Fragment) OS=Homo sapiens PE=2 SV=1	32587	664	153	40	82	0.23	0.93	4.043478	0	2	2	3	2	3	2	3	1.145199
sp|Q8TCS8|PNPT1_HUMAN	PNPT1	Polyribonucleotide nucleotidyltransferase 1, mitochondrial OS=Homo sapiens GN=PNPT1 PE=1 SV=2	86524	1056	543	27	52	0.04	0.16	4	0	1	1	4	1	3	3	4	1.882368
tr|B2RBM7|B2RBM7_HUMAN	tr|B2RBM7	cDNA, FLJ95595, highly similar to Homo sapiens proteasome (prosome, macropain) 26S subunit, non-ATPase, 13 (PSMD13), mRNA OS=Hom	43131	750	293	48	102	0.16	0.64	4	0	2	2	4	2	4	2	4	1.50809
tr|Q6IAV5|Q6IAV5_HUMAN	OXCT	Succinyl-CoA:3-ketoacid-coenzyme A transferase OS=Homo sapiens GN=OXCT PE=2 SV=1	56612	542	822	69	30	0.06	0.21	3.5	1	0	1	1	1	1	1	1	0.659333
tr|H3BRG4|H3BRG4_HUMAN	UQCRC2	Cytochrome b-c1 complex subunit 2, mitochondrial OS=Homo sapiens GN=UQCRC2 PE=3 SV=1	44778	460	264	84	112	0.07	0.24	3.428571	1	1	1	3	1	3	2	4	1.882368
sp|O00116|ADAS_HUMAN	AGPS	Alkyldihydroxyacetonephosphate synthase, peroxisomal OS=Homo sapiens GN=AGPS PE=1 SV=1	73664	648	854	56	28	0.05	0.17	3.4	1	0	1	1	1	1	2	1	0.659333
tr|B4DFG0|B4DFG0_HUMAN	DEK	Protein DEK OS=Homo sapiens GN=DEK PE=2 SV=1	39704	1032	364	28	80	0.08	0.27	3.375	0	2	1	3	2	3	2	3	1.145199
tr|B4DWI8|B4DWI8_HUMAN	tr|B4DWI8	cDNA FLJ57805, highly similar to Homo sapiens paraspeckle component 1 (PSPC1), transcript variant alpha, mRNA OS=Homo sapiens PE=2 S	52660	1137	666	22	41	0.06	0.2	3.333333	0	0	1	3	2	3	2	3	1.145199
tr|H0YH87|H0YH87_HUMAN	ATXN2	Ataxin-2 (Fragment) OS=Homo sapiens GN=ATXN2 PE=4 SV=1	98626	490	285	78	105	0.03	0.1	3.333333	1	1	1	3	1	3	1	3	1.882368
tr|C9JRL4|C9JRL4_HUMAN	MDH1	Malate dehydrogenase, cytoplasmic (Fragment) OS=Homo sapiens GN=MDH1 PE=2 SV=1	13207	352	738	84	35	0.3	0.99	3.3	1	0	2	3	2	2	2	3	0.65945
tr|B1ANK7|B1ANK7_HUMAN	FH	Fumarate hydratase OS=Homo sapiens GN=FH PE=2 SV=1	54773	790	255	44	119	0.06	0.19	3.166667	1	3	1	3	1	3	1	3	1.882368
tr|D6RG13|D6RG13_HUMAN	RPS3A	40S ribosomal protein S3a (Fragment) OS=Homo sapiens GN=RPS3A PE=2 SV=1	25819	608	269	60	111	0.27	0.83	3.074074	2	5	2	5	3	6	3	6	1.553618
sp|P46013|KI67_HUMAN	MKI67	Antigen KI-67 OS=Homo sapiens GN=MKI67 PE=1 SV=2	360698	872	524	38	54	0.01	0.03	3	1	1	1	3	2	2	3	3	0.65945
tr|B4DNL5|B4DNL5_HUMAN	tr|B4DNL5	cDNA FLJ59361, highly similar to Protein disulfide-isomerase (EC 5.3.4.1) OS=Homo sapiens PE=2 SV=1	55746	466	237	83	115	0.12	0.36	3	1	2	2	4	3	4	4	4	1.02246
tr|B4DJT7|B4DJT7_HUMAN	tr|B4DJT7	cDNA FLJ56511, highly similar to Neurofilament triplet M protein OS=Homo sapiens PE=2 SV=1	78895	616	114	60	247	0.08	0.23	2.875	2	5	2	5	2	6	3	6	2.039248
tr|B4DT31|B4DT31_HUMAN	FUBP1	Far upstream element-binding protein 1 OS=Homo sapiens GN=FUBP1 PE=2 SV=1	69993	478	220	81	139	0.1	0.26	2.6	1	2	2	5	2	5	3	6	1.797919
sp|Q15019|SEPT2_HUMAN	Sep-02	Septin-2 OS=Homo sapiens GN=SEPT2 PE=1 SV=1	41689	553	207	68	146	0.16	0.41	2.5625	1	3	2	4	2	4	2	4	1.50809
tr|E7EQT4|E7EQT4_HUMAN	ACIN1	Apoptotic chromatin condensation inducer in the nucleus OS=Homo sapiens GN=ACIN1 PE=2 SV=2	147674	896	954	36	22	0.02	0.05	2.5	1	0	1	1	1	1	3	2	0.659333
sp|Q9UBB4|ATX10_HUMAN	ATXN10	Ataxin-10 OS=Homo sapiens GN=ATXN10 PE=1 SV=1	54196	769	460	46	64	0.06	0.15	2.5	1	1	1	1	1	1	1	1	0.659333
tr|B4DH17|B4DH17_HUMAN	tr|B4DH17	cDNA FLJ59298, highly similar to Eukaryotic translation initiation factor 4 gamma 3 OS=Homo sapiens PE=2 SV=1	134921	716	368	51	80	0.02	0.05	2.5	1	1	1	2	3	4	3	5	1.02246
tr|B4DGN5|B4DGN5_HUMAN	GLUD1	Glutamate dehydrogenase OS=Homo sapiens GN=GLUD1 PE=2 SV=1	46945	664	167	54	186	0.23	0.56	2.434783	0	5	3	6	3	6	4	7	1.553618
sp|P40925|MDHC_HUMAN	MDH1	Malate dehydrogenase, cytoplasmic OS=Homo sapiens GN=MDH1 PE=1 SV=4	36631	352	430	116	67	0.3	0.71	2.366667	2	1	3	4	3	4	3	4	1.02246
sp|P49756|RBM25_HUMAN	RBM25	RNA-binding protein 25 OS=Homo sapiens GN=RBM25 PE=1 SV=3	100467	1083	648	25	42	0.03	0.07	2.333333	1	1	1	2	2	2	2	2	0.65945
tr|B4DZR0|B4DZR0_HUMAN	tr|B4DZR0	cDNA FLJ55529, highly similar to Heat shock 70 kDa protein 4L OS=Homo sapiens PE=2 SV=1	98526	347	251	116	120	0.07	0.16	2.285714	2	3	2	4	2	4	6	6	1.50809
tr|E5RI99|E5RI99_HUMAN	RPL30	60S ribosomal protein L30 (Fragment) OS=Homo sapiens GN=RPL30 PE=2 SV=1	12819	976	292	32	102	0.27	0.61	2.259259	0	2	1	2	1	2	1	2	1.39662
tr|B3KU62|B3KU62_HUMAN	tr|B3KU62	cDNA FLJ39243 fis, clone OCBBF2008283, highly similar to Protein NDRG1 OS=Homo sapiens PE=2 SV=1	38948	795	752	44	34	0.08	0.18	2.25	0	0	2	2	2	2	2	2	0.65945
tr|H0YJA2|H0YJA2_HUMAN	ZC3H14	Zinc finger CCCH domain-containing protein 14 (Fragment) OS=Homo sapiens GN=ZC3H14 PE=4 SV=1	74241	809	541	43	52	0.04	0.09	2.25	1	1	1	2	1	1	2	2	0.659333
tr|B2RDN4|B2RDN4_HUMAN	tr|B2RDN4	cDNA, FLJ96693, highly similar to Homo sapiens block of proliferation 1 (BOP1), mRNA OS=Homo sapiens PE=2 SV=1	84219	1125	435	23	35	0.04	0.09	2.25	0	1	1	1	1	1	1	2	0.659333
sp|Q7Z4V5|HDGR2_HUMAN	HDGFRP2	Hepatoma-derived growth factor-related protein 2 OS=Homo sapiens GN=HDGFRP2 PE=1 SV=1	74443	699	321	52	91	0.04	0.09	2.25	1	2	1	2	1	2	1	2	1.39662
tr|Q5CAQ5|Q5CAQ5_HUMAN	TRA1	Tumor rejection antigen (Gp96) 1 OS=Homo sapiens GN=TRA1 PE=2 SV=1	92567	189	51	211	446	0.28	0.63	2.25	6	11	7	14	7	15	9	18	1.709923
tr|G3V359|G3V359_HUMAN	APEX1	DNA-(apurinic or apyrimidinic site) lyase (Fragment) OS=Homo sapiens GN=APEX1 PE=2 SV=1	19384	1043	566	28	43	0.17	0.38	2.235294	0	1	1	1	1	1	1	1	0.659333
sp|P62280|RS11_HUMAN	RPS11	40S ribosomal protein S11 OS=Homo sapiens GN=RPS11 PE=1 SV=3	18590	940	560	34	50	0.18	0.4	2.222222	0	1	1	2	1	2	2	3	1.39662
tr|D3DVQ1|D3DVQ1_HUMAN	LETM1	Leucine zipper-EF-hand containing transmembrane protein 1, isoform CRA_a OS=Homo sapiens GN=LETM1 PE=2 SV=1	63730	462	372	84	79	0.05	0.11	2.2	1	2	1	2	1	2	1	2	1.39662
sp|P46060|RAGP1_HUMAN	RANGAP1	Ran GTPase-activating protein 1 OS=Homo sapiens GN=RANGAP1 PE=1 SV=1	63958	331	929	123	23	0.11	0.24	2.181818	2	0	2	1	5	1	5	1	-1.21595
sp|P28074|PSB5_HUMAN	PSMB5	Proteasome subunit beta type-5 OS=Homo sapiens GN=PSMB5 PE=1 SV=3	28633	844	620	40	45	0.12	0.26	2.166667	0	1	1	1	1	1	1	1	0.659333
tr|A4D2J6|A4D2J6_HUMAN	PGAM2	Phosphoglycerate mutase OS=Homo sapiens GN=PGAM2 PE=3 SV=1	28544	726	352	50	25	0.12	0.26	2.166667	1	0	1	1	1	1	1	1	0.659333
sp|O60884|DNJA2_HUMAN	DNAJA2	DnaJ homolog subfamily A member 2 OS=Homo sapiens GN=DNAJA2 PE=1 SV=1	46344	1136	779	22	33	0.07	0.15	2.142857	0	0	1	2	1	2	2	3	1.39662
tr|B3KMZ6|B3KMZ6_HUMAN	tr|B3KMZ6	cDNA FLJ13058 fis, clone NT2RP3001587, highly similar to Ubiquitin-like 1-activating enzyme E1B OS=Homo sapiens PE=2 SV=1	71688	325	229	125	107	0.14	0.3	2.142857	2	3	3	4	5	4	6	4	0.369976
tr|A4ZU86|A4ZU86_HUMAN	NPM1	Truncated nucleolar phosphoprotein B23 OS=Homo sapiens GN=NPM1 PE=2 SV=1	30074	271	169	147	172	0.37	0.79	2.135135	3	5	3	5	4	7	4	8	1.397617
sp|O75367|H2AY_HUMAN	H2AFY	Core histone macro-H2A.1 OS=Homo sapiens GN=H2AFY PE=1 SV=4	39764	561	343	66	86	0.08	0.17	2.125	1	1	1	2	1	2	1	2	1.39662
tr|F8WE84|F8WE84_HUMAN	ACTR3	Actin-related protein 3 OS=Homo sapiens GN=ACTR3 PE=2 SV=1	4471	411	545	34	52	0.4	0.84	2.1	0	1	1	1	1	1	1	1	0.659333
sp|P31946|1433B_HUMAN	YWHAB	14-3-3 protein beta/alpha OS=Homo sapiens GN=YWHAB PE=1 SV=3	28179	643	471	57	62	0.12	0.25	2.083333	1	1	1	2	3	2	3	3	0.17382
sp|P08236|BGLR_HUMAN	GUSB	Beta-glucuronidase OS=Homo sapiens GN=GUSB PE=1 SV=2	75027	496	943	77	22	0.04	0.08	2	1	0	1	1	2	1	2	1	-0.07784
tr|D3DVA5|D3DVA5_HUMAN	ARHGEF2	Rho/rac guanine nucleotide exchange factor (GEF) 2, isoform CRA_a OS=Homo sapiens GN=ARHGEF2 PE=2 SV=1	116954	866	737	38	0	0.03	0.06	2	1	0	1	0	1	0	1	1	-0.92595
sp|P12270|TPR_HUMAN	TPR	Nucleoprotein TPR OS=Homo sapiens GN=TPR PE=1 SV=3	267530	818	728	42	36	0.01	0.02	2	1	0	1	2	1	3	3	5	1.882368
tr|A8K1Z2|A8K1Z2_HUMAN	tr|A8K1Z2	cDNA FLJ78475, highly similar to Homo sapiens glycerol-3-phosphate dehydrogenase 2 (mitochondrial) (GPD2), mRNA OS=Homo sapiens PE	81197	578	708	64	37	0.04	0.08	2	1	1	1	1	1	1	3	1	0.659333
sp|P45954|ACDSB_HUMAN	ACADSB	Short/branched chain specific acyl-CoA dehydrogenase, mitochondrial OS=Homo sapiens GN=ACADSB PE=1 SV=1	47797	719	696	50	38	0.09	0.18	2	1	1	1	1	1	1	2	1	0.659333
tr|B4DQ15|B4DQ15_HUMAN	tr|B4DQ15	cDNA FLJ51525, highly similar to Homo sapiens aldehyde dehydrogenase 16 family, member A1 (ALDH16A1), mRNA OS=Homo sapiens PE=2	67899	1205	668	16	41	0.05	0.1	2	0	1	1	2	0	1	1	2	2.244499
tr|Q05CP8|Q05CP8_HUMAN	CCDC6	CCDC6 protein (Fragment) OS=Homo sapiens GN=CCDC6 PE=2 SV=1	38254	1081	591	25	48	0.09	0.18	2	0	1	1	2	1	2	1	2	1.39662
tr|A8MW61|A8MW61_HUMA	PLRG1	Pleiotropic regulator 1 OS=Homo sapiens GN=PLRG1 PE=2 SV=1	57545	898	590	36	48	0.06	0.12	2	1	1	1	2	1	2	2	2	1.39662
tr|J3KQ32|J3KQ32_HUMAN	OLA1	Obg-like ATPase 1 OS=Homo sapiens GN=OLA1 PE=4 SV=1	47136	1162	585	21	48	0.07	0.14	2	0	1	1	2	1	1	1	2	0.659333
tr|D3DWL9|D3DWL9_HUMAN	CPSF1	Cleavage and polyadenylation specific factor 1, 160kDa, isoform CRA_a OS=Homo sapiens GN=CPSF1 PE=2 SV=1	153087	990	584	31	49	0.02	0.04	2	0	1	1	2	1	2	4	3	1.39662
tr|K7EKE5|K7EKE5_HUMAN	TUBG1	Tubulin gamma-1 chain OS=Homo sapiens GN=TUBG1 PE=4 SV=1	15277	1129	563	22	50	0.11	0.22	2	0	1	1	1	1	1	1	1	0.659333
tr|H0Y2W2|H0Y2W2_HUMAN	ATAD3A	ATPase family AAA domain-containing protein 3A (Fragment) OS=Homo sapiens GN=ATAD3A PE=4 SV=1	64600	964	556	33	37	0.05	0.1	2	0	1	1	1	2	1	2	1	-0.07784
tr|F8W930|F8W930_HUMAN	IGF2BP2	Insulin-like growth factor 2 mRNA-binding protein 2 OS=Homo sapiens GN=IGF2BP2 PE=2 SV=1	66859	1093	499	25	58	0.05	0.1	2	0	2	2	2	1	2	3	2	1.39662
tr|B3KXU8|B3KXU8_HUMAN	tr|B3KXU8	cDNA FLJ46087 fis, clone TESTI2009497, highly similar to GPI-anchored protein p137 OS=Homo sapiens PE=2 SV=1	70489	1061	406	26	70	0.05	0.1	2	0	1	1	2	1	1	1	3	0.659333
sp|O43837|IDH3B_HUMAN	IDH3B	Isocitrate dehydrogenase [NAD] subunit beta, mitochondrial OS=Homo sapiens GN=IDH3B PE=1 SV=2	42442	622	391	59	74	0.08	0.16	2	1	1	1	2	1	1	1	2	0.659333
tr|G3V5E1|G3V5E1_HUMAN	CCNK	Cyclin-K OS=Homo sapiens GN=CCNK PE=2 SV=1	43776	877	280	38	106	0.08	0.16	2	1	2	1	2	1	2	1	2	1.39662
tr|B4DWM5|B4DWM5_HUMA	tr|B4DWM	cDNA FLJ61436, highly similar to Melanoma-associated antigen D2 OS=Homo sapiens PE=2 SV=1	26229	606	96	61	75	0.13	0.26	2	1	1	1	1	1	1	1	1	0.659333
tr|B2ZZ89|B2ZZ89_HUMAN	SPTBN1	Spectrin beta non-erythrocytic 1 OS=Homo sapiens GN=SPTBN1 PE=2 SV=1	275237	73	19	474	810	0.22	0.42	1.909091	13	17	17	30	17	30	19	35	1.466877
tr|B4DJV2|B4DJV2_HUMAN	CS	Citrate synthase OS=Homo sapiens GN=CS PE=2 SV=1	50628	868	651	38	42	0.07	0.13	1.857143	1	1	1	2	1	3	3	4	1.882368
tr|B4DP52|B4DP52_HUMAN	DDX39B	HCG2005638, isoform CRA_c OS=Homo sapiens GN=DDX39B PE=2 SV=1	40893	311	165	128	187	0.55	1.01	1.836364	3	4	7	9	10	10	10	13	0.660395
tr|B4DDM6|B4DDM6_HUMAI	BUB3	Mitotic checkpoint protein BUB3 OS=Homo sapiens GN=BUB3 PE=2 SV=1	28620	998	772	31	33	0.12	0.22	1.833333	0	0	1	2	3	2	3	2	0.17382
tr|E7ESY4|E7ESY4_HUMAN	MTA1	Metastasis-associated protein MTA1 OS=Homo sapiens GN=MTA1 PE=2 SV=1	80008	824	276	41	108	0.06	0.11	1.833333	1	2	1	2	2	2	2	2	0.65945
sp|P12956|XRCC6_HUMAN	XRCC6	X-ray repair cross-complementing protein 6 OS=Homo sapiens GN=XRCC6 PE=1 SV=2	70084	127	60	303	386	0.66	1.21	1.833333	7	9	11	17	17	17	21	20	0.661224
tr|B4DEX8|B4DEX8_HUMAN	MAT2A	S-adenosylmethionine synthase OS=Homo sapiens GN=MAT2A PE=2 SV=1	40027	299	151	132	202	0.27	0.49	1.814815	3	4	4	5	4	5	6	6	0.949515
tr|B4DPZ3|B4DPZ3_HUMAN	tr|B4DPZ3	cDNA FLJ53290, highly similar to Cytoplasmic dynein 1 intermediate chain 2 OS=Homo sapiens PE=2 SV=1	70869	552	425	68	68	0.05	0.09	1.8	1	1	1	2	1	2	1	2	1.39662
tr|F8VQ10|F8VQ10_HUMAN	DDX39B	Spliceosome RNA helicase DDX39B OS=Homo sapiens GN=DDX39B PE=4 SV=1	51226	311	133	128	219	0.55	0.99	1.8	3	5	7	11	11	13	11	15	0.892483
sp|O00148|DX39A_HUMAN	DDX39A	ATP-dependent RNA helicase DDX39A OS=Homo sapiens GN=DDX39A PE=1 SV=2	49611	342	234	118	131	0.38	0.67	1.763158	3	4	5	8	8	10	9	11	0.965657
tr|H0YNT2|H0YNT2_HUMAN	TLE3	Transducin-like enhancer protein 3 OS=Homo sapiens GN=TLE3 PE=4 SV=1	77590	1005	647	30	42	0.04	0.07	1.75	0	1	1	1	1	1	1	1	0.659333
tr|B4DR68|B4DR68_HUMAN	tr|B4DR68	cDNA FLJ58608, highly similar to Heat shock protein 75 kDa, mitochondrial OS=Homo sapiens PE=2 SV=1	74408	124	102	323	254	0.35	0.61	1.742857	6	7	8	11	8	11	9	12	1.097224
tr|I6L9E8|I6L9E8_HUMAN	FAM98A	Family with sequence similarity 98, member A OS=Homo sapiens GN=FAM98A PE=2 SV=1	55595	315	222	127	138	0.19	0.33	1.736842	2	2	3	5	3	5	4	5	1.312288
sp|Q15046|SYK_HUMAN	KARS	Lysine--tRNA ligase OS=Homo sapiens GN=KARS PE=1 SV=3	68461	484	383	80	76	0.15	0.26	1.733333	2	2	3	5	4	6	4	7	1.190844
tr|Q5STZ8|Q5STZ8_HUMAN	ABCF1	ATP-binding cassette sub-family F member 1 (Fragment) OS=Homo sapiens GN=ABCF1 PE=4 SV=2	38353	187	104	167	266	0.23	0.39	1.695652	3	4	3	4	3	4	4	5	1.02246
sp|Q9BYX7|ACTBM_HUMAN	POTEKP	Putative beta-actin-like protein 3 OS=Homo sapiens GN=POTEKP PE=5 SV=1	42331	43	108	283	256	0.41	0.69	1.682927	7	6	12	14	13	13	14	18	0.66075
tr|F8WA43|F8WA43_HUMAN	CTNND1	Catenin delta-1 OS=Homo sapiens GN=CTNND1 PE=2 SV=1	106284	1095	681	25	40	0.03	0.05	1.666667	0	1	1	1	1	1	1	1	0.659333
tr|Q96L67|Q96L67_HUMAN	NRD1	Nardilysin OS=Homo sapiens GN=NRD1 PE=2 SV=1	109243	513	622	74	0	0.03	0.05	1.666667	1	0	1	0	1	0	2	1	-0.92595
tr|H7C5W9|H7C5W9_HUMAN	ATP2A2	Sarcoplasmic/endoplasmic reticulum calcium ATPase 2 (Fragment) OS=Homo sapiens GN=ATP2A2 PE=3 SV=1	104121	463	267	83	111	0.06	0.1	1.666667	2	2	2	3	2	3	4	3	1.145199
tr|B2RDW1|B2RDW1_HUMAN	RPS27A	Ribosomal protein S27a, isoform CRA_c OS=Homo sapiens GN=RPS27A PE=2 SV=1	18296	213	295	184	101	0.4	0.66	1.65	5	2	7	5	11	6	11	6	-0.16422
tr|C9JA17|C9JA17_HUMAN	ATP6V1A	V-type proton ATPase catalytic subunit A (Fragment) OS=Homo sapiens GN=ATP6V1A PE=2 SV=1	20286	793	182	44	66	0.17	0.28	1.647059	1	1	1	1	2	1	2	1	-0.07784
tr|B4DKM5|B4DKM5_HUMAN	VDAC2	Voltage-dependent anion-selective channel protein 2 OS=Homo sapiens GN=VDAC2 PE=2 SV=1	27804	709	337	51	87	0.25	0.41	1.64	1	1	2	3	3	2	3	3	0.17382
tr|B4DUL5|B4DUL5_HUMAN	tr|B4DUL5	cDNA FLJ51625, highly similar to Ubiquinol-cytochrome-c reductase complex core protein I, mitochondrial (EC 1.10.2.2) OS=Homo sapiens P	40917	870	362	38	79	0.08	0.13	1.625	1	1	1	1	1	1	2	2	0.659333
tr|F8WJN3|F8WJN3_HUMAN	CPSF6	Cleavage and polyadenylation-specificity factor subunit 6 OS=Homo sapiens GN=CPSF6 PE=2 SV=1	52409	403	442	101	66	0.13	0.21	1.615385	2	1	2	2	2	3	2	3	1.145199
tr|A8K639|A8K639_HUMAN	GMPS	Guanine monphosphate synthetase, isoform CRA_b OS=Homo sapiens GN=GMPS PE=2 SV=1	77408	204	157	193	196	0.23	0.37	1.608696	5	5	6	7	7	9	8	9	1.001721
tr|Q96CV8|Q96CV8_HUMAN	THOP1	Thimet oligopeptidase 1 OS=Homo sapiens GN=THOP1 PE=2 SV=1	79686	371	574	110	49	0.1	0.16	1.6	2	1	2	1	2	1	3	1	-0.07784
tr|B7Z4U7|B7Z4U7_HUMAN	SCFD1	Sec1 family domain containing 1, isoform CRA_b OS=Homo sapiens GN=SCFD1 PE=2 SV=1	65362	516	252	74	120	0.1	0.16	1.6	1	3	2	3	1	3	2	4	1.882368
sp|P38919|IF4A3_HUMAN	EIF4A3	Eukaryotic initiation factor 4A-III OS=Homo sapiens GN=EIF4A3 PE=1 SV=4	47126	152	132	265	220	0.61	0.97	1.590164	7	6	8	11	9	11	9	11	0.936555
sp|P22059|OSBP1_HUMAN	OSBP	Oxysterol-binding protein 1 OS=Homo sapiens GN=OSBP PE=1 SV=1	90220	850	328	40	88	0.07	0.11	1.571429	1	1	2	3	1	2	2	3	1.39662
sp|Q8NI27|THOC2_HUMAN	THOC2	THO complex subunit 2 OS=Homo sapiens GN=THOC2 PE=1 SV=2	184541	250	183	162	172	0.07	0.11	1.571429	3	4	4	6	4	6	5	6	1.190844
sp|Q13151|ROA0_HUMAN	HNRNPA0	Heterogeneous nuclear ribonucleoprotein A0 OS=Homo sapiens GN=HNRNPA0 PE=1 SV=1	30993	346	227	117	136	0.23	0.36	1.565217	2	3	2	3	4	3	5	4	0.296795
tr|H0Y3Y4|H0Y3Y4_HUMAN	Sep-07	Septin-7 (Fragment) OS=Homo sapiens GN=SEPT7 PE=3 SV=1	43294	576	283	64	105	0.16	0.25	1.5625	3	3	3	3	3	4	3	4	1.02246
sp|O76094|SRP72_HUMAN	SRP72	Signal recognition particle 72 kDa protein OS=Homo sapiens GN=SRP72 PE=1 SV=3	75130	661	253	54	120	0.09	0.14	1.555556	2	2	2	3	2	3	2	5	1.145199
sp|P51116|FXR2_HUMAN	FXR2	Fragile X mental retardation syndrome-related protein 2 OS=Homo sapiens GN=FXR2 PE=1 SV=2	74520	437	223	91	138	0.09	0.14	1.555556	2	3	2	3	2	3	4	3	1.145199
tr|G5E9Q2|G5E9Q2_HUMAN	CSDE1	Cold shock domain containing E1, RNA-binding, isoform CRA_d OS=Homo sapiens GN=CSDE1 PE=2 SV=1	94539	257	134	157	217	0.15	0.23	1.533333	3	5	4	7	4	8	6	8	1.578511
tr|H0YEK7|H0YEK7_HUMAN	CKAP5	Cytoskeleton-associated protein 5 (Fragment) OS=Homo sapiens GN=CKAP5 PE=4 SV=1	40792	183	861	68	27	0.12	0.18	1.5	2	0	2	1	2	1	2	2	-0.07784
tr|B4DYY5|B4DYY5_HUMAN	tr|B4DYY5	cDNA FLJ55671, highly similar to Ubiquitin-associated protein 2-like OS=Homo sapiens PE=2 SV=1	113662	646	625	57	44	0.06	0.09	1.5	1	1	2	3	2	3	2	4	1.145199
tr|F5H8E5|F5H8E5_HUMAN	USP7	Ubiquitin carboxyl-terminal hydrolase OS=Homo sapiens GN=USP7 PE=2 SV=1	117951	677	474	53	61	0.06	0.09	1.5	1	1	2	3	3	3	6	3	0.659568
sp|Q9NX58|LYAR_HUMAN	LYAR	Cell growth-regulating nucleolar protein OS=Homo sapiens GN=LYAR PE=1 SV=2	44044	523	453	72	65	0.16	0.24	1.5	2	1	2	3	2	3	2	3	1.145199
tr|B4DFL1|B4DFL1_HUMAN	tr|B4DFL1	Dihydrolipoyl dehydrogenase OS=Homo sapiens PE=2 SV=1	49381	525	221	72	138	0.14	0.21	1.5	2	2	2	3	2	3	3	4	1.145199
tr|B4DGP8|B4DGP8_HUMAN	CANX	Calnexin OS=Homo sapiens GN=CANX PE=2 SV=1	71971	251	106	161	258	0.25	0.37	1.48	3	6	5	8	5	9	5	9	1.449587
tr|Q5T621|Q5T621_HUMAN	AKR1A1	Alcohol dehydrogenase [NADP(+)] (Fragment) OS=Homo sapiens GN=AKR1A1 PE=4 SV=1	16677	816	618	42	17	0.2	0.29	1.45	1	0	1	1	1	0	1	2	-0.92595
tr|E7ENU4|E7ENU4_HUMAN	ADAR	Double-stranded RNA-specific adenosine deaminase OS=Homo sapiens GN=ADAR PE=2 SV=1	142051	319	206	127	149	0.07	0.1	1.428571	2	4	3	4	4	4	4	4	0.659686
tr|H0Y8W1|H0Y8W1_HUMAN	ZFR	Zinc finger RNA-binding protein (Fragment) OS=Homo sapiens GN=ZFR PE=4 SV=1	20174	326	404	51	71	0.12	0.17	1.416667	1	1	1	1	1	2	1	2	1.39662
tr|B7Z7P8|B7Z7P8_HUMAN	ETF1	Eukaryotic peptide chain release factor subunit 1 OS=Homo sapiens GN=ETF1 PE=2 SV=1	47731	406	363	101	81	0.22	0.31	1.409091	2	1	3	4	3	3	5	6	0.659568
tr|H0Y7C0|H0Y7C0_HUMAN	USO1	General vesicular transport factor p115 (Fragment) OS=Homo sapiens GN=USO1 PE=4 SV=1	72345	681	317	53	92	0.05	0.07	1.4	1	1	1	1	1	1	1	1	0.659333
tr|Q0VGD6|Q0VGD6_HUMAN	HNRPR	HNRPR protein (Fragment) OS=Homo sapiens GN=HNRPR PE=2 SV=1	68218	146	73	274	333	0.6	0.84	1.4	10	11	12	15	11	17	17	17	1.268168
tr|F8W7C6|F8W7C6_HUMAN	RPL10	60S ribosomal protein L10 OS=Homo sapiens GN=RPL10 PE=2 SV=1	18808	485	291	59	103	0.28	0.39	1.392857	1	1	1	2	2	2	2	2	0.65945
tr|Q5H928|Q5H928_HUMAN	HSD17B10	3-hydroxyacyl-CoA dehydrogenase type-2 OS=Homo sapiens GN=HSD17B10 PE=4 SV=1	17498	782	409	45	70	0.19	0.26	1.368421	1	2	1	2	1	2	1	2	1.39662
tr|Q6ZSB8|Q6ZSB8_HUMAN	tr|Q6ZSB8	cDNA FLJ45654 fis, clone CTONG2012123, moderately similar to Mus musculus enabled homolog (Drosophila) (Enah) OS=Homo sapiens PE	22845	808	814	0	31	0.11	0.15	1.363636	0	0	0	1	0	1	1	2	2.244499
tr|B4DIJ6|B4DIJ6_HUMAN	DDX39B	Spliceosome RNA helicase DDX39B OS=Homo sapiens GN=DDX39B PE=2 SV=1	28167	311	387	48	76	0.55	0.75	1.363636	1	1	4	5	7	6	7	7	0.453268
tr|B3KRK4|B3KRK4_HUMAN	tr|B3KRK4	cDNA FLJ34459 fis, clone HLUNG2002916, highly similar to SRC SUBSTRATE CORTACTIN OS=Homo sapiens PE=2 SV=1	26264	495	235	77	73	0.13	0.17	1.307692	1	1	1	1	1	1	1	1	0.659333
sp|Q9UBU9|NXF1_HUMAN	NXF1	Nuclear RNA export factor 1 OS=Homo sapiens GN=NXF1 PE=1 SV=1	70652	683	988	53	19	0.1	0.13	1.3	1	0	2	1	2	0	2	1	-1.66312
sp|Q7L2E3|DHX30_HUMAN	DHX30	Putative ATP-dependent RNA helicase DHX30 OS=Homo sapiens GN=DHX30 PE=1 SV=1	134938	321	452	125	65	0.1	0.13	1.3	3	0	4	5	5	4	6	6	0.369976
tr|B4DRW3|B4DRW3_HUMAN	tr|B4DRW3	cDNA FLJ57180, highly similar to TAR DNA-binding protein 43 OS=Homo sapiens PE=2 SV=1	35596	308	290	130	90	0.19	0.24	1.263158	2	2	2	2	2	2	2	2	0.65945
tr|J3KSD8|J3KSD8_HUMAN	BLMH	Bleomycin hydrolase (Fragment) OS=Homo sapiens GN=BLMH PE=4 SV=1	30628	695	623	52	23	0.23	0.29	1.26087	1	0	2	2	2	1	2	2	-0.07784
sp|P54886|P5CS_HUMAN	ALDH18A1	Delta-1-pyrroline-5-carboxylate synthase OS=Homo sapiens GN=ALDH18A1 PE=1 SV=2	87989	195	131	204	225	0.44	0.55	1.25	7	7	11	12	13	13	14	14	0.66075
tr|B0AZQ4|B0AZQ4_HUMAN	tr|B0AZQ4	cDNA, FLJ79494, highly similar to Structural maintenance of chromosome 3 OS=Homo sapiens PE=2 SV=1	141841	267	84	150	310	0.12	0.15	1.25	3	5	5	6	7	6	7	6	0.453268
tr|Q59GY2|Q59GY2_HUMAN	tr|Q59GY2	Ribosomal protein L4 variant (Fragment) OS=Homo sapiens PE=2 SV=1	49307	143	70	276	336	1.32	1.64	1.242424	8	9	13	16	15	15	18	19	0.660987
sp|Q3ZCM7|TBB8_HUMAN	TUBB8	Tubulin beta-8 chain OS=Homo sapiens GN=TUBB8 PE=1 SV=2	50257	61	76	575	327	1.28	1.59	1.242188	26	15	28	20	30	22	34	24	0.2213
sp|P11021|GRP78_HUMAN	HSPA5	78 kDa glucose-regulated protein OS=Homo sapiens GN=HSPA5 PE=1 SV=2	72402	79	35	443	597	0.63	0.78	1.238095	12	13	13	15	13	14	13	16	0.764166
sp|P41250|SYG_HUMAN	GARS	Glycine--tRNA ligase OS=Homo sapiens GN=GARS PE=1 SV=3	83854	415	273	98	109	0.17	0.21	1.235294	3	2	4	5	4	4	6	5	0.659686
sp|O94776|MTA2_HUMAN	MTA2	Metastasis-associated protein MTA2 OS=Homo sapiens GN=MTA2 PE=1 SV=1	75717	216	159	182	194	0.24	0.29	1.208333	4	5	5	6	5	6	5	6	0.901134
tr|A6NEM2|A6NEM2_HUMAN	HCFC1	HCF N-terminal chain 5 OS=Homo sapiens GN=HCFC1 PE=2 SV=2	215338	465	455	83	65	0.05	0.06	1.2	2	2	3	4	3	3	4	5	0.659568
sp|Q15393|SF3B3_HUMAN	SF3B3	Splicing factor 3B subunit 3 OS=Homo sapiens GN=SF3B3 PE=1 SV=4	136575	120	123	331	235	0.21	0.25	1.190476	10	7	11	8	12	8	14	8	0.102951
tr|E2QRB3|E2QRB3_HUMAN	PYCR1	Pyrroline-5-carboxylate reductase 1, isoform CRA_c OS=Homo sapiens GN=PYCR1 PE=4 SV=2	30422	911	294	35	101	0.11	0.13	1.181818	0	1	1	1	1	1	2	1	0.659333
tr|Q6DC98|Q6DC98_HUMAN	LMNB1	LMNB1 protein (Fragment) OS=Homo sapiens GN=LMNB1 PE=2 SV=1	38289	381	158	85	195	0.33	0.39	1.181818	4	4	4	5	4	7	5	7	1.397617
sp|P49411|EFTU_HUMAN	TUFM	Elongation factor Tu, mitochondrial OS=Homo sapiens GN=TUFM PE=1 SV=2	49852	179	119	220	240	0.57	0.67	1.175439	5	4	7	8	8	8	9	9	0.660159
tr|B4DGB4|B4DGB4_HUMAN	PLS3	Plastin-3 OS=Homo sapiens GN=PLS3 PE=2 SV=1	69820	241	199	166	156	0.38	0.44	1.157895	5	4	7	8	9	9	11	9	0.660277
sp|P00338|LDHA_HUMAN	LDHA	L-lactate dehydrogenase A chain OS=Homo sapiens GN=LDHA PE=1 SV=2	36950	180	79	219	319	1.8	2.05	1.138889	8	9	13	15	17	17	20	19	0.661224
sp|P08670|VIME_HUMAN	VIM	Vimentin OS=Homo sapiens GN=VIM PE=1 SV=4	53676	128	54	293	413	1.04	1.17	1.125	9	10	12	14	14	16	15	17	0.847926
sp|Q9UHX1|PUF60_HUMAN	PUF60	Poly(U)-binding-splicing factor PUF60 OS=Homo sapiens GN=PUF60 PE=1 SV=1	60009	557	278	67	107	0.17	0.19	1.117647	2	2	3	2	3	2	3	3	0.17382
tr|B2R8R5|B2R8R5_HUMAN	tr|B2R8R5	cDNA, FLJ94025, highly similar to Homo sapiens tripartite motif-containing 28 (TRIM28), mRNA OS=Homo sapiens PE=2 SV=1	90271	137	82	284	312	0.43	0.48	1.116279	7	8	14	12	17	15	17	18	0.485493
tr|K9JA46|K9JA46_HUMAN	EL52	Epididymis luminal secretory protein 52 OS=Homo sapiens GN=EL52 PE=2 SV=1	85006	17	14	1239	1146	2	2.23	1.115	31	29	43	50	47	54	50	58	0.865404
tr|Q6P151|Q6P151_HUMAN	SNW1	SNW1 protein (Fragment) OS=Homo sapiens GN=SNW1 PE=2 SV=1	36026	975	676	32	41	0.09	0.1	1.111111	0	1	1	1	1	1	2	1	0.659333

tr|A8K6U7|A8K6U7_HUMAN	tr|A8K6U7	cDNA FLJ78252, highly similar to Homo sapiens heterogeneous nuclear ribonucleoprotein U-like 1 (HNRPUL1), transcript variant 1, mRNA O	96189	258	360	156	81	0.18	0.2	1.111111	3	2	5	5	5	6	5	6	0.901134
sp|P55072|TERA_HUMAN	VCP	Transitional endoplasmic reticulum ATPase OS=Homo sapiens GN=VCP PE=1 SV=4	89950	93	46	396	472	0.54	0.59	1.092593	9	8	12	13	11	14	14	16	0.995899
tr|B4DNN4|B4DNN4_HUMAN	tr|B4DNN4	Ribonucleoside-diphosphate reductase OS=Homo sapiens PE=2 SV=1	87252	294	388	134	75	0.12	0.13	1.083333	4	2	4	3	5	3	5	5	0.007085
sp|P51991|ROA3_HUMAN	HNRNPA3	Heterogeneous nuclear ribonucleoprotein A3 OS=Homo sapiens GN=HNRNPA3 PE=1 SV=2	39799	197	259	202	115	0.49	0.53	1.081633	4	2	5	5	10	6	11	7	-0.03277
tr|H0YHC3|H0YHC3_HUMAN	NAP1L1	Nucleosome assembly protein 1-like 1 (Fragment) OS=Homo sapiens GN=NAP1L1 PE=3 SV=1	23517	289	136	136	175	0.49	0.53	1.081633	3	3	4	5	4	6	4	6	1.190844
tr|H0YKD0|H0YKD0_HUMAN	IDH3A	Isocitrate dehydrogenase [NAD] subunit alpha, mitochondrial (Fragment) OS=Homo sapiens GN=IDH3A PE=3 SV=1	12184	710	835	51	24	0.28	0.3	1.071429	1	0	1	1	1	1	1	1	0.659333
tr|Q5SU16|Q5SU16_HUMAN	TUBB	Beta 5-tubulin OS=Homo sapiens GN=TUBB PE=2 SV=1	50095	2	7	1882	1323	15.54	16.63	1.070142	64	45	79	59	85	61	97	70	0.186212
tr|B4DJ30|B4DJ30_HUMAN	tr|B4DJ30	cDNA FLJ61290, highly similar to Neutral alpha-glucosidase AB OS=Homo sapiens PE=2 SV=1	113539	45	20	707	797	0.82	0.87	1.060976	15	18	24	29	22	29	30	35	1.054869
tr|B4DQX0|B4DQX0_HUMAN	tr|B4DQX0	cDNA FLJ59219, highly similar to Poly(A)-binding protein 1 OS=Homo sapiens PE=2 SV=1	67239	115	50	344	396	0.69	0.72	1.043478	8	9	11	12	12	14	14	16	0.875401
tr|B4DIW2|B4DIW2_HUMAN	tr|B4DIW2	cDNA FLJ54035, highly similar to Neutral alpha-glucosidase AB OS=Homo sapiens PE=2 SV=1	94218	45	22	697	750	0.82	0.85	1.036585	15	16	23	25	21	25	28	31	0.909151
sp|Q2NL82|TSR1_HUMAN	TSR1	Pre-rRNA-processing protein TSR1 homolog OS=Homo sapiens GN=TSR1 PE=1 SV=1	92151	1170	1017	20	16	0.04	0.04	1	0	0	1	1	1	0	1	3	-0.92595
tr|B4DVS2|B4DVS2_HUMAN	tr|B4DVS2	cDNA FLJ57617 OS=Homo sapiens PE=2 SV=1	89176	753	1015	48	16	0.04	0.04	1	1	0	1	1	1	0	1	1	-0.92595
sp|Q06830|PRDX1_HUMAN	PRDX1	Peroxiredoxin-1 OS=Homo sapiens GN=PRDX1 PE=1 SV=1	22324	949	1014	33	17	0.15	0.15	1	1	0	1	1	2	1	3	3	-0.07784
tr|B3KPL5|B3KPL5_HUMAN	tr|B3KPL5	cDNA FLJ31917 fis, clone NT2RP7004925, highly similar to Sprouty-related, EVH1 domain-containing protein 2 OS=Homo sapiens PE=2 SV=1	48711	1126	1009	23	17	0.07	0.07	1	0	0	1	1	8	4	15	13	-0.25867
tr|H0Y4J2|H0Y4J2_HUMAN	MRPL37	39S ribosomal protein L37, mitochondrial (Fragment) OS=Homo sapiens GN=MRPL37 PE=4 SV=1	29806	583	1007	64	17	0.11	0.11	1	1	0	1	1	1	0	1	1	-0.92595
tr|B3KRC6|B3KRC6_HUMAN	tr|B3KRC6	cDNA FLJ34004 fis, clone FCBBF1000232, highly similar to Cytochrome P450 51A1 (EC 1.14.13.70) OS=Homo sapiens PE=2 SV=1	51732	1107	996	24	18	0.06	0.06	1	0	0	1	1	1	0	1	1	-0.92595
tr|B4DI19|B4DI19_HUMAN	tr|B4DI19	cDNA FLJ59524, highly similar to Cysteinyl-tRNA synthetase (EC 6.1.1.16) OS=Homo sapiens PE=2 SV=1	76209	761	984	47	19	0.04	0.04	1	1	0	1	1	1	0	1	1	-0.92595
tr|B4E074|B4E074_HUMAN	tr|B4E074	cDNA FLJ58655, highly similar to Notchless homolog 1 OS=Homo sapiens PE=2 SV=1	51211	1054	980	27	20	0.06	0.06	1	0	0	1	1	1	0	1	1	-0.92595
tr|Q6AHZ7|Q6AHZ7_HUMAN	DKFZp686A	Putative uncharacterized protein DKFZp686A111 OS=Homo sapiens GN=DKFZp686A111 PE=2 SV=1	240988	707	969	51	21	0.01	0.01	1	1	0	1	1	1	1	3	2	0.659333
tr|G0ZJH6|G0ZJH6_HUMAN	Ube4B	UFD2A-III/UBE4B-III splice isoform OS=Homo sapiens GN=Ube4B PE=2 SV=1	152890	691	968	52	21	0.02	0.02	1	1	0	1	1	1	1	1	1	0.659333
sp|Q00653|NFKB2_HUMAN	NFKB2	Nuclear factor NF-kappa-B p100 subunit OS=Homo sapiens GN=NFKB2 PE=1 SV=4	97373	1101	967	24	21	0.03	0.03	1	0	0	1	1	1	1	2	2	0.659333
tr|Q0VAM0|Q0VAM0_HUMA	CTNNBL1	Catenin, beta like 1 OS=Homo sapiens GN=CTNNBL1 PE=2 SV=1	65560	1064	963	26	21	0.05	0.05	1	0	0	1	1	1	1	1	1	0.659333
tr|B4DRK5|B4DRK5_HUMAN	tr|B4DRK5	cDNA FLJ59584, highly similar to Mitochondrial-processing peptidase alpha subunit, mitochondrial (EC 3.4.24.64) OS=Homo sapiens PE=2 S	28622	945	960	33	21	0.12	0.12	1	1	0	1	1	1	1	2	1	0.659333
tr|B4DL79|B4DL79_HUMAN	KIF20A	Kinesin-like protein KIF20A OS=Homo sapiens GN=KIF20A PE=2 SV=1	99066	895	956	36	22	0.03	0.03	1	1	0	1	1	1	1	1	3	0.659333
tr|Q5JXH7|Q5JXH7_HUMAN	FHL1	Four and a half LIM domains 1 (Fragment) OS=Homo sapiens GN=FHL1 PE=4 SV=1	24735	1100	934	24	23	0.14	0.14	1	1	0	1	1	1	1	1	1	0.659333
tr|B4DFQ5|B4DFQ5_HUMAN	tr|B4DFQ5	cDNA FLJ58134, highly similar to Cleavage stimulation factor 77 kDa subunit OS=Homo sapiens PE=2 SV=1	72538	902	926	36	23	0.05	0.05	1	1	0	1	1	1	1	1	1	0.659333
tr|B5MBZ0|B5MBZ0_HUMAN	EML4	Echinoderm microtubule-associated protein-like 4 OS=Homo sapiens GN=EML4 PE=2 SV=3	111192	971	917	32	24	0.03	0.03	1	0	0	1	1	1	2	3	2	1.39662
sp|Q9NQH7|XPP3_HUMAN	XPNPEP3	Probable Xaa-Pro aminopeptidase 3 OS=Homo sapiens GN=XPNPEP3 PE=1 SV=1	57624	933	914	34	24	0.06	0.06	1	1	0	2	1	2	1	2	1	-0.07784
tr|Q6UWE9|Q6UWE9_HUMA	UNQ2550	SFVP2550 OS=Homo sapiens GN=UNQ2550 PE=4 SV=1	11890	1172	907	20	25	0.29	0.29	1	1	1	1	1	0	1	1	1	2.244499
tr|G3V3E6|G3V3E6_HUMAN	ERO1L	ERO1-like protein alpha OS=Homo sapiens GN=ERO1L PE=4 SV=1	30351	700	904	52	25	0.11	0.11	1	1	0	1	1	1	1	1	1	0.659333
tr|B4DJE3|B4DJE3_HUMAN	tr|B4DJE3	cDNA FLJ52929, highly similar to Dolichyl-diphosphooligosaccharide--proteinglycosyltransferase 48 kDa subunit (EC2.4.1.119) OS=Homo sa	46620	996	900	31	25	0.07	0.07	1	1	0	1	1	1	2	1	4	1.39662
tr|G3V2H3|G3V2H3_HUMAN	PNP	Purine nucleoside phosphorylase (Fragment) OS=Homo sapiens GN=PNP PE=2 SV=1	10579	1121	897	23	25	0.33	0.33	1	0	0	1	1	1	1	1	1	0.659333
tr|C0JXB8|C0JXB8_HUMAN	AIP	Aryl hydrocarbon receptor interacting protein OS=Homo sapiens GN=AIP PE=2 SV=1	38041	1158	894	21	25	0.09	0.09	1	0	0	1	1	1	1	1	1	0.659333
tr|B2R4C0|B2R4C0_HUMAN	tr|B2R4C0	60S ribosomal protein L18a OS=Homo sapiens PE=2 SV=1	21024	755	892	47	26	0.16	0.16	1	1	0	1	1	2	2	2	2	0.65945
tr|B0AZR9|B0AZR9_HUMAN	GNAS	GNAS complex locus, isoform CRA_b OS=Homo sapiens GN=GNAS PE=2 SV=1	39373	1085	884	25	26	0.08	0.08	1	0	0	1	1	1	1	1	1	0.659333
tr|B2R713|B2R713_HUMAN	tr|B2R713	cDNA, FLJ93224 OS=Homo sapiens PE=2 SV=1	82921	878	871	38	27	0.04	0.04	1	1	0	1	1	1	1	1	1	0.659333
sp|P0CW22|RS17L_HUMAN	RPS17L	40S ribosomal protein S17-like OS=Homo sapiens GN=RPS17L PE=3 SV=1	15597	819	870	42	27	0.22	0.22	1	1	0	1	1	1	1	1	1	0.659333
tr|F2Z303|F2Z303_HUMAN	UMPS	Orotate phosphoribosyltransferase OS=Homo sapiens GN=UMPS PE=4 SV=1	7082	751	868	48	27	0.5	0.5	1	1	0	1	1	1	1	1	1	0.659333
tr|B4DFK6|B4DFK6_HUMAN	CNN3	Calponin-3 OS=Homo sapiens GN=CNN3 PE=2 SV=1	31889	1011	857	30	27	0.1	0.1	1	0	0	1	1	1	1	1	1	0.659333
tr|I6L9B1|I6L9B1_HUMAN	EPB41L2	EPB41L2 protein OS=Homo sapiens GN=EPB41L2 PE=2 SV=1	71998	823	843	41	28	0.05	0.05	1	1	0	1	1	1	1	3	1	0.659333
tr|B7Z611|B7Z611_HUMAN	tr|B7Z611	cDNA FLJ50848, highly similar to Homo sapiens suppressor of var1, 3-like 1 (S. cerevisiae) (SUPV3L1), mRNA (Fragment) OS=Homo sapiens F	71856	659	841	55	29	0.05	0.05	1	1	0	1	1	1	1	1	1	0.659333
tr|B2RBM8|B2RBM8_HUMAN	tr|B2RBM8	cDNA, FLJ95596, highly similar to Homo sapiens activity-dependent neuroprotector (ADNP), mRNA OS=Homo sapiens PE=2 SV=1	124850	956	840	33	29	0.03	0.03	1	0	0	1	1	1	1	2	1	0.659333
sp|Q9BVI4|NOC4L_HUMAN	NOC4L	Nucleolar complex protein 4 homolog OS=Homo sapiens GN=NOC4L PE=1 SV=1	58830	1169	838	20	29	0.06	0.06	1	0	0	1	1	1	1	1	2	0.659333
tr|F8VS81|F8VS81_HUMAN	TWF1	Twinfilin-1 (Fragment) OS=Homo sapiens GN=TWF1 PE=2 SV=1	33615	680	836	53	29	0.1	0.1	1	1	0	1	1	1	1	1	1	0.659333
tr|H0Y8B6|H0Y8B6_HUMAN	NVL	Nuclear valosin-containing protein-like (Fragment) OS=Homo sapiens GN=NVL PE=3 SV=1	82802	901	827	36	30	0.04	0.04	1	1	0	1	1	1	1	1	1	0.659333
tr|B4DZM3|B4DZM3_HUMAN	tr|B4DZM3	cDNA FLJ61500, highly similar to NNP-1 protein OS=Homo sapiens PE=2 SV=1	46521	701	821	52	30	0.07	0.07	1	1	0	1	1	1	1	1	1	0.659333
tr|A2VCT2|A2VCT2_HUMAN	OGDH	OGDH protein (Fragment) OS=Homo sapiens GN=OGDH PE=2 SV=1	72973	886	817	37	31	0.05	0.05	1	1	0	1	1	1	1	1	2	0.659333
tr|B4DZ67|B4DZ67_HUMAN	NUP107	Nuclear pore complex protein Nup107 OS=Homo sapiens GN=NUP107 PE=2 SV=1	103851	986	815	31	0	0.03	0.03	1	0	0	1	0	1	0	2	1	-0.92595
tr|B1ANM7|B1ANM7_HUMA	FAF1	FAS-associated factor 1 OS=Homo sapiens GN=FAF1 PE=4 SV=1	56305	1037	808	28	31	0.06	0.06	1	0	0	1	1	1	1	1	1	0.659333
tr|B3KMC2|B3KMC2_HUMAN	tr|B3KMC2	cDNA FLJ10676 fis, clone NT2RP2006464, highly similar to WD repeat and HMG-box DNA-binding protein 1 OS=Homo sapiens PE=2 SV=1	73526	507	803	75	32	0.04	0.04	1	1	1	1	1	1	1	1	1	0.659333
sp|Q96IJ6|GMPPA_HUMAN	GMPPA	Mannose-1-phosphate guanyltransferase alpha OS=Homo sapiens GN=GMPPA PE=1 SV=1	46604	1013	801	30	32	0.07	0.07	1	0	0	1	1	1	1	1	1	0.659333
tr|A2A2Q9|A2A2Q9_HUMAN	C20orf4	Chromosome 20 open reading frame 4 OS=Homo sapiens GN=C20orf4 PE=4 SV=1	45575	1074	795	26	32	0.07	0.07	1	0	0	1	1	1	1	1	1	0.659333
tr|A5D904|A5D904_HUMAN	RPS9	RPS9 protein (Fragment) OS=Homo sapiens GN=RPS9 PE=2 SV=1	13210	662	793	54	32	0.26	0.26	1	1	0	1	1	2	1	2	4	-0.07784
tr|B4DME9|B4DME9_HUMAN	KLC4	Kinesin light chain 4 OS=Homo sapiens GN=KLC4 PE=2 SV=1	60003	1025	789	29	32	0.05	0.05	1	0	0	1	1	1	1	1	1	0.659333
tr|H0YF33|H0YF33_HUMAN	NASP	Nuclear autoantigenic sperm protein (Fragment) OS=Homo sapiens GN=NASP PE=4 SV=1	31883	1059	776	27	33	0.1	0.1	1	0	0	1	1	1	1	2	1	0.659333
tr|H0Y449|H0Y449_HUMAN	YBX1	Nuclease-sensitive element-binding protein 1 (Fragment) OS=Homo sapiens GN=YBX1 PE=4 SV=1	42333	439	775	90	33	0.16	0.16	1	1	1	2	2	2	1	2	2	-0.07784
tr|B7Z3P1|B7Z3P1_HUMAN	TBCE	Tubulin-specific chaperone E OS=Homo sapiens GN=TBCE PE=2 SV=1	65666	864	771	39	33	0.05	0.05	1	1	1	1	1	1	1	1	1	0.659333
sp|Q16222|UAP1_HUMAN	UAP1	UDP-N-acetylhexosamine pyrophosphorylase OS=Homo sapiens GN=UAP1 PE=1 SV=3	59131	846	765	40	33	0.06	0.06	1	1	0	1	1	1	1	2	2	0.659333
tr|B5BUB5|B5BUB5_HUMAN	SSB	Autoantigen La (Fragment) OS=Homo sapiens GN=SSB PE=2 SV=1	47009	623	764	59	33	0.07	0.07	1	1	1	1	1	1	2	2	4	1.39662
tr|D3DVR8|D3DVR8_HUMAN	HD	Huntingtin (Huntington disease), isoform CRA_a OS=Homo sapiens GN=HD PE=2 SV=1	291559	907	758	35	33	0.01	0.01	1	0	0	1	1	1	1	2	1	0.659333
tr|B7Z438|B7Z438_HUMAN	tr|B7Z438	cDNA FLJ56352, highly similar to Succinyl-CoA ligase (GDP-forming) subunit alpha, mitochondrial (EC 6.2.1.4) OS=Homo sapiens PE=2 SV=1	26801	894	753	36	34	0.26	0.26	1	1	0	2	2	2	1	2	2	-0.07784
tr|Q0IJ56|Q0IJ56_HUMAN	ST13	ST13 protein (Fragment) OS=Homo sapiens GN=ST13 PE=2 SV=1	35164	577	741	64	35	0.09	0.09	1	1	1	1	1	2	1	2	1	-0.07784
tr|B2RD24|B2RD24_HUMAN	tr|B2RD24	cDNA, FLJ96424 OS=Homo sapiens PE=2 SV=1	80216	738	732	49	35	0.04	0.04	1	1	1	1	1	2	1	2	1	-0.07784
tr|B4DGU4|B4DGU4_HUMAN	CTNNB1	Catenin beta-1 OS=Homo sapiens GN=CTNNB1 PE=2 SV=1	85339	1052	727	27	36	0.04	0.04	1	1	0	1	1	2	2	2	3	0.65945
tr|B4DS71|B4DS71_HUMAN	tr|B4DS71	cDNA FLJ57081, moderately similar to WD repeat protein 1 OS=Homo sapiens PE=2 SV=1	22638	777	722	45	36	0.15	0.15	1	1	0	1	1	1	1	1	1	0.659333
tr|D3YTB1|D3YTB1_HUMAN	RPL32	60S ribosomal protein L32 (Fragment) OS=Homo sapiens GN=RPL32 PE=2 SV=1	15721	647	721	56	36	0.22	0.22	1	1	1	1	1	1	1	1	1	0.659333
tr|J3QL54|J3QL54_HUMAN	NUP85	Nuclear pore complex protein Nup85 OS=Homo sapiens GN=NUP85 PE=4 SV=1	62746	880	718	37	36	0.05	0.05	1	1	1	1	1	1	1	1	1	0.659333
tr|F6U211|F6U211_HUMAN	RPS10	40S ribosomal protein S10 OS=Homo sapiens GN=RPS10 PE=2 SV=1	19912	670	716	54	37	0.17	0.17	1	1	1	1	1	1	1	1	1	0.659333
tr|F5H4C2|F5H4C2_HUMAN	RANBP3	Ran-binding protein 3 OS=Homo sapiens GN=RANBP3 PE=2 SV=1	47206	1070	707	26	37	0.07	0.07	1	0	1	1	1	1	1	2	1	0.659333
tr|B4DTX6|B4DTX6_HUMAN	tr|B4DTX6	cDNA FLJ54311, highly similar to Ran-binding protein 6 OS=Homo sapiens PE=2 SV=1	79646	1187	704	18	37	0.04	0.04	1	0	1	1	1	1	1	3	1	0.659333
tr|B4DLY2|B4DLY2_HUMAN	tr|B4DLY2	cDNA FLJ56600, highly similar to Large proline-rich protein BAT2 OS=Homo sapiens PE=2 SV=1	146160	778	693	45	39	0.05	0.05	1	0	1	2	2	2	1	2	2	-0.07784
tr|B3KNC3|B3KNC3_HUMAN	tr|B3KNC3	cDNA FLJ14222 fis, clone NT2RP3003992, highly similar to Nucleolar complex protein 2 homolog OS=Homo sapiens PE=2 SV=1	85719	451	690	86	39	0.08	0.08	1	2	0	2	2	3	1	3	2	-0.56347
sp|Q6PKG0|LARP1_HUMAN	LARP1	La-related protein 1 OS=Homo sapiens GN=LARP1 PE=1 SV=2	123833	1024	673	29	41	0.03	0.03	1	0	1	1	1	1	1	1	3	0.659333
tr|H0Y9G6|H0Y9G6_HUMAN	MRPL3	39S ribosomal protein L3, mitochondrial (Fragment) OS=Homo sapiens GN=MRPL3 PE=3 SV=1	40898	828	671	41	41	0.08	0.08	1	1	1	1	1	2	1	2	1	-0.07784
tr|D3DQA6|D3DQA6_HUMAN	DDX46	DEAD (Asp-Glu-Ala-Asp) box polypeptide 46, isoform CRA_a OS=Homo sapiens GN=DDX46 PE=3 SV=1	100958	1040	667	28	41	0.03	0.03	1	0	1	1	1	1	1	1	2	0.659333
tr|H0Y8R1|H0Y8R1_HUMAN	GRSF1	G-rich sequence factor 1 (Fragment) OS=Homo sapiens GN=GRSF1 PE=4 SV=1	47637	586	649	63	42	0.07	0.07	1	1	1	1	1	1	1	1	1	0.659333
sp|P27635|RL10_HUMAN	RPL10	60S ribosomal protein L10 OS=Homo sapiens GN=RPL10 PE=1 SV=4	25044	485	641	80	43	0.28	0.28	1	2	0	2	2	4	2	4	2	-0.18895
sp|Q9P2D3|HTR5B_HUMAN	HEATR5B	HEAT repeat-containing protein 5B OS=Homo sapiens GN=HEATR5B PE=1 SV=2	226839	788	633	44	44	0.01	0.01	1	1	1	1	1	1	1	1	2	0.659333
tr|Q6FHM2|Q6FHM2_HUMA	GNB2	GNB2 protein OS=Homo sapiens GN=GNB2 PE=2 SV=1	38048	617	617	59	0	0.09	0.09	1	1	0	1	0	1	0	1	1	-0.92595
tr|B4DZE5|B4DZE5_HUMAN	tr|B4DZE5	cDNA FLJ58806, highly similar to DNA-directed RNA polymerase, mitochondrial (EC 2.7.7.6) OS=Homo sapiens PE=2 SV=1	106234	714	616	51	45	0.03	0.03	1	1	1	1	1	2	2	3	3	0.65945
tr|B4DZ87|B4DZ87_HUMAN	tr|B4DZ87	cDNA FLJ57240, highly similar to Mitochondrial proteins import receptor OS=Homo sapiens PE=2 SV=1	57579	565	612	66	46	0.12	0.12	1	1	1	2	1	2	1	3	1	-0.07784
tr|B3KPC8|B3KPC8_HUMAN	tr|B3KPC8	cDNA FLJ31621 fis, clone NT2RI2003205, highly similar to Transportin-2 OS=Homo sapiens PE=2 SV=1	79954	1173	611	20	46	0.04	0.04	1	0	1	1	1	1	1	4	2	0.659333
tr|K7EKG4|K7EKG4_HUMAN	TOMM40	Mitochondrial import receptor subunit TOM40 homolog (Fragment) OS=Homo sapiens GN=TOMM40 PE=4 SV=1	18328	1078	609	26	46	0.18	0.18	1	0	1	1	1	2	1	2	1	-0.07784
tr|B4DFK7|B4DFK7_HUMAN	CBR1	Carbonyl reductase 1, isoform CRA_c OS=Homo sapiens GN=CBR1 PE=2 SV=1	19092	508	606	75	46	0.18	0.18	1	1	1	1	1	1	1	1	1	0.659333
tr|B2RAU8|B2RAU8_HUMAN	tr|B2RAU8	cDNA, FLJ95131, highly similar to Homo sapiens nucleolar and coiled-body phosphoprotein 1 (NOLC1), mRNA OS=Homo sapiens PE=2 SV=1	73561	1110	597	24	47	0.04	0.04	1	0	1	1	2	1	2	1	2	1.39662
tr|Q5T1D1|Q5T1D1_HUMAN	RP11-632C	Ribosomal protein L29 (RPL29) pseudogene OS=Homo sapiens GN=RP11-632C17__A.1-001 PE=2 SV=2	17471	883	594	37	48	0.19	0.19	1	1	1	1	1	1	1	2	1	0.659333
tr|A0N4V7|A0N4V7_HUMAN	Tcr-alpha	HCG2039797 (Fragment) OS=Homo sapiens GN=Tcr-alpha PE=4 SV=1	2269	781	581	45	49	1.81	1.81	1	2	2	2	2	4	2	4	2	-0.18895
tr|H7C4F6|H7C4F6_HUMAN	KPNA4	Importin subunit alpha-4 (Fragment) OS=Homo sapiens GN=KPNA4 PE=4 SV=1	19041	985	578	31	49	0.18	0.18	1	0	1	1	1	1	1	1	1	0.659333
tr|A1L3A7|A1L3A7_HUMAN	NUFIP2	Nuclear fragile X mental retardation protein interacting protein 2 OS=Homo sapiens GN=NUFIP2 PE=2 SV=1	76132	969	573	33	49	0.04	0.04	1	0	1	1	1	1	1	1	1	0.659333
tr|B4DKI9|B4DKI9_HUMAN	tr|B4DKI9	cDNA FLJ54799, highly similar to Mitochondrial 28S ribosomal protein S27 OS=Homo sapiens PE=2 SV=1	22078	815	565	42	50	0.15	0.15	1	1	1	1	1	1	1	2	2	0.659333
sp|Q96I24|FUBP3_HUMAN	FUBP3	Far upstream element-binding protein 3 OS=Homo sapiens GN=FUBP3 PE=1 SV=2	61944	927	555	35	51	0.05	0.05	1	0	1	1	1	2	1	4	1	-0.07784
sp|P23381|SYWC_HUMAN	WARS	Tryptophan--tRNA ligase, cytoplasmic OS=Homo sapiens GN=WARS PE=1 SV=2	53474	373	542	109	52	0.2	0.2	1	3	1	3	3	3	2	4	4	0.17382
tr|A8K670|A8K670_HUMAN	tr|A8K670	cDNA FLJ75703, highly similar to Homo sapiens nitric oxide synthase interacting protein (NOSIP), mRNA OS=Homo sapiens PE=2 SV=1	33676	743	537	48	53	0.1	0.1	1	1	1	1	1	2	2	3	2	0.65945
tr|J3KR44|J3KR44_HUMAN	OTUB1	Ubiquitin thioesterase OTUB1 OS=Homo sapiens GN=OTUB1 PE=4 SV=1	35649	456	530	85	53	0.09	0.09	1	1	1	1	1	1	1	1	1	0.659333
tr|G1UI17|G1UI17_HUMAN	AGL	Glycogen debranching enzyme (Fragment) OS=Homo sapiens GN=AGL PE=2 SV=1	145569	1017	528	29	53	0.02	0.02	1	0	1	1	1	1	1	1	1	0.659333
sp|Q9NYU2|UGGG1_HUMAN	UGGT1	UDP-glucose:glycoprotein glucosyltransferase 1 OS=Homo sapiens GN=UGGT1 PE=1 SV=3	177819	669	522	54	54	0.06	0.06	1	1	1	3	3	3	4	6	6	1.02246
tr|B3KTA3|B3KTA3_HUMAN	tr|B3KTA3	Fascin OS=Homo sapiens PE=2 SV=1	52814	559	519	67	55	0.06	0.06	1	1	1	1	1	1	1	1	1	0.659333
tr|Q6FI97|Q6FI97_HUMAN	BAF53A	BAF53A protein OS=Homo sapiens GN=BAF53A PE=2 SV=1	47864	718	517	51	55	0.14	0.14	1	1	1	2	2	2	2	2	2	0.65945
tr|C9JDR0|C9JDR0_HUMAN	NSDHL	Sterol-4-alpha-carboxylate 3-dehydrogenase, decarboxylating (Fragment) OS=Homo sapiens GN=NSDHL PE=2 SV=1	28356	950	516	33	55	0.12	0.12	1	0	1	1	1	1	1	1	2	0.659333
sp|Q96P70|IPO9_HUMAN	IPO9	Importin-9 OS=Homo sapiens GN=IPO9 PE=1 SV=3	116858	773	513	45	56	0.06	0.06	1	1	1	2	2	1	2	3	2	1.39662
sp|Q9Y2W1|TR150_HUMAN	THRAP3	Thyroid hormone receptor-associated protein 3 OS=Homo sapiens GN=THRAP3 PE=1 SV=2	108658	1080	509	26	56	0.03	0.03	1	0	1	1	1	2	1	2	1	1.39662
tr|Q6FG59|Q6FG59_HUMAN	CDC37	CDC37 protein OS=Homo sapiens GN=CDC37 PE=2 SV=1	44938	473	498	82	58	0.15	0.15	1	2	2	2	2	2	2	3	2	0.65945
sp|Q9H9J2|RM44_HUMAN	MRPL44	39S ribosomal protein L44, mitochondrial OS=Homo sapiens GN=MRPL44 PE=1 SV=1	37854	968	487	33	60	0.09	0.09	1	0	1	1	1	1	1	1	1	0.659333
tr|Q2TA84|Q2TA84_HUMAN	FKBP5	FK506 binding protein 5 OS=Homo sapiens GN=FKBP5 PE=2 SV=1	51659	713	476	51	61	0.13	0.13	1	1	2	2	2	2	2	2	2	0.65945
tr|B5BU24|B5BU24_HUMAN	YWHAB	14-3-3 protein beta/alpha OS=Homo sapiens GN=YWHAB PE=2 SV=1	28209	643	469	57	63	0.12	0.12	1	1	1	1	1	3	2	3	2	0.17382
sp|Q9Y266|NUDC_HUMAN	NUDC	Nuclear migration protein nudC OS=Homo sapiens GN=NUDC PE=1 SV=1	38276	982	466	32	63	0.09	0.09	1	0	1	1	1	1	1	1	1	0.659333
tr|B4DM84|B4DM84_HUMAN	tr|B4DM84	cDNA FLJ60694, highly similar to Deubiquitinating protein VCIP135 (EC 3.4.22.-) OS=Homo sapiens PE=2 SV=1	87230	832	457	41	64	0.04	0.04	1	1	1	1	1	1	1	1	1	0.659333
tr|B0AZM4|B0AZM4_HUMAN	tr|B0AZM4	cDNA, FLJ79464, highly similar to Homo sapiens aquarius (Fragment) OS=Homo sapiens PE=2 SV=1	157764	947	447	33	66	0.02	0.02	1	1	1	1	1	2	1	4	3	-0.07784
tr|B2R5U7|B2R5U7_HUMAN	tr|B2R5U7	cDNA, FLJ92633, highly similar to Homo sapiens CCAAT-box-binding transcription factor (CBF2), mRNA OS=Homo sapiens PE=2 SV=1	114755	551	439	68	66	0.06	0.06	1	2	1	2	2	2	2	3	2	0.65945
tr|Q6IAM0|Q6IAM0_HUMAN	EIF3S4	Eukaryotic translation initiation factor 3 subunit G OS=Homo sapiens GN=EIF3S4 PE=2 SV=1	35864	703	437	51	66	0.19	0.19	1	1	2	2	2	3	2	3	3	1.145199
tr|Q9BS14|Q9BS14_HUMAN	GANAB	GANAB protein (Fragment) OS=Homo sapiens GN=GANAB PE=2 SV=1	34786	588	434	63	66	0.2	0.2	1	1	2	2	2	4	3	5	4	0.296795
tr|B3KY11|B3KY11_HUMAN	tr|B3KY11	cDNA FLJ46571 fis, clone THYMU3041428, highly similar to Probable ATP-dependent RNA helicase DDX23 (EC 3.6.1.-) OS=Homo sapiens PE	93519	1122	431	23	67	0.04	0.04	1	0	1	1	1	1	1	1	1	0.659333
tr|D0PQI2|D0PQI2_HUMAN	ENAH	Enabled-like protein variant hMenaDv6 OS=Homo sapiens GN=ENAH PE=2 SV=1	60426	808	429	43	67	0.11	0.11	1	1	1	2	2	2	2	3	2	0.65945
tr|H7C1W5|H7C1W5_HUMAN	MMS19	MMS19 nucleotide excision repair protein homolog (Fragment) OS=Homo sapiens GN=MMS19 PE=4 SV=1	16833	649	424	56	68	0.2	0.2	1	1	1	1	1	1	1	1	2	0.659333
sp|Q32MZ4|LRRF1_HUMAN	LRRFIP1	Leucine-rich repeat flightless-interacting protein 1 OS=Homo sapiens GN=LRRFIP1 PE=1 SV=2	89826	1155	407	21	70	0.04	0.04	1	0	1	1	1	1	1	1	1	0.659333
tr|Q59FA0|Q59FA0_HUMAN	tr|Q59FA0	DNA polymerase (Fragment) OS=Homo sapiens PE=2 SV=1	113271	510	403	75	71	0.06	0.06	1	2	2	2	2	2	2	3	3	0.65945
tr|H0YH81|H0YH81_HUMAN	ATP5B	ATP synthase subunit beta (Fragment) OS=Homo sapiens GN=ATP5B PE=3 SV=1	38226	873	396	38	72	0.09	0.09	1	1	1	1	1	1	1	1	3	0.659333
tr|B4DKU3|B4DKU3_HUMAN	tr|B4DKU3	cDNA FLJ53449, highly similar to rRNA methyltransferase 3 (EC 2.1.1.-) OS=Homo sapiens PE=2 SV=1	82984	430	395	93	72	0.08	0.08	1	1	1	2	2	2	1	2	2	-0.07784
sp|O14929|HAT1_HUMAN	HAT1	Histone acetyltransferase type B catalytic subunit OS=Homo sapiens GN=HAT1 PE=1 SV=1	49880	278	382	142	77	0.14	0.14	1	2	2	2	2	2	2	2	2	0.65945
tr|Q58FF4|Q58FF4_HUMAN	HSP90Bf	Heat shock protein 90Bf OS=Homo sapiens GN=HSP90Bf PE=1 SV=1	41833	841	381	40	77	0.16	0.16	1	1	4	2	4	3	4	3	4	1.02246
sp|Q9UHR4|BI2L1_HUMAN	BAIAP2L1	Brain-specific angiogenesis inhibitor 1-associated protein 2-like protein 1 OS=Homo sapiens GN=BAIAP2L1 PE=1 SV=2	57189	641	378	57	77	0.06	0.06	1	1	1	1	1	1	1	2	1	0.659333
tr|J3KTE4|J3KTE4_HUMAN	RPL19	Ribosomal protein L19 OS=Homo sapiens GN=RPL19 PE=3 SV=1	23347	349	376	116	78	0.31	0.31	1	2	1	2	2	2	3	3	3	1.145199
tr|B3KSQ7|B3KSQ7_HUMAN	DBN1	Drebrin 1, isoform CRA_d OS=Homo sapiens GN=DBN1 PE=2 SV=1	66053	638	369	57	80	0.05	0.05	1	1	1	1	1	1	1	1	1	0.659333
tr|B3KRR1|B3KRR1_HUMAN	tr|B3KRR1	cDNA FLJ34725 fis, clone MESAN2005958, highly similar to RNA-binding protein Luc7-like 2 OS=Homo sapiens PE=2 SV=1	40752	595	357	62	82	0.08	0.08	1	1	1	1	1	1	1	1	1	0.659333
tr|B4DK79|B4DK79_HUMAN	tr|B4DK79	cDNA FLJ58742, highly similar to Leucine-rich repeat-containing protein 41 OS=Homo sapiens PE=2 SV=1	74302	452	341	85	86	0.04	0.04	1	1	1	1	1	1	1	1	1	0.659333
tr|B4DR45|B4DR45_HUMAN	tr|B4DR45	cDNA FLJ61339, highly similar to Alanyl-tRNA synthetase (EC 6.1.1.7) OS=Homo sapiens PE=2 SV=1	110017	798	336	44	87	0.12	0.12	1	0	3	4	4	2	5	5	7	1.797919
tr|G8IFA7|G8IFA7_HUMAN	tr|G8IFA7	p90 OS=Homo sapiens PE=2 SV=1	59481	363	327	114	89	0.06	0.06	1	1	1	1	1	1	1	1	2	0.659333
tr|Q5T0F3|Q5T0F3_HUMAN	GNL2	Guanine nucleotide binding protein-like 2 (Nucleolar) OS=Homo sapiens GN=GNL2 PE=2 SV=1	83831	791	326	44	89	0.04	0.04	1	1	1	1	1	1	1	1	1	0.659333
tr|J3KMW7|J3KMW7_HUMAN	UBR5	E3 ubiquitin-protein ligase UBR5 OS=Homo sapiens GN=UBR5 PE=4 SV=1	312224	1049	320	27	92	0.01	0.01	1	0	1	1	1	2	1	2	2	-0.07784
tr|Q5U0Q1|Q5U0Q1_HUMAN	DKFZp686L	Putative uncharacterized protein DKFZp686L1159 OS=Homo sapiens GN=DKFZp686L1159 PE=2 SV=1	52189	418	305	97	98	0.13	0.13	1	2	2	2	2	2	2	3	2	0.65945
tr|B2RBS8|B2RBS8_HUMAN	tr|B2RBS8	cDNA, FLJ95666, highly similar to Homo sapiens albumin (ALB), mRNA OS=Homo sapiens PE=2 SV=1	71343	292	304	135	98	0.2	0.2	1	3	3	4	4	5	4	6	4	0.369976
tr|Q5TGM0|Q5TGM0_HUMA	VTA1	Vacuolar protein sorting-associated protein VTA1 homolog OS=Homo sapiens GN=VTA1 PE=4 SV=1	27485	327	298	125	100	0.12	0.12	1	1	1	1	1	1	1	1	1	0.659333
tr|B3KWR2|B3KWR2_HUMAN	tr|B3KWR2	cDNA FLJ43638 fis, clone SPLEN2039845, highly similar to IMPORTIN ALPHA-1 SUBUNIT OS=Homo sapiens PE=2 SV=1	38087	435	270	66	67	0.17	0.17	1	2	1	2	1	2	1	2	1	-0.07784
sp|Q9H3P7|GCP60_HUMAN	ACBD3	Golgi resident protein GCP60 OS=Homo sapiens GN=ACBD3 PE=1 SV=4	60841	417	245	98	123	0.11	0.11	1	2	2	2	2	2	2	2	2	0.65945
tr|B3KQT9|B3KQT9_HUMAN	tr|B3KQT9	cDNA PSEC0175 fis, clone OVARC1000169, highly similar to Protein disulfide-isomerase A3 (EC 5.3.4.1) OS=Homo sapiens PE=2 SV=1	54468	515	236	74	129	0.26	0.26	1	2	3	4	4	3	4	5	4	1.02246
tr|B4DM97|B4DM97_HUMAN	tr|B4DM97	cDNA FLJ55002, highly similar to Alpha-centractin OS=Homo sapiens PE=2 SV=1	38365	358	233	114	132	0.28	0.28	1	2	2	3	3	3	3	3	4	0.659568
tr|B4DY09|B4DY09_HUMAN	tr|B4DY09	cDNA FLJ51660, highly similar to Interleukin enhancer-binding factor 2 OS=Homo sapiens PE=2 SV=1	39057	273	232	146	134	0.5	0.5	1	2	3	5	5	5	5	5	6	0.659804
tr|Q6FHF5|Q6FHF5_HUMAN	PCNA	Proliferating cell nuclear antigen (Fragment) OS=Homo sapiens GN=PCNA PE=2 SV=1	29029	487	224	79	137	0.38	0.38	1	2	2	3	3	3	3	5	3	0.659568
tr|B4DVB8|B4DVB8_HUMAN	ELAVL1	ELAV-like protein 1 OS=Homo sapiens GN=ELAVL1 PE=2 SV=1	39200	362	218	114	140	0.5	0.5	1	4	2	5	5	5	5	5	5	0.659804
tr|B4DQJ8|B4DQJ8_HUMAN	PGD	6-phosphogluconate dehydrogenase, decarboxylating OS=Homo sapiens GN=PGD PE=2 SV=1	52352	193	213	209	143	0.53	0.53	1	4	3	7	7	6	7	8	7	0.866695
tr|A6NN80|A6NN80_HUMAN	ANXA6	Annexin OS=Homo sapiens GN=ANXA6 PE=3 SV=3	75571	446	201	88	153	0.24	0.24	1	2	3	5	5	5	5	7	7	0.659804
tr|A8K4Z4|A8K4Z4_HUMAN	tr|A8K4Z4	cDNA FLJ75549, highly similar to Homo sapiens ribosomal protein, large, P0 (RPLP0), transcript variant 1, mRNA OS=Homo sapiens PE=2 SV	34389	393	188	103	169	0.45	0.45	1	2	5	5	5	6	6	6	7	0.659923
sp|P25705|ATPA_HUMAN	ATP5A1	ATP synthase subunit alpha, mitochondrial OS=Homo sapiens GN=ATP5A1 PE=1 SV=1	59828	99	187	369	170	0.62	0.62	1	9	4	10	9	10	7	11	10	0.174003
tr|B3KM80|B3KM80_HUMAN	NCL	Nucleolin, isoform CRA_c OS=Homo sapiens GN=NCL PE=2 SV=1	58576	222	180	179	173	0.39	0.39	1	3	7	6	7	7	9	8	11	1.001721
tr|H0YCK7|H0YCK7_HUMAN	EEF1D	Elongation factor 1-delta (Fragment) OS=Homo sapiens GN=EEF1D PE=4 SV=1	23105	286	179	138	174	0.31	0.31	1	2	2	2	2	2	3	2	4	1.145199
tr|B2R8Z8|B2R8Z8_HUMAN	tr|B2R8Z8	cDNA, FLJ94136, highly similar to Homo sapiens synaptotagmin binding, cytoplasmic RNA interacting protein (SYNCRIP), mRNA OS=Homo sa	69787	185	141	215	208	0.51	0.51	1	6	4	9	10	9	11	12	11	0.936555
tr|Q59EG8|Q59EG8_HUMAN	tr|Q59EG8	Proteasome 26S non-ATPase subunit 2 variant (Fragment) OS=Homo sapiens PE=2 SV=1	101322	385	139	106	210	0.33	0.33	1	1	6	9	9	10	10	13	13	0.660395
tr|B3KS31|B3KS31_HUMAN	TUBB6	Tubulin, beta 6, isoform CRA_a OS=Homo sapiens GN=TUBB6 PE=2 SV=1	42324	81	126	442	230	1.66	1.66	1	17	11	18	16	23	16	28	18	0.149483
tr|B2R858|B2R858_HUMAN	tr|B2R858	cDNA, FLJ93750, Homo sapiens DEAD (Asp-Glu-Ala-Asp) box polypeptide 6 (DDX6), mRNA OS=Homo sapiens PE=2 SV=1	53582	191	125	209	232	0.52	0.52	1	6	5	7	7	7	8	8	8	0.840935
sp|Q9P258|RCC2_HUMAN	RCC2	Protein RCC2 OS=Homo sapiens GN=RCC2 PE=1 SV=2	56790	383	116	107	245	0.4	0.4	1	4	6	6	6	8	7	11	7	0.479265
sp|P20700|LMNB1_HUMAN	LMNB1	Lamin-B1 OS=Homo sapiens GN=LMNB1 PE=1 SV=2	66653	381	105	107	262	0.33	0.33	1	5	6	6	7	6	8	7	8	1.047589
tr|E7EN95|E7EN95_HUMAN	FLNB	Filamin-B OS=Homo sapiens GN=FLNB PE=2 SV=1	258059	192	75	209	329	0.13	0.13	1	5	8	11	10	14	11	17	12	0.325483
sp|P11142|HSP7C_HUMAN	HSPA8	Heat shock cognate 71 kDa protein OS=Homo sapiens GN=HSPA8 PE=1 SV=1	71082	57	18	602	937	1.7	1.7	1	16	25	27	30	26	33	30	38	1.002726
sp|Q9BVA1|TBB2B_HUMAN	TUBB2B	Tubulin beta-2B chain OS=Homo sapiens GN=TUBB2B PE=1 SV=1	50377	15	16	1357	1067	8.18	8.18	1	50	36	62	45	69	45	77	53	0.048493
tr|F5H335|F5H335_HUMAN	EIF3A	Eukaryotic translation initiation factor 3 subunit A OS=Homo sapiens GN=EIF3A PE=2 SV=1	162936	74	120	473	236	0.49	0.47	0.959184	14	6	21	9	25	9	27	12	-0.76748
tr|Q2L6I2|Q2L6I2_HUMAN	ABCF1	ABC50 protein OS=Homo sapiens GN=ABCF1 PE=3 SV=1	92080	187	55	214	352	0.23	0.22	0.956522	4	6	6	7	6	7	7	7	0.866695
sp|Q96C36|P5CR2_HUMAN	PYCR2	Pyrroline-5-carboxylate reductase 2 OS=Homo sapiens GN=PYCR2 PE=1 SV=1	33958	425	408	94	70	0.2	0.19	0.95	2	1	2	2	2	3	2	3	1.145199
tr|Q8IWP6|Q8IWP6_HUMAN	tr|Q8IWP6	Class IVb beta tubulin OS=Homo sapiens PE=2 SV=1	50177	5	12	1694	1231	14.43	13.48	0.934165	59	41	75	53	80	57	88	65	0.17585
sp|P08243|ASNS_HUMAN	ASNS	Asparagine synthetase [glutamine-hydrolyzing] OS=Homo sapiens GN=ASNS PE=1 SV=4	64899	291	217	136	128	0.28	0.26	0.928571	4	2	5	4	7	4	7	4	-0.07789
tr|B4DF50|B4DF50_HUMAN	tr|B4DF50	cDNA FLJ60458, highly similar to SWI/SNF-related matrix-associated actin-dependent regulator of chromatin subfamily D member 1 OS=Ho	28346	413	595	36	48	0.13	0.12	0.923077	0	1	1	1	1	1	1	1	0.659333
tr|B7ZB91|B7ZB91_HUMAN	TNRC6B	Trinucleotide repeat containing 6B (Fragment) OS=Homo sapiens GN=TNRC6B PE=4 SV=1	81825	480	477	80	61	0.13	0.12	0.923077	3	2	3	2	3	2	3	3	0.17382
sp|Q9BYG3|MK67I_HUMAN	MKI67IP	MKI67 FHA domain-interacting nucleolar phosphoprotein OS=Homo sapiens GN=MKI67IP PE=1 SV=1	34372	599	949	61	22	0.32	0.29	0.90625	1	0	3	1	2	1	3	1	-0.07784
sp|P04406|G3P_HUMAN	GAPDH	Glyceraldehyde-3-phosphate dehydrogenase OS=Homo sapiens GN=GAPDH PE=1 SV=3	36201	13	4	1386	1459	6.5	5.87	0.903077	29	28	40	48	42	51	46	56	0.944233
tr|F2Z393|F2Z393_HUMAN	TALDO1	Transaldolase OS=Homo sapiens GN=TALDO1 PE=2 SV=1	35535	365	375	111	44	0.2	0.18	0.9	2	1	2	1	2	2	3	3	0.65945
tr|B7ZKM0|B7ZKM0_HUMAN	SART3	SART3 protein OS=Homo sapiens GN=SART3 PE=2 SV=1	106372	171	156	225	196	0.27	0.24	0.888889	8	3	8	4	9	4	11	4	-0.41933

sp|O43776|SYNC_HUMAN	NARS	Asparagine--tRNA ligase, cytoplasmic OS=Homo sapiens GN=NARS PE=1 SV=1	63758	110	81	349	318	0.74	0.65	0.878378	8	8	11	11	11	14	11	16	0.995899
tr|B3KS36|B3KS36_HUMAN	tr|B3KS36	cDNA FLJ35376 fis, clone SKMUS2004044, highly similar to Homo sapiens ribosomal protein L3 (RPL3), transcript variant 2, mRNA OS=Homo	40234	113	63	345	369	1.8	1.58	0.877778	12	10	13	13	14	12	14	13	0.446099
tr|Q8IZ29|Q8IZ29_HUMAN	TUBB2C	Tubulin, beta 2C OS=Homo sapiens GN=TUBB2C PE=2 SV=1	50264	4	10	1730	1236	15.34	13.39	0.872881	59	41	75	53	80	55	87	62	0.124125
tr|Q6IAX2|Q6IAX2_HUMAN	RPL21	RPL21 protein OS=Homo sapiens GN=RPL21 PE=2 SV=1	18610	364	318	111	93	0.94	0.82	0.87234	3	2	4	2	4	2	4	2	-0.18895
tr|F8VRQ1|F8VRQ1_HUMAN	HNRNPA1	Heterogeneous nuclear ribonucleoprotein A1 OS=Homo sapiens GN=HNRNPA1 PE=2 SV=1	33249	49	53	660	415	2.13	1.84	0.86385	16	10	21	15	22	17	28	20	0.297634
tr|E9PKU7|E9PKU7_HUMAN	GANAB	Neutral alpha-glucosidase AB OS=Homo sapiens GN=GANAB PE=2 SV=1	96768	45	31	660	656	0.82	0.7	0.853659	14	15	22	21	20	22	27	26	0.796527
tr|G8JLB6|G8JLB6_HUMAN	HNRNPH1	Heterogeneous nuclear ribonucleoprotein H OS=Homo sapiens GN=HNRNPH1 PE=4 SV=1	51482	83	74	426	332	0.75	0.64	0.853333	10	7	12	11	13	11	14	12	0.42878
tr|Q1RMG2|Q1RMG2_HUMA	AHCY	Adenosylhomocysteinase OS=Homo sapiens GN=AHCY PE=2 SV=1	34188	142	175	229	178	1.07	0.91	0.850467	6	7	9	8	9	7	12	8	0.318596
tr|B0YJ88|B0YJ88_HUMAN	RDX	Radixin OS=Homo sapiens GN=RDX PE=2 SV=1	68635	262	303	153	98	0.32	0.27	0.84375	5	2	6	4	6	4	7	4	0.128765
tr|B2RBD5|B2RBD5_HUMAN	tr|B2RBD5	cDNA, FLJ95457, highly similar to Homo sapiens tubulin, beta, 4 (TUBB4), mRNA OS=Homo sapiens PE=2 SV=1	50828	32	30	913	658	2.98	2.51	0.842282	26	21	35	26	41	28	46	37	0.117724
tr|Q2Q9B7|Q2Q9B7_HUMAN	G6PD	Glucose-6-phosphate 1-dehydrogenase (Fragment) OS=Homo sapiens GN=G6PD PE=2 SV=1	55221	472	511	82	0	0.12	0.1	0.833333	2	0	2	0	3	0	6	4	-2.14875
tr|B7Z463|B7Z463_HUMAN	NPEPPS	Puromycin-sensitive aminopeptidase OS=Homo sapiens GN=NPEPPS PE=2 SV=1	95083	511	324	74	67	0.18	0.15	0.833333	2	2	5	3	4	3	6	3	0.296795
tr|Q5TCU6|Q5TCU6_HUMAN	TLN1	Talin-1 OS=Homo sapiens GN=TLN1 PE=4 SV=1	260030	186	101	214	270	0.12	0.1	0.833333	6	5	9	8	9	6	11	9	0.111824
tr|B4DF96|B4DF96_HUMAN	EIF2A	Eukaryotic translation initiation factor 2A OS=Homo sapiens GN=EIF2A PE=2 SV=1	58357	293	189	135	167	0.47	0.39	0.829787	2	4	7	6	7	6	8	10	0.453268
tr|G5E9M5|G5E9M5_HUMAN	ILF3	Interleukin enhancer binding factor 3, 90kDa, isoform CRA_b OS=Homo sapiens GN=ILF3 PE=2 SV=1	96147	62	45	550	479	1.23	1.02	0.829268	19	14	29	22	30	24	31	26	0.344802
sp|Q9BXP5|SRRT_HUMAN	SRRT	Serrate RNA effector molecule homolog OS=Homo sapiens GN=SRRT PE=1 SV=1	101060	377	262	108	113	0.17	0.14	0.823529	2	3	5	4	7	4	8	5	-0.07789
tr|I3L1L3|I3L1L3_HUMAN	MYBBP1A	Myb-binding protein 1A (Fragment) OS=Homo sapiens GN=MYBBP1A PE=4 SV=1	141124	328	231	124	135	0.17	0.14	0.823529	3	5	7	7	6	6	9	10	0.659923
sp|Q9Y230|RUVB2_HUMAN	RUVBL2	RuvB-like 2 OS=Homo sapiens GN=RUVBL2 PE=1 SV=3	51296	174	240	223	126	0.55	0.45	0.818182	6	4	7	6	9	7	9	8	0.318596
tr|B4DMA2|B4DMA2_HUMAN	tr|B4DMA2	cDNA FLJ54023, highly similar to Heat shock protein HSP 90-beta OS=Homo sapiens PE=2 SV=1	79487	7	2	1628	1555	4.25	3.46	0.814118	40	44	55	56	61	61	69	66	0.666483
tr|B2RBA6|B2RBA6_HUMAN	tr|B2RBA6	cDNA, FLJ95407, highly similar to Homo sapiens MCM7 minichromosome maintenance deficient 7 (S. cerevisiae) (MCM7), mRNA OS=Homo	81856	236	215	168	142	0.37	0.3	0.810811	5	3	8	6	10	5	10	7	-0.2741
sp|P35527|K1C9_HUMAN	KRT9	Keratin, type I cytoskeletal 9 OS=Homo sapiens GN=KRT9 PE=1 SV=3	62255	214	124	183	235	0.36	0.29	0.805556	4	3	6	5	7	5	9	7	0.211938
tr|A8K5Y7|A8K5Y7_HUMAN	tr|A8K5Y7	cDNA FLJ78655, highly similar to Homo sapiens exportin 5 (XPO5), mRNA OS=Homo sapiens PE=2 SV=1	138331	202	355	195	82	0.15	0.12	0.8	4	3	6	5	7	7	9	7	0.660041
tr|J3KN36|J3KN36_HUMAN	NOMO3	Nodal modulator 3 OS=Homo sapiens GN=NOMO3 PE=4 SV=1	140376	530	1002	71	18	0.05	0.04	0.8	2	0	2	1	2	0	2	3	-1.66312
tr|A2A274|A2A274_HUMAN	ACO2	Aconitase 2, mitochondrial OS=Homo sapiens GN=ACO2 PE=4 SV=1	88563	182	254	216	119	0.2	0.16	0.8	5	2	5	4	7	4	7	4	-0.07789
tr|Q53ET4|Q53ET4_HUMAN	tr|Q53ET4	Serine hydroxymethyltransferase (Fragment) OS=Homo sapiens PE=2 SV=1	56395	86	100	416	270	1.17	0.93	0.794872	12	7	15	11	16	10	17	14	0.007096
tr|B1AH89|B1AH89_HUMAN	TTLL12	Tubulin tyrosine ligase-like family, member 12 OS=Homo sapiens GN=TTLL12 PE=4 SV=1	74185	235	209	168	146	0.24	0.19	0.791667	5	3	5	4	5	6	7	8	0.901134
tr|B7Z9L0|B7Z9L0_HUMAN	CCT4	T-complex protein 1 subunit delta OS=Homo sapiens GN=CCT4 PE=2 SV=1	52811	95	149	389	204	1.2	0.95	0.791667	8	5	15	11	18	10	20	16	-0.15837
tr|Q5U077|Q5U077_HUMAN	LDHB	L-lactate dehydrogenase OS=Homo sapiens GN=LDHB PE=2 SV=1	36900	90	48	408	459	2.96	2.34	0.790541	13	11	18	18	21	21	23	25	0.661699
tr|B4E043|B4E043_HUMAN	KHDRBS1	KH domain containing, RNA binding, signal transduction associated 1, isoform CRA_b OS=Homo sapiens GN=KHDRBS1 PE=2 SV=1	38772	514	497	74	59	0.28	0.22	0.785714	2	1	3	3	3	4	3	4	1.02246
tr|B5BUB1|B5BUB1_HUMAN	RUVBL1	RuvB-like 1 (Fragment) OS=Homo sapiens GN=RUVBL1 PE=2 SV=1	50526	196	266	203	112	0.56	0.44	0.785714	5	2	7	4	8	4	9	4	-0.25867
tr|E9PPU1|E9PPU1_HUMAN	RPS3	40S ribosomal protein S3 OS=Homo sapiens GN=RPS3 PE=2 SV=1	17397	269	244	149	91	1.03	0.8	0.776699	3	2	5	6	5	6	6	6	0.901134
sp|O75533|SF3B1_HUMAN	SF3B1	Splicing factor 3B subunit 1 OS=Homo sapiens GN=SF3B1 PE=1 SV=3	146479	145	140	275	209	0.39	0.3	0.769231	8	5	15	12	13	10	19	14	0.297214
tr|Q6IBN0|Q6IBN0_HUMAN	PSMD3	PSMD3 protein OS=Homo sapiens GN=PSMD3 PE=2 SV=1	61022	221	438	179	66	0.3	0.23	0.766667	5	1	5	4	7	7	10	8	0.660041
tr|B7Z213|B7Z213_HUMAN	tr|B7Z213	cDNA FLJ50130, highly similar to Heterogeneous nuclear ribonucleoprotein Q OS=Homo sapiens PE=2 SV=1	28561	185	587	42	48	0.51	0.39	0.764706	0	0	2	3	2	3	2	3	1.145199
tr|B4DLC0|B4DLC0_HUMAN	PCBP2	Poly(rC)-binding protein 2 OS=Homo sapiens GN=PCBP2 PE=2 SV=1	32601	132	91	221	216	1.26	0.95	0.753968	5	5	7	5	7	5	8	5	0.211938
sp|P04075|ALDOA_HUMAN	ALDOA	Fructose-bisphosphate aldolase A OS=Homo sapiens GN=ALDOA PE=1 SV=2	39851	14	1	1366	1644	6.34	4.78	0.753943	28	33	43	44	47	49	54	57	0.724951
sp|Q14684|RRP1B_HUMAN	RRP1B	Ribosomal RNA processing protein 1 homolog B OS=Homo sapiens GN=RRP1B PE=1 SV=3	84774	375	397	108	72	0.16	0.12	0.75	3	1	4	3	5	4	6	4	0.369976
tr|B2RNR6|B2RNR6_HUMAN	ZFR	Zinc finger RNA binding protein OS=Homo sapiens GN=ZFR PE=2 SV=1	118081	326	184	125	172	0.12	0.09	0.75	2	3	4	3	4	3	4	3	0.296795
tr|Q53HE2|Q53HE2_HUMAN	tr|Q53HE2	Triosephosphate isomerase (Fragment) OS=Homo sapiens PE=2 SV=1	26981	297	390	133	74	0.79	0.59	0.746835	3	1	5	4	4	4	5	5	0.659686
sp|O43175|SERA_HUMAN	PHGDH	D-3-phosphoglycerate dehydrogenase OS=Homo sapiens GN=PHGDH PE=1 SV=4	57356	154	103	261	268	0.75	0.56	0.746667	7	4	11	8	11	10	12	11	0.528947
sp|Q9UMS4|PRP19_HUMAN	PRPF19	Pre-mRNA-processing factor 19 OS=Homo sapiens GN=PRPF19 PE=1 SV=1	55603	139	115	279	246	0.78	0.58	0.74359	9	8	10	8	10	9	14	12	0.515683
tr|B4DE36|B4DE36_HUMAN	tr|B4DE36	Glucose-6-phosphate isomerase OS=Homo sapiens PE=2 SV=1	60376	92	142	397	199	0.89	0.66	0.741573	12	7	13	10	13	13	13	13	0.66075
tr|B2R6D0|B2R6D0_HUMAN	tr|B2R6D0	cDNA, FLJ92896, highly similar to Homo sapiens proteasome (prosome, macropain) 26S subunit, non-ATPase, 1 (PSMD1), mRNA OS=Homo	106797	302	152	131	198	0.27	0.2	0.740741	2	5	8	6	10	6	10	6	-0.03277
tr|B4DHX4|B4DHX4_HUMAN	tr|B4DHX4	cDNA FLJ52902, highly similar to Rab GDP dissociation inhibitor alpha OS=Homo sapiens PE=2 SV=1	47505	254	198	159	144	0.5	0.37	0.74	5	4	6	4	6	4	7	5	0.128765
tr|B0QY89|B0QY89_HUMAN	EIF3EIP	Eukaryotic translation initiation factor 3 subunit L OS=Homo sapiens GN=EIF3EIP PE=4 SV=1	71085	114	196	345	160	0.5	0.37	0.74	9	2	9	7	11	7	11	7	0.042554
tr|B4DHK9|B4DHK9_HUMAN	tr|B4DHK9	cDNA FLJ51656, highly similar to Actin-like protein 2 OS=Homo sapiens PE=2 SV=1	39175	296	335	133	87	0.38	0.28	0.736842	3	2	4	3	5	3	5	3	0.007085
tr|B2RDD7|B2RDD7_HUMAN	tr|B2RDD7	cDNA, FLJ96564, highly similar to Homo sapiens SKB1 homolog (S. pombe) (SKB1), mRNA OS=Homo sapiens PE=2 SV=1	73348	275	340	145	86	0.3	0.22	0.733333	4	1	6	2	6	2	7	3	-0.71988
tr|B3KX11|B3KX11_HUMAN	tr|B3KX11	cDNA FLJ44436 fis, clone UTERU2019706, highly similar to T-complex protein 1 subunit gamma OS=Homo sapiens PE=2 SV=1	58479	76	77	466	325	1.27	0.93	0.732283	10	6	15	14	18	13	23	15	0.205165
tr|H3BLZ8|H3BLZ8_HUMAN	DDX17	Probable ATP-dependent RNA helicase DDX17 OS=Homo sapiens GN=DDX17 PE=3 SV=1	81073	96	164	381	187	0.67	0.49	0.731343	13	8	15	10	16	13	23	14	0.370632
tr|F5H5D3|F5H5D3_HUMAN	TUBA1C	Tubulin alpha-1C chain OS=Homo sapiens GN=TUBA1C PE=2 SV=1	58606	19	13	1165	1191	2.71	1.98	0.730627	31	34	45	44	48	50	54	56	0.723869
tr|Q562M3|Q562M3_HUMAN	ACT	Actin-like protein (Fragment) OS=Homo sapiens GN=ACT PE=2 SV=1	11529	161	155	239	196	5.21	3.79	0.727447	9	6	16	12	20	13	24	17	0.056658
tr|B4DE59|B4DE59_HUMAN	tr|B4DE59	cDNA FLJ60424, highly similar to Junction plakoglobin OS=Homo sapiens PE=2 SV=1	62862	674	661	54	37	0.11	0.08	0.727273	2	1	2	1	3	1	4	2	-0.56347
sp|Q8N1G4|LRC47_HUMAN	LRRC47	Leucine-rich repeat-containing protein 47 OS=Homo sapiens GN=LRRC47 PE=1 SV=1	64004	313	191	127	164	0.22	0.16	0.727273	4	3	4	4	5	4	6	4	0.369976
sp|P42166|LAP2A_HUMAN	TMPO	Lamina-associated polypeptide 2, isoform alpha OS=Homo sapiens GN=TMPO PE=1 SV=2	76016	233	211	170	144	0.4	0.29	0.725	4	3	9	6	9	6	11	8	0.111824
sp|Q58FF8|H90B2_HUMAN	HSP90AB2I	Putative heat shock protein HSP 90-beta 2 OS=Homo sapiens GN=HSP90AB2P PE=1 SV=2	44492	169	117	228	241	0.9	0.65	0.722222	6	8	9	9	10	9	11	10	0.515683
tr|A8K2X8|A8K2X8_HUMAN	tr|A8K2X8	cDNA FLJ78433, highly similar to Homo sapiens chaperonin containing TCP1, subunit 5 (epsilon) (CCT5), mRNA OS=Homo sapiens PE=2 SV=1	60148	134	112	287	247	1.22	0.88	0.721311	8	8	15	11	18	10	22	16	-0.15837
tr|A8K3K1|A8K3K1_HUMAN	tr|A8K3K1	cDNA FLJ78096, highly similar to Homo sapiens actin, alpha, cardiac muscle (ACTC), mRNA OS=Homo sapiens PE=2 SV=1	42362	65	85	516	310	2.32	1.67	0.719828	19	11	27	18	30	20	32	25	0.086353
sp|P26038|MOES_HUMAN	MSN	Moesin OS=Homo sapiens GN=MSN PE=1 SV=3	67892	226	311	176	96	0.46	0.33	0.717391	6	3	8	6	8	6	10	6	0.272492
tr|B2RDF2|B2RDF2_HUMAN	PES1	Pescadillo homolog 1 OS=Homo sapiens GN=PES1 PE=2 SV=1	68359	534	436	71	66	0.21	0.15	0.714286	1	2	4	3	4	3	4	4	0.296795
sp|P13489|RINI_HUMAN	RNH1	Ribonuclease inhibitor OS=Homo sapiens GN=RNH1 PE=1 SV=2	51766	312	426	128	68	0.28	0.2	0.714286	2	2	4	3	4	3	5	3	0.296795
tr|B3KRS5|B3KRS5_HUMAN	HDAC2	Histone deacetylase OS=Homo sapiens GN=HDAC2 PE=2 SV=1	52479	300	316	132	94	0.28	0.2	0.714286	4	2	4	3	5	4	6	4	0.369976
tr|A8K6Q8|A8K6Q8_HUMAN	tr|A8K6Q8	cDNA FLJ75881, highly similar to Homo sapiens transferrin receptor (p90, CD71) (TFRC), mRNA OS=Homo sapiens PE=2 SV=1	85276	184	162	215	188	0.35	0.25	0.714286	6	4	8	6	9	4	9	6	-0.41933
tr|B2R5U1|B2R5U1_HUMAN	tr|B2R5U1	cDNA, FLJ92620, highly similar to Homo sapiens staphylococcal nuclease domain containing 1 (SND1),mRNA OS=Homo sapiens PE=2 SV=1	100294	102	129	360	226	0.52	0.37	0.711538	9	5	14	11	14	9	15	14	0.049205
tr|Q2NKY5|Q2NKY5_HUMAN	TUBB6	TUBB6 protein OS=Homo sapiens GN=TUBB6 PE=2 SV=1	50514	55	69	617	345	1.42	1	0.704225	18	12	21	16	26	17	28	19	0.06075
tr|F8VSC4|F8VSC4_HUMAN	METAP2	Methionine aminopeptidase 2 (Fragment) OS=Homo sapiens GN=METAP2 PE=2 SV=1	17286	686	393	52	46	0.43	0.3	0.697674	1	1	2	1	2	1	2	1	-0.07784
sp|Q92945|FUBP2_HUMAN	KHSRP	Far upstream element-binding protein 2 OS=Homo sapiens GN=KHSRP PE=1 SV=4	73355	91	64	405	369	0.69	0.48	0.695652	9	8	14	11	16	11	16	11	0.138662
tr|B4DTG2|B4DTG2_HUMAN	EEF1G	Elongation factor 1-gamma OS=Homo sapiens GN=EEF1G PE=2 SV=1	56456	48	21	667	787	1.93	1.34	0.694301	18	18	25	21	28	22	33	24	0.319556
sp|Q6P2Q9|PRP8_HUMAN	PRPF8	Pre-mRNA-processing-splicing factor 8 OS=Homo sapiens GN=PRPF8 PE=1 SV=2	274738	46	37	699	566	0.39	0.27	0.692308	23	16	29	21	33	24	40	28	0.208838
tr|F8VTY8|F8VTY8_HUMAN	PA2G4	Proliferation-associated protein 2G4 OS=Homo sapiens GN=PA2G4 PE=2 SV=1	42062	159	86	242	308	1.88	1.3	0.691489	8	10	14	12	15	12	18	13	0.34968
sp|Q9NZI8|IF2B1_HUMAN	IGF2BP1	Insulin-like growth factor 2 mRNA-binding protein 1 OS=Homo sapiens GN=IGF2BP1 PE=1 SV=2	63783	190	414	210	70	0.42	0.29	0.690476	5	1	8	6	9	6	12	7	0.111824
sp|P34931|HS71L_HUMAN	HSPA1L	Heat shock 70 kDa protein 1-like OS=Homo sapiens GN=HSPA1L PE=1 SV=2	70730	26	24	981	731	1.16	0.8	0.689655	23	19	33	30	33	27	37	35	0.376457
sp|P49327|FAS_HUMAN	FASN	Fatty acid synthase OS=Homo sapiens GN=FASN PE=1 SV=3	275877	3	6	1817	1420	0.93	0.64	0.688172	46	35	59	49	65	50	78	56	0.286873
sp|Q08J23|NSUN2_HUMAN	NSUN2	tRNA (cytosine(34)-C(5))-methyltransferase OS=Homo sapiens GN=NSUN2 PE=1 SV=2	87214	290	470	136	62	0.16	0.11	0.6875	2	1	4	2	4	2	5	2	-0.18895
tr|F5GWX5|F5GWX5_HUMAN	CHD4	Chromodomain-helicase-DNA-binding protein 4 OS=Homo sapiens GN=CHD4 PE=2 SV=1	218449	150	194	268	162	0.16	0.11	0.6875	7	5	10	7	9	8	12	9	0.49949
tr|E7EQV9|E7EQV9_HUMAN	RPL15	Ribosomal protein L15 (Fragment) OS=Homo sapiens GN=RPL15 PE=2 SV=1	20611	380	461	107	64	0.83	0.57	0.686747	3	1	4	3	5	4	5	4	0.369976
tr|B4DLA3|B4DLA3_HUMAN	tr|B4DLA3	cDNA FLJ58509, highly similar to Cullin-1 OS=Homo sapiens PE=2 SV=1	82091	263	257	152	117	0.22	0.15	0.681818	4	3	5	4	5	5	6	6	0.659804
tr|B4DVZ8|B4DVZ8_HUMAN	tr|B4DVZ8	cDNA FLJ52432, highly similar to Leukotriene A-4 hydrolase (EC 3.3.2.6) OS=Homo sapiens PE=2 SV=1	64246	268	181	150	121	0.22	0.15	0.681818	3	2	4	2	4	2	5	2	-0.18895
tr|B2RU06|B2RU06_HUMAN	EIF4G1	EIF4G1 protein OS=Homo sapiens GN=EIF4G1 PE=2 SV=1	176807	103	147	357	205	0.22	0.15	0.681818	8	4	11	7	12	9	16	11	0.263737
tr|B4DSK9|B4DSK9_HUMAN	tr|B4DSK9	cDNA FLJ60860, highly similar to Histone deacetylase 1 OS=Homo sapiens PE=2 SV=1	27596	300	286	45	55	0.28	0.19	0.678571	1	1	1	1	1	2	2	2	1.39662
tr|B2R7C5|B2R7C5_HUMAN	tr|B2R7C5	cDNA, FLJ93378, highly similar to Homo sapiens MCM3 minichromosome maintenance deficient 3 (S. cerevisiae) (MCM3), mRNA OS=Homo	91579	265	203	152	150	0.28	0.19	0.678571	5	4	7	5	9	6	9	6	0.111824
tr|A2RUM7|A2RUM7_HUMAN	RPL5	Ribosomal protein L5 OS=Homo sapiens GN=RPL5 PE=2 SV=1	34569	149	166	270	186	1.08	0.73	0.675926	8	4	9	7	10	6	11	8	-0.03277
tr|F5GY55|F5GY55_HUMAN	DDB1	DNA damage-binding protein 1 OS=Homo sapiens GN=DDB1 PE=4 SV=1	122891	581	985	64	19	0.06	0.04	0.666667	1	0	2	1	2	0	2	1	-1.66312
sp|Q9BWU0|NADAP_HUMAN	SLC4A1AP	Kanadaptin OS=Homo sapiens GN=SLC4A1AP PE=1 SV=1	89500	1183	944	19	22	0.06	0.04	0.666667	0	0	1	1	0	1	1	1	2.244499
sp|Q13619|CUL4A_HUMAN	CUL4A	Cullin-4A OS=Homo sapiens GN=CUL4A PE=1 SV=3	88138	629	920	41	24	0.06	0.04	0.666667	1	0	1	1	2	1	2	1	-0.07784
sp|A6NHR9|SMHD1_HUMAN	SMCHD1	Structural maintenance of chromosomes flexible hinge domain-containing protein 1 OS=Homo sapiens GN=SMCHD1 PE=1 SV=2	227942	467	686	83	40	0.03	0.02	0.666667	2	1	2	1	2	1	3	1	-0.07784
sp|Q14651|PLSI_HUMAN	PLS1	Plastin-1 OS=Homo sapiens GN=PLS1 PE=1 SV=2	70608	498	302	77	98	0.15	0.1	0.666667	2	2	3	2	4	2	5	2	-0.18895
tr|J3KN16|J3KN16_HUMAN	KIAA0368	Protein KIAA0368 OS=Homo sapiens GN=KIAA0368 PE=4 SV=1	225491	678	300	53	100	0.06	0.04	0.666667	1	2	4	2	3	2	6	2	0.17382
tr|D6RER5|D6RER5_HUMAN	Sep-11	Septin-11 OS=Homo sapiens GN=SEPT11 PE=2 SV=1	50119	441	275	89	108	0.21	0.14	0.666667	3	2	4	2	4	2	4	2	-0.18895
sp|Q562R1|ACTBL_HUMAN	ACTBL2	Beta-actin-like protein 2 OS=Homo sapiens GN=ACTBL2 PE=1 SV=2	42318	156	160	260	192	0.69	0.46	0.666667	7	5	12	7	14	9	17	13	0.049205
tr|H0YER0|H0YER0_HUMAN	PABPC4	Polyadenylate-binding protein 4 (Fragment) OS=Homo sapiens GN=PABPC4 PE=4 SV=1	7211	115	94	51	72	0.69	0.46	0.666667	1	1	1	2	1	3	1	3	1.882368
sp|Q8WWM7|ATX2L_HUMAN	ATXN2L	Ataxin-2-like protein OS=Homo sapiens GN=ATXN2L PE=1 SV=2	113589	533	876	71	26	0.09	0.06	0.666667	1	0	3	2	4	2	7	2	-0.18895
tr|F6SBX2|F6SBX2_HUMAN	IARS2	Isoleucine--tRNA ligase, mitochondrial OS=Homo sapiens GN=IARS2 PE=2 SV=1	106884	529	478	71	61	0.09	0.06	0.666667	1	1	3	2	4	2	5	2	-0.18895
tr|A6NDY9|A6NDY9_HUMAN	FLNA	Filamin A OS=Homo sapiens GN=FLNA PE=2 SV=4	248165	125	89	321	293	0.18	0.12	0.666667	6	7	13	9	11	10	15	10	0.528947
sp|P07814|SYEP_HUMAN	EPRS	Bifunctional glutamate/proline--tRNA ligase OS=Homo sapiens GN=EPRS PE=1 SV=5	172080	30	39	932	554	0.72	0.48	0.666667	23	16	31	22	34	20	39	25	-0.09225
tr|A8K5I0|A8K5I0_HUMAN	HSPA1A	Heat shock 70kDa protein 1A OS=Homo sapiens GN=HSPA1A PE=2 SV=1	70294	11	8	1514	1252	4.42	2.94	0.665158	37	34	62	57	63	53	68	61	0.416252
sp|P13010|XRCC5_HUMAN	XRCC5	X-ray repair cross-complementing protein 5 OS=Homo sapiens GN=XRCC5 PE=1 SV=3	83222	108	122	350	236	0.47	0.31	0.659574	8	4	10	9	11	10	14	12	0.528947
sp|P22314|UBA1_HUMAN	UBA1	Ubiquitin-like modifier-activating enzyme 1 OS=Homo sapiens GN=UBA1 PE=1 SV=3	118858	51	38	636	559	0.82	0.54	0.658537	17	12	24	18	23	19	27	19	0.391459
sp|Q6S8J3|POTEE_HUMAN	POTEE	POTE ankyrin domain family member E OS=Homo sapiens GN=POTEE PE=1 SV=3	122882	43	28	714	708	0.41	0.27	0.658537	17	14	28	33	28	32	31	40	0.853303
tr|B3KPS3|B3KPS3_HUMAN	tr|B3KPS3	cDNA FLJ32131 fis, clone PEBLM2000267, highly similar to Tubulin alpha-ubiquitous chain OS=Homo sapiens PE=2 SV=1	46725	18	9	1209	1241	5.31	3.49	0.65725	33	35	50	47	52	52	60	59	0.665401
tr|B4DZC0|B4DZC0_HUMAN	tr|B4DZC0	cDNA FLJ51771, highly similar to SWI/SNF-related matrix-associatedactin-dependent regulator of chromatin subfamily A member5 (EC 3.6.	117367	208	214	189	142	0.32	0.21	0.65625	5	3	10	7	11	7	13	7	0.042554
sp|Q02790|FKBP4_HUMAN	FKBP4	Peptidyl-prolyl cis-trans isomerase FKBP4 OS=Homo sapiens GN=FKBP4 PE=1 SV=3	52057	157	118	249	241	0.96	0.63	0.65625	5	6	11	8	11	8	12	8	0.223449
sp|P23526|SAHH_HUMAN	AHCY	Adenosylhomocysteinase OS=Homo sapiens GN=AHCY PE=1 SV=4	48255	142	176	277	177	1.07	0.7	0.654206	7	7	13	9	13	7	17	9	-0.18918
tr|G5E9B2|G5E9B2_HUMAN	CCT8	Chaperonin containing TCP1, subunit 8 (Theta), isoform CRA_a OS=Homo sapiens GN=CCT8 PE=2 SV=1	60011	69	109	487	251	1.23	0.8	0.650407	14	6	16	11	19	12	19	16	0.017659
tr|E9PHA6|E9PHA6_HUMAN	MSH2	DNA mismatch repair protein Msh2 OS=Homo sapiens GN=MSH2 PE=2 SV=1	103977	188	377	212	77	0.2	0.13	0.65	4	3	6	3	9	3	10	4	-0.78223
tr|B2RCS5|B2RCS5_HUMAN	ACTN2	Actinin, alpha 2, isoform CRA_b OS=Homo sapiens GN=ACTN2 PE=2 SV=1	104425	298	204	133	150	0.2	0.13	0.65	4	2	6	3	7	3	7	4	-0.44078
tr|B2RWP9|B2RWP9_HUMAN	MYH10	MYH10 protein OS=Homo sapiens GN=MYH10 PE=2 SV=1	230858	121	97	331	272	0.2	0.13	0.65	10	6	14	9	16	9	19	11	-0.13762
tr|Q5BKZ2|Q5BKZ2_HUMAN	KPNA1	Importin subunit alpha OS=Homo sapiens GN=KPNA1 PE=2 SV=1	60955	435	359	91	82	0.17	0.11	0.647059	3	1	3	2	4	2	4	3	-0.18895
tr|B2RDV7|B2RDV7_HUMAN	tr|B2RDV7	cDNA, FLJ96789, highly similar to Homo sapiens dihydrouridine synthase 3-like (S. cerevisiae) (DUS3L), mRNA OS=Homo sapiens PE=2 SV=1	73714	440	333	90	87	0.14	0.09	0.642857	2	1	3	2	3	2	3	2	0.17382
tr|A8K525|A8K525_HUMAN	tr|A8K525	cDNA FLJ76817, highly similar to Homo sapiens non-POU domain containing, octamer-binding (NONO), mRNA OS=Homo sapiens PE=2 SV=1	54367	75	41	467	513	1.42	0.91	0.640845	13	13	19	14	20	16	22	17	0.347132
tr|Q6LC01|Q6LC01_HUMAN	tr|Q6LC01	MRNA encoding beta-tubulin. (from clone D-beta-1) (Fragment) OS=Homo sapiens PE=2 SV=1	49362	2	15	1668	1072	15.54	9.92	0.638353	59	39	67	49	73	51	82	59	0.147594
sp|P45974|UBP5_HUMAN	USP5	Ubiquitin carboxyl-terminal hydrolase 5 OS=Homo sapiens GN=USP5 PE=1 SV=2	96638	426	428	94	67	0.11	0.07	0.636364	2	1	3	2	4	2	4	2	-0.18895
sp|P46087|NOP2_HUMAN	NOP2	Putative ribosomal RNA methyltransferase NOP2 OS=Homo sapiens GN=NOP2 PE=1 SV=2	89589	419	313	97	95	0.11	0.07	0.636364	2	1	3	2	4	2	5	4	-0.18895
tr|B4E022|B4E022_HUMAN	TKT	Transketolase OS=Homo sapiens GN=TKT PE=2 SV=1	63410	201	145	197	206	0.66	0.42	0.636364	4	5	11	9	13	10	15	11	0.297214
sp|Q9Y3I0|RTCB_HUMAN	C22orf28	tRNA-splicing ligase RtcB homolog OS=Homo sapiens GN=C22orf28 PE=1 SV=1	55688	305	246	130	122	0.41	0.26	0.634146	5	3	6	4	6	4	7	5	0.128765
sp|Q96SI9|STRBP_HUMAN	STRBP	Spermatid perinuclear RNA-binding protein OS=Homo sapiens GN=STRBP PE=1 SV=1	74290	279	417	142	69	0.3	0.19	0.633333	5	2	8	4	8	4	11	4	-0.25867
tr|D2CFK9|D2CFK9_HUMAN	GTPBP4	Nucleolar GTP-binding protein 1 OS=Homo sapiens GN=GTPBP4 PE=2 SV=1	74317	355	338	115	86	0.3	0.19	0.633333	3	1	6	4	6	4	7	5	0.128765
sp|P11177|ODPB_HUMAN	PDHB	Pyruvate dehydrogenase E1 component subunit beta, mitochondrial OS=Homo sapiens GN=PDHB PE=1 SV=3	39550	573	444	65	66	0.27	0.17	0.62963	2	1	3	2	3	2	4	2	0.17382
tr|B4DLV7|B4DLV7_HUMAN	tr|B4DLV7	cDNA FLJ60299, highly similar to Rab GDP dissociation inhibitor beta OS=Homo sapiens PE=2 SV=1	51577	98	98	374	272	0.86	0.54	0.627907	10	7	10	7	10	8	11	9	0.354897
tr|B5BTY4|B5BTY4_HUMAN	DDX3X	ATP-dependent RNA helicase DDX3X OS=Homo sapiens GN=DDX3X PE=2 SV=1	73525	60	95	582	279	1.1	0.69	0.627273	16	8	19	12	18	13	21	17	0.205165
tr|B4DH29|B4DH29_HUMAN	tr|B4DH29	DNA-directed RNA polymerase OS=Homo sapiens PE=2 SV=1	126564	499	532	77	53	0.08	0.05	0.625	2	2	3	2	2	2	5	3	0.65945
tr|F5H1Y3|F5H1Y3_HUMAN	FEN1	Flap endonuclease 1 (Fragment) OS=Homo sapiens GN=FEN1 PE=2 SV=1	18377	400	226	101	104	0.4	0.25	0.625	2	1	2	1	2	1	2	2	-0.07784
sp|Q1KMD3|HNRL2_HUMAN	HNRNPUL2	Heterogeneous nuclear ribonucleoprotein U-like protein 2 OS=Homo sapiens GN=HNRNPUL2 PE=1 SV=1	85622	228	154	174	198	0.4	0.25	0.625	6	5	9	6	8	6	10	6	0.272492
tr|Q6NTA2|Q6NTA2_HUMAN	HNRNPL	HNRNPL protein (Fragment) OS=Homo sapiens GN=HNRNPL PE=2 SV=2	62515	63	29	539	698	1.52	0.95	0.625	18	17	20	22	23	25	28	27	0.780419
sp|P78371|TCPB_HUMAN	CCT2	T-complex protein 1 subunit beta OS=Homo sapiens GN=CCT2 PE=1 SV=4	57794	29	26	936	719	2.77	1.71	0.617329	22	16	29	22	32	23	34	26	0.192319
tr|Q6NZ55|Q6NZ55_HUMAN	RPL13	60S ribosomal protein L13 OS=Homo sapiens GN=RPL13 PE=2 SV=1	24308	402	551	101	52	0.47	0.29	0.617021	2	2	3	2	4	2	6	3	-0.18895
tr|B4DVY1|B4DVY1_HUMAN	EIF3D	Eukaryotic translation initiation factor 3 subunit D OS=Homo sapiens GN=EIF3D PE=2 SV=1	58731	303	250	131	121	0.39	0.24	0.615385	3	3	6	4	7	4	9	5	-0.07789
sp|P12268|IMDH2_HUMAN	IMPDH2	Inosine-5~-monophosphate dehydrogenase 2 OS=Homo sapiens GN=IMPDH2 PE=1 SV=2	56226	66	72	513	333	1.22	0.75	0.614754	12	6	14	10	14	11	14	11	0.325483
tr|B2R5W3|B2R5W3_HUMAN	tr|B2R5W3	cDNA, FLJ92658, highly similar to Homo sapiens poly (ADP-ribose) polymerase family, member 1 (PARP1), mRNA OS=Homo sapiens PE=2 SV	113813	89	127	411	229	0.49	0.3	0.612245	13	6	15	10	16	12	17	14	0.259279
tr|Q53G71|Q53G71_HUMAN	tr|Q53G71	Calreticulin variant (Fragment) OS=Homo sapiens PE=2 SV=1	47061	635	568	57	50	0.23	0.14	0.608696	1	1	3	1	3	1	4	2	-0.56347
tr|Q59GF8|Q59GF8_HUMAN	tr|Q59GF8	Heat shock 70kDa protein 4 isoform a variant (Fragment) OS=Homo sapiens PE=2 SV=1	88805	53	42	626	510	1.3	0.79	0.607692	15	13	23	17	24	17	25	20	0.174369
tr|Q5BJF5|Q5BJF5_HUMAN	SHMT2	Serine hydroxymethyltransferase (Fragment) OS=Homo sapiens GN=SHMT2 PE=2 SV=1	53561	86	99	416	217	1.17	0.71	0.606838	12	6	15	9	16	9	17	12	-0.13762
tr|Q0P607|Q0P607_HUMAN	SFRS15	Splicing factor, arginine/serine-rich 15 OS=Homo sapiens GN=SFRS15 PE=2 SV=1	126004	859	891	39	26	0.05	0.03	0.6	0	0	2	1	2	1	2	1	-0.07784
tr|B2R6H7|B2R6H7_HUMAN	tr|B2R6H7	cDNA, FLJ92955, highly similar to Homo sapiens transportin-SR (TRN-SR), mRNA OS=Homo sapiens PE=2 SV=1	105989	461	760	84	33	0.1	0.06	0.6	1	0	3	2	4	2	5	2	-0.18895
tr|G5E988|G5E988_HUMAN	AP3D1	AP-3 complex subunit delta-1 OS=Homo sapiens GN=AP3D1 PE=2 SV=1	126074	684	554	53	51	0.05	0.03	0.6	1	1	2	1	2	1	3	2	-0.07784
sp|Q7KZ85|SPT6H_HUMAN	SUPT6H	Transcription elongation factor SPT6 OS=Homo sapiens GN=SUPT6H PE=1 SV=2	200203	630	514	58	55	0.05	0.03	0.6	1	1	3	2	3	2	6	2	0.17382
sp|Q9NZB2|F120A_HUMAN	FAM120A	Constitutive coactivator of PPAR-gamma-like protein 1 OS=Homo sapiens GN=FAM120A PE=1 SV=2	123008	569	441	65	66	0.05	0.03	0.6	1	1	2	1	3	2	4	3	0.17382
tr|F2Z2C0|F2Z2C0_HUMAN	DIS3	Exosome complex exonuclease RRP44 OS=Homo sapiens GN=DIS3 PE=2 SV=1	64376	666	419	54	53	0.1	0.06	0.6	2	1	2	1	2	2	2	2	0.65945
tr|Q9BSV4|Q9BSV4_HUMAN	SFPQ	SFPQ protein (Fragment) OS=Homo sapiens GN=SFPQ PE=2 SV=2	68702	122	88	330	303	0.75	0.45	0.6	10	10	14	11	14	12	17	14	0.446099
tr|B2R959|B2R959_HUMAN	tr|B2R959	cDNA, FLJ94229, highly similar to Homo sapiens heterogeneous nuclear ribonucleoprotein L (HNRPL),mRNA OS=Homo sapiens PE=2 SV=1	60838	94	49	396	454	1.32	0.78	0.590909	14	12	16	17	19	23	24	25	0.931939
tr|B4DFM5|B4DFM5_HUMAN	tr|B4DFM5	cDNA FLJ51779, highly similar to Vacuolar ATP synthase subunit B, brain isoform (EC 3.6.3.14) OS=Homo sapiens PE=2 SV=1	43446	433	652	92	0	0.34	0.2	0.588235	2	0	4	0	4	0	4	2	-2.51152
tr|B3KY28|B3KY28_HUMAN	tr|B3KY28	cDNA FLJ46689 fis, clone TRACH3012106, highly similar to Band 4.1-like protein 3 OS=Homo sapiens PE=2 SV=1	85088	424	344	94	45	0.12	0.07	0.583333	1	1	3	1	3	1	3	2	-0.56347
tr|B2RDX5|B2RDX5_HUMAN	tr|B2RDX5	cDNA, FLJ96812, highly similar to Homo sapiens threonyl-tRNA synthetase (TARS), mRNA OS=Homo sapiens PE=2 SV=1	82974	135	170	286	185	0.72	0.42	0.583333	10	7	16	9	20	9	21	13	-0.45159
tr|B4DSI9|B4DSI9_HUMAN	tr|B4DSI9	cDNA FLJ56483, highly similar to Eukaryotic translation initiation factor 4 gamma 1 OS=Homo sapiens PE=2 SV=1	167220	104	148	357	205	0.24	0.14	0.583333	8	4	11	7	12	9	16	11	0.263737
sp|P15121|ALDR_HUMAN	AKR1B1	Aldose reductase OS=Homo sapiens GN=AKR1B1 PE=1 SV=3	36230	593	512	62	56	0.19	0.11	0.578947	2	1	2	1	2	1	2	2	-0.07784
sp|P52597|HNRPF_HUMAN	HNRNPF	Heterogeneous nuclear ribonucleoprotein F OS=Homo sapiens GN=HNRNPF PE=1 SV=3	45985	111	110	349	251	1.14	0.66	0.578947	9	4	12	8	12	8	14	8	0.102951
tr|G3V2B8|G3V2B8_HUMAN	MTHFD1	C-1-tetrahydrofolate synthase, cytoplasmic OS=Homo sapiens GN=MTHFD1 PE=2 SV=1	102152	107	93	351	282	0.71	0.41	0.577465	13	8	18	11	22	11	24	12	-0.31002
sp|P54136|SYRC_HUMAN	RARS	Arginine--tRNA ligase, cytoplasmic OS=Homo sapiens GN=RARS PE=1 SV=2	76129	284	195	140	162	0.4	0.23	0.575	6	4	8	5	8	5	10	6	0.031162
sp|O00232|PSD12_HUMAN	PSMD12	26S proteasome non-ATPase regulatory subunit 12 OS=Homo sapiens GN=PSMD12 PE=1 SV=3	53270	307	239	130	127	0.35	0.2	0.571429	3	2	5	3	5	3	7	3	0.007085
sp|Q92621|NU205_HUMAN	NUP205	Nuclear pore complex protein Nup205 OS=Homo sapiens GN=NUP205 PE=1 SV=3	230171	324	384	125	76	0.07	0.04	0.571429	4	2	5	3	5	3	8	3	0.007085
tr|B4DP06|B4DP06_HUMAN	tr|B4DP06	cDNA FLJ57133, highly similar to Bifunctional purine biosynthesis protein PURH OS=Homo sapiens PE=2 SV=1	59138	116	146	343	205	0.81	0.46	0.567901	8	5	11	7	11	7	11	10	0.042554
tr|B3KTJ9|B3KTJ9_HUMAN	tr|B3KTJ9	cDNA FLJ38393 fis, clone FEBRA2007212 OS=Homo sapiens PE=2 SV=1	103493	131	177	290	176	0.37	0.21	0.567568	6	2	10	6	10	6	10	6	-0.03277
tr|J3KPE3|J3KPE3_HUMAN	GNB2L1	Guanine nucleotide-binding protein subunit beta-2-like 1 OS=Homo sapiens GN=GNB2L1 PE=4 SV=1	30541	100	56	368	405	1.54	0.87	0.564935	7	9	10	9	11	10	11	10	0.528947
sp|P60842|IF4A1_HUMAN	EIF4A1	Eukaryotic initiation factor 4A-I OS=Homo sapiens GN=EIF4A1 PE=1 SV=1	46353	23	25	1052	722	6.86	3.87	0.56414	30	21	40	30	43	32	46	32	0.240182
tr|A8K2Y2|A8K2Y2_HUMAN	tr|A8K2Y2	cDNA FLJ78120, highly similar to Homo sapiens eukaryotic translation initiation factor 2, subunit 3 gamma, 52kDa (EIF2S3), mRNA OS=Hom	51648	356	346	115	85	0.36	0.2	0.555556	2	1	5	3	6	3	7	4	-0.23413
tr|B2RCM2|B2RCM2_HUMAN	tr|B2RCM2	cDNA, FLJ96156, highly similar to Homo sapiens leucyl-tRNA synthetase (LARS), mRNA OS=Homo sapiens PE=2 SV=1	135550	281	265	142	112	0.27	0.15	0.555556	5	2	10	6	10	5	12	7	-0.2741
sp|O43707|ACTN4_HUMAN	ACTN4	Alpha-actinin-4 OS=Homo sapiens GN=ACTN4 PE=1 SV=2	105245	41	32	729	640	1.43	0.79	0.552448	19	15	29	20	31	21	34	22	0.108642
tr|Q1KLZ0|Q1KLZ0_HUMAN	PS1TP5BP1	HCG15971, isoform CRA_a OS=Homo sapiens GN=PS1TP5BP1 PE=2 SV=1	42052	6	3	1655	1511	11.13	6.14	0.551662	43	33	67	63	73	65	79	76	0.499073
tr|A4D210|A4D210_HUMAN	EIF3S9	Eukaryotic translation initiation factor 3, subunit 9 eta, 116kDa OS=Homo sapiens GN=EIF3S9 PE=4 SV=1	89024	200	272	200	109	0.2	0.11	0.55	5	3	5	3	5	3	5	4	0.007085
sp|P13929|ENOB_HUMAN	ENO3	Beta-enolase OS=Homo sapiens GN=ENO3 PE=1 SV=5	47299	144	197	275	160	0.4	0.22	0.55	5	3	7	5	8	5	8	6	0.031162
sp|P54577|SYYC_HUMAN	YARS	Tyrosine--tRNA ligase, cytoplasmic OS=Homo sapiens GN=YARS PE=1 SV=4	59448	584	496	64	59	0.11	0.06	0.545455	1	1	2	1	2	1	3	1	-0.07784
tr|A8K492|A8K492_HUMAN	tr|A8K492	cDNA FLJ76789, highly similar to Homo sapiens methionine-tRNA synthetase (MARS), mRNA OS=Homo sapiens PE=2 SV=1	102277	80	288	442	104	0.55	0.3	0.545455	12	3	14	6	14	6	14	7	-0.49925
tr|B2R6X5|B2R6X5_HUMAN	tr|B2R6X5	cDNA, FLJ93166, highly similar to Homo sapiens heat shock 70kDa protein 6 (HSP70B~) (HSPA6), mRNA OS=Homo sapiens PE=2 SV=1	71370	105	92	356	296	0.57	0.31	0.54386	11	8	15	8	16	9	18	10	-0.13762
tr|B4DJ60|B4DJ60_HUMAN	tr|B4DJ60	cDNA FLJ55072, highly similar to Succinate dehydrogenase (ubiquinone) flavoprotein subunit, mitochondrial (EC1.3.5.1) OS=Homo sapiens	68233	219	210	180	112	0.46	0.25	0.543478	4	2	8	3	8	4	8	4	-0.25867
sp|O14654|IRS4_HUMAN	IRS4	Insulin receptor substrate 4 OS=Homo sapiens GN=IRS4 PE=1 SV=1	134711	133	163	287	188	0.24	0.13	0.541667	8	4	9	5	9	6	10	6	0.111824
tr|G5E9H4|G5E9H4_HUMAN	DCTN1	Dynactin 1 (P150, glued homolog, Drosophila), isoform CRA_a OS=Homo sapiens GN=DCTN1 PE=2 SV=1	127339	350	310	116	97	0.19	0.1	0.526316	4	2	7	4	8	3	9	5	-0.62156

tr|Q6IAX5|Q6IAX5_HUMAN	EIF3S6	Eukaryotic translation initiation factor 3 subunit E OS=Homo sapiens GN=EIF3S6 PE=2 SV=1	52572	210	66	186	354	0.84	0.44	0.52381	6	6	10	6	12	7	13	7	-0.07794
tr|A8MUD9|A8MUD9_HUMA	RPL7	60S ribosomal protein L7 OS=Homo sapiens GN=RPL7 PE=3 SV=1	24474	317	485	127	60	0.9	0.47	0.522222	4	2	5	3	7	3	7	4	-0.44078
tr|K7EIG1|K7EIG1_HUMAN	CLUH	Clustered mitochondria protein homolog OS=Homo sapiens GN=CLUH PE=3 SV=1	140903	178	190	221	165	0.23	0.12	0.521739	5	4	9	5	10	5	12	6	-0.2741
sp|P22626|ROA2_HUMAN	HNRNPA2E	Heterogeneous nuclear ribonucleoproteins A2/B1 OS=Homo sapiens GN=HNRNPA2B1 PE=1 SV=2	37464	40	33	746	634	2.56	1.33	0.519531	15	12	18	15	23	18	27	23	0.315187
sp|Q15366|PCBP2_HUMAN	PCBP2	Poly(rC)-binding protein 2 OS=Homo sapiens GN=PCBP2 PE=1 SV=1	38955	132	113	289	248	1.26	0.64	0.507937	6	7	10	8	10	8	11	8	0.354897
tr|B3KUZ8|B3KUZ8_HUMAN	tr|B3KUZ8	Aspartate aminotransferase OS=Homo sapiens PE=2 SV=1	41699	438	982	90	19	0.16	0.08	0.5	2	0	2	1	2	0	2	2	-1.66312
tr|B4DKR1|B4DKR1_HUMAN	tr|B4DKR1	cDNA FLJ51884, highly similar to Mitochondrial inner membrane protein OS=Homo sapiens PE=2 SV=1	82864	704	930	51	23	0.08	0.04	0.5	1	0	2	1	2	2	3	5	0.65945
tr|B2RTR1|B2RTR1_HUMAN	CLASP2	CLASP2 protein OS=Homo sapiens GN=CLASP2 PE=2 SV=1	166835	739	886	49	26	0.04	0.02	0.5	1	0	2	1	2	1	3	1	-0.07784
sp|Q14690|RRP5_HUMAN	PDCD11	Protein RRP5 homolog OS=Homo sapiens GN=PDCD11 PE=1 SV=3	209939	563	839	66	29	0.08	0.04	0.5	2	0	5	1	5	1	6	1	-1.21595
tr|Q4LE64|Q4LE64_HUMAN	NUMA1	NUMA1 variant protein (Fragment) OS=Homo sapiens GN=NUMA1 variant protein PE=2 SV=1	239798	253	837	160	29	0.06	0.03	0.5	4	0	4	1	4	1	5	2	-0.92624
sp|Q6L8Q7|PDE12_HUMAN	PDE12	2~,5~-phosphodiesterase 12 OS=Homo sapiens GN=PDE12 PE=1 SV=2	68221	752	829	48	29	0.1	0.05	0.5	1	0	2	1	3	1	3	1	-0.56347
tr|B4DMU7|B4DMU7_HUMA	tr|B4DMU	cDNA FLJ59351, highly similar to Striatin-4 OS=Homo sapiens PE=2 SV=1	69271	727	826	50	30	0.1	0.05	0.5	2	0	2	1	2	1	2	1	-0.07784
tr|B3KML1|B3KML1_HUMAN	tr|B3KML1	cDNA FLJ11308 fis, clone PLACE1010074, highly similar to Sorting nexin-2 OS=Homo sapiens PE=2 SV=1	58550	892	782	36	33	0.12	0.06	0.5	0	0	2	1	2	1	2	1	-0.07784
sp|Q9BYZ2|LDH6B_HUMAN	LDHAL6B	L-lactate dehydrogenase A-like 6B OS=Homo sapiens GN=LDHAL6B PE=1 SV=3	42372	536	761	71	33	0.16	0.08	0.5	2	1	2	1	2	1	3	1	-0.07784
tr|A2A3R6|A2A3R6_HUMAN	RPS6	40S ribosomal protein S6 OS=Homo sapiens GN=RPS6 PE=2 SV=1	28834	457	698	84	38	0.24	0.12	0.5	2	1	2	1	3	1	4	2	-0.56347
tr|B0YIW5|B0YIW5_HUMAN	ARCN1	Archain 1, isoform CRA_b OS=Homo sapiens GN=ARCN1 PE=2 SV=1	57630	825	692	41	39	0.12	0.06	0.5	1	1	2	1	2	1	4	1	-0.07784
tr|B2RAH7|B2RAH7_HUMAN	tr|B2RAH7	cDNA, FLJ94921, highly similar to Homo sapiens prolyl endopeptidase (PREP), mRNA OS=Homo sapiens PE=2 SV=1	81594	928	660	35	42	0.08	0.04	0.5	0	1	2	1	2	1	2	1	-0.07784
sp|A0AVT1|UBA6_HUMAN	UBA6	Ubiquitin-like modifier-activating enzyme 6 OS=Homo sapiens GN=UBA6 PE=1 SV=1	119207	721	638	50	43	0.06	0.03	0.5	2	1	2	1	2	1	2	1	-0.07784
tr|B0QYK0|B0QYK0_HUMAN	EWSR1	Ewing sarcoma breakpoint region 1 OS=Homo sapiens GN=EWSR1 PE=4 SV=1	65174	776	604	45	47	0.1	0.05	0.5	1	1	2	1	2	1	2	1	-0.07784
tr|Q5HYM2|Q5HYM2_HUMA	DKFZp686C	Putative uncharacterized protein DKFZp686O2462 (Fragment) OS=Homo sapiens GN=DKFZp686O2462 PE=2 SV=1	72627	697	600	52	47	0.14	0.07	0.5	1	1	3	2	3	2	3	2	0.17382
tr|H0YBW4|H0YBW4_HUMAN	PLAA	Phospholipase A-2-activating protein (Fragment) OS=Homo sapiens GN=PLAA PE=4 SV=1	31645	566	582	65	49	0.22	0.11	0.5	1	1	2	1	2	1	2	1	-0.07784
tr|B4DHG3|B4DHG3_HUMAN	tr|B4DHG3	cDNA FLJ55329, highly similar to Procollagen-lysine,2-oxoglutarate5-dioxygenase 2 (EC 1.14.11.4) OS=Homo sapiens PE=2 SV=1	81716	444	571	89	49	0.08	0.04	0.5	1	1	2	1	2	1	2	1	-0.07784
tr|B0LM41|B0LM41_HUMAN	RBM14/RB	Protein RBM14-RBM4 OS=Homo sapiens GN=RBM14/RBM4 fusion PE=2 SV=1	37410	247	491	164	59	0.18	0.09	0.5	2	1	2	1	3	2	3	2	0.17382
tr|F5H4R9|F5H4R9_HUMAN	DNM2	Dynamin-2 OS=Homo sapiens GN=DNM2 PE=2 SV=1	98218	335	484	120	60	0.14	0.07	0.5	3	1	4	2	4	2	5	3	-0.18895
tr|Q05DV5|Q05DV5_HUMAN	ZC3HAV1	ZC3HAV1 protein (Fragment) OS=Homo sapiens GN=ZC3HAV1 PE=2 SV=1	49964	366	472	111	62	0.14	0.07	0.5	1	1	2	1	2	1	2	1	-0.07784
tr|B4DXL9|B4DXL9_HUMAN	tr|B4DXL9	cDNA FLJ53677, highly similar to Drebrin-like protein OS=Homo sapiens PE=2 SV=1	42985	503	465	76	63	0.16	0.08	0.5	1	1	2	1	3	1	4	1	-0.56347
sp|Q9UID3|VPS51_HUMAN	VPS51	Vacuolar protein sorting-associated protein 51 homolog OS=Homo sapiens GN=VPS51 PE=1 SV=2	86901	494	410	78	70	0.08	0.04	0.5	1	1	2	1	2	1	2	1	-0.07784
tr|B4DZJ7|B4DZJ7_HUMAN	tr|B4DZJ7	cDNA FLJ55629, highly similar to Transcription elongation factor SPT5 OS=Homo sapiens PE=2 SV=1	118511	568	322	65	91	0.06	0.03	0.5	1	1	2	1	2	1	3	1	-0.07784
sp|Q7Z6Z7|HUWE1_HUMAN	HUWE1	E3 ubiquitin-protein ligase HUWE1 OS=Homo sapiens GN=HUWE1 PE=1 SV=3	485523	106	212	353	143	0.08	0.04	0.5	7	4	11	6	12	7	19	9	-0.07794
tr|E9KL44|E9KL44_HUMAN	tr|E9KL44	Epididymis tissue sperm binding protein Li 14m OS=Homo sapiens PE=2 SV=1	83688	167	200	231	155	0.26	0.13	0.5	5	3	6	4	8	4	9	4	-0.25867
sp|O00410|IPO5_HUMAN	IPO5	Importin-5 OS=Homo sapiens GN=IPO5 PE=1 SV=4	125032	126	121	310	237	0.4	0.2	0.5	8	6	13	7	13	6	16	8	-0.39595
sp|P17987|TCPA_HUMAN	TCP1	T-complex protein 1 subunit alpha OS=Homo sapiens GN=TCP1 PE=1 SV=1	60819	56	144	617	207	1.58	0.78	0.493671	14	6	18	11	20	10	24	13	-0.30688
tr|A8MXP9|A8MXP9_HUMAN	MATR3	Matrin-3 OS=Homo sapiens GN=MATR3 PE=4 SV=1	100419	117	130	336	226	0.67	0.33	0.492537	11	6	16	9	19	9	20	10	-0.37924
sp|O00425|IF2B3_HUMAN	IGF2BP3	Insulin-like growth factor 2 mRNA-binding protein 3 OS=Homo sapiens GN=IGF2BP3 PE=1 SV=2	64008	173	186	224	170	0.57	0.28	0.491228	8	3	10	5	9	6	10	6	0.111824
tr|B3KX19|B3KX19_HUMAN	tr|B3KX19	cDNA FLJ44500 fis, clone UTERU3000828, highly similar to 116 kDa U5 small nuclear ribonucleoprotein component OS=Homo sapiens PE=2	109111	138	193	280	163	0.47	0.23	0.489362	7	4	13	7	13	7	15	9	-0.18918
tr|F1T0B3|F1T0B3_HUMAN	DDX1	ATP-dependent RNA helicase DDX1 OS=Homo sapiens GN=DDX1 PE=2 SV=1	74838	176	174	222	180	0.6	0.29	0.483333	6	5	11	6	12	7	13	7	-0.07794
tr|B4DW52|B4DW52_HUMAN	tr|B4DW52	cDNA FLJ55253, highly similar to Actin, cytoplasmic 1 OS=Homo sapiens PE=2 SV=1	38950	10	5	1559	1456	12.58	6.08	0.483307	40	32	63	61	68	68	75	80	0.667328
sp|P40222|TXLNA_HUMAN	TXLNA	Alpha-taxilin OS=Homo sapiens GN=TXLNA PE=1 SV=3	62195	301	243	132	123	0.23	0.11	0.478261	2	1	4	2	4	3	5	3	0.296795
tr|B2RCV1|B2RCV1_HUMAN	tr|B2RCV1	cDNA, FLJ96310, highly similar to Homo sapiens solute carrier family 25 (mitochondrial carrier; adenine nucleotide translocator), member 5	33062	632	910	57	25	0.21	0.1	0.47619	1	0	2	1	3	1	3	2	-0.56347
tr|A6NKB8|A6NKB8_HUMAN	RNPEP	Aminopeptidase B OS=Homo sapiens GN=RNPEP PE=2 SV=1	68752	502	284	76	105	0.21	0.1	0.47619	1	2	4	2	3	2	4	2	0.17382
tr|J3KTA4|J3KTA4_HUMAN	DDX5	Probable ATP-dependent RNA helicase DDX5 OS=Homo sapiens GN=DDX5 PE=3 SV=1	69557	88	107	412	258	1.4	0.66	0.471429	15	9	21	11	23	12	28	14	-0.25234
tr|B2RE88|B2RE88_HUMAN	tr|B2RE88	cDNA, FLJ96465, highly similar to Homo sapiens solute carrier family 25 (mitochondrial carrier;phosphate carrier), member 3 (SLC25A3), nu	40379	921	706	35	37	0.17	0.08	0.470588	0	1	2	1	2	1	2	1	-0.07784
tr|B3KWY7|B3KWY7_HUMAN	tr|B3KWY7	cDNA FLJ44270 fis, clone TLIVE2008229, highly similar to SIGNAL RECOGNITION PARTICLE 68 kDa PROTEIN OS=Homo sapiens PE=2 SV=1	66524	272	329	146	88	0.34	0.16	0.470588	5	2	6	3	7	5	8	5	0.211938
sp|P53396|ACLY_HUMAN	ACLY	ATP-citrate synthase OS=Homo sapiens GN=ACLY PE=1 SV=3	121674	20	43	1097	502	1.53	0.72	0.470588	30	15	44	22	46	22	56	25	-0.39039
tr|B3KY60|B3KY60_HUMAN	tr|B3KY60	cDNA FLJ16777 fis, clone BRHIP2029567, highly similar to Cell division cycle 5-like protein OS=Homo sapiens PE=2 SV=1	92448	344	906	117	25	0.15	0.07	0.466667	2	0	4	2	4	1	6	2	-0.92624
tr|B4DSP0|B4DSP0_HUMAN	hCG_16427	HCG1642748, isoform CRA_b OS=Homo sapiens GN=hCG_1642748 PE=2 SV=1	45559	390	637	104	43	0.15	0.07	0.466667	2	1	2	1	2	1	2	1	-0.07784
sp|P53621|COPA_HUMAN	COPA	Coatomer subunit alpha OS=Homo sapiens GN=COPA PE=1 SV=2	139797	396	448	103	66	0.15	0.07	0.466667	4	2	6	3	6	2	7	3	-0.71988
tr|B4DPJ2|B4DPJ2_HUMAN	tr|B4DPJ2	Annexin OS=Homo sapiens PE=2 SV=1	46025	486	296	79	101	0.15	0.07	0.466667	1	1	2	1	2	1	2	1	-0.07784
tr|H7BZT7|H7BZT7_HUMAN	ESD	S-formylglutathione hydrolase (Fragment) OS=Homo sapiens GN=ESD PE=4 SV=1	25806	1076	1016	26	16	0.28	0.13	0.464286	0	0	2	1	1	0	2	1	-0.92595
tr|B2RE56|B2RE56_HUMAN	tr|B2RE56	Peptidyl-prolyl cis-trans isomerase OS=Homo sapiens PE=2 SV=1	18225	800	715	43	37	0.41	0.19	0.463415	0	0	2	1	4	2	5	2	-0.18895
tr|J3KQE5|J3KQE5_HUMAN	RAN	GTP-binding nuclear protein Ran (Fragment) OS=Homo sapiens GN=RAN PE=4 SV=1	27027	728	825	50	30	0.26	0.12	0.461538	0	0	2	1	2	1	3	2	-0.07784
tr|Q56VW8|Q56VW8_HUMA	tr|Q56VW	Lung cancer-related protein 9 OS=Homo sapiens PE=2 SV=1	52186	944	562	33	50	0.13	0.06	0.461538	1	1	2	1	2	2	2	4	0.65945
tr|Q0VAB1|Q0VAB1_HUMAN	TIMM50	Translocase of inner mitochondrial membrane 50 homolog (S. cerevisiae) OS=Homo sapiens GN=TIMM50 PE=2 SV=1	50960	369	445	110	66	0.13	0.06	0.461538	2	1	2	1	2	1	2	1	-0.07784
tr|J3KMX2|J3KMX2_HUMAN	SMARCD2	SWI/SNF-related matrix-associated actin-dependent regulator of chromatin subfamily D member 2 OS=Homo sapiens GN=SMARCD2 PE=4 S	52433	413	287	100	104	0.13	0.06	0.461538	1	2	2	2	2	2	2	3	0.65945
tr|B4DH52|B4DH52_HUMAN	tr|B4DH52	cDNA FLJ55916, highly similar to General transcription factor II-I OS=Homo sapiens PE=2 SV=1	112359	234	230	169	135	0.26	0.12	0.461538	5	3	8	5	10	7	12	9	0.174003
tr|A4UCU0|A4UCU0_HUMAN	tr|A4UCU0	DEAD box polypeptide 47 isoform 1 variant (Fragment) OS=Homo sapiens PE=2 SV=1	19904	491	936	78	23	0.37	0.17	0.459459	2	0	2	1	2	1	2	1	-0.07784
sp|Q15645|PCH2_HUMAN	TRIP13	Pachytene checkpoint protein 2 homolog OS=Homo sapiens GN=TRIP13 PE=1 SV=2	48863	401	634	101	44	0.48	0.22	0.458333	3	0	6	3	7	4	8	5	-0.07789
tr|Q4ZG57|Q4ZG57_HUMAN	MCM6	Putative uncharacterized protein MCM6 (Fragment) OS=Homo sapiens GN=MCM6 PE=2 SV=1	89803	357	463	114	63	0.24	0.11	0.458333	3	2	6	3	7	3	7	4	-0.44078
tr|Q5LJA0|Q5LJA0_HUMAN	TROVE2	60 kDa SS-A/Ro ribonucleoprotein OS=Homo sapiens GN=TROVE2 PE=4 SV=1	59916	360	432	114	67	0.11	0.05	0.454545	2	1	2	1	2	1	2	2	-0.07784
tr|Q59FI9|Q59FI9_HUMAN	tr|Q59FI9	Ribosomal protein L12 variant (Fragment) OS=Homo sapiens PE=2 SV=1	21755	621	488	59	60	0.33	0.15	0.454545	2	1	2	1	3	1	3	1	-0.56347
tr|C9J9K3|C9J9K3_HUMAN	RPSA	40S ribosomal protein SA (Fragment) OS=Homo sapiens GN=RPSA PE=2 SV=1	29601	447	433	88	67	0.53	0.24	0.45283	1	2	4	2	4	2	4	2	-0.18895
tr|A8K781|A8K781_HUMAN	tr|A8K781	cDNA FLJ75299, highly similar to Xenopus laevis proteasome (prosome, macropain) 26S subunit, ATPase 3, mRNA OS=Homo sapiens PE=2 S	47578	548	493	68	59	0.31	0.14	0.451613	2	1	4	2	4	2	6	2	-0.18895
tr|B4DUP5|B4DUP5_HUMAN	tr|B4DUP5	cDNA FLJ60165, highly similar to Translation initiation factor eIF-2B subunit delta OS=Homo sapiens PE=2 SV=1	34946	488	828	79	30	0.2	0.09	0.45	2	0	2	1	2	1	2	1	-0.07784
tr|B4DI28|B4DI28_HUMAN	PTBP2	Polypyrimidine tract-binding protein 2 OS=Homo sapiens GN=PTBP2 PE=2 SV=1	34947	758	626	47	44	0.2	0.09	0.45	1	1	2	1	2	1	2	2	-0.07784
tr|H3BLU7|H3BLU7_HUMAN	AKR7A2	Aflatoxin B1 aldehyde reductase member 2 (Fragment) OS=Homo sapiens GN=AKR7A2 PE=4 SV=1	35004	535	576	71	49	0.2	0.09	0.45	2	1	2	1	2	1	2	1	-0.07784
sp|O94906|PRP6_HUMAN	PRPF6	Pre-mRNA-processing factor 6 OS=Homo sapiens GN=PRPF6 PE=1 SV=1	107656	280	274	142	109	0.2	0.09	0.45	3	2	6	3	5	3	7	3	0.007085
tr|J3KT29|J3KT29_HUMAN	RPL23	60S ribosomal protein L23 OS=Homo sapiens GN=RPL23 PE=3 SV=1	13241	489	374	79	79	0.58	0.26	0.448276	1	1	2	1	2	1	2	2	-0.07784
tr|B0V0F8|B0V0F8_HUMAN	DHX16	Putative pre-mRNA-splicing factor ATP-dependent RNA helicase DHX16 (Fragment) OS=Homo sapiens GN=DHX16 PE=4 SV=1	77228	772	847	45	28	0.09	0.04	0.444444	1	0	2	1	2	1	3	2	-0.07784
tr|J3KRX5|J3KRX5_HUMAN	RPL17	60S ribosomal protein L17 (Fragment) OS=Homo sapiens GN=RPL17 PE=3 SV=1	20386	521	689	73	39	0.36	0.16	0.444444	2	1	2	1	2	1	2	1	-0.07784
tr|A7E2E1|A7E2E1_HUMAN	SMARCA4	SWI/SNF related, matrix associated, actin dependent regulator of chromatin, subfamily a, member 4 OS=Homo sapiens GN=SMARCA4 PE=2	185100	345	451	117	65	0.09	0.04	0.444444	3	1	5	1	6	1	9	2	-1.45716
tr|B3KRE0|B3KRE0_HUMAN	tr|B3KRE0	cDNA FLJ34086 fis, clone FCBBF3005320, highly similar to Double-stranded RNA-binding protein Staufen homolog 1 OS=Homo sapiens PE=2	40204	423	567	95	35	0.27	0.12	0.444444	1	0	3	1	3	1	3	1	-0.56347
sp|Q14240|IF4A2_HUMAN	EIF4A2	Eukaryotic initiation factor 4A-II OS=Homo sapiens GN=EIF4A2 PE=1 SV=2	46601	64	67	519	347	1.15	0.51	0.443478	11	8	14	11	16	12	17	13	0.259279
tr|B7TY16|B7TY16_HUMAN	ACTN1	Actinin alpha 1 isoform 3 OS=Homo sapiens GN=ACTN1 PE=2 SV=1	107644	109	168	349	185	0.52	0.23	0.442308	11	3	14	7	16	8	18	10	-0.2984
tr|Q3B7A7|Q3B7A7_HUMAN	GART	Phosphoribosylglycinamide formyltransferase, phosphoribosylglycinamide synthetase, phosphoribosylaminoimidazole synthetase OS=Hom	108909	50	47	653	463	0.98	0.43	0.438776	14	9	23	13	24	13	30	14	-0.20132
tr|D0PNI1|D0PNI1_HUMAN	YWHAZ	Tyrosine 3-monooxygenase/tryptophan 5-monooxygenase activation protein zeta polypeptide OS=Homo sapiens GN=YWHAZ PE=2 SV=1	27899	329	307	124	98	0.57	0.25	0.438596	4	1	4	2	5	3	6	3	0.007085
sp|Q9H853|TBA4B_HUMAN	TUBA4B	Putative tubulin-like protein alpha-4B OS=Homo sapiens GN=TUBA4B PE=5 SV=2	27819	205	192	191	164	0.57	0.25	0.438596	3	5	7	6	6	6	7	7	0.659923
tr|E9PK47|E9PK47_HUMAN	PYGL	Phosphorylase OS=Homo sapiens GN=PYGL PE=2 SV=1	94410	388	579	105	49	0.23	0.1	0.434783	3	0	6	3	5	2	7	3	-0.47866
tr|Q9UJU1|Q9UJU1_HUMAN	VIL2	Cytovillin 2 (Fragment) OS=Homo sapiens GN=VIL2 PE=2 SV=1	16294	226	241	84	55	0.46	0.2	0.434783	2	1	3	3	3	2	3	3	0.17382
tr|B4DLR3|B4DLR3_HUMAN	tr|B4DLR3	cDNA FLJ54020, highly similar to Heterogeneous nuclear ribonucleoprotein U OS=Homo sapiens PE=2 SV=1	87548	12	40	1423	534	2.49	1.08	0.433735	37	17	54	27	56	29	61	31	-0.28041
tr|Q5JR94|Q5JR94_HUMAN	RPS8	40S ribosomal protein S8 OS=Homo sapiens GN=RPS8 PE=2 SV=1	24475	215	423	183	68	0.67	0.29	0.432836	5	1	5	2	6	2	7	2	-0.71988
tr|B4DUQ1|B4DUQ1_HUMAN	tr|B4DUQ1	cDNA FLJ54552, highly similar to Heterogeneous nuclear ribonucleoprotein K OS=Homo sapiens PE=2 SV=1	48708	77	62	459	371	1.85	0.8	0.432432	15	10	21	13	21	13	23	19	-0.01226
tr|E5KNY5|E5KNY5_HUMAN	LRPPRC	Leucine-rich PPR-motif containing OS=Homo sapiens GN=LRPPRC PE=2 SV=1	159003	52	59	634	387	0.77	0.33	0.428571	21	10	28	14	29	20	33	22	0.134652
sp|Q8TDD1|DDX54_HUMAN	DDX54	ATP-dependent RNA helicase DDX54 OS=Homo sapiens GN=DDX54 PE=1 SV=2	98819	580	888	64	26	0.07	0.03	0.428571	2	0	2	1	2	1	2	2	-0.07784
tr|B7Z5S9|B7Z5S9_HUMAN	tr|B7Z5S9	cDNA FLJ61359, highly similar to Adapter-relatedprotein complex 2 alpha- 2 subunit OS=Homo sapiens PE=2 SV=1	103962	475	529	81	53	0.14	0.06	0.428571	2	1	3	2	3	1	3	2	-0.56347
tr|B4E3Q9|B4E3Q9_HUMAN	tr|B4E3Q9	cDNA FLJ59659, highly similar to Vinculin OS=Homo sapiens PE=2 SV=1	101217	601	510	61	56	0.07	0.03	0.428571	2	1	2	1	2	1	4	2	-0.07784
tr|H0Y8C6|H0Y8C6_HUMAN	IPO5	Importin-5 (Fragment) OS=Homo sapiens GN=IPO5 PE=4 SV=1	125319	126	161	249	191	0.4	0.17	0.425	6	5	11	6	11	5	14	8	-0.40555
tr|B4DLT2|B4DLT2_HUMAN	tr|B4DLT2	cDNA FLJ56637, highly similar to Nuclear pore complex protein Nup155 OS=Homo sapiens PE=2 SV=1	152734	153	216	263	141	0.26	0.11	0.423077	6	3	11	5	11	6	11	6	-0.16422
tr|H7C3T4|H7C3T4_HUMAN	PRDX4	Peroxiredoxin-4 (Fragment) OS=Homo sapiens GN=PRDX4 PE=4 SV=1	18485	246	518	164	55	0.95	0.4	0.421053	4	1	4	2	5	3	5	3	0.007085
tr|J3KR24|J3KR24_HUMAN	IARS	Isoleucine--tRNA ligase, cytoplasmic OS=Homo sapiens GN=IARS PE=4 SV=1	132820	151	205	266	150	0.31	0.13	0.419355	6	3	11	5	16	4	18	6	-1.21723
tr|B7Z4V2|B7Z4V2_HUMAN	tr|B7Z4V2	cDNA FLJ51907, highly similar to Stress-70 protein, mitochondrial OS=Homo sapiens PE=2 SV=1	72641	97	172	378	183	0.86	0.36	0.418605	10	4	17	7	17	6	20	7	-0.77116
tr|Q60FE2|Q60FE2_HUMAN	MYH9	MYH9 variant protein OS=Homo sapiens GN=MYH9 PE=2 SV=1	227646	33	57	911	393	0.53	0.22	0.415094	22	9	31	14	38	15	44	19	-0.6563
tr|B4E1E0|B4E1E0_HUMAN	tr|B4E1E0	cDNA FLJ58563, highly similar to CTP synthase 1 (EC 6.3.4.2) OS=Homo sapiens PE=2 SV=1	50233	338	268	119	111	0.46	0.19	0.413043	3	2	6	3	8	4	8	4	-0.25867
tr|G3XAB4|G3XAB4_HUMAN	ACAT1	Acetyl-CoA acetyltransferase, mitochondrial OS=Homo sapiens GN=ACAT1 PE=2 SV=1	17449	231	185	172	101	1.03	0.42	0.407767	4	2	4	2	4	2	4	2	-0.18895
sp|P26599|PTBP1_HUMAN	PTBP1	Polypyrimidine tract-binding protein 1 OS=Homo sapiens GN=PTBP1 PE=1 SV=1	57357	47	78	672	323	1.18	0.48	0.40678	12	7	15	7	15	7	18	8	-0.3889
sp|Q9ULT8|HECD1_HUMAN	HECTD1	E3 ubiquitin-protein ligase HECTD1 OS=Homo sapiens GN=HECTD1 PE=1 SV=3	292225	232	893	170	26	0.05	0.02	0.4	4	0	4	1	5	1	7	1	-1.21595
tr|Q1L838|Q1L838_HUMAN	GTBP	GTBP-ALT OS=Homo sapiens GN=GTBP PE=2 SV=1	122267	212	345	184	85	0.2	0.08	0.4	5	2	7	3	9	5	10	5	-0.12951
sp|P13639|EF2_HUMAN	EEF2	Elongation factor 2 OS=Homo sapiens GN=EEF2 PE=1 SV=4	96246	1	11	2167	1232	6.45	2.57	0.39845	54	35	92	60	95	61	111	77	0.024575
sp|Q9BVP2|GNL3_HUMAN	GNL3	Guanine nucleotide-binding protein-like 3 OS=Homo sapiens GN=GNL3 PE=1 SV=2	62468	333	655	121	42	0.43	0.17	0.395349	3	0	7	3	7	3	8	4	-0.44078
sp|P52272|HNRPM_HUMAN	HNRNPM	Heterogeneous nuclear ribonucleoprotein M OS=Homo sapiens GN=HNRNPM PE=1 SV=3	77749	37	44	862	483	2.18	0.86	0.394495	26	11	34	18	39	22	44	24	-0.15358
tr|B2RBE5|B2RBE5_HUMAN	tr|B2RBE5	cDNA, FLJ95468, highly similar to Homo sapiens transcriptional coactivator tubedown-100 (TBDN100),transcript variant 1, mRNA OS=Homo	102490	368	399	110	72	0.33	0.13	0.393939	3	1	9	4	9	4	12	4	-0.41933
sp|Q9NR30|DDX21_HUMAN	DDX21	Nucleolar RNA helicase 2 OS=Homo sapiens GN=DDX21 PE=1 SV=5	87804	58	71	591	334	1.41	0.55	0.390071	18	10	28	12	27	12	34	13	-0.47993
tr|B3KX96|B3KX96_HUMAN	tr|B3KX96	cDNA FLJ45003 fis, clone BRAWH3011623, highly similar to Heterogeneous nuclear ribonucleoproteins C OS=Homo sapiens PE=2 SV=1	32368	255	137	158	209	1.18	0.46	0.389831	6	5	8	6	7	6	8	7	0.453268
tr|E9PN50|E9PN50_HUMAN	PSMC3	26S protease regulatory subunit 6A (Fragment) OS=Homo sapiens GN=PSMC3 PE=2 SV=1	26737	548	482	36	61	0.31	0.12	0.387097	1	1	2	1	2	1	3	1	-0.07784
tr|B4DM78|B4DM78_HUMAN	tr|B4DM78	cDNA FLJ58199, highly similar to Fragile X mental retardation syndrome-related protein 1 OS=Homo sapiens PE=2 SV=1	63280	361	992	114	19	0.13	0.05	0.384615	2	0	2	1	2	0	3	1	-1.66312
tr|Q6IBR0|Q6IBR0_HUMAN	RPN1	RPN1 protein OS=Homo sapiens GN=RPN1 PE=2 SV=1	68678	429	279	93	106	0.26	0.1	0.384615	3	2	5	2	5	3	7	3	0.007085
tr|D6R9I9|D6R9I9_HUMAN	ABCE1	ATP-binding cassette sub-family E member 1 OS=Homo sapiens GN=ABCE1 PE=2 SV=1	47919	448	281	88	106	0.39	0.15	0.384615	1	2	5	3	4	2	6	3	-0.18895
tr|A8K690|A8K690_HUMAN	tr|A8K690	cDNA FLJ76863, highly similar to Homo sapiens stress-induced-phosphoprotein 1 (Hsp70/Hsp90-organizing protein) (STIP1), mRNA OS=Hon	63243	78	68	445	346	1.75	0.67	0.382857	13	7	20	11	21	13	28	15	-0.01226
sp|P04181|OAT_HUMAN	OAT	Ornithine aminotransferase, mitochondrial OS=Homo sapiens GN=OAT PE=1 SV=1	48846	162	598	236	47	0.58	0.22	0.37931	7	1	7	3	7	2	7	3	-0.92653
tr|B2RAM6|B2RAM6_HUMAN	tr|B2RAM6	cDNA, FLJ95005, highly similar to Homo sapiens kinesin family member 11 (KIF11), mRNA OS=Homo sapiens PE=2 SV=1	120084	392	991	104	19	0.08	0.03	0.375	2	0	3	1	3	1	3	2	-0.56347
tr|A0PJ61|A0PJ61_HUMAN	RSL1D1	RSL1D1 protein (Fragment) OS=Homo sapiens GN=RSL1D1 PE=2 SV=1	49688	468	905	82	25	0.38	0.14	0.368421	2	0	5	2	6	1	7	3	-1.45716
sp|Q14258|TRI25_HUMAN	TRIM25	E3 ubiquitin/ISG15 ligase TRIM25 OS=Homo sapiens GN=TRIM25 PE=1 SV=2	72581	394	624	103	45	0.19	0.07	0.368421	3	1	4	1	5	1	5	1	-1.21595
tr|G3V529|G3V529_HUMAN	DDX24	ATP-dependent RNA helicase DDX24 OS=Homo sapiens GN=DDX24 PE=2 SV=1	91994	1036	941	28	23	0.11	0.04	0.363636	0	0	3	1	2	1	4	1	-0.07784
tr|B3KMS0|B3KMS0_HUMAN	tr|B3KMS0	cDNA FLJ12449 fis, clone NT2RM1000314, highly similar to Condensin complex subunit 1 OS=Homo sapiens PE=2 SV=1	158464	248	361	163	81	0.11	0.04	0.363636	3	1	5	2	6	2	13	2	-0.71988
sp|Q14204|DYHC1_HUMAN	DYNC1H1	Cytoplasmic dynein 1 heavy chain 1 OS=Homo sapiens GN=DYNC1H1 PE=1 SV=5	534809	44	80	711	318	0.22	0.08	0.363636	23	8	33	13	39	12	48	17	-1.00481
tr|Q6IPS9|Q6IPS9_HUMAN	EEF1A1	Elongation factor 1-alpha OS=Homo sapiens GN=EEF1A1 PE=2 SV=1	50451	59	128	587	226	2.12	0.77	0.363208	18	6	28	11	31	13	39	13	-0.56532
sp|Q58FF6|H90B4_HUMAN	HSP90AB4F	Putative heat shock protein HSP 90-beta 4 OS=Homo sapiens GN=HSP90AB4P PE=5 SV=1	58855	322	797	125	32	0.31	0.11	0.354839	3	0	6	2	6	3	7	5	-0.23413
tr|Q53XC0|Q53XC0_HUMAN	EIF2S1	Eukaryotic translation initiation factor 2, subunit 1 alpha, 35kDa, isoform CRA_a OS=Homo sapiens GN=EIF2S1 PE=2 SV=1	36374	130	319	292	92	1.19	0.42	0.352941	7	2	9	4	9	4	10	5	-0.41933
tr|B3KPS9|B3KPS9_HUMAN	tr|B3KPS9	cDNA FLJ32140 fis, clone PLACE5000058, highly similar to Importin-4 OS=Homo sapiens PE=2 SV=1	104593	436	654	91	42	0.17	0.06	0.352941	3	2	5	2	6	3	7	3	-0.23413
tr|E9PKZ0|E9PKZ0_HUMAN	RPL8	60S ribosomal protein L8 OS=Homo sapiens GN=RPL8 PE=4 SV=1	22603	320	639	125	43	0.51	0.18	0.352941	3	1	3	1	4	1	5	1	-0.92624
tr|K7ENG2|K7ENG2_HUMAN	U2AF2	Splicing factor U2AF 65 kDa subunit OS=Homo sapiens GN=U2AF2 PE=4 SV=1	34222	209	225	186	137	0.91	0.32	0.351648	5	3	7	3	7	3	7	3	-0.44078
tr|Q05D80|Q05D80_HUMAN	RNMT	mRNA cap guanine-N7 methyltransferase (Fragment) OS=Homo sapiens GN=RNMT PE=2 SV=1	47947	930	780	34	33	0.2	0.07	0.35	0	0	2	1	2	1	2	1	-0.07784
sp|P61158|ARP3_HUMAN	ACTR3	Actin-related protein 3 OS=Homo sapiens GN=ACTR3 PE=1 SV=3	47797	411	553	100	51	0.4	0.14	0.35	2	1	5	2	6	3	6	3	-0.23413
tr|Q8N5A0|Q8N5A0_HUMAN	EIF5B	Eukaryotic translation initiation factor 5B OS=Homo sapiens GN=EIF5B PE=2 SV=1	139172	304	526	130	54	0.2	0.07	0.35	4	1	8	3	8	3	8	5	-0.62156
sp|Q12904|AIMP1_HUMAN	AIMP1	Aminoacyl tRNA synthase complex-interacting multifunctional protein 1 OS=Homo sapiens GN=AIMP1 PE=1 SV=2	34616	652	330	56	88	0.58	0.2	0.344828	0	1	5	2	5	3	6	3	0.007085
sp|Q00610|CLH1_HUMAN	CLTC	Clathrin heavy chain 1 OS=Homo sapiens GN=CLTC PE=1 SV=5	193260	25	36	1034	596	1.02	0.35	0.343137	24	13	42	19	44	20	50	24	-0.46151
tr|H0Y8G5|H0Y8G5_HUMAN	HNRNPD	Heterogeneous nuclear ribonucleoprotein D0 (Fragment) OS=Homo sapiens GN=HNRNPD PE=4 SV=1	29877	266	527	150	54	0.7	0.24	0.342857	4	1	6	2	7	2	8	2	-0.92653
tr|Q1L6K4|Q1L6K4_HUMAN	PDCD8	Apoptosis-inducing factor 1, mitochondrial OS=Homo sapiens GN=PDCD8 PE=2 SV=1	26131	420	720	96	0	0.44	0.15	0.340909	2	0	3	0	3	2	3	2	0.17382
tr|B4DN01|B4DN01_HUMAN	tr|B4DN01	cDNA FLJ57332, highly similar to COP9 signalosome complex subunit 3 OS=Homo sapiens PE=2 SV=1	33950	343	351	117	83	0.59	0.2	0.338983	3	1	5	2	5	2	5	2	-0.47866
tr|B4DTA2|B4DTA2_HUMAN	tr|B4DTA2	cDNA FLJ60148, highly similar to Homo sapiens heterogeneous nuclear ribonucleoprotein D-like (HNRPDL), transcript variant 2, mRNA OS=H	30367	276	845	144	28	0.68	0.23	0.338235	4	0	6	2	7	2	7	2	-0.92653
tr|H6VRF8|H6VRF8_HUMAN	KRT1	Keratin 1 OS=Homo sapiens GN=KRT1 PE=3 SV=1	66184	31	87	917	304	1.64	0.55	0.335366	25	10	27	12	28	13	31	15	-0.42031
sp|P01023|A2MG_HUMAN	A2M	Alpha-2-macroglobulin OS=Homo sapiens GN=A2M PE=1 SV=3	164613	474	863	81	27	0.06	0.02	0.333333	3	0	4	1	4	1	5	1	-0.92624
tr|J3KPM9|J3KPM9_HUMAN	STAT1	Signal transducer and activator of transcription 1-alpha/beta OS=Homo sapiens GN=STAT1 PE=4 SV=1	83878	734	791	49	32	0.12	0.04	0.333333	1	0	3	1	3	1	3	1	-0.56347
tr|B4DYQ1|B4DYQ1_HUMAN	tr|B4DYQ1	cDNA FLJ57671, highly similar to SWI/SNF-related matrix-associated actin-dependent regulator of chromatin subfamily C member 1 OS=Ho	112027	378	653	107	42	0.15	0.05	0.333333	3	1	6	1	6	1	6	1	-1.45716
tr|Q96F88|Q96F88_HUMAN	POP1	Processing of 1, ribonuclease P/MRP subunit (S. cerevisiae) OS=Homo sapiens GN=POP1 PE=2 SV=1	116318	453	912	85	25	0.09	0.03	0.333333	2	0	3	1	3	1	4	2	-0.56347
tr|B3KXH0|B3KXH0_HUMAN	tr|B3KXH0	cDNA FLJ45395 fis, clone BRHIP3027191, highly similar to 150 kDa oxygen-regulated protein (Orp150) OS=Homo sapiens PE=2 SV=1	110601	492	380	78	77	0.09	0.03	0.333333	1	1	3	1	3	1	3	2	-0.56347
sp|O14980|XPO1_HUMAN	XPO1	Exportin-1 OS=Homo sapiens GN=XPO1 PE=1 SV=1	124447	166	173	234	182	0.33	0.11	0.333333	6	4	11	4	13	6	18	9	-0.39595
sp|Q08211|DHX9_HUMAN	DHX9	ATP-dependent RNA helicase A OS=Homo sapiens GN=DHX9 PE=1 SV=4	142181	27	58	964	387	1.43	0.47	0.328671	25	10	44	18	47	21	54	24	-0.48721
tr|B2R4K7|B2R4K7_HUMAN	tr|B2R4K7	60S ribosomal protein L6 OS=Homo sapiens PE=2 SV=1	32739	147	366	273	80	1.89	0.62	0.328042	7	1	11	3	12	3	12	4	-1.17877
tr|H0YJS4|H0YJS4_HUMAN	EIF2S1	Eukaryotic translation initiation factor 2 subunit 1 (Fragment) OS=Homo sapiens GN=EIF2S1 PE=4 SV=1	29243	130	550	217	52	1.19	0.38	0.319328	5	1	7	3	7	2	8	4	-0.92653
tr|A3KFI9|A3KFI9_HUMAN	CSTF1	Cleavage stimulation factor subunit 1 (Fragment) OS=Homo sapiens GN=CSTF1 PE=4 SV=1	15755	840	557	40	50	0.22	0.07	0.318182	1	1	1	1	1	1	1	1	0.659333
tr|Q53F48|Q53F48_HUMAN	tr|Q53F48	Heterogeneous nuclear ribonucleoprotein H3 isoform a variant (Fragment) OS=Homo sapiens PE=2 SV=1	36959	386	788	105	32	0.41	0.13	0.317073	3	0	4	1	3	1	5	1	-0.56347
tr|B0V043|B0V043_HUMAN	VARS	Valine--tRNA ligase OS=Homo sapiens GN=VARS PE=3 SV=1	141632	72	143	481	207	0.38	0.12	0.315789	11	4	16	6	16	6	17	7	-0.68607
sp|O75643|U520_HUMAN	SNRNP200	U5 small nuclear ribonucleoprotein 200 kDa helicase OS=Homo sapiens GN=SNRNP200 PE=1 SV=2	246006	21	83	1089	311	0.54	0.17	0.314815	28	8	35	13	40	13	44	16	-0.92973
sp|P06733|ENOA_HUMAN	ENO1	Alpha-enolase OS=Homo sapiens GN=ENO1 PE=1 SV=2	47481	22	34	1078	599	5.55	1.74	0.313514	28	17	39	26	43	27	48	31	-0.00244
tr|Q7RTQ4|Q7RTQ4_HUMAN	KNS2	Kinesin light chain 1M OS=Homo sapiens GN=KNS2 PE=2 SV=1	71354	316	848	127	28	0.16	0.05	0.3125	2	0	3	1	3	1	4	2	-0.56347
tr|B4DGB3|B4DGB3_HUMAN	tr|B4DGB3	cDNA FLJ61233, highly similar to Kinesin light chain 1 OS=Homo sapiens PE=2 SV=1	65883	316	846	127	28	0.16	0.05	0.3125	2	0	3	1	3	1	5	2	-0.56347
tr|B2R659|B2R659_HUMAN	tr|B2R659	cDNA, FLJ92803, highly similar to Homo sapiens hydroxysteroid (17-beta) dehydrogenase 4 (HSD17B4), mRNA OS=Homo sapiens PE=2 SV=1	80018	395	842	103	28	0.13	0.04	0.307692	2	0	3	1	3	1	8	3	-0.56347
sp|Q92900|RENT1_HUMAN	UPF1	Regulator of nonsense transcripts 1 OS=Homo sapiens GN=UPF1 PE=1 SV=2	125578	256	282	157	105	0.26	0.08	0.307692	4	2	9	3	11	4	12	4	-0.69538
tr|B2RBR9|B2RBR9_HUMAN	tr|B2RBR9	cDNA, FLJ95650, highly similar to Homo sapiens karyopherin (importin) beta 1 (KPNB1), mRNA OS=Homo sapiens PE=2 SV=1	98434	54	90	623	299	0.99	0.3	0.30303	14	5	21	8	24	8	26	10	-0.87035
tr|B2R9K8|B2R9K8_HUMAN	tr|B2R9K8	cDNA, FLJ94440, highly similar to Homo sapiens chaperonin containing TCP1, subunit 6A (zeta 1)(CCT6A), mRNA OS=Homo sapiens PE=2 SV	58386	141	289	277	104	0.64	0.19	0.296875	8	2	11	5	16	6	19	11	-0.68607
sp|O75131|CPNE3_HUMAN	CPNE3	Copine-3 OS=Homo sapiens GN=CPNE3 PE=1 SV=1	60947	628	717	58	37	0.17	0.05	0.294118	2	1	3	1	3	1	4	1	-0.56347
tr|Q53ET2|Q53ET2_HUMAN	tr|Q53ET2	Dihydropyrimidinase-like 2 variant (Fragment) OS=Homo sapiens PE=2 SV=1	62688	450	494	86	59	0.17	0.05	0.294118	2	1	3	1	3	1	4	2	-0.56347
tr|B4E0S6|B4E0S6_HUMAN	tr|B4E0S6	cDNA FLJ55635, highly similar to pre-mRNA-splicing factorATP-dependent RNA helicase DHX15 (EC 3.6.1.-) OS=Homo sapiens PE=2 SV=1	90289	218	480	181	61	0.24	0.07	0.291667	5	1	6	2	8	2	11	4	-1.10731
tr|G1UI16|G1UI16_HUMAN	PDS5A	SCC-112 protein, isoform CRA_b OS=Homo sapiens GN=PDS5A PE=2 SV=1	152274	434	812	92	31	0.07	0.02	0.285714	2	0	3	1	4	2	4	3	-0.18895
sp|O94822|LTN1_HUMAN	LTN1	E3 ubiquitin-protein ligase listerin OS=Homo sapiens GN=LTN1 PE=1 SV=6	203103	657	767	55	33	0.07	0.02	0.285714	1	0	4	1	4	1	5	1	-0.92624
tr|B4E063|B4E063_HUMAN	tr|B4E063	cDNA FLJ54292, highly similar to Kinesin-like protein KIFC1 OS=Homo sapiens PE=2 SV=1	73571	626	644	58	43	0.14	0.04	0.285714	1	1	3	1	2	1	3	1	-0.07784
tr|C9IZE4|C9IZE4_HUMAN	PSMD6	26S proteasome non-ATPase regulatory subunit 6 OS=Homo sapiens GN=PSMD6 PE=4 SV=1	52346	341	615	118	45	0.28	0.08	0.285714	3	1	4	1	3	1	5	1	-0.56347
tr|G3V198|G3V198_HUMAN	NUP160	Nuclear pore complex protein Nup160 OS=Homo sapiens GN=NUP160 PE=2 SV=1	142365	571	593	65	48	0.07	0.02	0.285714	2	1	3	1	4	1	4	1	-0.92624
tr|B7Z1Q4|B7Z1Q4_HUMAN	tr|B7Z1Q4	cDNA FLJ57955, highly similar to Adapter-relatedprotein complex 2 alpha- 2 subunit OS=Homo sapiens PE=2 SV=1	75682	475	523	81	54	0.14	0.04	0.285714	2	1	3	1	3	1	3	1	-0.56347
tr|E7EW20|E7EW20_HUMAN	MYO6	Unconventional myosin-VI OS=Homo sapiens GN=MYO6 PE=2 SV=1	150111	650	418	56	69	0.07	0.02	0.285714	2	1	3	1	3	1	3	2	-0.56347
tr|B3GQS7|B3GQS7_HUMAN	HSPD1	Mitochondrial heat shock 60kD protein 1 variant 1 OS=Homo sapiens GN=HSPD1 PE=2 SV=1	60813	85	61	423	375	1.58	0.45	0.28481	15	7	18	9	19	11	23	14	-0.10296
tr|G3V203|G3V203_HUMAN	RPL18	60S ribosomal protein L18 OS=Homo sapiens GN=RPL18 PE=2 SV=1	18859	589	1011	63	17	0.64	0.18	0.28125	2	0	3	1	4	0	6	1	-2.51152
sp|Q9UHD1|CHRD1_HUMAN	CHORDC1	Cysteine and histidine-rich domain-containing protein 1 OS=Homo sapiens GN=CHORDC1 PE=1 SV=2	38264	422	473	96	62	0.64	0.18	0.28125	2	1	6	2	7	2	7	2	-0.92653
sp|Q14683|SMC1A_HUMAN	SMC1A	Structural maintenance of chromosomes protein 1A OS=Homo sapiens GN=SMC1A PE=1 SV=2	143771	155	260	260	114	0.25	0.07	0.28	7	3	10	3	12	5	14	6	-0.52605
tr|H0Y4X3|H0Y4X3_HUMAN	RBM39	RNA-binding protein 39 (Fragment) OS=Homo sapiens GN=RBM39 PE=4 SV=1	36959	306	695	130	38	0.29	0.08	0.275862	3	1	3	1	3	1	3	1	-0.56347

tr|A4QPB0|A4QPB0_HUMAN	IQGAP1	IQ motif containing GTPase activating protein 1 OS=Homo sapiens GN=IQGAP1 PE=2 SV=1	189789	249	599	162	47	0.11	0.03	0.272727	4	1	6	2	7	3	11	4	-0.44078
tr|F5H3A1|F5H3A1_HUMAN	ATP1A1	Sodium/potassium-transporting ATPase subunit alpha-1 OS=Homo sapiens GN=ATP1A1 PE=2 SV=1	114239	170	354	227	82	0.33	0.09	0.272727	6	2	10	3	11	3	13	4	-1.05827
tr|B4DWJ2|B4DWJ2_HUMAN	QARS	Glutamine--tRNA ligase OS=Homo sapiens GN=QARS PE=2 SV=1	87437	217	261	181	114	0.44	0.12	0.272727	4	3	10	3	11	3	16	4	-1.05827
sp|Q8WUM4|PDC6I_HUMAN	PDCD6IP	Programmed cell death 6-interacting protein OS=Homo sapiens GN=PDCD6IP PE=1 SV=1	96590	194	277	204	107	0.26	0.07	0.269231	5	2	7	2	7	2	7	3	-0.92653
tr|Q6NVW7|Q6NVW7_HUMA	KPNA2	Importin subunit alpha OS=Homo sapiens GN=KPNA2 PE=2 SV=1	58271	118	258	335	116	0.93	0.25	0.268817	8	3	12	4	12	4	12	5	-0.81587
tr|Q6NZ52|Q6NZ52_HUMAN	RPL27A	Ribosomal protein L27a OS=Homo sapiens GN=RPL27A PE=2 SV=1	16582	560	858	67	27	0.75	0.2	0.266667	1	0	3	1	3	2	3	2	0.17382
sp|P62424|RL7A_HUMAN	RPL7A	60S ribosomal protein L7a OS=Homo sapiens GN=RPL7A PE=1 SV=2	30148	283	691	140	39	0.87	0.23	0.264368	3	1	6	2	5	3	6	5	0.007085
tr|Q0D2Q6|Q0D2Q6_HUMAN	PGAM1	Phosphoglycerate mutase 1 (Brain) OS=Homo sapiens GN=PGAM1 PE=2 SV=1	28888	731	347	20	84	0.46	0.12	0.26087	0	1	1	1	1	1	1	1	0.659333
sp|Q14008|CKAP5_HUMAN	CKAP5	Cytoskeleton-associated protein 5 OS=Homo sapiens GN=CKAP5 PE=1 SV=3	227062	183	860	216	27	0.12	0.03	0.25	5	0	8	1	9	1	10	2	-2.00526
tr|D3DWB6|D3DWB6_HUMAI	USP9X	Ubiquitin carboxyl-terminal hydrolase OS=Homo sapiens GN=USP9X PE=2 SV=1	274331	554	745	67	34	0.04	0.01	0.25	2	0	3	1	5	1	5	1	-1.21595
tr|B3KXX5|B3KXX5_HUMAN	tr|B3KXX5	Structural maintenance of chromosomes protein OS=Homo sapiens PE=2 SV=1	145050	277	680	142	40	0.12	0.03	0.25	4	1	5	1	7	1	10	4	-1.66382
sp|Q96EK5|KBP_HUMAN	KIAA1279	KIF1-binding protein OS=Homo sapiens GN=KIAA1279 PE=1 SV=1	72453	181	636	219	44	0.36	0.09	0.25	4	1	7	2	6	3	8	3	-0.23413
sp|Q9UG63|ABCF2_HUMAN	ABCF2	ATP-binding cassette sub-family F member 2 OS=Homo sapiens GN=ABCF2 PE=1 SV=2	71815	538	416	70	69	0.2	0.05	0.25	1	1	4	1	4	1	5	1	-0.92624
sp|P07737|PROF1_HUMAN	PFN1	Profilin-1 OS=Homo sapiens GN=PFN1 PE=1 SV=2	15216	230	658	173	42	1.24	0.3	0.241935	5	1	5	1	5	1	7	2	-1.21595
sp|P53004|BIEA_HUMAN	BLVRA	Biliverdin reductase A OS=Homo sapiens GN=BLVRA PE=1 SV=2	33692	207	459	189	64	1.33	0.32	0.240602	5	2	10	3	10	3	12	3	-0.92682
tr|Q6IBT3|Q6IBT3_HUMAN	CCT7	CCT7 protein OS=Homo sapiens GN=CCT7 PE=2 SV=1	59804	34	135	901	215	1.62	0.38	0.234568	18	4	23	6	23	6	24	7	-1.19769
tr|B4E3M6|B4E3M6_HUMAN	tr|B4E3M6	cDNA FLJ55446, highly similar to Superkiller viralicidic activity 2-like 2 (EC 3.6.1.-) OS=Homo sapiens PE=2 SV=1	107714	575	796	64	32	0.13	0.03	0.230769	0	0	4	1	5	1	6	1	-1.21595
tr|Q548W1|Q548W1_HUMAI	OGT	HRNT1 OS=Homo sapiens GN=OGT PE=2 SV=1	104029	354	773	115	33	0.13	0.03	0.230769	3	1	4	1	3	1	4	1	-0.56347
sp|O75844|FACE1_HUMAN	ZMPSTE24	CAAX prenyl protease 1 homolog OS=Homo sapiens GN=ZMPSTE24 PE=1 SV=2	55063	675	602	54	47	0.26	0.06	0.230769	1	1	2	1	2	1	2	1	-0.07784
sp|Q9Y678|COPG1_HUMAN	COPG1	Coatomer subunit gamma-1 OS=Homo sapiens GN=COPG1 PE=1 SV=1	98967	261	881	155	26	0.22	0.05	0.227273	3	0	6	1	6	1	7	2	-1.45716
tr|B3KXI2|B3KXI2_HUMAN	SEC23A	Protein transport protein Sec23A OS=Homo sapiens GN=SEC23A PE=2 SV=1	63574	555	570	67	49	0.22	0.05	0.227273	1	1	4	1	3	1	5	1	-0.56347
tr|Q2VPA0|Q2VPA0_HUMAN	LONP1	Lon protease homolog (Fragment) OS=Homo sapiens GN=LONP1 PE=2 SV=1	95436	211	492	185	59	0.31	0.07	0.225806	5	2	8	2	8	2	10	3	-1.10731
tr|F5GX68|F5GX68_HUMAN	DNMT1	Cytosine-specific methyltransferase OS=Homo sapiens GN=DNMT1 PE=2 SV=1	185605	382	866	107	27	0.09	0.02	0.222222	3	0	5	2	6	1	9	4	-1.45716
sp|Q06210|GFPT1_HUMAN	GFPT1	Glutamine--fructose-6-phosphate aminotransferase [isomerizing] 1 OS=Homo sapiens GN=GFPT1 PE=1 SV=3	79555	431	702	92	38	0.18	0.04	0.222222	2	1	4	1	4	1	6	2	-0.92624
tr|B3KN82|B3KN82_HUMAN	tr|B3KN82	cDNA FLJ13913 fis, clone Y79AA1000231, highly similar to Nucleolar protein NOP5 OS=Homo sapiens PE=2 SV=1	52769	602	734	61	35	0.27	0.06	0.222222	2	0	4	1	3	1	5	1	-0.56347
tr|D3DU83|D3DU83_HUMAN	RPS2	Ribosomal protein S2, isoform CRA_c OS=Homo sapiens GN=RPS2 PE=4 SV=1	19208	698	701	52	0	0.5	0.11	0.22	1	0	1	0	1	1	1	1	0.659333
tr|Q5T081|Q5T081_HUMAN	RCC1	CHC1 protein OS=Homo sapiens GN=RCC1 PE=2 SV=1	45397	497	370	77	79	0.32	0.07	0.21875	3	1	4	1	4	2	4	2	-0.18895
tr|B4E0W4|B4E0W4_HUMAN	tr|B4E0W4	cDNA FLJ61020, highly similar to Heterogeneous nuclear ribonucleoprotein D0 OS=Homo sapiens PE=2 SV=1	23062	266	495	76	59	0.7	0.15	0.214286	1	1	2	1	3	1	3	1	-0.56347
tr|D3DPU2|D3DPU2_HUMAN	CAP1	Adenylyl cyclase-associated protein OS=Homo sapiens GN=CAP1 PE=3 SV=1	51926	288	559	137	50	0.28	0.06	0.214286	3	1	4	1	4	2	4	3	-0.18895
tr|B3KMX0|B3KMX0_HUMAN	tr|B3KMX0	cDNA FLJ12837 fis, clone NT2RP2003228, highly similar to DNA replication licensing factor MCM4 OS=Homo sapiens PE=2 SV=1	97114	493	547	78	52	0.14	0.03	0.214286	2	1	4	1	4	1	5	1	-0.92624
sp|P22234|PUR6_HUMAN	PAICS	Multifunctional protein ADE2 OS=Homo sapiens GN=PAICS PE=1 SV=3	47790	123	630	330	44	1.08	0.23	0.212963	7	2	11	3	13	3	16	4	-1.29
tr|A8K6V7|A8K6V7_HUMAN	tr|A8K6V7	cDNA FLJ76053, highly similar to Homo sapiens Ras-GTPase activating protein SH3 domain-binding protein 2 (G3BP2), transcript variant 3, r	50842	618	852	59	28	0.29	0.06	0.206897	1	0	4	1	4	1	5	1	-0.92624
tr|B4DJX1|B4DJX1_HUMAN	tr|B4DJX1	cDNA FLJ50978, highly similar to Dihydrolipoyllysine-residue acetyltransferasecomponent of pyruvate dehydrogenase complex, mitochondi	63057	240	450	166	65	0.29	0.06	0.206897	4	1	5	1	6	1	6	3	-1.45716
tr|B4DS27|B4DS27_HUMAN	tr|B4DS27	cDNA FLJ51288, highly similar to Kinesin heavy chain OS=Homo sapiens PE=2 SV=1	50233	168	723	117	36	0.34	0.07	0.205882	3	1	4	1	6	1	6	2	-1.45716
tr|Q59GS3|Q59GS3_HUMAN	tr|Q59GS3	Proteasome 26S ATPase subunit 5 variant (Fragment) OS=Homo sapiens PE=2 SV=1	38907	309	392	129	73	0.39	0.08	0.205128	3	1	4	1	4	1	4	2	-0.92624
sp|P11498|PYC_HUMAN	PC	Pyruvate carboxylase, mitochondrial OS=Homo sapiens GN=PC PE=1 SV=2	130293	101	365	367	80	0.49	0.1	0.204082	11	1	16	4	18	4	20	5	-1.38269
sp|P24928|RPB1_HUMAN	POLR2A	DNA-directed RNA polymerase II subunit RPB1 OS=Homo sapiens GN=POLR2A PE=1 SV=2	218408	348	830	116	29	0.05	0.01	0.2	2	0	3	1	3	2	3	4	0.17382
tr|J3KPF3|J3KPF3_HUMAN	SLC3A2	4F2 cell-surface antigen heavy chain OS=Homo sapiens GN=SLC3A2 PE=4 SV=1	68230	245	538	164	53	0.26	0.05	0.192308	4	1	5	1	5	1	6	2	-1.21595
sp|P61011|SRP54_HUMAN	SRP54	Signal recognition particle 54 kDa protein OS=Homo sapiens GN=SRP54 PE=1 SV=1	55953	988	490	31	59	0.77	0.14	0.181818	0	1	1	2	1	2	1	5	1.39662
tr|C9J2Z7|C9J2Z7_HUMAN	EIF4G1	Eukaryotic translation initiation factor 4 gamma 1 (Fragment) OS=Homo sapiens GN=EIF4G1 PE=2 SV=1	89370	103	464	225	63	0.22	0.04	0.181818	5	1	5	1	5	2	5	4	-0.47866
sp|P62249|RS16_HUMAN	RPS16	40S ribosomal protein S16 OS=Homo sapiens GN=RPS16 PE=1 SV=2	16549	531	978	71	20	1.11	0.2	0.18018	4	1	4	1	4	1	4	1	-0.92624
sp|O95373|IPO7_HUMAN	IPO7	Importin-7 OS=Homo sapiens GN=IPO7 PE=1 SV=1	120751	70	297	485	100	0.62	0.11	0.177419	14	2	18	4	19	4	21	7	-1.45885
tr|D3DRX6|D3DRX6_HUMAN	KIF5B	Kinesin family member 5B, isoform CRA_a OS=Homo sapiens GN=KIF5B PE=3 SV=1	110331	168	747	229	34	0.34	0.06	0.176471	7	1	10	2	12	1	14	3	-2.4018
tr|E1NZA1|E1NZA1_HUMAN	PRIC295	Peroxisome proliferator activated receptor interacting complex protein OS=Homo sapiens GN=PRIC295 PE=2 SV=1	294953	163	247	236	121	0.23	0.04	0.173913	7	2	19	4	20	5	25	7	-1.24137
tr|Q5T8U3|Q5T8U3_HUMAN	RPL7A	60S ribosomal protein L7a (Fragment) OS=Homo sapiens GN=RPL7A PE=4 SV=1	21702	283	664	135	41	0.87	0.15	0.172414	3	1	5	1	4	1	5	3	-0.92624
tr|F8VPD4|F8VPD4_HUMAN	CAD	CAD protein OS=Homo sapiens GN=CAD PE=2 SV=1	238097	82	314	433	94	0.35	0.06	0.171429	11	2	22	5	22	4	27	5	-1.66591
tr|B2R5V9|B2R5V9_HUMAN	tr|B2R5V9	cDNA, FLJ92652, highly similar to Homo sapiens high density lipoprotein binding protein (vigilin)(HDLBP), mRNA OS=Homo sapiens PE=2 SV	141919	353	605	115	47	0.12	0.02	0.166667	2	1	5	1	5	2	6	3	-0.47866
tr|H0Y3P2|H0Y3P2_HUMAN	EIF4G2	Eukaryotic translation initiation factor 4 gamma 2 OS=Homo sapiens GN=EIF4G2 PE=4 SV=1	98569	421	325	96	90	0.18	0.03	0.166667	2	1	5	1	5	1	8	1	-1.21595
tr|B7Z9W6|B7Z9W6_HUMAN	tr|B7Z9W6	cDNA, FLJ78980, highly similar to DnaJ homolog subfamily C member 7 OS=Homo sapiens PE=2 SV=1	50776	282	456	141	64	0.37	0.06	0.162162	3	1	5	1	4	1	5	1	-0.92624
tr|B7ZA86|B7ZA86_HUMAN	tr|B7ZA86	cDNA, FLJ79100, highly similar to 14-3-3 protein epsilon (14-3-3E) OS=Homo sapiens PE=2 SV=1	19138	227	356	175	82	1.65	0.26	0.157576	6	2	6	4	8	3	8	4	-0.62156
tr|H7BZF2|H7BZF2_HUMAN	DKC1	H/ACA ribonucleoprotein complex subunit 4 (Fragment) OS=Homo sapiens GN=DKC1 PE=4 SV=1	8803	524	679	72	38	1.74	0.27	0.155172	3	1	3	1	3	1	3	1	-0.56347
sp|P13645|K1C10_HUMAN	KRT10	Keratin, type I cytoskeletal 10 OS=Homo sapiens GN=KRT10 PE=1 SV=6	59020	28	171	956	184	2.48	0.38	0.153226	23	4	25	6	27	7	28	8	-1.21851
tr|B3KNB4|B3KNB4_HUMAN	tr|B3KNB4	cDNA FLJ14168 fis, clone NT2RP2001440, highly similar to 14-3-3 protein gamma OS=Homo sapiens PE=2 SV=1	28453	227	371	57	79	1.65	0.25	0.151515	1	2	1	2	2	3	2	3	1.145199
tr|B2R6N6|B2R6N6_HUMAN	tr|B2R6N6	cDNA, FLJ93036, highly similar to Homo sapiens tyrosine 3-monooxygenase/tryptophan 5-monooxygenaseactivation protein, eta polypepti	28274	227	256	57	0	1.65	0.25	0.151515	1	0	1	0	2	1	2	1	-0.07784
tr|B2R5P6|B2R5P6_HUMAN	TXNRD1	Thioredoxin reductase 1, cytoplasmic OS=Homo sapiens GN=TXNRD1 PE=2 SV=1	55253	206	386	190	76	0.41	0.06	0.146341	6	1	6	1	6	3	7	3	-0.23413
sp|A5YKK6|CNOT1_HUMAN	CNOT1	CCR4-NOT transcription complex subunit 1 OS=Homo sapiens GN=CNOT1 PE=1 SV=2	269106	389	575	105	49	0.07	0.01	0.142857	4	1	6	1	4	1	7	4	-0.92624
sp|P49792|RBP2_HUMAN	RANBP2	E3 SUMO-protein ligase RanBP2 OS=Homo sapiens GN=RANBP2 PE=1 SV=2	362365	367	540	111	52	0.07	0.01	0.142857	4	1	8	1	8	3	11	4	-0.62156
sp|Q10567|AP1B1_HUMAN	AP1B1	AP-1 complex subunit beta-1 OS=Homo sapiens GN=AP1B1 PE=1 SV=2	105482	136	508	208	56	0.44	0.06	0.136364	5	1	7	2	9	1	9	3	-2.00526
tr|Q68DI0|Q68DI0_HUMAN	DKFZp781K	Putative uncharacterized protein DKFZp781K0743 OS=Homo sapiens GN=DKFZp781K0743 PE=2 SV=1	106527	136	312	285	96	0.44	0.06	0.136364	8	2	12	2	14	3	16	4	-1.3933
tr|A1KYQ7|A1KYQ7_HUMAN	tr|A1KYQ7	Cell migration-inducing protein 17 OS=Homo sapiens PE=2 SV=1	106022	87	228	415	135	0.44	0.06	0.136364	12	3	12	3	14	3	15	5	-1.3933
tr|B4E3C3|B4E3C3_HUMAN	tr|B4E3C3	cDNA FLJ55610, highly similar to Protein arginine N-methyltransferase 1 (EC 2.1.1.-) OS=Homo sapiens PE=2 SV=1	41150	416	501	98	58	0.59	0.08	0.135593	2	1	6	1	6	1	7	3	-1.45716
tr|Q0QF37|Q0QF37_HUMAN	MDH2	Malate dehydrogenase (Fragment) OS=Homo sapiens GN=MDH2 PE=2 SV=1	32405	84	238	423	127	2.55	0.34	0.133333	12	2	13	3	13	3	14	3	-1.29
sp|Q96PK6|RBM14_HUMAN	RBM14	RNA-binding protein 14 OS=Homo sapiens GN=RBM14 PE=1 SV=2	69620	177	889	222	26	0.45	0.06	0.133333	3	0	8	1	9	1	9	1	-2.00526
sp|P04259|K2C6B_HUMAN	KRT6B	Keratin, type II cytoskeletal 6B OS=Homo sapiens GN=KRT6B PE=1 SV=5	60315	252	572	160	49	0.38	0.05	0.131579	6	1	7	1	8	2	10	2	-1.10731
sp|Q92973|TNPO1_HUMAN	TNPO1	Transportin-1 OS=Homo sapiens GN=TNPO1 PE=1 SV=2	103771	314	916	127	24	0.24	0.03	0.125	3	0	7	1	7	1	9	3	-1.66382
tr|B4DZI8|B4DZI8_HUMAN	COPB2	Coatomer protein complex, subunit beta 2 (Beta prime), isoform CRA_b OS=Homo sapiens GN=COPB2 PE=2 SV=1	99839	244	735	165	35	0.25	0.03	0.12	6	0	7	1	7	1	7	1	-1.66382
tr|Q6NUR1|Q6NUR1_HUMAN	NCAPG	Non-SMC condensin I complex, subunit G OS=Homo sapiens GN=NCAPG PE=2 SV=1	115205	239	564	166	50	0.25	0.03	0.12	3	1	8	1	8	1	8	1	-1.84459
tr|A8K8U1|A8K8U1_HUMAN	tr|A8K8U1	cDNA FLJ77762, highly similar to Homo sapiens cullin-associated and neddylation-dissociated 1 (CAND1), mRNA OS=Homo sapiens PE=2 SV	137941	67	421	501	69	0.6	0.07	0.116667	15	2	22	3	24	4	25	5	-1.78918
tr|B4DMY3|B4DMY3_HUMAN	tr|B4DMY3	cDNA FLJ60713, highly similar to Homo sapiens heterogeneous nuclear ribonucleoprotein A/B (HNRPAB), transcript variant 1, mRNA OS=Ho	35101	220	619	180	45	0.88	0.09	0.102273	7	1	9	1	10	1	10	1	-2.14985
tr|B1AKZ1|B1AKZ1_HUMAN	ATP1A4	ATPase, Na+/K+ transporting, alpha 4 polypeptide (Fragment) OS=Homo sapiens GN=ATP1A4 PE=2 SV=1	51810	170	940	25	0	0.33	0.03	0.090909	0	0	1	0	1	0	1	1	-0.92595
sp|P35908|K22E_HUMAN	KRT2	Keratin, type II cytoskeletal 2 epidermal OS=Homo sapiens GN=KRT2 PE=1 SV=2	65678	35	249	895	121	2.07	0.16	0.077295	20	2	27	3	30	3	34	4	-2.46932
tr|O14729|O14729_HUMAN	tr|O14729	Smooth muscle myosin heavy chain SM2 (Fragment) OS=Homo sapiens PE=2 SV=1	68203	33	589	98	37	0.53	0.04	0.075472	2	1	2	1	2	1	2	2	-0.07784
sp|P63241|IF5A1_HUMAN	EIF5A	Eukaryotic translation initiation factor 5A-1 OS=Homo sapiens GN=EIF5A PE=1 SV=2	17049	379	703	107	37	3.24	0.21	0.064815	2	0	8	1	7	1	8	1	-1.66382
tr|K0J110|K0J110_HUMAN	mcdrh	Malignant cell derived RNA helicase OS=Homo sapiens GN=mcdrh PE=2 SV=1	82499	58	549	79	52	1.41	0.09	0.06383	4	1	4	2	4	2	4	2	-0.18895
tr|B3KXZ4|B3KXZ4_HUMAN	tr|B3KXZ4	cDNA FLJ46429 fis, clone THYMU3014372, highly similar to DNA replication licensing factor MCM2 OS=Homo sapiens PE=2 SV=1	91898	172	506	224	56	0.47	0.03	0.06383	7	1	11	1	10	3	12	4	-0.92682
tr|F8VS61|F8VS61_HUMAN	KRT77	Keratin, type II cytoskeletal 1b OS=Homo sapiens GN=KRT77 PE=2 SV=1	25704	35	339	32	0	2.07	0.11	0.05314	0	0	1	0	1	0	1	1	-0.92595
tr|B4DUC5|B4DUC5_HUMAN	CSE1L	Exportin-2 OS=Homo sapiens GN=CSE1L PE=2 SV=1	86045	129	958	292	21	0.57	0.03	0.052632	4	0	12	1	12	1	14	1	-2.4018
tr|B7Z3K7|B7Z3K7_HUMAN	tr|B7Z3K7	cDNA FLJ50972, highly similar to Fructose-bisphosphate aldolase C (EC 4.1.2.13) OS=Homo sapiens PE=2 SV=1	31427	14	52	99	82	6.34	0.3	0.047319	2	2	8	3	10	3	11	6	-0.92682
sp|O14556|G3PT_HUMAN	GAPDHS	Glyceraldehyde-3-phosphate dehydrogenase, testis-specific OS=Homo sapiens GN=GAPDHS PE=1 SV=2	44815	13	0	41	0	6.5	0	0	1	0	1	0	1	0	1	0	-0.92595
tr|Q5T7D5|Q5T7D5_HUMAN	ALDOB	Fructose-bisphosphate aldolase B OS=Homo sapiens GN=ALDOB PE=4 SV=1	24512	14	0	39	0	6.34	0	0	1	0	1	0	1	0	1	0	-0.92595
tr|D3DX41|D3DX41_HUMAN	MGC16703	Alpha tubulin-like, isoform CRA_a OS=Homo sapiens GN=MGC16703 PE=3 SV=1	17215	18	0	0	0	5.31	0	0	0	0	0	0	1	0	1	0	-0.92595
tr|K7EIE7|K7EIE7_HUMAN	ACLY	ATP-citrate synthase (Fragment) OS=Homo sapiens GN=ACLY PE=4 SV=1	14168	20	0	65	0	1.53	0	0	1	0	2	0	2	0	3	0	-1.66312
tr|Q5ZF01|Q5ZF01_HUMAN	ASCC3L1	Activating signal cointegrator 1 complex subunit 3-like 1 (Fragment) OS=Homo sapiens GN=ASCC3L1 PE=2 SV=1	8524	21	0	79	0	0.54	0	0	2	0	2	0	2	0	2	0	-1.66312
tr|F5H5N6|F5H5N6_HUMAN	CLTCL1	Clathrin heavy chain 2 (Fragment) OS=Homo sapiens GN=CLTCL1 PE=2 SV=1	18833	25	0	31	0	1.02	0	0	0	0	1	0	1	0	1	0	-0.92595
tr|K7ERE3|K7ERE3_HUMAN	KRT13	Keratin, type I cytoskeletal 13 OS=Homo sapiens GN=KRT13 PE=3 SV=1	45574	28	0	77	0	2.48	0	0	2	0	2	0	2	0	3	0	-1.66312
tr|Q9UMJ0|Q9UMJ0_HUMAN	tr|Q9UMJ0	Myosin (Fragment) OS=Homo sapiens PE=2 SV=1	4239	33	0	32	0	0.53	0	0	0	0	1	0	2	0	2	0	-1.66312
sp|Q01546|K22O_HUMAN	KRT76	Keratin, type II cytoskeletal 2 oral OS=Homo sapiens GN=KRT76 PE=1 SV=2	66370	35	0	88	0	2.07	0	0	4	0	5	0	5	0	5	0	-2.80123
tr|G3V5M4|G3V5M4_HUMAN	ACTN1	Alpha-actinin-1 (Fragment) OS=Homo sapiens GN=ACTN1 PE=2 SV=1	14086	41	0	74	0	1.43	0	0	3	0	3	0	3	0	3	0	-2.14875
tr|B4DSR6|B4DSR6_HUMAN	tr|B4DSR6	cDNA FLJ52684, highly similar to Dynein heavy chain, cytosolic OS=Homo sapiens PE=2 SV=1	28436	44	0	47	0	0.22	0	0	1	0	1	0	1	0	1	0	-0.92595
tr|A6NLN1|A6NLN1_HUMAN	PTBP1	Polypyrimidine tract binding protein 1, isoform CRA_e OS=Homo sapiens GN=PTBP1 PE=4 SV=3	21899	47	0	118	0	1.18	0	0	3	0	3	0	3	0	5	0	-2.14875
tr|Q712V1|Q712V1_HUMAN	UBE1	Ubiquitin activating enzyme E1 (Fragment) OS=Homo sapiens GN=UBE1 PE=2 SV=1	4483	51	0	31	0	0.82	0	0	0	0	1	0	1	0	1	0	-0.92595
tr|J3QRG4|J3QRG4_HUMAN	KPNB1	Importin subunit beta-1 (Fragment) OS=Homo sapiens GN=KPNB1 PE=4 SV=1	6734	54	0	126	0	0.99	0	0	2	0	2	0	2	0	2	0	-1.66312
tr|K7EL29|K7EL29_HUMAN	TUBB6	Tubulin beta-6 chain (Fragment) OS=Homo sapiens GN=TUBB6 PE=4 SV=1	14526	55	0	0	0	1.42	0	0	0	0	0	0	1	0	1	0	-0.92595
tr|F5GYL4|F5GYL4_HUMAN	TCP1	T-complex protein 1 subunit alpha OS=Homo sapiens GN=TCP1 PE=2 SV=1	7059	56	0	120	0	1.58	0	0	2	0	2	0	3	0	3	0	-2.14875
tr|Q6NS35|Q6NS35_HUMAN	EEF1A1	EEF1A1 protein OS=Homo sapiens GN=EEF1A1 PE=4 SV=1	7639	59	0	99	0	2.12	0	0	3	0	3	0	4	0	5	0	-2.51152
tr|B4E3C4|B4E3C4_HUMAN	DDX3X	ATP-dependent RNA helicase DDX3X OS=Homo sapiens GN=DDX3X PE=2 SV=1	34057	60	0	88	0	1.1	0	0	1	0	2	0	2	0	2	0	-1.66312
tr|K7EQ75|K7EQ75_HUMAN	ILF3	Interleukin enhancer-binding factor 3 (Fragment) OS=Homo sapiens GN=ILF3 PE=4 SV=1	6139	62	0	30	0	1.23	0	0	0	0	1	0	1	0	1	0	-0.92595
tr|Q562S2|Q562S2_HUMAN	ACT	Actin-like protein (Fragment) OS=Homo sapiens GN=ACT PE=2 SV=1	11529	65	0	37	0	2.32	0	0	1	0	1	0	2	0	2	0	-1.66312
tr|B3KM30|B3KM30_HUMAN	tr|B3KM30	cDNA FLJ10114 fis, clone HEMBA1002770, highly similar to Cullin-associated NEDD8-dissociated protein 1 OS=Homo sapiens PE=2 SV=1	18641	67	0	65	0	0.6	0	0	2	0	3	0	3	0	4	0	-2.14875
tr|F5H2V6|F5H2V6_HUMAN	MARS	Methionine--tRNA ligase, cytoplasmic OS=Homo sapiens GN=MARS PE=2 SV=2	15629	80	0	74	0	0.55	0	0	1	0	1	0	1	0	1	0	-0.92595
tr|Q96CK3|Q96CK3_HUMAN	CAD	CAD protein (Fragment) OS=Homo sapiens GN=CAD PE=2 SV=2	94161	82	0	147	0	0.35	0	0	2	0	4	0	5	0	5	0	-2.80123
tr|B7Z532|B7Z532_HUMAN	tr|B7Z532	cDNA FLJ51028, highly similar to 60 kDa heat shock protein, mitochondrial OS=Homo sapiens PE=2 SV=1	26726	85	0	110	0	1.58	0	0	2	0	4	0	4	0	5	0	-2.51152
tr|H3BPE3|H3BPE3_HUMAN	EIF3C	Eukaryotic translation initiation factor 3 subunit C (Fragment) OS=Homo sapiens GN=EIF3C PE=4 SV=1	15394	87	0	36	0	0.44	0	0	1	0	1	0	1	0	1	0	-0.92595
tr|Q59G78|Q59G78_HUMAN	tr|Q59G78	Lactate dehydrogenase B variant (Fragment) OS=Homo sapiens PE=2 SV=1	15109	90	0	68	0	2.96	0	0	1	0	1	0	2	0	2	0	-1.66312
tr|C9IZA5|C9IZA5_HUMAN	VCP	Transitional endoplasmic reticulum ATPase (Fragment) OS=Homo sapiens GN=VCP PE=2 SV=1	18590	93	0	71	0	0.54	0	0	2	0	2	0	2	0	2	0	-1.66312
tr|H0YBG6|H0YBG6_HUMAN	HSPA9	Stress-70 protein, mitochondrial (Fragment) OS=Homo sapiens GN=HSPA9 PE=4 SV=1	18491	97	0	35	0	0.86	0	0	0	0	2	0	2	0	2	0	-1.66312
tr|H0YJ11|H0YJ11_HUMAN	ACTN1	Alpha-actinin-1 (Fragment) OS=Homo sapiens GN=ACTN1 PE=4 SV=1	24359	109	0	83	0	0.52	0	0	3	0	3	0	3	0	3	0	-2.14875
tr|D6R8Z5|D6R8Z5_HUMAN	MATR3	Matrin-3 (Fragment) OS=Homo sapiens GN=MATR3 PE=2 SV=1	10976	117	0	58	0	0.67	0	0	1	0	1	0	1	0	1	0	-0.92595
tr|Q86TQ3|Q86TQ3_HUMAN	tr|Q86TQ3	Similar to filamin C, gamma (Actin binding protein 280) (Fragment) OS=Homo sapiens PE=2 SV=1	19163	125	0	37	0	0.18	0	0	0	0	2	0	1	0	2	0	-0.92595
tr|B4DM67|B4DM67_HUMAN	tr|B4DM67	cDNA FLJ59343, highly similar to Exportin-2 OS=Homo sapiens PE=2 SV=1	69098	129	0	181	0	0.57	0	0	2	0	7	0	7	0	7	0	-3.2491
tr|B4DM12|B4DM12_HUMAN	tr|B4DM12	cDNA FLJ51242, moderately similar to Eukaryotic translation initiation factor 2 subunit 1 OS=Homo sapiens PE=2 SV=1	15318	130	0	61	0	1.19	0	0	1	0	1	0	1	0	1	0	-0.92595
tr|H0YC58|H0YC58_HUMAN	KIAA1967	DBIRD complex subunit KIAA1967 (Fragment) OS=Homo sapiens GN=KIAA1967 PE=4 SV=1	19387	131	0	75	0	0.37	0	0	1	0	2	0	2	0	2	0	-1.66312
tr|H0Y914|H0Y914_HUMAN	CCT5	T-complex protein 1 subunit epsilon (Fragment) OS=Homo sapiens GN=CCT5 PE=4 SV=1	6745	134	0	0	0	1.22	0	0	0	0	0	0	1	0	1	0	-0.92595
tr|D6RCS6|D6RCS6_HUMAN	TARS	Threonine--tRNA ligase, cytoplasmic OS=Homo sapiens GN=TARS PE=2 SV=1	6912	135	0	0	0	0.72	0	0	0	0	0	0	2	0	2	0	-1.66312
tr|H7C034|H7C034_HUMAN	AP1B1	AP-1 complex subunit beta-1 (Fragment) OS=Homo sapiens GN=AP1B1 PE=4 SV=1	19502	136	0	54	0	0.44	0	0	1	0	2	0	2	0	2	0	-1.66312
tr|J3KRI6|J3KRI6_HUMAN	CCT6B	T-complex protein 1 subunit zeta-2 (Fragment) OS=Homo sapiens GN=CCT6B PE=4 SV=1	14807	141	0	31	0	0.64	0	0	0	0	1	0	1	0	1	0	-0.92595
tr|Q9NTB4|Q9NTB4_HUMAN	DKFZp434M	Putative uncharacterized protein DKFZp434M052 (Fragment) OS=Homo sapiens GN=DKFZp434M052 PE=2 SV=1	16648	145	0	38	0	0.39	0	0	1	0	1	0	1	0	1	0	-0.92595
tr|F8VR69|F8VR69_HUMAN	RPL6	60S ribosomal protein L6 (Fragment) OS=Homo sapiens GN=RPL6 PE=2 SV=1	7285	147	0	47	0	1.89	0	0	1	0	1	0	1	0	1	0	-0.92595
tr|Q9BUV4|Q9BUV4_HUMAN	RPL5	RPL5 protein OS=Homo sapiens GN=RPL5 PE=2 SV=1	12182	149	0	0	0	1.08	0	0	0	0	0	0	0	0	1	0	0.659215
tr|B1AK51|B1AK51_HUMAN	CHD5	Uncharacterized protein OS=Homo sapiens GN=CHD5 PE=4 SV=1	136766	150	0	106	0	0.16	0	0	2	0	2	0	2	0	2	0	-1.66312
tr|Q5TCC5|Q5TCC5_HUMAN	IARS	Isoleucine--tRNA ligase, cytoplasmic (Fragment) OS=Homo sapiens GN=IARS PE=4 SV=1	19001	151	0	93	0	0.31	0	0	1	0	2	0	3	0	3	0	-2.14875
tr|H0Y7K8|H0Y7K8_HUMAN	SMC1A	Structural maintenance of chromosomes protein 1A (Fragment) OS=Homo sapiens GN=SMC1A PE=4 SV=1	33648	155	0	107	0	0.25	0	0	2	0	4	0	4	0	6	0	-2.51152
sp|P0CB38|PAB4L_HUMAN	PABPC4L	Polyadenylate-binding protein 4-like OS=Homo sapiens GN=PABPC4L PE=2 SV=1	42056	160	0	42	0	0.46	0	0	1	0	1	0	1	0	1	0	-0.92595
sp|P53618|COPB_HUMAN	COPB1	Coatomer subunit beta OS=Homo sapiens GN=COPB1 PE=1 SV=3	108214	165	0	235	0	0.27	0	0	8	0	8	0	9	0	10	0	-3.59054
tr|H0YFD6|H0YFD6_HUMAN	HADHA	Trifunctional enzyme subunit alpha, mitochondrial (Fragment) OS=Homo sapiens GN=HADHA PE=4 SV=1	27376	167	0	91	0	0.26	0	0	2	0	2	0	2	0	2	0	-1.66312
tr|B2RCP3|B2RCP3_HUMAN	tr|B2RCP3	cDNA, FLJ96188, highly similar to Homo sapiens kinesin family member 5A (KIF5A), mRNA OS=Homo sapiens PE=2 SV=1	118189	168	0	0	0	0.34	0	0	0	0	0	0	0	0	1	0	0.659215
tr|B1AKY9|B1AKY9_HUMAN	ATP1A2	ATPase, Na+/K+ transporting, alpha 2 (+) polypeptide (Fragment) OS=Homo sapiens GN=ATP1A2 PE=3 SV=1	112046	170	0	62	0	0.33	0	0	3	0	4	0	4	0	4	0	-2.51152
tr|H0YHU8|H0YHU8_HUMAN	SART3	Squamous cell carcinoma antigen recognized by T-cells 3 (Fragment) OS=Homo sapiens GN=SART3 PE=4 SV=1	16246	171	0	49	0	0.27	0	0	1	0	1	0	2	0	2	0	-0.92595
tr|F8WDM3|F8WDM3_HUMAN	MCM2	DNA replication licensing factor MCM2 OS=Homo sapiens GN=MCM2 PE=2 SV=1	16182	172	0	0	0	0.47	0	0	0	0	0	0	0	0	1	0	0.659215
tr|B4DNG4|B4DNG4_HUMAN	tr|B4DNG4	cDNA FLJ59137, highly similar to RNA-binding protein 14 OS=Homo sapiens PE=2 SV=1	22679	177	0	137	0	0.45	0	0	2	0	4	0	4	0	4	0	-2.51152
tr|Q6P4R2|Q6P4R2_HUMAN	PCDHAC2	Protocadherin alpha subfamily C, 2 OS=Homo sapiens GN=PCDHAC2 PE=2 SV=1	110037	178	0	52	0	0.23	0	0	1	0	1	0	1	0	1	0	-0.92595
tr|H0YDX5|H0YDX5_HUMAN	CKAP5	Cytoskeleton-associated protein 5 (Fragment) OS=Homo sapiens GN=CKAP5 PE=4 SV=1	23027	183	0	120	0	0.12	0	0	2	0	3	0	3	0	3	0	-2.14875
tr|H7C3V5|H7C3V5_HUMAN	TFRC	Transferrin receptor protein 1, serum form (Fragment) OS=Homo sapiens GN=TFRC PE=4 SV=1	13662	184	0	0	0	0.35	0	0	0	0	0	0	1	0	1	0	-0.92595
tr|Q0ZAJ1|Q0ZAJ1_HUMAN	MSH2	MSH2-Ex5 isoform (Fragment) OS=Homo sapiens GN=MSH2 PE=2 SV=1	11803	188	0	73	0	0.2	0	0	1	0	1	0	3	0	3	0	-2.14875
tr|H7C5L4|H7C5L4_HUMAN	FLNB	Filamin-B (Fragment) OS=Homo sapiens GN=FLNB PE=4 SV=1	14361	192	0	18	0	0.13	0	0	1	0	1	0	1	0	3	0	-0.92595
tr|H7C4I3|H7C4I3_HUMAN	RUVBL1	RuvB-like 1 (Fragment) OS=Homo sapiens GN=RUVBL1 PE=4 SV=1	25136	196	0	37	0	0.56	0	0	1	0	1	0	1	0	1	0	-0.92595
tr|B4E036|B4E036_HUMAN	tr|B4E036	cDNA FLJ59080, highly similar to Heterogeneous nuclear ribonucleoprotein A3 OS=Homo sapiens PE=2 SV=1	14031	197	0	121	0	0.49	0	0	1	0	2	0	3	0	3	0	-2.14875
tr|Q59FS8|Q59FS8_HUMAN	tr|Q59FS8	Eukaryotic translation initiation factor 3, subunit 9 eta, 116kDa isoform b variant (Fragment) OS=Homo sapiens PE=2 SV=1	19913	200	0	80	0	0.2	0	0	2	0	2	0	2	0	2	0	-1.66312
tr|B2R491|B2R491_HUMAN	RPS4X	Ribosomal protein S4, X-linked, isoform CRA_c OS=Homo sapiens GN=RPS4X PE=2 SV=1	29807	203	0	193	0	0.7	0	0	3	0	5	0	5	0	7	0	-2.80123
tr|B3KUQ5|B3KUQ5_HUMAN	tr|B3KUQ5	cDNA FLJ40389 fis, clone TESTI2036691, highly similar to Thioredoxin reductase 2, mitochondrial (EC 1.8.1.9) OS=Homo sapiens PE=2 SV=1	50486	206	0	55	0	0.41	0	0	1	0	1	0	1	0	1	0	-0.92595
tr|C9J1E1|C9J1E1_HUMAN	BLVRA	Biliverdin reductase A (Fragment) OS=Homo sapiens GN=BLVRA PE=2 SV=1	11343	207	0	70	0	1.33	0	0	1	0	1	0	1	0	1	0	-0.92595
tr|Q4W5H1|Q4W5H1_HUMAN	SMARCA5	Putative uncharacterized protein SMARCA5 (Fragment) OS=Homo sapiens GN=SMARCA5 PE=2 SV=1	43245	208	0	44	0	0.32	0	0	1	0	3	0	3	0	4	0	-2.14875
tr|E5RIT4|E5RIT4_HUMAN	EIF3E	Eukaryotic translation initiation factor 3 subunit E (Fragment) OS=Homo sapiens GN=EIF3E PE=4 SV=1	8862	210	0	48	0	0.84	0	0	2	0	2	0	2	0	2	0	-1.66312
tr|F8WAX8|F8WAX8_HUMAN	MSH6	DNA mismatch repair protein Msh6 OS=Homo sapiens GN=MSH6 PE=2 SV=1	17412	212	0	48	0	0.2	0	0	1	0	1	0	1	0	1	0	-0.92595
tr|Q9BS10|Q9BS10_HUMAN	tr|Q9BS10	Similar to ribosomal protein S8 (Fragment) OS=Homo sapiens PE=2 SV=1	9391	215	0	93	0	0.67	0	0	2	0	2	0	2	0	3	0	-1.66312
tr|H0YGV8|H0YGV8_HUMAN	PSMD3	26S proteasome non-ATPase regulatory subunit 3 (Fragment) OS=Homo sapiens GN=PSMD3 PE=4 SV=2	13091	221	0	0	0	0.2	0	0	0	0	1	0	1	0	1	0	-0.92595
sp|P13647|K2C5_HUMAN	KRT5	Keratin, type II cytoskeletal 5 OS=Homo sapiens GN=KRT5 PE=1 SV=3	62568	224	0	178	0	0.43	0	0	5	0	7	0	8	0	9	0	-3.42988
tr|E9PNV3|E9PNV3_HUMAN	RDX	Radixin (Fragment) OS=Homo sapiens GN=RDX PE=2 SV=1	17329	226	0	35	0	0.46	0	0	1	0	1	0	1	0	1	0	-0.92595
tr|B4DJB0|B4DJB0_HUMAN	tr|B4DJB0	cDNA FLJ60092, highly similar to 14-3-3 protein epsilon (14-3-3E) OS=Homo sapiens PE=2 SV=1	16088	227	0	64	0	1.65	0	0	1	0	1	0	1	0	1	0	-0.92595
tr|I3L3D5|I3L3D5_HUMAN	PFN1	Profilin-1 (Fragment) OS=Homo sapiens GN=PFN1 PE=4 SV=1	17620	230	0	85	0	1.24	0	0	2	0	2	0	2	0	2	0	-1.66312
tr|Q2KQ74|Q2KQ74_HUMAN	tr|Q2KQ74	HECTD1 (Fragment) OS=Homo sapiens PE=2 SV=1	136749	232	0	75	0	0.05	0	0	1	0	1	0	1	0	3	0	-0.92595
tr|B4DM07|B4DM07_HUMAN	tr|B4DM07	cDNA FLJ56355, highly similar to General transcription factor II-I OS=Homo sapiens PE=2 SV=1	20937	234	0	21	0	0.26	0	0	0	0	1	0	1	0	1	0	-0.92595
sp|P14868|SYDC_HUMAN	DARS	Aspartate--tRNA ligase, cytoplasmic OS=Homo sapiens GN=DARS PE=1 SV=2	57499	238	0	167	0	0.4	0	0	4	0	6	0	8	0	8	0	-3.42988
tr|H0Y9Z8|H0Y9Z8_HUMAN	NCAPG	Condensin complex subunit 3 (Fragment) OS=Homo sapiens GN=NCAPG PE=4 SV=1	30654	239	0	93	0	0.25	0	0	1	0	3	0	3	0	3	0	-2.14875
tr|Q16791|Q16791_HUMAN	tr|Q16791	Mammary dihydrolipoamide acetyltransferase, mature sequence (Fragment) OS=Homo sapiens PE=2 SV=1	29551	240	0	36	0	0.29	0	0	1	0	1	0	1	0	1	0	-0.92595
tr|C9JAM8|C9JAM8_HUMAN	PLS1	Plastin-1 (Fragment) OS=Homo sapiens GN=PLS1 PE=2 SV=1	29616	241	0	35	0	0.38	0	0	1	0	1	0	2	0	2	0	-1.66312
tr|Q2NLA0|Q2NLA0_HUMAN	COPG2	Coatomer subunit gamma OS=Homo sapiens GN=COPG2 PE=2 SV=1	82400	243	0	165	0	0.12	0	0	3	0	3	0	3	0	3	0	-2.14875
tr|B4E2C9|B4E2C9_HUMAN	tr|B4E2C9	cDNA FLJ61545, highly similar to Coatomer subunit beta OS=Homo sapiens PE=2 SV=1	30965	244	0	77	0	0.25	0	0	3	0	3	0	3	0	3	0	-2.14875
tr|F5H0E2|F5H0E2_HUMAN	SLC3A2	4F2 cell-surface antigen heavy chain (Fragment) OS=Homo sapiens GN=SLC3A2 PE=4 SV=1	15744	245	0	93	0	0.26	0	0	1	0	1	0	1	0	1	0	-0.92595
tr|A6NJJ0|A6NJJ0_HUMAN	PRDX4	Peroxiredoxin-4 (Fragment) OS=Homo sapiens GN=PRDX4 PE=2 SV=1	21403	246	0	100	0	0.95	0	0	2	0	2	0	2	0	2	0	-1.66312
tr|A0PJ76|A0PJ76_HUMAN	NCAPD2	NCAPD2 protein (Fragment) OS=Homo sapiens GN=NCAPD2 PE=2 SV=1	44957	248	0	0	0	0.11	0	0	0	0	0	0	0	0	1	0	0.659215
tr|Q96PA3|Q96PA3_HUMAN	tr|Q96PA3	IQGAP1 protein (Fragment) OS=Homo sapiens PE=2 SV=1	10782	249	0	73	0	0.11	0	0	1	0	1	0	1	0	1	0	-0.92595
tr|Q3SYK8|Q3SYK8_HUMAN	NUMA1	NUMA1 protein (Fragment) OS=Homo sapiens GN=NUMA1 PE=2 SV=1	78666	253	0	84	0	0.06	0	0	2	0	2	0	2	0	3	0	-1.66312
tr|E9PHF7|E9PHF7_HUMAN	MCCC1	Methylcrotonoyl-CoA carboxylase subunit alpha, mitochondrial OS=Homo sapiens GN=MCCC1 PE=2 SV=1	68745	259	0	155	0	0.39	0	0	4	0	7	0	6	0	8	0	-3.04245
tr|H0Y8G2|H0Y8G2_HUMAN	COPG1	Coatomer subunit gamma-1 (Fragment) OS=Homo sapiens GN=COPG1 PE=4 SV=1	8747	261	0	15	0	0.22	0	0	0	0	1	0	0	0	1	0	0.659215

tr|E9PKN5|E9PKN5_HUMAN	RDX	Radixin OS=Homo sapiens GN=RDX PE=2 SV=1	6040	262	0	43	0	0.32	0	0	1	0	1	0	1	0	1	0	-0.92595
tr|Q0VGA5|Q0VGA5_HUMAN	SARS	SARS protein OS=Homo sapiens GN=SARS PE=2 SV=1	58883	264	0	152	0	0.24	0	0	4	0	4	0	4	0	4	0	-2.51152
tr|D6RD83|D6RD83_HUMAN	HNRNPD	Heterogeneous nuclear ribonucleoprotein D0 (Fragment) OS=Homo sapiens GN=HNRNPD PE=2 SV=1	9992	266	0	73	0	0.7	0	0	2	0	3	0	3	0	4	0	-2.14875
tr|Q504R3|Q504R3_HUMAN	CLEC2D	Alternative protein CLEC2D OS=Homo sapiens GN=CLEC2D PE=2 SV=1	23902	271	0	0	0	0.37	0	0	0	0	0	0	1	0	1	0	-0.92595
tr|H0YJD3|H0YJD3_HUMAN	PRMT5	Protein arginine N-methyltransferase 5 (Fragment) OS=Homo sapiens GN=PRMT5 PE=4 SV=1	20375	275	0	43	0	0.3	0	0	1	0	1	0	1	0	1	0	-0.92595
tr|Q6NSG3|Q6NSG3_HUMAN	SMC4	SMC4 protein (Fragment) OS=Homo sapiens GN=SMC4 PE=2 SV=1	52835	277	0	54	0	0.12	0	0	1	0	2	0	2	0	2	0	-1.66312
tr|Q9NVP8|Q9NVP8_HUMAN	tr|Q9NVP8	cDNA FLJ10595 fis, clone NT2RP2004791, moderately similar to PUTATIVE LEUCYL-TRNA SYNTHETASE, CYTOPLASMIC (EC 6.1.1.4) OS=Homo	13706	281	0	48	0	0.27	0	0	1	0	2	0	2	0	2	0	-1.66312
tr|B4DKK3|B4DKK3_HUMAN	tr|B4DKK3	cDNA FLJ54791, highly similar to DnaJ homolog subfamily C member 7 OS=Homo sapiens PE=2 SV=1	42561	282	0	105	0	0.37	0	0	2	0	4	0	3	0	4	0	-2.14875
tr|D6RAS3|D6RAS3_HUMAN	NSUN2	tRNA (cytosine(34)-C(5))-methyltransferase OS=Homo sapiens GN=NSUN2 PE=2 SV=1	23483	290	0	34	0	0.16	0	0	0	0	1	0	1	0	2	0	-0.92595
tr|C9J057|C9J057_HUMAN	ASNS	Asparagine synthetase [glutamine-hydrolyzing] (Fragment) OS=Homo sapiens GN=ASNS PE=2 SV=1	24946	291	0	48	0	0.28	0	0	1	0	2	0	3	0	3	0	-2.14875
tr|H7C013|H7C013_HUMAN	ALB	Serum albumin (Fragment) OS=Homo sapiens GN=ALB PE=4 SV=1	23414	292	0	23	0	0.2	0	0	0	0	1	0	1	0	1	0	-0.92595
tr|E5RK19|E5RK19_HUMAN	HDAC2	Histone deacetylase 2 (Fragment) OS=Homo sapiens GN=HDAC2 PE=2 SV=1	6474	300	0	0	0	0.28	0	0	0	0	0	0	0	0	1	0	0.659215
tr|B0QYA4|B0QYA4_HUMAN	EIF3D	Eukaryotic translation initiation factor 3 subunit D (Fragment) OS=Homo sapiens GN=EIF3D PE=4 SV=1	19404	303	0	37	0	0.39	0	0	1	0	1	0	1	0	1	0	-0.92595
tr|B4DNG5|B4DNG5_HUMAN	tr|B4DNG5	cDNA FLJ57752 OS=Homo sapiens PE=2 SV=1	19309	305	0	0	0	0.41	0	0	0	0	0	0	0	0	1	0	0.659215
tr|Q5QP23|Q5QP23_HUMAN	RBM39	RNA binding motif protein 39 (Fragment) OS=Homo sapiens GN=RBM39 PE=4 SV=1	27122	306	0	67	0	0.29	0	0	2	0	2	0	2	0	2	0	-1.66312
tr|E9PIK5|E9PIK5_HUMAN	RNH1	Ribonuclease inhibitor (Fragment) OS=Homo sapiens GN=RNH1 PE=2 SV=1	20014	312	0	64	0	0.28	0	0	1	0	2	0	2	0	2	0	-1.66312
tr|H0YG16|H0YG16_HUMAN	KLC1	Kinesin light chain 1 (Fragment) OS=Homo sapiens GN=KLC1 PE=4 SV=1	30009	316	0	45	0	0.16	0	0	1	0	1	0	1	0	2	0	-0.92595
tr|C9JIJ5|C9JIJ5_HUMAN	RPL7	60S ribosomal protein L7 (Fragment) OS=Homo sapiens GN=RPL7 PE=2 SV=1	11885	317	0	29	0	0.9	0	0	2	0	2	0	3	0	3	0	-2.14875
tr|E9PKU4|E9PKU4_HUMAN	RPL8	60S ribosomal protein L8 (Fragment) OS=Homo sapiens GN=RPL8 PE=2 SV=1	25806	320	0	64	0	0.51	0	0	1	0	1	0	2	0	2	0	-1.66312
tr|A8K8N7|A8K8N7_HUMAN	PFAS	Phosphoribosylformylglycinamidine synthase (FGAR amidotransferase), isoform CRA_b OS=Homo sapiens GN=PFAS PE=2 SV=1	146286	323	0	125	0	0.17	0	0	3	0	7	0	6	0	7	0	-3.04245
tr|F2Z2D0|F2Z2D0_HUMAN	NUP205	Nuclear pore complex protein Nup205 OS=Homo sapiens GN=NUP205 PE=4 SV=1	23590	324	0	55	0	0.07	0	0	1	0	1	0	1	0	1	0	-0.92595
tr|Q05D65|Q05D65_HUMAN	ZFR	ZFR protein OS=Homo sapiens GN=ZFR PE=2 SV=1	19907	326	0	31	0	0.12	0	0	0	0	1	0	1	0	1	0	-0.92595
tr|B4E0U0|B4E0U0_HUMAN	tr|B4E0U0	cDNA FLJ61018, highly similar to Ran GTPase-activating protein 1 OS=Homo sapiens PE=2 SV=1	41040	331	0	123	0	0.11	0	0	2	0	2	0	4	0	4	0	-2.51152
sp|P27694|RFA1_HUMAN	RPA1	Replication protein A 70 kDa DNA-binding subunit OS=Homo sapiens GN=RPA1 PE=1 SV=2	68723	332	0	122	0	0.21	0	0	3	0	4	0	5	0	5	0	-2.80123
tr|B4DMU5|B4DMU5_HUMA	GNL3	Guanine nucleotide-binding protein-like 3 OS=Homo sapiens GN=GNL3 PE=2 SV=1	16668	333	0	0	0	0.43	0	0	0	0	0	0	0	0	1	0	0.659215
tr|Q5T2B7|Q5T2B7_HUMAN	CUL2	Cullin-2 OS=Homo sapiens GN=CUL2 PE=4 SV=1	80893	334	0	120	0	0.17	0	0	3	0	4	0	4	0	4	0	-2.51152
tr|Q6ZSZ9|Q6ZSZ9_HUMAN	tr|Q6ZSZ9	cDNA FLJ45089 fis, clone BRAWH3029385, highly similar to Dynamin 2 (EC 3.6.1.50) OS=Homo sapiens PE=2 SV=1	44901	335	0	32	0	0.14	0	0	0	0	1	0	1	0	1	0	-0.92595
tr|Q0D2M2|Q0D2M2_HUMA	HIST1H2BC	HIST1H2BC protein OS=Homo sapiens GN=HIST1H2BC PE=2 SV=1	13826	337	0	119	0	0.93	0	0	2	0	3	0	3	0	3	0	-2.14875
tr|B2RE46|B2RE46_HUMAN	tr|B2RE46	cDNA, FLJ96923, highly similar to Homo sapiens ribophorin II (RPN2), mRNA OS=Homo sapiens PE=2 SV=1	69405	340	0	118	0	0.32	0	0	4	0	6	0	6	0	6	0	-3.04245
tr|C9J7B7|C9J7B7_HUMAN	PSMD6	26S proteasome non-ATPase regulatory subunit 6 (Fragment) OS=Homo sapiens GN=PSMD6 PE=2 SV=1	16725	341	0	53	0	0.28	0	0	1	0	1	0	1	0	2	0	-0.92595
tr|A5D6X0|A5D6X0_HUMAN	SMARCA4	SMARCA4 protein (Fragment) OS=Homo sapiens GN=SMARCA4 PE=2 SV=1	63022	345	0	58	0	0.09	0	0	1	0	2	0	2	0	4	0	-1.66312
tr|C9JLV6|C9JLV6_HUMAN	MDH1	Malate dehydrogenase, cytoplasmic (Fragment) OS=Homo sapiens GN=MDH1 PE=2 SV=1	12411	352	0	35	0	0.3	0	0	0	0	1	0	1	0	1	0	-0.92595
tr|C9J5E5|C9J5E5_HUMAN	HDLBP	Vigilin (Fragment) OS=Homo sapiens GN=HDLBP PE=2 SV=1	24497	353	0	64	0	0.12	0	0	1	0	1	0	1	0	1	0	-0.92595
tr|B4DGH9|B4DGH9_HUMAN	tr|B4DGH9	cDNA FLJ58772, highly similar to Fragile X mental retardation syndrome-related protein 1 OS=Homo sapiens PE=2 SV=1	51203	361	0	114	0	0.13	0	0	2	0	2	0	2	0	3	0	-1.66312
tr|C9J6P4|C9J6P4_HUMAN	ZC3HAV1	Zinc finger CCCH-type antiviral protein 1 OS=Homo sapiens GN=ZC3HAV1 PE=2 SV=1	115838	366	0	111	0	0.14	0	0	1	0	2	0	2	0	2	0	-1.66312
tr|Q13073|Q13073_HUMAN	tr|Q13073	Ran binding protein (Fragment) OS=Homo sapiens PE=2 SV=1	24853	367	0	47	0	0.07	0	0	1	0	1	0	1	0	1	0	-0.92595
tr|A4FU51|A4FU51_HUMAN	NARG1L	NMDA receptor regulated 1-like OS=Homo sapiens GN=NARG1L PE=2 SV=1	102424	368	0	56	0	0.33	0	0	1	0	2	0	4	0	4	0	-2.51152
sp|Q92878|RAD50_HUMAN	RAD50	DNA repair protein RAD50 OS=Homo sapiens GN=RAD50 PE=1 SV=1	154823	370	0	110	0	0.11	0	0	3	0	5	0	7	0	9	0	-3.2491
tr|K7EP46|K7EP46_HUMAN	THOP1	Thimet oligopeptidase OS=Homo sapiens GN=THOP1 PE=3 SV=1	65876	371	0	110	0	0.1	0	0	2	0	2	0	2	0	3	0	-1.66312
sp|P35998|PRS7_HUMAN	PSMC2	26S protease regulatory subunit 7 OS=Homo sapiens GN=PSMC2 PE=1 SV=3	49002	372	0	109	0	0.22	0	0	3	0	4	0	4	0	5	0	-2.51152
tr|G3V277|G3V277_HUMAN	WARS	T1-TrpRS (Fragment) OS=Homo sapiens GN=WARS PE=2 SV=1	23928	373	0	0	0	0.2	0	0	0	0	0	0	0	0	1	0	0.659215
tr|Q15244|Q15244_HUMAN	tr|Q15244	Phosphoribosylpyrophosphate synthetase isoform I (Fragment) OS=Homo sapiens PE=2 SV=1	4443	374	0	56	0	0.88	0	0	2	0	2	0	2	0	2	0	-1.66312
tr|Q6PJM8|Q6PJM8_HUMAN	RRP1B	RRP1B protein (Fragment) OS=Homo sapiens GN=RRP1B PE=2 SV=1	47217	375	0	63	0	0.16	0	0	1	0	2	0	3	0	4	0	-2.14875
tr|F5GZS0|F5GZS0_HUMAN	DHX36	Probable ATP-dependent RNA helicase DHX36 OS=Homo sapiens GN=DHX36 PE=2 SV=1	113928	376	0	108	0	0.09	0	0	3	0	3	0	4	0	4	0	-2.51152
tr|H7C1K0|H7C1K0_HUMAN	SRRT	Serrate RNA effector molecule homolog (Fragment) OS=Homo sapiens GN=SRRT PE=4 SV=1	5606	377	0	55	0	0.17	0	0	1	0	1	0	2	0	2	0	-1.66312
sp|Q6IS14|IF5AL_HUMAN	EIF5AL1	Eukaryotic translation initiation factor 5A-1-like OS=Homo sapiens GN=EIF5AL1 PE=1 SV=2	16990	379	0	84	0	3.24	0	0	1	0	5	0	5	0	5	0	-2.80123
tr|B4DFR3|B4DFR3_HUMAN	tr|B4DFR3	cDNA FLJ55771, highly similar to Lamin-A/C OS=Homo sapiens PE=2 SV=1	25697	381	0	49	0	0.33	0	0	1	0	1	0	1	0	1	0	-0.92595
tr|K7EJL0|K7EJL0_HUMAN	DNMT1	DNA (cytosine-5)-methyltransferase 1 (Fragment) OS=Homo sapiens GN=DNMT1 PE=4 SV=1	28503	382	0	34	0	0.09	0	0	1	0	1	0	1	0	1	0	-0.92595
tr|A8MX29|A8MX29_HUMAN	KLC2	Kinesin light chain 2 OS=Homo sapiens GN=KLC2 PE=2 SV=1	36716	384	0	21	0	0.2	0	0	0	0	1	0	1	0	1	0	-0.92595
sp|P46063|RECQ1_HUMAN	RECQL	ATP-dependent DNA helicase Q1 OS=Homo sapiens GN=RECQL PE=1 SV=3	74436	387	0	105	0	0.3	0	0	3	0	6	0	6	0	6	0	-3.04245
tr|Q6P1L4|Q6P1L4_HUMAN	PYGL	PYGL protein (Fragment) OS=Homo sapiens GN=PYGL PE=2 SV=1	28787	388	0	38	0	0.23	0	0	1	0	1	0	1	0	2	0	-0.92595
tr|H3BMH0|H3BMH0_HUMA	CNOT1	CCR4-NOT transcription complex subunit 1 (Fragment) OS=Homo sapiens GN=CNOT1 PE=4 SV=1	29701	389	0	39	0	0.07	0	0	1	0	1	0	1	0	1	0	-0.92595
tr|A0PJ92|A0PJ92_HUMAN	NOP56	NOP56 protein (Fragment) OS=Homo sapiens GN=NOP56 PE=2 SV=1	50068	397	0	102	0	0.21	0	0	2	0	3	0	3	0	6	0	-2.14875
tr|Q59GM9|Q59GM9_HUMA	tr|Q59GM	Phosphorylase (Fragment) OS=Homo sapiens PE=2 SV=1	99508	398	0	102	0	0.18	0	0	2	0	5	0	5	0	6	0	-2.80123
tr|B4E1L0|B4E1L0_HUMAN	tr|B4E1L0	Adenylosuccinate synthetase OS=Homo sapiens PE=2 SV=1	48308	399	0	101	0	0.22	0	0	1	0	3	0	4	0	5	0	-2.51152
tr|J3QSB4|J3QSB4_HUMAN	RPL13	60S ribosomal protein L13 (Fragment) OS=Homo sapiens GN=RPL13 PE=3 SV=1	14740	402	0	101	0	0.47	0	0	2	0	2	0	2	0	2	0	-1.66312
tr|B7ZKR9|B7ZKR9_HUMAN	POLR1A	DNA-directed RNA polymerase OS=Homo sapiens GN=POLR1A PE=2 SV=1	196658	405	0	101	0	0.07	0	0	3	0	4	0	4	0	4	0	-2.51152
tr|B4DSN3|B4DSN3_HUMAN	tr|B4DSN3	cDNA FLJ60345, highly similar to Protein transport protein Sec24C OS=Homo sapiens PE=2 SV=1	107974	410	0	100	0	0.13	0	0	3	0	5	0	5	0	5	0	-2.80123
tr|B3KM55|B3KM55_HUMAN	tr|B3KM55	cDNA FLJ10336 fis, clone NT2RM2000691, highly similar to Homo sapiens ARP3 actin-related protein 3 homolog B (yeast) (ACTR3B), mRNA	14619	411	0	66	0	0.4	0	0	1	0	1	0	1	0	1	0	-0.92595
tr|B3KXL9|B3KXL9_HUMAN	tr|B3KXL9	cDNA FLJ45690 fis, clone FCBBF5000384, highly similar to SWI/SNF-related matrix-associated actin-dependent regulator of chromatin subfa	50398	413	0	36	0	0.13	0	0	0	0	1	0	1	0	1	0	-0.92595
tr|Q6ZP53|Q6ZP53_HUMAN	tr|Q6ZP53	CDNA FLJ26493 fis, clone KDN06317, highly similar to Ras-GTPase-activating protein binding protein 1 OS=Homo sapiens PE=2 SV=1	34554	418	0	51	0	0.13	0	0	1	0	1	0	1	0	2	0	-0.92595
tr|Q2TU89|Q2TU89_HUMAN	AAG1	Aging-associated protein 1 OS=Homo sapiens GN=AAG1 PE=2 SV=1	79962	421	0	49	0	0.18	0	0	1	0	4	0	4	0	6	0	-2.51152
tr|E9PHZ2|E9PHZ2_HUMAN	CHORDC1	Cysteine and histidine-rich domain-containing protein 1 OS=Homo sapiens GN=CHORDC1 PE=4 SV=1	16742	422	0	30	0	0.64	0	0	0	0	1	0	2	0	2	0	-1.66312
tr|Q5TE93|Q5TE93_HUMAN	PYCR2	Pyrroline-5-carboxylate reductase family, member 2 OS=Homo sapiens GN=PYCR2 PE=2 SV=1	19775	425	0	51	0	0.2	0	0	1	0	1	0	1	0	1	0	-0.92595
tr|F5H571|F5H571_HUMAN	USP5	Ubiquitin carboxyl-terminal hydrolase 5 (Fragment) OS=Homo sapiens GN=USP5 PE=2 SV=1	15755	426	0	40	0	0.11	0	0	1	0	1	0	1	0	1	0	-0.92595
tr|B2RB90|B2RB90_HUMAN	tr|B2RB90	cDNA, FLJ95375, highly similar to Homo sapiens glutamine-fructose-6-phosphate transaminase 2 (GFPT2), mRNA OS=Homo sapiens PE=2 SV	77651	431	0	41	0	0.18	0	0	1	0	1	0	1	0	2	0	-0.92595
tr|B4DQI9|B4DQI9_HUMAN	tr|B4DQI9	cDNA FLJ53854, highly similar to Vacuolar ATP synthase subunit B, brain isoform (EC 3.6.3.14) OS=Homo sapiens PE=2 SV=1	31796	433	0	92	0	0.34	0	0	2	0	4	0	4	0	4	0	-2.51152
tr|H0Y9L6|H0Y9L6_HUMAN	PDS5A	Sister chromatid cohesion protein PDS5 homolog A (Fragment) OS=Homo sapiens GN=PDS5A PE=4 SV=1	14071	434	0	24	0	0.07	0	0	0	0	1	0	1	0	1	0	-0.92595
tr|B4DJA6|B4DJA6_HUMAN	tr|B4DJA6	Aspartate aminotransferase OS=Homo sapiens PE=2 SV=1	43302	438	0	59	0	0.16	0	0	1	0	1	0	1	0	1	0	-0.92595
tr|Q05D43|Q05D43_HUMAN	YBX1	YBX1 protein (Fragment) OS=Homo sapiens GN=YBX1 PE=2 SV=1	29487	439	0	83	0	0.16	0	0	1	0	1	0	1	0	1	0	-0.92595
tr|D6W636|D6W636_HUMAN	DUS3L	Dihydrouridine synthase 3-like (S. cerevisiae), isoform CRA_a OS=Homo sapiens GN=DUS3L PE=2 SV=1	36425	440	0	25	0	0.14	0	0	0	0	1	0	1	0	1	0	-0.92595
tr|B1AMS2|B1AMS2_HUMAN	Sep-06	Septin 6 OS=Homo sapiens GN=SEPT6 PE=3 SV=1	49614	441	0	39	0	0.21	0	0	2	0	2	0	2	0	2	0	-1.66312
tr|B4DUR9|B4DUR9_HUMAN	AHSA1	Activator of 90 kDa heat shock protein ATPase homolog 1 OS=Homo sapiens GN=AHSA1 PE=2 SV=1	32490	442	0	89	0	0.48	0	0	3	0	4	0	4	0	4	0	-2.51152
tr|A8K9E1|A8K9E1_HUMAN	tr|A8K9E1	cDNA FLJ77957, highly similar to Homo sapiens HBS1-like (S. cerevisiae) (HBS1L), mRNA OS=Homo sapiens PE=2 SV=1	75969	443	0	89	0	0.14	0	0	2	0	3	0	3	0	5	0	-2.14875
tr|B3KWS3|B3KWS3_HUMAN	PLOD2	Procollagen-lysine,2-oxoglutarate 5-dioxygenase 2 OS=Homo sapiens GN=PLOD2 PE=2 SV=1	49567	444	0	20	0	0.08	0	0	0	0	1	0	1	0	1	0	-0.92595
tr|B0AZS5|B0AZS5_HUMAN	tr|B0AZS5	cDNA, FLJ79515, highly similar to Kinesin-like protein KIF2 OS=Homo sapiens PE=2 SV=1	75666	449	0	86	0	0.18	0	0	3	0	4	0	4	0	4	0	-2.51152
tr|H0YBT4|H0YBT4_HUMAN	DPYSL3	Dihydropyrimidinase-related protein 3 (Fragment) OS=Homo sapiens GN=DPYSL3 PE=4 SV=1	22001	450	0	45	0	0.17	0	0	1	0	1	0	1	0	1	0	-0.92595
tr|B4E205|B4E205_HUMAN	tr|B4E205	cDNA FLJ61651, highly similar to Protein transport protein Sec24A OS=Homo sapiens PE=2 SV=1	96953	455	0	85	0	0.14	0	0	2	0	4	0	4	0	4	0	-2.51152
tr|B4DYP7|B4DYP7_HUMAN	tr|B4DYP7	cDNA FLJ55435, highly similar to Gamma-tubulin complex component 3 OS=Homo sapiens PE=2 SV=1	103306	464	0	83	0	0.1	0	0	2	0	3	0	4	0	4	0	-2.51152
tr|I3L3P5|I3L3P5_HUMAN	P4HB	Protein disulfide-isomerase (Fragment) OS=Homo sapiens GN=P4HB PE=4 SV=1	16923	466	0	0	0	0.12	0	0	0	0	0	0	0	0	1	0	0.659215
tr|J3QSH1|J3QSH1_HUMAN	SMCHD1	Structural maintenance of chromosomes flexible hinge domain-containing protein 1 (Fragment) OS=Homo sapiens GN=SMCHD1 PE=4 SV=1	16870	467	0	0	0	0.03	0	0	0	0	0	0	0	0	1	0	0.659215
tr|B4DJ58|B4DJ58_HUMAN	RSL1D1	Ribosomal L1 domain-containing protein 1 OS=Homo sapiens GN=RSL1D1 PE=2 SV=1	30336	468	0	65	0	0.38	0	0	1	0	3	0	4	0	4	0	-2.51152
tr|B3KSU9|B3KSU9_HUMAN	tr|B3KSU9	cDNA FLJ37014 fis, clone BRACE2010203, highly similar to Zinc phosphodiesterase ELAC protein 2 (EC 3.1.26.11) OS=Homo sapiens PE=2 SV	66302	470	0	82	0	0.16	0	0	3	0	3	0	3	0	4	0	-2.14875
tr|Q53G58|Q53G58_HUMAN	tr|Q53G58	Coronin (Fragment) OS=Homo sapiens PE=2 SV=1	53931	471	0	82	0	0.19	0	0	2	0	3	0	3	0	4	0	-2.14875
tr|Q0PHS2|Q0PHS2_HUMAN	G6PD	Glucose-6-phosphate dehydrogenase (Fragment) OS=Homo sapiens GN=G6PD PE=2 SV=1	5747	472	0	0	0	0.12	0	0	0	0	0	0	0	0	1	0	0.659215
tr|F8VSH0|F8VSH0_HUMAN	ATP2A2	Sarcoplasmic/endoplasmic reticulum calcium ATPase 2 (Fragment) OS=Homo sapiens GN=ATP2A2 PE=2 SV=1	6277	476	0	15	0	0.09	0	0	0	0	1	0	0	0	1	0	0.659215
tr|Q8N9K4|Q8N9K4_HUMAN	tr|Q8N9K4	cDNA FLJ36998 fis, clone BRACE2007295, highly similar to ALPHA-ADAPTIN A OS=Homo sapiens PE=2 SV=1	52284	477	0	81	0	0.2	0	0	2	0	3	0	3	0	3	0	-2.14875
tr|B4DUM2|B4DUM2_HUMA	tr|B4DUM	cDNA FLJ53891, highly similar to Adenylosuccinate lyase (EC 4.3.2.2) OS=Homo sapiens PE=2 SV=1	34875	480	0	32	0	0.13	0	0	1	0	1	0	1	0	1	0	-0.92595
tr|H3BQQ2|H3BQQ2_HUMAN	ZNF598	Zinc finger protein 598 OS=Homo sapiens GN=ZNF598 PE=4 SV=1	94712	481	0	80	0	0.07	0	0	2	0	2	0	2	0	2	0	-1.66312
tr|B3KQH2|B3KQH2_HUMAN	tr|B3KQH2	cDNA FLJ90454 fis, clone NT2RP3001560, highly similar to Cleft lip and palate transmembrane protein 1 OS=Homo sapiens PE=2 SV=1	76247	482	0	80	0	0.04	0	0	1	0	1	0	1	0	1	0	-0.92595
sp|Q96L21|RL10L_HUMAN	RPL10L	60S ribosomal protein L10-like OS=Homo sapiens GN=RPL10L PE=1 SV=3	24959	485	0	0	0	0.28	0	0	0	0	0	0	2	0	2	0	-1.66312
tr|Q5T0G9|Q5T0G9_HUMAN	ANXA11	Annexin A11 (Fragment) OS=Homo sapiens GN=ANXA11 PE=4 SV=1	20474	486	0	21	0	0.15	0	0	0	0	1	0	1	0	1	0	-0.92595
tr|Q7Z6A1|Q7Z6A1_HUMAN	PCNA	Proliferating cell nuclear antigen (Fragment) OS=Homo sapiens GN=PCNA PE=2 SV=1	8271	487	0	0	0	0.38	0	0	0	0	0	0	0	0	1	0	0.659215
tr|F8W8L6|F8W8L6_HUMAN	EIF2B4	Eukaryotic translation initiation factor 2B subunit 4 delta transcript variant 4 OS=Homo sapiens GN=EIF2B4 PE=2 SV=1	25601	488	0	40	0	0.2	0	0	1	0	1	0	1	0	1	0	-0.92595
tr|F5H1N9|F5H1N9_HUMAN	DDX47	Probable ATP-dependent RNA helicase DDX47 (Fragment) OS=Homo sapiens GN=DDX47 PE=2 SV=1	22190	491	0	37	0	0.37	0	0	1	0	1	0	1	0	1	0	-0.92595
tr|E5RG31|E5RG31_HUMAN	MCM4	DNA replication licensing factor MCM4 (Fragment) OS=Homo sapiens GN=MCM4 PE=2 SV=1	31785	493	0	54	0	0.14	0	0	1	0	2	0	2	0	2	0	-1.66312
tr|H0YDX9|H0YDX9_HUMAN	VPS51	Vacuolar protein sorting-associated protein 51 homolog (Fragment) OS=Homo sapiens GN=VPS51 PE=4 SV=1	7976	494	0	25	0	0.08	0	0	0	0	1	0	1	0	1	0	-0.92595
tr|E9PKG3|E9PKG3_HUMAN	CTTN	Src substrate cortactin (Fragment) OS=Homo sapiens GN=CTTN PE=2 SV=1	5789	495	0	77	0	0.13	0	0	1	0	1	0	1	0	1	0	-0.92595
tr|B2R6X2|B2R6X2_HUMAN	tr|B2R6X2	cDNA, FLJ93161, highly similar to Homo sapiens glucuronidase, beta (GUSB), mRNA OS=Homo sapiens PE=2 SV=1	75001	496	0	77	0	0.04	0	0	1	0	1	0	2	0	2	0	-1.66312
tr|Q6I7N8|Q6I7N8_HUMAN	HELLS	Lymphoid specific helicase variant9 OS=Homo sapiens GN=HELLS PE=2 SV=1	94530	500	0	77	0	0.15	0	0	1	0	4	0	4	0	6	0	-2.51152
tr|B4DFN6|B4DFN6_HUMAN	CNP	2~,3~-cyclic-nucleotide 3~-phosphodiesterase OS=Homo sapiens GN=CNP PE=2 SV=1	20461	501	0	77	0	0.16	0	0	1	0	1	0	1	0	1	0	-0.92595
tr|C9JMZ3|C9JMZ3_HUMAN	RNPEP	Aminopeptidase B (Fragment) OS=Homo sapiens GN=RNPEP PE=2 SV=1	39600	502	0	19	0	0.21	0	0	0	0	1	0	0	0	1	0	0.659215
tr|B4DDP6|B4DDP6_HUMAN	DBNL	Drebrin-like protein OS=Homo sapiens GN=DBNL PE=2 SV=1	43242	503	0	29	0	0.16	0	0	0	0	1	0	2	0	3	0	-1.66312
tr|D3DTH7|D3DTH7_HUMAN	MYO1C	Myosin IC, isoform CRA_a OS=Homo sapiens GN=MYO1C PE=2 SV=1	99613	506	0	75	0	0.14	0	0	1	0	4	0	3	0	4	0	-2.14875
tr|B2RDE0|B2RDE0_HUMAN	tr|B2RDE0	cDNA, FLJ96567, highly similar to Homo sapiens propionyl Coenzyme A carboxylase, alpha polypeptide(PCCA), mRNA OS=Homo sapiens PE=	77992	509	0	75	0	0.09	0	0	1	0	2	0	2	0	3	0	-1.66312
tr|H0YDG0|H0YDG0_HUMAN	NPEPPS	Puromycin-sensitive aminopeptidase (Fragment) OS=Homo sapiens GN=NPEPPS PE=4 SV=1	19154	511	0	33	0	0.18	0	0	0	0	1	0	1	0	1	0	-0.92595
tr|K7EQ02|K7EQ02_HUMAN	DAZAP1	DAZ-associated protein 1 (Fragment) OS=Homo sapiens GN=DAZAP1 PE=4 SV=1	24960	512	0	74	0	0.29	0	0	1	0	2	0	2	0	2	0	-1.66312
tr|B4DYV0|B4DYV0_HUMAN	tr|B4DYV0	cDNA FLJ59785, highly similar to Nardilysin (EC 3.4.24.61) OS=Homo sapiens PE=2 SV=1	86730	513	0	0	0	0.03	0	0	0	0	0	0	0	0	1	0	0.659215
tr|B4DJ98|B4DJ98_HUMAN	tr|B4DJ98	cDNA FLJ53558, highly similar to Protein disulfide-isomerase A3 (EC 5.3.4.1) OS=Homo sapiens PE=2 SV=1	51874	515	0	21	0	0.26	0	0	0	0	1	0	1	0	1	0	-0.92595
sp|Q9H0A0|NAT10_HUMAN	NAT10	N-acetyltransferase 10 OS=Homo sapiens GN=NAT10 PE=1 SV=2	116569	518	0	73	0	0.12	0	0	1	0	4	0	6	0	6	0	-3.04245
sp|O00231|PSD11_HUMAN	PSMD11	26S proteasome non-ATPase regulatory subunit 11 OS=Homo sapiens GN=PSMD11 PE=1 SV=3	47719	520	0	73	0	0.49	0	0	2	0	6	0	6	0	8	0	-3.04245
tr|J3KRB3|J3KRB3_HUMAN	RPL17	60S ribosomal protein L17 (Fragment) OS=Homo sapiens GN=RPL17 PE=3 SV=1	12356	521	0	36	0	0.36	0	0	1	0	1	0	1	0	1	0	-0.92595
tr|J3KR78|J3KR78_HUMAN	QTRTD1	Queuine tRNA-ribosyltransferase subunit QTRTD1 OS=Homo sapiens GN=QTRTD1 PE=4 SV=1	49085	522	0	72	0	0.07	0	0	1	0	1	0	1	0	1	0	-0.92595
sp|Q13425|SNTB2_HUMAN	SNTB2	Beta-2-syntrophin OS=Homo sapiens GN=SNTB2 PE=1 SV=1	58369	527	0	72	0	0.06	0	0	1	0	1	0	1	0	2	0	-0.92595
tr|H3BSK9|H3BSK9_HUMAN	ATXN2L	Ataxin-2-like protein (Fragment) OS=Homo sapiens GN=ATXN2L PE=4 SV=1	37229	533	0	35	0	0.09	0	0	0	0	1	0	1	0	2	0	-0.92595
tr|H0YHX9|H0YHX9_HUMAN	NACA	Nascent polypeptide-associated complex subunit alpha (Fragment) OS=Homo sapiens GN=NACA PE=4 SV=1	22930	537	0	70	0	0.15	0	0	1	0	1	0	1	0	2	0	-0.92595
tr|Q53G25|Q53G25_HUMAN	tr|Q53G25	Ribosomal protein S5 variant (Fragment) OS=Homo sapiens PE=2 SV=1	23121	539	0	70	0	0.15	0	0	1	0	1	0	1	0	1	0	-0.92595
tr|B2RCT6|B2RCT6_HUMAN	tr|B2RCT6	cDNA, FLJ96276, highly similar to Homo sapiens G1 to S phase transition 1 (GSPT1), mRNA OS=Homo sapiens PE=2 SV=1	56320	540	0	70	0	0.26	0	0	3	0	4	0	4	0	5	0	-2.51152
sp|Q9NSD9|SYFB_HUMAN	FARSB	Phenylalanine--tRNA ligase beta subunit OS=Homo sapiens GN=FARSB PE=1 SV=3	66701	541	0	70	0	0.1	0	0	2	0	2	0	2	0	3	0	-1.66312
tr|B3KWV6|B3KWV6_HUMAN	tr|B3KWV6	cDNA FLJ43948 fis, clone TESTI4014924, highly similar to Homo sapiens cytoplasmic FMR1 interacting protein 1 (CYFIP1), transcript variant	147082	543	0	69	0	0.04	0	0	2	0	2	0	4	0	4	0	-2.51152
tr|F2Z2G2|F2Z2G2_HUMAN	EEF1B2	Elongation factor 1-beta OS=Homo sapiens GN=EEF1B2 PE=4 SV=1	7611	544	0	69	0	0.46	0	0	1	0	1	0	1	0	1	0	-0.92595
tr|B5BUD2|B5BUD2_HUMAN	RFC2	Replication factor C 2 isoform 1 (Fragment) OS=Homo sapiens GN=RFC2 PE=2 SV=1	39616	545	0	68	0	0.08	0	0	1	0	1	0	1	0	1	0	-0.92595
tr|G5E9H0|G5E9H0_HUMAN	USP39	U4/U6.U5 tri-snRNP-associated protein 2 OS=Homo sapiens GN=USP39 PE=2 SV=1	56722	546	0	68	0	0.33	0	0	2	0	5	0	5	0	5	0	-2.80123
tr|Q6IBS5|Q6IBS5_HUMAN	DLST	DLST protein OS=Homo sapiens GN=DLST PE=2 SV=1	49131	547	0	68	0	0.14	0	0	2	0	2	0	2	0	2	0	-1.66312
tr|E9PRB8|E9PRB8_HUMAN	PSMC3	26S protease regulatory subunit 6A OS=Homo sapiens GN=PSMC3 PE=2 SV=1	13661	548	0	38	0	0.31	0	0	1	0	1	0	1	0	1	0	-0.92595
tr|F8WB65|F8WB65_HUMAN	Sep-02	Septin-2 OS=Homo sapiens GN=SEPT2 PE=2 SV=1	8930	553	0	30	0	0.16	0	0	0	0	1	0	1	0	1	0	-0.92595
tr|E9PFG1|E9PFG1_HUMAN	USP9X	Probable ubiquitin carboxyl-terminal hydrolase FAF-X OS=Homo sapiens GN=USP9X PE=2 SV=1	117432	554	0	50	0	0.04	0	0	1	0	2	0	4	0	4	0	-2.51152
tr|J3QQX0|J3QQX0_HUMAN	GPS1	COP9 signalosome complex subunit 1 (Fragment) OS=Homo sapiens GN=GPS1 PE=4 SV=1	8703	558	0	67	0	0.4	0	0	1	0	1	0	1	0	1	0	-0.92595
tr|E9PLX7|E9PLX7_HUMAN	RPL27A	60S ribosomal protein L27a OS=Homo sapiens GN=RPL27A PE=2 SV=1	12526	560	0	30	0	0.75	0	0	0	0	1	0	1	0	1	0	-0.92595
tr|B4E2H2|B4E2H2_HUMAN	tr|B4E2H2	cDNA FLJ56559, highly similar to FKBP12-rapamycin complex-associated protein OS=Homo sapiens PE=2 SV=1	196418	562	0	66	0	0.02	0	0	1	0	1	0	1	0	2	0	-0.92595
tr|B4DR88|B4DR88_HUMAN	tr|B4DR88	cDNA FLJ58252, highly similar to Homo sapiens DEAH (Asp-Glu-Ala-His) box polypeptide 40 (DHX40), mRNA OS=Homo sapiens PE=2 SV=1	73228	564	0	66	0	0.09	0	0	2	0	2	0	2	0	2	0	-1.66312
tr|H7C0T0|H7C0T0_HUMAN	FAM120A	Constitutive coactivator of PPAR-gamma-like protein 1 (Fragment) OS=Homo sapiens GN=FAM120A PE=4 SV=1	54253	569	0	0	0	0.05	0	0	0	0	0	0	1	0	1	0	-0.92595
tr|B1AHB0|B1AHB0_HUMAN	MCM5	MCM5 minichromosome maintenance deficient 5, cell division cycle 46 (S. cerevisiae), isoform CRA_a OS=Homo sapiens GN=MCM5 PE=2 S	83031	570	0	65	0	0.08	0	0	1	0	2	0	3	0	5	0	-2.14875
tr|E9PSI3|E9PSI3_HUMAN	NUP160	Nuclear pore complex protein Nup160 OS=Homo sapiens GN=NUP160 PE=2 SV=1	30585	571	0	0	0	0.07	0	0	0	0	0	0	1	0	1	0	-0.92595
tr|H3BM93|H3BM93_HUMAN	NUP93	Nuclear pore complex protein Nup93 (Fragment) OS=Homo sapiens GN=NUP93 PE=4 SV=1	19858	572	0	65	0	0.17	0	0	1	0	1	0	1	0	1	0	-0.92595
tr|F8WF02|F8WF02_HUMAN	PDHB	Pyruvate dehydrogenase E1 component subunit beta, mitochondrial OS=Homo sapiens GN=PDHB PE=2 SV=1	27804	573	0	16	0	0.27	0	0	0	0	1	0	1	0	2	0	-0.92595
sp|Q9Y5B9|SP16H_HUMAN	SUPT16H	FACT complex subunit SPT16 OS=Homo sapiens GN=SUPT16H PE=1 SV=1	120409	574	0	65	0	0.06	0	0	2	0	2	0	3	0	4	0	-2.14875
tr|H0YAC4|H0YAC4_HUMAN	SKIV2L2	Superkiller viralicidic activity 2-like 2 (Fragment) OS=Homo sapiens GN=SKIV2L2 PE=4 SV=1	8470	575	0	0	0	0.13	0	0	0	0	0	0	0	0	1	0	0.659215
tr|B1AHN5|B1AHN5_HUMAN	ST13	Suppression of tumorigenicity 13 (Colon carcinoma) (Hsp70 interacting protein) (Fragment) OS=Homo sapiens GN=ST13 PE=2 SV=1	18131	577	0	64	0	0.09	0	0	1	0	1	0	2	0	2	0	-1.66312
sp|Q9Y6G9|DC1L1_HUMAN	DYNC1LI1	Cytoplasmic dynein 1 light intermediate chain 1 OS=Homo sapiens GN=DYNC1LI1 PE=1 SV=3	56829	579	0	64	0	0.12	0	0	1	0	2	0	3	0	3	0	-2.14875
tr|B4DSA8|B4DSA8_HUMAN	tr|B4DSA8	cDNA FLJ51067, highly similar to DNA damage-binding protein 1 OS=Homo sapiens PE=2 SV=1	112974	581	0	64	0	0.06	0	0	1	0	2	0	2	0	2	0	-1.66312
tr|B2R7X3|B2R7X3_HUMAN	tr|B2R7X3	cDNA, FLJ93645, highly similar to Homo sapiens chromatin assembly factor 1, subunit B (p60) (CHAF1B),mRNA OS=Homo sapiens PE=2 SV=	61936	585	0	64	0	0.17	0	0	2	0	3	0	3	0	4	0	-2.14875
tr|H0YAM1|H0YAM1_HUMAN	GRSF1	G-rich sequence factor 1 (Fragment) OS=Homo sapiens GN=GRSF1 PE=4 SV=1	13410	586	0	63	0	0.07	0	0	1	0	1	0	1	0	1	0	-0.92595
tr|H0YMV8|H0YMV8_HUMAN	RPS27L	40S ribosomal protein S27 OS=Homo sapiens GN=RPS27L PE=3 SV=1	11851	587	0	63	0	0.66	0	0	2	0	2	0	2	0	2	0	-1.66312
tr|E9PNA1|E9PNA1_HUMAN	B3GAT3	Galactosylgalactosylxylosylprotein 3-beta-glucuronosyltransferase 3 (Fragment) OS=Homo sapiens GN=B3GAT3 PE=2 SV=1	24581	588	0	0	0	0.2	0	0	0	0	0	0	2	0	2	0	-1.66312
tr|B4DDY5|B4DDY5_HUMAN	tr|B4DDY5	60S ribosomal protein L18 OS=Homo sapiens PE=2 SV=1	16341	589	0	56	0	0.64	0	0	1	0	2	0	3	0	5	0	-2.14875
tr|B0QZ65|B0QZ65_HUMAN	GAPVD1	GTPase-activating protein and VPS9 domain-containing protein 1 OS=Homo sapiens GN=GAPVD1 PE=4 SV=1	56996	591	0	62	0	0.12	0	0	1	0	2	0	2	0	4	0	-1.66312
tr|B2RCD8|B2RCD8_HUMAN	tr|B2RCD8	cDNA, FLJ96012, highly similar to Homo sapiens interferon regulatory factor 2 binding protein 1 (IRF2BP1), mRNA OS=Homo sapiens PE=2 S	62587	592	0	62	0	0.11	0	0	1	0	2	0	2	0	2	0	-1.66312
tr|A4D1P0|A4D1P0_HUMAN	LOC402299	Uncharacterized protein OS=Homo sapiens GN=LOC402299 PE=4 SV=1	42109	593	0	52	0	0.19	0	0	1	0	1	0	1	0	1	0	-0.92595
tr|Q0VAS5|Q0VAS5_HUMAN	HIST1H4H	Histone H4 OS=Homo sapiens GN=HIST1H4H PE=2 SV=1	11364	594	0	62	0	0.7	0	0	1	0	2	0	2	0	2	0	-1.66312
tr|D3DNL3|D3DNL3_HUMAN	GFM1	G elongation factor, mitochondrial 1, isoform CRA_c OS=Homo sapiens GN=GFM1 PE=2 SV=1	46282	596	0	62	0	0.23	0	0	1	0	3	0	2	0	3	0	-1.66312
tr|B2R8Y6|B2R8Y6_HUMAN	tr|B2R8Y6	cDNA, FLJ94121, highly similar to Homo sapiens stromal antigen 2 (STAG2), mRNA OS=Homo sapiens PE=2 SV=1	135116	598	0	62	0	0.05	0	0	1	0	2	0	3	0	3	0	-2.14875
tr|C9J6C5|C9J6C5_HUMAN	MKI67IP	MKI67 FHA domain-interacting nucleolar phosphoprotein (Fragment) OS=Homo sapiens GN=MKI67IP PE=2 SV=1	19598	599	0	36	0	0.32	0	0	0	0	1	0	1	0	1	0	-0.92595
tr|B0QZK4|B0QZK4_HUMAN	HP1BP3	Heterochromatin protein 1-binding protein 3 (Fragment) OS=Homo sapiens GN=HP1BP3 PE=4 SV=1	28725	600	0	61	0	0.25	0	0	2	0	2	0	2	0	2	0	-1.66312
tr|B4DKC9|B4DKC9_HUMAN	tr|B4DKC9	cDNA FLJ55698, highly similar to Vinculin OS=Homo sapiens PE=2 SV=1	54529	601	0	35	0	0.07	0	0	1	0	1	0	1	0	2	0	-0.92595
tr|B4DUY3|B4DUY3_HUMAN	tr|B4DUY3	cDNA FLJ56668, highly similar to Nucleolar protein NOP5 OS=Homo sapiens PE=2 SV=1	16345	602	0	23	0	0.27	0	0	0	0	1	0	1	0	1	0	-0.92595
tr|I4AY87|I4AY87_HUMAN	tr|I4AY87|	Macrophage migration inhibitory factor (Fragment) OS=Homo sapiens PE=2 SV=1	12639	603	0	61	0	0.27	0	0	1	0	1	0	1	0	1	0	-0.92595
tr|I3L0N3|I3L0N3_HUMAN	NSF	Vesicle-fusing ATPase OS=Homo sapiens GN=NSF PE=3 SV=1	82553	604	0	61	0	0.08	0	0	2	0	2	0	3	0	3	0	-2.14875
tr|H0YJF9|H0YJF9_HUMAN	DLST	Dihydrolipoyllysine-residue succinyltransferase component of 2-oxoglutarate dehydrogenase complex, mitochondrial (Fragment) OS=Homo	8710	607	0	60	0	0.4	0	0	1	0	1	0	1	0	2	0	-0.92595
tr|I3L2U8|I3L2U8_HUMAN	PAFAH1B1	Platelet-activating factor acetylhydrolase IB subunit alpha OS=Homo sapiens GN=PAFAH1B1 PE=4 SV=1	39493	610	0	60	0	0.17	0	0	2	0	2	0	2	0	2	0	-1.66312
sp|Q86UE4|LYRIC_HUMAN	MTDH	Protein LYRIC OS=Homo sapiens GN=MTDH PE=1 SV=2	63856	612	0	60	0	0.11	0	0	1	0	2	0	2	0	3	0	-1.66312
sp|Q7Z5L9|I2BP2_HUMAN	IRF2BP2	Interferon regulatory factor 2-binding protein 2 OS=Homo sapiens GN=IRF2BP2 PE=1 SV=2	61728	613	0	60	0	0.05	0	0	1	0	1	0	1	0	2	0	-0.92595
tr|B4E2W0|B4E2W0_HUMAN	HADHB	3-ketoacyl-CoA thiolase OS=Homo sapiens GN=HADHB PE=2 SV=1	49076	614	0	60	0	0.07	0	0	1	0	1	0	1	0	2	0	-0.92595
sp|P62263|RS14_HUMAN	RPS14	40S ribosomal protein S14 OS=Homo sapiens GN=RPS14 PE=1 SV=3	16434	615	0	60	0	0.45	0	0	1	0	2	0	2	0	3	0	-1.66312
sp|Q5T4S7|UBR4_HUMAN	UBR4	E3 ubiquitin-protein ligase UBR4 OS=Homo sapiens GN=UBR4 PE=1 SV=1	580547	619	0	59	0	0.02	0	0	2	0	4	0	3	0	5	0	-2.14875

tr|Q6IPN0|Q6IPN0_HUMAN	RTN4	RTN4 protein OS=Homo sapiens GN=RTN4 PE=2 SV=1	36952	620	0	59	0	0.29	0	0	1	0	3	0	3	0	3	0	-2.14875
tr|D3DS95|D3DS95_HUMAN	hCG_2117	HCG21173, isoform CRA_a OS=Homo sapiens GN=hCG_21173 PE=2 SV=1	10174	621	0	35	0	0.33	0	0	1	0	1	0	1	0	1	0	-0.92595
sp|Q69YN2|C19L1_HUMAN	CWF19L1	CWF19-like protein 1 OS=Homo sapiens GN=CWF19L1 PE=1 SV=2	61322	624	0	59	0	0.11	0	0	2	0	2	0	2	0	2	0	-1.66312
sp|P42695|CNDD3_HUMAN	NCAPD3	Condensin-2 complex subunit D3 OS=Homo sapiens GN=NCAPD3 PE=1 SV=2	170950	625	0	59	0	0.02	0	0	1	0	1	0	1	0	2	0	-0.92595
tr|B4DMA8|B4DMA8_HUMAN	tr|B4DMA8	cDNA FLJ53841, highly similar to Kinesin-like protein KIFC1 OS=Homo sapiens PE=2 SV=1	60989	626	0	52	0	0.14	0	0	1	0	2	0	1	0	2	0	-0.92595
tr|G3V1U5|G3V1U5_HUMAN	GOLT1B	Golgi transport 1 homolog B (S. cerevisiae), isoform CRA_c OS=Homo sapiens GN=GOLT1B PE=2 SV=1	8208	627	0	58	0	0.43	0	0	1	0	1	0	1	0	1	0	-0.92595
tr|H0YB26|H0YB26_HUMAN	CPNE3	Copine-3 (Fragment) OS=Homo sapiens GN=CPNE3 PE=4 SV=1	29941	628	0	38	0	0.17	0	0	1	0	1	0	1	0	2	0	-0.92595
tr|K4DI93|K4DI93_HUMAN	CUL4B	Cullin 4B, isoform CRA_e OS=Homo sapiens GN=CUL4B PE=3 SV=1	103318	629	0	58	0	0.06	0	0	2	0	2	0	3	0	3	0	-2.14875
tr|Q59GH5|Q59GH5_HUMAN	tr|Q59GH5	COP9 signalosome subunit 5 variant (Fragment) OS=Homo sapiens PE=2 SV=1	31467	631	0	58	0	0.11	0	0	1	0	1	0	1	0	1	0	-0.92595
tr|A8K787|A8K787_HUMAN	tr|A8K787	cDNA FLJ75273, highly similar to Homo sapiens solute carrier family 25 (mitochondrial carrier; adenine nucleotide translocator), member 4,	33287	632	0	53	0	0.21	0	0	1	0	1	0	2	0	2	0	-1.66312
tr|H0Y6E7|H0Y6E7_HUMAN	RBMX	RNA-binding motif protein, X chromosome, N-terminally processed (Fragment) OS=Homo sapiens GN=RBMX PE=4 SV=2	31837	633	0	57	0	0.1	0	0	1	0	1	0	1	0	1	0	-0.92595
tr|D3DSQ7|D3DSQ7_HUMAN	XPO7	Exportin 7, isoform CRA_c OS=Homo sapiens GN=XPO7 PE=2 SV=1	100370	634	0	57	0	0.07	0	0	1	0	2	0	2	0	3	0	-1.66312
tr|D3DUW6|D3DUW6_HUMA	DNM1L	Dynamin 1-like, isoform CRA_f OS=Homo sapiens GN=DNM1L PE=2 SV=1	86007	636	0	57	0	0.12	0	0	1	0	3	0	3	0	4	0	-2.14875
tr|E7ETD6|E7ETD6_HUMAN	BPTF	Nucleosome-remodeling factor subunit BPTF OS=Homo sapiens GN=BPTF PE=2 SV=3	310562	637	0	57	0	0.04	0	0	1	0	4	0	3	0	4	0	-2.14875
tr|D6R9Q9|D6R9Q9_HUMAN	DBN1	Drebrin OS=Homo sapiens GN=DBN1 PE=2 SV=1	41878	638	0	57	0	0.05	0	0	1	0	1	0	1	0	1	0	-0.92595
tr|B2R7M3|B2R7M3_HUMAN	tr|B2R7M3	cDNA, FLJ93510, highly similar to Homo sapiens JTV1 gene (JTV1), mRNA OS=Homo sapiens PE=2 SV=1	35767	639	0	57	0	0.43	0	0	1	0	4	0	4	0	4	0	-2.51152
tr|H0YEN0|H0YEN0_HUMAN	RIC8A	Synembryn-A (Fragment) OS=Homo sapiens GN=RIC8A PE=4 SV=1	32826	640	0	57	0	0.1	0	0	1	0	1	0	1	0	1	0	-0.92595
tr|H0YFF5|H0YFF5_HUMAN	CLPB	Caseinolytic peptidase B protein homolog (Fragment) OS=Homo sapiens GN=CLPB PE=4 SV=1	37480	642	0	57	0	0.09	0	0	1	0	1	0	1	0	1	0	-0.92595
tr|B3KQ12|B3KQ12_HUMAN	tr|B3KQ12	cDNA FLJ32606 fis, clone STOMA1000186, highly similar to Homo sapiens acyl-CoA thioesterase 7 (ACOT7), transcript variant hBACHa, mRN	40814	644	0	57	0	0.08	0	0	1	0	1	0	1	0	2	0	-0.92595
tr|J3QKW7|J3QKW7_HUMAN	DRG2	Developmentally-regulated GTP-binding protein 2 OS=Homo sapiens GN=DRG2 PE=4 SV=1	26892	645	0	57	0	0.12	0	0	1	0	1	0	1	0	1	0	-0.92595
tr|Q5VU77|Q5VU77_HUMAN	UBAP2L	Ubiquitin associated protein 2-like (Fragment) OS=Homo sapiens GN=UBAP2L PE=1 SV=1	38109	646	0	51	0	0.06	0	0	1	0	1	0	1	0	1	0	-0.92595
tr|B7Z3Q4|B7Z3Q4_HUMAN	tr|B7Z3Q4	cDNA FLJ51873, highly similar to Alkyldihydroxyacetonephosphate synthase, peroxisomal (EC 2.5.1.26) OS=Homo sapiens PE=2 SV=1	64547	648	0	56	0	0.05	0	0	1	0	1	0	1	0	2	0	-0.92595
tr|Q14784|Q14784_HUMAN	tr|Q14784	Myosin (Fragment) OS=Homo sapiens PE=2 SV=1	9580	650	0	45	0	0.07	0	0	1	0	1	0	1	0	1	0	-0.92595
sp|Q9UJS0|CMC2_HUMAN	SLC25A13	Calcium-binding mitochondrial carrier protein Aralar2 OS=Homo sapiens GN=SLC25A13 PE=1 SV=2	74528	651	0	56	0	0.14	0	0	1	0	3	0	3	0	3	0	-2.14875
tr|D6R937|D6R937_HUMAN	AIMP1	Endothelial monocyte-activating polypeptide 2 (Fragment) OS=Homo sapiens GN=AIMP1 PE=2 SV=1	16568	652	0	39	0	0.58	0	0	0	0	2	0	2	0	2	0	-1.66312
sp|Q6UXN9|WDR82_HUMAN	WDR82	WD repeat-containing protein 82 OS=Homo sapiens GN=WDR82 PE=1 SV=1	35456	653	0	56	0	0.09	0	0	1	0	1	0	1	0	5	0	-0.92595
tr|G3V3F1|G3V3F1_HUMAN	NEDD1	Neural cell expressed, developmentally down-regulated 1, isoform CRA_c OS=Homo sapiens GN=NEDD1 PE=2 SV=1	73292	655	0	55	0	0.04	0	0	1	0	1	0	1	0	1	0	-0.92595
tr|Q8TEN0|Q8TEN0_HUMAN	FLJ00163	FLJ00163 protein (Fragment) OS=Homo sapiens GN=FLJ00163 PE=2 SV=1	56335	656	0	55	0	0.06	0	0	1	0	1	0	1	0	1	0	-0.92595
tr|H7BYG8|H7BYG8_HUMAN	LTN1	E3 ubiquitin-protein ligase listerin OS=Homo sapiens GN=LTN1 PE=4 SV=1	92487	657	0	35	0	0.07	0	0	0	0	2	0	2	0	2	0	-1.66312
tr|H0YL11|H0YL11_HUMAN	IDH2	Isocitrate dehydrogenase [NADP], mitochondrial OS=Homo sapiens GN=IDH2 PE=3 SV=1	35536	658	0	55	0	0.09	0	0	1	0	1	0	1	0	2	0	-0.92595
tr|Q5TD90|Q5TD90_HUMAN	KPNA5	Importin subunit alpha-6 (Fragment) OS=Homo sapiens GN=KPNA5 PE=4 SV=1	16812	660	0	0	0	0.11	0	0	0	0	0	0	1	0	1	0	-0.92595
tr|A5PHJ9|A5PHJ9_HUMAN	PDHA1/LO	PDHA1/LOC79064 protein (Fragment) OS=Homo sapiens GN=PDHA1/LOC79064 PE=2 SV=1	34113	665	0	54	0	0.1	0	0	1	0	1	0	2	0	2	0	-1.66312
tr|Q1WWM3|Q1WWM3_HU	EPB41	EPB41 protein (Fragment) OS=Homo sapiens GN=EPB41 PE=2 SV=1	83618	668	0	54	0	0.04	0	0	1	0	1	0	1	0	1	0	-0.92595
tr|Q7Z3K9|Q7Z3K9_HUMAN	DKFZp781L	Putative uncharacterized protein DKFZp781L0540 (Fragment) OS=Homo sapiens GN=DKFZp781L0540 PE=2 SV=1	90081	671	0	54	0	0.07	0	0	2	0	2	0	2	0	2	0	-1.66312
tr|Q6IB71|Q6IB71_HUMAN	PSMA3	Proteasome subunit alpha type OS=Homo sapiens GN=PSMA3 PE=2 SV=1	28625	672	0	54	0	0.12	0	0	1	0	1	0	1	0	1	0	-0.92595
sp|Q9Y5Q9|TF3C3_HUMAN	GTF3C3	General transcription factor 3C polypeptide 3 OS=Homo sapiens GN=GTF3C3 PE=1 SV=1	102006	673	0	54	0	0.03	0	0	1	0	1	0	1	0	2	0	-0.92595
tr|B3KVF5|B3KVF5_HUMAN	tr|B3KVF5	cDNA FLJ16494 fis, clone CTONG3004576, highly similar to Keratin, type I cytoskeletal 15 OS=Homo sapiens PE=2 SV=1	49307	674	0	40	0	0.11	0	0	1	0	1	0	1	0	3	0	-0.92595
tr|B3KNM6|B3KNM6_HUMAN	tr|B3KNM6	cDNA FLJ14968 fis, clone THYRO1000288, highly similar to CAAX prenyl protease 1 homolog (EC 3.4.24.84) OS=Homo sapiens PE=2 SV=1	27279	675	0	54	0	0.26	0	0	1	0	2	0	2	0	2	0	-1.66312
tr|B7Z6F7|B7Z6F7_HUMAN	tr|B7Z6F7	cDNA FLJ61705, highly similar to Symplekin OS=Homo sapiens PE=2 SV=1	141898	676	0	53	0	0.05	0	0	1	0	2	0	3	0	4	0	-2.14875
tr|B7Z842|B7Z842_HUMAN	tr|B7Z842	Ubiquitin carboxyl-terminal hydrolase OS=Homo sapiens PE=2 SV=1	59074	677	0	0	0	0.06	0	0	0	0	0	0	0	0	1	0	0.659215
tr|J3QST3|J3QST3_HUMAN	KRT3	Keratin, type II cytoskeletal 3 OS=Homo sapiens GN=KRT3 PE=3 SV=1	64636	679	0	53	0	0.16	0	0	2	0	3	0	3	0	4	0	-2.14875
tr|H3BNB9|H3BNB9_HUMAN	ARIH1	E3 ubiquitin-protein ligase ARIH1 (Fragment) OS=Homo sapiens GN=ARIH1 PE=4 SV=1	22769	682	0	53	0	0.15	0	0	1	0	1	0	1	0	1	0	-0.92595
tr|Q68CW9|Q68CW9_HUMA	DKFZp667	Putative uncharacterized protein DKFZp667O0311 (Fragment) OS=Homo sapiens GN=DKFZp667O0311 PE=2 SV=1	31790	683	0	33	0	0.1	0	0	0	0	1	0	1	0	1	0	-0.92595
tr|Q53T70|Q53T70_HUMAN	RAB10	Putative uncharacterized protein RAB10 (Fragment) OS=Homo sapiens GN=RAB10 PE=2 SV=1	17970	690	0	52	0	0.41	0	0	1	0	2	0	2	0	2	0	-1.66312
tr|B0I1S9|B0I1S9_HUMAN	MYO1B	MYO1B variant protein OS=Homo sapiens GN=MYO1B PE=2 SV=1	132928	692	0	52	0	0.02	0	0	1	0	1	0	1	0	2	0	-0.92595
tr|B2R643|B2R643_HUMAN	tr|B2R643	cDNA, FLJ92777, highly similar to Homo sapiens myosin IA (MYO1A), mRNA OS=Homo sapiens PE=2 SV=1	119252	693	0	52	0	0.03	0	0	1	0	1	0	2	0	2	0	-1.66312
tr|B3KN30|B3KN30_HUMAN	tr|B3KN30	cDNA FLJ13372 fis, clone PLACE1000706, highly similar to Transcription intermediary factor 1-gamma OS=Homo sapiens PE=2 SV=1	84930	694	0	52	0	0.04	0	0	1	0	1	0	2	0	2	0	-1.66312
sp|P62851|RS25_HUMAN	RPS25	40S ribosomal protein S25 OS=Homo sapiens GN=RPS25 PE=1 SV=1	13791	696	0	52	0	0.25	0	0	1	0	1	0	1	0	1	0	-0.92595
tr|H0YE27|H0YE27_HUMAN	RPS2	40S ribosomal protein S2 (Fragment) OS=Homo sapiens GN=RPS2 PE=4 SV=1	7140	698	0	52	0	0.5	0	0	1	0	1	0	1	0	1	0	-0.92595
tr|B4DK32|B4DK32_HUMAN	tr|B4DK32	cDNA FLJ61095, highly similar to Ubiquitin conjugation factor E4 A (Fragment) OS=Homo sapiens PE=2 SV=1	120816	702	0	52	0	0.05	0	0	1	0	2	0	2	0	2	0	-1.66312
tr|A8K7N0|A8K7N0_HUMAN	tr|A8K7N0	cDNA FLJ75556, highly similar to Homo sapiens ribosomal protein L14, mRNA OS=Homo sapiens PE=2 SV=1	23745	705	0	51	0	0.3	0	0	1	0	2	0	2	0	2	0	-1.66312
sp|P11388|TOP2A_HUMAN	TOP2A	DNA topoisomerase 2-alpha OS=Homo sapiens GN=TOP2A PE=1 SV=3	175017	706	0	51	0	0.04	0	0	1	0	2	0	2	0	3	0	-1.66312
tr|A0AV56|A0AV56_HUMAN	SAFB	SAFB protein OS=Homo sapiens GN=SAFB PE=2 SV=1	103249	708	0	51	0	0.06	0	0	1	0	2	0	2	0	2	0	-1.66312
tr|B4DLG2|B4DLG2_HUMAN	tr|B4DLG2	cDNA FLJ58196, highly similar to Zinc finger CCCH domain-containing protein 11A OS=Homo sapiens PE=2 SV=1	83177	712	0	51	0	0.04	0	0	1	0	1	0	1	0	1	0	-0.92595
sp|A2A3N6|PIPSL_HUMAN	PIPSL	Putative PIP5K1A and PSMD4-like protein OS=Homo sapiens GN=PIPSL PE=5 SV=1	95729	715	0	51	0	0.03	0	0	1	0	1	0	1	0	1	0	-0.92595
tr|B3KY12|B3KY12_HUMAN	tr|B3KY12	cDNA FLJ46581 fis, clone THYMU3043200, highly similar to Splicing factor 3A subunit 3 OS=Homo sapiens PE=2 SV=1	59082	717	0	51	0	0.06	0	0	1	0	1	0	1	0	1	0	-0.92595
tr|D3DNS0|D3DNS0_HUMAN	ACTL6A	Actin-like 6A, isoform CRA_b OS=Homo sapiens GN=ACTL6A PE=2 SV=1	25781	718	0	31	0	0.14	0	0	0	0	1	0	1	0	1	0	-0.92595
tr|B4DQ51|B4DQ51_HUMAN	ACADSB	Short/branched chain-specific acyl-CoA dehydrogenase, mitochondrial OS=Homo sapiens GN=ACADSB PE=2 SV=1	36287	719	0	50	0	0.09	0	0	1	0	1	0	1	0	2	0	-0.92595
tr|Q2M1V9|Q2M1V9_HUMA	BTAF1	BTAF1 RNA polymerase II, B-TFIID transcription factor-associated, 170kDa OS=Homo sapiens GN=BTAF1 PE=2 SV=1	209037	720	0	50	0	0.03	0	0	2	0	2	0	2	0	3	0	-1.66312
tr|B7ZLC9|B7ZLC9_HUMAN	GEMIN5	GEMIN5 protein OS=Homo sapiens GN=GEMIN5 PE=2 SV=1	170664	722	0	50	0	0.02	0	0	1	0	1	0	1	0	1	0	-0.92595
tr|B3KUP9|B3KUP9_HUMAN	tr|B3KUP9	cDNA FLJ40361 fis, clone TESTI2034407, highly similar to NEDD8-activating enzyme E1 regulatory subunit OS=Homo sapiens PE=2 SV=1	60951	723	0	50	0	0.05	0	0	1	0	1	0	1	0	1	0	-0.92595
tr|F6S0T5|F6S0T5_HUMAN	KDM1A	Lysine-specific histone demethylase 1A OS=Homo sapiens GN=KDM1A PE=2 SV=1	95677	724	0	50	0	0.07	0	0	2	0	2	0	2	0	2	0	-1.66312
tr|B3KM73|B3KM73_HUMAN	tr|B3KM73	cDNA FLJ10419 fis, clone NT2RP1000163, highly similar to Homo sapiens transforming growth factor beta regulator 4 (TBRG4), transcript va	55418	725	0	50	0	0.06	0	0	1	0	1	0	1	0	1	0	-0.92595
tr|B3KPF6|B3KPF6_HUMAN	tr|B3KPF6	cDNA FLJ31732 fis, clone NT2RI2006856, highly similar to Striatin-4 OS=Homo sapiens PE=2 SV=1	39506	727	0	35	0	0.1	0	0	1	0	1	0	1	0	1	0	-0.92595
tr|H0YFC6|H0YFC6_HUMAN	RAN	GTP-binding nuclear protein Ran (Fragment) OS=Homo sapiens GN=RAN PE=4 SV=1	11786	728	0	0	0	0.26	0	0	0	0	0	0	0	0	1	0	0.659215
sp|P02533|K1C14_HUMAN	KRT14	Keratin, type I cytoskeletal 14 OS=Homo sapiens GN=KRT14 PE=1 SV=4	51872	729	0	49	0	0.13	0	0	2	0	2	0	3	0	4	0	-2.14875
tr|H7C4X8|H7C4X8_HUMAN	CLASP2	CLIP-associating protein 2 (Fragment) OS=Homo sapiens GN=CLASP2 PE=4 SV=1	20981	730	0	49	0	0.16	0	0	1	0	1	0	1	0	2	0	-0.92595
tr|Q1JQ76|Q1JQ76_HUMAN	RPL10A	Ribosomal protein (Fragment) OS=Homo sapiens GN=RPL10A PE=2 SV=1	23692	732	0	49	0	0.14	0	0	1	0	1	0	1	0	1	0	-0.92595
tr|D2KFR9|D2KFR9_HUMAN	STAT1	Signal transducer and activator of transcription 1-alpha/beta OS=Homo sapiens GN=STAT1 PE=2 SV=1	23360	734	0	38	0	0.12	0	0	1	0	1	0	1	0	1	0	-0.92595
tr|Q3BDU5|Q3BDU5_HUMAN	tr|Q3BDU5	Rhabdomyosarcoma antigen MU-RMS-40.12 OS=Homo sapiens PE=2 SV=1	55605	735	0	49	0	0.06	0	0	1	0	1	0	1	0	2	0	-0.92595
tr|A0JNT2|A0JNT2_HUMAN	KRT83	KRT83 protein OS=Homo sapiens GN=KRT83 PE=2 SV=1	50978	736	0	49	0	0.06	0	0	1	0	1	0	1	0	1	0	-0.92595
sp|O00507|USP9Y_HUMAN	USP9Y	Probable ubiquitin carboxyl-terminal hydrolase FAF-Y OS=Homo sapiens GN=USP9Y PE=1 SV=2	294370	737	0	49	0	0.01	0	0	1	0	1	0	3	0	3	0	-2.14875
tr|Q4VXM0|Q4VXM0_HUMA	MTHFD1L	Methylenetetrahydrofolate dehydrogenase (NADP+ dependent) 1-like (Fragment) OS=Homo sapiens GN=MTHFD1L PE=4 SV=1	10005	738	0	0	0	0.04	0	0	0	0	0	0	1	0	1	0	-0.92595
tr|Q6N029|Q6N029_HUMAN	DKFZp686	Putative uncharacterized protein DKFZp686D11262 (Fragment) OS=Homo sapiens GN=DKFZp686D11262 PE=2 SV=1	79409	739	0	49	0	0.04	0	0	1	0	2	0	2	0	3	0	-1.66312
tr|H0YDT5|H0YDT5_HUMAN	ACADM	Medium-chain-specific acyl-CoA dehydrogenase, mitochondrial (Fragment) OS=Homo sapiens GN=ACADM PE=4 SV=1	7051	740	0	49	0	0.5	0	0	1	0	1	0	1	0	1	0	-0.92595
sp|Q9Y3Z3|SAMH1_HUMAN	SAMHD1	SAM domain and HD domain-containing protein 1 OS=Homo sapiens GN=SAMHD1 PE=1 SV=2	72896	741	0	49	0	0.09	0	0	1	0	2	0	2	0	4	0	-1.66312
tr|Q59ES3|Q59ES3_HUMAN	tr|Q59ES3	Solute carrier family 1 (Neutral amino acid transporter), member 5 variant (Fragment) OS=Homo sapiens PE=2 SV=1	57810	742	0	48	0	0.06	0	0	1	0	1	0	1	0	1	0	-0.92595
tr|B4DUN3|B4DUN3_HUMAN	tr|B4DUN3	cDNA FLJ54756, moderately similar to Homo sapiens nitric oxide synthase interacting protein (NOSIP), mRNA OS=Homo sapiens PE=2 SV=1	18325	743	0	0	0	0.1	0	0	0	0	0	0	0	0	1	0	0.659215
tr|Q59H95|Q59H95_HUMAN	tr|Q59H95	Flightless I homolog variant (Fragment) OS=Homo sapiens PE=2 SV=1	126396	744	0	48	0	0.05	0	0	1	0	2	0	2	0	3	0	-1.66312
tr|B4DJ92|B4DJ92_HUMAN	tr|B4DJ92	cDNA FLJ57843, highly similar to Homo sapiens neurochondrin (NCDN), transcript variant 3, mRNA OS=Homo sapiens PE=2 SV=1	20030	746	0	48	0	0.17	0	0	1	0	1	0	1	0	1	0	-0.92595
tr|H0YK49|H0YK49_HUMAN	ETFA	Electron transfer flavoprotein subunit alpha, mitochondrial OS=Homo sapiens GN=ETFA PE=4 SV=1	24259	748	0	48	0	0.29	0	0	1	0	2	0	1	0	2	0	-0.92595
tr|B3KVR1|B3KVR1_HUMAN	SNRPN	Small nuclear ribonucleoprotein-associated protein OS=Homo sapiens GN=SNRPN PE=2 SV=1	25230	749	0	48	0	0.13	0	0	1	0	1	0	1	0	1	0	-0.92595
tr|I3L2C7|I3L2C7_HUMAN	GEMIN4	Gem-associated protein 4 OS=Homo sapiens GN=GEMIN4 PE=4 SV=1	120456	754	0	47	0	0.06	0	0	2	0	2	0	2	0	2	0	-1.66312
tr|K7EJT5|K7EJT5_HUMAN	RPL22	60S ribosomal protein L22 (Fragment) OS=Homo sapiens GN=RPL22 PE=4 SV=1	5080	756	0	47	0	0.72	0	0	1	0	1	0	1	0	1	0	-0.92595
tr|Q68E00|Q68E00_HUMAN	DKFZp686	Putative uncharacterized protein DKFZp686G2045 (Fragment) OS=Homo sapiens GN=DKFZp686G2045 PE=2 SV=2	32787	759	0	47	0	0.1	0	0	1	0	1	0	1	0	1	0	-0.92595
tr|E9PCI9|E9PCI9_HUMAN	FDPS	Farnesyl pyrophosphate synthase OS=Homo sapiens GN=FDPS PE=2 SV=1	40849	760	0	47	0	0.08	0	0	1	0	1	0	1	0	1	0	-0.92595
tr|A8KA19|A8KA19_HUMAN	tr|A8KA19	cDNA FLJ75831, highly similar to Homo sapiens exportin, tRNA (nuclear export receptor for tRNAs) (XPOT), mRNA OS=Homo sapiens PE=2 S	111104	762	0	47	0	0.06	0	0	1	0	2	0	2	0	4	0	-1.66312
tr|A2AB15|A2AB15_HUMAN	DHX16	Putative pre-mRNA-splicing factor ATP-dependent RNA helicase DHX16 (Fragment) OS=Homo sapiens GN=DHX16 PE=4 SV=1	34860	763	0	47	0	0.1	0	0	1	0	1	0	1	0	1	0	-0.92595
tr|A0AV58|A0AV58_HUMAN	STRN3	Striatin, calmodulin binding protein 3 OS=Homo sapiens GN=STRN3 PE=2 SV=1	78069	765	0	46	0	0.09	0	0	2	0	2	0	2	0	2	0	-1.66312
tr|B5BU72|B5BU72_HUMAN	PICALM	Phosphatidylinositol-binding clathrin assembly protein isoform 2 OS=Homo sapiens GN=PICALM PE=2 SV=1	66523	766	0	46	0	0.05	0	0	1	0	1	0	1	0	1	0	-0.92595
sp|Q8NFW8|NEUA_HUMAN	CMAS	N-acylneuraminate cytidylyltransferase OS=Homo sapiens GN=CMAS PE=1 SV=2	49033	767	0	46	0	0.07	0	0	1	0	1	0	1	0	1	0	-0.92595
tr|B4DM41|B4DM41_HUMAN	RFC4	Replication factor C (Activator 1) 4, 37kDa, isoform CRA_b OS=Homo sapiens GN=RFC4 PE=2 SV=1	33996	768	0	46	0	0.1	0	0	1	0	1	0	2	0	2	0	-1.66312
tr|Q7Z524|Q7Z524_HUMAN	tr|Q7Z524	HUMEEP OS=Homo sapiens PE=2 SV=1	51391	769	0	46	0	0.06	0	0	1	0	1	0	1	0	1	0	-0.92595
tr|B3KSI3|B3KSI3_HUMAN	BCAT2	Branched-chain-amino-acid aminotransferase OS=Homo sapiens GN=BCAT2 PE=2 SV=1	40232	770	0	46	0	0.08	0	0	1	0	1	0	1	0	1	0	-0.92595
tr|B4E206|B4E206_HUMAN	tr|B4E206	cDNA FLJ55441, highly similar to Nuclear pore complex protein Nup133 OS=Homo sapiens PE=2 SV=1	128349	771	0	46	0	0.05	0	0	1	0	2	0	2	0	2	0	-1.66312
sp|A9QM74|IMA8_HUMAN	KPNA7	Importin subunit alpha-8 OS=Homo sapiens GN=KPNA7 PE=1 SV=1	57529	774	0	45	0	0.06	0	0	1	0	1	0	1	0	1	0	-0.92595
tr|C9J1F6|C9J1F6_HUMAN	PRRC2A	Protein PRRC2A OS=Homo sapiens GN=PRRC2A PE=2 SV=1	227467	778	0	36	0	0.05	0	0	0	0	1	0	1	0	1	0	-0.92595
tr|A8MX94|A8MX94_HUMAN	GSTP1	Glutathione S-transferase P OS=Homo sapiens GN=GSTP1 PE=2 SV=1	19696	779	0	45	0	0.17	0	0	1	0	1	0	1	0	1	0	-0.92595
sp|Q6PI48|SYDM_HUMAN	DARS2	Aspartate--tRNA ligase, mitochondrial OS=Homo sapiens GN=DARS2 PE=1 SV=1	74086	780	0	45	0	0.04	0	0	1	0	1	0	2	0	2	0	-1.66312
tr|B3KSI7|B3KSI7_HUMAN	tr|B3KSI7	cDNA FLJ36374 fis, clone THYMU2008185, highly similar to Xaa-Pro aminopeptidase 1 (EC 3.4.11.9) OS=Homo sapiens PE=2 SV=1	62680	783	0	45	0	0.11	0	0	0	0	2	0	2	0	3	0	-1.66312
sp|Q9Y295|DRG1_HUMAN	DRG1	Developmentally-regulated GTP-binding protein 1 OS=Homo sapiens GN=DRG1 PE=1 SV=1	40802	784	0	44	0	0.08	0	0	1	0	1	0	1	0	1	0	-0.92595
tr|B2RB52|B2RB52_HUMAN	tr|B2RB52	cDNA, FLJ95314, highly similar to Homo sapiens transducin (beta)-like 2 (TBL2), transcript variant 1, mRNA OS=Homo sapiens PE=2 SV=1	50423	785	0	44	0	0.07	0	0	1	0	1	0	1	0	1	0	-0.92595
tr|B4DKE6|B4DKE6_HUMAN	tr|B4DKE6	cDNA FLJ60629, highly similar to Replication factor C subunit 3 OS=Homo sapiens PE=2 SV=1	35551	786	0	44	0	0.09	0	0	1	0	1	0	1	0	1	0	-0.92595
sp|Q8N1G2|MTR1_HUMAN	FTSJD2	Cap-specific mRNA (nucleoside-2~-O-)-methyltransferase 1 OS=Homo sapiens GN=FTSJD2 PE=1 SV=1	96172	787	0	44	0	0.03	0	0	1	0	1	0	1	0	1	0	-0.92595
sp|Q7RTS7|K2C74_HUMAN	KRT74	Keratin, type II cytoskeletal 74 OS=Homo sapiens GN=KRT74 PE=1 SV=2	58229	789	0	44	0	0.12	0	0	1	0	2	0	2	0	4	0	-1.66312
tr|Q6DCA8|Q6DCA8_HUMAN	BCLAF1	BCLAF1 protein (Fragment) OS=Homo sapiens GN=BCLAF1 PE=2 SV=1	77958	792	0	44	0	0.04	0	0	1	0	1	0	2	0	3	0	-1.66312
sp|Q9Y2A7|NCKP1_HUMAN	NCKAP1	Nck-associated protein 1 OS=Homo sapiens GN=NCKAP1 PE=1 SV=1	130018	797	0	44	0	0.03	0	0	1	0	1	0	1	0	2	0	-0.92595
tr|Q4ZG72|Q4ZG72_HUMAN	DDX18	Putative uncharacterized protein DDX18 (Fragment) OS=Homo sapiens GN=DDX18 PE=2 SV=1	61898	799	0	43	0	0.11	0	0	1	0	2	0	2	0	3	0	-1.66312
sp|Q9Y536|PAL4A_HUMAN	PPIAL4A	Peptidyl-prolyl cis-trans isomerase A-like 4A/B/C OS=Homo sapiens GN=PPIAL4A PE=2 SV=1	18398	800	0	0	0	0.41	0	0	0	0	0	0	0	0	1	0	0.659215
tr|A1L377|A1L377_HUMAN	CABC1	CABC1 protein OS=Homo sapiens GN=CABC1 PE=2 SV=1	44564	801	0	43	0	0.07	0	0	1	0	1	0	1	0	1	0	-0.92595
tr|E9PDG5|E9PDG5_HUMAN	LARP4	La-related protein 4 OS=Homo sapiens GN=LARP4 PE=2 SV=1	50319	802	0	43	0	0.14	0	0	1	0	2	0	2	0	2	0	-1.66312
tr|B3KPF2|B3KPF2_HUMAN	tr|B3KPF2	cDNA FLJ31717 fis, clone NT2RI2006640, highly similar to Dynamin-3 (EC 3.6.5.5) OS=Homo sapiens PE=2 SV=1	63057	803	0	43	0	0.05	0	0	1	0	1	0	1	0	2	0	-0.92595
sp|Q5JVF3|PCID2_HUMAN	PCID2	PCI domain-containing protein 2 OS=Homo sapiens GN=PCID2 PE=1 SV=2	46627	804	0	43	0	0.23	0	0	1	0	3	0	3	0	3	0	-2.14875
tr|B2RN89|B2RN89_HUMAN	PCDHA8	Protocadherin alpha 8 OS=Homo sapiens GN=PCDHA8 PE=2 SV=1	89242	805	0	43	0	0.04	0	0	1	0	1	0	1	0	1	0	-0.92595
sp|Q5JTZ9|SYAM_HUMAN	AARS2	Alanine--tRNA ligase, mitochondrial OS=Homo sapiens GN=AARS2 PE=1 SV=1	108299	806	0	43	0	0.03	0	0	1	0	1	0	1	0	3	0	-0.92595
sp|Q93034|CUL5_HUMAN	CUL5	Cullin-5 OS=Homo sapiens GN=CUL5 PE=1 SV=4	91468	807	0	43	0	0.04	0	0	1	0	1	0	2	0	3	0	-1.66312
tr|Q5SRT3|Q5SRT3_HUMAN	CLIC1	Chloride intracellular channel 1, isoform CRA_a OS=Homo sapiens GN=CLIC1 PE=2 SV=2	27248	810	0	43	0	0.12	0	0	1	0	1	0	1	0	1	0	-0.92595
tr|Q5VZU9|Q5VZU9_HUMAN	TPP2	Tripeptidyl-peptidase 2 OS=Homo sapiens GN=TPP2 PE=4 SV=1	141160	811	0	43	0	0.05	0	0	1	0	2	0	2	0	3	0	-1.66312
tr|A0RZB8|A0RZB8_HUMAN	DIAPH1	Diaphanous-1 OS=Homo sapiens GN=DIAPH1 PE=2 SV=1	140767	813	0	42	0	0.05	0	0	1	0	2	0	3	0	4	0	-2.14875
tr|Q6FHJ5|Q6FHJ5_HUMAN	SCAMP3	SCAMP3 protein (Fragment) OS=Homo sapiens GN=SCAMP3 PE=2 SV=1	38674	814	0	42	0	0.18	0	0	1	0	2	0	2	0	2	0	-1.66312
tr|B2ZZ90|B2ZZ90_HUMAN	ACACA	Acetyl-Coenzyme A carboxylase alpha OS=Homo sapiens GN=ACACA PE=2 SV=1	267095	817	0	42	0	0.04	0	0	0	0	3	0	3	0	4	0	-2.14875
tr|Q5VVC8|Q5VVC8_HUMAN	RPL11	60S ribosomal protein L11 (Fragment) OS=Homo sapiens GN=RPL11 PE=4 SV=1	20167	820	0	42	0	0.17	0	0	1	0	1	0	1	0	1	0	-0.92595
tr|B1AMX9|B1AMX9_HUMAN	TRAF2	TNF receptor-associated factor 2 OS=Homo sapiens GN=TRAF2 PE=4 SV=1	47848	821	0	42	0	0.07	0	0	1	0	1	0	1	0	1	0	-0.92595
tr|B2RBE0|B2RBE0_HUMAN	tr|B2RBE0	cDNA, FLJ95462, highly similar to Homo sapiens fatty-acid-Coenzyme A ligase, long-chain 3 (FACL3),mRNA OS=Homo sapiens PE=2 SV=1	81266	822	0	42	0	0.04	0	0	1	0	1	0	1	0	1	0	-0.92595
tr|H0Y5B0|H0Y5B0_HUMAN	EPB41L2	Band 4.1-like protein 2 (Fragment) OS=Homo sapiens GN=EPB41L2 PE=4 SV=1	42169	823	0	0	0	0.05	0	0	0	0	0	0	0	0	1	0	0.659215
tr|F8W9Y9|F8W9Y9_HUMAN	MTA1	Metastasis-associated protein MTA1 OS=Homo sapiens GN=MTA1 PE=2 SV=1	28772	824	0	0	0	0.06	0	0	0	0	0	0	1	0	1	0	-0.92595
tr|Q6P1Q5|Q6P1Q5_HUMAN	ARCN1	ARCN1 protein OS=Homo sapiens GN=ARCN1 PE=2 SV=1	22011	825	0	26	0	0.12	0	0	0	0	1	0	1	0	2	0	-0.92595
tr|Q14DU5|Q14DU5_HUMAN	ROCK2	ROCK2 protein OS=Homo sapiens GN=ROCK2 PE=2 SV=1	82724	826	0	41	0	0.04	0	0	1	0	1	0	1	0	3	0	-0.92595
sp|O60287|NPA1P_HUMAN	URB1	Nucleolar pre-ribosomal-associated protein 1 OS=Homo sapiens GN=URB1 PE=1 SV=4	256508	827	0	41	0	0.01	0	0	1	0	1	0	1	0	2	0	-0.92595
tr|B4DW56|B4DW56_HUMAN	tr|B4DW56	cDNA FLJ50890, highly similar to Mitochondrial 39S ribosomal protein L3 OS=Homo sapiens PE=2 SV=1	27262	828	0	0	0	0.08	0	0	0	0	0	0	1	0	1	0	-0.92595
tr|B4DP32|B4DP32_HUMAN	tr|B4DP32	cDNA FLJ56786, moderately similar to 40S ribosomal protein S16 OS=Homo sapiens PE=2 SV=1	13727	829	0	41	0	0.25	0	0	1	0	1	0	1	0	2	0	-0.92595
tr|B7Z871|B7Z871_HUMAN	tr|B7Z871	cDNA FLJ50930, highly similar to Lipoma-preferred partner OS=Homo sapiens PE=2 SV=1	49854	831	0	41	0	0.07	0	0	1	0	1	0	1	0	1	0	-0.92595
sp|A3KN83|SBNO1_HUMAN	SBNO1	Protein strawberry notch homolog 1 OS=Homo sapiens GN=SBNO1 PE=1 SV=1	154900	833	0	41	0	0.02	0	0	1	0	1	0	3	0	3	0	-2.14875
tr|K7ES28|K7ES28_HUMAN	SBNO2	Protein strawberry notch homolog 2 OS=Homo sapiens GN=SBNO2 PE=4 SV=1	150363	834	0	41	0	0.02	0	0	1	0	1	0	2	0	2	0	-1.66312
tr|B4DHJ9|B4DHJ9_HUMAN	tr|B4DHJ9	cDNA FLJ61262, highly similar to Ubiquitin-protein ligase E3C (EC 6.3.2.-) OS=Homo sapiens PE=2 SV=1	107426	835	0	41	0	0.03	0	0	1	0	1	0	1	0	1	0	-0.92595
tr|B3KNJ4|B3KNJ4_HUMAN	SAE1	SUMO-1 activating enzyme subunit 1, isoform CRA_a OS=Homo sapiens GN=SAE1 PE=2 SV=1	33705	836	0	40	0	0.21	0	0	1	0	2	0	1	0	2	0	-0.92595
sp|Q14739|LBR_HUMAN	LBR	Lamin-B receptor OS=Homo sapiens GN=LBR PE=1 SV=2	71057	837	0	40	0	0.05	0	0	1	0	1	0	1	0	1	0	-0.92595
sp|O76003|GLRX3_HUMAN	GLRX3	Glutaredoxin-3 OS=Homo sapiens GN=GLRX3 PE=1 SV=2	37693	838	0	40	0	0.09	0	0	1	0	1	0	2	0	2	0	-1.66312
sp|P35251|RFC1_HUMAN	RFC1	Replication factor C subunit 1 OS=Homo sapiens GN=RFC1 PE=1 SV=4	128688	839	0	40	0	0.03	0	0	1	0	1	0	3	0	3	0	-2.14875
tr|Q5QTS3|Q5QTS3_HUMAN	tr|Q5QTS3	FWP004 OS=Homo sapiens PE=2 SV=1	23704	843	0	40	0	0.3	0	0	1	0	2	0	3	0	3	0	-2.14875
tr|G3V1S7|G3V1S7_HUMAN	HAS1	Hyaluronan synthase 1 OS=Homo sapiens GN=HAS1 PE=2 SV=1	65517	845	0	40	0	0.05	0	0	1	0	1	0	1	0	1	0	-0.92595
tr|B1AR80|B1AR80_HUMAN	UAP1	UDP-N-acetylhexosamine pyrophosphorylase (Fragment) OS=Homo sapiens GN=UAP1 PE=4 SV=1	25341	846	0	0	0	0.06	0	0	0	0	0	0	0	0	1	0	0.659215
sp|P51398|RT29_HUMAN	DAP3	28S ribosomal protein S29, mitochondrial OS=Homo sapiens GN=DAP3 PE=1 SV=1	45880	847	0	40	0	0.07	0	0	1	0	1	0	1	0	1	0	-0.92595
tr|B7ZCA0|B7ZCA0_HUMAN	SYNGAP1	Ras GTPase-activating protein SynGAP OS=Homo sapiens GN=SYNGAP1 PE=2 SV=1	143744	849	0	40	0	0.02	0	0	1	0	1	0	1	0	2	0	-0.92595
tr|H3BND9|H3BND9_HUMAN	PCBP2	Poly(rC)-binding protein 2 (Fragment) OS=Homo sapiens GN=PCBP2 PE=4 SV=1	12425	851	0	40	0	0.28	0	0	1	0	1	0	1	0	3	0	-0.92595
tr|B3KPZ0|B3KPZ0_HUMAN	tr|B3KPZ0	cDNA FLJ32485 fis, clone SKNMC2001596, highly similar to Ankyrin repeat and FYVE domain-containing protein 1 (Fragment) OS=Homo sap	121468	853	0	39	0	0.03	0	0	1	0	1	0	1	0	1	0	-0.92595
sp|Q6ZU15|SEP14_HUMAN	Sep-14	Septin-14 OS=Homo sapiens GN=SEPT14 PE=1 SV=2	50449	854	0	39	0	0.07	0	0	2	0	2	0	2	0	3	0	-1.66312
sp|Q7Z3U7|MON2_HUMAN	MON2	Protein MON2 homolog OS=Homo sapiens GN=MON2 PE=1 SV=2	192475	855	0	39	0	0.03	0	0	1	0	2	0	2	0	3	0	-1.66312
tr|E5RHH7|E5RHH7_HUMAN	ARFGAP1	ADP-ribosylation factor GTPase-activating protein 1 (Fragment) OS=Homo sapiens GN=ARFGAP1 PE=2 SV=1	16483	856	0	39	0	0.21	0	0	1	0	1	0	1	0	1	0	-0.92595
tr|Q4LE79|Q4LE79_HUMAN	DSP	DSP variant protein (Fragment) OS=Homo sapiens GN=DSP variant protein PE=2 SV=1	267367	857	0	39	0	0.01	0	0	1	0	1	0	1	0	2	0	-0.92595
tr|B4DJP0|B4DJP0_HUMAN	tr|B4DJP0	cDNA FLJ60601, highly similar to Methylosome protein 50 OS=Homo sapiens PE=2 SV=1	12271	860	0	39	0	0.28	0	0	1	0	1	0	1	0	1	0	-0.92595
tr|A6NKV8|A6NKV8_HUMAN	PAIP1	Polyadenylate-binding protein-interacting protein 1 OS=Homo sapiens GN=PAIP1 PE=2 SV=1	42379	861	0	39	0	0.08	0	0	1	0	1	0	1	0	1	0	-0.92595
tr|Q6FGY5|Q6FGY5_HUMAN	CKAP1	CKAP1 protein OS=Homo sapiens GN=CKAP1 PE=2 SV=1	21959	862	0	39	0	0.15	0	0	1	0	1	0	1	0	1	0	-0.92595
tr|B7Z8Z8|B7Z8Z8_HUMAN	tr|B7Z8Z8	cDNA FLJ61702, highly similar to 80 kDa MCM3-associated protein OS=Homo sapiens PE=2 SV=1	181264	863	0	39	0	0.02	0	0	1	0	1	0	4	0	5	0	-2.51152
tr|B0AZM0|B0AZM0_HUMAN	tr|B0AZM0	cDNA, FLJ79460, highly similar to Voltage-gated potassium channel subunitbeta-2 OS=Homo sapiens PE=2 SV=1	39027	865	0	39	0	0.08	0	0	1	0	1	0	1	0	1	0	-0.92595
sp|Q96RW7|HMCN1_HUMAN	HMCN1	Hemicentin-1 OS=Homo sapiens GN=HMCN1 PE=1 SV=2	623265	867	0	38	0	0.01	0	0	1	0	1	0	3	0	4	0	-2.14875
tr|A0MNN4|A0MNN4_HUMA	SMU1	CDW3/SMU1 OS=Homo sapiens GN=SMU1 PE=2 SV=1	58134	869	0	38	0	0.18	0	0	0	0	3	0	4	0	5	0	-2.51152
tr|F8VQY0|F8VQY0_HUMAN	ATP5B	ATP synthase subunit beta, mitochondrial (Fragment) OS=Homo sapiens GN=ATP5B PE=2 SV=1	23397	873	0	38	0	0.09	0	0	1	0	1	0	1	0	1	0	-0.92595
sp|Q9UKX7|NUP50_HUMAN	NUP50	Nuclear pore complex protein Nup50 OS=Homo sapiens GN=NUP50 PE=1 SV=2	50512	874	0	38	0	0.07	0	0	1	0	1	0	1	0	1	0	-0.92595
tr|A8K6K7|A8K6K7_HUMAN	tr|A8K6K7	cDNA FLJ76881, highly similar to Homo sapiens glycogen synthase 1 (muscle) (GYS1), mRNA OS=Homo sapiens PE=2 SV=1	84559	875	0	38	0	0.04	0	0	1	0	1	0	1	0	1	0	-0.92595
tr|B4DZ88|B4DZ88_HUMAN	KTN1	KTN1 protein OS=Homo sapiens GN=KTN1 PE=2 SV=1	153179	876	0	38	0	0.02	0	0	1	0	1	0	2	0	2	0	-1.66312
tr|F6VDE0|F6VDE0_HUMAN	SMARCA2	Probable global transcription activator SNF2L2 (Fragment) OS=Homo sapiens GN=SMARCA2 PE=2 SV=1	61946	879	0	0	0	0.03	0	0	0	0	0	0	1	0	3	0	-0.92595
tr|J3KT10|J3KT10_HUMAN	NUP85	Nuclear pore complex protein Nup85 OS=Homo sapiens GN=NUP85 PE=4 SV=1	70931	880	0	37	0	0.05	0	0	1	0	1	0	1	0	1	0	-0.92595
tr|B4DUV6|B4DUV6_HUMAN	TRMT6	cDNA FLJ51319, highly similar to tRNA (adenine-N(1)-)-methyltransferase non-catalytic subunit TRM6 OS=Homo sapiens GN=TRMT6 PE=2 S	36701	881	0	37	0	0.09	0	0	1	0	1	0	1	0	2	0	-0.92595
tr|Q5VXN0|Q5VXN0_HUMAN	BXDC1	Brix domain containing 1 (Fragment) OS=Homo sapiens GN=BXDC1 PE=4 SV=1	24760	882	0	37	0	0.14	0	0	1	0	1	0	2	0	3	0	-1.66312

sp|Q9ULD0|OGDHL_HUMAN	OGDHL	2-oxoglutarate dehydrogenase-like, mitochondrial OS=Homo sapiens GN=OGDHL PE=1 SV=3	115264	886	0	37	0	0.05	0	0	1	0	1	0	1	0	1	0	-0.92595
sp|Q96JM3|CHAP1_HUMAN	CHAMP1	Chromosome alignment-maintaining phosphoprotein 1 OS=Homo sapiens GN=CHAMP1 PE=1 SV=2	90012	888	0	37	0	0.04	0	0	1	0	1	0	1	0	1	0	-0.92595
tr|Q6ZPD6|Q6ZPD6_HUMAN	tr|Q6ZPD6	cDNA FLJ25992 fis, clone DMC03508 OS=Homo sapiens PE=2 SV=1	41924	890	0	37	0	0.08	0	0	1	0	1	0	1	0	2	0	-0.92595
sp|Q9P0N8|MARH2_HUMAN	Mar-02	E3 ubiquitin-protein ligase MARCH2 OS=Homo sapiens GN=MARCH2 PE=1 SV=1	27947	891	0	37	0	0.12	0	0	1	0	1	0	1	0	1	0	-0.92595
tr|B2R4C1|B2R4C1_HUMAN	tr|B2R4C1	cDNA, FLJ92036, highly similar to Homo sapiens ribosomal protein L31 (RPL31), mRNA OS=Homo sapiens PE=2 SV=1	14486	893	0	36	0	0.23	0	0	1	0	1	0	1	0	1	0	-0.92595
tr|B4DQZ7|B4DQZ7_HUMAN	tr|B4DQZ7	cDNA FLJ50838, highly similar to Apoptotic chromatin condensation inducer in thenucleus (Fragment) OS=Homo sapiens PE=2 SV=1	122827	896	0	36	0	0.02	0	0	1	0	1	0	1	0	2	0	-0.92595
sp|Q9BPU6|DPYL5_HUMAN	DPYSL5	Dihydropyrimidinase-related protein 5 OS=Homo sapiens GN=DPYSL5 PE=1 SV=1	61952	897	0	36	0	0.05	0	0	0	0	1	0	2	0	3	0	-1.66312
tr|D6RA26|D6RA26_HUMAN	PLRG1	Pleiotropic regulator 1 (Fragment) OS=Homo sapiens GN=PLRG1 PE=2 SV=1	23562	898	0	36	0	0.06	0	0	1	0	1	0	1	0	1	0	-0.92595
sp|Q9UI26|IPO11_HUMAN	IPO11	Importin-11 OS=Homo sapiens GN=IPO11 PE=1 SV=1	113489	899	0	36	0	0.06	0	0	0	0	2	0	3	0	4	0	-2.14875
tr|Q96HI4|Q96HI4_HUMAN	NMT1	Glycylpeptide N-tetradecanoyltransferase (Fragment) OS=Homo sapiens GN=NMT1 PE=2 SV=2	56910	900	0	36	0	0.06	0	0	0	0	1	0	1	0	2	0	-0.92595
tr|H0YNU5|H0YNU5_HUMAN	BLM	Bloom syndrome protein OS=Homo sapiens GN=BLM PE=4 SV=1	145978	904	0	36	0	0.02	0	0	1	0	1	0	1	0	1	0	-0.92595
tr|H3BM14|H3BM14_HUMAN	NUB1	NEDD8 ultimate buster 1 OS=Homo sapiens GN=NUB1 PE=4 SV=1	72732	906	0	36	0	0.05	0	0	0	0	1	0	1	0	2	0	-0.92595
tr|A8K3C9|A8K3C9_HUMAN	tr|A8K3C9	cDNA FLJ76709, highly similar to Homo sapiens Huntington disease-associated protein (HD) mRNA OS=Homo sapiens PE=2 SV=1	65604	907	0	0	0	0.01	0	0	0	0	0	0	0	0	1	0	0.659215
sp|Q9NYK5|RM39_HUMAN	MRPL39	39S ribosomal protein L39, mitochondrial OS=Homo sapiens GN=MRPL39 PE=1 SV=3	39200	908	0	35	0	0.08	0	0	1	0	1	0	1	0	1	0	-0.92595
tr|H0YJ30|H0YJ30_HUMAN	GPHN	Molybdopterin molybdenumtransferase (Fragment) OS=Homo sapiens GN=GPHN PE=4 SV=1	30436	909	0	35	0	0.11	0	0	0	0	1	0	1	0	2	0	-0.92595
tr|B3KMV5|B3KMV5_HUMAN	tr|B3KMV5	cDNA FLJ12728 fis, clone NT2RP2000040, highly similar to Protein FAM62A OS=Homo sapiens PE=2 SV=1	123342	912	0	35	0	0.03	0	0	0	0	1	0	1	0	2	0	-0.92595
tr|J3KP72|J3KP72_HUMAN	PCMT1	Protein-L-isoaspartate O-methyltransferase OS=Homo sapiens GN=PCMT1 PE=3 SV=1	30524	913	0	35	0	0.11	0	0	0	0	1	0	1	0	1	0	-0.92595
tr|B3KRZ3|B3KRZ3_HUMAN	IMPDH	Inosine-5~-monophosphate dehydrogenase OS=Homo sapiens GN=IMPDH PE=2 SV=1	56729	914	0	35	0	0.06	0	0	1	0	1	0	1	0	1	0	-0.92595
tr|Q5T7U4|Q5T7U4_HUMAN	GTF3C5	General transcription factor 3C polypeptide 5 (Fragment) OS=Homo sapiens GN=GTF3C5 PE=4 SV=1	28666	915	0	35	0	0.12	0	0	0	0	1	0	1	0	1	0	-0.92595
tr|B4DDH9|B4DDH9_HUMAN	tr|B4DDH9	cDNA FLJ53820, highly similar to U4/U6.U5 tri-snRNP-associated protein 1 OS=Homo sapiens PE=2 SV=1	73566	916	0	35	0	0.04	0	0	0	0	1	0	2	0	2	0	-1.66312
tr|D3DS96|D3DS96_HUMAN	BAZ1A	Bromodomain adjacent to zinc finger domain, 1A, isoform CRA_c OS=Homo sapiens GN=BAZ1A PE=2 SV=1	180117	917	0	35	0	0.04	0	0	0	0	2	0	2	0	3	0	-1.66312
tr|B4E2E1|B4E2E1_HUMAN	tr|B4E2E1	cDNA FLJ54322, highly similar to Protein transport protein Sec24B OS=Homo sapiens PE=2 SV=1	97141	919	0	35	0	0.14	0	0	0	0	4	0	4	0	4	0	-2.51152
sp|Q14160|SCRIB_HUMAN	SCRIB	Protein scribble homolog OS=Homo sapiens GN=SCRIB PE=1 SV=4	175748	920	0	35	0	0.04	0	0	1	0	2	0	1	0	2	0	-0.92595
tr|A0JLS5|A0JLS5_HUMAN	TBL3	TBL3 protein (Fragment) OS=Homo sapiens GN=TBL3 PE=2 SV=1	64079	922	0	35	0	0.05	0	0	1	0	1	0	1	0	1	0	-0.92595
tr|H3BP78|H3BP78_HUMAN	FANCI	Fanconi anemia group I protein (Fragment) OS=Homo sapiens GN=FANCI PE=4 SV=1	124065	923	0	35	0	0.08	0	0	0	0	3	0	4	0	5	0	-2.51152
tr|Q2YD88|Q2YD88_HUMAN	FAM129B	FAM129B protein (Fragment) OS=Homo sapiens GN=FAM129B PE=2 SV=1	35264	924	0	35	0	0.09	0	0	0	0	1	0	1	0	1	0	-0.92595
tr|Q2TAM5|Q2TAM5_HUMAN	RELA	RELA protein OS=Homo sapiens GN=RELA PE=2 SV=1	43355	925	0	35	0	0.08	0	0	0	0	1	0	1	0	1	0	-0.92595
tr|Q5JY65|Q5JY65_HUMAN	CRNKL1	Crooked neck-like protein 1 OS=Homo sapiens GN=CRNKL1 PE=4 SV=1	99625	929	0	34	0	0.03	0	0	0	0	1	0	1	0	1	0	-0.92595
tr|K7EP06|K7EP06_HUMAN	RNMT	mRNA cap guanine-N7 methyltransferase OS=Homo sapiens GN=RNMT PE=4 SV=1	35330	930	0	34	0	0.2	0	0	0	0	2	0	2	0	2	0	-1.66312
tr|B4DH39|B4DH39_HUMAN	tr|B4DH39	cDNA FLJ57028, highly similar to Long-chain-fatty-acid--CoA ligase 4 (EC 6.2.1.3) OS=Homo sapiens PE=2 SV=1	73123	931	0	34	0	0.09	0	0	1	0	2	0	2	0	3	0	-1.66312
sp|P0C0S8|H2A1_HUMAN	HIST1H2AG	Histone H2A type 1 OS=Homo sapiens GN=HIST1H2AG PE=1 SV=2	14083	932	0	34	0	0.24	0	0	1	0	1	0	1	0	1	0	-0.92595
tr|Q5U0I6|Q5U0I6_HUMAN	RAB1A	RAB1A protein OS=Homo sapiens GN=RAB1A PE=2 SV=1	22891	934	0	34	0	0.15	0	0	0	0	1	0	1	0	1	0	-0.92595
tr|Q6AWA7|Q6AWA7_HUMA	DKFZp686J	Putative uncharacterized protein DKFZp686J1525 (Fragment) OS=Homo sapiens GN=DKFZp686J1525 PE=2 SV=1	52127	935	0	34	0	0.06	0	0	0	0	1	0	1	0	1	0	-0.92595
tr|B3KPX5|B3KPX5_HUMAN	tr|B3KPX5	cDNA FLJ32418 fis, clone SKMUS2000863, weakly similar to PROBABLE ASPARAGINYL-TRNA SYNTHETASE (EC 6.1.1.22) OS=Homo sapiens PE	54523	936	0	34	0	0.06	0	0	1	0	1	0	1	0	1	0	-0.92595
tr|F8W1U3|F8W1U3_HUMAN	KRT8	Keratin, type II cytoskeletal 8 (Fragment) OS=Homo sapiens GN=KRT8 PE=2 SV=1	32586	937	0	34	0	0.21	0	0	1	0	2	0	2	0	2	0	-1.66312
tr|E7ETK0|E7ETK0_HUMAN	RPS24	40S ribosomal protein S24 OS=Homo sapiens GN=RPS24 PE=2 SV=1	15187	938	0	34	0	0.22	0	0	0	0	1	0	1	0	1	0	-0.92595
tr|K7ES11|K7ES11_HUMAN	UBE2O	Ubiquitin-conjugating enzyme E2 O (Fragment) OS=Homo sapiens GN=UBE2O PE=4 SV=1	89434	939	0	34	0	0.07	0	0	1	0	2	0	2	0	4	0	-1.66312
tr|B5BU44|B5BU44_HUMAN	CRADD	CASP2 and RIPK1 domain containing adaptor with death domain OS=Homo sapiens GN=CRADD PE=2 SV=1	22774	941	0	34	0	0.15	0	0	0	0	1	0	1	0	1	0	-0.92595
tr|B4DE91|B4DE91_HUMAN	tr|B4DE91	cDNA FLJ55534, highly similar to 4-trimethylaminobutyraldehyde dehydrogenase (EC 1.2.1.47) OS=Homo sapiens PE=2 SV=1	53648	942	0	34	0	0.06	0	0	0	0	1	0	2	0	3	0	-1.66312
tr|Q5SXN0|Q5SXN0_HUMAN	PMPCA	Mitochondrial-processing peptidase subunit alpha OS=Homo sapiens GN=PMPCA PE=4 SV=1	15995	945	0	0	0	0.12	0	0	0	0	0	0	0	0	1	0	0.659215
sp|A6NJG6|ARGFX_HUMAN	ARGFX	Arginine-fifty homeobox OS=Homo sapiens GN=ARGFX PE=2 SV=1	35766	946	0	33	0	0.09	0	0	1	0	1	0	1	0	1	0	-0.92595
tr|H0YH15|H0YH15_HUMAN	AQR	Aquarius homolog (Mouse), isoform CRA_a OS=Homo sapiens GN=AQR PE=4 SV=1	76390	947	0	0	0	0.02	0	0	0	0	0	0	1	0	2	0	-0.92595
sp|Q86VV8|RTTN_HUMAN	RTTN	Rotatin OS=Homo sapiens GN=RTTN PE=1 SV=3	252290	948	0	33	0	0.01	0	0	1	0	1	0	2	0	2	0	-1.66312
tr|J3QQY1|J3QQY1_HUMAN	CDK5RAP3	CDK5 regulatory subunit-associated protein 3 (Fragment) OS=Homo sapiens GN=CDK5RAP3 PE=4 SV=1	32228	951	0	33	0	0.1	0	0	1	0	1	0	2	0	2	0	-1.66312
tr|K7EN59|K7EN59_HUMAN	CCDC151	Coiled-coil domain-containing protein 151 OS=Homo sapiens GN=CCDC151 PE=4 SV=1	62098	952	0	33	0	0.06	0	0	1	0	1	0	1	0	2	0	-0.92595
tr|Q8TCA8|Q8TCA8_HUMAN	ANKRD5	Ankyrin repeat domain 5 OS=Homo sapiens GN=ANKRD5 PE=2 SV=1	87252	953	0	33	0	0.04	0	0	1	0	1	0	1	0	2	0	-0.92595
sp|Q13057|COASY_HUMAN	COASY	Bifunctional coenzyme A synthase OS=Homo sapiens GN=COASY PE=1 SV=4	62632	954	0	33	0	0.05	0	0	1	0	1	0	1	0	2	0	-0.92595
tr|J3KRE2|J3KRE2_HUMAN	ARHGDIA	Rho GDP-dissociation inhibitor 1 OS=Homo sapiens GN=ARHGDIA PE=4 SV=1	14805	957	0	33	0	0.23	0	0	0	0	1	0	1	0	1	0	-0.92595
tr|D3YTE0|D3YTE0_HUMAN	KRI1	Protein KRI1 homolog OS=Homo sapiens GN=KRI1 PE=2 SV=1	83005	958	0	33	0	0.04	0	0	0	0	1	0	1	0	1	0	-0.92595
tr|Q13380|Q13380_HUMAN	RBM	RNA binding motif (Fragment) OS=Homo sapiens GN=RBM PE=4 SV=1	4015	959	0	33	0	0.97	0	0	1	0	1	0	1	0	1	0	-0.92595
tr|O00370|O00370_HUMAN	tr|O00370	Putative p150 OS=Homo sapiens PE=2 SV=1	150000	960	0	33	0	0.02	0	0	0	0	1	0	1	0	1	0	-0.92595
sp|Q9UK61|F208A_HUMAN	FAM208A	Protein FAM208A OS=Homo sapiens GN=FAM208A PE=1 SV=3	190453	962	0	33	0	0.02	0	0	0	0	1	0	1	0	2	0	-0.92595
tr|F2Z2Q4|F2Z2Q4_HUMAN	TRMT11	tRNA (guanine(10)-N2)-methyltransferase homolog OS=Homo sapiens GN=TRMT11 PE=2 SV=1	39270	963	0	33	0	0.08	0	0	0	0	1	0	1	0	2	0	-0.92595
tr|Q5SV16|Q5SV16_HUMAN	ATAD3A	ATPase family AAA domain-containing protein 3A (Fragment) OS=Homo sapiens GN=ATAD3A PE=4 SV=1	22896	964	0	33	0	0.05	0	0	0	0	1	0	1	0	1	0	-0.92595
sp|Q5T2N8|ATD3C_HUMAN	ATAD3C	ATPase family AAA domain-containing protein 3C OS=Homo sapiens GN=ATAD3C PE=2 SV=2	46635	965	0	33	0	0.07	0	0	0	0	1	0	1	0	2	0	-0.92595
sp|P52948|NUP98_HUMAN	NUP98	Nuclear pore complex protein Nup98-Nup96 OS=Homo sapiens GN=NUP98 PE=1 SV=4	198597	966	0	33	0	0.03	0	0	0	0	2	0	3	0	3	0	-2.14875
tr|B7ZMI1|B7ZMI1_HUMAN	MED12L	MED12L protein OS=Homo sapiens GN=MED12L PE=2 SV=1	206075	970	0	32	0	0.02	0	0	0	0	1	0	1	0	1	0	-0.92595
tr|B3KUU8|B3KUU8_HUMAN	tr|B3KUU8	cDNA FLJ40670 fis, clone THYMU2020959, highly similar to Dynamin-2 OS=Homo sapiens PE=2 SV=1	67964	972	0	32	0	0.05	0	0	0	0	1	0	2	0	2	0	-1.66312
tr|Q6ZWH1|Q6ZWH1_HUMA	tr|Q6ZWH	cDNA FLJ41081 fis, clone ADRGL2009755, highly similar to Homo sapiens brain and reproductive organ-expressed protein (BRE) mRNA OS=I	45824	973	0	32	0	0.07	0	0	0	0	1	0	1	0	1	0	-0.92595
tr|B4DX96|B4DX96_HUMAN	tr|B4DX96	cDNA FLJ57646, highly similar to Zinc finger protein 38 OS=Homo sapiens PE=2 SV=1	51790	974	0	32	0	0.06	0	0	0	0	1	0	1	0	1	0	-0.92595
tr|B2RAG9|B2RAG9_HUMAN	tr|B2RAG9	cDNA, FLJ94908, highly similar to Homo sapiens PPAR binding protein (PPARBP), mRNA OS=Homo sapiens PE=2 SV=1	167569	977	0	32	0	0.02	0	0	1	0	1	0	1	0	1	0	-0.92595
tr|J3QT54|J3QT54_HUMAN	CPSF7	Cleavage and polyadenylation-specificity factor subunit 7 (Fragment) OS=Homo sapiens GN=CPSF7 PE=4 SV=1	22762	978	0	32	0	0.15	0	0	1	0	1	0	1	0	1	0	-0.92595
tr|E9PC74|E9PC74_HUMAN	EIF2B5	Translation initiation factor eIF-2B subunit epsilon OS=Homo sapiens GN=EIF2B5 PE=2 SV=1	79096	979	0	32	0	0.04	0	0	1	0	1	0	1	0	2	0	-0.92595
tr|A4FVC0|A4FVC0_HUMAN	EIF2C2	EIF2C2 protein (Fragment) OS=Homo sapiens GN=EIF2C2 PE=2 SV=1	92636	980	0	32	0	0.04	0	0	0	0	1	0	1	0	1	0	-0.92595
tr|Q9UPE4|Q9UPE4_HUMAN	hTIM44	Putative mitochondrial inner membrane protein import receptor OS=Homo sapiens GN=hTIM44 PE=2 SV=1	47508	981	0	32	0	0.07	0	0	0	0	1	0	1	0	2	0	-0.92595
tr|B7Z5S1|B7Z5S1_HUMAN	tr|B7Z5S1	cDNA FLJ61699, highly similar to Homo sapiens androgen-induced proliferation inhibitor (APRIN), transcript variant 1, mRNA OS=Homo sap	149900	984	0	32	0	0.02	0	0	1	0	1	0	1	0	1	0	-0.92595
sp|Q8N122|RPTOR_HUMAN	RPTOR	Regulatory-associated protein of mTOR OS=Homo sapiens GN=RPTOR PE=1 SV=1	150653	987	0	31	0	0.02	0	0	0	0	1	0	1	0	3	0	-0.92595
tr|G3V346|G3V346_HUMAN	SRP54	Signal recognition particle 54 kDa protein (Fragment) OS=Homo sapiens GN=SRP54 PE=2 SV=1	4785	988	0	31	0	0.77	0	0	0	0	1	0	1	0	1	0	-0.92595
tr|B4DEF4|B4DEF4_HUMAN	tr|B4DEF4	cDNA FLJ60434, highly similar to Cleavage and polyadenylation specificity factor 160 kDa subunit OS=Homo sapiens PE=2 SV=1	36825	990	0	0	0	0.02	0	0	0	0	0	0	0	0	1	0	0.659215
sp|Q7L0Y3|MRRP1_HUMAN	TRMT10C	Mitochondrial ribonuclease P protein 1 OS=Homo sapiens GN=TRMT10C PE=1 SV=2	47602	991	0	31	0	0.07	0	0	0	0	1	0	2	0	2	0	-1.66312
sp|Q92526|TCPW_HUMAN	CCT6B	T-complex protein 1 subunit zeta-2 OS=Homo sapiens GN=CCT6B PE=1 SV=5	58355	992	0	31	0	0.06	0	0	0	0	1	0	3	0	4	0	-2.14875
tr|K7EIG7|K7EIG7_HUMAN	MYO1D	Unconventional myosin-Id OS=Homo sapiens GN=MYO1D PE=4 SV=1	106738	993	0	31	0	0.06	0	0	0	0	2	0	2	0	3	0	-1.66312
tr|B2R6G5|B2R6G5_HUMAN	tr|B2R6G5	cDNA, FLJ92942, highly similar to Homo sapiens T-box, brain, 1 (TBR1), mRNA OS=Homo sapiens PE=2 SV=1	74530	994	0	31	0	0.04	0	0	0	0	1	0	1	0	1	0	-0.92595
tr|Q05DL3|Q05DL3_HUMAN	CSNK1A1	CSNK1A1 protein (Fragment) OS=Homo sapiens GN=CSNK1A1 PE=2 SV=1	26799	995	0	31	0	0.12	0	0	1	0	1	0	1	0	1	0	-0.92595
tr|F5GZI5|F5GZI5_HUMAN	ZAN	Zonadhesin OS=Homo sapiens GN=ZAN PE=2 SV=1	299445	997	0	31	0	0.01	0	0	0	0	1	0	1	0	3	0	-0.92595
sp|Q9NSB4|KRT82_HUMAN	KRT82	Keratin, type II cuticular Hb2 OS=Homo sapiens GN=KRT82 PE=1 SV=3	57985	999	0	31	0	0.06	0	0	0	0	1	0	1	0	1	0	-0.92595
tr|J3KSL8|J3KSL8_HUMAN	TRAPPC8	Trafficking protein particle complex subunit 8 OS=Homo sapiens GN=TRAPPC8 PE=4 SV=1	105091	1000	0	31	0	0.03	0	0	0	0	1	0	1	0	2	0	-0.92595
tr|B7ZKT9|B7ZKT9_HUMAN	KIAA1033	KIAA1033 protein OS=Homo sapiens GN=KIAA1033 PE=2 SV=1	137456	1003	0	30	0	0.02	0	0	0	0	1	0	1	0	1	0	-0.92595
tr|B4DS83|B4DS83_HUMAN	tr|B4DS83	cDNA FLJ53179, highly similar to Nucleolar protein 10 OS=Homo sapiens PE=2 SV=1	74879	1004	0	30	0	0.04	0	0	0	0	1	0	1	0	1	0	-0.92595
tr|G1UI18|G1UI18_HUMAN	PSME4	Proteasome activator complex subunit 4 (Fragment) OS=Homo sapiens GN=PSME4 PE=2 SV=1	130054	1006	0	30	0	0.03	0	0	1	0	1	0	1	0	1	0	-0.92595
tr|B4DGY8|B4DGY8_HUMAN	tr|B4DGY8	cDNA FLJ53528 OS=Homo sapiens PE=2 SV=1	22981	1007	0	30	0	0.15	0	0	0	0	1	0	1	0	1	0	-0.92595
tr|B0QZ18|B0QZ18_HUMAN	CPNE1	Copine I OS=Homo sapiens GN=CPNE1 PE=2 SV=1	60307	1008	0	30	0	0.05	0	0	0	0	1	0	2	0	3	0	-1.66312
tr|H0Y5B5|H0Y5B5_HUMAN	PBRM1	Protein polybromo-1 (Fragment) OS=Homo sapiens GN=PBRM1 PE=4 SV=1	127011	1009	0	30	0	0.03	0	0	0	0	1	0	1	0	1	0	-0.92595
tr|E9PDU6|E9PDU6_HUMAN	CNN3	Calponin-3 (Fragment) OS=Homo sapiens GN=CNN3 PE=2 SV=1	20309	1011	0	30	0	0.1	0	0	0	0	1	0	1	0	1	0	-0.92595
tr|H0YJC0|H0YJC0_HUMAN	PSMC6	26S protease regulatory subunit 10B (Fragment) OS=Homo sapiens GN=PSMC6 PE=4 SV=1	29919	1012	0	30	0	0.11	0	0	0	0	1	0	1	0	1	0	-0.92595
tr|B4E0H3|B4E0H3_HUMAN	tr|B4E0H3	cDNA FLJ53386, highly similar to Signal recognition particle receptor subunit alpha OS=Homo sapiens PE=2 SV=1	66987	1014	0	30	0	0.1	0	0	0	0	2	0	2	0	2	0	-1.66312
sp|Q9BTE3|MCMBP_HUMAN	MCMBP	Mini-chromosome maintenance complex-binding protein OS=Homo sapiens GN=MCMBP PE=1 SV=2	73789	1015	0	30	0	0.04	0	0	0	0	1	0	1	0	1	0	-0.92595
tr|H7C441|H7C441_HUMAN	THOC5	THO complex subunit 5 homolog (Fragment) OS=Homo sapiens GN=THOC5 PE=4 SV=1	22943	1016	0	29	0	0.15	0	0	0	0	1	0	3	0	3	0	-2.14875
tr|B4DFM1|B4DFM1_HUMAN	tr|B4DFM1	cDNA FLJ53034, highly similar to AP-2 complex subunit mu-1 OS=Homo sapiens PE=2 SV=1	44226	1018	0	29	0	0.07	0	0	0	0	1	0	1	0	1	0	-0.92595
tr|Q4G0X8|Q4G0X8_HUMAN	BRD4	BRD4 protein OS=Homo sapiens GN=BRD4 PE=2 SV=1	89032	1019	0	29	0	0.04	0	0	0	0	1	0	1	0	2	0	-0.92595
tr|B4DEF9|B4DEF9_HUMAN	tr|B4DEF9	cDNA FLJ59241, highly similar to Transducin-like enhancer protein 1 OS=Homo sapiens PE=2 SV=1	82574	1020	0	20	0	0.14	0	0	0	0	1	0	1	0	1	0	-0.92595
tr|J3KNI9|J3KNI9_HUMAN	TBC1D15	TBC1 domain family member 15 OS=Homo sapiens GN=TBC1D15 PE=4 SV=1	79491	1021	0	29	0	0.08	0	0	0	0	2	0	2	0	2	0	-1.66312
tr|H3BQ23|H3BQ23_HUMAN	CIAPIN1	Anamorsin (Fragment) OS=Homo sapiens GN=CIAPIN1 PE=4 SV=1	13738	1022	0	29	0	0.25	0	0	0	0	1	0	1	0	1	0	-0.92595
tr|B4DXG8|B4DXG8_HUMAN	tr|B4DXG8	cDNA FLJ55260, highly similar to Exosome complex exonuclease RRP45 (EC 3.1.13.-) OS=Homo sapiens PE=2 SV=1	44161	1023	0	29	0	0.07	0	0	0	0	1	0	1	0	1	0	-0.92595
tr|H7C4M1|H7C4M1_HUMAN	KLC4	Kinesin light chain 4 (Fragment) OS=Homo sapiens GN=KLC4 PE=4 SV=1	18173	1025	0	29	0	0.05	0	0	0	0	1	0	1	0	1	0	-0.92595
tr|B2R4D8|B2R4D8_HUMAN	tr|B2R4D8	60S ribosomal protein L27 OS=Homo sapiens PE=2 SV=1	15760	1027	0	29	0	0.22	0	0	0	0	1	0	1	0	3	0	-0.92595
tr|H3BS86|H3BS86_HUMAN	STUB1	E3 ubiquitin-protein ligase CHIP (Fragment) OS=Homo sapiens GN=STUB1 PE=4 SV=1	24812	1028	0	29	0	0.13	0	0	0	0	1	0	1	0	1	0	-0.92595
tr|Q5TBP5|Q5TBP5_HUMAN	TAF4	TAF4 RNA polymerase II, TATA box binding protein (TBP)-associated factor, 135kDa (Fragment) OS=Homo sapiens GN=TAF4 PE=4 SV=1	97594	1029	0	29	0	0.03	0	0	0	0	1	0	1	0	1	0	-0.92595
tr|J3QSX4|J3QSX4_HUMAN	BUB3	Mitotic checkpoint protein BUB3 OS=Homo sapiens GN=BUB3 PE=4 SV=1	16498	1030	0	18	0	0.22	0	0	0	0	1	0	0	0	2	0	0.659215
sp|P49750|YLPM1_HUMAN	YLPM1	YLP motif-containing protein 1 OS=Homo sapiens GN=YLPM1 PE=1 SV=3	220077	1031	0	28	0	0.01	0	0	0	0	1	0	2	0	2	0	-1.66312
tr|H0Y8X0|H0Y8X0_HUMAN	DEK	Protein DEK (Fragment) OS=Homo sapiens GN=DEK PE=4 SV=1	18768	1032	0	0	0	0.08	0	0	0	0	0	0	1	0	1	0	-0.92595
sp|Q9H9A6|LRC40_HUMAN	LRRC40	Leucine-rich repeat-containing protein 40 OS=Homo sapiens GN=LRRC40 PE=1 SV=1	68720	1033	0	28	0	0.05	0	0	0	0	1	0	2	0	2	0	-1.66312
tr|Q7Z4W0|Q7Z4W0_HUMAN	tr|Q7Z4W0	ATP-dependent RNA helicase OS=Homo sapiens PE=2 SV=1	53113	1034	0	28	0	0.06	0	0	0	0	1	0	2	0	2	0	-1.66312
tr|H0Y5B4|H0Y5B4_HUMAN	RPL36A	60S ribosomal protein L36a (Fragment) OS=Homo sapiens GN=RPL36A PE=3 SV=1	15576	1035	0	28	0	0.22	0	0	0	0	1	0	1	0	1	0	-0.92595
tr|H0YE81|H0YE81_HUMAN	NDUFV1	NADH dehydrogenase [ubiquinone] flavoprotein 1, mitochondrial (Fragment) OS=Homo sapiens GN=NDUFV1 PE=4 SV=1	14494	1039	0	28	0	0.23	0	0	0	0	1	0	1	0	1	0	-0.92595
sp|Q5D862|FILA2_HUMAN	FLG2	Filaggrin-2 OS=Homo sapiens GN=FLG2 PE=1 SV=1	249296	1041	0	28	0	0.01	0	0	1	0	1	0	1	0	1	0	-0.92595
tr|B4DUS9|B4DUS9_HUMAN	BPNT1	3~(2~),5~-bisphosphate nucleotidase 1 OS=Homo sapiens GN=BPNT1 PE=2 SV=1	29455	1042	0	28	0	0.11	0	0	0	0	1	0	1	0	1	0	-0.92595
tr|Q3MNE1|Q3MNE1_HUMA	tr|Q3MNE	Adaptor-related protein complex 3 beta 1 subunit (Fragment) OS=Homo sapiens PE=2 SV=1	89828	1045	0	28	0	0.04	0	0	0	0	1	0	1	0	1	0	-0.92595
tr|B4DYZ7|B4DYZ7_HUMAN	tr|B4DYZ7	cDNA FLJ54316, highly similar to DNA polymerase subunit delta 3 OS=Homo sapiens PE=2 SV=1	51595	1046	0	27	0	0.06	0	0	0	0	1	0	1	0	1	0	-0.92595
tr|J3QS89|J3QS89_HUMAN	FA2H	Fatty acid 2-hydroxylase (Fragment) OS=Homo sapiens GN=FA2H PE=4 SV=1	22573	1047	0	27	0	0.15	0	0	0	0	1	0	1	0	1	0	-0.92595
tr|B3KQF0|B3KQF0_HUMAN	tr|B3KQF0	cDNA FLJ90354 fis, clone NT2RP2003390, highly similar to Translocation protein SEC63 homolog OS=Homo sapiens PE=2 SV=1	88343	1048	0	27	0	0.04	0	0	0	0	1	0	1	0	1	0	-0.92595
tr|E5RFK7|E5RFK7_HUMAN	UBR5	E3 ubiquitin-protein ligase UBR5 (Fragment) OS=Homo sapiens GN=UBR5 PE=2 SV=1	19270	1049	0	27	0	0.01	0	0	0	0	1	0	1	0	1	0	-0.92595
tr|B7Z4A9|B7Z4A9_HUMAN	tr|B7Z4A9	cDNA FLJ60125, highly similar to Pre-B-cell leukemia transcription factor 3 OS=Homo sapiens PE=2 SV=1	46743	1050	0	27	0	0.07	0	0	0	0	1	0	1	0	1	0	-0.92595
tr|B4DYE3|B4DYE3_HUMAN	tr|B4DYE3	cDNA FLJ58091, highly similar to Chloride channel protein 2 OS=Homo sapiens PE=2 SV=1	50506	1051	0	27	0	0.07	0	0	0	0	1	0	1	0	1	0	-0.92595
sp|P80217|IN35_HUMAN	IFI35	Interferon-induced 35 kDa protein OS=Homo sapiens GN=IFI35 PE=1 SV=5	31698	1053	0	27	0	0.1	0	0	0	0	1	0	1	0	1	0	-0.92595
tr|B4E0D5|B4E0D5_HUMAN	tr|B4E0D5	cDNA FLJ54218, highly similar to Acidic leucine-rich nuclear phosphoprotein 32 family member E OS=Homo sapiens PE=2 SV=1	19540	1055	0	27	0	0.17	0	0	0	0	1	0	1	0	1	0	-0.92595
sp|P19623|SPEE_HUMAN	SRM	Spermidine synthase OS=Homo sapiens GN=SRM PE=1 SV=1	34373	1057	0	27	0	0.2	0	0	0	0	2	0	1	0	2	0	-0.92595
tr|J3KSP2|J3KSP2_HUMAN	RPL38	60S ribosomal protein L38 (Fragment) OS=Homo sapiens GN=RPL38 PE=3 SV=1	2557	1058	0	27	0	1.53	0	0	0	0	1	0	1	0	1	0	-0.92595
tr|H0YDS9|H0YDS9_HUMAN	NASP	Nuclear autoantigenic sperm protein (Fragment) OS=Homo sapiens GN=NASP PE=4 SV=1	18974	1059	0	0	0	0.1	0	0	0	0	0	0	0	0	1	0	0.659215
tr|H0YIK6|H0YIK6_HUMAN	SCYL2	SCY1-like protein 2 (Fragment) OS=Homo sapiens GN=SCYL2 PE=4 SV=1	13212	1060	0	27	0	0.26	0	0	0	0	1	0	1	0	1	0	-0.92595
tr|B5MCP9|B5MCP9_HUMAN	RPS7	40S ribosomal protein S7 OS=Homo sapiens GN=RPS7 PE=2 SV=1	21299	1062	0	26	0	0.16	0	0	0	0	1	0	1	0	1	0	-0.92595
sp|P0DJD0|RGPD1_HUMAN	RGPD1	RANBP2-like and GRIP domain-containing protein 1 OS=Homo sapiens GN=RGPD1 PE=2 SV=1	198022	1063	0	26	0	0.03	0	0	0	0	2	0	1	0	3	0	-0.92595
sp|Q8NB90|SPAT5_HUMAN	SPATA5	Spermatogenesis-associated protein 5 OS=Homo sapiens GN=SPATA5 PE=1 SV=3	98470	1065	0	26	0	0.03	0	0	0	0	1	0	2	0	2	0	-1.66312
sp|P23368|MAOM_HUMAN	ME2	NAD-dependent malic enzyme, mitochondrial OS=Homo sapiens GN=ME2 PE=1 SV=1	66029	1066	0	26	0	0.05	0	0	0	0	1	0	1	0	1	0	-0.92595
tr|H3BP71|H3BP71_HUMAN	RNF40	E3 ubiquitin-protein ligase BRE1B OS=Homo sapiens GN=RNF40 PE=4 SV=1	109696	1067	0	26	0	0.03	0	0	0	0	1	0	2	0	2	0	-1.66312
tr|E9PR18|E9PR18_HUMAN	FAM111A	Protein FAM111A (Fragment) OS=Homo sapiens GN=FAM111A PE=2 SV=1	21407	1068	0	26	0	0.16	0	0	0	0	1	0	1	0	1	0	-0.92595
tr|B4DW54|B4DW54_HUMAN	tr|B4DW54	cDNA FLJ50888, highly similar to Aldehyde dehydrogenase, mitochondrial (EC 1.2.1.3) OS=Homo sapiens PE=2 SV=1	51467	1069	0	26	0	0.06	0	0	0	0	1	0	1	0	1	0	-0.92595
tr|Q53GE1|Q53GE1_HUMAN	RANBP3	RAN binding protein 3 isoform RANBP3-a variant (Fragment) OS=Homo sapiens GN=RANBP3 PE=2 SV=1	59924	1070	0	26	0	0.07	0	0	0	0	1	0	1	0	2	0	-0.92595
sp|Q5T8P6|RBM26_HUMAN	RBM26	RNA-binding protein 26 OS=Homo sapiens GN=RBM26 PE=1 SV=3	113927	1071	0	26	0	0.03	0	0	0	0	1	0	1	0	2	0	-0.92595
tr|D3DQF6|D3DQF6_HUMAN	POU4F3	POU domain, class 4, transcription factor 3, isoform CRA_a OS=Homo sapiens GN=POU4F3 PE=4 SV=1	84396	1072	0	26	0	0.04	0	0	0	0	1	0	1	0	2	0	-0.92595
sp|Q86UY8|NT5D3_HUMAN	NT5DC3	5~-nucleotidase domain-containing protein 3 OS=Homo sapiens GN=NT5DC3 PE=2 SV=1	63721	1073	0	26	0	0.05	0	0	0	0	1	0	1	0	1	0	-0.92595
tr|Q6IBT1|Q6IBT1_HUMAN	PSMB7	Proteasome subunit beta type OS=Homo sapiens GN=PSMB7 PE=2 SV=1	30260	1075	0	26	0	0.23	0	0	0	0	2	0	2	0	2	0	-1.66312
tr|B4DN25|B4DN25_HUMAN	UGDH	UDP-glucose 6-dehydrogenase OS=Homo sapiens GN=UGDH PE=2 SV=1	44786	1077	0	26	0	0.07	0	0	0	0	1	0	1	0	1	0	-0.92595
sp|Q2TAC2|CCD57_HUMAN	CCDC57	Coiled-coil domain-containing protein 57 OS=Homo sapiens GN=CCDC57 PE=2 SV=2	103390	1079	0	26	0	0.03	0	0	0	0	1	0	1	0	1	0	-0.92595
tr|K7EMD6|K7EMD6_HUMAN	SGTA	Small glutamine-rich tetratricopeptide repeat-containing protein alpha (Fragment) OS=Homo sapiens GN=SGTA PE=4 SV=1	18009	1082	0	25	0	0.19	0	0	0	0	1	0	1	0	1	0	-0.92595
tr|Q16083|Q16083_HUMAN	tr|Q16083	U1 small nuclear ribonucleoprotein 1SNRP homolog protein (Fragment) OS=Homo sapiens PE=2 SV=1	11203	1083	0	25	0	0.03	0	0	1	0	1	0	1	0	1	0	-0.92595
tr|B4DPX9|B4DPX9_HUMAN	tr|B4DPX9	cDNA FLJ56221, highly similar to YTH domain protein 3 OS=Homo sapiens PE=2 SV=1	64596	1084	0	25	0	0.05	0	0	0	0	1	0	1	0	1	0	-0.92595
tr|H0YH61|H0YH61_HUMAN	MUC19	Mucin-19 (Fragment) OS=Homo sapiens GN=MUC19 PE=4 SV=1	11135	1086	0	25	0	0.31	0	0	0	0	1	0	1	0	1	0	-0.92595
sp|Q4KMP7|TB10B_HUMAN	TBC1D10B	TBC1 domain family member 10B OS=Homo sapiens GN=TBC1D10B PE=1 SV=3	87658	1087	0	25	0	0.04	0	0	1	0	1	0	1	0	1	0	-0.92595
tr|B0QYR0|B0QYR0_HUMAN	BTBD3	BTB/POZ domain-containing protein 3 (Fragment) OS=Homo sapiens GN=BTBD3 PE=4 SV=1	11110	1088	0	25	0	0.31	0	0	0	0	1	0	1	0	1	0	-0.92595
tr|G0XQ39|G0XQ39_HUMAN	STIM1	STIM1L OS=Homo sapiens GN=STIM1 PE=2 SV=1	88933	1089	0	25	0	0.04	0	0	0	0	1	0	1	0	2	0	-0.92595
tr|G3V5V3|G3V5V3_HUMAN	NEMF	Nuclear export mediator factor NEMF (Fragment) OS=Homo sapiens GN=NEMF PE=2 SV=1	114348	1090	0	25	0	0.03	0	0	0	0	1	0	1	0	1	0	-0.92595
tr|A8MV66|A8MV66_HUMAN	GPR123	Probable G-protein-coupled receptor 123 OS=Homo sapiens GN=GPR123 PE=3 SV=1	51296	1091	0	25	0	0.06	0	0	0	0	1	0	1	0	1	0	-0.92595
tr|H0Y792|H0Y792_HUMAN	DOPEY2	Protein dopey-2 (Fragment) OS=Homo sapiens GN=DOPEY2 PE=4 SV=1	5545	1092	0	25	0	0.7	0	0	0	0	1	0	1	0	1	0	-0.92595
tr|B4DKT5|B4DKT5_HUMAN	tr|B4DKT5	cDNA FLJ53054, highly similar to Insulin-like growth factor 2 mRNA-binding protein 2 OS=Homo sapiens PE=2 SV=1	33360	1093	0	0	0	0.05	0	0	0	0	0	0	0	0	1	0	0.659215
tr|Q59H97|Q59H97_HUMAN	tr|Q59H97	Zinc finger protein ZNF-U69274 variant (Fragment) OS=Homo sapiens PE=2 SV=1	81027	1094	0	25	0	0.04	0	0	0	0	1	0	1	0	2	0	-0.92595
tr|E9PRE2|E9PRE2_HUMAN	CTNND1	Catenin delta-1 (Fragment) OS=Homo sapiens GN=CTNND1 PE=2 SV=1	29211	1095	0	25	0	0.03	0	0	0	0	1	0	1	0	1	0	-0.92595
tr|H0Y7P1|H0Y7P1_HUMAN	MED12	Mediator of RNA polymerase II transcription subunit 12 (Fragment) OS=Homo sapiens GN=MED12 PE=4 SV=1	240034	1096	0	25	0	0.01	0	0	1	0	1	0	1	0	1	0	-0.92595
tr|B2RCJ6|B2RCJ6_HUMAN	tr|B2RCJ6	cDNA, FLJ96114, highly similar to Homo sapiens bromodomain and WD repeat domain containing 2 (BRWD2), mRNA OS=Homo sapiens PE=	138515	1097	0	24	0	0.02	0	0	0	0	1	0	1	0	1	0	-0.92595
tr|I6L959|I6L959_HUMAN	TBCD	TBCD protein OS=Homo sapiens GN=TBCD PE=2 SV=1	84715	1098	0	24	0	0.02	0	0	1	0	1	0	1	0	1	0	-0.92595
tr|H0YKD8|H0YKD8_HUMAN	RPL28	60S ribosomal protein L28 OS=Homo sapiens GN=RPL28 PE=4 SV=1	19174	1099	0	24	0	0.18	0	0	0	0	1	0	1	0	1	0	-0.92595
tr|B7Z9A1|B7Z9A1_HUMAN	tr|B7Z9A1	cDNA FLJ55274, highly similar to Four and a half LIM domains protein 1 OS=Homo sapiens PE=2 SV=1	38231	1100	0	24	0	0.14	0	0	1	0	1	0	1	0	1	0	-0.92595
tr|F5GX80|F5GX80_HUMAN	ZCCHC8	Zinc finger CCHC domain-containing protein 8 (Fragment) OS=Homo sapiens GN=ZCCHC8 PE=2 SV=1	23636	1103	0	24	0	0.14	0	0	0	0	1	0	1	0	1	0	-0.92595
tr|B2R5M8|B2R5M8_HUMAN	tr|B2R5M8	Isocitrate dehydrogenase [NADP] OS=Homo sapiens PE=2 SV=1	46905	1104	0	24	0	0.07	0	0	0	0	1	0	2	0	3	0	-1.66312
sp|Q5VSL9|STRP1_HUMAN	STRIP1	Striatin-interacting protein 1 OS=Homo sapiens GN=STRIP1 PE=1 SV=1	96256	1105	0	24	0	0.03	0	0	0	0	1	0	1	0	2	0	-0.92595
tr|B4DHC6|B4DHC6_HUMAN	tr|B4DHC6	cDNA FLJ57928, highly similar to Excitatory amino acid transporter 1 OS=Homo sapiens PE=2 SV=1	47096	1106	0	24	0	0.07	0	0	0	0	1	0	1	0	1	0	-0.92595
tr|H7C0D0|H7C0D0_HUMAN	CYP51A1	Lanosterol 14-alpha demethylase (Fragment) OS=Homo sapiens GN=CYP51A1 PE=3 SV=1	25860	1107	0	24	0	0.06	0	0	0	0	1	0	1	0	1	0	-0.92595
tr|I3L2F4|I3L2F4_HUMAN	EDC4	Enhancer of mRNA-decapping protein 4 (Fragment) OS=Homo sapiens GN=EDC4 PE=4 SV=1	14939	1108	0	24	0	0.23	0	0	0	0	1	0	1	0	2	0	-0.92595
sp|Q9H2M9|RBGPR_HUMAN	RAB3GAP2	Rab3 GTPase-activating protein non-catalytic subunit OS=Homo sapiens GN=RAB3GAP2 PE=1 SV=1	157482	1109	0	24	0	0.02	0	0	0	0	1	0	1	0	1	0	-0.92595
sp|O14686|MLL2_HUMAN	MLL2	Histone-lysine N-methyltransferase MLL2 OS=Homo sapiens GN=MLL2 PE=1 SV=2	599575	1111	0	24	0	0.01	0	0	0	0	1	0	1	0	4	0	-0.92595
tr|B2R9G9|B2R9G9_HUMAN	tr|B2R9G9	cDNA, FLJ94385, highly similar to Homo sapiens cofactor required for Sp1 transcriptional activation, subunit 7, 70kDa (CRSP7), mRNA OS=H	65805	1112	0	24	0	0.05	0	0	0	0	1	0	1	0	1	0	-0.92595
tr|B7WPF4|B7WPF4_HUMAN	USP24	Ubiquitin carboxyl-terminal hydrolase OS=Homo sapiens GN=USP24 PE=2 SV=2	280147	1113	0	24	0	0.01	0	0	0	0	1	0	3	0	3	0	-2.14875
tr|J3KTG2|J3KTG2_HUMAN	DHX38	Pre-mRNA-splicing factor ATP-dependent RNA helicase PRP16 (Fragment) OS=Homo sapiens GN=DHX38 PE=4 SV=1	13784	1114	0	23	0	0.25	0	0	0	0	1	0	1	0	1	0	-0.92595
tr|H0Y955|H0Y955_HUMAN	METAP1	Methionine aminopeptidase 1 (Fragment) OS=Homo sapiens GN=METAP1 PE=4 SV=1	19559	1115	0	23	0	0.17	0	0	0	0	1	0	1	0	1	0	-0.92595
tr|A0AUM2|A0AUM2_HUMA	FOLR2	FOLR2 protein (Fragment) OS=Homo sapiens GN=FOLR2 PE=2 SV=1	27870	1116	0	23	0	0.12	0	0	0	0	1	0	1	0	1	0	-0.92595

tr|H0YI90|H0YI90_HUMAN	DDI2	Protein DDI1 homolog 2 (Fragment) OS=Homo sapiens GN=DDI2 PE=4 SV=1	11954	1117	0	23	0	0.29	0	0	0	0	1	0	1	0	1	0	-0.92595
tr|F5H2U1|F5H2U1_HUMAN	CBS	Cystathionine beta-synthase OS=Homo sapiens GN=CBS PE=2 SV=1	51641	1118	0	23	0	0.06	0	0	0	0	1	0	1	0	1	0	-0.92595
tr|B4DUS0|B4DUS0_HUMAN	tr|B4DUS0	cDNA FLJ60898, highly similar to Homo sapiens BRCA2 and CDKN1A interacting protein (BCCIP), transcript variant C, mRNA OS=Homo sapie	33249	1119	0	23	0	0.1	0	0	0	0	1	0	2	0	2	0	-1.66312
sp|Q14669|TRIPC_HUMAN	TRIP12	E3 ubiquitin-protein ligase TRIP12 OS=Homo sapiens GN=TRIP12 PE=1 SV=1	222234	1123	0	23	0	0.01	0	0	0	0	1	0	2	0	2	0	-1.66312
tr|E9PBB1|E9PBB1_HUMAN	CLIP4	CAP-Gly domain-containing linker protein 4 OS=Homo sapiens GN=CLIP4 PE=2 SV=1	11112	1124	0	23	0	0.31	0	0	0	0	1	0	1	0	1	0	-0.92595
tr|H0YAT9|H0YAT9_HUMAN	FGF20	Fibroblast growth factor 20 (Fragment) OS=Homo sapiens GN=FGF20 PE=4 SV=1	12983	1127	0	23	0	0.26	0	0	0	0	1	0	1	0	1	0	-0.92595
tr|B4DJ81|B4DJ81_HUMAN	NDUFS1	NADH-ubiquinone oxidoreductase 75 kDa subunit, mitochondrial OS=Homo sapiens GN=NDUFS1 PE=2 SV=1	67563	1128	0	22	0	0.05	0	0	0	0	1	0	1	0	1	0	-0.92595
tr|K7EIS0|K7EIS0_HUMAN	TUBG1	Tubulin gamma chain (Fragment) OS=Homo sapiens GN=TUBG1 PE=3 SV=1	29797	1129	0	22	0	0.11	0	0	0	0	1	0	1	0	1	0	-0.92595
tr|H3BMZ1|H3BMZ1_HUMAN	PTPLAD1	Very-long-chain (3R)-3-hydroxyacyl-[acyl-carrier protein] dehydratase 3 (Fragment) OS=Homo sapiens GN=PTPLAD1 PE=4 SV=1	16267	1130	0	22	0	0.22	0	0	0	0	1	0	1	0	1	0	-0.92595
tr|G5E933|G5E933_HUMAN	SBF1	Myotubularin-related protein 5 OS=Homo sapiens GN=SBF1 PE=2 SV=1	210422	1131	0	22	0	0.02	0	0	0	0	1	0	1	0	1	0	-0.92595
tr|B2WTI3|B2WTI3_HUMAN	JMJD6	Bifunctional arginine demethylase and lysyl-hydroxylase JMJD6 OS=Homo sapiens GN=JMJD6 PE=2 SV=1	39331	1132	0	22	0	0.08	0	0	0	0	1	0	1	0	1	0	-0.92595
tr|F5H6D0|F5H6D0_HUMAN	MOGS	Mannosyl-oligosaccharide glucosidase OS=Homo sapiens GN=MOGS PE=2 SV=1	80824	1133	0	22	0	0.04	0	0	0	0	1	0	1	0	1	0	-0.92595
tr|A7BI36|A7BI36_HUMAN	RRBP1	p180/ribosome receptor OS=Homo sapiens GN=RRBP1 PE=2 SV=2	166048	1134	0	22	0	0.02	0	0	0	0	1	0	1	0	2	0	-0.92595
sp|Q6IA86|ELP2_HUMAN	ELP2	Elongator complex protein 2 OS=Homo sapiens GN=ELP2 PE=1 SV=2	94266	1135	0	22	0	0.03	0	0	0	0	1	0	1	0	1	0	-0.92595
tr|B7Z6H4|B7Z6H4_HUMAN	tr|B7Z6H4	DNA-directed RNA polymerase OS=Homo sapiens PE=2 SV=1	155407	1138	0	22	0	0.02	0	0	0	0	1	0	2	0	2	0	-1.66312
tr|J3KR91|J3KR91_HUMAN	IQGAP2	Ras GTPase-activating-like protein IQGAP2 OS=Homo sapiens GN=IQGAP2 PE=4 SV=1	123072	1140	0	22	0	0.03	0	0	0	0	1	0	1	0	1	0	-0.92595
tr|H7C2X7|H7C2X7_HUMAN	LSG1	Large subunit GTPase 1 homolog (Fragment) OS=Homo sapiens GN=LSG1 PE=4 SV=1	32482	1141	0	22	0	0.1	0	0	0	0	1	0	1	0	1	0	-0.92595
tr|J3KN33|J3KN33_HUMAN	CNTLN	Centlein OS=Homo sapiens GN=CNTLN PE=4 SV=1	162069	1142	0	22	0	0.02	0	0	0	0	1	0	1	0	2	0	-0.92595
tr|B4DQR2|B4DQR2_HUMAN	tr|B4DQR2	cDNA FLJ57562, highly similar to Cleavage and polyadenylation specificityfactor 73 kDa subunit OS=Homo sapiens PE=2 SV=1	46241	1143	0	22	0	0.07	0	0	0	0	1	0	1	0	1	0	-0.92595
tr|B4E126|B4E126_HUMAN	tr|B4E126	cDNA FLJ61199, highly similar to UBX domain-containing protein 7 OS=Homo sapiens PE=2 SV=1	39093	1144	0	22	0	0.08	0	0	0	0	1	0	1	0	1	0	-0.92595
tr|H3BTN2|H3BTN2_HUMAN	MGA	MAX gene-associated protein (Fragment) OS=Homo sapiens GN=MGA PE=4 SV=1	9116	1145	0	22	0	0.38	0	0	0	0	1	0	1	0	1	0	-0.92595
sp|Q15061|WDR43_HUMAN	WDR43	WD repeat-containing protein 43 OS=Homo sapiens GN=WDR43 PE=1 SV=3	75813	1146	0	22	0	0.04	0	0	0	0	1	0	2	0	3	0	-1.66312
tr|H3BST5|H3BST5_HUMAN	RHOT2	Mitochondrial Rho GTPase 2 (Fragment) OS=Homo sapiens GN=RHOT2 PE=4 SV=2	23300	1147	0	21	0	0.14	0	0	0	0	1	0	1	0	1	0	-0.92595
sp|Q9Y3L5|RAP2C_HUMAN	RAP2C	Ras-related protein Rap-2c OS=Homo sapiens GN=RAP2C PE=1 SV=1	20959	1148	0	21	0	0.16	0	0	0	0	1	0	1	0	1	0	-0.92595
tr|Q75MH1|Q75MH1_HUMAI	RPS26	HCG1745083 OS=Homo sapiens GN=RPS26 PE=2 SV=1	13319	1150	0	21	0	0.26	0	0	0	0	1	0	1	0	1	0	-0.92595
tr|B4DPV3|B4DPV3_HUMAN	tr|B4DPV3	cDNA FLJ58109, highly similar to Protein diaphanous homolog 3 (Fragment) OS=Homo sapiens PE=2 SV=1	117640	1151	0	21	0	0.03	0	0	0	0	1	0	1	0	1	0	-0.92595
tr|K7EP82|K7EP82_HUMAN	RPRD1A	Regulation of nuclear pre-mRNA domain-containing protein 1A (Fragment) OS=Homo sapiens GN=RPRD1A PE=4 SV=1	13852	1152	0	21	0	0.25	0	0	0	0	1	0	1	0	1	0	-0.92595
tr|H0Y4P3|H0Y4P3_HUMAN	VPRBP	Protein VPRBP (Fragment) OS=Homo sapiens GN=VPRBP PE=4 SV=1	121870	1153	0	21	0	0.03	0	0	1	0	1	0	1	0	1	0	-0.92595
tr|F1T0J4|F1T0J4_HUMAN	HMX1	Homeobox protein HMX1 OS=Homo sapiens GN=HMX1 PE=2 SV=1	20170	1154	0	21	0	0.17	0	0	0	0	1	0	1	0	1	0	-0.92595
sp|Q8TBP0|TBC16_HUMAN	TBC1D16	TBC1 domain family member 16 OS=Homo sapiens GN=TBC1D16 PE=2 SV=1	87400	1156	0	21	0	0.04	0	0	0	0	1	0	1	0	1	0	-0.92595
tr|Q2TAM4|Q2TAM4_HUMAI	POLR1B	DNA-directed RNA polymerase OS=Homo sapiens GN=POLR1B PE=2 SV=1	108996	1157	0	21	0	0.03	0	0	0	0	1	0	1	0	1	0	-0.92595
tr|D2CVN5|D2CVN5_HUMAN	AIP	Aryl hydrocarbon receptor-interacting protein AIP722 OS=Homo sapiens GN=AIP PE=4 SV=1	31094	1158	0	21	0	0.09	0	0	0	0	1	0	1	0	1	0	-0.92595
sp|Q9NVV4|PAPD1_HUMAN	MTPAP	Poly(A) RNA polymerase, mitochondrial OS=Homo sapiens GN=MTPAP PE=1 SV=1	66985	1159	0	21	0	0.05	0	0	0	0	1	0	1	0	1	0	-0.92595
tr|D2DGW5|D2DGW5_HUMA	DTNA	Alpha-dystrobrevin (Fragment) OS=Homo sapiens GN=DTNA PE=2 SV=1	28310	1160	0	21	0	0.12	0	0	0	0	1	0	1	0	1	0	-0.92595
tr|Q5XJ20|Q5XJ20_HUMAN	BCR	BCR protein (Fragment) OS=Homo sapiens GN=BCR PE=2 SV=1	6741	1161	0	21	0	0.53	0	0	0	0	1	0	1	0	1	0	-0.92595
tr|E7ESZ7|E7ESZ7_HUMAN	NDUFA10	NADH dehydrogenase [ubiquinone] 1 alpha subcomplex subunit 10, mitochondrial OS=Homo sapiens GN=NDUFA10 PE=2 SV=1	45052	1165	0	20	0	0.07	0	0	0	0	1	0	1	0	1	0	-0.92595
sp|Q8NI36|WDR36_HUMAN	WDR36	WD repeat-containing protein 36 OS=Homo sapiens GN=WDR36 PE=1 SV=1	106282	1166	0	20	0	0.03	0	0	0	0	1	0	2	0	2	0	-1.66312
tr|A4D0W4|A4D0W4_HUMAI	AASS	Aminoadipate-semialdehyde synthase OS=Homo sapiens GN=AASS PE=2 SV=1	102808	1167	0	20	0	0.03	0	0	0	0	1	0	1	0	1	0	-0.92595
tr|A0MNP2|A0MNP2_HUMAI	WDR57	CDW11/WDR57 OS=Homo sapiens GN=WDR57 PE=2 SV=1	39742	1168	0	20	0	0.08	0	0	0	0	1	0	2	0	6	0	-1.66312
tr|Q5JSY8|Q5JSY8_HUMAN	TLE4	Transducin-like enhancer protein 4 (Fragment) OS=Homo sapiens GN=TLE4 PE=4 SV=1	22150	1171	0	20	0	0.15	0	0	0	0	1	0	1	0	2	0	-0.92595
sp|Q13224|NMDE2_HUMAN	GRIN2B	Glutamate receptor ionotropic, NMDA 2B OS=Homo sapiens GN=GRIN2B PE=1 SV=3	167972	1174	0	20	0	0.02	0	0	0	0	1	0	0	0	1	0	0.659215
tr|A6QKW0|A6QKW0_HUMAI	SHINC3	SHINC3 OS=Homo sapiens GN=SHINC3 PE=2 SV=1	28275	1175	0	20	0	0.12	0	0	0	0	1	0	0	0	1	0	0.659215
tr|B3KM97|B3KM97_HUMAN	tr|B3KM97	cDNA FLJ10554 fis, clone NT2RP2002385, highly similar to Synaptic glycoprotein SC2 OS=Homo sapiens PE=2 SV=1	36429	1176	0	20	0	0.09	0	0	0	0	1	0	0	0	2	0	0.659215
tr|B3KNB0|B3KNB0_HUMAN	tr|B3KNB0	cDNA FLJ14159 fis, clone NT2RM2001360, highly similar to Homo sapiens clone C40 unknown mRNA OS=Homo sapiens PE=2 SV=1	36422	1177	0	19	0	0.09	0	0	0	0	1	0	0	0	1	0	0.659215
tr|Q05CP4|Q05CP4_HUMAN	EIF2AK2	EIF2AK2 protein (Fragment) OS=Homo sapiens GN=EIF2AK2 PE=2 SV=1	40940	1178	0	19	0	0.08	0	0	0	0	1	0	0	0	1	0	0.659215
tr|B4DH46|B4DH46_HUMAN	tr|B4DH46	cDNA FLJ60519, highly similar to WD repeat protein 68 OS=Homo sapiens PE=2 SV=1	19542	1179	0	19	0	0.17	0	0	0	0	1	0	0	0	1	0	0.659215
tr|B7ZBM3|B7ZBM3_HUMAN	FOXP4	Forkhead box P4 OS=Homo sapiens GN=FOXP4 PE=4 SV=1	72641	1180	0	19	0	0.05	0	0	0	0	1	0	0	0	1	0	0.659215
sp|Q8NBJ5|GT251_HUMAN	GLT25D1	Procollagen galactosyltransferase 1 OS=Homo sapiens GN=GLT25D1 PE=1 SV=1	71933	1181	0	19	0	0.05	0	0	0	0	1	0	2	0	4	0	-1.66312
tr|H0YJU4|H0YJU4_HUMAN	YY1	Transcriptional repressor protein YY1 (Fragment) OS=Homo sapiens GN=YY1 PE=4 SV=1	13138	1182	0	19	0	0.26	0	0	0	0	1	0	0	0	1	0	0.659215
tr|B4DV74|B4DV74_HUMAN	tr|B4DV74	cDNA FLJ53855, highly similar to Kanadaptin OS=Homo sapiens PE=2 SV=1	56134	1183	0	19	0	0.06	0	0	0	0	1	0	0	0	1	0	0.659215
tr|Q5VSQ6|Q5VSQ6_HUMAN	P4HA1	Procollagen-proline, 2-oxoglutarate 4-dioxygenase (Proline 4-hydroxylase), alpha polypeptide I variant (Fragment) OS=Homo sapiens GN=P4	61214	1184	0	19	0	0.05	0	0	0	0	1	0	0	0	1	0	0.659215
tr|B4E2N1|B4E2N1_HUMAN	tr|B4E2N1	cDNA FLJ56560, highly similar to Caspase recruitment domain-containing protein 6 OS=Homo sapiens PE=2 SV=1	55261	1185	0	18	0	0.06	0	0	1	0	1	0	0	0	1	0	0.659215
tr|H0YDT4|H0YDT4_HUMAN	NDUFS8	NADH dehydrogenase [ubiquinone] iron-sulfur protein 8, mitochondrial (Fragment) OS=Homo sapiens GN=NDUFS8 PE=4 SV=1	10209	1188	0	18	0	0.34	0	0	0	0	1	0	0	0	1	0	0.659215
tr|A1A508|A1A508_HUMAN	PRSS3	PRSS3 protein OS=Homo sapiens GN=PRSS3 PE=2 SV=1	27040	1189	0	18	0	0.12	0	0	0	0	1	0	0	0	1	0	0.659215
tr|Q0VF99|Q0VF99_HUMAN	OSBP2	Oxysterol-binding protein OS=Homo sapiens GN=OSBP2 PE=2 SV=1	102085	1190	0	18	0	0.03	0	0	0	0	1	0	1	0	2	0	-0.92595
tr|B2RAR3|B2RAR3_HUMAN	tr|B2RAR3	Queuine tRNA-ribosyltransferase OS=Homo sapiens PE=2 SV=1	44645	1191	0	18	0	0.07	0	0	0	0	1	0	0	0	1	0	0.659215
sp|Q7Z4Q2|HEAT3_HUMAN	HEATR3	HEAT repeat-containing protein 3 OS=Homo sapiens GN=HEATR3 PE=1 SV=2	75789	1192	0	18	0	0.04	0	0	0	0	1	0	0	0	1	0	0.659215
tr|B4DJU4|B4DJU4_HUMAN	tr|B4DJU4	cDNA FLJ53344, highly similar to Splicing factor 1 OS=Homo sapiens PE=2 SV=1	67535	1193	0	18	0	0.05	0	0	0	0	1	0	0	0	2	0	0.659215
sp|Q6ZMV8|ZN730_HUMAN	ZNF730	Putative zinc finger protein 730 OS=Homo sapiens GN=ZNF730 PE=2 SV=1	60769	1194	0	18	0	0.06	0	0	0	0	1	0	0	0	1	0	0.659215
tr|K7EQQ8|K7EQQ8_HUMAN	TRMT1	tRNA (guanine(26)-N(2))-dimethyltransferase (Fragment) OS=Homo sapiens GN=TRMT1 PE=4 SV=1	32946	1197	0	17	0	0.1	0	0	0	0	1	0	1	0	3	0	-0.92595
tr|F4MHH8|F4MHH8_HUMAI	UTY	Ubiquitously transcribed tetratricopeptide repeat protein Y-linked transcript variant 187 OS=Homo sapiens GN=UTY PE=2 SV=1	121442	1198	0	17	0	0.03	0	0	0	0	1	0	0	0	1	0	0.659215
tr|H0YAQ8|H0YAQ8_HUMAN	PPIL6	Peptidyl-prolyl cis-trans isomerase-like 6 (Fragment) OS=Homo sapiens GN=PPIL6 PE=3 SV=1	19590	1199	0	17	0	0.17	0	0	0	0	1	0	0	0	1	0	0.659215
sp|Q6UWZ7|F175A_HUMAN	FAM175A	BRCA1-A complex subunit Abraxas OS=Homo sapiens GN=FAM175A PE=1 SV=2	47033	1201	0	17	0	0.07	0	0	0	0	1	0	0	0	1	0	0.659215
tr|H0UI08|H0UI08_HUMAN	IMPG1	Interphotoreceptor matrix proteoglycan 1, isoform CRA_a OS=Homo sapiens GN=IMPG1 PE=4 SV=1	64359	1202	0	16	0	0.05	0	0	0	0	1	0	0	0	1	0	0.659215
tr|A4UCS6|A4UCS6_HUMAN	tr|A4UCS6	Peroxiredoxin 6 (Fragment) OS=Homo sapiens PE=2 SV=1	9011	1204	0	16	0	0.39	0	0	0	0	1	0	0	0	1	0	0.659215
sp|Q5C9Z4|NOM1_HUMAN	NOM1	Nucleolar MIF4G domain-containing protein 1 OS=Homo sapiens GN=NOM1 PE=1 SV=1	96768	1206	0	16	0	0.03	0	0	0	0	1	0	0	0	1	0	0.659215
tr|B4DJQ5|B4DJQ5_HUMAN	tr|B4DJQ5	cDNA FLJ59211, highly similar to Glucosidase 2 subunit beta OS=Homo sapiens PE=2 SV=1	61066	1207	0	15	0	0.05	0	0	0	0	1	0	0	0	1	0	0.659215
